# Metabolic Consequences of TGFβ Stimulation in Cultured Primary Mouse Hepatocytes Screened from Transcript Data with ModeScore 

**DOI:** 10.3390/metabo2040983

**Published:** 2012-11-21

**Authors:** Andreas Hoppe, Iryna Ilkavets, Steven Dooley, Hermann-Georg Holzhütter

**Affiliations:** 1 Institute for Biochemistry, Charité University Medicine Berlin, Charitéplatz 1/Virchowweg 6, 10117 Berlin, Germany; Email: hergo@charite.de (H.-G.H.); 2 Molecular Hepatology and Alcohol Associated Diseases, Medical Clinic II, Medical Faculty Mannheim at Heidelberg University, Theodor-Kutzer-Ufer 1-3, H42 E4, 68167 Mannheim, Germany; Email: iryna.ilkavets@medma.uni-heidelberg.de (I.I.); steven.dooley@medma.uni-heidelberg.de (S.D.)

**Keywords:** hepatocyte, transforming growth factor beta, transcriptomics, metabolic network, VirtualLiver Network

## Abstract

TGFβ signaling plays a major role in the reorganization of liver tissue upon injury and is an important driver of chronic liver disease. This is achieved by a deep impact on a cohort of cellular functions. To comprehensively assess the full range of affected *metabolic* functions, transcript changes of cultured mouse hepatocytes were analyzed with a novel method (ModeScore), which predicts the activity of metabolic functions by scoring transcript expression changes with 987 reference flux distributions, which yielded the following hypotheses. TGFβ multiplies down-regulation of most metabolic functions occurring in culture stressed controls. This is especially pronounced for tyrosine degradation, urea synthesis, glucuronization capacity, and cholesterol synthesis. Ethanol degradation and creatine synthesis are down-regulated only in TGFβ treated hepatocytes, but not in the control. Among the few TGFβ dependently up-regulated functions, synthesis of various collagens is most pronounced. Further interesting findings include: down-regulation of glucose export is postponed by TGFβ, TGFβ up-regulates the synthesis capacity of ketone bodies only as an early response, TGFβ suppresses the strong up-regulation of Vanin, and TGFβ induces re-formation of ceramides and sphingomyelin.

## 1. Introduction

TGFβ signaling is central in the late stages of liver regeneration [1]. Increased levels of TGFβ are an intermediate driver of chronic liver diseases [2] and represent a critical positive feedback loop in alcoholic liver disease [3]. Although besides hepatocytes also Kupffer cells and stellate cells are affected by TGFβ, we here have enfolded its role towards hepatocytes, the dominant cell type of the liver. We found that hepatocytes subjected to elevated TGFβ levels undergo substantial changes including its metabolic functions [1]. 

Primary isolates of hepatocytes can be very reliably and reproducibly cultured on a collagen layer [4,5]. In particular, the metabolism of these hepatocytes resembles the *in vivo* situation better than immortalized (*i.e*., cancer) cells [6]. Freshly isolated hepatocytes suffer from an immediate loss of function due to culture stress, which can partly be restored by a calf embryo medium and attachment to the collagen layer. Still, the metabolism of mouse hepatocytes in culture differs quantitatively and also qualitative aspects from hepatocytes *in vivo* [7,8], and the cytokine TGFβ is involved in this process [9]. Hepatocytes in culture are in a non-steady state, which is characterized by permanent functional changes, especially loss of metabolic functions, and the purpose of this study was to identify if and how the effects of TGFβ on hepatocytes in culture account for such outcome. 

Therefore, a set of transcript profiles of primary mouse hepatocytes (3 time points, 1 h, 6 h, and 24 h, control *versus* TGFβ stimulation, 3 repeats, which have been analyzed before [9,10,11]) was screened for remarkable alterations of metabolic function. Various approaches have been proposed to relate RNA expression levels to metabolic function [12]. As the majority of metabolic enzymes remains active in cultured hepatocytes (although at a reduced level), the most widely-used on/off model to relate transcripts to metabolic networks [13,14] is not suitable here. Therefore, gene changes are analyzed without an *a priori* threshold (amplitude or significance level). Gene changes with a lesser amplitude may be ignored (if the metabolic function scores low in the rankings), but may also become integrated if complementary genes with respect to a particular function occur with a higher amplitude. In this study, ModeScore [15] was applied for a plethora of metabolic functions—a novel approach, which relates RNA differences to functional flux distributions [16] computed in the stoichiometric network of hepatocyte metabolism [17]. 

## 2. Results

### 2.1. General Observations

**Figure 1:**
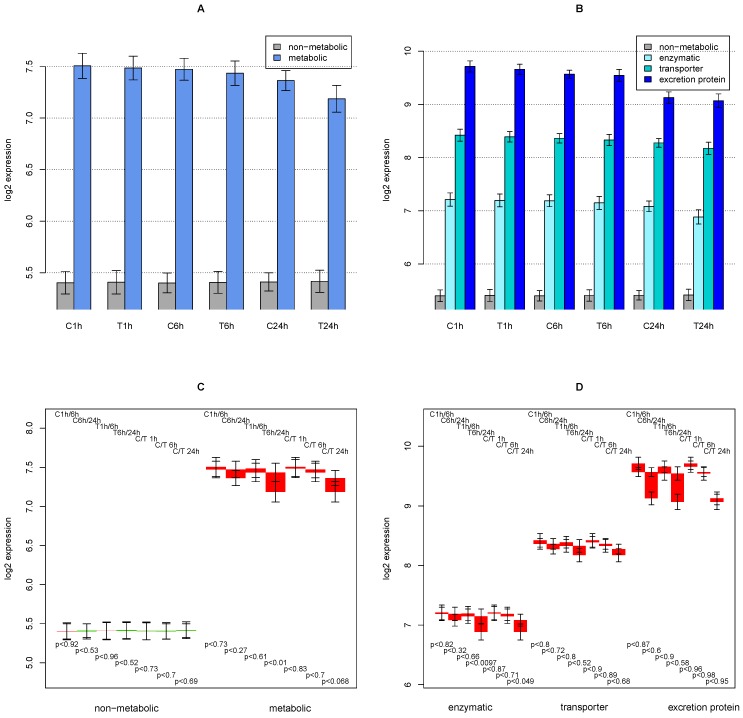
**A** Average expression values of metabolic genes (upon mapping to HepatoNet1) *vs.* all other genes, by expression profile (C = control, T = TGFβ treated). In **B**, the set of metabolic genes is split into genes encoding enzymes, transporters, and selected excretion proteins. Error bars indicate average standard deviation of the 3 repeat experiments. **C** and **D** show differences of average expressions. Red bars indicate down-regulation and green bars indicate up-regulation. Either 2 time points in the control experiment are compared (e.g., C1 h/24 h) or the same time point of control *vs.* treated sample (e.g., C/T 24 h). P-values refer to the probability that the averages of the two series are equal, determined with the Welch’s t test [18] and the average RNA abundance values. The error bars refer to the average of standard deviation of the repeats, not the standard deviation of the gene abundances (which would be huge in this diagram).

The average expression of genes associated to metabolic functions shows a 4-fold (2 on log_2_ scale) higher expression as compared with the rest of the genes (see Figure 1A). The difference between the average expression of metabolic *versus* non-metabolic genes is smaller for later time points and for TGFβ treated examples. 

When comparing transcript profiles, two types are evaluated—changes in the time course and changes induced by TGFβ treatment. For changes with time, differences between transcript abundances from 1 h to 24 h, 1 h to 6 h, and 6 h to 24 h in the control experiment (**C1 h/24 h**, **C1h/6 h**, and **C6h/24 h**) and the TGFβ treated hepatocytes (**T1h/24 h**, **T1h/6 h**, and **T6 h/24 h**) are considered. For treatment-induced changes, the differences between abundances of the control and TGFβ treated hepatocytes at 6 h and 24 h (**C/T 6 h** and **C/T 24 h**) are evaluated. The difference at 1 h is negligible and not considered. 

In Figure 1C, a difference analysis of the average expression is presented. A Welch’s t-test [18] was performed to assert whether the averages differ significantly. For the non-metabolic genes, there is no significant difference. For the metabolic genes, the averages of T6 h/24 h and C/T 24 h comparisons are considerably different with high significance, whereas the averages of the C6 h/24 h comparison are of low significant differences. 

Figure 1B presents a finer distinction in classes of genes associated to HepatoNet1. Excretion proteins such as albumin, haptoglobin, and collagens display the highest expression (14-fold higher than the non-metabolic genes—4.3 in log_2_ scale), which decreases with time but is not affected by TGFβ treatment. Transporters show the 2^nd ^highest expression also decreasing with time and further decreasing upon TGFβ treatment. Expression of enzymes is at a lower level, but still more than threefold (1.8 in log_2_ scale) higher than the rest of the genes. TGFβ treatment decreases the average expression of this group of genes at 24 h stronger than transporters or excretion proteins. 

The difference analysis in Figure 1D shows that considerable deviations only occur at the 24 h time point—longer bars occur at C6 h/24 h, T6h/24 h, and C/T 24 h. Down-regulation of the average for excretion proteins is larger than for enzymes, and is quite small for transporters. Interestingly, TGFβ treatment seems to play a minor role for excretion proteins, while for enzymes (and for the transporters at a lower degree) the difference induced by TGFβ treatment (C/T 24 h) is larger than for comparison of time points (e.g., C6 h/24 h). The significance of these average differences is low for excretion proteins (due to their low number and their large deviations), low for transport proteins (due to the small difference), and is high only for enzymes (T6 h/24 h and C/T 24 h comparisons). 

### 2.2. ModeScore Analysis

Metabolism is represented by a modified version of HepatoNet1 [17] (see Section 4.2 and Supplementary file 2) and 987 reference functions (see Section 4.3 and Supplementary file 3) for which flux distributions have been computed using FASIMU [19], see Supplementary file 4. In the ModeScore method ([15], see also Section 4.5) a regulation amplitude for each reference flux distribution and each pair of transcript profiles is computed. This value is compatible to the change in log_2_ abundances, *viz.* if only one gene is assigned to the flux distribution, then the score is equal to the difference of both log_2_ abundances. These scores are shown in Supplementary file 5. Additionally, for each gene assigned to the flux distribution, a contribution score is computed regarding how much the gene reflects the evaluation of the flux distribution. A score of 1 is computed for those genes whose difference in abundances is equal to the flux distribution amplitude. A score of 0 is given to a difference far from the flux distribution score. For more details, see the methods section. These component scores are shown in the Supplementary file 6 in the column “Score”. 

For each pair of transcript profiles, the functions with the highest up-regulation or down-regulation are inspected, and those functional units with the most remarkable pattern were selected—see Supplemen­tary file 1 for a detailed account of this process. For a functional unit, the relevant genes with a consistent pattern (see Supplementary file 6 for all genes) have been selected for the functional interpretation as follows. 

### 2.3. Tyrosine Degradation

**Figure 2:**
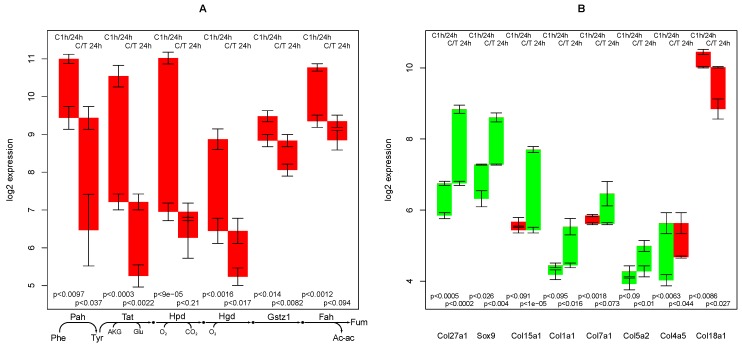
(**A**) Regulation of the degradation cascade of phenylalanine and tyrosine; (**B**) Regulation of selected collagens and a promoter. Red bars indicate down-regulation and green bars indicate up-regulation. Either 2 time points in the control experiment (e.g., C1h/24 h) or the same time point of control *vs.* TGFβ treated sample (e.g., C/T 24 h) are compared. Error bars indicate average standard deviation of 3 independent experiments. P-values refer to the probability that there is equal expression of two respective probe values, as determined with the Welch’s t test [18] in 3 independent experiments.

Three hepatic functions are closely connected—degradation of tyrosine, conversion of phenylala­nine to tyrosine (consisting of a single intracellular reaction), and degradation of phenylalanine (a combination of the other two)—and thus are treated in combination. These functions are among the most down-regulated functions with time (comparing 24 h and 1 h in both TGFβ treated and untreated cells) and among those with the highest down-regulating effect of TGFβ. See Supplementary file 1 for more details, in particular Section 2.4 and Table 1 for the ranking of selected functions, furthermore Supplementary file 5 for the full ranked lists. The specific part consists of 6 enzymes—the reaction chain from phenylalanine to acetoacetate and fumarate, see Figure 2A and Supplementary file 6. 

Degradation of tyrosine is among the most critical liver functions for the organism. Liver damage accompanied by a deranged tyrosine degradation capacity may lead to accumulation of false neurotransmitters, a main factor for hepatic encephalopathy [20]. The particularly intensive drop in the expression of RNAs encoding for enzymes of this pathway documents the loss of hepatic functions during hepatocyte culture. 

Note that although the length of the bars in Figure 2A (resp. the fold-change of the mRNA) is largely different, a clear common pattern can be recognized. Some of the gene changes (e.g., Hpd, C/T 24 h) would likely be excluded by the often applied thresholds (less than 2-fold, p-value 0.21) but it cannot be denied that this gene’s change follows the identified pattern. Retaining also the lesser changed genes is in accordance with the finding that the amount of RNA change is not well correlated with flux changes [12] and that the typical range for relevant RNA changes differs considerably for different genes [21]. 

To sum it up, based on the analyzed expression profiles, it can be hypothesized that (i) hepatocytes in culture lose the ability to degrade tyrosine; (ii) TGFβ increases this effect; and (iii) genes associated with phenylalanine/tyrosine are commonly regulated (e.g., by the same transcription factor). 

### 2.4. Collagen Regulation

Macroscopically, cultured hepatocytes undergo a dedifferentiation, which is accompanied by an in­crease of fibers. Thus, the regulation of collagen proteins was analyzed. In the ModeScore analysis, the most remarkable regulation is observed for the following collagens (see Figure 2B): 

Collagens XXVII_α_1__ (CORA1 in Supplementary file 5) and XV_α_1__ (COFA1) show the strongest up-regulating effect of TGFβ (top 2 scorers in the treatment/control comparison at 24 h, Table 1 in Supplementary file 5). Thus, a specific accumulation of these collagens in the TGFβ treated culture can be expected. In fetal liver tissue, a high concentration of RNA encoding collagen XXVII_α_1__ and a low concentration of the corresponding protein was found [22], indicating that export is possible. SOX9 is an activator of the collagen XXVII_α_1__ gene [23] and indeed, the respective gene as well is up-regulated (see Figure 2B). Other collagens with a high up-regulation upon TGFβ are collagen I_α_1__, VII_α_1__, and V_α_2__(see Figure 1 in Supplementary file 1 and Table 1 in Supplementary file 5). Interestingly, collagen XXVII_α_1__ is also considerably up-regulated in the control experiment, while the other collagens mentioned above are down-regulated (VII_α_1__ and XV_α_1__ ) or only slightly up-regulated (I_α_1__ and V_α_2__) in this setting. 

Collagen IV_α_5__ (CO4A5) is highly up-regulated in the control experiment (most of all collagens, 4th of all functions) but much less upon TGFβ treatment. Thus, TGFβ suppresses the up-regulation of this protein. 

Collagen XVIII_α_1__ represents the strongest down-regulated collagen in the TGFβ treated sample, whereas there is only a mild down-regulation for this gene in the control sample—it is among the top 10% of down-regulated functions in C/T 24 h (rank 66 of 987, in Table 1 in Supplementary file 5). Functionally, this collagen is an endostatin precursor [24] and cannot be regarded as a fibrogenic collagen in hepatocytes. Its expression is liver-specific [25] and it plays a negative regulatory role in angionesis during liver regeneration [26,27]. Its down-regulation is in agreement with the general loss of liver-specific functions. 

As these collagens are exceptionally rich in proline, we analyzed whether the proline synthesis/transamination pathway would also be up-regulated, but found that it is relatively constant (see Supplementary file 1). 

### 2.5. Ethanol Degradation

**Figure 3:**
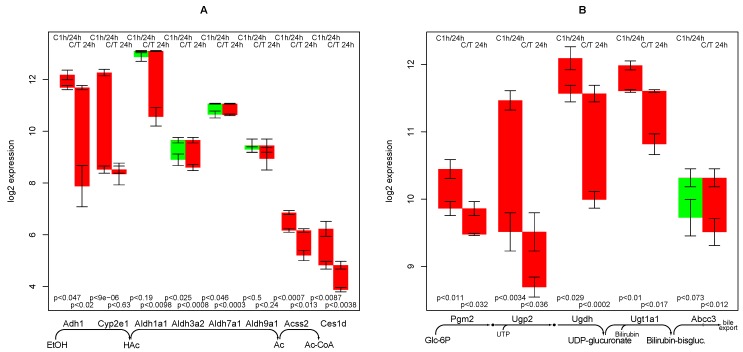
Regulation of genes involved in ethanol degradation (**A**); and bilirubin conjugation (**B**). Among the genes encoding alcohol and aldehyde dehydrogenase only those are selected that show a sufficient expression in hepatocytes and activity on ethanol/ethanal.

All relevant genes involved in the main degradation pathway of alcohol are relatively constant in the control experiment but strongly down-regulated by TGFβ, see Figure 3A. Cuiclan *et al.* [11] confirmed that TGFβ induces down-regulation of Adh1 (encoding alcohol dehydrogenase) on RNA and protein level. The 4 most relevant forms of aldehyde dehydrogenase are even (slightly) up-regulated in the control experiment but down-regulated upon TGFβ treatment, Aldh1a1 especially strong. The final reaction, acetyl-CoA synthase, is down-regulated in the control experiment but even more so upon TGFβ treatment. 

Interestingly, the microsomal ethanol degradation pathway (indicated by the Cyp2e1 gene) is also strongly down-regulated in the control culture, independently of TGFβ. The pathological ethanol esteration in the absence of the enzymes for proper degradation (by fatty acid ethyl ester synthase, gene Ces1d) has a low expression, is down-regulated in time and further down-regulated by TGFβ. This confirms an assumption that alcohol and TGFβ are factors in a positive feedback loop [3,28]. 

### 2.6. Bilirubin Conjugation

The specific reactions of bilirubin conjugation are UDP glucuronosyltransferase 1 (Ugt1, several isoforms) and a specific transporter of conjugated bilirubin (Abcc3), the first of which shows a considerable down-regulation in TGFβ treated cells, see Figure 3B. 

Reactions steps with less specificity for bilirubin, but nevertheless involved in functions supplying UDP-glucuronate are UDP-glucose dehydrogenase (Ugdh), Phosphoglucomutase (Pgm2), and UDP-glucose pyrophosphorylase (Ugp2), all of which show a stronger down-regulation, especially in TGFβ treated hepatocytes. 

It can be summarized that there is a loss of glucuronization capacity, which is enhanced by TGFβ treatment. The loss affects bilirubin conjugation but is not particularly specific to bilirubin. 

In pig liver, inhibition of TGFβ did not display a strong impact on bilirubin levels as compared with other liver function markers (Figure 2 in [1]). This finding is in concordance with the lack of a short term response in the bilirubin conjugation here. 

### 2.7. Urea Synthesis

**Figure 4:**
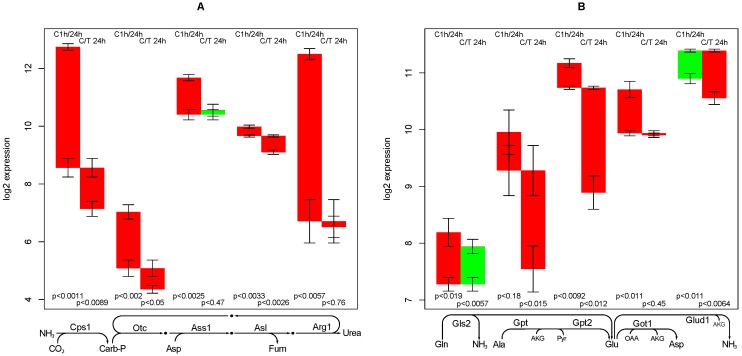
Regulation of genes involved in urea synthesis. Panel (**A**) shows genes essential for urea synthesis, whereas panel (**B**) shows genes used for the supply of precursors.

Urea can be synthesized from a range of nitrogen-containing metabolites, however, the final reaction steps are common—urea cycle and carbamoyl synthase—Cps1, Otc, Ass1, Asl, Arg1 (see Figure 4A). A set of facultative reactions are often used to supply the necessary precursors (aspartate, ammonium, and carbon dioxide) from typical substrates as alanine, glutamine, glutamate, and pyruvate: Got1, Gpt/Gpt2, Glud1, and Gls2, see Figure 4B. 

Arginase is one of the most down-regulated genes in the dataset. Apparently, it is switched off from a highly abundant state and TGFβ treatment makes no difference. Ornithine transcarbamylase (Otc) is down-regulated at a moderate quantity, and a further down-regulation is achieved upon TGFβ treatment. Argininosuccinate synthase (Ass1) is moderately down-regulated but constant in TGFβ treated hepatocytes. Argininosuccinate lyase (Asl) is down-regulated by a considerable amount only in TGFβ treated hepatocytes. Carbamoyl synthetase (Cps1) is sharply down-regulated, even more so in the TGFβ treated group. Apparently, the urea cycle is not regulated in a synchronized manner. It can be predicted that hepatocytes in culture lose most of their capacity to synthesize urea, and TGFβ leads to an additional down-regulation. The amount of this effect is less clear, and an estimation would depend on the information which enzymes are rate-limiting in the pathway. 

Among the facultative reactions, both forms of alanine aminotransferase (Gpt and Gpt2) are possibly commonly regulated. A strong decline can be found only in TGFβ treated hepatocytes. Glutamate oxaloacetate transaminase (Got) has only a slight down-regulation with time. Since these genes are also involved in many other functions, it is apparent that their regulation is very likely decoupled from the function of urea synthesis. Interestingly, reactions providing NH3 are oppositely regulated—in the control experiment, Gls2 (providing NH_3_ from glutamine) is up-regulated and Glud1 (providing NH3 from glutamate) is down-regulated, while upon TGFβ treatment, Gls2 is constant and Glud1 is down-regulated. 

### 2.8. Cholesterol Synthesis

**Figure 5:**
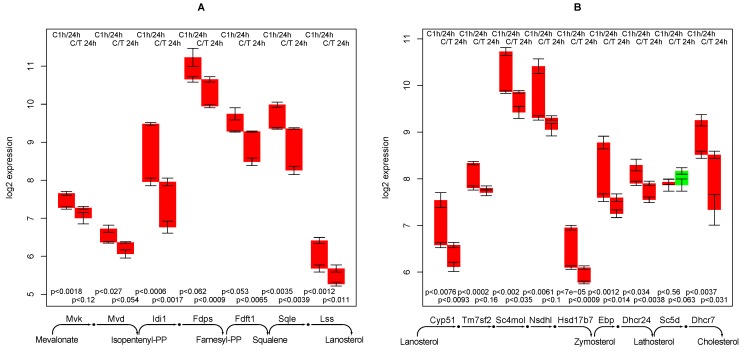
Regulation of genes involved in cholesterol synthesis.

The reaction steps from mevalonate to farnesyl-diphosphate are down-regulated with time, and further down-regulated by TGFβ treatment, see Figure 5A. The 12 reaction steps from Farnesyl-diphosphate show a similar pattern (see Figure 5A and B), which is a considerable down-regulation with time and further down-regulation by TGFβ treatment, with a single exception—lathosterol oxidase (Sc5d) is up-regulated by TGFβ. However, this multi-specific enzyme (alternative substrates are for example δ_7_-avenasterol and episterol) is also involved in other relevant functions. 

Conclusively, cholesterol synthesis can be predicted as down-regulated in the control experiment and even more down-regulated in TGFβ treated hepatocytes. This is not surprising as cholesterol synthesis (for bile acids and lipoprotein particles export) is a typical liver function. 

### 2.9. Glucose Release from Glycogen

**Figure 6:**
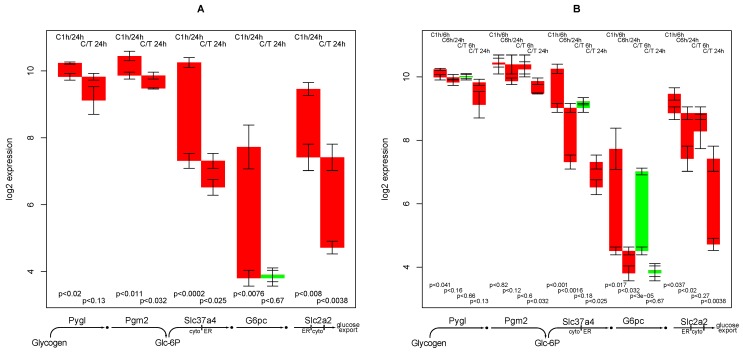
Regulation of genes involved in glucose release from glycogen.

The reactions involved in the hepatic release of glucose from glycogen storage can be grouped in two—split of activated glucose from the glycogen polysaccharide structure and dephosphorylation and export of glucose. As can be seen from Figure 6A, there is only a slight down-regulation of the first group of genes (Pygl, Pgm2) while the second group (Slc37a4, G6pc, Slc2a2) is sharply down-regulated with time, in particular in TGFβ treated hepatocytes. This is agreement with macroscopic observations— degradation of the cell’s glycogen storage is a universal function of human cells while the actual export of glucose is specific for hepatocytes. In particular, dephosphorylation of glucose-6-phosphate (G6pc) (a reaction only needed for glucose export) switches from a clearly *on* to an *off* status. 

Glucose-6-phosphatase shows an early down-regulation in the control experiment (from 1 h to 6 h), while in TGFβ treated sample, down-regulation occurs later, *i.e*., in the interval between 6 h and 24 h (Figure 6B). From this result, it can be hypothesized that loss of glucose export capability is delayed in the TGFβ treated hepatocytes. 

### 2.10. Supply of β-hydroxybutyrate

**Figure 7:**
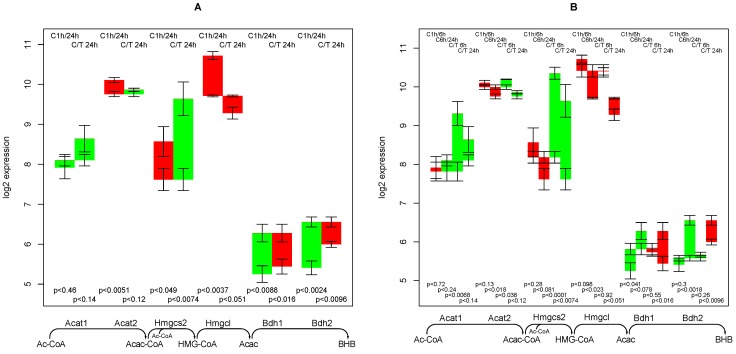
Regulation of genes involved in synthesis of β-hydroxybutyrate. Panel A shows comparisons of the 1 h and 24 h time points only, panel B shows comparisons involving the 6 h time point.

Genes involved in the β-hydroxybutyrate synthesis pathway of show an inconclusive regulation when comparing 1 h to 24 h. While mitochondrial HMG-CoA synthase (Hmgcs2) is down-regulated in the control experiments and up-regulated upon TGFβ treatment, both types of 3-hydroxybutyrate dehy­drogenase (Bdh1/Bdh2) are up-regulated in the control experiment and unchanged in TGFβ treated hepatocytes. Intriguingly, the two genes Acat1 and Hmgcs2 show a rare pattern in TGFβ treated sample, that is they are up-regulated at the 6 h time point and then down-regulated again (see Figure 7B). Thus, TGFβ apparently induces an early response of increasing the production of ketone bodies. 

In a rat proximal tubule cell line β-hydroxybutyrate induced TGFβ expression [29]. Thus, a positive feedback of the two parameters can be hypothesized. TGFβ may mediate elevation of ketone bodies by exercise, as TGFβ application to murine brains mobilizes ketone bodies [30], while inhibition of TGFβ by an antibody [31] reduces it. 

### 2.11. Creatine Synthesis

**Figure 8:**
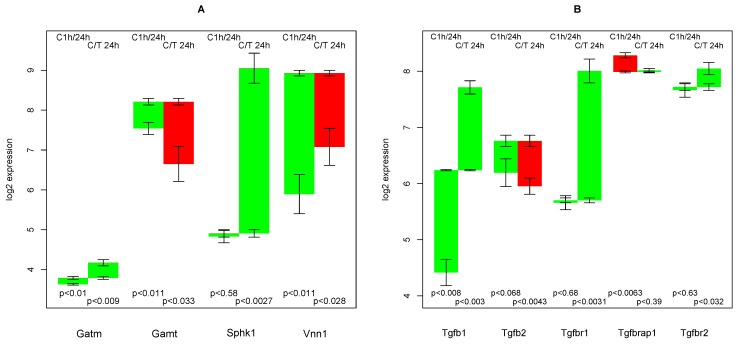
(**A**) Regulation of creatine synthesis and of some solitude genes; (**B**) Regulation of selected genes encoding TGFβ, its receptors and related proteins.

Guanidinoacetate methyltransferase (Gamt), the final step in creatine synthesis, shows the rare pattern, up-regulation in untreated hepatocytes and down-regulation in TGFβ treated hepatocytes (see Figure 8A, second group of bars). The respective transcript changes are moderate (+0.67,−1.56), but statistically highly significant (p < 0.011, p < 0.033). This fact is especially remarkable, as there is endogenous TGFβ production (see Section 2.13) in untreated cultured hepatocytes. Apparently, there is a threshold switch, which suppresses the effect of moderate TGFβ levels to Gamt. 

The penultimate step, glycine amidinotransferase (Gatm), shows only a slight up-regulation in both experiments (see Figure 8A, first group of bars). The absolute expression is also low, but as creatine is known to be exported by hepatocytes and glycine amidinotransferase is essential for this function, we assume that sufficient RNA is present in the cell to synthesize glycine amidinotransferase. Apparently, this seems to be the case, where low affinity of the chip’s probes lead to a low luminescence. The low deviation in the three repeats supports this assumption. 

It can be concluded that creatine synthesis is sensitive to TGFβ. Creatine supplementation prevents NAFLD in rat [32]. Thus, down-regulation of Gamt by TGFβ and a decrease in internal creatine production will possibly increase fat accumulation in hepatocytes. 

This result is in concordance with the finding in pig livers (Figure 2 in [1]), in which there is a strong creatine excretion into the serum of control livers (with elevated TGFβ levels) that does not occur when TGFβ is inhibited. 

As ornithine is a byproduct of Gatm, creatine synthesis is coupled to reactions of the urea cycle (see Supplementary file 6 for these reactions and Section 2.7). As urea synthesis is the function with a much higher reaction flux in hepatocytes, it is not feasible to associate transcript changes of enzymes such as arginase to creatine synthesis. 

### 2.12. Solitude Genes with a Remarkable Pattern

In this subsection, genes with a remarkable regulation not accompanied by a similar pattern of other genes belonging to the same functional context are considered. 

Sphingosine kinase (Sphk1) is constant in the control group and heavily up-regulated in TGFβ treated sample, see Figure 8A, third group of bars. As the genes encoding enzymes adjacent in the network are not regulated in this way, the only conclusive explanation is that the re-formation of phospholipids that depend on sphingosine kinase, such as sphingomyelin and the various ceramides, is enhanced by TGFβ. 

It has been found that Vanin (Vnn1), besides the enzymatic function as pantetheinases, plays a role in inflammation, oxidative stress, cell migration, and numerous diseases [33]. Vanin is strongly up-regulated in the control culture and the effect is attenuated by TGFβ. 

### 2.13. Endogenous TGFβ Production

It has been noted that hepatocytes in monolayer culture start to produce TGFβ [10]. Indeed, the RNA encoding TGFβ_1_ increases threefold in the control experiment, see Figure 8B. Thus, even in the control TGFβ is not completely absent. Interestingly, in TGFβ treated group, there is an even larger increase in endogenous TGFβ production, which additionally accelerates the effects sensitive to TGFβ. Furthermore, the expression of the main TGFβ receptor Tgfbr1, while constantly low in the control experiment, increases dramatically in TGFβ treated hepatocytes. Thus, in TGFβ treated hepatocytes, an amplified effect of TGFβ is observed. The other TGFβ form, TGFβ_2_, the alternative receptor Tgfbr2, and the associated protein Tgfbrap1 are relatively constant throughout both culture conditions. 

## 3. Discussion

As the metabolism of primary hepatocytes changes during culture even without the addition of TGFβ, the question arises as for which conclusions can be drawn from TGFβ treated cultures for hepatocytes in vivo. It must be assumed that changes induced by TGFβ are independent from the changes induced by culture stress, at least regarding metabolic liver functions. Endogenous production of TGFβ in the control culture is taken into account in Section 2.13. Consequently, the main problem is to distinguish whether the TGFβ treatment just leads to an acceleration of processes that would have occurred later in the control or whether there is a specific regulation that would not have occurred. To account for this, the findings in the results section have been related to studies of metabolic functions in hepatocytes and also other cell types. 

Although fetal calf serum is provided for 3 hours directly after extraction from mouse liver to facilitate the survival from extraction stress, in the presented course of the experiment a serum-free medium is used [4]. Apparently, this condition differs from the *in vivo* state where the hepatocyte is confronted with a constantly changing metabolic load. It is commonly accepted that each metabolic challenge of hepatocytes *in vivo* stimulates a response of transcription, while a lack of new responses leads to a gradual loss of enzymatic capacity by protein degradation [34]. Thus, a reduction in the overall metabolic capacity can be expected, which is reflected in the general down-regulation of all enzymes by average, see Figure 1. Consequently, the interesting cases are those that deviate from the trivial pattern, either by a stronger than normal down-regulation, by an up-regulation, or by a specific effect of TGFβ. 

Would it have been possible to obtain the same results with alternative approaches that relate transcript data to metabolic functions? Often, metabolic functions are represented by metabolic subsystems defined for instance by GO terms [35] or KEGG maps [36], e.g., tyrosine degradation is represented in the KEGG map 00350. But this map also includes synthesis of thyridoxine, tyramine, hydroxyphenylacetate, dopaquinone, eumelanine, adrenaline, metanephrine, 3-methoxy-4-hydroxy­mandelate, 3-methoxy-4-hydroxy-phenylethylene-glycol, and homovanillate (only genes present in mice are considered). Most of these synthesis pathways are specific to other cell types and are switched *off* in hepatocytes. Thus, tyrosine degradation is represented by only 5 out of 37 genes related to this KEGG map. So the down-regulation of genes involved in tyrosine degradation would not be particularly remarkable considering the fact that many metabolic genes are down-regulated (see Section 2.1). 

The system of KEGG modules [37] represents small functional pathways. The module M00044 for example would indeed represent the 5 genes for tyrosine degradation. However, the set of KEGG modules is a fixed and restricted set of pathways and does not cover comprehensively all relevant metabolic functions, considering that the 260 modules cover pathways in cells in all kingdoms of life. For instance, hepatic ethanol degradation is not such a module. 

Both classification schemes would ignore any transporters (which is for instance very relevant for glucose release), supply of initial substrates, and disposal of side products (see for instance the urea synthesis, several reactions are apparently coupled to the urea cycle although belonging to amino acid metabolism in the first place). 

Gene ontology annotations are available from various sources. For instance, the gene ontology term *tyrosine catabolic process* GO:0006572 would have recovered the five genes using the annotation of the Brainarray chip definition file. For this pathway, the GO term *tyrosine catabolic process to fumarate* (GO:0019445) would even be more appropriate, but this annotation is not present in the data annotation deposited at Ensemble/BioMART. For several appropriate GO terms of ethanol detoxification, no genes are annotated to the enzymes involved (alcohol dehydrogenase and aldehyde dehydrogenase) as they are multi-specific. The GO annotation provides only a set of genes connected to the function, whereas a specific role is not asserted but would be given by the flux distribution annotated with genes. 

Often, to assess the regulation of such a subsystem, the number of gene changes above a certain threshold (usually 2-fold) is counted. Looking at Figure 2A, there is a convincing and remarkably complete pattern of down-regulation. However, in fact, 4 of the bars are just below 2-fold, thus, only 8 from 12 comparisons would be considered as down-regulated, obscuring the pattern that is obvious when looking at the data directly. ModeScore, however, considers the data continuously. 

Another alternative approach to screen for remarkable regulations would be to rank the genes by their transcript change. In this way, the huge drop in arginase (see Section 2.7) is found as the top down-regulated gene, and homogentisate 1,2-dioxygenase from tyrosine degradation would also be considered. However, this approach is biased towards single genes with large transcript changes and quite consistent regulations with a lesser magnitude such as in the cholesterol synthesis would be disregarded. 

An additional approach would be the computation of gene correlations and subsequent restriction of metabolic genes. It can be expected that many genes have a higher correlation with each other than the 6 genes in phenylalanine degradation, as the fold change ranges from 16-fold to 1.6-fold. The correlation coefficient would be rather low and probably obscured by the noise. The convincing regulation pattern in Figure 2A does not come from an extremely high correlation but *from a combination of the position in a pathway and the correlation.*

Elementary flux modes would be an alternative to the reference solutions computed by FBA. As the set of all elementary flux modes is too large, the set of the k-shortest should be used instead [38]. The elementary flux modes computed from the whole network would be considerably larger than the functional flux distributions used here, because in most of the functional definitions the supply of intermediate substrates (such as pyruvate) and energy carriers (ATP, NADH) is allowed. Beside the intermediates, most of the reference flux distributions used in ModeScore also satisfy the conditions of an elementary flux mode, caused by the application of the flux minimization principle [39]. To cover the same functionality with a complete set of k-shortest elementary flux modes would require many more modes since all alternative routes are considered while here only a single flux distribution for each function is used. The completeness of alternative routes would be traded for a larger set of flux distribution, and could be worthwhile in a future analysis. 

The underlying metabolic network is of critical importance for the ModeScore application. As the aim of the study is a broad screening, only genome-scale networks are suitable. The only published alternative to HepatoNet1 [17] is the network by Jerby *et al*. [40], based on Recon1 [41] inferred from expression data [42]. The big advantage of HepatoNet1 is the manual curation of individual reactions based on biochemical literature and the comprehensive testing: computed flux distributions for specified liver functions have been tested to represent experimentally confirmed biochemical pathways. In fact, the functions used in ModeScore have been derived from the functions used to test HepatoNet1. 

## 4. Methods and Materials

### 4.1. Affymetrix Chip Experiment

Primary hepatocytes were isolated from livers of male C57/BL-6 mice (100–150 g) using collagenase perfusion. Hepatocytes were plated on collagen coated 6-well plates at a density of 3 × 105 cells/well in Williams’ medium E supplemented with 10% fetal bovine serum, 2 mM L-glutamine, 1% penicillin/streptomycin and 100 nM dexamethasone. Hepatocytes were allowed to attach, and medium was exchanged after 4 h with Williams medium E supplemented with 2 mM L-glutamine and 1% penicillin/streptomycin. 5 ng/mL recombinant TGFβ_1_ was added to the serum-free culture medium for 1, 6 and 24 hours. Control conditions included cells maintained for the same period in serum-free medium without TGFβ_1_. Total RNA was collected at each time point and purified with the RNeasy Mini kit (Qiagen, Hilden, Germany), and the integrity was verified by denaturating agarose electrophoresis. A total of 5 g RNA was transcribed into cDNA by oligo dT primers, reverse transcribed to biotinylated complementary RNA with the Gene Chip IVT Labeling Kit (Affymetrix, High Wycombe, England), and hybridized to arrays of type moe430_2 from Affymetrix (Santa Clara, CA). The data is publicly available with Acc. No. E-MEXP-1176 at ArrayExpress [46]. 

### 4.2. Network and Gene Assignments

HepatoNet1 [17] was chosen as a tested metabolic model for the human hepatocyte. It is applicable to murine hepatocytes except for the gene assignments that are obtained from the following resources: (i) Metabolic reactions with a KEGG reaction annotation in HepatoNet1 can be mapped to a human gene using KEGG; (ii) Other metabolic functions with an EC number can also be mapped to a human gene using KEGG; (iii) Transporters with an annotation in TCDB can be mapped to a mammalian enzyme (UniProt nomenclature) using the TCDB; (iv) Protein synthesis reactions have been mapped to the protein directly, which makes sense for this work as the RNA is the most specific requisite for the synthesis reaction; (v) Proteins have been mapped to their encoding gene using ENSEMBL/BioMART; (vi) Genes of the different species have been mapped to mouse genes (ENSEMBL nomenclature) using the computed homologies contained in ENSEMBL/BioMART database; (vii) Ensemble annotations have been given by the Affymetrix software HepatoNet1 was enlarged by the synthesis reactions of collagens. If a reaction is catalyzed by more than one enzyme and the gene transcript abundances indicate that some of the isozymes are always off, it is removed from the annotation [43,44]. See Supplementary file 2 for the final network, which has also been deposited in BioModels [47] under the identifier MODEL1208060000. 

### 4.3. Reference Solutions

The simulations published for HepatoNet1 [17] have been redesigned for the use of ModeScore. There are 3 categories of functions: (i) regeneration of internal building blocks; (ii) hepatic functions towards the other organism of the body; (iii) replication of all internal metabolites, totaling 992 simulations. The solutions have been obtained with 5 different parameters settings, all of which include thermodynamic realizability [45] with concentration bound hard bounds. The first solution series is computed without any implied scoring scheme, the second with flux minimization, the rest additionally with soft bounds for the concentrations. Among the different solutions, the one with the fewest used reactions is used. Reference flux distributions with no associated genes are removed, 987 remain. After the computation each mode *M_k_* = (*m_i_*^(*k*)^)_*i* = 1...*n*_ is normalized, *i.e*., divided by Σ*_i _*_∊ *I_k_*_ |*m_i_*^(*k*)^| , where *m_i_*^(*k*)^ is the flux rate through reaction *i*, and *n* the number of reactions. 

### 4.4. ModeScore Method

The core of the ModeScore method [15] is the calculation of an amplitude value for a reference mode and a pair of transcript profiles, and additionally for each gene a contribution score, as follows: Let the k-th reference mode be denoted by *M_k_* = (*m_i_*^(*k*)^)_*i* = 1...*n*_ and the relative expression profile by *V* = (*v_i_*)_*i* = 1...*n*_, where *v_i_* is the difference of the log_2_ values of the transcript abundances of one state and a reference state. Then the score of the mode is 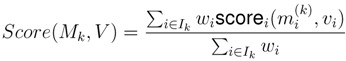 where 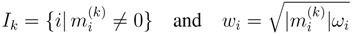 and 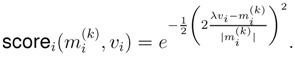


*λ* is chosen such that *Score*(*M_k_*, *V*) is maximal, see [15] for details on the optimization procedure. The non-negative numbers *ω_i_* are the weight adjustments to increase the influence of reactions with stoichiometric factors larger than one (except protons). For lumped reactions, *ω_i_* is set to the number of individual conversions. For spontaneous reactions, it is set to zero. For a complete list, see Supplementary file 7. 

To evaluate a function (*i.e*., a reference flux distribution) with respect to two expression profiles, the amplitude, calculated as 1/*λ* is used. The amplitude is compatible to the log_2_ expression change, *i.e*., if all genes are changed by the same amount α the amplitude would be *α*. Rather than averaging the transcript changes of all genes related to a function, the amplitude shows the most consistent pattern of synchronous regulation. The *Score*(*M_k_*, *V*) is of lesser importance; it serves as a significance score where values close to 1 indicate an unequivocal decision and low values less than 0.2 show an ambigu­ous pattern. 

To evaluate the contributions of the individual genes, the score_i_(*m_i_*^(*k*)^, *v_i_*) values are used, where a high score_i_(*m_i_*^(*k*)^,*v_i_*) shows a high influence of the gene on the whole function’s evaluation. The table in Supplementary file 5 are sorted by the combined score_i_(*m_i_*^(*k*)^, *v_i_*) adjusted with the weight parameters *w_i_*. 

### 4.5. ModeScore Analysis

The functions with very high and low amplitudes (about 20 each) of the profile comparisons 1 h *vs.* 24 h for each untreated (A) and treated samples (B) and the control *vs.* TGFβ comparison for 24 h (C) have been analyzed for their functional relevance, see Supplementary file 5. Functions related to intermediates are replaced by their super-ordinate function (e.g., for tyrosine degradation); similar functions have been collected (collagens). 

For each of these functions, the scores for individual reactions (see Supplementary file 6) have been analyzed for the set of genes that are responsible to get the function in question to appear with a particular regulation pattern. For a complete account of the selection process, see Supplementary file 1. 

## 5. Conclusions

ModeScore analysis of RNA transcripts from cultured mouse hepatocytes treated with TGFβ *vs.* untreated controls has yielded hypotheses for the regulation of metabolic functions worth elucidating *in vitro* and *in vivo*, in mouse and man. The main conclusions are:

- the high sensitivity of the phenylalanine/tyrosine degradation capacity to TGFβ. Even though there is a strong down-regulation in the control culture, the large additional impact of TGFβ makes a specific effect very likely. The inhibition of TGFβ production or its signaling pathway could be a starting point for treatment of an imminent danger of brain failure in patients with critical liver diseases [20].
- the strong negative impact of TGFβ on ethanol degradation capacity, a fact that has been experimentally confirmed [11]. As it is not found in the control culture, a specific mechanism is highly likely.
- the collagens that are up-regulated most by TGFβ treatment (XXVII_α_1__, XV_α_1__,I_α_1__, VII_α_1__,V_α_2__). They can subsequently be related to the predominant fiber proteins in liver fibrosis and lead to a therapeutic starting point to estimate how much a particular cirrhotic disease process is related to hepatocyte dysfunction and sensitive to TGFβ,
- down-regulation of glucose export is postponed by TGFβ while for most other functions TGFβ accelerates the down-regulation,
- creatine synthesis, glucuronization capacity, urea synthesis, and cholesterol synthesis are negatively affected by TGFβ,
- an early and short-term up-regulating response to TGFβ regarding the synthesis capacity of ketone bodies
- that TGFβ suppresses the strong culture stress induced up-regulation of Vanin, and
- that TGFβ induces the re-formation of ceramides and sphingomyelin.

## Supplementary Files

Supplementary File 1 Complete account of the selection process (PDF, 2112 KB)

Supplementary File 2 Supplemental File (ZIP, 168 KB)

Supplementary File 3 Definition of the simulations and solution summary (PDF, 792 KB)

Supplementary File 4 Solutions (PDF, 3957 KB)

Supplementary File 5 Scores and amplitudes tables (PDF, 994 KB)

Supplementary File 6 Detailed reaction scores (PDF, 617 KB)

Supplementary File 7 Setting of the weights (PDF, 80 KB)
