# Supplementary material for: Metabolic Consequences of TGFβ Stimulation in Cultured Primary Mouse Hepatocytes Screened from Transcript Data with ModeScore
Source: Metabolites. 2012 Nov 21;2(4):983–1003. doi: 10.3390/metabo2040983 (PMC3901234; doi:10.3390/metabo2040983)
Supplement: Supplementary File 1 — Complete account of the selection process (PDF, 2112 KB) [file metabolites-02-00983-s001.pdf]

Complete account of the selection process

Andreas Hoppe.

October 17, 2012

Contents

1 Principles **1**
1.1 Selection of functions 1
1.2 Selection of genes 2

2 Selection in detail **3**
2.1 Collagens 4
2.2 Ethanol degradation 5
2.3 Sphinganine-1P synthesis 6
2.4 Tyrosine degradation 7
2.5 Panthetheine synthesis 8
2.6 Inositol synthesis 9
2.7 Urea synthesis 9
2.8 Bilirubin conjugation 10
2.9 Cholesterol synthesis 10
2.10 Bile acid synthesis and excretion 12
2.11 β-hydroxybutyrate 13
2.12 Glycolysis 13
2.13 Triglycerides 14

1 Principles

1.1 Selection of functions

The selection process was based on the result of the ModeScore computation of the most regulated metabolic functions, in particular (with decreasing order of importance)

- the comparison of the control and the TGFβ series at the 24h time point (`C/T 24h`),
- the comparison of the 1h to the 24h time point in the control series (`C1h/24h`),
- the comparison of the 1h to the 24h time point in the TGFβ series (`T1h/24h`),
- the comparison of the 1h to the 6h time point in the control series (`C1h/6h`) in conjunction with the comparison of the 6h to the 24h time point in the control series (`C6h/24h`), and
- the comparison of the control and the TGFβ series at the 6h time point (`C/T 6h`),
- the comparison of the 1h to the 6h time point in the control series (`T1h/24h`) in conjunction with the comparison of the 6h to the 24h time point in the TGFβ series (`T6h/24h`) — not shown in the diagrams below.

For each the top and the bottom 20 of the list have been used. For each of this functions, a detailed reaction chart was produced. These charts have been manually analyzed to select the most relevant functions with the following criteria:

1. central, well-known liver functions are favored to more peripheral functions (synthesis or degradation of of low-amount metabolites)
2. if a function can be seen as a subfunction of another, more central function, check if the superfunction is also a good candidate (e.g. the synthesis of homogentisate from tyrosine can be seen as a subfunction of tyrosine degradation, the more important function)
3. prefer functions which deviates in the amount the regulation from the average (see bar graphs in the main manuscript) — up-regulation or strong down-regulation
4. prefer functions which are top/bottom scorer in more than one of the comparisons
5. prefer functions which show a plausible pattern of genes regulated in a consistent manner.
6. prefer functions which have already been noted as specifically sensitive to TGFβ or the monolayer cell culture
7. prefer functions which contain genes with highly remarkable regulation amplitude or pattern
8. prefer functions whose pattern is not based on a single gene regulation

It must be noted that this selection process takes into account the available domain knowledge.

1.2 Selection of genes

Once the functions have been selected, the list of the relevant genes which led to the amplitude estimation of the whole function are selected. This is also a manual process which takes into account several information sources and is guided by the following criteria:

- prefer genes with large transcript value changes,
- prefer genes with large absolute values,
- prefer genes associated to reactions which are adjacent to reactions whose genes have already been selected,
- prefer genes of highly specific enzymes or transporters,
- prefer genes of reactions which are most important for the function under regard and not for any more important function,
- close gaps in an otherwise preferable reaction path.

Mostly, a connected reaction path (as a subset for the whole flux distribution) appears to be responsible for the regulation. Transporters are also integrated if consistent with the regulation of the enzymes. This is not often the case but some cases it seems to be highly relevant. This aspect is noteworthy in comparison to the PathRanker concept [1] which ignores transporters altogether. Another important aspect in comparison to PathRanker (based on the KEGG MODULE reaction paths [2]) is that the definition of the relevant paths is not predefined (and fixed) but flexible and dependent on the actual expression values of the experiment. Often, they are in accordance with the well-known reaction paths but in some cases there is a unique and noteworthy different selection of genes forming a functional unit.

2 Selection in detail

Function	C/T 24h		C1h/24h		T1h/24h		C1h/6h		C6h/24h		C/T 6h		T1h/24h		T6h/24h	
	pos	neg	pos	neg	pos	neg	pos	neg	pos	neg	pos	neg	pos	neg	pos	neg
Tyrosine degr	854	134	978	10	980	8	955	33	982	6	505	483	795	193	979	9
Phenylalanine degr	792	196	975	13	979	9	959	29	980	8	479	509	850	138	980	8
Tyrosine	986	2	915	73	977	11	373	615	882	106	221	767	900	88	982	6
Collagen CORA1 c synthesis	2	986	5	983	1	987	347	641	4	984	3	985	1	987	4	984
Collagen COIA1 c synthesis	922	66	546	442	859	129	326	662	725	263	322	666	145	843	901	87
Collagen COFA1 c synthesis	1	987	418	570	2	986	718	270	279	709	4	984	5	983	19	969
Collagen CO4A5 c synthesis	872	116	4	984	18	970	290	698	1	987	388	600	130	858	28	960
Ethanol degr	987	1	597	391	954	34	271	717	679	309	984	4	801	187	974	14
Bilirubin conjugation	851	137	545	443	900	88	10	978	807	181	423	565	225	763	922	66
Urea from alanine	970	18	655	333	928	60	801	187	985	3	493	495	937	51	908	80
Cholesterol	525	463	829	159	760	228	904	84	629	359	894	94	945	43	605	383
(R)-Mevalonate	13	975	777	211	761	227	919	69	442	546	532	456	870	118	550	438
Farnesyl-PP	722	266	672	316	662	326	923	65	397	591	366	622	857	131	523	465
Creatine	944	44	19	969	556	432	416	572	921	67	335	653	318	670	578	410
Glycogen glucose release	249	739	973	15	960	28	329	659	954	34	102	886	982	6	955	33
(R)-3-Hydroxybutanoate	747	241	863	125	77	911	796	192	822	166	234	754	4	984	701	287
Glutamine	859	129	513	475	472	516	570	418	459	529	509	479	602	386	450	538
Inositol	192	796	3	985	4	984	33	955	3	985	350	638	49	939	2	986
PI	201	787	416	572	695	293	283	705	581	407	355	633	56	932	777	211
Sphinganine-1P	3	985	223	765	9	979	540	448	156	832	13	975	37	951	9	979
4-Hydroxyphenylpyruvate	983	5	974	14	978	10	986	2	957	31	560	428	987	1	953	35
Saccharopine	984	4	940	48	951	37	982	6	869	119	550	438	986	2	947	41
Tyrosine	986	2	915	73	977	11	373	615	882	106	221	767	900	88	982	6
Urea from NH3	971	17	656	332	929	59	802	186	986	2	494	494	938	50	909	79
Pantetheine	979	9	1	987	204	784	909	79	175	813	10	978	8	980	36	952
Glucose-6P	985	3	18	970	984	4	60	928	26	962	983	5	983	5	978	10
Alanine	968	20	25	963	939	49	12	976	728	260	468	520	46	942	928	60
Proline degr	628	360	984	4	507	481	729	259	369	619	521	467	840	148	421	567
beta-Alanine degr	778	210	986	2	985	3	970	18	193	795	308	680	926	62	526	462
Aspartate degr	866	122	987	1	987	1	972	16	987	1	300	688	950	38	987	1
PEP	274	714	983	5	950	38	987	1	951	37	6	982	985	3	949	39
Lysine degr	700	288	926	62	652	336	976	12	883	105	293	695	321	667	658	330
Proline	478	510	460	528	363	625	721	267	291	697	720	268	392	596	311	677
CoA	305	683	166	822	584	404	90	898	148	840	619	369	503	485	606	382
Anaerobic rephosph of ATP	323	665	741	247	637	351	797	191	736	252	970	18	294	694	667	321
Proline	478	510	460	528	363	625	721	267	291	697	720	268	392	596	311	677
Triacylglycerol	723	265	419	569	667	321	223	765	587	401	288	700	67	921	738	250
Choloyl-CoA	559	429	791	197	798	190	856	132	701	287	882	106	885	103	672	316
Gly-CD-cholate(b)	781	207	797	191	784	204	870	118	633	355	853	135	935	53	673	315

Table 1: Ranks of selected functions. The respective first number is the rank from above (up-regulation) or below (down-regulation). At the blank ranking space, no score could be computed.

For the following functions, the respective ranks are summarized in Table 1. Note that not all functions have been selected upon the rank but on other reasons described below. Other high-ranked functions have been omitted as they are obviously subfunctions of other selected functions.

2.1 Collagens

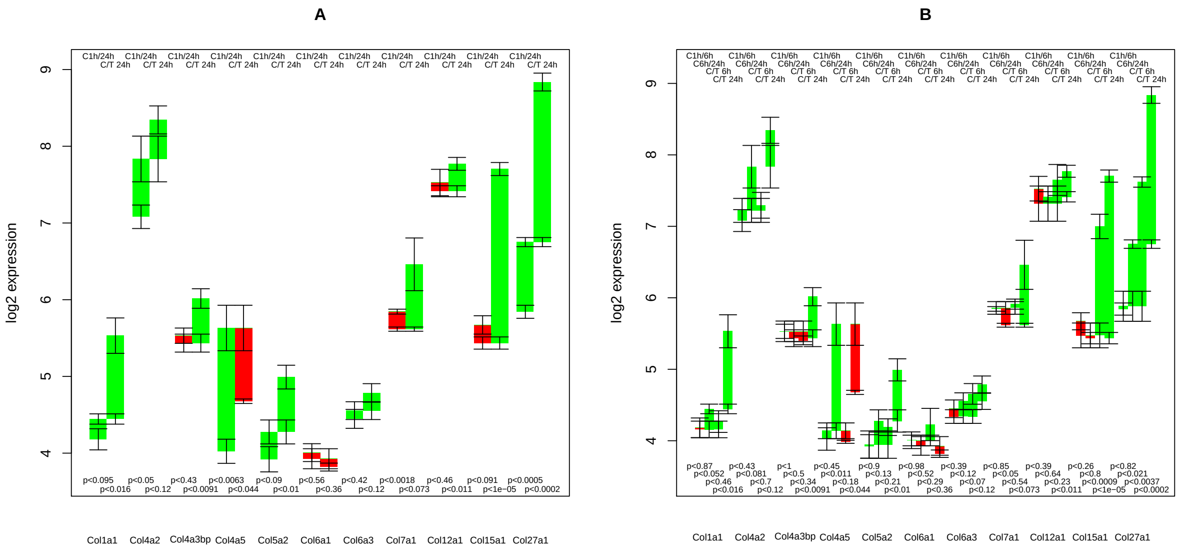

Figure 1: Collagen up-regulation genes, **A** full time period, **B** 1h/6h and 6h/24h comparisons.

The top ranks of the several function lists are dominated by some collagens. These are dominated by a single gene (the coding gene of itself) and therefore not in the focus of a pathway-oriented analysis. They are dealt with in a separate section in the main manuscript. Most of the collagens are up-regulated but to complement the section also the collagen with the highest down-regulation is used.

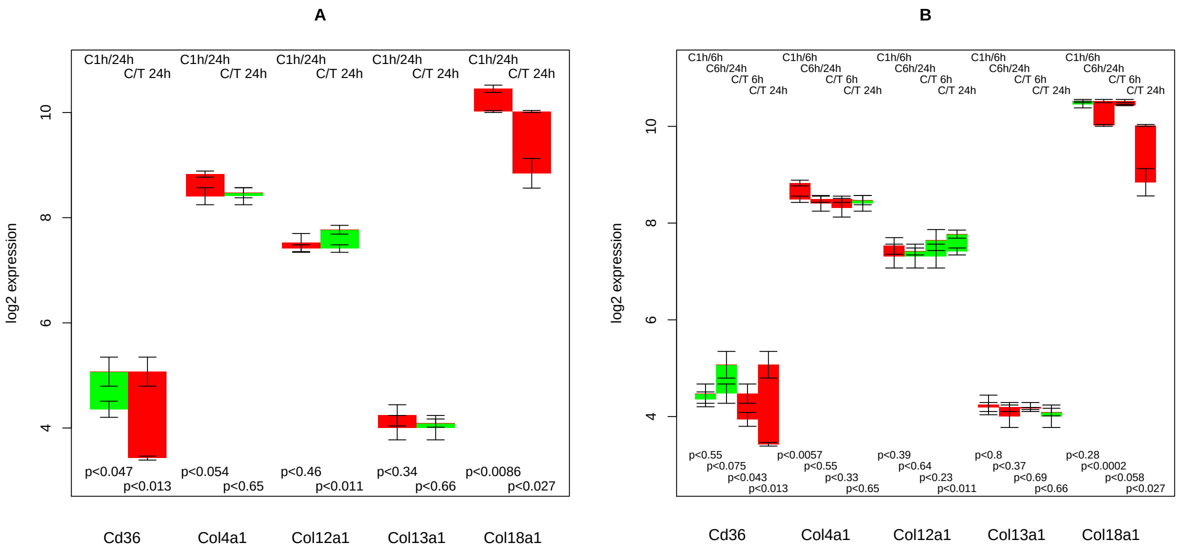

Figure 2: Collagens down-regulation genes, **A** full time period, **B** 1h/6h and 6h/24h comparisons.

It has been checked if other metabolic genes involved in the formation of collagens are suitable to be combined with the collagen synthesis but that does not seem to be the case. The genes coding for amino acid

transport are quite constant in their expression and are furthermore at a relatively low rate. This might be due to the fact that the dominant share of the amino acids required for the protein synthesis are recruited from protein degradation.

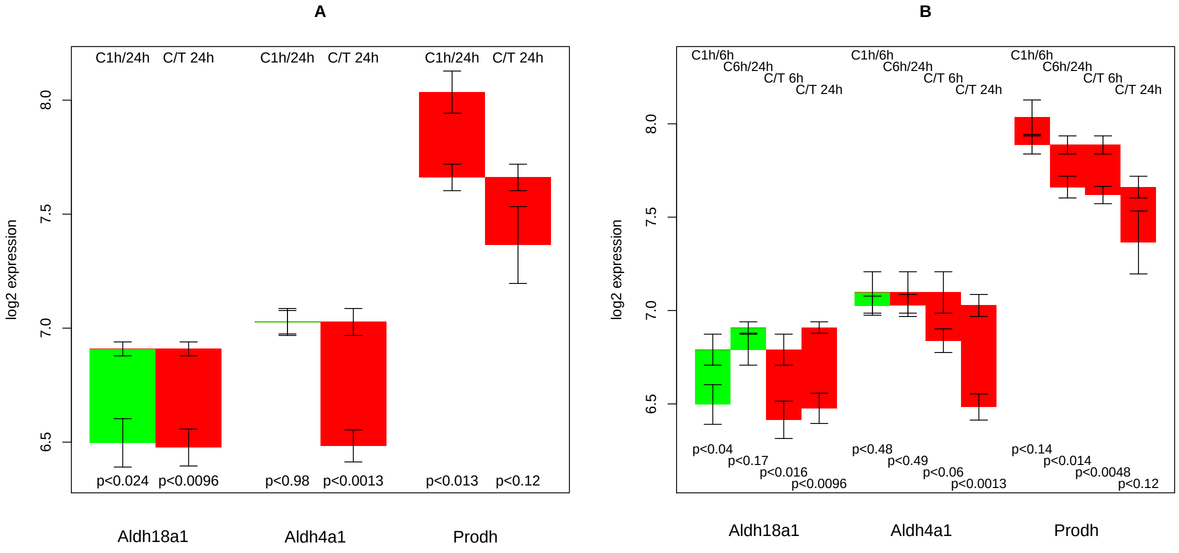

Figure 3: Proline synthesis genes, **A** full time period, **B** 1h/6h and 6h/24h comparisons.

As most fibrogenic collagens are immensely proline-rich, it has also been checked if the transamination paths to proline are up-regulated, however, they appear to be quite constantly expressed. As the only useful addition to the otherwise single-gene functions Sox9 [3] was selected as a collagen promoter (which is not based on the ModeScore analysis as Sox9 is not a metabolic gene).

2.2 Ethanol degradation

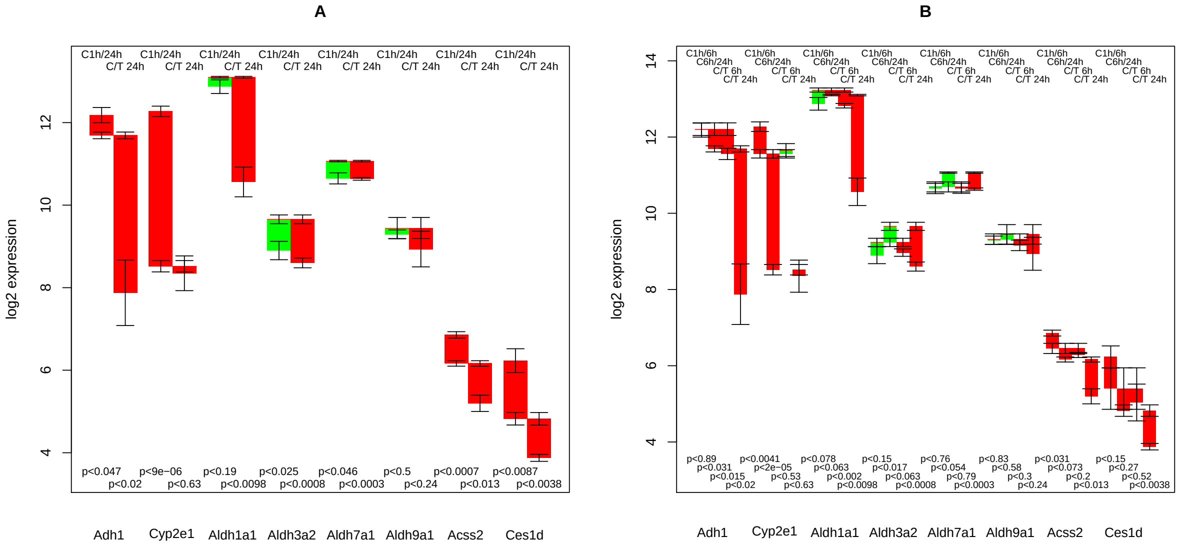

Figure 4: Ethanol degradation genes, **A** full time period, **B** 1h/6h and 6h/24h comparisons.

Ethanol degradation is the negative top scorer of C/T 24h and is selected as it has already been observed that there is a strong relation to TGFβ [4] and is therefore selected for the main manuscript.

2.3 Sphinganine-1P synthesis

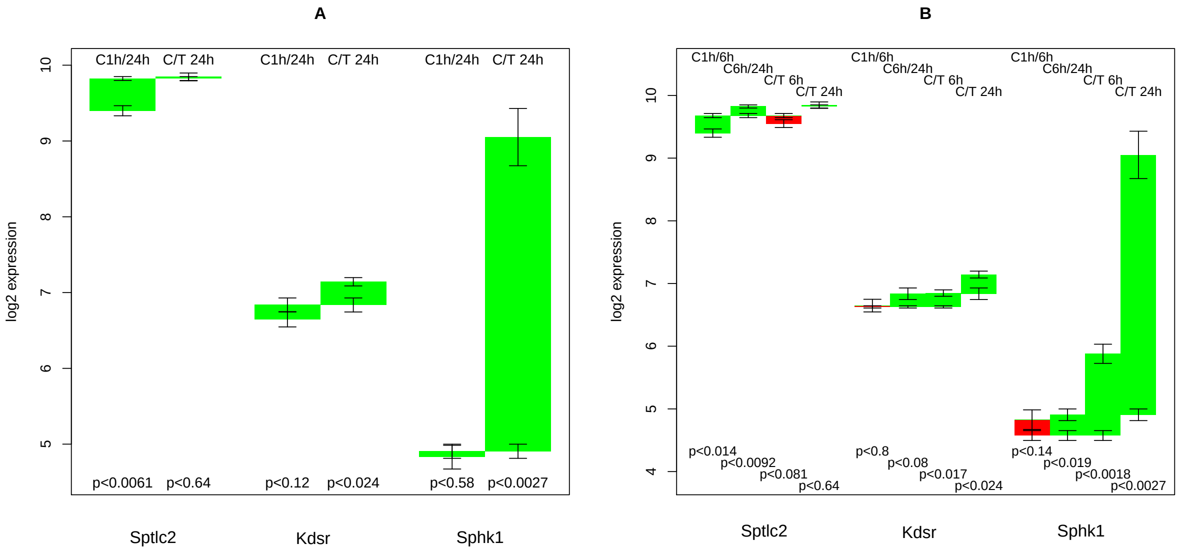

Figure 5: Sphinganine-1P synthesis genes, **A** full time period, **B** 1h/6h and 6h/24h comparisons.

The positive top scorer in C/T 24h not considering collagens is the synthesis of Sphinganine-1P. However, the gene coding sphingosine kinase is the only one up-regulated in the TGFβ series. As this is remarkable anyway it has been added to the solitude genes section.

2.4 Tyrosine degradation

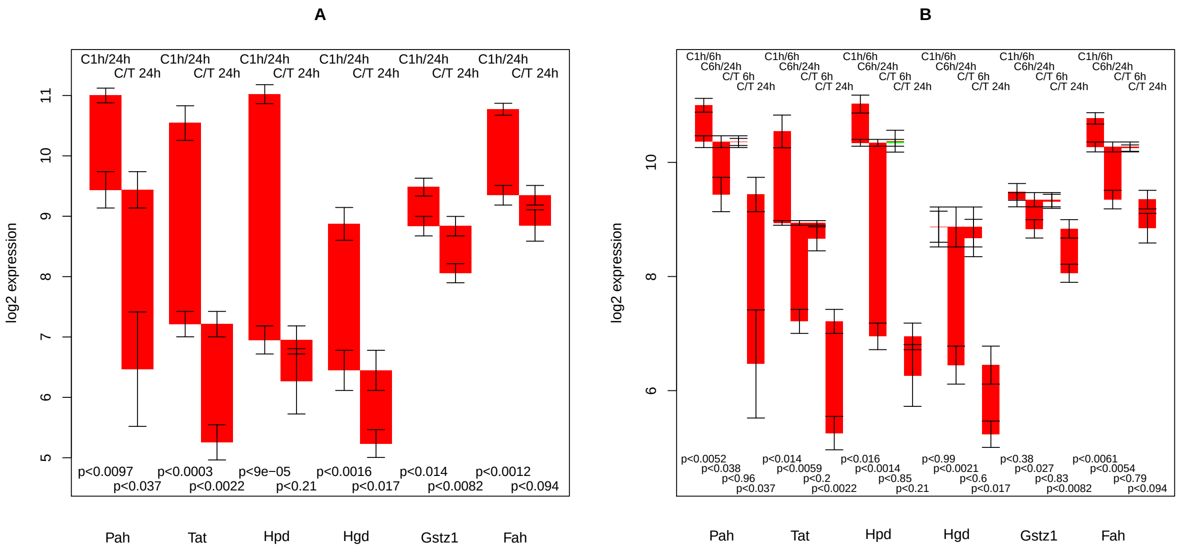

Figure 6: Tyrosine degradation genes, **A** full time period, **B** 1h/6h and 6h/24h comparisons.

Among the negative top 10 of `C/T 24h`, `T1h/24h`, and `C6h/24h`. Additionally, several functions can be found which are in fact subfunctions of the Phenylalanine/Tyrosine degradation pathway: synthesis 4-hydroxyphenylpyruvate, homogentisate, 4-maleoylacetoacetate, fumarylacetoacetate, several transamination functions from tyrosine and phenylalanine. Indeed, each of the 6 involved enzymes in the degradation pathway (starting from phenylalanine) is regulated in a similar way: heavily down-regulated in control and even more so in the TGFβ treated experiment. The amount the down-regulation and the consistent pattern throughout a complete reaction path makes it the top result in the ModeScore ranking.

See Supplementary File 4 for a full acount of rankings. In Table 1.1 (comparing TGFβ treated and control at 24h), column “bottom up”, ranks 2,5,7,8,14,19 are occupied by functions related to phenylalanine degradation. In Table 1.2 (comparing 1h and 24h in the control) the ranks are 7,10,11,12,13,14,16-25. In Table 1.3 (comparing 1h and 24h in the treated cells) the ranks are 6,8-23.

2.5 Panthetheine synthesis

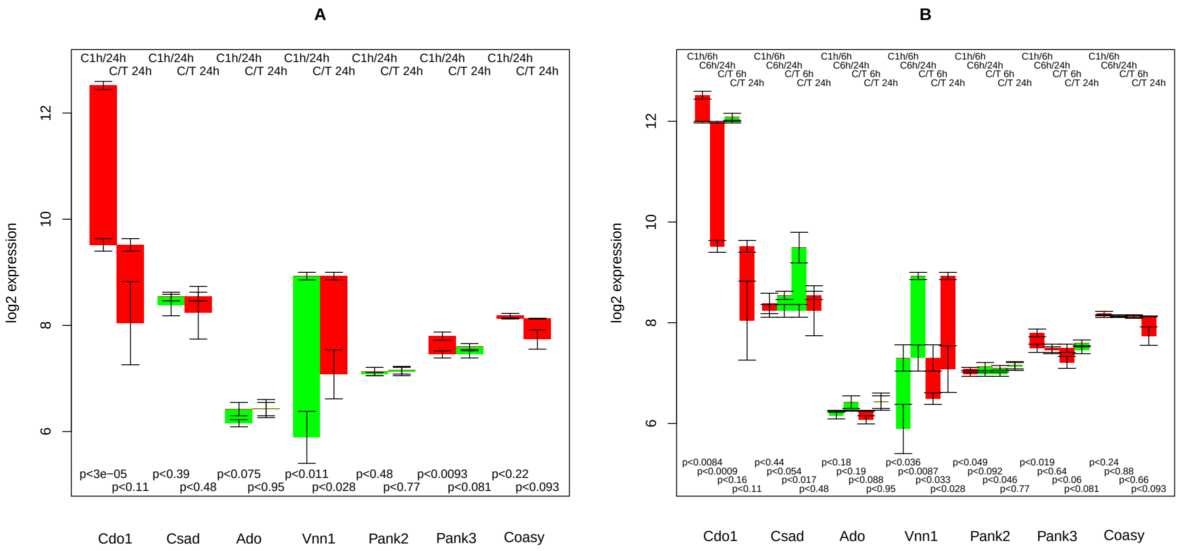

Figure 7: Panthetheine synthesis genes, **A** full time period, **B** 1h/6h and 6h/24h comparisons.

Top positive scorer in C1h/24h and rank 10 of negative scorers in list C/T 24h is Panthetheine synthesis. Only Vanin 1 (Vnn1) which is directly involved in the CoA synthesis pathway, shows this intriguing pattern. Cysteine dioxygenase 1 (Cdo1) which is also involved in this flux distribution shows an opposite pattern, down-regulated in time, aggravated by TGFβ, a more average pattern. The other genes show a constant and rather low expression. In fact, cysteine dioxygenase is also involved in cysteine degradation. As this is presumably the process with a higher flux rate, its regulation should not be interpreted with respect to CoA synthesis. The full flux distribution of CoA synthesis (see Supplementary File 4) shows that other enzymes involved in CoA pathway post panthetheine are remarkably constant. Thus, Vanin 1, will be kept as a solitude gene.

2.6 Inositol synthesis

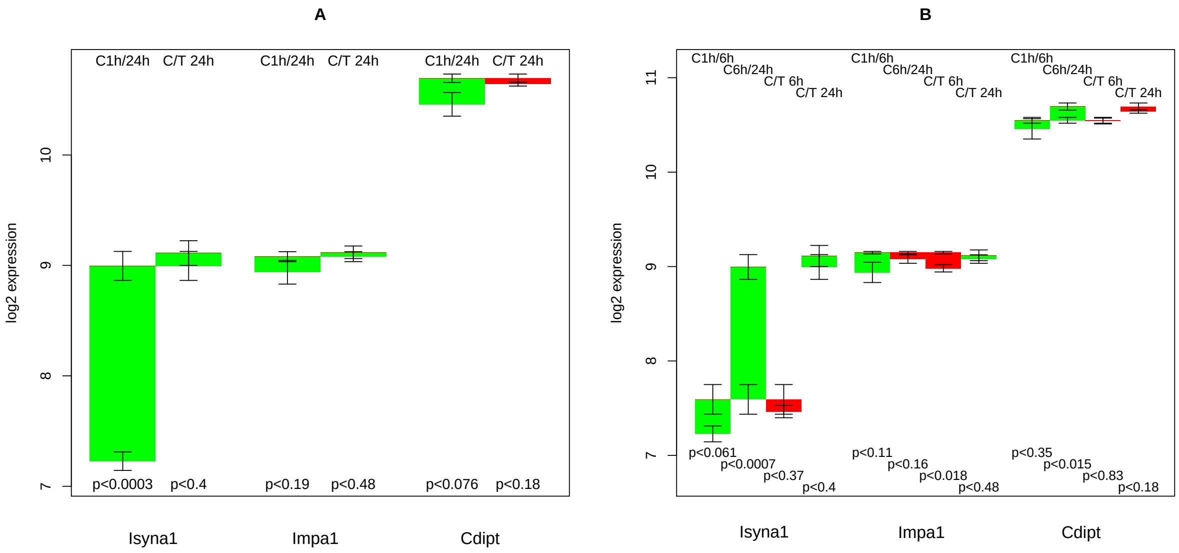

Figure 8: Inositol synthesis genes, **A** full time period, **B** 1h/6h and 6h/24h comparisons.

Inositol synthesis is the top positive 2 ranker in list `C1h/24h` and `C6h/24h`. Apparently, the only gene which accentuates this behavior is Inositol-1P synthase (Isyna1). Presumably, the up-regulation shows a larger activity in phosphoinositol in later time points, but Isyna1 is the only information in the transcripts on which this hypothesis can be observed. Also the gene coding the enzyme to the binding of inositol into the CDP-diacylglycerol (Cdipt) is expressed constantly. Thus, it is also kept as a solitude gene.

2.7 Urea synthesis

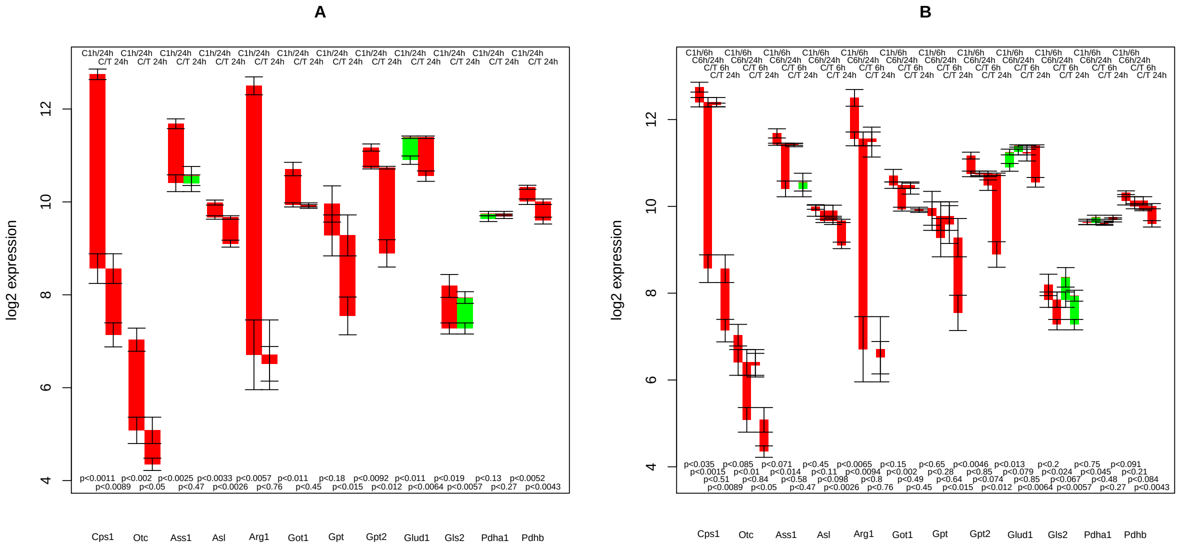

Figure 9: Urea synthesis genes, **A** full time period, **B** 1h/6h and 6h/24h comparisons.

Urea synthesis appears at rank 3 of negative scorers in C6h/24h and is also among the negative top 20 in C/T 24h.

Subfunctions such as ornithine synthesis, aspartate degradation, and alanine degradation also appear as high negative scorers. It is also selected because it is such a central hepatocyte function. One involved gene, arginase 1, is the top down-regulated gene in the comparison C1h/24h and also the top down-regulated metabolic gene in T1h/24h (just short of cytochrome p450 2b10). It can be observed that the different enzymes in the urea cycle and further supporting enzymes are regulated in a rather irregular pattern.

2.8 Bilirubin conjugation

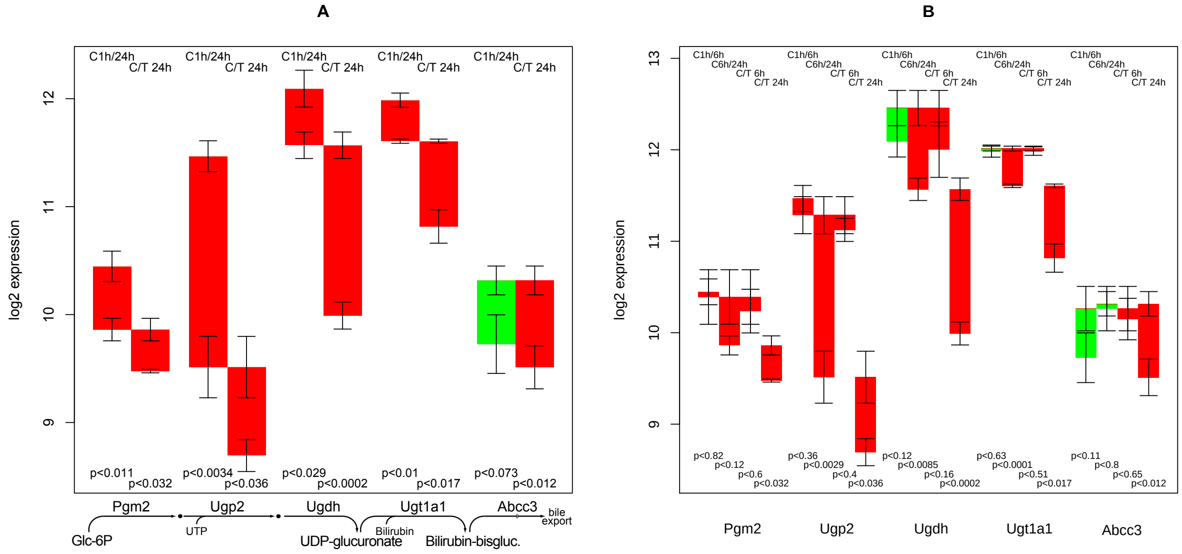

Figure 10: Bilirubin conjugation genes, **A** full time period, **B** 1h/6h and 6h/24h comparisons.

Bilirubin conjugation is an important clinical indicator of liver status and is regarded here even though the function's ModeScore evaluation is not as spectacular. However, the supply of UDP-glucuronate is considerably down-regulated in comparison to the glucuronate transfer to bilirubin. There is one issue, however, which complicates the evaluation of this function. On the Affymetrix chip Mouse430_2 there is no way to uniquely identify each of the different isoforms of glucuronosyltransferase 1, the probes do not allow to distinguish between them. Thus, the probeset allows an average assessment for all isoforms which could blur a more specific regulation.

2.9 Cholesterol synthesis

Cholesterol is synthesized from acetyl-CoA in a rather long chain of reactions which are considered in several blocks, mainly because certain intermediates of this chain are also used as building blocks for other cellular constituents, namely mevalonate and farnesyl-diphosphate.

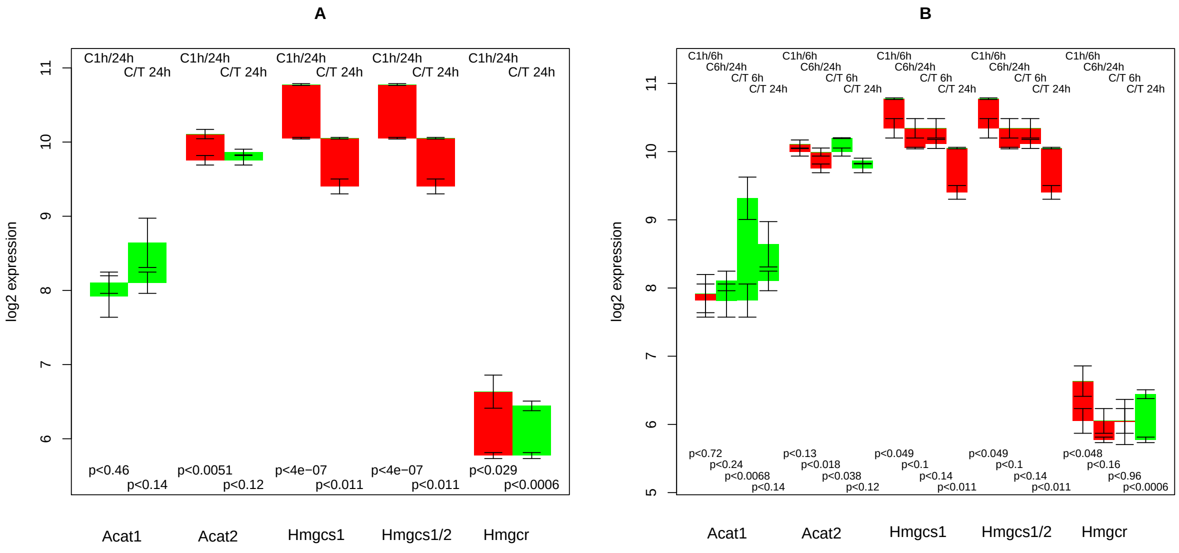

Figure 11: Mevalonate from acetyl-CoA genes, **A** full time period, **B** 1h/6h and 6h/24h comparisons.

The reactions up to mevalonate are regulated inconclusively. Worth noting is that the Acetoacetyl-CoA synthase (Acat1) and the HMG-CoA-Reductase are up-regulated in the treated group, in the case of Acat1even as an early response (6h). HMG-CoA-synthase appears twice as two transcripts are defined for the gene, however, the probes on the chip are not sufficient to distinguish between them, so the actual values are identical.

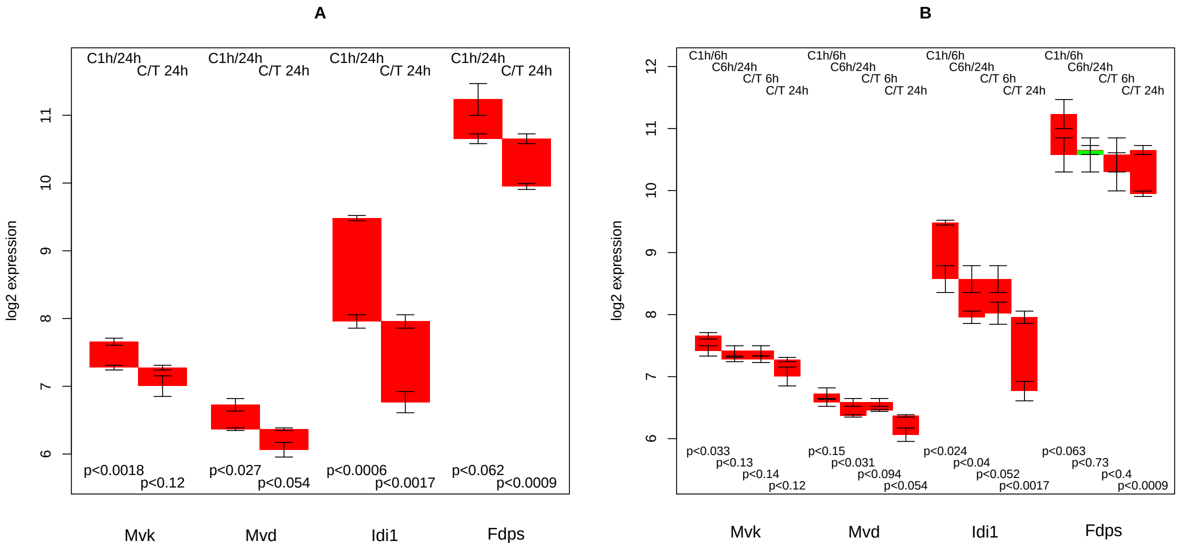

Figure 12: Farnesyl-diphosphate from mevalonate genes, **A** full time period, **B** 1h/6h and 6h/24h comparisons.

The rest of the cholesterol synthesis pathway seems to be regulated in a very consistent way: down-regulated in the control group, aggravated by TGFβ. This is meaningful as this down-regulation occurs at a higher amplitude than the average gene.

An interesting feature in the reactions towards farnesyl-diphosphate is that Idi1 and Fdps show an early

down-regulation, see Figure 12B. For most other genes the largest amplitude of change occurs between 6h and 24h and the change between 1h and 6h is low.

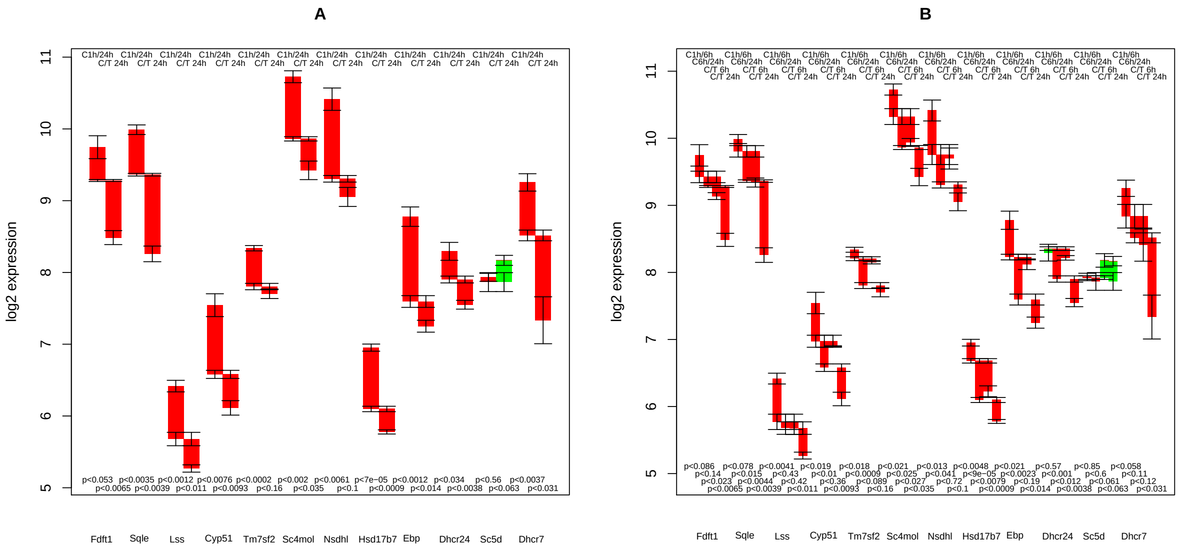

Figure 13: Cholesterol from farnesyl-diphosphate genes, **A** full time period, **B** 1h/6h and 6h/24h comparisons.

The pathway is selected for the main manuscript and dealt with in more detail there.

2.10 Bile acid synthesis and excretion

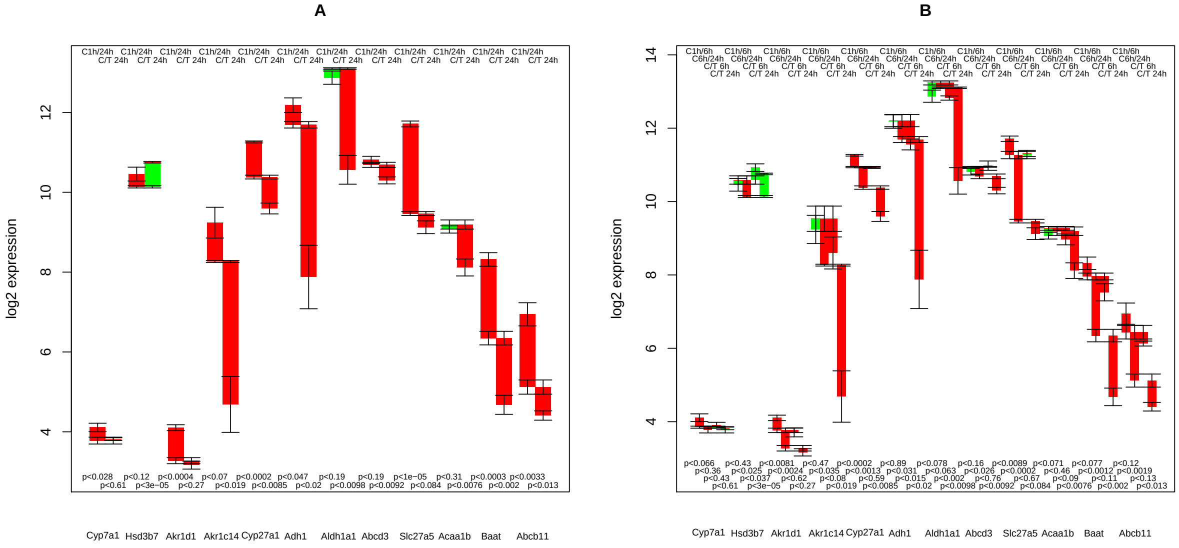

Figure 14: Glycochenodeoxycholate from cholesterol genes, **A** full time period, **B** 1h/6h and 6h/24h comparisons.

Several of genes show down-regulation with high amplitude only in the treated group, some other are strongly down-regulated also in the control group and thus are not sensitive to TGFβ treatment. Which of

the groups of genes dominates could only be decided upon the knowledge which are the rate-limiting steps in the pathway.

2.11 β-hydroxybutyrate

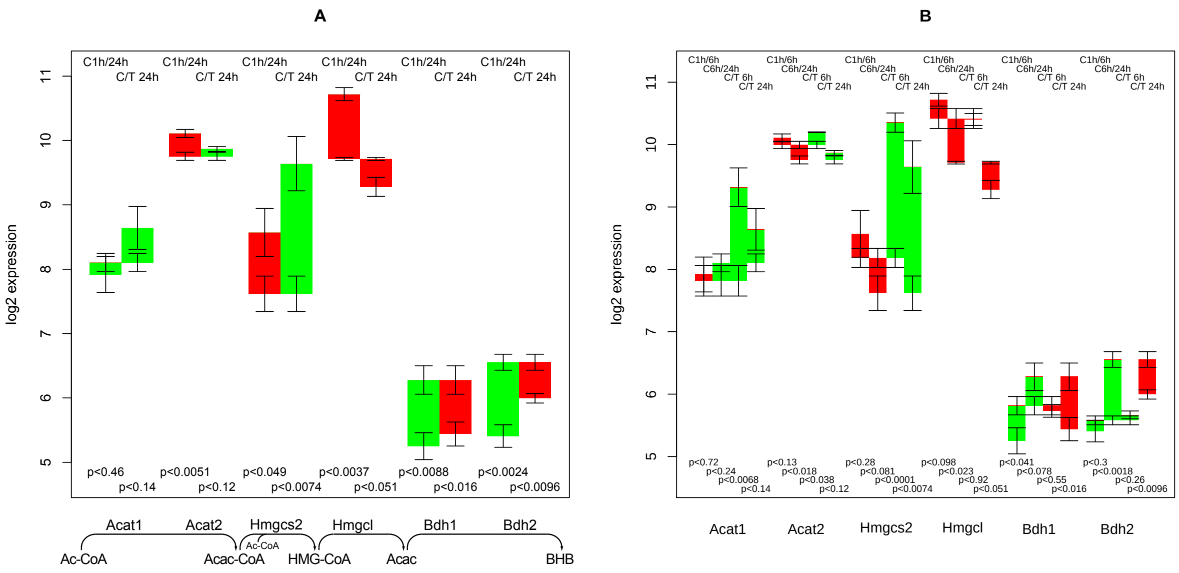

Figure 15: β-hydroxybutyratefrom Acetyl-CoA genes, **A** full time period, **B** 1h/6h and 6h/24h comparisons.

This pathway is selected as there is an intriguing early up-regulation in the mitochondrial HMG-CoA synthase.

2.12 Glycolysis

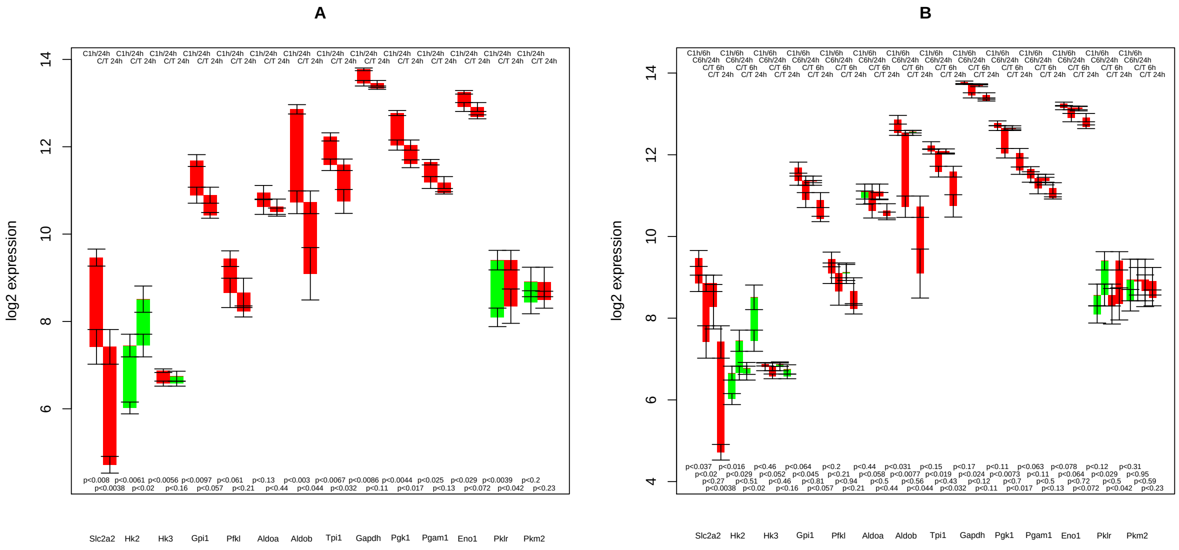

Figure 16: Glycolysis genes, **A** full time period, **B** 1h/6h and 6h/24h comparisons.

The function called "Anaerobic rephosph of ATP" in Table 1 does not show any particularly strong regulation and is dealt with only because its cellular importance. The strongest down-regulation shows the glucose transporter gene. As the same protein is integrated in the plasma membrane and the membrane of the endoplasmatic reticulum (ER), it is not possible to assign a unique purpose for this regulation.

There is a straightforward interpretation of the regulation of hexokinases. Apparently, the hexokinase 2 with a broad substrate specificity is up-regulated, allowing the use of imported sugars for the own energy supply. The glucokinase which has a high affinity to glucose is related to the homeostatic task of the liver to reduce the blood glucose higher than a threshold very efficiently. This enzyme is down-regulated, in other words, the hexokinase activity is switched from the Glc to Hk2, indicating a loss of specific liver function of the hepatocytes.

2.13 Triglycerides

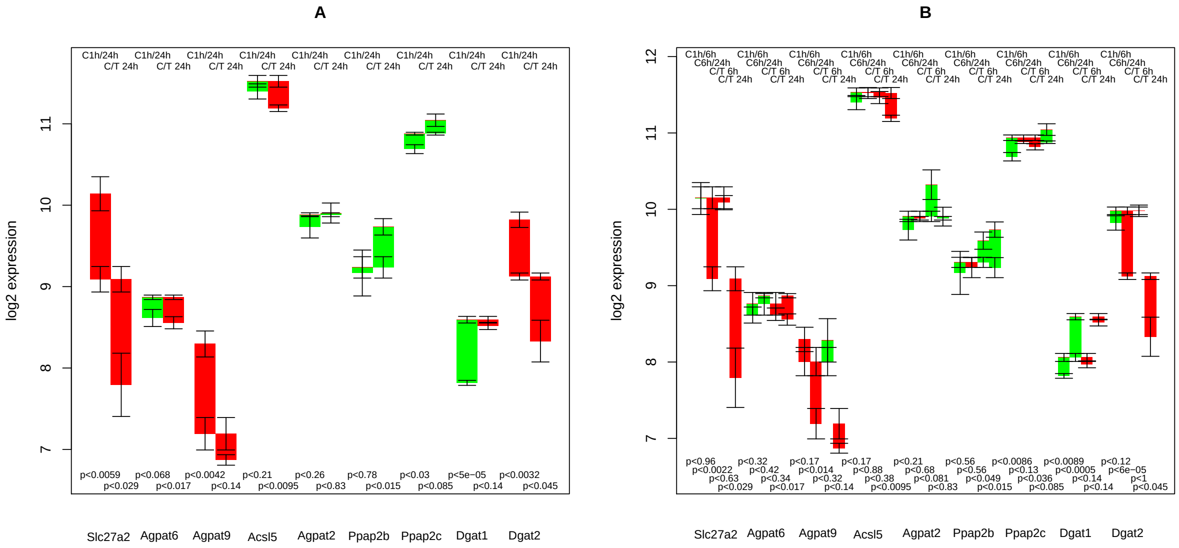

Figure 17: Triacylglycerol synthesis genes, **A** full time period, **B** 1h/6h and 6h/24h comparisons.

This function also does not show any particularly strong regulation and is dealt with because there is an experimental indication that the cells lose their internal triglycerides in the culture. The strongest down-regulation, aggravated by TGFβ can be found in the fatty acid transporter (Slc27a2), 1-acylglycerol-3-phosphate acyltransferase (Agpat9), and diacylglycerol acyltransferase (Dgat2). The other isozyme diacylglycerol acyltransferase (Dgat1) is up-regulated, independently of treatment. Thus, a redistribution of isozyme can be hypothesized.

References

[1] Hancock T, Takigawa I, Mamitsuka H: **Mining metabolic pathways through gene expression.** *Bioinformatics* 2010, **26**(17):2128–2135, [http://dx.doi.org/10.1093/bioinformatics/btq344].

[2] Kanehisa M, Araki M, Goto S, Hattori M, Hirakawa M, Itoh M, Katayama T, Kawashima S, Okuda S, Tokimatsu T, Yamanishi Y: **KEGG for linking genomes to life and the environment.** *Nucleic Acids Res* 2008, **36**(Database issue):D480–D484, [http://dx.doi.org/10.1093/nar/gkm882].

[3] Jenkins E, Moss JB, Pace JM, Bridgewater LC: **The new collagen gene COL27A1 contains SOX9-responsive enhancer elements.** *Matrix Biol* 2005, **24**(3):177–184, [http://dx.doi.org/10.1016/j.matbio.2005.02.004].

[4] Ciuclan L, Ehnert S, Ilkavets I, Weng HL, Gaitantzi H, Tsukamoto H, Ueberham E, Meindl-Beinker NM, Singer MV, Breitkopf K, Dooley S: **TGF-beta enhances alcohol dependent hepatocyte damage via down-regulation of alcohol dehydrogenase I.** *J Hepatol* 2010, **52**(3):407–416, [http://dx.doi.org/10.1016/j.jhep.2009.12.003].
