# Supplementary material for: Metabolic Consequences of TGFβ Stimulation in Cultured Primary Mouse Hepatocytes Screened from Transcript Data with ModeScore
Source: Metabolites. 2012 Nov 21;2(4):983–1003. doi: 10.3390/metabo2040983 (PMC3901234; doi:10.3390/metabo2040983)
Supplement: Supplementary File 3 — Definition of the simulations and solution summary (PDF, 792 KB) [file metabolites-02-00983-s003.pdf]

Definition of the simulations and solution summary

Contents

1 Definitions and specification of exchange sets **2**

2 A. Maintenance functions **4**

2.1 A1. pure energy 4

2.2 A2. Membrane gradients 9

2.3 A3. Small groups activated 9

2.4 A4. Activated sugars 12

2.5 A5. Important intermediates 13

2.6 A6. Salvage 15

2.7 A7. Detoxification of internally emerging substances 16

3 B. Functions of organismic duty **16**

3.1 B1. Storage/Release 16

3.2 B2. Turnover functions 17

3.3 B3. Excretion of de novo synthesized substances 56

3.4 B4. clearence " ... degr" simulations 86

4 C. Proliferative functions **95**

4.1 C1. Cofactors 95

4.2 C2. Precursors for macromolecules 100

4.3 C3. Constituents of cellular structures 105

4.4 C4. Other building blocks 108

4.5 C5. Comprehensive list 109

1 Definitions and specification of exchange sets

List of compartments

Identifer	compartment	comment
s	sinusoid (blood)	external
b	bile	external
c	cytosol	
m	mitochondrium	
r	ER and Golgi	combined
p	peroxysome	
l	lysosome	
n	nucleus	

Symbols in *Constraints* column

Symbol	meaning
$-$	uptake allowed
$+$	excretion allowed
$=$	any exchange allowed
MCES/AAA/ALFA/PIPES	use exchange set (see below)

Maintained cell exchange set: MCES

Metabolite	compartment	direction
ATP-energy	c	both
ATP-energy	m	both
Activated methyl group	c	both
THF-activated methyl group	c	both
NADH-redox-potential	r	both
NADH-redox-potential	c	both
NADH-redox-potential	m	both
NADH-redox-potential	p	both
NADPH-redox-potential	r	both
NADPH-redox-potential	c	both
NADPH-redox-potential	m	both
NADPH-redox-potential	p	both
FADH-redox-potential	c	both
Adenosylmethioninamine-potential	c	both
Proton-gradient	c	both
Proton-gradient	m	both
activated-sulphur	c	both
Glucose-6P	c	both
Pyruvate	c	both
Pyruvate	m	both
Farnesyl-PP	r	both
Farnesyl-PP	p	both
CoA-activated acetyl group	c	both
CoA-activated acetyl group	m	both
CoA-activated acetyl group	p	both
CoA-activated acetyl group	r	both
Na-gradient	c	both
P_i	c	both
H_2O	s	both
CO_2	s	export
O_2	s	import

All amino acids: AAA

Metabolite	compartment	direction
Histidine	s	both
Isoleucine	s	both
Leucine	s	both
Lysine	s	both
Methionine	s	both
Phenylalanine	s	both
Threonine	s	both
Tryptophan	s	both
Valine	s	both
Alanine	s	both
Arginine	s	both
Asparagine	s	both
Aspartate	s	both
Cysteine	s	both
Cystine	s	both
Glutamate	s	both
Glycine	s	both
Glutamine	s	both
Proline	s	both
Serine	s	both
Tyrosine	s	both

All lipid-relevant fatty acids and group donors: ALFA

Metabolite	compartment	direction
Linoleate	c	both
Palmitate	c	both
Stearate	c	both
Oleate	c	both
Arachidonate	c	both
Palmitolate	c	both
gamma-Linolenate	c	both
Ethanolamine	c	both
Choline	c	both

Physiological exchange set: PIPES

Metabolite	compartment	direction
Glucose	s	both
O_2	s	import
NH_3	s	import
Sulfate	s	both
P_i	s	both
Fe^{2+}	s	both
Nicotinamide	s	import
Folate	s	import
Histidine	s	both
Isoleucine	s	both
Leucine	s	both
Lysine	s	both
Methionine	s	both
Phenylalanine	s	both
Threonine	s	both
Tryptophan	s	both
Valine	s	both
Pantothenate	s	import
CO_2	s	export
Urea	s	export
Urate	s	export
Ethanolamine	c	both
Choline	c	both
Linoleate	s	both
Pyridoxine	s	import
Riboflavin	s	import
H_2O	s	both
L-Lactate	s	both
Alanine	s	both
Arginine	s	both
Asparagine	s	both
Aspartate	s	both
Cysteine	s	both
Cystine	s	both
Glutamate	s	both
Glycine	s	both
Glutamine	s	both
Proline	s	both
Serine	s	both
Tyrosine	s	both
Palmitate	s	both
Stearate	s	both
Oleate	s	both
Arachidonate	s	both
Palmitolate	s	both
Cholesterol	b	export
H_2S	s	export

2 A. Maintenance functions

2.1 A1. pure energy

Table 1: A1. pure energy

	Simulation	Definition			Solution													
					exchanges		reactions								transport			Prot
		Objective	Contraints	Comment	imports	exports	c	m	r	p	l	n	s	b	s-c	b-c	intra	syn
	**A1.1. NTP rephosphorylation**																	
1	Aerobic ATP rephosph (FA)	ATP-energy(c)	−Palmitate +CO_2 −O_2 =H_2O	Aerobic rephosphorylation of cytosolic ATP from a fatty acid	0.22 O_2(s) 0.01 Palmitate(s)	0.15 H_2O(s) 0.15 CO_2(s) 1 ATP-energy(c)	6	38	-	-	-	-	-	-	4	-	11	-
2	Aerobic ATP rephosph (gluc)	ATP-energy(c)	−Glucose +CO_2 −O_2 =H_2O	Aerobic rephosphorylation of cytosolic ATP from glucose	0.19 O_2(s) 0.03 Glucose(s)	0.19 H_2O(s) 0.19 CO_2(s) 1 ATP-energy(c)	13	11	-	-	-	-	-	-	4	-	11	-
3	Anaerobic rephosph of ATP	ATP-energy(c)	−Glucose +L-Lactate +CO_2 =H_2O =Proton-gradient(c)	Anaerobic rephosphorylation of cytosolic ATP from glucose	0.5 Glucose(s)	1 L-Lactate(s) 1 ATP-energy(c) 1 Proton-gradient(c)	12	-	-	-	-	-	-	-	3	-	-	-
4	Aerobic rephosph of GTP	GTP(c)	−Palmitate −P_i(c) −GDP(c) +CO_2 −O_2 =H_2O	Aerobic rephosphorylation of cytosolic GTP from glucose	0.22 O_2(s) 1 P_i(c) 1 GDP(c) 0.01 Palmitate(s)	1.15 H_2O(s) 0.15 CO_2(s) 1 GTP(c)	5	38	-	-	-	-	-	-	4	-	11	-
5	Aerobic rephosph of CTP	CTP(c)	−Palmitate −P_i(c) −UTP(c) −CDP(c) =Glutamine =Glutamate +CO_2 −O_2 =H_2O	Aerobic rephosphorylation of cytosolic CTP from glucose	0.15 O_2(s) 0.8 P_i(c) 0.1 UTP(c) 0.95 CDP(c)	0.85 H_2O(s) 0.2 CO_2(s) 0.05 Glutamate(s) 1 CTP(c)	29	13	-	-	-	-	-	-	4	-	15	-
6	Aerobic rephosph of UDP	UDP(c)	−Palmitate −P_i(c) −UMP(c) +CO_2 −O_2 =H_2O	Aerobic rephosphorylation of cytosolic UDP from glucose	0.22 O_2(s) 1 P_i(c) 1 UMP(c) 0.01 Palmitate(s)	1.15 H_2O(s) 0.15 CO_2(s) 1 UDP(c)	6	38	-	-	-	-	-	-	4	-	11	-

Continued on next page

A1. pure energy – continued.

Simulation	Definition: Objective	Definition: Contraints	Definition: Comment	Solution: exchanges: imports	Solution: exchanges: exports	reactions: c	m	r	p	l	n	s	b	transport: s-c	b-c	intra	Prot syn
7 Aerobic rephosph of UTP	UTP(c)	−Palmitate $-P_i$(c) −UDP(c) $+CO_2$ $-O_2$ $=H_2O$	Aerobic rephosphorylation of cytosolic UTP from glucose	0.22 O_2(s) 1 P_i(c) 1 UDP(c) 0.01 Palmitate(s)	1.15 H_2O(s) 0.15 CO_2(s) 1 UTP(c)	6	38	-	-	-	-	-	-	4	-	11	-
8 Anaerobic rephosph of GTP	GTP(c)	−Glucose $-P_i$(c) −GDP(c) +L-Lactate $+CO_2$ $=H_2O$ =Proton-gradient(c)	Anaerobic rephosphorylation of cytosolic GTP from glucose	1 P_i(c) 0.5 Glucose(s) 1 GDP(c)	1 H_2O(s) 1 GTP(c) 1 L-Lactate(s) 1 Proton-gradient(c)	12	-	-	-	-	-	-	-	4	-	-	-
9 Anaerobic rephosph of CTP	CTP(c)	−Glucose $-P_i$(c) −CDP(c) +L-Lactate $+CO_2$ $=H_2O$ =Proton-gradient(c)	Anaerobic rephosphorylation of cytosolic CTP from glucose	1 P_i(c) 0.5 Glucose(s) 1 CDP(c)	1 H_2O(s) 1 CTP(c) 1 L-Lactate(s) 1 Proton-gradient(c)	12	-	-	-	-	-	-	-	4	-	-	-
10 Anaerobic rephosph of UTP	UTP(c)	−Glucose $-P_i$(c) −UDP(c) +L-Lactate $+CO_2$ $=H_2O$ =Proton-gradient(c)	Anaerobic rephosphorylation of cytosolic UTP from glucose	1 P_i(c) 1 UDP(c) 0.5 Glucose(s)	1 H_2O(s) 1 UTP(c) 1 L-Lactate(s) 1 Proton-gradient(c)	12	-	-	-	-	-	-	-	4	-	-	-
11 ATP from NADH(m)	ATP-energy(m)	=NADH-redox-potential(m) $=H_2O$ $-O_2$ %r0205 %r0820 %r0083	test that NADH/NAD^+ potential can be transformed to ATP/ADP potential	0.15 O_2(s) 0.3 NADH-redox-potential(m)	0.3 H_2O(s) 1 ATP-energy(m)	-	2	-	-	-	-	-	-	2	-	6	-
12 ATP from NADH	ATP-energy(c)	=NADH-redox-potential(c) $=H_2O$ $-O_2$	test that NADH/NAD^+ potential can be transformed to ATP/ADP potential	0.33 O_2(s) 0.67 NADH-redox-potential(c)	0.67 H_2O(s) 1 ATP-energy(c)	3	-	-	-	-	-	-	-	2	-	8	-

Continued on next page

Simulation	Definition			Solution													
				exchanges		reactions								transport			Prot
	Objective	Contraints	Comment	imports	exports	c	m	r	p	l	n	s	b	s-c	b-c	intra	syn
13 NADH potential transport	NADH-redox-potential(m)	=NADH-redox-potential(c) $=H_2O$ $-O_2$	NADH/NAD^+ potential transport from the cytosol into the mitochondrium	0.08 O_2(s) 1.17 NADH-redox-potential(c)	0.17 H_2O(s) 1 NADH-redox-potential(m)	4	3	-	-	-	-	-	-	2	-	7	-
14 Ubiquinol-to-ATP	ATP(c)	−ADP(c) $-P_i$(c) +Ubiquinone(m) −Ubiquinol(m) $=H_2O$(m) $-O_2$(m)	test that mitochondrial ATP/ADP potential without any exchange of substrates	0.33 O_2(m) 1 ADP(c) 1 P_i(c) 0.67 Ubiquinol(m)	1.67 H_2O(m) 1 ATP(c) 0.67 Ubiquinone(m)	-	-	-	-	-	-	-	-	-	-	5	-
15 Dephosphorylation of ATP	-1 ATP-energy(c)	−Palmitate $+CO_2$ $-O_2$ $=H_2O$	Dephosphorylation of ATP, release of energy	1 ATP-energy(c)		3	-	-	-	-	-	-	-	-	-	-	-
**A1.2. NAD(P)H, FADH**																	
16 Aerobic reduction of NAD^+ (FA)	NADH-redox-potential(c)	−Palmitate $+CO_2$ $-O_2$ $=H_2O$	Aerobic reduction of NAD^+	0.48 H_2O(s) 0.24 O_2(s) 0.03 Palmitate(s)	0.52 CO_2(s) 1 NADH-redox-potential(c)	16	38	-	-	-	-	-	-	4	-	14	-
17 Aerobic reduction of NAD^+ (gluc)	NADH-redox-potential(c)	−Glucose $+CO_2$ $-O_2$ $=H_2O$	Aerobic reduction of NAD^+	0.49 H_2O(s) 0.01 O_2(s) 0.09 Glucose(s)	0.51 CO_2(s) 1 NADH-redox-potential(c)	27	8	-	-	-	-	-	-	4	-	13	-
18 Aerobic reduction of $NADP^+$ (FA)	NADPH-redox-potential(c)	−Palmitate $+CO_2$ $-O_2$ $=H_2O$	Aerobic reduction of $NADP^+$	0.48 H_2O(s) 0.24 O_2(s) 0.03 Palmitate(s)	0.52 CO_2(s) 1 NADPH-redox-potential(c)	12	38	-	-	-	-	-	-	4	-	14	-
19 Aerobic reduction of $NADP^+$ (gluc)	NADPH-redox-potential(c)	−Glucose $+CO_2$ $-O_2$ $=H_2O$	Aerobic reduction of $NADP^+$	0.49 H_2O(s) 0.01 O_2(s) 0.09 Glucose(s)	0.51 CO_2(s) 1 NADPH-redox-potential(c)	24	10	-	-	-	-	-	-	4	-	12	-
20 Aerobic reduction of FAD (gluc)	FADH-redox-potential(c)	−Glucose $+CO_2$ $-O_2$ $=H_2O$	Aerobic reduction of FAD	0.88 O_2(s) 0.1 Glucose(s)	0.13 H_2O(s) 1.13 CO_2(s) 1 FADH-redox-potential(c)	65	50	-	-	-	-	-	-	4	-	19	-
21 Aerobic reduction of FAD (FA)	FADH-redox-potential(c)	−Palmitate $+CO_2$ $-O_2$ $=H_2O$	Aerobic reduction of FAD	1.04 O_2(s) 0.03 Palmitate(s)	0.05 H_2O(s) 1.05 CO_2(s) 1 FADH-redox-potential(c)	56	50	-	-	-	-	-	-	4	-	19	-

Continued on next page

Simulation	Definition			Solution													
				exchanges		reactions								transport			Prot
	Objective	Contraints	Comment	imports	exports	c	m	r	p	l	n	s	b	s-c	b-c	intra	syn
22 Oxidation of NADH	-1 NADH-redox-potential(c)	−Palmitate +CO_2 −O_2 =H_2O %r1414 %r0201	Oxydation of NADH, release of energy	0.5 O_2(s) 1 NADH-redox-potential(c)	1 H_2O(s)	3	6	-	-	-	-	-	-	2	-	8	-
23 Oxidation of NADPH	-1 NADPH-redox-potential(c)	−Palmitate +CO_2 −O_2 =H_2O %r1414 %r0201	Oxydation of NADPH, release of energy	0.5 O_2(s) 1 NADPH-redox-potential(c)	1 H_2O(s)	3	6	-	-	-	-	-	-	2	-	8	-
24 NADH redox potential into mito	NADH-redox-potential(m)	−NADH-redox-potential(c) %r0750	transport of redox potential	1 NADH-redox-potential(c)	1 NADH-redox-potential(m)	5	3	-	-	-	-	-	-	-	-	2	-
25 NADH redox potential into peroxy	NADH-redox-potential(p)	−NADH-redox-potential(c)	transport of redox potential	1 NADH-redox-potential(c)	1 NADH-redox-potential(p)	2	-	-	2	-	-	-	-	-	-	2	-
26 NADPH redox potential into mito	NADPH-redox-potential(m)	−NADPH-redox-potential(c)	transport of redox potential	1 NADPH-redox-potential(c)	1 NADPH-redox-potential(m)	3	3	-	-	-	-	-	-	-	-	2	-
27 NADPH redox potential into peroxy	NADPH-redox-potential(p)	−NADPH-redox-potential(c)	transport of redox potential	1 NADPH-redox-potential(c)	1 NADPH-redox-potential(p)	3	-	-	3	-	-	-	-	-	-	2	-
28 NADPH to NADH transhydrogenase	NADH-redox-potential(c)	−NADPH-redox-potential(c) %r0427 %r0750 %r0746 %r0236		1 NADPH-redox-potential(c)	1 NADH-redox-potential(c)	4	-	-	-	-	-	-	-	-	-	-	-
29 NADH to NADPH transhydrogenase	NADPH-redox-potential(c)	−NADH-redox-potential(c) %r0427 %r0750 %r0746 %r0236		1 NADH-redox-potential(c)	1 NADPH-redox-potential(c)	4	-	-	-	-	-	-	-	-	-	-	-
30 NADPH to NADH transhydrogenase in mito	NADH-redox-potential(m)	−NADPH-redox-potential(m) %r0525		1 NADPH-redox-potential(m)	1 NADH-redox-potential(m)	-	4	-	-	-	-	-	-	-	-	-	-
31 NADH to NADPH transhydrogenase in mito	NADPH-redox-potential(m)	−NADH-redox-potential(m) %r0525		1 NADH-redox-potential(m)	1 NADPH-redox-potential(m)	-	4	-	-	-	-	-	-	-	-	-	-
**A1.3. Other redox equivalents**																	

Continued on next page

Simulation	Definition			Solution													
				exchanges		reactions								transport			Prot
	Objective	Contraints	Comment	imports	exports	c	m	r	p	l	n	s	b	s-c	b-c	intra	syn
32 Thioredoxin(m) reduction	Thioredoxin-redox-potential(m)	−NADPH-redox-potential(m)	test that cytosolic thioredoxin can be reduced using the minimal exchange set	1 NADPH-redox-potential(m)	1 Thioredoxin-redox-potential(m)	-	3	-	-	-	-	-	-	-	-	-	-
33 Thioredoxin(m) oxidation	-1 Thioredoxin-redox-potential(m)	=NADPH-redox-potential(m) −Palmitate +CO_2 −O_2 =H_2O	test that cytosolic thioredoxin can be reduced using the minimal exchange set	1 Thioredoxin-redox-potential(m)	1 NADPH-redox-potential(m)	-	3	-	-	-	-	-	-	-	-	-	-
34 Thioredoxin(c) reduction	Thioredoxin-redox-potential(c)	=NADH-redox-potential(c) −Palmitate +CO_2 −O_2 =H_2O	test that cytosolic thioredoxin can be reduced using the minimal exchange set	1 NADH-redox-potential(c)	1 Thioredoxin-redox-potential(c)	5	-	-	-	-	-	-	-	-	-	-	-
35 Thioredoxin(c) oxidation	-1 Thioredoxin-redox-potential(c)	=NADH-redox-potential(c) −Palmitate +CO_2 −O_2 =H_2O	test that cytosolic thioredoxin can be reduced using the minimal exchange set	1 Thioredoxin-redox-potential(c)	1 NADH-redox-potential(c)	5	-	-	-	-	-	-	-	-	-	-	-
36 GSH reduction using NADH redox potential	GSH-potential(c)	=NADH-redox-potential(c)	Regeneration of Gluthatione redox potential	0.5 NADH-redox-potential(c)	1 GSH-potential(c)	5	-	-	-	-	-	-	-	-	-	-	-
37 GSH reduction using NADPH redox potential	GSH-potential(c)	=NADPH-redox-potential(c)	Regeneration of Gluthatione redox potential	0.5 NADPH-redox-potential(c)	1 GSH-potential(c)	3	-	-	-	-	-	-	-	-	-	-	-
38 GSH oxidation	-1 GSH-potential(c)	=ATP-energy(c) =NADH-redox-potential(c) =NADPH-redox-potential(c)	Oxidation of Gluthatione	1 GSH-potential(c)	0.5 NADPH-redox-potential(c)	3	-	-	-	-	-	-	-	-	-	-	-

2.2 A2. Membrane gradients

Table 2: A2. Membrane gradients

Simulation	Definition			Solution													
				exchanges		reactions								transport			Prot
	Objective	Contraints	Comment	imports	exports	c	m	r	p	l	n	s	b	s-c	b-c	intra	syn
**A2.1. Mitochondrial protons**																	
39 Proton-gradient(m) build up	Proton-gradient(m)	−Palmitate $+CO_2$ $-O_2$ $=H_2O$	mitochondrial Proton-gradient build up, protons pumped from the mitochondrial matrix into the cytosol	0.06 O_2(s) 0.003 Palmitate(s)	0.04 H_2O(s) 0.04 CO_2(s) 1 Proton-gradient(m)	4	41	-	-	-	-	-	-	4	-	10	-
**A2.2. Plasma membrane protons**																	
40 Proton-gradient(c) build up	Proton-gradient(m)	−Palmitate $+CO_2$ $-O_2$ $=H_2O$	apical membrane Proton-gradient build up, protons pumped from the sinusouid into the cytosol	0.06 O_2(s) 0.003 Palmitate(s)	0.04 H_2O(s) 0.04 CO_2(s) 1 Proton-gradient(m)	4	41	-	-	-	-	-	-	4	-	10	-
**A2.3. Sodium gradients**																	
41 Na+ importgradient	Na+(s)	−Na+(c) −Palmitate $-O_2$ $+CO_2$ $+H_2O$	tests that natrium can be pumped out the cell	0.22 O_2(s) 0.01 Palmitate(s) 1 Na+(c)	0.15 H_2O(s) 0.15 CO_2(s) 1 Na+(s)	5	38	-	-	-	-	-	-	8	-	11	-
42 Na+ exportgradient	Na+(c)	−Na+(s) −Palmitate $-O_2$ $+CO_2$ $+H_2O$	tests that natrium can be pumped into of the cell	1 Na+(s)	1 Na+(c)	-	-	-	-	-	-	-	-	2	-	-	-

2.3 A3. Small groups activated

Table 3: A3. Small groups activated

Simulation	Definition			Solution													
				exchanges		reactions								transport			Prot
	Objective	Contraints	Comment	imports	exports	c	m	r	p	l	n	s	b	s-c	b-c	intra	syn
**A3.1. Methyl(ene) group**																	
43 Activated methyl group (SAM)	Activated methyl group(c)	−Serine +Glycine −ATP-energy(c) =NADPH-redox-potential(c) $=H_2O$	de novo synthesis of cytosolic Activated methyl group	1 Serine(s) 3 ATP-energy(c) 1 NADPH-redox-potential(c)	1 H_2O(s) 1 Glycine(s) 1 Activated methyl group(c)	11	-	-	-	-	-	-	-	2	-	-	-
44 Activated methyl group (THF)	THF-activated methyl group(c)	−Serine +Glycine −ATP-energy(c) =NADPH-redox-potential(c) $=H_2O$	de novo synthesis of cytosolic Activated methyl group	1 Serine(s) 1 NADPH-redox-potential(c)	1 H_2O(s) 1 Glycine(s) 1 THF-activated methyl group(c)	4	-	-	-	-	-	-	-	2	-	-	-
45 Activated methylene group from Try	THF-activated methylene group(m)	−Tryptophan +Urea −Palmitate $+CO_2$ $-O_2$ $=H_2O$	de novo synthesis of mitochondrial Activated methyl group	10.5 O_2(s) 1 Tryptophan(s)	4 H_2O(s) 9 CO_2(s) 1 Urea(s) 1 THF-activated methylene group(m)	30	23	-	-	-	-	-	-	5	-	18	-
46 Activated methyl group from Histidine	THF-activated methyl group(c)	−Histidine +Urea −ATP-energy(c) =NADH-redox-potential(c) =NADPH-redox-potential(c) $=H_2O$ $-O_2$ $+CO_2$ $-NH_3$ %r1414	de novo synthesis of cytosolic Activated methyl group	5.34 H_2O(s) 0.58 O_2(s) 1 Histidine(s) 4.92 ATP-energy(c)	3.5 CO_2(s) 1.5 Urea(s) 0.5 NADH-redox-potential(c) 5.34 NADPH-redox-potential(c) 1 THF-activated methyl group(c)	41	12	-	-	-	-	-	-	6	-	15	-
**A3.2. Formyl group**																	
47 Formylgroup(c)	THF-activated formyl group(c)	−Serine +Glycine −ATP-energy(c) =NADPH-redox-potential(c) $=H_2O$	test that the methyl group on THF can be regenerated from the minimal exchange set	1 Serine(s)	1 Glycine(s) 1 NADPH-redox-potential(c) 1 THF-activated formyl group(c)	5	-	-	-	-	-	-	-	1	-	-	-

Continued on next page

A3. Small groups activated – continued.

Simulation	Definition			Solution													
				exchanges		reactions								transport			Prot
	Objective	Contraints	Comment	imports	exports	c	m	r	p	l	n	s	b	s-c	b-c	intra	syn
48 Formylgroup(m)	THF-activated formyl group(m)	−Serine +Glycine −ATP-energy(c) =NADPH-redox-potential(c) $=H_2O$ +Urea −Palmitate $-O_2$ $+CO_2$ +Glutamine(c) +Glutamate(c) −Tryptophan(c)	test that the methyl group on THF can be regenerated from the minimal exchange set	1.8 H_2O(s) 3.6 O_2(s) 1 Serine(s) 1 Tryptophan(c) 1 ATP-energy(c)	3 CO_2(s) 1 Glutamate(c) 1 Glutamine(c) 1.8 NADPH-redox-potential(c) 1 THF-activated formyl group(m)	17	16	-	-	-	-	-	-	4	-	15	-
**A3.3. Acetyl group**																	
49 Acetyl group(c)	CoA-activated acetyl group(c)	−Palmitate $-O_2$ $=H_2O$ =Proton-gradient(m) +ATP-energy(m) =ATP-energy(c) =NADH-redox-potential(m)	Regeneration of Acetyl-CoA minus CoA	0.44 O_2(s) 0.13 Palmitate(s) 2.25 ATP-energy(c)	0.13 H_2O(s) 0.88 NADH-redox-potential(m) 5.25 Proton-gradient(m) 1 CoA-activated acetyl group(c)	7	31	-	-	-	-	-	-	3	-	7	-
50 Acetyl group(m)	CoA-activated acetyl group(m)	−Palmitate $-O_2$ $=H_2O$ =Proton-gradient(m) +ATP-energy(m) =ATP-energy(c) =NADH-redox-potential(m)	Regeneration of Acetyl-CoA minus CoA	0.44 O_2(s) 0.13 Palmitate(s) 0.25 ATP-energy(c)	0.13 H_2O(s) 0.88 NADH-redox-potential(m) 5.25 Proton-gradient(m) 1 CoA-activated acetyl group(m)	5	31	-	-	-	-	-	-	3	-	5	-
51 Acetyl group(p)	CoA-activated acetyl group(p)	−Palmitate $-O_2$ $=H_2O$ =Proton-gradient(m) +ATP-energy(m) =ATP-energy(c) =NADH-redox-potential(m)	Regeneration of Acetyl-CoA minus CoA	0.44 O_2(s) 0.13 Palmitate(s) 2.25 ATP-energy(c)	0.13 H_2O(s) 0.88 NADH-redox-potential(m) 5.25 Proton-gradient(m) 1 CoA-activated acetyl group(p)	7	31	-	2	-	-	-	-	3	-	8	-
52 Acetyl group(r)	CoA-activated acetyl group(r)	−Palmitate $-O_2$ $=H_2O$ =Proton-gradient(m) +ATP-energy(m) =ATP-energy(c) =NADH-redox-potential(m)	Regeneration of Acetyl-CoA minus CoA	0.44 O_2(s) 0.13 Palmitate(s) 2.25 ATP-energy(c)	0.13 H_2O(s) 0.88 NADH-redox-potential(m) 5.25 Proton-gradient(m) 1 CoA-activated acetyl group(r)	6	31	1	-	-	-	-	-	3	-	9	-
**A3.4. Sulfate group**																	

Continued on next page

A3. Small groups activated – continued.

Simulation	Objective	Contraints	Comment	imports	exports	c	m	r	p	l	n	s	b	s-c	b-c	intra	Prot syn
53 activated sulphur	activated-sulphur(c)	−Sulfate(c) =ATP-energy(c) =NADH-redox-potential(c) =NADPH-redox-potential(c) $-O_2$ $+CO_2$ $=H_2O$	Regeneration of cytosolic activated-sulphur	1 Sulfate(c) 3 ATP-energy(c)	1 H_2O(s) 1 activated-sulphur(c)	7	-	-	-	-	-	-	-	1	-	-	-

2.4 A4. Activated sugars

Table 4: A4. Activated sugars

Simulation	Objective	Contraints	Comment	imports	exports	c	m	r	p	l	n	s	b	s-c	b-c	intra	Prot syn
**A4.1. Glucose-6-P**																	
54 Glucose-6P	Glucose-6P(c)	−Glucose =ATP-energy(c) $=P_i$(c) $=H_2O$	Synthesis of Glucose-6P	1 P_i(c) 1 Glucose(s) 1 ATP-energy(c)	1 H_2O(s) 1 Glucose-6P(c)	2	-	-	-	-	-	-	-	2	-	-	-
**A4.2. UDP-activated sugars**																	
55 UDP-activated glucose	UDP-activated-glucose(c)	−Glucose-6P(c) =ATP-energy(c) =NADH-redox-potential(c) =NADPH-redox-potential(c) $-O_2$ $+CO_2$ $+H_2O$ $=P_i$(c)	Regeneration of UDP glucose minus UDP	1 Glucose-6P(c) 1 ATP-energy(c)	1 P_i(c) 1 UDP-activated-glucose(c)	6	-	-	-	-	-	-	-	-	-	-	-
56 UDP-activated glucuronate	UDP-activated-glucuronate(c)	−Glucose-6P(c) =ATP-energy(c) =Proton-gradient(m) $-O_2$ $+CO_2$ $+H_2O$ $=P_i$(c) %r1239 %r1414	Regeneration of UDP glucuronate minus UDP	1 O_2(s) 1 Glucose-6P(c) 1 ATP-energy(c)	1 H_2O(s) 1 P_i(c) 12 Proton-gradient(m) 1 UDP-activated-glucuronate(c)	8	-	-	-	-	-	-	-	2	-	6	-
57 UDP-activated galactose	UDP-activated-galactose(c)	−Glucose-6P(c) =ATP-energy(c) =NADH-redox-potential(c) $=H_2O$ $=P_i$(c) =NADPH-redox-potential(c)	Regeneration of UDP-activated galactose	1 Glucose-6P(c) 1 ATP-energy(c)	1 P_i(c) 1 UDP-activated-galactose(c)	7	-	-	-	-	-	-	-	-	-	-	-

Continued on next page

A4. Activated sugars – continued.

Simulation	Definition: Objective	Definition: Contraints	Definition: Comment	Solution: exchanges: imports	Solution: exchanges: exports	reactions: c	m	r	p	l	n	s	b	transport: s-c	b-c	intra	Prot syn
**A4.3. Other nucleosides-activated sugars**																	
58 GDP-activated fucose	GDP-activated-fucose(c)	−Glucose-6P(c) =ATP-energy(c) =NADH-redox-potential(c) =H_2O =P_i(c) =NADPH-redox-potential(c)	Regeneration of GDP-activated fucose	1 Glucose-6P(c) 1 ATP-energy(c) 1 NADH-redox-potential(c)	1 H_2O(s) 1 P_i(c) 1 GDP-activated-fucose(c)	11	-	-	-	-	-	-	-	1	-	-	-
59 GDP-activated mannose	GDP-activated-mannose(c)	−Glucose-6P(c) =ATP-energy(c) =NADH-redox-potential(c) =H_2O =P_i(c) =NADPH-redox-potential(c)	Regeneration of GDP-activated mannose	1 Glucose-6P(c) 1 ATP-energy(c)	1 P_i(c) 1 GDP-activated-mannose(c)	8	-	-	-	-	-	-	-	-	-	-	-

2.5 A5. Important intermediates

Table 5: A5. Important intermediates

Simulation	Definition: Objective	Definition: Contraints	Definition: Comment	Solution: exchanges: imports	Solution: exchanges: exports	reactions: c	m	r	p	l	n	s	b	transport: s-c	b-c	intra	Prot syn
**A5.1. Pyruvate**																	
60 Pyruvate	Pyruvate(c)	−Glucose-6P(c) =ATP-energy(c) =NADH-redox-potential(c) =H_2O =P_i(c) =NADPH-redox-potential(c)	Synthesis of Pyruvate	0.5 H_2O(s) 0.5 Glucose-6P(c)	0.5 P_i(c) 1 Pyruvate(c) 1.5 ATP-energy(c) 1 NADH-redox-potential(c)	11	-	-	-	-	-	-	-	1	-	-	-
**A5.2. AKG**																	

Continued on next page

A5. Important intermediates – continued.

Simulation	Definition			Solution													
				exchanges		reactions								transport			Prot
	Objective	Contraints	Comment	imports	exports	c	m	r	p	l	n	s	b	s-c	b-c	intra	syn
61 AKG	AKG(c)	−Glucose-6P(c) =ATP-energy(c) =NADH-redox-potential(c) =NADH-redox-potential(m) =H_2O =P_i(c) =NADPH-redox-potential(m) =NADPH-redox-potential(c) =CO_2(m) =CO_2(c) =CoA-activated acetyl group(m)	Synthesis of AKG	1.5 H_2O(s) 1 CO_2(c) 0.5 Glucose-6P(c) 1 NADPH-redox-potential(c) 1 CoA-activated acetyl group(m)	0.5 P_i(c) 1 CO_2(m) 1 AKG(c) 1.5 ATP-energy(c) 1 NADH-redox-potential(c) 1 NADH-redox-potential(m) 1 NADPH-redox-potential(m)	13	8	-	-	-	-	-	-	1	-	2	-
**A5.3. Terpenoids**																	
62 Isopentenyl-PP	Isopentenyl-PP(p)	−Acetyl-CoA(c) +CoA(c) =ATP-energy(c) =NADH-redox-potential(c) =NADPH-redox-potential(c) =H_2O =P_i(c) =CO_2	synthesis of peroxysomal Isopentenyl-PP	2 P_i(c) 3 Acetyl-CoA(c) 3 ATP-energy(c) 2 NADPH-redox-potential(c)	2 H_2O(s) 3 CoA(c) 1 CO_2(s) 1 Isopentenyl-PP(p)	5	-	-	3	-	-	-	-	2	-	4	-
63 Farnesyl-PP	Farnesyl-PP(p)	−Acetyl-CoA(c) +CoA(c) =ATP-energy(c) =NADH-redox-potential(c) =NADPH-redox-potential(c) =H_2O =P_i(c) =CO_2	de novo synthesis of peroxysomal Farnesyl-PP	2 P_i(c) 9 Acetyl-CoA(c) 9 ATP-energy(c) 6 NADPH-redox-potential(c)	4 H_2O(s) 9 CoA(c) 3 CO_2(s) 1 Farnesyl-PP(p)	6	-	-	6	-	-	-	-	2	-	5	-
**A5.4. Aminosugars**																	

Continued on next page

A5. Important intermediates – continued.

Simulation	Definition: Objective	Definition: Contraints	Definition: Comment	Solution: exchanges: imports	Solution: exchanges: exports	reactions: c	m	r	p	l	n	s	b	transport: s-c	b-c	intra	Prot syn
64 Glucosamine-6P	Glucosamine-6P(c)	−CoA-activated acetyl group(c) +Glutamate =Na-gradient(c) −Glutamine −Glucose-6P(c) =ATP-energy(c) =NADH-redox-potential(c) =NADPH-redox-potential(c) $=H_2O$ $=P_i(c)$ $=CO_2$	synthesis of D-Glucosamine 6-phosphate from activated acetyl and Glutamine minus Glutamate	1 Glutamine(s) 1 Glucose-6P(c)	1 Glutamate(s) 1 Glucosamine-6P(c)	2	-	-	-	-	-	-	-	2	-	-	-

2.6 A6. Salvage

Table 6: A6. Salvage

Simulation	Definition: Objective	Definition: Contraints	Definition: Comment	Solution: exchanges: imports	Solution: exchanges: exports	reactions: c	m	r	p	l	n	s	b	transport: s-c	b-c	intra	Prot syn
65 ATP salvage from Adenosine	ATP(c)	−Adenosine(c) =ATP-energy(c) =NADH-redox-potential(c) $-P_i(c)$ $+CO_2$ $-O_2$ $=H_2O$	ATP salvage from Adenosine	3 $P_i(c)$ 1 Adenosine(c) 3 ATP-energy(c)	3 $H_2O(s)$ 1 ATP(c)	3	-	-	-	-	-	-	-	1	-	-	-
66 ATP salvage from Hypoxanthine	ATP(c)	−Hypoxanthine(c) −Glucose-6P(c) −Pyruvate(c) =ATP-energy(c) =NADH-redox-potential(c) =NADPH-redox-potential(c) =NADH-redox-potential(m) =NADPH-redox-potential(m) $-P_i(c)$ −Glutamine(c) $+CO_2$ $-O_2$ $=H_2O$	ATP salvage from Hypoxanthine	0.25 $O_2(s)$ 2.42 $P_i(c)$ 0.5 Glutamine(c) 0.58 Glucose-6P(c) 1 Hypoxanthine(c) 5.42 ATP-energy(c)	2.92 $H_2O(s)$ 1 ATP(c) 1 $CO_2(s)$ 0.5 NADH-redox-potential(c) 0.5 NADH-redox-potential(m)	26	9	-	-	-	-	-	-	3	-	11	-

Continued on next page

A6. Salvage – continued.

Simulation	Definition			Solution													
				exchanges		reactions								transport			Prot
	Objective	Contraints	Comment	imports	exports	c	m	r	p	l	n	s	b	s-c	b-c	intra	syn
67 dTTP salvage from Thymine	dTTP(c)	−Thymine(c) −Glucose-6P(c) =ATP-energy(c) =NADH-redox-potential(c) =NADPH-redox-potential(c) =NADH-redox-potential(m) =NADPH-redox-potential(m) $-P_i$(c) $+CO_2$ $-O_2$ $=H_2O$	dTTP salvage from Thymine	2.17 P_i(c) 0.83 Glucose-6P(c) 1 Thymine(c) 5.17 ATP-energy(c) 1 NADPH-redox-potential(c)	4.17 H_2O(s) 1 dTTP(c)	24	-	-	-	-	-	-	-	1	-	-	-

2.7 A7. Detoxification of internally emerging substances

Table 7: A7. Detoxification of internally emerging substances

Simulation	Definition			Solution													
				exchanges		reactions								transport			Prot
	Objective	Contraints	Comment	imports	exports	c	m	r	p	l	n	s	b	s-c	b-c	intra	syn
68 O_2.-(c) degr	-1 O_2.-(c)	MCES $=O_2$	degradation of cytosolic O_2.-	1 O_2.-(c)	0.5 H_2O(s) 0.75 O_2(s)	2	-	-	-	-	-	-	-	2	-	-	-
69 Formaldehyde degr	-1 Formaldehyde	MCES	degradation of external formaldehyde	1 Formaldehyde(s) 1 ATP-energy(c)	0.5 CO_2(s) 0.5 NADH-redox-potential(c) 0.5 THF-activated methyl group(c)	11	-	-	-	-	-	-	-	2	-	-	-
70 Formate degr	-1 Formate	MCES	degradation of external formate	1 Formate(s) 1 ATP-energy(c) 1 Proton-gradient(c)	1 CO_2(s) 1 NADPH-redox-potential(c)	4	-	-	-	-	-	-	-	4	-	-	-

3 B. Functions of organismic duty

3.1 B1. Storage/Release

Table 8: B1. Storage/Release

Simulation	Definition: Objective	Definition: Contraints	Definition: Comment	Solution: exchanges: imports	Solution: exchanges: exports	reactions: c	m	r	p	l	n	s	b	transport: s-c	b-c	intra	Prot syn
**B1.1. Glycogen**																	
71 Glycogen glucose storage	Glycogenin-G4G7(c)	−Glycogenin(c) −UDP-activated-glucose(c)	test the storage of glucose in a glycogen component	1 Glycogenin(c) 11 UDP-activated-glucose(c)	1 Glycogenin-G4G7(c)	4	-	-	-	-	-	-	-	-	-	-	-
72 Glycogen glucose release	Glucose	−Glycogenin-G4G7(c) +Glycogenin(c) −Glucose $-O_2$ $+CO_2$ $=H_2O$	test the release of glucose from a glycogen component	1 H_2O(s) 0.09 Glycogenin-G4G7(c)	1 Glucose(s) 0.09 Glycogenin(c)	5	-	1	-	-	-	-	-	2	-	3	-

3.2 B2. Turnover functions

Table 9: B2. Turnover functions

Simulation	Definition: Objective	Definition: Contraints	Definition: Comment	Solution: exchanges: imports	Solution: exchanges: exports	reactions: c	m	r	p	l	n	s	b	transport: s-c	b-c	intra	Prot syn
**B2.1. Gluconeogenesis**																	
73 Gluconeogen from Lactate	Glucose	−L-Lactate(c) −ATP-energy(m) −ATP-energy(c) =NADH-redox-potential(c) =NADPH-redox-potential(c) =NADH-redox-potential(m) =NADPH-redox-potential(m) =Proton-gradient(m) $=H_2O$ $=CO_2$ $=O_2$	Gluconeogenese from Lactate	2 L-Lactate(c) 2 ATP-energy(c) 2 ATP-energy(m) 2 NADPH-redox-potential(m) 2 Proton-gradient(m)	1 Glucose(s) 2 NADH-redox-potential(m)	10	7	1	-	-	-	-	-	1	-	9	-

Continued on next page

#	Simulation	Definition: Objective	Definition: Contraints	Definition: Comment	Solution: exchanges: imports	Solution: exchanges: exports	reactions: c	reactions: m	reactions: r	reactions: p	reactions: l	reactions: n	reactions: s	reactions: b	transport: s-c	transport: b-c	transport: intra	Prot syn
74	Gluconeogen from Glycerol	Glucose	−Glycerol −ATP-energy(m) −ATP-energy(c) =NADH-redox-potential(c) =NADPH-redox-potential(c) =Proton-gradient(m) $=H_2O$ $=CO_2$ $=O_2$ %r0530	Gluconeogenese from Glycerol	1 O_2(s) 2 Glycerol(s) 2 ATP-energy(c)	2 H_2O(s) 1 Glucose(s) 12 Proton-gradient(m)	6	-	1	-	-	-	-	-	4	-	9	-
75	Gluconeogen from Alanine	Glucose	−Alanine −ATP-energy(m) −ATP-energy(c) =NADH-redox-potential(c) =NADPH-redox-potential(c) =Proton-gradient(m) $=H_2O$ $=CO_2$ $=O_2$ +Urea	Gluconeogenese from Alanine	1 H_2O(s) 1 CO_2(s) 2 Alanine(s) 5 ATP-energy(c) 3 ATP-energy(m) 1 NADPH-redox-potential(c)	1 Glucose(s) 1 Urea(s) 1 NADH-redox-potential(c)	23	7	1	-	-	-	-	-	5	-	9	-
	**B2.2. Amino acid nxn**																	
76	Alanine from Arginine	Alanine	−Arginine MCES +Urea	Transamination to Alanine using Arginine	1.5 H_2O(s) 0.25 O_2(s) 0.5 Pyruvate(m) 0.5 Arginine(s) 0.5 NADPH-redox-potential(m)	1 CO_2(s) 1 Alanine(s) 0.5 Urea(s) 0.5 ATP-energy(m) 1 NADH-redox-potential(m) 1 NADPH-redox-potential(c) 1.5 Proton-gradient(m)	4	11	-	-	-	-	-	-	7	-	10	-
77	Alanine from Asparagine	Alanine	−Asparagine MCES +Urea	Transamination to Alanine using Asparagine	0.5 Pyruvate(c) 0.5 Asparagine(s) 0.5 NADH-redox-potential(c) 0.5 NADPH-redox-potential(m) 1 Proton-gradient(m)	0.5 CO_2(s) 1 Alanine(s) 0.5 NADPH-redox-potential(c)	7	2	-	-	-	-	-	-	4	-	7	-
78	Alanine from Aspartate	Alanine	−Aspartate MCES +Urea	Transamination to Alanine using Aspartate	1 Aspartate(s) 1 NADH-redox-potential(c)	1 CO_2(s) 1 Alanine(s) 1 NADPH-redox-potential(c) 1 Na-gradient(c)	6	-	-	-	-	-	-	-	4	-	-	-
79	Alanine from Cysteine	Alanine	−Cysteine MCES +Urea $+H_2S$	Transamination to Alanine using Cysteine	1 Cysteine(s) 1 NADH-redox-potential(m) 2 Proton-gradient(m)	1 Alanine(s) 1 H_2S(s) 1 Na-gradient(c)	2	2	-	-	-	-	-	-	4	-	7	-

Continued on next page

B2. Turnover functions – continued.

Simulation		Definition			Solution													
					exchanges		reactions								transport			Prot
		Objective	Contraints	Comment	imports	exports	c	m	r	p	l	n	s	b	s-c	b-c	intra	syn
80	Alanine from Cystine	Alanine	−Cystine MCES +Urea +H_2S	Transamination to Alanine using Cystine	0.5 Cystine(s) 1 NADH-redox-potential(m) 0.5 NADPH-redox-potential(c) 2 Proton-gradient(m)	1 Alanine(s) 1 H_2S(s) 0.5 Na-gradient(c)	4	2	-	-	-	-	-	-	5	-	7	-
81	Alanine from Glutamate	Alanine	−Glutamate MCES +Urea	Transamination to Alanine using Glutamate	1 H_2O(s) 0.5 O_2(s) 1 Glutamate(s)	2 CO_2(s) 1 Alanine(s) 1 ATP-energy(m) 1 NADH-redox-potential(m) 1 NADPH-redox-potential(c) 6 Proton-gradient(m) 1 Na-gradient(c)	3	7	-	-	-	-	-	-	6	-	7	-
82	Alanine from Glycine	Alanine	−Glycine MCES +Urea	Transamination to Alanine using Glycine	1 Glycine(s) 1 THF-activated methyl group(c)	1 Alanine(s)	9	-	-	-	-	-	-	-	1	-	-	-
83	Alanine from Histidine	Alanine	−Histidine MCES +Urea	Transamination to Alanine using Histidine	0.33 H_2O(s) 0.17 O_2(s) 0.33 Pyruvate(c) 0.33 Pyruvate(m) 0.33 Histidine(s) 0.33 NADH-redox-potential(c) 0.33 NADH-redox-potential(m) 0.33 NADPH-redox-potential(m)	0.67 CO_2(s) 1 Alanine(s) 0.33 ATP-energy(m) 0.33 NADPH-redox-potential(c) 1 Proton-gradient(m) 0.33 THF-activated methyl group(c)	12	10	-	-	-	-	-	-	6	-	11	-
84	Alanine from Isoleucine	Alanine	−Isoleucine MCES +Urea	Transamination to Alanine using Isoleucine	1 H_2O(s) 1 O_2(s) 1 Pyruvate(c) 1 Isoleucine(s)	2 CO_2(s) 1 Alanine(s) 4 NADH-redox-potential(m) 9 Proton-gradient(m) 2 CoA-activated acetyl group(m)	1	13	-	-	-	-	-	-	5	-	11	-
85	Alanine from Glutamine	Alanine	−Glutamine MCES +Urea	Transamination to Alanine using Glutamine	0.5 H_2O(s) 0.25 O_2(s) 0.5 Pyruvate(c) 0.5 Glutamine(s) 0.5 NADPH-redox-potential(m)	1 CO_2(s) 1 Alanine(s) 0.5 ATP-energy(m) 0.5 NADH-redox-potential(m) 0.5 NADPH-redox-potential(c) 2 Proton-gradient(m)	5	9	-	-	-	-	-	-	5	-	11	-
86	Alanine from Leucine	Alanine	−Leucine MCES +Urea	Transamination to Alanine using Leucine	0.5 O_2(s) 1 Pyruvate(c) 1 Leucine(s) 1 NADPH-redox-potential(m)	1 H_2O(s) 1 Alanine(s) 2 NADH-redox-potential(m) 4 Proton-gradient(m) 1 Na-gradient(c) 3 CoA-activated acetyl group(m)	1	13	-	-	-	-	-	-	5	-	10	-
87	Alanine from Lysine	Alanine	−Lysine MCES +Urea	Transamination to Alanine using Lysine	0.5 H_2O(s) 0.25 O_2(s) 1 Pyruvate(c) 0.5 Lysine(s)	1 CO_2(s) 1 Alanine(s) 0.5 NADH-redox-potential(c) 1 NADH-redox-potential(m) 1 Proton-gradient(m) 1 CoA-activated acetyl group(m)	3	10	-	-	-	-	-	-	5	-	11	-

Continued on next page

B2. Turnover functions – continued.

Simulation		Definition			Solution													
					exchanges		reactions								transport			Prot
		Objective	Contraints	Comment	imports	exports	c	m	r	p	l	n	s	b	s-c	b-c	intra	syn
88	Alanine from Methionine	Alanine	−Methionine MCES +Urea +H_2S	Transamination to Alanine using Methionine	1.5 H_2O(s) 0.5 O_2(s) 1 Methionine(s) 0.5 Glucose-6P(c)	0.5 P_i(c) 2 CO_2(s) 1 Alanine(s) 1 H_2S(s) 0.5 ATP-energy(c) 2 NADH-redox-potential(c) 1 NADH-redox-potential(m) 1 Proton-gradient(m) 1 Na-gradient(c) 1 THF-activated methyl group(c) 1 CoA-activated acetyl group(m)	17	9	-	-	-	-	-	-	7	-	12	-
89	Alanine from Phenylalanine	Alanine	−Phenylalanine MCES +Urea	Transamination to Alanine using Phenylalanine	3 O_2(s) 1 Phenylalanine(s) 2 ATP-energy(c)	2 CO_2(s) 1 Alanine(s) 2 CoA-activated acetyl group(c)	16	-	-	-	-	-	-	-	4	-	-	-
90	Alanine from Proline	Alanine	−Proline MCES +Urea	Transamination to Alanine using Proline	4 H_2O(s) 1 Proline(s)	2 CO_2(s) 1 Alanine(s) 1 ATP-energy(m) 2 NADH-redox-potential(m) 1 NADPH-redox-potential(c) 1 Na-gradient(c)	3	10	-	-	-	-	-	-	5	-	4	-
91	Alanine from Serine	Alanine	−Serine MCES +Urea	Transamination to Alanine using Serine	1 Serine(s) 1 NADPH-redox-potential(c)	1 H_2O(s) 1 Alanine(s)	7	-	-	-	-	-	-	-	3	-	-	-
92	Alanine from Threonine	Alanine	−Threonine MCES +Urea	Transamination to Alanine using Threonine	0.5 O_2(s) 1 Pyruvate(c) 1 Threonine(s)	2 CO_2(s) 1 Alanine(s) 2 NADH-redox-potential(m) 3 Proton-gradient(m) 1 CoA-activated acetyl group(m)	2	9	-	-	-	-	-	-	4	-	11	-
93	Alanine from Tryptophan	Alanine	−Tryptophan MCES +Urea	Transamination to Alanine using Tryptophan	1.75 O_2(s) 0.5 Pyruvate(c) 0.5 Tryptophan(s) 0.5 ATP-energy(c) 1.5 NADPH-redox-potential(c) 0.5 NADPH-redox-potential(m)	1.5 CO_2(s) 1 Alanine(s) 1 NADH-redox-potential(m) 1.5 Proton-gradient(m) 0.5 THF-activated methyl group(c) 1 CoA-activated acetyl group(m)	16	9	-	-	-	-	-	-	5	-	12	-
94	Alanine from Tyrosine	Alanine	−Tyrosine MCES +Urea	Transamination to Alanine using Tyrosine	1 H_2O(s) 2 O_2(s) 1 Tyrosine(s) 2 ATP-energy(c)	2 CO_2(s) 1 Alanine(s) 1 NADPH-redox-potential(c) 1 Na-gradient(c) 2 CoA-activated acetyl group(c)	15	-	-	-	-	-	-	-	6	-	-	-
95	Alanine from Valine	Alanine	−Valine MCES +Urea	Transamination to Alanine using Valine	2 H_2O(s) 1 O_2(s) 1 Valine(s)	2 CO_2(s) 1 Alanine(s) 1 ATP-energy(m) 3 NADH-redox-potential(m) 1 NADPH-redox-potential(c) 10 Proton-gradient(m)	3	14	-	-	-	-	-	-	5	-	10	-

Continued on next page

B2. Turnover functions – continued.

	Simulation	Definition: Objective	Definition: Contraints	Definition: Comment	Solution: exchanges: imports	Solution: exchanges: exports	reactions: c	reactions: m	reactions: r	reactions: p	reactions: l	reactions: n	reactions: s	reactions: b	transport: s-c	transport: b-c	transport: intra	Prot syn
96	Alanine from beta-Alanine	Alanine	−beta-Alanine MCES +Urea	Transamination to Alanine using beta-Alanine	1 Pyruvate(c) 1 beta-Alanine(s) 2 Proton-gradient(m)	1 CO_2(s) 1 Alanine(s) 1 NADH-redox-potential(m) 1 CoA-activated acetyl group(m)	1	4	-	-	-	-	-	-	3	-	7	-
97	Arginine from Alanine	Arginine	−Alanine MCES +Urea	Transamination to Arginine using Alanine	4 Alanine(s) 2 ATP-energy(c) 3 ATP-energy(m) 2 NADPH-redox-potential(m) 1 Proton-gradient(m)	1 Pyruvate(c) 1 Pyruvate(m) 1 Arginine(s) 1 NADH-redox-potential(c) 4 NADH-redox-potential(m) 1 Na-gradient(c)	10	17	-	-	-	-	-	-	4	-	3	-
98	Arginine from Asparagine	Arginine	−Asparagine MCES +Urea	Transamination to Arginine using Asparagine	2 Asparagine(s) 3 ATP-energy(c) 3 ATP-energy(m) 1 NADH-redox-potential(c) 2 NADPH-redox-potential(m) 1 Proton-gradient(m) 1 CoA-activated acetyl group(m)	1 CO_2(s) 1 Pyruvate(c) 1 Arginine(s) 2 NADH-redox-potential(m) 1 NADPH-redox-potential(c) 1 Proton-gradient(c) 2 Na-gradient(c)	13	16	-	-	-	-	-	-	6	-	6	-
99	Arginine from Aspartate	Arginine	−Aspartate MCES +Urea	Transamination to Arginine using Aspartate	4 Aspartate(s) 2 ATP-energy(c) 3 ATP-energy(m) 1 NADH-redox-potential(c) 2 Proton-gradient(m)	5 CO_2(s) 1 Pyruvate(c) 1 Arginine(s) 3 NADH-redox-potential(m) 2 NADPH-redox-potential(c) 4 Na-gradient(c) 1 CoA-activated acetyl group(m)	14	14	-	-	-	-	-	-	4	-	6	-
100	Arginine from Cysteine	Arginine	−Cysteine MCES +Urea +H_2S	Transamination to Arginine using Cysteine	2 Pyruvate(m) 4 Cysteine(s) 2 ATP-energy(c) 3 ATP-energy(m) 2 NADPH-redox-potential(m) 3 Proton-gradient(m)	4 Pyruvate(c) 1 Arginine(s) 4 H_2S(s) 1 NADH-redox-potential(c) 4 Na-gradient(c)	10	15	-	-	-	-	-	-	5	-	8	-
101	Arginine from Cystine	Arginine	−Cystine MCES +Urea +H_2S	Transamination to Arginine using Cystine	2 Pyruvate(m) 2 Cystine(s) 2 ATP-energy(c) 3 ATP-energy(m) 2 NADPH-redox-potential(c) 2 NADPH-redox-potential(m) 3 Proton-gradient(m)	4 Pyruvate(c) 1 Arginine(s) 4 H_2S(s) 1 NADH-redox-potential(c) 2 Na-gradient(c)	12	15	-	-	-	-	-	-	5	-	8	-
102	Arginine from Glutamate	Arginine	−Glutamate MCES +Urea	Transamination to Arginine using Glutamate	2 H_2O(s) 1.5 O_2(s) 4 Glutamate(s) 2 ATP-energy(c)	5 CO_2(s) 1 Pyruvate(c) 2 Pyruvate(m) 1 Arginine(s) 1 ATP-energy(m) 1 NADH-redox-potential(c) 2 NADH-redox-potential(m) 1 NADPH-redox-potential(c) 3 NADPH-redox-potential(m) 14 Proton-gradient(m) 4 Na-gradient(c)	11	14	-	-	-	-	-	-	6	-	10	-

Continued on next page

B2. Turnover functions – continued.

Simulation	Definition			Solution													
				exchanges		reactions								transport			Prot
	Objective	Contraints	Comment	imports	exports	c	m	r	p	l	n	s	b	s-c	b-c	intra	syn
103 Arginine from Glycine	Arginine	−Glycine MCES +Urea	Transamination to Arginine using Glycine	2 Pyruvate(m) 4 Glycine(s) 2 ATP-energy(c) 3 ATP-energy(m) 4 NADPH-redox-potential(m) 1 Proton-gradient(m) 4 THF-activated methyl group(c)	4 Pyruvate(c) 1 Arginine(s) 1 NADH-redox-potential(c) 3 NADH-redox-potential(m) 3 NADPH-redox-potential(c) 4 Na-gradient(c)	21	16	-	-	-	-	-	-	3	-	4	-
104 Arginine from Histidine	Arginine	−Histidine MCES +Urea	Transamination to Arginine using Histidine	0.17 O_2(s) 1.33 Histidine(s) 2 ATP-energy(c) 1.67 ATP-energy(m) 0.67 NADH-redox-potential(m) 0.33 NADPH-redox-potential(c) 1.67 NADPH-redox-potential(m)	0.33 Pyruvate(c) 1 Arginine(s) 0.33 Na-gradient(c) 1 THF-activated methyl group(c)	20	13	-	-	-	-	-	-	5	-	11	-
105 Arginine from Isoleucine	Arginine	−Isoleucine MCES +Urea	Transamination to Arginine using Isoleucine	2 H_2O(s) 3.5 O_2(s) 1 Pyruvate(m) 4 Isoleucine(s) 2 ATP-energy(c) 3 ATP-energy(m) 3 NADPH-redox-potential(m)	4 CO_2(s) 1 Pyruvate(c) 1 Arginine(s) 16 NADH-redox-potential(m) 1 NADPH-redox-potential(c) 35 Proton-gradient(m) 1 Na-gradient(c) 7 CoA-activated acetyl group(m)	8	29	-	-	-	-	-	-	7	-	11	-
106 Arginine from Glutamine	Arginine	−Glutamine MCES +Urea	Transamination to Arginine using Glutamine	0.5 O_2(s) 2 Glutamine(s) 2 ATP-energy(c) 1 ATP-energy(m) 1 NADPH-redox-potential(m)	1 CO_2(s) 1 Pyruvate(c) 1 Arginine(s) 1 NADH-redox-potential(c) 1 NADPH-redox-potential(c) 4 Proton-gradient(m) 1 Na-gradient(c)	11	13	-	-	-	-	-	-	6	-	9	-
107 Arginine from Leucine	Arginine	−Leucine MCES +Urea	Transamination to Arginine using Leucine	2 O_2(s) 2 Pyruvate(m) 4 Leucine(s) 2 ATP-energy(c) 3 ATP-energy(m) 6 NADPH-redox-potential(m)	4 H_2O(s) 1 Arginine(s) 1 NADH-redox-potential(c) 12 NADH-redox-potential(m) 21 Proton-gradient(m) 4 Na-gradient(c) 12 CoA-activated acetyl group(m)	9	25	-	-	-	-	-	-	6	-	11	-
108 Arginine from Lysine	Arginine	−Lysine MCES +Urea	Transamination to Arginine using Lysine	2 H_2O(s) 1 O_2(s) 2 Pyruvate(m) 2 Lysine(s) 2 ATP-energy(c) 3 ATP-energy(m) 3 NADPH-redox-potential(m)	3 CO_2(s) 1 Pyruvate(c) 1 Arginine(s) 2 NADH-redox-potential(c) 8 NADH-redox-potential(m) 1 NADPH-redox-potential(c) 9 Proton-gradient(m) 3 CoA-activated acetyl group(m)	10	25	-	-	-	-	-	-	5	-	11	-

Continued on next page

B2. Turnover functions – continued.

Simulation	Definition: Objective	Definition: Contraints	Definition: Comment	Solution: exchanges: imports	Solution: exchanges: exports	reactions: c	reactions: m	reactions: r	reactions: p	reactions: l	reactions: n	reactions: s	reactions: b	transport: s-c	transport: b-c	transport: intra	Prot syn
109 Arginine from Methionine	Arginine	−Methionine MCES +Urea +H_2S	Transamination to Arginine using Methionine	4 H_2O(s) 1.5 O_2(s) 1 Pyruvate(m) 4 Methionine(s) 2 Glucose-6P(c) 3 ATP-energy(m) 3 NADPH-redox-potential(m)	2 P_i(c) 4 CO_2(s) 5 Pyruvate(c) 1 Arginine(s) 4 H_2S(s) 8 NADH-redox-potential(c) 4 NADH-redox-potential(m) 1 NADPH-redox-potential(c) 3 Proton-gradient(m) 4 Na-gradient(c) 4 THF-activated methyl group(c) 3 CoA-activated acetyl group(m)	22	24	-	-	-	-	-	-	8	-	14	-
110 Arginine from Phenylalanine	Arginine	−Phenylalanine MCES +Urea	Transamination to Arginine using Phenylalanine	12 O_2(s) 4 Phenylalanine(s) 10 ATP-energy(c) 3 ATP-energy(m) 2 NADPH-redox-potential(c) 4 Proton-gradient(m) 1 CoA-activated acetyl group(m)	7 CO_2(s) 1 Pyruvate(c) 2 Pyruvate(m) 1 Arginine(s) 1 NADH-redox-potential(c) 2 NADH-redox-potential(m) 1 NADPH-redox-potential(m) 1 Na-gradient(c) 8 CoA-activated acetyl group(c)	24	13	-	-	-	-	-	-	6	-	6	-
111 Arginine from Proline	Arginine	−Proline MCES +Urea	Transamination to Arginine using Proline	13 H_2O(s) 4 Proline(s) 2 ATP-energy(c)	5 CO_2(s) 1 Pyruvate(c) 2 Pyruvate(m) 1 Arginine(s) 1 ATP-energy(m) 1 NADH-redox-potential(c) 5 NADH-redox-potential(m) 1 NADPH-redox-potential(c) 3 NADPH-redox-potential(m) 1 Na-gradient(c)	11	16	-	-	-	-	-	-	6	-	8	-
112 Arginine from Serine	Arginine	−Serine MCES +Urea	Transamination to Arginine using Serine	2 Pyruvate(m) 4 Serine(s) 2 ATP-energy(c) 3 ATP-energy(m) 1 NADPH-redox-potential(c) 4 NADPH-redox-potential(m) 1 Proton-gradient(m)	4 H_2O(s) 4 Pyruvate(c) 1 Arginine(s) 1 NADH-redox-potential(c) 3 NADH-redox-potential(m) 4 Proton-gradient(c) 4 Na-gradient(c)	18	16	-	-	-	-	-	-	5	-	4	-
113 Arginine from Threonine	Arginine	−Threonine MCES +Urea	Transamination to Arginine using Threonine	1.5 O_2(s) 1 Pyruvate(m) 4 Threonine(s) 2 ATP-energy(c) 3 ATP-energy(m) 3 NADPH-redox-potential(m)	2 H_2O(s) 4 CO_2(s) 1 Pyruvate(c) 1 Arginine(s) 8 NADH-redox-potential(m) 1 NADPH-redox-potential(c) 11 Proton-gradient(m) 1 Na-gradient(c) 3 CoA-activated acetyl group(m)	9	24	-	-	-	-	-	-	7	-	12	-

Continued on next page

B2. Turnover functions – continued.

Simulation	Definition			Solution													
				exchanges		reactions								transport			Prot
	Objective	Contraints	Comment	imports	exports	c	m	r	p	l	n	s	b	s-c	b-c	intra	syn
114 Arginine from Tryptophan	Arginine	−Tryptophan MCES +Urea	Transamination to Arginine using Tryptophan	7 O_2(s) 2 Pyruvate(m) 2 Tryptophan(s) 5 ATP-energy(c) 3 ATP-energy(m) 4 NADPH-redox-potential(c) 4 NADPH-redox-potential(m)	5 CO_2(s) 3 Pyruvate(c) 1 Arginine(s) 6 NADH-redox-potential(m) 10 Proton-gradient(m) 1 Na-gradient(c) 2 THF-activated methyl group(c) 3 CoA-activated acetyl group(m)	26	20	-	-	-	-	-	-	6	-	12	-
115 Arginine from Tyrosine	Arginine	−Tyrosine MCES +Urea	Transamination to Arginine using Tyrosine	4 H_2O(s) 8 O_2(s) 4 Tyrosine(s) 10 ATP-energy(c) 3 ATP-energy(m) 4 Proton-gradient(m) 1 CoA-activated acetyl group(m)	7 CO_2(s) 1 Pyruvate(c) 2 Pyruvate(m) 1 Arginine(s) 1 NADH-redox-potential(c) 2 NADH-redox-potential(m) 2 NADPH-redox-potential(c) 1 NADPH-redox-potential(m) 4 Na-gradient(c) 8 CoA-activated acetyl group(c)	22	13	-	-	-	-	-	-	6	-	6	-
116 Arginine from Valine	Arginine	−Valine MCES +Urea	Transamination to Arginine using Valine	6 H_2O(s) 3.5 O_2(s) 4 Valine(s) 2 ATP-energy(c)	5 CO_2(s) 1 Pyruvate(c) 2 Pyruvate(m) 1 Arginine(s) 1 NADH-redox-potential(c) 12 NADH-redox-potential(m) 1 NADPH-redox-potential(c) 1 NADPH-redox-potential(m) 36 Proton-gradient(m) 4 Na-gradient(c)	11	22	-	-	-	-	-	-	7	-	11	-
117 Arginine from beta-Alanine	Arginine	−beta-Alanine MCES +Urea	Transamination to Arginine using beta-Alanine	2 Pyruvate(m) 4 beta-Alanine(s) 2 ATP-energy(c) 3 ATP-energy(m) 2 NADPH-redox-potential(m) 3 Proton-gradient(m)	3 CO_2(s) 1 Pyruvate(c) 1 Arginine(s) 6 NADH-redox-potential(m) 2 NADPH-redox-potential(c) 3 CoA-activated acetyl group(m)	10	17	-	-	-	-	-	-	3	-	8	-
118 Asparagine from Alanine	Asparagine	−Alanine MCES +Urea	Transamination to Asparagine using Alanine	1 Pyruvate(m) 2 Alanine(s) 2 ATP-energy(c) 1 ATP-energy(m) 1 NADPH-redox-potential(m)	1 Pyruvate(c) 1 Asparagine(s) 1 NADH-redox-potential(c) 2 NADH-redox-potential(m) 1 Na-gradient(c) 1 CoA-activated acetyl group(m)	9	8	-	-	-	-	-	-	4	-	4	-
119 Asparagine from Arginine	Asparagine	−Arginine MCES +Urea	Transamination to Asparagine using Arginine	3 H_2O(s) 0.5 O_2(s) 1 Arginine(s) 3 ATP-energy(c)	1 CO_2(s) 1 Urea(s) 1 Asparagine(s) 1 ATP-energy(m) 1 NADH-redox-potential(c) 3 NADH-redox-potential(m) 4 Proton-gradient(m)	9	10	-	-	-	-	-	-	6	-	12	-

Continued on next page

B2. Turnover functions – continued.

Simulation	Definition			Solution													
				exchanges		reactions								transport			Prot
	Objective	Contraints	Comment	imports	exports	c	m	r	p	l	n	s	b	s-c	b-c	intra	syn
120 Asparagine from Aspartate	Asparagine	−Aspartate MCES +Urea	Transamination to Asparagine using Aspartate	2 Aspartate(s) 4 ATP-energy(c)	1 CO_2(s) 1 Pyruvate(c) 1 Asparagine(s) 1 NADPH-redox-potential(c) 2 Na-gradient(c)	12	-	-	-	-	-	-	-	4	-	-	-
121 Asparagine from Cysteine	Asparagine	−Cysteine MCES +Urea +H_2S	Transamination to Asparagine using Cysteine	2 Pyruvate(m) 2 Cysteine(s) 3 ATP-energy(c) 1 NADPH-redox-potential(m) 2 Proton-gradient(m)	2 Pyruvate(c) 1 Asparagine(s) 2 H_2S(s) 1 NADH-redox-potential(m) 2 Na-gradient(c) 1 CoA-activated acetyl group(m)	6	8	-	-	-	-	-	-	5	-	6	-
122 Asparagine from Cystine	Asparagine	−Cystine MCES +Urea +H_2S	Transamination to Asparagine using Cystine	2 Pyruvate(m) 1 Cystine(s) 3 ATP-energy(c) 1 NADPH-redox-potential(c) 1 NADPH-redox-potential(m) 2 Proton-gradient(m)	2 Pyruvate(c) 1 Asparagine(s) 2 H_2S(s) 1 NADH-redox-potential(m) 1 Na-gradient(c) 1 CoA-activated acetyl group(m)	8	8	-	-	-	-	-	-	5	-	6	-
123 Asparagine from Glutamate	Asparagine	−Glutamate MCES +Urea	Transamination to Asparagine using Glutamate	2 H_2O(s) 1 O_2(s) 2 Glutamate(s) 3 ATP-energy(c)	3 CO_2(s) 1 Pyruvate(m) 1 Asparagine(s) 2 ATP-energy(m) 3 NADH-redox-potential(m) 2 NADPH-redox-potential(m) 10 Proton-gradient(m) 2 Na-gradient(c)	5	12	-	-	-	-	-	-	6	-	9	-
124 Asparagine from Glycine	Asparagine	−Glycine MCES +Urea	Transamination to Asparagine using Glycine	2 Pyruvate(m) 2 Glycine(s) 3 ATP-energy(c) 2 NADPH-redox-potential(m) 2 Proton-gradient(m) 2 THF-activated methyl group(c)	2 Pyruvate(c) 1 Asparagine(s) 2 NADH-redox-potential(m) 2 NADPH-redox-potential(c) 1 Na-gradient(c) 1 CoA-activated acetyl group(m)	10	8	-	-	-	-	-	-	3	-	6	-
125 Asparagine from Histidine	Asparagine	−Histidine MCES +Urea	Transamination to Asparagine using Histidine	0.67 H_2O(s) 0.33 O_2(s) 0.33 Pyruvate(m) 0.67 Histidine(s) 3 ATP-energy(c) 0.67 NADPH-redox-potential(c) 0.33 NADPH-redox-potential(m)	0.33 CO_2(s) 1 Asparagine(s) 0.67 ATP-energy(m) 0.33 NADH-redox-potential(c) 0.33 NADH-redox-potential(m) 3.67 Proton-gradient(m) 0.67 THF-activated methyl group(c)	17	10	-	-	-	-	-	-	6	-	10	-
126 Asparagine from Isoleucine	Asparagine	−Isoleucine MCES +Urea	Transamination to Asparagine using Isoleucine	2 H_2O(s) 2 O_2(s) 1 Pyruvate(m) 2 Isoleucine(s) 3 ATP-energy(c) 1 NADPH-redox-potential(m)	3 CO_2(s) 1 Asparagine(s) 10 NADH-redox-potential(m) 20 Proton-gradient(m) 1 Na-gradient(c) 4 CoA-activated acetyl group(m)	5	18	-	-	-	-	-	-	7	-	11	-

Continued on next page

B2. Turnover functions – continued.

Simulation	Definition			Solution													
				exchanges		reactions								transport			Prot
	Objective	Contraints	Comment	imports	exports	c	m	r	p	l	n	s	b	s-c	b-c	intra	syn
127 Asparagine from Glutamine	Asparagine	−Glutamine MCES +Urea	Transamination to Asparagine using Glutamine	1 H_2O(s) 0.5 O_2(s) 1 Glutamine(s) 2 ATP-energy(c)	1 CO_2(s) 1 Asparagine(s) 1 ATP-energy(m) 2 NADH-redox-potential(m) 5 Proton-gradient(m)	4	9	-	-	-	-	-	-	5	-	7	-
128 Asparagine from Leucine	Asparagine	−Leucine MCES +Urea	Transamination to Asparagine using Leucine	1 O_2(s) 2 Pyruvate(m) 2 Leucine(s) 3 ATP-energy(c) 3 NADPH-redox-potential(m)	2 H_2O(s) 1 Asparagine(s) 7 NADH-redox-potential(m) 10 Proton-gradient(m) 2 Na-gradient(c) 7 CoA-activated acetyl group(m)	5	16	-	-	-	-	-	-	5	-	10	-
129 Asparagine from Lysine	Asparagine	−Lysine MCES +Urea	Transamination to Asparagine using Lysine	1 H_2O(s) 0.5 O_2(s) 1 Pyruvate(m) 1 Lysine(s) 3 ATP-energy(c) 1 NADPH-redox-potential(m)	1 CO_2(s) 1 Asparagine(s) 2 NADH-redox-potential(c) 3 NADH-redox-potential(m) 5 Proton-gradient(m) 2 CoA-activated acetyl group(m)	9	13	-	-	-	-	-	-	4	-	12	-
130 Asparagine from Methionine	Asparagine	−Methionine MCES +Urea +H_2S	Transamination to Asparagine using Methionine	3 H_2O(s) 1 O_2(s) 1 Pyruvate(m) 2 Methionine(s) 1 Glucose-6P(c) 2 ATP-energy(c) 1 NADPH-redox-potential(m)	1 P_i(c) 3 CO_2(s) 2 Pyruvate(c) 1 Asparagine(s) 2 H_2S(s) 5 NADH-redox-potential(c) 3 NADH-redox-potential(m) 5 Proton-gradient(m) 2 Na-gradient(c) 2 THF-activated methyl group(c) 2 CoA-activated acetyl group(m)	22	11	-	-	-	-	-	-	7	-	13	-
131 Asparagine from Phenylalanine	Asparagine	−Phenylalanine MCES +Urea	Transamination to Asparagine using Phenylalanine	6 O_2(s) 2 Phenylalanine(s) 6 ATP-energy(c) 1 ATP-energy(m) 1 NADPH-redox-potential(c) 1 Proton-gradient(m)	3 CO_2(s) 1 Pyruvate(c) 1 Asparagine(s) 1 NADH-redox-potential(c) 1 NADH-redox-potential(m) 1 Na-gradient(c) 4 CoA-activated acetyl group(c)	21	3	-	-	-	-	-	-	6	-	5	-
132 Asparagine from Proline	Asparagine	−Proline MCES +Urea	Transamination to Asparagine using Proline	8 H_2O(s) 2 Proline(s) 3 ATP-energy(c) 1 Proton-gradient(m)	3 CO_2(s) 1 Pyruvate(m) 1 Asparagine(s) 2 ATP-energy(m) 1 NADH-redox-potential(c) 4 NADH-redox-potential(m) 2 NADPH-redox-potential(m)	8	13	-	-	-	-	-	-	4	-	7	-

Continued on next page

B2. Turnover functions – continued.

Simulation	Definition			Solution													
				exchanges		reactions								transport			Prot
	Objective	Contraints	Comment	imports	exports	c	m	r	p	l	n	s	b	s-c	b-c	intra	syn
133 Asparagine from Serine	Asparagine	−Serine MCES +Urea	Transamination to Asparagine using Serine	2 Pyruvate(m) 2 Serine(s) 3 ATP-energy(c) 2 NADPH-redox-potential(m) 1 Proton-gradient(m)	2 H_2O(s) 2 Pyruvate(c) 1 Asparagine(s) 1 NADH-redox-potential(c) 1 NADH-redox-potential(m) 2 Proton-gradient(c) 2 Na-gradient(c) 1 CoA-activated acetyl group(m)	9	6	-	-	-	-	-	-	5	-	6	-
134 Asparagine from Threonine	Asparagine	−Threonine MCES +Urea	Transamination to Asparagine using Threonine	1 O_2(s) 1 Pyruvate(m) 2 Threonine(s) 3 ATP-energy(c) 1 NADPH-redox-potential(m)	3 CO_2(s) 1 Asparagine(s) 6 NADH-redox-potential(m) 8 Proton-gradient(m) 2 CoA-activated acetyl group(m)	6	13	-	-	-	-	-	-	4	-	11	-
135 Asparagine from Tryptophan	Asparagine	−Tryptophan MCES +Urea	Transamination to Asparagine using Tryptophan	3.5 O_2(s) 1 Pyruvate(m) 1 Tryptophan(s) 4 ATP-energy(c) 3 NADPH-redox-potential(c) 1 NADPH-redox-potential(m)	2 CO_2(s) 1 Pyruvate(c) 1 Asparagine(s) 1 NADH-redox-potential(c) 2 NADH-redox-potential(m) 6 Proton-gradient(m) 1 THF-activated methyl group(c) 2 CoA-activated acetyl group(m)	23	9	-	-	-	-	-	-	4	-	7	-
136 Asparagine from Tyrosine	Asparagine	−Tyrosine MCES +Urea	Transamination to Asparagine using Tyrosine	2 H_2O(s) 4 O_2(s) 2 Tyrosine(s) 6 ATP-energy(c) 1 ATP-energy(m) 1 Proton-gradient(m)	3 CO_2(s) 1 Pyruvate(c) 1 Asparagine(s) 1 NADH-redox-potential(c) 1 NADH-redox-potential(m) 1 NADPH-redox-potential(c) 2 Na-gradient(c) 4 CoA-activated acetyl group(c)	19	3	-	-	-	-	-	-	7	-	5	-
137 Asparagine from Valine	Asparagine	−Valine MCES +Urea	Transamination to Asparagine using Valine	4 H_2O(s) 2 O_2(s) 2 Valine(s) 3 ATP-energy(c)	3 CO_2(s) 1 Pyruvate(m) 1 Asparagine(s) 2 ATP-energy(m) 1 NADH-redox-potential(c) 7 NADH-redox-potential(m) 1 NADPH-redox-potential(m) 21 Proton-gradient(m) 1 Na-gradient(c)	8	17	-	-	-	-	-	-	6	-	11	-
138 Asparagine from beta-Alanine	Asparagine	−beta-Alanine MCES +Urea	Transamination to Asparagine using beta-Alanine	1 Pyruvate(m) 2 beta-Alanine(s) 3 ATP-energy(c) 2 Proton-gradient(m)	1 CO_2(s) 1 Asparagine(s) 3 NADH-redox-potential(m) 2 CoA-activated acetyl group(m)	5	8	-	-	-	-	-	-	3	-	8	-

Continued on next page

B2. Turnover functions – continued.

Simulation	Definition			Solution													
				exchanges		reactions								transport			Prot
	Objective	Contraints	Comment	imports	exports	c	m	r	p	l	n	s	b	s-c	b-c	intra	syn
139 Aspartate from Alanine	Aspartate	−Alanine MCES +Urea	Transamination to Aspartate using Alanine	1 Pyruvate(m) 1 Alanine(s) 1 NADPH-redox-potential(c)	1 Aspartate(s) 1 NADH-redox-potential(c) 1 NADH-redox-potential(m) 1 CoA-activated acetyl group(m)	6	3	-	-	-	-	-	-	2	-	1	-
140 Aspartate from Arginine	Aspartate	−Arginine MCES +Urea	Transamination to Aspartate using Arginine	1.5 H_2O(s) 0.25 O_2(s) 0.5 Pyruvate(m) 0.5 Arginine(s) 0.5 NADPH-redox-potential(m)	1 Aspartate(s) 0.5 Urea(s) 0.5 ATP-energy(m) 2 NADH-redox-potential(m) 0.5 Proton-gradient(m)	1	13	-	-	-	-	-	-	5	-	10	-
141 Aspartate from Asparagine	Aspartate	−Asparagine MCES +Urea	Transamination to Aspartate using Asparagine	1 Pyruvate(m) 0.5 Asparagine(s) 0.5 NADPH-redox-potential(m) 1 Proton-gradient(m)	1 Aspartate(s) 0.5 NADH-redox-potential(m) 0.5 Proton-gradient(c) 0.5 Na-gradient(c) 0.5 CoA-activated acetyl group(m)	1	8	-	-	-	-	-	-	4	-	6	-
142 Aspartate from Cysteine	Aspartate	−Cysteine MCES +Urea +H_2S	Transamination to Aspartate using Cysteine	2 Pyruvate(m) 1 Cysteine(s) 2 NADPH-redox-potential(m) 2 Proton-gradient(m)	1 Pyruvate(c) 1 Aspartate(s) 1 H_2S(s) 2 NADH-redox-potential(m) 1 Na-gradient(c) 1 CoA-activated acetyl group(m)	1	8	-	-	-	-	-	-	4	-	6	-
143 Aspartate from Cystine	Aspartate	−Cystine MCES +Urea +H_2S	Transamination to Aspartate using Cystine	2 Pyruvate(m) 0.5 Cystine(s) 0.5 NADPH-redox-potential(c) 2 NADPH-redox-potential(m) 1 Proton-gradient(m)	1 Pyruvate(c) 1 Aspartate(s) 1 H_2S(s) 1 NADH-redox-potential(c) 1 NADH-redox-potential(m) 0.5 Na-gradient(c) 1 CoA-activated acetyl group(m)	6	6	-	-	-	-	-	-	5	-	6	-
144 Aspartate from Glutamate	Aspartate	−Glutamate MCES +Urea	Transamination to Aspartate using Glutamate	1 H_2O(s) 0.5 O_2(s) 1 Glutamate(s)	1 CO_2(s) 1 Aspartate(s) 1 ATP-energy(m) 2 NADH-redox-potential(m) 5 Proton-gradient(m) 1 Na-gradient(c)	-	9	-	-	-	-	-	-	6	-	7	-
145 Aspartate from Glycine	Aspartate	−Glycine MCES +Urea	Transamination to Aspartate using Glycine	2 Pyruvate(m) 1 Glycine(s) 2 NADPH-redox-potential(m) 2 Proton-gradient(m) 1 THF-activated methyl group(c)	1 Pyruvate(c) 1 Aspartate(s) 2 NADH-redox-potential(m) 1 NADPH-redox-potential(c) 1 Na-gradient(c) 1 CoA-activated acetyl group(m)	5	8	-	-	-	-	-	-	3	-	6	-

Continued on next page

Simulation	Definition			Solution													
				exchanges		reactions								transport			Prot
	Objective	Contraints	Comment	imports	exports	c	m	r	p	l	n	s	b	s-c	b-c	intra	syn
146 Aspartate from Histidine	Aspartate	−Histidine MCES +Urea	Transamination to Aspartate using Histidine	0.33 H_2O(s) 0.17 O_2(s) 1 Pyruvate(m) 0.33 Histidine(s) 0.33 NADPH-redox-potential(c) 0.67 NADPH-redox-potential(m)	1 Aspartate(s) 0.33 ATP-energy(m) 0.67 NADH-redox-potential(c) 0.33 Proton-gradient(c) 1.33 Proton-gradient(m) 0.33 Na-gradient(c) 0.33 THF-activated methyl group(c) 0.33 CoA-activated acetyl group(m)	12	11	-	-	-	-	-	-	6	-	9	-
147 Aspartate from Isoleucine	Aspartate	−Isoleucine MCES +Urea	Transamination to Aspartate using Isoleucine	1 H_2O(s) 1 O_2(s) 1 Pyruvate(m) 1 Isoleucine(s) 1 NADPH-redox-potential(m)	1 CO_2(s) 1 Aspartate(s) 5 NADH-redox-potential(m) 9 Proton-gradient(m) 2 CoA-activated acetyl group(m)	-	17	-	-	-	-	-	-	5	-	10	-
148 Aspartate from Glutamine	Aspartate	−Glutamine MCES +Urea	Transamination to Aspartate using Glutamine	0.5 H_2O(s) 0.25 O_2(s) 0.5 Pyruvate(m) 0.5 Glutamine(s) 0.5 NADPH-redox-potential(m)	1 Aspartate(s) 0.5 ATP-energy(m) 1 NADH-redox-potential(m) 1.5 Proton-gradient(m)	2	12	-	-	-	-	-	-	4	-	8	-
149 Aspartate from Leucine	Aspartate	−Leucine MCES +Urea	Transamination to Aspartate using Leucine	0.5 O_2(s) 2 Pyruvate(m) 1 Leucine(s) 2 NADPH-redox-potential(m)	1 H_2O(s) 1 Aspartate(s) 4 NADH-redox-potential(m) 4 Proton-gradient(m) 1 Na-gradient(c) 4 CoA-activated acetyl group(m)	-	15	-	-	-	-	-	-	5	-	9	-
150 Aspartate from Lysine	Aspartate	−Lysine MCES +Urea	Transamination to Aspartate using Lysine	0.5 H_2O(s) 0.25 O_2(s) 1 Pyruvate(m) 0.5 Lysine(s) 1 NADPH-redox-potential(m)	1 Aspartate(s) 0.5 NADH-redox-potential(c) 2 NADH-redox-potential(m) 1 Proton-gradient(m) 1 CoA-activated acetyl group(m)	2	14	-	-	-	-	-	-	4	-	9	-
151 Aspartate from Methionine	Aspartate	−Methionine MCES +Urea +H_2S	Transamination to Aspartate using Methionine	1.5 H_2O(s) 0.5 O_2(s) 1 Pyruvate(m) 1 Methionine(s) 0.5 Glucose-6P(c) 1 NADPH-redox-potential(m)	0.5 P_i(c) 1 CO_2(s) 1 Pyruvate(c) 1 Aspartate(s) 1 H_2S(s) 0.5 ATP-energy(c) 2 NADH-redox-potential(c) 2 NADH-redox-potential(m) 1 Proton-gradient(m) 1 Na-gradient(c) 1 THF-activated methyl group(c) 1 CoA-activated acetyl group(m)	16	13	-	-	-	-	-	-	7	-	13	-

Continued on next page

B2. Turnover functions – continued.

Simulation	Objective	Contraints	Comment	imports	exports	c	m	r	p	l	n	s	b	s-c	b-c	intra	Prot syn
				exchanges		reactions								transport			
152 Aspartate from Phenylalanine	Aspartate	−Phenylalanine MCES +Urea	Transamination to Aspartate using Phenylalanine	3 O_2(s) 1 Phenylalanine(s) 2 ATP-energy(c) 1 NADPH-redox-potential(c)	1 CO_2(s) 1 Aspartate(s) 1 NADH-redox-potential(c) 1 Na-gradient(c) 2 CoA-activated acetyl group(c)	18	-	-	-	-	-	-	-	5	-	-	-
153 Aspartate from Proline	Aspartate	−Proline MCES +Urea	Transamination to Aspartate using Proline	4 H_2O(s) 1 Proline(s)	1 CO_2(s) 1 Aspartate(s) 1 ATP-energy(m) 1 NADH-redox-potential(c) 2 NADH-redox-potential(m)	3	10	-	-	-	-	-	-	4	-	4	-
154 Aspartate from Serine	Aspartate	−Serine MCES +Urea	Transamination to Aspartate using Serine	2 Pyruvate(m) 1 Serine(s) 1 NADPH-redox-potential(m) 1 Proton-gradient(m)	1 H_2O(s) 1 Pyruvate(c) 1 Aspartate(s) 1 NADH-redox-potential(c) 1 Proton-gradient(c) 1 Na-gradient(c) 1 CoA-activated acetyl group(m)	4	5	-	-	-	-	-	-	5	-	6	-
155 Aspartate from Threonine	Aspartate	−Threonine MCES +Urea	Transamination to Aspartate using Threonine	0.5 O_2(s) 1 Pyruvate(m) 1 Threonine(s) 1 NADPH-redox-potential(m)	1 CO_2(s) 1 Aspartate(s) 1 NADH-redox-potential(c) 2 NADH-redox-potential(m) 4 Proton-gradient(m) 1 CoA-activated acetyl group(m)	4	11	-	-	-	-	-	-	4	-	10	-
156 Aspartate from Tryptophan	Aspartate	−Tryptophan MCES +Urea	Transamination to Aspartate using Tryptophan	1.75 O_2(s) 1 Pyruvate(m) 0.5 Tryptophan(s) 0.5 ATP-energy(c) 1.5 NADPH-redox-potential(c) 1.5 NADPH-redox-potential(m)	0.5 CO_2(s) 0.5 Pyruvate(c) 1 Aspartate(s) 1 NADH-redox-potential(c) 1 NADH-redox-potential(m) 2.5 Proton-gradient(m) 0.5 THF-activated methyl group(c) 1 CoA-activated acetyl group(m)	19	10	-	-	-	-	-	-	4	-	10	-
157 Aspartate from Tyrosine	Aspartate	−Tyrosine MCES +Urea	Transamination to Aspartate using Tyrosine	1 H_2O(s) 2 O_2(s) 1 Tyrosine(s) 2 ATP-energy(c)	1 CO_2(s) 1 Aspartate(s) 1 NADH-redox-potential(c) 1 Na-gradient(c) 2 CoA-activated acetyl group(c)	15	-	-	-	-	-	-	-	6	-	-	-
158 Aspartate from Valine	Aspartate	−Valine MCES +Urea	Transamination to Aspartate using Valine	2 H_2O(s) 1 O_2(s) 1 Valine(s)	1 CO_2(s) 1 Aspartate(s) 1 ATP-energy(m) 1 NADH-redox-potential(c) 3 NADH-redox-potential(m) 10 Proton-gradient(m) 1 Na-gradient(c)	3	14	-	-	-	-	-	-	6	-	10	-

Continued on next page

B2. Turnover functions – continued.

Simulation	Definition			Solution													
				exchanges		reactions								transport			Prot
	Objective	Contraints	Comment	imports	exports	c	m	r	p	l	n	s	b	s-c	b-c	intra	syn
159 Aspartate from beta-Alanine	Aspartate	−beta-Alanine MCES +Urea	Transamination to Aspartate using beta-Alanine	1 Pyruvate(m) 1 beta-Alanine(s) 1 NADPH-redox-potential(m) 2 Proton-gradient(m)	1 Aspartate(s) 2 NADH-redox-potential(m) 1 CoA-activated acetyl group(m)	-	8	-	-	-	-	-	-	2	-	5	-
160 Cysteine from Cystine	Cysteine	−Cystine MCES +Urea	Transamination to Cysteine using Cystine	0.5 Cystine(s) 0.5 NADPH-redox-potential(c)	1 Cysteine(s) 0.5 Na-gradient(c)	2	-	-	-	-	-	-	-	4	-	-	-
161 Cysteine from Methionine	Cysteine	−Methionine MCES +Urea	Transamination to Cysteine using Methionine	1.5 H_2O(s) 0.5 O_2(s) 1 Methionine(s) 0.5 Glucose-6P(c)	0.5 P_i(c) 2 CO_2(s) 1 Cysteine(s) 0.5 ATP-energy(c) 2 NADH-redox-potential(c) 2 NADH-redox-potential(m) 3 Proton-gradient(m) 2 Na-gradient(c) 1 THF-activated methyl group(c) 1 CoA-activated acetyl group(m)	15	9	-	-	-	-	-	-	6	-	11	-
162 Cystine from Cysteine	Cystine	−Cysteine MCES +Urea	Transamination to Cystine using Cysteine	2 Cysteine(s)	1 Cystine(s) 1 NADPH-redox-potential(c)	2	-	-	-	-	-	-	-	3	-	-	-
163 Cystine from Methionine	Cystine	−Methionine MCES +Urea	Transamination to Cystine using Methionine	3 H_2O(s) 1 O_2(s) 2 Methionine(s) 1 Glucose-6P(c)	1 P_i(c) 4 CO_2(s) 1 Cystine(s) 1 ATP-energy(c) 4 NADH-redox-potential(c) 4 NADH-redox-potential(m) 1 NADPH-redox-potential(c) 6 Proton-gradient(m) 2 Na-gradient(c) 2 THF-activated methyl group(c) 2 CoA-activated acetyl group(m)	17	9	-	-	-	-	-	-	7	-	11	-
164 Glutamate from Alanine	Glutamate	−Alanine MCES +Urea	Transamination to Glutamate using Alanine	1 H_2O(s) 1 Pyruvate(m) 1 Alanine(s) 1 ATP-energy(m) 1 NADPH-redox-potential(m) 1 CoA-activated acetyl group(m)	1 Pyruvate(c) 1 Glutamate(s) 1 ATP-energy(c) 2 NADH-redox-potential(m)	2	10	-	-	-	-	-	-	3	-	2	-
165 Glutamate from Arginine	Glutamate	−Arginine MCES +Urea	Transamination to Glutamate using Arginine	1.5 H_2O(s) 0.5 Pyruvate(m) 0.5 Arginine(s) 0.5 NADPH-redox-potential(m) 1.5 Proton-gradient(m) 0.5 CoA-activated acetyl group(m)	1 Glutamate(s) 0.5 Urea(s) 1.5 NADH-redox-potential(m)	1	11	-	-	-	-	-	-	4	-	6	-

Continued on next page

B2. Turnover functions – continued.

Simulation	Definition			Solution													
				exchanges		reactions								transport			Prot
	Objective	Contraints	Comment	imports	exports	c	m	r	p	l	n	s	b	s-c	b-c	intra	syn
166 Glutamate from Asparagine	Glutamate	−Asparagine MCES +Urea	Transamination to Glutamate using Asparagine	1 H_2O(s) 0.5 Pyruvate(m) 0.5 Asparagine(s) 0.5 NADH-redox-potential(c) 1 NADPH-redox-potential(m) 0.5 Proton-gradient(m) 1 CoA-activated acetyl group(m)	0.5 CO_2(s) 1 Glutamate(s) 1.5 NADH-redox-potential(m) 0.5 NADPH-redox-potential(c) 0.5 Proton-gradient(c) 0.5 Na-gradient(c)	7	10	-	-	-	-	-	-	6	-	6	-
167 Glutamate from Aspartate	Glutamate	−Aspartate MCES +Urea	Transamination to Glutamate using Aspartate	1 H_2O(s) 1 Aspartate(s) 1 NADH-redox-potential(c) 1 CoA-activated acetyl group(m)	1 CO_2(s) 1 Glutamate(s) 1 NADH-redox-potential(m) 1 NADPH-redox-potential(c) 1 Na-gradient(c)	6	5	-	-	-	-	-	-	5	-	2	-
168 Glutamate from Cysteine	Glutamate	−Cysteine MCES +Urea +H_2S	Transamination to Glutamate using Cysteine	1 H_2O(s) 1 Pyruvate(m) 1 Cysteine(s) 1 NADPH-redox-potential(m) 1 Proton-gradient(m) 1 CoA-activated acetyl group(m)	1 Pyruvate(c) 1 Glutamate(s) 1 H_2S(s) 1 NADH-redox-potential(m) 1 Na-gradient(c)	1	10	-	-	-	-	-	-	5	-	4	-
169 Glutamate from Cystine	Glutamate	−Cystine MCES +Urea +H_2S	Transamination to Glutamate using Cystine	1 H_2O(s) 1 Pyruvate(m) 0.5 Cystine(s) 0.5 NADPH-redox-potential(c) 1 NADPH-redox-potential(m) 1 Proton-gradient(m) 1 CoA-activated acetyl group(m)	1 Pyruvate(c) 1 Glutamate(s) 1 H_2S(s) 1 NADH-redox-potential(m)	3	10	-	-	-	-	-	-	4	-	4	-
170 Glutamate from Glycine	Glutamate	−Glycine MCES +Urea	Transamination to Glutamate using Glycine	1 H_2O(s) 1 Pyruvate(m) 1 Glycine(s) 2 NADPH-redox-potential(m) 1 Proton-gradient(m) 1 THF-activated methyl group(c) 1 CoA-activated acetyl group(m)	1 Pyruvate(c) 1 Glutamate(s) 2 NADH-redox-potential(m) 1 NADPH-redox-potential(c) 1 Na-gradient(c)	5	10	-	-	-	-	-	-	4	-	4	-
171 Glutamate from Histidine	Glutamate	−Histidine MCES +Urea	Transamination to Glutamate using Histidine	0.67 H_2O(s) 0.67 Pyruvate(m) 0.33 Histidine(s) 0.33 NADH-redox-potential(c) 0.33 NADPH-redox-potential(c) 0.67 NADPH-redox-potential(m) 0.67 Proton-gradient(m) 0.67 CoA-activated acetyl group(m)	1 Glutamate(s) 0.67 NADH-redox-potential(m) 0.33 Na-gradient(c) 0.33 THF-activated methyl group(c)	10	10	-	-	-	-	-	-	4	-	4	-

Continued on next page

B2. Turnover functions – continued.

Simulation		Definition			Solution													
					exchanges		reactions								transport			Prot
		Objective	Contraints	Comment	imports	exports	c	m	r	p	l	n	s	b	s-c	b-c	intra	syn
172	Glutamate from Isoleucine	Glutamate	−Isoleucine MCES +Urea	Transamination to Glutamate using Isoleucine	0.5 O_2(s) 1 Pyruvate(m) 1 Isoleucine(s) 1 NADPH-redox-potential(m)	1 Glutamate(s) 3 NADH-redox-potential(m) 4 Proton-gradient(m) 2 CoA-activated acetyl group(m)	-	17	-	-	-	-	-	-	3	-	7	-
173	Glutamate from Glutamine	Glutamate	−Glutamine MCES +Urea	Transamination to Glutamate using Glutamine	0.5 H_2O(s) 0.5 Pyruvate(m) 0.5 Glutamine(s) 1 NADPH-redox-potential(m) 0.5 Proton-gradient(m) 0.5 CoA-activated acetyl group(m)	1 Glutamate(s) 1 NADH-redox-potential(m)	2	10	-	-	-	-	-	-	3	-	4	-
174	Glutamate from Leucine	Glutamate	−Leucine MCES +Urea	Transamination to Glutamate using Leucine	0.5 O_2(s) 1 Pyruvate(m) 1 Leucine(s) 2 NADPH-redox-potential(m)	1 Glutamate(s) 4 NADH-redox-potential(m) 5 Proton-gradient(m) 1 Na-gradient(c) 2 CoA-activated acetyl group(m)	-	17	-	-	-	-	-	-	4	-	7	-
175	Glutamate from Lysine	Glutamate	−Lysine MCES +Urea	Transamination to Glutamate using Lysine	1.5 H_2O(s) 0.25 O_2(s) 1 Pyruvate(m) 0.5 Lysine(s) 1 NADPH-redox-potential(m)	1 CO_2(s) 1 Glutamate(s) 0.5 NADH-redox-potential(c) 2 NADH-redox-potential(m) 1 NADPH-redox-potential(c) 2 Proton-gradient(m)	6	13	-	-	-	-	-	-	5	-	10	-
176	Glutamate from Methionine	Glutamate	−Methionine MCES +Urea +H_2S	Transamination to Glutamate using Methionine	0.5 H_2O(s) 1 Pyruvate(m) 1 Methionine(s) 0.5 Glucose-6P(c) 1 NADPH-redox-potential(m) 4 Proton-gradient(m)	0.5 P_i(c) 1 Pyruvate(c) 1 Glutamate(s) 1 H_2S(s) 0.5 ATP-energy(c) 2 NADH-redox-potential(c) 1 Na-gradient(c) 1 THF-activated methyl group(c) 1 CoA-activated acetyl group(m)	16	12	-	-	-	-	-	-	5	-	8	-
177	Glutamate from Phenylalanine	Glutamate	−Phenylalanine MCES +Urea	Transamination to Glutamate using Phenylalanine	1 H_2O(s) 3 O_2(s) 1 Phenylalanine(s) 2 ATP-energy(c) 1 CoA-activated acetyl group(m)	2 CO_2(s) 1 Glutamate(s) 1 NADH-redox-potential(m) 2 CoA-activated acetyl group(c)	17	4	-	-	-	-	-	-	5	-	2	-
178	Glutamate from Proline	Glutamate	−Proline MCES +Urea	Transamination to Glutamate using Proline	2 H_2O(s) 1 Proline(s)	1 Glutamate(s) 4 Proton-gradient(m)	-	3	-	-	-	-	-	-	3	-	4	-
179	Glutamate from Serine	Glutamate	−Serine MCES +Urea	Transamination to Glutamate using Serine	1 Pyruvate(m) 1 Serine(s) 2 NADPH-redox-potential(m) 1 Proton-gradient(m) 1 CoA-activated acetyl group(m)	1 Pyruvate(c) 1 Glutamate(s) 2 NADH-redox-potential(m) 1 Proton-gradient(c) 1 Na-gradient(c)	1	10	-	-	-	-	-	-	4	-	4	-

Continued on next page

B2. Turnover functions – continued.

	Simulation	Definition			Solution													
					exchanges		reactions								transport			Prot
		Objective	Contraints	Comment	imports	exports	c	m	r	p	l	n	s	b	s-c	b-c	intra	syn
180	Glutamate from Threonine	Glutamate	−Threonine MCES +Urea	Transamination to Glutamate using Threonine	0.17 O_2(s) 0.33 Pyruvate(m) 1 Threonine(s) 1 NADPH-redox-potential(m)	0.33 H_2O(s) 1 Glutamate(s) 1.33 NADH-redox-potential(m)	1	20	-	-	-	-	-	-	4	-	8	-
181	Glutamate from Tryptophan	Glutamate	−Tryptophan MCES +Urea	Transamination to Glutamate using Tryptophan	1 H_2O(s) 1.75 O_2(s) 1 Pyruvate(m) 0.5 Tryptophan(s) 0.5 ATP-energy(c) 0.5 NADPH-redox-potential(c) 1.5 NADPH-redox-potential(m)	1.5 CO_2(s) 0.5 Pyruvate(c) 1 Glutamate(s) 2 NADH-redox-potential(m) 2.5 Proton-gradient(m) 0.5 THF-activated methyl group(c)	19	11	-	-	-	-	-	-	5	-	10	-
182	Glutamate from Tyrosine	Glutamate	−Tyrosine MCES +Urea	Transamination to Glutamate using Tyrosine	2 H_2O(s) 2 O_2(s) 1 Tyrosine(s) 2 ATP-energy(c) 1 CoA-activated acetyl group(m)	2 CO_2(s) 1 Glutamate(s) 1 NADH-redox-potential(m) 1 NADPH-redox-potential(c) 1 Na-gradient(c) 2 CoA-activated acetyl group(c)	16	4	-	-	-	-	-	-	6	-	2	-
183	Glutamate from Valine	Glutamate	−Valine MCES +Urea	Transamination to Glutamate using Valine	1 H_2O(s) 0.5 O_2(s) 1 Valine(s)	1 Glutamate(s) 2 NADH-redox-potential(m) 4 Proton-gradient(m) 1 Na-gradient(c)	-	10	-	-	-	-	-	-	5	-	8	-
184	Glutamate from beta-Alanine	Glutamate	−beta-Alanine MCES +Urea	Transamination to Glutamate using beta-Alanine	1 H_2O(s) 1 Pyruvate(m) 1 beta-Alanine(s) 1 NADPH-redox-potential(m) 1 Proton-gradient(m)	1 CO_2(s) 1 Glutamate(s) 2 NADH-redox-potential(m) 1 NADPH-redox-potential(c)	3	8	-	-	-	-	-	-	4	-	6	-
185	Glycine from Alanine	Glycine	−Alanine MCES +Urea	Transamination to Glycine using Alanine	0.5 H_2O(s) 1 Alanine(s) 0.5 Glucose-6P(c)	0.5 P_i(c) 1 Pyruvate(c) 1 Glycine(s) 0.5 ATP-energy(c) 1 NADH-redox-potential(c) 1 Na-gradient(c) 1 THF-activated methyl group(c)	15	-	-	-	-	-	-	-	3	-	-	-
186	Glycine from Arginine	Glycine	−Arginine MCES +Urea	Transamination to Glycine using Arginine	2 H_2O(s) 0.25 O_2(s) 0.5 Arginine(s) 0.5 Glucose-6P(c)	0.5 P_i(c) 1 CO_2(s) 0.5 Pyruvate(c) 1 Glycine(s) 0.5 Urea(s) 0.5 ATP-energy(c) 0.5 ATP-energy(m) 1 NADH-redox-potential(c) 1 NADH-redox-potential(m) 0.5 NADPH-redox-potential(c) 1 Proton-gradient(m) 1 THF-activated methyl group(c)	17	9	-	-	-	-	-	-	6	-	13	-

Continued on next page

B2. Turnover functions – continued.

Simulation	Definition			Solution													
				exchanges		reactions								transport			Prot
	Objective	Contraints	Comment	imports	exports	c	m	r	p	l	n	s	b	s-c	b-c	intra	syn
187 Glycine from Asparagine	Glycine	−Asparagine MCES +Urea	Transamination to Glycine using Asparagine	0.5 H_2O(s) 0.5 Glucose-6P(c) 0.5 Asparagine(s) 0.5 NADPH-redox-potential(c) 0.5 NADPH-redox-potential(m) 1 Proton-gradient(m)	0.5 P_i(c) 0.5 CO_2(s) 0.5 Pyruvate(c) 1 Glycine(s) 0.5 ATP-energy(c) 1.5 NADH-redox-potential(c) 0.5 Na-gradient(c) 1 THF-activated methyl group(c)	19	2	-	-	-	-	-	-	5	-	7	-
188 Glycine from Aspartate	Glycine	−Aspartate MCES +Urea	Transamination to Glycine using Aspartate	0.5 H_2O(s) 1 Aspartate(s) 0.5 Glucose-6P(c)	0.5 P_i(c) 1 CO_2(s) 1 Pyruvate(c) 1 Glycine(s) 0.5 ATP-energy(c) 1 NADPH-redox-potential(c) 1 Na-gradient(c) 1 THF-activated methyl group(c)	17	-	-	-	-	-	-	-	5	-	-	-
189 Glycine from Cysteine	Glycine	−Cysteine MCES +Urea +H_2S	Transamination to Glycine using Cysteine	0.5 H_2O(s) 0.5 Glucose-6P(c) 1 Cysteine(s) 1 NADH-redox-potential(m) 1 NADPH-redox-potential(c) 2 Proton-gradient(m)	0.5 P_i(c) 1 Pyruvate(c) 1 Glycine(s) 1 H_2S(s) 0.5 ATP-energy(c) 2 NADH-redox-potential(c) 1 Na-gradient(c) 1 THF-activated methyl group(c)	16	2	-	-	-	-	-	-	4	-	7	-
190 Glycine from Cystine	Glycine	−Cystine MCES +Urea +H_2S	Transamination to Glycine using Cystine	0.5 H_2O(s) 0.5 Glucose-6P(c) 0.5 Cystine(s) 1 NADH-redox-potential(m) 1.5 NADPH-redox-potential(c) 2 Proton-gradient(m)	0.5 P_i(c) 1 Pyruvate(c) 1 Glycine(s) 1 H_2S(s) 0.5 ATP-energy(c) 2 NADH-redox-potential(c) 0.5 Na-gradient(c) 1 THF-activated methyl group(c)	17	2	-	-	-	-	-	-	6	-	7	-
191 Glycine from Glutamate	Glycine	−Glutamate MCES +Urea	Transamination to Glycine using Glutamate	1.5 H_2O(s) 0.5 O_2(s) 1 Glutamate(s) 0.5 Glucose-6P(c)	0.5 P_i(c) 2 CO_2(s) 1 Pyruvate(c) 1 Glycine(s) 0.5 ATP-energy(c) 1 ATP-energy(m) 1 NADH-redox-potential(c) 1 NADH-redox-potential(m) 1 NADPH-redox-potential(c) 6 Proton-gradient(m) 1 Na-gradient(c) 1 THF-activated methyl group(c)	16	7	-	-	-	-	-	-	6	-	7	-
192 Glycine from Histidine	Glycine	−Histidine MCES +Urea	Transamination to Glycine using Histidine	0.83 H_2O(s) 0.17 O_2(s) 0.5 Glucose-6P(c) 0.33 Histidine(s) 0.33 NADH-redox-potential(m)	0.5 P_i(c) 0.67 CO_2(s) 0.33 Pyruvate(c) 1 Glycine(s) 0.5 ATP-energy(c) 0.33 ATP-energy(m) 0.67 NADH-redox-potential(c) 0.67 Proton-gradient(m) 1.33 THF-activated methyl group(c)	21	8	-	-	-	-	-	-	5	-	12	-

Continued on next page

Simulation		Definition			Solution													
					exchanges		reactions								transport			Prot
		Objective	Contraints	Comment	imports	exports	c	m	r	p	l	n	s	b	s-c	b-c	intra	syn
193	Glycine from Isoleucine	Glycine	−Isoleucine MCES +Urea	Transamination to Glycine using Isoleucine	1.5 H_2O(s) 1 O_2(s) 0.5 Glucose-6P(c) 1 Isoleucine(s)	0.5 P_i(c) 2 CO_2(s) 1 Glycine(s) 0.5 ATP-energy(c) 1 NADH-redox-potential(c) 4 NADH-redox-potential(m) 9 Proton-gradient(m) 1 THF-activated methyl group(c) 2 CoA-activated acetyl group(m)	14	13	-	-	-	-	-	-	4	-	11	-
194	Glycine from Glutamine	Glycine	−Glutamine MCES +Urea	Transamination to Glycine using Glutamine	1 H_2O(s) 0.25 O_2(s) 0.5 Glutamine(s) 0.5 Glucose-6P(c) 0.5 NADPH-redox-potential(m)	0.5 P_i(c) 1 CO_2(s) 0.5 Pyruvate(c) 1 Glycine(s) 0.5 ATP-energy(c) 0.5 ATP-energy(m) 1 NADH-redox-potential(c) 0.5 NADH-redox-potential(m) 0.5 NADPH-redox-potential(c) 2 Proton-gradient(m) 1 THF-activated methyl group(c)	18	9	-	-	-	-	-	-	5	-	11	-
195	Glycine from Leucine	Glycine	−Leucine MCES +Urea	Transamination to Glycine using Leucine	0.5 O_2(s) 0.5 Glucose-6P(c) 1 Leucine(s) 1 NADPH-redox-potential(m)	0.5 H_2O(s) 0.5 P_i(c) 1 Glycine(s) 0.5 ATP-energy(c) 1 NADH-redox-potential(c) 2 NADH-redox-potential(m) 4 Proton-gradient(m) 1 Na-gradient(c) 1 THF-activated methyl group(c) 3 CoA-activated acetyl group(m)	14	13	-	-	-	-	-	-	4	-	10	-
196	Glycine from Lysine	Glycine	−Lysine MCES +Urea	Transamination to Glycine using Lysine	1 H_2O(s) 0.25 O_2(s) 0.5 Lysine(s) 0.5 Glucose-6P(c) 0.5 NADPH-redox-potential(m)	0.5 P_i(c) 1 CO_2(s) 1 Glycine(s) 0.5 ATP-energy(c) 1.5 NADH-redox-potential(c) 1.5 NADH-redox-potential(m) 1 Proton-gradient(m) 1 THF-activated methyl group(c) 1 CoA-activated acetyl group(m)	15	11	-	-	-	-	-	-	5	-	11	-

Continued on next page

B2. Turnover functions – continued.

Simulation	Definition			Solution													
				exchanges		reactions								transport			Prot
	Objective	Contraints	Comment	imports	exports	c	m	r	p	l	n	s	b	s-c	b-c	intra	syn
197 Glycine from Methionine	Glycine	−Methionine MCES +Urea $+H_2S$	Transamination to Glycine using Methionine	2 H_2O(s) 0.5 O_2(s) 1 Methionine(s) 1 Glucose-6P(c) 1 NADPH-redox-potential(c)	1 P_i(c) 2 CO_2(s) 1 Pyruvate(c) 1 Glycine(s) 1 H_2S(s) 1 ATP-energy(c) 4 NADH-redox-potential(c) 1 NADH-redox-potential(m) 1 Proton-gradient(m) 2 THF-activated methyl group(c) 1 CoA-activated acetyl group(m)	19	9	-	-	-	-	-	-	5	-	12	-
198 Glycine from Phenylalanine	Glycine	−Phenylalanine MCES +Urea	Transamination to Glycine using Phenylalanine	0.5 H_2O(s) 3 O_2(s) 1 Phenylalanine(s) 0.5 Glucose-6P(c) 1.5 ATP-energy(c)	0.5 P_i(c) 2 CO_2(s) 1 Pyruvate(c) 1 Glycine(s) 1 NADH-redox-potential(c) 1 THF-activated methyl group(c) 2 CoA-activated acetyl group(c)	28	-	-	-	-	-	-	-	4	-	-	-
199 Glycine from Proline	Glycine	−Proline MCES +Urea	Transamination to Glycine using Proline	4.5 H_2O(s) 0.5 Glucose-6P(c) 1 Proline(s)	0.5 P_i(c) 2 CO_2(s) 1 Pyruvate(c) 1 Glycine(s) 0.5 ATP-energy(c) 1 ATP-energy(m) 1 NADH-redox-potential(c) 2 NADH-redox-potential(m) 1 NADPH-redox-potential(c) 1 THF-activated methyl group(c)	16	10	-	-	-	-	-	-	3	-	4	-
200 Glycine from Serine	Glycine	−Serine MCES +Urea	Transamination to Glycine using Serine	1 Serine(s) 1 NADH-redox-potential(c)	1 H_2O(s) 1 Glycine(s) 1 THF-activated methyl group(c)	4	-	-	-	-	-	-	-	2	-	-	-
201 Glycine from Threonine	Glycine	−Threonine MCES +Urea	Transamination to Glycine using Threonine	0.5 H_2O(s) 0.5 O_2(s) 0.5 Glucose-6P(c) 1 Threonine(s)	0.5 P_i(c) 2 CO_2(s) 1 Glycine(s) 0.5 ATP-energy(c) 1 NADH-redox-potential(c) 2 NADH-redox-potential(m) 3 Proton-gradient(m) 1 THF-activated methyl group(c) 1 CoA-activated acetyl group(m)	15	9	-	-	-	-	-	-	4	-	11	-

Continued on next page

B2. Turnover functions – continued.

Simulation	Definition			Solution													
				exchanges		reactions								transport			Prot
	Objective	Contraints	Comment	imports	exports	c	m	r	p	l	n	s	b	s-c	b-c	intra	syn
202 Glycine from Tryptophan	Glycine	−Tryptophan MCES +Urea	Transamination to Glycine using Tryptophan	0.5 H_2O(s) 1.75 O_2(s) 0.5 Tryptophan(s) 0.5 Glucose-6P(c) 1.5 NADPH-redox-potential(c) 0.5 NADPH-redox-potential(m)	0.5 P_i(c) 1.5 CO_2(s) 0.5 Pyruvate(c) 1 Glycine(s) 1 NADH-redox-potential(c) 1 NADH-redox-potential(m) 1.5 Proton-gradient(m) 0.5 Na-gradient(c) 1.5 THF-activated methyl group(c) 1 CoA-activated acetyl group(m)	26	9	-	-	-	-	-	-	6	-	12	-
203 Glycine from Tyrosine	Glycine	−Tyrosine MCES +Urea	Transamination to Glycine using Tyrosine	1.5 H_2O(s) 2 O_2(s) 1 Tyrosine(s) 0.5 Glucose-6P(c) 1.5 ATP-energy(c)	0.5 P_i(c) 2 CO_2(s) 1 Pyruvate(c) 1 Glycine(s) 2 NADH-redox-potential(c) 1 THF-activated methyl group(c) 2 CoA-activated acetyl group(c)	26	-	-	-	-	-	-	-	4	-	-	-
204 Glycine from Valine	Glycine	−Valine MCES +Urea	Transamination to Glycine using Valine	2.5 H_2O(s) 1 O_2(s) 0.5 Glucose-6P(c) 1 Valine(s)	0.5 P_i(c) 2 CO_2(s) 1 Pyruvate(c) 1 Glycine(s) 0.5 ATP-energy(c) 1 ATP-energy(m) 2 NADH-redox-potential(c) 3 NADH-redox-potential(m) 10 Proton-gradient(m) 1 THF-activated methyl group(c)	15	14	-	-	-	-	-	-	4	-	10	-
205 Glycine from beta-Alanine	Glycine	−beta-Alanine MCES +Urea	Transamination to Glycine using beta-Alanine	0.5 H_2O(s) 0.5 Glucose-6P(c) 1 beta-Alanine(s) 1 NADPH-redox-potential(c) 2 Proton-gradient(m)	0.5 P_i(c) 1 CO_2(s) 1 Glycine(s) 0.5 ATP-energy(c) 2 NADH-redox-potential(c) 1 NADH-redox-potential(m) 1 THF-activated methyl group(c) 1 CoA-activated acetyl group(m)	15	4	-	-	-	-	-	-	4	-	7	-
206 Glutamine from Alanine	Glutamine	−Alanine MCES +Urea	Transamination to Glutamine using Alanine	1 H_2O(s) 2 Alanine(s) 1 ATP-energy(m) 1 NADPH-redox-potential(m) 1 CoA-activated acetyl group(m)	1 Pyruvate(c) 1 Glutamine(s) 3 NADH-redox-potential(m)	2	12	-	-	-	-	-	-	3	-	5	-
207 Glutamine from Arginine	Glutamine	−Arginine MCES +Urea	Transamination to Glutamine using Arginine	2 H_2O(s) 1 Arginine(s) 1 ATP-energy(c) 2 Proton-gradient(m)	1 Glutamine(s) 1 Urea(s) 2 NADH-redox-potential(m)	3	4	-	-	-	-	-	-	4	-	7	-
208 Glutamine from Asparagine	Glutamine	−Asparagine MCES +Urea	Transamination to Glutamine using Asparagine	1 H_2O(s) 1 Asparagine(s) 1 ATP-energy(c) 1 NADH-redox-potential(c) 1 CoA-activated acetyl group(m)	1 CO_2(s) 1 Glutamine(s) 1 NADH-redox-potential(m) 1 NADPH-redox-potential(c)	9	5	-	-	-	-	-	-	4	-	2	-

Continued on next page

B2. Turnover functions – continued.

Simulation		Definition			Solution													
					exchanges		reactions								transport			Prot
		Objective	Contraints	Comment	imports	exports	c	m	r	p	l	n	s	b	s-c	b-c	intra	syn
209	Glutamine from Aspartate	Glutamine	−Aspartate MCES +Urea	Transamination to Glutamine using Aspartate	1 H_2O(s) 2 Aspartate(s) 2 ATP-energy(c) 1 NADH-redox-potential(c) 1 CoA-activated acetyl group(m)	2 CO_2(s) 1 Pyruvate(c) 1 Glutamine(s) 1 NADH-redox-potential(m) 2 NADPH-redox-potential(c) 2 Na-gradient(c)	14	5	-	-	-	-	-	-	5	-	2	-
210	Glutamine from Cysteine	Glutamine	−Cysteine MCES +Urea +H_2S	Transamination to Glutamine using Cysteine	1 H_2O(s) 1 Pyruvate(m) 2 Cysteine(s) 1 ATP-energy(c) 1 NADPH-redox-potential(m) 1 Proton-gradient(m) 1 CoA-activated acetyl group(m)	2 Pyruvate(c) 1 Glutamine(s) 2 H_2S(s) 1 NADH-redox-potential(m) 2 Na-gradient(c)	3	10	-	-	-	-	-	-	6	-	4	-
211	Glutamine from Cystine	Glutamine	−Cystine MCES +Urea +H_2S	Transamination to Glutamine using Cystine	1 H_2O(s) 1 Pyruvate(m) 1 Cystine(s) 1 ATP-energy(c) 1 NADPH-redox-potential(c) 1 NADPH-redox-potential(m) 1 Proton-gradient(m) 1 CoA-activated acetyl group(m)	2 Pyruvate(c) 1 Glutamine(s) 2 H_2S(s) 1 NADH-redox-potential(m) 1 Na-gradient(c)	5	10	-	-	-	-	-	-	6	-	4	-
212	Glutamine from Glutamate	Glutamine	−Glutamate MCES +Urea	Transamination to Glutamine using Glutamate	1 H_2O(s) 0.5 O_2(s) 2 Glutamate(s) 1 ATP-energy(c)	2 CO_2(s) 1 Pyruvate(m) 1 Glutamine(s) 1 ATP-energy(m) 2 NADH-redox-potential(m) 1 NADPH-redox-potential(m) 5 Proton-gradient(m) 2 Na-gradient(c)	2	10	-	-	-	-	-	-	6	-	8	-
213	Glutamine from Glycine	Glutamine	−Glycine MCES +Urea	Transamination to Glutamine using Glycine	1 H_2O(s) 1 Pyruvate(m) 2 Glycine(s) 1 ATP-energy(c) 2 NADPH-redox-potential(m) 1 Proton-gradient(m) 2 THF-activated methyl group(c) 1 CoA-activated acetyl group(m)	2 Pyruvate(c) 1 Glutamine(s) 2 NADH-redox-potential(m) 2 NADPH-redox-potential(c) 2 Na-gradient(c)	7	10	-	-	-	-	-	-	4	-	4	-
214	Glutamine from Histidine	Glutamine	−Histidine MCES +Urea	Transamination to Glutamine using Histidine	0.33 H_2O(s) 0.33 Pyruvate(m) 0.67 Histidine(s) 1 ATP-energy(c) 0.67 NADH-redox-potential(c) 0.67 NADPH-redox-potential(c) 0.33 NADPH-redox-potential(m) 0.33 Proton-gradient(m) 0.33 CoA-activated acetyl group(m)	1 Glutamine(s) 0.33 NADH-redox-potential(m) 0.67 THF-activated methyl group(c)	12	10	-	-	-	-	-	-	4	-	4	-

Continued on next page

B2. Turnover functions – continued.

Simulation		Definition			Solution													
					exchanges		reactions								transport			Prot
		Objective	Contraints	Comment	imports	exports	c	m	r	p	l	n	s	b	s-c	b-c	intra	syn
215	Glutamine from Isoleucine	Glutamine	−Isoleucine MCES +Urea	Transamination to Glutamine using Isoleucine	1 H_2O(s) 1.5 O_2(s) 2 Isoleucine(s) 1 ATP-energy(c) 1 ATP-energy(m)	1 CO_2(s) 1 Glutamine(s) 6 NADH-redox-potential(m) 15 Proton-gradient(m) 3 CoA-activated acetyl group(m)	4	19	-	-	-	-	-	-	6	-	11	-
216	Glutamine from Leucine	Glutamine	−Leucine MCES +Urea	Transamination to Glutamine using Leucine	1 O_2(s) 1 Pyruvate(m) 2 Leucine(s) 1 ATP-energy(c) 3 NADPH-redox-potential(m)	1 H_2O(s) 1 Glutamine(s) 7 NADH-redox-potential(m) 11 Proton-gradient(m) 2 Na-gradient(c) 5 CoA-activated acetyl group(m)	2	18	-	-	-	-	-	-	6	-	8	-
217	Glutamine from Lysine	Glutamine	−Lysine MCES +Urea	Transamination to Glutamine using Lysine	2 H_2O(s) 0.5 O_2(s) 1 Pyruvate(m) 1 Lysine(s) 1 ATP-energy(c) 1 NADPH-redox-potential(m)	2 CO_2(s) 1 Glutamine(s) 1 NADH-redox-potential(c) 5 NADH-redox-potential(m) 5 Proton-gradient(m) 1 CoA-activated acetyl group(m)	4	18	-	-	-	-	-	-	4	-	11	-
218	Glutamine from Methionine	Glutamine	−Methionine MCES +Urea +H_2S	Transamination to Glutamine using Methionine	2 H_2O(s) 0.5 O_2(s) 2 Methionine(s) 1 Glucose-6P(c) 1 NADPH-redox-potential(m) 1 Proton-gradient(m)	1 P_i(c) 1 CO_2(s) 2 Pyruvate(c) 1 Glutamine(s) 2 H_2S(s) 4 NADH-redox-potential(c) 1 NADH-redox-potential(m) 1 Na-gradient(c) 2 THF-activated methyl group(c) 1 CoA-activated acetyl group(m)	16	17	-	-	-	-	-	-	8	-	12	-
219	Glutamine from Phenylalanine	Glutamine	−Phenylalanine MCES +Urea	Transamination to Glutamine using Phenylalanine	1 H_2O(s) 6 O_2(s) 2 Phenylalanine(s) 4 ATP-energy(c) 1 ATP-energy(m) 1 Proton-gradient(m) 1 CoA-activated acetyl group(m)	4 CO_2(s) 1 Pyruvate(c) 1 Glutamine(s) 1 NADH-redox-potential(m) 1 NADPH-redox-potential(m) 1 Na-gradient(c) 4 CoA-activated acetyl group(c)	19	7	-	-	-	-	-	-	7	-	7	-
220	Glutamine from Proline	Glutamine	−Proline MCES +Urea	Transamination to Glutamine using Proline	6 H_2O(s) 2 Proline(s) 1 ATP-energy(c)	2 CO_2(s) 1 Pyruvate(m) 1 Glutamine(s) 1 ATP-energy(m) 2 NADH-redox-potential(m) 2 NADPH-redox-potential(m) 3 Proton-gradient(m) 1 Na-gradient(c)	2	13	-	-	-	-	-	-	6	-	7	-
221	Glutamine from Serine	Glutamine	−Serine MCES +Urea	Transamination to Glutamine using Serine	1 Pyruvate(m) 2 Serine(s) 1 ATP-energy(c) 2 NADPH-redox-potential(m) 1 Proton-gradient(m) 1 CoA-activated acetyl group(m)	1 H_2O(s) 2 Pyruvate(c) 1 Glutamine(s) 2 NADH-redox-potential(m) 1 Proton-gradient(c) 1 Na-gradient(c)	3	10	-	-	-	-	-	-	5	-	4	-

Continued on next page

B2. Turnover functions – continued.

Simulation	Definition			Solution													
				exchanges		reactions								transport			Prot
	Objective	Contraints	Comment	imports	exports	c	m	r	p	l	n	s	b	s-c	b-c	intra	syn
222 Glutamine from Threonine	Glutamine	−Threonine MCES +Urea	Transamination to Glutamine using Threonine	0.5 O_2(s) 2 Threonine(s) 1 ATP-energy(c) 1 NADPH-redox-potential(m)	1 H_2O(s) 1 CO_2(s) 1 Glutamine(s) 3 NADH-redox-potential(m) 3 Proton-gradient(m) 1 CoA-activated acetyl group(m)	3	17	-	-	-	-	-	-	6	-	9	-
223 Glutamine from Tryptophan	Glutamine	−Tryptophan MCES +Urea	Transamination to Glutamine using Tryptophan	1 H_2O(s) 3.5 O_2(s) 1 Pyruvate(m) 1 Tryptophan(s) 2 ATP-energy(c) 2 NADPH-redox-potential(c) 1 NADPH-redox-potential(m)	3 CO_2(s) 1 Pyruvate(c) 1 Glutamine(s) 3 NADH-redox-potential(m) 6 Proton-gradient(m) 1 Na-gradient(c) 1 THF-activated methyl group(c) 1 CoA-activated acetyl group(m)	20	11	-	-	-	-	-	-	6	-	8	-
224 Glutamine from Tyrosine	Glutamine	−Tyrosine MCES +Urea	Transamination to Glutamine using Tyrosine	3 H_2O(s) 4 O_2(s) 2 Tyrosine(s) 4 ATP-energy(c) 1 ATP-energy(m) 1 Proton-gradient(m) 1 CoA-activated acetyl group(m)	4 CO_2(s) 1 Pyruvate(c) 1 Glutamine(s) 2 NADH-redox-potential(m) 2 NADPH-redox-potential(c) 2 Na-gradient(c) 4 CoA-activated acetyl group(c)	18	6	-	-	-	-	-	-	7	-	7	-
225 Glutamine from Valine	Glutamine	−Valine MCES +Urea	Transamination to Glutamine using Valine	3 H_2O(s) 1.5 O_2(s) 2 Valine(s)	2 CO_2(s) 1 Pyruvate(c) 1 Glutamine(s) 6 NADH-redox-potential(m) 1 NADPH-redox-potential(c) 15 Proton-gradient(m) 2 Na-gradient(c)	4	14	-	-	-	-	-	-	7	-	13	-
226 Glutamine from beta-Alanine	Glutamine	−beta-Alanine MCES +Urea	Transamination to Glutamine using beta-Alanine	1 H_2O(s) 1 Pyruvate(m) 2 beta-Alanine(s) 1 ATP-energy(c) 1 Proton-gradient(m)	2 CO_2(s) 1 Glutamine(s) 3 NADH-redox-potential(m) 1 NADPH-redox-potential(c) 1 CoA-activated acetyl group(m)	5	9	-	-	-	-	-	-	4	-	8	-
227 Methionine from Cysteine	Methionine	−Cysteine MCES +Urea	Transamination to Methionine using Cysteine	1 H_2O(s) 1 Pyruvate(m) 1 Cysteine(s) 1 NADPH-redox-potential(m) 1 Proton-gradient(m) 1 THF-activated methyl group(c) 1 CoA-activated acetyl group(m)	1 CO_2(s) 1 Pyruvate(c) 1 Methionine(s) 1 NADH-redox-potential(m) 1 NADPH-redox-potential(c) 1 Na-gradient(c)	8	12	-	-	-	-	-	-	5	-	4	-

Continued on next page

B2. Turnover functions – continued.

Simulation		Definition			Solution													
					exchanges		reactions								transport			Prot
		Objective	Contraints	Comment	imports	exports	c	m	r	p	l	n	s	b	s-c	b-c	intra	syn
228	Methionine from Cystine	Methionine	−Cystine MCES +Urea	Transamination to Methionine using Cystine	1 H_2O(s) 1 Pyruvate(m) 0.5 Cystine(s) 0.5 NADPH-redox-potential(c) 1 NADPH-redox-potential(m) 1 Proton-gradient(m) 1 THF-activated methyl group(c) 1 CoA-activated acetyl group(m)	1 CO_2(s) 1 Pyruvate(c) 1 Methionine(s) 2 NADH-redox-potential(m) 0.5 Na-gradient(c)	7	14	-	-	-	-	-	-	6	-	4	-
229	Proline from Alanine	Proline	−Alanine MCES +Urea	Transamination to Proline using Alanine	0.5 O_2(s) 1 Alanine(s) 1 ATP-energy(m) 2 NADPH-redox-potential(m) 1 CoA-activated acetyl group(m)	2 H_2O(s) 1 Proline(s) 2 NADH-redox-potential(m) 5 Proton-gradient(m) 1 Na-gradient(c)	-	15	-	-	-	-	-	-	5	-	7	-
230	Proline from Arginine	Proline	−Arginine MCES +Urea	Transamination to Proline using Arginine	0.5 O_2(s) 0.5 Pyruvate(m) 0.5 Arginine(s) 0.5 ATP-energy(m) 1 NADPH-redox-potential(m) 0.5 CoA-activated acetyl group(m)	1.5 H_2O(s) 0.5 Urea(s) 1 Proline(s) 1 NADH-redox-potential(m) 4.5 Proton-gradient(m)	1	15	-	-	-	-	-	-	6	-	8	-
231	Proline from Asparagine	Proline	−Asparagine MCES +Urea	Transamination to Proline using Asparagine	0.5 O_2(s) 0.5 Pyruvate(m) 0.5 Asparagine(s) 1 ATP-energy(m) 0.5 NADH-redox-potential(c) 2 NADPH-redox-potential(m) 1 CoA-activated acetyl group(m)	2 H_2O(s) 0.5 CO_2(s) 1 Proline(s) 1.5 NADH-redox-potential(m) 0.5 NADPH-redox-potential(c) 5.5 Proton-gradient(m)	7	15	-	-	-	-	-	-	5	-	9	-
232	Proline from Aspartate	Proline	−Aspartate MCES +Urea	Transamination to Proline using Aspartate	0.5 O_2(s) 1 Aspartate(s) 1 ATP-energy(m) 1 NADH-redox-potential(c) 1 NADPH-redox-potential(m) 1 CoA-activated acetyl group(m)	2 H_2O(s) 1 CO_2(s) 1 Proline(s) 1 NADH-redox-potential(m) 1 NADPH-redox-potential(c) 6 Proton-gradient(m) 1 Na-gradient(c)	6	11	-	-	-	-	-	-	6	-	7	-
233	Proline from Cysteine	Proline	−Cysteine MCES +Urea +H_2S	Transamination to Proline using Cysteine	0.5 O_2(s) 1 Pyruvate(m) 1 Cysteine(s) 1 ATP-energy(m) 2 NADPH-redox-potential(m) 1 CoA-activated acetyl group(m)	2 H_2O(s) 1 Pyruvate(c) 1 Proline(s) 1 H_2S(s) 1 NADH-redox-potential(m) 5 Proton-gradient(m)	1	15	-	-	-	-	-	-	5	-	7	-
234	Proline from Cystine	Proline	−Cystine MCES +Urea +H_2S	Transamination to Proline using Cystine	0.5 O_2(s) 1 Pyruvate(m) 0.5 Cystine(s) 1 ATP-energy(m) 0.5 NADPH-redox-potential(c) 3 NADPH-redox-potential(m) 1 CoA-activated acetyl group(m)	2 H_2O(s) 1 Pyruvate(c) 1 Proline(s) 1 H_2S(s) 2 NADH-redox-potential(m) 5 Proton-gradient(m) 0.5 Na-gradient(c)	3	15	-	-	-	-	-	-	7	-	7	-

Continued on next page

B2. Turnover functions – continued.

Simulation	Definition			Solution													
				exchanges		reactions								transport			Prot
	Objective	Contraints	Comment	imports	exports	c	m	r	p	l	n	s	b	s-c	b-c	intra	syn
235 Proline from Glutamate	Proline	−Glutamate MCES +Urea	Transamination to Proline using Glutamate	0.5 O_2(s) 1 Glutamate(s) 1 NADH-redox-potential(m)	3 H_2O(s) 1 Proline(s) 6 Proton-gradient(m) 1 Na-gradient(c)	-	4	-	-	-	-	-	-	5	-	6	-
236 Proline from Glycine	Proline	−Glycine MCES +Urea	Transamination to Proline using Glycine	0.5 O_2(s) 1 Pyruvate(m) 1 Glycine(s) 1 ATP-energy(m) 3 NADPH-redox-potential(m) 1 THF-activated methyl group(c) 1 CoA-activated acetyl group(m)	2 H_2O(s) 1 Pyruvate(c) 1 Proline(s) 2 NADH-redox-potential(m) 1 NADPH-redox-potential(c) 5 Proton-gradient(m) 1 Na-gradient(c)	5	15	-	-	-	-	-	-	4	-	7	-
237 Proline from Histidine	Proline	−Histidine MCES +Urea	Transamination to Proline using Histidine	0.5 O_2(s) 0.67 Pyruvate(m) 0.33 Histidine(s) 1 ATP-energy(m) 1.67 NADPH-redox-potential(m) 0.67 CoA-activated acetyl group(m)	1.67 H_2O(s) 0.33 CO_2(s) 1 Proline(s) 0.67 NADH-redox-potential(m) 0.33 NADPH-redox-potential(c) 5.33 Proton-gradient(m) 0.33 Na-gradient(c)	8	15	-	-	-	-	-	-	7	-	8	-
238 Proline from Isoleucine	Proline	−Isoleucine MCES +Urea	Transamination to Proline using Isoleucine	1.17 O_2(s) 0.33 Pyruvate(m) 1 Isoleucine(s) 1 ATP-energy(m) 2 NADPH-redox-potential(m)	2.33 H_2O(s) 1 Proline(s) 3.33 NADH-redox-potential(m) 12 Proton-gradient(m) 1 Na-gradient(c) 1 CoA-activated acetyl group(m)	-	30	-	-	-	-	-	-	5	-	7	-
239 Proline from Glutamine	Proline	−Glutamine MCES +Urea	Transamination to Proline using Glutamine	0.5 O_2(s) 0.5 Pyruvate(m) 0.5 Glutamine(s) 1 ATP-energy(m) 2 NADPH-redox-potential(m) 0.5 CoA-activated acetyl group(m)	2.5 H_2O(s) 1 Proline(s) 1 NADH-redox-potential(m) 5 Proton-gradient(m) 0.5 Na-gradient(c)	-	16	-	-	-	-	-	-	6	-	7	-
240 Proline from Leucine	Proline	−Leucine MCES +Urea	Transamination to Proline using Leucine	1 O_2(s) 1 Pyruvate(m) 1 Leucine(s) 1 ATP-energy(m) 3 NADPH-redox-potential(m)	3 H_2O(s) 1 Proline(s) 4 NADH-redox-potential(m) 11 Proton-gradient(m) 2 CoA-activated acetyl group(m)	-	22	-	-	-	-	-	-	3	-	7	-
241 Proline from Lysine	Proline	−Lysine MCES +Urea	Transamination to Proline using Lysine	0.75 O_2(s) 1 Pyruvate(m) 0.5 Lysine(s) 1 ATP-energy(m) 2.5 NADPH-redox-potential(m)	1.5 H_2O(s) 1 CO_2(s) 1 Proline(s) 0.5 NADH-redox-potential(c) 2.5 NADH-redox-potential(m) 1 NADPH-redox-potential(c) 8 Proton-gradient(m)	6	18	-	-	-	-	-	-	5	-	9	-

Continued on next page

B2. Turnover functions – continued.

Simulation	Definition			Solution													
				exchanges		reactions								transport			Prot
	Objective	Contraints	Comment	imports	exports	c	m	r	p	l	n	s	b	s-c	b-c	intra	syn
242 Proline from Methionine	Proline	−Methionine MCES +Urea +H_2S	Transamination to Proline using Methionine	0.67 O_2(s) 0.33 Pyruvate(m) 1 Methionine(s) 0.5 Glucose-6P(c) 1 ATP-energy(m) 2 NADPH-redox-potential(m)	1.83 H_2O(s) 0.5 P_i(c) 1 Pyruvate(c) 1 Proline(s) 1 H_2S(s) 0.5 ATP-energy(c) 2 NADH-redox-potential(c) 0.33 NADH-redox-potential(m) 4 Proton-gradient(m) 1 Na-gradient(c) 1 THF-activated methyl group(c)	16	25	-	-	-	-	-	-	6	-	11	-
243 Proline from Phenylalanine	Proline	−Phenylalanine MCES +Urea	Transamination to Proline using Phenylalanine	3.5 O_2(s) 1 Phenylalanine(s) 2 ATP-energy(c) 1 ATP-energy(m) 1 NADPH-redox-potential(m) 1 CoA-activated acetyl group(m)	2 H_2O(s) 2 CO_2(s) 1 Proline(s) 1 NADH-redox-potential(m) 6 Proton-gradient(m) 1 Na-gradient(c) 2 CoA-activated acetyl group(c)	17	10	-	-	-	-	-	-	6	-	7	-
244 Proline from Serine	Proline	−Serine MCES +Urea	Transamination to Proline using Serine	0.5 O_2(s) 1 Pyruvate(m) 1 Serine(s) 1 ATP-energy(m) 3 NADPH-redox-potential(m) 1 CoA-activated acetyl group(m)	3 H_2O(s) 1 Pyruvate(c) 1 Proline(s) 2 NADH-redox-potential(m) 1 Proton-gradient(c) 5 Proton-gradient(m) 1 Na-gradient(c)	1	15	-	-	-	-	-	-	6	-	7	-
245 Proline from Threonine	Proline	−Threonine MCES +Urea	Transamination to Proline using Threonine	0.67 O_2(s) 0.33 Pyruvate(m) 1 Threonine(s) 1 ATP-energy(m) 2 NADPH-redox-potential(m)	3.33 H_2O(s) 1 Proline(s) 1.33 NADH-redox-potential(m) 6 Proton-gradient(m)	1	25	-	-	-	-	-	-	4	-	8	-
246 Proline from Tryptophan	Proline	−Tryptophan MCES +Urea	Transamination to Proline using Tryptophan	2.25 O_2(s) 1 Pyruvate(m) 0.5 Tryptophan(s) 0.5 ATP-energy(c) 1 ATP-energy(m) 2.5 NADPH-redox-potential(m)	1 H_2O(s) 2 CO_2(s) 0.5 Pyruvate(c) 1 Proline(s) 0.5 NADH-redox-potential(c) 2 NADH-redox-potential(m) 0.5 NADPH-redox-potential(c) 8.5 Proton-gradient(m) 0.5 Na-gradient(c)	17	16	-	-	-	-	-	-	7	-	10	-
247 Proline from Tyrosine	Proline	−Tyrosine MCES +Urea	Transamination to Proline using Tyrosine	2.5 O_2(s) 1 Tyrosine(s) 2 ATP-energy(c) 1 ATP-energy(m) 1 NADPH-redox-potential(m) 1 CoA-activated acetyl group(m)	1 H_2O(s) 2 CO_2(s) 1 Proline(s) 1 NADH-redox-potential(m) 1 NADPH-redox-potential(c) 6 Proton-gradient(m) 1 Na-gradient(c) 2 CoA-activated acetyl group(c)	15	11	-	-	-	-	-	-	6	-	7	-
248 Proline from Valine	Proline	−Valine MCES +Urea	Transamination to Proline using Valine	1 O_2(s) 1 Valine(s) 1 ATP-energy(m) 1 NADPH-redox-potential(m)	2 H_2O(s) 1 Proline(s) 2 NADH-redox-potential(m) 10 Proton-gradient(m)	-	16	-	-	-	-	-	-	4	-	7	-

Continued on next page

B2. Turnover functions – continued.

Simulation		Definition			Solution													
					exchanges		reactions								transport			Prot
		Objective	Contraints	Comment	imports	exports	c	m	r	p	l	n	s	b	s-c	b-c	intra	syn
249	Proline from beta-Alanine	Proline	−beta-Alanine MCES +Urea	Transamination to Proline using beta-Alanine	0.5 O_2(s) 1 Pyruvate(m) 1 beta-Alanine(s) 1 ATP-energy(m) 2 NADPH-redox-potential(m)	2 H_2O(s) 1 CO_2(s) 1 Proline(s) 2 NADH-redox-potential(m) 1 NADPH-redox-potential(c) 5 Proton-gradient(m)	3	13	-	-	-	-	-	-	5	-	8	-
250	Serine from Alanine	Serine	−Alanine MCES +Urea	Transamination to Serine using Alanine	1.5 H_2O(s) 1 Alanine(s) 0.5 Glucose-6P(c)	0.5 P_i(c) 1 Pyruvate(c) 1 Serine(s) 0.5 ATP-energy(c) 2 NADH-redox-potential(c) 1 Na-gradient(c)	12	-	-	-	-	-	-	-	4	-	-	-
251	Serine from Arginine	Serine	−Arginine MCES +Urea	Transamination to Serine using Arginine	3 H_2O(s) 0.25 O_2(s) 0.5 Pyruvate(m) 0.5 Arginine(s) 0.5 Glucose-6P(c) 0.5 NADPH-redox-potential(m)	0.5 P_i(c) 1 CO_2(s) 1 Pyruvate(c) 1 Serine(s) 0.5 Urea(s) 0.5 ATP-energy(c) 0.5 ATP-energy(m) 2 NADH-redox-potential(c) 1 NADH-redox-potential(m) 1 NADPH-redox-potential(c) 1.5 Proton-gradient(m)	14	11	-	-	-	-	-	-	7	-	10	-
252	Serine from Asparagine	Serine	−Asparagine MCES +Urea	Transamination to Serine using Asparagine	1.5 H_2O(s) 0.5 Glucose-6P(c) 0.5 Asparagine(s) 0.5 NADPH-redox-potential(m) 1 Proton-gradient(m)	0.5 P_i(c) 0.5 CO_2(s) 0.5 Pyruvate(c) 1 Serine(s) 0.5 ATP-energy(c) 1.5 NADH-redox-potential(c) 0.5 NADPH-redox-potential(c)	16	2	-	-	-	-	-	-	5	-	7	-
253	Serine from Aspartate	Serine	−Aspartate MCES +Urea	Transamination to Serine using Aspartate	1.5 H_2O(s) 1 Aspartate(s) 0.5 Glucose-6P(c)	0.5 P_i(c) 1 CO_2(s) 1 Pyruvate(c) 1 Serine(s) 0.5 ATP-energy(c) 1 NADH-redox-potential(c) 1 NADPH-redox-potential(c) 1 Na-gradient(c)	15	-	-	-	-	-	-	-	5	-	-	-
254	Serine from Cysteine	Serine	−Cysteine MCES +Urea +H_2S	Transamination to Serine using Cysteine	1.5 H_2O(s) 0.5 Glucose-6P(c) 1 Cysteine(s) 1 NADH-redox-potential(m) 2 Proton-gradient(m)	0.5 P_i(c) 1 Pyruvate(c) 1 Serine(s) 1 H_2S(s) 0.5 ATP-energy(c) 2 NADH-redox-potential(c) 1 Na-gradient(c)	12	2	-	-	-	-	-	-	5	-	7	-
255	Serine from Cystine	Serine	−Cystine MCES +Urea +H_2S	Transamination to Serine using Cystine	1.5 H_2O(s) 0.5 Glucose-6P(c) 0.5 Cystine(s) 1 NADH-redox-potential(m) 0.5 NADPH-redox-potential(c) 2 Proton-gradient(m)	0.5 P_i(c) 1 Pyruvate(c) 1 Serine(s) 1 H_2S(s) 0.5 ATP-energy(c) 2 NADH-redox-potential(c) 0.5 Na-gradient(c)	14	2	-	-	-	-	-	-	6	-	7	-

Continued on next page

B2. Turnover functions – continued.

Simulation		Definition			Solution													
					exchanges		reactions								transport			Prot
		Objective	Contraints	Comment	imports	exports	c	m	r	p	l	n	s	b	s-c	b-c	intra	syn
256	Serine from Glutamate	Serine	−Glutamate MCES +Urea	Transamination to Serine using Glutamate	2.5 H_2O(s) 0.5 O_2(s) 1 Glutamate(s) 0.5 Glucose-6P(c)	0.5 P_i(c) 2 CO_2(s) 1 Pyruvate(c) 1 Serine(s) 0.5 ATP-energy(c) 1 ATP-energy(m) 2 NADH-redox-potential(c) 1 NADH-redox-potential(m) 1 NADPH-redox-potential(c) 6 Proton-gradient(m) 1 Na-gradient(c)	13	7	-	-	-	-	-	-	6	-	7	-
257	Serine from Glycine	Serine	−Glycine MCES +Urea	Transamination to Serine using Glycine	1 H_2O(s) 1 Glycine(s) 1 THF-activated methyl group(c)	1 Serine(s) 1 NADPH-redox-potential(c) 1 Na-gradient(c)	4	-	-	-	-	-	-	-	3	-	-	-
258	Serine from Histidine	Serine	−Histidine MCES +Urea	Transamination to Serine using Histidine	1.83 H_2O(s) 0.17 O_2(s) 0.5 Glucose-6P(c) 0.33 Histidine(s) 0.33 NADH-redox-potential(m)	0.5 P_i(c) 0.67 CO_2(s) 0.33 Pyruvate(c) 1 Serine(s) 0.5 ATP-energy(c) 0.33 ATP-energy(m) 1.67 NADH-redox-potential(c) 0.67 Proton-gradient(m) 0.33 THF-activated methyl group(c)	20	8	-	-	-	-	-	-	6	-	12	-
259	Serine from Isoleucine	Serine	−Isoleucine MCES +Urea	Transamination to Serine using Isoleucine	2.5 H_2O(s) 1 O_2(s) 0.5 Glucose-6P(c) 1 Isoleucine(s)	0.5 P_i(c) 2 CO_2(s) 1 Serine(s) 0.5 ATP-energy(c) 2 NADH-redox-potential(c) 4 NADH-redox-potential(m) 9 Proton-gradient(m) 2 CoA-activated acetyl group(m)	11	13	-	-	-	-	-	-	5	-	11	-
260	Serine from Glutamine	Serine	−Glutamine MCES +Urea	Transamination to Serine using Glutamine	2 H_2O(s) 0.25 O_2(s) 0.5 Glutamine(s) 0.5 Glucose-6P(c) 0.5 NADPH-redox-potential(m)	0.5 P_i(c) 1 CO_2(s) 0.5 Pyruvate(c) 1 Serine(s) 0.5 ATP-energy(c) 0.5 ATP-energy(m) 2 NADH-redox-potential(c) 0.5 NADH-redox-potential(m) 0.5 NADPH-redox-potential(c) 2 Proton-gradient(m)	15	9	-	-	-	-	-	-	5	-	11	-
261	Serine from Leucine	Serine	−Leucine MCES +Urea	Transamination to Serine using Leucine	0.5 H_2O(s) 0.5 O_2(s) 0.5 Glucose-6P(c) 1 Leucine(s) 1 NADPH-redox-potential(m)	0.5 P_i(c) 1 Serine(s) 0.5 ATP-energy(c) 2 NADH-redox-potential(c) 2 NADH-redox-potential(m) 4 Proton-gradient(m) 1 Na-gradient(c) 3 CoA-activated acetyl group(m)	11	13	-	-	-	-	-	-	5	-	10	-

Continued on next page

B2. Turnover functions – continued.

Simulation		Definition			Solution													
					exchanges		reactions								transport			Prot
		Objective	Contraints	Comment	imports	exports	c	m	r	p	l	n	s	b	s-c	b-c	intra	syn
262	Serine from Lysine	Serine	−Lysine MCES +Urea	Transamination to Serine using Lysine	2 H_2O(s) 0.25 O_2(s) 0.5 Lysine(s) 0.5 Glucose-6P(c) 0.5 NADPH-redox-potential(m)	0.5 P_i(c) 1 CO_2(s) 1 Serine(s) 0.5 ATP-energy(c) 2.5 NADH-redox-potential(c) 1.5 NADH-redox-potential(m) 1 Proton-gradient(m) 1 CoA-activated acetyl group(m)	12	11	-	-	-	-	-	-	5	-	11	-
263	Serine from Methionine	Serine	−Methionine MCES +Urea +H_2S	Transamination to Serine using Methionine	3 H_2O(s) 0.5 O_2(s) 1 Methionine(s) 1 Glucose-6P(c)	1 P_i(c) 2 CO_2(s) 1 Pyruvate(c) 1 Serine(s) 1 H_2S(s) 1 ATP-energy(c) 4 NADH-redox-potential(c) 1 NADH-redox-potential(m) 1 Proton-gradient(m) 1 THF-activated methyl group(c) 1 CoA-activated acetyl group(m)	16	9	-	-	-	-	-	-	5	-	12	-
264	Serine from Phenylalanine	Serine	−Phenylalanine MCES +Urea	Transamination to Serine using Phenylalanine	1.5 H_2O(s) 3 O_2(s) 1 Phenylalanine(s) 0.5 Glucose-6P(c) 1.5 ATP-energy(c)	0.5 P_i(c) 2 CO_2(s) 1 Pyruvate(c) 1 Serine(s) 2 NADH-redox-potential(c) 2 CoA-activated acetyl group(c)	25	-	-	-	-	-	-	-	5	-	-	-
265	Serine from Proline	Serine	−Proline MCES +Urea	Transamination to Serine using Proline	5.5 H_2O(s) 0.5 Glucose-6P(c) 1 Proline(s)	0.5 P_i(c) 2 CO_2(s) 1 Pyruvate(c) 1 Serine(s) 0.5 ATP-energy(c) 1 ATP-energy(m) 2 NADH-redox-potential(c) 2 NADH-redox-potential(m) 1 NADPH-redox-potential(c) 1 Na-gradient(c)	13	10	-	-	-	-	-	-	5	-	4	-
266	Serine from Threonine	Serine	−Threonine MCES +Urea	Transamination to Serine using Threonine	1.5 H_2O(s) 0.5 O_2(s) 0.5 Glucose-6P(c) 1 Threonine(s)	0.5 P_i(c) 2 CO_2(s) 1 Serine(s) 0.5 ATP-energy(c) 2 NADH-redox-potential(c) 2 NADH-redox-potential(m) 3 Proton-gradient(m) 1 CoA-activated acetyl group(m)	12	9	-	-	-	-	-	-	5	-	11	-

Continued on next page

B2. Turnover functions – continued.

Simulation		Definition: Objective	Definition: Contraints	Definition: Comment	Solution: exchanges: imports	Solution: exchanges: exports	reactions: c	reactions: m	reactions: r	reactions: p	reactions: l	reactions: n	reactions: s	reactions: b	transport: s-c	transport: b-c	transport: intra	Prot syn
267	Serine from Tryptophan	Serine	−Tryptophan MCES +Urea	Transamination to Serine using Tryptophan	1.5 H_2O(s) 1.75 O_2(s) 0.5 Tryptophan(s) 0.5 Glucose-6P(c) 1.5 NADPH-redox-potential(c) 0.5 NADPH-redox-potential(m)	0.5 P_i(c) 1.5 CO_2(s) 0.5 Pyruvate(c) 1 Serine(s) 2 NADH-redox-potential(c) 1 NADH-redox-potential(m) 1.5 Proton-gradient(m) 0.5 THF-activated methyl group(c) 1 CoA-activated acetyl group(m)	25	9	-	-	-	-	-	-	6	-	12	-
268	Serine from Tyrosine	Serine	−Tyrosine MCES +Urea	Transamination to Serine using Tyrosine	2.5 H_2O(s) 2 O_2(s) 1 Tyrosine(s) 0.5 Glucose-6P(c) 1.5 ATP-energy(c)	0.5 P_i(c) 2 CO_2(s) 1 Pyruvate(c) 1 Serine(s) 2 NADH-redox-potential(c) 1 NADPH-redox-potential(c) 1 Na-gradient(c) 2 CoA-activated acetyl group(c)	24	-	-	-	-	-	-	-	6	-	-	-
269	Serine from Valine	Serine	−Valine MCES +Urea	Transamination to Serine using Valine	3.5 H_2O(s) 1 O_2(s) 0.5 Glucose-6P(c) 1 Valine(s)	0.5 P_i(c) 2 CO_2(s) 1 Pyruvate(c) 1 Serine(s) 0.5 ATP-energy(c) 1 ATP-energy(m) 2 NADH-redox-potential(c) 3 NADH-redox-potential(m) 1 NADPH-redox-potential(c) 10 Proton-gradient(m) 1 Na-gradient(c)	13	14	-	-	-	-	-	-	6	-	10	-
270	Serine from beta-Alanine	Serine	−beta-Alanine MCES +Urea	Transamination to Serine using beta-Alanine	1.5 H_2O(s) 0.5 Glucose-6P(c) 1 beta-Alanine(s) 2 Proton-gradient(m)	0.5 P_i(c) 1 CO_2(s) 1 Serine(s) 0.5 ATP-energy(c) 2 NADH-redox-potential(c) 1 NADH-redox-potential(m) 1 CoA-activated acetyl group(m)	11	4	-	-	-	-	-	-	4	-	7	-
271	Tyrosine from Phenylalanine	Tyrosine	−Phenylalanine MCES +Urea	Transamination to Tyrosine using Phenylalanine	1 O_2(s) 1 Phenylalanine(s) 1 NADPH-redox-potential(c)	1 H_2O(s) 1 Tyrosine(s)	3	-	-	-	-	-	-	-	3	-	-	-
272	beta-Alanine from Alanine	beta-Alanine	−Alanine MCES +Urea	Transamination to beta-Alanine using Alanine	1 Alanine(s) 1 NADPH-redox-potential(c)	1 beta-Alanine(s) 1 NADH-redox-potential(c)	7	-	-	-	-	-	-	-	2	-	-	-

Continued on next page

B2. Turnover functions – continued.

Simulation		Definition			Solution													
					exchanges		reactions								transport			Prot
		Objective	Contraints	Comment	imports	exports	c	m	r	p	l	n	s	b	s-c	b-c	intra	syn
273	beta-Alanine from Arginine	beta-Alanine	−Arginine MCES +Urea	Transamination to beta-Alanine using Arginine	1.5 H_2O(s) 0.25 O_2(s) 0.5 Pyruvate(m) 0.5 Arginine(s) 0.5 NADPH-redox-potential(m)	1 CO_2(s) 0.5 Urea(s) 1 beta-Alanine(s) 0.5 ATP-energy(m) 1 NADH-redox-potential(c) 1 NADH-redox-potential(m) 1.5 Proton-gradient(m)	5	11	-	-	-	-	-	-	6	-	10	-
274	beta-Alanine from Asparagine	beta-Alanine	−Asparagine MCES +Urea	Transamination to beta-Alanine using Asparagine	0.5 Pyruvate(c) 0.5 Asparagine(s) 0.5 NADPH-redox-potential(c) 0.5 NADPH-redox-potential(m) 1 Proton-gradient(m)	0.5 CO_2(s) 1 beta-Alanine(s) 0.5 NADH-redox-potential(c)	7	2	-	-	-	-	-	-	3	-	7	-
275	beta-Alanine from Aspartate	beta-Alanine	−Aspartate MCES +Urea	Transamination to beta-Alanine using Aspartate	1 Aspartate(s)	1 CO_2(s) 1 beta-Alanine(s) 1 Na-gradient(c)	1	-	-	-	-	-	-	-	4	-	-	-
276	beta-Alanine from Cysteine	beta-Alanine	−Cysteine MCES +Urea +H_2S	Transamination to beta-Alanine using Cysteine	1 Pyruvate(m) 1 Cysteine(s) 2 NADPH-redox-potential(m) 1 Proton-gradient(m)	1 Pyruvate(c) 1 beta-Alanine(s) 1 H_2S(s) 1 NADH-redox-potential(c)	5	3	-	-	-	-	-	-	3	-	7	-
277	beta-Alanine from Cystine	beta-Alanine	−Cystine MCES +Urea +H_2S	Transamination to beta-Alanine using Cystine	0.5 Cystine(s) 1.5 NADPH-redox-potential(c) 1 NADPH-redox-potential(m) 2 Proton-gradient(m)	1 beta-Alanine(s) 1 H_2S(s) 1 NADH-redox-potential(c) 0.5 Na-gradient(c)	8	2	-	-	-	-	-	-	5	-	7	-
278	beta-Alanine from Glutamate	beta-Alanine	−Glutamate MCES +Urea	Transamination to beta-Alanine using Glutamate	1 H_2O(s) 0.5 O_2(s) 1 Glutamate(s)	2 CO_2(s) 1 beta-Alanine(s) 1 ATP-energy(m) 1 NADH-redox-potential(c) 1 NADH-redox-potential(m) 6 Proton-gradient(m) 1 Na-gradient(c)	4	7	-	-	-	-	-	-	6	-	7	-
279	beta-Alanine from Glycine	beta-Alanine	−Glycine MCES +Urea	Transamination to beta-Alanine using Glycine	1 Glycine(s) 1 NADPH-redox-potential(c) 1 THF-activated methyl group(c)	1 beta-Alanine(s) 1 NADH-redox-potential(c) 1 Na-gradient(c)	16	-	-	-	-	-	-	-	3	-	-	-
280	beta-Alanine from Histidine	beta-Alanine	−Histidine MCES +Urea	Transamination to beta-Alanine using Histidine	0.33 H_2O(s) 0.17 O_2(s) 0.67 Pyruvate(m) 0.33 Histidine(s) 0.67 NADPH-redox-potential(c) 1.33 NADPH-redox-potential(m)	0.67 CO_2(s) 1 beta-Alanine(s) 0.67 NADH-redox-potential(c) 0.67 NADH-redox-potential(m) 1.67 Proton-gradient(m) 0.33 Na-gradient(c) 0.33 THF-activated methyl group(c)	13	13	-	-	-	-	-	-	6	-	10	-

Continued on next page

B2. Turnover functions – continued.

Simulation		Definition			Solution													
					exchanges		reactions								transport			Prot
		Objective	Contraints	Comment	imports	exports	c	m	r	p	l	n	s	b	s-c	b-c	intra	syn
281	beta-Alanine from Isoleucine	beta-Alanine	−Isoleucine MCES +Urea	Transamination to beta-Alanine using Isoleucine	1 H_2O(s) 1 O_2(s) 1 Isoleucine(s)	1 CO_2(s) 1 beta-Alanine(s) 3 NADH-redox-potential(m) 11 Proton-gradient(m) 1 CoA-activated acetyl group(m)	-	13	-	-	-	-	-	-	5	-	8	-
282	beta-Alanine from Glutamine	beta-Alanine	−Glutamine MCES +Urea	Transamination to beta-Alanine using Glutamine	0.5 H_2O(s) 0.25 O_2(s) 0.5 Pyruvate(m) 0.5 Glutamine(s) 0.5 NADPH-redox-potential(m)	1 CO_2(s) 1 beta-Alanine(s) 0.5 ATP-energy(m) 1 NADH-redox-potential(c) 0.5 Proton-gradient(c) 2.5 Proton-gradient(m) 0.5 Na-gradient(c)	6	9	-	-	-	-	-	-	7	-	8	-
283	beta-Alanine from Leucine	beta-Alanine	−Leucine MCES +Urea	Transamination to beta-Alanine using Leucine	0.5 O_2(s) 1 Pyruvate(m) 1 Leucine(s) 2 NADPH-redox-potential(m)	1 H_2O(s) 1 beta-Alanine(s) 3 NADH-redox-potential(m) 3 Proton-gradient(m) 1 Na-gradient(c) 3 CoA-activated acetyl group(m)	1	14	-	-	-	-	-	-	5	-	10	-
284	beta-Alanine from Lysine	beta-Alanine	−Lysine MCES +Urea	Transamination to beta-Alanine using Lysine	0.5 H_2O(s) 0.25 O_2(s) 1 Pyruvate(m) 0.5 Lysine(s) 1.5 NADPH-redox-potential(m)	1 CO_2(s) 1 beta-Alanine(s) 0.5 NADH-redox-potential(c) 2.5 NADH-redox-potential(m) 1 Proton-gradient(m) 1 CoA-activated acetyl group(m)	3	14	-	-	-	-	-	-	5	-	9	-
285	beta-Alanine from Methionine	beta-Alanine	−Methionine MCES +Urea +H_2S	Transamination to beta-Alanine using Methionine	1.5 H_2O(s) 0.5 O_2(s) 1 Pyruvate(m) 1 Methionine(s) 0.5 Glucose-6P(c) 1 NADPH-redox-potential(m)	0.5 P_i(c) 1 CO_2(s) 2 Pyruvate(c) 1 beta-Alanine(s) 1 H_2S(s) 0.5 ATP-energy(c) 2 NADH-redox-potential(c) 1 NADPH-redox-potential(c) 4 Proton-gradient(m) 1 Na-gradient(c) 1 THF-activated methyl group(c)	18	9	-	-	-	-	-	-	7	-	11	-
286	beta-Alanine from Phenylalanine	beta-Alanine	−Phenylalanine MCES +Urea	Transamination to beta-Alanine using Phenylalanine	3 O_2(s) 1 Phenylalanine(s) 2 ATP-energy(c)	2 CO_2(s) 1 beta-Alanine(s) 2 CoA-activated acetyl group(c)	17	-	-	-	-	-	-	-	4	-	-	-
287	beta-Alanine from Proline	beta-Alanine	−Proline MCES +Urea	Transamination to beta-Alanine using Proline	4 H_2O(s) 1 Proline(s)	2 CO_2(s) 1 beta-Alanine(s) 1 ATP-energy(m) 1 NADH-redox-potential(c) 2 NADH-redox-potential(m) 1 Na-gradient(c)	4	10	-	-	-	-	-	-	5	-	4	-

Continued on next page

B2. Turnover functions – continued.

Simulation		Definition			Solution													
					exchanges		reactions								transport			Prot
		Objective	Contraints	Comment	imports	exports	c	m	r	p	l	n	s	b	s-c	b-c	intra	syn
288	beta-Alanine from Serine	beta-Alanine	−Serine MCES +Urea	Transamination to beta-Alanine using Serine	1 Serine(s) 2 NADPH-redox-potential(c)	1 H_2O(s) 1 beta-Alanine(s) 1 NADH-redox-potential(c) 1 Proton-gradient(c) 1 Na-gradient(c)	13	-	-	-	-	-	-	-	5	-	-	-
289	beta-Alanine from Threonine	beta-Alanine	−Threonine MCES +Urea	Transamination to beta-Alanine using Threonine	0.5 O_2(s) 1 Threonine(s)	1 CO_2(s) 1 beta-Alanine(s) 1 NADH-redox-potential(m) 5 Proton-gradient(m)	1	8	-	-	-	-	-	-	4	-	8	-
290	beta-Alanine from Tryptophan	beta-Alanine	−Tryptophan MCES +Urea	Transamination to beta-Alanine using Tryptophan	1.75 O_2(s) 1 Pyruvate(m) 0.5 Tryptophan(s) 0.5 ATP-energy(c) 2 NADPH-redox-potential(c) 1 NADPH-redox-potential(m)	1.5 CO_2(s) 0.5 Pyruvate(c) 1 beta-Alanine(s) 1.5 NADH-redox-potential(c) 0.5 NADH-redox-potential(m) 2.5 Proton-gradient(m) 0.5 Na-gradient(c) 0.5 THF-activated methyl group(c) 1 CoA-activated acetyl group(m)	20	10	-	-	-	-	-	-	5	-	10	-
291	beta-Alanine from Tyrosine	beta-Alanine	−Tyrosine MCES +Urea	Transamination to beta-Alanine using Tyrosine	1 H_2O(s) 2 O_2(s) 1 Tyrosine(s) 2 ATP-energy(c)	2 CO_2(s) 1 beta-Alanine(s) 1 NADH-redox-potential(c) 1 Na-gradient(c) 2 CoA-activated acetyl group(c)	16	-	-	-	-	-	-	-	6	-	-	-
292	beta-Alanine from Valine	beta-Alanine	−Valine MCES +Urea	Transamination to beta-Alanine using Valine	2 H_2O(s) 1 O_2(s) 1 Valine(s)	2 CO_2(s) 1 beta-Alanine(s) 4 NADH-redox-potential(m) 11 Proton-gradient(m)	-	13	-	-	-	-	-	-	5	-	8	-
293	Homocysteine from Methionine	Homocysteine(c)	−Methionine(c) MCES	test that cytosolic Homocysteine can be produced from Methionine	1 Methionine(c)	1 Homocysteine(c) 1 THF-activated methyl group(c)	2	-	-	-	-	-	-	-	-	-	-	-
294	Taurine from Methionine	Taurine	−Methionine MCES	Conversion of methionine in taurine	2.5 H_2O(s) 1.5 O_2(s) 1 Methionine(s) 0.5 Glucose-6P(c)	0.5 P_i(c) 3 CO_2(s) 1 Taurine(s) 0.5 ATP-energy(c) 3 NADH-redox-potential(c) 2 NADH-redox-potential(m) 3 Proton-gradient(m) 1 Na-gradient(c) 1 THF-activated methyl group(c) 1 CoA-activated acetyl group(m)	18	9	-	-	-	-	-	-	6	-	11	-

Continued on next page

B2. Turnover functions – continued.

Simulation	Definition			Solution													
				exchanges		reactions								transport			Prot
	Objective	Contraints	Comment	imports	exports	c	m	r	p	l	n	s	b	s-c	b-c	intra	syn
295 Taurine from Cysteine	Taurine	−Cysteine MCES	Conversion of methionine in taurine	1 H_2O(s) 1 O_2(s) 1 Cysteine(s)	1 CO_2(s) 1 Taurine(s) 1 NADH-redox-potential(c)	4	-	-	-	-	-	-	-	5	-	-	-
**B2.3. Fatty acid conversion**																	
**B2.3.1. Saturated**																	
296 Stearate from Palmitate	Stearate	−Palmitate MCES	Conversion of Palmitate into Stearate	1 Palmitate(s) 3 ATP-energy(c) 1 NADH-redox-potential(m) 1 FADH-redox-potential(c) 1 CoA-activated acetyl group(m)	1 H_2O(s) 1 Stearate(s)	8	6	-	-	-	-	-	-	3	-	4	-
**B2.3.2. Omega-3-Fatty acids**																	
297 Palmitate from Palmitolate	Palmitate	−Palmitolate MCES	Conversion of Palmitolate into Palmitate	1 Palmitolate(s) 4 ATP-energy(c) 2 NADPH-redox-potential(c)	1 Palmitate(s) 1 NADH-redox-potential(c)	17	-	-	-	-	-	-	-	2	-	-	-
298 Palmitolate from Palmitate	Palmitolate	−Palmitate MCES	Conversion of Palmitate into Palmitolate	1 O_2(s) 1 Palmitate(s) 1 ATP-energy(c) 1 NADH-redox-potential(c)	2 H_2O(s) 1 Palmitolate(s)	6	-	-	-	-	-	-	-	4	-	-	-
299 Oleate from Stearate	Oleate	−Stearate MCES	Conversion of Stearate into Oleate	1 O_2(s) 1 Stearate(s) 3 ATP-energy(c) 1 NADH-redox-potential(c)	2 H_2O(s) 1 Oleate(s)	7	-	-	-	-	-	-	-	4	-	-	-
300 Stearate from Oleate	Stearate	−Oleate MCES	Conversion of into Stearate	1 Oleate(s) 4 ATP-energy(c) 2 NADPH-redox-potential(c)	1 Stearate(s) 1 NADH-redox-potential(c)	16	-	-	-	-	-	-	-	2	-	-	-
301 Palmitolate from Arachidonate	Palmitolate	−Arachidonate MCES	Conversion of from Arachidonate	1 O_2(s) 8 ATP-energy(c) 1 NADH-redox-potential(c) 14 NADPH-redox-potential(c) 8 CoA-activated acetyl group(c)	8 H_2O(s) 1 Palmitolate(s)	42	-	-	-	-	-	-	-	3	-	-	-
**B2.3.3. Omega-6-Fatty acids**																	
302 gamma-Linolenate from Linoleate	gamma-Linolenate	−Linoleate MCES	Conversion of Linoleate into gamma-Linolenate	1 O_2(s) 1 Linoleate(s) 3 ATP-energy(c) 1 NADH-redox-potential(c)	1 H_2O(s) 1 gamma-Linolenate(s)	7	-	-	-	-	-	-	-	4	-	-	-
303 Dihomo-gamma-linolenate from Linoleate	Dihomo-gamma-linolenate	−Linoleate MCES	Conversion of Linoleate into Dihomo-gamma-linolenate	2 O_2(s) 1 Linoleate(s) 4 ATP-energy(c) 1 NADH-redox-potential(c) 4 NADPH-redox-potential(r) 1 CoA-activated acetyl group(c)	4 H_2O(s) 1 Dihomo-gamma-linolenate(s)	12	-	3	-	-	-	-	-	4	-	6	-

Continued on next page

B2. Turnover functions – continued.

Simulation		Definition			Solution												
					exchanges		reactions								transport		Prot
		Objective	Contraints	Comment	imports	exports	c	m	r	p	l	n	s	b	s-c	b-c	intra	syn
304	Arachidonate from Linoleate	Arachidonate	−Linoleate MCES	Conversion of Linoleate into Arachidonate	3 O_2(s) 1 Linoleate(s) 4 ATP-energy(c) 2 NADH-redox-potential(c) 4 NADPH-redox-potential(r) 1 CoA-activated acetyl group(c)	5 H_2O(s) 1 Arachidonate(s)	13	-	3	-	-	-	-	-	4	-	6	-
305	Dihomo-gamma-linolenate from gamma-Linolenate	Dihomo-gamma-linolenate	−gamma-Linolenate MCES	Conversion of gamma-Linolenate into Dihomo-gamma-linolenate	1 O_2(s) 1 gamma-Linolenate(s) 4 ATP-energy(c) 4 NADPH-redox-potential(r) 1 CoA-activated acetyl group(c)	3 H_2O(s) 1 Dihomo-gamma-linolenate(s)	10	-	3	-	-	-	-	-	4	-	6	-
306	Arachidonate from gamma-Linolenate	Arachidonate	−gamma-Linolenate MCES	Conversion of gamma-Linolenate into Arachidonate	2 O_2(s) 1 gamma-Linolenate(s) 4 ATP-energy(c) 1 NADH-redox-potential(c) 4 NADPH-redox-potential(r) 1 CoA-activated acetyl group(c)	4 H_2O(s) 1 Arachidonate(s)	12	-	3	-	-	-	-	-	4	-	6	-
307	Arachidonate from Dihomo-gamma-linolenate	Arachidonate	−Dihomo-gamma-linolenate MCES	Conversion of Dihomo-gamma-linolenate into Arachidonate	1 O_2(s) 1 Dihomo-gamma-linolenate(s) 3 ATP-energy(c) 1 NADH-redox-potential(c)	1 H_2O(s) 1 Arachidonate(s)	7	-	-	-	-	-	-	-	4	-	-	-
	**B2.4. Lipoproteins**																	

Continued on next page

B2. Turnover functions – continued.

Simulation	Definition			Solution												
				exchanges		reactions							transport			Prot
	Objective	Contraints	Comment	imports	exports	c	m	r	p	l	n	s	b	s-c	b-c	intra	syn
308 VLDL from LDL	VLDL	−LDL MCES =Cysteine +Methionine =Leucine =Isoleucine =Histidine =Asparagine =Valine =Tryptophan =Lysine =Phenylalanine =Threonine =Tyrosine =Valine =Ethanolamine(c) =Choline(c) ALFA	Lipoprotein conversion VLDL from LDL	381.4 Pyruvate(m) 99.1 Lysine(s) 56.8 Tryptophan(s) 33.1 Phenylalanine(s) 31.4 Tyrosine(s) 6074 Glucose-6P(c) 695.2 Cysteine(s) 2489 Choline(c) 299.1 Leucine(s) 16.6 Histidine(s) 917.6 Asparagine(s) 173.7 Valine(s) 771.4 Threonine(s) 311.4 Ethanolamine(c) 1277 Arachidonate(c) 11891 Palmitate(c) 20.5 Isoleucine(s) 12595 Oleate(c) 2193 Stearate(c) 8299 Linoleate(c) 0.98 LDL(s) $1.2{\cdot}10^5$ ATP-energy(c) 784.1 ATP-energy(m) 12485 NADH-redox-potential(c) 508.6 NADPH-redox-potential(c) 1332 NADPH-redox-potential(m) 649.3 Proton-gradient(m) 549.9 THF-activated methyl group(c) 709.8 CoA-activated acetyl group(m)	34610 H_2O(s) 3643 P_i(c) 1239 CO_2(s) 107.5 Pyruvate(c) 622.8 Methionine(s) 1 VLDL(s) 1440 NADH-redox-potential(m) 450.8 Proton-gradient(c) 1318 Na-gradient(c)	94	21	17	-	-	-	-	-	18	-	63	5

Continued on next page

Simulation		Definition			Solution													
					exchanges		reactions								transport			Prot
		Objective	Contraints	Comment	imports	exports	c	m	r	p	l	n	s	b	s-c	b-c	intra	syn
309	VLDL from HDL	VLDL	−HDL MCES =Cysteine +Methionine =Leucine =Isoleucine =Histidine =Asparagine =Valine =Tryptophan =Lysine =Phenylalanine =Threonine =Tyrosine =Valine =Ethanolamine(c) =Choline(c) ALFA	Lipoprotein conversion VLDL from HDL	49 Pyruvate(m) 73.8 Phenylalanine(s) 23.1 Tyrosine(s) 5626 Glucose-6P(c) 84.9 Cysteine(s) 1247 Choline(c) 47.3 Asparagine(s) 85.9 Valine(s) 116.7 Threonine(s) 4817 Arachidonate(c) 10414 Palmitate(c) 299 Isoleucine(s) 3845 Farnesyl-PP(r) 14488 Oleate(c) 3419 Linoleate(c) 1344 gamma-Linolenate(c) 11.1 HDL(s) $1.2{\cdot}10^5$ ATP-energy(c) 18.7 ATP-energy(m) 35.3 NADH-redox-potential(m) 5767 NADPH-redox-potential(r) 49 NADPH-redox-potential(m) 67.6 Proton-gradient(m)	18479 H_2O(s) 12494 P_i(c) 5674 CO_2(s) 167.4 Pyruvate(c) 42 Lysine(s) 18.4 Tryptophan(s) 93.3 Leucine(s) 4.65 Histidine(s) 93.4 Ethanolamine(c) 205.6 Stearate(c) 1 VLDL(s) 1922 NADH-redox-potential(r) 194 NADH-redox-potential(c) 1959 NADPH-redox-potential(c) 47.3 Proton-gradient(c) 627.3 Na-gradient(c) 42.1 THF-activated methyl group(c) 3845 CoA-activated acetyl group(c)	83	16	33	-	-	-	-	-	17	-	64	5
**B2.5. Bilirubin**																		
310	Bilirubin conjugation	Bilirubin-bisglucuronoside(b)	MCES −Bilirubin	Bilirubin import, conjugation, and export	2 H_2O(s) 2 Glucose-6P(c) 1 Bilirubin(s) 5 ATP-energy(c)	2 P_i(c) 1 Bilirubin-bisglucuronoside(b) 4 NADH-redox-potential(c)	8	-	2	-	-	-	-	-	2	1	5	-
**B2.6. Urea**																		
311	Urea from glutamine	Urea	MCES −Glutamine	Urea from glutamine	2 H_2O(s) 0.5 O_2(s) 1 Glutamine(s) 2 ATP-energy(c) 1 ATP-energy(m)	1 CO_2(s) 1 Pyruvate(c) 1 Urea(s) 1 NADH-redox-potential(c) 1 NADH-redox-potential(m) 1 NADPH-redox-potential(c) 1 Proton-gradient(c) 6 Proton-gradient(m) 1 Na-gradient(c)	14	9	-	-	-	-	-	-	7	-	7	-
312	Urea from alanine	Urea	MCES −Alanine	Urea from alanine	1 H_2O(s) 2 Alanine(s) 2 ATP-energy(c) 2 ATP-energy(m)	1 Pyruvate(c) 1 Urea(s) 1 NADH-redox-potential(c) 1 NADH-redox-potential(m) 1 NADPH-redox-potential(m) 1 CoA-activated acetyl group(m)	11	9	-	-	-	-	-	-	3	-	2	-

Continued on next page

B2. Turnover functions – continued.

Simulation	Definition			Solution													
				exchanges		reactions								transport			Prot
	Objective	Contraints	Comment	imports	exports	c	m	r	p	l	n	s	b	s-c	b-c	intra	syn
313 Urea from NH_3	Urea	MCES $-NH_3$	Urea from alanine	1 H_2O(s) 2 Alanine(s) 2 ATP-energy(c) 2 ATP-energy(m)	1 Pyruvate(c) 1 Urea(s) 1 NADH-redox-potential(c) 1 NADH-redox-potential(m) 1 NADPH-redox-potential(m) 1 CoA-activated acetyl group(m)	11	9	-	-	-	-	-	-	3	-	2	-
**B2.7. Creatine**																	
314 Creatine	Creatine(c)	MCES −Arginine −Glycine	de novo synthesis of cytosolic Creatine	1 H_2O(s) 0.25 O_2(s) 1 Glycine(s) 0.5 Arginine(s) 1 Activated methyl group(c) 1 ATP-energy(c) 0.5 ATP-energy(m)	0.5 CO_2(s) 0.5 Pyruvate(c) 1 Creatine(c) 2 NADH-redox-potential(m) 0.5 NADPH-redox-potential(c) 1.5 Proton-gradient(m)	11	14	-	-	-	-	-	-	5	-	11	-

3.3 B3. Excretion of de novo synthesized substances

Table 10: B3. Excretion of de novo synthesized substances

Simulation	Definition			Solution													
				exchanges		reactions								transport			Prot
	Objective	Contraints	Comment	imports	exports	c	m	r	p	l	n	s	b	s-c	b-c	intra	syn
**B3.1. Bile excretion**																	
**B3.1.1. Bile acids**																	
315 Glycocholate(b)	Glycocholate(b)	+Urea −Alanine −Serine MCES	de novo synthesis and bile export of Glycocholate	12 O_2(s) 1 Serine(s) 2 Farnesyl-PP(r) 5 ATP-energy(c) 12 NADPH-redox-potential(r) 1 NADPH-redox-potential(c) 1 Proton-gradient(c)	10 H_2O(s) 4 P_i(c) 4 CO_2(s) 1 Glycocholate(b) 1 NADH-redox-potential(r) 2 NADH-redox-potential(m) 1 NADH-redox-potential(p) 6 Proton-gradient(m) 1 THF-activated methyl group(c) 1 CoA-activated acetyl group(m)	15	7	19	7	-	-	-	-	5	1	21	-

Continued on next page

B3. Excretion of de novo synthesized substances – continued.

Simulation	Definition			Solution													
				exchanges		reactions								transport			Prot
	Objective	Contraints	Comment	imports	exports	c	m	r	p	l	n	s	b	s-c	b-c	intra	syn
316 Gly-CD-cholate(b)	Glycocheno-deoxycholate(b)	+Urea −Alanine −Serine MCES	de novo synthesis and bile export of Glycocheno-deoxycholate from Palmitate, Alanine, Serine, excreting urea and CO_2	10.5 O_2(s) 1 Serine(s) 2 Farnesyl-PP(r) 5 ATP-energy(c) 11 NADPH-redox-potential(r) 1 NADPH-redox-potential(m) 1 Proton-gradient(c)	8 H_2O(s) 4 P_i(c) 4 CO_2(s) 1 Glycochenodeoxycholate(b) 1 NADH-redox-potential(r) 2 NADH-redox-potential(m) 6 Proton-gradient(m) 1 THF-activated methyl group(c) 1 CoA-activated acetyl group(m)	13	9	18	2	-	-	-	-	5	1	22	-
317 Taurocholate(b)	Taurocholate(b)	+Urea −Alanine −Serine −Methionine MCES	de novo synthesis and bile export of Taurocholate	13.5 O_2(s) 1 Serine(s) 1 Methionine(s) 2 Farnesyl-PP(r) 6 ATP-energy(c) 1 ATP-energy(m) 12 NADPH-redox-potential(r) 2 NADPH-redox-potential(c)	7.5 H_2O(s) 4 P_i(c) 6.5 CO_2(s) 0.5 Urea(s) 1 Taurocholate(b) 1 NADH-redox-potential(r) 3.5 NADH-redox-potential(c) 4.5 NADH-redox-potential(m) 1 NADH-redox-potential(p) 1 Proton-gradient(c) 10 Proton-gradient(m) 2 Na-gradient(c) 1 THF-activated methyl group(c) 2 CoA-activated acetyl group(m)	26	12	19	7	-	-	-	-	8	1	28	-
318 tcdchola(b)	Taurochenode-oxycholate(b)	+Urea −Alanine −Serine −Methionine MCES	de novo synthesis and bile export of Taurocheno-deoxycholate	12 O_2(s) 1 Serine(s) 1 Methionine(s) 2 Farnesyl-PP(r) 7 ATP-energy(c) 1 ATP-energy(m) 11 NADPH-redox-potential(r) 1 NADPH-redox-potential(c) 1 NADPH-redox-potential(m)	5.5 H_2O(s) 4 P_i(c) 6.5 CO_2(s) 0.5 Urea(s) 1 Taurocheno-deoxycholate(b) 1 NADH-redox-potential(r) 3.5 NADH-redox-potential(c) 4.5 NADH-redox-potential(m) 10 Proton-gradient(m) 1 Na-gradient(c) 1 THF-activated methyl group(c) 2 CoA-activated acetyl group(m)	25	14	18	2	-	-	-	-	7	1	29	-
319 Chenodiol(b)	Chenodiol(b)	+Urea −Alanine −Serine MCES	de novo synthesis and bile export of Chenodiol	10.5 O_2(s) 2 Farnesyl-PP(r) 4 ATP-energy(c) 11 NADPH-redox-potential(r) 1 NADPH-redox-potential(m)	6 H_2O(s) 4 P_i(c) 4 CO_2(s) 1 Chenodiol(b) 1 NADH-redox-potential(r) 1 NADH-redox-potential(c) 2 NADH-redox-potential(m) 6 Proton-gradient(m) 1 CoA-activated acetyl group(m)	10	9	18	2	-	-	-	-	3	1	22	-
**B3.1.2. Phospholipids**																	

Continued on next page

B3. Excretion of de novo synthesized substances – continued.

Simulation	Definition			Solution													
				exchanges		reactions								transport			Prot
	Objective	Contraints	Comment	imports	exports	c	m	r	p	l	n	s	b	s-c	b-c	intra	syn
320 Bile-PC(b)	Bile-PC-pool(b)	MCES ALFA	de novo synthesis and bile export of Bile-PC-pool	0.5 P_i(c) 0.5 Glucose-6P(c) 1 Choline(c) 0.11 Arachidonate(c) 0.83 Palmitate(c) 0.24 Oleate(c) 0.11 Stearate(c) 0.66 Linoleate(c) 0.05 Palmitolate(c) 8.5 ATP-energy(c) 1 NADH-redox-potential(c)	3.5 H_2O(s) 1 Bile-PC-pool(b)	30	-	-	-	-	-	-	-	1	1	-	-
321 SM(b)	SM-pool(b)	MCES ALFA	de novo synthesis and bile export of SM-pool	0.5 P_i(c) 0.5 Glucose-6P(c) 1 Choline(c) 1 Ethanolamine(c) 0.02 Arachidonate(c) 0.91 Palmitate(c) 0.24 Oleate(c) 0.03 gamma-Linolenate(c) 0.8 Palmitolate(c) 10.5 ATP-energy(c) 1 NADPH-redox-potential(c) 1 NADPH-redox-potential(m) 2 Proton-gradient(m)	3.53 H_2O(s) 1 CO_2(s) 1 SM-pool(b) 3 NADH-redox-potential(c) 1 CoA-activated acetyl group(c)	37	2	1	-	-	-	-	-	2	1	11	-
322 PS(b)	PS-VLDL-pool(b)	MCES ALFA	de novo synthesis and bile export of PS-VLDL-pool	0.5 P_i(c) 1 Pyruvate(m) 0.5 Glucose-6P(c) 1 Ethanolamine(c) 0.53 Arachidonate(c) 0.09 Palmitate(c) 0.09 Oleate(c) 1.25 Stearate(c) 0.05 Linoleate(c) 7.5 ATP-energy(c) 1 NADH-redox-potential(c)	3.5 H_2O(s) 1 PS-VLDL-pool(b) 1 NADH-redox-potential(m) 1 CoA-activated acetyl group(m)	29	3	-	-	-	-	-	-	1	1	1	-
323 PE(b)	PE-VLDL-pool(b)	MCES ALFA	de novo synthesis and bile export of PE-VLDL-pool	0.5 P_i(c) 0.5 Glucose-6P(c) 1 Ethanolamine(c) 0.6 Arachidonate(c) 0.34 Palmitate(c) 0.17 Oleate(c) 0.54 Stearate(c) 0.36 Linoleate(c) 8.5 ATP-energy(c) 1 NADH-redox-potential(c)	3.5 H_2O(s) 1 PE-VLDL-pool(b)	28	-	-	-	-	-	-	-	1	1	-	-
**B3.1.3. Cholesterol**																	
324 Cholesterol(b)	Cholesterol(b)	MCES	de novo synthesis and bile export of Cholesterol	8 O_2(s) 2 Farnesyl-PP(r) 3 ATP-energy(c) 10 NADPH-redox-potential(r)	7 H_2O(s) 4 P_i(c) 3 CO_2(s) 1 Cholesterol(b) 1 NADPH-redox-potential(c)	5	-	15	-	-	-	-	-	3	1	7	-
**B3.2. blood excretion**																	
**B3.2.1. Plasma proteins**																	

Continued on next page

B3. Excretion of de novo synthesized substances – continued.

Simulation	Definition			Solution													
				exchanges		reactions								transport			Prot
	Objective	Contraints	Comment	imports	exports	c	m	r	p	l	n	s	b	s-c	b-c	intra	syn
325 Albumin	Albumin	MCES AAA	de novo synthesis and export of Albumin	62 Glutamate(s) 13 Glycine(s) 63 Alanine(s) 60 Lysine(s) 36 Aspartate(s) 27 Arginine(s) 20 Glutamine(s) 28 Serine(s) 7 Methionine(s) 2 Tryptophan(s) 35 Phenylalanine(s) 19 Tyrosine(s) 35 Cysteine(s) 64 Leucine(s) 16 Histidine(s) 24 Proline(s) 17 Asparagine(s) 43 Valine(s) 29 Threonine(s) 9 Isoleucine(s) 3045 ATP-energy(c) 46 Proton-gradient(c)	1 Albumin(s) 239 Na-gradient(c)	3	-	-	-	-	-	-	-	23	-	-	1
326 Fibrinogen	Fibrinogen	MCES AAA	de novo synthesis and export of Fibrinogen	186 Glutamate(s) 262 Glycine(s) 134 Alanine(s) 154 Lysine(s) 176 Aspartate(s) 134 Arginine(s) 110 Glutamine(s) 278 Serine(s) 48 Methionine(s) 60 Tryptophan(s) 98 Phenylalanine(s) 98 Tyrosine(s) 48 Cysteine(s) 166 Leucine(s) 62 Histidine(s) 114 Proline(s) 138 Asparagine(s) 122 Valine(s) 182 Threonine(s) 104 Isoleucine(s) 13370 ATP-energy(c)	1 Fibrinogen(s) 4 Proton-gradient(c) 1044 Na-gradient(c)	3	-	-	-	-	-	-	-	23	-	-	1
327 Antichymotrypsin	Antichymotrypsin	MCES AAA	de novo synthesis and export of Antichymotrypsin	29 Glutamate(s) 16 Glycine(s) 34 Alanine(s) 26 Lysine(s) 25 Aspartate(s) 16 Arginine(s) 17 Glutamine(s) 30 Serine(s) 14 Methionine(s) 3 Tryptophan(s) 25 Phenylalanine(s) 9 Tyrosine(s) 3 Cysteine(s) 59 Leucine(s) 9 Histidine(s) 16 Proline(s) 19 Asparagine(s) 24 Valine(s) 29 Threonine(s) 20 Isoleucine(s) 2115 ATP-energy(c) 15 Proton-gradient(c)	1 Antichymotrypsin(s) 163 Na-gradient(c)	3	-	-	-	-	-	-	-	23	-	-	1

Continued on next page

B3. Excretion of de novo synthesized substances – continued.

Simulation	Definition			Solution													
				exchanges		reactions								transport			Prot
	Objective	Contraints	Comment	imports	exports	c	m	r	p	l	n	s	b	s-c	b-c	intra	syn
328 Antitrypsin	Antitrypsin	MCES AAA	de novo synthesis and export of Antitrypsin	32 Glutamate(s) 24 Glycine(s) 26 Alanine(s) 34 Lysine(s) 24 Aspartate(s) 7 Arginine(s) 18 Glutamine(s) 25 Serine(s) 10 Methionine(s) 3 Tryptophan(s) 27 Phenylalanine(s) 6 Tyrosine(s) 3 Cysteine(s) 51 Leucine(s) 13 Histidine(s) 19 Proline(s) 19 Asparagine(s) 27 Valine(s) 30 Threonine(s) 20 Isoleucine(s) 2090 ATP-energy(c) 7 Proton-gradient(c)	1 Antitrypsin(s) 171 Na-gradient(c)	3	-	-	-	-	-	-	-	23	-	-	1
329 ApoA1	ApoA1	MCES AAA	de novo synthesis and export of ApoA1	30 Glutamate(s) 11 Glycine(s) 23 Alanine(s) 22 Lysine(s) 16 Aspartate(s) 17 Arginine(s) 19 Glutamine(s) 16 Serine(s) 4 Methionine(s) 5 Tryptophan(s) 8 Phenylalanine(s) 7 Tyrosine(s) 41 Leucine(s) 6 Histidine(s) 10 Proline(s) 5 Asparagine(s) 15 Valine(s) 12 Threonine(s) 1335 ATP-energy(c)	1 ApoA1(s) 8 Proton-gradient(c) 122 Na-gradient(c)	3	-	-	-	-	-	-	-	21	-	-	1
330 ApoB100(r)	ApoB100(r)	MCES AAA	de novo synthesis of ApoB100 in ER/Golgi	298 Glutamate(s) 207 Glycine(s) 275 Alanine(s) 357 Lysine(s) 233 Aspartate(s) 150 Arginine(s) 230 Glutamine(s) 392 Serine(s) 79 Methionine(s) 37 Tryptophan(s) 224 Phenylalanine(s) 151 Tyrosine(s) 25 Cysteine(s) 533 Leucine(s) 115 Histidine(s) 171 Proline(s) 247 Asparagine(s) 252 Valine(s) 300 Threonine(s) 285 Isoleucine(s) 22805 ATP-energy(c)	1 ApoB100(r) 40 Proton-gradient(c) 2259 Na-gradient(c)	3	-	-	-	-	-	-	-	22	-	1	1

Continued on next page

B3. Excretion of de novo synthesized substances – continued.

Simulation	Definition			Solution													
				exchanges		reactions								transport			Prot
	Objective	Contraints	Comment	imports	exports	c	m	r	p	l	n	s	b	s-c	b-c	intra	syn
331 ApoC1(c)	ApoC1(c)	MCES AAA	de novo synthesis of cytosolic ApoC1	8 Glutamate(s) 3 Glycine(s) 5 Alanine(s) 9 Lysine(s) 4 Aspartate(s) 4 Arginine(s) 3 Glutamine(s) 9 Serine(s) 2 Methionine(s) 1 Tryptophan(s) 4 Phenylalanine(s) 12 Leucine(s) 4 Proline(s) 1 Asparagine(s) 7 Valine(s) 3 Threonine(s) 4 Isoleucine(s) 415 ATP-energy(c) 2 Proton-gradient(c)	1 ApoC1(c) 38 Na-gradient(c)	3	-	-	-	-	-	-	-	19	-	-	1
332 ApoC2(c)	ApoC2(c)	MCES AAA	de novo synthesis of cytosolic ApoC2	8 Glutamate(s) 5 Glycine(s) 7 Alanine(s) 6 Lysine(s) 4 Aspartate(s) 2 Arginine(s) 8 Glutamine(s) 9 Serine(s) 3 Methionine(s) 1 Tryptophan(s) 4 Phenylalanine(s) 5 Tyrosine(s) 15 Leucine(s) 5 Proline(s) 1 Asparagine(s) 7 Valine(s) 10 Threonine(s) 1 Isoleucine(s) 505 ATP-energy(c) 4 Proton-gradient(c)	1 ApoC2(c) 40 Na-gradient(c)	3	-	-	-	-	-	-	-	20	-	-	1
333 ApoC3(c)	ApoC3(c)	MCES AAA	de novo synthesis of cytosolic ApoC3	5 Glutamate(s) 3 Glycine(s) 15 Alanine(s) 6 Lysine(s) 7 Aspartate(s) 4 Arginine(s) 6 Glutamine(s) 12 Serine(s) 3 Methionine(s) 3 Tryptophan(s) 4 Phenylalanine(s) 2 Tyrosine(s) 11 Leucine(s) 1 Histidine(s) 3 Proline(s) 9 Valine(s) 5 Threonine(s) 495 ATP-energy(c) 3 Proton-gradient(c)	1 ApoC3(c) 43 Na-gradient(c)	3	-	-	-	-	-	-	-	19	-	-	1

Continued on next page

B3. Excretion of de novo synthesized substances – continued.

Simulation	Definition			Solution													
				exchanges		reactions								transport			Prot
	Objective	Contraints	Comment	imports	exports	c	m	r	p	l	n	s	b	s-c	b-c	intra	syn
334 ApoE(c)	ApoE(c)	MCES AAA	de novo synthesis of cytosolic ApoE	40 Glutamate(s) 18 Glycine(s) 39 Alanine(s) 13 Lysine(s) 11 Aspartate(s) 34 Arginine(s) 32 Glutamine(s) 14 Serine(s) 8 Methionine(s) 8 Tryptophan(s) 4 Phenylalanine(s) 4 Tyrosine(s) 2 Cysteine(s) 41 Leucine(s) 2 Histidine(s) 8 Proline(s) 1 Asparagine(s) 24 Valine(s) 12 Threonine(s) 2 Isoleucine(s) 1585 ATP-energy(c) 17 Proton-gradient(c)	1 ApoE(c) 112 Na-gradient(c)	3	-	-	-	-	-	-	-	22	-	-	1
335 Plasminogen	Plasminogen	MCES AAA	de novo synthesis and export of Plasminogen	56 Glutamate(s) 62 Glycine(s) 37 Alanine(s) 49 Lysine(s) 36 Aspartate(s) 42 Arginine(s) 31 Glutamine(s) 56 Serine(s) 11 Methionine(s) 19 Tryptophan(s) 21 Phenylalanine(s) 30 Tyrosine(s) 48 Cysteine(s) 48 Leucine(s) 24 Histidine(s) 69 Proline(s) 40 Asparagine(s) 48 Valine(s) 61 Threonine(s) 22 Isoleucine(s) 4050 ATP-energy(c)	1 Plasminogen(s) 3 Proton-gradient(c) 296 Na-gradient(c)	3	-	-	-	-	-	-	-	23	-	-	1
336 Prothrombin	Prothrombin	MCES AAA	de novo synthesis and export of Prothrombin	51 Glutamate(s) 49 Glycine(s) 42 Alanine(s) 29 Lysine(s) 35 Aspartate(s) 44 Arginine(s) 26 Glutamine(s) 38 Serine(s) 9 Methionine(s) 14 Tryptophan(s) 21 Phenylalanine(s) 21 Tyrosine(s) 26 Cysteine(s) 51 Leucine(s) 13 Histidine(s) 33 Proline(s) 25 Asparagine(s) 37 Valine(s) 36 Threonine(s) 22 Isoleucine(s) 3110 ATP-energy(c) 17 Proton-gradient(c)	1 Prothrombin(s) 239 Na-gradient(c)	3	-	-	-	-	-	-	-	23	-	-	1

Continued on next page

B3. Excretion of de novo synthesized substances – continued.

Simulation	Definition			Solution													
				exchanges		reactions								transport			Prot
	Objective	Contraints	Comment	imports	exports	c	m	r	p	l	n	s	b	s-c	b-c	intra	syn
337 ApoTransferin	ApoTransferin	MCES AAA	de novo synthesis and export of ApoTransferin	42 Glutamate(s) 52 Glycine(s) 61 Alanine(s) 58 Lysine(s) 45 Aspartate(s) 27 Arginine(s) 17 Glutamine(s) 41 Serine(s) 10 Methionine(s) 8 Tryptophan(s) 28 Phenylalanine(s) 26 Tyrosine(s) 40 Cysteine(s) 65 Leucine(s) 19 Histidine(s) 32 Proline(s) 34 Asparagine(s) 48 Valine(s) 30 Threonine(s) 15 Isoleucine(s) 3490 ATP-energy(c) 27 Proton-gradient(c)	1 ApoTransferin(s) 265 Na-gradient(c)	3	-	-	-	-	-	-	-	23	-	-	1
338 Haptoglobin	Haptoglobin	MCES AAA	de novo synthesis and export of Haptoglobin	25 Glutamate(s) 31 Glycine(s) 30 Alanine(s) 35 Lysine(s) 25 Aspartate(s) 9 Arginine(s) 17 Glutamine(s) 18 Serine(s) 5 Methionine(s) 8 Tryptophan(s) 8 Phenylalanine(s) 21 Tyrosine(s) 12 Cysteine(s) 31 Leucine(s) 13 Histidine(s) 21 Proline(s) 21 Asparagine(s) 36 Valine(s) 22 Threonine(s) 18 Isoleucine(s) 2030 ATP-energy(c) 9 Proton-gradient(c)	1 Haptoglobin(s) 175 Na-gradient(c)	3	-	-	-	-	-	-	-	23	-	-	1
339 Collagen ADIPO(c) synthesis	Collagen ADIPO(c)	MCES AAA	test synthesis of Collagen ADIPO c	12 Glutamate(s) 39 Glycine(s) 12 Alanine(s) 12 Lysine(s) 15 Aspartate(s) 7 Arginine(s) 10 Glutamine(s) 7 Serine(s) 5 Methionine(s) 2 Tryptophan(s) 10 Phenylalanine(s) 14 Tyrosine(s) 2 Cysteine(s) 23 Leucine(s) 8 Histidine(s) 17 Proline(s) 11 Asparagine(s) 14 Valine(s) 15 Threonine(s) 9 Isoleucine(s) 1220 ATP-energy(c) 28 Proton-gradient(c)	1 Collagen ADIPO(c) 104 Na-gradient(c)	4	-	-	-	-	-	-	-	22	-	-	-

Continued on next page

B3. Excretion of de novo synthesized substances – continued.

Simulation		Definition			Solution													
					exchanges		reactions								transport			Prot
		Objective	Contraints	Comment	imports	exports	c	m	r	p	l	n	s	b	s-c	b-c	intra	syn
340	Collagen BGH3(c) synthesis	Collagen BGH3(c)	MCES AAA	test synthesis of Collagen BGH3 c	42 Glutamate(s) 48 Glycine(s) 61 Alanine(s) 33 Lysine(s) 27 Aspartate(s) 37 Arginine(s) 22 Glutamine(s) 41 Serine(s) 14 Methionine(s) 2 Tryptophan(s) 16 Phenylalanine(s) 19 Tyrosine(s) 11 Cysteine(s) 87 Leucine(s) 19 Histidine(s) 31 Proline(s) 40 Asparagine(s) 47 Valine(s) 46 Threonine(s) 40 Isoleucine(s) 3415 ATP-energy(c) 8 Proton-gradient(c)	1 Collagen BGH3(c) 304 Na-gradient(c)	4	-	-	-	-	-	-	-	22	-	-	-
341	Collagen C43BP(c) synthesis	Collagen C43BP(c)	MCES AAA	test synthesis of Collagen C43BP c	54 Glutamate(s) 33 Glycine(s) 37 Alanine(s) 40 Lysine(s) 45 Aspartate(s) 34 Arginine(s) 23 Glutamine(s) 58 Serine(s) 10 Methionine(s) 14 Tryptophan(s) 22 Phenylalanine(s) 19 Tyrosine(s) 12 Cysteine(s) 38 Leucine(s) 19 Histidine(s) 24 Proline(s) 29 Asparagine(s) 49 Valine(s) 35 Threonine(s) 29 Isoleucine(s) 3120 ATP-energy(c) 4 Proton-gradient(c)	1 Collagen C43BP(c) 350 Na-gradient(c)	4	-	-	-	-	-	-	-	22	-	-	-
342	Collagen CCBE1(c) synthesis	Collagen CCBE1(c)	MCES AAA	test synthesis of Collagen CCBE1 c	26 Glutamate(s) 52 Glycine(s) 20 Alanine(s) 23 Lysine(s) 24 Aspartate(s) 29 Arginine(s) 13 Glutamine(s) 23 Serine(s) 8 Methionine(s) 1 Tryptophan(s) 10 Phenylalanine(s) 17 Tyrosine(s) 21 Cysteine(s) 32 Leucine(s) 8 Histidine(s) 47 Proline(s) 9 Asparagine(s) 9 Valine(s) 22 Threonine(s) 12 Isoleucine(s) 2030 ATP-energy(c) 43 Proton-gradient(c)	1 Collagen CCBE1(c) 160 Na-gradient(c)	4	-	-	-	-	-	-	-	22	-	-	-

Continued on next page

B3. Excretion of de novo synthesized substances – continued.

Simulation	Definition			Solution													
				exchanges		reactions								transport			Prot
	Objective	Contraints	Comment	imports	exports	c	m	r	p	l	n	s	b	s-c	b-c	intra	syn
343 Collagen CD36(c) synthesis	Collagen CD36(c)	MCES AAA	test synthesis of Collagen CD36 c	24 Glutamate(s) 28 Glycine(s) 25 Alanine(s) 30 Lysine(s) 20 Aspartate(s) 17 Arginine(s) 19 Glutamine(s) 33 Serine(s) 11 Methionine(s) 5 Tryptophan(s) 27 Phenylalanine(s) 20 Tyrosine(s) 10 Cysteine(s) 41 Leucine(s) 4 Histidine(s) 23 Proline(s) 31 Asparagine(s) 44 Valine(s) 25 Threonine(s) 35 Isoleucine(s) 2360 ATP-energy(c)	3 Proton-gradient(c) 1 Collagen CD36(c) 253 Na-gradient(c)	4	-	-	-	-	-	-	-	22	-	-	-
344 Collagen CO1A1(c) synthesis	Collagen CO1A1(c)	MCES AAA	test synthesis of Collagen CO1A1 c	75 Glutamate(s) 391 Glycine(s) 139 Alanine(s) 57 Lysine(s) 66 Aspartate(s) 71 Arginine(s) 49 Glutamine(s) 60 Serine(s) 13 Methionine(s) 6 Tryptophan(s) 27 Phenylalanine(s) 13 Tyrosine(s) 18 Cysteine(s) 48 Leucine(s) 9 Histidine(s) 278 Proline(s) 28 Asparagine(s) 47 Valine(s) 45 Threonine(s) 24 Isoleucine(s) 7320 ATP-energy(c) 363 Proton-gradient(c)	1 Collagen CO1A1(c) 373 Na-gradient(c)	4	-	-	-	-	-	-	-	22	-	-	-
345 Collagen CO1A2(c) synthesis	Collagen CO1A2(c)	MCES AAA	test synthesis of Collagen CO1A2 c	66 Glutamate(s) 381 Glycine(s) 129 Alanine(s) 50 Lysine(s) 43 Aspartate(s) 72 Arginine(s) 33 Glutamine(s) 52 Serine(s) 10 Methionine(s) 5 Tryptophan(s) 22 Phenylalanine(s) 16 Tyrosine(s) 9 Cysteine(s) 61 Leucine(s) 15 Histidine(s) 232 Proline(s) 41 Asparagine(s) 55 Valine(s) 42 Threonine(s) 32 Isoleucine(s) 6830 ATP-energy(c) 340 Proton-gradient(c)	1 Collagen CO1A2(c) 356 Na-gradient(c)	4	-	-	-	-	-	-	-	22	-	-	-

Continued on next page

B3. Excretion of de novo synthesized substances – continued.

Simulation	Definition			Solution													
				exchanges		reactions								transport			Prot
	Objective	Contraints	Comment	imports	exports	c	m	r	p	l	n	s	b	s-c	b-c	intra	syn
346 Collagen CO_2A1(c) synthesis	Collagen CO_2A1(c)	MCES AAA	test synthesis of Collagen CO_2A1 c	79 Glutamate(s) 406 Glycine(s) 134 Alanine(s) 67 Lysine(s) 62 Aspartate(s) 72 Arginine(s) 60 Glutamine(s) 48 Serine(s) 16 Methionine(s) 7 Tryptophan(s) 25 Phenylalanine(s) 10 Tyrosine(s) 19 Cysteine(s) 56 Leucine(s) 8 Histidine(s) 270 Proline(s) 32 Asparagine(s) 38 Valine(s) 44 Threonine(s) 34 Isoleucine(s) 7435 ATP-energy(c) 374 Proton-gradient(c)	1 Collagen CO_2A1(c) 362 Na-gradient(c)	4	-	-	-	-	-	-	-	22	-	-	-
347 Collagen CO3A1(c) synthesis	Collagen CO3A1(c)	MCES AAA	test synthesis of Collagen CO3A1 c	74 Glutamate(s) 413 Glycine(s) 115 Alanine(s) 62 Lysine(s) 55 Aspartate(s) 60 Arginine(s) 42 Glutamine(s) 73 Serine(s) 17 Methionine(s) 7 Tryptophan(s) 23 Phenylalanine(s) 15 Tyrosine(s) 22 Cysteine(s) 48 Leucine(s) 15 Histidine(s) 281 Proline(s) 41 Asparagine(s) 36 Valine(s) 31 Threonine(s) 36 Isoleucine(s) 7330 ATP-energy(c) 372 Proton-gradient(c)	1 Collagen CO3A1(c) 397 Na-gradient(c)	4	-	-	-	-	-	-	-	22	-	-	-
348 Collagen CO4A1(c) synthesis	Collagen CO4A1(c)	MCES AAA	test synthesis of Collagen CO4A1 c	70 Glutamate(s) 478 Glycine(s) 58 Alanine(s) 93 Lysine(s) 58 Aspartate(s) 45 Arginine(s) 73 Glutamine(s) 72 Serine(s) 31 Methionine(s) 6 Tryptophan(s) 46 Phenylalanine(s) 18 Tyrosine(s) 20 Cysteine(s) 92 Leucine(s) 16 Histidine(s) 324 Proline(s) 16 Asparagine(s) 51 Valine(s) 44 Threonine(s) 58 Isoleucine(s) 8345 ATP-energy(c) 462 Proton-gradient(c)	1 Collagen CO4A1(c) 431 Na-gradient(c)	4	-	-	-	-	-	-	-	22	-	-	-

Continued on next page

B3. Excretion of de novo synthesized substances – continued.

Simulation	Definition			Solution													
				exchanges		reactions								transport			Prot
	Objective	Contraints	Comment	imports	exports	c	m	r	p	l	n	s	b	s-c	b-c	intra	syn
349 Collagen CO4A2(c) synthesis	Collagen CO4A2(c)	MCES AAA	test synthesis of Collagen CO4A2 c	60 Glutamate(s) 473 Glycine(s) 82 Alanine(s) 81 Lysine(s) 82 Aspartate(s) 79 Arginine(s) 62 Glutamine(s) 64 Serine(s) 25 Methionine(s) 9 Tryptophan(s) 57 Phenylalanine(s) 28 Tyrosine(s) 21 Cysteine(s) 102 Leucine(s) 19 Histidine(s) 286 Proline(s) 18 Asparagine(s) 50 Valine(s) 51 Threonine(s) 63 Isoleucine(s) 8560 ATP-energy(c) 455 Proton-gradient(c)	1 Collagen CO4A2(c) 471 Na-gradient(c)	4	-	-	-	-	-	-	-	22	-	-	-
350 Collagen CO4A3(c) synthesis	Collagen CO4A3(c)	MCES AAA	test synthesis of Collagen CO4A3 c	67 Glutamate(s) 455 Glycine(s) 69 Alanine(s) 89 Lysine(s) 53 Aspartate(s) 65 Arginine(s) 48 Glutamine(s) 78 Serine(s) 29 Methionine(s) 6 Tryptophan(s) 46 Phenylalanine(s) 18 Tyrosine(s) 24 Cysteine(s) 106 Leucine(s) 19 Histidine(s) 320 Proline(s) 28 Asparagine(s) 40 Valine(s) 59 Threonine(s) 51 Isoleucine(s) 8350 ATP-energy(c) 427 Proton-gradient(c)	1 Collagen CO4A3(c) 430 Na-gradient(c)	4	-	-	-	-	-	-	-	22	-	-	-
351 Collagen CO4A4(c) synthesis	Collagen CO4A4(c)	MCES AAA	test synthesis of Collagen CO4A4 c	58 Glutamate(s) 464 Glycine(s) 69 Alanine(s) 83 Lysine(s) 72 Aspartate(s) 70 Arginine(s) 48 Glutamine(s) 76 Serine(s) 24 Methionine(s) 9 Tryptophan(s) 45 Phenylalanine(s) 23 Tyrosine(s) 32 Cysteine(s) 101 Leucine(s) 33 Histidine(s) 345 Proline(s) 15 Asparagine(s) 50 Valine(s) 30 Threonine(s) 43 Isoleucine(s) 8450 ATP-energy(c) 449 Proton-gradient(c)	1 Collagen CO4A4(c) 456 Na-gradient(c)	4	-	-	-	-	-	-	-	22	-	-	-

Continued on next page

B3. Excretion of de novo synthesized substances – continued.

Simulation	Definition			Solution													
				exchanges		reactions								transport			Prot
	Objective	Contraints	Comment	imports	exports	c	m	r	p	l	n	s	b	s-c	b-c	intra	syn
352 Collagen CO4A5(c) synthesis	Collagen CO4A5(c)	MCES AAA	test synthesis of Collagen CO4A5 c	61 Glutamate(s) 478 Glycine(s) 47 Alanine(s) 80 Lysine(s) 54 Aspartate(s) 37 Arginine(s) 73 Glutamine(s) 64 Serine(s) 26 Methionine(s) 5 Tryptophan(s) 41 Phenylalanine(s) 13 Tyrosine(s) 20 Cysteine(s) 112 Leucine(s) 13 Histidine(s) 391 Proline(s) 32 Asparagine(s) 31 Valine(s) 38 Threonine(s) 69 Isoleucine(s) 8425 ATP-energy(c) 446 Proton-gradient(c)	1 Collagen CO4A5(c) 403 Na-gradient(c)	4	-	-	-	-	-	-	-	22	-	-	-
353 Collagen CO4A6(c) synthesis	Collagen CO4A6(c)	MCES AAA	test synthesis of Collagen CO4A6 c	57 Glutamate(s) 463 Glycine(s) 60 Alanine(s) 96 Lysine(s) 52 Aspartate(s) 47 Arginine(s) 65 Glutamine(s) 98 Serine(s) 25 Methionine(s) 8 Tryptophan(s) 55 Phenylalanine(s) 15 Tyrosine(s) 21 Cysteine(s) 140 Leucine(s) 17 Histidine(s) 279 Proline(s) 26 Asparagine(s) 51 Valine(s) 53 Threonine(s) 63 Isoleucine(s) 8455 ATP-energy(c) 437 Proton-gradient(c)	1 Collagen CO4A6(c) 463 Na-gradient(c)	4	-	-	-	-	-	-	-	22	-	-	-
354 Collagen CO5A1(c) synthesis	Collagen CO5A1(c)	MCES AAA	test synthesis of Collagen CO5A1 c	120 Glutamate(s) 429 Glycine(s) 92 Alanine(s) 98 Lysine(s) 105 Aspartate(s) 70 Arginine(s) 74 Glutamine(s) 70 Serine(s) 23 Methionine(s) 7 Tryptophan(s) 39 Phenylalanine(s) 40 Tyrosine(s) 12 Cysteine(s) 98 Leucine(s) 17 Histidine(s) 334 Proline(s) 34 Asparagine(s) 54 Valine(s) 70 Threonine(s) 52 Isoleucine(s) 9190 ATP-energy(c) 395 Proton-gradient(c)	1 Collagen CO5A1(c) 550 Na-gradient(c)	4	-	-	-	-	-	-	-	22	-	-	-

Continued on next page

B3. Excretion of de novo synthesized substances – continued.

Simulation	Definition			Solution													
				exchanges		reactions								transport			Prot
	Objective	Contraints	Comment	imports	exports	c	m	r	p	l	n	s	b	s-c	b-c	intra	syn
355 Collagen CO5A2(c) synthesis	Collagen CO5A2(c)	MCES AAA	test synthesis of Collagen CO5A2 c	76 Glutamate(s) 402 Glycine(s) 83 Alanine(s) 61 Lysine(s) 69 Aspartate(s) 74 Arginine(s) 60 Glutamine(s) 63 Serine(s) 24 Methionine(s) 6 Tryptophan(s) 20 Phenylalanine(s) 11 Tyrosine(s) 17 Cysteine(s) 62 Leucine(s) 17 Histidine(s) 270 Proline(s) 36 Asparagine(s) 58 Valine(s) 51 Threonine(s) 39 Isoleucine(s) 7495 ATP-energy(c) 366 Proton-gradient(c)	1 Collagen CO5A2(c) 412 Na-gradient(c)	4	-	-	-	-	-	-	-	22	-	-	-
356 Collagen CO5A3(c) synthesis	Collagen CO5A3(c)	MCES AAA	test synthesis of Collagen CO5A3 c	101 Glutamate(s) 429 Glycine(s) 102 Alanine(s) 86 Lysine(s) 76 Aspartate(s) 84 Arginine(s) 78 Glutamine(s) 69 Serine(s) 12 Methionine(s) 8 Tryptophan(s) 46 Phenylalanine(s) 11 Tyrosine(s) 12 Cysteine(s) 122 Leucine(s) 23 Histidine(s) 290 Proline(s) 24 Asparagine(s) 67 Valine(s) 67 Threonine(s) 38 Isoleucine(s) 8725 ATP-energy(c) 405 Proton-gradient(c)	1 Collagen CO5A3(c) 475 Na-gradient(c)	4	-	-	-	-	-	-	-	22	-	-	-
357 Collagen CO6A1(c) synthesis	Collagen CO6A1(c)	MCES AAA	test synthesis of Collagen CO6A1 c	68 Glutamate(s) 156 Glycine(s) 77 Alanine(s) 54 Lysine(s) 71 Aspartate(s) 60 Arginine(s) 42 Glutamine(s) 54 Serine(s) 11 Methionine(s) 4 Tryptophan(s) 33 Phenylalanine(s) 28 Tyrosine(s) 20 Cysteine(s) 71 Leucine(s) 14 Histidine(s) 89 Proline(s) 28 Asparagine(s) 65 Valine(s) 41 Threonine(s) 42 Isoleucine(s) 5140 ATP-energy(c) 128 Proton-gradient(c)	1 Collagen CO6A1(c) 427 Na-gradient(c)	4	-	-	-	-	-	-	-	22	-	-	-

Continued on next page

B3. Excretion of de novo synthesized substances – continued.

Simulation		Definition			Solution													
					exchanges		reactions								transport			Prot
		Objective	Contraints	Comment	imports	exports	c	m	r	p	l	n	s	b	s-c	b-c	intra	syn
358	Collagen CO6A2(c) synthesis	Collagen CO6A2(c)	MCES AAA	test synthesis of Collagen CO6A2 c	65 Glutamate(s) 155 Glycine(s) 60 Alanine(s) 53 Lysine(s) 71 Aspartate(s) 68 Arginine(s) 45 Glutamine(s) 54 Serine(s) 13 Methionine(s) 5 Tryptophan(s) 39 Phenylalanine(s) 18 Tyrosine(s) 21 Cysteine(s) 66 Leucine(s) 20 Histidine(s) 89 Proline(s) 30 Asparagine(s) 60 Valine(s) 45 Threonine(s) 42 Isoleucine(s) 5095 ATP-energy(c) 125 Proton-gradient(c)	1 Collagen CO6A2(c) 425 Na-gradient(c)	4	-	-	-	-	-	-	-	22	-	-	-
359	Collagen CO6A3(c) synthesis	Collagen CO6A3(c)	MCES AAA	test synthesis of Collagen CO6A3 c	183 Glutamate(s) 303 Glycine(s) 235 Alanine(s) 158 Lysine(s) 183 Aspartate(s) 189 Arginine(s) 147 Glutamine(s) 217 Serine(s) 37 Methionine(s) 6 Tryptophan(s) 149 Phenylalanine(s) 62 Tyrosine(s) 30 Cysteine(s) 282 Leucine(s) 45 Histidine(s) 209 Proline(s) 130 Asparagine(s) 292 Valine(s) 167 Threonine(s) 153 Isoleucine(s) 15885 ATP-energy(c) 173 Proton-gradient(c)	1 Collagen CO6A3(c) 1450 Na-gradient(c)	4	-	-	-	-	-	-	-	22	-	-	-
360	Collagen CO6A5(c) synthesis	Collagen CO6A5(c)	MCES AAA	test synthesis of Collagen CO6A5 c	154 Glutamate(s) 244 Glycine(s) 138 Alanine(s) 179 Lysine(s) 164 Aspartate(s) 124 Arginine(s) 143 Glutamine(s) 188 Serine(s) 55 Methionine(s) 12 Tryptophan(s) 132 Phenylalanine(s) 71 Tyrosine(s) 36 Cysteine(s) 223 Leucine(s) 58 Histidine(s) 108 Proline(s) 130 Asparagine(s) 160 Valine(s) 135 Threonine(s) 161 Isoleucine(s) 13075 ATP-energy(c) 114 Proton-gradient(c)	1 Collagen CO6A5(c) 1266 Na-gradient(c)	4	-	-	-	-	-	-	-	22	-	-	-

Continued on next page

B3. Excretion of de novo synthesized substances – continued.

Simulation	Definition: Objective	Definition: Contraints	Definition: Comment	Solution: exchanges: imports	Solution: exchanges: exports	reactions: c	reactions: m	reactions: r	reactions: p	reactions: l	reactions: n	reactions: s	reactions: b	transport: s-c	transport: b-c	transport: intra	Prot syn
361 Collagen CO6A6(c) synthesis	Collagen CO6A6(c)	MCES AAA	test synthesis of Collagen CO6A6 c	150 Glutamate(s) 230 Glycine(s) 149 Alanine(s) 152 Lysine(s) 137 Aspartate(s) 121 Arginine(s) 95 Glutamine(s) 170 Serine(s) 47 Methionine(s) 6 Tryptophan(s) 109 Phenylalanine(s) 47 Tyrosine(s) 29 Cysteine(s) 180 Leucine(s) 48 Histidine(s) 108 Proline(s) 92 Asparagine(s) 157 Valine(s) 103 Threonine(s) 133 Isoleucine(s) 11315 ATP-energy(c) 138 Proton-gradient(c)	1 Collagen CO6A6(c) 1078 Na-gradient(c)	4	-	-	-	-	-	-	-	22	-	-	-
362 Collagen CO7A1(c) synthesis	Collagen CO7A1(c)	MCES AAA	test synthesis of Collagen CO7A1 c	187 Glutamate(s) 627 Glycine(s) 193 Alanine(s) 93 Lysine(s) 145 Aspartate(s) 217 Arginine(s) 105 Glutamine(s) 166 Serine(s) 18 Methionine(s) 19 Tryptophan(s) 39 Phenylalanine(s) 36 Tyrosine(s) 17 Cysteine(s) 212 Leucine(s) 28 Histidine(s) 426 Proline(s) 27 Asparagine(s) 201 Valine(s) 132 Threonine(s) 56 Isoleucine(s) 14720 ATP-energy(c) 600 Proton-gradient(c)	1 Collagen CO7A1(c) 921 Na-gradient(c)	4	-	-	-	-	-	-	-	22	-	-	-
363 Collagen CO8A1(c) synthesis	Collagen CO8A1(c)	MCES AAA	test synthesis of Collagen CO8A1 c	26 Glutamate(s) 190 Glycine(s) 33 Alanine(s) 47 Lysine(s) 11 Aspartate(s) 13 Arginine(s) 38 Glutamine(s) 11 Serine(s) 23 Methionine(s) 1 Tryptophan(s) 17 Phenylalanine(s) 22 Tyrosine(s) 2 Cysteine(s) 53 Leucine(s) 11 Histidine(s) 167 Proline(s) 8 Asparagine(s) 30 Valine(s) 8 Threonine(s) 33 Isoleucine(s) 3720 ATP-energy(c) 182 Proton-gradient(c)	1 Collagen CO8A1(c) 172 Na-gradient(c)	4	-	-	-	-	-	-	-	22	-	-	-

Continued on next page

B3. Excretion of de novo synthesized substances – continued.

Simulation	Definition			Solution													
				exchanges		reactions								transport			Prot
	Objective	Contraints	Comment	imports	exports	c	m	r	p	l	n	s	b	s-c	b-c	intra	syn
364 Collagen CO8A2(c) synthesis	Collagen CO8A2(c)	MCES AAA	test synthesis of Collagen CO8A2 c	24 Glutamate(s) 193 Glycine(s) 42 Alanine(s) 28 Lysine(s) 18 Aspartate(s) 21 Arginine(s) 25 Glutamine(s) 22 Serine(s) 9 Methionine(s) 2 Tryptophan(s) 14 Phenylalanine(s) 16 Tyrosine(s) 3 Cysteine(s) 59 Leucine(s) 8 Histidine(s) 145 Proline(s) 9 Asparagine(s) 31 Valine(s) 22 Threonine(s) 12 Isoleucine(s) 3515 ATP-energy(c) 184 Proton-gradient(c)	1 Collagen CO8A2(c) 159 Na-gradient(c)	4	-	-	-	-	-	-	-	22	-	-	-
365 Collagen CO9A1(c) synthesis	Collagen CO9A1(c)	MCES AAA	test synthesis of Collagen CO9A1 c	47 Glutamate(s) 217 Glycine(s) 47 Alanine(s) 42 Lysine(s) 39 Aspartate(s) 54 Arginine(s) 39 Glutamine(s) 42 Serine(s) 12 Methionine(s) 6 Tryptophan(s) 22 Phenylalanine(s) 6 Tyrosine(s) 11 Cysteine(s) 57 Leucine(s) 8 Histidine(s) 153 Proline(s) 20 Asparagine(s) 37 Valine(s) 26 Threonine(s) 36 Isoleucine(s) 4605 ATP-energy(c) 197 Proton-gradient(c)	1 Collagen CO9A1(c) 274 Na-gradient(c)	4	-	-	-	-	-	-	-	22	-	-	-
366 Collagen CO9A2(c) synthesis	Collagen CO9A2(c)	MCES AAA	test synthesis of Collagen CO9A2 c	31 Glutamate(s) 203 Glycine(s) 42 Alanine(s) 38 Lysine(s) 24 Aspartate(s) 27 Arginine(s) 34 Glutamine(s) 18 Serine(s) 11 Methionine(s) 4 Phenylalanine(s) 6 Tyrosine(s) 4 Cysteine(s) 28 Leucine(s) 8 Histidine(s) 138 Proline(s) 6 Asparagine(s) 29 Valine(s) 15 Threonine(s) 23 Isoleucine(s) 3445 ATP-energy(c) 197 Proton-gradient(c)	1 Collagen CO9A2(c) 153 Na-gradient(c)	4	-	-	-	-	-	-	-	21	-	-	-

Continued on next page

B3. Excretion of de novo synthesized substances – continued.

Simulation	Definition			Solution													
				exchanges		reactions								transport			Prot
	Objective	Contraints	Comment	imports	exports	c	m	r	p	l	n	s	b	s-c	b-c	intra	syn
367 Collagen CO9A3(c) synthesis	Collagen CO9A3(c)	MCES AAA	test synthesis of Collagen CO9A3 c	32 Glutamate(s) 210 Glycine(s) 45 Alanine(s) 29 Lysine(s) 28 Aspartate(s) 32 Arginine(s) 25 Glutamine(s) 25 Serine(s) 6 Methionine(s) 4 Phenylalanine(s) 2 Tyrosine(s) 6 Cysteine(s) 49 Leucine(s) 4 Histidine(s) 137 Proline(s) 5 Asparagine(s) 20 Valine(s) 11 Threonine(s) 14 Isoleucine(s) 3420 ATP-energy(c) 205 Proton-gradient(c)	1 Collagen CO9A3(c) 140 Na-gradient(c)	4	-	-	-	-	-	-	-	21	-	-	-
368 Collagen COAA1(c) synthesis	Collagen COAA1(c)	MCES AAA	test synthesis of Collagen COAA1 c	22 Glutamate(s) 175 Glycine(s) 36 Alanine(s) 35 Lysine(s) 12 Aspartate(s) 19 Arginine(s) 23 Glutamine(s) 27 Serine(s) 11 Methionine(s) 2 Tryptophan(s) 15 Phenylalanine(s) 21 Tyrosine(s) 1 Cysteine(s) 35 Leucine(s) 10 Histidine(s) 145 Proline(s) 14 Asparagine(s) 26 Valine(s) 24 Threonine(s) 27 Isoleucine(s) 3400 ATP-energy(c) 161 Proton-gradient(c)	1 Collagen COAA1(c) 177 Na-gradient(c)	4	-	-	-	-	-	-	-	22	-	-	-
369 Collagen COBA1(c) synthesis	Collagen COBA1(c)	MCES AAA	test synthesis of Collagen COBA1 c	123 Glutamate(s) 422 Glycine(s) 98 Alanine(s) 106 Lysine(s) 99 Aspartate(s) 68 Arginine(s) 81 Glutamine(s) 73 Serine(s) 24 Methionine(s) 9 Tryptophan(s) 47 Phenylalanine(s) 36 Tyrosine(s) 11 Cysteine(s) 80 Leucine(s) 17 Histidine(s) 287 Proline(s) 37 Asparagine(s) 63 Valine(s) 69 Threonine(s) 56 Isoleucine(s) 9030 ATP-energy(c) 385 Proton-gradient(c)	1 Collagen COBA1(c) 571 Na-gradient(c)	4	-	-	-	-	-	-	-	22	-	-	-

Continued on next page

B3. Excretion of de novo synthesized substances – continued.

Simulation	Definition			Solution													
				exchanges		reactions								transport			Prot
	Objective	Contraints	Comment	imports	exports	c	m	r	p	l	n	s	b	s-c	b-c	intra	syn
370 Collagen COBA2(c) synthesis	Collagen COBA2(c)	MCES AAA	test synthesis of Collagen COBA2 c	110 Glutamate(s) 428 Glycine(s) 98 Alanine(s) 72 Lysine(s) 79 Aspartate(s) 101 Arginine(s) 87 Glutamine(s) 65 Serine(s) 16 Methionine(s) 3 Tryptophan(s) 31 Phenylalanine(s) 23 Tyrosine(s) 14 Cysteine(s) 111 Leucine(s) 20 Histidine(s) 299 Proline(s) 15 Asparagine(s) 74 Valine(s) 60 Threonine(s) 30 Isoleucine(s) 8680 ATP-energy(c) 413 Proton-gradient(c)	1 Collagen COBA2(c) 464 Na-gradient(c)	4	-	-	-	-	-	-	-	22	-	-	-
371 Collagen COCA1(c) synthesis	Collagen COCA1(c)	MCES AAA	test synthesis of Collagen COCA1 c	194 Glutamate(s) 281 Glycine(s) 159 Alanine(s) 156 Lysine(s) 172 Aspartate(s) 157 Arginine(s) 104 Glutamine(s) 241 Serine(s) 45 Methionine(s) 30 Tryptophan(s) 91 Phenylalanine(s) 113 Tyrosine(s) 21 Cysteine(s) 203 Leucine(s) 31 Histidine(s) 263 Proline(s) 118 Asparagine(s) 270 Valine(s) 266 Threonine(s) 148 Isoleucine(s) 15315 ATP-energy(c) 163 Proton-gradient(c)	1 Collagen COCA1(c) 1429 Na-gradient(c)	4	-	-	-	-	-	-	-	22	-	-	-
372 Collagen CODA1(c) synthesis	Collagen CODA1(c)	MCES AAA	test synthesis of Collagen CODA1 c	44 Glutamate(s) 184 Glycine(s) 49 Alanine(s) 46 Lysine(s) 23 Aspartate(s) 35 Arginine(s) 28 Glutamine(s) 24 Serine(s) 13 Methionine(s) 2 Tryptophan(s) 5 Phenylalanine(s) 3 Tyrosine(s) 8 Cysteine(s) 53 Leucine(s) 11 Histidine(s) 125 Proline(s) 11 Asparagine(s) 17 Valine(s) 17 Threonine(s) 19 Isoleucine(s) 3585 ATP-energy(c) 173 Proton-gradient(c)	1 Collagen CODA1(c) 167 Na-gradient(c)	4	-	-	-	-	-	-	-	22	-	-	-

Continued on next page

B3. Excretion of de novo synthesized substances – continued.

Simulation	Definition			Solution													
				exchanges		reactions								transport			Prot
	Objective	Contraints	Comment	imports	exports	c	m	r	p	l	n	s	b	s-c	b-c	intra	syn
373 Collagen COEA1(c) synthesis	Collagen COEA1(c)	MCES AAA	test synthesis of Collagen COEA1 c	118 Glutamate(s) 195 Glycine(s) 94 Alanine(s) 86 Lysine(s) 93 Aspartate(s) 74 Arginine(s) 71 Glutamine(s) 133 Serine(s) 33 Methionine(s) 18 Tryptophan(s) 60 Phenylalanine(s) 53 Tyrosine(s) 19 Cysteine(s) 129 Leucine(s) 29 Histidine(s) 156 Proline(s) 58 Asparagine(s) 142 Valine(s) 136 Threonine(s) 99 Isoleucine(s) 8980 ATP-energy(c) 137 Proton-gradient(c)	1 Collagen COEA1(c) 822 Na-gradient(c)	4	-	-	-	-	-	-	-	22	-	-	-
374 Collagen COFA1(c) synthesis	Collagen COFA1(c)	MCES AAA	test synthesis of Collagen COFA1 c	97 Glutamate(s) 221 Glycine(s) 108 Alanine(s) 52 Lysine(s) 58 Aspartate(s) 47 Arginine(s) 40 Glutamine(s) 99 Serine(s) 30 Methionine(s) 10 Tryptophan(s) 43 Phenylalanine(s) 14 Tyrosine(s) 10 Cysteine(s) 112 Leucine(s) 25 Histidine(s) 191 Proline(s) 38 Asparagine(s) 65 Valine(s) 74 Threonine(s) 54 Isoleucine(s) 6940 ATP-energy(c) 183 Proton-gradient(c)	1 Collagen COFA1(c) 513 Na-gradient(c)	4	-	-	-	-	-	-	-	22	-	-	-
375 Collagen COGA1(c) synthesis	Collagen COGA1(c)	MCES AAA	test synthesis of Collagen COGA1 c	92 Glutamate(s) 391 Glycine(s) 85 Alanine(s) 87 Lysine(s) 52 Aspartate(s) 63 Arginine(s) 79 Glutamine(s) 76 Serine(s) 26 Methionine(s) 8 Tryptophan(s) 30 Phenylalanine(s) 13 Tyrosine(s) 32 Cysteine(s) 99 Leucine(s) 19 Histidine(s) 282 Proline(s) 23 Asparagine(s) 63 Valine(s) 43 Threonine(s) 41 Isoleucine(s) 8020 ATP-energy(c) 368 Proton-gradient(c)	1 Collagen COGA1(c) 449 Na-gradient(c)	4	-	-	-	-	-	-	-	22	-	-	-

Continued on next page

B3. Excretion of de novo synthesized substances – continued.

Simulation	Definition			Solution													
				exchanges		reactions								transport			Prot
	Objective	Contraints	Comment	imports	exports	c	m	r	p	l	n	s	b	s-c	b-c	intra	syn
376 Collagen COHA1(c) synthesis	Collagen COHA1(c)	MCES AAA	test synthesis of Collagen COHA1 c	63 Glutamate(s) 282 Glycine(s) 85 Alanine(s) 60 Lysine(s) 54 Aspartate(s) 68 Arginine(s) 48 Glutamine(s) 178 Serine(s) 33 Methionine(s) 10 Tryptophan(s) 25 Phenylalanine(s) 36 Tyrosine(s) 7 Cysteine(s) 107 Leucine(s) 23 Histidine(s) 204 Proline(s) 31 Asparagine(s) 58 Valine(s) 84 Threonine(s) 41 Isoleucine(s) 7485 ATP-energy(c) 251 Proton-gradient(c)	1 Collagen COHA1(c) 526 Na-gradient(c)	4	-	-	-	-	-	-	-	22	-	-	-
377 Collagen COIA1(c) synthesis	Collagen COIA1(c)	MCES AAA	test synthesis of Collagen COIA1 c	90 Glutamate(s) 302 Glycine(s) 140 Alanine(s) 42 Lysine(s) 74 Aspartate(s) 91 Arginine(s) 67 Glutamine(s) 121 Serine(s) 15 Methionine(s) 21 Tryptophan(s) 57 Phenylalanine(s) 19 Tyrosine(s) 23 Cysteine(s) 148 Leucine(s) 36 Histidine(s) 296 Proline(s) 27 Asparagine(s) 87 Valine(s) 69 Threonine(s) 29 Isoleucine(s) 8770 ATP-energy(c) 275 Proton-gradient(c)	1 Collagen COIA1(c) 584 Na-gradient(c)	4	-	-	-	-	-	-	-	22	-	-	-
378 Collagen COJA1(c) synthesis	Collagen COJA1(c)	MCES AAA	test synthesis of Collagen COJA1 c	67 Glutamate(s) 261 Glycine(s) 52 Alanine(s) 77 Lysine(s) 49 Aspartate(s) 47 Arginine(s) 46 Glutamine(s) 53 Serine(s) 18 Methionine(s) 7 Tryptophan(s) 25 Phenylalanine(s) 16 Tyrosine(s) 14 Cysteine(s) 71 Leucine(s) 16 Histidine(s) 173 Proline(s) 30 Asparagine(s) 33 Valine(s) 30 Threonine(s) 57 Isoleucine(s) 5710 ATP-energy(c) 231 Proton-gradient(c)	1 Collagen COJA1(c) 367 Na-gradient(c)	4	-	-	-	-	-	-	-	22	-	-	-

Continued on next page

B3. Excretion of de novo synthesized substances – continued.

Simulation	Definition			Solution													
				exchanges		reactions								transport			Prot
	Objective	Contraints	Comment	imports	exports	c	m	r	p	l	n	s	b	s-c	b-c	intra	syn
379 Collagen COKA1(c) synthesis	Collagen COKA1(c)	MCES AAA	test synthesis of Collagen COKA1 c	73 Glutamate(s) 148 Glycine(s) 114 Alanine(s) 38 Lysine(s) 46 Aspartate(s) 85 Arginine(s) 57 Glutamine(s) 107 Serine(s) 14 Methionine(s) 17 Tryptophan(s) 35 Phenylalanine(s) 26 Tyrosine(s) 13 Cysteine(s) 137 Leucine(s) 28 Histidine(s) 128 Proline(s) 11 Asparagine(s) 98 Valine(s) 86 Threonine(s) 23 Isoleucine(s) 6420 ATP-energy(c) 137 Proton-gradient(c)	1 Collagen COKA1(c) 477 Na-gradient(c)	4	-	-	-	-	-	-	-	22	-	-	-
380 Collagen COLA1(c) synthesis	Collagen COLA1(c)	MCES AAA	test synthesis of Collagen COLA1 c	50 Glutamate(s) 181 Glycine(s) 40 Alanine(s) 68 Lysine(s) 48 Aspartate(s) 38 Arginine(s) 51 Glutamine(s) 50 Serine(s) 12 Methionine(s) 4 Tryptophan(s) 29 Phenylalanine(s) 22 Tyrosine(s) 14 Cysteine(s) 66 Leucine(s) 11 Histidine(s) 106 Proline(s) 26 Asparagine(s) 53 Valine(s) 34 Threonine(s) 54 Isoleucine(s) 4785 ATP-energy(c) 155 Proton-gradient(c)	1 Collagen COLA1(c) 361 Na-gradient(c)	4	-	-	-	-	-	-	-	22	-	-	-
381 Collagen COLQ(c) synthesis	Collagen COLQ(c)	MCES AAA	test synthesis of Collagen COLQ c	21 Glutamate(s) 86 Glycine(s) 9 Alanine(s) 22 Lysine(s) 21 Aspartate(s) 25 Arginine(s) 19 Glutamine(s) 27 Serine(s) 14 Methionine(s) 2 Tryptophan(s) 15 Phenylalanine(s) 14 Tyrosine(s) 15 Cysteine(s) 36 Leucine(s) 6 Histidine(s) 69 Proline(s) 11 Asparagine(s) 15 Valine(s) 16 Threonine(s) 12 Isoleucine(s) 2275 ATP-energy(c) 75 Proton-gradient(c)	1 Collagen COLQ(c) 159 Na-gradient(c)	4	-	-	-	-	-	-	-	22	-	-	-

Continued on next page

B3. Excretion of de novo synthesized substances – continued.

Simulation	Definition			Solution													
				exchanges		reactions								transport			Prot
	Objective	Contraints	Comment	imports	exports	c	m	r	p	l	n	s	b	s-c	b-c	intra	syn
382 Collagen COMA1(c) synthesis	Collagen COMA1(c)	MCES AAA	test synthesis of Collagen COMA1 c	104 Glutamate(s) 395 Glycine(s) 94 Alanine(s) 87 Lysine(s) 70 Aspartate(s) 85 Arginine(s) 62 Glutamine(s) 66 Serine(s) 20 Methionine(s) 6 Tryptophan(s) 34 Phenylalanine(s) 16 Tyrosine(s) 18 Cysteine(s) 101 Leucine(s) 19 Histidine(s) 267 Proline(s) 25 Asparagine(s) 70 Valine(s) 42 Threonine(s) 45 Isoleucine(s) 8130 ATP-energy(c) 370 Proton-gradient(c)	1 Collagen COMA1(c) 473 Na-gradient(c)	4	-	-	-	-	-	-	-	22	-	-	-
383 Collagen CONA1(c) synthesis	Collagen CONA1(c)	MCES AAA	test synthesis of Collagen CONA1 c	36 Glutamate(s) 147 Glycine(s) 43 Alanine(s) 35 Lysine(s) 29 Aspartate(s) 30 Arginine(s) 17 Glutamine(s) 17 Serine(s) 5 Methionine(s) 2 Tryptophan(s) 2 Phenylalanine(s) 2 Tyrosine(s) 6 Cysteine(s) 40 Leucine(s) 3 Histidine(s) 93 Proline(s) 2 Asparagine(s) 15 Valine(s) 8 Threonine(s) 8 Isoleucine(s) 2700 ATP-energy(c) 145 Proton-gradient(c)	1 Collagen CONA1(c) 122 Na-gradient(c)	4	-	-	-	-	-	-	-	22	-	-	-
384 Collagen COOA1(c) synthesis	Collagen COOA1(c)	MCES AAA	test synthesis of Collagen COOA1 c	102 Glutamate(s) 365 Glycine(s) 58 Alanine(s) 104 Lysine(s) 60 Aspartate(s) 66 Arginine(s) 90 Glutamine(s) 90 Serine(s) 24 Methionine(s) 5 Tryptophan(s) 42 Phenylalanine(s) 30 Tyrosine(s) 15 Cysteine(s) 128 Leucine(s) 38 Histidine(s) 207 Proline(s) 54 Asparagine(s) 69 Valine(s) 79 Threonine(s) 88 Isoleucine(s) 8570 ATP-energy(c) 311 Proton-gradient(c)	1 Collagen COOA1(c) 593 Na-gradient(c)	4	-	-	-	-	-	-	-	22	-	-	-

Continued on next page

B3. Excretion of de novo synthesized substances – continued.

Simulation	Definition			Solution													
				exchanges		reactions								transport			Prot
	Objective	Contraints	Comment	imports	exports	c	m	r	p	l	n	s	b	s-c	b-c	intra	syn
385 Collagen COPA1(c) synthesis	Collagen COPA1(c)	MCES AAA	test synthesis of Collagen COPA1 c	45 Glutamate(s) 167 Glycine(s) 33 Alanine(s) 50 Lysine(s) 28 Aspartate(s) 28 Arginine(s) 32 Glutamine(s) 20 Serine(s) 17 Methionine(s) 1 Tryptophan(s) 5 Phenylalanine(s) 4 Tyrosine(s) 7 Cysteine(s) 41 Leucine(s) 10 Histidine(s) 108 Proline(s) 9 Asparagine(s) 13 Valine(s) 16 Threonine(s) 20 Isoleucine(s) 3270 ATP-energy(c) 158 Proton-gradient(c)	1 Collagen COPA1(c) 162 Na-gradient(c)	4	-	-	-	-	-	-	-	22	-	-	-
386 Collagen CORA1(c) synthesis	Collagen CORA1(c)	MCES AAA	test synthesis of Collagen CORA1 c	67 Glutamate(s) 397 Glycine(s) 110 Alanine(s) 95 Lysine(s) 69 Aspartate(s) 101 Arginine(s) 96 Glutamine(s) 106 Serine(s) 38 Methionine(s) 10 Tryptophan(s) 49 Phenylalanine(s) 17 Tyrosine(s) 15 Cysteine(s) 134 Leucine(s) 31 Histidine(s) 303 Proline(s) 21 Asparagine(s) 70 Valine(s) 87 Threonine(s) 44 Isoleucine(s) 9300 ATP-energy(c) 376 Proton-gradient(c)	1 Collagen CORA1(c) 499 Na-gradient(c)	4	-	-	-	-	-	-	-	22	-	-	-
387 Collagen COSA1(c) synthesis	Collagen COSA1(c)	MCES AAA	test synthesis of Collagen COSA1 c	73 Glutamate(s) 211 Glycine(s) 42 Alanine(s) 91 Lysine(s) 63 Aspartate(s) 40 Arginine(s) 58 Glutamine(s) 69 Serine(s) 14 Methionine(s) 5 Tryptophan(s) 38 Phenylalanine(s) 18 Tyrosine(s) 15 Cysteine(s) 66 Leucine(s) 7 Histidine(s) 129 Proline(s) 27 Asparagine(s) 53 Valine(s) 45 Threonine(s) 61 Isoleucine(s) 5625 ATP-energy(c) 184 Proton-gradient(c)	1 Collagen COSA1(c) 429 Na-gradient(c)	4	-	-	-	-	-	-	-	22	-	-	-

Continued on next page

B3. Excretion of de novo synthesized substances – continued.

Simulation	Definition: Objective	Definition: Contraints	Definition: Comment	Solution: exchanges: imports	Solution: exchanges: exports	reactions: c	reactions: m	reactions: r	reactions: p	reactions: l	reactions: n	reactions: s	reactions: b	transport: s-c	transport: b-c	transport: intra	Prot syn
388 Collagen CTHR1(c) synthesis	Collagen CTHR1(c)	MCES AAA	test synthesis of Collagen CTHR1 c	15 Glutamate(s) 29 Glycine(s) 15 Alanine(s) 11 Lysine(s) 8 Aspartate(s) 15 Arginine(s) 10 Glutamine(s) 23 Serine(s) 4 Methionine(s) 5 Tryptophan(s) 6 Phenylalanine(s) 6 Tyrosine(s) 10 Cysteine(s) 24 Leucine(s) 1 Histidine(s) 18 Proline(s) 10 Asparagine(s) 9 Valine(s) 9 Threonine(s) 15 Isoleucine(s) 1215 ATP-energy(c) 19 Proton-gradient(c)	1 Collagen CTHR1(c) 108 Na-gradient(c)	4	-	-	-	-	-	-	-	22	-	-	-
389 Collagen EMID2(c) synthesis	Collagen EMID2(c)	MCES AAA	test synthesis of Collagen EMID2 c	24 Glutamate(s) 65 Glycine(s) 38 Alanine(s) 12 Lysine(s) 16 Aspartate(s) 28 Arginine(s) 18 Glutamine(s) 29 Serine(s) 6 Methionine(s) 5 Tryptophan(s) 4 Phenylalanine(s) 8 Tyrosine(s) 13 Cysteine(s) 39 Leucine(s) 7 Histidine(s) 68 Proline(s) 10 Asparagine(s) 21 Valine(s) 23 Threonine(s) 7 Isoleucine(s) 2205 ATP-energy(c) 55 Proton-gradient(c)	1 Collagen EMID2(c) 144 Na-gradient(c)	4	-	-	-	-	-	-	-	22	-	-	-
390 Collagen FCN1(c) synthesis	Collagen FCN1(c)	MCES AAA	test synthesis of Collagen FCN1 c	16 Glutamate(s) 46 Glycine(s) 29 Alanine(s) 21 Lysine(s) 21 Aspartate(s) 14 Arginine(s) 10 Glutamine(s) 21 Serine(s) 7 Methionine(s) 7 Tryptophan(s) 13 Phenylalanine(s) 12 Tyrosine(s) 8 Cysteine(s) 29 Leucine(s) 7 Histidine(s) 15 Proline(s) 15 Asparagine(s) 18 Valine(s) 11 Threonine(s) 6 Isoleucine(s) 1630 ATP-energy(c) 31 Proton-gradient(c)	1 Collagen FCN1(c) 144 Na-gradient(c)	4	-	-	-	-	-	-	-	22	-	-	-

Continued on next page

B3. Excretion of de novo synthesized substances – continued.

Simulation	Definition			Solution													
				exchanges		reactions								transport			Prot
	Objective	Contraints	Comment	imports	exports	c	m	r	p	l	n	s	b	s-c	b-c	intra	syn
391 Collagen FCN2(c) synthesis	Collagen FCN2(c)	MCES AAA	test synthesis of Collagen FCN2 c	13 Glutamate(s) 43 Glycine(s) 23 Alanine(s) 16 Lysine(s) 21 Aspartate(s) 16 Arginine(s) 9 Glutamine(s) 16 Serine(s) 6 Methionine(s) 8 Tryptophan(s) 14 Phenylalanine(s) 11 Tyrosine(s) 8 Cysteine(s) 29 Leucine(s) 6 Histidine(s) 19 Proline(s) 17 Asparagine(s) 17 Valine(s) 17 Threonine(s) 4 Isoleucine(s) 1565 ATP-energy(c) 26 Proton-gradient(c)	1 Collagen FCN2(c) 135 Na-gradient(c)	4	-	-	-	-	-	-	-	22	-	-	-
392 Collagen FCN3(c) synthesis	Collagen FCN3(c)	MCES AAA	test synthesis of Collagen FCN3 c	18 Glutamate(s) 38 Glycine(s) 20 Alanine(s) 7 Lysine(s) 13 Aspartate(s) 20 Arginine(s) 10 Glutamine(s) 23 Serine(s) 5 Methionine(s) 10 Tryptophan(s) 11 Phenylalanine(s) 10 Tyrosine(s) 9 Cysteine(s) 35 Leucine(s) 10 Histidine(s) 23 Proline(s) 12 Asparagine(s) 13 Valine(s) 9 Threonine(s) 3 Isoleucine(s) 1495 ATP-energy(c) 26 Proton-gradient(c)	1 Collagen FCN3(c) 132 Na-gradient(c)	4	-	-	-	-	-	-	-	22	-	-	-
393 Collagen FMOD(c) synthesis	Collagen FMOD(c)	MCES AAA	test synthesis of Collagen FMOD c	19 Glutamate(s) 17 Glycine(s) 15 Alanine(s) 10 Lysine(s) 20 Aspartate(s) 20 Arginine(s) 18 Glutamine(s) 32 Serine(s) 7 Methionine(s) 4 Tryptophan(s) 12 Phenylalanine(s) 25 Tyrosine(s) 6 Cysteine(s) 55 Leucine(s) 11 Histidine(s) 29 Proline(s) 30 Asparagine(s) 18 Valine(s) 15 Threonine(s) 13 Isoleucine(s) 1880 ATP-energy(c)	13 Proton-gradient(c) 1 Collagen FMOD(c) 190 Na-gradient(c)	4	-	-	-	-	-	-	-	22	-	-	-

Continued on next page

B3. Excretion of de novo synthesized substances – continued.

Simulation	Definition			Solution													
				exchanges		reactions								transport			Prot
	Objective	Contraints	Comment	imports	exports	c	m	r	p	l	n	s	b	s-c	b-c	intra	syn
394 Collagen ITA1(c) synthesis	Collagen ITA1(c)	MCES AAA	test synthesis of Collagen ITA1 c	73 Glutamate(s) 81 Glycine(s) 60 Alanine(s) 72 Lysine(s) 60 Aspartate(s) 46 Arginine(s) 42 Glutamine(s) 101 Serine(s) 26 Methionine(s) 9 Tryptophan(s) 50 Phenylalanine(s) 41 Tyrosine(s) 27 Cysteine(s) 96 Leucine(s) 22 Histidine(s) 47 Proline(s) 71 Asparagine(s) 90 Valine(s) 86 Threonine(s) 79 Isoleucine(s) 5895 ATP-energy(c) 10 Proton-gradient(c)	1 Collagen ITA1(c) 623 Na-gradient(c)	4	-	-	-	-	-	-	-	22	-	-	-
395 Collagen ITA2(c) synthesis	Collagen ITA2(c)	MCES AAA	test synthesis of Collagen ITA2 c	60 Glutamate(s) 88 Glycine(s) 79 Alanine(s) 59 Lysine(s) 60 Aspartate(s) 32 Arginine(s) 63 Glutamine(s) 102 Serine(s) 21 Methionine(s) 9 Tryptophan(s) 57 Phenylalanine(s) 40 Tyrosine(s) 22 Cysteine(s) 98 Leucine(s) 17 Histidine(s) 47 Proline(s) 75 Asparagine(s) 89 Valine(s) 79 Threonine(s) 84 Isoleucine(s) 5905 ATP-energy(c) 13 Proton-gradient(c)	1 Collagen ITA2(c) 615 Na-gradient(c)	4	-	-	-	-	-	-	-	22	-	-	-
396 Collagen LPP3(c) synthesis	Collagen LPP3(c)	MCES AAA	test synthesis of Collagen LPP3 c	8 Glutamate(s) 18 Glycine(s) 22 Alanine(s) 17 Lysine(s) 13 Aspartate(s) 18 Arginine(s) 10 Glutamine(s) 25 Serine(s) 8 Methionine(s) 1 Tryptophan(s) 20 Phenylalanine(s) 16 Tyrosine(s) 11 Cysteine(s) 32 Leucine(s) 8 Histidine(s) 14 Proline(s) 14 Asparagine(s) 17 Valine(s) 14 Threonine(s) 25 Isoleucine(s) 1555 ATP-energy(c) 4 Proton-gradient(c)	1 Collagen LPP3(c) 158 Na-gradient(c)	4	-	-	-	-	-	-	-	22	-	-	-

Continued on next page

B3. Excretion of de novo synthesized substances – continued.

Simulation	Definition			Solution													
				exchanges		reactions								transport			Prot
	Objective	Contraints	Comment	imports	exports	c	m	r	p	l	n	s	b	s-c	b-c	intra	syn
397 Collagen PCOTH(c) synthesis	Collagen PCOTH(c)	MCES AAA	test synthesis of Collagen PCOTH c	2 Glutamate(s) 16 Glycine(s) 2 Alanine(s) 4 Lysine(s) 3 Arginine(s) 1 Glutamine(s) 11 Serine(s) 4 Methionine(s) 2 Tryptophan(s) 7 Phenylalanine(s) 1 Cysteine(s) 10 Leucine(s) 26 Proline(s) 2 Asparagine(s) 6 Valine(s) 6 Threonine(s) 4 Isoleucine(s) 535 ATP-energy(c) 14 Proton-gradient(c)	1 Collagen PCOTH(c) 35 Na-gradient(c)	4	-	-	-	-	-	-	-	19	-	-	-
398 Collagen SCRB1(c) synthesis	Collagen SCRB1(c)	MCES AAA	test synthesis of Collagen SCRB1 c	25 Glutamate(s) 45 Glycine(s) 39 Alanine(s) 24 Lysine(s) 14 Aspartate(s) 21 Arginine(s) 17 Glutamine(s) 45 Serine(s) 23 Methionine(s) 11 Tryptophan(s) 32 Phenylalanine(s) 16 Tyrosine(s) 14 Cysteine(s) 59 Leucine(s) 13 Histidine(s) 38 Proline(s) 24 Asparagine(s) 41 Valine(s) 27 Threonine(s) 24 Isoleucine(s) 2760 ATP-energy(c) 21 Proton-gradient(c)	1 Collagen SCRB1(c) 259 Na-gradient(c)	4	-	-	-	-	-	-	-	22	-	-	-
399 Collagen SERPH(c) synthesis	Collagen SERPH(c)	MCES AAA	test synthesis of Collagen SERPH c	27 Glutamate(s) 24 Glycine(s) 43 Alanine(s) 34 Lysine(s) 24 Aspartate(s) 21 Arginine(s) 13 Glutamine(s) 33 Serine(s) 14 Methionine(s) 4 Tryptophan(s) 16 Phenylalanine(s) 10 Tyrosine(s) 2 Cysteine(s) 54 Leucine(s) 14 Histidine(s) 15 Proline(s) 11 Asparagine(s) 28 Valine(s) 19 Threonine(s) 12 Isoleucine(s) 2090 ATP-energy(c) 13 Proton-gradient(c)	1 Collagen SERPH(c) 181 Na-gradient(c)	4	-	-	-	-	-	-	-	22	-	-	-

Continued on next page

B3. Excretion of de novo synthesized substances – continued.

Simulation	Definition			Solution													
				exchanges		reactions								transport			Prot
	Objective	Contraints	Comment	imports	exports	c	m	r	p	l	n	s	b	s-c	b-c	intra	syn
400 Collagen VWA2(c) synthesis	Collagen VWA2(c)	MCES AAA	test synthesis of Collagen VWA2 c	44 Glutamate(s) 66 Glycine(s) 66 Alanine(s) 25 Lysine(s) 32 Aspartate(s) 58 Arginine(s) 32 Glutamine(s) 56 Serine(s) 12 Methionine(s) 7 Tryptophan(s) 31 Phenylalanine(s) 13 Tyrosine(s) 27 Cysteine(s) 81 Leucine(s) 17 Histidine(s) 47 Proline(s) 15 Asparagine(s) 72 Valine(s) 36 Threonine(s) 18 Isoleucine(s) 3775 ATP-energy(c) 51 Proton-gradient(c)	1 Collagen VWA2(c) 332 Na-gradient(c)	4	-	-	-	-	-	-	-	22	-	-	-
**B3.2.2. Lipoproteins**																	
401 VLDL	VLDL	MCES AAA ALFA	de novo synthesis and export of VLDL	578 Glutamate(s) 333 Glycine(s) 548 Alanine(s) 448 Lysine(s) 310 Aspartate(s) 388 Arginine(s) 454 Glutamine(s) 1060 Serine(s) 135 Methionine(s) 93 Tryptophan(s) 252 Phenylalanine(s) 179 Tyrosine(s) 6452 Glucose-6P(c) 39 Cysteine(s) 3085 Choline(c) 820 Leucine(s) 129 Histidine(s) 227 Proline(s) 254 Asparagine(s) 420 Valine(s) 384 Threonine(s) 370 Ethanolamine(c) 5800 Arachidonate(c) 12708 Palmitate(c) 299 Isoleucine(s) 4290 Farnesyl-PP(r) 15589 Oleate(c) 25.1 Stearate(c) 3107 Linoleate(c) 1949 gamma-Linolenate(c) $1.3{\cdot}10^5$ ATP-energy(c) 6435 NADPH-redox-potential(r) 829.9 CoA-activated acetyl group(r)	26201 H_2O(s) 11761 P_i(c) 7005 CO_2(s) 1 VLDL(s) 2145 NADH-redox-potential(r) 724 NADH-redox-potential(c) 635 NADPH-redox-potential(c) 179 Proton-gradient(c) 4115 Na-gradient(c) 5120 CoA-activated acetyl group(c)	66	-	35	-	-	-	-	-	25	-	25	3

Continued on next page

B3. Excretion of de novo synthesized substances – continued.

Simulation	Definition			Solution													
				exchanges		reactions								transport			Prot
	Objective	Contraints	Comment	imports	exports	c	m	r	p	l	n	s	b	s-c	b-c	intra	syn
402 HDL	HDL	MCES AAA ALFA	de novo synthesis and export of HDL	67.5 P_i(c) 60 Glutamate(s) 22 Glycine(s) 46 Alanine(s) 44 Lysine(s) 32 Aspartate(s) 34 Arginine(s) 38 Glutamine(s) 82 Serine(s) 8 Methionine(s) 10 Tryptophan(s) 16 Phenylalanine(s) 14 Tyrosine(s) 72.5 Glucose-6P(c) 165 Choline(c) 82 Leucine(s) 12 Histidine(s) 20 Proline(s) 10 Asparagine(s) 30 Valine(s) 24 Threonine(s) 80 Ethanolamine(c) 85.7 Arachidonate(c) 196.4 Palmitate(c) 40 Farnesyl-PP(r) 143.5 Oleate(c) 7.93 Linoleate(c) 6.38 gamma-Linolenate(c) 4303 ATP-energy(c) 60 NADPH-redox-potential(r) 130 NADPH-redox-potential(c)	673.9 H_2O(s) 80 CO_2(s) 1 HDL(s) 20 NADH-redox-potential(r) 50 NADH-redox-potential(c) 14 Proton-gradient(c) 260 Na-gradient(c) 40 CoA-activated acetyl group(c)	51	-	19	-	-	-	-	-	23	-	14	2
**B3.2.3. Ketone bodies**																	
403 Acetoacetate	Acetoacetate	MCES	de novo synthesis and export of Acetoacetate from Palmitate, excreting CO_2	1 H_2O(s) 1 Pyruvate(c) 2 CoA-activated acetyl group(m)	1 Pyruvate(m) 1 Acetoacetate(s) 1 Proton-gradient(c)	-	4	-	-	-	-	-	-	3	-	2	-
404 (R)-3-Hydroxybutanoate	(R)-3-Hydroxy-butanoate(c)	MCES	de novo synthesis and export of (R)-3-Hydroxybutanoate from Palmitate, excreting CO_2	1 H_2O(s) 1 NADH-redox-potential(m) 1 Proton-gradient(m) 2 CoA-activated acetyl group(m)	1 (R)-3-Hydroxybutanoate(c)	-	6	-	-	-	-	-	-	1	-	3	-

Continued on next page

B3. Excretion of de novo synthesized substances – continued.

Simulation	Definition			Solution													
				exchanges		reactions								transport			Prot
	Objective	Contraints	Comment	imports	exports	c	m	r	p	l	n	s	b	s-c	b-c	intra	syn
405 Acetone	Acetone	MCES	de novo synthesis and export of Acetone	1 H_2O(s) 2 CoA-activated acetyl group(m)	1 CO_2(s) 1 Acetone(s)	-	5	-	-	-	-	-	-	3	-	2	-
**B3.2.4. Other**																	
406 Heme	Heme(c)	MCES AAA $-Fe^{2+}+$	de novo synthesis of cytosolic Heme	2.5 O_2(s) 4 Pyruvate(m) 10.5 Glutamate(s) 4 Serine(s) 1.5 Proline(s) 1 Fe^{2+}+(s) 4 ATP-energy(c) 1 Proton-gradient(c) 2 Proton-gradient(m)	14 H_2O(s) 14 CO_2(s) 1 Heme(c) 4 Aspartate(s) 4 Glutamine(s) 8 NADH-redox-potential(m) 10.5 Na-gradient(c)	7	14	-	-	-	-	-	-	10	-	12	-

3.4 B4. clearence " ... degr" simulations

Table 11: B4. clearence " ... degr" simulations

Simulation	Definition			Solution													
				exchanges		reactions								transport			Prot
	Objective	Contraints	Comment	imports	exports	c	m	r	p	l	n	s	b	s-c	b-c	intra	syn
**B4.1. amino acids**																	
407 Alanine degr	-1 Alanine(c)	MCES +Urea	Alanine degradation using palmitate excreting urea	0.5 H_2O(s) 1 Alanine(c) 1 ATP-energy(c) 1 ATP-energy(m)	0.5 Pyruvate(c) 0.5 Urea(s) 0.5 NADH-redox-potential(c) 0.5 NADH-redox-potential(m) 0.5 NADPH-redox-potential(m) 0.5 CoA-activated acetyl group(m)	11	9	-	-	-	-	-	-	2	-	2	-
408 Arginine degr	-1 Arginine(c)	MCES +Urea	Arginine degradation using palmitate excreting urea	4 H_2O(s) 0.5 O_2(s) 1 Arginine(c) 2 ATP-energy(c) 1 ATP-energy(m)	1 CO_2(s) 1 Pyruvate(c) 2 Urea(s) 3 NADH-redox-potential(m) 1 NADPH-redox-potential(c) 1 NADPH-redox-potential(m) 3 Proton-gradient(m)	9	15	-	-	-	-	-	-	4	-	11	-
409 Asparagine degr	-1 Asparagine(c)	MCES +Urea	Asparagine degradation using palmitate excreting urea	1 H_2O(s) 1 Asparagine(c) 2 ATP-energy(c) 2 ATP-energy(m)	1 Pyruvate(c) 1 Urea(s) 1 NADPH-redox-potential(c)	10	3	-	-	-	-	-	-	2	-	4	-

Continued on next page

B4. clearence " ... degr" simulations – continued.

Simulation	Definition			Solution													
				exchanges		reactions								transport			Prot
	Objective	Contraints	Comment	imports	exports	c	m	r	p	l	n	s	b	s-c	b-c	intra	syn
410 Aspartate degr	-1 Aspartate(c)	MCES +Urea	Aspartate degradation using palmitate excreting urea	0.5 H_2O(s) 1 Aspartate(c) 1 ATP-energy(c) 1 ATP-energy(m)	1 CO_2(s) 0.5 Pyruvate(c) 0.5 Urea(s) 0.5 NADH-redox-potential(m) 0.5 NADPH-redox-potential(c) 0.5 NADPH-redox-potential(m) 0.5 CoA-activated acetyl group(m)	10	9	-	-	-	-	-	-	3	-	2	-
411 Cysteine degr	-1 Cysteine(c)	MCES +Urea +H_2S	Cysteine degradation using palmitate excreting urea	0.5 H_2O(s) 0.5 Pyruvate(m) 1 Cysteine(c) 1 ATP-energy(c) 1 ATP-energy(m) 1 Proton-gradient(m)	1 Pyruvate(c) 0.5 Urea(s) 1 H_2S(s) 0.5 NADH-redox-potential(c) 0.5 CoA-activated acetyl group(m)	11	6	-	-	-	-	-	-	3	-	8	-
412 Glutamate degr	-1 Glutamate(c)	MCES +Urea	Glutamate degradation using palmitate excreting urea	1.5 H_2O(s) 0.5 O_2(s) 1 Glutamate(c) 1 ATP-energy(c)	1.5 CO_2(s) 0.5 Pyruvate(c) 0.5 Pyruvate(m) 0.5 Urea(s) 1.5 NADH-redox-potential(m) 0.5 NADPH-redox-potential(c) 1 NADPH-redox-potential(m) 5 Proton-gradient(m)	9	13	-	-	-	-	-	-	4	-	9	-
413 Histidine degr	-1 Histidine(c)	MCES +Urea	Histidine degradation using palmitate excreting urea	2.5 H_2O(s) 0.5 O_2(s) 1 Histidine(c) 3 ATP-energy(c) 2 ATP-energy(m) 0.5 NADPH-redox-potential(c)	0.5 CO_2(s) 0.5 Pyruvate(c) 0.5 Pyruvate(m) 1.5 Urea(s) 0.5 NADH-redox-potential(c) 1 NADH-redox-potential(m) 4.5 Proton-gradient(m) 1 THF-activated methyl group(c)	20	11	-	-	-	-	-	-	4	-	12	-
414 Glutamine degr	-1 Glutamine(c)	MCES +Urea	Glutamine degradation using palmitate excreting urea	2 H_2O(s) 0.5 O_2(s) 1 Glutamine(c) 2 ATP-energy(c) 1 ATP-energy(m)	1 CO_2(s) 1 Pyruvate(c) 1 Urea(s) 2 NADH-redox-potential(m) 1 NADPH-redox-potential(c) 5 Proton-gradient(m)	11	11	-	-	-	-	-	-	4	-	7	-
415 Leucine degr	-1 Leucine(c)	MCES +Urea	Leucine degradation using palmitate excreting urea	0.5 O_2(s) 0.5 Pyruvate(m) 1 Leucine(c) 1 ATP-energy(c) 1 ATP-energy(m) 1 NADPH-redox-potential(m)	0.5 H_2O(s) 0.5 Urea(s) 0.5 NADH-redox-potential(c) 3 NADH-redox-potential(m) 4 Proton-gradient(m) 3.5 CoA-activated acetyl group(m)	10	18	-	-	-	-	-	-	3	-	11	-

Continued on next page

B4. clearence " ... degr" simulations – continued.

Simulation	Definition			Solution													
				exchanges		reactions								transport			Prot
	Objective	Contraints	Comment	imports	exports	c	m	r	p	l	n	s	b	s-c	b-c	intra	syn
416 Lysine degr	-1 Lysine(c)	MCES +Urea	Lysine degradation using palmitate excreting urea	2 H_2O(s) 0.5 O_2(s) 1 Pyruvate(m) 1 Lysine(c) 2 ATP-energy(c) 2 ATP-energy(m) 2 NADPH-redox-potential(m)	1 CO_2(s) 1 Pyruvate(c) 1 Urea(s) 1 NADH-redox-potential(c) 5 NADH-redox-potential(m) 1 NADPH-redox-potential(c) 4 Proton-gradient(m) 2 CoA-activated acetyl group(m)	11	18	-	-	-	-	-	-	4	-	10	-
417 Methionine degr	-1 Methionine(c)	MCES +Urea −Serine +H_2S	Methionine degradation using palmitate excreting urea	1 H_2O(s) 0.5 O_2(s) 1 Serine(s) 1 Methionine(c) 2 ATP-energy(c) 2 ATP-energy(m)	1 CO_2(s) 1 Pyruvate(c) 1 Urea(s) 1 H_2S(s) 1 NADH-redox-potential(c) 2 NADH-redox-potential(m) 3 Proton-gradient(m) 1 THF-activated methyl group(c) 1 CoA-activated acetyl group(m)	15	12	-	-	-	-	-	-	6	-	13	-
418 Phenylalanine degr	-1 Phenylalanine(c)	MCES +Urea	Phenylalanine degradation using palmitate excreting urea	0.5 H_2O(s) 3 O_2(s) 1 Phenylalanine(c) 3 ATP-energy(c) 1 ATP-energy(m) 0.5 NADPH-redox-potential(c) 0.5 Proton-gradient(m)	1.5 CO_2(s) 0.5 Pyruvate(c) 0.5 Pyruvate(m) 0.5 Urea(s) 0.5 NADH-redox-potential(c) 1 NADPH-redox-potential(m) 2 CoA-activated acetyl group(c)	22	6	-	-	-	-	-	-	4	-	4	-
419 Proline degr	-1 Proline(c)	MCES +Urea	Proline degradation using palmitate excreting urea	4.5 H_2O(s) 1 Proline(c) 1 ATP-energy(c) 0.5 Proton-gradient(m)	1.5 CO_2(s) 0.5 Pyruvate(c) 0.5 Pyruvate(m) 0.5 Urea(s) 0.5 NADH-redox-potential(c) 2 NADH-redox-potential(m) 0.5 NADPH-redox-potential(c) 1 NADPH-redox-potential(m)	12	14	-	-	-	-	-	-	3	-	7	-
420 Serine degr	-1 Serine(c)	MCES +Urea	Serine degradation using palmitate excreting urea	0.5 Pyruvate(m) 1 Serine(c) 1 ATP-energy(c) 1 ATP-energy(m) 0.5 NADPH-redox-potential(c)	0.5 H_2O(s) 1 Pyruvate(c) 0.5 Urea(s) 0.5 NADH-redox-potential(c) 0.5 NADH-redox-potential(m) 0.5 CoA-activated acetyl group(m)	19	6	-	-	-	-	-	-	2	-	3	-
421 Threonine degr	-1 Threonine(c)	MCES +Urea	Threonine degradation using palmitate excreting urea	0.5 H_2O(s) 0.5 O_2(s) 1 Threonine(c) 1 ATP-energy(c) 1 ATP-energy(m) 0.5 NADPH-redox-potential(m)	1.5 CO_2(s) 0.5 Urea(s) 0.5 NADH-redox-potential(c) 3 NADH-redox-potential(m) 4 Proton-gradient(m) 1 CoA-activated acetyl group(m)	11	13	-	-	-	-	-	-	4	-	12	-

Continued on next page

B4. clearence " ... degr" simulations – continued.

Simulation	Definition			Solution													
				exchanges		reactions								transport			Prot
	Objective	Contraints	Comment	imports	exports	c	m	r	p	l	n	s	b	s-c	b-c	intra	syn
422 Tryptophan degr	-1 Tryptophan(c)	MCES +Urea	Tryptophan degradation using palmitate excreting urea	1 H_2O(s) 3.5 O_2(s) 1 Pyruvate(m) 1 Tryptophan(c) 3 ATP-energy(c) 2 ATP-energy(m) 2 NADPH-redox-potential(c) 1 NADPH-redox-potential(m)	2 CO_2(s) 2 Pyruvate(c) 1 Urea(s) 3 NADH-redox-potential(m) 5 Proton-gradient(m) 1 THF-activated methyl group(c) 2 CoA-activated acetyl group(m)	23	14	-	-	-	-	-	-	4	-	9	-
423 Valine degr	-1 Valine(c)	MCES +Urea	Valine degradation using palmitate excreting urea	2.5 H_2O(s) 1 O_2(s) 1 Valine(c) 1 ATP-energy(c)	1.5 CO_2(s) 0.5 Pyruvate(c) 0.5 Pyruvate(m) 0.5 Urea(s) 0.5 NADH-redox-potential(c) 3 NADH-redox-potential(m) 0.5 NADPH-redox-potential(c) 1 NADPH-redox-potential(m) 11.5 Proton-gradient(m)	12	18	-	-	-	-	-	-	4	-	11	-
424 Glycine degr	-1 Glycine(c)	MCES +Urea −Methionine +H_2S	Glycine degradation using palmitate excreting urea	0.5 H_2O(s) 0.5 Pyruvate(m) 1 Glycine(c) 1 ATP-energy(c) 1 ATP-energy(m) 1 THF-activated methyl group(c)	1 Pyruvate(c) 0.5 Urea(s) 0.5 NADH-redox-potential(c) 0.5 NADH-redox-potential(m) 0.5 NADPH-redox-potential(c) 0.5 CoA-activated acetyl group(m)	22	6	-	-	-	-	-	-	2	-	3	-
425 Isoleucine degr	-1 Isoleucine(c)	MCES +Urea	Isoleucine degradation using palmitate excreting urea	1.5 H_2O(s) 1 O_2(s) 1 Isoleucine(c) 1 ATP-energy(c) 1 ATP-energy(m)	1.5 CO_2(s) 0.5 Urea(s) 0.5 NADH-redox-potential(c) 4 NADH-redox-potential(m) 0.5 NADPH-redox-potential(m) 10 Proton-gradient(m) 2 CoA-activated acetyl group(m)	10	18	-	-	-	-	-	-	4	-	12	-
426 Tyrosine degr	-1 Tyrosine(c)	MCES +Urea	Tyrosine degradation using palmitate excreting urea	1.5 H_2O(s) 2 O_2(s) 1 Tyrosine(c) 3 ATP-energy(c) 1 ATP-energy(m) 0.5 Proton-gradient(m)	1.5 CO_2(s) 0.5 Pyruvate(c) 0.5 Pyruvate(m) 0.5 Urea(s) 0.5 NADH-redox-potential(c) 0.5 NADPH-redox-potential(c) 1 NADPH-redox-potential(m) 2 CoA-activated acetyl group(c)	20	6	-	-	-	-	-	-	4	-	4	-
427 Homocysteine degr	-1 Homocysteine(c)	MCES +Urea −Serine +H_2S	Homocysteine degradation using palmitate excreting urea	1 H_2O(s) 0.5 O_2(s) 1 Serine(s) 1 Homocysteine(c) 2 ATP-energy(c) 2 ATP-energy(m)	1 CO_2(s) 1 Pyruvate(c) 1 Urea(s) 1 H_2S(s) 1 NADH-redox-potential(c) 2 NADH-redox-potential(m) 3 Proton-gradient(m) 1 CoA-activated acetyl group(m)	13	12	-	-	-	-	-	-	6	-	13	-

Continued on next page

Simulation		Definition			Solution													
					exchanges		reactions								transport			Prot
		Objective	Contraints	Comment	imports	exports	c	m	r	p	l	n	s	b	s-c	b-c	intra	syn
428	beta-Alanine degr	-1 beta-Alanine(c)	MCES +Urea	beta-Alanine degradation using palmitate excreting urea	0.5 H_2O(s) 1 beta-Alanine(c) 1 ATP-energy(c) 1 ATP-energy(m) 1 Proton-gradient(m)	0.5 CO_2(s) 0.5 Urea(s) 0.5 NADH-redox-potential(c) 1 NADH-redox-potential(m) 0.5 NADPH-redox-potential(m) 1 CoA-activated acetyl group(m)	10	9	-	-	-	-	-	-	3	-	8	-
429	Ornithine degr	-1 Ornithine(c)	MCES +Urea	Ornithine degradation using palmitate excreting urea	3 H_2O(s) 0.5 O_2(s) 1 Ornithine(c) 2 ATP-energy(c) 1 ATP-energy(m)	1 CO_2(s) 1 Pyruvate(c) 1 Urea(s) 3 NADH-redox-potential(m) 1 NADPH-redox-potential(c) 1 NADPH-redox-potential(m) 3 Proton-gradient(m)	9	15	-	-	-	-	-	-	4	-	11	-
**B4.2. lipids, fatty acids, lipoproteins**																		
430	LDL degr	-1 LDL	MCES AAA ALFA +Cholesterol(b)	degradation of LDL	4259 H_2O(s) 119 O_2(s) 1 LDL(s) 5395 ATP-energy(c) 110 NADH-redox-potential(c)	473.3 P_i(c) 60 CO_2(s) 207 Glycine(s) 275 Alanine(s) 357 Lysine(s) 233 Aspartate(s) 150 Arginine(s) 560 Glutamine(s) 552 Serine(s) 79 Methionine(s) 37 Tryptophan(s) 224 Phenylalanine(s) 151 Tyrosine(s) 386.7 Glucose-6P(c) 25 Cysteine(s) 610 Choline(c) 533 Leucine(s) 115 Histidine(s) 139 Proline(s) 247 Asparagine(s) 252 Valine(s) 2195 Cholesterol(b) 300 Threonine(s) 30 Ethanolamine(c) 240.1 Arachidonate(c) 843.9 Palmitate(c) 285 Isoleucine(s) 510.2 Oleate(c) 316 Stearate(c) 1080 Linoleate(c) 252 NADH-redox-potential(m) 320 NADPH-redox-potential(c) 3348 Proton-gradient(m) 110 CoA-activated acetyl group(m)	48	8	2	-	8	-	-	-	23	1	50	2

Continued on next page

Simulation	Definition			Solution													
				exchanges		reactions								transport			Prot
	Objective	Contraints	Comment	imports	exports	c	m	r	p	l	n	s	b	s-c	b-c	intra	syn
431 HDL degr	-1 HDL	MCES AAA ALFA +Cholesterol(b)	degradation of HDL	747.5 H_2O(s) 75 Pyruvate(m) 1 HDL(s) 491 ATP-energy(c) 2.5 NADH-redox-potential(c)	147.5 P_i(c) 60 Pyruvate(c) 22 Glycine(s) 46 Alanine(s) 44 Lysine(s) 32 Aspartate(s) 34 Arginine(s) 98 Glutamine(s) 77 Serine(s) 8 Methionine(s) 10 Tryptophan(s) 16 Phenylalanine(s) 14 Tyrosine(s) 72.5 Glucose-6P(c) 165 Choline(c) 82 Leucine(s) 12 Histidine(s) 20 Proline(s) 10 Asparagine(s) 30 Valine(s) 20 Cholesterol(b) 24 Threonine(s) 25 Ethanolamine(c) 49.3 Arachidonate(c) 207.6 Palmitate(c) 39.5 Oleate(c) 69.7 Stearate(c) 71.4 Linoleate(c) 2.5 Palmitolate(c) 75 NADH-redox-potential(m) 150 NADPH-redox-potential(c) 870 Proton-gradient(m) 75 CoA-activated acetyl group(m)	31	3	1	-	-	-	-	-	19	1	39	2
432 Oleate degr	-1 Oleate(c)	MCES	degradation of cytosolic oleate	2 H_2O(s) 2.5 O_2(s) 1 Oleate(c) 2 ATP-energy(c)	1 NADH-redox-potential(c) 8 NADH-redox-potential(m) 1 FADH-redox-potential(c) 42 Proton-gradient(m) 9 CoA-activated acetyl group(m)	9	35	-	-	-	-	-	-	2	-	9	-
433 Stearate degr	-1 Stearate(c)	MCES	degradation of cytosolic stearate	3.5 O_2(s) 1 Stearate(c) 2 ATP-energy(c)	8 NADH-redox-potential(m) 1 FADH-redox-potential(c) 42 Proton-gradient(m) 9 CoA-activated acetyl group(m)	8	35	-	-	-	-	-	-	1	-	9	-
**B4.3. sugars**																	
434 Fructose degr	-1 Fructose	MCES	Fructose degradation	1 P_i(c) 1 Fructose(s) 2 ATP-energy(c)	1 H_2O(s) 1 Glucose-6P(c)	7	-	-	-	-	-	-	-	2	-	-	-
435 Galactose degr	-1 Galactose	MCES	Galactose degradation	1 P_i(c) 1 Galactose(s) 1 ATP-energy(c)	1 H_2O(s) 1 Glucose-6P(c)	5	-	-	-	-	-	-	-	2	-	-	-
436 Mannose degr	-1 Mannose	MCES	Mannose degradation	1 P_i(c) 1 Mannose(s) 1 ATP-energy(c)	1 H_2O(s) 1 Glucose-6P(c)	4	-	-	-	-	-	-	-	2	-	-	-
**B4.4. Proteins**																	

Continued on next page

B4. clearence " ... degr" simulations – continued.

Simulation	Definition			Solution													
				exchanges		reactions								transport			Prot
	Objective	Contraints	Comment	imports	exports	c	m	r	p	l	n	s	b	s-c	b-c	intra	syn
437 Albumin degr	-1 Albumin	MCES AAA	degradation of Albumin	1 Albumin(s) 304 ATP-energy(c)	62 Glutamate(s) 13 Glycine(s) 63 Alanine(s) 60 Lysine(s) 36 Aspartate(s) 27 Arginine(s) 20 Glutamine(s) 28 Serine(s) 7 Methionine(s) 2 Tryptophan(s) 35 Phenylalanine(s) 19 Tyrosine(s) 35 Cysteine(s) 64 Leucine(s) 16 Histidine(s) 24 Proline(s) 17 Asparagine(s) 43 Valine(s) 29 Threonine(s) 9 Isoleucine(s)	1	-	-	-	-	-	-	-	21	-	-	1
438 Antichymotrypsin degr	-1 Antichymotrypsin	MCES AAA	degradation of Antichymotrypsin	1 Antichymotrypsin(s) 211 ATP-energy(c)	29 Glutamate(s) 16 Glycine(s) 34 Alanine(s) 26 Lysine(s) 25 Aspartate(s) 16 Arginine(s) 17 Glutamine(s) 30 Serine(s) 14 Methionine(s) 3 Tryptophan(s) 25 Phenylalanine(s) 9 Tyrosine(s) 3 Cysteine(s) 59 Leucine(s) 9 Histidine(s) 16 Proline(s) 19 Asparagine(s) 24 Valine(s) 29 Threonine(s) 20 Isoleucine(s)	1	-	-	-	-	-	-	-	21	-	-	1
439 Antitrypsin degr	-1 Antitrypsin	MCES AAA	degradation of Antitrypsin	1 Antitrypsin(s) 209 ATP-energy(c)	32 Glutamate(s) 24 Glycine(s) 26 Alanine(s) 34 Lysine(s) 24 Aspartate(s) 7 Arginine(s) 18 Glutamine(s) 25 Serine(s) 10 Methionine(s) 3 Tryptophan(s) 27 Phenylalanine(s) 6 Tyrosine(s) 3 Cysteine(s) 51 Leucine(s) 13 Histidine(s) 19 Proline(s) 19 Asparagine(s) 27 Valine(s) 30 Threonine(s) 20 Isoleucine(s)	1	-	-	-	-	-	-	-	21	-	-	1

Continued on next page

B4. clearence " ... degr" simulations – continued.

Simulation	Definition			Solution													
				exchanges		reactions								transport			Prot
	Objective	Contraints	Comment	imports	exports	c	m	r	p	l	n	s	b	s-c	b-c	intra	syn
440 ApoA1 degr	-1 ApoA1	MCES AAA	degradation of ApoA1	1 ApoA1(s) 133 ATP-energy(c)	30 Glutamate(s) 11 Glycine(s) 23 Alanine(s) 22 Lysine(s) 16 Aspartate(s) 17 Arginine(s) 19 Glutamine(s) 16 Serine(s) 4 Methionine(s) 5 Tryptophan(s) 8 Phenylalanine(s) 7 Tyrosine(s) 41 Leucine(s) 6 Histidine(s) 10 Proline(s) 5 Asparagine(s) 15 Valine(s) 12 Threonine(s)	1	-	-	-	-	-	-	-	19	-	-	1
441 Fibrinogen degr	-1 Fibrinogen	MCES AAA	degradation of Fibrinogen	1 Fibrinogen(s) 1337 ATP-energy(c)	186 Glutamate(s) 262 Glycine(s) 134 Alanine(s) 154 Lysine(s) 176 Aspartate(s) 134 Arginine(s) 110 Glutamine(s) 278 Serine(s) 48 Methionine(s) 60 Tryptophan(s) 98 Phenylalanine(s) 98 Tyrosine(s) 48 Cysteine(s) 166 Leucine(s) 62 Histidine(s) 114 Proline(s) 138 Asparagine(s) 122 Valine(s) 182 Threonine(s) 104 Isoleucine(s)	1	-	-	-	-	-	-	-	21	-	-	1
442 Haptoglobin degr	-1 Haptoglobin	MCES AAA	degradation of Haptoglobin	1 Haptoglobin(s) 203 ATP-energy(c)	25 Glutamate(s) 31 Glycine(s) 30 Alanine(s) 35 Lysine(s) 25 Aspartate(s) 9 Arginine(s) 17 Glutamine(s) 18 Serine(s) 5 Methionine(s) 8 Tryptophan(s) 8 Phenylalanine(s) 21 Tyrosine(s) 12 Cysteine(s) 31 Leucine(s) 13 Histidine(s) 21 Proline(s) 21 Asparagine(s) 36 Valine(s) 22 Threonine(s) 18 Isoleucine(s)	1	-	-	-	-	-	-	-	21	-	-	1

Continued on next page

B4. clearence " ... degr" simulations – continued.

Simulation	Definition			Solution													
				exchanges		reactions								transport			Prot
	Objective	Contraints	Comment	imports	exports	c	m	r	p	l	n	s	b	s-c	b-c	intra	syn
443 Plasminogen degr	-1 Plasminogen	MCES AAA	degradation of Plasminogen	1 Plasminogen(s) 405 ATP-energy(c)	56 Glutamate(s) 62 Glycine(s) 37 Alanine(s) 49 Lysine(s) 36 Aspartate(s) 42 Arginine(s) 31 Glutamine(s) 56 Serine(s) 11 Methionine(s) 19 Tryptophan(s) 21 Phenylalanine(s) 30 Tyrosine(s) 48 Cysteine(s) 48 Leucine(s) 24 Histidine(s) 69 Proline(s) 40 Asparagine(s) 48 Valine(s) 61 Threonine(s) 22 Isoleucine(s)	1	-	-	-	-	-	-	-	21	-	-	1
444 Prothrombin degr	-1 Prothrombin	MCES AAA	degradation of Prothrombin	1 Prothrombin(s) 311 ATP-energy(c)	51 Glutamate(s) 49 Glycine(s) 42 Alanine(s) 29 Lysine(s) 35 Aspartate(s) 44 Arginine(s) 26 Glutamine(s) 38 Serine(s) 9 Methionine(s) 14 Tryptophan(s) 21 Phenylalanine(s) 21 Tyrosine(s) 26 Cysteine(s) 51 Leucine(s) 13 Histidine(s) 33 Proline(s) 25 Asparagine(s) 37 Valine(s) 36 Threonine(s) 22 Isoleucine(s)	1	-	-	-	-	-	-	-	21	-	-	1
445 ApoTransferin degr	-1 ApoTransferin	MCES AAA	degradation of ApoTransferin	1 ApoTransferin(s) 349 ATP-energy(c)	42 Glutamate(s) 52 Glycine(s) 61 Alanine(s) 58 Lysine(s) 45 Aspartate(s) 27 Arginine(s) 17 Glutamine(s) 41 Serine(s) 10 Methionine(s) 8 Tryptophan(s) 28 Phenylalanine(s) 26 Tyrosine(s) 40 Cysteine(s) 65 Leucine(s) 19 Histidine(s) 32 Proline(s) 34 Asparagine(s) 48 Valine(s) 30 Threonine(s) 15 Isoleucine(s)	1	-	-	-	-	-	-	-	21	-	-	1

Continued on next page

B4. clearence " ... degr" simulations – continued.

Simulation	Definition			Solution													
				exchanges		reactions								transport			Prot
	Objective	Contraints	Comment	imports	exports	c	m	r	p	l	n	s	b	s-c	b-c	intra	syn
446 ApoB100 degr	-1 ApoB100(l)	MCES AAA	degradation of lysosomal ApoB100	1 ApoB100(l) 2280 ATP-energy(c)	298 Glutamate(s) 207 Glycine(s) 275 Alanine(s) 357 Lysine(s) 233 Aspartate(s) 150 Arginine(s) 230 Glutamine(s) 392 Serine(s) 79 Methionine(s) 37 Tryptophan(s) 224 Phenylalanine(s) 151 Tyrosine(s) 25 Cysteine(s) 533 Leucine(s) 115 Histidine(s) 171 Proline(s) 247 Asparagine(s) 252 Valine(s) 300 Threonine(s) 285 Isoleucine(s)	1	-	-	-	-	-	-	-	20	-	23	1
447 Glycogenin degr	-1 Glycogenin(c)	MCES AAA	degradation of cytosolic Glycogenin	175 ATP-energy(c) 1 Glycogenin(c)	15 Glutamate(s) 18 Glycine(s) 22 Alanine(s) 18 Lysine(s) 26 Aspartate(s) 12 Arginine(s) 14 Glutamine(s) 31 Serine(s) 8 Methionine(s) 7 Tryptophan(s) 20 Phenylalanine(s) 13 Tyrosine(s) 6 Cysteine(s) 39 Leucine(s) 9 Histidine(s) 15 Proline(s) 12 Asparagine(s) 28 Valine(s) 25 Threonine(s) 12 Isoleucine(s)	1	-	-	-	-	-	-	-	20	-	-	1
**B4.5. Ethanol**																	
448 Ethanol degr	-1 Ethanol	MCES	degradation of Ethanol	1 Ethanol(s) 2 ATP-energy(c)	2 NADH-redox-potential(c) 1 CoA-activated acetyl group(c)	8	-	-	-	-	-	-	-	1	-	-	-
**B4.6. Other**																	
449 Chitin-component degr	-1 Chitin-component	MCES +Urea	degradation of Chitin-component	0.5 H_2O(s) 1 P_i(c) 0.5 Pyruvate(m) 4 ATP-energy(c) 1 ATP-energy(m) 1 Proton-gradient(m) 1 Chitin-component(s)	0.5 Urea(s) 1 Glucose-6P(c) 0.5 NADH-redox-potential(c) 1 CoA-activated acetyl group(c) 0.5 CoA-activated acetyl group(m)	16	6	-	-	1	-	1	-	2	-	10	-

4 C. Proliferative functions

4.1 C1. Cofactors

Table 12: C1. Cofactors

Simulation	Definition			Solution													
				exchanges		reactions								transport			Prot
	Objective	Contraints	Comment	imports	exports	c	m	r	p	l	n	s	b	s-c	b-c	intra	syn
450 CoA	CoA(c)	MCES −Pantothenate AAA	de novo synthesis of cytosolic CoA	2.17 P_i(c) 1 Glycine(s) 2 Aspartate(s) 2 Glutamine(s) 0.83 Glucose-6P(c) 0.5 Cystine(s) 1 Pantothenate(s) 11.2 ATP-energy(c) 2 THF-activated methyl group(c)	5.17 H_2O(s) 1 CoA(c) 2 CO_2(s) 2 Pyruvate(c) 2 Glutamate(s) 2 NADH-redox-potential(c) 3.5 NADPH-redox-potential(c) 2 Proton-gradient(c) 5 Na-gradient(c)	42	-	-	-	-	-	-	-	10	-	-	-
451 NAD^+	NAD^+(c)	−Nicotinamide MCES AAA	de novo synthesis of cytosolic NAD^+	0.33 P_i(c) 1 Glycine(s) 1.67 Glucose-6P(c) 2 Asparagine(s) 1 Nicotinamide(s) 16.3 ATP-energy(c) 2 THF-activated methyl group(c)	3.33 H_2O(s) 1 NAD^+(c) 1 CO_2(s) 2 Pyruvate(c) 2 NADH-redox-potential(c) 4 NADPH-redox-potential(c) 1 Proton-gradient(c) 2 Na-gradient(c)	40	-	-	-	-	-	-	-	7	-	-	-
452 $NADP^+$	$NADP^+$(c)	−Nicotinamide MCES AAA	de novo synthesis of cytosolic $NADP^+$	1.33 P_i(c) 1 Glycine(s) 1.67 Glucose-6P(c) 2 Asparagine(s) 1 Nicotinamide(s) 17.3 ATP-energy(c) 2 THF-activated methyl group(c)	4.33 H_2O(s) 1 $NADP^+$(c) 1 CO_2(s) 2 Pyruvate(c) 2 NADH-redox-potential(c) 4 NADPH-redox-potential(c) 1 Proton-gradient(c) 2 Na-gradient(c)	41	-	-	-	-	-	-	-	7	-	-	-
453 FAD	FAD(c)	−Riboflavin MCES AAA	de novo synthesis of cytosolic FAD	1.17 P_i(c) 1 Glycine(s) 2 Aspartate(s) 2 Glutamine(s) 0.83 Glucose-6P(c) 1 Riboflavin(s) 10.2 ATP-energy(c) 2 THF-activated methyl group(c)	3.17 H_2O(s) 1 CO_2(s) 1 FAD(c) 2 Pyruvate(c) 2 Glutamate(s) 2 NADH-redox-potential(c) 4 NADPH-redox-potential(c) 2 Na-gradient(c)	36	-	-	-	-	-	-	-	8	-	-	-
454 Glycogenin	Glycogenin(c)	MCES AAA	de novo synthesis of cytosolic Glycogenin	15 Glutamate(s) 18 Glycine(s) 22 Alanine(s) 18 Lysine(s) 26 Aspartate(s) 12 Arginine(s) 14 Glutamine(s) 31 Serine(s) 8 Methionine(s) 7 Tryptophan(s) 20 Phenylalanine(s) 13 Tyrosine(s) 6 Cysteine(s) 39 Leucine(s) 9 Histidine(s) 15 Proline(s) 12 Asparagine(s) 28 Valine(s) 25 Threonine(s) 12 Isoleucine(s) 1750 ATP-energy(c) 6 Proton-gradient(c)	1 Glycogenin(c) 179 Na-gradient(c)	3	-	-	-	-	-	-	-	22	-	-	1

Continued on next page

Simulation	Definition			Solution													
				exchanges		reactions								transport			Prot
	Objective	Contraints	Comment	imports	exports	c	m	r	p	l	n	s	b	s-c	b-c	intra	syn
455 Thioredoxin	Thioredoxin(c)	MCES AAA	de novo synthesis of cytosolic Thioredoxin	10 Glutamate(s) 5 Glycine(s) 8 Alanine(s) 12 Lysine(s) 7 Aspartate(s) 5 Glutamine(s) 7 Serine(s) 3 Methionine(s) 1 Tryptophan(s) 9 Phenylalanine(s) 1 Tyrosine(s) 5 Cysteine(s) 6 Leucine(s) 1 Histidine(s) 3 Proline(s) 3 Asparagine(s) 11 Valine(s) 4 Threonine(s) 4 Isoleucine(s) 525 ATP-energy(c) 2 Proton-gradient(c)	1 Thioredoxin(c) 59 Na-gradient(c)	3	-	-	-	-	-	-	-	21	-	-	1
456 Thioredoxin(m)	mitoThioredoxin(m)	MCES AAA	de novo synthesis of mitochondrial mitoThioredoxin	6 Glutamate(s) 10 Glycine(s) 11 Alanine(s) 11 Lysine(s) 12 Aspartate(s) 9 Arginine(s) 10 Glutamine(s) 8 Serine(s) 4 Methionine(s) 2 Tryptophan(s) 6 Phenylalanine(s) 2 Tyrosine(s) 3 Cysteine(s) 15 Leucine(s) 3 Histidine(s) 12 Proline(s) 4 Asparagine(s) 17 Valine(s) 12 Threonine(s) 9 Isoleucine(s) 830 ATP-energy(c) 6 Proton-gradient(c)	1 mitoThioredoxin(m) 72 Na-gradient(c)	3	-	-	-	-	-	-	-	22	-	1	1
457 Apo-ACP	Apo-ACP(c)	MCES AAA	de novo synthesis of cytosolic Apo-ACP	5 Glutamate(s) 6 Glycine(s) 11 Alanine(s) 4 Lysine(s) 8 Aspartate(s) 9 Arginine(s) 7 Glutamine(s) 8 Serine(s) 5 Methionine(s) 3 Phenylalanine(s) 4 Tyrosine(s) 2 Cysteine(s) 20 Leucine(s) 1 Histidine(s) 9 Proline(s) 1 Asparagine(s) 11 Valine(s) 6 Threonine(s) 4 Isoleucine(s) 620 ATP-energy(c) 5 Proton-gradient(c)	1 Apo-ACP(c) 47 Na-gradient(c)	3	-	-	-	-	-	-	-	21	-	-	1

Continued on next page

C1. Cofactors – continued.

Simulation	Definition			Solution													
				exchanges		reactions								transport			Prot
	Objective	Contraints	Comment	imports	exports	c	m	r	p	l	n	s	b	s-c	b-c	intra	syn
458 Apo-ACP(m)	mitoApo-ACP(m)	MCES AAA	de novo synthesis of mitochondrial mitoApo-ACP	22 Pyruvate(m) 8.5 Alanine(s) 7 Lysine(s) 1 Arginine(s) 12 Glutamine(s) 4.5 Serine(s) 5 Methionine(s) 3 Phenylalanine(s) 4 Tyrosine(s) 1 Cysteine(s) 10 Leucine(s) 1 Histidine(s) 5 Proline(s) 1 Asparagine(s) 6 Valine(s) 1 Threonine(s) 8 Isoleucine(s) 435 ATP-energy(m) 13 NADPH-redox-potential(m) 34 Proton-gradient(m)	18.5 NADH-redox-potential(m) 8 Proton-gradient(c) 1 mitoApo-ACP(m) 46.5 Na-gradient(c) 14.5 CoA-activated acetyl group(m)	-	15	-	-	-	-	-	-	18	-	17	1
459 THF	THF(c)	−Folate MCES	de novo synthesis of cytosolic THF	1 Folate(s) 1 NADH-redox-potential(c) 1 NADPH-redox-potential(c)	1 THF(c)	4	-	-	-	-	-	-	-	1	-	-	-
460 Pyridoxal-P	Pyridoxal-P(c)	−Pyridoxine MCES	de novo synthesis of cytosolic Pyridoxal-P	0.5 O_2(s) 1 P_i(c) 1 Pyridoxine(s) 1 ATP-energy(c)	2 H_2O(s) 1 Pyridoxal-P(c)	4	-	-	-	-	-	-	-	3	-	-	-
461 4ppan	D-4-Phosphopantothenate(c)	−Pantothenate MCES	de novo synthesis of cytosolic D-4-Phosphopantothenate	1 P_i(c) 1 Pantothenate(s) 1 ATP-energy(c)	1 H_2O(s) 1 D-4-Phosphopantothenate(c) 1 Na-gradient(c)	2	-	-	-	-	-	-	-	3	-	-	-
462 thbpt	Tetrahydrobiopterin(c)	−Glutamine(c) MCES	de novo synthesis of cytosolic Tetrahydrobiopterin	1.83 H_2O(s) 1.25 O_2(s) 2.5 Glutamine(c) 1.33 Glucose-6P(c) 9.67 ATP-energy(c)	1.33 P_i(c) 4 CO_2(s) 2.5 Pyruvate(c) 1 Tetrahydrobiopterin(c) 1.5 ATP-energy(m) 4 NADH-redox-potential(c) 2.5 NADH-redox-potential(m) 1.5 NADPH-redox-potential(c) 0.5 NADPH-redox-potential(m) 14 Proton-gradient(m)	47	9	-	-	-	-	-	-	3	-	12	-

Continued on next page

Simulation	Definition			Solution													
				exchanges		reactions								transport			Prot
	Objective	Contraints	Comment	imports	exports	c	m	r	p	l	n	s	b	s-c	b-c	intra	syn
463 dhbpt	Dihydrobiopterin(c)	−Glutamine(c) MCES	de novo synthesis of cytosolic Dihydrobiopterin	1.83 H_2O(s) 1.25 O_2(s) 2.5 Glutamine(c) 1.33 Glucose-6P(c) 9.67 ATP-energy(c)	1.33 P_i(c) 4 CO_2(s) 2.5 Pyruvate(c) 1 Dihydrobiopterin(c) 1.5 ATP-energy(m) 4 NADH-redox-potential(c) 4 NADH-redox-potential(m) 1.5 NADPH-redox-potential(c) 13 Proton-gradient(m)	46	10	-	-	-	-	-	-	3	-	13	-
464 mlthf	5,10-Methylene-THF(c)	−Folate MCES	de novo synthesis of cytosolic 5,10-Methylene-THF	1 Folate(s) 1 NADPH-redox-potential(c) 1 THF-activated methyl group(c)	1 5,10-Methylene-THF(c)	5	-	-	-	-	-	-	-	1	-	-	-
465 acgam6p	N-Acetylglucosamine-6P(c)	MCES AAA	de novo synthesis of cytosolic N-Acetylglucosamine-6P	1 Glutamine(s) 1 Glucose-6P(c) 1 CoA-activated acetyl group(c)	1 Glutamate(s) 1 N-Acetylglucosamine-6P(c)	4	-	-	-	-	-	-	-	2	-	-	-
466 THF(m)	THF(m)	−Folate MCES	de novo synthesis of mitochondrial THF	1 Folate(s) 2 NADPH-redox-potential(m)	1 THF(m)	-	3	-	-	-	-	-	-	1	-	1	-
467 1fthf	10-Formyl-THF(c)	−Folate MCES	de novo synthesis of cytosolic 10-Formyl-THF	1 H_2O(s) 1 Folate(s) 1 THF-activated methyl group(c)	1 10-Formyl-THF(c)	6	-	-	-	-	-	-	-	2	-	-	-
468 PAPS	PAPS(c)	−Glycine(c) MCES	de novo synthesis of cytosolic PAPS	1 P_i(c) 5 Glycine(c) 1 Glucose-6P(c) 12 ATP-energy(c) 1 activated-sulphur(c) 6 THF-activated methyl group(c)	1 H_2O(s) 4 Pyruvate(c) 1 PAPS(c) 8 NADH-redox-potential(c) 2 NADPH-redox-potential(c)	45	-	-	-	-	-	-	-	1	-	-	-
469 ametam	S-Adenosylmethioninamine(c)	−Methionine(c) −Glutamine(c) +Glutamate(c) MCES	de novo synthesis of cytosolic S-Adenosylmethioninamine	5 Glutamine(c) 1 Methionine(c) 1.33 Glucose-6P(c) 1 Activated methyl group(c) 8.67 ATP-energy(c) 3 NADPH-redox-potential(m) 4 Proton-gradient(m)	0.67 H_2O(s) 1.33 P_i(c) 5 Glutamate(c) 1 S-Adenosylmethioninamine(c) 2 ATP-energy(m) 4 NADH-redox-potential(c) 3 NADPH-redox-potential(c)	46	5	-	-	-	-	-	-	1	-	8	-

Continued on next page

C1. Cofactors – continued.

Simulation	Objective	Contraints	Comment	imports	exports	c	m	r	p	l	n	s	b	s-c	b-c	intra	Prot syn
	Definition			Solution: exchanges		reactions								transport			
470 GSH	GSH(c)	−Cysteine(c) −Glutamine(c) +Glutamate(c) MCES	de novo synthesis of cytosolic GSH	1 Glutamine(c) 0.5 Glucose-6P(c) 1 Cysteine(c) 1.5 ATP-energy(c) 1 NADH-redox-potential(m) 4 Proton-gradient(m)	1.5 H_2O(s) 0.5 P_i(c) 1 GSH(c) 1 NADH-redox-potential(c) 1 THF-activated methyl group(c)	16	3	-	-	-	-	-	-	1	-	6	-
471 Homocysteine	Homocysteine(c)	−Cysteine(c) MCES	de novo synthesis of cytosolic Homocysteine	1 H_2O(s) 1 Pyruvate(m) 1 Cysteine(c) 1 NADPH-redox-potential(m) 1 Proton-gradient(m) 1 CoA-activated acetyl group(m)	1 CO_2(s) 1 Pyruvate(c) 1 Homocysteine(c) 1 NADH-redox-potential(m) 1 NADPH-redox-potential(c)	7	11	-	-	-	-	-	-	2	-	4	-

4.2 C2. Precursors for macromolecules

Table 13: C2. Precursors for macromolecules

Simulation	Objective	Contraints	Comment	imports	exports	c	m	r	p	l	n	s	b	s-c	b-c	intra	Prot syn
	Definition			Solution: exchanges		reactions								transport			
**C2.1. For DNA**																	
472 dATP	dATP(c)	−Glutamine(c) +Glutamate(c) −Aspartate(c) MCES	de novo synthesis of cytosolic dATP	1.67 P_i(c) 3 Aspartate(c) 2 Glutamine(c) 1.33 Glucose-6P(c) 8.67 ATP-energy(c) 1 THF-activated methyl group(c)	3.67 H_2O(s) 2 CO_2(s) 3 Pyruvate(c) 2 Glutamate(c) 1 dATP(c) 1 NADH-redox-potential(c) 5 NADPH-redox-potential(c)	44	-	-	-	-	-	-	-	2	-	-	-
473 dCTP	dCTP(c)	−Glutamine(c) +Glutamate(c) −Aspartate(c) −NH_3(c) MCES	de novo synthesis of cytosolic dCTP	0.5 O_2(s) 2.17 P_i(c) 1 Aspartate(c) 2 Glutamine(c) 0.83 Glucose-6P(c) 7.17 ATP-energy(c) 1 NADPH-redox-potential(c)	6.17 H_2O(s) 2 Glutamate(c) 1 dCTP(c) 6 Proton-gradient(m)	26	-	-	-	-	-	-	-	2	-	6	-
474 dGTP	dGTP(c)	−Glutamine(c) +Glutamate(c) −Aspartate(c) MCES	de novo synthesis of cytosolic dGTP	1.67 P_i(c) 2 Aspartate(c) 3 Glutamine(c) 1.33 Glucose-6P(c) 9.67 ATP-energy(c) 1 THF-activated methyl group(c)	2.67 H_2O(s) 1 CO_2(s) 2 Pyruvate(c) 3 Glutamate(c) 1 dGTP(c) 2 NADH-redox-potential(c) 4 NADPH-redox-potential(c)	45	-	-	-	-	-	-	-	2	-	-	-

Continued on next page

C2. Precursors for macromolecules – continued.

Simulation	Definition			Solution													
				exchanges		reactions								transport			Prot
	Objective	Contraints	Comment	imports	exports	c	m	r	p	l	n	s	b	s-c	b-c	intra	syn
475 dTTP	dTTP(c)	−Glutamine(c) +Glutamate(c) −Aspartate(c) MCES	de novo synthesis of cytosolic dTTP	0.5 O_2(s) 2.17 P_i(c) 1 Aspartate(c) 1 Glutamine(c) 0.83 Glucose-6P(c) 7.17 ATP-energy(c) 1 ATP-energy(m) 1 NADH-redox-potential(c) 1 THF-activated methyl group(c)	6.17 H_2O(s) 1 Glutamate(c) 1 dTTP(c) 6 Proton-gradient(m)	30	4	-	-	-	-	-	-	2	-	8	-
**C2.2. For RNA**																	
476 ATP	ATP(c)	−Aspartate(c) +Glutamate(c) −Glutamine(c) MCES	de novo synthesis of cytosolic ATP	1.67 P_i(c) 3 Aspartate(c) 2 Glutamine(c) 1.33 Glucose-6P(c) 8.67 ATP-energy(c) 1 THF-activated methyl group(c)	2.67 H_2O(s) 1 ATP(c) 2 CO_2(s) 3 Pyruvate(c) 2 Glutamate(c) 1 NADH-redox-potential(c) 6 NADPH-redox-potential(c)	42	-	-	-	-	-	-	-	2	-	-	-
477 CTP	CTP(c)	−Glutamine(c) +Glutamate(c) −Aspartate(c) −NH_3(c) MCES	de novo synthesis of cytosolic CTP	0.5 O_2(s) 2.17 P_i(c) 1 Aspartate(c) 2 Glutamine(c) 0.83 Glucose-6P(c) 7.17 ATP-energy(c)	5.17 H_2O(s) 2 Glutamate(c) 1 CTP(c) 6 Proton-gradient(m)	23	-	-	-	-	-	-	-	2	-	6	-
478 GTP	GTP(c)	−Glutamine(c) +Glutamate(c) −Aspartate(c) MCES	de novo synthesis of cytosolic GTP	1.67 P_i(c) 2 Aspartate(c) 3 Glutamine(c) 1.33 Glucose-6P(c) 9.67 ATP-energy(c) 1 THF-activated methyl group(c)	1.67 H_2O(s) 1 CO_2(s) 2 Pyruvate(c) 3 Glutamate(c) 1 GTP(c) 2 NADH-redox-potential(c) 5 NADPH-redox-potential(c)	43	-	-	-	-	-	-	-	2	-	-	-
479 UTP	UTP(c)	−Glutamine(c) +Glutamate(c) −Aspartate(c) MCES	de novo synthesis of cytosolic UTP	0.5 O_2(s) 2.17 P_i(c) 1 Aspartate(c) 1 Glutamine(c) 0.83 Glucose-6P(c) 6.17 ATP-energy(c)	5.17 H_2O(s) 1 Glutamate(c) 1 UTP(c) 6 Proton-gradient(m)	22	-	-	-	-	-	-	-	2	-	6	-
**C2.3. For proteins**																	
480 Alanine	Alanine(c)	−NH_3(c) MCES	de novo synthesis of cytosolic Alanine	1 NH_3(c) 1 Pyruvate(c) 1 NADH-redox-potential(m) 2 Proton-gradient(m)	1 H_2O(s) 1 Alanine(c)	1	2	-	-	-	-	-	-	1	-	7	-
481 Arginine	Arginine(c)	−NH_3(c) MCES	de novo synthesis of cytosolic Arginine	4 NH_3(c) 2 Pyruvate(m) 2 ATP-energy(c) 3 ATP-energy(m) 2 NADPH-redox-potential(m) 3 Proton-gradient(m)	4 H_2O(s) 1 Arginine(c) 1 NADH-redox-potential(c)	9	15	-	-	-	-	-	-	1	-	8	-

Continued on next page

C2. Precursors for macromolecules – continued.

Simulation	Definition			Solution													
				exchanges		reactions								transport			Prot
	Objective	Contraints	Comment	imports	exports	c	m	r	p	l	n	s	b	s-c	b-c	intra	syn
482 Asparagine	Asparagine(c)	−Histidine(c) MCES	de novo synthesis of cytosolic Asparagine	0.67 H_2O(s) 0.33 O_2(s) 0.33 Pyruvate(m) 0.67 Histidine(c) 3 ATP-energy(c) 0.67 NADPH-redox-potential(c) 0.33 NADPH-redox-potential(m)	0.33 CO_2(s) 1 Asparagine(c) 0.67 ATP-energy(m) 0.33 NADH-redox-potential(c) 0.33 NADH-redox-potential(m) 3.67 Proton-gradient(m) 0.67 THF-activated methyl group(c)	17	10	-	-	-	-	-	-	3	-	10	-
483 Aspartate	Aspartate(c)	−Histidine(c) MCES	de novo synthesis of cytosolic Aspartate	0.33 H_2O(s) 0.17 O_2(s) 1 Pyruvate(m) 0.33 Histidine(c) 0.33 NADPH-redox-potential(c) 0.67 NADPH-redox-potential(m)	1 Aspartate(c) 0.33 ATP-energy(m) 0.67 NADH-redox-potential(c) 1.33 Proton-gradient(m) 0.33 THF-activated methyl group(c) 0.33 CoA-activated acetyl group(m)	12	11	-	-	-	-	-	-	2	-	9	-
484 Glutamate	Glutamate(c)	$-NH_3$(c) MCES	de novo synthesis of cytosolic Glutamate	1 NH_3(c) 1 Pyruvate(m) 1 NADPH-redox-potential(m) 1 Proton-gradient(m) 1 CoA-activated acetyl group(m)	1 Glutamate(c) 1 NADH-redox-potential(m)	-	10	-	-	-	-	-	-	-	-	4	-
485 Glycine	Glycine(c)	$-NH_3$(c) MCES	de novo synthesis of cytosolic Glycine	1 NH_3(c) 0.5 Glucose-6P(c) 1 NADH-redox-potential(m) 2 Proton-gradient(m)	0.5 H_2O(s) 0.5 P_i(c) 1 Glycine(c) 0.5 ATP-energy(c) 1 NADH-redox-potential(c) 1 THF-activated methyl group(c)	14	2	-	-	-	-	-	-	1	-	7	-
486 Glutamine	Glutamine(c)	$-NH_3$(c) MCES	de novo synthesis of cytosolic Glutamine	2 NH_3(c) 1 Pyruvate(m) 1 ATP-energy(c) 1 NADPH-redox-potential(m) 1 Proton-gradient(m) 1 CoA-activated acetyl group(m)	1 H_2O(s) 1 Glutamine(c) 1 NADH-redox-potential(m)	2	10	-	-	-	-	-	-	1	-	4	-
487 Proline	Proline(c)	−Valine(c) MCES	de novo synthesis of cytosolic Proline	1 O_2(s) 1 Valine(c) 1 ATP-energy(m) 1 NADPH-redox-potential(m)	2 H_2O(s) 1 Proline(c) 2 NADH-redox-potential(m) 10 Proton-gradient(m)	-	16	-	-	-	-	-	-	2	-	7	-
488 Serine	Serine(c)	$-NH_3$(c) MCES	de novo synthesis of cytosolic Serine	0.5 H_2O(s) 1 NH_3(c) 0.5 Glucose-6P(c) 1 NADH-redox-potential(m) 2 Proton-gradient(m)	0.5 P_i(c) 1 Serine(c) 0.5 ATP-energy(c) 2 NADH-redox-potential(c)	11	2	-	-	-	-	-	-	1	-	7	-
489 Tyrosine	Tyrosine(c)	−Phenylalanine(c) MCES	de novo synthesis of cytosolic Tyrosine	1 O_2(s) 1 Phenylalanine(c) 1 NADH-redox-potential(c)	1 H_2O(s) 1 Tyrosine(c)	3	-	-	-	-	-	-	-	2	-	-	-
**C2.4. For lipids**																	

Continued on next page

C2. Precursors for macromolecules – continued.

Simulation	Definition			Solution													
				exchanges		reactions								transport			Prot
	Objective	Contraints	Comment	imports	exports	c	m	r	p	l	n	s	b	s-c	b-c	intra	syn
490 Palmitate	Palmitate(c)	MCES	de novo synthesis of cytosolic Palmitate	7 ATP-energy(c) 14 NADPH-redox-potential(c) 8 CoA-activated acetyl group(c)	6 H_2O(s) 1 Palmitate(c)	37	-	-	-	-	-	-	-	1	-	-	-
491 Glycerol	Glycerol(c)	MCES	de novo synthesis of cytosolic Glycerol	0.5 H_2O(s) 0.5 Glucose-6P(c) 4.5 ATP-energy(c) 1 NADH-redox-potential(c)	0.5 P_i(c) 1 Glycerol(c)	26	-	-	-	-	-	-	-	1	-	-	-
492 Oleate	Oleate(c)	MCES	de novo synthesis of cytosolic Oleate	1 O_2(s) 10 ATP-energy(c) 1 NADH-redox-potential(c) 16 NADPH-redox-potential(c) 9 CoA-activated acetyl group(c)	9 H_2O(s) 1 Oleate(c)	47	-	-	-	-	-	-	-	2	-	-	-
493 Stearate	Stearate(c)	MCES	de novo synthesis of cytosolic Stearate	8 ATP-energy(c) 16 NADPH-redox-potential(c) 9 CoA-activated acetyl group(c)	7 H_2O(s) 1 Stearate(c)	41	-	-	-	-	-	-	-	1	-	-	-
**C2.5. Activated sugars**																	
494 Mannose-1P	Mannose-1P(c)	MCES	de novo synthesis of cytosolic Mannose-1P	1 Glucose-6P(c)	1 Mannose-1P(c)	3	-	-	-	-	-	-	-	-	-	-	-
495 Mannose-6P	Mannose-6P(c)	MCES	de novo synthesis of cytosolic Mannose-6P	1 Glucose-6P(c)	1 Mannose-6P(c)	2	-	-	-	-	-	-	-	-	-	-	-
496 Fructose-6P	Fructose-6P(c)	MCES	de novo synthesis of cytosolic Fructose-6P	1 Glucose-6P(c)	1 Fructose-6P(c)	1	-	-	-	-	-	-	-	-	-	-	-
497 UDP-xylose	UDP-xylose(c)	−Glutamine(c) +Glutamate(c) −Aspartate(c) MCES	de novo synthesis of cytosolic UDP-xylose	0.5 O_2(s) 0.17 P_i(c) 1 Aspartate(c) 1 Glutamine(c) 1.83 Glucose-6P(c) 6.17 ATP-energy(c)	3.17 H_2O(s) 1 CO_2(s) 1 Glutamate(c) 1 UDP-xylose(c) 2 NADH-redox-potential(c) 6 Proton-gradient(m)	26	-	1	-	-	-	-	-	3	-	9	-
498 UDP-glucose	UDP-glucose(c)	MCES −Glutamine(c) +Glutamate(c) −Aspartate(c)	de novo synthesis of cytosolic UDP-glucose	0.5 O_2(s) 0.17 P_i(c) 1 Aspartate(c) 1 Glutamine(c) 1.83 Glucose-6P(c) 6.17 ATP-energy(c)	4.17 H_2O(s) 1 Glutamate(c) 1 UDP-glucose(c) 6 Proton-gradient(m)	24	-	-	-	-	-	-	-	2	-	6	-

Continued on next page

Simulation	Definition			Solution													
				exchanges		reactions								transport			Prot
	Objective	Contraints	Comment	imports	exports	c	m	r	p	l	n	s	b	s-c	b-c	intra	syn
499 udpgal	UDP-galactose(c)	MCES −Glutamine(c) +Glutamate(c) −Aspartate(c)	de novo synthesis of cytosolic UDP-galactose	0.5 O_2(s) 0.17 P_i(c) 1 Aspartate(c) 1 Glutamine(c) 1.83 Glucose-6P(c) 6.17 ATP-energy(c)	4.17 H_2O(s) 1 Glutamate(c) 1 UDP-galactose(c) 6 Proton-gradient(m)	25	-	-	-	-	-	-	-	2	-	6	-
500 udpglcur	UDP-glucuronate(c)	MCES −Glutamine(c) +Glutamate(c) −Aspartate(c)	de novo synthesis of cytosolic UDP-glucuronate	0.5 O_2(s) 0.17 P_i(c) 1 Aspartate(c) 1 Glutamine(c) 1.83 Glucose-6P(c) 6.17 ATP-energy(c)	3.17 H_2O(s) 1 Glutamate(c) 1 UDP-glucuronate(c) 2 NADH-redox-potential(c) 6 Proton-gradient(m)	26	-	-	-	-	-	-	-	2	-	6	-
501 GDP-L-fucose	GDP-L-fucose(c)	MCES −Glutamine(c) +Glutamate(c) −Aspartate(c)	de novo synthesis of cytosolic GDP-L-fucose	2 Aspartate(c) 3 Glutamine(c) 2.33 Glucose-6P(c) 9.67 ATP-energy(c) 1 THF-activated methyl group(c)	1.67 H_2O(s) 0.33 P_i(c) 1 CO_2(s) 2 Pyruvate(c) 3 Glutamate(c) 1 GDP-L-fucose(c) 2 NADH-redox-potential(c) 4 NADPH-redox-potential(c)	48	-	-	-	-	-	-	-	2	-	-	-
502 GDP-mannose	GDP-mannose(c)	MCES −Glutamine(c) +Glutamate(c) −Aspartate(c)	de novo synthesis of cytosolic GDP-mannose	2 Aspartate(c) 3 Glutamine(c) 2.33 Glucose-6P(c) 9.67 ATP-energy(c) 1 THF-activated methyl group(c)	0.67 H_2O(s) 0.33 P_i(c) 1 CO_2(s) 2 Pyruvate(c) 3 Glutamate(c) 1 GDP-mannose(c) 2 NADH-redox-potential(c) 5 NADPH-redox-potential(c)	46	-	-	-	-	-	-	-	2	-	-	-
**C2.6. Other**																	
503 IMP	IMP(c)	−Glutamine(c) +Glutamate(c) −Aspartate(c) MCES	de novo synthesis of cytosolic IMP	2 Aspartate(c) 2 Glutamine(c) 1.33 Glucose-6P(c) 5.67 ATP-energy(c) 1 THF-activated methyl group(c)	0.67 H_2O(s) 0.33 P_i(c) 1 CO_2(s) 2 Pyruvate(c) 2 Glutamate(c) 1 IMP(c) 1 NADH-redox-potential(c) 5 NADPH-redox-potential(c)	39	-	-	-	-	-	-	-	2	-	-	-
504 XMP	Xanthosine-5P(c)	−Glutamine(c) +Glutamate(c) −Aspartate(c) MCES	de novo synthesis of cytosolic Xanthosine-5P	0.33 H_2O(s) 2 Aspartate(c) 2 Glutamine(c) 1.33 Glucose-6P(c) 5.67 ATP-energy(c) 1 THF-activated methyl group(c)	0.33 P_i(c) 1 CO_2(s) 2 Pyruvate(c) 2 Glutamate(c) 1 Xanthosine-5P(c) 2 NADH-redox-potential(c) 5 NADPH-redox-potential(c)	40	-	-	-	-	-	-	-	2	-	-	-
505 Uracil	Uracil(c)	−Glutamine(c) +Glutamate(c) −Aspartate(c) MCES	de novo synthesis of cytosolic Uracil	0.5 O_2(s) 1 Aspartate(c) 1 Glutamine(c) 3 ATP-energy(c)	2 H_2O(s) 1 Glutamate(c) 1 Uracil(c) 6 Proton-gradient(m)	14	-	-	-	-	-	-	-	2	-	6	-
506 Uridine	Uridine(c)	−Glutamine(c) +Glutamate(c) −Aspartate(c) MCES	de novo synthesis of cytosolic Uridine	0.5 O_2(s) 1 Aspartate(c) 1 Glutamine(c) 0.83 Glucose-6P(c) 3.17 ATP-energy(c)	2.17 H_2O(s) 0.83 P_i(c) 1 Glutamate(c) 1 Uridine(c) 6 Proton-gradient(m)	21	-	-	-	-	-	-	-	2	-	6	-

Continued on next page

C2. Precursors for macromolecules – continued.

Simulation	Definition			Solution													
				exchanges		reactions								transport			Prot
	Objective	Contraints	Comment	imports	exports	c	m	r	p	l	n	s	b	s-c	b-c	intra	syn
507 Cytidine	Cytidine(c)	−Glutamine(c) +Glutamate(c) −Aspartate(c) $-NH_3$(c) MCES	de novo synthesis of cytosolic Cytidine	0.5 O_2(s) 1 Aspartate(c) 2 Glutamine(c) 0.83 Glucose-6P(c) 5.17 ATP-energy(c)	2.17 H_2O(s) 0.83 P_i(c) 2 Glutamate(c) 1 Cytidine(c) 6 Proton-gradient(m)	26	-	-	-	-	-	-	-	2	-	6	-
508 Xanthine	Xanthine(c)	−Glutamine(c) +Glutamate(c) −Aspartate(c) MCES	de novo synthesis of cytosolic Xanthine	1.5 H_2O(s) 2 Aspartate(c) 2 Glutamine(c) 0.5 Glucose-6P(c) 5.5 ATP-energy(c) 1 THF-activated methyl group(c)	0.5 P_i(c) 1 CO_2(s) 2 Pyruvate(c) 2 Glutamate(c) 1 Xanthine(c) 3 NADH-redox-potential(c) 4 NADPH-redox-potential(c)	38	-	-	-	-	-	-	-	2	-	-	-
509 Guanine	Guanine(c)	−Glutamine(c) +Glutamate(c) −Aspartate(c) MCES	de novo synthesis of cytosolic Guanine	1.5 H_2O(s) 2 Aspartate(c) 3 Glutamine(c) 0.5 Glucose-6P(c) 7.5 ATP-energy(c) 1 THF-activated methyl group(c)	0.5 P_i(c) 1 CO_2(s) 2 Pyruvate(c) 3 Glutamate(c) 1 Guanine(c) 2 NADH-redox-potential(c) 5 NADPH-redox-potential(c)	39	-	-	-	-	-	-	-	2	-	-	-
510 Adenosine	Adenosine(c)	−Glutamine(c) +Glutamate(c) −Aspartate(c) MCES	de novo synthesis of cytosolic Guanine	0.33 H_2O(s) 3 Aspartate(c) 2 Glutamine(c) 1.33 Glucose-6P(c) 5.67 ATP-energy(c) 1 THF-activated methyl group(c)	1.33 P_i(c) 2 CO_2(s) 3 Pyruvate(c) 2 Glutamate(c) 1 Adenosine(c) 1 NADH-redox-potential(c) 6 NADPH-redox-potential(c)	43	-	-	-	-	-	-	-	2	-	-	-
511 UDP-N-acetylgalactosamine	UDP-N-acetyl-D-galactosamine(c)	MCES AAA	de novo synthesis of cytosolic UDP-N-acetyl-D-galactosamine	0.33 O_2(s) 0.17 P_i(c) 1 Glutamine(s) 1.83 Glucose-6P(c) 0.33 Proline(s) 1 Asparagine(s) 6.17 ATP-energy(c) 1 CoA-activated acetyl group(c)	3.17 H_2O(s) 1.33 Glutamate(s) 1 UDP-N-acetyl-D-galactosamine(c) 0.33 NADH-redox-potential(m) 1 Proton-gradient(c) 4 Proton-gradient(m) 1.33 Na-gradient(c)	29	4	-	-	-	-	-	-	8	-	6	-
512 N-acglucam	N-Acetylglucosamine(c)	MCES AAA	de novo synthesis of cytosolic N-Acetylglucosamine	1 Serine(s) 1 Glucose-6P(c) 1 ATP-energy(c) 1 CoA-activated acetyl group(c)	1 P_i(c) 1 Pyruvate(c) 1 N-Acetylglucosamine(c)	12	-	-	-	-	-	-	-	1	-	-	-
513 CMP-N-acetylneuraminate	CMP-N-acetylneuraminate(n)	MCES −CMP(c) +Glutamate −Glutamine =Asparagine =Proline =Cystine $=H_2S$ $=NH_3$	Synthesis of CMP-N-acetylneuraminate in nucleus	1 Pyruvate(c) 1 CMP(c) 1 Glutamine(s) 1 Glucose-6P(c) 5 ATP-energy(c) 1 NADPH-redox-potential(c) 1 CoA-activated acetyl group(c)	1 P_i(c) 1 Glutamate(s) 1 CMP-N-acetylneuraminate(n) 1 NADH-redox-potential(c)	21	-	-	-	-	1	-	-	2	-	3	-

4.3 C3. Constituents of cellular structures

Table 14: C3. Constituents of cellular structures

Simulation	Definition			Solution													
				exchanges		reactions								transport			Prot
	Objective	Contraints	Comment	imports	exports	c	m	r	p	l	n	s	b	s-c	b-c	intra	syn
**C3.1. Lipids**																	
514 PC	PC-VLDL-pool(c)	MCES ALFA	de novo synthesis of cytosolic PC-VLDL-pool	0.5 P_i(c) 0.5 Glucose-6P(c) 1 Choline(c) 0.17 Arachidonate(c) 0.7 Palmitate(c) 0.27 Oleate(c) 0.3 Stearate(c) 0.55 Linoleate(c) 7.5 ATP-energy(c) 1 NADH-redox-potential(c)	3.5 H_2O(s) 1 PC-VLDL-pool(c)	28	-	-	-	-	-	-	-	1	-	-	-
515 PE	PE-VLDL-pool(c)	MCES ALFA	de novo synthesis of cytosolic PE-VLDL-pool	0.5 P_i(c) 0.5 Glucose-6P(c) 1 Ethanolamine(c) 0.6 Arachidonate(c) 0.34 Palmitate(c) 0.17 Oleate(c) 0.54 Stearate(c) 0.36 Linoleate(c) 7.5 ATP-energy(c) 1 NADH-redox-potential(c)	3.5 H_2O(s) 1 PE-VLDL-pool(c)	28	-	-	-	-	-	-	-	1	-	-	-
516 PS	PS-VLDL-pool(c)	MCES ALFA	de novo synthesis of cytosolic PS-VLDL-pool	0.5 P_i(c) 1 Pyruvate(m) 0.5 Glucose-6P(c) 1 Ethanolamine(c) 0.53 Arachidonate(c) 0.09 Palmitate(c) 0.09 Oleate(c) 1.25 Stearate(c) 0.05 Linoleate(c) 7.5 ATP-energy(c) 1 NADH-redox-potential(c)	3.5 H_2O(s) 1 PS-VLDL-pool(c) 1 NADH-redox-potential(m) 1 CoA-activated acetyl group(m)	29	3	-	-	-	-	-	-	1	-	1	-
517 PI	PI-pool(c)	MCES ALFA	de novo synthesis of cytosolic PI-pool	1.5 Glucose-6P(c) 0.51 Arachidonate(c) 0.16 Palmitate(c) 0.32 Oleate(c) 0.85 Stearate(c) 0.16 Linoleate(c) 6.5 ATP-energy(c) 1 NADH-redox-potential(c)	2.5 H_2O(s) 0.5 P_i(c) 1 PI-pool(c)	28	-	-	-	-	-	-	-	1	-	-	-
518 CL	CL-pool(m)	MCES ALFA	de novo synthesis of mitochondrial CL-pool	2 Glucose-6P(c) 0.17 Palmitate(c) 0.56 Oleate(c) 0.08 Stearate(c) 3 Linoleate(c) 0.19 Palmitolate(c) 10 ATP-energy(c) 4 ATP-energy(m) 4 NADH-redox-potential(c) 1 Proton-gradient(m)	6 H_2O(s) 1 P_i(c) 1 CL-pool(m)	15	10	-	-	-	-	-	-	1	-	5	-

Continued on next page

Simulation	Definition			Solution													
				exchanges		reactions								transport			Prot
	Objective	Contraints	Comment	imports	exports	c	m	r	p	l	n	s	b	s-c	b-c	intra	syn
519 LacCer	LacCer-pool(r)	MCES ALFA	de novo synthesis of LacCer-pool in ER/Golgi	2.5 Glucose-6P(c) 1 Ethanolamine(c) 0.02 Arachidonate(c) 0.91 Palmitate(c) 0.24 Oleate(c) 0.03 gamma-Linolenate(c) 0.8 Palmitolate(c) 9.5 ATP-energy(c) 1 NADH-redox-potential(m) 1 NADPH-redox-potential(c) 2 Proton-gradient(m)	1.53 H_2O(s) 2.5 P_i(c) 1 CO_2(s) 3 NADH-redox-potential(c) 1 LacCer-pool(r) 1 CoA-activated acetyl group(c)	39	2	2	-	-	-	-	-	2	-	11	-
520 SM	SM-pool(c)	MCES ALFA	de novo synthesis of cytosolic SM-pool	0.5 P_i(c) 0.5 Glucose-6P(c) 1 Choline(c) 1 Ethanolamine(c) 0.02 Arachidonate(c) 0.91 Palmitate(c) 0.24 Oleate(c) 0.03 gamma-Linolenate(c) 0.8 Palmitolate(c) 9.5 ATP-energy(c) 1 NADPH-redox-potential(c) 1 NADPH-redox-potential(m) 2 Proton-gradient(m)	3.53 H_2O(s) 1 CO_2(s) 1 SM-pool(c) 3 NADH-redox-potential(c) 1 CoA-activated acetyl group(c)	37	2	1	-	-	-	-	-	2	-	11	-
521 Ceramide	Ceramide-pool(c)	MCES ALFA	de novo synthesis of cytosolic Ceramide-pool	0.5 Glucose-6P(c) 1 Ethanolamine(c) 0.02 Arachidonate(c) 0.91 Palmitate(c) 0.24 Oleate(c) 0.03 gamma-Linolenate(c) 0.8 Palmitolate(c) 6.5 ATP-energy(c) 1 NADPH-redox-potential(c) 1 NADPH-redox-potential(m) 2 Proton-gradient(m)	1.53 H_2O(s) 0.5 P_i(c) 1 CO_2(s) 1 Ceramide-pool(c) 3 NADH-redox-potential(c) 1 CoA-activated acetyl group(c)	33	2	-	-	-	-	-	-	2	-	7	-
522 Triacylglycerol	Triacylglycerol-VLDL-pool(r)	MCES ALFA	de novo synthesis of Triacylglycerol-VLDL-pool in ER/Golgi	0.5 Glucose-6P(c) 0.09 Arachidonate(c) 0.92 Palmitate(c) 1.1 Oleate(c) 0.2 Stearate(c) 0.69 Linoleate(c) 6.5 ATP-energy(c) 1 NADH-redox-potential(c)	2.5 H_2O(s) 0.5 P_i(c) 1 Triacylglycerol-VLDL-pool(r)	25	-	-	-	-	-	-	-	1	-	1	-
523 Cholesterol	Cholesterol(r)	MCES	de novo synthesis of Cholesterol in ER/Golgi	8 O_2(s) 2 Farnesyl-PP(r) 1 ATP-energy(c) 11 NADPH-redox-potential(r)	7 H_2O(s) 4 P_i(c) 3 CO_2(s) 1 Cholesterol(r) 1 NADH-redox-potential(r) 1 NADPH-redox-potential(c)	5	-	16	-	-	-	-	-	3	-	5	-

4.4 C4. Other building blocks

Table 15: C4. Other building blocks

Simulation	Definition			Solution													
				exchanges		reactions								transport			Prot
	Objective	Contraints	Comment	imports	exports	c	m	r	p	l	n	s	b	s-c	b-c	intra	syn
524 gdpddman	GDP-4-dehydro-6-deoxy-D-mannose(c)	MCES −Glutamine(c) +Glutamate(c) −Aspartate(c)	de novo synthesis of cytosolic GDP-4-dehydro-6-deoxy-D-mannose	2 Aspartate(c) 3 Glutamine(c) 2.33 Glucose-6P(c) 9.67 ATP-energy(c) 1 THF-activated methyl group(c)	1.67 H_2O(s) 0.33 P_i(c) 1 CO_2(s) 2 Pyruvate(c) 3 Glutamate(c) 1 GDP-4-dehydro-6-deoxy-D-mannose(c) 2 NADH-redox-potential(c) 5 NADPH-redox-potential(c)	47	-	-	-	-	-	-	-	2	-	-	-
525 beta-Alanine	beta-Alanine(c)	MCES −Glutamine(c) +Glutamate(c)	de novo synthesis of cytosolic beta-Alanine	0.33 H_2O(s) 0.17 O_2(s) 0.67 Pyruvate(m) 0.67 Glutamine(c) 1.33 NADPH-redox-potential(m)	0.67 CO_2(s) 0.33 Glutamate(c) 1 beta-Alanine(c) 0.67 NADH-redox-potential(c) 0.67 NADH-redox-potential(m) 1.67 Proton-gradient(m)	6	13	-	-	-	-	-	-	3	-	10	-
526 Putrescine	Putrescine(c)	MCES −Glutamine(c) +Glutamate(c)	de novo synthesis of cytosolic Putrescine	1 Glutamine(c) 1 NADH-redox-potential(m) 1 NADPH-redox-potential(m) 2 Proton-gradient(m)	1 H_2O(s) 1 CO_2(s) 1 Putrescine(c)	1	6	-	-	-	-	-	-	2	-	4	-
527 Spermidine	Spermidine	MCES +Methylthioribose-1P(c) −Glutamine(c) +Glutamate(c) −Methionine(c)	test that cytosolic Spermidine can be produced from the minimal exchange set while the cytosolic dump of Methylthioribose-1P is allowed	0.17 P_i(c) 1 Glutamine(c) 1 Methionine(c) 0.83 Glucose-6P(c) 4.17 ATP-energy(c) 1 NADH-redox-potential(m) 1 NADPH-redox-potential(m) 1 Proton-gradient(m)	2.17 H_2O(s) 2 CO_2(s) 1 Spermidine(s) 1 Methylthioribose-1P(c)	19	6	-	-	-	-	-	-	3	-	4	-

Continued on next page

C4. Other building blocks – continued.

Simulation	Definition			Solution													
				exchanges		reactions								transport			Prot
	Objective	Contraints	Comment	imports	exports	c	m	r	p	l	n	s	b	s-c	b-c	intra	syn
528 Spermine	Spermine	MCES +Methylthioribose-1P(c) −Glutamine(c) +Glutamate(c) −Methionine(c)	test that cytosolic Spermine can be produced from the minimal exchange set while the cytosolic dump of Methylthioribose-1P is allowed	0.33 P_i(c) 1 Glutamine(c) 2 Methionine(c) 1.67 Glucose-6P(c) 8.33 ATP-energy(c) 2 NADH-redox-potential(m) 1 Proton-gradient(m)	3.33 H_2O(s) 3 CO_2(s) 1 Spermine(s) 2 Methylthioribose-1P(c)	20	5	-	-	-	-	-	-	3	-	4	-
529 Methylthioribose-1P	Methylthioribose-1P(c)	+Spermidine MCES −Glutamine(c) +Glutamate(c) −Methionine(c)	test that cytosolic Methylthioribose-1P can be produced from the minimal exchange set while the cytosolic dump of Spermidine is allowed	0.17 P_i(c) 1 Methionine(c) 0.83 Glucose-6P(c) 4.17 ATP-energy(c)	1.17 H_2O(s) 1 CO_2(s) 1 Adenosylmethioninamine-potential(c) 1 Methylthioribose-1P(c)	18	-	-	-	-	-	-	-	2	-	-	-

4.5 C5. Comprehensive list

Table 16: C5. Comprehensive list

Simulation	Definition			Solution													
				exchanges		reactions								transport			Prot
	Objective	Contraints	Comment	imports	exports	c	m	r	p	l	n	s	b	s-c	b-c	intra	syn
530 (2E)-Decenoyl-ACP	(2E)-Decenoyl-ACP(c)	PIPES MCES	synthesis of cytosolic (2E)-Decenoyl-ACP	1 P_i(c) 5 Glutamate(s) 6 Glycine(s) 11 Alanine(s) 4 Lysine(s) 8 Aspartate(s) 9 Arginine(s) 7 Glutamine(s) 8 Serine(s) 5 Methionine(s) 3 Phenylalanine(s) 4 Tyrosine(s) 3 Cysteine(s) 20 Leucine(s) 1 Histidine(s) 9 Proline(s) 1 Asparagine(s) 11 Valine(s) 6 Threonine(s) 4 Isoleucine(s) 1 Pantothenate(s) 628 ATP-energy(c) 7 NADPH-redox-potential(c) 3 Proton-gradient(c) 5 CoA-activated acetyl group(c)	7 H_2O(s) 1 CO_2(s) 1 (2E)-Decenoyl-ACP(c) 66 Na-gradient(c)	34	-	-	-	-	-	-	-	24	-	-	1
531 (2E)-Decenoyl-CoA	(2E)-Decenoyl-CoA(m)	PIPES MCES	synthesis of mitochondrial (2E)-Decenoyl-CoA	0.5 O_2(s) 2.17 P_i(c) 1 Glycine(s) 2 Aspartate(s) 2 Glutamine(s) 0.83 Glucose-6P(c) 0.5 Cystine(s) 1 Pantothenate(s) 1 Palmitolate(s) 13.2 ATP-energy(c) 1 Proton-gradient(c) 2 THF-activated methyl group(c)	3.17 H_2O(s) 2 CO_2(s) 2 Pyruvate(c) 2 Glutamate(s) 1 (2E)-Decenoyl-CoA(m) 2 NADH-redox-potential(c) 4 NADH-redox-potential(m) 3.5 NADPH-redox-potential(c) 3 FADH-redox-potential(c) 6 Proton-gradient(m) 3 Na-gradient(c) 4 CoA-activated acetyl group(m)	44	19	-	-	-	-	-	-	12	-	7	-

Continued on next page

Simulation	Definition			Solution													
				exchanges		reactions								transport			Prot
	Objective	Contraints	Comment	imports	exports	c	m	r	p	l	n	s	b	s-c	b-c	intra	syn
532 (2E)-Dodecenoyl-ACP	(2E)-Dodecenoyl-ACP(c)	PIPES MCES	synthesis of cytosolic (2E)-Dodecenoyl-ACP	1 P_i(c) 5 Glutamate(s) 6 Glycine(s) 11 Alanine(s) 4 Lysine(s) 8 Aspartate(s) 9 Arginine(s) 7 Glutamine(s) 8 Serine(s) 5 Methionine(s) 3 Phenylalanine(s) 4 Tyrosine(s) 3 Cysteine(s) 20 Leucine(s) 1 Histidine(s) 9 Proline(s) 1 Asparagine(s) 11 Valine(s) 6 Threonine(s) 4 Isoleucine(s) 1 Pantothenate(s) 629 ATP-energy(c) 9 NADPH-redox-potential(c) 3 Proton-gradient(c) 6 CoA-activated acetyl group(c)	8 H_2O(s) 1 CO_2(s) 1 (2E)-Dodecenoyl-ACP(c) 66 Na-gradient(c)	38	-	-	-	-	-	-	-	24	-	-	1
533 (2E)-Dodecenoyl-CoA	(2E)-Dodecenoyl-CoA(m)	PIPES MCES	synthesis of mitochondrial (2E)-Dodecenoyl-CoA	2.17 P_i(c) 1 Glycine(s) 2 Aspartate(s) 2 Glutamine(s) 0.83 Glucose-6P(c) 0.5 Cystine(s) 1 Pantothenate(s) 1 Palmitolate(s) 13.2 ATP-energy(c) 1 Proton-gradient(c) 2 THF-activated methyl group(c)	3.17 H_2O(s) 2 CO_2(s) 2 Pyruvate(c) 2 Glutamate(s) 1 (2E)-Dodecenoyl-CoA(m) 2 NADH-redox-potential(c) 3 NADH-redox-potential(m) 3.5 NADPH-redox-potential(c) 3 FADH-redox-potential(c) 3 Na-gradient(c) 3 CoA-activated acetyl group(m)	44	15	-	-	-	-	-	-	11	-	3	-
534 (2E)-Hexadecenoyl-ACP	(2E)-Hexadecenoyl-ACP(c)	PIPES MCES	synthesis of cytosolic (2E)-Hexadecenoyl-ACP	1 P_i(c) 5 Glutamate(s) 6 Glycine(s) 11 Alanine(s) 4 Lysine(s) 8 Aspartate(s) 9 Arginine(s) 7 Glutamine(s) 8 Serine(s) 5 Methionine(s) 3 Phenylalanine(s) 4 Tyrosine(s) 3 Cysteine(s) 20 Leucine(s) 1 Histidine(s) 9 Proline(s) 1 Asparagine(s) 11 Valine(s) 6 Threonine(s) 4 Isoleucine(s) 1 Pantothenate(s) 631 ATP-energy(c) 13 NADPH-redox-potential(c) 3 Proton-gradient(c) 8 CoA-activated acetyl group(c)	10 H_2O(s) 1 CO_2(s) 1 (2E)-Hexadecenoyl-ACP(c) 66 Na-gradient(c)	46	-	-	-	-	-	-	-	24	-	-	1

Continued on next page

Simulation	Definition			Solution													
				exchanges		reactions								transport			Prot
	Objective	Contraints	Comment	imports	exports	c	m	r	p	l	n	s	b	s-c	b-c	intra	syn
535 (2E)-Hexadecenoyl-CoA	(2E)-Hexadecenoyl-CoA(c)	PIPES MCES	synthesis of cytosolic (2E)-Hexadecenoyl-CoA	2.17 P_i(c) 1 Glycine(s) 2 Aspartate(s) 2 Glutamine(s) 0.83 Glucose-6P(c) 0.5 Cystine(s) 1 Pantothenate(s) 1 Palmitolate(s) 13.2 ATP-energy(c) 1 Proton-gradient(c) 2 THF-activated methyl group(c)	6.17 H_2O(s) 2 CO_2(s) 2 Pyruvate(c) 2 Glutamate(s) 1 (2E)-Hexadecenoyl-CoA(c) 2 NADH-redox-potential(c) 3.5 NADPH-redox-potential(c) 3 Na-gradient(c)	43	-	-	-	-	-	-	-	11	-	-	-
536 (2E)-Hexenoyl-ACP	(2E)-Hexenoyl-ACP(c)	PIPES MCES	synthesis of cytosolic (2E)-Hexenoyl-ACP	1 P_i(c) 5 Glutamate(s) 6 Glycine(s) 11 Alanine(s) 4 Lysine(s) 8 Aspartate(s) 9 Arginine(s) 7 Glutamine(s) 8 Serine(s) 5 Methionine(s) 3 Phenylalanine(s) 4 Tyrosine(s) 3 Cysteine(s) 20 Leucine(s) 1 Histidine(s) 9 Proline(s) 1 Asparagine(s) 11 Valine(s) 6 Threonine(s) 4 Isoleucine(s) 1 Pantothenate(s) 626 ATP-energy(c) 3 NADPH-redox-potential(c) 3 Proton-gradient(c) 3 CoA-activated acetyl group(c)	5 H_2O(s) 1 CO_2(s) 1 (2E)-Hexenoyl-ACP(c) 66 Na-gradient(c)	26	-	-	-	-	-	-	-	24	-	-	1
537 (2E)-Hexenoyl-CoA	(2E)-Hexenoyl-CoA(m)	PIPES MCES	synthesis of mitochondrial (2E)-Hexenoyl-CoA	1.5 O_2(s) 2.17 P_i(c) 1 Glycine(s) 2 Aspartate(s) 2 Glutamine(s) 0.83 Glucose-6P(c) 0.5 Cystine(s) 1 Pantothenate(s) 1 Palmitolate(s) 13.2 ATP-energy(c) 2 THF-activated methyl group(c)	3.17 H_2O(s) 2 CO_2(s) 2 Pyruvate(c) 2 Glutamate(s) 1 (2E)-Hexenoyl-CoA(m) 2 NADH-redox-potential(c) 6 NADH-redox-potential(m) 3.5 NADPH-redox-potential(c) 3 FADH-redox-potential(c) 2 Proton-gradient(c) 18 Proton-gradient(m) 5 Na-gradient(c) 6 CoA-activated acetyl group(m)	44	27	-	-	-	-	-	-	12	-	7	-

Continued on next page

C5. Comprehensive list – continued.

Simulation	Definition			Solution													
				exchanges		reactions								transport			Prot
	Objective	Contraints	Comment	imports	exports	c	m	r	p	l	n	s	b	s-c	b-c	intra	syn
538 (2E)-Octadecenoyl-ACP	(2E)-Octadecenoyl-ACP(c)	PIPES MCES	synthesis of cytosolic (2E)-Octadecenoyl-ACP	1 P_i(c) 5 Glutamate(s) 6 Glycine(s) 11 Alanine(s) 4 Lysine(s) 8 Aspartate(s) 9 Arginine(s) 7 Glutamine(s) 8 Serine(s) 5 Methionine(s) 3 Phenylalanine(s) 4 Tyrosine(s) 3 Cysteine(s) 20 Leucine(s) 1 Histidine(s) 9 Proline(s) 1 Asparagine(s) 11 Valine(s) 6 Threonine(s) 4 Isoleucine(s) 1 Pantothenate(s) 632 ATP-energy(c) 15 NADPH-redox-potential(c) 3 Proton-gradient(c) 9 CoA-activated acetyl group(c)	11 H_2O(s) 1 CO_2(s) 1 (2E)-Octadecenoyl-ACP(c) 66 Na-gradient(c)	50	-	-	-	-	-	-	-	24	-	-	1
539 (2E)-Octenoyl-ACP	(2E)-Octenoyl-ACP(c)	PIPES MCES	synthesis of cytosolic (2E)-Octenoyl-ACP	1 P_i(c) 5 Glutamate(s) 6 Glycine(s) 11 Alanine(s) 4 Lysine(s) 8 Aspartate(s) 9 Arginine(s) 7 Glutamine(s) 8 Serine(s) 5 Methionine(s) 3 Phenylalanine(s) 4 Tyrosine(s) 3 Cysteine(s) 20 Leucine(s) 1 Histidine(s) 9 Proline(s) 1 Asparagine(s) 11 Valine(s) 6 Threonine(s) 4 Isoleucine(s) 1 Pantothenate(s) 627 ATP-energy(c) 5 NADPH-redox-potential(c) 3 Proton-gradient(c) 4 CoA-activated acetyl group(c)	6 H_2O(s) 1 CO_2(s) 1 (2E)-Octenoyl-ACP(c) 66 Na-gradient(c)	30	-	-	-	-	-	-	-	24	-	-	1

Continued on next page

C5. Comprehensive list – continued.

Simulation	Definition			Solution													
				exchanges		reactions								transport			Prot
	Objective	Contraints	Comment	imports	exports	c	m	r	p	l	n	s	b	s-c	b-c	intra	syn
540 (2E)-Octenoyl-CoA	(2E)-Octenoyl-CoA(m)	PIPES MCES	synthesis of mitochondrial (2E)-Octenoyl-CoA	1 O_2(s) 2.17 P_i(c) 1 Glycine(s) 2 Aspartate(s) 2 Glutamine(s) 0.83 Glucose-6P(c) 0.5 Cystine(s) 1 Pantothenate(s) 1 Palmitolate(s) 13.2 ATP-energy(c) 2 THF-activated methyl group(c)	3.17 H_2O(s) 2 CO_2(s) 2 Pyruvate(c) 2 Glutamate(s) 1 (2E)-Octenoyl-CoA(m) 2 NADH-redox-potential(c) 5 NADH-redox-potential(m) 3.5 NADPH-redox-potential(c) 3 FADH-redox-potential(c) 2 Proton-gradient(c) 12 Proton-gradient(m) 5 Na-gradient(c) 5 CoA-activated acetyl group(m)	44	23	-	-	-	-	-	-	12	-	7	-
541 (2E)-Tetradecenoyl-ACP	(2E)-Tetradecenoyl-ACP(c)	PIPES MCES	synthesis of cytosolic (2E)-Tetradecenoyl-ACP	1 P_i(c) 5 Glutamate(s) 6 Glycine(s) 11 Alanine(s) 4 Lysine(s) 8 Aspartate(s) 9 Arginine(s) 7 Glutamine(s) 8 Serine(s) 5 Methionine(s) 3 Phenylalanine(s) 4 Tyrosine(s) 3 Cysteine(s) 20 Leucine(s) 1 Histidine(s) 9 Proline(s) 1 Asparagine(s) 11 Valine(s) 6 Threonine(s) 4 Isoleucine(s) 1 Pantothenate(s) 630 ATP-energy(c) 11 NADPH-redox-potential(c) 3 Proton-gradient(c) 7 CoA-activated acetyl group(c)	9 H_2O(s) 1 CO_2(s) 1 (2E)-Tetradecenoyl-ACP(c) 66 Na-gradient(c)	42	-	-	-	-	-	-	-	24	-	-	1
542 (2E)-Tetradecenoyl-CoA	(2E)-Tetradecenoyl-CoA(m)	PIPES MCES	synthesis of mitochondrial (2E)-Tetradecenoyl-CoA	2.17 P_i(c) 1 Glycine(s) 2 Aspartate(s) 2 Glutamine(s) 0.83 Glucose-6P(c) 0.33 Proline(s) 0.17 Palmitate(s) 0.5 Cystine(s) 1 Pantothenate(s) 0.83 Palmitolate(s) 13.2 ATP-energy(c) 2 THF-activated methyl group(c)	4.5 H_2O(s) 2 CO_2(s) 2 Pyruvate(c) 2.33 Glutamate(s) 1 (2E)-Tetradecenoyl-CoA(m) 2.83 NADH-redox-potential(c) 1.33 NADH-redox-potential(m) 3.5 NADPH-redox-potential(c) 2 Proton-gradient(c) 10 Proton-gradient(m) 5.33 Na-gradient(c) 1 CoA-activated acetyl group(m)	47	11	-	-	-	-	-	-	13	-	7	-

Continued on next page

C5. Comprehensive list – continued.

Simulation		Definition			Solution													
					exchanges		reactions								transport			Prot
		Objective	Contraints	Comment	imports	exports	c	m	r	p	l	n	s	b	s-c	b-c	intra	syn
543	(3Z)-Dodecenoyl-CoA	(3Z)-Dodecenoyl-CoA(m)	PIPES MCES	synthesis of mitochondrial (3Z)-Dodecenoyl-CoA	2.17 P_i(c) 1 Glycine(s) 2 Aspartate(s) 2 Glutamine(s) 0.83 Glucose-6P(c) 0.5 Cystine(s) 1 Pantothenate(s) 1 Palmitolate(s) 13.2 ATP-energy(c) 1 Proton-gradient(c) 2 THF-activated methyl group(c)	3.17 H_2O(s) 2 CO_2(s) 2 Pyruvate(c) 2 Glutamate(s) 1 (3Z)-Dodecenoyl-CoA(m) 2 NADH-redox-potential(c) 3 NADH-redox-potential(m) 3.5 NADPH-redox-potential(c) 3 FADH-redox-potential(c) 3 Na-gradient(c) 3 CoA-activated acetyl group(m)	44	14	-	-	-	-	-	-	11	-	3	-
544	(R)-3-Hydroxybutanoyl-ACP	(R)-3-Hydroxybutanoyl-ACP(c)	PIPES MCES	synthesis of cytosolic (R)-3-Hydroxybutanoyl-ACP	1 P_i(c) 5 Glutamate(s) 6 Glycine(s) 11 Alanine(s) 4 Lysine(s) 8 Aspartate(s) 9 Arginine(s) 7 Glutamine(s) 8 Serine(s) 5 Methionine(s) 3 Phenylalanine(s) 4 Tyrosine(s) 3 Cysteine(s) 20 Leucine(s) 1 Histidine(s) 9 Proline(s) 1 Asparagine(s) 11 Valine(s) 6 Threonine(s) 4 Isoleucine(s) 1 Pantothenate(s) 625 ATP-energy(c) 1 NADPH-redox-potential(c) 2 CoA-activated acetyl group(c)	3 H_2O(s) 1 CO_2(s) 1 (R)-3-Hydroxybutanoyl-ACP(c) 2 Proton-gradient(c) 82 Na-gradient(c)	21	-	-	-	-	-	-	-	24	-	-	1
545	(R)-3-Hydroxydecanoyl-ACP	(R)-3-Hydroxydecanoyl-ACP(c)	PIPES MCES	synthesis of cytosolic (R)-3-Hydroxydecanoyl-ACP	1 P_i(c) 5 Glutamate(s) 6 Glycine(s) 11 Alanine(s) 4 Lysine(s) 8 Aspartate(s) 9 Arginine(s) 7 Glutamine(s) 8 Serine(s) 5 Methionine(s) 3 Phenylalanine(s) 4 Tyrosine(s) 3 Cysteine(s) 20 Leucine(s) 1 Histidine(s) 9 Proline(s) 1 Asparagine(s) 11 Valine(s) 6 Threonine(s) 4 Isoleucine(s) 1 Pantothenate(s) 628 ATP-energy(c) 7 NADPH-redox-potential(c) 3 Proton-gradient(c) 5 CoA-activated acetyl group(c)	6 H_2O(s) 1 CO_2(s) 1 (R)-3-Hydroxydecanoyl-ACP(c) 66 Na-gradient(c)	33	-	-	-	-	-	-	-	24	-	-	1

Continued on next page

C5. Comprehensive list – continued.

Simulation		Definition			Solution: exchanges		reactions								transport			Prot
		Objective	Contraints	Comment	imports	exports	c	m	r	p	l	n	s	b	s-c	b-c	intra	syn
546	(R)-3-Hydroxyoctanoyl-ACP	(R)-3-Hydroxyoctanoyl-ACP(c)	PIPES MCES	synthesis of cytosolic (R)-3-Hydroxyoctanoyl-ACP	1 P_i(c) 5 Glutamate(s) 6 Glycine(s) 11 Alanine(s) 4 Lysine(s) 8 Aspartate(s) 9 Arginine(s) 7 Glutamine(s) 8 Serine(s) 5 Methionine(s) 3 Phenylalanine(s) 4 Tyrosine(s) 3 Cysteine(s) 20 Leucine(s) 1 Histidine(s) 9 Proline(s) 1 Asparagine(s) 11 Valine(s) 6 Threonine(s) 4 Isoleucine(s) 1 Pantothenate(s) 627 ATP-energy(c) 5 NADPH-redox-potential(c) 3 Proton-gradient(c) 4 CoA-activated acetyl group(c)	5 H_2O(s) 1 CO_2(s) 1 (R)-3-Hydroxyoctanoyl-ACP(c) 66 Na-gradient(c)	29	-	-	-	-	-	-	-	24	-	-	1
547	(R)-3-Hydroxypalmitoyl-ACP	(R)-3-Hydroxypalmitoyl-ACP(c)	PIPES MCES	synthesis of cytosolic (R)-3-Hydroxypalmitoyl-ACP	1 P_i(c) 5 Glutamate(s) 6 Glycine(s) 11 Alanine(s) 4 Lysine(s) 8 Aspartate(s) 9 Arginine(s) 7 Glutamine(s) 8 Serine(s) 5 Methionine(s) 3 Phenylalanine(s) 4 Tyrosine(s) 3 Cysteine(s) 20 Leucine(s) 1 Histidine(s) 9 Proline(s) 1 Asparagine(s) 11 Valine(s) 6 Threonine(s) 4 Isoleucine(s) 1 Pantothenate(s) 631 ATP-energy(c) 13 NADPH-redox-potential(c) 3 Proton-gradient(c) 8 CoA-activated acetyl group(c)	9 H_2O(s) 1 CO_2(s) 1 (R)-3-Hydroxypalmitoyl-ACP(c) 66 Na-gradient(c)	45	-	-	-	-	-	-	-	24	-	-	1
548	(R)-4-Phosphopantothenoyl-cysteine	(R)-4-Phosphopantothenoyl-cysteine(c)	PIPES MCES	synthesis of cytosolic (R)-4-Phosphopantothenoyl-cysteine	1 P_i(c) 1 Cysteine(s) 1 Pantothenate(s) 3 ATP-energy(c)	2 H_2O(s) 1 (R)-4-Phosphopantothenoyl-cysteine(c) 1 Na-gradient(c)	6	-	-	-	-	-	-	-	4	-	-	-
549	(R)-5-Diphosphomevalonate	(R)-5-Diphosphomevalonate(p)	PIPES MCES	synthesis of peroxysomal (R)-5-Diphosphomevalonate	2 P_i(c) 2 ATP-energy(c) 2 NADPH-redox-potential(c) 3 CoA-activated acetyl group(c)	1 H_2O(s) 1 (R)-5-Diphosphomevalonate(p)	6	-	-	2	-	-	-	-	1	-	2	-

Continued on next page

C5. Comprehensive list – continued.

Simulation	Definition			Solution													
				exchanges		reactions								transport			Prot
	Objective	Contraints	Comment	imports	exports	c	m	r	p	l	n	s	b	s-c	b-c	intra	syn
550 (R)-5-Phosphomevalonate	(R)-5-Phosphomevalonate(p)	PIPES MCES	synthesis of peroxysomal (R)-5-Phosphomevalonate	1 P_i(c) 1 ATP-energy(c) 2 NADPH-redox-potential(c) 3 CoA-activated acetyl group(c)	1 (R)-5-Phosphomevalonate(p)	6	-	-	1	-	-	-	-	-	-	2	-
551 (R)-Methylmalonyl-CoA	(R)-Methylmalonyl-CoA(m)	PIPES MCES	synthesis of mitochondrial (R)-Methylmalonyl-CoA	2.17 P_i(c) 1 Glycine(s) 1 Aspartate(s) 2 Glutamine(s) 0.83 Glucose-6P(c) 0.5 Cystine(s) 1 Pantothenate(s) 11.2 ATP-energy(c) 1 Proton-gradient(c) 1 Proton-gradient(m) 2 THF-activated methyl group(c)	5.17 H_2O(s) 2 CO_2(s) 1 Pyruvate(c) 1 Glutamate(s) 1 (R)-Methylmalonyl-CoA(m) 3 NADH-redox-potential(c) 1 NADH-redox-potential(m) 2.5 NADPH-redox-potential(c) 4 Na-gradient(c)	44	3	-	-	-	-	-	-	10	-	5	-
552 (R)-Mevalonate	(R)-Mevalonate(c)	PIPES MCES	synthesis of cytosolic (R)-Mevalonate	1 H_2O(s) 2 NADPH-redox-potential(c) 3 CoA-activated acetyl group(c)	1 (R)-Mevalonate(c)	5	-	-	-	-	-	-	-	1	-	-	-
553 (S)-3-Hydroxy-2-methylbutyryl-CoA	(S)-3-Hydroxy-2-methylbutyryl-CoA(m)	PIPES MCES	synthesis of mitochondrial (S)-3-Hydroxy-2-methylbutyryl-CoA	2.17 P_i(c) 1 Glycine(s) 1.5 Aspartate(s) 0.5 Glutamine(s) 0.83 Glucose-6P(c) 0.5 Asparagine(s) 1 Threonine(s) 0.5 Cystine(s) 1 Pantothenate(s) 12.7 ATP-energy(c) 1 Proton-gradient(m) 2 THF-activated methyl group(c) 1 CoA-activated acetyl group(m)	6.17 H_2O(s) 3 CO_2(s) 2 Pyruvate(c) 0.5 Glutamate(s) 1 (S)-3-Hydroxy-2-methylbutyryl-CoA(m) 2 NADH-redox-potential(c) 3.5 NADPH-redox-potential(c) 3.5 Na-gradient(c)	45	4	-	-	-	-	-	-	10	-	4	-
554 (S)-3-Hydroxybutyryl-CoA	(S)-3-Hydroxybutyryl-CoA(m)	PIPES MCES	synthesis of mitochondrial (S)-3-Hydroxybutyryl-CoA	2.17 P_i(c) 1 Glycine(s) 2 Aspartate(s) 2 Glutamine(s) 0.83 Glucose-6P(c) 0.5 Cystine(s) 1 Pantothenate(s) 11.2 ATP-energy(c) 1 NADH-redox-potential(m) 1 Proton-gradient(c) 2 THF-activated methyl group(c) 2 CoA-activated acetyl group(m)	5.17 H_2O(s) 2 CO_2(s) 2 Pyruvate(c) 2 Glutamate(s) 1 (S)-3-Hydroxybutyryl-CoA(m) 2 NADH-redox-potential(c) 3.5 NADPH-redox-potential(c) 3 Na-gradient(c)	42	4	-	-	-	-	-	-	10	-	1	-

Continued on next page

C5. Comprehensive list – continued.

Simulation		Definition			Solution													
					exchanges		reactions								transport			Prot
		Objective	Contraints	Comment	imports	exports	c	m	r	p	l	n	s	b	s-c	b-c	intra	syn
555	(S)-3-Hydroxydodecanoyl-CoA	(S)-3-Hydroxydodecanoyl-CoA(m)	PIPES MCES	synthesis of mitochondrial (S)-3-Hydroxydodecanoyl-CoA	2.17 P_i(c) 1 Glycine(s) 2 Aspartate(s) 2 Glutamine(s) 0.83 Glucose-6P(c) 0.5 Cystine(s) 1 Pantothenate(s) 1 Palmitolate(s) 13.2 ATP-energy(c) 1 Proton-gradient(c) 2 THF-activated methyl group(c)	2.17 H_2O(s) 2 CO_2(s) 2 Pyruvate(c) 2 Glutamate(s) 1 (S)-3-Hydroxydodecanoyl-CoA(m) 2 NADH-redox-potential(c) 3 NADH-redox-potential(m) 3.5 NADPH-redox-potential(c) 3 FADH-redox-potential(c) 3 Na-gradient(c) 3 CoA-activated acetyl group(m)	44	16	-	-	-	-	-	-	11	-	3	-
556	(S)-3-Hydroxyhexadecanoyl-CoA	(S)-3-Hydroxyhexadecanoyl-CoA(m)	PIPES MCES	synthesis of mitochondrial (S)-3-Hydroxyhexadecanoyl-CoA	2.17 P_i(c) 1 Glycine(s) 2 Aspartate(s) 2 Glutamine(s) 0.83 Glucose-6P(c) 0.5 Palmitate(s) 0.5 Cystine(s) 1 Pantothenate(s) 0.5 Palmitolate(s) 13.2 ATP-energy(c) 1 Proton-gradient(c) 2 THF-activated methyl group(c)	5.17 H_2O(s) 2 CO_2(s) 2 Pyruvate(c) 2 Glutamate(s) 1 (S)-3-Hydroxyhexadecanoyl-CoA(m) 0.5 NADH-redox-potential(c) 5.5 NADPH-redox-potential(c) 6 Proton-gradient(m) 3 Na-gradient(c)	47	3	-	-	-	-	-	-	12	-	6	-
557	(S)-3-Hydroxytetradecanoyl-CoA	(S)-3-Hydroxytetradecanoyl-CoA(m)	PIPES MCES	synthesis of mitochondrial (S)-3-Hydroxytetradecanoyl-CoA	2.17 P_i(c) 1 Glycine(s) 0.5 Aspartate(s) 0.5 Glutamine(s) 0.83 Glucose-6P(c) 1.5 Asparagine(s) 0.5 Cystine(s) 1 Pantothenate(s) 1 Palmitolate(s) 14.7 ATP-energy(c) 2 THF-activated methyl group(c)	4.17 H_2O(s) 2 CO_2(s) 2 Pyruvate(c) 0.5 Glutamate(s) 1 (S)-3-Hydroxytetradecanoyl-CoA(m) 1 NADH-redox-potential(c) 1 NADH-redox-potential(m) 5.5 NADPH-redox-potential(c) 12 Proton-gradient(m) 2.5 Na-gradient(c) 1 CoA-activated acetyl group(m)	48	9	-	-	-	-	-	-	10	-	6	-
558	(S)-3-hydroxyoleyleoyl-CoA	(S)-3-hydroxyoleyleoyl-CoA(m)	PIPES MCES	synthesis of mitochondrial (S)-3-hydroxyoleyleoyl-CoA	2.17 P_i(c) 1 Glycine(s) 2 Aspartate(s) 2 Glutamine(s) 0.83 Glucose-6P(c) 0.5 Cystine(s) 1 Pantothenate(s) 1 Palmitolate(s) 13.2 ATP-energy(c) 1 NADH-redox-potential(m) 1 Proton-gradient(c) 2 THF-activated methyl group(c) 1 CoA-activated acetyl group(m)	6.17 H_2O(s) 2 CO_2(s) 2 Pyruvate(c) 2 Glutamate(s) 2 NADH-redox-potential(c) 3.5 NADPH-redox-potential(c) 1 (S)-3-hydroxyoleyleoyl-CoA(m) 3 Na-gradient(c)	43	4	-	-	-	-	-	-	11	-	1	-

Continued on next page

Simulation		Definition			Solution													
					exchanges		reactions								transport			Prot
		Objective	Contraints	Comment	imports	exports	c	m	r	p	l	n	s	b	s-c	b-c	intra	syn
559	(S)-3-hydroxypalmitoleoyl-CoA	(S)-3-hydroxypalmitoleoyl-CoA(m)	PIPES MCES	synthesis of mitochondrial (S)-3-hydroxypalmitoleoyl-CoA	2.17 P_i(c) 1 Glycine(s) 2 Aspartate(s) 2 Glutamine(s) 0.83 Glucose-6P(c) 0.5 Cystine(s) 1 Pantothenate(s) 1 Palmitolate(s) 13.2 ATP-energy(c) 1 Proton-gradient(c) 2 THF-activated methyl group(c)	5.17 H_2O(s) 2 CO_2(s) 2 Pyruvate(c) 2 Glutamate(s) 2 NADH-redox-potential(c) 3.5 NADPH-redox-potential(c) 1 FADH-redox-potential(c) 1 (S)-3-hydroxypalmitoleoyl-CoA(m) 3 Na-gradient(c)	44	2	-	-	-	-	-	-	11	-	3	-
560	(S)-Dihydroorotate	(S)-Dihydroorotate(c)	PIPES MCES	synthesis of cytosolic (S)-Dihydroorotate	0.5 Pyruvate(m) 1 Asparagine(s) 3 ATP-energy(c) 0.5 THF-activated methyl group(c)	1 (S)-Dihydroorotate(c) 0.5 NADH-redox-potential(c) 0.5 NADH-redox-potential(m) 1 NADPH-redox-potential(c) 0.5 CoA-activated acetyl group(m)	15	3	-	-	-	-	-	-	1	-	1	-
561	(S)-Hydroxydecanoyl-CoA	(S)-Hydroxydecanoyl-CoA(m)	PIPES MCES	synthesis of mitochondrial (S)-Hydroxydecanoyl-CoA	0.5 O_2(s) 2.17 P_i(c) 1 Glycine(s) 2 Aspartate(s) 2 Glutamine(s) 0.83 Glucose-6P(c) 0.5 Cystine(s) 1 Pantothenate(s) 1 Palmitolate(s) 13.2 ATP-energy(c) 1 Proton-gradient(c) 2 THF-activated methyl group(c)	2.17 H_2O(s) 2 CO_2(s) 2 Pyruvate(c) 2 Glutamate(s) 1 (S)-Hydroxydecanoyl-CoA(m) 2 NADH-redox-potential(c) 4 NADH-redox-potential(m) 3.5 NADPH-redox-potential(c) 3 FADH-redox-potential(c) 6 Proton-gradient(m) 3 Na-gradient(c) 4 CoA-activated acetyl group(m)	44	20	-	-	-	-	-	-	12	-	7	-
562	(S)-Hydroxyhexanoyl-CoA	(S)-Hydroxyhexanoyl-CoA(m)	PIPES MCES	synthesis of mitochondrial (S)-Hydroxyhexanoyl-CoA	1.5 O_2(s) 2.17 P_i(c) 1 Glycine(s) 0.5 Aspartate(s) 0.5 Glutamine(s) 0.83 Glucose-6P(c) 1.5 Asparagine(s) 0.5 Cystine(s) 1 Pantothenate(s) 1 Palmitolate(s) 14.7 ATP-energy(c) 2 THF-activated methyl group(c)	2.17 H_2O(s) 2 CO_2(s) 2 Pyruvate(c) 0.5 Glutamate(s) 1 (S)-Hydroxyhexanoyl-CoA(m) 2 NADH-redox-potential(c) 6 NADH-redox-potential(m) 3.5 NADPH-redox-potential(c) 3 FADH-redox-potential(c) 0.5 Proton-gradient(c) 18 Proton-gradient(m) 2 Na-gradient(c) 6 CoA-activated acetyl group(m)	46	28	-	-	-	-	-	-	12	-	7	-

Continued on next page

Simulation		Definition: Objective	Definition: Contraints	Definition: Comment	Solution: exchanges: imports	Solution: exchanges: exports	reactions: c	reactions: m	reactions: r	reactions: p	reactions: l	reactions: n	reactions: s	reactions: b	transport: s-c	transport: b-c	transport: intra	Prot syn
563	(S)-Hydroxyoctanoyl-CoA	(S)-Hydroxyoctanoyl-CoA(m)	PIPES MCES	synthesis of mitochondrial (S)-Hydroxyoctanoyl-CoA	1 O_2(s) 2.17 P_i(c) 1 Glycine(s) 2 Aspartate(s) 2 Glutamine(s) 0.83 Glucose-6P(c) 0.5 Cystine(s) 1 Pantothenate(s) 1 Palmitolate(s) 13.2 ATP-energy(c) 2 THF-activated methyl group(c)	2.17 H_2O(s) 2 CO_2(s) 2 Pyruvate(c) 2 Glutamate(s) 1 (S)-Hydroxyoctanoyl-CoA(m) 2 NADH-redox-potential(c) 5 NADH-redox-potential(m) 3.5 NADPH-redox-potential(c) 3 FADH-redox-potential(c) 12 Proton-gradient(m) 3 Na-gradient(c) 5 CoA-activated acetyl group(m)	44	24	-	-	-	-	-	-	11	-	7	-
564	1,2-Diacylglycerol-Bile-PC-pool	1,2-Diacylglycerol-Bile-PC-pool(c)	PIPES MCES	synthesis of cytosolic 1,2-Diacylglycerol-Bile-PC-pool	0.5 Glucose-6P(c) 0.11 Arachidonate(s) 0.83 Palmitate(s) 0.24 Oleate(s) 0.11 Stearate(s) 0.66 Linoleate(s) 0.05 Palmitolate(s) 4.5 ATP-energy(c) 1 NADH-redox-potential(c)	1.5 H_2O(s) 0.5 P_i(c) 1 1,2-Diacylglycerol-Bile-PC-pool(c)	25	-	-	-	-	-	-	-	7	-	-	-
565	1,2-Diacylglycerol-VLDL-PC-pool	1,2-Diacylglycerol-VLDL-PC-pool(c)	PIPES MCES	synthesis of cytosolic 1,2-Diacylglycerol-VLDL-PC-pool	0.5 Glucose-6P(c) 0.17 Arachidonate(s) 0.7 Palmitate(s) 0.27 Oleate(s) 0.3 Stearate(s) 0.55 Linoleate(s) 4.5 ATP-energy(c) 1 NADH-redox-potential(c)	1.5 H_2O(s) 0.5 P_i(c) 1 1,2-Diacylglycerol-VLDL-PC-pool(c)	23	-	-	-	-	-	-	-	6	-	-	-
566	1,2-Diacylglycerol-VLDL-PE-pool	1,2-Diacylglycerol-VLDL-PE-pool(c)	PIPES MCES	synthesis of cytosolic 1,2-Diacylglycerol-VLDL-PE-pool	0.5 Glucose-6P(c) 0.6 Arachidonate(s) 0.34 Palmitate(s) 0.17 Oleate(s) 0.54 Stearate(s) 0.36 Linoleate(s) 4.5 ATP-energy(c) 1 NADH-redox-potential(c)	1.5 H_2O(s) 0.5 P_i(c) 1 1,2-Diacylglycerol-VLDL-PE-pool(c)	23	-	-	-	-	-	-	-	6	-	-	-
567	1,2-Diacylglycerol-VLDL-PI-pool	1,2-Diacylglycerol-VLDL-PI-pool(c)	PIPES MCES	synthesis of cytosolic 1,2-Diacylglycerol-VLDL-PI-pool	0.5 Glucose-6P(c) 0.51 Arachidonate(s) 0.16 Palmitate(s) 0.32 Oleate(s) 0.85 Stearate(s) 0.16 Linoleate(s) 4.5 ATP-energy(c) 1 NADH-redox-potential(c)	1.5 H_2O(s) 0.5 P_i(c) 1 1,2-Diacylglycerol-VLDL-PI-pool(c)	23	-	-	-	-	-	-	-	6	-	-	-
568	1,2-Diacylglycerol-VLDL-PS-pool	1,2-Diacylglycerol-VLDL-PS-pool(c)	PIPES MCES	synthesis of cytosolic 1,2-Diacylglycerol-VLDL-PS-pool	0.5 Glucose-6P(c) 0.53 Arachidonate(s) 0.09 Palmitate(s) 0.09 Oleate(s) 1.25 Stearate(s) 0.05 Linoleate(s) 4.5 ATP-energy(c) 1 NADH-redox-potential(c)	1.5 H_2O(s) 0.5 P_i(c) 1 1,2-Diacylglycerol-VLDL-PS-pool(c)	23	-	-	-	-	-	-	-	6	-	-	-

Continued on next page

C5. Comprehensive list – continued.

Simulation		Definition			Solution														
					exchanges		reactions								transport			Prot
		Objective	Contraints	Comment	imports	exports	c	m	r	p	l	n	s	b	s-c	b-c	intra	syn
569	1,2-Diacylglycerol-VLDL-SM-pool	1,2-Diacylglycerol-VLDL-SM-pool(c)	PIPES MCES	synthesis of cytosolic 1,2-Diacylglycerol-VLDL-SM-pool	0.5 Glucose-6P(c) 0.03 Arachidonate(s) 1.42 Palmitate(s) 0.15 Oleate(s) 0.34 Stearate(s) 0.06 Linoleate(s) 4.5 ATP-energy(c) 1 NADH-redox-potential(c)	1.5 H_2O(s) 0.5 P_i(c) 1 1,2-Diacylglycerol-VLDL-SM-pool(c)	23	-	-	-	-	-	-	-	6	-	-	-
570	1,2-Diacylglycerol-VLDL-TG-pool	1,2-Diacylglycerol-VLDL-TG-pool(c)	PIPES MCES	synthesis of cytosolic 1,2-Diacylglycerol-VLDL-TG-pool	0.5 Glucose-6P(c) 0.06 Arachidonate(s) 0.83 Palmitate(s) 0.6 Oleate(s) 0.13 Stearate(s) 0.39 Linoleate(s) 4.5 ATP-energy(c) 1 NADH-redox-potential(c)	1.5 H_2O(s) 0.5 P_i(c) 1 1,2-Diacylglycerol-VLDL-TG-pool(c)	23	-	-	-	-	-	-	-	6	-	-	-
571	1,3DPG	1,3DPG(c)	PIPES MCES	synthesis of cytosolic 1,3DPG	1.5 P_i(c) 0.5 Glucose-6P(c) 0.5 ATP-energy(c)	0.5 H_2O(s) 1 1,3DPG(c) 1 NADH-redox-potential(c)	7	-	-	-	-	-	-	-	1	-	-	-
572	1-Acylglycerol-3P-Bile-PC-pool	1-Acylglycerol-3P-Bile-PC-pool(c)	PIPES MCES	synthesis of cytosolic 1-Acylglycerol-3P-Bile-PC-pool	0.5 P_i(c) 0.5 Glucose-6P(c) 0.06 Arachidonate(s) 0.41 Palmitate(s) 0.12 Oleate(s) 0.05 Stearate(s) 0.33 Linoleate(s) 0.03 Palmitolate(s) 2.5 ATP-energy(c) 1 NADH-redox-potential(c)	1.5 H_2O(s) 1 1-Acylglycerol-3P-Bile-PC-pool(c)	22	-	-	-	-	-	-	-	7	-	-	-
573	1-Acylglycerol-3P-CL-pool	1-Acylglycerol-3P-CL-pool(m)	PIPES MCES	synthesis of mitochondrial 1-Acylglycerol-3P-CL-pool	0.5 P_i(c) 0.5 Glucose-6P(c) 0.04 Palmitate(s) 0.14 Oleate(s) 0.02 Stearate(s) 0.75 Linoleate(s) 0.05 Palmitolate(s) 2.5 ATP-energy(c) 1 NADH-redox-potential(c) 1 Proton-gradient(m)	1.5 H_2O(s) 1 1-Acylglycerol-3P-CL-pool(m)	15	1	-	-	-	-	-	-	6	-	5	-
574	1-Acylglycerol-3P-VLDL-PC-pool	1-Acylglycerol-3P-VLDL-PC-pool(c)	PIPES MCES	synthesis of cytosolic 1-Acylglycerol-3P-VLDL-PC-pool	0.5 P_i(c) 0.5 Glucose-6P(c) 0.08 Arachidonate(s) 0.35 Palmitate(s) 0.14 Oleate(s) 0.15 Stearate(s) 0.27 Linoleate(s) 2.5 ATP-energy(c) 1 NADH-redox-potential(c)	1.5 H_2O(s) 1 1-Acylglycerol-3P-VLDL-PC-pool(c)	20	-	-	-	-	-	-	-	6	-	-	-

Continued on next page

C5. Comprehensive list – continued.

Simulation		Definition			Solution													
					exchanges		reactions								transport			Prot
		Objective	Contraints	Comment	imports	exports	c	m	r	p	l	n	s	b	s-c	b-c	intra	syn
575	1-Acylglycerol-3P-VLDL-PE-pool	1-Acylglycerol-3P-VLDL-PE-pool(c)	PIPES MCES	synthesis of cytosolic 1-Acylglycerol-3P-VLDL-PE-pool	0.5 P_i(c) 0.5 Glucose-6P(c) 0.3 Arachidonate(s) 0.17 Palmitate(s) 0.08 Oleate(s) 0.27 Stearate(s) 0.18 Linoleate(s) 2.5 ATP-energy(c) 1 NADH-redox-potential(c)	1.5 H_2O(s) 1 1-Acylglycerol-3P-VLDL-PE-pool(c)	20	-	-	-	-	-	-	-	6	-	-	-
576	1-Acylglycerol-3P-VLDL-PI-pool	1-Acylglycerol-3P-VLDL-PI-pool(c)	PIPES MCES	synthesis of cytosolic 1-Acylglycerol-3P-VLDL-PI-pool	0.5 P_i(c) 0.5 Glucose-6P(c) 0.25 Arachidonate(s) 0.08 Palmitate(s) 0.16 Oleate(s) 0.43 Stearate(s) 0.08 Linoleate(s) 2.5 ATP-energy(c) 1 NADH-redox-potential(c)	1.5 H_2O(s) 1 1-Acylglycerol-3P-VLDL-PI-pool(c)	20	-	-	-	-	-	-	-	6	-	-	-
577	1-Acylglycerol-3P-VLDL-PS-pool	1-Acylglycerol-3P-VLDL-PS-pool(c)	PIPES MCES	synthesis of cytosolic 1-Acylglycerol-3P-VLDL-PS-pool	0.5 P_i(c) 0.5 Glucose-6P(c) 0.27 Arachidonate(s) 0.04 Palmitate(s) 0.04 Oleate(s) 0.62 Stearate(s) 0.02 Linoleate(s) 2.5 ATP-energy(c) 1 NADH-redox-potential(c)	1.5 H_2O(s) 1 1-Acylglycerol-3P-VLDL-PS-pool(c)	20	-	-	-	-	-	-	-	6	-	-	-
578	1-Acylglycerol-3P-VLDL-SM-pool	1-Acylglycerol-3P-VLDL-SM-pool(c)	PIPES MCES	synthesis of cytosolic 1-Acylglycerol-3P-VLDL-SM-pool	0.5 P_i(c) 0.5 Glucose-6P(c) 0.02 Arachidonate(s) 0.71 Palmitate(s) 0.07 Oleate(s) 0.17 Stearate(s) 0.03 Linoleate(s) 2.5 ATP-energy(c) 1 NADH-redox-potential(c)	1.5 H_2O(s) 1 1-Acylglycerol-3P-VLDL-SM-pool(c)	20	-	-	-	-	-	-	-	6	-	-	-
579	1-Acylglycerol-3P-VLDL-TG1-pool	1-Acylglycerol-3P-VLDL-TG1-pool(c)	PIPES MCES	synthesis of cytosolic 1-Acylglycerol-3P-VLDL-TG1-pool	0.5 P_i(c) 0.5 Glucose-6P(c) 0.003 Arachidonate(s) 0.7 Palmitate(s) 0.13 Oleate(s) 0.09 Stearate(s) 0.08 Linoleate(s) 2.5 ATP-energy(c) 1 NADH-redox-potential(c)	1.5 H_2O(s) 1 1-Acylglycerol-3P-VLDL-TG1-pool(c)	20	-	-	-	-	-	-	-	6	-	-	-
580	1-Acylglycerol-3P-arach	1-Acylglycerol-3P-arach(c)	PIPES MCES	synthesis of cytosolic 1-Acylglycerol-3P-arach	0.5 P_i(c) 0.5 Glucose-6P(c) 1 Arachidonate(s) 2.5 ATP-energy(c) 1 NADH-redox-potential(c)	1.5 H_2O(s) 1 1-Acylglycerol-3P-arach(c)	11	-	-	-	-	-	-	-	2	-	-	-

Continued on next page

C5. Comprehensive list – continued.

Simulation		Definition			Solution													
					exchanges		reactions								transport			Prot
		Objective	Contraints	Comment	imports	exports	c	m	r	p	l	n	s	b	s-c	b-c	intra	syn
581	1-Acylglycerol-3P-lin	1-Acylglycerol-3P-lin(c)	PIPES MCES	synthesis of cytosolic 1-Acylglycerol-3P-lin	0.5 P_i(c) 0.5 Glucose-6P(c) 1 Linoleate(s) 2.5 ATP-energy(c) 1 NADH-redox-potential(c)	1.5 H_2O(s) 1 1-Acylglycerol-3P-lin(c)	11	-	-	-	-	-	-	-	2	-	-	-
582	1-Acylglycerol-3P-ol	1-Acylglycerol-3P-ol(c)	PIPES MCES	synthesis of cytosolic 1-Acylglycerol-3P-ol	0.5 P_i(c) 0.5 Glucose-6P(c) 1 Oleate(s) 2.5 ATP-energy(c) 1 NADH-redox-potential(c)	1.5 H_2O(s) 1 1-Acylglycerol-3P-ol(c)	11	-	-	-	-	-	-	-	2	-	-	-
583	1-Acylglycerol-3P-palm	1-Acylglycerol-3P-palm(c)	PIPES MCES	synthesis of cytosolic 1-Acylglycerol-3P-palm	0.5 P_i(c) 0.5 Glucose-6P(c) 1 Palmitate(s) 2.5 ATP-energy(c) 1 NADH-redox-potential(c)	1.5 H_2O(s) 1 1-Acylglycerol-3P-palm(c)	11	-	-	-	-	-	-	-	2	-	-	-
584	1-Acylglycerol-3P-palmn	1-Acylglycerol-3P-palmn(c)	PIPES MCES	synthesis of cytosolic 1-Acylglycerol-3P-palmn	0.5 P_i(c) 0.5 Glucose-6P(c) 1 Palmitolate(s) 2.5 ATP-energy(c) 1 NADH-redox-potential(c)	1.5 H_2O(s) 1 1-Acylglycerol-3P-palmn(c)	11	-	-	-	-	-	-	-	2	-	-	-
585	1-Acylglycerol-3P-stea	1-Acylglycerol-3P-stea(c)	PIPES MCES	synthesis of cytosolic 1-Acylglycerol-3P-stea	0.5 P_i(c) 0.5 Glucose-6P(c) 1 Stearate(s) 2.5 ATP-energy(c) 1 NADH-redox-potential(c)	1.5 H_2O(s) 1 1-Acylglycerol-3P-stea(c)	11	-	-	-	-	-	-	-	2	-	-	-
586	1-Acylglycerol-VLDL-PC-pool	1-Acylglycerol-VLDL-PC-pool(c)	PIPES MCES	synthesis of cytosolic 1-Acylglycerol-VLDL-PC-pool	0.5 Glucose-6P(c) 0.08 Arachidonate(s) 0.35 Palmitate(s) 0.14 Oleate(s) 0.15 Stearate(s) 0.27 Linoleate(s) 4.5 ATP-energy(c) 1 NADH-redox-potential(c)	0.5 H_2O(s) 0.5 P_i(c) 1 1-Acylglycerol-VLDL-PC-pool(c)	25	-	-	-	-	-	-	-	6	-	-	-
587	1-Acylglycerol-VLDL-PE-pool	1-Acylglycerol-VLDL-PE-pool(c)	PIPES MCES	synthesis of cytosolic 1-Acylglycerol-VLDL-PE-pool	0.5 Glucose-6P(c) 0.3 Arachidonate(s) 0.17 Palmitate(s) 0.08 Oleate(s) 0.27 Stearate(s) 0.18 Linoleate(s) 4.5 ATP-energy(c) 1 NADH-redox-potential(c)	0.5 H_2O(s) 0.5 P_i(c) 1 1-Acylglycerol-VLDL-PE-pool(c)	25	-	-	-	-	-	-	-	6	-	-	-
588	1-Acylglycerol-VLDL-PI-pool	1-Acylglycerol-VLDL-PI-pool(c)	PIPES MCES	synthesis of cytosolic 1-Acylglycerol-VLDL-PI-pool	0.5 Glucose-6P(c) 0.25 Arachidonate(s) 0.08 Palmitate(s) 0.16 Oleate(s) 0.43 Stearate(s) 0.08 Linoleate(s) 4.5 ATP-energy(c) 1 NADH-redox-potential(c)	0.5 H_2O(s) 0.5 P_i(c) 1 1-Acylglycerol-VLDL-PI-pool(c)	25	-	-	-	-	-	-	-	6	-	-	-

Continued on next page

C5. Comprehensive list – continued.

Simulation		Definition: Objective	Definition: Contraints	Definition: Comment	Solution – exchanges: imports	Solution – exchanges: exports	reactions: c	m	r	p	l	n	s	b	transport: s-c	b-c	intra	Prot syn
589	1-Acylglycerol-VLDL-PS-pool	1-Acylglycerol-VLDL-PS-pool(c)	PIPES MCES	synthesis of cytosolic 1-Acylglycerol-VLDL-PS-pool	0.5 Glucose-6P(c) 0.27 Arachidonate(s) 0.04 Palmitate(s) 0.04 Oleate(s) 0.62 Stearate(s) 0.02 Linoleate(s) 4.5 ATP-energy(c) 1 NADH-redox-potential(c)	0.5 H_2O(s) 0.5 P_i(c) 1 1-Acylglycerol-VLDL-PS-pool(c)	25	-	-	-	-	-	-	-	6	-	-	-
590	1-Acylglycerol-VLDL-SM-pool	1-Acylglycerol-VLDL-SM-pool(c)	PIPES MCES	synthesis of cytosolic 1-Acylglycerol-VLDL-SM-pool	0.5 Glucose-6P(c) 0.02 Arachidonate(s) 0.71 Palmitate(s) 0.07 Oleate(s) 0.17 Stearate(s) 0.03 Linoleate(s) 4.5 ATP-energy(c) 1 NADH-redox-potential(c)	0.5 H_2O(s) 0.5 P_i(c) 1 1-Acylglycerol-VLDL-SM-pool(c)	25	-	-	-	-	-	-	-	6	-	-	-
591	1-Acylglycerol-VLDL-TG1-pool	1-Acylglycerol-VLDL-TG1-pool(c)	PIPES MCES	synthesis of cytosolic 1-Acylglycerol-VLDL-TG1-pool	0.5 Glucose-6P(c) 0.003 Arachidonate(s) 0.7 Palmitate(s) 0.13 Oleate(s) 0.09 Stearate(s) 0.08 Linoleate(s) 4.5 ATP-energy(c) 1 NADH-redox-potential(c)	0.5 H_2O(s) 0.5 P_i(c) 1 1-Acylglycerol-VLDL-TG1-pool(c)	25	-	-	-	-	-	-	-	6	-	-	-
592	1-Pyrroline-5-carboxylate	1-Pyrroline-5-carboxylate(c)	PIPES MCES	synthesis of cytosolic 1-Pyrroline-5-carboxylate	1 Proline(s)	1 1-Pyrroline-5-carboxylate(c) 1 NADPH-redox-potential(c) 1 Na-gradient(c)	2	-	-	-	-	-	-	-	2	-	-	-
593	14-Demethyllanosterol	14-Demethyllanosterol(r)	PIPES MCES	synthesis of Golgi/ER 14-Demethyllanosterol	4 O_2(s) 2 Farnesyl-PP(r) 1 ATP-energy(c) 6 NADPH-redox-potential(r)	3 H_2O(s) 4 P_i(c) 1 CO_2(s) 1 14-Demethyllanosterol(r) 1 NADPH-redox-potential(c)	5	-	7	-	-	-	-	-	3	-	4	-
594	2,5-Diamino-6-(5-triphosphoryl-3,4-trihydroxy-2-oxopentyl)- amino-4-oxopyrimidine	2,5-Diamino-6-(5-triphosphoryl-3,4-trihydroxy-2-oxopentyl)-amino-4-oxopyrimidine(c)	PIPES MCES	synthesis of cytosolic 2,5-Diamino-6-(5-triphosphoryl-3,4-trihydroxy-2-oxopentyl)-amino-4-oxopyrimidine	2.17 P_i(c) 1 Glycine(s) 2 Glutamine(s) 0.83 Glucose-6P(c) 1 Asparagine(s) 11.2 ATP-energy(c) 1 Proton-gradient(c) 1 THF-activated methyl group(c)	2.17 H_2O(s) 1 Pyruvate(c) 2 Glutamate(s) 1 2,5-Diamino-6-(5-triphosphoryl-3,4-trihydroxy-2-oxopentyl)- amino-4-oxopyrimidine(c) 2 NADH-redox-potential(c) 2 NADPH-redox-potential(c)	40	-	-	-	-	-	-	-	6	-	-	-
595	2,5-Diaminopyrimidine(n)leoside triphosphate	2,5-Diaminopyrimidine nleoside triphosphate(c)	PIPES MCES	synthesis of cytosolic 2,5-Diaminopyrimidine nleoside triphosphate	2.17 P_i(c) 1 Glycine(s) 2 Glutamine(s) 0.83 Glucose-6P(c) 1 Asparagine(s) 11.2 ATP-energy(c) 1 Proton-gradient(c) 1 THF-activated methyl group(c)	2.17 H_2O(s) 1 Pyruvate(c) 2 Glutamate(s) 1 2,5-Diaminopyrimidine nleoside triphosphate(c) 2 NADH-redox-potential(c) 2 NADPH-redox-potential(c)	39	-	-	-	-	-	-	-	6	-	-	-

Continued on next page

C5. Comprehensive list – continued.

Simulation		Definition			Solution													
					exchanges		reactions								transport			Prot
		Objective	Contraints	Comment	imports	exports	c	m	r	p	l	n	s	b	s-c	b-c	intra	syn
596	2-Amino-3-carboxymuconate(s)emialdehyde	2-Amino-3-carboxymuconate semialdehyde(c)	PIPES MCES	synthesis of cytosolic 2-Amino-3-carboxymuconate semialdehyde	3 O_2(s) 1 Tryptophan(s) 1 ATP-energy(c) 1.5 NADPH-redox-potential(c)	0.5 CO_2(s) 1 Alanine(s) 1 2-Amino-3-carboxymuconate semialdehyde(c) 1 Na-gradient(c) 0.5 THF-activated methyl group(c)	13	-	-	-	-	-	-	-	5	-	-	-
597	2-Amino-3-oxoadipate	2-Amino-3-oxoadipate(m)	PIPES MCES	synthesis of mitochondrial 2-Amino-3-oxoadipate	0.5 Pyruvate(m) 1 Glutamate(s) 0.5 Serine(s) 0.5 NADPH-redox-potential(m) 0.5 Proton-gradient(m)	0.5 Aspartate(s) 1 2-Amino-3-oxoadipate(m) 0.5 NADH-redox-potential(c) 1 NADH-redox-potential(m) 0.5 Proton-gradient(c) 1.5 Na-gradient(c)	3	10	-	-	-	-	-	-	5	-	4	-
598	2-Aminomuconate	2-Aminomuconate(c)	PIPES MCES	synthesis of cytosolic 2-Aminomuconate	3 O_2(s) 1 Tryptophan(s) 1 ATP-energy(c) 3 NADPH-redox-potential(c)	1 CO_2(s) 1 Alanine(s) 1 2-Aminomuconate(c) 1 NADH-redox-potential(c) 1 Na-gradient(c) 1 THF-activated methyl group(c)	15	-	-	-	-	-	-	-	5	-	-	-
599	2-Aminomuconate(s)emialdehyde	2-Aminomuconate semialdehyde(c)	PIPES MCES	synthesis of cytosolic 2-Aminomuconate semialdehyde	3 O_2(s) 1 Tryptophan(s) 1 ATP-energy(c) 1.5 NADPH-redox-potential(c)	1.5 CO_2(s) 1 Alanine(s) 1 2-Aminomuconate semialdehyde(c) 1 Na-gradient(c) 0.5 THF-activated methyl group(c)	14	-	-	-	-	-	-	-	5	-	-	-
600	2-Deoxy-D-ribose-1P	2-Deoxy-D-ribose-1P(c)	PIPES MCES	synthesis of cytosolic 2-Deoxy-D-ribose-1P	0.5 P_i(c) 1 Glutamate(s) 0.5 Glucose-6P(c) 1 Ethanolamine(c) 2.5 ATP-energy(c)	1.5 H_2O(s) 1 Glutamine(s) 1 2-Deoxy-D-ribose-1P(c) 1 Na-gradient(c)	10	-	-	-	-	-	-	-	4	-	-	-
601	2-Deoxy-D-ribose-5P	2-Deoxy-D-ribose-5P(c)	PIPES MCES	synthesis of cytosolic 2-Deoxy-D-ribose-5P	0.5 P_i(c) 1 Glutamate(s) 0.5 Glucose-6P(c) 1 Ethanolamine(c) 2.5 ATP-energy(c)	1.5 H_2O(s) 1 Glutamine(s) 1 2-Deoxy-D-ribose-5P(c) 1 Na-gradient(c)	9	-	-	-	-	-	-	-	4	-	-	-
602	2-Lysolecithin-pool	2-Lysolecithin-pool(c)	PIPES MCES	synthesis of cytosolic 2-Lysolecithin-pool	0.5 P_i(c) 0.5 Glucose-6P(c) 1 Choline(c) 0.08 Arachidonate(s) 0.35 Palmitate(s) 0.14 Oleate(s) 0.15 Stearate(s) 0.27 Linoleate(s) 7.5 ATP-energy(c) 1 NADH-redox-potential(c)	2.5 H_2O(s) 1 2-Lysolecithin-pool(c)	30	-	-	-	-	-	-	-	6	-	-	-

Continued on next page

Simulation		Definition			Solution													
					exchanges		reactions								transport			Prot
		Objective	Contraints	Comment	imports	exports	c	m	r	p	l	n	s	b	s-c	b-c	intra	syn
603	2-Methylacetoacetyl-CoA	2-Methylacetoacetyl-CoA(m)	PIPES MCES	synthesis of mitochondrial 2-Methylacetoacetyl-CoA	2.17 P_i(c) 1 Glycine(s) 2 Aspartate(s) 1 Glutamine(s) 0.83 Glucose-6P(c) 1 Threonine(s) 0.5 Cystine(s) 1 Pantothenate(s) 12.2 ATP-energy(c) 1 Proton-gradient(m) 2 THF-activated methyl group(c) 1 CoA-activated acetyl group(m)	6.17 H_2O(s) 3 CO_2(s) 2 Pyruvate(c) 1 Glutamate(s) 1 2-Methylacetoacetyl-CoA(m) 2 NADH-redox-potential(c) 1 NADH-redox-potential(m) 3.5 NADPH-redox-potential(c) 5 Na-gradient(c)	44	4	-	-	-	-	-	-	10	-	4	-
604	2-Methylbutyryl-CoA	2-Methylbutyryl-CoA(m)	PIPES MCES	synthesis of mitochondrial 2-Methylbutyryl-CoA	2.17 P_i(c) 1 Glycine(s) 2 Aspartate(s) 1 Glutamine(s) 0.83 Glucose-6P(c) 1 Isoleucine(s) 0.5 Cystine(s) 1 Pantothenate(s) 12.2 ATP-energy(c) 1 Proton-gradient(m) 2 THF-activated methyl group(c)	5.17 H_2O(s) 3 CO_2(s) 2 Pyruvate(c) 1 Glutamate(s) 1 2-Methylbutyryl-CoA(m) 1 NADH-redox-potential(m) 5.5 NADPH-redox-potential(c) 1 NADPH-redox-potential(m) 5 Na-gradient(c)	42	5	-	-	-	-	-	-	10	-	6	-
605	2-Oxo-3-methylvalerate	2-Oxo-3-methylvalerate(m)	PIPES MCES	synthesis of mitochondrial 2-Oxo-3-methylvalerate	1 Glutamate(s) 1 Isoleucine(s) 1 ATP-energy(c) 1 Proton-gradient(m)	1 Glutamine(s) 1 2-Oxo-3-methylvalerate(m) 1 NADH-redox-potential(m) 2 Na-gradient(c)	2	3	-	-	-	-	-	-	4	-	4	-
606	2-Oxoadipate	2-Oxoadipate(c)	PIPES MCES	synthesis of cytosolic 2-Oxoadipate	1 Pyruvate(c) 1 Pyruvate(m) 0.5 Lysine(s) 0.5 NADH-redox-potential(m)	1 Alanine(s) 1 2-Oxoadipate(c) 0.5 NADH-redox-potential(c) 1 Proton-gradient(m)	3	10	-	-	-	-	-	-	2	-	8	-
607	2-Oxobutyrate	2-Oxobutyrate(c)	PIPES MCES	synthesis of cytosolic 2-Oxobutyrate	1 Glutamate(s) 1 Threonine(s) 1 ATP-energy(c)	1 H_2O(s) 1 Glutamine(s) 1 2-Oxobutyrate(c) 2 Na-gradient(c)	3	-	-	-	-	-	-	-	5	-	-	-
608	2-Oxoglutaramate	2-Oxoglutaramate(c)	PIPES MCES	synthesis of cytosolic 2-Oxoglutaramate	1 Pyruvate(c) 1 Glutamine(s)	1 Alanine(s) 1 2-Oxoglutaramate(c)	1	-	-	-	-	-	-	-	1	-	-	-
609	2PG	2PG(c)	PIPES MCES	synthesis of cytosolic 2PG	1 P_i(c) 1 Pyruvate(c) 1 ATP-energy(c) 1 NADPH-redox-potential(c)	1 2PG(c) 1 NADH-redox-potential(c)	8	-	-	-	-	-	-	-	-	-	-	-

Continued on next page

Simulation		Definition			Solution													
					exchanges		reactions								transport			Prot
		Objective	Contraints	Comment	imports	exports	c	m	r	p	l	n	s	b	s-c	b-c	intra	syn
610	3(S)-3-hydroxydodecen-(5Z)-oyl-CoA	3(S)-3-hydroxydodecen-(5Z)-oyl-CoA(m)	PIPES MCES	synthesis of mitochondrial 3(S)-3-hydroxydodecen-(5Z)-oyl-CoA	2.17 P_i(c) 1 Glycine(s) 2 Aspartate(s) 2 Glutamine(s) 0.83 Glucose-6P(c) 0.5 Cystine(s) 1 Pantothenate(s) 1 Palmitolate(s) 13.2 ATP-energy(c) 1 Proton-gradient(c) 2 THF-activated methyl group(c)	3.17 H_2O(s) 2 CO_2(s) 2 Pyruvate(c) 2 Glutamate(s) 2 NADH-redox-potential(c) 2 NADH-redox-potential(m) 3.5 NADPH-redox-potential(c) 3 FADH-redox-potential(c) 1 3(S)-3-hydroxydodecen-(5Z)-oyl-CoA(m) 3 Na-gradient(c) 2 CoA-activated acetyl group(m)	44	12	-	-	-	-	-	-	11	-	3	-
611	3-Dehydrosphinganine	3-Dehydrosphinganine(c)	PIPES MCES	synthesis of cytosolic 3-Dehydrosphinganine	1 Serine(s) 1 Palmitate(s) 2 ATP-energy(c)	1 H_2O(s) 1 CO_2(s) 1 3-Dehydrosphinganine(c) 1 Proton-gradient(c) 1 Na-gradient(c)	5	-	-	-	-	-	-	-	6	-	-	-
612	3-Hydroxy-L-kynurenine	3-Hydroxy-L-kynurenine(c)	PIPES MCES	synthesis of cytosolic 3-Hydroxy-L-kynurenine	2 O_2(s) 1 Tryptophan(s) 1 ATP-energy(c)	1 CO_2(s) 1 3-Hydroxy-L-kynurenine(c) 1 Na-gradient(c)	6	-	-	-	-	-	-	-	4	-	-	-
613	3-Hydroxyanthranilate	3-Hydroxyanthranilate(c)	PIPES MCES	synthesis of cytosolic 3-Hydroxyanthranilate	2 O_2(s) 1 Tryptophan(s) 1 ATP-energy(c) 1.5 NADPH-redox-potential(c)	0.5 CO_2(s) 1 Alanine(s) 1 3-Hydroxyanthranilate(c) 1 Na-gradient(c) 0.5 THF-activated methyl group(c)	12	-	-	-	-	-	-	-	5	-	-	-
614	3-Hydroxyisobutyrate	3-Hydroxyisobutyrate(m)	PIPES MCES	synthesis of mitochondrial 3-Hydroxyisobutyrate	0.67 H_2O(s) 0.5 O_2(s) 0.33 Glutamate(s) 1 Valine(s) 0.67 ATP-energy(c) 0.67 ATP-energy(m)	0.67 CO_2(s) 0.33 Arginine(s) 1 3-Hydroxyisobutyrate(m) 0.33 NADH-redox-potential(c) 1 NADH-redox-potential(m) 4.33 Proton-gradient(m) 1.33 Na-gradient(c)	9	12	-	-	-	-	-	-	7	-	10	-
615	3-Hydroxyisobutyryl-CoA	3-Hydroxyisobutyryl-CoA(m)	PIPES MCES	synthesis of mitochondrial 3-Hydroxyisobutyryl-CoA	0.5 O_2(s) 2.17 P_i(c) 1 Glycine(s) 2 Aspartate(s) 1 Glutamine(s) 0.83 Glucose-6P(c) 1 Valine(s) 0.5 Cystine(s) 1 Pantothenate(s) 12.2 ATP-energy(c) 1 Proton-gradient(c) 2 THF-activated methyl group(c)	5.17 H_2O(s) 3 CO_2(s) 2 Pyruvate(c) 1 Glutamate(s) 1 3-Hydroxyisobutyryl-CoA(m) 2 NADH-redox-potential(c) 2 NADH-redox-potential(m) 3.5 NADPH-redox-potential(c) 5 Proton-gradient(m) 4 Na-gradient(c)	43	6	-	-	-	-	-	-	12	-	9	-

Continued on next page

C5. Comprehensive list – continued.

Simulation		Definition			Solution													
					exchanges		reactions								transport			Prot
		Objective	Contraints	Comment	imports	exports	c	m	r	p	l	n	s	b	s-c	b-c	intra	syn
616	3-Hydroxypropionyl-CoA	3-Hydroxypropionyl-CoA(m)	PIPES MCES	synthesis of mitochondrial 3-Hydroxypropionyl-CoA	0.5 O_2(s) 2.17 P_i(c) 1 Glycine(s) 2 Aspartate(s) 1 Glutamine(s) 0.83 Glucose-6P(c) 1 Threonine(s) 0.5 Cystine(s) 1 Pantothenate(s) 12.2 ATP-energy(c) 1 Proton-gradient(c) 2 THF-activated methyl group(c)	6.17 H_2O(s) 3 CO_2(s) 2 Pyruvate(c) 1 Glutamate(s) 1 3-Hydroxypropionyl-CoA(m) 2 NADH-redox-potential(c) 1 NADH-redox-potential(m) 3.5 NADPH-redox-potential(c) 5 Proton-gradient(m) 4 Na-gradient(c)	44	4	-	-	-	-	-	-	12	-	7	-
617	3-Hydroxystearoyl-ACP	3-Hydroxystearoyl-ACP(c)	PIPES MCES	synthesis of cytosolic 3-Hydroxystearoyl-ACP	1 P_i(c) 5 Glutamate(s) 6 Glycine(s) 11 Alanine(s) 4 Lysine(s) 8 Aspartate(s) 9 Arginine(s) 7 Glutamine(s) 8 Serine(s) 5 Methionine(s) 3 Phenylalanine(s) 4 Tyrosine(s) 3 Cysteine(s) 20 Leucine(s) 1 Histidine(s) 9 Proline(s) 1 Asparagine(s) 11 Valine(s) 6 Threonine(s) 4 Isoleucine(s) 1 Pantothenate(s) 632 ATP-energy(c) 15 NADPH-redox-potential(c) 3 Proton-gradient(c) 9 CoA-activated acetyl group(c)	10 H_2O(s) 1 CO_2(s) 1 3-Hydroxystearoyl-ACP(c) 66 Na-gradient(c)	49	-	-	-	-	-	-	-	24	-	-	1
618	3-Keto-4-methylzymosterol	3-Keto-4-methylzymosterol(r)	PIPES MCES	synthesis of Golgi/ER 3-Keto-4-methylzymosterol	7 O_2(s) 2 Farnesyl-PP(r) 1 ATP-energy(c) 9 NADPH-redox-potential(r)	7 H_2O(s) 4 P_i(c) 2 CO_2(s) 1 3-Keto-4-methylzymosterol(r) 1 NADH-redox-potential(r) 1 NADPH-redox-potential(c)	5	-	10	-	-	-	-	-	3	-	5	-
619	3-Methyl-2-oxobutyrate	3-Methyl-2-oxobutyrate(m)	PIPES MCES	synthesis of mitochondrial 3-Methyl-2-oxobutyrate	1 Pyruvate(c) 1 Valine(s) 3 Proton-gradient(m)	1 Alanine(s) 1 3-Methyl-2-oxobutyrate(m) 2 Na-gradient(c)	1	1	-	-	-	-	-	-	3	-	6	-
620	3-Methylcrotonyl-CoA	3-Methylcrotonyl-CoA(m)	PIPES MCES	synthesis of mitochondrial 3-Methylcrotonyl-CoA	2.17 P_i(c) 1 Glycine(s) 2 Aspartate(s) 2 Glutamine(s) 0.83 Glucose-6P(c) 0.5 Cystine(s) 1 Pantothenate(s) 11.2 ATP-energy(c) 2 THF-activated methyl group(c) 3 CoA-activated acetyl group(m)	5.17 H_2O(s) 3 CO_2(s) 2 Pyruvate(c) 2 Glutamate(s) 1 3-Methylcrotonyl-CoA(m) 2 NADH-redox-potential(c) 3.5 NADPH-redox-potential(c) 1 Proton-gradient(c) 5 Na-gradient(c)	42	7	-	-	-	-	-	-	10	-	2	-

Continued on next page

C5. Comprehensive list – continued.

Simulation		Definition			Solution													
					exchanges		reactions								transport			Prot
		Objective	Contraints	Comment	imports	exports	c	m	r	p	l	n	s	b	s-c	b-c	intra	syn
621	3-Methylglutaconyl-CoA	3-Methylglutaconyl-CoA(m)	PIPES MCES	synthesis of mitochondrial 3-Methylglutaconyl-CoA	2.17 P_i(c) 1 Glycine(s) 2 Aspartate(s) 2 Glutamine(s) 0.83 Glucose-6P(c) 0.5 Cystine(s) 1 Pantothenate(s) 11.2 ATP-energy(c) 2 THF-activated methyl group(c) 3 CoA-activated acetyl group(m)	5.17 H_2O(s) 2 CO_2(s) 2 Pyruvate(c) 2 Glutamate(s) 1 3-Methylglutaconyl-CoA(m) 2 NADH-redox-potential(c) 3.5 NADPH-redox-potential(c) 1 Proton-gradient(c) 5 Na-gradient(c)	42	4	-	-	-	-	-	-	10	-	1	-
622	3-Oxodecanoyl-ACP	3-Oxodecanoyl-ACP(c)	PIPES MCES	synthesis of cytosolic 3-Oxodecanoyl-ACP	1 P_i(c) 5 Glutamate(s) 6 Glycine(s) 11 Alanine(s) 4 Lysine(s) 8 Aspartate(s) 9 Arginine(s) 7 Glutamine(s) 8 Serine(s) 5 Methionine(s) 3 Phenylalanine(s) 4 Tyrosine(s) 3 Cysteine(s) 20 Leucine(s) 1 Histidine(s) 9 Proline(s) 1 Asparagine(s) 11 Valine(s) 6 Threonine(s) 4 Isoleucine(s) 1 Pantothenate(s) 628 ATP-energy(c) 6 NADPH-redox-potential(c) 3 Proton-gradient(c) 5 CoA-activated acetyl group(c)	6 H_2O(s) 1 CO_2(s) 1 3-Oxodecanoyl-ACP(c) 66 Na-gradient(c)	32	-	-	-	-	-	-	-	24	-	-	1
623	3-Oxodecanoyl-CoA	3-Oxodecanoyl-CoA(m)	PIPES MCES	synthesis of mitochondrial 3-Oxodecanoyl-CoA	0.5 O_2(s) 2.17 P_i(c) 1 Glycine(s) 2 Aspartate(s) 2 Glutamine(s) 0.83 Glucose-6P(c) 0.5 Cystine(s) 1 Pantothenate(s) 1 Palmitolate(s) 13.2 ATP-energy(c) 1 Proton-gradient(c) 2 THF-activated methyl group(c)	2.17 H_2O(s) 2 CO_2(s) 2 Pyruvate(c) 2 Glutamate(s) 1 3-Oxodecanoyl-CoA(m) 2 NADH-redox-potential(c) 5 NADH-redox-potential(m) 3.5 NADPH-redox-potential(c) 3 FADH-redox-potential(c) 6 Proton-gradient(m) 3 Na-gradient(c) 4 CoA-activated acetyl group(m)	44	21	-	-	-	-	-	-	12	-	7	-

Continued on next page

C5. Comprehensive list – continued.

Simulation		Definition			Solution													
					exchanges		reactions								transport			Prot
		Objective	Contraints	Comment	imports	exports	c	m	r	p	l	n	s	b	s-c	b-c	intra	syn
624	3-Oxododecanoyl-ACP	3-Oxododecanoyl-ACP(c)	PIPES MCES	synthesis of cytosolic 3-Oxododecanoyl-ACP	1 P_i(c) 5 Glutamate(s) 6 Glycine(s) 11 Alanine(s) 4 Lysine(s) 8 Aspartate(s) 9 Arginine(s) 7 Glutamine(s) 8 Serine(s) 5 Methionine(s) 3 Phenylalanine(s) 4 Tyrosine(s) 3 Cysteine(s) 20 Leucine(s) 1 Histidine(s) 9 Proline(s) 1 Asparagine(s) 11 Valine(s) 6 Threonine(s) 4 Isoleucine(s) 1 Pantothenate(s) 629 ATP-energy(c) 8 NADPH-redox-potential(c) 3 Proton-gradient(c) 6 CoA-activated acetyl group(c)	7 H_2O(s) 1 CO_2(s) 1 3-Oxododecanoyl-ACP(c) 66 Na-gradient(c)	36	-	-	-	-	-	-	-	24	-	-	1
625	3-Oxododecanoyl-CoA	3-Oxododecanoyl-CoA(m)	PIPES MCES	synthesis of mitochondrial 3-Oxododecanoyl-CoA	2.17 P_i(c) 1 Glycine(s) 2 Aspartate(s) 2 Glutamine(s) 0.83 Glucose-6P(c) 0.5 Cystine(s) 1 Pantothenate(s) 1 Palmitolate(s) 13.2 ATP-energy(c) 1 Proton-gradient(c) 2 THF-activated methyl group(c)	2.17 H_2O(s) 2 CO_2(s) 2 Pyruvate(c) 2 Glutamate(s) 1 3-Oxododecanoyl-CoA(m) 2 NADH-redox-potential(c) 4 NADH-redox-potential(m) 3.5 NADPH-redox-potential(c) 3 FADH-redox-potential(c) 3 Na-gradient(c) 3 CoA-activated acetyl group(m)	44	17	-	-	-	-	-	-	11	-	3	-
626	3-Oxohexadecanoyl-ACP	3-Oxohexadecanoyl-ACP(c)	PIPES MCES	synthesis of cytosolic 3-Oxohexadecanoyl-ACP	1 P_i(c) 5 Glutamate(s) 6 Glycine(s) 11 Alanine(s) 4 Lysine(s) 8 Aspartate(s) 9 Arginine(s) 7 Glutamine(s) 8 Serine(s) 5 Methionine(s) 3 Phenylalanine(s) 4 Tyrosine(s) 3 Cysteine(s) 20 Leucine(s) 1 Histidine(s) 9 Proline(s) 1 Asparagine(s) 11 Valine(s) 6 Threonine(s) 4 Isoleucine(s) 1 Pantothenate(s) 631 ATP-energy(c) 12 NADPH-redox-potential(c) 3 Proton-gradient(c) 8 CoA-activated acetyl group(c)	9 H_2O(s) 1 CO_2(s) 1 3-Oxohexadecanoyl-ACP(c) 66 Na-gradient(c)	44	-	-	-	-	-	-	-	24	-	-	1

Continued on next page

Simulation	Definition			Solution													
				exchanges		reactions								transport			Prot
	Objective	Contraints	Comment	imports	exports	c	m	r	p	l	n	s	b	s-c	b-c	intra	syn
627 3-Oxohexanoyl-ACP	3-Oxohexanoyl-ACP(c)	PIPES MCES	synthesis of cytosolic 3-Oxohexanoyl-ACP	1 P_i(c) 5 Glutamate(s) 6 Glycine(s) 11 Alanine(s) 4 Lysine(s) 8 Aspartate(s) 9 Arginine(s) 7 Glutamine(s) 8 Serine(s) 5 Methionine(s) 3 Phenylalanine(s) 4 Tyrosine(s) 3 Cysteine(s) 20 Leucine(s) 1 Histidine(s) 9 Proline(s) 1 Asparagine(s) 11 Valine(s) 6 Threonine(s) 4 Isoleucine(s) 1 Pantothenate(s) 626 ATP-energy(c) 2 NADPH-redox-potential(c) 3 CoA-activated acetyl group(c)	4 H_2O(s) 1 CO_2(s) 1 3-Oxohexanoyl-ACP(c) 2 Proton-gradient(c) 82 Na-gradient(c)	24	-	-	-	-	-	-	-	24	-	-	1
628 3-Oxohexanoyl-CoA	3-Oxohexanoyl-CoA(m)	PIPES MCES	synthesis of mitochondrial 3-Oxohexanoyl-CoA	1.5 O_2(s) 2.17 P_i(c) 1 Glycine(s) 2 Aspartate(s) 2 Glutamine(s) 0.83 Glucose-6P(c) 0.5 Cystine(s) 1 Pantothenate(s) 1 Palmitolate(s) 13.2 ATP-energy(c) 2 THF-activated methyl group(c)	2.17 H_2O(s) 2 CO_2(s) 2 Pyruvate(c) 2 Glutamate(s) 1 3-Oxohexanoyl-CoA(m) 2 NADH-redox-potential(c) 7 NADH-redox-potential(m) 3.5 NADPH-redox-potential(c) 3 FADH-redox-potential(c) 1 Proton-gradient(c) 18 Proton-gradient(m) 5 Na-gradient(c) 6 CoA-activated acetyl group(m)	44	29	-	-	-	-	-	-	12	-	7	-
629 3-Oxooctanoyl-ACP	3-Oxooctanoyl-ACP(c)	PIPES MCES	synthesis of cytosolic 3-Oxooctanoyl-ACP	1 P_i(c) 5 Glutamate(s) 6 Glycine(s) 11 Alanine(s) 4 Lysine(s) 8 Aspartate(s) 9 Arginine(s) 7 Glutamine(s) 8 Serine(s) 5 Methionine(s) 3 Phenylalanine(s) 4 Tyrosine(s) 3 Cysteine(s) 20 Leucine(s) 1 Histidine(s) 9 Proline(s) 1 Asparagine(s) 11 Valine(s) 6 Threonine(s) 4 Isoleucine(s) 1 Pantothenate(s) 627 ATP-energy(c) 4 NADPH-redox-potential(c) 3 Proton-gradient(c) 4 CoA-activated acetyl group(c)	5 H_2O(s) 1 CO_2(s) 1 3-Oxooctanoyl-ACP(c) 66 Na-gradient(c)	28	-	-	-	-	-	-	-	24	-	-	1

Continued on next page

C5. Comprehensive list – continued.

Simulation	Definition			Solution													
				exchanges		reactions								transport			Prot
	Objective	Contraints	Comment	imports	exports	c	m	r	p	l	n	s	b	s-c	b-c	intra	syn
630 3-Oxooctanoyl-CoA	3-Oxooctanoyl-CoA(m)	PIPES MCES	synthesis of mitochondrial 3-Oxooctanoyl-CoA	1 O_2(s) 2.17 P_i(c) 1 Glycine(s) 2 Aspartate(s) 2 Glutamine(s) 0.83 Glucose-6P(c) 0.5 Cystine(s) 1 Pantothenate(s) 1 Palmitolate(s) 13.2 ATP-energy(c) 2 THF-activated methyl group(c)	2.17 H_2O(s) 2 CO_2(s) 2 Pyruvate(c) 2 Glutamate(s) 1 3-Oxooctanoyl-CoA(m) 2 NADH-redox-potential(c) 6 NADH-redox-potential(m) 3.5 NADPH-redox-potential(c) 3 FADH-redox-potential(c) 12 Proton-gradient(m) 3 Na-gradient(c) 5 CoA-activated acetyl group(m)	44	25	-	-	-	-	-	-	11	-	7	-
631 3-Oxopalmitoyl-CoA	3-Oxopalmitoyl-CoA(m)	PIPES MCES	synthesis of mitochondrial 3-Oxopalmitoyl-CoA	2.17 P_i(c) 1 Glycine(s) 0.5 Aspartate(s) 0.5 Glutamine(s) 0.83 Glucose-6P(c) 1.5 Asparagine(s) 0.5 Palmitate(s) 0.5 Cystine(s) 1 Pantothenate(s) 0.5 Palmitolate(s) 14.7 ATP-energy(c) 1 Proton-gradient(c) 2 THF-activated methyl group(c)	5.17 H_2O(s) 2 CO_2(s) 2 Pyruvate(c) 0.5 Glutamate(s) 1 3-Oxopalmitoyl-CoA(m) 2.5 NADH-redox-potential(c) 1 NADH-redox-potential(m) 3.5 NADPH-redox-potential(c) 6 Proton-gradient(m) 1.5 Na-gradient(c)	49	5	-	-	-	-	-	-	12	-	6	-
632 3-Oxopropanoate	3-Oxopropanoate(m)	PIPES MCES	synthesis of mitochondrial 3-Oxopropanoate	1 Pyruvate(c) 1 NADPH-redox-potential(c) 2 Proton-gradient(m)	1 3-Oxopropanoate(m) 1 NADH-redox-potential(c)	6	1	-	-	-	-	-	-	-	-	6	-
633 3-Oxostearoyl-ACP	3-Oxostearoyl-ACP(c)	PIPES MCES	synthesis of cytosolic 3-Oxostearoyl-ACP	1 P_i(c) 5 Glutamate(s) 6 Glycine(s) 11 Alanine(s) 4 Lysine(s) 8 Aspartate(s) 9 Arginine(s) 7 Glutamine(s) 8 Serine(s) 5 Methionine(s) 3 Phenylalanine(s) 4 Tyrosine(s) 3 Cysteine(s) 20 Leucine(s) 1 Histidine(s) 9 Proline(s) 1 Asparagine(s) 11 Valine(s) 6 Threonine(s) 4 Isoleucine(s) 1 Pantothenate(s) 632 ATP-energy(c) 14 NADPH-redox-potential(c) 3 Proton-gradient(c) 9 CoA-activated acetyl group(c)	10 H_2O(s) 1 CO_2(s) 1 3-Oxostearoyl-ACP(c) 66 Na-gradient(c)	48	-	-	-	-	-	-	-	24	-	-	1

Continued on next page

Simulation		Definition			Solution													
					exchanges		reactions								transport			Prot
		Objective	Contraints	Comment	imports	exports	c	m	r	p	l	n	s	b	s-c	b-c	intra	syn
634	3-Oxotetradecanoyl-ACP	3-Oxotetradecanoyl-ACP(c)	PIPES MCES	synthesis of cytosolic 3-Oxotetradecanoyl-ACP	1 P_i(c) 5 Glutamate(s) 6 Glycine(s) 11 Alanine(s) 4 Lysine(s) 8 Aspartate(s) 9 Arginine(s) 7 Glutamine(s) 8 Serine(s) 5 Methionine(s) 3 Phenylalanine(s) 4 Tyrosine(s) 3 Cysteine(s) 20 Leucine(s) 1 Histidine(s) 9 Proline(s) 1 Asparagine(s) 11 Valine(s) 6 Threonine(s) 4 Isoleucine(s) 1 Pantothenate(s) 630 ATP-energy(c) 10 NADPH-redox-potential(c) 3 Proton-gradient(c) 7 CoA-activated acetyl group(c)	8 H_2O(s) 1 CO_2(s) 1 3-Oxotetradecanoyl-ACP(c) 66 Na-gradient(c)	40	-	-	-	-	-	-	-	24	-	-	1
635	3-Oxotetradecanoyl-CoA	3-Oxotetradecanoyl-CoA(m)	PIPES MCES	synthesis of mitochondrial 3-Oxotetradecanoyl-CoA	2.17 P_i(c) 1 Glycine(s) 0.5 Aspartate(s) 0.5 Glutamine(s) 0.83 Glucose-6P(c) 1.5 Asparagine(s) 0.5 Cystine(s) 1 Pantothenate(s) 1 Palmitolate(s) 14.7 ATP-energy(c) 2 THF-activated methyl group(c)	4.17 H_2O(s) 2 CO_2(s) 2 Pyruvate(c) 0.5 Glutamate(s) 1 3-Oxotetradecanoyl-CoA(m) 3 NADH-redox-potential(c) 2 NADH-redox-potential(m) 3.5 NADPH-redox-potential(c) 12 Proton-gradient(m) 1.5 Na-gradient(c) 1 CoA-activated acetyl group(m)	48	10	-	-	-	-	-	-	10	-	6	-
636	3-Phosphonooxypyruvate	3-Phosphonooxypyruvate(c)	PIPES MCES	synthesis of cytosolic 3-Phosphonooxypyruvate	0.67 P_i(c) 0.33 Pyruvate(c) 0.33 Serine(s) 0.33 Glucose-6P(c) 0.33 NADPH-redox-potential(c)	0.33 Alanine(s) 1 3-Phosphonooxypyruvate(c) 1.67 NADH-redox-potential(c) 0.33 Proton-gradient(c) 0.33 Na-gradient(c)	12	-	-	-	-	-	-	-	4	-	-	-
637	3-Phosphoserine	3-Phosphoserine(c)	PIPES MCES	synthesis of cytosolic 3-Phosphoserine	0.67 P_i(c) 0.67 Alanine(s) 0.33 Serine(s) 0.33 Glucose-6P(c) 0.33 NADPH-redox-potential(c) 0.33 Proton-gradient(c)	0.67 Pyruvate(c) 1 3-Phosphoserine(c) 1.67 NADH-redox-potential(c) 0.33 Na-gradient(c)	14	-	-	-	-	-	-	-	4	-	-	-
638	3-Sulfinoalanine	3-Sulfinoalanine(c)	PIPES MCES	synthesis of cytosolic 3-Sulfinoalanine	1 O_2(s) 1 Cysteine(s)	1 3-Sulfinoalanine(c)	1	-	-	-	-	-	-	-	2	-	-	-

Continued on next page

Simulation		Definition			Solution													
					exchanges		reactions								transport			Prot
		Objective	Contraints	Comment	imports	exports	c	m	r	p	l	n	s	b	s-c	b-c	intra	syn
639	3-Ureidoisobutyrate	3-Ureidoisobutyrate(c)	PIPES MCES	synthesis of cytosolic 3-Ureidoisobutyrate	0.33 O_2(s) 1 Aspartate(s) 1 Glutamine(s) 0.33 Proline(s) 4 ATP-energy(c) 2 NADH-redox-potential(c) 1 THF-activated methyl group(c)	1.33 Glutamate(s) 1 3-Ureidoisobutyrate(c) 0.33 NADH-redox-potential(m) 1 NADPH-redox-potential(c) 1 Proton-gradient(c) 4 Proton-gradient(m) 2.33 Na-gradient(c)	26	4	-	-	-	-	-	-	7	-	6	-
640	3-Ureidopropionate	3-Ureidopropionate(c)	PIPES MCES	synthesis of cytosolic 3-Ureidopropionate	0.33 O_2(s) 1 Aspartate(s) 1 Glutamine(s) 0.33 Proline(s) 3 ATP-energy(c) 1 NADPH-redox-potential(c)	1.33 Glutamate(s) 1 3-Ureidopropionate(c) 0.33 NADH-redox-potential(m) 4 Proton-gradient(m) 1.33 Na-gradient(c)	17	4	-	-	-	-	-	-	6	-	6	-
641	3-oxolaur-cis-5-enoyl-CoA	3-oxolaur-cis-5-enoyl-CoA(m)	PIPES MCES	synthesis of mitochondrial 3-oxolaur-cis-5-enoyl-CoA	2.17 P_i(c) 1 Glycine(s) 2 Aspartate(s) 2 Glutamine(s) 0.83 Glucose-6P(c) 0.5 Cystine(s) 1 Pantothenate(s) 1 Palmitolate(s) 13.2 ATP-energy(c) 1 Proton-gradient(c) 2 THF-activated methyl group(c)	3.17 H_2O(s) 2 CO_2(s) 2 Pyruvate(c) 2 Glutamate(s) 2 NADH-redox-potential(c) 3 NADH-redox-potential(m) 3.5 NADPH-redox-potential(c) 3 FADH-redox-potential(c) 1 3-oxolaur-cis-5-enoyl-CoA(m) 3 Na-gradient(c) 2 CoA-activated acetyl group(m)	44	13	-	-	-	-	-	-	11	-	3	-
642	3-oxomyrist-7-enoyl-CoA	3-oxomyrist-7-enoyl-CoA(m)	PIPES MCES	synthesis of mitochondrial 3-oxomyrist-7-enoyl-CoA	2.17 P_i(c) 1 Glycine(s) 2 Aspartate(s) 2 Glutamine(s) 0.83 Glucose-6P(c) 0.5 Cystine(s) 1 Pantothenate(s) 1 Palmitolate(s) 13.2 ATP-energy(c) 1 Proton-gradient(c) 2 THF-activated methyl group(c)	4.17 H_2O(s) 2 CO_2(s) 2 Pyruvate(c) 2 Glutamate(s) 2 NADH-redox-potential(c) 2 NADH-redox-potential(m) 3.5 NADPH-redox-potential(c) 2 FADH-redox-potential(c) 1 3-oxomyrist-7-enoyl-CoA(m) 3 Na-gradient(c) 1 CoA-activated acetyl group(m)	44	9	-	-	-	-	-	-	11	-	3	-
643	3-oxooleoyl-CoA	3-oxooleoyl-CoA(m)	PIPES MCES	synthesis of mitochondrial 3-oxooleoyl-CoA	2.17 P_i(c) 1 Glycine(s) 2 Aspartate(s) 2 Glutamine(s) 0.83 Glucose-6P(c) 0.5 Cystine(s) 1 Pantothenate(s) 1 Palmitolate(s) 13.2 ATP-energy(c) 1 Proton-gradient(c) 2 THF-activated methyl group(c) 1 CoA-activated acetyl group(m)	6.17 H_2O(s) 2 CO_2(s) 2 Pyruvate(c) 2 Glutamate(s) 2 NADH-redox-potential(c) 3.5 NADPH-redox-potential(c) 1 3-oxooleoyl-CoA(m) 3 Na-gradient(c)	43	2	-	-	-	-	-	-	11	-	1	-

Continued on next page

C5. Comprehensive list – continued.

Simulation		Definition			Solution													
					exchanges		reactions								transport			Prot
		Objective	Contraints	Comment	imports	exports	c	m	r	p	l	n	s	b	s-c	b-c	intra	syn
644	3-oxopalmitoleoyl-CoA	3-oxopalmitoleoyl-CoA(m)	PIPES MCES	synthesis of mitochondrial 3-oxopalmitoleoyl-CoA	2.17 P_i(c) 1 Glycine(s) 2 Aspartate(s) 2 Glutamine(s) 0.83 Glucose-6P(c) 0.5 Cystine(s) 1 Pantothenate(s) 1 Palmitolate(s) 13.2 ATP-energy(c) 1 Proton-gradient(c) 2 THF-activated methyl group(c)	5.17 H_2O(s) 2 CO_2(s) 2 Pyruvate(c) 2 Glutamate(s) 2 NADH-redox-potential(c) 1 NADH-redox-potential(m) 3.5 NADPH-redox-potential(c) 1 FADH-redox-potential(c) 1 3-oxopalmitoleoyl-CoA(m) 3 Na-gradient(c)	44	4	-	-	-	-	-	-	11	-	3	-
645	3PG	3PG(c)	PIPES MCES	synthesis of cytosolic 3PG	0.67 P_i(c) 0.33 Pyruvate(c) 0.33 Serine(s) 0.33 Glucose-6P(c) 0.33 NADPH-redox-potential(c)	0.33 Alanine(s) 1 3PG(c) 0.67 NADH-redox-potential(c) 0.33 Proton-gradient(c) 0.33 Na-gradient(c)	11	-	-	-	-	-	-	-	4	-	-	-
646	3alpha,7alpha,12alpha,24-Tetrahydroxy-5beta-cholestanoyl-CoA	3alpha,7alpha,12alpha,24-Tetrahydroxy-5beta-cholestanoyl-CoA(p)	PIPES MCES	synthesis of peroxysomal 3alpha,7alpha,12alpha,24-Tetrahydroxy-5beta-cholestanoyl-CoA	11.5 O_2(s) 1 Glycine(s) 2 Aspartate(s) 2 Glutamine(s) 0.83 Glucose-6P(c) 2 Farnesyl-PP(r) 0.5 Cystine(s) 1 Pantothenate(s) 15.2 ATP-energy(c) 12 NADPH-redox-potential(r) 0.5 NADPH-redox-potential(c) 1 Proton-gradient(c) 1 THF-activated methyl group(c)	17.2 H_2O(s) 1.83 P_i(c) 4 CO_2(s) 2 Pyruvate(c) 2 Glutamate(s) 1 3alpha,7alpha,12alpha,24-Tetrahydroxy-5beta-cholestanoyl-CoA(p) 1 NADH-redox-potential(r) 3 NADH-redox-potential(c) 5 Na-gradient(c)	48	-	19	4	-	-	-	-	11	-	11	-
647	3alpha,7alpha,12alpha-Trihydroxy-5beta-24-oxocholestanoyl-CoA	3alpha,7alpha,12alpha-Trihydroxy-5beta-24-oxocholestanoyl-CoA(p)	PIPES MCES	synthesis of peroxysomal 3alpha,7alpha,12alpha-Trihydroxy-5beta-24-oxocholestanoyl-CoA	11.5 O_2(s) 1 Glycine(s) 2 Aspartate(s) 2 Glutamine(s) 0.83 Glucose-6P(c) 2 Farnesyl-PP(r) 0.5 Cystine(s) 1 Pantothenate(s) 15.2 ATP-energy(c) 12 NADPH-redox-potential(r) 0.5 NADPH-redox-potential(c) 1 Proton-gradient(c) 1 THF-activated methyl group(c)	17.2 H_2O(s) 1.83 P_i(c) 4 CO_2(s) 2 Pyruvate(c) 2 Glutamate(s) 1 3alpha,7alpha,12alpha-Trihydroxy-5beta-24-oxocholestanoyl-CoA(p) 1 NADH-redox-potential(r) 3 NADH-redox-potential(c) 1 NADH-redox-potential(p) 5 Na-gradient(c)	48	-	19	6	-	-	-	-	11	-	11	-

Continued on next page

C5. Comprehensive list – continued.

Simulation		Definition			Solution													
					exchanges		reactions								transport			Prot
		Objective	Contraints	Comment	imports	exports	c	m	r	p	l	n	s	b	s-c	b-c	intra	syn
648	3alpha,7alpha,12alpha-Trihydroxy-5beta-cholest-24-enoyl-CoA	3alpha,7alpha,12alpha-Trihydroxy-5beta-cholest-24-enoyl-CoA(p)	PIPES MCES	synthesis of peroxysomal 3alpha,7alpha,12alpha-Trihydroxy-5beta-cholest-24-enoyl-CoA	11.5 O_2(s) 1 Glycine(s) 2 Aspartate(s) 2 Glutamine(s) 0.83 Glucose-6P(c) 2 Farnesyl-PP(r) 0.5 Cystine(s) 1 Pantothenate(s) 15.2 ATP-energy(c) 12 NADPH-redox-potential(r) 0.5 NADPH-redox-potential(c) 1 Proton-gradient(c) 1 THF-activated methyl group(c)	18.2 H_2O(s) 1.83 P_i(c) 4 CO_2(s) 2 Pyruvate(c) 2 Glutamate(s) 1 3alpha,7alpha,12alpha-Trihydroxy-5beta-cholest-24-enoyl-CoA(p) 1 NADH-redox-potential(r) 3 NADH-redox-potential(c) 3 Na-gradient(c)	48	-	19	4	-	-	-	-	11	-	11	-
649	3alpha,7alpha,12alpha-Trihydroxy-5beta-cholestan-26-al	3alpha,7alpha,12alpha-Trihydroxy-5beta-cholestan-26-al(c)	PIPES MCES	synthesis of cytosolic 3alpha,7alpha,12alpha-Trihydroxy-5beta-cholestan-26-al	11 O_2(s) 2 Farnesyl-PP(r) 1 ATP-energy(c) 12 NADPH-redox-potential(r) 2 NADPH-redox-potential(c)	10 H_2O(s) 4 P_i(c) 3 CO_2(s) 1 3alpha,7alpha,12alpha-Trihydroxy-5beta-cholestan-26-al(c) 1 NADH-redox-potential(r) 1 NADH-redox-potential(c)	10	-	19	-	-	-	-	-	3	-	6	-
650	3alpha,7alpha,12alpha-Trihydroxy-5beta-cholestanate	3alpha,7alpha,12alpha-Trihydroxy-5beta-cholestanate(c)	PIPES MCES	synthesis of cytosolic 3alpha,7alpha,12alpha-Trihydroxy-5beta-cholestanate	11 O_2(s) 2 Farnesyl-PP(r) 1 ATP-energy(c) 12 NADPH-redox-potential(r) 2 NADPH-redox-potential(c)	9 H_2O(s) 4 P_i(c) 3 CO_2(s) 1 3alpha,7alpha,12alpha-Trihydroxy-5beta-cholestanate(c) 1 NADH-redox-potential(r) 2 NADH-redox-potential(c)	11	-	19	-	-	-	-	-	3	-	6	-
651	3alpha,7alpha,12alpha-Trihydroxy-5beta-cholestanoyl-CoA	3alpha,7alpha,12alpha-Trihydroxy-5beta-cholestanoyl-CoA(r)	PIPES MCES	synthesis of Golgi/ER 3alpha,7alpha,12alpha-Trihydroxy-5beta-cholestanoyl-CoA	11 O_2(s) 1 Glycine(s) 2 Aspartate(s) 2 Glutamine(s) 0.83 Glucose-6P(c) 2 Farnesyl-PP(r) 0.5 Cystine(s) 1 Pantothenate(s) 14.2 ATP-energy(c) 12 NADPH-redox-potential(r) 1 Proton-gradient(c) 1 THF-activated methyl group(c)	17.2 H_2O(s) 1.83 P_i(c) 4 CO_2(s) 2 Pyruvate(c) 2 Glutamate(s) 1 3alpha,7alpha,12alpha-Trihydroxy-5beta-cholestanoyl-CoA(r) 1 NADH-redox-potential(r) 2 NADH-redox-potential(c) 0.5 NADPH-redox-potential(c) 3 Na-gradient(c)	48	-	20	-	-	-	-	-	11	-	9	-
652	3alpha,7alpha,12alpha-Trihydroxycoprostane	3alpha,7alpha,12alpha-Trihydroxycoprostane(c)	PIPES MCES	synthesis of cytosolic 3alpha,7alpha,12alpha-Trihydroxycoprostane	10 O_2(s) 2 Farnesyl-PP(r) 1 ATP-energy(c) 12 NADPH-redox-potential(r) 1 NADPH-redox-potential(c)	9 H_2O(s) 4 P_i(c) 3 CO_2(s) 1 3alpha,7alpha,12alpha-Trihydroxycoprostane(c) 1 NADH-redox-potential(r)	7	-	19	-	-	-	-	-	3	-	6	-

Continued on next page

C5. Comprehensive list – continued.

Simulation		Definition			Solution													
					exchanges		reactions								transport			Prot
		Objective	Contraints	Comment	imports	exports	c	m	r	p	l	n	s	b	s-c	b-c	intra	syn
653	3alpha,7alpha-Dihydroxy-5beta-cholestan-26-al	3alpha,7alpha-Dihydroxy-5beta-cholestan-26-al(c)	PIPES MCES	synthesis of cytosolic 3alpha,7alpha-Dihydroxy-5beta-cholestan-26-al	10 O_2(s) 2 Farnesyl-PP(r) 1 ATP-energy(c) 11 NADPH-redox-potential(r) 1 NADPH-redox-potential(c) 1 NADPH-redox-potential(m)	9 H_2O(s) 4 P_i(c) 3 CO_2(s) 1 3alpha,7alpha-Dihydroxy-5beta-cholestan-26-al(c) 1 NADH-redox-potential(r) 1 NADH-redox-potential(c)	9	2	18	-	-	-	-	-	3	-	10	-
654	3alpha,7alpha-Dihydroxy-5beta-cholestanate	3alpha,7alpha-Dihydroxy-5beta-cholestanate(c)	PIPES MCES	synthesis of cytosolic 3alpha,7alpha-Dihydroxy-5beta-cholestanate	10 O_2(s) 2 Farnesyl-PP(r) 1 ATP-energy(c) 11 NADPH-redox-potential(r) 1 NADPH-redox-potential(c) 1 NADPH-redox-potential(m)	8 H_2O(s) 4 P_i(c) 3 CO_2(s) 1 3alpha,7alpha-Dihydroxy-5beta-cholestanate(c) 1 NADH-redox-potential(r) 2 NADH-redox-potential(c)	10	2	18	-	-	-	-	-	3	-	10	-
655	3alpha,7alpha-Dihydroxy-5beta-cholestanoyl-CoA	3alpha,7alpha-Dihydroxy-5beta-cholestanoyl-CoA(r)	PIPES MCES	synthesis of Golgi/ER 3alpha,7alpha-Dihydroxy-5beta-cholestanoyl-CoA	10 O_2(s) 1 Glycine(s) 2 Aspartate(s) 2 Glutamine(s) 0.83 Glucose-6P(c) 2 Farnesyl-PP(r) 0.5 Cystine(s) 1 Pantothenate(s) 14.2 ATP-energy(c) 11 NADPH-redox-potential(r) 1 NADPH-redox-potential(m) 1 Proton-gradient(c) 1 THF-activated methyl group(c)	16.2 H_2O(s) 1.83 P_i(c) 4 CO_2(s) 2 Pyruvate(c) 2 Glutamate(s) 1 3alpha,7alpha-Dihydroxy-5beta-cholestanoyl-CoA(r) 1 NADH-redox-potential(r) 3 NADH-redox-potential(c) 0.5 NADPH-redox-potential(c) 3 Na-gradient(c)	47	2	19	-	-	-	-	-	11	-	13	-
656	4,4-Dimethyl-5alpha-cholesta-8,14,24-trien-3beta-ol	4,4-Dimethyl-5alpha-cholesta-8,14,24-trien-3beta-ol(r)	PIPES MCES	synthesis of Golgi/ER 4,4-Dimethyl-5alpha-cholesta-8,14,24-trien-3beta-ol	4 O_2(s) 2 Farnesyl-PP(r) 1 ATP-energy(c) 5 NADPH-redox-potential(r)	3 H_2O(s) 4 P_i(c) 1 CO_2(s) 1 4,4-Dimethyl-5alpha-cholesta-8,14,24-trien-3beta-ol(r) 1 NADPH-redox-potential(c)	5	-	6	-	-	-	-	-	3	-	4	-
657	4-(2-Amino-3-hydroxyphenyl)-2,4-dioxobutanoate	4-(2-Amino-3-hydroxyphenyl)-2,4-dioxobutanoate(c)	PIPES MCES	synthesis of cytosolic 4-(2-Amino-3-hydroxyphenyl)-2,4-dioxobutanoate	2 O_2(s) 1 Pyruvate(c) 1 Tryptophan(s) 1 ATP-energy(c)	1 CO_2(s) 1 Alanine(s) 1 4-(2-Amino-3-hydroxyphenyl)-2,4-dioxobutanoate(c) 1 Na-gradient(c)	8	-	-	-	-	-	-	-	5	-	-	-

Continued on next page

C5. Comprehensive list – continued.

Simulation		Definition			Solution													
					exchanges		reactions								transport			Prot
		Objective	Contraints	Comment	imports	exports	c	m	r	p	l	n	s	b	s-c	b-c	intra	syn
658	4-Hydroxyphenylpyruvate	4-Hydroxyphenylpyruvate(c)	PIPES MCES	synthesis of cytosolic 4-Hydroxyphenylpyruvate	1 Pyruvate(c) 1 Tyrosine(s)	1 Alanine(s) 1 4-Hydroxyphenylpyruvate(c) 1 Na-gradient(c)	2	-	-	-	-	-	-	-	3	-	-	-
659	4-Imidazolone-5-propanoate	4-Imidazolone-5-propanoate(c)	PIPES MCES	synthesis of cytosolic 4-Imidazolone-5-propanoate	1 Glutamate(s) 1 Histidine(s) 1 ATP-energy(c)	1 Glutamine(s) 1 4-Imidazolone-5-propanoate(c) 1 Na-gradient(c)	4	-	-	-	-	-	-	-	4	-	-	-
660	4-Maleylacetoacetate	4-Maleylacetoacetate(c)	PIPES MCES	synthesis of cytosolic 4-Maleylacetoacetate	2 O_2(s) 1 Pyruvate(c) 1 Tyrosine(s)	1 CO_2(s) 1 Alanine(s) 1 4-Maleylacetoacetate(c) 1 Na-gradient(c)	4	-	-	-	-	-	-	-	5	-	-	-
661	4-Methyl-2-oxopentanoate	4-Methyl-2-oxopentanoate(m)	PIPES MCES	synthesis of mitochondrial 4-Methyl-2-oxopentanoate	0.4 Pyruvate(m) 0.6 Leucine(s) 0.2 ATP-energy(c) 0.6 NADH-redox-potential(m) 0.2 Proton-gradient(m) 1.6 CoA-activated acetyl group(m)	0.2 Glutamine(s) 0.2 Proline(s) 1 4-Methyl-2-oxopentanoate(m) 0.4 NADPH-redox-potential(m) 0.6 Na-gradient(c)	2	19	-	-	-	-	-	-	5	-	7	-
662	4alpha-Methylzymosterol	4alpha-Methylzymosterol(r)	PIPES MCES	synthesis of Golgi/ER 4alpha-Methylzymosterol	7 O_2(s) 2 Farnesyl-PP(r) 1 ATP-energy(c) 8 NADPH-redox-potential(r)	7 H_2O(s) 4 P_i(c) 2 CO_2(s) 1 4alpha-Methylzymosterol(r) 1 NADH-redox-potential(r) 1 NADPH-redox-potential(c)	5	-	11	-	-	-	-	-	3	-	5	-
663	4alpha-Methylzymosterol-4-carboxylate	4alpha-Methylzymosterol-4-carboxylate(r)	PIPES MCES	synthesis of Golgi/ER 4alpha-Methylzymosterol-4-carboxylate	7 O_2(s) 2 Farnesyl-PP(r) 1 ATP-energy(c) 9 NADPH-redox-potential(r)	7 H_2O(s) 4 P_i(c) 1 CO_2(s) 1 4alpha-Methylzymosterol-4-carboxylate(r) 1 NADPH-redox-potential(c)	5	-	8	-	-	-	-	-	3	-	4	-
664	5,10-Methenyl-THF	5,10-Methenyl-THF(c)	PIPES MCES	synthesis of cytosolic 5,10-Methenyl-THF	1 Folate(s) 1 NADPH-redox-potential(c) 1 THF-activated methyl group(c)	1 5,10-Methenyl-THF(c) 1 NADH-redox-potential(c)	7	-	-	-	-	-	-	-	1	-	-	-
665	5,6-Dihydrouracil	5,6-Dihydrouracil(c)	PIPES MCES	synthesis of cytosolic 5,6-Dihydrouracil	0.33 O_2(s) 1 Aspartate(s) 1 Glutamine(s) 0.33 Proline(s) 3 ATP-energy(c) 1 NADPH-redox-potential(c)	1 H_2O(s) 1.33 Glutamate(s) 1 5,6-Dihydrouracil(c) 0.33 NADH-redox-potential(m) 4 Proton-gradient(m) 1.33 Na-gradient(c)	16	4	-	-	-	-	-	-	7	-	6	-

Continued on next page

C5. Comprehensive list – continued.

	Simulation	Definition			Solution													
					exchanges		reactions								transport			Prot
		Objective	Contraints	Comment	imports	exports	c	m	r	p	l	n	s	b	s-c	b-c	intra	syn
666	5-Aminolevulinate	5-Aminolevulinate(c)	PIPES MCES	synthesis of cytosolic 5-Aminolevulinate	0.5 Pyruvate(m) 1 Glutamate(s) 0.5 Serine(s) 1 Proton-gradient(m)	1 CO_2(s) 0.5 Aspartate(s) 1 5-Aminolevulinate(c) 1 NADH-redox-potential(m) 1 Na-gradient(c)	-	9	-	-	-	-	-	-	5	-	6	-
667	5-Formiminotetrahydrofolate	5-Formiminotetrahydrofolate(c)	PIPES MCES	synthesis of cytosolic 5-Formiminotetrahydrofolate	1 NH_3(s) 1 Folate(s) 1 NADPH-redox-potential(c) 1 THF-activated methyl group(c)	1 5-Formiminotetrahydrofolate(c) 1 NADH-redox-potential(c)	8	-	-	-	-	-	-	-	2	-	-	-
668	5-Formyl-THF	5-Formyl-THF(m)	PIPES MCES	synthesis of mitochondrial 5-Formyl-THF	1 H_2O(s) 3.5 O_2(s) 1 Glutamate(s) 1 Tryptophan(s) 1 Folate(s) 1 ATP-energy(c) 1 ATP-energy(m) 2 NADPH-redox-potential(c) 2 NADPH-redox-potential(m)	3 CO_2(s) 1 Alanine(s) 1 Glutamine(s) 1 5-Formyl-THF(m) 1 NADH-redox-potential(c) 2 NADH-redox-potential(m) 5 Proton-gradient(m) 2 Na-gradient(c) 2 CoA-activated acetyl group(m)	12	14	-	-	-	-	-	-	9	-	11	-
669	5-Hydroxy-L-tryptophan	5-Hydroxy-L-tryptophan(c)	PIPES MCES	synthesis of cytosolic 5-Hydroxy-L-tryptophan	1 O_2(s) 1 Tryptophan(s) 1 NADPH-redox-potential(c)	1 H_2O(s) 1 5-Hydroxy-L-tryptophan(c) 1 Na-gradient(c)	3	-	-	-	-	-	-	-	4	-	-	-
670	5-Methyl-THF	5-Methyl-THF(c)	PIPES MCES	synthesis of cytosolic 5-Methyl-THF	1 Folate(s) 2 NADPH-redox-potential(c) 1 THF-activated methyl group(c)	1 5-Methyl-THF(c)	4	-	-	-	-	-	-	-	1	-	-	-
671	5-Methylthioadenosine	5-Methylthioadenosine(c)	PIPES MCES	synthesis of cytosolic 5-Methylthioadenosine	1 Glycine(s) 2 Aspartate(s) 2 Glutamine(s) 1 Methionine(s) 0.83 Glucose-6P(c) 9.17 ATP-energy(c) 1 Proton-gradient(c) 2 THF-activated methyl group(c)	1.17 H_2O(s) 0.83 P_i(c) 2 CO_2(s) 2 Pyruvate(c) 2 Glutamate(s) 1 5-Methylthioadenosine(c) 2 NADH-redox-potential(c) 4 NADPH-redox-potential(c) 1 Adenosylmethioninamine-potential(c) 3 Na-gradient(c)	37	-	-	-	-	-	-	-	9	-	-	-
672	5-Oxoproline	5-Oxoproline(c)	PIPES MCES	synthesis of cytosolic 5-Oxoproline	1 Glutamate(s) 1 ATP-energy(c)	1 H_2O(s) 1 5-Oxoproline(c) 1 Na-gradient(c)	3	-	-	-	-	-	-	-	3	-	-	-
673	5-Phosphoribosyl-4-carboxy-5-aminoimidazole	5-Phosphoribosyl-4-carboxy-5-aminoimidazole(c)	PIPES MCES	synthesis of cytosolic 5-Phosphoribosyl-4-carboxy-5-aminoimidazole	1 Glycine(s) 2 Glutamine(s) 1 Glucose-6P(c) 5 ATP-energy(c) 1 Proton-gradient(c) 1 THF-activated methyl group(c)	2 Glutamate(s) 1 5-Phosphoribosyl-4-carboxy-5-aminoimidazole(c) 1 NADH-redox-potential(c) 3 NADPH-redox-potential(c)	20	-	-	-	-	-	-	-	4	-	-	-

Continued on next page

	Simulation	Definition			Solution													
					exchanges		reactions								transport			Prot
		Objective	Contraints	Comment	imports	exports	c	m	r	p	l	n	s	b	s-c	b-c	intra	syn
674	5-Phosphoribosylamine	5-Phosphoribosylamine(c)	PIPES MCES	synthesis of cytosolic 5-Phosphoribosylamine	0.17 P_i(c) 1 Glutamine(s) 0.83 Glucose-6P(c) 2.17 ATP-energy(c)	0.17 H_2O(s) 1 Glutamate(s) 1 5-Phosphoribosylamine(c)	14	-	-	-	-	-	-	-	3	-	-	-
675	5-Phosphoribosylformylglycinamidine	5-Phosphoribosylformylglycinamidine(c)	PIPES MCES	synthesis of cytosolic 5-Phosphoribosylformylglycinamidine	0.17 P_i(c) 1 Glycine(s) 2 Glutamine(s) 0.83 Glucose-6P(c) 4.17 ATP-energy(c) 1 Proton-gradient(c) 1 THF-activated methyl group(c)	0.17 H_2O(s) 2 Glutamate(s) 1 5-Phosphoribosylformylglycinamidine(c) 1 NADH-redox-potential(c) 1 NADPH-redox-potential(c)	23	-	-	-	-	-	-	-	5	-	-	-
676	5alpha-Cholesta-7,24-dien-3beta-ol	5alpha-Cholesta-7,24-dien-3beta-ol(r)	PIPES MCES	synthesis of Golgi/ER 5alpha-Cholesta-7,24-dien-3beta-ol	7 O_2(s) 2 Farnesyl-PP(r) 1 ATP-energy(c) 8 NADPH-redox-potential(r)	5 H_2O(s) 4 P_i(c) 3 CO_2(s) 1 5alpha-Cholesta-7,24-dien-3beta-ol(r) 1 NADH-redox-potential(r) 1 NADPH-redox-potential(c)	5	-	13	-	-	-	-	-	3	-	5	-
677	5beta-Cholestane-3alpha,7alpha,12alpha,26-tetrol	5beta-Cholestane-3alpha,7alpha,12alpha,26-tetrol(c)	PIPES MCES	synthesis of cytosolic 5beta-Cholestane-3alpha,7alpha,12alpha,26-tetrol	11 O_2(s) 2 Farnesyl-PP(r) 1 ATP-energy(c) 12 NADPH-redox-potential(r) 2 NADPH-redox-potential(c)	10 H_2O(s) 4 P_i(c) 3 CO_2(s) 1 5beta-Cholestane-3alpha,7alpha,12alpha,26-tetrol(c) 1 NADH-redox-potential(r)	8	-	19	-	-	-	-	-	3	-	6	-
678	5beta-Cholestane-3alpha,7alpha,26-triol	5beta-Cholestane-3alpha,7alpha,26-triol(c)	PIPES MCES	synthesis of cytosolic 5beta-Cholestane-3alpha,7alpha,26-triol	10 O_2(s) 2 Farnesyl-PP(r) 1 ATP-energy(c) 11 NADPH-redox-potential(r) 1 NADPH-redox-potential(c) 1 NADPH-redox-potential(m)	9 H_2O(s) 4 P_i(c) 3 CO_2(s) 1 5beta-Cholestane-3alpha,7alpha,26-triol(c) 1 NADH-redox-potential(r)	6	2	19	-	-	-	-	-	3	-	10	-
679	5beta-Cholestane-3alpha,7alpha-diol	5beta-Cholestane-3alpha,7alpha-diol(c)	PIPES MCES	synthesis of cytosolic 5beta-Cholestane-3alpha,7alpha-diol	9 O_2(s) 2 Farnesyl-PP(r) 1 ATP-energy(c) 11 NADPH-redox-potential(r) 1 NADPH-redox-potential(c)	8 H_2O(s) 4 P_i(c) 3 CO_2(s) 1 5beta-Cholestane-3alpha,7alpha-diol(c) 1 NADH-redox-potential(r)	7	-	18	-	-	-	-	-	3	-	6	-
680	6-Phospho-D-gluconate	6-Phospho-D-gluconate(c)	PIPES MCES	synthesis of cytosolic 6-Phospho-D-gluconate	1 H_2O(s) 1 Glucose-6P(c)	1 6-Phospho-D-gluconate(c) 1 NADPH-redox-potential(c)	3	-	-	-	-	-	-	-	1	-	-	-

Continued on next page

Simulation		Definition			Solution													
					exchanges		reactions								transport			Prot
		Objective	Contraints	Comment	imports	exports	c	m	r	p	l	n	s	b	s-c	b-c	intra	syn
681	6-Pyruvoyltetrahydropterin	6-Pyruvoyltetrahydropterin(c)	PIPES MCES	synthesis of cytosolic 6-Pyruvoyltetrahydropterin	1 Glycine(s) 2 Glutamine(s) 0.83 Glucose-6P(c) 1 Asparagine(s) 11.2 ATP-energy(c) 1 Proton-gradient(c) 1 THF-activated methyl group(c)	1.17 H_2O(s) 0.83 P_i(c) 1 Pyruvate(c) 2 Glutamate(s) 1 6-Pyruvoyltetrahydropterin(c) 2 NADH-redox-potential(c) 2 NADPH-redox-potential(c)	40	-	-	-	-	-	-	-	6	-	-	-
682	7-Dehydrodesmosterol	7-Dehydrodesmosterol(r)	PIPES MCES	synthesis of Golgi/ER 7-Dehydrodesmosterol	8 O_2(s) 2 Farnesyl-PP(r) 1 ATP-energy(c) 9 NADPH-redox-potential(r)	7 H_2O(s) 4 P_i(c) 3 CO_2(s) 1 7-Dehydrodesmosterol(r) 1 NADH-redox-potential(r) 1 NADPH-redox-potential(c)	5	-	14	-	-	-	-	-	3	-	5	-
683	7alpha,12alpha-Dihydroxy-5beta-cholestan-3-one	7alpha,12alpha-Dihydroxy-5beta-cholestan-3-one(c)	PIPES MCES	synthesis of cytosolic 7alpha,12alpha-Dihydroxy-5beta-cholestan-3-one	10 O_2(s) 2 Farnesyl-PP(r) 1 ATP-energy(c) 12 NADPH-redox-potential(r)	9 H_2O(s) 4 P_i(c) 3 CO_2(s) 1 7alpha,12alpha-Dihydroxy-5beta-cholestan-3-one(c) 1 NADH-redox-potential(r)	5	-	19	-	-	-	-	-	3	-	6	-
684	7alpha,12alpha-Dihydroxycholest-4-en-3-one	7alpha,12alpha-Dihydroxycholest-4-en-3-one(c)	PIPES MCES	synthesis of cytosolic 7alpha,12alpha-Dihydroxycholest-4-en-3-one	10 O_2(s) 2 Farnesyl-PP(r) 1 ATP-energy(c) 12 NADPH-redox-potential(r)	9 H_2O(s) 4 P_i(c) 3 CO_2(s) 1 7alpha,12alpha-Dihydroxycholest-4-en-3-one(c) 1 NADH-redox-potential(r) 1 NADPH-redox-potential(c)	5	-	19	-	-	-	-	-	3	-	6	-
685	7alpha-Hydroxy-5beta-cholestan-3-one	7alpha-Hydroxy-5beta-cholestan-3-one(c)	PIPES MCES	synthesis of cytosolic 7alpha-Hydroxy-5beta-cholestan-3-one	9 O_2(s) 2 Farnesyl-PP(r) 1 ATP-energy(c) 11 NADPH-redox-potential(r)	8 H_2O(s) 4 P_i(c) 3 CO_2(s) 1 7alpha-Hydroxy-5beta-cholestan-3-one(c) 1 NADH-redox-potential(r)	5	-	18	-	-	-	-	-	3	-	6	-
686	7alpha-Hydroxycholest-4-en-3-one	7alpha-Hydroxycholest-4-en-3-one(c)	PIPES MCES	synthesis of cytosolic 7alpha-Hydroxycholest-4-en-3-one	9 O_2(s) 2 Farnesyl-PP(r) 1 ATP-energy(c) 11 NADPH-redox-potential(r)	8 H_2O(s) 4 P_i(c) 3 CO_2(s) 1 7alpha-Hydroxycholest-4-en-3-one(c) 1 NADH-redox-potential(r) 1 NADPH-redox-potential(c)	5	-	18	-	-	-	-	-	3	-	6	-
687	7alpha-Hydroxycholesterol	7alpha-Hydroxycholesterol(r)	PIPES MCES	synthesis of Golgi/ER 7alpha-Hydroxycholesterol	9 O_2(s) 2 Farnesyl-PP(r) 1 ATP-energy(c) 12 NADPH-redox-potential(r)	8 H_2O(s) 4 P_i(c) 3 CO_2(s) 1 7alpha-Hydroxycholesterol(r) 1 NADH-redox-potential(r) 1 NADPH-redox-potential(c)	5	-	17	-	-	-	-	-	3	-	5	-

Continued on next page

C5. Comprehensive list – continued.

Simulation	Definition			Solution													
				exchanges		reactions								transport			Prot
	Objective	Contraints	Comment	imports	exports	c	m	r	p	l	n	s	b	s-c	b-c	intra	syn
688 ACP	ACP(c)	PIPES MCES	synthesis of cytosolic ACP	1 P_i(c) 5 Glutamate(s) 6 Glycine(s) 11 Alanine(s) 4 Lysine(s) 8 Aspartate(s) 9 Arginine(s) 7 Glutamine(s) 8 Serine(s) 5 Methionine(s) 3 Phenylalanine(s) 4 Tyrosine(s) 3 Cysteine(s) 20 Leucine(s) 1 Histidine(s) 9 Proline(s) 1 Asparagine(s) 11 Valine(s) 6 Threonine(s) 4 Isoleucine(s) 1 Pantothenate(s) 624 ATP-energy(c) 9 Proton-gradient(c)	3 H_2O(s) 1 CO_2(s) 1 ACP(c) 82 Na-gradient(c)	12	-	-	-	-	-	-	-	24	-	-	1
689 ADP	ADP(c)	PIPES MCES	synthesis of ADP	1.17 P_i(c) 1 Glycine(s) 0.83 Glucose-6P(c) 2 Asparagine(s) 10.2 ATP-energy(c) 2 THF-activated methyl group(c)	2.17 H_2O(s) 1 ADP(c) 1 CO_2(s) 2 Pyruvate(c) 2 NADH-redox-potential(c) 4 NADPH-redox-potential(c) 1 Proton-gradient(c) 2 Na-gradient(c)	36	-	-	-	-	-	-	-	6	-	-	-
690 AICAR	AICAR(c)	PIPES MCES	synthesis of cytosolic AICAR	0.17 P_i(c) 1 Glycine(s) 1 Aspartate(s) 2 Glutamine(s) 0.83 Glucose-6P(c) 6.17 ATP-energy(c) 1 THF-activated methyl group(c)	1.17 H_2O(s) 1 Pyruvate(c) 2 Glutamate(s) 1 AICAR(c) 1 NADH-redox-potential(c) 2 NADPH-redox-potential(c) 2 Proton-gradient(c) 3 Na-gradient(c)	29	-	-	-	-	-	-	-	7	-	-	-
691 AIR	AIR(c)	PIPES MCES	synthesis of cytosolic AIR	0.17 P_i(c) 1 Glycine(s) 2 Glutamine(s) 0.83 Glucose-6P(c) 5.17 ATP-energy(c) 1 Proton-gradient(c) 1 THF-activated methyl group(c)	1.17 H_2O(s) 2 Glutamate(s) 1 AIR(c) 1 NADH-redox-potential(c) 1 NADPH-redox-potential(c)	24	-	-	-	-	-	-	-	5	-	-	-
692 AMP	AMP(c)	PIPES MCES	synthesis of AMP	0.17 P_i(c) 1 Glycine(s) 0.83 Glucose-6P(c) 2 Asparagine(s) 9.17 ATP-energy(c) 2 THF-activated methyl group(c)	1.17 H_2O(s) 1 CO_2(s) 1 AMP(c) 2 Pyruvate(c) 2 NADH-redox-potential(c) 4 NADPH-redox-potential(c) 1 Proton-gradient(c) 2 Na-gradient(c)	36	-	-	-	-	-	-	-	6	-	-	-
693 Acetaldehyde	Acetaldehyde(s)	PIPES MCES	synthesis of Acetaldehyde and blood export	1 Glutamate(s) 1 Ethanolamine(c) 2 ATP-energy(c)	1 H_2O(s) 1 Glutamine(s) 1 Acetaldehyde(s) 1 Na-gradient(c)	4	-	-	-	-	-	-	-	5	-	-	-

Continued on next page

C5. Comprehensive list – continued.

Simulation	Definition			Solution													
				exchanges		reactions								transport			Prot
	Objective	Contraints	Comment	imports	exports	c	m	r	p	l	n	s	b	s-c	b-c	intra	syn
694 Acetate	Acetate(s)	PIPES MCES	synthesis of Acetate and blood export	1 H_2O(s) 1 CoA-activated acetyl group(c)	1 Acetate(s) 1 Proton-gradient(c)	2	-	-	-	-	-	-	-	3	-	-	-
695 Acetoacetyl-ACP	Acetoacetyl-ACP(c)	PIPES MCES	synthesis of cytosolic Acetoacetyl-ACP	1 P_i(c) 5 Glutamate(s) 6 Glycine(s) 11 Alanine(s) 4 Lysine(s) 8 Aspartate(s) 9 Arginine(s) 7 Glutamine(s) 8 Serine(s) 5 Methionine(s) 3 Phenylalanine(s) 4 Tyrosine(s) 3 Cysteine(s) 20 Leucine(s) 1 Histidine(s) 9 Proline(s) 1 Asparagine(s) 11 Valine(s) 6 Threonine(s) 4 Isoleucine(s) 1 Pantothenate(s) 625 ATP-energy(c) 9 Proton-gradient(c) 2 CoA-activated acetyl group(c)	3 H_2O(s) 1 CO_2(s) 1 Acetoacetyl-ACP(c) 82 Na-gradient(c)	19	-	-	-	-	-	-	-	24	-	-	1
696 Acetoacetyl-CoA	Acetoacetyl-CoA(c)	PIPES MCES	synthesis of cytosolic Acetoacetyl-CoA	2.17 P_i(c) 1 Glycine(s) 2 Aspartate(s) 2 Glutamine(s) 0.83 Glucose-6P(c) 0.5 Cystine(s) 1 Pantothenate(s) 11.2 ATP-energy(c) 1 Proton-gradient(c) 2 THF-activated methyl group(c) 2 CoA-activated acetyl group(c)	5.17 H_2O(s) 2 CO_2(s) 2 Pyruvate(c) 2 Glutamate(s) 1 Acetoacetyl-CoA(c) 2 NADH-redox-potential(c) 3.5 NADPH-redox-potential(c) 3 Na-gradient(c)	44	-	-	-	-	-	-	-	10	-	-	-
697 Acetyl-ACP	Acetyl-ACP(c)	PIPES MCES	synthesis of cytosolic Acetyl-ACP	1 P_i(c) 5 Glutamate(s) 6 Glycine(s) 11 Alanine(s) 4 Lysine(s) 8 Aspartate(s) 9 Arginine(s) 7 Glutamine(s) 8 Serine(s) 5 Methionine(s) 3 Phenylalanine(s) 4 Tyrosine(s) 3 Cysteine(s) 20 Leucine(s) 1 Histidine(s) 9 Proline(s) 1 Asparagine(s) 11 Valine(s) 6 Threonine(s) 4 Isoleucine(s) 1 Pantothenate(s) 624 ATP-energy(c) 9 Proton-gradient(c) 1 CoA-activated acetyl group(c)	3 H_2O(s) 1 CO_2(s) 1 Acetyl-ACP(c) 82 Na-gradient(c)	14	-	-	-	-	-	-	-	24	-	-	1

Continued on next page

C5. Comprehensive list – continued.

Simulation	Definition			Solution													
				exchanges		reactions								transport			Prot
	Objective	Contraints	Comment	imports	exports	c	m	r	p	l	n	s	b	s-c	b-c	intra	syn
698 Acetyl-CoA	Acetyl-CoA(c)	PIPES MCES	synthesis of cytosolic Acetyl-CoA	2.17 P_i(c) 1 Glycine(s) 2 Aspartate(s) 2 Glutamine(s) 0.83 Glucose-6P(c) 0.5 Cystine(s) 1 Pantothenate(s) 11.2 ATP-energy(c) 1 Proton-gradient(c) 2 THF-activated methyl group(c) 1 CoA-activated acetyl group(c)	5.17 H_2O(s) 2 CO_2(s) 2 Pyruvate(c) 1 Acetyl-CoA(c) 2 Glutamate(s) 2 NADH-redox-potential(c) 3.5 NADPH-redox-potential(c) 3 Na-gradient(c)	43	-	-	-	-	-	-	-	10	-	-	-
699 Acrylyl-CoA	Acrylyl-CoA(m)	PIPES MCES	synthesis of mitochondrial Acrylyl-CoA	0.33 O_2(s) 2.17 P_i(c) 1 Glycine(s) 2 Aspartate(s) 1 Glutamine(s) 0.83 Glucose-6P(c) 0.33 Proline(s) 1 Threonine(s) 0.5 Cystine(s) 1 Pantothenate(s) 12.2 ATP-energy(c) 1 Proton-gradient(c) 2 THF-activated methyl group(c)	6.17 H_2O(s) 3 CO_2(s) 2 Pyruvate(c) 1.33 Glutamate(s) 1 Acrylyl-CoA(m) 2 NADH-redox-potential(c) 1.33 NADH-redox-potential(m) 3.5 NADPH-redox-potential(c) 3 Proton-gradient(m) 4.33 Na-gradient(c)	44	6	-	-	-	-	-	-	13	-	8	-
700 Acyl-CoA-Bile-PC-pool	Acyl-CoA-Bile-PC-pool(c)	PIPES MCES	synthesis of cytosolic Acyl-CoA-Bile-PC-pool	2.17 P_i(c) 1 Glycine(s) 2 Aspartate(s) 2 Glutamine(s) 0.83 Glucose-6P(c) 0.06 Arachidonate(s) 0.41 Palmitate(s) 0.5 Cystine(s) 0.12 Oleate(s) 1 Pantothenate(s) 0.05 Stearate(s) 0.33 Linoleate(s) 0.03 Palmitolate(s) 13.2 ATP-energy(c) 1 Proton-gradient(c) 2 THF-activated methyl group(c)	6.17 H_2O(s) 2 CO_2(s) 2 Pyruvate(c) 2 Glutamate(s) 1 Acyl-CoA-Bile-PC-pool(c) 2 NADH-redox-potential(c) 3.5 NADPH-redox-potential(c) 3 Na-gradient(c)	49	-	-	-	-	-	-	-	16	-	-	-
701 Acyl-CoA-CL-pool	Acyl-CoA-CL-pool(c)	PIPES MCES	synthesis of cytosolic Acyl-CoA-CL-pool	2.17 P_i(c) 1 Glycine(s) 2 Aspartate(s) 2 Glutamine(s) 0.83 Glucose-6P(c) 0.04 Palmitate(s) 0.5 Cystine(s) 0.14 Oleate(s) 1 Pantothenate(s) 0.02 Stearate(s) 0.75 Linoleate(s) 0.05 Palmitolate(s) 13.2 ATP-energy(c) 1 Proton-gradient(c) 2 THF-activated methyl group(c)	6.17 H_2O(s) 2 CO_2(s) 2 Pyruvate(c) 2 Glutamate(s) 1 Acyl-CoA-CL-pool(c) 2 NADH-redox-potential(c) 3.5 NADPH-redox-potential(c) 3 Na-gradient(c)	48	-	-	-	-	-	-	-	15	-	-	-

Continued on next page

C5. Comprehensive list – continued.

Simulation	Definition			Solution													
				exchanges		reactions								transport			Prot
	Objective	Contraints	Comment	imports	exports	c	m	r	p	l	n	s	b	s-c	b-c	intra	syn
702 Acyl-CoA-VLDL-PC-pool	Acyl-CoA-VLDL-PC-pool(c)	PIPES MCES	synthesis of cytosolic Acyl-CoA-VLDL-PC-pool	2.17 P_i(c) 1 Glycine(s) 2 Aspartate(s) 2 Glutamine(s) 0.83 Glucose-6P(c) 0.08 Arachidonate(s) 0.35 Palmitate(s) 0.5 Cystine(s) 0.14 Oleate(s) 1 Pantothenate(s) 0.15 Stearate(s) 0.27 Linoleate(s) 13.2 ATP-energy(c) 1 Proton-gradient(c) 2 THF-activated methyl group(c)	6.17 H_2O(s) 2 CO_2(s) 2 Pyruvate(c) 2 Glutamate(s) 1 Acyl-CoA-VLDL-PC-pool(c) 2 NADH-redox-potential(c) 3.5 NADPH-redox-potential(c) 3 Na-gradient(c)	48	-	-	-	-	-	-	-	15	-	-	-
703 Acyl-CoA-VLDL-PE-pool	Acyl-CoA-VLDL-PE-pool(c)	PIPES MCES	synthesis of cytosolic Acyl-CoA-VLDL-PE-pool	2.17 P_i(c) 1 Glycine(s) 2 Aspartate(s) 2 Glutamine(s) 0.83 Glucose-6P(c) 0.3 Arachidonate(s) 0.17 Palmitate(s) 0.5 Cystine(s) 0.08 Oleate(s) 1 Pantothenate(s) 0.27 Stearate(s) 0.18 Linoleate(s) 13.2 ATP-energy(c) 1 Proton-gradient(c) 2 THF-activated methyl group(c)	6.17 H_2O(s) 2 CO_2(s) 2 Pyruvate(c) 2 Glutamate(s) 1 Acyl-CoA-VLDL-PE-pool(c) 2 NADH-redox-potential(c) 3.5 NADPH-redox-potential(c) 3 Na-gradient(c)	48	-	-	-	-	-	-	-	15	-	-	-
704 Acyl-CoA-VLDL-PI-pool	Acyl-CoA-VLDL-PI-pool(c)	PIPES MCES	synthesis of cytosolic Acyl-CoA-VLDL-PI-pool	2.17 P_i(c) 1 Glycine(s) 2 Aspartate(s) 2 Glutamine(s) 0.83 Glucose-6P(c) 0.25 Arachidonate(s) 0.08 Palmitate(s) 0.5 Cystine(s) 0.16 Oleate(s) 1 Pantothenate(s) 0.43 Stearate(s) 0.08 Linoleate(s) 13.2 ATP-energy(c) 1 Proton-gradient(c) 2 THF-activated methyl group(c)	6.17 H_2O(s) 2 CO_2(s) 2 Pyruvate(c) 2 Glutamate(s) 1 Acyl-CoA-VLDL-PI-pool(c) 2 NADH-redox-potential(c) 3.5 NADPH-redox-potential(c) 3 Na-gradient(c)	48	-	-	-	-	-	-	-	15	-	-	-
705 Acyl-CoA-VLDL-PS-pool	Acyl-CoA-VLDL-PS-pool(c)	PIPES MCES	synthesis of cytosolic Acyl-CoA-VLDL-PS-pool	2.17 P_i(c) 1 Glycine(s) 2 Aspartate(s) 2 Glutamine(s) 0.83 Glucose-6P(c) 0.27 Arachidonate(s) 0.04 Palmitate(s) 0.5 Cystine(s) 0.04 Oleate(s) 1 Pantothenate(s) 0.62 Stearate(s) 0.02 Linoleate(s) 13.2 ATP-energy(c) 1 Proton-gradient(c) 2 THF-activated methyl group(c)	6.17 H_2O(s) 2 CO_2(s) 2 Pyruvate(c) 2 Glutamate(s) 1 Acyl-CoA-VLDL-PS-pool(c) 2 NADH-redox-potential(c) 3.5 NADPH-redox-potential(c) 3 Na-gradient(c)	48	-	-	-	-	-	-	-	15	-	-	-

Continued on next page

Simulation	Definition			Solution													
				exchanges		reactions								transport			Prot
	Objective	Contraints	Comment	imports	exports	c	m	r	p	l	n	s	b	s-c	b-c	intra	syn
706 Acyl-CoA-VLDL-SM-pool	Acyl-CoA-VLDL-SM-pool(c)	PIPES MCES	synthesis of cytosolic Acyl-CoA-VLDL-SM-pool	2.17 P_i(c) 1 Glycine(s) 2 Aspartate(s) 2 Glutamine(s) 0.83 Glucose-6P(c) 0.02 Arachidonate(s) 0.71 Palmitate(s) 0.5 Cystine(s) 0.07 Oleate(s) 1 Pantothenate(s) 0.17 Stearate(s) 0.03 Linoleate(s) 13.2 ATP-energy(c) 1 Proton-gradient(c) 2 THF-activated methyl group(c)	6.17 H_2O(s) 2 CO_2(s) 2 Pyruvate(c) 2 Glutamate(s) 1 Acyl-CoA-VLDL-SM-pool(c) 2 NADH-redox-potential(c) 3.5 NADPH-redox-potential(c) 3 Na-gradient(c)	48	-	-	-	-	-	-	-	15	-	-	-
707 Acyl-CoA-VLDL-TG2-pool	Acyl-CoA-VLDL-TG2-pool(c)	PIPES MCES	synthesis of cytosolic Acyl-CoA-VLDL-TG2-pool	2.17 P_i(c) 1 Glycine(s) 2 Aspartate(s) 2 Glutamine(s) 0.83 Glucose-6P(c) 0.06 Arachidonate(s) 0.13 Palmitate(s) 0.5 Cystine(s) 0.46 Oleate(s) 1 Pantothenate(s) 0.04 Stearate(s) 0.31 Linoleate(s) 13.2 ATP-energy(c) 1 Proton-gradient(c) 2 THF-activated methyl group(c)	6.17 H_2O(s) 2 CO_2(s) 2 Pyruvate(c) 2 Glutamate(s) 1 Acyl-CoA-VLDL-TG2-pool(c) 2 NADH-redox-potential(c) 3.5 NADPH-redox-potential(c) 3 Na-gradient(c)	48	-	-	-	-	-	-	-	15	-	-	-
708 Acyl-CoA-VLDL-TG3-pool	Acyl-CoA-VLDL-TG3-pool(c)	PIPES MCES	synthesis of cytosolic Acyl-CoA-VLDL-TG3-pool	2.17 P_i(c) 1 Glycine(s) 2 Aspartate(s) 2 Glutamine(s) 0.83 Glucose-6P(c) 0.03 Arachidonate(s) 0.09 Palmitate(s) 0.5 Cystine(s) 0.51 Oleate(s) 1 Pantothenate(s) 0.06 Stearate(s) 0.31 Linoleate(s) 13.2 ATP-energy(c) 1 Proton-gradient(c) 2 THF-activated methyl group(c)	6.17 H_2O(s) 2 CO_2(s) 2 Pyruvate(c) 2 Glutamate(s) 1 Acyl-CoA-VLDL-TG3-pool(c) 2 NADH-redox-potential(c) 3.5 NADPH-redox-potential(c) 3 Na-gradient(c)	48	-	-	-	-	-	-	-	15	-	-	-
709 Adenine	Adenine(s)	PIPES MCES	synthesis of Adenine and blood export	1 Glycine(s) 2 Asparagine(s) 10 ATP-energy(c) 2 THF-activated methyl group(c)	1 CO_2(s) 2 Pyruvate(c) 1 Adenine(s) 2 NADH-redox-potential(c) 4 NADPH-redox-potential(c) 1 Proton-gradient(c) 2 Na-gradient(c)	39	-	-	-	-	-	-	-	6	-	-	-
710 Adenylosuccinate	Adenylosuccinate(c)	PIPES MCES	synthesis of cytosolic Adenylosuccinate	0.17 P_i(c) 1 Glycine(s) 2 Aspartate(s) 2 Glutamine(s) 0.83 Glucose-6P(c) 7.17 ATP-energy(c) 1 Proton-gradient(c) 2 THF-activated methyl group(c)	2.17 H_2O(s) 1 Pyruvate(c) 2 Glutamate(s) 1 Adenylosuccinate(c) 2 NADH-redox-potential(c) 3 NADPH-redox-potential(c) 2 Na-gradient(c)	33	-	-	-	-	-	-	-	7	-	-	-

Continued on next page

C5. Comprehensive list – continued.

Simulation	Definition			Solution													
				exchanges		reactions								transport			Prot
	Objective	Contraints	Comment	imports	exports	c	m	r	p	l	n	s	b	s-c	b-c	intra	syn
711 Adenylyl(s)ulfate	Adenylyl sulfate(c)	PIPES MCES	synthesis of cytosolic Adenylyl sulfate	0.17 P_i(c) 1 Glycine(s) 2 Aspartate(s) 2 Glutamine(s) 0.83 Glucose-6P(c) 10.2 ATP-energy(c) 1 Proton-gradient(c) 1 activated-sulphur(c) 2 THF-activated methyl group(c)	1.17 H_2O(s) 1 CO_2(s) 2 Pyruvate(c) 2 Glutamate(s) 1 Adenylyl sulfate(c) 2 NADH-redox-potential(c) 4 NADPH-redox-potential(c) 2 Na-gradient(c)	40	-	-	-	-	-	-	-	8	-	-	-
712 Agmatine	Agmatine(m)	PIPES MCES	synthesis of mitochondrial Agmatine	1 Arginine(s) 1 Proton-gradient(m)	1 CO_2(s) 1 Agmatine(m)	-	1	-	-	-	-	-	-	2	-	3	-
713 Anthranilate	Anthranilate(c)	PIPES MCES	synthesis of cytosolic Anthranilate	1 O_2(s) 1 Tryptophan(s) 1 ATP-energy(c) 2 NADPH-redox-potential(c)	1 Alanine(s) 1 Anthranilate(c) 1 Na-gradient(c) 1 THF-activated methyl group(c)	10	-	-	-	-	-	-	-	4	-	-	-
714 Arachidonyl-Carnitine	Arachidonyl-Carnitine(c)	PIPES MCES −L-Carnitine(c)	synthesis of cytosolic Arachidonyl-Carnitine	1 Arachidonate(s) 1 L-Carnitine(c) 2 ATP-energy(c)	1 H_2O(s) 1 Arachidonyl-Carnitine(c)	5	-	-	-	-	-	-	-	2	-	-	-
715 Arachidonyl-CoA	Arachidonyl-CoA(c)	PIPES MCES	synthesis of cytosolic Arachidonyl-CoA	2.17 P_i(c) 1 Glycine(s) 2 Aspartate(s) 2 Glutamine(s) 0.83 Glucose-6P(c) 1 Arachidonate(s) 0.5 Cystine(s) 1 Pantothenate(s) 13.2 ATP-energy(c) 1 Proton-gradient(c) 2 THF-activated methyl group(c)	6.17 H_2O(s) 2 CO_2(s) 2 Pyruvate(c) 2 Glutamate(s) 1 Arachidonyl-CoA(c) 2 NADH-redox-potential(c) 3.5 NADPH-redox-potential(c) 3 Na-gradient(c)	43	-	-	-	-	-	-	-	11	-	-	-
716 Argininosuccinate	Argininosuccinate(c)	PIPES MCES	synthesis of cytosolic Argininosuccinate	1 Pyruvate(c) 1 Pyruvate(m) 1 Arginine(s) 1 NADPH-redox-potential(c)	1 H_2O(s) 1 Argininosuccinate(c) 1 NADH-redox-potential(m) 1 CoA-activated acetyl group(m)	4	3	-	-	-	-	-	-	2	-	1	-

Continued on next page

Simulation	Definition			Solution													
				exchanges		reactions								transport			Prot
	Objective	Contraints	Comment	imports	exports	c	m	r	p	l	n	s	b	s-c	b-c	intra	syn
717 But-2-enoyl-ACP	But-2-enoyl-ACP(c)	PIPES MCES	synthesis of cytosolic But-2-enoyl-ACP	1 P_i(c) 5 Glutamate(s) 6 Glycine(s) 11 Alanine(s) 4 Lysine(s) 8 Aspartate(s) 9 Arginine(s) 7 Glutamine(s) 8 Serine(s) 5 Methionine(s) 3 Phenylalanine(s) 4 Tyrosine(s) 3 Cysteine(s) 20 Leucine(s) 1 Histidine(s) 9 Proline(s) 1 Asparagine(s) 11 Valine(s) 6 Threonine(s) 4 Isoleucine(s) 1 Pantothenate(s) 625 ATP-energy(c) 1 NADPH-redox-potential(c) 2 CoA-activated acetyl group(c)	4 H_2O(s) 1 CO_2(s) 1 But-2-enoyl-ACP(c) 2 Proton-gradient(c) 82 Na-gradient(c)	22	-	-	-	-	-	-	-	24	-	-	1
718 Butyryl-ACP	Butyryl-ACP(c)	PIPES MCES	synthesis of cytosolic Butyryl-ACP	1 P_i(c) 5 Glutamate(s) 6 Glycine(s) 11 Alanine(s) 4 Lysine(s) 8 Aspartate(s) 9 Arginine(s) 7 Glutamine(s) 8 Serine(s) 5 Methionine(s) 3 Phenylalanine(s) 4 Tyrosine(s) 3 Cysteine(s) 20 Leucine(s) 1 Histidine(s) 9 Proline(s) 1 Asparagine(s) 11 Valine(s) 6 Threonine(s) 4 Isoleucine(s) 1 Pantothenate(s) 625 ATP-energy(c) 2 NADPH-redox-potential(c) 2 CoA-activated acetyl group(c)	4 H_2O(s) 1 CO_2(s) 1 Butyryl-ACP(c) 2 Proton-gradient(c) 82 Na-gradient(c)	23	-	-	-	-	-	-	-	24	-	-	1
719 Butyryl-CoA	Butyryl-CoA(c)	PIPES MCES	synthesis of cytosolic Butyryl-CoA	1.5 O_2(s) 2.17 P_i(c) 1 Glycine(s) 2 Aspartate(s) 2 Glutamine(s) 0.83 Glucose-6P(c) 0.5 Cystine(s) 1 Pantothenate(s) 1 Palmitolate(s) 13.2 ATP-energy(c) 2 THF-activated methyl group(c)	2.17 H_2O(s) 2 CO_2(s) 2 Pyruvate(c) 2 Glutamate(s) 1 Butyryl-CoA(c) 7 NADH-redox-potential(m) 5.5 NADPH-redox-potential(c) 3 FADH-redox-potential(c) 2 Proton-gradient(c) 18 Proton-gradient(m) 5 Na-gradient(c) 7 CoA-activated acetyl group(m)	43	30	-	-	-	-	-	-	12	-	10	-

Continued on next page

Simulation	Definition			Solution													
				exchanges		reactions								transport			Prot
	Objective	Contraints	Comment	imports	exports	c	m	r	p	l	n	s	b	s-c	b-c	intra	syn
720 CDP	CDP(c)	PIPES MCES	synthesis of CDP and blood export	0.33 O_2(s) 1.17 P_i(c) 1 Glutamine(s) 0.83 Glucose-6P(c) 0.33 Proline(s) 1 Asparagine(s) 6.17 ATP-energy(c)	3.17 H_2O(s) 1.33 Glutamate(s) 1 CDP(c) 0.33 NADH-redox-potential(m) 4 Proton-gradient(m) 0.33 Na-gradient(c)	25	4	-	-	-	-	-	-	7	-	6	-
721 CDP-choline	CDP-choline(c)	PIPES MCES	synthesis of cytosolic CDP-choline	0.33 O_2(s) 1.17 P_i(c) 1 Glutamine(s) 0.83 Glucose-6P(c) 1 Choline(c) 0.33 Proline(s) 1 Asparagine(s) 8.17 ATP-energy(c)	4.17 H_2O(s) 1.33 Glutamate(s) 1 CDP-choline(c) 0.33 NADH-redox-potential(m) 4 Proton-gradient(m) 0.33 Na-gradient(c)	26	4	-	-	-	-	-	-	7	-	6	-
722 CDP-ethanolamine	CDP-ethanolamine(c)	PIPES MCES	synthesis of cytosolic CDP-ethanolamine	0.33 O_2(s) 1.17 P_i(c) 1 Glutamine(s) 0.83 Glucose-6P(c) 0.33 Proline(s) 1 Asparagine(s) 1 Ethanolamine(c) 8.17 ATP-energy(c)	4.17 H_2O(s) 1.33 Glutamate(s) 1 CDP-ethanolamine(c) 0.33 NADH-redox-potential(m) 4 Proton-gradient(m) 0.33 Na-gradient(c)	26	4	-	-	-	-	-	-	7	-	6	-
723 CMP	CMP(c)	PIPES MCES	synthesis of CMP and blood export	0.33 O_2(s) 0.17 P_i(c) 1 Glutamine(s) 0.83 Glucose-6P(c) 0.33 Proline(s) 1 Asparagine(s) 5.17 ATP-energy(c)	2.17 H_2O(s) 1.33 Glutamate(s) 1 CMP(c) 0.33 NADH-redox-potential(m) 4 Proton-gradient(m) 0.33 Na-gradient(c)	26	4	-	-	-	-	-	-	7	-	6	-
724 CMP-NeuNGc	CMP-NeuNGc(c)	PIPES MCES	synthesis of cytosolic CMP-NeuNGc	1.33 O_2(s) 1 Pyruvate(c) 2 Glutamine(s) 1.83 Glucose-6P(c) 0.33 Proline(s) 1 Asparagine(s) 10.2 ATP-energy(c) 1 NADPH-redox-potential(c) 1 CoA-activated acetyl group(c)	3.17 H_2O(s) 0.83 P_i(c) 2.33 Glutamate(s) 1 CMP-NeuNGc(c) 2 NADH-redox-potential(c) 0.33 NADH-redox-potential(m) 4 Proton-gradient(m) 0.33 Na-gradient(c)	41	4	-	-	-	-	-	-	7	-	6	-
725 CMP-activated-N-acetylneuraminate	CMP-activated-N-acetylneuraminate(n)	PIPES MCES	synthesis of nucleous CMP-activated-N-acetylneuraminate	1 Pyruvate(c) 1 Glutamine(s) 1 Glucose-6P(c) 5 ATP-energy(c) 1 NADPH-redox-potential(c) 1 CoA-activated acetyl group(c)	1 P_i(c) 1 Glutamate(s) 1 NADH-redox-potential(c) 1 CMP-activated-N-acetylneuraminate(n)	21	-	-	-	-	2	-	-	2	-	4	-
726 Carbamoyl-P	Carbamoyl-P(c)	PIPES MCES	synthesis of cytosolic Carbamoyl-P	1 P_i(c) 1 Pyruvate(m) 1 Glutamine(s) 2 ATP-energy(c)	1 Glutamate(s) 1 Carbamoyl-P(c) 1 NADH-redox-potential(m) 1 CoA-activated acetyl group(m)	4	3	-	-	-	-	-	-	2	-	1	-
727 Carbonate	Carbonate(s)	PIPES MCES	synthesis of Carbonate and blood export	1 H_2O(s) 1 Pyruvate(m)	1 NADH-redox-potential(m) 1 Carbonate(s) 1 CoA-activated acetyl group(m)	-	3	-	-	-	-	1	-	1	-	1	-

Continued on next page

C5. Comprehensive list – continued.

Simulation	Definition			Solution													
				exchanges		reactions								transport			Prot
	Objective	Contraints	Comment	imports	exports	c	m	r	p	l	n	s	b	s-c	b-c	intra	syn
728 Carnosine	Carnosine(c)	PIPES MCES	synthesis of cytosolic Carnosine	1 Aspartate(s) 1 Histidine(s) 2 ATP-energy(c)	1 H_2O(s) 1 CO_2(s) 1 Carnosine(c) 2 Na-gradient(c)	5	-	-	-	-	-	-	-	5	-	-	-
729 Ceramide-1P-pool	Ceramide-1P-pool(c)	PIPES MCES	synthesis of cytosolic Ceramide-1P-pool	1 P_i(c) 1 Serine(s) 0.02 Arachidonate(s) 0.88 Palmitate(s) 0.24 Oleate(s) 0.03 Linoleate(s) 0.83 Palmitolate(s) 5 ATP-energy(c) 2 NADPH-redox-potential(c)	3 H_2O(s) 1 CO_2(s) 1 NADH-redox-potential(c) 1 Proton-gradient(c) 1 Ceramide-1P-pool(c) 1 Na-gradient(c)	19	-	-	-	-	-	-	-	10	-	-	-
730 Chenodeoxycholoyl-CoA	Chenodeoxycholoyl-CoA(c)	PIPES MCES	synthesis of cytosolic Chenodeoxycholoyl-CoA	10 O_2(s) 0.75 Glycine(s) 1.25 Aspartate(s) 2 Glutamine(s) 0.83 Glucose-6P(c) 0.25 Proline(s) 0.75 Asparagine(s) 2 Farnesyl-PP(r) 0.88 Cystine(s) 1 Pantothenate(s) 15.2 ATP-energy(c) 11 NADPH-redox-potential(r) 1 NADPH-redox-potential(m) 0.75 Proton-gradient(m) 1.5 THF-activated methyl group(c)	13.7 H_2O(s) 1.83 P_i(c) 3.5 CO_2(s) 2 Pyruvate(c) 2.25 Glutamate(s) 0.5 Serine(s) 0.75 Methionine(s) 1 Chenodeoxycholoyl-CoA(c) 1 NADH-redox-potential(r) 2.75 NADH-redox-potential(c) 0.13 NADPH-redox-potential(c) 1.5 Proton-gradient(c) 4.5 Na-gradient(c) 0.25 CoA-activated acetyl group(m)	52	12	18	2	-	-	-	-	14	-	22	-
731 Cholate	Cholate(s)	PIPES MCES	synthesis of Cholate and blood export	11.8 O_2(s) 0.5 Cysteine(s) 2 Farnesyl-PP(r) 4 ATP-energy(c) 12 NADPH-redox-potential(r) 2 NADPH-redox-potential(c) 0.5 THF-activated methyl group(c)	8.5 H_2O(s) 4 P_i(c) 3 CO_2(s) 0.5 Pyruvate(c) 0.5 Methionine(s) 1 Cholate(s) 1 NADH-redox-potential(r) 2 NADH-redox-potential(c) 0.5 NADH-redox-potential(m) 1 NADH-redox-potential(p) 2.5 Proton-gradient(m) 0.5 Na-gradient(c) 0.5 CoA-activated acetyl group(m)	15	8	22	6	-	-	-	-	9	-	23	-
732 Cholestenol	Cholestenol(r)	PIPES MCES	synthesis of Golgi/ER Cholestenol	7 O_2(s) 2 Farnesyl-PP(r) 1 ATP-energy(c) 9 NADPH-redox-potential(r)	5 H_2O(s) 4 P_i(c) 3 CO_2(s) 1 NADH-redox-potential(r) 1 NADPH-redox-potential(c) 1 Cholestenol(r)	5	-	13	-	-	-	-	-	3	-	5	-
733 Cholesterol-ester-arach	Cholesterol-ester-arach(r)	PIPES MCES	synthesis of Golgi/ER Cholesterol-ester-arach	8 O_2(s) 1 Arachidonate(s) 2 Farnesyl-PP(r) 3 ATP-energy(c) 11 NADPH-redox-potential(r)	8 H_2O(s) 4 P_i(c) 3 CO_2(s) 1 Cholesterol-ester-arach(r) 1 NADH-redox-potential(r) 1 NADPH-redox-potential(c)	8	-	18	-	-	-	-	-	4	-	6	-

Continued on next page

C5. Comprehensive list – continued.

Simulation	Definition			Solution													
				exchanges		reactions								transport			Prot
	Objective	Contraints	Comment	imports	exports	c	m	r	p	l	n	s	b	s-c	b-c	intra	syn
734 Cholesterol-ester-gla	Cholesterol-ester-gla(r)	PIPES MCES	synthesis of Golgi/ER Cholesterol-ester-gla	6 O_2(s) 1 Arachidonate(s) 2 Farnesyl-PP(r) 3 ATP-energy(c) 7 NADPH-redox-potential(r)	4 H_2O(s) 4 P_i(c) 3 CO_2(s) 1 Cholesterol-ester-gla(r) 1 NADH-redox-potential(r) 1 NADH-redox-potential(c) 1 NADPH-redox-potential(c) 1 CoA-activated acetyl group(c)	12	-	19	-	-	-	-	-	4	-	8	-
735 Cholesterol-ester-lin	Cholesterol-ester-lin(r)	PIPES MCES	synthesis of Golgi/ER Cholesterol-ester-lin	5 O_2(s) 1 Arachidonate(s) 2 Farnesyl-PP(r) 3 ATP-energy(c) 7 NADPH-redox-potential(r)	3 H_2O(s) 4 P_i(c) 3 CO_2(s) 1 Cholesterol-ester-lin(r) 1 NADH-redox-potential(r) 2 NADH-redox-potential(c) 1 NADPH-redox-potential(c) 1 CoA-activated acetyl group(c)	13	-	19	-	-	-	-	-	4	-	10	-
736 Cholesterol-ester-ol	Cholesterol-ester-ol(r)	PIPES MCES	synthesis of Golgi/ER Cholesterol-ester-ol	8 O_2(s) 2 Farnesyl-PP(r) 1 Oleate(s) 3 ATP-energy(c) 11 NADPH-redox-potential(r)	8 H_2O(s) 4 P_i(c) 3 CO_2(s) 1 Cholesterol-ester-ol(r) 1 NADH-redox-potential(r) 1 NADPH-redox-potential(c)	6	-	18	-	-	-	-	-	4	-	8	-
737 Cholesterol-ester-palm	Cholesterol-ester-palm(r)	PIPES MCES	synthesis of Golgi/ER Cholesterol-ester-palm	7 O_2(s) 2 Farnesyl-PP(r) 1 Palmitolate(s) 3 ATP-energy(c) 11 NADPH-redox-potential(r)	6 H_2O(s) 4 P_i(c) 3 CO_2(s) 1 Cholesterol-ester-palm(r) 1 NADH-redox-potential(r) 1 NADH-redox-potential(c) 1 NADPH-redox-potential(c)	11	-	18	-	-	-	-	-	4	-	6	-
738 Cholesterol-ester-palmn	Cholesterol-ester-palmn(r)	PIPES MCES	synthesis of Golgi/ER Cholesterol-ester-palmn	8 O_2(s) 2 Farnesyl-PP(r) 1 Palmitolate(s) 3 ATP-energy(c) 11 NADPH-redox-potential(r)	8 H_2O(s) 4 P_i(c) 3 CO_2(s) 1 Cholesterol-ester-palmn(r) 1 NADH-redox-potential(r) 1 NADPH-redox-potential(c)	6	-	18	-	-	-	-	-	4	-	8	-
739 Cholesterol-ester-pool	Cholesterol-ester-pool(r)	PIPES MCES	synthesis of Golgi/ER Cholesterol-ester-pool	6.25 O_2(s) 0.59 Arachidonate(s) 2 Farnesyl-PP(r) 0.22 Oleate(s) 0.03 Stearate(s) 0.15 Palmitolate(s) 3 ATP-energy(c) 8.87 NADPH-redox-potential(r)	5.03 H_2O(s) 4 P_i(c) 3 CO_2(s) 1 Cholesterol-ester-pool(r) 1 NADH-redox-potential(r) 1.22 NADH-redox-potential(c) 1 NADPH-redox-potential(c) 0.53 CoA-activated acetyl group(c)	18	-	28	-	-	-	-	-	7	-	16	-
740 Cholesterol-ester-stea	Cholesterol-ester-stea(r)	PIPES MCES	synthesis of Golgi/ER Cholesterol-ester-stea	8 O_2(s) 2 Farnesyl-PP(r) 1 Stearate(s) 3 ATP-energy(c) 11 NADPH-redox-potential(r)	8 H_2O(s) 4 P_i(c) 3 CO_2(s) 1 Cholesterol-ester-stea(r) 1 NADH-redox-potential(r) 1 NADPH-redox-potential(c)	6	-	18	-	-	-	-	-	4	-	8	-

Continued on next page

Simulation	Definition			Solution													
				exchanges		reactions								transport			Prot
	Objective	Contraints	Comment	imports	exports	c	m	r	p	l	n	s	b	s-c	b-c	intra	syn
741 Choloyl-CoA	Choloyl-CoA(c)	PIPES MCES	synthesis of cytosolic Choloyl-CoA	11.5 O_2(s) 1 Glycine(s) 1 Glutamine(s) 0.83 Glucose-6P(c) 2 Asparagine(s) 2 Farnesyl-PP(r) 1 Cystine(s) 1 Pantothenate(s) 16.2 ATP-energy(c) 1 NADH-redox-potential(m) 12 NADPH-redox-potential(r) 1 NADPH-redox-potential(c) 1 Proton-gradient(m) 2 THF-activated methyl group(c)	16.2 H_2O(s) 1.83 P_i(c) 3 CO_2(s) 2 Pyruvate(c) 1 Glutamate(s) 1 Serine(s) 1 Methionine(s) 1 Choloyl-CoA(c) 1 NADH-redox-potential(r) 3 NADH-redox-potential(c) 1 NADH-redox-potential(p) 2 Na-gradient(c)	53	2	19	7	-	-	-	-	9	-	18	-
742 Citrate	Citrate(s)	PIPES MCES	synthesis of Citrate and blood export	1 H_2O(s) 1 Pyruvate(c) 1 Pyruvate(m) 1 NADPH-redox-potential(c)	1 Citrate(s) 2 NADH-redox-potential(m) 1 Proton-gradient(c)	2	4	-	-	-	-	-	-	3	-	3	-
743 Citrulline	Citrulline(s)	PIPES MCES	synthesis of Citrulline and blood export	0.6 Pyruvate(c) 0.8 Arginine(s) 0.2 Glutamine(s) 0.8 ATP-energy(m) 0.2 NADH-redox-potential(c) 0.4 NADPH-redox-potential(c) 0.2 Proton-gradient(m)	0.2 Alanine(s) 0.2 Urea(s) 1 Citrulline(s) 0.2 NADH-redox-potential(m) 0.2 NADPH-redox-potential(m) 0.4 CoA-activated acetyl group(m)	13	12	-	-	-	-	-	-	4	-	6	-
744 CoproporphyrinogenIII	CoproporphyrinogenIII(c)	PIPES MCES	synthesis of cytosolic CoproporphyrinogenIII	4 Pyruvate(m) 12 Glutamate(s) 4 Serine(s) 4 ATP-energy(c) 4 NADPH-redox-potential(m) 4 Proton-gradient(m)	12 H_2O(s) 12 CO_2(s) 4 Aspartate(s) 4 Glutamine(s) 1 CoproporphyrinogenIII(c) 4 NADH-redox-potential(c) 8 NADH-redox-potential(m) 12 Na-gradient(c)	9	8	-	-	-	-	-	-	6	-	6	-
745 Crotonyl-CoA	Crotonyl-CoA(m)	PIPES MCES	synthesis of mitochondrial Crotonyl-CoA	2.17 P_i(c) 1 Glycine(s) 2 Aspartate(s) 2 Glutamine(s) 0.83 Glucose-6P(c) 0.5 Cystine(s) 1 Pantothenate(s) 11.2 ATP-energy(c) 1 NADH-redox-potential(m) 1 Proton-gradient(c) 2 THF-activated methyl group(c) 2 CoA-activated acetyl group(m)	6.17 H_2O(s) 2 CO_2(s) 2 Pyruvate(c) 2 Glutamate(s) 1 Crotonyl-CoA(m) 2 NADH-redox-potential(c) 3.5 NADPH-redox-potential(c) 3 Na-gradient(c)	42	5	-	-	-	-	-	-	10	-	2	-
746 Cys-Gly	Cys-Gly(s)	PIPES MCES	synthesis of Cys-Gly and blood export	1 Glycine(s) 1 Cysteine(s)	1 H_2O(s) 1 Cys-Gly(s)	-	-	-	-	-	-	1	-	-	-	-	-

Continued on next page

Simulation	Definition			Solution													
				exchanges		reactions								transport			Prot
	Objective	Contraints	Comment	imports	exports	c	m	r	p	l	n	s	b	s-c	b-c	intra	syn
747 Cysteamine	Cysteamine(c)	PIPES MCES	synthesis of cytosolic Cysteamine	1 Cysteine(s)	1 CO_2(s) 1 Cysteamine(c)	3	-	-	-	-	-	-	-	2	-	-	-
748 D-3-Amino-isobutanoate	D-3-Amino-isobutanoate(s)	PIPES MCES	synthesis of D-3-Amino-isobutanoate and blood export	0.33 O_2(s) 1 Aspartate(s) 0.33 Proline(s) 5 ATP-energy(c) 2 NADH-redox-potential(c) 0.33 Proton-gradient(c) 1 THF-activated methyl group(c)	1 CO_2(s) 0.33 Glutamate(s) 1 D-3-Amino-isobutanoate(s) 0.33 NADH-redox-potential(m) 1 NADPH-redox-potential(c) 4 Proton-gradient(m) 1 Na-gradient(c)	28	4	-	-	-	-	-	-	8	-	6	-
749 D-3-Hydroxydodecanoyl-ACP	D-3-Hydroxydodecanoyl-ACP(c)	PIPES MCES	synthesis of cytosolic D-3-Hydroxydodecanoyl-ACP	1 P_i(c) 5 Glutamate(s) 6 Glycine(s) 11 Alanine(s) 4 Lysine(s) 8 Aspartate(s) 9 Arginine(s) 7 Glutamine(s) 8 Serine(s) 5 Methionine(s) 3 Phenylalanine(s) 4 Tyrosine(s) 3 Cysteine(s) 20 Leucine(s) 1 Histidine(s) 9 Proline(s) 1 Asparagine(s) 11 Valine(s) 6 Threonine(s) 4 Isoleucine(s) 1 Pantothenate(s) 629 ATP-energy(c) 9 NADPH-redox-potential(c) 3 Proton-gradient(c) 6 CoA-activated acetyl group(c)	7 H_2O(s) 1 CO_2(s) 1 D-3-Hydroxydodecanoyl-ACP(c) 66 Na-gradient(c)	37	-	-	-	-	-	-	-	24	-	-	1
750 D-3-Hydroxyhexanoyl-ACP	D-3-Hydroxyhexanoyl-ACP(c)	PIPES MCES	synthesis of cytosolic D-3-Hydroxyhexanoyl-ACP	1 P_i(c) 5 Glutamate(s) 6 Glycine(s) 11 Alanine(s) 4 Lysine(s) 8 Aspartate(s) 9 Arginine(s) 7 Glutamine(s) 8 Serine(s) 5 Methionine(s) 3 Phenylalanine(s) 4 Tyrosine(s) 3 Cysteine(s) 20 Leucine(s) 1 Histidine(s) 9 Proline(s) 1 Asparagine(s) 11 Valine(s) 6 Threonine(s) 4 Isoleucine(s) 1 Pantothenate(s) 626 ATP-energy(c) 3 NADPH-redox-potential(c) 3 CoA-activated acetyl group(c)	4 H_2O(s) 1 CO_2(s) 1 D-3-Hydroxyhexanoyl-ACP(c) 2 Proton-gradient(c) 82 Na-gradient(c)	25	-	-	-	-	-	-	-	24	-	-	1

Continued on next page

C5. Comprehensive list – continued.

Simulation	Definition			Solution													
				exchanges		reactions								transport			Prot
	Objective	Contraints	Comment	imports	exports	c	m	r	p	l	n	s	b	s-c	b-c	intra	syn
751 D-Xylulose-5P	D-Xylulose-5P(c)	PIPES MCES	synthesis of cytosolic D-Xylulose-5P	0.17 P_i(c) 0.83 Glucose-6P(c) 0.17 ATP-energy(c)	0.17 H_2O(s) 1 D-Xylulose-5P(c)	10	-	-	-	-	-	-	-	1	-	-	-
752 DHAP	DHAP(c)	PIPES MCES	synthesis of DHAP and blood export	0.5 P_i(c) 0.5 Glucose-6P(c) 0.5 ATP-energy(c)	0.5 H_2O(s) 1 DHAP(c)	5	-	-	-	-	-	-	-	1	-	-	-
753 Deamido-NAD	Deamido-NAD(c)	PIPES MCES	synthesis of cytosolic Deamido-NAD	0.33 P_i(c) 1 Glycine(s) 1 Aspartate(s) 1.67 Glucose-6P(c) 1 Asparagine(s) 1 Nicotinamide(s) 13.3 ATP-energy(c) 1 Proton-gradient(c) 2 THF-activated methyl group(c)	3.33 H_2O(s) 1 CO_2(s) 2 Pyruvate(c) 1 Deamido-NAD(c) 2 NADH-redox-potential(c) 4 NADPH-redox-potential(c) 1 Na-gradient(c)	39	-	-	-	-	-	-	-	8	-	-	-
754 Decanoyl-ACP	Decanoyl-ACP(c)	PIPES MCES	synthesis of cytosolic Decanoyl-ACP	1 P_i(c) 5 Glutamate(s) 6 Glycine(s) 11 Alanine(s) 4 Lysine(s) 8 Aspartate(s) 9 Arginine(s) 7 Glutamine(s) 8 Serine(s) 5 Methionine(s) 3 Phenylalanine(s) 4 Tyrosine(s) 3 Cysteine(s) 20 Leucine(s) 1 Histidine(s) 9 Proline(s) 1 Asparagine(s) 11 Valine(s) 6 Threonine(s) 4 Isoleucine(s) 1 Pantothenate(s) 628 ATP-energy(c) 8 NADPH-redox-potential(c) 3 Proton-gradient(c) 5 CoA-activated acetyl group(c)	7 H_2O(s) 1 CO_2(s) 1 Decanoyl-ACP(c) 66 Na-gradient(c)	35	-	-	-	-	-	-	-	24	-	-	1
755 Decanoyl-CoA	Decanoyl-CoA(m)	PIPES MCES	synthesis of mitochondrial Decanoyl-CoA	2.17 P_i(c) 1 Glycine(s) 2 Aspartate(s) 2 Glutamine(s) 0.83 Glucose-6P(c) 0.5 Cystine(s) 1 Pantothenate(s) 1 Palmitolate(s) 13.2 ATP-energy(c) 1 Proton-gradient(c) 2 THF-activated methyl group(c)	2.17 H_2O(s) 2 CO_2(s) 2 Pyruvate(c) 2 Glutamate(s) 1 Decanoyl-CoA(m) 2 NADH-redox-potential(c) 4 NADH-redox-potential(m) 3.5 NADPH-redox-potential(c) 3 FADH-redox-potential(c) 3 Na-gradient(c) 4 CoA-activated acetyl group(m)	44	18	-	-	-	-	-	-	11	-	3	-

Continued on next page

C5. Comprehensive list – continued.

Simulation	Definition			Solution													
				exchanges		reactions								transport			Prot
	Objective	Contraints	Comment	imports	exports	c	m	r	p	l	n	s	b	s-c	b-c	intra	syn
756 Dehydroalanine	Dehydroalanine(c)	PIPES MCES	synthesis of cytosolic Dehydroalanine	1 Serine(s)	1 H_2O(s) 1 Dehydroalanine(c) 1 Proton-gradient(c) 1 Na-gradient(c)	1	-	-	-	-	-	-	-	4	-	-	-
757 Deoxyadenosine	Deoxyadenosine(s)	PIPES MCES	synthesis of Deoxyadenosine and blood export	1 Glycine(s) 0.83 Glucose-6P(c) 2 Asparagine(s) 9.17 ATP-energy(c) 2 THF-activated methyl group(c)	1.17 H_2O(s) 0.83 P_i(c) 1 CO_2(s) 2 Pyruvate(c) 1 Deoxyadenosine(s) 2 NADH-redox-potential(c) 3 NADPH-redox-potential(c) 1 Proton-gradient(c) 2 Na-gradient(c)	40	-	-	-	-	-	-	-	7	-	-	-
758 Deoxycytidine	Deoxycytidine(s)	PIPES MCES	synthesis of Deoxycytidine and blood export	0.33 O_2(s) 1 Glutamine(s) 0.83 Glucose-6P(c) 0.33 Proline(s) 1 Asparagine(s) 6.17 ATP-energy(c) 1 NADPH-redox-potential(c)	2.17 H_2O(s) 0.83 P_i(c) 1.33 Glutamate(s) 1 Deoxycytidine(s) 0.33 NADH-redox-potential(m) 4 Proton-gradient(m) 0.33 Na-gradient(c)	30	4	-	-	-	-	-	-	8	-	6	-
759 Deoxyguanosine	Deoxyguanosine(s)	PIPES MCES	synthesis of Deoxyguanosine and blood export	1 Glycine(s) 2 Glutamine(s) 0.83 Glucose-6P(c) 1 Asparagine(s) 8.17 ATP-energy(c) 2 THF-activated methyl group(c)	0.17 H_2O(s) 0.83 P_i(c) 1 Pyruvate(c) 2 Glutamate(s) 1 Deoxyguanosine(s) 3 NADH-redox-potential(c) 2 NADPH-redox-potential(c)	39	-	-	-	-	-	-	-	6	-	-	-
760 Deoxyinosine	Deoxyinosine(s)	PIPES MCES	synthesis of Deoxyinosine and blood export	1 Glycine(s) 1 Aspartate(s) 1 Glutamine(s) 0.5 Glucose-6P(c) 1 Ethanolamine(c) 8.5 ATP-energy(c) 2 THF-activated methyl group(c)	1.5 H_2O(s) 0.5 P_i(c) 1 Pyruvate(c) 1 Glutamate(s) 1 Deoxyinosine(s) 2 NADH-redox-potential(c) 3 NADPH-redox-potential(c) 1 Na-gradient(c)	35	-	-	-	-	-	-	-	7	-	-	-
761 Deoxyuridine	Deoxyuridine(s)	PIPES MCES	synthesis of Deoxyuridine and blood export	0.33 O_2(s) 1 Aspartate(s) 0.5 Glucose-6P(c) 0.33 Proline(s) 1 Ethanolamine(c) 5.5 ATP-energy(c)	2.5 H_2O(s) 0.5 P_i(c) 0.33 Glutamate(s) 1 Deoxyuridine(s) 0.33 NADH-redox-potential(m) 4 Proton-gradient(m) 1.33 Na-gradient(c)	24	4	-	-	-	-	-	-	7	-	6	-
762 Dephospho-CoA	Dephospho-CoA(c)	PIPES MCES	synthesis of cytosolic Dephospho-CoA	1.17 P_i(c) 1 Glycine(s) 0.5 Aspartate(s) 0.5 Glutamine(s) 0.83 Glucose-6P(c) 1.5 Asparagine(s) 0.5 Cystine(s) 1 Pantothenate(s) 11.7 ATP-energy(c) 1 Proton-gradient(c) 2 THF-activated methyl group(c)	4.17 H_2O(s) 2 CO_2(s) 2 Pyruvate(c) 0.5 Glutamate(s) 1 Dephospho-CoA(c) 2 NADH-redox-potential(c) 3.5 NADPH-redox-potential(c) 1.5 Na-gradient(c)	43	-	-	-	-	-	-	-	10	-	-	-

Continued on next page

Simulation	Definition			Solution													
				exchanges		reactions								transport			Prot
	Objective	Contraints	Comment	imports	exports	c	m	r	p	l	n	s	b	s-c	b-c	intra	syn
763 Desmosterol	Desmosterol(r)	PIPES MCES	synthesis of Golgi/ER Desmosterol	8 O_2(s) 2 Farnesyl-PP(r) 1 ATP-energy(c) 10 NADPH-redox-potential(r)	7 H_2O(s) 4 P_i(c) 3 CO_2(s) 1 Desmosterol(r) 1 NADH-redox-potential(r) 1 NADPH-redox-potential(c)	5	-	15	-	-	-	-	-	3	-	5	-
764 Dihomo-gamma-linolenoyl-CoA	Dihomo-gamma-linolenoyl-CoA(c)	PIPES MCES	synthesis of cytosolic Dihomo-gamma-linolenoyl-CoA	2.17 P_i(c) 1 Glycine(s) 2 Aspartate(s) 2 Glutamine(s) 1 Phenylalanine(s) 0.83 Glucose-6P(c) 1 Arachidonate(s) 0.5 Cystine(s) 1 Pantothenate(s) 13.2 ATP-energy(c) 2 THF-activated methyl group(c)	6.17 H_2O(s) 2 CO_2(s) 2 Pyruvate(c) 2 Glutamate(s) 1 Tyrosine(s) 1 Dihomo-gamma-linolenoyl-CoA(c) 3 NADH-redox-potential(c) 2.5 NADPH-redox-potential(c) 3 Na-gradient(c)	46	-	-	-	-	-	-	-	11	-	-	-
765 Dihydroceramide-pool	Dihydroceramide-pool(c)	PIPES MCES	synthesis of cytosolic Dihydroceramide-pool	1 Serine(s) 0.02 Arachidonate(s) 1.71 Palmitate(s) 0.07 Oleate(s) 0.17 Stearate(s) 0.03 Linoleate(s) 4 ATP-energy(c) 1 NADPH-redox-potential(c)	2 H_2O(s) 1 CO_2(s) 1 Proton-gradient(c) 1 Dihydroceramide-pool(c) 1 Na-gradient(c)	13	-	-	-	-	-	-	-	10	-	-	-
766 Dihydrofolate	Dihydrofolate(c)	PIPES MCES	synthesis of cytosolic Dihydrofolate	1 Folate(s) 1 NADPH-redox-potential(c)	1 Dihydrofolate(c)	2	-	-	-	-	-	-	-	1	-	-	-
767 Dihydrothymine	Dihydrothymine(c)	PIPES MCES	synthesis of cytosolic Dihydrothymine	0.33 O_2(s) 1 Aspartate(s) 1 Glutamine(s) 0.33 Proline(s) 4 ATP-energy(c) 1 NADH-redox-potential(c) 1 THF-activated methyl group(c)	1 H_2O(s) 1.33 Glutamate(s) 1 Dihydrothymine(c) 0.33 NADH-redox-potential(m) 1 Proton-gradient(c) 4 Proton-gradient(m) 2.33 Na-gradient(c)	24	4	-	-	-	-	-	-	8	-	6	-
768 Dimethylallyl-PP	Dimethylallyl-PP(p)	PIPES MCES	synthesis of peroxysomal Dimethylallyl-PP	2 P_i(c) 3 ATP-energy(c) 2 NADPH-redox-potential(c) 3 CoA-activated acetyl group(c)	2 H_2O(s) 1 CO_2(s) 1 Dimethylallyl-PP(p)	6	-	-	4	-	-	-	-	2	-	4	-

Continued on next page

C5. Comprehensive list – continued.

Simulation	Definition			Solution													
				exchanges		reactions								transport			Prot
	Objective	Contraints	Comment	imports	exports	c	m	r	p	l	n	s	b	s-c	b-c	intra	syn
769 Dodecanoyl-ACP	Dodecanoyl-ACP(c)	PIPES MCES	synthesis of cytosolic Dodecanoyl-ACP	1 P_i(c) 5 Glutamate(s) 6 Glycine(s) 11 Alanine(s) 4 Lysine(s) 8 Aspartate(s) 9 Arginine(s) 7 Glutamine(s) 8 Serine(s) 5 Methionine(s) 3 Phenylalanine(s) 4 Tyrosine(s) 3 Cysteine(s) 20 Leucine(s) 1 Histidine(s) 9 Proline(s) 1 Asparagine(s) 11 Valine(s) 6 Threonine(s) 4 Isoleucine(s) 1 Pantothenate(s) 629 ATP-energy(c) 10 NADPH-redox-potential(c) 3 Proton-gradient(c) 6 CoA-activated acetyl group(c)	8 H_2O(s) 1 CO_2(s) 1 Dodecanoyl-ACP(c) 66 Na-gradient(c)	39	-	-	-	-	-	-	-	24	-	-	1
770 Erythrose-4P	Erythrose-4P(c)	PIPES MCES	synthesis of cytosolic Erythrose-4P	0.33 P_i(c) 0.67 Glucose-6P(c) 0.33 ATP-energy(c)	0.33 H_2O(s) 1 Erythrose-4P(c)	9	-	-	-	-	-	-	-	1	-	-	-
771 Ethanolamine-P	Ethanolamine-P(c)	PIPES MCES	synthesis of cytosolic Ethanolamine-P	1 P_i(c) 1 Ethanolamine(c) 1 ATP-energy(c)	1 H_2O(s) 1 Ethanolamine-P(c)	2	-	-	-	-	-	-	-	1	-	-	-
772 FADH2	FADH2(c)	PIPES MCES	synthesis of cytosolic FADH2	1.17 P_i(c) 1 Glycine(s) 0.83 Glucose-6P(c) 2 Asparagine(s) 1 Riboflavin(s) 12.2 ATP-energy(c) 1 FADH-redox-potential(c) 1 Proton-gradient(c) 2 THF-activated methyl group(c)	3.17 H_2O(s) 1 CO_2(s) 2 Pyruvate(c) 1 FADH2(c) 2 NADH-redox-potential(c) 4 NADPH-redox-potential(c)	39	-	-	-	-	-	-	-	6	-	-	-
773 FAICAR	FAICAR(c)	PIPES MCES	synthesis of cytosolic FAICAR	0.17 P_i(c) 1 Glycine(s) 1 Aspartate(s) 2 Glutamine(s) 0.83 Glucose-6P(c) 6.17 ATP-energy(c) 2 THF-activated methyl group(c)	0.17 H_2O(s) 1 Pyruvate(c) 2 Glutamate(s) 1 FAICAR(c) 2 NADH-redox-potential(c) 3 NADPH-redox-potential(c) 1 Proton-gradient(c) 3 Na-gradient(c)	30	-	-	-	-	-	-	-	7	-	-	-
774 FMN	FMN(c)	PIPES MCES	synthesis of cytosolic FMN	1 P_i(c) 1 Riboflavin(s) 1 ATP-energy(c)	1 H_2O(s) 1 FMN(c)	2	-	-	-	-	-	-	-	2	-	-	-

Continued on next page

C5. Comprehensive list – continued.

Simulation	Definition			Solution													
				exchanges		reactions								transport			Prot
	Objective	Contraints	Comment	imports	exports	c	m	r	p	l	n	s	b	s-c	b-c	intra	syn
775 Fatty-acid-VLDL-PC-pool	Fatty-acid-VLDL-PC-pool(c)	PIPES MCES	synthesis of cytosolic Fatty-acid-VLDL-PC-pool	0.08 Arachidonate(s) 0.35 Palmitate(s) 0.14 Oleate(s) 0.15 Stearate(s) 0.27 Linoleate(s)	1 Fatty-acid-VLDL-PC-pool(c)	1	-	-	-	-	-	-	-	5	-	-	-
776 Fatty-acid-VLDL-PE-pool	Fatty-acid-VLDL-PE-pool(c)	PIPES MCES	synthesis of cytosolic Fatty-acid-VLDL-PE-pool	0.3 Arachidonate(s) 0.17 Palmitate(s) 0.08 Oleate(s) 0.27 Stearate(s) 0.18 Linoleate(s) 2.5 ATP-energy(c)	1 Fatty-acid-VLDL-PE-pool(c)	20	-	-	-	-	-	-	-	5	-	-	-
777 Fatty-acid-VLDL-PI-pool	Fatty-acid-VLDL-PI-pool(c)	PIPES MCES	synthesis of cytosolic Fatty-acid-VLDL-PI-pool	0.25 Arachidonate(s) 0.08 Palmitate(s) 0.16 Oleate(s) 0.43 Stearate(s) 0.08 Linoleate(s) 2.5 ATP-energy(c)	1 Fatty-acid-VLDL-PI-pool(c)	20	-	-	-	-	-	-	-	5	-	-	-
778 Fatty-acid-VLDL-PS-pool	Fatty-acid-VLDL-PS-pool(c)	PIPES MCES	synthesis of cytosolic Fatty-acid-VLDL-PS-pool	0.27 Arachidonate(s) 0.04 Palmitate(s) 0.04 Oleate(s) 0.62 Stearate(s) 0.02 Linoleate(s) 2.5 ATP-energy(c)	1 Fatty-acid-VLDL-PS-pool(c)	20	-	-	-	-	-	-	-	5	-	-	-
779 Fatty-acid-VLDL-SM-pool	Fatty-acid-VLDL-SM-pool(c)	PIPES MCES	synthesis of cytosolic Fatty-acid-VLDL-SM-pool	0.02 Arachidonate(s) 0.71 Palmitate(s) 0.07 Oleate(s) 0.17 Stearate(s) 0.03 Linoleate(s)	1 Fatty-acid-VLDL-SM-pool(c)	1	-	-	-	-	-	-	-	5	-	-	-
780 Fatty-acid-VLDL-TG1-pool	Fatty-acid-VLDL-TG1-pool(c)	PIPES MCES	synthesis of cytosolic Fatty-acid-VLDL-TG1-pool	0.003 Arachidonate(s) 0.7 Palmitate(s) 0.13 Oleate(s) 0.09 Stearate(s) 0.08 Linoleate(s) 5 ATP-energy(c)	1 Fatty-acid-VLDL-TG1-pool(c)	21	-	-	-	-	-	-	-	5	-	-	-
781 Fatty-acid-VLDL-TG2-pool	Fatty-acid-VLDL-TG2-pool(c)	PIPES MCES	synthesis of cytosolic Fatty-acid-VLDL-TG2-pool	0.06 Arachidonate(s) 0.13 Palmitate(s) 0.46 Oleate(s) 0.04 Stearate(s) 0.31 Linoleate(s) 5 ATP-energy(c)	1 Fatty-acid-VLDL-TG2-pool(c)	21	-	-	-	-	-	-	-	5	-	-	-

Continued on next page

C5. Comprehensive list – continued.

Simulation		Definition: Objective	Definition: Contraints	Definition: Comment	Solution: exchanges: imports	Solution: exchanges: exports	reactions: c	m	r	p	l	n	s	b	transport: s-c	b-c	intra	Prot syn
782	Fatty-acid-VLDL-TG3-pool	Fatty-acid-VLDL-TG3-pool(c)	PIPES MCES	synthesis of cytosolic Fatty-acid-VLDL-TG3-pool	0.03 Arachidonate(s) 0.09 Palmitate(s) 0.51 Oleate(s) 0.06 Stearate(s) 0.31 Linoleate(s) 2 ATP-energy(c)	1 Fatty-acid-VLDL-TG3-pool(c)	11	-	-	-	-	-	-	-	5	-	-	-
783	Formamidopyrimidine(nleoside) triphosphate	Formamidopyrimidine nleoside triphosphate(c)	PIPES MCES	synthesis of cytosolic Formamidopyrimidine nleoside triphosphate	2.17 P_i(c) 1 Glycine(s) 2 Glutamine(s) 0.83 Glucose-6P(c) 1 Asparagine(s) 10.2 ATP-energy(c) 1 Proton-gradient(c) 2 THF-activated methyl group(c)	1.17 H_2O(s) 1 Pyruvate(c) 2 Glutamate(s) 1 Formamidopyrimidine nleoside triphosphate(c) 3 NADH-redox-potential(c) 3 NADPH-redox-potential(c)	37	-	-	-	-	-	-	-	6	-	-	-
784	Formylanthranilate	Formylanthranilate(c)	PIPES MCES	synthesis of cytosolic Formylanthranilate	1 H_2O(s) 1 O_2(s) 1 Tryptophan(s)	1 Alanine(s) 1 Formylanthranilate(c) 2 Na-gradient(c)	2	-	-	-	-	-	-	-	5	-	-	-
785	Fructose-1,6PP	Fructose-1,6PP(c)	PIPES MCES	synthesis of cytosolic Fructose-1,6PP	1 P_i(c) 1 Glucose-6P(c) 1 ATP-energy(c)	1 H_2O(s) 1 Fructose-1,6PP(c)	3	-	-	-	-	-	-	-	1	-	-	-
786	Fructose-2,6PP	Fructose-2,6PP(c)	PIPES MCES	synthesis of cytosolic Fructose-2,6PP	1 P_i(c) 1 Glucose-6P(c) 1 ATP-energy(c)	1 H_2O(s) 1 Fructose-2,6PP(c)	3	-	-	-	-	-	-	-	1	-	-	-
787	Fumarate	Fumarate(c)	PIPES MCES	synthesis of cytosolic Fumarate	1 Pyruvate(c) 1 Pyruvate(m) 1 NADPH-redox-potential(c)	1 H_2O(s) 1 Fumarate(c) 1 NADH-redox-potential(m) 1 CoA-activated acetyl group(m)	3	3	-	-	-	-	-	-	1	-	1	-
788	Fumarylacetoacetate	Fumarylacetoacetate(c)	PIPES MCES	synthesis of cytosolic Fumarylacetoacetate	2 O_2(s) 1 Pyruvate(c) 1 Tyrosine(s)	1 CO_2(s) 1 Alanine(s) 1 Fumarylacetoacetate(c) 1 Na-gradient(c)	5	-	-	-	-	-	-	-	5	-	-	-
789	GAP	GAP(c)	PIPES MCES	synthesis of cytosolic GAP	0.5 P_i(c) 0.5 Glucose-6P(c) 0.5 ATP-energy(c)	0.5 H_2O(s) 1 GAP(c)	5	-	-	-	-	-	-	-	1	-	-	-
790	GAR	GAR(c)	PIPES MCES	synthesis of cytosolic GAR	0.17 P_i(c) 1 Glycine(s) 1 Glutamine(s) 0.83 Glucose-6P(c) 3.17 ATP-energy(c) 1 Proton-gradient(c)	1.17 H_2O(s) 1 Glutamate(s) 1 GAR(c)	15	-	-	-	-	-	-	-	5	-	-	-

Continued on next page

Simulation	Definition			Solution													
				exchanges		reactions								transport			Prot
	Objective	Contraints	Comment	imports	exports	c	m	r	p	l	n	s	b	s-c	b-c	intra	syn
791 GDP	GDP(c)	PIPES MCES	synthesis of GDP	1.17 P_i(c) 1 Glycine(s) 2 Glutamine(s) 0.83 Glucose-6P(c) 1 Asparagine(s) 9.17 ATP-energy(c) 1 Proton-gradient(c) 2 THF-activated methyl group(c)	1.17 H_2O(s) 1 Pyruvate(c) 2 Glutamate(s) 1 GDP(c) 3 NADH-redox-potential(c) 3 NADPH-redox-potential(c)	35	-	-	-	-	-	-	-	6	-	-	-
792 GMP	GMP(c)	PIPES MCES	synthesis of GMP	0.17 P_i(c) 1 Glycine(s) 2 Glutamine(s) 0.83 Glucose-6P(c) 1 Asparagine(s) 8.17 ATP-energy(c) 1 Proton-gradient(c) 2 THF-activated methyl group(c)	0.17 H_2O(s) 1 Pyruvate(c) 2 Glutamate(s) 1 GMP(c) 3 NADH-redox-potential(c) 3 NADPH-redox-potential(c)	34	-	-	-	-	-	-	-	6	-	-	-
793 GSSG	GSSG(c)	PIPES MCES	synthesis of GSSG	2 Glutamate(s) 2 Glycine(s) 2 Cysteine(s) 4 ATP-energy(c) 2 Proton-gradient(c)	4 H_2O(s) 1 GSSG(c) 1 NADPH-redox-potential(c) 2 Na-gradient(c)	5	-	-	-	-	-	-	-	6	-	-	-
794 Galactose-1P	Galactose-1P(c)	PIPES MCES	synthesis of cytosolic Galactose-1P	1 Glucose-6P(c)	1 Galactose-1P(c)	3	-	-	-	-	-	-	-	-	-	-	-
795 Geranyl-PP	Geranyl-PP(p)	PIPES MCES	synthesis of peroxysomal Geranyl-PP	2 P_i(c) 6 ATP-energy(c) 4 NADPH-redox-potential(c) 6 CoA-activated acetyl group(c)	3 H_2O(s) 2 CO_2(s) 1 Geranyl-PP(p)	7	-	-	5	-	-	-	-	2	-	5	-
796 Glucono-1,5-lactone-6P	Glucono-1,5-lactone-6P(c)	PIPES MCES	synthesis of cytosolic Glucono-1,5-lactone-6P	1 Glucose-6P(c)	1 Glucono-1,5-lactone-6P(c) 1 NADPH-redox-potential(c)	2	-	-	-	-	-	-	-	-	-	-	-
797 Glucose-1P	Glucose-1P(c)	PIPES MCES	synthesis of Glucose-1P	1 Glucose-6P(c)	1 Glucose-1P(c)	1	-	-	-	-	-	-	-	-	-	-	-
798 Glucosylceramide-pool	Glucosylceramide-pool(c)	PIPES MCES	synthesis of Glucosylceramide-pool	1 Serine(s) 1 Glucose-6P(c) 0.02 Arachidonate(s) 0.88 Palmitate(s) 0.24 Oleate(s) 0.03 Linoleate(s) 0.83 Palmitolate(s) 5 ATP-energy(c) 2 NADPH-redox-potential(c)	2 H_2O(s) 1 P_i(c) 1 CO_2(s) 1 Glucosylceramide-pool(c) 1 NADH-redox-potential(c)	22	-	-	-	-	-	-	-	8	-	-	-
799 Glucuronate	Glucuronate(c)	PIPES MCES	synthesis of cytosolic Glucuronate	1 O_2(s) 1 Glucose-6P(c)	1 P_i(c) 1 Glucuronate(c)	3	-	-	-	-	-	-	-	1	-	-	-
800 Glutamyl-5P	Glutamyl-5P(m)	PIPES MCES	synthesis of mitochondrial Glutamyl-5P	1 P_i(c) 0.5 Glutamate(s) 0.5 Proline(s) 1 ATP-energy(m)	1 Glutamyl-5P(m) 1 Na-gradient(c)	-	5	-	-	-	-	-	-	3	-	4	-

Continued on next page

Simulation	Definition			Solution													
				exchanges		reactions								transport			Prot
	Objective	Contraints	Comment	imports	exports	c	m	r	p	l	n	s	b	s-c	b-c	intra	syn
801 Glutaryl-CoA	Glutaryl-CoA(m)	PIPES MCES	synthesis of mitochondrial Glutaryl-CoA	2.17 P_i(c) 1 Glycine(s) 2 Aspartate(s) 2 Glutamine(s) 0.83 Glucose-6P(c) 0.5 Cystine(s) 1 Pantothenate(s) 11.2 ATP-energy(c) 2 NADH-redox-potential(m) 1 Proton-gradient(c) 2 THF-activated methyl group(c) 2 CoA-activated acetyl group(m)	6.17 H_2O(s) 1 CO_2(s) 2 Pyruvate(c) 2 Glutamate(s) 1 Glutaryl-CoA(m) 2 NADH-redox-potential(c) 3.5 NADPH-redox-potential(c) 4 Proton-gradient(m) 3 Na-gradient(c)	42	6	-	-	-	-	-	-	10	-	5	-
802 Glycerate	Glycerate(c)	PIPES MCES	synthesis of cytosolic Glycerate	1 Pyruvate(c) 1 Serine(s) 1 NADPH-redox-potential(c)	1 Alanine(s) 1 Glycerate(c) 2 Na-gradient(c)	3	-	-	-	-	-	-	-	3	-	-	-
803 Glycogenin-G11	Glycogenin-G11(c)	PIPES MCES	synthesis of cytosolic Glycogenin-G11	15 Glutamate(s) 18 Glycine(s) 22 Alanine(s) 18 Lysine(s) 26 Aspartate(s) 12 Arginine(s) 14 Glutamine(s) 31 Serine(s) 8 Methionine(s) 7 Tryptophan(s) 20 Phenylalanine(s) 13 Tyrosine(s) 11 Glucose-6P(c) 6 Cysteine(s) 39 Leucine(s) 9 Histidine(s) 15 Proline(s) 12 Asparagine(s) 28 Valine(s) 25 Threonine(s) 12 Isoleucine(s) 1761 ATP-energy(c)	11 P_i(c) 3 Proton-gradient(c) 1 Glycogenin-G11(c) 246 Na-gradient(c)	8	-	-	-	-	-	-	-	22	-	-	1
804 Glycogenin-G4G4	Glycogenin-G4G4(c)	PIPES MCES	synthesis of cytosolic Glycogenin-G4G4	15 Glutamate(s) 18 Glycine(s) 22 Alanine(s) 18 Lysine(s) 26 Aspartate(s) 12 Arginine(s) 14 Glutamine(s) 31 Serine(s) 8 Methionine(s) 7 Tryptophan(s) 20 Phenylalanine(s) 13 Tyrosine(s) 8 Glucose-6P(c) 6 Cysteine(s) 39 Leucine(s) 9 Histidine(s) 15 Proline(s) 12 Asparagine(s) 28 Valine(s) 25 Threonine(s) 12 Isoleucine(s) 1761 ATP-energy(c)	8 P_i(c) 3 Proton-gradient(c) 1 Glycogenin-G4G4(c) 246 Na-gradient(c)	10	-	-	-	-	-	-	-	22	-	-	1

Continued on next page

C5. Comprehensive list – continued.

Simulation	Definition			Solution													
				exchanges		reactions								transport			Prot
	Objective	Contraints	Comment	imports	exports	c	m	r	p	l	n	s	b	s-c	b-c	intra	syn
805 Glycogenin-G7	Glycogenin-G7(c)	PIPES MCES	synthesis of cytosolic Glycogenin-G7	15 Glutamate(s) 18 Glycine(s) 22 Alanine(s) 18 Lysine(s) 26 Aspartate(s) 12 Arginine(s) 14 Glutamine(s) 31 Serine(s) 8 Methionine(s) 7 Tryptophan(s) 20 Phenylalanine(s) 13 Tyrosine(s) 7 Glucose-6P(c) 6 Cysteine(s) 39 Leucine(s) 9 Histidine(s) 15 Proline(s) 12 Asparagine(s) 28 Valine(s) 25 Threonine(s) 12 Isoleucine(s) 1762 ATP-energy(c)	7 P_i(c) 3 Proton-gradient(c) 1 Glycogenin-G7(c) 246 Na-gradient(c)	13	-	-	-	-	-	-	-	22	-	-	1
806 Glycogenin-G7G1	Glycogenin-G7G1(c)	PIPES MCES	synthesis of cytosolic Glycogenin-G7G1	15 Glutamate(s) 18 Glycine(s) 22 Alanine(s) 18 Lysine(s) 26 Aspartate(s) 12 Arginine(s) 14 Glutamine(s) 31 Serine(s) 8 Methionine(s) 7 Tryptophan(s) 20 Phenylalanine(s) 13 Tyrosine(s) 8 Glucose-6P(c) 6 Cysteine(s) 39 Leucine(s) 9 Histidine(s) 15 Proline(s) 12 Asparagine(s) 28 Valine(s) 25 Threonine(s) 12 Isoleucine(s) 1761 ATP-energy(c)	8 P_i(c) 3 Proton-gradient(c) 1 Glycogenin-G7G1(c) 246 Na-gradient(c)	11	-	-	-	-	-	-	-	22	-	-	1
807 Glycogenin-G8	Glycogenin-G8(c)	PIPES MCES	synthesis of cytosolic Glycogenin-G8	15 Glutamate(s) 18 Glycine(s) 22 Alanine(s) 18 Lysine(s) 26 Aspartate(s) 12 Arginine(s) 14 Glutamine(s) 31 Serine(s) 8 Methionine(s) 7 Tryptophan(s) 20 Phenylalanine(s) 13 Tyrosine(s) 8 Glucose-6P(c) 6 Cysteine(s) 39 Leucine(s) 9 Histidine(s) 15 Proline(s) 12 Asparagine(s) 28 Valine(s) 25 Threonine(s) 12 Isoleucine(s) 1758 ATP-energy(c)	8 P_i(c) 3 Proton-gradient(c) 1 Glycogenin-G8(c) 246 Na-gradient(c)	7	-	-	-	-	-	-	-	22	-	-	1

Continued on next page

C5. Comprehensive list – continued.

Simulation	Definition			Solution													
				exchanges		reactions								transport			Prot
	Objective	Contraints	Comment	imports	exports	c	m	r	p	l	n	s	b	s-c	b-c	intra	syn
808 Guanidinoacetate	Guanidinoacetate(c)	PIPES MCES	synthesis of cytosolic Guanidinoacetate	0.08 O_2(s) 1 Glycine(s) 0.5 Aspartate(s) 0.5 Arginine(s) 1 ATP-energy(c) 1 ATP-energy(m) 0.83 Proton-gradient(c)	0.5 Pyruvate(c) 0.33 Glutamate(s) 0.17 Proline(s) 1 Guanidinoacetate(c) 0.33 NADH-redox-potential(m) 0.5 NADPH-redox-potential(c) 0.5 NADPH-redox-potential(m) 0.67 Na-gradient(c)	9	10	-	-	-	-	-	-	8	-	9	-
809 Guanosine	Guanosine(s)	PIPES MCES	synthesis of Guanosine and blood export	0.17 Glycine(s) 2 Glutamine(s) 0.83 Serine(s) 0.83 Glucose-6P(c) 1 Asparagine(s) 8.17 ATP-energy(c) 1.17 THF-activated methyl group(c)	0.83 P_i(c) 1 Pyruvate(c) 2 Glutamate(s) 1 Guanosine(s) 2.17 NADH-redox-potential(c) 3 NADPH-redox-potential(c) 2.83 Proton-gradient(c) 2.83 Na-gradient(c)	36	-	-	-	-	-	-	-	8	-	-	-
810 H_2O_2	H_2O_2(c)	PIPES MCES	synthesis of H_2O_2 and blood export	1 O_2(s) 1 NH_3(s) 1 Aspartate(s) 1 Glutamine(s) 1 Serine(s) 8 ATP-energy(c) 1 THF-activated methyl group(c)	1 Pyruvate(c) 1 Glutamate(s) 1 H_2O_2(c) 1 Urate(s) 2 NADH-redox-potential(c) 3 NADPH-redox-potential(c) 1 Proton-gradient(c) 2 Na-gradient(c)	29	-	-	-	-	-	-	-	9	-	-	-
811 H_2S	H_2S(s)	PIPES MCES	synthesis of H_2S and blood export	1 Glutamate(s) 1 Cysteine(s) 1 ATP-energy(c)	1 Pyruvate(c) 1 Glutamine(s) 1 H_2S(s) 1 Na-gradient(c)	3	-	-	-	-	-	-	-	5	-	-	-
812 HCO3-	HCO3-(s)	PIPES MCES	synthesis of HCO3- and blood export	1 H_2O(s) 1 Pyruvate(m)	1 HCO3-(s) 1 NADH-redox-potential(m) 1 CoA-activated acetyl group(m)	-	3	-	-	-	-	2	-	1	-	1	-

Continued on next page

Simulation	Definition			Solution													
				exchanges		reactions								transport			Prot
	Objective	Contraints	Comment	imports	exports	c	m	r	p	l	n	s	b	s-c	b-c	intra	syn
813 HMA	HMA(c)	PIPES MCES	synthesis of cytosolic HMA	1 P_i(c) 5 Glutamate(s) 6 Glycine(s) 11 Alanine(s) 4 Lysine(s) 8 Aspartate(s) 9 Arginine(s) 7 Glutamine(s) 8 Serine(s) 5 Methionine(s) 3 Phenylalanine(s) 4 Tyrosine(s) 3 Cysteine(s) 20 Leucine(s) 1 Histidine(s) 9 Proline(s) 1 Asparagine(s) 11 Valine(s) 6 Threonine(s) 4 Isoleucine(s) 1 Pantothenate(s) 630 ATP-energy(c) 11 NADPH-redox-potential(c) 3 Proton-gradient(c) 7 CoA-activated acetyl group(c)	8 H_2O(s) 1 CO_2(s) 1 HMA(c) 66 Na-gradient(c)	41	-	-	-	-	-	-	-	24	-	-	1
814 HMG-CoA	HMG-CoA(c)	PIPES MCES	synthesis of cytosolic HMG-CoA	2.17 P_i(c) 1 Glycine(s) 2 Aspartate(s) 2 Glutamine(s) 0.83 Glucose-6P(c) 0.5 Cystine(s) 1 Pantothenate(s) 11.2 ATP-energy(c) 1 Proton-gradient(c) 2 THF-activated methyl group(c) 3 CoA-activated acetyl group(c)	4.17 H_2O(s) 2 CO_2(s) 2 Pyruvate(c) 2 Glutamate(s) 1 HMG-CoA(c) 2 NADH-redox-potential(c) 3.5 NADPH-redox-potential(c) 3 Na-gradient(c)	45	-	-	-	-	-	-	-	10	-	-	-
815 Hexadecanal	Hexadecanal(c)	PIPES MCES	synthesis of cytosolic Hexadecanal	1 Serine(s) 1 Palmitolate(s) 3 ATP-energy(c) 3 NADPH-redox-potential(c)	1 H_2O(s) 1 CO_2(s) 1 Ethanolamine(c) 1 Hexadecanal(c) 1 NADH-redox-potential(c) 1 Proton-gradient(c) 1 Na-gradient(c)	17	-	-	-	-	-	-	-	6	-	-	-

Continued on next page

C5. Comprehensive list – continued.

Simulation	Definition			Solution													
				exchanges		reactions								transport			Prot
	Objective	Contraints	Comment	imports	exports	c	m	r	p	l	n	s	b	s-c	b-c	intra	syn
816 Hexadecanoyl-ACP	Hexadecanoyl-ACP(c)	PIPES MCES	synthesis of cytosolic Hexadecanoyl-ACP	1 P_i(c) 5 Glutamate(s) 6 Glycine(s) 11 Alanine(s) 4 Lysine(s) 8 Aspartate(s) 9 Arginine(s) 7 Glutamine(s) 8 Serine(s) 5 Methionine(s) 3 Phenylalanine(s) 4 Tyrosine(s) 3 Cysteine(s) 20 Leucine(s) 1 Histidine(s) 9 Proline(s) 1 Asparagine(s) 11 Valine(s) 6 Threonine(s) 4 Isoleucine(s) 1 Pantothenate(s) 631 ATP-energy(c) 14 NADPH-redox-potential(c) 3 Proton-gradient(c) 8 CoA-activated acetyl group(c)	10 H_2O(s) 1 CO_2(s) 1 Hexadecanoyl-ACP(c) 66 Na-gradient(c)	47	-	-	-	-	-	-	-	24	-	-	1
817 Hexadecenal	Hexadecenal(c)	PIPES MCES	synthesis of cytosolic Hexadecenal	1 Serine(s) 1 Palmitolate(s) 3 ATP-energy(c) 2 NADPH-redox-potential(c)	1 H_2O(s) 1 CO_2(s) 1 Ethanolamine(c) 1 NADH-redox-potential(c) 1 Proton-gradient(c) 1 Hexadecenal(c) 1 Na-gradient(c)	16	-	-	-	-	-	-	-	6	-	-	-
818 Hexanoyl-ACP	Hexanoyl-ACP(c)	PIPES MCES	synthesis of cytosolic Hexanoyl-ACP	1 P_i(c) 5 Glutamate(s) 6 Glycine(s) 11 Alanine(s) 4 Lysine(s) 8 Aspartate(s) 9 Arginine(s) 7 Glutamine(s) 8 Serine(s) 5 Methionine(s) 3 Phenylalanine(s) 4 Tyrosine(s) 3 Cysteine(s) 20 Leucine(s) 1 Histidine(s) 9 Proline(s) 1 Asparagine(s) 11 Valine(s) 6 Threonine(s) 4 Isoleucine(s) 1 Pantothenate(s) 626 ATP-energy(c) 4 NADPH-redox-potential(c) 3 Proton-gradient(c) 3 CoA-activated acetyl group(c)	5 H_2O(s) 1 CO_2(s) 1 Hexanoyl-ACP(c) 66 Na-gradient(c)	27	-	-	-	-	-	-	-	24	-	-	1

Continued on next page

Simulation	Definition			Solution													
				exchanges		reactions								transport			Prot
	Objective	Contraints	Comment	imports	exports	c	m	r	p	l	n	s	b	s-c	b-c	intra	syn
819 Hexanoyl-CoA	Hexanoyl-CoA(m)	PIPES MCES	synthesis of mitochondrial Hexanoyl-CoA	1 O_2(s) 2.17 P_i(c) 1 Glycine(s) 2 Aspartate(s) 2 Glutamine(s) 0.83 Glucose-6P(c) 0.5 Cystine(s) 1 Pantothenate(s) 1 Palmitolate(s) 13.2 ATP-energy(c) 1 Proton-gradient(c) 2 THF-activated methyl group(c)	2.17 H_2O(s) 2 CO_2(s) 2 Pyruvate(c) 2 Glutamate(s) 1 Hexanoyl-CoA(m) 2 NADH-redox-potential(c) 6 NADH-redox-potential(m) 3.5 NADPH-redox-potential(c) 3 FADH-redox-potential(c) 12 Proton-gradient(m) 3 Na-gradient(c) 6 CoA-activated acetyl group(m)	44	26	-	-	-	-	-	-	12	-	7	-
820 Histamine	Histamine(s)	PIPES MCES	synthesis of Histamine and blood export	1 Histidine(s)	1 CO_2(s) 1 Histamine(s) 1 Na-gradient(c)	1	-	-	-	-	-	-	-	4	-	-	-
821 Homogentisate	Homogentisate(c)	PIPES MCES	synthesis of cytosolic Homogentisate	1 O_2(s) 1 Pyruvate(c) 1 Tyrosine(s)	1 CO_2(s) 1 Alanine(s) 1 Homogentisate(c) 1 Na-gradient(c)	3	-	-	-	-	-	-	-	5	-	-	-
822 Homovanillate	Homovanillate(m)	PIPES MCES -3-Methoxy-4-hydroxyphenylacetaldehyde(m)	synthesis of mitochondrial Homovanillate using 3-Methoxy-4-hydroxyphenylacetaldehyde	1 H_2O(s) 1 3-Methoxy-4-hydroxyphenylacetaldehyde(m)	1 Homovanillate(m) 1 NADH-redox-potential(m)	-	2	-	-	-	-	-	-	1	-	1	-
823 Hydracrylate	Hydracrylate(m)	PIPES MCES	synthesis of mitochondrial Hydracrylate	0.5 O_2(s) 1 Glutamate(s) 1 Threonine(s) 1 ATP-energy(c)	1 CO_2(s) 1 Glutamine(s) 1 Hydracrylate(m) 1 NADH-redox-potential(m) 5 Proton-gradient(m) 2 Na-gradient(c)	3	5	-	-	-	-	-	-	6	-	7	-
824 Hydroxymethylbilane	Hydroxymethylbilane(c)	PIPES MCES	synthesis of cytosolic Hydroxymethylbilane	4 Pyruvate(m) 12 Glutamate(s) 4 Serine(s) 4 ATP-energy(c) 4 NADPH-redox-potential(m) 4 Proton-gradient(m)	11 H_2O(s) 8 CO_2(s) 4 Aspartate(s) 4 Glutamine(s) 1 Hydroxymethylbilane(c) 4 NADH-redox-potential(c) 8 NADH-redox-potential(m) 12 Na-gradient(c)	7	8	-	-	-	-	-	-	6	-	6	-
825 Hydroxypyruvate	Hydroxypyruvate(c)	−Pyruvate(c) −Serine(c) +Alanine(c)	synthesis of cytosolic Hydroxypyruvate	1 Pyruvate(c) 1 Serine(c)	1 Alanine(c) 1 Hydroxypyruvate(c)	1	-	-	-	-	-	-	-	-	-	-	-

Continued on next page

Simulation	Definition			Solution													
				exchanges		reactions								transport			Prot
	Objective	Contraints	Comment	imports	exports	c	m	r	p	l	n	s	b	s-c	b-c	intra	syn
826 Hypotaurine	Hypotaurine(c)	PIPES MCES	synthesis of cytosolic Hypotaurine	1 O_2(s) 1 Cysteine(s)	1 CO_2(s) 1 Hypotaurine(c)	2	-	-	-	-	-	-	-	3	-	-	-
827 Hypoxanthine	Hypoxanthine(s)	PIPES MCES	synthesis of Hypoxanthine and blood export	1 Glycine(s) 1 Glutamine(s) 1 Asparagine(s) 7 ATP-energy(c) 1 Proton-gradient(c) 2 THF-activated methyl group(c)	1 Pyruvate(c) 1 Glutamate(s) 1 Hypoxanthine(s) 2 NADH-redox-potential(c) 3 NADPH-redox-potential(c)	27	-	-	-	-	-	-	-	6	-	-	-
828 Inosine	Inosine(s)	PIPES MCES	synthesis of Inosine and blood export	1 Glycine(s) 1 Glutamine(s) 0.83 Glucose-6P(c) 1 Asparagine(s) 7.17 ATP-energy(c) 2 THF-activated methyl group(c)	0.17 H_2O(s) 0.83 P_i(c) 1 Pyruvate(c) 1 Glutamate(s) 1 Inosine(s) 2 NADH-redox-potential(c) 3 NADPH-redox-potential(c) 1 Na-gradient(c)	34	-	-	-	-	-	-	-	7	-	-	-
829 Inositol	Inositol(s)	PIPES MCES	synthesis of Inositol and blood export	1 H_2O(s) 1 Glucose-6P(c)	1 P_i(c) 1 Inositol(s)	2	-	-	-	-	-	-	-	2	-	-	-
830 Inositol-1P	Inositol-1P(c)	PIPES MCES	synthesis of cytosolic Inositol-1P	1 Glucose-6P(c)	1 Inositol-1P(c)	1	-	-	-	-	-	-	-	-	-	-	-
831 Isobutyryl-CoA	Isobutyryl-CoA(m)	PIPES MCES	synthesis of mitochondrial Isobutyryl-CoA	2.17 P_i(c) 1 Glycine(s) 2 Aspartate(s) 1 Glutamine(s) 0.83 Glucose-6P(c) 1 Valine(s) 0.5 Cystine(s) 1 Pantothenate(s) 12.2 ATP-energy(c) 1 Proton-gradient(c) 1 Proton-gradient(m) 2 THF-activated methyl group(c)	5.17 H_2O(s) 3 CO_2(s) 2 Pyruvate(c) 1 Glutamate(s) 1 Isobutyryl-CoA(m) 2 NADH-redox-potential(c) 1 NADH-redox-potential(m) 3.5 NADPH-redox-potential(c) 1 NADPH-redox-potential(m) 4 Na-gradient(c)	43	5	-	-	-	-	-	-	11	-	6	-
832 Isocitrate	Isocitrate(c)	PIPES MCES	synthesis of cytosolic Isocitrate	1 Pyruvate(c) 1 Pyruvate(m) 1 Glutamate(s) 1 NADPH-redox-potential(c)	1 Alanine(s) 1 Isocitrate(c) 1 NADH-redox-potential(m) 1 Na-gradient(c) 1 CoA-activated acetyl group(m)	4	3	-	-	-	-	-	-	3	-	1	-

Continued on next page

C5. Comprehensive list – continued.

Simulation	Definition			Solution													
				exchanges		reactions								transport			Prot
	Objective	Contraints	Comment	imports	exports	c	m	r	p	l	n	s	b	s-c	b-c	intra	syn
833 Isovaleryl-CoA	Isovaleryl-CoA(m)	PIPES MCES	synthesis of mitochondrial Isovaleryl-CoA	2.17 P_i(c) 0.25 Pyruvate(m) 1 Glycine(s) 1.75 Aspartate(s) 1.5 Glutamine(s) 0.83 Glucose-6P(c) 0.75 Leucine(s) 0.5 Cystine(s) 1 Pantothenate(s) 11.7 ATP-energy(c) 1.25 Proton-gradient(m) 2 THF-activated methyl group(c) 0.75 CoA-activated acetyl group(m)	5.67 H_2O(s) 2.75 CO_2(s) 2 Pyruvate(c) 1.25 Glutamate(s) 0.25 Proline(s) 1 Isovaleryl-CoA(m) 2 NADH-redox-potential(c) 0.5 NADH-redox-potential(m) 3.5 NADPH-redox-potential(c) 0.5 NADPH-redox-potential(m) 3.75 Na-gradient(c)	43	16	-	-	-	-	-	-	11	-	7	-
834 Kynurenine	Kynurenine(c)	PIPES MCES	synthesis of cytosolic Kynurenine	1 O_2(s) 1 Tryptophan(s) 1 ATP-energy(c) 0.5 NADPH-redox-potential(c)	0.5 CO_2(s) 1 Kynurenine(c) 1 Na-gradient(c) 0.5 THF-activated methyl group(c)	10	-	-	-	-	-	-	-	4	-	-	-
835 L-1-Pyrroline-3-hydroxy-5-carboxylate	L-1-Pyrroline-3-hydroxy-5-carboxylate(c)	PIPES MCES −trans-4-Hydroxy-L-proline(c)	synthesis of cytosolic L-1-Pyrroline-3-hydroxy-5-carboxylate	1 trans-4-Hydroxy-L-proline(c)	1 L-1-Pyrroline-3-hydroxy-5-carboxylate(c) 1 NADPH-redox-potential(c)	2	-	-	-	-	-	-	-	-	-	-	-
836 L-2-Aminoadipate	L-2-Aminoadipate(c)	PIPES MCES -2-Aminoadipate 6-semialdehyde(c)	synthesis of cytosolic L-2-Aminoadipate	1 H_2O(s) 1 2-Aminoadipate 6-semialdehyde(c)	1 L-2-Aminoadipate(c) 1 NADH-redox-potential(c)	2	-	-	-	-	-	-	-	1	-	-	-
837 L-Cystathionine	L-Cystathionine(c)	PIPES MCES	synthesis of cytosolic L-Cystathionine	1 Serine(s) 1 Methionine(s)	1 H_2O(s) 1 L-Cystathionine(c) 1 Proton-gradient(c) 2 Na-gradient(c) 1 THF-activated methyl group(c)	3	-	-	-	-	-	-	-	5	-	-	-
838 L-Formylkynurenine	L-Formylkynurenine(c)	PIPES MCES	synthesis of cytosolic L-Formylkynurenine	1 O_2(s) 1 Tryptophan(s)	1 L-Formylkynurenine(c) 1 Na-gradient(c)	1	-	-	-	-	-	-	-	3	-	-	-
839 L-Fucose-1P	L-Fucose-1P(c)	PIPES MCES	synthesis of cytosolic L-Fucose-1P	1 Glucose-6P(c) 1 NADH-redox-potential(c)	1 H_2O(s) 1 L-Fucose-1P(c)	8	-	-	-	-	-	-	-	1	-	-	-
840 L-Glutamate 5-semialdehyde	L-Glutamate 5-semialdehyde(m)	PIPES MCES	synthesis of mitochondrial L-Glutamate 5-semialdehyde	0.5 Glutamate(s) 0.5 Proline(s) 1 NADH-redox-potential(m)	1 L-Glutamate 5-semialdehyde(m) 1 Proton-gradient(m) 1 Na-gradient(c)	-	4	-	-	-	-	-	-	3	-	4	-

Continued on next page

Simulation	Definition			Solution													
				exchanges		reactions								transport			Prot
	Objective	Contraints	Comment	imports	exports	c	m	r	p	l	n	s	b	s-c	b-c	intra	syn
841 L-Lactate	L-Lactate(s)	PIPES MCES	synthesis of L-Lactate and blood export	1 Pyruvate(c) 1 NADH-redox-potential(c)	1 L-Lactate(s) 1 Proton-gradient(c)	2	-	-	-	-	-	-	-	2	-	-	-
842 L-Octanoylcarnitine	L-Octanoylcarnitine(c)	PIPES MCES −L-Carnitine(c)	synthesis of cytosolic L-Octanoylcarnitine	2.67 H_2O(s) 0.33 Arachidonate(s) 1 L-Carnitine(c) 1 Palmitolate(s) 4 ATP-energy(c)	0.33 Linoleate(s) 1 L-Octanoylcarnitine(c) 1.67 NADH-redox-potential(c) 4 NADH-redox-potential(p) 1.33 NADPH-redox-potential(r) 0.33 CoA-activated acetyl group(c) 4 CoA-activated acetyl group(p)	14	-	3	20	-	-	-	-	4	-	10	-
843 L-Oleoylcarnitine	L-Oleoylcarnitine(c)	PIPES MCES −L-Carnitine(c)	synthesis of cytosolic L-Oleoylcarnitine	1 L-Carnitine(c) 1 Oleate(s) 2 ATP-energy(c)	1 H_2O(s) 1 L-Oleoylcarnitine(c)	5	-	-	-	-	-	-	-	2	-	-	-
844 L-Palmitoylcarnitine	L-Palmitoylcarnitine(c)	PIPES MCES −L-Carnitine(c)	synthesis of cytosolic L-Palmitoylcarnitine	1 Palmitate(s) 1 L-Carnitine(c) 2 ATP-energy(c)	1 H_2O(s) 1 L-Palmitoylcarnitine(c)	5	-	-	-	-	-	-	-	2	-	-	-
845 Lanosterol	Lanosterol(r)	PIPES MCES	synthesis of Golgi/ER Lanosterol	1 H_2O(s) 1 O_2(s) 2 Farnesyl-PP(r) 2 NADPH-redox-potential(r)	4 P_i(c) 1 Lanosterol(r)	1	-	5	-	-	-	-	-	2	-	3	-
846 Lathosterol	Lathosterol(r)	PIPES MCES	synthesis of Golgi/ER Lathosterol	7 O_2(s) 2 Farnesyl-PP(r) 1 ATP-energy(c) 9 NADPH-redox-potential(r)	5 H_2O(s) 4 P_i(c) 3 CO_2(s) 1 Lathosterol(r) 1 NADH-redox-potential(r) 1 NADPH-redox-potential(c)	5	-	14	-	-	-	-	-	3	-	5	-
847 Lauroyl-CoA	Lauroyl-CoA(m)	PIPES MCES	synthesis of mitochondrial Lauroyl-CoA	2.17 P_i(c) 1 Glycine(s) 2 Aspartate(s) 2 Glutamine(s) 0.83 Glucose-6P(c) 0.5 Cystine(s) 1 Pantothenate(s) 1 Palmitolate(s) 13.2 ATP-energy(c) 2 THF-activated methyl group(c)	4.17 H_2O(s) 2 CO_2(s) 2 Pyruvate(c) 2 Glutamate(s) 1 Lauroyl-CoA(m) 3 NADH-redox-potential(c) 2 NADH-redox-potential(m) 3.5 NADPH-redox-potential(c) 2 Proton-gradient(c) 12 Proton-gradient(m) 5 Na-gradient(c) 2 CoA-activated acetyl group(m)	46	11	-	-	-	-	-	-	11	-	6	-

Continued on next page

C5. Comprehensive list – continued.

Simulation	Definition			Solution													
				exchanges		reactions								transport			Prot
	Objective	Contraints	Comment	imports	exports	c	m	r	p	l	n	s	b	s-c	b-c	intra	syn
848 Linoleate	Linoleate(s)	PIPES MCES	synthesis of Linoleate and blood export	1.67 H_2O(s) 0.33 Phenylalanine(s) 1 Arachidonate(s) 0.67 Farnesyl-PP(r) 3.67 ATP-energy(c) 1 NADPH-redox-potential(c)	1.33 P_i(c) 0.67 CO_2(s) 0.33 Tyrosine(s) 0.33 Cholesterol(b) 1 Linoleate(s) 0.33 NADH-redox-potential(r) 2 NADH-redox-potential(c) 0.33 NADPH-redox-potential(r) 0.33 THF-activated methyl group(c) 1 CoA-activated acetyl group(c)	19	-	18	-	-	-	-	-	5	1	9	-
849 Linoleoyl-CoA	Linoleoyl-CoA(c)	PIPES MCES	synthesis of cytosolic Linoleoyl-CoA	2.17 P_i(c) 1 Glycine(s) 2 Aspartate(s) 2 Glutamine(s) 0.83 Glucose-6P(c) 0.5 Cystine(s) 1 Pantothenate(s) 1 Linoleate(s) 13.2 ATP-energy(c) 1 Proton-gradient(c) 2 THF-activated methyl group(c)	6.17 H_2O(s) 2 CO_2(s) 2 Pyruvate(c) 2 Glutamate(s) 1 Linoleoyl-CoA(c) 2 NADH-redox-potential(c) 3.5 NADPH-redox-potential(c) 3 Na-gradient(c)	43	-	-	-	-	-	-	-	11	-	-	-
850 Malate	Malate(c)	PIPES MCES	synthesis of cytosolic Malate	1 Pyruvate(c) 1 Pyruvate(m) 1 NADPH-redox-potential(c)	1 Malate(c) 1 NADH-redox-potential(m) 1 CoA-activated acetyl group(m)	2	3	-	-	-	-	-	-	-	-	1	-
851 Malonyl-ACP	Malonyl-ACP(c)	PIPES MCES	synthesis of cytosolic Malonyl-ACP	1 P_i(c) 5 Glutamate(s) 6 Glycine(s) 11 Alanine(s) 4 Lysine(s) 8 Aspartate(s) 9 Arginine(s) 7 Glutamine(s) 8 Serine(s) 5 Methionine(s) 3 Phenylalanine(s) 4 Tyrosine(s) 3 Cysteine(s) 20 Leucine(s) 1 Histidine(s) 9 Proline(s) 1 Asparagine(s) 11 Valine(s) 6 Threonine(s) 4 Isoleucine(s) 1 Pantothenate(s) 625 ATP-energy(c) 9 Proton-gradient(c) 1 CoA-activated acetyl group(c)	3 H_2O(s) 1 Malonyl-ACP(c) 82 Na-gradient(c)	17	-	-	-	-	-	-	-	23	-	-	1
852 Malonyl-Carnitin	Malonyl-Carnitin(c)	PIPES MCES –L-Carnitine(c)	synthesis of cytosolic Malonyl-Carnitin	1 Pyruvate(m) 1 L-Carnitine(c) 1 ATP-energy(c) 1 CoA-activated acetyl group(c)	1 NADH-redox-potential(m) 1 Malonyl-Carnitin(c) 1 CoA-activated acetyl group(m)	6	3	-	-	-	-	-	-	-	-	1	-

Continued on next page

C5. Comprehensive list – continued.

Simulation	Definition			Solution													
				exchanges		reactions								transport			Prot
	Objective	Contraints	Comment	imports	exports	c	m	r	p	l	n	s	b	s-c	b-c	intra	syn
853 Malonyl-CoA	Malonyl-CoA(c)	PIPES MCES	synthesis of cytosolic Malonyl-CoA	2.17 P_i(c) 1 Glycine(s) 2 Aspartate(s) 2 Glutamine(s) 0.83 Glucose-6P(c) 0.5 Cystine(s) 1 Pantothenate(s) 12.2 ATP-energy(c) 1 Proton-gradient(c) 2 THF-activated methyl group(c) 1 CoA-activated acetyl group(c)	5.17 H_2O(s) 1 CO_2(s) 2 Pyruvate(c) 2 Glutamate(s) 1 Malonyl-CoA(c) 2 NADH-redox-potential(c) 3.5 NADPH-redox-potential(c) 3 Na-gradient(c)	46	-	-	-	-	-	-	-	10	-	-	-
854 Maltose	Maltose(s)	PIPES MCES	synthesis of Maltose and blood export	2 Glucose(s)	1 H_2O(s) 1 Maltose(s)	-	-	-	-	-	-	1	-	-	-	-	-
855 Mercaptopyruvate	Mercaptopyruvate(c)	PIPES MCES	synthesis of cytosolic Mercaptopyruvate	1 Pyruvate(c) 1 Cysteine(s)	1 Alanine(s) 1 Mercaptopyruvate(c) 1 Na-gradient(c)	2	-	-	-	-	-	-	-	3	-	-	-
856 Methacrylyl-CoA	Methacrylyl-CoA(m)	PIPES MCES	synthesis of mitochondrial Methacrylyl-CoA	0.5 O_2(s) 2.17 P_i(c) 1 Glycine(s) 2 Aspartate(s) 1 Glutamine(s) 0.83 Glucose-6P(c) 1 Valine(s) 0.5 Cystine(s) 1 Pantothenate(s) 12.2 ATP-energy(c) 2 THF-activated methyl group(c)	6.17 H_2O(s) 3 CO_2(s) 2 Pyruvate(c) 1 Glutamate(s) 1 Methacrylyl-CoA(m) 2 NADH-redox-potential(c) 1 NADH-redox-potential(m) 3.5 NADPH-redox-potential(c) 1 NADPH-redox-potential(m) 5 Proton-gradient(m) 5 Na-gradient(c)	43	6	-	-	-	-	-	-	11	-	8	-
857 Methylmalonyl-CoA	Methylmalonyl-CoA(m)	PIPES MCES	synthesis of mitochondrial Methylmalonyl-CoA	2.17 P_i(c) 1 Pyruvate(m) 1 Glycine(s) 1 Aspartate(s) 2 Glutamine(s) 0.83 Glucose-6P(c) 0.5 Cystine(s) 1 Pantothenate(s) 11.2 ATP-energy(c) 1 NADPH-redox-potential(m) 1 Proton-gradient(m) 2 THF-activated methyl group(c)	5.17 H_2O(s) 2 CO_2(s) 2 Pyruvate(c) 1 Glutamate(s) 1 Methylmalonyl-CoA(m) 2 NADH-redox-potential(c) 2 NADH-redox-potential(m) 3.5 NADPH-redox-potential(c) 2 Proton-gradient(c) 4 Na-gradient(c)	42	8	-	-	-	-	-	-	10	-	3	-
858 Myristoyl-CoA	Myristoyl-CoA(m)	PIPES MCES	synthesis of mitochondrial Myristoyl-CoA	2.17 P_i(c) 1 Glycine(s) 2 Aspartate(s) 2 Glutamine(s) 0.83 Glucose-6P(c) 0.5 Palmitate(s) 0.5 Cystine(s) 1 Pantothenate(s) 0.5 Palmitolate(s) 13.2 ATP-energy(c) 1 Proton-gradient(c) 2 THF-activated methyl group(c)	5.17 H_2O(s) 2 CO_2(s) 2 Pyruvate(c) 2 Glutamate(s) 1 Myristoyl-CoA(m) 2.5 NADH-redox-potential(c) 1 NADH-redox-potential(m) 3.5 NADPH-redox-potential(c) 6 Proton-gradient(m) 3 Na-gradient(c) 1 CoA-activated acetyl group(m)	47	7	-	-	-	-	-	-	12	-	6	-

Continued on next page

Simulation	Definition			Solution													
				exchanges		reactions								transport			Prot
	Objective	Contraints	Comment	imports	exports	c	m	r	p	l	n	s	b	s-c	b-c	intra	syn
859 N-(omega)-Hydroxyarginine	N-(omega)-Hydroxyarginine(c)	PIPES MCES	synthesis of cytosolic N-(omega)-Hydroxyarginine	1 O_2(s) 1 Arginine(s) 1 NADPH-redox-potential(c)	1 H_2O(s) 1 N-(omega)-Hydroxyarginine(c)	2	-	-	-	-	-	-	-	3	-	-	-
860 N-Acetyl-D-mannosamine	N-Acetyl-D-mannosamine(s)	PIPES MCES	synthesis of N-Acetyl-D-mannosamine and blood export	1 NH_3(s) 1 Glucose-6P(c) 2 ATP-energy(c) 1 CoA-activated acetyl group(c)	1 P_i(c) 1 N-Acetyl-D-mannosamine(s)	10	-	2	-	-	-	-	-	2	-	3	-
861 N-Acetylglucosamine-1P	N-Acetylglucosamine-1P(c)	PIPES MCES	synthesis of cytosolic N-Acetylglucosamine-1P	1 Glutamine(s) 1 Glucose-6P(c) 1 CoA-activated acetyl group(c)	1 Glutamate(s) 1 N-Acetylglucosamine-1P(c)	5	-	-	-	-	-	-	-	2	-	-	-
862 N-Acetylmannosamine-6P	N-Acetylmannosamine-6P(c)	PIPES MCES	synthesis of cytosolic N-Acetylmannosamine-6P	1 Glutamine(s) 1 Glucose-6P(c) 2 ATP-energy(c) 1 CoA-activated acetyl group(c)	1 Glutamate(s) 1 N-Acetylmannosamine-6P(c)	11	-	-	-	-	-	-	-	2	-	-	-
863 N-Acetylneuraminate	N-Acetylneuraminate(c)	PIPES MCES	synthesis of cytosolic N-Acetylneuraminate	1 NH_3(s) 1 Pyruvate(c) 1 Glucose-6P(c) 3 ATP-energy(c) 1 NADPH-redox-potential(c) 1 CoA-activated acetyl group(c)	1 P_i(c) 1 N-Acetylneuraminate(c) 1 NADH-redox-potential(c)	19	-	-	-	-	-	-	-	1	-	-	-
864 N-Acetylneuraminate-9P	N-Acetylneuraminate-9P(c)	PIPES MCES	synthesis of cytosolic N-Acetylneuraminate-9P	1 Pyruvate(c) 1 Glutamine(s) 1 Glucose-6P(c) 3 ATP-energy(c) 1 NADPH-redox-potential(c) 1 CoA-activated acetyl group(c)	1 Glutamate(s) 1 N-Acetylneuraminate-9P(c) 1 NADH-redox-potential(c)	18	-	-	-	-	-	-	-	2	-	-	-
865 N-Carbamoyl-L-aspartate	N-Carbamoyl-L-aspartate(c)	PIPES MCES	synthesis of cytosolic N-Carbamoyl-L-aspartate	1 Pyruvate(m) 1 Asparagine(s) 3 ATP-energy(c)	1 N-Carbamoyl-L-aspartate(c) 1 NADH-redox-potential(m) 1 CoA-activated acetyl group(m)	7	3	-	-	-	-	-	-	1	-	1	-
866 N-Formimino-L-glutamate	N-Formimino-L-glutamate(c)	PIPES MCES	synthesis of cytosolic N-Formimino-L-glutamate	1 H_2O(s) 0.5 Glutamate(s) 0.5 Histidine(s) 0.5 THF-activated methyl group(c)	1 N-Formimino-L-glutamate(c) 0.5 NADH-redox-potential(c) 0.5 NADPH-redox-potential(c) 1 Na-gradient(c)	10	-	-	-	-	-	-	-	4	-	-	-

Continued on next page

Simulation		Definition			Solution													
					exchanges		reactions								transport			Prot
		Objective	Contraints	Comment	imports	exports	c	m	r	p	l	n	s	b	s-c	b-c	intra	syn
867	N-Formyl-GAR	N-Formyl-GAR(c)	PIPES MCES	synthesis of cytosolic N-Formyl-GAR	0.17 P_i(c) 1 Glycine(s) 1 Glutamine(s) 0.83 Glucose-6P(c) 3.17 ATP-energy(c) 1 Proton-gradient(c) 1 THF-activated methyl group(c)	0.17 H_2O(s) 1 Glutamate(s) 1 N-Formyl-GAR(c) 1 NADH-redox-potential(c) 1 NADPH-redox-potential(c)	22	-	-	-	-	-	-	-	5	-	-	-
868	N-Methylethanolamine-P	N-Methylethanolamine-P(c)	PIPES MCES	synthesis of cytosolic N-Methylethanolamine-P	1 P_i(c) 1 Ethanolamine(c) 1 Activated methyl group(c) 1 ATP-energy(c)	1 H_2O(s) 1 N-Methylethanolamine-P(c)	4	-	-	-	-	-	-	-	1	-	-	-
869	N-Pantothenoylcysteine	N-Pantothenoylcysteine(c)	PIPES MCES	synthesis of cytosolic N-Pantothenoylcysteine	1 Cysteine(s) 1 Pantothenate(s) 2 ATP-energy(c)	1 H_2O(s) 1 N-Pantothenoylcysteine(c) 1 Na-gradient(c)	7	-	-	-	-	-	-	-	4	-	-	-
870	NADH	NADH(c)	PIPES MCES	synthesis of cytosolic NADH	0.33 P_i(c) 1 Glycine(s) 1.67 Glucose-6P(c) 2 Asparagine(s) 1 Nicotinamide(s) 16.3 ATP-energy(c) 1 Proton-gradient(c) 2 THF-activated methyl group(c)	3.33 H_2O(s) 1 NADH(c) 1 CO_2(s) 2 Pyruvate(c) 1 NADH-redox-potential(c) 4 NADPH-redox-potential(c)	40	-	-	-	-	-	-	-	6	-	-	-
871	NADPH	NADPH(c)	PIPES MCES	synthesis of cytosolic NADPH	1.33 P_i(c) 1 Glycine(s) 1.67 Glucose-6P(c) 2 Asparagine(s) 1 Nicotinamide(s) 17.3 ATP-energy(c) 1 Proton-gradient(c) 2 THF-activated methyl group(c)	4.33 H_2O(s) 1 NADPH(c) 1 CO_2(s) 2 Pyruvate(c) 2 NADH-redox-potential(c) 3 NADPH-redox-potential(c)	41	-	-	-	-	-	-	-	6	-	-	-
872	NeuNGc	NeuNGc(c)	PIPES MCES	synthesis of cytosolic NeuNGc	1 O_2(s) 1 Pyruvate(c) 1 Glutamine(s) 1 Glucose-6P(c) 3 ATP-energy(c) 1 NADPH-redox-potential(c) 1 CoA-activated acetyl group(c)	1 P_i(c) 1 Glutamate(s) 1 NeuNGc(c) 2 NADH-redox-potential(c)	20	-	-	-	-	-	-	-	3	-	-	-
873	Nicotinamide D-ribonucleotide	Nicotinamide D-ribonucleotide(c)	PIPES MCES	synthesis of cytosolic Nicotinamide D-ribonucleotide	0.17 P_i(c) 0.83 Glucose-6P(c) 1 Nicotinamide(s) 2.17 ATP-energy(c)	1.17 H_2O(s) 1 Nicotinamide D-ribonucleotide(c)	14	-	-	-	-	-	-	-	2	-	-	-
874	Nicotinate	Nicotinate(s)	PIPES MCES	synthesis of Nicotinate and blood export	1 Glutamate(s) 1 Nicotinamide(s) 1 ATP-energy(c)	1 Glutamine(s) 1 Nicotinate(s) 1 Na-gradient(c)	5	-	-	-	-	-	-	-	5	-	-	-

Continued on next page

C5. Comprehensive list – continued.

Simulation	Definition			Solution													
				exchanges		reactions								transport			Prot
	Objective	Contraints	Comment	imports	exports	c	m	r	p	l	n	s	b	s-c	b-c	intra	syn
875 Nicotinate D-ribonucleoside	Nicotinate D-ribonucleoside(c)	PIPES MCES	synthesis of cytosolic Nicotinate D-ribonucleoside	1 Glutamate(s) 0.83 Glucose-6P(c) 1 Nicotinamide(s) 3.17 ATP-energy(c)	0.17 H_2O(s) 0.83 P_i(c) 1 Glutamine(s) 1 Nicotinate D-ribonucleoside(c) 1 Na-gradient(c)	17	-	-	-	-	-	-	-	5	-	-	-
876 Nicotinate(r)ibonucleotide	Nicotinate ribonucleotide(c)	PIPES MCES	synthesis of cytosolic Nicotinate ribonucleotide	0.17 P_i(c) 1 Glutamate(s) 0.83 Glucose-6P(c) 1 Nicotinamide(s) 3.17 ATP-energy(c)	1.17 H_2O(s) 1 Glutamine(s) 1 Nicotinate ribonucleotide(c) 1 Na-gradient(c)	16	-	-	-	-	-	-	-	5	-	-	-
877 O-Acetylcarnitine	O-Acetylcarnitine(c)	PIPES MCES –L-Carnitine(c)	synthesis of cytosolic O-Acetylcarnitine	1 L-Carnitine(c) 1 CoA-activated acetyl group(c)	1 O-Acetylcarnitine(c)	2	-	-	-	-	-	-	-	-	-	-	-
878 O-Butanoylcarnitine	O-Butanoylcarnitine(c)	PIPES MCES –L-Carnitine(c)	synthesis of cytosolic O-Butanoylcarnitine	2.63 O_2(s) 0.75 Palmitate(s) 1 L-Carnitine(c) 0.25 Palmitolate(s) 2 ATP-energy(c)	6.25 NADH-redox-potential(m) 0.75 FADH-redox-potential(c) 31.5 Proton-gradient(m) 1 O-Butanoylcarnitine(c) 6.25 CoA-activated acetyl group(m)	8	40	-	-	-	-	-	-	3	-	12	-
879 O-Propanoylcarnitine	O-Propanoylcarnitine(c)	PIPES MCES –L-Carnitine(c)	synthesis of cytosolic O-Propanoylcarnitine	1 Glutamate(s) 1 Threonine(s) 1 L-Carnitine(c) 1 ATP-energy(c) 1 Proton-gradient(m)	1 H_2O(s) 1 CO_2(s) 1 Glutamine(s) 1 NADH-redox-potential(m) 1 O-Propanoylcarnitine(c) 2 Na-gradient(c)	4	2	-	-	-	-	-	-	6	-	5	-
880 OAA	OAA(c)	PIPES MCES	synthesis of cytosolic OAA	1 Pyruvate(c) 1 Pyruvate(m) 1 NADPH-redox-potential(c)	1 OAA(c) 1 NADH-redox-potential(c) 1 NADH-redox-potential(m) 1 CoA-activated acetyl group(m)	4	3	-	-	-	-	-	-	-	-	1	-

Continued on next page

C5. Comprehensive list – continued.

Simulation	Definition			Solution													
				exchanges		reactions								transport			Prot
	Objective	Contraints	Comment	imports	exports	c	m	r	p	l	n	s	b	s-c	b-c	intra	syn
881 Octanoyl-ACP	Octanoyl-ACP(c)	PIPES MCES	synthesis of cytosolic Octanoyl-ACP	1 P_i(c) 5 Glutamate(s) 6 Glycine(s) 11 Alanine(s) 4 Lysine(s) 8 Aspartate(s) 9 Arginine(s) 7 Glutamine(s) 8 Serine(s) 5 Methionine(s) 3 Phenylalanine(s) 4 Tyrosine(s) 3 Cysteine(s) 20 Leucine(s) 1 Histidine(s) 9 Proline(s) 1 Asparagine(s) 11 Valine(s) 6 Threonine(s) 4 Isoleucine(s) 1 Pantothenate(s) 627 ATP-energy(c) 6 NADPH-redox-potential(c) 3 Proton-gradient(c) 4 CoA-activated acetyl group(c)	6 H_2O(s) 1 CO_2(s) 1 Octanoyl-ACP(c) 66 Na-gradient(c)	31	-	-	-	-	-	-	-	24	-	-	1
882 Octanoyl-CoA	Octanoyl-CoA(m)	PIPES MCES	synthesis of mitochondrial Octanoyl-CoA	0.5 O_2(s) 2.17 P_i(c) 1 Glycine(s) 2 Aspartate(s) 2 Glutamine(s) 0.83 Glucose-6P(c) 0.5 Cystine(s) 1 Pantothenate(s) 1 Palmitolate(s) 13.2 ATP-energy(c) 1 Proton-gradient(c) 2 THF-activated methyl group(c)	2.17 H_2O(s) 2 CO_2(s) 2 Pyruvate(c) 2 Glutamate(s) 1 Octanoyl-CoA(m) 2 NADH-redox-potential(c) 5 NADH-redox-potential(m) 3.5 NADPH-redox-potential(c) 3 FADH-redox-potential(c) 6 Proton-gradient(m) 3 Na-gradient(c) 5 CoA-activated acetyl group(m)	44	22	-	-	-	-	-	-	12	-	7	-
883 Oleoyl-CoA	Oleoyl-CoA(c)	PIPES MCES	synthesis of cytosolic Oleoyl-CoA	2.17 P_i(c) 1 Glycine(s) 2 Aspartate(s) 2 Glutamine(s) 0.83 Glucose-6P(c) 0.5 Cystine(s) 1 Oleate(s) 1 Pantothenate(s) 13.2 ATP-energy(c) 1 Proton-gradient(c) 2 THF-activated methyl group(c)	6.17 H_2O(s) 2 CO_2(s) 2 Pyruvate(c) 2 Glutamate(s) 1 Oleoyl-CoA(c) 2 NADH-redox-potential(c) 3.5 NADPH-redox-potential(c) 3 Na-gradient(c)	43	-	-	-	-	-	-	-	11	-	-	-
884 Orotate	Orotate(s)	PIPES MCES	synthesis of Orotate and blood export	0.33 O_2(s) 0.5 Pyruvate(m) 1 Aspartate(s) 1 Glutamine(s) 0.33 Proline(s) 2 ATP-energy(c) 0.5 THF-activated methyl group(c)	1.33 Glutamate(s) 1 Orotate(s) 0.5 NADH-redox-potential(c) 0.83 NADH-redox-potential(m) 1 NADPH-redox-potential(c) 4 Proton-gradient(m) 1.33 Na-gradient(c) 0.5 CoA-activated acetyl group(m)	13	6	-	-	-	-	-	-	7	-	7	-

Continued on next page

C5. Comprehensive list – continued.

Simulation	Definition			Solution													
				exchanges		reactions								transport			Prot
	Objective	Contraints	Comment	imports	exports	c	m	r	p	l	n	s	b	s-c	b-c	intra	syn
885 Orotidine-5P	Orotidine-5P(c)	PIPES MCES	synthesis of cytosolic Orotidine-5P	0.33 O_2(s) 1 Aspartate(s) 1 Glutamine(s) 1 Glucose-6P(c) 0.33 Proline(s) 4 ATP-energy(c)	1 H_2O(s) 1.33 Glutamate(s) 1 Orotidine-5P(c) 0.33 NADH-redox-potential(m) 2 NADPH-redox-potential(c) 1 Proton-gradient(c) 4 Proton-gradient(m) 2.33 Na-gradient(c)	15	4	-	-	-	-	-	-	8	-	6	-
886 Oxalosuccinate	Oxalosuccinate(c)	PIPES MCES	synthesis of cytosolic Oxalosuccinate	1 Pyruvate(c) 1 Pyruvate(m) 1 Glutamate(s)	1 Alanine(s) 1 Oxalosuccinate(c) 1 NADH-redox-potential(m) 1 Na-gradient(c) 1 CoA-activated acetyl group(m)	2	3	-	-	-	-	-	-	3	-	1	-
887 Oxidized thioredoxin	Oxidized thioredoxin(c)	PIPES MCES	synthesis of cytosolic Oxidized thioredoxin	10 Glutamate(s) 5 Glycine(s) 8 Alanine(s) 12 Lysine(s) 7 Aspartate(s) 5 Glutamine(s) 7 Serine(s) 3 Methionine(s) 1 Tryptophan(s) 9 Phenylalanine(s) 1 Tyrosine(s) 5 Cysteine(s) 6 Leucine(s) 1 Histidine(s) 3 Proline(s) 3 Asparagine(s) 11 Valine(s) 4 Threonine(s) 4 Isoleucine(s) 525 ATP-energy(c) 6 Proton-gradient(c)	1 Oxidized thioredoxin(c) 1 NADPH-redox-potential(c) 58 Na-gradient(c)	5	-	-	-	-	-	-	-	21	-	-	1
888 PAP	PAP(c)	PIPES MCES	synthesis of cytosolic PAP	1.17 P_i(c) 1 Glycine(s) 0.83 Glucose-6P(c) 2 Asparagine(s) 12.2 ATP-energy(c) 2 THF-activated methyl group(c)	2.17 H_2O(s) 1 CO_2(s) 2 Pyruvate(c) 1 PAP(c) 2 NADH-redox-potential(c) 4 NADPH-redox-potential(c) 1 Proton-gradient(c) 2 Na-gradient(c)	40	-	-	-	-	-	-	-	6	-	-	-
889 PE-PS-VLDL-pool	PE-PS-VLDL-pool(c)	PIPES MCES	synthesis of cytosolic PE-PS-VLDL-pool	0.5 P_i(c) 0.5 Glucose-6P(c) 1 Ethanolamine(c) 0.53 Arachidonate(s) 0.09 Palmitate(s) 0.09 Oleate(s) 1.25 Stearate(s) 0.05 Linoleate(s) 7.5 ATP-energy(c) 1 NADH-redox-potential(c)	3.5 H_2O(s) 1 PE-PS-VLDL-pool(c)	28	-	-	-	-	-	-	-	6	-	-	-
890 PEP	PEP(c)	PIPES MCES	synthesis of cytosolic PEP	1 P_i(c) 1 Pyruvate(c) 1 ATP-energy(c) 1 NADPH-redox-potential(c)	1 H_2O(s) 1 PEP(c) 1 NADH-redox-potential(c)	7	-	-	-	-	-	-	-	1	-	-	-

Continued on next page

C5. Comprehensive list – continued.

Simulation	Definition			Solution													
				exchanges		reactions								transport			Prot
	Objective	Contraints	Comment	imports	exports	c	m	r	p	l	n	s	b	s-c	b-c	intra	syn
891 PG-CL-pool	PG-CL-pool(m)	PIPES MCES	synthesis of mitochondrial PG-CL-pool	1 Glucose-6P(c) 0.09 Palmitate(s) 0.28 Oleate(s) 0.04 Stearate(s) 1.5 Linoleate(s) 0.1 Palmitolate(s) 5 ATP-energy(c) 2 ATP-energy(m) 2 NADH-redox-potential(c) 1 Proton-gradient(m)	3 H_2O(s) 1 PG-CL-pool(m)	15	9	-	-	-	-	-	-	6	-	5	-
892 PGP-CL-pool	PGP-CL-pool(m)	PIPES MCES	synthesis of mitochondrial PGP-CL-pool	1 P_i(c) 1 Glucose-6P(c) 0.09 Palmitate(s) 0.28 Oleate(s) 0.04 Stearate(s) 1.5 Linoleate(s) 0.1 Palmitolate(s) 5 ATP-energy(c) 2 ATP-energy(m) 2 NADH-redox-potential(c) 2 Proton-gradient(m)	4 H_2O(s) 1 PGP-CL-pool(m)	15	8	-	-	-	-	-	-	6	-	6	-
893 PP_i	PP_i(c)	PIPES MCES	synthesis of PP_i	1.91 P_i(c) 0.09 Glucose-6P(c) 1 ATP-energy(c)	0.91 H_2O(s) 1 PP_i(c) 0.09 Glucose(s)	11	-	-	-	-	-	-	-	2	-	-	-
894 PRPP	PRPP(c)	PIPES MCES	synthesis of PRPP	2.17 P_i(c) 0.83 Glucose-6P(c) 2.17 ATP-energy(c)	2.17 H_2O(s) 1 PRPP(c)	12	-	-	-	-	-	-	-	1	-	-	-
895 Palmitoyl-CoA	Palmitoyl-CoA(c)	PIPES MCES	synthesis of cytosolic Palmitoyl-CoA	2.17 P_i(c) 1 Glycine(s) 2 Aspartate(s) 2 Glutamine(s) 0.83 Glucose-6P(c) 1 Palmitate(s) 0.5 Cystine(s) 1 Pantothenate(s) 13.2 ATP-energy(c) 1 Proton-gradient(c) 2 THF-activated methyl group(c)	6.17 H_2O(s) 2 CO_2(s) 2 Pyruvate(c) 2 Glutamate(s) 1 Palmitoyl-CoA(c) 2 NADH-redox-potential(c) 3.5 NADPH-redox-potential(c) 3 Na-gradient(c)	43	-	-	-	-	-	-	-	11	-	-	-
896 Pantetheine	Pantetheine(c)	PIPES MCES	synthesis of cytosolic Pantetheine	1 Cysteine(s) 1 Pantothenate(s)	1 H_2O(s) 1 CO_2(s) 1 Pantetheine(c) 1 Na-gradient(c)	4	-	-	-	-	-	-	-	5	-	-	-
897 Phosphatidate-Bile-PC-pool	Phosphatidate-Bile-PC-pool(c)	PIPES MCES	synthesis of cytosolic Phosphatidate-Bile-PC-pool	0.5 P_i(c) 0.5 Glucose-6P(c) 0.11 Arachidonate(s) 0.83 Palmitate(s) 0.24 Oleate(s) 0.11 Stearate(s) 0.66 Linoleate(s) 0.05 Palmitolate(s) 4.5 ATP-energy(c) 1 NADH-redox-potential(c)	2.5 H_2O(s) 1 Phosphatidate-Bile-PC-pool(c)	24	-	-	-	-	-	-	-	7	-	-	-

Continued on next page

C5. Comprehensive list – continued.

Simulation	Definition: Objective	Definition: Contraints	Definition: Comment	Solution – exchanges: imports	Solution – exchanges: exports	reactions: c	reactions: m	reactions: r	reactions: p	reactions: l	reactions: n	reactions: s	reactions: b	transport: s-c	transport: b-c	transport: intra	Prot syn
898 Phosphatidate-CL-pool	Phosphatidate-CL-pool(m)	PIPES MCES	synthesis of mitochondrial Phosphatidate-CL-pool	0.5 P_i(c) 0.5 Glucose-6P(c) 0.09 Palmitate(s) 0.28 Oleate(s) 0.04 Stearate(s) 1.5 Linoleate(s) 0.1 Palmitolate(s) 4.5 ATP-energy(c) 1 NADH-redox-potential(c) 1 Proton-gradient(m)	2.5 H_2O(s) 1 Phosphatidate-CL-pool(m)	15	2	-	-	-	-	-	-	6	-	5	-
899 Phosphatidate-VLDL-PC-pool	Phosphatidate-VLDL-PC-pool(c)	PIPES MCES	synthesis of cytosolic Phosphatidate-VLDL-PC-pool	0.5 P_i(c) 0.5 Glucose-6P(c) 0.17 Arachidonate(s) 0.7 Palmitate(s) 0.27 Oleate(s) 0.3 Stearate(s) 0.55 Linoleate(s) 4.5 ATP-energy(c) 1 NADH-redox-potential(c)	2.5 H_2O(s) 1 Phosphatidate-VLDL-PC-pool(c)	22	-	-	-	-	-	-	-	6	-	-	-
900 Phosphatidate-VLDL-PE-pool	Phosphatidate-VLDL-PE-pool(c)	PIPES MCES	synthesis of cytosolic Phosphatidate-VLDL-PE-pool	0.5 P_i(c) 0.5 Glucose-6P(c) 0.6 Arachidonate(s) 0.34 Palmitate(s) 0.17 Oleate(s) 0.54 Stearate(s) 0.36 Linoleate(s) 4.5 ATP-energy(c) 1 NADH-redox-potential(c)	2.5 H_2O(s) 1 Phosphatidate-VLDL-PE-pool(c)	22	-	-	-	-	-	-	-	6	-	-	-
901 Phosphatidate-VLDL-PI-pool	Phosphatidate-VLDL-PI-pool(c)	PIPES MCES	synthesis of cytosolic Phosphatidate-VLDL-PI-pool	0.5 P_i(c) 0.5 Glucose-6P(c) 0.51 Arachidonate(s) 0.16 Palmitate(s) 0.32 Oleate(s) 0.85 Stearate(s) 0.16 Linoleate(s) 4.5 ATP-energy(c) 1 NADH-redox-potential(c)	2.5 H_2O(s) 1 Phosphatidate-VLDL-PI-pool(c)	22	-	-	-	-	-	-	-	6	-	-	-
902 Phosphatidate-VLDL-PS-pool	Phosphatidate-VLDL-PS-pool(c)	PIPES MCES	synthesis of cytosolic Phosphatidate-VLDL-PS-pool	0.5 P_i(c) 0.5 Glucose-6P(c) 0.53 Arachidonate(s) 0.09 Palmitate(s) 0.09 Oleate(s) 1.25 Stearate(s) 0.05 Linoleate(s) 4.5 ATP-energy(c) 1 NADH-redox-potential(c)	2.5 H_2O(s) 1 Phosphatidate-VLDL-PS-pool(c)	22	-	-	-	-	-	-	-	6	-	-	-
903 Phosphatidate-VLDL-SM-pool	Phosphatidate-VLDL-SM-pool(c)	PIPES MCES	synthesis of cytosolic Phosphatidate-VLDL-SM-pool	0.5 P_i(c) 0.5 Glucose-6P(c) 0.03 Arachidonate(s) 1.42 Palmitate(s) 0.15 Oleate(s) 0.34 Stearate(s) 0.06 Linoleate(s) 4.5 ATP-energy(c) 1 NADH-redox-potential(c)	2.5 H_2O(s) 1 Phosphatidate-VLDL-SM-pool(c)	22	-	-	-	-	-	-	-	6	-	-	-

Continued on next page

Simulation	Definition			Solution													
				exchanges		reactions								transport			Prot
	Objective	Contraints	Comment	imports	exports	c	m	r	p	l	n	s	b	s-c	b-c	intra	syn
904 Phosphatidate-VLDL-TG-pool	Phosphatidate-VLDL-TG-pool(c)	PIPES MCES	synthesis of cytosolic Phosphatidate-VLDL-TG-pool	0.5 P_i(c) 0.5 Glucose-6P(c) 0.06 Arachidonate(s) 0.83 Palmitate(s) 0.6 Oleate(s) 0.13 Stearate(s) 0.39 Linoleate(s) 4.5 ATP-energy(c) 1 NADH-redox-potential(c)	2.5 H_2O(s) 1 Phosphatidate-VLDL-TG-pool(c)	22	-	-	-	-	-	-	-	6	-	-	-
905 Phosphocholine	Phosphocholine(c)	PIPES MCES	synthesis of cytosolic Phosphocholine	1 P_i(c) 1 Choline(c) 1 ATP-energy(c)	1 H_2O(s) 1 Phosphocholine(c)	2	-	-	-	-	-	-	-	1	-	-	-
906 Phosphodimethylethanolamine	Phosphodimethylethanolamine(c)	PIPES MCES	synthesis of cytosolic Phospho-dimethylethanolamine	1 P_i(c) 1 Choline(c) 1 ATP-energy(c)	1 H_2O(s) 1 Activated methyl group(c) 1 Phospho-dimethylethanolamine(c)	4	-	-	-	-	-	-	-	1	-	-	-
907 Phosphopantetheine	Phosphopantetheine(c)	PIPES MCES	synthesis of cytosolic Phosphopantetheine	1 P_i(c) 1 Cysteine(s) 1 Pantothenate(s) 1 ATP-energy(c)	2 H_2O(s) 1 CO_2(s) 1 Phosphopantetheine(c) 1 Na-gradient(c)	6	-	-	-	-	-	-	-	5	-	-	-
908 Porphobilinogen	Porphobilinogen(c)	PIPES MCES	synthesis of cytosolic Porphobilinogen	1 Pyruvate(m) 2 Glutamate(s) 1 Serine(s) 2 Proton-gradient(m)	2 H_2O(s) 2 CO_2(s) 1 Aspartate(s) 1 Porphobilinogen(c) 2 NADH-redox-potential(m) 1 Proton-gradient(c) 3 Na-gradient(c)	1	9	-	-	-	-	-	-	7	-	6	-
909 Presqualene-PP	Presqualene-PP(r)	PIPES MCES	synthesis of Golgi/ER Presqualene-PP	1 H_2O(s) 2 Farnesyl-PP(r)	2 P_i(c) 1 Presqualene-PP(r)	1	-	1	-	-	-	-	-	1	-	1	-
910 Propanoate	Propanoate(s)	PIPES MCES	synthesis of Propanoate and blood export	1 Aspartate(s) 1 Threonine(s) 1 ATP-energy(c) 1 Proton-gradient(m)	1 CO_2(s) 1 Asparagine(s) 1 Propanoate(s) 1 NADH-redox-potential(m) 1 Proton-gradient(c) 2 Na-gradient(c)	5	2	-	-	-	-	-	-	8	-	5	-

Continued on next page

C5. Comprehensive list – continued.

Simulation	Definition			Solution													
				exchanges		reactions								transport			Prot
	Objective	Contraints	Comment	imports	exports	c	m	r	p	l	n	s	b	s-c	b-c	intra	syn
911 Propanoyl-CoA	Propanoyl-CoA(c)	PIPES MCES	synthesis of cytosolic Propanoyl-CoA	2.17 P_i(c) 1 Glycine(s) 1.5 Aspartate(s) 0.5 Glutamine(s) 0.83 Glucose-6P(c) 0.5 Asparagine(s) 1 Threonine(s) 0.5 Cystine(s) 1 Pantothenate(s) 12.7 ATP-energy(c) 1 Proton-gradient(m) 2 THF-activated methyl group(c)	6.17 H_2O(s) 3 CO_2(s) 2 Pyruvate(c) 0.5 Glutamate(s) 1 Propanoyl-CoA(c) 1 NADH-redox-potential(m) 5.5 NADPH-redox-potential(c) 4.5 Na-gradient(c)	44	2	-	-	-	-	-	-	10	-	5	-
912 Protein(l)ysine	Protein lysine(c)	PIPES MCES	synthesis of cytosolic Protein lysine	1 Lysine(s)	1 Protein lysine(c)	1	-	-	-	-	-	-	-	1	-	-	-
913 Protoporphyrin	Protoporphyrin(m)	PIPES MCES	synthesis of mitochondrial Protoporphyrin	2.5 O_2(s) 4 Pyruvate(m) 10.5 Glutamate(s) 4 Serine(s) 1.5 Proline(s) 4 ATP-energy(c)	14 H_2O(s) 14 CO_2(s) 4 Aspartate(s) 4 Glutamine(s) 1 Protoporphyrin(m) 4 NADH-redox-potential(c) 4 NADH-redox-potential(m) 2 Proton-gradient(m) 10.5 Na-gradient(c)	10	11	-	-	-	-	-	-	8	-	10	-
914 Protoporphyrinogen IX	Protoporphyrinogen IX(c)	PIPES MCES	synthesis of cytosolic Protoporphyrinogen IX	1 O_2(s) 4 Pyruvate(m) 12 Glutamate(s) 4 Serine(s) 4 ATP-energy(c) 4 NADPH-redox-potential(m) 4 Proton-gradient(m)	14 H_2O(s) 14 CO_2(s) 4 Aspartate(s) 4 Glutamine(s) 1 Protoporphyrinogen IX(c) 4 NADH-redox-potential(c) 8 NADH-redox-potential(m) 12 Na-gradient(c)	10	8	-	-	-	-	-	-	7	-	6	-
915 Provitamin D3	Provitamin D3(r)	PIPES MCES	synthesis of Golgi/ER Provitamin D3	8 O_2(s) 2 Farnesyl-PP(r) 1 ATP-energy(c) 10 NADPH-redox-potential(r)	7 H_2O(s) 4 P_i(c) 3 CO_2(s) 1 Provitamin D3(r) 1 NADH-redox-potential(r) 1 NADPH-redox-potential(c)	5	-	15	-	-	-	-	-	3	-	5	-
916 Pyridoxal	Pyridoxal(s)	PIPES MCES	synthesis of Pyridoxal and blood export	0.5 O_2(s) 1 Pyridoxine(s)	1 H_2O(s) 1 Pyridoxal(s)	2	-	-	-	-	-	-	-	4	-	-	-
917 Pyridoxine-P	Pyridoxine-P(c)	PIPES MCES	synthesis of cytosolic Pyridoxine-P	1 P_i(c) 1 Pyridoxine(s) 1 ATP-energy(c)	1 H_2O(s) 1 Pyridoxine-P(c)	2	-	-	-	-	-	-	-	2	-	-	-
918 Quinolinate	Quinolinate(c)	PIPES MCES	synthesis of cytosolic Quinolinate	1 Pyruvate(m) 1 Glutamate(s) 1 Nicotinamide(s) 1 ATP-energy(c)	1 Glutamine(s) 1 Quinolinate(c) 1 NADH-redox-potential(m) 1 Na-gradient(c) 1 CoA-activated acetyl group(m)	5	3	-	-	-	-	-	-	4	-	1	-

Continued on next page

C5. Comprehensive list – continued.

Simulation	Definition			Solution													
				exchanges		reactions								transport			Prot
	Objective	Contraints	Comment	imports	exports	c	m	r	p	l	n	s	b	s-c	b-c	intra	syn
919 Ribose-1P	Ribose-1P(c)	PIPES MCES	synthesis of cytosolic Ribose-1P	0.17 P_i(c) 0.83 Glucose-6P(c) 0.17 ATP-energy(c)	0.17 H_2O(s) 1 Ribose-1P(c)	11	-	-	-	-	-	-	-	1	-	-	-
920 Ribose-5P	Ribose-5P(c)	PIPES MCES	synthesis of cytosolic Ribose-5P	0.17 P_i(c) 0.83 Glucose-6P(c) 0.17 ATP-energy(c)	0.17 H_2O(s) 1 Ribose-5P(c)	10	-	-	-	-	-	-	-	1	-	-	-
921 Ribulose-5P	Ribulose-5P(c)	PIPES MCES	synthesis of cytosolic Ribulose-5P	0.14 P_i(c) 0.86 Glucose-6P(c) 0.14 ATP-energy(c)	0.14 CO_2(s) 1 Ribulose-5P(c) 0.29 NADPH-redox-potential(c)	14	-	-	-	-	-	-	-	1	-	-	-
922 SAH	SAH(c)	PIPES MCES	synthesis of cytosolic SAH	1 Glycine(s) 2 Aspartate(s) 2 Glutamine(s) 1 Methionine(s) 0.83 Glucose-6P(c) 9.17 ATP-energy(c) 1 Proton-gradient(c) 2 THF-activated methyl group(c)	1.17 H_2O(s) 0.83 P_i(c) 1 CO_2(s) 1 SAH(c) 2 Pyruvate(c) 2 Glutamate(s) 1 Activated methyl group(c) 2 NADH-redox-potential(c) 4 NADPH-redox-potential(c) 3 Na-gradient(c)	36	-	-	-	-	-	-	-	9	-	-	-
923 SAICAR	SAICAR(c)	PIPES MCES	synthesis of cytosolic SAICAR	1 Glycine(s) 1 Aspartate(s) 2 Glutamine(s) 1 Glucose-6P(c) 6 ATP-energy(c) 1 THF-activated methyl group(c)	1 H_2O(s) 2 Glutamate(s) 1 SAICAR(c) 1 NADH-redox-potential(c) 3 NADPH-redox-potential(c) 1 Proton-gradient(c) 3 Na-gradient(c)	21	-	-	-	-	-	-	-	7	-	-	-
924 SAM	SAM(c)	PIPES MCES	synthesis of cytosolic SAM	1 Glycine(s) 2 Aspartate(s) 2 Glutamine(s) 1 Methionine(s) 0.83 Glucose-6P(c) 9.17 ATP-energy(c) 1 Proton-gradient(c) 2 THF-activated methyl group(c)	1.17 H_2O(s) 0.83 P_i(c) 1 CO_2(s) 1 SAM(c) 2 Pyruvate(c) 2 Glutamate(s) 2 NADH-redox-potential(c) 4 NADPH-redox-potential(c) 3 Na-gradient(c)	35	-	-	-	-	-	-	-	9	-	-	-
925 Saccharopine	Saccharopine(m)	PIPES MCES	synthesis of mitochondrial Saccharopine	1 Pyruvate(c) 1 Glutamate(s) 1 Lysine(s) 1 NADPH-redox-potential(m) 1 Proton-gradient(m)	1 H_2O(s) 1 Alanine(s) 1 Saccharopine(m) 1 Na-gradient(c)	1	2	-	-	-	-	-	-	4	-	5	-
926 Sarcosine	Sarcosine(s)	PIPES MCES	synthesis of Sarcosine and blood export	1 Glycine(s) 1 Activated methyl group(c) 1 Proton-gradient(c)	1 Sarcosine(s)	2	-	-	-	-	-	-	-	3	-	-	-
927 Sedoheptulose-1,7PP	Sedoheptulose-1,7PP(c)	PIPES MCES	synthesis of cytosolic Sedoheptulose-1,7PP	0.83 P_i(c) 1.17 Glucose-6P(c) 0.83 ATP-energy(c)	0.83 H_2O(s) 1 Sedoheptulose-1,7PP(c)	10	-	-	-	-	-	-	-	1	-	-	-

Continued on next page

C5. Comprehensive list – continued.

Simulation	Definition: Objective	Definition: Contraints	Definition: Comment	Solution: exchanges: imports	Solution: exchanges: exports	reactions: c	reactions: m	reactions: r	reactions: p	reactions: l	reactions: n	reactions: s	reactions: b	transport: s-c	transport: b-c	transport: intra	Prot syn
928 Sedoheptulose-7P	Sedoheptulose-7P(c)	PIPES MCES	synthesis of cytosolic Sedoheptulose-7P	0.17 H_2O(s) 1.17 Glucose-6P(c)	0.17 P_i(c) 1 Sedoheptulose-7P(c) 0.17 ATP-energy(c)	10	-	-	-	-	-	-	-	1	-	-	-
929 Serotonin	Serotonin(s)	PIPES MCES	synthesis of Serotonin and blood export	1 O_2(s) 1 Tryptophan(s) 1 NADPH-redox-potential(c)	1 H_2O(s) 1 CO_2(s) 1 Serotonin(s) 1 Na-gradient(c)	4	-	-	-	-	-	-	-	6	-	-	-
930 Sphinganine	Sphinganine(c)	PIPES MCES	synthesis of cytosolic Sphinganine	1 Serine(s) 1 Palmitate(s) 2 ATP-energy(c) 1 NADPH-redox-potential(c)	1 H_2O(s) 1 CO_2(s) 1 Sphinganine(c) 1 Proton-gradient(c) 1 Na-gradient(c)	7	-	-	-	-	-	-	-	6	-	-	-
931 Sphinganine-1P	Sphinganine-1P(c)	PIPES MCES	synthesis of cytosolic Sphinganine-1P	1 P_i(c) 1 Serine(s) 1 Palmitate(s) 3 ATP-energy(c) 1 NADPH-redox-potential(c)	2 H_2O(s) 1 CO_2(s) 1 Sphinganine-1P(c) 1 Proton-gradient(c) 1 Na-gradient(c)	8	-	-	-	-	-	-	-	6	-	-	-
932 Sphingosine	Sphingosine(c)	PIPES MCES	synthesis of cytosolic Sphingosine	1 Serine(s) 1 Palmitolate(s) 2 ATP-energy(c) 2 NADPH-redox-potential(c)	1 H_2O(s) 1 CO_2(s) 1 Sphingosine(c) 1 NADH-redox-potential(c) 1 Proton-gradient(c) 1 Na-gradient(c)	13	-	-	-	-	-	-	-	6	-	-	-
933 Sphingosine-1P	Sphingosine-1P(c)	PIPES MCES	synthesis of cytosolic Sphingosine-1P	1 P_i(c) 1 Serine(s) 1 Palmitolate(s) 3 ATP-energy(c) 2 NADPH-redox-potential(c)	2 H_2O(s) 1 CO_2(s) 1 Sphingosine-1P(c) 1 NADH-redox-potential(c) 1 Proton-gradient(c) 1 Na-gradient(c)	14	-	-	-	-	-	-	-	6	-	-	-
934 Squalene	Squalene(r)	PIPES MCES	synthesis of Golgi/ER Squalene	2 H_2O(s) 2 Farnesyl-PP(r) 1 NADPH-redox-potential(r)	4 P_i(c) 1 Squalene(r)	1	-	3	-	-	-	-	-	1	-	1	-
935 Squalene 2,3-oxide	Squalene 2,3-oxide(r)	PIPES MCES	synthesis of Golgi/ER Squalene 2,3-oxide	1 H_2O(s) 1 O_2(s) 2 Farnesyl-PP(r) 2 NADPH-redox-potential(r)	4 P_i(c) 1 Squalene 2,3-oxide(r)	1	-	4	-	-	-	-	-	2	-	3	-

Continued on next page

C5. Comprehensive list – continued.

Simulation	Definition			Solution													
				exchanges		reactions								transport			Prot
	Objective	Contraints	Comment	imports	exports	c	m	r	p	l	n	s	b	s-c	b-c	intra	syn
936 Stearoyl-ACP	Stearoyl-ACP(c)	PIPES MCES	synthesis of cytosolic Stearoyl-ACP	1 P_i(c) 5 Glutamate(s) 6 Glycine(s) 11 Alanine(s) 4 Lysine(s) 8 Aspartate(s) 9 Arginine(s) 7 Glutamine(s) 8 Serine(s) 5 Methionine(s) 3 Phenylalanine(s) 4 Tyrosine(s) 3 Cysteine(s) 20 Leucine(s) 1 Histidine(s) 9 Proline(s) 1 Asparagine(s) 11 Valine(s) 6 Threonine(s) 4 Isoleucine(s) 1 Pantothenate(s) 632 ATP-energy(c) 16 NADPH-redox-potential(c) 3 Proton-gradient(c) 9 CoA-activated acetyl group(c)	11 H_2O(s) 1 CO_2(s) 1 Stearoyl-ACP(c) 66 Na-gradient(c)	51	-	-	-	-	-	-	-	24	-	-	1
937 Stearoyl-CoA	Stearoyl-CoA(c)	PIPES MCES	synthesis of cytosolic Stearoyl-CoA	2.17 P_i(c) 1 Glycine(s) 2 Aspartate(s) 2 Glutamine(s) 0.83 Glucose-6P(c) 0.5 Cystine(s) 1 Pantothenate(s) 1 Stearate(s) 13.2 ATP-energy(c) 1 Proton-gradient(c) 2 THF-activated methyl group(c)	6.17 H_2O(s) 2 CO_2(s) 2 Pyruvate(c) 2 Glutamate(s) 1 Stearoyl-CoA(c) 2 NADH-redox-potential(c) 3.5 NADPH-redox-potential(c) 3 Na-gradient(c)	43	-	-	-	-	-	-	-	11	-	-	-
938 Succinate	Succinate(c)	PIPES MCES	synthesis of Succinate and blood export	1 H_2O(s) 1 Pyruvate(c) 1 Glutamate(s)	1 CO_2(s) 1 Alanine(s) 1 Succinate(c) 1 ATP-energy(m) 1 NADH-redox-potential(m) 1 Na-gradient(c)	1	5	-	-	-	-	-	-	5	-	3	-
939 Succinyl-CoA	Succinyl-CoA(m)	PIPES MCES	synthesis of mitochondrial Succinyl-CoA	2.17 P_i(c) 1 Pyruvate(m) 1 Glycine(s) 1 Aspartate(s) 2 Glutamine(s) 0.83 Glucose-6P(c) 0.5 Cystine(s) 1 Pantothenate(s) 11.2 ATP-energy(c) 1 NADPH-redox-potential(m) 1 Proton-gradient(c) 1 Proton-gradient(m) 2 THF-activated methyl group(c)	5.17 H_2O(s) 2 CO_2(s) 2 Pyruvate(c) 1 Glutamate(s) 1 Succinyl-CoA(m) 2 NADH-redox-potential(c) 2 NADH-redox-potential(m) 3.5 NADPH-redox-potential(c) 2 Na-gradient(c)	42	6	-	-	-	-	-	-	10	-	3	-
940 Sulfite degr	-1 Sulfite(c)	PIPES MCES +Sulfate(c)	synthesis of cytosolic Sulfite	0.5 O_2(s) 1 Sulfite(c)	1 Sulfate(c) 2 Proton-gradient(m)	-	-	-	-	-	-	-	-	1	-	5	-

Continued on next page

C5. Comprehensive list – continued.

Simulation	Definition			Solution													
				exchanges		reactions								transport			Prot
	Objective	Contraints	Comment	imports	exports	c	m	r	p	l	n	s	b	s-c	b-c	intra	syn
941 THF-hexaglutamate	THF-hexaglutamate(c)	PIPES MCES	synthesis of cytosolic THF-hexaglutamate	6 Glutamate(s) 1 Folate(s) 6 ATP-energy(c) 1 NADH-redox-potential(c) 1 NADPH-redox-potential(c)	1 THF-hexaglutamate(c) 6 Na-gradient(c)	6	-	-	-	-	-	-	-	3	-	-	-
942 Tetradecanoyl-ACP	Tetradecanoyl-ACP(c)	PIPES MCES	synthesis of cytosolic Tetradecanoyl-ACP	1 P_i(c) 5 Glutamate(s) 6 Glycine(s) 11 Alanine(s) 4 Lysine(s) 8 Aspartate(s) 9 Arginine(s) 7 Glutamine(s) 8 Serine(s) 5 Methionine(s) 3 Phenylalanine(s) 4 Tyrosine(s) 3 Cysteine(s) 20 Leucine(s) 1 Histidine(s) 9 Proline(s) 1 Asparagine(s) 11 Valine(s) 6 Threonine(s) 4 Isoleucine(s) 1 Pantothenate(s) 630 ATP-energy(c) 12 NADPH-redox-potential(c) 3 Proton-gradient(c) 7 CoA-activated acetyl group(c)	9 H_2O(s) 1 CO_2(s) 1 Tetradecanoyl-ACP(c) 66 Na-gradient(c)	43	-	-	-	-	-	-	-	24	-	-	1
943 Thymidine	Thymidine(s)	PIPES MCES	synthesis of Thymidine and blood export	0.33 O_2(s) 1 Aspartate(s) 0.5 Glucose-6P(c) 0.33 Proline(s) 1 Ethanolamine(c) 6.5 ATP-energy(c) 1 THF-activated methyl group(c)	2.5 H_2O(s) 0.5 P_i(c) 0.33 Glutamate(s) 1 Thymidine(s) 0.33 NADH-redox-potential(m) 4 Proton-gradient(m) 1.33 Na-gradient(c)	30	4	-	-	-	-	-	-	7	-	6	-
944 Thymine	Thymine(s)	PIPES MCES	synthesis of Thymine and blood export	0.33 O_2(s) 1 Aspartate(s) 1 Glutamine(s) 0.33 Proline(s) 4 ATP-energy(c) 1 NADH-redox-potential(c) 1 THF-activated methyl group(c)	1 H_2O(s) 1.33 Glutamate(s) 1 Thymine(s) 0.33 NADH-redox-potential(m) 1 NADPH-redox-potential(c) 1 Proton-gradient(c) 4 Proton-gradient(m) 2.33 Na-gradient(c)	24	4	-	-	-	-	-	-	9	-	6	-

Continued on next page

Simulation	Definition			Solution													
				exchanges		reactions								transport			Prot
	Objective	Contraints	Comment	imports	exports	c	m	r	p	l	n	s	b	s-c	b-c	intra	syn
945 Tiglyl-CoA	Tiglyl-CoA(m)	PIPES MCES	synthesis of mitochondrial Tiglyl-CoA	2.17 P_i(c) 1 Glycine(s) 1.25 Aspartate(s) 0.25 Glutamine(s) 0.83 Glucose-6P(c) 0.25 Proline(s) 0.75 Asparagine(s) 0.75 Threonine(s) 0.25 Isoleucine(s) 0.5 Cystine(s) 1 Pantothenate(s) 12.9 ATP-energy(c) 1 Proton-gradient(m) 2 THF-activated methyl group(c) 0.75 CoA-activated acetyl group(m)	6.17 H_2O(s) 3 CO_2(s) 2 Pyruvate(c) 0.5 Glutamate(s) 1 Tiglyl-CoA(m) 2 NADH-redox-potential(c) 0.75 NADH-redox-potential(m) 3.5 NADPH-redox-potential(c) 4.25 Na-gradient(c)	45	13	-	-	-	-	-	-	12	-	7	-
946 Trehalose	Trehalose(s)	PIPES MCES	synthesis of Trehalose and blood export	2 Glucose(s)	1 H_2O(s) 1 Trehalose(s)	-	-	-	-	-	-	1	-	-	-	-	-
947 Triphosphate degr	-1 Triphosphate(c)	PIPES MCES	degradation of cytosolic Triphosphate	2 H_2O(s) 1 Triphosphate(c)	3 P_i(c)	2	-	-	-	-	-	-	-	1	-	-	-
948 UDP-N-acetylglucosamine	UDP-N-acetylglucosamine(c)	PIPES MCES	synthesis of cytosolic UDP-N-acetylglucosamine	0.33 O_2(s) 0.17 P_i(c) 1 Glutamine(s) 1.83 Glucose-6P(c) 0.33 Proline(s) 1 Asparagine(s) 6.17 ATP-energy(c) 1 CoA-activated acetyl group(c)	3.17 H_2O(s) 1.33 Glutamate(s) 1 UDP-N-acetylglucosamine(c) 0.33 NADH-redox-potential(m) 4 Proton-gradient(m) 0.33 Na-gradient(c)	28	4	-	-	-	-	-	-	7	-	6	-
949 UDP-activated-N-acetyl-D-galactosamine	UDP-activated-N-acetyl-D-galactosamine(c)	PIPES MCES	synthesis of cytosolic UDP-activated-N-acetyl-D-galactosamine	1 Glutamine(s) 1 Glucose-6P(c) 1 ATP-energy(c) 1 CoA-activated acetyl group(c)	1 P_i(c) 1 Glutamate(s) 1 UDP-activated-N-acetyl-D-galactosamine(c)	11	-	-	-	-	-	-	-	2	-	-	-
950 UDP-activated-xylose	UDP-activated-xylose(c)	PIPES MCES	synthesis of cytosolic UDP-activated-xylose	1 H_2O(s) 1 Glucose-6P(c) 1 ATP-energy(c)	1 P_i(c) 1 CO_2(s) 2 NADH-redox-potential(c) 1 UDP-activated-xylose(c)	8	-	1	-	-	-	-	-	2	-	3	-
951 UMP	UMP(s)	PIPES MCES	synthesis of UMP and blood export	0.33 O_2(s) 2.17 P_i(c) 0.83 Glucose-6P(c) 0.33 Proline(s) 1 Asparagine(s) 7.17 ATP-energy(c)	2.17 H_2O(s) 2 P_i(s) 0.33 Glutamate(s) 1 UMP(s) 0.33 NADH-redox-potential(m) 1 Proton-gradient(c) 4 Proton-gradient(m) 1.33 Na-gradient(c)	24	4	-	-	-	-	2	-	8	-	6	-

Continued on next page

C5. Comprehensive list – continued.

Simulation	Definition			Solution													
				exchanges		reactions								transport			Prot
	Objective	Contraints	Comment	imports	exports	c	m	r	p	l	n	s	b	s-c	b-c	intra	syn
952 Urate	Urate(s)	PIPES MCES	synthesis of Urate and blood export	1 NH_3(s) 1 Aspartate(s) 1 Glutamine(s) 1 Serine(s) 8 ATP-energy(c) 1 THF-activated methyl group(c)	1 Pyruvate(c) 1 Glutamate(s) 1 Urate(s) 3 NADH-redox-potential(c) 3 NADPH-redox-potential(c) 1 Proton-gradient(c) 2 Na-gradient(c)	29	-	-	-	-	-	-	-	8	-	-	-
953 Urocanate	Urocanate(c)	PIPES MCES	synthesis of cytosolic Urocanate	1 Glutamate(s) 1 Histidine(s) 1 ATP-energy(c)	1 H_2O(s) 1 Glutamine(s) 1 Urocanate(c) 2 Na-gradient(c)	3	-	-	-	-	-	-	-	5	-	-	-
954 UroporphyrinogenIII	UroporphyrinogenIII(c)	PIPES MCES	synthesis of cytosolic UroporphyrinogenIII	4 Pyruvate(m) 12 Glutamate(s) 4 Serine(s) 4 ATP-energy(c) 4 NADPH-redox-potential(m) 4 Proton-gradient(m)	12 H_2O(s) 8 CO_2(s) 4 Aspartate(s) 4 Glutamine(s) 1 UroporphyrinogenIII(c) 4 NADH-redox-potential(c) 8 NADH-redox-potential(m) 12 Na-gradient(c)	8	8	-	-	-	-	-	-	6	-	6	-
955 Xanthosine	Xanthosine(c)	PIPES MCES	synthesis of cytosolic Xanthosine	0.83 NH_3(s) 1 Glycine(s) 0.17 Glutamine(s) 0.83 Glucose-6P(c) 1 Asparagine(s) 8 ATP-energy(c) 2 THF-activated methyl group(c)	0.83 P_i(c) 1 Pyruvate(c) 0.17 Glutamate(s) 1 Xanthosine(c) 3 NADH-redox-potential(c) 3 NADPH-redox-potential(c) 1.17 Proton-gradient(c) 1.17 Na-gradient(c)	35	-	-	-	-	-	-	-	7	-	-	-
956 Xanthurenate	Xanthurenate(s)	PIPES MCES	synthesis of Xanthurenate and blood export	2 O_2(s) 1 Pyruvate(c) 1 Tryptophan(s) 1 ATP-energy(c)	1 H_2O(s) 1 CO_2(s) 1 Alanine(s) 1 Xanthurenate(s) 1 Na-gradient(c)	9	-	-	-	-	-	-	-	7	-	-	-
957 Zymosterol	Zymosterol(r)	PIPES MCES	synthesis of Golgi/ER Zymosterol	7 O_2(s) 2 Farnesyl-PP(r) 1 ATP-energy(c) 8 NADPH-redox-potential(r)	5 H_2O(s) 4 P_i(c) 3 CO_2(s) 1 Zymosterol(r) 1 NADH-redox-potential(r) 1 NADPH-redox-potential(c)	5	-	12	-	-	-	-	-	3	-	5	-
958 cAMP	cAMP(c)	PIPES MCES	synthesis of cytosolic cAMP	0.17 P_i(c) 1 Glycine(s) 0.83 Glucose-6P(c) 2 Asparagine(s) 11.2 ATP-energy(c) 2 THF-activated methyl group(c)	2.17 H_2O(s) 1 CO_2(s) 2 Pyruvate(c) 1 cAMP(c) 2 NADH-redox-potential(c) 4 NADPH-redox-potential(c) 1 Proton-gradient(c) 2 Na-gradient(c)	37	-	-	-	-	-	-	-	6	-	-	-
959 cGMP	cGMP(c)	PIPES MCES	synthesis of cytosolic cGMP	0.17 P_i(c) 1 Glycine(s) 2 Glutamine(s) 0.83 Glucose-6P(c) 1 Asparagine(s) 10.2 ATP-energy(c) 1 Proton-gradient(c) 2 THF-activated methyl group(c)	1.17 H_2O(s) 1 Pyruvate(c) 2 Glutamate(s) 1 cGMP(c) 3 NADH-redox-potential(c) 3 NADPH-redox-potential(c)	37	-	-	-	-	-	-	-	6	-	-	-

Continued on next page

C5. Comprehensive list – continued.

Simulation		Definition			Solution													
					exchanges		reactions								transport			Prot
		Objective	Contraints	Comment	imports	exports	c	m	r	p	l	n	s	b	s-c	b-c	intra	syn
960	cis-(3S)-hydroxytetradec-7-enoyl-CoA	cis-(3S)-hydroxytetradec-7-enoyl-CoA(m)	PIPES MCES	synthesis of mitochondrial cis-(3S)-hydroxytetradec-7-enoyl-CoA	2.17 P_i(c) 1 Glycine(s) 2 Aspartate(s) 2 Glutamine(s) 0.83 Glucose-6P(c) 0.5 Cystine(s) 1 Pantothenate(s) 1 Palmitolate(s) 13.2 ATP-energy(c) 1 Proton-gradient(c) 2 THF-activated methyl group(c)	4.17 H_2O(s) 2 CO_2(s) 2 Pyruvate(c) 2 Glutamate(s) 2 NADH-redox-potential(c) 1 NADH-redox-potential(m) 3.5 NADPH-redox-potential(c) 2 FADH-redox-potential(c) 1 cis-(3S)-hydroxytetradec-7-enoyl-CoA(m) 3 Na-gradient(c) 1 CoA-activated acetyl group(m)	44	8	-	-	-	-	-	-	11	-	3	-
961	cis-Aconitate	cis-Aconitate(c)	PIPES MCES	synthesis of cytosolic cis-Aconitate	1 Pyruvate(c) 1 Pyruvate(m) 1 NADPH-redox-potential(c)	1 cis-Aconitate(c) 2 NADH-redox-potential(m)	2	5	-	-	-	-	-	-	-	-	2	-
962	cis-laur-5-enoyl-CoA	cis-laur-5-enoyl-CoA(m)	PIPES MCES	synthesis of mitochondrial cis-laur-5-enoyl-CoA	2.17 P_i(c) 1 Glycine(s) 2 Aspartate(s) 2 Glutamine(s) 0.83 Glucose-6P(c) 0.5 Cystine(s) 1 Pantothenate(s) 1 Palmitolate(s) 13.2 ATP-energy(c) 1 Proton-gradient(c) 2 THF-activated methyl group(c)	4.17 H_2O(s) 2 CO_2(s) 2 Pyruvate(c) 2 Glutamate(s) 2 NADH-redox-potential(c) 2 NADH-redox-potential(m) 3.5 NADPH-redox-potential(c) 2 FADH-redox-potential(c) 1 cis-laur-5-enoyl-CoA(m) 3 Na-gradient(c) 2 CoA-activated acetyl group(m)	44	10	-	-	-	-	-	-	11	-	3	-
963	cis-myrist-7-enoyl-CoA	cis-myrist-7-enoyl-CoA(m)	PIPES MCES	synthesis of mitochondrial cis-myrist-7-enoyl-CoA	2.17 P_i(c) 1 Glycine(s) 2 Aspartate(s) 2 Glutamine(s) 0.83 Glucose-6P(c) 0.5 Cystine(s) 1 Pantothenate(s) 1 Palmitolate(s) 13.2 ATP-energy(c) 1 Proton-gradient(c) 2 THF-activated methyl group(c)	5.17 H_2O(s) 2 CO_2(s) 2 Pyruvate(c) 2 Glutamate(s) 2 NADH-redox-potential(c) 1 NADH-redox-potential(m) 3.5 NADPH-redox-potential(c) 1 FADH-redox-potential(c) 1 cis-myrist-7-enoyl-CoA(m) 3 Na-gradient(c) 1 CoA-activated acetyl group(m)	44	6	-	-	-	-	-	-	11	-	3	-
964	dADP	dADP(c)	PIPES MCES	synthesis of cytosolic dADP	1.17 P_i(c) 1 Glycine(s) 0.83 Glucose-6P(c) 2 Asparagine(s) 10.2 ATP-energy(c) 2 THF-activated methyl group(c)	3.17 H_2O(s) 1 CO_2(s) 2 Pyruvate(c) 1 dADP(c) 2 NADH-redox-potential(c) 3 NADPH-redox-potential(c) 1 Proton-gradient(c) 2 Na-gradient(c)	38	-	-	-	-	-	-	-	6	-	-	-

Continued on next page

C5. Comprehensive list – continued.

Simulation	Definition			Solution													
				exchanges		reactions								transport			Prot
	Objective	Contraints	Comment	imports	exports	c	m	r	p	l	n	s	b	s-c	b-c	intra	syn
965 dAMP	dAMP(c)	PIPES MCES	synthesis of cytosolic dAMP	0.17 P_i(c) 1 Glycine(s) 2 Aspartate(s) 2 Glutamine(s) 0.83 Glucose-6P(c) 7.17 ATP-energy(c) 1 Proton-gradient(c) 2 THF-activated methyl group(c)	2.17 H_2O(s) 1 CO_2(s) 2 Pyruvate(c) 2 Glutamate(s) 1 dAMP(c) 2 NADH-redox-potential(c) 3 NADPH-redox-potential(c) 2 Na-gradient(c)	37	-	-	-	-	-	-	-	8	-	-	-
966 dCDP	dCDP(c)	PIPES MCES	synthesis of cytosolic dCDP	0.33 O_2(s) 1.17 P_i(c) 1 Glutamine(s) 0.83 Glucose-6P(c) 0.33 Proline(s) 1 Asparagine(s) 6.17 ATP-energy(c) 1 NADPH-redox-potential(c)	4.17 H_2O(s) 1.33 Glutamate(s) 1 dCDP(c) 0.33 NADH-redox-potential(m) 4 Proton-gradient(m) 0.33 Na-gradient(c)	28	4	-	-	-	-	-	-	7	-	6	-
967 dCMP	dCMP(c)	PIPES MCES	synthesis of cytosolic dCMP	0.33 O_2(s) 0.17 P_i(c) 1 Glutamine(s) 0.83 Glucose-6P(c) 0.33 Proline(s) 1 Asparagine(s) 6.17 ATP-energy(c) 1 NADPH-redox-potential(c)	3.17 H_2O(s) 1.33 Glutamate(s) 1 dCMP(c) 0.33 NADH-redox-potential(m) 4 Proton-gradient(m) 0.33 Na-gradient(c)	29	4	-	-	-	-	-	-	7	-	6	-
968 dGDP	dGDP(c)	PIPES MCES	synthesis of cytosolic dGDP	1.17 P_i(c) 1 Glycine(s) 2 Glutamine(s) 0.83 Glucose-6P(c) 1 Asparagine(s) 9.17 ATP-energy(c) 1 Proton-gradient(c) 2 THF-activated methyl group(c)	2.17 H_2O(s) 1 Pyruvate(c) 2 Glutamate(s) 1 dGDP(c) 3 NADH-redox-potential(c) 2 NADPH-redox-potential(c)	37	-	-	-	-	-	-	-	6	-	-	-
969 dGMP	dGMP(c)	PIPES MCES	synthesis of dGMP	0.17 P_i(c) 1 Glycine(s) 2 Glutamine(s) 0.83 Glucose-6P(c) 1 Asparagine(s) 8.17 ATP-energy(c) 1 Proton-gradient(c) 2 THF-activated methyl group(c)	1.17 H_2O(s) 1 Pyruvate(c) 2 Glutamate(s) 1 dGMP(c) 3 NADH-redox-potential(c) 2 NADPH-redox-potential(c)	38	-	-	-	-	-	-	-	6	-	-	-
970 dTDP	dTDP(c)	PIPES MCES	synthesis of dTDP	0.33 O_2(s) 1.5 P_i(c) 1 Aspartate(s) 0.5 Glucose-6P(c) 0.33 Proline(s) 1 Ethanolamine(c) 7.5 ATP-energy(c) 1 THF-activated methyl group(c)	4.5 H_2O(s) 0.33 Glutamate(s) 1 dTDP(c) 0.33 NADH-redox-potential(m) 4 Proton-gradient(m) 1.33 Na-gradient(c)	30	4	-	-	-	-	-	-	6	-	6	-
971 dTMP	dTMP(c)	PIPES MCES	synthesis of dTMP	0.33 O_2(s) 0.5 P_i(c) 1 Aspartate(s) 0.5 Glucose-6P(c) 0.33 Proline(s) 1 Ethanolamine(c) 6.5 ATP-energy(c) 1 NADPH-redox-potential(c) 1 THF-activated methyl group(c)	3.5 H_2O(s) 0.33 Glutamate(s) 1 dTMP(c) 1 NADH-redox-potential(c) 0.33 NADH-redox-potential(m) 4 Proton-gradient(m) 1.33 Na-gradient(c)	31	4	-	-	-	-	-	-	6	-	6	-

Continued on next page

Simulation	Definition			Solution													
				exchanges		reactions								transport			Prot
	Objective	Contraints	Comment	imports	exports	c	m	r	p	l	n	s	b	s-c	b-c	intra	syn
972 dUDP	dUDP(c)	PIPES MCES	synthesis of cytosolic dUDP	0.33 O_2(s) 1.17 P_i(c) 0.83 Glucose-6P(c) 0.33 Proline(s) 1 Asparagine(s) 6.17 ATP-energy(c) 1 NADPH-redox-potential(c)	4.17 H_2O(s) 0.33 Glutamate(s) 1 dUDP(c) 0.33 NADH-redox-potential(m) 1 Proton-gradient(c) 4 Proton-gradient(m) 1.33 Na-gradient(c)	26	4	-	-	-	-	-	-	7	-	6	-
973 dUMP	dUMP(c)	PIPES MCES	synthesis of cytosolic dUMP	0.33 O_2(s) 0.5 P_i(c) 1 Aspartate(s) 0.5 Glucose-6P(c) 0.33 Proline(s) 1 Ethanolamine(c) 6.5 ATP-energy(c)	3.5 H_2O(s) 0.33 Glutamate(s) 1 dUMP(c) 0.33 NADH-redox-potential(m) 4 Proton-gradient(m) 1.33 Na-gradient(c)	25	4	-	-	-	-	-	-	6	-	6	-
974 dUTP	dUTP(c)	PIPES MCES	synthesis of cytosolic dUTP	0.33 O_2(s) 2.17 P_i(c) 0.83 Glucose-6P(c) 0.33 Proline(s) 1 Asparagine(s) 7.17 ATP-energy(c) 1 NADPH-redox-potential(c)	5.17 H_2O(s) 0.33 Glutamate(s) 1 dUTP(c) 0.33 NADH-redox-potential(m) 1 Proton-gradient(c) 4 Proton-gradient(m) 1.33 Na-gradient(c)	27	4	-	-	-	-	-	-	7	-	6	-
975 gamma-Glutamyl-cysteine	gamma-Glutamyl-cysteine(c)	PIPES MCES	synthesis of cytosolic gamma-Glutamyl-cysteine	1 Glutamate(s) 1 Cysteine(s) 1 ATP-energy(c)	1 H_2O(s) 1 gamma-Glutamyl-cysteine(c) 1 Na-gradient(c)	2	-	-	-	-	-	-	-	4	-	-	-
976 gamma-Linolenoyl-CoA	gamma-Linolenoyl-CoA(c)	PIPES MCES	synthesis of cytosolic gamma-Linolenoyl-CoA	1 O_2(s) 2.17 P_i(c) 1 Glycine(s) 2 Aspartate(s) 2 Glutamine(s) 0.83 Glucose-6P(c) 0.5 Cystine(s) 1 Pantothenate(s) 1 Linoleate(s) 13.2 ATP-energy(c) 1 Proton-gradient(c) 2 THF-activated methyl group(c)	7.17 H_2O(s) 2 CO_2(s) 2 Pyruvate(c) 2 Glutamate(s) 1 gamma-Linolenoyl-CoA(c) 1 NADH-redox-potential(c) 3.5 NADPH-redox-potential(c) 3 Na-gradient(c)	44	-	-	-	-	-	-	-	12	-	-	-
977 linoleic-Carnitine	linoleic-Carnitine(c)	PIPES MCES −L-Carnitine(c)	synthesis of cytosolic linoleic-Carnitine	1 L-Carnitine(c) 1 Linoleate(s) 2 ATP-energy(c)	1 H_2O(s) 1 linoleic-Carnitine(c)	5	-	-	-	-	-	-	-	2	-	-	-

Continued on next page

C5. Comprehensive list – continued.

Simulation	Definition			Solution													
				exchanges		reactions								transport			Prot
	Objective	Contraints	Comment	imports	exports	c	m	r	p	l	n	s	b	s-c	b-c	intra	syn
978 mitoACP	mitoACP(m)	PIPES MCES	synthesis of mitochondrial mitoACP	1 P_i(c) 16.8 Pyruvate(m) 3 Alanine(s) 7 Lysine(s) 2.75 Aspartate(s) 1 Arginine(s) 12 Glutamine(s) 4.5 Serine(s) 5 Methionine(s) 3 Phenylalanine(s) 4 Tyrosine(s) 2 Cysteine(s) 10 Leucine(s) 1 Histidine(s) 5.75 Proline(s) 2 Asparagine(s) 6 Valine(s) 1 Threonine(s) 8 Isoleucine(s) 1 Pantothenate(s) 435.3 ATP-energy(m) 3.75 NADH-redox-potential(c) 17 NADPH-redox-potential(m) 1 Proton-gradient(c) 23.5 Proton-gradient(m)	21.2 NADH-redox-potential(m) 1 mitoACP(m) 24.8 Na-gradient(c) 8.25 CoA-activated acetyl group(m)	8	24	-	-	-	-	-	-	20	-	23	1
979 mitoOxidizedThioredoxin	mitoOxidizedThioredoxin(m)	PIPES MCES	synthesis of mitochondrial mitoOxidizedThioredoxin	6 Glutamate(s) 10 Glycine(s) 11 Alanine(s) 11 Lysine(s) 12 Aspartate(s) 9 Arginine(s) 10 Glutamine(s) 8 Serine(s) 4 Methionine(s) 2 Tryptophan(s) 6 Phenylalanine(s) 2 Tyrosine(s) 3 Cysteine(s) 15 Leucine(s) 3 Histidine(s) 12 Proline(s) 4 Asparagine(s) 17 Valine(s) 12 Threonine(s) 9 Isoleucine(s) 830 ATP-energy(c) 2 Proton-gradient(c)	1 NADPH-redox-potential(m) 1 mitoOxidizedThioredoxin(m) 102 Na-gradient(c)	3	2	-	-	-	-	-	-	22	-	1	1
980 palmitoleoyl-Carnitine	palmitoleoyl-Carnitine(c)	PIPES MCES −L-Carnitine(c)	synthesis of cytosolic palmitoleoyl-Carnitine	1 L-Carnitine(c) 1 Palmitolate(s) 2 ATP-energy(c)	1 H_2O(s) 1 palmitoleoyl-Carnitine(c)	5	-	-	-	-	-	-	-	2	-	-	-

Continued on next page

Simulation	Definition			Solution													
				exchanges		reactions								transport			Prot
	Objective	Contraints	Comment	imports	exports	c	m	r	p	l	n	s	b	s-c	b-c	intra	syn
981 palmitoleoyl-CoA	palmitoleoyl-CoA(c)	PIPES MCES	synthesis of cytosolic palmitoleoyl-CoA	2.17 P_i(c) 1 Glycine(s) 2 Aspartate(s) 2 Glutamine(s) 0.83 Glucose-6P(c) 0.5 Cystine(s) 1 Pantothenate(s) 1 Palmitolate(s) 13.2 ATP-energy(c) 1 Proton-gradient(c) 2 THF-activated methyl group(c)	6.17 H_2O(s) 2 CO_2(s) 2 Pyruvate(c) 2 Glutamate(s) 2 NADH-redox-potential(c) 3.5 NADPH-redox-potential(c) 1 palmitoleoyl-CoA(c) 3 Na-gradient(c)	43	-	-	-	-	-	-	-	11	-	-	-
982 sn-Glycerol-3P	sn-Glycerol-3P(c)	PIPES MCES	synthesis of cytosolic sn-Glycerol-3P	0.5 P_i(c) 0.5 Glucose-6P(c) 0.5 ATP-energy(c) 1 NADH-redox-potential(c)	0.5 H_2O(s) 1 sn-Glycerol-3P(c)	7	-	-	-	-	-	-	-	1	-	-	-
983 trans,cis-dodeca-2,5-dienoyl-CoA	trans,cis-dodeca-2,5-dienoyl-CoA(m)	PIPES MCES	synthesis of mitochondrial trans,cis-dodeca-2,5-dienoyl-CoA	2.17 P_i(c) 1 Glycine(s) 2 Aspartate(s) 2 Glutamine(s) 0.83 Glucose-6P(c) 0.5 Cystine(s) 1 Pantothenate(s) 1 Palmitolate(s) 13.2 ATP-energy(c) 1 Proton-gradient(c) 2 THF-activated methyl group(c)	4.17 H_2O(s) 2 CO_2(s) 2 Pyruvate(c) 2 Glutamate(s) 2 NADH-redox-potential(c) 2 NADH-redox-potential(m) 3.5 NADPH-redox-potential(c) 3 FADH-redox-potential(c) 1 trans,cis-dodeca-2,5-dienoyl-CoA(m) 3 Na-gradient(c) 2 CoA-activated acetyl group(m)	44	11	-	-	-	-	-	-	11	-	3	-
984 trans,cis-hexadeca-2,9-dienoyl-CoA	trans,cis-hexadeca-2,9-dienoyl-CoA(m)	PIPES MCES	synthesis of mitochondrial trans,cis-hexadeca-2,9-dienoyl-CoA	2.17 P_i(c) 1 Glycine(s) 2 Aspartate(s) 2 Glutamine(s) 0.83 Glucose-6P(c) 0.5 Cystine(s) 1 Pantothenate(s) 1 Palmitolate(s) 13.2 ATP-energy(c) 1 Proton-gradient(c) 2 THF-activated methyl group(c)	6.17 H_2O(s) 2 CO_2(s) 2 Pyruvate(c) 2 Glutamate(s) 2 NADH-redox-potential(c) 3.5 NADPH-redox-potential(c) 1 FADH-redox-potential(c) 1 trans,cis-hexadeca-2,9-dienoyl-CoA(m) 3 Na-gradient(c)	44	1	-	-	-	-	-	-	11	-	2	-
985 trans,cis-myristo-2,7-dienoyl-CoA	trans,cis-myristo-2,7-dienoyl-CoA(m)	PIPES MCES	synthesis of mitochondrial trans,cis-myristo-2,7-dienoyl-CoA	2.17 P_i(c) 1 Glycine(s) 2 Aspartate(s) 2 Glutamine(s) 0.83 Glucose-6P(c) 0.5 Cystine(s) 1 Pantothenate(s) 1 Palmitolate(s) 13.2 ATP-energy(c) 1 Proton-gradient(c) 2 THF-activated methyl group(c)	5.17 H_2O(s) 2 CO_2(s) 2 Pyruvate(c) 2 Glutamate(s) 2 NADH-redox-potential(c) 1 NADH-redox-potential(m) 3.5 NADPH-redox-potential(c) 2 FADH-redox-potential(c) 1 trans,cis-myristo-2,7-dienoyl-CoA(m) 3 Na-gradient(c) 1 CoA-activated acetyl group(m)	44	7	-	-	-	-	-	-	11	-	3	-

Continued on next page

C5. Comprehensive list – continued.

Simulation		Definition			Solution													
					exchanges		reactions								transport			Prot
		Objective	Contraints	Comment	imports	exports	c	m	r	p	l	n	s	b	s-c	b-c	intra	syn
986	trans,cis-octadeca-2,9-dienoyl-CoA	trans,cis-octadeca-2,9-dienoyl-CoA(m)	PIPES MCES	synthesis of mitochondrial trans,cis-octadeca-2,9-dienoyl-CoA	2.17 P_i(c) 1 Glycine(s) 2 Aspartate(s) 2 Glutamine(s) 0.83 Glucose-6P(c) 0.5 Cystine(s) 1 Oleate(s) 1 Pantothenate(s) 13.2 ATP-energy(c) 1 Proton-gradient(c) 2 THF-activated methyl group(c)	6.17 H_2O(s) 2 CO_2(s) 2 Pyruvate(c) 2 Glutamate(s) 2 NADH-redox-potential(c) 3.5 NADPH-redox-potential(c) 1 FADH-redox-potential(c) 1 trans,cis-octadeca-2,9-dienoyl-CoA(m) 3 Na-gradient(c)	44	1	-	-	-	-	-	-	11	-	2	-
987	trans-4-Hydroxy-L-proline	trans-4-Hydroxy-L-proline(c)	PIPES MCES −L-1-Pyrroline-3-hydroxy-5-carboxylate(c)	synthesis of cytosolic trans-4-Hydroxy-L-proline	1 L-1-Pyrroline-3-hydroxy-5-carboxylate(c) 1 NADH-redox-potential(c)	1 trans-4-Hydroxy-L-proline(c)	2	-	-	-	-	-	-	-	-	-	-	-
