# Supplementary material for: Metabolic Consequences of TGFβ Stimulation in Cultured Primary Mouse Hepatocytes Screened from Transcript Data with ModeScore
Source: Metabolites. 2012 Nov 21;2(4):983–1003. doi: 10.3390/metabo2040983 (PMC3901234; doi:10.3390/metabo2040983)
Supplement: Supplementary File 5 — Scores and amplitudes tables (PDF, 994 KB) [file metabolites-02-00983-s005.pdf]

Scores and amplitudes tables

October 17, 2012

Contents

1 Relative expression scoring sorted by amplitudes **1**

1.1 Matches relating TGFβ/contr 24h . . . 2
1.2 Matches relating full time control . . . 11
1.3 Matches relating full time TGFβ . . . 20
1.4 Matches relating control 1/6h vs. 6/24h . . . 29
1.5 Matches relating TGFβ/contr 6h vs. 24h . . . 46
1.6 Matches relating TGFβ/contr 6h . . . 63

2 Amplitude differences in relative expression scoring **72**

2.1 Amplitude differences: Treatment difference . . . 72
2.2 Amplitude differences: Treatment difference . . . 89
2.3 Amplitude differences: Period difference, control . . . 106
2.4 Amplitude differences: TGFβ/contr 6h vs. 24h . . . 123

3 Amplitudes with opposite signs **140**

3.1 Amplitude reversal of Treatment difference . . . 140
3.2 Amplitude reversal of Period difference, control . . . 146
3.3 Amplitude reversal of Period difference, TGFβ . . . 152

1 Relative expression scoring sorted by amplitudes

The scores in this chapter are based on the difference of two expression profiles. As opposed to the absolute expression profiles these profiles contain negative and positive values. Thus, the sign of the fluxes in the reference profile also matters.

Critical to the scoring of the relative profiles is the estimation of the scaling factor between the flux profile and the reference flux distribution. This is completely different from absolute scoring, there is an threshold range below which a gene is turned off, above which it is turned on. Consequently, the scaling factor is fixed for the absolute scoring. For the relative scoring, it must be flexible. The biochemically most important distinction is between positive and negative scaling factors: a negative scaling represents a downregulation of the respective function, whereas a positive scaling factor represents upregulation. Also the absolute value of the scaling factor can be telling. The larger it is, the larger the amount of up resp. downregulation with respect to a unit flux distribution. Therefore it is called amplitude here — it is a measure of the strength of the regulation. The tables show the simulations ordered by the amplitudes: in the left panel the top half of the functions in descending order, in the right panel the other half in ascending order.

List of compartments

Identifer	compartment	comment
s	sinusoid (blood)	external
b	bile	external
c	cytosol	
m	mitochondrium	
r	ER and Golgi	combined
p	peroxysome	
l	lysosome	
n	nucleus	

1.1 Matches relating TGFβ/contr 24h

Table 1: Matches relating TGFβ/contr 24h

Top down, T24h higher Simulation	ampl	score	Bottom up, C24h higher Simulation	ampl	score
374 Collagen COFA1(c) synthesis	2.27	1	448 Ethanol degr	-3.56	0.54
386 Collagen CORA1(c) synthesis	2.09	1	489 Tyrosine	-2.86	0.65
931 Sphinganine-1P	1.29	0.36	54 Glucose-6P	-2.74	0.52
344 Collagen CO1A1(c) synthesis	1.09	1	925 Saccharopine	-1.92	0.63
619 3-Methyl-2-oxobutyrate	1.04	0.37	658 4-Hydroxyphenylpyruvate	-1.87	0.73
802 Glycerate	0.9	0.55	667 5-Formiminotetrahydrofolate	-1.82	0.46
207 Glutamine from Arginine	0.88	0.27	821 Homogentisate	-1.76	0.65
362 Collagen CO7A1(c) synthesis	0.84	1	89 Alanine from Phenylalanine	-1.74	0.47
355 Collagen CO5A2(c) synthesis	0.71	1	896 Pantetheine	-1.68	0.52
600 2-Deoxy-D-ribose-1P	0.7	0.41	907 Phosphopantetheine	-1.67	0.45
941 THF-hexaglutamate	0.69	0.87	272 beta-Alanine from Alanine	-1.67	0.29
15 Dephosphorylation of ATP	0.63	0.71	78 Alanine from Aspartate	-1.65	0.43
552 (R)-Mevalonate	0.62	0.54	343 Collagen CD36(c) synthesis	-1.64	1
341 Collagen C43BP(c) synthesis	0.58	1	660 4-Maleylacetoacetate	-1.62	0.7
872 NeuNGc	0.56	0.31	118 Asparagine from Alanine	-1.6	0.39
760 Deoxyinosine	0.56	0.29	407 Alanine degr	-1.54	0.43
825 Hydroxypyruvate	0.55	1	313 Urea from NH3	-1.54	0.43
96 Alanine from beta-Alanine	0.54	0.33	312 Urea from alanine	-1.54	0.43
120 Asparagine from Aspartate	0.54	0.37	788 Fumarylacetoacetate	-1.51	0.68
632 3-Oxopropanoate	0.52	0.33	480 Alanine	-1.51	0.67
349 Collagen CO4A2(c) synthesis	0.51	1	206 Glutamine from Alanine	-1.5	0.51
396 Collagen LPP3(c) synthesis	0.5	1	97 Arginine from Alanine	-1.49	0.42
796 Glucono-1,5-lactone-6P	0.45	1	250 Serine from Alanine	-1.49	0.49
680 6-Phospho-D-gluconate	0.43	0.63	185 Glycine from Alanine	-1.47	0.57
436 Mannose degr	0.43	0.42	637 3-Phosphoserine	-1.46	0.52
863 N-Acetylneuraminate	0.42	0.32	638 3-Sulfinoalanine	-1.46	0.73
827 Hypoxanthine	0.41	0.29	747 Cysteamine	-1.46	0.39
605 2-Oxo-3-methylvalerate	0.41	0.49	826 Hypotaurine	-1.46	0.5
345 Collagen CO1A2(c) synthesis	0.38	1	295 Taurine from Cysteine	-1.46	0.5
560 (S)-Dihydroorotate	0.38	0.32	196 Glycine from Lysine	-1.44	0.47
371 Collagen COCA1(c) synthesis	0.36	1	886 Oxalosuccinate	-1.44	0.59
327 Antichymotrypsin	0.33	0.97	257 Serine from Glycine	-1.42	0.43
394 Collagen ITA1(c) synthesis	0.33	0.28	94 Alanine from Tyrosine	-1.42	0.49
399 Collagen SERPH(c) synthesis	0.3	1	950 UDP-activated-xylose	-1.41	0.44
330 ApoB100(r)	0.28	0.99	79 Alanine from Cysteine	-1.4	0.5
309 VLDL from HDL	0.28	0.68	832 Isocitrate	-1.35	0.61
401 VLDL	0.28	0.68	205 Glycine from beta-Alanine	-1.33	0.45
308 VLDL from LDL	0.28	0.71	192 Glycine from Histidine	-1.33	0.48
930 Sphinganine	0.28	0.38	188 Glycine from Aspartate	-1.32	0.5
817 Hexadecenal	0.28	0.26	80 Alanine from Cystine	-1.31	0.66
815 Hexadecanal	0.28	0.26	44 Activated methyl group (THF)	-1.31	0.58
725 CMP-activated-N-acetylneuraminate	0.28	0.3	200 Glycine from Serine	-1.31	0.58
513 CMP-N-acetylneuraminate	0.28	0.3	88 Alanine from Methionine	-1.28	0.52
391 Collagen FCN2(c) synthesis	0.26	1	314 Creatine	-1.27	0.39
370 Collagen COBA2(c) synthesis	0.26	1	187 Glycine from Asparagine	-1.27	0.47
799 Glucuronate	0.24	0.57	189 Glycine from Cysteine	-1.27	0.58
669 5-Hydroxy-L-tryptophan	0.24	0.61	294 Taurine from Methionine	-1.27	0.51
58 GDP-activated fucose	0.24	0.48	485 Glycine	-1.26	0.59
839 L-Fucose-1P	0.24	0.42	811 H2S	-1.26	0.51
494 Mannose-1P	0.23	0.5	926 Sarcosine	-1.26	0.86
359 Collagen CO6A3(c) synthesis	0.23	1	203 Glycine from Tyrosine	-1.25	0.53
712 Agmatine	0.23	0.67	497 UDP-xylose	-1.23	0.3
41 Na+ importgradient	0.23	0.47	197 Glycine from Methionine	-1.23	0.55
53 activated sulphur	0.22	0.67	190 Glycine from Cystine	-1.23	0.61
395 Collagen ITA2(c) synthesis	0.21	1	268 Serine from Tyrosine	-1.22	0.5
797 Glucose-1P	0.2	1	164 Glutamate from Alanine	-1.22	0.49

Continued on next page

Matches relating TGFβ/contr 24h – continued

Top down, T24h higher			Bottom up, C24h higher		
Simulation	ampl	score	Simulation	ampl	score
59 GDP-activated mannose	0.2	0.49	227 Methionine from Cysteine	-1.21	0.39
375 Collagen COGA1(c) synthesis	0.2	1	929 Serotonin	-1.21	0.53
919 Ribose-1P	0.19	0.4	500 udpglcur	-1.21	0.34
350 Collagen CO4A3(c) synthesis	0.19	1	161 Cysteine from Methionine	-1.21	0.49
949 UDP-activated-N-acetyl-D-galactosamine	0.18	0.44	198 Glycine from Phenylalanine	-1.21	0.5
354 Collagen CO5A1(c) synthesis	0.18	1	228 Methionine from Cystine	-1.21	0.43
526 Putrescine	0.18	0.52	607 2-Oxobutyrate	-1.2	0.38
346 Collagen CO2A1(c) synthesis	0.17	1	254 Serine from Cysteine	-1.19	0.57
342 Collagen CCBE1(c) synthesis	0.17	1	285 beta-Alanine from Methionine	-1.19	0.46
353 Collagen CO4A6(c) synthesis	0.16	1	377 Collagen COIA1(c) synthesis	-1.18	1
512 N-acglucam	0.16	0.32	263 Serine from Methionine	-1.17	0.54
382 Collagen COMA1(c) synthesis	0.13	1	163 Cystine from Methionine	-1.17	0.51
946 Trehalose	0.13	1	213 Glutamine from Glycine	-1.16	0.46
364 Collagen CO8A2(c) synthesis	0.12	1	279 beta-Alanine from Glycine	-1.15	0.42
830 Inositol-1P	0.12	1	202 Glycine from Tryptophan	-1.15	0.49
385 Collagen COPA1(c) synthesis	0.11	1	151 Aspartate from Methionine	-1.15	0.48
64 Glucosamine-6P	0.11	0.63	424 Glycine degr	-1.14	0.4
275 beta-Alanine from Aspartate	0.11	0.68	255 Serine from Cystine	-1.14	0.6
274 beta-Alanine from Asparagine	0.11	0.3	784 Formylanthranilate	-1.14	0.48
861 N-Acetylglucosamine-1P	0.1	0.55	471 Homocysteine	-1.14	0.47
877 O-Acetylcarnitine	0.1	1	980 palmitoleoyl-Carnitine	-1.13	0.49
351 Collagen CO4A4(c) synthesis	0.09	1	130 Asparagine from Methionine	-1.13	0.48
389 Collagen EMID2(c) synthesis	0.09	1	56 UDP-activated glucuronate	-1.12	0.53
611 3-Dehydrosphinganine	0.09	0.44	170 Glutamate from Glycine	-1.12	0.55
372 Collagen CODA1(c) synthesis	0.09	1	417 Methionine degr	-1.11	0.43
384 Collagen COOA1(c) synthesis	0.08	1	470 GSH	-1.11	0.63
348 Collagen CO4A1(c) synthesis	0.07	1	291 beta-Alanine from Tyrosine	-1.11	0.38
332 ApoC2(c)	0.05	0.95	427 Homocysteine degr	-1.11	0.45
383 Collagen CONA1(c) synthesis	0.04	1	47 Formylgroup(c)	-1.1	0.71
358 Collagen CO6A2(c) synthesis	0.04	1	103 Arginine from Glycine	-1.09	0.45
293 Homocysteine from Methionine	0.04	1	411 Cysteine degr	-1.09	0.43
837 L-Cystathionine	0.04	0.48	465 acgam6p	-1.09	0.52
855 Mercaptopyruvate	0.03	0.38	276 beta-Alanine from Cysteine	-1.08	0.36
376 Collagen COHA1(c) synthesis	0.03	1	124 Asparagine from Glycine	-1.07	0.43
367 Collagen CO9A3(c) synthesis	0.03	1	210 Glutamine from Cysteine	-1.07	0.44
774 FMN	0.03	1	211 Glutamine from Cystine	-1.03	0.56
334 ApoE(c)	0.03	0.95	168 Glutamate from Cysteine	-1.03	0.57
887 Oxidized thioredoxin	0.03	0.93	446 ApoB100 degr	-1.02	0.51
455 Thioredoxin	0.03	0.94	31 NADH to NADPH transhydrogenase in mito	-1.02	1
368 Collagen COAA1(c) synthesis	0.02	1	657 4-(2-Amino-3-hydroxyphenyl)-2,4-dioxobutanoate	-1.02	0.5
400 Collagen VWA2(c) synthesis	0.02	1	956 Xanthurenate	-1.02	0.5
912 Protein(l)ysine	0.01	1	143 Aspartate from Cystine	-1.01	0.48
756 Dehydroalanine	0.002	0.62	613 3-Hydroxyanthranilate	-1.01	0.52
728 Carnosine	0.001	0.46	277 beta-Alanine from Cystine	-1.01	0.41
379 Collagen COKA1(c) synthesis	0.001	1	169 Glutamate from Cystine	-1.01	0.62
363 Collagen CO8A1(c) synthesis	-0.0003	1	584 1-Acylglycerol-3P-palmn	-1	0.59
333 ApoC3(c)	-0.004	0.93	793 GSSG	-1	0.71
859 N-(omega)-Hydroxyarginine	-0.01	0.77	157 Aspartate from Tyrosine	-0.99	0.44
331 ApoC1(c)	-0.01	0.92	100 Arginine from Cysteine	-0.99	0.45
940 Sulfite degr	-0.01	1	975 gamma-Glutamyl-cysteine	-0.99	0.87
495 Mannose-6P	-0.01	0.78	121 Asparagine from Cysteine	-0.99	0.38
373 Collagen COEA1(c) synthesis	-0.02	1	596 2-Amino-3-carboxymuconate(s)emialdehyde	-0.98	0.5
822 Homovanillate	-0.02	1	599 2-Aminomuconate(s)emialdehyde	-0.98	0.5
37 GSH reduction using NADPH redox potential	-0.02	1	101 Arginine from Cystine	-0.98	0.51
36 GSH reduction using NADH redox potential	-0.02	0.5	409 Asparagine degr	-0.98	0.34
38 GSH oxidation	-0.02	1	449 Chitin-component degr	-0.97	0.41
365 Collagen CO9A1(c) synthesis	-0.02	1	26 NADPH redox potential into mito	-0.96	0.78
325 Albumin	-0.02	0.97	598 2-Aminomuconate	-0.96	0.54
369 Collagen COBA1(c) synthesis	-0.02	1	122 Asparagine from Cystine	-0.96	0.48

Continued on next page

Top down, T24h higher			Bottom up, C24h higher		
Simulation	ampl	score	Simulation	ampl	score
388 Collagen CTHR1(c) synthesis	-0.02	1	352 Collagen CO4A5(c) synthesis	-0.96	1
366 Collagen CO9A2(c) synthesis	-0.02	1	935 Squalene 2,3-oxide	-0.95	0.84
347 Collagen CO3A1(c) synthesis	-0.02	1	430 LDL degr	-0.93	0.43
393 Collagen FMOD(c) synthesis	-0.02	1	27 NADPH redox potential into peroxy	-0.93	1
340 Collagen BGH3(c) synthesis	-0.04	1	24 NADH redox potential into mito	-0.92	0.61
32 Thioredoxin(m) reduction	-0.06	1	713 Anthranilate	-0.9	0.48
33 Thioredoxin(m) oxidation	-0.06	1	410 Aspartate degr	-0.9	0.34
786 Fructose-2,6PP	-0.06	0.84	320 Bile-PC(b)	-0.9	0.44
339 Collagen ADIPO(c) synthesis	-0.07	1	160 Cysteine from Cystine	-0.89	0.51
356 Collagen CO5A3(c) synthesis	-0.07	1	162 Cystine from Cysteine	-0.89	0.51
378 Collagen COJA1(c) synthesis	-0.07	1	845 Lanosterol	-0.89	0.77
688 ACP	-0.07	0.86	173 Glutamate from Glutamine	-0.88	0.39
851 Malonyl-ACP	-0.07	0.84	573 1-Acylglycerol-3P-CL-pool	-0.88	0.54
697 Acetyl-ACP	-0.07	0.85	486 Glutamine	-0.88	0.51
457 Apo-ACP	-0.07	0.9	179 Glutamate from Serine	-0.88	0.47
695 Acetoacetyl-ACP	-0.07	0.84	484 Glutamate	-0.88	0.58
544 (R)-3-Hydroxybutanoyl-ACP	-0.07	0.84	234 Proline from Cystine	-0.87	0.54
717 But-2-enoyl-ACP	-0.07	0.84	898 Phosphatidate-CL-pool	-0.87	0.46
718 Butyryl-ACP	-0.07	0.84	426 Tyrosine degr	-0.87	0.44
627 3-Oxohexanoyl-ACP	-0.07	0.83	236 Proline from Glycine	-0.87	0.44
750 D-3-Hydroxyhexanoyl-ACP	-0.07	0.83	166 Glutamate from Asparagine	-0.87	0.47
536 (2E)-Hexenoyl-ACP	-0.07	0.83	310 Bilirubin conjugation	-0.86	0.62
818 Hexanoyl-ACP	-0.07	0.83	491 Glycerol	-0.86	0.56
629 3-Oxooctanoyl-ACP	-0.07	0.82	612 3-Hydroxy-L-kynurenine	-0.86	0.44
546 (R)-3-Hydroxyoctanoyl-ACP	-0.07	0.82	982 sn-Glycerol-3P	-0.85	0.84
539 (2E)-Octenoyl-ACP	-0.07	0.82	307 Arachidonate from Dihomo-gamma-linolenate	-0.85	0.6
881 Octanoyl-ACP	-0.07	0.82	431 HDL degr	-0.84	0.41
622 3-Oxodecanoyl-ACP	-0.07	0.82	290 beta-Alanine from Tryptophan	-0.84	0.39
545 (R)-3-Hydroxydecanoyl-ACP	-0.07	0.82	30 NADPH to NADH transhydrogenase in mito	-0.84	1
530 (2E)-Decenoyl-ACP	-0.07	0.81	422 Tryptophan degr	-0.83	0.42
754 Decanoyl-ACP	-0.07	0.81	856 Methacrylyl-CoA	-0.82	0.32
624 3-Oxododecanoyl-ACP	-0.07	0.81	743 Citrulline	-0.82	0.31
749 D-3-Hydroxydodecanoyl-ACP	-0.07	0.81	221 Glutamine from Serine	-0.82	0.43
532 (2E)-Dodecenoyl-ACP	-0.07	0.81	654 3alpha,7alpha-Dihydroxy-5beta-cholestanate	-0.82	0.56
769 Dodecanoyl-ACP	-0.07	0.81	156 Aspartate from Tryptophan	-0.82	0.44
634 3-Oxotetradecanoyl-ACP	-0.07	0.81	48 Formylgroup(m)	-0.82	0.52
813 HMA	-0.07	0.81	226 Glutamine from beta-Alanine	-0.81	0.35
541 (2E)-Tetradecenoyl-ACP	-0.07	0.81	780 Fatty-acid-VLDL-TG1-pool	-0.81	0.56
942 Tetradecanoyl-ACP	-0.07	0.81	781 Fatty-acid-VLDL-TG2-pool	-0.81	0.56
626 3-Oxohexadecanoyl-ACP	-0.07	0.8	653 3alpha,7alpha-Dihydroxy-5beta-cholestan-26-al	-0.8	0.53
547 (R)-3-Hydroxypalmitoyl-ACP	-0.07	0.8	165 Glutamate from Arginine	-0.8	0.42
534 (2E)-Hexadecenoyl-ACP	-0.07	0.8	874 Nicotinate	-0.8	0.56
816 Hexadecanoyl-ACP	-0.07	0.8	589 1-Acylglycerol-VLDL-PS-pool	-0.8	0.5
633 3-Oxostearoyl-ACP	-0.07	0.79	184 Glutamate from beta-Alanine	-0.8	0.37
617 3-Hydroxystearoyl-ACP	-0.07	0.79	182 Glutamate from Tyrosine	-0.8	0.53
538 (2E)-Octadecenoyl-ACP	-0.07	0.79	588 1-Acylglycerol-VLDL-PI-pool	-0.8	0.5
936 Stearoyl-ACP	-0.07	0.79	587 1-Acylglycerol-VLDL-PE-pool	-0.8	0.5
458 Apo-ACP(m)	-0.07	0.7	778 Fatty-acid-VLDL-PS-pool	-0.8	0.56
978 mitoACP	-0.07	0.68	777 Fatty-acid-VLDL-PI-pool	-0.8	0.56
507 Cytidine	-0.08	0.37	768 Dimethylallyl-PP	-0.8	0.52
672 5-Oxoproline	-0.08	0.55	586 1-Acylglycerol-VLDL-PC-pool	-0.8	0.49
461 4ppan	-0.08	1	590 1-Acylglycerol-VLDL-SM-pool	-0.8	0.49
947 Triphosphate degr	-0.09	1	591 1-Acylglycerol-VLDL-TG1-pool	-0.8	0.49
11 ATP from NADH(m)	-0.09	0.8	776 Fatty-acid-VLDL-PE-pool	-0.8	0.56
14 Ubiquinol-to-ATP	-0.09	0.62	905 Phosphocholine	-0.8	1
944 Thymine	-0.09	0.42	906 Phosphodimethylethanolamine	-0.8	1
505 Uracil	-0.09	0.32	181 Glutamate from Tryptophan	-0.8	0.51
748 D-3-Amino-isobutanoate	-0.09	0.35	82 Alanine from Glycine	-0.8	0.5
34 Thioredoxin(c) reduction	-0.09	0.92	135 Asparagine from Tryptophan	-0.79	0.44

Continued on next page

Top down, T24h higher Simulation	ampl	score	Bottom up, C24h higher Simulation	ampl	score
767 Dihydrothymine	-0.1	0.39	112 Arginine from Serine	-0.79	0.43
639 3-Ureidoisobutyrate	-0.1	0.37	55 UDP-activated glucose	-0.79	0.53
464 mlthf	-0.1	0.8	842 L-Octanoylcarnitine	-0.79	0.64
664 5,10-Methenyl-THF	-0.1	0.65	909 Presqualene-PP	-0.79	0.83
467 1fthf	-0.1	0.54	934 Squalene	-0.79	0.83
466 THF(m)	-0.1	1	892 PGP-CL-pool	-0.79	0.44
459 THF	-0.1	1	891 PG-CL-pool	-0.79	0.44
29 NADH to NADPH transhydrogenase	-0.1	1	115 Arginine from Tyrosine	-0.79	0.47
766 Dihydrofolate	-0.1	1	233 Proline from Cysteine	-0.79	0.55
670 5-Methyl-THF	-0.1	1	167 Glutamate from Aspartate	-0.78	0.47
916 Pyridoxal	-0.11	0.66	518 CL	-0.78	0.42
1 Aerobic ATP rephosph (FA)	-0.11	0.45	668 5-Formyl-THF	-0.78	0.43
357 Collagen CO6A1(c) synthesis	-0.11	1	481 Arginine	-0.77	0.46
869 N-Pantothenoylcysteine	-0.11	0.48	286 beta-Alanine from Phenylalanine	-0.77	0.39
6 Aerobic rephosph of UDP	-0.11	0.52	152 Aspartate from Phenylalanine	-0.77	0.43
548 (R)-4-Phosphopantothenoyl-cysteine	-0.11	0.45	99 Arginine from Aspartate	-0.77	0.38
23 Oxidation of NADPH	-0.12	0.47	223 Glutamine from Tryptophan	-0.77	0.47
829 Inositol	-0.12	0.5	136 Asparagine from Tyrosine	-0.77	0.46
953 Urocanate	-0.12	0.56	319 Chenodiol(b)	-0.76	0.6
460 Pyridoxal-P	-0.12	0.76	318 tcdchola(b)	-0.76	0.52
865 N-Carbamoyl-L-aspartate	-0.13	0.4	224 Glutamine from Tyrosine	-0.76	0.48
726 Carbamoyl-P	-0.14	0.58	418 Phenylalanine degr	-0.76	0.43
917 Pyridoxine-P	-0.14	1	12 ATP from NADH	-0.75	0.47
875 Nicotinate D-ribonucleoside	-0.14	0.42	177 Glutamate from Phenylalanine	-0.75	0.52
454 Glycogenin	-0.14	0.96	655 3alpha,7alpha-Dihydroxy-5beta-cholestanoyl-CoA	-0.75	0.47
807 Glycogenin-G8	-0.14	0.95	57 UDP-activated galactose	-0.74	0.45
517 PI	-0.14	0.47	514 PC	-0.74	0.4
803 Glycogenin-G11	-0.14	0.95	131 Asparagine from Phenylalanine	-0.74	0.44
804 Glycogenin-G4G4	-0.14	0.94	420 Serine degr	-0.74	0.39
806 Glycogenin-G7G1	-0.14	0.94	267 Serine from Tryptophan	-0.74	0.5
805 Glycogenin-G7	-0.14	0.94	808 Guanidinoacetate	-0.74	0.36
326 Fibrinogen	-0.14	0.3	171 Glutamate from Histidine	-0.74	0.5
765 Dihydroceramide-pool	-0.14	0.36	316 Gly-CD-cholate(b)	-0.73	0.57
794 Galactose-1P	-0.15	0.66	93 Alanine from Tryptophan	-0.73	0.5
154 Aspartate from Serine	-0.15	0.39	321 SM(b)	-0.73	0.47
139 Aspartate from Alanine	-0.15	0.53	428 beta-Alanine degr	-0.73	0.34
2 Aerobic ATP rephosph (gluc)	-0.15	0.44	61 AKG	-0.73	0.57
880 OAA	-0.16	0.76	247 Proline from Tyrosine	-0.72	0.47
336 Prothrombin	-0.16	0.98	117 Arginine from beta-Alanine	-0.72	0.39
212 Glutamine from Glutamate	-0.16	0.33	110 Arginine from Phenylalanine	-0.72	0.45
328 Antitrypsin	-0.17	0.3	468 PAPS	-0.72	0.33
435 Galactose degr	-0.17	0.59	217 Glutamine from Lysine	-0.72	0.45
22 Oxidation of NADH	-0.17	0.53	65 ATP salvage from Adenosine	-0.71	0.88
338 Haptoglobin	-0.18	0.97	219 Glutamine from Phenylalanine	-0.71	0.48
854 Maltose	-0.18	1	288 beta-Alanine from Serine	-0.71	0.38
71 Glycogen glucose storage	-0.19	0.75	602 2-Lysolecithin-pool	-0.71	0.39
183 Glutamate from Valine	-0.19	0.41	580 1-Acylglycerol-3P-arach	-0.71	0.57
360 Collagen CO6A5(c) synthesis	-0.2	0.28	581 1-Acylglycerol-3P-lin	-0.71	0.57
361 Collagen CO6A6(c) synthesis	-0.21	0.28	582 1-Acylglycerol-3P-ol	-0.71	0.57
397 Collagen PCOTH(c) synthesis	-0.21	0.32	585 1-Acylglycerol-3P-stea	-0.71	0.57
392 Collagen FCN3(c) synthesis	-0.21	0.29	583 1-Acylglycerol-3P-palm	-0.7	0.57
390 Collagen FCN1(c) synthesis	-0.22	0.29	114 Arginine from Tryptophan	-0.7	0.44
841 L-Lactate	-0.22	0.94	782 Fatty-acid-VLDL-TG3-pool	-0.7	0.66
809 Guanosine	-0.22	0.35	60 Pyruvate	-0.7	0.55
952 Urate	-0.22	0.29	133 Asparagine from Serine	-0.7	0.37
810 H2O2	-0.22	0.29	488 Serine	-0.69	0.57
145 Aspartate from Glycine	-0.22	0.44	74 Gluconeogen from Glycerol	-0.69	0.51
142 Aspartate from Cysteine	-0.22	0.54	614 3-Hydroxyisobutyrate	-0.69	0.36
381 Collagen COLQ(c) synthesis	-0.22	0.31	208 Glutamine from Asparagine	-0.68	0.42

Continued on next page

Matches relating TGFβ/contr 24h – continued

Top down, T24h higher Simulation	ampl	score	Bottom up, C24h higher Simulation	ampl	score
141 Aspartate from Asparagine	-0.22	0.54	138 Asparagine from beta-Alanine	-0.68	0.33
380 Collagen COLA1(c) synthesis	-0.22	0.31	42 Na+ exportgradient	-0.67	1
387 Collagen COSA1(c) synthesis	-0.22	0.32	91 Alanine from Serine	-0.67	0.56
123 Asparagine from Glutamate	-0.23	0.42	43 Activated methyl group (SAM)	-0.67	0.41
893 PPi	-0.24	0.71	231 Proline from Asparagine	-0.67	0.5
140 Aspartate from Arginine	-0.24	0.48	656 4,4-Dimethyl-5alpha-cholesta-8,14,24-trien-3beta-ol	-0.67	0.68
148 Aspartate from Glutamine	-0.24	0.49	264 Serine from Phenylalanine	-0.66	0.51
159 Aspartate from beta-Alanine	-0.24	0.49	404 (R)-3-Hydroxybutanoate	-0.66	0.58
987 trans-4-Hydroxy-L-proline	-0.24	1	98 Arginine from Asparagine	-0.66	0.38
835 L-1-Pyrroline-3-hydroxy-5-carboxylate	-0.24	1	730 Chenodeoxycholoyl-CoA	-0.66	0.48
592 1-Pyrroline-5-carboxylate	-0.24	0.76	593 14-Demethyllanosterol	-0.66	0.63
144 Aspartate from Glutamate	-0.25	0.64	636 3-Phosphonooxypyruvate	-0.65	0.51
456 Thioredoxin(m)	-0.25	0.95	440 ApoA1 degr	-0.65	0.48
979 mitoOxidizedThioredoxin	-0.25	0.94	129 Asparagine from Lysine	-0.65	0.46
923 SAICAR	-0.25	0.41	244 Proline from Serine	-0.65	0.48
72 Glycogen glucose release	-0.25	0.45	795 Geranyl-PP	-0.65	0.5
787 Fumarate	-0.26	0.74	214 Glutamine from Histidine	-0.64	0.41
673 5-Phosphoribosyl-4-carboxy-5-aminoimidazole	-0.26	0.35	746 Cys-Gly	-0.64	1
933 Sphingosine-1P	-0.26	0.32	444 Prothrombin degr	-0.64	0.5
932 Sphingosine	-0.26	0.37	119 Asparagine from Arginine	-0.64	0.42
609 2PG	-0.27	0.65	175 Glutamate from Lysine	-0.64	0.51
729 Ceramide-1P-pool	-0.27	0.39	243 Proline from Phenylalanine	-0.63	0.52
329 ApoA1	-0.27	0.97	45 Activated methylene group from Try	-0.63	0.39
798 Glucosylceramide-pool	-0.27	0.37	75 Gluconeogen from Alanine	-0.63	0.35
297 Palmitate from Palmitolate	-0.27	0.43	640 3-Ureidopropionate	-0.63	0.43
597 2-Amino-3-oxoadipate	-0.27	0.56	498 UDP-glucose	-0.62	0.36
209 Glutamine from Aspartate	-0.28	0.41	910 Propanoate	-0.62	0.59
402 HDL	-0.28	0.78	549 (R)-5-Diphosphomevalonate	-0.62	0.56
924 SAM	-0.28	0.43	17 Aerobic reduction of NAD+ (gluc)	-0.61	0.45
922 SAH	-0.28	0.43	716 Argininosuccinate	-0.61	0.66
300 Stearate from Oleate	-0.29	0.52	879 O-Propanoylcarnitine	-0.61	0.53
529 Methylthioribose-1P	-0.29	0.56	522 Triacylglycerol	-0.61	0.5
492 Oleate	-0.29	0.68	63 Farnesyl-PP	-0.61	0.51
690 AICAR	-0.29	0.45	510 Adenosine	-0.61	0.43
493 Stearate	-0.3	0.67	108 Arginine from Lysine	-0.61	0.45
475 dTTP	-0.3	0.49	104 Arginine from Histidine	-0.6	0.42
939 Succinyl-CoA	-0.3	0.42	438 Antichymotrypsin degr	-0.6	0.45
671 5-Methylthioadenosine	-0.3	0.48	85 Alanine from Glutamine	-0.6	0.58
850 Malate	-0.31	0.7	16 Aerobic reduction of NAD+ (FA)	-0.6	0.4
301 Palmitolate from Arachidonate	-0.31	0.64	237 Proline from Histidine	-0.59	0.49
890 PEP	-0.31	0.54	289 beta-Alanine from Threonine	-0.58	0.47
870 NADH	-0.31	0.34	443 Plasminogen degr	-0.58	0.49
490 Palmitate	-0.31	0.63	437 Albumin degr	-0.58	0.47
918 Quinolinate	-0.31	0.63	520 SM	-0.58	0.47
66 ATP salvage from Hypoxanthine	-0.31	0.44	482 Asparagine	-0.58	0.47
5 Aerobic rephosph of CTP	-0.32	0.38	752 DHAP	-0.58	0.85
298 Palmitolate from Palmitate	-0.32	0.7	789 GAP	-0.58	0.85
621 3-Methylglutaconyl-CoA	-0.32	0.37	525 beta-Alanine	-0.58	0.33
25 NADH redox potential into peroxy	-0.32	0.83	499 udpgal	-0.58	0.36
527 Spermidine	-0.32	0.43	125 Asparagine from Histidine	-0.58	0.43
961 cis-Aconitate	-0.32	0.67	441 Fibrinogen degr	-0.58	0.49
753 Deamido-NAD	-0.32	0.37	28 NADPH to NADH transhydrogenase	-0.58	1
149 Aspartate from Leucine	-0.32	0.45	421 Threonine degr	-0.58	0.49
322 PS(b)	-0.32	0.49	62 Isopentenyl-PP	-0.58	0.57
516 PS	-0.32	0.49	416 Lysine degr	-0.58	0.43
773 FAICAR	-0.32	0.43	102 Arginine from Glutamate	-0.58	0.4
857 Methylmalonyl-CoA	-0.32	0.46	134 Asparagine from Threonine	-0.57	0.54
876 Nicotinate(r)ibonucleotide	-0.32	0.41	35 Thioredoxin(c) oxidation	-0.57	0.77
299 Oleate from Stearate	-0.33	0.83	764 Dihomo-gamma-linolenoyl-CoA	-0.57	0.39

Continued on next page

Top down, T24h higher Simulation	ampl	score	Bottom up, C24h higher Simulation	ampl	score
666 5-Aminolevulinate	-0.33	0.61	266 Serine from Threonine	-0.57	0.58
790 GAR	-0.33	0.5	106 Arginine from Glutamine	-0.57	0.36
772 FADH2	-0.33	0.33	834 Kynurenine	-0.57	0.43
515 PE	-0.33	0.54	73 Gluconeogen from Lactate	-0.57	0.51
323 PE(b)	-0.33	0.48	280 beta-Alanine from Histidine	-0.57	0.41
451 NAD+	-0.33	0.33	645 3PG	-0.56	0.59
986 trans,cis-octadeca-2,9-dienoyl-CoA	-0.33	0.4	823 Hydracrylate	-0.56	0.49
889 PE-PS-VLDL-pool	-0.33	0.57	92 Alanine from Threonine	-0.56	0.56
283 beta-Alanine from Leucine	-0.33	0.39	831 Isobutyryl-CoA	-0.56	0.34
10 Anaerobic rephosph of UTP	-0.33	0.45	721 CDP-choline	-0.56	0.37
8 Anaerobic rephosph of GTP	-0.33	0.45	201 Glycine from Threonine	-0.56	0.56
9 Anaerobic rephosph of CTP	-0.33	0.45	814 HMG-CoA	-0.56	0.39
450 CoA	-0.34	0.39	425 Isoleucine degr	-0.56	0.52
742 Citrate	-0.34	0.71	866 N-Formimino-L-glutamate	-0.56	0.49
302 gamma-Linolenate from Linoleate	-0.34	0.56	469 ametam	-0.55	0.32
693 Acetaldehyde	-0.34	0.61	262 Serine from Lysine	-0.55	0.48
595 2,5-Diaminopyrimidine(n)leoside triphosphate	-0.34	0.31	155 Aspartate from Threonine	-0.55	0.55
594 2,5-Diamino-6-(5-triphosphoryl-3,4-trihydroxy-2-oxopentyl)- amino-4-oxopyrimidine	-0.34	0.31	615 3-Hydroxyisobutyryl-CoA	-0.55	0.32
559 (S)-3-hydroxypalmitoleoyl-CoA	-0.34	0.35	413 Histidine degr	-0.55	0.43
13 NADH potential transport	-0.34	0.59	508 Xanthine	-0.55	0.34
337 ApoTransferin	-0.34	0.98	519 LacCer	-0.55	0.43
984 trans,cis-hexadeca-2,9-dienoyl-CoA	-0.34	0.36	317 Taurocholate(b)	-0.55	0.51
700 Acyl-CoA-Bile-PC-pool	-0.34	0.4	126 Asparagine from Isoleucine	-0.55	0.55
779 Fatty-acid-VLDL-SM-pool	-0.34	1	973 dUMP	-0.55	0.33
775 Fatty-acid-VLDL-PC-pool	-0.34	1	46 Activated methyl group from Histidine	-0.55	0.38
706 Acyl-CoA-VLDL-SM-pool	-0.34	0.4	503 IMP	-0.54	0.39
702 Acyl-CoA-VLDL-PC-pool	-0.34	0.4	504 XMP	-0.54	0.38
19 Aerobic reduction of NADP+ (gluc)	-0.34	0.31	109 Arginine from Methionine	-0.54	0.48
873 Nicotinamide D-ribonucleotide	-0.34	0.46	270 Serine from beta-Alanine	-0.54	0.42
703 Acyl-CoA-VLDL-PE-pool	-0.34	0.4	571 1,3DPG	-0.54	0.63
3 Anaerobic rephosph of ATP	-0.34	0.42	649 3alpha,7alpha,12alpha-Trihydroxy-5beta-cholestan-26-al	-0.54	0.49
707 Acyl-CoA-VLDL-TG2-pool	-0.34	0.4	650 3alpha,7alpha,12alpha-Trihydroxy-5beta-cholestanate	-0.54	0.49
439 Antitrypsin degr	-0.34	0.44	677 5beta-Cholestane-3alpha,7alpha,12alpha,26-tetrol	-0.54	0.49
708 Acyl-CoA-VLDL-TG3-pool	-0.34	0.4	258 Serine from Histidine	-0.54	0.53
704 Acyl-CoA-VLDL-PI-pool	-0.34	0.4	509 Guanine	-0.54	0.33
128 Asparagine from Leucine	-0.34	0.38	884 Orotate	-0.54	0.43
705 Acyl-CoA-VLDL-PS-pool	-0.35	0.4	252 Serine from Asparagine	-0.54	0.52
701 Acyl-CoA-CL-pool	-0.35	0.4	292 beta-Alanine from Valine	-0.54	0.37
674 5-Phosphoribosylamine	-0.35	0.51	678 5beta-Cholestane-3alpha,7alpha,26-triol	-0.53	0.52
67 dTTP salvage from Thymine	-0.35	0.47	87 Alanine from Lysine	-0.53	0.44
895 Palmitoyl-CoA	-0.35	0.4	315 Glycocholate(b)	-0.53	0.55
570 1,2-Diacylglycerol-VLDL-TG-pool	-0.35	0.47	843 L-Oleoylcarnitine	-0.53	0.7
564 1,2-Diacylglycerol-Bile-PC-pool	-0.35	0.46	18 Aerobic reduction of NADP+ (FA)	-0.53	0.37
569 1,2-Diacylglycerol-VLDL-SM-pool	-0.35	0.47	271 Tyrosine from Phenylalanine	-0.53	0.59
565 1,2-Diacylglycerol-VLDL-PC-pool	-0.35	0.47	83 Alanine from Histidine	-0.53	0.43
937 Stearoyl-CoA	-0.35	0.4	971 dTMP	-0.53	0.31
883 Oleoyl-CoA	-0.35	0.4	259 Serine from Isoleucine	-0.53	0.59
849 Linoleoyl-CoA	-0.35	0.4	663 4alpha-Methylzymosterol-4-carboxylate	-0.53	0.69
715 Arachidonyl-CoA	-0.35	0.4	867 N-Formyl-GAR	-0.53	0.45
904 Phosphatidate-VLDL-TG-pool	-0.35	0.52	943 Thymidine	-0.53	0.29
897 Phosphatidate-Bile-PC-pool	-0.35	0.5	691 AIR	-0.53	0.41
903 Phosphatidate-VLDL-SM-pool	-0.35	0.52	675 5-Phosphoribosylformylglycinamidine	-0.53	0.42
899 Phosphatidate-VLDL-PC-pool	-0.35	0.52	483 Aspartate	-0.53	0.47
976 gamma-Linolenoyl-CoA	-0.35	0.38	218 Glutamine from Methionine	-0.53	0.5
864 N-Acetylneuraminate-9P	-0.35	0.34	281 beta-Alanine from Isoleucine	-0.53	0.5
868 N-Methylethanolamine-P	-0.35	1	21 Aerobic reduction of FAD (FA)	-0.53	0.52
771 Ethanolamine-P	-0.35	1	193 Glycine from Isoleucine	-0.52	0.57

Continued on next page

Top down, T24h higher Simulation	ampl	score	Bottom up, C24h higher Simulation	ampl	score
871 NADPH	-0.35	0.39	137 Asparagine from Valine	-0.52	0.42
566 1,2-Diacylglycerol-VLDL-PE-pool	-0.35	0.47	132 Asparagine from Proline	-0.52	0.58
900 Phosphatidate-VLDL-PE-pool	-0.35	0.52	606 2-Oxoadipate	-0.52	0.41
535 (2E)-Hexadecenoyl-CoA	-0.35	0.36	429 Ornithine degr	-0.51	0.42
7 Aerobic rephosph of UTP	-0.35	0.48	502 GDP-mannose	-0.51	0.32
567 1,2-Diacylglycerol-VLDL-PI-pool	-0.35	0.47	69 Formaldehyde degr	-0.51	0.38
901 Phosphatidate-VLDL-PI-pool	-0.35	0.51	194 Glycine from Glutamine	-0.51	0.44
4 Aerobic rephosph of GTP	-0.35	0.47	478 GTP	-0.51	0.37
711 Adenylyl(s)ulfate	-0.35	0.45	260 Serine from Glutamine	-0.51	0.48
568 1,2-Diacylglycerol-VLDL-PS-pool	-0.35	0.47	253 Serine from Aspartate	-0.51	0.49
902 Phosphatidate-VLDL-PS-pool	-0.36	0.51	419 Proline degr	-0.51	0.5
551 (R)-Methylmalonyl-CoA	-0.36	0.38	659 4-Imidazolone-5-propanoate	-0.51	0.35
604 2-Methylbutyryl-CoA	-0.36	0.35	553 (S)-3-Hydroxy-2-methylbutyryl-CoA	-0.51	0.39
620 3-Methylcrotonyl-CoA	-0.36	0.45	146 Aspartate from Histidine	-0.51	0.48
783 Formamidopyrimidine(n)leoside triphosphate	-0.36	0.32	113 Arginine from Threonine	-0.51	0.54
556 (S)-3-Hydroxyhexadecanoyl-CoA	-0.36	0.41	84 Alanine from Isoleucine	-0.51	0.62
452 NADP+	-0.36	0.38	665 5,6-Dihydrouracil	-0.51	0.41
709 Adenine	-0.36	0.31	618 3-Keto-4-methylzymosterol	-0.51	0.68
862 N-Acetylmannosamine-6P	-0.36	0.36	423 Valine degr	-0.51	0.4
959 cGMP	-0.36	0.31	408 Arginine degr	-0.51	0.42
885 Orotidine-5P	-0.36	0.37	476 ATP	-0.51	0.4
174 Glutamate from Leucine	-0.37	0.39	105 Arginine from Isoleucine	-0.5	0.55
981 palmitoleoyl-CoA	-0.37	0.35	215 Glutamine from Isoleucine	-0.5	0.64
572 1-Acylglycerol-3P-Bile-PC-pool	-0.37	0.55	242 Proline from Methionine	-0.5	0.53
579 1-Acylglycerol-3P-VLDL-TG1-pool	-0.37	0.57	955 Xanthosine	-0.5	0.31
578 1-Acylglycerol-3P-VLDL-SM-pool	-0.37	0.57	770 Erythrose-4P	-0.5	0.64
574 1-Acylglycerol-3P-VLDL-PC-pool	-0.37	0.57	180 Glutamate from Threonine	-0.5	0.56
575 1-Acylglycerol-3P-VLDL-PE-pool	-0.37	0.57	241 Proline from Lysine	-0.5	0.54
576 1-Acylglycerol-3P-VLDL-PI-pool	-0.37	0.57	550 (R)-5-Phosphomevalonate	-0.5	0.49
453 FAD	-0.37	0.35	699 Acrylyl-CoA	-0.5	0.39
434 Fructose degr	-0.37	0.44	111 Arginine from Proline	-0.5	0.48
577 1-Acylglycerol-3P-VLDL-PS-pool	-0.37	0.57	521 Ceramide	-0.5	0.48
70 Formate degr	-0.37	0.61	848 Linoleate	-0.5	0.62
762 Dephospho-CoA	-0.37	0.33	731 Cholate	-0.5	0.52
758 Deoxycytidine	-0.37	0.43	20 Aerobic reduction of FAD (gluc)	-0.5	0.48
150 Aspartate from Lysine	-0.38	0.49	176 Glutamate from Methionine	-0.5	0.47
631 3-Oxopalmitoyl-CoA	-0.38	0.41	533 (2E)-Dodecenoyl-CoA	-0.49	0.44
698 Acetyl-CoA	-0.38	0.38	51 Acetyl group(p)	-0.49	0.47
696 Acetoacetyl-CoA	-0.38	0.36	555 (S)-3-Hydroxydodecanoyl-CoA	-0.49	0.43
335 Plasminogen	-0.38	0.98	501 GDP-L-fucose	-0.49	0.31
282 beta-Alanine from Glutamine	-0.38	0.32	524 gdpddman	-0.49	0.32
284 beta-Alanine from Lysine	-0.38	0.4	186 Glycine from Arginine	-0.49	0.42
216 Glutamine from Leucine	-0.38	0.38	755 Decanoyl-CoA	-0.49	0.45
724 CMP-NeuNGc	-0.38	0.29	554 (S)-3-Hydroxybutyryl-CoA	-0.49	0.38
967 dCMP	-0.38	0.45	927 Sedoheptulose-1,7PP	-0.49	0.64
442 Haptoglobin degr	-0.38	0.53	49 Acetyl group(c)	-0.49	0.58
694 Acetate	-0.38	0.97	52 Acetyl group(r)	-0.49	0.58
722 CDP-ethanolamine	-0.39	0.46	147 Aspartate from Isoleucine	-0.49	0.58
86 Alanine from Leucine	-0.39	0.35	222 Glutamine from Threonine	-0.49	0.6
447 Glycogenin degr	-0.39	0.5	928 Sedoheptulose-7P	-0.49	0.55
68 O2.-(c) degr	-0.39	0.72	625 3-Oxododecanoyl-CoA	-0.49	0.45
403 Acetoacetate	-0.39	0.57	970 dTDP	-0.48	0.34
296 Stearate from Palmitate	-0.39	0.7	563 (S)-Hydroxyoctanoyl-CoA	-0.48	0.44
39 Proton-gradient(m) build up	-0.39	0.45	662 4alpha-Methylzymosterol	-0.48	0.69
40 Proton-gradient(c) build up	-0.39	0.45	957 Zymosterol	-0.48	0.69
445 ApoTransferin degr	-0.39	0.48	945 Tiglyl-CoA	-0.48	0.4
278 beta-Alanine from Glutamate	-0.4	0.39	251 Serine from Arginine	-0.48	0.44
542 (2E)-Tetradecenoyl-CoA	-0.4	0.44	630 3-Oxooctanoyl-CoA	-0.48	0.44
921 Ribulose-5P	-0.4	0.37	531 (2E)-Decenoyl-CoA	-0.48	0.44

Continued on next page

Top down, T24h higher Simulation	ampl	score	Bottom up, C24h higher Simulation	ampl	score
81 Alanine from Glutamate	-0.4	0.51	561 (S)-Hydroxydecanoyl-CoA	-0.48	0.44
406 Heme	-0.4	0.59	474 dGTP	-0.48	0.39
888 PAP	-0.4	0.38	116 Arginine from Valine	-0.48	0.41
757 Deoxyadenosine	-0.4	0.36	882 Octanoyl-CoA	-0.48	0.45
847 Lauroyl-CoA	-0.4	0.44	828 Inosine	-0.48	0.32
477 CTP	-0.4	0.36	920 Ribose-5P	-0.48	0.43
240 Proline from Leucine	-0.4	0.5	623 3-Oxodecanoyl-CoA	-0.48	0.44
838 L-Formylkynurenine	-0.4	0.69	265 Serine from Proline	-0.48	0.56
230 Proline from Arginine	-0.41	0.41	819 Hexanoyl-CoA	-0.48	0.45
858 Myristoyl-CoA	-0.41	0.44	472 dATP	-0.48	0.42
158 Aspartate from Valine	-0.41	0.42	751 D-Xylulose-5P	-0.48	0.47
681 6-Pyruvoyltetrahydropterin	-0.41	0.38	562 (S)-Hydroxyhexanoyl-CoA	-0.48	0.42
853 Malonyl-CoA	-0.41	0.42	220 Glutamine from Proline	-0.48	0.46
965 dAMP	-0.41	0.45	269 Serine from Valine	-0.47	0.44
479 UTP	-0.41	0.38	245 Proline from Threonine	-0.47	0.61
801 Glutaryl-CoA	-0.41	0.42	616 3-Hydroxypropionyl-CoA	-0.47	0.39
528 Spermine	-0.41	0.41	199 Glycine from Proline	-0.47	0.6
894 PRPP	-0.41	0.48	235 Proline from Glutamate	-0.47	0.57
473 dCTP	-0.41	0.41	191 Glycine from Glutamate	-0.47	0.48
723 CMP	-0.41	0.38	256 Serine from Glutamate	-0.47	0.54
833 Isovaleryl-CoA	-0.41	0.37	741 Choloyl-CoA	-0.47	0.47
644 3-oxopalmitoleoyl-CoA	-0.41	0.37	763 Desmosterol	-0.47	0.62
232 Proline from Aspartate	-0.41	0.53	540 (2E)-Octenoyl-CoA	-0.47	0.44
557 (S)-3-Hydroxytetradecanoyl-CoA	-0.41	0.38	273 beta-Alanine from Arginine	-0.47	0.34
635 3-Oxotetradecanoyl-CoA	-0.41	0.4	840 L-Glutamate 5-semialdehyde	-0.47	0.42
720 CDP	-0.42	0.38	311 Urea from glutamine	-0.47	0.37
966 dCDP	-0.42	0.42	537 (2E)-Hexenoyl-CoA	-0.47	0.45
305 Dihomo-gamma-linolenate from gamma-Linolenate	-0.42	0.81	238 Proline from Isoleucine	-0.47	0.61
692 AMP	-0.42	0.35	204 Glycine from Valine	-0.47	0.47
433 Stearate degr	-0.42	0.59	558 (S)-3-hydroxyoleyleoyl-CoA	-0.47	0.4
964 dADP	-0.42	0.38	682 7-Dehydrodesmosterol	-0.47	0.66
107 Arginine from Leucine	-0.42	0.37	732 Cholestenol	-0.47	0.7
689 ADP	-0.42	0.34	676 5alpha-Cholesta-7,24-dien-3beta-ol	-0.47	0.7
50 Acetyl group(m)	-0.42	0.57	77 Alanine from Asparagine	-0.47	0.44
432 Oleate degr	-0.42	0.57	246 Proline from Tryptophan	-0.47	0.51
229 Proline from Alanine	-0.42	0.42	412 Glutamate degr	-0.47	0.4
661 4-Methyl-2-oxopentanoate	-0.42	0.4	543 (3Z)-Dodecenoyl-CoA	-0.47	0.41
601 2-Deoxy-D-ribose-5P	-0.42	0.44	792 GMP	-0.46	0.35
913 Protoporphyrin	-0.42	0.58	727 Carbonate	-0.46	0.68
76 Alanine from Arginine	-0.42	0.38	812 HCO3-	-0.46	0.68
95 Alanine from Valine	-0.42	0.49	496 Fructose-6P	-0.46	1
398 Collagen SCRB1(c) synthesis	-0.43	1	962 cis-laur-5-enoyl-CoA	-0.46	0.41
740 Cholesterol-ester-stea	-0.43	0.62	791 GDP	-0.46	0.33
736 Cholesterol-ester-ol	-0.43	0.62	641 3-oxolaur-cis-5-enoyl-CoA	-0.46	0.41
844 L-Palmitoylcarnitine	-0.43	0.68	178 Glutamate from Proline	-0.46	0.62
977 linoleic-Carnitine	-0.43	0.67	878 O-Butanoylcarnitine	-0.46	0.59
714 Arachidonyl-Carnitine	-0.43	0.67	983 trans,cis-dodeca-2,5-dienoyl-CoA	-0.46	0.4
974 dUTP	-0.43	0.4	643 3-oxooleoyl-CoA	-0.46	0.37
914 Protoporphyrinogen IX	-0.43	0.58	610 3(S)-3-hydroxydodecen-(5Z)-oyl-CoA	-0.46	0.4
153 Aspartate from Proline	-0.43	0.64	684 7alpha,12alpha-Dihydroxycholest-4-en-3-one	-0.46	0.53
127 Asparagine from Glutamine	-0.43	0.56	648 3alpha,7alpha,12alpha-Trihydroxy-5beta-cholest-24-enoyl-CoA	-0.46	0.48
414 Glutamine degr	-0.43	0.38	908 Porphobilinogen	-0.46	0.56
303 Dihomo-gamma-linolenate from Linoleate	-0.43	0.68	686 7alpha-Hydroxycholest-4-en-3-one	-0.46	0.57
306 Arachidonate from gamma-Linolenate	-0.43	0.68	687 7alpha-Hydroxycholesterol	-0.46	0.6
969 dGMP	-0.43	0.37	523 Cholesterol	-0.45	0.63
415 Leucine degr	-0.43	0.37	963 cis-myrist-7-enoyl-CoA	-0.45	0.39
968 dGDP	-0.43	0.37	651 3alpha,7alpha,12alpha-Trihydroxy-5beta-cholestanoyl-CoA	-0.45	0.45

Continued on next page

Matches relating TGFβ/contr 24h – continued

Top down, T24h higher Simulation	ampl	score	Bottom up, C24h higher Simulation	ampl	score
462 thbpt	-0.43	0.44	506 Uridine	-0.45	0.32
759 Deoxyguanosine	-0.43	0.34	745 Crotonyl-CoA	-0.45	0.4
405 Acetone	-0.43	0.45	915 Provitamin D3	-0.45	0.67
836 L-2-Aminoadipate	-0.43	1	642 3-oxomyrist-7-enoyl-CoA	-0.45	0.4
738 Cholesterol-ester-palmn	-0.43	0.59	628 3-Oxohexanoyl-CoA	-0.45	0.46
249 Proline from beta-Alanine	-0.43	0.44	846 Lathosterol	-0.45	0.71
710 Adenylosuccinate	-0.43	0.4	239 Proline from Glutamine	-0.45	0.5
761 Deoxyuridine	-0.43	0.29	800 Glutamyl-5P	-0.45	0.54
603 2-Methylacetoacetyl-CoA	-0.44	0.4	652 3alpha,7alpha,12alpha-Trihydroxycoprostane	-0.45	0.5
463 dhbpt	-0.44	0.46	683 7alpha,12alpha-Dihydroxy-5beta-cholestan-3-one	-0.45	0.52
820 Histamine	-0.44	0.67	679 5beta-Cholestane-3alpha,7alpha-diol	-0.45	0.52
737 Cholesterol-ester-palm	-0.44	0.59	225 Glutamine from Valine	-0.45	0.38
487 Proline	-0.44	0.48	685 7alpha-Hydroxy-5beta-cholestan-3-one	-0.45	0.55
287 beta-Alanine from Proline	-0.44	0.49	646 3alpha,7alpha,12alpha,24-Tetrahydroxy-5beta-cholestanoyl-CoA	-0.45	0.49
744 CoproporphyrinogenIII	-0.44	0.59	985 trans,cis-myristo-2,7-dienoyl-CoA	-0.45	0.39
739 Cholesterol-ester-pool	-0.44	0.61	960 cis-(3S)-hydroxytetradec-7-enoyl-CoA	-0.45	0.39
248 Proline from Valine	-0.44	0.53	735 Cholesterol-ester-lin	-0.45	0.62
958 cAMP	-0.44	0.31	860 N-Acetyl-D-mannosamine	-0.45	0.34
90 Alanine from Proline	-0.44	0.54	852 Malonyl-Carnitin	-0.45	0.8
948 UDP-N-acetylglucosamine	-0.44	0.29	911 Propanoyl-CoA	-0.45	0.34
951 UMP	-0.44	0.44	647 3alpha,7alpha,12alpha-Trihydroxy-5beta-24-oxocholestanoyl-CoA	-0.45	0.5
972 dUDP	-0.44	0.39	261 Serine from Leucine	-0.44	0.4
824 Hydroxymethylbilane	-0.44	0.64	719 Butyryl-CoA	-0.44	0.47
324 Cholesterol(b)	-0.44	0.61	172 Glutamate from Isoleucine	-0.44	0.57
304 Arachidonate from Linoleate	-0.44	0.64	734 Cholesterol-ester-gla	-0.44	0.63
954 UroporphyrinogenIII	-0.44	0.61	195 Glycine from Leucine	-0.44	0.39
608 2-Oxoglutaramate	-0.44	1	785 Fructose-1,6PP	-0.44	1
733 Cholesterol-ester-arach	-0.44	0.64	938 Succinate	-0.44	0.56
511 UDP-N-acetylgalactosamine	-0.44	0.28			

1.2 Matches relating full time control

Table 2: Matches relating full time control

Top down, C24h higher Simulation	ampl	score	Bottom up, C1h higher Simulation	ampl	score
896 Pantetheine	3	0.31	410 Aspartate degr	-4.7	0.32
830 Inositol-1P	1.77	1	428 beta-Alanine degr	-4.66	0.31
829 Inositol	1.72	0.62	409 Asparagine degr	-4.6	0.38
352 Collagen CO4A5(c) synthesis	1.61	1	419 Proline degr	-4.6	0.25
386 Collagen CORA1(c) synthesis	0.91	1	890 PEP	-3.96	0.51
349 Collagen CO4A2(c) synthesis	0.75	1	609 2PG	-3.9	0.42
343 Collagen CD36(c) synthesis	0.71	1	821 Homogentisate	-3.67	0.65
300 Stearate from Oleate	0.68	0.32	926 Sarcosine	-3.61	0.82
592 1-Pyrroline-5-carboxylate	0.68	0.77	75 Gluconeogen from Alanine	-3.52	0.4
987 trans-4-Hydroxy-L-proline	0.68	1	426 Tyrosine degr	-3.5	0.41
835 L-1-Pyrroline-3-hydroxy-5-carboxylate	0.68	1	660 4-Maleylacetoacetate	-3.38	0.69
449 Chitin-component degr	0.67	0.39	788 Fumarylacetoacetate	-3.32	0.61
297 Palmitate from Palmitolate	0.66	0.33	418 Phenylalanine degr	-3.23	0.41
347 Collagen CO3A1(c) synthesis	0.66	1	658 4-Hydroxyphenylpyruvate	-3.2	0.53
399 Collagen SERPH(c) synthesis	0.65	1	72 Glycogen glucose release	-3.18	0.45
24 NADH redox potential into mito	0.64	0.53	182 Glutamate from Tyrosine	-3.18	0.37
85 Alanine from Glutamine	0.63	0.43	224 Glutamine from Tyrosine	-3.17	0.34
54 Glucose-6P	0.61	0.51	94 Alanine from Tyrosine	-3.14	0.45
314 Creatine	0.6	0.27	157 Aspartate from Tyrosine	-3.14	0.46
875 Nicotinate D-ribonucleoside	0.55	0.3	247 Proline from Tyrosine	-3.14	0.31
529 Methylthioribose-1P	0.53	0.38	136 Asparagine from Tyrosine	-3.14	0.37
865 N-Carbamoyl-L-aspartate	0.53	0.33	291 beta-Alanine from Tyrosine	-3.12	0.41
874 Nicotinate	0.52	0.59	115 Arginine from Tyrosine	-3.08	0.38
220 Glutamine from Proline	0.51	0.29	203 Glycine from Tyrosine	-3.05	0.35
480 Alanine	0.5	0.55	268 Serine from Tyrosine	-3.04	0.38
469 ametam	0.49	0.31	747 Cysteamine	-3.02	0.37
30 NADPH to NADH transhydrogenase in mito	0.49	1	638 3-Sulfinoalanine	-2.99	0.72
293 Homocysteine from Methionine	0.48	1	826 Hypotaurine	-2.99	0.49
436 Mannose degr	0.48	0.64	295 Taurine from Cysteine	-2.99	0.49
79 Alanine from Cysteine	0.46	0.4	74 Gluconeogen from Glycerol	-2.98	0.47
938 Succinate	0.44	0.38	243 Proline from Phenylalanine	-2.93	0.32
71 Glycogen glucose storage	0.44	0.65	177 Glutamate from Phenylalanine	-2.93	0.38
807 Glycogenin-G8	0.44	0.95	219 Glutamine from Phenylalanine	-2.92	0.35
454 Glycogenin	0.44	0.96	89 Alanine from Phenylalanine	-2.9	0.45
803 Glycogenin-G11	0.44	0.95	152 Aspartate from Phenylalanine	-2.89	0.45
805 Glycogenin-G7	0.44	0.94	808 Guanidinoacetate	-2.88	0.29
804 Glycogenin-G4G4	0.44	0.95	131 Asparagine from Phenylalanine	-2.88	0.38
806 Glycogenin-G7G1	0.44	0.94	110 Arginine from Phenylalanine	-2.87	0.37
67 dTTP salvage from Thymine	0.42	0.33	286 beta-Alanine from Phenylalanine	-2.87	0.41
619 3-Methyl-2-oxobutyrate	0.42	0.46	198 Glycine from Phenylalanine	-2.78	0.36
836 L-2-Aminoadipate	0.42	1	264 Serine from Phenylalanine	-2.77	0.38
974 dUTP	0.4	0.28	73 Gluconeogen from Lactate	-2.54	0.39
96 Alanine from beta-Alanine	0.4	0.39	775 Fatty-acid-VLDL-PC-pool	-2.25	1
615 3-Hydroxyisobutyryl-CoA	0.4	0.37	779 Fatty-acid-VLDL-SM-pool	-2.25	1
856 Methacrylyl-CoA	0.4	0.36	335 Plasminogen	-2.1	0.98
90 Alanine from Proline	0.4	0.32	838 L-Formylkynurenine	-2.07	0.75
893 PPi	0.39	0.66	834 Kynurenine	-2.07	0.34
894 PRPP	0.39	0.39	925 Saccharopine	-1.92	0.48
236 Proline from Glycine	0.38	0.34	694 Acetate	-1.89	0.79
979 mitoOxidizedThioredoxin	0.38	0.94	55 UDP-activated glucose	-1.88	0.55
456 Thioredoxin(m)	0.38	0.95	307 Arachidonate from Dihomo-gamma-linolenate	-1.84	0.39
632 3-Oxopropanoate	0.37	0.41	57 UDP-activated galactose	-1.83	0.43
462 thbpt	0.37	0.35	302 gamma-Linolenate from Linoleate	-1.79	0.37
681 6-Pyruvoyltetrahydropterin	0.37	0.46	298 Palmitolate from Palmitate	-1.79	0.34
463 dhbpt	0.36	0.32	613 3-Hydroxyanthranilate	-1.78	0.35
241 Proline from Lysine	0.36	0.29	598 2-Aminomuconate	-1.76	0.44

Continued on next page

Matches relating full time control – continued

Top down, C24h higher Simulation	ampl	score	Bottom up, C1h higher Simulation	ampl	score
709 Adenine	0.36	0.46	612 3-Hydroxy-L-kynurenine	-1.76	0.45
475 dTTP	0.36	0.5	596 2-Amino-3-carboxymuconate(s)emialdehyde	-1.75	0.44
355 Collagen CO5A2(c) synthesis	0.36	1	599 2-Aminomuconate(s)emialdehyde	-1.75	0.44
784 Formylanthranilate	0.35	0.67	299 Oleate from Stearate	-1.74	0.44
58 GDP-activated fucose	0.35	0.59	48 Formylgroup(m)	-1.7	0.32
790 GAR	0.34	0.47	416 Lysine degr	-1.7	0.33
246 Proline from Tryptophan	0.34	0.32	668 5-Formyl-THF	-1.69	0.31
674 5-Phosphoribosylamine	0.34	0.35	93 Alanine from Tryptophan	-1.67	0.3
723 CMP	0.34	0.36	43 Activated methyl group (SAM)	-1.65	0.45
720 CDP	0.34	0.37	798 Glucosylceramide-pool	-1.64	0.35
507 Cytidine	0.34	0.41	327 Antichymotrypsin	-1.63	0.97
839 L-Fucose-1P	0.34	0.46	296 Stearate from Palmitate	-1.6	0.33
435 Galactose degr	0.33	0.5	422 Tryptophan degr	-1.59	0.35
966 dCDP	0.33	0.35	135 Asparagine from Tryptophan	-1.58	0.31
831 Isobutyryl-CoA	0.33	0.38	156 Aspartate from Tryptophan	-1.57	0.32
604 2-Methylbutyryl-CoA	0.33	0.37	108 Arginine from Lysine	-1.53	0.32
477 CTP	0.33	0.44	489 Tyrosine	-1.51	0.65
722 CDP-ethanolamine	0.33	0.31	271 Tyrosine from Phenylalanine	-1.5	0.52
867 N-Formyl-GAR	0.33	0.43	114 Arginine from Tryptophan	-1.49	0.33
473 dCTP	0.33	0.4	429 Ornithine degr	-1.38	0.31
17 Aerobic reduction of NAD+ (gluc)	0.32	0.33	408 Arginine degr	-1.38	0.3
958 cAMP	0.32	0.4	65 ATP salvage from Adenosine	-1.38	0.89
479 UTP	0.32	0.36	412 Glutamate degr	-1.37	0.28
689 ADP	0.32	0.44	202 Glycine from Tryptophan	-1.36	0.3
692 AMP	0.32	0.44	729 Ceramide-1P-pool	-1.36	0.3
888 PAP	0.32	0.42	929 Serotonin	-1.35	0.61
713 Anthranilate	0.32	0.51	411 Cysteine degr	-1.34	0.34
721 CDP-choline	0.32	0.34	267 Serine from Tryptophan	-1.33	0.31
498 UDP-glucose	0.32	0.3	111 Arginine from Proline	-1.3	0.25
595 2,5-Diaminopyrimidine(n)leoside triphosphate	0.31	0.42	481 Arginine	-1.28	0.3
594 2,5-Diamino-6-(5-triphosphoryl-3,4-trihydroxy-2-oxopentyl)- amino-4-oxopyrimidine	0.31	0.41	975 gamma-Glutamyl-cysteine	-1.24	0.86
783 Formamidopyrimidine(n)leoside triphosphate	0.31	0.43	953 Urocanate	-1.23	0.51
959 cGMP	0.31	0.43	434 Fructose degr	-1.23	0.6
551 (R)-Methylmalonyl-CoA	0.31	0.37	140 Aspartate from Arginine	-1.18	0.31
964 dADP	0.31	0.41	413 Histidine degr	-1.18	0.31
257 Serine from Glycine	0.31	0.67	100 Arginine from Cysteine	-1.17	0.35
501 GDP-L-fucose	0.31	0.43	106 Arginine from Glutamine	-1.17	0.34
945 Tiglyl-CoA	0.31	0.31	102 Arginine from Glutamate	-1.16	0.28
762 Dephospho-CoA	0.3	0.39	142 Aspartate from Cysteine	-1.15	0.47
616 3-Hydroxypropionyl-CoA	0.3	0.32	101 Arginine from Cystine	-1.14	0.36
699 Acrylyl-CoA	0.3	0.32	908 Porphobilinogen	-1.12	0.52
969 dGMP	0.3	0.44	666 5-Aminolevulinate	-1.11	0.61
506 Uridine	0.3	0.46	811 H2S	-1.11	0.5
603 2-Methylacetoacetyl-CoA	0.3	0.33	933 Sphingosine-1P	-1.11	0.32
791 GDP	0.3	0.46	932 Sphingosine	-1.11	0.34
675 5-Phosphoribosylformylglycinamidine	0.3	0.45	99 Arginine from Aspartate	-1.1	0.29
967 dCMP	0.3	0.39	825 Hydroxypyruvate	-1.09	1
691 AIR	0.3	0.47	406 Heme	-1.09	0.32
711 Adenylyl(s)ulfate	0.3	0.46	104 Arginine from Histidine	-1.08	0.37
833 Isovaleryl-CoA	0.3	0.34	659 4-Imidazolone-5-propanoate	-1.08	0.49
345 Collagen CO1A2(c) synthesis	0.3	1	13 NADH potential transport	-1.07	0.41
968 dGDP	0.3	0.43	842 L-Octanoylcarnitine	-1.06	0.38
710 Adenylosuccinate	0.3	0.51	331 ApoC1(c)	-1.06	0.94
853 Malonyl-CoA	0.3	0.38	802 Glycerate	-1.05	0.45
814 HMG-CoA	0.3	0.38	744 CoproporphyrinogenIII	-1.03	0.37
696 Acetoacetyl-CoA	0.3	0.4	141 Aspartate from Asparagine	-1.02	0.42
924 SAM	0.3	0.47	914 Protoporphyrinogen IX	-1.02	0.36
922 SAH	0.3	0.47	954 UroporphyrinogenIII	-1.02	0.39

Continued on next page

Matches relating full time control – continued

Top down, C24h higher Simulation	ampl	score	Bottom up, C1h higher Simulation	ampl	score
628 3-Oxohexanoyl-CoA	0.3	0.32	528 Spermine	-1.02	0.29
698 Acetyl-CoA	0.29	0.42	913 Protoporphyrin	-1.02	0.32
223 Glutamine from Tryptophan	0.29	0.31	824 Hydroxymethylbilane	-1.02	0.4
965 dAMP	0.29	0.47	526 Putrescine	-1.02	0.45
643 3-oxooleoyl-CoA	0.29	0.37	424 Glycine degr	-1	0.38
981 palmitoleoyl-CoA	0.29	0.38	597 2-Amino-3-oxoadipate	-1	0.43
706 Acyl-CoA-VLDL-SM-pool	0.29	0.38	98 Arginine from Asparagine	-1	0.35
700 Acyl-CoA-Bile-PC-pool	0.29	0.38	420 Serine degr	-0.99	0.41
895 Palmitoyl-CoA	0.29	0.39	145 Aspartate from Glycine	-0.99	0.45
702 Acyl-CoA-VLDL-PC-pool	0.29	0.38	82 Alanine from Glycine	-0.99	0.39
703 Acyl-CoA-VLDL-PE-pool	0.29	0.38	404 (R)-3-Hydroxybutanoate	-0.98	0.52
707 Acyl-CoA-VLDL-TG2-pool	0.29	0.38	405 Acetone	-0.98	0.82
708 Acyl-CoA-VLDL-TG3-pool	0.29	0.38	403 Acetoacetate	-0.98	0.64
704 Acyl-CoA-VLDL-PI-pool	0.29	0.38	91 Alanine from Serine	-0.98	0.43
705 Acyl-CoA-VLDL-PS-pool	0.29	0.38	112 Arginine from Serine	-0.96	0.4
701 Acyl-CoA-CL-pool	0.29	0.38	103 Arginine from Glycine	-0.95	0.4
976 gamma-Linolenoyl-CoA	0.29	0.37	470 GSH	-0.94	0.47
937 Stearoyl-CoA	0.29	0.39	417 Methionine degr	-0.94	0.37
883 Oleoyl-CoA	0.29	0.39	143 Aspartate from Cystine	-0.94	0.39
849 Linoleoyl-CoA	0.29	0.39	427 Homocysteine degr	-0.93	0.39
715 Arachidonyl-CoA	0.29	0.39	866 N-Formimino-L-glutamate	-0.93	0.52
535 (2E)-Hexadecenoyl-CoA	0.29	0.38	124 Asparagine from Glycine	-0.92	0.38
671 5-Methylthioadenosine	0.29	0.45	80 Alanine from Cystine	-0.92	0.47
478 GTP	0.29	0.38	527 Spermidine	-0.91	0.28
621 3-Methylglutaconyl-CoA	0.29	0.36	324 Cholesterol(b)	-0.91	0.79
559 (S)-3-hydroxypalmitoleoyl-CoA	0.29	0.37	66 ATP salvage from Hypoxanthine	-0.91	0.38
984 trans,cis-hexadeca-2,9-dienoyl-CoA	0.29	0.38	279 beta-Alanine from Glycine	-0.9	0.45
986 trans,cis-octadeca-2,9-dienoyl-CoA	0.29	0.39	737 Cholesterol-ester-palm	-0.89	0.69
505 Uracil	0.29	0.39	676 5alpha-Cholesta-7,24-dien-3beta-ol	-0.89	0.82
558 (S)-3-hydroxyoleyleoyl-CoA	0.29	0.39	682 7-Dehydrodesmosterol	-0.89	0.78
474 dGTP	0.29	0.36	288 beta-Alanine from Serine	-0.89	0.44
181 Glutamate from Tryptophan	0.29	0.3	731 Cholate	-0.88	0.6
939 Succinyl-CoA	0.29	0.37	657 4-(2-Amino-3-hydroxyphenyl)-2,4-dioxobutanoate	-0.88	0.46
911 Propanoyl-CoA	0.29	0.33	956 Xanthurenate	-0.88	0.46
556 (S)-3-Hydroxyhexadecanoyl-CoA	0.29	0.31	846 Lathosterol	-0.88	0.8
452 NADP+	0.29	0.37	763 Desmosterol	-0.88	0.79
801 Glutaryl-CoA	0.29	0.38	915 Provitamin D3	-0.88	0.76
871 NADPH	0.29	0.38	684 7alpha,12alpha-Dihydroxycholest-4-en-3-one	-0.88	0.7
451 NAD+	0.29	0.4	636 3-Phosphonooxypyruvate	-0.88	0.61
870 NADH	0.28	0.41	645 3PG	-0.88	0.71
757 Deoxyadenosine	0.28	0.41	333 ApoC3(c)	-0.87	0.95
745 Crotonyl-CoA	0.28	0.41	468 PAPS	-0.87	0.31
554 (S)-3-Hydroxybutyryl-CoA	0.28	0.42	738 Cholesterol-ester-palmn	-0.87	0.73
719 Butyryl-CoA	0.28	0.31	683 7alpha,12alpha-Dihydroxy-5beta-cholestan-3-one	-0.87	0.71
753 Deamido-NAD	0.28	0.45	523 Cholesterol	-0.87	0.77
963 cis-myrist-7-enoyl-CoA	0.28	0.38	739 Cholesterol-ester-pool	-0.86	0.68
644 3-oxopalmitoleoyl-CoA	0.28	0.39	649 3alpha,7alpha,12alpha-Trihydroxy-5beta-cholestan-26-al	-0.86	0.71
758 Deoxycytidine	0.28	0.39	650 3alpha,7alpha,12alpha-Trihydroxy-5beta-cholestanate	-0.86	0.71
960 cis-(3S)-hydroxytetradec-7-enoyl-CoA	0.28	0.38	677 5beta-Cholestane-3alpha,7alpha,12alpha,26-tetrol	-0.86	0.71
985 trans,cis-myristo-2,7-dienoyl-CoA	0.28	0.38	652 3alpha,7alpha,12alpha-Trihydroxycoprostane	-0.86	0.7
764 Dihomo-gamma-linolenoyl-CoA	0.28	0.31	651 3alpha,7alpha,12alpha-Trihydroxy-5beta-cholestanoyl-CoA	-0.86	0.46
450 CoA	0.28	0.38	980 palmitoleoyl-Carnitine	-0.86	0.57
962 cis-laur-5-enoyl-CoA	0.28	0.38	648 3alpha,7alpha,12alpha-Trihydroxy-5beta-cholest-24-enoyl-CoA	-0.86	0.42
642 3-oxomyrist-7-enoyl-CoA	0.28	0.39	848 Linoleate	-0.86	0.59
631 3-Oxopalmitoyl-CoA	0.28	0.3	618 3-Keto-4-methylzymosterol	-0.86	0.81
759 Deoxyguanosine	0.28	0.43	662 4alpha-Methylzymosterol	-0.86	0.82

Continued on next page

Matches relating full time control – continued

Top down, C24h higher Simulation	ampl	score	Bottom up, C1h higher Simulation	ampl	score
773 FAICAR	0.28	0.48	957 Zymosterol	-0.86	0.82
772 FADH2	0.28	0.43	687 7alpha-Hydroxycholesterol	-0.86	0.75
610 3(S)-3-hydroxydodecen-(5Z)-oyl-CoA	0.28	0.38	317 Taurocholate(b)	-0.86	0.48
983 trans,cis-dodeca-2,5-dienoyl-CoA	0.28	0.38	736 Cholesterol-ester-ol	-0.86	0.69
792 GMP	0.28	0.42	740 Cholesterol-ester-stea	-0.86	0.69
555 (S)-3-Hydroxydodecanoyl-CoA	0.28	0.36	646 3alpha,7alpha,12alpha,24-Tetrahydroxy-5beta-cholestanoyl-CoA	-0.85	0.42
533 (2E)-Dodecenoyl-CoA	0.28	0.36	607 2-Oxobutyrate	-0.85	0.66
641 3-oxolaur-cis-5-enoyl-CoA	0.28	0.38	647 3alpha,7alpha,12alpha-Trihydroxy-5beta-24-oxocholestanoyl-CoA	-0.85	0.42
543 (3Z)-Dodecenoyl-CoA	0.28	0.38	315 Glycocholate(b)	-0.85	0.51
620 3-Methylcrotonyl-CoA	0.28	0.36	686 7alpha-Hydroxycholest-4-en-3-one	-0.85	0.74
858 Myristoyl-CoA	0.28	0.31	483 Aspartate	-0.85	0.35
553 (S)-3-Hydroxy-2-methylbutyryl-CoA	0.27	0.34	685 7alpha-Hydroxy-5beta-cholestan-3-one	-0.84	0.75
524 gdpddman	0.27	0.42	732 Cholestenol	-0.84	0.8
673 5-Phosphoribosyl-4-carboxy-5-aminoimidazole	0.27	0.38	318 tcdchola(b)	-0.84	0.48
857 Methylmalonyl-CoA	0.27	0.32	735 Cholesterol-ester-lin	-0.84	0.7
561 (S)-Hydroxydecanoyl-CoA	0.27	0.34	189 Glycine from Cysteine	-0.84	0.38
531 (2E)-Decenoyl-CoA	0.27	0.35	678 5beta-Cholestane-3alpha,7alpha,26-triol	-0.84	0.75
476 ATP	0.27	0.38	734 Cholesterol-ester-gla	-0.84	0.69
510 Adenosine	0.27	0.36	679 5beta-Cholestane-3alpha,7alpha-diol	-0.84	0.73
755 Decanoyl-CoA	0.27	0.37	733 Cholesterol-ester-arach	-0.84	0.7
625 3-Oxododecanoyl-CoA	0.27	0.37	316 Gly-CD-cholate(b)	-0.83	0.51
560 (S)-Dihydroorotate	0.27	0.3	841 L-Lactate	-0.83	0.87
690 AICAR	0.27	0.45	654 3alpha,7alpha-Dihydroxy-5beta-cholestanate	-0.83	0.7
882 Octanoyl-CoA	0.27	0.35	653 3alpha,7alpha-Dihydroxy-5beta-cholestan-26-al	-0.82	0.74
623 3-Oxodecanoyl-CoA	0.27	0.35	656 4,4-Dimethyl-5alpha-cholesta-8,14,24-trien-3beta-ol	-0.82	0.74
472 dATP	0.27	0.37	637 3-Phosphoserine	-0.82	0.59
819 Hexanoyl-CoA	0.26	0.34	741 Choloyl-CoA	-0.82	0.45
344 Collagen CO1A1(c) synthesis	0.26	1	655 3alpha,7alpha-Dihydroxy-5beta-cholestanoyl-CoA	-0.82	0.46
502 GDP-mannose	0.26	0.39	663 4alpha-Methylzymosterol-4-carboxylate	-0.82	0.81
955 Xanthosine	0.26	0.39	402 HDL	-0.82	0.79
923 SAICAR	0.26	0.35	254 Serine from Cysteine	-0.82	0.45
828 Inosine	0.26	0.45	482 Asparagine	-0.82	0.34
497 UDP-xylose	0.26	0.31	329 ApoA1	-0.82	0.96
563 (S)-Hydroxyoctanoyl-CoA	0.26	0.33	491 Glycerol	-0.82	0.53
453 FAD	0.26	0.48	107 Arginine from Leucine	-0.82	0.34
540 (2E)-Octenoyl-CoA	0.26	0.32	879 O-Propanoylcarnitine	-0.81	0.53
635 3-Oxotetradecanoyl-CoA	0.26	0.28	125 Asparagine from Histidine	-0.81	0.32
562 (S)-Hydroxyhexanoyl-CoA	0.26	0.3	319 Chenodiol(b)	-0.81	0.54
537 (2E)-Hexenoyl-CoA	0.25	0.32	109 Arginine from Methionine	-0.8	0.42
630 3-Oxooctanoyl-CoA	0.25	0.34	415 Leucine degr	-0.8	0.33
504 XMP	0.25	0.37	552 (R)-Mevalonate	-0.8	0.82
503 IMP	0.25	0.39	190 Glycine from Cystine	-0.8	0.41
15 Dephosphorylation of ATP	0.24	0.51	730 Chenodeoxycholoyl-CoA	-0.8	0.45
827 Hypoxanthine	0.23	0.48	496 Fructose-6P	-0.79	1
809 Guanosine	0.23	0.4	593 14-Demethyllanosterol	-0.79	0.75
508 Xanthine	0.23	0.36	25 NADH redox potential into peroxy	-0.79	0.74
509 Guanine	0.23	0.35	83 Alanine from Histidine	-0.79	0.33
952 Urate	0.23	0.38	255 Serine from Cystine	-0.79	0.46
810 H2O2	0.23	0.38	785 Fructose-1,6PP	-0.79	1
338 Haptoglobin	0.22	0.97	584 1-Acylglycerol-3P-palmn	-0.79	0.61
760 Deoxyinosine	0.21	0.39	756 Dehydroalanine	-0.78	0.7
930 Sphinganine	0.18	0.32	186 Glycine from Arginine	-0.78	0.29
931 Sphinganine-1P	0.18	0.28	778 Fatty-acid-VLDL-PS-pool	-0.78	0.48
31 NADH to NADPH transhydrogenase in mito	0.17	1	777 Fatty-acid-VLDL-PI-pool	-0.78	0.48
200 Glycine from Serine	0.16	0.52	776 Fatty-acid-VLDL-PE-pool	-0.78	0.48
44 Activated methyl group (THF)	0.16	0.52	780 Fatty-acid-VLDL-TG1-pool	-0.78	0.49
47 Formylgroup(c)	0.16	0.42	781 Fatty-acid-VLDL-TG2-pool	-0.78	0.49

Continued on next page

Matches relating full time control – continued

Top down, C24h higher Simulation	ampl	score	Bottom up, C1h higher Simulation	ampl	score
916 Pyridoxal	0.16	0.68	431 HDL degr	-0.77	0.41
672 5-Oxoproline	0.16	0.55	151 Aspartate from Methionine	-0.77	0.47
854 Maltose	0.15	1	251 Serine from Arginine	-0.77	0.3
495 Mannose-6P	0.15	0.78	920 Ribose-5P	-0.77	0.38
26 NADPH redox potential into mito	0.15	0.69	557 (S)-3-Hydroxytetradecanoyl-CoA	-0.77	0.28
877 O-Acetylcarnitine	0.15	1	751 D-Xylulose-5P	-0.77	0.42
59 GDP-activated mannose	0.15	0.58	146 Aspartate from Histidine	-0.76	0.37
947 Triphosphate degr	0.15	1	946 Trehalose	-0.76	1
887 Oxidized thioredoxin	0.13	0.93	154 Aspartate from Serine	-0.76	0.4
455 Thioredoxin	0.13	0.94	752 DHAP	-0.75	0.99
843 L-Oleoylcarnitine	0.13	0.54	789 GAP	-0.75	0.99
746 Cys-Gly	0.13	1	748 D-3-Amino-isobutanoate	-0.75	0.34
832 Isocitrate	0.12	0.41	213 Glutamine from Glycine	-0.75	0.37
27 NADPH redox potential into peroxy	0.12	1	471 Homocysteine	-0.75	0.49
906 Phosphodimethylethanolamine	0.11	1	768 Dimethylallyl-PP	-0.74	0.6
905 Phosphocholine	0.11	1	550 (R)-5-Phosphomevalonate	-0.74	0.64
41 Na+ importgradient	0.11	0.52	9 Anaerobic rephosph of CTP	-0.74	0.43
548 (R)-4-Phosphopantothenoyl-cysteine	0.11	0.45	8 Anaerobic rephosph of GTP	-0.74	0.43
359 Collagen CO6A3(c) synthesis	0.11	1	10 Anaerobic rephosph of UTP	-0.74	0.43
375 Collagen COGA1(c) synthesis	0.08	1	3 Anaerobic rephosph of ATP	-0.74	0.45
396 Collagen LPP3(c) synthesis	0.07	1	304 Arachidonate from Linoleate	-0.73	0.48
373 Collagen COEA1(c) synthesis	0.06	1	170 Glutamate from Glycine	-0.73	0.43
918 Quinolinate	0.05	0.38	512 N-acglucam	-0.73	0.44
339 Collagen ADIPO(c) synthesis	0.04	1	88 Alanine from Methionine	-0.73	0.47
378 Collagen COJA1(c) synthesis	0.04	1	197 Glycine from Methionine	-0.73	0.45
369 Collagen COBA1(c) synthesis	0.03	1	263 Serine from Methionine	-0.73	0.48
53 activated sulphur	0.03	0.65	133 Asparagine from Serine	-0.72	0.37
400 Collagen VWA2(c) synthesis	0.02	1	862 N-Acetylmannosamine-6P	-0.72	0.55
712 Agmatine	0.01	0.68	192 Glycine from Histidine	-0.72	0.36
354 Collagen CO5A1(c) synthesis	0.01	1	864 N-Acetylneuraminate-9P	-0.72	0.39
388 Collagen CTHR1(c) synthesis	0.01	1	863 N-Acetylneuraminate	-0.72	0.35
14 Ubiquinol-to-ATP	0.002	0.54	285 beta-Alanine from Methionine	-0.72	0.43
11 ATP from NADH(m)	0.002	0.48	130 Asparagine from Methionine	-0.72	0.46
365 Collagen CO9A1(c) synthesis	-0.0005	1	665 5,6-Dihydrouracil	-0.72	0.39
822 Homovanillate	-0.01	1	823 Hydracrylate	-0.72	0.58
382 Collagen COMA1(c) synthesis	-0.02	1	258 Serine from Histidine	-0.72	0.38
787 Fumarate	-0.03	0.7	113 Arginine from Threonine	-0.72	0.41
716 Argininosuccinate	-0.03	0.39	982 sn-Glycerol-3P	-0.72	0.71
608 2-Oxoglutaramate	-0.03	1	78 Alanine from Aspartate	-0.71	0.58
340 Collagen BGH3(c) synthesis	-0.03	1	549 (R)-5-Diphosphomevalonate	-0.71	0.71
376 Collagen COHA1(c) synthesis	-0.03	1	139 Aspartate from Alanine	-0.71	0.49
70 Formate degr	-0.03	0.56	46 Activated methyl group from Histidine	-0.71	0.3
850 Malate	-0.04	0.64	161 Cysteine from Methionine	-0.71	0.46
393 Collagen FMOD(c) synthesis	-0.04	1	176 Glutamate from Methionine	-0.7	0.48
395 Collagen ITA2(c) synthesis	-0.04	1	421 Threonine degr	-0.7	0.39
358 Collagen CO6A2(c) synthesis	-0.05	1	77 Alanine from Asparagine	-0.7	0.47
42 Na+ exportgradient	-0.06	1	86 Alanine from Leucine	-0.7	0.39
880 OAA	-0.06	0.61	446 ApoB100 degr	-0.7	0.6
940 Sulfite degr	-0.07	1	294 Taurine from Methionine	-0.7	0.46
356 Collagen CO5A3(c) synthesis	-0.07	1	921 Ribulose-5P	-0.7	0.35
153 Aspartate from Proline	-0.08	0.29	188 Glycine from Aspartate	-0.7	0.45
81 Alanine from Glutamate	-0.08	0.37	195 Glycine from Leucine	-0.7	0.4
119 Asparagine from Arginine	-0.08	0.32	571 1,3DPG	-0.69	0.81
688 ACP	-0.08	0.88	430 LDL degr	-0.69	0.4
697 Acetyl-ACP	-0.08	0.88	253 Serine from Aspartate	-0.69	0.52
457 Apo-ACP	-0.08	0.92	218 Glutamine from Methionine	-0.69	0.47
458 Apo-ACP(m)	-0.08	0.72	187 Glycine from Asparagine	-0.69	0.42
978 mitoACP	-0.08	0.71	261 Serine from Leucine	-0.69	0.41
851 Malonyl-ACP	-0.08	0.87	2 Aerobic ATP rephosph (gluc)	-0.69	0.3

Continued on next page

Matches relating full time control – continued

Top down, C24h higher			Bottom up, C1h higher		
Simulation	ampl	score	Simulation	ampl	score
695 Acetoacetyl-ACP	-0.08	0.87	252 Serine from Asparagine	-0.69	0.48
717 But-2-enoyl-ACP	-0.08	0.87	943 Thymidine	-0.68	0.35
544 (R)-3-Hydroxybutanoyl-ACP	-0.08	0.87	163 Cystine from Methionine	-0.68	0.49
718 Butyryl-ACP	-0.08	0.87	62 Isopentenyl-PP	-0.68	0.69
818 Hexanoyl-ACP	-0.08	0.86	185 Glycine from Alanine	-0.68	0.47
536 (2E)-Hexenoyl-ACP	-0.08	0.86	485 Glycine	-0.67	0.38
750 D-3-Hydroxyhexanoyl-ACP	-0.08	0.86	837 L-Cystathionine	-0.67	0.42
627 3-Oxohexanoyl-ACP	-0.08	0.86	886 Oxalosuccinate	-0.67	0.37
629 3-Oxooctanoyl-ACP	-0.08	0.85	306 Arachidonate from gamma-Linolenate	-0.67	0.46
546 (R)-3-Hydroxyoctanoyl-ACP	-0.08	0.85	303 Dihomo-gamma-linolenate from Linoleate	-0.67	0.46
539 (2E)-Octenoyl-ACP	-0.08	0.85	227 Methionine from Cysteine	-0.67	0.52
881 Octanoyl-ACP	-0.08	0.85	640 3-Ureidopropionate	-0.67	0.44
622 3-Oxodecanoyl-ACP	-0.08	0.85	488 Serine	-0.67	0.45
545 (R)-3-Hydroxydecanoyl-ACP	-0.08	0.85	589 1-Acylglycerol-VLDL-PS-pool	-0.67	0.47
530 (2E)-Decenoyl-ACP	-0.08	0.85	928 Sedoheptulose-7P	-0.67	0.44
754 Decanoyl-ACP	-0.08	0.85	588 1-Acylglycerol-VLDL-PI-pool	-0.67	0.47
749 D-3-Hydroxydodecanoyl-ACP	-0.08	0.84	795 Geranyl-PP	-0.67	0.61
624 3-Oxododecanoyl-ACP	-0.08	0.85	590 1-Acylglycerol-VLDL-SM-pool	-0.67	0.46
532 (2E)-Dodecenoyl-ACP	-0.08	0.84	587 1-Acylglycerol-VLDL-PE-pool	-0.67	0.47
769 Dodecanoyl-ACP	-0.08	0.84	250 Serine from Alanine	-0.67	0.51
813 HMA	-0.08	0.84	586 1-Acylglycerol-VLDL-PC-pool	-0.67	0.47
634 3-Oxotetradecanoyl-ACP	-0.08	0.84	591 1-Acylglycerol-VLDL-TG1-pool	-0.67	0.47
942 Tetradecanoyl-ACP	-0.08	0.84	155 Aspartate from Threonine	-0.66	0.51
541 (2E)-Tetradecenoyl-ACP	-0.08	0.84	191 Glycine from Glutamate	-0.66	0.3
816 Hexadecanoyl-ACP	-0.08	0.84	228 Methionine from Cystine	-0.66	0.55
626 3-Oxohexadecanoyl-ACP	-0.08	0.84	855 Mercaptopyruvate	-0.66	0.4
547 (R)-3-Hydroxypalmitoyl-ACP	-0.08	0.84	201 Glycine from Threonine	-0.66	0.47
534 (2E)-Hexadecenoyl-ACP	-0.08	0.84	407 Alanine degr	-0.66	0.33
633 3-Oxostearoyl-ACP	-0.08	0.83	60 Pyruvate	-0.66	0.49
617 3-Hydroxystearoyl-ACP	-0.08	0.83	63 Farnesyl-PP	-0.66	0.62
936 Stearoyl-ACP	-0.08	0.83	256 Serine from Glutamate	-0.66	0.33
538 (2E)-Octadecenoyl-ACP	-0.08	0.83	266 Serine from Threonine	-0.65	0.53
357 Collagen CO6A1(c) synthesis	-0.08	1	910 Propanoate	-0.65	0.48
370 Collagen COBA2(c) synthesis	-0.08	1	724 CMP-NeuNGc	-0.65	0.29
212 Glutamine from Glutamate	-0.08	0.33	92 Alanine from Threonine	-0.65	0.55
311 Urea from glutamine	-0.08	0.26	205 Glycine from beta-Alanine	-0.65	0.35
76 Alanine from Arginine	-0.08	0.33	919 Ribose-1P	-0.65	0.5
207 Glutamine from Arginine	-0.08	0.3	242 Proline from Methionine	-0.65	0.42
235 Proline from Glutamate	-0.08	0.46	194 Glycine from Glutamine	-0.65	0.28
129 Asparagine from Lysine	-0.08	0.29	661 4-Methyl-2-oxopentanoate	-0.65	0.35
812 HCO3-	-0.09	0.71	270 Serine from beta-Alanine	-0.65	0.4
727 Carbonate	-0.09	0.71	196 Glycine from Lysine	-0.64	0.33
368 Collagen COAA1(c) synthesis	-0.09	1	5 Aerobic rephosph of CTP	-0.64	0.32
884 Orotate	-0.09	0.38	262 Serine from Lysine	-0.64	0.36
37 GSH reduction using NADPH redox potential	-0.09	1	216 Glutamine from Leucine	-0.64	0.36
38 GSH oxidation	-0.09	1	313 Urea from NH3	-0.64	0.32
726 Carbamoyl-P	-0.09	0.5	312 Urea from alanine	-0.64	0.32
341 Collagen C43BP(c) synthesis	-0.1	1	221 Glutamine from Serine	-0.63	0.4
325 Albumin	-0.11	0.98	289 beta-Alanine from Threonine	-0.63	0.46
120 Asparagine from Aspartate	-0.11	0.44	972 dUDP	-0.63	0.26
743 Citrulline	-0.11	0.36	199 Glycine from Proline	-0.63	0.31
371 Collagen COCA1(c) synthesis	-0.11	1	606 2-Oxoadipate	-0.63	0.33
494 Mannose-1P	-0.12	0.45	770 Erythrose-4P	-0.63	0.54
728 Carnosine	-0.12	0.87	87 Alanine from Lysine	-0.62	0.32
384 Collagen COOA1(c) synthesis	-0.12	1	845 Lanosterol	-0.62	0.84
326 Fibrinogen	-0.12	0.49	265 Serine from Proline	-0.62	0.32
351 Collagen CO4A4(c) synthesis	-0.12	1	944 Thymine	-0.61	0.33
278 beta-Alanine from Glutamate	-0.12	0.47	767 Dihydrothymine	-0.61	0.34
328 Antitrypsin	-0.12	0.49	222 Glutamine from Threonine	-0.61	0.51

Continued on next page

Matches relating full time control – continued

Top down, C24h higher Simulation	ampl	score	Bottom up, C1h higher Simulation	ampl	score
381 Collagen COLQ(c) synthesis	-0.12	0.44	572 1-Acylglycerol-3P-Bile-PC-pool	-0.61	0.52
387 Collagen COSA1(c) synthesis	-0.12	0.44	564 1,2-Diacylglycerol-Bile-PC-pool	-0.6	0.38
380 Collagen COLA1(c) synthesis	-0.12	0.45	583 1-Acylglycerol-3P-palm	-0.6	0.52
390 Collagen FCN1(c) synthesis	-0.12	0.46	897 Phosphatidate-Bile-PC-pool	-0.59	0.43
392 Collagen FCN3(c) synthesis	-0.13	0.47	580 1-Acylglycerol-3P-arach	-0.59	0.53
360 Collagen CO6A5(c) synthesis	-0.13	0.47	581 1-Acylglycerol-3P-lin	-0.59	0.53
397 Collagen PCOTH(c) synthesis	-0.13	0.47	582 1-Acylglycerol-3P-ol	-0.59	0.53
361 Collagen CO6A6(c) synthesis	-0.13	0.46	585 1-Acylglycerol-3P-stea	-0.59	0.53
394 Collagen ITA1(c) synthesis	-0.13	0.49	134 Asparagine from Threonine	-0.59	0.49
273 beta-Alanine from Arginine	-0.13	0.4	180 Glutamate from Threonine	-0.58	0.53
367 Collagen CO9A3(c) synthesis	-0.13	1	639 3-Ureidoisobutyrate	-0.58	0.39
342 Collagen CCBE1(c) synthesis	-0.13	1	577 1-Acylglycerol-3P-VLDL-PS-pool	-0.58	0.53
290 beta-Alanine from Tryptophan	-0.14	0.32	927 Sedoheptulose-1,7PP	-0.58	0.52
209 Glutamine from Aspartate	-0.14	0.36	208 Glutamine from Asparagine	-0.58	0.43
272 beta-Alanine from Alanine	-0.14	0.53	576 1-Acylglycerol-3P-VLDL-PI-pool	-0.58	0.52
287 beta-Alanine from Proline	-0.14	0.39	578 1-Acylglycerol-3P-VLDL-SM-pool	-0.58	0.52
282 beta-Alanine from Glutamine	-0.14	0.45	579 1-Acylglycerol-3P-VLDL-TG1-pool	-0.58	0.52
274 beta-Alanine from Asparagine	-0.14	0.49	521 Ceramide	-0.58	0.37
276 beta-Alanine from Cysteine	-0.15	0.51	575 1-Acylglycerol-3P-VLDL-PE-pool	-0.58	0.52
275 beta-Alanine from Aspartate	-0.15	0.95	574 1-Acylglycerol-3P-VLDL-PC-pool	-0.58	0.52
907 Phosphopantetheine	-0.15	0.3	61 AKG	-0.57	0.44
64 Glucosamine-6P	-0.15	0.77	179 Glutamate from Serine	-0.57	0.45
774 FMN	-0.16	1	786 Fructose-2,6PP	-0.57	0.94
158 Aspartate from Valine	-0.16	0.41	568 1,2-Diacylglycerol-VLDL-PS-pool	-0.57	0.38
383 Collagen CONA1(c) synthesis	-0.16	1	567 1,2-Diacylglycerol-VLDL-PI-pool	-0.56	0.39
95 Alanine from Valine	-0.16	0.35	569 1,2-Diacylglycerol-VLDL-SM-pool	-0.56	0.38
364 Collagen CO8A2(c) synthesis	-0.16	1	566 1,2-Diacylglycerol-VLDL-PE-pool	-0.56	0.39
225 Glutamine from Valine	-0.16	0.37	902 Phosphatidate-VLDL-PS-pool	-0.56	0.44
292 beta-Alanine from Valine	-0.17	0.45	245 Proline from Threonine	-0.56	0.47
869 N-Pantothenoylcysteine	-0.17	0.45	761 Deoxyuridine	-0.55	0.36
499 udpgal	-0.17	0.3	500 udpglcur	-0.55	0.31
876 Nicotinate(r)ibonucleotide	-0.17	0.33	565 1,2-Diacylglycerol-VLDL-PC-pool	-0.55	0.39
461 4ppan	-0.17	1	570 1,2-Diacylglycerol-VLDL-TG-pool	-0.55	0.38
765 Dihydroceramide-pool	-0.17	0.4	901 Phosphatidate-VLDL-PI-pool	-0.55	0.44
873 Nicotinamide D-ribonucleotide	-0.17	0.35	903 Phosphatidate-VLDL-SM-pool	-0.55	0.43
951 UMP	-0.17	0.38	973 dUMP	-0.55	0.36
12 ATP from NADH	-0.18	0.34	900 Phosphatidate-VLDL-PE-pool	-0.55	0.44
423 Valine degr	-0.18	0.33	118 Asparagine from Alanine	-0.55	0.38
611 3-Dehydrosphinganine	-0.18	0.38	899 Phosphatidate-VLDL-PC-pool	-0.55	0.43
379 Collagen COKA1(c) synthesis	-0.18	1	904 Phosphatidate-VLDL-TG-pool	-0.55	0.43
389 Collagen EMID2(c) synthesis	-0.18	1	935 Squalene 2,3-oxide	-0.55	0.83
116 Arginine from Valine	-0.18	0.33	332 ApoC2(c)	-0.54	0.95
859 N-(omega)-Hydroxyarginine	-0.18	0.77	820 Histamine	-0.54	0.9
460 Pyridoxal-P	-0.19	0.44	166 Glutamate from Asparagine	-0.53	0.44
385 Collagen COPA1(c) synthesis	-0.19	1	346 Collagen CO2A1(c) synthesis	-0.52	1
917 Pyridoxine-P	-0.19	1	448 Ethanol degr	-0.52	0.64
363 Collagen CO8A1(c) synthesis	-0.19	1	800 Glutamyl-5P	-0.52	0.37
137 Asparagine from Valine	-0.19	0.42	840 L-Glutamate 5-semialdehyde	-0.52	0.49
183 Glutamate from Valine	-0.19	0.48	309 VLDL from HDL	-0.52	0.81
614 3-Hydroxyisobutyrate	-0.19	0.29	948 UDP-N-acetylglucosamine	-0.52	0.34
277 beta-Alanine from Cystine	-0.2	0.4	330 ApoB100(r)	-0.52	0.99
605 2-Oxo-3-methylvalerate	-0.2	0.54	401 VLDL	-0.51	0.81
799 Glucuronate	-0.2	0.41	511 UDP-N-acetylgalactosamine	-0.51	0.34
353 Collagen CO4A6(c) synthesis	-0.2	1	950 UDP-activated-xylose	-0.51	0.41
260 Serine from Glutamine	-0.2	0.31	193 Glycine from Isoleucine	-0.51	0.39
366 Collagen CO9A2(c) synthesis	-0.21	1	970 dTDP	-0.51	0.32
891 PG-CL-pool	-0.21	0.34	971 dTMP	-0.51	0.35
892 PGP-CL-pool	-0.21	0.34	259 Serine from Isoleucine	-0.5	0.43
872 NeuNGc	-0.21	0.34	308 VLDL from LDL	-0.5	0.81

Continued on next page

Matches relating full time control – continued

Top down, C24h higher			Bottom up, C1h higher		
Simulation	ampl	score	Simulation	ampl	score
782 Fatty-acid-VLDL-TG3-pool	-0.22	0.53	844 L-Palmitoylcarnitine	-0.5	0.48
898 Phosphatidate-CL-pool	-0.22	0.43	465 acgam6p	-0.5	0.64
793 GSSG	-0.22	0.48	217 Glutamine from Lysine	-0.49	0.31
269 Serine from Valine	-0.22	0.38	714 Arachidonyl-Carnitine	-0.49	0.51
573 1-Acylglycerol-3P-CL-pool	-0.22	0.46	977 linoleic-Carnitine	-0.49	0.51
669 5-Hydroxy-L-tryptophan	-0.22	0.73	336 Prothrombin	-0.49	0.98
601 2-Deoxy-D-ribose-5P	-0.23	0.55	912 Protein(l)ysine	-0.49	1
362 Collagen CO7A1(c) synthesis	-0.23	1	164 Glutamate from Alanine	-0.49	0.49
69 Formaldehyde degr	-0.23	0.47	97 Arginine from Alanine	-0.48	0.36
204 Glycine from Valine	-0.23	0.35	442 Haptoglobin degr	-0.48	0.67
372 Collagen CODA1(c) synthesis	-0.23	1	797 Glucose-1P	-0.48	1
517 PI	-0.23	0.29	445 ApoTransferin degr	-0.48	0.63
518 CL	-0.24	0.4	437 Albumin degr	-0.48	0.62
374 Collagen COFA1(c) synthesis	-0.24	1	441 Fibrinogen degr	-0.48	0.61
522 Triacylglycerol	-0.24	0.51	443 Plasminogen degr	-0.48	0.6
860 N-Acetyl-D-mannosamine	-0.24	0.53	447 Glycogenin degr	-0.48	0.62
36 GSH reduction using NADH redox potential	-0.24	0.73	440 ApoA1 degr	-0.48	0.59
725 CMP-activated-N-acetylneuraminate	-0.24	0.46	444 Prothrombin degr	-0.48	0.57
513 CMP-N-acetylneuraminate	-0.24	0.46	438 Antichymotrypsin degr	-0.47	0.6
334 ApoE(c)	-0.24	0.97	56 UDP-activated glucuronate	-0.47	0.4
680 6-Phospho-D-gluconate	-0.25	0.83	439 Antitrypsin degr	-0.47	0.62
226 Glutamine from beta-Alanine	-0.25	0.42	150 Aspartate from Lysine	-0.47	0.37
301 Palmitolate from Arachidonate	-0.25	0.65	852 Malonyl-Carnitin	-0.47	0.62
138 Asparagine from beta-Alanine	-0.26	0.47	909 Presqualene-PP	-0.47	0.79
32 Thioredoxin(m) reduction	-0.26	1	934 Squalene	-0.47	0.79
33 Thioredoxin(m) oxidation	-0.26	1	84 Alanine from Isoleucine	-0.47	0.41
693 Acetaldehyde	-0.26	0.6	520 SM	-0.46	0.36
23 Oxidation of NADPH	-0.26	0.4	51 Acetyl group(p)	-0.46	0.34
492 Oleate	-0.26	0.74	175 Glutamate from Lysine	-0.46	0.32
742 Citrate	-0.26	0.59	305 Dihomo-gamma-linolenate from gamma-Linolenate	-0.46	0.47
490 Palmitate	-0.26	0.8	206 Glutamine from Alanine	-0.46	0.47
493 Stearate	-0.26	0.81	49 Acetyl group(c)	-0.45	0.43
121 Asparagine from Cysteine	-0.27	0.39	52 Acetyl group(r)	-0.45	0.43
162 Cystine from Cysteine	-0.27	0.87	231 Proline from Asparagine	-0.45	0.37
160 Cysteine from Cystine	-0.27	0.87	280 beta-Alanine from Histidine	-0.45	0.4
350 Collagen CO4A3(c) synthesis	-0.27	1	391 Collagen FCN2(c) synthesis	-0.44	1
868 N-Methylethanolamine-P	-0.27	1	941 THF-hexaglutamate	-0.43	0.98
771 Ethanolamine-P	-0.27	1	377 Collagen COIA1(c) synthesis	-0.43	1
283 beta-Alanine from Leucine	-0.27	0.44	310 Bilirubin conjugation	-0.43	0.54
148 Aspartate from Glutamine	-0.27	0.37	167 Glutamate from Aspartate	-0.42	0.48
122 Asparagine from Cystine	-0.28	0.49	847 Lauroyl-CoA	-0.42	0.3
19 Aerobic reduction of NADP+ (gluc)	-0.28	0.31	348 Collagen CO4A1(c) synthesis	-0.42	1
35 Thioredoxin(c) oxidation	-0.28	0.99	398 Collagen SCRB1(c) synthesis	-0.41	1
159 Aspartate from beta-Alanine	-0.28	0.51	519 LacCer	-0.41	0.34
127 Asparagine from Glutamine	-0.28	0.39	542 (2E)-Tetradecenoyl-CoA	-0.41	0.31
414 Glutamine degr	-0.28	0.27	284 beta-Alanine from Lysine	-0.41	0.37
132 Asparagine from Proline	-0.28	0.29	18 Aerobic reduction of NADP+ (FA)	-0.41	0.36
128 Asparagine from Leucine	-0.29	0.4	525 beta-Alanine	-0.4	0.34
796 Glucono-1,5-lactone-6P	-0.29	1	425 Isoleucine degr	-0.4	0.34
28 NADPH to NADH transhydrogenase	-0.29	1	794 Galactose-1P	-0.39	0.48
117 Arginine from beta-Alanine	-0.29	0.33	244 Proline from Serine	-0.39	0.38
123 Asparagine from Glutamate	-0.29	0.34	861 N-Acetylglucosamine-1P	-0.39	0.65
248 Proline from Valine	-0.29	0.38	16 Aerobic reduction of NAD+ (FA)	-0.39	0.37
184 Glutamate from beta-Alanine	-0.29	0.49	238 Proline from Isoleucine	-0.39	0.43
602 2-Lysolecithin-pool	-0.29	0.38	229 Proline from Alanine	-0.38	0.46
487 Proline	-0.29	0.39	214 Glutamine from Histidine	-0.38	0.38
22 Oxidation of NADH	-0.3	0.45	105 Arginine from Isoleucine	-0.38	0.39
6 Aerobic rephosph of UDP	-0.3	0.37	281 beta-Alanine from Isoleucine	-0.38	0.42
949 UDP-activated-N-acetyl-D-galactosamine	-0.3	0.62	171 Glutamate from Histidine	-0.37	0.47

Continued on next page

Matches relating full time control – continued

Top down, C24h higher Simulation	ampl	score	Bottom up, C1h higher Simulation	ampl	score
149 Aspartate from Leucine	-0.3	0.4	240 Proline from Leucine	-0.37	0.38
514 PC	-0.31	0.4	147 Aspartate from Isoleucine	-0.37	0.49
144 Aspartate from Glutamate	-0.31	0.34	889 PE-PS-VLDL-pool	-0.37	0.43
34 Thioredoxin(c) reduction	-0.31	0.98	215 Glutamine from Isoleucine	-0.37	0.43
249 Proline from beta-Alanine	-0.31	0.42	232 Proline from Aspartate	-0.37	0.4
600 2-Deoxy-D-ribose-1P	-0.31	0.59	432 Oleate degr	-0.37	0.42
320 Bile-PC(b)	-0.31	0.47	433 Stearate degr	-0.36	0.43
173 Glutamate from Glutamine	-0.32	0.43	20 Aerobic reduction of FAD (gluc)	-0.36	0.36
68 O2.-(c) degr	-0.32	0.74	516 PS	-0.36	0.4
467 1fthf	-0.32	0.59	322 PS(b)	-0.36	0.4
211 Glutamine from Cystine	-0.32	0.45	484 Glutamate	-0.36	0.51
466 THF(m)	-0.33	1	486 Glutamine	-0.36	0.45
459 THF	-0.33	1	50 Acetyl group(m)	-0.36	0.47
29 NADH to NADPH transhydrogenase	-0.33	1	178 Glutamate from Proline	-0.36	0.37
766 Dihydrofolate	-0.33	1	515 PE	-0.36	0.47
670 5-Methyl-THF	-0.33	1	7 Aerobic rephosph of UTP	-0.36	0.31
464 mlthf	-0.33	0.78	4 Aerobic rephosph of GTP	-0.36	0.31
664 5,10-Methenyl-THF	-0.33	0.64	1 Aerobic ATP rephosph (FA)	-0.36	0.33
210 Glutamine from Cysteine	-0.33	0.33	885 Orotidine-5P	-0.36	0.33
169 Glutamate from Cystine	-0.33	0.5	126 Asparagine from Isoleucine	-0.36	0.44
39 Proton-gradient(m) build up	-0.34	0.41	239 Proline from Glutamine	-0.36	0.32
40 Proton-gradient(c) build up	-0.34	0.41	233 Proline from Cysteine	-0.36	0.35
234 Proline from Cystine	-0.34	0.42	667 5-Formiminotetrahydrofolate	-0.35	0.75
817 Hexadecenal	-0.34	0.32	172 Glutamate from Isoleucine	-0.35	0.46
815 Hexadecanal	-0.34	0.32	174 Glutamate from Leucine	-0.35	0.41
230 Proline from Arginine	-0.34	0.33	165 Glutamate from Arginine	-0.35	0.31
45 Activated methylene group from Try	-0.34	0.26	321 SM(b)	-0.35	0.4
21 Aerobic reduction of FAD (FA)	-0.34	0.36	337 ApoTransferin	-0.35	0.98
878 O-Butanoylcarnitine	-0.34	0.42	168 Glutamate from Cysteine	-0.35	0.44
323 PE(b)	-0.34	0.52	961 cis-Aconitate	-0.35	0.59
237 Proline from Histidine	-0.35	0.42			

1.3 Matches relating full time TGFβ

Table 3: Matches relating full time TGFβ

Top down, T24h higher Simulation	ampl	score	Bottom up, T1h higher Simulation	ampl	score
386 Collagen CORA1(c) synthesis	3.03	1	410 Aspartate degr	-5.14	0.34
374 Collagen COFA1(c) synthesis	2	1	409 Asparagine degr	-5.11	0.4
830 Inositol-1P	1.85	1	428 beta-Alanine degr	-5.11	0.33
829 Inositol	1.83	0.59	54 Glucose-6P	-4.62	0.51
344 Collagen CO1A1(c) synthesis	1.41	1	411 Cysteine degr	-4.58	0.38
619 3-Methyl-2-oxobutyrate	1.29	0.35	821 Homogentisate	-4.47	0.74
257 Serine from Glycine	1.28	0.4	638 3-Sulfinoalanine	-4.43	0.72
349 Collagen CO4A2(c) synthesis	1.24	1	426 Tyrosine degr	-4.41	0.45
931 Sphinganine-1P	1.21	0.38	418 Phenylalanine degr	-4.38	0.47
817 Hexadecenal	1.11	0.29	658 4-Hydroxyphenylpyruvate	-4.36	0.63
815 Hexadecanal	1.11	0.29	489 Tyrosine	-4.27	0.65
300 Stearate from Oleate	1.1	0.35	660 4-Maleylacetoacetate	-4.27	0.79
297 Palmitate from Palmitolate	1.09	0.34	152 Aspartate from Phenylalanine	-4.15	0.52
355 Collagen CO5A2(c) synthesis	1.09	1	788 Fumarylacetoacetate	-4.13	0.72
399 Collagen SERPH(c) synthesis	0.83	1	286 beta-Alanine from Phenylalanine	-4.13	0.47
347 Collagen CO3A1(c) synthesis	0.78	1	110 Arginine from Phenylalanine	-4.12	0.42
436 Mannose degr	0.76	0.64	131 Asparagine from Phenylalanine	-4.11	0.43
352 Collagen CO4A5(c) synthesis	0.68	1	157 Aspartate from Tyrosine	-4.1	0.49
341 Collagen C43BP(c) synthesis	0.58	1	219 Glutamine from Phenylalanine	-4.09	0.41
67 dTTP salvage from Thymine	0.57	0.32	291 beta-Alanine from Tyrosine	-4.08	0.43
345 Collagen CO1A2(c) synthesis	0.55	1	177 Glutamate from Phenylalanine	-4.07	0.46
371 Collagen COCA1(c) synthesis	0.54	1	89 Alanine from Phenylalanine	-4.04	0.55
827 Hypoxanthine	0.49	0.3	136 Asparagine from Tyrosine	-4.04	0.4
15 Dephosphorylation of ATP	0.48	0.67	743 Citrulline	-4.03	0.31
396 Collagen LPP3(c) synthesis	0.47	1	224 Glutamine from Tyrosine	-4.02	0.37
760 Deoxyinosine	0.47	0.33	115 Arginine from Tyrosine	-4.02	0.4
435 Galactose degr	0.47	0.37	243 Proline from Phenylalanine	-4.01	0.38
71 Glycogen glucose storage	0.46	0.65	72 Glycogen glucose release	-4.01	0.5
805 Glycogenin-G7	0.46	0.94	182 Glutamate from Tyrosine	-4	0.42
807 Glycogenin-G8	0.46	0.96	94 Alanine from Tyrosine	-3.96	0.52
803 Glycogenin-G11	0.46	0.95	264 Serine from Phenylalanine	-3.87	0.47
806 Glycogenin-G7G1	0.46	0.94	75 Gluconeogen from Alanine	-3.82	0.48
804 Glycogenin-G4G4	0.46	0.94	198 Glycine from Phenylalanine	-3.81	0.46
454 Glycogenin	0.46	0.96	448 Ethanol degr	-3.79	0.55
709 Adenine	0.46	0.36	268 Serine from Tyrosine	-3.74	0.44
120 Asparagine from Aspartate	0.46	0.32	74 Gluconeogen from Glycerol	-3.72	0.54
362 Collagen CO7A1(c) synthesis	0.45	1	925 Saccharopine	-3.66	0.57
952 Urate	0.45	0.29	890 PEP	-3.64	0.53
810 H2O2	0.45	0.29	609 2PG	-3.54	0.45
680 6-Phospho-D-gluconate	0.43	0.51	73 Gluconeogen from Lactate	-3.42	0.42
796 Glucono-1,5-lactone-6P	0.43	1	87 Alanine from Lysine	-3.06	0.35
973 dUMP	0.41	0.33	606 2-Oxoadipate	-3.04	0.35
293 Homocysteine from Methionine	0.4	1	55 UDP-activated glucose	-2.52	0.52
673 5-Phosphoribosyl-4-carboxy-5-aminoimidazole	0.4	0.34	775 Fatty-acid-VLDL-PC-pool	-2.48	1
837 L-Cystathionine	0.39	0.53	779 Fatty-acid-VLDL-SM-pool	-2.48	1
923 SAICAR	0.39	0.31	855 Mercaptopyruvate	-2.45	0.39
592 1-Pyrroline-5-carboxylate	0.37	1	929 Serotonin	-2.43	0.57
987 trans-4-Hydroxy-L-proline	0.37	1	838 L-Formylkynurenine	-2.42	0.71
835 L-1-Pyrroline-3-hydroxy-5-carboxylate	0.37	1	480 Alanine	-2.41	0.51
41 Na+ importgradient	0.36	0.66	811 H2S	-2.4	0.49
971 dTMP	0.36	0.32	335 Plasminogen	-2.4	0.98
921 Ribulose-5P	0.36	0.36	79 Alanine from Cysteine	-2.39	0.45
375 Collagen COGA1(c) synthesis	0.35	1	938 Succinate	-2.34	0.37
675 5-Phosphoribosylformylglycinamidine	0.35	0.31	950 UDP-activated-xylose	-2.3	0.5
924 SAM	0.34	0.33	80 Alanine from Cystine	-2.29	0.56
922 SAH	0.34	0.33	612 3-Hydroxy-L-kynurenine	-2.25	0.5

Continued on next page

Matches relating full time TGFβ – continued

Top down, T24h higher Simulation	ampl	score	Bottom up, T1h higher Simulation	ampl	score
691 AIR	0.33	0.33	886 Oxalosuccinate	-2.21	0.48
792 GMP	0.33	0.3	407 Alanine degr	-2.21	0.42
955 Xanthosine	0.33	0.28	313 Urea from NH3	-2.2	0.41
710 Adenylosuccinate	0.33	0.34	312 Urea from alanine	-2.2	0.41
453 FAD	0.33	0.29	975 gamma-Glutamyl-cysteine	-2.19	0.86
506 Uridine	0.33	0.4	798 Glucosylceramide-pool	-2.17	0.36
828 Inosine	0.32	0.3	657 4-(2-Amino-3-hydroxyphenyl)-2,4-dioxobutanoate	-2.16	0.59
761 Deoxyuridine	0.32	0.32	956 Xanthurenate	-2.16	0.59
965 dAMP	0.32	0.3	784 Formylanthranilate	-2.15	0.5
689 ADP	0.31	0.3	500 udpglcur	-2.14	0.35
692 AMP	0.31	0.3	307 Arachidonate from Dihomo-gamma-linolenate	-2.12	0.52
968 dGDP	0.31	0.31	694 Acetate	-2.07	0.86
958 cAMP	0.31	0.28	302 gamma-Linolenate from Linoleate	-2.07	0.49
791 GDP	0.31	0.34	118 Asparagine from Alanine	-2.07	0.38
759 Deoxyguanosine	0.3	0.31	65 ATP salvage from Adenosine	-2.06	0.88
783 Formamidopyrimidine(n)leoside triphosphate	0.3	0.31	613 3-Hydroxyanthranilate	-2.05	0.48
969 dGMP	0.3	0.33	56 UDP-activated glucuronate	-2.03	0.52
696 Acetoacetyl-CoA	0.3	0.29	446 ApoB100 degr	-2.03	0.48
894 PRPP	0.29	0.36	97 Arginine from Alanine	-2.01	0.42
920 Ribose-5P	0.29	0.45	596 2-Amino-3-carboxymuconate(s)emialdehyde	-2.01	0.56
404 (R)-3-Hydroxybutanoate	0.28	0.5	599 2-Aminomuconate(s)emialdehyde	-2.01	0.56
970 dTDP	0.28	0.35	417 Methionine degr	-2	0.4
753 Deamido-NAD	0.28	0.3	427 Homocysteine degr	-1.99	0.42
681 6-Pyruvoyltetrahydropterin	0.27	0.34	598 2-Aminomuconate	-1.99	0.58
790 GAR	0.27	0.37	980 palmitoleoyl-Carnitine	-1.97	0.54
502 GDP-mannose	0.27	0.37	93 Alanine from Tryptophan	-1.97	0.45
53 activated sulphur	0.27	0.68	844 L-Palmitoylcarnitine	-1.95	0.54
959 cGMP	0.26	0.38	83 Alanine from Histidine	-1.95	0.42
501 GDP-L-fucose	0.26	0.38	714 Arachidonyl-Carnitine	-1.94	0.54
524 gdpddman	0.26	0.4	977 linoleic-Carnitine	-1.94	0.54
834 Kynurenine	0.25	0.4	156 Aspartate from Tryptophan	-1.94	0.43
59 GDP-activated mannose	0.24	0.84	310 Bilirubin conjugation	-1.93	0.53
494 Mannose-1P	0.24	0.88	290 beta-Alanine from Tryptophan	-1.93	0.39
395 Collagen ITA2(c) synthesis	0.24	1	88 Alanine from Methionine	-1.92	0.49
495 Mannose-6P	0.24	0.78	729 Ceramide-1P-pool	-1.91	0.31
497 UDP-xylose	0.24	0.34	298 Palmitolate from Palmitate	-1.91	0.37
839 L-Fucose-1P	0.24	0.58	422 Tryptophan degr	-1.9	0.42
58 GDP-activated fucose	0.23	0.69	135 Asparagine from Tryptophan	-1.89	0.43
966 dCDP	0.21	0.33	299 Oleate from Stearate	-1.89	0.46
723 CMP	0.21	0.36	668 5-Formyl-THF	-1.86	0.41
720 CDP	0.21	0.37	793 GSSG	-1.85	0.61
473 dCTP	0.21	0.38	43 Activated methyl group (SAM)	-1.84	0.56
477 CTP	0.2	0.44	637 3-Phosphoserine	-1.81	0.58
507 Cytidine	0.2	0.41	285 beta-Alanine from Methionine	-1.79	0.44
479 UTP	0.2	0.37	267 Serine from Tryptophan	-1.77	0.49
498 UDP-glucose	0.2	0.3	778 Fatty-acid-VLDL-PS-pool	-1.77	0.59
877 O-Acetylcarnitine	0.2	1	777 Fatty-acid-VLDL-PI-pool	-1.77	0.58
359 Collagen CO6A3(c) synthesis	0.19	1	776 Fatty-acid-VLDL-PE-pool	-1.77	0.58
382 Collagen COMA1(c) synthesis	0.19	1	933 Sphingosine-1P	-1.77	0.28
475 dTTP	0.17	0.44	254 Serine from Cysteine	-1.77	0.51
869 N-Pantothenoylcysteine	0.17	0.45	250 Serine from Alanine	-1.76	0.5
548 (R)-4-Phosphopantothenoyl-cysteine	0.17	0.48	932 Sphingosine	-1.76	0.32
672 5-Oxoproline	0.15	0.55	51 Acetyl group(p)	-1.75	0.37
455 Thioredoxin	0.14	0.94	202 Glycine from Tryptophan	-1.74	0.49
887 Oxidized thioredoxin	0.14	0.93	294 Taurine from Methionine	-1.73	0.46
941 THF-hexaglutamate	0.12	0.86	49 Acetyl group(c)	-1.72	0.45
370 Collagen COBA2(c) synthesis	0.1	1	52 Acetyl group(r)	-1.72	0.45
893 PPi	0.09	0.39	161 Cysteine from Methionine	-1.72	0.47
712 Agmatine	0.08	0.8	866 N-Formimino-L-glutamate	-1.71	0.64

Continued on next page

Matches relating full time TGFβ – continued

Top down, T24h higher Simulation	ampl	score	Bottom up, T1h higher Simulation	ampl	score
342 Collagen CCBE1(c) synthesis	0.08	1	780 Fatty-acid-VLDL-TG1-pool	-1.7	0.58
947 Triphosphate degr	0.07	1	781 Fatty-acid-VLDL-TG2-pool	-1.7	0.58
926 Sarcosine	0.07	0.2	607 2-Oxobutyrate	-1.69	0.49
930 Sphinganine	0.07	0.4	263 Serine from Methionine	-1.69	0.51
611 3-Dehydrosphinganine	0.07	0.54	304 Arachidonate from Linoleate	-1.69	0.54
397 Collagen PCOTH(c) synthesis	0.07	0.33	130 Asparagine from Methionine	-1.67	0.47
387 Collagen COSA1(c) synthesis	0.07	0.35	255 Serine from Cystine	-1.65	0.54
381 Collagen COLQ(c) synthesis	0.07	0.35	306 Arachidonate from gamma-Linolenate	-1.65	0.51
380 Collagen COLA1(c) synthesis	0.07	0.35	303 Dihomo-gamma-linolenate from Linoleate	-1.65	0.51
392 Collagen FCN3(c) synthesis	0.07	0.34	279 beta-Alanine from Glycine	-1.65	0.44
361 Collagen CO6A6(c) synthesis	0.07	0.33	185 Glycine from Alanine	-1.65	0.56
390 Collagen FCN1(c) synthesis	0.07	0.35	189 Glycine from Cysteine	-1.64	0.52
360 Collagen CO6A5(c) synthesis	0.07	0.33	163 Cystine from Methionine	-1.64	0.5
328 Antitrypsin	0.07	0.33	377 Collagen COIA1(c) synthesis	-1.63	1
326 Fibrinogen	0.07	0.32	197 Glycine from Methionine	-1.62	0.52
394 Collagen ITA1(c) synthesis	0.07	0.33	564 1,2-Diacylglycerol-Bile-PC-pool	-1.6	0.48
354 Collagen CO5A1(c) synthesis	0.06	1	897 Phosphatidate-Bile-PC-pool	-1.59	0.54
456 Thioredoxin(m)	0.06	0.96	782 Fatty-acid-VLDL-TG3-pool	-1.58	0.61
979 mitoOxidizedThioredoxin	0.06	0.95	470 GSH	-1.58	0.6
669 5-Hydroxy-L-tryptophan	0.04	0.52	151 Aspartate from Methionine	-1.57	0.5
384 Collagen COOA1(c) synthesis	0.03	1	36 GSH reduction using NADH redox potential	-1.55	0.6
836 L-2-Aminoadipate	0.02	1	190 Glycine from Cystine	-1.54	0.55
372 Collagen CODA1(c) synthesis	0.02	1	491 Glycerol	-1.53	0.6
338 Haptoglobin	0.01	0.96	296 Stearate from Palmitate	-1.51	0.4
64 Glucosamine-6P	-0.001	0.63	510 Adenosine	-1.5	0.39
388 Collagen CTHR1(c) synthesis	-0.02	1	431 HDL degr	-1.5	0.4
799 Glucuronate	-0.04	0.41	430 LDL degr	-1.48	0.42
916 Pyridoxal	-0.06	0.66	976 gamma-Linolenoyl-CoA	-1.47	0.27
340 Collagen BGH3(c) synthesis	-0.06	1	203 Glycine from Tyrosine	-1.47	0.44
822 Homovanillate	-0.07	1	584 1-Acylglycerol-3P-palmn	-1.46	0.67
369 Collagen COBA1(c) synthesis	-0.08	1	589 1-Acylglycerol-VLDL-PS-pool	-1.44	0.56
457 Apo-ACP	-0.08	0.91	588 1-Acylglycerol-VLDL-PI-pool	-1.44	0.56
851 Malonyl-ACP	-0.08	0.84	587 1-Acylglycerol-VLDL-PE-pool	-1.44	0.56
697 Acetyl-ACP	-0.08	0.85	590 1-Acylglycerol-VLDL-SM-pool	-1.43	0.55
695 Acetoacetyl-ACP	-0.08	0.84	586 1-Acylglycerol-VLDL-PC-pool	-1.43	0.55
688 ACP	-0.08	0.86	591 1-Acylglycerol-VLDL-TG1-pool	-1.43	0.55
750 D-3-Hydroxyhexanoyl-ACP	-0.08	0.83	568 1,2-Diacylglycerol-VLDL-PS-pool	-1.43	0.49
718 Butyryl-ACP	-0.08	0.84	465 acgam6p	-1.41	0.58
717 But-2-enoyl-ACP	-0.08	0.84	567 1,2-Diacylglycerol-VLDL-PI-pool	-1.41	0.49
627 3-Oxohexanoyl-ACP	-0.08	0.83	902 Phosphatidate-VLDL-PS-pool	-1.41	0.54
544 (R)-3-Hydroxybutanoyl-ACP	-0.08	0.84	566 1,2-Diacylglycerol-VLDL-PE-pool	-1.4	0.48
942 Tetradecanoyl-ACP	-0.08	0.8	949 UDP-activated-N-acetyl-D-galactosamine	-1.39	0.36
936 Stearoyl-ACP	-0.08	0.79	317 Taurocholate(b)	-1.39	0.52
881 Octanoyl-ACP	-0.08	0.82	901 Phosphatidate-VLDL-PI-pool	-1.39	0.54
818 Hexanoyl-ACP	-0.08	0.82	569 1,2-Diacylglycerol-VLDL-SM-pool	-1.39	0.48
816 Hexadecanoyl-ACP	-0.08	0.8	861 N-Acetylglucosamine-1P	-1.39	0.47
813 HMA	-0.08	0.8	565 1,2-Diacylglycerol-VLDL-PC-pool	-1.38	0.48
769 Dodecanoyl-ACP	-0.08	0.81	570 1,2-Diacylglycerol-VLDL-TG-pool	-1.38	0.48
754 Decanoyl-ACP	-0.08	0.81	935 Squalene 2,3-oxide	-1.38	0.83
749 D-3-Hydroxydodecanoyl-ACP	-0.08	0.81	900 Phosphatidate-VLDL-PE-pool	-1.38	0.54
634 3-Oxotetradecanoyl-ACP	-0.08	0.8	434 Fructose degr	-1.38	0.57
633 3-Oxostearoyl-ACP	-0.08	0.79	903 Phosphatidate-VLDL-SM-pool	-1.37	0.54
629 3-Oxooctanoyl-ACP	-0.08	0.82	899 Phosphatidate-VLDL-PC-pool	-1.36	0.54
626 3-Oxohexadecanoyl-ACP	-0.08	0.8	649 3alpha,7alpha,12alpha-Trihydroxy-5beta-cholestan-26-al	-1.36	0.73
624 3-Oxododecanoyl-ACP	-0.08	0.81	650 3alpha,7alpha,12alpha-Trihydroxy-5beta-cholestanate	-1.36	0.73
622 3-Oxodecanoyl-ACP	-0.08	0.81	677 5beta-Cholestane-3alpha,7alpha,12alpha,26-tetrol	-1.36	0.73
617 3-Hydroxystearoyl-ACP	-0.08	0.79	904 Phosphatidate-VLDL-TG-pool	-1.36	0.54

Continued on next page

Top down, T24h higher			Bottom up, T1h higher		
Simulation	ampl	score	Simulation	ampl	score
547 (R)-3-Hydroxypalmitoyl-ACP	-0.08	0.8	405 Acetone	-1.36	0.46
546 (R)-3-Hydroxyoctanoyl-ACP	-0.08	0.82	572 1-Acylglycerol-3P-Bile-PC-pool	-1.35	0.65
545 (R)-3-Hydroxydecanoyl-ACP	-0.08	0.81	651 3alpha,7alpha,12alpha-Trihydroxy-5beta-cholestanoyl-CoA	-1.34	0.51
541 (2E)-Tetradecenoyl-ACP	-0.08	0.8	652 3alpha,7alpha,12alpha-Trihydroxycoprostane	-1.33	0.72
539 (2E)-Octenoyl-ACP	-0.08	0.82	318 tcdchola(b)	-1.33	0.53
538 (2E)-Octadecenoyl-ACP	-0.08	0.79	889 PE-PS-VLDL-pool	-1.33	0.49
536 (2E)-Hexenoyl-ACP	-0.08	0.82	684 7alpha,12alpha-Dihydroxycholest-4-en-3-one	-1.33	0.7
534 (2E)-Hexadecenoyl-ACP	-0.08	0.8	848 Linoleate	-1.33	0.66
532 (2E)-Dodecenoyl-ACP	-0.08	0.81	315 Glycocholate(b)	-1.32	0.6
530 (2E)-Decenoyl-ACP	-0.08	0.81	666 5-Aminolevulinate	-1.32	0.61
367 Collagen CO9A3(c) synthesis	-0.08	1	731 Cholate	-1.31	0.62
458 Apo-ACP(m)	-0.08	0.73	192 Glycine from Histidine	-1.31	0.53
978 mitoACP	-0.08	0.72	908 Porphobilinogen	-1.31	0.55
11 ATP from NADH(m)	-0.08	0.98	583 1-Acylglycerol-3P-palm	-1.31	0.67
14 Ubiquinol-to-ATP	-0.08	0.91	653 3alpha,7alpha-Dihydroxy-5beta-cholestan-26-al	-1.3	0.75
12 ATP from NADH	-0.08	0.5	739 Cholesterol-ester-pool	-1.3	0.71
358 Collagen CO6A2(c) synthesis	-0.09	1	648 3alpha,7alpha,12alpha-Trihydroxy-5beta-cholest-24-enoyl-CoA	-1.3	0.49
854 Maltose	-0.09	1	741 Choloyl-CoA	-1.3	0.48
400 Collagen VWA2(c) synthesis	-0.09	1	580 1-Acylglycerol-3P-arach	-1.3	0.67
353 Collagen CO4A6(c) synthesis	-0.09	1	581 1-Acylglycerol-3P-lin	-1.3	0.67
383 Collagen CONA1(c) synthesis	-0.1	1	582 1-Acylglycerol-3P-ol	-1.3	0.67
940 Sulfite degr	-0.1	1	585 1-Acylglycerol-3P-stea	-1.3	0.67
774 FMN	-0.1	1	678 5beta-Cholestane-3alpha,7alpha,26-triol	-1.3	0.73
365 Collagen CO9A1(c) synthesis	-0.1	1	683 7alpha,12alpha-Dihydroxy-5beta-cholestan-3-one	-1.3	0.71
364 Collagen CO8A2(c) synthesis	-0.1	1	735 Cholesterol-ester-lin	-1.3	0.72
350 Collagen CO4A3(c) synthesis	-0.11	1	205 Glycine from beta-Alanine	-1.3	0.47
393 Collagen FMOD(c) synthesis	-0.11	1	982 sn-Glycerol-3P	-1.3	0.97
366 Collagen CO9A2(c) synthesis	-0.11	1	898 Phosphatidate-CL-pool	-1.3	0.46
81 Alanine from Glutamate	-0.11	0.41	403 Acetoacetate	-1.29	0.42
295 Taurine from Cysteine	-0.12	0.6	577 1-Acylglycerol-3P-VLDL-PS-pool	-1.29	0.67
826 Hypotaurine	-0.12	0.6	654 3alpha,7alpha-Dihydroxy-5beta-cholestanate	-1.29	0.74
896 Pantetheine	-0.12	0.33	316 Gly-CD-cholate(b)	-1.28	0.61
747 Cysteamine	-0.12	0.43	576 1-Acylglycerol-3P-VLDL-PI-pool	-1.28	0.66
351 Collagen CO4A4(c) synthesis	-0.12	1	845 Lanosterol	-1.28	0.85
907 Phosphopantetheine	-0.12	0.45	196 Glycine from Lysine	-1.28	0.44
275 beta-Alanine from Aspartate	-0.13	0.96	646 3alpha,7alpha,12alpha,24-Tetrahydroxy-5beta-cholestanoyl-CoA	-1.28	0.49
278 beta-Alanine from Glutamate	-0.13	0.53	270 Serine from beta-Alanine	-1.28	0.44
461 4ppan	-0.13	1	575 1-Acylglycerol-3P-VLDL-PE-pool	-1.28	0.66
96 Alanine from beta-Alanine	-0.13	0.39	485 Glycine	-1.28	0.59
379 Collagen COKA1(c) synthesis	-0.14	1	578 1-Acylglycerol-3P-VLDL-SM-pool	-1.28	0.66
378 Collagen COJA1(c) synthesis	-0.14	1	579 1-Acylglycerol-3P-VLDL-TG1-pool	-1.28	0.66
325 Albumin	-0.15	0.98	574 1-Acylglycerol-3P-VLDL-PC-pool	-1.27	0.66
376 Collagen COHA1(c) synthesis	-0.15	1	734 Cholesterol-ester-gla	-1.27	0.72
273 beta-Alanine from Arginine	-0.15	0.4	656 4,4-Dimethyl-5alpha-cholesta-8,14,24-trien-3beta-ol	-1.27	0.79
728 Carnosine	-0.15	0.72	763 Desmosterol	-1.27	0.79
272 beta-Alanine from Alanine	-0.15	0.64	647 3alpha,7alpha,12alpha-Trihydroxy-5beta-24-oxocholestanoyl-CoA	-1.27	0.49
282 beta-Alanine from Glutamine	-0.15	0.41	737 Cholesterol-ester-palm	-1.27	0.71
274 beta-Alanine from Asparagine	-0.15	0.6	323 PE(b)	-1.27	0.57
373 Collagen COEA1(c) synthesis	-0.15	1	655 3alpha,7alpha-Dihydroxy-5beta-cholestanoyl-CoA	-1.27	0.52
850 Malate	-0.16	0.97	262 Serine from Lysine	-1.27	0.42
339 Collagen ADIPO(c) synthesis	-0.16	1	679 5beta-Cholestane-3alpha,7alpha-diol	-1.26	0.72
632 3-Oxopropanoate	-0.16	0.45	892 PGP-CL-pool	-1.26	0.36
276 beta-Alanine from Cysteine	-0.16	0.53	891 PG-CL-pool	-1.26	0.37
37 GSH reduction using NADPH redox potential	-0.16	1	738 Cholesterol-ester-palmn	-1.26	0.72
38 GSH oxidation	-0.16	1	552 (R)-Mevalonate	-1.25	0.45
139 Aspartate from Alanine	-0.17	0.57	523 Cholesterol	-1.25	0.79

Continued on next page

Top down, T24h higher Simulation	ampl	score	Bottom up, T1h higher Simulation	ampl	score
389 Collagen EMID2(c) synthesis	-0.17	1	862 N-Acetylmannosamine-6P	-1.25	0.5
277 beta-Alanine from Cystine	-0.17	0.46	686 7alpha-Hydroxycholest-4-en-3-one	-1.25	0.71
391 Collagen FCN2(c) synthesis	-0.17	1	687 7alpha-Hydroxycholesterol	-1.25	0.75
880 OAA	-0.17	0.96	744 CoproporphyrinogenIII	-1.24	0.42
78 Alanine from Aspartate	-0.18	0.47	736 Cholesterol-ester-ol	-1.24	0.72
787 Fumarate	-0.2	0.85	740 Cholesterol-ester-stea	-1.24	0.72
716 Argininosuccinate	-0.2	0.47	863 N-Acetylneuraminate	-1.24	0.32
330 ApoB100(r)	-0.2	0.99	872 NeuNGc	-1.24	0.3
309 VLDL from HDL	-0.2	0.77	954 UroporphyrinogenIII	-1.24	0.44
401 VLDL	-0.2	0.77	914 Protoporphyrinogen IX	-1.24	0.4
308 VLDL from LDL	-0.2	0.81	488 Serine	-1.24	0.56
334 ApoE(c)	-0.22	0.96	593 14-Demethyllanosterol	-1.24	0.77
917 Pyridoxine-P	-0.23	1	864 N-Acetylneuraminate-9P	-1.24	0.36
797 Glucose-1P	-0.24	1	824 Hydroxymethylbilane	-1.24	0.46
555 (S)-3-Hydroxydodecanoyl-CoA	-0.24	0.35	324 Cholesterol(b)	-1.24	0.81
363 Collagen CO8A1(c) synthesis	-0.24	1	733 Cholesterol-ester-arach	-1.23	0.74
641 3-oxolaur-cis-5-enoyl-CoA	-0.24	0.36	685 7alpha-Hydroxy-5beta-cholestan-3-one	-1.22	0.72
543 (3Z)-Dodecenoyl-CoA	-0.24	0.36	513 CMP-N-acetylneuraminate	-1.22	0.31
533 (2E)-Dodecenoyl-CoA	-0.24	0.34	725 CMP-activated-N-acetylneuraminate	-1.22	0.31
610 3(S)-3-hydroxydodecen-(5Z)-oyl-CoA	-0.24	0.35	319 Chenodiol(b)	-1.22	0.65
983 trans,cis-dodeca-2,5-dienoyl-CoA	-0.24	0.35	645 3PG	-1.21	0.66
642 3-oxomyrist-7-enoyl-CoA	-0.25	0.35	682 7-Dehydrodesmosterol	-1.21	0.8
962 cis-laur-5-enoyl-CoA	-0.25	0.35	469 ametam	-1.21	0.34
960 cis-(3S)-hydroxytetradec-7-enoyl-CoA	-0.25	0.34	676 5alpha-Cholesta-7,24-dien-3beta-ol	-1.21	0.85
985 trans,cis-myristo-2,7-dienoyl-CoA	-0.25	0.34	512 N-acglucam	-1.21	0.39
644 3-oxopalmitoleoyl-CoA	-0.25	0.35	636 3-Phosphonooxypyruvate	-1.21	0.59
963 cis-myrist-7-enoyl-CoA	-0.25	0.33	573 1-Acylglycerol-3P-CL-pool	-1.21	0.54
356 Collagen CO5A3(c) synthesis	-0.25	1	730 Chenodeoxycholoyl-CoA	-1.21	0.52
625 3-Oxododecanoyl-CoA	-0.26	0.36	258 Serine from Histidine	-1.21	0.5
755 Decanoyl-CoA	-0.26	0.36	618 3-Keto-4-methylzymosterol	-1.21	0.84
559 (S)-3-hydroxypalmitoleoyl-CoA	-0.26	0.32	188 Glycine from Aspartate	-1.21	0.59
597 2-Amino-3-oxoadipate	-0.26	0.5	516 PS	-1.2	0.45
859 N-(omega)-Hydroxyarginine	-0.26	0.77	322 PS(b)	-1.2	0.45
143 Aspartate from Cystine	-0.26	0.44	768 Dimethylallyl-PP	-1.2	0.53
919 Ribose-1P	-0.26	0.39	515 PE	-1.2	0.52
711 Adenylyl(s)ulfate	-0.26	0.31	752 DHAP	-1.19	0.97
984 trans,cis-hexadeca-2,9-dienoyl-CoA	-0.27	0.3	789 GAP	-1.19	0.97
561 (S)-Hydroxydecanoyl-CoA	-0.27	0.37	915 Provitamin D3	-1.19	0.8
531 (2E)-Decenoyl-CoA	-0.27	0.37	662 4alpha-Methylzymosterol	-1.19	0.84
558 (S)-3-hydroxyoleyleoyl-CoA	-0.27	0.31	957 Zymosterol	-1.19	0.84
623 3-Oxodecanoyl-CoA	-0.27	0.38	191 Glycine from Glutamate	-1.19	0.45
882 Octanoyl-CoA	-0.27	0.38	846 Lathosterol	-1.19	0.85
698 Acetyl-CoA	-0.27	0.28	663 4alpha-Methylzymosterol-4-carboxylate	-1.19	0.82
563 (S)-Hydroxyoctanoyl-CoA	-0.27	0.39	187 Glycine from Asparagine	-1.19	0.52
772 FADH2	-0.28	0.27	659 4-Imidazolone-5-propanoate	-1.19	0.54
385 Collagen COPA1(c) synthesis	-0.28	1	44 Activated methyl group (THF)	-1.19	0.62
981 palmitoleoyl-CoA	-0.28	0.3	200 Glycine from Serine	-1.19	0.62
630 3-Oxooctanoyl-CoA	-0.28	0.39	327 Antichymotrypsin	-1.18	0.97
615 3-Hydroxyisobutyryl-CoA	-0.28	0.3	732 Cholestenol	-1.17	0.84
819 Hexanoyl-CoA	-0.28	0.39	145 Aspartate from Glycine	-1.17	0.59
671 5-Methylthioadenosine	-0.28	0.31	722 CDP-ethanolamine	-1.17	0.32
918 Quinolinate	-0.28	0.77	194 Glycine from Glutamine	-1.15	0.43
562 (S)-Hydroxyhexanoyl-CoA	-0.28	0.4	511 UDP-N-acetylgalactosamine	-1.15	0.28
529 Methylthioribose-1P	-0.29	0.44	860 N-Acetyl-D-mannosamine	-1.15	0.43
856 Methacrylyl-CoA	-0.29	0.28	948 UDP-N-acetylglucosamine	-1.15	0.29
762 Dephospho-CoA	-0.29	0.29	521 Ceramide	-1.15	0.47
719 Butyryl-CoA	-0.29	0.39	320 Bile-PC(b)	-1.15	0.5
870 NADH	-0.3	0.34	549 (R)-5-Diphosphomevalonate	-1.15	0.58
794 Galactose-1P	-0.3	0.66	321 SM(b)	-1.15	0.49

Continued on next page

Top down, T24h higher Simulation	ampl	score	Bottom up, T1h higher Simulation	ampl	score
831 Isobutyryl-CoA	-0.3	0.3	751 D-Xylulose-5P	-1.15	0.45
368 Collagen COAA1(c) synthesis	-0.3	1	909 Presqualene-PP	-1.14	0.81
292 beta-Alanine from Valine	-0.3	0.4	934 Squalene	-1.14	0.81
451 NAD+	-0.3	0.33	953 Urocanate	-1.13	0.48
757 Deoxyadenosine	-0.3	0.27	724 CMP-NeuNGc	-1.13	0.26
986 trans,cis-octadeca-2,9-dienoyl-CoA	-0.3	0.29	517 PI	-1.12	0.34
964 dADP	-0.3	0.28	674 5-Phosphoribosylamine	-1.12	0.31
57 UDP-activated galactose	-0.31	0.55	186 Glycine from Arginine	-1.11	0.42
32 Thioredoxin(m) reduction	-0.31	1	571 1,3DPG	-1.11	0.77
33 Thioredoxin(m) oxidation	-0.31	1	640 3-Ureidopropionate	-1.11	0.41
876 Nicotinate(r)ibonucleotide	-0.31	0.36	406 Heme	-1.11	0.42
875 Nicotinate D-ribonucleoside	-0.31	0.32	402 HDL	-1.1	0.81
801 Glutaryl-CoA	-0.31	0.31	253 Serine from Aspartate	-1.1	0.57
895 Palmitoyl-CoA	-0.31	0.28	496 Fructose-6P	-1.09	1
873 Nicotinamide D-ribonucleotide	-0.31	0.4	329 ApoA1	-1.09	0.96
888 PAP	-0.32	0.3	503 IMP	-1.09	0.4
937 Stearoyl-CoA	-0.32	0.28	478 GTP	-1.08	0.35
883 Oleoyl-CoA	-0.32	0.28	504 XMP	-1.08	0.39
849 Linoleoyl-CoA	-0.32	0.28	820 Histamine	-1.07	0.73
715 Arachidonyl-CoA	-0.32	0.28	785 Fructose-1,6PP	-1.07	1
535 (2E)-Hexadecenoyl-CoA	-0.32	0.28	476 ATP	-1.07	0.38
499 udpgal	-0.32	0.35	600 2-Deoxy-D-ribose-1P	-1.06	0.32
701 Acyl-CoA-CL-pool	-0.32	0.28	331 ApoC1(c)	-1.06	0.93
705 Acyl-CoA-VLDL-PS-pool	-0.32	0.28	518 CL	-1.06	0.4
706 Acyl-CoA-VLDL-SM-pool	-0.33	0.29	474 dGTP	-1.06	0.34
704 Acyl-CoA-VLDL-PI-pool	-0.33	0.29	508 Xanthine	-1.06	0.36
708 Acyl-CoA-VLDL-TG3-pool	-0.33	0.29	509 Guanine	-1.06	0.34
460 Pyridoxal-P	-0.33	0.58	62 Isopentenyl-PP	-1.06	0.61
707 Acyl-CoA-VLDL-TG2-pool	-0.33	0.29	256 Serine from Glutamate	-1.06	0.43
703 Acyl-CoA-VLDL-PE-pool	-0.33	0.29	795 Geranyl-PP	-1.05	0.53
635 3-Oxotetradecanoyl-CoA	-0.33	0.37	601 2-Deoxy-D-ribose-5P	-1.05	0.41
557 (S)-3-Hydroxytetradecanoyl-CoA	-0.33	0.35	472 dATP	-1.04	0.37
700 Acyl-CoA-Bile-PC-pool	-0.33	0.29	60 Pyruvate	-1.04	0.51
702 Acyl-CoA-VLDL-PC-pool	-0.33	0.29	522 Triacylglycerol	-1.04	0.59
554 (S)-3-Hydroxybutyryl-CoA	-0.33	0.28	814 HMG-CoA	-1.04	0.29
631 3-Oxopalmitoyl-CoA	-0.33	0.36	124 Asparagine from Glycine	-1.04	0.58
605 2-Oxo-3-methylvalerate	-0.33	0.5	519 LacCer	-1.04	0.38
556 (S)-3-Hydroxyhexadecanoyl-CoA	-0.33	0.32	424 Glycine degr	-1.04	0.41
858 Myristoyl-CoA	-0.34	0.33	63 Farnesyl-PP	-1.03	0.54
17 Aerobic reduction of NAD+ (gluc)	-0.35	0.45	47 Formylgroup(c)	-1.02	0.77
433 Stearate degr	-0.36	0.52	520 SM	-1.02	0.47
432 Oleate degr	-0.36	0.5	201 Glycine from Threonine	-1.02	0.63
357 Collagen CO6A1(c) synthesis	-0.36	1	150 Aspartate from Lysine	-1.02	0.44
878 O-Butanoylcarnitine	-0.36	0.56	911 Propanoyl-CoA	-1.01	0.31
183 Glutamate from Valine	-0.37	0.53	160 Cysteine from Cystine	-1.01	0.57
50 Acetyl group(m)	-0.37	0.54	162 Cystine from Cysteine	-1.01	0.57
765 Dihydroceramide-pool	-0.37	0.31	928 Sedoheptulose-7P	-1.01	0.5
225 Glutamine from Valine	-0.38	0.49	550 (R)-5-Phosphomevalonate	-1.01	0.49
19 Aerobic reduction of NADP+ (gluc)	-0.38	0.35	416 Lysine degr	-1	0.36
912 Protein(l)ysine	-0.38	1	770 Erythrose-4P	-0.99	0.6
842 L-Octanoylcarnitine	-0.38	0.54	482 Asparagine	-0.99	0.48
758 Deoxycytidine	-0.39	0.29	260 Serine from Glutamine	-0.99	0.41
70 Formate degr	-0.4	0.65	214 Glutamine from Histidine	-0.99	0.51
348 Collagen CO4A1(c) synthesis	-0.4	1	413 Histidine degr	-0.98	0.4
967 dCMP	-0.4	0.31	284 beta-Alanine from Lysine	-0.98	0.39
865 N-Carbamoyl-L-aspartate	-0.4	0.41	910 Propanoate	-0.98	0.64
269 Serine from Valine	-0.4	0.42	867 N-Formyl-GAR	-0.98	0.32
34 Thioredoxin(c) reduction	-0.41	0.96	125 Asparagine from Histidine	-0.98	0.44
423 Valine degr	-0.41	0.42	199 Glycine from Proline	-0.97	0.52

Continued on next page

Matches relating full time TGFβ – continued

Top down, T24h higher Simulation	ampl	score	Bottom up, T1h higher Simulation	ampl	score
158 Aspartate from Valine	-0.42	0.5	266 Serine from Threonine	-0.97	0.62
116 Arginine from Valine	-0.42	0.47	9 Anaerobic rephosph of CTP	-0.97	0.46
212 Glutamine from Glutamate	-0.42	0.39	8 Anaerobic rephosph of GTP	-0.97	0.46
346 Collagen CO2A1(c) synthesis	-0.42	1	10 Anaerobic rephosph of UTP	-0.97	0.46
871 NADPH	-0.43	0.34	3 Anaerobic rephosph of ATP	-0.97	0.47
30 NADPH to NADH transhydrogenase in mito	-0.43	1	823 Hydracrylate	-0.97	0.58
95 Alanine from Valine	-0.43	0.53	514 PC	-0.97	0.46
464 mlthf	-0.43	0.8	129 Asparagine from Lysine	-0.97	0.36
466 THF(m)	-0.43	1	195 Glycine from Leucine	-0.96	0.42
459 THF	-0.43	1	449 Chitin-component degr	-0.96	0.37
29 NADH to NADPH transhydrogenase	-0.43	1	104 Arginine from Histidine	-0.96	0.44
766 Dihydrofolate	-0.43	1	252 Serine from Asparagine	-0.96	0.54
670 5-Methyl-THF	-0.43	1	809 Guanosine	-0.96	0.32
76 Alanine from Arginine	-0.43	0.36	602 2-Lysolecithin-pool	-0.96	0.42
972 dUDP	-0.44	0.32	616 3-Hydroxypropionyl-CoA	-0.95	0.34
974 dUTP	-0.44	0.31	483 Aspartate	-0.94	0.45
487 Proline	-0.44	0.53	699 Acrylyl-CoA	-0.93	0.34
137 Asparagine from Valine	-0.44	0.49	122 Asparagine from Cystine	-0.93	0.5
452 NADP+	-0.44	0.33	193 Glycine from Isoleucine	-0.93	0.56
68 O2.-(c) degr	-0.44	1	46 Activated methyl group from Histidine	-0.93	0.35
843 L-Oleoylcarnitine	-0.45	0.69	305 Dihomo-gamma-linolenate from gamma-Linolenate	-0.93	0.47
207 Glutamine from Arginine	-0.45	0.41	665 5,6-Dihydrouracil	-0.93	0.36
528 Spermine	-0.45	0.33	603 2-Methylacetoacetyl-CoA	-0.92	0.36
505 Uracil	-0.45	0.33	25 NADH redox potential into peroxy	-0.92	0.82
726 Carbamoyl-P	-0.46	0.41	146 Aspartate from Histidine	-0.92	0.49
204 Glycine from Valine	-0.46	0.43	280 beta-Alanine from Histidine	-0.91	0.45
248 Proline from Valine	-0.46	0.57	343 Collagen CD36(c) synthesis	-0.91	1
812 HCO3-	-0.46	0.66	847 Lauroyl-CoA	-0.91	0.37
727 Carbonate	-0.46	0.66	109 Arginine from Methionine	-0.91	0.47
667 5-Formiminotetrahydrofolate	-0.47	0.56	251 Serine from Arginine	-0.9	0.39
664 5,10-Methenyl-THF	-0.47	0.82	927 Sedoheptulose-1,7PP	-0.9	0.58
85 Alanine from Glutamine	-0.47	0.37	13 NADH potential transport	-0.9	0.62
946 Trehalose	-0.47	1	134 Asparagine from Threonine	-0.9	0.68
913 Protoporphyrin	-0.47	0.39	690 AICAR	-0.9	0.36
526 Putrescine	-0.48	0.45	333 ApoC3(c)	-0.89	0.94
693 Acetaldehyde	-0.48	0.58	171 Glutamate from Histidine	-0.89	0.63
868 N-Methylethanolamine-P	-0.48	1	773 FAICAR	-0.89	0.35
771 Ethanolamine-P	-0.48	1	463 dhbpt	-0.89	0.38
874 Nicotinate	-0.49	0.71	421 Threonine degr	-0.88	0.53
6 Aerobic rephosph of UDP	-0.49	0.46	553 (S)-3-Hydroxy-2-methylbutyryl-CoA	-0.88	0.34
77 Alanine from Asparagine	-0.5	0.52	138 Asparagine from beta-Alanine	-0.88	0.44
69 Formaldehyde degr	-0.5	0.45	121 Asparagine from Cysteine	-0.88	0.41
492 Oleate	-0.51	0.85	852 Malonyl-Carnitin	-0.88	0.64
301 Palmitolate from Arachidonate	-0.51	0.76	939 Succinyl-CoA	-0.88	0.32
742 Citrate	-0.51	0.71	159 Aspartate from beta-Alanine	-0.87	0.47
493 Stearate	-0.51	0.94	92 Alanine from Threonine	-0.87	0.58
608 2-Oxoglutaramate	-0.52	1	414 Glutamine degr	-0.87	0.35
961 cis-Aconitate	-0.52	0.76	879 O-Propanoylcarnitine	-0.87	0.54
825 Hydroxypyruvate	-0.52	1	542 (2E)-Tetradecenoyl-CoA	-0.87	0.38
490 Palmitate	-0.52	0.97	259 Serine from Isoleucine	-0.87	0.55
560 (S)-Dihydroorotate	-0.53	0.35	213 Glutamine from Glycine	-0.87	0.55
721 CDP-choline	-0.54	0.39	841 L-Lactate	-0.87	0.92
141 Aspartate from Asparagine	-0.54	0.56	289 beta-Alanine from Threonine	-0.86	0.56
527 Spermidine	-0.54	0.4	462 thbpt	-0.86	0.34
220 Glutamine from Proline	-0.55	0.5	218 Glutamine from Methionine	-0.86	0.48
746 Cys-Gly	-0.55	1	66 ATP salvage from Hypoxanthine	-0.86	0.45
172 Glutamate from Isoleucine	-0.56	0.65	31 NADH to NADPH transhydrogenase in mito	-0.86	1
90 Alanine from Proline	-0.56	0.44	450 CoA	-0.85	0.32
332 ApoC2(c)	-0.56	0.95	621 3-Methylglutaconyl-CoA	-0.85	0.29

Continued on next page

Matches relating full time TGFβ – continued

Top down, T24h higher Simulation	ampl	score	Bottom up, T1h higher Simulation	ampl	score
467 1fthf	-0.57	0.73	61 AKG	-0.85	0.58
215 Glutamine from Isoleucine	-0.57	0.65	265 Serine from Proline	-0.85	0.47
337 ApoTransferin	-0.57	0.98	155 Aspartate from Threonine	-0.84	0.64
336 Prothrombin	-0.58	0.98	639 3-Ureidoisobutyrate	-0.84	0.35
91 Alanine from Serine	-0.59	0.56	748 D-3-Amino-isobutanoate	-0.84	0.29
800 Glutamyl-5P	-0.59	0.44	28 NADPH to NADH transhydrogenase	-0.84	1
226 Glutamine from beta-Alanine	-0.59	0.55	643 3-oxooleoyl-CoA	-0.83	0.29
140 Aspartate from Arginine	-0.59	0.46	764 Dihomo-gamma-linolenoyl-CoA	-0.83	0.3
18 Aerobic reduction of NADP+ (FA)	-0.59	0.29	176 Glutamate from Methionine	-0.83	0.47
943 Thymidine	-0.6	0.33	311 Urea from glutamine	-0.82	0.34
42 Na+ exportgradient	-0.6	1	713 Anthranilate	-0.81	0.44
1 Aerobic ATP rephosph (FA)	-0.6	0.44	142 Aspartate from Cysteine	-0.81	0.47
4 Aerobic rephosph of GTP	-0.6	0.42	108 Arginine from Lysine	-0.81	0.43
7 Aerobic rephosph of UTP	-0.6	0.42	412 Glutamate degr	-0.81	0.38
614 3-Hydroxyisobutyrate	-0.6	0.38	261 Serine from Leucine	-0.81	0.41
249 Proline from beta-Alanine	-0.6	0.59	604 2-Methylbutyryl-CoA	-0.81	0.3
86 Alanine from Leucine	-0.61	0.37	237 Proline from Histidine	-0.81	0.55
287 beta-Alanine from Proline	-0.61	0.49	2 Aerobic ATP rephosph (gluc)	-0.81	0.41
184 Glutamate from beta-Alanine	-0.61	0.64	103 Arginine from Glycine	-0.8	0.47
208 Glutamine from Asparagine	-0.61	0.77	35 Thioredoxin(c) oxidation	-0.8	0.84
288 beta-Alanine from Serine	-0.61	0.42	857 Methylmalonyl-CoA	-0.8	0.39
235 Proline from Glutamate	-0.61	0.64	127 Asparagine from Glutamine	-0.8	0.56
241 Proline from Lysine	-0.61	0.47	113 Arginine from Threonine	-0.8	0.6
786 Fructose-2,6PP	-0.61	0.87	5 Aerobic rephosph of CTP	-0.8	0.36
239 Proline from Glutamine	-0.61	0.59	945 Tiglyl-CoA	-0.79	0.36
178 Glutamate from Proline	-0.62	0.64	745 Crotonyl-CoA	-0.79	0.3
230 Proline from Arginine	-0.62	0.52	314 Creatine	-0.79	0.47
238 Proline from Isoleucine	-0.62	0.66	170 Glutamate from Glycine	-0.79	0.65
802 Glycerate	-0.62	0.66	123 Asparagine from Glutamate	-0.78	0.44
247 Proline from Tyrosine	-0.62	0.38	133 Asparagine from Serine	-0.78	0.5
82 Alanine from Glycine	-0.62	0.48	24 NADH redox potential into mito	-0.78	0.55
233 Proline from Cysteine	-0.62	0.66	101 Arginine from Cystine	-0.77	0.47
39 Proton-gradient(m) build up	-0.62	0.47	551 (R)-Methylmalonyl-CoA	-0.77	0.29
40 Proton-gradient(c) build up	-0.62	0.47	228 Methionine from Cystine	-0.77	0.54
840 L-Glutamate 5-semialdehyde	-0.62	0.48	242 Proline from Methionine	-0.77	0.52
175 Glutamate from Lysine	-0.62	0.47	26 NADPH redox potential into mito	-0.77	0.88
884 Orotate	-0.62	0.4	429 Ornithine degr	-0.77	0.43
153 Aspartate from Proline	-0.62	0.57	27 NADPH redox potential into peroxy	-0.77	1
209 Glutamine from Aspartate	-0.62	0.52	408 Arginine degr	-0.77	0.41
165 Glutamate from Arginine	-0.63	0.57	425 Isoleucine degr	-0.76	0.5
231 Proline from Asparagine	-0.63	0.71	540 (2E)-Octenoyl-CoA	-0.76	0.38
216 Glutamine from Leucine	-0.63	0.49	808 Guanidinoacetate	-0.76	0.33
206 Glutamine from Alanine	-0.63	0.51	620 3-Methylcrotonyl-CoA	-0.76	0.33
906 Phosphodimethylethanolamine	-0.63	1	628 3-Oxohexanoyl-CoA	-0.76	0.37
905 Phosphocholine	-0.63	1	420 Serine degr	-0.76	0.41
240 Proline from Leucine	-0.64	0.57	126 Asparagine from Isoleucine	-0.76	0.58
232 Proline from Aspartate	-0.64	0.7	537 (2E)-Hexenoyl-CoA	-0.75	0.38
439 Antitrypsin degr	-0.64	0.5	144 Aspartate from Glutamate	-0.75	0.5
246 Proline from Tryptophan	-0.64	0.46	114 Arginine from Tryptophan	-0.75	0.41
217 Glutamine from Lysine	-0.64	0.43	271 Tyrosine from Phenylalanine	-0.75	0.56
229 Proline from Alanine	-0.64	0.53	147 Aspartate from Isoleucine	-0.75	0.62
661 4-Methyl-2-oxopentanoate	-0.64	0.53	22 Oxidation of NADH	-0.75	0.4
210 Glutamine from Cysteine	-0.65	0.47	833 Isovaleryl-CoA	-0.74	0.32
102 Arginine from Glutamate	-0.65	0.38	832 Isocitrate	-0.74	0.49
84 Alanine from Isoleucine	-0.65	0.57	756 Dehydroalanine	-0.74	0.66
167 Glutamate from Aspartate	-0.65	0.84	595 2,5-Diaminopyrimidine(n)leoside triphosphate	-0.73	0.31
132 Asparagine from Proline	-0.65	0.56	211 Glutamine from Cystine	-0.73	0.56
111 Arginine from Proline	-0.65	0.47	398 Collagen SCRB1(c) synthesis	-0.73	1

Continued on next page

Matches relating full time TGFβ – continued

Top down, T24h higher Simulation	ampl	score	Bottom up, T1h higher Simulation	ampl	score
119 Asparagine from Arginine	-0.66	0.39	594 2,5-Diamino-6-(5-triphosphoryl-3,4-trihydroxy-2-oxopentyl)- amino-4-oxopyrimidine	-0.73	0.33
885 Orotidine-5P	-0.66	0.33	222 Glutamine from Threonine	-0.73	0.69
174 Glutamate from Leucine	-0.66	0.53	442 Haptoglobin degr	-0.73	0.56
166 Glutamate from Asparagine	-0.66	0.76	48 Formylgroup(m)	-0.72	0.5
283 beta-Alanine from Leucine	-0.66	0.44	180 Glutamate from Threonine	-0.72	0.73
244 Proline from Serine	-0.66	0.67	444 Prothrombin degr	-0.72	0.48
951 UMP	-0.66	0.41	481 Arginine	-0.72	0.53
173 Glutamate from Glutamine	-0.66	0.55	443 Plasminogen degr	-0.72	0.5
486 Glutamine	-0.66	0.73	447 Glycogenin degr	-0.72	0.52
484 Glutamate	-0.66	0.83	441 Fibrinogen degr	-0.72	0.5
168 Glutamate from Cysteine	-0.67	0.63	471 Homocysteine	-0.72	0.54
154 Aspartate from Serine	-0.67	0.53	128 Asparagine from Leucine	-0.72	0.44
234 Proline from Cystine	-0.67	0.6	169 Glutamate from Cystine	-0.71	0.66
221 Glutamine from Serine	-0.67	0.74	23 Oxidation of NADPH	-0.71	0.45
149 Aspartate from Leucine	-0.67	0.45	445 ApoTransferin degr	-0.71	0.52
107 Arginine from Leucine	-0.67	0.44	236 Proline from Glycine	-0.7	0.61
227 Methionine from Cysteine	-0.67	0.54	117 Arginine from beta-Alanine	-0.7	0.47
105 Arginine from Isoleucine	-0.68	0.58	419 Proline degr	-0.7	0.44
438 Antichymotrypsin degr	-0.68	0.48	415 Leucine degr	-0.7	0.39
944 Thymine	-0.68	0.35	100 Arginine from Cysteine	-0.7	0.45
21 Aerobic reduction of FAD (FA)	-0.68	0.52	853 Malonyl-CoA	-0.7	0.31
767 Dihydrothymine	-0.68	0.36	468 PAPS	-0.7	0.36
281 beta-Alanine from Isoleucine	-0.68	0.51	16 Aerobic reduction of NAD+ (FA)	-0.7	0.32
45 Activated methylene group from Try	-0.68	0.34	245 Proline from Threonine	-0.7	0.69
99 Arginine from Aspartate	-0.68	0.46	437 Albumin degr	-0.7	0.51
164 Glutamate from Alanine	-0.68	0.62	112 Arginine from Serine	-0.7	0.51
179 Glutamate from Serine	-0.69	0.79	223 Glutamine from Tryptophan	-0.69	0.41
181 Glutamate from Tryptophan	-0.69	0.46	106 Arginine from Glutamine	-0.69	0.38
20 Aerobic reduction of FAD (gluc)	-0.69	0.5	440 ApoA1 degr	-0.69	0.48
525 beta-Alanine	-0.69	0.38	148 Aspartate from Glutamine	-0.69	0.43
98 Arginine from Asparagine	-0.69	0.48			

1.4 Matches relating control 1/6h vs. 6/24h

Table 4: Matches relating control 1/6h vs. 6/24h

Simulation	control 1/6h		control 6/24h	
	ampl	score	ampl	score
830 Inositol-1P	0.37	1	1.4	1
829 Inositol	0.34	0.7	1.38	0.6
352 Collagen CO4A5(c) synthesis	0.11	1	1.49	1
386 Collagen CORA1(c) synthesis	0.04	1	0.87	1
85 Alanine from Glutamine	0.3	0.35	0.49	0.36
349 Collagen CO4A2(c) synthesis	0.14	1	0.61	1
449 Chitin-component degr	0.21	0.3	0.52	0.39
343 Collagen CD36(c) synthesis	0.12	1	0.6	1
987 trans-4-Hydroxy-L-proline	0.2	1	0.47	1
835 L-1-Pyrroline-3-hydroxy-5-carboxylate	0.2	1	0.47	1
347 Collagen CO3A1(c) synthesis	0.11	1	0.55	1
592 1-Pyrroline-5-carboxylate	0.2	0.76	0.46	0.88
300 Stearate from Oleate	0.42	0.36	0.23	0.37
399 Collagen SERPH(c) synthesis	0.17	1	0.48	1
24 NADH redox potential into mito	0.2	0.64	0.45	0.52
893 PPi	0.08	0.45	0.56	0.49
297 Palmitate from Palmitolate	0.42	0.36	0.22	0.39
54 Glucose-6P	0.25	0.51	0.36	0.51
15 Dephosphorylation of ATP	0.34	0.57	0.23	0.51
18 Aerobic reduction of NADP+ (FA)	0.33	0.35	0.23	0.32
529 Methylthioribose-1P	0.36	0.54	0.15	0.36
475 dTTP	0.24	0.41	0.26	0.41
30 NADPH to NADH transhydrogenase in mito	0.35	1	0.14	1
293 Homocysteine from Methionine	0.13	1	0.34	1
506 Uridine	0.25	0.43	0.21	0.33
71 Glycogen glucose storage	-0.23	0.74	0.67	0.65
605 2-Oxo-3-methylvalerate	0.28	0.42	0.17	0.37
713 Anthranilate	0.12	0.44	0.32	0.44
807 Glycogenin-G8	-0.23	0.96	0.67	0.95
803 Glycogenin-G11	-0.23	0.96	0.67	0.95
454 Glycogenin	-0.24	0.96	0.67	0.96
806 Glycogenin-G7G1	-0.23	0.95	0.67	0.94
804 Glycogenin-G4G4	-0.23	0.95	0.67	0.94
805 Glycogenin-G7	-0.23	0.95	0.67	0.94
828 Inosine	0.26	0.44	0.17	0.32
721 CDP-choline	0.2	0.38	0.23	0.31
96 Alanine from beta-Alanine	0.35	0.36	0.08	0.45
874 Nicotinate	0.32	0.61	0.11	0.5
632 3-Oxopropanoate	0.35	0.33	0.08	0.42
620 3-Methylcrotonyl-CoA	0.21	0.36	0.21	0.29
709 Adenine	0.23	0.48	0.19	0.39
827 Hypoxanthine	0.25	0.43	0.16	0.33
836 L-2-Aminoadipate	0.04	1	0.38	1
671 5-Methylthioadenosine	0.23	0.49	0.19	0.36
67 dTTP salvage from Thymine	0.25	0.38	0.16	0.33
477 CTP	0.23	0.46	0.17	0.33
757 Deoxyadenosine	0.22	0.46	0.19	0.28
952 Urate	0.25	0.41	0.15	0.27
810 H2O2	0.25	0.41	0.15	0.27
604 2-Methylbutyryl-CoA	0.22	0.45	0.18	0.33
773 FAICAR	0.22	0.53	0.17	0.37
473 dCTP	0.22	0.44	0.17	0.28
450 CoA	0.22	0.44	0.17	0.31
759 Deoxyguanosine	0.24	0.46	0.15	0.33
621 3-Methylglutaconyl-CoA	0.21	0.41	0.17	0.31
58 GDP-activated fucose	0.13	0.67	0.25	0.62

Continued on next page

Matches relating control 1/6h vs. 6/24h – continued

Simulation	control 1/6h		control 6/24h	
	ampl	score	ampl	score
720 CDP	0.2	0.44	0.18	0.3
857 Methylmalonyl-CoA	0.22	0.35	0.16	0.31
501 GDP-L-fucose	0.16	0.46	0.22	0.36
839 L-Fucose-1P	0.13	0.53	0.25	0.49
966 dCDP	0.2	0.43	0.18	0.27
554 (S)-3-Hydroxybutyryl-CoA	0.2	0.42	0.18	0.34
456 Thioredoxin(m)	0.26	0.95	0.12	0.96
979 mitoOxidizedThioredoxin	0.26	0.94	0.12	0.95
745 Crotonyl-CoA	0.2	0.41	0.18	0.33
615 3-Hydroxyisobutyryl-CoA	0.18	0.41	0.19	0.31
690 AICAR	0.21	0.53	0.16	0.4
833 Isovaleryl-CoA	0.19	0.34	0.18	0.29
831 Isobutyryl-CoA	0.19	0.42	0.18	0.35
967 dCMP	0.19	0.49	0.18	0.26
965 dAMP	0.18	0.49	0.19	0.35
710 Adenylosuccinate	0.18	0.5	0.18	0.39
801 Glutaryl-CoA	0.2	0.38	0.16	0.34
257 Serine from Glycine	-0.02	0.46	0.38	0.46
524 gdpddman	0.17	0.42	0.2	0.33
924 SAM	0.17	0.48	0.19	0.39
922 SAH	0.17	0.48	0.19	0.39
856 Methacrylyl-CoA	0.18	0.4	0.18	0.3
59 GDP-activated mannose	0.12	0.65	0.24	0.4
478 GTP	0.2	0.4	0.16	0.29
355 Collagen CO5A2(c) synthesis	0.02	1	0.33	1
698 Acetyl-CoA	0.18	0.42	0.18	0.35
435 Galactose degr	-0.02	0.58	0.38	0.5
964 dADP	0.17	0.48	0.18	0.31
706 Acyl-CoA-VLDL-SM-pool	0.17	0.39	0.18	0.32
702 Acyl-CoA-VLDL-PC-pool	0.17	0.39	0.18	0.32
703 Acyl-CoA-VLDL-PE-pool	0.17	0.39	0.18	0.32
707 Acyl-CoA-VLDL-TG2-pool	0.17	0.39	0.18	0.32
700 Acyl-CoA-Bile-PC-pool	0.17	0.39	0.18	0.32
708 Acyl-CoA-VLDL-TG3-pool	0.17	0.4	0.18	0.32
704 Acyl-CoA-VLDL-PI-pool	0.17	0.4	0.18	0.32
705 Acyl-CoA-VLDL-PS-pool	0.17	0.4	0.18	0.32
895 Palmitoyl-CoA	0.17	0.4	0.18	0.32
976 gamma-Linolenoyl-CoA	0.17	0.38	0.18	0.31
937 Stearoyl-CoA	0.17	0.4	0.18	0.32
883 Oleoyl-CoA	0.17	0.4	0.18	0.32
849 Linoleoyl-CoA	0.17	0.4	0.18	0.32
715 Arachidonyl-CoA	0.17	0.4	0.18	0.32
701 Acyl-CoA-CL-pool	0.17	0.39	0.18	0.32
643 3-oxooleoyl-CoA	0.17	0.38	0.18	0.31
551 (R)-Methylmalonyl-CoA	0.18	0.35	0.18	0.35
981 palmitoleoyl-CoA	0.17	0.4	0.18	0.32
535 (2E)-Hexadecenoyl-CoA	0.17	0.4	0.18	0.32
711 Adenylyl(s)ulfate	0.17	0.49	0.18	0.36
853 Malonyl-CoA	0.18	0.39	0.18	0.33
692 AMP	0.17	0.49	0.18	0.35
939 Succinyl-CoA	0.17	0.37	0.18	0.32
814 HMG-CoA	0.18	0.38	0.18	0.33
696 Acetoacetyl-CoA	0.18	0.41	0.18	0.35
689 ADP	0.17	0.49	0.18	0.34
558 (S)-3-hydroxyoleyleoyl-CoA	0.18	0.41	0.17	0.31
888 PAP	0.17	0.47	0.18	0.33
958 cAMP	0.17	0.44	0.18	0.33
502 GDP-mannose	0.17	0.43	0.18	0.3
559 (S)-3-hydroxypalmitoleoyl-CoA	0.17	0.4	0.18	0.3

Continued on next page

Matches relating control 1/6h vs. 6/24h – continued

Simulation	control 1/6h		control 6/24h	
	ampl	score	ampl	score
969 dGMP	0.19	0.47	0.16	0.36
986 trans,cis-octadeca-2,9-dienoyl-CoA	0.17	0.41	0.18	0.31
762 Dephospho-CoA	0.17	0.43	0.18	0.32
984 trans,cis-hexadeca-2,9-dienoyl-CoA	0.17	0.41	0.18	0.31
955 Xanthosine	0.19	0.41	0.16	0.3
968 dGDP	0.18	0.46	0.16	0.35
963 cis-myrist-7-enoyl-CoA	0.17	0.41	0.17	0.3
644 3-oxopalmitoleoyl-CoA	0.17	0.42	0.17	0.31
791 GDP	0.18	0.47	0.16	0.39
960 cis-(3S)-hydroxytetradec-7-enoyl-CoA	0.17	0.4	0.17	0.29
985 trans,cis-myristo-2,7-dienoyl-CoA	0.17	0.41	0.17	0.29
962 cis-laur-5-enoyl-CoA	0.17	0.41	0.17	0.29
642 3-oxomyrist-7-enoyl-CoA	0.17	0.41	0.17	0.3
610 3(S)-3-hydroxydodecen-(5Z)-oyl-CoA	0.17	0.41	0.17	0.29
983 trans,cis-dodeca-2,5-dienoyl-CoA	0.17	0.41	0.17	0.29
543 (3Z)-Dodecenoyl-CoA	0.17	0.41	0.17	0.29
641 3-oxolaur-cis-5-enoyl-CoA	0.17	0.41	0.17	0.29
555 (S)-3-Hydroxydodecanoyl-CoA	0.17	0.4	0.17	0.27
691 AIR	0.18	0.51	0.16	0.37
533 (2E)-Dodecenoyl-CoA	0.17	0.4	0.17	0.28
675 5-Phosphoribosylformylglycinamidine	0.17	0.5	0.17	0.36
387 Collagen COSA1(c) synthesis	0.13	0.37	0.2	0.43
381 Collagen COLQ(c) synthesis	0.13	0.37	0.2	0.42
380 Collagen COLA1(c) synthesis	0.13	0.37	0.2	0.43
783 Formamidopyrimidine(n)leoside triphosphate	0.17	0.48	0.17	0.35
681 6-Pyruvoyltetrahydropterin	0.17	0.47	0.17	0.33
595 2,5-Diaminopyrimidine(n)leoside triphosphate	0.16	0.49	0.17	0.34
397 Collagen PCOTH(c) synthesis	0.13	0.36	0.2	0.44
959 cGMP	0.16	0.48	0.17	0.35
390 Collagen FCN1(c) synthesis	0.13	0.35	0.2	0.42
463 dhbpt	0.19	0.31	0.14	0.28
594 2,5-Diamino-6-(5-triphosphoryl-3,4-trihydroxy-2-oxopentyl)- amino-4-oxopyrimidine	0.16	0.49	0.17	0.34
392 Collagen FCN3(c) synthesis	0.13	0.35	0.2	0.43
792 GMP	0.16	0.44	0.17	0.34
462 thbpt	0.19	0.33	0.14	0.31
790 GAR	0.15	0.5	0.18	0.44
361 Collagen CO6A6(c) synthesis	0.13	0.33	0.19	0.42
753 Deamido-NAD	0.15	0.51	0.17	0.39
772 FADH2	0.15	0.48	0.17	0.33
453 FAD	0.17	0.47	0.16	0.37
867 N-Formyl-GAR	0.15	0.49	0.17	0.38
870 NADH	0.14	0.52	0.17	0.35
451 NAD+	0.14	0.51	0.17	0.35
345 Collagen CO1A2(c) synthesis	0.03	1	0.26	1
436 Mannose degr	-0.07	0.64	0.35	0.83
894 PRPP	0.12	0.4	0.17	0.47
674 5-Phosphoribosylamine	0.12	0.45	0.16	0.42
344 Collagen CO1A1(c) synthesis	-0.02	1	0.29	1
920 Ribose-5P	0.1	0.36	0.14	0.44
338 Haptoglobin	-0.01	0.96	0.23	0.98
800 Glutamyl-5P	0.07	0.34	0.13	0.37
837 L-Cystathionine	-0.14	0.51	0.32	0.48
31 NADH to NADPH transhydrogenase in mito	-0.03	1	0.2	1
672 5-Oxoproline	0.16	0.55	0.001	0.55
854 Maltose	0.03	1	0.12	1
495 Mannose-6P	-0.07	0.78	0.22	0.78
877 O-Acetylcarnitine	0.14	1	0.01	1
916 Pyridoxal	0.29	0.81	-0.15	0.99
947 Triphosphate degr	0.04	1	0.1	1

Continued on next page

Matches relating control 1/6h vs. 6/24h – continued

Simulation	control 1/6h		control 6/24h	
	ampl	score	ampl	score
887 Oxidized thioredoxin	0.14	0.94	-0.01	0.91
455 Thioredoxin	0.14	0.95	-0.01	0.93
225 Glutamine from Valine	-0.23	0.31	0.36	0.29
746 Cys-Gly	0.11	1	0.02	1
843 L-Oleoylcarnitine	-0.11	0.52	0.24	0.51
27 NADPH redox potential into peroxy	0.18	1	-0.06	1
869 N-Pantothenoylcysteine	0.35	0.45	-0.22	0.55
613 3-Hydroxyanthranilate	-0.15	0.38	0.27	0.37
548 (R)-4-Phosphopantothenoyl-cysteine	0.35	0.48	-0.23	0.57
906 Phosphodimethylethanolamine	-0.19	1	0.3	1
905 Phosphocholine	-0.19	1	0.3	1
799 Glucuronate	0.33	0.48	-0.22	0.41
359 Collagen CO6A3(c) synthesis	-0.11	1	0.22	1
479 UTP	0.26	0.41	-0.16	0.35
41 Na+ importgradient	0.05	0.49	0.06	0.59
69 Formaldehyde degr	0.22	0.43	-0.12	0.9
809 Guanosine	0.26	0.48	-0.16	0.31
460 Pyridoxal-P	0.29	0.58	-0.19	0.91
375 Collagen COGA1(c) synthesis	-0.15	1	0.23	1
787 Fumarate	0.04	0.51	0.04	0.48
248 Proline from Valine	0.21	0.35	-0.13	0.32
507 Cytidine	0.22	0.43	-0.15	0.31
396 Collagen LPP3(c) synthesis	0.14	1	-0.07	1
200 Glycine from Serine	0.2	0.67	-0.13	0.57
44 Activated methyl group (THF)	0.2	0.67	-0.13	0.57
972 dUDP	0.23	0.35	-0.17	0.28
373 Collagen COEA1(c) synthesis	0.1	1	-0.04	1
47 Formylgroup(c)	0.18	0.71	-0.12	0.76
938 Succinate	-0.18	0.36	0.23	0.33
931 Sphinganine-1P	-0.11	0.4	0.17	0.41
974 dUTP	0.22	0.36	-0.17	0.27
758 Deoxycytidine	0.23	0.47	-0.18	0.29
918 Quinolinate	0	0.38	0.05	0.6
81 Alanine from Glutamate	-0.15	0.34	0.19	0.32
339 Collagen ADIPO(c) synthesis	-0.02	1	0.06	1
378 Collagen COJA1(c) synthesis	0.09	1	-0.05	1
948 UDP-N-acetylglucosamine	0.23	0.34	-0.19	0.39
723 CMP	0.2	0.43	-0.16	0.31
53 activated sulphur	0.07	0.87	-0.03	0.65
369 Collagen COBA1(c) synthesis	0.06	1	-0.02	1
469 ametam	0.26	0.39	-0.22	0.29
907 Phosphopantetheine	-0.12	0.39	0.16	0.29
95 Alanine from Valine	0.13	0.33	-0.1	0.33
236 Proline from Glycine	0.29	0.3	-0.27	0.39
400 Collagen VWA2(c) synthesis	0.05	1	-0.03	1
712 Agmatine	0.02	0.6	-0.01	0.6
354 Collagen CO5A1(c) synthesis	0.001	1	0.01	1
388 Collagen CTHR1(c) synthesis	-0.07	1	0.08	1
117 Arginine from beta-Alanine	0.24	0.31	-0.24	0.42
365 Collagen CO9A1(c) synthesis	-0.04	1	0.04	1
526 Putrescine	0.06	0.37	-0.07	0.5
14 Ubiquinol-to-ATP	-0.04	0.8	0.04	0.56
11 ATP from NADH(m)	-0.05	0.83	0.04	0.68
898 Phosphatidate-CL-pool	0.43	0.32	-0.44	0.47
249 Proline from beta-Alanine	0.24	0.37	-0.25	0.47
19 Aerobic reduction of NADP+ (gluc)	0.4	0.34	-0.42	0.48
822 Homovanillate	-0.04	1	0.03	1
12 ATP from NADH	-0.04	0.44	0.03	0.53
2 Aerobic ATP rephosph (gluc)	-0.07	0.45	0.05	0.33

Continued on next page

Matches relating control 1/6h vs. 6/24h – continued

Simulation	control 1/6h		control 6/24h	
	ampl	score	ampl	score
70 Formate degr	0.16	0.71	-0.18	0.85
241 Proline from Lysine	0.22	0.33	-0.25	0.31
382 Collagen COMA1(c) synthesis	0.04	1	-0.06	1
885 Orotidine-5P	0.34	0.48	-0.37	0.43
505 Uracil	0.24	0.38	-0.27	0.37
226 Glutamine from beta-Alanine	0.22	0.37	-0.26	0.47
608 2-Oxoglutaramate	0.11	1	-0.15	1
340 Collagen BGH3(c) synthesis	0.03	1	-0.07	1
376 Collagen COHA1(c) synthesis	0.02	1	-0.06	1
573 1-Acylglycerol-3P-CL-pool	0.43	0.38	-0.47	0.54
850 Malate	-0.08	0.65	0.04	0.64
393 Collagen FMOD(c) synthesis	0.03	1	-0.07	1
395 Collagen ITA2(c) synthesis	-0.16	1	0.11	1
358 Collagen CO6A2(c) synthesis	0.09	1	-0.14	1
880 OAA	-0.1	0.7	0.04	0.75
42 Na+ exportgradient	-0.32	1	0.26	1
891 PG-CL-pool	0.39	0.3	-0.45	0.38
892 PGP-CL-pool	0.39	0.3	-0.45	0.38
184 Glutamate from beta-Alanine	0.2	0.4	-0.26	0.58
36 GSH reduction using NADH redox potential	0.43	0.51	-0.49	0.63
940 Sulfite degr	-0.04	1	-0.03	1
356 Collagen CO5A3(c) synthesis	-0.07	1	0.005	1
458 Apo-ACP(m)	-0.11	0.75	0.03	0.71
978 mitoACP	-0.11	0.75	0.03	0.68
697 Acetyl-ACP	-0.11	0.87	0.03	0.84
688 ACP	-0.11	0.87	0.03	0.85
457 Apo-ACP	-0.11	0.91	0.03	0.89
695 Acetoacetyl-ACP	-0.11	0.86	0.03	0.83
851 Malonyl-ACP	-0.11	0.86	0.03	0.83
718 Butyryl-ACP	-0.11	0.87	0.03	0.83
717 But-2-enoyl-ACP	-0.11	0.87	0.03	0.83
544 (R)-3-Hydroxybutanoyl-ACP	-0.11	0.87	0.03	0.83
818 Hexanoyl-ACP	-0.11	0.87	0.03	0.82
536 (2E)-Hexenoyl-ACP	-0.11	0.87	0.03	0.82
750 D-3-Hydroxyhexanoyl-ACP	-0.11	0.87	0.03	0.82
627 3-Oxohexanoyl-ACP	-0.11	0.87	0.03	0.82
881 Octanoyl-ACP	-0.11	0.87	0.03	0.81
539 (2E)-Octenoyl-ACP	-0.11	0.87	0.03	0.81
546 (R)-3-Hydroxyoctanoyl-ACP	-0.11	0.87	0.03	0.81
629 3-Oxooctanoyl-ACP	-0.11	0.87	0.03	0.81
754 Decanoyl-ACP	-0.11	0.86	0.03	0.81
530 (2E)-Decenoyl-ACP	-0.11	0.86	0.03	0.81
545 (R)-3-Hydroxydecanoyl-ACP	-0.11	0.86	0.03	0.81
622 3-Oxodecanoyl-ACP	-0.11	0.86	0.03	0.81
769 Dodecanoyl-ACP	-0.11	0.86	0.03	0.8
532 (2E)-Dodecenoyl-ACP	-0.11	0.86	0.03	0.8
749 D-3-Hydroxydodecanoyl-ACP	-0.11	0.86	0.03	0.8
624 3-Oxododecanoyl-ACP	-0.11	0.86	0.03	0.8
942 Tetradecanoyl-ACP	-0.11	0.86	0.03	0.8
541 (2E)-Tetradecenoyl-ACP	-0.11	0.86	0.03	0.8
936 Stearoyl-ACP	-0.11	0.86	0.03	0.78
538 (2E)-Octadecenoyl-ACP	-0.11	0.86	0.03	0.78
813 HMA	-0.11	0.86	0.03	0.8
617 3-Hydroxystearoyl-ACP	-0.11	0.86	0.03	0.78
634 3-Oxotetradecanoyl-ACP	-0.11	0.86	0.03	0.8
633 3-Oxostearoyl-ACP	-0.11	0.86	0.03	0.79
816 Hexadecanoyl-ACP	-0.11	0.86	0.03	0.79
534 (2E)-Hexadecenoyl-ACP	-0.11	0.86	0.03	0.79
547 (R)-3-Hydroxypalmitoyl-ACP	-0.11	0.86	0.03	0.8

Continued on next page

Matches relating control 1/6h vs. 6/24h – continued

Simulation	control 1/6h ampl	control 1/6h score	control 6/24h ampl	control 6/24h score
626 3-Oxohexadecanoyl-ACP	-0.11	0.86	0.03	0.8
357 Collagen CO6A1(c) synthesis	-0.003	1	-0.08	1
724 CMP-NeuNGc	0.17	0.37	-0.26	0.35
764 Dihomo-gamma-linolenoyl-CoA	0.17	0.35	-0.25	0.29
370 Collagen COBA2(c) synthesis	0.06	1	-0.14	1
178 Glutamate from Proline	-0.14	0.52	0.05	0.43
812 HCO3-	-0.14	0.79	0.05	0.92
727 Carbonate	-0.14	0.79	0.05	0.92
611 3-Dehydrosphinganine	0.09	0.39	-0.17	0.45
726 Carbamoyl-P	-0.14	0.55	0.05	0.62
368 Collagen COAA1(c) synthesis	-0.03	1	-0.07	1
439 Antitrypsin degr	-0.15	0.55	0.05	0.38
944 Thymine	0.27	0.34	-0.36	0.36
619 3-Methyl-2-oxobutyrate	0.37	0.35	-0.46	0.5
37 GSH reduction using NADPH redox potential	0.42	1	-0.51	1
38 GSH oxidation	0.42	1	-0.51	1
728 Carnosine	-0.01	0.46	-0.09	0.74
480 Alanine	0.41	0.54	-0.51	0.44
341 Collagen C43BP(c) synthesis	0	1	-0.1	1
560 (S)-Dihydroorotate	-0.15	0.42	0.06	0.29
865 N-Carbamoyl-L-aspartate	-0.15	0.47	0.05	0.38
133 Asparagine from Serine	-0.15	0.44	0.05	0.36
760 Deoxyinosine	0.27	0.33	-0.37	0.34
154 Aspartate from Serine	-0.15	0.39	0.05	0.48
90 Alanine from Proline	0.11	0.32	-0.22	0.32
325 Albumin	0.01	0.97	-0.12	0.97
51 Acetyl group(p)	0.16	0.36	-0.27	0.36
863 N-Acetylneuraminate	0.13	0.3	-0.24	0.48
326 Fibrinogen	-0.29	0.33	0.18	0.39
328 Antitrypsin	-0.29	0.34	0.19	0.41
371 Collagen COCA1(c) synthesis	-0.21	1	0.1	1
886 Oxalosuccinate	-0.16	0.55	0.04	0.39
199 Glycine from Proline	0.1	0.3	-0.22	0.35
360 Collagen CO6A5(c) synthesis	-0.31	0.33	0.19	0.42
970 dTDP	0.26	0.29	-0.38	0.36
719 Butyryl-CoA	0.2	0.4	-0.31	0.29
537 (2E)-Hexenoyl-CoA	0.2	0.39	-0.32	0.3
384 Collagen COOA1(c) synthesis	0.07	1	-0.19	1
351 Collagen CO4A4(c) synthesis	-0.02	1	-0.1	1
540 (2E)-Octenoyl-CoA	0.2	0.4	-0.32	0.3
394 Collagen ITA1(c) synthesis	-0.31	0.35	0.19	0.42
628 3-Oxohexanoyl-CoA	0.19	0.42	-0.32	0.29
139 Aspartate from Alanine	-0.17	0.72	0.04	0.47
367 Collagen CO9A3(c) synthesis	0.05	1	-0.18	1
630 3-Oxooctanoyl-CoA	0.18	0.38	-0.31	0.31
209 Glutamine from Aspartate	0.17	0.35	-0.31	0.43
562 (S)-Hydroxyhexanoyl-CoA	0.19	0.38	-0.32	0.29
342 Collagen CCBE1(c) synthesis	0.04	1	-0.18	1
819 Hexanoyl-CoA	0.18	0.39	-0.31	0.31
755 Decanoyl-CoA	0.18	0.41	-0.31	0.28
563 (S)-Hydroxyoctanoyl-CoA	0.18	0.38	-0.31	0.31
639 3-Ureidoisobutyrate	0.23	0.29	-0.37	0.39
882 Octanoyl-CoA	0.18	0.39	-0.31	0.3
625 3-Oxododecanoyl-CoA	0.18	0.42	-0.31	0.27
623 3-Oxodecanoyl-CoA	0.18	0.4	-0.31	0.29
26 NADPH redox potential into mito	-0.04	0.51	-0.1	0.52
669 5-Hydroxy-L-tryptophan	0.11	0.55	-0.25	0.84
498 UDP-glucose	0.26	0.34	-0.4	0.3
561 (S)-Hydroxydecanoyl-CoA	0.18	0.39	-0.32	0.29

Continued on next page

Matches relating control 1/6h vs. 6/24h – continued

Simulation	control 1/6h		control 6/24h	
	ampl	score	ampl	score
531 (2E)-Decenoyl-CoA	0.18	0.39	-0.32	0.29
265 Serine from Proline	0.11	0.3	-0.25	0.37
499 udpgal	0.22	0.38	-0.37	0.37
774 FMN	-0.04	1	-0.12	1
485 Glycine	0.35	0.31	-0.5	0.41
767 Dihydrothymine	0.23	0.31	-0.39	0.35
383 Collagen CONA1(c) synthesis	-0.13	1	-0.03	1
275 beta-Alanine from Aspartate	-0.04	0.89	-0.12	0.87
364 Collagen CO8A2(c) synthesis	0.05	1	-0.21	1
274 beta-Alanine from Asparagine	-0.03	0.38	-0.13	0.37
494 Mannose-1P	0.13	0.46	-0.29	0.48
278 beta-Alanine from Glutamate	-0.06	0.41	-0.11	0.38
120 Asparagine from Aspartate	0.03	0.4	-0.2	0.46
64 Glucosamine-6P	-0.08	0.64	-0.09	0.63
461 4ppan	-0.13	1	-0.04	1
525 beta-Alanine	-0.09	0.32	-0.08	0.34
208 Glutamine from Asparagine	0.15	0.38	-0.32	0.45
282 beta-Alanine from Glutamine	-0.07	0.36	-0.11	0.35
379 Collagen COKA1(c) synthesis	-0.16	1	-0.02	1
389 Collagen EMID2(c) synthesis	-0.07	1	-0.11	1
859 N-(omega)-Hydroxyarginine	-0.06	0.77	-0.13	0.77
982 sn-Glycerol-3P	0.41	0.47	-0.59	0.93
385 Collagen COPA1(c) synthesis	-0.06	1	-0.13	1
917 Pyridoxine-P	0.04	1	-0.23	1
363 Collagen CO8A1(c) synthesis	-0.01	1	-0.18	1
527 Spermidine	0.31	0.45	-0.5	0.4
930 Sphinganine	-0.02	0.4	-0.17	0.34
765 Dihydroceramide-pool	-0.02	0.52	-0.17	0.42
951 UMP	0.22	0.44	-0.42	0.37
353 Collagen CO4A6(c) synthesis	0.004	1	-0.2	1
235 Proline from Glutamate	-0.14	0.51	-0.06	0.44
673 5-Phosphoribosyl-4-carboxy-5-aminoimidazole	0.17	0.46	-0.37	0.39
366 Collagen CO9A2(c) synthesis	-0.04	1	-0.17	1
310 Bilirubin conjugation	0.41	0.46	-0.62	0.5
832 Isocitrate	-0.16	0.48	-0.06	0.4
292 beta-Alanine from Valine	0.18	0.35	-0.4	0.34
362 Collagen CO7A1(c) synthesis	0.01	1	-0.24	1
372 Collagen CODA1(c) synthesis	-0.04	1	-0.19	1
871 NADPH	0.14	0.55	-0.38	0.35
452 NADP+	0.14	0.55	-0.38	0.34
374 Collagen COFA1(c) synthesis	-0.2	1	-0.04	1
162 Cystine from Cysteine	0.46	0.57	-0.69	0.68
160 Cysteine from Cystine	0.46	0.57	-0.69	0.68
153 Aspartate from Proline	-0.16	0.38	-0.07	0.31
884 Orotate	-0.14	0.41	-0.09	0.36
205 Glycine from beta-Alanine	0.23	0.31	-0.47	0.37
873 Nicotinamide D-ribonucleotide	0.13	0.56	-0.37	0.48
334 ApoE(c)	-0.01	0.95	-0.24	0.96
876 Nicotinate(r)ibonucleotide	0.13	0.47	-0.37	0.4
468 PAPS	0.35	0.33	-0.59	0.35
680 6-Phospho-D-gluconate	0.12	0.64	-0.36	0.77
875 Nicotinate D-ribonucleoside	0.12	0.45	-0.37	0.36
273 beta-Alanine from Arginine	-0.11	0.29	-0.14	0.37
32 Thioredoxin(m) reduction	0.19	1	-0.45	1
33 Thioredoxin(m) oxidation	0.19	1	-0.45	1
23 Oxidation of NADPH	-0.08	0.56	-0.18	0.35
522 Triacylglycerol	0.17	0.38	-0.43	0.61
509 Guanine	0.2	0.33	-0.46	0.31
508 Xanthine	0.2	0.34	-0.46	0.33

Continued on next page

Matches relating control 1/6h vs. 6/24h – continued

Simulation		control 1/6h ampl	control 1/6h score	control 6/24h ampl	control 6/24h score
472	dATP	0.19	0.42	-0.45	0.32
474	dGTP	0.2	0.4	-0.46	0.29
476	ATP	0.19	0.42	-0.45	0.32
504	XMP	0.2	0.38	-0.46	0.32
287	beta-Alanine from Proline	-0.16	0.32	-0.11	0.39
350	Collagen CO4A3(c) synthesis	-0.14	1	-0.13	1
868	N-Methylethanolamine-P	0	1	-0.27	1
771	Ethanolamine-P	0	1	-0.27	1
57	UDP-activated galactose	0.12	0.49	-0.39	0.54
269	Serine from Valine	0.13	0.3	-0.4	0.32
487	Proline	-0.2	0.34	-0.07	0.31
298	Palmitolate from Palmitate	-0.47	0.36	0.2	0.36
204	Glycine from Valine	0.15	0.3	-0.42	0.31
923	SAICAR	0.29	0.52	-0.56	0.38
138	Asparagine from beta-Alanine	-0.05	0.36	-0.23	0.48
438	Antichymotrypsin degr	-0.33	0.5	0.05	0.35
584	1-Acylglycerol-3P-palmn	0.4	0.27	-0.68	0.66
855	Mercaptopyruvate	0.14	0.4	-0.42	0.55
528	Spermine	0.32	0.41	-0.6	0.37
127	Asparagine from Glutamine	-0.06	0.46	-0.22	0.45
667	5-Formiminotetrahydrofolate	0.12	0.57	-0.4	0.75
699	Acrylyl-CoA	0.17	0.33	-0.46	0.28
503	IMP	0.2	0.39	-0.48	0.31
782	Fatty-acid-VLDL-TG3-pool	0.18	0.4	-0.47	0.66
796	Glucono-1,5-lactone-6P	0.12	1	-0.41	1
490	Palmitate	-0.14	0.85	-0.15	0.74
28	NADPH to NADH transhydrogenase	-0.01	1	-0.28	1
493	Stearate	-0.14	0.85	-0.15	0.74
123	Asparagine from Glutamate	-0.06	0.38	-0.23	0.33
159	Aspartate from beta-Alanine	-0.05	0.38	-0.25	0.54
301	Palmitolate from Arachidonate	-0.14	0.62	-0.15	0.61
447	Glycogenin degr	-0.35	0.54	0.05	0.34
518	CL	0.11	0.32	-0.41	0.45
492	Oleate	-0.14	0.73	-0.16	0.67
158	Aspartate from Valine	-0.23	0.38	-0.07	0.32
34	Thioredoxin(c) reduction	0.14	0.88	-0.44	1
423	Valine degr	-0.23	0.34	-0.08	0.27
928	Sedoheptulose-7P	0.11	0.33	-0.42	0.47
517	PI	0.12	0.42	-0.42	0.36
68	O2.-(c) degr	-0.12	0.68	-0.18	0.94
17	Aerobic reduction of NAD+ (gluc)	0.1	0.32	-0.42	0.36
716	Argininosuccinate	-0.08	0.53	-0.23	0.33
511	UDP-N-acetylgalactosamine	-0.13	0.36	-0.19	0.38
921	Ribulose-5P	0.11	0.4	-0.43	0.56
616	3-Hydroxypropionyl-CoA	0.17	0.33	-0.49	0.29
467	1fthf	0.11	0.74	-0.43	0.62
148	Aspartate from Glutamine	-0.06	0.42	-0.25	0.33
119	Asparagine from Arginine	-0.14	0.32	-0.18	0.28
664	5,10-Methenyl-THF	0.12	0.83	-0.44	0.68
834	Kynurenine	-0.63	0.42	0.31	0.35
183	Glutamate from Valine	-0.25	0.39	-0.07	0.31
742	Citrate	-0.06	0.58	-0.27	0.52
512	N-acglucam	-0.11	0.5	-0.22	0.46
144	Aspartate from Glutamate	-0.06	0.48	-0.27	0.32
466	THF(m)	0.11	1	-0.44	1
459	THF	0.11	1	-0.44	1
29	NADH to NADPH transhydrogenase	0.11	1	-0.44	1
766	Dihydrofolate	0.11	1	-0.44	1
670	5-Methyl-THF	0.11	1	-0.44	1

Continued on next page

Matches relating control 1/6h vs. 6/24h – continued

Simulation	control 1/6h ampl	control 1/6h score	control 6/24h ampl	control 6/24h score
862 N-Acetylmannosamine-6P	-0.11	0.54	-0.22	0.66
464 mlthf	0.11	0.79	-0.44	0.78
864 N-Acetylneuraminate-9P	-0.11	0.42	-0.22	0.45
484 Glutamate	-0.03	0.39	-0.3	0.58
116 Arginine from Valine	-0.25	0.31	-0.08	0.26
519 LacCer	0.1	0.28	-0.43	0.39
448 Ethanol degr	0.13	0.44	-0.47	0.54
132 Asparagine from Proline	-0.15	0.33	-0.19	0.39
186 Glycine from Arginine	-0.11	0.27	-0.24	0.33
76 Alanine from Arginine	-0.15	0.26	-0.2	0.29
251 Serine from Arginine	-0.11	0.28	-0.24	0.34
337 ApoTransferin	-0.03	0.97	-0.32	0.98
6 Aerobic rephosph of UDP	-0.06	0.55	-0.29	0.32
220 Glutamine from Proline	-0.14	0.33	-0.21	0.36
35 Thioredoxin(c) oxidation	-0.01	0.71	-0.34	0.9
896 Pantetheine	-0.51	0.33	0.15	0.35
747 Cysteamine	-0.51	0.42	0.15	0.45
949 UDP-activated-N-acetyl-D-galactosamine	-0.11	0.7	-0.25	0.56
137 Asparagine from Valine	-0.21	0.34	-0.15	0.29
21 Aerobic reduction of FAD (FA)	-0.11	0.33	-0.25	0.32
872 NeuNGc	-0.13	0.42	-0.23	0.45
553 (S)-3-Hydroxy-2-methylbutyryl-CoA	0.2	0.36	-0.57	0.28
39 Proton-gradient(m) build up	-0.07	0.5	-0.29	0.38
40 Proton-gradient(c) build up	-0.07	0.5	-0.29	0.38
246 Proline from Tryptophan	-0.15	0.32	-0.22	0.28
919 Ribose-1P	0.11	0.28	-0.48	0.55
230 Proline from Arginine	-0.12	0.32	-0.25	0.44
229 Proline from Alanine	-0.11	0.27	-0.27	0.44
173 Glutamate from Glutamine	-0.09	0.38	-0.29	0.43
784 Formylanthranilate	-0.71	0.4	0.33	0.58
961 cis-Aconitate	-0.08	0.59	-0.3	0.59
1 Aerobic ATP rephosph (FA)	-0.06	0.49	-0.32	0.3
4 Aerobic rephosph of GTP	-0.06	0.46	-0.32	0.29
7 Aerobic rephosph of UTP	-0.06	0.46	-0.32	0.28
858 Myristoyl-CoA	0.18	0.34	-0.56	0.28
945 Tiglyl-CoA	0.21	0.37	-0.6	0.28
244 Proline from Serine	-0.12	0.35	-0.27	0.42
430 LDL degr	0.17	0.32	-0.57	0.44
111 Arginine from Proline	-0.19	0.29	-0.2	0.32
179 Glutamate from Serine	-0.11	0.34	-0.29	0.52
878 O-Butanoylcarnitine	-0.1	0.37	-0.3	0.41
725 CMP-activated-N-acetylneuraminate	-0.1	0.35	-0.29	0.47
513 CMP-N-acetylneuraminate	-0.1	0.35	-0.29	0.47
121 Asparagine from Cysteine	-0.15	0.36	-0.24	0.42
793 GSSG	0.39	0.58	-0.78	0.74
221 Glutamine from Serine	-0.13	0.32	-0.27	0.48
22 Oxidation of NADH	-0.09	0.56	-0.31	0.38
603 2-Methylacetoacetyl-CoA	0.2	0.37	-0.6	0.29
231 Proline from Asparagine	-0.13	0.32	-0.28	0.4
233 Proline from Cysteine	-0.14	0.32	-0.27	0.43
751 D-Xylulose-5P	0.11	0.37	-0.52	0.43
911 Propanoyl-CoA	0.17	0.36	-0.59	0.28
794 Galactose-1P	-0.02	0.64	-0.4	0.59
398 Collagen SCRB1(c) synthesis	-0.38	1	-0.04	1
56 UDP-activated glucuronate	0.38	0.38	-0.8	0.41
419 Proline degr	-0.21	0.3	-0.2	0.28
348 Collagen CO4A1(c) synthesis	-0.34	1	-0.08	1
861 N-Acetylglucosamine-1P	-0.12	0.75	-0.3	0.61
50 Acetyl group(m)	-0.1	0.36	-0.33	0.44

Continued on next page

Matches relating control 1/6h vs. 6/24h – continued

Simulation	control 1/6h		control 6/24h	
	ampl	score	ampl	score
941 THF-hexaglutamate	0.34	0.91	-0.77	0.95
237 Proline from Histidine	-0.13	0.35	-0.3	0.47
377 Collagen COIA1(c) synthesis	0.07	1	-0.5	1
486 Glutamine	-0.13	0.38	-0.3	0.51
247 Proline from Tyrosine	-0.12	0.27	-0.32	0.31
693 Acetaldehyde	-0.16	0.54	-0.27	0.54
126 Asparagine from Isoleucine	-0.09	0.43	-0.34	0.44
432 Oleate degr	-0.1	0.32	-0.34	0.4
860 N-Acetyl-D-mannosamine	-0.1	0.38	-0.34	0.51
433 Stearate degr	-0.1	0.32	-0.34	0.41
391 Collagen FCN2(c) synthesis	0.08	1	-0.52	1
556 (S)-3-Hydroxyhexadecanoyl-CoA	0.17	0.33	-0.61	0.28
232 Proline from Aspartate	-0.14	0.4	-0.31	0.42
166 Glutamate from Asparagine	-0.11	0.36	-0.33	0.44
45 Activated methylene group from Try	-0.11	0.31	-0.34	0.26
542 (2E)-Tetradecenoyl-CoA	0.2	0.34	-0.65	0.32
295 Taurine from Cysteine	-0.5	0.57	0.05	0.6
826 Hypotaurine	-0.5	0.57	0.05	0.6
210 Glutamine from Cysteine	-0.17	0.38	-0.29	0.4
147 Aspartate from Isoleucine	-0.09	0.47	-0.36	0.49
234 Proline from Cystine	-0.14	0.35	-0.31	0.38
497 UDP-xylose	0.29	0.45	-0.74	0.3
105 Arginine from Isoleucine	-0.1	0.35	-0.36	0.38
847 Lauroyl-CoA	0.21	0.35	-0.67	0.33
171 Glutamate from Histidine	-0.12	0.31	-0.34	0.54
638 3-Sulfinoalanine	-0.51	0.79	0.05	0.33
934 Squalene	-0.32	0.8	-0.14	0.79
909 Presqualene-PP	-0.32	0.8	-0.14	0.79
168 Glutamate from Cysteine	-0.16	0.36	-0.3	0.44
167 Glutamate from Aspartate	-0.11	0.41	-0.36	0.55
557 (S)-3-Hydroxytetradecanoyl-CoA	0.17	0.32	-0.64	0.32
239 Proline from Glutamine	-0.12	0.28	-0.35	0.37
281 beta-Alanine from Isoleucine	-0.1	0.45	-0.38	0.41
515 PE	-0.07	0.32	-0.4	0.51
761 Deoxyuridine	-0.14	0.29	-0.33	0.44
797 Glucose-1P	-0.02	1	-0.46	1
510 Adenosine	0.19	0.39	-0.67	0.32
977 linoleic-Carnitine	0.05	0.42	-0.53	0.51
714 Arachidonyl-Carnitine	0.05	0.42	-0.53	0.51
214 Glutamine from Histidine	-0.15	0.35	-0.34	0.49
465 acgam6p	-0.13	0.74	-0.35	0.62
912 Protein(l)ysine	-0.07	1	-0.41	1
927 Sedoheptulose-1,7PP	-0.13	0.42	-0.36	0.56
336 Prothrombin	-0.13	0.98	-0.36	0.98
635 3-Oxotetradecanoyl-CoA	0.17	0.34	-0.66	0.31
844 L-Palmitoylcarnitine	0.05	0.42	-0.54	0.49
5 Aerobic rephosph of CTP	-0.1	0.36	-0.39	0.35
973 dUMP	-0.14	0.31	-0.36	0.39
61 AKG	-0.1	0.38	-0.4	0.46
748 D-3-Amino-isobutanoate	-0.15	0.32	-0.35	0.36
425 Isoleucine degr	-0.1	0.4	-0.4	0.35
215 Glutamine from Isoleucine	-0.14	0.45	-0.36	0.34
786 Fructose-2,6PP	-0.4	0.99	-0.1	0.84
631 3-Oxopalmitoyl-CoA	0.17	0.36	-0.68	0.29
193 Glycine from Isoleucine	-0.1	0.4	-0.41	0.45
238 Proline from Isoleucine	-0.16	0.39	-0.35	0.4
308 VLDL from LDL	-0.19	0.74	-0.32	0.86
84 Alanine from Isoleucine	-0.11	0.42	-0.41	0.47
322 PS(b)	-0.07	0.32	-0.44	0.44

Continued on next page

Matches relating control 1/6h vs. 6/24h – continued

Simulation	control 1/6h		control 6/24h	
	ampl	score	ampl	score
516 PS	-0.07	0.32	-0.44	0.44
164 Glutamate from Alanine	-0.15	0.41	-0.37	0.56
330 ApoB100(r)	-0.19	0.99	-0.33	0.99
240 Proline from Leucine	-0.13	0.39	-0.39	0.45
401 VLDL	-0.19	0.71	-0.33	0.86
309 VLDL from HDL	-0.19	0.72	-0.33	0.87
971 dTMP	-0.14	0.28	-0.38	0.39
346 Collagen CO2A1(c) synthesis	-0.21	1	-0.31	1
481 Arginine	-0.24	0.28	-0.28	0.34
259 Serine from Isoleucine	-0.1	0.46	-0.43	0.46
770 Erythrose-4P	-0.13	0.39	-0.4	0.55
125 Asparagine from Histidine	-0.15	0.35	-0.38	0.37
174 Glutamate from Leucine	-0.12	0.42	-0.42	0.48
820 Histamine	-0.002	0.64	-0.54	0.82
602 2-Lysolecithin-pool	-0.15	0.27	-0.39	0.41
332 ApoC2(c)	-0.14	0.95	-0.4	0.95
840 L-Glutamate 5-semialdehyde	0.07	0.46	-0.61	0.39
216 Glutamine from Leucine	-0.13	0.41	-0.42	0.43
194 Glycine from Glutamine	-0.09	0.31	-0.47	0.3
260 Serine from Glutamine	-0.09	0.34	-0.47	0.31
46 Activated methyl group from Histidine	-0.12	0.27	-0.44	0.38
258 Serine from Histidine	-0.12	0.31	-0.44	0.42
20 Aerobic reduction of FAD (gluc)	-0.13	0.3	-0.44	0.38
722 CDP-ethanolamine	0.2	0.38	-0.77	0.32
283 beta-Alanine from Leucine	-0.11	0.4	-0.46	0.46
661 4-Methyl-2-oxopentanoate	-0.13	0.42	-0.43	0.41
192 Glycine from Histidine	-0.12	0.29	-0.45	0.4
514 PC	-0.15	0.31	-0.42	0.43
600 2-Deoxy-D-ribose-1P	-0.17	0.49	-0.4	0.57
107 Arginine from Leucine	-0.13	0.36	-0.43	0.39
482 Asparagine	-0.15	0.36	-0.42	0.38
172 Glutamate from Isoleucine	-0.19	0.5	-0.38	0.37
212 Glutamine from Glutamate	0.06	0.33	-0.63	0.26
261 Serine from Leucine	-0.12	0.38	-0.45	0.48
118 Asparagine from Alanine	-0.17	0.38	-0.41	0.34
601 2-Deoxy-D-ribose-5P	-0.16	0.62	-0.41	0.44
128 Asparagine from Leucine	-0.12	0.43	-0.46	0.43
83 Alanine from Histidine	-0.16	0.31	-0.43	0.38
191 Glycine from Glutamate	-0.09	0.32	-0.49	0.31
195 Glycine from Leucine	-0.12	0.38	-0.47	0.45
866 N-Formimino-L-glutamate	-0.16	0.33	-0.43	0.53
256 Serine from Glutamate	-0.09	0.35	-0.5	0.32
428 beta-Alanine degr	-0.68	0.32	0.09	0.32
270 Serine from beta-Alanine	-0.11	0.31	-0.48	0.41
86 Alanine from Leucine	-0.15	0.41	-0.45	0.48
149 Aspartate from Leucine	-0.11	0.43	-0.49	0.46
323 PE(b)	-0.07	0.28	-0.53	0.56
201 Glycine from Threonine	-0.1	0.3	-0.51	0.45
289 beta-Alanine from Threonine	-0.09	0.29	-0.52	0.39
250 Serine from Alanine	-0.14	0.37	-0.47	0.55
488 Serine	-0.11	0.33	-0.51	0.46
60 Pyruvate	-0.11	0.41	-0.5	0.48
415 Leucine degr	-0.15	0.4	-0.46	0.36
845 Lanosterol	-0.49	0.64	-0.13	0.59
817 Hexadecenal	0.08	0.35	-0.7	0.34
815 Hexadecanal	0.08	0.35	-0.7	0.34
280 beta-Alanine from Histidine	-0.2	0.31	-0.42	0.41
483 Aspartate	-0.15	0.35	-0.47	0.37
320 Bile-PC(b)	-0.15	0.28	-0.47	0.48

Continued on next page

Matches relating control 1/6h vs. 6/24h – continued

Simulation	control 1/6h		control 6/24h	
	ampl	score	ampl	score
245 Proline from Threonine	-0.19	0.39	-0.43	0.38
134 Asparagine from Threonine	-0.1	0.35	-0.53	0.46
266 Serine from Threonine	-0.11	0.35	-0.52	0.48
52 Acetyl group(r)	-0.37	0.41	-0.27	0.43
49 Acetyl group(c)	-0.37	0.41	-0.27	0.43
185 Glycine from Alanine	-0.14	0.36	-0.5	0.48
175 Glutamate from Lysine	-0.09	0.31	-0.56	0.35
272 beta-Alanine from Alanine	-0.17	0.56	-0.49	0.44
155 Aspartate from Threonine	-0.14	0.33	-0.53	0.45
180 Glutamate from Threonine	-0.21	0.42	-0.46	0.42
92 Alanine from Threonine	-0.14	0.35	-0.52	0.47
188 Glycine from Aspartate	-0.16	0.39	-0.52	0.45
253 Serine from Aspartate	-0.16	0.45	-0.52	0.5
935 Squalene 2,3-oxide	-0.29	0.78	-0.39	0.58
222 Glutamine from Threonine	-0.21	0.41	-0.48	0.41
206 Glutamine from Alanine	-0.35	0.34	-0.35	0.42
217 Glutamine from Lysine	-0.12	0.36	-0.59	0.36
16 Aerobic reduction of NAD+ (FA)	0.35	0.33	-1.06	0.32
78 Alanine from Aspartate	-0.18	0.78	-0.53	0.54
520 SM	-0.39	0.27	-0.32	0.41
521 Ceramide	-0.42	0.27	-0.29	0.4
879 O-Propanoylcarnitine	-0.13	0.51	-0.58	0.34
910 Propanoate	-0.53	0.33	-0.19	0.67
252 Serine from Asparagine	-0.14	0.32	-0.58	0.46
852 Malonyl-Carnitin	-0.17	0.61	-0.55	0.42
889 PE-PS-VLDL-pool	-0.31	0.31	-0.41	0.47
187 Glycine from Asparagine	-0.14	0.31	-0.59	0.41
550 (R)-5-Phosphomevalonate	-0.48	0.61	-0.25	0.78
294 Taurine from Methionine	-0.12	0.38	-0.6	0.45
161 Cysteine from Methionine	-0.13	0.38	-0.6	0.46
640 3-Ureidopropionate	-0.35	0.37	-0.38	0.48
431 HDL degr	-0.15	0.34	-0.58	0.39
62 Isopentenyl-PP	-0.5	0.63	-0.23	0.77
242 Proline from Methionine	-0.18	0.39	-0.55	0.4
97 Arginine from Alanine	-0.38	0.38	-0.35	0.31
197 Glycine from Methionine	-0.13	0.35	-0.6	0.46
263 Serine from Methionine	-0.13	0.37	-0.61	0.49
99 Arginine from Aspartate	0.24	0.3	-0.98	0.32
437 Albumin degr	-0.34	0.51	-0.4	0.36
445 ApoTransferin degr	-0.35	0.53	-0.39	0.35
163 Cystine from Methionine	-0.13	0.35	-0.61	0.51
932 Sphingosine	0.07	0.34	-0.82	0.37
442 Haptoglobin degr	-0.36	0.57	-0.39	0.36
285 beta-Alanine from Methionine	-0.14	0.33	-0.61	0.44
549 (R)-5-Diphosphomevalonate	-0.51	0.68	-0.24	0.72
130 Asparagine from Methionine	-0.15	0.41	-0.6	0.46
88 Alanine from Methionine	-0.15	0.38	-0.6	0.48
146 Aspartate from Histidine	-0.15	0.33	-0.6	0.38
729 Ceramide-1P-pool	0.08	0.38	-0.83	0.31
933 Sphingosine-1P	0.07	0.3	-0.83	0.33
196 Glycine from Lysine	-0.11	0.33	-0.64	0.4
262 Serine from Lysine	-0.11	0.36	-0.64	0.43
946 Trehalose	-0.04	1	-0.72	1
63 Farnesyl-PP	-0.53	0.63	-0.23	0.6
151 Aspartate from Methionine	-0.13	0.36	-0.64	0.5
129 Asparagine from Lysine	-0.13	0.36	-0.64	0.35
571 1,3DPG	-0.3	0.49	-0.47	0.79
227 Methionine from Cysteine	-0.22	0.34	-0.55	0.45
284 beta-Alanine from Lysine	-0.08	0.39	-0.7	0.44

Continued on next page

Matches relating control 1/6h vs. 6/24h – continued

Simulation	control 1/6h		control 6/24h	
	ampl	score	ampl	score
150 Aspartate from Lysine	-0.09	0.39	-0.68	0.47
756 Dehydroalanine	-0.66	0.72	-0.11	0.63
279 beta-Alanine from Glycine	-0.1	0.37	-0.68	0.42
795 Geranyl-PP	-0.55	0.62	-0.23	0.6
665 5,6-Dihydrouracil	-0.38	0.35	-0.4	0.43
637 3-Phosphoserine	-0.13	0.43	-0.65	0.57
606 2-Oxoadipate	-0.13	0.44	-0.66	0.39
785 Fructose-1,6PP	-0.33	1	-0.46	1
218 Glutamine from Methionine	-0.19	0.42	-0.6	0.44
87 Alanine from Lysine	-0.14	0.38	-0.65	0.4
496 Fructose-6P	-0.32	1	-0.48	1
228 Methionine from Cystine	-0.19	0.34	-0.61	0.49
552 (R)-Mevalonate	-0.51	0.82	-0.28	0.8
77 Alanine from Asparagine	-0.18	0.41	-0.62	0.46
402 HDL	-0.14	0.72	-0.67	0.79
781 Fatty-acid-VLDL-TG2-pool	-0.23	0.4	-0.57	0.54
780 Fatty-acid-VLDL-TG1-pool	-0.23	0.4	-0.57	0.54
141 Aspartate from Asparagine	-0.08	0.38	-0.73	0.49
303 Dihomo-gamma-linolenate from Linoleate	-0.59	0.36	-0.22	0.42
306 Arachidonate from gamma-Linolenate	-0.59	0.36	-0.22	0.42
980 palmitoleoyl-Carnitine	0.05	0.45	-0.86	0.59
329 ApoA1	-0.14	0.96	-0.68	0.97
841 L-Lactate	-0.3	0.85	-0.53	0.92
645 3PG	-0.11	0.57	-0.72	0.67
636 3-Phosphonooxypyruvate	-0.11	0.49	-0.72	0.57
288 beta-Alanine from Serine	-0.11	0.46	-0.71	0.42
176 Glutamate from Methionine	-0.21	0.47	-0.62	0.42
446 ApoB100 degr	-0.46	0.59	-0.37	0.51
304 Arachidonate from Linoleate	-0.62	0.39	-0.22	0.46
305 Dihomo-gamma-linolenate from gamma-Linolenate	-0.32	0.31	-0.52	0.38
124 Asparagine from Glycine	-0.59	0.36	-0.25	0.49
189 Glycine from Cysteine	-0.15	0.32	-0.69	0.39
190 Glycine from Cystine	-0.16	0.32	-0.69	0.46
255 Serine from Cystine	-0.16	0.36	-0.69	0.5
3 Anaerobic rephosph of ATP	-0.33	0.34	-0.52	0.46
776 Fatty-acid-VLDL-PE-pool	-0.28	0.4	-0.57	0.53
321 SM(b)	-0.39	0.26	-0.46	0.42
591 1-Acylglycerol-VLDL-TG1-pool	-0.33	0.3	-0.53	0.55
169 Glutamate from Cystine	-0.15	0.33	-0.7	0.48
586 1-Acylglycerol-VLDL-PC-pool	-0.33	0.3	-0.53	0.55
471 Homocysteine	-0.21	0.31	-0.64	0.42
777 Fatty-acid-VLDL-PI-pool	-0.28	0.4	-0.57	0.53
587 1-Acylglycerol-VLDL-PE-pool	-0.33	0.3	-0.53	0.55
590 1-Acylglycerol-VLDL-SM-pool	-0.33	0.3	-0.53	0.55
778 Fatty-acid-VLDL-PS-pool	-0.28	0.4	-0.58	0.53
588 1-Acylglycerol-VLDL-PI-pool	-0.33	0.3	-0.53	0.56
589 1-Acylglycerol-VLDL-PS-pool	-0.33	0.3	-0.53	0.56
25 NADH redox potential into peroxy	-0.3	0.52	-0.56	0.81
108 Arginine from Lysine	-0.1	0.31	-0.76	0.35
768 Dimethylallyl-PP	-0.63	0.6	-0.23	0.65
109 Arginine from Methionine	-0.21	0.35	-0.65	0.42
491 Glycerol	-0.25	0.39	-0.62	0.62
307 Arachidonate from Dihomo-gamma-linolenate	-0.64	0.49	-0.22	0.4
904 Phosphatidate-VLDL-TG-pool	-0.39	0.32	-0.47	0.53
899 Phosphatidate-VLDL-PC-pool	-0.39	0.32	-0.47	0.53
302 gamma-Linolenate from Linoleate	-0.64	0.43	-0.22	0.5
10 Anaerobic rephosph of UTP	-0.35	0.33	-0.52	0.44
8 Anaerobic rephosph of GTP	-0.35	0.33	-0.52	0.44
9 Anaerobic rephosph of CTP	-0.35	0.33	-0.52	0.44

Continued on next page

Matches relating control 1/6h vs. 6/24h – continued

Simulation	control 1/6h		control 6/24h	
	ampl	score	ampl	score
900 Phosphatidate-VLDL-PE-pool	-0.39	0.32	-0.48	0.53
903 Phosphatidate-VLDL-SM-pool	-0.39	0.32	-0.48	0.52
901 Phosphatidate-VLDL-PI-pool	-0.39	0.32	-0.48	0.53
570 1,2-Diacylglycerol-VLDL-TG-pool	-0.4	0.28	-0.47	0.47
565 1,2-Diacylglycerol-VLDL-PC-pool	-0.4	0.28	-0.47	0.47
574 1-Acylglycerol-3P-VLDL-PC-pool	-0.38	0.35	-0.5	0.63
902 Phosphatidate-VLDL-PS-pool	-0.39	0.32	-0.48	0.53
489 Tyrosine	-0.01	0.48	-0.86	0.71
566 1,2-Diacylglycerol-VLDL-PE-pool	-0.4	0.28	-0.48	0.48
575 1-Acylglycerol-3P-VLDL-PE-pool	-0.38	0.35	-0.5	0.64
569 1,2-Diacylglycerol-VLDL-SM-pool	-0.4	0.28	-0.48	0.47
579 1-Acylglycerol-3P-VLDL-TG1-pool	-0.38	0.35	-0.5	0.63
333 ApoC3(c)	-0.14	0.95	-0.74	0.95
578 1-Acylglycerol-3P-VLDL-SM-pool	-0.38	0.35	-0.5	0.63
145 Aspartate from Glycine	-0.59	0.43	-0.29	0.47
897 Phosphatidate-Bile-PC-pool	-0.39	0.31	-0.48	0.51
91 Alanine from Serine	-0.09	0.43	-0.79	0.44
576 1-Acylglycerol-3P-VLDL-PI-pool	-0.38	0.35	-0.5	0.64
567 1,2-Diacylglycerol-VLDL-PI-pool	-0.4	0.28	-0.48	0.48
577 1-Acylglycerol-3P-VLDL-PS-pool	-0.38	0.35	-0.5	0.64
82 Alanine from Glycine	-0.09	0.39	-0.79	0.4
315 Glycocholate(b)	-0.44	0.51	-0.44	0.57
568 1,2-Diacylglycerol-VLDL-PS-pool	-0.4	0.29	-0.48	0.48
564 1,2-Diacylglycerol-Bile-PC-pool	-0.4	0.28	-0.48	0.46
572 1-Acylglycerol-3P-Bile-PC-pool	-0.38	0.34	-0.5	0.62
585 1-Acylglycerol-3P-stea	-0.38	0.36	-0.5	0.65
582 1-Acylglycerol-3P-ol	-0.38	0.36	-0.5	0.65
581 1-Acylglycerol-3P-lin	-0.38	0.36	-0.5	0.65
580 1-Acylglycerol-3P-arach	-0.38	0.36	-0.5	0.65
103 Arginine from Glycine	-0.58	0.33	-0.31	0.34
789 GAP	-0.31	0.66	-0.58	0.88
752 DHAP	-0.31	0.66	-0.58	0.88
583 1-Acylglycerol-3P-palm	-0.38	0.36	-0.51	0.63
317 Taurocholate(b)	-0.44	0.49	-0.46	0.5
647 3alpha,7alpha,12alpha-Trihydroxy-5beta-24-oxocholestanoyl-CoA	-0.46	0.39	-0.44	0.48
646 3alpha,7alpha,12alpha,24-Tetrahydroxy-5beta-cholestanoyl-CoA	-0.46	0.4	-0.45	0.47
663 4alpha-Methylzymosterol-4-carboxylate	-0.49	0.72	-0.41	0.76
211 Glutamine from Cystine	-0.16	0.42	-0.75	0.46
316 Gly-CD-cholate(b)	-0.46	0.49	-0.45	0.55
500 udpglcur	0.31	0.4	-1.23	0.32
319 Chenodiol(b)	-0.46	0.46	-0.45	0.6
648 3alpha,7alpha,12alpha-Trihydroxy-5beta-cholest-24-enoyl-CoA	-0.46	0.4	-0.45	0.46
652 3alpha,7alpha,12alpha-Trihydroxycoprostane	-0.46	0.66	-0.45	0.69
677 5beta-Cholestane-3alpha,7alpha,12alpha,26-tetrol	-0.45	0.66	-0.46	0.7
650 3alpha,7alpha,12alpha-Trihydroxy-5beta-cholestanate	-0.45	0.66	-0.46	0.7
649 3alpha,7alpha,12alpha-Trihydroxy-5beta-cholestan-26-al	-0.45	0.66	-0.46	0.7
66 ATP salvage from Hypoxanthine	-0.31	0.32	-0.6	0.42
593 14-Demethyllanosterol	-0.53	0.65	-0.38	0.69
213 Glutamine from Glycine	-0.62	0.32	-0.3	0.44
122 Asparagine from Cystine	-0.15	0.39	-0.77	0.5
651 3alpha,7alpha,12alpha-Trihydroxy-5beta-cholestanoyl-CoA	-0.46	0.44	-0.46	0.48
656 4,4-Dimethyl-5alpha-cholesta-8,14,24-trien-3beta-ol	-0.54	0.69	-0.38	0.66
683 7alpha,12alpha-Dihydroxy-5beta-cholestan-3-one	-0.47	0.67	-0.45	0.71
678 5beta-Cholestane-3alpha,7alpha,26-triol	-0.46	0.65	-0.46	0.74
679 5beta-Cholestane-3alpha,7alpha-diol	-0.47	0.64	-0.45	0.73
653 3alpha,7alpha-Dihydroxy-5beta-cholestan-26-al	-0.46	0.62	-0.46	0.75
654 3alpha,7alpha-Dihydroxy-5beta-cholestanate	-0.46	0.58	-0.46	0.71
170 Glutamate from Glycine	-0.62	0.32	-0.31	0.54
741 Choloyl-CoA	-0.44	0.37	-0.49	0.48

Continued on next page

Matches relating control 1/6h vs. 6/24h – continued

Simulation	control 1/6h		control 6/24h	
	ampl	score	ampl	score
607 2-Oxobutyrate	-0.6	0.75	-0.32	0.45
684 7alpha,12alpha-Dihydroxycholest-4-en-3-one	-0.48	0.66	-0.45	0.69
655 3alpha,7alpha-Dihydroxy-5beta-cholestanoyl-CoA	-0.46	0.41	-0.46	0.5
318 tcdchola(b)	-0.45	0.47	-0.48	0.49
254 Serine from Cysteine	-0.24	0.39	-0.69	0.43
685 7alpha-Hydroxy-5beta-cholestan-3-one	-0.48	0.65	-0.45	0.75
740 Cholesterol-ester-stea	-0.49	0.63	-0.44	0.69
736 Cholesterol-ester-ol	-0.49	0.63	-0.44	0.69
618 3-Keto-4-methylzymosterol	-0.51	0.73	-0.42	0.78
102 Arginine from Glutamate	0.06	0.3	-1	0.3
738 Cholesterol-ester-palmn	-0.5	0.6	-0.44	0.69
687 7alpha-Hydroxycholesterol	-0.49	0.68	-0.45	0.72
686 7alpha-Hydroxycholest-4-en-3-one	-0.49	0.64	-0.45	0.74
957 Zymosterol	-0.5	0.72	-0.44	0.77
662 4alpha-Methylzymosterol	-0.5	0.72	-0.44	0.77
731 Cholate	-0.44	0.54	-0.5	0.59
732 Cholestenol	-0.5	0.68	-0.44	0.79
277 beta-Alanine from Cystine	-0.18	0.37	-0.77	0.42
763 Desmosterol	-0.5	0.72	-0.45	0.74
614 3-Hydroxyisobutyrate	0.13	0.29	-1.08	0.27
523 Cholesterol	-0.5	0.68	-0.45	0.76
276 beta-Alanine from Cysteine	-0.18	0.46	-0.77	0.36
143 Aspartate from Cystine	-0.18	0.43	-0.77	0.47
943 Thymidine	-0.6	0.26	-0.36	0.39
733 Cholesterol-ester-arach	-0.49	0.6	-0.46	0.7
79 Alanine from Cysteine	-0.18	0.41	-0.77	0.41
80 Alanine from Cystine	-0.18	0.48	-0.78	0.57
734 Cholesterol-ester-gla	-0.52	0.59	-0.45	0.68
682 7-Dehydrodesmosterol	-0.51	0.7	-0.46	0.73
403 Acetoacetate	-0.34	0.63	-0.63	0.68
915 Provitamin D3	-0.51	0.66	-0.46	0.75
676 5alpha-Cholesta-7,24-dien-3beta-ol	-0.51	0.74	-0.46	0.77
846 Lathosterol	-0.51	0.7	-0.46	0.79
730 Chenodeoxycholoyl-CoA	-0.46	0.34	-0.51	0.48
739 Cholesterol-ester-pool	-0.52	0.58	-0.45	0.65
735 Cholesterol-ester-lin	-0.53	0.59	-0.44	0.67
470 GSH	-0.29	0.37	-0.69	0.56
324 Cholesterol(b)	-0.48	0.7	-0.5	0.73
429 Ornithine degr	-0.75	0.34	-0.23	0.3
842 L-Octanoylcarnitine	0.26	0.54	-1.25	0.37
737 Cholesterol-ester-palm	-0.5	0.56	-0.49	0.65
112 Arginine from Serine	-0.11	0.33	-0.88	0.35
405 Acetone	-0.33	0.85	-0.66	0.79
404 (R)-3-Hydroxybutanoate	-0.33	0.54	-0.66	0.5
406 Heme	-0.12	0.32	-0.88	0.34
181 Glutamate from Tryptophan	0.17	0.3	-1.17	0.3
408 Arginine degr	-0.78	0.35	-0.23	0.29
427 Homocysteine degr	-0.18	0.43	-0.84	0.41
417 Methionine degr	-0.18	0.4	-0.84	0.39
848 Linoleate	-0.56	0.49	-0.46	0.6
666 5-Aminolevulinate	-0.11	0.48	-0.91	0.63
908 Porphobilinogen	-0.11	0.43	-0.92	0.54
802 Glycerate	-0.32	0.51	-0.71	0.44
914 Protoporphyrinogen IX	-0.13	0.36	-0.92	0.37
824 Hydroxymethylbilane	-0.14	0.36	-0.91	0.42
954 UroporphyrinogenIII	-0.13	0.36	-0.91	0.4
744 CoproporphyrinogenIII	-0.14	0.34	-0.91	0.38
913 Protoporphyrin	-0.13	0.36	-0.92	0.32
331 ApoC1(c)	-0.14	0.94	-0.92	0.94

Continued on next page

Matches relating control 1/6h vs. 6/24h – continued

Simulation	control 1/6h		control 6/24h	
	ampl	score	ampl	score
98 Arginine from Asparagine	-0.29	0.31	-0.78	0.34
597 2-Amino-3-oxoadipate	-0.16	0.49	-0.91	0.43
823 Hydracrylate	-0.59	0.38	-0.49	0.49
414 Glutamine degr	-0.08	0.33	-1	0.27
13 NADH potential transport	-0.45	0.34	-0.64	0.5
825 Hydroxypyruvate	-0.35	1	-0.74	1
950 UDP-activated-xylose	0.3	0.52	-1.39	0.48
311 Urea from glutamine	-0.1	0.29	-0.99	0.27
743 Citrulline	-1.01	0.3	-0.09	0.39
113 Arginine from Threonine	-0.57	0.32	-0.53	0.37
811 H2S	-0.17	0.99	-0.93	0.48
424 Glycine degr	-0.25	0.36	-0.88	0.35
420 Serine degr	-0.25	0.39	-0.88	0.37
207 Glutamine from Arginine	-0.93	0.36	-0.2	0.36
808 Guanidinoacetate	0.07	0.4	-1.22	0.28
101 Arginine from Cystine	-0.21	0.3	-0.94	0.41
104 Arginine from Histidine	-0.22	0.29	-0.94	0.36
956 Xanthurenate	-0.15	0.6	-1.02	0.49
657 4-(2-Amino-3-hydroxyphenyl)-2,4-dioxobutanoate	-0.15	0.6	-1.02	0.49
314 Creatine	-0.06	0.32	-1.11	0.25
267 Serine from Tryptophan	-0.15	0.35	-1.03	0.34
140 Aspartate from Arginine	-0.95	0.34	-0.24	0.37
100 Arginine from Cysteine	-0.25	0.33	-0.94	0.33
106 Arginine from Glutamine	-0.32	0.35	-0.87	0.3
202 Glycine from Tryptophan	-0.15	0.34	-1.05	0.32
413 Histidine degr	-0.2	0.32	-1.01	0.33
659 4-Imidazolone-5-propanoate	-0.17	0.46	-1.05	0.47
296 Stearate from Palmitate	-0.46	0.35	-0.78	0.32
142 Aspartate from Cysteine	-0.43	0.4	-0.8	0.48
43 Activated methyl group (SAM)	0.15	0.37	-1.39	0.43
975 gamma-Glutamyl-cysteine	-0.24	0.96	-1	0.85
411 Cysteine degr	-0.21	0.43	-1.03	0.36
798 Glucosylceramide-pool	0.08	0.35	-1.37	0.35
434 Fructose degr	-0.55	0.51	-0.75	0.67
264 Serine from Phenylalanine	-0.58	0.33	-0.74	0.39
198 Glycine from Phenylalanine	-0.58	0.31	-0.74	0.37
421 Threonine degr	-0.66	0.34	-0.68	0.35
165 Glutamate from Arginine	-1.07	0.37	-0.27	0.45
290 beta-Alanine from Tryptophan	-0.19	0.37	-1.16	0.31
114 Arginine from Tryptophan	-0.24	0.3	-1.1	0.33
135 Asparagine from Tryptophan	-0.17	0.43	-1.18	0.32
422 Tryptophan degr	-0.21	0.34	-1.14	0.36
223 Glutamine from Tryptophan	-0.17	0.32	-1.19	0.29
612 3-Hydroxy-L-kynurenine	-0.15	0.5	-1.21	0.47
156 Aspartate from Tryptophan	-0.18	0.4	-1.18	0.33
65 ATP salvage from Adenosine	-0.47	0.87	-0.9	0.91
72 Glycogen glucose release	0.07	0.42	-1.46	0.44
74 Gluconeogen from Glycerol	-0.1	0.36	-1.3	0.51
668 5-Formyl-THF	-0.18	0.35	-1.21	0.34
93 Alanine from Tryptophan	-0.18	0.34	-1.23	0.33
929 Serotonin	-0.31	0.56	-1.1	0.6
609 2PG	-0.11	0.45	-1.34	0.42
48 Formylgroup(m)	-0.14	0.32	-1.32	0.32
271 Tyrosine from Phenylalanine	-0.64	0.46	-0.84	0.59
953 Urocanate	-0.27	0.48	-1.25	0.48
412 Glutamate degr	-0.63	0.29	-0.91	0.31
599 2-Aminomuconate(s)emialdehyde	-0.24	0.42	-1.31	0.45
596 2-Amino-3-carboxymuconate(s)emialdehyde	-0.24	0.42	-1.31	0.45
598 2-Aminomuconate	-0.26	0.38	-1.31	0.45

Continued on next page

Matches relating control 1/6h vs. 6/24h – continued

Simulation	control 1/6h		control 6/24h	
	ampl	score	ampl	score
110 Arginine from Phenylalanine	-0.62	0.32	-0.95	0.35
440 ApoA1 degr	-0.34	0.49	-1.29	0.36
327 Antichymotrypsin	-0.46	0.97	-1.17	0.97
441 Fibrinogen degr	-0.35	0.53	-1.28	0.34
443 Plasminogen degr	-0.35	0.53	-1.29	0.34
444 Prothrombin degr	-0.35	0.51	-1.32	0.36
75 Gluconeogen from Alanine	-0.5	0.32	-1.23	0.43
73 Gluconeogen from Lactate	-0.41	0.32	-1.33	0.43
416 Lysine degr	-0.9	0.33	-0.87	0.37
299 Oleate from Stearate	-0.47	0.47	-1.36	0.41
55 UDP-activated glucose	-0.18	0.49	-1.69	0.56
694 Acetate	-0.39	0.69	-1.49	0.83
925 Saccharopine	-1.12	0.42	-0.78	0.6
838 L-Formylkynurenine	-0.66	0.89	-1.39	0.72
335 Plasminogen	-0.49	0.98	-1.61	0.98
779 Fatty-acid-VLDL-SM-pool	-0.44	1	-1.8	1
775 Fatty-acid-VLDL-PC-pool	-0.44	1	-1.8	1
224 Glutamine from Tyrosine	0.13	0.26	-2.45	0.33
131 Asparagine from Phenylalanine	-0.59	0.33	-2.18	0.36
286 beta-Alanine from Phenylalanine	-0.58	0.37	-2.24	0.38
219 Glutamine from Phenylalanine	-0.6	0.29	-2.22	0.33
152 Aspartate from Phenylalanine	-0.57	0.42	-2.27	0.42
89 Alanine from Phenylalanine	-0.59	0.39	-2.27	0.42
243 Proline from Phenylalanine	-0.6	0.26	-2.25	0.3
268 Serine from Tyrosine	-0.51	0.33	-2.36	0.36
177 Glutamate from Phenylalanine	-0.61	0.31	-2.27	0.36
203 Glycine from Tyrosine	-0.54	0.27	-2.34	0.34
136 Asparagine from Tyrosine	-0.54	0.3	-2.44	0.35
291 beta-Alanine from Tyrosine	-0.52	0.35	-2.47	0.38
157 Aspartate from Tyrosine	-0.53	0.38	-2.49	0.43
115 Arginine from Tyrosine	-0.6	0.3	-2.42	0.36
94 Alanine from Tyrosine	-0.53	0.37	-2.49	0.42
182 Glutamate from Tyrosine	-0.56	0.28	-2.49	0.36
658 4-Hydroxyphenylpyruvate	-1.53	0.54	-1.64	0.54
418 Phenylalanine degr	-0.62	0.38	-2.74	0.38
426 Tyrosine degr	-0.61	0.35	-2.97	0.39
926 Sarcosine	-0.97	0.82	-2.64	0.82
788 Fumarylacetoacetate	-1.32	0.41	-2.62	0.58
890 PEP	-2.56	0.52	-1.39	0.49
660 4-Maleylacetoacetate	-1.35	0.46	-2.68	0.65
821 Homogentisate	-1.35	0.55	-2.88	0.55
312 Urea from alanine	-0.34	0.46	-4.03	0.3
313 Urea from NH3	-0.34	0.46	-4.03	0.3
407 Alanine degr	-0.36	0.45	-4.03	0.31
409 Asparagine degr	-0.72	0.38	-4.03	0.38
410 Aspartate degr	-0.72	0.33	-4.12	0.32

1.5 Matches relating TGFβ/contr 6h vs. 24h

Table 5: Matches relating TGFβ/contr 6h vs. 24h

Simulation	TGFβ/C 6h		TGFβ/C 24h	
	ampl	score	ampl	score
386 Collagen CORA1(c) synthesis	1.74	1	2.09	1
374 Collagen COFA1(c) synthesis	1.52	1	2.27	1
931 Sphinganine-1P	0.38	0.47	1.29	0.36
403 Acetoacetate	2	0.41	-0.39	0.57
405 Acetone	1.99	0.52	-0.43	0.45
344 Collagen CO1A1(c) synthesis	0.11	1	1.09	1
802 Glycerate	0.2	0.81	0.9	0.55
756 Dehydroalanine	1.06	0.72	0.002	0.62
609 2PG	1.23	0.41	-0.27	0.65
355 Collagen CO5A2(c) synthesis	0.24	1	0.71	1
890 PEP	1.23	0.52	-0.31	0.54
362 Collagen CO7A1(c) synthesis	0.05	1	0.84	1
619 3-Methyl-2-oxobutyrate	-0.2	0.65	1.04	0.37
207 Glutamine from Arginine	-0.09	0.39	0.88	0.27
396 Collagen LPP3(c) synthesis	0.28	1	0.5	1
796 Glucono-1,5-lactone-6P	0.3	1	0.45	1
632 3-Oxopropanoate	0.17	0.38	0.52	0.33
371 Collagen COCA1(c) synthesis	0.33	1	0.36	1
600 2-Deoxy-D-ribose-1P	-0.05	0.33	0.7	0.41
825 Hydroxypyruvate	0.08	1	0.55	1
327 Antichymotrypsin	0.25	0.97	0.33	0.97
349 Collagen CO4A2(c) synthesis	0.07	1	0.51	1
436 Mannose degr	0.15	0.48	0.43	0.42
941 THF-hexaglutamate	-0.14	0.87	0.69	0.87
359 Collagen CO6A3(c) synthesis	0.32	1	0.23	1
930 Sphinganine	0.21	0.45	0.28	0.38
15 Dephosphorylation of ATP	-0.14	0.57	0.63	0.71
345 Collagen CO1A2(c) synthesis	0.1	1	0.38	1
341 Collagen C43BP(c) synthesis	-0.13	1	0.58	1
346 Collagen CO2A1(c) synthesis	0.28	1	0.17	1
375 Collagen COGA1(c) synthesis	0.25	1	0.2	1
712 Agmatine	0.2	0.8	0.23	0.67
552 (R)-Mevalonate	-0.23	0.46	0.62	0.54
872 NeuNGc	-0.2	0.38	0.56	0.31
879 O-Propanoylcarnitine	0.97	0.35	-0.61	0.53
385 Collagen COPA1(c) synthesis	0.24	1	0.11	1
96 Alanine from beta-Alanine	-0.19	0.43	0.54	0.33
395 Collagen ITA2(c) synthesis	0.1	1	0.21	1
680 6-Phospho-D-gluconate	-0.13	0.5	0.43	0.63
53 activated sulphur	0.07	0.66	0.22	0.67
399 Collagen SERPH(c) synthesis	-0.02	1	0.3	1
382 Collagen COMA1(c) synthesis	0.13	1	0.13	1
379 Collagen COKA1(c) synthesis	0.26	1	0.001	1
353 Collagen CO4A6(c) synthesis	0.09	1	0.16	1
330 ApoB100(r)	-0.03	0.99	0.28	0.99
309 VLDL from HDL	-0.03	0.7	0.28	0.68
401 VLDL	-0.03	0.7	0.28	0.68
308 VLDL from LDL	-0.03	0.72	0.28	0.71
370 Collagen COBA2(c) synthesis	-0.01	1	0.26	1
120 Asparagine from Aspartate	-0.3	0.45	0.54	0.37
389 Collagen EMID2(c) synthesis	0.15	1	0.09	1
354 Collagen CO5A1(c) synthesis	0.05	1	0.18	1
365 Collagen CO9A1(c) synthesis	0.25	1	-0.02	1
877 O-Acetylcarnitine	0.12	1	0.1	1
351 Collagen CO4A4(c) synthesis	0.13	1	0.09	1
760 Deoxyinosine	-0.34	0.38	0.56	0.29

Continued on next page

Matches relating TGFβ/contr 6h vs. 24h – continued

Simulation	TGFβ/C 6h		TGFβ/C 24h	
	ampl	score	ampl	score
863 N-Acetylneuraminate	-0.2	0.36	0.42	0.32
605 2-Oxo-3-methylvalerate	-0.2	0.4	0.41	0.49
400 Collagen VWA2(c) synthesis	0.19	1	0.02	1
797 Glucose-1P	-0.04	1	0.2	1
332 ApoC2(c)	0.11	0.95	0.05	0.95
342 Collagen CCBE1(c) synthesis	-0.01	1	0.17	1
394 Collagen ITA1(c) synthesis	-0.18	0.37	0.33	0.28
58 GDP-activated fucose	-0.09	0.69	0.24	0.48
919 Ribose-1P	-0.04	0.53	0.19	0.4
839 L-Fucose-1P	-0.09	0.57	0.24	0.42
275 beta-Alanine from Aspartate	0.04	0.52	0.11	0.68
799 Glucuronate	-0.11	0.51	0.24	0.57
364 Collagen CO8A2(c) synthesis	0.01	1	0.12	1
669 5-Hydroxy-L-tryptophan	-0.12	0.86	0.24	0.61
383 Collagen CONA1(c) synthesis	0.08	1	0.04	1
350 Collagen CO4A3(c) synthesis	-0.07	1	0.19	1
357 Collagen CO6A1(c) synthesis	0.22	1	-0.11	1
827 Hypoxanthine	-0.3	0.41	0.41	0.29
494 Mannose-1P	-0.13	0.66	0.23	0.5
933 Sphingosine-1P	0.36	0.32	-0.26	0.32
358 Collagen CO6A2(c) synthesis	0.06	1	0.04	1
946 Trehalose	-0.04	1	0.13	1
59 GDP-activated mannose	-0.11	0.66	0.2	0.49
822 Homovanillate	0.1	1	-0.02	1
725 CMP-activated-N-acetylneuraminate	-0.19	0.49	0.28	0.3
513 CMP-N-acetylneuraminate	-0.19	0.49	0.28	0.3
774 FMN	0.05	1	0.03	1
388 Collagen CTHR1(c) synthesis	0.1	1	-0.02	1
728 Carnosine	0.07	0.64	0.001	0.46
356 Collagen CO5A3(c) synthesis	0.14	1	-0.07	1
817 Hexadecenal	-0.21	0.38	0.28	0.26
815 Hexadecanal	-0.21	0.38	0.28	0.26
274 beta-Alanine from Asparagine	-0.04	0.34	0.11	0.3
391 Collagen FCN2(c) synthesis	-0.19	1	0.26	1
765 Dihydroceramide-pool	0.21	0.34	-0.14	0.36
339 Collagen ADIPO(c) synthesis	0.13	1	-0.07	1
526 Putrescine	-0.12	0.47	0.18	0.52
372 Collagen CODA1(c) synthesis	-0.03	1	0.09	1
916 Pyridoxal	0.16	0.88	-0.11	0.66
41 Na+ importgradient	-0.18	0.62	0.23	0.47
368 Collagen COAA1(c) synthesis	0.03	1	0.02	1
373 Collagen COEA1(c) synthesis	0.06	1	-0.02	1
333 ApoC3(c)	0.04	0.94	-0.004	0.93
611 3-Dehydrosphinganine	-0.05	0.33	0.09	0.44
949 UDP-activated-N-acetyl-D-galactosamine	-0.15	0.55	0.18	0.44
460 Pyridoxal-P	0.16	0.53	-0.12	0.76
729 Ceramide-1P-pool	0.3	0.35	-0.27	0.39
334 ApoE(c)	0.001	0.95	0.03	0.95
293 Homocysteine from Methionine	-0.02	1	0.04	1
340 Collagen BGH3(c) synthesis	0.06	1	-0.04	1
837 L-Cystathionine	-0.02	0.47	0.04	0.48
560 (S)-Dihydroorotate	-0.36	0.44	0.38	0.32
940 Sulfite degr	0.02	1	-0.01	1
859 N-(omega)-Hydroxyarginine	0.01	0.77	-0.01	0.77
393 Collagen FMOD(c) synthesis	0.02	1	-0.02	1
331 ApoC1(c)	0.01	0.92	-0.01	0.92
830 Inositol-1P	-0.13	1	0.12	1
367 Collagen CO9A3(c) synthesis	-0.04	1	0.03	1
366 Collagen CO9A2(c) synthesis	0.01	1	-0.02	1

Continued on next page

Matches relating TGFβ/contr 6h vs. 24h – continued

Simulation	TGFβ/C 6h		TGFβ/C 24h	
	ampl	score	ampl	score
838 L-Formylkynurenine	0.39	0.73	-0.4	0.69
325 Albumin	-0.001	0.97	-0.02	0.97
887 Oxidized thioredoxin	-0.05	0.93	0.03	0.93
455 Thioredoxin	-0.05	0.94	0.03	0.94
369 Collagen COBA1(c) synthesis	0.0002	1	-0.02	1
376 Collagen COHA1(c) synthesis	-0.05	1	0.03	1
378 Collagen COJA1(c) synthesis	0.04	1	-0.07	1
512 N-acglucam	-0.22	0.31	0.16	0.32
495 Mannose-6P	-0.04	0.91	-0.01	0.78
861 N-Acetylglucosamine-1P	-0.16	0.48	0.1	0.55
688 ACP	0.001	0.83	-0.07	0.86
851 Malonyl-ACP	0.001	0.82	-0.07	0.84
697 Acetyl-ACP	0.001	0.83	-0.07	0.85
457 Apo-ACP	0.001	0.88	-0.07	0.9
695 Acetoacetyl-ACP	0.001	0.81	-0.07	0.84
544 (R)-3-Hydroxybutanoyl-ACP	0.001	0.81	-0.07	0.84
717 But-2-enoyl-ACP	0.001	0.81	-0.07	0.84
718 Butyryl-ACP	0.001	0.81	-0.07	0.84
627 3-Oxohexanoyl-ACP	0.001	0.81	-0.07	0.83
750 D-3-Hydroxyhexanoyl-ACP	0.001	0.8	-0.07	0.83
536 (2E)-Hexenoyl-ACP	0.001	0.8	-0.07	0.83
818 Hexanoyl-ACP	0.001	0.8	-0.07	0.83
629 3-Oxooctanoyl-ACP	0.001	0.79	-0.07	0.82
546 (R)-3-Hydroxyoctanoyl-ACP	0.001	0.79	-0.07	0.82
539 (2E)-Octenoyl-ACP	0.001	0.79	-0.07	0.82
881 Octanoyl-ACP	0.001	0.79	-0.07	0.82
622 3-Oxodecanoyl-ACP	0.001	0.79	-0.07	0.82
545 (R)-3-Hydroxydecanoyl-ACP	0.001	0.79	-0.07	0.82
530 (2E)-Decenoyl-ACP	0.001	0.79	-0.07	0.81
754 Decanoyl-ACP	0.001	0.79	-0.07	0.81
624 3-Oxododecanoyl-ACP	0.001	0.78	-0.07	0.81
749 D-3-Hydroxydodecanoyl-ACP	0.001	0.78	-0.07	0.81
532 (2E)-Dodecenoyl-ACP	0.001	0.78	-0.07	0.81
769 Dodecanoyl-ACP	0.001	0.78	-0.07	0.81
634 3-Oxotetradecanoyl-ACP	0.001	0.78	-0.07	0.81
813 HMA	0.001	0.78	-0.07	0.81
541 (2E)-Tetradecenoyl-ACP	0.001	0.78	-0.07	0.81
942 Tetradecanoyl-ACP	0.001	0.78	-0.07	0.81
626 3-Oxohexadecanoyl-ACP	0.001	0.78	-0.07	0.8
547 (R)-3-Hydroxypalmitoyl-ACP	0.001	0.78	-0.07	0.8
534 (2E)-Hexadecenoyl-ACP	0.001	0.78	-0.07	0.8
816 Hexadecanoyl-ACP	0.001	0.78	-0.07	0.8
633 3-Oxostearoyl-ACP	0.001	0.77	-0.07	0.79
617 3-Hydroxystearoyl-ACP	0.001	0.77	-0.07	0.79
538 (2E)-Octadecenoyl-ACP	0.001	0.77	-0.07	0.79
936 Stearoyl-ACP	0.001	0.77	-0.07	0.79
458 Apo-ACP(m)	0.001	0.66	-0.07	0.7
978 mitoACP	0.001	0.63	-0.07	0.68
786 Fructose-2,6PP	-0.02	0.99	-0.06	0.84
64 Glucosamine-6P	-0.19	0.67	0.11	0.63
694 Acetate	0.3	0.66	-0.38	0.97
384 Collagen COOA1(c) synthesis	-0.17	1	0.08	1
34 Thioredoxin(c) reduction	0.01	0.71	-0.09	0.92
363 Collagen CO8A1(c) synthesis	-0.09	1	-0.0003	1
912 Protein(l)ysine	-0.1	1	0.01	1
464 mlthf	0.01	0.77	-0.1	0.8
664 5,10-Methenyl-THF	0.01	0.62	-0.1	0.65
467 1fthf	0.01	0.51	-0.1	0.54
466 THF(m)	0.01	1	-0.1	1

Continued on next page

Matches relating TGFβ/contr 6h vs. 24h – continued

Simulation	TGFβ/C 6h		TGFβ/C 24h	
	ampl	score	ampl	score
459 THF	0.01	1	-0.1	1
29 NADH to NADPH transhydrogenase	0.01	1	-0.1	1
766 Dihydrofolate	0.01	1	-0.1	1
670 5-Methyl-THF	0.01	1	-0.1	1
302 gamma-Linolenate from Linoleate	0.23	0.4	-0.34	0.56
348 Collagen CO4A1(c) synthesis	-0.17	1	0.07	1
299 Oleate from Stearate	0.2	0.55	-0.33	0.83
11 ATP from NADH(m)	-0.04	0.78	-0.09	0.8
14 Ubiquinol-to-ATP	-0.04	0.62	-0.09	0.62
336 Prothrombin	0.03	0.97	-0.16	0.98
855 Mercaptopyruvate	-0.17	0.52	0.03	0.38
672 5-Oxoproline	-0.06	0.72	-0.08	0.55
37 GSH reduction using NADPH redox potential	-0.13	1	-0.02	1
38 GSH oxidation	-0.13	1	-0.02	1
434 Fructose degr	0.22	0.56	-0.37	0.44
1 Aerobic ATP rephosph (FA)	-0.04	0.43	-0.11	0.45
338 Haptoglobin	0.02	0.96	-0.18	0.97
6 Aerobic rephosph of UDP	-0.05	0.49	-0.11	0.52
869 N-Pantothenoylcysteine	-0.06	0.69	-0.11	0.48
548 (R)-4-Phosphopantothenoyl-cysteine	-0.06	0.72	-0.11	0.45
72 Glycogen glucose release	0.07	0.42	-0.25	0.45
305 Dihomo-gamma-linolenate from gamma-Linolenate	0.23	0.31	-0.42	0.81
303 Dihomo-gamma-linolenate from Linoleate	0.24	0.27	-0.43	0.68
306 Arachidonate from gamma-Linolenate	0.24	0.27	-0.43	0.68
2 Aerobic ATP rephosph (gluc)	-0.04	0.42	-0.15	0.44
212 Glutamine from Glutamate	-0.04	0.29	-0.16	0.33
461 4ppan	-0.12	1	-0.08	1
841 L-Lactate	0.01	0.85	-0.22	0.94
347 Collagen CO3A1(c) synthesis	-0.2	1	-0.02	1
329 ApoA1	0.04	0.95	-0.27	0.97
439 Antitrypsin degr	0.11	0.47	-0.34	0.44
36 GSH reduction using NADH redox potential	-0.21	0.83	-0.02	0.5
829 Inositol	-0.11	0.85	-0.12	0.5
52 Acetyl group(r)	0.25	0.38	-0.49	0.58
49 Acetyl group(c)	0.25	0.38	-0.49	0.58
854 Maltose	-0.05	1	-0.18	1
607 2-Oxobutyrate	0.97	0.57	-1.2	0.38
402 HDL	0.04	0.67	-0.28	0.78
834 Kynurenine	0.32	0.46	-0.57	0.43
917 Pyridoxine-P	-0.12	1	-0.14	1
517 PI	-0.12	0.44	-0.14	0.47
880 OAA	-0.1	0.54	-0.16	0.76
447 Glycogenin degr	0.12	0.45	-0.39	0.5
445 ApoTransferin degr	0.12	0.44	-0.39	0.48
298 Palmitolate from Palmitate	0.03	0.5	-0.32	0.7
296 Stearate from Palmitate	0.1	0.51	-0.39	0.7
51 Acetyl group(p)	0.2	0.43	-0.49	0.47
154 Aspartate from Serine	-0.14	0.44	-0.15	0.39
435 Galactose degr	-0.12	0.52	-0.17	0.59
50 Acetyl group(m)	0.12	0.38	-0.42	0.57
779 Fatty-acid-VLDL-SM-pool	0.04	1	-0.34	1
775 Fatty-acid-VLDL-PC-pool	0.04	1	-0.34	1
947 Triphosphate degr	-0.21	1	-0.09	1
139 Aspartate from Alanine	-0.15	0.54	-0.15	0.53
807 Glycogenin-G8	-0.16	0.97	-0.14	0.95
454 Glycogenin	-0.16	0.97	-0.14	0.96
803 Glycogenin-G11	-0.16	0.97	-0.14	0.95
804 Glycogenin-G4G4	-0.16	0.96	-0.14	0.94
806 Glycogenin-G7G1	-0.16	0.96	-0.14	0.94

Continued on next page

Simulation	TGFβ/C 6h ampl	TGFβ/C 6h score	TGFβ/C 24h ampl	TGFβ/C 24h score
805 Glycogenin-G7	-0.16	0.96	-0.14	0.94
569 1,2-Diacylglycerol-VLDL-SM-pool	0.05	0.44	-0.35	0.47
570 1,2-Diacylglycerol-VLDL-TG-pool	0.04	0.44	-0.35	0.47
564 1,2-Diacylglycerol-Bile-PC-pool	0.04	0.43	-0.35	0.46
592 1-Pyrroline-5-carboxylate	-0.06	0.87	-0.24	0.76
903 Phosphatidate-VLDL-SM-pool	0.05	0.5	-0.35	0.52
565 1,2-Diacylglycerol-VLDL-PC-pool	0.04	0.45	-0.35	0.47
904 Phosphatidate-VLDL-TG-pool	0.04	0.51	-0.35	0.52
897 Phosphatidate-Bile-PC-pool	0.04	0.49	-0.35	0.5
899 Phosphatidate-VLDL-PC-pool	0.04	0.51	-0.35	0.52
123 Asparagine from Glutamate	-0.07	0.31	-0.23	0.42
987 trans-4-Hydroxy-L-proline	-0.06	1	-0.24	1
835 L-1-Pyrroline-3-hydroxy-5-carboxylate	-0.06	1	-0.24	1
566 1,2-Diacylglycerol-VLDL-PE-pool	0.05	0.45	-0.35	0.47
22 Oxidation of NADH	-0.13	0.42	-0.17	0.53
900 Phosphatidate-VLDL-PE-pool	0.05	0.51	-0.35	0.52
433 Stearate degr	0.11	0.42	-0.42	0.59
567 1,2-Diacylglycerol-VLDL-PI-pool	0.05	0.45	-0.35	0.47
901 Phosphatidate-VLDL-PI-pool	0.05	0.51	-0.35	0.51
568 1,2-Diacylglycerol-VLDL-PS-pool	0.05	0.45	-0.35	0.47
432 Oleate degr	0.11	0.4	-0.42	0.57
902 Phosphatidate-VLDL-PS-pool	0.05	0.51	-0.36	0.51
579 1-Acylglycerol-3P-VLDL-TG1-pool	0.06	0.41	-0.37	0.57
578 1-Acylglycerol-3P-VLDL-SM-pool	0.06	0.41	-0.37	0.57
572 1-Acylglycerol-3P-Bile-PC-pool	0.06	0.41	-0.37	0.55
159 Aspartate from beta-Alanine	-0.07	0.43	-0.24	0.49
574 1-Acylglycerol-3P-VLDL-PC-pool	0.06	0.42	-0.37	0.57
575 1-Acylglycerol-3P-VLDL-PE-pool	0.06	0.42	-0.37	0.57
398 Collagen SCRB1(c) synthesis	0.11	1	-0.43	1
576 1-Acylglycerol-3P-VLDL-PI-pool	0.06	0.42	-0.37	0.57
577 1-Acylglycerol-3P-VLDL-PS-pool	0.06	0.42	-0.37	0.57
144 Aspartate from Glutamate	-0.07	0.47	-0.25	0.64
953 Urocanate	-0.2	0.49	-0.12	0.56
142 Aspartate from Cysteine	-0.1	0.49	-0.22	0.54
326 Fibrinogen	-0.18	0.36	-0.14	0.3
145 Aspartate from Glycine	-0.11	0.36	-0.22	0.44
141 Aspartate from Asparagine	-0.1	0.46	-0.22	0.54
140 Aspartate from Arginine	-0.09	0.41	-0.24	0.48
23 Oxidation of NADPH	-0.23	0.46	-0.12	0.47
878 O-Butanoylcarnitine	0.11	0.35	-0.46	0.59
335 Plasminogen	0.03	0.97	-0.38	0.98
328 Antitrypsin	-0.18	0.38	-0.17	0.3
71 Glycogen glucose storage	-0.17	0.97	-0.19	0.75
172 Glutamate from Isoleucine	0.09	0.41	-0.44	0.57
787 Fumarate	-0.11	0.84	-0.26	0.74
5 Aerobic rephosph of CTP	-0.05	0.4	-0.32	0.38
10 Anaerobic rephosph of UTP	-0.04	0.3	-0.33	0.45
8 Anaerobic rephosph of GTP	-0.04	0.3	-0.33	0.45
9 Anaerobic rephosph of CTP	-0.04	0.3	-0.33	0.45
148 Aspartate from Glutamine	-0.14	0.37	-0.24	0.49
66 ATP salvage from Hypoxanthine	-0.06	0.34	-0.31	0.44
25 NADH redox potential into peroxy	-0.06	0.52	-0.32	0.83
360 Collagen CO6A5(c) synthesis	-0.18	0.38	-0.2	0.28
361 Collagen CO6A6(c) synthesis	-0.18	0.38	-0.21	0.28
515 PE	-0.06	0.34	-0.33	0.54
323 PE(b)	-0.06	0.31	-0.33	0.48
944 Thymine	-0.3	0.44	-0.09	0.42
397 Collagen PCOTH(c) synthesis	-0.18	0.41	-0.21	0.32
68 O2.-(c) degr	-0.002	0.68	-0.39	0.72

Continued on next page

Matches relating TGFβ/contr 6h vs. 24h – continued

Simulation	TGFβ/C 6h ampl	TGFβ/C 6h score	TGFβ/C 24h ampl	TGFβ/C 24h score
844 L-Palmitoylcarnitine	0.04	0.49	-0.43	0.68
889 PE-PS-VLDL-pool	-0.06	0.32	-0.33	0.57
977 linoleic-Carnitine	0.04	0.54	-0.43	0.67
714 Arachidonyl-Carnitine	0.04	0.54	-0.43	0.67
322 PS(b)	-0.07	0.34	-0.32	0.49
516 PS	-0.07	0.34	-0.32	0.49
645 3PG	0.17	0.36	-0.56	0.59
147 Aspartate from Isoleucine	0.09	0.43	-0.49	0.58
392 Collagen FCN3(c) synthesis	-0.18	0.37	-0.21	0.29
390 Collagen FCN1(c) synthesis	-0.18	0.4	-0.22	0.29
7 Aerobic rephosph of UTP	-0.04	0.46	-0.35	0.48
4 Aerobic rephosph of GTP	-0.04	0.46	-0.35	0.47
767 Dihydrothymine	-0.3	0.45	-0.1	0.39
639 3-Ureidoisobutyrate	-0.3	0.42	-0.1	0.37
387 Collagen COSA1(c) synthesis	-0.18	0.42	-0.22	0.32
381 Collagen COLQ(c) synthesis	-0.18	0.41	-0.22	0.31
380 Collagen COLA1(c) synthesis	-0.18	0.42	-0.22	0.31
893 PPi	-0.16	0.51	-0.24	0.71
32 Thioredoxin(m) reduction	-0.34	1	-0.06	1
33 Thioredoxin(m) oxidation	-0.34	1	-0.06	1
507 Cytidine	-0.32	0.46	-0.08	0.37
748 D-3-Amino-isobutanoate	-0.31	0.38	-0.09	0.35
215 Glutamine from Isoleucine	0.09	0.39	-0.5	0.64
281 beta-Alanine from Isoleucine	0.11	0.46	-0.53	0.5
850 Malate	-0.1	0.79	-0.31	0.7
961 cis-Aconitate	-0.09	0.71	-0.32	0.67
13 NADH potential transport	-0.07	0.41	-0.34	0.59
84 Alanine from Isoleucine	0.09	0.4	-0.51	0.62
105 Arginine from Isoleucine	0.09	0.34	-0.5	0.55
283 beta-Alanine from Leucine	-0.08	0.36	-0.33	0.39
794 Galactose-1P	-0.27	0.61	-0.15	0.66
597 2-Amino-3-oxoadipate	-0.15	0.59	-0.27	0.56
742 Citrate	-0.09	0.67	-0.34	0.71
785 Fructose-1,6PP	0.02	0.52	-0.44	1
149 Aspartate from Leucine	-0.1	0.42	-0.32	0.45
183 Glutamate from Valine	-0.24	0.31	-0.19	0.41
39 Proton-gradient(m) build up	-0.04	0.31	-0.39	0.45
40 Proton-gradient(c) build up	-0.04	0.31	-0.39	0.45
505 Uracil	-0.34	0.53	-0.09	0.32
259 Serine from Isoleucine	0.09	0.34	-0.53	0.59
456 Thioredoxin(m)	-0.19	0.96	-0.25	0.95
979 mitoOxidizedThioredoxin	-0.19	0.95	-0.25	0.94
70 Formate degr	-0.06	0.75	-0.37	0.61
820 Histamine	-0.003	0.64	-0.44	0.67
875 Nicotinate D-ribonucleoside	-0.3	0.44	-0.14	0.42
798 Glucosylceramide-pool	-0.17	0.42	-0.27	0.37
284 beta-Alanine from Lysine	-0.07	0.34	-0.38	0.4
150 Aspartate from Lysine	-0.07	0.36	-0.38	0.49
128 Asparagine from Leucine	-0.11	0.35	-0.34	0.38
126 Asparagine from Isoleucine	0.09	0.36	-0.55	0.55
932 Sphingosine	-0.2	0.36	-0.26	0.37
441 Fibrinogen degr	0.12	0.46	-0.58	0.49
304 Arachidonate from Linoleate	-0.02	0.31	-0.44	0.64
278 beta-Alanine from Glutamate	-0.06	0.36	-0.4	0.39
923 SAICAR	-0.21	0.58	-0.25	0.41
666 5-Aminolevulinate	-0.13	0.54	-0.33	0.61
443 Plasminogen degr	0.12	0.46	-0.58	0.49
437 Albumin degr	0.12	0.44	-0.58	0.47
442 Haptoglobin degr	-0.08	0.43	-0.38	0.53

Continued on next page

Matches relating TGFβ/contr 6h vs. 24h – continued

Simulation	TGFβ/C 6h		TGFβ/C 24h	
	ampl	score	ampl	score
425 Isoleucine degr	0.09	0.38	-0.56	0.52
836 L-2-Aminoadipate	-0.04	1	-0.43	1
297 Palmitate from Palmitolate	-0.2	0.49	-0.27	0.43
438 Antichymotrypsin degr	0.12	0.47	-0.6	0.45
601 2-Deoxy-D-ribose-5P	-0.05	0.43	-0.42	0.44
496 Fructose-6P	-0.02	1	-0.46	1
300 Stearate from Oleate	-0.21	0.51	-0.29	0.52
823 Hydracrylate	0.07	0.29	-0.56	0.49
726 Carbamoyl-P	-0.36	0.49	-0.14	0.58
414 Glutamine degr	-0.07	0.4	-0.43	0.38
865 N-Carbamoyl-L-aspartate	-0.37	0.38	-0.13	0.4
790 GAR	-0.17	0.57	-0.33	0.5
809 Guanosine	-0.28	0.47	-0.22	0.35
444 Prothrombin degr	0.13	0.49	-0.64	0.5
19 Aerobic reduction of NADP+ (gluc)	-0.17	0.42	-0.34	0.31
673 5-Phosphoribosyl-4-carboxy-5-aminoimidazole	-0.26	0.53	-0.26	0.35
415 Leucine degr	-0.09	0.39	-0.43	0.37
440 ApoA1 degr	0.13	0.47	-0.65	0.48
42 Na+ exportgradient	0.15	1	-0.67	1
674 5-Phosphoribosylamine	-0.18	0.47	-0.35	0.51
412 Glutamate degr	-0.07	0.42	-0.47	0.4
174 Glutamate from Leucine	-0.17	0.42	-0.37	0.39
843 L-Oleoylcarnitine	-0.01	0.51	-0.53	0.7
216 Glutamine from Leucine	-0.16	0.35	-0.38	0.38
282 beta-Alanine from Glutamine	-0.17	0.4	-0.38	0.32
86 Alanine from Leucine	-0.16	0.4	-0.39	0.35
69 Formaldehyde degr	-0.04	0.45	-0.51	0.38
927 Sedoheptulose-1,7PP	-0.07	0.65	-0.49	0.64
594 2,5-Diamino-6-(5-triphosphoryl-3,4-trihydroxy-2-oxopentyl)- amino-4-oxopyrimidine	-0.22	0.49	-0.34	0.31
921 Ribulose-5P	-0.16	0.55	-0.4	0.37
690 AICAR	-0.27	0.54	-0.29	0.45
595 2,5-Diaminopyrimidine(n)leoside triphosphate	-0.23	0.49	-0.34	0.31
952 Urate	-0.34	0.46	-0.22	0.29
810 H2O2	-0.34	0.46	-0.22	0.29
864 N-Acetylneuraminate-9P	-0.22	0.41	-0.35	0.34
770 Erythrose-4P	-0.06	0.7	-0.5	0.64
812 HCO3-	-0.1	0.66	-0.46	0.68
727 Carbonate	-0.1	0.66	-0.46	0.68
894 PRPP	-0.16	0.57	-0.41	0.48
928 Sedoheptulose-7P	-0.08	0.6	-0.49	0.55
209 Glutamine from Aspartate	-0.3	0.44	-0.28	0.41
222 Glutamine from Threonine	-0.09	0.25	-0.49	0.6
862 N-Acetylmannosamine-6P	-0.22	0.58	-0.36	0.36
81 Alanine from Glutamate	-0.18	0.4	-0.4	0.51
939 Succinyl-CoA	-0.27	0.45	-0.3	0.42
857 Methylmalonyl-CoA	-0.26	0.46	-0.32	0.46
107 Arginine from Leucine	-0.16	0.39	-0.42	0.37
408 Arginine degr	-0.07	0.44	-0.51	0.42
475 dTTP	-0.28	0.49	-0.3	0.49
924 SAM	-0.3	0.59	-0.28	0.43
922 SAH	-0.3	0.59	-0.28	0.43
571 1,3DPG	-0.05	0.69	-0.54	0.63
621 3-Methylglutaconyl-CoA	-0.27	0.45	-0.32	0.37
852 Malonyl-Carnitin	-0.14	0.56	-0.45	0.8
751 D-Xylulose-5P	-0.11	0.53	-0.48	0.47
429 Ornithine degr	-0.07	0.44	-0.51	0.42
113 Arginine from Threonine	-0.08	0.31	-0.51	0.54
773 FAICAR	-0.27	0.58	-0.32	0.43
908 Porphobilinogen	-0.14	0.46	-0.46	0.56

Continued on next page

Matches relating TGFβ/contr 6h vs. 24h – continued

Simulation	TGFβ/C 6h ampl	TGFβ/C 6h score	TGFβ/C 24h ampl	TGFβ/C 24h score
76 Alanine from Arginine	-0.17	0.43	-0.42	0.38
870 NADH	-0.29	0.49	-0.31	0.34
492 Oleate	-0.31	0.58	-0.29	0.68
273 beta-Alanine from Arginine	-0.13	0.34	-0.47	0.34
918 Quinolinate	-0.29	0.54	-0.31	0.63
620 3-Methylcrotonyl-CoA	-0.25	0.49	-0.36	0.45
783 Formamidopyrimidine(n)leoside triphosphate	-0.25	0.47	-0.36	0.32
671 5-Methylthioadenosine	-0.3	0.62	-0.3	0.48
450 CoA	-0.27	0.5	-0.34	0.39
493 Stearate	-0.31	0.66	-0.3	0.67
67 dTTP salvage from Thymine	-0.26	0.51	-0.35	0.47
307 Arachidonate from Dihomo-gamma-linolenate	0.24	0.44	-0.85	0.6
158 Aspartate from Valine	-0.21	0.34	-0.41	0.42
986 trans,cis-octadeca-2,9-dienoyl-CoA	-0.28	0.45	-0.33	0.4
559 (S)-3-hydroxypalmitoleoyl-CoA	-0.28	0.44	-0.34	0.35
984 trans,cis-hexadeca-2,9-dienoyl-CoA	-0.28	0.45	-0.34	0.36
604 2-Methylbutyryl-CoA	-0.26	0.5	-0.36	0.35
451 NAD+	-0.29	0.49	-0.33	0.33
772 FADH2	-0.29	0.41	-0.33	0.33
753 Deamido-NAD	-0.3	0.5	-0.32	0.37
700 Acyl-CoA-Bile-PC-pool	-0.29	0.45	-0.34	0.4
706 Acyl-CoA-VLDL-SM-pool	-0.29	0.45	-0.34	0.4
702 Acyl-CoA-VLDL-PC-pool	-0.29	0.45	-0.34	0.4
871 NADPH	-0.28	0.52	-0.35	0.39
261 Serine from Leucine	-0.18	0.33	-0.44	0.4
703 Acyl-CoA-VLDL-PE-pool	-0.29	0.46	-0.34	0.4
289 beta-Alanine from Threonine	-0.04	0.37	-0.58	0.47
707 Acyl-CoA-VLDL-TG2-pool	-0.29	0.46	-0.34	0.4
708 Acyl-CoA-VLDL-TG3-pool	-0.29	0.46	-0.34	0.4
529 Methylthioribose-1P	-0.34	0.71	-0.29	0.56
704 Acyl-CoA-VLDL-PI-pool	-0.29	0.46	-0.34	0.4
701 Acyl-CoA-CL-pool	-0.29	0.46	-0.35	0.4
876 Nicotinate(r)ibonucleotide	-0.31	0.48	-0.32	0.41
705 Acyl-CoA-VLDL-PS-pool	-0.29	0.46	-0.35	0.4
490 Palmitate	-0.32	0.67	-0.31	0.63
301 Palmitolate from Arachidonate	-0.32	0.49	-0.31	0.64
938 Succinate	-0.19	0.52	-0.44	0.56
551 (R)-Methylmalonyl-CoA	-0.28	0.45	-0.36	0.38
895 Palmitoyl-CoA	-0.29	0.46	-0.35	0.4
711 Adenylyl(s)ulfate	-0.28	0.55	-0.35	0.45
535 (2E)-Hexadecenoyl-CoA	-0.28	0.47	-0.35	0.36
937 Stearoyl-CoA	-0.29	0.46	-0.35	0.4
883 Oleoyl-CoA	-0.29	0.46	-0.35	0.4
849 Linoleoyl-CoA	-0.29	0.46	-0.35	0.4
715 Arachidonyl-CoA	-0.29	0.46	-0.35	0.4
976 gamma-Linolenoyl-CoA	-0.29	0.44	-0.35	0.38
452 NADP+	-0.28	0.52	-0.36	0.38
920 Ribose-5P	-0.16	0.52	-0.48	0.43
959 cGMP	-0.27	0.42	-0.36	0.31
416 Lysine degr	-0.06	0.37	-0.58	0.43
681 6-Pyruvoyltetrahydropterin	-0.23	0.46	-0.41	0.38
421 Threonine degr	-0.07	0.37	-0.58	0.49
789 GAP	-0.06	0.55	-0.58	0.85
752 DHAP	-0.06	0.55	-0.58	0.85
556 (S)-3-Hydroxyhexadecanoyl-CoA	-0.28	0.37	-0.36	0.41
95 Alanine from Valine	-0.22	0.36	-0.42	0.49
583 1-Acylglycerol-3P-palm	0.06	0.4	-0.7	0.57
311 Urea from glutamine	-0.18	0.41	-0.47	0.37
873 Nicotinamide D-ribonucleotide	-0.31	0.57	-0.34	0.46

Continued on next page

Matches relating TGFβ/contr 6h vs. 24h – continued

Simulation	TGFβ/C 6h		TGFβ/C 24h	
	ampl	score	ampl	score
724 CMP-NeuNGc	-0.26	0.4	-0.38	0.29
885 Orotidine-5P	-0.28	0.59	-0.36	0.37
981 palmitoleoyl-CoA	-0.28	0.46	-0.37	0.35
585 1-Acylglycerol-3P-stea	0.06	0.42	-0.71	0.57
582 1-Acylglycerol-3P-ol	0.06	0.42	-0.71	0.57
581 1-Acylglycerol-3P-lin	0.06	0.42	-0.71	0.57
580 1-Acylglycerol-3P-arach	0.06	0.42	-0.71	0.57
860 N-Acetyl-D-mannosamine	-0.2	0.47	-0.45	0.34
709 Adenine	-0.29	0.33	-0.36	0.31
225 Glutamine from Valine	-0.21	0.32	-0.45	0.38
661 4-Methyl-2-oxopentanoate	-0.24	0.4	-0.42	0.4
762 Dephospho-CoA	-0.29	0.45	-0.37	0.33
90 Alanine from Proline	-0.22	0.52	-0.44	0.54
631 3-Oxopalmitoyl-CoA	-0.29	0.34	-0.38	0.41
698 Acetyl-CoA	-0.29	0.49	-0.38	0.38
696 Acetoacetyl-CoA	-0.29	0.47	-0.38	0.36
153 Aspartate from Proline	-0.24	0.48	-0.43	0.64
337 ApoTransferin	-0.33	0.98	-0.34	0.98
287 beta-Alanine from Proline	-0.23	0.41	-0.44	0.49
522 Triacylglycerol	-0.06	0.32	-0.61	0.5
758 Deoxycytidine	-0.3	0.46	-0.37	0.43
542 (2E)-Tetradecenoyl-CoA	-0.27	0.37	-0.4	0.44
73 Gluconeogen from Lactate	-0.1	0.34	-0.57	0.51
453 FAD	-0.3	0.45	-0.37	0.35
180 Glutamate from Threonine	-0.17	0.32	-0.5	0.56
659 4-Imidazolone-5-propanoate	-0.17	0.45	-0.51	0.35
602 2-Lysolecithin-pool	0.04	0.28	-0.71	0.39
847 Lauroyl-CoA	-0.27	0.34	-0.4	0.44
18 Aerobic reduction of NADP+ (FA)	-0.15	0.4	-0.53	0.37
550 (R)-5-Phosphomevalonate	-0.18	0.53	-0.5	0.49
146 Aspartate from Histidine	-0.17	0.52	-0.51	0.48
853 Malonyl-CoA	-0.28	0.48	-0.41	0.42
230 Proline from Arginine	-0.28	0.49	-0.41	0.41
833 Isovaleryl-CoA	-0.27	0.44	-0.41	0.37
967 dCMP	-0.3	0.49	-0.38	0.45
404 (R)-3-Hydroxybutanoate	-0.02	0.57	-0.66	0.58
858 Myristoyl-CoA	-0.29	0.35	-0.41	0.44
888 PAP	-0.29	0.46	-0.4	0.38
801 Glutaryl-CoA	-0.28	0.42	-0.41	0.42
644 3-oxopalmitoleoyl-CoA	-0.28	0.42	-0.41	0.37
527 Spermidine	-0.38	0.52	-0.32	0.43
757 Deoxyadenosine	-0.29	0.44	-0.4	0.36
483 Aspartate	-0.17	0.49	-0.53	0.47
240 Proline from Leucine	-0.3	0.45	-0.4	0.5
557 (S)-3-Hydroxytetradecanoyl-CoA	-0.29	0.31	-0.41	0.38
635 3-Oxotetradecanoyl-CoA	-0.29	0.31	-0.41	0.4
229 Proline from Alanine	-0.28	0.64	-0.42	0.42
423 Valine degr	-0.2	0.35	-0.51	0.4
719 Butyryl-CoA	-0.26	0.32	-0.44	0.47
628 3-Oxohexanoyl-CoA	-0.26	0.34	-0.45	0.46
603 2-Methylacetoacetyl-CoA	-0.27	0.43	-0.44	0.4
965 dAMP	-0.3	0.54	-0.41	0.45
220 Glutamine from Proline	-0.23	0.42	-0.48	0.46
406 Heme	-0.31	0.43	-0.4	0.59
692 AMP	-0.29	0.48	-0.42	0.35
910 Propanoate	-0.09	0.39	-0.62	0.59
689 ADP	-0.29	0.47	-0.42	0.34
83 Alanine from Histidine	-0.18	0.5	-0.53	0.43
62 Isopentenyl-PP	-0.14	0.62	-0.58	0.57

Continued on next page

Matches relating TGFβ/contr 6h vs. 24h – continued

Simulation	TGFβ/C 6h ampl	TGFβ/C 6h score	TGFβ/C 24h ampl	TGFβ/C 24h score
28 NADPH to NADH transhydrogenase	-0.14	1	-0.58	1
35 Thioredoxin(c) oxidation	-0.14	0.71	-0.57	0.77
46 Activated methyl group from Histidine	-0.17	0.39	-0.55	0.38
759 Deoxyguanosine	-0.29	0.42	-0.43	0.34
463 dhbpt	-0.28	0.4	-0.44	0.46
116 Arginine from Valine	-0.24	0.34	-0.48	0.41
969 dGMP	-0.29	0.45	-0.43	0.37
178 Glutamate from Proline	-0.26	0.64	-0.46	0.62
111 Arginine from Proline	-0.22	0.45	-0.5	0.48
968 dGDP	-0.29	0.45	-0.43	0.37
964 dADP	-0.3	0.46	-0.42	0.38
723 CMP	-0.31	0.47	-0.41	0.38
606 2-Oxoadipate	-0.21	0.37	-0.52	0.41
782 Fatty-acid-VLDL-TG3-pool	-0.03	0.52	-0.7	0.66
60 Pyruvate	-0.03	0.41	-0.7	0.55
840 L-Glutamate 5-semialdehyde	-0.26	0.49	-0.47	0.42
593 14-Demethyllanosterol	-0.07	0.58	-0.66	0.63
960 cis-(3S)-hydroxytetradec-7-enoyl-CoA	-0.28	0.4	-0.45	0.39
462 thbpt	-0.3	0.41	-0.43	0.44
985 trans,cis-myristo-2,7-dienoyl-CoA	-0.28	0.41	-0.45	0.39
419 Proline degr	-0.22	0.44	-0.51	0.5
913 Protoporphyrin	-0.31	0.49	-0.42	0.58
232 Proline from Aspartate	-0.32	0.59	-0.41	0.53
710 Adenylosuccinate	-0.3	0.56	-0.43	0.4
537 (2E)-Hexenoyl-CoA	-0.26	0.35	-0.47	0.45
958 cAMP	-0.29	0.43	-0.44	0.31
720 CDP	-0.32	0.47	-0.42	0.38
430 LDL degr	0.2	0.34	-0.93	0.43
65 ATP salvage from Adenosine	-0.02	0.9	-0.71	0.88
540 (2E)-Octenoyl-CoA	-0.26	0.36	-0.47	0.44
477 CTP	-0.33	0.49	-0.4	0.36
722 CDP-ethanolamine	-0.35	0.47	-0.39	0.46
487 Proline	-0.3	0.45	-0.44	0.48
642 3-oxomyrist-7-enoyl-CoA	-0.28	0.4	-0.45	0.4
235 Proline from Glutamate	-0.26	0.61	-0.47	0.57
963 cis-myrist-7-enoyl-CoA	-0.28	0.41	-0.45	0.39
413 Histidine degr	-0.18	0.43	-0.55	0.43
911 Propanoyl-CoA	-0.29	0.4	-0.45	0.34
63 Farnesyl-PP	-0.13	0.6	-0.61	0.51
791 GDP	-0.28	0.46	-0.46	0.33
745 Crotonyl-CoA	-0.28	0.44	-0.45	0.4
966 dCDP	-0.32	0.46	-0.42	0.42
610 3(S)-3-hydroxydodecen-(5Z)-oyl-CoA	-0.28	0.39	-0.46	0.4
983 trans,cis-dodeca-2,5-dienoyl-CoA	-0.28	0.39	-0.46	0.4
951 UMP	-0.3	0.46	-0.44	0.44
138 Asparagine from beta-Alanine	-0.06	0.31	-0.68	0.33
641 3-oxolaur-cis-5-enoyl-CoA	-0.28	0.38	-0.46	0.41
643 3-oxooleoyl-CoA	-0.28	0.44	-0.46	0.37
656 4,4-Dimethyl-5alpha-cholesta-8,14,24-trien-3beta-ol	-0.08	0.54	-0.67	0.68
792 GMP	-0.28	0.48	-0.46	0.35
473 dCTP	-0.33	0.48	-0.41	0.41
962 cis-laur-5-enoyl-CoA	-0.28	0.39	-0.46	0.41
743 Citrulline	0.08	0.37	-0.82	0.31
479 UTP	-0.34	0.51	-0.41	0.38
562 (S)-Hydroxyhexanoyl-CoA	-0.27	0.32	-0.48	0.42
543 (3Z)-Dodecenoyl-CoA	-0.28	0.38	-0.47	0.41
914 Protoporphyrinogen IX	-0.32	0.49	-0.43	0.58
528 Spermine	-0.34	0.52	-0.41	0.41
948 UDP-N-acetylglucosamine	-0.31	0.41	-0.44	0.29

Continued on next page

Matches relating TGFβ/contr 6h vs. 24h – continued

Simulation	TGFβ/C 6h		TGFβ/C 24h	
	ampl	score	ampl	score
249 Proline from beta-Alanine	-0.31	0.55	-0.43	0.44
102 Arginine from Glutamate	-0.17	0.36	-0.58	0.4
16 Aerobic reduction of NAD+ (FA)	-0.15	0.42	-0.6	0.4
511 UDP-N-acetylgalactosamine	-0.31	0.4	-0.44	0.28
558 (S)-3-hydroxyoleyleoyl-CoA	-0.28	0.43	-0.47	0.4
819 Hexanoyl-CoA	-0.27	0.34	-0.48	0.45
590 1-Acylglycerol-VLDL-SM-pool	0.05	0.36	-0.8	0.49
623 3-Oxodecanoyl-CoA	-0.27	0.35	-0.48	0.44
591 1-Acylglycerol-VLDL-TG1-pool	0.05	0.37	-0.8	0.49
586 1-Acylglycerol-VLDL-PC-pool	0.05	0.37	-0.8	0.49
587 1-Acylglycerol-VLDL-PE-pool	0.05	0.37	-0.8	0.5
882 Octanoyl-CoA	-0.27	0.35	-0.48	0.45
588 1-Acylglycerol-VLDL-PI-pool	0.05	0.37	-0.8	0.5
589 1-Acylglycerol-VLDL-PS-pool	0.05	0.37	-0.8	0.5
531 (2E)-Decenoyl-CoA	-0.27	0.35	-0.48	0.44
561 (S)-Hydroxydecanoyl-CoA	-0.27	0.35	-0.48	0.44
866 N-Formimino-L-glutamate	-0.2	0.57	-0.56	0.49
974 dUTP	-0.33	0.45	-0.43	0.4
744 CoproporphyrinogenIII	-0.32	0.51	-0.44	0.59
616 3-Hydroxypropionyl-CoA	-0.28	0.4	-0.47	0.39
106 Arginine from Glutamine	-0.19	0.32	-0.57	0.36
155 Aspartate from Threonine	-0.2	0.34	-0.55	0.55
945 Tiglyl-CoA	-0.27	0.4	-0.48	0.4
20 Aerobic reduction of FAD (gluc)	-0.26	0.38	-0.5	0.48
87 Alanine from Lysine	-0.23	0.37	-0.53	0.44
625 3-Oxododecanoyl-CoA	-0.27	0.37	-0.49	0.45
824 Hydroxymethylbilane	-0.32	0.55	-0.44	0.64
954 UroporphyrinogenIII	-0.32	0.52	-0.44	0.61
755 Decanoyl-CoA	-0.27	0.37	-0.49	0.45
630 3-Oxooctanoyl-CoA	-0.28	0.33	-0.48	0.44
245 Proline from Threonine	-0.29	0.4	-0.47	0.61
828 Inosine	-0.28	0.45	-0.48	0.32
17 Aerobic reduction of NAD+ (gluc)	-0.15	0.51	-0.61	0.45
563 (S)-Hydroxyoctanoyl-CoA	-0.28	0.33	-0.48	0.44
280 beta-Alanine from Histidine	-0.2	0.38	-0.57	0.41
972 dUDP	-0.33	0.46	-0.44	0.39
85 Alanine from Glutamine	-0.17	0.49	-0.6	0.58
533 (2E)-Dodecenoyl-CoA	-0.27	0.38	-0.49	0.44
555 (S)-3-Hydroxydodecanoyl-CoA	-0.27	0.38	-0.49	0.43
292 beta-Alanine from Valine	-0.23	0.29	-0.54	0.37
800 Glutamyl-5P	-0.32	0.55	-0.45	0.54
519 LacCer	-0.22	0.42	-0.55	0.43
554 (S)-3-Hydroxybutyryl-CoA	-0.29	0.45	-0.49	0.38
269 Serine from Valine	-0.3	0.35	-0.47	0.44
320 Bile-PC(b)	0.12	0.32	-0.9	0.44
795 Geranyl-PP	-0.13	0.58	-0.65	0.5
955 Xanthosine	-0.28	0.46	-0.5	0.31
549 (R)-5-Diphosphomevalonate	-0.16	0.55	-0.62	0.56
699 Acrylyl-CoA	-0.28	0.42	-0.5	0.39
525 beta-Alanine	-0.2	0.34	-0.58	0.33
137 Asparagine from Valine	-0.26	0.34	-0.52	0.42
92 Alanine from Threonine	-0.22	0.37	-0.56	0.56
238 Proline from Isoleucine	-0.31	0.37	-0.47	0.61
239 Proline from Glutamine	-0.33	0.53	-0.45	0.5
761 Deoxyuridine	-0.35	0.41	-0.43	0.29
651 3alpha,7alpha,12alpha-Trihydroxy-5beta-cholestanoyl-CoA	-0.33	0.46	-0.45	0.45
265 Serine from Proline	-0.31	0.45	-0.48	0.56
647 3alpha,7alpha,12alpha-Trihydroxy-5beta-24-oxocholestanoyl-CoA	-0.34	0.43	-0.45	0.5
246 Proline from Tryptophan	-0.32	0.5	-0.47	0.51

Continued on next page

Matches relating TGFβ/contr 6h vs. 24h – continued

Simulation	TGFβ/C 6h ampl	TGFβ/C 6h score	TGFβ/C 24h ampl	TGFβ/C 24h score
248 Proline from Valine	-0.35	0.46	-0.44	0.53
12 ATP from NADH	-0.04	0.43	-0.75	0.47
646 3alpha,7alpha,12alpha,24-Tetrahydroxy-5beta-cholestanoyl-CoA	-0.34	0.44	-0.45	0.49
506 Uridine	-0.34	0.49	-0.45	0.32
867 N-Formyl-GAR	-0.27	0.55	-0.53	0.45
125 Asparagine from Histidine	-0.22	0.42	-0.58	0.43
104 Arginine from Histidine	-0.2	0.44	-0.6	0.42
251 Serine from Arginine	-0.32	0.37	-0.48	0.44
740 Cholesterol-ester-stea	-0.37	0.49	-0.43	0.62
736 Cholesterol-ester-ol	-0.37	0.49	-0.43	0.62
553 (S)-3-Hydroxy-2-methylbutyryl-CoA	-0.29	0.38	-0.51	0.39
241 Proline from Lysine	-0.3	0.53	-0.5	0.54
739 Cholesterol-ester-pool	-0.36	0.45	-0.44	0.61
21 Aerobic reduction of FAD (FA)	-0.28	0.37	-0.53	0.52
428 beta-Alanine degr	-0.07	0.37	-0.73	0.34
648 3alpha,7alpha,12alpha-Trihydroxy-5beta-cholest-24-enoyl-CoA	-0.35	0.44	-0.46	0.48
501 GDP-L-fucose	-0.31	0.45	-0.49	0.31
132 Asparagine from Proline	-0.28	0.47	-0.52	0.58
524 gdpddman	-0.31	0.46	-0.49	0.32
734 Cholesterol-ester-gla	-0.36	0.47	-0.44	0.63
784 Formylanthranilate	0.33	0.59	-1.14	0.48
716 Argininosuccinate	-0.19	0.55	-0.61	0.66
195 Glycine from Leucine	-0.36	0.34	-0.44	0.39
738 Cholesterol-ester-palmn	-0.37	0.5	-0.43	0.59
482 Asparagine	-0.22	0.44	-0.58	0.47
735 Cholesterol-ester-lin	-0.36	0.47	-0.45	0.62
693 Acetaldehyde	-0.47	0.54	-0.34	0.61
737 Cholesterol-ester-palm	-0.37	0.43	-0.44	0.59
679 5beta-Cholestane-3alpha,7alpha-diol	-0.36	0.51	-0.45	0.52
117 Arginine from beta-Alanine	-0.09	0.39	-0.72	0.39
652 3alpha,7alpha,12alpha-Trihydroxycoprostane	-0.36	0.48	-0.45	0.5
324 Cholesterol(b)	-0.37	0.51	-0.44	0.61
846 Lathosterol	-0.36	0.58	-0.45	0.71
915 Provitamin D3	-0.36	0.55	-0.45	0.67
3 Anaerobic rephosph of ATP	-0.47	0.28	-0.34	0.42
733 Cholesterol-ester-arach	-0.37	0.47	-0.44	0.64
970 dTDP	-0.33	0.39	-0.48	0.34
868 N-Methylethanolamine-P	-0.47	1	-0.35	1
771 Ethanolamine-P	-0.47	1	-0.35	1
685 7alpha-Hydroxy-5beta-cholestan-3-one	-0.37	0.49	-0.45	0.55
683 7alpha,12alpha-Dihydroxy-5beta-cholestan-3-one	-0.37	0.46	-0.45	0.52
472 dATP	-0.35	0.53	-0.48	0.42
75 Gluconeogen from Alanine	-0.2	0.27	-0.63	0.35
474 dGTP	-0.34	0.48	-0.48	0.39
523 Cholesterol	-0.37	0.57	-0.45	0.63
687 7alpha-Hydroxycholesterol	-0.37	0.54	-0.46	0.6
686 7alpha-Hydroxycholest-4-en-3-one	-0.37	0.51	-0.46	0.57
615 3-Hydroxyisobutyryl-CoA	-0.28	0.43	-0.55	0.32
684 7alpha,12alpha-Dihydroxycholest-4-en-3-one	-0.37	0.47	-0.46	0.53
732 Cholestenol	-0.36	0.59	-0.47	0.7
665 5,6-Dihydrouracil	-0.32	0.48	-0.51	0.41
127 Asparagine from Glutamine	-0.4	0.37	-0.43	0.56
256 Serine from Glutamate	-0.36	0.37	-0.47	0.54
814 HMG-CoA	-0.27	0.5	-0.56	0.39
676 5alpha-Cholesta-7,24-dien-3beta-ol	-0.37	0.59	-0.47	0.7
682 7-Dehydrodesmosterol	-0.37	0.55	-0.47	0.66
214 Glutamine from Histidine	-0.19	0.51	-0.64	0.41
502 GDP-mannose	-0.32	0.46	-0.51	0.32
691 AIR	-0.31	0.54	-0.53	0.41

Continued on next page

Matches relating TGFβ/contr 6h vs. 24h – continued

Simulation	TGFβ/C 6h		TGFβ/C 24h	
	ampl	score	ampl	score
741 Choloyl-CoA	-0.37	0.4	-0.47	0.47
831 Isobutyryl-CoA	-0.28	0.48	-0.56	0.34
898 Phosphatidate-CL-pool	0.03	0.38	-0.87	0.46
134 Asparagine from Threonine	-0.27	0.31	-0.57	0.54
675 5-Phosphoribosylformylglycinamidine	-0.32	0.54	-0.53	0.42
521 Ceramide	-0.34	0.37	-0.5	0.48
45 Activated methylene group from Try	-0.22	0.45	-0.63	0.39
763 Desmosterol	-0.38	0.58	-0.47	0.62
476 ATP	-0.35	0.55	-0.51	0.4
478 GTP	-0.34	0.49	-0.51	0.37
514 PC	-0.11	0.3	-0.74	0.4
848 Linoleate	-0.36	0.42	-0.5	0.62
957 Zymosterol	-0.37	0.6	-0.48	0.69
662 4alpha-Methylzymosterol	-0.37	0.6	-0.48	0.69
204 Glycine from Valine	-0.39	0.39	-0.47	0.47
199 Glycine from Proline	-0.39	0.51	-0.47	0.6
618 3-Keto-4-methylzymosterol	-0.35	0.58	-0.51	0.68
186 Glycine from Arginine	-0.38	0.39	-0.49	0.42
237 Proline from Histidine	-0.28	0.56	-0.59	0.49
30 NADPH to NADH transhydrogenase in mito	-0.03	1	-0.84	1
731 Cholate	-0.37	0.39	-0.5	0.52
108 Arginine from Lysine	-0.26	0.37	-0.61	0.45
77 Alanine from Asparagine	-0.4	0.38	-0.47	0.44
746 Cys-Gly	-0.23	1	-0.64	1
191 Glycine from Glutamate	-0.4	0.39	-0.47	0.48
469 ametam	-0.32	0.46	-0.55	0.32
764 Dihomo-gamma-linolenoyl-CoA	-0.3	0.43	-0.57	0.39
982 sn-Glycerol-3P	-0.02	0.57	-0.85	0.84
258 Serine from Histidine	-0.34	0.42	-0.54	0.53
884 Orotate	-0.34	0.54	-0.54	0.43
242 Proline from Methionine	-0.38	0.44	-0.5	0.53
943 Thymidine	-0.35	0.33	-0.53	0.29
971 dTMP	-0.35	0.36	-0.53	0.31
119 Asparagine from Arginine	-0.25	0.33	-0.64	0.42
663 4alpha-Methylzymosterol-4-carboxylate	-0.36	0.61	-0.53	0.69
504 XMP	-0.34	0.54	-0.54	0.38
260 Serine from Glutamine	-0.38	0.4	-0.51	0.48
509 Guanine	-0.35	0.46	-0.54	0.33
262 Serine from Lysine	-0.34	0.39	-0.55	0.48
176 Glutamate from Methionine	-0.4	0.35	-0.5	0.47
678 5beta-Cholestane-3alpha,7alpha,26-triol	-0.36	0.49	-0.53	0.52
499 udpgal	-0.32	0.56	-0.58	0.36
503 IMP	-0.35	0.54	-0.54	0.39
253 Serine from Aspartate	-0.39	0.42	-0.51	0.49
973 dUMP	-0.35	0.39	-0.55	0.33
677 5beta-Cholestane-3alpha,7alpha,12alpha,26-tetrol	-0.36	0.46	-0.54	0.49
650 3alpha,7alpha,12alpha-Trihydroxy-5beta-cholestanate	-0.36	0.46	-0.54	0.49
649 3alpha,7alpha,12alpha-Trihydroxy-5beta-cholestan-26-al	-0.36	0.46	-0.54	0.49
508 Xanthine	-0.35	0.48	-0.55	0.34
315 Glycocholate(b)	-0.37	0.44	-0.53	0.55
721 CDP-choline	-0.35	0.5	-0.56	0.37
614 3-Hydroxyisobutyrate	-0.22	0.42	-0.69	0.36
636 3-Phosphonooxypyruvate	-0.26	0.36	-0.65	0.51
266 Serine from Threonine	-0.34	0.37	-0.57	0.58
270 Serine from beta-Alanine	-0.37	0.4	-0.54	0.42
317 Taurocholate(b)	-0.36	0.39	-0.55	0.51
129 Asparagine from Lysine	-0.27	0.34	-0.65	0.46
175 Glutamate from Lysine	-0.28	0.45	-0.64	0.51
194 Glycine from Glutamine	-0.41	0.42	-0.51	0.44

Continued on next page

Matches relating TGFβ/contr 6h vs. 24h – continued

Simulation	TGFβ/C 6h		TGFβ/C 24h	
	ampl	score	ampl	score
243 Proline from Phenylalanine	-0.29	0.53	-0.63	0.52
446 ApoB100 degr	0.1	0.49	-1.02	0.51
193 Glycine from Isoleucine	-0.4	0.39	-0.52	0.57
109 Arginine from Methionine	-0.38	0.38	-0.54	0.48
608 2-Oxoglutaramate	-0.5	1	-0.44	1
171 Glutamate from Histidine	-0.2	0.6	-0.74	0.5
768 Dimethylallyl-PP	-0.14	0.53	-0.8	0.52
498 UDP-glucose	-0.32	0.5	-0.62	0.36
218 Glutamine from Methionine	-0.42	0.38	-0.53	0.5
244 Proline from Serine	-0.3	0.53	-0.65	0.48
808 Guanidinoacetate	-0.21	0.35	-0.74	0.36
133 Asparagine from Serine	-0.25	0.35	-0.7	0.37
640 3-Ureidopropionate	-0.32	0.43	-0.63	0.43
252 Serine from Asparagine	-0.41	0.45	-0.54	0.52
510 Adenosine	-0.34	0.52	-0.61	0.43
110 Arginine from Phenylalanine	-0.23	0.45	-0.72	0.45
55 UDP-activated glucose	-0.16	0.66	-0.79	0.53
520 SM	-0.37	0.39	-0.58	0.47
219 Glutamine from Phenylalanine	-0.24	0.45	-0.71	0.48
418 Phenylalanine degr	-0.2	0.44	-0.76	0.43
217 Glutamine from Lysine	-0.25	0.36	-0.72	0.45
288 beta-Alanine from Serine	-0.25	0.33	-0.71	0.38
776 Fatty-acid-VLDL-PE-pool	-0.17	0.36	-0.8	0.56
777 Fatty-acid-VLDL-PI-pool	-0.17	0.36	-0.8	0.56
131 Asparagine from Phenylalanine	-0.22	0.42	-0.74	0.44
778 Fatty-acid-VLDL-PS-pool	-0.17	0.36	-0.8	0.56
420 Serine degr	-0.23	0.37	-0.74	0.39
410 Aspartate degr	-0.07	0.37	-0.9	0.34
98 Arginine from Asparagine	-0.31	0.37	-0.66	0.38
201 Glycine from Threonine	-0.41	0.42	-0.56	0.56
781 Fatty-acid-VLDL-TG2-pool	-0.17	0.37	-0.81	0.56
780 Fatty-acid-VLDL-TG1-pool	-0.17	0.37	-0.81	0.56
61 AKG	-0.25	0.47	-0.73	0.57
271 Tyrosine from Phenylalanine	-0.45	0.44	-0.53	0.59
286 beta-Alanine from Phenylalanine	-0.21	0.41	-0.77	0.39
152 Aspartate from Phenylalanine	-0.21	0.46	-0.77	0.43
431 HDL degr	-0.13	0.33	-0.84	0.41
518 CL	-0.2	0.59	-0.78	0.42
891 PG-CL-pool	-0.2	0.52	-0.79	0.44
892 PGP-CL-pool	-0.2	0.52	-0.79	0.44
231 Proline from Asparagine	-0.32	0.57	-0.67	0.5
177 Glutamate from Phenylalanine	-0.25	0.52	-0.75	0.52
730 Chenodeoxycholoyl-CoA	-0.34	0.49	-0.66	0.48
264 Serine from Phenylalanine	-0.34	0.45	-0.66	0.51
975 gamma-Glutamyl-cysteine	-0.02	0.83	-0.99	0.87
247 Proline from Tyrosine	-0.29	0.53	-0.72	0.47
481 Arginine	-0.24	0.5	-0.77	0.46
57 UDP-activated galactose	-0.27	0.66	-0.74	0.45
165 Glutamate from Arginine	-0.21	0.5	-0.8	0.42
468 PAPS	-0.3	0.45	-0.72	0.33
224 Glutamine from Tyrosine	-0.26	0.46	-0.76	0.48
114 Arginine from Tryptophan	-0.32	0.39	-0.7	0.44
136 Asparagine from Tyrosine	-0.25	0.41	-0.77	0.46
115 Arginine from Tyrosine	-0.25	0.47	-0.79	0.47
310 Bilirubin conjugation	-0.17	0.53	-0.86	0.62
491 Glycerol	-0.18	0.33	-0.86	0.56
409 Asparagine degr	-0.07	0.42	-0.98	0.34
747 Cysteamine	0.41	0.43	-1.46	0.39
91 Alanine from Serine	-0.38	0.4	-0.67	0.56

Continued on next page

Matches relating TGFβ/contr 6h vs. 24h – continued

Simulation	TGFβ/C 6h		TGFβ/C 24h	
	ampl	score	ampl	score
112 Arginine from Serine	-0.26	0.41	-0.79	0.43
182 Glutamate from Tyrosine	-0.26	0.54	-0.8	0.53
584 1-Acylglycerol-3P-palmn	-0.06	0.38	-1	0.59
488 Serine	-0.37	0.5	-0.69	0.57
934 Squalene	-0.27	0.98	-0.79	0.83
909 Presqualene-PP	-0.27	0.98	-0.79	0.83
655 3alpha,7alpha-Dihydroxy-5beta-cholestanoyl-CoA	-0.32	0.5	-0.75	0.47
208 Glutamine from Asparagine	-0.38	0.46	-0.68	0.42
99 Arginine from Aspartate	-0.3	0.37	-0.77	0.38
426 Tyrosine degr	-0.21	0.43	-0.87	0.44
173 Glutamate from Glutamine	-0.2	0.56	-0.88	0.39
316 Gly-CD-cholate(b)	-0.35	0.54	-0.73	0.57
842 L-Octanoylcarnitine	-0.3	0.34	-0.79	0.64
43 Activated methyl group (SAM)	-0.42	0.43	-0.67	0.41
484 Glutamate	-0.22	0.63	-0.88	0.58
167 Glutamate from Aspartate	-0.31	0.54	-0.78	0.47
179 Glutamate from Serine	-0.22	0.47	-0.88	0.47
486 Glutamine	-0.22	0.55	-0.88	0.51
980 palmitoleoyl-Carnitine	0.04	0.35	-1.13	0.49
93 Alanine from Tryptophan	-0.37	0.39	-0.73	0.5
184 Glutamate from beta-Alanine	-0.3	0.48	-0.8	0.37
181 Glutamate from Tryptophan	-0.31	0.46	-0.8	0.51
321 SM(b)	-0.37	0.37	-0.73	0.47
223 Glutamine from Tryptophan	-0.33	0.42	-0.77	0.47
422 Tryptophan degr	-0.27	0.34	-0.83	0.42
135 Asparagine from Tryptophan	-0.31	0.39	-0.79	0.44
318 tcdchola(b)	-0.35	0.46	-0.76	0.52
856 Methacrylyl-CoA	-0.28	0.42	-0.82	0.32
48 Formylgroup(m)	-0.29	0.41	-0.82	0.52
226 Glutamine from beta-Alanine	-0.3	0.38	-0.81	0.35
156 Aspartate from Tryptophan	-0.29	0.37	-0.82	0.44
352 Collagen CO4A5(c) synthesis	-0.15	1	-0.96	1
573 1-Acylglycerol-3P-CL-pool	-0.23	0.45	-0.88	0.54
267 Serine from Tryptophan	-0.38	0.43	-0.74	0.5
668 5-Formyl-THF	-0.34	0.31	-0.78	0.43
290 beta-Alanine from Tryptophan	-0.29	0.34	-0.84	0.39
612 3-Hydroxy-L-kynurenine	-0.28	0.37	-0.86	0.44
874 Nicotinate	-0.33	0.48	-0.8	0.56
319 Chenodiol(b)	-0.37	0.49	-0.76	0.6
449 Chitin-component degr	-0.17	0.49	-0.97	0.41
166 Glutamate from Asparagine	-0.28	0.49	-0.87	0.47
653 3alpha,7alpha-Dihydroxy-5beta-cholestan-26-al	-0.35	0.52	-0.8	0.53
654 3alpha,7alpha-Dihydroxy-5beta-cholestanate	-0.34	0.53	-0.82	0.56
221 Glutamine from Serine	-0.34	0.39	-0.82	0.43
411 Cysteine degr	-0.08	0.4	-1.09	0.43
233 Proline from Cysteine	-0.38	0.64	-0.79	0.55
24 NADH redox potential into mito	-0.26	0.59	-0.92	0.61
257 Serine from Glycine	0.24	0.6	-1.42	0.43
427 Homocysteine degr	-0.08	0.38	-1.11	0.45
417 Methionine degr	-0.08	0.38	-1.11	0.43
82 Alanine from Glycine	-0.4	0.41	-0.8	0.5
236 Proline from Glycine	-0.33	0.52	-0.87	0.44
74 Gluconeogen from Glycerol	-0.53	0.36	-0.69	0.51
157 Aspartate from Tyrosine	-0.22	0.46	-0.99	0.44
234 Proline from Cystine	-0.34	0.6	-0.87	0.54
31 NADH to NADPH transhydrogenase in mito	-0.21	1	-1.02	1
926 Sarcosine	0.02	0.8	-1.26	0.86
26 NADPH redox potential into mito	-0.27	0.76	-0.96	0.78
845 Lanosterol	-0.35	0.72	-0.89	0.77

Continued on next page

Simulation	TGFβ/C 6h ampl	TGFβ/C 6h score	TGFβ/C 24h ampl	TGFβ/C 24h score
27 NADPH redox potential into peroxy	-0.32	1	-0.93	1
377 Collagen COIA1(c) synthesis	-0.08	1	-1.18	1
896 Pantetheine	0.41	0.33	-1.68	0.52
956 Xanthurenate	-0.26	0.38	-1.02	0.5
657 4-(2-Amino-3-hydroxyphenyl)-2,4-dioxobutanoate	-0.26	0.38	-1.02	0.5
906 Phosphodimethylethanolamine	-0.48	1	-0.8	1
905 Phosphocholine	-0.48	1	-0.8	1
929 Serotonin	-0.1	0.78	-1.21	0.53
227 Methionine from Cysteine	-0.1	0.41	-1.21	0.39
598 2-Aminomuconate	-0.35	0.47	-0.96	0.54
935 Squalene 2,3-oxide	-0.37	0.86	-0.95	0.84
291 beta-Alanine from Tyrosine	-0.22	0.4	-1.11	0.38
713 Anthranilate	-0.43	0.45	-0.9	0.48
599 2-Aminomuconate(s)emialdehyde	-0.35	0.44	-0.98	0.5
596 2-Amino-3-carboxymuconate(s)emialdehyde	-0.35	0.44	-0.98	0.5
101 Arginine from Cystine	-0.36	0.47	-0.98	0.51
295 Taurine from Cysteine	0.1	0.6	-1.46	0.5
826 Hypotaurine	0.1	0.6	-1.46	0.5
638 3-Sulfinoalanine	0.1	1	-1.46	0.73
100 Arginine from Cysteine	-0.38	0.46	-0.99	0.45
314 Creatine	-0.1	0.41	-1.27	0.39
613 3-Hydroxyanthranilate	-0.36	0.45	-1.01	0.52
793 GSSG	-0.38	0.51	-1	0.71
121 Asparagine from Cysteine	-0.4	0.37	-0.99	0.38
169 Glutamate from Cystine	-0.39	0.56	-1.01	0.62
168 Glutamate from Cysteine	-0.37	0.54	-1.03	0.57
103 Arginine from Glycine	-0.31	0.39	-1.09	0.45
424 Glycine degr	-0.26	0.34	-1.14	0.4
122 Asparagine from Cystine	-0.46	0.44	-0.96	0.48
164 Glutamate from Alanine	-0.2	0.72	-1.22	0.49
279 beta-Alanine from Glycine	-0.3	0.32	-1.15	0.42
170 Glutamate from Glycine	-0.34	0.42	-1.12	0.55
210 Glutamine from Cysteine	-0.39	0.43	-1.07	0.44
471 Homocysteine	-0.34	0.45	-1.14	0.47
211 Glutamine from Cystine	-0.45	0.51	-1.03	0.56
124 Asparagine from Glycine	-0.42	0.37	-1.07	0.43
130 Asparagine from Methionine	-0.38	0.41	-1.13	0.48
143 Aspartate from Cystine	-0.5	0.45	-1.01	0.48
268 Serine from Tyrosine	-0.29	0.44	-1.22	0.5
470 GSH	-0.41	0.49	-1.11	0.63
276 beta-Alanine from Cysteine	-0.44	0.32	-1.08	0.36
202 Glycine from Tryptophan	-0.38	0.42	-1.15	0.49
213 Glutamine from Glycine	-0.37	0.35	-1.16	0.46
151 Aspartate from Methionine	-0.39	0.38	-1.15	0.48
228 Methionine from Cystine	-0.33	0.38	-1.21	0.43
47 Formylgroup(c)	-0.44	0.94	-1.1	0.71
950 UDP-activated-xylose	-0.14	0.56	-1.41	0.44
277 beta-Alanine from Cystine	-0.55	0.36	-1.01	0.41
163 Cystine from Methionine	-0.39	0.41	-1.17	0.51
198 Glycine from Phenylalanine	-0.36	0.44	-1.21	0.5
500 udpglcur	-0.36	0.53	-1.21	0.34
285 beta-Alanine from Methionine	-0.39	0.37	-1.19	0.46
56 UDP-activated glucuronate	-0.46	0.52	-1.12	0.53
497 UDP-xylose	-0.35	0.52	-1.23	0.3
79 Alanine from Cysteine	-0.18	0.43	-1.4	0.5
255 Serine from Cystine	-0.44	0.53	-1.14	0.6
263 Serine from Methionine	-0.41	0.46	-1.17	0.54
161 Cysteine from Methionine	-0.39	0.38	-1.21	0.49
254 Serine from Cysteine	-0.42	0.5	-1.19	0.57

Continued on next page

Matches relating TGFβ/contr 6h vs. 24h – continued

Simulation	TGFβ/C 6h		TGFβ/C 24h	
	ampl	score	ampl	score
162 Cystine from Cysteine	-0.73	0.61	-0.89	0.51
160 Cysteine from Cystine	-0.73	0.61	-0.89	0.51
832 Isocitrate	-0.28	0.71	-1.35	0.61
203 Glycine from Tyrosine	-0.37	0.47	-1.25	0.53
94 Alanine from Tyrosine	-0.22	0.5	-1.42	0.49
294 Taurine from Methionine	-0.39	0.37	-1.27	0.51
197 Glycine from Methionine	-0.42	0.47	-1.23	0.55
485 Glycine	-0.41	0.52	-1.26	0.59
187 Glycine from Asparagine	-0.41	0.41	-1.27	0.47
190 Glycine from Cystine	-0.45	0.54	-1.23	0.61
192 Glycine from Histidine	-0.36	0.42	-1.33	0.48
88 Alanine from Methionine	-0.41	0.43	-1.28	0.52
886 Oxalosuccinate	-0.26	0.68	-1.44	0.59
189 Glycine from Cysteine	-0.44	0.51	-1.27	0.58
480 Alanine	-0.2	0.74	-1.51	0.67
205 Glycine from beta-Alanine	-0.41	0.43	-1.33	0.45
188 Glycine from Aspartate	-0.42	0.45	-1.32	0.5
788 Fumarylacetoacetate	-0.23	0.54	-1.51	0.68
97 Arginine from Alanine	-0.25	0.53	-1.49	0.42
206 Glutamine from Alanine	-0.25	0.62	-1.5	0.51
312 Urea from alanine	-0.21	0.46	-1.54	0.43
313 Urea from NH3	-0.21	0.46	-1.54	0.43
407 Alanine degr	-0.21	0.48	-1.54	0.43
637 3-Phosphoserine	-0.29	0.44	-1.46	0.52
811 H2S	-0.52	0.46	-1.26	0.51
200 Glycine from Serine	-0.49	0.77	-1.31	0.58
44 Activated methyl group (THF)	-0.49	0.77	-1.31	0.58
196 Glycine from Lysine	-0.37	0.4	-1.44	0.47
465 acgam6p	-0.74	0.55	-1.09	0.52
78 Alanine from Aspartate	-0.17	0.46	-1.65	0.43
80 Alanine from Cystine	-0.51	0.55	-1.31	0.66
907 Phosphopantetheine	-0.16	0.34	-1.67	0.45
272 beta-Alanine from Alanine	-0.17	0.33	-1.67	0.29
118 Asparagine from Alanine	-0.24	0.53	-1.6	0.39
660 4-Maleylacetoacetate	-0.23	0.61	-1.62	0.7
250 Serine from Alanine	-0.36	0.46	-1.49	0.49
185 Glycine from Alanine	-0.4	0.5	-1.47	0.57
89 Alanine from Phenylalanine	-0.21	0.5	-1.74	0.47
821 Homogentisate	-0.25	0.52	-1.76	0.65
667 5-Formiminotetrahydrofolate	-0.23	0.45	-1.82	0.46
658 4-Hydroxyphenylpyruvate	-0.25	0.7	-1.87	0.73
925 Saccharopine	-0.24	0.76	-1.92	0.63
343 Collagen CD36(c) synthesis	-0.53	1	-1.64	1
489 Tyrosine	-0.005	0.52	-2.86	0.65
54 Glucose-6P	-0.58	0.54	-2.74	0.52
448 Ethanol degr	-0.59	0.57	-3.56	0.54

1.6 Matches relating TGFβ/contr 6h

Table 6: Matches relating TGFβ/contr 6h

Top down, T6h higher Simulation	ampl	score	Bottom up, C6h higher Simulation	ampl	score
403 Acetoacetate	2	0.41	465 acgam6p	-0.74	0.55
405 Acetone	1.99	0.52	160 Cysteine from Cystine	-0.73	0.61
386 Collagen CORA1(c) synthesis	1.74	1	162 Cystine from Cysteine	-0.73	0.61
374 Collagen COFA1(c) synthesis	1.52	1	448 Ethanol degr	-0.59	0.57
609 2PG	1.23	0.41	54 Glucose-6P	-0.58	0.54
890 PEP	1.23	0.52	277 beta-Alanine from Cystine	-0.55	0.36
756 Dehydroalanine	1.06	0.72	343 Collagen CD36(c) synthesis	-0.53	1
879 O-Propanoylcarnitine	0.97	0.35	74 Gluconeogen from Glycerol	-0.53	0.36
607 2-Oxobutyrate	0.97	0.57	811 H2S	-0.52	0.46
896 Pantetheine	0.41	0.33	80 Alanine from Cystine	-0.51	0.55
747 Cysteamine	0.41	0.43	143 Aspartate from Cystine	-0.5	0.45
838 L-Formylkynurenine	0.39	0.73	608 2-Oxoglutaramate	-0.5	1
931 Sphinganine-1P	0.38	0.47	44 Activated methyl group (THF)	-0.49	0.77
933 Sphingosine-1P	0.36	0.32	200 Glycine from Serine	-0.49	0.77
784 Formylanthranilate	0.33	0.59	905 Phosphocholine	-0.48	1
371 Collagen COCA1(c) synthesis	0.33	1	906 Phosphodimethylethanolamine	-0.48	1
834 Kynurenine	0.32	0.46	693 Acetaldehyde	-0.47	0.54
359 Collagen CO6A3(c) synthesis	0.32	1	3 Anaerobic rephosph of ATP	-0.47	0.28
729 Ceramide-1P-pool	0.3	0.35	771 Ethanolamine-P	-0.47	1
694 Acetate	0.3	0.66	868 N-Methylethanolamine-P	-0.47	1
796 Glucono-1,5-lactone-6P	0.3	1	56 UDP-activated glucuronate	-0.46	0.52
346 Collagen CO2A1(c) synthesis	0.28	1	122 Asparagine from Cystine	-0.46	0.44
396 Collagen LPP3(c) synthesis	0.28	1	190 Glycine from Cystine	-0.45	0.54
379 Collagen COKA1(c) synthesis	0.26	1	271 Tyrosine from Phenylalanine	-0.45	0.44
52 Acetyl group(r)	0.25	0.38	211 Glutamine from Cystine	-0.45	0.51
49 Acetyl group(c)	0.25	0.38	255 Serine from Cystine	-0.44	0.53
327 Antichymotrypsin	0.25	0.97	276 beta-Alanine from Cysteine	-0.44	0.32
375 Collagen COGA1(c) synthesis	0.25	1	47 Formylgroup(c)	-0.44	0.94
365 Collagen CO9A1(c) synthesis	0.25	1	189 Glycine from Cysteine	-0.44	0.51
355 Collagen CO5A2(c) synthesis	0.24	1	713 Anthranilate	-0.43	0.45
257 Serine from Glycine	0.24	0.6	197 Glycine from Methionine	-0.42	0.47
307 Arachidonate from Dihomo-gamma-linolenate	0.24	0.44	254 Serine from Cysteine	-0.42	0.5
385 Collagen COPA1(c) synthesis	0.24	1	43 Activated methyl group (SAM)	-0.42	0.43
303 Dihomo-gamma-linolenate from Linoleate	0.24	0.27	218 Glutamine from Methionine	-0.42	0.38
306 Arachidonate from gamma-Linolenate	0.24	0.27	188 Glycine from Aspartate	-0.42	0.45
302 gamma-Linolenate from Linoleate	0.23	0.4	124 Asparagine from Glycine	-0.42	0.37
305 Dihomo-gamma-linolenate from gamma-Linolenate	0.23	0.31	263 Serine from Methionine	-0.41	0.46
434 Fructose degr	0.22	0.56	252 Serine from Asparagine	-0.41	0.45
357 Collagen CO6A1(c) synthesis	0.22	1	187 Glycine from Asparagine	-0.41	0.41
930 Sphinganine	0.21	0.45	470 GSH	-0.41	0.49
765 Dihydroceramide-pool	0.21	0.34	201 Glycine from Threonine	-0.41	0.42
51 Acetyl group(p)	0.2	0.43	205 Glycine from beta-Alanine	-0.41	0.43
299 Oleate from Stearate	0.2	0.55	485 Glycine	-0.41	0.52
802 Glycerate	0.2	0.81	88 Alanine from Methionine	-0.41	0.43
430 LDL degr	0.2	0.34	194 Glycine from Glutamine	-0.41	0.42
712 Agmatine	0.2	0.8	185 Glycine from Alanine	-0.4	0.5
400 Collagen VWA2(c) synthesis	0.19	1	127 Asparagine from Glutamine	-0.4	0.37
645 3PG	0.17	0.36	77 Alanine from Asparagine	-0.4	0.38
632 3-Oxopropanoate	0.17	0.38	121 Asparagine from Cysteine	-0.4	0.37
460 Pyridoxal-P	0.16	0.53	193 Glycine from Isoleucine	-0.4	0.39
916 Pyridoxal	0.16	0.88	82 Alanine from Glycine	-0.4	0.41
436 Mannose degr	0.15	0.48	191 Glycine from Glutamate	-0.4	0.39
389 Collagen EMID2(c) synthesis	0.15	1	176 Glutamate from Methionine	-0.4	0.35
42 Na+ exportgradient	0.15	1	163 Cystine from Methionine	-0.39	0.41
356 Collagen CO5A3(c) synthesis	0.14	1	210 Glutamine from Cysteine	-0.39	0.43
382 Collagen COMA1(c) synthesis	0.13	1	285 beta-Alanine from Methionine	-0.39	0.37

Continued on next page

Top down, T6h higher Simulation	ampl	score	Bottom up, C6h higher Simulation	ampl	score
339 Collagen ADIPO(c) synthesis	0.13	1	204 Glycine from Valine	-0.39	0.39
444 Prothrombin degr	0.13	0.49	253 Serine from Aspartate	-0.39	0.42
351 Collagen CO4A4(c) synthesis	0.13	1	199 Glycine from Proline	-0.39	0.51
440 ApoA1 degr	0.13	0.47	151 Aspartate from Methionine	-0.39	0.38
443 Plasminogen degr	0.12	0.46	161 Cysteine from Methionine	-0.39	0.38
441 Fibrinogen degr	0.12	0.46	294 Taurine from Methionine	-0.39	0.37
437 Albumin degr	0.12	0.44	169 Glutamate from Cystine	-0.39	0.56
50 Acetyl group(m)	0.12	0.38	208 Glutamine from Asparagine	-0.38	0.46
877 O-Acetylcarnitine	0.12	1	233 Proline from Cysteine	-0.38	0.64
445 ApoTransferin degr	0.12	0.44	109 Arginine from Methionine	-0.38	0.38
438 Antichymotrypsin degr	0.12	0.47	100 Arginine from Cysteine	-0.38	0.46
447 Glycogenin degr	0.12	0.45	793 GSSG	-0.38	0.51
320 Bile-PC(b)	0.12	0.32	267 Serine from Tryptophan	-0.38	0.43
439 Antitrypsin degr	0.11	0.47	130 Asparagine from Methionine	-0.38	0.41
433 Stearate degr	0.11	0.42	260 Serine from Glutamine	-0.38	0.4
281 beta-Alanine from Isoleucine	0.11	0.46	763 Desmosterol	-0.38	0.58
432 Oleate degr	0.11	0.4	91 Alanine from Serine	-0.38	0.4
398 Collagen SCRB1(c) synthesis	0.11	1	202 Glycine from Tryptophan	-0.38	0.42
332 ApoC2(c)	0.11	0.95	527 Spermidine	-0.38	0.52
878 O-Butanoylcarnitine	0.11	0.35	186 Glycine from Arginine	-0.38	0.39
344 Collagen CO1A1(c) synthesis	0.11	1	242 Proline from Methionine	-0.38	0.44
822 Homovanillate	0.1	1	315 Glycocholate(b)	-0.37	0.44
295 Taurine from Cysteine	0.1	0.6	196 Glycine from Lysine	-0.37	0.4
826 Hypotaurine	0.1	0.6	321 SM(b)	-0.37	0.37
638 3-Sulfinoalanine	0.1	1	270 Serine from beta-Alanine	-0.37	0.4
446 ApoB100 degr	0.1	0.49	319 Chenodiol(b)	-0.37	0.49
296 Stearate from Palmitate	0.1	0.51	733 Cholesterol-ester-arach	-0.37	0.47
388 Collagen CTHR1(c) synthesis	0.1	1	736 Cholesterol-ester-ol	-0.37	0.49
345 Collagen CO1A2(c) synthesis	0.1	1	740 Cholesterol-ester-stea	-0.37	0.49
395 Collagen ITA2(c) synthesis	0.1	1	488 Serine	-0.37	0.5
259 Serine from Isoleucine	0.09	0.34	684 7alpha,12alpha-Dihydroxycholest-4-en-3-one	-0.37	0.47
353 Collagen CO4A6(c) synthesis	0.09	1	686 7alpha-Hydroxycholest-4-en-3-one	-0.37	0.51
84 Alanine from Isoleucine	0.09	0.4	687 7alpha-Hydroxycholesterol	-0.37	0.54
126 Asparagine from Isoleucine	0.09	0.36	324 Cholesterol(b)	-0.37	0.51
215 Glutamine from Isoleucine	0.09	0.39	203 Glycine from Tyrosine	-0.37	0.47
147 Aspartate from Isoleucine	0.09	0.43	213 Glutamine from Glycine	-0.37	0.35
172 Glutamate from Isoleucine	0.09	0.41	737 Cholesterol-ester-palm	-0.37	0.43
105 Arginine from Isoleucine	0.09	0.34	523 Cholesterol	-0.37	0.57
425 Isoleucine degr	0.09	0.38	662 4alpha-Methylzymosterol	-0.37	0.6
825 Hydroxypyruvate	0.08	1	957 Zymosterol	-0.37	0.6
743 Citrulline	0.08	0.37	520 SM	-0.37	0.39
383 Collagen CONA1(c) synthesis	0.08	1	683 7alpha,12alpha-Dihydroxy-5beta-cholestan-3-one	-0.37	0.46
728 Carnosine	0.07	0.64	685 7alpha-Hydroxy-5beta-cholestan-3-one	-0.37	0.49
823 Hydracrylate	0.07	0.29	738 Cholesterol-ester-palmn	-0.37	0.5
349 Collagen CO4A2(c) synthesis	0.07	1	731 Cholate	-0.37	0.39
72 Glycogen glucose release	0.07	0.42	935 Squalene 2,3-oxide	-0.37	0.86
53 activated sulphur	0.07	0.66	865 N-Carbamoyl-L-aspartate	-0.37	0.38
373 Collagen COEA1(c) synthesis	0.06	1	93 Alanine from Tryptophan	-0.37	0.39
583 1-Acylglycerol-3P-palm	0.06	0.4	682 7-Dehydrodesmosterol	-0.37	0.55
358 Collagen CO6A2(c) synthesis	0.06	1	741 Choloyl-CoA	-0.37	0.4
585 1-Acylglycerol-3P-stea	0.06	0.42	676 5alpha-Cholesta-7,24-dien-3beta-ol	-0.37	0.59
582 1-Acylglycerol-3P-ol	0.06	0.42	168 Glutamate from Cysteine	-0.37	0.54
581 1-Acylglycerol-3P-lin	0.06	0.42	732 Cholestenol	-0.36	0.59
580 1-Acylglycerol-3P-arach	0.06	0.42	317 Taurocholate(b)	-0.36	0.39
577 1-Acylglycerol-3P-VLDL-PS-pool	0.06	0.42	613 3-Hydroxyanthranilate	-0.36	0.45
578 1-Acylglycerol-3P-VLDL-SM-pool	0.06	0.41	560 (S)-Dihydroorotate	-0.36	0.44
579 1-Acylglycerol-3P-VLDL-TG1-pool	0.06	0.41	739 Cholesterol-ester-pool	-0.36	0.45
576 1-Acylglycerol-3P-VLDL-PI-pool	0.06	0.42	500 udpglcur	-0.36	0.53
575 1-Acylglycerol-3P-VLDL-PE-pool	0.06	0.42	256 Serine from Glutamate	-0.36	0.37

Continued on next page

Top down, T6h higher Simulation	ampl	score	Bottom up, C6h higher Simulation	ampl	score
572 1-Acylglycerol-3P-Bile-PC-pool	0.06	0.41	726 Carbamoyl-P	-0.36	0.49
574 1-Acylglycerol-3P-VLDL-PC-pool	0.06	0.42	198 Glycine from Phenylalanine	-0.36	0.44
340 Collagen BGH3(c) synthesis	0.06	1	915 Provitamin D3	-0.36	0.55
362 Collagen CO7A1(c) synthesis	0.05	1	195 Glycine from Leucine	-0.36	0.34
774 FMN	0.05	1	846 Lathosterol	-0.36	0.58
354 Collagen CO5A1(c) synthesis	0.05	1	652 3alpha,7alpha,12alpha-Trihydroxycoprostane	-0.36	0.48
589 1-Acylglycerol-VLDL-PS-pool	0.05	0.37	679 5beta-Cholestane-3alpha,7alpha-diol	-0.36	0.51
588 1-Acylglycerol-VLDL-PI-pool	0.05	0.37	101 Arginine from Cystine	-0.36	0.47
590 1-Acylglycerol-VLDL-SM-pool	0.05	0.36	649 3alpha,7alpha,12alpha-Trihydroxy-5beta-cholestan-26-al	-0.36	0.46
587 1-Acylglycerol-VLDL-PE-pool	0.05	0.37	650 3alpha,7alpha,12alpha-Trihydroxy-5beta-cholestanate	-0.36	0.46
586 1-Acylglycerol-VLDL-PC-pool	0.05	0.37	677 5beta-Cholestane-3alpha,7alpha,12alpha,26-tetrol	-0.36	0.46
591 1-Acylglycerol-VLDL-TG1-pool	0.05	0.37	678 5beta-Cholestane-3alpha,7alpha,26-triol	-0.36	0.49
902 Phosphatidate-VLDL-PS-pool	0.05	0.51	734 Cholesterol-ester-gla	-0.36	0.47
568 1,2-Diacylglycerol-VLDL-PS-pool	0.05	0.45	735 Cholesterol-ester-lin	-0.36	0.47
567 1,2-Diacylglycerol-VLDL-PI-pool	0.05	0.45	250 Serine from Alanine	-0.36	0.46
901 Phosphatidate-VLDL-PI-pool	0.05	0.51	848 Linoleate	-0.36	0.42
569 1,2-Diacylglycerol-VLDL-SM-pool	0.05	0.44	663 4alpha-Methylzymosterol-4-carboxylate	-0.36	0.61
903 Phosphatidate-VLDL-SM-pool	0.05	0.5	192 Glycine from Histidine	-0.36	0.42
900 Phosphatidate-VLDL-PE-pool	0.05	0.51	845 Lanosterol	-0.35	0.72
566 1,2-Diacylglycerol-VLDL-PE-pool	0.05	0.45	316 Gly-CD-cholate(b)	-0.35	0.54
897 Phosphatidate-Bile-PC-pool	0.04	0.49	618 3-Keto-4-methylzymosterol	-0.35	0.58
564 1,2-Diacylglycerol-Bile-PC-pool	0.04	0.43	973 dUMP	-0.35	0.39
899 Phosphatidate-VLDL-PC-pool	0.04	0.51	503 IMP	-0.35	0.54
565 1,2-Diacylglycerol-VLDL-PC-pool	0.04	0.45	248 Proline from Valine	-0.35	0.46
904 Phosphatidate-VLDL-TG-pool	0.04	0.51	508 Xanthine	-0.35	0.48
570 1,2-Diacylglycerol-VLDL-TG-pool	0.04	0.44	509 Guanine	-0.35	0.46
329 ApoA1	0.04	0.95	971 dTMP	-0.35	0.36
402 HDL	0.04	0.67	653 3alpha,7alpha-Dihydroxy-5beta-cholestan-26-al	-0.35	0.52
333 ApoC3(c)	0.04	0.94	497 UDP-xylose	-0.35	0.52
779 Fatty-acid-VLDL-SM-pool	0.04	1	943 Thymidine	-0.35	0.33
775 Fatty-acid-VLDL-PC-pool	0.04	1	598 2-Aminomuconate	-0.35	0.47
378 Collagen COJA1(c) synthesis	0.04	1	761 Deoxyuridine	-0.35	0.41
275 beta-Alanine from Aspartate	0.04	0.52	722 CDP-ethanolamine	-0.35	0.47
977 linoleic-Carnitine	0.04	0.54	596 2-Amino-3-carboxymuconate(s)emialdehyde	-0.35	0.44
714 Arachidonyl-Carnitine	0.04	0.54	599 2-Aminomuconate(s)emialdehyde	-0.35	0.44
980 palmitoleoyl-Carnitine	0.04	0.35	721 CDP-choline	-0.35	0.5
602 2-Lysolecithin-pool	0.04	0.28	318 tcdchola(b)	-0.35	0.46
844 L-Palmitoylcarnitine	0.04	0.49	472 dATP	-0.35	0.53
898 Phosphatidate-CL-pool	0.03	0.38	476 ATP	-0.35	0.55
298 Palmitolate from Palmitate	0.03	0.5	648 3alpha,7alpha,12alpha-Trihydroxy-5beta-cholest-24-enoyl-CoA	-0.35	0.44
335 Plasminogen	0.03	0.97	504 XMP	-0.34	0.54
368 Collagen COAA1(c) synthesis	0.03	1	510 Adenosine	-0.34	0.52
336 Prothrombin	0.03	0.97	234 Proline from Cystine	-0.34	0.6
926 Sarcosine	0.02	0.8	170 Glutamate from Glycine	-0.34	0.42
393 Collagen FMOD(c) synthesis	0.02	1	33 Thioredoxin(m) oxidation	-0.34	1
338 Haptoglobin	0.02	0.96	32 Thioredoxin(m) reduction	-0.34	1
785 Fructose-1,6PP	0.02	0.52	221 Glutamine from Serine	-0.34	0.39
940 Sulfite degr	0.02	1	760 Deoxyinosine	-0.34	0.38
859 N-(omega)-Hydroxyarginine	0.01	0.77	646 3alpha,7alpha,12alpha,24-Tetrahydroxy-5beta-cholestanoyl-CoA	-0.34	0.44
366 Collagen CO9A2(c) synthesis	0.01	1	521 Ceramide	-0.34	0.37
331 ApoC1(c)	0.01	0.92	647 3alpha,7alpha,12alpha-Trihydroxy-5beta-24-oxocholestanoyl-CoA	-0.34	0.43
364 Collagen CO8A2(c) synthesis	0.01	1	474 dGTP	-0.34	0.48
464 mlthf	0.01	0.77	266 Serine from Threonine	-0.34	0.37
34 Thioredoxin(c) reduction	0.01	0.71	478 GTP	-0.34	0.49
466 THF(m)	0.01	1	506 Uridine	-0.34	0.49

Continued on next page

Matches relating TGFβ/contr 6h – continued

Top down, T6h higher Simulation	ampl	score	Bottom up, C6h higher Simulation	ampl	score
459 THF	0.01	1	810 H2O2	-0.34	0.46
29 NADH to NADPH transhydrogenase	0.01	1	952 Urate	-0.34	0.46
766 Dihydrofolate	0.01	1	529 Methylthioribose-1P	-0.34	0.71
670 5-Methyl-THF	0.01	1	264 Serine from Phenylalanine	-0.34	0.45
664 5,10-Methenyl-THF	0.01	0.62	654 3alpha,7alpha-Dihydroxy-5beta-cholestanate	-0.34	0.53
467 1fthf	0.01	0.51	884 Orotate	-0.34	0.54
841 L-Lactate	0.01	0.85	528 Spermine	-0.34	0.52
978 mitoACP	0.001	0.63	668 5-Formyl-THF	-0.34	0.31
942 Tetradecanoyl-ACP	0.001	0.78	258 Serine from Histidine	-0.34	0.42
936 Stearoyl-ACP	0.001	0.77	730 Chenodeoxycholoyl-CoA	-0.34	0.49
881 Octanoyl-ACP	0.001	0.79	262 Serine from Lysine	-0.34	0.39
851 Malonyl-ACP	0.001	0.82	479 UTP	-0.34	0.51
818 Hexanoyl-ACP	0.001	0.8	471 Homocysteine	-0.34	0.45
816 Hexadecanoyl-ACP	0.001	0.78	505 Uracil	-0.34	0.53
813 HMA	0.001	0.78	223 Glutamine from Tryptophan	-0.33	0.42
769 Dodecanoyl-ACP	0.001	0.78	874 Nicotinate	-0.33	0.48
754 Decanoyl-ACP	0.001	0.79	651 3alpha,7alpha,12alpha-Trihydroxy-5beta-cholestanoyl-CoA	-0.33	0.46
750 D-3-Hydroxyhexanoyl-ACP	0.001	0.8	473 dCTP	-0.33	0.48
749 D-3-Hydroxydodecanoyl-ACP	0.001	0.78	236 Proline from Glycine	-0.33	0.52
718 Butyryl-ACP	0.001	0.81	239 Proline from Glutamine	-0.33	0.53
717 But-2-enoyl-ACP	0.001	0.81	970 dTDP	-0.33	0.39
458 Apo-ACP(m)	0.001	0.66	228 Methionine from Cystine	-0.33	0.38
457 Apo-ACP	0.001	0.88	477 CTP	-0.33	0.49
697 Acetyl-ACP	0.001	0.83	337 ApoTransferin	-0.33	0.98
695 Acetoacetyl-ACP	0.001	0.81	972 dUDP	-0.33	0.46
688 ACP	0.001	0.83	974 dUTP	-0.33	0.45
634 3-Oxotetradecanoyl-ACP	0.001	0.78	640 3-Ureidopropionate	-0.32	0.43
633 3-Oxostearoyl-ACP	0.001	0.77	507 Cytidine	-0.32	0.46
629 3-Oxooctanoyl-ACP	0.001	0.79	665 5,6-Dihydrouracil	-0.32	0.48
627 3-Oxohexanoyl-ACP	0.001	0.81	246 Proline from Tryptophan	-0.32	0.5
626 3-Oxohexadecanoyl-ACP	0.001	0.78	231 Proline from Asparagine	-0.32	0.57
624 3-Oxododecanoyl-ACP	0.001	0.78	27 NADPH redox potential into peroxy	-0.32	1
622 3-Oxodecanoyl-ACP	0.001	0.79	502 GDP-mannose	-0.32	0.46
617 3-Hydroxystearoyl-ACP	0.001	0.77	301 Palmitolate from Arachidonate	-0.32	0.49
547 (R)-3-Hydroxypalmitoyl-ACP	0.001	0.78	655 3alpha,7alpha-Dihydroxy-5beta-cholestanoyl-CoA	-0.32	0.5
546 (R)-3-Hydroxyoctanoyl-ACP	0.001	0.79	966 dCDP	-0.32	0.46
545 (R)-3-Hydroxydecanoyl-ACP	0.001	0.79	114 Arginine from Tryptophan	-0.32	0.39
544 (R)-3-Hydroxybutanoyl-ACP	0.001	0.81	490 Palmitate	-0.32	0.67
541 (2E)-Tetradecenoyl-ACP	0.001	0.78	954 UroporphyrinogenIII	-0.32	0.52
539 (2E)-Octenoyl-ACP	0.001	0.79	824 Hydroxymethylbilane	-0.32	0.55
538 (2E)-Octadecenoyl-ACP	0.001	0.77	232 Proline from Aspartate	-0.32	0.59
536 (2E)-Hexenoyl-ACP	0.001	0.8	914 Protoporphyrinogen IX	-0.32	0.49
534 (2E)-Hexadecenoyl-ACP	0.001	0.78	469 ametam	-0.32	0.46
532 (2E)-Dodecenoyl-ACP	0.001	0.78	744 CoproporphyrinogenIII	-0.32	0.51
530 (2E)-Decenoyl-ACP	0.001	0.79	800 Glutamyl-5P	-0.32	0.55
334 ApoE(c)	0.001	0.95	251 Serine from Arginine	-0.32	0.37
369 Collagen COBA1(c) synthesis	0.0002	1	498 UDP-glucose	-0.32	0.5
325 Albumin	-0.001	0.97	720 CDP	-0.32	0.47
68 O2.-(c) degr	-0.002	0.68	499 udpgal	-0.32	0.56
820 Histamine	-0.003	0.64	675 5-Phosphoribosylformylglycinamidine	-0.32	0.54
489 Tyrosine	-0.005	0.52	723 CMP	-0.31	0.47
843 L-Oleoylcarnitine	-0.01	0.51	524 gdpddman	-0.31	0.46
342 Collagen CCBE1(c) synthesis	-0.01	1	238 Proline from Isoleucine	-0.31	0.37
370 Collagen COBA2(c) synthesis	-0.01	1	249 Proline from beta-Alanine	-0.31	0.55
65 ATP salvage from Adenosine	-0.02	0.9	493 Stearate	-0.31	0.66
975 gamma-Glutamyl-cysteine	-0.02	0.83	501 GDP-L-fucose	-0.31	0.45
293 Homocysteine from Methionine	-0.02	1	492 Oleate	-0.31	0.58
304 Arachidonate from Linoleate	-0.02	0.31	748 D-3-Amino-isobutanoate	-0.31	0.38

Continued on next page

Matches relating TGFβ/contr 6h – continued

Top down, T6h higher Simulation	ampl	score	Bottom up, C6h higher Simulation	ampl	score
786 Fructose-2,6PP	-0.02	0.99	406 Heme	-0.31	0.43
399 Collagen SERPH(c) synthesis	-0.02	1	691 AIR	-0.31	0.54
837 L-Cystathionine	-0.02	0.47	135 Asparagine from Tryptophan	-0.31	0.39
982 sn-Glycerol-3P	-0.02	0.57	511 UDP-N-acetylgalactosamine	-0.31	0.4
496 Fructose-6P	-0.02	1	167 Glutamate from Aspartate	-0.31	0.54
404 (R)-3-Hydroxybutanoate	-0.02	0.57	948 UDP-N-acetylglucosamine	-0.31	0.41
782 Fatty-acid-VLDL-TG3-pool	-0.03	0.52	265 Serine from Proline	-0.31	0.45
30 NADPH to NADH transhydrogenase in mito	-0.03	1	103 Arginine from Glycine	-0.31	0.39
372 Collagen CODA1(c) synthesis	-0.03	1	876 Nicotinate(r)ibonucleotide	-0.31	0.48
60 Pyruvate	-0.03	0.41	913 Protoporphyrin	-0.31	0.49
330 ApoB100(r)	-0.03	0.99	181 Glutamate from Tryptophan	-0.31	0.46
308 VLDL from LDL	-0.03	0.72	873 Nicotinamide D-ribonucleotide	-0.31	0.57
309 VLDL from HDL	-0.03	0.7	98 Arginine from Asparagine	-0.31	0.37
401 VLDL	-0.03	0.7	99 Arginine from Aspartate	-0.3	0.37
274 beta-Alanine from Asparagine	-0.04	0.34	279 beta-Alanine from Glycine	-0.3	0.32
836 L-2-Aminoadipate	-0.04	1	671 5-Methylthioadenosine	-0.3	0.62
39 Proton-gradient(m) build up	-0.04	0.31	753 Deamido-NAD	-0.3	0.5
40 Proton-gradient(c) build up	-0.04	0.31	764 Dihomo-gamma-linolenoyl-CoA	-0.3	0.43
797 Glucose-1P	-0.04	1	120 Asparagine from Aspartate	-0.3	0.45
946 Trehalose	-0.04	1	639 3-Ureidoisobutyrate	-0.3	0.42
12 ATP from NADH	-0.04	0.43	767 Dihydrothymine	-0.3	0.45
69 Formaldehyde degr	-0.04	0.45	967 dCMP	-0.3	0.49
919 Ribose-1P	-0.04	0.53	965 dAMP	-0.3	0.54
11 ATP from NADH(m)	-0.04	0.78	964 dADP	-0.3	0.46
212 Glutamine from Glutamate	-0.04	0.29	951 UMP	-0.3	0.46
2 Aerobic ATP rephosph (gluc)	-0.04	0.42	875 Nicotinate D-ribonucleoside	-0.3	0.44
10 Anaerobic rephosph of UTP	-0.04	0.3	241 Proline from Lysine	-0.3	0.53
8 Anaerobic rephosph of GTP	-0.04	0.3	453 FAD	-0.3	0.45
9 Anaerobic rephosph of CTP	-0.04	0.3	269 Serine from Valine	-0.3	0.35
14 Ubiquinol-to-ATP	-0.04	0.62	922 SAH	-0.3	0.59
1 Aerobic ATP rephosph (FA)	-0.04	0.43	924 SAM	-0.3	0.59
4 Aerobic rephosph of GTP	-0.04	0.46	209 Glutamine from Aspartate	-0.3	0.44
7 Aerobic rephosph of UTP	-0.04	0.46	184 Glutamate from beta-Alanine	-0.3	0.48
367 Collagen CO9A3(c) synthesis	-0.04	1	944 Thymine	-0.3	0.44
495 Mannose-6P	-0.04	0.91	462 thbpt	-0.3	0.41
289 beta-Alanine from Threonine	-0.04	0.37	226 Glutamine from beta-Alanine	-0.3	0.38
571 1,3DPG	-0.05	0.69	244 Proline from Serine	-0.3	0.53
455 Thioredoxin	-0.05	0.94	240 Proline from Leucine	-0.3	0.45
887 Oxidized thioredoxin	-0.05	0.93	827 Hypoxanthine	-0.3	0.41
854 Maltose	-0.05	1	487 Proline	-0.3	0.45
5 Aerobic rephosph of CTP	-0.05	0.4	468 PAPS	-0.3	0.45
6 Aerobic rephosph of UDP	-0.05	0.49	842 L-Octanoylcarnitine	-0.3	0.34
611 3-Dehydrosphinganine	-0.05	0.33	710 Adenylosuccinate	-0.3	0.56
376 Collagen COHA1(c) synthesis	-0.05	1	758 Deoxycytidine	-0.3	0.46
601 2-Deoxy-D-ribose-5P	-0.05	0.43	637 3-Phosphoserine	-0.29	0.44
600 2-Deoxy-D-ribose-1P	-0.05	0.33	757 Deoxyadenosine	-0.29	0.44
548 (R)-4-Phosphopantothenoyl-cysteine	-0.06	0.72	772 FADH2	-0.29	0.41
869 N-Pantothenoylcysteine	-0.06	0.69	709 Adenine	-0.29	0.33
672 5-Oxoproline	-0.06	0.72	958 cAMP	-0.29	0.43
323 PE(b)	-0.06	0.31	268 Serine from Tyrosine	-0.29	0.44
515 PE	-0.06	0.34	692 AMP	-0.29	0.48
889 PE-PS-VLDL-pool	-0.06	0.32	689 ADP	-0.29	0.47
278 beta-Alanine from Glutamate	-0.06	0.36	243 Proline from Phenylalanine	-0.29	0.53
138 Asparagine from beta-Alanine	-0.06	0.31	48 Formylgroup(m)	-0.29	0.41
584 1-Acylglycerol-3P-palmn	-0.06	0.38	888 PAP	-0.29	0.46
592 1-Pyrroline-5-carboxylate	-0.06	0.87	553 (S)-3-Hydroxy-2-methylbutyryl-CoA	-0.29	0.38
789 GAP	-0.06	0.55	870 NADH	-0.29	0.49
752 DHAP	-0.06	0.55	451 NAD+	-0.29	0.49
25 NADH redox potential into peroxy	-0.06	0.52	156 Aspartate from Tryptophan	-0.29	0.37

Continued on next page

Top down, T6h higher			Bottom up, C6h higher		
Simulation	ampl	score	Simulation	ampl	score
522 Triacylglycerol	-0.06	0.32	918 Quinolinate	-0.29	0.54
66 ATP salvage from Hypoxanthine	-0.06	0.34	557 (S)-3-Hydroxytetradecanoyl-CoA	-0.29	0.31
987 trans-4-Hydroxy-L-proline	-0.06	1	911 Propanoyl-CoA	-0.29	0.4
835 L-1-Pyrroline-3-hydroxy-5-carboxylate	-0.06	1	635 3-Oxotetradecanoyl-CoA	-0.29	0.31
70 Formate degr	-0.06	0.75	968 dGDP	-0.29	0.45
416 Lysine degr	-0.06	0.37	969 dGMP	-0.29	0.45
770 Erythrose-4P	-0.06	0.7	245 Proline from Threonine	-0.29	0.4
284 beta-Alanine from Lysine	-0.07	0.34	290 beta-Alanine from Tryptophan	-0.29	0.34
421 Threonine degr	-0.07	0.37	554 (S)-3-Hydroxybutyryl-CoA	-0.29	0.45
927 Sedoheptulose-1,7PP	-0.07	0.65	759 Deoxyguanosine	-0.29	0.42
412 Glutamate degr	-0.07	0.42	702 Acyl-CoA-VLDL-PC-pool	-0.29	0.45
414 Glutamine degr	-0.07	0.4	703 Acyl-CoA-VLDL-PE-pool	-0.29	0.46
410 Aspartate degr	-0.07	0.37	706 Acyl-CoA-VLDL-SM-pool	-0.29	0.45
159 Aspartate from beta-Alanine	-0.07	0.43	707 Acyl-CoA-VLDL-TG2-pool	-0.29	0.46
409 Asparagine degr	-0.07	0.42	708 Acyl-CoA-VLDL-TG3-pool	-0.29	0.46
144 Aspartate from Glutamate	-0.07	0.47	704 Acyl-CoA-VLDL-PI-pool	-0.29	0.46
322 PS(b)	-0.07	0.34	705 Acyl-CoA-VLDL-PS-pool	-0.29	0.46
516 PS	-0.07	0.34	715 Arachidonyl-CoA	-0.29	0.46
350 Collagen CO4A3(c) synthesis	-0.07	1	849 Linoleoyl-CoA	-0.29	0.46
123 Asparagine from Glutamate	-0.07	0.31	883 Oleoyl-CoA	-0.29	0.46
428 beta-Alanine degr	-0.07	0.37	937 Stearoyl-CoA	-0.29	0.46
593 14-Demethyllanosterol	-0.07	0.58	762 Dephospho-CoA	-0.29	0.45
13 NADH potential transport	-0.07	0.41	895 Palmitoyl-CoA	-0.29	0.46
150 Aspartate from Lysine	-0.07	0.36	696 Acetoacetyl-CoA	-0.29	0.47
408 Arginine degr	-0.07	0.44	698 Acetyl-CoA	-0.29	0.49
429 Ornithine degr	-0.07	0.44	858 Myristoyl-CoA	-0.29	0.35
417 Methionine degr	-0.08	0.38	700 Acyl-CoA-Bile-PC-pool	-0.29	0.45
427 Homocysteine degr	-0.08	0.38	247 Proline from Tyrosine	-0.29	0.53
656 4,4-Dimethyl-5alpha-cholesta-8,14,24-trien-3beta-ol	-0.08	0.54	701 Acyl-CoA-CL-pool	-0.29	0.46
442 Haptoglobin degr	-0.08	0.43	976 gamma-Linolenoyl-CoA	-0.29	0.44
411 Cysteine degr	-0.08	0.4	631 3-Oxopalmitoyl-CoA	-0.29	0.34
283 beta-Alanine from Leucine	-0.08	0.36	556 (S)-3-Hydroxyhexadecanoyl-CoA	-0.28	0.37
113 Arginine from Threonine	-0.08	0.31	558 (S)-3-hydroxyoleyleoyl-CoA	-0.28	0.43
928 Sedoheptulose-7P	-0.08	0.6	885 Orotidine-5P	-0.28	0.59
377 Collagen COIA1(c) synthesis	-0.08	1	745 Crotonyl-CoA	-0.28	0.44
742 Citrate	-0.09	0.67	229 Proline from Alanine	-0.28	0.64
117 Arginine from beta-Alanine	-0.09	0.39	809 Guanosine	-0.28	0.47
415 Leucine degr	-0.09	0.39	643 3-oxooleoyl-CoA	-0.28	0.44
363 Collagen CO8A1(c) synthesis	-0.09	1	986 trans,cis-octadeca-2,9-dienoyl-CoA	-0.28	0.45
58 GDP-activated fucose	-0.09	0.69	801 Glutaryl-CoA	-0.28	0.42
910 Propanoate	-0.09	0.39	616 3-Hydroxypropionyl-CoA	-0.28	0.4
207 Glutamine from Arginine	-0.09	0.39	981 palmitoleoyl-CoA	-0.28	0.46
222 Glutamine from Threonine	-0.09	0.25	475 dTTP	-0.28	0.49
140 Aspartate from Arginine	-0.09	0.41	535 (2E)-Hexadecenoyl-CoA	-0.28	0.47
839 L-Fucose-1P	-0.09	0.57	828 Inosine	-0.28	0.45
961 cis-Aconitate	-0.09	0.71	463 dhbpt	-0.28	0.4
929 Serotonin	-0.1	0.78	963 cis-myrist-7-enoyl-CoA	-0.28	0.41
314 Creatine	-0.1	0.41	962 cis-laur-5-enoyl-CoA	-0.28	0.39
912 Protein(l)ysine	-0.1	1	644 3-oxopalmitoleoyl-CoA	-0.28	0.42
227 Methionine from Cysteine	-0.1	0.41	642 3-oxomyrist-7-enoyl-CoA	-0.28	0.4
142 Aspartate from Cysteine	-0.1	0.49	543 (3Z)-Dodecenoyl-CoA	-0.28	0.38
850 Malate	-0.1	0.79	856 Methacrylyl-CoA	-0.28	0.42
149 Aspartate from Leucine	-0.1	0.42	641 3-oxolaur-cis-5-enoyl-CoA	-0.28	0.38
880 OAA	-0.1	0.54	132 Asparagine from Proline	-0.28	0.47
73 Gluconeogen from Lactate	-0.1	0.34	960 cis-(3S)-hydroxytetradec-7-enoyl-CoA	-0.28	0.4
141 Aspartate from Asparagine	-0.1	0.46	699 Acrylyl-CoA	-0.28	0.42
812 HCO3-	-0.1	0.66	610 3(S)-3-hydroxydodecen-(5Z)-oyl-CoA	-0.28	0.39
727 Carbonate	-0.1	0.66	985 trans,cis-myristo-2,7-dienoyl-CoA	-0.28	0.41
145 Aspartate from Glycine	-0.11	0.36	175 Glutamate from Lysine	-0.28	0.45

Continued on next page

Top down, T6h higher Simulation	ampl	score	Bottom up, C6h higher Simulation	ampl	score
787 Fumarate	-0.11	0.84	983 trans,cis-dodeca-2,5-dienoyl-CoA	-0.28	0.39
128 Asparagine from Leucine	-0.11	0.35	559 (S)-3-hydroxypalmitoleoyl-CoA	-0.28	0.44
59 GDP-activated mannose	-0.11	0.66	984 trans,cis-hexadeca-2,9-dienoyl-CoA	-0.28	0.45
829 Inositol	-0.11	0.85	711 Adenylyl(s)ulfate	-0.28	0.55
799 Glucuronate	-0.11	0.51	630 3-Oxooctanoyl-CoA	-0.28	0.33
751 D-Xylulose-5P	-0.11	0.53	563 (S)-Hydroxyoctanoyl-CoA	-0.28	0.33
514 PC	-0.11	0.3	237 Proline from Histidine	-0.28	0.56
526 Putrescine	-0.12	0.47	792 GMP	-0.28	0.48
517 PI	-0.12	0.44	832 Isocitrate	-0.28	0.71
669 5-Hydroxy-L-tryptophan	-0.12	0.86	230 Proline from Arginine	-0.28	0.49
917 Pyridoxine-P	-0.12	1	166 Glutamate from Asparagine	-0.28	0.49
435 Galactose degr	-0.12	0.52	871 NADPH	-0.28	0.52
461 4ppan	-0.12	1	452 NADP+	-0.28	0.52
37 GSH reduction using NADPH redox potential	-0.13	1	551 (R)-Methylmalonyl-CoA	-0.28	0.45
38 GSH oxidation	-0.13	1	612 3-Hydroxy-L-kynurenine	-0.28	0.37
341 Collagen C43BP(c) synthesis	-0.13	1	615 3-Hydroxyisobutyryl-CoA	-0.28	0.43
680 6-Phospho-D-gluconate	-0.13	0.5	21 Aerobic reduction of FAD (FA)	-0.28	0.37
494 Mannose-1P	-0.13	0.66	853 Malonyl-CoA	-0.28	0.48
830 Inositol-1P	-0.13	1	831 Isobutyryl-CoA	-0.28	0.48
63 Farnesyl-PP	-0.13	0.6	791 GDP	-0.28	0.46
795 Geranyl-PP	-0.13	0.58	955 Xanthosine	-0.28	0.46
22 Oxidation of NADH	-0.13	0.42	959 cGMP	-0.27	0.42
273 beta-Alanine from Arginine	-0.13	0.34	450 CoA	-0.27	0.5
666 5-Aminolevulinate	-0.13	0.54	814 HMG-CoA	-0.27	0.5
431 HDL degr	-0.13	0.33	939 Succinyl-CoA	-0.27	0.45
950 UDP-activated-xylose	-0.14	0.56	909 Presqualene-PP	-0.27	0.98
15 Dephosphorylation of ATP	-0.14	0.57	934 Squalene	-0.27	0.98
148 Aspartate from Glutamine	-0.14	0.37	26 NADPH redox potential into mito	-0.27	0.76
62 Isopentenyl-PP	-0.14	0.62	945 Tiglyl-CoA	-0.27	0.4
768 Dimethylallyl-PP	-0.14	0.53	755 Decanoyl-CoA	-0.27	0.37
941 THF-hexaglutamate	-0.14	0.87	625 3-Oxododecanoyl-CoA	-0.27	0.37
908 Porphobilinogen	-0.14	0.46	603 2-Methylacetoacetyl-CoA	-0.27	0.43
28 NADPH to NADH transhydrogenase	-0.14	1	847 Lauroyl-CoA	-0.27	0.34
852 Malonyl-Carnitin	-0.14	0.56	819 Hexanoyl-CoA	-0.27	0.34
154 Aspartate from Serine	-0.14	0.44	882 Octanoyl-CoA	-0.27	0.35
35 Thioredoxin(c) oxidation	-0.14	0.71	794 Galactose-1P	-0.27	0.61
597 2-Amino-3-oxoadipate	-0.15	0.59	623 3-Oxodecanoyl-CoA	-0.27	0.35
18 Aerobic reduction of NADP+ (FA)	-0.15	0.4	833 Isovaleryl-CoA	-0.27	0.44
949 UDP-activated-N-acetyl-D-galactosamine	-0.15	0.55	555 (S)-3-Hydroxydodecanoyl-CoA	-0.27	0.38
139 Aspartate from Alanine	-0.15	0.54	561 (S)-Hydroxydecanoyl-CoA	-0.27	0.35
17 Aerobic reduction of NAD+ (gluc)	-0.15	0.51	533 (2E)-Dodecenoyl-CoA	-0.27	0.38
352 Collagen CO4A5(c) synthesis	-0.15	1	531 (2E)-Decenoyl-CoA	-0.27	0.35
16 Aerobic reduction of NAD+ (FA)	-0.15	0.42	542 (2E)-Tetradecenoyl-CoA	-0.27	0.37
549 (R)-5-Diphosphomevalonate	-0.16	0.55	621 3-Methylglutaconyl-CoA	-0.27	0.45
907 Phosphopantetheine	-0.16	0.34	562 (S)-Hydroxyhexanoyl-CoA	-0.27	0.32
894 PRPP	-0.16	0.57	422 Tryptophan degr	-0.27	0.34
107 Arginine from Leucine	-0.16	0.39	773 FAICAR	-0.27	0.58
920 Ribose-5P	-0.16	0.52	57 UDP-activated galactose	-0.27	0.66
803 Glycogenin-G11	-0.16	0.97	867 N-Formyl-GAR	-0.27	0.55
807 Glycogenin-G8	-0.16	0.97	690 AICAR	-0.27	0.54
454 Glycogenin	-0.16	0.97	129 Asparagine from Lysine	-0.27	0.34
806 Glycogenin-G7G1	-0.16	0.96	134 Asparagine from Threonine	-0.27	0.31
804 Glycogenin-G4G4	-0.16	0.96	67 dTTP salvage from Thymine	-0.26	0.51
805 Glycogenin-G7	-0.16	0.96	604 2-Methylbutyryl-CoA	-0.26	0.5
216 Glutamine from Leucine	-0.16	0.35	724 CMP-NeuNGc	-0.26	0.4
55 UDP-activated glucose	-0.16	0.66	235 Proline from Glutamate	-0.26	0.61
921 Ribulose-5P	-0.16	0.55	886 Oxalosuccinate	-0.26	0.68
86 Alanine from Leucine	-0.16	0.4	20 Aerobic reduction of FAD (gluc)	-0.26	0.38
893 PPi	-0.16	0.51	719 Butyryl-CoA	-0.26	0.32

Continued on next page

Matches relating TGFβ/contr 6h – continued

Top down, T6h higher			Bottom up, C6h higher		
Simulation	ampl	score	Simulation	ampl	score
861 N-Acetylglucosamine-1P	-0.16	0.48	537 (2E)-Hexenoyl-CoA	-0.26	0.35
449 Chitin-component degr	-0.17	0.49	540 (2E)-Octenoyl-CoA	-0.26	0.36
71 Glycogen glucose storage	-0.17	0.97	108 Arginine from Lysine	-0.26	0.37
781 Fatty-acid-VLDL-TG2-pool	-0.17	0.37	424 Glycine degr	-0.26	0.34
780 Fatty-acid-VLDL-TG1-pool	-0.17	0.37	178 Glutamate from Proline	-0.26	0.64
778 Fatty-acid-VLDL-PS-pool	-0.17	0.36	636 3-Phosphonooxypyruvate	-0.26	0.36
777 Fatty-acid-VLDL-PI-pool	-0.17	0.36	224 Glutamine from Tyrosine	-0.26	0.46
776 Fatty-acid-VLDL-PE-pool	-0.17	0.36	182 Glutamate from Tyrosine	-0.26	0.54
659 4-Imidazolone-5-propanoate	-0.17	0.45	137 Asparagine from Valine	-0.26	0.34
282 beta-Alanine from Glutamine	-0.17	0.4	673 5-Phosphoribosyl-4-carboxy-5-aminoimidazole	-0.26	0.53
272 beta-Alanine from Alanine	-0.17	0.33	840 L-Glutamate 5-semialdehyde	-0.26	0.49
174 Glutamate from Leucine	-0.17	0.42	24 NADH redox potential into mito	-0.26	0.59
384 Collagen COOA1(c) synthesis	-0.17	1	628 3-Oxohexanoyl-CoA	-0.26	0.34
855 Mercaptopyruvate	-0.17	0.52	112 Arginine from Serine	-0.26	0.41
19 Aerobic reduction of NADP+ (gluc)	-0.17	0.42	857 Methylmalonyl-CoA	-0.26	0.46
798 Glucosylceramide-pool	-0.17	0.42	657 4-(2-Amino-3-hydroxyphenyl)-2,4-dioxobutanoate	-0.26	0.38
85 Alanine from Glutamine	-0.17	0.49	956 Xanthurenate	-0.26	0.38
310 Bilirubin conjugation	-0.17	0.53	136 Asparagine from Tyrosine	-0.25	0.41
76 Alanine from Arginine	-0.17	0.43	133 Asparagine from Serine	-0.25	0.35
46 Activated methyl group from Histidine	-0.17	0.39	821 Homogentisate	-0.25	0.52
180 Glutamate from Threonine	-0.17	0.32	288 beta-Alanine from Serine	-0.25	0.33
146 Aspartate from Histidine	-0.17	0.52	620 3-Methylcrotonyl-CoA	-0.25	0.49
483 Aspartate	-0.17	0.49	658 4-Hydroxyphenylpyruvate	-0.25	0.7
790 GAR	-0.17	0.57	783 Formamidopyrimidine(n)leoside triphosphate	-0.25	0.47
102 Arginine from Glutamate	-0.17	0.36	97 Arginine from Alanine	-0.25	0.53
78 Alanine from Aspartate	-0.17	0.46	177 Glutamate from Phenylalanine	-0.25	0.52
348 Collagen CO4A1(c) synthesis	-0.17	1	119 Asparagine from Arginine	-0.25	0.33
387 Collagen COSA1(c) synthesis	-0.18	0.42	217 Glutamine from Lysine	-0.25	0.36
41 Na+ importgradient	-0.18	0.62	61 AKG	-0.25	0.47
311 Urea from glutamine	-0.18	0.41	115 Arginine from Tyrosine	-0.25	0.47
380 Collagen COLA1(c) synthesis	-0.18	0.42	206 Glutamine from Alanine	-0.25	0.62
491 Glycerol	-0.18	0.33	219 Glutamine from Phenylalanine	-0.24	0.45
381 Collagen COLQ(c) synthesis	-0.18	0.41	925 Saccharopine	-0.24	0.76
81 Alanine from Glutamate	-0.18	0.4	481 Arginine	-0.24	0.5
390 Collagen FCN1(c) synthesis	-0.18	0.4	118 Asparagine from Alanine	-0.24	0.53
397 Collagen PCOTH(c) synthesis	-0.18	0.41	661 4-Methyl-2-oxopentanoate	-0.24	0.4
361 Collagen CO6A6(c) synthesis	-0.18	0.38	116 Arginine from Valine	-0.24	0.34
360 Collagen CO6A5(c) synthesis	-0.18	0.38	183 Glutamate from Valine	-0.24	0.31
328 Antitrypsin	-0.18	0.38	153 Aspartate from Proline	-0.24	0.48
394 Collagen ITA1(c) synthesis	-0.18	0.37	681 6-Pyruvoyltetrahydropterin	-0.23	0.46
550 (R)-5-Phosphomevalonate	-0.18	0.53	573 1-Acylglycerol-3P-CL-pool	-0.23	0.45
79 Alanine from Cysteine	-0.18	0.43	110 Arginine from Phenylalanine	-0.23	0.45
392 Collagen FCN3(c) synthesis	-0.18	0.37	220 Glutamine from Proline	-0.23	0.42
326 Fibrinogen	-0.18	0.36	292 beta-Alanine from Valine	-0.23	0.29
413 Histidine degr	-0.18	0.43	287 beta-Alanine from Proline	-0.23	0.41
674 5-Phosphoribosylamine	-0.18	0.47	660 4-Maleylacetoacetate	-0.23	0.61
83 Alanine from Histidine	-0.18	0.5	746 Cys-Gly	-0.23	1
261 Serine from Leucine	-0.18	0.33	788 Fumarylacetoacetate	-0.23	0.54
456 Thioredoxin(m)	-0.19	0.96	420 Serine degr	-0.23	0.37
979 mitoOxidizedThioredoxin	-0.19	0.95	23 Oxidation of NADPH	-0.23	0.46
106 Arginine from Glutamine	-0.19	0.32	552 (R)-Mevalonate	-0.23	0.46
938 Succinate	-0.19	0.52	595 2,5-Diaminopyrimidine(n)leoside triphosphate	-0.23	0.49
391 Collagen FCN2(c) synthesis	-0.19	1	87 Alanine from Lysine	-0.23	0.37
725 CMP-activated-N-acetylneuraminate	-0.19	0.49	667 5-Formiminotetrahydrofolate	-0.23	0.45
513 CMP-N-acetylneuraminate	-0.19	0.49	131 Asparagine from Phenylalanine	-0.22	0.42
64 Glucosamine-6P	-0.19	0.67	482 Asparagine	-0.22	0.44
716 Argininosuccinate	-0.19	0.55	90 Alanine from Proline	-0.22	0.52
96 Alanine from beta-Alanine	-0.19	0.43	614 3-Hydroxyisobutyrate	-0.22	0.42
214 Glutamine from Histidine	-0.19	0.51	519 LacCer	-0.22	0.42

Continued on next page

Matches relating TGFβ/contr 6h – continued

Top down, T6h higher Simulation	ampl	score	Bottom up, C6h higher Simulation	ampl	score
891 PG-CL-pool	-0.2	0.52	157 Aspartate from Tyrosine	-0.22	0.46
892 PGP-CL-pool	-0.2	0.52	111 Arginine from Proline	-0.22	0.45
953 Urocanate	-0.2	0.49	419 Proline degr	-0.22	0.44
480 Alanine	-0.2	0.74	291 beta-Alanine from Tyrosine	-0.22	0.4
280 beta-Alanine from Histidine	-0.2	0.38	94 Alanine from Tyrosine	-0.22	0.5
932 Sphingosine	-0.2	0.36	95 Alanine from Valine	-0.22	0.36
75 Gluconeogen from Alanine	-0.2	0.27	45 Activated methylene group from Try	-0.22	0.45
866 N-Formimino-L-glutamate	-0.2	0.57	92 Alanine from Threonine	-0.22	0.37
423 Valine degr	-0.2	0.35	594 2,5-Diamino-6-(5-triphosphoryl-3,4-trihydroxy-2-oxopentyl)- amino-4-oxopyrimidine	-0.22	0.49
872 NeuNGc	-0.2	0.38	125 Asparagine from Histidine	-0.22	0.42
104 Arginine from Histidine	-0.2	0.44	864 N-Acetylneuraminate-9P	-0.22	0.41
525 beta-Alanine	-0.2	0.34	862 N-Acetylmannosamine-6P	-0.22	0.58
518 CL	-0.2	0.59	179 Glutamate from Serine	-0.22	0.47
347 Collagen CO3A1(c) synthesis	-0.2	1	484 Glutamate	-0.22	0.63
418 Phenylalanine degr	-0.2	0.44	486 Glutamine	-0.22	0.55
619 3-Methyl-2-oxobutyrate	-0.2	0.65	512 N-acglucam	-0.22	0.31
605 2-Oxo-3-methylvalerate	-0.2	0.4	947 Triphosphate degr	-0.21	1
173 Glutamate from Glutamine	-0.2	0.56	36 GSH reduction using NADH redox potential	-0.21	0.83
171 Glutamate from Histidine	-0.2	0.6	426 Tyrosine degr	-0.21	0.43
860 N-Acetyl-D-mannosamine	-0.2	0.47	225 Glutamine from Valine	-0.21	0.32
297 Palmitate from Palmitolate	-0.2	0.49	923 SAICAR	-0.21	0.58
155 Aspartate from Threonine	-0.2	0.34	808 Guanidinoacetate	-0.21	0.35
863 N-Acetylneuraminate	-0.2	0.36	165 Glutamate from Arginine	-0.21	0.5
164 Glutamate from Alanine	-0.2	0.72	606 2-Oxoadipate	-0.21	0.37
817 Hexadecenal	-0.21	0.38	89 Alanine from Phenylalanine	-0.21	0.5
815 Hexadecanal	-0.21	0.38	158 Aspartate from Valine	-0.21	0.34
407 Alanine degr	-0.21	0.48	31 NADH to NADPH transhydrogenase in mito	-0.21	1
300 Stearate from Oleate	-0.21	0.51	152 Aspartate from Phenylalanine	-0.21	0.46
312 Urea from alanine	-0.21	0.46	286 beta-Alanine from Phenylalanine	-0.21	0.41
313 Urea from NH3	-0.21	0.46			

2 Amplitude differences in relative expression scoring

The tables are sorted by the differences of amplitudes.

2.1 Amplitude differences: Treatment difference

Table 7: Amplitude differences: Treatment difference

Simulation	Δampl	TGFβ/C 24h		control 1/24h	
		ampl	score	ampl	score
896 Pantetheine	4.68	-1.68	0.52	3	0.31
54 Glucose-6P	3.35	-2.74	0.52	0.61	0.51
448 Ethanol degr	3.04	-3.56	0.54	-0.52	0.64
352 Collagen CO4A5(c) synthesis	2.56	-0.96	1	1.61	1
343 Collagen CD36(c) synthesis	2.36	-1.64	1	0.71	1
480 Alanine	2	-1.51	0.67	0.5	0.55
314 Creatine	1.87	-1.27	0.39	0.6	0.27
79 Alanine from Cysteine	1.86	-1.4	0.5	0.46	0.4
829 Inositol	1.84	-0.12	0.5	1.72	0.62
257 Serine from Glycine	1.73	-1.42	0.43	0.31	0.67
830 Inositol-1P	1.65	0.12	1	1.77	1
449 Chitin-component degr	1.64	-0.97	0.41	0.67	0.39
24 NADH redox potential into mito	1.56	-0.92	0.61	0.64	0.53
272 beta-Alanine from Alanine	1.53	-1.67	0.29	-0.14	0.53
907 Phosphopantetheine	1.52	-1.67	0.45	-0.15	0.3
497 UDP-xylose	1.49	-1.23	0.3	0.26	0.31
784 Formylanthranilate	1.48	-1.14	0.48	0.35	0.67
200 Glycine from Serine	1.47	-1.31	0.58	0.16	0.52
44 Activated methyl group (THF)	1.47	-1.31	0.58	0.16	0.52
832 Isocitrate	1.47	-1.35	0.61	0.12	0.41
667 5-Formiminotetrahydrofolate	1.46	-1.82	0.46	-0.35	0.75
489 Tyrosine	1.35	-2.86	0.65	-1.51	0.65
30 NADPH to NADH transhydrogenase in mito	1.33	-0.84	1	0.49	1
874 Nicotinate	1.33	-0.8	0.56	0.52	0.59
47 Formylgroup(c)	1.26	-1.1	0.71	0.16	0.42
236 Proline from Glycine	1.25	-0.87	0.44	0.38	0.34
85 Alanine from Glutamine	1.22	-0.6	0.58	0.63	0.43
856 Methacrylyl-CoA	1.22	-0.82	0.32	0.4	0.36
713 Anthranilate	1.22	-0.9	0.48	0.32	0.51
31 NADH to NADPH transhydrogenase in mito	1.19	-1.02	1	0.17	1
26 NADPH redox potential into mito	1.12	-0.96	0.78	0.15	0.69
181 Glutamate from Tryptophan	1.08	-0.8	0.51	0.29	0.3
223 Glutamine from Tryptophan	1.06	-0.77	0.47	0.29	0.31
27 NADPH redox potential into peroxy	1.05	-0.93	1	0.12	1
118 Asparagine from Alanine	1.05	-1.6	0.39	-0.55	0.38
469 ametam	1.05	-0.55	0.32	0.49	0.31
206 Glutamine from Alanine	1.04	-1.5	0.51	-0.46	0.47
97 Arginine from Alanine	1.01	-1.49	0.42	-0.48	0.36
220 Glutamine from Proline	0.98	-0.48	0.46	0.51	0.29
300 Stearate from Oleate	0.97	-0.29	0.52	0.68	0.32
615 3-Hydroxyisobutyryl-CoA	0.95	-0.55	0.32	0.4	0.37
498 UDP-glucose	0.94	-0.62	0.36	0.32	0.3
17 Aerobic reduction of NAD+ (gluc)	0.94	-0.61	0.45	0.32	0.33
297 Palmitate from Palmitolate	0.94	-0.27	0.43	0.66	0.33
78 Alanine from Aspartate	0.94	-1.65	0.43	-0.71	0.58
276 beta-Alanine from Cysteine	0.93	-1.08	0.36	-0.15	0.51
592 1-Pyrroline-5-carboxylate	0.92	-0.24	0.76	0.68	0.77
987 trans-4-Hydroxy-L-proline	0.92	-0.24	1	0.68	1
835 L-1-Pyrroline-3-hydroxy-5-carboxylate	0.92	-0.24	1	0.68	1
906 Phosphodimethylethanolamine	0.91	-0.8	1	0.11	1
905 Phosphocholine	0.91	-0.8	1	0.11	1
312 Urea from alanine	0.9	-1.54	0.43	-0.64	0.32

Continued on next page

Amplitude differences: Treatment difference – continued

Simulation	Δampl	TGFβ/C 24h		control 1/24h	
		ampl	score	ampl	score
313 Urea from NH3	0.9	-1.54	0.43	-0.64	0.32
950 UDP-activated-xylose	0.9	-1.41	0.44	-0.51	0.41
831 Isobutyryl-CoA	0.9	-0.56	0.34	0.33	0.38
938 Succinate	0.89	-0.44	0.56	0.44	0.38
407 Alanine degr	0.89	-1.54	0.43	-0.66	0.33
510 Adenosine	0.88	-0.61	0.43	0.27	0.36
721 CDP-choline	0.88	-0.56	0.37	0.32	0.34
241 Proline from Lysine	0.86	-0.5	0.54	0.36	0.29
867 N-Formyl-GAR	0.86	-0.53	0.45	0.33	0.43
814 HMG-CoA	0.85	-0.56	0.39	0.3	0.38
836 L-2-Aminoadipate	0.85	-0.43	1	0.42	1
764 Dihomo-gamma-linolenoyl-CoA	0.85	-0.57	0.39	0.28	0.31
90 Alanine from Proline	0.84	-0.44	0.54	0.4	0.32
974 dUTP	0.83	-0.43	0.4	0.4	0.28
675 5-Phosphoribosylformylglycinamidine	0.83	-0.53	0.42	0.3	0.45
691 AIR	0.83	-0.53	0.41	0.3	0.47
250 Serine from Alanine	0.83	-1.49	0.49	-0.67	0.51
529 Methylthioribose-1P	0.82	-0.29	0.56	0.53	0.38
277 beta-Alanine from Cystine	0.81	-1.01	0.41	-0.2	0.4
246 Proline from Tryptophan	0.81	-0.47	0.51	0.34	0.32
699 Acrylyl-CoA	0.8	-0.5	0.39	0.3	0.32
478 GTP	0.8	-0.51	0.37	0.29	0.38
462 thbpt	0.8	-0.43	0.44	0.37	0.35
463 dhbpt	0.8	-0.44	0.46	0.36	0.32
894 PRPP	0.8	-0.41	0.48	0.39	0.39
501 GDP-L-fucose	0.8	-0.49	0.31	0.31	0.43
196 Glycine from Lysine	0.8	-1.44	0.47	-0.64	0.33
185 Glycine from Alanine	0.79	-1.47	0.57	-0.68	0.47
945 Tiglyl-CoA	0.79	-0.48	0.4	0.31	0.31
503 IMP	0.79	-0.54	0.39	0.25	0.39
504 XMP	0.79	-0.54	0.38	0.25	0.37
793 GSSG	0.78	-1	0.71	-0.22	0.48
553 (S)-3-Hydroxy-2-methylbutyryl-CoA	0.78	-0.51	0.39	0.27	0.34
508 Xanthine	0.78	-0.55	0.34	0.23	0.36
616 3-Hydroxypropionyl-CoA	0.78	-0.47	0.39	0.3	0.32
502 GDP-mannose	0.78	-0.51	0.32	0.26	0.39
476 ATP	0.78	-0.51	0.4	0.27	0.38
681 6-Pyruvoyltetrahydropterin	0.77	-0.41	0.38	0.37	0.46
533 (2E)-Dodecenoyl-CoA	0.77	-0.49	0.44	0.28	0.36
555 (S)-3-Hydroxydodecanoyl-CoA	0.77	-0.49	0.43	0.28	0.36
554 (S)-3-Hydroxybutyryl-CoA	0.77	-0.49	0.38	0.28	0.42
474 dGTP	0.77	-0.48	0.39	0.29	0.36
67 dTTP salvage from Thymine	0.77	-0.35	0.47	0.42	0.33
746 Cys-Gly	0.77	-0.64	1	0.13	1
509 Guanine	0.77	-0.54	0.33	0.23	0.35
886 Oxalosuccinate	0.76	-1.44	0.59	-0.67	0.37
955 Xanthosine	0.76	-0.5	0.31	0.26	0.39
791 GDP	0.76	-0.46	0.33	0.3	0.46
524 gdpddman	0.76	-0.49	0.32	0.27	0.42
958 cAMP	0.76	-0.44	0.31	0.32	0.4
755 Decanoyl-CoA	0.76	-0.49	0.45	0.27	0.37
506 Uridine	0.76	-0.45	0.32	0.3	0.46
558 (S)-3-hydroxyoleyleoyl-CoA	0.76	-0.47	0.4	0.29	0.39
625 3-Oxododecanoyl-CoA	0.75	-0.49	0.45	0.27	0.37
720 CDP	0.75	-0.42	0.38	0.34	0.37
643 3-oxooleoyl-CoA	0.75	-0.46	0.37	0.29	0.37
531 (2E)-Decenoyl-CoA	0.75	-0.48	0.44	0.27	0.35
561 (S)-Hydroxydecanoyl-CoA	0.75	-0.48	0.44	0.27	0.34
966 dCDP	0.75	-0.42	0.42	0.33	0.35

Continued on next page

Amplitude differences: Treatment difference – continued

Simulation	Δampl	TGFβ/C 24h		control 1/24h	
		ampl	score	ampl	score
723 CMP	0.75	-0.41	0.38	0.34	0.36
882 Octanoyl-CoA	0.75	-0.48	0.45	0.27	0.35
628 3-Oxohexanoyl-CoA	0.75	-0.45	0.46	0.3	0.32
623 3-Oxodecanoyl-CoA	0.75	-0.48	0.44	0.27	0.35
377 Collagen COIA1(c) synthesis	0.74	-1.18	1	-0.43	1
819 Hexanoyl-CoA	0.74	-0.48	0.45	0.26	0.34
472 dATP	0.74	-0.48	0.42	0.27	0.37
543 (3Z)-Dodecenoyl-CoA	0.74	-0.47	0.41	0.28	0.38
792 GMP	0.74	-0.46	0.35	0.28	0.42
563 (S)-Hydroxyoctanoyl-CoA	0.74	-0.48	0.44	0.26	0.33
962 cis-laur-5-enoyl-CoA	0.74	-0.46	0.41	0.28	0.38
828 Inosine	0.74	-0.48	0.32	0.26	0.45
689 ADP	0.74	-0.42	0.34	0.32	0.44
641 3-oxolaur-cis-5-enoyl-CoA	0.74	-0.46	0.41	0.28	0.38
692 AMP	0.74	-0.42	0.35	0.32	0.44
745 Crotonyl-CoA	0.74	-0.45	0.4	0.28	0.41
603 2-Methylacetoacetyl-CoA	0.74	-0.44	0.4	0.3	0.33
963 cis-myrist-7-enoyl-CoA	0.74	-0.45	0.39	0.28	0.38
983 trans,cis-dodeca-2,5-dienoyl-CoA	0.74	-0.46	0.4	0.28	0.38
630 3-Oxooctanoyl-CoA	0.74	-0.48	0.44	0.25	0.34
610 3(S)-3-hydroxydodecen-(5Z)-oyl-CoA	0.74	-0.46	0.4	0.28	0.38
473 dCTP	0.74	-0.41	0.41	0.33	0.4
210 Glutamine from Cysteine	0.74	-1.07	0.44	-0.33	0.33
477 CTP	0.74	-0.4	0.36	0.33	0.44
969 dGMP	0.73	-0.43	0.37	0.3	0.44
911 Propanoyl-CoA	0.73	-0.45	0.34	0.29	0.33
642 3-oxomyrist-7-enoyl-CoA	0.73	-0.45	0.4	0.28	0.39
164 Glutamate from Alanine	0.73	-1.22	0.49	-0.49	0.49
562 (S)-Hydroxyhexanoyl-CoA	0.73	-0.48	0.42	0.26	0.3
964 dADP	0.73	-0.42	0.38	0.31	0.41
710 Adenylosuccinate	0.73	-0.43	0.4	0.3	0.51
985 trans,cis-myristo-2,7-dienoyl-CoA	0.73	-0.45	0.39	0.28	0.38
960 cis-(3S)-hydroxytetradec-7-enoyl-CoA	0.73	-0.45	0.39	0.28	0.38
968 dGDP	0.73	-0.43	0.37	0.3	0.43
719 Butyryl-CoA	0.73	-0.44	0.47	0.28	0.31
540 (2E)-Octenoyl-CoA	0.73	-0.47	0.44	0.26	0.32
479 UTP	0.73	-0.41	0.38	0.32	0.36
537 (2E)-Hexenoyl-CoA	0.72	-0.47	0.45	0.25	0.32
121 Asparagine from Cysteine	0.72	-0.99	0.38	-0.27	0.39
888 PAP	0.72	-0.4	0.38	0.32	0.42
709 Adenine	0.72	-0.36	0.31	0.36	0.46
722 CDP-ethanolamine	0.72	-0.39	0.46	0.33	0.31
759 Deoxyguanosine	0.71	-0.43	0.34	0.28	0.43
743 Citrulline	0.71	-0.82	0.31	-0.11	0.36
211 Glutamine from Cystine	0.71	-1.03	0.56	-0.32	0.45
833 Isovaleryl-CoA	0.71	-0.41	0.37	0.3	0.34
290 beta-Alanine from Tryptophan	0.71	-0.84	0.39	-0.14	0.32
853 Malonyl-CoA	0.7	-0.41	0.42	0.3	0.38
965 dAMP	0.7	-0.41	0.45	0.29	0.47
644 3-oxopalmitoleoyl-CoA	0.69	-0.41	0.37	0.28	0.39
801 Glutaryl-CoA	0.69	-0.41	0.42	0.29	0.38
604 2-Methylbutyryl-CoA	0.69	-0.36	0.35	0.33	0.37
875 Nicotinate D-ribonucleoside	0.69	-0.14	0.42	0.55	0.3
757 Deoxyadenosine	0.69	-0.4	0.36	0.28	0.41
347 Collagen CO3A1(c) synthesis	0.69	-0.02	1	0.66	1
674 5-Phosphoribosylamine	0.69	-0.35	0.51	0.34	0.35
967 dCMP	0.68	-0.38	0.45	0.3	0.39
858 Myristoyl-CoA	0.68	-0.41	0.44	0.28	0.31
168 Glutamate from Cysteine	0.68	-1.03	0.57	-0.35	0.44

Continued on next page

Simulation	Δampl	TGFβ/C 24h ampl	TGFβ/C 24h score	control 1/24h ampl	control 1/24h score
762 Dephospho-CoA	0.68	-0.37	0.33	0.3	0.39
122 Asparagine from Cystine	0.68	-0.96	0.48	-0.28	0.49
205 Glycine from beta-Alanine	0.68	-1.33	0.45	-0.65	0.35
959 cGMP	0.68	-0.36	0.31	0.31	0.43
696 Acetoacetyl-CoA	0.67	-0.38	0.36	0.3	0.4
698 Acetyl-CoA	0.67	-0.38	0.38	0.29	0.42
169 Glutamate from Cystine	0.67	-1.01	0.62	-0.33	0.5
783 Formamidopyrimidine(n)leoside triphosphate	0.67	-0.36	0.32	0.31	0.43
790 GAR	0.67	-0.33	0.5	0.34	0.47
635 3-Oxotetradecanoyl-CoA	0.67	-0.41	0.4	0.26	0.28
551 (R)-Methylmalonyl-CoA	0.67	-0.36	0.38	0.31	0.37
843 L-Oleoylcarnitine	0.66	-0.53	0.7	0.13	0.54
981 palmitoleoyl-CoA	0.66	-0.37	0.35	0.29	0.38
865 N-Carbamoyl-L-aspartate	0.66	-0.13	0.4	0.53	0.33
573 1-Acylglycerol-3P-CL-pool	0.66	-0.88	0.54	-0.22	0.46
758 Deoxycytidine	0.66	-0.37	0.43	0.28	0.39
631 3-Oxopalmitoyl-CoA	0.66	-0.38	0.41	0.28	0.3
475 dTTP	0.66	-0.3	0.49	0.36	0.5
898 Phosphatidate-CL-pool	0.66	-0.87	0.46	-0.22	0.43
500 udpglcur	0.66	-1.21	0.34	-0.55	0.31
711 Adenylyl(s)ulfate	0.65	-0.35	0.45	0.3	0.46
594 2,5-Diamino-6-(5-triphosphoryl-3,4-trihydroxy-2-oxopentyl)- amino-4-oxopyrimidine	0.65	-0.34	0.31	0.31	0.41
595 2,5-Diaminopyrimidine(n)leoside triphosphate	0.65	-0.34	0.31	0.31	0.42
56 UDP-activated glucuronate	0.65	-1.12	0.53	-0.47	0.4
535 (2E)-Hexadecenoyl-CoA	0.65	-0.35	0.36	0.29	0.38
556 (S)-3-Hydroxyhexadecanoyl-CoA	0.64	-0.36	0.41	0.29	0.31
452 NADP+	0.64	-0.36	0.38	0.29	0.37
976 gamma-Linolenoyl-CoA	0.64	-0.35	0.38	0.29	0.37
937 Stearoyl-CoA	0.64	-0.35	0.4	0.29	0.39
883 Oleoyl-CoA	0.64	-0.35	0.4	0.29	0.39
849 Linoleoyl-CoA	0.64	-0.35	0.4	0.29	0.39
715 Arachidonyl-CoA	0.64	-0.35	0.4	0.29	0.39
895 Palmitoyl-CoA	0.64	-0.35	0.4	0.29	0.39
637 3-Phosphoserine	0.64	-1.46	0.52	-0.82	0.59
701 Acyl-CoA-CL-pool	0.64	-0.35	0.4	0.29	0.38
705 Acyl-CoA-VLDL-PS-pool	0.64	-0.35	0.4	0.29	0.38
704 Acyl-CoA-VLDL-PI-pool	0.64	-0.34	0.4	0.29	0.38
708 Acyl-CoA-VLDL-TG3-pool	0.64	-0.34	0.4	0.29	0.38
707 Acyl-CoA-VLDL-TG2-pool	0.64	-0.34	0.4	0.29	0.38
703 Acyl-CoA-VLDL-PE-pool	0.64	-0.34	0.4	0.29	0.38
871 NADPH	0.64	-0.35	0.39	0.29	0.38
702 Acyl-CoA-VLDL-PC-pool	0.64	-0.34	0.4	0.29	0.38
706 Acyl-CoA-VLDL-SM-pool	0.64	-0.34	0.4	0.29	0.38
700 Acyl-CoA-Bile-PC-pool	0.64	-0.34	0.4	0.29	0.38
620 3-Methylcrotonyl-CoA	0.63	-0.36	0.45	0.28	0.36
984 trans,cis-hexadeca-2,9-dienoyl-CoA	0.63	-0.34	0.36	0.29	0.38
71 Glycogen glucose storage	0.63	-0.19	0.75	0.44	0.65
453 FAD	0.63	-0.37	0.35	0.26	0.48
893 PPi	0.63	-0.24	0.71	0.39	0.66
559 (S)-3-hydroxypalmitoleoyl-CoA	0.63	-0.34	0.35	0.29	0.37
188 Glycine from Aspartate	0.63	-1.32	0.5	-0.7	0.45
979 mitoOxidizedThioredoxin	0.62	-0.25	0.94	0.38	0.94
456 Thioredoxin(m)	0.62	-0.25	0.95	0.38	0.95
162 Cystine from Cysteine	0.62	-0.89	0.51	-0.27	0.87
160 Cysteine from Cystine	0.62	-0.89	0.51	-0.27	0.87
986 trans,cis-octadeca-2,9-dienoyl-CoA	0.62	-0.33	0.4	0.29	0.39
451 NAD+	0.62	-0.33	0.33	0.29	0.4
42 Na+ exportgradient	0.62	-0.67	1	-0.06	1
450 CoA	0.62	-0.34	0.39	0.28	0.38

Continued on next page

Amplitude differences: Treatment difference – continued

Simulation	Δampl	TGFβ/C 24h		control 1/24h	
		ampl	score	ampl	score
772 FADH2	0.61	-0.33	0.33	0.28	0.43
621 3-Methylglutaconyl-CoA	0.61	-0.32	0.37	0.29	0.36
192 Glycine from Histidine	0.6	-1.33	0.48	-0.72	0.36
753 Deamido-NAD	0.6	-0.32	0.37	0.28	0.45
773 FAICAR	0.6	-0.32	0.43	0.28	0.48
671 5-Methylthioadenosine	0.6	-0.3	0.48	0.29	0.45
870 NADH	0.6	-0.31	0.34	0.28	0.41
857 Methylmalonyl-CoA	0.6	-0.32	0.46	0.27	0.32
939 Succinyl-CoA	0.59	-0.3	0.42	0.29	0.37
485 Glycine	0.59	-1.26	0.59	-0.67	0.38
465 acgam6p	0.59	-1.09	0.52	-0.5	0.64
320 Bile-PC(b)	0.58	-0.9	0.44	-0.31	0.47
805 Glycogenin-G7	0.58	-0.14	0.94	0.44	0.94
804 Glycogenin-G4G4	0.58	-0.14	0.94	0.44	0.95
803 Glycogenin-G11	0.58	-0.14	0.95	0.44	0.95
806 Glycogenin-G7G1	0.58	-0.14	0.94	0.44	0.94
807 Glycogenin-G8	0.58	-0.14	0.95	0.44	0.95
454 Glycogenin	0.58	-0.14	0.96	0.44	0.96
187 Glycine from Asparagine	0.58	-1.27	0.47	-0.69	0.42
924 SAM	0.58	-0.28	0.43	0.3	0.47
922 SAH	0.58	-0.28	0.43	0.3	0.47
12 ATP from NADH	0.58	-0.75	0.47	-0.18	0.34
716 Argininosuccinate	0.58	-0.61	0.66	-0.03	0.39
892 PGP-CL-pool	0.58	-0.79	0.44	-0.21	0.34
891 PG-CL-pool	0.58	-0.79	0.44	-0.21	0.34
294 Taurine from Methionine	0.57	-1.27	0.51	-0.7	0.46
173 Glutamate from Glutamine	0.56	-0.88	0.39	-0.32	0.43
129 Asparagine from Lysine	0.56	-0.65	0.46	-0.08	0.29
690 AICAR	0.56	-0.29	0.45	0.27	0.45
226 Glutamine from beta-Alanine	0.56	-0.81	0.35	-0.25	0.42
119 Asparagine from Arginine	0.56	-0.64	0.42	-0.08	0.32
88 Alanine from Methionine	0.56	-1.28	0.52	-0.73	0.47
518 CL	0.55	-0.78	0.42	-0.24	0.4
228 Methionine from Cystine	0.54	-1.21	0.43	-0.66	0.55
227 Methionine from Cysteine	0.54	-1.21	0.39	-0.67	0.52
234 Proline from Cystine	0.54	-0.87	0.54	-0.34	0.42
673 5-Phosphoribosyl-4-carboxy-5-aminoimidazole	0.53	-0.26	0.35	0.27	0.38
486 Glutamine	0.52	-0.88	0.51	-0.36	0.45
484 Glutamate	0.51	-0.88	0.58	-0.36	0.51
184 Glutamate from beta-Alanine	0.51	-0.8	0.37	-0.29	0.49
923 SAICAR	0.51	-0.25	0.41	0.26	0.35
435 Galactose degr	0.51	-0.17	0.59	0.33	0.5
161 Cysteine from Methionine	0.5	-1.21	0.49	-0.71	0.46
197 Glycine from Methionine	0.5	-1.23	0.55	-0.73	0.45
614 3-Hydroxyisobutyrate	0.49	-0.69	0.36	-0.19	0.29
163 Cystine from Methionine	0.49	-1.17	0.51	-0.68	0.49
782 Fatty-acid-VLDL-TG3-pool	0.48	-0.7	0.66	-0.22	0.53
285 beta-Alanine from Methionine	0.47	-1.19	0.46	-0.72	0.43
809 Guanosine	0.45	-0.22	0.35	0.23	0.4
165 Glutamate from Arginine	0.45	-0.8	0.42	-0.35	0.31
952 Urate	0.45	-0.22	0.29	0.23	0.38
810 H2O2	0.45	-0.22	0.29	0.23	0.38
884 Orotate	0.44	-0.54	0.43	-0.09	0.38
263 Serine from Methionine	0.44	-1.17	0.54	-0.73	0.48
310 Bilirubin conjugation	0.44	-0.86	0.62	-0.43	0.54
293 Homocysteine from Methionine	0.44	0.04	1	0.48	1
117 Arginine from beta-Alanine	0.43	-0.72	0.39	-0.29	0.33
514 PC	0.43	-0.74	0.4	-0.31	0.4
233 Proline from Cysteine	0.43	-0.79	0.55	-0.36	0.35

Continued on next page

Amplitude differences: Treatment difference – continued

Simulation	Δampl	TGFβ/C 24h		control 1/24h	
		ampl	score	ampl	score
190 Glycine from Cystine	0.43	-1.23	0.61	-0.8	0.41
138 Asparagine from beta-Alanine	0.43	-0.68	0.33	-0.26	0.47
189 Glycine from Cysteine	0.42	-1.27	0.58	-0.84	0.38
602 2-Lysolecithin-pool	0.42	-0.71	0.39	-0.29	0.38
507 Cytidine	0.42	-0.08	0.37	0.34	0.41
499 udpgal	0.41	-0.58	0.36	-0.17	0.3
130 Asparagine from Methionine	0.41	-1.13	0.48	-0.72	0.46
213 Glutamine from Glycine	0.41	-1.16	0.46	-0.75	0.37
608 2-Oxoglutaramate	0.41	-0.44	1	-0.03	1
935 Squalene 2,3-oxide	0.4	-0.95	0.84	-0.55	0.83
338 Haptoglobin	0.4	-0.18	0.97	0.22	0.97
80 Alanine from Cystine	0.4	-1.31	0.66	-0.92	0.47
471 Homocysteine	0.39	-1.14	0.47	-0.75	0.49
235 Proline from Glutamate	0.39	-0.47	0.57	-0.08	0.46
311 Urea from glutamine	0.39	-0.47	0.37	-0.08	0.26
170 Glutamate from Glycine	0.38	-1.12	0.55	-0.73	0.43
505 Uracil	0.38	-0.09	0.32	0.29	0.39
321 SM(b)	0.38	-0.73	0.47	-0.35	0.4
151 Aspartate from Methionine	0.38	-1.15	0.48	-0.77	0.47
812 HCO3-	0.37	-0.46	0.68	-0.09	0.71
727 Carbonate	0.37	-0.46	0.68	-0.09	0.71
254 Serine from Cysteine	0.37	-1.19	0.57	-0.82	0.45
522 Triacylglycerol	0.37	-0.61	0.5	-0.24	0.51
292 beta-Alanine from Valine	0.37	-0.54	0.37	-0.17	0.45
918 Quinolinate	0.36	-0.31	0.63	0.05	0.38
171 Glutamate from Histidine	0.36	-0.74	0.5	-0.37	0.47
167 Glutamate from Aspartate	0.36	-0.78	0.47	-0.42	0.48
607 2-Oxobutyrate	0.35	-1.2	0.38	-0.85	0.66
153 Aspartate from Proline	0.35	-0.43	0.64	-0.08	0.29
255 Serine from Cystine	0.35	-1.14	0.6	-0.79	0.46
399 Collagen SERPH(c) synthesis	0.34	0.3	1	0.65	1
76 Alanine from Arginine	0.34	-0.42	0.38	-0.08	0.33
273 beta-Alanine from Arginine	0.34	-0.47	0.34	-0.13	0.4
166 Glutamate from Asparagine	0.34	-0.87	0.47	-0.53	0.44
70 Formate degr	0.34	-0.37	0.61	-0.03	0.56
854 Maltose	0.34	-0.18	1	0.15	1
137 Asparagine from Valine	0.33	-0.52	0.42	-0.19	0.42
423 Valine degr	0.33	-0.51	0.4	-0.18	0.33
934 Squalene	0.33	-0.79	0.83	-0.47	0.79
909 Presqualene-PP	0.33	-0.79	0.83	-0.47	0.79
446 ApoB100 degr	0.32	-1.02	0.51	-0.7	0.6
81 Alanine from Glutamate	0.32	-0.4	0.51	-0.08	0.37
179 Glutamate from Serine	0.31	-0.88	0.47	-0.57	0.45
260 Serine from Glutamine	0.31	-0.51	0.48	-0.2	0.31
116 Arginine from Valine	0.3	-0.48	0.41	-0.18	0.33
287 beta-Alanine from Proline	0.3	-0.44	0.49	-0.14	0.39
35 Thioredoxin(c) oxidation	0.29	-0.57	0.77	-0.28	0.99
225 Glutamine from Valine	0.29	-0.45	0.38	-0.16	0.37
28 NADPH to NADH transhydrogenase	0.29	-0.58	1	-0.29	1
45 Activated methylene group from Try	0.29	-0.63	0.39	-0.34	0.26
69 Formaldehyde degr	0.28	-0.51	0.38	-0.23	0.47
278 beta-Alanine from Glutamate	0.28	-0.4	0.39	-0.12	0.47
980 palmitoleoyl-Carnitine	0.28	-1.13	0.49	-0.86	0.57
850 Malate	0.27	-0.31	0.7	-0.04	0.64
845 Lanosterol	0.27	-0.89	0.77	-0.62	0.84
951 UMP	0.26	-0.44	0.44	-0.17	0.38
95 Alanine from Valine	0.26	-0.42	0.49	-0.16	0.35
916 Pyridoxal	0.26	-0.11	0.66	0.16	0.68
214 Glutamine from Histidine	0.26	-0.64	0.41	-0.38	0.38

Continued on next page

Amplitude differences: Treatment difference – continued

Simulation	Δampl	TGFβ/C 24h		control 1/24h	
		ampl	score	ampl	score
279 beta-Alanine from Glycine	0.26	-1.15	0.42	-0.9	0.45
244 Proline from Serine	0.26	-0.65	0.48	-0.39	0.38
269 Serine from Valine	0.26	-0.47	0.44	-0.22	0.38
158 Aspartate from Valine	0.25	-0.41	0.42	-0.16	0.41
349 Collagen CO4A2(c) synthesis	0.24	0.51	1	0.75	1
430 LDL degr	0.24	-0.93	0.43	-0.69	0.4
237 Proline from Histidine	0.24	-0.59	0.49	-0.35	0.42
132 Asparagine from Proline	0.24	-0.52	0.58	-0.28	0.29
282 beta-Alanine from Glutamine	0.24	-0.38	0.32	-0.14	0.45
672 5-Oxoproline	0.24	-0.08	0.55	0.16	0.55
204 Glycine from Valine	0.24	-0.47	0.47	-0.23	0.35
947 Triphosphate degr	0.23	-0.09	1	0.15	1
548 (R)-4-Phosphopantothenoyl-cysteine	0.23	-0.11	0.45	0.11	0.45
787 Fumarate	0.22	-0.26	0.74	-0.03	0.7
217 Glutamine from Lysine	0.22	-0.72	0.45	-0.49	0.31
231 Proline from Asparagine	0.22	-0.67	0.5	-0.45	0.37
584 1-Acylglycerol-3P-palmn	0.21	-1	0.59	-0.79	0.61
16 Aerobic reduction of NAD+ (FA)	0.21	-0.6	0.4	-0.39	0.37
860 N-Acetyl-D-mannosamine	0.21	-0.45	0.34	-0.24	0.53
601 2-Deoxy-D-ribose-5P	0.2	-0.42	0.44	-0.23	0.55
221 Glutamine from Serine	0.19	-0.82	0.43	-0.63	0.4
126 Asparagine from Isoleucine	0.19	-0.55	0.55	-0.36	0.44
21 Aerobic reduction of FAD (FA)	0.18	-0.53	0.52	-0.34	0.36
525 beta-Alanine	0.18	-0.58	0.33	-0.4	0.34
175 Glutamate from Lysine	0.17	-0.64	0.51	-0.46	0.32
427 Homocysteine degr	0.17	-1.11	0.45	-0.93	0.39
417 Methionine degr	0.17	-1.11	0.43	-0.94	0.37
440 ApoA1 degr	0.17	-0.65	0.48	-0.48	0.59
873 Nicotinamide D-ribonucleotide	0.17	-0.34	0.46	-0.17	0.35
470 GSH	0.16	-1.11	0.63	-0.94	0.47
495 Mannose-6P	0.16	-0.01	0.78	0.15	0.78
444 Prothrombin degr	0.16	-0.64	0.5	-0.48	0.57
61 AKG	0.16	-0.73	0.57	-0.57	0.44
876 Nicotinate(r)ibonucleotide	0.16	-0.32	0.41	-0.17	0.33
425 Isoleucine degr	0.16	-0.56	0.52	-0.4	0.34
811 H2S	0.16	-1.26	0.51	-1.11	0.5
124 Asparagine from Glycine	0.15	-1.07	0.43	-0.92	0.38
248 Proline from Valine	0.15	-0.44	0.53	-0.29	0.38
414 Glutamine degr	0.15	-0.43	0.38	-0.28	0.27
127 Asparagine from Glutamine	0.15	-0.43	0.56	-0.28	0.39
281 beta-Alanine from Isoleucine	0.15	-0.53	0.5	-0.38	0.42
487 Proline	0.14	-0.44	0.48	-0.29	0.39
424 Glycine degr	0.14	-1.14	0.4	-1	0.38
209 Glutamine from Aspartate	0.14	-0.28	0.41	-0.14	0.36
519 LacCer	0.14	-0.55	0.43	-0.41	0.34
982 sn-Glycerol-3P	0.14	-0.85	0.84	-0.72	0.71
103 Arginine from Glycine	0.14	-1.09	0.45	-0.95	0.4
215 Glutamine from Isoleucine	0.14	-0.5	0.64	-0.37	0.43
587 1-Acylglycerol-VLDL-PE-pool	0.13	-0.8	0.5	-0.67	0.47
586 1-Acylglycerol-VLDL-PC-pool	0.13	-0.8	0.49	-0.67	0.47
588 1-Acylglycerol-VLDL-PI-pool	0.13	-0.8	0.5	-0.67	0.47
591 1-Acylglycerol-VLDL-TG1-pool	0.13	-0.8	0.49	-0.67	0.47
589 1-Acylglycerol-VLDL-PS-pool	0.13	-0.8	0.5	-0.67	0.47
956 Xanthurenate	0.13	-1.02	0.5	-0.88	0.46
657 4-(2-Amino-3-hydroxyphenyl)-2,4-dioxobutanoate	0.13	-1.02	0.5	-0.88	0.46
590 1-Acylglycerol-VLDL-SM-pool	0.13	-0.8	0.49	-0.67	0.46
20 Aerobic reduction of FAD (gluc)	0.13	-0.5	0.48	-0.36	0.36
18 Aerobic reduction of NADP+ (FA)	0.12	-0.53	0.37	-0.41	0.36
105 Arginine from Isoleucine	0.12	-0.5	0.55	-0.38	0.39

Continued on next page

Amplitude differences: Treatment difference – continued

Simulation	Δampl	TGFβ/C 24h		control 1/24h	
		ampl	score	ampl	score
249 Proline from beta-Alanine	0.12	-0.43	0.44	-0.31	0.42
438 Antichymotrypsin degr	0.12	-0.6	0.45	-0.47	0.6
585 1-Acylglycerol-3P-stea	0.12	-0.71	0.57	-0.59	0.53
582 1-Acylglycerol-3P-ol	0.12	-0.71	0.57	-0.59	0.53
581 1-Acylglycerol-3P-lin	0.12	-0.71	0.57	-0.59	0.53
580 1-Acylglycerol-3P-arach	0.12	-0.71	0.57	-0.59	0.53
147 Aspartate from Isoleucine	0.12	-0.49	0.58	-0.37	0.49
520 SM	0.12	-0.58	0.47	-0.46	0.36
280 beta-Alanine from Histidine	0.12	-0.57	0.41	-0.45	0.4
878 O-Butanoylcarnitine	0.12	-0.46	0.59	-0.34	0.42
339 Collagen ADIPO(c) synthesis	0.11	-0.07	1	0.04	1
378 Collagen COJA1(c) synthesis	0.11	-0.07	1	0.04	1
583 1-Acylglycerol-3P-palm	0.11	-0.7	0.57	-0.6	0.52
455 Thioredoxin	0.11	0.03	0.94	0.13	0.94
887 Oxidized thioredoxin	0.11	0.03	0.93	0.13	0.93
443 Plasminogen degr	0.11	-0.58	0.49	-0.48	0.6
437 Albumin degr	0.11	-0.58	0.47	-0.48	0.62
58 GDP-activated fucose	0.1	0.24	0.48	0.35	0.59
208 Glutamine from Asparagine	0.1	-0.68	0.42	-0.58	0.43
441 Fibrinogen degr	0.1	-0.58	0.49	-0.48	0.61
387 Collagen COSA1(c) synthesis	0.1	-0.22	0.32	-0.12	0.44
380 Collagen COLA1(c) synthesis	0.1	-0.22	0.31	-0.12	0.45
381 Collagen COLQ(c) synthesis	0.1	-0.22	0.31	-0.12	0.44
880 OAA	0.1	-0.16	0.76	-0.06	0.61
178 Glutamate from Proline	0.1	-0.46	0.62	-0.36	0.37
839 L-Fucose-1P	0.1	0.24	0.42	0.34	0.46
239 Proline from Glutamine	0.09	-0.45	0.5	-0.36	0.32
390 Collagen FCN1(c) synthesis	0.09	-0.22	0.29	-0.12	0.46
172 Glutamate from Isoleucine	0.09	-0.44	0.57	-0.35	0.46
14 Ubiquinol-to-ATP	0.09	-0.09	0.62	0.002	0.54
11 ATP from NADH(m)	0.09	-0.09	0.8	0.002	0.48
392 Collagen FCN3(c) synthesis	0.09	-0.21	0.29	-0.13	0.47
397 Collagen PCOTH(c) synthesis	0.09	-0.21	0.32	-0.13	0.47
868 N-Methylethanolamine-P	0.08	-0.35	1	-0.27	1
771 Ethanolamine-P	0.08	-0.35	1	-0.27	1
212 Glutamine from Glutamate	0.08	-0.16	0.33	-0.08	0.33
361 Collagen CO6A6(c) synthesis	0.08	-0.21	0.28	-0.13	0.46
238 Proline from Isoleucine	0.08	-0.47	0.61	-0.39	0.43
360 Collagen CO6A5(c) synthesis	0.08	-0.2	0.28	-0.13	0.47
693 Acetaldehyde	0.08	-0.34	0.61	-0.26	0.6
143 Aspartate from Cystine	0.08	-1.01	0.48	-0.94	0.39
373 Collagen COEA1(c) synthesis	0.08	-0.02	1	0.06	1
742 Citrate	0.07	-0.34	0.71	-0.26	0.59
431 HDL degr	0.07	-0.84	0.41	-0.77	0.41
68 O2.-(c) degr	0.07	-0.39	0.72	-0.32	0.74
230 Proline from Arginine	0.07	-0.41	0.41	-0.34	0.33
19 Aerobic reduction of NADP+ (gluc)	0.06	-0.34	0.31	-0.28	0.31
283 beta-Alanine from Leucine	0.06	-0.33	0.39	-0.27	0.44
301 Palmitolate from Arachidonate	0.06	-0.31	0.64	-0.25	0.65
768 Dimethylallyl-PP	0.06	-0.8	0.52	-0.74	0.6
128 Asparagine from Leucine	0.06	-0.34	0.38	-0.29	0.4
50 Acetyl group(m)	0.06	-0.42	0.57	-0.36	0.47
369 Collagen COBA1(c) synthesis	0.06	-0.02	1	0.03	1
432 Oleate degr	0.06	-0.42	0.57	-0.37	0.42
433 Stearate degr	0.06	-0.42	0.59	-0.36	0.43
39 Proton-gradient(m) build up	0.05	-0.39	0.45	-0.34	0.41
40 Proton-gradient(c) build up	0.05	-0.39	0.45	-0.34	0.41
877 O-Acetylcarnitine	0.05	0.1	1	0.15	1
328 Antitrypsin	0.05	-0.17	0.3	-0.12	0.49

Continued on next page

Simulation	Δampl	TGFβ/C 24h		control 1/24h	
		ampl	score	ampl	score
490 Palmitate	0.05	-0.31	0.63	-0.26	0.8
436 Mannose degr	0.05	0.43	0.42	0.48	0.64
232 Proline from Aspartate	0.05	-0.41	0.53	-0.37	0.4
491 Glycerol	0.05	-0.86	0.56	-0.82	0.53
84 Alanine from Isoleucine	0.04	-0.51	0.62	-0.47	0.41
726 Carbamoyl-P	0.04	-0.14	0.58	-0.09	0.5
229 Proline from Alanine	0.04	-0.42	0.42	-0.38	0.46
60 Pyruvate	0.04	-0.7	0.55	-0.66	0.49
493 Stearate	0.03	-0.3	0.67	-0.26	0.81
51 Acetyl group(p)	0.03	-0.49	0.47	-0.46	0.34
240 Proline from Leucine	0.03	-0.4	0.5	-0.37	0.38
388 Collagen CTHR1(c) synthesis	0.03	-0.02	1	0.01	1
52 Acetyl group(r)	0.03	-0.49	0.58	-0.45	0.43
49 Acetyl group(c)	0.03	-0.49	0.58	-0.45	0.43
492 Oleate	0.03	-0.29	0.68	-0.26	0.74
357 Collagen CO6A1(c) synthesis	0.03	-0.11	1	-0.08	1
781 Fatty-acid-VLDL-TG2-pool	0.03	-0.81	0.56	-0.78	0.49
780 Fatty-acid-VLDL-TG1-pool	0.03	-0.81	0.56	-0.78	0.49
259 Serine from Isoleucine	0.02	-0.53	0.59	-0.5	0.43
971 dTMP	0.02	-0.53	0.31	-0.51	0.35
488 Serine	0.02	-0.69	0.57	-0.67	0.45
326 Fibrinogen	0.02	-0.14	0.3	-0.12	0.49
776 Fatty-acid-VLDL-PE-pool	0.02	-0.8	0.56	-0.78	0.48
365 Collagen CO9A1(c) synthesis	0.02	-0.02	1	-0.0005	1
149 Aspartate from Leucine	0.02	-0.32	0.45	-0.3	0.4
777 Fatty-acid-VLDL-PI-pool	0.02	-0.8	0.56	-0.78	0.48
778 Fatty-acid-VLDL-PS-pool	0.02	-0.8	0.56	-0.78	0.48
174 Glutamate from Leucine	0.01	-0.37	0.39	-0.35	0.41
193 Glycine from Isoleucine	0.01	-0.52	0.57	-0.51	0.39
398 Collagen SCRB1(c) synthesis	0.01	-0.43	1	-0.41	1
885 Orotidine-5P	0.005	-0.36	0.37	-0.36	0.33
822 Homovanillate	0.003	-0.02	1	-0.01	1
340 Collagen BGH3(c) synthesis	0.001	-0.04	1	-0.03	1
925 Saccharopine	0.001	-1.92	0.63	-1.92	0.48
356 Collagen CO5A3(c) synthesis	0.0004	-0.07	1	-0.07	1
400 Collagen VWA2(c) synthesis	-0.0001	0.02	1	0.02	1
183 Glutamate from Valine	-0.002	-0.19	0.41	-0.19	0.48
978 mitoACP	-0.004	-0.07	0.68	-0.08	0.71
458 Apo-ACP(m)	-0.005	-0.07	0.7	-0.08	0.72
457 Apo-ACP	-0.01	-0.07	0.9	-0.08	0.92
697 Acetyl-ACP	-0.01	-0.07	0.85	-0.08	0.88
688 ACP	-0.01	-0.07	0.86	-0.08	0.88
818 Hexanoyl-ACP	-0.01	-0.07	0.83	-0.08	0.86
536 (2E)-Hexenoyl-ACP	-0.01	-0.07	0.83	-0.08	0.86
695 Acetoacetyl-ACP	-0.01	-0.07	0.84	-0.08	0.87
881 Octanoyl-ACP	-0.01	-0.07	0.82	-0.08	0.85
718 Butyryl-ACP	-0.01	-0.07	0.84	-0.08	0.87
539 (2E)-Octenoyl-ACP	-0.01	-0.07	0.82	-0.08	0.85
973 dUMP	-0.01	-0.55	0.33	-0.55	0.36
851 Malonyl-ACP	-0.01	-0.07	0.84	-0.08	0.87
546 (R)-3-Hydroxyoctanoyl-ACP	-0.01	-0.07	0.82	-0.08	0.85
717 But-2-enoyl-ACP	-0.01	-0.07	0.84	-0.08	0.87
629 3-Oxooctanoyl-ACP	-0.01	-0.07	0.82	-0.08	0.85
544 (R)-3-Hydroxybutanoyl-ACP	-0.01	-0.07	0.84	-0.08	0.87
754 Decanoyl-ACP	-0.01	-0.07	0.81	-0.08	0.85
530 (2E)-Decenoyl-ACP	-0.01	-0.07	0.81	-0.08	0.85
545 (R)-3-Hydroxydecanoyl-ACP	-0.01	-0.07	0.82	-0.08	0.85
622 3-Oxodecanoyl-ACP	-0.01	-0.07	0.82	-0.08	0.85
769 Dodecanoyl-ACP	-0.01	-0.07	0.81	-0.08	0.84

Continued on next page

Simulation		Δampl	TGFβ/C 24h ampl	TGFβ/C 24h score	control 1/24h ampl	control 1/24h score
532	(2E)-Dodecenoyl-ACP	-0.01	-0.07	0.81	-0.08	0.84
749	D-3-Hydroxydodecanoyl-ACP	-0.01	-0.07	0.81	-0.08	0.84
750	D-3-Hydroxyhexanoyl-ACP	-0.01	-0.07	0.83	-0.08	0.86
624	3-Oxododecanoyl-ACP	-0.01	-0.07	0.81	-0.08	0.85
627	3-Oxohexanoyl-ACP	-0.01	-0.07	0.83	-0.08	0.86
942	Tetradecanoyl-ACP	-0.01	-0.07	0.81	-0.08	0.84
541	(2E)-Tetradecenoyl-ACP	-0.01	-0.07	0.81	-0.08	0.84
813	HMA	-0.01	-0.07	0.81	-0.08	0.84
634	3-Oxotetradecanoyl-ACP	-0.01	-0.07	0.81	-0.08	0.84
816	Hexadecanoyl-ACP	-0.01	-0.07	0.8	-0.08	0.84
534	(2E)-Hexadecenoyl-ACP	-0.01	-0.07	0.8	-0.08	0.84
547	(R)-3-Hydroxypalmitoyl-ACP	-0.01	-0.07	0.8	-0.08	0.84
626	3-Oxohexadecanoyl-ACP	-0.01	-0.07	0.8	-0.08	0.84
936	Stearoyl-ACP	-0.01	-0.07	0.79	-0.08	0.83
538	(2E)-Octadecenoyl-ACP	-0.01	-0.07	0.79	-0.08	0.83
617	3-Hydroxystearoyl-ACP	-0.01	-0.07	0.79	-0.08	0.83
633	3-Oxostearoyl-ACP	-0.01	-0.07	0.79	-0.08	0.83
654	3alpha,7alpha-Dihydroxy-5beta-cholestanate	-0.01	-0.82	0.56	-0.83	0.7
4	Aerobic rephosph of GTP	-0.01	-0.35	0.47	-0.36	0.31
337	ApoTransferin	-0.01	-0.34	0.98	-0.35	0.98
7	Aerobic rephosph of UTP	-0.01	-0.35	0.48	-0.36	0.31
134	Asparagine from Threonine	-0.01	-0.57	0.54	-0.59	0.49
542	(2E)-Tetradecenoyl-CoA	-0.01	-0.4	0.44	-0.41	0.31
323	PE(b)	-0.01	-0.33	0.48	-0.34	0.52
393	Collagen FMOD(c) synthesis	-0.02	-0.02	1	-0.04	1
653	3alpha,7alpha-Dihydroxy-5beta-cholestan-26-al	-0.02	-0.8	0.53	-0.82	0.74
847	Lauroyl-CoA	-0.02	-0.4	0.44	-0.42	0.3
795	Geranyl-PP	-0.02	-0.65	0.5	-0.67	0.61
970	dTDP	-0.02	-0.48	0.34	-0.51	0.32
852	Malonyl-Carnitin	-0.02	-0.45	0.8	-0.47	0.62
284	beta-Alanine from Lysine	-0.03	-0.38	0.4	-0.41	0.37
133	Asparagine from Serine	-0.03	-0.7	0.37	-0.72	0.37
961	cis-Aconitate	-0.03	-0.32	0.67	-0.35	0.59
765	Dihydroceramide-pool	-0.03	-0.14	0.36	-0.17	0.4
910	Propanoate	-0.03	-0.62	0.59	-0.65	0.48
515	PE	-0.03	-0.33	0.54	-0.36	0.47
889	PE-PS-VLDL-pool	-0.03	-0.33	0.57	-0.37	0.43
148	Aspartate from Glutamine	-0.04	-0.24	0.49	-0.27	0.37
159	Aspartate from beta-Alanine	-0.04	-0.24	0.49	-0.28	0.51
322	PS(b)	-0.04	-0.32	0.49	-0.36	0.4
516	PS	-0.04	-0.32	0.49	-0.36	0.4
305	Dihomo-gamma-linolenate from gamma-Linolenate	-0.04	-0.42	0.81	-0.46	0.47
319	Chenodiol(b)	-0.04	-0.76	0.6	-0.81	0.54
640	3-Ureidopropionate	-0.04	-0.63	0.43	-0.67	0.44
289	beta-Alanine from Threonine	-0.04	-0.58	0.47	-0.63	0.46
917	Pyridoxine-P	-0.05	-0.14	1	-0.19	1
63	Farnesyl-PP	-0.05	-0.61	0.51	-0.66	0.62
840	L-Glutamate 5-semialdehyde	-0.05	-0.47	0.42	-0.52	0.49
869	N-Pantothenoylcysteine	-0.05	-0.11	0.48	-0.17	0.45
59	GDP-activated mannose	-0.05	0.2	0.49	0.15	0.58
940	Sulfite degr	-0.06	-0.01	1	-0.07	1
123	Asparagine from Glutamate	-0.06	-0.23	0.42	-0.29	0.34
460	Pyridoxal-P	-0.06	-0.12	0.76	-0.19	0.44
977	linoleic-Carnitine	-0.06	-0.43	0.67	-0.49	0.51
714	Arachidonyl-Carnitine	-0.06	-0.43	0.67	-0.49	0.51
144	Aspartate from Glutamate	-0.06	-0.25	0.64	-0.31	0.34
376	Collagen COHA1(c) synthesis	-0.06	0.03	1	-0.03	1
844	L-Palmitoylcarnitine	-0.07	-0.43	0.68	-0.5	0.48
511	UDP-N-acetylgalactosamine	-0.07	-0.44	0.28	-0.51	0.34

Continued on next page

Amplitude differences: Treatment difference – continued

Simulation	Δampl	TGFβ/C 24h		control 1/24h	
		ampl	score	ampl	score
800 Glutamyl-5P	-0.07	-0.45	0.54	-0.52	0.37
37 GSH reduction using NADPH redox potential	-0.08	-0.02	1	-0.09	1
38 GSH oxidation	-0.08	-0.02	1	-0.09	1
655 3alpha,7alpha-Dihydroxy-5beta-cholestanoyl-CoA	-0.08	-0.75	0.47	-0.82	0.46
948 UDP-N-acetylglucosamine	-0.08	-0.44	0.29	-0.52	0.34
521 Ceramide	-0.08	-0.5	0.48	-0.58	0.37
180 Glutamate from Threonine	-0.08	-0.5	0.56	-0.58	0.53
245 Proline from Threonine	-0.08	-0.47	0.61	-0.56	0.47
445 ApoTransferin degr	-0.08	-0.39	0.48	-0.48	0.63
318 tcdchola(b)	-0.08	-0.76	0.52	-0.84	0.48
262 Serine from Lysine	-0.08	-0.55	0.48	-0.64	0.36
345 Collagen CO1A2(c) synthesis	-0.09	0.38	1	0.3	1
266 Serine from Threonine	-0.09	-0.57	0.58	-0.65	0.53
461 4ppan	-0.09	-0.08	1	-0.17	1
325 Albumin	-0.09	-0.02	0.97	-0.11	0.98
92 Alanine from Threonine	-0.09	-0.56	0.56	-0.65	0.55
447 Glycogenin degr	-0.09	-0.39	0.5	-0.48	0.62
87 Alanine from Lysine	-0.09	-0.53	0.44	-0.62	0.32
517 PI	-0.09	-0.14	0.47	-0.23	0.29
549 (R)-5-Diphosphomevalonate	-0.09	-0.62	0.56	-0.71	0.71
358 Collagen CO6A2(c) synthesis	-0.09	0.04	1	-0.05	1
930 Sphinganine	-0.09	0.28	0.38	0.18	0.32
927 Sedoheptulose-1,7PP	-0.09	-0.49	0.64	-0.58	0.52
442 Haptoglobin degr	-0.1	-0.38	0.53	-0.48	0.67
150 Aspartate from Lysine	-0.1	-0.38	0.49	-0.47	0.37
201 Glycine from Threonine	-0.1	-0.56	0.56	-0.66	0.47
62 Isopentenyl-PP	-0.1	-0.58	0.57	-0.68	0.69
316 Gly-CD-cholate(b)	-0.1	-0.73	0.57	-0.83	0.51
820 Histamine	-0.1	-0.44	0.67	-0.54	0.9
270 Serine from beta-Alanine	-0.11	-0.54	0.42	-0.65	0.4
606 2-Oxoadipate	-0.11	-0.52	0.41	-0.63	0.33
155 Aspartate from Threonine	-0.11	-0.55	0.55	-0.66	0.51
560 (S)-Dihydroorotate	-0.11	0.38	0.32	0.27	0.3
368 Collagen COAA1(c) synthesis	-0.11	0.02	1	-0.09	1
41 Na+ importgradient	-0.12	0.23	0.47	0.11	0.52
728 Carnosine	-0.12	0.001	0.46	-0.12	0.87
375 Collagen COGA1(c) synthesis	-0.12	0.2	1	0.08	1
761 Deoxyuridine	-0.12	-0.43	0.29	-0.55	0.36
222 Glutamine from Threonine	-0.12	-0.49	0.6	-0.61	0.51
22 Oxidation of NADH	-0.12	-0.17	0.53	-0.3	0.45
359 Collagen CO6A3(c) synthesis	-0.12	0.23	1	0.11	1
421 Threonine degr	-0.12	-0.58	0.49	-0.7	0.39
770 Erythrose-4P	-0.12	-0.5	0.64	-0.63	0.54
439 Antitrypsin degr	-0.13	-0.34	0.44	-0.47	0.62
194 Glycine from Glutamine	-0.14	-0.51	0.44	-0.65	0.28
265 Serine from Proline	-0.14	-0.48	0.56	-0.62	0.32
593 14-Demethyllanosterol	-0.14	-0.66	0.63	-0.79	0.75
730 Chenodeoxycholoyl-CoA	-0.14	-0.66	0.48	-0.8	0.45
96 Alanine from beta-Alanine	-0.14	0.54	0.33	0.4	0.39
929 Serotonin	-0.14	-1.21	0.53	-1.35	0.61
23 Oxidation of NADPH	-0.14	-0.12	0.47	-0.26	0.4
242 Proline from Methionine	-0.15	-0.5	0.53	-0.65	0.42
252 Serine from Asparagine	-0.15	-0.54	0.52	-0.69	0.48
632 3-Oxopropanoate	-0.15	0.52	0.33	0.37	0.41
823 Hydracrylate	-0.15	-0.56	0.49	-0.72	0.58
571 1,3DPG	-0.15	-0.54	0.63	-0.69	0.81
468 PAPS	-0.15	-0.72	0.33	-0.87	0.31
199 Glycine from Proline	-0.15	-0.47	0.6	-0.63	0.31
382 Collagen COMA1(c) synthesis	-0.16	0.13	1	-0.02	1

Continued on next page

Amplitude differences: Treatment difference – continued

Simulation		Δampl	TGFβ/C 24h		control 1/24h	
			ampl	score	ampl	score
943	Thymidine	-0.16	-0.53	0.29	-0.68	0.35
367	Collagen CO9A3(c) synthesis	-0.16	0.03	1	-0.13	1
656	4,4-Dimethyl-5alpha-cholesta-8,14,24-trien-3beta-ol	-0.16	-0.67	0.68	-0.82	0.74
112	Arginine from Serine	-0.16	-0.79	0.43	-0.96	0.4
46	Activated methyl group from Histidine	-0.16	-0.55	0.38	-0.71	0.3
101	Arginine from Cystine	-0.16	-0.98	0.51	-1.14	0.36
218	Glutamine from Methionine	-0.17	-0.53	0.5	-0.69	0.47
354	Collagen CO5A1(c) synthesis	-0.17	0.18	1	0.01	1
789	GAP	-0.17	-0.58	0.85	-0.75	0.99
752	DHAP	-0.17	-0.58	0.85	-0.75	0.99
859	N-(omega)-Hydroxyarginine	-0.17	-0.01	0.77	-0.18	0.77
288	beta-Alanine from Serine	-0.17	-0.71	0.38	-0.89	0.44
827	Hypoxanthine	-0.18	0.41	0.29	0.23	0.48
258	Serine from Histidine	-0.18	-0.54	0.53	-0.72	0.38
379	Collagen COKA1(c) synthesis	-0.18	0.001	1	-0.18	1
253	Serine from Aspartate	-0.18	-0.51	0.49	-0.69	0.52
928	Sedoheptulose-7P	-0.18	-0.49	0.55	-0.67	0.44
100	Arginine from Cysteine	-0.18	-0.99	0.45	-1.17	0.35
774	FMN	-0.18	0.03	1	-0.16	1
366	Collagen CO9A2(c) synthesis	-0.18	-0.02	1	-0.21	1
256	Serine from Glutamate	-0.19	-0.47	0.54	-0.66	0.33
6	Aerobic rephosph of UDP	-0.19	-0.11	0.52	-0.3	0.37
363	Collagen CO8A1(c) synthesis	-0.19	-0.0003	1	-0.19	1
972	dUDP	-0.19	-0.44	0.39	-0.63	0.26
191	Glycine from Glutamate	-0.19	-0.47	0.48	-0.66	0.3
82	Alanine from Glycine	-0.19	-0.8	0.5	-0.99	0.39
53	activated sulphur	-0.2	0.22	0.67	0.03	0.65
899	Phosphatidate-VLDL-PC-pool	-0.2	-0.35	0.52	-0.55	0.43
904	Phosphatidate-VLDL-TG-pool	-0.2	-0.35	0.52	-0.55	0.43
900	Phosphatidate-VLDL-PE-pool	-0.2	-0.35	0.52	-0.55	0.44
879	O-Propanoylcarnitine	-0.2	-0.61	0.53	-0.81	0.53
32	Thioredoxin(m) reduction	-0.2	-0.06	1	-0.26	1
33	Thioredoxin(m) oxidation	-0.2	-0.06	1	-0.26	1
901	Phosphatidate-VLDL-PI-pool	-0.2	-0.35	0.51	-0.55	0.44
384	Collagen COOA1(c) synthesis	-0.2	0.08	1	-0.12	1
902	Phosphatidate-VLDL-PS-pool	-0.2	-0.36	0.51	-0.56	0.44
903	Phosphatidate-VLDL-SM-pool	-0.2	-0.35	0.52	-0.55	0.43
383	Collagen CONA1(c) synthesis	-0.2	0.04	1	-0.16	1
565	1,2-Diacylglycerol-VLDL-PC-pool	-0.21	-0.35	0.47	-0.55	0.39
570	1,2-Diacylglycerol-VLDL-TG-pool	-0.21	-0.35	0.47	-0.55	0.38
566	1,2-Diacylglycerol-VLDL-PE-pool	-0.21	-0.35	0.47	-0.56	0.39
202	Glycine from Tryptophan	-0.21	-1.15	0.49	-1.36	0.3
113	Arginine from Threonine	-0.21	-0.51	0.54	-0.72	0.41
176	Glutamate from Methionine	-0.21	-0.5	0.47	-0.7	0.48
567	1,2-Diacylglycerol-VLDL-PI-pool	-0.21	-0.35	0.47	-0.56	0.39
575	1-Acylglycerol-3P-VLDL-PE-pool	-0.21	-0.37	0.57	-0.58	0.52
574	1-Acylglycerol-3P-VLDL-PC-pool	-0.21	-0.37	0.57	-0.58	0.52
576	1-Acylglycerol-3P-VLDL-PI-pool	-0.21	-0.37	0.57	-0.58	0.52
577	1-Acylglycerol-3P-VLDL-PS-pool	-0.21	-0.37	0.57	-0.58	0.53
665	5,6-Dihydrouracil	-0.21	-0.51	0.41	-0.72	0.39
568	1,2-Diacylglycerol-VLDL-PS-pool	-0.21	-0.35	0.47	-0.57	0.38
569	1,2-Diacylglycerol-VLDL-SM-pool	-0.21	-0.35	0.47	-0.56	0.38
579	1-Acylglycerol-3P-VLDL-TG1-pool	-0.21	-0.37	0.57	-0.58	0.52
578	1-Acylglycerol-3P-VLDL-SM-pool	-0.21	-0.37	0.57	-0.58	0.52
351	Collagen CO4A4(c) synthesis	-0.21	0.09	1	-0.12	1
34	Thioredoxin(c) reduction	-0.22	-0.09	0.92	-0.31	0.98
712	Agmatine	-0.22	0.23	0.67	0.01	0.68
467	1fthf	-0.22	-0.1	0.54	-0.32	0.59
36	GSH reduction using NADH redox potential	-0.22	-0.02	0.5	-0.24	0.73

Continued on next page

Amplitude differences: Treatment difference – continued

Simulation		Δampl	TGFβ/C 24h ampl	TGFβ/C 24h score	control 1/24h ampl	control 1/24h score
661	4-Methyl-2-oxopentanoate	-0.22	-0.42	0.4	-0.65	0.35
466	THF(m)	-0.23	-0.1	1	-0.33	1
459	THF	-0.23	-0.1	1	-0.33	1
29	NADH to NADPH transhydrogenase	-0.23	-0.1	1	-0.33	1
766	Dihydrofolate	-0.23	-0.1	1	-0.33	1
670	5-Methyl-THF	-0.23	-0.1	1	-0.33	1
636	3-Phosphonooxypyruvate	-0.23	-0.65	0.51	-0.88	0.61
464	mlthf	-0.23	-0.1	0.8	-0.33	0.78
125	Asparagine from Histidine	-0.23	-0.58	0.43	-0.81	0.32
664	5,10-Methenyl-THF	-0.23	-0.1	0.65	-0.33	0.64
77	Alanine from Asparagine	-0.23	-0.47	0.44	-0.7	0.47
482	Asparagine	-0.24	-0.58	0.47	-0.82	0.34
550	(R)-5-Phosphomevalonate	-0.24	-0.5	0.49	-0.74	0.64
572	1-Acylglycerol-3P-Bile-PC-pool	-0.24	-0.37	0.55	-0.61	0.52
303	Dihomo-gamma-linolenate from Linoleate	-0.24	-0.43	0.68	-0.67	0.46
306	Arachidonate from gamma-Linolenate	-0.24	-0.43	0.68	-0.67	0.46
897	Phosphatidate-Bile-PC-pool	-0.24	-0.35	0.5	-0.59	0.43
261	Serine from Leucine	-0.24	-0.44	0.4	-0.69	0.41
794	Galactose-1P	-0.25	-0.15	0.66	-0.39	0.48
274	beta-Alanine from Asparagine	-0.25	0.11	0.3	-0.14	0.49
411	Cysteine degr	-0.25	-1.09	0.43	-1.34	0.34
195	Glycine from Leucine	-0.25	-0.44	0.39	-0.7	0.4
564	1,2-Diacylglycerol-Bile-PC-pool	-0.25	-0.35	0.46	-0.6	0.38
216	Glutamine from Leucine	-0.25	-0.38	0.38	-0.64	0.36
1	Aerobic ATP rephosph (FA)	-0.25	-0.11	0.45	-0.36	0.33
975	gamma-Glutamyl-cysteine	-0.25	-0.99	0.87	-1.24	0.86
146	Aspartate from Histidine	-0.25	-0.51	0.48	-0.76	0.37
420	Serine degr	-0.26	-0.74	0.39	-0.99	0.41
395	Collagen ITA2(c) synthesis	-0.26	0.21	1	-0.04	1
275	beta-Alanine from Aspartate	-0.26	0.11	0.68	-0.15	0.95
109	Arginine from Methionine	-0.26	-0.54	0.48	-0.8	0.42
64	Glucosamine-6P	-0.26	0.11	0.63	-0.15	0.77
83	Alanine from Histidine	-0.26	-0.53	0.43	-0.79	0.33
611	3-Dehydrosphinganine	-0.27	0.09	0.44	-0.18	0.38
842	L-Octanoylcarnitine	-0.27	-0.79	0.64	-1.06	0.38
724	CMP-NeuNGc	-0.27	-0.38	0.29	-0.65	0.29
389	Collagen EMID2(c) synthesis	-0.27	0.09	1	-0.18	1
334	ApoE(c)	-0.27	0.03	0.95	-0.24	0.97
364	Collagen CO8A2(c) synthesis	-0.28	0.12	1	-0.16	1
251	Serine from Arginine	-0.29	-0.48	0.44	-0.77	0.3
751	D-Xylulose-5P	-0.29	-0.48	0.47	-0.77	0.42
920	Ribose-5P	-0.29	-0.48	0.43	-0.77	0.38
304	Arachidonate from Linoleate	-0.29	-0.44	0.64	-0.73	0.48
663	4alpha-Methylzymosterol-4-carboxylate	-0.29	-0.53	0.69	-0.82	0.81
186	Glycine from Arginine	-0.3	-0.49	0.42	-0.78	0.29
921	Ribulose-5P	-0.3	-0.4	0.37	-0.7	0.35
385	Collagen COPA1(c) synthesis	-0.3	0.11	1	-0.19	1
342	Collagen CCBE1(c) synthesis	-0.3	0.17	1	-0.13	1
317	Taurocholate(b)	-0.31	-0.55	0.51	-0.86	0.48
678	5beta-Cholestane-3alpha,7alpha,26-triol	-0.31	-0.53	0.52	-0.84	0.75
91	Alanine from Serine	-0.31	-0.67	0.56	-0.98	0.43
645	3PG	-0.31	-0.56	0.59	-0.88	0.71
86	Alanine from Leucine	-0.31	-0.39	0.35	-0.7	0.39
315	Glycocholate(b)	-0.31	-0.53	0.55	-0.85	0.51
372	Collagen CODA1(c) synthesis	-0.32	0.09	1	-0.23	1
483	Aspartate	-0.32	-0.53	0.47	-0.85	0.35
404	(R)-3-Hydroxybutanoate	-0.32	-0.66	0.58	-0.98	0.52
5	Aerobic rephosph of CTP	-0.32	-0.32	0.38	-0.64	0.32
677	5beta-Cholestane-3alpha,7alpha,12alpha,26-tetrol	-0.32	-0.54	0.49	-0.86	0.71

Continued on next page

Simulation	Δampl	TGFβ/C 24h ampl	TGFβ/C 24h score	control 1/24h ampl	control 1/24h score
650 3alpha,7alpha,12alpha-Trihydroxy-5beta-cholestanate	-0.32	-0.54	0.49	-0.86	0.71
649 3alpha,7alpha,12alpha-Trihydroxy-5beta-cholestan-26-al	-0.32	-0.54	0.49	-0.86	0.71
336 Prothrombin	-0.33	-0.16	0.98	-0.49	0.98
99 Arginine from Aspartate	-0.33	-0.77	0.38	-1.1	0.29
496 Fructose-6P	-0.33	-0.46	1	-0.79	1
98 Arginine from Asparagine	-0.33	-0.66	0.38	-1	0.35
370 Collagen COBA2(c) synthesis	-0.34	0.26	1	-0.08	1
760 Deoxyinosine	-0.34	0.56	0.29	0.21	0.39
785 Fructose-1,6PP	-0.35	-0.44	1	-0.79	1
494 Mannose-1P	-0.35	0.23	0.5	-0.12	0.45
618 3-Keto-4-methylzymosterol	-0.35	-0.51	0.68	-0.86	0.81
741 Choloyl-CoA	-0.35	-0.47	0.47	-0.82	0.45
557 (S)-3-Hydroxytetradecanoyl-CoA	-0.35	-0.41	0.38	-0.77	0.28
355 Collagen CO5A2(c) synthesis	-0.36	0.71	1	0.36	1
848 Linoleate	-0.36	-0.5	0.62	-0.86	0.59
862 N-Acetylmannosamine-6P	-0.36	-0.36	0.36	-0.72	0.55
353 Collagen CO4A6(c) synthesis	-0.36	0.16	1	-0.2	1
415 Leucine degr	-0.37	-0.43	0.37	-0.8	0.33
864 N-Acetylneuraminate-9P	-0.37	-0.35	0.34	-0.72	0.39
957 Zymosterol	-0.37	-0.48	0.69	-0.86	0.82
662 4alpha-Methylzymosterol	-0.37	-0.48	0.69	-0.86	0.82
866 N-Formimino-L-glutamate	-0.37	-0.56	0.49	-0.93	0.52
732 Cholestenol	-0.38	-0.47	0.7	-0.84	0.8
15 Dephosphorylation of ATP	-0.38	0.63	0.71	0.24	0.51
679 5beta-Cholestane-3alpha,7alpha-diol	-0.39	-0.45	0.52	-0.84	0.73
731 Cholate	-0.39	-0.5	0.52	-0.88	0.6
686 7alpha-Hydroxycholest-4-en-3-one	-0.39	-0.46	0.57	-0.85	0.74
3 Anaerobic rephosph of ATP	-0.39	-0.34	0.42	-0.74	0.45
733 Cholesterol-ester-arach	-0.39	-0.44	0.64	-0.84	0.7
735 Cholesterol-ester-lin	-0.39	-0.45	0.62	-0.84	0.7
685 7alpha-Hydroxy-5beta-cholestan-3-one	-0.39	-0.45	0.55	-0.84	0.75
107 Arginine from Leucine	-0.39	-0.42	0.37	-0.82	0.34
734 Cholesterol-ester-gla	-0.4	-0.44	0.63	-0.84	0.69
646 3alpha,7alpha,12alpha,24-Tetrahydroxy-5beta-cholestanoyl-CoA	-0.4	-0.45	0.49	-0.85	0.42
647 3alpha,7alpha,12alpha-Trihydroxy-5beta-24-oxocholestanoyl-CoA	-0.4	-0.45	0.5	-0.85	0.42
687 7alpha-Hydroxycholesterol	-0.4	-0.46	0.6	-0.86	0.75
648 3alpha,7alpha,12alpha-Trihydroxy-5beta-cholest-24-enoyl-CoA	-0.4	-0.46	0.48	-0.86	0.42
10 Anaerobic rephosph of UTP	-0.4	-0.33	0.45	-0.74	0.43
8 Anaerobic rephosph of GTP	-0.4	-0.33	0.45	-0.74	0.43
9 Anaerobic rephosph of CTP	-0.4	-0.33	0.45	-0.74	0.43
651 3alpha,7alpha,12alpha-Trihydroxy-5beta-cholestanoyl-CoA	-0.4	-0.45	0.45	-0.86	0.46
763 Desmosterol	-0.41	-0.47	0.62	-0.88	0.79
652 3alpha,7alpha,12alpha-Trihydroxycoprostane	-0.41	-0.45	0.5	-0.86	0.7
523 Cholesterol	-0.41	-0.45	0.63	-0.87	0.77
684 7alpha,12alpha-Dihydroxycholest-4-en-3-one	-0.42	-0.46	0.53	-0.88	0.7
683 7alpha,12alpha-Dihydroxy-5beta-cholestan-3-one	-0.42	-0.45	0.52	-0.87	0.71
682 7-Dehydrodesmosterol	-0.42	-0.47	0.66	-0.89	0.78
739 Cholesterol-ester-pool	-0.43	-0.44	0.61	-0.86	0.68
676 5alpha-Cholesta-7,24-dien-3beta-ol	-0.43	-0.47	0.7	-0.89	0.82
915 Provitamin D3	-0.43	-0.45	0.67	-0.88	0.76
846 Lathosterol	-0.43	-0.45	0.71	-0.88	0.8
396 Collagen LPP3(c) synthesis	-0.43	0.5	1	0.07	1
740 Cholesterol-ester-stea	-0.43	-0.43	0.62	-0.86	0.69
736 Cholesterol-ester-ol	-0.43	-0.43	0.62	-0.86	0.69
738 Cholesterol-ester-palmn	-0.44	-0.43	0.59	-0.87	0.73
799 Glucuronate	-0.44	0.24	0.57	-0.2	0.41
350 Collagen CO4A3(c) synthesis	-0.46	0.19	1	-0.27	1
737 Cholesterol-ester-palm	-0.46	-0.44	0.59	-0.89	0.69
394 Collagen ITA1(c) synthesis	-0.46	0.33	0.28	-0.13	0.49

Continued on next page

Amplitude differences: Treatment difference – continued

Simulation	Δampl	TGFβ/C 24h ampl	TGFβ/C 24h score	control 1/24h ampl	control 1/24h score
324 Cholesterol(b)	-0.47	-0.44	0.61	-0.91	0.79
669 5-Hydroxy-L-tryptophan	-0.47	0.24	0.61	-0.22	0.73
371 Collagen COCA1(c) synthesis	-0.47	0.36	1	-0.11	1
25 NADH redox potential into peroxy	-0.47	-0.32	0.83	-0.79	0.74
949 UDP-activated-N-acetyl-D-galactosamine	-0.48	0.18	0.44	-0.3	0.62
104 Arginine from Histidine	-0.48	-0.6	0.42	-1.08	0.37
348 Collagen CO4A1(c) synthesis	-0.49	0.07	1	-0.42	1
639 3-Ureidoisobutyrate	-0.49	-0.1	0.37	-0.58	0.39
861 N-Acetylglucosamine-1P	-0.49	0.1	0.55	-0.39	0.65
912 Protein(l)ysine	-0.49	0.01	1	-0.49	1
481 Arginine	-0.51	-0.77	0.46	-1.28	0.3
786 Fructose-2,6PP	-0.51	-0.06	0.84	-0.57	0.94
767 Dihydrothymine	-0.51	-0.1	0.39	-0.61	0.34
944 Thymine	-0.52	-0.09	0.42	-0.61	0.33
725 CMP-activated-N-acetylneuraminate	-0.52	0.28	0.3	-0.24	0.46
513 CMP-N-acetylneuraminate	-0.52	0.28	0.3	-0.24	0.46
2 Aerobic ATP rephosph (gluc)	-0.53	-0.15	0.44	-0.69	0.3
402 HDL	-0.54	-0.28	0.78	-0.82	0.79
329 ApoA1	-0.55	-0.27	0.97	-0.82	0.96
405 Acetone	-0.55	-0.43	0.45	-0.98	0.82
139 Aspartate from Alanine	-0.56	-0.15	0.53	-0.71	0.49
659 4-Imidazolone-5-propanoate	-0.57	-0.51	0.35	-1.08	0.49
102 Arginine from Glutamate	-0.58	-0.58	0.4	-1.16	0.28
824 Hydroxymethylbilane	-0.58	-0.44	0.64	-1.02	0.4
954 UroporphyrinogenIII	-0.58	-0.44	0.61	-1.02	0.39
744 CoproporphyrinogenIII	-0.59	-0.44	0.59	-1.03	0.37
332 ApoC2(c)	-0.59	0.05	0.95	-0.54	0.95
527 Spermidine	-0.59	-0.32	0.43	-0.91	0.28
66 ATP salvage from Hypoxanthine	-0.59	-0.31	0.44	-0.91	0.38
403 Acetoacetate	-0.59	-0.39	0.57	-0.98	0.64
267 Serine from Tryptophan	-0.59	-0.74	0.5	-1.33	0.31
914 Protoporphyrinogen IX	-0.59	-0.43	0.58	-1.02	0.36
913 Protoporphyrin	-0.6	-0.42	0.58	-1.02	0.32
106 Arginine from Glutamine	-0.6	-0.57	0.36	-1.17	0.34
605 2-Oxo-3-methylvalerate	-0.61	0.41	0.49	-0.2	0.54
154 Aspartate from Serine	-0.61	-0.15	0.39	-0.76	0.4
841 L-Lactate	-0.61	-0.22	0.94	-0.83	0.87
528 Spermine	-0.61	-0.41	0.41	-1.02	0.29
817 Hexadecenal	-0.62	0.28	0.26	-0.34	0.32
815 Hexadecanal	-0.62	0.28	0.26	-0.34	0.32
619 3-Methyl-2-oxobutyrate	-0.62	1.04	0.37	0.42	0.46
413 Histidine degr	-0.63	-0.55	0.43	-1.18	0.31
120 Asparagine from Aspartate	-0.65	0.54	0.37	-0.11	0.44
748 D-3-Amino-isobutanoate	-0.66	-0.09	0.35	-0.75	0.34
908 Porphobilinogen	-0.66	-0.46	0.56	-1.12	0.52
65 ATP salvage from Adenosine	-0.67	-0.71	0.88	-1.38	0.89
341 Collagen C43BP(c) synthesis	-0.68	0.58	1	-0.1	1
680 6-Phospho-D-gluconate	-0.68	0.43	0.63	-0.25	0.83
797 Glucose-1P	-0.68	0.2	1	-0.48	1
406 Heme	-0.69	-0.4	0.59	-1.09	0.32
346 Collagen CO2A1(c) synthesis	-0.69	0.17	1	-0.52	1
855 Mercaptopyruvate	-0.69	0.03	0.38	-0.66	0.4
391 Collagen FCN2(c) synthesis	-0.7	0.26	1	-0.44	1
837 L-Cystathionine	-0.71	0.04	0.48	-0.67	0.42
597 2-Amino-3-oxoadipate	-0.72	-0.27	0.56	-1	0.43
13 NADH potential transport	-0.73	-0.34	0.59	-1.07	0.41
796 Glucono-1,5-lactone-6P	-0.74	0.45	1	-0.29	1
422 Tryptophan degr	-0.75	-0.83	0.42	-1.59	0.35
156 Aspartate from Tryptophan	-0.75	-0.82	0.44	-1.57	0.32

Continued on next page

Simulation	Δampl	TGFβ/C 24h ampl	TGFβ/C 24h score	control 1/24h ampl	control 1/24h score
599 2-Aminomuconate(s)emialdehyde	-0.76	-0.98	0.5	-1.75	0.44
596 2-Amino-3-carboxymuconate(s)emialdehyde	-0.76	-0.98	0.5	-1.75	0.44
145 Aspartate from Glycine	-0.77	-0.22	0.44	-0.99	0.45
613 3-Hydroxyanthranilate	-0.77	-1.01	0.52	-1.78	0.35
872 NeuNGc	-0.78	0.56	0.31	-0.21	0.34
135 Asparagine from Tryptophan	-0.78	-0.79	0.44	-1.58	0.31
756 Dehydroalanine	-0.79	0.002	0.62	-0.78	0.7
666 5-Aminolevulinate	-0.79	-0.33	0.61	-1.11	0.61
308 VLDL from LDL	-0.79	0.28	0.71	-0.5	0.81
114 Arginine from Tryptophan	-0.79	-0.7	0.44	-1.49	0.33
598 2-Aminomuconate	-0.8	-0.96	0.54	-1.76	0.44
401 VLDL	-0.8	0.28	0.68	-0.51	0.81
111 Arginine from Proline	-0.8	-0.5	0.48	-1.3	0.25
330 ApoB100(r)	-0.8	0.28	0.99	-0.52	0.99
141 Aspartate from Asparagine	-0.8	-0.22	0.54	-1.02	0.42
309 VLDL from HDL	-0.8	0.28	0.68	-0.52	0.81
344 Collagen CO1A1(c) synthesis	-0.82	1.09	1	0.26	1
919 Ribose-1P	-0.84	0.19	0.4	-0.65	0.5
932 Sphingosine	-0.85	-0.26	0.37	-1.11	0.34
933 Sphingosine-1P	-0.85	-0.26	0.32	-1.11	0.32
434 Fructose degr	-0.85	-0.37	0.44	-1.23	0.6
333 ApoC3(c)	-0.87	-0.004	0.93	-0.87	0.95
429 Ornithine degr	-0.87	-0.51	0.42	-1.38	0.31
408 Arginine degr	-0.88	-0.51	0.42	-1.38	0.3
48 Formylgroup(m)	-0.89	-0.82	0.52	-1.7	0.32
946 Trehalose	-0.89	0.13	1	-0.76	1
512 N-acglucam	-0.89	0.16	0.32	-0.73	0.44
612 3-Hydroxy-L-kynurenine	-0.9	-0.86	0.44	-1.76	0.45
412 Glutamate degr	-0.9	-0.47	0.4	-1.37	0.28
668 5-Formyl-THF	-0.9	-0.78	0.43	-1.69	0.31
108 Arginine from Lysine	-0.93	-0.61	0.45	-1.53	0.32
142 Aspartate from Cysteine	-0.93	-0.22	0.54	-1.15	0.47
93 Alanine from Tryptophan	-0.94	-0.73	0.5	-1.67	0.3
140 Aspartate from Arginine	-0.95	-0.24	0.48	-1.18	0.31
207 Glutamine from Arginine	-0.96	0.88	0.27	-0.08	0.3
271 Tyrosine from Phenylalanine	-0.97	-0.53	0.59	-1.5	0.52
43 Activated methyl group (SAM)	-0.98	-0.67	0.41	-1.65	0.45
307 Arachidonate from Dihomo-gamma-linolenate	-0.99	-0.85	0.6	-1.84	0.39
600 2-Deoxy-D-ribose-1P	-1.01	0.7	0.41	-0.31	0.59
331 ApoC1(c)	-1.04	-0.01	0.92	-1.06	0.94
362 Collagen CO7A1(c) synthesis	-1.07	0.84	1	-0.23	1
55 UDP-activated glucose	-1.08	-0.79	0.53	-1.88	0.55
729 Ceramide-1P-pool	-1.09	-0.27	0.39	-1.36	0.3
57 UDP-activated galactose	-1.09	-0.74	0.45	-1.83	0.43
953 Urocanate	-1.11	-0.12	0.56	-1.23	0.51
931 Sphinganine-1P	-1.11	1.29	0.36	0.18	0.28
941 THF-hexaglutamate	-1.12	0.69	0.87	-0.43	0.98
416 Lysine degr	-1.13	-0.58	0.43	-1.7	0.33
863 N-Acetylneuraminate	-1.14	0.42	0.32	-0.72	0.35
89 Alanine from Phenylalanine	-1.16	-1.74	0.47	-2.9	0.45
386 Collagen CORA1(c) synthesis	-1.18	2.09	1	0.91	1
526 Putrescine	-1.2	0.18	0.52	-1.02	0.45
296 Stearate from Palmitate	-1.21	-0.39	0.7	-1.6	0.33
658 4-Hydroxyphenylpyruvate	-1.33	-1.87	0.73	-3.2	0.53
798 Glucosylceramide-pool	-1.37	-0.27	0.37	-1.64	0.35
299 Oleate from Stearate	-1.41	-0.33	0.83	-1.74	0.44
552 (R)-Mevalonate	-1.42	0.62	0.54	-0.8	0.82
302 gamma-Linolenate from Linoleate	-1.46	-0.34	0.56	-1.79	0.37
298 Palmitolate from Palmitate	-1.47	-0.32	0.7	-1.79	0.34

Continued on next page

Amplitude differences: Treatment difference – continued

Simulation	Δampl	TGFβ/C 24h		control 1/24h	
		ampl	score	ampl	score
834 Kynurenine	-1.5	-0.57	0.43	-2.07	0.34
694 Acetate	-1.5	-0.38	0.97	-1.89	0.79
295 Taurine from Cysteine	-1.53	-1.46	0.5	-2.99	0.49
826 Hypotaurine	-1.53	-1.46	0.5	-2.99	0.49
638 3-Sulfinoalanine	-1.53	-1.46	0.73	-2.99	0.72
747 Cysteamine	-1.56	-1.46	0.39	-3.02	0.37
198 Glycine from Phenylalanine	-1.57	-1.21	0.5	-2.78	0.36
825 Hydroxypyruvate	-1.64	0.55	1	-1.09	1
838 L-Formylkynurenine	-1.67	-0.4	0.69	-2.07	0.75
335 Plasminogen	-1.72	-0.38	0.98	-2.1	0.98
94 Alanine from Tyrosine	-1.73	-1.42	0.49	-3.14	0.45
660 4-Maleylacetoacetate	-1.76	-1.62	0.7	-3.38	0.69
203 Glycine from Tyrosine	-1.8	-1.25	0.53	-3.05	0.35
788 Fumarylacetoacetate	-1.81	-1.51	0.68	-3.32	0.61
268 Serine from Tyrosine	-1.82	-1.22	0.5	-3.04	0.38
779 Fatty-acid-VLDL-SM-pool	-1.9	-0.34	1	-2.25	1
775 Fatty-acid-VLDL-PC-pool	-1.9	-0.34	1	-2.25	1
821 Homogentisate	-1.92	-1.76	0.65	-3.67	0.65
802 Glycerate	-1.95	0.9	0.55	-1.05	0.45
327 Antichymotrypsin	-1.97	0.33	0.97	-1.63	0.97
73 Gluconeogen from Lactate	-1.97	-0.57	0.51	-2.54	0.39
291 beta-Alanine from Tyrosine	-2.01	-1.11	0.38	-3.12	0.41
286 beta-Alanine from Phenylalanine	-2.1	-0.77	0.39	-2.87	0.41
264 Serine from Phenylalanine	-2.11	-0.66	0.51	-2.77	0.38
152 Aspartate from Phenylalanine	-2.12	-0.77	0.43	-2.89	0.45
131 Asparagine from Phenylalanine	-2.14	-0.74	0.44	-2.88	0.38
157 Aspartate from Tyrosine	-2.15	-0.99	0.44	-3.14	0.46
808 Guanidinoacetate	-2.15	-0.74	0.36	-2.88	0.29
110 Arginine from Phenylalanine	-2.15	-0.72	0.45	-2.87	0.37
177 Glutamate from Phenylalanine	-2.18	-0.75	0.52	-2.93	0.38
219 Glutamine from Phenylalanine	-2.21	-0.71	0.48	-2.92	0.35
115 Arginine from Tyrosine	-2.29	-0.79	0.47	-3.08	0.38
74 Gluconeogen from Glycerol	-2.29	-0.69	0.51	-2.98	0.47
243 Proline from Phenylalanine	-2.3	-0.63	0.52	-2.93	0.32
926 Sarcosine	-2.35	-1.26	0.86	-3.61	0.82
136 Asparagine from Tyrosine	-2.37	-0.77	0.46	-3.14	0.37
182 Glutamate from Tyrosine	-2.37	-0.8	0.53	-3.18	0.37
247 Proline from Tyrosine	-2.41	-0.72	0.47	-3.14	0.31
224 Glutamine from Tyrosine	-2.41	-0.76	0.48	-3.17	0.34
418 Phenylalanine degr	-2.47	-0.76	0.43	-3.23	0.41
374 Collagen COFA1(c) synthesis	-2.5	2.27	1	-0.24	1
426 Tyrosine degr	-2.63	-0.87	0.44	-3.5	0.41
75 Gluconeogen from Alanine	-2.89	-0.63	0.35	-3.52	0.4
72 Glycogen glucose release	-2.93	-0.25	0.45	-3.18	0.45
409 Asparagine degr	-3.62	-0.98	0.34	-4.6	0.38
609 2PG	-3.64	-0.27	0.65	-3.9	0.42
890 PEP	-3.65	-0.31	0.54	-3.96	0.51
410 Aspartate degr	-3.8	-0.9	0.34	-4.7	0.32
428 beta-Alanine degr	-3.93	-0.73	0.34	-4.66	0.31
419 Proline degr	-4.09	-0.51	0.5	-4.6	0.25

2.2 Amplitude differences: Treatment difference

Table 8: Amplitude differences: Treatment difference

Simulation	Δampl	control 1/24h		TGFβ 1/24h	
		ampl	score	ampl	score
419 Proline degr	3.89	-4.6	0.25	-0.7	0.44
926 Sarcosine	3.68	-3.61	0.82	0.07	0.2
747 Cysteamine	2.9	-3.02	0.37	-0.12	0.43
295 Taurine from Cysteine	2.87	-2.99	0.49	-0.12	0.6
826 Hypotaurine	2.87	-2.99	0.49	-0.12	0.6
247 Proline from Tyrosine	2.52	-3.14	0.31	-0.62	0.38
834 Kynurenine	2.32	-2.07	0.34	0.25	0.4
374 Collagen COFA1(c) synthesis	2.24	-0.24	1	2	1
808 Guanidinoacetate	2.12	-2.88	0.29	-0.76	0.33
386 Collagen CORA1(c) synthesis	2.12	0.91	1	3.03	1
203 Glycine from Tyrosine	1.58	-3.05	0.35	-1.47	0.44
57 UDP-activated galactose	1.53	-1.83	0.43	-0.31	0.55
817 Hexadecenal	1.44	-0.34	0.32	1.11	0.29
815 Hexadecanal	1.44	-0.34	0.32	1.11	0.29
404 (R)-3-Hydroxybutanoate	1.26	-0.98	0.52	0.28	0.5
344 Collagen CO1A1(c) synthesis	1.15	0.26	1	1.41	1
837 L-Cystathionine	1.06	-0.67	0.42	0.39	0.53
921 Ribulose-5P	1.05	-0.7	0.35	0.36	0.36
920 Ribose-5P	1.05	-0.77	0.38	0.29	0.45
931 Sphinganine-1P	1.02	0.18	0.28	1.21	0.38
48 Formylgroup(m)	0.98	-1.7	0.32	-0.72	0.5
257 Serine from Glycine	0.97	0.31	0.67	1.28	0.4
973 dUMP	0.96	-0.55	0.36	0.41	0.33
761 Deoxyuridine	0.88	-0.55	0.36	0.32	0.32
619 3-Methyl-2-oxobutyrate	0.87	0.42	0.46	1.29	0.35
971 dTMP	0.86	-0.51	0.35	0.36	0.32
970 dTDP	0.78	-0.51	0.32	0.28	0.35
271 Tyrosine from Phenylalanine	0.75	-1.5	0.52	-0.75	0.56
114 Arginine from Tryptophan	0.74	-1.49	0.33	-0.75	0.41
597 2-Amino-3-oxoadipate	0.74	-1	0.43	-0.26	0.5
355 Collagen CO5A2(c) synthesis	0.73	0.36	1	1.09	1
108 Arginine from Lysine	0.72	-1.53	0.32	-0.81	0.43
796 Glucono-1,5-lactone-6P	0.71	-0.29	1	0.43	1
416 Lysine degr	0.71	-1.7	0.33	-1	0.36
680 6-Phospho-D-gluconate	0.68	-0.25	0.83	0.43	0.51
341 Collagen C43BP(c) synthesis	0.68	-0.1	1	0.58	1
362 Collagen CO7A1(c) synthesis	0.68	-0.23	1	0.45	1
842 L-Octanoylcarnitine	0.68	-1.06	0.38	-0.38	0.54
143 Aspartate from Cystine	0.67	-0.94	0.39	-0.26	0.44
371 Collagen COCA1(c) synthesis	0.65	-0.11	1	0.54	1
111 Arginine from Proline	0.64	-1.3	0.25	-0.65	0.47
429 Ornithine degr	0.62	-1.38	0.31	-0.77	0.43
408 Arginine degr	0.62	-1.38	0.3	-0.77	0.41
140 Aspartate from Arginine	0.6	-1.18	0.31	-0.59	0.46
825 Hydroxypyruvate	0.57	-1.09	1	-0.52	1
528 Spermine	0.57	-1.02	0.29	-0.45	0.33
120 Asparagine from Aspartate	0.57	-0.11	0.44	0.46	0.32
481 Arginine	0.56	-1.28	0.3	-0.72	0.53
412 Glutamate degr	0.56	-1.37	0.28	-0.81	0.38
941 THF-hexaglutamate	0.55	-0.43	0.98	0.12	0.86
913 Protoporphyrin	0.55	-1.02	0.32	-0.47	0.39
139 Aspartate from Alanine	0.54	-0.71	0.49	-0.17	0.57
526 Putrescine	0.54	-1.02	0.45	-0.48	0.45
78 Alanine from Aspartate	0.53	-0.71	0.58	-0.18	0.47
102 Arginine from Glutamate	0.51	-1.16	0.28	-0.65	0.38
349 Collagen CO4A2(c) synthesis	0.49	0.75	1	1.24	1

Continued on next page

Amplitude differences: Treatment difference – continued

Simulation	Δampl	control 1/24h		TGFβ 1/24h	
		ampl	score	ampl	score
141 Aspartate from Asparagine	0.48	-1.02	0.42	-0.54	0.56
106 Arginine from Glutamine	0.48	-1.17	0.34	-0.69	0.38
100 Arginine from Cysteine	0.47	-1.17	0.35	-0.7	0.45
327 Antichymotrypsin	0.45	-1.63	0.97	-1.18	0.97
557 (S)-3-Hydroxytetradecanoyl-CoA	0.44	-0.77	0.28	-0.33	0.35
297 Palmitate from Palmitolate	0.43	0.66	0.33	1.09	0.34
802 Glycerate	0.43	-1.05	0.45	-0.62	0.66
300 Stearate from Oleate	0.42	0.68	0.32	1.1	0.35
99 Arginine from Aspartate	0.41	-1.1	0.29	-0.68	0.46
396 Collagen LPP3(c) synthesis	0.41	0.07	1	0.47	1
91 Alanine from Serine	0.4	-0.98	0.43	-0.59	0.56
919 Ribose-1P	0.39	-0.65	0.5	-0.26	0.39
101 Arginine from Cystine	0.37	-1.14	0.36	-0.77	0.47
82 Alanine from Glycine	0.37	-0.99	0.39	-0.62	0.48
527 Spermidine	0.36	-0.91	0.28	-0.54	0.4
609 2PG	0.36	-3.9	0.42	-3.54	0.45
494 Mannose-1P	0.36	-0.12	0.45	0.24	0.88
142 Aspartate from Cysteine	0.34	-1.15	0.47	-0.81	0.47
869 N-Pantothenoylcysteine	0.33	-0.17	0.45	0.17	0.45
330 ApoB100(r)	0.32	-0.52	0.99	-0.2	0.99
309 VLDL from HDL	0.32	-0.52	0.81	-0.2	0.77
890 PEP	0.31	-3.96	0.51	-3.64	0.53
401 VLDL	0.31	-0.51	0.81	-0.2	0.77
98 Arginine from Asparagine	0.31	-1	0.35	-0.69	0.48
308 VLDL from LDL	0.3	-0.5	0.81	-0.2	0.81
946 Trehalose	0.29	-0.76	1	-0.47	1
436 Mannose degr	0.28	0.48	0.64	0.76	0.64
395 Collagen ITA2(c) synthesis	0.28	-0.04	1	0.24	1
288 beta-Alanine from Serine	0.27	-0.89	0.44	-0.61	0.42
391 Collagen FCN2(c) synthesis	0.27	-0.44	1	-0.17	1
375 Collagen COGA1(c) synthesis	0.27	0.08	1	0.35	1
669 5-Hydroxy-L-tryptophan	0.26	-0.22	0.73	0.04	0.52
112 Arginine from Serine	0.26	-0.96	0.4	-0.7	0.51
760 Deoxyinosine	0.26	0.21	0.39	0.47	0.33
827 Hypoxanthine	0.26	0.23	0.48	0.49	0.3
345 Collagen CO1A2(c) synthesis	0.25	0.3	1	0.55	1
372 Collagen CODA1(c) synthesis	0.25	-0.23	1	0.02	1
41 Na+ importgradient	0.25	0.11	0.52	0.36	0.66
611 3-Dehydrosphinganine	0.25	-0.18	0.38	0.07	0.54
797 Glucose-1P	0.24	-0.48	1	-0.24	1
53 activated sulphur	0.24	0.03	0.65	0.27	0.68
420 Serine degr	0.24	-0.99	0.41	-0.76	0.41
15 Dephosphorylation of ATP	0.24	0.24	0.51	0.48	0.67
952 Urate	0.22	0.23	0.38	0.45	0.29
810 H2O2	0.22	0.23	0.38	0.45	0.29
342 Collagen CCBE1(c) synthesis	0.21	-0.13	1	0.08	1
382 Collagen COMA1(c) synthesis	0.21	-0.02	1	0.19	1
77 Alanine from Asparagine	0.2	-0.7	0.47	-0.5	0.52
413 Histidine degr	0.2	-1.18	0.31	-0.98	0.4
397 Collagen PCOTH(c) synthesis	0.2	-0.13	0.47	0.07	0.33
361 Collagen CO6A6(c) synthesis	0.2	-0.13	0.46	0.07	0.33
394 Collagen ITA1(c) synthesis	0.2	-0.13	0.49	0.07	0.33
360 Collagen CO6A5(c) synthesis	0.2	-0.13	0.47	0.07	0.33
392 Collagen FCN3(c) synthesis	0.2	-0.13	0.47	0.07	0.34
380 Collagen COLA1(c) synthesis	0.2	-0.12	0.45	0.07	0.35
387 Collagen COSA1(c) synthesis	0.19	-0.12	0.44	0.07	0.35
390 Collagen FCN1(c) synthesis	0.19	-0.12	0.46	0.07	0.35
381 Collagen COLQ(c) synthesis	0.19	-0.12	0.44	0.07	0.35
972 dUDP	0.19	-0.63	0.26	-0.44	0.32

Continued on next page

Amplitude differences: Treatment difference – continued

Simulation	Δampl	control 1/24h		TGFβ 1/24h	
		ampl	score	ampl	score
328 Antitrypsin	0.19	-0.12	0.49	0.07	0.33
326 Fibrinogen	0.19	-0.12	0.49	0.07	0.32
399 Collagen SERPH(c) synthesis	0.18	0.65	1	0.83	1
370 Collagen COBA2(c) synthesis	0.18	-0.08	1	0.1	1
468 PAPS	0.17	-0.87	0.31	-0.7	0.36
13 NADH potential transport	0.17	-1.07	0.41	-0.9	0.62
350 Collagen CO4A3(c) synthesis	0.16	-0.27	1	-0.11	1
799 Glucuronate	0.16	-0.2	0.41	-0.04	0.41
103 Arginine from Glycine	0.15	-0.95	0.4	-0.8	0.47
384 Collagen COOA1(c) synthesis	0.15	-0.12	1	0.03	1
64 Glucosamine-6P	0.15	-0.15	0.77	-0.001	0.63
67 dTTP salvage from Thymine	0.14	0.42	0.33	0.57	0.32
107 Arginine from Leucine	0.14	-0.82	0.34	-0.67	0.44
435 Galactose degr	0.13	0.33	0.5	0.47	0.37
923 SAICAR	0.13	0.26	0.35	0.39	0.31
673 5-Phosphoribosyl-4-carboxy-5-aminoimidazole	0.12	0.27	0.38	0.4	0.34
104 Arginine from Histidine	0.12	-1.08	0.37	-0.96	0.44
347 Collagen CO3A1(c) synthesis	0.12	0.66	1	0.78	1
829 Inositol	0.12	1.72	0.62	1.83	0.59
912 Protein(l)ysine	0.11	-0.49	1	-0.38	1
353 Collagen CO4A6(c) synthesis	0.11	-0.2	1	-0.09	1
953 Urocanate	0.1	-1.23	0.51	-1.13	0.48
346 Collagen CO2A1(c) synthesis	0.1	-0.52	1	-0.42	1
415 Leucine degr	0.1	-0.8	0.33	-0.7	0.39
709 Adenine	0.1	0.36	0.46	0.46	0.36
59 GDP-activated mannose	0.1	0.15	0.58	0.24	0.84
366 Collagen CO9A2(c) synthesis	0.1	-0.21	1	-0.11	1
86 Alanine from Leucine	0.09	-0.7	0.39	-0.61	0.37
794 Galactose-1P	0.09	-0.39	0.48	-0.3	0.66
296 Stearate from Palmitate	0.09	-1.6	0.33	-1.51	0.4
12 ATP from NADH	0.09	-0.18	0.34	-0.08	0.5
154 Aspartate from Serine	0.09	-0.76	0.4	-0.67	0.53
943 Thymidine	0.09	-0.68	0.35	-0.6	0.33
359 Collagen CO6A3(c) synthesis	0.09	0.11	1	0.19	1
830 Inositol-1P	0.09	1.77	1	1.85	1
495 Mannose-6P	0.09	0.15	0.78	0.24	0.78
453 FAD	0.07	0.26	0.48	0.33	0.29
955 Xanthosine	0.07	0.26	0.39	0.33	0.28
712 Agmatine	0.07	0.01	0.68	0.08	0.8
828 Inosine	0.06	0.26	0.45	0.32	0.3
383 Collagen CONA1(c) synthesis	0.06	-0.16	1	-0.1	1
364 Collagen CO8A2(c) synthesis	0.06	-0.16	1	-0.1	1
548 (R)-4-Phosphopantothenoyl-cysteine	0.06	0.11	0.45	0.17	0.48
774 FMN	0.06	-0.16	1	-0.1	1
792 GMP	0.05	0.28	0.42	0.33	0.3
354 Collagen CO5A1(c) synthesis	0.05	0.01	1	0.06	1
367 Collagen CO9A3(c) synthesis	0.05	-0.13	1	-0.08	1
877 O-Acetylcarnitine	0.05	0.15	1	0.2	1
675 5-Phosphoribosylformylglycinamidine	0.05	0.3	0.45	0.35	0.31
66 ATP salvage from Hypoxanthine	0.05	-0.91	0.38	-0.86	0.45
756 Dehydroalanine	0.04	-0.78	0.7	-0.74	0.66
379 Collagen COKA1(c) synthesis	0.04	-0.18	1	-0.14	1
924 SAM	0.04	0.3	0.47	0.34	0.33
922 SAH	0.04	0.3	0.47	0.34	0.33
461 4ppan	0.04	-0.17	1	-0.13	1
691 AIR	0.03	0.3	0.47	0.33	0.33
710 Adenylosuccinate	0.03	0.3	0.51	0.33	0.34
965 dAMP	0.03	0.29	0.47	0.32	0.3
277 beta-Alanine from Cystine	0.03	-0.2	0.4	-0.17	0.46

Continued on next page

Amplitude differences: Treatment difference – continued

Simulation	Δampl	control 1/24h ampl	control 1/24h score	TGFβ 1/24h ampl	TGFβ 1/24h score
907 Phosphopantetheine	0.03	-0.15	0.3	-0.12	0.45
471 Homocysteine	0.03	-0.75	0.49	-0.72	0.54
759 Deoxyguanosine	0.02	0.28	0.43	0.3	0.31
348 Collagen CO4A1(c) synthesis	0.02	-0.42	1	-0.4	1
506 Uridine	0.02	0.3	0.46	0.33	0.4
275 beta-Alanine from Aspartate	0.02	-0.15	0.95	-0.13	0.96
806 Glycogenin-G7G1	0.02	0.44	0.94	0.46	0.94
71 Glycogen glucose storage	0.02	0.44	0.65	0.46	0.65
804 Glycogenin-G4G4	0.02	0.44	0.95	0.46	0.94
805 Glycogenin-G7	0.02	0.44	0.94	0.46	0.94
803 Glycogenin-G11	0.02	0.44	0.95	0.46	0.95
807 Glycogenin-G8	0.02	0.44	0.95	0.46	0.96
454 Glycogenin	0.02	0.44	0.96	0.46	0.96
334 ApoE(c)	0.02	-0.24	0.97	-0.22	0.96
389 Collagen EMID2(c) synthesis	0.01	-0.18	1	-0.17	1
968 dGDP	0.01	0.3	0.43	0.31	0.31
455 Thioredoxin	0.01	0.13	0.94	0.14	0.94
887 Oxidized thioredoxin	0.01	0.13	0.93	0.14	0.93
502 GDP-mannose	0.01	0.26	0.39	0.27	0.37
216 Glutamine from Leucine	0.01	-0.64	0.36	-0.63	0.49
791 GDP	0.01	0.3	0.46	0.31	0.34
433 Stearate degr	0.004	-0.36	0.43	-0.36	0.52
432 Oleate degr	0.004	-0.37	0.42	-0.36	0.5
661 4-Methyl-2-oxopentanoate	0.002	-0.65	0.35	-0.64	0.53
696 Acetoacetyl-CoA	0.001	0.3	0.4	0.3	0.29
936 Stearoyl-ACP	0.001	-0.08	0.83	-0.08	0.79
538 (2E)-Octadecenoyl-ACP	0.001	-0.08	0.83	-0.08	0.79
633 3-Oxostearoyl-ACP	0.001	-0.08	0.83	-0.08	0.79
617 3-Hydroxystearoyl-ACP	0.001	-0.08	0.83	-0.08	0.79
816 Hexadecanoyl-ACP	0.001	-0.08	0.84	-0.08	0.8
626 3-Oxohexadecanoyl-ACP	0.001	-0.08	0.84	-0.08	0.8
547 (R)-3-Hydroxypalmitoyl-ACP	0.001	-0.08	0.84	-0.08	0.8
534 (2E)-Hexadecenoyl-ACP	0.001	-0.08	0.84	-0.08	0.8
942 Tetradecanoyl-ACP	0.001	-0.08	0.84	-0.08	0.8
541 (2E)-Tetradecenoyl-ACP	0.001	-0.08	0.84	-0.08	0.8
813 HMA	0.001	-0.08	0.84	-0.08	0.8
634 3-Oxotetradecanoyl-ACP	0.001	-0.08	0.84	-0.08	0.8
769 Dodecanoyl-ACP	0.001	-0.08	0.84	-0.08	0.81
749 D-3-Hydroxydodecanoyl-ACP	0.001	-0.08	0.84	-0.08	0.81
624 3-Oxododecanoyl-ACP	0.001	-0.08	0.85	-0.08	0.81
532 (2E)-Dodecenoyl-ACP	0.001	-0.08	0.84	-0.08	0.81
754 Decanoyl-ACP	0.001	-0.08	0.85	-0.08	0.81
622 3-Oxodecanoyl-ACP	0.001	-0.08	0.85	-0.08	0.81
545 (R)-3-Hydroxydecanoyl-ACP	0.001	-0.08	0.85	-0.08	0.81
530 (2E)-Decenoyl-ACP	0.001	-0.08	0.85	-0.08	0.81
750 D-3-Hydroxyhexanoyl-ACP	0.001	-0.08	0.86	-0.08	0.83
627 3-Oxohexanoyl-ACP	0.001	-0.08	0.86	-0.08	0.83
881 Octanoyl-ACP	0.001	-0.08	0.85	-0.08	0.82
629 3-Oxooctanoyl-ACP	0.001	-0.08	0.85	-0.08	0.82
546 (R)-3-Hydroxyoctanoyl-ACP	0.001	-0.08	0.85	-0.08	0.82
539 (2E)-Octenoyl-ACP	0.001	-0.08	0.85	-0.08	0.82
695 Acetoacetyl-ACP	0.001	-0.08	0.87	-0.08	0.84
851 Malonyl-ACP	0.001	-0.08	0.87	-0.08	0.84
457 Apo-ACP	0.001	-0.08	0.92	-0.08	0.91
718 Butyryl-ACP	0.001	-0.08	0.87	-0.08	0.84
717 But-2-enoyl-ACP	0.001	-0.08	0.87	-0.08	0.84
544 (R)-3-Hydroxybutanoyl-ACP	0.001	-0.08	0.87	-0.08	0.84
818 Hexanoyl-ACP	0.001	-0.08	0.86	-0.08	0.82
536 (2E)-Hexenoyl-ACP	0.001	-0.08	0.86	-0.08	0.82

Continued on next page

Amplitude differences: Treatment difference – continued

Simulation	Δampl	control 1/24h		TGFβ 1/24h	
		ampl	score	ampl	score
697 Acetyl-ACP	0.001	-0.08	0.88	-0.08	0.85
688 ACP	0.001	-0.08	0.88	-0.08	0.86
458 Apo-ACP(m)	-0.0004	-0.08	0.72	-0.08	0.73
978 mitoACP	-0.001	-0.08	0.71	-0.08	0.72
351 Collagen CO4A4(c) synthesis	-0.002	-0.12	1	-0.12	1
969 dGMP	-0.002	0.3	0.44	0.3	0.33
227 Methionine from Cysteine	-0.003	-0.67	0.52	-0.67	0.54
672 5-Oxoproline	-0.01	0.16	0.55	0.15	0.55
689 ADP	-0.01	0.32	0.44	0.31	0.3
692 AMP	-0.01	0.32	0.44	0.31	0.3
331 ApoC1(c)	-0.01	-1.06	0.94	-1.06	0.93
753 Deamido-NAD	-0.01	0.28	0.45	0.28	0.3
50 Acetyl group(m)	-0.01	-0.36	0.47	-0.37	0.54
783 Formamidopyrimidine(n)leoside triphosphate	-0.01	0.31	0.43	0.3	0.31
278 beta-Alanine from Glutamate	-0.01	-0.12	0.47	-0.13	0.53
282 beta-Alanine from Glutamine	-0.01	-0.14	0.45	-0.15	0.41
958 cAMP	-0.01	0.32	0.4	0.31	0.28
274 beta-Alanine from Asparagine	-0.01	-0.14	0.49	-0.15	0.6
272 beta-Alanine from Alanine	-0.01	-0.14	0.53	-0.15	0.64
524 gdpddman	-0.02	0.27	0.42	0.26	0.4
276 beta-Alanine from Cysteine	-0.02	-0.15	0.51	-0.16	0.53
406 Heme	-0.02	-1.09	0.32	-1.11	0.42
273 beta-Alanine from Arginine	-0.02	-0.13	0.4	-0.15	0.4
333 ApoC3(c)	-0.02	-0.87	0.95	-0.89	0.94
878 O-Butanoylcarnitine	-0.02	-0.34	0.42	-0.36	0.56
497 UDP-xylose	-0.02	0.26	0.31	0.24	0.34
332 ApoC2(c)	-0.02	-0.54	0.95	-0.56	0.95
388 Collagen CTHR1(c) synthesis	-0.03	0.01	1	-0.02	1
340 Collagen BGH3(c) synthesis	-0.03	-0.03	1	-0.06	1
208 Glutamine from Asparagine	-0.03	-0.58	0.43	-0.61	0.77
940 Sulfite degr	-0.03	-0.07	1	-0.1	1
728 Carnosine	-0.03	-0.12	0.87	-0.15	0.72
841 L-Lactate	-0.03	-0.83	0.87	-0.87	0.92
424 Glycine degr	-0.03	-1	0.38	-1.04	0.41
358 Collagen CO6A2(c) synthesis	-0.04	-0.05	1	-0.09	1
81 Alanine from Glutamate	-0.04	-0.08	0.37	-0.11	0.41
325 Albumin	-0.04	-0.11	0.98	-0.15	0.98
917 Pyridoxine-P	-0.04	-0.19	1	-0.23	1
221 Glutamine from Serine	-0.04	-0.63	0.4	-0.67	0.74
501 GDP-L-fucose	-0.05	0.31	0.43	0.26	0.38
32 Thioredoxin(m) reduction	-0.05	-0.26	1	-0.31	1
33 Thioredoxin(m) oxidation	-0.05	-0.26	1	-0.31	1
786 Fructose-2,6PP	-0.05	-0.57	0.94	-0.61	0.87
959 cGMP	-0.05	0.31	0.43	0.26	0.38
363 Collagen CO8A1(c) synthesis	-0.05	-0.19	1	-0.24	1
170 Glutamate from Glycine	-0.06	-0.73	0.43	-0.79	0.65
133 Asparagine from Serine	-0.06	-0.72	0.37	-0.78	0.5
822 Homovanillate	-0.06	-0.01	1	-0.07	1
800 Glutamyl-5P	-0.06	-0.52	0.37	-0.59	0.44
393 Collagen FMOD(c) synthesis	-0.06	-0.04	1	-0.11	1
879 O-Propanoylcarnitine	-0.06	-0.81	0.53	-0.87	0.54
944 Thymine	-0.07	-0.61	0.33	-0.68	0.35
37 GSH reduction using NADPH redox potential	-0.07	-0.09	1	-0.16	1
38 GSH oxidation	-0.07	-0.09	1	-0.16	1
790 GAR	-0.07	0.34	0.47	0.27	0.37
293 Homocysteine from Methionine	-0.07	0.48	1	0.4	1
767 Dihydrothymine	-0.07	-0.61	0.34	-0.68	0.36
947 Triphosphate degr	-0.07	0.15	1	0.07	1
859 N-(omega)-Hydroxyarginine	-0.08	-0.18	0.77	-0.26	0.77

Continued on next page

Simulation	Δampl	control 1/24h ampl	control 1/24h score	TGFβ 1/24h ampl	TGFβ 1/24h score
113 Arginine from Threonine	-0.08	-0.72	0.41	-0.8	0.6
11 ATP from NADH(m)	-0.08	0.002	0.48	-0.08	0.98
748 D-3-Amino-isobutanoate	-0.08	-0.75	0.34	-0.84	0.29
14 Ubiquinol-to-ATP	-0.09	0.002	0.54	-0.08	0.91
336 Prothrombin	-0.09	-0.49	0.98	-0.58	0.98
385 Collagen COPA1(c) synthesis	-0.09	-0.19	1	-0.28	1
681 6-Pyruvoyltetrahydropterin	-0.09	0.37	0.46	0.27	0.34
34 Thioredoxin(c) reduction	-0.09	-0.31	0.98	-0.41	0.96
483 Aspartate	-0.1	-0.85	0.35	-0.94	0.45
840 L-Glutamate 5-semialdehyde	-0.1	-0.52	0.49	-0.62	0.48
19 Aerobic reduction of NADP+ (gluc)	-0.1	-0.28	0.31	-0.38	0.35
365 Collagen CO9A1(c) synthesis	-0.1	-0.0005	1	-0.1	1
839 L-Fucose-1P	-0.1	0.34	0.46	0.24	0.58
894 PRPP	-0.1	0.39	0.39	0.29	0.36
464 mlthf	-0.1	-0.33	0.78	-0.43	0.8
109 Arginine from Methionine	-0.1	-0.8	0.42	-0.91	0.47
466 THF(m)	-0.1	-0.33	1	-0.43	1
459 THF	-0.1	-0.33	1	-0.43	1
29 NADH to NADPH transhydrogenase	-0.1	-0.33	1	-0.43	1
766 Dihydrofolate	-0.1	-0.33	1	-0.43	1
670 5-Methyl-THF	-0.1	-0.33	1	-0.43	1
400 Collagen VWA2(c) synthesis	-0.11	0.02	1	-0.09	1
369 Collagen COBA1(c) synthesis	-0.11	0.03	1	-0.08	1
659 4-Imidazolone-5-propanoate	-0.11	-1.08	0.49	-1.19	0.54
228 Methionine from Cystine	-0.11	-0.66	0.55	-0.77	0.54
376 Collagen COHA1(c) synthesis	-0.11	-0.03	1	-0.15	1
124 Asparagine from Glycine	-0.11	-0.92	0.38	-1.04	0.58
58 GDP-activated fucose	-0.11	0.35	0.59	0.23	0.69
880 OAA	-0.11	-0.06	0.61	-0.17	0.96
667 5-Formiminotetrahydrofolate	-0.11	-0.35	0.75	-0.47	0.56
930 Sphinganine	-0.11	0.18	0.32	0.07	0.4
498 UDP-glucose	-0.12	0.32	0.3	0.2	0.3
179 Glutamate from Serine	-0.12	-0.57	0.45	-0.69	0.79
2 Aerobic ATP rephosph (gluc)	-0.12	-0.69	0.3	-0.81	0.41
479 UTP	-0.12	0.32	0.36	0.2	0.37
222 Glutamine from Threonine	-0.12	-0.61	0.51	-0.73	0.69
966 dCDP	-0.12	0.33	0.35	0.21	0.33
473 dCTP	-0.12	0.33	0.4	0.21	0.38
850 Malate	-0.12	-0.04	0.64	-0.16	0.97
213 Glutamine from Glycine	-0.12	-0.75	0.37	-0.87	0.55
261 Serine from Leucine	-0.12	-0.69	0.41	-0.81	0.41
242 Proline from Methionine	-0.12	-0.65	0.42	-0.77	0.52
298 Palmitolate from Palmitate	-0.13	-1.79	0.34	-1.91	0.37
68 O2.-(c) degr	-0.13	-0.32	0.74	-0.44	1
25 NADH redox potential into peroxy	-0.13	-0.79	0.74	-0.92	0.82
720 CDP	-0.13	0.34	0.37	0.21	0.37
723 CMP	-0.13	0.34	0.36	0.21	0.36
477 CTP	-0.13	0.33	0.44	0.2	0.44
176 Glutamate from Methionine	-0.13	-0.7	0.48	-0.83	0.47
166 Glutamate from Asparagine	-0.13	-0.53	0.44	-0.66	0.76
605 2-Oxo-3-methylvalerate	-0.13	-0.2	0.54	-0.33	0.5
292 beta-Alanine from Valine	-0.13	-0.17	0.45	-0.3	0.4
251 Serine from Arginine	-0.14	-0.77	0.3	-0.9	0.39
507 Cytidine	-0.14	0.34	0.41	0.2	0.41
664 5,10-Methenyl-THF	-0.14	-0.33	0.64	-0.47	0.82
873 Nicotinamide D-ribonucleotide	-0.14	-0.17	0.35	-0.31	0.4
876 Nicotinate(r)ibonucleotide	-0.14	-0.17	0.33	-0.31	0.36
180 Glutamate from Threonine	-0.14	-0.58	0.53	-0.72	0.73
245 Proline from Threonine	-0.14	-0.56	0.47	-0.7	0.69

Continued on next page

Amplitude differences: Treatment difference – continued

Simulation	Δampl	control 1/24h ampl	control 1/24h score	TGFβ 1/24h ampl	TGFβ 1/24h score
460 Pyridoxal-P	-0.14	-0.19	0.44	-0.33	0.58
487 Proline	-0.14	-0.29	0.39	-0.44	0.53
217 Glutamine from Lysine	-0.15	-0.49	0.31	-0.64	0.43
434 Fructose degr	-0.15	-1.23	0.6	-1.38	0.57
299 Oleate from Stearate	-0.15	-1.74	0.44	-1.89	0.46
146 Aspartate from Histidine	-0.15	-0.76	0.37	-0.92	0.49
499 udpgal	-0.16	-0.17	0.3	-0.32	0.35
5 Aerobic rephosph of CTP	-0.16	-0.64	0.32	-0.8	0.36
175 Glutamate from Lysine	-0.16	-0.46	0.32	-0.62	0.47
787 Fumarate	-0.16	-0.03	0.7	-0.2	0.85
716 Argininosuccinate	-0.16	-0.03	0.39	-0.2	0.47
125 Asparagine from Histidine	-0.17	-0.81	0.32	-0.98	0.44
439 Antitrypsin degr	-0.17	-0.47	0.62	-0.64	0.5
482 Asparagine	-0.17	-0.82	0.34	-0.99	0.48
218 Glutamine from Methionine	-0.17	-0.69	0.47	-0.86	0.48
961 cis-Aconitate	-0.17	-0.35	0.59	-0.52	0.76
248 Proline from Valine	-0.17	-0.29	0.38	-0.46	0.57
206 Glutamine from Alanine	-0.18	-0.46	0.47	-0.63	0.51
183 Glutamate from Valine	-0.18	-0.19	0.48	-0.37	0.53
668 5-Formyl-THF	-0.18	-1.69	0.31	-1.86	0.41
155 Aspartate from Threonine	-0.18	-0.66	0.51	-0.84	0.64
145 Aspartate from Glycine	-0.18	-0.99	0.45	-1.17	0.59
231 Proline from Asparagine	-0.18	-0.45	0.37	-0.63	0.71
378 Collagen COJA1(c) synthesis	-0.18	0.04	1	-0.14	1
18 Aerobic reduction of NADP+ (FA)	-0.18	-0.41	0.36	-0.59	0.29
84 Alanine from Isoleucine	-0.18	-0.47	0.41	-0.65	0.57
421 Threonine degr	-0.18	-0.7	0.39	-0.88	0.53
356 Collagen CO5A3(c) synthesis	-0.18	-0.07	1	-0.25	1
475 dTTP	-0.19	0.36	0.5	0.17	0.44
694 Acetate	-0.19	-1.89	0.79	-2.07	0.86
269 Serine from Valine	-0.19	-0.22	0.38	-0.4	0.42
43 Activated methyl group (SAM)	-0.19	-1.65	0.45	-1.84	0.56
6 Aerobic rephosph of UDP	-0.19	-0.3	0.37	-0.49	0.46
908 Porphobilinogen	-0.19	-1.12	0.52	-1.31	0.55
164 Glutamate from Alanine	-0.2	-0.49	0.49	-0.68	0.62
765 Dihydroceramide-pool	-0.2	-0.17	0.4	-0.37	0.31
215 Glutamine from Isoleucine	-0.2	-0.37	0.43	-0.57	0.65
339 Collagen ADIPO(c) synthesis	-0.2	0.04	1	-0.16	1
438 Antichymotrypsin degr	-0.2	-0.47	0.6	-0.68	0.48
172 Glutamate from Isoleucine	-0.2	-0.35	0.46	-0.56	0.65
368 Collagen COAA1(c) synthesis	-0.21	-0.09	1	-0.3	1
665 5,6-Dihydrouracil	-0.21	-0.72	0.39	-0.93	0.36
338 Haptoglobin	-0.21	0.22	0.97	0.01	0.96
666 5-Aminolevulinate	-0.21	-1.11	0.61	-1.32	0.61
225 Glutamine from Valine	-0.21	-0.16	0.37	-0.38	0.49
824 Hydroxymethylbilane	-0.21	-1.02	0.4	-1.24	0.46
440 ApoA1 degr	-0.22	-0.48	0.59	-0.69	0.48
868 N-Methylethanolamine-P	-0.22	-0.27	1	-0.48	1
771 Ethanolamine-P	-0.22	-0.27	1	-0.48	1
373 Collagen COEA1(c) synthesis	-0.22	0.06	1	-0.15	1
693 Acetaldehyde	-0.22	-0.26	0.6	-0.48	0.58
914 Protoporphyrinogen IX	-0.22	-1.02	0.36	-1.24	0.4
954 UroporphyrinogenIII	-0.22	-1.02	0.39	-1.24	0.44
744 CoproporphyrinogenIII	-0.22	-1.03	0.37	-1.24	0.42
437 Albumin degr	-0.22	-0.48	0.62	-0.7	0.51
916 Pyridoxal	-0.22	0.16	0.68	-0.06	0.66
46 Activated methyl group from Histidine	-0.22	-0.71	0.3	-0.93	0.35
92 Alanine from Threonine	-0.22	-0.65	0.55	-0.87	0.58
337 ApoTransferin	-0.22	-0.35	0.98	-0.57	0.98

Continued on next page

Simulation	Δampl	control 1/24h ampl	control 1/24h score	TGFβ 1/24h ampl	TGFβ 1/24h score
423 Valine degr	-0.23	-0.18	0.33	-0.41	0.42
238 Proline from Isoleucine	-0.23	-0.39	0.43	-0.62	0.66
167 Glutamate from Aspartate	-0.23	-0.42	0.48	-0.65	0.84
265 Serine from Proline	-0.23	-0.62	0.32	-0.85	0.47
598 2-Aminomuconate	-0.23	-1.76	0.44	-1.99	0.58
445 ApoTransferin degr	-0.23	-0.48	0.63	-0.71	0.52
204 Glycine from Valine	-0.23	-0.23	0.35	-0.46	0.43
116 Arginine from Valine	-0.23	-0.18	0.33	-0.42	0.47
779 Fatty-acid-VLDL-SM-pool	-0.23	-2.25	1	-2.48	1
775 Fatty-acid-VLDL-PC-pool	-0.23	-2.25	1	-2.48	1
289 beta-Alanine from Threonine	-0.24	-0.63	0.46	-0.86	0.56
3 Anaerobic rephosph of ATP	-0.24	-0.74	0.45	-0.97	0.47
10 Anaerobic rephosph of UTP	-0.24	-0.74	0.43	-0.97	0.46
8 Anaerobic rephosph of GTP	-0.24	-0.74	0.43	-0.97	0.46
9 Anaerobic rephosph of CTP	-0.24	-0.74	0.43	-0.97	0.46
1 Aerobic ATP rephosph (FA)	-0.24	-0.36	0.33	-0.6	0.44
4 Aerobic rephosph of GTP	-0.24	-0.36	0.31	-0.6	0.42
7 Aerobic rephosph of UTP	-0.24	-0.36	0.31	-0.6	0.42
441 Fibrinogen degr	-0.24	-0.48	0.61	-0.72	0.5
447 Glycogenin degr	-0.24	-0.48	0.62	-0.72	0.52
443 Plasminogen degr	-0.24	-0.48	0.6	-0.72	0.5
467 1fthf	-0.24	-0.32	0.59	-0.57	0.73
854 Maltose	-0.24	0.15	1	-0.09	1
442 Haptoglobin degr	-0.25	-0.48	0.67	-0.73	0.56
444 Prothrombin degr	-0.25	-0.48	0.57	-0.72	0.48
137 Asparagine from Valine	-0.25	-0.19	0.42	-0.44	0.49
492 Oleate	-0.25	-0.26	0.74	-0.51	0.85
493 Stearate	-0.25	-0.26	0.81	-0.51	0.94
742 Citrate	-0.25	-0.26	0.59	-0.51	0.71
178 Glutamate from Proline	-0.25	-0.36	0.37	-0.62	0.64
823 Hydracrylate	-0.26	-0.72	0.58	-0.97	0.58
239 Proline from Glutamine	-0.26	-0.36	0.32	-0.61	0.59
158 Aspartate from Valine	-0.26	-0.16	0.41	-0.42	0.5
490 Palmitate	-0.26	-0.26	0.8	-0.52	0.97
639 3-Ureidoisobutyrate	-0.26	-0.58	0.39	-0.84	0.35
301 Palmitolate from Arachidonate	-0.26	-0.25	0.65	-0.51	0.76
229 Proline from Alanine	-0.26	-0.38	0.46	-0.64	0.53
233 Proline from Cysteine	-0.26	-0.36	0.35	-0.62	0.66
599 2-Aminomuconate(s)emialdehyde	-0.26	-1.75	0.44	-2.01	0.56
596 2-Amino-3-carboxymuconate(s)emialdehyde	-0.26	-1.75	0.44	-2.01	0.56
240 Proline from Leucine	-0.26	-0.37	0.38	-0.64	0.57
95 Alanine from Valine	-0.27	-0.16	0.35	-0.43	0.53
613 3-Hydroxyanthranilate	-0.27	-1.78	0.35	-2.05	0.48
550 (R)-5-Phosphomevalonate	-0.27	-0.74	0.64	-1.01	0.49
195 Glycine from Leucine	-0.27	-0.7	0.4	-0.96	0.42
69 Formaldehyde degr	-0.27	-0.23	0.47	-0.5	0.45
329 ApoA1	-0.27	-0.82	0.96	-1.09	0.96
232 Proline from Aspartate	-0.27	-0.37	0.4	-0.64	0.7
244 Proline from Serine	-0.27	-0.39	0.38	-0.66	0.67
307 Arachidonate from Dihomo-gamma-linolenate	-0.27	-1.84	0.39	-2.12	0.52
165 Glutamate from Arginine	-0.27	-0.35	0.31	-0.63	0.57
252 Serine from Asparagine	-0.27	-0.69	0.48	-0.96	0.54
230 Proline from Arginine	-0.28	-0.34	0.33	-0.62	0.52
302 gamma-Linolenate from Linoleate	-0.28	-1.79	0.37	-2.07	0.49
785 Fructose-1,6PP	-0.28	-0.79	1	-1.07	1
61 AKG	-0.28	-0.57	0.44	-0.85	0.58
39 Proton-gradient(m) build up	-0.28	-0.34	0.41	-0.62	0.47
40 Proton-gradient(c) build up	-0.28	-0.34	0.41	-0.62	0.47
357 Collagen CO6A1(c) synthesis	-0.28	-0.08	1	-0.36	1

Continued on next page

Amplitude differences: Treatment difference – continued

Simulation	Δampl	control 1/24h		TGFβ 1/24h	
		ampl	score	ampl	score
402 HDL	-0.28	-0.82	0.79	-1.1	0.81
525 beta-Alanine	-0.28	-0.4	0.34	-0.69	0.38
249 Proline from beta-Alanine	-0.29	-0.31	0.42	-0.6	0.59
75 Gluconeogen from Alanine	-0.29	-3.52	0.4	-3.82	0.48
93 Alanine from Tryptophan	-0.3	-1.67	0.3	-1.97	0.45
885 Orotidine-5P	-0.3	-0.36	0.33	-0.66	0.33
105 Arginine from Isoleucine	-0.3	-0.38	0.39	-0.68	0.58
496 Fructose-6P	-0.3	-0.79	1	-1.09	1
335 Plasminogen	-0.3	-2.1	0.98	-2.4	0.98
486 Glutamine	-0.3	-0.36	0.45	-0.66	0.73
484 Glutamate	-0.3	-0.36	0.51	-0.66	0.83
281 beta-Alanine from Isoleucine	-0.31	-0.38	0.42	-0.68	0.51
174 Glutamate from Leucine	-0.31	-0.35	0.41	-0.66	0.53
987 trans-4-Hydroxy-L-proline	-0.31	0.68	1	0.37	1
835 L-1-Pyrroline-3-hydroxy-5-carboxylate	-0.31	0.68	1	0.37	1
893 PPi	-0.31	0.39	0.66	0.09	0.39
592 1-Pyrroline-5-carboxylate	-0.31	0.68	0.77	0.37	1
403 Acetoacetate	-0.31	-0.98	0.64	-1.29	0.42
134 Asparagine from Threonine	-0.31	-0.59	0.49	-0.9	0.68
16 Aerobic reduction of NAD+ (FA)	-0.31	-0.39	0.37	-0.7	0.32
456 Thioredoxin(m)	-0.31	0.38	0.95	0.06	0.96
979 mitoOxidizedThioredoxin	-0.31	0.38	0.94	0.06	0.95
210 Glutamine from Cysteine	-0.31	-0.33	0.33	-0.65	0.47
846 Lathosterol	-0.31	-0.88	0.8	-1.19	0.85
422 Tryptophan degr	-0.31	-1.59	0.35	-1.9	0.42
135 Asparagine from Tryptophan	-0.32	-1.58	0.31	-1.89	0.43
915 Provitamin D3	-0.32	-0.88	0.76	-1.19	0.8
398 Collagen SCRB1(c) synthesis	-0.32	-0.41	1	-0.73	1
168 Glutamate from Cysteine	-0.32	-0.35	0.44	-0.67	0.63
184 Glutamate from beta-Alanine	-0.32	-0.29	0.49	-0.61	0.64
266 Serine from Threonine	-0.32	-0.65	0.53	-0.97	0.62
676 5alpha-Cholesta-7,24-dien-3beta-ol	-0.32	-0.89	0.82	-1.21	0.85
927 Sedoheptulose-1,7PP	-0.32	-0.58	0.52	-0.9	0.58
20 Aerobic reduction of FAD (gluc)	-0.32	-0.36	0.36	-0.69	0.5
682 7-Dehydrodesmosterol	-0.32	-0.89	0.78	-1.21	0.8
910 Propanoate	-0.32	-0.65	0.48	-0.98	0.64
186 Glycine from Arginine	-0.33	-0.78	0.29	-1.11	0.42
732 Cholestenol	-0.33	-0.84	0.8	-1.17	0.84
324 Cholesterol(b)	-0.33	-0.91	0.79	-1.24	0.81
234 Proline from Cystine	-0.33	-0.34	0.42	-0.67	0.6
918 Quinolinate	-0.33	0.05	0.38	-0.28	0.77
636 3-Phosphonooxypyruvate	-0.33	-0.88	0.61	-1.21	0.59
957 Zymosterol	-0.34	-0.86	0.82	-1.19	0.84
662 4alpha-Methylzymosterol	-0.34	-0.86	0.82	-1.19	0.84
226 Glutamine from beta-Alanine	-0.34	-0.25	0.42	-0.59	0.55
212 Glutamine from Glutamate	-0.34	-0.08	0.33	-0.42	0.39
21 Aerobic reduction of FAD (FA)	-0.34	-0.34	0.36	-0.68	0.52
645 3PG	-0.34	-0.88	0.71	-1.21	0.66
928 Sedoheptulose-7P	-0.34	-0.67	0.44	-1.01	0.5
45 Activated methylene group from Try	-0.34	-0.34	0.26	-0.68	0.34
173 Glutamate from Glutamine	-0.34	-0.32	0.43	-0.66	0.55
199 Glycine from Proline	-0.35	-0.63	0.31	-0.97	0.52
76 Alanine from Arginine	-0.35	-0.08	0.33	-0.43	0.36
618 3-Keto-4-methylzymosterol	-0.35	-0.86	0.81	-1.21	0.84
838 L-Formylkynurenine	-0.35	-2.07	0.75	-2.42	0.71
201 Glycine from Threonine	-0.36	-0.66	0.47	-1.02	0.63
70 Formate degr	-0.36	-0.03	0.56	-0.4	0.65
156 Aspartate from Tryptophan	-0.36	-1.57	0.32	-1.94	0.43
425 Isoleucine degr	-0.36	-0.4	0.34	-0.76	0.5

Continued on next page

Amplitude differences: Treatment difference – continued

Simulation	Δampl	control 1/24h		TGFβ 1/24h	
		ampl	score	ampl	score
259 Serine from Isoleucine	-0.36	-0.5	0.43	-0.87	0.55
726 Carbamoyl-P	-0.36	-0.09	0.5	-0.46	0.41
770 Erythrose-4P	-0.37	-0.63	0.54	-0.99	0.6
663 4alpha-Methylzymosterol-4-carboxylate	-0.37	-0.82	0.81	-1.19	0.82
207 Glutamine from Arginine	-0.37	-0.08	0.3	-0.45	0.41
149 Aspartate from Leucine	-0.37	-0.3	0.4	-0.67	0.45
132 Asparagine from Proline	-0.37	-0.28	0.29	-0.65	0.56
405 Acetone	-0.37	-0.98	0.82	-1.36	0.46
63 Farnesyl-PP	-0.37	-0.66	0.62	-1.03	0.54
812 HCO3-	-0.38	-0.09	0.71	-0.46	0.66
727 Carbonate	-0.38	-0.09	0.71	-0.46	0.66
737 Cholesterol-ester-palm	-0.38	-0.89	0.69	-1.27	0.71
169 Glutamate from Cystine	-0.38	-0.33	0.5	-0.71	0.66
685 7alpha-Hydroxy-5beta-cholestan-3-one	-0.38	-0.84	0.75	-1.22	0.72
751 D-Xylulose-5P	-0.38	-0.77	0.42	-1.15	0.45
202 Glycine from Tryptophan	-0.38	-1.36	0.3	-1.74	0.49
795 Geranyl-PP	-0.38	-0.67	0.61	-1.05	0.53
147 Aspartate from Isoleucine	-0.38	-0.37	0.49	-0.75	0.62
523 Cholesterol	-0.38	-0.87	0.77	-1.25	0.79
60 Pyruvate	-0.38	-0.66	0.49	-1.04	0.51
62 Isopentenyl-PP	-0.38	-0.68	0.69	-1.06	0.61
283 beta-Alanine from Leucine	-0.38	-0.27	0.44	-0.66	0.44
740 Cholesterol-ester-stea	-0.39	-0.86	0.69	-1.24	0.72
736 Cholesterol-ester-ol	-0.39	-0.86	0.69	-1.24	0.72
738 Cholesterol-ester-palmn	-0.39	-0.87	0.73	-1.26	0.72
687 7alpha-Hydroxycholesterol	-0.39	-0.86	0.75	-1.25	0.75
763 Desmosterol	-0.39	-0.88	0.79	-1.27	0.79
126 Asparagine from Isoleucine	-0.4	-0.36	0.44	-0.76	0.58
733 Cholesterol-ester-arach	-0.4	-0.84	0.7	-1.23	0.74
836 L-2-Aminoadipate	-0.4	0.42	1	0.02	1
256 Serine from Glutamate	-0.4	-0.66	0.33	-1.06	0.43
686 7alpha-Hydroxycholest-4-en-3-one	-0.4	-0.85	0.74	-1.25	0.71
852 Malonyl-Carnitin	-0.41	-0.47	0.62	-0.88	0.64
211 Glutamine from Cystine	-0.41	-0.32	0.45	-0.73	0.56
614 3-Hydroxyisobutyrate	-0.41	-0.19	0.29	-0.6	0.38
319 Chenodiol(b)	-0.41	-0.81	0.54	-1.22	0.65
730 Chenodeoxycholoyl-CoA	-0.41	-0.8	0.45	-1.21	0.52
253 Serine from Aspartate	-0.41	-0.69	0.52	-1.1	0.57
117 Arginine from beta-Alanine	-0.41	-0.29	0.33	-0.7	0.47
148 Aspartate from Glutamine	-0.42	-0.27	0.37	-0.69	0.43
571 1,3DPG	-0.42	-0.69	0.81	-1.11	0.77
193 Glycine from Isoleucine	-0.42	-0.51	0.39	-0.93	0.56
647 3alpha,7alpha,12alpha-Trihydroxy-5beta-24-oxocholestanoyl-CoA	-0.42	-0.85	0.42	-1.27	0.49
679 5beta-Cholestane-3alpha,7alpha-diol	-0.43	-0.84	0.73	-1.26	0.72
731 Cholate	-0.43	-0.88	0.6	-1.31	0.62
128 Asparagine from Leucine	-0.43	-0.29	0.4	-0.72	0.44
683 7alpha,12alpha-Dihydroxy-5beta-cholestan-3-one	-0.43	-0.87	0.71	-1.3	0.71
646 3alpha,7alpha,12alpha,24-Tetrahydroxy-5beta-cholestanoyl-CoA	-0.43	-0.85	0.42	-1.28	0.49
549 (R)-5-Diphosphomevalonate	-0.43	-0.71	0.71	-1.15	0.58
734 Cholesterol-ester-gla	-0.43	-0.84	0.69	-1.27	0.72
640 3-Ureidopropionate	-0.44	-0.67	0.44	-1.11	0.41
789 GAP	-0.44	-0.75	0.99	-1.19	0.97
752 DHAP	-0.44	-0.75	0.99	-1.19	0.97
739 Cholesterol-ester-pool	-0.44	-0.86	0.68	-1.3	0.71
144 Aspartate from Glutamate	-0.44	-0.31	0.34	-0.75	0.5
267 Serine from Tryptophan	-0.44	-1.33	0.31	-1.77	0.49
410 Aspartate degr	-0.44	-4.7	0.32	-5.14	0.34
593 14-Demethyllanosterol	-0.44	-0.79	0.75	-1.24	0.77
655 3alpha,7alpha-Dihydroxy-5beta-cholestanoyl-CoA	-0.44	-0.82	0.46	-1.27	0.52

Continued on next page

Amplitude differences: Treatment difference – continued

Simulation	Δampl	control 1/24h		TGFβ 1/24h	
		ampl	score	ampl	score
648 3alpha,7alpha,12alpha-Trihydroxy-5beta-cholest-24-enoyl-CoA	-0.45	-0.86	0.42	-1.3	0.49
656 4,4-Dimethyl-5alpha-cholesta-8,14,24-trien-3beta-ol	-0.45	-0.82	0.74	-1.27	0.79
428 beta-Alanine degr	-0.45	-4.66	0.31	-5.11	0.33
22 Oxidation of NADH	-0.45	-0.3	0.45	-0.75	0.4
316 Gly-CD-cholate(b)	-0.45	-0.83	0.51	-1.28	0.61
684 7alpha,12alpha-Dihydroxycholest-4-en-3-one	-0.45	-0.88	0.7	-1.33	0.7
23 Oxidation of NADPH	-0.45	-0.26	0.4	-0.71	0.45
552 (R)-Mevalonate	-0.45	-0.8	0.82	-1.25	0.45
735 Cholesterol-ester-lin	-0.46	-0.84	0.7	-1.3	0.72
678 5beta-Cholestane-3alpha,7alpha,26-triol	-0.46	-0.84	0.75	-1.3	0.73
542 (2E)-Tetradecenoyl-CoA	-0.46	-0.41	0.31	-0.87	0.38
654 3alpha,7alpha-Dihydroxy-5beta-cholestanate	-0.46	-0.83	0.7	-1.29	0.74
237 Proline from Histidine	-0.46	-0.35	0.42	-0.81	0.55
768 Dimethylallyl-PP	-0.46	-0.74	0.6	-1.2	0.53
280 beta-Alanine from Histidine	-0.46	-0.45	0.4	-0.91	0.45
287 beta-Alanine from Proline	-0.47	-0.14	0.39	-0.61	0.49
305 Dihomo-gamma-linolenate from gamma-Linolenate	-0.47	-0.46	0.47	-0.93	0.47
848 Linoleate	-0.47	-0.86	0.59	-1.33	0.66
652 3alpha,7alpha,12alpha-Trihydroxycoprostane	-0.47	-0.86	0.7	-1.33	0.72
724 CMP-NeuNGc	-0.47	-0.65	0.29	-1.13	0.26
315 Glycocholate(b)	-0.48	-0.85	0.51	-1.32	0.6
741 Choloyl-CoA	-0.48	-0.82	0.45	-1.3	0.48
653 3alpha,7alpha-Dihydroxy-5beta-cholestan-26-al	-0.48	-0.82	0.74	-1.3	0.75
512 N-acglucam	-0.48	-0.73	0.44	-1.21	0.39
651 3alpha,7alpha,12alpha-Trihydroxy-5beta-cholestanoyl-CoA	-0.48	-0.86	0.46	-1.34	0.51
847 Lauroyl-CoA	-0.48	-0.42	0.3	-0.91	0.37
608 2-Oxoglutaramate	-0.48	-0.03	1	-0.52	1
951 UMP	-0.48	-0.17	0.38	-0.66	0.41
209 Glutamine from Aspartate	-0.49	-0.14	0.36	-0.62	0.52
318 tcdchola(b)	-0.49	-0.84	0.48	-1.33	0.53
612 3-Hydroxy-L-kynurenine	-0.49	-1.76	0.45	-2.25	0.5
258 Serine from Histidine	-0.49	-0.72	0.38	-1.21	0.5
123 Asparagine from Glutamate	-0.49	-0.29	0.34	-0.78	0.44
187 Glycine from Asparagine	-0.5	-0.69	0.42	-1.19	0.52
677 5beta-Cholestane-3alpha,7alpha,12alpha,26-tetrol	-0.5	-0.86	0.71	-1.36	0.73
650 3alpha,7alpha,12alpha-Trihydroxy-5beta-cholestanate	-0.5	-0.86	0.71	-1.36	0.73
649 3alpha,7alpha,12alpha-Trihydroxy-5beta-cholestan-26-al	-0.5	-0.86	0.71	-1.36	0.73
194 Glycine from Glutamine	-0.51	-0.65	0.28	-1.15	0.43
188 Glycine from Aspartate	-0.51	-0.7	0.45	-1.21	0.59
409 Asparagine degr	-0.51	-4.6	0.38	-5.11	0.4
864 N-Acetylneuraminate-9P	-0.51	-0.72	0.39	-1.24	0.36
171 Glutamate from Histidine	-0.52	-0.37	0.47	-0.89	0.63
127 Asparagine from Glutamine	-0.52	-0.28	0.39	-0.8	0.56
555 (S)-3-Hydroxydodecanoyl-CoA	-0.52	0.28	0.36	-0.24	0.35
35 Thioredoxin(c) oxidation	-0.52	-0.28	0.99	-0.8	0.84
641 3-oxolaur-cis-5-enoyl-CoA	-0.52	0.28	0.38	-0.24	0.36
543 (3Z)-Dodecenoyl-CoA	-0.52	0.28	0.38	-0.24	0.36
533 (2E)-Dodecenoyl-CoA	-0.52	0.28	0.36	-0.24	0.34
610 3(S)-3-hydroxydodecen-(5Z)-oyl-CoA	-0.52	0.28	0.38	-0.24	0.35
863 N-Acetylneuraminate	-0.52	-0.72	0.35	-1.24	0.32
983 trans,cis-dodeca-2,5-dienoyl-CoA	-0.52	0.28	0.38	-0.24	0.35
862 N-Acetylmannosamine-6P	-0.53	-0.72	0.55	-1.25	0.5
625 3-Oxododecanoyl-CoA	-0.53	0.27	0.37	-0.26	0.36
755 Decanoyl-CoA	-0.53	0.27	0.37	-0.26	0.36
642 3-oxomyrist-7-enoyl-CoA	-0.53	0.28	0.39	-0.25	0.35
962 cis-laur-5-enoyl-CoA	-0.53	0.28	0.38	-0.25	0.35
798 Glucosylceramide-pool	-0.53	-1.64	0.35	-2.17	0.36
235 Proline from Glutamate	-0.53	-0.08	0.46	-0.61	0.64
884 Orotate	-0.53	-0.09	0.38	-0.62	0.4

Continued on next page

Amplitude differences: Treatment difference – continued

Simulation	Δampl	control 1/24h ampl	control 1/24h score	TGFβ 1/24h ampl	TGFβ 1/24h score
960 cis-(3S)-hydroxytetradec-7-enoyl-CoA	-0.53	0.28	0.38	-0.25	0.34
191 Glycine from Glutamate	-0.53	-0.66	0.3	-1.19	0.45
563 (S)-Hydroxyoctanoyl-CoA	-0.53	0.26	0.33	-0.27	0.39
630 3-Oxooctanoyl-CoA	-0.53	0.25	0.34	-0.28	0.39
632 3-Oxopropanoate	-0.53	0.37	0.41	-0.16	0.45
820 Histamine	-0.53	-0.54	0.9	-1.07	0.73
985 trans,cis-myristo-2,7-dienoyl-CoA	-0.53	0.28	0.38	-0.25	0.34
96 Alanine from beta-Alanine	-0.53	0.4	0.39	-0.13	0.39
644 3-oxopalmitoleoyl-CoA	-0.54	0.28	0.39	-0.25	0.35
963 cis-myrist-7-enoyl-CoA	-0.54	0.28	0.38	-0.25	0.33
561 (S)-Hydroxydecanoyl-CoA	-0.54	0.27	0.34	-0.27	0.37
317 Taurocholate(b)	-0.54	-0.86	0.48	-1.39	0.52
623 3-Oxodecanoyl-CoA	-0.54	0.27	0.35	-0.27	0.38
882 Octanoyl-CoA	-0.54	0.27	0.35	-0.27	0.38
531 (2E)-Decenoyl-CoA	-0.54	0.27	0.35	-0.27	0.37
562 (S)-Hydroxyhexanoyl-CoA	-0.54	0.26	0.3	-0.28	0.4
42 Na+ exportgradient	-0.54	-0.06	1	-0.6	1
819 Hexanoyl-CoA	-0.54	0.26	0.34	-0.28	0.39
28 NADPH to NADH transhydrogenase	-0.55	-0.29	1	-0.84	1
559 (S)-3-hydroxypalmitoleoyl-CoA	-0.55	0.29	0.37	-0.26	0.32
150 Aspartate from Lysine	-0.55	-0.47	0.37	-1.02	0.44
153 Aspartate from Proline	-0.55	-0.08	0.29	-0.62	0.57
772 FADH2	-0.55	0.28	0.43	-0.28	0.27
729 Ceramide-1P-pool	-0.56	-1.36	0.3	-1.91	0.31
984 trans,cis-hexadeca-2,9-dienoyl-CoA	-0.56	0.29	0.38	-0.27	0.3
520 SM	-0.56	-0.46	0.36	-1.02	0.47
558 (S)-3-hydroxyoleyleoyl-CoA	-0.56	0.29	0.39	-0.27	0.31
711 Adenylyl(s)ulfate	-0.56	0.3	0.46	-0.26	0.31
698 Acetyl-CoA	-0.57	0.29	0.42	-0.27	0.28
488 Serine	-0.57	-0.67	0.45	-1.24	0.56
981 palmitoleoyl-CoA	-0.57	0.29	0.38	-0.28	0.3
521 Ceramide	-0.57	-0.58	0.37	-1.15	0.47
284 beta-Alanine from Lysine	-0.57	-0.41	0.37	-0.98	0.39
671 5-Methylthioadenosine	-0.57	0.29	0.45	-0.28	0.31
719 Butyryl-CoA	-0.57	0.28	0.31	-0.29	0.39
843 L-Oleoylcarnitine	-0.58	0.13	0.54	-0.45	0.69
119 Asparagine from Arginine	-0.58	-0.08	0.32	-0.66	0.39
870 NADH	-0.58	0.28	0.41	-0.3	0.34
982 sn-Glycerol-3P	-0.58	-0.72	0.71	-1.3	0.97
635 3-Oxotetradecanoyl-CoA	-0.58	0.26	0.28	-0.33	0.37
757 Deoxyadenosine	-0.59	0.28	0.41	-0.3	0.27
451 NAD+	-0.59	0.29	0.4	-0.3	0.33
192 Glycine from Histidine	-0.59	-0.72	0.36	-1.31	0.53
414 Glutamine degr	-0.59	-0.28	0.27	-0.87	0.35
986 trans,cis-octadeca-2,9-dienoyl-CoA	-0.59	0.29	0.39	-0.3	0.29
762 Dephospho-CoA	-0.59	0.3	0.39	-0.29	0.29
159 Aspartate from beta-Alanine	-0.59	-0.28	0.51	-0.87	0.47
801 Glutaryl-CoA	-0.59	0.29	0.38	-0.31	0.31
485 Glycine	-0.6	-0.67	0.38	-1.28	0.59
895 Palmitoyl-CoA	-0.6	0.29	0.39	-0.31	0.28
214 Glutamine from Histidine	-0.6	-0.38	0.38	-0.99	0.51
858 Myristoyl-CoA	-0.61	0.28	0.31	-0.34	0.33
631 3-Oxopalmitoyl-CoA	-0.61	0.28	0.3	-0.33	0.36
121 Asparagine from Cysteine	-0.61	-0.27	0.39	-0.88	0.41
535 (2E)-Hexadecenoyl-CoA	-0.61	0.29	0.38	-0.32	0.28
937 Stearoyl-CoA	-0.61	0.29	0.39	-0.32	0.28
883 Oleoyl-CoA	-0.61	0.29	0.39	-0.32	0.28
849 Linoleoyl-CoA	-0.61	0.29	0.39	-0.32	0.28
715 Arachidonyl-CoA	-0.61	0.29	0.39	-0.32	0.28

Continued on next page

Amplitude differences: Treatment difference – continued

Simulation	Δampl	control 1/24h ampl	control 1/24h score	TGFβ 1/24h ampl	TGFβ 1/24h score
554 (S)-3-Hydroxybutyryl-CoA	-0.61	0.28	0.42	-0.33	0.28
964 dADP	-0.62	0.31	0.41	-0.3	0.28
701 Acyl-CoA-CL-pool	-0.62	0.29	0.38	-0.32	0.28
705 Acyl-CoA-VLDL-PS-pool	-0.62	0.29	0.38	-0.32	0.28
706 Acyl-CoA-VLDL-SM-pool	-0.62	0.29	0.38	-0.33	0.29
704 Acyl-CoA-VLDL-PI-pool	-0.62	0.29	0.38	-0.33	0.29
708 Acyl-CoA-VLDL-TG3-pool	-0.62	0.29	0.38	-0.33	0.29
556 (S)-3-Hydroxyhexadecanoyl-CoA	-0.62	0.29	0.31	-0.33	0.32
707 Acyl-CoA-VLDL-TG2-pool	-0.62	0.29	0.38	-0.33	0.29
519 LacCer	-0.62	-0.41	0.34	-1.04	0.38
703 Acyl-CoA-VLDL-PE-pool	-0.62	0.29	0.38	-0.33	0.29
700 Acyl-CoA-Bile-PC-pool	-0.62	0.29	0.38	-0.33	0.29
702 Acyl-CoA-VLDL-PC-pool	-0.62	0.29	0.38	-0.33	0.29
138 Asparagine from beta-Alanine	-0.62	-0.26	0.47	-0.88	0.44
262 Serine from Lysine	-0.63	-0.64	0.36	-1.27	0.42
831 Isobutyryl-CoA	-0.63	0.33	0.38	-0.3	0.3
948 UDP-N-acetylglucosamine	-0.63	-0.52	0.34	-1.15	0.29
270 Serine from beta-Alanine	-0.63	-0.65	0.4	-1.28	0.44
888 PAP	-0.63	0.32	0.42	-0.32	0.3
470 GSH	-0.64	-0.94	0.47	-1.58	0.6
511 UDP-N-acetylgalactosamine	-0.64	-0.51	0.34	-1.15	0.28
196 Glycine from Lysine	-0.64	-0.64	0.33	-1.28	0.44
55 UDP-activated glucose	-0.65	-1.88	0.55	-2.52	0.52
205 Glycine from beta-Alanine	-0.65	-0.65	0.35	-1.3	0.47
122 Asparagine from Cystine	-0.65	-0.28	0.49	-0.93	0.5
932 Sphingosine	-0.65	-1.11	0.34	-1.76	0.32
933 Sphingosine-1P	-0.66	-1.11	0.32	-1.77	0.28
514 PC	-0.66	-0.31	0.4	-0.97	0.46
845 Lanosterol	-0.66	-0.62	0.84	-1.28	0.85
602 2-Lysolecithin-pool	-0.67	-0.29	0.38	-0.96	0.42
615 3-Hydroxyisobutyryl-CoA	-0.67	0.4	0.37	-0.28	0.3
584 1-Acylglycerol-3P-palmn	-0.68	-0.79	0.61	-1.46	0.67
758 Deoxycytidine	-0.68	0.28	0.39	-0.39	0.29
934 Squalene	-0.68	-0.47	0.79	-1.14	0.81
909 Presqualene-PP	-0.68	-0.47	0.79	-1.14	0.81
17 Aerobic reduction of NAD+ (gluc)	-0.68	0.32	0.33	-0.35	0.45
65 ATP salvage from Adenosine	-0.68	-1.38	0.89	-2.06	0.88
746 Cys-Gly	-0.68	0.13	1	-0.55	1
856 Methacrylyl-CoA	-0.69	0.4	0.36	-0.29	0.28
579 1-Acylglycerol-3P-VLDL-TG1-pool	-0.7	-0.58	0.52	-1.28	0.66
578 1-Acylglycerol-3P-VLDL-SM-pool	-0.7	-0.58	0.52	-1.28	0.66
574 1-Acylglycerol-3P-VLDL-PC-pool	-0.7	-0.58	0.52	-1.27	0.66
967 dCMP	-0.7	0.3	0.39	-0.4	0.31
268 Serine from Tyrosine	-0.7	-3.04	0.38	-3.74	0.44
575 1-Acylglycerol-3P-VLDL-PE-pool	-0.7	-0.58	0.52	-1.28	0.66
576 1-Acylglycerol-3P-VLDL-PI-pool	-0.7	-0.58	0.52	-1.28	0.66
577 1-Acylglycerol-3P-VLDL-PS-pool	-0.71	-0.58	0.53	-1.29	0.67
491 Glycerol	-0.71	-0.82	0.53	-1.53	0.6
583 1-Acylglycerol-3P-palm	-0.71	-0.6	0.52	-1.31	0.67
871 NADPH	-0.71	0.29	0.38	-0.43	0.34
585 1-Acylglycerol-3P-stea	-0.71	-0.59	0.53	-1.3	0.67
582 1-Acylglycerol-3P-ol	-0.71	-0.59	0.53	-1.3	0.67
581 1-Acylglycerol-3P-lin	-0.71	-0.59	0.53	-1.3	0.67
580 1-Acylglycerol-3P-arach	-0.71	-0.59	0.53	-1.3	0.67
431 HDL degr	-0.72	-0.77	0.41	-1.5	0.4
452 NADP+	-0.73	0.29	0.37	-0.44	0.33
74 Gluconeogen from Glycerol	-0.74	-2.98	0.47	-3.72	0.54
572 1-Acylglycerol-3P-Bile-PC-pool	-0.74	-0.61	0.52	-1.35	0.65
311 Urea from glutamine	-0.74	-0.08	0.26	-0.82	0.34

Continued on next page

Amplitude differences: Treatment difference – continued

Simulation	Δampl	control 1/24h		TGFβ 1/24h	
		ampl	score	ampl	score
505 Uracil	-0.74	0.29	0.39	-0.45	0.33
190 Glycine from Cystine	-0.74	-0.8	0.41	-1.54	0.55
162 Cystine from Cysteine	-0.74	-0.27	0.87	-1.01	0.57
160 Cysteine from Cystine	-0.74	-0.27	0.87	-1.01	0.57
906 Phosphodimethylethanolamine	-0.75	0.11	1	-0.63	1
905 Phosphocholine	-0.75	0.11	1	-0.63	1
600 2-Deoxy-D-ribose-1P	-0.75	-0.31	0.59	-1.06	0.32
279 beta-Alanine from Glycine	-0.76	-0.9	0.45	-1.65	0.44
591 1-Acylglycerol-VLDL-TG1-pool	-0.77	-0.67	0.47	-1.43	0.55
586 1-Acylglycerol-VLDL-PC-pool	-0.77	-0.67	0.47	-1.43	0.55
590 1-Acylglycerol-VLDL-SM-pool	-0.77	-0.67	0.46	-1.43	0.55
587 1-Acylglycerol-VLDL-PE-pool	-0.77	-0.67	0.47	-1.44	0.56
588 1-Acylglycerol-VLDL-PI-pool	-0.77	-0.67	0.47	-1.44	0.56
589 1-Acylglycerol-VLDL-PS-pool	-0.77	-0.67	0.47	-1.44	0.56
866 N-Formimino-L-glutamate	-0.78	-0.93	0.52	-1.71	0.64
430 LDL degr	-0.78	-0.69	0.4	-1.48	0.42
260 Serine from Glutamine	-0.79	-0.2	0.31	-0.99	0.41
821 Homogentisate	-0.79	-3.67	0.65	-4.47	0.74
321 SM(b)	-0.8	-0.35	0.4	-1.15	0.49
560 (S)-Dihydroorotate	-0.8	0.27	0.3	-0.53	0.35
189 Glycine from Cysteine	-0.8	-0.84	0.38	-1.64	0.52
151 Aspartate from Methionine	-0.8	-0.77	0.47	-1.57	0.5
522 Triacylglycerol	-0.8	-0.24	0.51	-1.04	0.59
94 Alanine from Tyrosine	-0.81	-3.14	0.45	-3.96	0.52
904 Phosphatidate-VLDL-TG-pool	-0.81	-0.55	0.43	-1.36	0.54
788 Fumarylacetoacetate	-0.82	-3.32	0.61	-4.13	0.72
899 Phosphatidate-VLDL-PC-pool	-0.82	-0.55	0.43	-1.36	0.54
529 Methylthioribose-1P	-0.82	0.53	0.38	-0.29	0.44
903 Phosphatidate-VLDL-SM-pool	-0.82	-0.55	0.43	-1.37	0.54
601 2-Deoxy-D-ribose-5P	-0.82	-0.23	0.55	-1.05	0.41
182 Glutamate from Tyrosine	-0.82	-3.18	0.37	-4	0.42
900 Phosphatidate-VLDL-PE-pool	-0.83	-0.55	0.44	-1.38	0.54
72 Glycogen glucose release	-0.83	-3.18	0.45	-4.01	0.5
570 1,2-Diacylglycerol-VLDL-TG-pool	-0.83	-0.55	0.38	-1.38	0.48
518 CL	-0.83	-0.24	0.4	-1.06	0.4
565 1,2-Diacylglycerol-VLDL-PC-pool	-0.83	-0.55	0.39	-1.38	0.48
569 1,2-Diacylglycerol-VLDL-SM-pool	-0.83	-0.56	0.38	-1.39	0.48
320 Bile-PC(b)	-0.84	-0.31	0.47	-1.15	0.5
935 Squalene 2,3-oxide	-0.84	-0.55	0.83	-1.38	0.83
515 PE	-0.84	-0.36	0.47	-1.2	0.52
901 Phosphatidate-VLDL-PI-pool	-0.84	-0.55	0.44	-1.39	0.54
566 1,2-Diacylglycerol-VLDL-PE-pool	-0.84	-0.56	0.39	-1.4	0.48
322 PS(b)	-0.84	-0.36	0.4	-1.2	0.45
516 PS	-0.84	-0.36	0.4	-1.2	0.45
974 dUTP	-0.84	0.4	0.28	-0.44	0.31
607 2-Oxobutyrate	-0.85	-0.85	0.66	-1.69	0.49
902 Phosphatidate-VLDL-PS-pool	-0.85	-0.56	0.44	-1.41	0.54
224 Glutamine from Tyrosine	-0.85	-3.17	0.34	-4.02	0.37
721 CDP-choline	-0.85	0.32	0.34	-0.54	0.39
567 1,2-Diacylglycerol-VLDL-PI-pool	-0.85	-0.56	0.39	-1.41	0.49
875 Nicotinate D-ribonucleoside	-0.86	0.55	0.3	-0.31	0.32
568 1,2-Diacylglycerol-VLDL-PS-pool	-0.86	-0.57	0.38	-1.43	0.49
255 Serine from Cystine	-0.86	-0.79	0.46	-1.65	0.54
832 Isocitrate	-0.87	0.12	0.41	-0.74	0.49
129 Asparagine from Lysine	-0.88	-0.08	0.29	-0.97	0.36
73 Gluconeogen from Lactate	-0.88	-2.54	0.39	-3.42	0.42
660 4-Maleylacetoacetate	-0.89	-3.38	0.69	-4.27	0.79
27 NADPH redox potential into peroxy	-0.89	0.12	1	-0.77	1
197 Glycine from Methionine	-0.89	-0.73	0.45	-1.62	0.52

Continued on next page

Amplitude differences: Treatment difference – continued

Simulation	Δampl	control 1/24h		TGFβ 1/24h	
		ampl	score	ampl	score
517 PI	-0.89	-0.23	0.29	-1.12	0.34
136 Asparagine from Tyrosine	-0.9	-3.14	0.37	-4.04	0.4
860 N-Acetyl-D-mannosamine	-0.91	-0.24	0.53	-1.15	0.43
426 Tyrosine degr	-0.91	-3.5	0.41	-4.41	0.45
465 acgam6p	-0.92	-0.5	0.64	-1.41	0.58
30 NADPH to NADH transhydrogenase in mito	-0.92	0.49	1	-0.43	1
26 NADPH redox potential into mito	-0.92	0.15	0.69	-0.77	0.88
323 PE(b)	-0.92	-0.34	0.52	-1.27	0.57
781 Fatty-acid-VLDL-TG2-pool	-0.92	-0.78	0.49	-1.7	0.58
780 Fatty-acid-VLDL-TG1-pool	-0.92	-0.78	0.49	-1.7	0.58
352 Collagen CO4A5(c) synthesis	-0.92	1.61	1	0.68	1
865 N-Carbamoyl-L-aspartate	-0.93	0.53	0.33	-0.4	0.41
115 Arginine from Tyrosine	-0.94	-3.08	0.38	-4.02	0.4
254 Serine from Cysteine	-0.95	-0.82	0.45	-1.77	0.51
975 gamma-Glutamyl-cysteine	-0.95	-1.24	0.86	-2.19	0.86
130 Asparagine from Methionine	-0.95	-0.72	0.46	-1.67	0.47
157 Aspartate from Tyrosine	-0.95	-3.14	0.46	-4.1	0.49
90 Alanine from Proline	-0.96	0.4	0.32	-0.56	0.44
304 Arachidonate from Linoleate	-0.96	-0.73	0.48	-1.69	0.54
163 Cystine from Methionine	-0.96	-0.68	0.49	-1.64	0.5
291 beta-Alanine from Tyrosine	-0.96	-3.12	0.41	-4.08	0.43
889 PE-PS-VLDL-pool	-0.96	-0.37	0.43	-1.33	0.49
263 Serine from Methionine	-0.97	-0.73	0.48	-1.69	0.51
185 Glycine from Alanine	-0.97	-0.68	0.47	-1.65	0.56
181 Glutamate from Tryptophan	-0.97	0.29	0.3	-0.69	0.46
241 Proline from Lysine	-0.98	0.36	0.29	-0.61	0.47
725 CMP-activated-N-acetylneuraminate	-0.98	-0.24	0.46	-1.22	0.31
513 CMP-N-acetylneuraminate	-0.98	-0.24	0.46	-1.22	0.31
303 Dihomo-gamma-linolenate from Linoleate	-0.98	-0.67	0.46	-1.65	0.51
306 Arachidonate from gamma-Linolenate	-0.98	-0.67	0.46	-1.65	0.51
246 Proline from Tryptophan	-0.99	0.34	0.32	-0.64	0.46
776 Fatty-acid-VLDL-PE-pool	-0.99	-0.78	0.48	-1.77	0.58
777 Fatty-acid-VLDL-PI-pool	-0.99	-0.78	0.48	-1.77	0.58
637 3-Phosphoserine	-0.99	-0.82	0.59	-1.81	0.58
778 Fatty-acid-VLDL-PS-pool	-0.99	-0.78	0.48	-1.77	0.59
573 1-Acylglycerol-3P-CL-pool	-0.99	-0.22	0.46	-1.21	0.54
223 Glutamine from Tryptophan	-0.99	0.29	0.31	-0.69	0.41
897 Phosphatidate-Bile-PC-pool	-0.99	-0.59	0.43	-1.59	0.54
853 Malonyl-CoA	-1	0.3	0.38	-0.7	0.31
564 1,2-Diacylglycerol-Bile-PC-pool	-1	-0.6	0.38	-1.6	0.48
861 N-Acetylglucosamine-1P	-1	-0.39	0.65	-1.39	0.47
537 (2E)-Hexenoyl-CoA	-1.01	0.25	0.32	-0.75	0.38
161 Cysteine from Methionine	-1.01	-0.71	0.46	-1.72	0.47
874 Nicotinate	-1.01	0.52	0.59	-0.49	0.71
540 (2E)-Octenoyl-CoA	-1.02	0.26	0.32	-0.76	0.38
198 Glycine from Phenylalanine	-1.03	-2.78	0.36	-3.81	0.46
294 Taurine from Methionine	-1.03	-0.7	0.46	-1.73	0.46
872 NeuNGc	-1.03	-0.21	0.34	-1.24	0.3
31 NADH to NADPH transhydrogenase in mito	-1.03	0.17	1	-0.86	1
620 3-Methylcrotonyl-CoA	-1.03	0.28	0.36	-0.76	0.33
594 2,5-Diamino-6-(5-triphosphoryl-3,4-trihydroxy-2-oxopentyl)- amino-4-oxopyrimidine	-1.04	0.31	0.41	-0.73	0.33
833 Isovaleryl-CoA	-1.04	0.3	0.34	-0.74	0.32
891 PG-CL-pool	-1.05	-0.21	0.34	-1.26	0.37
892 PGP-CL-pool	-1.05	-0.21	0.34	-1.26	0.36
595 2,5-Diaminopyrimidine(n)leoside triphosphate	-1.05	0.31	0.42	-0.73	0.31
628 3-Oxohexanoyl-CoA	-1.05	0.3	0.32	-0.76	0.37
220 Glutamine from Proline	-1.05	0.51	0.29	-0.55	0.5
427 Homocysteine degr	-1.06	-0.93	0.39	-1.99	0.42
417 Methionine degr	-1.06	-0.94	0.37	-2	0.4

Continued on next page

Amplitude differences: Treatment difference – continued

Simulation	Δampl	control 1/24h		TGFβ 1/24h	
		ampl	score	ampl	score
285 beta-Alanine from Methionine	-1.07	-0.72	0.43	-1.79	0.44
857 Methylmalonyl-CoA	-1.07	0.27	0.32	-0.8	0.39
929 Serotonin	-1.08	-1.35	0.61	-2.43	0.57
745 Crotonyl-CoA	-1.08	0.28	0.41	-0.79	0.3
898 Phosphatidate-CL-pool	-1.08	-0.22	0.43	-1.3	0.46
243 Proline from Phenylalanine	-1.08	-2.93	0.32	-4.01	0.38
236 Proline from Glycine	-1.09	0.38	0.34	-0.7	0.61
551 (R)-Methylmalonyl-CoA	-1.09	0.31	0.37	-0.77	0.29
949 UDP-activated-N-acetyl-D-galactosamine	-1.09	-0.3	0.62	-1.39	0.36
85 Alanine from Glutamine	-1.1	0.63	0.43	-0.47	0.37
264 Serine from Phenylalanine	-1.1	-2.77	0.38	-3.87	0.47
250 Serine from Alanine	-1.1	-0.67	0.51	-1.76	0.5
945 Tiglyl-CoA	-1.1	0.31	0.31	-0.79	0.36
980 palmitoleoyl-Carnitine	-1.11	-0.86	0.57	-1.97	0.54
764 Dihomo-gamma-linolenoyl-CoA	-1.11	0.28	0.31	-0.83	0.3
643 3-oxooleoyl-CoA	-1.13	0.29	0.37	-0.83	0.29
713 Anthranilate	-1.13	0.32	0.51	-0.81	0.44
450 CoA	-1.14	0.28	0.38	-0.85	0.32
604 2-Methylbutyryl-CoA	-1.14	0.33	0.37	-0.81	0.3
177 Glutamate from Phenylalanine	-1.14	-2.93	0.38	-4.07	0.46
621 3-Methylglutaconyl-CoA	-1.14	0.29	0.36	-0.85	0.29
89 Alanine from Phenylalanine	-1.14	-2.9	0.45	-4.04	0.55
418 Phenylalanine degr	-1.14	-3.23	0.41	-4.38	0.47
83 Alanine from Histidine	-1.15	-0.79	0.33	-1.95	0.42
553 (S)-3-Hydroxy-2-methylbutyryl-CoA	-1.16	0.27	0.34	-0.88	0.34
658 4-Hydroxyphenylpyruvate	-1.16	-3.2	0.53	-4.36	0.63
690 AICAR	-1.16	0.27	0.45	-0.9	0.36
939 Succinyl-CoA	-1.16	0.29	0.37	-0.88	0.32
773 FAICAR	-1.17	0.28	0.48	-0.89	0.35
219 Glutamine from Phenylalanine	-1.17	-2.92	0.35	-4.09	0.41
47 Formylgroup(c)	-1.18	0.16	0.42	-1.02	0.77
88 Alanine from Methionine	-1.19	-0.73	0.47	-1.92	0.49
809 Guanosine	-1.19	0.23	0.4	-0.96	0.32
377 Collagen COIA1(c) synthesis	-1.2	-0.43	1	-1.63	1
603 2-Methylacetoacetyl-CoA	-1.22	0.3	0.33	-0.92	0.36
131 Asparagine from Phenylalanine	-1.23	-2.88	0.38	-4.11	0.43
462 thbpt	-1.23	0.37	0.35	-0.86	0.34
699 Acrylyl-CoA	-1.24	0.3	0.32	-0.93	0.34
110 Arginine from Phenylalanine	-1.25	-2.87	0.37	-4.12	0.42
463 dhbpt	-1.25	0.36	0.32	-0.89	0.38
152 Aspartate from Phenylalanine	-1.26	-2.89	0.45	-4.15	0.52
616 3-Hydroxypropionyl-CoA	-1.26	0.3	0.32	-0.95	0.34
286 beta-Alanine from Phenylalanine	-1.26	-2.87	0.41	-4.13	0.47
52 Acetyl group(r)	-1.26	-0.45	0.43	-1.72	0.45
49 Acetyl group(c)	-1.26	-0.45	0.43	-1.72	0.45
956 Xanthurenate	-1.27	-0.88	0.46	-2.16	0.59
657 4-(2-Amino-3-hydroxyphenyl)-2,4-dioxobutanoate	-1.27	-0.88	0.46	-2.16	0.59
509 Guanine	-1.29	0.23	0.35	-1.06	0.34
508 Xanthine	-1.29	0.23	0.36	-1.06	0.36
51 Acetyl group(p)	-1.29	-0.46	0.34	-1.75	0.37
811 H2S	-1.3	-1.11	0.5	-2.4	0.49
911 Propanoyl-CoA	-1.3	0.29	0.33	-1.01	0.31
867 N-Formyl-GAR	-1.3	0.33	0.43	-0.98	0.32
472 dATP	-1.31	0.27	0.37	-1.04	0.37
36 GSH reduction using NADH redox potential	-1.31	-0.24	0.73	-1.55	0.6
504 XMP	-1.32	0.25	0.37	-1.08	0.39
446 ApoB100 degr	-1.33	-0.7	0.6	-2.03	0.48
814 HMG-CoA	-1.33	0.3	0.38	-1.04	0.29
503 IMP	-1.33	0.25	0.39	-1.09	0.4

Continued on next page

Amplitude differences: Treatment difference – continued

Simulation	∆ampl	control 1/24h		TGFβ 1/24h	
		ampl	score	ampl	score
476 ATP	-1.34	0.27	0.38	-1.07	0.38
200 Glycine from Serine	-1.35	0.16	0.52	-1.19	0.62
44 Activated methyl group (THF)	-1.35	0.16	0.52	-1.19	0.62
474 dGTP	-1.35	0.29	0.36	-1.06	0.34
782 Fatty-acid-VLDL-TG3-pool	-1.37	-0.22	0.53	-1.58	0.61
80 Alanine from Cystine	-1.37	-0.92	0.47	-2.29	0.56
478 GTP	-1.38	0.29	0.38	-1.08	0.35
314 Creatine	-1.39	0.6	0.27	-0.79	0.47
24 NADH redox potential into mito	-1.42	0.64	0.53	-0.78	0.55
638 3-Sulfinoalanine	-1.43	-2.99	0.72	-4.43	0.72
844 L-Palmitoylcarnitine	-1.45	-0.5	0.48	-1.95	0.54
977 linoleic-Carnitine	-1.45	-0.49	0.51	-1.94	0.54
714 Arachidonyl-Carnitine	-1.45	-0.49	0.51	-1.94	0.54
674 5-Phosphoribosylamine	-1.46	0.34	0.35	-1.12	0.31
722 CDP-ethanolamine	-1.5	0.33	0.31	-1.17	0.32
310 Bilirubin conjugation	-1.5	-0.43	0.54	-1.93	0.53
118 Asparagine from Alanine	-1.52	-0.55	0.38	-2.07	0.38
97 Arginine from Alanine	-1.53	-0.48	0.36	-2.01	0.42
886 Oxalosuccinate	-1.54	-0.67	0.37	-2.21	0.48
407 Alanine degr	-1.55	-0.66	0.33	-2.21	0.42
56 UDP-activated glucuronate	-1.56	-0.47	0.4	-2.03	0.52
312 Urea from alanine	-1.57	-0.64	0.32	-2.2	0.41
313 Urea from NH3	-1.57	-0.64	0.32	-2.2	0.41
500 udpglcur	-1.58	-0.55	0.31	-2.14	0.35
343 Collagen CD36(c) synthesis	-1.63	0.71	1	-0.91	1
449 Chitin-component degr	-1.63	0.67	0.39	-0.96	0.37
793 GSSG	-1.63	-0.22	0.48	-1.85	0.61
469 ametam	-1.71	0.49	0.31	-1.21	0.34
925 Saccharopine	-1.74	-1.92	0.48	-3.66	0.57
976 gamma-Linolenoyl-CoA	-1.77	0.29	0.37	-1.47	0.27
510 Adenosine	-1.77	0.27	0.36	-1.5	0.39
855 Mercaptopyruvate	-1.79	-0.66	0.4	-2.45	0.39
950 UDP-activated-xylose	-1.79	-0.51	0.41	-2.3	0.5
290 beta-Alanine from Tryptophan	-1.79	-0.14	0.32	-1.93	0.39
606 2-Oxoadipate	-2.42	-0.63	0.33	-3.04	0.35
87 Alanine from Lysine	-2.43	-0.62	0.32	-3.06	0.35
784 Formylanthranilate	-2.5	0.35	0.67	-2.15	0.5
489 Tyrosine	-2.76	-1.51	0.65	-4.27	0.65
938 Succinate	-2.78	0.44	0.38	-2.34	0.37
79 Alanine from Cysteine	-2.85	0.46	0.4	-2.39	0.45
480 Alanine	-2.9	0.5	0.55	-2.41	0.51
896 Pantetheine	-3.12	3	0.31	-0.12	0.33
411 Cysteine degr	-3.24	-1.34	0.34	-4.58	0.38
448 Ethanol degr	-3.27	-0.52	0.64	-3.79	0.55
743 Citrulline	-3.91	-0.11	0.36	-4.03	0.31
54 Glucose-6P	-5.23	0.61	0.51	-4.62	0.51

2.3 Amplitude differences: Period difference, control

Table 9: Amplitude differences: Period difference, control

Simulation	Δampl	control 1/6h		control 6/24h	
		ampl	score	ampl	score
352 Collagen CO4A5(c) synthesis	1.38	0.11	1	1.49	1
890 PEP	1.18	-2.56	0.52	-1.39	0.49
784 Formylanthranilate	1.04	-0.71	0.4	0.33	0.58
829 Inositol	1.04	0.34	0.7	1.38	0.6
830 Inositol-1P	1.04	0.37	1	1.4	1
834 Kynurenine	0.94	-0.63	0.42	0.31	0.35
743 Citrulline	0.92	-1.01	0.3	-0.09	0.39
454 Glycogenin	0.91	-0.24	0.96	0.67	0.96
807 Glycogenin-G8	0.91	-0.23	0.96	0.67	0.95
803 Glycogenin-G11	0.91	-0.23	0.96	0.67	0.95
806 Glycogenin-G7G1	0.91	-0.23	0.95	0.67	0.94
804 Glycogenin-G4G4	0.91	-0.23	0.95	0.67	0.94
805 Glycogenin-G7	0.91	-0.23	0.95	0.67	0.94
71 Glycogen glucose storage	0.91	-0.23	0.74	0.67	0.65
386 Collagen CORA1(c) synthesis	0.83	0.04	1	0.87	1
165 Glutamate from Arginine	0.8	-1.07	0.37	-0.27	0.45
428 beta-Alanine degr	0.77	-0.68	0.32	0.09	0.32
207 Glutamine from Arginine	0.73	-0.93	0.36	-0.2	0.36
140 Aspartate from Arginine	0.71	-0.95	0.34	-0.24	0.37
298 Palmitolate from Palmitate	0.67	-0.47	0.36	0.2	0.36
896 Pantetheine	0.66	-0.51	0.33	0.15	0.35
747 Cysteamine	0.66	-0.51	0.42	0.15	0.45
42 Na+ exportgradient	0.58	-0.32	1	0.26	1
225 Glutamine from Valine	0.58	-0.23	0.31	0.36	0.29
638 3-Sulfinoalanine	0.57	-0.51	0.79	0.05	0.33
295 Taurine from Cysteine	0.55	-0.5	0.57	0.05	0.6
826 Hypotaurine	0.55	-0.5	0.57	0.05	0.6
756 Dehydroalanine	0.55	-0.66	0.72	-0.11	0.63
408 Arginine degr	0.55	-0.78	0.35	-0.23	0.29
429 Ornithine degr	0.52	-0.75	0.34	-0.23	0.3
360 Collagen CO6A5(c) synthesis	0.5	-0.31	0.33	0.19	0.42
394 Collagen ITA1(c) synthesis	0.5	-0.31	0.35	0.19	0.42
906 Phosphodimethylethanolamine	0.49	-0.19	1	0.3	1
905 Phosphocholine	0.49	-0.19	1	0.3	1
343 Collagen CD36(c) synthesis	0.48	0.12	1	0.6	1
328 Antitrypsin	0.48	-0.29	0.34	0.19	0.41
893 PPi	0.48	0.08	0.45	0.56	0.49
326 Fibrinogen	0.47	-0.29	0.33	0.18	0.39
349 Collagen CO4A2(c) synthesis	0.47	0.14	1	0.61	1
837 L-Cystathionine	0.45	-0.14	0.51	0.32	0.48
347 Collagen CO3A1(c) synthesis	0.44	0.11	1	0.55	1
613 3-Hydroxyanthranilate	0.42	-0.15	0.38	0.27	0.37
436 Mannose degr	0.42	-0.07	0.64	0.35	0.83
307 Arachidonate from Dihomo-gamma-linolenate	0.42	-0.64	0.49	-0.22	0.4
302 gamma-Linolenate from Linoleate	0.42	-0.64	0.43	-0.22	0.5
938 Succinate	0.41	-0.18	0.36	0.23	0.33
257 Serine from Glycine	0.4	-0.02	0.46	0.38	0.46
304 Arachidonate from Linoleate	0.4	-0.62	0.39	-0.22	0.46
447 Glycogenin degr	0.4	-0.35	0.54	0.05	0.34
435 Galactose degr	0.4	-0.02	0.58	0.38	0.5
768 Dimethylallyl-PP	0.39	-0.63	0.6	-0.23	0.65
438 Antichymotrypsin degr	0.38	-0.33	0.5	0.05	0.35
375 Collagen COGA1(c) synthesis	0.38	-0.15	1	0.23	1
303 Dihomo-gamma-linolenate from Linoleate	0.37	-0.59	0.36	-0.22	0.42
306 Arachidonate from gamma-Linolenate	0.37	-0.59	0.36	-0.22	0.42
845 Lanosterol	0.36	-0.49	0.64	-0.13	0.59

Continued on next page

Amplitude differences: Period difference, control – continued

Simulation	Δampl	control 1/6h		control 6/24h	
		ampl	score	ampl	score
124 Asparagine from Glycine	0.35	-0.59	0.36	-0.25	0.49
843 L-Oleoylcarnitine	0.35	-0.11	0.52	0.24	0.51
81 Alanine from Glutamate	0.34	-0.15	0.34	0.19	0.32
925 Saccharopine	0.34	-1.12	0.42	-0.78	0.6
910 Propanoate	0.34	-0.53	0.33	-0.19	0.67
398 Collagen SCRB1(c) synthesis	0.34	-0.38	1	-0.04	1
836 L-2-Aminoadipate	0.33	0.04	1	0.38	1
359 Collagen CO6A3(c) synthesis	0.32	-0.11	1	0.22	1
213 Glutamine from Glycine	0.32	-0.62	0.32	-0.3	0.44
449 Chitin-component degr	0.31	0.21	0.3	0.52	0.39
170 Glutamate from Glycine	0.31	-0.62	0.32	-0.31	0.54
795 Geranyl-PP	0.31	-0.55	0.62	-0.23	0.6
344 Collagen CO1A1(c) synthesis	0.31	-0.02	1	0.29	1
399 Collagen SERPH(c) synthesis	0.31	0.17	1	0.48	1
355 Collagen CO5A2(c) synthesis	0.31	0.02	1	0.33	1
371 Collagen COCA1(c) synthesis	0.3	-0.21	1	0.1	1
145 Aspartate from Glycine	0.3	-0.59	0.43	-0.29	0.47
786 Fructose-2,6PP	0.3	-0.4	0.99	-0.1	0.84
63 Farnesyl-PP	0.29	-0.53	0.63	-0.23	0.6
495 Mannose-6P	0.28	-0.07	0.78	0.22	0.78
931 Sphinganine-1P	0.28	-0.11	0.4	0.17	0.41
907 Phosphopantetheine	0.28	-0.12	0.39	0.16	0.29
607 2-Oxobutyrate	0.28	-0.6	0.75	-0.32	0.45
549 (R)-5-Diphosphomevalonate	0.27	-0.51	0.68	-0.24	0.72
103 Arginine from Glycine	0.27	-0.58	0.33	-0.31	0.34
395 Collagen ITA2(c) synthesis	0.27	-0.16	1	0.11	1
987 trans-4-Hydroxy-L-proline	0.27	0.2	1	0.47	1
835 L-1-Pyrroline-3-hydroxy-5-carboxylate	0.27	0.2	1	0.47	1
62 Isopentenyl-PP	0.27	-0.5	0.63	-0.23	0.77
348 Collagen CO4A1(c) synthesis	0.25	-0.34	1	-0.08	1
592 1-Pyrroline-5-carboxylate	0.25	0.2	0.76	0.46	0.88
24 NADH redox potential into mito	0.25	0.2	0.64	0.45	0.52
943 Thymidine	0.24	-0.6	0.26	-0.36	0.39
338 Haptoglobin	0.24	-0.01	0.96	0.23	0.98
552 (R)-Mevalonate	0.23	-0.51	0.82	-0.28	0.8
345 Collagen CO1A2(c) synthesis	0.23	0.03	1	0.26	1
550 (R)-5-Phosphomevalonate	0.23	-0.48	0.61	-0.25	0.78
31 NADH to NADPH transhydrogenase in mito	0.22	-0.03	1	0.2	1
139 Aspartate from Alanine	0.22	-0.17	0.72	0.04	0.47
293 Homocysteine from Methionine	0.21	0.13	1	0.34	1
560 (S)-Dihydroorotate	0.21	-0.15	0.42	0.06	0.29
713 Anthranilate	0.21	0.12	0.44	0.32	0.44
865 N-Carbamoyl-L-aspartate	0.2	-0.15	0.47	0.05	0.38
886 Oxalosuccinate	0.2	-0.16	0.55	0.04	0.39
439 Antitrypsin degr	0.2	-0.15	0.55	0.05	0.38
726 Carbamoyl-P	0.2	-0.14	0.55	0.05	0.62
154 Aspartate from Serine	0.2	-0.15	0.39	0.05	0.48
133 Asparagine from Serine	0.19	-0.15	0.44	0.05	0.36
812 HCO3-	0.19	-0.14	0.79	0.05	0.92
727 Carbonate	0.19	-0.14	0.79	0.05	0.92
178 Glutamate from Proline	0.19	-0.14	0.52	0.05	0.43
85 Alanine from Glutamine	0.19	0.3	0.35	0.49	0.36
183 Glutamate from Valine	0.18	-0.25	0.39	-0.07	0.31
934 Squalene	0.18	-0.32	0.8	-0.14	0.79
909 Presqualene-PP	0.18	-0.32	0.8	-0.14	0.79
116 Arginine from Valine	0.17	-0.25	0.31	-0.08	0.26
656 4,4-Dimethyl-5alpha-cholesta-8,14,24-trien-3beta-ol	0.16	-0.54	0.69	-0.38	0.66
374 Collagen COFA1(c) synthesis	0.16	-0.2	1	-0.04	1
158 Aspartate from Valine	0.15	-0.23	0.38	-0.07	0.32

Continued on next page

Amplitude differences: Period difference, control – continued

Simulation		Δampl	control 1/6h ampl	control 1/6h score	control 6/24h ampl	control 6/24h score
423	Valine degr	0.15	-0.23	0.34	-0.08	0.27
388	Collagen CTHR1(c) synthesis	0.15	-0.07	1	0.08	1
593	14-Demethyllanosterol	0.15	-0.53	0.65	-0.38	0.69
379	Collagen COKA1(c) synthesis	0.14	-0.16	1	-0.02	1
626	3-Oxohexadecanoyl-ACP	0.14	-0.11	0.86	0.03	0.8
547	(R)-3-Hydroxypalmitoyl-ACP	0.14	-0.11	0.86	0.03	0.8
534	(2E)-Hexadecenoyl-ACP	0.14	-0.11	0.86	0.03	0.79
816	Hexadecanoyl-ACP	0.14	-0.11	0.86	0.03	0.79
633	3-Oxostearoyl-ACP	0.14	-0.11	0.86	0.03	0.79
617	3-Hydroxystearoyl-ACP	0.14	-0.11	0.86	0.03	0.78
538	(2E)-Octadecenoyl-ACP	0.14	-0.11	0.86	0.03	0.78
936	Stearoyl-ACP	0.14	-0.11	0.86	0.03	0.78
634	3-Oxotetradecanoyl-ACP	0.14	-0.11	0.86	0.03	0.8
813	HMA	0.14	-0.11	0.86	0.03	0.8
541	(2E)-Tetradecenoyl-ACP	0.14	-0.11	0.86	0.03	0.8
942	Tetradecanoyl-ACP	0.14	-0.11	0.86	0.03	0.8
624	3-Oxododecanoyl-ACP	0.14	-0.11	0.86	0.03	0.8
749	D-3-Hydroxydodecanoyl-ACP	0.14	-0.11	0.86	0.03	0.8
532	(2E)-Dodecenoyl-ACP	0.14	-0.11	0.86	0.03	0.8
769	Dodecanoyl-ACP	0.14	-0.11	0.86	0.03	0.8
622	3-Oxodecanoyl-ACP	0.14	-0.11	0.86	0.03	0.81
545	(R)-3-Hydroxydecanoyl-ACP	0.14	-0.11	0.86	0.03	0.81
530	(2E)-Decenoyl-ACP	0.14	-0.11	0.86	0.03	0.81
754	Decanoyl-ACP	0.14	-0.11	0.86	0.03	0.81
629	3-Oxooctanoyl-ACP	0.14	-0.11	0.87	0.03	0.81
546	(R)-3-Hydroxyoctanoyl-ACP	0.14	-0.11	0.87	0.03	0.81
539	(2E)-Octenoyl-ACP	0.14	-0.11	0.87	0.03	0.81
881	Octanoyl-ACP	0.14	-0.11	0.87	0.03	0.81
978	mitoACP	0.14	-0.11	0.75	0.03	0.68
627	3-Oxohexanoyl-ACP	0.14	-0.11	0.87	0.03	0.82
750	D-3-Hydroxyhexanoyl-ACP	0.14	-0.11	0.87	0.03	0.82
536	(2E)-Hexenoyl-ACP	0.14	-0.11	0.87	0.03	0.82
818	Hexanoyl-ACP	0.14	-0.11	0.87	0.03	0.82
544	(R)-3-Hydroxybutanoyl-ACP	0.14	-0.11	0.87	0.03	0.83
717	But-2-enoyl-ACP	0.14	-0.11	0.87	0.03	0.83
718	Butyryl-ACP	0.14	-0.11	0.87	0.03	0.83
851	Malonyl-ACP	0.14	-0.11	0.86	0.03	0.83
695	Acetoacetyl-ACP	0.14	-0.11	0.86	0.03	0.83
457	Apo-ACP	0.14	-0.11	0.91	0.03	0.89
688	ACP	0.14	-0.11	0.87	0.03	0.85
697	Acetyl-ACP	0.14	-0.11	0.87	0.03	0.84
880	OAA	0.14	-0.1	0.7	0.04	0.75
458	Apo-ACP(m)	0.14	-0.11	0.75	0.03	0.71
487	Proline	0.13	-0.2	0.34	-0.07	0.31
521	Ceramide	0.13	-0.42	0.27	-0.29	0.4
58	GDP-activated fucose	0.12	0.13	0.67	0.25	0.62
839	L-Fucose-1P	0.12	0.13	0.53	0.25	0.49
850	Malate	0.12	-0.08	0.65	0.04	0.64
2	Aerobic ATP rephosph (gluc)	0.11	-0.07	0.45	0.05	0.33
59	GDP-activated mannose	0.11	0.12	0.65	0.24	0.4
52	Acetyl group(r)	0.1	-0.37	0.41	-0.27	0.43
49	Acetyl group(c)	0.1	-0.37	0.41	-0.27	0.43
54	Glucose-6P	0.1	0.25	0.51	0.36	0.51
832	Isocitrate	0.1	-0.16	0.48	-0.06	0.4
848	Linoleate	0.1	-0.56	0.49	-0.46	0.6
383	Collagen CONA1(c) synthesis	0.1	-0.13	1	-0.03	1
823	Hydracrylate	0.1	-0.59	0.38	-0.49	0.49
854	Maltose	0.1	0.03	1	0.12	1
11	ATP from NADH(m)	0.1	-0.05	0.83	0.04	0.68

Continued on next page

Simulation	Δampl	control 1/6h		control 6/24h	
		ampl	score	ampl	score
618 3-Keto-4-methylzymosterol	0.1	-0.51	0.73	-0.42	0.78
153 Aspartate from Proline	0.09	-0.16	0.38	-0.07	0.31
446 ApoB100 degr	0.09	-0.46	0.59	-0.37	0.51
735 Cholesterol-ester-lin	0.08	-0.53	0.59	-0.44	0.67
461 4ppan	0.08	-0.13	1	-0.04	1
356 Collagen CO5A3(c) synthesis	0.08	-0.07	1	0.005	1
365 Collagen CO9A1(c) synthesis	0.08	-0.04	1	0.04	1
339 Collagen ADIPO(c) synthesis	0.08	-0.02	1	0.06	1
14 Ubiquinol-to-ATP	0.08	-0.04	0.8	0.04	0.56
12 ATP from NADH	0.07	-0.04	0.44	0.03	0.53
663 4alpha-Methylzymosterol-4-carboxylate	0.07	-0.49	0.72	-0.41	0.76
235 Proline from Glutamate	0.07	-0.14	0.51	-0.06	0.44
734 Cholesterol-ester-gla	0.07	-0.52	0.59	-0.45	0.68
387 Collagen COSA1(c) synthesis	0.07	0.13	0.37	0.2	0.43
381 Collagen COLQ(c) synthesis	0.07	0.13	0.37	0.2	0.42
380 Collagen COLA1(c) synthesis	0.07	0.13	0.37	0.2	0.43
520 SM	0.07	-0.39	0.27	-0.32	0.41
397 Collagen PCOTH(c) synthesis	0.07	0.13	0.36	0.2	0.44
822 Homovanillate	0.07	-0.04	1	0.03	1
739 Cholesterol-ester-pool	0.07	-0.52	0.58	-0.45	0.65
390 Collagen FCN1(c) synthesis	0.07	0.13	0.35	0.2	0.42
957 Zymosterol	0.06	-0.5	0.72	-0.44	0.77
662 4alpha-Methylzymosterol	0.06	-0.5	0.72	-0.44	0.77
732 Cholestenol	0.06	-0.5	0.68	-0.44	0.79
392 Collagen FCN3(c) synthesis	0.06	0.13	0.35	0.2	0.43
947 Triphosphate degr	0.06	0.04	1	0.1	1
800 Glutamyl-5P	0.06	0.07	0.34	0.13	0.37
361 Collagen CO6A6(c) synthesis	0.06	0.13	0.33	0.19	0.42
137 Asparagine from Valine	0.06	-0.21	0.34	-0.15	0.29
738 Cholesterol-ester-palmn	0.06	-0.5	0.6	-0.44	0.69
740 Cholesterol-ester-stea	0.06	-0.49	0.63	-0.44	0.69
736 Cholesterol-ester-ol	0.06	-0.49	0.63	-0.44	0.69
501 GDP-L-fucose	0.05	0.16	0.46	0.22	0.36
915 Provitamin D3	0.05	-0.51	0.66	-0.46	0.75
523 Cholesterol	0.05	-0.5	0.68	-0.45	0.76
763 Desmosterol	0.05	-0.5	0.72	-0.45	0.74
846 Lathosterol	0.05	-0.51	0.7	-0.46	0.79
682 7-Dehydrodesmosterol	0.05	-0.51	0.7	-0.46	0.73
884 Orotate	0.05	-0.14	0.41	-0.09	0.36
676 5alpha-Cholesta-7,24-dien-3beta-ol	0.05	-0.51	0.74	-0.46	0.77
894 PRPP	0.05	0.12	0.4	0.17	0.47
287 beta-Alanine from Proline	0.05	-0.16	0.32	-0.11	0.39
918 Quinolinate	0.05	0	0.38	0.05	0.6
686 7alpha-Hydroxycholest-4-en-3-one	0.04	-0.49	0.64	-0.45	0.74
687 7alpha-Hydroxycholesterol	0.04	-0.49	0.68	-0.45	0.72
113 Arginine from Threonine	0.04	-0.57	0.32	-0.53	0.37
674 5-Phosphoribosylamine	0.04	0.12	0.45	0.16	0.42
733 Cholesterol-ester-arach	0.04	-0.49	0.6	-0.46	0.7
920 Ribose-5P	0.04	0.1	0.36	0.14	0.44
721 CDP-choline	0.03	0.2	0.38	0.23	0.31
684 7alpha,12alpha-Dihydroxycholest-4-en-3-one	0.03	-0.48	0.66	-0.45	0.69
524 gdpddman	0.03	0.17	0.42	0.2	0.33
97 Arginine from Alanine	0.03	-0.38	0.38	-0.35	0.31
790 GAR	0.03	0.15	0.5	0.18	0.44
416 Lysine degr	0.03	-0.9	0.33	-0.87	0.37
870 NADH	0.03	0.14	0.52	0.17	0.35
685 7alpha-Hydroxy-5beta-cholestan-3-one	0.03	-0.48	0.65	-0.45	0.75
451 NAD+	0.03	0.14	0.51	0.17	0.35
679 5beta-Cholestane-3alpha,7alpha-diol	0.02	-0.47	0.64	-0.45	0.73

Continued on next page

Simulation	Δampl	control 1/6h		control 6/24h	
		ampl	score	ampl	score
689 ADP	0.02	0.17	0.49	0.18	0.34
753 Deamido-NAD	0.02	0.15	0.51	0.17	0.39
772 FADH2	0.02	0.15	0.48	0.17	0.33
940 Sulfite degr	0.02	-0.04	1	-0.03	1
692 AMP	0.02	0.17	0.49	0.18	0.35
888 PAP	0.02	0.17	0.47	0.18	0.33
683 7alpha,12alpha-Dihydroxy-5beta-cholestan-3-one	0.02	-0.47	0.67	-0.45	0.71
615 3-Hydroxyisobutyryl-CoA	0.02	0.18	0.41	0.19	0.31
711 Adenylyl(s)ulfate	0.02	0.17	0.49	0.18	0.36
958 cAMP	0.02	0.17	0.44	0.18	0.33
867 N-Formyl-GAR	0.02	0.15	0.49	0.17	0.38
647 3alpha,7alpha,12alpha-Trihydroxy-5beta-24-oxocholestanoyl-CoA	0.02	-0.46	0.39	-0.44	0.48
924 SAM	0.01	0.17	0.48	0.19	0.39
922 SAH	0.01	0.17	0.48	0.19	0.39
762 Dephospho-CoA	0.01	0.17	0.43	0.18	0.32
964 dADP	0.01	0.17	0.48	0.18	0.31
559 (S)-3-hydroxypalmitoleoyl-CoA	0.01	0.17	0.4	0.18	0.3
984 trans,cis-hexadeca-2,9-dienoyl-CoA	0.01	0.17	0.41	0.18	0.31
652 3alpha,7alpha,12alpha-Trihydroxycoprostane	0.01	-0.46	0.66	-0.45	0.69
986 trans,cis-octadeca-2,9-dienoyl-CoA	0.01	0.17	0.41	0.18	0.31
475 dTTP	0.01	0.24	0.41	0.26	0.41
646 3alpha,7alpha,12alpha,24-Tetrahydroxy-5beta-cholestanoyl-CoA	0.01	-0.46	0.4	-0.45	0.47
350 Collagen CO4A3(c) synthesis	0.01	-0.14	1	-0.13	1
419 Proline degr	0.01	-0.21	0.3	-0.2	0.28
594 2,5-Diamino-6-(5-triphosphoryl-3,4-trihydroxy-2-oxopentyl)- amino-4-oxopyrimidine	0.01	0.16	0.49	0.17	0.34
319 Chenodiol(b)	0.01	-0.46	0.46	-0.45	0.6
354 Collagen CO5A1(c) synthesis	0.01	0.001	1	0.01	1
316 Gly-CD-cholate(b)	0.01	-0.46	0.49	-0.45	0.55
41 Na+ importgradient	0.01	0.05	0.49	0.06	0.59
315 Glycocholate(b)	0.01	-0.44	0.51	-0.44	0.57
737 Cholesterol-ester-palm	0.01	-0.5	0.56	-0.49	0.65
502 GDP-mannose	0.01	0.17	0.43	0.18	0.3
595 2,5-Diaminopyrimidine(n)leoside triphosphate	0.01	0.16	0.49	0.17	0.34
648 3alpha,7alpha,12alpha-Trihydroxy-5beta-cholest-24-enoyl-CoA	0.01	-0.46	0.4	-0.45	0.46
535 (2E)-Hexadecenoyl-CoA	0.01	0.17	0.4	0.18	0.32
981 palmitoleoyl-CoA	0.01	0.17	0.4	0.18	0.32
965 dAMP	0.01	0.18	0.49	0.19	0.35
681 6-Pyruvoyltetrahydropterin	0.01	0.17	0.47	0.17	0.33
643 3-oxooleoyl-CoA	0.01	0.17	0.38	0.18	0.31
960 cis-(3S)-hydroxytetradec-7-enoyl-CoA	0.01	0.17	0.4	0.17	0.29
698 Acetyl-CoA	0.01	0.18	0.42	0.18	0.35
985 trans,cis-myristo-2,7-dienoyl-CoA	0.005	0.17	0.41	0.17	0.29
701 Acyl-CoA-CL-pool	0.005	0.17	0.39	0.18	0.32
976 gamma-Linolenoyl-CoA	0.005	0.17	0.38	0.18	0.31
937 Stearoyl-CoA	0.005	0.17	0.4	0.18	0.32
883 Oleoyl-CoA	0.005	0.17	0.4	0.18	0.32
849 Linoleoyl-CoA	0.005	0.17	0.4	0.18	0.32
715 Arachidonyl-CoA	0.005	0.17	0.4	0.18	0.32
959 cGMP	0.005	0.16	0.48	0.17	0.35
895 Palmitoyl-CoA	0.005	0.17	0.4	0.18	0.32
705 Acyl-CoA-VLDL-PS-pool	0.005	0.17	0.4	0.18	0.32
704 Acyl-CoA-VLDL-PI-pool	0.005	0.17	0.4	0.18	0.32
700 Acyl-CoA-Bile-PC-pool	0.005	0.17	0.39	0.18	0.32
708 Acyl-CoA-VLDL-TG3-pool	0.005	0.17	0.4	0.18	0.32
707 Acyl-CoA-VLDL-TG2-pool	0.005	0.17	0.39	0.18	0.32
703 Acyl-CoA-VLDL-PE-pool	0.005	0.17	0.39	0.18	0.32
702 Acyl-CoA-VLDL-PC-pool	0.005	0.17	0.39	0.18	0.32
706 Acyl-CoA-VLDL-SM-pool	0.005	0.17	0.39	0.18	0.32
525 beta-Alanine	0.004	-0.09	0.32	-0.08	0.34

Continued on next page

Amplitude differences: Period difference, control – continued

Simulation	Δampl	control 1/6h		control 6/24h	
		ampl	score	ampl	score
792 GMP	0.004	0.16	0.44	0.17	0.34
939 Succinyl-CoA	0.003	0.17	0.37	0.18	0.32
610 3(S)-3-hydroxydodecen-(5Z)-oyl-CoA	0.003	0.17	0.41	0.17	0.29
983 trans,cis-dodeca-2,5-dienoyl-CoA	0.002	0.17	0.41	0.17	0.29
644 3-oxopalmitoleoyl-CoA	0.002	0.17	0.42	0.17	0.31
963 cis-myrist-7-enoyl-CoA	0.002	0.17	0.41	0.17	0.3
787 Fumarate	0.002	0.04	0.51	0.04	0.48
710 Adenylosuccinate	0.002	0.18	0.5	0.18	0.39
853 Malonyl-CoA	0.001	0.18	0.39	0.18	0.33
642 3-oxomyrist-7-enoyl-CoA	0.001	0.17	0.41	0.17	0.3
962 cis-laur-5-enoyl-CoA	0.001	0.17	0.41	0.17	0.29
783 Formamidopyrimidine(n)leoside triphosphate	0.001	0.17	0.48	0.17	0.35
620 3-Methylcrotonyl-CoA	0.001	0.21	0.36	0.21	0.29
678 5beta-Cholestane-3alpha,7alpha,26-triol	0.0004	-0.46	0.65	-0.46	0.74
655 3alpha,7alpha-Dihydroxy-5beta-cholestanoyl-CoA	0.0004	-0.46	0.41	-0.46	0.5
654 3alpha,7alpha-Dihydroxy-5beta-cholestanate	0	-0.46	0.58	-0.46	0.71
814 HMG-CoA	-0.0003	0.18	0.38	0.18	0.33
696 Acetoacetyl-CoA	-0.0003	0.18	0.41	0.18	0.35
555 (S)-3-Hydroxydodecanoyl-CoA	-0.0003	0.17	0.4	0.17	0.27
533 (2E)-Dodecenoyl-CoA	-0.0004	0.17	0.4	0.17	0.28
551 (R)-Methylmalonyl-CoA	-0.0005	0.18	0.35	0.18	0.35
653 3alpha,7alpha-Dihydroxy-5beta-cholestan-26-al	-0.001	-0.46	0.62	-0.46	0.75
641 3-oxolaur-cis-5-enoyl-CoA	-0.001	0.17	0.41	0.17	0.29
543 (3Z)-Dodecenoyl-CoA	-0.001	0.17	0.41	0.17	0.29
206 Glutamine from Alanine	-0.004	-0.35	0.34	-0.35	0.42
490 Palmitate	-0.004	-0.14	0.85	-0.15	0.74
558 (S)-3-hydroxyoleyleoyl-CoA	-0.004	0.18	0.41	0.17	0.31
856 Methacrylyl-CoA	-0.01	0.18	0.4	0.18	0.3
833 Isovaleryl-CoA	-0.01	0.19	0.34	0.18	0.29
675 5-Phosphoribosylformylglycinamidine	-0.01	0.17	0.5	0.17	0.36
651 3alpha,7alpha,12alpha-Trihydroxy-5beta-cholestanoyl-CoA	-0.01	-0.46	0.44	-0.46	0.48
64 Glucosamine-6P	-0.01	-0.08	0.64	-0.09	0.63
677 5beta-Cholestane-3alpha,7alpha,12alpha,26-tetrol	-0.01	-0.45	0.66	-0.46	0.7
650 3alpha,7alpha,12alpha-Trihydroxy-5beta-cholestanate	-0.01	-0.45	0.66	-0.46	0.7
649 3alpha,7alpha,12alpha-Trihydroxy-5beta-cholestan-26-al	-0.01	-0.45	0.66	-0.46	0.7
111 Arginine from Proline	-0.01	-0.19	0.29	-0.2	0.32
453 FAD	-0.01	0.17	0.47	0.16	0.37
967 dCMP	-0.01	0.19	0.49	0.18	0.26
301 Palmitolate from Arachidonate	-0.01	-0.14	0.62	-0.15	0.61
831 Isobutyryl-CoA	-0.01	0.19	0.42	0.18	0.35
966 dCDP	-0.01	0.2	0.43	0.18	0.27
493 Stearate	-0.01	-0.14	0.85	-0.15	0.74
691 AIR	-0.02	0.18	0.51	0.16	0.37
720 CDP	-0.02	0.2	0.44	0.18	0.3
421 Threonine degr	-0.02	-0.66	0.34	-0.68	0.35
324 Cholesterol(b)	-0.02	-0.48	0.7	-0.5	0.73
745 Crotonyl-CoA	-0.02	0.2	0.41	0.18	0.33
492 Oleate	-0.02	-0.14	0.73	-0.16	0.67
791 GDP	-0.02	0.18	0.47	0.16	0.39
968 dGDP	-0.02	0.18	0.46	0.16	0.35
665 5,6-Dihydrouracil	-0.02	-0.38	0.35	-0.4	0.43
554 (S)-3-Hydroxybutyryl-CoA	-0.02	0.2	0.42	0.18	0.34
317 Taurocholate(b)	-0.03	-0.44	0.49	-0.46	0.5
712 Agmatine	-0.03	0.02	0.6	-0.01	0.6
969 dGMP	-0.03	0.19	0.47	0.16	0.36
757 Deoxyadenosine	-0.03	0.22	0.46	0.19	0.28
318 tcdchola(b)	-0.03	-0.45	0.47	-0.48	0.49
442 Haptoglobin degr	-0.03	-0.36	0.57	-0.39	0.36
640 3-Ureidopropionate	-0.03	-0.35	0.37	-0.38	0.48

Continued on next page

Amplitude differences: Period difference, control – continued

Simulation	Δampl	control 1/6h		control 6/24h	
		ampl	score	ampl	score
955 Xanthosine	-0.03	0.19	0.41	0.16	0.3
801 Glutaryl-CoA	-0.03	0.2	0.38	0.16	0.34
273 beta-Alanine from Arginine	-0.03	-0.11	0.29	-0.14	0.37
478 GTP	-0.04	0.2	0.4	0.16	0.29
132 Asparagine from Proline	-0.04	-0.15	0.33	-0.19	0.39
621 3-Methylglutaconyl-CoA	-0.04	0.21	0.41	0.17	0.31
119 Asparagine from Arginine	-0.04	-0.14	0.32	-0.18	0.28
604 2-Methylbutyryl-CoA	-0.04	0.22	0.45	0.18	0.33
368 Collagen COAA1(c) synthesis	-0.04	-0.03	1	-0.07	1
389 Collagen EMID2(c) synthesis	-0.04	-0.07	1	-0.11	1
481 Arginine	-0.04	-0.24	0.28	-0.28	0.34
671 5-Methylthioadenosine	-0.04	0.23	0.49	0.19	0.36
506 Uridine	-0.04	0.25	0.43	0.21	0.33
709 Adenine	-0.04	0.23	0.48	0.19	0.39
282 beta-Alanine from Glutamine	-0.04	-0.07	0.36	-0.11	0.35
473 dCTP	-0.05	0.22	0.44	0.17	0.28
463 dhbpt	-0.05	0.19	0.31	0.14	0.28
445 ApoTransferin degr	-0.05	-0.35	0.53	-0.39	0.35
462 thbpt	-0.05	0.19	0.33	0.14	0.31
278 beta-Alanine from Glutamate	-0.05	-0.06	0.41	-0.11	0.38
741 Choloyl-CoA	-0.05	-0.44	0.37	-0.49	0.48
450 CoA	-0.05	0.22	0.44	0.17	0.31
730 Chenodeoxycholoyl-CoA	-0.05	-0.46	0.34	-0.51	0.48
773 FAICAR	-0.05	0.22	0.53	0.17	0.37
76 Alanine from Arginine	-0.05	-0.15	0.26	-0.2	0.29
437 Albumin degr	-0.05	-0.34	0.51	-0.4	0.36
690 AICAR	-0.05	0.21	0.53	0.16	0.4
477 CTP	-0.06	0.23	0.46	0.17	0.33
26 NADPH redox potential into mito	-0.06	-0.04	0.51	-0.1	0.52
857 Methylmalonyl-CoA	-0.06	0.22	0.35	0.16	0.31
731 Cholate	-0.06	-0.44	0.54	-0.5	0.59
511 UDP-N-acetylgalactosamine	-0.06	-0.13	0.36	-0.19	0.38
68 O2.-(c) degr	-0.06	-0.12	0.68	-0.18	0.94
246 Proline from Tryptophan	-0.07	-0.15	0.32	-0.22	0.28
385 Collagen COPA1(c) synthesis	-0.07	-0.06	1	-0.13	1
321 SM(b)	-0.07	-0.39	0.26	-0.46	0.42
220 Glutamine from Proline	-0.07	-0.14	0.33	-0.21	0.36
859 N-(omega)-Hydroxyarginine	-0.07	-0.06	0.77	-0.13	0.77
357 Collagen CO6A1(c) synthesis	-0.07	-0.003	1	-0.08	1
400 Collagen VWA2(c) synthesis	-0.08	0.05	1	-0.03	1
728 Carnosine	-0.08	-0.01	0.46	-0.09	0.74
565 1,2-Diacylglycerol-VLDL-PC-pool	-0.08	-0.4	0.28	-0.47	0.47
570 1,2-Diacylglycerol-VLDL-TG-pool	-0.08	-0.4	0.28	-0.47	0.47
566 1,2-Diacylglycerol-VLDL-PE-pool	-0.08	-0.4	0.28	-0.48	0.48
351 Collagen CO4A4(c) synthesis	-0.08	-0.02	1	-0.1	1
569 1,2-Diacylglycerol-VLDL-SM-pool	-0.08	-0.4	0.28	-0.48	0.47
899 Phosphatidate-VLDL-PC-pool	-0.08	-0.39	0.32	-0.47	0.53
904 Phosphatidate-VLDL-TG-pool	-0.08	-0.39	0.32	-0.47	0.53
567 1,2-Diacylglycerol-VLDL-PI-pool	-0.08	-0.4	0.28	-0.48	0.48
376 Collagen COHA1(c) synthesis	-0.08	0.02	1	-0.06	1
900 Phosphatidate-VLDL-PE-pool	-0.08	-0.39	0.32	-0.48	0.53
568 1,2-Diacylglycerol-VLDL-PS-pool	-0.08	-0.4	0.29	-0.48	0.48
369 Collagen COBA1(c) synthesis	-0.08	0.06	1	-0.02	1
903 Phosphatidate-VLDL-SM-pool	-0.08	-0.39	0.32	-0.48	0.52
67 dTTP salvage from Thymine	-0.08	0.25	0.38	0.16	0.33
901 Phosphatidate-VLDL-PI-pool	-0.08	-0.39	0.32	-0.48	0.53
759 Deoxyguanosine	-0.09	0.24	0.46	0.15	0.33
902 Phosphatidate-VLDL-PS-pool	-0.09	-0.39	0.32	-0.48	0.53
774 FMN	-0.09	-0.04	1	-0.12	1

Continued on next page

Amplitude differences: Period difference, control – continued

Simulation	∆ampl	control 1/6h		control 6/24h	
		ampl	score	ampl	score
564 1,2-Diacylglycerol-Bile-PC-pool	-0.09	-0.4	0.28	-0.48	0.46
897 Phosphatidate-Bile-PC-pool	-0.09	-0.39	0.31	-0.48	0.51
827 Hypoxanthine	-0.09	0.25	0.43	0.16	0.33
275 beta-Alanine from Aspartate	-0.09	-0.04	0.89	-0.12	0.87
121 Asparagine from Cysteine	-0.09	-0.15	0.36	-0.24	0.42
274 beta-Alanine from Asparagine	-0.09	-0.03	0.38	-0.13	0.37
746 Cys-Gly	-0.09	0.11	1	0.02	1
952 Urate	-0.09	0.25	0.41	0.15	0.27
810 H2O2	-0.09	0.25	0.41	0.15	0.27
828 Inosine	-0.09	0.26	0.44	0.17	0.32
340 Collagen BGH3(c) synthesis	-0.1	0.03	1	-0.07	1
23 Oxidation of NADPH	-0.1	-0.08	0.56	-0.18	0.35
341 Collagen C43BP(c) synthesis	-0.1	0	1	-0.1	1
889 PE-PS-VLDL-pool	-0.1	-0.31	0.31	-0.41	0.47
382 Collagen COMA1(c) synthesis	-0.1	0.04	1	-0.06	1
872 NeuNGc	-0.1	-0.13	0.42	-0.23	0.45
346 Collagen CO2A1(c) synthesis	-0.1	-0.21	1	-0.31	1
18 Aerobic reduction of NADP+ (FA)	-0.1	0.33	0.35	0.23	0.32
935 Squalene 2,3-oxide	-0.1	-0.29	0.78	-0.39	0.58
658 4-Hydroxyphenylpyruvate	-0.1	-1.53	0.54	-1.64	0.54
393 Collagen FMOD(c) synthesis	-0.11	0.03	1	-0.07	1
53 activated sulphur	-0.11	0.07	0.87	-0.03	0.65
693 Acetaldehyde	-0.11	-0.16	0.54	-0.27	0.54
15 Dephosphorylation of ATP	-0.11	0.34	0.57	0.23	0.51
864 N-Acetylneuraminate-9P	-0.11	-0.11	0.42	-0.22	0.45
862 N-Acetylmannosamine-6P	-0.11	-0.11	0.54	-0.22	0.66
605 2-Oxo-3-methylvalerate	-0.11	0.28	0.42	0.17	0.37
512 N-acglucam	-0.11	-0.11	0.5	-0.22	0.46
574 1-Acylglycerol-3P-VLDL-PC-pool	-0.12	-0.38	0.35	-0.5	0.63
575 1-Acylglycerol-3P-VLDL-PE-pool	-0.12	-0.38	0.35	-0.5	0.64
210 Glutamine from Cysteine	-0.12	-0.17	0.38	-0.29	0.4
579 1-Acylglycerol-3P-VLDL-TG1-pool	-0.12	-0.38	0.35	-0.5	0.63
578 1-Acylglycerol-3P-VLDL-SM-pool	-0.12	-0.38	0.35	-0.5	0.63
576 1-Acylglycerol-3P-VLDL-PI-pool	-0.12	-0.38	0.35	-0.5	0.64
577 1-Acylglycerol-3P-VLDL-PS-pool	-0.12	-0.38	0.35	-0.5	0.64
233 Proline from Cysteine	-0.12	-0.14	0.32	-0.27	0.43
585 1-Acylglycerol-3P-stea	-0.12	-0.38	0.36	-0.5	0.65
582 1-Acylglycerol-3P-ol	-0.12	-0.38	0.36	-0.5	0.65
581 1-Acylglycerol-3P-lin	-0.12	-0.38	0.36	-0.5	0.65
580 1-Acylglycerol-3P-arach	-0.12	-0.38	0.36	-0.5	0.65
572 1-Acylglycerol-3P-Bile-PC-pool	-0.13	-0.38	0.34	-0.5	0.62
366 Collagen CO9A2(c) synthesis	-0.13	-0.04	1	-0.17	1
877 O-Acetylcarnitine	-0.13	0.14	1	0.01	1
186 Glycine from Arginine	-0.13	-0.11	0.27	-0.24	0.33
230 Proline from Arginine	-0.13	-0.12	0.32	-0.25	0.44
251 Serine from Arginine	-0.13	-0.11	0.28	-0.24	0.34
583 1-Acylglycerol-3P-palm	-0.13	-0.38	0.36	-0.51	0.63
785 Fructose-1,6PP	-0.13	-0.33	1	-0.46	1
373 Collagen COEA1(c) synthesis	-0.13	0.1	1	-0.04	1
526 Putrescine	-0.13	0.06	0.37	-0.07	0.5
308 VLDL from LDL	-0.13	-0.19	0.74	-0.32	0.86
325 Albumin	-0.14	0.01	0.97	-0.12	0.97
309 VLDL from HDL	-0.14	-0.19	0.72	-0.33	0.87
401 VLDL	-0.14	-0.19	0.71	-0.33	0.86
949 UDP-activated-N-acetyl-D-galactosamine	-0.14	-0.11	0.7	-0.25	0.56
330 ApoB100(r)	-0.14	-0.19	0.99	-0.33	0.99
221 Glutamine from Serine	-0.14	-0.13	0.32	-0.27	0.48
168 Glutamate from Cysteine	-0.14	-0.16	0.36	-0.3	0.44
21 Aerobic reduction of FAD (FA)	-0.14	-0.11	0.33	-0.25	0.32

Continued on next page

Simulation	Δampl	control 1/6h		control 6/24h	
		ampl	score	ampl	score
979 mitoOxidizedThioredoxin	-0.14	0.26	0.94	0.12	0.95
456 Thioredoxin(m)	-0.14	0.26	0.95	0.12	0.96
378 Collagen COJA1(c) synthesis	-0.15	0.09	1	-0.05	1
372 Collagen CODA1(c) synthesis	-0.15	-0.04	1	-0.19	1
716 Argininosuccinate	-0.15	-0.08	0.53	-0.23	0.33
244 Proline from Serine	-0.15	-0.12	0.35	-0.27	0.42
231 Proline from Asparagine	-0.15	-0.13	0.32	-0.28	0.4
765 Dihydroceramide-pool	-0.15	-0.02	0.52	-0.17	0.42
930 Sphinganine	-0.15	-0.02	0.4	-0.17	0.34
455 Thioredoxin	-0.15	0.14	0.95	-0.01	0.93
887 Oxidized thioredoxin	-0.15	0.14	0.94	-0.01	0.91
672 5-Oxoproline	-0.15	0.16	0.55	0.001	0.55
264 Serine from Phenylalanine	-0.16	-0.58	0.33	-0.74	0.39
496 Fructose-6P	-0.16	-0.32	1	-0.48	1
198 Glycine from Phenylalanine	-0.16	-0.58	0.31	-0.74	0.37
237 Proline from Histidine	-0.16	-0.13	0.35	-0.3	0.47
127 Asparagine from Glutamine	-0.16	-0.06	0.46	-0.22	0.45
229 Proline from Alanine	-0.17	-0.11	0.27	-0.27	0.44
234 Proline from Cystine	-0.17	-0.14	0.35	-0.31	0.38
10 Anaerobic rephosph of UTP	-0.17	-0.35	0.33	-0.52	0.44
8 Anaerobic rephosph of GTP	-0.17	-0.35	0.33	-0.52	0.44
9 Anaerobic rephosph of CTP	-0.17	-0.35	0.33	-0.52	0.44
571 1,3DPG	-0.17	-0.3	0.49	-0.47	0.79
486 Glutamine	-0.17	-0.13	0.38	-0.3	0.51
232 Proline from Aspartate	-0.17	-0.14	0.4	-0.31	0.42
363 Collagen CO8A1(c) synthesis	-0.18	-0.01	1	-0.18	1
123 Asparagine from Glutamate	-0.18	-0.06	0.38	-0.23	0.33
138 Asparagine from beta-Alanine	-0.18	-0.05	0.36	-0.23	0.48
13 NADH potential transport	-0.18	-0.45	0.34	-0.64	0.5
3 Anaerobic rephosph of ATP	-0.18	-0.33	0.34	-0.52	0.46
179 Glutamate from Serine	-0.18	-0.11	0.34	-0.29	0.52
861 N-Acetylglucosamine-1P	-0.18	-0.12	0.75	-0.3	0.61
238 Proline from Isoleucine	-0.19	-0.16	0.39	-0.35	0.4
725 CMP-activated-N-acetylneuraminate	-0.19	-0.1	0.35	-0.29	0.47
513 CMP-N-acetylneuraminate	-0.19	-0.1	0.35	-0.29	0.47
148 Aspartate from Glutamine	-0.19	-0.06	0.42	-0.25	0.33
761 Deoxyuridine	-0.19	-0.14	0.29	-0.33	0.44
300 Stearate from Oleate	-0.19	0.42	0.36	0.23	0.37
172 Glutamate from Isoleucine	-0.19	-0.19	0.5	-0.38	0.37
214 Glutamine from Histidine	-0.19	-0.15	0.35	-0.34	0.49
878 O-Butanoylcarnitine	-0.19	-0.1	0.37	-0.3	0.41
297 Palmitate from Palmitolate	-0.19	0.42	0.36	0.22	0.39
434 Fructose degr	-0.2	-0.55	0.51	-0.75	0.67
159 Aspartate from beta-Alanine	-0.2	-0.05	0.38	-0.25	0.54
247 Proline from Tyrosine	-0.2	-0.12	0.27	-0.32	0.31
305 Dihomo-gamma-linolenate from gamma-Linolenate	-0.2	-0.32	0.31	-0.52	0.38
586 1-Acylglycerol-VLDL-PC-pool	-0.2	-0.33	0.3	-0.53	0.55
591 1-Acylglycerol-VLDL-TG1-pool	-0.2	-0.33	0.3	-0.53	0.55
587 1-Acylglycerol-VLDL-PE-pool	-0.2	-0.33	0.3	-0.53	0.55
271 Tyrosine from Phenylalanine	-0.2	-0.64	0.46	-0.84	0.59
588 1-Acylglycerol-VLDL-PI-pool	-0.2	-0.33	0.3	-0.53	0.56
590 1-Acylglycerol-VLDL-SM-pool	-0.2	-0.33	0.3	-0.53	0.55
589 1-Acylglycerol-VLDL-PS-pool	-0.2	-0.33	0.3	-0.53	0.56
529 Methylthioribose-1P	-0.2	0.36	0.54	0.15	0.36
742 Citrate	-0.21	-0.06	0.58	-0.27	0.52
173 Glutamate from Glutamine	-0.21	-0.09	0.38	-0.29	0.43
748 D-3-Amino-isobutanoate	-0.21	-0.15	0.32	-0.35	0.36
396 Collagen LPP3(c) synthesis	-0.21	0.14	1	-0.07	1
370 Collagen COBA2(c) synthesis	-0.21	0.06	1	-0.14	1

Continued on next page

Amplitude differences: Period difference, control – continued

Simulation	Δampl	control 1/6h		control 6/24h	
		ampl	score	ampl	score
144 Aspartate from Glutamate	-0.21	-0.06	0.48	-0.27	0.32
353 Collagen CO4A6(c) synthesis	-0.21	0.004	1	-0.2	1
874 Nicotinate	-0.21	0.32	0.61	0.11	0.5
973 dUMP	-0.21	-0.14	0.31	-0.36	0.39
30 NADPH to NADH transhydrogenase in mito	-0.21	0.35	1	0.14	1
215 Glutamine from Isoleucine	-0.22	-0.14	0.45	-0.36	0.34
465 acgam6p	-0.22	-0.13	0.74	-0.35	0.62
164 Glutamate from Alanine	-0.22	-0.15	0.41	-0.37	0.56
39 Proton-gradient(m) build up	-0.22	-0.07	0.5	-0.29	0.38
40 Proton-gradient(c) build up	-0.22	-0.07	0.5	-0.29	0.38
280 beta-Alanine from Histidine	-0.22	-0.2	0.31	-0.42	0.41
22 Oxidation of NADH	-0.22	-0.09	0.56	-0.31	0.38
367 Collagen CO9A3(c) synthesis	-0.22	0.05	1	-0.18	1
171 Glutamate from Histidine	-0.22	-0.12	0.31	-0.34	0.54
342 Collagen CCBE1(c) synthesis	-0.22	0.04	1	-0.18	1
166 Glutamate from Asparagine	-0.22	-0.11	0.36	-0.33	0.44
45 Activated methylene group from Try	-0.22	-0.11	0.31	-0.34	0.26
6 Aerobic rephosph of UDP	-0.22	-0.06	0.55	-0.29	0.32
961 cis-Aconitate	-0.22	-0.08	0.59	-0.3	0.59
95 Alanine from Valine	-0.22	0.13	0.33	-0.1	0.33
336 Prothrombin	-0.22	-0.13	0.98	-0.36	0.98
927 Sedoheptulose-1,7PP	-0.23	-0.13	0.42	-0.36	0.56
841 L-Lactate	-0.23	-0.3	0.85	-0.53	0.92
358 Collagen CO6A2(c) synthesis	-0.23	0.09	1	-0.14	1
334 ApoE(c)	-0.23	-0.01	0.95	-0.24	0.96
239 Proline from Glutamine	-0.23	-0.12	0.28	-0.35	0.37
50 Acetyl group(m)	-0.23	-0.1	0.36	-0.33	0.44
125 Asparagine from Histidine	-0.23	-0.15	0.35	-0.38	0.37
600 2-Deoxy-D-ribose-1P	-0.23	-0.17	0.49	-0.4	0.57
971 dTMP	-0.23	-0.14	0.28	-0.38	0.39
120 Asparagine from Aspartate	-0.23	0.03	0.4	-0.2	0.46
245 Proline from Threonine	-0.23	-0.19	0.39	-0.43	0.38
27 NADPH redox potential into peroxy	-0.24	0.18	1	-0.06	1
602 2-Lysolecithin-pool	-0.24	-0.15	0.27	-0.39	0.41
860 N-Acetyl-D-mannosamine	-0.24	-0.1	0.38	-0.34	0.51
432 Oleate degr	-0.24	-0.1	0.32	-0.34	0.4
167 Glutamate from Aspartate	-0.24	-0.11	0.41	-0.36	0.55
433 Stearate degr	-0.24	-0.1	0.32	-0.34	0.41
118 Asparagine from Alanine	-0.25	-0.17	0.38	-0.41	0.34
601 2-Deoxy-D-ribose-5P	-0.25	-0.16	0.62	-0.41	0.44
126 Asparagine from Isoleucine	-0.25	-0.09	0.43	-0.34	0.44
180 Glutamate from Threonine	-0.25	-0.21	0.42	-0.46	0.42
384 Collagen COOA1(c) synthesis	-0.25	0.07	1	-0.19	1
332 ApoC2(c)	-0.25	-0.14	0.95	-0.4	0.95
362 Collagen CO7A1(c) synthesis	-0.26	0.01	1	-0.24	1
105 Arginine from Isoleucine	-0.26	-0.1	0.35	-0.36	0.38
1 Aerobic ATP rephosph (FA)	-0.26	-0.06	0.49	-0.32	0.3
4 Aerobic rephosph of GTP	-0.26	-0.06	0.46	-0.32	0.29
7 Aerobic rephosph of UTP	-0.26	-0.06	0.46	-0.32	0.28
608 2-Oxoglutaramate	-0.26	0.11	1	-0.15	1
611 3-Dehydrosphinganine	-0.26	0.09	0.39	-0.17	0.45
770 Erythrose-4P	-0.26	-0.13	0.39	-0.4	0.55
632 3-Oxopropanoate	-0.26	0.35	0.33	0.08	0.42
364 Collagen CO8A2(c) synthesis	-0.26	0.05	1	-0.21	1
96 Alanine from beta-Alanine	-0.26	0.35	0.36	0.08	0.45
917 Pyridoxine-P	-0.27	0.04	1	-0.23	1
28 NADPH to NADH transhydrogenase	-0.27	-0.01	1	-0.28	1
789 GAP	-0.27	-0.31	0.66	-0.58	0.88
752 DHAP	-0.27	-0.31	0.66	-0.58	0.88

Continued on next page

Simulation	Δampl	control 1/6h		control 6/24h	
		ampl	score	ampl	score
25 NADH redox potential into peroxy	-0.27	-0.3	0.52	-0.56	0.81
240 Proline from Leucine	-0.27	-0.13	0.39	-0.39	0.45
868 N-Methylethanolamine-P	-0.27	0	1	-0.27	1
771 Ethanolamine-P	-0.27	0	1	-0.27	1
482 Asparagine	-0.27	-0.15	0.36	-0.42	0.38
147 Aspartate from Isoleucine	-0.27	-0.09	0.47	-0.36	0.49
514 PC	-0.27	-0.15	0.31	-0.42	0.43
83 Alanine from Histidine	-0.27	-0.16	0.31	-0.43	0.38
484 Glutamate	-0.27	-0.03	0.39	-0.3	0.58
866 N-Formimino-L-glutamate	-0.28	-0.16	0.33	-0.43	0.53
222 Glutamine from Threonine	-0.28	-0.21	0.41	-0.48	0.41
281 beta-Alanine from Isoleucine	-0.28	-0.1	0.45	-0.38	0.41
412 Glutamate degr	-0.28	-0.63	0.29	-0.91	0.31
337 ApoTransferin	-0.29	-0.03	0.97	-0.32	0.98
5 Aerobic rephosph of CTP	-0.29	-0.1	0.36	-0.39	0.35
66 ATP salvage from Hypoxanthine	-0.29	-0.31	0.32	-0.6	0.42
216 Glutamine from Leucine	-0.29	-0.13	0.41	-0.42	0.43
61 AKG	-0.29	-0.1	0.38	-0.4	0.46
776 Fatty-acid-VLDL-PE-pool	-0.29	-0.28	0.4	-0.57	0.53
403 Acetoacetate	-0.29	-0.34	0.63	-0.63	0.68
425 Isoleucine degr	-0.29	-0.1	0.4	-0.4	0.35
777 Fatty-acid-VLDL-PI-pool	-0.3	-0.28	0.4	-0.57	0.53
47 Formylgroup(c)	-0.3	0.18	0.71	-0.12	0.76
778 Fatty-acid-VLDL-PS-pool	-0.3	-0.28	0.4	-0.58	0.53
661 4-Methyl-2-oxopentanoate	-0.3	-0.13	0.42	-0.43	0.41
84 Alanine from Isoleucine	-0.3	-0.11	0.42	-0.41	0.47
174 Glutamate from Leucine	-0.3	-0.12	0.42	-0.42	0.48
107 Arginine from Leucine	-0.3	-0.13	0.36	-0.43	0.39
86 Alanine from Leucine	-0.3	-0.15	0.41	-0.45	0.48
20 Aerobic reduction of FAD (gluc)	-0.31	-0.13	0.3	-0.44	0.38
46 Activated methyl group from Histidine	-0.31	-0.12	0.27	-0.44	0.38
199 Glycine from Proline	-0.31	0.1	0.3	-0.22	0.35
415 Leucine degr	-0.32	-0.15	0.4	-0.46	0.36
272 beta-Alanine from Alanine	-0.32	-0.17	0.56	-0.49	0.44
193 Glycine from Isoleucine	-0.32	-0.1	0.4	-0.41	0.45
483 Aspartate	-0.32	-0.15	0.35	-0.47	0.37
296 Stearate from Palmitate	-0.32	-0.46	0.35	-0.78	0.32
405 Acetone	-0.32	-0.33	0.85	-0.66	0.79
404 (R)-3-Hydroxybutanoate	-0.32	-0.33	0.54	-0.66	0.5
250 Serine from Alanine	-0.32	-0.14	0.37	-0.47	0.55
259 Serine from Isoleucine	-0.32	-0.1	0.46	-0.43	0.46
200 Glycine from Serine	-0.33	0.2	0.67	-0.13	0.57
44 Activated methyl group (THF)	-0.33	0.2	0.67	-0.13	0.57
320 Bile-PC(b)	-0.33	-0.15	0.28	-0.47	0.48
258 Serine from Histidine	-0.33	-0.12	0.31	-0.44	0.42
35 Thioredoxin(c) oxidation	-0.33	-0.01	0.71	-0.34	0.9
515 PE	-0.33	-0.07	0.32	-0.4	0.51
227 Methionine from Cysteine	-0.33	-0.22	0.34	-0.55	0.45
90 Alanine from Proline	-0.33	0.11	0.32	-0.22	0.32
128 Asparagine from Leucine	-0.33	-0.12	0.43	-0.46	0.43
110 Arginine from Phenylalanine	-0.33	-0.62	0.32	-0.95	0.35
192 Glycine from Histidine	-0.33	-0.12	0.29	-0.45	0.4
261 Serine from Leucine	-0.33	-0.12	0.38	-0.45	0.48
70 Formate degr	-0.34	0.16	0.71	-0.18	0.85
248 Proline from Valine	-0.34	0.21	0.35	-0.13	0.32
781 Fatty-acid-VLDL-TG2-pool	-0.34	-0.23	0.4	-0.57	0.54
780 Fatty-acid-VLDL-TG1-pool	-0.34	-0.23	0.4	-0.57	0.54
912 Protein(l)ysine	-0.34	-0.07	1	-0.41	1
69 Formaldehyde degr	-0.34	0.22	0.43	-0.12	0.9

Continued on next page

Amplitude differences: Period difference, control – continued

Simulation	Δampl	control 1/6h		control 6/24h	
		ampl	score	ampl	score
78 Alanine from Aspartate	-0.35	-0.18	0.78	-0.53	0.54
195 Glycine from Leucine	-0.35	-0.12	0.38	-0.47	0.45
283 beta-Alanine from Leucine	-0.35	-0.11	0.4	-0.46	0.46
185 Glycine from Alanine	-0.35	-0.14	0.36	-0.5	0.48
669 5-Hydroxy-L-tryptophan	-0.36	0.11	0.55	-0.25	0.84
265 Serine from Proline	-0.36	0.11	0.3	-0.25	0.37
723 CMP	-0.36	0.2	0.43	-0.16	0.31
188 Glycine from Aspartate	-0.36	-0.16	0.39	-0.52	0.45
253 Serine from Aspartate	-0.36	-0.16	0.45	-0.52	0.5
270 Serine from beta-Alanine	-0.37	-0.11	0.31	-0.48	0.41
863 N-Acetylneuraminate	-0.37	0.13	0.3	-0.24	0.48
142 Aspartate from Cysteine	-0.37	-0.43	0.4	-0.8	0.48
322 PS(b)	-0.37	-0.07	0.32	-0.44	0.44
516 PS	-0.37	-0.07	0.32	-0.44	0.44
491 Glycerol	-0.37	-0.25	0.39	-0.62	0.62
242 Proline from Methionine	-0.37	-0.18	0.39	-0.55	0.4
507 Cytidine	-0.37	0.22	0.43	-0.15	0.31
794 Galactose-1P	-0.38	-0.02	0.64	-0.4	0.59
852 Malonyl-Carnitin	-0.38	-0.17	0.61	-0.55	0.42
194 Glycine from Glutamine	-0.38	-0.09	0.31	-0.47	0.3
149 Aspartate from Leucine	-0.38	-0.11	0.43	-0.49	0.46
92 Alanine from Threonine	-0.38	-0.14	0.35	-0.52	0.47
260 Serine from Glutamine	-0.39	-0.09	0.34	-0.47	0.31
974 dUTP	-0.39	0.22	0.36	-0.17	0.27
802 Glycerate	-0.39	-0.32	0.51	-0.71	0.44
155 Aspartate from Threonine	-0.39	-0.14	0.33	-0.53	0.45
60 Pyruvate	-0.39	-0.11	0.41	-0.5	0.48
825 Hydroxypyruvate	-0.39	-0.35	1	-0.74	1
972 dUDP	-0.4	0.23	0.35	-0.17	0.28
191 Glycine from Glutamate	-0.4	-0.09	0.32	-0.49	0.31
488 Serine	-0.4	-0.11	0.33	-0.51	0.46
218 Glutamine from Methionine	-0.4	-0.19	0.42	-0.6	0.44
470 GSH	-0.4	-0.29	0.37	-0.69	0.56
256 Serine from Glutamate	-0.4	-0.09	0.35	-0.5	0.32
176 Glutamate from Methionine	-0.41	-0.21	0.47	-0.62	0.42
758 Deoxycytidine	-0.41	0.23	0.47	-0.18	0.29
201 Glycine from Threonine	-0.41	-0.1	0.3	-0.51	0.45
948 UDP-N-acetylglucosamine	-0.41	0.23	0.34	-0.19	0.39
266 Serine from Threonine	-0.41	-0.11	0.35	-0.52	0.48
809 Guanosine	-0.42	0.26	0.48	-0.16	0.31
479 UTP	-0.42	0.26	0.41	-0.16	0.35
228 Methionine from Cystine	-0.42	-0.19	0.34	-0.61	0.49
494 Mannose-1P	-0.42	0.13	0.46	-0.29	0.48
289 beta-Alanine from Threonine	-0.42	-0.09	0.29	-0.52	0.39
764 Dihomo-gamma-linolenoyl-CoA	-0.43	0.17	0.35	-0.25	0.29
51 Acetyl group(p)	-0.43	0.16	0.36	-0.27	0.36
471 Homocysteine	-0.43	-0.21	0.31	-0.64	0.42
724 CMP-NeuNGc	-0.43	0.17	0.37	-0.26	0.35
134 Asparagine from Threonine	-0.43	-0.1	0.35	-0.53	0.46
65 ATP salvage from Adenosine	-0.43	-0.47	0.87	-0.9	0.91
109 Arginine from Methionine	-0.44	-0.21	0.35	-0.65	0.42
431 HDL degr	-0.44	-0.15	0.34	-0.58	0.39
252 Serine from Asparagine	-0.44	-0.14	0.32	-0.58	0.46
77 Alanine from Asparagine	-0.44	-0.18	0.41	-0.62	0.46
916 Pyridoxal	-0.44	0.29	0.81	-0.15	0.99
797 Glucose-1P	-0.44	-0.02	1	-0.46	1
879 O-Propanoylcarnitine	-0.45	-0.13	0.51	-0.58	0.34
146 Aspartate from Histidine	-0.45	-0.15	0.33	-0.6	0.38
187 Glycine from Asparagine	-0.45	-0.14	0.31	-0.59	0.41

Continued on next page

Simulation	Δampl	control 1/6h ampl	control 1/6h score	control 6/24h ampl	control 6/24h score
323 PE(b)	-0.46	-0.07	0.28	-0.53	0.56
254 Serine from Cysteine	-0.46	-0.24	0.39	-0.69	0.43
130 Asparagine from Methionine	-0.46	-0.15	0.41	-0.6	0.46
184 Glutamate from beta-Alanine	-0.46	0.2	0.4	-0.26	0.58
88 Alanine from Methionine	-0.46	-0.15	0.38	-0.6	0.48
175 Glutamate from Lysine	-0.47	-0.09	0.31	-0.56	0.35
241 Proline from Lysine	-0.47	0.22	0.33	-0.25	0.31
217 Glutamine from Lysine	-0.47	-0.12	0.36	-0.59	0.36
197 Glycine from Methionine	-0.47	-0.13	0.35	-0.6	0.46
460 Pyridoxal-P	-0.47	0.29	0.58	-0.19	0.91
208 Glutamine from Asparagine	-0.47	0.15	0.38	-0.32	0.45
161 Cysteine from Methionine	-0.47	-0.13	0.38	-0.6	0.46
263 Serine from Methionine	-0.47	-0.13	0.37	-0.61	0.49
209 Glutamine from Aspartate	-0.48	0.17	0.35	-0.31	0.43
680 6-Phospho-D-gluconate	-0.48	0.12	0.64	-0.36	0.77
117 Arginine from beta-Alanine	-0.48	0.24	0.31	-0.24	0.42
294 Taurine from Methionine	-0.48	-0.12	0.38	-0.6	0.45
226 Glutamine from beta-Alanine	-0.48	0.22	0.37	-0.26	0.47
285 beta-Alanine from Methionine	-0.48	-0.14	0.33	-0.61	0.44
469 ametam	-0.48	0.26	0.39	-0.22	0.29
755 Decanoyl-CoA	-0.48	0.18	0.41	-0.31	0.28
163 Cystine from Methionine	-0.49	-0.13	0.35	-0.61	0.51
625 3-Oxododecanoyl-CoA	-0.49	0.18	0.42	-0.31	0.27
249 Proline from beta-Alanine	-0.49	0.24	0.37	-0.25	0.47
882 Octanoyl-CoA	-0.49	0.18	0.39	-0.31	0.3
561 (S)-Hydroxydecanoyl-CoA	-0.49	0.18	0.39	-0.32	0.29
531 (2E)-Decenoyl-CoA	-0.49	0.18	0.39	-0.32	0.29
623 3-Oxodecanoyl-CoA	-0.49	0.18	0.4	-0.31	0.29
819 Hexanoyl-CoA	-0.49	0.18	0.39	-0.31	0.31
875 Nicotinate D-ribonucleoside	-0.49	0.12	0.45	-0.37	0.36
98 Arginine from Asparagine	-0.49	-0.29	0.31	-0.78	0.34
563 (S)-Hydroxyoctanoyl-CoA	-0.49	0.18	0.38	-0.31	0.31
630 3-Oxooctanoyl-CoA	-0.5	0.18	0.38	-0.31	0.31
873 Nicotinamide D-ribonucleotide	-0.5	0.13	0.56	-0.37	0.48
876 Nicotinate(r)ibonucleotide	-0.5	0.13	0.47	-0.37	0.4
562 (S)-Hydroxyhexanoyl-CoA	-0.5	0.19	0.38	-0.32	0.29
129 Asparagine from Lysine	-0.51	-0.13	0.36	-0.64	0.35
151 Aspartate from Methionine	-0.51	-0.13	0.36	-0.64	0.5
719 Butyryl-CoA	-0.51	0.2	0.4	-0.31	0.29
628 3-Oxohexanoyl-CoA	-0.51	0.19	0.42	-0.32	0.29
87 Alanine from Lysine	-0.51	-0.14	0.38	-0.65	0.4
667 5-Formiminotetrahydrofolate	-0.51	0.12	0.57	-0.4	0.75
637 3-Phosphoserine	-0.52	-0.13	0.43	-0.65	0.57
57 UDP-activated galactose	-0.52	0.12	0.49	-0.39	0.54
505 Uracil	-0.52	0.24	0.38	-0.27	0.37
537 (2E)-Hexenoyl-CoA	-0.52	0.2	0.39	-0.32	0.3
518 CL	-0.52	0.11	0.32	-0.41	0.45
540 (2E)-Octenoyl-CoA	-0.52	0.2	0.4	-0.32	0.3
17 Aerobic reduction of NAD+ (gluc)	-0.52	0.1	0.32	-0.42	0.36
871 NADPH	-0.52	0.14	0.55	-0.38	0.35
452 NADP+	-0.52	0.14	0.55	-0.38	0.34
606 2-Oxoadipate	-0.53	-0.13	0.44	-0.66	0.39
402 HDL	-0.53	-0.14	0.72	-0.67	0.79
519 LacCer	-0.53	0.1	0.28	-0.43	0.39
196 Glycine from Lysine	-0.53	-0.11	0.33	-0.64	0.4
796 Glucono-1,5-lactone-6P	-0.53	0.12	1	-0.41	1
190 Glycine from Cystine	-0.53	-0.16	0.32	-0.69	0.46
262 Serine from Lysine	-0.53	-0.11	0.36	-0.64	0.43
255 Serine from Cystine	-0.53	-0.16	0.36	-0.69	0.5

Continued on next page

Amplitude differences: Period difference, control – continued

Simulation	Δampl	control 1/6h		control 6/24h	
		ampl	score	ampl	score
928 Sedoheptulose-7P	-0.53	0.11	0.33	-0.42	0.47
269 Serine from Valine	-0.54	0.13	0.3	-0.4	0.32
820 Histamine	-0.54	-0.002	0.64	-0.54	0.82
673 5-Phosphoribosyl-4-carboxy-5-aminoimidazole	-0.54	0.17	0.46	-0.37	0.39
921 Ribulose-5P	-0.54	0.11	0.4	-0.43	0.56
189 Glycine from Cysteine	-0.54	-0.15	0.32	-0.69	0.39
517 PI	-0.54	0.12	0.42	-0.42	0.36
467 1fthf	-0.54	0.11	0.74	-0.43	0.62
329 ApoA1	-0.55	-0.14	0.96	-0.68	0.97
169 Glutamate from Cystine	-0.55	-0.15	0.33	-0.7	0.48
466 THF(m)	-0.55	0.11	1	-0.44	1
459 THF	-0.55	0.11	1	-0.44	1
29 NADH to NADPH transhydrogenase	-0.55	0.11	1	-0.44	1
766 Dihydrofolate	-0.55	0.11	1	-0.44	1
670 5-Methyl-THF	-0.55	0.11	1	-0.44	1
799 Glucuronate	-0.55	0.33	0.48	-0.22	0.41
664 5,10-Methenyl-THF	-0.55	0.12	0.83	-0.44	0.68
464 mlthf	-0.55	0.11	0.79	-0.44	0.78
106 Arginine from Glutamine	-0.56	-0.32	0.35	-0.87	0.3
236 Proline from Glycine	-0.56	0.29	0.3	-0.27	0.39
855 Mercaptopyruvate	-0.57	0.14	0.4	-0.42	0.55
204 Glycine from Valine	-0.57	0.15	0.3	-0.42	0.31
869 N-Pantothenoylcysteine	-0.57	0.35	0.45	-0.22	0.55
548 (R)-4-Phosphopantothenoyl-cysteine	-0.57	0.35	0.48	-0.23	0.57
377 Collagen COIA1(c) synthesis	-0.58	0.07	1	-0.5	1
292 beta-Alanine from Valine	-0.58	0.18	0.35	-0.4	0.34
977 linoleic-Carnitine	-0.58	0.05	0.42	-0.53	0.51
714 Arachidonyl-Carnitine	-0.58	0.05	0.42	-0.53	0.51
279 beta-Alanine from Glycine	-0.58	-0.1	0.37	-0.68	0.42
34 Thioredoxin(c) reduction	-0.58	0.14	0.88	-0.44	1
211 Glutamine from Cystine	-0.59	-0.16	0.42	-0.75	0.46
844 L-Palmitoylcarnitine	-0.59	0.05	0.42	-0.54	0.49
277 beta-Alanine from Cystine	-0.59	-0.18	0.37	-0.77	0.42
499 udpgal	-0.59	0.22	0.38	-0.37	0.37
150 Aspartate from Lysine	-0.59	-0.09	0.39	-0.68	0.47
919 Ribose-1P	-0.59	0.11	0.28	-0.48	0.55
522 Triacylglycerol	-0.59	0.17	0.38	-0.43	0.61
79 Alanine from Cysteine	-0.59	-0.18	0.41	-0.77	0.41
333 ApoC3(c)	-0.6	-0.14	0.95	-0.74	0.95
143 Aspartate from Cystine	-0.6	-0.18	0.43	-0.77	0.47
288 beta-Alanine from Serine	-0.6	-0.11	0.46	-0.71	0.42
276 beta-Alanine from Cysteine	-0.6	-0.18	0.46	-0.77	0.36
80 Alanine from Cystine	-0.6	-0.18	0.48	-0.78	0.57
391 Collagen FCN2(c) synthesis	-0.6	0.08	1	-0.52	1
639 3-Ureidoisobutyrate	-0.6	0.23	0.29	-0.37	0.39
448 Ethanol degr	-0.6	0.13	0.44	-0.47	0.54
636 3-Phosphonooxypyruvate	-0.61	-0.11	0.49	-0.72	0.57
645 3PG	-0.61	-0.11	0.57	-0.72	0.67
122 Asparagine from Cystine	-0.61	-0.15	0.39	-0.77	0.5
284 beta-Alanine from Lysine	-0.62	-0.08	0.39	-0.7	0.44
944 Thymine	-0.62	0.27	0.34	-0.36	0.36
767 Dihydrothymine	-0.62	0.23	0.31	-0.39	0.35
699 Acrylyl-CoA	-0.63	0.17	0.33	-0.46	0.28
424 Glycine degr	-0.63	-0.25	0.36	-0.88	0.35
420 Serine degr	-0.63	-0.25	0.39	-0.88	0.37
751 D-Xylulose-5P	-0.63	0.11	0.37	-0.52	0.43
32 Thioredoxin(m) reduction	-0.64	0.19	1	-0.45	1
33 Thioredoxin(m) oxidation	-0.64	0.19	1	-0.45	1
970 dTDP	-0.64	0.26	0.29	-0.38	0.36

Continued on next page

Simulation	Δampl	control 1/6h ampl	control 1/6h score	control 6/24h ampl	control 6/24h score
472 dATP	-0.64	0.19	0.42	-0.45	0.32
476 ATP	-0.64	0.19	0.42	-0.45	0.32
760 Deoxyinosine	-0.64	0.27	0.33	-0.37	0.34
951 UMP	-0.65	0.22	0.44	-0.42	0.37
782 Fatty-acid-VLDL-TG3-pool	-0.65	0.18	0.4	-0.47	0.66
141 Aspartate from Asparagine	-0.65	-0.08	0.38	-0.73	0.49
474 dGTP	-0.65	0.2	0.4	-0.46	0.29
504 XMP	-0.66	0.2	0.38	-0.46	0.32
509 Guanine	-0.66	0.2	0.33	-0.46	0.31
427 Homocysteine degr	-0.66	-0.18	0.43	-0.84	0.41
508 Xanthine	-0.66	0.2	0.34	-0.46	0.33
616 3-Hydroxypropionyl-CoA	-0.66	0.17	0.33	-0.49	0.29
417 Methionine degr	-0.66	-0.18	0.4	-0.84	0.39
498 UDP-glucose	-0.67	0.26	0.34	-0.4	0.3
108 Arginine from Lysine	-0.67	-0.1	0.31	-0.76	0.35
840 L-Glutamate 5-semialdehyde	-0.68	0.07	0.46	-0.61	0.39
946 Trehalose	-0.68	-0.04	1	-0.72	1
212 Glutamine from Glutamate	-0.68	0.06	0.33	-0.63	0.26
503 IMP	-0.68	0.2	0.39	-0.48	0.31
100 Arginine from Cysteine	-0.69	-0.25	0.33	-0.94	0.33
91 Alanine from Serine	-0.7	-0.09	0.43	-0.79	0.44
82 Alanine from Glycine	-0.7	-0.09	0.39	-0.79	0.4
205 Glycine from beta-Alanine	-0.71	0.23	0.31	-0.47	0.37
327 Antichymotrypsin	-0.71	-0.46	0.97	-1.17	0.97
885 Orotidine-5P	-0.71	0.34	0.48	-0.37	0.43
104 Arginine from Histidine	-0.72	-0.22	0.29	-0.94	0.36
838 L-Formylkynurenine	-0.73	-0.66	0.89	-1.39	0.72
75 Gluconeogen from Alanine	-0.73	-0.5	0.32	-1.23	0.43
101 Arginine from Cystine	-0.74	-0.21	0.3	-0.94	0.41
858 Myristoyl-CoA	-0.74	0.18	0.34	-0.56	0.28
430 LDL degr	-0.74	0.17	0.32	-0.57	0.44
597 2-Amino-3-oxoadipate	-0.74	-0.16	0.49	-0.91	0.43
975 gamma-Glutamyl-cysteine	-0.76	-0.24	0.96	-1	0.85
911 Propanoyl-CoA	-0.76	0.17	0.36	-0.59	0.28
811 H2S	-0.76	-0.17	0.99	-0.93	0.48
553 (S)-3-Hydroxy-2-methylbutyryl-CoA	-0.77	0.2	0.36	-0.57	0.28
406 Heme	-0.77	-0.12	0.32	-0.88	0.34
112 Arginine from Serine	-0.77	-0.11	0.33	-0.88	0.35
331 ApoC1(c)	-0.77	-0.14	0.94	-0.92	0.94
824 Hydroxymethylbilane	-0.77	-0.14	0.36	-0.91	0.42
954 UroporphyrinogenIII	-0.78	-0.13	0.36	-0.91	0.4
556 (S)-3-Hydroxyhexadecanoyl-CoA	-0.78	0.17	0.33	-0.61	0.28
744 CoproporphyrinogenIII	-0.78	-0.14	0.34	-0.91	0.38
817 Hexadecenal	-0.78	0.08	0.35	-0.7	0.34
815 Hexadecanal	-0.78	0.08	0.35	-0.7	0.34
914 Protoporphyrinogen IX	-0.79	-0.13	0.36	-0.92	0.37
929 Serotonin	-0.79	-0.31	0.56	-1.1	0.6
913 Protoporphyrin	-0.79	-0.13	0.36	-0.92	0.32
666 5-Aminolevulinate	-0.8	-0.11	0.48	-0.91	0.63
945 Tiglyl-CoA	-0.8	0.21	0.37	-0.6	0.28
908 Porphobilinogen	-0.81	-0.11	0.43	-0.92	0.54
603 2-Methylacetoacetyl-CoA	-0.81	0.2	0.37	-0.6	0.29
557 (S)-3-Hydroxytetradecanoyl-CoA	-0.81	0.17	0.32	-0.64	0.32
413 Histidine degr	-0.81	-0.2	0.32	-1.01	0.33
411 Cysteine degr	-0.82	-0.21	0.43	-1.03	0.36
527 Spermidine	-0.82	0.31	0.45	-0.5	0.4
19 Aerobic reduction of NADP+ (gluc)	-0.82	0.4	0.34	-0.42	0.48
619 3-Methyl-2-oxobutyrate	-0.83	0.37	0.35	-0.46	0.5
635 3-Oxotetradecanoyl-CoA	-0.84	0.17	0.34	-0.66	0.31

Continued on next page

Amplitude differences: Period difference, control – continued

Simulation	Δampl	control 1/6h		control 6/24h	
		ampl	score	ampl	score
891 PG-CL-pool	-0.84	0.39	0.3	-0.45	0.38
892 PGP-CL-pool	-0.84	0.39	0.3	-0.45	0.38
631 3-Oxopalmitoyl-CoA	-0.85	0.17	0.36	-0.68	0.29
485 Glycine	-0.85	0.35	0.31	-0.5	0.41
489 Tyrosine	-0.85	-0.01	0.48	-0.86	0.71
923 SAICAR	-0.86	0.29	0.52	-0.56	0.38
542 (2E)-Tetradecenoyl-CoA	-0.86	0.2	0.34	-0.65	0.32
114 Arginine from Tryptophan	-0.86	-0.24	0.3	-1.1	0.33
510 Adenosine	-0.86	0.19	0.39	-0.67	0.32
898 Phosphatidate-CL-pool	-0.86	0.43	0.32	-0.44	0.47
956 Xanthurenate	-0.87	-0.15	0.6	-1.02	0.49
657 4-(2-Amino-3-hydroxyphenyl)-2,4-dioxobutanoate	-0.87	-0.15	0.6	-1.02	0.49
847 Lauroyl-CoA	-0.87	0.21	0.35	-0.67	0.33
267 Serine from Tryptophan	-0.88	-0.15	0.35	-1.03	0.34
659 4-Imidazolone-5-propanoate	-0.88	-0.17	0.46	-1.05	0.47
299 Oleate from Stearate	-0.89	-0.47	0.47	-1.36	0.41
202 Glycine from Tryptophan	-0.89	-0.15	0.34	-1.05	0.32
932 Sphingosine	-0.89	0.07	0.34	-0.82	0.37
311 Urea from glutamine	-0.9	-0.1	0.29	-0.99	0.27
573 1-Acylglycerol-3P-CL-pool	-0.9	0.43	0.38	-0.47	0.54
933 Sphingosine-1P	-0.9	0.07	0.3	-0.83	0.33
729 Ceramide-1P-pool	-0.91	0.08	0.38	-0.83	0.31
980 palmitoleoyl-Carnitine	-0.91	0.05	0.45	-0.86	0.59
480 Alanine	-0.91	0.41	0.54	-0.51	0.44
36 GSH reduction using NADH redox potential	-0.92	0.43	0.51	-0.49	0.63
73 Gluconeogen from Lactate	-0.92	-0.41	0.32	-1.33	0.43
528 Spermine	-0.92	0.32	0.41	-0.6	0.37
414 Glutamine degr	-0.93	-0.08	0.33	-1	0.27
422 Tryptophan degr	-0.93	-0.21	0.34	-1.14	0.36
441 Fibrinogen degr	-0.93	-0.35	0.53	-1.28	0.34
37 GSH reduction using NADPH redox potential	-0.94	0.42	1	-0.51	1
38 GSH oxidation	-0.94	0.42	1	-0.51	1
468 PAPS	-0.94	0.35	0.33	-0.59	0.35
443 Plasminogen degr	-0.94	-0.35	0.53	-1.29	0.34
440 ApoA1 degr	-0.95	-0.34	0.49	-1.29	0.36
722 CDP-ethanolamine	-0.97	0.2	0.38	-0.77	0.32
444 Prothrombin degr	-0.97	-0.35	0.51	-1.32	0.36
290 beta-Alanine from Tryptophan	-0.97	-0.19	0.37	-1.16	0.31
953 Urocanate	-0.99	-0.27	0.48	-1.25	0.48
156 Aspartate from Tryptophan	-1	-0.18	0.4	-1.18	0.33
135 Asparagine from Tryptophan	-1	-0.17	0.43	-1.18	0.32
982 sn-Glycerol-3P	-1	0.41	0.47	-0.59	0.93
223 Glutamine from Tryptophan	-1.02	-0.17	0.32	-1.19	0.29
668 5-Formyl-THF	-1.03	-0.18	0.35	-1.21	0.34
497 UDP-xylose	-1.03	0.29	0.45	-0.74	0.3
310 Bilirubin conjugation	-1.03	0.41	0.46	-0.62	0.5
314 Creatine	-1.04	-0.06	0.32	-1.11	0.25
598 2-Aminomuconate	-1.05	-0.26	0.38	-1.31	0.45
93 Alanine from Tryptophan	-1.05	-0.18	0.34	-1.23	0.33
612 3-Hydroxy-L-kynurenine	-1.06	-0.15	0.5	-1.21	0.47
102 Arginine from Glutamate	-1.06	0.06	0.3	-1	0.3
599 2-Aminomuconate(s)emialdehyde	-1.07	-0.24	0.42	-1.31	0.45
596 2-Amino-3-carboxymuconate(s)emialdehyde	-1.07	-0.24	0.42	-1.31	0.45
584 1-Acylglycerol-3P-palmn	-1.08	0.4	0.27	-0.68	0.66
694 Acetate	-1.1	-0.39	0.69	-1.49	0.83
941 THF-hexaglutamate	-1.11	0.34	0.91	-0.77	0.95
335 Plasminogen	-1.12	-0.49	0.98	-1.61	0.98
162 Cystine from Cysteine	-1.15	0.46	0.57	-0.69	0.68
160 Cysteine from Cystine	-1.15	0.46	0.57	-0.69	0.68

Continued on next page

Amplitude differences: Period difference, control – continued

Simulation	Δampl	control 1/6h		control 6/24h	
		ampl	score	ampl	score
793 GSSG	-1.17	0.39	0.58	-0.78	0.74
48 Formylgroup(m)	-1.18	-0.14	0.32	-1.32	0.32
56 UDP-activated glucuronate	-1.18	0.38	0.38	-0.8	0.41
74 Gluconeogen from Glycerol	-1.19	-0.1	0.36	-1.3	0.51
614 3-Hydroxyisobutyrate	-1.22	0.13	0.29	-1.08	0.27
99 Arginine from Aspartate	-1.23	0.24	0.3	-0.98	0.32
609 2PG	-1.24	-0.11	0.45	-1.34	0.42
808 Guanidinoacetate	-1.3	0.07	0.4	-1.22	0.28
788 Fumarylacetoacetate	-1.3	-1.32	0.41	-2.62	0.58
660 4-Maleylacetoacetate	-1.34	-1.35	0.46	-2.68	0.65
181 Glutamate from Tryptophan	-1.34	0.17	0.3	-1.17	0.3
779 Fatty-acid-VLDL-SM-pool	-1.36	-0.44	1	-1.8	1
775 Fatty-acid-VLDL-PC-pool	-1.36	-0.44	1	-1.8	1
16 Aerobic reduction of NAD+ (FA)	-1.41	0.35	0.33	-1.06	0.32
798 Glucosylceramide-pool	-1.45	0.08	0.35	-1.37	0.35
842 L-Octanoylcarnitine	-1.51	0.26	0.54	-1.25	0.37
55 UDP-activated glucose	-1.51	-0.18	0.49	-1.69	0.56
72 Glycogen glucose release	-1.52	0.07	0.42	-1.46	0.44
821 Homogentisate	-1.53	-1.35	0.55	-2.88	0.55
500 udpglcur	-1.54	0.31	0.4	-1.23	0.32
43 Activated methyl group (SAM)	-1.54	0.15	0.37	-1.39	0.43
131 Asparagine from Phenylalanine	-1.6	-0.59	0.33	-2.18	0.36
219 Glutamine from Phenylalanine	-1.61	-0.6	0.29	-2.22	0.33
243 Proline from Phenylalanine	-1.65	-0.6	0.26	-2.25	0.3
286 beta-Alanine from Phenylalanine	-1.66	-0.58	0.37	-2.24	0.38
177 Glutamate from Phenylalanine	-1.66	-0.61	0.31	-2.27	0.36
89 Alanine from Phenylalanine	-1.68	-0.59	0.39	-2.27	0.42
926 Sarcosine	-1.68	-0.97	0.82	-2.64	0.82
950 UDP-activated-xylose	-1.69	0.3	0.52	-1.39	0.48
152 Aspartate from Phenylalanine	-1.7	-0.57	0.42	-2.27	0.42
203 Glycine from Tyrosine	-1.8	-0.54	0.27	-2.34	0.34
115 Arginine from Tyrosine	-1.82	-0.6	0.3	-2.42	0.36
268 Serine from Tyrosine	-1.85	-0.51	0.33	-2.36	0.36
136 Asparagine from Tyrosine	-1.9	-0.54	0.3	-2.44	0.35
182 Glutamate from Tyrosine	-1.93	-0.56	0.28	-2.49	0.36
291 beta-Alanine from Tyrosine	-1.94	-0.52	0.35	-2.47	0.38
94 Alanine from Tyrosine	-1.96	-0.53	0.37	-2.49	0.42
157 Aspartate from Tyrosine	-1.96	-0.53	0.38	-2.49	0.43
418 Phenylalanine degr	-2.12	-0.62	0.38	-2.74	0.38
426 Tyrosine degr	-2.36	-0.61	0.35	-2.97	0.39
224 Glutamine from Tyrosine	-2.58	0.13	0.26	-2.45	0.33
409 Asparagine degr	-3.31	-0.72	0.38	-4.03	0.38
410 Aspartate degr	-3.4	-0.72	0.33	-4.12	0.32
407 Alanine degr	-3.67	-0.36	0.45	-4.03	0.31
312 Urea from alanine	-3.69	-0.34	0.46	-4.03	0.3
313 Urea from NH3	-3.69	-0.34	0.46	-4.03	0.3

2.4 Amplitude differences: TGFβ/contr 6h vs. 24h

Table 10: Amplitude differences: TGFβ/contr 6h vs. 24h

Simulation	Δampl	TGFβ/C 6h		TGFβ/C 24h	
		ampl	score	ampl	score
619 3-Methyl-2-oxobutyrate	1.24	-0.2	0.65	1.04	0.37
344 Collagen CO1A1(c) synthesis	0.98	0.11	1	1.09	1
207 Glutamine from Arginine	0.97	-0.09	0.39	0.88	0.27
931 Sphinganine-1P	0.92	0.38	0.47	1.29	0.36
760 Deoxyinosine	0.9	-0.34	0.38	0.56	0.29
552 (R)-Mevalonate	0.85	-0.23	0.46	0.62	0.54
120 Asparagine from Aspartate	0.84	-0.3	0.45	0.54	0.37
941 THF-hexaglutamate	0.83	-0.14	0.87	0.69	0.87
362 Collagen CO7A1(c) synthesis	0.79	0.05	1	0.84	1
15 Dephosphorylation of ATP	0.76	-0.14	0.57	0.63	0.71
872 NeuNGc	0.76	-0.2	0.38	0.56	0.31
600 2-Deoxy-D-ribose-1P	0.75	-0.05	0.33	0.7	0.41
374 Collagen COFA1(c) synthesis	0.74	1.52	1	2.27	1
560 (S)-Dihydroorotate	0.74	-0.36	0.44	0.38	0.32
96 Alanine from beta-Alanine	0.73	-0.19	0.43	0.54	0.33
341 Collagen C43BP(c) synthesis	0.71	-0.13	1	0.58	1
827 Hypoxanthine	0.71	-0.3	0.41	0.41	0.29
802 Glycerate	0.7	0.2	0.81	0.9	0.55
863 N-Acetylneuraminate	0.62	-0.2	0.36	0.42	0.32
605 2-Oxo-3-methylvalerate	0.61	-0.2	0.4	0.41	0.49
680 6-Phospho-D-gluconate	0.56	-0.13	0.5	0.43	0.63
394 Collagen ITA1(c) synthesis	0.51	-0.18	0.37	0.33	0.28
817 Hexadecenal	0.48	-0.21	0.38	0.28	0.26
815 Hexadecanal	0.48	-0.21	0.38	0.28	0.26
355 Collagen CO5A2(c) synthesis	0.47	0.24	1	0.71	1
725 CMP-activated-N-acetylneuraminate	0.47	-0.19	0.49	0.28	0.3
513 CMP-N-acetylneuraminate	0.47	-0.19	0.49	0.28	0.3
825 Hydroxypyruvate	0.47	0.08	1	0.55	1
391 Collagen FCN2(c) synthesis	0.45	-0.19	1	0.26	1
349 Collagen CO4A2(c) synthesis	0.44	0.07	1	0.51	1
41 Na+ importgradient	0.4	-0.18	0.62	0.23	0.47
512 N-acglucam	0.38	-0.22	0.31	0.16	0.32
669 5-Hydroxy-L-tryptophan	0.36	-0.12	0.86	0.24	0.61
494 Mannose-1P	0.36	-0.13	0.66	0.23	0.5
799 Glucuronate	0.36	-0.11	0.51	0.24	0.57
632 3-Oxopropanoate	0.35	0.17	0.38	0.52	0.33
386 Collagen CORA1(c) synthesis	0.35	1.74	1	2.09	1
839 L-Fucose-1P	0.33	-0.09	0.57	0.24	0.42
58 GDP-activated fucose	0.33	-0.09	0.69	0.24	0.48
949 UDP-activated-N-acetyl-D-galactosamine	0.33	-0.15	0.55	0.18	0.44
399 Collagen SERPH(c) synthesis	0.32	-0.02	1	0.3	1
330 ApoB100(r)	0.32	-0.03	0.99	0.28	0.99
401 VLDL	0.32	-0.03	0.7	0.28	0.68
309 VLDL from HDL	0.32	-0.03	0.7	0.28	0.68
308 VLDL from LDL	0.32	-0.03	0.72	0.28	0.71
59 GDP-activated mannose	0.31	-0.11	0.66	0.2	0.49
64 Glucosamine-6P	0.3	-0.19	0.67	0.11	0.63
526 Putrescine	0.29	-0.12	0.47	0.18	0.52
345 Collagen CO1A2(c) synthesis	0.29	0.1	1	0.38	1
32 Thioredoxin(m) reduction	0.29	-0.34	1	-0.06	1
33 Thioredoxin(m) oxidation	0.29	-0.34	1	-0.06	1
436 Mannose degr	0.28	0.15	0.48	0.43	0.42
370 Collagen COBA2(c) synthesis	0.27	-0.01	1	0.26	1
861 N-Acetylglucosamine-1P	0.27	-0.16	0.48	0.1	0.55
350 Collagen CO4A3(c) synthesis	0.26	-0.07	1	0.19	1
384 Collagen COOA1(c) synthesis	0.25	-0.17	1	0.08	1

Continued on next page

Simulation	Δampl	TGFβ/C 6h		TGFβ/C 24h	
		ampl	score	ampl	score
830 Inositol-1P	0.24	-0.13	1	0.12	1
507 Cytidine	0.24	-0.32	0.46	-0.08	0.37
505 Uracil	0.24	-0.34	0.53	-0.09	0.32
797 Glucose-1P	0.24	-0.04	1	0.2	1
348 Collagen CO4A1(c) synthesis	0.24	-0.17	1	0.07	1
865 N-Carbamoyl-L-aspartate	0.24	-0.37	0.38	-0.13	0.4
919 Ribose-1P	0.23	-0.04	0.53	0.19	0.4
726 Carbamoyl-P	0.22	-0.36	0.49	-0.14	0.58
748 D-3-Amino-isobutanoate	0.22	-0.31	0.38	-0.09	0.35
396 Collagen LPP3(c) synthesis	0.21	0.28	1	0.5	1
944 Thymine	0.21	-0.3	0.44	-0.09	0.42
639 3-Ureidoisobutyrate	0.21	-0.3	0.42	-0.1	0.37
767 Dihydrothymine	0.21	-0.3	0.45	-0.1	0.39
855 Mercaptopyruvate	0.2	-0.17	0.52	0.03	0.38
36 GSH reduction using NADH redox potential	0.2	-0.21	0.83	-0.02	0.5
342 Collagen CCBE1(c) synthesis	0.18	-0.01	1	0.17	1
347 Collagen CO3A1(c) synthesis	0.18	-0.2	1	-0.02	1
946 Trehalose	0.17	-0.04	1	0.13	1
875 Nicotinate D-ribonucleoside	0.16	-0.3	0.44	-0.14	0.42
53 activated sulphur	0.16	0.07	0.66	0.22	0.67
796 Glucono-1,5-lactone-6P	0.15	0.3	1	0.45	1
274 beta-Alanine from Asparagine	0.14	-0.04	0.34	0.11	0.3
611 3-Dehydrosphinganine	0.14	-0.05	0.33	0.09	0.44
693 Acetaldehyde	0.13	-0.47	0.54	-0.34	0.61
354 Collagen CO5A1(c) synthesis	0.13	0.05	1	0.18	1
947 Triphosphate degr	0.13	-0.21	1	-0.09	1
794 Galactose-1P	0.13	-0.27	0.61	-0.15	0.66
3 Anaerobic rephosph of ATP	0.13	-0.47	0.28	-0.34	0.42
952 Urate	0.12	-0.34	0.46	-0.22	0.29
810 H2O2	0.12	-0.34	0.46	-0.22	0.29
395 Collagen ITA2(c) synthesis	0.12	0.1	1	0.21	1
868 N-Methylethanolamine-P	0.12	-0.47	1	-0.35	1
771 Ethanolamine-P	0.12	-0.47	1	-0.35	1
372 Collagen CODA1(c) synthesis	0.12	-0.03	1	0.09	1
364 Collagen CO8A2(c) synthesis	0.11	0.01	1	0.12	1
23 Oxidation of NADPH	0.11	-0.23	0.46	-0.12	0.47
37 GSH reduction using NADPH redox potential	0.11	-0.13	1	-0.02	1
38 GSH oxidation	0.11	-0.13	1	-0.02	1
912 Protein(l)ysine	0.1	-0.1	1	0.01	1
363 Collagen CO8A1(c) synthesis	0.09	-0.09	1	-0.0003	1
327 Antichymotrypsin	0.09	0.25	0.97	0.33	0.97
376 Collagen COHA1(c) synthesis	0.08	-0.05	1	0.03	1
887 Oxidized thioredoxin	0.07	-0.05	0.93	0.03	0.93
455 Thioredoxin	0.07	-0.05	0.94	0.03	0.94
367 Collagen CO9A3(c) synthesis	0.07	-0.04	1	0.03	1
953 Urocanate	0.07	-0.2	0.49	-0.12	0.56
353 Collagen CO4A6(c) synthesis	0.07	0.09	1	0.16	1
930 Sphinganine	0.07	0.21	0.45	0.28	0.38
275 beta-Alanine from Aspartate	0.07	0.04	0.52	0.11	0.68
809 Guanosine	0.06	-0.28	0.47	-0.22	0.35
293 Homocysteine from Methionine	0.06	-0.02	1	0.04	1
837 L-Cystathionine	0.06	-0.02	0.47	0.04	0.48
527 Spermidine	0.06	-0.38	0.52	-0.32	0.43
608 2-Oxoglutaramate	0.06	-0.5	1	-0.44	1
529 Methylthioribose-1P	0.05	-0.34	0.71	-0.29	0.56
183 Glutamate from Valine	0.05	-0.24	0.31	-0.19	0.41
326 Fibrinogen	0.04	-0.18	0.36	-0.14	0.3
461 4ppan	0.04	-0.12	1	-0.08	1
495 Mannose-6P	0.03	-0.04	0.91	-0.01	0.78

Continued on next page

Simulation	Δampl	TGFβ/C 6h ampl	TGFβ/C 6h score	TGFβ/C 24h ampl	TGFβ/C 24h score
712 Agmatine	0.03	0.2	0.8	0.23	0.67
371 Collagen COCA1(c) synthesis	0.03	0.33	1	0.36	1
334 ApoE(c)	0.03	0.001	0.95	0.03	0.95
209 Glutamine from Aspartate	0.02	-0.3	0.44	-0.28	0.41
492 Oleate	0.02	-0.31	0.58	-0.29	0.68
454 Glycogenin	0.02	-0.16	0.97	-0.14	0.96
807 Glycogenin-G8	0.02	-0.16	0.97	-0.14	0.95
803 Glycogenin-G11	0.02	-0.16	0.97	-0.14	0.95
804 Glycogenin-G4G4	0.02	-0.16	0.96	-0.14	0.94
805 Glycogenin-G7	0.02	-0.16	0.96	-0.14	0.94
806 Glycogenin-G7G1	0.02	-0.16	0.96	-0.14	0.94
924 SAM	0.02	-0.3	0.59	-0.28	0.43
922 SAH	0.02	-0.3	0.59	-0.28	0.43
493 Stearate	0.01	-0.31	0.66	-0.3	0.67
301 Palmitolate from Arachidonate	0.01	-0.32	0.49	-0.31	0.64
328 Antitrypsin	0.01	-0.18	0.38	-0.17	0.3
490 Palmitate	0.01	-0.32	0.67	-0.31	0.63
673 5-Phosphoribosyl-4-carboxy-5-aminoimidazole	0.001	-0.26	0.53	-0.26	0.35
671 5-Methylthioadenosine	0	-0.3	0.62	-0.3	0.48
382 Collagen COMA1(c) synthesis	-0.002	0.13	1	0.13	1
139 Aspartate from Alanine	-0.003	-0.15	0.54	-0.15	0.53
154 Aspartate from Serine	-0.005	-0.14	0.44	-0.15	0.39
368 Collagen COAA1(c) synthesis	-0.01	0.03	1	0.02	1
829 Inositol	-0.01	-0.11	0.85	-0.12	0.5
337 ApoTransferin	-0.01	-0.33	0.98	-0.34	0.98
358 Collagen CO6A2(c) synthesis	-0.02	0.06	1	0.04	1
753 Deamido-NAD	-0.02	-0.3	0.5	-0.32	0.37
475 dTTP	-0.02	-0.28	0.49	-0.3	0.49
331 ApoC1(c)	-0.02	0.01	0.92	-0.01	0.92
917 Pyridoxine-P	-0.02	-0.12	1	-0.14	1
876 Nicotinate(r)ibonucleotide	-0.02	-0.31	0.48	-0.32	0.41
325 Albumin	-0.02	-0.001	0.97	-0.02	0.97
870 NADH	-0.02	-0.29	0.49	-0.31	0.34
877 O-Acetylcarnitine	-0.02	0.12	1	0.1	1
517 PI	-0.02	-0.12	0.44	-0.14	0.47
774 FMN	-0.02	0.05	1	0.03	1
369 Collagen COBA1(c) synthesis	-0.02	0.0002	1	-0.02	1
859 N-(omega)-Hydroxyarginine	-0.02	0.01	0.77	-0.01	0.77
71 Glycogen glucose storage	-0.02	-0.17	0.97	-0.19	0.75
672 5-Oxoproline	-0.02	-0.06	0.72	-0.08	0.55
918 Quinolinate	-0.02	-0.29	0.54	-0.31	0.63
360 Collagen CO6A5(c) synthesis	-0.03	-0.18	0.38	-0.2	0.28
690 AICAR	-0.03	-0.27	0.54	-0.29	0.45
127 Asparagine from Glutamine	-0.03	-0.4	0.37	-0.43	0.56
940 Sulfite degr	-0.03	0.02	1	-0.01	1
939 Succinyl-CoA	-0.03	-0.27	0.45	-0.3	0.42
361 Collagen CO6A6(c) synthesis	-0.03	-0.18	0.38	-0.21	0.28
392 Collagen FCN3(c) synthesis	-0.03	-0.18	0.37	-0.21	0.29
366 Collagen CO9A2(c) synthesis	-0.03	0.01	1	-0.02	1
351 Collagen CO4A4(c) synthesis	-0.03	0.13	1	0.09	1
397 Collagen PCOTH(c) synthesis	-0.03	-0.18	0.41	-0.21	0.32
383 Collagen CONA1(c) synthesis	-0.03	0.08	1	0.04	1
772 FADH2	-0.03	-0.29	0.41	-0.33	0.33
873 Nicotinamide D-ribonucleotide	-0.04	-0.31	0.57	-0.34	0.46
722 CDP-ethanolamine	-0.04	-0.35	0.47	-0.39	0.46
22 Oxidation of NADH	-0.04	-0.13	0.42	-0.17	0.53
390 Collagen FCN1(c) synthesis	-0.04	-0.18	0.4	-0.22	0.29
786 Fructose-2,6PP	-0.04	-0.02	0.99	-0.06	0.84
451 NAD+	-0.04	-0.29	0.49	-0.33	0.33

Continued on next page

Amplitude differences: TGFβ/contr 6h vs. 24h – continued

Simulation	Δampl	TGFβ/C 6h ampl	TGFβ/C 6h score	TGFβ/C 24h ampl	TGFβ/C 24h score
923 SAICAR	-0.04	-0.21	0.58	-0.25	0.41
14 Ubiquinol-to-ATP	-0.05	-0.04	0.62	-0.09	0.62
381 Collagen COLQ(c) synthesis	-0.05	-0.18	0.41	-0.22	0.31
11 ATP from NADH(m)	-0.05	-0.04	0.78	-0.09	0.8
621 3-Methylglutaconyl-CoA	-0.05	-0.27	0.45	-0.32	0.37
380 Collagen COLA1(c) synthesis	-0.05	-0.18	0.42	-0.22	0.31
333 ApoC3(c)	-0.05	0.04	0.94	-0.004	0.93
387 Collagen COSA1(c) synthesis	-0.05	-0.18	0.42	-0.22	0.32
393 Collagen FMOD(c) synthesis	-0.05	0.02	1	-0.02	1
375 Collagen COGA1(c) synthesis	-0.05	0.25	1	0.2	1
986 trans,cis-octadeca-2,9-dienoyl-CoA	-0.05	-0.28	0.45	-0.33	0.4
435 Galactose degr	-0.05	-0.12	0.52	-0.17	0.59
740 Cholesterol-ester-stea	-0.05	-0.37	0.49	-0.43	0.62
736 Cholesterol-ester-ol	-0.05	-0.37	0.49	-0.43	0.62
773 FAICAR	-0.05	-0.27	0.58	-0.32	0.43
869 N-Pantothenoylcysteine	-0.05	-0.06	0.69	-0.11	0.48
880 OAA	-0.05	-0.1	0.54	-0.16	0.76
706 Acyl-CoA-VLDL-SM-pool	-0.06	-0.29	0.45	-0.34	0.4
702 Acyl-CoA-VLDL-PC-pool	-0.06	-0.29	0.45	-0.34	0.4
700 Acyl-CoA-Bile-PC-pool	-0.06	-0.29	0.45	-0.34	0.4
703 Acyl-CoA-VLDL-PE-pool	-0.06	-0.29	0.46	-0.34	0.4
707 Acyl-CoA-VLDL-TG2-pool	-0.06	-0.29	0.46	-0.34	0.4
708 Acyl-CoA-VLDL-TG3-pool	-0.06	-0.29	0.46	-0.34	0.4
559 (S)-3-hydroxypalmitoleoyl-CoA	-0.06	-0.28	0.44	-0.34	0.35
704 Acyl-CoA-VLDL-PI-pool	-0.06	-0.29	0.46	-0.34	0.4
389 Collagen EMID2(c) synthesis	-0.06	0.15	1	0.09	1
548 (R)-4-Phosphopantothenoyl-cysteine	-0.06	-0.06	0.72	-0.11	0.45
705 Acyl-CoA-VLDL-PS-pool	-0.06	-0.29	0.46	-0.35	0.4
932 Sphingosine	-0.06	-0.2	0.36	-0.26	0.37
701 Acyl-CoA-CL-pool	-0.06	-0.29	0.46	-0.35	0.4
450 CoA	-0.06	-0.27	0.5	-0.34	0.39
984 trans,cis-hexadeca-2,9-dienoyl-CoA	-0.06	-0.28	0.45	-0.34	0.36
895 Palmitoyl-CoA	-0.06	-0.29	0.46	-0.35	0.4
979 mitoOxidizedThioredoxin	-0.06	-0.19	0.95	-0.25	0.94
456 Thioredoxin(m)	-0.06	-0.19	0.96	-0.25	0.95
937 Stearoyl-CoA	-0.06	-0.29	0.46	-0.35	0.4
883 Oleoyl-CoA	-0.06	-0.29	0.46	-0.35	0.4
849 Linoleoyl-CoA	-0.06	-0.29	0.46	-0.35	0.4
715 Arachidonyl-CoA	-0.06	-0.29	0.46	-0.35	0.4
738 Cholesterol-ester-palmn	-0.06	-0.37	0.5	-0.43	0.59
332 ApoC2(c)	-0.06	0.11	0.95	0.05	0.95
6 Aerobic rephosph of UDP	-0.06	-0.05	0.49	-0.11	0.52
976 gamma-Linolenoyl-CoA	-0.06	-0.29	0.44	-0.35	0.38
1 Aerobic ATP rephosph (FA)	-0.06	-0.04	0.43	-0.11	0.45
737 Cholesterol-ester-palm	-0.06	-0.37	0.43	-0.44	0.59
77 Alanine from Asparagine	-0.06	-0.4	0.38	-0.47	0.44
324 Cholesterol(b)	-0.07	-0.37	0.51	-0.44	0.61
709 Adenine	-0.07	-0.29	0.33	-0.36	0.31
733 Cholesterol-ester-arach	-0.07	-0.37	0.47	-0.44	0.64
297 Palmitate from Palmitolate	-0.07	-0.2	0.49	-0.27	0.43
857 Methylmalonyl-CoA	-0.07	-0.26	0.46	-0.32	0.46
535 (2E)-Hexadecenoyl-CoA	-0.07	-0.28	0.47	-0.35	0.36
528 Spermine	-0.07	-0.34	0.52	-0.41	0.41
728 Carnosine	-0.07	0.07	0.64	0.001	0.46
893 PPi	-0.07	-0.16	0.51	-0.24	0.71
453 FAD	-0.07	-0.3	0.45	-0.37	0.35
479 UTP	-0.07	-0.34	0.51	-0.41	0.38
191 Glycine from Glutamate	-0.07	-0.4	0.39	-0.47	0.48
556 (S)-3-Hydroxyhexadecanoyl-CoA	-0.07	-0.28	0.37	-0.36	0.41

Continued on next page

Amplitude differences: TGFβ/contr 6h vs. 24h – continued

Simulation	Δampl	TGFβ/C 6h		TGFβ/C 24h	
		ampl	score	ampl	score
871 NADPH	-0.07	-0.28	0.52	-0.35	0.39
688 ACP	-0.07	0.001	0.83	-0.07	0.86
851 Malonyl-ACP	-0.07	0.001	0.82	-0.07	0.84
697 Acetyl-ACP	-0.07	0.001	0.83	-0.07	0.85
457 Apo-ACP	-0.07	0.001	0.88	-0.07	0.9
695 Acetoacetyl-ACP	-0.07	0.001	0.81	-0.07	0.84
544 (R)-3-Hydroxybutanoyl-ACP	-0.07	0.001	0.81	-0.07	0.84
717 But-2-enoyl-ACP	-0.07	0.001	0.81	-0.07	0.84
718 Butyryl-ACP	-0.07	0.001	0.81	-0.07	0.84
627 3-Oxohexanoyl-ACP	-0.07	0.001	0.81	-0.07	0.83
750 D-3-Hydroxyhexanoyl-ACP	-0.07	0.001	0.8	-0.07	0.83
536 (2E)-Hexenoyl-ACP	-0.07	0.001	0.8	-0.07	0.83
818 Hexanoyl-ACP	-0.07	0.001	0.8	-0.07	0.83
629 3-Oxooctanoyl-ACP	-0.07	0.001	0.79	-0.07	0.82
546 (R)-3-Hydroxyoctanoyl-ACP	-0.07	0.001	0.79	-0.07	0.82
539 (2E)-Octenoyl-ACP	-0.07	0.001	0.79	-0.07	0.82
881 Octanoyl-ACP	-0.07	0.001	0.79	-0.07	0.82
622 3-Oxodecanoyl-ACP	-0.07	0.001	0.79	-0.07	0.82
545 (R)-3-Hydroxydecanoyl-ACP	-0.07	0.001	0.79	-0.07	0.82
530 (2E)-Decenoyl-ACP	-0.07	0.001	0.79	-0.07	0.81
754 Decanoyl-ACP	-0.07	0.001	0.79	-0.07	0.81
624 3-Oxododecanoyl-ACP	-0.07	0.001	0.78	-0.07	0.81
749 D-3-Hydroxydodecanoyl-ACP	-0.07	0.001	0.78	-0.07	0.81
532 (2E)-Dodecenoyl-ACP	-0.07	0.001	0.78	-0.07	0.81
769 Dodecanoyl-ACP	-0.07	0.001	0.78	-0.07	0.81
634 3-Oxotetradecanoyl-ACP	-0.07	0.001	0.78	-0.07	0.81
813 HMA	-0.07	0.001	0.78	-0.07	0.81
541 (2E)-Tetradecenoyl-ACP	-0.07	0.001	0.78	-0.07	0.81
942 Tetradecanoyl-ACP	-0.07	0.001	0.78	-0.07	0.81
626 3-Oxohexadecanoyl-ACP	-0.07	0.001	0.78	-0.07	0.8
547 (R)-3-Hydroxypalmitoyl-ACP	-0.07	0.001	0.78	-0.07	0.8
534 (2E)-Hexadecenoyl-ACP	-0.07	0.001	0.78	-0.07	0.8
816 Hexadecanoyl-ACP	-0.07	0.001	0.78	-0.07	0.8
633 3-Oxostearoyl-ACP	-0.07	0.001	0.77	-0.07	0.79
617 3-Hydroxystearoyl-ACP	-0.07	0.001	0.77	-0.07	0.79
538 (2E)-Octadecenoyl-ACP	-0.07	0.001	0.77	-0.07	0.79
936 Stearoyl-ACP	-0.07	0.001	0.77	-0.07	0.79
458 Apo-ACP(m)	-0.07	0.001	0.66	-0.07	0.7
477 CTP	-0.07	-0.33	0.49	-0.4	0.36
978 mitoACP	-0.07	0.001	0.63	-0.07	0.68
711 Adenylyl(s)ulfate	-0.08	-0.28	0.55	-0.35	0.45
739 Cholesterol-ester-pool	-0.08	-0.36	0.45	-0.44	0.61
373 Collagen COEA1(c) synthesis	-0.08	0.06	1	-0.02	1
473 dCTP	-0.08	-0.33	0.48	-0.41	0.41
551 (R)-Methylmalonyl-CoA	-0.08	-0.28	0.45	-0.36	0.38
685 7alpha-Hydroxy-5beta-cholestan-3-one	-0.08	-0.37	0.49	-0.45	0.55
204 Glycine from Valine	-0.08	-0.39	0.39	-0.47	0.47
758 Deoxycytidine	-0.08	-0.3	0.46	-0.37	0.43
300 Stearate from Oleate	-0.08	-0.21	0.51	-0.29	0.52
683 7alpha,12alpha-Dihydroxy-5beta-cholestan-3-one	-0.08	-0.37	0.46	-0.45	0.52
885 Orotidine-5P	-0.08	-0.28	0.59	-0.36	0.37
452 NADP+	-0.08	-0.28	0.52	-0.36	0.38
67 dTTP salvage from Thymine	-0.08	-0.26	0.51	-0.35	0.47
967 dCMP	-0.08	-0.3	0.49	-0.38	0.45
523 Cholesterol	-0.08	-0.37	0.57	-0.45	0.63
686 7alpha-Hydroxycholest-4-en-3-one	-0.08	-0.37	0.51	-0.46	0.57
687 7alpha-Hydroxycholesterol	-0.08	-0.37	0.54	-0.46	0.6
195 Glycine from Leucine	-0.08	-0.36	0.34	-0.44	0.39
981 palmitoleoyl-CoA	-0.08	-0.28	0.46	-0.37	0.35

Continued on next page

Amplitude differences: TGFβ/contr 6h vs. 24h – continued

Simulation	Δampl	TGFβ/C 6h		TGFβ/C 24h	
		ampl	score	ampl	score
271 Tyrosine from Phenylalanine	-0.08	-0.45	0.44	-0.53	0.59
734 Cholesterol-ester-gla	-0.08	-0.36	0.47	-0.44	0.63
684 7alpha,12alpha-Dihydroxycholest-4-en-3-one	-0.08	-0.37	0.47	-0.46	0.53
359 Collagen CO6A3(c) synthesis	-0.09	0.32	1	0.23	1
761 Deoxyuridine	-0.09	-0.35	0.41	-0.43	0.29
248 Proline from Valine	-0.09	-0.35	0.46	-0.44	0.53
199 Glycine from Proline	-0.09	-0.39	0.51	-0.47	0.6
735 Cholesterol-ester-lin	-0.09	-0.36	0.47	-0.45	0.62
762 Dephospho-CoA	-0.09	-0.29	0.45	-0.37	0.33
959 cGMP	-0.09	-0.27	0.42	-0.36	0.31
679 5beta-Cholestane-3alpha,7alpha-diol	-0.09	-0.36	0.51	-0.45	0.52
406 Heme	-0.09	-0.31	0.43	-0.4	0.59
340 Collagen BGH3(c) synthesis	-0.09	0.06	1	-0.04	1
652 3alpha,7alpha,12alpha-Trihydroxycoprostane	-0.09	-0.36	0.48	-0.45	0.5
604 2-Methylbutyryl-CoA	-0.09	-0.26	0.5	-0.36	0.35
631 3-Oxopalmitoyl-CoA	-0.09	-0.29	0.34	-0.38	0.41
846 Lathosterol	-0.09	-0.36	0.58	-0.45	0.71
915 Provitamin D3	-0.09	-0.36	0.55	-0.45	0.67
698 Acetyl-CoA	-0.09	-0.29	0.49	-0.38	0.38
232 Proline from Aspartate	-0.09	-0.32	0.59	-0.41	0.53
696 Acetoacetyl-CoA	-0.09	-0.29	0.47	-0.38	0.36
763 Desmosterol	-0.09	-0.38	0.58	-0.47	0.62
723 CMP	-0.1	-0.31	0.47	-0.41	0.38
966 dCDP	-0.1	-0.32	0.46	-0.42	0.42
176 Glutamate from Methionine	-0.1	-0.4	0.35	-0.5	0.47
798 Glucosylceramide-pool	-0.1	-0.17	0.42	-0.27	0.37
676 5alpha-Cholesta-7,24-dien-3beta-ol	-0.1	-0.37	0.59	-0.47	0.7
720 CDP	-0.1	-0.32	0.47	-0.42	0.38
682 7-Dehydrodesmosterol	-0.1	-0.37	0.55	-0.47	0.66
148 Aspartate from Glutamine	-0.1	-0.14	0.37	-0.24	0.49
34 Thioredoxin(c) reduction	-0.1	0.01	0.71	-0.09	0.92
732 Cholestenol	-0.1	-0.36	0.59	-0.47	0.7
974 dUTP	-0.1	-0.33	0.45	-0.43	0.4
647 3alpha,7alpha,12alpha-Trihydroxy-5beta-24-oxocholestanoyl-CoA	-0.1	-0.34	0.43	-0.45	0.5
741 Choloyl-CoA	-0.1	-0.37	0.4	-0.47	0.47
646 3alpha,7alpha,12alpha,24-Tetrahydroxy-5beta-cholestanoyl-CoA	-0.11	-0.34	0.44	-0.45	0.49
194 Glycine from Glutamine	-0.11	-0.41	0.42	-0.51	0.44
240 Proline from Leucine	-0.11	-0.3	0.45	-0.4	0.5
620 3-Methylcrotonyl-CoA	-0.11	-0.25	0.49	-0.36	0.45
464 mlthf	-0.11	0.01	0.77	-0.1	0.8
664 5,10-Methenyl-THF	-0.11	0.01	0.62	-0.1	0.65
467 1fthf	-0.11	0.01	0.51	-0.1	0.54
466 THF(m)	-0.11	0.01	1	-0.1	1
459 THF	-0.11	0.01	1	-0.1	1
29 NADH to NADPH transhydrogenase	-0.11	0.01	1	-0.1	1
766 Dihydrofolate	-0.11	0.01	1	-0.1	1
670 5-Methyl-THF	-0.11	0.01	1	-0.1	1
965 dAMP	-0.11	-0.3	0.54	-0.41	0.45
757 Deoxyadenosine	-0.11	-0.29	0.44	-0.4	0.36
783 Formamidopyrimidine(n)leoside triphosphate	-0.11	-0.25	0.47	-0.36	0.32
218 Glutamine from Methionine	-0.11	-0.42	0.38	-0.53	0.5
888 PAP	-0.11	-0.29	0.46	-0.4	0.38
256 Serine from Glutamate	-0.11	-0.36	0.37	-0.47	0.54
378 Collagen COJA1(c) synthesis	-0.11	0.04	1	-0.07	1
914 Protoporphyrinogen IX	-0.11	-0.32	0.49	-0.43	0.58
506 Uridine	-0.11	-0.34	0.49	-0.45	0.32
2 Aerobic ATP rephosph (gluc)	-0.11	-0.04	0.42	-0.15	0.44
648 3alpha,7alpha,12alpha-Trihydroxy-5beta-cholest-24-enoyl-CoA	-0.11	-0.35	0.44	-0.46	0.48
972 dUDP	-0.11	-0.33	0.46	-0.44	0.39

Continued on next page

Amplitude differences: TGFβ/contr 6h vs. 24h – continued

Simulation	Δampl	TGFβ/C 6h		TGFβ/C 24h	
		ampl	score	ampl	score
186 Glycine from Arginine	-0.11	-0.38	0.39	-0.49	0.42
595 2,5-Diaminopyrimidine(n)leoside triphosphate	-0.11	-0.23	0.49	-0.34	0.31
957 Zymosterol	-0.11	-0.37	0.6	-0.48	0.69
662 4alpha-Methylzymosterol	-0.11	-0.37	0.6	-0.48	0.69
346 Collagen CO2A1(c) synthesis	-0.12	0.28	1	0.17	1
145 Aspartate from Glycine	-0.12	-0.11	0.36	-0.22	0.44
913 Protoporphyrin	-0.12	-0.31	0.49	-0.42	0.58
141 Aspartate from Asparagine	-0.12	-0.1	0.46	-0.22	0.54
824 Hydroxymethylbilane	-0.12	-0.32	0.55	-0.44	0.64
724 CMP-NeuNGc	-0.12	-0.26	0.4	-0.38	0.29
964 dADP	-0.12	-0.3	0.46	-0.42	0.38
954 UroporphyrinogenIII	-0.12	-0.32	0.52	-0.44	0.61
744 CoproporphyrinogenIII	-0.12	-0.32	0.51	-0.44	0.59
858 Myristoyl-CoA	-0.12	-0.29	0.35	-0.41	0.44
142 Aspartate from Cysteine	-0.12	-0.1	0.49	-0.22	0.54
822 Homovanillate	-0.12	0.1	1	-0.02	1
249 Proline from beta-Alanine	-0.12	-0.31	0.55	-0.43	0.44
651 3alpha,7alpha,12alpha-Trihydroxy-5beta-cholestanoyl-CoA	-0.12	-0.33	0.46	-0.45	0.45
239 Proline from Glutamine	-0.12	-0.33	0.53	-0.45	0.5
594 2,5-Diamino-6-(5-triphosphoryl-3,4-trihydroxy-2-oxopentyl)- amino-4-oxopyrimidine	-0.12	-0.22	0.49	-0.34	0.31
212 Glutamine from Glutamate	-0.12	-0.04	0.29	-0.16	0.33
253 Serine from Aspartate	-0.12	-0.39	0.42	-0.51	0.49
557 (S)-3-Hydroxytetradecanoyl-CoA	-0.12	-0.29	0.31	-0.41	0.38
385 Collagen COPA1(c) synthesis	-0.12	0.24	1	0.11	1
252 Serine from Asparagine	-0.12	-0.41	0.45	-0.54	0.52
635 3-Oxotetradecanoyl-CoA	-0.12	-0.29	0.31	-0.41	0.4
193 Glycine from Isoleucine	-0.12	-0.4	0.39	-0.52	0.57
801 Glutaryl-CoA	-0.13	-0.28	0.42	-0.41	0.42
731 Cholate	-0.13	-0.37	0.39	-0.5	0.52
388 Collagen CTHR1(c) synthesis	-0.13	0.1	1	-0.02	1
597 2-Amino-3-oxoadipate	-0.13	-0.15	0.59	-0.27	0.56
230 Proline from Arginine	-0.13	-0.28	0.49	-0.41	0.41
692 AMP	-0.13	-0.29	0.48	-0.42	0.35
242 Proline from Methionine	-0.13	-0.38	0.44	-0.5	0.53
542 (2E)-Tetradecenoyl-CoA	-0.13	-0.27	0.37	-0.4	0.44
689 ADP	-0.13	-0.29	0.47	-0.42	0.34
847 Lauroyl-CoA	-0.13	-0.27	0.34	-0.4	0.44
644 3-oxopalmitoleoyl-CoA	-0.13	-0.28	0.42	-0.41	0.37
948 UDP-N-acetylglucosamine	-0.13	-0.31	0.41	-0.44	0.29
853 Malonyl-CoA	-0.13	-0.28	0.48	-0.41	0.42
260 Serine from Glutamine	-0.13	-0.38	0.4	-0.51	0.48
472 dATP	-0.13	-0.35	0.53	-0.48	0.42
462 thbpt	-0.13	-0.3	0.41	-0.43	0.44
511 UDP-N-acetylgalactosamine	-0.13	-0.31	0.4	-0.44	0.28
864 N-Acetylneuraminate-9P	-0.13	-0.22	0.41	-0.35	0.34
800 Glutamyl-5P	-0.13	-0.32	0.55	-0.45	0.54
854 Maltose	-0.13	-0.05	1	-0.18	1
833 Isovaleryl-CoA	-0.14	-0.27	0.44	-0.41	0.37
229 Proline from Alanine	-0.14	-0.28	0.64	-0.42	0.42
951 UMP	-0.14	-0.3	0.46	-0.44	0.44
710 Adenylosuccinate	-0.14	-0.3	0.56	-0.43	0.4
848 Linoleate	-0.14	-0.36	0.42	-0.5	0.62
474 dGTP	-0.14	-0.34	0.48	-0.48	0.39
487 Proline	-0.14	-0.3	0.45	-0.44	0.48
968 dGDP	-0.14	-0.29	0.45	-0.43	0.37
246 Proline from Tryptophan	-0.14	-0.32	0.5	-0.47	0.51
969 dGMP	-0.14	-0.29	0.45	-0.43	0.37
862 N-Acetylmannosamine-6P	-0.14	-0.22	0.58	-0.36	0.36
958 cAMP	-0.15	-0.29	0.43	-0.44	0.31

Continued on next page

Simulation	Δampl	TGFβ/C 6h ampl	TGFβ/C 6h score	TGFβ/C 24h ampl	TGFβ/C 24h score
759 Deoxyguanosine	-0.15	-0.29	0.42	-0.43	0.34
140 Aspartate from Arginine	-0.15	-0.09	0.41	-0.24	0.48
787 Fumarate	-0.15	-0.11	0.84	-0.26	0.74
201 Glycine from Threonine	-0.15	-0.41	0.42	-0.56	0.56
790 GAR	-0.15	-0.17	0.57	-0.33	0.5
618 3-Keto-4-methylzymosterol	-0.15	-0.35	0.58	-0.51	0.68
238 Proline from Isoleucine	-0.15	-0.31	0.37	-0.47	0.61
970 dTDP	-0.15	-0.33	0.39	-0.48	0.34
463 dhbpt	-0.15	-0.28	0.4	-0.44	0.46
521 Ceramide	-0.16	-0.34	0.37	-0.5	0.48
911 Propanoyl-CoA	-0.16	-0.29	0.4	-0.45	0.34
109 Arginine from Methionine	-0.16	-0.38	0.38	-0.54	0.48
315 Glycocholate(b)	-0.16	-0.37	0.44	-0.53	0.55
123 Asparagine from Glutamate	-0.16	-0.07	0.31	-0.23	0.42
74 Gluconeogen from Glycerol	-0.16	-0.53	0.36	-0.69	0.51
476 ATP	-0.16	-0.35	0.55	-0.51	0.4
603 2-Methylacetoacetyl-CoA	-0.16	-0.27	0.43	-0.44	0.4
674 5-Phosphoribosylamine	-0.16	-0.18	0.47	-0.35	0.51
162 Cystine from Cysteine	-0.16	-0.73	0.61	-0.89	0.51
160 Cysteine from Cystine	-0.16	-0.73	0.61	-0.89	0.51
251 Serine from Arginine	-0.17	-0.32	0.37	-0.48	0.44
270 Serine from beta-Alanine	-0.17	-0.37	0.4	-0.54	0.42
960 cis-(3S)-hydroxytetradec-7-enoyl-CoA	-0.17	-0.28	0.4	-0.45	0.39
985 trans,cis-myristo-2,7-dienoyl-CoA	-0.17	-0.28	0.41	-0.45	0.39
745 Crotonyl-CoA	-0.17	-0.28	0.44	-0.45	0.4
478 GTP	-0.17	-0.34	0.49	-0.51	0.37
400 Collagen VWA2(c) synthesis	-0.17	0.19	1	0.02	1
265 Serine from Proline	-0.17	-0.31	0.45	-0.48	0.56
19 Aerobic reduction of NADP+ (gluc)	-0.17	-0.17	0.42	-0.34	0.31
642 3-oxomyrist-7-enoyl-CoA	-0.17	-0.28	0.4	-0.45	0.4
963 cis-myrist-7-enoyl-CoA	-0.17	-0.28	0.41	-0.45	0.39
681 6-Pyruvoyltetrahydropterin	-0.17	-0.23	0.46	-0.41	0.38
159 Aspartate from beta-Alanine	-0.17	-0.07	0.43	-0.24	0.49
663 4alpha-Methylzymosterol-4-carboxylate	-0.17	-0.36	0.61	-0.53	0.69
524 gdpddman	-0.17	-0.31	0.46	-0.49	0.32
269 Serine from Valine	-0.17	-0.3	0.35	-0.47	0.44
678 5beta-Cholestane-3alpha,7alpha,26-triol	-0.17	-0.36	0.49	-0.53	0.52
643 3-oxooleoyl-CoA	-0.18	-0.28	0.44	-0.46	0.37
943 Thymidine	-0.18	-0.35	0.33	-0.53	0.29
610 3(S)-3-hydroxydodecen-(5Z)-oyl-CoA	-0.18	-0.28	0.39	-0.46	0.4
983 trans,cis-dodeca-2,5-dienoyl-CoA	-0.18	-0.28	0.39	-0.46	0.4
144 Aspartate from Glutamate	-0.18	-0.07	0.47	-0.25	0.64
971 dTMP	-0.18	-0.35	0.36	-0.53	0.31
501 GDP-L-fucose	-0.18	-0.31	0.45	-0.49	0.31
641 3-oxolaur-cis-5-enoyl-CoA	-0.18	-0.28	0.38	-0.46	0.41
987 trans-4-Hydroxy-L-proline	-0.18	-0.06	1	-0.24	1
835 L-1-Pyrroline-3-hydroxy-5-carboxylate	-0.18	-0.06	1	-0.24	1
677 5beta-Cholestane-3alpha,7alpha,12alpha,26-tetrol	-0.18	-0.36	0.46	-0.54	0.49
650 3alpha,7alpha,12alpha-Trihydroxy-5beta-cholestanate	-0.18	-0.36	0.46	-0.54	0.49
649 3alpha,7alpha,12alpha-Trihydroxy-5beta-cholestan-26-al	-0.18	-0.36	0.46	-0.54	0.49
962 cis-laur-5-enoyl-CoA	-0.18	-0.28	0.39	-0.46	0.41
592 1-Pyrroline-5-carboxylate	-0.18	-0.06	0.87	-0.24	0.76
719 Butyryl-CoA	-0.18	-0.26	0.32	-0.44	0.47
558 (S)-3-hydroxyoleyleoyl-CoA	-0.18	-0.28	0.43	-0.47	0.4
665 5,6-Dihydrouracil	-0.18	-0.32	0.48	-0.51	0.41
543 (3Z)-Dodecenoyl-CoA	-0.18	-0.28	0.38	-0.47	0.41
317 Taurocholate(b)	-0.18	-0.36	0.39	-0.55	0.51
245 Proline from Threonine	-0.19	-0.29	0.4	-0.47	0.61
661 4-Methyl-2-oxopentanoate	-0.19	-0.24	0.4	-0.42	0.4

Continued on next page

Amplitude differences: TGFβ/contr 6h vs. 24h – continued

Simulation	Δampl	TGFβ/C 6h		TGFβ/C 24h	
		ampl	score	ampl	score
336 Prothrombin	-0.19	0.03	0.97	-0.16	0.98
791 GDP	-0.19	-0.28	0.46	-0.46	0.33
792 GMP	-0.19	-0.28	0.48	-0.46	0.35
509 Guanine	-0.19	-0.35	0.46	-0.54	0.33
616 3-Hydroxypropionyl-CoA	-0.19	-0.28	0.4	-0.47	0.39
666 5-Aminolevulinate	-0.19	-0.13	0.54	-0.33	0.61
503 IMP	-0.19	-0.35	0.54	-0.54	0.39
502 GDP-mannose	-0.19	-0.32	0.46	-0.51	0.32
973 dUMP	-0.19	-0.35	0.39	-0.55	0.33
153 Aspartate from Proline	-0.19	-0.24	0.48	-0.43	0.64
628 3-Oxohexanoyl-CoA	-0.2	-0.26	0.34	-0.45	0.46
504 XMP	-0.2	-0.34	0.54	-0.54	0.38
828 Inosine	-0.2	-0.28	0.45	-0.48	0.32
174 Glutamate from Leucine	-0.2	-0.17	0.42	-0.37	0.39
158 Aspartate from Valine	-0.2	-0.21	0.34	-0.41	0.42
884 Orotate	-0.2	-0.34	0.54	-0.54	0.43
178 Glutamate from Proline	-0.2	-0.26	0.64	-0.46	0.62
339 Collagen ADIPO(c) synthesis	-0.2	0.13	1	-0.07	1
508 Xanthine	-0.2	-0.35	0.48	-0.55	0.34
241 Proline from Lysine	-0.2	-0.3	0.53	-0.5	0.54
554 (S)-3-Hydroxybutyryl-CoA	-0.2	-0.29	0.45	-0.49	0.38
338 Haptoglobin	-0.2	0.02	0.96	-0.18	0.97
258 Serine from Histidine	-0.2	-0.34	0.42	-0.54	0.53
630 3-Oxooctanoyl-CoA	-0.2	-0.28	0.33	-0.48	0.44
850 Malate	-0.21	-0.1	0.79	-0.31	0.7
287 beta-Alanine from Proline	-0.21	-0.23	0.41	-0.44	0.49
819 Hexanoyl-CoA	-0.21	-0.27	0.34	-0.48	0.45
562 (S)-Hydroxyhexanoyl-CoA	-0.21	-0.27	0.32	-0.48	0.42
95 Alanine from Valine	-0.21	-0.22	0.36	-0.42	0.49
623 3-Oxodecanoyl-CoA	-0.21	-0.27	0.35	-0.48	0.44
563 (S)-Hydroxyoctanoyl-CoA	-0.21	-0.28	0.33	-0.48	0.44
882 Octanoyl-CoA	-0.21	-0.27	0.35	-0.48	0.45
537 (2E)-Hexenoyl-CoA	-0.21	-0.26	0.35	-0.47	0.45
235 Proline from Glutamate	-0.21	-0.26	0.61	-0.47	0.57
540 (2E)-Octenoyl-CoA	-0.21	-0.26	0.36	-0.47	0.44
561 (S)-Hydroxydecanoyl-CoA	-0.21	-0.27	0.35	-0.48	0.44
531 (2E)-Decenoyl-CoA	-0.21	-0.27	0.35	-0.48	0.44
945 Tiglyl-CoA	-0.21	-0.27	0.4	-0.48	0.4
520 SM	-0.21	-0.37	0.39	-0.58	0.47
840 L-Glutamate 5-semialdehyde	-0.21	-0.26	0.49	-0.47	0.42
625 3-Oxododecanoyl-CoA	-0.21	-0.27	0.37	-0.49	0.45
675 5-Phosphoribosylformylglycinamidine	-0.21	-0.32	0.54	-0.53	0.42
721 CDP-choline	-0.21	-0.35	0.5	-0.56	0.37
755 Decanoyl-CoA	-0.21	-0.27	0.37	-0.49	0.45
282 beta-Alanine from Glutamine	-0.21	-0.17	0.4	-0.38	0.32
356 Collagen CO5A3(c) synthesis	-0.21	0.14	1	-0.07	1
90 Alanine from Proline	-0.22	-0.22	0.52	-0.44	0.54
149 Aspartate from Leucine	-0.22	-0.1	0.42	-0.32	0.45
691 AIR	-0.22	-0.31	0.54	-0.53	0.41
262 Serine from Lysine	-0.22	-0.34	0.39	-0.55	0.48
699 Acrylyl-CoA	-0.22	-0.28	0.42	-0.5	0.39
553 (S)-3-Hydroxy-2-methylbutyryl-CoA	-0.22	-0.29	0.38	-0.51	0.39
555 (S)-3-Hydroxydodecanoyl-CoA	-0.22	-0.27	0.38	-0.49	0.43
533 (2E)-Dodecenoyl-CoA	-0.22	-0.27	0.38	-0.49	0.44
86 Alanine from Leucine	-0.22	-0.16	0.4	-0.39	0.35
216 Glutamine from Leucine	-0.22	-0.16	0.35	-0.38	0.38
81 Alanine from Glutamate	-0.22	-0.18	0.4	-0.4	0.51
961 cis-Aconitate	-0.22	-0.09	0.71	-0.32	0.67
841 L-Lactate	-0.23	0.01	0.85	-0.22	0.94

Continued on next page

Amplitude differences: TGFβ/contr 6h vs. 24h – continued

Simulation		Δampl	TGFβ/C 6h		TGFβ/C 24h	
			ampl	score	ampl	score
266	Serine from Threonine	-0.23	-0.34	0.37	-0.57	0.58
955	Xanthosine	-0.23	-0.28	0.46	-0.5	0.31
20	Aerobic reduction of FAD (gluc)	-0.23	-0.26	0.38	-0.5	0.48
469	ametam	-0.24	-0.32	0.46	-0.55	0.32
128	Asparagine from Leucine	-0.24	-0.11	0.35	-0.34	0.38
921	Ribulose-5P	-0.24	-0.16	0.55	-0.4	0.37
132	Asparagine from Proline	-0.24	-0.28	0.47	-0.52	0.58
225	Glutamine from Valine	-0.24	-0.21	0.32	-0.45	0.38
220	Glutamine from Proline	-0.24	-0.23	0.42	-0.48	0.46
860	N-Acetyl-D-mannosamine	-0.24	-0.2	0.47	-0.45	0.34
116	Arginine from Valine	-0.24	-0.24	0.34	-0.48	0.41
43	Activated methyl group (SAM)	-0.25	-0.42	0.43	-0.67	0.41
21	Aerobic reduction of FAD (FA)	-0.25	-0.28	0.37	-0.53	0.52
742	Citrate	-0.25	-0.09	0.67	-0.34	0.71
283	beta-Alanine from Leucine	-0.25	-0.08	0.36	-0.33	0.39
66	ATP salvage from Hypoxanthine	-0.25	-0.06	0.34	-0.31	0.44
894	PRPP	-0.25	-0.16	0.57	-0.41	0.48
76	Alanine from Arginine	-0.25	-0.17	0.43	-0.42	0.38
322	PS(b)	-0.25	-0.07	0.34	-0.32	0.49
516	PS	-0.25	-0.07	0.34	-0.32	0.49
938	Succinate	-0.25	-0.19	0.52	-0.44	0.56
379	Collagen COKA1(c) synthesis	-0.26	0.26	1	0.001	1
25	NADH redox potential into peroxy	-0.26	-0.06	0.52	-0.32	0.83
261	Serine from Leucine	-0.26	-0.18	0.33	-0.44	0.4
867	N-Formyl-GAR	-0.26	-0.27	0.55	-0.53	0.45
107	Arginine from Leucine	-0.26	-0.16	0.39	-0.42	0.37
916	Pyridoxal	-0.26	0.16	0.88	-0.11	0.66
510	Adenosine	-0.26	-0.34	0.52	-0.61	0.43
764	Dihomo-gamma-linolenoyl-CoA	-0.27	-0.3	0.43	-0.57	0.39
137	Asparagine from Valine	-0.27	-0.26	0.34	-0.52	0.42
499	udpgal	-0.27	-0.32	0.56	-0.58	0.36
13	NADH potential transport	-0.27	-0.07	0.41	-0.34	0.59
365	Collagen CO9A1(c) synthesis	-0.27	0.25	1	-0.02	1
5	Aerobic rephosph of CTP	-0.27	-0.05	0.4	-0.32	0.38
515	PE	-0.27	-0.06	0.34	-0.33	0.54
323	PE(b)	-0.27	-0.06	0.31	-0.33	0.48
889	PE-PS-VLDL-pool	-0.27	-0.06	0.32	-0.33	0.57
615	3-Hydroxyisobutyryl-CoA	-0.28	-0.28	0.43	-0.55	0.32
111	Arginine from Proline	-0.28	-0.22	0.45	-0.5	0.48
460	Pyridoxal-P	-0.28	0.16	0.53	-0.12	0.76
814	HMG-CoA	-0.28	-0.27	0.5	-0.56	0.39
831	Isobutyryl-CoA	-0.29	-0.28	0.48	-0.56	0.34
419	Proline degr	-0.29	-0.22	0.44	-0.51	0.5
10	Anaerobic rephosph of UTP	-0.29	-0.04	0.3	-0.33	0.45
8	Anaerobic rephosph of GTP	-0.29	-0.04	0.3	-0.33	0.45
9	Anaerobic rephosph of CTP	-0.29	-0.04	0.3	-0.33	0.45
311	Urea from glutamine	-0.29	-0.18	0.41	-0.47	0.37
91	Alanine from Serine	-0.3	-0.38	0.4	-0.67	0.56
208	Glutamine from Asparagine	-0.3	-0.38	0.46	-0.68	0.42
150	Aspartate from Lysine	-0.3	-0.07	0.36	-0.38	0.49
640	3-Ureidopropionate	-0.3	-0.32	0.43	-0.63	0.43
292	beta-Alanine from Valine	-0.3	-0.23	0.29	-0.54	0.37
442	Haptoglobin degr	-0.3	-0.08	0.43	-0.38	0.53
852	Malonyl-Carnitin	-0.3	-0.14	0.56	-0.45	0.8
87	Alanine from Lysine	-0.31	-0.23	0.37	-0.53	0.44
237	Proline from Histidine	-0.31	-0.28	0.56	-0.59	0.49
423	Valine degr	-0.31	-0.2	0.35	-0.51	0.4
498	UDP-glucose	-0.31	-0.32	0.5	-0.62	0.36
134	Asparagine from Threonine	-0.31	-0.27	0.31	-0.57	0.54

Continued on next page

Amplitude differences: TGFβ/contr 6h vs. 24h – continued

Simulation		Δampl	TGFβ/C 6h		TGFβ/C 24h	
			ampl	score	ampl	score
7	Aerobic rephosph of UTP	-0.31	-0.04	0.46	-0.35	0.48
606	2-Oxoadipate	-0.31	-0.21	0.37	-0.52	0.41
70	Formate degr	-0.31	-0.06	0.75	-0.37	0.61
4	Aerobic rephosph of GTP	-0.31	-0.04	0.46	-0.35	0.47
329	ApoA1	-0.31	0.04	0.95	-0.27	0.97
906	Phosphodimethylethanolamine	-0.32	-0.48	1	-0.8	1
905	Phosphocholine	-0.32	-0.48	1	-0.8	1
284	beta-Alanine from Lysine	-0.32	-0.07	0.34	-0.38	0.4
908	Porphobilinogen	-0.32	-0.14	0.46	-0.46	0.56
550	(R)-5-Phosphomevalonate	-0.32	-0.18	0.53	-0.5	0.49
488	Serine	-0.32	-0.37	0.5	-0.69	0.57
920	Ribose-5P	-0.32	-0.16	0.52	-0.48	0.43
72	Glycogen glucose release	-0.32	0.07	0.42	-0.25	0.45
730	Chenodeoxycholoyl-CoA	-0.32	-0.34	0.49	-0.66	0.48
264	Serine from Phenylalanine	-0.32	-0.34	0.45	-0.66	0.51
402	HDL	-0.33	0.04	0.67	-0.28	0.78
180	Glutamate from Threonine	-0.33	-0.17	0.32	-0.5	0.56
519	LacCer	-0.33	-0.22	0.42	-0.55	0.43
357	Collagen CO6A1(c) synthesis	-0.33	0.22	1	-0.11	1
146	Aspartate from Histidine	-0.34	-0.17	0.52	-0.51	0.48
243	Proline from Phenylalanine	-0.34	-0.29	0.53	-0.63	0.52
273	beta-Alanine from Arginine	-0.34	-0.13	0.34	-0.47	0.34
278	beta-Alanine from Glutamate	-0.34	-0.06	0.36	-0.4	0.39
415	Leucine degr	-0.34	-0.09	0.39	-0.43	0.37
659	4-Imidazolone-5-propanoate	-0.34	-0.17	0.45	-0.51	0.35
108	Arginine from Lysine	-0.34	-0.26	0.37	-0.61	0.45
231	Proline from Asparagine	-0.35	-0.32	0.57	-0.67	0.5
83	Alanine from Histidine	-0.35	-0.18	0.5	-0.53	0.43
92	Alanine from Threonine	-0.35	-0.22	0.37	-0.56	0.56
465	acgam6p	-0.35	-0.74	0.55	-1.09	0.52
298	Palmitolate from Palmitate	-0.35	0.03	0.5	-0.32	0.7
155	Aspartate from Threonine	-0.35	-0.2	0.34	-0.55	0.55
244	Proline from Serine	-0.35	-0.3	0.53	-0.65	0.48
765	Dihydroceramide-pool	-0.35	0.21	0.34	-0.14	0.36
39	Proton-gradient(m) build up	-0.35	-0.04	0.31	-0.39	0.45
40	Proton-gradient(c) build up	-0.35	-0.04	0.31	-0.39	0.45
483	Aspartate	-0.35	-0.17	0.49	-0.53	0.47
175	Glutamate from Lysine	-0.35	-0.28	0.45	-0.64	0.51
321	SM(b)	-0.36	-0.37	0.37	-0.73	0.47
98	Arginine from Asparagine	-0.36	-0.31	0.37	-0.66	0.38
482	Asparagine	-0.36	-0.22	0.44	-0.58	0.47
267	Serine from Tryptophan	-0.36	-0.38	0.43	-0.74	0.5
866	N-Formimino-L-glutamate	-0.36	-0.2	0.57	-0.56	0.49
812	HCO3-	-0.36	-0.1	0.66	-0.46	0.68
727	Carbonate	-0.36	-0.1	0.66	-0.46	0.68
93	Alanine from Tryptophan	-0.36	-0.37	0.39	-0.73	0.5
414	Glutamine degr	-0.36	-0.07	0.4	-0.43	0.38
125	Asparagine from Histidine	-0.36	-0.22	0.42	-0.58	0.43
751	D-Xylulose-5P	-0.37	-0.11	0.53	-0.48	0.47
413	Histidine degr	-0.37	-0.18	0.43	-0.55	0.43
280	beta-Alanine from Histidine	-0.37	-0.2	0.38	-0.57	0.41
601	2-Deoxy-D-ribose-5P	-0.37	-0.05	0.43	-0.42	0.44
46	Activated methyl group from Histidine	-0.37	-0.17	0.39	-0.55	0.38
316	Gly-CD-cholate(b)	-0.38	-0.35	0.54	-0.73	0.57
106	Arginine from Glutamine	-0.38	-0.19	0.32	-0.57	0.36
114	Arginine from Tryptophan	-0.38	-0.32	0.39	-0.7	0.44
129	Asparagine from Lysine	-0.38	-0.27	0.34	-0.65	0.46
525	beta-Alanine	-0.38	-0.2	0.34	-0.58	0.33
779	Fatty-acid-VLDL-SM-pool	-0.38	0.04	1	-0.34	1

Continued on next page

Simulation	Δampl	TGFβ/C 6h ampl	TGFβ/C 6h score	TGFβ/C 24h ampl	TGFβ/C 24h score
775 Fatty-acid-VLDL-PC-pool	-0.38	0.04	1	-0.34	1
18 Aerobic reduction of NADP+ (FA)	-0.39	-0.15	0.4	-0.53	0.37
68 O2.-(c) degr	-0.39	-0.002	0.68	-0.39	0.72
119 Asparagine from Arginine	-0.39	-0.25	0.33	-0.64	0.42
636 3-Phosphonooxypyruvate	-0.39	-0.26	0.36	-0.65	0.51
319 Chenodiol(b)	-0.39	-0.37	0.49	-0.76	0.6
570 1,2-Diacylglycerol-VLDL-TG-pool	-0.39	0.04	0.44	-0.35	0.47
564 1,2-Diacylglycerol-Bile-PC-pool	-0.39	0.04	0.43	-0.35	0.46
565 1,2-Diacylglycerol-VLDL-PC-pool	-0.39	0.04	0.45	-0.35	0.47
904 Phosphatidate-VLDL-TG-pool	-0.39	0.04	0.51	-0.35	0.52
897 Phosphatidate-Bile-PC-pool	-0.39	0.04	0.49	-0.35	0.5
569 1,2-Diacylglycerol-VLDL-SM-pool	-0.39	0.05	0.44	-0.35	0.47
899 Phosphatidate-VLDL-PC-pool	-0.39	0.04	0.51	-0.35	0.52
903 Phosphatidate-VLDL-SM-pool	-0.39	0.05	0.5	-0.35	0.52
222 Glutamine from Threonine	-0.39	-0.09	0.25	-0.49	0.6
836 L-2-Aminoadipate	-0.4	-0.04	1	-0.43	1
566 1,2-Diacylglycerol-VLDL-PE-pool	-0.4	0.05	0.45	-0.35	0.47
900 Phosphatidate-VLDL-PE-pool	-0.4	0.05	0.51	-0.35	0.52
82 Alanine from Glycine	-0.4	-0.4	0.41	-0.8	0.5
104 Arginine from Histidine	-0.4	-0.2	0.44	-0.6	0.42
567 1,2-Diacylglycerol-VLDL-PI-pool	-0.4	0.05	0.45	-0.35	0.47
412 Glutamate degr	-0.4	-0.07	0.42	-0.47	0.4
901 Phosphatidate-VLDL-PI-pool	-0.4	0.05	0.51	-0.35	0.51
568 1,2-Diacylglycerol-VLDL-PS-pool	-0.4	0.05	0.45	-0.35	0.47
928 Sedoheptulose-7P	-0.4	-0.08	0.6	-0.49	0.55
902 Phosphatidate-VLDL-PS-pool	-0.4	0.05	0.51	-0.36	0.51
102 Arginine from Glutamate	-0.4	-0.17	0.36	-0.58	0.4
233 Proline from Cysteine	-0.4	-0.38	0.64	-0.79	0.55
746 Cys-Gly	-0.41	-0.23	1	-0.64	1
45 Activated methylene group from Try	-0.41	-0.22	0.45	-0.63	0.39
335 Plasminogen	-0.41	0.03	0.97	-0.38	0.98
318 tcdchola(b)	-0.41	-0.35	0.46	-0.76	0.52
716 Argininosuccinate	-0.42	-0.19	0.55	-0.61	0.66
927 Sedoheptulose-1,7PP	-0.42	-0.07	0.65	-0.49	0.64
304 Arachidonate from Linoleate	-0.42	-0.02	0.31	-0.44	0.64
572 1-Acylglycerol-3P-Bile-PC-pool	-0.42	0.06	0.41	-0.37	0.55
574 1-Acylglycerol-3P-VLDL-PC-pool	-0.42	0.06	0.42	-0.37	0.57
468 PAPS	-0.42	-0.3	0.45	-0.72	0.33
579 1-Acylglycerol-3P-VLDL-TG1-pool	-0.42	0.06	0.41	-0.37	0.57
578 1-Acylglycerol-3P-VLDL-SM-pool	-0.42	0.06	0.41	-0.37	0.57
85 Alanine from Glutamine	-0.42	-0.17	0.49	-0.6	0.58
655 3alpha,7alpha-Dihydroxy-5beta-cholestanoyl-CoA	-0.42	-0.32	0.5	-0.75	0.47
575 1-Acylglycerol-3P-VLDL-PE-pool	-0.42	0.06	0.42	-0.37	0.57
113 Arginine from Threonine	-0.42	-0.08	0.31	-0.51	0.54
576 1-Acylglycerol-3P-VLDL-PI-pool	-0.43	0.06	0.42	-0.37	0.57
35 Thioredoxin(c) oxidation	-0.43	-0.14	0.71	-0.57	0.77
75 Gluconeogen from Alanine	-0.43	-0.2	0.27	-0.63	0.35
577 1-Acylglycerol-3P-VLDL-PS-pool	-0.43	0.06	0.42	-0.37	0.57
408 Arginine degr	-0.43	-0.07	0.44	-0.51	0.42
820 Histamine	-0.43	-0.003	0.64	-0.44	0.67
223 Glutamine from Tryptophan	-0.43	-0.33	0.42	-0.77	0.47
28 NADPH to NADH transhydrogenase	-0.44	-0.14	1	-0.58	1
62 Isopentenyl-PP	-0.44	-0.14	0.62	-0.58	0.57
770 Erythrose-4P	-0.44	-0.06	0.7	-0.5	0.64
247 Proline from Tyrosine	-0.44	-0.29	0.53	-0.72	0.47
496 Fructose-6P	-0.44	-0.02	1	-0.46	1
429 Ornithine degr	-0.44	-0.07	0.44	-0.51	0.42
16 Aerobic reduction of NAD+ (FA)	-0.44	-0.15	0.42	-0.6	0.4
133 Asparagine from Serine	-0.44	-0.25	0.35	-0.7	0.37

Continued on next page

Amplitude differences: TGFβ/contr 6h vs. 24h – continued

Simulation	Δampl	TGFβ/C 6h ampl	TGFβ/C 6h score	TGFβ/C 24h ampl	TGFβ/C 24h score
668 5-Formyl-THF	-0.44	-0.34	0.31	-0.78	0.43
214 Glutamine from Histidine	-0.45	-0.19	0.51	-0.64	0.41
653 3alpha,7alpha-Dihydroxy-5beta-cholestan-26-al	-0.45	-0.35	0.52	-0.8	0.53
439 Antitrypsin degr	-0.46	0.11	0.47	-0.34	0.44
277 beta-Alanine from Cystine	-0.46	-0.55	0.36	-1.01	0.41
288 beta-Alanine from Serine	-0.46	-0.25	0.33	-0.71	0.38
17 Aerobic reduction of NAD+ (gluc)	-0.46	-0.15	0.51	-0.61	0.45
844 L-Palmitoylcarnitine	-0.46	0.04	0.49	-0.43	0.68
785 Fructose-1,6PP	-0.46	0.02	0.52	-0.44	1
73 Gluconeogen from Lactate	-0.46	-0.1	0.34	-0.57	0.51
99 Arginine from Aspartate	-0.46	-0.3	0.37	-0.77	0.38
614 3-Hydroxyisobutyrate	-0.46	-0.22	0.42	-0.69	0.36
549 (R)-5-Diphosphomevalonate	-0.46	-0.16	0.55	-0.62	0.56
977 linoleic-Carnitine	-0.47	0.04	0.54	-0.43	0.67
714 Arachidonyl-Carnitine	-0.47	0.04	0.54	-0.43	0.67
713 Anthranilate	-0.47	-0.43	0.45	-0.9	0.48
217 Glutamine from Lysine	-0.47	-0.25	0.36	-0.72	0.45
219 Glutamine from Phenylalanine	-0.47	-0.24	0.45	-0.71	0.48
874 Nicotinate	-0.47	-0.33	0.48	-0.8	0.56
69 Formaldehyde degr	-0.47	-0.04	0.45	-0.51	0.38
167 Glutamate from Aspartate	-0.47	-0.31	0.54	-0.78	0.47
57 UDP-activated galactose	-0.48	-0.27	0.66	-0.74	0.45
221 Glutamine from Serine	-0.48	-0.34	0.39	-0.82	0.43
63 Farnesyl-PP	-0.48	-0.13	0.6	-0.61	0.51
654 3alpha,7alpha-Dihydroxy-5beta-cholestanate	-0.48	-0.34	0.53	-0.82	0.56
61 AKG	-0.48	-0.25	0.47	-0.73	0.57
135 Asparagine from Tryptophan	-0.48	-0.31	0.39	-0.79	0.44
110 Arginine from Phenylalanine	-0.48	-0.23	0.45	-0.72	0.45
181 Glutamate from Tryptophan	-0.49	-0.31	0.46	-0.8	0.51
296 Stearate from Palmitate	-0.49	0.1	0.51	-0.39	0.7
571 1,3DPG	-0.49	-0.05	0.69	-0.54	0.63
842 L-Octanoylcarnitine	-0.5	-0.3	0.34	-0.79	0.64
177 Glutamate from Phenylalanine	-0.5	-0.25	0.52	-0.75	0.52
224 Glutamine from Tyrosine	-0.5	-0.26	0.46	-0.76	0.48
122 Asparagine from Cystine	-0.5	-0.46	0.44	-0.96	0.48
184 Glutamate from beta-Alanine	-0.5	-0.3	0.48	-0.8	0.37
447 Glycogenin degr	-0.51	0.12	0.45	-0.39	0.5
420 Serine degr	-0.51	-0.23	0.37	-0.74	0.39
421 Threonine degr	-0.51	-0.07	0.37	-0.58	0.49
416 Lysine degr	-0.51	-0.06	0.37	-0.58	0.43
136 Asparagine from Tyrosine	-0.51	-0.25	0.41	-0.77	0.46
226 Glutamine from beta-Alanine	-0.51	-0.3	0.38	-0.81	0.35
143 Aspartate from Cystine	-0.51	-0.5	0.45	-1.01	0.48
131 Asparagine from Phenylalanine	-0.52	-0.22	0.42	-0.74	0.44
445 ApoTransferin degr	-0.52	0.12	0.44	-0.39	0.48
795 Geranyl-PP	-0.52	-0.13	0.58	-0.65	0.5
934 Squalene	-0.52	-0.27	0.98	-0.79	0.83
909 Presqualene-PP	-0.52	-0.27	0.98	-0.79	0.83
789 GAP	-0.52	-0.06	0.55	-0.58	0.85
752 DHAP	-0.52	-0.06	0.55	-0.58	0.85
48 Formylgroup(m)	-0.52	-0.29	0.41	-0.82	0.52
843 L-Oleoylcarnitine	-0.53	-0.01	0.51	-0.53	0.7
299 Oleate from Stearate	-0.53	0.2	0.55	-0.33	0.83
808 Guanidinoacetate	-0.53	-0.21	0.35	-0.74	0.36
156 Aspartate from Tryptophan	-0.53	-0.29	0.37	-0.82	0.44
845 Lanosterol	-0.53	-0.35	0.72	-0.89	0.77
234 Proline from Cystine	-0.53	-0.34	0.6	-0.87	0.54
172 Glutamate from Isoleucine	-0.53	0.09	0.41	-0.44	0.57
910 Propanoate	-0.53	-0.09	0.39	-0.62	0.59

Continued on next page

Amplitude differences: TGFβ/contr 6h vs. 24h – continued

Simulation	Δampl	TGFβ/C 6h ampl	TGFβ/C 6h score	TGFβ/C 24h ampl	TGFβ/C 24h score
171 Glutamate from Histidine	-0.53	-0.2	0.6	-0.74	0.5
433 Stearate degr	-0.53	0.11	0.42	-0.42	0.59
432 Oleate degr	-0.53	0.11	0.4	-0.42	0.57
481 Arginine	-0.53	-0.24	0.5	-0.77	0.46
236 Proline from Glycine	-0.54	-0.33	0.52	-0.87	0.44
398 Collagen SCRB1(c) synthesis	-0.54	0.11	1	-0.43	1
112 Arginine from Serine	-0.54	-0.26	0.41	-0.79	0.43
289 beta-Alanine from Threonine	-0.54	-0.04	0.37	-0.58	0.47
115 Arginine from Tyrosine	-0.54	-0.25	0.47	-0.79	0.47
856 Methacrylyl-CoA	-0.54	-0.28	0.42	-0.82	0.32
50 Acetyl group(m)	-0.54	0.12	0.38	-0.42	0.57
182 Glutamate from Tyrosine	-0.54	-0.26	0.54	-0.8	0.53
522 Triacylglycerol	-0.54	-0.06	0.32	-0.61	0.5
418 Phenylalanine degr	-0.56	-0.2	0.44	-0.76	0.43
290 beta-Alanine from Tryptophan	-0.56	-0.29	0.34	-0.84	0.39
152 Aspartate from Phenylalanine	-0.56	-0.21	0.46	-0.77	0.43
286 beta-Alanine from Phenylalanine	-0.56	-0.21	0.41	-0.77	0.39
422 Tryptophan degr	-0.57	-0.27	0.34	-0.83	0.42
302 gamma-Linolenate from Linoleate	-0.57	0.23	0.4	-0.34	0.56
878 O-Butanoylcarnitine	-0.57	0.11	0.35	-0.46	0.59
729 Ceramide-1P-pool	-0.57	0.3	0.35	-0.27	0.39
935 Squalene 2,3-oxide	-0.58	-0.37	0.86	-0.95	0.84
147 Aspartate from Isoleucine	-0.58	0.09	0.43	-0.49	0.58
593 14-Demethyllanosterol	-0.58	-0.07	0.58	-0.66	0.63
518 CL	-0.58	-0.2	0.59	-0.78	0.42
612 3-Hydroxy-L-kynurenine	-0.58	-0.28	0.37	-0.86	0.44
121 Asparagine from Cysteine	-0.58	-0.4	0.37	-0.99	0.38
211 Glutamine from Cystine	-0.59	-0.45	0.51	-1.03	0.56
656 4,4-Dimethyl-5alpha-cholesta-8,14,24-trien-3beta-ol	-0.59	-0.08	0.54	-0.67	0.68
166 Glutamate from Asparagine	-0.59	-0.28	0.49	-0.87	0.47
105 Arginine from Isoleucine	-0.59	0.09	0.34	-0.5	0.55
891 PG-CL-pool	-0.59	-0.2	0.52	-0.79	0.44
892 PGP-CL-pool	-0.59	-0.2	0.52	-0.79	0.44
215 Glutamine from Isoleucine	-0.6	0.09	0.39	-0.5	0.64
165 Glutamate from Arginine	-0.6	-0.21	0.5	-0.8	0.42
434 Fructose degr	-0.6	0.22	0.56	-0.37	0.44
84 Alanine from Isoleucine	-0.6	0.09	0.4	-0.51	0.62
27 NADPH redox potential into peroxy	-0.6	-0.32	1	-0.93	1
100 Arginine from Cysteine	-0.61	-0.38	0.46	-0.99	0.45
598 2-Aminomuconate	-0.61	-0.35	0.47	-0.96	0.54
933 Sphingosine-1P	-0.62	0.36	0.32	-0.26	0.32
101 Arginine from Cystine	-0.62	-0.36	0.47	-0.98	0.51
138 Asparagine from beta-Alanine	-0.62	-0.06	0.31	-0.68	0.33
793 GSSG	-0.62	-0.38	0.51	-1	0.71
169 Glutamate from Cystine	-0.62	-0.39	0.56	-1.01	0.62
259 Serine from Isoleucine	-0.62	0.09	0.34	-0.53	0.59
514 PC	-0.63	-0.11	0.3	-0.74	0.4
55 UDP-activated glucose	-0.63	-0.16	0.66	-0.79	0.53
599 2-Aminomuconate(s)emialdehyde	-0.63	-0.35	0.44	-0.98	0.5
596 2-Amino-3-carboxymuconate(s)emialdehyde	-0.63	-0.35	0.44	-0.98	0.5
776 Fatty-acid-VLDL-PE-pool	-0.63	-0.17	0.36	-0.8	0.56
276 beta-Alanine from Cysteine	-0.63	-0.44	0.32	-1.08	0.36
777 Fatty-acid-VLDL-PI-pool	-0.63	-0.17	0.36	-0.8	0.56
117 Arginine from beta-Alanine	-0.64	-0.09	0.39	-0.72	0.39
778 Fatty-acid-VLDL-PS-pool	-0.64	-0.17	0.36	-0.8	0.56
823 Hydracrylate	-0.64	0.07	0.29	-0.56	0.49
126 Asparagine from Isoleucine	-0.64	0.09	0.36	-0.55	0.55
281 beta-Alanine from Isoleucine	-0.64	0.11	0.46	-0.53	0.5
781 Fatty-acid-VLDL-TG2-pool	-0.64	-0.17	0.37	-0.81	0.56

Continued on next page

Amplitude differences: TGFβ/contr 6h vs. 24h – continued

Simulation	Δampl	TGFβ/C 6h ampl	TGFβ/C 6h score	TGFβ/C 24h ampl	TGFβ/C 24h score
780 Fatty-acid-VLDL-TG1-pool	-0.64	-0.17	0.37	-0.81	0.56
404 (R)-3-Hydroxybutanoate	-0.64	-0.02	0.57	-0.66	0.58
425 Isoleucine degr	-0.64	0.09	0.38	-0.56	0.52
573 1-Acylglycerol-3P-CL-pool	-0.65	-0.23	0.45	-0.88	0.54
613 3-Hydroxyanthranilate	-0.65	-0.36	0.45	-1.01	0.52
305 Dihomo-gamma-linolenate from gamma-Linolenate	-0.65	0.23	0.31	-0.42	0.81
428 beta-Alanine degr	-0.66	-0.07	0.37	-0.73	0.34
47 Formylgroup(c)	-0.66	-0.44	0.94	-1.1	0.71
24 NADH redox potential into mito	-0.66	-0.26	0.59	-0.92	0.61
124 Asparagine from Glycine	-0.66	-0.42	0.37	-1.07	0.43
768 Dimethylallyl-PP	-0.66	-0.14	0.53	-0.8	0.52
426 Tyrosine degr	-0.66	-0.21	0.43	-0.87	0.44
484 Glutamate	-0.66	-0.22	0.63	-0.88	0.58
179 Glutamate from Serine	-0.66	-0.22	0.47	-0.88	0.47
486 Glutamine	-0.66	-0.22	0.55	-0.88	0.51
168 Glutamate from Cysteine	-0.66	-0.37	0.54	-1.03	0.57
56 UDP-activated glucuronate	-0.66	-0.46	0.52	-1.12	0.53
303 Dihomo-gamma-linolenate from Linoleate	-0.67	0.24	0.27	-0.43	0.68
306 Arachidonate from gamma-Linolenate	-0.67	0.24	0.27	-0.43	0.68
60 Pyruvate	-0.67	-0.03	0.41	-0.7	0.55
782 Fatty-acid-VLDL-TG3-pool	-0.67	-0.03	0.52	-0.7	0.66
210 Glutamine from Cysteine	-0.68	-0.39	0.43	-1.07	0.44
173 Glutamate from Glutamine	-0.68	-0.2	0.56	-0.88	0.39
694 Acetate	-0.68	0.3	0.66	-0.38	0.97
491 Glycerol	-0.69	-0.18	0.33	-0.86	0.56
26 NADPH redox potential into mito	-0.69	-0.27	0.76	-0.96	0.78
310 Bilirubin conjugation	-0.69	-0.17	0.53	-0.86	0.62
255 Serine from Cystine	-0.7	-0.44	0.53	-1.14	0.6
65 ATP salvage from Adenosine	-0.7	-0.02	0.9	-0.71	0.88
51 Acetyl group(p)	-0.7	0.2	0.43	-0.49	0.47
470 GSH	-0.7	-0.41	0.49	-1.11	0.63
441 Fibrinogen degr	-0.7	0.12	0.46	-0.58	0.49
437 Albumin degr	-0.71	0.12	0.44	-0.58	0.47
443 Plasminogen degr	-0.71	0.12	0.46	-0.58	0.49
431 HDL degr	-0.71	-0.13	0.33	-0.84	0.41
12 ATP from NADH	-0.71	-0.04	0.43	-0.75	0.47
438 Antichymotrypsin degr	-0.72	0.12	0.47	-0.6	0.45
645 3PG	-0.74	0.17	0.36	-0.56	0.59
52 Acetyl group(r)	-0.74	0.25	0.38	-0.49	0.58
49 Acetyl group(c)	-0.74	0.25	0.38	-0.49	0.58
811 H2S	-0.75	-0.52	0.46	-1.26	0.51
602 2-Lysolecithin-pool	-0.75	0.04	0.28	-0.71	0.39
130 Asparagine from Methionine	-0.75	-0.38	0.41	-1.13	0.48
263 Serine from Methionine	-0.76	-0.41	0.46	-1.17	0.54
444 Prothrombin degr	-0.76	0.13	0.49	-0.64	0.5
151 Aspartate from Methionine	-0.76	-0.39	0.38	-1.15	0.48
956 Xanthurenate	-0.76	-0.26	0.38	-1.02	0.5
657 4-(2-Amino-3-hydroxyphenyl)-2,4-dioxobutanoate	-0.76	-0.26	0.38	-1.02	0.5
583 1-Acylglycerol-3P-palm	-0.76	0.06	0.4	-0.7	0.57
585 1-Acylglycerol-3P-stea	-0.76	0.06	0.42	-0.71	0.57
582 1-Acylglycerol-3P-ol	-0.76	0.06	0.42	-0.71	0.57
581 1-Acylglycerol-3P-lin	-0.76	0.06	0.42	-0.71	0.57
580 1-Acylglycerol-3P-arach	-0.76	0.06	0.42	-0.71	0.57
254 Serine from Cysteine	-0.77	-0.42	0.5	-1.19	0.57
170 Glutamate from Glycine	-0.77	-0.34	0.42	-1.12	0.55
157 Aspartate from Tyrosine	-0.77	-0.22	0.46	-0.99	0.44
190 Glycine from Cystine	-0.77	-0.45	0.54	-1.23	0.61
202 Glycine from Tryptophan	-0.77	-0.38	0.42	-1.15	0.49
440 ApoA1 degr	-0.77	0.13	0.47	-0.65	0.48

Continued on next page

Simulation	Δampl	TGFβ/C 6h ampl	TGFβ/C 6h score	TGFβ/C 24h ampl	TGFβ/C 24h score
163 Cystine from Methionine	-0.77	-0.39	0.41	-1.17	0.51
213 Glutamine from Glycine	-0.78	-0.37	0.35	-1.16	0.46
103 Arginine from Glycine	-0.78	-0.31	0.39	-1.09	0.45
838 L-Formylkynurenine	-0.79	0.39	0.73	-0.4	0.69
285 beta-Alanine from Methionine	-0.8	-0.39	0.37	-1.19	0.46
471 Homocysteine	-0.8	-0.34	0.45	-1.14	0.47
352 Collagen CO4A5(c) synthesis	-0.8	-0.15	1	-0.96	1
197 Glycine from Methionine	-0.8	-0.42	0.47	-1.23	0.55
80 Alanine from Cystine	-0.8	-0.51	0.55	-1.31	0.66
449 Chitin-component degr	-0.81	-0.17	0.49	-0.97	0.41
30 NADPH to NADH transhydrogenase in mito	-0.81	-0.03	1	-0.84	1
31 NADH to NADPH transhydrogenase in mito	-0.81	-0.21	1	-1.02	1
42 Na+ exportgradient	-0.82	0.15	1	-0.67	1
161 Cysteine from Methionine	-0.82	-0.39	0.38	-1.21	0.49
200 Glycine from Serine	-0.83	-0.49	0.77	-1.31	0.58
44 Activated methyl group (THF)	-0.83	-0.49	0.77	-1.31	0.58
189 Glycine from Cysteine	-0.83	-0.44	0.51	-1.27	0.58
410 Aspartate degr	-0.83	-0.07	0.37	-0.9	0.34
982 sn-Glycerol-3P	-0.83	-0.02	0.57	-0.85	0.84
591 1-Acylglycerol-VLDL-TG1-pool	-0.85	0.05	0.37	-0.8	0.49
586 1-Acylglycerol-VLDL-PC-pool	-0.85	0.05	0.37	-0.8	0.49
590 1-Acylglycerol-VLDL-SM-pool	-0.85	0.05	0.36	-0.8	0.49
198 Glycine from Phenylalanine	-0.85	-0.36	0.44	-1.21	0.5
587 1-Acylglycerol-VLDL-PE-pool	-0.85	0.05	0.37	-0.8	0.5
500 udpglcur	-0.85	-0.36	0.53	-1.21	0.34
588 1-Acylglycerol-VLDL-PI-pool	-0.85	0.05	0.37	-0.8	0.5
589 1-Acylglycerol-VLDL-PS-pool	-0.85	0.05	0.37	-0.8	0.5
279 beta-Alanine from Glycine	-0.85	-0.3	0.32	-1.15	0.42
485 Glycine	-0.86	-0.41	0.52	-1.26	0.59
187 Glycine from Asparagine	-0.86	-0.41	0.41	-1.27	0.47
228 Methionine from Cystine	-0.88	-0.33	0.38	-1.21	0.43
497 UDP-xylose	-0.88	-0.35	0.52	-1.23	0.3
88 Alanine from Methionine	-0.88	-0.41	0.43	-1.28	0.52
294 Taurine from Methionine	-0.88	-0.39	0.37	-1.27	0.51
203 Glycine from Tyrosine	-0.88	-0.37	0.47	-1.25	0.53
424 Glycine degr	-0.88	-0.26	0.34	-1.14	0.4
291 beta-Alanine from Tyrosine	-0.89	-0.22	0.4	-1.11	0.38
834 Kynurenine	-0.89	0.32	0.46	-0.57	0.43
743 Citrulline	-0.9	0.08	0.37	-0.82	0.31
188 Glycine from Aspartate	-0.9	-0.42	0.45	-1.32	0.5
898 Phosphatidate-CL-pool	-0.91	0.03	0.38	-0.87	0.46
409 Asparagine degr	-0.91	-0.07	0.42	-0.98	0.34
205 Glycine from beta-Alanine	-0.92	-0.41	0.43	-1.33	0.45
268 Serine from Tyrosine	-0.93	-0.29	0.44	-1.22	0.5
584 1-Acylglycerol-3P-palmn	-0.94	-0.06	0.38	-1	0.59
975 gamma-Glutamyl-cysteine	-0.97	-0.02	0.83	-0.99	0.87
192 Glycine from Histidine	-0.97	-0.36	0.42	-1.33	0.48
411 Cysteine degr	-1	-0.08	0.4	-1.09	0.43
164 Glutamate from Alanine	-1.01	-0.2	0.72	-1.22	0.49
320 Bile-PC(b)	-1.01	0.12	0.32	-0.9	0.44
427 Homocysteine degr	-1.03	-0.08	0.38	-1.11	0.45
417 Methionine degr	-1.03	-0.08	0.38	-1.11	0.43
756 Dehydroalanine	-1.06	1.06	0.72	0.002	0.62
196 Glycine from Lysine	-1.07	-0.37	0.4	-1.44	0.47
185 Glycine from Alanine	-1.07	-0.4	0.5	-1.47	0.57
832 Isocitrate	-1.07	-0.28	0.71	-1.35	0.61
307 Arachidonate from Dihomo-gamma-linolenate	-1.09	0.24	0.44	-0.85	0.6
377 Collagen COIA1(c) synthesis	-1.09	-0.08	1	-1.18	1
343 Collagen CD36(c) synthesis	-1.11	-0.53	1	-1.64	1

Continued on next page

Amplitude differences: TGFβ/contr 6h vs. 24h – continued

Simulation	Δampl	TGFβ/C 6h		TGFβ/C 24h	
		ampl	score	ampl	score
227 Methionine from Cysteine	-1.11	-0.1	0.41	-1.21	0.39
929 Serotonin	-1.11	-0.1	0.78	-1.21	0.53
446 ApoB100 degr	-1.13	0.1	0.49	-1.02	0.51
250 Serine from Alanine	-1.13	-0.36	0.46	-1.49	0.49
430 LDL degr	-1.14	0.2	0.34	-0.93	0.43
980 palmitoleoyl-Carnitine	-1.17	0.04	0.35	-1.13	0.49
637 3-Phosphoserine	-1.17	-0.29	0.44	-1.46	0.52
886 Oxalosuccinate	-1.17	-0.26	0.68	-1.44	0.59
314 Creatine	-1.18	-0.1	0.41	-1.27	0.39
94 Alanine from Tyrosine	-1.2	-0.22	0.5	-1.42	0.49
79 Alanine from Cysteine	-1.22	-0.18	0.43	-1.4	0.5
97 Arginine from Alanine	-1.24	-0.25	0.53	-1.49	0.42
206 Glutamine from Alanine	-1.25	-0.25	0.62	-1.5	0.51
950 UDP-activated-xylose	-1.28	-0.14	0.56	-1.41	0.44
788 Fumarylacetoacetate	-1.28	-0.23	0.54	-1.51	0.68
926 Sarcosine	-1.28	0.02	0.8	-1.26	0.86
480 Alanine	-1.31	-0.2	0.74	-1.51	0.67
312 Urea from alanine	-1.33	-0.21	0.46	-1.54	0.43
313 Urea from NH3	-1.33	-0.21	0.46	-1.54	0.43
407 Alanine degr	-1.34	-0.21	0.48	-1.54	0.43
118 Asparagine from Alanine	-1.36	-0.24	0.53	-1.6	0.39
660 4-Maleylacetoacetate	-1.38	-0.23	0.61	-1.62	0.7
784 Formylanthranilate	-1.47	0.33	0.59	-1.14	0.48
78 Alanine from Aspartate	-1.48	-0.17	0.46	-1.65	0.43
609 2PG	-1.5	1.23	0.41	-0.27	0.65
272 beta-Alanine from Alanine	-1.5	-0.17	0.33	-1.67	0.29
821 Homogentisate	-1.51	-0.25	0.52	-1.76	0.65
907 Phosphopantetheine	-1.51	-0.16	0.34	-1.67	0.45
89 Alanine from Phenylalanine	-1.53	-0.21	0.5	-1.74	0.47
890 PEP	-1.54	1.23	0.52	-0.31	0.54
295 Taurine from Cysteine	-1.56	0.1	0.6	-1.46	0.5
826 Hypotaurine	-1.56	0.1	0.6	-1.46	0.5
638 3-Sulfinoalanine	-1.57	0.1	1	-1.46	0.73
879 O-Propanoylcarnitine	-1.58	0.97	0.35	-0.61	0.53
667 5-Formiminotetrahydrofolate	-1.59	-0.23	0.45	-1.82	0.46
658 4-Hydroxyphenylpyruvate	-1.62	-0.25	0.7	-1.87	0.73
257 Serine from Glycine	-1.66	0.24	0.6	-1.42	0.43
925 Saccharopine	-1.68	-0.24	0.76	-1.92	0.63
747 Cysteamine	-1.87	0.41	0.43	-1.46	0.39
896 Pantetheine	-2.09	0.41	0.33	-1.68	0.52
54 Glucose-6P	-2.16	-0.58	0.54	-2.74	0.52
607 2-Oxobutyrate	-2.17	0.97	0.57	-1.2	0.38
403 Acetoacetate	-2.39	2	0.41	-0.39	0.57
405 Acetone	-2.43	1.99	0.52	-0.43	0.45
489 Tyrosine	-2.85	-0.005	0.52	-2.86	0.65
448 Ethanol degr	-2.97	-0.59	0.57	-3.56	0.54

3 Amplitudes with opposite signs

The tables show only those functions which show a opposite sign of the amplitudes. They are sorted by the combined score (not shown) in descending order.

3.1 Amplitude reversal of Treatment difference

Table 11: Amplitude reversal of Treatment difference

Simulation	TGFβ/C 24h		control 1/24h	
	ampl	score	ampl	score
987 trans-4-Hydroxy-L-proline	-0.24	1	0.68	1
947 Triphosphate degr	-0.09	1	0.15	1
946 Trehalose	0.13	1	-0.76	1
912 Protein(l)ysine	0.01	1	-0.49	1
906 Phosphodimethylethanolamine	-0.8	1	0.11	1
905 Phosphocholine	-0.8	1	0.11	1
30 NADPH to NADH transhydrogenase in mito	-0.84	1	0.49	1
27 NADPH redox potential into peroxy	-0.93	1	0.12	1
31 NADH to NADPH transhydrogenase in mito	-1.02	1	0.17	1
854 Maltose	-0.18	1	0.15	1
836 L-2-Aminoadipate	-0.43	1	0.42	1
835 L-1-Pyrroline-3-hydroxy-5-carboxylate	-0.24	1	0.68	1
825 Hydroxypyruvate	0.55	1	-1.09	1
797 Glucose-1P	0.2	1	-0.48	1
796 Glucono-1,5-lactone-6P	0.45	1	-0.29	1
774 FMN	0.03	1	-0.16	1
746 Cys-Gly	-0.64	1	0.13	1
371 Collagen COCA1(c) synthesis	0.36	1	-0.11	1
362 Collagen CO7A1(c) synthesis	0.84	1	-0.23	1
384 Collagen COOA1(c) synthesis	0.08	1	-0.12	1
351 Collagen CO4A4(c) synthesis	0.09	1	-0.12	1
370 Collagen COBA2(c) synthesis	0.26	1	-0.08	1
348 Collagen CO4A1(c) synthesis	0.07	1	-0.42	1
353 Collagen CO4A6(c) synthesis	0.16	1	-0.2	1
350 Collagen CO4A3(c) synthesis	0.19	1	-0.27	1
352 Collagen CO4A5(c) synthesis	-0.96	1	1.61	1
374 Collagen COFA1(c) synthesis	2.27	1	-0.24	1
346 Collagen CO2A1(c) synthesis	0.17	1	-0.52	1
379 Collagen COKA1(c) synthesis	0.001	1	-0.18	1
382 Collagen COMA1(c) synthesis	0.13	1	-0.02	1
369 Collagen COBA1(c) synthesis	-0.02	1	0.03	1
347 Collagen CO3A1(c) synthesis	-0.02	1	0.66	1
373 Collagen COEA1(c) synthesis	-0.02	1	0.06	1
376 Collagen COHA1(c) synthesis	0.03	1	-0.03	1
395 Collagen ITA2(c) synthesis	0.21	1	-0.04	1
364 Collagen CO8A2(c) synthesis	0.12	1	-0.16	1
341 Collagen C43BP(c) synthesis	0.58	1	-0.1	1
378 Collagen COJA1(c) synthesis	-0.07	1	0.04	1
358 Collagen CO6A2(c) synthesis	0.04	1	-0.05	1
367 Collagen CO9A3(c) synthesis	0.03	1	-0.13	1
372 Collagen CODA1(c) synthesis	0.09	1	-0.23	1
368 Collagen COAA1(c) synthesis	0.02	1	-0.09	1
385 Collagen COPA1(c) synthesis	0.11	1	-0.19	1
383 Collagen CONA1(c) synthesis	0.04	1	-0.16	1
389 Collagen EMID2(c) synthesis	0.09	1	-0.18	1
342 Collagen CCBE1(c) synthesis	0.17	1	-0.13	1
343 Collagen CD36(c) synthesis	-1.64	1	0.71	1
391 Collagen FCN2(c) synthesis	0.26	1	-0.44	1
339 Collagen ADIPO(c) synthesis	-0.07	1	0.04	1
388 Collagen CTHR1(c) synthesis	-0.02	1	0.01	1
330 ApoB100(r)	0.28	0.99	-0.52	0.99

Continued on next page

Amplitude reversal of Treatment difference – continued

Simulation	TGFβ/C 24h		control 1/24h	
	ampl	score	ampl	score
327 Antichymotrypsin	0.33	0.97	-1.63	0.97
338 Haptoglobin	-0.18	0.97	0.22	0.97
334 ApoE(c)	0.03	0.95	-0.24	0.97
454 Glycogenin	-0.14	0.96	0.44	0.96
807 Glycogenin-G8	-0.14	0.95	0.44	0.95
456 Thioredoxin(m)	-0.25	0.95	0.38	0.95
332 ApoC2(c)	0.05	0.95	-0.54	0.95
803 Glycogenin-G11	-0.14	0.95	0.44	0.95
804 Glycogenin-G4G4	-0.14	0.94	0.44	0.95
806 Glycogenin-G7G1	-0.14	0.94	0.44	0.94
805 Glycogenin-G7	-0.14	0.94	0.44	0.94
979 mitoOxidizedThioredoxin	-0.25	0.94	0.38	0.94
941 THF-hexaglutamate	0.69	0.87	-0.43	0.98
275 beta-Alanine from Aspartate	0.11	0.68	-0.15	0.95
495 Mannose-6P	-0.01	0.78	0.15	0.78
592 1-Pyrroline-5-carboxylate	-0.24	0.76	0.68	0.77
308 VLDL from LDL	0.28	0.71	-0.5	0.81
309 VLDL from HDL	0.28	0.68	-0.52	0.81
401 VLDL	0.28	0.68	-0.51	0.81
26 NADPH redox potential into mito	-0.96	0.78	0.15	0.69
680 6-Phospho-D-gluconate	0.43	0.63	-0.25	0.83
71 Glycogen glucose storage	-0.19	0.75	0.44	0.65
64 Glucosamine-6P	0.11	0.63	-0.15	0.77
893 PPi	-0.24	0.71	0.39	0.66
552 (R)-Mevalonate	0.62	0.54	-0.8	0.82
916 Pyridoxal	-0.11	0.66	0.16	0.68
669 5-Hydroxy-L-tryptophan	0.24	0.61	-0.22	0.73
728 Carnosine	0.001	0.46	-0.12	0.87
756 Dehydroalanine	0.002	0.62	-0.78	0.7
11 ATP from NADH(m)	-0.09	0.8	0.002	0.48
843 L-Oleoylcarnitine	-0.53	0.7	0.13	0.54
480 Alanine	-1.51	0.67	0.5	0.55
861 N-Acetylglucosamine-1P	0.1	0.55	-0.39	0.65
14 Ubiquinol-to-ATP	-0.09	0.62	0.002	0.54
784 Formylanthranilate	-1.14	0.48	0.35	0.67
874 Nicotinate	-0.8	0.56	0.52	0.59
24 NADH redox potential into mito	-0.92	0.61	0.64	0.53
47 Formylgroup(c)	-1.1	0.71	0.16	0.42
829 Inositol	-0.12	0.5	1.72	0.62
672 5-Oxoproline	-0.08	0.55	0.16	0.55
257 Serine from Glycine	-1.42	0.43	0.31	0.67
200 Glycine from Serine	-1.31	0.58	0.16	0.52
44 Activated methyl group (THF)	-1.31	0.58	0.16	0.52
435 Galactose degr	-0.17	0.59	0.33	0.5
949 UDP-activated-N-acetyl-D-galactosamine	0.18	0.44	-0.3	0.62
54 Glucose-6P	-2.74	0.52	0.61	0.51
832 Isocitrate	-1.35	0.61	0.12	0.41
605 2-Oxo-3-methylvalerate	0.41	0.49	-0.2	0.54
918 Quinolinate	-0.31	0.63	0.05	0.38
85 Alanine from Glutamine	-0.6	0.58	0.63	0.43
802 Glycerate	0.9	0.55	-1.05	0.45
600 2-Deoxy-D-ribose-1P	0.7	0.41	-0.31	0.59
713 Anthranilate	-0.9	0.48	0.32	0.51
475 dTTP	-0.3	0.49	0.36	0.5
799 Glucuronate	0.24	0.57	-0.2	0.41
790 GAR	-0.33	0.5	0.34	0.47
526 Putrescine	0.18	0.52	-1.02	0.45
529 Methylthioribose-1P	-0.29	0.56	0.53	0.38
494 Mannose-1P	0.23	0.5	-0.12	0.45

Continued on next page

Amplitude reversal of Treatment difference – continued

Simulation	TGFβ/C 24h		control 1/24h	
	ampl	score	ampl	score
938 Succinate	-0.44	0.56	0.44	0.38
671 5-Methylthioadenosine	-0.3	0.48	0.29	0.45
965 dAMP	-0.41	0.45	0.29	0.47
773 FAICAR	-0.32	0.43	0.28	0.48
710 Adenylosuccinate	-0.43	0.4	0.3	0.51
711 Adenylyl(s)ulfate	-0.35	0.45	0.3	0.46
924 SAM	-0.28	0.43	0.3	0.47
922 SAH	-0.28	0.43	0.3	0.47
79 Alanine from Cysteine	-1.4	0.5	0.46	0.4
548 (R)-4-Phosphopantothenoyl-cysteine	-0.11	0.45	0.11	0.45
690 AICAR	-0.29	0.45	0.27	0.45
837 L-Cystathionine	0.04	0.48	-0.67	0.42
919 Ribose-1P	0.19	0.4	-0.65	0.5
867 N-Formyl-GAR	-0.53	0.45	0.33	0.43
691 AIR	-0.53	0.41	0.3	0.47
894 PRPP	-0.41	0.48	0.39	0.39
675 5-Phosphoribosylformylglycinamidine	-0.53	0.42	0.3	0.45
90 Alanine from Proline	-0.44	0.54	0.4	0.32
674 5-Phosphoribosylamine	-0.35	0.51	0.34	0.35
300 Stearate from Oleate	-0.29	0.52	0.68	0.32
967 dCMP	-0.38	0.45	0.3	0.39
681 6-Pyruvoyltetrahydropterin	-0.41	0.38	0.37	0.46
246 Proline from Tryptophan	-0.47	0.51	0.34	0.32
453 FAD	-0.37	0.35	0.26	0.48
241 Proline from Lysine	-0.5	0.54	0.36	0.29
896 Pantetheine	-1.68	0.52	3	0.31
755 Decanoyl-CoA	-0.49	0.45	0.27	0.37
625 3-Oxododecanoyl-CoA	-0.49	0.45	0.27	0.37
753 Deamido-NAD	-0.32	0.37	0.28	0.45
758 Deoxycytidine	-0.37	0.43	0.28	0.39
611 3-Dehydrosphinganine	0.09	0.44	-0.18	0.38
181 Glutamate from Tryptophan	-0.8	0.51	0.29	0.3
120 Asparagine from Aspartate	0.54	0.37	-0.11	0.44
473 dCTP	-0.41	0.41	0.33	0.4
969 dGMP	-0.43	0.37	0.3	0.44
745 Crotonyl-CoA	-0.45	0.4	0.28	0.41
449 Chitin-component degr	-0.97	0.41	0.67	0.39
554 (S)-3-Hydroxybutyryl-CoA	-0.49	0.38	0.28	0.42
67 dTTP salvage from Thymine	-0.35	0.47	0.42	0.33
968 dGDP	-0.43	0.37	0.3	0.43
620 3-Methylcrotonyl-CoA	-0.36	0.45	0.28	0.36
533 (2E)-Dodecenoyl-CoA	-0.49	0.44	0.28	0.36
853 Malonyl-CoA	-0.41	0.42	0.3	0.38
888 PAP	-0.4	0.38	0.32	0.42
555 (S)-3-Hydroxydodecanoyl-CoA	-0.49	0.43	0.28	0.36
477 CTP	-0.4	0.36	0.33	0.44
543 (3Z)-Dodecenoyl-CoA	-0.47	0.41	0.28	0.38
964 dADP	-0.42	0.38	0.31	0.41
558 (S)-3-hydroxyoleyleoyl-CoA	-0.47	0.4	0.29	0.39
274 beta-Alanine from Asparagine	0.11	0.3	-0.14	0.49
882 Octanoyl-CoA	-0.48	0.45	0.27	0.35
641 3-oxolaur-cis-5-enoyl-CoA	-0.46	0.41	0.28	0.38
623 3-Oxodecanoyl-CoA	-0.48	0.44	0.27	0.35
801 Glutaryl-CoA	-0.41	0.42	0.29	0.38
698 Acetyl-CoA	-0.38	0.38	0.29	0.42
791 GDP	-0.46	0.33	0.3	0.46
472 dATP	-0.48	0.42	0.27	0.37
510 Adenosine	-0.61	0.43	0.27	0.36
692 AMP	-0.42	0.35	0.32	0.44

Continued on next page

Amplitude reversal of Treatment difference – continued

Simulation	TGFβ/C 24h		control 1/24h	
	ampl	score	ampl	score
986 trans,cis-octadeca-2,9-dienoyl-CoA	-0.33	0.4	0.29	0.39
895 Palmitoyl-CoA	-0.35	0.4	0.29	0.39
937 Stearoyl-CoA	-0.35	0.4	0.29	0.39
883 Oleoyl-CoA	-0.35	0.4	0.29	0.39
849 Linoleoyl-CoA	-0.35	0.4	0.29	0.39
715 Arachidonyl-CoA	-0.35	0.4	0.29	0.39
819 Hexanoyl-CoA	-0.48	0.45	0.26	0.34
939 Succinyl-CoA	-0.3	0.42	0.29	0.37
463 dhbpt	-0.44	0.46	0.36	0.32
531 (2E)-Decenoyl-CoA	-0.48	0.44	0.27	0.35
962 cis-laur-5-enoyl-CoA	-0.46	0.41	0.28	0.38
983 trans,cis-dodeca-2,5-dienoyl-CoA	-0.46	0.4	0.28	0.38
705 Acyl-CoA-VLDL-PS-pool	-0.35	0.4	0.29	0.38
704 Acyl-CoA-VLDL-PI-pool	-0.34	0.4	0.29	0.38
708 Acyl-CoA-VLDL-TG3-pool	-0.34	0.4	0.29	0.38
707 Acyl-CoA-VLDL-TG2-pool	-0.34	0.4	0.29	0.38
703 Acyl-CoA-VLDL-PE-pool	-0.34	0.4	0.29	0.38
462 thbpt	-0.43	0.44	0.37	0.35
706 Acyl-CoA-VLDL-SM-pool	-0.34	0.4	0.29	0.38
702 Acyl-CoA-VLDL-PC-pool	-0.34	0.4	0.29	0.38
642 3-oxomyrist-7-enoyl-CoA	-0.45	0.4	0.28	0.39
689 ADP	-0.42	0.34	0.32	0.44
503 IMP	-0.54	0.39	0.25	0.39
561 (S)-Hydroxydecanoyl-CoA	-0.48	0.44	0.27	0.34
628 3-Oxohexanoyl-CoA	-0.45	0.46	0.3	0.32
507 Cytidine	-0.08	0.37	0.34	0.41
236 Proline from Glycine	-0.87	0.44	0.38	0.34
476 ATP	-0.51	0.4	0.27	0.38
855 Mercaptopyruvate	0.03	0.38	-0.66	0.4
610 3(S)-3-hydroxydodecen-(5Z)-oyl-CoA	-0.46	0.4	0.28	0.38
700 Acyl-CoA-Bile-PC-pool	-0.34	0.4	0.29	0.38
701 Acyl-CoA-CL-pool	-0.35	0.4	0.29	0.38
506 Uridine	-0.45	0.32	0.3	0.46
828 Inosine	-0.48	0.32	0.26	0.45
630 3-Oxooctanoyl-CoA	-0.48	0.44	0.25	0.34
857 Methylmalonyl-CoA	-0.32	0.46	0.27	0.32
17 Aerobic reduction of NAD+ (gluc)	-0.61	0.45	0.32	0.33
722 CDP-ethanolamine	-0.39	0.46	0.33	0.31
719 Butyryl-CoA	-0.44	0.47	0.28	0.31
223 Glutamine from Tryptophan	-0.77	0.47	0.29	0.31
963 cis-myrist-7-enoyl-CoA	-0.45	0.39	0.28	0.38
757 Deoxyadenosine	-0.4	0.36	0.28	0.41
985 trans,cis-myristo-2,7-dienoyl-CoA	-0.45	0.39	0.28	0.38
450 CoA	-0.34	0.39	0.28	0.38
709 Adenine	-0.36	0.31	0.36	0.46
792 GMP	-0.46	0.35	0.28	0.42
394 Collagen ITA1(c) synthesis	0.33	0.28	-0.13	0.49
759 Deoxyguanosine	-0.43	0.34	0.28	0.43
814 HMG-CoA	-0.56	0.39	0.3	0.38
563 (S)-Hydroxyoctanoyl-CoA	-0.48	0.44	0.26	0.33
966 dCDP	-0.42	0.42	0.33	0.35
644 3-oxopalmitoleoyl-CoA	-0.41	0.37	0.28	0.39
871 NADPH	-0.35	0.39	0.29	0.38
540 (2E)-Octenoyl-CoA	-0.47	0.44	0.26	0.32
923 SAICAR	-0.25	0.41	0.26	0.35
696 Acetoacetyl-CoA	-0.38	0.36	0.3	0.4
960 cis-(3S)-hydroxytetradec-7-enoyl-CoA	-0.45	0.39	0.28	0.38
725 CMP-activated-N-acetylneuraminate	0.28	0.3	-0.24	0.46
513 CMP-N-acetylneuraminate	0.28	0.3	-0.24	0.46

Continued on next page

Amplitude reversal of Treatment difference – continued

Simulation	TGFβ/C 24h		control 1/24h	
	ampl	score	ampl	score
772 FADH2	-0.33	0.33	0.28	0.43
537 (2E)-Hexenoyl-CoA	-0.47	0.45	0.25	0.32
512 N-acglucam	0.16	0.32	-0.73	0.44
474 dGTP	-0.48	0.39	0.29	0.36
297 Palmitate from Palmitolate	-0.27	0.43	0.66	0.33
720 CDP	-0.42	0.38	0.34	0.37
452 NADP+	-0.36	0.38	0.29	0.37
870 NADH	-0.31	0.34	0.28	0.41
858 Myristoyl-CoA	-0.41	0.44	0.28	0.31
551 (R)-Methylmalonyl-CoA	-0.36	0.38	0.31	0.37
783 Formamidopyrimidine(n)leoside triphosphate	-0.36	0.32	0.31	0.43
535 (2E)-Hexadecenoyl-CoA	-0.35	0.36	0.29	0.38
504 XMP	-0.54	0.38	0.25	0.37
809 Guanosine	-0.22	0.35	0.23	0.4
220 Glutamine from Proline	-0.48	0.46	0.51	0.29
959 cGMP	-0.36	0.31	0.31	0.43
478 GTP	-0.51	0.37	0.29	0.38
976 gamma-Linolenoyl-CoA	-0.35	0.38	0.29	0.37
479 UTP	-0.41	0.38	0.32	0.36
524 gdpddman	-0.49	0.32	0.27	0.42
723 CMP	-0.41	0.38	0.34	0.36
643 3-oxooleoyl-CoA	-0.46	0.37	0.29	0.37
984 trans,cis-hexadeca-2,9-dienoyl-CoA	-0.34	0.36	0.29	0.38
501 GDP-L-fucose	-0.49	0.31	0.31	0.43
595 2,5-Diaminopyrimidine(n)leoside triphosphate	-0.34	0.31	0.31	0.42
981 palmitoleoyl-CoA	-0.37	0.35	0.29	0.38
451 NAD+	-0.33	0.33	0.29	0.4
603 2-Methylacetoacetyl-CoA	-0.44	0.4	0.3	0.33
553 (S)-3-Hydroxy-2-methylbutyryl-CoA	-0.51	0.39	0.27	0.34
621 3-Methylglutaconyl-CoA	-0.32	0.37	0.29	0.36
865 N-Carbamoyl-L-aspartate	-0.13	0.4	0.53	0.33
562 (S)-Hydroxyhexanoyl-CoA	-0.48	0.42	0.26	0.3
673 5-Phosphoribosyl-4-carboxy-5-aminoimidazole	-0.26	0.35	0.27	0.38
831 Isobutyryl-CoA	-0.56	0.34	0.33	0.38
875 Nicotinate D-ribonucleoside	-0.14	0.42	0.55	0.3
556 (S)-3-Hydroxyhexadecanoyl-CoA	-0.36	0.41	0.29	0.31
559 (S)-3-hydroxypalmitoleoyl-CoA	-0.34	0.35	0.29	0.37
762 Dephospho-CoA	-0.37	0.33	0.3	0.39
604 2-Methylbutyryl-CoA	-0.36	0.35	0.33	0.37
594 2,5-Diamino-6-(5-triphosphoryl-3,4-trihydroxy-2-oxopentyl)-amino-4-oxopyrimidine	-0.34	0.31	0.31	0.41
958 cAMP	-0.44	0.31	0.32	0.4
505 Uracil	-0.09	0.32	0.29	0.39
721 CDP-choline	-0.56	0.37	0.32	0.34
833 Isovaleryl-CoA	-0.41	0.37	0.3	0.34
945 Tiglyl-CoA	-0.48	0.4	0.31	0.31
699 Acrylyl-CoA	-0.5	0.39	0.3	0.32
502 GDP-mannose	-0.51	0.32	0.26	0.39
764 Dihomo-gamma-linolenoyl-CoA	-0.57	0.39	0.28	0.31
616 3-Hydroxypropionyl-CoA	-0.47	0.39	0.3	0.32
631 3-Oxopalmitoyl-CoA	-0.38	0.41	0.28	0.3
955 Xanthosine	-0.5	0.31	0.26	0.39
508 Xanthine	-0.55	0.34	0.23	0.36
615 3-Hydroxyisobutyryl-CoA	-0.55	0.32	0.4	0.37
635 3-Oxotetradecanoyl-CoA	-0.41	0.4	0.26	0.28
974 dUTP	-0.43	0.4	0.4	0.28
863 N-Acetylneuraminate	0.42	0.32	-0.72	0.35
509 Guanine	-0.54	0.33	0.23	0.35
856 Methacrylyl-CoA	-0.82	0.32	0.4	0.36

Continued on next page

Amplitude reversal of Treatment difference – continued

Simulation	TGFβ/C 24h		control 1/24h	
	ampl	score	ampl	score
911 Propanoyl-CoA	-0.45	0.34	0.29	0.33
952 Urate	-0.22	0.29	0.23	0.38
810 H2O2	-0.22	0.29	0.23	0.38
498 UDP-glucose	-0.62	0.36	0.32	0.3
314 Creatine	-1.27	0.39	0.6	0.27
872 NeuNGc	0.56	0.31	-0.21	0.34
469 ametam	-0.55	0.32	0.49	0.31
497 UDP-xylose	-1.23	0.3	0.26	0.31
817 Hexadecenal	0.28	0.26	-0.34	0.32
815 Hexadecanal	0.28	0.26	-0.34	0.32
207 Glutamine from Arginine	0.88	0.27	-0.08	0.3

3.2 Amplitude reversal of Period difference, control

Table 12: Amplitude reversal of Period difference, control

Simulation	control 1/6h		control 6/24h	
	ampl	score	ampl	score
32 Thioredoxin(m) reduction	0.19	1	-0.45	1
33 Thioredoxin(m) oxidation	0.19	1	-0.45	1
466 THF(m)	0.11	1	-0.44	1
459 THF	0.11	1	-0.44	1
917 Pyridoxine-P	0.04	1	-0.23	1
906 Phosphodimethylethanolamine	-0.19	1	0.3	1
905 Phosphocholine	-0.19	1	0.3	1
42 Na+ exportgradient	-0.32	1	0.26	1
27 NADPH redox potential into peroxy	0.18	1	-0.06	1
31 NADH to NADPH transhydrogenase in mito	-0.03	1	0.2	1
29 NADH to NADPH transhydrogenase	0.11	1	-0.44	1
868 N-Methylethanolamine-P	0	1	-0.27	1
822 Homovanillate	-0.04	1	0.03	1
796 Glucono-1,5-lactone-6P	0.12	1	-0.41	1
37 GSH reduction using NADPH redox potential	0.42	1	-0.51	1
38 GSH oxidation	0.42	1	-0.51	1
771 Ethanolamine-P	0	1	-0.27	1
766 Dihydrofolate	0.11	1	-0.44	1
670 5-Methyl-THF	0.11	1	-0.44	1
608 2-Oxoglutaramate	0.11	1	-0.15	1
359 Collagen CO6A3(c) synthesis	-0.11	1	0.22	1
371 Collagen COCA1(c) synthesis	-0.21	1	0.1	1
362 Collagen CO7A1(c) synthesis	0.01	1	-0.24	1
375 Collagen COGA1(c) synthesis	-0.15	1	0.23	1
377 Collagen COIA1(c) synthesis	0.07	1	-0.5	1
395 Collagen ITA2(c) synthesis	-0.16	1	0.11	1
373 Collagen COEA1(c) synthesis	0.1	1	-0.04	1
370 Collagen COBA2(c) synthesis	0.06	1	-0.14	1
384 Collagen COOA1(c) synthesis	0.07	1	-0.19	1
344 Collagen CO1A1(c) synthesis	-0.02	1	0.29	1
369 Collagen COBA1(c) synthesis	0.06	1	-0.02	1
353 Collagen CO4A6(c) synthesis	0.004	1	-0.2	1
356 Collagen CO5A3(c) synthesis	-0.07	1	0.005	1
382 Collagen COMA1(c) synthesis	0.04	1	-0.06	1
376 Collagen COHA1(c) synthesis	0.02	1	-0.06	1
378 Collagen COJA1(c) synthesis	0.09	1	-0.05	1
358 Collagen CO6A2(c) synthesis	0.09	1	-0.14	1
364 Collagen CO8A2(c) synthesis	0.05	1	-0.21	1
365 Collagen CO9A1(c) synthesis	-0.04	1	0.04	1
367 Collagen CO9A3(c) synthesis	0.05	1	-0.18	1
400 Collagen VWA2(c) synthesis	0.05	1	-0.03	1
340 Collagen BGH3(c) synthesis	0.03	1	-0.07	1
341 Collagen C43BP(c) synthesis	0	1	-0.1	1
342 Collagen CCBE1(c) synthesis	0.04	1	-0.18	1
391 Collagen FCN2(c) synthesis	0.08	1	-0.52	1
393 Collagen FMOD(c) synthesis	0.03	1	-0.07	1
396 Collagen LPP3(c) synthesis	0.14	1	-0.07	1
388 Collagen CTHR1(c) synthesis	-0.07	1	0.08	1
339 Collagen ADIPO(c) synthesis	-0.02	1	0.06	1
338 Haptoglobin	-0.01	0.96	0.23	0.98
325 Albumin	0.01	0.97	-0.12	0.97
454 Glycogenin	-0.24	0.96	0.67	0.96
807 Glycogenin-G8	-0.23	0.96	0.67	0.95
803 Glycogenin-G11	-0.23	0.96	0.67	0.95
804 Glycogenin-G4G4	-0.23	0.95	0.67	0.94
806 Glycogenin-G7G1	-0.23	0.95	0.67	0.94

Continued on next page

Amplitude reversal of Period difference, control – continued

Simulation	control 1/6h		control 6/24h	
	ampl	score	ampl	score
805 Glycogenin-G7	-0.23	0.95	0.67	0.94
34 Thioredoxin(c) reduction	0.14	0.88	-0.44	1
455 Thioredoxin	0.14	0.95	-0.01	0.93
941 THF-hexaglutamate	0.34	0.91	-0.77	0.95
887 Oxidized thioredoxin	0.14	0.94	-0.01	0.91
916 Pyridoxal	0.29	0.81	-0.15	0.99
457 Apo-ACP	-0.11	0.91	0.03	0.89
688 ACP	-0.11	0.87	0.03	0.85
812 HCO3-	-0.14	0.79	0.05	0.92
727 Carbonate	-0.14	0.79	0.05	0.92
697 Acetyl-ACP	-0.11	0.87	0.03	0.84
544 (R)-3-Hydroxybutanoyl-ACP	-0.11	0.87	0.03	0.83
717 But-2-enoyl-ACP	-0.11	0.87	0.03	0.83
718 Butyryl-ACP	-0.11	0.87	0.03	0.83
851 Malonyl-ACP	-0.11	0.86	0.03	0.83
695 Acetoacetyl-ACP	-0.11	0.86	0.03	0.83
627 3-Oxohexanoyl-ACP	-0.11	0.87	0.03	0.82
750 D-3-Hydroxyhexanoyl-ACP	-0.11	0.87	0.03	0.82
536 (2E)-Hexenoyl-ACP	-0.11	0.87	0.03	0.82
818 Hexanoyl-ACP	-0.11	0.87	0.03	0.82
629 3-Oxooctanoyl-ACP	-0.11	0.87	0.03	0.81
546 (R)-3-Hydroxyoctanoyl-ACP	-0.11	0.87	0.03	0.81
539 (2E)-Octenoyl-ACP	-0.11	0.87	0.03	0.81
881 Octanoyl-ACP	-0.11	0.87	0.03	0.81
622 3-Oxodecanoyl-ACP	-0.11	0.86	0.03	0.81
545 (R)-3-Hydroxydecanoyl-ACP	-0.11	0.86	0.03	0.81
530 (2E)-Decenoyl-ACP	-0.11	0.86	0.03	0.81
754 Decanoyl-ACP	-0.11	0.86	0.03	0.81
624 3-Oxododecanoyl-ACP	-0.11	0.86	0.03	0.8
749 D-3-Hydroxydodecanoyl-ACP	-0.11	0.86	0.03	0.8
532 (2E)-Dodecenoyl-ACP	-0.11	0.86	0.03	0.8
769 Dodecanoyl-ACP	-0.11	0.86	0.03	0.8
634 3-Oxotetradecanoyl-ACP	-0.11	0.86	0.03	0.8
813 HMA	-0.11	0.86	0.03	0.8
541 (2E)-Tetradecenoyl-ACP	-0.11	0.86	0.03	0.8
942 Tetradecanoyl-ACP	-0.11	0.86	0.03	0.8
626 3-Oxohexadecanoyl-ACP	-0.11	0.86	0.03	0.8
547 (R)-3-Hydroxypalmitoyl-ACP	-0.11	0.86	0.03	0.8
534 (2E)-Hexadecenoyl-ACP	-0.11	0.86	0.03	0.79
816 Hexadecanoyl-ACP	-0.11	0.86	0.03	0.79
633 3-Oxostearoyl-ACP	-0.11	0.86	0.03	0.79
617 3-Hydroxystearoyl-ACP	-0.11	0.86	0.03	0.78
538 (2E)-Octadecenoyl-ACP	-0.11	0.86	0.03	0.78
936 Stearoyl-ACP	-0.11	0.86	0.03	0.78
464 mlthf	0.11	0.79	-0.44	0.78
495 Mannose-6P	-0.07	0.78	0.22	0.78
70 Formate degr	0.16	0.71	-0.18	0.85
53 activated sulphur	0.07	0.87	-0.03	0.65
11 ATP from NADH(m)	-0.05	0.83	0.04	0.68
664 5,10-Methenyl-THF	0.12	0.83	-0.44	0.68
460 Pyridoxal-P	0.29	0.58	-0.19	0.91
436 Mannose degr	-0.07	0.64	0.35	0.83
47 Formylgroup(c)	0.18	0.71	-0.12	0.76
458 Apo-ACP(m)	-0.11	0.75	0.03	0.71
880 OAA	-0.1	0.7	0.04	0.75
978 mitoACP	-0.11	0.75	0.03	0.68
680 6-Phospho-D-gluconate	0.12	0.64	-0.36	0.77
982 sn-Glycerol-3P	0.41	0.47	-0.59	0.93
669 5-Hydroxy-L-tryptophan	0.11	0.55	-0.25	0.84

Continued on next page

Amplitude reversal of Period difference, control – continued

Simulation	control 1/6h		control 6/24h	
	ampl	score	ampl	score
71 Glycogen glucose storage	-0.23	0.74	0.67	0.65
14 Ubiquinol-to-ATP	-0.04	0.8	0.04	0.56
467 1fthf	0.11	0.74	-0.43	0.62
69 Formaldehyde degr	0.22	0.43	-0.12	0.9
793 GSSG	0.39	0.58	-0.78	0.74
667 5-Formiminotetrahydrofolate	0.12	0.57	-0.4	0.75
850 Malate	-0.08	0.65	0.04	0.64
162 Cystine from Cysteine	0.46	0.57	-0.69	0.68
160 Cysteine from Cystine	0.46	0.57	-0.69	0.68
200 Glycine from Serine	0.2	0.67	-0.13	0.57
44 Activated methyl group (THF)	0.2	0.67	-0.13	0.57
712 Agmatine	0.02	0.6	-0.01	0.6
139 Aspartate from Alanine	-0.17	0.72	0.04	0.47
726 Carbamoyl-P	-0.14	0.55	0.05	0.62
295 Taurine from Cysteine	-0.5	0.57	0.05	0.6
826 Hypotaurine	-0.5	0.57	0.05	0.6
36 GSH reduction using NADH redox potential	0.43	0.51	-0.49	0.63
638 3-Sulfinoalanine	-0.51	0.79	0.05	0.33
435 Galactose degr	-0.02	0.58	0.38	0.5
782 Fatty-acid-VLDL-TG3-pool	0.18	0.4	-0.47	0.66
980 palmitoleoyl-Carnitine	0.05	0.45	-0.86	0.59
548 (R)-4-Phosphopantothenoyl-cysteine	0.35	0.48	-0.23	0.57
873 Nicotinamide D-ribonucleotide	0.13	0.56	-0.37	0.48
843 L-Oleoylcarnitine	-0.11	0.52	0.24	0.51
57 UDP-activated galactose	0.12	0.49	-0.39	0.54
869 N-Pantothenoylcysteine	0.35	0.45	-0.22	0.55
837 L-Cystathionine	-0.14	0.51	0.32	0.48
950 UDP-activated-xylose	0.3	0.52	-1.39	0.48
522 Triacylglycerol	0.17	0.38	-0.43	0.61
448 Ethanol degr	0.13	0.44	-0.47	0.54
184 Glutamate from beta-Alanine	0.2	0.4	-0.26	0.58
784 Formylanthranilate	-0.71	0.4	0.33	0.58
480 Alanine	0.41	0.54	-0.51	0.44
12 ATP from NADH	-0.04	0.44	0.03	0.53
310 Bilirubin conjugation	0.41	0.46	-0.62	0.5
921 Ribulose-5P	0.11	0.4	-0.43	0.56
178 Glutamate from Proline	-0.14	0.52	0.05	0.43
855 Mercaptopyruvate	0.14	0.4	-0.42	0.55
494 Mannose-1P	0.13	0.46	-0.29	0.48
439 Antitrypsin degr	-0.15	0.55	0.05	0.38
977 linoleic-Carnitine	0.05	0.42	-0.53	0.51
714 Arachidonyl-Carnitine	0.05	0.42	-0.53	0.51
886 Oxalosuccinate	-0.16	0.55	0.04	0.39
584 1-Acylglycerol-3P-palmn	0.4	0.27	-0.68	0.66
573 1-Acylglycerol-3P-CL-pool	0.43	0.38	-0.47	0.54
257 Serine from Glycine	-0.02	0.46	0.38	0.46
885 Orotidine-5P	0.34	0.48	-0.37	0.43
842 L-Octanoylcarnitine	0.26	0.54	-1.25	0.37
844 L-Palmitoylcarnitine	0.05	0.42	-0.54	0.49
923 SAICAR	0.29	0.52	-0.56	0.38
871 NADPH	0.14	0.55	-0.38	0.35
799 Glucuronate	0.33	0.48	-0.22	0.41
452 NADP+	0.14	0.55	-0.38	0.34
447 Glycogenin degr	-0.35	0.54	0.05	0.34
747 Cysteamine	-0.51	0.42	0.15	0.45
526 Putrescine	0.06	0.37	-0.07	0.5
876 Nicotinate(r)ibonucleotide	0.13	0.47	-0.37	0.4
154 Aspartate from Serine	-0.15	0.39	0.05	0.48
72 Glycogen glucose release	0.07	0.42	-1.46	0.44

Continued on next page

Amplitude reversal of Period difference, control – continued

Simulation	control 1/6h		control 6/24h	
	ampl	score	ampl	score
120 Asparagine from Aspartate	0.03	0.4	-0.2	0.46
673 5-Phosphoribosyl-4-carboxy-5-aminoimidazole	0.17	0.46	-0.37	0.39
438 Antichymotrypsin degr	-0.33	0.5	0.05	0.35
619 3-Methyl-2-oxobutyrate	0.37	0.35	-0.46	0.5
527 Spermidine	0.31	0.45	-0.5	0.4
840 L-Glutamate 5-semialdehyde	0.07	0.46	-0.61	0.39
249 Proline from beta-Alanine	0.24	0.37	-0.25	0.47
865 N-Carbamoyl-L-aspartate	-0.15	0.47	0.05	0.38
226 Glutamine from beta-Alanine	0.22	0.37	-0.26	0.47
611 3-Dehydrosphinganine	0.09	0.39	-0.17	0.45
208 Glutamine from Asparagine	0.15	0.38	-0.32	0.45
919 Ribose-1P	0.11	0.28	-0.48	0.55
19 Aerobic reduction of NADP+ (gluc)	0.4	0.34	-0.42	0.48
931 Sphinganine-1P	-0.11	0.4	0.17	0.41
951 UMP	0.22	0.44	-0.42	0.37
875 Nicotinate D-ribonucleoside	0.12	0.45	-0.37	0.36
133 Asparagine from Serine	-0.15	0.44	0.05	0.36
43 Activated methyl group (SAM)	0.15	0.37	-1.39	0.43
928 Sedoheptulose-7P	0.11	0.33	-0.42	0.47
751 D-Xylulose-5P	0.11	0.37	-0.52	0.43
56 UDP-activated glucuronate	0.38	0.38	-0.8	0.41
809 Guanosine	0.26	0.48	-0.16	0.31
898 Phosphatidate-CL-pool	0.43	0.32	-0.44	0.47
2 Aerobic ATP rephosph (gluc)	-0.07	0.45	0.05	0.33
209 Glutamine from Aspartate	0.17	0.35	-0.31	0.43
528 Spermine	0.32	0.41	-0.6	0.37
517 PI	0.12	0.42	-0.42	0.36
863 N-Acetylneuraminate	0.13	0.3	-0.24	0.48
394 Collagen ITA1(c) synthesis	-0.31	0.35	0.19	0.42
518 CL	0.11	0.32	-0.41	0.45
834 Kynurenine	-0.63	0.42	0.31	0.35
758 Deoxycytidine	0.23	0.47	-0.18	0.29
479 UTP	0.26	0.41	-0.16	0.35
430 LDL degr	0.17	0.32	-0.57	0.44
505 Uracil	0.24	0.38	-0.27	0.37
613 3-Hydroxyanthranilate	-0.15	0.38	0.27	0.37
360 Collagen CO6A5(c) synthesis	-0.31	0.33	0.19	0.42
497 UDP-xylose	0.29	0.45	-0.74	0.3
328 Antitrypsin	-0.29	0.34	0.19	0.41
499 udpgal	0.22	0.38	-0.37	0.37
476 ATP	0.19	0.42	-0.45	0.32
507 Cytidine	0.22	0.43	-0.15	0.31
723 CMP	0.2	0.43	-0.16	0.31
472 dATP	0.19	0.42	-0.45	0.32
117 Arginine from beta-Alanine	0.24	0.31	-0.24	0.42
948 UDP-N-acetylglucosamine	0.23	0.34	-0.19	0.39
298 Palmitolate from Palmitate	-0.47	0.36	0.2	0.36
485 Glycine	0.35	0.31	-0.5	0.41
51 Acetyl group(p)	0.16	0.36	-0.27	0.36
500 udpglcur	0.31	0.4	-1.23	0.32
326 Fibrinogen	-0.29	0.33	0.18	0.39
560 (S)-Dihydroorotate	-0.15	0.42	0.06	0.29
724 CMP-NeuNGc	0.17	0.37	-0.26	0.35
932 Sphingosine	0.07	0.34	-0.82	0.37
510 Adenosine	0.19	0.39	-0.67	0.32
628 3-Oxohexanoyl-CoA	0.19	0.42	-0.32	0.29
944 Thymine	0.27	0.34	-0.36	0.36
798 Glucosylceramide-pool	0.08	0.35	-1.37	0.35
729 Ceramide-1P-pool	0.08	0.38	-0.83	0.31

Continued on next page

Amplitude reversal of Period difference, control – continued

Simulation	control 1/6h		control 6/24h	
	ampl	score	ampl	score
503 IMP	0.2	0.39	-0.48	0.31
504 XMP	0.2	0.38	-0.46	0.32
537 (2E)-Hexenoyl-CoA	0.2	0.39	-0.32	0.3
722 CDP-ethanolamine	0.2	0.38	-0.77	0.32
719 Butyryl-CoA	0.2	0.4	-0.31	0.29
819 Hexanoyl-CoA	0.18	0.39	-0.31	0.31
292 beta-Alanine from Valine	0.18	0.35	-0.4	0.34
817 Hexadecenal	0.08	0.35	-0.7	0.34
815 Hexadecanal	0.08	0.35	-0.7	0.34
540 (2E)-Octenoyl-CoA	0.2	0.4	-0.32	0.3
236 Proline from Glycine	0.29	0.3	-0.27	0.39
882 Octanoyl-CoA	0.18	0.39	-0.31	0.3
623 3-Oxodecanoyl-CoA	0.18	0.4	-0.31	0.29
755 Decanoyl-CoA	0.18	0.41	-0.31	0.28
474 dGTP	0.2	0.4	-0.46	0.29
630 3-Oxooctanoyl-CoA	0.18	0.38	-0.31	0.31
938 Succinate	-0.18	0.36	0.23	0.33
469 ametam	0.26	0.39	-0.22	0.29
625 3-Oxododecanoyl-CoA	0.18	0.42	-0.31	0.27
531 (2E)-Decenoyl-CoA	0.18	0.39	-0.32	0.29
907 Phosphopantetheine	-0.12	0.39	0.16	0.29
205 Glycine from beta-Alanine	0.23	0.31	-0.47	0.37
563 (S)-Hydroxyoctanoyl-CoA	0.18	0.38	-0.31	0.31
561 (S)-Hydroxydecanoyl-CoA	0.18	0.39	-0.32	0.29
891 PG-CL-pool	0.39	0.3	-0.45	0.38
639 3-Ureidoisobutyrate	0.23	0.29	-0.37	0.39
892 PGP-CL-pool	0.39	0.3	-0.45	0.38
847 Lauroyl-CoA	0.21	0.35	-0.67	0.33
17 Aerobic reduction of NAD+ (gluc)	0.1	0.32	-0.42	0.36
468 PAPS	0.35	0.33	-0.59	0.35
519 LacCer	0.1	0.28	-0.43	0.39
896 Pantetheine	-0.51	0.33	0.15	0.35
808 Guanidinoacetate	0.07	0.4	-1.22	0.28
562 (S)-Hydroxyhexanoyl-CoA	0.19	0.38	-0.32	0.29
265 Serine from Proline	0.11	0.3	-0.25	0.37
760 Deoxyinosine	0.27	0.33	-0.37	0.34
508 Xanthine	0.2	0.34	-0.46	0.33
248 Proline from Valine	0.21	0.35	-0.13	0.32
81 Alanine from Glutamate	-0.15	0.34	0.19	0.32
603 2-Methylacetoacetyl-CoA	0.2	0.37	-0.6	0.29
542 (2E)-Tetradecenoyl-CoA	0.2	0.34	-0.65	0.32
95 Alanine from Valine	0.13	0.33	-0.1	0.33
767 Dihydrothymine	0.23	0.31	-0.39	0.35
199 Glycine from Proline	0.1	0.3	-0.22	0.35
16 Aerobic reduction of NAD+ (FA)	0.35	0.33	-1.06	0.32
970 dTDP	0.26	0.29	-0.38	0.36
635 3-Oxotetradecanoyl-CoA	0.17	0.34	-0.66	0.31
90 Alanine from Proline	0.11	0.32	-0.22	0.32
241 Proline from Lysine	0.22	0.33	-0.25	0.31
631 3-Oxopalmitoyl-CoA	0.17	0.36	-0.68	0.29
945 Tiglyl-CoA	0.21	0.37	-0.6	0.28
509 Guanine	0.2	0.33	-0.46	0.31
557 (S)-3-Hydroxytetradecanoyl-CoA	0.17	0.32	-0.64	0.32
764 Dihomo-gamma-linolenoyl-CoA	0.17	0.35	-0.25	0.29
428 beta-Alanine degr	-0.68	0.32	0.09	0.32
498 UDP-glucose	0.26	0.34	-0.4	0.3
553 (S)-3-Hydroxy-2-methylbutyryl-CoA	0.2	0.36	-0.57	0.28
911 Propanoyl-CoA	0.17	0.36	-0.59	0.28
972 dUDP	0.23	0.35	-0.17	0.28

Continued on next page

Amplitude reversal of Period difference, control – continued

Simulation	control 1/6h		control 6/24h	
	**ampl**	**score**	**ampl**	**score**
974 dUTP	0.22	0.36	-0.17	0.27
933 Sphingosine-1P	0.07	0.3	-0.83	0.33
616 3-Hydroxypropionyl-CoA	0.17	0.33	-0.49	0.29
858 Myristoyl-CoA	0.18	0.34	-0.56	0.28
99 Arginine from Aspartate	0.24	0.3	-0.98	0.32
269 Serine from Valine	0.13	0.3	-0.4	0.32
204 Glycine from Valine	0.15	0.3	-0.42	0.31
556 (S)-3-Hydroxyhexadecanoyl-CoA	0.17	0.33	-0.61	0.28
699 Acrylyl-CoA	0.17	0.33	-0.46	0.28
102 Arginine from Glutamate	0.06	0.3	-1	0.3
225 Glutamine from Valine	-0.23	0.31	0.36	0.29
181 Glutamate from Tryptophan	0.17	0.3	-1.17	0.3
224 Glutamine from Tyrosine	0.13	0.26	-2.45	0.33
212 Glutamine from Glutamate	0.06	0.33	-0.63	0.26
614 3-Hydroxyisobutyrate	0.13	0.29	-1.08	0.27

3.3 Amplitude reversal of Period difference, TGFβ

Table 13: Amplitude reversal of Period difference, TGFβ

Simulation	TGFβ 1/6h		TGFβ 6/24h	
	ampl	score	ampl	score
947 Triphosphate degr	-0.16	1	0.23	1
946 Trehalose	0.08	1	-0.55	1
466 THF(m)	0.11	1	-0.55	1
459 THF	0.11	1	-0.55	1
917 Pyridoxine-P	0.02	1	-0.24	1
877 O-Acetylcarnitine	0.21	1	-0.01	1
30 NADPH to NADH transhydrogenase in mito	0.24	1	-0.67	1
29 NADH to NADPH transhydrogenase	0.11	1	-0.55	1
836 L-2-Aminoadipate	0.04	1	-0.02	1
822 Homovanillate	0.02	1	-0.09	1
796 Glucono-1,5-lactone-6P	0.68	1	-0.26	1
37 GSH reduction using NADPH redox potential	0.24	1	-0.41	1
38 GSH oxidation	0.24	1	-0.41	1
774 FMN	0.04	1	-0.14	1
766 Dihydrofolate	0.11	1	-0.55	1
670 5-Methyl-THF	0.11	1	-0.55	1
362 Collagen CO7A1(c) synthesis	-0.1	1	0.55	1
354 Collagen CO5A1(c) synthesis	-0.08	1	0.14	1
350 Collagen CO4A3(c) synthesis	-0.24	1	0.13	1
348 Collagen CO4A1(c) synthesis	-0.55	1	0.16	1
379 Collagen COKA1(c) synthesis	0.14	1	-0.27	1
353 Collagen CO4A6(c) synthesis	0.04	1	-0.13	1
376 Collagen COHA1(c) synthesis	-0.17	1	0.02	1
351 Collagen CO4A4(c) synthesis	0.01	1	-0.13	1
352 Collagen CO4A5(c) synthesis	-0.01	1	0.69	1
370 Collagen COBA2(c) synthesis	-0.03	1	0.13	1
365 Collagen CO9A1(c) synthesis	0.13	1	-0.23	1
382 Collagen COMA1(c) synthesis	0.25	1	-0.06	1
346 Collagen CO2A1(c) synthesis	0.004	1	-0.43	1
358 Collagen CO6A2(c) synthesis	0.07	1	-0.16	1
400 Collagen VWA2(c) synthesis	0.11	1	-0.2	1
384 Collagen COOA1(c) synthesis	-0.03	1	0.06	1
378 Collagen COJA1(c) synthesis	0.02	1	-0.16	1
366 Collagen CO9A2(c) synthesis	0.09	1	-0.2	1
357 Collagen CO6A1(c) synthesis	0.05	1	-0.41	1
340 Collagen BGH3(c) synthesis	0.09	1	-0.16	1
372 Collagen CODA1(c) synthesis	0.09	1	-0.07	1
341 Collagen C43BP(c) synthesis	-0.03	1	0.61	1
367 Collagen CO9A3(c) synthesis	0.03	1	-0.1	1
389 Collagen EMID2(c) synthesis	0.001	1	-0.17	1
393 Collagen FMOD(c) synthesis	0.02	1	-0.12	1
388 Collagen CTHR1(c) synthesis	0.03	1	-0.05	1
454 Glycogenin	-0.23	0.97	0.69	0.96
338 Haptoglobin	-0.02	0.96	0.03	0.96
807 Glycogenin-G8	-0.23	0.97	0.69	0.96
803 Glycogenin-G11	-0.23	0.96	0.69	0.95
804 Glycogenin-G4G4	-0.23	0.96	0.69	0.94
806 Glycogenin-G7G1	-0.23	0.96	0.69	0.94
805 Glycogenin-G7	-0.23	0.95	0.69	0.94
916 Pyridoxal	0.34	0.97	-0.57	0.87
464 mlthf	0.13	0.96	-0.53	0.86
664 5,10-Methenyl-THF	0.13	0.78	-0.51	0.85
495 Mannose-6P	-0.01	0.78	0.25	0.78
34 Thioredoxin(c) reduction	0.11	0.71	-0.53	0.82
467 1fthf	0.13	0.64	-0.51	0.88
756 Dehydroalanine	0.43	0.81	-1.19	0.7

Continued on next page

Amplitude reversal of Period difference, TGFβ – continued

Simulation	TGFβ 1/6h		TGFβ 6/24h	
	ampl	score	ampl	score
64 Glucosamine-6P	-0.21	0.82	0.22	0.63
71 Glycogen glucose storage	-0.23	0.77	0.69	0.65
982 sn-Glycerol-3P	0.16	0.49	-1.37	0.92
460 Pyridoxal-P	0.34	0.65	-0.47	0.75
405 Acetone	1.58	0.5	-0.95	0.86
70 Formate degr	0.11	0.57	-0.43	0.71
607 2-Oxobutyrate	0.46	0.61	-1.42	0.64
669 5-Hydroxy-L-tryptophan	-0.12	0.7	0.09	0.52
490 Palmitate	-0.51	0.74	0.04	0.48
403 Acetoacetate	1.56	0.42	-0.87	0.79
726 Carbamoyl-P	-0.11	0.82	0.29	0.37
475 dTTP	-0.12	0.62	0.23	0.54
802 Glycerate	0.08	0.66	-0.69	0.5
949 UDP-activated-N-acetyl-D-galactosamine	-0.19	0.66	0.2	0.49
435 Galactose degr	-0.11	0.63	0.31	0.52
506 Uridine	-0.12	0.59	0.27	0.55
434 Fructose degr	0.3	0.44	-1.08	0.69
667 5-Formiminotetrahydrofolate	0.13	0.53	-0.51	0.58
479 UTP	-0.11	0.67	0.28	0.43
799 Glucuronate	0.03	0.7	-0.08	0.41
439 Antitrypsin degr	0.07	0.38	-0.33	0.72
672 5-Oxoproline	0.17	0.55	-0.02	0.55
473 dCTP	-0.13	0.62	0.25	0.48
477 CTP	-0.11	0.61	0.26	0.49
874 Nicotinate	0.07	0.53	-0.6	0.56
480 Alanine	0.21	0.51	-1.9	0.57
41 Na+ importgradient	-0.05	0.51	0.4	0.57
497 UDP-xylose	-0.1	0.67	0.3	0.39
404 (R)-3-Hydroxybutanoate	1.45	0.43	-0.93	0.62
680 6-Phospho-D-gluconate	0.69	0.52	-0.26	0.53
498 UDP-glucose	-0.11	0.67	0.28	0.36
505 Uracil	-0.1	0.55	0.25	0.49
782 Fatty-acid-VLDL-TG3-pool	0.08	0.35	-1.44	0.66
507 Cytidine	-0.11	0.56	0.26	0.43
837 L-Cystathionine	-0.23	0.51	0.46	0.47
842 L-Octanoylcarnitine	0.26	0.4	-0.54	0.58
295 Taurine from Cysteine	0.3	0.46	-0.4	0.51
826 Hypotaurine	0.3	0.46	-0.4	0.51
548 (R)-4-Phosphopantothenoyl-cysteine	0.24	0.46	-0.15	0.52
966 dCDP	-0.14	0.56	0.25	0.4
50 Acetyl group(m)	0.29	0.35	-0.68	0.61
433 Stearate degr	0.28	0.35	-0.75	0.6
257 Serine from Glycine	0.12	0.48	-0.98	0.46
432 Oleate degr	0.28	0.36	-0.72	0.58
953 Urocanate	0.03	0.46	-1.16	0.48
967 dCMP	-0.13	0.57	0.25	0.37
862 N-Acetylmannosamine-6P	-0.75	0.56	0.2	0.38
869 N-Pantothenoylcysteine	0.24	0.43	-0.15	0.5
972 dUDP	-0.14	0.59	0.27	0.34
720 CDP	-0.13	0.53	0.27	0.4
723 CMP	-0.12	0.53	0.27	0.39
834 Kynurenine	-0.24	0.56	0.18	0.36
600 2-Deoxy-D-ribose-1P	-0.26	0.47	0.28	0.44
974 dUTP	-0.14	0.57	0.27	0.34
878 O-Butanoylcarnitine	0.28	0.33	-0.61	0.57
605 2-Oxo-3-methylvalerate	-0.18	0.49	0.7	0.4
973 dUMP	-0.24	0.39	0.25	0.49
971 dTMP	-0.14	0.39	0.25	0.49
522 Triacylglycerol	0.13	0.32	-1.02	0.56

Continued on next page

Amplitude reversal of Period difference, TGFβ – continued

Simulation		TGFβ 1/6h		TGFβ 6/24h	
		ampl	score	ampl	score
970	dTDP	-0.14	0.37	0.24	0.51
721	CDP-choline	-0.12	0.52	0.27	0.35
51	Acetyl group(p)	0.24	0.42	-0.75	0.46
790	GAR	-0.18	0.46	0.27	0.42
529	Methylthioribose-1P	-0.2	0.55	0.6	0.32
948	UDP-N-acetylglucosamine	-0.15	0.53	0.26	0.33
560	(S)-Dihydroorotate	-0.11	0.5	0.28	0.35
133	Asparagine from Serine	0.41	0.39	-0.87	0.45
511	UDP-N-acetylgalactosamine	-0.15	0.52	0.26	0.32
761	Deoxyuridine	-0.24	0.39	0.26	0.44
924	SAM	-0.19	0.49	0.36	0.35
922	SAH	-0.19	0.49	0.36	0.35
674	5-Phosphoribosylamine	-0.18	0.5	0.33	0.33
758	Deoxycytidine	-0.13	0.5	0.25	0.33
665	5,6-Dihydrouracil	-0.12	0.46	0.26	0.36
154	Aspartate from Serine	0.38	0.38	-0.94	0.45
52	Acetyl group(r)	0.29	0.27	-0.75	0.54
49	Acetyl group(c)	0.29	0.27	-0.75	0.54
965	dAMP	-0.18	0.48	0.31	0.34
671	5-Methylthioadenosine	-0.18	0.48	0.36	0.32
722	CDP-ethanolamine	-0.13	0.46	0.27	0.34
943	Thymidine	-0.33	0.36	0.25	0.43
867	N-Formyl-GAR	-0.22	0.44	0.26	0.35
96	Alanine from beta-Alanine	0.29	0.42	-0.18	0.36
691	AIR	-0.21	0.43	0.29	0.34
300	Stearate from Oleate	-0.2	0.44	0.89	0.33
767	Dihydrothymine	-0.12	0.35	0.24	0.41
907	Phosphopantetheine	-0.31	0.4	0.51	0.36
932	Sphingosine	-0.2	0.44	0.05	0.32
675	5-Phosphoribosylformylglycinamidine	-0.21	0.44	0.37	0.32
430	LDL degr	0.34	0.33	-1.55	0.42
67	dTTP salvage from Thymine	-0.19	0.35	0.22	0.4
710	Adenylosuccinate	-0.19	0.41	0.34	0.34
619	3-Methyl-2-oxobutyrate	-0.38	0.39	1.21	0.35
528	Spermine	-0.21	0.46	0.48	0.28
120	Asparagine from Aspartate	-0.29	0.34	0.45	0.4
632	3-Oxopropanoate	0.3	0.35	-0.18	0.39
831	Isobutyryl-CoA	-0.18	0.44	0.48	0.3
524	gdpddman	-0.29	0.34	0.24	0.39
512	N-acglucam	-0.77	0.38	0.68	0.35
856	Methacrylyl-CoA	-0.21	0.42	0.45	0.31
748	D-3-Amino-isobutanoate	-0.13	0.35	0.24	0.37
698	Acetyl-CoA	-0.19	0.42	0.39	0.3
615	3-Hydroxyisobutyryl-CoA	-0.18	0.41	0.44	0.31
719	Butyryl-CoA	0.26	0.28	-0.68	0.43
944	Thymine	-0.12	0.33	0.24	0.39
964	dADP	-0.18	0.42	0.33	0.29
969	dGMP	-0.18	0.38	0.27	0.33
639	3-Ureidoisobutyrate	-0.12	0.33	0.24	0.38
968	dGDP	-0.18	0.39	0.28	0.32
502	GDP-mannose	-0.29	0.35	0.25	0.36
760	Deoxyinosine	-0.25	0.32	0.3	0.39
753	Deamido-NAD	-0.2	0.4	0.34	0.3
808	Guanidinoacetate	-0.52	0.39	0.18	0.31
297	Palmitate from Palmitolate	-0.18	0.39	0.89	0.31
765	Dihydroceramide-pool	-0.25	0.38	0.05	0.31
759	Deoxyguanosine	0.12	0.37	-0.18	0.32
501	GDP-L-fucose	-0.29	0.32	0.24	0.37
863	N-Acetylneuraminate	-0.86	0.42	0.98	0.27

Continued on next page

Amplitude reversal of Period difference, TGFβ – continued

**Simulation**	**TGFβ 1/6h**		**TGFβ 6/24h**	
	**ampl**	**score**	**ampl**	**score**
517 PI	0.16	0.37	-1.11	0.32
762 Dephospho-CoA	-0.19	0.39	0.47	0.29
809 Guanosine	0.14	0.33	-0.82	0.35
18 Aerobic reduction of NADP+ (FA)	0.07	0.37	-0.58	0.3
933 Sphingosine-1P	-0.2	0.39	0.05	0.27
872 NeuNGc	-0.85	0.39	0.85	0.27
16 Aerobic reduction of NAD+ (FA)	0.07	0.32	-0.63	0.34
692 AMP	-0.18	0.36	0.47	0.3
611 3-Dehydrosphinganine	0.02	0.32	-0.34	0.33
689 ADP	-0.18	0.35	0.47	0.3
681 6-Pyruvoyltetrahydropterin	-0.17	0.35	0.29	0.3
827 Hypoxanthine	0.34	0.32	-0.2	0.32
791 GDP	-0.18	0.32	0.3	0.32
792 GMP	-0.18	0.35	0.34	0.29
952 Urate	0.29	0.32	-0.49	0.31
810 H2O2	0.29	0.32	-0.49	0.31
828 Inosine	0.15	0.32	-0.2	0.3
958 cAMP	-0.18	0.33	0.47	0.28
207 Glutamine from Arginine	-0.71	0.35	1.06	0.25
724 CMP-NeuNGc	-0.15	0.33	0.75	0.27
