# Supplementary material for: Metabolic Consequences of TGFβ Stimulation in Cultured Primary Mouse Hepatocytes Screened from Transcript Data with ModeScore
Source: Metabolites. 2012 Nov 21;2(4):983–1003. doi: 10.3390/metabo2040983 (PMC3901234; doi:10.3390/metabo2040983)
Supplement: Supplementary File 6 — Detailed reaction scores (PDF, 617 KB) [file metabolites-02-00983-s006.pdf]

Detailed reaction scores

Contents

1 Introduction 2

2 Reaction scores for Tyrosine degr (426), series control 1/24h, TGFβ/C 24h, and TGFβ 1/24h 3

3 Reaction scores for Phenylalanine degr (418), series control 1/24h, TGFβ/C 24h, and TGFβ 1/24h 5

4 Reaction scores for Tyrosine (489), series control 1/24h, TGFβ/C 24h, and TGFβ 1/24h 7

5 Reaction scores for Collagen CORA1(c) synthesis (386), series control 1/24h, TGFβ/C 24h, and TGFβ 1/24h 8

6 Reaction scores for Collagen COIA1(c) synthesis (377), series control 1/24h, TGFβ/C 24h, and TGFβ 1/24h 9

7 Reaction scores for Collagen COFA1(c) synthesis (374), series control 1/24h, TGFβ/C 24h, and TGFβ 1/24h 10

8 Reaction scores for Collagen CO4A5(c) synthesis (352), series control 1/24h, TGFβ/C 24h, and TGFβ 1/24h 11

9 Reaction scores for Ethanol degr (448), series control 1/24h, TGFβ/C 24h, and TGFβ 1/24h 12

10 Reaction scores for Bilirubin conjugation (310), series control 1/24h, TGFβ/C 24h, and TGFβ 1/24h 13

11 Reaction scores for Urea from alanine (312), series control 1/24h, TGFβ/C 24h, and TGFβ 1/24h 14

12 Reaction scores for Cholesterol (523), series control 1/24h, TGFβ/C 24h, and TGFβ 1/24h 15

13 Reaction scores for (R)-Mevalonate (552), series control 1/24h, TGFβ/C 24h, and TGFβ 1/24h 16

14 Reaction scores for Farnesyl-PP (63), series control 1/24h, TGFβ/C 24h, and TGFβ 1/24h 17

15 Reaction scores for Creatine (314), series control 1/24h, TGFβ/C 24h, and TGFβ 1/24h 18

16 Reaction scores for Glycogen glucose release (72), series control 1/24h, TGFβ/C 24h, and TGFβ 1/24h 20

17 Reaction scores for (R)-3-Hydroxybutanoate (404), series control 1/24h, TGFβ/C 24h, and TGFβ 1/24h 21

18 Reaction scores for Glutamine (486), series control 1/24h, TGFβ/C 24h, and TGFβ 1/24h 22

19 Reaction scores for Inositol (829), series control 1/24h, TGFβ/C 24h, and TGFβ 1/24h 23

20 Reaction scores for PI (517), series control 1/24h, TGFβ/C 24h, and TGFβ 1/24h 24

21 Reaction scores for Sphinganine-1P (931), series control 1/24h, TGFβ/C 24h, and TGFβ 1/24h 26

22 Reaction scores for 4-Hydroxyphenylpyruvate (658), series control 1/24h, TGFβ/C 24h, and TGFβ 1/24h 27

23 Reaction scores for Saccharopine (925), series control 1/24h, TGFβ/C 24h, and TGFβ 1/24h 28

24 Reaction scores for Tyrosine (489), series control 1/24h, TGFβ/C 24h, and TGFβ 1/24h 29

25 Reaction scores for Urea from NH3 (313), series control 1/24h, TGFβ/C 24h, and TGFβ 1/24h 30

26 Reaction scores for Pantetheine (896), series control 1/24h, TGFβ/C 24h, and TGFβ 1/24h 31

27 Reaction scores for Glucose-6P (54), series control 1/24h, TGFβ/C 24h, and TGFβ 1/24h 32

28 Reaction scores for Alanine (480), series control 1/24h, TGFβ/C 24h, and TGFβ 1/24h 33

29 Reaction scores for Proline degr (419), series control 1/24h, TGFβ/C 24h, and TGFβ 1/24h 34

30 Reaction scores for beta-Alanine degr (428), series control 1/24h, TGFβ/C 24h, and TGFβ 1/24h 36

31 Reaction scores for Aspartate degr (410), series control 1/24h, TGFβ/C 24h, and TGFβ 1/24h 37

32 Reaction scores for PEP (890), series control 1/24h, TGFβ/C 24h, and TGFβ 1/24h 38

33 Reaction scores for Lysine degr (416), series control 1/24h, TGFβ/C 24h, and TGFβ 1/24h 39

34 Reaction scores for Proline (487), series control 1/24h, TGFβ/C 24h, and TGFβ 1/24h 41

35 Reaction scores for CoA (450), series control 1/24h, TGFβ/C 24h, and TGFβ 1/24h 42

36 Reaction scores for Anaerobic rephosph of ATP (3), series control 1/24h, TGFβ/C 24h, and TGFβ 1/24h 44

37 Reaction scores for Proline (487), series control 1/24h, TGFβ/C 24h, and TGFβ 1/24h 45

38 Reaction scores for Triacylglycerol (522), series control 1/24h, TGFβ/C 24h, and TGFβ 1/24h 46

39 Reaction scores for Choloyl-CoA (741), series control 1/24h, TGFβ/C 24h, and TGFβ 1/24h 47

40 Reaction scores for Gly-CD-cholate(b) (316), series control 1/24h, TGFβ/C 24h, and TGFβ 1/24h 50

1 Introduction

The following tables document how the final score for a reference flux distribution emerges (as a weighted average) from the individual reaction scores. The scores of individual reactions (identifier listed in column **Rea ID**) are listed in the column **Scre**. In the header of this column also the total score value is given.

The scaling parameter λ respectively the amplitude $(1/\lambda)$ can freely be chosen to maximize the score. If a λ would exist such that the score would be perfect (value 1) then also the score for each individual reaction would pe perfect, in other words, the relative expression profile would perfectly meet a flux distribution. Otherwise, the chosen λ is a compromise beetween the fit of the reactions — for some reactions the fit is very good, score is near to one, for other reactions the fit is bad and the score is near to zero. In other words, the compromise λ is fixed to the favor of the fit of some reactions and, thus, the following tables also document why a particular match of the relative expression profile and the reference flux distribution is chosen.

The table also lists the absolute expression values for the two considered samples, they are marked with the respective sample identifer. The column **Expr** shows the difference of these two expression values. The column **Expr*<value of λ>** shows the scaled relative expression which is the actual value compared to the flux value in the reference flux distribution (Shown in column **Ref value**). These two values are the function arguments for the scoring function whose results are shown in column **Score**.

The final score for the flux distribution is a *weighted* sum of individual scores. The respective weights are also displayed in the table. The weights depend on the absolute flux value and on the stoichiometric coefficients in the reaction. This weighting scheme is based on the fact that the reactions carrying a high flux play a greater role in the flux distribution and mostly also more improtant and critical to respective metabolic function. The weight is also modified by the number of occurrences of a reaction throughout all flux solutions: less often used reactions have a higher weight that more frequent reactions to account for specificity.

Below is a line with more information on the reaction: a comprehensive name, one or more pathways (KEGG maps), and a EC identifier for enzymes and a TCDB identifier for transporters where available.

Here, also a second type of rows is shown, the data of the genes assigned to the reaction above. In the first (combined) column the gene name (from ENSEMBL data base) is printed. If there is more than one spot assigned to this gene, it is indicated here. If this is the case, averaged numbers are shown.

(arithmetic mean of the ld values, which is equivalent to geometric mean of the absolute values).

In the second column, below the header **Expr** the quotient between the logarithmic expression values (ld) is shown. Note that the calculation is different from the value assigned to the reaction (the difference between absolute expression values). It is shown because this ratio is commonly used in the interpretation of gene expression data. In the columns below the absolute expression values the logarithmic expression value (ld) is shown.In the (combined) column below the reaction, the ENSEMBL identifier of the gene is shown together with the gene description in this database.

The lines below the main reaction line (which contain entries for score and impact) also show entries for the expression but they are shown as they appear in the data source (as opposed to the values in the main reaction line which contain transformed values).

List of compartments

Identifer	compartment	comment
s	sinusoid (blood)	external
b	bile	external
c	cytosol	
m	mitochondrium	
r	ER and Golgi	combined
p	peroxysome	
l	lysosome	
n	nucleus	

2 Reaction scores for Tyrosine degr (426), series control 1/24h, TGFβ/C 24h, and TGFβ 1/24h

The amplitudes of the mode are -3.5, -0.87 and -4.41.

Simulation	Definition			Solution													
				exchanges		reactions								transp			Prot
	Objective	Constraints	Comment	imports	exports	c	m	r	p	l	n	s	b	s-c	b-c	intra	synth
426 Tyrosine degr	-1 Tyrosine(c)	MCES +Urea	Tyrosine degradation using palmitate excreting urea	1.5 H_2O(s) 2 O_2(s) 1 Tyrosine(c) 3 ATP-energy(c) 1 ATP-energy(m) 0.5 Proton-gradient(m)	1.5 CO_2(s) 0.5 Pyruvate(c) 0.5 Pyruvate(m) 0.5 Urea(s) 0.5 NADH-redox-potential(c) 0.5 NADPH-redox-potential(c) 1 NADPH-redox-potential(m) 2 CoA-activated acetyl group(c)	20	6	-	-	-	-	-	-	4	-	4	-

Table 1: Reaction scores for Tyrosine degr (426), series control 1/24h, TGFβ/C 24h, and TGFβ 1/24h

Rea ID	control 1/24h Score *0.41*	Expr Δ	Expr C1h	Expr C24h	TGFβ/C 24h Score *0.44*	Expr Δ	TGFβ 1/24h Score *0.45*	Expr Δ	Expr T1h	Expr T24h	Wght	Reaction
r0544	0.95	-4.07	11	6.95	0.91	-0.69	1	-4.59	10.9	6.27	12.9	4-Hydroxyphenylpyruvate(c) + O2(c) ⇌ Homogentisate(c) + CO2(c). 4-Hydroxyphenylpyruvate:oxygen oxidoreductase (hydroxylating,decarboxylating) Tyrosine metabolism EC:1.13.11.27
Hpd												ENSMUSG00000029445 4-hydroxyphenylpyruvic acid dioxygenase
r0183	1	-3.33	10.5	7.21	0.04	-1.96	0.98	-4.86	10.1	5.26	12.8	Tyrosine(c) + AKG(c) ⇌ 4-Hydroxyphenylpyruvate(c) + Glutamate(c). L-Tyrosine:2-oxoglutarate aminotransferase Tyrosine metabolism / Phenylalanine, tyrosine and tryptophan biosynthesis EC:2.6.1.5 EC:2.6.1.57
Tat												ENSMUSG00000001670 tyrosine aminotransferase
r0543	0.83	-2.43	8.87	6.45	0.74	-1.21	0.95	-3.74	8.97	5.24	13	Homogentisate(c) + O2(c) ⇌ 4-Maleylacetoacetate(c). Homogentisate:oxygen 1,2-oxidoreductase (decyclizing) Tyrosine metabolism / Styrene degradation EC:1.13.11.5
Hgd												ENSMUSG00000022821 homogentisate 1, 2-dioxygenase
r0320	0.11	0.16	6.45	6.61	0.19	-0.08	0.13	0.07	6.46	6.53	13	ATP(c) + CoA(c) + Acetoacetate(c) → AMP(c) + PPi(c) + Acetoacetyl-CoA(c). Acetoacetate:CoA ligase (AMP-forming) Butanoate metabolism EC:6.2.1.16 (3 sp.)
Aacs (3 sp.)												ENSMUSG00000029482 acetoacetyl-CoA synthetase
r0605	0.26	-0.65	9.48	8.84	0.98	-0.78	0.4	-1.44	9.49	8.06	13.2	4-Maleylacetoacetate(c) ⇌ Fumarylacetoacetate(c). 4-Maleylacetoacetate cis-trans-isomerase Tyrosine metabolism / Styrene degradation EC:5.2.1.2
Gstz1												ENSMUSG00000021033 glutathione transferase zeta 1 (maleylacetoacetate isomerase)
r0069	0.14	-0.03	8.79	8.76	0.02	0.37	0.1	0.28	8.85	9.13	8.65	CoA(c) + Acetoacetyl-CoA(c) ⇌ 2 Acetyl-CoA(c). Acetyl-CoA:acetyl-CoA C-acetyltransferase Synthesis and degradation of ketone bodies EC:2.3.1.9 (2 genes, 5 sp.)
Acat1 (3 sp.)		0.19	7.92	8.1		0.54		0.67	7.97	8.64		ENSMUSG00000032047 acetyl-Coenzyme A acetyltransferase 1
Acat2 (2 sp.)		-0.35	10.1	9.75		0.11		-0.3	10.2	9.87		ENSMUSG00000023832 acetyl-Coenzyme A acetyltransferase 2
r0875	0.25	-0.6	8.06	7.46	0.001	0.77	0.14	-0.03	8.26	8.23	8.88	Glutamate(c) + H+(PG)(c) → Glutamate(m) + H+(PG)(m). Mitochondrial Carrier (MC) TCDB:2.A.29.14.3
Slc25a22												ENSMUSG00000019082 solute carrier family 25 (mitochondrial carrier, glutamate), member 22
r0329	0.68	-1.95	7.03	5.08	0.95	-0.73	0.71	-2.58	6.93	4.35	7.16	Carbamoyl-P(m) + Ornithine(m) ⇌ Pi(m) + Citrulline(m). Carbamoyl-phosphate:L-ornithine carbamoyltransferase Urea cycle and metabolism of amino groups / Arginine and proline metabolism EC:2.1.3.3 (3 sp.)
Otc (3 sp.)												ENSMUSG00000031173 ornithine transcarbamylase
r0896	0.15	-0.08	3.58	3.5	0.14	-0.004	0.14	-0.04	3.54	3.5	7.96	Urea(c) ⇌ Urea(s). Urea Transporter (UT) TCDB:1.A.28.1.3 (2 sp.)
Slc14a1 (2 sp.)												ENSMUSG00000059336 solute carrier family 14 (urea transporter), member 1
r0034	0.93	-4.18	12.7	8.56	0.44	-1.43	0.88	-5.55	12.7	7.14	7.16	2 ATP(m) + CO2(m) + H2O(m) + NH3(m) ⇌ 2 ADP(m) + Pi(m) + Carbamoyl-P(m). Carbon-dioxide:ammonia ligase (ADP-forming,carbamate-phosphorylating) Urea cycle and metabolism of amino groups / Nitrogen metabolism EC:6.3.4.16
Cps1												ENSMUSG00000025991 carbamoyl-phosphate synthetase 1
r0947	0.14	-0.01	3.1	3.08	0.08	0.11	0.14	-0.02	3.21	3.2	6.68	Citrulline(m) + Ornithine(c) ⇌ Citrulline(c) + Ornithine(m). Mitochondrial Carrier (MC) TCDB:2.A.29.19.1
Slc25a2												ENSMUSG00000050304 solute carrier family 25 (mitochondrial carrier, ornithine transporter) member 2
r0261	0.19	-0.32	9.99	9.66	0.78	-0.56	0.28	-0.88	9.98	9.1	7.12	Argininosuccinate(c) ⇌ Fumarate(c) + Arginine(c). N-(L-Argininosuccinate) arginie-lyase Urea cycle and metabolism of amino groups / Alanine and aspartate metabolism / Arginine and proline metabolism EC:4.3.2.1
Asl												ENSMUSG00000025533 argininosuccinate lyase
r0324	0.5	-1.42	10.8	9.35	0.7	-0.5	0.49	-1.79	10.6	8.85	13.3	Fumarylacetoacetate(c) + H2O(c) ⇌ Fumarate(c) + Acetoacetate(c). 4-Fumarylacetoacetate fumarylhydrola Tyrosine metabolism / Styrene degradation EC:3.7.1.2
Fah												ENSMUSG00000030630 fumarylacetoacetate hydrolase
r0075	0.07	0.49	10.9	11.4	1	-0.84	0.2	-0.43	11	10.6	4.48	Glutamate(m) + H2O(m) + NADP+(m) ⇌ AKG(m) + NADPH(m) + NH3(m). L-Glutamate:NADP+ oxidoreductase (deaminating) Glutamate metabolism EC:1.4.1.3 EC:1.4.1.4 (3 sp.)
Glud1 (3 sp.)												ENSMUSG00000021794 glutamate dehydrogenase 1
r0258	0.14	-0.03	10.4	10.3	0.31	-0.2	0.17	-0.28	10.4	10.1	6.36	Fumarate(c) + H2O(c) ⇌ Malate(c). (S)-Malate hydro-lyase Citrate cycle (TCA cycle) EC:4.2.1.2
Fh1												ENSMUSG00000026526 fumarate hydratase 1
r0820	0.07	0.5	7.47	7.97	0.65	-0.46	0.14	-0.04	7.54	7.51	4.91	AKG(m) + Malate(c) ⇌ AKG(c) + Malate(m). Mitochondrial Carrier (MC) TCDB:2.A.29.2.2 (2 sp.)
Slc25a10 (2 sp.)												ENSMUSG00000025792 solute carrier family 25 (mitochondrial carrier, dicarboxylate transporter), member 10
r0144	0.42	-5.79	12.5	6.71	0.3	-0.19	0.82	-5.79	12.3	6.51	7.96	Arginine(c) + H2O(c) ⇌ Urea(c) + Ornithine(c). L-Arginine amidinohydrolase Urea cycle and metabolism of amino groups / Arginine and proline metabolism EC:3.5.3.1
Arg1												ENSMUSG00000019987 arginase, liver
r0059	0.16	-0.17	4.52	4.35	0.17	-0.05	0.17	-0.23	4.53	4.3	5.06	Malate(m) + NADP+(m) ⇌ Pyruvate(m) + CO2(m) + NADPH(m). (S)-Malate:NADP+ oxidoreductase(oxaloacetate-decarboxylating) Pyruvate metabolism EC:1.1.1.40
Me3												ENSMUSG00000030621 malic enzyme 3, NADP(+)-dependent, mitochondrial
r0453	0.45	-1.28	11.7	10.4	0.06	0.16	0.31	-1.03	11.6	10.6	7.16	ATP(c) + Citrulline(c) + Aspartate(c) ⇌ AMP(c) + PPi(c) + Argininosuccinate(c). L-Citrulline:L-aspartate ligase (AMP-forming) Urea cycle and metabolism of amino groups / Alanine and aspartate metabolism / Arginine and proline metabolism EC:6.3.4.5
Ass1												ENSMUSG00000076441 argininosuccinate synthetase 1
r0104	0.15	-0.07	10.8	10.8	0.26	-0.16	0.16	-0.2	10.8	10.6	5.34	NAD+(c) + Malate(c) ⇌ OAA(c) + NADH(c). (S)-malate:NAD+ oxidoreductase Citrate cycle (TCA cycle) EC:1.1.1.37 (3 sp.)
Mdh1 (3 sp.)												ENSMUSG00000020321 malate dehydrogenase 1, NAD (soluble)
r0030	0.15	-0.09	6.05	5.96	0.1	0.06	0.14	-0.05	6.06	6.02	3.81	ATP(c) + AMP(c) ⇌ 2 ADP(c). ATP:AMP phosphotransferase Purine metabolism EC:2.7.4.3 (5 genes, 11 sp.)
Ak1 (2 sp.)		-0.07	3.97	3.89		0.76		0.59	4.07	4.66		ENSMUSG00000026817 adenylate kinase 1
Ak2 (2 sp.)		0.08	9.35	9.43		0.13		0.15	9.41	9.55		ENSMUSG00000028792 adenylate kinase 2
Ak4 (4 sp.)		-0.36	6.41	6.05		-0.23		-0.45	6.27	5.82		ENSMUSG00000028527 adenylate kinase 4
Ak5 (2 sp.)		-0.17	4.72	4.56		0.12		-0.12	4.79	4.68		ENSMUSG00000039058 adenylate kinase 5
Ak7		0.74	4.79	5.53		-0.39		0.06	5.08	5.14		ENSMUSG00000041323 adenylate kinase 7
r0109	0.3	-0.77	10.7	9.94	0.16	-0.03	0.26	-0.79	10.7	9.9	5.57	OAA(c) + Glutamate(c) ⇌ AKG(c) + Aspartate(c). L-Aspartate:2-oxoglutarate aminotransferase Glutamate metabolism / Alanine and aspartate metabolism EC:2.6.1.1
Got1												ENSMUSG00000025190 glutamate oxaloacetate transaminase 1, soluble
r0007	0.11	0.15	10.1	10.2	0.2	-0.09	0.13	0.07	10.1	10.1	3.45	PPi(c) + H2O(c) → 2 Pi(c). Pyrophosphate phosphohydrolase EC:3.6.1.1 (2 genes, 2 sp.)
Ppa1		-0.26	11.1	10.8		0.18		-0.14	11.1	11		ENSMUSG00000020089 pyrophosphatase (inorganic) 1

Continued on next page

	**control 1/24h**				**TGFβ/C 24h**		**TGFβ 1/24h**					
**Rea ID**	**Score** *0.41*	**Expr** Δ	**Expr** C1h	**Expr** C24h	**Score** *0.44*	**Expr** Δ	**Score** *0.45*	**Expr** Δ	**Expr** T1h	**Expr** T24h	**Wght**	**Reaction**
Ppa2		0.56	9.07	9.63		-0.35		0.28	9	9.28	ENSMUSG00000028013 pyrophosphatase (inorganic) 2	
r0058	0.1	0.23	10.9	11.1	0.54	-0.38	0.16	-0.18	10.9	10.7	3.99	Malate(c) + NADP+(c) ⇌ Pyruvate(c) + CO2(c) + NADPH(c). (S)-Malate:NADP+ oxidoreductase(oxaloacetate-decarboxylating) Pyruvate metabolism EC:1.1.1.40
Me1											ENSMUSG00000032418 malic enzyme 1, NADP(+)-dependent, cytosolic	

3 Reaction scores for Phenylalanine degr (418), series control 1/24h, TGFβ/C 24h, and TGFβ 1/24h

The amplitudes of the mode are -3.23, -0.76 and -4.38.

Simulation	Definition			Solution													
				exchanges		reactions								transp			Prot
	Objective	Constraints	Comment	imports	exports	c	m	r	p	l	n	s	b	s-c	b-c	intra	synth
418 Phenylalanine degr	-1 Phenylalanine(c)	MCES +Urea	Phenylalanine degradation using palmitate excreting urea	0.5 H_2O(s) 3 O_2(s) 1 Phenylalanine(c) 3 ATP-energy(c) 1 ATP-energy(m) 0.5 NADPH-redox-potential(c) 0.5 Proton-gradient(m)	1.5 CO_2(s) 0.5 Pyruvate(c) 0.5 Pyruvate(m) 0.5 Urea(s) 0.5 NADH-redox-potential(c) 1 NADPH-redox-potential(m) 2 CoA-activated acetyl group(c)	22	6	-	-	-	-	-	-	4	-	4	-

Table 2: Reaction scores for Phenylalanine degr (418), series control 1/24h, TGFβ/C 24h, and TGFβ 1/24h

	control 1/24h				TGFβ/C 24h		TGFβ 1/24h					
Rea ID	Score *0.41*	Expr Δ	Expr C1h	Expr C24h	Score *0.43*	Expr Δ	Score *0.47*	Expr Δ	Expr T1h	Expr T24h	Wght	Reaction
r0544	0.87	-4.07	11	6.95	0.98	-0.69	1	-4.59	10.9	6.27	12.7	4-Hydroxyphenylpyruvate(c) + O2(c) ⇌ Homogentisate(c) + CO2(c). 4-Hydroxyphenylpyruvate:oxygen oxidoreductase (hydroxylating,decarboxylating) Tyrosine metabolism EC:1.13.11.27
Hpd												ENSMUSG00000029445 4-hydroxyphenylpyruvic acid dioxygenase
r0183	1	-3.33	10.5	7.21	0.01	-1.96	0.98	-4.86	10.1	5.26	12.6	Tyrosine(c) + AKG(c) ⇌ 4-Hydroxyphenylpyruvate(c) + Glutamate(c). L-Tyrosine:2-oxoglutarate aminotransferase Tyrosine metabolism / Phenylalanine, tyrosine and tryptophan biosynthesis EC:2.6.1.5 EC:2.6.1.57
Tat												ENSMUSG00000001670 tyrosine aminotransferase
r0399	0.59	-1.56	11	9.44	0	-2.97	1	-4.43	10.9	6.47	14.3	Tetrahydrobiopterin(c) + Phenylalanine(c) + O2(c) ⇌ Dihydrobiopterin(c) + Tyrosine(c) + H2O(c). L-Phenylalanine,tetrahydrobiopterin:oxygen oxidoreductase (4-hydroxylating) Phenylalanine, tyrosine and tryptophan biosynthesis EC:1.14.16.1
Pah												ENSMUSG00000020051 phenylalanine hydroxylase
r0543	0.88	-2.43	8.87	6.45	0.49	-1.21	0.96	-3.74	8.97	5.24	12.8	Homogentisate(c) + O2(c) ⇌ 4-Maleylacetoacetate(c). Homogentisate:oxygen 1,2-oxidoreductase (decyclizing) Tyrosine metabolism / Styrene degradation EC:1.13.11.5
Hgd												ENSMUSG00000022821 homogentisate 1, 2-dioxygenase
r0398	0.19	-0.29	10.2	9.92	0.89	-0.58	0.27	-0.84	10.2	9.34	13.1	Dihydrobiopterin(c) + NADPH(c) ⇌ Tetrahydrobiopterin(c) + NADP+(c). NADPH:6,7-dihydropteridine oxidoreductase Folate biosynthesis EC:1.5.1.34 (2 sp.)
Qdpr (2 sp.)												ENSMUSG00000015806 quinoid dihydropteridine reductase
r0320	0.11	0.16	6.45	6.61	0.2	-0.08	0.13	0.07	6.46	6.53	12.8	ATP(c) + CoA(c) + Acetoacetate(c) → AMP(c) + PPi(c) + Acetoacetyl-CoA(c). Acetoacetate:CoA ligase (AMP-forming) Butanoate metabolism EC:6.2.1.16 (3 sp.)
Aacs (3 sp.)												ENSMUSG00000029482 acetoacetyl-CoA synthetase
r0605	0.28	-0.65	9.48	8.84	1	-0.78	0.41	-1.44	9.49	8.06	12.9	4-Maleylacetoacetate(c) ⇌ Fumarylacetoacetate(c). 4-Maleylacetoacetate cis-trans-isomerase Tyrosine metabolism / Styrene degradation EC:5.2.1.2
Gstz1												ENSMUSG00000021033 glutathione transferase zeta 1 (maleylacetoacetate isomerase)
r0069	0.14	-0.03	8.79	8.76	0.01	0.37	0.1	0.28	8.85	9.13	8.49	CoA(c) + Acetoacetyl-CoA(c) ⇌ 2 Acetyl-CoA(c). Acetyl-CoA:acetyl-CoA C-acetyltransferase Synthesis and degradation of ketone bodies EC:2.3.1.9 (2 genes, 5 sp.)
Acat1 (3 sp.)		0.19	7.92	8.1		0.54		0.67	7.97	8.64		ENSMUSG00000032047 acetyl-Coenzyme A acetyltransferase 1
Acat2 (2 sp.)		-0.35	10.1	9.75		0.11		-0.3	10.2	9.87		ENSMUSG00000023832 acetyl-Coenzyme A acetyltransferase 2
r0329	0.73	-1.95	7.03	5.08	1	-0.73	0.71	-2.58	6.93	4.35	7.02	Carbamoyl-P(m) + Ornithine(m) ⇌ Pi(m) + Citrulline(m). Carbamoyl-phosphate:L-ornithine carbamoyltransferase Urea cycle and metabolism of amino groups / Arginine and proline metabolism EC:2.1.3.3 (3 sp.)
Otc (3 sp.)												ENSMUSG00000031173 ornithine transcarbamylase
r0875	0.27	-0.6	8.06	7.46	0.0003	0.77	0.14	-0.03	8.26	8.23	8.71	Glutamate(c) + H+(PG)(c) → Glutamate(m) + H+(PG)(m). Mitochondrial Carrier (MC) TCDB:2.A.29.14.3
Slc25a22												ENSMUSG00000019082 solute carrier family 25 (mitochondrial carrier, glutamate), member 22
r0034	0.84	-4.18	12.7	8.56	0.21	-1.43	0.87	-5.55	12.7	7.14	7.02	2 ATP(m) + CO2(m) + H2O(m) + NH3(m) ⇌ 2 ADP(m) + Pi(m) + Carbamoyl-P(m). Carbon-dioxide:ammonia ligase (ADP-forming,carbamate-phosphorylating) Urea cycle and metabolism of amino groups / Nitrogen metabolism EC:6.3.4.16
Cps1												ENSMUSG00000025991 carbamoyl-phosphate synthetase 1
r0896	0.15	-0.08	3.58	3.5	0.14	-0.004	0.14	-0.04	3.54	3.5	7.81	Urea(c) ⇌ Urea(s). Urea Transporter (UT) TCDB:1.A.28.1.3 (2 sp.)
Slc14a1 (2 sp.)												ENSMUSG00000059336 solute carrier family 14 (urea transporter), member 1
r0324	0.53	-1.42	10.8	9.35	0.79	-0.5	0.5	-1.79	10.6	8.85	13.1	Fumarylacetoacetate(c) + H2O(c) ⇌ Fumarate(c) + Acetoacetate(c). 4-Fumarylacetoacetate fumarylhydrola Tyrosine metabolism / Styrene degradation EC:3.7.1.2
Fah												ENSMUSG00000030630 fumarylacetoacetate hydrolase
r0947	0.14	-0.01	3.1	3.08	0.07	0.11	0.14	-0.02	3.21	3.2	6.56	Citrulline(m) + Ornithine(c) ⇌ Citrulline(c) + Ornithine(m). Mitochondrial Carrier (MC) TCDB:2.A.29.19.1
Slc25a2												ENSMUSG00000050304 solute carrier family 25 (mitochondrial carrier, ornithine transporter) member 2
r0261	0.2	-0.32	9.99	9.66	0.88	-0.56	0.28	-0.88	9.98	9.1	6.99	Argininosuccinate(c) ⇌ Fumarate(c) + Arginine(c). N-(L-Argininosuccinate) arginie-lyase Urea cycle and metabolism of amino groups / Alanine and aspartate metabolism / Arginine and proline metabolism EC:4.3.2.1
Asl												ENSMUSG00000025533 argininosuccinate lyase
r0075	0.07	0.49	10.9	11.4	0.98	-0.84	0.2	-0.43	11	10.6	4.4	Glutamate(m) + H2O(m) + NADP+(m) ⇌ AKG(m) + NADPH(m) + NH3(m). L-Glutamate:NADP+ oxidoreductase (deaminating) Glutamate metabolism EC:1.4.1.3 EC:1.4.1.4 (3 sp.)
Glud1 (3 sp.)												ENSMUSG00000021794 glutamate dehydrogenase 1
r0820	0.07	0.5	7.47	7.97	0.74	-0.46	0.14	-0.04	7.54	7.51	4.82	AKG(m) + Malate(c) ⇌ AKG(c) + Malate(m). Mitochondrial Carrier (MC) TCDB:2.A.29.2.2 (2 sp.)
Slc25a10 (2 sp.)												ENSMUSG00000025792 solute carrier family 25 (mitochondrial carrier, dicarboxylate transporter), member 10
r0144	0.29	-5.79	12.5	6.71	0.33	-0.19	0.81	-5.79	12.3	6.51	7.81	Arginine(c) + H2O(c) ⇌ Urea(c) + Ornithine(c). L-Arginine amidinohydrolase Urea cycle and metabolism of amino groups / Arginine and proline metabolism EC:3.5.3.1
Arg1												ENSMUSG00000019987 arginase, liver
r0453	0.48	-1.28	11.7	10.4	0.05	0.16	0.31	-1.03	11.6	10.6	7.02	ATP(c) + Citrulline(c) + Aspartate(c) ⇌ AMP(c) + PPi(c) + Argininosuccinate(c). L-Citrulline:L-aspartate ligase (AMP-forming) Urea cycle and metabolism of amino groups / Alanine and aspartate metabolism / Arginine and proline metabolism EC:6.3.4.5
Ass1												ENSMUSG00000076441 argininosuccinate synthetase 1
r0258	0.14	-0.03	10.4	10.3	0.34	-0.2	0.17	-0.28	10.4	10.1	6.24	Fumarate(c) + H2O(c) ⇌ Malate(c). (S)-Malate hydro-lyase Citrate cycle (TCA cycle) EC:4.2.1.2
Fh1												ENSMUSG00000026526 fumarate hydratase 1
r0059	0.17	-0.17	4.52	4.35	0.17	-0.05	0.17	-0.23	4.53	4.3	4.96	Malate(m) + NADP+(m) ⇌ Pyruvate(m) + CO2(m) + NADPH(m). (S)-Malate:NADP+ oxidoreductase(oxaloacetate-decarboxylating) Pyruvate metabolism EC:1.1.1.40
Me3												ENSMUSG00000030621 malic enzyme 3, NADP(+)-dependent, mitochondrial
r0104	0.15	-0.07	10.8	10.8	0.29	-0.16	0.16	-0.2	10.8	10.6	5.24	NAD+(c) + Malate(c) ⇌ OAA(c) + NADH(c). (S)-malate:NAD+ oxidoreductase Citrate cycle (TCA cycle) EC:1.1.1.37 (3 sp.)
Mdh1 (3 sp.)												ENSMUSG00000020321 malate dehydrogenase 1, NAD (soluble)
r0030	0.15	-0.09	6.05	5.96	0.1	0.06	0.14	-0.05	6.06	6.02	3.74	ATP(c) + AMP(c) ⇌ 2 ADP(c). ATP:AMP phosphotransferase Purine metabolism EC:2.7.4.3 (5 genes, 11 sp.)
Ak1 (2 sp.)		-0.07	3.97	3.89		0.76		0.59	4.07	4.66		ENSMUSG00000026817 adenylate kinase 1
Ak2 (2 sp.)		0.08	9.35	9.43		0.13		0.15	9.41	9.55		ENSMUSG00000028792 adenylate kinase 2
Ak4 (4 sp.)		-0.36	6.41	6.05		-0.23		-0.45	6.27	5.82		ENSMUSG00000028527 adenylate kinase 4
Ak5 (2 sp.)		-0.17	4.72	4.56		0.12		-0.12	4.79	4.68		ENSMUSG00000039058 adenylate kinase 5
Ak7		0.74	4.79	5.53		-0.39		0.06	5.08	5.14		ENSMUSG00000041323 adenylate kinase 7

Continued on next page

Rea ID	control 1/24h Score *0.41*	Expr Δ	Expr C1h	Expr C24h	TGFβ/C 24h Score *0.43*	Expr Δ	TGFβ 1/24h Score *0.47*	Expr Δ	Expr T1h	Expr T24h	Wght	Reaction
r0109	0.31	-0.77	10.7	9.94	0.16	-0.03	0.26	-0.79	10.7	9.9	5.47	OAA(c) + Glutamate(c) ⇌ AKG(c) + Aspartate(c). L-Aspartate:2-oxoglutarate aminotransferase Glutamate metabolism / Alanine and aspartate metabolism EC:2.6.1.1
Got1												ENSMUSG00000025190 glutamate oxaloacetate transaminase 1, soluble
r0058	0.1	0.23	10.9	11.1	0.61	-0.38	0.16	-0.18	10.9	10.7	3.91	Malate(c) + NADP+(c) ⇌ Pyruvate(c) + CO2(c) + NADPH(c). (S)-Malate:NADP+ oxidoreductase(oxaloacetate-decarboxylating) Pyruvate metabolism EC:1.1.1.40
Me1												ENSMUSG00000032418 malic enzyme 1, NADP(+)-dependent, cytosolic
r0007	0.11	0.15	10.1	10.2	0.21	-0.09	0.13	0.07	10.1	10.1	3.39	PPi(c) + H2O(c) → 2 Pi(c). Pyrophosphate phosphohydrolase EC:3.6.1.1 (2 genes, 2 sp.)
Ppa1		-0.26	11.1	10.8		0.18		-0.14	11.1	11		ENSMUSG00000020089 pyrophosphatase (inorganic) 1
Ppa2		0.56	9.07	9.63		-0.35		0.28	9	9.28		ENSMUSG00000028013 pyrophosphatase (inorganic) 2

4 Reaction scores for Tyrosine (489), series control 1/24h, TGFβ/C 24h, and TGFβ 1/24h

The amplitudes of the mode are -1.51, -2.86 and -4.27.

Simulation	Definition			Solution													
				exchanges		reactions								transp			Prot
	Objective	Constraints	Comment	imports	exports	c	m	r	p	l	n	s	b	s-c	b-c	intra	synth
489 Tyrosine	Tyrosine(c)	−Phenylalanine(c) MCES	de novo synthesis of cytosolic Tyrosine	1 O_2(s) 1 Phenylalanine(c) 1 NADH-redox-potential(c)	1 H_2O(s) 1 Tyrosine(c)	3	-	-	-	-	-	-	-	2	-	-	-

Table 3: Reaction scores for Tyrosine (489), series control 1/24h, TGFβ/C 24h, and TGFβ 1/24h

	control 1/24h				TGFβ/C 24h		TGFβ 1/24h					
Rea ID	Score *0.65*	Expr Δ	Expr C1h	Expr C24h	Score *0.65*	Expr Δ	Score *0.65*	Expr Δ	Expr T1h	Expr T24h	Wght	Reaction
r0397	0.27	-0.29	10.2	9.92	0.28	-0.58	0.27	-0.84	10.2	9.34	11.5	Dihydrobiopterin(c) + NADH(c) ⇌ Tetrahydrobiopterin(c) + NAD+(c). NADH:6,7-dihydropteridine oxidoreductase Folate biosynthesis EC:1.5.1.34 (2 sp.)
Qdpr (2 sp.)												ENSMUSG00000015806 quinoid dihydropteridine reductase
r0399	1	-1.56	11	9.44	1	-2.97	1	-4.43	10.9	6.47	12.5	Tetrahydrobiopterin(c) + Phenylalanine(c) + O2(c) ⇌ Dihydrobiopterin(c) + Tyrosine(c) + H2O(c). L-Phenylalanine,tetrahydrobiopterin:oxygen oxidoreductase (4-hydroxylating) Phenylalanine, tyrosine and tryptophan biosynthesis EC:1.14.16.1
Pah												ENSMUSG00000020051 phenylalanine hydroxylase

5 Reaction scores for Collagen CORA1(c) synthesis (386), series control 1/24h, TGFβ/C 24h, and TGFβ 1/24h

The amplitudes of the mode are 0.91, 2.09 and 3.03.

Simulation	Definition			Solution													
				exchanges		reactions								transp			Prot
	Objective	Constraints	Comment	imports	exports	c	m	r	p	l	n	s	b	s-c	b-c	intra	synth
386 Collagen CORA1(c) synthesis	Collagen CORA1(c)	MCES AAA	test synthesis of Collagen CORA1 c	67 Glutamate(s) 397 Glycine(s) 110 Alanine(s) 95 Lysine(s) 69 Aspartate(s) 101 Arginine(s) 96 Glutamine(s) 106 Serine(s) 38 Methionine(s) 10 Tryptophan(s) 49 Phenylalanine(s) 17 Tyrosine(s) 15 Cysteine(s) 134 Leucine(s) 31 Histidine(s) 303 Proline(s) 21 Asparagine(s) 70 Valine(s) 87 Threonine(s) 44 Isoleucine(s) 9300 ATP-energy(c) 376 Proton-gradient(c)	1 Collagen CORA1(c) 499 Na-gradient(c)	4	-	-	-	-	-	-	-	22	-	-	-

Table 4: Reaction scores for Collagen CORA1(c) synthesis (386), series control 1/24h, TGFβ/C 24h, and TGFβ 1/24h

	control 1/24h				TGFβ/C 24h		TGFβ 1/24h					
Rea ID	Score *1*	Expr Δ	Expr C1h	Expr C24h	Score *1*	Expr Δ	Score *1*	Expr Δ	Expr T1h	Expr T24h	Wght	Reaction
r2634	1	0.91	5.84	6.75	1	2.09	1	3.03	5.81	8.84	$7.4{\cdot}10^5$	110 Alanine(c) + 15 Cysteine(c) + 69 Aspartate(c) + 67 Glutamate(c) + 49 Phenylalanine(c) + 397 Glycine(c) + 31 Histidine(c) + 44 Isoleucine(c) + 95 Lysine(c) + 134 Leucine(c) + 38 Methionine(c) + 21 Asparagine(c) + 303 Proline(c) + 96 Glutamine(c) + 101 Arginine(c) + 106 Serine(c) + 87 Threonine(c) + 70 Valine(c) + 10 Tryptophan(c) + 17 Tyrosine(c) + 7440 ATP(c) + 7440 H2O(c) → Collagen CORA1(c) + 5580 ADP(c) + 5580 Pi(c) + 1860 AMP(c) + 1860 PPi(c). Collagen alpha-1(XXVII) chain Protein assembly (2 sp.)
Col27a1 (2 sp.)											ENSMUSG00000045672 collagen, type XXVII, alpha 1	
r0030	0.09	-0.09	6.05	5.96	0.15	0.06	0.13	-0.05	6.06	6.02	114.5	ATP(c) + AMP(c) ⇌ 2 ADP(c). ATP:AMP phosphotransferase Purine metabolism EC:2.7.4.3 (5 genes, 11 sp.)
Ak1 (2 sp.)		-0.07	3.97	3.89		0.76		0.59	4.07	4.66	ENSMUSG00000026817 adenylate kinase 1	
Ak2 (2 sp.)		0.08	9.35	9.43		0.13		0.15	9.41	9.55	ENSMUSG00000028792 adenylate kinase 2	
Ak4 (4 sp.)		-0.36	6.41	6.05		-0.23		-0.45	6.27	5.82	ENSMUSG00000028527 adenylate kinase 4	
Ak5 (2 sp.)		-0.17	4.72	4.56		0.12		-0.12	4.79	4.68	ENSMUSG00000039058 adenylate kinase 5	
Ak7		0.74	4.79	5.53		-0.39		0.06	5.08	5.14	ENSMUSG00000041323 adenylate kinase 7	
r0007	0.24	0.15	10.1	10.2	0.11	-0.09	0.15	0.07	10.1	10.1	103.7	PPi(c) + H2O(c) → 2 Pi(c). Pyrophosphate phosphohydrolase EC:3.6.1.1 (2 genes, 2 sp.)
Ppa1		-0.26	11.1	10.8		0.18		-0.14	11.1	11	ENSMUSG00000020089 pyrophosphatase (inorganic) 1	
Ppa2		0.56	9.07	9.63		-0.35		0.28	9	9.28	ENSMUSG00000028013 pyrophosphatase (inorganic) 2	
C0219	0.08	-0.11	5.07	4.96	0.1	-0.14	0.11	-0.12	4.94	4.82	87	several reactions. Major Facilitator(MFS) TCDB:2.A.1.44.1
Slc43a1											ENSMUSG00000027075 solute carrier family 43, member 1	
C0163	0.06	-0.16	9.26	9.1	0.25	0.34	0.22	0.38	9.06	9.44	90.4	several reactions. Major Facilitator(MFS) TCDB:2.A.18.6.5 (1 genes, 2 sp.)
Slc38a2 (2 sp.)											ENSMUSG00000022462 solute carrier family 38, member 2	
r2468	0.52	0.39	6.41	6.8	0.08	-0.28	0.15	0.07	6.45	6.52	96.9	Glycine(s) + H+(PG)(s) → Glycine(c) + H+(PG)(c). Major Facilitator(MFS) TCDB:2.A.18.8.1
Slc36a1											ENSMUSG00000020261 solute carrier family 36 (proton/amino acid symporter), member 1	
C0153	0.01	-0.49	6.56	6.08	0.14	0.01	0.08	-0.38	6.46	6.08	71.1	several reactions. solute carrier family 22 (organic cation/carnitine transporter), member 5 TCDB:2.A.1.19.1 TCDB:2.A.1.19.6 TCDB:2.A.1.19.3 (2 genes, 4 sp.)
Slc22a21 (2 sp.)		-0.4	5.83	5.43		0.04		-0.25	5.72	5.47	ENSMUSG00000063652 solute carrier family 22 (organic cation transporter), member 21	
Slc22a5 (2 sp.)		-0.58	7.3	6.72		-0.03		-0.5	7.2	6.69	ENSMUSG00000018900 solute carrier family 22 (organic cation transporter), member 5	
r1058	0	-2.86	8.24	5.38	0.02	-0.84	0.0001	-3.34	7.88	4.54	60.7	Arginine(s) ⇌ Arginine(c). Amino Acid-Polyamine-Organocation (APC) TCDB:2.A.3.3.2 (4 sp.)
Slc7a2 (4 sp.)											ENSMUSG00000031596 solute carrier family 7 (cationic amino acid transporter, y+ system), member 2	
r2479	0.09	-0.09	3.38	3.3	0.14	0.03	0.15	0.06	3.26	3.32	26.4	Asparagine(s) + Na+(s) + H+(PG)(c) → Asparagine(c) + Na+(c) + H+(PG)(s). Major Facilitator(MFS) TCDB:2.A.18.6.8
Slc38a5											ENSMUSG00000031170 solute carrier family 38, member 5	

6 Reaction scores for Collagen COIA1(c) synthesis (377), series control 1/24h, TGFβ/C 24h, and TGFβ 1/24h

The amplitudes of the mode are -0.43, -1.18 and -1.63.

Simulation	Definition			Solution														
				exchanges		reactions								transp			Prot
	Objective	Constraints	Comment	imports	exports	c	m	r	p	l	n	s	b	s-c	b-c	intra	synth
377 Collagen COIA1(c) synthesis	Collagen COIA1(c)	MCES AAA	test synthesis of Collagen COIA1 c	90 Glutamate(s) 302 Glycine(s) 140 Alanine(s) 42 Lysine(s) 74 Aspartate(s) 91 Arginine(s) 67 Glutamine(s) 121 Serine(s) 15 Methionine(s) 21 Tryptophan(s) 57 Phenylalanine(s) 19 Tyrosine(s) 23 Cysteine(s) 148 Leucine(s) 36 Histidine(s) 296 Proline(s) 27 Asparagine(s) 87 Valine(s) 69 Threonine(s) 29 Isoleucine(s) 8770 ATP-energy(c) 275 Proton-gradient(c)	1 Collagen COIA1(c) 584 Na-gradient(c)	4	-	-	-	-	-	-	-	22	-	-	-

Table 5: Reaction scores for Collagen COIA1(c) synthesis (377), series control 1/24h, TGFβ/C 24h, and TGFβ 1/24h

	control 1/24h				TGFβ/C 24h		TGFβ 1/24h					
Rea ID	Score *1*	Expr Δ	Expr C1h	Expr C24h	Score *1*	Expr Δ	Score *1*	Expr Δ	Expr T1h	Expr T24h	Wght	Reaction
r2616	1	-0.43	10.5	10	1	-1.18	1	-1.63	10.5	8.84	$6.9 \cdot 10^5$	140 Alanine(c) + 23 Cysteine(c) + 74 Aspartate(c) + 90 Glutamate(c) + 57 Phenylalanine(c) + 302 Glycine(c) + 36 Histidine(c) + 29 Isoleucine(c) + 42 Lysine(c) + 148 Leucine(c) + 15 Methionine(c) + 27 Asparagine(c) + 296 Proline(c) + 67 Glutamine(c) + 91 Arginine(c) + 121 Serine(c) + 69 Threonine(c) + 87 Valine(c) + 21 Tryptophan(c) + 19 Tyrosine(c) + 7016 ATP(c) + 7016 H2O(c) → Collagen COIA1(c) + 5262 ADP(c) + 5262 Pi(c) + 1754 AMP(c) + 1754 PPi(c). collagen, type XVIII, alpha 1 Protein assembly (2 sp.)
Col18a1 (2 sp.)												ENSMUSG00000001435 collagen, type XVIII, alpha 1
r0030	0.29	-0.09	6.05	5.96	0.11	0.06	0.15	-0.05	6.06	6.02	111.2	ATP(c) + AMP(c) ⇌ 2 ADP(c). ATP:AMP phosphotransferase Purine metabolism EC:2.7.4.3 (5 genes, 11 sp.)
Ak1 (2 sp.)		-0.07	3.97	3.89		0.76		0.59	4.07	4.66		ENSMUSG00000026817 adenylate kinase 1
Ak2 (2 sp.)		0.08	9.35	9.43		0.13		0.15	9.41	9.55		ENSMUSG00000028792 adenylate kinase 2
Ak4 (4 sp.)		-0.36	6.41	6.05		-0.23		-0.45	6.27	5.82		ENSMUSG00000028527 adenylate kinase 4
Ak5 (2 sp.)		-0.17	4.72	4.56		0.12		-0.12	4.79	4.68		ENSMUSG00000039058 adenylate kinase 5
Ak7		0.74	4.79	5.53		-0.39		0.06	5.08	5.14		ENSMUSG00000041323 adenylate kinase 7
r0007	0.03	0.15	10.1	10.2	0.18	-0.09	0.11	0.07	10.1	10.1	100.7	PPi(c) + H2O(c) → 2 Pi(c). Pyrophosphate phosphohydrolase EC:3.6.1.1 (2 genes, 2 sp.)
Ppa1		-0.26	11.1	10.8		0.18		-0.14	11.1	11		ENSMUSG00000020089 pyrophosphatase (inorganic) 1
Ppa2		0.56	9.07	9.63		-0.35		0.28	9	9.28		ENSMUSG00000028013 pyrophosphatase (inorganic) 2
C0163	0.46	-0.16	9.26	9.1	0.04	0.34	0.05	0.38	9.06	9.44	96.9	several reactions. Major Facilitator(MFS) TCDB:2.A.18.6.5 (1 genes, 2 sp.)
Slc38a2 (2 sp.)												ENSMUSG00000022462 solute carrier family 38, member 2
r2468	0.001	0.39	6.41	6.8	0.31	-0.28	0.11	0.07	6.45	6.52	84.5	Glycine(s) + H+(PG)(s) → Glycine(c) + H+(PG)(c). Major Facilitator(MFS) TCDB:2.A.18.8.1
Slc36a1												ENSMUSG00000020261 solute carrier family 36 (proton/amino acid symporter), member 1
C0219	0.32	-0.11	5.07	4.96	0.21	-0.14	0.18	-0.12	4.94	4.82	86.4	several reactions. Major Facilitator(MFS) TCDB:2.A.1.44.1
Slc43a1												ENSMUSG00000027075 solute carrier family 43, member 1
r1058	0	-2.86	8.24	5.38	0.85	-0.84	0.11	-3.34	7.88	4.54	57.7	Arginine(s) ⇌ Arginine(c). Amino Acid-Polyamine-Organocation (APC) TCDB:2.A.3.3.2 (4 sp.)
Slc7a2 (4 sp.)												ENSMUSG00000031596 solute carrier family 7 (cationic amino acid transporter, y+ system), member 2
r2479	0.28	-0.09	3.38	3.3	0.12	0.03	0.12	0.06	3.26	3.32	29.9	Asparagine(s) + Na+(s) + H+(PG)(c) → Asparagine(c) + Na+(c) + H+(PG)(s). Major Facilitator(MFS) TCDB:2.A.18.6.8
Slc38a5												ENSMUSG00000031170 solute carrier family 38, member 5
C0153	0.97	-0.49	6.56	6.08	0.13	0.01	0.31	-0.38	6.46	6.08	46.6	several reactions. solute carrier family 22 (organic cation/carnitine transporter), member 5 TCDB:2.A.1.19.1 TCDB:2.A.1.19.6 TCDB:2.A.1.19.3 (2 genes, 4 sp.)
Slc22a21 (2 sp.)		-0.4	5.83	5.43		0.04		-0.25	5.72	5.47		ENSMUSG00000063652 solute carrier family 22 (organic cation transporter), member 21
Slc22a5 (2 sp.)		-0.58	7.3	6.72		-0.03		-0.5	7.2	6.69		ENSMUSG00000018900 solute carrier family 22 (organic cation transporter), member 5

7 Reaction scores for Collagen COFA1(c) synthesis (374), series control 1/24h, TGFβ/C 24h, and TGFβ 1/24h

The amplitudes of the mode are -0.24, 2.27 and 2.

Simulation	Definition			Solution														
				exchanges		reactions								transp			Prot
	Objective	Constraints	Comment	imports	exports	c	m	r	p	l	n	s	b	s-c	b-c	intra	synth
374 Collagen COFA1(c) synthesis	Collagen COFA1(c)	MCES AAA	test synthesis of Collagen COFA1 c	97 Glutamate(s) 221 Glycine(s) 108 Alanine(s) 52 Lysine(s) 58 Aspartate(s) 47 Arginine(s) 40 Glutamine(s) 99 Serine(s) 30 Methionine(s) 10 Tryptophan(s) 43 Phenylalanine(s) 14 Tyrosine(s) 10 Cysteine(s) 112 Leucine(s) 25 Histidine(s) 191 Proline(s) 38 Asparagine(s) 65 Valine(s) 74 Threonine(s) 54 Isoleucine(s) 6940 ATP-energy(c) 183 Proton-gradient(c)	1 Collagen COFA1(c) 513 Na-gradient(c)	4	-	-	-	-	-	-	-	22	-	-	-

Table 6: Reaction scores for Collagen COFA1(c) synthesis (374), series control 1/24h, TGFβ/C 24h, and TGFβ 1/24h

	control 1/24h				TGFβ/C 24h		TGFβ 1/24h					
Rea ID	Score *1*	Expr Δ	Expr C1h	Expr C24h	Score *1*	Expr Δ	Score *1*	Expr Δ	Expr T1h	Expr T24h	Wght	Reaction
r2610	1	-0.24	5.67	5.44	1	2.27	1	2	5.7	7.7	$5.5{\cdot}10^5$	108 Alanine(c) + 10 Cysteine(c) + 58 Aspartate(c) + 97 Glutamate(c) + 43 Phenylalanine(c) + 221 Glycine(c) + 25 Histidine(c) + 54 Isoleucine(c) + 52 Lysine(c) + 112 Leucine(c) + 30 Methionine(c) + 38 Asparagine(c) + 191 Proline(c) + 40 Glutamine(c) + 47 Arginine(c) + 99 Serine(c) + 74 Threonine(c) + 65 Valine(c) + 10 Tryptophan(c) + 14 Tyrosine(c) + 5552 ATP(c) + 5552 H2O(c) → Collagen COFA1(c) + 4164 ADP(c) + 4164 Pi(c) + 1388 AMP(c) + 1388 PPi(c). Collagen alpha-1(XV) chain Protein assembly
Col15a1												ENSMUSG00000028339 collagen, type XV, alpha 1
r0007	0.01	0.15	10.1	10.2	0.12	-0.09	0.16	0.07	10.1	10.1	89.6	PPi(c) + H2O(c) → 2 Pi(c). Pyrophosphate phosphohydrolase EC:3.6.1.1 (2 genes, 2 sp.)
Ppa1		-0.26	11.1	10.8		0.18		-0.14	11.1	11		ENSMUSG00000020089 pyrophosphatase (inorganic) 1
Ppa2		0.56	9.07	9.63		-0.35		0.28	9	9.28		ENSMUSG00000028013 pyrophosphatase (inorganic) 2
r0030	0.48	-0.09	6.05	5.96	0.15	0.06	0.12	-0.05	6.06	6.02	98.9	ATP(c) + AMP(c) ⇌ 2 ADP(c). ATP:AMP phosphotransferase Purine metabolism EC:2.7.4.3 (5 genes, 11 sp.)
Ak1 (2 sp.)		-0.07	3.97	3.89		0.76		0.59	4.07	4.66		ENSMUSG00000026817 adenylate kinase 1
Ak2 (2 sp.)		0.08	9.35	9.43		0.13		0.15	9.41	9.55		ENSMUSG00000028792 adenylate kinase 2
Ak4 (4 sp.)		-0.36	6.41	6.05		-0.23		-0.45	6.27	5.82		ENSMUSG00000028527 adenylate kinase 4
Ak5 (2 sp.)		-0.17	4.72	4.56		0.12		-0.12	4.79	4.68		ENSMUSG00000039058 adenylate kinase 5
Ak7		0.74	4.79	5.53		-0.39		0.06	5.08	5.14		ENSMUSG00000041323 adenylate kinase 7
r2468	0	0.39	6.41	6.8	0.08	-0.28	0.16	0.07	6.45	6.52	72.3	Glycine(s) + H+(PG)(s) → Glycine(c) + H+(PG)(c). Major Facilitator(MFS) TCDB:2.A.18.8.1
Slc36a1												ENSMUSG00000020261 solute carrier family 36 (proton/amino acid symporter), member 1
C0219	0.55	-0.11	5.07	4.96	0.1	-0.14	0.11	-0.12	4.94	4.82	73.8	several reactions. Major Facilitator(MFS) TCDB:2.A.1.44.1
Slc43a1												ENSMUSG00000027075 solute carrier family 43, member 1
C0153	0.1	-0.49	6.56	6.08	0.14	0.01	0.06	-0.38	6.46	6.08	55.9	several reactions. solute carrier family 22 (organic cation/carnitine transporter), member 5 TCDB:2.A.1.19.1 TCDB:2.A.1.19.6 TCDB:2.A.1.19.3 (2 genes, 4 sp.)
Slc22a21 (2 sp.)		-0.4	5.83	5.43		0.04		-0.25	5.72	5.47		ENSMUSG00000063652 solute carrier family 22 (organic cation transporter), member 21
Slc22a5 (2 sp.)		-0.58	7.3	6.72		-0.03		-0.5	7.2	6.69		ENSMUSG00000018900 solute carrier family 22 (organic cation transporter), member 5
C0163	0.83	-0.16	9.26	9.1	0.24	0.34	0.27	0.38	9.06	9.44	87.4	several reactions. Major Facilitator(MFS) TCDB:2.A.18.6.5 (1 genes, 2 sp.)
Slc38a2 (2 sp.)												ENSMUSG00000022462 solute carrier family 38, member 2
r1058	0	-2.86	8.24	5.38	0.02	-0.84	0	-3.34	7.88	4.54	41.4	Arginine(s) ⇌ Arginine(c). Amino Acid-Polyamine-Organocation (APC) TCDB:2.A.3.3.2 (4 sp.)
Slc7a2 (4 sp.)												ENSMUSG00000031596 solute carrier family 7 (cationic amino acid transporter, y+ system), member 2
r2479	0.44	-0.09	3.38	3.3	0.14	0.03	0.15	0.06	3.26	3.32	35.5	Asparagine(s) + Na+(s) + H+(PG)(c) → Asparagine(c) + Na+(c) + H+(PG)(s). Major Facilitator(MFS) TCDB:2.A.18.6.8
Slc38a5												ENSMUSG00000031170 solute carrier family 38, member 5

8 Reaction scores for Collagen CO4A5(c) synthesis (352), series control 1/24h, TGFβ/C 24h, and TGFβ 1/24h

The amplitudes of the mode are 1.61, -0.96 and 0.68.

Simulation	Definition			Solution													
				exchanges		reactions								transp			Prot
	Objective	Constraints	Comment	imports	exports	c	m	r	p	l	n	s	b	s-c	b-c	intra	synth
352 Collagen CO4A5(c) synthesis	Collagen CO4A5(c)	MCES AAA	test synthesis of Collagen CO4A5 c	61 Glutamate(s) 478 Glycine(s) 47 Alanine(s) 80 Lysine(s) 54 Aspartate(s) 37 Arginine(s) 73 Glutamine(s) 64 Serine(s) 26 Methionine(s) 5 Tryptophan(s) 41 Phenylalanine(s) 13 Tyrosine(s) 20 Cysteine(s) 112 Leucine(s) 13 Histidine(s) 391 Proline(s) 32 Asparagine(s) 31 Valine(s) 38 Threonine(s) 69 Isoleucine(s) 8425 ATP-energy(c) 446 Proton-gradient(c)	1 Collagen CO4A5(c) 403 Na-gradient(c)	4	-	-	-	-	-	-	-	22	-	-	-

Table 7: Reaction scores for Collagen CO4A5(c) synthesis (352), series control 1/24h, TGFβ/C 24h, and TGFβ 1/24h

	control 1/24h				TGFβ/C 24h		TGFβ 1/24h					
Rea ID	Score *1*	Expr Δ	Expr C1h	Expr C24h	Score *1*	Expr Δ	Score *1*	Expr Δ	Expr T1h	Expr T24h	Wght	Reaction
r2566	1	1.61	4.02	5.63	1	-0.96	1	0.68	3.99	4.68	$6.7 \cdot 10^5$	47 Alanine(c) + 20 Cysteine(c) + 54 Aspartate(c) + 61 Glutamate(c) + 41 Phenylalanine(c) + 478 Glycine(c) + 13 Histidine(c) + 69 Isoleucine(c) + 80 Lysine(c) + 112 Leucine(c) + 26 Methionine(c) + 32 Asparagine(c) + 391 Proline(c) + 73 Glutamine(c) + 37 Arginine(c) + 64 Serine(c) + 38 Threonine(c) + 31 Valine(c) + 5 Tryptophan(c) + 13 Tyrosine(c) + 6740 ATP(c) + 6740 H2O(c) → Collagen CO4A5(c) + 5055 ADP(c) + 5055 Pi(c) + 1685 AMP(c) + 1685 PPi(c). Collagen alpha-5(IV) chain Protein assembly (2 sp.)
Col4a5 (2 sp.)											ENSMUSG00000031274 collagen, type IV, alpha 5	
r0030	0.11	-0.09	6.05	5.96	0.1	0.06	0.1	-0.05	6.06	6.02	109	ATP(c) + AMP(c) ⇌ 2 ADP(c). ATP:AMP phosphotransferase Purine metabolism EC:2.7.4.3 (5 genes, 11 sp.)
Ak1 (2 sp.)		-0.07	3.97	3.89		0.76		0.59	4.07	4.66	ENSMUSG00000026817 adenylate kinase 1	
Ak2 (2 sp.)		0.08	9.35	9.43		0.13		0.15	9.41	9.55	ENSMUSG00000028792 adenylate kinase 2	
Ak4 (4 sp.)		-0.36	6.41	6.05		-0.23		-0.45	6.27	5.82	ENSMUSG00000028527 adenylate kinase 4	
Ak5 (2 sp.)		-0.17	4.72	4.56		0.12		-0.12	4.79	4.68	ENSMUSG00000039058 adenylate kinase 5	
Ak7		0.74	4.79	5.53		-0.39		0.06	5.08	5.14	ENSMUSG00000041323 adenylate kinase 7	
r0007	0.19	0.15	10.1	10.2	0.19	-0.09	0.2	0.07	10.1	10.1	98.7	PPi(c) + H2O(c) → 2 Pi(c). Pyrophosphate phosphohydrolase EC:3.6.1.1 (2 genes, 2 sp.)
Ppa1		-0.26	11.1	10.8		0.18		-0.14	11.1	11	ENSMUSG00000020089 pyrophosphatase (inorganic) 1	
Ppa2		0.56	9.07	9.63		-0.35		0.28	9	9.28	ENSMUSG00000028013 pyrophosphatase (inorganic) 2	
r2468	0.32	0.39	6.41	6.8	0.36	-0.28	0.2	0.07	6.45	6.52	106.3	Glycine(s) + H+(PG)(s) → Glycine(c) + H+(PG)(c). Major Facilitator(MFS) TCDB:2.A.18.8.1
Slc36a1											ENSMUSG00000020261 solute carrier family 36 (proton/amino acid symporter), member 1	
C0219	0.1	-0.11	5.07	4.96	0.24	-0.14	0.06	-0.12	4.94	4.82	82.8	several reactions. Major Facilitator(MFS) TCDB:2.A.1.44.1
Slc43a1											ENSMUSG00000027075 solute carrier family 43, member 1	
C0153	0.03	-0.49	6.56	6.08	0.13	0.01	0.01	-0.38	6.46	6.08	63.5	several reactions. solute carrier family 22 (organic cation/carnitine transporter), member 5 TCDB:2.A.1.19.1 TCDB:2.A.1.19.6 TCDB:2.A.1.19.3 (2 genes, 4 sp.)
Slc22a21 (2 sp.)		-0.4	5.83	5.43		0.04		-0.25	5.72	5.47	ENSMUSG00000063652 solute carrier family 22 (organic cation transporter), member 21	
Slc22a5 (2 sp.)		-0.58	7.3	6.72		-0.03		-0.5	7.2	6.69	ENSMUSG00000018900 solute carrier family 22 (organic cation transporter), member 5	
C0163	0.09	-0.16	9.26	9.1	0.02	0.34	0.68	0.38	9.06	9.44	78.2	several reactions. Major Facilitator(MFS) TCDB:2.A.18.6.5 (1 genes, 2 sp.)
Slc38a2 (2 sp.)											ENSMUSG00000022462 solute carrier family 38, member 2	
r2479	0.11	-0.09	3.38	3.3	0.12	0.03	0.19	0.06	3.26	3.32	32.6	Asparagine(s) + Na+(s) + H+(PG)(c) → Asparagine(c) + Na+(c) + H+(PG)(s). Major Facilitator(MFS) TCDB:2.A.18.6.8
Slc38a5											ENSMUSG00000031170 solute carrier family 38, member 5	
r1058	0	-2.86	8.24	5.38	0.97	-0.84	0	-3.34	7.88	4.54	36.8	Arginine(s) ⇌ Arginine(c). Amino Acid-Polyamine-Organocation (APC) TCDB:2.A.3.3.2 (4 sp.)
Slc7a2 (4 sp.)											ENSMUSG00000031596 solute carrier family 7 (cationic amino acid transporter, y+ system), member 2	

9 Reaction scores for Ethanol degr (448), series control 1/24h, TGFβ/C 24h, and TGFβ 1/24h

The amplitudes of the mode are -0.52, -3.56 and -3.79.

Simulation	Definition			Solution														
				exchanges		reactions								transp				Prot
	Objective	Constraints	Comment	imports	exports	c	m	r	p	l	n	s	b	s-c	b-c	intra		synth
448 Ethanol degr	-1 Ethanol	MCES	degradation of Ethanol	1 Ethanol(s) 2 ATP-energy(c)	2 NADH-redox-potential(c) 1 CoA-activated acetyl group(c)	8	-	-	-	-	-	-	-	1	-	-		-

Table 8: Reaction scores for Ethanol degr (448), series control 1/24h, TGFβ/C 24h, and TGFβ 1/24h

Rea ID	control 1/24h Score *0.64*	Expr Δ	Expr C1h	Expr C24h	TGFβ/C 24h Score *0.54*	Expr Δ	TGFβ 1/24h Score *0.55*	Expr Δ	Expr T1h	Expr T24h	Wght	Reaction
r0187	0.99	-0.49	12.2	11.7	0.99	-3.81	0.99	-4.08	12	7.88	20	Ethanol(c) + NAD+(c) ⇌ Acetaldehyde(c) + NADH(c). Ethanol:NAD+ oxidoreductase Glycolysis / Gluconeogenesis EC:1.1.1.1 EC:1.1.1.71
Adh1												ENSMUSG00000074207 alcohol dehydrogenase 1 (class I)
r0176	0.004	0.34	9.32	9.66	0.32	-0.86	0.23	-0.56	9.36	8.8	14.6	Acetaldehyde(c) + NAD+(c) + H2O(c) → Acetate(c) + NADH(c). Acetaldehyde:NAD+ oxidoreductase Glycolysis / Gluconeogenesis / Pyruvate metabolism EC:1.2.1.3 EC:1.2.1.5 (5 genes, 6 sp.)
Aldh1a1		0.23	12.9	13.1		-2.54		-2.38	12.9	10.6		ENSMUSG00000053279 aldehyde dehydrogenase family 1, subfamily A1
Aldh1b1		0.35	4.91	5.27		-0.12		0.16	4.98	5.14		ENSMUSG00000035561 aldehyde dehydrogenase 1 family, member B1
Aldh3a2		0.76	8.9	9.66		-1.06		-0.42	9.03	8.6		ENSMUSG00000010025 aldehyde dehydrogenase family 3, subfamily A2
Aldh7a1		0.42	10.6	11.1		-0.43		0.02	10.6	10.6		ENSMUSG00000053644 aldehyde dehydrogenase family 7, member A1
Aldh9a1 (2 sp.)		0.15	9.29	9.45		-0.51		-0.37	9.31	8.94		ENSMUSG00000026687 aldehyde dehydrogenase 9, subfamily A1
r0066	0.99	-0.56	6	5.44	0.29	-0.76	0.39	-1.2	5.88	4.68	13.7	ATP(c) + Acetate(c) + CoA(c) → AMP(c) + PPi(c) + Acetyl-CoA(c). Acetate:CoA ligase (AMP-forming) Glycolysis / Gluconeogenesis EC:6.2.1.1 (2 genes, 4 sp.)
Acss1		-0.18	3.44	3.27		-0.13		-0.08	3.22	3.13		ENSMUSG00000027452 acyl-CoA synthetase short-chain family member 1
Acss2 (3 sp.)		-0.69	6.86	6.16		-0.97		-1.57	6.77	5.2		ENSMUSG00000027605 acyl-CoA synthetase short-chain family member 2
r0007	0.04	0.15	10.1	10.2	0.15	-0.09	0.13	0.07	10.1	10.1	2.43	PPi(c) + H2O(c) → 2 Pi(c). Pyrophosphate phosphohydrolase EC:3.6.1.1 (2 genes, 2 sp.)
Ppa1		-0.26	11.1	10.8		0.18		-0.14	11.1	11		ENSMUSG00000020089 pyrophosphatase (inorganic) 1
Ppa2		0.56	9.07	9.63		-0.35		0.28	9	9.28		ENSMUSG00000028013 pyrophosphatase (inorganic) 2
r0030	0.26	-0.09	6.05	5.96	0.13	0.06	0.14	-0.05	6.06	6.02	2.68	ATP(c) + AMP(c) ⇌ 2 ADP(c). ATP:AMP phosphotransferase Purine metabolism EC:2.7.4.3 (5 genes, 11 sp.)
Ak1 (2 sp.)		-0.07	3.97	3.89		0.76		0.59	4.07	4.66		ENSMUSG00000026817 adenylate kinase 1
Ak2 (2 sp.)		0.08	9.35	9.43		0.13		0.15	9.41	9.55		ENSMUSG00000028792 adenylate kinase 2
Ak4 (4 sp.)		-0.36	6.41	6.05		-0.23		-0.45	6.27	5.82		ENSMUSG00000028527 adenylate kinase 4
Ak5 (2 sp.)		-0.17	4.72	4.56		0.12		-0.12	4.79	4.68		ENSMUSG00000039058 adenylate kinase 5
Ak7		0.74	4.79	5.53		-0.39		0.06	5.08	5.14		ENSMUSG00000041323 adenylate kinase 7

10 Reaction scores for Bilirubin conjugation (310), series control 1/24h, TGFβ/C 24h, and TGFβ 1/24h

The amplitudes of the mode are -0.43, -0.86 and -1.93.

Simulation	Definition			Solution													
				exchanges		reactions								transp			Prot
	Objective	Constraints	Comment	imports	exports	c	m	r	p	l	n	s	b	s-c	b-c	intra	synth
310 Bilirubin conjugation	Bilirubin-bisglucuronoside(b)	MCES −Bilirubin	Bilirubin import, conjugation, and export	2 H_2O(s) 2 Glucose-6P(c) 1 Bilirubin(s) 5 ATP-energy(c)	2 P_i(c) 1 Bilirubin-bisglucuronoside(b) 4 NADH-redox-potential(c)	8	-	2	-	-	-	-	-	2	1	5	-

Table 9: Reaction scores for Bilirubin conjugation (310), series control 1/24h, TGFβ/C 24h, and TGFβ 1/24h

	control 1/24h				TGFβ/C 24h		TGFβ 1/24h					
Rea ID	Score *0.54*	Expr Δ	Expr C1h	Expr C24h	Score *0.62*	Expr Δ	Score *0.53*	Expr Δ	Expr T1h	Expr T24h	Wght	Reaction
r0092	0.9	-0.52	12.1	11.6	0.26	-1.58	0.99	-2.06	12	9.99	17.2	UDP-glucose(c) + H2O(c) + 2 NAD+(c) → 2 NADH(c) + UDP-glucuronate(c). UDPglucose:NAD+ oxidoreductase Pentose and glucuronate interconversions / Nucleotide sugars metabolism EC:1.1.1.22
Ugdh												ENSMUSG00000029201 UDP-glucose dehydrogenase
r0813	0	0.59	9.73	10.3	0.99	-0.8	0.24	-0.3	9.81	9.51	15.6	Bilirubin-bisglucuronoside(r) + ATP(r) + H2O(r) → Bilirubin-bisglucuronoside(b) + ADP(r) + Pi(r). ATP-binding Cassette (ABC) TCDB:3.A.1.208.9
Abcc3												ENSMUSG00000020865 ATP-binding cassette, sub-family C (CFTR/MRP), member 3
r0094	0	-1.95	11.5	9.51	0.99	-0.82	0.81	-2.55	11.2	8.69	13.5	UTP(c) + Glucose-1P(c) → PPi(c) + UDP-glucose(c). UTP:alpha-D-glucose-1-phosphate uridylyltransferase Pentose and glucuronate interconversions / Nucleotide sugars metabolism EC:2.7.7.9 (5 sp.)
Ugp2 (5 sp.)												ENSMUSG00000001891 UDP-glucose pyrophosphorylase 2
r0532	0.98	-0.38	12	11.6	0.99	-0.79	0.69	-1.1	11.9	10.8	15.6	Bilirubin(r) + 2 UDP-glucuronate(r) → 2 UDP(r) + Bilirubin-bisglucuronoside(r). UDPglucuronate beta-D-glucuronosyltransferase(acceptor-unspecific) Porphyrin and chlorophyll metabolism EC:2.4.1.17 (9 genes, 27 sp.)
Ugt1a1 (3 sp.)		-0.38	12	11.6		-0.79		-1.1	11.9	10.8		ENSMUSG00000089960 UDP glucuronosyltransferase 1 family, polypeptide A1
Ugt1a10 (3 sp.)		-0.38	12	11.6		-0.79		-1.1	11.9	10.8		ENSMUSG00000090165 UDP glycosyltransferase 1 family, polypeptide A10
Ugt1a2 (3 sp.)		-0.38	12	11.6		-0.79		-1.1	11.9	10.8		ENSMUSG00000090171 UDP glucuronosyltransferase 1 family, polypeptide A2
Ugt1a5 (3 sp.)		-0.38	12	11.6		-0.79		-1.1	11.9	10.8		ENSMUSG00000089943 UDP glucuronosyltransferase 1 family, polypeptide A5
Ugt1a6a (3 sp.)		-0.38	12	11.6		-0.79		-1.1	11.9	10.8		ENSMUSG00000054545 UDP glucuronosyltransferase 1 family, polypeptide A6A
Ugt1a6b (3 sp.)		-0.38	12	11.6		-0.79		-1.1	11.9	10.8		ENSMUSG00000090145 UDP glucuronosyltransferase 1 family, polypeptide A6B
Ugt1a7c (3 sp.)		-0.38	12	11.6		-0.79		-1.1	11.9	10.8		ENSMUSG00000090124 UDP glucuronosyltransferase 1 family, polypeptide A7C
Ugt1a8 (3 sp.)		-0.38	12	11.6		-0.79		-1.1	11.9	10.8		ENSMUSG00000089675 UDP glucuronosyltransferase 1 family, polypeptide A8
Ugt1a9 (3 sp.)		-0.38	12	11.6		-0.79		-1.1	11.9	10.8		ENSMUSG00000090175 UDP glucuronosyltransferase 1 family, polypeptide A9
r0621	0.97	-0.48	9.17	8.69	0.05	0.2	0.22	-0.24	9.14	8.9	10.6	Glucose-6P(c) ⇌ Glucose-1P(c). alpha-D-Glucose 1-phosphate 1,6-phosphomutase Glycolysis / Gluconeogenesis / Starch and sucrose metabolism EC:5.4.2.2 EC:5.4.2.6 (2 genes, 2 sp.)
Pgm1		-0.37	7.9	7.52		0.79		0.34	7.97	8.32		ENSMUSG00000029171 phosphoglucomutase 1
Pgm2		-0.59	10.4	9.86		-0.38		-0.82	10.3	9.48		ENSMUSG00000025791 phosphoglucomutase 2
r0038	0.49	-0.17	7.52	7.35	0.23	-0.12	0.22	-0.25	7.48	7.23	10.5	ATP(c) + UMP(c) ⇌ ADP(c) + UDP(c). ATP:nucleoside-phosphate phosphotransferase Pyrimidine metabolism EC:2.7.4.4 (2 sp.)
Cmpk1 (2 sp.)												ENSMUSG00000028719 cytidine monophosphate (UMP-CMP) kinase 1
r0037	0.0004	0.42	8.17	8.59	0.43	-0.31	0.12	0.05	8.23	8.28	5.07	ATP(c) + UDP(c) ⇌ ADP(c) + UTP(c). ATP:nucleoside-diphosphate phosphatransferase Pyrimidine metabolism EC:2.7.4.6 (7 genes, 7 sp.)
Nme1		0.44	11.4	11.8		-0.3		0.11	11.4	11.5		ENSMUSG00000037601 non-metastatic cells 1, protein (NM23A) expressed in
Nme2		0.09	13	13.1		0.04		0.14	13	13.2		ENSMUSG00000020857 non-metastatic cells 2, protein (NM23B) expressed in
Nme3		0.86	6.83	7.69		-1.4		-0.66	6.96	6.29		ENSMUSG00000073435 non-metastatic cells 3, protein expressed in
Nme4		0.95	6.48	7.42		-0.82		-0.06	6.67	6.6		ENSMUSG00000024177 non-metastatic cells 4, protein expressed in
Nme5		0.01	4.78	4.79		0.08		-0.02	4.88	4.87		ENSMUSG00000035984 non-metastatic cells 5, protein expressed in (nucleoside-diphosphate kinase)
Nme6		-0.01	7.51	7.5		0.11		0.03	7.58	7.61		ENSMUSG00000032478 non-metastatic cells 6, protein expressed in (nucleoside-diphosphate kinase)
Nme7		0.57	7.21	7.78		0.14		0.85	7.08	7.92		ENSMUSG00000026575 non-metastatic cells 7, protein expressed in (nucleoside-diphosphate kinase)
r0035	0.7	-0.25	6.99	6.75	0.72	-0.51	0.52	-0.82	7.06	6.24	17.9	UDP(r) + H2O(r) → Pi(r) + UMP(r). UDP diphosphohydrolase Pyrimidine metabolism EC:3.6.1.6 (6 genes, 13 sp.)
Entpd1 (3 sp.)		0.01	3.2	3.21		-0.02		-0.16	3.34	3.18		ENSMUSG00000048120 ectonucleoside triphosphate diphosphohydrolase 1
Entpd3		-0.12	3.85	3.74		0.04		-0.21	3.98	3.78		ENSMUSG00000041608 ectonucleoside triphosphate diphosphohydrolase 3
Entpd4 (2 sp.)		0.42	8.02	8.44		0.08		0.43	8.09	8.52		ENSMUSG00000022066 ectonucleoside triphosphate diphosphohydrolase 4
Entpd5 (5 sp.)		-0.7	9.44	8.74		-1.17		-1.9	9.47	7.57		ENSMUSG00000021236 ectonucleoside triphosphate diphosphohydrolase 5
Entpd6		0.18	7.32	7.5		0.004		0.11	7.4	7.5		ENSMUSG00000033068 ectonucleoside triphosphate diphosphohydrolase 6
Entpd8		-0.62	6.91	6.29		-0.94		-1.47	6.82	5.35		ENSMUSG00000036813 ectonucleoside triphosphate diphosphohydrolase 8
r0007	0.03	0.15	10.1	10.2	0.2	-0.09	0.12	0.07	10.1	10.1	2.69	PPi(c) + H2O(c) → 2 Pi(c). Pyrophosphate phosphohydrolase EC:3.6.1.1 (2 genes, 2 sp.)
Ppa1		-0.26	11.1	10.8		0.18		-0.14	11.1	11		ENSMUSG00000020089 pyrophosphatase (inorganic) 1
Ppa2		0.56	9.07	9.63		-0.35		0.28	9	9.28		ENSMUSG00000028013 pyrophosphatase (inorganic) 2

11 Reaction scores for Urea from alanine (312), series control 1/24h, TGFβ/C 24h, and TGFβ 1/24h

The amplitudes of the mode are -0.64, -1.54 and -2.2.

Simulation	Definition			Solution													
				exchanges		reactions								transp			Prot
	Objective	Constraints	Comment	imports	exports	c	m	r	p	l	n	s	b	s-c	b-c	intra	synth
312 Urea from alanine	Urea	MCES −Alanine	Urea from alanine	1 H_2O(s) 2 Alanine(s) 2 ATP-energy(c) 2 ATP-energy(m)	1 Pyruvate(c) 1 Urea(s) 1 NADH-redox-potential(c) 1 NADH-redox-potential(m) 1 NADPH-redox-potential(m) 1 CoA-activated acetyl group(m)	11	9	-	-	-	-	-	-	3	-	2	-

Table 10: Reaction scores for Urea from alanine (312), series control 1/24h, TGFβ/C 24h, and TGFβ 1/24h

	control 1/24h				TGFβ/C 24h		TGFβ 1/24h					
Rea ID	Score *0.32*	Expr Δ	Expr C1h	Expr C24h	Score *0.43*	Expr Δ	Score *0.41*	Expr Δ	Expr T1h	Expr T24h	Wght	Reaction
r0081	0.82	-0.43	11.2	10.7	0.92	-1.85	1	-2.24	11.1	8.89	13.8	Alanine(m) + AKG(m) ⇌ Pyruvate(m) + Glutamate(m). L-Alanine:2-oxoglutarate aminotransferase Glutamate metabolism / Alanine and aspartate metabolism EC:2.6.1.2 (4 sp.)
Gpt2 (4 sp.)												ENSMUSG00000031700 glutamic pyruvate transaminase (alanine aminotransferase) 2
r0080	0.99	-0.68	9.96	9.28	0.97	-1.73	0.97	-2.47	10	7.55	8.31	Alanine(c) + AKG(c) ⇌ Pyruvate(c) + Glutamate(c). L-Alanine:2-oxoglutarate aminotransferase Glutamate metabolism / Alanine and aspartate metabolism EC:2.6.1.2
Gpt												ENSMUSG00000022546 glutamic pyruvic transaminase, soluble
r0034	0	-4.18	12.7	8.56	0.99	-1.43	0.01	-5.55	12.7	7.14	8.61	2 ATP(m) + CO2(m) + H2O(m) + NH3(m) ⇌ 2 ADP(m) + Pi(m) + Carbamoyl-P(m). Carbon-dioxide:ammonia ligase (ADP-forming,carbamate-phosphorylating) Urea cycle and metabolism of amino groups / Nitrogen metabolism EC:6.3.4.16
Cps1												ENSMUSG00000025991 carbamoyl-phosphate synthetase 1
r0144	0	-5.79	12.5	6.71	0.22	-0.19	0.005	-5.79	12.3	6.51	9.58	Arginine(c) + H2O(c) ⇌ Urea(c) + Ornithine(c). L-Arginine amidinohydrolase Urea cycle and metabolism of amino groups / Arginine and proline metabolism EC:3.5.3.1
Arg1												ENSMUSG00000019987 arginase, liver
r0329	0.0002	-1.95	7.03	5.08	0.58	-0.73	0.94	-2.58	6.93	4.35	8.61	Carbamoyl-P(m) + Ornithine(m) ⇌ Pi(m) + Citrulline(m). Carbamoyl-phosphate:L-ornithine carbamoyltransferase Urea cycle and metabolism of amino groups / Arginine and proline metabolism EC:2.1.3.3 (3 sp.)
Otc (3 sp.)												ENSMUSG00000031173 ornithine transcarbamylase
r0896	0.21	-0.08	3.58	3.5	0.14	-0.004	0.14	-0.04	3.54	3.5	9.58	Urea(c) ⇌ Urea(s). Urea Transporter (UT) TCDB:1.A.28.1.3 (2 sp.)
Slc14a1 (2 sp.)												ENSMUSG00000059336 solute carrier family 14 (urea transporter), member 1
r0947	0.15	-0.01	3.1	3.08	0.1	0.11	0.14	-0.02	3.21	3.2	8.04	Citrulline(m) + Ornithine(c) ⇌ Citrulline(c) + Ornithine(m). Mitochondrial Carrier (MC) TCDB:2.A.29.19.1
Slc25a2												ENSMUSG00000050304 solute carrier family 25 (mitochondrial carrier, ornithine transporter) member 2
r0109	0.91	-0.77	10.7	9.94	0.15	-0.03	0.44	-0.79	10.7	9.9	6.71	OAA(c) + Glutamate(c) ⇌ AKG(c) + Aspartate(c). L-Aspartate:2-oxoglutarate aminotransferase Glutamate metabolism / Alanine and aspartate metabolism EC:2.6.1.1
Got1												ENSMUSG00000025190 glutamate oxaloacetate transaminase 1, soluble
r0453	0.13	-1.28	11.7	10.4	0.09	0.16	0.57	-1.03	11.6	10.6	8.61	ATP(c) + Citrulline(c) + Aspartate(c) ⇌ AMP(c) + PPi(c) + Argininosuccinate(c). L-Citrulline:L-aspartate ligase (AMP-forming) Urea cycle and metabolism of amino groups / Alanine and aspartate metabolism / Arginine and proline metabolism EC:6.3.4.5
Ass1												ENSMUSG00000076441 argininosuccinate synthetase 1
r0056	0.17	-0.04	8.14	8.1	0.2	-0.15	0.17	-0.14	8.09	7.95	8.61	Pyruvate(m) + CoA(m) + NAD+(m) → Acetyl-CoA(m) + CO2(m) + NADH(m). pyruvate:NAD+ 2-oxidoreductase (CoA-acetylating) Complex of EC:1.2.4.1 EC:2.3.1.12 EC:1.8.1.4 (5 genes, 8 sp.)
Dlat (3 sp.)		0.08	7.7	7.78		-0.16		-0.04	7.66	7.61		ENSMUSG00000000168 dihydrolipoamide S-acetyltransferase (E2 component of pyruvate dehydrogenase complex)
Dld		-0.21	8.88	8.66		-0.16		-0.22	8.73	8.51		ENSMUSG00000020664 dihydrolipoamide dehydrogenase
Pdha1 (2 sp.)		0.11	9.64	9.75		-0.06		0.08	9.61	9.69		ENSMUSG00000031299 pyruvate dehydrogenase E1 alpha 1
Pdha2		-0.22	3.53	3.31		-0.06		-0.25	3.51	3.26		ENSMUSG00000047674 pyruvate dehydrogenase E1 alpha 2
Pdhb		-0.31	10.3	10		-0.41		-0.68	10.3	9.6		ENSMUSG00000021748 pyruvate dehydrogenase (lipoamide) beta
r0075	0.002	0.49	10.9	11.4	0.66	-0.84	0.27	-0.43	11	10.6	5.4	Glutamate(m) + H2O(m) + NADP+(m) ⇌ AKG(m) + NADPH(m) + NH3(m). L-Glutamate:NADP+ oxidoreductase (deaminating) Glutamate metabolism EC:1.4.1.3 EC:1.4.1.4 (3 sp.)
Glud1 (3 sp.)												ENSMUSG00000021794 glutamate dehydrogenase 1
r0104	0.21	-0.07	10.8	10.8	0.2	-0.16	0.19	-0.2	10.8	10.6	6.43	NAD+(c) + Malate(c) ⇌ OAA(c) + NADH(c). (S)-malate:NAD+ oxidoreductase Citrate cycle (TCA cycle) EC:1.1.1.37 (3 sp.)
Mdh1 (3 sp.)												ENSMUSG00000020321 malate dehydrogenase 1, NAD (soluble)
r0261	0.61	-0.32	9.99	9.66	0.45	-0.56	0.49	-0.88	9.98	9.1	8.57	Argininosuccinate(c) ⇌ Fumarate(c) + Arginine(c). N-(L-Argininosuccinate) arginie-lyase Urea cycle and metabolism of amino groups / Alanine and aspartate metabolism / Arginine and proline metabolism EC:4.3.2.1
Asl												ENSMUSG00000025533 argininosuccinate lyase
r0258	0.16	-0.03	10.4	10.3	0.22	-0.2	0.22	-0.28	10.4	10.1	4.42	Fumarate(c) + H2O(c) ⇌ Malate(c). (S)-Malate hydro-lyase Citrate cycle (TCA cycle) EC:4.2.1.2
Fh1												ENSMUSG00000026526 fumarate hydratase 1
r2524	0.25	-0.11	5.07	4.96	0.19	-0.14	0.17	-0.12	4.94	4.82	4.55	Alanine(s) ⇌ Alanine(c). Major Facilitator(MFS) TCDB:2.A.1.44.1
Slc43a1												ENSMUSG00000027075 solute carrier family 43, member 1
r0007	0.05	0.15	10.1	10.2	0.17	-0.09	0.12	0.07	10.1	10.1	2.4	PPi(c) + H2O(c) → 2 Pi(c). Pyrophosphate phosphohydrolase EC:3.6.1.1 (2 genes, 2 sp.)
Ppa1		-0.26	11.1	10.8		0.18		-0.14	11.1	11		ENSMUSG00000020089 pyrophosphatase (inorganic) 1
Ppa2		0.56	9.07	9.63		-0.35		0.28	9	9.28		ENSMUSG00000028013 pyrophosphatase (inorganic) 2
r0030	0.23	-0.09	6.05	5.96	0.11	0.06	0.15	-0.05	6.06	6.02	2.65	ATP(c) + AMP(c) ⇌ 2 ADP(c). ATP:AMP phosphotransferase Purine metabolism EC:2.7.4.3 (5 genes, 11 sp.)
Ak1 (2 sp.)		-0.07	3.97	3.89		0.76		0.59	4.07	4.66		ENSMUSG00000026817 adenylate kinase 1
Ak2 (2 sp.)		0.08	9.35	9.43		0.13		0.15	9.41	9.55		ENSMUSG00000028792 adenylate kinase 2
Ak4 (4 sp.)		-0.36	6.41	6.05		-0.23		-0.45	6.27	5.82		ENSMUSG00000028527 adenylate kinase 4
Ak5 (2 sp.)		-0.17	4.72	4.56		0.12		-0.12	4.79	4.68		ENSMUSG00000039058 adenylate kinase 5
Ak7		0.74	4.79	5.53		-0.39		0.06	5.08	5.14		ENSMUSG00000041323 adenylate kinase 7

12 Reaction scores for Cholesterol (523), series control 1/24h, TGFβ/C 24h, and TGFβ 1/24h

The amplitudes of the mode are -0.87, -0.45 and -1.25.

Simulation	Definition			Solution													
				exchanges		reactions								transp			Prot
	Objective	Constraints	Comment	imports	exports	c	m	r	p	l	n	s	b	s-c	b-c	intra	synth
523 Cholesterol	Cholesterol(r)	MCES	de novo synthesis of Cholesterol in ER/Golgi	8 O_2(s) 2 Farnesyl-PP(r) 1 ATP-energy(c) 11 NADPH-redox-potential(r)	7 H_2O(s) 4 P_i(c) 3 CO_2(s) 1 Cholesterol(r) 1 NADH-redox-potential(r) 1 NADPH-redox-potential(c)	5	-	16	-	-	-	-	-	3	-	5	-

Table 11: Reaction scores for Cholesterol (523), series control 1/24h, TGFβ/C 24h, and TGFβ 1/24h

Rea ID	control 1/24h Score *0.77*	Expr Δ	Expr C1h	Expr C24h	TGFβ/C 24h Score *0.63*	Expr Δ	TGFβ 1/24h Score *0.79*	Expr Δ	Expr T1h	Expr T24h	Wght	Reaction
r1134	1	-0.87	10.7	9.86	1	-0.44	0.97	-1.08	10.5	9.42	26.8	14-Demethyllanosterol(r) + 3 NADPH(r) + 3 O2(r) ⇌ 4 H2O(r) + 4alpha-Methylzymosterol-4-carboxylate(r) + 3 NADP+(r). methylsterol monooxygenase Biosynthesis of steroids EC:1.14.13.72
Sc4mol												ENSMUSG00000031604 sterol-C4-methyl oxidase-like
r0781	0.98	-0.96	7.54	6.58	1	-0.47	1	-1.29	7.4	6.11	26.5	3 O2(r) + 3 NADPH(r) + Lanosterol(r) ⇌ 4,4-Dimethyl-5alpha-cholesta-8,14,24-trien-3beta-ol(r) + Formate(r) + 4 H2O(r) + 3 NADP+(r). Lanosterol,NADPH:oxygen oxidoreductase (14-methyl cleaving) Biosynthesis of steroids EC:1.14.13.70 (3 sp.)
Cyp51 (3 sp.)												ENSMUSG00000001467 cytochrome P450, family 51
r0793	0.18	-0.07	7.93	7.87	0.004	0.3	0.06	0.24	7.93	8.17	9.33	Lathosterol(r) + NADPH(r) + O2(r) ⇌ Provitamin D3(r) + 2 H2O(r) + NADP+(r). 5alpha-cholest-7-en-3beta-ol, NADPH:oxygen 5-oxidoreductase Biosynthesis of steroids EC:1.14.21.6 (2 sp.)
Sc5d (2 sp.)												ENSMUSG00000032018 sterol-C5-desaturase (fungal ERG3, delta-5-desaturase) homolog (S. cerevisae)
r0227	0.4	-0.28	6.69	6.41	0.004	0.3	0.08	0.16	6.55	6.71	8.53	10-Formyl-THF(c) + H2O(c) + NADP+(c) → THF(c) + CO2(c) + NADPH(c). 10-formyltetrahydrofolate:NADP+ oxidoreductase One carbon pool by folate EC:1.5.1.6 (2 genes, 3 sp.)
Aldh1l1 (2 sp.)		-0.49	7.57	7.07		0.64		0.31	7.41	7.72		ENSMUSG00000030088 aldehyde dehydrogenase 1 family, member L1
Aldh1l2		0.16	4.93	5.09		-0.4		-0.15	4.84	4.69		ENSMUSG00000020256 aldehyde dehydrogenase 1 family, member L2
r0333	0.96	-0.74	9.25	8.52	0.01	-1.18	0.57	-1.92	9.25	7.33	9.45	Provitamin D3(r) + NADPH(r) ⇌ Cholesterol(r) + NADP+(r). Cholesterol:NADP+ delta7-oxidoreductase Biosynthesis of steroids EC:1.3.1.21
Dhcr7												ENSMUSG00000058454 7-dehydrocholesterol reductase
C0104	0.65	-0.46	9.74	9.28	0.33	-0.8	0.99	-1.14	9.63	8.49	12.2	several reactions. Farnesyl-diphosphate:farnesyl-diphosphate farnesyltransferase Biosynthesis of steroids EC:2.5.1.21 (1 genes, 2 sp.)
Fdft1 (2 sp.)												ENSMUSG00000021273 farnesyl diphosphate farnesyl transferase 1
r0576	0.86	-0.63	9.99	9.36	0.02	-1.1	0.84	-1.62	9.87	8.26	8.72	Squalene(r) + NADPH(r) + O2(r) ⇌ H2O(r) + Squalene 2,3-oxide(r) + NADP+(r). Squalene,hydrogen-donor:oxygen oxidoreductase (2,3-epoxidizing) Biosynthesis of steroids EC:1.14.99.7
Sqle												ENSMUSG00000022351 squalene epoxidase
r0607	0.96	-0.74	6.42	5.68	0.98	-0.41	0.93	-1.01	6.28	5.27	8.77	Squalene 2,3-oxide(r) ⇌ Lanosterol(r). (S)-2,3-Epoxysqualene mutase (cyclizing, lanosterol-forming) Biosynthesis of steroids EC:5.4.99.7 (2 sp.)
Lss (2 sp.)												ENSMUSG00000033105 lanosterol synthase
r0007	0.07	0.15	10.1	10.2	0.27	-0.09	0.11	0.07	10.1	10.1	3.33	PPi(c) + H2O(c) → 2 Pi(c). Pyrophosphate phosphohydrolase EC:3.6.1.1 (2 genes, 2 sp.)
Ppa1		-0.26	11.1	10.8		0.18		-0.14	11.1	11		ENSMUSG00000020089 pyrophosphatase (inorganic) 1
Ppa2		0.56	9.07	9.63		-0.35		0.28	9	9.28		ENSMUSG00000028013 pyrophosphatase (inorganic) 2
r0228	0.1	0.07	7.92	7.98	0.84	-0.59	0.55	-0.57	7.96	7.39	4.77	THF(c) + Formate(c) + ATP(c) → ADP(c) + Pi(c) + 10-Formyl-THF(c). Formate:tetrahydrofolate ligase (ADP-forming) Glyoxylate and dicarboxylate metabolism / One carbon pool by folate EC:6.3.4.3 (3 sp.)
Mthfd1 (3 sp.)												ENSMUSG00000021048 methylenetetrahydrofolate dehydrogenase (NADP+ dependent), methenyltetrahydrofolate cyclohydrolase, formyltetrahydrofolate synthase
r1135	1	-0.85	6.95	6.1	0.84	-0.32	0.94	-1.02	6.8	5.78	9.03	3-Keto-4-methylzymosterol(r) + NADP+(r) ⇌ NADPH(r) + 4alpha-Methylzymosterol(r). hydroxysteroid (17-beta) dehydrogenase 7 Biosynthesis of steroids EC:1.1.1.270 (4 sp.)
Hsd17b7 (4 sp.)												ENSMUSG00000026675 hydroxysteroid (17-beta) dehydrogenase 7
r0783	0.55	-0.39	8.29	7.9	0.9	-0.35	0.8	-0.83	8.38	7.55	9.33	5alpha-Cholesta-7,24-dien-3beta-ol(r) + NADPH(r) ⇌ Lathosterol(r) + NADP+(r). lanosterol D24-reductase Biosynthesis of steroids EC:1.3.1.72 (3 sp.)
Dhcr24 (3 sp.)												ENSMUSG00000034926 24-dehydrocholesterol reductase
r0780	0.74	-0.53	8.34	7.8	0.3	-0.1	0.63	-0.65	8.35	7.7	8.87	4,4-Dimethyl-5alpha-cholesta-8,14,24-trien-3beta-ol(r) + NADPH(r) ⇌ 14-Demethyllanosterol(r) + NADP+(r). 4,4-dimethyl-5a-cholesta-8,24-dien-3b-ol:NADP+ D14-oxidoreductase Biosynthesis of steroids EC:1.3.1.70 (2 sp.)
Tm7sf2 (2 sp.)												ENSMUSG00000024799 transmembrane 7 superfamily member 2
r0738	0.77	-1.18	8.78	7.6	0.89	-0.34	0.98	-1.38	8.63	7.25	9.21	Zymosterol(r) ⇌ 5alpha-Cholesta-7,24-dien-3beta-ol(r). delta8,24-Cholestadien-3beta-ol delta7-delta8-isomerase Biosynthesis of steroids EC:5.3.3.5 (2 sp.)
Ebp (2 sp.)												ENSMUSG00000031168 phenylalkylamine Ca2+ antagonist (emopamil) binding protein
r1137	0.86	-1.11	10.4	9.3	0.67	-0.25	0.99	-1.36	10.4	9.05	8.98	NAD+(r) + 4alpha-Methylzymosterol-4-carboxylate(r) ⇌ NADH(r) + CO2(r) + 3-Keto-4-methylzymosterol(r). NAD(P) dependent steroid dehydrogenase-like EC:1.1.1.170
Nsdhl												ENSMUSG00000031349 NAD(P) dependent steroid dehydrogenase-like

13 Reaction scores for (R)-Mevalonate (552), series control 1/24h, TGFβ/C 24h, and TGFβ 1/24h

The amplitudes of the mode are -0.8, 0.62 and -1.25.

Simulation	Definition			Solution														
				exchanges		reactions								transp			Prot
	Objective	Constraints	Comment	imports	exports	c	m	r	p	l	n	s	b	s-c	b-c	intra	synth
552 (R)-Mevalonate	(R)-Mevalonate(c)	PIPES MCES	synthesis of cytosolic (R)-Mevalonate	1 H_2O(s) 2 NADPH-redox-potential(c) 3 CoA-activated acetyl group(c)	1 (R)-Mevalonate(c)	5	-	-	-	-	-	-	-	1	-	-	-

Table 12: Reaction scores for (R)-Mevalonate (552), series control 1/24h, TGFβ/C 24h, and TGFβ 1/24h

Rea ID	control 1/24h Score *0.82*	Expr Δ	Expr C1h	Expr C24h	TGFβ/C 24h Score *0.54*	Expr Δ	TGFβ 1/24h Score *0.45*	Expr Δ	Expr T1h	Expr T24h	Wght	Reaction
r0461	0.98	-0.72	10.8	10.1	0.0002	-0.65	1	-1.24	10.6	9.4	14.3	Acetyl-CoA(c) + H2O(c) + Acetoacetyl-CoA(c) ⇌ HMG-CoA(c) + CoA(c). (S)-3-Hydroxy-3-methylglutaryl-CoA acetoacetyl-CoA-lyase (CoA-acetylating) Synthesis and degradation of ketone bodies / Valine, leucine and isoleucine degradation / Butanoate metabolism EC:2.3.3.10 (2 genes, 8 sp.)
Hmgcs1 (4 sp.)		-0.72	10.8	10.1		-0.65		-1.24	10.6	9.4		ENSMUSG00000079301 3-hydroxy-3-methylglutaryl-Coenzyme A synthase 1
Hmgcs1 (4 sp.)		-0.72	10.8	10.1		-0.65		-1.24	10.6	9.4		ENSMUSG00000079828 3-hydroxy-3-methylglutaryl-Coenzyme A synthase 1
r0488	0.99	-0.86	6.63	5.77	0.99	0.67	0.13	0.02	6.42	6.44	14.6	HMG-CoA(c) + 2 NADPH(c) ⇌ (R)-Mevalonate(c) + CoA(c) + 2 NADP+(c). (R)-Mevalonate:NADP+ oxidoreductase (CoA acylating) Biosynthesis of steroids EC:1.1.1.34
Hmgcr												ENSMUSG00000021670 3-hydroxy-3-methylglutaryl-Coenzyme A reductase
r0069	0.16	-0.03	8.79	8.76	0.72	0.37	0.05	0.28	8.85	9.13	7.46	2 Acetyl-CoA(c) ⇌ CoA(c) + Acetoacetyl-CoA(c). Acetyl-CoA:acetyl-CoA C-acetyltransferase Synthesis and degradation of ketone bodies EC:2.3.1.9 (2 genes, 5 sp.)
Acat1 (3 sp.)		0.19	7.92	8.1		0.54		0.67	7.97	8.64		ENSMUSG00000032047 acetyl-Coenzyme A acetyltransferase 1
Acat2 (2 sp.)		-0.35	10.1	9.75		0.11		-0.3	10.2	9.87		ENSMUSG00000023832 acetyl-Coenzyme A acetyltransferase 2

14 Reaction scores for Farnesyl-PP (63), series control 1/24h, TGFβ/C 24h, and TGFβ 1/24h

The amplitudes of the mode are -0.66, -0.61 and -1.03.

Simulation	Definition			Solution													
				exchanges		reactions								transp			Prot
	Objective	Constraints	Comment	imports	exports	c	m	r	p	l	n	s	b	s-c	b-c	intra	synth
63 Farnesyl-PP	Farnesyl-PP(p)	−Acetyl-CoA(c) +CoA(c) =ATP-energy(c) =NADH-redox-potential(c) =NADPH-redox-potential(c) =H_2O =P_i(c) =CO_2	de novo synthesis of peroxysomal Farnesyl-PP	2 P_i(c) 9 Acetyl-CoA(c) 9 ATP-energy(c) 6 NADPH-redox-potential(c)	4 H_2O(s) 9 CoA(c) 3 CO_2(s) 1 Farnesyl-PP(p)	6	-	-	6	-	-	-	-	2	-	5	-

Table 13: Reaction scores for Farnesyl-PP (63), series control 1/24h, TGFβ/C 24h, and TGFβ 1/24h

Rea	control 1/24h				TGFβ/C 24h		TGFβ 1/24h				Wght	Reaction
ID	Score *0.62*	Expr Δ	Expr C1h	Expr C24h	Score *0.51*	Expr Δ	Score *0.54*	Expr Δ	Expr T1h	Expr T24h		
r0612	1	-0.67	7.95	7.28	0.91	-0.74	0.88	-1.29	7.83	6.54	16.8	ATP(p) + (R)-5-Phosphomevalonate(p) ⇌ ADP(p) + (R)-5-Diphosphomevalonate(p). ATP:(R)-5-phosphomevalonate phosphotransferase Biosynthesis of steroids EC:2.7.4.2
Pmvk												ENSMUSG00000027952 phosphomevalonate kinase
r0461	0.98	-0.72	10.8	10.1	0.99	-0.65	0.92	-1.24	10.6	9.4	15.4	Acetyl-CoA(c) + H2O(c) + Acetoacetyl-CoA(c) ⇌ HMG-CoA(c) + CoA(c). (S)-3-Hydroxy-3-methylglutaryl-CoA acetoacetyl-CoA-lyase (CoA-acetylating) Synthesis and degradation of ketone bodies / Valine, leucine and isoleucine degradation / Butanoate metabolism EC:2.3.3.10 (2 genes, 8 sp.)
Hmgcs1 (4 sp.)		-0.72	10.8	10.1		-0.65		-1.24	10.6	9.4		ENSMUSG00000079301 3-hydroxy-3-methylglutaryl-Coenzyme A synthase 1
Hmgcs1 (4 sp.)		-0.72	10.8	10.1		-0.65		-1.24	10.6	9.4		ENSMUSG00000079828 3-hydroxy-3-methylglutaryl-Coenzyme A synthase 1
r0488	0.82	-0.86	6.63	5.77	0.0001	0.67	0.12	0.02	6.42	6.44	15.8	HMG-CoA(c) + 2 NADPH(c) ⇌ (R)-Mevalonate(c) + CoA(c) + 2 NADP+(c). (R)-Mevalonate:NADP+ oxidoreductase (CoA acylating) Biosynthesis of steroids EC:1.1.1.34
Hmgcr												ENSMUSG00000021670 3-hydroxy-3-methylglutaryl-Coenzyme A reductase
r0271	0.03	-1.53	9.48	7.96	0.16	-1.19	0.02	-2.44	9.2	6.77	10.5	Isopentenyl-PP(p) ⇌ Dimethylallyl-PP(p). Isopentenyl-diphosphate delta3-delta2-isomerase Biosynthesis of steroids / Terpenoid biosynthesis EC:5.3.3.2 (2 sp.)
Idi1 (2 sp.)												ENSMUSG00000058258 isopentenyl-diphosphate delta isomerase
r0069	0.16	-0.03	8.79	8.76	0.01	0.37	0.04	0.28	8.85	9.13	8.03	2 Acetyl-CoA(c) ⇌ CoA(c) + Acetoacetyl-CoA(c). Acetyl-CoA:acetyl-CoA C-acetyltransferase Synthesis and degradation of ketone bodies EC:2.3.1.9 (2 genes, 5 sp.)
Acat1 (3 sp.)		0.19	7.92	8.1		0.54		0.67	7.97	8.64		ENSMUSG00000032047 acetyl-Coenzyme A acetyltransferase 1
Acat2 (2 sp.)		-0.35	10.1	9.75		0.11		-0.3	10.2	9.87		ENSMUSG00000023832 acetyl-Coenzyme A acetyltransferase 2
r0954	0.17	-0.03	6.62	6.59	0.62	-0.31	0.4	-0.33	6.61	6.28	13.5	(R)-Mevalonate(c) ⇌ (R)-Mevalonate(p). MCT 1 Transport reaction (2 sp.)
Slc16a1 (2 sp.)												ENSMUSG00000032902 solute carrier family 16 (monocarboxylic acid transporters), member 1
r0270	0.66	-0.36	6.73	6.37	0.61	-0.3	0.82	-0.7	6.77	6.06	17.4	ATP(p) + (R)-5-Diphosphomevalonate(p) ⇌ CO2(p) + Isopentenyl-PP(p) + ADP(p) + Pi(p). ATP:(R)-5-diphosphomevalonate carboxy-lyase (dehydrating) Biosynthesis of steroids EC:4.1.1.33 (2 sp.)
Mvd (2 sp.)												ENSMUSG00000006517 mevalonate (diphospho) decarboxylase
r0515	0.7	-0.38	7.66	7.28	0.55	-0.27	0.73	-0.62	7.63	7	16.2	ATP(p) + (R)-Mevalonate(p) ⇌ ADP(p) + (R)-5-Phosphomevalonate(p). ATP:(R)-mevalonate 5-phosphotransferase Biosynthesis of steroids EC:2.7.1.36 (2 sp.)
Mvk (2 sp.)												ENSMUSG00000041939 mevalonate kinase
C0087	0.61	-0.33	8.09	7.77	0.38	-0.18	0.49	-0.42	8	7.58	15.9	several reactions. Dimethylallyl-diphosphate:isopentenyl-diphosphate dimethylallyltranstransferase Biosynthesis of steroids / Terpenoid biosynthesis EC:2.5.1.1 (2 genes, 3 sp.)
Fdps		-0.58	11.2	10.7		-0.7		-1.2	11.2	9.95		ENSMUSG00000059743 farnesyl diphosphate synthetase
Ggps1 (2 sp.)		-0.2	6.53	6.32		0.08		-0.03	6.42	6.4		ENSMUSG00000021302 geranylgeranyl diphosphate synthase 1
r0007	0.05	0.15	10.1	10.2	0.23	-0.09	0.1	0.07	10.1	10.1	2.14	PPi(c) + H2O(c) → 2 Pi(c). Pyrophosphate phosphohydrolase EC:3.6.1.1 (2 genes, 2 sp.)
Ppa1		-0.26	11.1	10.8		0.18		-0.14	11.1	11		ENSMUSG00000020089 pyrophosphatase (inorganic) 1
Ppa2		0.56	9.07	9.63		-0.35		0.28	9	9.28		ENSMUSG00000028013 pyrophosphatase (inorganic) 2

15 Reaction scores for Creatine (314), series control 1/24h, TGFβ/C 24h, and TGFβ 1/24h

The amplitudes of the mode are 0.6, -1.27 and -0.79.

Simulation	Definition			Solution													
				exchanges		reactions								transp			Prot
	Objective	Constraints	Comment	imports	exports	c	m	r	p	l	n	s	b	s-c	b-c	intra	synth
314 Creatine	Creatine(c)	MCES −Arginine −Glycine	de novo synthesis of cytosolic Creatine	1 H_2O(s) 0.25 O_2(s) 1 Glycine(s) 0.5 Arginine(s) 1 Activated methyl group(c) 1 ATP-energy(c) 0.5 ATP-energy(m)	0.5 CO_2(s) 0.5 Pyruvate(c) 1 Creatine(c) 2 NADH-redox-potential(m) 0.5 NADPH-redox-potential(c) 1.5 Proton-gradient(m)	11	14	-	-	-	-	-	-	5	-	11	-

Table 14: Reaction scores for Creatine (314), series control 1/24h, TGFβ/C 24h, and TGFβ 1/24h

	control 1/24h				TGFβ/C 24h		TGFβ 1/24h					
Rea ID	Score *0.27*	Expr Δ	Expr C1h	Expr C24h	Score *0.39*	Expr Δ	Score *0.47*	Expr Δ	Expr T1h	Expr T24h	Wght	Reaction
r0420	0.97	0.67	7.54	8.21	0.9	-1.56	1	-0.81	7.46	6.65	25.3	SAM(c) + Guanidinoacetate(c) ⇌ SAH(c) + Creatine(c). S-Adenosyl-L-methionine:guanidinoacetate N-methyltransferase Glycine, serine and threonine metabolism / Arginine and proline metabolism EC:2.1.1.2
Gamt												ENSMUSG00000020150 guanidinoacetate methyltransferase
r0147	0.34	0.16	3.63	3.79	0.03	0.38	0.02	0.34	3.83	4.17	22.9	Arginine(c) + Glycine(c) ⇌ Guanidinoacetate(c) + Ornithine(c). L-Arginine:glycine amidinotransferase Glycine, serine and threonine metabolism / Arginine and proline metabolism EC:2.1.4.1
Gatm												ENSMUSG00000027199 glycine amidinotransferase (L-arginine:glycine amidinotransferase)
r0034	0	-4.18	12.7	8.56	0.97	-1.43	0	-5.55	12.7	7.14	7.8	2 ATP(m) + CO2(m) + H2O(m) + NH3(m) ⇌ 2 ADP(m) + Pi(m) + Carbamoyl-P(m). Carbon-dioxide:ammonia ligase (ADP-forming,carbamate-phosphorylating) Urea cycle and metabolism of amino groups / Nitrogen metabolism EC:6.3.4.16
Cps1												ENSMUSG00000025991 carbamoyl-phosphate synthetase 1
r0329	0	-1.95	7.03	5.08	0.69	-0.73	0	-2.58	6.93	4.35	7.8	Carbamoyl-P(m) + Ornithine(m) ⇌ Pi(m) + Citrulline(m). Carbamoyl-phosphate:L-ornithine carbamoyl-transferase Urea cycle and metabolism of amino groups / Arginine and proline metabolism EC:2.1.3.3 (3 sp.)
Otc (3 sp.)												ENSMUSG00000031173 ornithine transcarbamylase
C0147	0.12	-0.01	3.1	3.08	0.09	0.11	0.15	-0.02	3.21	3.2	10.3	several reactions. Mitochondrial Carrier (MC) TCDB:2.A.29.19.1
Slc25a2												ENSMUSG00000050304 solute carrier family 25 (mitochondrial carrier, ornithine transporter) member 2
r0453	0	-1.28	11.7	10.4	0.08	0.16	0.83	-1.03	11.6	10.6	7.8	ATP(c) + Citrulline(c) + Aspartate(c) ⇌ AMP(c) + PPi(c) + Argininosuccinate(c). L-Citrulline:L-aspartate ligase (AMP-forming) Urea cycle and metabolism of amino groups / Alanine and aspartate metabolism / Arginine and proline metabolism EC:6.3.4.5
Ass1												ENSMUSG00000076441 argininosuccinate synthetase 1
r0167	0	-1.14	8.85	7.71	0.07	0.21	0.71	-0.46	8.39	7.92	8.68	AKG(m) + Ornithine(m) ⇌ L-Glutamate 5-semialdehyde(m) + Glutamate(m). L-Ornithine:2-oxo-acid aminotransferase Arginine and proline metabolism EC:2.6.1.13
Oat												ENSMUSG00000030934 ornithine aminotransferase
r0261	0.01	-0.32	9.99	9.66	0.54	-0.56	0.97	-0.88	9.98	9.1	7.76	Argininosuccinate(c) ⇌ Fumarate(c) + Arginine(c). N-(L-Argininosuccinate) arginie-lyase Urea cycle and metabolism of amino groups / Alanine and aspartate metabolism / Arginine and proline metabolism EC:4.3.2.1
Asl												ENSMUSG00000025533 argininosuccinate lyase
r0110	0.01	-0.31	11.4	11	0.27	-0.24	0.87	-0.58	11.4	10.8	8.4	OAA(m) + Glutamate(m) → AKG(m) + Aspartate(m). L-Aspartate:2-oxoglutarate aminotransferase Glutamate metabolism / Alanine and aspartate metabolism EC:2.6.1.1 (2 sp.)
Got2 (2 sp.)												ENSMUSG00000031672 glutamate oxaloacetate transaminase 2, mitochondrial
r1058	0	-2.86	8.24	5.38	0.79	-0.84	0	-3.34	7.88	4.54	5.46	Arginine(s) ⇌ Arginine(c). Amino Acid-Polyamine-Organocation (APC) TCDB:2.A.3.3.2 (4 sp.)
Slc7a2 (4 sp.)												ENSMUSG00000031596 solute carrier family 7 (cationic amino acid transporter, y+ system), member 2
r0073	0.94	0.49	10.9	11.4	0.79	-0.84	0.65	-0.43	11	10.6	4.89	Glutamate(m) + NAD+(m) + H2O(m) ⇌ AKG(m) + NADH(m) + NH3(m). L-Glutamate:NAD+ oxidoreductase (deaminating) Glutamate metabolism EC:1.4.1.3 EC:1.4.1.2 (3 sp.)
Glud1 (3 sp.)												ENSMUSG00000021794 glutamate dehydrogenase 1
r0876	0	-1.34	9.88	8.54	0.23	-0.18	0.62	-1.18	9.53	8.36	8.51	Glutamate(c) + H+(PG)(c) + Aspartate(m) → Glutamate(m) + H+(PG)(m) + Aspartate(c). Mitochondrial Carrier (MC) TCDB:2.A.29.14.2
Slc25a13												ENSMUSG00000015112 solute carrier family 25 (mitochondrial carrier, adenine nucleotide translocator), member 13
r0125	0.08	-0.07	9.76	9.7	0.48	-0.5	0.86	-0.57	9.77	9.2	7.25	GDP(m) + Pi(m) + Succinyl-CoA(m) ⇌ GTP(m) + Succinate(m) + CoA(m). Succinate:CoA ligase (GDP-forming) Citrate cycle (TCA cycle) / Propanoate metabolism EC:6.2.1.4 (2 genes, 3 sp.)
Suclg1		0.18	10.1	10.2		-0.06		0.07	10.1	10.2		ENSMUSG00000052738 succinate-CoA ligase, GDP-forming, alpha subunit
Suclg2 (2 sp.)		-0.19	9.62	9.43		-0.72		-0.89	9.6	8.71		ENSMUSG00000061838 succinate-Coenzyme A ligase, GDP-forming, beta subunit
r0105	0.01	-0.35	11.8	11.4	0.31	-0.3	0.93	-0.64	11.7	11.1	5.66	NAD+(m) + Malate(m) ⇌ OAA(m) + NADH(m). (S)-malate:NAD+ oxidoreductase Citrate cycle (TCA cycle) EC:1.1.1.37 (2 sp.)
Mdh2 (2 sp.)												ENSMUSG00000019179 malate dehydrogenase 2, NAD (mitochondrial)
r0017	0.08	-0.07	10	9.95	0.14	-0.01	0.22	-0.1	10	9.93	6.17	O2(m) + 4 H+(PG)(m) + 4 Ferrocytochrome C(m) → 4 Ferricytochrome C(m) + 2 H2O(m) + 4 H+(PG)(c). Ferrocytochrome-c:oxygen oxidoreductase Oxidative phosphorylation EC:1.9.3.1 (16 genes, 26 sp.)
Cox4i1		0.04	12.5	12.6		0.03		0.03	12.6	12.6		ENSMUSG00000031818 cytochrome c oxidase subunit IV isoform 1
Cox4i2		-0.02	4.73	4.71		-0.01		-0.1	4.8	4.71		ENSMUSG00000009876 cytochrome c oxidase subunit IV isoform 2
Cox5a (3 sp.)		-0.09	11.6	11.5		-0.03		-0.07	11.5	11.4		ENSMUSG00000000088 cytochrome c oxidase, subunit Va
Cox5b (4 sp.)		-0.09	11.9	11.8		-0.07		-0.12	11.9	11.8		ENSMUSG00000061518 cytochrome c oxidase, subunit Vb
Cox6a1 (2 sp.)		0.01	12.4	12.5		-0.03		-0.04	12.5	12.4		ENSMUSG00000041697 cytochrome c oxidase, subunit VI a, polypeptide 1
Cox6a2		-0.05	6.85	6.8		-0.08		-0.14	6.86	6.72		ENSMUSG00000030785 cytochrome c oxidase, subunit VI a, polypeptide 2
Cox6b1 (2 sp.)		0.06	12	12.1		-0.04		0.05	12	12		ENSMUSG00000036751 cytochrome c oxidase, subunit VIb polypeptide 1
Cox6b2		0.06	5.02	5.08		0.16		0.15	5.09	5.24		ENSMUSG00000051811 cytochrome c oxidase subunit VIb polypeptide 2
Cox6c (2 sp.)		-0.1	11.4	11.3		-0.01		-0.14	11.4	11.2		ENSMUSG00000014313 cytochrome c oxidase, subunit VIc
Cox7a1		-0.19	4.49	4.3		0.21		-0.2	4.71	4.51		ENSMUSG00000074218 cytochrome c oxidase, subunit VIIa 1
Cox7a2 (2 sp.)		-0.31	12.2	11.9		-0.12		-0.38	12.2	11.8		ENSMUSG00000032330 cytochrome c oxidase, subunit VIIa 2
Cox7b2		-0.01	2.91	2.9		0.19		-0.07	3.15	3.09		ENSMUSG00000049387 cytochrome c oxidase subunit VIIb2
Cox7c		-0.11	11.7	11.6		0.01		-0.1	11.7	11.6		ENSMUSG00000017778 cytochrome c oxidase, subunit VIIc
Cox8a (2 sp.)		-0.08	10.4	10.3		-0.05		-0.13	10.4	10.3		ENSMUSG00000035885 cytochrome c oxidase, subunit VIIIa
Cox8b		0.01	5.54	5.54		-0.01		-0.14	5.67	5.53		ENSMUSG00000025488 cytochrome c oxidase, subunit VIIIb
Cox8c		-0.06	7.29	7.23		0.05		-0.04	7.32	7.28		ENSMUSG00000043319 cytochrome c oxidase, subunit VIIIc
r0100	0.84	0.42	8.17	8.59	0.32	-0.31	0.1	0.05	8.23	8.28	4.1	ADP(m) + GTP(m) ⇌ ATP(m) + GDP(m). ATP:nucleoside-diphosphate phosphatransferase Purine metabolism EC:2.7.4.6 (7 genes, 7 sp.)
Nme1		0.44	11.4	11.8		-0.3		0.11	11.4	11.5		ENSMUSG00000037601 non-metastatic cells 1, protein (NM23A) expressed in
Nme2		0.09	13	13.1		0.04		0.14	13	13.2		ENSMUSG00000020857 non-metastatic cells 2, protein (NM23B) expressed in
Nme3		0.86	6.83	7.69		-1.4		-0.66	6.96	6.29		ENSMUSG00000073435 non-metastatic cells 3, protein expressed in
Nme4		0.95	6.48	7.42		-0.82		-0.06	6.67	6.6		ENSMUSG00000024177 non-metastatic cells 4, protein expressed in
Nme5		0.01	4.78	4.79		0.08		-0.02	4.88	4.87		ENSMUSG00000035984 non-metastatic cells 5, protein expressed in (nucleoside-diphosphate kinase)
Nme6		-0.01	7.51	7.5		0.11		0.03	7.58	7.61		ENSMUSG00000032478 non-metastatic cells 6, protein expressed in (nucleoside-diphosphate kinase)
Nme7		0.57	7.21	7.78		0.14		0.85	7.08	7.92		ENSMUSG00000026575 non-metastatic cells 7, protein expressed in (nucleoside-diphosphate kinase)
r0074	0.14	0.001	7.03	7.03	0.52	-0.54	0.81	-0.53	7.02	6.48	6.75	L-Glutamate 5-semialdehyde(m) + NAD+(m) + H2O(m) ⇌ Glutamate(m) + NADH(m). L-Glutamate 5-semialdehyde:NAD+ oxidoreductase Arginine and proline metabolism EC:1.5.1.12

Continued on next page

Rea ID	control 1/24h Score *0.27*	Expr Δ	Expr C1h	Expr C24h	TGFβ/C 24h Score *0.39*	Expr Δ	TGFβ 1/24h Score *0.47*	Expr Δ	Expr T1h	Expr T24h	Wght	Reaction
Aldh4a1											ENSMUSG00000028737 aldehyde dehydrogenase 4 family, member A1	
r0509	0.14	0.001	9.86	9.86	0.21	-0.15	0.22	-0.1	9.81	9.71	7.28	Ubiquinone(m) + Succinate(m) → Ubiquinol(m) + Fumarate(m). Succinate:ubiquinone oxidoreductase Citrate cycle (TCA cycle) EC:1.3.5.1 (4 genes, 7 sp.)
Sdha (2 sp.)		-0.14	10.7	10.5		-0.16		-0.27	10.6	10.4	ENSMUSG00000021577 succinate dehydrogenase complex, subunit A, flavoprotein (Fp)	
Sdhb		0.06	11.3	11.4		-0.24		-0.17	11.3	11.1	ENSMUSG00000009863 succinate dehydrogenase complex, subunit B, iron sulfur (Ip)	
Sdhc (2 sp.)		0.04	9.29	9.33		-0.07		0.02	9.24	9.26	ENSMUSG00000058076 succinate dehydrogenase complex, subunit C, integral membrane protein	
Sdhd (2 sp.)		0.07	8.89	8.96		-0.17		-0.02	8.81	8.79	ENSMUSG00000000171 succinate dehydrogenase complex, subunit D, integral membrane protein	
r1114	0.34	0.16	7.56	7.71	0.18	-0.1	0.11	0.03	7.58	7.62	5.95	AKG(m) + CoA(m) + NAD+(m) ⇌ NADH(m) + CO2(m) + Succinyl-CoA(m). EC:1.2.4.2 (2 sp.)
Ogdh (2 sp.)											ENSMUSG00000020456 oxoglutarate dehydrogenase (lipoamide)	
r0507	0.07	-0.08	10.5	10.4	0.14	-0.02	0.2	-0.08	10.5	10.4	4.38	Ubiquinol(m) + 2 Ferricytochrome C(m) + 4 H+(PG)(m) → Ubiquinone(m) + 4 H+(PG)(c) + 2 Ferrocytochrome C(m). Ubiquinol:ferricytochrome-c oxidoreductase Oxidative phosphorylation EC:1.10.2.2 (8 genes, 13 sp.)
Uqcr10 (2 sp.)		-0.53	9.74	9.21		-0.04		-0.46	9.63	9.18	ENSMUSG00000059534 ubiquinol-cytochrome c reductase, complex III subunit X	
Uqcr11		-0.13	11.1	11		-0.13		-0.24	11.1	10.8	ENSMUSG00000020163 ubiquinol-cytochrome c reductase, complex III subunit XI	
Uqcrb (2 sp.)		0.001	12	12		-0.05		0.04	11.9	11.9	ENSMUSG00000021520 ubiquinol-cytochrome c reductase binding protein	
Uqcrc1		0.11	10.8	10.9		0.06		0.11	10.9	11	ENSMUSG00000025651 ubiquinol-cytochrome c reductase core protein 1	
Uqcrc2 (2 sp.)		-0.04	10.6	10.5		0.05		0.08	10.5	10.6	ENSMUSG00000030884 ubiquinol cytochrome c reductase core protein 2	
Uqcrfs1		-0.01	11.4	11.4		-0.07		-0.1	11.4	11.3	ENSMUSG00000038462 ubiquinol-cytochrome c reductase, Rieske iron-sulfur polypeptide 1	
Uqcrh (2 sp.)		0.06	11.1	11.2		0.003		0.11	11.1	11.2	ENSMUSG00000063882 ubiquinol-cytochrome c reductase hinge protein	
Uqcrq (2 sp.)		-0.01	8.07	8.06		-0.03		-0.15	8.18	8.03	ENSMUSG00000044894 ubiquinol-cytochrome c reductase, complex III subunit VII	
r2523	0.06	-0.11	5.07	4.96	0.21	-0.14	0.24	-0.12	4.94	4.82	4.12	Glycine(s) ⇌ Glycine(c). Major Facilitator(MFS) TCDB:2.A.1.44.1
Slc43a1											ENSMUSG00000027075 solute carrier family 43, member 1	
C0047	0.11	-0.03	10.4	10.3	0.24	-0.2	0.43	-0.28	10.4	10.1	5.65	several reactions. (S)-Malate hydro-lyase Citrate cycle (TCA cycle) EC:4.2.1.2
Fh1											ENSMUSG00000026526 fumarate hydratase 1	
r0030	0.07	-0.09	6.05	5.96	0.11	0.06	0.17	-0.05	6.06	6.02	2.4	ATP(c) + AMP(c) ⇌ 2 ADP(c). ATP:AMP phosphotransferase Purine metabolism EC:2.7.4.3 (5 genes, 11 sp.)
Ak1 (2 sp.)		-0.07	3.97	3.89		0.76		0.59	4.07	4.66	ENSMUSG00000026817 adenylate kinase 1	
Ak2 (2 sp.)		0.08	9.35	9.43		0.13		0.15	9.41	9.55	ENSMUSG00000028792 adenylate kinase 2	
Ak4 (4 sp.)		-0.36	6.41	6.05		-0.23		-0.45	6.27	5.82	ENSMUSG00000028527 adenylate kinase 4	
Ak5 (2 sp.)		-0.17	4.72	4.56		0.12		-0.12	4.79	4.68	ENSMUSG00000039058 adenylate kinase 5	
Ak7		0.74	4.79	5.53		-0.39		0.06	5.08	5.14	ENSMUSG00000041323 adenylate kinase 7	
r0058	0.47	0.23	10.9	11.1	0.38	-0.38	0.3	-0.18	10.9	10.7	4.35	Malate(c) + NADP+(c) ⇌ Pyruvate(c) + CO2(c) + NADPH(c). (S)-Malate:NADP+ oxidoreductase(oxaloacetate-decarboxylating) Pyruvate metabolism EC:1.1.1.40
Me1											ENSMUSG00000032418 malic enzyme 1, NADP(+)-dependent, cytosolic	
r0007	0.32	0.15	10.1	10.2	0.18	-0.09	0.09	0.07	10.1	10.1	2.17	PPi(c) + H2O(c) → 2 Pi(c). Pyrophosphate phosphohydrolase EC:3.6.1.1 (2 genes, 2 sp.)
Ppa1		-0.26	11.1	10.8		0.18		-0.14	11.1	11	ENSMUSG00000020089 pyrophosphatase (inorganic) 1	
Ppa2		0.56	9.07	9.63		-0.35		0.28	9	9.28	ENSMUSG00000028013 pyrophosphatase (inorganic) 2	

16 Reaction scores for Glycogen glucose release (72), series control 1/24h, TGFβ/C 24h, and TGFβ 1/24h

The amplitudes of the mode are -3.18, -0.25 and -4.01.

Simulation	Definition			Solution													
				exchanges		reactions								transp			Prot
	Objective	Constraints	Comment	imports	exports	c	m	r	p	l	n	s	b	s-c	b-c	intra	synth
72 Glycogen glucose release	Glucose	−Glycogenin-G4G7(c) +Glycogenin(c) −Glucose −O_2 +CO_2 =H_2O	test the release of glucose from a glycogen component	1 H_2O(s) 0.09 Glycogenin-G4G7(c)	1 Glucose(s) 0.09 Glycogenin(c)	5	-	1	-	-	-	-	-	2	-	3	-

Table 15: Reaction scores for Glycogen glucose release (72), series control 1/24h, TGFβ/C 24h, and TGFβ 1/24h

Rea ID	control 1/24h Score *0.45*	Expr Δ	Expr C1h	Expr C24h	TGFβ/C 24h Score *0.45*	Expr Δ	TGFβ 1/24h Score *0.5*	Expr Δ	Expr T1h	Expr T24h	Wght	Reaction
C0181	0.09	0.35	6.77	7.12	0.99	-0.23	0.12	0.09	6.8	6.89	67.5	several reactions. EC:2.4.1.1 (3 genes, 3 sp.)
Pygb		1.48	6.28	7.76		-0.08		1.27	6.4	7.68		ENSMUSG00000033059 brain glycogen phosphorylase
Pygl		-0.41	10.2	9.83		-0.71		-0.99	10.1	9.12		ENSMUSG00000021069 liver glycogen phosphorylase
Pygm		-0.03	3.8	3.77		0.09		-0.02	3.89	3.86		ENSMUSG00000032648 muscle glycogen phosphorylase
r2473	0.99	-2.94	10.3	7.31	0.0001	-0.79	0.97	-3.55	10.1	6.52	22.4	Glucose-6P(c) + Pi(r) → Glucose-6P(r) + Pi(c). Major Facilitator(MFS) TCDB:2.A.1.4.5
Slc37a4												ENSMUSG00000032114 solute carrier family 37 (glucose-6-phosphate transporter), member 4
C0155	0.77	-2.04	9.46	7.42	0	-2.7	0.96	-4.55	9.27	4.72	25.6	several reactions. Major Facilitator(MFS) TCDB:2.A.1.1.29
Slc2a2												ENSMUSG00000027690 solute carrier family 2 (facilitated glucose transporter), member 2
r0396	0.9	-3.92	7.73	3.8	0.02	0.1	0.99	-3.72	7.63	3.91	22.4	H2O(r) + Glucose-6P(r) → Glucose(r) + Pi(r). D-Glucose-6-phosphate phosphohydrolase EC:3.1.3.9
G6pc												ENSMUSG00000078650 glucose-6-phosphatase, catalytic
r0621	0.24	-0.48	9.17	8.69	0.002	0.2	0.17	-0.24	9.14	8.9	13.3	Glucose-1P(c) ⇌ Glucose-6P(c). alpha-D-Glucose 1-phosphate 1,6-phosphomutase Glycolysis / Gluconeogenesis / Starch and sucrose metabolism EC:5.4.2.2 EC:5.4.2.6 (2 genes, 2 sp.)
Pgm1		-0.37	7.9	7.52		0.79		0.34	7.97	8.32		ENSMUSG00000029171 phosphoglucomutase 1
Pgm2		-0.59	10.4	9.86		-0.38		-0.82	10.3	9.48		ENSMUSG00000025791 phosphoglucomutase 2
C0180	0.12	0.09	8.43	8.52	0.58	-0.38	0.18	-0.28	8.42	8.13	10	several reactions. amylo-1,6-glucosidase, 4-alpha-glucanotransferase EC:2.4.1.25 (1 genes, 3 sp.)
Agl (3 sp.)												ENSMUSG00000033400 amylo-1,6-glucosidase, 4-alpha-glucanotransferase

17 Reaction scores for (R)-3-Hydroxybutanoate (404), series control 1/24h, TGFβ/C 24h, and TGFβ 1/24h

The amplitudes of the mode are -0.98, -0.66 and 0.28.

Simulation	Definition			Solution													
				exchanges		reactions								transp			Prot
	Objective	Constraints	Comment	imports	exports	c	m	r	p	l	n	s	b	s-c	b-c	intra	synth
404 (R)-3-Hydroxybutanoate	(R)-3-Hydroxybutanoate(c)	MCES	de novo synthesis and export of (R)-3-Hydroxybutanoate from Palmitate, excreting CO_2	1 H_2O(s) 1 NADH-redox-potential(m) 1 Proton-gradient(m) 2 CoA-activated acetyl group(m)	1 (R)-3-Hydroxybutanoate(c)	-	6	-	-	-	-	-	-	1	-	3	-

Table 16: Reaction scores for (R)-3-Hydroxybutanoate (404), series control 1/24h, TGFβ/C 24h, and TGFβ 1/24h

	control 1/24h				TGFβ/C 24h		TGFβ 1/24h					
Rea ID	Score *0.52*	Expr Δ	Expr C1h	Expr C24h	Score *0.58*	Expr Δ	Score *0.5*	Expr Δ	Expr T1h	Expr T24h	Wght	Reaction
r0323	0.0002	1.07	5.3	6.37	0.97	-0.75	1	0.28	5.35	5.62	20	NADH(m) + Acetoacetate(m) ⇌ NAD+(m) + (R)-3-Hydroxybutanoate(m). (R)-3-Hydroxybutanoate:NAD+ oxidoreductase Synthesis and degradation of ketone bodies / Butanoate metabolism EC:1.1.1.30 (2 genes, 3 sp.)
Bdh1 (2 sp.)		1.03	5.25	6.28		-0.84		0.17	5.27	5.44	ENSMUSG00000046598 3-hydroxybutyrate dehydrogenase, type 1	
Bdh2		1.15	5.41	6.56		-0.56		0.5	5.49	5.99	ENSMUSG00000028167 3-hydroxybutyrate dehydrogenase, type 2	
r0322	1	-1.01	10.7	9.71	0.78	-0.43	0	-1.34	10.6	9.28	15.6	HMG-CoA(m) ⇌ Acetyl-CoA(m) + Acetoacetate(m). (S)-3-Hydroxy-3-methylglutaryl-CoA acetoacetate-lyase Synthesis and degradation of ketone bodies / Valine, leucine and isoleucine degradation / Butanoate metabolism EC:4.1.3.4
Hmgcl											ENSMUSG00000028672 3-hydroxy-3-methylglutaryl-Coenzyme A lyase	
r0462	1	-0.95	8.57	7.62	0	2.02	0	0.99	8.65	9.64	11.8	Acetyl-CoA(m) + H2O(m) + Acetoacetyl-CoA(m) ⇌ HMG-CoA(m) + CoA(m). (S)-3-Hydroxy-3-methylglutaryl-CoA acetoacetyl-CoA-lyase (CoA-acetylating) Synthesis and degradation of ketone bodies / Valine, leucine and isoleucine degradation / Butanoate metabolism EC:2.3.3.10 (2 sp.)
Hmgcs2 (2 sp.)											ENSMUSG00000027875 3-hydroxy-3-methylglutaryl-Coenzyme A synthase 2	
r0070	0.15	-0.03	8.79	8.76	0.01	0.37	1	0.28	8.85	9.13	7.46	2 Acetyl-CoA(m) ⇌ CoA(m) + Acetoacetyl-CoA(m). Acetyl-CoA:acetyl-CoA C-acetyltransferase Synthesis and degradation of ketone bodies EC:2.3.1.9 (2 genes, 5 sp.)
Acat1 (3 sp.)		0.19	7.92	8.1		0.54		0.67	7.97	8.64	ENSMUSG00000032047 acetyl-Coenzyme A acetyltransferase 1	
Acat2 (2 sp.)		-0.35	10.1	9.75		0.11		-0.3	10.2	9.87	ENSMUSG00000023832 acetyl-Coenzyme A acetyltransferase 2	

18 Reaction scores for Glutamine (486), series control 1/24h, TGFβ/C 24h, and TGFβ 1/24h

The amplitudes of the mode are -0.36, -0.88 and -0.66.

Simulation	Definition			Solution													
				exchanges		reactions								transp			Prot
	Objective	Constraints	Comment	imports	exports	c	m	r	p	l	n	s	b	s-c	b-c	intra	synth
486 Glutamine	Glutamine(c)	$-NH_3$(c) MCES	de novo synthesis of cytosolic Glutamine	2 NH_3(c) 1 Pyruvate(m) 1 ATP-energy(c) 1 NADPH-redox-potential(m) 1 Proton-gradient(m) 1 CoA-activated acetyl group(m)	1 H_2O(s) 1 Glutamine(c) 1 NADH-redox-potential(m)	2	10	-	-	-	-	-	-	1	-	4	-

Table 17: Reaction scores for Glutamine (486), series control 1/24h, TGFβ/C 24h, and TGFβ 1/24h

Rea	control 1/24h				TGFβ/C 24h		TGFβ 1/24h					
Rea ID	Score *0.45*	Expr Δ	Expr C1h	Expr C24h	Score *0.51*	Expr Δ	Score *0.73*	Expr Δ	Expr T1h	Expr T24h	Wght	Reaction
C0078	0.01	0.17	9.03	9.2	0.95	-1.02	0.84	-0.86	9.04	8.18	11.1	several reactions. Oxalosuccinate:NADP+ oxidoreductase (decarboxylating) Citrate cycle (TCA cycle) EC:1.1.1.42
Idh2												ENSMUSG00000030541 isocitrate dehydrogenase 2 (NADP+), mitochondrial
r0077	0	0.66	8.34	9	0.01	0.47	0	1.2	8.28	9.48	6.27	ATP(c) + Glutamate(c) + NH3(c) → ADP(c) + Pi(c) + Glutamine(c). L-Glutamate:ammonia ligase (ADP-forming) Glutamate metabolism / Nitrogen metabolism EC:6.3.1.2 (2 sp.)
Glul (2 sp.)												ENSMUSG00000026473 glutamate-ammonia ligase (glutamine synthetase)
r0316	0.88	-0.46	10.4	9.98	0.23	-0.12	0.93	-0.53	10.4	9.86	7.59	Citrate(m) ⇌ Isocitrate(m). citrate hydro-lyase Citrate cycle (TCA cycle) EC:4.2.1.3 (2 sp.)
Aco2 (2 sp.)												ENSMUSG00000022477 aconitase 2, mitochondrial
r0105	1	-0.35	11.8	11.4	0.42	-0.3	1	-0.64	11.7	11.1	5.85	NAD+(m) + Malate(m) ⇌ OAA(m) + NADH(m). (S)-malate:NAD+ oxidoreductase Citrate cycle (TCA cycle) EC:1.1.1.37 (2 sp.)
Mdh2 (2 sp.)												ENSMUSG00000019179 malate dehydrogenase 2, NAD (mitochondrial)
r0073	0	0.49	10.9	11.4	1	-0.84	0.77	-0.43	11	10.6	5.05	AKG(m) + NADH(m) + NH3(m) ⇌ Glutamate(m) + NAD+(m) + H2O(m). L-Glutamate:NAD+ oxidoreductase (deaminating) Glutamate metabolism EC:1.4.1.3 EC:1.4.1.2 (3 sp.)
Glud1 (3 sp.)												ENSMUSG00000021794 glutamate dehydrogenase 1
r0107	0.82	-0.25	9.95	9.71	0.57	-0.41	0.96	-0.57	9.87	9.3	6.97	Acetyl-CoA(m) + H2O(m) + OAA(m) ⇌ Citrate(m) + CoA(m). Citrate oxaloacetate-lyase ((pro-3S)-CH2COO- -> acetyl-CoA) Citrate cycle (TCA cycle) EC:2.3.3.3 (3 sp.)
Cs (3 sp.)												ENSMUSG00000005683 citrate synthase
r0059	0.58	-0.17	4.52	4.35	0.17	-0.05	0.42	-0.23	4.53	4.3	5.69	Pyruvate(m) + CO2(m) + NADPH(m) ⇌ Malate(m) + NADP+(m). (S)-Malate:NADP+ oxidoreductase(oxaloacetate-decarboxylating) Pyruvate metabolism EC:1.1.1.40
Me3												ENSMUSG00000030621 malic enzyme 3, NADP(+)-dependent, mitochondrial

19 Reaction scores for Inositol (829), series control 1/24h, TGFβ/C 24h, and TGFβ 1/24h

The amplitudes of the mode are 1.72, -0.12 and 1.83.

Simulation	Definition			Solution														
				exchanges		reactions								transp			Prot
	Objective	Constraints	Comment	imports	exports	c	m	r	p	l	n	s	b	s-c	b-c	intra	synth
829 Inositol	Inositol(s)	PIPES MCES	synthesis of Inositol and blood export	1 H_2O(s) 1 Glucose-6P(c)	1 P_i(c) 1 Inositol(s)	2	-	-	-	-	-	-	-	2	-	-	-

Table 18: Reaction scores for Inositol (829), series control 1/24h, TGFβ/C 24h, and TGFβ 1/24h

Rea ID	control 1/24h Score *0.62*	Expr Δ	Expr C1h	Expr C24h	TGFβ/C 24h Score *0.5*	Expr Δ	TGFβ 1/24h Score *0.59*	Expr Δ	Expr T1h	Expr T24h	Wght	Reaction
r0200	1	1.77	7.23	9	0.0004	0.12	1	1.85	7.26	9.11	14.6	Glucose-6P(c) ⇌ Inositol-1P(c). 1L-myo-Inositol-1-phosphate lyase (isomerizing) Streptomycin biosynthesis / Inositol phosphate metabolism EC:5.5.1.4
Isyna1												ENSMUSG00000019139 myo-inositol 1-phosphate synthase A1
r0289	0.23	0.25	6.93	7.18	1	-0.12	0.18	0.12	6.94	7.06	14.6	Inositol-1P(c) + H2O(c) → Inositol(c) + Pi(c). myo-Inositol 1-phosphate phosphahydrolase Streptomycin biosynthesis / Inositol phosphate metabolism / Phosphatidylinositol signaling system EC:3.1.3.25 (2 genes, 4 sp.)
Impa1 (2 sp.)		0.14	8.94	9.08		0.04		0.25	8.87	9.12		ENSMUSG00000027531 inositol (myo)-1(or 4)-monophosphatase 1
Impa2 (2 sp.)		0.37	4.91	5.28		-0.28		-0.001	5	5		ENSMUSG00000024525 inositol (myo)-1(or 4)-monophosphatase 2

20 Reaction scores for PI (517), series control 1/24h, TGFβ/C 24h, and TGFβ 1/24h

The amplitudes of the mode are -0.23, -0.14 and -1.12.

Simulation	Definition			Solution													
				exchanges		reactions								transp			Prot
	Objective	Constraints	Comment	imports	exports	c	m	r	p	l	n	s	b	s-c	b-c	intra	synth
517 PI	PI-pool(c)	MCES ALFA	de novo synthesis of cytosolic PI-pool	1.5 Glucose-6P(c) 0.51 Arachidonate(c) 0.16 Palmitate(c) 0.32 Oleate(c) 0.85 Stearate(c) 0.16 Linoleate(c) 6.5 ATP-energy(c) 1 NADH-redox-potential(c)	2.5 H_2O(s) 0.5 P_i(c) 1 PI-pool(c)	28	-	-	-	-	-	-	-	1	-	-	-

Table 19: Reaction scores for PI (517), series control 1/24h, TGFβ/C 24h, and TGFβ 1/24h

Rea ID	control 1/24h Score *0.29*	Expr Δ	Expr C1h	Expr C24h	TGFβ/C 24h Score *0.47*	Expr Δ	TGFβ 1/24h Score *0.34*	Expr Δ	Expr T1h	Expr T24h	Wght	Reaction
r0200	0	1.77	7.23	9	0.001	0.12	0	1.85	7.26	9.11	14.7	Glucose-6P(c) ⇌ Inositol-1P(c). 1L-myo-Inositol-1-phosphate lyase (isomerizing) Streptomycin biosynthesis / Inositol phosphate metabolism EC:5.5.1.4
Isyna1												ENSMUSG00000019139 myo-inositol 1-phosphate synthase A1
r0289	0.0002	0.25	6.93	7.18	0.96	-0.12	0.09	0.12	6.94	7.06	14.7	Inositol-1P(c) + H2O(c) → Inositol(c) + Pi(c). myo-Inositol 1-phosphate phosphahydrolase Streptomycin biosynthesis / Inositol phosphate metabolism / Phosphatidylinositol signaling system EC:3.1.3.25 (2 genes, 4 sp.)
Impa1 (2 sp.)		0.14	8.94	9.08		0.04		0.25	8.87	9.12		ENSMUSG00000027531 inositol (myo)-1(or 4)-monophosphatase 1
Impa2 (2 sp.)		0.37	4.91	5.28		-0.28		-0.001	5	5		ENSMUSG00000024525 inositol (myo)-1(or 4)-monophosphatase 2
r0201	0.96	-0.2	8.56	8.36	0	-0.98	0.89	-1.4	8.78	7.38	8.89	DHAP(c) + NADH(c) ⇌ NAD+(c) + sn-Glycerol-3P(c). sn-Glycerol-3-phosphate:NAD+ 2-oxidoreductase Glycerophospholipid metabolism EC:1.1.1.8 (2 sp.)
Gpd1 (2 sp.)												ENSMUSG00000023019 glycerol-3-phosphate dehydrogenase 1 (soluble)
C0174	0.99	-0.25	6.94	6.69	0.98	-0.16	0.47	-0.43	6.97	6.54	9.73	several reactions. EC:6.2.1.3 (8 genes, 14 sp.)
Acsbg1		-0.07	4.21	4.13		1.05		0.85	4.34	5.19		ENSMUSG00000032281 acyl-CoA synthetase bubblegum family member 1
Acsbg2		-0.05	3.84	3.79		-0.15		-0.14	3.78	3.64		ENSMUSG00000024207 acyl-CoA synthetase bubblegum family member 2
Acsl1 (3 sp.)		-0.85	8.72	7.87		-0.25		-1.03	8.64	7.62		ENSMUSG00000018796 acyl-CoA synthetase long-chain family member 1
Acsl3 (3 sp.)		-0.29	4.68	4.39		0.6		0.12	4.88	4.99		ENSMUSG00000032883 acyl-CoA synthetase long-chain family member 3
Acsl4 (3 sp.)		0.42	8.12	8.54		-0.91		-0.44	8.07	7.63		ENSMUSG00000031278 acyl-CoA synthetase long-chain family member 4
Acsl5		0.12	11.4	11.5		-0.33		-0.24	11.4	11.2		ENSMUSG00000024981 acyl-CoA synthetase long-chain family member 5
Acsl6		-0.21	3	2.79		0.2		-0.12	3.12	2.99		ENSMUSG00000020333 acyl-CoA synthetase long-chain family member 6
Slc27a2		-1.05	10.1	9.09		-1.3		-2.36	10.2	7.79		ENSMUSG00000027359 solute carrier family 27 (fatty acid transporter), member 2
r0136	0.86	-0.17	7.52	7.35	0.96	-0.12	0.3	-0.25	7.48	7.23	9.48	ATP(c) + CMP(c) ⇌ ADP(c) + CDP(c). ATP:CMP phosphotransferase Pyrimidine metabolism EC:2.7.4.14 (2 sp.)
Cmpk1 (2 sp.)												ENSMUSG00000028719 cytidine monophosphate (UMP-CMP) kinase 1
C0167	0.27	-0.42	8.46	8.03	0.05	-0.32	0.96	-0.96	8.67	7.71	9.09	several reactions. EC:2.3.1.15 (2 genes, 2 sp.)
Agpat6		0.25	8.61	8.87		-0.31		-0.07	8.63	8.56		ENSMUSG00000031545 1-acylglycerol-3-phosphate O-acyltransferase 6 (lysophosphatidic acid acyltransferase, zeta)
Agpat9		-1.1	8.3	7.19		-0.32		-1.85	8.72	6.87		ENSMUSG00000029314 1-acylglycerol-3-phosphate O-acyltransferase 9
r1197	0.0003	0.24	10.5	10.7	0.46	-0.05	0.06	0.19	10.5	10.6	15.6	CDP-diacylglycerol-VLDL-PI-pool(c) + Inositol(c) ⇌ PI-pool(c) + CMP(c). CDPdiacylglycerol:myo-inositol 3-phosphatidyltransferase Glycerophospholipid metabolism / Phosphatidylinositol signaling system EC:2.7.8.11 (2 sp.)
Cdipt (2 sp.)												ENSMUSG00000030682 CDP-diacylglycerol–inositol 3-phosphatidyltransferase (phosphatidylinositol synthase)
r1238	0.28	-0.05	7.84	7.8	0.97	-0.16	0.24	-0.17	7.81	7.64	14.3	Phosphatidate-VLDL-PI-pool(c) + CTP(c) ⇌ CDP-diacylglycerol-VLDL-PI-pool(c) + PPi(c). CTP:phosphatidate cytidyltransferase Glycerophospholipid metabolism / Phosphatidylinositol signaling system EC:2.7.7.41 (2 genes, 8 sp.)
Cds1 (2 sp.)		-0.07	4.51	4.44		0.09		0.15	4.38	4.53		ENSMUSG00000029330 CDP-diacylglycerol synthase 1
Cds2 (6 sp.)		-0.04	8.95	8.91		-0.24		-0.28	8.95	8.67		ENSMUSG00000058793 CDP-diacylglycerol synthase (phosphatidate cytidylyltransferase) 2
r1215	0.15	-0.01	6.36	6.35	0.21	-0.02	0.17	-0.06	6.39	6.33	9.87	1-Acylglycerol-3P-VLDL-PI-pool(c) + Acyl-CoA-VLDL-PI-pool(c) → Phosphatidate-VLDL-PI-pool(c) + CoA(c). Glycerolipid metabolism / Glycerophospholipid metabolism EC:2.3.1.51 (6 genes, 11 sp.)
Agpat1 (2 sp.)		-0.16	5.77	5.6		0.22		0.04	5.78	5.83		ENSMUSG00000034254 1-acylglycerol-3-phosphate O-acyltransferase 1 (lysophosphatidic acid acyltransferase, alpha)
Agpat2		0.15	9.73	9.88		0.02		0.01	9.89	9.9		ENSMUSG00000026922 1-acylglycerol-3-phosphate O-acyltransferase 2 (lysophosphatidic acid acyltransferase, beta)
Agpat3 (4 sp.)		-0.33	7.84	7.5		0.2		-0.11	7.81	7.71		ENSMUSG00000001211 1-acylglycerol-3-phosphate O-acyltransferase 3
Agpat4 (2 sp.)		0.03	4.47	4.5		-0.03		-0.04	4.5	4.47		ENSMUSG00000023827 1-acylglycerol-3-phosphate O-acyltransferase 4 (lysophosphatidic acid acyltransferase, delta)
Mboat1		1.52	4.36	5.89		-1.51		-0.12	4.5	4.38		ENSMUSG00000038732 membrane bound O-acyltransferase domain containing 1
Mboat2		-0.16	4	3.84		0.11		-0.17	4.13	3.95		ENSMUSG00000020646 membrane bound O-acyltransferase domain containing 2
r0150	0	0.42	8.17	8.59	0.07	-0.31	0.11	0.05	8.23	8.28	4.6	ATP(c) + CDP(c) ⇌ ADP(c) + CTP(c). ATP:nucleoside-diphosphate phosphatransferase Pyrimidine metabolism EC:2.7.4.6 (7 genes, 7 sp.)
Nme1		0.44	11.4	11.8		-0.3		0.11	11.4	11.5		ENSMUSG00000037601 non-metastatic cells 1, protein (NM23A) expressed in
Nme2		0.09	13	13.1		0.04		0.14	13	13.2		ENSMUSG00000020857 non-metastatic cells 2, protein (NM23B) expressed in
Nme3		0.86	6.83	7.69		-1.4		-0.66	6.96	6.29		ENSMUSG00000073435 non-metastatic cells 3, protein expressed in
Nme4		0.95	6.48	7.42		-0.82		-0.06	6.67	6.6		ENSMUSG00000024177 non-metastatic cells 4, protein expressed in
Nme5		0.01	4.78	4.79		0.08		-0.02	4.88	4.87		ENSMUSG00000035984 non-metastatic cells 5, protein expressed in (nucleoside-diphosphate kinase)
Nme6		-0.01	7.51	7.5		0.11		0.03	7.58	7.61		ENSMUSG00000032478 non-metastatic cells 6, protein expressed in (nucleoside-diphosphate kinase)
Nme7		0.57	7.21	7.78		0.14		0.85	7.08	7.92		ENSMUSG00000026575 non-metastatic cells 7, protein expressed in (nucleoside-diphosphate kinase)
r0736	0	-0.78	9.44	8.65	0.0004	-0.42	0.99	-1.05	9.28	8.23	2.97	ATP(c) + Fructose-6P(c) → ADP(c) + Fructose-1,6PP(c). ATP:D-fructose-6-phosphate 1-phosphotransferase EC:2.7.1.11 (2 sp.)
Pfkl (2 sp.)												ENSMUSG00000020277 phosphofructokinase, liver, B-type
r0256	0	-0.78	11.4	10.7	0	-0.5	0.99	-1.18	11.3	10.2	2.89	Fructose-1,6PP(c) ⇌ DHAP(c) + GAP(c). D-Fructose-1,6-bisphosphate D-glyceraldehyde-3-phosphate-lyase Carbon fixation EC:4.1.2.13 (2 genes, 4 sp.)
Aldoa (3 sp.)		-0.32	11	10.6		-0.12		-0.36	10.9	10.5		ENSMUSG00000030695 aldolase A, fructose-bisphosphate
Aldob		-2.13	12.9	10.7		-1.64		-3.66	12.7	9.09		ENSMUSG00000028307 aldolase B, fructose-bisphosphate
r0192	0	-0.79	11.7	10.9	0	-0.46	1	-1.09	11.5	10.4	2.76	Glucose-6P(c) ⇌ Fructose-6P(c). D-Glucose-6-phosphate ketol-isomerase EC:5.3.1.9 (3 sp.)
Gpi1 (3 sp.)												ENSMUSG00000036427 glucose phosphate isomerase 1
r0243	0.003	-0.64	12.2	11.6	0	-0.84	0.89	-1.4	12.1	10.7	2.89	GAP(c) ⇌ DHAP(c). D-Glyceraldehyde-3-phosphate ketol-isomerase Glycolysis / Gluconeogenesis EC:5.3.1.1 (3 sp.)
Tpi1 (3 sp.)												ENSMUSG00000023456 triosephosphate isomerase 1
r0312	0.97	-0.21	7.18	6.97	0.95	-0.16	0.44	-0.4	7.21	6.81	2.99	Palmitate(c) + ATP(c) + CoA(c) → Palmitoyl-CoA(c) + AMP(c) + PPi(c). Palmitate:CoA ligase (AMP-forming) Fatty acid metabolism EC:6.2.1.3 (5 genes, 11 sp.)
Acsl1 (3 sp.)		-0.85	8.72	7.87		-0.25		-1.03	8.64	7.62		ENSMUSG00000018796 acyl-CoA synthetase long-chain family member 1
Acsl3 (3 sp.)		-0.29	4.68	4.39		0.6		0.12	4.88	4.99		ENSMUSG00000032883 acyl-CoA synthetase long-chain family member 3
Acsl4 (3 sp.)		0.42	8.12	8.54		-0.91		-0.44	8.07	7.63		ENSMUSG00000031278 acyl-CoA synthetase long-chain family member 4
Acsl5		0.12	11.4	11.5		-0.33		-0.24	11.4	11.2		ENSMUSG00000024981 acyl-CoA synthetase long-chain family member 5
Acsl6		-0.21	3	2.79		0.2		-0.12	3.12	2.99		ENSMUSG00000020333 acyl-CoA synthetase long-chain family member 6

Continued on next page

Rea ID	control 1/24h Score *0.29*	Expr Δ	Expr C1h	Expr C24h	TGFβ/C 24h Score *0.47*	Expr Δ	TGFβ 1/24h Score *0.34*	Expr Δ	Expr T1h	Expr T24h	Wght	Reaction
r0007	0.01	0.15	10.1	10.2	0.74	-0.09	0.1	0.07	10.1	10.1	4.22	PPi(c) + H2O(c) → 2 Pi(c). Pyrophosphate phosphohydrolase EC:3.6.1.1 (2 genes, 2 sp.)
Ppa1		-0.26	11.1	10.8		0.18		-0.14	11.1	11		ENSMUSG00000020089 pyrophosphatase (inorganic) 1
Ppa2		0.56	9.07	9.63		-0.35		0.28	9	9.28		ENSMUSG00000028013 pyrophosphatase (inorganic) 2
r0030	0.48	-0.09	6.05	5.96	0.02	0.06	0.16	-0.05	6.06	6.02	3.81	ATP(c) + AMP(c) ⇌ 2 ADP(c). ATP:AMP phosphotransferase Purine metabolism EC:2.7.4.3 (5 genes, 11 sp.)
Ak1 (2 sp.)		-0.07	3.97	3.89		0.76		0.59	4.07	4.66		ENSMUSG00000026817 adenylate kinase 1
Ak2 (2 sp.)		0.08	9.35	9.43		0.13		0.15	9.41	9.55		ENSMUSG00000028792 adenylate kinase 2
Ak4 (4 sp.)		-0.36	6.41	6.05		-0.23		-0.45	6.27	5.82		ENSMUSG00000028527 adenylate kinase 4
Ak5 (2 sp.)		-0.17	4.72	4.56		0.12		-0.12	4.79	4.68		ENSMUSG00000039058 adenylate kinase 5
Ak7		0.74	4.79	5.53		-0.39		0.06	5.08	5.14		ENSMUSG00000041323 adenylate kinase 7

21 Reaction scores for Sphinganine-1P (931), series control 1/24h, TGFβ/C 24h, and TGFβ 1/24h

The amplitudes of the mode are 0.18, 1.29 and 1.21.

Simulation	Definition			Solution														
				exchanges		reactions								transp			Prot
	Objective	Constraints	Comment	imports	exports	c	m	r	p	l	n	s	b	s-c	b-c	intra	synth
931 Sphinganine-1P	Sphinganine-1P(c)	PIPES MCES	synthesis of cytosolic Sphinganine-1P	1 P_i(c) 1 Serine(s) 1 Palmitate(s) 3 ATP-energy(c) 1 NADPH-redox-potential(c)	2 H_2O(s) 1 CO_2(s) 1 Sphinganine-1P(c) 1 Proton-gradient(c) 1 Na-gradient(c)	8	-	-	-	-	-	-	-	6	-	-	-

Table 20: Reaction scores for Sphinganine-1P (931), series control 1/24h, TGFβ/C 24h, and TGFβ 1/24h

	control 1/24h				TGFβ/C 24h		TGFβ 1/24h					
Rea ID	Score *0.28*	Expr Δ	Expr C1h	Expr C24h	Score *0.36*	Expr Δ	Score *0.38*	Expr Δ	Expr T1h	Expr T24h	Wght	Reaction
r0582	0.14	0.001	5.51	5.51	1	1.35	0.98	1.32	5.54	6.86	14.6	ATP(c) + Sphinganine(c) → ADP(c) + Sphinganine-1P(c). ATP:sphinganine 1-phosphotransferase Sphingolipid metabolism EC:2.7.1.91 (2 genes, 3 sp.)
Sphk1		0.08	4.83	4.91		4.15		4.03	5.02	9.05		ENSMUSG00000061878 sphingosine kinase 1
Sphk2 (2 sp.)		-0.04	5.85	5.81		-0.05		-0.04	5.8	5.76		ENSMUSG00000057342 sphingosine kinase 2
r0583	1	0.19	6.65	6.84	0.31	0.31	0.48	0.47	6.67	7.14	11.9	3-Dehydrosphinganine(c) + NADPH(c) ⇌ Sphinganine(c) + NADP+(c). Sphinganine:NADP+ 3-oxidoreductase Sphingolipid metabolism EC:1.1.1.102 (2 sp.)
Kdsr (2 sp.)												ENSMUSG00000009905 3-ketodihydrosphingosine reductase
r0312	0.0001	-0.21	7.18	6.97	0.08	-0.16	0.03	-0.4	7.21	6.81	7.4	Palmitate(c) + ATP(c) + CoA(c) → Palmitoyl-CoA(c) + AMP(c) + PPi(c). Palmitate:CoA ligase (AMP-forming) Fatty acid metabolism EC:6.2.1.3 (5 genes, 11 sp.)
Acsl1 (3 sp.)		-0.85	8.72	7.87		-0.25		-1.03	8.64	7.62		ENSMUSG00000018796 acyl-CoA synthetase long-chain family member 1
Acsl3 (3 sp.)		-0.29	4.68	4.39		0.6		0.12	4.88	4.99		ENSMUSG00000032883 acyl-CoA synthetase long-chain family member 3
Acsl4 (3 sp.)		0.42	8.12	8.54		-0.91		-0.44	8.07	7.63		ENSMUSG00000031278 acyl-CoA synthetase long-chain family member 4
Acsl5		0.12	11.4	11.5		-0.33		-0.24	11.4	11.2		ENSMUSG00000024981 acyl-CoA synthetase long-chain family member 5
Acsl6		-0.21	3	2.79		0.2		-0.12	3.12	2.99		ENSMUSG00000020333 acyl-CoA synthetase long-chain family member 6
r0935	0	-2.25	11.7	9.47	0.04	-0.34	0	-2.48	11.6	9.13	6.7	Palmitate(s) → Palmitate(c). solute carrier family 27 (fatty acid transporter), member 5 TCDB:4.C.1.1.5 TCDB:4.C.1.1.8
Slc27a5												ENSMUSG00000030382 solute carrier family 27 (fatty acid transporter), member 5
r0313	0.12	-0.004	6.27	6.27	0.18	0.09	0.17	0.08	6.29	6.36	11.8	Serine(c) + Palmitoyl-CoA(c) ⇌ 3-Dehydrosphinganine(c) + CoA(c) + CO2(c). Palmitoyl-CoA:L-serine C-palmitoyltransferase (decarboxylating) Sphingolipid metabolism EC:2.3.1.50 (3 genes, 6 sp.)
Sptlc1 (3 sp.)		-0.05	6.75	6.7		0.1		0.08	6.72	6.8		ENSMUSG00000021468 serine palmitoyltransferase, long chain base subunit 1
Sptlc2		0.42	9.4	9.82		0.02		0.35	9.5	9.85		ENSMUSG00000021036 serine palmitoyltransferase, long chain base subunit 2
Sptlc3 (2 sp.)		-0.15	3.99	3.84		0.12		-0.07	4.03	3.96		ENSMUSG00000039092 serine palmitoyltransferase, long chain base subunit 3
r2478	0.01	-0.09	3.38	3.3	0.15	0.03	0.16	0.06	3.26	3.32	5.83	Serine(s) + Na+(s) + H+(PG)(c) → Serine(c) + Na+(c) + H+(PG)(s). Major Facilitator(MFS) TCDB:2.A.18.6.8
Slc38a5												ENSMUSG00000031170 solute carrier family 38, member 5
r0007	0.92	0.15	10.1	10.2	0.1	-0.09	0.17	0.07	10.1	10.1	2.43	PPi(c) + H2O(c) → 2 Pi(c). Pyrophosphate phosphohydrolase EC:3.6.1.1 (2 genes, 2 sp.)
Ppa1		-0.26	11.1	10.8		0.18		-0.14	11.1	11		ENSMUSG00000020089 pyrophosphatase (inorganic) 1
Ppa2		0.56	9.07	9.63		-0.35		0.28	9	9.28		ENSMUSG00000028013 pyrophosphatase (inorganic) 2
r0030	0.01	-0.09	6.05	5.96	0.16	0.06	0.12	-0.05	6.06	6.02	2.68	ATP(c) + AMP(c) ⇌ 2 ADP(c). ATP:AMP phosphotransferase Purine metabolism EC:2.7.4.3 (5 genes, 11 sp.)
Ak1 (2 sp.)		-0.07	3.97	3.89		0.76		0.59	4.07	4.66		ENSMUSG00000026817 adenylate kinase 1
Ak2 (2 sp.)		0.08	9.35	9.43		0.13		0.15	9.41	9.55		ENSMUSG00000028792 adenylate kinase 2
Ak4 (4 sp.)		-0.36	6.41	6.05		-0.23		-0.45	6.27	5.82		ENSMUSG00000028527 adenylate kinase 4
Ak5 (2 sp.)		-0.17	4.72	4.56		0.12		-0.12	4.79	4.68		ENSMUSG00000039058 adenylate kinase 5
Ak7		0.74	4.79	5.53		-0.39		0.06	5.08	5.14		ENSMUSG00000041323 adenylate kinase 7

22 Reaction scores for 4-Hydroxyphenylpyruvate (658), series control 1/24h, TGFβ/C 24h, and TGFβ 1/24h

The amplitudes of the mode are -3.2, -1.87 and -4.36.

Simulation	Definition			Solution														
				exchanges		reactions								transp			Prot
	Objective	Constraints	Comment	imports	exports	c	m	r	p	l	n	s	b	s-c	b-c	intra	synth
658 4-Hydroxyphenylpyruvate	4-Hydroxyphenylpyruvate(c)	PIPES MCES	synthesis of cytosolic 4-Hydroxyphenylpyruvate	1 Pyruvate(c) 1 Tyrosine(s)	1 Alanine(s) 1 4-Hydroxyphenylpyruvate(c) 1 Na-gradient(c)	2	-	-	-	-	-	-	-	3	-	-	-

Table 21: Reaction scores for 4-Hydroxyphenylpyruvate (658), series control 1/24h, TGFβ/C 24h, and TGFβ 1/24h

Rea ID	control 1/24h Score *0.53*	Expr Δ	Expr C1h	Expr C24h	TGFβ/C 24h Score *0.73*	Expr Δ	TGFβ 1/24h Score *0.63*	Expr Δ	Expr T1h	Expr T24h	Wght	Reaction
r0183	1	-3.33	10.5	7.21	1	-1.96	0.97	-4.86	10.1	5.26	11	Tyrosine(c) + AKG(c) ⇌ 4-Hydroxyphenylpyruvate(c) + Glutamate(c). L-Tyrosine:2-oxoglutarate aminotransferase Tyrosine metabolism / Phenylalanine, tyrosine and tryptophan biosynthesis EC:2.6.1.5 EC:2.6.1.57
Tat												ENSMUSG00000001670 tyrosine aminotransferase
r1125	0.17	-0.16	9.26	9.1	0.06	0.34	0.09	0.38	9.06	9.44	4.94	Tyrosine(s) + Na+(s) → Tyrosine(c) + Na+(c). Major Facilitator(MFS) TCDB:2.A.18.6.5 (2 sp.)
Slc38a2 (2 sp.)												ENSMUSG00000022462 solute carrier family 38, member 2
r0080	0.29	-0.68	9.96	9.28	0.99	-1.73	0.69	-2.47	10	7.55	8.42	Pyruvate(c) + Glutamate(c) ⇌ Alanine(c) + AKG(c). L-Alanine:2-oxoglutarate aminotransferase Glutamate metabolism / Alanine and aspartate metabolism EC:2.6.1.2
Gpt												ENSMUSG00000022546 glutamic pyruvic transaminase, soluble
r2524	0.15	-0.11	5.07	4.96	0.18	-0.14	0.15	-0.12	4.94	4.82	3.26	Alanine(c) ⇌ Alanine(s). Major Facilitator(MFS) TCDB:2.A.1.44.1
Slc43a1												ENSMUSG00000027075 solute carrier family 43, member 1

23 Reaction scores for Saccharopine (925), series control 1/24h, TGFβ/C 24h, and TGFβ 1/24h

The amplitudes of the mode are -1.92, -1.92 and -3.66.

Simulation	Definition			Solution													
				exchanges		reactions								transp			Prot
	Objective	Constraints	Comment	imports	exports	c	m	r	p	l	n	s	b	s-c	b-c	intra	synth
925 Saccharopine	Saccharopine(m)	PIPES MCES	synthesis of mitochondrial Saccharopine	1 Pyruvate(c) 1 Glutamate(s) 1 Lysine(s) 1 NADPH-redox-potential(m) 1 Proton-gradient(m)	1 H_2O(s) 1 Alanine(s) 1 Saccharopine(m) 1 Na-gradient(c)	1	2	-	-	-	-	-	-	4	-	5	-

Table 22: Reaction scores for Saccharopine (925), series control 1/24h, TGFβ/C 24h, and TGFβ 1/24h

Rea ID	control 1/24h Score *0.48*	Expr Δ	Expr C1h	Expr C24h	TGFβ/C 24h Score *0.63*	Expr Δ	TGFβ 1/24h Score *0.57*	Expr Δ	Expr T1h	Expr T24h	Wght	Reaction
r0180	0.99	-2.03	8.97	6.94	0.99	-2.07	0.97	-4.08	8.95	4.87	12.9	AKG(m) + NADPH(m) + Lysine(m) ⇌ H2O(m) + Saccharopine(m) + NADP+(m). N6-(L-1,3-Dicarboxypropyl)-L-lysine:NADP+ oxidoreductase (L-lysine-forming) Lysine biosynthesis / Lysine degradation EC:1.5.1.8
Aass												ENSMUSG00000029695 aminoadipate-semialdehyde synthase
r1993	0.16	-0.09	3.61	3.52	0.13	0.03	0.17	-0.2	3.75	3.55	9.98	Lysine(s) + Alanine(c) → Lysine(c) + Alanine(s). Amino Acid-Polyamine-Organocation (APC) TCDB:2.A.3.8.15
Slc7a9												ENSMUSG00000030492 solute carrier family 7 (cationic amino acid transporter, y+ system), member 9
r2419	0.04	0.5	7.47	7.97	0.32	-0.46	0.14	-0.04	7.54	7.51	5.99	AKG(c) + Pi(m) ⇌ AKG(m) + Pi(c). Mitochondrial Carrier (MC) TCDB:2.A.29.2.7 (2 sp.)
Slc25a10 (2 sp.)												ENSMUSG00000025792 solute carrier family 25 (mitochondrial carrier, dicarboxylate transporter), member 10
r0080	0.43	-0.68	9.96	9.28	0.98	-1.73	0.81	-2.47	10	7.55	8.42	Pyruvate(c) + Glutamate(c) ⇌ Alanine(c) + AKG(c). L-Alanine:2-oxoglutarate aminotransferase Glutamate metabolism / Alanine and aspartate metabolism EC:2.6.1.2
Gpt												ENSMUSG00000022546 glutamic pyruvic transaminase, soluble
r1047	0.13	0.004	12.3	12.3	0.12	0.06	0.13	0.06	12.3	12.3	1.43	Pi(c) + H+(PG)(c) → Pi(m) + H+(PG)(m). Mitochondrial Carrier (MC) TCDB:2.A.29.4.1
Slc25a3												ENSMUSG00000061904 solute carrier family 25 (mitochondrial carrier, phosphate carrier), member 3

24 Reaction scores for Tyrosine (489), series control 1/24h, TGFβ/C 24h, and TGFβ 1/24h

The amplitudes of the mode are -1.51, -2.86 and -4.27.

Simulation	Definition			Solution														
				exchanges		reactions								transp			Prot
	Objective	Constraints	Comment	imports	exports	c	m	r	p	l	n	s	b	s-c	b-c	intra	synth
489 Tyrosine	Tyrosine(c)	−Phenylalanine(c) MCES	de novo synthesis of cytosolic Tyrosine	1 O_2(s) 1 Phenylalanine(c) 1 NADH-redox-potential(c)	1 H_2O(s) 1 Tyrosine(c)	3	-	-	-	-	-	-	-	2	-	-	-

Table 23: Reaction scores for Tyrosine (489), series control 1/24h, TGFβ/C 24h, and TGFβ 1/24h

	control 1/24h				TGFβ/C 24h		TGFβ 1/24h					
Rea ID	Score *0.65*	Expr Δ	Expr C1h	Expr C24h	Score *0.65*	Expr Δ	Score *0.65*	Expr Δ	Expr T1h	Expr T24h	Wght	Reaction
r0397	0.27	-0.29	10.2	9.92	0.28	-0.58	0.27	-0.84	10.2	9.34	11.5	Dihydrobiopterin(c) + NADH(c) ⇌ Tetrahydrobiopterin(c) + NAD+(c). NADH:6,7-dihydropteridine oxidoreductase Folate biosynthesis EC:1.5.1.34 (2 sp.)
Qdpr (2 sp.)												ENSMUSG00000015806 quinoid dihydropteridine reductase
r0399	1	-1.56	11	9.44	1	-2.97	1	-4.43	10.9	6.47	12.5	Tetrahydrobiopterin(c) + Phenylalanine(c) + O2(c) ⇌ Dihydrobiopterin(c) + Tyrosine(c) + H2O(c). L-Phenylalanine,tetrahydrobiopterin:oxygen oxidoreductase (4-hydroxylating) Phenylalanine, tyrosine and tryptophan biosynthesis EC:1.14.16.1
Pah												ENSMUSG00000020051 phenylalanine hydroxylase

25 Reaction scores for Urea from NH3 (313), series control 1/24h, TGFβ/C 24h, and TGFβ 1/24h

The amplitudes of the mode are -0.64, -1.54 and -2.2.

Simulation	Definition			Solution													
				exchanges		reactions								transp			Prot
	Objective	Constraints	Comment	imports	exports	c	m	r	p	l	n	s	b	s-c	b-c	intra	synth
313 Urea from NH_3	Urea	MCES $-NH_3$	Urea from alanine	1 H_2O(s) 2 Alanine(s) 2 ATP-energy(c) 2 ATP-energy(m)	1 Pyruvate(c) 1 Urea(s) 1 NADH-redox-potential(c) 1 NADH-redox-potential(m) 1 NADPH-redox-potential(m) 1 CoA-activated acetyl group(m)	11	9	-	-	-	-	-	-	3	-	2	-

Table 24: Reaction scores for Urea from NH3 (313), series control 1/24h, TGFβ/C 24h, and TGFβ 1/24h

	control 1/24h				TGFβ/C 24h		TGFβ 1/24h					
Rea ID	Score *0.32*	Expr Δ	Expr C1h	Expr C24h	Score *0.43*	Expr Δ	Score *0.41*	Expr Δ	Expr T1h	Expr T24h	Wght	Reaction
r0081	0.82	-0.43	11.2	10.7	0.92	-1.85	1	-2.24	11.1	8.89	13.8	Alanine(m) + AKG(m) ⇌ Pyruvate(m) + Glutamate(m). L-Alanine:2-oxoglutarate aminotransferase Glutamate metabolism / Alanine and aspartate metabolism EC:2.6.1.2 (4 sp.)
Gpt2 (4 sp.)												ENSMUSG00000031700 glutamic pyruvate transaminase (alanine aminotransferase) 2
r0080	0.99	-0.68	9.96	9.28	0.97	-1.73	0.97	-2.47	10	7.55	8.31	Alanine(c) + AKG(c) ⇌ Pyruvate(c) + Glutamate(c). L-Alanine:2-oxoglutarate aminotransferase Glutamate metabolism / Alanine and aspartate metabolism EC:2.6.1.2
Gpt												ENSMUSG00000022546 glutamic pyruvic transaminase, soluble
r0034	0	-4.18	12.7	8.56	0.99	-1.43	0.01	-5.55	12.7	7.14	8.61	2 ATP(m) + CO2(m) + H2O(m) + NH3(m) ⇌ 2 ADP(m) + Pi(m) + Carbamoyl-P(m). Carbon-dioxide:ammonia ligase (ADP-forming,carbamate-phosphorylating) Urea cycle and metabolism of amino groups / Nitrogen metabolism EC:6.3.4.16
Cps1												ENSMUSG00000025991 carbamoyl-phosphate synthetase 1
r0144	0	-5.79	12.5	6.71	0.22	-0.19	0.005	-5.79	12.3	6.51	9.58	Arginine(c) + H2O(c) ⇌ Urea(c) + Ornithine(c). L-Arginine amidinohydrolase Urea cycle and metabolism of amino groups / Arginine and proline metabolism EC:3.5.3.1
Arg1												ENSMUSG00000019987 arginase, liver
r0329	0.0002	-1.95	7.03	5.08	0.58	-0.73	0.94	-2.58	6.93	4.35	8.61	Carbamoyl-P(m) + Ornithine(m) ⇌ Pi(m) + Citrulline(m). Carbamoyl-phosphate:L-ornithine carbamoyltransferase Urea cycle and metabolism of amino groups / Arginine and proline metabolism EC:2.1.3.3 (3 sp.)
Otc (3 sp.)												ENSMUSG00000031173 ornithine transcarbamylase
r0896	0.21	-0.08	3.58	3.5	0.14	-0.004	0.14	-0.04	3.54	3.5	9.58	Urea(c) ⇌ Urea(s). Urea Transporter (UT) TCDB:1.A.28.1.3 (2 sp.)
Slc14a1 (2 sp.)												ENSMUSG00000059336 solute carrier family 14 (urea transporter), member 1
r0947	0.15	-0.01	3.1	3.08	0.1	0.11	0.14	-0.02	3.21	3.2	8.04	Citrulline(m) + Ornithine(c) ⇌ Citrulline(c) + Ornithine(m). Mitochondrial Carrier (MC) TCDB:2.A.29.19.1
Slc25a2												ENSMUSG00000050304 solute carrier family 25 (mitochondrial carrier, ornithine transporter) member 2
r0109	0.91	-0.77	10.7	9.94	0.15	-0.03	0.44	-0.79	10.7	9.9	6.71	OAA(c) + Glutamate(c) ⇌ AKG(c) + Aspartate(c). L-Aspartate:2-oxoglutarate aminotransferase Glutamate metabolism / Alanine and aspartate metabolism EC:2.6.1.1
Got1												ENSMUSG00000025190 glutamate oxaloacetate transaminase 1, soluble
r0453	0.13	-1.28	11.7	10.4	0.09	0.16	0.57	-1.03	11.6	10.6	8.61	ATP(c) + Citrulline(c) + Aspartate(c) ⇌ AMP(c) + PPi(c) + Argininosuccinate(c). L-Citrulline:L-aspartate ligase (AMP-forming) Urea cycle and metabolism of amino groups / Alanine and aspartate metabolism / Arginine and proline metabolism EC:6.3.4.5
Ass1												ENSMUSG00000076441 argininosuccinate synthetase 1
r0056	0.17	-0.04	8.14	8.1	0.2	-0.15	0.17	-0.14	8.09	7.95	8.61	Pyruvate(m) + CoA(m) + NAD+(m) → Acetyl-CoA(m) + CO2(m) + NADH(m). pyruvate:NAD+ 2-oxidoreductase (CoA-acetylating) Complex of EC:1.2.4.1 EC:2.3.1.12 EC:1.8.1.4 (5 genes, 8 sp.)
Dlat (3 sp.)		0.08	7.7	7.78		-0.16		-0.04	7.66	7.61		ENSMUSG00000000168 dihydrolipoamide S-acetyltransferase (E2 component of pyruvate dehydrogenase complex)
Dld		-0.21	8.88	8.66		-0.16		-0.22	8.73	8.51		ENSMUSG00000020664 dihydrolipoamide dehydrogenase
Pdha1 (2 sp.)		0.11	9.64	9.75		-0.06		0.08	9.61	9.69		ENSMUSG00000031299 pyruvate dehydrogenase E1 alpha 1
Pdha2		-0.22	3.53	3.31		-0.06		-0.25	3.51	3.26		ENSMUSG00000047674 pyruvate dehydrogenase E1 alpha 2
Pdhb		-0.31	10.3	10		-0.41		-0.68	10.3	9.6		ENSMUSG00000021748 pyruvate dehydrogenase (lipoamide) beta
r0075	0.002	0.49	10.9	11.4	0.66	-0.84	0.27	-0.43	11	10.6	5.4	Glutamate(m) + H2O(m) + NADP+(m) ⇌ AKG(m) + NADPH(m) + NH3(m). L-Glutamate:NADP+ oxidoreductase (deaminating) Glutamate metabolism EC:1.4.1.3 EC:1.4.1.4 (3 sp.)
Glud1 (3 sp.)												ENSMUSG00000021794 glutamate dehydrogenase 1
r0104	0.21	-0.07	10.8	10.8	0.2	-0.16	0.19	-0.2	10.8	10.6	6.43	NAD+(c) + Malate(c) ⇌ OAA(c) + NADH(c). (S)-malate:NAD+ oxidoreductase Citrate cycle (TCA cycle) EC:1.1.1.37 (3 sp.)
Mdh1 (3 sp.)												ENSMUSG00000020321 malate dehydrogenase 1, NAD (soluble)
r0261	0.61	-0.32	9.99	9.66	0.45	-0.56	0.49	-0.88	9.98	9.1	8.57	Argininosuccinate(c) ⇌ Fumarate(c) + Arginine(c). N-(L-Argininosuccinate) arginie-lyase Urea cycle and metabolism of amino groups / Alanine and aspartate metabolism / Arginine and proline metabolism EC:4.3.2.1
Asl												ENSMUSG00000025533 argininosuccinate lyase
r0258	0.16	-0.03	10.4	10.3	0.22	-0.2	0.22	-0.28	10.4	10.1	4.42	Fumarate(c) + H2O(c) ⇌ Malate(c). (S)-Malate hydro-lyase Citrate cycle (TCA cycle) EC:4.2.1.2
Fh1												ENSMUSG00000026526 fumarate hydratase 1
r2524	0.25	-0.11	5.07	4.96	0.19	-0.14	0.17	-0.12	4.94	4.82	4.55	Alanine(s) ⇌ Alanine(c). Major Facilitator(MFS) TCDB:2.A.1.44.1
Slc43a1												ENSMUSG00000027075 solute carrier family 43, member 1
r0007	0.05	0.15	10.1	10.2	0.17	-0.09	0.12	0.07	10.1	10.1	2.4	PPi(c) + H2O(c) → 2 Pi(c). Pyrophosphate phosphohydrolase EC:3.6.1.1 (2 genes, 2 sp.)
Ppa1		-0.26	11.1	10.8		0.18		-0.14	11.1	11		ENSMUSG00000020089 pyrophosphatase (inorganic) 1
Ppa2		0.56	9.07	9.63		-0.35		0.28	9	9.28		ENSMUSG00000028013 pyrophosphatase (inorganic) 2
r0030	0.23	-0.09	6.05	5.96	0.11	0.06	0.15	-0.05	6.06	6.02	2.65	ATP(c) + AMP(c) ⇌ 2 ADP(c). ATP:AMP phosphotransferase Purine metabolism EC:2.7.4.3 (5 genes, 11 sp.)
Ak1 (2 sp.)		-0.07	3.97	3.89		0.76		0.59	4.07	4.66		ENSMUSG00000026817 adenylate kinase 1
Ak2 (2 sp.)		0.08	9.35	9.43		0.13		0.15	9.41	9.55		ENSMUSG00000028792 adenylate kinase 2
Ak4 (4 sp.)		-0.36	6.41	6.05		-0.23		-0.45	6.27	5.82		ENSMUSG00000028527 adenylate kinase 4
Ak5 (2 sp.)		-0.17	4.72	4.56		0.12		-0.12	4.79	4.68		ENSMUSG00000039058 adenylate kinase 5
Ak7		0.74	4.79	5.53		-0.39		0.06	5.08	5.14		ENSMUSG00000041323 adenylate kinase 7

26 Reaction scores for Pantetheine (896), series control 1/24h, TGFβ/C 24h, and TGFβ 1/24h

The amplitudes of the mode are 3, -1.68 and -0.12.

Simulation	Definition			Solution													
				exchanges		reactions								transp			Prot
	Objective	Constraints	Comment	imports	exports	c	m	r	p	l	n	s	b	s-c	b-c	intra	synth
896 Pantetheine	Pantetheine(c)	PIPES MCES	synthesis of cytosolic Pantetheine	1 Cysteine(s) 1 Pantothenate(s)	1 H_2O(s) 1 CO_2(s) 1 Pantetheine(c) 1 Na-gradient(c)	4	-	-	-	-	-	-	-	5	-	-	-

Table 25: Reaction scores for Pantetheine (896), series control 1/24h, TGFβ/C 24h, and TGFβ 1/24h

	control 1/24h				TGFβ/C 24h		TGFβ 1/24h					
Rea ID	Score *0.31*	Expr Δ	Expr C1h	Expr C24h	Score *0.52*	Expr Δ	Score *0.33*	Expr Δ	Expr T1h	Expr T24h	Wght	Reaction
r0581	1	3.04	5.89	8.93	0.98	-1.85	0	1.18	5.9	7.08	6.68	Pantothenate(c) + Cysteamine(c) ⇌ H2O(c) + Pantetheine(c). (R)-pantetheine amidohydrolase Pantothenate and CoA biosynthesis EC:3.5.1.- (2 sp.)
Vnn1 (2 sp.)												ENSMUSG00000037440 vanin 1
r0538	0.13	-0.03	5.09	5.06	0.14	-0.03	1	-0.12	5.15	5.03	6.55	3-Sulfinoalanine(c) ⇌ CO2(c) + Hypotaurine(c). 3-Sulfino-L-alanine carboxy-lyase Taurine and hypotaurine metabolism EC:4.1.1.29 (3 genes, 6 sp.)
Csad (2 sp.)		0.16	8.38	8.54		-0.31		-0.14	8.38	8.24		ENSMUSG00000023044 cysteine sulfinic acid decarboxylase
Gad1 (2 sp.)		-0.12	3.37	3.25		0.08		-0.14	3.48	3.33		ENSMUSG00000070880 glutamic acid decarboxylase 1
Gad2 (2 sp.)		-0.13	3.51	3.38		0.14		-0.07	3.6	3.53		ENSMUSG00000026787 glutamic acid decarboxylase 2
r0211	0.0003	-3	12.5	9.52	0.97	-1.47	0	-4.44	12.5	8.04	6.53	Cysteine(c) + O2(c) ⇌ 3-Sulfinoalanine(c). L-Cysteine:oxygen oxidoreductase Cysteine metabolism / Taurine and hypotaurine metabolism EC:1.13.11.20
Cdo1												ENSMUSG00000033022 cysteine dioxygenase 1, cytosolic
r0539	0.19	0.27	6.16	6.42	0.13	0.01	0	0.14	6.29	6.43	6.66	Hypotaurine(c) ⇌ Cysteamine(c) + O2(c). Cysteamine:oxygen oxidoreductase Taurine and hypotaurine metabolism EC:1.13.11.19
Ado												ENSMUSG00000057134 2-aminoethanethiol (cysteamine) dioxygenase
r2529	0.12	-0.11	5.07	4.96	0.19	-0.14	1	-0.12	4.94	4.82	3.26	Cysteine(s) ⇌ Cysteine(c). Major Facilitator(MFS) TCDB:2.A.1.44.1
Slc43a1												ENSMUSG00000027075 solute carrier family 43, member 1

27 Reaction scores for Glucose-6P (54), series control 1/24h, TGFβ/C 24h, and TGFβ 1/24h

The amplitudes of the mode are 0.61, -2.74 and -4.62.

Simulation	Definition			Solution														
				exchanges		reactions								transsp			Prot
	Objective	Constraints	Comment	imports	exports	c	m	r	p	l	n	s	b	s-c	b-c	intra	synth
54 Glucose-6P	Glucose-6P(c)	−Glucose =ATP-energy(c) =P_i(c) =H_2O	Synthesis of Glucose-6P	1 P_i(c) 1 Glucose(s) 1 ATP-energy(c)	1 H_2O(s) 1 Glucose-6P(c)	2	-	-	-	-	-	-	-	2	-	-	-

Table 26: Reaction scores for Glucose-6P (54), series control 1/24h, TGFβ/C 24h, and TGFβ 1/24h

Rea ID	control 1/24h Score *0.51*	Expr Δ	Expr C1h	Expr C24h	TGFβ/C 24h Score *0.52*	Expr Δ	TGFβ 1/24h Score *0.51*	Expr Δ	Expr T1h	Expr T24h	Wght	Reaction
r0353	1	0.61	5.5	6.11	0.07	0.45	0.05	0.97	5.59	6.56	13.4	Glucose(c) + ATP(c) → Glucose-6P(c) + ADP(c). Glycolysis / Glyconeogenesis EC:2.7.1.1 (3 genes, 4 sp.)
Hk1 (2 sp.)		0.66	4.55	5.21		0.29		0.87	4.63	5.5	ENSMUSG00000037012 hexokinase 1	
Hk2		1.43	6.02	7.45		1.06		2.41	6.1	8.51	ENSMUSG00000000628 hexokinase 2	
Hk3		-0.3	6.87	6.58		0.17		-0.25	6.99	6.74	ENSMUSG00000025877 hexokinase 3	
r1032	0	-2.04	9.46	7.42	1	-2.7	1	-4.55	9.27	4.72	12.7	Glucose(s) ⇌ Glucose(c). Major Facilitator(MFS) TCDB:2.A.1.1.29
Slc2a2											ENSMUSG00000027690 solute carrier family 2 (facilitated glucose transporter), member 2	

28 Reaction scores for Alanine (480), series control 1/24h, TGFβ/C 24h, and TGFβ 1/24h

The amplitudes of the mode are 0.5, -1.51 and -2.41.

Simulation	Definition			Solution													
				exchanges		reactions								transp			Prot
	Objective	Constraints	Comment	imports	exports	c	m	r	p	l	n	s	b	s-c	b-c	intra	synth
480 Alanine	Alanine(c)	$-NH_3$(c) MCES	de novo synthesis of cytosolic Alanine	1 NH_3(c) 1 Pyruvate(c) 1 NADH-redox-potential(m) 2 Proton-gradient(m)	1 H_2O(s) 1 Alanine(c)	1	2	-	-	-	-	-	-	1	-	7	-

Table 27: Reaction scores for Alanine (480), series control 1/24h, TGFβ/C 24h, and TGFβ 1/24h

Rea ID	control 1/24h Score *0.55*	Expr Δ	Expr C1h	Expr C24h	TGFβ/C 24h Score *0.67*	Expr Δ	TGFβ 1/24h Score *0.51*	Expr Δ	Expr T1h	Expr T24h	Wght	Reaction
r0080	0	-0.68	9.96	9.28	0.96	-1.73	1	-2.47	10	7.55	8.42	Pyruvate(c) + Glutamate(c) ⇌ Alanine(c) + AKG(c). L-Alanine:2-oxoglutarate aminotransferase Glutamate metabolism / Alanine and aspartate metabolism EC:2.6.1.2
Gpt												ENSMUSG00000022546 glutamic pyruvic transaminase, soluble
r2419	1	0.5	7.47	7.97	0.38	-0.46	0.14	-0.04	7.54	7.51	5.99	AKG(c) + Pi(m) ⇌ AKG(m) + Pi(c). Mitochondrial Carrier (MC) TCDB:2.A.29.2.7 (2 sp.)
Slc25a10 (2 sp.)												ENSMUSG00000025792 solute carrier family 25 (mitochondrial carrier, dicarboxylate transporter), member 10
r0073	1	0.49	10.9	11.4	0.67	-0.84	0.26	-0.43	11	10.6	5.47	AKG(m) + NADH(m) + NH3(m) ⇌ Glutamate(m) + NAD+(m) + H2O(m). L-Glutamate:NAD+ oxidoreductase (deaminating) Glutamate metabolism EC:1.4.1.3 EC:1.4.1.2 (3 sp.)
Glud1 (3 sp.)												ENSMUSG00000021794 glutamate dehydrogenase 1
r1047	0.14	0.004	12.3	12.3	0.11	0.06	0.12	0.06	12.3	12.3	1.43	Pi(c) + H+(PG)(c) → Pi(m) + H+(PG)(m). Mitochondrial Carrier (MC) TCDB:2.A.29.4.1
Slc25a3												ENSMUSG00000061904 solute carrier family 25 (mitochondrial carrier, phosphate carrier), member 3

29 Reaction scores for Proline degr (419), series control 1/24h, TGFβ/C 24h, and TGFβ 1/24h

The amplitudes of the mode are -4.6, -0.51 and -0.7.

Simulation	Definition			Solution														
				exchanges		reactions								transp			Prot
	Objective	Constraints	Comment	imports	exports	c	m	r	p	l	n	s	b	s-c	b-c	intra	synth
419 Proline degr	-1 Proline(c)	MCES +Urea	Proline degradation using palmitate excreting urea	4.5 H_2O(s) 1 Proline(c) 1 ATP-energy(c) 0.5 Proton-gradient(m)	1.5 CO_2(s) 0.5 Pyruvate(c) 0.5 Pyruvate(m) 0.5 Urea(s) 0.5 NADH-redox-potential(c) 2 NADH-redox-potential(m) 0.5 NADPH-redox-potential(c) 1 NADPH-redox-potential(m)	12	14	-	-	-	-	-	-	3	-	7	-

Table 28: Reaction scores for Proline degr (419), series control 1/24h, TGFβ/C 24h, and TGFβ 1/24h

	control 1/24h				TGFβ/C 24h		TGFβ 1/24h					
Rea ID	Score *0.25*	Expr Δ	Expr C1h	Expr C24h	Score *0.5*	Expr Δ	Score *0.44*	Expr Δ	Expr T1h	Expr T24h	Wght	Reaction
C0034	0.14	0.001	7.03	7.03	0.99	-0.54	0.89	-0.53	7.02	6.48	12.6	several reactions. L-Glutamate 5-semialdehyde:NAD+ oxidoreductase Arginine and proline metabolism EC:1.5.1.12
Aldh4a1												ENSMUSG00000028737 aldehyde dehydrogenase 4 family, member A1
r0034	0.98	-4.18	12.7	8.56	0.002	-1.43	0	-5.55	12.7	7.14	7.26	2 ATP(m) + CO2(m) + H2O(m) + NH3(m) ⇌ 2 ADP(m) + Pi(m) + Carbamoyl-P(m). Carbon-dioxide:ammonia ligase (ADP-forming,carbamate-phosphorylating) Urea cycle and metabolism of amino groups / Nitrogen metabolism EC:6.3.4.16
Cps1												ENSMUSG00000025991 carbamoyl-phosphate synthetase 1
r0125	0.14	-0.07	9.76	9.7	1	-0.5	0.93	-0.57	9.77	9.2	9.54	GDP(m) + Pi(m) + Succinyl-CoA(m) ⇌ GTP(m) + Succinate(m) + CoA(m). Succinate:CoA ligase (GDP-forming) Citrate cycle (TCA cycle) / Propanoate metabolism EC:6.2.1.4 (2 genes, 3 sp.)
Suclg1		0.18	10.1	10.2		-0.06		0.07	10.1	10.2		ENSMUSG00000052738 succinate-CoA ligase, GDP-forming, alpha subunit
Suclg2 (2 sp.)		-0.19	9.62	9.43		-0.72		-0.89	9.6	8.71		ENSMUSG00000061838 succinate-Coenzyme A ligase, GDP-forming, beta subunit
r0144	0.87	-5.79	12.5	6.71	0.47	-0.19	0	-5.79	12.3	6.51	8.07	Arginine(c) + H2O(c) ⇌ Urea(c) + Ornithine(c). L-Arginine amidinohydrolase Urea cycle and metabolism of amino groups / Arginine and proline metabolism EC:3.5.3.1
Arg1												ENSMUSG00000019987 arginase, liver
r1453	0.18	-0.37	8.04	7.66	0.7	-0.3	1	-0.72	8.09	7.36	9.63	Proline(m) + Ubiquinol(m) ⇌ 1-Pyrroline-5-carboxylate(m) + Ubiquinone(m). L-proline:(acceptor) oxidoreductase Arginine and proline metabolism EC:1.5.99.8
Prodh												ENSMUSG00000003526 proline dehydrogenase
r0261	0.18	-0.32	9.99	9.66	0.98	-0.56	0.88	-0.88	9.98	9.1	7.22	Argininosuccinate(c) ⇌ Fumarate(c) + Arginine(c). N-(L-Argininosuccinate) arginie-lyase Urea cycle and metabolism of amino groups / Alanine and aspartate metabolism / Arginine and proline metabolism EC:4.3.2.1
Asl												ENSMUSG00000025533 argininosuccinate lyase
r0329	0.52	-1.95	7.03	5.08	0.69	-0.73	0	-2.58	6.93	4.35	7.26	Carbamoyl-P(m) + Ornithine(m) ⇌ Pi(m) + Citrulline(m). Carbamoyl-phosphate:L-ornithine carbamoyltransferase Urea cycle and metabolism of amino groups / Arginine and proline metabolism EC:2.1.3.3 (3 sp.)
Otc (3 sp.)												ENSMUSG00000031173 ornithine transcarbamylase
r0896	0.14	-0.08	3.58	3.5	0.14	-0.004	0.17	-0.04	3.54	3.5	8.07	Urea(c) ⇌ Urea(s). Urea Transporter (UT) TCDB:1.A.28.1.3 (2 sp.)
Slc14a1 (2 sp.)												ENSMUSG00000059336 solute carrier family 14 (urea transporter), member 1
r0947	0.14	-0.01	3.1	3.08	0.05	0.11	0.15	-0.02	3.21	3.2	6.78	Citrulline(m) + Ornithine(c) ⇌ Citrulline(c) + Ornithine(m). Mitochondrial Carrier (MC) TCDB:2.A.29.19.1
Slc25a2												ENSMUSG00000050304 solute carrier family 25 (mitochondrial carrier, ornithine transporter) member 2
r0453	0.35	-1.28	11.7	10.4	0.03	0.16	0.64	-1.03	11.6	10.6	7.26	ATP(c) + Citrulline(c) + Aspartate(c) ⇌ AMP(c) + PPi(c) + Argininosuccinate(c). L-Citrulline:L-aspartate ligase (AMP-forming) Urea cycle and metabolism of amino groups / Alanine and aspartate metabolism / Arginine and proline metabolism EC:6.3.4.5
Ass1												ENSMUSG00000076441 argininosuccinate synthetase 1
r1114	0.12	0.16	7.56	7.71	0.27	-0.1	0.11	0.03	7.58	7.62	7.83	AKG(m) + CoA(m) + NAD+(m) ⇌ NADH(m) + CO2(m) + Succinyl-CoA(m). EC:1.2.4.2 (2 sp.)
Ogdh (2 sp.)												ENSMUSG00000020456 oxoglutarate dehydrogenase (lipoamide)
r0109	0.25	-0.77	10.7	9.94	0.18	-0.03	0.97	-0.79	10.7	9.9	5.65	OAA(c) + Glutamate(c) ⇌ AKG(c) + Aspartate(c). L-Aspartate:2-oxoglutarate aminotransferase Glutamate metabolism / Alanine and aspartate metabolism EC:2.6.1.1
Got1												ENSMUSG00000025190 glutamate oxaloacetate transaminase 1, soluble
r0820	0.09	0.5	7.47	7.97	0.98	-0.46	0.17	-0.04	7.54	7.51	4.98	AKG(c) + Malate(m) ⇌ AKG(m) + Malate(c). Mitochondrial Carrier (MC) TCDB:2.A.29.2.2 (2 sp.)
Slc25a10 (2 sp.)												ENSMUSG00000025792 solute carrier family 25 (mitochondrial carrier, dicarboxylate transporter), member 10
r0509	0.14	0.001	9.86	9.86	0.37	-0.15	0.23	-0.1	9.81	9.71	9.58	Ubiquinone(m) + Succinate(m) → Ubiquinol(m) + Fumarate(m). Succinate:ubiquinone oxidoreductase Citrate cycle (TCA cycle) EC:1.3.5.1 (4 genes, 7 sp.)
Sdha (2 sp.)		-0.14	10.7	10.5		-0.16		-0.27	10.6	10.4		ENSMUSG00000021577 succinate dehydrogenase complex, subunit A, flavoprotein (Fp)
Sdhb		0.06	11.3	11.4		-0.24		-0.17	11.3	11.1		ENSMUSG00000009863 succinate dehydrogenase complex, subunit B, iron sulfur (Ip)
Sdhc (2 sp.)		0.04	9.29	9.33		-0.07		0.02	9.24	9.26		ENSMUSG00000058076 succinate dehydrogenase complex, subunit C, integral membrane protein
Sdhd (2 sp.)		0.07	8.89	8.96		-0.17		-0.02	8.81	8.79		ENSMUSG00000000171 succinate dehydrogenase complex, subunit D, integral membrane protein
r0100	0.09	0.42	8.17	8.59	0.73	-0.31	0.1	0.05	8.23	8.28	5.39	ADP(m) + GTP(m) ⇌ ATP(m) + GDP(m). ATP:nucleoside-diphosphate phosphatransferase Purine metabolism EC:2.7.4.6 (7 genes, 7 sp.)
Nme1		0.44	11.4	11.8		-0.3		0.11	11.4	11.5		ENSMUSG00000037601 non-metastatic cells 1, protein (NM23A) expressed in
Nme2		0.09	13	13.1		0.04		0.14	13	13.2		ENSMUSG00000020857 non-metastatic cells 2, protein (NM23B) expressed in
Nme3		0.86	6.83	7.69		-1.4		-0.66	6.96	6.29		ENSMUSG00000073435 non-metastatic cells 3, protein expressed in
Nme4		0.95	6.48	7.42		-0.82		-0.06	6.67	6.6		ENSMUSG00000024177 non-metastatic cells 4, protein expressed in
Nme5		0.01	4.78	4.79		0.08		-0.02	4.88	4.87		ENSMUSG00000035984 non-metastatic cells 5, protein expressed in (nucleoside-diphosphate kinase)
Nme6		-0.01	7.51	7.5		0.11		0.03	7.58	7.61		ENSMUSG00000032478 non-metastatic cells 6, protein expressed in (nucleoside-diphosphate kinase)
Nme7		0.57	7.21	7.78		0.14		0.85	7.08	7.92		ENSMUSG00000026575 non-metastatic cells 7, protein expressed in (nucleoside-diphosphate kinase)
r0058	0.11	0.23	10.9	11.1	0.89	-0.38	0.33	-0.18	10.9	10.7	4.04	Malate(c) + NADP+(c) ⇌ Pyruvate(c) + CO2(c) + NADPH(c). (S)-Malate:NADP+ oxidoreductase(oxaloacetate-decarboxylating) Pyruvate metabolism EC:1.1.1.40
Me1												ENSMUSG00000032418 malic enzyme 1, NADP(+)-dependent, cytosolic
r0075	0.09	0.49	10.9	11.4	0.44	-0.84	0.73	-0.43	11	10.6	4.55	Glutamate(m) + H2O(m) + NADP+(m) ⇌ AKG(m) + NADPH(m) + NH3(m). L-Glutamate:NADP+ oxidoreductase (deaminating) Glutamate metabolism EC:1.4.1.3 EC:1.4.1.4 (3 sp.)
Glud1 (3 sp.)												ENSMUSG00000021794 glutamate dehydrogenase 1
r0059	0.16	-0.17	4.52	4.35	0.19	-0.05	0.4	-0.23	4.53	4.3	5.13	Malate(m) + NADP+(m) ⇌ Pyruvate(m) + CO2(m) + NADPH(m). (S)-Malate:NADP+ oxidoreductase(oxaloacetate-decarboxylating) Pyruvate metabolism EC:1.1.1.40
Me3												ENSMUSG00000030621 malic enzyme 3, NADP(+)-dependent, mitochondrial
r0030	0.15	-0.09	6.05	5.96	0.08	0.06	0.17	-0.05	6.06	6.02	2.23	ATP(c) + AMP(c) ⇌ 2 ADP(c). ATP:AMP phosphotransferase Purine metabolism EC:2.7.4.3 (5 genes, 11 sp.)
Ak1 (2 sp.)		-0.07	3.97	3.89		0.76		0.59	4.07	4.66		ENSMUSG00000026817 adenylate kinase 1
Ak2 (2 sp.)		0.08	9.35	9.43		0.13		0.15	9.41	9.55		ENSMUSG00000028792 adenylate kinase 2
Ak4 (4 sp.)		-0.36	6.41	6.05		-0.23		-0.45	6.27	5.82		ENSMUSG00000028527 adenylate kinase 4
Ak5 (2 sp.)		-0.17	4.72	4.56		0.12		-0.12	4.79	4.68		ENSMUSG00000039058 adenylate kinase 5
Ak7		0.74	4.79	5.53		-0.39		0.06	5.08	5.14		ENSMUSG00000041323 adenylate kinase 7
r0104	0.14	-0.07	10.8	10.8	0.39	-0.16	0.36	-0.2	10.8	10.6	5.42	NAD+(c) + Malate(c) ⇌ OAA(c) + NADH(c). (S)-malate:NAD+ oxidoreductase Citrate cycle (TCA cycle) EC:1.1.1.37 (3 sp.)

Continued on next page

Rea ID	control 1/24h Score *0.25*	Expr Δ	Expr C1h	Expr C24h	TGFβ/C 24h Score *0.5*	Expr Δ	TGFβ 1/24h Score *0.44*	Expr Δ	Expr T1h	Expr T24h	Wght	Reaction
Mdh1 (3 sp.)												ENSMUSG00000020321 malate dehydrogenase 1, NAD (soluble)
r0007	0.12	0.15	10.1	10.2	0.25	-0.09	0.09	0.07	10.1	10.1	2.02	PPi(c) + H2O(c) → 2 Pi(c). Pyrophosphate phosphohydrolase EC:3.6.1.1 (2 genes, 2 sp.)
Ppa1		-0.26	11.1	10.8		0.18		-0.14	11.1	11		ENSMUSG00000020089 pyrophosphatase (inorganic) 1
Ppa2		0.56	9.07	9.63		-0.35		0.28	9	9.28		ENSMUSG00000028013 pyrophosphatase (inorganic) 2
C0047	0.14	-0.03	10.4	10.3	0.48	-0.2	0.48	-0.28	10.4	10.1	6.44	several reactions. (S)-Malate hydro-lyase Citrate cycle (TCA cycle) EC:4.2.1.2
Fh1												ENSMUSG00000026526 fumarate hydratase 1

30 Reaction scores for beta-Alanine degr (428), series control 1/24h, TGFβ/C 24h, and TGFβ 1/24h

The amplitudes of the mode are -4.66, -0.73 and -5.11.

Simulation	Definition			Solution													
				exchanges		reactions								transp			Prot
	Objective	Constraints	Comment	imports	exports	c	m	r	p	l	n	s	b	s-c	b-c	intra	synth
428 beta-Alanine degr	-1 beta-Alanine(c)	MCES +Urea	beta-Alanine degradation using palmitate excreting urea	0.5 H_2O(s) 1 beta-Alanine(c) 1 ATP-energy(c) 1 ATP-energy(m) 1 Proton-gradient(m)	0.5 CO_2(s) 0.5 Urea(s) 0.5 NADH-redox-potential(c) 1 NADH-redox-potential(m) 0.5 NADPH-redox-potential(m) 1 CoA-activated acetyl group(m)	10	9	-	-	-	-	-	-	3	-	8	-

Table 29: Reaction scores for beta-Alanine degr (428), series control 1/24h, TGFβ/C 24h, and TGFβ 1/24h

	control 1/24h				TGFβ/C 24h		TGFβ 1/24h					
Rea ID	Score *0.31*	Expr Δ	Expr C1h	Expr C24h	Score *0.34*	Expr Δ	Score *0.33*	Expr Δ	Expr T1h	Expr T24h	Wght	Reaction
r0034	0.98	-4.18	12.7	8.56	0.16	-1.43	0.99	-5.55	12.7	7.14	8.31	2 ATP(m) + CO2(m) + H2O(m) + NH3(m) ⇌ 2 ADP(m) + Pi(m) + Carbamoyl-P(m). Carbon-dioxide:ammonia ligase (ADP-forming,carbamate-phosphorylating) Urea cycle and metabolism of amino groups / Nitrogen metabolism EC:6.3.4.16
Cps1												ENSMUSG00000025991 carbamoyl-phosphate synthetase 1
r0217	0.1	0.31	7.11	7.41	0.002	0.54	0.07	0.77	7.19	7.95	15.3	beta-Alanine(m) + AKG(m) ⇌ Glutamate(m) + 3-Oxopropanoate(m). 3-Aminopropanoate:2-oxoglutarate aminotransferase Alanine and aspartate metabolism / beta-Alanine metabolism / Propanoate metabolism EC:2.6.1.55 EC:2.6.1.19
Abat												ENSMUSG00000057880 4-aminobutyrate aminotransferase
r0144	0.89	-5.79	12.5	6.71	0.34	-0.19	0.96	-5.79	12.3	6.51	9.24	Arginine(c) + H2O(c) ⇌ Urea(c) + Ornithine(c). L-Arginine amidinohydrolase Urea cycle and metabolism of amino groups / Arginine and proline metabolism EC:3.5.3.1
Arg1												ENSMUSG00000019987 arginase, liver
r0329	0.51	-1.95	7.03	5.08	1	-0.73	0.61	-2.58	6.93	4.35	8.31	Carbamoyl-P(m) + Ornithine(m) ⇌ Pi(m) + Citrulline(m). Carbamoyl-phosphate:L-ornithine carbamoyltransferase Urea cycle and metabolism of amino groups / Arginine and proline metabolism EC:2.1.3.3 (3 sp.)
Otc (3 sp.)												ENSMUSG00000031173 ornithine transcarbamylase
r0261	0.18	-0.32	9.99	9.66	0.9	-0.56	0.25	-0.88	9.98	9.1	8.26	Argininosuccinate(c) ⇌ Fumarate(c) + Arginine(c). N-(L-Argininosuccinate) arginie-lyase Urea cycle and metabolism of amino groups / Alanine and aspartate metabolism / Arginine and proline metabolism EC:4.3.2.1
Asl												ENSMUSG00000025533 argininosuccinate lyase
r0896	0.14	-0.08	3.58	3.5	0.14	-0.004	0.14	-0.04	3.54	3.5	9.24	Urea(c) ⇌ Urea(s). Urea Transporter (UT) TCDB:1.A.28.1.3 (2 sp.)
Slc14a1 (2 sp.)												ENSMUSG00000059336 solute carrier family 14 (urea transporter), member 1
r0075	0.09	0.49	10.9	11.4	0.96	-0.84	0.19	-0.43	11	10.6	5.2	Glutamate(m) + H2O(m) + NADP+(m) ⇌ AKG(m) + NADPH(m) + NH3(m). L-Glutamate:NADP+ oxidoreductase (deaminating) Glutamate metabolism EC:1.4.1.3 EC:1.4.1.4 (3 sp.)
Glud1 (3 sp.)												ENSMUSG00000021794 glutamate dehydrogenase 1
r0947	0.14	-0.01	3.1	3.08	0.07	0.11	0.14	-0.02	3.21	3.2	7.76	Citrulline(m) + Ornithine(c) ⇌ Citrulline(c) + Ornithine(m). Mitochondrial Carrier (MC) TCDB:2.A.29.19.1
Slc25a2												ENSMUSG00000050304 solute carrier family 25 (mitochondrial carrier, ornithine transporter) member 2
r2419	0.09	0.5	7.47	7.97	0.77	-0.46	0.14	-0.04	7.54	7.51	5.7	AKG(c) + Pi(m) ⇌ AKG(m) + Pi(c). Mitochondrial Carrier (MC) TCDB:2.A.29.2.7 (2 sp.)
Slc25a10 (2 sp.)												ENSMUSG00000025792 solute carrier family 25 (mitochondrial carrier, dicarboxylate transporter), member 10
r0453	0.35	-1.28	11.7	10.4	0.05	0.16	0.28	-1.03	11.6	10.6	8.31	ATP(c) + Citrulline(c) + Aspartate(c) ⇌ AMP(c) + PPi(c) + Argininosuccinate(c). L-Citrulline:L-aspartate ligase (AMP-forming) Urea cycle and metabolism of amino groups / Alanine and aspartate metabolism / Arginine and proline metabolism EC:6.3.4.5
Ass1												ENSMUSG00000076441 argininosuccinate synthetase 1
r0104	0.14	-0.07	10.8	10.8	0.29	-0.16	0.16	-0.2	10.8	10.6	6.2	NAD+(c) + Malate(c) ⇌ OAA(c) + NADH(c). (S)-malate:NAD+ oxidoreductase Citrate cycle (TCA cycle) EC:1.1.1.37 (3 sp.)
Mdh1 (3 sp.)												ENSMUSG00000020321 malate dehydrogenase 1, NAD (soluble)
r0109	0.25	-0.77	10.7	9.94	0.16	-0.03	0.24	-0.79	10.7	9.9	6.47	OAA(c) + Glutamate(c) ⇌ AKG(c) + Aspartate(c). L-Aspartate:2-oxoglutarate aminotransferase Glutamate metabolism / Alanine and aspartate metabolism EC:2.6.1.1
Got1												ENSMUSG00000025190 glutamate oxaloacetate transaminase 1, soluble
r0030	0.15	-0.09	6.05	5.96	0.09	0.06	0.14	-0.05	6.06	6.02	2.56	ATP(c) + AMP(c) ⇌ 2 ADP(c). ATP:AMP phosphotransferase Purine metabolism EC:2.7.4.3 (5 genes, 11 sp.)
Ak1 (2 sp.)		-0.07	3.97	3.89		0.76		0.59	4.07	4.66		ENSMUSG00000026817 adenylate kinase 1
Ak2 (2 sp.)		0.08	9.35	9.43		0.13		0.15	9.41	9.55		ENSMUSG00000028792 adenylate kinase 2
Ak4 (4 sp.)		-0.36	6.41	6.05		-0.23		-0.45	6.27	5.82		ENSMUSG00000028527 adenylate kinase 4
Ak5 (2 sp.)		-0.17	4.72	4.56		0.12		-0.12	4.79	4.68		ENSMUSG00000039058 adenylate kinase 5
Ak7		0.74	4.79	5.53		-0.39		0.06	5.08	5.14		ENSMUSG00000041323 adenylate kinase 7
r0258	0.14	-0.03	10.4	10.3	0.35	-0.2	0.17	-0.28	10.4	10.1	4.26	Fumarate(c) + H2O(c) ⇌ Malate(c). (S)-Malate hydro-lyase Citrate cycle (TCA cycle) EC:4.2.1.2
Fh1												ENSMUSG00000026526 fumarate hydratase 1
r0007	0.12	0.15	10.1	10.2	0.21	-0.09	0.13	0.07	10.1	10.1	2.31	PPi(c) + H2O(c) → 2 Pi(c). Pyrophosphate phosphohydrolase EC:3.6.1.1 (2 genes, 2 sp.)
Ppa1		-0.26	11.1	10.8		0.18		-0.14	11.1	11		ENSMUSG00000020089 pyrophosphatase (inorganic) 1
Ppa2		0.56	9.07	9.63		-0.35		0.28	9	9.28		ENSMUSG00000028013 pyrophosphatase (inorganic) 2
r1047	0.13	0.004	12.3	12.3	0.1	0.06	0.13	0.06	12.3	12.3	1.36	Pi(c) + H+(PG)(c) → Pi(m) + H+(PG)(m). Mitochondrial Carrier (MC) TCDB:2.A.29.4.1
Slc25a3												ENSMUSG00000061904 solute carrier family 25 (mitochondrial carrier, phosphate carrier), member 3

31 Reaction scores for Aspartate degr (410), series control 1/24h, TGFβ/C 24h, and TGFβ 1/24h

The amplitudes of the mode are -4.7, -0.9 and -5.14.

Simulation	Definition			Solution													
				exchanges		reactions								transp			Prot
	Objective	Constraints	Comment	imports	exports	c	m	r	p	l	n	s	b	s-c	b-c	intra	synth
410 Aspartate degr	-1 Aspartate(c)	MCES +Urea	Aspartate degradation using palmitate excreting urea	0.5 H_2O(s) 1 Aspartate(c) 1 ATP-energy(c) 1 ATP-energy(m)	1 CO_2(s) 0.5 Pyruvate(c) 0.5 Urea(s) 0.5 NADH-redox-potential(m) 0.5 NADPH-redox-potential(c) 0.5 NADPH-redox-potential(m) 0.5 CoA-activated acetyl group(m)	10	9	-	-	-	-	-	-	3	-	2	-

Table 30: Reaction scores for Aspartate degr (410), series control 1/24h, TGFβ/C 24h, and TGFβ 1/24h

	control 1/24h				TGFβ/C 24h		TGFβ 1/24h					
**Rea ID**	**Score** *0.32*	**Expr** Δ	**Expr** C1h	**Expr** C24h	**Score** *0.34*	**Expr** Δ	**Score** *0.34*	**Expr** Δ	**Expr** T1h	**Expr** T24h	**Wght**	**Reaction**
r0034	0.98	-4.18	12.7	8.56	0.5	-1.43	0.99	-5.55	12.7	7.14	8.73	2 ATP(m) + CO2(m) + H2O(m) + NH3(m) ⇌ 2 ADP(m) + Pi(m) + Carbamoyl-P(m). Carbon-dioxide:ammonia ligase (ADP-forming,carbamate-phosphorylating) Urea cycle and metabolism of amino groups / Nitrogen metabolism EC:6.3.4.16
Cps1											ENSMUSG00000025991 carbamoyl-phosphate synthetase 1	
r0144	0.9	-5.79	12.5	6.71	0.29	-0.19	0.97	-5.79	12.3	6.51	9.71	Arginine(c) + H2O(c) ⇌ Urea(c) + Ornithine(c). L-Arginine amidinohydrolase Urea cycle and metabolism of amino groups / Arginine and proline metabolism EC:3.5.3.1
Arg1											ENSMUSG00000019987 arginase, liver	
r0217	0.1	0.31	7.11	7.41	0.01	0.54	0.07	0.77	7.19	7.95	11.4	beta-Alanine(m) + AKG(m) ⇌ Glutamate(m) + 3-Oxopropanoate(m). 3-Aminopropanoate:2-oxoglutarate aminotransferase Alanine and aspartate metabolism / beta-Alanine metabolism / Propanoate metabolism EC:2.6.1.55 EC:2.6.1.19
Abat											ENSMUSG00000057880 4-aminobutyrate aminotransferase	
r0329	0.51	-1.95	7.03	5.08	0.93	-0.73	0.61	-2.58	6.93	4.35	8.73	Carbamoyl-P(m) + Ornithine(m) ⇌ Pi(m) + Citrulline(m). Carbamoyl-phosphate:L-ornithine carbamoyltransferase Urea cycle and metabolism of amino groups / Arginine and proline metabolism EC:2.1.3.3 (3 sp.)
Otc (3 sp.)											ENSMUSG00000031173 ornithine transcarbamylase	
r0128	0.15	-0.12	3.44	3.32	0.08	0.11	0.15	-0.11	3.54	3.43	11.4	Aspartate(c) ⇌ beta-Alanine(c) + CO2(c). L-Aspartate 1-carboxy-lyase Alanine and aspartate metabolism / beta-Alanine metabolism EC:4.1.1.11 (2 genes, 4 sp.)
Gad1 (2 sp.)		-0.12	3.37	3.25		0.08		-0.14	3.48	3.33	ENSMUSG00000070880 glutamic acid decarboxylase 1	
Gad2 (2 sp.)		-0.13	3.51	3.38		0.14		-0.07	3.6	3.53	ENSMUSG00000026787 glutamic acid decarboxylase 2	
r0075	0.09	0.49	10.9	11.4	0.99	-0.84	0.19	-0.43	11	10.6	5.47	Glutamate(m) + H2O(m) + NADP+(m) ⇌ AKG(m) + NADPH(m) + NH3(m). L-Glutamate:NADP+ oxidoreductase (deaminating) Glutamate metabolism EC:1.4.1.3 EC:1.4.1.4 (3 sp.)
Glud1 (3 sp.)											ENSMUSG00000021794 glutamate dehydrogenase 1	
r0261	0.18	-0.32	9.99	9.66	0.76	-0.56	0.25	-0.88	9.98	9.1	8.68	Argininosuccinate(c) ⇌ Fumarate(c) + Arginine(c). N-(L-Argininosuccinate) arginie-lyase Urea cycle and metabolism of amino groups / Alanine and aspartate metabolism / Arginine and proline metabolism EC:4.3.2.1
Asl											ENSMUSG00000025533 argininosuccinate lyase	
r0896	0.14	-0.08	3.58	3.5	0.14	-0.004	0.14	-0.04	3.54	3.5	9.71	Urea(c) ⇌ Urea(s). Urea Transporter (UT) TCDB:1.A.28.1.3 (2 sp.)
Slc14a1 (2 sp.)											ENSMUSG00000059336 solute carrier family 14 (urea transporter), member 1	
r0947	0.14	-0.01	3.1	3.08	0.08	0.11	0.14	-0.02	3.21	3.2	8.15	Citrulline(m) + Ornithine(c) ⇌ Citrulline(c) + Ornithine(m). Mitochondrial Carrier (MC) TCDB:2.A.29.19.1
Slc25a2											ENSMUSG00000050304 solute carrier family 25 (mitochondrial carrier, ornithine transporter) member 2	
r0453	0.35	-1.28	11.7	10.4	0.06	0.16	0.28	-1.03	11.6	10.6	8.73	ATP(c) + Citrulline(c) + Aspartate(c) ⇌ AMP(c) + PPi(c) + Argininosuccinate(c). L-Citrulline:L-aspartate ligase (AMP-forming) Urea cycle and metabolism of amino groups / Alanine and aspartate metabolism / Arginine and proline metabolism EC:6.3.4.5
Ass1											ENSMUSG00000076441 argininosuccinate synthetase 1	
r0058	0.11	0.23	10.9	11.1	0.52	-0.38	0.16	-0.18	10.9	10.7	4.86	Malate(c) + NADP+(c) ⇌ Pyruvate(c) + CO2(c) + NADPH(c). (S)-Malate:NADP+ oxidoreductase(oxaloacetate-decarboxylating) Pyruvate metabolism EC:1.1.1.40
Me1											ENSMUSG00000032418 malic enzyme 1, NADP(+)-dependent, cytosolic	
r0258	0.14	-0.03	10.4	10.3	0.3	-0.2	0.17	-0.28	10.4	10.1	4.47	Fumarate(c) + H2O(c) ⇌ Malate(c). (S)-Malate hydro-lyase Citrate cycle (TCA cycle) EC:4.2.1.2
Fh1											ENSMUSG00000026526 fumarate hydratase 1	
r0030	0.15	-0.09	6.05	5.96	0.1	0.06	0.14	-0.05	6.06	6.02	2.68	ATP(c) + AMP(c) ⇌ 2 ADP(c). ATP:AMP phosphotransferase Purine metabolism EC:2.7.4.3 (5 genes, 11 sp.)
Ak1 (2 sp.)		-0.07	3.97	3.89		0.76		0.59	4.07	4.66	ENSMUSG00000026817 adenylate kinase 1	
Ak2 (2 sp.)		0.08	9.35	9.43		0.13		0.15	9.41	9.55	ENSMUSG00000028792 adenylate kinase 2	
Ak4 (4 sp.)		-0.36	6.41	6.05		-0.23		-0.45	6.27	5.82	ENSMUSG00000028527 adenylate kinase 4	
Ak5 (2 sp.)		-0.17	4.72	4.56		0.12		-0.12	4.79	4.68	ENSMUSG00000039058 adenylate kinase 5	
Ak7		0.74	4.79	5.53		-0.39		0.06	5.08	5.14	ENSMUSG00000041323 adenylate kinase 7	
r0007	0.12	0.15	10.1	10.2	0.2	-0.09	0.13	0.07	10.1	10.1	2.43	PPi(c) + H2O(c) → 2 Pi(c). Pyrophosphate phosphohydrolase EC:3.6.1.1 (2 genes, 2 sp.)
Ppa1		-0.26	11.1	10.8		0.18		-0.14	11.1	11	ENSMUSG00000020089 pyrophosphatase (inorganic) 1	
Ppa2		0.56	9.07	9.63		-0.35		0.28	9	9.28	ENSMUSG00000028013 pyrophosphatase (inorganic) 2	

32 Reaction scores for PEP (890), series control 1/24h, TGFβ/C 24h, and TGFβ 1/24h

The amplitudes of the mode are -3.96, -0.31 and -3.64.

Simulation	Definition			Solution														
				exchanges		reactions								transp			Prot
	Objective	Constraints	Comment	imports	exports	c	m	r	p	l	n	s	b	s-c	b-c	intra	synth
890 PEP	PEP(c)	PIPES MCES	synthesis of cytosolic PEP	1 P_i(c) 1 Pyruvate(c) 1 ATP-energy(c) 1 NADPH-redox-potential(c)	1 H_2O(s) 1 PEP(c) 1 NADH-redox-potential(c)	7	-	-	-	-	-	-	-	1	-	-	-

Table 31: Reaction scores for PEP (890), series control 1/24h, TGFβ/C 24h, and TGFβ 1/24h

	control 1/24h				TGFβ/C 24h		TGFβ 1/24h					
Rea ID	Score *0.51*	Expr Δ	Expr C1h	Expr C24h	Score *0.54*	Expr Δ	Score *0.53*	Expr Δ	Expr T1h	Expr T24h	Wght	Reaction
r0123	1	-3.94	7.86	3.92	0.2	-0.03	1	-3.67	7.56	3.89	12.5	GTP(c) + OAA(c) → GDP(c) + PEP(c) + CO2(c). GTP:oxaloacetate carboxy-lyase (transphosphorylating) Citrate cycle (TCA cycle) / Pyruvate metabolism EC:4.1.1.32 (2 sp.)
Pck1 (2 sp.)											ENSMUSG00000027513 phosphoenolpyruvate carboxykinase 1, cytosolic	
r0099	0.09	0.42	8.17	8.59	1	-0.31	0.13	0.05	8.23	8.28	4.58	ATP(c) + GDP(c) ⇌ ADP(c) + GTP(c). ATP:nucleoside-diphosphate phosphatransferase Purine metabolism EC:2.7.4.6 (7 genes, 7 sp.)
Nme1		0.44	11.4	11.8		-0.3		0.11	11.4	11.5	ENSMUSG00000037601 non-metastatic cells 1, protein (NM23A) expressed in	
Nme2		0.09	13	13.1		0.04		0.14	13	13.2	ENSMUSG00000020857 non-metastatic cells 2, protein (NM23B) expressed in	
Nme3		0.86	6.83	7.69		-1.4		-0.66	6.96	6.29	ENSMUSG00000073435 non-metastatic cells 3, protein expressed in	
Nme4		0.95	6.48	7.42		-0.82		-0.06	6.67	6.6	ENSMUSG00000024177 non-metastatic cells 4, protein expressed in	
Nme5		0.01	4.78	4.79		0.08		-0.02	4.88	4.87	ENSMUSG00000035984 non-metastatic cells 5, protein expressed in (nucleoside-diphosphate kinase)	
Nme6		-0.01	7.51	7.5		0.11		0.03	7.58	7.61	ENSMUSG00000032478 non-metastatic cells 6, protein expressed in (nucleoside-diphosphate kinase)	
Nme7		0.57	7.21	7.78		0.14		0.85	7.08	7.92	ENSMUSG00000026575 non-metastatic cells 7, protein expressed in (nucleoside-diphosphate kinase)	
r0058	0.11	0.23	10.9	11.1	0.89	-0.38	0.16	-0.18	10.9	10.7	4.86	Pyruvate(c) + CO2(c) + NADPH(c) ⇌ Malate(c) + NADP+(c). (S)-Malate:NADP+ oxidoreductase(oxaloacetate-decarboxylating) Pyruvate metabolism EC:1.1.1.40
Me1											ENSMUSG00000032418 malic enzyme 1, NADP(+)-dependent, cytosolic	
r0104	0.15	-0.07	10.8	10.8	0.62	-0.16	0.17	-0.2	10.8	10.6	6.51	NAD+(c) + Malate(c) ⇌ OAA(c) + NADH(c). (S)-malate:NAD+ oxidoreductase Citrate cycle (TCA cycle) EC:1.1.1.37 (3 sp.)
Mdh1 (3 sp.)											ENSMUSG00000020321 malate dehydrogenase 1, NAD (soluble)	

33 Reaction scores for Lysine degr (416), series control 1/24h, TGFβ/C 24h, and TGFβ 1/24h

The amplitudes of the mode are -1.7, -0.58 and -1.

Simulation	Definition			Solution													
				exchanges		reactions								transp			Prot
	Objective	Constraints	Comment	imports	exports	c	m	r	p	l	n	s	b	s-c	b-c	intra	synth
416 Lysine degr	-1 Lysine(c)	MCES +Urea	Lysine degradation using palmitate excreting urea	2 H_2O(s) 0.5 O_2(s) 1 Pyruvate(m) 1 Lysine(c) 2 ATP-energy(c) 2 ATP-energy(m) 2 NADPH-redox-potential(m)	1 CO_2(s) 1 Pyruvate(c) 1 Urea(s) 1 NADH-redox-potential(c) 5 NADH-redox-potential(m) 1 NADPH-redox-potential(c) 4 Proton-gradient(m) 2 CoA-activated acetyl group(m)	11	18	-	-	-	-	-	-	4	-	10	-

Table 32: Reaction scores for Lysine degr (416), series control 1/24h, TGFβ/C 24h, and TGFβ 1/24h

	control 1/24h				TGFβ/C 24h		TGFβ 1/24h					
Rea ID	Score *0.33*	Expr Δ	Expr C1h	Expr C24h	Score *0.43*	Expr Δ	Score *0.36*	Expr Δ	Expr T1h	Expr T24h	Wght	Reaction
C0097	0.93	-2.03	8.97	6.94	0	-2.07	0	-4.08	8.95	4.87	18	several reactions. N6-(L-1,3-Dicarboxypropyl)-L-lysine:NADP+ oxidoreductase (L-lysine-forming) Lysine biosynthesis / Lysine degradation EC:1.5.1.8
Aass												ENSMUSG00000029695 aminoadipate-semialdehyde synthase
r0453	0.88	-1.28	11.7	10.4	0.04	0.16	1	-1.03	11.6	10.6	8.56	ATP(c) + Citrulline(c) + Aspartate(c) ⇌ AMP(c) + PPi(c) + Argininosuccinate(c). L-Citrulline:L-aspartate ligase (AMP-forming) Urea cycle and metabolism of amino groups / Alanine and aspartate metabolism / Arginine and proline metabolism EC:6.3.4.5
Ass1												ENSMUSG00000076441 argininosuccinate synthetase 1
r0594	0.04	0.42	10.6	11.1	0.89	-0.43	0.12	0.02	10.6	10.6	12.7	2-Aminoadipate 6-semialdehyde(c) + NAD+(c) + H2O(c) ⇌ NADH(c) + L-2-Aminoadipate(c). L-2-Aminoadipate-6-semialdehyde:NAD+ 6-oxidoreductase Lysine degradation EC:1.2.1.31
Aldh7a1												ENSMUSG00000053644 aldehyde dehydrogenase family 7, member A1
r0329	0.96	-1.95	7.03	5.08	0.87	-0.73	0.01	-2.58	6.93	4.35	8.56	Carbamoyl-P(m) + Ornithine(m) ⇌ Pi(m) + Citrulline(m). Carbamoyl-phosphate:L-ornithine carbamoyltransferase Urea cycle and metabolism of amino groups / Arginine and proline metabolism EC:2.1.3.3 (3 sp.)
Otc (3 sp.)												ENSMUSG00000031173 ornithine transcarbamylase
r0876	0.91	-1.34	9.88	8.54	0.4	-0.18	0.94	-1.18	9.53	8.36	9.34	Glutamate(c) + H+(PG)(c) + Aspartate(m) → Glutamate(m) + H+(PG)(m) + Aspartate(c). Mitochondrial Carrier (MC) TCDB:2.A.29.14.2
Slc25a13												ENSMUSG00000015112 solute carrier family 25 (mitochondrial carrier, adenine nucleotide translocator), member 13
r0261	0.27	-0.32	9.99	9.66	1	-0.56	0.97	-0.88	9.98	9.1	8.52	Argininosuccinate(c) ⇌ Fumarate(c) + Arginine(c). N-(L-Argininosuccinate) arginie-lyase Urea cycle and metabolism of amino groups / Alanine and aspartate metabolism / Arginine and proline metabolism EC:4.3.2.1
Asl												ENSMUSG00000025533 argininosuccinate lyase
r0034	0.01	-4.18	12.7	8.56	0.01	-1.43	0	-5.55	12.7	7.14	8.56	2 ATP(m) + CO2(m) + H2O(m) + NH3(m) ⇌ 2 ADP(m) + Pi(m) + Carbamoyl-P(m). Carbon-dioxide:ammonia ligase (ADP-forming,carbamate-phosphorylating) Urea cycle and metabolism of amino groups / Nitrogen metabolism EC:6.3.4.16
Cps1												ENSMUSG00000025991 carbamoyl-phosphate synthetase 1
r0460	0.08	0.2	8.7	8.9	1	-0.56	0.53	-0.43	8.77	8.34	8.61	(S)-3-Hydroxybutyryl-CoA(m) + NAD+(m) ⇌ NADH(m) + Acetoacetyl-CoA(m). (S)-3-Hydroxybutanoyl-CoA:NAD+ oxidoreductase Fatty acid metabolism / Lysine degradation / Tryptophan metabolism / Butanoate metabolism EC:1.1.1.35 (2 genes, 4 sp.)
Ehhadh		-1.1	8.51	7.41		-1		-2.08	8.49	6.41		ENSMUSG00000022853 enoyl-Coenzyme A, hydratase/3-hydroxyacyl Coenzyme A dehydrogenase
Hadh (3 sp.)		0.63	8.77	9.4		-0.42		0.12	8.87	8.98		ENSMUSG00000027984 hydroxyacyl-Coenzyme A dehydrogenase
r0450	0.48	-0.67	7.92	7.26	0.63	-0.85	0.59	-1.5	7.91	6.4	12.5	AKG(m) + L-2-Aminoadipate(m) ⇌ 2-Oxoadipate(m) + Glutamate(m). L-2-Aminoadipate:2-oxoglutarate aminotransferase Lysine biosynthesis / Lysine degradation EC:2.6.1.39
Aadat												ENSMUSG00000057228 aminoadipate aminotransferase
r0070	0.15	-0.03	8.79	8.76	0.005	0.37	0.04	0.28	8.85	9.13	7.32	CoA(m) + Acetoacetyl-CoA(m) ⇌ 2 Acetyl-CoA(m). Acetyl-CoA:acetyl-CoA C-acetyltransferase Synthesis and degradation of ketone bodies EC:2.3.1.9 (2 genes, 5 sp.)
Acat1 (3 sp.)		0.19	7.92	8.1		0.54		0.67	7.97	8.64		ENSMUSG00000032047 acetyl-Coenzyme A acetyltransferase 1
Acat2 (2 sp.)		-0.35	10.1	9.75		0.11		-0.3	10.2	9.87		ENSMUSG00000023832 acetyl-Coenzyme A acetyltransferase 2
r0144	0	-5.79	12.5	6.71	0.42	-0.19	0	-5.79	12.3	6.51	9.53	Arginine(c) + H2O(c) ⇌ Urea(c) + Ornithine(c). L-Arginine amidinohydrolase Urea cycle and metabolism of amino groups / Arginine and proline metabolism EC:3.5.3.1
Arg1												ENSMUSG00000019987 arginase, liver
r0588	0.34	-0.46	9.55	9.09	0.74	-0.35	0.92	-0.79	9.53	8.74	7.41	Crotonyl-CoA(m) + H2O(m) ⇌ (S)-3-Hydroxybutyryl-CoA(m). (S)-3-Hydroxybutanoyl-CoA hydro-lyase Fatty acid metabolism / Lysine degradation / Tryptophan metabolism / Benzoate degradation via CoA ligation / Butanoate metabolism EC:4.2.1.17 (3 genes, 3 sp.)
Echs1		-0.24	10.3	10		-0.15		-0.37	10.2	9.87		ENSMUSG00000025465 enoyl Coenzyme A hydratase, short chain, 1, mitochondrial
Ehhadh		-1.1	8.51	7.41		-1		-2.08	8.49	6.41		ENSMUSG00000022853 enoyl-Coenzyme A, hydratase/3-hydroxyacyl Coenzyme A dehydrogenase
Hadha		-0.03	9.87	9.84		0.1		0.08	9.86	9.94		ENSMUSG00000025745 hydroxyacyl-Coenzyme A dehydrogenase/3-ketoacyl-Coenzyme A thiolase/enoyl-Coenzyme A hydratase (trifunctional protein), alpha subunit
r0896	0.16	-0.08	3.58	3.5	0.14	-0.004	0.16	-0.04	3.54	3.5	9.53	Urea(c) ⇌ Urea(s). Urea Transporter (UT) TCDB:1.A.28.1.3 (2 sp.)
Slc14a1 (2 sp.)												ENSMUSG00000059336 solute carrier family 14 (urea transporter), member 1
r0947	0.14	-0.01	3.1	3.08	0.06	0.11	0.14	-0.02	3.21	3.2	7.99	Citrulline(m) + Ornithine(c) ⇌ Citrulline(c) + Ornithine(m). Mitochondrial Carrier (MC) TCDB:2.A.29.19.1
Slc25a2												ENSMUSG00000050304 solute carrier family 25 (mitochondrial carrier, ornithine transporter) member 2
r0541	0.16	-0.08	8.43	8.34	0.73	-0.35	0.35	-0.28	8.27	8	10.7	Glutaryl-CoA(m) + Ubiquinone(m) ⇌ Crotonyl-CoA(m) + CO2(m) + Ubiquinol(m). Glutaryl-CoA:(acceptor) 2,3-oxidoreductase (decarboxylating) Fatty acid metabolism EC:1.3.99.7
Gcdh												ENSMUSG00000003809 glutaryl-Coenzyme A dehydrogenase
r0110	0.26	-0.31	11.4	11	0.51	-0.24	0.7	-0.58	11.4	10.8	9.22	OAA(m) + Glutamate(m) → AKG(m) + Aspartate(m). L-Aspartate:2-oxoglutarate aminotransferase Glutamate metabolism / Alanine and aspartate metabolism EC:2.6.1.1 (2 sp.)
Got2 (2 sp.)												ENSMUSG00000031672 glutamate oxaloacetate transaminase 2, mitochondrial
r0448	0.09	0.16	7.56	7.71	0.25	-0.1	0.12	0.03	7.58	7.62	6.54	2-Oxoadipate(m) + CoA(m) + NAD+(m) ⇌ Glutaryl-CoA(m) + CO2(m) + NADH(m). 2-Oxoadipate:lipoamde 2-oxidoreductase(decarboxylating and acceptor-succinylating) Tryptophan metabolism EC:1.2.4.2 (2 sp.)
Ogdh (2 sp.)												ENSMUSG00000020456 oxoglutarate dehydrogenase (lipoamide)
r0017	0.16	-0.07	10	9.95	0.15	-0.01	0.2	-0.1	10	9.93	6.78	O2(m) + 4 H+(PG)(m) + 4 Ferrocytochrome C(m) → 4 Ferricytochrome C(m) + 2 H2O(m) + 4 H+(PG)(c). Ferrocytochrome-c:oxygen oxidoreductase Oxidative phosphorylation EC:1.9.3.1 (16 genes, 26 sp.)
Cox4i1		0.04	12.5	12.6		0.03		0.03	12.6	12.6		ENSMUSG00000031818 cytochrome c oxidase subunit IV isoform 1
Cox4i2		-0.02	4.73	4.71		-0.01		-0.1	4.8	4.71		ENSMUSG00000009876 cytochrome c oxidase subunit IV isoform 2
Cox5a (3 sp.)		-0.09	11.6	11.5		-0.03		-0.07	11.5	11.4		ENSMUSG00000000088 cytochrome c oxidase, subunit Va
Cox5b (4 sp.)		-0.09	11.9	11.8		-0.07		-0.12	11.9	11.8		ENSMUSG00000061518 cytochrome c oxidase, subunit Vb
Cox6a1 (2 sp.)		0.01	12.4	12.5		-0.03		-0.04	12.5	12.4		ENSMUSG00000041697 cytochrome c oxidase, subunit VI a, polypeptide 1
Cox6a2		-0.05	6.85	6.8		-0.08		-0.14	6.86	6.72		ENSMUSG00000030785 cytochrome c oxidase, subunit VI a, polypeptide 2
Cox6b1 (2 sp.)		0.06	12	12.1		-0.04		0.05	12	12		ENSMUSG00000036751 cytochrome c oxidase subunit VIb polypeptide 1
Cox6b2		0.06	5.02	5.08		0.16		0.15	5.09	5.24		ENSMUSG00000051811 cytochrome c oxidase subunit VIb polypeptide 2
Cox6c (2 sp.)		-0.1	11.4	11.3		-0.01		-0.14	11.4	11.2		ENSMUSG00000014313 cytochrome c oxidase, subunit VIc
Cox7a1		-0.19	4.49	4.3		0.21		-0.2	4.71	4.51		ENSMUSG00000074218 cytochrome c oxidase, subunit VIIa 1

Continued on next page

	control 1/24h				TGFβ/C 24h		TGFβ 1/24h					
**Rea ID**	**Score** *0.33*	**Expr** Δ	**Expr** C1h	**Expr** C24h	**Score** *0.43*	**Expr** Δ	**Score** *0.36*	**Expr** Δ	**Expr** T1h	**Expr** T24h	**Wght**	**Reaction**
Cox7a2 (2 sp.)		-0.31	12.2	11.9		-0.12		-0.38	12.2	11.8		ENSMUSG00000032330 cytochrome c oxidase, subunit VIIa 2
Cox7b2		-0.01	2.91	2.9		0.19		-0.07	3.15	3.09		ENSMUSG00000049387 cytochrome c oxidase subunit VIIb2
Cox7c		-0.11	11.7	11.6		0.01		-0.1	11.7	11.6		ENSMUSG00000017778 cytochrome c oxidase, subunit VIIc
Cox8a (2 sp.)		-0.08	10.4	10.3		-0.05		-0.13	10.4	10.3		ENSMUSG00000035885 cytochrome c oxidase, subunit VIIIa
Cox8b		0.01	5.54	5.54		-0.01		-0.14	5.67	5.53		ENSMUSG00000025488 cytochrome c oxidase, subunit VIIIb
Cox8c		-0.06	7.29	7.23		0.05		-0.04	7.32	7.28		ENSMUSG00000043319 cytochrome c oxidase, subunit VIIIc
r0105	0.28	-0.35	11.8	11.4	0.63	-0.3	0.78	-0.64	11.7	11.1	6.21	NAD+(m) + Malate(m) ⇌ OAA(m) + NADH(m). (S)-malate:NAD+ oxidoreductase Citrate cycle (TCA cycle) EC:1.1.1.37 (2 sp.)
Mdh2 (2 sp.)												ENSMUSG00000019179 malate dehydrogenase 2, NAD (mitochondrial)
r0073	0.04	0.49	10.9	11.4	0.66	-0.84	0.52	-0.43	11	10.6	5.36	Glutamate(m) + NAD+(m) + H2O(m) ⇌ AKG(m) + NADH(m) + NH3(m). L-Glutamate:NAD+ oxidoreductase (deaminating) Glutamate metabolism EC:1.4.1.3 EC:1.4.1.2 (3 sp.)
Glud1 (3 sp.)												ENSMUSG00000021794 glutamate dehydrogenase 1
r0058	0.08	0.23	10.9	11.1	0.8	-0.38	0.26	-0.18	10.9	10.7	4.77	Malate(c) + NADP+(c) ⇌ Pyruvate(c) + CO2(c) + NADPH(c). (S)-Malate:NADP+ oxidoreductase(oxaloacetate-decarboxylating) Pyruvate metabolism EC:1.1.1.40
Me1												ENSMUSG00000032418 malic enzyme 1, NADP(+)-dependent, cytosolic
r0507	0.16	-0.08	10.5	10.4	0.16	-0.02	0.18	-0.08	10.5	10.4	4.8	Ubiquinol(m) + 2 Ferricytochrome C(m) + 4 H+(PG)(m) → Ubiquinone(m) + 4 H+(PG)(c) + 2 Ferrocytochrome C(m). Ubiquinol:ferricytochrome-c oxidoreductase Oxidative phosphorylation EC:1.10.2.2 (8 genes, 13 sp.)
Uqcr10 (2 sp.)		-0.53	9.74	9.21		-0.04		-0.46	9.63	9.18		ENSMUSG00000059534 ubiquinol-cytochrome c reductase, complex III subunit X
Uqcr11		-0.13	11.1	11		-0.13		-0.24	11.1	10.8		ENSMUSG00000020163 ubiquinol-cytochrome c reductase, complex III subunit XI
Uqcrb (2 sp.)		0.001	12	12		-0.05		0.04	11.9	11.9		ENSMUSG00000021520 ubiquinol-cytochrome c reductase binding protein
Uqcrc1		0.11	10.8	10.9		0.06		0.11	10.9	11		ENSMUSG00000025651 ubiquinol-cytochrome c reductase core protein 1
Uqcrc2 (2 sp.)		-0.04	10.6	10.5		0.05		0.08	10.5	10.6		ENSMUSG00000030884 ubiquinol cytochrome c reductase core protein 2
Uqcrfs1		-0.01	11.4	11.4		-0.07		-0.1	11.4	11.3		ENSMUSG00000038462 ubiquinol-cytochrome c reductase, Rieske iron-sulfur polypeptide 1
Uqcrh (2 sp.)		0.06	11.1	11.2		0.003		0.11	11.1	11.2		ENSMUSG00000063882 ubiquinol-cytochrome c reductase hinge protein
Uqcrq (2 sp.)		-0.01	8.07	8.06		-0.03		-0.15	8.18	8.03		ENSMUSG00000044894 ubiquinol-cytochrome c reductase, complex III subunit VII
r0059	0.2	-0.17	4.52	4.35	0.18	-0.05	0.3	-0.23	4.53	4.3	6.05	Pyruvate(m) + CO2(m) + NADPH(m) ⇌ Malate(m) + NADP+(m). (S)-Malate:NADP+ oxidoreductase(oxaloacetate-decarboxylating) Pyruvate metabolism EC:1.1.1.40
Me3												ENSMUSG00000030621 malic enzyme 3, NADP(+)-dependent, mitochondrial
r0030	0.17	-0.09	6.05	5.96	0.09	0.06	0.16	-0.05	6.06	6.02	2.63	ATP(c) + AMP(c) ⇌ 2 ADP(c). ATP:AMP phosphotransferase Purine metabolism EC:2.7.4.3 (5 genes, 11 sp.)
Ak1 (2 sp.)		-0.07	3.97	3.89		0.76		0.59	4.07	4.66		ENSMUSG00000026817 adenylate kinase 1
Ak2 (2 sp.)		0.08	9.35	9.43		0.13		0.15	9.41	9.55		ENSMUSG00000028792 adenylate kinase 2
Ak4 (4 sp.)		-0.36	6.41	6.05		-0.23		-0.45	6.27	5.82		ENSMUSG00000028527 adenylate kinase 4
Ak5 (2 sp.)		-0.17	4.72	4.56		0.12		-0.12	4.79	4.68		ENSMUSG00000039058 adenylate kinase 5
Ak7		0.74	4.79	5.53		-0.39		0.06	5.08	5.14		ENSMUSG00000041323 adenylate kinase 7
r0007	0.09	0.15	10.1	10.2	0.24	-0.09	0.1	0.07	10.1	10.1	2.39	PPi(c) + H2O(c) → 2 Pi(c). Pyrophosphate phosphohydrolase EC:3.6.1.1 (2 genes, 2 sp.)
Ppa1		-0.26	11.1	10.8		0.18		-0.14	11.1	11		ENSMUSG00000020089 pyrophosphatase (inorganic) 1
Ppa2		0.56	9.07	9.63		-0.35		0.28	9	9.28		ENSMUSG00000028013 pyrophosphatase (inorganic) 2
r0258	0.14	-0.03	10.4	10.3	0.43	-0.2	0.35	-0.28	10.4	10.1	4.39	Fumarate(c) + H2O(c) ⇌ Malate(c). (S)-Malate hydro-lyase Citrate cycle (TCA cycle) EC:4.2.1.2
Fh1												ENSMUSG00000026526 fumarate hydratase 1

34 Reaction scores for Proline (487), series control 1/24h, TGFβ/C 24h, and TGFβ 1/24h

The amplitudes of the mode are -0.29, -0.44 and -0.44.

Simulation	Definition			Solution														
				exchanges		reactions								transp			Prot
	Objective	Constraints	Comment	imports	exports	c	m	r	p	l	n	s	b	s-c	b-c	intra	synth
487 Proline	Proline(c)	−Valine(c) MCES	de novo synthesis of cytosolic Proline	1 O_2(s) 1 Valine(c) 1 ATP-energy(m) 1 NADPH-redox-potential(m)	2 H_2O(s) 1 Proline(c) 2 NADH-redox-potential(m) 10 Proton-gradient(m)	-	16	-	-	-	-	-	-	2	-	7	-

Table 33: Reaction scores for Proline (487), series control 1/24h, TGFβ/C 24h, and TGFβ 1/24h

	control 1/24h				TGFβ/C 24h		TGFβ 1/24h					
Rea ID	Score *0.39*	Expr Δ	Expr C1h	Expr C24h	Score *0.48*	Expr Δ	Score *0.53*	Expr Δ	Expr T1h	Expr T24h	Wght	Reaction
r0560	0	0.52	8.94	9.46	0.19	-0.04	0.002	0.32	9.1	9.42	11.8	Isobutyryl-CoA(m) + Ubiquinone(m) → Methacrylyl-CoA(m) + Ubiquinol(m). 2-Methylpropanoyl-CoA:oxygen 2,3-oxidoreductase Valine, leucine and isoleucine degradation EC:1.3.99.2
Acads												ENSMUSG00000029545 acyl-Coenzyme A dehydrogenase, short chain
C0112	0	0.41	6.5	6.91	1	-0.43	0.45	-0.16	6.64	6.48	13	several reactions. ATP:L-glutamate 5-phosphotransferase Urea cycle and metabolism of amino groups EC:2.7.2.11 (1 genes, 4 sp.)
Aldh18a1 (4 sp.)												ENSMUSG00000025007 aldehyde dehydrogenase 18 family, member A1
r0263	0.89	-0.22	4.56	4.33	0.04	0.11	0.76	-0.27	4.72	4.45	9	Valine(m) + AKG(m) → Glutamate(m) + 3-Methyl-2-oxobutyrate(m). L-Leucine:2-oxoglutarate aminotransferase Valine, leucine and isoleucine degradation / Valine, leucine and isoleucine biosynthesis EC:2.6.1.42
Bcat2												ENSMUSG00000030826 branched chain aminotransferase 2, mitochondrial
r0197	0.85	-0.38	9.18	8.8	0.54	-0.19	1	-0.43	9.03	8.6	9.9	(R)-Methylmalonyl-CoA(m) ⇌ Succinyl-CoA(m). (R)-2-Methyl-3-oxopropanoyl-CoA CoA-carbonylmutase Valine, leucine and isoleucine degradation / Propanoate metabolism EC:5.4.99.2
Mut												ENSMUSG00000023921 methylmalonyl-Coenzyme A mutase
r0779	0.1	0.02	6.56	6.58	0.74	-0.61	0.95	-0.51	6.48	5.97	8.28	3-Hydroxyisobutyryl-CoA(m) + H2O(m) → 3-Hydroxyisobutyrate(m) + CoA(m). 3-Hydroxy-2-methylpropanoyl-CoA hydrolase EC:3.1.2.4 (2 sp.)
Hibch (2 sp.)												ENSMUSG00000041426 3-hydroxyisobutyryl-Coenzyme A hydrolase
r0482	0.67	-0.16	10.5	10.4	0.59	-0.21	0.78	-0.29	10.4	10.2	12.4	3-Hydroxyisobutyrate(m) + NAD+(m) → 2-Methyl-3-oxopropanoate(m) + NADH(m). 3-Hydroxy-2-methylpropanoate:NAD+ oxidoreductase EC:1.1.1.31 (2 sp.)
Hibadh (2 sp.)												ENSMUSG00000029776 3-hydroxyisobutyrate dehydrogenase
r0175	0.13	0.001	7.03	7.03	0.89	-0.54	0.91	-0.53	7.02	6.48	7.09	L-Glutamate 5-semialdehyde(m) ⇌ 1-Pyrroline-5-carboxylate(m) + H2O(m). L-1-Pyrroline-5-carboxylate:NAD+ oxidoreductase Glutamate metabolism / Arginine and proline metabolism EC:1.5.1.12
Aldh4a1												ENSMUSG00000028737 aldehyde dehydrogenase 4 family, member A1
r1453	0.86	-0.37	8.04	7.66	0.81	-0.3	0.43	-0.72	8.09	7.36	7.69	1-Pyrroline-5-carboxylate(m) + Ubiquinone(m) ⇌ Proline(m) + Ubiquinol(m). L-proline:(acceptor) oxidoreductase Arginine and proline metabolism EC:1.5.99.8
Prodh												ENSMUSG00000003526 proline dehydrogenase
r1114	0.01	0.16	7.56	7.71	0.3	-0.1	0.1	0.03	7.58	7.62	6.25	NADH(m) + CO2(m) + Succinyl-CoA(m) ⇌ AKG(m) + CoA(m) + NAD+(m). EC:1.2.4.2 (2 sp.)
Ogdh (2 sp.)												ENSMUSG00000020456 oxoglutarate dehydrogenase (lipoamide)
r0017	0.31	-0.07	10	9.95	0.15	-0.01	0.3	-0.1	10	9.93	9.17	O2(m) + 4 H+(PG)(m) + 4 Ferrocytochrome C(m) → 4 Ferricytochrome C(m) + 2 H2O(m) + 4 H+(PG)(c). Ferrocytochrome-c:oxygen oxidoreductase Oxidative phosphorylation EC:1.9.3.1 (16 genes, 26 sp.)
Cox4i1		0.04	12.5	12.6		0.03		0.03	12.6	12.6		ENSMUSG00000031818 cytochrome c oxidase subunit IV isoform 1
Cox4i2		-0.02	4.73	4.71		-0.01		-0.1	4.8	4.71		ENSMUSG00000009876 cytochrome c oxidase subunit IV isoform 2
Cox5a (3 sp.)		-0.09	11.6	11.5		-0.03		-0.07	11.5	11.4		ENSMUSG00000000088 cytochrome c oxidase, subunit Va
Cox5b (4 sp.)		-0.09	11.9	11.8		-0.07		-0.12	11.9	11.8		ENSMUSG00000061518 cytochrome c oxidase, subunit Vb
Cox6a1 (2 sp.)		0.01	12.4	12.5		-0.03		-0.04	12.5	12.4		ENSMUSG00000041697 cytochrome c oxidase, subunit VI a, polypeptide 1
Cox6a2		-0.05	6.85	6.8		-0.08		-0.14	6.86	6.72		ENSMUSG00000030785 cytochrome c oxidase, subunit VI a, polypeptide 2
Cox6b1 (2 sp.)		0.06	12	12.1		-0.04		0.05	12	12		ENSMUSG00000036751 cytochrome c oxidase, subunit VIb polypeptide 1
Cox6b2		0.06	5.02	5.08		0.16		0.15	5.09	5.24		ENSMUSG00000051811 cytochrome c oxidase subunit VIb polypeptide 2
Cox6c (2 sp.)		-0.1	11.4	11.3		-0.01		-0.14	11.4	11.2		ENSMUSG00000014313 cytochrome c oxidase, subunit VIc
Cox7a1		-0.19	4.49	4.3		0.21		-0.2	4.71	4.51		ENSMUSG00000074218 cytochrome c oxidase, subunit VIIa 1
Cox7a2 (2 sp.)		-0.31	12.2	11.9		-0.12		-0.38	12.2	11.8		ENSMUSG00000032330 cytochrome c oxidase, subunit VIIa 2
Cox7b2		-0.01	2.91	2.9		0.19		-0.07	3.15	3.09		ENSMUSG00000049387 cytochrome c oxidase subunit VIIb2
Cox7c		-0.11	11.7	11.6		0.01		-0.1	11.7	11.6		ENSMUSG00000017778 cytochrome c oxidase, subunit VIIc
Cox8a (2 sp.)		-0.08	10.4	10.3		-0.05		-0.13	10.4	10.3		ENSMUSG00000035885 cytochrome c oxidase, subunit VIIIa
Cox8b		0.01	5.54	5.54		-0.01		-0.14	5.67	5.53		ENSMUSG00000025488 cytochrome c oxidase, subunit VIIIb
Cox8c		-0.06	7.29	7.23		0.05		-0.04	7.32	7.28		ENSMUSG00000043319 cytochrome c oxidase, subunit VIIIc
r0669	0.56	-0.14	10.1	9.93	0.17	-0.03	0.41	-0.15	10.1	9.91	7.48	Methacrylyl-CoA(m) + H2O(m) → 3-Hydroxyisobutyryl-CoA(m). (S)-3-Hydroxyisobutyryl-CoA hydro-lyase Valine, leucine and isoleucine degradation EC:4.2.1.17 (2 genes, 2 sp.)
Echs1		-0.24	10.3	10		-0.15		-0.37	10.2	9.87		ENSMUSG00000025465 enoyl Coenzyme A hydratase, short chain, 1, mitochondrial
Hadha		-0.03	9.87	9.84		0.1		0.08	9.86	9.94		ENSMUSG00000025745 hydroxyacyl-Coenzyme A dehydrogenase/3-ketoacyl-Coenzyme A thiolase/enoyl-Coenzyme A hydratase (trifunctional protein), alpha subunit
r0507	0.36	-0.08	10.5	10.4	0.16	-0.02	0.26	-0.08	10.5	10.4	6.5	Ubiquinol(m) + 2 Ferricytochrome C(m) + 4 H+(PG)(m) → Ubiquinone(m) + 4 H+(PG)(c) + 2 Ferrocytochrome C(m). Ubiquinol:ferricytochrome-c oxidoreductase Oxidative phosphorylation EC:1.10.2.2 (8 genes, 13 sp.)
Uqcr10 (2 sp.)		-0.53	9.74	9.21		-0.04		-0.46	9.63	9.18		ENSMUSG00000059534 ubiquinol-cytochrome c reductase, complex III subunit X
Uqcr11		-0.13	11.1	11		-0.13		-0.24	11.1	10.8		ENSMUSG00000020163 ubiquinol-cytochrome c reductase, complex III subunit XI
Uqcrb (2 sp.)		0.001	12	12		-0.05		0.04	11.9	11.9		ENSMUSG00000021520 ubiquinol-cytochrome c reductase binding protein
Uqcrc1		0.11	10.8	10.9		0.06		0.11	10.9	11		ENSMUSG00000025651 ubiquinol-cytochrome c reductase core protein 1
Uqcrc2 (2 sp.)		-0.04	10.6	10.5		0.05		0.08	10.5	10.6		ENSMUSG00000030884 ubiquinol cytochrome c reductase core protein 2
Uqcrfs1		-0.01	11.4	11.4		-0.07		-0.1	11.4	11.3		ENSMUSG00000038462 ubiquinol-cytochrome c reductase, Rieske iron-sulfur polypeptide 1
Uqcrh (2 sp.)		0.06	11.1	11.2		0.003		0.11	11.1	11.2		ENSMUSG00000063882 ubiquinol-cytochrome c reductase hinge protein
Uqcrq (2 sp.)		-0.01	8.07	8.06		-0.03		-0.15	8.18	8.03		ENSMUSG00000044894 ubiquinol-cytochrome c reductase, complex III subunit VII

35 Reaction scores for CoA (450), series control 1/24h, TGFβ/C 24h, and TGFβ 1/24h

The amplitudes of the mode are 0.28, -0.34 and -0.85.

Simulation	Definition			Solution													
				exchanges		reactions								transp			Prot
	Objective	Constraints	Comment	imports	exports	c	m	r	p	l	n	s	b	s-c	b-c	intra	synth
450 CoA	CoA(c)	MCES −Pantothenate AAA	de novo synthesis of cytosolic CoA	2.17 P_i(c) 1 Glycine(s) 2 Aspartate(s) 2 Glutamine(s) 0.83 Glucose-6P(c) 0.5 Cystine(s) 1 Pantothenate(s) 11.2 ATP-energy(c) 2 THF-activated methyl group(c)	5.17 H_2O(s) 1 CoA(c) 2 CO_2(s) 2 Pyruvate(c) 2 Glutamate(s) 2 NADH-redox-potential(c) 3.5 NADPH-redox-potential(c) 2 Proton-gradient(c) 5 Na-gradient(c)	42	-	-	-	-	-	-	-	10	-	-	-

Table 34: Reaction scores for CoA (450), series control 1/24h, TGFβ/C 24h, and TGFβ 1/24h

	control 1/24h				TGFβ/C 24h		TGFβ 1/24h					
Rea ID	Score *0.38*	Expr Δ	Expr C1h	Expr C24h	Score *0.39*	Expr Δ	Score *0.32*	Expr Δ	Expr T1h	Expr T24h	Wght	Reaction
r2477	0	-0.52	7.87	7.35	0.82	-0.23	1	-0.85	7.97	7.12	13.5	Glutamine(s) + Na+(s) + H+(PG)(c) → Glutamine(c) + Na+(c) + H+(PG)(s). Major Facilitator(MFS) TCDB:2.A.18.6.2 (2 sp.)
Slc38a3 (2 sp.)												ENSMUSG00000010064 solute carrier family 38, member 3
C0125	1	0.29	6.91	7.2	0.13	0.004	0.03	0.26	6.94	7.21	9.56	several reactions. 5-Phospho-D-ribosylamine:glycine ligase (ADP-forming) Purine metabolism EC:6.3.4.13 (1 genes, 4 sp.)
Gart (4 sp.)												ENSMUSG00000022962 phosphoribosylglycinamide formyltransferase
C0129	0.94	0.23	8.52	8.75	0.003	0.23	0.01	0.4	8.58	8.98	7.97	several reactions. IMP 1,2-hydrolase (decyclizing) Purine metabolism EC:3.5.4.10 (1 genes, 2 sp.)
Atic (2 sp.)												ENSMUSG00000026192 5-aminoimidazole-4-carboxamide ribonucleotide formyltransferase/IMP cyclohydrolase
r0371	0.03	-0.08	8.16	8.07	0.03	-0.78	1	-0.87	8.17	7.29	7.25	5,10-Methenyl-THF(c) + H2O(c) ⇌ 10-Formyl-THF(c). 5,10-Methenyltetrahydrofolate 5-hydrolase(decyclizing) Glyoxylate and dicarboxylate metabolism / One carbon pool by folate EC:3.5.4.9 (2 genes, 5 sp.)
Mthfd1 (3 sp.)		0.07	7.92	7.98		-0.59		-0.57	7.96	7.39		ENSMUSG00000021048 methylenetetrahydrofolate dehydrogenase (NADP+ dependent), methenyltetrahydrofolate cyclohydrolase, formyltetrahydrofolate synthase
Mthfd2 (2 sp.)		-0.31	8.51	8.21		-1.07		-1.34	8.47	7.13		ENSMUSG00000005667 methylenetetrahydrofolate dehydrogenase (NAD+ dependent), methenyltetrahydrofolate cyclohydrolase
C0006	0.06	-0.05	8.18	8.13	0.94	-0.39	0.46	-0.32	8.06	7.74	8.38	several reactions. ATP:dephospho-CoA 3'-phosphotransferase Pantothenate and CoA biosynthesis EC:2.7.1.24 (1 genes, 2 sp.)
Coasy (2 sp.)												ENSMUSG00000001755 Coenzyme A synthase
r0058	0.93	0.23	10.9	11.1	0.96	-0.38	0.29	-0.18	10.9	10.7	6.1	Malate(c) + NADP+(c) ⇌ Pyruvate(c) + CO2(c) + NADPH(c). (S)-Malate:NADP+ oxidoreductase(oxaloacetate-decarboxylating) Pyruvate metabolism EC:1.1.1.40
Me1												ENSMUSG00000032418 malic enzyme 1, NADP(+)-dependent, cytosolic
C0128	0.84	0.36	7.6	7.96	0.54	-0.15	0.05	0.18	7.63	7.81	7.88	several reactions. N6-(1,2-Dicarboxyethyl)AMP AMP-lyase Purine metabolism / Alanine and aspartate metabolism EC:4.3.2.2
Adsl												ENSMUSG00000022407 adenylosuccinate lyase
r0687	0.91	0.22	6.01	6.23	0.01	0.18	0.005	0.54	5.87	6.4	5.55	ATP(c) + N-Formyl-GAR(c) + Glutamine(c) + H2O(c) ⇌ ADP(c) + Pi(c) + Glutamate(c) + 5-Phosphoribosylformylglycinamidine(c). 5'-Phosphoribosylformylglycinamide:L-glutamine amido-ligase (ADP-forming) Purine metabolism EC:6.3.5.3
Pfas												ENSMUSG00000020899 phosphoribosylformylglycinamidine synthase (FGAR amidotransferase)
r0539	0.99	0.27	6.16	6.42	0.12	0.01	0.07	0.14	6.29	6.43	5.91	Hypotaurine(c) ⇌ Cysteamine(c) + O2(c). Cysteamine:oxygen oxidoreductase Taurine and hypotaurine metabolism EC:1.13.11.19
Ado												ENSMUSG00000057134 2-aminoethanethiol (cysteamine) dioxygenase
r0247	0.94	0.23	8.11	8.34	1	-0.33	0.16	-0.03	8.04	8.01	4.86	ATP(c) + Ribose-5P(c) → AMP(c) + PRPP(c). ATP:D-ribose-5-phosphate pyrophosphotransferase Pentose phosphate pathway / Purine metabolism EC:2.7.6.1 (2 genes, 5 sp.)
Prps1 (2 sp.)		-0.58	10.3	9.71		-0.35		-0.88	10.2	9.36		ENSMUSG00000031432 phosphoribosyl pyrophosphate synthetase 1
Prps2 (3 sp.)		0.77	6.66	7.43		-0.32		0.54	6.57	7.11		ENSMUSG00000025742 phosphoribosyl pyrophosphate synthetase 2
r0581	0	3.04	5.89	8.93	0	-1.85	0	1.18	5.9	7.08	5.93	Pantothenate(c) + Cysteamine(c) ⇌ H2O(c) + Pantetheine(c). (R)-pantetheine amidohydrolase Pantothenate and CoA biosynthesis EC:3.5.1.- (2 sp.)
Vnn1 (2 sp.)												ENSMUSG00000037440 vanin 1
r0211	0	-3	12.5	9.52	0	-1.47	0	-4.44	12.5	8.04	5.8	Cysteine(c) + O2(c) ⇌ 3-Sulfinoalanine(c). L-Cysteine:oxygen oxidoreductase Cysteine metabolism / Taurine and hypotaurine metabolism EC:1.13.11.20
Cdo1												ENSMUSG00000033022 cysteine dioxygenase 1, cytosolic
r0877	0.0002	-0.29	5.35	5.06	0.002	-0.92	0.85	-1.09	5.23	4.14	4.56	Glutamate(c) + Cystine(s) ⇌ Glutamate(s) + Cystine(c). Amino Acid-Polyamine-Organocation (APC) TCDB:2.A.3.8.5 (2 sp.)
Slc7a11 (2 sp.)												ENSMUSG00000027737 solute carrier family 7 (cationic amino acid transporter, y+ system), member 11
C0130	0.76	0.18	10.1	10.3	0.31	-0.08	0.06	0.15	10	10.2	7.9	several reactions. 1-(5-Phospho-D-ribosyl)-5-amino-4-imidazolecarboxylate carboxy-lyase Purine metabolism EC:4.1.1.21 (1 genes, 3 sp.)
Paics (3 sp.)												ENSMUSG00000029247 phosphoribosylaminoimidazole carboxylase, phosphoribosylaminoribosylaminoimidazole, succinocarboxamide synthetase
r0099	0.62	0.42	8.17	8.59	0.99	-0.31	0.1	0.05	8.23	8.28	4.07	ATP(c) + GDP(c) ⇌ ADP(c) + GTP(c). ATP:nucleoside-diphosphate phosphatransferase Purine metabolism EC:2.7.4.6 (7 genes, 7 sp.)
Nme1		0.44	11.4	11.8		-0.3		0.11	11.4	11.5		ENSMUSG00000037601 non-metastatic cells 1, protein (NM23A) expressed in
Nme2		0.09	13	13.1		0.04		0.14	13	13.2		ENSMUSG00000020857 non-metastatic cells 2, protein (NM23B) expressed in
Nme3		0.86	6.83	7.69		-1.4		-0.66	6.96	6.29		ENSMUSG00000073435 non-metastatic cells 3, protein expressed in
Nme4		0.95	6.48	7.42		-0.82		-0.06	6.67	6.6		ENSMUSG00000024177 non-metastatic cells 4, protein expressed in
Nme5		0.01	4.78	4.79		0.08		-0.02	4.88	4.87		ENSMUSG00000035984 non-metastatic cells 5, protein expressed in (nucleoside-diphosphate kinase)
Nme6		-0.01	7.51	7.5		0.11		0.03	7.58	7.61		ENSMUSG00000032478 non-metastatic cells 6, protein expressed in (nucleoside-diphosphate kinase)
Nme7		0.57	7.21	7.78		0.14		0.85	7.08	7.92		ENSMUSG00000026575 non-metastatic cells 7, protein expressed in (nucleoside-diphosphate kinase)
r0192	0	-0.79	11.7	10.9	0.75	-0.46	0.86	-1.09	11.5	10.4	3.15	Glucose-6P(c) ⇌ Fructose-6P(c). D-Glucose-6-phosphate ketol-isomerase EC:5.3.1.9 (3 sp.)
Gpi1 (3 sp.)												ENSMUSG00000036427 glucose phosphate isomerase 1
r0258	0.09	-0.03	10.4	10.3	0.72	-0.2	0.4	-0.28	10.4	10.1	5.61	Fumarate(c) + H2O(c) ⇌ Malate(c). (S)-Malate hydro-lyase Citrate cycle (TCA cycle) EC:4.2.1.2
Fh1												ENSMUSG00000026526 fumarate hydratase 1
r0293	0.31	0.07	7.92	7.98	0.31	-0.59	0.8	-0.57	7.96	7.39	6.19	5,10-Methylene-THF(c) + NADP+(c) ⇌ 5,10-Methenyl-THF(c) + NADPH(c). 5,10-methylenetetrahydrofolate:NADP+ oxidoreductase Glyoxylate and dicarboxylate metabolism / One carbon pool by folate EC:1.5.1.5 (3 sp.)
Mthfd1 (3 sp.)												ENSMUSG00000021048 methylenetetrahydrofolate dehydrogenase (NADP+ dependent), methenyltetrahydrofolate cyclohydrolase, formyltetrahydrofolate synthase
r0344	0.75	0.18	6.37	6.55	0.14	-0.01	0.04	0.22	6.33	6.54	4.06	D-Xylulose-5P(c) ⇌ Ribulose-5P(c). D-Ribulose-5-phosphate 3-epimerase Pentose phosphate pathway EC:5.1.3.1 (3 sp.)
Rpe (3 sp.)												ENSMUSG00000026005 ribulose-5-phosphate-3-epimerase
r0257	0.29	0.06	6.91	6.97	0.85	-0.24	0.24	-0.13	6.86	6.73	5.5	Glutamine(c) + PRPP(c) + H2O(c) ⇌ 5-Phosphoribosylamine(c) + PPi(c) + Glutamate(c). 5-Phosphoribosylamine:pyrophosphate phosphoribosyltransferase (glutamate-amidating) Purine metabolism / Glutamate metabolism EC:2.4.2.14 (2 sp.)
Ppat (2 sp.)												ENSMUSG00000029246 phosphoribosyl pyrophosphate amidotransferase
r0250	0.17	0.55	6.88	7.43	0.03	0.11	0.03	0.27	7.27	7.54	4.02	Ribulose-5P(c) ⇌ Ribose-5P(c). D-Ribose-5-phosphate ketol-isomerase Pentose phosphate pathway EC:5.3.1.6

Continued on next page

Rea ID	control 1/24h Score *0.38*	Expr Δ	Expr C1h	Expr C24h	TGFβ/C 24h Score *0.39*	Expr Δ	TGFβ 1/24h Score *0.32*	Expr Δ	Expr T1h	Expr T24h	Wght	Reaction
Rpia												ENSMUSG00000053604 ribose 5-phosphate isomerase A
r0538	0.09	-0.03	5.09	5.06	0.18	-0.03	0.23	-0.12	5.15	5.03	5.82	3-Sulfinoalanine(c) ⇌ CO2(c) + Hypotaurine(c). 3-Sulfino-L-alanine carboxy-lyase Taurine and hypotaurine metabolism EC:4.1.1.29 (3 genes, 6 sp.)
Csad (2 sp.)		0.16	8.38	8.54		-0.31		-0.14	8.38	8.24		ENSMUSG00000023044 cysteine sulfinic acid decarboxylase
Gad1 (2 sp.)		-0.12	3.37	3.25		0.08		-0.14	3.48	3.33		ENSMUSG00000070880 glutamic acid decarboxylase 1
Gad2 (2 sp.)		-0.13	3.51	3.38		0.14		-0.07	3.6	3.53		ENSMUSG00000026787 glutamic acid decarboxylase 2
r0792	0.28	0.06	6.46	6.51	0.14	-0.005	0.15	-0.02	6.53	6.51	6.32	5-Methyl-THF(c) + NAD+(c) ⇌ 5,10-Methylene-THF(c) + NADH(c). 5-methyltetrahydrofolate:NAD+ oxidoreductase One carbon pool by folate / Methane metabolism EC:1.5.1.20 (3 sp.)
Mthfr (3 sp.)												ENSMUSG00000029009 5,10-methylenetetrahydrofolate reductase
r0406	0.93	0.34	10.4	10.8	0.12	0.01	0.02	0.36	10.4	10.8	2.9	Fructose-6P(c) + Erythrose-4P(c) ⇌ Sedoheptulose-7P(c) + GAP(c). Sedoheptulose-7-phosphate:D-glyceraldehyde-3-phosphate glyceronetransferase Pentose phosphate pathway EC:2.2.1.2
Taldo1												ENSMUSG00000025503 transaldolase 1
C0067	0.05	-0.07	9.41	9.34	0.72	-0.2	0.44	-0.31	9.45	9.14	4.06	several reactions. D-Fructose 6-phosphate:D-glyceraldehyde-3-phosphate glycolaldehyde transferase Carbon fixation EC:2.2.1.1 (1 genes, 3 sp.)
Tkt (3 sp.)												ENSMUSG00000021957 transketolase
r0578	0.01	-0.17	6.93	6.76	0.33	-0.08	0.24	-0.13	6.81	6.68	5.89	ATP(c) + Pantetheine(c) ⇌ ADP(c) + Phosphopantetheine(c). ATP:pantothenate 4'-phosphotransferase Pantothenate and CoA biosynthesis EC:2.7.1.33 (4 genes, 8 sp.)
Pank1 (3 sp.)		-0.28	6.13	5.85		-0.35		-0.6	6.1	5.5		ENSMUSG00000033610 pantothenate kinase 1
Pank2 (2 sp.)		0.05	7.08	7.13		0.02		0.29	6.87	7.16		ENSMUSG00000037514 pantothenate kinase 2
Pank3 (2 sp.)		-0.34	7.8	7.45		0.15		0.01	7.59	7.6		ENSMUSG00000018846 pantothenate kinase 3
Pank4		0.07	7.31	7.38		0.03		0.16	7.25	7.41		ENSMUSG00000029056 pantothenate kinase 4
r0030	0.03	-0.09	6.05	5.96	0.06	0.06	0.17	-0.05	6.06	6.02	3.37	ATP(c) + AMP(c) ⇌ 2 ADP(c). ATP:AMP phosphotransferase Purine metabolism EC:2.7.4.3 (5 genes, 11 sp.)
Ak1 (2 sp.)		-0.07	3.97	3.89		0.76		0.59	4.07	4.66		ENSMUSG00000026817 adenylate kinase 1
Ak2 (2 sp.)		0.08	9.35	9.43		0.13		0.15	9.41	9.55		ENSMUSG00000028792 adenylate kinase 2
Ak4 (4 sp.)		-0.36	6.41	6.05		-0.23		-0.45	6.27	5.82		ENSMUSG00000028527 adenylate kinase 4
Ak5 (2 sp.)		-0.17	4.72	4.56		0.12		-0.12	4.79	4.68		ENSMUSG00000039058 adenylate kinase 5
Ak7		0.74	4.79	5.53		-0.39		0.06	5.08	5.14		ENSMUSG00000041323 adenylate kinase 7
r0277	0.01	-0.13	10.2	10.1	0.36	-0.1	0.39	-0.27	10.2	9.97	6.13	GTP(c) + IMP(c) + Aspartate(c) → GDP(c) + Pi(c) + Adenylosuccinate(c). IMP:L-aspartate ligase (GDP-forming) Purine metabolism / Alanine and aspartate metabolism EC:6.3.4.4
Adss												ENSMUSG00000015961 adenylosuccinate synthetase, non muscle
r0027	0.001	-0.26	7.88	7.62	0.26	-0.06	0.44	-0.31	7.86	7.56	4.22	NADPH(c) + Cystine(c) ⇌ 2 Cysteine(c) + NADP+(c). NADPH:CoA-glutathione oxidoreductase EC:1.8.1.9 (3 genes, 9 sp.)
Txnrd1 (3 sp.)		-0.4	9.09	8.69		-0.39		-0.74	9.04	8.3		ENSMUSG00000020250 thioredoxin reductase 1
Txnrd2 (3 sp.)		-0.03	7.7	7.68		0.01		-0.07	7.76	7.68		ENSMUSG00000075704 thioredoxin reductase 2
Txnrd3 (3 sp.)		-0.34	6.83	6.49		0.21		-0.1	6.79	6.69		ENSMUSG00000000811 thioredoxin reductase 3
r0736	0	-0.78	9.44	8.65	0.87	-0.42	0.9	-1.05	9.28	8.23	1.52	ATP(c) + Fructose-6P(c) → ADP(c) + Fructose-1,6PP(c). ATP:D-fructose-6-phosphate 1-phosphotransferase EC:2.7.1.11 (2 sp.)
Pfkl (2 sp.)												ENSMUSG00000020277 phosphofructokinase, liver, B-type
r2523	0.02	-0.11	5.07	4.96	0.52	-0.14	0.23	-0.12	4.94	4.82	2.89	Glycine(s) ⇌ Glycine(c). Major Facilitator(MFS) TCDB:2.A.1.44.1
Slc43a1												ENSMUSG00000027075 solute carrier family 43, member 1
r0007	0.63	0.15	10.1	10.2	0.33	-0.09	0.1	0.07	10.1	10.1	3.05	PPi(c) + H2O(c) → 2 Pi(c). Pyrophosphate phosphohydrolase EC:3.6.1.1 (2 genes, 2 sp.)
Ppa1		-0.26	11.1	10.8		0.18		-0.14	11.1	11		ENSMUSG00000020089 pyrophosphatase (inorganic) 1
Ppa2		0.56	9.07	9.63		-0.35		0.28	9	9.28		ENSMUSG00000028013 pyrophosphatase (inorganic) 2
r0256	0	-0.78	11.4	10.7	0.61	-0.5	0.75	-1.18	11.3	10.2	1.48	Fructose-1,6PP(c) ⇌ DHAP(c) + GAP(c). D-Fructose-1,6-bisphosphate D-glyceraldehyde-3-phosphate-lyase Carbon fixation EC:4.1.2.13 (2 genes, 4 sp.)
Aldoa (3 sp.)		-0.32	11	10.6		-0.12		-0.36	10.9	10.5		ENSMUSG00000030695 aldolase A, fructose-bisphosphate
Aldob		-2.13	12.9	10.7		-1.64		-3.66	12.7	9.09		ENSMUSG00000028307 aldolase B, fructose-bisphosphate
r0243	0	-0.64	12.2	11.6	0.01	-0.84	0.44	-1.4	12.1	10.7	1.48	DHAP(c) ⇌ GAP(c). D-Glyceraldehyde-3-phosphate ketol-isomerase Glycolysis / Gluconeogenesis EC:5.3.1.1 (3 sp.)
Tpi1 (3 sp.)												ENSMUSG00000023456 triosephosphate isomerase 1

36 Reaction scores for Anaerobic rephosph of ATP (3), series control 1/24h, TGFβ/C 24h, and TGFβ 1/24h

The amplitudes of the mode are -0.74, -0.34 and -0.97.

Simulation	Definition			Solution													
				exchanges		reactions								transp			Prot
	Objective	Constraints	Comment	imports	exports	c	m	r	p	l	n	s	b	s-c	b-c	intra	synth
3 Anaerobic rephosph of ATP	ATP-energy(c)	−Glucose +L-Lactate +CO_2 =H_2O =Proton-gradient(c)	Anaerobic rephosphorylation of cytosolic ATP from glucose	0.5 Glucose(s)	1 L-Lactate(s) 1 ATP-energy(c) 1 Proton-gradient(c)	12	-	-	-	-	-	-	-	3	-	-	-

Table 35: Reaction scores for Anaerobic rephosph of ATP (3), series control 1/24h, TGFβ/C 24h, and TGFβ 1/24h

	control 1/24h				TGFβ/C 24h		TGFβ 1/24h					
Rea ID	Score *0.45*	Expr Δ	Expr C1h	Expr C24h	Score *0.42*	Expr Δ	Score *0.47*	Expr Δ	Expr T1h	Expr T24h	Wght	Reaction
r0171	0.97	-0.83	12.7	11.9	0.71	-0.2	0.98	-0.88	12.5	11.7	15.6	Pyruvate(c) + NADH(c) ⇌ L-Lactate(c) + NAD+(c). (S)-Lactate:NAD+ oxidoreductase Glycolysis / Gluconeogenesis / Pyruvate metabolism EC:1.1.1.27
Ldha												ENSMUSG00000063229 lactate dehydrogenase A
r0054	0	1.1	8.18	9.28	0.01	-0.89	0.05	0.21	8.18	8.39	12.8	ADP(c) + PEP(c) → ATP(c) + Pyruvate(c). ATP:pyruvate O2-phosphotransferase Glycolysis / Gluconeogenesis / Purine metabolism / Pyruvate metabolism EC:2.7.1.40 (2 genes, 4 sp.)
Pklr (3 sp.)		1.31	8.09	9.4		-1.05		0.17	8.18	8.35		ENSMUSG00000041237 pyruvate kinase liver and red blood cell
Pkm2		0.46	8.44	8.9		-0.41		0.33	8.17	8.5		ENSMUSG00000032294 pyruvate kinase, muscle
r0353	0.001	0.61	5.5	6.11	0	0.45	0.0003	0.97	5.59	6.56	10.6	Glucose(c) + ATP(c) → Glucose-6P(c) + ADP(c). Glycolysis / Glyconeogenesis EC:2.7.1.1 (3 genes, 4 sp.)
Hk1 (2 sp.)		0.66	4.55	5.21		0.29		0.87	4.63	5.5		ENSMUSG00000037012 hexokinase 1
Hk2		1.43	6.02	7.45		1.06		2.41	6.1	8.51		ENSMUSG00000000628 hexokinase 2
Hk3		-0.3	6.87	6.58		0.17		-0.25	6.99	6.74		ENSMUSG00000025877 hexokinase 3
r1032	0.002	-2.04	9.46	7.42	0	-2.7	0	-4.55	9.27	4.72	10	Glucose(s) ⇌ Glucose(c). Major Facilitator(MFS) TCDB:2.A.1.1.29
Slc2a2												ENSMUSG00000027690 solute carrier family 2 (facilitated glucose transporter), member 2
r0341	0.04	0.2	7.6	7.8	0.2	-0.04	0.07	0.15	7.62	7.77	12.7	3PG(c) ⇌ 2PG(c). 2-Phospho-D-glycerate 2,3-phosphomutase Glycolysis / Gluconeogenesis EC:5.4.2.1 (2 genes, 4 sp.)
Bpgm (3 sp.)		0.42	6.25	6.68		0.03		0.4	6.31	6.71		ENSMUSG00000038871 2,3-bisphosphoglycerate mutase
Pgam1		-0.47	11.6	11.2		-0.24		-0.59	11.5	10.9		ENSMUSG00000011752 phosphoglycerate mutase 1
r0337	1	-0.73	12.8	12	0.89	-0.43	0.94	-1.14	12.8	11.6	8.45	ADP(c) + 1,3DPG(c) ⇌ ATP(c) + 3PG(c). ATP:3-phospho-D-glycerate 1-phosphotransferase Glycolysis / Gluconeogenesis EC:2.7.2.3
Pgk1												ENSMUSG00000062070 phosphoglycerate kinase 1
r0164	0.56	-0.34	13.2	12.9	0.79	-0.22	0.7	-0.56	13.2	12.7	12.5	2PG(c) ⇌ PEP(c) + H2O(c). 2-Phospho-D-glucerate hydro-lyase Glycolysis / Gluconeogenesis EC:4.2.1.11 (3 sp.)
Eno1 (3 sp.)												ENSMUSG00000063524 enolase 1, alpha non-neuron
r0736	0.99	-0.78	9.44	8.65	0.9	-0.42	0.99	-1.05	9.28	8.23	3.31	ATP(c) + Fructose-6P(c) → ADP(c) + Fructose-1,6PP(c). ATP:D-fructose-6-phosphate 1-phosphotransferase EC:2.7.1.11 (2 sp.)
Pfkl (2 sp.)												ENSMUSG00000020277 phosphofructokinase, liver, B-type
r0192	0.99	-0.79	11.7	10.9	0.79	-0.46	0.97	-1.09	11.5	10.4	3.07	Glucose-6P(c) ⇌ Fructose-6P(c). D-Glucose-6-phosphate ketol-isomerase EC:5.3.1.9 (3 sp.)
Gpi1 (3 sp.)												ENSMUSG00000036427 glucose phosphate isomerase 1
r0256	0.99	-0.78	11.4	10.7	0.65	-0.5	0.91	-1.18	11.3	10.2	3.21	Fructose-1,6PP(c) ⇌ DHAP(c) + GAP(c). D-Fructose-1,6-bisphosphate D-glyceraldehyde-3-phosphate-lyase Carbon fixation EC:4.1.2.13 (2 genes, 4 sp.)
Aldoa (3 sp.)		-0.32	11	10.6		-0.12		-0.36	10.9	10.5		ENSMUSG00000030695 aldolase A, fructose-bisphosphate
Aldob		-2.13	12.9	10.7		-1.64		-3.66	12.7	9.09		ENSMUSG00000028307 aldolase B, fructose-bisphosphate
r0243	0.97	-0.64	12.2	11.6	0.02	-0.84	0.68	-1.4	12.1	10.7	3.22	DHAP(c) ⇌ GAP(c). D-Glyceraldehyde-3-phosphate ketol-isomerase Glycolysis / Gluconeogenesis EC:5.3.1.1 (3 sp.)
Tpi1 (3 sp.)												ENSMUSG00000023456 triosephosphate isomerase 1
r2078	0.16	-0.03	6.62	6.59	0.98	-0.31	0.42	-0.33	6.61	6.28	2.8	H+(PG)(c) + L-Lactate(c) ⇌ H+(PG)(s) + L-Lactate(s). Major Facilitator(MFS) TCDB:2.A.1.13.1 (2 sp.)
Slc16a1 (2 sp.)												ENSMUSG00000032902 solute carrier family 16 (monocarboxylic acid transporters), member 1
r0253	0.53	-0.32	13.8	13.5	0.42	-0.12	0.53	-0.43	13.8	13.3	8.42	GAP(c) + Pi(c) + NAD+(c) ⇌ 1,3DPG(c) + NADH(c). D-Glyceraldehyde-3-phosphate:NAD+ oxidoreductase(phosphorylating) Glycolysis / Gluconeogenesis EC:1.2.1.12 (4 sp.)
Gapdh (4 sp.)												ENSMUSG00000057666 glyceraldehyde-3-phosphate dehydrogenase

37 Reaction scores for Proline (487), series control 1/24h, TGFβ/C 24h, and TGFβ 1/24h

The amplitudes of the mode are -0.29, -0.44 and -0.44.

Simulation	Definition			Solution													
				exchanges		reactions								transp			Prot
	Objective	Constraints	Comment	imports	exports	c	m	r	p	l	n	s	b	s-c	b-c	intra	synth
487 Proline	Proline(c)	−Valine(c) MCES	de novo synthesis of cytosolic Proline	1 O_2(s) 1 Valine(c) 1 ATP-energy(m) 1 NADPH-redox-potential(m)	2 H_2O(s) 1 Proline(c) 2 NADH-redox-potential(m) 10 Proton-gradient(m)	-	16	-	-	-	-	-	-	2	-	7	-

Table 36: Reaction scores for Proline (487), series control 1/24h, TGFβ/C 24h, and TGFβ 1/24h

Rea ID	control 1/24h Score *0.39*	Expr Δ	Expr C1h	Expr C24h	TGFβ/C 24h Score *0.48*	Expr Δ	TGFβ 1/24h Score *0.53*	Expr Δ	Expr T1h	Expr T24h	Wght	Reaction
r0560	0	0.52	8.94	9.46	0.19	-0.04	0.002	0.32	9.1	9.42	11.8	Isobutyryl-CoA(m) + Ubiquinone(m) → Methacrylyl-CoA(m) + Ubiquinol(m). 2-Methylpropanoyl-CoA:oxygen 2,3-oxidoreductase Valine, leucine and isoleucine degradation EC:1.3.99.2
Acads												ENSMUSG00000029545 acyl-Coenzyme A dehydrogenase, short chain
C0112	0	0.41	6.5	6.91	1	-0.43	0.45	-0.16	6.64	6.48	13	several reactions. ATP:L-glutamate 5-phosphotransferase Urea cycle and metabolism of amino groups EC:2.7.2.11 (1 genes, 4 sp.)
Aldh18a1 (4 sp.)												ENSMUSG00000025007 aldehyde dehydrogenase 18 family, member A1
r0263	0.89	-0.22	4.56	4.33	0.04	0.11	0.76	-0.27	4.72	4.45	9	Valine(m) + AKG(m) → Glutamate(m) + 3-Methyl-2-oxobutyrate(m). L-Leucine:2-oxoglutarate aminotransferase Valine, leucine and isoleucine degradation / Valine, leucine and isoleucine biosynthesis EC:2.6.1.42
Bcat2												ENSMUSG00000030826 branched chain aminotransferase 2, mitochondrial
r0197	0.85	-0.38	9.18	8.8	0.54	-0.19	1	-0.43	9.03	8.6	9.9	(R)-Methylmalonyl-CoA(m) ⇌ Succinyl-CoA(m). (R)-2-Methyl-3-oxopropanoyl-CoA CoA-carbonylmutase Valine, leucine and isoleucine degradation / Propanoate metabolism EC:5.4.99.2
Mut												ENSMUSG00000023921 methylmalonyl-Coenzyme A mutase
r0779	0.1	0.02	6.56	6.58	0.74	-0.61	0.95	-0.51	6.48	5.97	8.28	3-Hydroxyisobutyryl-CoA(m) + H2O(m) → 3-Hydroxyisobutyrate(m) + CoA(m). 3-Hydroxy-2-methylpropanoyl-CoA hydrolase EC:3.1.2.4 (2 sp.)
Hibch (2 sp.)												ENSMUSG00000041426 3-hydroxyisobutyryl-Coenzyme A hydrolase
r0482	0.67	-0.16	10.5	10.4	0.59	-0.21	0.78	-0.29	10.4	10.2	12.4	3-Hydroxyisobutyrate(m) + NAD+(m) → 2-Methyl-3-oxopropanoate(m) + NADH(m). 3-Hydroxy-2-methylpropanoate:NAD+ oxidoreductase EC:1.1.1.31 (2 sp.)
Hibadh (2 sp.)												ENSMUSG00000029776 3-hydroxyisobutyrate dehydrogenase
r0175	0.13	0.001	7.03	7.03	0.89	-0.54	0.91	-0.53	7.02	6.48	7.09	L-Glutamate 5-semialdehyde(m) ⇌ 1-Pyrroline-5-carboxylate(m) + H2O(m). L-1-Pyrroline-5-carboxylate:NAD+ oxidoreductase Glutamate metabolism / Arginine and proline metabolism EC:1.5.1.12
Aldh4a1												ENSMUSG00000028737 aldehyde dehydrogenase 4 family, member A1
r1453	0.86	-0.37	8.04	7.66	0.81	-0.3	0.43	-0.72	8.09	7.36	7.69	1-Pyrroline-5-carboxylate(m) + Ubiquinone(m) ⇌ Proline(m) + Ubiquinol(m). L-proline:(acceptor) oxidoreductase Arginine and proline metabolism EC:1.5.99.8
Prodh												ENSMUSG00000003526 proline dehydrogenase
r1114	0.01	0.16	7.56	7.71	0.3	-0.1	0.1	0.03	7.58	7.62	6.25	NADH(m) + CO2(m) + Succinyl-CoA(m) ⇌ AKG(m) + CoA(m) + NAD+(m). EC:1.2.4.2 (2 sp.)
Ogdh (2 sp.)												ENSMUSG00000020456 oxoglutarate dehydrogenase (lipoamide)
r0017	0.31	-0.07	10	9.95	0.15	-0.01	0.3	-0.1	10	9.93	9.17	O2(m) + 4 H+(PG)(m) + 4 Ferrocytochrome C(m) → 4 Ferricytochrome C(m) + 2 H2O(m) + 4 H+(PG)(c). Ferrocytochrome-c:oxygen oxidoreductase Oxidative phosphorylation EC:1.9.3.1 (16 genes, 26 sp.)
Cox4i1		0.04	12.5	12.6		0.03		0.03	12.6	12.6		ENSMUSG00000031818 cytochrome c oxidase subunit IV isoform 1
Cox4i2		-0.02	4.73	4.71		-0.01		-0.1	4.8	4.71		ENSMUSG00000009876 cytochrome c oxidase subunit IV isoform 2
Cox5a (3 sp.)		-0.09	11.6	11.5		-0.03		-0.07	11.5	11.4		ENSMUSG00000000088 cytochrome c oxidase, subunit Va
Cox5b (4 sp.)		-0.09	11.9	11.8		-0.07		-0.12	11.9	11.8		ENSMUSG00000061518 cytochrome c oxidase, subunit Vb
Cox6a1 (2 sp.)		0.01	12.4	12.5		-0.03		-0.04	12.5	12.4		ENSMUSG00000041697 cytochrome c oxidase, subunit VI a, polypeptide 1
Cox6a2		-0.05	6.85	6.8		-0.08		-0.14	6.86	6.72		ENSMUSG00000030785 cytochrome c oxidase, subunit VI a, polypeptide 2
Cox6b1 (2 sp.)		0.06	12	12.1		-0.04		0.05	12	12		ENSMUSG00000036751 cytochrome c oxidase, subunit VIb polypeptide 1
Cox6b2		0.06	5.02	5.08		0.16		0.15	5.09	5.24		ENSMUSG00000051811 cytochrome c oxidase subunit VIb polypeptide 2
Cox6c (2 sp.)		-0.1	11.4	11.3		-0.01		-0.14	11.4	11.2		ENSMUSG00000014313 cytochrome c oxidase, subunit VIc
Cox7a1		-0.19	4.49	4.3		0.21		-0.2	4.71	4.51		ENSMUSG00000074218 cytochrome c oxidase, subunit VIIa 1
Cox7a2 (2 sp.)		-0.31	12.2	11.9		-0.12		-0.38	12.2	11.8		ENSMUSG00000032330 cytochrome c oxidase, subunit VIIa 2
Cox7b2		-0.01	2.91	2.9		0.19		-0.07	3.15	3.09		ENSMUSG00000049387 cytochrome c oxidase subunit VIIb2
Cox7c		-0.11	11.7	11.6		0.01		-0.1	11.7	11.6		ENSMUSG00000017778 cytochrome c oxidase, subunit VIIc
Cox8a (2 sp.)		-0.08	10.4	10.3		-0.05		-0.13	10.4	10.3		ENSMUSG00000035885 cytochrome c oxidase, subunit VIIIa
Cox8b		0.01	5.54	5.54		-0.01		-0.14	5.67	5.53		ENSMUSG00000025488 cytochrome c oxidase, subunit VIIIb
Cox8c		-0.06	7.29	7.23		0.05		-0.04	7.32	7.28		ENSMUSG00000043319 cytochrome c oxidase, subunit VIIIc
r0669	0.56	-0.14	10.1	9.93	0.17	-0.03	0.41	-0.15	10.1	9.91	7.48	Methacrylyl-CoA(m) + H2O(m) → 3-Hydroxyisobutyryl-CoA(m). (S)-3-Hydroxyisobutyryl-CoA hydro-lyase Valine, leucine and isoleucine degradation EC:4.2.1.17 (2 genes, 2 sp.)
Echs1		-0.24	10.3	10		-0.15		-0.37	10.2	9.87		ENSMUSG00000025465 enoyl Coenzyme A hydratase, short chain, 1, mitochondrial
Hadha		-0.03	9.87	9.84		0.1		0.08	9.86	9.94		ENSMUSG00000025745 hydroxyacyl-Coenzyme A dehydrogenase/3-ketoacyl-Coenzyme A thiolase/enoyl-Coenzyme A hydratase (trifunctional protein), alpha subunit
r0507	0.36	-0.08	10.5	10.4	0.16	-0.02	0.26	-0.08	10.5	10.4	6.5	Ubiquinol(m) + 2 Ferricytochrome C(m) + 4 H+(PG)(m) → Ubiquinone(m) + 4 H+(PG)(c) + 2 Ferrocytochrome C(m). Ubiquinol:ferricytochrome-c oxidoreductase Oxidative phosphorylation EC:1.10.2.2 (8 genes, 13 sp.)
Uqcr10 (2 sp.)		-0.53	9.74	9.21		-0.04		-0.46	9.63	9.18		ENSMUSG00000059534 ubiquinol-cytochrome c reductase, complex III subunit X
Uqcr11		-0.13	11.1	11		-0.13		-0.24	11.1	10.8		ENSMUSG00000020163 ubiquinol-cytochrome c reductase, complex III subunit XI
Uqcrb (2 sp.)		0.001	12	12		-0.05		0.04	11.9	11.9		ENSMUSG00000021520 ubiquinol-cytochrome c reductase binding protein
Uqcrc1		0.11	10.8	10.9		0.06		0.11	10.9	11		ENSMUSG00000025651 ubiquinol-cytochrome c reductase core protein 1
Uqcrc2 (2 sp.)		-0.04	10.6	10.5		0.05		0.08	10.5	10.6		ENSMUSG00000030884 ubiquinol cytochrome c reductase core protein 2
Uqcrfs1		-0.01	11.4	11.4		-0.07		-0.1	11.4	11.3		ENSMUSG00000038462 ubiquinol-cytochrome c reductase, Rieske iron-sulfur polypeptide 1
Uqcrh (2 sp.)		0.06	11.1	11.2		0.003		0.11	11.1	11.2		ENSMUSG00000063882 ubiquinol-cytochrome c reductase hinge protein
Uqcrq (2 sp.)		-0.01	8.07	8.06		-0.03		-0.15	8.18	8.03		ENSMUSG00000044894 ubiquinol-cytochrome c reductase, complex III subunit VII

38 Reaction scores for Triacylglycerol (522), series control 1/24h, TGFβ/C 24h, and TGFβ 1/24h

The amplitudes of the mode are -0.24, -0.61 and -1.04.

Simulation	Definition			Solution													
				exchanges		reactions								transp			Prot
	Objective	Constraints	Comment	imports	exports	c	m	r	p	l	n	s	b	s-c	b-c	intra	synth
522 Triacylglycerol	Triacylglycerol-VLDL-pool(r)	MCES ALFA	de novo synthesis of Triacylglycerol-VLDL-pool in ER/Golgi	0.5 Glucose-6P(c) 0.09 Arachidonate(c) 0.92 Palmitate(c) 1.1 Oleate(c) 0.2 Stearate(c) 0.69 Linoleate(c) 6.5 ATP-energy(c) 1 NADH-redox-potential(c)	2.5 H_2O(s) 0.5 P_i(c) 1 Triacylglycerol-VLDL-pool(r)	25	-	-	-	-	-	-	-	1	-	1	-

Table 37: Reaction scores for Triacylglycerol (522), series control 1/24h, TGFβ/C 24h, and TGFβ 1/24h

	control 1/24h				TGFβ/C 24h		TGFβ 1/24h					
Rea ID	Score *0.51*	Expr Δ	Expr C1h	Expr C24h	Score *0.5*	Expr Δ	Score *0.59*	Expr Δ	Expr T1h	Expr T24h	Wght	Reaction
r1223	0.97	-0.21	9.15	8.95	0.99	-0.55	0.93	-0.84	9.24	8.39	14.8	Acyl-CoA-VLDL-TG3-pool(c) + 1,2-Diacylglycerol-VLDL-TG-pool(c) → Triacylglycerol-VLDL-pool(c) + CoA(c). Glycerolipid metabolism EC:2.3.1.20 (2 genes, 3 sp.)
Dgat1		0.78	7.82	8.59		-0.08		0.72	7.8	8.52		ENSMUSG00000022555 diacylglycerol O-acyltransferase 1
Dgat2 (2 sp.)		-0.7	9.82	9.12		-0.79		-1.62	9.95	8.33		ENSMUSG00000030747 diacylglycerol O-acyltransferase 2
r1217	0.01	0.14	8.74	8.87	0.14	-0.01	0.09	0.09	8.78	8.87	9.8	Phosphatidate-VLDL-TG-pool(c) + H2O(c) → 1,2-Diacylglycerol-VLDL-TG-pool(c) + Pi(c). 1,2-Diacyl-sn-glycerol 3-phosphate phosphohydrolase Glycerolipid metabolism / Glycerophospholipid metabolism EC:3.1.3.4 (3 genes, 5 sp.)
Ppap2a (2 sp.)		0.18	7.33	7.51		-0.6		-0.39	7.31	6.91		ENSMUSG00000021759 phosphatidic acid phosphatase type 2A
Ppap2b (2 sp.)		0.07	9.17	9.24		0.5		0.47	9.26	9.73		ENSMUSG00000028517 phosphatidic acid phosphatase type 2B
Ppap2c		0.19	10.7	10.9		0.17		0.29	10.8	11		ENSMUSG00000052151 phosphatidic acid phosphatase type 2C
r1211	0.15	-0.01	6.36	6.35	0.15	-0.02	0.17	-0.06	6.39	6.33	9.37	Acyl-CoA-VLDL-TG2-pool(c) + 1-Acylglycerol-3P-VLDL-TG1-pool(c) → Phosphatidate-VLDL-TG-pool(c) + CoA(c). Glycerolipid metabolism / Glycerophospholipid metabolism EC:2.3.1.51 (6 genes, 11 sp.)
Agpat1 (2 sp.)		-0.16	5.77	5.6		0.22		0.04	5.78	5.83		ENSMUSG00000034254 1-acylglycerol-3-phosphate O-acyltransferase 1 (lysophosphatidic acid acyltransferase, alpha)
Agpat2		0.15	9.73	9.88		0.02		0.01	9.89	9.9		ENSMUSG00000026922 1-acylglycerol-3-phosphate O-acyltransferase 2 (lysophosphatidic acid acyltransferase, beta)
Agpat3 (4 sp.)		-0.33	7.84	7.5		0.2		-0.11	7.81	7.71		ENSMUSG00000001211 1-acylglycerol-3-phosphate O-acyltransferase 3
Agpat4 (2 sp.)		0.03	4.47	4.5		-0.03		-0.04	4.5	4.47		ENSMUSG00000023827 1-acylglycerol-3-phosphate O-acyltransferase 4 (lysophosphatidic acid acyltransferase, delta)
Mboat1		1.52	4.36	5.89		-1.51		-0.12	4.5	4.38		ENSMUSG00000038732 membrane bound O-acyltransferase domain containing 1
Mboat2		-0.16	4	3.84		0.11		-0.17	4.13	3.95		ENSMUSG00000020646 membrane bound O-acyltransferase domain containing 2
C0174	1	-0.25	6.94	6.69	0.33	-0.16	0.51	-0.43	6.97	6.54	9.83	several reactions. EC:6.2.1.3 (8 genes, 14 sp.)
Acsbg1		-0.07	4.21	4.13		1.05		0.85	4.34	5.19		ENSMUSG00000032281 acyl-CoA synthetase bubblegum family member 1
Acsbg2		-0.05	3.84	3.79		-0.15		-0.14	3.78	3.64		ENSMUSG00000024207 acyl-CoA synthetase bubblegum family member 2
Acsl1 (3 sp.)		-0.85	8.72	7.87		-0.25		-1.03	8.64	7.62		ENSMUSG00000018796 acyl-CoA synthetase long-chain family member 1
Acsl3 (3 sp.)		-0.29	4.68	4.39		0.6		0.12	4.88	4.99		ENSMUSG00000032883 acyl-CoA synthetase long-chain family member 3
Acsl4 (3 sp.)		0.42	8.12	8.54		-0.91		-0.44	8.07	7.63		ENSMUSG00000031278 acyl-CoA synthetase long-chain family member 4
Acsl5		0.12	11.4	11.5		-0.33		-0.24	11.4	11.2		ENSMUSG00000024981 acyl-CoA synthetase long-chain family member 5
Acsl6		-0.21	3	2.79		0.2		-0.12	3.12	2.99		ENSMUSG00000020333 acyl-CoA synthetase long-chain family member 6
Slc27a2		-1.05	10.1	9.09		-1.3		-2.36	10.2	7.79		ENSMUSG00000027359 solute carrier family 27 (fatty acid transporter), member 2
C0167	0.29	-0.42	8.46	8.03	0.63	-0.32	0.99	-0.96	8.67	7.71	8.63	several reactions. EC:2.3.1.15 (2 genes, 2 sp.)
Agpat6		0.25	8.61	8.87		-0.31		-0.07	8.63	8.56		ENSMUSG00000031545 1-acylglycerol-3-phosphate O-acyltransferase 6 (lysophosphatidic acid acyltransferase, zeta)
Agpat9		-1.1	8.3	7.19		-0.32		-1.85	8.72	6.87		ENSMUSG00000029314 1-acylglycerol-3-phosphate O-acyltransferase 9
r0201	0.95	-0.2	8.56	8.36	0.47	-0.98	0.79	-1.4	8.78	7.38	8.44	DHAP(c) + NADH(c) ⇌ NAD+(c) + sn-Glycerol-3P(c). sn-Glycerol-3-phosphate:NAD+ 2-oxidoreductase Glycerophospholipid metabolism EC:1.1.1.8 (2 sp.)
Gpd1 (2 sp.)												ENSMUSG00000023019 glycerol-3-phosphate dehydrogenase 1 (soluble)
r0007	0.01	0.15	10.1	10.2	0.23	-0.09	0.1	0.07	10.1	10.1	4.01	PPi(c) + H2O(c) → 2 Pi(c). Pyrophosphate phosphohydrolase EC:3.6.1.1 (2 genes, 2 sp.)
Ppa1		-0.26	11.1	10.8		0.18		-0.14	11.1	11		ENSMUSG00000020089 pyrophosphatase (inorganic) 1
Ppa2		0.56	9.07	9.63		-0.35		0.28	9	9.28		ENSMUSG00000028013 pyrophosphatase (inorganic) 2
r0312	0.97	-0.21	7.18	6.97	0.34	-0.16	0.47	-0.4	7.21	6.81	6.76	Palmitate(c) + ATP(c) + CoA(c) → Palmitoyl-CoA(c) + AMP(c) + PPi(c). Palmitate:CoA ligase (AMP-forming) Fatty acid metabolism EC:6.2.1.3 (5 genes, 11 sp.)
Acsl1 (3 sp.)		-0.85	8.72	7.87		-0.25		-1.03	8.64	7.62		ENSMUSG00000018796 acyl-CoA synthetase long-chain family member 1
Acsl3 (3 sp.)		-0.29	4.68	4.39		0.6		0.12	4.88	4.99		ENSMUSG00000032883 acyl-CoA synthetase long-chain family member 3
Acsl4 (3 sp.)		0.42	8.12	8.54		-0.91		-0.44	8.07	7.63		ENSMUSG00000031278 acyl-CoA synthetase long-chain family member 4
Acsl5		0.12	11.4	11.5		-0.33		-0.24	11.4	11.2		ENSMUSG00000024981 acyl-CoA synthetase long-chain family member 5
Acsl6		-0.21	3	2.79		0.2		-0.12	3.12	2.99		ENSMUSG00000020333 acyl-CoA synthetase long-chain family member 6
r0030	0.47	-0.09	6.05	5.96	0.09	0.06	0.16	-0.05	6.06	6.02	4.43	ATP(c) + AMP(c) ⇌ 2 ADP(c). ATP:AMP phosphotransferase Purine metabolism EC:2.7.4.3 (5 genes, 11 sp.)
Ak1 (2 sp.)		-0.07	3.97	3.89		0.76		0.59	4.07	4.66		ENSMUSG00000026817 adenylate kinase 1
Ak2 (2 sp.)		0.08	9.35	9.43		0.13		0.15	9.41	9.55		ENSMUSG00000028792 adenylate kinase 2
Ak4 (4 sp.)		-0.36	6.41	6.05		-0.23		-0.45	6.27	5.82		ENSMUSG00000028527 adenylate kinase 4
Ak5 (2 sp.)		-0.17	4.72	4.56		0.12		-0.12	4.79	4.68		ENSMUSG00000039058 adenylate kinase 5
Ak7		0.74	4.79	5.53		-0.39		0.06	5.08	5.14		ENSMUSG00000041323 adenylate kinase 7
r0256	0	-0.78	11.4	10.7	0.94	-0.5	0.96	-1.18	11.3	10.2	2.74	Fructose-1,6PP(c) ⇌ DHAP(c) + GAP(c). D-Fructose-1,6-bisphosphate D-glyceraldehyde-3-phosphate-lyase Carbon fixation EC:4.1.2.13 (2 genes, 4 sp.)
Aldoa (3 sp.)		-0.32	11	10.6		-0.12		-0.36	10.9	10.5		ENSMUSG00000030695 aldolase A, fructose-bisphosphate
Aldob		-2.13	12.9	10.7		-1.64		-3.66	12.7	9.09		ENSMUSG00000028307 aldolase B, fructose-bisphosphate
r0736	0	-0.78	9.44	8.65	0.83	-0.42	1	-1.05	9.28	8.23	2.82	ATP(c) + Fructose-6P(c) → ADP(c) + Fructose-1,6PP(c). ATP:D-fructose-6-phosphate 1-phosphotransferase EC:2.7.1.11 (2 sp.)
Pfkl (2 sp.)												ENSMUSG00000020277 phosphofructokinase, liver, B-type
r0192	0	-0.79	11.7	10.9	0.89	-0.46	0.99	-1.09	11.5	10.4	2.62	Glucose-6P(c) ⇌ Fructose-6P(c). D-Glucose-6-phosphate ketol-isomerase EC:5.3.1.9 (3 sp.)
Gpi1 (3 sp.)												ENSMUSG00000036427 glucose phosphate isomerase 1
r0243	0.003	-0.64	12.2	11.6	0.75	-0.84	0.79	-1.4	12.1	10.7	2.75	GAP(c) ⇌ DHAP(c). D-Glyceraldehyde-3-phosphate ketol-isomerase Glycolysis / Gluconeogenesis EC:5.3.1.1 (3 sp.)
Tpi1 (3 sp.)												ENSMUSG00000023456 triosephosphate isomerase 1

39 Reaction scores for Choloyl-CoA (741), series control 1/24h, TGFβ/C 24h, and TGFβ 1/24h

The amplitudes of the mode are -0.82, -0.47 and -1.3.

Simulation	Definition			Solution													
				exchanges		reactions								transp			Prot
	Objective	Constraints	Comment	imports	exports	c	m	r	p	l	n	s	b	s-c	b-c	intra	synth
741 Choloyl-CoA	Choloyl-CoA(c)	PIPES MCES	synthesis of cytosolic Choloyl-CoA	11.5 O_2(s) 1 Glycine(s) 1 Glutamine(s) 0.83 Glucose-6P(c) 2 Asparagine(s) 2 Farnesyl-PP(r) 1 Cystine(s) 1 Pantothenate(s) 16.2 ATP-energy(c) 1 NADH-redox-potential(m) 12 NADPH-redox-potential(r) 1 NADPH-redox-potential(c) 1 Proton-gradient(m) 2 THF-activated methyl group(c)	16.2 H_2O(s) 1.83 P_i(c) 3 CO_2(s) 2 Pyruvate(c) 1 Glutamate(s) 1 Serine(s) 1 Methionine(s) 1 Choloyl-CoA(c) 1 NADH-redox-potential(r) 3 NADH-redox-potential(c) 1 NADH-redox-potential(p) 2 Na-gradient(c)	53	2	19	7	-	-	-	-	9	-	18	-

Table 38: Reaction scores for Choloyl-CoA (741), series control 1/24h, TGFβ/C 24h, and TGFβ 1/24h

Rea	control 1/24h				TGFβ/C 24h		TGFβ 1/24h				Wght	Reaction
	Score	Expr	Expr	Expr	Score	Expr	Score	Expr	Expr	Expr		
ID	*0.45*	Δ	C1h	C24h	*0.47*	Δ	*0.48*	Δ	T1h	T24h		
r1134	0.99	-0.87	10.7	9.86	0.99	-0.44	0.95	-1.08	10.5	9.42	26	14-Demethyllanosterol(r) + 3 NADPH(r) + 3 O2(r) ⇌ 4 H2O(r) + 4alpha-Methylzymosterol-4-carboxylate(r) + 3 NADP+(r). methylsterol monooxygenase Biosynthesis of steroids EC:1.14.13.72
Sc4mol												ENSMUSG00000031604 sterol-C4-methyl oxidase-like
r0781	0.94	-0.96	7.54	6.58	1	-0.47	1	-1.29	7.4	6.11	25.7	3 O2(r) + 3 NADPH(r) + Lanosterol(r) ⇌ 4,4-Dimethyl-5alpha-cholesta-8,14,24-trien-3beta-ol(r) + Formate(r) + 4 H2O(r) + 3 NADP+(r). Lanosterol,NADPH:oxygen oxidoreductase (14-methyl cleaving) Biosynthesis of steroids EC:1.14.13.70 (3 sp.)
Cyp51 (3 sp.)												ENSMUSG00000001467 cytochrome P450, family 51
r0628	0.94	-0.67	12.3	11.6	0.89	-0.59	0.97	-1.15	12.1	11	15.2	CoA(p) + 3alpha,7alpha,12alpha-Trihydroxy-5beta-24-oxocholestanoyl-CoA(p) → Propanoyl-CoA(p) + Choloyl-CoA(p). Propanoyl-CoA:acetyl-CoA C-acyltransferase Bile acid biosynthesis EC:2.3.1.16 (2 sp.)
Scp2 (2 sp.)												ENSMUSG00000028603 sterol carrier protein 2, liver
r0706	0.04	0.2	6.11	6.31	0.13	0.003	0.07	0.2	6.12	6.32	13.7	O2(p) + 3alpha,7alpha,12alpha-Trihydroxy-5beta-cholestanoyl-CoA(p) → 3alpha,7alpha,12alpha-Trihydroxy-5beta-cholest-24-enoyl-CoA(p) + H2O2(p). acyl-Coenzyme A dehydrogenase family, member 9 Bile acid biosynthesis EC:1.3.3.6 (3 sp.)
Acad9 (3 sp.)												ENSMUSG00000027710 acyl-Coenzyme A dehydrogenase family, member 9
C0135	0.32	-0.2	10.4	10.2	0.94	-0.39	0.57	-0.61	10.4	9.83	20	several reactions. hydroxysteroid (17-beta) dehydrogenase 4 Bile acid biosynthesis EC:1.1.1.35 (1 genes, 2 sp.)
Hsd17b4 (2 sp.)												ENSMUSG00000024507 hydroxysteroid (17-beta) dehydrogenase 4
r1454	0.96	-0.7	7.02	6.32	0.99	-0.44	0.95	-1.1	6.98	5.88	8.88	2-Oxobutyrate(m) + H+(PG)(c) → 2-Oxobutyrate(c) + H+(PG)(m). TCDB:2.A.1.13.5 TCDB:2.A.1.13.1 (2 genes, 3 sp.)
Slc16a1 (2 sp.)		-0.03	6.62	6.59		-0.31		-0.33	6.61	6.28		ENSMUSG00000032902 solute carrier family 16 (monocarboxylic acid transporters), member 1
Slc16a7		-2.03	7.82	5.79		-0.7		-2.62	7.71	5.09		ENSMUSG00000020102 solute carrier family 16 (monocarboxylic acid transporters), member 7
r0232	0.01	0.48	5.06	5.54	0.09	0.04	0.03	0.4	5.17	5.58	10.3	5-Methyl-THF(c) + Homocysteine(c) ⇌ THF(c) + Methionine(c). 5-Methyltetrahydrofolate:L-homocysteine S-methyltransferase Methionine metabolism / One carbon pool by folate EC:2.1.1.13 (2 sp.)
Mtr (2 sp.)												ENSMUSG00000021311 5-methyltetrahydrofolate-homocysteine methyltransferase
r0812	0.07	0.13	8.82	8.95	1	-0.47	0.37	-0.39	8.86	8.47	12.8	ATP(c) + AMP(p) ⇌ ATP(p) + AMP(c). Mitochondrial Carrier (MC) TCDB:2.A.29.20.1
Slc25a17												ENSMUSG00000022404 solute carrier family 25 (mitochondrial carrier, peroxisomal membrane protein), member 17
r0127	0.95	-0.69	6.93	6.25	0.004	0.31	0.32	-0.33	6.88	6.55	11.3	H2O(c) + Asparagine(c) → Aspartate(c) + NH3(c). L-Asparagine amidohydrolase Alanine and aspartate metabolism / Cyanoamino acid metabolism / Nitrogen metabolism EC:3.5.1.1 EC:3.5.1.38
Aspg												ENSMUSG00000037686 asparaginase homolog (S. cerevisiae)
r0314	0.95	-0.7	9.45	8.76	0	-1.69	0.24	-2.4	9.46	7.06	10.4	H2O(c) + L-Cystathionine(c) ⇌ Serine(c) + Homocysteine(c). L-Serine hydro-lyase (adding homocysteine) Glycine, serine and threonine metabolism / Methionine metabolism EC:4.2.1.22 (2 sp.)
Cbs (2 sp.)												ENSMUSG00000024039 cystathionine beta-synthase
r0607	0.98	-0.74	6.42	5.68	0.97	-0.41	0.9	-1.01	6.28	5.27	8.51	Squalene 2,3-oxide(r) ⇌ Lanosterol(r). (S)-2,3-Epoxysqualene mutase (cyclizing, lanosterol-forming) Biosynthesis of steroids EC:5.4.99.7 (2 sp.)
Lss (2 sp.)												ENSMUSG00000033105 lanosterol synthase
C0125	0.03	0.29	6.91	7.2	0.13	0.004	0.06	0.26	6.94	7.21	10.1	several reactions. 5-Phospho-D-ribosylamine:glycine ligase (ADP-forming) Purine metabolism EC:6.3.4.13 (1 genes, 4 sp.)
Gart (4 sp.)												ENSMUSG00000022962 phosphoribosylglycinamide formyltransferase
r1135	1	-0.85	6.95	6.1	0.81	-0.32	0.91	-1.02	6.8	5.78	8.77	3-Keto-4-methylzymosterol(r) + NADP+(r) ⇌ NADPH(r) + 4alpha-Methylzymosterol(r). hydroxysteroid (17-beta) dehydrogenase 7 Biosynthesis of steroids EC:1.1.1.270 (4 sp.)
Hsd17b7 (4 sp.)												ENSMUSG00000026675 hydroxysteroid (17-beta) dehydrogenase 7
r0741	0.99	-0.88	11.3	10.4	0.41	-0.79	0.9	-1.6	11.2	9.59	11.2	3alpha,7alpha,12alpha-Trihydroxycoprostane(c) + NADPH(c) + O2(c) ⇌ 5beta-Cholestane-3alpha,7alpha,12alpha,26-tetrol(c) + H2O(c) + NADP+(c). 5beta-Cholestane-3alpha,7alpha,12alpha-triol,NADPH:oxygen oxidoreductase (26-hydroxylating) Bile acid biosynthesis EC:1.14.13.15
Cyp27a1												ENSMUSG00000026170 cytochrome P450, family 27, subfamily a, polypeptide 1
C0129	0.04	0.23	8.52	8.75	0.01	0.23	0.03	0.4	8.58	8.98	8.43	several reactions. IMP 1,2-hydrolase (decyclizing) Purine metabolism EC:3.5.4.10 (1 genes, 2 sp.)
Atic (2 sp.)												ENSMUSG00000026192 5-aminoimidazole-4-carboxamide ribonucleotide formyltransferase/IMP cyclohydrolase
r0751	0.07	-1.76	7.07	5.3	0.06	0.09	0.62	-1.94	7.33	5.39	12	7alpha-Hydroxycholest-4-en-3-one(r) + O2(r) + NADPH(r) → 7alpha,12alpha-Dihydroxycholest-4-en-3-one(r) + H2O(r) + NADP+(r). 7alpha-hydroxycholest-4-en-3-one,NADPH:oxygen oxidoreductase(12alpha-hydroxylating) Bile acid biosynthesis EC:1.14.-.-
Cyp8b1												ENSMUSG00000050445 cytochrome P450, family 8, subfamily b, polypeptide 1
r0576	0.89	-0.63	9.99	9.36	0.03	-1.1	0.89	-1.62	9.87	8.26	8.46	Squalene(r) + NADPH(r) + O2(r) ⇌ H2O(r) + Squalene 2,3-oxide(r) + NADP+(r). Squalene,hydrogen-donor:oxygen oxidoreductase (2,3-epoxidizing) Biosynthesis of steroids EC:1.14.99.7
Sqle												ENSMUSG00000022351 squalene epoxidase
r1675	0.02	0.37	4.1	4.47	0	1.05	0.0004	1.28	4.25	5.52	7.65	Glycine(s) + Methionine(c) + Na+(s) → Glycine(c) + Methionine(s) + Na+(c). Y+LAT2 Utilized transport (3 sp.)
Slc7a6 (3 sp.)												ENSMUSG00000031904 solute carrier family 7 (cationic amino acid transporter, y+ system), member 6
r0748	1	-0.83	4.1	3.28	0.31	-0.11	0.76	-0.82	3.99	3.16	10.7	7alpha,12alpha-Dihydroxycholest-4-en-3-one(c) + NADPH(c) → 7alpha,12alpha-Dihydroxy-5beta-cholestan-3-one(c) + NADP+(c). 7alpha,12alpha-Dihydroxy-5beta-cholestan-3-one:(acceptor) delta4-oxidoreductase; 7alpha,12alpha-Dihydroxy-5beta-cholestan-3-one:NADP+ delta4-oxidoreductase Bile acid biosynthesis EC:1.3.1.3 (2 sp.)
Akr1d1 (2 sp.)												ENSMUSG00000038641 aldo-keto reductase family 1, member D1
r0632	0.98	-0.74	9.25	8.52	0.01	-1.18	0.64	-1.92	9.25	7.33	9.17	7-Dehydrodesmosterol(r) + NADPH(r) ⇌ Desmosterol(r) + NADP+(r). 7-dehydrocholesterol reductase Biosynthesis of steroids EC:1.3.1.21
Dhcr7												ENSMUSG00000058454 7-dehydrocholesterol reductase
r0711	0.18	-0.07	7.93	7.87	0.005	0.3	0.06	0.24	7.93	8.17	9.05	5alpha-Cholesta-7,24-dien-3beta-ol(r) + NADPH(r) + O2(r) ⇌ 7-Dehydrodesmosterol(r) + 2 H2O(r) + NADP+(r). sterol-C5-desaturase (fungal ERG3, delta-5-desaturase) homolog (S. cerevisae) Biosynthesis of steroids EC:1.14.21.6 (2 sp.)

Continued on next page

	control 1/24h				TGFβ/C 24h		TGFβ 1/24h					
Rea ID	Score *0.45*	Expr Δ	Expr C1h	Expr C24h	Score *0.47*	Expr Δ	Score *0.48*	Expr Δ	Expr T1h	Expr T24h	Wght	Reaction
Sc5d (2 sp.)												ENSMUSG00000032018 sterol-C5-desaturase (fungal ERG3, delta-5-desaturase) homolog (S. cerevisae)
r0750	0.4	-0.26	6.05	5.78	0.001	-1.36	0.91	-1.58	6	4.42	10.8	NADPH(c) + 7alpha,12alpha-Dihydroxy-5beta-cholestan-3-one(c) ⇌ 3alpha,7alpha,12alpha-Trihydroxycoprostane(c) + NADP+(c). 3alpha,7alpha,12alpha-Trihydroxy-5beta-cholestane:NADP+ oxidoreductase (B-specific); 3alpha,7alpha,12alpha-Trihydroxy-5beta-cholestane:NADP+ oxidoreductase Bile acid biosynthesis EC:1.1.1.50 (6 genes, 7 sp.)
Akr1c14		-0.97	9.24	8.27		-3.58		-4.56	9.24	4.69		ENSMUSG00000033715 aldo-keto reductase family 1, member C14
Akr1c18		0.13	5.73	5.86		-0.31		-0.22	5.78	5.55		ENSMUSG00000021214 aldo-keto reductase family 1, member C18
Akr1c20 (2 sp.)		-0.02	5.88	5.86		-1.82		-1.78	5.82	4.04		ENSMUSG00000054757 aldo-keto reductase family 1, member C20
Akr1c21		-0.27	4.87	4.6		-0.02		-0.29	4.87	4.59		ENSMUSG00000021207 aldo-keto reductase family 1, member C21
Akr1c6		-0.78	6.81	6.03		-1.98		-2.28	6.33	4.05		ENSMUSG00000021210 aldo-keto reductase family 1, member C6
Akr1cl		0.07	3.94	4.01		-0.04		-0.17	4.15	3.97		ENSMUSG00000025955 aldo-keto reductase family 1, member C-like
r0738	0.68	-1.18	8.78	7.6	0.87	-0.34	0.99	-1.38	8.63	7.25	8.93	Zymosterol(r) ⇌ 5alpha-Cholesta-7,24-dien-3beta-ol(r). delta8,24-Cholestadien-3beta-ol delta7-delta8-isomerase Biosynthesis of steroids EC:5.3.3.5 (2 sp.)
Ebp (2 sp.)												ENSMUSG00000031168 phenylalkylamine Ca2+ antagonist (emopamil) binding protein
C0104	0.68	-0.46	9.74	9.28	0.39	-0.8	0.97	-1.14	9.63	8.49	11.8	several reactions. Farnesyl-diphosphate:farnesyl-diphosphate farnesyltransferase Biosynthesis of steroids EC:2.5.1.21 (1 genes, 2 sp.)
Fdft1 (2 sp.)												ENSMUSG00000021273 farnesyl diphosphate farnesyl transferase 1
r0672	0.47	-0.32	10.5	10.1	0	0.62	0.07	0.19	10.6	10.8	10.5	7alpha-Hydroxycholesterol(r) + NADP+(r) ⇌ 7alpha-Hydroxycholest-4-en-3-one(r) + NADPH(r). Cholest-5-ene-3beta,7alpha-diol:NAD+ 3-oxidoreductase Bile acid biosynthesis EC:1.1.1.181
Hsd3b7												ENSMUSG00000042289 hydroxy-delta-5-steroid dehydrogenase, 3 beta- and steroid delta-isomerase 7
r0241	0.78	-1.11	10.1	8.99	0.003	-1.27	0.24	-2.4	10.1	7.73	8.82	Cysteine(c) + NH3(c) + 2-Oxobutyrate(c) ⇌ H2O(c) + L-Cystathionine(c). L-Cystathionine Lysteine-lyase (deaminating) Glycine, serine and threonine metabolism / Methionine metabolism / Nitrogen metabolism EC:4.4.1.1
Cth												ENSMUSG00000028179 cystathionase (cystathionine gamma-lyase)
r0077	0.001	0.66	8.34	9	0.0003	0.47	0.001	1.2	8.28	9.48	6.38	ATP(c) + Glutamate(c) + NH3(c) → ADP(c) + Pi(c) + Glutamine(c). L-Glutamate:ammonia ligase (ADP-forming) Glutamate metabolism / Nitrogen metabolism EC:6.3.1.2 (2 sp.)
Glul (2 sp.)												ENSMUSG00000026473 glutamate-ammonia ligase (glutamine synthetase)
r1137	0.79	-1.11	10.4	9.3	0.65	-0.25	1	-1.36	10.4	9.05	8.71	NAD+(r) + 4alpha-Methylzymosterol-4-carboxylate(r) ⇌ NADH(r) + CO2(r) + 3-Keto-4-methylzymosterol(r). NAD(P) dependent steroid dehydrogenase-like EC:1.1.1.170
Nsdhl												ENSMUSG00000031349 NAD(P) dependent steroid dehydrogenase-like
r0581	0	3.04	5.89	8.93	0	-1.85	0.001	1.18	5.9	7.08	6.27	Pantothenate(c) + Cysteamine(c) ⇌ H2O(c) + Pantetheine(c). (R)-pantetheine amidohydrolase Pantothenate and CoA biosynthesis EC:3.5.1.- (2 sp.)
Vnn1 (2 sp.)												ENSMUSG00000037440 vanin 1
r2517	0.24	-0.13	10.8	10.7	0.94	-0.39	0.49	-0.52	10.8	10.3	12.8	3alpha,7alpha,12alpha-Trihydroxy-5beta-cholestanate(c) + ATP(c) + H2O(c) ⇌ 3alpha,7alpha,12alpha-Trihydroxy-5beta-cholestanate(p) + ADP(c) + Pi(c). ATP-binding Cassette (ABC) TCDB:3.A.1.203.1
Abcd3												ENSMUSG00000028127 ATP-binding cassette, sub-family D (ALD), member 3
r0211	0	-3	12.5	9.52	0.0001	-1.47	0	-4.44	12.5	8.04	6.13	Cysteine(c) + O2(c) ⇌ 3-Sulfinoalanine(c). L-Cysteine:oxygen oxidoreductase Cysteine metabolism / Taurine and hypotaurine metabolism EC:1.13.11.20
Cdo1												ENSMUSG00000033022 cysteine dioxygenase 1, cytosolic
C0130	0.05	0.18	10.1	10.3	0.25	-0.08	0.08	0.15	10	10.2	8.36	several reactions. 1-(5-Phospho-D-ribosyl)-5-amino-4-imidazolecarboxylate carboxy-lyase Purine metabolism EC:4.1.1.21 (1 genes, 3 sp.)
Paics (3 sp.)												ENSMUSG00000029247 phosphoribosylaminoimidazole carboxylase, phosphoribosylaminoribosylaminoimidazole, succinocarboxamide synthetase
C0006	0.17	-0.05	8.18	8.13	0.95	-0.39	0.32	-0.32	8.06	7.74	8.87	several reactions. ATP:dephospho-CoA 3'-phosphotransferase Pantothenate and CoA biosynthesis EC:2.7.1.24 (1 genes, 2 sp.)
Coasy (2 sp.)												ENSMUSG00000001755 Coenzyme A synthase
r0687	0.04	0.22	6.01	6.23	0.02	0.18	0.02	0.54	5.87	6.4	5.87	ATP(c) + N-Formyl-GAR(c) + Glutamine(c) + H2O(c) ⇌ ADP(c) + Pi(c) + Glutamate(c) + 5-Phosphoribosylformylglycinamidine(c). 5'-Phosphoribosylformylglycinamide:L-glutamine amido-ligase (ADP-forming) Purine metabolism EC:6.3.5.3
Pfas												ENSMUSG00000020899 phosphoribosylformylglycinamidine synthase (FGAR amidotransferase)
C0128	0.02	0.36	7.6	7.96	0.39	-0.15	0.07	0.18	7.63	7.81	8.33	several reactions. N6-(1,2-Dicarboxyethyl)AMP AMP-lyase Purine metabolism / Alanine and aspartate metabolism EC:4.3.2.2
Adsl												ENSMUSG00000022407 adenylosuccinate lyase
r0012	0.15	-0.02	11.8	11.8	0.98	-0.52	0.44	-0.46	11.7	11.2	8.89	2 H2O2(p) → O2(p) + 2 H2O(p). hydrogen-peroxide:hydrogen-peroxide oxidoreductase EC:1.11.1.6 (2 sp.)
Cat (2 sp.)												ENSMUSG00000027187 catalase
r0334	0.58	-0.39	8.29	7.9	0.88	-0.35	0.77	-0.83	8.38	7.55	9.05	NADPH(r) + Desmosterol(r) ⇌ Cholesterol(r) + NADP+(r). delta24-sterol reductase Biosynthesis of steroids EC:1.3.1.72 (3 sp.)
Dhcr24 (3 sp.)												ENSMUSG00000034926 24-dehydrocholesterol reductase
r0539	0.03	0.27	6.16	6.42	0.12	0.01	0.09	0.14	6.29	6.43	6.25	Hypotaurine(c) ⇌ Cysteamine(c) + O2(c). Cysteamine:oxygen oxidoreductase Taurine and hypotaurine metabolism EC:1.13.11.19
Ado												ENSMUSG00000057134 2-aminoethanethiol (cysteamine) dioxygenase
r0058	0.04	0.23	10.9	11.1	0.93	-0.38	0.23	-0.18	10.9	10.7	6.45	Malate(c) + NADP+(c) ⇌ Pyruvate(c) + CO2(c) + NADPH(c). (S)-Malate:NADP+ oxidoreductase(oxaloacetate-decarboxylating) Pyruvate metabolism EC:1.1.1.40
Me1												ENSMUSG00000032418 malic enzyme 1, NADP(+)-dependent, cytosolic
r0335	0.49	-0.33	4.11	3.78	0.1	0.04	0.21	-0.14	3.96	3.82	10.4	Cholesterol(r) + O2(r) + NADPH(r) ⇌ 7alpha-Hydroxycholesterol(r) + H2O(r) + NADP+(r). Cholesterol,NADPH:oxygen oxidoreductase (7alpha-hydroxylating) Bile acid biosynthesis EC:1.14.13.17 (2 sp.)
Cyp7a1 (2 sp.)												ENSMUSG00000028240 cytochrome P450, family 7, subfamily a, polypeptide 1
r0704	0.003	-2.25	11.7	9.47	0.86	-0.34	0.2	-2.48	11.6	9.13	6.29	ATP(p) + 3alpha,7alpha,12alpha-Trihydroxy-5beta-cholestanate(p) + CoA(p) ⇌ AMP(p) + PPi(p) + 3alpha,7alpha,12alpha-Trihydroxy-5beta-cholestanoyl-CoA(p). 3alpha,7alpha,12alpha-Trihydroxy-5beta-cholestanate:CoA ligase (AMP-forming) Bile acid biosynthesis EC:6.2.1.7
Slc27a5												ENSMUSG00000030382 solute carrier family 27 (fatty acid transporter), member 5
r1572	0.16	-0.03	6.92	6.89	0.99	-0.44	0.48	-0.52	6.96	6.45	7.58	Glutamine(s) + Serine(c) ⇌ Glutamine(c) + Serine(s). Amino Acid-Polyamine-Organocation (APC) TCDB:2.A.3.8.1
Slc7a5												ENSMUSG00000040010 solute carrier family 7 (cationic amino acid transporter, y+ system), member 5
r0247	0.04	0.23	8.11	8.34	0.84	-0.33	0.15	-0.03	8.04	8.01	5.14	ATP(c) + Ribose-5P(c) → AMP(c) + PRPP(c). ATP:D-ribose-5-phosphate pyrophosphotransferase Pentose phosphate pathway / Purine metabolism EC:2.7.6.1 (2 genes, 5 sp.)
Prps1 (2 sp.)		-0.58	10.3	9.71		-0.35		-0.88	10.2	9.36		ENSMUSG00000031432 phosphoribosyl pyrophosphate synthetase 1
Prps2 (3 sp.)		0.77	6.66	7.43		-0.32		0.54	6.57	7.11		ENSMUSG00000025742 phosphoribosyl pyrophosphate synthetase 2
r0250	0.004	0.55	6.88	7.43	0.05	0.11	0.05	0.27	7.27	7.54	4.25	Ribulose-5P(c) ⇌ Ribose-5P(c). D-Ribose-5-phosphate ketol-isomerase Pentose phosphate pathway EC:5.3.1.6
Rpia												ENSMUSG00000053604 ribose 5-phosphate isomerase A
r0538	0.16	-0.03	5.09	5.06	0.17	-0.03	0.19	-0.12	5.15	5.03	6.15	3-Sulfinoalanine(c) ⇌ CO2(c) + Hypotaurine(c). 3-Sulfino-L-alanine carboxy-lyase Taurine and hypotaurine metabolism EC:4.1.1.29 (3 genes, 6 sp.)
Csad (2 sp.)		0.16	8.38	8.54		-0.31		-0.14	8.38	8.24		ENSMUSG00000023044 cysteine sulfinic acid decarboxylase
Gad1 (2 sp.)		-0.12	3.37	3.25		0.08		-0.14	3.48	3.33		ENSMUSG00000070880 glutamic acid decarboxylase 1
Gad2 (2 sp.)		-0.13	3.51	3.38		0.14		-0.07	3.6	3.53		ENSMUSG00000026787 glutamic acid decarboxylase 2
r0780	0.78	-0.53	8.34	7.8	0.29	-0.1	0.6	-0.65	8.35	7.7	8.61	4,4-Dimethyl-5alpha-cholesta-8,14,24-trien-3beta-ol(r) + NADPH(r) ⇌ 14-Demethyllanosterol(r) + NADP+(r). 4,4-dimethyl-5a-cholesta-8,24-dien-3b-ol:NADP+ D14-oxidoreductase Biosynthesis of steroids EC:1.3.1.70 (2 sp.)
Tm7sf2 (2 sp.)												ENSMUSG00000024799 transmembrane 7 superfamily member 2
r0877	0.43	-0.29	5.35	5.06	0.17	-0.92	0.95	-1.09	5.23	4.14	6.82	Glutamate(c) + Cystine(s) ⇌ Glutamate(s) + Cystine(c). Amino Acid-Polyamine-Organocation (APC) TCDB:2.A.3.8.5 (2 sp.)
Slc7a11 (2 sp.)												ENSMUSG00000027737 solute carrier family 7 (cationic amino acid transporter, y+ system), member 11

Continued on next page

	control 1/24h				TGFβ/C 24h		TGFβ 1/24h					
**Rea ID**	**Score** *0.45*	**Expr** Δ	**Expr** C1h	**Expr** C24h	**Score** *0.47*	**Expr** Δ	**Score** *0.48*	**Expr** Δ	**Expr** T1h	**Expr** T24h	**Wght**	**Reaction**
C0043	0.1	0.07	7.92	7.98	0.88	-0.59	0.53	-0.57	7.96	7.39	6.55	several reactions. Formate:tetrahydrofolate ligase (ADP-forming) Glyoxylate and dicarboxylate metabolism / One carbon pool by folate EC:6.3.4.3 (1 genes, 3 sp.)
Mthfd1 (3 sp.)												ENSMUSG00000021048 methylenetetrahydrofolate dehydrogenase (NADP+ dependent), methenyltetrahydrofolate cyclohydrolase, formyltetrahydrofolate synthase
r0192	1	-0.79	11.7	10.9	1	-0.46	0.95	-1.09	11.5	10.4	3.34	Glucose-6P(c) ⇌ Fructose-6P(c). D-Glucose-6-phosphate ketol-isomerase EC:5.3.1.9 (3 sp.)
Gpi1 (3 sp.)												ENSMUSG00000036427 glucose phosphate isomerase 1
r0007	0.06	0.15	10.1	10.2	0.26	-0.09	0.11	0.07	10.1	10.1	5.1	PPi(c) + H2O(c) → 2 Pi(c). Pyrophosphate phosphohydrolase EC:3.6.1.1 (2 genes, 2 sp.)
Ppa1		-0.26	11.1	10.8		0.18		-0.14	11.1	11		ENSMUSG00000020089 pyrophosphatase (inorganic) 1
Ppa2		0.56	9.07	9.63		-0.35		0.28	9	9.28		ENSMUSG00000028013 pyrophosphatase (inorganic) 2
r0344	0.05	0.18	6.37	6.55	0.14	-0.01	0.07	0.22	6.33	6.54	4.3	D-Xylulose-5P(c) ⇌ Ribulose-5P(c). D-Ribulose-5-phosphate 3-epimerase Pentose phosphate pathway EC:5.1.3.1 (3 sp.)
Rpe (3 sp.)												ENSMUSG00000026005 ribulose-5-phosphate-3-epimerase
r0792	0.1	0.06	6.46	6.51	0.14	-0.005	0.14	-0.02	6.53	6.51	4.73	5-Methyl-THF(c) + NAD+(c) ⇌ 5,10-Methylene-THF(c) + NADH(c). 5-methyltetrahydrofolate:NAD+ oxidoreductase One carbon pool by folate / Methane metabolism EC:1.5.1.20 (3 sp.)
Mthfr (3 sp.)												ENSMUSG00000029009 5,10-methylenetetrahydrofolate reductase
r0099	0.01	0.42	8.17	8.59	0.78	-0.31	0.11	0.05	8.23	8.28	4.3	ATP(c) + GDP(c) ⇌ ADP(c) + GTP(c). ATP:nucleoside-diphosphate phosphatransferase Purine metabolism EC:2.7.4.6 (7 genes, 7 sp.)
Nme1		0.44	11.4	11.8		-0.3		0.11	11.4	11.5		ENSMUSG00000037601 non-metastatic cells 1, protein (NM23A) expressed in
Nme2		0.09	13	13.1		0.04		0.14	13	13.2		ENSMUSG00000020857 non-metastatic cells 2, protein (NM23B) expressed in
Nme3		0.86	6.83	7.69		-1.4		-0.66	6.96	6.29		ENSMUSG00000073435 non-metastatic cells 3, protein expressed in
Nme4		0.95	6.48	7.42		-0.82		-0.06	6.67	6.6		ENSMUSG00000024177 non-metastatic cells 4, protein expressed in
Nme5		0.01	4.78	4.79		0.08		-0.02	4.88	4.87		ENSMUSG00000035984 non-metastatic cells 5, protein expressed in (nucleoside-diphosphate kinase)
Nme6		-0.01	7.51	7.5		0.11		0.03	7.58	7.61		ENSMUSG00000032478 non-metastatic cells 6, protein expressed in (nucleoside-diphosphate kinase)
Nme7		0.57	7.21	7.78		0.14		0.85	7.08	7.92		ENSMUSG00000026575 non-metastatic cells 7, protein expressed in (nucleoside-diphosphate kinase)
r0257	0.1	0.06	6.91	6.97	0.62	-0.24	0.2	-0.13	6.86	6.73	5.82	Glutamine(c) + PRPP(c) + H2O(c) ⇌ 5-Phosphoribosylamine(c) + PPi(c) + Glutamate(c). 5-Phosphoribosylamine:pyrophosphate phosphoribosyltransferase (glutamate-amidating) Purine metabolism / Glutamate metabolism EC:2.4.2.14 (2 sp.)
Ppat (2 sp.)												ENSMUSG00000029246 phosphoribosyl pyrophosphate amidotransferase
r0030	0.21	-0.09	6.05	5.96	0.08	0.06	0.16	-0.05	6.06	6.02	4.36	ATP(c) + AMP(c) ⇌ 2 ADP(c). ATP:AMP phosphotransferase Purine metabolism EC:2.7.4.3 (5 genes, 11 sp.)
Ak1 (2 sp.)		-0.07	3.97	3.89		0.76		0.59	4.07	4.66		ENSMUSG00000026817 adenylate kinase 1
Ak2 (2 sp.)		0.08	9.35	9.43		0.13		0.15	9.41	9.55		ENSMUSG00000028792 adenylate kinase 2
Ak4 (4 sp.)		-0.36	6.41	6.05		-0.23		-0.45	6.27	5.82		ENSMUSG00000028527 adenylate kinase 4
Ak5 (2 sp.)		-0.17	4.72	4.56		0.12		-0.12	4.79	4.68		ENSMUSG00000039058 adenylate kinase 5
Ak7		0.74	4.79	5.53		-0.39		0.06	5.08	5.14		ENSMUSG00000041323 adenylate kinase 7
r0578	0.28	-0.17	6.93	6.76	0.26	-0.08	0.2	-0.13	6.81	6.68	6.23	ATP(c) + Pantetheine(c) ⇌ ADP(c) + Phosphopantetheine(c). ATP:pantothenate 4'-phosphotransferase Pantothenate and CoA biosynthesis EC:2.7.1.33 (4 genes, 8 sp.)
Pank1 (3 sp.)		-0.28	6.13	5.85		-0.35		-0.6	6.1	5.5		ENSMUSG00000033610 pantothenate kinase 1
Pank2 (2 sp.)		0.05	7.08	7.13		0.02		0.29	6.87	7.16		ENSMUSG00000037514 pantothenate kinase 2
Pank3 (2 sp.)		-0.34	7.8	7.45		0.15		0.01	7.59	7.6		ENSMUSG00000018846 pantothenate kinase 3
Pank4		0.07	7.31	7.38		0.03		0.16	7.25	7.41		ENSMUSG00000029056 pantothenate kinase 4
r0277	0.24	-0.13	10.2	10.1	0.28	-0.1	0.28	-0.27	10.2	9.97	6.49	GTP(c) + IMP(c) + Aspartate(c) → GDP(c) + Pi(c) + Adenylosuccinate(c). IMP:L-aspartate ligase (GDP-forming) Purine metabolism / Alanine and aspartate metabolism EC:6.3.4.4
Adss												ENSMUSG00000015961 adenylosuccinate synthetase, non muscle
r0406	0.02	0.34	10.4	10.8	0.12	0.01	0.04	0.36	10.4	10.8	3.06	Fructose-6P(c) + Erythrose-4P(c) ⇌ Sedoheptulose-7P(c) + GAP(c). Sedoheptulose-7-phosphate:D-glyceraldehyde-3-phosphate glyceronetransferase Pentose phosphate pathway EC:2.2.1.2
Taldo1												ENSMUSG00000025503 transaldolase 1
r0371	0.2	-0.08	8.16	8.07	0.42	-0.78	0.81	-0.87	8.17	7.29	5.42	5,10-Methenyl-THF(c) + H2O(c) ⇌ 10-Formyl-THF(c). 5,10-Methenyltetrahydrofolate 5-hydrolase(decyclizing) Glyoxylate and dicarboxylate metabolism / One carbon pool by folate EC:3.5.4.9 (2 genes, 5 sp.)
Mthfd1 (3 sp.)		0.07	7.92	7.98		-0.59		-0.57	7.96	7.39		ENSMUSG00000021048 methylenetetrahydrofolate dehydrogenase (NADP+ dependent), methenyltetrahydrofolate cyclohydrolase, formyltetrahydrofolate synthase
Mthfd2 (2 sp.)		-0.31	8.51	8.21		-1.07		-1.34	8.47	7.13		ENSMUSG00000005667 methylenetetrahydrofolate dehydrogenase (NAD+ dependent), methenyltetrahydrofolate cyclohydrolase
r0258	0.15	-0.03	10.4	10.3	0.52	-0.2	0.29	-0.28	10.4	10.1	5.94	Fumarate(c) + H2O(c) ⇌ Malate(c). (S)-Malate hydro-lyase Citrate cycle (TCA cycle) EC:4.2.1.2
Fh1												ENSMUSG00000026526 fumarate hydratase 1
r0027	0.39	-0.26	7.88	7.62	0.22	-0.06	0.31	-0.31	7.86	7.56	6.31	NADPH(c) + Cystine(c) ⇌ 2 Cysteine(c) + NADP+(c). NADPH:CoA-glutathione oxidoreductase EC:1.8.1.9 (3 genes, 9 sp.)
Txnrd1 (3 sp.)		-0.4	9.09	8.69		-0.39		-0.74	9.04	8.3		ENSMUSG00000020250 thioredoxin reductase 1
Txnrd2 (3 sp.)		-0.03	7.7	7.68		0.01		-0.07	7.76	7.68		ENSMUSG00000075704 thioredoxin reductase 2
Txnrd3 (3 sp.)		-0.34	6.83	6.49		0.21		-0.1	6.79	6.69		ENSMUSG00000000811 thioredoxin reductase 3
r2532	0.22	-0.11	5.07	4.96	0.38	-0.14	0.19	-0.12	4.94	4.82	4.32	Asparagine(s) ⇌ Asparagine(c). Major Facilitator(MFS) TCDB:2.A.1.44.1
Slc43a1												ENSMUSG00000027075 solute carrier family 43, member 1
r0256	0.99	-0.78	11.4	10.7	0.99	-0.5	0.98	-1.18	11.3	10.2	1.56	Fructose-1,6PP(c) ⇌ DHAP(c) + GAP(c). D-Fructose-1,6-bisphosphate D-glyceraldehyde-3-phosphate-lyase Carbon fixation EC:4.1.2.13 (2 genes, 4 sp.)
Aldoa (3 sp.)		-0.32	11	10.6		-0.12		-0.36	10.9	10.5		ENSMUSG00000030695 aldolase A, fructose-bisphosphate
Aldob		-2.13	12.9	10.7		-1.64		-3.66	12.7	9.09		ENSMUSG00000028307 aldolase B, fructose-bisphosphate
r0736	1	-0.78	9.44	8.65	0.98	-0.42	0.93	-1.05	9.28	8.23	1.61	ATP(c) + Fructose-6P(c) → ADP(c) + Fructose-1,6PP(c). ATP:D-fructose-6-phosphate 1-phosphotransferase EC:2.7.1.11 (2 sp.)
Pfkl (2 sp.)												ENSMUSG00000020277 phosphofructokinase, liver, B-type
C0067	0.18	-0.07	9.41	9.34	0.51	-0.2	0.31	-0.31	9.45	9.14	4.3	several reactions. D-Fructose 6-phosphate:D-glyceraldehyde-3-phosphate glycolaldehyde transferase Carbon fixation EC:2.2.1.1 (1 genes, 3 sp.)
Tkt (3 sp.)												ENSMUSG00000021957 transketolase
r0243	0.91	-0.64	12.2	11.6	0.3	-0.84	0.99	-1.4	12.1	10.7	1.56	DHAP(c) ⇌ GAP(c). D-Glyceraldehyde-3-phosphate ketol-isomerase Glycolysis / Gluconeogenesis EC:5.3.1.1 (3 sp.)
Tpi1 (3 sp.)												ENSMUSG00000023456 triosephosphate isomerase 1

40 Reaction scores for Gly-CD-cholate(b) (316), series control 1/24h, TGFβ/C 24h, and TGFβ 1/24h

The amplitudes of the mode are -0.83, -0.73 and -1.28.

Simulation	Definition			Solution												
				exchanges		reactions								transp		Prot
	Objective	Constraints	Comment	imports	exports	c	m	r	p	l	n	s	b	s-c	b-c	intra synth
316 Gly-CD-cholate(b)	Glycochenodeoxy-cholate(b)	+Urea −Alanine −Serine MCES	de novo synthesis and bile export of Glycochenodeoxycholate from Palmitate, Alanine, Serine, excreting urea and CO_2	10.5 O_2(s) 1 Serine(s) 2 Farnesyl-PP(r) 5 ATP-energy(c) 11 NADPH-redox-potential(r) 1 NADPH-redox-potential(m) 1 Proton-gradient(c)	8 H_2O(s) 4 P_i(c) 4 CO_2(s) 1 Gly-cochenodeoxycholate(b) 1 NADH-redox-potential(r) 2 NADH-redox-potential(m) 6 Proton-gradient(m) 1 THF-activated methyl group(c) 1 CoA-activated acetyl group(m)	13	9	18	2	-	-	-	-	5	1	22	-

Table 39: Reaction scores for Gly-CD-cholate(b) (316), series control 1/24h, TGFβ/C 24h, and TGFβ 1/24h

	control 1/24h				TGFβ/C 24h		TGFβ 1/24h					
Rea ID	Score *0.51*	Expr Δ	Expr C1h	Expr C24h	Score *0.57*	Expr Δ	Score *0.61*	Expr Δ	Expr T1h	Expr T24h	Wght	Reaction
r0781	0.95	-0.96	7.54	6.58	0.77	-0.47	1	-1.29	7.4	6.11	27.1	3 O2(r) + 3 NADPH(r) + Lanosterol(r) ⇌ 4,4-Dimethyl-5alpha-cholesta-8,14,24-trien-3beta-ol(r) + Formate(r) + 4 H2O(r) + 3 NADP+(r). Lanosterol,NADPH:oxygen oxidoreductase (14-methyl cleaving) Biosynthesis of steroids EC:1.14.13.70 (3 sp.)
Cyp51 (3 sp.)												ENSMUSG00000001467 cytochrome P450, family 51
r1134	1	-0.87	10.7	9.86	0.73	-0.44	0.95	-1.08	10.5	9.42	27.4	14-Demethyllanosterol(r) + 3 NADPH(r) + 3 O2(r) ⇌ 4 H2O(r) + 4alpha-Methylzymosterol-4-carboxylate(r) + 3 NADP+(r). methylsterol monooxygenase Biosynthesis of steroids EC:1.14.13.72
Sc4mol												ENSMUSG00000031604 sterol-C4-methyl oxidase-like
r0797	0.02	-1.97	8.32	6.35	0.04	-1.67	0.002	-3.59	8.27	4.68	16	Chenodeoxycholoyl-CoA(c) + Glycine(c) ⇌ CoA(c) + Glycochenodeoxycholate(c). glycine N-choloyltransferase EC:2.3.1.65 (2 sp.)
Baat (2 sp.)												ENSMUSG00000039653 bile acid-Coenzyme A: amino acid N-acyltransferase
r1507	0.06	-1.82	6.94	5.12	1	-0.71	0.18	-2.47	6.88	4.41	16	Glycochenodeoxycholate(c) + ATP(c) + H2O(c) → Glycochenodeoxycholate(b) + ADP(c) + Pi(c). ATP-binding Cassette (ABC) TCDB:3.A.1.201.2
Abcb11												ENSMUSG00000027048 ATP-binding cassette, sub-family B (MDR/TAP), member 11
r0698	0.05	0.19	8.62	8.81	1	-0.72	0.41	-0.43	8.52	8.09	15.4	CoA(p) + 3alpha,7alpha-Dihydroxy-5beta-cholestanoyl-CoA(p) + H2O(p) ⇌ Propanoyl-CoA(p) + Chenodeoxycholoyl-CoA(p). Propanoyl-CoA:acetyl-CoA C-acyltransferase Bile acid biosynthesis EC:2.3.1.16 (2 genes, 6 sp.)
Acaa1a (3 sp.)		0.26	8.16	8.42		-0.36		-0.06	8.12	8.06		ENSMUSG00000036138 acetyl-Coenzyme A acyltransferase 1A
Acaa1b (3 sp.)		0.12	9.07	9.19		-1.08		-0.79	8.91	8.12		ENSMUSG00000010651 acetyl-Coenzyme A acyltransferase 1B
r0740	0.99	-0.88	11.3	10.4	0.99	-0.79	0.89	-1.6	11.2	9.59	11.8	5beta-Cholestane-3alpha,7alpha-diol(m) + NADPH(m) + O2(m) ⇌ 5beta-Cholestane-3alpha,7alpha,26-triol(m) + H2O(m) + NADP+(m). 5beta-cholestane-3alpha,7alpha-diol,NADPH:oxygen oxidoreductase (26-hydroxylating) Bile acid biosynthesis EC:1.14.13.15
Cyp27a1												ENSMUSG00000026170 cytochrome P450, family 27, subfamily a, polypeptide 1
r0688	0.02	0.34	9.32	9.66	0.94	-0.86	0.53	-0.56	9.36	8.8	14.5	3alpha,7alpha-Dihydroxy-5beta-cholestan-26-al(c) + NAD+(c) + H2O(c) ⇌ NADH(c) + 3alpha,7alpha-Dihydroxy-5beta-cholestanate(c). 3alpha,7alpha-Dihydroxy-5beta-cholestan-26-al:NAD+ oxidoreductase Bile acid biosynthesis EC:1.2.1.3 (5 genes, 6 sp.)
Aldh1a1		0.23	12.9	13.1		-2.54		-2.38	12.9	10.6		ENSMUSG00000053279 aldehyde dehydrogenase family 1, subfamily A1
Aldh1b1		0.35	4.91	5.27		-0.12		0.16	4.98	5.14		ENSMUSG00000035561 aldehyde dehydrogenase 1 family, member B1
Aldh3a2		0.76	8.9	9.66		-1.06		-0.42	9.03	8.6		ENSMUSG00000010025 aldehyde dehydrogenase family 3, subfamily A2
Aldh7a1		0.42	10.6	11.1		-0.43		0.02	10.6	10.6		ENSMUSG00000053644 aldehyde dehydrogenase family 7, member A1
Aldh9a1 (2 sp.)		0.15	9.29	9.45		-0.51		-0.37	9.31	8.94		ENSMUSG00000026687 aldehyde dehydrogenase 9, subfamily A1
r0793	0.18	-0.07	7.93	7.87	0.02	0.3	0.06	0.24	7.93	8.17	9.54	Lathosterol(r) + NADPH(r) + O2(r) ⇌ Provitamin D3(r) + 2 H2O(r) + NADP+(r). 5alpha-cholest-7-en-3beta-ol, NADPH:oxygen 5-oxidoreductase Biosynthesis of steroids EC:1.14.21.6 (2 sp.)
Sc5d (2 sp.)												ENSMUSG00000032018 sterol-C5-desaturase (fungal ERG3, delta-5-desaturase) homolog (S. cerevisae)
r0672	0.47	-0.32	10.5	10.1	0.001	0.62	0.07	0.19	10.6	10.8	11	7alpha-Hydroxycholesterol(r) + NADP+(r) ⇌ 7alpha-Hydroxycholest-4-en-3-one(r) + NADPH(r). Cholest-5-ene-3beta,7alpha-diol:NAD+ 3-oxidoreductase Bile acid biosynthesis EC:1.1.1.181
Hsd3b7												ENSMUSG00000042289 hydroxy-delta-5-steroid dehydrogenase, 3 beta- and steroid delta-isomerase 7
C0104	0.68	-0.46	9.74	9.28	0.99	-0.8	0.98	-1.14	9.63	8.49	12.5	several reactions. Farnesyl-diphosphate:farnesyl-diphosphate farnesyltransferase Biosynthesis of steroids EC:2.5.1.21 (1 genes, 2 sp.)
Fdft1 (2 sp.)												ENSMUSG00000021273 farnesyl diphosphate farnesyl transferase 1
r0812	0.07	0.13	8.82	8.95	0.78	-0.47	0.38	-0.39	8.86	8.47	13.5	ATP(c) + AMP(p) ⇌ ATP(p) + AMP(c). Mitochondrial Carrier (MC) TCDB:2.A.29.20.1
Slc25a17												ENSMUSG00000022404 solute carrier family 25 (mitochondrial carrier, peroxisomal membrane protein), member 17
r0739	0.69	-0.47	8.15	7.68	0.8	-0.98	1	-1.34	8.05	6.7	14.5	5beta-Cholestane-3alpha,7alpha,26-triol(c) + NAD+(c) ⇌ 3alpha,7alpha-Dihydroxy-5beta-cholestan-26-al(c) + NADH(c). alcohol dehydrogenase Bile acid biosynthesis EC:1.1.1.1 (4 genes, 5 sp.)
Adh1		-0.49	12.2	11.7		-3.81		-4.08	12	7.88		ENSMUSG00000074207 alcohol dehydrogenase 1 (class I)
Adh4		-0.88	7.59	6.7		-0.57		-1.44	7.58	6.14		ENSMUSG00000037797 alcohol dehydrogenase 4 (class II), pi polypeptide
Adh5		-0.21	11.1	10.9		-0.27		-0.4	11	10.6		ENSMUSG00000028138 alcohol dehydrogenase 5 (class III), chi polypeptide
Adh7 (2 sp.)		-0.38	4.97	4.58		-0.12		-0.4	4.86	4.46		ENSMUSG00000055301 alcohol dehydrogenase 7 (class IV), mu or sigma polypeptide
r0745	1	-0.83	4.1	3.28	0.24	-0.11	0.77	-0.82	3.99	3.16	11.2	7alpha-Hydroxycholest-4-en-3-one(c) + NADPH(c) ⇌ 7alpha-Hydroxy-5beta-cholestan-3-one(c) + NADP+(c). 7alpha-Hydroxy-5beta-cholestan-3-one:(acceptor) delta4-oxidoreductase; 7alpha-Hydroxy-5beta-cholestan-3-one:NADP+ delta4-oxidoreductase Bile acid biosynthesis EC:1.3.99.6 (2 sp.)
Akr1d1 (2 sp.)												ENSMUSG00000038641 aldo-keto reductase family 1, member D1
r0230	0.06	0.17	7.65	7.82	0.17	-1.42	1	-1.32	7.72	6.4	8.63	THF(c) + Serine(c) ⇌ 5,10-Methylene-THF(c) + Glycine(c) + H2O(c). 5,10-Methylenetetrahydrofolate:glycine hydroxymethyltransferase Glycine, serine and threonine metabolism / One carbon pool by folate EC:2.1.2.1 (4 sp.)
Shmt1 (4 sp.)												ENSMUSG00000020534 serine hydroxymethyltransferase 1 (soluble)
r0227	0.41	-0.28	6.69	6.41	0.02	0.3	0.08	0.16	6.55	6.71	8.72	10-Formyl-THF(c) + H2O(c) + NADP+(c) → THF(c) + CO2(c) + NADPH(c). 10-formyltetrahydrofolate:NADP+ oxidoreductase One carbon pool by folate EC:1.5.1.6 (2 genes, 3 sp.)
Aldh1l1 (2 sp.)		-0.49	7.57	7.07		0.64		0.31	7.41	7.72		ENSMUSG00000030088 aldehyde dehydrogenase 1 family, member L1
Aldh1l2		0.16	4.93	5.09		-0.4		-0.15	4.84	4.69		ENSMUSG00000020256 aldehyde dehydrogenase 1 family, member L2
r0335	0.48	-0.33	4.11	3.78	0.11	0.04	0.21	-0.14	3.96	3.82	10.9	Cholesterol(r) + O2(r) + NADPH(r) ⇌ 7alpha-Hydroxycholesterol(r) + H2O(r) + NADP+(r). Cholesterol,NADPH:oxygen oxidoreductase (7alpha-hydroxylating) Bile acid biosynthesis EC:1.14.13.17 (2 sp.)
Cyp7a1 (2 sp.)												ENSMUSG00000028240 cytochrome P450, family 7, subfamily a, polypeptide 1
r0746	0.39	-0.26	6.05	5.78	0.22	-1.36	0.9	-1.58	6	4.42	11.4	7alpha-Hydroxy-5beta-cholestan-3-one(c) + NADH(c) ⇌ 5beta-Cholestane-3alpha,7alpha-diol(c) + NAD+(c). 3alpha,7alpha-Dihydroxy-5beta-cholestane:NAD+ oxidoreductase (B-specific); 3alpha,7alpha-Dihydroxy-5beta-cholestane:NAD+ oxidoreductase Bile acid biosynthesis EC:1.1.1.50 (6 genes, 7 sp.)
Akr1c14		-0.97	9.24	8.27		-3.58		-4.56	9.24	4.69		ENSMUSG00000033715 aldo-keto reductase family 1, member C14
Akr1c18		0.13	5.73	5.86		-0.31		-0.22	5.78	5.55		ENSMUSG00000021214 aldo-keto reductase family 1, member C18
Akr1c20 (2 sp.)		-0.02	5.88	5.86		-1.82		-1.78	5.82	4.04		ENSMUSG00000054757 aldo-keto reductase family 1, member C20
Akr1c21		-0.27	4.87	4.6		-0.02		-0.29	4.87	4.59		ENSMUSG00000021207 aldo-keto reductase family 1, member C21
Akr1c6		-0.78	6.81	6.03		-1.98		-2.28	6.33	4.05		ENSMUSG00000021210 aldo-keto reductase family 1, member C6
Akr1cl		0.07	3.94	4.01		-0.04		-0.17	4.15	3.97		ENSMUSG00000025955 aldo-keto reductase family 1, member C-like

Continued on next page

Rea ID	control 1/24h Score 0.51	Expr Δ	Expr C1h	Expr C24h	TGFβ/C 24h Score 0.57	Expr Δ	TGFβ 1/24h Score 0.61	Expr Δ	Expr T1h	Expr T24h	Wght	Reaction
r0017	0.19	-0.07	10	9.95	0.14	-0.01	0.18	-0.1	10	9.93	6.83	O2(m) + 4 H+(PG)(m) + 4 Ferrocytochrome C(m) → 4 Ferricytochrome C(m) + 2 H2O(m) + 4 H+(PG)(c). Ferrocytochrome-c:oxygen oxidoreductase Oxidative phosphorylation EC:1.9.3.1 (16 genes, 26 sp.)
Cox4i1		0.04	12.5	12.6		0.03		0.03	12.6	12.6		ENSMUSG00000031818 cytochrome c oxidase subunit IV isoform 1
Cox4i2		-0.02	4.73	4.71		-0.01		-0.1	4.8	4.71		ENSMUSG00000009876 cytochrome c oxidase subunit IV isoform 2
Cox5a (3 sp.)		-0.09	11.6	11.5		-0.03		-0.07	11.5	11.4		ENSMUSG00000000088 cytochrome c oxidase, subunit Va
Cox5b (4 sp.)		-0.09	11.9	11.8		-0.07		-0.12	11.9	11.8		ENSMUSG00000061518 cytochrome c oxidase, subunit Vb
Cox6a1 (2 sp.)		0.01	12.4	12.5		-0.03		-0.04	12.5	12.4		ENSMUSG00000041697 cytochrome c oxidase, subunit VI a, polypeptide 1
Cox6a2		-0.05	6.85	6.8		-0.08		-0.14	6.86	6.72		ENSMUSG00000030785 cytochrome c oxidase, subunit VI a, polypeptide 2
Cox6b1 (2 sp.)		0.06	12	12.1		-0.04		0.05	12	12		ENSMUSG00000036751 cytochrome c oxidase, subunit VIb polypeptide 1
Cox6b2		0.06	5.02	5.08		0.16		0.15	5.09	5.24		ENSMUSG00000051811 cytochrome c oxidase subunit VIb polypeptide 2
Cox6c (2 sp.)		-0.1	11.4	11.3		-0.01		-0.14	11.4	11.2		ENSMUSG00000014313 cytochrome c oxidase, subunit VIc
Cox7a1		-0.19	4.49	4.3		0.21		-0.2	4.71	4.51		ENSMUSG00000074218 cytochrome c oxidase, subunit VIIa 1
Cox7a2 (2 sp.)		-0.31	12.2	11.9		-0.12		-0.38	12.2	11.8		ENSMUSG00000032330 cytochrome c oxidase, subunit VIIa 2
Cox7b2		-0.01	2.91	2.9		0.19		-0.07	3.15	3.09		ENSMUSG00000049387 cytochrome c oxidase subunit VIIb2
Cox7c		-0.11	11.7	11.6		0.01		-0.1	11.7	11.6		ENSMUSG00000017778 cytochrome c oxidase, subunit VIIc
Cox8a (2 sp.)		-0.08	10.4	10.3		-0.05		-0.13	10.4	10.3		ENSMUSG00000035885 cytochrome c oxidase, subunit VIIIa
Cox8b		0.01	5.54	5.54		-0.01		-0.14	5.67	5.53		ENSMUSG00000025488 cytochrome c oxidase, subunit VIIIb
Cox8c		-0.06	7.29	7.23		0.05		-0.04	7.32	7.28		ENSMUSG00000043319 cytochrome c oxidase, subunit VIIIc
r0607	0.97	-0.74	6.42	5.68	0.68	-0.41	0.91	-1.01	6.28	5.27	8.97	Squalene 2,3-oxide(r) ⇌ Lanosterol(r). (S)-2,3-Epoxysqualene mutase (cyclizing, lanosterol-forming) Biosynthesis of steroids EC:5.4.99.7 (2 sp.)
Lss (2 sp.)												ENSMUSG00000033105 lanosterol synthase
r0683	0.67	-0.46	11.7	11.3	0.94	-0.61	0.92	-1.02	11.7	10.7	9.24	Propanoyl-CoA(m) + Ubiquinone(m) → Ubiquinol(m) + Acrylyl-CoA(m). Propanoyl-CoA:(acceptor) 2,3-oxidoreductase beta-Alanine metabolism EC:1.3.3.6 EC:1.3.99.3
Acadm												ENSMUSG00000062908 acyl-Coenzyme A dehydrogenase, medium chain
r0596	0.12	0.02	6.56	6.58	0.94	-0.61	0.48	-0.51	6.48	5.97	8.72	3-Hydroxypropionyl-CoA(m) + H2O(m) → Hydracrylate(m) + CoA(m). 3-hydroxyisobutyryl-CoA hydrolase beta-Alanine metabolism / Propanoate metabolism EC:3.1.2.4 (2 sp.)
Hibch (2 sp.)												ENSMUSG00000041426 3-hydroxyisobutyryl-Coenzyme A hydrolase
r1135	1	-0.85	6.95	6.1	0.53	-0.32	0.92	-1.02	6.8	5.78	9.24	3-Keto-4-methylzymosterol(r) + NADP+(r) ⇌ NADPH(r) + 4alpha-Methylzymosterol(r). hydroxysteroid (17-beta) dehydrogenase 7 Biosynthesis of steroids EC:1.1.1.270 (4 sp.)
Hsd17b7 (4 sp.)												ENSMUSG00000026675 hydroxysteroid (17-beta) dehydrogenase 7
r1137	0.8	-1.11	10.4	9.3	0.42	-0.25	0.99	-1.36	10.4	9.05	9.18	NAD+(r) + 4alpha-Methylzymosterol-4-carboxylate(r) ⇌ NADH(r) + CO2(r) + 3-Keto-4-methylzymosterol(r). NAD(P) dependent steroid dehydrogenase-like EC:1.1.1.170
Nsdhl												ENSMUSG00000031349 NAD(P) dependent steroid dehydrogenase-like
r0792	0.1	0.06	6.46	6.51	0.14	-0.005	0.14	-0.02	6.53	6.51	4.98	5,10-Methylene-THF(c) + NADH(c) ⇌ 5-Methyl-THF(c) + NAD+(c). 5-methyltetrahydrofolate:NAD+ oxidoreductase One carbon pool by folate / Methane metabolism EC:1.5.1.20 (3 sp.)
Mthfr (3 sp.)												ENSMUSG00000029009 5,10-methylenetetrahydrofolate reductase
r2518	0.24	-0.13	10.8	10.7	0.64	-0.39	0.49	-0.52	10.8	10.3	13.5	3alpha,7alpha-Dihydroxy-5beta-cholestanate(c) + ATP(c) + H2O(c) ⇌ 3alpha,7alpha-Dihydroxy-5beta-cholestanate(p) + ADP(c) + Pi(c). ATP-binding Cassette (ABC) TCDB:3.A.1.203.1
Abcd3												ENSMUSG00000028127 ATP-binding cassette, sub-family D (ALD), member 3
r0690	0.003	-2.25	11.7	9.47	0.57	-0.34	0.18	-2.48	11.6	9.13	6.63	ATP(p) + CoA(p) + 3alpha,7alpha-Dihydroxy-5beta-cholestanate(p) ⇌ AMP(p) + PPi(p) + 3alpha,7alpha-Dihydroxy-5beta-cholestanoyl-CoA(p). 3alpha,7alpha-Dihydroxy-5beta-cholestanate:CoA ligase (AMP-forming) Bile acid biosynthesis EC:6.2.1.7
Slc27a5												ENSMUSG00000030382 solute carrier family 27 (fatty acid transporter), member 5
r0576	0.88	-0.63	9.99	9.36	0.6	-1.1	0.88	-1.62	9.87	8.26	8.92	Squalene(r) + NADPH(r) + O2(r) ⇌ H2O(r) + Squalene 2,3-oxide(r) + NADP+(r). Squalene,hydrogen-donor:oxygen oxidoreductase (2,3-epoxidizing) Biosynthesis of steroids EC:1.14.99.7
Sqle												ENSMUSG00000022351 squalene epoxidase
r0507	0.2	-0.08	10.5	10.4	0.15	-0.02	0.17	-0.08	10.5	10.4	4.84	Ubiquinol(m) + 2 Ferricytochrome C(m) + 4 H+(PG)(m) → Ubiquinone(m) + 4 H+(PG)(c) + 2 Ferrocytochrome C(m). Ubiquinol:ferricytochrome-c oxidoreductase Oxidative phosphorylation EC:1.10.2.2 (8 genes, 13 sp.)
Uqcr10 (2 sp.)		-0.53	9.74	9.21		-0.04		-0.46	9.63	9.18		ENSMUSG00000059534 ubiquinol-cytochrome c reductase, complex III subunit X
Uqcr11		-0.13	11.1	11		-0.13		-0.24	11.1	10.8		ENSMUSG00000020163 ubiquinol-cytochrome c reductase, complex III subunit XI
Uqcrb (2 sp.)		0.001	12	12		-0.05		0.04	11.9	11.9		ENSMUSG00000021520 ubiquinol-cytochrome c reductase binding protein
Uqcrc1		0.11	10.8	10.9		0.06		0.11	10.9	11		ENSMUSG00000025651 ubiquinol-cytochrome c reductase core protein 1
Uqcrc2 (2 sp.)		-0.04	10.6	10.5		0.05		0.08	10.5	10.6		ENSMUSG00000030884 ubiquinol cytochrome c reductase core protein 2
Uqcrfs1		-0.01	11.4	11.4		-0.07		-0.1	11.4	11.3		ENSMUSG00000038462 ubiquinol-cytochrome c reductase, Rieske iron-sulfur polypeptide 1
Uqcrh (2 sp.)		0.06	11.1	11.2		0.003		0.11	11.1	11.2		ENSMUSG00000063882 ubiquinol-cytochrome c reductase hinge protein
Uqcrq (2 sp.)		-0.01	8.07	8.06		-0.03		-0.15	8.18	8.03		ENSMUSG00000044894 ubiquinol-cytochrome c reductase, complex III subunit VII
r0780	0.77	-0.53	8.34	7.8	0.23	-0.1	0.61	-0.65	8.35	7.7	9.07	4,4-Dimethyl-5alpha-cholesta-8,14,24-trien-3beta-ol(r) + NADPH(r) ⇌ 14-Demethyllanosterol(r) + NADP+(r). 4,4-dimethyl-5a-cholesta-8,24-dien-3b-ol:NADP+ D14-oxidoreductase Biosynthesis of steroids EC:1.3.1.70 (2 sp.)
Tm7sf2 (2 sp.)												ENSMUSG00000024799 transmembrane 7 superfamily member 2
r0333	0.97	-0.74	9.25	8.52	0.47	-1.18	0.62	-1.92	9.25	7.33	9.67	Provitamin D3(r) + NADPH(r) ⇌ Cholesterol(r) + NADP+(r). Cholesterol:NADP+ delta7-oxidoreductase Biosynthesis of steroids EC:1.3.1.21
Dhcr7												ENSMUSG00000058454 7-dehydrocholesterol reductase
r0738	0.7	-1.18	8.78	7.6	0.57	-0.34	0.99	-1.38	8.63	7.25	9.41	Zymosterol(r) ⇌ 5alpha-Cholesta-7,24-dien-3beta-ol(r). delta8,24-Cholestadien-3beta-ol delta7-delta8-isomerase Biosynthesis of steroids EC:5.3.3.5 (2 sp.)
Ebp (2 sp.)												ENSMUSG00000031168 phenylalkylamine Ca2+ antagonist (emopamil) binding protein
r0007	0.06	0.15	10.1	10.2	0.21	-0.09	0.11	0.07	10.1	10.1	4.16	PPi(c) + H2O(c) → 2 Pi(c). Pyrophosphate phosphohydrolase EC:3.6.1.1 (2 genes, 2 sp.)
Ppa1		-0.26	11.1	10.8		0.18		-0.14	11.1	11		ENSMUSG00000020089 pyrophosphatase (inorganic) 1
Ppa2		0.56	9.07	9.63		-0.35		0.28	9	9.28		ENSMUSG00000028013 pyrophosphatase (inorganic) 2
r2471	0.01	0.39	6.41	6.8	0.46	-0.28	0.11	0.07	6.45	6.52	4.86	Serine(s) + H+(PG)(s) → Serine(c) + H+(PG)(c). Major Facilitator(MFS) TCDB:2.A.18.8.1
Slc36a1												ENSMUSG00000020261 solute carrier family 36 (proton/amino acid symporter), member 1
r0228	0.1	0.07	7.92	7.98	0.93	-0.59	0.53	-0.57	7.96	7.39	4.88	THF(c) + Formate(c) + ATP(c) → ADP(c) + Pi(c) + 10-Formyl-THF(c). Formate:tetrahydrofolate ligase (ADP-forming) Glyoxylate and dicarboxylate metabolism / One carbon pool by folate EC:6.3.4.3 (3 sp.)
Mthfd1 (3 sp.)												ENSMUSG00000021048 methylenetetrahydrofolate dehydrogenase (NADP+ dependent), methenyltetrahydrofolate cyclohydrolase, formyltetrahydrofolate synthase
r0030	0.21	-0.09	6.05	5.96	0.09	0.06	0.16	-0.05	6.06	6.02	2.66	ATP(c) + AMP(c) ⇌ 2 ADP(c). ATP:AMP phosphotransferase Purine metabolism EC:2.7.4.3 (5 genes, 11 sp.)
Ak1 (2 sp.)		-0.07	3.97	3.89		0.76		0.59	4.07	4.66		ENSMUSG00000026817 adenylate kinase 1
Ak2 (2 sp.)		0.08	9.35	9.43		0.13		0.15	9.41	9.55		ENSMUSG00000028792 adenylate kinase 2
Ak4 (4 sp.)		-0.36	6.41	6.05		-0.23		-0.45	6.27	5.82		ENSMUSG00000028527 adenylate kinase 4
Ak5 (2 sp.)		-0.17	4.72	4.56		0.12		-0.12	4.79	4.68		ENSMUSG00000039058 adenylate kinase 5
Ak7		0.74	4.79	5.53		-0.39		0.06	5.08	5.14		ENSMUSG00000041323 adenylate kinase 7
r0783	0.57	-0.39	8.29	7.9	0.58	-0.35	0.78	-0.83	8.38	7.55	9.54	5alpha-Cholesta-7,24-dien-3beta-ol(r) + NADPH(r) ⇌ Lathosterol(r) + NADP+(r). lanosterol D24-reductase Biosynthesis of steroids EC:1.3.1.72 (3 sp.)
Dhcr24 (3 sp.)												ENSMUSG00000034926 24-dehydrocholesterol reductase
r0592	0.67	-0.46	9.55	9.09	0.58	-0.35	0.75	-0.79	9.53	8.74	7.46	H2O(m) + Acrylyl-CoA(m) → 3-Hydroxypropionyl-CoA(m). 3-Hydroxypropionyl-CoA hydro-lyase beta-Alanine metabolism / Propanoate metabolism EC:4.2.1.17 (3 genes, 3 sp.)
Echs1		-0.24	10.3	10		-0.15		-0.37	10.2	9.87		ENSMUSG00000025465 enoyl Coenzyme A hydratase, short chain, 1, mitochondrial
Ehhadh		-1.1	8.51	7.41		-1		-2.08	8.49	6.41		ENSMUSG00000022853 enoyl-Coenzyme A, hydratase/3-hydroxyacyl Coenzyme A dehydrogenase

Continued on next page

Rea ID	control 1/24h Score *0.51*	Expr Δ	Expr C1h	Expr C24h	TGFβ/C 24h Score *0.57*	Expr Δ	TGFβ 1/24h Score *0.61*	Expr Δ	Expr T1h	Expr T24h	Wght	Reaction
Hadha		-0.03	9.87	9.84		0.1		0.08	9.86	9.94		ENSMUSG00000025745 hydroxyacyl-Coenzyme A dehydrogenase/3-ketoacyl-Coenzyme A thiolase/enoyl-Coenzyme A hydratase (trifunctional protein), alpha subunit
