# Supplementary material for: Metabolic Consequences of TGFβ Stimulation in Cultured Primary Mouse Hepatocytes Screened from Transcript Data with ModeScore
Source: Metabolites. 2012 Nov 21;2(4):983–1003. doi: 10.3390/metabo2040983 (PMC3901234; doi:10.3390/metabo2040983)
Supplement: Supplementary File 7 — Setting of the weights (PDF, 80 KB) [file metabolites-02-00983-s007.pdf]

Setting of the weights

Andreas Hoppe

Definition of the weights adjustment ω_i, see main manuscript.

Reaction	weight adjust
r0003	242
r0004	242
r0016	2
r0017	2
r0018	4
r0019	1
r0507	1
r0508	1
r0609	2
r0707	3
r0781	3
r0861	3
r0878	3
r1092	609
r1093	423
r1094	418
r1095	4561
r1096	122
r1097	82
r1098	81
r1099	98
r1100	98
r1101	2674
r1102	406
r1103	810
r1104	622
r1105	698
r1112	316
r1113	267
r1134	3
r1158	2945
r1192	9172
r1193	9172
r1198	9248
r1199	8556
r1200	7448
r1201	7728
r1202	8558
r1203	8823
r1204	9175
r1205	9047
r1206	8556
r1207	7448
r1208	7728
r1209	8558

Reaction	weight adjust
r1210	8823
r1241	9248
r1242	9175
r1243	9047
r1244	8556
r1245	8556
r1246	8556
r1247	7448
r1248	7728
r1249	8558
r1250	8823
r1280	15807
r1281	15807
r1284	10014
r1285	10014
r1293	102
r1294	166
r1305	985
r1333	609
r1334	609
r1335	423
r1336	423
r1337	418
r1338	418
r1339	267
r1340	267
r1341	4561
r1342	4561
r1343	81
r1344	98
r1345	98
r1346	316
r1347	316
r1348	2674
r1349	2674
r1350	406
r1351	406
r1352	810
r1353	810
r1354	622
r1355	622
r1356	698
r1357	698
r1358	122
r1359	82

Reaction	weight adjust
r1382	6
r1383	6
r1387	8
r1388	3
r1390	3
r1393	7
r1402	350
r1403	350
r1466	4
r1489	3
r1537	3
r2075	3
r2076	3
r2077	4
r1047	0.2
r2465	0.05
r2073	0.05
r2078	0.2
r2540	4880
r2541	488
r2542	13660
r2543	1366
r2544	12480
r2545	1248
r2546	8120
r2547	812
r2548	9440
r2549	944
r2550	29280
r2551	2928
r2552	27320
r2553	2732
r2554	29740
r2555	2974
r2556	29320
r2557	2932
r2558	33380
r2559	3338
r2560	34240
r2561	3424
r2562	33400
r2563	3340
r2564	33800
r2565	3380
r2566	33700

Reaction	weight adjust
r2567	3370
r2568	33820
r2569	3382
r2570	36760
r2571	3676
r2572	29980
r2573	2998
r2574	34900
r2575	3490
r2576	20560
r2577	2056
r2578	20380
r2579	2038
r2580	63540
r2581	6354
r2582	52300
r2583	5230
r2584	45260
r2585	4526
r2586	58880
r2587	5888
r2588	14880
r2589	1488
r2590	14060
r2591	1406
r2592	18420
r2593	1842
r2594	13780
r2595	1378
r2596	13680
r2597	1368
r2598	13600
r2599	1360
r2600	36120
r2601	3612
r2602	34720
r2603	3472
r2604	61260
r2605	6126
r2606	14340
r2607	1434
r2608	35920
r2609	3592
r2610	27760
r2611	2776

Reaction	weight adjust
r2612	32080
r2613	3208
r2614	29940
r2615	2994
r2616	35080
r2617	3508
r2618	22840
r2619	2284
r2620	25680
r2621	2568
r2622	19140
r2623	1914
r2624	9100
r2625	910
r2626	32520
r2627	3252
r2628	10800
r2629	1080
r2630	34280
r2631	3428
r2632	13080
r2633	1308
r2634	37200
r2635	3720
r2636	22500
r2637	2250
r2638	4860
r2639	486
r2640	8820
r2641	882
r2642	6520
r2643	652
r2644	6260
r2645	626
r2646	5980
r2647	598
r2648	7520
r2649	752
r2650	23580
r2651	2358
r2652	23620
r2653	2362
r2654	6220
r2655	622
r2656	2140

Reaction	weight adjust
r2657	214
r2658	11040
r2659	1104
r2660	8360
r2661	836
r2662	15100
r2663	1510
r2664	0
r2665	0
r2666	0
r2667	0
r2668	0
r2669	0
r2670	0
r2671	0
r2672	0
r2673	0
r2674	0
r2675	0
r2676	0
r2677	0
r2678	0
r2679	0
r2680	0
r2681	0
r2682	0
r2683	0
r2684	0
