# Supplementary material for: Metabolic Consequences of TGFβ Stimulation in Cultured Primary Mouse Hepatocytes Screened from Transcript Data with ModeScore
Source: Metabolites. 2012 Nov 21;2(4):983–1003. doi: 10.3390/metabo2040983 (PMC3901234; doi:10.3390/metabo2040983)
Supplement: Supplementary File 4 — Solutions (PDF, 3957 KB) [file metabolites-02-00983-s004.pdf]

Solutions

October 17, 2012

Contents

1 Definitions and specification of exchange sets **2**

2 A. Maintenance functions **4**

2.1 A1. pure energy 4

2.2 A2. Membrane gradients 32

2.3 A3. Small groups activated 37

2.4 A4. Activated sugars 49

2.5 A5. Important intermediates 51

2.6 A6. Salvage 55

2.7 A7. Detoxification of internally emerging substances 58

3 B. Functions of organismic duty **59**

3.1 B1. Storage/Release 59

3.2 B2. Turnover functions 60

3.3 B3. Excretion of de novo synthesized substances 260

3.4 B4. clearence " ... degr" simulations (only obvious end products) 356

4 C. Proliferative functions **396**

4.1 C1. Cofactors 396

4.2 C2. Precursors for macromolecules 415

4.3 C3. Constituents of cellular structures 449

4.4 C4. Other building blocks 459

4.5 C5. Comprehensive list 464

1 Definitions and specification of exchange sets

List of compartments

Identifer	compartment	comment
s	sinusoid (blood)	external
b	bile	external
c	cytosol	
m	mitochondrium	
r	ER and Golgi	combined
p	peroxysome	
l	lysosome	
n	nucleus	

Symbols in *Constraints* column

Symbol	meaning
−	uptake allowed
+	excretion allowed
=	any exchange allowed
MCES/AAA/ALFA/PIPES	use exchange set (see below)

Maintained cell exchange set: MCES

Metabolite	compartment	direction
ATP-energy	c	both
ATP-energy	m	both
Activated methyl group	c	both
THF-activated methyl group	c	both
NADH-redox-potential	r	both
NADH-redox-potential	c	both
NADH-redox-potential	m	both
NADH-redox-potential	p	both
NADPH-redox-potential	r	both
NADPH-redox-potential	c	both
NADPH-redox-potential	m	both
NADPH-redox-potential	p	both
FADH-redox-potential	c	both
Adenosylmethioninamine-potential	c	both
Proton-gradient	c	both
Proton-gradient	m	both
activated-sulphur	c	both
Glucose-6P	c	both
Pyruvate	c	both
Pyruvate	m	both
Farnesyl-PP	r	both
Farnesyl-PP	p	both
CoA-activated acetyl group	c	both
CoA-activated acetyl group	m	both
CoA-activated acetyl group	p	both
CoA-activated acetyl group	r	both
Na-gradient	c	both
P_i	c	both
H_2O	s	both
CO_2	s	export
O_2	s	import

All amino acids: AAA

Metabolite	compartment	direction
Histidine	s	both
Isoleucine	s	both
Leucine	s	both
Lysine	s	both
Methionine	s	both
Phenylalanine	s	both
Threonine	s	both
Tryptophan	s	both
Valine	s	both
Alanine	s	both
Arginine	s	both
Asparagine	s	both
Aspartate	s	both
Cysteine	s	both
Cystine	s	both
Glutamate	s	both
Glycine	s	both
Glutamine	s	both
Proline	s	both
Serine	s	both
Tyrosine	s	both

All lipid-relevant fatty acids and group donors: ALFA

Metabolite	compartment	direction
Linoleate	c	both
Palmitate	c	both
Stearate	c	both
Oleate	c	both
Arachidonate	c	both
Palmitolate	c	both
gamma-Linolenate	c	both
Ethanolamine	c	both
Choline	c	both

Physiological exchange set: PIPES

Metabolite	compartment	direction
Glucose	s	both
O_2	s	import
NH_3	s	import
Sulfate	s	both
P_i	s	both
Fe^{2+}	s	both
Nicotinamide	s	import
Folate	s	import
Histidine	s	both
Isoleucine	s	both
Leucine	s	both
Lysine	s	both
Methionine	s	both
Phenylalanine	s	both
Threonine	s	both
Tryptophan	s	both
Valine	s	both
Pantothenate	s	import
CO_2	s	export
Urea	s	export
Urate	s	export
Ethanolamine	c	both
Choline	c	both
Linoleate	s	both
Pyridoxine	s	import
Riboflavin	s	import
H_2O	s	both
L-Lactate	s	both
Alanine	s	both
Arginine	s	both
Asparagine	s	both
Aspartate	s	both
Cysteine	s	both
Cystine	s	both
Glutamate	s	both
Glycine	s	both
Glutamine	s	both
Proline	s	both
Serine	s	both
Tyrosine	s	both
Palmitate	s	both
Stearate	s	both
Oleate	s	both
Arachidonate	s	both
Palmitolate	s	both
Cholesterol	b	export
H_2S	s	export

2 A. Maintenance functions

2.1 A1. pure energy

A1.1. NTP rephosphorylation

Aerobic ATP rephosph (FA) (1)

Definition			Solution												
			exchanges		reactions								transp		
Objective	Constraints	Comment	imports	exports	c	m	r	p	l	n	s	b	s-c	b-c	int
ATP-energy(c)	−Palmitate +CO_2 −O_2 =H_2O	Aerobic rephosphorylation of cytosolic ATP from a fatty acid	0.22 O_2(s) 0.01 Palmitate(s)	0.15 H_2O(s) 0.15 CO_2(s) 1 ATP-energy(c)	6	38	-	-	-	-	-	-	4	-	11

ID	flux	reaction
r2435	0.01	L-Carnitine(m) + L-Palmitoylcarnitine(c) → L-Carnitine(c) + L-Palmitoylcarnitine(m)
r0125	-0.08	GDP(m) + Pi(m) + Succinyl-CoA(m) ⇌ GTP(m) + Succinate(m) + CoA(m)
r0855	0.22	O2(s) ⇌ O2(c)
r0856	-0.22	O2(m) ⇌ O2(c)
r0720	-0.01	(S)-3-Hydroxytetradecanoyl-CoA(m) ← (2E)-Tetradecenoyl-CoA(m) + H2O(m)
r0425	0.08	Isocitrate(m) + NAD+(m) ⇌ Oxalosuccinate(m) + NADH(m)
r0722	0.01	(S)-3-Hydroxydodecanoyl-CoA(m) + NAD+(m) ⇌ 3-Oxododecanoyl-CoA(m) + NADH(m)
r0724	-0.01	Decanoyl-CoA(m) + Acetyl-CoA(m) ⇌ CoA(m) + 3-Oxododecanoyl-CoA(m)
r0726	0.01	(S)-Hydroxydecanoyl-CoA(m) + NAD+(m) ⇌ 3-Oxodecanoyl-CoA(m) + NADH(m)
r0728	-0.01	(S)-Hydroxydecanoyl-CoA(m) ⇌ (2E)-Decenoyl-CoA(m) + H2O(m)
r0460	0.01	(S)-3-Hydroxybutyryl-CoA(m) + NAD+(m) ⇌ NADH(m) + Acetoacetyl-CoA(m)
r0310	0.01	Palmitoyl-CoA(m) + Ubiquinone(m) → (2E)-Hexadecenoyl-CoA(m) + Ubiquinol(m)
r0017	0.22	O2(m) + 4 H+(PG)(m) + 4 Ferrocytochrome C(m) → 4 Ferricytochrome C(m) + 2 H2O(m) + 4 H+(PG)(c)
r0312	0.01	Palmitate(c) + ATP(c) + CoA(c) → Palmitoyl-CoA(c) + AMP(c) + PPi(c)
r0030	0.01	ATP(c) + AMP(c) ⇌ 2 ADP(c)
r0019	-0.94	ADP(m) + Pi(m) + 3 H+(PG)(c) → ATP(m) + H2O(m) + 3 H+(PG)(m)
r0316	0.08	Citrate(m) ⇌ Isocitrate(m)
r0070	-0.01	CoA(m) + Acetoacetyl-CoA(m) ⇌ 2 Acetyl-CoA(m)
r0634	-0.01	Acetyl-CoA(m) + Octanoyl-CoA(m) ⇌ CoA(m) + 3-Oxodecanoyl-CoA(m)
r0639	-0.01	Lauroyl-CoA(m) + Acetyl-CoA(m) ← CoA(m) + 3-Oxotetradecanoyl-CoA(m)
r0935	0.01	Palmitate(s) → Palmitate(c)
r0653	-0.01	Myristoyl-CoA(m) + Acetyl-CoA(m) ← CoA(m) + 3-Oxopalmitoyl-CoA(m)
r1446	0.01	Ubiquinone(m) + Butyryl-CoA(m) → Crotonyl-CoA(m) + Ubiquinol(m)
r1447	0.01	Ubiquinone(m) + Lauroyl-CoA(m) → (2E)-Dodecenoyl-CoA(m) + Ubiquinol(m)
r1448	0.01	Ubiquinone(m) + Octanoyl-CoA(m) → (2E)-Octenoyl-CoA(m) + Ubiquinol(m)
r1449	0.01	Ubiquinone(m) + Myristoyl-CoA(m) → (2E)-Tetradecenoyl-CoA(m) + Ubiquinol(m)
r0801	-0.08	GTP(c) + GDP(m) ⇌ GTP(m) + GDP(c)
r0507	0.44	Ubiquinol(m) + 2 Ferricytochrome C(m) + 4 H+(PG)(m) → Ubiquinone(m) + 4 H+(PG)(c) + 2 Ferrocytochrome C(m)
r0508	-0.3	Ubiquinone(m) + NADH(m) + 4 H+(PG)(m) → Ubiquinol(m) + NAD+(m) + 4 H+(PG)(c)
r0509	0.08	Ubiquinone(m) + Succinate(m) → Ubiquinol(m) + Fumarate(m)
r0099	-0.08	ADP(c) + GTP(c) ⇌ ATP(c) + GDP(c)
r1319	1	ATP(c) + H2O(c) ⇌ ADP(c) + Pi(c) + ATP-energy(c)
r0849	-0.15	H2O(s) ⇌ H2O(c)
r0287	-0.01	CoA(m) + 3-Oxohexanoyl-CoA(m) → Acetyl-CoA(m) + Butyryl-CoA(m)
r0714	0.01	NAD+(m) + (S)-3-Hydroxyhexadecanoyl-CoA(m) → 3-Oxopalmitoyl-CoA(m) + NADH(m)
r0867	0.15	CO2(c) ⇌ CO2(s)
r0868	-0.15	CO2(c) ⇌ CO2(m)
r0716	-0.01	(S)-3-Hydroxyhexadecanoyl-CoA(m) ← (2E)-Hexadecenoyl-CoA(m) + H2O(m)
r0435	0.01	Palmitoyl-CoA(c) + L-Carnitine(c) ⇌ CoA(c) + L-Palmitoylcarnitine(c)
r0730	0.01	(S)-Hydroxyoctanoyl-CoA(m) + NAD+(m) → 3-Oxooctanoyl-CoA(m) + NADH(m)
r0588	-0.01	Crotonyl-CoA(m) + H2O(m) ⇌ (S)-3-Hydroxybutyryl-CoA(m)
r0718	0.01	(S)-3-Hydroxytetradecanoyl-CoA(m) + NAD+(m) → 3-Oxotetradecanoyl-CoA(m) + NADH(m)
r0436	-0.01	Palmitoyl-CoA(m) + L-Carnitine(m) ← CoA(m) + L-Palmitoylcarnitine(m)

Continued on next page

Aerobic ATP rephosph (FA) (1)– continued

ID	flux	reaction
r0731	-0.01	(S)-Hydroxyoctanoyl-CoA(m) ← (2E)-Octenoyl-CoA(m) + H2O(m)
r0732	-0.01	Hexanoyl-CoA(m) + Acetyl-CoA(m) ← CoA(m) + 3-Oxooctanoyl-CoA(m)
r0733	0.01	(S)-Hydroxyhexanoyl-CoA(m) + NAD+(m) → 3-Oxohexanoyl-CoA(m) + NADH(m)
r0734	-0.01	(S)-Hydroxyhexanoyl-CoA(m) ← (2E)-Hexenoyl-CoA(m) + H2O(m)
r0007	0.01	PPi(c) + H2O(c) → 2 Pi(c)
r1114	0.08	AKG(m) + CoA(m) + NAD+(m) ⇌ NADH(m) + CO2(m) + Succinyl-CoA(m)
r1450	0.01	Ubiquinone(m) + Hexanoyl-CoA(m) → (2E)-Hexenoyl-CoA(m) + Ubiquinol(m)
r1451	0.01	Ubiquinone(m) + Decanoyl-CoA(m) → (2E)-Decenoyl-CoA(m) + Ubiquinol(m)
r0660	-0.01	(S)-3-Hydroxydodecanoyl-CoA(m) ⇌ (2E)-Dodecenoyl-CoA(m) + H2O(m)
r0083	0.08	Oxalosuccinate(m) ⇌ AKG(m) + CO2(m)
r1047	1.02	Pi(c) + H+(PG)(c) → Pi(m) + H+(PG)(m)
r0105	0.08	NAD+(m) + Malate(m) ⇌ OAA(m) + NADH(m)
r2418	0.94	ADP(c) + ATP(m) → ADP(m) + ATP(c)
r0107	-0.08	Acetyl-CoA(m) + H2O(m) + OAA(m) ⇌ Citrate(m) + CoA(m)
r0259	-0.08	Fumarate(m) + H2O(m) ⇌ Malate(m)
r0851	1.16	H2O(m) ⇌ H2O(c)

Aerobic ATP rephosph (gluc) (2)

Definition			Solution												
			exchanges		reactions								transp		
Objective	Constraints	Comment	imports	exports	c	m	r	p	l	n	s	b	s-c	b-c	int
ATP-energy(c)	−Glucose +CO_2 −O_2 =H_2O	Aerobic rephosphorylation of cytosolic ATP from glucose	0.19 O_2(s) 0.03 Glucose(s)	0.19 H_2O(s) 0.19 CO_2(s) 1 ATP-energy(c)	13	11	-	-	-	-	-	-	4	-	11

ID	flux	reaction
r0125	-0.06	GDP(m) + Pi(m) + Succinyl-CoA(m) ⇌ GTP(m) + Succinate(m) + CoA(m)
r0855	0.19	O2(s) ⇌ O2(c)
r0856	-0.19	O2(m) ⇌ O2(c)
r0425	0.06	Isocitrate(m) + NAD+(m) ⇌ Oxalosuccinate(m) + NADH(m)
r0164	0.06	2PG(c) ⇌ PEP(c) + H2O(c)
r0017	0.19	O2(m) + 4 H+(PG)(m) + 4 Ferrocytochrome C(m) → 4 Ferricytochrome C(m) + 2 H2O(m) + 4 H+(PG)(c)
r0019	-0.87	ADP(m) + Pi(m) + 3 H+(PG)(c) → ATP(m) + H2O(m) + 3 H+(PG)(m)
r0316	0.06	Citrate(m) ⇌ Isocitrate(m)
r0054	-0.06	ADP(c) + PEP(c) → ATP(c) + Pyruvate(c)
r1422	0.06	L-Lactate(c) + H+(PG)(c) → L-Lactate(m) + H+(PG)(m)
r0337	-0.06	ADP(c) + 1,3DPG(c) ⇌ ATP(c) + 3PG(c)
r0056	0.06	Pyruvate(m) + CoA(m) + NAD+(m) → Acetyl-CoA(m) + CO2(m) + NADH(m)
r0353	0.03	Glucose(c) + ATP(c) → Glucose-6P(c) + ADP(c)
r0801	-0.06	GTP(c) + GDP(m) ⇌ GTP(m) + GDP(c)
r0507	0.39	Ubiquinol(m) + 2 Ferricytochrome C(m) + 4 H+(PG)(m) → Ubiquinone(m) + 4 H+(PG)(c) + 2 Ferrocytochrome C(m)
r0508	-0.32	Ubiquinone(m) + NADH(m) + 4 H+(PG)(m) → Ubiquinol(m) + NAD+(m) + 4 H+(PG)(c)
r0509	0.06	Ubiquinone(m) + Succinate(m) → Ubiquinol(m) + Fumarate(m)
r1032	0.03	Glucose(s) ⇌ Glucose(c)
r0099	-0.06	ADP(c) + GTP(c) ⇌ ATP(c) + GDP(c)
r0243	-0.03	DHAP(c) ⇌ GAP(c)
r1319	1	ATP(c) + H2O(c) ⇌ ADP(c) + Pi(c) + ATP-energy(c)
r0849	-0.19	H2O(s) ⇌ H2O(c)
r0867	0.19	CO2(c) ⇌ CO2(s)
r0868	-0.19	CO2(c) ⇌ CO2(m)
r0171	-0.06	L-Lactate(c) + NAD+(c) ⇌ Pyruvate(c) + NADH(c)
r0736	0.03	ATP(c) + Fructose-6P(c) → ADP(c) + Fructose-1,6PP(c)
r0172	0.06	L-Lactate(m) + NAD+(m) → Pyruvate(m) + NADH(m)

Continued on next page

Aerobic ATP rephosph (gluc) (2)– continued

ID	flux	reaction
r0192	0.03	Glucose-6P(c) ⇌ Fructose-6P(c)
r1114	0.06	AKG(m) + CoA(m) + NAD+(m) ⇌ NADH(m) + CO2(m) + Succinyl-CoA(m)
r0341	-0.06	3PG(c) ⇌ 2PG(c)
r0083	0.06	Oxalosuccinate(m) ⇌ AKG(m) + CO2(m)
r0253	0.06	GAP(c) + Pi(c) + NAD+(c) ⇌ 1,3DPG(c) + NADH(c)
r0256	0.03	Fructose-1,6PP(c) ⇌ DHAP(c) + GAP(c)
r1047	0.94	Pi(c) + H+(PG)(c) → Pi(m) + H+(PG)(m)
r0105	0.06	NAD+(m) + Malate(m) ⇌ OAA(m) + NADH(m)
r2418	0.87	ADP(c) + ATP(m) → ADP(m) + ATP(c)
r0107	-0.06	Acetyl-CoA(m) + H2O(m) + OAA(m) ⇌ Citrate(m) + CoA(m)
r0259	-0.06	Fumarate(m) + H2O(m) ⇌ Malate(m)
r0851	1.13	H2O(m) ⇌ H2O(c)

Anaerobic rephosph of ATP (3)

Definition			Solution												
			exchanges		reactions								transp		
Objective	Constraints	Comment	imports	exports	c	m	r	p	l	n	s	b	s-c	b-c	int
ATP-energy(c)	−Glucose +L-Lactate $+CO_2$ $=H_2O$ =Proton-gradient(c)	Anaerobic rephosphorylation of cytosolic ATP from glucose	0.5 Glucose(s)	1 L-Lactate(s) 1 ATP-energy(c) 1 Proton-gradient(c)	12	-	-	-	-	-	-	-	3	-	-

ID	flux	reaction
r0253	1	GAP(c) + Pi(c) + NAD+(c) ⇌ 1,3DPG(c) + NADH(c)
r0353	0.5	Glucose(c) + ATP(c) → Glucose-6P(c) + ADP(c)
r0164	1	2PG(c) ⇌ PEP(c) + H2O(c)
r0192	0.5	Glucose-6P(c) ⇌ Fructose-6P(c)
r0337	-1	ADP(c) + 1,3DPG(c) ⇌ ATP(c) + 3PG(c)
r0256	0.5	Fructose-1,6PP(c) ⇌ DHAP(c) + GAP(c)
r1319	1	ATP(c) + H2O(c) ⇌ ADP(c) + Pi(c) + ATP-energy(c)
r0736	0.5	ATP(c) + Fructose-6P(c) → ADP(c) + Fructose-1,6PP(c)
r2078	-1	H+(PG)(c) + L-Lactate(c) ⇌ H+(PG)(s) + L-Lactate(s)
r1032	0.5	Glucose(s) ⇌ Glucose(c)
r1331	1	H+(PG)(s) ⇌ H+(PG)(c) + Proton-gradient(c)
r0341	-1	3PG(c) ⇌ 2PG(c)
r0243	-0.5	DHAP(c) ⇌ GAP(c)
r0171	-1	Pyruvate(c) + NADH(c) ⇌ L-Lactate(c) + NAD+(c)
r0054	-1	ADP(c) + PEP(c) → ATP(c) + Pyruvate(c)

Aerobic rephosph of GTP (4)

Definition			Solution												
			exchanges		reactions								transp		
Objective	Constraints	Comment	imports	exports	c	m	r	p	l	n	s	b	s-c	b-c	int
GTP(c)	−Palmitate $-P_i(c)$ −GDP(c) $+CO_2$ $-O_2$ $=H_2O$	Aerobic rephosphorylation of cytosolic GTP from glucose	0.22 $O_2(s)$ 1 $P_i(c)$ 1 GDP(c) 0.01 Palmitate(s)	1.15 $H_2O(s)$ 0.15 $CO_2(s)$ 1 GTP(c)	5	38	-	-	-	-	-	-	4	-	11

ID	flux	reaction
r2435	0.01	L-Carnitine(m) + L-Palmitoylcarnitine(c) → L-Carnitine(c) + L-Palmitoylcarnitine(m)

Continued on next page

Aerobic rephosph of GTP (4)– continued

ID	flux	reaction
r0125	-0.08	GDP(m) + Pi(m) + Succinyl-CoA(m) ⇌ GTP(m) + Succinate(m) + CoA(m)
r0855	0.22	O2(s) ⇌ O2(c)
r0856	-0.22	O2(m) ⇌ O2(c)
r0720	-0.01	(S)-3-Hydroxytetradecanoyl-CoA(m) ← (2E)-Tetradecenoyl-CoA(m) + H2O(m)
r0425	0.08	Isocitrate(m) + NAD+(m) ⇌ Oxalosuccinate(m) + NADH(m)
r0722	0.01	(S)-3-Hydroxydodecanoyl-CoA(m) + NAD+(m) ⇌ 3-Oxododecanoyl-CoA(m) + NADH(m)
r0724	-0.01	Decanoyl-CoA(m) + Acetyl-CoA(m) ⇌ CoA(m) + 3-Oxododecanoyl-CoA(m)
r0726	0.01	(S)-Hydroxydecanoyl-CoA(m) + NAD+(m) ⇌ 3-Oxodecanoyl-CoA(m) + NADH(m)
r0728	-0.01	(S)-Hydroxydecanoyl-CoA(m) ⇌ (2E)-Decenoyl-CoA(m) + H2O(m)
r0460	0.01	(S)-3-Hydroxybutyryl-CoA(m) + NAD+(m) ⇌ NADH(m) + Acetoacetyl-CoA(m)
r0310	0.01	Palmitoyl-CoA(m) + Ubiquinone(m) → (2E)-Hexadecenoyl-CoA(m) + Ubiquinol(m)
r0017	0.22	O2(m) + 4 H+(PG)(m) + 4 Ferrocytochrome C(m) → 4 Ferricytochrome C(m) + 2 H2O(m) + 4 H+(PG)(c)
r0312	0.01	Palmitate(c) + ATP(c) + CoA(c) → Palmitoyl-CoA(c) + AMP(c) + PPi(c)
r0030	0.01	ATP(c) + AMP(c) ⇌ 2 ADP(c)
r0019	-0.94	ADP(m) + Pi(m) + 3 H+(PG)(c) → ATP(m) + H2O(m) + 3 H+(PG)(m)
r0316	0.08	Citrate(m) ⇌ Isocitrate(m)
r0070	-0.01	CoA(m) + Acetoacetyl-CoA(m) ⇌ 2 Acetyl-CoA(m)
r0634	-0.01	Acetyl-CoA(m) + Octanoyl-CoA(m) ⇌ CoA(m) + 3-Oxodecanoyl-CoA(m)
r0639	-0.01	Lauroyl-CoA(m) + Acetyl-CoA(m) ← CoA(m) + 3-Oxotetradecanoyl-CoA(m)
r0935	0.01	Palmitate(s) → Palmitate(c)
r0653	-0.01	Myristoyl-CoA(m) + Acetyl-CoA(m) ← CoA(m) + 3-Oxopalmitoyl-CoA(m)
r1446	0.01	Ubiquinone(m) + Butyryl-CoA(m) → Crotonyl-CoA(m) + Ubiquinol(m)
r1447	0.01	Ubiquinone(m) + Lauroyl-CoA(m) → (2E)-Dodecenoyl-CoA(m) + Ubiquinol(m)
r1448	0.01	Ubiquinone(m) + Octanoyl-CoA(m) → (2E)-Octenoyl-CoA(m) + Ubiquinol(m)
r1449	0.01	Ubiquinone(m) + Myristoyl-CoA(m) → (2E)-Tetradecenoyl-CoA(m) + Ubiquinol(m)
r0801	-0.08	GTP(c) + GDP(m) ⇌ GTP(m) + GDP(c)
r0507	0.44	Ubiquinol(m) + 2 Ferricytochrome C(m) + 4 H+(PG)(m) → Ubiquinone(m) + 4 H+(PG)(c) + 2 Ferrocytochrome C(m)
r0508	-0.3	Ubiquinone(m) + NADH(m) + 4 H+(PG)(m) → Ubiquinol(m) + NAD+(m) + 4 H+(PG)(c)
r0509	0.08	Ubiquinone(m) + Succinate(m) → Ubiquinol(m) + Fumarate(m)
r0099	0.92	ATP(c) + GDP(c) ⇌ ADP(c) + GTP(c)
r0849	-1.15	H2O(s) ⇌ H2O(c)
r0287	-0.01	CoA(m) + 3-Oxohexanoyl-CoA(m) → Acetyl-CoA(m) + Butyryl-CoA(m)
r0714	0.01	NAD+(m) + (S)-3-Hydroxyhexadecanoyl-CoA(m) → 3-Oxopalmitoyl-CoA(m) + NADH(m)
r0867	0.15	CO2(c) ⇌ CO2(s)
r0868	-0.15	CO2(c) ⇌ CO2(m)
r0716	-0.01	(S)-3-Hydroxyhexadecanoyl-CoA(m) ← (2E)-Hexadecenoyl-CoA(m) + H2O(m)
r0435	0.01	Palmitoyl-CoA(c) + L-Carnitine(c) ⇌ CoA(c) + L-Palmitoylcarnitine(c)
r0730	0.01	(S)-Hydroxyoctanoyl-CoA(m) + NAD+(m) → 3-Oxooctanoyl-CoA(m) + NADH(m)
r0588	-0.01	Crotonyl-CoA(m) + H2O(m) ⇌ (S)-3-Hydroxybutyryl-CoA(m)
r0718	0.01	(S)-3-Hydroxytetradecanoyl-CoA(m) + NAD+(m) → 3-Oxotetradecanoyl-CoA(m) + NADH(m)
r0436	-0.01	Palmitoyl-CoA(m) + L-Carnitine(m) ← CoA(m) + L-Palmitoylcarnitine(m)
r0731	-0.01	(S)-Hydroxyoctanoyl-CoA(m) ← (2E)-Octenoyl-CoA(m) + H2O(m)
r0732	-0.01	Hexanoyl-CoA(m) + Acetyl-CoA(m) ← CoA(m) + 3-Oxooctanoyl-CoA(m)
r0733	0.01	(S)-Hydroxyhexanoyl-CoA(m) + NAD+(m) → 3-Oxohexanoyl-CoA(m) + NADH(m)
r0734	-0.01	(S)-Hydroxyhexanoyl-CoA(m) ← (2E)-Hexenoyl-CoA(m) + H2O(m)
r0007	0.01	PPi(c) + H2O(c) → 2 Pi(c)
r1114	0.08	AKG(m) + CoA(m) + NAD+(m) ⇌ NADH(m) + CO2(m) + Succinyl-CoA(m)
r1450	0.01	Ubiquinone(m) + Hexanoyl-CoA(m) → (2E)-Hexenoyl-CoA(m) + Ubiquinol(m)
r1451	0.01	Ubiquinone(m) + Decanoyl-CoA(m) → (2E)-Decenoyl-CoA(m) + Ubiquinol(m)
r0660	-0.01	(S)-3-Hydroxydodecanoyl-CoA(m) ⇌ (2E)-Dodecenoyl-CoA(m) + H2O(m)
r0083	0.08	Oxalosuccinate(m) ⇌ AKG(m) + CO2(m)
r1047	1.02	Pi(c) + H+(PG)(c) → Pi(m) + H+(PG)(m)
r0105	0.08	NAD+(m) + Malate(m) ⇌ OAA(m) + NADH(m)
r2418	0.94	ADP(c) + ATP(m) → ADP(m) + ATP(c)
r0107	-0.08	Acetyl-CoA(m) + H2O(m) + OAA(m) ⇌ Citrate(m) + CoA(m)
r0259	-0.08	Fumarate(m) + H2O(m) ⇌ Malate(m)

Continued on next page

ID	flux	reaction
r0851	1.16	H2O(m) ⇌ H2O(c)

Aerobic rephosph of CTP (5)

Definition			Solution												
			exchanges		reactions								transp		
Objective	Constraints	Comment	imports	exports	c	m	r	p	l	n	s	b	s-c	b-c	int
CTP(c)	−Palmitate −P_i(c) −UTP(c) −CDP(c) =Glutamine =Glutamate +CO_2 −O_2 =H_2O	Aerobic rephosphorylation of cytosolic CTP from glucose	0.15 O_2(s) 0.8 P_i(c) 0.1 UTP(c) 0.95 CDP(c)	0.85 H_2O(s) 0.2 CO_2(s) 0.05 Glutamate(s) 1 CTP(c)	29	13	-	-	-	-	-	-	4	-	15

ID	flux	reaction
r0125	-0.03	GDP(m) + Pi(m) + Succinyl-CoA(m) ⇌ GTP(m) + Succinate(m) + CoA(m)
r0407	0.03	Sedoheptulose-1,7PP(c) ⇌ DHAP(c) + Erythrose-4P(c)
r0855	0.15	O2(s) ⇌ O2(c)
r0856	-0.15	O2(m) ⇌ O2(c)
r0409	0.03	UTP(c) + Sedoheptulose-7P(c) ⇌ UDP(c) + Sedoheptulose-1,7PP(c)
r0425	0.08	Isocitrate(m) + NAD+(m) ⇌ Oxalosuccinate(m) + NADH(m)
r0164	0.08	2PG(c) ⇌ PEP(c) + H2O(c)
r0017	0.15	O2(m) + 4 H+(PG)(m) + 4 Ferrocytochrome C(m) → 4 Ferricytochrome C(m) + 2 H2O(m) + 4 H+(PG)(c)
r0019	-0.7	ADP(m) + Pi(m) + 3 H+(PG)(c) → ATP(m) + H2O(m) + 3 H+(PG)(m)
r0316	0.08	Citrate(m) ⇌ Isocitrate(m)
r0037	-0.03	ATP(c) + UDP(c) ⇌ ADP(c) + UTP(c)
r0038	-0.05	ADP(c) + UDP(c) ⇌ ATP(c) + UMP(c)
r0911	0.05	Proline(c) + Glutamate(m) ⇌ Proline(m) + Glutamate(c)
r1422	0.03	L-Lactate(c) + H+(PG)(c) → L-Lactate(m) + H+(PG)(m)
r0054	-0.08	ADP(c) + PEP(c) → ATP(c) + Pyruvate(c)
r0337	-0.08	ADP(c) + 1,3DPG(c) ⇌ ATP(c) + 3PG(c)
r0056	0.03	Pyruvate(m) + CoA(m) + NAD+(m) → Acetyl-CoA(m) + CO2(m) + NADH(m)
r1426	0.05	Proline(m) + H+(PG)(c) → Proline(c) + H+(PG)(m)
r0058	-0.05	Pyruvate(c) + CO2(c) + NADPH(c) ⇌ Malate(c) + NADP+(c)
r0370	-0.03	Sedoheptulose-7P(c) + GAP(c) ⇌ Ribose-5P(c) + D-Xylulose-5P(c)
r2685	0.05	Glutamate(c) ⇌ Glutamate(s)
r0801	-0.03	GTP(c) + GDP(m) ⇌ GTP(m) + GDP(c)
r0507	0.3	Ubiquinol(m) + 2 Ferricytochrome C(m) + 4 H+(PG)(m) → Ubiquinone(m) + 4 H+(PG)(c) + 2 Ferrocytochrome C(m)
r0508	-0.28	Ubiquinone(m) + NADH(m) + 4 H+(PG)(m) → Ubiquinol(m) + NAD+(m) + 4 H+(PG)(c)
r0509	0.03	Ubiquinone(m) + Succinate(m) → Ubiquinol(m) + Fumarate(m)
r0099	-0.03	ATP(c) + GDP(c) ⇌ ADP(c) + GTP(c)
r0243	-0.03	DHAP(c) ⇌ GAP(c)
r0849	-0.85	H2O(s) ⇌ H2O(c)
r0418	0.05	Uridine(c) + Pi(c) ⇌ Uracil(c) + Ribose-1P(c)
r0150	0.95	ATP(c) + CDP(c) ⇌ ADP(c) + CTP(c)
r0867	0.2	CO2(c) ⇌ CO2(s)
r0868	-0.18	CO2(c) ⇌ CO2(m)
r0152	0.05	ATP(c) + UTP(c) + NH3(c) → ADP(c) + Pi(c) + CTP(c)
r0171	-0.03	L-Lactate(c) + NAD+(c) ⇌ Pyruvate(c) + NADH(c)
r0172	0.03	L-Lactate(m) + NAD+(m) → Pyruvate(m) + NADH(m)
r0174	0.05	CoA(m) + NAD+(m) + 3-Oxopropanoate(m) → Acetyl-CoA(m) + CO2(m) + NADH(m)
r0192	-0.03	Fructose-6P(c) ⇌ Glucose-6P(c)
r1114	0.03	AKG(m) + CoA(m) + NAD+(m) ⇌ NADH(m) + CO2(m) + Succinyl-CoA(m)
r0900	0.05	beta-Alanine(c) ⇌ beta-Alanine(m)

Continued on next page

ID	flux	reaction
r1115	-0.05	ADP(c) + UMP(c) ⇌ ATP(c) + Uridine(c)
r0479	0.03	Glucono-1,5-lactone-6P(c) + H2O(c) ⇌ 6-Phospho-D-gluconate(c)
r0341	-0.08	3PG(c) ⇌ 2PG(c)
r0199	0.03	Glucose-6P(c) + NADP+(c) ⇌ NADPH(c) + Glucono-1,5-lactone-6P(c)
r0343	0.03	6-Phospho-D-gluconate(c) + NADP+(c) ⇌ NADPH(c) + CO2(c) + Ribulose-5P(c)
r0344	0.05	Ribulose-5P(c) ⇌ D-Xylulose-5P(c)
r0083	0.08	Oxalosuccinate(m) ⇌ AKG(m) + CO2(m)
r0216	0.05	H2O(c) + 3-Ureidopropionate(c) → beta-Alanine(c) + CO2(c) + NH3(c)
r0217	0.05	beta-Alanine(m) + AKG(m) ⇌ Glutamate(m) + 3-Oxopropanoate(m)
r0516	0.05	5,6-Dihydrouracil(c) + H2O(c) → 3-Ureidopropionate(c)
r0236	-0.05	5,6-Dihydrouracil(c) + NAD+(c) ⇌ Uracil(c) + NADH(c)
r0250	0.03	Ribose-5P(c) ⇌ Ribulose-5P(c)
r0251	0.05	Ribose-1P(c) ⇌ Ribose-5P(c)
r0253	0.08	GAP(c) + Pi(c) + NAD+(c) ⇌ 1,3DPG(c) + NADH(c)
r0831	0.05	Malate(c) + Pi(m) ⇌ Malate(m) + Pi(c)
r0255	-0.03	Fructose-6P(c) + GAP(c) ⇌ D-Xylulose-5P(c) + Erythrose-4P(c)
r1047	0.77	Pi(c) + H+(PG)(c) → Pi(m) + H+(PG)(m)
r0105	0.08	NAD+(m) + Malate(m) ⇌ OAA(m) + NADH(m)
r2418	0.7	ADP(c) + ATP(m) → ADP(m) + ATP(c)
r0107	-0.08	Acetyl-CoA(m) + H2O(m) + OAA(m) ⇌ Citrate(m) + CoA(m)
r0259	-0.03	Fumarate(m) + H2O(m) ⇌ Malate(m)
r0851	0.9	H2O(m) ⇌ H2O(c)

Aerobic rephosph of UDP (6)

Definition			Solution													
			exchanges		reactions								transp		
Objective	Constraints	Comment	imports	exports	c	m	r	p	l	n	s	b	s-c	b-c	int
UDP(c)	$-$Palmitate $-P_i(c)$ $-$UMP(c) $+CO_2$ $-O_2$ $=H_2O$	Aerobic rephosphorylation of cytosolic UDP from glucose	0.22 O_2(s) 1 P_i(c) 1 UMP(c) 0.01 Palmitate(s)	1.15 H_2O(s) 0.15 CO_2(s) 1 UDP(c)	6	38	-	-	-	-	-	-	4	-	11

ID	flux	reaction
r2435	0.01	L-Carnitine(m) + L-Palmitoylcarnitine(c) → L-Carnitine(c) + L-Palmitoylcarnitine(m)
r0125	-0.08	GDP(m) + Pi(m) + Succinyl-CoA(m) ⇌ GTP(m) + Succinate(m) + CoA(m)
r0855	0.22	O2(s) ⇌ O2(c)
r0856	-0.22	O2(m) ⇌ O2(c)
r0720	-0.01	(S)-3-Hydroxytetradecanoyl-CoA(m) ← (2E)-Tetradecenoyl-CoA(m) + H2O(m)
r0425	0.08	Isocitrate(m) + NAD+(m) ⇌ Oxalosuccinate(m) + NADH(m)
r0722	0.01	(S)-3-Hydroxydodecanoyl-CoA(m) + NAD+(m) ⇌ 3-Oxododecanoyl-CoA(m) + NADH(m)
r0724	-0.01	Decanoyl-CoA(m) + Acetyl-CoA(m) ⇌ CoA(m) + 3-Oxododecanoyl-CoA(m)
r0726	0.01	(S)-Hydroxydecanoyl-CoA(m) + NAD+(m) ⇌ 3-Oxodecanoyl-CoA(m) + NADH(m)
r0728	-0.01	(S)-Hydroxydecanoyl-CoA(m) ⇌ (2E)-Decenoyl-CoA(m) + H2O(m)
r0460	0.01	(S)-3-Hydroxybutyryl-CoA(m) + NAD+(m) ⇌ NADH(m) + Acetoacetyl-CoA(m)
r0310	0.01	Palmitoyl-CoA(m) + Ubiquinone(m) → (2E)-Hexadecenoyl-CoA(m) + Ubiquinol(m)
r0017	0.22	O2(m) + 4 H+(PG)(m) + 4 Ferrocytochrome C(m) → 4 Ferricytochrome C(m) + 2 H2O(m) + 4 H+(PG)(c)
r0312	0.01	Palmitate(c) + ATP(c) + CoA(c) → Palmitoyl-CoA(c) + AMP(c) + PPi(c)
r0030	0.01	ATP(c) + AMP(c) ⇌ 2 ADP(c)
r0019	-0.94	ADP(m) + Pi(m) + 3 H+(PG)(c) → ATP(m) + H2O(m) + 3 H+(PG)(m)
r0316	0.08	Citrate(m) ⇌ Isocitrate(m)
r0038	1	ATP(c) + UMP(c) ⇌ ADP(c) + UDP(c)
r0070	-0.01	CoA(m) + Acetoacetyl-CoA(m) ⇌ 2 Acetyl-CoA(m)
r0634	-0.01	Acetyl-CoA(m) + Octanoyl-CoA(m) ⇌ CoA(m) + 3-Oxodecanoyl-CoA(m)

Continued on next page

ID	flux	reaction
r0639	-0.01	Lauroyl-CoA(m) + Acetyl-CoA(m) ← CoA(m) + 3-Oxotetradecanoyl-CoA(m)
r0935	0.01	Palmitate(s) → Palmitate(c)
r0653	-0.01	Myristoyl-CoA(m) + Acetyl-CoA(m) ← CoA(m) + 3-Oxopalmitoyl-CoA(m)
r1446	0.01	Ubiquinone(m) + Butyryl-CoA(m) → Crotonyl-CoA(m) + Ubiquinol(m)
r1447	0.01	Ubiquinone(m) + Lauroyl-CoA(m) → (2E)-Dodecenoyl-CoA(m) + Ubiquinol(m)
r1448	0.01	Ubiquinone(m) + Octanoyl-CoA(m) → (2E)-Octenoyl-CoA(m) + Ubiquinol(m)
r1449	0.01	Ubiquinone(m) + Myristoyl-CoA(m) → (2E)-Tetradecenoyl-CoA(m) + Ubiquinol(m)
r0801	-0.08	GTP(c) + GDP(m) ⇌ GTP(m) + GDP(c)
r0507	0.44	Ubiquinol(m) + 2 Ferricytochrome C(m) + 4 H+(PG)(m) → Ubiquinone(m) + 4 H+(PG)(c) + 2 Ferrocytochrome C(m)
r0508	-0.3	Ubiquinone(m) + NADH(m) + 4 H+(PG)(m) → Ubiquinol(m) + NAD+(m) + 4 H+(PG)(c)
r0509	0.08	Ubiquinone(m) + Succinate(m) → Ubiquinol(m) + Fumarate(m)
r0099	-0.08	ADP(c) + GTP(c) ⇌ ATP(c) + GDP(c)
r0849	-1.15	H2O(s) ⇌ H2O(c)
r0287	-0.01	CoA(m) + 3-Oxohexanoyl-CoA(m) → Acetyl-CoA(m) + Butyryl-CoA(m)
r0714	0.01	NAD+(m) + (S)-3-Hydroxyhexadecanoyl-CoA(m) → 3-Oxopalmitoyl-CoA(m) + NADH(m)
r0867	0.15	CO2(c) ⇌ CO2(s)
r0868	-0.15	CO2(c) ⇌ CO2(m)
r0716	-0.01	(S)-3-Hydroxyhexadecanoyl-CoA(m) ← (2E)-Hexadecenoyl-CoA(m) + H2O(m)
r0435	0.01	Palmitoyl-CoA(c) + L-Carnitine(c) ⇌ CoA(c) + L-Palmitoylcarnitine(c)
r0730	0.01	(S)-Hydroxyoctanoyl-CoA(m) + NAD+(m) → 3-Oxooctanoyl-CoA(m) + NADH(m)
r0588	-0.01	Crotonyl-CoA(m) + H2O(m) ⇌ (S)-3-Hydroxybutyryl-CoA(m)
r0718	0.01	(S)-3-Hydroxytetradecanoyl-CoA(m) + NAD+(m) → 3-Oxotetradecanoyl-CoA(m) + NADH(m)
r0436	-0.01	Palmitoyl-CoA(m) + L-Carnitine(m) ← CoA(m) + L-Palmitoylcarnitine(m)
r0731	-0.01	(S)-Hydroxyoctanoyl-CoA(m) ← (2E)-Octenoyl-CoA(m) + H2O(m)
r0732	-0.01	Hexanoyl-CoA(m) + Acetyl-CoA(m) ← CoA(m) + 3-Oxooctanoyl-CoA(m)
r0733	0.01	(S)-Hydroxyhexanoyl-CoA(m) + NAD+(m) → 3-Oxohexanoyl-CoA(m) + NADH(m)
r0734	-0.01	(S)-Hydroxyhexanoyl-CoA(m) ← (2E)-Hexenoyl-CoA(m) + H2O(m)
r0007	0.01	PPi(c) + H2O(c) → 2 Pi(c)
r1114	0.08	AKG(m) + CoA(m) + NAD+(m) ⇌ NADH(m) + CO2(m) + Succinyl-CoA(m)
r1450	0.01	Ubiquinone(m) + Hexanoyl-CoA(m) → (2E)-Hexenoyl-CoA(m) + Ubiquinol(m)
r1451	0.01	Ubiquinone(m) + Decanoyl-CoA(m) → (2E)-Decenoyl-CoA(m) + Ubiquinol(m)
r0660	-0.01	(S)-3-Hydroxydodecanoyl-CoA(m) ⇌ (2E)-Dodecenoyl-CoA(m) + H2O(m)
r0083	0.08	Oxalosuccinate(m) ⇌ AKG(m) + CO2(m)
r1047	1.02	Pi(c) + H+(PG)(c) → Pi(m) + H+(PG)(m)
r0105	0.08	NAD+(m) + Malate(m) ⇌ OAA(m) + NADH(m)
r2418	0.94	ADP(c) + ATP(m) → ADP(m) + ATP(c)
r0107	-0.08	Acetyl-CoA(m) + H2O(m) + OAA(m) ⇌ Citrate(m) + CoA(m)
r0259	-0.08	Fumarate(m) + H2O(m) ⇌ Malate(m)
r0851	1.16	H2O(m) ⇌ H2O(c)

Aerobic rephosph of UTP (7)

Definition			Solution												
			exchanges		reactions								transp		
Objective	Constraints	Comment	imports	exports	c	m	r	p	l	n	s	b	s-c	b-c	int
UTP(c)	−Palmitate −P_i(c) −UDP(c) +CO_2 −O_2 =H_2O	Aerobic rephosphorylation of cytosolic UTP from glucose	0.22 O_2(s) 1 P_i(c) 1 UDP(c) 0.01 Palmitate(s)	1.15 H_2O(s) 0.15 CO_2(s) 1 UTP(c)	6	38	-	-	-	-	-	-	4	-	11

ID	flux	reaction
r2435	0.01	L-Carnitine(m) + L-Palmitoylcarnitine(c) → L-Carnitine(c) + L-Palmitoylcarnitine(m)
r0125	-0.08	GDP(m) + Pi(m) + Succinyl-CoA(m) ⇌ GTP(m) + Succinate(m) + CoA(m)
r0855	0.22	O2(s) ⇌ O2(c)

Continued on next page

ID	flux	reaction
r0856	-0.22	O2(m) ⇌ O2(c)
r0720	-0.01	(S)-3-Hydroxytetradecanoyl-CoA(m) ← (2E)-Tetradecenoyl-CoA(m) + H2O(m)
r0425	0.08	Isocitrate(m) + NAD+(m) ⇌ Oxalosuccinate(m) + NADH(m)
r0722	0.01	(S)-3-Hydroxydodecanoyl-CoA(m) + NAD+(m) ⇌ 3-Oxododecanoyl-CoA(m) + NADH(m)
r0724	-0.01	Decanoyl-CoA(m) + Acetyl-CoA(m) ⇌ CoA(m) + 3-Oxododecanoyl-CoA(m)
r0726	0.01	(S)-Hydroxydecanoyl-CoA(m) + NAD+(m) ⇌ 3-Oxodecanoyl-CoA(m) + NADH(m)
r0728	-0.01	(S)-Hydroxydecanoyl-CoA(m) ⇌ (2E)-Decenoyl-CoA(m) + H2O(m)
r0460	0.01	(S)-3-Hydroxybutyryl-CoA(m) + NAD+(m) ⇌ NADH(m) + Acetoacetyl-CoA(m)
r0310	0.01	Palmitoyl-CoA(m) + Ubiquinone(m) → (2E)-Hexadecenoyl-CoA(m) + Ubiquinol(m)
r0017	0.22	O2(m) + 4 H+(PG)(m) + 4 Ferrocytochrome C(m) → 4 Ferricytochrome C(m) + 2 H2O(m) + 4 H+(PG)(c)
r0312	0.01	Palmitate(c) + ATP(c) + CoA(c) → Palmitoyl-CoA(c) + AMP(c) + PPi(c)
r0030	0.01	ATP(c) + AMP(c) ⇌ 2 ADP(c)
r0019	-0.94	ADP(m) + Pi(m) + 3 H+(PG)(c) → ATP(m) + H2O(m) + 3 H+(PG)(m)
r0316	0.08	Citrate(m) ⇌ Isocitrate(m)
r0037	1	ATP(c) + UDP(c) ⇌ ADP(c) + UTP(c)
r0070	-0.01	CoA(m) + Acetoacetyl-CoA(m) ⇌ 2 Acetyl-CoA(m)
r0634	-0.01	Acetyl-CoA(m) + Octanoyl-CoA(m) ⇌ CoA(m) + 3-Oxodecanoyl-CoA(m)
r0639	-0.01	Lauroyl-CoA(m) + Acetyl-CoA(m) ← CoA(m) + 3-Oxotetradecanoyl-CoA(m)
r0935	0.01	Palmitate(s) → Palmitate(c)
r0653	-0.01	Myristoyl-CoA(m) + Acetyl-CoA(m) ← CoA(m) + 3-Oxopalmitoyl-CoA(m)
r1446	0.01	Ubiquinone(m) + Butyryl-CoA(m) → Crotonyl-CoA(m) + Ubiquinol(m)
r1447	0.01	Ubiquinone(m) + Lauroyl-CoA(m) → (2E)-Dodecenoyl-CoA(m) + Ubiquinol(m)
r1448	0.01	Ubiquinone(m) + Octanoyl-CoA(m) → (2E)-Octenoyl-CoA(m) + Ubiquinol(m)
r1449	0.01	Ubiquinone(m) + Myristoyl-CoA(m) → (2E)-Tetradecenoyl-CoA(m) + Ubiquinol(m)
r0801	-0.08	GTP(c) + GDP(m) ⇌ GTP(m) + GDP(c)
r0507	0.44	Ubiquinol(m) + 2 Ferricytochrome C(m) + 4 H+(PG)(m) → Ubiquinone(m) + 4 H+(PG)(c) + 2 Ferrocytochrome C(m)
r0508	-0.3	Ubiquinone(m) + NADH(m) + 4 H+(PG)(m) → Ubiquinol(m) + NAD+(m) + 4 H+(PG)(c)
r0509	0.08	Ubiquinone(m) + Succinate(m) → Ubiquinol(m) + Fumarate(m)
r0099	-0.08	ATP(c) + GDP(c) ⇌ ADP(c) + GTP(c)
r0849	-1.15	H2O(s) ⇌ H2O(c)
r0287	-0.01	CoA(m) + 3-Oxohexanoyl-CoA(m) → Acetyl-CoA(m) + Butyryl-CoA(m)
r0714	0.01	NAD+(m) + (S)-3-Hydroxyhexadecanoyl-CoA(m) → 3-Oxopalmitoyl-CoA(m) + NADH(m)
r0867	0.15	CO2(c) ⇌ CO2(s)
r0868	-0.15	CO2(c) ⇌ CO2(m)
r0716	-0.01	(S)-3-Hydroxyhexadecanoyl-CoA(m) ← (2E)-Hexadecenoyl-CoA(m) + H2O(m)
r0435	0.01	Palmitoyl-CoA(c) + L-Carnitine(c) ⇌ CoA(c) + L-Palmitoylcarnitine(c)
r0730	0.01	(S)-Hydroxyoctanoyl-CoA(m) + NAD+(m) → 3-Oxooctanoyl-CoA(m) + NADH(m)
r0588	-0.01	Crotonyl-CoA(m) + H2O(m) ⇌ (S)-3-Hydroxybutyryl-CoA(m)
r0718	0.01	(S)-3-Hydroxytetradecanoyl-CoA(m) + NAD+(m) → 3-Oxotetradecanoyl-CoA(m) + NADH(m)
r0436	-0.01	Palmitoyl-CoA(m) + L-Carnitine(m) ← CoA(m) + L-Palmitoylcarnitine(m)
r0731	-0.01	(S)-Hydroxyoctanoyl-CoA(m) ← (2E)-Octenoyl-CoA(m) + H2O(m)
r0732	-0.01	Hexanoyl-CoA(m) + Acetyl-CoA(m) ← CoA(m) + 3-Oxooctanoyl-CoA(m)
r0733	0.01	(S)-Hydroxyhexanoyl-CoA(m) + NAD+(m) → 3-Oxohexanoyl-CoA(m) + NADH(m)
r0734	-0.01	(S)-Hydroxyhexanoyl-CoA(m) ← (2E)-Hexenoyl-CoA(m) + H2O(m)
r0007	0.01	PPi(c) + H2O(c) → 2 Pi(c)
r1114	0.08	AKG(m) + CoA(m) + NAD+(m) ⇌ NADH(m) + CO2(m) + Succinyl-CoA(m)
r1450	0.01	Ubiquinone(m) + Hexanoyl-CoA(m) → (2E)-Hexenoyl-CoA(m) + Ubiquinol(m)
r1451	0.01	Ubiquinone(m) + Decanoyl-CoA(m) → (2E)-Decenoyl-CoA(m) + Ubiquinol(m)
r0660	-0.01	(S)-3-Hydroxydodecanoyl-CoA(m) ⇌ (2E)-Dodecenoyl-CoA(m) + H2O(m)
r0083	0.08	Oxalosuccinate(m) ⇌ AKG(m) + CO2(m)
r1047	1.02	Pi(c) + H+(PG)(c) → Pi(m) + H+(PG)(m)
r0105	0.08	NAD+(m) + Malate(m) ⇌ OAA(m) + NADH(m)
r2418	0.94	ADP(c) + ATP(m) → ADP(m) + ATP(c)
r0107	-0.08	Acetyl-CoA(m) + H2O(m) + OAA(m) ⇌ Citrate(m) + CoA(m)
r0259	-0.08	Fumarate(m) + H2O(m) ⇌ Malate(m)

Continued on next page

Aerobic rephosph of UTP (7)– continued

ID	flux	reaction
r0851	1.16	H2O(m) ⇌ H2O(c)

Anaerobic rephosph of GTP (8)

Definition			Solution												
			exchanges		reactions								transp		
Objective	Constraints	Comment	imports	exports	c	m	r	p	l	n	s	b	s-c	b-c	int
GTP(c)	−Glucose −P_i(c) −GDP(c) +L-Lactate +CO_2 =H_2O =Proton-gradient(c)	Anaerobic rephosphorylation of cytosolic GTP from glucose	1 P_i(c) 0.5 Glucose(s) 1 GDP(c)	1 H_2O(s) 1 GTP(c) 1 L-Lactate(s) 1 Proton-gradient(c)	12	-	-	-	-	-	-	-	4	-	-

ID	flux	reaction
r0253	1	GAP(c) + Pi(c) + NAD+(c) ⇌ 1,3DPG(c) + NADH(c)
r0353	0.5	Glucose(c) + ATP(c) → Glucose-6P(c) + ADP(c)
r0164	1	2PG(c) ⇌ PEP(c) + H2O(c)
r0192	0.5	Glucose-6P(c) ⇌ Fructose-6P(c)
r0337	-1	ADP(c) + 1,3DPG(c) ⇌ ATP(c) + 3PG(c)
r0256	0.5	Fructose-1,6PP(c) ⇌ DHAP(c) + GAP(c)
r0736	0.5	ATP(c) + Fructose-6P(c) → ADP(c) + Fructose-1,6PP(c)
r2078	-1	H+(PG)(c) + L-Lactate(c) ⇌ H+(PG)(s) + L-Lactate(s)
r0099	1	ATP(c) + GDP(c) ⇌ ADP(c) + GTP(c)
r1032	0.5	Glucose(s) ⇌ Glucose(c)
r1331	1	H+(PG)(s) ⇌ H+(PG)(c) + Proton-gradient(c)
r0849	-1	H2O(s) ⇌ H2O(c)
r0341	-1	3PG(c) ⇌ 2PG(c)
r0243	-0.5	DHAP(c) ⇌ GAP(c)
r0171	-1	Pyruvate(c) + NADH(c) ⇌ L-Lactate(c) + NAD+(c)
r0054	-1	ADP(c) + PEP(c) → ATP(c) + Pyruvate(c)

Anaerobic rephosph of CTP (9)

Definition			Solution												
			exchanges		reactions								transp		
Objective	Constraints	Comment	imports	exports	c	m	r	p	l	n	s	b	s-c	b-c	int
CTP(c)	−Glucose −P_i(c) −CDP(c) +L-Lactate +CO_2 =H_2O =Proton-gradient(c)	Anaerobic rephosphorylation of cytosolic CTP from glucose	1 P_i(c) 0.5 Glucose(s) 1 CDP(c)	1 H_2O(s) 1 CTP(c) 1 L-Lactate(s) 1 Proton-gradient(c)	12	-	-	-	-	-	-	-	4	-	-

ID	flux	reaction
r0253	1	GAP(c) + Pi(c) + NAD+(c) ⇌ 1,3DPG(c) + NADH(c)
r0353	0.5	Glucose(c) + ATP(c) → Glucose-6P(c) + ADP(c)
r0164	1	2PG(c) ⇌ PEP(c) + H2O(c)
r0192	0.5	Glucose-6P(c) ⇌ Fructose-6P(c)
r0337	-1	ADP(c) + 1,3DPG(c) ⇌ ATP(c) + 3PG(c)
r0256	0.5	Fructose-1,6PP(c) ⇌ DHAP(c) + GAP(c)
r0736	0.5	ATP(c) + Fructose-6P(c) → ADP(c) + Fructose-1,6PP(c)
r2078	-1	H+(PG)(c) + L-Lactate(c) ⇌ H+(PG)(s) + L-Lactate(s)

Continued on next page

ID	flux	reaction
r1032	0.5	Glucose(s) ⇌ Glucose(c)
r0150	1	ATP(c) + CDP(c) ⇌ ADP(c) + CTP(c)
r1331	1	H+(PG)(s) ⇌ H+(PG)(c) + Proton-gradient(c)
r0849	-1	H2O(s) ⇌ H2O(c)
r0341	-1	3PG(c) ⇌ 2PG(c)
r0243	-0.5	DHAP(c) ⇌ GAP(c)
r0171	-1	Pyruvate(c) + NADH(c) ⇌ L-Lactate(c) + NAD+(c)
r0054	-1	ADP(c) + PEP(c) → ATP(c) + Pyruvate(c)

Anaerobic rephosph of UTP (10)

Definition			Solution												
			exchanges		reactions								transp		
Objective	Constraints	Comment	imports	exports	c	m	r	p	l	n	s	b	s-c	b-c	int
UTP(c)	−Glucose −P_i(c) −UDP(c) +L-Lactate +CO_2 =H_2O =Proton-gradient(c)	Anaerobic rephosphorylation of cytosolic UTP from glucose	1 P_i(c) 1 UDP(c) 0.5 Glucose(s)	1 H_2O(s) 1 UTP(c) 1 L-Lactate(s) 1 Proton-gradient(c)	12	-	-	-	-	-	-	-	4	-	-

ID	flux	reaction
r0253	1	GAP(c) + Pi(c) + NAD+(c) ⇌ 1,3DPG(c) + NADH(c)
r0037	1	ATP(c) + UDP(c) ⇌ ADP(c) + UTP(c)
r0353	0.5	Glucose(c) + ATP(c) → Glucose-6P(c) + ADP(c)
r0164	1	2PG(c) ⇌ PEP(c) + H2O(c)
r0192	0.5	Glucose-6P(c) ⇌ Fructose-6P(c)
r0337	-1	ADP(c) + 1,3DPG(c) ⇌ ATP(c) + 3PG(c)
r0256	0.5	Fructose-1,6PP(c) ⇌ DHAP(c) + GAP(c)
r0736	0.5	ATP(c) + Fructose-6P(c) → ADP(c) + Fructose-1,6PP(c)
r2078	-1	H+(PG)(c) + L-Lactate(c) ⇌ H+(PG)(s) + L-Lactate(s)
r1032	0.5	Glucose(s) ⇌ Glucose(c)
r1331	1	H+(PG)(s) ⇌ H+(PG)(c) + Proton-gradient(c)
r0849	-1	H2O(s) ⇌ H2O(c)
r0341	-1	3PG(c) ⇌ 2PG(c)
r0243	-0.5	DHAP(c) ⇌ GAP(c)
r0171	-1	Pyruvate(c) + NADH(c) ⇌ L-Lactate(c) + NAD+(c)
r0054	-1	ADP(c) + PEP(c) → ATP(c) + Pyruvate(c)

ATP from NADH(m) (11)

Definition			Solution												
			exchanges		reactions								transp		
Objective	Constraints	Comment	imports	exports	c	m	r	p	l	n	s	b	s-c	b-c	int
ATP-energy(m)	=NADH-redox-potential(m) =H_2O −O_2 %r0205 %r0820 %r0083	test that NADH/NAD^+ potential can be transformed to ATP/ADP potential	0.15 O_2(s) 0.3 NADH-redox-potential(m)	0.3 H_2O(s) 1 ATP-energy(m)	-	2	-	-	-	-	-	-	2	-	6

ID	flux	reaction
r0019	-1	ADP(m) + Pi(m) + 3 H+(PG)(c) → ATP(m) + H2O(m) + 3 H+(PG)(m)

Continued on next page

ID	flux	reaction
r0507	0.3	Ubiquinol(m) + 2 Ferricytochrome C(m) + 4 H+(PG)(m) → Ubiquinone(m) + 4 H+(PG)(c) + 2 Ferrocytochrome C(m)
r0508	-0.3	Ubiquinone(m) + NADH(m) + 4 H+(PG)(m) → Ubiquinol(m) + NAD+(m) + 4 H+(PG)(c)
r0851	0.3	H2O(m) ⇌ H2O(c)
r0855	0.15	O2(s) ⇌ O2(c)
r1320	1	ATP(m) + H2O(m) ⇌ ADP(m) + Pi(m) + ATP-energy(m)
r0856	-0.15	O2(m) ⇌ O2(c)
r0849	-0.3	H2O(s) ⇌ H2O(c)
r0017	0.15	O2(m) + 4 H+(PG)(m) + 4 Ferrocytochrome C(m) → 4 Ferricytochrome C(m) + 2 H2O(m) + 4 H+(PG)(c)
r1323	-0.3	NADH(m) ⇌ NAD+(m) + NADH-redox-potential(m)

ATP from NADH (12)

Definition			Solution												
			exchanges		reactions								transp		
Objective	Constraints	Comment	imports	exports	c	m	r	p	l	n	s	b	s-c	b-c	int
ATP-energy(c)	=NADH-redox-potential(c) =H_2O $-O_2$	test that NADH/NAD^+ potential can be transformed to ATP/ADP potential	0.33 O_2(s) 0.67 NADH-redox-potential(c)	0.67 H_2O(s) 1 ATP-energy(c)	3	-	-	-	-	-	-	-	2	-	8

ID	flux	reaction
r0019	-1	ADP(m) + Pi(m) + 3 H+(PG)(c) → ATP(m) + H2O(m) + 3 H+(PG)(m)
r1047	1	Pi(c) + H+(PG)(c) → Pi(m) + H+(PG)(m)
r0507	0.67	Ubiquinol(m) + 2 Ferricytochrome C(m) + 4 H+(PG)(m) → Ubiquinone(m) + 4 H+(PG)(c) + 2 Ferrocytochrome C(m)
r1319	1	ATP(c) + H2O(c) ⇌ ADP(c) + Pi(c) + ATP-energy(c)
r0851	1.67	H2O(m) ⇌ H2O(c)
r2418	1	ADP(c) + ATP(m) → ADP(m) + ATP(c)
r0201	-0.67	DHAP(c) + NADH(c) ⇌ NAD+(c) + sn-Glycerol-3P(c)
r0855	0.33	O2(s) ⇌ O2(c)
r0856	-0.33	O2(m) ⇌ O2(c)
r0205	0.67	Ubiquinone(m) + sn-Glycerol-3P(c) ⇌ DHAP(c) + Ubiquinol(m)
r1322	-0.67	NADH(c) ⇌ NAD+(c) + NADH-redox-potential(c)
r0849	-0.67	H2O(s) ⇌ H2O(c)
r0017	0.33	O2(m) + 4 H+(PG)(m) + 4 Ferrocytochrome C(m) → 4 Ferricytochrome C(m) + 2 H2O(m) + 4 H+(PG)(c)

NADH potential transport (13)

Definition			Solution												
			exchanges		reactions								transp		
Objective	Constraints	Comment	imports	exports	c	m	r	p	l	n	s	b	s-c	b-c	int
NADH-redox-potential(m)	=NADH-redox-potential(c) =H_2O $-O_2$	NADH/NAD^+ potential transport from the cytosol into the mitochondrium	0.08 O_2(s) 1.17 NADH-redox-potential(c)	0.17 H_2O(s) 1 NADH-redox-potential(m)	4	3	-	-	-	-	-	-	2	-	7

ID	flux	reaction
r0820	-1	AKG(m) + Malate(c) ⇌ AKG(c) + Malate(m)
r0109	1	AKG(c) + Aspartate(c) ⇌ OAA(c) + Glutamate(c)
r0507	0.17	Ubiquinol(m) + 2 Ferricytochrome C(m) + 4 H+(PG)(m) → Ubiquinone(m) + 4 H+(PG)(c) + 2 Ferrocytochrome C(m)
r0851	0.17	H2O(m) ⇌ H2O(c)
r0110	-1	OAA(m) + Glutamate(m) → AKG(m) + Aspartate(m)

Continued on next page

ID	flux	reaction
r0201	-0.17	DHAP(c) + NADH(c) ⇌ NAD+(c) + sn-Glycerol-3P(c)
r0855	0.08	O2(s) ⇌ O2(c)
r0104	-1	OAA(c) + NADH(c) ⇌ NAD+(c) + Malate(c)
r0856	-0.08	O2(m) ⇌ O2(c)
r0105	1	NAD+(m) + Malate(m) ⇌ OAA(m) + NADH(m)
r0205	0.17	Ubiquinone(m) + sn-Glycerol-3P(c) ⇌ DHAP(c) + Ubiquinol(m)
r1322	-1.17	NADH(c) ⇌ NAD+(c) + NADH-redox-potential(c)
r0876	1	Glutamate(c) + H+(PG)(c) + Aspartate(m) → Glutamate(m) + H+(PG)(m) + Aspartate(c)
r0849	-0.17	H2O(s) ⇌ H2O(c)
r0017	0.08	O2(m) + 4 H+(PG)(m) + 4 Ferrocytochrome C(m) → 4 Ferricytochrome C(m) + 2 H2O(m) + 4 H+(PG)(c)
r1323	1	NADH(m) ⇌ NAD+(m) + NADH-redox-potential(m)

Ubiquinol-to-ATP (14)

Definition			Solution												
			exchanges		reactions								transp		
Objective	Constraints	Comment	imports	exports	c	m	r	p	l	n	s	b	s-c	b-c	int
ATP(c)	−ADP(c) −P_i(c) +Ubiquinone(m) −Ubiquinol(m) =H_2O(m) −O_2(m)	test that mitochondrial ATP/ADP potential without any exchange of substrates	0.33 O_2(m) 1 ADP(c) 1 P_i(c) 0.67 Ubiquinol(m)	1.67 H_2O(m) 1 ATP(c) 0.67 Ubiquinone(m)	-	-	-	-	-	-	-	-	-	-	5

ID	flux	reaction
r0019	-1	ADP(m) + Pi(m) + 3 H+(PG)(c) → ATP(m) + H2O(m) + 3 H+(PG)(m)
r1047	1	Pi(c) + H+(PG)(c) → Pi(m) + H+(PG)(m)
r0507	0.67	Ubiquinol(m) + 2 Ferricytochrome C(m) + 4 H+(PG)(m) → Ubiquinone(m) + 4 H+(PG)(c) + 2 Ferrocytochrome C(m)
r2418	1	ADP(c) + ATP(m) → ADP(m) + ATP(c)
r0017	0.33	O2(m) + 4 H+(PG)(m) + 4 Ferrocytochrome C(m) → 4 Ferricytochrome C(m) + 2 H2O(m) + 4 H+(PG)(c)

Dephosphorylation of ATP (15)

Definition			Solution												
			exchanges		reactions								transp		
Objective	Constraints	Comment	imports	exports	c	m	r	p	l	n	s	b	s-c	b-c	int
-1 ATP-energy(c)	−Palmitate +CO_2 −O_2 =H_2O	Dephosphorylation of ATP, release of energy	1 ATP-energy(c)		3	-	-	-	-	-	-	-	-	-	-

ID	flux	reaction
r1382	0.17	6 Glutamate(c) + THF(c) + 6 ATP(c) + 6 H2O(c) → 6 Pi(c) + 6 ADP(c) + THF-hexaglutamate(c)
r1319	-1	ATP(c) + H2O(c) ⇌ ADP(c) + Pi(c) + ATP-energy(c)
r1383	0.17	THF-hexaglutamate(c) → 6 Glutamate(c) + THF(c)

A1.2. NAD(P)H, FADH

Aerobic reduction of NAD+ (FA) (16)

Definition			Solution												
			exchanges		reactions								transp		
Objective	Constraints	Comment	imports	exports	c	m	r	p	l	n	s	b	s-c	b-c	int
NADH-redox-potential(c)	−Palmitate $+CO_2$ $-O_2$ $=H_2O$	Aerobic reduction of NAD^+	0.48 H_2O(s) 0.24 O_2(s) 0.03 Palmitate(s)	0.52 CO_2(s) 1 NADH-redox-potential(c)	16	38	-	-	-	-	-	-	4	-	14

ID	flux	reaction
r2435	0.03	L-Carnitine(m) + L-Palmitoylcarnitine(c) → L-Carnitine(c) + L-Palmitoylcarnitine(m)
r0125	-0.26	GDP(m) + Pi(m) + Succinyl-CoA(m) ⇌ GTP(m) + Succinate(m) + CoA(m)
r0855	0.24	O2(s) ⇌ O2(c)
r0856	-0.24	O2(m) ⇌ O2(c)
r0422	0.26	Isocitrate(c) + NADP+(c) ⇌ Oxalosuccinate(c) + NADPH(c)
r0872	0.74	Pyruvate(c) + H+(PG)(c) → Pyruvate(m) + H+(PG)(m)
r0720	-0.03	(S)-3-Hydroxytetradecanoyl-CoA(m) ← (2E)-Tetradecenoyl-CoA(m) + H2O(m)
r0425	-0.01	Isocitrate(m) + NAD+(m) ⇌ Oxalosuccinate(m) + NADH(m)
r1382	0.11	6 Glutamate(c) + THF(c) + 6 ATP(c) + 6 H2O(c) → 6 Pi(c) + 6 ADP(c) + THF-hexaglutamate(c)
r1383	0.11	THF-hexaglutamate(c) → 6 Glutamate(c) + THF(c)
r0722	0.03	(S)-3-Hydroxydodecanoyl-CoA(m) + NAD+(m) ⇌ 3-Oxododecanoyl-CoA(m) + NADH(m)
r1385	-0.74	NADPH(m) + NAD+(m) + H+(PG)(m) ← NADH(m) + NADP+(m) + H+(PG)(c)
r0724	-0.03	Decanoyl-CoA(m) + Acetyl-CoA(m) ⇌ CoA(m) + 3-Oxododecanoyl-CoA(m)
r0726	0.03	(S)-Hydroxydecanoyl-CoA(m) + NAD+(m) ⇌ 3-Oxodecanoyl-CoA(m) + NADH(m)
r0728	-0.03	(S)-Hydroxydecanoyl-CoA(m) ⇌ (2E)-Decenoyl-CoA(m) + H2O(m)
r0460	0.03	(S)-3-Hydroxybutyryl-CoA(m) + NAD+(m) ⇌ NADH(m) + Acetoacetyl-CoA(m)
r0310	0.03	Palmitoyl-CoA(m) + Ubiquinone(m) → (2E)-Hexadecenoyl-CoA(m) + Ubiquinol(m)
r0017	0.24	O2(m) + 4 H+(PG)(m) + 4 Ferrocytochrome C(m) → 4 Ferricytochrome C(m) + 2 H2O(m) + 4 H+(PG)(c)
r0312	0.03	Palmitate(c) + ATP(c) + CoA(c) → Palmitoyl-CoA(c) + AMP(c) + PPi(c)
r0030	0.03	ATP(c) + AMP(c) ⇌ 2 ADP(c)
r0019	-0.48	ADP(m) + Pi(m) + 3 H+(PG)(c) → ATP(m) + H2O(m) + 3 H+(PG)(m)
r0315	0.26	Citrate(c) ⇌ Isocitrate(c)
r0054	-0.26	ADP(c) + PEP(c) → ATP(c) + Pyruvate(c)
r0070	-0.03	CoA(m) + Acetoacetyl-CoA(m) ⇌ 2 Acetyl-CoA(m)
r0634	-0.03	Acetyl-CoA(m) + Octanoyl-CoA(m) ⇌ CoA(m) + 3-Oxodecanoyl-CoA(m)
r0058	0.48	Malate(c) + NADP+(c) ⇌ Pyruvate(c) + CO2(c) + NADPH(c)
r0059	-0.74	Pyruvate(m) + CO2(m) + NADPH(m) ⇌ Malate(m) + NADP+(m)
r1147	0.01	AKG(c) + Isocitrate(m) ⇌ AKG(m) + Isocitrate(c)
r0639	-0.03	Lauroyl-CoA(m) + Acetyl-CoA(m) ← CoA(m) + 3-Oxotetradecanoyl-CoA(m)
r0935	0.03	Palmitate(s) → Palmitate(c)
r0653	-0.03	Myristoyl-CoA(m) + Acetyl-CoA(m) ← CoA(m) + 3-Oxopalmitoyl-CoA(m)
r1446	0.03	Ubiquinone(m) + Butyryl-CoA(m) → Crotonyl-CoA(m) + Ubiquinol(m)
r1447	0.03	Ubiquinone(m) + Lauroyl-CoA(m) → (2E)-Dodecenoyl-CoA(m) + Ubiquinol(m)
r1448	0.03	Ubiquinone(m) + Octanoyl-CoA(m) → (2E)-Octenoyl-CoA(m) + Ubiquinol(m)
r1449	0.03	Ubiquinone(m) + Myristoyl-CoA(m) → (2E)-Tetradecenoyl-CoA(m) + Ubiquinol(m)
r0801	-0.26	GTP(c) + GDP(m) ⇌ GTP(m) + GDP(c)
r0507	0.48	Ubiquinol(m) + 2 Ferricytochrome C(m) + 4 H+(PG)(m) → Ubiquinone(m) + 4 H+(PG)(c) + 2 Ferrocytochrome C(m)
r0509	0.26	Ubiquinone(m) + Succinate(m) → Ubiquinol(m) + Fumarate(m)
r0397	-0.74	Dihydrobiopterin(c) + NADH(c) ⇌ Tetrahydrobiopterin(c) + NAD+(c)
r0398	0.74	Dihydrobiopterin(c) + NADPH(c) ⇌ Tetrahydrobiopterin(c) + NADP+(c)
r0849	0.48	H2O(s) ⇌ H2O(c)
r0287	-0.03	CoA(m) + 3-Oxohexanoyl-CoA(m) → Acetyl-CoA(m) + Butyryl-CoA(m)
r0714	0.03	NAD+(m) + (S)-3-Hydroxyhexadecanoyl-CoA(m) → 3-Oxopalmitoyl-CoA(m) + NADH(m)
r0867	0.52	CO2(c) ⇌ CO2(s)
r0868	0.48	CO2(c) ⇌ CO2(m)
r0716	-0.03	(S)-3-Hydroxyhexadecanoyl-CoA(m) ← (2E)-Hexadecenoyl-CoA(m) + H2O(m)
r0730	0.03	(S)-Hydroxyoctanoyl-CoA(m) + NAD+(m) → 3-Oxooctanoyl-CoA(m) + NADH(m)

Continued on next page

Aerobic reduction of NAD+ (FA) (16)– continued

ID	flux	reaction
r0435	0.03	Palmitoyl-CoA(c) + L-Carnitine(c) ⇌ CoA(c) + L-Palmitoylcarnitine(c)
r0731	-0.03	(S)-Hydroxyoctanoyl-CoA(m) ← (2E)-Octenoyl-CoA(m) + H2O(m)
r0588	-0.03	Crotonyl-CoA(m) + H2O(m) ⇌ (S)-3-Hydroxybutyryl-CoA(m)
r0718	0.03	(S)-3-Hydroxytetradecanoyl-CoA(m) + NAD+(m) → 3-Oxotetradecanoyl-CoA(m) + NADH(m)
r0436	-0.03	Palmitoyl-CoA(m) + L-Carnitine(m) ← CoA(m) + L-Palmitoylcarnitine(m)
r0732	-0.03	Hexanoyl-CoA(m) + Acetyl-CoA(m) ← CoA(m) + 3-Oxooctanoyl-CoA(m)
r0733	0.03	(S)-Hydroxyhexanoyl-CoA(m) + NAD+(m) → 3-Oxohexanoyl-CoA(m) + NADH(m)
r0734	-0.03	(S)-Hydroxyhexanoyl-CoA(m) ← (2E)-Hexenoyl-CoA(m) + H2O(m)
r0007	0.03	PPi(c) + H2O(c) → 2 Pi(c)
r1114	0.26	AKG(m) + CoA(m) + NAD+(m) ⇌ NADH(m) + CO2(m) + Succinyl-CoA(m)
r2373	0.26	Citrate(m) + AKG(c) ⇌ Citrate(c) + AKG(m)
r1450	0.03	Ubiquinone(m) + Hexanoyl-CoA(m) → (2E)-Hexenoyl-CoA(m) + Ubiquinol(m)
r0082	0.26	Oxalosuccinate(c) ⇌ AKG(c) + CO2(c)
r1451	0.03	Ubiquinone(m) + Decanoyl-CoA(m) → (2E)-Decenoyl-CoA(m) + Ubiquinol(m)
r0660	-0.03	(S)-3-Hydroxydodecanoyl-CoA(m) ⇌ (2E)-Dodecenoyl-CoA(m) + H2O(m)
r0083	-0.01	Oxalosuccinate(m) ⇌ AKG(m) + CO2(m)
r1322	1	NADH(c) ⇌ NAD+(c) + NADH-redox-potential(c)
r0831	-0.74	Malate(m) + Pi(c) ⇌ Malate(c) + Pi(m)
r0104	0.26	NAD+(c) + Malate(c) ⇌ OAA(c) + NADH(c)
r0105	0.26	NAD+(m) + Malate(m) ⇌ OAA(m) + NADH(m)
r2418	0.48	ADP(c) + ATP(m) → ADP(m) + ATP(c)
r0107	-0.26	Acetyl-CoA(m) + H2O(m) + OAA(m) ⇌ Citrate(m) + CoA(m)
r0259	-0.26	Fumarate(m) + H2O(m) ⇌ Malate(m)
r0851	0.22	H2O(m) ⇌ H2O(c)
r0123	0.26	GTP(c) + OAA(c) → GDP(c) + PEP(c) + CO2(c)

Aerobic reduction of NAD+ (gluc) (17)

Definition			Solution												
			exchanges		reactions								transp		
Objective	Constraints	Comment	imports	exports	c	m	r	p	l	n	s	b	s-c	b-c	int
NADH-redox-potential(c)	$-$Glucose $+CO_2$ $-O_2$ $=H_2O$	Aerobic reduction of NAD^+	0.49 H_2O(s) 0.01 O_2(s) 0.09 Glucose(s)	0.51 CO_2(s) 1 NADH-redox-potential(c)	27	8	-	-	-	-	-	-	4	-	13

ID	flux	reaction
r0406	0.15	Sedoheptulose-7P(c) + GAP(c) ⇌ Fructose-6P(c) + Erythrose-4P(c)
r0125	-0.02	GDP(m) + Pi(m) + Succinyl-CoA(m) ⇌ GTP(m) + Succinate(m) + CoA(m)
r0855	0.01	O2(s) ⇌ O2(c)
r0856	-0.01	O2(m) ⇌ O2(c)
r0422	0.02	Isocitrate(c) + NADP+(c) ⇌ Oxalosuccinate(c) + NADPH(c)
r0872	0.07	Pyruvate(c) + H+(PG)(c) → Pyruvate(m) + H+(PG)(m)
r0427	-0.96	L-Xylulose(c) + NADPH(c) ⇌ Xylitol(c) + NADP+(c)
r1385	-0.05	NADPH(m) + NAD+(m) + H+(PG)(m) ← NADH(m) + NADP+(m) + H+(PG)(c)
r0164	0.02	2PG(c) ⇌ PEP(c) + H2O(c)
r0017	0.01	O2(m) + 4 H+(PG)(m) + 4 Ferrocytochrome C(m) → 4 Ferricytochrome C(m) + 2 H2O(m) + 4 H+(PG)(c)
r0019	-0.03	ADP(m) + Pi(m) + 3 H+(PG)(c) → ATP(m) + H2O(m) + 3 H+(PG)(m)
r0315	0.02	Citrate(c) ⇌ Isocitrate(c)
r0487	0.07	Fructose-1,6PP(c) + H2O(c) → Fructose-6P(c) + Pi(c)
r0054	-0.04	ADP(c) + PEP(c) → ATP(c) + Pyruvate(c)
r0784	0.96	Xylitol(c) + NAD+(c) ⇌ L-Xylulose(c) + NADH(c)
r0337	-0.02	ADP(c) + 1,3DPG(c) ⇌ ATP(c) + 3PG(c)
r0056	0.02	Pyruvate(m) + CoA(m) + NAD+(m) → Acetyl-CoA(m) + CO2(m) + NADH(m)
r0058	0.03	Malate(c) + NADP+(c) ⇌ Pyruvate(c) + CO2(c) + NADPH(c)
r0353	0.09	Glucose(c) + ATP(c) → Glucose-6P(c) + ADP(c)

Continued on next page

ID	flux	reaction
r0059	-0.05	Pyruvate(m) + CO2(m) + NADPH(m) ⇌ Malate(m) + NADP+(m)
r0370	-0.15	Sedoheptulose-7P(c) + GAP(c) ⇌ Ribose-5P(c) + D-Xylulose-5P(c)
r0801	-0.02	GTP(c) + GDP(m) ⇌ GTP(m) + GDP(c)
r0507	0.03	Ubiquinol(m) + 2 Ferricytochrome C(m) + 4 H+(PG)(m) → Ubiquinone(m) + 4 H+(PG)(c) + 2 Ferrocytochrome C(m)
r0508	-0.01	Ubiquinone(m) + NADH(m) + 4 H+(PG)(m) → Ubiquinol(m) + NAD+(m) + 4 H+(PG)(c)
r0509	0.02	Ubiquinone(m) + Succinate(m) → Ubiquinol(m) + Fumarate(m)
r1032	0.09	Glucose(s) ⇌ Glucose(c)
r0243	0.07	GAP(c) ⇌ DHAP(c)
r0849	0.49	H2O(s) ⇌ H2O(c)
r0867	0.51	CO2(c) ⇌ CO2(s)
r0868	0.01	CO2(c) ⇌ CO2(m)
r0192	-0.37	Fructose-6P(c) ⇌ Glucose-6P(c)
r1114	0.02	AKG(m) + CoA(m) + NAD+(m) ⇌ NADH(m) + CO2(m) + Succinyl-CoA(m)
r0479	0.46	Glucono-1,5-lactone-6P(c) + H2O(c) ⇌ 6-Phospho-D-gluconate(c)
r0341	-0.02	3PG(c) ⇌ 2PG(c)
r0199	0.46	Glucose-6P(c) + NADP+(c) ⇌ NADPH(c) + Glucono-1,5-lactone-6P(c)
r0343	0.46	6-Phospho-D-gluconate(c) + NADP+(c) ⇌ NADPH(c) + CO2(c) + Ribulose-5P(c)
r2373	0.02	Citrate(m) + AKG(c) ⇌ Citrate(c) + AKG(m)
r0344	0.3	Ribulose-5P(c) ⇌ D-Xylulose-5P(c)
r0082	0.02	Oxalosuccinate(c) ⇌ AKG(c) + CO2(c)
r1322	1	NADH(c) ⇌ NAD+(c) + NADH-redox-potential(c)
r0250	-0.15	Ribulose-5P(c) ⇌ Ribose-5P(c)
r0253	0.02	GAP(c) + Pi(c) + NAD+(c) ⇌ 1,3DPG(c) + NADH(c)
r0831	-0.05	Malate(m) + Pi(c) ⇌ Malate(c) + Pi(m)
r0255	-0.15	Fructose-6P(c) + GAP(c) ⇌ D-Xylulose-5P(c) + Erythrose-4P(c)
r0104	0.02	NAD+(c) + Malate(c) ⇌ OAA(c) + NADH(c)
r0256	-0.07	DHAP(c) + GAP(c) ⇌ Fructose-1,6PP(c)
r0105	0.02	NAD+(m) + Malate(m) ⇌ OAA(m) + NADH(m)
r2418	0.03	ADP(c) + ATP(m) → ADP(m) + ATP(c)
r0107	-0.02	Acetyl-CoA(m) + H2O(m) + OAA(m) ⇌ Citrate(m) + CoA(m)
r0259	-0.02	Fumarate(m) + H2O(m) ⇌ Malate(m)
r0851	0.02	H2O(m) ⇌ H2O(c)
r0123	0.02	GTP(c) + OAA(c) → GDP(c) + PEP(c) + CO2(c)

Aerobic reduction of NADP+ (FA) (18)

Definition			Solution												
			exchanges		reactions								transp		
Objective	Constraints	Comment	imports	exports	c	m	r	p	l	n	s	b	s-c	b-c	int
NADPH-redox-potential(c)	$-$Palmitate $+CO_2$ $-O_2$ $=H_2O$	Aerobic reduction of $NADP^+$	0.48 H_2O(s) 0.24 O_2(s) 0.03 Palmitate(s)	0.52 CO_2(s) 1 NADPH-redox-potential(c)	12	38	-	-	-	-	-	-	4	-	14

ID	flux	reaction
r2435	0.03	L-Carnitine(m) + L-Palmitoylcarnitine(c) → L-Carnitine(c) + L-Palmitoylcarnitine(m)
r0125	-0.26	GDP(m) + Pi(m) + Succinyl-CoA(m) ⇌ GTP(m) + Succinate(m) + CoA(m)
r0855	0.24	O2(s) ⇌ O2(c)
r0856	-0.24	O2(m) ⇌ O2(c)
r0422	0.26	Isocitrate(c) + NADP+(c) ⇌ Oxalosuccinate(c) + NADPH(c)
r0872	0.74	Pyruvate(c) + H+(PG)(c) → Pyruvate(m) + H+(PG)(m)
r0720	-0.03	(S)-3-Hydroxytetradecanoyl-CoA(m) ← (2E)-Tetradecenoyl-CoA(m) + H2O(m)
r0425	-0.01	Isocitrate(m) + NAD+(m) ⇌ Oxalosuccinate(m) + NADH(m)
r1382	0.11	6 Glutamate(c) + THF(c) + 6 ATP(c) + 6 H2O(c) → 6 Pi(c) + 6 ADP(c) + THF-hexaglutamate(c)
r1383	0.11	THF-hexaglutamate(c) → 6 Glutamate(c) + THF(c)
r0722	0.03	(S)-3-Hydroxydodecanoyl-CoA(m) + NAD+(m) ⇌ 3-Oxododecanoyl-CoA(m) + NADH(m)

Continued on next page

Aerobic reduction of NADP+ (FA) (18)– continued

ID	flux	reaction
r1385	-0.74	NADPH(m) + NAD+(m) + H+(PG)(m) ← NADH(m) + NADP+(m) + H+(PG)(c)
r0724	-0.03	Decanoyl-CoA(m) + Acetyl-CoA(m) ⇌ CoA(m) + 3-Oxododecanoyl-CoA(m)
r0726	0.03	(S)-Hydroxydecanoyl-CoA(m) + NAD+(m) ⇌ 3-Oxodecanoyl-CoA(m) + NADH(m)
r0728	-0.03	(S)-Hydroxydecanoyl-CoA(m) ⇌ (2E)-Decenoyl-CoA(m) + H2O(m)
r0460	0.03	(S)-3-Hydroxybutyryl-CoA(m) + NAD+(m) ⇌ NADH(m) + Acetoacetyl-CoA(m)
r0310	0.03	Palmitoyl-CoA(m) + Ubiquinone(m) → (2E)-Hexadecenoyl-CoA(m) + Ubiquinol(m)
r0017	0.24	O2(m) + 4 H+(PG)(m) + 4 Ferrocytochrome C(m) → 4 Ferricytochrome C(m) + 2 H2O(m) + 4 H+(PG)(c)
r0312	0.03	Palmitate(c) + ATP(c) + CoA(c) → Palmitoyl-CoA(c) + AMP(c) + PPi(c)
r0030	0.03	ATP(c) + AMP(c) ⇌ 2 ADP(c)
r0019	-0.48	ADP(m) + Pi(m) + 3 H+(PG)(c) → ATP(m) + H2O(m) + 3 H+(PG)(m)
r0315	0.26	Citrate(c) ⇌ Isocitrate(c)
r0070	-0.03	CoA(m) + Acetoacetyl-CoA(m) ⇌ 2 Acetyl-CoA(m)
r0634	-0.03	Acetyl-CoA(m) + Octanoyl-CoA(m) ⇌ CoA(m) + 3-Oxodecanoyl-CoA(m)
r0058	0.74	Malate(c) + NADP+(c) ⇌ Pyruvate(c) + CO2(c) + NADPH(c)
r0059	-0.74	Pyruvate(m) + CO2(m) + NADPH(m) ⇌ Malate(m) + NADP+(m)
r1147	0.01	AKG(c) + Isocitrate(m) ⇌ AKG(m) + Isocitrate(c)
r0639	-0.03	Lauroyl-CoA(m) + Acetyl-CoA(m) ← CoA(m) + 3-Oxotetradecanoyl-CoA(m)
r0935	0.03	Palmitate(s) → Palmitate(c)
r0653	-0.03	Myristoyl-CoA(m) + Acetyl-CoA(m) ← CoA(m) + 3-Oxopalmitoyl-CoA(m)
r1446	0.03	Ubiquinone(m) + Butyryl-CoA(m) → Crotonyl-CoA(m) + Ubiquinol(m)
r1447	0.03	Ubiquinone(m) + Lauroyl-CoA(m) → (2E)-Dodecenoyl-CoA(m) + Ubiquinol(m)
r1448	0.03	Ubiquinone(m) + Octanoyl-CoA(m) → (2E)-Octenoyl-CoA(m) + Ubiquinol(m)
r1449	0.03	Ubiquinone(m) + Myristoyl-CoA(m) → (2E)-Tetradecenoyl-CoA(m) + Ubiquinol(m)
r0801	-0.26	GTP(c) + GDP(m) ⇌ GTP(m) + GDP(c)
r0507	0.48	Ubiquinol(m) + 2 Ferricytochrome C(m) + 4 H+(PG)(m) → Ubiquinone(m) + 4 H+(PG)(c) + 2 Ferrocytochrome C(m)
r0509	0.26	Ubiquinone(m) + Succinate(m) → Ubiquinol(m) + Fumarate(m)
r0099	-0.26	ADP(c) + GTP(c) ⇌ ATP(c) + GDP(c)
r0849	0.48	H2O(s) ⇌ H2O(c)
r0287	-0.03	CoA(m) + 3-Oxohexanoyl-CoA(m) → Acetyl-CoA(m) + Butyryl-CoA(m)
r0714	0.03	NAD+(m) + (S)-3-Hydroxyhexadecanoyl-CoA(m) → 3-Oxopalmitoyl-CoA(m) + NADH(m)
r0867	0.52	CO2(c) ⇌ CO2(s)
r0868	0.48	CO2(c) ⇌ CO2(m)
r0716	-0.03	(S)-3-Hydroxyhexadecanoyl-CoA(m) ← (2E)-Hexadecenoyl-CoA(m) + H2O(m)
r0730	0.03	(S)-Hydroxyoctanoyl-CoA(m) + NAD+(m) → 3-Oxooctanoyl-CoA(m) + NADH(m)
r0435	0.03	Palmitoyl-CoA(c) + L-Carnitine(c) ⇌ CoA(c) + L-Palmitoylcarnitine(c)
r0731	-0.03	(S)-Hydroxyoctanoyl-CoA(m) ← (2E)-Octenoyl-CoA(m) + H2O(m)
r0588	-0.03	Crotonyl-CoA(m) + H2O(m) ⇌ (S)-3-Hydroxybutyryl-CoA(m)
r0718	0.03	(S)-3-Hydroxytetradecanoyl-CoA(m) + NAD+(m) → 3-Oxotetradecanoyl-CoA(m) + NADH(m)
r0436	-0.03	Palmitoyl-CoA(m) + L-Carnitine(m) ← CoA(m) + L-Palmitoylcarnitine(m)
r0732	-0.03	Hexanoyl-CoA(m) + Acetyl-CoA(m) ← CoA(m) + 3-Oxooctanoyl-CoA(m)
r0733	0.03	(S)-Hydroxyhexanoyl-CoA(m) + NAD+(m) → 3-Oxohexanoyl-CoA(m) + NADH(m)
r0734	-0.03	(S)-Hydroxyhexanoyl-CoA(m) ← (2E)-Hexenoyl-CoA(m) + H2O(m)
r0007	0.03	PPi(c) + H2O(c) → 2 Pi(c)
r1114	0.26	AKG(m) + CoA(m) + NAD+(m) ⇌ NADH(m) + CO2(m) + Succinyl-CoA(m)
r2373	0.26	Citrate(m) + AKG(c) ⇌ Citrate(c) + AKG(m)
r1450	0.03	Ubiquinone(m) + Hexanoyl-CoA(m) → (2E)-Hexenoyl-CoA(m) + Ubiquinol(m)
r0082	0.26	Oxalosuccinate(c) ⇌ AKG(c) + CO2(c)
r1451	0.03	Ubiquinone(m) + Decanoyl-CoA(m) → (2E)-Decenoyl-CoA(m) + Ubiquinol(m)
r0660	-0.03	(S)-3-Hydroxydodecanoyl-CoA(m) ⇌ (2E)-Dodecenoyl-CoA(m) + H2O(m)
r0083	-0.01	Oxalosuccinate(m) ⇌ AKG(m) + CO2(m)
r1326	1	NADPH(c) ⇌ NADPH-redox-potential(c) + NADP+(c)
r0831	-0.74	Malate(m) + Pi(c) ⇌ Malate(c) + Pi(m)
r0105	0.26	NAD+(m) + Malate(m) ⇌ OAA(m) + NADH(m)
r2418	0.48	ADP(c) + ATP(m) → ADP(m) + ATP(c)
r0107	-0.26	Acetyl-CoA(m) + H2O(m) + OAA(m) ⇌ Citrate(m) + CoA(m)
r0259	-0.26	Fumarate(m) + H2O(m) ⇌ Malate(m)

Continued on next page

ID	flux	reaction
r0851	0.22	H2O(m) ⇌ H2O(c)

Aerobic reduction of NADP+ (gluc) (19)

Definition			Solution												
			exchanges		reactions								transp		
Objective	Constraints	Comment	imports	exports	c	m	r	p	l	n	s	b	s-c	b-c	int
NADPH-redox-potential(c)	−Glucose +CO_2 −O_2 =H_2O	Aerobic reduction of $NADP^+$	0.49 H_2O(s) 0.01 O_2(s) 0.09 Glucose(s)	0.51 CO_2(s) 1 NADPH-redox-potential(c)	24	10	-	-	-	-	-	-	4	-	12

ID	flux	reaction
r0406	0.16	Sedoheptulose-7P(c) + GAP(c) ⇌ Fructose-6P(c) + Erythrose-4P(c)
r0125	-0.01	GDP(m) + Pi(m) + Succinyl-CoA(m) ⇌ GTP(m) + Succinate(m) + CoA(m)
r0855	0.01	O2(s) ⇌ O2(c)
r0856	-0.01	O2(m) ⇌ O2(c)
r0422	0.01	Isocitrate(c) + NADP+(c) ⇌ Oxalosuccinate(c) + NADPH(c)
r0872	0.04	Pyruvate(c) + H+(PG)(c) → Pyruvate(m) + H+(PG)(m)
r1385	-0.03	NADPH(m) + NAD+(m) + H+(PG)(m) ← NADH(m) + NADP+(m) + H+(PG)(c)
r0164	0.01	2PG(c) ⇌ PEP(c) + H2O(c)
r0017	0.01	O2(m) + 4 H+(PG)(m) + 4 Ferrocytochrome C(m) → 4 Ferricytochrome C(m) + 2 H2O(m) + 4 H+(PG)(c)
r0019	-0.04	ADP(m) + Pi(m) + 3 H+(PG)(c) → ATP(m) + H2O(m) + 3 H+(PG)(m)
r0316	0.01	Citrate(m) ⇌ Isocitrate(m)
r0487	0.07	Fructose-1,6PP(c) + H2O(c) → Fructose-6P(c) + Pi(c)
r0054	-0.01	ADP(c) + PEP(c) → ATP(c) + Pyruvate(c)
r0337	-0.01	ADP(c) + 1,3DPG(c) ⇌ ATP(c) + 3PG(c)
r0056	0.01	Pyruvate(m) + CoA(m) + NAD+(m) → Acetyl-CoA(m) + CO2(m) + NADH(m)
r0058	0.03	Malate(c) + NADP+(c) ⇌ Pyruvate(c) + CO2(c) + NADPH(c)
r0353	0.09	Glucose(c) + ATP(c) → Glucose-6P(c) + ADP(c)
r0059	-0.03	Pyruvate(m) + CO2(m) + NADPH(m) ⇌ Malate(m) + NADP+(m)
r1147	0.01	AKG(c) + Isocitrate(m) ⇌ AKG(m) + Isocitrate(c)
r0370	-0.16	Sedoheptulose-7P(c) + GAP(c) ⇌ Ribose-5P(c) + D-Xylulose-5P(c)
r0507	0.03	Ubiquinol(m) + 2 Ferricytochrome C(m) + 4 H+(PG)(m) → Ubiquinone(m) + 4 H+(PG)(c) + 2 Ferrocytochrome C(m)
r0508	-0.01	Ubiquinone(m) + NADH(m) + 4 H+(PG)(m) → Ubiquinol(m) + NAD+(m) + 4 H+(PG)(c)
r0509	0.01	Ubiquinone(m) + Succinate(m) → Ubiquinol(m) + Fumarate(m)
r1032	0.09	Glucose(s) ⇌ Glucose(c)
r0243	0.07	GAP(c) ⇌ DHAP(c)
r0849	0.49	H2O(s) ⇌ H2O(c)
r0867	0.51	CO2(c) ⇌ CO2(s)
r0192	-0.39	Fructose-6P(c) ⇌ Glucose-6P(c)
r1114	0.01	AKG(m) + CoA(m) + NAD+(m) ⇌ NADH(m) + CO2(m) + Succinyl-CoA(m)
r0479	0.47	Glucono-1,5-lactone-6P(c) + H2O(c) ⇌ 6-Phospho-D-gluconate(c)
r0341	-0.01	3PG(c) ⇌ 2PG(c)
r0199	0.47	Glucose-6P(c) + NADP+(c) ⇌ NADPH(c) + Glucono-1,5-lactone-6P(c)
r0343	0.47	6-Phospho-D-gluconate(c) + NADP+(c) ⇌ NADPH(c) + CO2(c) + Ribulose-5P(c)
r0344	0.31	Ribulose-5P(c) ⇌ D-Xylulose-5P(c)
r0082	0.01	Oxalosuccinate(c) ⇌ AKG(c) + CO2(c)
r0512	-0.01	Dihydrofolate(c) + NAD+(c) ⇌ Folate(c) + NADH(c)
r0513	0.01	Dihydrofolate(c) + NADP+(c) ⇌ Folate(c) + NADPH(c)
r0250	-0.16	Ribulose-5P(c) ⇌ Ribose-5P(c)
r0100	-0.01	ADP(m) + GTP(m) ⇌ ATP(m) + GDP(m)
r1326	1	NADPH(c) ⇌ NADPH-redox-potential(c) + NADP+(c)
r0253	0.01	GAP(c) + Pi(c) + NAD+(c) ⇌ 1,3DPG(c) + NADH(c)
r0831	-0.03	Malate(m) + Pi(c) ⇌ Malate(c) + Pi(m)
r0255	-0.16	Fructose-6P(c) + GAP(c) ⇌ D-Xylulose-5P(c) + Erythrose-4P(c)

Continued on next page

ID	flux	reaction
r1047	0.03	Pi(c) + H+(PG)(c) → Pi(m) + H+(PG)(m)
r0256	-0.07	DHAP(c) + GAP(c) ⇌ Fructose-1,6PP(c)
r0105	0.01	NAD+(m) + Malate(m) ⇌ OAA(m) + NADH(m)
r2418	0.06	ADP(c) + ATP(m) → ADP(m) + ATP(c)
r0107	-0.01	Acetyl-CoA(m) + H2O(m) + OAA(m) ⇌ Citrate(m) + CoA(m)
r0259	-0.01	Fumarate(m) + H2O(m) ⇌ Malate(m)
r0851	0.04	H2O(m) ⇌ H2O(c)

Aerobic reduction of FAD (gluc) (20)

Definition			Solution												
			exchanges		reactions								transp		
Objective	Constraints	Comment	imports	exports	c	m	r	p	l	n	s	b	s-c	b-c	int
FADH-redox-potential(c)	−Glucose +CO_2 −O_2 =H_2O	Aerobic reduction of FAD	0.88 O_2(s) 0.1 Glucose(s)	0.13 H_2O(s) 1.13 CO_2(s) 1 FADH-redox-potential(c)	65	50	-	-	-	-	-	-	4	-	19

ID	flux	reaction
r0701	-0.25	HMA(c) + NADP+(c) ⇌ 3-Oxotetradecanoyl-ACP(c) + NADPH(c)
r0406	0.21	Sedoheptulose-7P(c) + GAP(c) ⇌ Fructose-6P(c) + Erythrose-4P(c)
r0702	0.25	HMA(c) ⇌ H2O(c) + (2E)-Tetradecenoyl-ACP(c)
r0125	-0.25	GDP(m) + Pi(m) + Succinyl-CoA(m) ⇌ GTP(m) + Succinate(m) + CoA(m)
r0855	0.88	O2(s) ⇌ O2(c)
r0856	-0.63	O2(m) ⇌ O2(c)
r0422	2.15	Isocitrate(c) + NADP+(c) ⇌ Oxalosuccinate(c) + NADPH(c)
r0872	0.73	Pyruvate(c) + H+(PG)(c) → Pyruvate(m) + H+(PG)(m)
r0425	-1.9	Isocitrate(m) + NAD+(m) ⇌ Oxalosuccinate(m) + NADH(m)
r0722	0.25	(S)-3-Hydroxydodecanoyl-CoA(m) + NAD+(m) ⇌ 3-Oxododecanoyl-CoA(m) + NADH(m)
r1385	-1.8	NADPH(m) + NAD+(m) + H+(PG)(m) ← NADH(m) + NADP+(m) + H+(PG)(c)
r0724	-0.25	Decanoyl-CoA(m) + Acetyl-CoA(m) ⇌ CoA(m) + 3-Oxododecanoyl-CoA(m)
r0726	0.25	(S)-Hydroxydecanoyl-CoA(m) + NAD+(m) ⇌ 3-Oxodecanoyl-CoA(m) + NADH(m)
r0728	-0.25	(S)-Hydroxydecanoyl-CoA(m) ⇌ (2E)-Decenoyl-CoA(m) + H2O(m)
r0460	0.25	(S)-3-Hydroxybutyryl-CoA(m) + NAD+(m) ⇌ NADH(m) + Acetoacetyl-CoA(m)
r0462	-0.13	Acetyl-CoA(m) + H2O(m) + Acetoacetyl-CoA(m) ⇌ HMG-CoA(m) + CoA(m)
r1254	0.25	Stearate(c) + ATP(c) + CoA(c) → Stearoyl-CoA(c) + AMP(c) + PPi(c)
r0017	0.63	O2(m) + 4 H+(PG)(m) + 4 Ferrocytochrome C(m) → 4 Ferricytochrome C(m) + 2 H2O(m) + 4 H+(PG)(c)
r0030	0.38	ATP(c) + AMP(c) ⇌ 2 ADP(c)
r0760	0.25	Butyryl-ACP(c) + Malonyl-ACP(c) ⇌ CO2(c) + ACP(c) + 3-Oxohexanoyl-ACP(c)
r0761	-0.25	D-3-Hydroxyhexanoyl-ACP(c) + NADP+(c) ⇌ NADPH(c) + 3-Oxohexanoyl-ACP(c)
r0019	-1.66	ADP(m) + Pi(m) + 3 H+(PG)(c) → ATP(m) + H2O(m) + 3 H+(PG)(m)
r0762	0.25	D-3-Hydroxyhexanoyl-ACP(c) ⇌ H2O(c) + (2E)-Hexenoyl-ACP(c)
r0315	0.25	Citrate(c) ⇌ Isocitrate(c)
r0185	2	ATP(m) + Acetyl-CoA(m) + HCO3-(m) → ADP(m) + Pi(m) + Malonyl-CoA(m)
r0763	-0.25	Hexanoyl-ACP(c) + NADP+(c) ⇌ NADPH(c) + (2E)-Hexenoyl-ACP(c)
r0764	0.25	Malonyl-ACP(c) + Hexanoyl-ACP(c) ⇌ 3-Oxooctanoyl-ACP(c) + CO2(c) + ACP(c)
r0765	-0.25	Octanoyl-ACP(c) + NADP+(c) ⇌ (2E)-Octenoyl-ACP(c) + NADPH(c)
r0766	0.25	Octanoyl-ACP(c) + Malonyl-ACP(c) ⇌ 3-Oxodecanoyl-ACP(c) + CO2(c) + ACP(c)
r0767	-0.25	Decanoyl-ACP(c) + NADP+(c) ⇌ (2E)-Decenoyl-ACP(c) + NADPH(c)
r1290	1	FAD(c) + FADH2(m) ⇌ FAD(m) + FADH2(c)
r0768	0.25	Decanoyl-ACP(c) + Malonyl-ACP(c) ⇌ 3-Oxododecanoyl-ACP(c) + CO2(c) + ACP(c)
r0769	-0.25	D-3-Hydroxydodecanoyl-ACP(c) + NADP+(c) ⇌ 3-Oxododecanoyl-ACP(c) + NADPH(c)
r0487	0.1	Fructose-1,6PP(c) + H2O(c) → Fructose-6P(c) + Pi(c)
r0054	-0.25	ADP(c) + PEP(c) → ATP(c) + Pyruvate(c)
r0634	-0.25	Acetyl-CoA(m) + Octanoyl-CoA(m) ⇌ CoA(m) + 3-Oxodecanoyl-CoA(m)
r0070	-0.13	2 Acetyl-CoA(m) ⇌ CoA(m) + Acetoacetyl-CoA(m)

Continued on next page

ID	flux	reaction
r0058	0.6	Malate(c) + NADP+(c) ⇌ Pyruvate(c) + CO2(c) + NADPH(c)
r0353	0.1	Glucose(c) + ATP(c) → Glucose-6P(c) + ADP(c)
r0059	-1.8	Pyruvate(m) + CO2(m) + NADPH(m) ⇌ Malate(m) + NADP+(m)
r1147	1.9	AKG(c) + Isocitrate(m) ⇌ AKG(m) + Isocitrate(c)
r0370	-0.21	Sedoheptulose-7P(c) + GAP(c) ⇌ Ribose-5P(c) + D-Xylulose-5P(c)
r1446	0.25	Ubiquinone(m) + Butyryl-CoA(m) → Crotonyl-CoA(m) + Ubiquinol(m)
r1448	0.25	Ubiquinone(m) + Octanoyl-CoA(m) → (2E)-Octenoyl-CoA(m) + Ubiquinol(m)
r0657	0.25	(3Z)-Dodecenoyl-CoA(m) ⇌ (2E)-Dodecenoyl-CoA(m)
r0801	-0.25	GTP(c) + GDP(m) ⇌ GTP(m) + GDP(c)
r0507	1.25	Ubiquinol(m) + 2 Ferricytochrome C(m) + 4 H+(PG)(m) → Ubiquinone(m) + 4 H+(PG)(c) + 2 Ferrocytochrome C(m)
r1464	-0.13	Pyruvate(m) + Acetoacetate(c) ⇌ Pyruvate(c) + Acetoacetate(m)
r1465	-0.25	NADH(c) + O2(c) + Stearoyl-CoA(c) ⇌ 2 H2O(c) + NAD+(c) + Oleoyl-CoA(c)
r1313	0.25	Hexadecanoyl-ACP(c) + Malonyl-ACP(c) ⇌ 3-Oxostearoyl-ACP(c) + ACP(c) + CO2(c)
r0509	0.25	Ubiquinone(m) + Succinate(m) → Ubiquinol(m) + Fumarate(m)
r1314	0.25	3-Oxostearoyl-ACP(c) + NADPH(c) ⇌ 3-Hydroxystearoyl-ACP(c) + NADP+(c)
r1032	0.1	Glucose(s) ⇌ Glucose(c)
r1480	0.25	CoA(m) + 3-oxopalmitoleoyl-CoA(m) ⇌ Acetyl-CoA(m) + cis-myrist-7-enoyl-CoA(m)
r1467	0.25	Oleoyl-CoA(c) ⇌ Oleoyl-CoA(m)
r1315	0.25	3-Hydroxystearoyl-ACP(c) ⇌ (2E)-Octadecenoyl-ACP(c) + H2O(c)
r1481	0.25	CoA(m) + 3-oxooleoyl-CoA(m) ⇌ Acetyl-CoA(m) + palmitoleoyl-CoA(m)
r1316	0.25	(2E)-Octadecenoyl-ACP(c) + NADPH(c) ⇌ Stearoyl-ACP(c) + NADP+(c)
r0243	0.1	GAP(c) ⇌ DHAP(c)
r1482	-0.25	FADH2(m) + trans,cis-dodeca-2,5-dienoyl-CoA(m) ⇌ FAD(m) + cis-laur-5-enoyl-CoA(m)
r1317	0.25	H2O(c) + Stearoyl-ACP(c) → ACP(c) + Stearate(c)
r0691	-0.25	(R)-3-Hydroxybutanoyl-ACP(c) + NADP+(c) ⇌ NADPH(c) + Acetoacetyl-ACP(c)
r0678	0.25	Acetyl-ACP(c) + Malonyl-ACP(c) ⇌ CO2(c) + ACP(c) + Acetoacetyl-ACP(c)
r1483	0.25	H2O(m) + trans,cis-myristo-2,7-dienoyl-CoA(m) ⇌ cis-(3S)-hydroxytetradec-7-enoyl-CoA(m)
r0692	-0.25	(R)-3-Hydroxydecanoyl-ACP(c) + NADP+(c) ⇌ 3-Oxodecanoyl-ACP(c) + NADPH(c)
r1484	0.25	CoA(m) + 3-oxomyrist-7-enoyl-CoA(m) ⇌ Acetyl-CoA(m) + cis-laur-5-enoyl-CoA(m)
r0693	0.25	(R)-3-Hydroxydecanoyl-ACP(c) ⇌ (2E)-Decenoyl-ACP(c) + H2O(c)
r1485	0.25	H2O(m) + trans,cis-dodeca-2,5-dienoyl-CoA(m) ⇌ 3(S)-3-hydroxydodecen-(5Z)-oyl-CoA(m)
r0694	-0.25	(R)-3-Hydroxyoctanoyl-ACP(c) + NADP+(c) ⇌ 3-Oxooctanoyl-ACP(c) + NADPH(c)
r1486	0.25	NAD+(m) + 3(S)-3-hydroxydodecen-(5Z)-oyl-CoA(m) ⇌ 3-oxolaur-cis-5-enoyl-CoA(m) + NADH(m)
r0695	0.25	(R)-3-Hydroxyoctanoyl-ACP(c) ⇌ (2E)-Octenoyl-ACP(c) + H2O(c)
r0696	-0.25	(R)-3-Hydroxypalmitoyl-ACP(c) + NADP+(c) ⇌ 3-Oxohexadecanoyl-ACP(c) + NADPH(c)
r0697	0.25	(R)-3-Hydroxypalmitoyl-ACP(c) ⇌ (2E)-Hexadecenoyl-ACP(c) + H2O(c)
r0849	-0.13	H2O(s) ⇌ H2O(c)
r0863	-0.25	CoA(c) ⇌ CoA(m)
r0712	-0.25	Dodecanoyl-ACP(c) + NADP+(c) ⇌ (2E)-Dodecenoyl-ACP(c) + NADPH(c)
r0287	-0.25	CoA(m) + 3-Oxohexanoyl-CoA(m) → Acetyl-CoA(m) + Butyryl-CoA(m)
r0713	0.25	Dodecanoyl-ACP(c) + Malonyl-ACP(c) ⇌ 3-Oxotetradecanoyl-ACP(c) + CO2(c) + ACP(c)
r0431	-2	Malonyl-CoA(c) + L-Carnitine(c) ⇌ CoA(c) + Malonyl-Carnitin(c)
r0432	2	Malonyl-CoA(m) + L-Carnitine(m) ⇌ CoA(m) + Malonyl-Carnitin(m)
r0867	1.13	CO2(c) ⇌ CO2(s)
r0868	3.45	CO2(c) ⇌ CO2(m)
r0730	0.25	(S)-Hydroxyoctanoyl-CoA(m) + NAD+(m) → 3-Oxooctanoyl-CoA(m) + NADH(m)
r0731	-0.25	(S)-Hydroxyoctanoyl-CoA(m) ← (2E)-Octenoyl-CoA(m) + H2O(m)
r0588	-0.25	Crotonyl-CoA(m) + H2O(m) ⇌ (S)-3-Hydroxybutyryl-CoA(m)
r0732	-0.25	Hexanoyl-CoA(m) + Acetyl-CoA(m) ← CoA(m) + 3-Oxooctanoyl-CoA(m)
r0733	0.25	(S)-Hydroxyhexanoyl-CoA(m) + NAD+(m) → 3-Oxohexanoyl-CoA(m) + NADH(m)
r0734	-0.25	(S)-Hydroxyhexanoyl-CoA(m) ← (2E)-Hexenoyl-CoA(m) + H2O(m)
r0007	0.38	PPi(c) + H2O(c) → 2 Pi(c)
r0320	0.13	ATP(c) + CoA(c) + Acetoacetate(c) → AMP(c) + PPi(c) + Acetoacetyl-CoA(c)
r0322	0.13	HMG-CoA(m) ⇌ Acetyl-CoA(m) + Acetoacetate(m)
r0192	-0.52	Fructose-6P(c) ⇌ Glucose-6P(c)
r1114	0.25	AKG(m) + CoA(m) + NAD+(m) ⇌ NADH(m) + CO2(m) + Succinyl-CoA(m)

Continued on next page

Aerobic reduction of FAD (gluc) (20)– continued

ID	flux	reaction
r0770	0.25	D-3-Hydroxydodecanoyl-ACP(c) ⇌ (2E)-Dodecenoyl-ACP(c) + H2O(c)
r0771	-0.25	Tetradecanoyl-ACP(c) + NADP+(c) ⇌ NADPH(c) + (2E)-Tetradecenoyl-ACP(c)
r0772	0.25	Tetradecanoyl-ACP(c) + Malonyl-ACP(c) ⇌ 3-Oxohexadecanoyl-ACP(c) + CO2(c) + ACP(c)
r0773	-0.25	Hexadecanoyl-ACP(c) + NADP+(c) ⇌ (2E)-Hexadecenoyl-ACP(c) + NADPH(c)
r0479	0.63	Glucono-1,5-lactone-6P(c) + H2O(c) ⇌ 6-Phospho-D-gluconate(c)
r0199	0.63	Glucose-6P(c) + NADP+(c) ⇌ NADPH(c) + Glucono-1,5-lactone-6P(c)
r0343	0.63	6-Phospho-D-gluconate(c) + NADP+(c) ⇌ NADPH(c) + CO2(c) + Ribulose-5P(c)
r2373	0.25	Citrate(m) + AKG(c) ⇌ Citrate(c) + AKG(m)
r1417	-1.05	CO2(c) + H2O(c) ⇌ Carbonate(c)
r0344	0.42	Ribulose-5P(c) ⇌ D-Xylulose-5P(c)
r1419	1.05	Carbonate(c) ⇌ HCO3-(c)
r1450	0.25	Ubiquinone(m) + Hexanoyl-CoA(m) → (2E)-Hexenoyl-CoA(m) + Ubiquinol(m)
r0941	1.05	HCO3-(c) ⇌ HCO3-(m)
r0069	-0.13	2 Acetyl-CoA(c) ⇌ CoA(c) + Acetoacetyl-CoA(c)
r0082	2.15	Oxalosuccinate(c) ⇌ AKG(c) + CO2(c)
r1451	0.25	Ubiquinone(m) + Decanoyl-CoA(m) → (2E)-Decenoyl-CoA(m) + Ubiquinol(m)
r0660	-0.25	(S)-3-Hydroxydodecanoyl-CoA(m) ⇌ (2E)-Dodecenoyl-CoA(m) + H2O(m)
r0083	-1.9	Oxalosuccinate(m) ⇌ AKG(m) + CO2(m)
r1005	2	L-Carnitine(c) + Malonyl-Carnitin(m) ⇌ L-Carnitine(m) + Malonyl-Carnitin(c)
r0367	0.25	Acetyl-CoA(c) + ACP(c) ⇌ CoA(c) + Acetyl-ACP(c)
r0369	2	ACP(c) + Malonyl-CoA(c) ⇌ CoA(c) + Malonyl-ACP(c)
r1471	0.25	FAD(m) + cis-myrist-7-enoyl-CoA(m) ⇌ FADH2(m) + trans,cis-myristo-2,7-dienoyl-CoA(m)
r1472	0.25	FAD(m) + Oleoyl-CoA(m) ⇌ FADH2(m) + trans,cis-octadeca-2,9-dienoyl-CoA(m)
r0681	0.25	(R)-3-Hydroxybutanoyl-ACP(c) ⇌ But-2-enoyl-ACP(c) + H2O(c)
r1473	0.25	FAD(m) + palmitoleoyl-CoA(m) ⇌ FADH2(m) + trans,cis-hexadeca-2,9-dienoyl-CoA(m)
r0682	-0.25	Butyryl-ACP(c) + NADP+(c) ⇌ But-2-enoyl-ACP(c) + NADPH(c)
r1474	0.25	H2O(m) + trans,cis-octadeca-2,9-dienoyl-CoA(m) ⇌ (S)-3-hydroxyoleyleoyl-CoA(m)
r1475	0.25	H2O(m) + trans,cis-hexadeca-2,9-dienoyl-CoA(m) ⇌ (S)-3-hydroxypalmitoleoyl-CoA(m)
r0250	-0.21	Ribulose-5P(c) ⇌ Ribose-5P(c)
r1476	0.25	NAD+(m) + cis-(3S)-hydroxytetradec-7-enoyl-CoA(m) ⇌ 3-oxomyrist-7-enoyl-CoA(m) + NADH(m)
r1477	0.25	NAD+(m) + (S)-3-hydroxyoleyleoyl-CoA(m) ⇌ NADH(m) + 3-oxooleoyl-CoA(m)
r1478	0.25	NAD+(m) + (S)-3-hydroxypalmitoleoyl-CoA(m) ⇌ NADH(m) + 3-oxopalmitoleoyl-CoA(m)
r1479	0.25	CoA(m) + 3-oxolaur-cis-5-enoyl-CoA(m) ⇌ Acetyl-CoA(m) + (3Z)-Dodecenoyl-CoA(m)
r0831	-0.85	Malate(m) + Pi(c) ⇌ Malate(c) + Pi(m)
r0255	-0.21	Fructose-6P(c) + GAP(c) ⇌ D-Xylulose-5P(c) + Erythrose-4P(c)
r1329	1	FADH2(c) ⇌ FAD(c) + FADH-redox-potential(c)
r0104	0.25	NAD+(c) + Malate(c) ⇌ OAA(c) + NADH(c)
r0256	-0.1	DHAP(c) + GAP(c) ⇌ Fructose-1,6PP(c)
r0105	1.2	NAD+(m) + Malate(m) ⇌ OAA(m) + NADH(m)
r0106	-0.95	ADP(m) + Pi(m) + OAA(m) ⇌ ATP(m) + Pyruvate(m) + HCO3-(m)
r2418	0.6	ADP(c) + ATP(m) → ADP(m) + ATP(c)
r0107	-0.25	Acetyl-CoA(m) + H2O(m) + OAA(m) ⇌ Citrate(m) + CoA(m)
r0259	-0.25	Fumarate(m) + H2O(m) ⇌ Malate(m)
r0851	0.03	H2O(m) ⇌ H2O(c)
r0123	0.25	GTP(c) + OAA(c) → GDP(c) + PEP(c) + CO2(c)

Aerobic reduction of FAD (FA) (21)

Definition			Solution												
			exchanges		reactions								transp		
Objective	Constraints	Comment	imports	exports	c	m	r	p	l	n	s	b	s-c	b-c	int
FADH-redox-potential(c)	−Palmitate +CO_2 −O_2 =H_2O	Aerobic reduction of FAD	1.04 O_2(s) 0.03 Palmitate(s)	0.05 H_2O(s) 1.05 CO_2(s) 1 FADH-redox-potential(c)	56	50	-	-	-	-	-	-	4	-	19

ID	flux	reaction
r0701	-0.23	HMA(c) + NADP+(c) ⇌ 3-Oxotetradecanoyl-ACP(c) + NADPH(c)
r0702	0.23	HMA(c) ⇌ H2O(c) + (2E)-Tetradecenoyl-ACP(c)
r0125	-0.52	GDP(m) + Pi(m) + Succinyl-CoA(m) ⇌ GTP(m) + Succinate(m) + CoA(m)
r0855	1.04	O2(s) ⇌ O2(c)
r0856	-0.78	O2(m) ⇌ O2(c)
r0422	2.53	Isocitrate(c) + NADP+(c) ⇌ Oxalosuccinate(c) + NADPH(c)
r0872	1.07	Pyruvate(c) + H+(PG)(c) → Pyruvate(m) + H+(PG)(m)
r0425	-2.01	Isocitrate(m) + NAD+(m) ⇌ Oxalosuccinate(m) + NADH(m)
r0722	0.26	(S)-3-Hydroxydodecanoyl-CoA(m) + NAD+(m) ⇌ 3-Oxododecanoyl-CoA(m) + NADH(m)
r1385	-1.33	NADPH(m) + NAD+(m) + H+(PG)(m) ← NADH(m) + NADP+(m) + H+(PG)(c)
r0724	-0.26	Decanoyl-CoA(m) + Acetyl-CoA(m) ⇌ CoA(m) + 3-Oxododecanoyl-CoA(m)
r0726	0.26	(S)-Hydroxydecanoyl-CoA(m) + NAD+(m) ⇌ 3-Oxodecanoyl-CoA(m) + NADH(m)
r0728	-0.26	(S)-Hydroxydecanoyl-CoA(m) ⇌ (2E)-Decenoyl-CoA(m) + H2O(m)
r0460	0.26	(S)-3-Hydroxybutyryl-CoA(m) + NAD+(m) ⇌ NADH(m) + Acetoacetyl-CoA(m)
r0462	-0.26	Acetyl-CoA(m) + H2O(m) + Acetoacetyl-CoA(m) ⇌ HMG-CoA(m) + CoA(m)
r1254	0.23	Stearate(c) + ATP(c) + CoA(c) → Stearoyl-CoA(c) + AMP(c) + PPi(c)
r0017	0.78	O2(m) + 4 H+(PG)(m) + 4 Ferrocytochrome C(m) → 4 Ferricytochrome C(m) + 2 H2O(m) + 4 H+(PG)(c)
r0312	0.03	Palmitate(c) + ATP(c) + CoA(c) → Palmitoyl-CoA(c) + AMP(c) + PPi(c)
r0030	0.52	ATP(c) + AMP(c) ⇌ 2 ADP(c)
r0760	0.23	Butyryl-ACP(c) + Malonyl-ACP(c) ⇌ CO2(c) + ACP(c) + 3-Oxohexanoyl-ACP(c)
r0761	-0.23	D-3-Hydroxyhexanoyl-ACP(c) + NADP+(c) ⇌ NADPH(c) + 3-Oxohexanoyl-ACP(c)
r0184	0.29	ATP(c) + Acetyl-CoA(c) + HCO3-(c) → ADP(c) + Pi(c) + Malonyl-CoA(c)
r0019	-2.31	ADP(m) + Pi(m) + 3 H+(PG)(c) → ATP(m) + H2O(m) + 3 H+(PG)(m)
r0762	0.23	D-3-Hydroxyhexanoyl-ACP(c) ⇌ H2O(c) + (2E)-Hexenoyl-ACP(c)
r0185	1.51	ATP(m) + Acetyl-CoA(m) + HCO3-(m) → ADP(m) + Pi(m) + Malonyl-CoA(m)
r0763	-0.23	Hexanoyl-ACP(c) + NADP+(c) ⇌ NADPH(c) + (2E)-Hexenoyl-ACP(c)
r0316	0.52	Citrate(m) ⇌ Isocitrate(m)
r0764	0.23	Malonyl-ACP(c) + Hexanoyl-ACP(c) ⇌ 3-Oxooctanoyl-ACP(c) + CO2(c) + ACP(c)
r0765	-0.23	Octanoyl-ACP(c) + NADP+(c) ⇌ (2E)-Octenoyl-ACP(c) + NADPH(c)
r0766	0.23	Octanoyl-ACP(c) + Malonyl-ACP(c) ⇌ 3-Oxodecanoyl-ACP(c) + CO2(c) + ACP(c)
r0767	-0.23	Decanoyl-ACP(c) + NADP+(c) ⇌ (2E)-Decenoyl-ACP(c) + NADPH(c)
r1290	1	FAD(c) + FADH2(m) ⇌ FAD(m) + FADH2(c)
r0768	0.23	Decanoyl-ACP(c) + Malonyl-ACP(c) ⇌ 3-Oxododecanoyl-ACP(c) + CO2(c) + ACP(c)
r0769	-0.23	D-3-Hydroxydodecanoyl-ACP(c) + NADP+(c) ⇌ 3-Oxododecanoyl-ACP(c) + NADPH(c)
r0054	-0.26	ADP(c) + PEP(c) → ATP(c) + Pyruvate(c)
r0634	-0.26	Acetyl-CoA(m) + Octanoyl-CoA(m) ⇌ CoA(m) + 3-Oxodecanoyl-CoA(m)
r0070	0.001	2 Acetyl-CoA(m) ⇌ CoA(m) + Acetoacetyl-CoA(m)
r0058	1.07	Malate(c) + NADP+(c) ⇌ Pyruvate(c) + CO2(c) + NADPH(c)
r0059	-1.33	Pyruvate(m) + CO2(m) + NADPH(m) ⇌ Malate(m) + NADP+(m)
r1147	2.53	AKG(c) + Isocitrate(m) ⇌ AKG(m) + Isocitrate(c)
r0935	0.03	Palmitate(s) → Palmitate(c)
r1446	0.26	Ubiquinone(m) + Butyryl-CoA(m) → Crotonyl-CoA(m) + Ubiquinol(m)
r1448	0.26	Ubiquinone(m) + Octanoyl-CoA(m) → (2E)-Octenoyl-CoA(m) + Ubiquinol(m)
r0657	0.26	(3Z)-Dodecenoyl-CoA(m) ⇌ (2E)-Dodecenoyl-CoA(m)
r0801	-0.52	GTP(c) + GDP(m) ⇌ GTP(m) + GDP(c)
r0507	1.56	Ubiquinol(m) + 2 Ferricytochrome C(m) + 4 H+(PG)(m) → Ubiquinone(m) + 4 H+(PG)(c) + 2 Ferrocytochrome C(m)
r1464	-0.26	Pyruvate(m) + Acetoacetate(c) ⇌ Pyruvate(c) + Acetoacetate(m)
r1465	-0.23	NADH(c) + O2(c) + Stearoyl-CoA(c) ⇌ 2 H2O(c) + NAD+(c) + Oleoyl-CoA(c)
r1313	0.23	Hexadecanoyl-ACP(c) + Malonyl-ACP(c) ⇌ 3-Oxostearoyl-ACP(c) + ACP(c) + CO2(c)
r0509	0.52	Ubiquinone(m) + Succinate(m) → Ubiquinol(m) + Fumarate(m)
r1314	0.23	3-Oxostearoyl-ACP(c) + NADPH(c) ⇌ 3-Hydroxystearoyl-ACP(c) + NADP+(c)
r1480	0.26	CoA(m) + 3-oxopalmitoleoyl-CoA(m) ⇌ Acetyl-CoA(m) + cis-myrist-7-enoyl-CoA(m)
r1467	0.23	Oleoyl-CoA(c) ⇌ Oleoyl-CoA(m)
r1315	0.23	3-Hydroxystearoyl-ACP(c) ⇌ (2E)-Octadecenoyl-ACP(c) + H2O(c)
r0099	-0.26	ADP(c) + GTP(c) ⇌ ATP(c) + GDP(c)
r1481	0.23	CoA(m) + 3-oxooleoyl-CoA(m) ⇌ Acetyl-CoA(m) + palmitoleoyl-CoA(m)
r1468	0.03	palmitoleoyl-CoA(c) ⇌ palmitoleoyl-CoA(m)

Continued on next page

Aerobic reduction of FAD (FA) (21)– continued

ID	flux	reaction
r1316	0.23	(2E)-Octadecenoyl-ACP(c) + NADPH(c) ⇌ Stearoyl-ACP(c) + NADP+(c)
r1482	-0.26	FADH2(m) + trans,cis-dodeca-2,5-dienoyl-CoA(m) ⇌ FAD(m) + cis-laur-5-enoyl-CoA(m)
r1317	0.23	H2O(c) + Stearoyl-ACP(c) → ACP(c) + Stearate(c)
r0691	-0.23	(R)-3-Hydroxybutanoyl-ACP(c) + NADP+(c) ⇌ NADPH(c) + Acetoacetyl-ACP(c)
r0678	0.23	Acetyl-ACP(c) + Malonyl-ACP(c) ⇌ CO2(c) + ACP(c) + Acetoacetyl-ACP(c)
r1483	0.26	H2O(m) + trans,cis-myristo-2,7-dienoyl-CoA(m) ⇌ cis-(3S)-hydroxytetradec-7-enoyl-CoA(m)
r0692	-0.23	(R)-3-Hydroxydecanoyl-ACP(c) + NADP+(c) ⇌ 3-Oxodecanoyl-ACP(c) + NADPH(c)
r1484	0.26	CoA(m) + 3-oxomyrist-7-enoyl-CoA(m) ⇌ Acetyl-CoA(m) + cis-laur-5-enoyl-CoA(m)
r0693	0.23	(R)-3-Hydroxydecanoyl-ACP(c) ⇌ (2E)-Decenoyl-ACP(c) + H2O(c)
r1485	0.26	H2O(m) + trans,cis-dodeca-2,5-dienoyl-CoA(m) ⇌ 3(S)-3-hydroxydodecen-(5Z)-oyl-CoA(m)
r0694	-0.23	(R)-3-Hydroxyoctanoyl-ACP(c) + NADP+(c) ⇌ 3-Oxooctanoyl-ACP(c) + NADPH(c)
r1486	0.26	NAD+(m) + 3(S)-3-hydroxydodecen-(5Z)-oyl-CoA(m) ⇌ 3-oxolaur-cis-5-enoyl-CoA(m) + NADH(m)
r0695	0.23	(R)-3-Hydroxyoctanoyl-ACP(c) ⇌ (2E)-Octenoyl-ACP(c) + H2O(c)
r0696	-0.23	(R)-3-Hydroxypalmitoyl-ACP(c) + NADP+(c) ⇌ 3-Oxohexadecanoyl-ACP(c) + NADPH(c)
r0697	0.23	(R)-3-Hydroxypalmitoyl-ACP(c) ⇌ (2E)-Hexadecenoyl-ACP(c) + H2O(c)
r0849	-0.05	H2O(s) ⇌ H2O(c)
r0863	-0.26	CoA(c) ⇌ CoA(m)
r0712	-0.23	Dodecanoyl-ACP(c) + NADP+(c) ⇌ (2E)-Dodecenoyl-ACP(c) + NADPH(c)
r0287	-0.26	CoA(m) + 3-Oxohexanoyl-CoA(m) → Acetyl-CoA(m) + Butyryl-CoA(m)
r0713	0.23	Dodecanoyl-ACP(c) + Malonyl-ACP(c) ⇌ 3-Oxotetradecanoyl-ACP(c) + CO2(c) + ACP(c)
r0431	-1.51	Malonyl-CoA(c) + L-Carnitine(c) ⇌ CoA(c) + Malonyl-Carnitin(c)
r0432	1.51	Malonyl-CoA(m) + L-Carnitine(m) ⇌ CoA(m) + Malonyl-Carnitin(m)
r0867	1.05	CO2(c) ⇌ CO2(s)
r0868	2.81	CO2(c) ⇌ CO2(m)
r0730	0.26	(S)-Hydroxyoctanoyl-CoA(m) + NAD+(m) → 3-Oxooctanoyl-CoA(m) + NADH(m)
r0731	-0.26	(S)-Hydroxyoctanoyl-CoA(m) ← (2E)-Octenoyl-CoA(m) + H2O(m)
r0588	-0.26	Crotonyl-CoA(m) + H2O(m) ⇌ (S)-3-Hydroxybutyryl-CoA(m)
r0732	-0.26	Hexanoyl-CoA(m) + Acetyl-CoA(m) ← CoA(m) + 3-Oxooctanoyl-CoA(m)
r0733	0.26	(S)-Hydroxyhexanoyl-CoA(m) + NAD+(m) → 3-Oxohexanoyl-CoA(m) + NADH(m)
r0734	-0.26	(S)-Hydroxyhexanoyl-CoA(m) ← (2E)-Hexenoyl-CoA(m) + H2O(m)
r0007	0.52	PPi(c) + H2O(c) → 2 Pi(c)
r0023	0.03	NADH(c) + 2 Ferricytochrome b5(c) ⇌ NAD+(c) + 2 Ferrocytochrome b5(c)
r0320	0.26	ATP(c) + CoA(c) + Acetoacetate(c) → AMP(c) + PPi(c) + Acetoacetyl-CoA(c)
r0322	0.26	HMG-CoA(m) ⇌ Acetyl-CoA(m) + Acetoacetate(m)
r1114	0.52	AKG(m) + CoA(m) + NAD+(m) ⇌ NADH(m) + CO2(m) + Succinyl-CoA(m)
r0770	0.23	D-3-Hydroxydodecanoyl-ACP(c) ⇌ (2E)-Dodecenoyl-ACP(c) + H2O(c)
r0771	-0.23	Tetradecanoyl-ACP(c) + NADP+(c) ⇌ NADPH(c) + (2E)-Tetradecenoyl-ACP(c)
r0772	0.23	Tetradecanoyl-ACP(c) + Malonyl-ACP(c) ⇌ 3-Oxohexadecanoyl-ACP(c) + CO2(c) + ACP(c)
r0773	-0.23	Hexadecanoyl-ACP(c) + NADP+(c) ⇌ (2E)-Hexadecenoyl-ACP(c) + NADPH(c)
r1417	-1.8	CO2(c) + H2O(c) ⇌ Carbonate(c)
r1419	1.8	Carbonate(c) ⇌ HCO3-(c)
r1450	0.26	Ubiquinone(m) + Hexanoyl-CoA(m) → (2E)-Hexenoyl-CoA(m) + Ubiquinol(m)
r0941	1.51	HCO3-(c) ⇌ HCO3-(m)
r0069	-0.26	2 Acetyl-CoA(c) ⇌ CoA(c) + Acetoacetyl-CoA(c)
r0082	2.53	Oxalosuccinate(c) ⇌ AKG(c) + CO2(c)
r1451	0.26	Ubiquinone(m) + Decanoyl-CoA(m) → (2E)-Decenoyl-CoA(m) + Ubiquinol(m)
r0660	-0.26	(S)-3-Hydroxydodecanoyl-CoA(m) ⇌ (2E)-Dodecenoyl-CoA(m) + H2O(m)
r0083	-2.01	Oxalosuccinate(m) ⇌ AKG(m) + CO2(m)
r1005	1.51	L-Carnitine(c) + Malonyl-Carnitin(m) ⇌ L-Carnitine(m) + Malonyl-Carnitin(c)
r0510	0.03	Palmitoyl-CoA(c) + O2(c) + 2 Ferrocytochrome b5(c) ⇌ palmitoleoyl-CoA(c) + 2 Ferricytochrome b5(c) + 2 H2O(c)
r0367	0.23	Acetyl-CoA(c) + ACP(c) ⇌ CoA(c) + Acetyl-ACP(c)
r0369	1.8	ACP(c) + Malonyl-CoA(c) ⇌ CoA(c) + Malonyl-ACP(c)
r1471	0.26	FAD(m) + cis-myrist-7-enoyl-CoA(m) ⇌ FADH2(m) + trans,cis-myristo-2,7-dienoyl-CoA(m)
r1472	0.23	FAD(m) + Oleoyl-CoA(m) ⇌ FADH2(m) + trans,cis-octadeca-2,9-dienoyl-CoA(m)
r0681	0.23	(R)-3-Hydroxybutanoyl-ACP(c) ⇌ But-2-enoyl-ACP(c) + H2O(c)
r1473	0.26	FAD(m) + palmitoleoyl-CoA(m) ⇌ FADH2(m) + trans,cis-hexadeca-2,9-dienoyl-CoA(m)

Continued on next page

Aerobic reduction of FAD (FA) (21)– continued

ID	flux	reaction
r0682	-0.23	Butyryl-ACP(c) + NADP+(c) ⇌ But-2-enoyl-ACP(c) + NADPH(c)
r1474	0.23	H2O(m) + trans,cis-octadeca-2,9-dienoyl-CoA(m) ⇌ (S)-3-hydroxyoleyleoyl-CoA(m)
r1475	0.26	H2O(m) + trans,cis-hexadeca-2,9-dienoyl-CoA(m) ⇌ (S)-3-hydroxypalmitoleoyl-CoA(m)
r1476	0.26	NAD+(m) + cis-(3S)-hydroxytetradec-7-enoyl-CoA(m) ⇌ 3-oxomyrist-7-enoyl-CoA(m) + NADH(m)
r1477	0.23	NAD+(m) + (S)-3-hydroxyoleyleoyl-CoA(m) ⇌ NADH(m) + 3-oxooleoyl-CoA(m)
r1478	0.26	NAD+(m) + (S)-3-hydroxypalmitoleoyl-CoA(m) ⇌ NADH(m) + 3-oxopalmitoleoyl-CoA(m)
r1479	0.26	CoA(m) + 3-oxolaur-cis-5-enoyl-CoA(m) ⇌ Acetyl-CoA(m) + (3Z)-Dodecenoyl-CoA(m)
r0831	-1.33	Malate(m) + Pi(c) ⇌ Malate(c) + Pi(m)
r1329	1	FADH2(c) ⇌ FAD(c) + FADH-redox-potential(c)
r0104	0.26	NAD+(c) + Malate(c) ⇌ OAA(c) + NADH(c)
r0105	0.52	NAD+(m) + Malate(m) ⇌ OAA(m) + NADH(m)
r2418	0.81	ADP(c) + ATP(m) → ADP(m) + ATP(c)
r0107	-0.52	Acetyl-CoA(m) + H2O(m) + OAA(m) ⇌ Citrate(m) + CoA(m)
r0259	-0.52	Fumarate(m) + H2O(m) ⇌ Malate(m)
r0851	0.27	H2O(m) ⇌ H2O(c)
r0123	0.26	GTP(c) + OAA(c) → GDP(c) + PEP(c) + CO2(c)

Oxidation of NADH (22)

Definition			Solution												
			exchanges		reactions								transp		
Objective	Constraints	Comment	imports	exports	c	m	r	p	l	n	s	b	s-c	b-c	int
-1 NADH-redox-potential(c)	−Palmitate $+CO_2$ $-O_2$ $=H_2O$ %r1414 %r0201	Oxydation of NADH, release of energy	0.5 O_2(s) 1 NADH-redox-potential(c)	1 H_2O(s)	3	6	-	-	-	-	-	-	2	-	8

ID	flux	reaction
r0820	-1	AKG(m) + Malate(c) ⇌ AKG(c) + Malate(m)
r0523	1.33	ATP(m) + 5-Formyl-THF(m) → ADP(m) + Pi(m) + 5,10-Methenyl-THF(m)
r0109	1	AKG(c) + Aspartate(c) ⇌ OAA(c) + Glutamate(c)
r0019	-1.33	ADP(m) + Pi(m) + 3 H+(PG)(c) → ATP(m) + H2O(m) + 3 H+(PG)(m)
r0507	1	Ubiquinol(m) + 2 Ferricytochrome C(m) + 4 H+(PG)(m) → Ubiquinone(m) + 4 H+(PG)(c) + 2 Ferrocytochrome C(m)
r0851	1	H2O(m) ⇌ H2O(c)
r0110	-1	OAA(m) + Glutamate(m) → AKG(m) + Aspartate(m)
r1447	1	Ubiquinone(m) + Lauroyl-CoA(m) → (2E)-Dodecenoyl-CoA(m) + Ubiquinol(m)
r1385	-1	NADPH(m) + NAD+(m) + H+(PG)(m) ← NADH(m) + NADP+(m) + H+(PG)(c)
r0638	-1	(2E)-Dodecenoyl-CoA(m) + NADPH(m) ⇌ Lauroyl-CoA(m) + NADP+(m)
r0855	0.5	O2(s) ⇌ O2(c)
r0104	-1	OAA(c) + NADH(c) ⇌ NAD+(c) + Malate(c)
r0856	-0.5	O2(m) ⇌ O2(c)
r0105	1	NAD+(m) + Malate(m) ⇌ OAA(m) + NADH(m)
r1322	-1	NADH(c) ⇌ NAD+(c) + NADH-redox-potential(c)
r0876	1	Glutamate(c) + H+(PG)(c) + Aspartate(m) → Glutamate(m) + H+(PG)(m) + Aspartate(c)
r0849	-1	H2O(s) ⇌ H2O(c)
r0017	0.5	O2(m) + 4 H+(PG)(m) + 4 Ferrocytochrome C(m) → 4 Ferricytochrome C(m) + 2 H2O(m) + 4 H+(PG)(c)
r0522	-1.33	5-Formyl-THF(m) ← 5,10-Methenyl-THF(m) + H2O(m)

Oxidation of NADPH (23)

Definition			Solution												
			exchanges		reactions								transp		
Objective	Constraints	Comment	imports	exports	c	m	r	p	l	n	s	b	s-c	b-c	int
-1 NADPH-redox-potential(c)	$-$Palmitate $+CO_2$ $-O_2$ $=H_2O$ %r1414 %r0201	Oxydation of NADPH, release of energy	0.5 O_2(s) 1 NADPH-redox-potential(c)	1 H_2O(s)	3	6	-	-	-	-	-	-	2	-	8

ID	flux	reaction
r0523	1.33	ATP(m) + 5-Formyl-THF(m) → ADP(m) + Pi(m) + 5,10-Methenyl-THF(m)
r0082	-1	Oxalosuccinate(c) ⇌ AKG(c) + CO2(c)
r0019	-1.33	ADP(m) + Pi(m) + 3 H+(PG)(c) → ATP(m) + H2O(m) + 3 H+(PG)(m)
r0425	1	Isocitrate(m) + NAD+(m) ⇌ Oxalosuccinate(m) + NADH(m)
r0083	1	Oxalosuccinate(m) ⇌ AKG(m) + CO2(m)
r1326	-1	NADPH(c) ⇌ NADPH-redox-potential(c) + NADP+(c)
r0507	1	Ubiquinol(m) + 2 Ferricytochrome C(m) + 4 H+(PG)(m) → Ubiquinone(m) + 4 H+(PG)(c) + 2 Ferrocytochrome C(m)
r0652	-1	(2E)-Tetradecenoyl-CoA(m) + NADPH(m) ⇌ Myristoyl-CoA(m) + NADP+(m)
r0851	1	H2O(m) ⇌ H2O(c)
r1385	-1	NADPH(m) + NAD+(m) + H+(PG)(m) ← NADH(m) + NADP+(m) + H+(PG)(c)
r2385	-1	Isocitrate(c) + H+(PG)(c) + AKG(m) → Isocitrate(m) + H+(PG)(m) + AKG(c)
r1449	1	Ubiquinone(m) + Myristoyl-CoA(m) → (2E)-Tetradecenoyl-CoA(m) + Ubiquinol(m)
r0855	0.5	O2(s) ⇌ O2(c)
r0856	-0.5	O2(m) ⇌ O2(c)
r0849	-1	H2O(s) ⇌ H2O(c)
r0422	-1	Isocitrate(c) + NADP+(c) ⇌ Oxalosuccinate(c) + NADPH(c)
r0017	0.5	O2(m) + 4 H+(PG)(m) + 4 Ferrocytochrome C(m) → 4 Ferricytochrome C(m) + 2 H2O(m) + 4 H+(PG)(c)
r0868	-1	CO2(c) ⇌ CO2(m)
r0522	-1.33	5-Formyl-THF(m) ← 5,10-Methenyl-THF(m) + H2O(m)

NADH redox potential into mito (24)

Definition			Solution												
			exchanges		reactions								transp		
Objective	Constraints	Comment	imports	exports	c	m	r	p	l	n	s	b	s-c	b-c	int
NADH-redox-potential(m)	$-$NADH-redox-potential(c) %r0750	transport of redox potential	1 NADH-redox-potential(c)	1 NADH-redox-potential(m)	5	3	-	-	-	-	-	-	-	-	2

ID	flux	reaction
r0082	-1	Oxalosuccinate(c) ⇌ AKG(c) + CO2(c)
r0425	1	Isocitrate(m) + NAD+(m) ⇌ Oxalosuccinate(m) + NADH(m)
r0083	1	Oxalosuccinate(m) ⇌ AKG(m) + CO2(m)
r1147	-1	AKG(m) + Isocitrate(c) ⇌ AKG(c) + Isocitrate(m)
r0304	-1	Proline(c) + NAD+(c) ⇌ 1-Pyrroline-5-carboxylate(c) + NADH(c)
r1322	-1	NADH(c) ⇌ NAD+(c) + NADH-redox-potential(c)
r0422	-1	Isocitrate(c) + NADP+(c) ⇌ Oxalosuccinate(c) + NADPH(c)
r0305	1	Proline(c) + NADP+(c) ⇌ 1-Pyrroline-5-carboxylate(c) + NADPH(c)
r1323	1	NADH(m) ⇌ NAD+(m) + NADH-redox-potential(m)
r0868	-1	CO2(c) ⇌ CO2(m)

NADH redox potential into peroxy (25)

Definition			Solution												
			exchanges		reactions								transp		
Objective	Constraints	Comment	imports	exports	c	m	r	p	l	n	s	b	s-c	b-c	int
NADH-redox-potential(p)	−NADH-redox-potential(c)	transport of redox potential	1 NADH-redox-potential(c)	1 NADH-redox-potential(p)	2	-	-	2	-	-	-	-	-	-	2

ID	flux	reaction
r1324	1	NADH(p) ⇌ NAD+(p) + NADH-redox-potential(p)
r0173	1	L-Lactate(p) + NAD+(p) ⇌ Pyruvate(p) + NADH(p)
r2516	-1	H+(PG)(c) + L-Lactate(c) ⇌ H+(PG)(p) + L-Lactate(p)
r0874	1	Pyruvate(p) + H+(PG)(p) ⇌ Pyruvate(c) + H+(PG)(c)
r1322	-1	NADH(c) ⇌ NAD+(c) + NADH-redox-potential(c)
r0171	-1	L-Lactate(c) + NAD+(c) ⇌ Pyruvate(c) + NADH(c)

NADPH redox potential into mito (26)

Definition			Solution												
			exchanges		reactions								transp		
Objective	Constraints	Comment	imports	exports	c	m	r	p	l	n	s	b	s-c	b-c	int
NADPH-redox-potential(m)	−NADPH-redox-potential(c)	transport of redox potential	1 NADPH-redox-potential(c)	1 NADPH-redox-potential(m)	3	3	-	-	-	-	-	-	-	-	2

ID	flux	reaction
r0082	-1	Oxalosuccinate(c) ⇌ AKG(c) + CO2(c)
r0083	1	Oxalosuccinate(m) ⇌ AKG(m) + CO2(m)
r1326	-1	NADPH(c) ⇌ NADPH-redox-potential(c) + NADP+(c)
r1327	1	NADPH(m) ⇌ NADPH-redox-potential(m) + NADP+(m)
r1147	-1	AKG(m) + Isocitrate(c) ⇌ AKG(c) + Isocitrate(m)
r0422	-1	Isocitrate(c) + NADP+(c) ⇌ Oxalosuccinate(c) + NADPH(c)
r0868	-1	CO2(c) ⇌ CO2(m)
r0423	1	Isocitrate(m) + NADP+(m) ⇌ Oxalosuccinate(m) + NADPH(m)

NADPH redox potential into peroxy (27)

Definition			Solution												
			exchanges		reactions								transp		
Objective	Constraints	Comment	imports	exports	c	m	r	p	l	n	s	b	s-c	b-c	int
NADPH-redox-potential(p)	−NADPH-redox-potential(c)	transport of redox potential	1 NADPH-redox-potential(c)	1 NADPH-redox-potential(p)	3	-	-	3	-	-	-	-	-	-	2

ID	flux	reaction
r0869	1	CO2(p) ⇌ CO2(c)
r0424	1	Isocitrate(p) + NADP+(p) ⇌ Oxalosuccinate(p) + NADPH(p)
r0082	-1	Oxalosuccinate(c) ⇌ AKG(c) + CO2(c)
r1326	-1	NADPH(c) ⇌ NADPH-redox-potential(c) + NADP+(c)
r0084	1	Oxalosuccinate(p) ⇌ AKG(p) + CO2(p)
r1328	1	NADPH(p) ⇌ NADPH-redox-potential(p) + NADP+(p)
r1148	-1	AKG(c) + Isocitrate(p) ⇌ AKG(p) + Isocitrate(c)
r0422	-1	Isocitrate(c) + NADP+(c) ⇌ Oxalosuccinate(c) + NADPH(c)

NADPH to NADH transhydrogenase (28)

Definition			Solution												
			exchanges		reactions								transp		
Objective	Constraints	Comment	imports	exports	c	m	r	p	l	n	s	b	s-c	b-c	int
NADH-redox-potential(c)	−NADPH-redox-potential(c)<br>%r0427<br>%r0750<br>%r0746<br>%r0236		1 NADPH-redox-potential(c)	1 NADH-redox-potential(c)	4	-	-	-	-	-	-	-	-	-	-

ID	flux	reaction
r1326	-1	NADPH(c) ⇌ NADPH-redox-potential(c) + NADP+(c)
r0397	-1	Dihydrobiopterin(c) + NADH(c) ⇌ Tetrahydrobiopterin(c) + NAD+(c)
r0398	1	Dihydrobiopterin(c) + NADPH(c) ⇌ Tetrahydrobiopterin(c) + NADP+(c)
r1322	1	NADH(c) ⇌ NAD+(c) + NADH-redox-potential(c)

NADH to NADPH transhydrogenase (29)

Definition			Solution												
			exchanges		reactions								transp		
Objective	Constraints	Comment	imports	exports	c	m	r	p	l	n	s	b	s-c	b-c	int
NADPH-redox-potential(c)	−NADH-redox-potential(c)<br>%r0427<br>%r0750<br>%r0746<br>%r0236		1 NADH-redox-potential(c)	1 NADPH-redox-potential(c)	4	-	-	-	-	-	-	-	-	-	-

ID	flux	reaction
r1326	1	NADPH(c) ⇌ NADPH-redox-potential(c) + NADP+(c)
r1322	-1	NADH(c) ⇌ NAD+(c) + NADH-redox-potential(c)
r0512	-1	Dihydrofolate(c) + NAD+(c) ⇌ Folate(c) + NADH(c)
r0513	1	Dihydrofolate(c) + NADP+(c) ⇌ Folate(c) + NADPH(c)

NADPH to NADH transhydrogenase in mito (30)

Definition			Solution												
			exchanges		reactions								transp		
Objective	Constraints	Comment	imports	exports	c	m	r	p	l	n	s	b	s-c	b-c	int
NADH-redox-potential(m)	−NADPH-redox-potential(m)<br>%r0525		1 NADPH-redox-potential(m)	1 NADH-redox-potential(m)	-	4	-	-	-	-	-	-	-	-	-

ID	flux	reaction
r0073	1	Glutamate(m) + NAD+(m) + H2O(m) ⇌ AKG(m) + NADH(m) + NH3(m)
r0075	-1	Glutamate(m) + H2O(m) + NADP+(m) ⇌ AKG(m) + NADPH(m) + NH3(m)
r1327	-1	NADPH(m) ⇌ NADPH-redox-potential(m) + NADP+(m)
r1323	1	NADH(m) ⇌ NAD+(m) + NADH-redox-potential(m)

NADH to NADPH transhydrogenase in mito (31)

Definition			Solution												
			exchanges		reactions								transp		
Objective	Constraints	Comment	imports	exports	c	m	r	p	l	n	s	b	s-c	b-c	int
NADPH-redox-potential(m)	−NADH-redox-potential(m) %r0525		1 NADH-redox-potential(m)	1 NADPH-redox-potential(m)	-	4	-	-	-	-	-	-	-	-	-

ID	flux	reaction
r0425	-1	Isocitrate(m) + NAD+(m) ⇌ Oxalosuccinate(m) + NADH(m)
r1327	1	NADPH(m) ⇌ NADPH-redox-potential(m) + NADP+(m)
r1323	-1	NADH(m) ⇌ NAD+(m) + NADH-redox-potential(m)
r0423	1	Isocitrate(m) + NADP+(m) ⇌ Oxalosuccinate(m) + NADPH(m)

A1.3. Other redox equivalents

Thioredoxin(m) reduction (32)

Definition			Solution												
			exchanges		reactions								transp		
Objective	Constraints	Comment	imports	exports	c	m	r	p	l	n	s	b	s-c	b-c	int
Thioredoxin-redox-potential(m)	−NADPH-redox-potential(m)	test that cytosolic thioredoxin can be reduced using the minimal exchange set	1 NADPH-redox-potential(m)	1 Thioredoxin-redox-potential(m)	-	3	-	-	-	-	-	-	-	-	-

ID	flux	reaction
r1433	-1	mitoOxidizedThioredoxin(m) + NADPH(m) ⇌ mitoThioredoxin(m) + NADP+(m)
r1327	-1	NADPH(m) ⇌ NADPH-redox-potential(m) + NADP+(m)
r2683	1	mitoThioredoxin(m) ⇌ mitoOxidizedThioredoxin(m) + Thioredoxin-redox-potential(m)

Thioredoxin(m) oxidation (33)

Definition			Solution												
			exchanges		reactions								transp		
Objective	Constraints	Comment	imports	exports	c	m	r	p	l	n	s	b	s-c	b-c	int
-1 Thioredoxin-redox-potential(m)	=NADPH-redox-potential(m) −Palmitate $+CO_2$ $-O_2$ $=H_2O$	test that cytosolic thioredoxin can be reduced using the minimal exchange set	1 Thioredoxin-redox-potential(m)	1 NADPH-redox-potential(m)	-	3	-	-	-	-	-	-	-	-	-

ID	flux	reaction
r1433	1	mitoThioredoxin(m) + NADP+(m) ⇌ mitoOxidizedThioredoxin(m) + NADPH(m)
r1327	1	NADPH(m) ⇌ NADPH-redox-potential(m) + NADP+(m)
r2683	-1	mitoThioredoxin(m) ⇌ mitoOxidizedThioredoxin(m) + Thioredoxin-redox-potential(m)

Thioredoxin(c) reduction (34)

Definition			Solution													
			exchanges		reactions								transp			
Objective	Constraints	Comment	imports	exports	c	m	r	p	l	n	s	b	s-c	b-c	int
Thioredoxin-redox-potential(c)	=NADH-redox-potential(c) −Palmitate $+CO_2$ $-O_2$ $=H_2O$	test that cytosolic thioredoxin can be reduced using the minimal exchange set	1 NADH-redox-potential(c)	1 Thioredoxin-redox-potential(c)	5	-	-	-	-	-	-	-	-	-	-

ID	flux	reaction
r2682	1	Thioredoxin(c) ⇌ Oxidized thioredoxin(c) + Thioredoxin-redox-potential(c)
r0469	-1	Oxidized thioredoxin(c) + NADPH(c) ⇌ Thioredoxin(c) + NADP+(c)
r1322	-1	NADH(c) ⇌ NAD+(c) + NADH-redox-potential(c)
r0512	-1	Dihydrofolate(c) + NAD+(c) ⇌ Folate(c) + NADH(c)
r0513	1	Dihydrofolate(c) + NADP+(c) ⇌ Folate(c) + NADPH(c)

Thioredoxin(c) oxidation (35)

Definition			Solution												
			exchanges		reactions								transp		
Objective	Constraints	Comment	imports	exports	c	m	r	p	l	n	s	b	s-c	b-c	int
-1 Thioredoxin-redox-potential(c)	=NADH-redox-potential(c) −Palmitate $+CO_2$ $-O_2$ $=H_2O$	test that cytosolic thioredoxin can be reduced using the minimal exchange set	1 Thioredoxin-redox-potential(c)	1 NADH-redox-potential(c)	5	-	-	-	-	-	-	-	-	-	-

ID	flux	reaction
r2682	-1	Thioredoxin(c) ⇌ Oxidized thioredoxin(c) + Thioredoxin-redox-potential(c)
r0469	1	Thioredoxin(c) + NADP+(c) ⇌ Oxidized thioredoxin(c) + NADPH(c)
r0397	-1	Dihydrobiopterin(c) + NADH(c) ⇌ Tetrahydrobiopterin(c) + NAD+(c)
r0398	1	Dihydrobiopterin(c) + NADPH(c) ⇌ Tetrahydrobiopterin(c) + NADP+(c)
r1322	1	NADH(c) ⇌ NAD+(c) + NADH-redox-potential(c)

GSH reduction using NADH redox potential (36)

Definition			Solution												
			exchanges		reactions								transp		
Objective	Constraints	Comment	imports	exports	c	m	r	p	l	n	s	b	s-c	b-c	int
GSH-potential(c)	=NADH-redox-potential(c)	Regeneration of Gluthatione redox potential	0.5 NADH-redox-potential(c)	1 GSH-potential(c)	5	-	-	-	-	-	-	-	-	-	-

ID	flux	reaction
r0750	0.5	3alpha,7alpha,12alpha-Trihydroxycoprostane(c) + NADP+(c) ⇌ NADPH(c) + 7alpha,12alpha-Dihydroxy-5beta-cholestan-3-one(c)
r2681	0.5	2 GSH(c) ⇌ GSSG(c) + 2 GSH-potential(c)
r0749	-0.5	3alpha,7alpha,12alpha-Trihydroxycoprostane(c) + NAD+(c) ⇌ NADH(c) + 7alpha,12alpha-Dihydroxy-5beta-cholestan-3-one(c)
r0025	-0.5	GSSG(c) + NADPH(c) ⇌ 2 GSH(c) + NADP+(c)
r1322	-0.5	NADH(c) ⇌ NAD+(c) + NADH-redox-potential(c)

GSH reduction using NADPH redox potential (37)

Definition			Solution												
			exchanges		reactions								transp		
Objective	Constraints	Comment	imports	exports	c	m	r	p	l	n	s	b	s-c	b-c	int
GSH-potential(c)	=NADPH-redox-potential(c)	Regeneration of Gluthatione redox potential	0.5 NADPH-redox-potential(c)	1 GSH-potential(c)	3	-	-	-	-	-	-	-	-	-	-

ID	flux	reaction
r1326	-0.5	NADPH(c) ⇌ NADPH-redox-potential(c) + NADP+(c)
r2681	0.5	2 GSH(c) ⇌ GSSG(c) + 2 GSH-potential(c)
r0025	-0.5	GSSG(c) + NADPH(c) ⇌ 2 GSH(c) + NADP+(c)

GSH oxidation (38)

Definition			Solution												
			exchanges		reactions								transp		
Objective	Constraints	Comment	imports	exports	c	m	r	p	l	n	s	b	s-c	b-c	int
-1 GSH-potential(c)	=ATP-energy(c) =NADH-redox-potential(c) =NADPH-redox-potential(c)	Oxidation of Gluthatione	1 GSH-potential(c)	0.5 NADPH-redox-potential(c)	3	-	-	-	-	-	-	-	-	-	-

ID	flux	reaction
r1326	0.5	NADPH(c) ⇌ NADPH-redox-potential(c) + NADP+(c)
r2681	-0.5	2 GSH(c) ⇌ GSSG(c) + 2 GSH-potential(c)
r0025	0.5	2 GSH(c) + NADP+(c) ⇌ GSSG(c) + NADPH(c)

2.2 A2. Membrane gradients

A2.1. Mitochondrial protons

Proton-gradient(m) build up (39)

Definition			Solution												
			exchanges		reactions								transp		
Objective	Constraints	Comment	imports	exports	c	m	r	p	l	n	s	b	s-c	b-c	int
Proton-gradient(m)	−Palmitate $+CO_2$ $-O_2$ $=H_2O$	mitochondrial Proton-gradient build up, protons pumped from the mitochondrial matrix into the cytosol	0.06 O_2(s) 0.003 Palmitate(s)	0.04 H_2O(s) 0.04 CO_2(s) 1 Proton-gradient(m)	4	41	-	-	-	-	-	-	4	-	10

ID	flux	reaction
r2435	0.003	L-Carnitine(m) + L-Palmitoylcarnitine(c) → L-Carnitine(c) + L-Palmitoylcarnitine(m)
r0125	-0.02	GDP(m) + Pi(m) + Succinyl-CoA(m) ⇌ GTP(m) + Succinate(m) + CoA(m)
r0855	0.06	O2(s) ⇌ O2(c)
r0856	-0.06	O2(m) ⇌ O2(c)
r0720	-0.003	(S)-3-Hydroxytetradecanoyl-CoA(m) ← (2E)-Tetradecenoyl-CoA(m) + H2O(m)
r0425	0.02	Isocitrate(m) + NAD+(m) ⇌ Oxalosuccinate(m) + NADH(m)
r0722	0.003	(S)-3-Hydroxydodecanoyl-CoA(m) + NAD+(m) ⇌ 3-Oxododecanoyl-CoA(m) + NADH(m)

Continued on next page

ID	flux	reaction
r0724	-0.003	Decanoyl-CoA(m) + Acetyl-CoA(m) ⇌ CoA(m) + 3-Oxododecanoyl-CoA(m)
r0726	0.003	(S)-Hydroxydecanoyl-CoA(m) + NAD+(m) ⇌ 3-Oxodecanoyl-CoA(m) + NADH(m)
r0728	-0.003	(S)-Hydroxydecanoyl-CoA(m) ⇌ (2E)-Decenoyl-CoA(m) + H2O(m)
r0460	0.003	(S)-3-Hydroxybutyryl-CoA(m) + NAD+(m) ⇌ NADH(m) + Acetoacetyl-CoA(m)
r0310	0.003	Palmitoyl-CoA(m) + Ubiquinone(m) → (2E)-Hexadecenoyl-CoA(m) + Ubiquinol(m)
r0017	0.06	O2(m) + 4 H+(PG)(m) + 4 Ferrocytochrome C(m) → 4 Ferricytochrome C(m) + 2 H2O(m) + 4 H+(PG)(c)
r0312	0.003	Palmitate(c) + ATP(c) + CoA(c) → Palmitoyl-CoA(c) + AMP(c) + PPi(c)
r0030	0.003	ATP(c) + AMP(c) ⇌ 2 ADP(c)
r0316	0.02	Citrate(m) ⇌ Isocitrate(m)
r0070	-0.003	CoA(m) + Acetoacetyl-CoA(m) ⇌ 2 Acetyl-CoA(m)
r0634	-0.003	Acetyl-CoA(m) + Octanoyl-CoA(m) ⇌ CoA(m) + 3-Oxodecanoyl-CoA(m)
r0639	-0.003	Lauroyl-CoA(m) + Acetyl-CoA(m) ← CoA(m) + 3-Oxotetradecanoyl-CoA(m)
r0935	0.003	Palmitate(s) → Palmitate(c)
r0653	-0.003	Myristoyl-CoA(m) + Acetyl-CoA(m) ← CoA(m) + 3-Oxopalmitoyl-CoA(m)
r1446	0.003	Ubiquinone(m) + Butyryl-CoA(m) → Crotonyl-CoA(m) + Ubiquinol(m)
r1447	0.003	Ubiquinone(m) + Lauroyl-CoA(m) → (2E)-Dodecenoyl-CoA(m) + Ubiquinol(m)
r1448	0.003	Ubiquinone(m) + Octanoyl-CoA(m) → (2E)-Octenoyl-CoA(m) + Ubiquinol(m)
r1449	0.003	Ubiquinone(m) + Myristoyl-CoA(m) → (2E)-Tetradecenoyl-CoA(m) + Ubiquinol(m)
r0507	0.12	Ubiquinol(m) + 2 Ferricytochrome C(m) + 4 H+(PG)(m) → Ubiquinone(m) + 4 H+(PG)(c) + 2 Ferrocytochrome C(m)
r0508	-0.08	Ubiquinone(m) + NADH(m) + 4 H+(PG)(m) → Ubiquinol(m) + NAD+(m) + 4 H+(PG)(c)
r0522	-0.02	5-Formyl-THF(m) ← 5,10-Methenyl-THF(m) + H2O(m)
r0509	0.02	Ubiquinone(m) + Succinate(m) → Ubiquinol(m) + Fumarate(m)
r0523	0.02	ATP(m) + 5-Formyl-THF(m) → ADP(m) + Pi(m) + 5,10-Methenyl-THF(m)
r1330	1	H+(PG)(c) ⇌ H+(PG)(m) + Proton-gradient(m)
r0849	-0.04	H2O(s) ⇌ H2O(c)
r0287	-0.003	CoA(m) + 3-Oxohexanoyl-CoA(m) → Acetyl-CoA(m) + Butyryl-CoA(m)
r0714	0.003	NAD+(m) + (S)-3-Hydroxyhexadecanoyl-CoA(m) → 3-Oxopalmitoyl-CoA(m) + NADH(m)
r0867	0.04	CO2(c) ⇌ CO2(s)
r0868	-0.04	CO2(c) ⇌ CO2(m)
r0716	-0.003	(S)-3-Hydroxyhexadecanoyl-CoA(m) ← (2E)-Hexadecenoyl-CoA(m) + H2O(m)
r0435	0.003	Palmitoyl-CoA(c) + L-Carnitine(c) ⇌ CoA(c) + L-Palmitoylcarnitine(c)
r0730	0.003	(S)-Hydroxyoctanoyl-CoA(m) + NAD+(m) → 3-Oxooctanoyl-CoA(m) + NADH(m)
r0588	-0.003	Crotonyl-CoA(m) + H2O(m) ⇌ (S)-3-Hydroxybutyryl-CoA(m)
r0718	0.003	(S)-3-Hydroxytetradecanoyl-CoA(m) + NAD+(m) → 3-Oxotetradecanoyl-CoA(m) + NADH(m)
r0436	-0.003	Palmitoyl-CoA(m) + L-Carnitine(m) ← CoA(m) + L-Palmitoylcarnitine(m)
r0731	-0.003	(S)-Hydroxyoctanoyl-CoA(m) ← (2E)-Octenoyl-CoA(m) + H2O(m)
r0732	-0.003	Hexanoyl-CoA(m) + Acetyl-CoA(m) ← CoA(m) + 3-Oxooctanoyl-CoA(m)
r0733	0.003	(S)-Hydroxyhexanoyl-CoA(m) + NAD+(m) → 3-Oxohexanoyl-CoA(m) + NADH(m)
r0734	-0.003	(S)-Hydroxyhexanoyl-CoA(m) ← (2E)-Hexenoyl-CoA(m) + H2O(m)
r0007	0.003	PPi(c) + H2O(c) → 2 Pi(c)
r1114	0.02	AKG(m) + CoA(m) + NAD+(m) ⇌ NADH(m) + CO2(m) + Succinyl-CoA(m)
r1450	0.003	Ubiquinone(m) + Hexanoyl-CoA(m) → (2E)-Hexenoyl-CoA(m) + Ubiquinol(m)
r1451	0.003	Ubiquinone(m) + Decanoyl-CoA(m) → (2E)-Decenoyl-CoA(m) + Ubiquinol(m)
r0660	-0.003	(S)-3-Hydroxydodecanoyl-CoA(m) ⇌ (2E)-Dodecenoyl-CoA(m) + H2O(m)
r0083	0.02	Oxalosuccinate(m) ⇌ AKG(m) + CO2(m)
r0100	-0.02	ADP(m) + GTP(m) ⇌ ATP(m) + GDP(m)
r1047	0.01	Pi(c) + H+(PG)(c) → Pi(m) + H+(PG)(m)
r0105	0.02	NAD+(m) + Malate(m) ⇌ OAA(m) + NADH(m)
r2418	0.01	ADP(c) + ATP(m) → ADP(m) + ATP(c)
r0107	-0.02	Acetyl-CoA(m) + H2O(m) + OAA(m) ⇌ Citrate(m) + CoA(m)
r0259	-0.02	Fumarate(m) + H2O(m) ⇌ Malate(m)
r0851	0.04	H2O(m) ⇌ H2O(c)

Proton-gradient(m) waste (40)

Definition			Solution												
			exchanges		reactions								transp		
Objective	Constraints	Comment	imports	exports	c	m	r	p	l	n	s	b	s-c	b-c	int
-1 Proton-gradient(m)	$-$Palmitate $+CO_2$ $-O_2$ $=H_2O$	mitochondrial Proton-gradient release, protons released from the cytosol into the mitochondrium	1 Proton-gradient(m)		-	-	-	-	-	-	-	-	-	-	3

ID	flux	reaction
r1455	0.5	Phenylalanine(c) + H+(PG)(c) → Phenylalanine(m) + H+(PG)(m)
r1456	0.5	Phenylalanine(m) + H+(PG)(c) → Phenylalanine(c) + H+(PG)(m)
r1330	-1	H+(PG)(c) ⇌ H+(PG)(m) + Proton-gradient(m)

A2.2. Plasma membrane protons

Proton-gradient(c) build up (41)

Definition			Solution												
			exchanges		reactions								transp		
Objective	Constraints	Comment	imports	exports	c	m	r	p	l	n	s	b	s-c	b-c	int
Proton-gradient(m)	$-$Palmitate $+CO_2$ $-O_2$ $=H_2O$	apical membrane Proton-gradient build up, protons pumped from the sinusouid into the cytosol	0.06 O_2(s) 0.003 Palmitate(s)	0.04 H_2O(s) 0.04 CO_2(s) 1 Proton-gradient(m)	4	41	-	-	-	-	-	-	4	-	10

ID	flux	reaction
r2435	0.003	L-Carnitine(m) + L-Palmitoylcarnitine(c) → L-Carnitine(c) + L-Palmitoylcarnitine(m)
r0125	-0.02	GDP(m) + Pi(m) + Succinyl-CoA(m) ⇌ GTP(m) + Succinate(m) + CoA(m)
r0855	0.06	O2(s) ⇌ O2(c)
r0856	-0.06	O2(m) ⇌ O2(c)
r0720	-0.003	(S)-3-Hydroxytetradecanoyl-CoA(m) ← (2E)-Tetradecenoyl-CoA(m) + H2O(m)
r0425	0.02	Isocitrate(m) + NAD+(m) ⇌ Oxalosuccinate(m) + NADH(m)
r0722	0.003	(S)-3-Hydroxydodecanoyl-CoA(m) + NAD+(m) ⇌ 3-Oxododecanoyl-CoA(m) + NADH(m)
r0724	-0.003	Decanoyl-CoA(m) + Acetyl-CoA(m) ⇌ CoA(m) + 3-Oxododecanoyl-CoA(m)
r0726	0.003	(S)-Hydroxydecanoyl-CoA(m) + NAD+(m) ⇌ 3-Oxodecanoyl-CoA(m) + NADH(m)
r0728	-0.003	(S)-Hydroxydecanoyl-CoA(m) ⇌ (2E)-Decenoyl-CoA(m) + H2O(m)
r0460	0.003	(S)-3-Hydroxybutyryl-CoA(m) + NAD+(m) ⇌ NADH(m) + Acetoacetyl-CoA(m)
r0310	0.003	Palmitoyl-CoA(m) + Ubiquinone(m) → (2E)-Hexadecenoyl-CoA(m) + Ubiquinol(m)
r0017	0.06	O2(m) + 4 H+(PG)(m) + 4 Ferrocytochrome C(m) → 4 Ferricytochrome C(m) + 2 H2O(m) + 4 H+(PG)(c)
r0312	0.003	Palmitate(c) + ATP(c) + CoA(c) → Palmitoyl-CoA(c) + AMP(c) + PPi(c)
r0030	0.003	ATP(c) + AMP(c) ⇌ 2 ADP(c)
r0316	0.02	Citrate(m) ⇌ Isocitrate(m)
r0070	-0.003	CoA(m) + Acetoacetyl-CoA(m) ⇌ 2 Acetyl-CoA(m)
r0634	-0.003	Acetyl-CoA(m) + Octanoyl-CoA(m) ⇌ CoA(m) + 3-Oxodecanoyl-CoA(m)
r0639	-0.003	Lauroyl-CoA(m) + Acetyl-CoA(m) ← CoA(m) + 3-Oxotetradecanoyl-CoA(m)
r0935	0.003	Palmitate(s) → Palmitate(c)
r0653	-0.003	Myristoyl-CoA(m) + Acetyl-CoA(m) ← CoA(m) + 3-Oxopalmitoyl-CoA(m)
r1446	0.003	Ubiquinone(m) + Butyryl-CoA(m) → Crotonyl-CoA(m) + Ubiquinol(m)
r1447	0.003	Ubiquinone(m) + Lauroyl-CoA(m) → (2E)-Dodecenoyl-CoA(m) + Ubiquinol(m)
r1448	0.003	Ubiquinone(m) + Octanoyl-CoA(m) → (2E)-Octenoyl-CoA(m) + Ubiquinol(m)
r1449	0.003	Ubiquinone(m) + Myristoyl-CoA(m) → (2E)-Tetradecenoyl-CoA(m) + Ubiquinol(m)
r0507	0.12	Ubiquinol(m) + 2 Ferricytochrome C(m) + 4 H+(PG)(m) → Ubiquinone(m) + 4 H+(PG)(c) + 2 Ferrocytochrome C(m)

Continued on next page

Proton-gradient(c) build up (41)– continued

ID	flux	reaction
r0508	-0.08	Ubiquinone(m) + NADH(m) + 4 H+(PG)(m) → Ubiquinol(m) + NAD+(m) + 4 H+(PG)(c)
r0522	-0.02	5-Formyl-THF(m) ← 5,10-Methenyl-THF(m) + H2O(m)
r0509	0.02	Ubiquinone(m) + Succinate(m) → Ubiquinol(m) + Fumarate(m)
r0523	0.02	ATP(m) + 5-Formyl-THF(m) → ADP(m) + Pi(m) + 5,10-Methenyl-THF(m)
r1330	1	H+(PG)(c) ⇌ H+(PG)(m) + Proton-gradient(m)
r0849	-0.04	H2O(s) ⇌ H2O(c)
r0287	-0.003	CoA(m) + 3-Oxohexanoyl-CoA(m) → Acetyl-CoA(m) + Butyryl-CoA(m)
r0714	0.003	NAD+(m) + (S)-3-Hydroxyhexadecanoyl-CoA(m) → 3-Oxopalmitoyl-CoA(m) + NADH(m)
r0867	0.04	CO2(c) ⇌ CO2(s)
r0868	-0.04	CO2(c) ⇌ CO2(m)
r0716	-0.003	(S)-3-Hydroxyhexadecanoyl-CoA(m) ← (2E)-Hexadecenoyl-CoA(m) + H2O(m)
r0435	0.003	Palmitoyl-CoA(c) + L-Carnitine(c) ⇌ CoA(c) + L-Palmitoylcarnitine(c)
r0730	0.003	(S)-Hydroxyoctanoyl-CoA(m) + NAD+(m) → 3-Oxooctanoyl-CoA(m) + NADH(m)
r0588	-0.003	Crotonyl-CoA(m) + H2O(m) ⇌ (S)-3-Hydroxybutyryl-CoA(m)
r0718	0.003	(S)-3-Hydroxytetradecanoyl-CoA(m) + NAD+(m) → 3-Oxotetradecanoyl-CoA(m) + NADH(m)
r0436	-0.003	Palmitoyl-CoA(m) + L-Carnitine(m) ← CoA(m) + L-Palmitoylcarnitine(m)
r0731	-0.003	(S)-Hydroxyoctanoyl-CoA(m) ← (2E)-Octenoyl-CoA(m) + H2O(m)
r0732	-0.003	Hexanoyl-CoA(m) + Acetyl-CoA(m) ← CoA(m) + 3-Oxooctanoyl-CoA(m)
r0733	0.003	(S)-Hydroxyhexanoyl-CoA(m) + NAD+(m) → 3-Oxohexanoyl-CoA(m) + NADH(m)
r0734	-0.003	(S)-Hydroxyhexanoyl-CoA(m) ← (2E)-Hexenoyl-CoA(m) + H2O(m)
r0007	0.003	PPi(c) + H2O(c) → 2 Pi(c)
r1114	0.02	AKG(m) + CoA(m) + NAD+(m) ⇌ NADH(m) + CO2(m) + Succinyl-CoA(m)
r1450	0.003	Ubiquinone(m) + Hexanoyl-CoA(m) → (2E)-Hexenoyl-CoA(m) + Ubiquinol(m)
r1451	0.003	Ubiquinone(m) + Decanoyl-CoA(m) → (2E)-Decenoyl-CoA(m) + Ubiquinol(m)
r0660	-0.003	(S)-3-Hydroxydodecanoyl-CoA(m) ⇌ (2E)-Dodecenoyl-CoA(m) + H2O(m)
r0083	0.02	Oxalosuccinate(m) ⇌ AKG(m) + CO2(m)
r0100	-0.02	ADP(m) + GTP(m) ⇌ ATP(m) + GDP(m)
r1047	0.01	Pi(c) + H+(PG)(c) → Pi(m) + H+(PG)(m)
r0105	0.02	NAD+(m) + Malate(m) ⇌ OAA(m) + NADH(m)
r2418	0.01	ADP(c) + ATP(m) → ADP(m) + ATP(c)
r0107	-0.02	Acetyl-CoA(m) + H2O(m) + OAA(m) ⇌ Citrate(m) + CoA(m)
r0259	-0.02	Fumarate(m) + H2O(m) ⇌ Malate(m)
r0851	0.04	H2O(m) ⇌ H2O(c)

Proton-gradient(c) waste (42)

Definition			Solution												
			exchanges		reactions								transp		
Objective	Constraints	Comment	imports	exports	c	m	r	p	l	n	s	b	s-c	b-c	int
-1 Proton-gradient(m)	−Palmitate $+CO_2$ $-O_2$ $=H_2O$	apical membrane Proton-gradient release, protons released from the sinusoid into the cytosol	1 Proton-gradient(m)		-	-	-	-	-	-	-	-	-	-	3

ID	flux	reaction
r1455	0.5	Phenylalanine(c) + H+(PG)(c) → Phenylalanine(m) + H+(PG)(m)
r1456	0.5	Phenylalanine(m) + H+(PG)(c) → Phenylalanine(c) + H+(PG)(m)
r1330	-1	H+(PG)(c) ⇌ H+(PG)(m) + Proton-gradient(m)

A2.3. Sodium gradients

Na+ importgradient (43)

Definition			Solution												
			exchanges		reactions								transp		
Objective	Constraints	Comment	imports	exports	c	m	r	p	l	n	s	b	s-c	b-c	int
Na+(s)	−Na+(c) −Palmitate $-O_2$ $+CO_2$ $+H_2O$	tests that natrium can be pumped out the cell	0.22 O_2(s) 0.01 Palmitate(s) 1 Na+(c)	0.15 H_2O(s) 0.15 CO_2(s) 1 Na+(s)	5	38	-	-	-	-	-	-	8	-	11

ID	flux	reaction
r2435	0.01	L-Carnitine(m) + L-Palmitoylcarnitine(c) → L-Carnitine(c) + L-Palmitoylcarnitine(m)
r0125	-0.08	GDP(m) + Pi(m) + Succinyl-CoA(m) ⇌ GTP(m) + Succinate(m) + CoA(m)
r0855	0.22	O2(s) ⇌ O2(c)
r0856	-0.22	O2(m) ⇌ O2(c)
r0720	-0.01	(S)-3-Hydroxytetradecanoyl-CoA(m) ← (2E)-Tetradecenoyl-CoA(m) + H2O(m)
r0425	0.08	Isocitrate(m) + NAD+(m) ⇌ Oxalosuccinate(m) + NADH(m)
r0722	0.01	(S)-3-Hydroxydodecanoyl-CoA(m) + NAD+(m) ⇌ 3-Oxododecanoyl-CoA(m) + NADH(m)
r0724	-0.01	Decanoyl-CoA(m) + Acetyl-CoA(m) ⇌ CoA(m) + 3-Oxododecanoyl-CoA(m)
r0726	0.01	(S)-Hydroxydecanoyl-CoA(m) + NAD+(m) ⇌ 3-Oxodecanoyl-CoA(m) + NADH(m)
r0728	-0.01	(S)-Hydroxydecanoyl-CoA(m) ⇌ (2E)-Decenoyl-CoA(m) + H2O(m)
r0460	0.01	(S)-3-Hydroxybutyryl-CoA(m) + NAD+(m) ⇌ NADH(m) + Acetoacetyl-CoA(m)
r0310	0.01	Palmitoyl-CoA(m) + Ubiquinone(m) → (2E)-Hexadecenoyl-CoA(m) + Ubiquinol(m)
r0017	0.22	O2(m) + 4 H+(PG)(m) + 4 Ferrocytochrome C(m) → 4 Ferricytochrome C(m) + 2 H2O(m) + 4 H+(PG)(c)
r0312	0.01	Palmitate(c) + ATP(c) + CoA(c) → Palmitoyl-CoA(c) + AMP(c) + PPi(c)
r0030	0.01	ATP(c) + AMP(c) ⇌ 2 ADP(c)
r0019	-0.94	ADP(m) + Pi(m) + 3 H+(PG)(c) → ATP(m) + H2O(m) + 3 H+(PG)(m)
r0316	0.08	Citrate(m) ⇌ Isocitrate(m)
r0070	-0.01	CoA(m) + Acetoacetyl-CoA(m) ⇌ 2 Acetyl-CoA(m)
r0634	-0.01	Acetyl-CoA(m) + Octanoyl-CoA(m) ⇌ CoA(m) + 3-Oxodecanoyl-CoA(m)
r0639	-0.01	Lauroyl-CoA(m) + Acetyl-CoA(m) ← CoA(m) + 3-Oxotetradecanoyl-CoA(m)
r0935	0.01	Palmitate(s) → Palmitate(c)
r0653	-0.01	Myristoyl-CoA(m) + Acetyl-CoA(m) ← CoA(m) + 3-Oxopalmitoyl-CoA(m)
r1446	0.01	Ubiquinone(m) + Butyryl-CoA(m) → Crotonyl-CoA(m) + Ubiquinol(m)
r1447	0.01	Ubiquinone(m) + Lauroyl-CoA(m) → (2E)-Dodecenoyl-CoA(m) + Ubiquinol(m)
r1448	0.01	Ubiquinone(m) + Octanoyl-CoA(m) → (2E)-Octenoyl-CoA(m) + Ubiquinol(m)
r2687	2	Taurine(c) ⇌ Taurine(s)
r1449	0.01	Ubiquinone(m) + Myristoyl-CoA(m) → (2E)-Tetradecenoyl-CoA(m) + Ubiquinol(m)
r0801	-0.08	GTP(c) + GDP(m) ⇌ GTP(m) + GDP(c)
r0507	0.44	Ubiquinol(m) + 2 Ferricytochrome C(m) + 4 H+(PG)(m) → Ubiquinone(m) + 4 H+(PG)(c) + 2 Ferrocytochrome C(m)
r0508	-0.3	Ubiquinone(m) + NADH(m) + 4 H+(PG)(m) → Ubiquinol(m) + NAD+(m) + 4 H+(PG)(c)
r0509	0.08	Ubiquinone(m) + Succinate(m) → Ubiquinol(m) + Fumarate(m)
r0099	-0.08	ADP(c) + GTP(c) ⇌ ATP(c) + GDP(c)
r1489	1	ATP(c) + H2O(c) + 3 Na+(c) + 2 K+(s) → ADP(c) + Pi(c) + 3 Na+(s) + 2 K+(c)
r0849	-0.15	H2O(s) ⇌ H2O(c)
r0287	-0.01	CoA(m) + 3-Oxohexanoyl-CoA(m) → Acetyl-CoA(m) + Butyryl-CoA(m)
r0714	0.01	NAD+(m) + (S)-3-Hydroxyhexadecanoyl-CoA(m) → 3-Oxopalmitoyl-CoA(m) + NADH(m)
r0867	0.15	CO2(c) ⇌ CO2(s)
r0868	-0.15	CO2(c) ⇌ CO2(m)
r0716	-0.01	(S)-3-Hydroxyhexadecanoyl-CoA(m) ← (2E)-Hexadecenoyl-CoA(m) + H2O(m)
r0435	0.01	Palmitoyl-CoA(c) + L-Carnitine(c) ⇌ CoA(c) + L-Palmitoylcarnitine(c)
r0730	0.01	(S)-Hydroxyoctanoyl-CoA(m) + NAD+(m) → 3-Oxooctanoyl-CoA(m) + NADH(m)
r0588	-0.01	Crotonyl-CoA(m) + H2O(m) ⇌ (S)-3-Hydroxybutyryl-CoA(m)
r0718	0.01	(S)-3-Hydroxytetradecanoyl-CoA(m) + NAD+(m) → 3-Oxotetradecanoyl-CoA(m) + NADH(m)
r0436	-0.01	Palmitoyl-CoA(m) + L-Carnitine(m) ← CoA(m) + L-Palmitoylcarnitine(m)
r0731	-0.01	(S)-Hydroxyoctanoyl-CoA(m) ← (2E)-Octenoyl-CoA(m) + H2O(m)
r0732	-0.01	Hexanoyl-CoA(m) + Acetyl-CoA(m) ← CoA(m) + 3-Oxooctanoyl-CoA(m)
r0733	0.01	(S)-Hydroxyhexanoyl-CoA(m) + NAD+(m) → 3-Oxohexanoyl-CoA(m) + NADH(m)

Continued on next page

Na+ importgradient (43)– continued

ID	flux	reaction
r0734	-0.01	(S)-Hydroxyhexanoyl-CoA(m) ← (2E)-Hexenoyl-CoA(m) + H2O(m)
r1540	2	Taurine(s) + Na+(s) + Chloride(s) → Taurine(c) + Na+(c) + Chloride(c)
r0007	0.01	PPi(c) + H2O(c) → 2 Pi(c)
r1114	0.08	AKG(m) + CoA(m) + NAD+(m) ⇌ NADH(m) + CO2(m) + Succinyl-CoA(m)
r2074	2	K+(c) + Chloride(c) → K+(s) + Chloride(s)
r1450	0.01	Ubiquinone(m) + Hexanoyl-CoA(m) → (2E)-Hexenoyl-CoA(m) + Ubiquinol(m)
r1451	0.01	Ubiquinone(m) + Decanoyl-CoA(m) → (2E)-Decenoyl-CoA(m) + Ubiquinol(m)
r0660	-0.01	(S)-3-Hydroxydodecanoyl-CoA(m) ⇌ (2E)-Dodecenoyl-CoA(m) + H2O(m)
r0083	0.08	Oxalosuccinate(m) ⇌ AKG(m) + CO2(m)
r1047	1.02	Pi(c) + H+(PG)(c) → Pi(m) + H+(PG)(m)
r0105	0.08	NAD+(m) + Malate(m) ⇌ OAA(m) + NADH(m)
r2418	0.94	ADP(c) + ATP(m) → ADP(m) + ATP(c)
r0107	-0.08	Acetyl-CoA(m) + H2O(m) + OAA(m) ⇌ Citrate(m) + CoA(m)
r0259	-0.08	Fumarate(m) + H2O(m) ⇌ Malate(m)
r0851	1.16	H2O(m) ⇌ H2O(c)

Na+ exportgradient (44)

Definition			Solution												
			exchanges		reactions								transp		
Objective	Constraints	Comment	imports	exports	c	m	r	p	l	n	s	b	s-c	b-c	int
Na+(c)	−Na+(s) −Palmitate $-O_2$ $+CO_2$ $+H_2O$	tests that natrium can be pumped into of the cell	1 Na+(s)	1 Na+(c)	-	-	-	-	-	-	-	-	2	-	-

ID	flux	reaction
r1423	0.33	Pi(c) → Pi(s)
r0861	0.33	Pi(s) + 3 Na+(s) → Pi(c) + 3 Na+(c)

2.3 A3. Small groups activated

A3.1. Methyl(ene) group

Activated methyl group (SAM) (45)

Definition			Solution												
			exchanges		reactions								transp		
Objective	Constraints	Comment	imports	exports	c	m	r	p	l	n	s	b	s-c	b-c	int
Activated methyl group(c)	−Serine +Glycine −ATP-energy(c) =NADPH-redox-potential(c) $=H_2O$	de novo synthesis of cytosolic Activated methyl group	1 Serine(s) 3 ATP-energy(c) 1 NADPH-redox-potential(c)	1 H_2O(s) 1 Glycine(s) 1 Activated methyl group(c)	11	-	-	-	-	-	-	-	2	-	-

ID	flux	reaction
r1326	-1	NADPH(c) ⇌ NADPH-redox-potential(c) + NADP+(c)
r0048	1	ATP(c) + Adenosine(c) ⇌ ADP(c) + AMP(c)
r1319	-3	ATP(c) + H2O(c) ⇌ ADP(c) + Pi(c) + ATP-energy(c)
r1545	-1	Glycine(c) + Serine(s) ⇌ Glycine(s) + Serine(c)
r0030	1	ATP(c) + AMP(c) ⇌ 2 ADP(c)
r0296	-1	5,10-Methylene-THF(c) + NADPH(c) ⇌ 5-Methyl-THF(c) + NADP+(c)

Continued on next page

Activated methyl group (SAM) (45)– continued

ID	flux	reaction
r0230	-1	THF(c) + Serine(c) ⇌ 5,10-Methylene-THF(c) + Glycine(c) + H2O(c)
r0042	-1	ATP(c) + Methionine(c) + H2O(c) ⇌ Pi(c) + PPi(c) + SAM(c)
r0232	1	5-Methyl-THF(c) + Homocysteine(c) ⇌ THF(c) + Methionine(c)
r0007	1	PPi(c) + H2O(c) → 2 Pi(c)
r0849	-1	H2O(s) ⇌ H2O(c)
r0800	1	SAM(c) ⇌ SAH(c) + Activated methyl group(c)
r0053	1	SAH(c) + H2O(c) ⇌ Adenosine(c) + Homocysteine(c)

Activated methyl group (THF) (46)

Definition			Solution											
			exchanges		reactions								transp		
Objective	Constraints	Comment	imports	exports	c	m	r	p	l	n	s	b	s-c	b-c	int
THF-activated methyl group(c)	−Serine +Glycine −ATP-energy(c) =NADPH-redox-potential(c) $=H_2O$	de novo synthesis of cytosolic Activated methyl group	1 Serine(s) 1 NADPH-redox-potential(c)	1 H_2O(s) 1 Glycine(s) 1 THF-activated methyl group(c)	4	-	-	-	-	-	-	-	2	-	-

ID	flux	reaction
r1326	-1	NADPH(c) ⇌ NADPH-redox-potential(c) + NADP+(c)
r1545	-1	Glycine(c) + Serine(s) ⇌ Glycine(s) + Serine(c)
r0296	-1	5,10-Methylene-THF(c) + NADPH(c) ⇌ 5-Methyl-THF(c) + NADP+(c)
r0230	-1	THF(c) + Serine(c) ⇌ 5,10-Methylene-THF(c) + Glycine(c) + H2O(c)
r2676	1	5-Methyl-THF(c) ⇌ THF(c) + THF-activated methyl group(c)
r0849	-1	H2O(s) ⇌ H2O(c)

Activated methylene group from Try (47)

Definition			Solution											
			exchanges		reactions								transp		
Objective	Constraints	Comment	imports	exports	c	m	r	p	l	n	s	b	s-c	b-c	int
THF-activated methylene group(m)	−Tryptophan +Urea −Palmitate $+CO_2$ $-O_2$ $=H_2O$	de novo synthesis of mitochondrial Activated methyl group	10.5 O_2(s) 1 Tryptophan(s)	4 H_2O(s) 9 CO_2(s) 1 Urea(s) 1 THF-activated methylene group(m)	30	23	-	-	-	-	-	-	5	-	18

ID	flux	reaction
r0125	-3	GDP(m) + Pi(m) + Succinyl-CoA(m) ⇌ GTP(m) + Succinate(m) + CoA(m)
r0855	10.5	O2(s) ⇌ O2(c)
r0856	-3.13	O2(m) ⇌ O2(c)
r0422	3	Isocitrate(c) + NADP+(c) ⇌ Oxalosuccinate(c) + NADPH(c)
r0292	-1	5,10-Methenyl-THF(m) + NADH(m) ⇌ 5,10-Methylene-THF(m) + NAD+(m)
r0872	6.75	Pyruvate(c) + H+(PG)(c) → Pyruvate(m) + H+(PG)(m)
r0144	1	Arginine(c) + H2O(c) ⇌ Urea(c) + Ornithine(c)
r1385	-8	NADPH(m) + NAD+(m) + H+(PG)(m) ← NADH(m) + NADP+(m) + H+(PG)(c)
r0447	4.37	Sphingosine(c) + ATP(c) → Sphingosine-1P(c) + ADP(c)
r0460	1	(S)-3-Hydroxybutyryl-CoA(m) + NAD+(m) ⇌ NADH(m) + Acetoacetyl-CoA(m)
r1239	-4.37	Sphingosine(c) + Acyl-CoA-VLDL-SM-pool(c) ← CoA(c) + Ceramide-pool(c)
r0448	1	2-Oxoadipate(m) + CoA(m) + NAD+(m) ⇌ Glutaryl-CoA(m) + CO2(m) + NADH(m)
r0896	1	Urea(c) ⇌ Urea(s)

Continued on next page

Activated methylene group from Try (47)– continued

ID	flux	reaction
r0449	-1	2-Oxoadipate(c) + NH3(c) + NADP+(c) ⇌ 2-Aminomuconate(c) + NADPH(c) + H2O(c)
r0017	3.13	O2(m) + 4 H+(PG)(m) + 4 Ferrocytochrome C(m) → 4 Ferricytochrome C(m) + 2 H2O(m) + 4 H+(PG)(c)
r0030	1	ATP(c) + AMP(c) ⇌ 2 ADP(c)
r0169	1	Tryptophan(c) + O2(c) ⇌ L-Formylkynurenine(c)
r0019	-6.38	ADP(m) + Pi(m) + 3 H+(PG)(c) → ATP(m) + H2O(m) + 3 H+(PG)(m)
r0034	1	2 ATP(m) + CO2(m) + H2O(m) + NH3(m) ⇌ 2 ADP(m) + Pi(m) + Carbamoyl-P(m)
r0316	3	Citrate(m) ⇌ Isocitrate(m)
r0633	-2.25	(2E)-Octenoyl-CoA(m) + NADPH(m) ⇌ Octanoyl-CoA(m) + NADP+(m)
r0056	1	Pyruvate(m) + CoA(m) + NAD+(m) → Acetyl-CoA(m) + CO2(m) + NADH(m)
r0786	4.37	Sphingosine-1P(c) → Ethanolamine-P(c) + Hexadecenal(c)
r0070	-1	CoA(m) + Acetoacetyl-CoA(m) ⇌ 2 Acetyl-CoA(m)
r0787	4.37	H2O(c) + Sphinganine-1P(c) → Sphinganine(c) + Pi(c)
r0058	5.75	Malate(c) + NADP+(c) ⇌ Pyruvate(c) + CO2(c) + NADPH(c)
r0059	-5.75	Pyruvate(m) + CO2(m) + NADPH(m) ⇌ Malate(m) + NADP+(m)
r1147	3	AKG(c) + Isocitrate(m) ⇌ AKG(m) + Isocitrate(c)
r0372	-1	10-Formyl-THF(m) → 5,10-Methenyl-THF(m) + H2O(m)
r1448	2.25	Ubiquinone(m) + Octanoyl-CoA(m) → (2E)-Octenoyl-CoA(m) + Ubiquinol(m)
r0507	6.25	Ubiquinol(m) + 2 Ferricytochrome C(m) + 4 H+(PG)(m) → Ubiquinone(m) + 4 H+(PG)(c) + 2 Ferrocytochrome C(m)
r0509	3	Ubiquinone(m) + Succinate(m) → Ubiquinol(m) + Fumarate(m)
r0676	1	2-Amino-3-carboxymuconate semialdehyde(c) ⇌ 2-Aminomuconate semialdehyde(c) + CO2(c)
r0229	1	THF(m) + Formate(m) + ATP(m) → ADP(m) + Pi(m) + 10-Formyl-THF(m)
r0820	1	AKG(c) + Malate(m) ⇌ AKG(m) + Malate(c)
r0541	1	Glutaryl-CoA(m) + Ubiquinone(m) ⇌ Crotonyl-CoA(m) + CO2(m) + Ubiquinol(m)
r0261	1	Argininosuccinate(c) ⇌ Fumarate(c) + Arginine(c)
r0562	1	3-Hydroxyanthranilate(c) + O2(c) ⇌ 2-Amino-3-carboxymuconate semialdehyde(c)
r0563	1	H2O(c) + 3-Hydroxy-L-kynurenine(c) ⇌ 3-Hydroxyanthranilate(c) + Alanine(c)
r0849	-4	H2O(s) ⇌ H2O(c)
r0867	9	CO2(c) ⇌ CO2(s)
r0868	0.75	CO2(c) ⇌ CO2(m)
r0588	-1	Crotonyl-CoA(m) + H2O(m) ⇌ (S)-3-Hydroxybutyryl-CoA(m)
r0453	1	ATP(c) + Citrulline(c) + Aspartate(c) ⇌ AMP(c) + PPi(c) + Argininosuccinate(c)
r0888	1	Formate(c) → Formate(m)
r0007	1	PPi(c) + H2O(c) → 2 Pi(c)
r0454	1	L-Formylkynurenine(c) + H2O(c) ⇌ Formate(c) + Kynurenine(c)
r0455	1	Kynurenine(c) + O2(c) + NADPH(c) ⇌ H2O(c) + 3-Hydroxy-L-kynurenine(c) + NADP+(c)
r0304	-2	Proline(c) + NAD+(c) ⇌ 1-Pyrroline-5-carboxylate(c) + NADH(c)
r0305	2	Proline(c) + NADP+(c) ⇌ 1-Pyrroline-5-carboxylate(c) + NADPH(c)
r1114	3	AKG(m) + CoA(m) + NAD+(m) ⇌ NADH(m) + CO2(m) + Succinyl-CoA(m)
r1412	4.37	Sphinganine(c) + Acyl-CoA-VLDL-SM-pool(c) ⇌ Dihydroceramide-pool(c) + CoA(c)
r1414	4.37	Dihydroceramide-pool(c) + O2(c) + NADPH(c) ⇌ 2 H2O(c) + Ceramide-pool(c) + NADP+(c)
r0329	1	Carbamoyl-P(m) + Ornithine(m) ⇌ Pi(m) + Citrulline(m)
r0080	1	Alanine(c) + AKG(c) ⇌ Pyruvate(c) + Glutamate(c)
r0645	1	2-Aminomuconate semialdehyde(c) + NAD+(c) + H2O(c) ⇌ 2-Aminomuconate(c) + NADH(c)
r2675	1	5,10-Methylene-THF(m) ⇌ THF(m) + THF-activated methylene group(m)
r0082	3	Oxalosuccinate(c) ⇌ AKG(c) + CO2(c)
r2396	-1	Lysine(c) + Ornithine(m) + H+(PG)(m) ← Lysine(m) + Ornithine(c) + H+(PG)(c)
r2527	1	Tryptophan(s) ⇌ Tryptophan(c)
r0945	1	2-Oxoadipate(c) + AKG(m) ⇌ 2-Oxoadipate(m) + AKG(c)
r1457	-4.37	Hexadecanal(c) + NADP+(c) ← Hexadecenal(c) + NADPH(c)
r2410	-1	Citrulline(c) + Lysine(m) + H+(PG)(m) ← Citrulline(m) + Lysine(c) + H+(PG)(c)
r0100	-3	ADP(m) + GTP(m) ⇌ ATP(m) + GDP(m)
r0831	-4.75	Malate(c) + Pi(m) ⇌ Malate(m) + Pi(c)
r0537	-4.37	Sphinganine-1P(c) ← Ethanolamine-P(c) + Hexadecanal(c)
r1047	1.63	Pi(c) + H+(PG)(c) → Pi(m) + H+(PG)(m)
r0104	1	NAD+(c) + Malate(c) ⇌ OAA(c) + NADH(c)
r0105	3	NAD+(m) + Malate(m) ⇌ OAA(m) + NADH(m)

Continued on next page

Activated methylene group from Try (47)– continued

ID	flux	reaction
r0258	-1	Malate(c) ⇌ Fumarate(c) + H2O(c)
r2418	6.38	ADP(c) + ATP(m) → ADP(m) + ATP(c)
r0107	-3	Acetyl-CoA(m) + H2O(m) + OAA(m) ⇌ Citrate(m) + CoA(m)
r0259	-3	Malate(m) ⇌ Fumarate(m) + H2O(m)
r0851	5.63	H2O(m) ⇌ H2O(c)
r0838	1	NH3(c) ⇌ NH3(m)
r0109	-1	OAA(c) + Glutamate(c) ⇌ AKG(c) + Aspartate(c)

Activated methyl group from Histidine (48)

Definition			Solution												
			exchanges		reactions								transp		
Objective	Constraints	Comment	imports	exports	c	m	r	p	l	n	s	b	s-c	b-c	int
THF-activated methyl group(c)	−Histidine +Urea −ATP-energy(c) =NADH-redox-potential(c) =NADPH-redox-potential(c) $=H_2O$ $-O_2$ $+CO_2$ $-NH_3$ %r1414	de novo synthesis of cytosolic Activated methyl group	5.34 H_2O(s) 0.58 O_2(s) 1 Histidine(s) 4.92 ATP-energy(c)	3.5 CO_2(s) 1.5 Urea(s) 0.5 NADH-redox-potential(c) 5.34 NADPH-redox-potential(c) 1 THF-activated methyl group(c)	41	12	-	-	-	-	-	-	6	-	15

ID	flux	reaction
r0406	0.96	Sedoheptulose-7P(c) + GAP(c) ⇌ Fructose-6P(c) + Erythrose-4P(c)
r0125	-1.04	GDP(m) + Pi(m) + Succinyl-CoA(m) ⇌ GTP(m) + Succinate(m) + CoA(m)
r0855	0.58	O2(s) ⇌ O2(c)
r0856	-0.58	O2(m) ⇌ O2(c)
r0422	0.04	Isocitrate(c) + NADP+(c) ⇌ Oxalosuccinate(c) + NADPH(c)
r0293	-1	5,10-Methenyl-THF(c) + NADPH(c) ⇌ 5,10-Methylene-THF(c) + NADP+(c)
r0872	0.54	Pyruvate(c) + H+(PG)(c) → Pyruvate(m) + H+(PG)(m)
r0577	-1	Urocanate(c) + H2O(c) ⇌ 4-Imidazolone-5-propanoate(c)
r0144	1.5	Arginine(c) + H2O(c) ⇌ Urea(c) + Ornithine(c)
r1385	-0.5	NADPH(m) + NAD+(m) + H+(PG)(m) ← NADH(m) + NADP+(m) + H+(PG)(c)
r0164	-0.96	PEP(c) + H2O(c) ⇌ 2PG(c)
r0896	1.5	Urea(c) ⇌ Urea(s)
r0017	0.58	O2(m) + 4 H+(PG)(m) + 4 Ferrocytochrome C(m) → 4 Ferricytochrome C(m) + 2 H2O(m) + 4 H+(PG)(c)
r0030	1.5	ATP(c) + AMP(c) ⇌ 2 ADP(c)
r0019	-1.96	ADP(m) + Pi(m) + 3 H+(PG)(c) → ATP(m) + H2O(m) + 3 H+(PG)(m)
r0315	0.04	Citrate(c) ⇌ Isocitrate(c)
r0034	1.5	2 ATP(m) + CO2(m) + H2O(m) + NH3(m) ⇌ 2 ADP(m) + Pi(m) + Carbamoyl-P(m)
r0911	0.5	Proline(c) + Glutamate(m) ⇌ Proline(m) + Glutamate(c)
r0487	0.96	Fructose-1,6PP(c) + H2O(c) → Fructose-6P(c) + Pi(c)
r0337	0.96	ATP(c) + 3PG(c) ⇌ ADP(c) + 1,3DPG(c)
r0056	0.04	Pyruvate(m) + CoA(m) + NAD+(m) → Acetyl-CoA(m) + CO2(m) + NADH(m)
r1426	0.5	Proline(m) + H+(PG)(c) → Proline(c) + H+(PG)(m)
r0058	0.54	Malate(c) + NADP+(c) ⇌ Pyruvate(c) + CO2(c) + NADPH(c)
r0059	-0.5	Pyruvate(m) + CO2(m) + NADPH(m) ⇌ Malate(m) + NADP+(m)
r0073	-0.5	AKG(m) + NADH(m) + NH3(m) ⇌ Glutamate(m) + NAD+(m) + H2O(m)
r0370	-0.96	Sedoheptulose-7P(c) + GAP(c) ⇌ Ribose-5P(c) + D-Xylulose-5P(c)
r0507	1.16	Ubiquinol(m) + 2 Ferricytochrome C(m) + 4 H+(PG)(m) → Ubiquinone(m) + 4 H+(PG)(c) + 2 Ferrocytochrome C(m)
r0508	-0.12	Ubiquinone(m) + NADH(m) + 4 H+(PG)(m) → Ubiquinol(m) + NAD+(m) + 4 H+(PG)(c)
r0509	1.04	Ubiquinone(m) + Succinate(m) → Ubiquinol(m) + Fumarate(m)
r0524	1	5-Formiminotetrahydrofolate(c) ⇌ 5,10-Methenyl-THF(c) + NH3(c)

Continued on next page

ID	flux	reaction
r0099	0.96	ATP(c) + GDP(c) ⇌ ADP(c) + GTP(c)
r0820	1.5	AKG(c) + Malate(m) ⇌ AKG(m) + Malate(c)
r0243	0.96	GAP(c) ⇌ DHAP(c)
r1319	-4.92	ATP(c) + H2O(c) ⇌ ADP(c) + Pi(c) + ATP-energy(c)
r0261	1.5	Argininosuccinate(c) ⇌ Fumarate(c) + Arginine(c)
r0849	5.34	H2O(s) ⇌ H2O(c)
r0286	1	Histidine(c) ⇌ Urocanate(c) + NH3(c)
r1671	1	Histidine(s) + Citrulline(c) ⇌ Histidine(c) + Citrulline(s)
r0867	3.5	CO2(c) ⇌ CO2(s)
r0868	0.92	CO2(c) ⇌ CO2(m)
r0453	1.5	ATP(c) + Citrulline(c) + Aspartate(c) ⇌ AMP(c) + PPi(c) + Argininosuccinate(c)
r0007	1.5	PPi(c) + H2O(c) → 2 Pi(c)
r0192	-2.88	Fructose-6P(c) ⇌ Glucose-6P(c)
r1114	1.04	AKG(m) + CoA(m) + NAD+(m) ⇌ NADH(m) + CO2(m) + Succinyl-CoA(m)
r0479	2.88	Glucono-1,5-lactone-6P(c) + H2O(c) ⇌ 6-Phospho-D-gluconate(c)
r0341	0.96	2PG(c) ⇌ 3PG(c)
r0199	2.88	Glucose-6P(c) + NADP+(c) ⇌ NADPH(c) + Glucono-1,5-lactone-6P(c)
r0329	1.5	Carbamoyl-P(m) + Ornithine(m) ⇌ Pi(m) + Citrulline(m)
r0343	2.88	6-Phospho-D-gluconate(c) + NADP+(c) ⇌ NADPH(c) + CO2(c) + Ribulose-5P(c)
r2373	0.04	Citrate(m) + AKG(c) ⇌ Citrate(c) + AKG(m)
r0344	1.92	Ribulose-5P(c) ⇌ D-Xylulose-5P(c)
r0792	-1	5,10-Methylene-THF(c) + NADH(c) ⇌ 5-Methyl-THF(c) + NAD+(c)
r0082	0.04	Oxalosuccinate(c) ⇌ AKG(c) + CO2(c)
r2676	1	5-Methyl-THF(c) ⇌ THF(c) + THF-activated methyl group(c)
r0947	-1.5	Citrulline(m) + Ornithine(c) ⇌ Citrulline(c) + Ornithine(m)
r1322	0.5	NADH(c) ⇌ NAD+(c) + NADH-redox-potential(c)
r0518	-1	Glutamate(c) + 5-Formiminotetrahydrofolate(c) ⇌ THF(c) + N-Formimino-L-glutamate(c)
r0519	1	H2O(c) + 4-Imidazolone-5-propanoate(c) ⇌ N-Formimino-L-glutamate(c)
r0250	-0.96	Ribulose-5P(c) ⇌ Ribose-5P(c)
r0100	-1.04	ATP(m) + GDP(m) ⇌ ADP(m) + GTP(m)
r1326	5.34	NADPH(c) ⇌ NADPH-redox-potential(c) + NADP+(c)
r0817	-1	Citrulline(s) ⇌ Citrulline(c)
r0253	-0.96	1,3DPG(c) + NADH(c) ⇌ GAP(c) + Pi(c) + NAD+(c)
r0255	-0.96	Fructose-6P(c) + GAP(c) ⇌ D-Xylulose-5P(c) + Erythrose-4P(c)
r0104	2.46	NAD+(c) + Malate(c) ⇌ OAA(c) + NADH(c)
r0256	-0.96	DHAP(c) + GAP(c) ⇌ Fructose-1,6PP(c)
r0105	0.04	NAD+(m) + Malate(m) ⇌ OAA(m) + NADH(m)
r0258	-1.5	Malate(c) ⇌ Fumarate(c) + H2O(c)
r0107	-0.04	Acetyl-CoA(m) + H2O(m) + OAA(m) ⇌ Citrate(m) + CoA(m)
r0259	-1.04	Malate(m) ⇌ Fumarate(m) + H2O(m)
r0851	1.04	H2O(m) ⇌ H2O(c)
r0838	2	NH3(c) ⇌ NH3(m)
r0109	-1.5	OAA(c) + Glutamate(c) ⇌ AKG(c) + Aspartate(c)
r0123	0.96	GTP(c) + OAA(c) → GDP(c) + PEP(c) + CO2(c)

A3.2. Formyl group

Formylgroup(c) (49)

Definition			Solution												
			exchanges		reactions								transp		
Objective	Constraints	Comment	imports	exports	c	m	r	p	l	n	s	b	s-c	b-c	int
THF-activated formyl group(c)	−Serine +Glycine −ATP-energy(c) =NADPH-redox-potential(c) =H_2O	test that the methyl group on THF can be regenerated from the minimal exchange set	1 Serine(s)	1 Glycine(s) 1 NADPH-redox-potential(c) 1 THF-activated formyl group(c)	5	-	-	-	-	-	-	-	1	-	-

ID	flux	reaction
r0371	1	5,10-Methenyl-THF(c) + H2O(c) ⇌ 10-Formyl-THF(c)
r1326	1	NADPH(c) ⇌ NADPH-redox-potential(c) + NADP+(c)
r0293	1	5,10-Methylene-THF(c) + NADP+(c) ⇌ 5,10-Methenyl-THF(c) + NADPH(c)
r1545	-1	Glycine(c) + Serine(s) ⇌ Glycine(s) + Serine(c)
r0230	-1	THF(c) + Serine(c) ⇌ 5,10-Methylene-THF(c) + Glycine(c) + H2O(c)
r2674	1	10-Formyl-THF(c) ⇌ THF(c) + THF-activated formyl group(c)

Formylgroup(m) (50)

Definition			Solution												
			exchanges		reactions								transp		
Objective	Constraints	Comment	imports	exports	c	m	r	p	l	n	s	b	s-c	b-c	int
THF-activated formyl group(m)	−Serine +Glycine −ATP-energy(c) =NADPH-redox-potential(c) =H_2O +Urea −Palmitate −O_2 +CO_2 +Glutamine(c) +Glutamate(c) −Tryptophan(c)	test that the methyl group on THF can be regenerated from the minimal exchange set	1.8 H_2O(s) 3.6 O_2(s) 1 Serine(s) 1 Tryptophan(c) 1 ATP-energy(c)	3 CO_2(s) 1 Glutamate(c) 1 Glutamine(c) 1.8 NADPH-redox-potential(c) 1 THF-activated formyl group(m)	17	16	-	-	-	-	-	-	4	-	15

ID	flux	reaction
r0855	3.6	O2(s) ⇌ O2(c)
r0856	-0.6	O2(m) ⇌ O2(c)
r0422	2.8	Isocitrate(c) + NADP+(c) ⇌ Oxalosuccinate(c) + NADPH(c)
r0872	2	Pyruvate(c) + H+(PG)(c) → Pyruvate(m) + H+(PG)(m)
r0425	-0.8	Isocitrate(m) + NAD+(m) ⇌ Oxalosuccinate(m) + NADH(m)
r1385	-2	NADPH(m) + NAD+(m) + H+(PG)(m) ← NADH(m) + NADP+(m) + H+(PG)(c)
r0460	1	(S)-3-Hydroxybutyryl-CoA(m) + NAD+(m) ⇌ NADH(m) + Acetoacetyl-CoA(m)
r0448	1	2-Oxoadipate(m) + CoA(m) + NAD+(m) ⇌ Glutaryl-CoA(m) + CO2(m) + NADH(m)
r0449	-1	2-Oxoadipate(c) + NH3(c) + NADP+(c) ⇌ 2-Aminomuconate(c) + NADPH(c) + H2O(c)
r0017	0.6	O2(m) + 4 H+(PG)(m) + 4 Ferrocytochrome C(m) → 4 Ferricytochrome C(m) + 2 H2O(m) + 4 H+(PG)(c)
r0169	1	Tryptophan(c) + O2(c) ⇌ L-Formylkynurenine(c)
r0019	-1	ADP(m) + Pi(m) + 3 H+(PG)(c) → ATP(m) + H2O(m) + 3 H+(PG)(m)
r0316	2	Citrate(m) ⇌ Isocitrate(m)
r0911	1	Proline(c) + Glutamate(m) ⇌ Proline(m) + Glutamate(c)
r0070	-1	CoA(m) + Acetoacetyl-CoA(m) ⇌ 2 Acetyl-CoA(m)
r1426	1	Proline(m) + H+(PG)(c) → Proline(c) + H+(PG)(m)
r0059	-2	Pyruvate(m) + CO2(m) + NADPH(m) ⇌ Malate(m) + NADP+(m)

Continued on next page

Formylgroup(m) (50)– continued

ID	flux	reaction
r0073	-1	AKG(m) + NADH(m) + NH3(m) ⇌ Glutamate(m) + NAD+(m) + H2O(m)
r1147	2.8	AKG(c) + Isocitrate(m) ⇌ AKG(m) + Isocitrate(c)
r0077	1	ATP(c) + Glutamate(c) + NH3(c) → ADP(c) + Pi(c) + Glutamine(c)
r0372	-1	10-Formyl-THF(m) → 5,10-Methenyl-THF(m) + H2O(m)
r0507	1.2	Ubiquinol(m) + 2 Ferricytochrome C(m) + 4 H+(PG)(m) → Ubiquinone(m) + 4 H+(PG)(c) + 2 Ferrocytochrome C(m)
r0508	-0.2	Ubiquinone(m) + NADH(m) + 4 H+(PG)(m) → Ubiquinol(m) + NAD+(m) + 4 H+(PG)(c)
r0522	-1	5-Formyl-THF(m) ← 5,10-Methenyl-THF(m) + H2O(m)
r0676	1	2-Amino-3-carboxymuconate semialdehyde(c) ⇌ 2-Aminomuconate semialdehyde(c) + CO2(c)
r0229	1	THF(m) + Formate(m) + ATP(m) → ADP(m) + Pi(m) + 10-Formyl-THF(m)
r0397	1	Dihydrobiopterin(c) + NADH(c) ⇌ Tetrahydrobiopterin(c) + NAD+(c)
r1319	-1	ATP(c) + H2O(c) ⇌ ADP(c) + Pi(c) + ATP-energy(c)
r0398	-1	Dihydrobiopterin(c) + NADPH(c) ⇌ Tetrahydrobiopterin(c) + NADP+(c)
r0541	1	Glutaryl-CoA(m) + Ubiquinone(m) ⇌ Crotonyl-CoA(m) + CO2(m) + Ubiquinol(m)
r0562	1	3-Hydroxyanthranilate(c) + O2(c) ⇌ 2-Amino-3-carboxymuconate semialdehyde(c)
r0563	1	H2O(c) + 3-Hydroxy-L-kynurenine(c) ⇌ 3-Hydroxyanthranilate(c) + Alanine(c)
r0849	1.8	H2O(s) ⇌ H2O(c)
r0867	3	CO2(c) ⇌ CO2(s)
r0868	0.8	CO2(c) ⇌ CO2(m)
r0588	-1	Crotonyl-CoA(m) + H2O(m) ⇌ (S)-3-Hydroxybutyryl-CoA(m)
r0888	1	Formate(c) → Formate(m)
r0454	1	L-Formylkynurenine(c) + H2O(c) ⇌ Formate(c) + Kynurenine(c)
r0455	1	Kynurenine(c) + O2(c) + NADPH(c) ⇌ H2O(c) + 3-Hydroxy-L-kynurenine(c) + NADP+(c)
r0060	1	Serine(c) → Pyruvate(c) + NH3(c)
r2673	1	5-Formyl-THF(m) ⇌ THF(m) + THF-activated formyl group(m)
r0080	1	Alanine(c) + AKG(c) ⇌ Pyruvate(c) + Glutamate(c)
r0645	1	2-Aminomuconate semialdehyde(c) + NAD+(c) + H2O(c) ⇌ 2-Aminomuconate(c) + NADH(c)
r0082	2.8	Oxalosuccinate(c) ⇌ AKG(c) + CO2(c)
r0083	-0.8	Oxalosuccinate(m) ⇌ AKG(m) + CO2(m)
r2526	1	Serine(s) ⇌ Serine(c)
r0945	1	2-Oxoadipate(c) + AKG(m) ⇌ 2-Oxoadipate(m) + AKG(c)
r1326	1.8	NADPH(c) ⇌ NADPH-redox-potential(c) + NADP+(c)
r0105	2	NAD+(m) + Malate(m) ⇌ OAA(m) + NADH(m)
r0107	-2	Acetyl-CoA(m) + H2O(m) + OAA(m) ⇌ Citrate(m) + CoA(m)
r0838	1	NH3(c) ⇌ NH3(m)
r0851	0.2	H2O(m) ⇌ H2O(c)

A3.3. Acetyl group

Acetyl group(c) (51)

Definition			Solution												
			exchanges		reactions								transp		
Objective	Constraints	Comment	imports	exports	c	m	r	p	l	n	s	b	s-c	b-c	int
CoA-activated acetyl group(c)	−Palmitate −O_2 =H_2O =Proton-gradient(m) +ATP-energy(m) =ATP-energy(c) =NADH-redox-potential(m)	Regeneration of Acetyl-CoA minus CoA	0.44 O_2(s) 0.13 Palmitate(s) 2.25 ATP-energy(c)	0.13 H_2O(s) 0.88 NADH-redox-potential(m) 5.25 Proton-gradient(m) 1 CoA-activated acetyl group(c)	7	31	-	-	-	-	-	-	3	-	7

ID	flux	reaction
r2435	0.13	L-Carnitine(m) + L-Palmitoylcarnitine(c) → L-Carnitine(c) + L-Palmitoylcarnitine(m)

Continued on next page

Acetyl group(c) (51)– continued

ID	flux	reaction
r0855	0.44	O2(s) ⇌ O2(c)
r0856	-0.44	O2(m) ⇌ O2(c)
r0720	-0.13	(S)-3-Hydroxytetradecanoyl-CoA(m) ← (2E)-Tetradecenoyl-CoA(m) + H2O(m)
r1087	-1	Acetate(c) ⇌ Acetate(m)
r0722	0.13	(S)-3-Hydroxydodecanoyl-CoA(m) + NAD+(m) ⇌ 3-Oxododecanoyl-CoA(m) + NADH(m)
r0724	-0.13	Decanoyl-CoA(m) + Acetyl-CoA(m) ⇌ CoA(m) + 3-Oxododecanoyl-CoA(m)
r0726	0.13	(S)-Hydroxydecanoyl-CoA(m) + NAD+(m) ⇌ 3-Oxodecanoyl-CoA(m) + NADH(m)
r0728	-0.13	(S)-Hydroxydecanoyl-CoA(m) ⇌ (2E)-Decenoyl-CoA(m) + H2O(m)
r0460	0.13	(S)-3-Hydroxybutyryl-CoA(m) + NAD+(m) ⇌ NADH(m) + Acetoacetyl-CoA(m)
r0310	0.13	Palmitoyl-CoA(m) + Ubiquinone(m) → (2E)-Hexadecenoyl-CoA(m) + Ubiquinol(m)
r0017	0.44	O2(m) + 4 H+(PG)(m) + 4 Ferrocytochrome C(m) → 4 Ferricytochrome C(m) + 2 H2O(m) + 4 H+(PG)(c)
r0312	0.13	Palmitate(c) + ATP(c) + CoA(c) → Palmitoyl-CoA(c) + AMP(c) + PPi(c)
r0030	1.13	ATP(c) + AMP(c) ⇌ 2 ADP(c)
r0070	-0.13	CoA(m) + Acetoacetyl-CoA(m) ⇌ 2 Acetyl-CoA(m)
r0634	-0.13	Acetyl-CoA(m) + Octanoyl-CoA(m) ⇌ CoA(m) + 3-Oxodecanoyl-CoA(m)
r0639	-0.13	Lauroyl-CoA(m) + Acetyl-CoA(m) ← CoA(m) + 3-Oxotetradecanoyl-CoA(m)
r0935	0.13	Palmitate(s) → Palmitate(c)
r0653	-0.13	Myristoyl-CoA(m) + Acetyl-CoA(m) ← CoA(m) + 3-Oxopalmitoyl-CoA(m)
r1446	0.13	Ubiquinone(m) + Butyryl-CoA(m) → Crotonyl-CoA(m) + Ubiquinol(m)
r1447	0.13	Ubiquinone(m) + Lauroyl-CoA(m) → (2E)-Dodecenoyl-CoA(m) + Ubiquinol(m)
r1448	0.13	Ubiquinone(m) + Octanoyl-CoA(m) → (2E)-Octenoyl-CoA(m) + Ubiquinol(m)
r1449	0.13	Ubiquinone(m) + Myristoyl-CoA(m) → (2E)-Tetradecenoyl-CoA(m) + Ubiquinol(m)
r0507	0.88	Ubiquinol(m) + 2 Ferricytochrome C(m) + 4 H+(PG)(m) → Ubiquinone(m) + 4 H+(PG)(c) + 2 Ferrocytochrome C(m)
r1330	5.25	H+(PG)(c) ⇌ H+(PG)(m) + Proton-gradient(m)
r1319	-2.25	ATP(c) + H2O(c) ⇌ ADP(c) + Pi(c) + ATP-energy(c)
r0849	-0.13	H2O(s) ⇌ H2O(c)
r0287	-0.13	CoA(m) + 3-Oxohexanoyl-CoA(m) → Acetyl-CoA(m) + Butyryl-CoA(m)
r0714	0.13	NAD+(m) + (S)-3-Hydroxyhexadecanoyl-CoA(m) → 3-Oxopalmitoyl-CoA(m) + NADH(m)
r0716	-0.13	(S)-3-Hydroxyhexadecanoyl-CoA(m) ← (2E)-Hexadecenoyl-CoA(m) + H2O(m)
r0435	0.13	Palmitoyl-CoA(c) + L-Carnitine(c) ⇌ CoA(c) + L-Palmitoylcarnitine(c)
r0730	0.13	(S)-Hydroxyoctanoyl-CoA(m) + NAD+(m) → 3-Oxooctanoyl-CoA(m) + NADH(m)
r0588	-0.13	Crotonyl-CoA(m) + H2O(m) ⇌ (S)-3-Hydroxybutyryl-CoA(m)
r0718	0.13	(S)-3-Hydroxytetradecanoyl-CoA(m) + NAD+(m) → 3-Oxotetradecanoyl-CoA(m) + NADH(m)
r0436	-0.13	Palmitoyl-CoA(m) + L-Carnitine(m) ← CoA(m) + L-Palmitoylcarnitine(m)
r0731	-0.13	(S)-Hydroxyoctanoyl-CoA(m) ← (2E)-Octenoyl-CoA(m) + H2O(m)
r0732	-0.13	Hexanoyl-CoA(m) + Acetyl-CoA(m) ← CoA(m) + 3-Oxooctanoyl-CoA(m)
r0733	0.13	(S)-Hydroxyhexanoyl-CoA(m) + NAD+(m) → 3-Oxohexanoyl-CoA(m) + NADH(m)
r0734	-0.13	(S)-Hydroxyhexanoyl-CoA(m) ← (2E)-Hexenoyl-CoA(m) + H2O(m)
r0007	1.13	PPi(c) + H2O(c) → 2 Pi(c)
r0062	1	Acetyl-CoA(m) + H2O(m) → CoA(m) + Acetate(m)
r0066	1	ATP(c) + Acetate(c) + CoA(c) → AMP(c) + PPi(c) + Acetyl-CoA(c)
r1450	0.13	Ubiquinone(m) + Hexanoyl-CoA(m) → (2E)-Hexenoyl-CoA(m) + Ubiquinol(m)
r1451	0.13	Ubiquinone(m) + Decanoyl-CoA(m) → (2E)-Decenoyl-CoA(m) + Ubiquinol(m)
r0660	-0.13	(S)-3-Hydroxydodecanoyl-CoA(m) ⇌ (2E)-Dodecenoyl-CoA(m) + H2O(m)
r2677	1	Acetyl-CoA(c) ⇌ CoA(c) + CoA-activated acetyl group(c)
r1323	0.88	NADH(m) ⇌ NAD+(m) + NADH-redox-potential(m)
r0851	-1	H2O(m) ⇌ H2O(c)

Acetyl group(m) (52)

Definition			Solution												
			exchanges		reactions								transp		
Objective	Constraints	Comment	imports	exports	c	m	r	p	l	n	s	b	s-c	b-c	int
CoA-activated acetyl group(m)	−Palmitate −O_2 =H_2O =Proton-gradient(m) +ATP-energy(m) =ATP-energy(c) =NADH-redox-potential(m)	Regeneration of Acetyl-CoA minus CoA	0.44 O_2(s) 0.13 Palmitate(s) 0.25 ATP-energy(c)	0.13 H_2O(s) 0.88 NADH-redox-potential(m) 5.25 Proton-gradient(m) 1 CoA-activated acetyl group(m)	5	31	-	-	-	-	-	-	3	-	5

ID	flux	reaction
r2435	0.13	L-Carnitine(m) + L-Palmitoylcarnitine(c) → L-Carnitine(c) + L-Palmitoylcarnitine(m)
r0855	0.44	O2(s) ⇌ O2(c)
r0856	-0.44	O2(m) ⇌ O2(c)
r0720	-0.13	(S)-3-Hydroxytetradecanoyl-CoA(m) ← (2E)-Tetradecenoyl-CoA(m) + H2O(m)
r0722	0.13	(S)-3-Hydroxydodecanoyl-CoA(m) + NAD+(m) ⇌ 3-Oxododecanoyl-CoA(m) + NADH(m)
r0724	-0.13	Decanoyl-CoA(m) + Acetyl-CoA(m) ⇌ CoA(m) + 3-Oxododecanoyl-CoA(m)
r0726	0.13	(S)-Hydroxydecanoyl-CoA(m) + NAD+(m) ⇌ 3-Oxodecanoyl-CoA(m) + NADH(m)
r0728	-0.13	(S)-Hydroxydecanoyl-CoA(m) ⇌ (2E)-Decenoyl-CoA(m) + H2O(m)
r0460	0.13	(S)-3-Hydroxybutyryl-CoA(m) + NAD+(m) ⇌ NADH(m) + Acetoacetyl-CoA(m)
r0310	0.13	Palmitoyl-CoA(m) + Ubiquinone(m) → (2E)-Hexadecenoyl-CoA(m) + Ubiquinol(m)
r0017	0.44	O2(m) + 4 H+(PG)(m) + 4 Ferrocytochrome C(m) → 4 Ferricytochrome C(m) + 2 H2O(m) + 4 H+(PG)(c)
r0312	0.13	Palmitate(c) + ATP(c) + CoA(c) → Palmitoyl-CoA(c) + AMP(c) + PPi(c)
r0030	0.13	ATP(c) + AMP(c) ⇌ 2 ADP(c)
r0070	-0.13	CoA(m) + Acetoacetyl-CoA(m) ⇌ 2 Acetyl-CoA(m)
r0634	-0.13	Acetyl-CoA(m) + Octanoyl-CoA(m) ⇌ CoA(m) + 3-Oxodecanoyl-CoA(m)
r0639	-0.13	Lauroyl-CoA(m) + Acetyl-CoA(m) ← CoA(m) + 3-Oxotetradecanoyl-CoA(m)
r0935	0.13	Palmitate(s) → Palmitate(c)
r0653	-0.13	Myristoyl-CoA(m) + Acetyl-CoA(m) ← CoA(m) + 3-Oxopalmitoyl-CoA(m)
r1446	0.13	Ubiquinone(m) + Butyryl-CoA(m) → Crotonyl-CoA(m) + Ubiquinol(m)
r1447	0.13	Ubiquinone(m) + Lauroyl-CoA(m) → (2E)-Dodecenoyl-CoA(m) + Ubiquinol(m)
r1448	0.13	Ubiquinone(m) + Octanoyl-CoA(m) → (2E)-Octenoyl-CoA(m) + Ubiquinol(m)
r1449	0.13	Ubiquinone(m) + Myristoyl-CoA(m) → (2E)-Tetradecenoyl-CoA(m) + Ubiquinol(m)
r0507	0.88	Ubiquinol(m) + 2 Ferricytochrome C(m) + 4 H+(PG)(m) → Ubiquinone(m) + 4 H+(PG)(c) + 2 Ferrocytochrome C(m)
r1330	5.25	H+(PG)(c) ⇌ H+(PG)(m) + Proton-gradient(m)
r1319	-0.25	ATP(c) + H2O(c) ⇌ ADP(c) + Pi(c) + ATP-energy(c)
r0849	-0.13	H2O(s) ⇌ H2O(c)
r0287	-0.13	CoA(m) + 3-Oxohexanoyl-CoA(m) → Acetyl-CoA(m) + Butyryl-CoA(m)
r0714	0.13	NAD+(m) + (S)-3-Hydroxyhexadecanoyl-CoA(m) → 3-Oxopalmitoyl-CoA(m) + NADH(m)
r0716	-0.13	(S)-3-Hydroxyhexadecanoyl-CoA(m) ← (2E)-Hexadecenoyl-CoA(m) + H2O(m)
r0435	0.13	Palmitoyl-CoA(c) + L-Carnitine(c) ⇌ CoA(c) + L-Palmitoylcarnitine(c)
r0730	0.13	(S)-Hydroxyoctanoyl-CoA(m) + NAD+(m) → 3-Oxooctanoyl-CoA(m) + NADH(m)
r0588	-0.13	Crotonyl-CoA(m) + H2O(m) ⇌ (S)-3-Hydroxybutyryl-CoA(m)
r0718	0.13	(S)-3-Hydroxytetradecanoyl-CoA(m) + NAD+(m) → 3-Oxotetradecanoyl-CoA(m) + NADH(m)
r0436	-0.13	Palmitoyl-CoA(m) + L-Carnitine(m) ← CoA(m) + L-Palmitoylcarnitine(m)
r0731	-0.13	(S)-Hydroxyoctanoyl-CoA(m) ← (2E)-Octenoyl-CoA(m) + H2O(m)
r0732	-0.13	Hexanoyl-CoA(m) + Acetyl-CoA(m) ← CoA(m) + 3-Oxooctanoyl-CoA(m)
r0733	0.13	(S)-Hydroxyhexanoyl-CoA(m) + NAD+(m) → 3-Oxohexanoyl-CoA(m) + NADH(m)
r0734	-0.13	(S)-Hydroxyhexanoyl-CoA(m) ← (2E)-Hexenoyl-CoA(m) + H2O(m)
r0007	0.13	PPi(c) + H2O(c) → 2 Pi(c)
r1450	0.13	Ubiquinone(m) + Hexanoyl-CoA(m) → (2E)-Hexenoyl-CoA(m) + Ubiquinol(m)
r1451	0.13	Ubiquinone(m) + Decanoyl-CoA(m) → (2E)-Decenoyl-CoA(m) + Ubiquinol(m)
r0660	-0.13	(S)-3-Hydroxydodecanoyl-CoA(m) ⇌ (2E)-Dodecenoyl-CoA(m) + H2O(m)
r2678	1	Acetyl-CoA(m) ⇌ CoA(m) + CoA-activated acetyl group(m)

Continued on next page

ID	flux	reaction
r1323	0.88	NADH(m) ⇌ NAD+(m) + NADH-redox-potential(m)

Acetyl group(p) (53)

Definition			Solution												
			exchanges		reactions								transp		
Objective	Constraints	Comment	imports	exports	c	m	r	p	l	n	s	b	s-c	b-c	int
CoA-activated acetyl group(p)	−Palmitate −O_2 =H_2O =Proton-gradient(m) +ATP-energy(m) =ATP-energy(c) =NADH-redox-potential(m)	Regeneration of Acetyl-CoA minus CoA	0.44 O_2(s) 0.13 Palmitate(s) 2.25 ATP-energy(c)	0.13 H_2O(s) 0.88 NADH-redox-potential(m) 5.25 Proton-gradient(m) 1 CoA-activated acetyl group(p)	7	31	-	2	-	-	-	-	3	-	8

ID	flux	reaction
r2435	0.13	L-Carnitine(m) + L-Palmitoylcarnitine(c) → L-Carnitine(c) + L-Palmitoylcarnitine(m)
r0855	0.44	O2(s) ⇌ O2(c)
r0856	-0.44	O2(m) ⇌ O2(c)
r0720	-0.13	(S)-3-Hydroxytetradecanoyl-CoA(m) ← (2E)-Tetradecenoyl-CoA(m) + H2O(m)
r1087	-1	Acetate(c) ⇌ Acetate(m)
r0722	0.13	(S)-3-Hydroxydodecanoyl-CoA(m) + NAD+(m) ⇌ 3-Oxododecanoyl-CoA(m) + NADH(m)
r0724	-0.13	Decanoyl-CoA(m) + Acetyl-CoA(m) ⇌ CoA(m) + 3-Oxododecanoyl-CoA(m)
r0726	0.13	(S)-Hydroxydecanoyl-CoA(m) + NAD+(m) ⇌ 3-Oxodecanoyl-CoA(m) + NADH(m)
r0728	-0.13	(S)-Hydroxydecanoyl-CoA(m) ⇌ (2E)-Decenoyl-CoA(m) + H2O(m)
r0460	0.13	(S)-3-Hydroxybutyryl-CoA(m) + NAD+(m) ⇌ NADH(m) + Acetoacetyl-CoA(m)
r0310	0.13	Palmitoyl-CoA(m) + Ubiquinone(m) → (2E)-Hexadecenoyl-CoA(m) + Ubiquinol(m)
r0017	0.44	O2(m) + 4 H+(PG)(m) + 4 Ferrocytochrome C(m) → 4 Ferricytochrome C(m) + 2 H2O(m) + 4 H+(PG)(c)
r0312	0.13	Palmitate(c) + ATP(c) + CoA(c) → Palmitoyl-CoA(c) + AMP(c) + PPi(c)
r0030	1.13	ATP(c) + AMP(c) ⇌ 2 ADP(c)
r0070	-0.13	CoA(m) + Acetoacetyl-CoA(m) ⇌ 2 Acetyl-CoA(m)
r0634	-0.13	Acetyl-CoA(m) + Octanoyl-CoA(m) ⇌ CoA(m) + 3-Oxodecanoyl-CoA(m)
r0639	-0.13	Lauroyl-CoA(m) + Acetyl-CoA(m) ← CoA(m) + 3-Oxotetradecanoyl-CoA(m)
r0935	0.13	Palmitate(s) → Palmitate(c)
r0653	-0.13	Myristoyl-CoA(m) + Acetyl-CoA(m) ← CoA(m) + 3-Oxopalmitoyl-CoA(m)
r1446	0.13	Ubiquinone(m) + Butyryl-CoA(m) → Crotonyl-CoA(m) + Ubiquinol(m)
r1447	0.13	Ubiquinone(m) + Lauroyl-CoA(m) → (2E)-Dodecenoyl-CoA(m) + Ubiquinol(m)
r1448	0.13	Ubiquinone(m) + Octanoyl-CoA(m) → (2E)-Octenoyl-CoA(m) + Ubiquinol(m)
r1449	0.13	Ubiquinone(m) + Myristoyl-CoA(m) → (2E)-Tetradecenoyl-CoA(m) + Ubiquinol(m)
r0507	0.88	Ubiquinol(m) + 2 Ferricytochrome C(m) + 4 H+(PG)(m) → Ubiquinone(m) + 4 H+(PG)(c) + 2 Ferrocytochrome C(m)
r1330	5.25	H+(PG)(c) ⇌ H+(PG)(m) + Proton-gradient(m)
r1319	-2.25	ATP(c) + H2O(c) ⇌ ADP(c) + Pi(c) + ATP-energy(c)
r0996	-1	L-Carnitine(c) + O-Acetylcarnitine(p) ⇌ L-Carnitine(p) + O-Acetylcarnitine(c)
r0849	-0.13	H2O(s) ⇌ H2O(c)
r0287	-0.13	CoA(m) + 3-Oxohexanoyl-CoA(m) → Acetyl-CoA(m) + Butyryl-CoA(m)
r0714	0.13	NAD+(m) + (S)-3-Hydroxyhexadecanoyl-CoA(m) → 3-Oxopalmitoyl-CoA(m) + NADH(m)
r0716	-0.13	(S)-3-Hydroxyhexadecanoyl-CoA(m) ← (2E)-Hexadecenoyl-CoA(m) + H2O(m)
r0435	0.13	Palmitoyl-CoA(c) + L-Carnitine(c) ⇌ CoA(c) + L-Palmitoylcarnitine(c)
r0730	0.13	(S)-Hydroxyoctanoyl-CoA(m) + NAD+(m) → 3-Oxooctanoyl-CoA(m) + NADH(m)
r0588	-0.13	Crotonyl-CoA(m) + H2O(m) ⇌ (S)-3-Hydroxybutyryl-CoA(m)
r0718	0.13	(S)-3-Hydroxytetradecanoyl-CoA(m) + NAD+(m) → 3-Oxotetradecanoyl-CoA(m) + NADH(m)
r0436	-0.13	Palmitoyl-CoA(m) + L-Carnitine(m) ← CoA(m) + L-Palmitoylcarnitine(m)
r0731	-0.13	(S)-Hydroxyoctanoyl-CoA(m) ← (2E)-Octenoyl-CoA(m) + H2O(m)

Continued on next page

ID	flux	reaction
r0732	-0.13	Hexanoyl-CoA(m) + Acetyl-CoA(m) ← CoA(m) + 3-Oxooctanoyl-CoA(m)
r0733	0.13	(S)-Hydroxyhexanoyl-CoA(m) + NAD+(m) → 3-Oxohexanoyl-CoA(m) + NADH(m)
r0734	-0.13	(S)-Hydroxyhexanoyl-CoA(m) ← (2E)-Hexenoyl-CoA(m) + H2O(m)
r0007	1.13	PPi(c) + H2O(c) → 2 Pi(c)
r0062	1	Acetyl-CoA(m) + H2O(m) → CoA(m) + Acetate(m)
r0066	1	ATP(c) + Acetate(c) + CoA(c) → AMP(c) + PPi(c) + Acetyl-CoA(c)
r1450	0.13	Ubiquinone(m) + Hexanoyl-CoA(m) → (2E)-Hexenoyl-CoA(m) + Ubiquinol(m)
r1451	0.13	Ubiquinone(m) + Decanoyl-CoA(m) → (2E)-Decenoyl-CoA(m) + Ubiquinol(m)
r0660	-0.13	(S)-3-Hydroxydodecanoyl-CoA(m) ⇌ (2E)-Dodecenoyl-CoA(m) + H2O(m)
r2679	1	Acetyl-CoA(p) ⇌ CoA(p) + CoA-activated acetyl group(p)
r1323	0.88	NADH(m) ⇌ NAD+(m) + NADH-redox-potential(m)
r0534	1	Acetyl-CoA(c) + L-Carnitine(c) ⇌ CoA(c) + O-Acetylcarnitine(c)
r0536	-1	Acetyl-CoA(p) + L-Carnitine(p) ⇌ CoA(p) + O-Acetylcarnitine(p)
r0851	-1	H2O(m) ⇌ H2O(c)

Acetyl group(r) (54)

Definition			Solution												
			exchanges		reactions								transp		
Objective	Constraints	Comment	imports	exports	c	m	r	p	l	n	s	b	s-c	b-c	int
CoA-activated acetyl group(r)	−Palmitate −O_2 =H_2O =Proton-gradient(m) +ATP-energy(m) =ATP-energy(c) =NADH-redox-potential(m)	Regeneration of Acetyl-CoA minus CoA	0.44 O_2(s) 0.13 Palmitate(s) 2.25 ATP-energy(c)	0.13 H_2O(s) 0.88 NADH-redox-potential(m) 5.25 Proton-gradient(m) 1 CoA-activated acetyl group(r)	6	31	1	-	-	-	-	-	3	-	9

ID	flux	reaction
r2435	0.13	L-Carnitine(m) + L-Palmitoylcarnitine(c) → L-Carnitine(c) + L-Palmitoylcarnitine(m)
r0855	0.44	O2(s) ⇌ O2(c)
r0856	-0.44	O2(m) ⇌ O2(c)
r0720	-0.13	(S)-3-Hydroxytetradecanoyl-CoA(m) ← (2E)-Tetradecenoyl-CoA(m) + H2O(m)
r1087	-1	Acetate(c) ⇌ Acetate(m)
r0722	0.13	(S)-3-Hydroxydodecanoyl-CoA(m) + NAD+(m) ⇌ 3-Oxododecanoyl-CoA(m) + NADH(m)
r0724	-0.13	Decanoyl-CoA(m) + Acetyl-CoA(m) ⇌ CoA(m) + 3-Oxododecanoyl-CoA(m)
r0726	0.13	(S)-Hydroxydecanoyl-CoA(m) + NAD+(m) ⇌ 3-Oxodecanoyl-CoA(m) + NADH(m)
r0728	-0.13	(S)-Hydroxydecanoyl-CoA(m) ⇌ (2E)-Decenoyl-CoA(m) + H2O(m)
r0460	0.13	(S)-3-Hydroxybutyryl-CoA(m) + NAD+(m) ⇌ NADH(m) + Acetoacetyl-CoA(m)
r0310	0.13	Palmitoyl-CoA(m) + Ubiquinone(m) → (2E)-Hexadecenoyl-CoA(m) + Ubiquinol(m)
r0017	0.44	O2(m) + 4 H+(PG)(m) + 4 Ferrocytochrome C(m) → 4 Ferricytochrome C(m) + 2 H2O(m) + 4 H+(PG)(c)
r0312	0.13	Palmitate(c) + ATP(c) + CoA(c) → Palmitoyl-CoA(c) + AMP(c) + PPi(c)
r0030	1.13	ATP(c) + AMP(c) ⇌ 2 ADP(c)
r0070	-0.13	CoA(m) + Acetoacetyl-CoA(m) ⇌ 2 Acetyl-CoA(m)
r0634	-0.13	Acetyl-CoA(m) + Octanoyl-CoA(m) ⇌ CoA(m) + 3-Oxodecanoyl-CoA(m)
r2680	1	Acetyl-CoA(r) ⇌ CoA(r) + CoA-activated acetyl group(r)
r0639	-0.13	Lauroyl-CoA(m) + Acetyl-CoA(m) ← CoA(m) + 3-Oxotetradecanoyl-CoA(m)
r0935	0.13	Palmitate(s) → Palmitate(c)
r0653	-0.13	Myristoyl-CoA(m) + Acetyl-CoA(m) ← CoA(m) + 3-Oxopalmitoyl-CoA(m)
r1446	0.13	Ubiquinone(m) + Butyryl-CoA(m) → Crotonyl-CoA(m) + Ubiquinol(m)
r1447	0.13	Ubiquinone(m) + Lauroyl-CoA(m) → (2E)-Dodecenoyl-CoA(m) + Ubiquinol(m)
r1448	0.13	Ubiquinone(m) + Octanoyl-CoA(m) → (2E)-Octenoyl-CoA(m) + Ubiquinol(m)
r1449	0.13	Ubiquinone(m) + Myristoyl-CoA(m) → (2E)-Tetradecenoyl-CoA(m) + Ubiquinol(m)

Continued on next page

ID	flux	reaction
r0507	0.88	Ubiquinol(m) + 2 Ferricytochrome C(m) + 4 H+(PG)(m) → Ubiquinone(m) + 4 H+(PG)(c) + 2 Ferrocytochrome C(m)
r1330	5.25	H+(PG)(c) ⇌ H+(PG)(m) + Proton-gradient(m)
r1319	-2.25	ATP(c) + H2O(c) ⇌ ADP(c) + Pi(c) + ATP-energy(c)
r0849	-0.13	H2O(s) ⇌ H2O(c)
r0862	-1	CoA(c) ⇌ CoA(r)
r0287	-0.13	CoA(m) + 3-Oxohexanoyl-CoA(m) → Acetyl-CoA(m) + Butyryl-CoA(m)
r0714	0.13	NAD+(m) + (S)-3-Hydroxyhexadecanoyl-CoA(m) → 3-Oxopalmitoyl-CoA(m) + NADH(m)
r0716	-0.13	(S)-3-Hydroxyhexadecanoyl-CoA(m) ← (2E)-Hexadecenoyl-CoA(m) + H2O(m)
r0435	0.13	Palmitoyl-CoA(c) + L-Carnitine(c) ⇌ CoA(c) + L-Palmitoylcarnitine(c)
r0730	0.13	(S)-Hydroxyoctanoyl-CoA(m) + NAD+(m) → 3-Oxooctanoyl-CoA(m) + NADH(m)
r0588	-0.13	Crotonyl-CoA(m) + H2O(m) ⇌ (S)-3-Hydroxybutyryl-CoA(m)
r0718	0.13	(S)-3-Hydroxytetradecanoyl-CoA(m) + NAD+(m) → 3-Oxotetradecanoyl-CoA(m) + NADH(m)
r0436	-0.13	Palmitoyl-CoA(m) + L-Carnitine(m) ← CoA(m) + L-Palmitoylcarnitine(m)
r0731	-0.13	(S)-Hydroxyoctanoyl-CoA(m) ← (2E)-Octenoyl-CoA(m) + H2O(m)
r2466	1	Acetyl-CoA(c) ⇌ Acetyl-CoA(r)
r0732	-0.13	Hexanoyl-CoA(m) + Acetyl-CoA(m) ← CoA(m) + 3-Oxooctanoyl-CoA(m)
r0733	0.13	(S)-Hydroxyhexanoyl-CoA(m) + NAD+(m) → 3-Oxohexanoyl-CoA(m) + NADH(m)
r0734	-0.13	(S)-Hydroxyhexanoyl-CoA(m) ← (2E)-Hexenoyl-CoA(m) + H2O(m)
r0007	1.13	PPi(c) + H2O(c) → 2 Pi(c)
r0062	1	Acetyl-CoA(m) + H2O(m) → CoA(m) + Acetate(m)
r0066	1	ATP(c) + Acetate(c) + CoA(c) → AMP(c) + PPi(c) + Acetyl-CoA(c)
r1450	0.13	Ubiquinone(m) + Hexanoyl-CoA(m) → (2E)-Hexenoyl-CoA(m) + Ubiquinol(m)
r1451	0.13	Ubiquinone(m) + Decanoyl-CoA(m) → (2E)-Decenoyl-CoA(m) + Ubiquinol(m)
r0660	-0.13	(S)-3-Hydroxydodecanoyl-CoA(m) ⇌ (2E)-Dodecenoyl-CoA(m) + H2O(m)
r1323	0.88	NADH(m) ⇌ NAD+(m) + NADH-redox-potential(m)
r0851	-1	H2O(m) ⇌ H2O(c)

A3.4. Sulfate group

activated sulphur (55)

Definition			Solution												
			exchanges		reactions								transp		
Objective	Constraints	Comment	imports	exports	c	m	r	p	l	n	s	b	s-c	b-c	int
activated-sulphur(c)	−Sulfate(c) =ATP-energy(c) =NADH-redox-potential(c) =NADPH-redox-potential(c) $-O_2$ $+CO_2$ $=H_2O$	Regeneration of cytosolic activated-sulphur	1 Sulfate(c) 3 ATP-energy(c)	1 H_2O(s) 1 activated-sulphur(c)	7	-	-	-	-	-	-	-	1	-	-

ID	flux	reaction
r0049	1	PAP(c) + H2O(c) → AMP(c) + Pi(c)
r1319	-3	ATP(c) + H2O(c) ⇌ ADP(c) + Pi(c) + ATP-energy(c)
r0030	1	ATP(c) + AMP(c) ⇌ 2 ADP(c)
r0141	1	ATP(c) + Sulfate(c) → PPi(c) + Adenylyl sulfate(c)
r0133	1	ATP(c) + Adenylyl sulfate(c) → ADP(c) + PAPS(c)
r0007	1	PPi(c) + H2O(c) → 2 Pi(c)
r0849	-1	H2O(s) ⇌ H2O(c)
r1332	1	PAPS(c) ⇌ PAP(c) + activated-sulphur(c)

2.4 A4. Activated sugars

A4.1. Glucose-6-P

Glucose-6P (56)

Definition			Solution												
			exchanges		reactions								transp		
Objective	Constraints	Comment	imports	exports	c	m	r	p	l	n	s	b	s-c	b-c	int
Glucose-6P(c)	−Glucose =ATP-energy(c) =P_i(c) =H_2O	Synthesis of Glucose-6P	1 P_i(c) 1 Glucose(s) 1 ATP-energy(c)	1 H_2O(s) 1 Glucose-6P(c)	2	-	-	-	-	-	-	-	2	-	-

ID	flux	reaction
r0353	1	Glucose(c) + ATP(c) → Glucose-6P(c) + ADP(c)
r1319	-1	ATP(c) + H2O(c) ⇌ ADP(c) + Pi(c) + ATP-energy(c)
r1032	1	Glucose(s) ⇌ Glucose(c)
r0849	-1	H2O(s) ⇌ H2O(c)

A4.2. UDP-activated sugars

UDP-activated glucose (57)

Definition			Solution												
			exchanges		reactions								transp		
Objective	Constraints	Comment	imports	exports	c	m	r	p	l	n	s	b	s-c	b-c	int
UDP-activated-glucose(c)	−Glucose-6P(c) =ATP-energy(c) =NADH-redox-potential(c) =NADPH-redox-potential(c) −O_2 +CO_2 +H_2O =P_i(c)	Regeneration of UDP glucose minus UDP	1 Glucose-6P(c) 1 ATP-energy(c)	1 P_i(c) 1 UDP-activated-glucose(c)	6	-	-	-	-	-	-	-	-	-	-

ID	flux	reaction
r0037	1	ATP(c) + UDP(c) ⇌ ADP(c) + UTP(c)
r0094	1	UTP(c) + Glucose-1P(c) → PPi(c) + UDP-glucose(c)
r1319	-1	ATP(c) + H2O(c) ⇌ ADP(c) + Pi(c) + ATP-energy(c)
r2665	1	UDP-glucose(c) ⇌ UDP(c) + UDP-activated-glucose(c)
r0007	1	PPi(c) + H2O(c) → 2 Pi(c)
r0621	-1	Glucose-6P(c) ⇌ Glucose-1P(c)

UDP-activated glucuronate (58)

Definition			Solution												
			exchanges		reactions								transp		
Objective	Constraints	Comment	imports	exports	c	m	r	p	l	n	s	b	s-c	b-c	int
UDP-activated-glucuronate(c)	−Glucose-6P(c) =ATP-energy(c) =Proton-gradient(m) −O_2 +CO_2 +H_2O =P_i(c) %r1239 %r1414	Regeneration of UDP glucuronate minus UDP	1 O_2(s) 1 Glucose-6P(c) 1 ATP-energy(c)	1 H_2O(s) 1 P_i(c) 12 Proton-gradient(m) 1 UDP-activated-glucuronate(c)	8	-	-	-	-	-	-	-	2	-	6

ID	flux	reaction
r0037	1	ATP(c) + UDP(c) ⇌ ADP(c) + UTP(c)
r0092	1	UDP-glucose(c) + H2O(c) + 2 NAD+(c) → 2 NADH(c) + UDP-glucuronate(c)
r0507	2	Ubiquinol(m) + 2 Ferricytochrome C(m) + 4 H+(PG)(m) → Ubiquinone(m) + 4 H+(PG)(c) + 2 Ferrocytochrome C(m)
r0094	1	UTP(c) + Glucose-1P(c) → PPi(c) + UDP-glucose(c)
r1319	-1	ATP(c) + H2O(c) ⇌ ADP(c) + Pi(c) + ATP-energy(c)
r0851	2	H2O(m) ⇌ H2O(c)
r0201	-2	DHAP(c) + NADH(c) ⇌ NAD+(c) + sn-Glycerol-3P(c)
r0855	1	O2(s) ⇌ O2(c)
r0856	-1	O2(m) ⇌ O2(c)
r1330	12	H+(PG)(c) ⇌ H+(PG)(m) + Proton-gradient(m)
r0205	2	Ubiquinone(m) + sn-Glycerol-3P(c) ⇌ DHAP(c) + Ubiquinol(m)
r0007	1	PPi(c) + H2O(c) → 2 Pi(c)
r2667	1	UDP-glucuronate(c) ⇌ UDP(c) + UDP-activated-glucuronate(c)
r0849	-1	H2O(s) ⇌ H2O(c)
r0017	1	O2(m) + 4 H+(PG)(m) + 4 Ferrocytochrome C(m) → 4 Ferricytochrome C(m) + 2 H2O(m) + 4 H+(PG)(c)
r0621	-1	Glucose-6P(c) ⇌ Glucose-1P(c)

UDP-activated galactose (59)

Definition			Solution												
			exchanges		reactions								transp		
Objective	Constraints	Comment	imports	exports	c	m	r	p	l	n	s	b	s-c	b-c	int
UDP-activated-galactose(c)	−Glucose-6P(c) =ATP-energy(c) =NADH-redox-potential(c) =H_2O =P_i(c) =NADPH-redox-potential(c)	Regeneration of UDP-activated galactose	1 Glucose-6P(c) 1 ATP-energy(c)	1 P_i(c) 1 UDP-activated-galactose(c)	7	-	-	-	-	-	-	-	-	-	-

ID	flux	reaction
r0037	1	ATP(c) + UDP(c) ⇌ ADP(c) + UTP(c)
r0094	1	UTP(c) + Glucose-1P(c) → PPi(c) + UDP-glucose(c)
r1319	-1	ATP(c) + H2O(c) ⇌ ADP(c) + Pi(c) + ATP-energy(c)
r0095	1	UDP-glucose(c) ⇌ UDP-galactose(c)
r2666	1	UDP-galactose(c) ⇌ UDP(c) + UDP-activated-galactose(c)
r0007	1	PPi(c) + H2O(c) → 2 Pi(c)
r0621	-1	Glucose-6P(c) ⇌ Glucose-1P(c)

A4.3. Other nucleosides-activated sugars

GDP-activated fucose (60)

Definition			Solution												
			exchanges		reactions								transp		
Objective	Constraints	Comment	imports	exports	c	m	r	p	l	n	s	b	s-c	b-c	int
GDP-activated-fucose(c)	−Glucose-6P(c) =ATP-energy(c) =NADH-redox-potential(c) =H_2O =P_i(c) =NADPH-redox-potential(c)	Regeneration of GDP-activated fucose	1 Glucose-6P(c) 1 ATP-energy(c) 1 NADH-redox-potential(c)	1 H_2O(s) 1 P_i(c) 1 GDP-activated-fucose(c)	11	-	-	-	-	-	-	-	1	-	-

ID	flux	reaction
r2669	1	GDP-L-fucose(c) ⇌ GDP(c) + GDP-activated-fucose(c)
r0208	1	GTP(c) + Mannose-1P(c) ⇌ PPi(c) + GDP-mannose(c)
r0209	1	GDP-mannose(c) → GDP-4-dehydro-6-deoxy-D-mannose(c) + H2O(c)
r0192	1	Glucose-6P(c) ⇌ Fructose-6P(c)
r1319	-1	ATP(c) + H2O(c) ⇌ ADP(c) + Pi(c) + ATP-energy(c)
r0782	-1	GDP-4-dehydro-6-deoxy-D-mannose(c) + NADH(c) → GDP-L-fucose(c) + NAD+(c)
r0099	1	ATP(c) + GDP(c) ⇌ ADP(c) + GTP(c)
r0007	1	PPi(c) + H2O(c) → 2 Pi(c)
r1322	-1	NADH(c) ⇌ NAD+(c) + NADH-redox-potential(c)
r0849	-1	H2O(s) ⇌ H2O(c)
r0404	1	Mannose-6P(c) → Mannose-1P(c)
r0405	-1	Fructose-6P(c) ⇌ Mannose-6P(c)

GDP-activated mannose (61)

Definition			Solution												
			exchanges		reactions								transp		
Objective	Constraints	Comment	imports	exports	c	m	r	p	l	n	s	b	s-c	b-c	int
GDP-activated-mannose(c)	−Glucose-6P(c) =ATP-energy(c) =NADH-redox-potential(c) =H_2O =P_i(c) =NADPH-redox-potential(c)	Regeneration of GDP-activated mannose	1 Glucose-6P(c) 1 ATP-energy(c)	1 P_i(c) 1 GDP-activated-mannose(c)	8	-	-	-	-	-	-	-	-	-	-

ID	flux	reaction
r0208	1	GTP(c) + Mannose-1P(c) ⇌ PPi(c) + GDP-mannose(c)
r0192	1	Glucose-6P(c) ⇌ Fructose-6P(c)
r1319	-1	ATP(c) + H2O(c) ⇌ ADP(c) + Pi(c) + ATP-energy(c)
r2670	1	GDP-mannose(c) ⇌ GDP(c) + GDP-activated-mannose(c)
r0099	1	ATP(c) + GDP(c) ⇌ ADP(c) + GTP(c)
r0007	1	PPi(c) + H2O(c) → 2 Pi(c)
r0404	1	Mannose-6P(c) → Mannose-1P(c)
r0405	-1	Fructose-6P(c) ⇌ Mannose-6P(c)

A4.4. Other

2.5 A5. Important intermediates

A5.1. Pyruvate

Pyruvate (62)

Definition			Solution												
			exchanges		reactions								transp		
Objective	Constraints	Comment	imports	exports	c	m	r	p	l	n	s	b	s-c	b-c	int
Pyruvate(c)	−Glucose-6P(c) =ATP-energy(c) =NADH-redox-potential(c) =H_2O =P_i(c) =NADPH-redox-potential(c)	Synthesis of Pyruvate	0.5 H_2O(s) 0.5 Glucose-6P(c)	0.5 P_i(c) 1 Pyruvate(c) 1.5 ATP-energy(c) 1 NADH-redox-potential(c)	11	-	-	-	-	-	-	-	1	-	-

ID	flux	reaction
r0253	1	GAP(c) + Pi(c) + NAD+(c) ⇌ 1,3DPG(c) + NADH(c)
r0164	1	2PG(c) ⇌ PEP(c) + H2O(c)
r0192	0.5	Glucose-6P(c) ⇌ Fructose-6P(c)
r0337	-1	ADP(c) + 1,3DPG(c) ⇌ ATP(c) + 3PG(c)
r0256	0.5	Fructose-1,6PP(c) ⇌ DHAP(c) + GAP(c)
r1319	1.5	ATP(c) + H2O(c) ⇌ ADP(c) + Pi(c) + ATP-energy(c)
r0736	0.5	ATP(c) + Fructose-6P(c) → ADP(c) + Fructose-1,6PP(c)
r1322	1	NADH(c) ⇌ NAD+(c) + NADH-redox-potential(c)
r0849	0.5	H2O(s) ⇌ H2O(c)
r0341	-1	3PG(c) ⇌ 2PG(c)
r0243	-0.5	DHAP(c) ⇌ GAP(c)
r0054	-1	ADP(c) + PEP(c) → ATP(c) + Pyruvate(c)

A5.2. AKG

AKG (63)

Definition			Solution												
			exchanges		reactions								transp		
Objective	Constraints	Comment	imports	exports	c	m	r	p	l	n	s	b	s-c	b-c	int
AKG(c)	−Glucose-6P(c) =ATP-energy(c) =NADH-redox-potential(c) =NADH-redox-potential(m) =H_2O =P_i(c) =NADPH-redox-potential(m) =NADPH-redox-potential(c) =CO_2(m) =CO_2(c) =CoA-activated acetyl group(m)	Synthesis of AKG	1.5 H_2O(s) 1 CO_2(c) 0.5 Glucose-6P(c) 1 NADPH-redox-potential(c) 1 CoA-activated acetyl group(m)	0.5 P_i(c) 1 CO_2(m) 1 AKG(c) 1.5 ATP-energy(c) 1 NADH-redox-potential(c) 1 NADH-redox-potential(m) 1 NADPH-redox-potential(m)	13	8	-	-	-	-	-	-	1	-	2

ID	flux	reaction
r2678	-1	Acetyl-CoA(m) ⇌ CoA(m) + CoA-activated acetyl group(m)
r0820	-1	AKG(m) + Malate(c) ⇌ AKG(c) + Malate(m)
r0316	1	Citrate(m) ⇌ Isocitrate(m)
r0253	1	GAP(c) + Pi(c) + NAD+(c) ⇌ 1,3DPG(c) + NADH(c)
r0164	1	2PG(c) ⇌ PEP(c) + H2O(c)
r0083	1	Oxalosuccinate(m) ⇌ AKG(m) + CO2(m)
r1326	-1	NADPH(c) ⇌ NADPH-redox-potential(c) + NADP+(c)
r0192	0.5	Glucose-6P(c) ⇌ Fructose-6P(c)
r1327	1	NADPH(m) ⇌ NADPH-redox-potential(m) + NADP+(m)
r0337	-1	ADP(c) + 1,3DPG(c) ⇌ ATP(c) + 3PG(c)
r0256	0.5	Fructose-1,6PP(c) ⇌ DHAP(c) + GAP(c)
r0058	-1	Pyruvate(c) + CO2(c) + NADPH(c) ⇌ Malate(c) + NADP+(c)
r1319	1.5	ATP(c) + H2O(c) ⇌ ADP(c) + Pi(c) + ATP-energy(c)
r0851	-1	H2O(m) ⇌ H2O(c)
r0736	0.5	ATP(c) + Fructose-6P(c) → ADP(c) + Fructose-1,6PP(c)

Continued on next page

ID	flux	reaction
r0105	1	NAD+(m) + Malate(m) ⇌ OAA(m) + NADH(m)
r1322	1	NADH(c) ⇌ NAD+(c) + NADH-redox-potential(c)
r0849	1.5	H2O(s) ⇌ H2O(c)
r0341	-1	3PG(c) ⇌ 2PG(c)
r0107	-1	Acetyl-CoA(m) + H2O(m) + OAA(m) ⇌ Citrate(m) + CoA(m)
r1323	1	NADH(m) ⇌ NAD+(m) + NADH-redox-potential(m)
r0423	1	Isocitrate(m) + NADP+(m) ⇌ Oxalosuccinate(m) + NADPH(m)
r0243	-0.5	DHAP(c) ⇌ GAP(c)
r0054	-1	ADP(c) + PEP(c) → ATP(c) + Pyruvate(c)

A5.3. Terpenoids

Isopentenyl-PP (64)

Definition			Solution												
			exchanges		reactions								transp		
Objective	Constraints	Comment	imports	exports	c	m	r	p	l	n	s	b	s-c	b-c	int
Isopentenyl-PP(p)	−Acetyl-CoA(c) +CoA(c) =ATP-energy(c) =NADH-redox-potential(c) =NADPH-redox-potential(c) =H_2O =P_i(c) =CO_2	synthesis of peroxysomal Isopentenyl-PP	2 P_i(c) 3 Acetyl-CoA(c) 3 ATP-energy(c) 2 NADPH-redox-potential(c)	2 H_2O(s) 3 CoA(c) 1 CO_2(s) 1 Isopentenyl-PP(p)	5	-	-	3	-	-	-	-	2	-	4

ID	flux	reaction
r0869	1	CO2(p) ⇌ CO2(c)
r0515	1	ATP(p) + (R)-Mevalonate(p) ⇌ ADP(p) + (R)-5-Phosphomevalonate(p)
r0461	-1	Acetyl-CoA(c) + H2O(c) + Acetoacetyl-CoA(c) ⇌ HMG-CoA(c) + CoA(c)
r1326	-2	NADPH(c) ⇌ NADPH-redox-potential(c) + NADP+(c)
r1319	-3	ATP(c) + H2O(c) ⇌ ADP(c) + Pi(c) + ATP-energy(c)
r0860	-3	ADP(c) + ATP(p) ⇌ ADP(p) + ATP(c)
r0069	1	2 Acetyl-CoA(c) ⇌ CoA(c) + Acetoacetyl-CoA(c)
r0954	1	(R)-Mevalonate(c) ⇌ (R)-Mevalonate(p)
r0488	-1	HMG-CoA(c) + 2 NADPH(c) ⇌ (R)-Mevalonate(c) + CoA(c) + 2 NADP+(c)
r1151	-1	Pi(c) ⇌ Pi(p)
r0867	1	CO2(c) ⇌ CO2(s)
r0849	-2	H2O(s) ⇌ H2O(c)
r0612	1	ATP(p) + (R)-5-Phosphomevalonate(p) ⇌ ADP(p) + (R)-5-Diphosphomevalonate(p)
r0270	1	ATP(p) + (R)-5-Diphosphomevalonate(p) ⇌ CO2(p) + Isopentenyl-PP(p) + ADP(p) + Pi(p)

Farnesyl-PP (65)

Definition			Solution													
			exchanges		reactions								transp		
Objective	Constraints	Comment	imports	exports	c	m	r	p	l	n	s	b	s-c	b-c	int
Farnesyl-PP(p)	−Acetyl-CoA(c) +CoA(c) =ATP-energy(c) =NADH-redox-potential(c) =NADPH-redox-potential(c) $=H_2O$ $=P_i(c)$ $=CO_2$	de novo synthesis of peroxysomal Farnesyl-PP	2 P_i(c) 9 Acetyl-CoA(c) 9 ATP-energy(c) 6 NADPH-redox-potential(c)	4 H_2O(s) 9 CoA(c) 3 CO_2(s) 1 Farnesyl-PP(p)	6	-	-	6	-	-	-	-	2	-	5

ID	flux	reaction
r0869	3	CO2(p) ⇌ CO2(c)
r0271	1	Isopentenyl-PP(p) ⇌ Dimethylallyl-PP(p)
r0515	3	ATP(p) + (R)-Mevalonate(p) ⇌ ADP(p) + (R)-5-Phosphomevalonate(p)
r0461	-3	Acetyl-CoA(c) + H2O(c) + Acetoacetyl-CoA(c) ⇌ HMG-CoA(c) + CoA(c)
r1326	-6	NADPH(c) ⇌ NADPH-redox-potential(c) + NADP+(c)
r0373	1	Dimethylallyl-PP(p) + Isopentenyl-PP(p) ⇌ PPi(p) + Geranyl-PP(p)
r1319	-9	ATP(c) + H2O(c) ⇌ ADP(c) + Pi(c) + ATP-energy(c)
r0860	-9	ADP(c) + ATP(p) ⇌ ADP(p) + ATP(c)
r0069	3	2 Acetyl-CoA(c) ⇌ CoA(c) + Acetoacetyl-CoA(c)
r0954	3	(R)-Mevalonate(c) ⇌ (R)-Mevalonate(p)
r0468	1	Isopentenyl-PP(p) + Geranyl-PP(p) ⇌ PPi(p) + Farnesyl-PP(p)
r0488	-3	HMG-CoA(c) + 2 NADPH(c) ⇌ (R)-Mevalonate(c) + CoA(c) + 2 NADP+(c)
r0007	2	PPi(c) + H2O(c) → 2 Pi(c)
r1151	-3	Pi(c) ⇌ Pi(p)
r0867	3	CO2(c) ⇌ CO2(s)
r0849	-4	H2O(s) ⇌ H2O(c)
r1152	-2	PPi(c) ⇌ PPi(p)
r0612	3	ATP(p) + (R)-5-Phosphomevalonate(p) ⇌ ADP(p) + (R)-5-Diphosphomevalonate(p)
r0270	3	ATP(p) + (R)-5-Diphosphomevalonate(p) ⇌ CO2(p) + Isopentenyl-PP(p) + ADP(p) + Pi(p)

A5.4. Aminosugars

Glucosamine-6P (66)

Definition			Solution												
			exchanges		reactions								transp		
Objective	Constraints	Comment	imports	exports	c	m	r	p	l	n	s	b	s-c	b-c	int
Glucosamine-6P(c)	−CoA-activated acetyl group(c) +Glutamate =Na-gradient(c) −Glutamine −Glucose-6P(c) =ATP-energy(c) =NADH-redox-potential(c) =NADPH-redox-potential(c) $=H_2O$ $=P_i(c)$ $=CO_2$	synthesis of D-Glucosamine 6-phosphate from activated acetyl and Glutamine minus Glutamate	1 Glutamine(s) 1 Glucose-6P(c)	1 Glutamate(s) 1 Glucosamine-6P(c)	2	-	-	-	-	-	-	-	2	-	-

ID	flux	reaction
r0190	1	Glutamine(c) + Fructose-6P(c) → Glutamate(c) + Glucosamine-6P(c)
r0192	1	Glucose-6P(c) ⇌ Fructose-6P(c)
r2525	1	Glutamine(s) ⇌ Glutamine(c)
r2685	1	Glutamate(c) ⇌ Glutamate(s)

A5.99. Other

2.6 A6. Salvage

ATP salvage from Adenosine (67)

Definition			Solution												
			exchanges		reactions								transp		
Objective	Constraints	Comment	imports	exports	c	m	r	p	l	n	s	b	s-c	b-c	int
ATP(c)	−Adenosine(c) =ATP-energy(c) =NADH-redox-potential(c) $-P_i(c)$ $+CO_2$ $-O_2$ $=H_2O$	ATP salvage from Adenosine	3 $P_i(c)$ 1 Adenosine(c) 3 ATP-energy(c)	3 $H_2O(s)$ 1 ATP(c)	3	-	-	-	-	-	-	-	1	-	-

ID	flux	reaction
r0048	1	ATP(c) + Adenosine(c) ⇌ ADP(c) + AMP(c)
r1319	-3	ATP(c) + H2O(c) ⇌ ADP(c) + Pi(c) + ATP-energy(c)
r0030	1	ATP(c) + AMP(c) ⇌ 2 ADP(c)
r0849	-3	H2O(s) ⇌ H2O(c)

ATP salvage from Hypoxanthine (68)

Definition			Solution												
			exchanges		reactions								transp		
Objective	Constraints	Comment	imports	exports	c	m	r	p	l	n	s	b	s-c	b-c	int
ATP(c)	−Hypoxanthine(c) −Glucose-6P(c) −Pyruvate(c) =ATP-energy(c) =NADH-redox-potential(c) =NADPH-redox-potential(c) =NADH-redox-potential(m) =NADPH-redox-potential(m) $-P_i(c)$ −Glutamine(c) $+CO_2$ $-O_2$ $=H_2O$	ATP salvage from Hypoxanthine	0.25 $O_2(s)$ 2.42 $P_i(c)$ 0.5 Glutamine(c) 0.58 Glucose-6P(c) 1 Hypoxanthine(c) 5.42 ATP-energy(c)	2.92 $H_2O(s)$ 1 ATP(c) 1 $CO_2(s)$ 0.5 NADH-redox-potential(c) 0.5 NADH-redox-potential(m)	26	9	-	-	-	-	-	-	3	-	11

ID	flux	reaction
r0406	-0.25	Fructose-6P(c) + Erythrose-4P(c) ⇌ Sedoheptulose-7P(c) + GAP(c)
r0125	-0.5	GDP(m) + Pi(m) + Succinyl-CoA(m) ⇌ GTP(m) + Succinate(m) + CoA(m)
r0277	1	GTP(c) + IMP(c) + Aspartate(c) → GDP(c) + Pi(c) + Adenylosuccinate(c)
r0407	-0.08	DHAP(c) + Erythrose-4P(c) ⇌ Sedoheptulose-1,7PP(c)
r0855	0.25	O2(s) ⇌ O2(c)
r0408	-0.08	ADP(c) + Sedoheptulose-1,7PP(c) ⇌ ATP(c) + Sedoheptulose-7P(c)
r0856	-0.25	O2(m) ⇌ O2(c)
r0876	0.5	Glutamate(c) + H+(PG)(c) + Aspartate(m) → Glutamate(m) + H+(PG)(m) + Aspartate(c)
r0891	0.5	Glutamine(c) + H+(PG)(c) → Glutamine(m) + H+(PG)(m)
r0164	-0.5	PEP(c) + H2O(c) ⇌ 2PG(c)
r0017	0.25	O2(m) + 4 H+(PG)(m) + 4 Ferrocytochrome C(m) → 4 Ferricytochrome C(m) + 2 H2O(m) + 4 H+(PG)(c)
r0030	2	ATP(c) + AMP(c) ⇌ 2 ADP(c)
r0911	1	Proline(c) + Glutamate(m) ⇌ Proline(m) + Glutamate(c)
r0337	0.5	ATP(c) + 3PG(c) ⇌ ADP(c) + 1,3DPG(c)
r1426	1	Proline(m) + H+(PG)(c) → Proline(c) + H+(PG)(m)
r0073	-0.5	AKG(m) + NADH(m) + NH3(m) ⇌ Glutamate(m) + NAD+(m) + H2O(m)
r0370	0.33	Sedoheptulose-7P(c) + GAP(c) ⇌ Ribose-5P(c) + D-Xylulose-5P(c)
r0078	0.5	Glutamine(m) + H2O(m) → Glutamate(m) + NH3(m)
r0801	-0.5	GTP(c) + GDP(m) ⇌ GTP(m) + GDP(c)
r0507	0.5	Ubiquinol(m) + 2 Ferricytochrome C(m) + 4 H+(PG)(m) → Ubiquinone(m) + 4 H+(PG)(c) + 2 Ferrocytochrome C(m)
r0509	0.5	Ubiquinone(m) + Succinate(m) → Ubiquinol(m) + Fumarate(m)
r0099	1	ATP(c) + GDP(c) ⇌ ADP(c) + GTP(c)
r0243	0.08	GAP(c) ⇌ DHAP(c)
r1319	-5.42	ATP(c) + H2O(c) ⇌ ADP(c) + Pi(c) + ATP-energy(c)
r0260	1	Adenylosuccinate(c) → Fumarate(c) + AMP(c)
r0247	1	ATP(c) + Ribose-5P(c) → AMP(c) + PRPP(c)
r0110	-0.5	OAA(m) + Glutamate(m) → AKG(m) + Aspartate(m)
r0849	-2.92	H2O(s) ⇌ H2O(c)
r0867	1	CO2(c) ⇌ CO2(s)
r0868	-0.5	CO2(c) ⇌ CO2(m)
r0007	1	PPi(c) + H2O(c) → 2 Pi(c)
r0192	0.58	Glucose-6P(c) ⇌ Fructose-6P(c)
r1114	0.5	AKG(m) + CoA(m) + NAD+(m) ⇌ NADH(m) + CO2(m) + Succinyl-CoA(m)
r0341	0.5	2PG(c) ⇌ 3PG(c)
		Continued on next page

ID	flux	reaction
r0344	-0.67	D-Xylulose-5P(c) ⇌ Ribulose-5P(c)
r1322	0.5	NADH(c) ⇌ NAD+(c) + NADH-redox-potential(c)
r1323	0.5	NADH(m) ⇌ NAD+(m) + NADH-redox-potential(m)
r0250	-0.67	Ribulose-5P(c) ⇌ Ribose-5P(c)
r0253	-0.5	1,3DPG(c) + NADH(c) ⇌ GAP(c) + Pi(c) + NAD+(c)
r0255	0.33	Fructose-6P(c) + GAP(c) ⇌ D-Xylulose-5P(c) + Erythrose-4P(c)
r1047	1	Pi(c) + H+(PG)(c) → Pi(m) + H+(PG)(m)
r0104	1	NAD+(c) + Malate(c) ⇌ OAA(c) + NADH(c)
r0105	0.5	NAD+(m) + Malate(m) ⇌ OAA(m) + NADH(m)
r0258	-1	Malate(c) ⇌ Fumarate(c) + H2O(c)
r0259	-0.5	Malate(m) ⇌ Fumarate(m) + H2O(m)
r2419	0.5	AKG(c) + Pi(m) ⇌ AKG(m) + Pi(c)
r0109	-0.5	OAA(c) + Glutamate(c) ⇌ AKG(c) + Aspartate(c)
r0123	0.5	GTP(c) + OAA(c) → GDP(c) + PEP(c) + CO2(c)
r0275	-1	Hypoxanthine(c) + PRPP(c) ⇌ IMP(c) + PPi(c)

dTTP salvage from Thymine (69)

Definition			Solution												
			exchanges		reactions								transp		
Objective	Constraints	Comment	imports	exports	c	m	r	p	l	n	s	b	s-c	b-c	int
dTTP(c)	−Thymine(c) −Glucose-6P(c) =ATP-energy(c) =NADH-redox-potential(c) =NADPH-redox-potential(c) =NADH-redox-potential(m) =NADPH-redox-potential(m) −P_i(c) +CO_2 −O_2 =H_2O	dTTP salvage from Thymine	2.17 P_i(c) 0.83 Glucose-6P(c) 1 Thymine(c) 5.17 ATP-energy(c) 1 NADPH-redox-potential(c)	4.17 H_2O(s) 1 dTTP(c)	24	-	-	-	-	-	-	-	1	-	-

ID	flux	reaction
r0406	-0.33	Fructose-6P(c) + Erythrose-4P(c) ⇌ Sedoheptulose-7P(c) + GAP(c)
r0370	0.33	Sedoheptulose-7P(c) + GAP(c) ⇌ Ribose-5P(c) + D-Xylulose-5P(c)
r0352	-1	Thymine(c) + 2-Deoxy-D-ribose-1P(c) ⇌ Thymidine(c) + Pi(c)
r0551	1	Deoxyadenosine(c) + Pi(c) ⇌ 2-Deoxy-D-ribose-1P(c) + Adenine(c)
r0344	-0.67	D-Xylulose-5P(c) ⇌ Ribulose-5P(c)
r1326	-1	NADPH(c) ⇌ NADPH-redox-potential(c) + NADP+(c)
r0471	-1	Thioredoxin(c) + ADP(c) ⇌ dADP(c) + Oxidized thioredoxin(c) + H2O(c)
r0345	-1	ATP(c) + dAMP(c) ⇌ ADP(c) + dADP(c)
r0255	0.33	Fructose-6P(c) + GAP(c) ⇌ D-Xylulose-5P(c) + Erythrose-4P(c)
r0192	0.83	Glucose-6P(c) ⇌ Fructose-6P(c)
r0256	0.17	Fructose-1,6PP(c) ⇌ DHAP(c) + GAP(c)
r0247	1	ATP(c) + Ribose-5P(c) → AMP(c) + PRPP(c)
r1319	-5.17	ATP(c) + H2O(c) ⇌ ADP(c) + Pi(c) + ATP-energy(c)
r0492	1	dAMP(c) + H2O(c) → Deoxyadenosine(c) + Pi(c)
r0348	1	ATP(c) + Thymidine(c) → ADP(c) + dTMP(c)
r0736	0.17	ATP(c) + Fructose-6P(c) → ADP(c) + Fructose-1,6PP(c)
r0030	2	ATP(c) + AMP(c) ⇌ 2 ADP(c)

Continued on next page

ID	flux	reaction
r0495	1	ATP(c) + dTDP(c) → ADP(c) + dTTP(c)
r0050	-1	PRPP(c) + Adenine(c) ⇌ AMP(c) + PPi(c)
r0496	1	ATP(c) + dTMP(c) → ADP(c) + dTDP(c)
r0469	-1	Oxidized thioredoxin(c) + NADPH(c) ⇌ Thioredoxin(c) + NADP+(c)
r0250	-0.67	Ribulose-5P(c) ⇌ Ribose-5P(c)
r0007	1	PPi(c) + H2O(c) → 2 Pi(c)
r0849	-4.17	H2O(s) ⇌ H2O(c)
r0243	-0.17	DHAP(c) ⇌ GAP(c)

2.7 A7. Detoxification of internally emerging substances

O2.-(c) degr (70)

Definition			Solution												
			exchanges		reactions								transp		
Objective	Constraints	Comment	imports	exports	c	m	r	p	l	n	s	b	s-c	b-c	int
-1 O_2.-(c)	MCES =O_2	degradation of cytosolic O_2.-	1 O_2.-(c)	0.5 H_2O(s) 0.75 O_2(s)	2	-	-	-	-	-	-	-	2	-	-

ID	flux	reaction
r1452	0.5	2 O2.-(c) ⇌ H2O2(c) + O2(c)
r0010	0.25	2 H2O2(c) → O2(c) + 2 H2O(c)
r0855	-0.75	O2(s) ⇌ O2(c)
r0849	-0.5	H2O(s) ⇌ H2O(c)

Formaldehyde degr (71)

Definition			Solution												
			exchanges		reactions								transp		
Objective	Constraints	Comment	imports	exports	c	m	r	p	l	n	s	b	s-c	b-c	int
-1 Formaldehyde	MCES	degradation of external formaldehyde	1 Formaldehyde(s) 1 ATP-energy(c)	0.5 CO_2(s) 0.5 NADH-redox-potential(c) 0.5 THF-activated methyl group(c)	11	-	-	-	-	-	-	-	2	-	-

ID	flux	reaction
r0371	-0.5	10-Formyl-THF(c) ⇌ 5,10-Methenyl-THF(c) + H2O(c)
r0227	0.5	10-Formyl-THF(c) + H2O(c) + NADP+(c) → THF(c) + CO2(c) + NADPH(c)
r0228	1	THF(c) + Formate(c) + ATP(c) → ADP(c) + Pi(c) + 10-Formyl-THF(c)
r1319	-1	ATP(c) + H2O(c) ⇌ ADP(c) + Pi(c) + ATP-energy(c)
r0293	-0.5	5,10-Methylene-THF(c) + NADP+(c) ⇌ 5,10-Methenyl-THF(c) + NADPH(c)
r1377	1	GSH(c) + Formaldehyde(c) ⇌ Hydroxymethylglutathione(c)
r0792	-0.5	5,10-Methylene-THF(c) + NADH(c) ⇌ 5-Methyl-THF(c) + NAD+(c)
r1378	1	Hydroxymethylglutathione(c) + NAD+(c) ⇌ NADH(c) + Formylglutathione(c)
r1379	1	H2O(c) + Formylglutathione(c) ⇌ Formate(c) + GSH(c)
r2676	0.5	5-Methyl-THF(c) ⇌ THF(c) + THF-activated methyl group(c)
r1421	1	Formaldehyde(s) ⇌ Formaldehyde(c)
r1322	0.5	NADH(c) ⇌ NAD+(c) + NADH-redox-potential(c)
r0867	0.5	CO2(c) ⇌ CO2(s)

Formate degr (72)

Definition			Solution												
			exchanges		reactions								transp		
Objective	Constraints	Comment	imports	exports	c	m	r	p	l	n	s	b	s-c	b-c	int
-1 Formate	MCES	degradation of external formate	1 Formate(s) 1 ATP-energy(c) 1 Proton-gradient(c)	1 CO_2(s) 1 NADPH-redox-potential(c)	4	-	-	-	-	-	-	-	4	-	-

ID	flux	reaction
r0227	1	10-Formyl-THF(c) + H2O(c) + NADP+(c) → THF(c) + CO2(c) + NADPH(c)
r1326	1	NADPH(c) ⇌ NADPH-redox-potential(c) + NADP+(c)
r0228	1	THF(c) + Formate(c) + ATP(c) → ADP(c) + Pi(c) + 10-Formyl-THF(c)
r1319	-1	ATP(c) + H2O(c) ⇌ ADP(c) + Pi(c) + ATP-energy(c)
r2095	-1	Pyruvate(s) + Formate(c) ⇌ Pyruvate(c) + Formate(s)
r0873	1	Pyruvate(s) + H+(PG)(s) ⇌ Pyruvate(c) + H+(PG)(c)
r1331	-1	H+(PG)(s) ⇌ H+(PG)(c) + Proton-gradient(c)
r0867	1	CO2(c) ⇌ CO2(s)

3 B. Functions of organismic duty

3.1 B1. Storage/Release

B1.1. Glycogen

Glycogen glucose storage (73)

Definition			Solution												
			exchanges		reactions								transp		
Objective	Constraints	Comment	imports	exports	c	m	r	p	l	n	s	b	s-c	b-c	int
Glycogenin-G4G7(c)	−Glycogenin(c) −UDP-activated-glucose(c)	test the storage of glucose in a glycogen component	1 Glycogenin(c) 11 UDP-activated-glucose(c)	1 Glycogenin-G4G7(c)	4	-	-	-	-	-	-	-	-	-	-

ID	flux	reaction
r2665	-11	UDP-glucose(c) ⇌ UDP(c) + UDP-activated-glucose(c)
r1387	1	Glycogenin(c) + 8 UDP-glucose(c) → 8 UDP(c) + Glycogenin-G8(c)
r1388	1	3 UDP-glucose(c) + Glycogenin-G8(c) → Glycogenin-G11(c) + 3 UDP(c)
r1389	1	Glycogenin-G11(c) → Glycogenin-G4G7(c)

Glycogen glucose release (74)

Definition			Solution												
			exchanges		reactions								transp		
Objective	Constraints	Comment	imports	exports	c	m	r	p	l	n	s	b	s-c	b-c	int
Glucose	−Glycogenin-G4G7(c) +Glycogenin(c) −Glucose $-O_2$ $+CO_2$ $=H_2O$	test the release of glucose from a glycogen component	1 H_2O(s) 0.09 Glycogenin-G4G7(c)	1 Glucose(s) 0.09 Glycogenin(c)	5	-	1	-	-	-	-	-	2	-	3

ID	flux	reaction
r1390	0.09	Glycogenin-G4G7(c) + 3 Pi(c) → 3 Glucose-1P(c) + Glycogenin-G4G4(c)
r1391	0.09	Glycogenin-G4G4(c) → Glycogenin-G7G1(c)
r1392	0.09	H2O(c) + Glycogenin-G7G1(c) → Glycogenin-G7(c) + Glucose(c)

Continued on next page

Glycogen glucose release (74)– continued

ID	flux	reaction
r2473	0.91	Glucose-6P(c) + Pi(r) → Glucose-6P(r) + Pi(c)
r1393	0.09	Glycogenin-G7(c) + 7 Pi(c) → Glycogenin(c) + 7 Glucose-1P(c)
r1031	-0.91	Glucose(c) ← Glucose(r)
r0396	0.91	H2O(r) + Glucose-6P(r) → Glucose(r) + Pi(r)
r1032	-1	Glucose(s) ⇌ Glucose(c)
r0848	-0.91	H2O(r) ⇌ H2O(c)
r0849	1	H2O(s) ⇌ H2O(c)
r0621	0.91	Glucose-1P(c) ⇌ Glucose-6P(c)

3.2 B2. Turnover functions

B2.1. Gluconeogenesis

Gluconeogen from Lactate (75)

Definition			Solution												
			exchanges		reactions								transp		
Objective	Constraints	Comment	imports	exports	c	m	r	p	l	n	s	b	s-c	b-c	int
Glucose	−L-Lactate(c) −ATP-energy(m) −ATP-energy(c) =NADH-redox-potential(c) =NADPH-redox-potential(c) =NADH-redox-potential(m) =NADPH-redox-potential(m) =Proton-gradient(m) =H_2O =CO_2 =O_2	Gluconeogenese from Lactate	2 L-Lactate(c) 2 ATP-energy(c) 2 ATP-energy(m) 2 NADPH-redox-potential(m) 2 Proton-gradient(m)	1 Glucose(s) 2 NADH-redox-potential(m)	10	7	1	-	-	-	-	-	1	-	9

ID	flux	reaction
r0253	-2	1,3DPG(c) + NADH(c) ⇌ GAP(c) + Pi(c) + NAD+(c)
r0164	-2	PEP(c) + H2O(c) ⇌ 2PG(c)
r0831	-2	Malate(m) + Pi(c) ⇌ Malate(c) + Pi(m)
r0192	-1	Fructose-6P(c) ⇌ Glucose-6P(c)
r1327	-2	NADPH(m) ⇌ NADPH-redox-potential(m) + NADP+(m)
r0337	2	ATP(c) + 3PG(c) ⇌ ADP(c) + 1,3DPG(c)
r0256	-1	DHAP(c) + GAP(c) ⇌ Fructose-1,6PP(c)
r1319	-2	ATP(c) + H2O(c) ⇌ ADP(c) + Pi(c) + ATP-energy(c)
r0851	2	H2O(m) ⇌ H2O(c)
r0100	2	ATP(m) + GDP(m) ⇌ ADP(m) + GTP(m)
r0059	-2	Pyruvate(m) + CO2(m) + NADPH(m) ⇌ Malate(m) + NADP+(m)
r2473	1	Glucose-6P(c) + Pi(r) → Glucose-6P(r) + Pi(c)
r0872	2	Pyruvate(c) + H+(PG)(c) → Pyruvate(m) + H+(PG)(m)
r2394	-2	cis-Aconitate(m) + PEP(c) ⇌ cis-Aconitate(c) + PEP(m)
r1031	-1	Glucose(c) ← Glucose(r)
r0396	1	H2O(r) + Glucose-6P(r) → Glucose(r) + Pi(r)
r1320	-2	ATP(m) + H2O(m) ⇌ ADP(m) + Pi(m) + ATP-energy(m)
r1032	-1	Glucose(s) ⇌ Glucose(c)

Continued on next page

ID	flux	reaction
r0487	1	Fructose-1,6PP(c) + H2O(c) → Fructose-6P(c) + Pi(c)
r0105	2	NAD+(m) + Malate(m) ⇌ OAA(m) + NADH(m)
r1330	-2	H+(PG)(c) ⇌ H+(PG)(m) + Proton-gradient(m)
r0848	-1	H2O(r) ⇌ H2O(c)
r0124	2	GTP(m) + OAA(m) → GDP(m) + PEP(m) + CO2(m)
r2379	2	cis-Aconitate(m) + Malate(c) ⇌ cis-Aconitate(c) + Malate(m)
r0341	2	2PG(c) ⇌ 3PG(c)
r1323	2	NADH(m) ⇌ NAD+(m) + NADH-redox-potential(m)
r0243	1	GAP(c) ⇌ DHAP(c)
r0171	2	L-Lactate(c) + NAD+(c) ⇌ Pyruvate(c) + NADH(c)

Gluconeogen from Glycerol (76)

Definition			Solution												
			exchanges		reactions								transp		
Objective	Constraints	Comment	imports	exports	c	m	r	p	l	n	s	b	s-c	b-c	int
Glucose	−Glycerol −ATP-energy(m) −ATP-energy(c) =NADH-redox-potential(c) =NADPH-redox-potential(c) =Proton-gradient(m) =H_2O =CO_2 =O_2 %r0530	Gluconeogenese from Glycerol	1 O_2(s) 2 Glycerol(s) 2 ATP-energy(c)	2 H_2O(s) 1 Glucose(s) 12 Proton-gradient(m)	6	-	1	-	-	-	-	-	4	-	9

ID	flux	reaction
r0903	-2	Glycerol(c) ⇌ Glycerol(s)
r0507	2	Ubiquinol(m) + 2 Ferricytochrome C(m) + 4 H+(PG)(m) → Ubiquinone(m) + 4 H+(PG)(c) + 2 Ferrocytochrome C(m)
r0192	-1	Fructose-6P(c) ⇌ Glucose-6P(c)
r0256	-1	DHAP(c) + GAP(c) ⇌ Fructose-1,6PP(c)
r1319	-2	ATP(c) + H2O(c) ⇌ ADP(c) + Pi(c) + ATP-energy(c)
r0851	2	H2O(m) ⇌ H2O(c)
r2473	1	Glucose-6P(c) + Pi(r) → Glucose-6P(r) + Pi(c)
r1031	-1	Glucose(c) ← Glucose(r)
r0855	1	O2(s) ⇌ O2(c)
r0396	1	H2O(r) + Glucose-6P(r) → Glucose(r) + Pi(r)
r0203	2	ATP(c) + Glycerol(c) ⇌ ADP(c) + sn-Glycerol-3P(c)
r1032	-1	Glucose(s) ⇌ Glucose(c)
r0856	-1	O2(m) ⇌ O2(c)
r0487	1	Fructose-1,6PP(c) + H2O(c) → Fructose-6P(c) + Pi(c)
r1330	12	H+(PG)(c) ⇌ H+(PG)(m) + Proton-gradient(m)
r0848	-1	H2O(r) ⇌ H2O(c)
r0205	2	Ubiquinone(m) + sn-Glycerol-3P(c) ⇌ DHAP(c) + Ubiquinol(m)
r0849	-2	H2O(s) ⇌ H2O(c)
r0017	1	O2(m) + 4 H+(PG)(m) + 4 Ferrocytochrome C(m) → 4 Ferricytochrome C(m) + 2 H2O(m) + 4 H+(PG)(c)
r0243	-1	DHAP(c) ⇌ GAP(c)

Gluconeogen from Alanine (77)

Definition			Solution												
			exchanges		reactions								transp		
Objective	Constraints	Comment	imports	exports	c	m	r	p	l	n	s	b	s-c	b-c	int
Glucose	−Alanine −ATP-energy(m) −ATP-energy(c) =NADH-redox-potential(c) =NADPH-redox-potential(c) =Proton-gradient(m) =H_2O =CO_2 =O_2 +Urea	Gluconeogenese from Alanine	1 H_2O(s) 1 CO_2(s) 2 Alanine(s) 5 ATP-energy(c) 3 ATP-energy(m) 1 NADPH-redox-potential(c)	1 Glucose(s) 1 Urea(s) 1 NADH-redox-potential(c)	23	7	1	-	-	-	-	-	5	-	9

ID	flux	reaction
r0144	1	Arginine(c) + H2O(c) ⇌ Urea(c) + Ornithine(c)
r2473	1	Glucose-6P(c) + Pi(r) → Glucose-6P(r) + Pi(c)
r0164	-2	PEP(c) + H2O(c) ⇌ 2PG(c)
r0896	1	Urea(c) ⇌ Urea(s)
r0030	1	ATP(c) + AMP(c) ⇌ 2 ADP(c)
r0034	1	2 ATP(m) + CO2(m) + H2O(m) + NH3(m) ⇌ 2 ADP(m) + Pi(m) + Carbamoyl-P(m)
r0487	1	Fructose-1,6PP(c) + H2O(c) → Fructose-6P(c) + Pi(c)
r0337	2	ATP(c) + 3PG(c) ⇌ ADP(c) + 1,3DPG(c)
r0058	-1	Pyruvate(c) + CO2(c) + NADPH(c) ⇌ Malate(c) + NADP+(c)
r0059	-1	Pyruvate(m) + CO2(m) + NADPH(m) ⇌ Malate(m) + NADP+(m)
r0075	1	Glutamate(m) + H2O(m) + NADP+(m) ⇌ AKG(m) + NADPH(m) + NH3(m)
r0801	-1	GTP(c) + GDP(m) ⇌ GTP(m) + GDP(c)
r1031	-1	Glucose(c) ← Glucose(r)
r1032	-1	Glucose(s) ⇌ Glucose(c)
r0099	1	ATP(c) + GDP(c) ⇌ ADP(c) + GTP(c)
r0243	1	GAP(c) ⇌ DHAP(c)
r0396	1	H2O(r) + Glucose-6P(r) → Glucose(r) + Pi(r)
r1319	-5	ATP(c) + H2O(c) ⇌ ADP(c) + Pi(c) + ATP-energy(c)
r0261	1	Argininosuccinate(c) ⇌ Fumarate(c) + Arginine(c)
r0848	-1	H2O(r) ⇌ H2O(c)
r0849	1	H2O(s) ⇌ H2O(c)
r0867	-1	CO2(c) ⇌ CO2(s)
r0868	2	CO2(c) ⇌ CO2(m)
r0453	1	ATP(c) + Citrulline(c) + Aspartate(c) ⇌ AMP(c) + PPi(c) + Argininosuccinate(c)
r0007	1	PPi(c) + H2O(c) → 2 Pi(c)
r0192	-1	Fructose-6P(c) ⇌ Glucose-6P(c)
r0341	2	2PG(c) ⇌ 3PG(c)
r0329	1	Carbamoyl-P(m) + Ornithine(m) ⇌ Pi(m) + Citrulline(m)
r1434	1	Alanine(c) → Alanine(m)
r0080	1	Alanine(c) + AKG(c) ⇌ Pyruvate(c) + Glutamate(c)
r0081	1	Alanine(m) + AKG(m) ⇌ Pyruvate(m) + Glutamate(m)
r2524	2	Alanine(s) ⇌ Alanine(c)
r0947	-1	Citrulline(m) + Ornithine(c) ⇌ Citrulline(c) + Ornithine(m)
r1320	-3	ATP(m) + H2O(m) ⇌ ADP(m) + Pi(m) + ATP-energy(m)
r1322	1	NADH(c) ⇌ NAD+(c) + NADH-redox-potential(c)
r0100	1	ATP(m) + GDP(m) ⇌ ADP(m) + GTP(m)
r1326	-1	NADPH(c) ⇌ NADPH-redox-potential(c) + NADP+(c)
r0253	-2	1,3DPG(c) + NADH(c) ⇌ GAP(c) + Pi(c) + NAD+(c)
r0831	-1	Malate(m) + Pi(c) ⇌ Malate(c) + Pi(m)
		Continued on next page

ID	flux	reaction
r0104	3	NAD+(c) + Malate(c) ⇌ OAA(c) + NADH(c)
r0256	-1	DHAP(c) + GAP(c) ⇌ Fructose-1,6PP(c)
r0258	-1	Fumarate(c) + H2O(c) ⇌ Malate(c)
r0851	1	H2O(m) ⇌ H2O(c)
r0109	-1	OAA(c) + Glutamate(c) ⇌ AKG(c) + Aspartate(c)
r0123	2	GTP(c) + OAA(c) → GDP(c) + PEP(c) + CO2(c)

B2.2. Amino acid nxn

Alanine from Arginine (78)

Definition			Solution												
			exchanges		reactions								transp		
Objective	Constraints	Comment	imports	exports	c	m	r	p	l	n	s	b	s-c	b-c	int
Alanine	−Arginine MCES +Urea	Transamination to Alanine using Arginine	1.5 H_2O(s) 0.25 O_2(s) 0.5 Pyruvate(m) 0.5 Arginine(s) 0.5 NADPH-redox-potential(m)	1 CO_2(s) 1 Alanine(s) 0.5 Urea(s) 0.5 ATP-energy(m) 1 NADH-redox-potential(m) 1 NADPH-redox-potential(c) 1.5 Proton-gradient(m)	4	11	-	-	-	-	-	-	7	-	10

ID	flux	reaction
r0896	0.5	Urea(c) ⇌ Urea(s)
r0820	1	AKG(c) + Malate(m) ⇌ AKG(m) + Malate(c)
r0911	1	Proline(c) + Glutamate(m) ⇌ Proline(m) + Glutamate(c)
r0074	0.5	L-Glutamate 5-semialdehyde(m) + NAD+(m) + H2O(m) ⇌ Glutamate(m) + NADH(m)
r1326	1	NADPH(c) ⇌ NADPH-redox-potential(c) + NADP+(c)
r0507	0.5	Ubiquinol(m) + 2 Ferricytochrome C(m) + 4 H+(PG)(m) → Ubiquinone(m) + 4 H+(PG)(c) + 2 Ferrocytochrome C(m)
r2524	-0.5	Alanine(c) ⇌ Alanine(s)
r1426	1	Proline(m) + H+(PG)(c) → Proline(c) + H+(PG)(m)
r1327	-0.5	NADPH(m) ⇌ NADPH-redox-potential(m) + NADP+(m)
r0058	1	Malate(c) + NADP+(c) ⇌ Pyruvate(c) + CO2(c) + NADPH(c)
r0851	-1	H2O(m) ⇌ H2O(c)
r0509	0.5	Ubiquinone(m) + Succinate(m) → Ubiquinol(m) + Fumarate(m)
r0167	0.5	AKG(m) + Ornithine(m) ⇌ L-Glutamate 5-semialdehyde(m) + Glutamate(m)
r0100	-0.5	ADP(m) + GTP(m) ⇌ ATP(m) + GDP(m)
r0059	-0.5	Pyruvate(m) + CO2(m) + NADPH(m) ⇌ Malate(m) + NADP+(m)
r1663	-0.5	Lysine(c) + Arginine(s) ⇌ Lysine(s) + Arginine(c)
r0259	-0.5	Fumarate(m) + H2O(m) ⇌ Malate(m)
r0855	0.25	O2(s) ⇌ O2(c)
r1320	0.5	ATP(m) + H2O(m) ⇌ ADP(m) + Pi(m) + ATP-energy(m)
r0856	-0.25	O2(m) ⇌ O2(c)
r2396	-0.5	Lysine(m) + Ornithine(c) + H+(PG)(c) → Lysine(c) + Ornithine(m) + H+(PG)(m)
r1330	1.5	H+(PG)(c) ⇌ H+(PG)(m) + Proton-gradient(m)
r1114	0.5	AKG(m) + CoA(m) + NAD+(m) ⇌ NADH(m) + CO2(m) + Succinyl-CoA(m)
r1070	0.5	Lysine(c) ⇌ Lysine(m)
r0867	1	CO2(c) ⇌ CO2(s)
r0849	1.5	H2O(s) ⇌ H2O(c)
r0125	-0.5	GDP(m) + Pi(m) + Succinyl-CoA(m) ⇌ GTP(m) + Succinate(m) + CoA(m)
r0080	-1	Pyruvate(c) + Glutamate(c) ⇌ Alanine(c) + AKG(c)
r0017	0.25	O2(m) + 4 H+(PG)(m) + 4 Ferrocytochrome C(m) → 4 Ferricytochrome C(m) + 2 H2O(m) + 4 H+(PG)(c)
r1993	0.5	Lysine(s) + Alanine(c) → Lysine(c) + Alanine(s)
r1323	1	NADH(m) ⇌ NAD+(m) + NADH-redox-potential(m)
r0144	0.5	Arginine(c) + H2O(c) ⇌ Urea(c) + Ornithine(c)

Alanine from Asparagine (79)

Definition			Solution												
			exchanges		reactions								transp		
Objective	Constraints	Comment	imports	exports	c	m	r	p	l	n	s	b	s-c	b-c	int
Alanine	−Asparagine MCES +Urea	Transamination to Alanine using Asparagine	0.5 Pyruvate(c) 0.5 Asparagine(s) 0.5 NADH-redox-potential(c) 0.5 NADPH-redox-potential(m) 1 Proton-gradient(m)	0.5 CO_2(s) 1 Alanine(s) 0.5 NADPH-redox-potential(c)	7	2	-	-	-	-	-	-	4	-	7

ID	flux	reaction
r0127	0.5	H2O(c) + Asparagine(c) → Aspartate(c) + NH3(c)
r0109	0.5	AKG(c) + Aspartate(c) ⇌ OAA(c) + Glutamate(c)
r0911	0.5	Proline(c) + Glutamate(m) ⇌ Proline(m) + Glutamate(c)
r1326	0.5	NADPH(c) ⇌ NADPH-redox-potential(c) + NADP+(c)
r1047	0.5	Pi(c) + H+(PG)(c) → Pi(m) + H+(PG)(m)
r0075	-0.5	AKG(m) + NADPH(m) + NH3(m) ⇌ Glutamate(m) + H2O(m) + NADP+(m)
r2524	-0.5	Alanine(c) ⇌ Alanine(s)
r1543	0.5	Glycine(s) + Alanine(c) ⇌ Glycine(c) + Alanine(s)
r1426	0.5	Proline(m) + H+(PG)(c) → Proline(c) + H+(PG)(m)
r1327	-0.5	NADPH(m) ⇌ NADPH-redox-potential(m) + NADP+(m)
r0058	0.5	Malate(c) + NADP+(c) ⇌ Pyruvate(c) + CO2(c) + NADPH(c)
r1553	-0.5	Glycine(s) + Asparagine(c) ⇌ Glycine(c) + Asparagine(s)
r0851	0.5	H2O(m) ⇌ H2O(c)
r2419	0.5	AKG(c) + Pi(m) ⇌ AKG(m) + Pi(c)
r0104	-0.5	OAA(c) + NADH(c) ⇌ NAD+(c) + Malate(c)
r0838	0.5	NH3(c) ⇌ NH3(m)
r1330	-1	H+(PG)(c) ⇌ H+(PG)(m) + Proton-gradient(m)
r1322	-0.5	NADH(c) ⇌ NAD+(c) + NADH-redox-potential(c)
r0867	0.5	CO2(c) ⇌ CO2(s)
r0080	-1	Pyruvate(c) + Glutamate(c) ⇌ Alanine(c) + AKG(c)

Alanine from Aspartate (80)

Definition			Solution												
			exchanges		reactions								transp		
Objective	Constraints	Comment	imports	exports	c	m	r	p	l	n	s	b	s-c	b-c	int
Alanine	−Aspartate MCES +Urea	Transamination to Alanine using Aspartate	1 Aspartate(s) 1 NADH-redox-potential(c)	1 CO_2(s) 1 Alanine(s) 1 NADPH-redox-potential(c) 1 Na-gradient(c)	6	-	-	-	-	-	-	-	4	-	-

ID	flux	reaction
r0109	1	AKG(c) + Aspartate(c) ⇌ OAA(c) + Glutamate(c)
r1326	1	NADPH(c) ⇌ NADPH-redox-potential(c) + NADP+(c)
r2524	-1	Alanine(c) ⇌ Alanine(s)
r0058	1	Malate(c) + NADP+(c) ⇌ Pyruvate(c) + CO2(c) + NADPH(c)
r2664	1	Na+(c) ⇌ Na+(s) + Na-gradient(c)
r0104	-1	OAA(c) + NADH(c) ⇌ NAD+(c) + Malate(c)
r1322	-1	NADH(c) ⇌ NAD+(c) + NADH-redox-potential(c)
r0867	1	CO2(c) ⇌ CO2(s)
r0080	-1	Pyruvate(c) + Glutamate(c) ⇌ Alanine(c) + AKG(c)
r1143	-1	Na+(c) + Aspartate(c) ← Na+(s) + Aspartate(s)

Alanine from Cysteine (81)

Definition			Solution												
			exchanges		reactions								transp		
Objective	Constraints	Comment	imports	exports	c	m	r	p	l	n	s	b	s-c	b-c	int
Alanine	−Cysteine MCES +Urea +H_2S	Transamination to Alanine using Cysteine	1 Cysteine(s) 1 NADH-redox-potential(m) 2 Proton-gradient(m)	1 Alanine(s) 1 H_2S(s) 1 Na-gradient(c)	2	2	-	-	-	-	-	-	4	-	7

ID	flux	reaction
r0073	-1	AKG(m) + NADH(m) + NH3(m) ⇌ Glutamate(m) + NAD+(m) + H2O(m)
r0911	1	Proline(c) + Glutamate(m) ⇌ Proline(m) + Glutamate(c)
r1047	1	Pi(c) + H+(PG)(c) → Pi(m) + H+(PG)(m)
r1426	1	Proline(m) + H+(PG)(c) → Proline(c) + H+(PG)(m)
r0940	1	H2S(c) ⇌ H2S(s)
r0193	1	Cysteine(c) + H2O(c) ⇌ Pyruvate(c) + H2S(c) + NH3(c)
r0851	1	H2O(m) ⇌ H2O(c)
r2419	1	AKG(c) + Pi(m) ⇌ AKG(m) + Pi(c)
r1952	1	Cysteine(s) + Na+(s) + Lysine(c) → Cysteine(c) + Na+(c) + Lysine(s)
r2664	1	Na+(c) ⇌ Na+(s) + Na-gradient(c)
r0838	1	NH3(c) ⇌ NH3(m)
r1330	-2	H+(PG)(c) ⇌ H+(PG)(m) + Proton-gradient(m)
r0080	-1	Pyruvate(c) + Glutamate(c) ⇌ Alanine(c) + AKG(c)
r1993	1	Lysine(s) + Alanine(c) → Lysine(c) + Alanine(s)
r1323	-1	NADH(m) ⇌ NAD+(m) + NADH-redox-potential(m)

Alanine from Cystine (82)

Definition			Solution												
			exchanges		reactions								transp		
Objective	Constraints	Comment	imports	exports	c	m	r	p	l	n	s	b	s-c	b-c	int
Alanine	−Cystine MCES +Urea +H_2S	Transamination to Alanine using Cystine	0.5 Cystine(s) 1 NADH-redox-potential(m) 0.5 NADPH-redox-potential(c) 2 Proton-gradient(m)	1 Alanine(s) 1 H_2S(s) 0.5 Na-gradient(c)	4	2	-	-	-	-	-	-	5	-	7

ID	flux	reaction
r1144	-0.5	Glutamate(c) + Na+(c) ← Glutamate(s) + Na+(s)
r0073	-1	AKG(m) + NADH(m) + NH3(m) ⇌ Glutamate(m) + NAD+(m) + H2O(m)
r0911	1	Proline(c) + Glutamate(m) ⇌ Proline(m) + Glutamate(c)
r1326	-0.5	NADPH(c) ⇌ NADPH-redox-potential(c) + NADP+(c)
r1047	1	Pi(c) + H+(PG)(c) → Pi(m) + H+(PG)(m)
r2524	-1	Alanine(c) ⇌ Alanine(s)
r1426	1	Proline(m) + H+(PG)(c) → Proline(c) + H+(PG)(m)
r0940	1	H2S(c) ⇌ H2S(s)
r0193	1	Cysteine(c) + H2O(c) ⇌ Pyruvate(c) + H2S(c) + NH3(c)
r0851	1	H2O(m) ⇌ H2O(c)
r2419	1	AKG(c) + Pi(m) ⇌ AKG(m) + Pi(c)
r2664	0.5	Na+(c) ⇌ Na+(s) + Na-gradient(c)
r0838	1	NH3(c) ⇌ NH3(m)
r1330	-2	H+(PG)(c) ⇌ H+(PG)(m) + Proton-gradient(m)
r0080	-1	Pyruvate(c) + Glutamate(c) ⇌ Alanine(c) + AKG(c)
r1323	-1	NADH(m) ⇌ NAD+(m) + NADH-redox-potential(m)
r0877	0.5	Glutamate(c) + Cystine(s) ⇌ Glutamate(s) + Cystine(c)
r0027	-0.5	NADPH(c) + Cystine(c) ⇌ 2 Cysteine(c) + NADP+(c)

Alanine from Glutamate (83)

Definition			Solution												
			exchanges		reactions								transp		
Objective	Constraints	Comment	imports	exports	c	m	r	p	l	n	s	b	s-c	b-c	int
Alanine	−Glutamate MCES +Urea	Transamination to Alanine using Glutamate	1 H_2O(s) 0.5 O_2(s) 1 Glutamate(s)	2 CO_2(s) 1 Alanine(s) 1 ATP-energy(m) 1 NADH-redox-potential(m) 1 NADPH-redox-potential(c) 6 Proton-gradient(m) 1 Na-gradient(c)	3	7	-	-	-	-	-	-	6	-	7

ID	flux	reaction
r1144	-1	Glutamate(c) + Na+(c) ← Glutamate(s) + Na+(s)
r0820	1	AKG(c) + Malate(m) ⇌ AKG(m) + Malate(c)
r1326	1	NADPH(c) ⇌ NADPH-redox-potential(c) + NADP+(c)
r0507	1	Ubiquinol(m) + 2 Ferricytochrome C(m) + 4 H+(PG)(m) → Ubiquinone(m) + 4 H+(PG)(c) + 2 Ferrocytochrome C(m)
r2524	-1	Alanine(c) ⇌ Alanine(s)
r0058	1	Malate(c) + NADP+(c) ⇌ Pyruvate(c) + CO2(c) + NADPH(c)
r0851	-1	H2O(m) ⇌ H2O(c)
r0509	1	Ubiquinone(m) + Succinate(m) → Ubiquinol(m) + Fumarate(m)
r0100	-1	ADP(m) + GTP(m) ⇌ ATP(m) + GDP(m)
r0259	-1	Fumarate(m) + H2O(m) ⇌ Malate(m)
r2664	1	Na+(c) ⇌ Na+(s) + Na-gradient(c)
r0855	0.5	O2(s) ⇌ O2(c)
r1320	1	ATP(m) + H2O(m) ⇌ ADP(m) + Pi(m) + ATP-energy(m)
r0856	-0.5	O2(m) ⇌ O2(c)
r1330	6	H+(PG)(c) ⇌ H+(PG)(m) + Proton-gradient(m)
r1114	1	AKG(m) + CoA(m) + NAD+(m) ⇌ NADH(m) + CO2(m) + Succinyl-CoA(m)
r0867	2	CO2(c) ⇌ CO2(s)
r0849	1	H2O(s) ⇌ H2O(c)
r0125	-1	GDP(m) + Pi(m) + Succinyl-CoA(m) ⇌ GTP(m) + Succinate(m) + CoA(m)
r0080	-1	Pyruvate(c) + Glutamate(c) ⇌ Alanine(c) + AKG(c)
r0017	0.5	O2(m) + 4 H+(PG)(m) + 4 Ferrocytochrome C(m) → 4 Ferricytochrome C(m) + 2 H2O(m) + 4 H+(PG)(c)
r1323	1	NADH(m) ⇌ NAD+(m) + NADH-redox-potential(m)
r0868	-1	CO2(c) ⇌ CO2(m)

Alanine from Glycine (84)

Definition			Solution												
			exchanges		reactions								transp		
Objective	Constraints	Comment	imports	exports	c	m	r	p	l	n	s	b	s-c	b-c	int
Alanine	−Glycine MCES +Urea	Transamination to Alanine using Glycine	1 Glycine(s) 1 THF-activated methyl group(c)	1 Alanine(s)	9	-	-	-	-	-	-	-	1	-	-

ID	flux	reaction
r0164	1	2PG(c) ⇌ PEP(c) + H2O(c)
r0327	-1	Hydroxypyruvate(c) + NADPH(c) → Glycerate(c) + NADP+(c)
r1543	1	Glycine(s) + Alanine(c) ⇌ Glycine(c) + Alanine(s)
r0339	1	ATP(c) + Glycerate(c) → ADP(c) + 3PG(c)
r0296	1	5-Methyl-THF(c) + NADP+(c) ⇌ 5,10-Methylene-THF(c) + NADPH(c)
r0230	1	5,10-Methylene-THF(c) + Glycine(c) + H2O(c) ⇌ THF(c) + Serine(c)
r0160	1	Serine(c) + Pyruvate(c) → Hydroxypyruvate(c) + Alanine(c)
r2676	-1	5-Methyl-THF(c) ⇌ THF(c) + THF-activated methyl group(c)
r0341	-1	3PG(c) ⇌ 2PG(c)
r0054	-1	ADP(c) + PEP(c) → ATP(c) + Pyruvate(c)

Alanine from Histidine (85)

Definition			Solution													
			exchanges		reactions								transp			
Objective	Constraints	Comment	imports	exports	c	m	r	p	l	n	s	b	s-c	b-c	int
Alanine	−Histidine MCES +Urea	Transamination to Alanine using Histidine	0.33 H_2O(s) 0.17 O_2(s) 0.33 Pyruvate(c) 0.33 Pyruvate(m) 0.33 Histidine(s) 0.33 NADH-redox-potential(c) 0.33 NADH-redox-potential(m) 0.33 NADPH-redox-potential(m)	0.67 CO_2(s) 1 Alanine(s) 0.33 ATP-energy(m) 0.33 NADPH-redox-potential(c) 1 Proton-gradient(m) 0.33 THF-activated methyl group(c)	12	10	-	-	-	-	-	-	6	-	11

ID	flux	reaction
r0125	-0.33	GDP(m) + Pi(m) + Succinyl-CoA(m) ⇌ GTP(m) + Succinate(m) + CoA(m)
r0855	0.17	O2(s) ⇌ O2(c)
r0856	-0.17	O2(m) ⇌ O2(c)
r0293	-0.33	5,10-Methenyl-THF(c) + NADPH(c) ⇌ 5,10-Methylene-THF(c) + NADP+(c)
r0577	-0.33	Urocanate(c) + H2O(c) ⇌ 4-Imidazolone-5-propanoate(c)
r1665	-0.33	Lysine(c) + Histidine(s) ⇌ Lysine(s) + Histidine(c)
r0017	0.17	O2(m) + 4 H+(PG)(m) + 4 Ferrocytochrome C(m) → 4 Ferricytochrome C(m) + 2 H2O(m) + 4 H+(PG)(c)
r0911	0.67	Proline(c) + Glutamate(m) ⇌ Proline(m) + Glutamate(c)
r0058	0.67	Malate(c) + NADP+(c) ⇌ Pyruvate(c) + CO2(c) + NADPH(c)
r1426	0.67	Proline(m) + H+(PG)(c) → Proline(c) + H+(PG)(m)
r0059	-0.33	Pyruvate(m) + CO2(m) + NADPH(m) ⇌ Malate(m) + NADP+(m)
r0073	-0.67	AKG(m) + NADH(m) + NH3(m) ⇌ Glutamate(m) + NAD+(m) + H2O(m)
r0507	0.33	Ubiquinol(m) + 2 Ferricytochrome C(m) + 4 H+(PG)(m) → Ubiquinone(m) + 4 H+(PG)(c) + 2 Ferrocytochrome C(m)
r0509	0.33	Ubiquinone(m) + Succinate(m) → Ubiquinol(m) + Fumarate(m)
r0524	0.33	5-Formiminotetrahydrofolate(c) ⇌ 5,10-Methenyl-THF(c) + NH3(c)
r0820	0.67	AKG(c) + Malate(m) ⇌ AKG(m) + Malate(c)
r1330	1	H+(PG)(c) ⇌ H+(PG)(m) + Proton-gradient(m)
r0849	0.33	H2O(s) ⇌ H2O(c)
r0286	0.33	Histidine(c) ⇌ Urocanate(c) + NH3(c)
r0867	0.67	CO2(c) ⇌ CO2(s)
r1993	0.33	Lysine(s) + Alanine(c) → Lysine(c) + Alanine(s)
r1114	0.33	AKG(m) + CoA(m) + NAD+(m) ⇌ NADH(m) + CO2(m) + Succinyl-CoA(m)
r0792	-0.33	5,10-Methylene-THF(c) + NADH(c) ⇌ 5-Methyl-THF(c) + NAD+(c)
r0080	-1	Pyruvate(c) + Glutamate(c) ⇌ Alanine(c) + AKG(c)
r2676	0.33	5-Methyl-THF(c) ⇌ THF(c) + THF-activated methyl group(c)
r2524	-0.67	Alanine(c) ⇌ Alanine(s)
r1320	0.33	ATP(m) + H2O(m) ⇌ ADP(m) + Pi(m) + ATP-energy(m)
r1322	-0.33	NADH(c) ⇌ NAD+(c) + NADH-redox-potential(c)
r0518	-0.33	Glutamate(c) + 5-Formiminotetrahydrofolate(c) ⇌ THF(c) + N-Formimino-L-glutamate(c)
r1323	-0.33	NADH(m) ⇌ NAD+(m) + NADH-redox-potential(m)
r0519	0.33	H2O(c) + 4-Imidazolone-5-propanoate(c) ⇌ N-Formimino-L-glutamate(c)
r0100	-0.33	ADP(m) + GTP(m) ⇌ ATP(m) + GDP(m)
r1326	0.33	NADPH(c) ⇌ NADPH-redox-potential(c) + NADP+(c)
r1327	-0.33	NADPH(m) ⇌ NADPH-redox-potential(m) + NADP+(m)
r1047	0.33	Pi(c) + H+(PG)(c) → Pi(m) + H+(PG)(m)
r0259	-0.33	Fumarate(m) + H2O(m) ⇌ Malate(m)
r2419	0.33	AKG(c) + Pi(m) ⇌ AKG(m) + Pi(c)
r0838	0.67	NH3(c) ⇌ NH3(m)
r0851	0.33	H2O(m) ⇌ H2O(c)

Alanine from Isoleucine (86)

Definition			Solution												
			exchanges		reactions								transp		
Objective	Constraints	Comment	imports	exports	c	m	r	p	l	n	s	b	s-c	b-c	int
Alanine	−Isoleucine MCES +Urea	Transamination to Alanine using Isoleucine	1 H_2O(s) 1 O_2(s) 1 Pyruvate(c) 1 Isoleucine(s)	2 CO_2(s) 1 Alanine(s) 4 NADH-redox-potential(m) 9 Proton-gradient(m) 2 CoA-activated acetyl group(m)	1	13	-	-	-	-	-	-	5	-	11

ID	flux	reaction
r2678	2	Acetyl-CoA(m) ⇌ CoA(m) + CoA-activated acetyl group(m)
r0911	1	Proline(c) + Glutamate(m) ⇌ Proline(m) + Glutamate(c)
r1047	1	Pi(c) + H+(PG)(c) → Pi(m) + H+(PG)(m)
r0507	2	Ubiquinol(m) + 2 Ferricytochrome C(m) + 4 H+(PG)(m) → Ubiquinone(m) + 4 H+(PG)(c) + 2 Ferrocytochrome C(m)
r0264	1	Isoleucine(m) + AKG(m) ⇌ Glutamate(m) + 2-Oxo-3-methylvalerate(m)
r0174	1	CoA(m) + NAD+(m) + 3-Oxopropanoate(m) → Acetyl-CoA(m) + CO2(m) + NADH(m)
r1543	1	Glycine(s) + Alanine(c) ⇌ Glycine(c) + Alanine(s)
r1426	1	Proline(m) + H+(PG)(c) → Proline(c) + H+(PG)(m)
r0851	-1	H2O(m) ⇌ H2O(c)
r0365	1	Hydracrylate(m) + NAD+(m) → NADH(m) + 3-Oxopropanoate(m)
r2419	1	AKG(c) + Pi(m) ⇌ AKG(m) + Pi(c)
r0664	1	NAD+(m) + (S)-3-Hydroxy-2-methylbutyryl-CoA(m) ⇌ 2-Methylacetoacetyl-CoA(m) + NADH(m)
r0592	-1	H2O(m) + Acrylyl-CoA(m) → 3-Hydroxypropionyl-CoA(m)
r0953	1	Isoleucine(c) + H+(PG)(c) → Isoleucine(m) + H+(PG)(m)
r0683	1	Propanoyl-CoA(m) + Ubiquinone(m) → Ubiquinol(m) + Acrylyl-CoA(m)
r0665	-1	H2O(m) + Tiglyl-CoA(m) ⇌ (S)-3-Hydroxy-2-methylbutyryl-CoA(m)
r1557	-1	Glycine(s) + Isoleucine(c) ⇌ Glycine(c) + Isoleucine(s)
r0855	1	O2(s) ⇌ O2(c)
r0856	-1	O2(m) ⇌ O2(c)
r0222	-1	CoA(m) + 2-Methylacetoacetyl-CoA(m) ⇌ Propanoyl-CoA(m) + Acetyl-CoA(m)
r1330	9	H+(PG)(c) ⇌ H+(PG)(m) + Proton-gradient(m)
r0596	-1	3-Hydroxypropionyl-CoA(m) + H2O(m) → Hydracrylate(m) + CoA(m)
r0867	2	CO2(c) ⇌ CO2(s)
r0849	1	H2O(s) ⇌ H2O(c)
r0602	1	2-Oxo-3-methylvalerate(m) + CoA(m) + NAD+(m) ⇌ 2-Methylbutyryl-CoA(m) + CO2(m) + NADH(m)
r0080	-1	Pyruvate(c) + Glutamate(c) ⇌ Alanine(c) + AKG(c)
r0017	1	O2(m) + 4 H+(PG)(m) + 4 Ferrocytochrome C(m) → 4 Ferricytochrome C(m) + 2 H2O(m) + 4 H+(PG)(c)
r1323	4	NADH(m) ⇌ NAD+(m) + NADH-redox-potential(m)
r0868	-2	CO2(c) ⇌ CO2(m)
r0603	1	2-Methylbutyryl-CoA(m) + Ubiquinone(m) ⇌ Tiglyl-CoA(m) + Ubiquinol(m)

Alanine from Glutamine (87)

Definition			Solution												
			exchanges		reactions								transp		
Objective	Constraints	Comment	imports	exports	c	m	r	p	l	n	s	b	s-c	b-c	int
Alanine	−Glutamine MCES +Urea	Transamination to Alanine using Glutamine	0.5 H_2O(s) 0.25 O_2(s) 0.5 Pyruvate(c) 0.5 Glutamine(s) 0.5 NADPH-redox-potential(m)	1 CO_2(s) 1 Alanine(s) 0.5 ATP-energy(m) 0.5 NADH-redox-potential(m) 0.5 NADPH-redox-potential(c) 2 Proton-gradient(m)	5	9	-	-	-	-	-	-	5	-	11

ID	flux	reaction
r0820	0.5	AKG(c) + Malate(m) ⇌ AKG(m) + Malate(c)
r0911	0.5	Proline(c) + Glutamate(m) ⇌ Proline(m) + Glutamate(c)
r1326	0.5	NADPH(c) ⇌ NADPH-redox-potential(c) + NADP+(c)
r1047	0.5	Pi(c) + H+(PG)(c) → Pi(m) + H+(PG)(m)
		Continued on next page

ID	flux	reaction
r0507	0.5	Ubiquinol(m) + 2 Ferricytochrome C(m) + 4 H+(PG)(m) → Ubiquinone(m) + 4 H+(PG)(c) + 2 Ferrocytochrome C(m)
r0156	0.5	Glutamine(c) + Pyruvate(c) → 2-Oxoglutaramate(c) + Alanine(c)
r0075	-0.5	AKG(m) + NADPH(m) + NH3(m) ⇌ Glutamate(m) + H2O(m) + NADP+(m)
r2524	-0.5	Alanine(c) ⇌ Alanine(s)
r1426	0.5	Proline(m) + H+(PG)(c) → Proline(c) + H+(PG)(m)
r1327	-0.5	NADPH(m) ⇌ NADPH-redox-potential(m) + NADP+(m)
r0085	0.5	2-Oxoglutaramate(c) + H2O(c) → AKG(c) + NH3(c)
r0058	0.5	Malate(c) + NADP+(c) ⇌ Pyruvate(c) + CO2(c) + NADPH(c)
r0509	0.5	Ubiquinone(m) + Succinate(m) → Ubiquinol(m) + Fumarate(m)
r0100	-0.5	ADP(m) + GTP(m) ⇌ ATP(m) + GDP(m)
r2419	0.5	AKG(c) + Pi(m) ⇌ AKG(m) + Pi(c)
r0259	-0.5	Fumarate(m) + H2O(m) ⇌ Malate(m)
r0855	0.25	O2(s) ⇌ O2(c)
r1558	-0.5	Alanine(c) + Glutamine(s) ⇌ Alanine(s) + Glutamine(c)
r1320	0.5	ATP(m) + H2O(m) ⇌ ADP(m) + Pi(m) + ATP-energy(m)
r0856	-0.25	O2(m) ⇌ O2(c)
r0838	0.5	NH3(c) ⇌ NH3(m)
r1330	2	H+(PG)(c) ⇌ H+(PG)(m) + Proton-gradient(m)
r1114	0.5	AKG(m) + CoA(m) + NAD+(m) ⇌ NADH(m) + CO2(m) + Succinyl-CoA(m)
r0867	1	CO2(c) ⇌ CO2(s)
r0849	0.5	H2O(s) ⇌ H2O(c)
r0125	-0.5	GDP(m) + Pi(m) + Succinyl-CoA(m) ⇌ GTP(m) + Succinate(m) + CoA(m)
r0080	-0.5	Pyruvate(c) + Glutamate(c) ⇌ Alanine(c) + AKG(c)
r0017	0.25	O2(m) + 4 H+(PG)(m) + 4 Ferrocytochrome C(m) → 4 Ferricytochrome C(m) + 2 H2O(m) + 4 H+(PG)(c)
r1323	0.5	NADH(m) ⇌ NAD+(m) + NADH-redox-potential(m)
r0868	-0.5	CO2(c) ⇌ CO2(m)

Alanine from Leucine (88)

Definition			Solution												
			exchanges		reactions								transp		
Objective	Constraints	Comment	imports	exports	c	m	r	p	l	n	s	b	s-c	b-c	int
Alanine	−Leucine MCES +Urea	Transamination to Alanine using Leucine	0.5 O_2(s) 1 Pyruvate(c) 1 Leucine(s) 1 NADPH-redox-potential(m)	1 H_2O(s) 1 Alanine(s) 2 NADH-redox-potential(m) 4 Proton-gradient(m) 1 Na-gradient(c) 3 CoA-activated acetyl group(m)	1	13	-	-	-	-	-	-	5	-	10

ID	flux	reaction
r2678	3	Acetyl-CoA(m) ⇌ CoA(m) + CoA-activated acetyl group(m)
r0262	1	Leucine(m) + AKG(m) ⇌ Glutamate(m) + 4-Methyl-2-oxopentanoate(m)
r0911	1	Proline(c) + Glutamate(m) ⇌ Proline(m) + Glutamate(c)
r1047	1	Pi(c) + H+(PG)(c) → Pi(m) + H+(PG)(m)
r0507	1	Ubiquinol(m) + 2 Ferricytochrome C(m) + 4 H+(PG)(m) → Ubiquinone(m) + 4 H+(PG)(c) + 2 Ferrocytochrome C(m)
r0462	1	HMG-CoA(m) + CoA(m) ⇌ Acetyl-CoA(m) + H2O(m) + Acetoacetyl-CoA(m)
r1426	1	Proline(m) + H+(PG)(c) → Proline(c) + H+(PG)(m)
r1327	-1	NADPH(m) ⇌ NADPH-redox-potential(m) + NADP+(m)
r0490	-1	3-Methylglutaconyl-CoA(m) + H2O(m) ⇌ HMG-CoA(m)
r0851	1	H2O(m) ⇌ H2O(c)
r0059	-1	Pyruvate(m) + CO2(m) + NADPH(m) ⇌ Malate(m) + NADP+(m)
r2419	1	AKG(c) + Pi(m) ⇌ AKG(m) + Pi(c)
r1438	1	Leucine(c) → Leucine(m)
r0655	1	Isovaleryl-CoA(m) + Ubiquinone(m) ⇌ 3-Methylcrotonyl-CoA(m) + Ubiquinol(m)
r2664	1	Na+(c) ⇌ Na+(s) + Na-gradient(c)
r0855	0.5	O2(s) ⇌ O2(c)
r0856	-0.5	O2(m) ⇌ O2(c)

Continued on next page

ID	flux	reaction
r0658	1	ATP(m) + 3-Methylcrotonyl-CoA(m) + HCO3-(m) ⇌ ADP(m) + Pi(m) + 3-Methylglutaconyl-CoA(m)
r0105	1	NAD+(m) + Malate(m) ⇌ OAA(m) + NADH(m)
r1330	4	H+(PG)(c) ⇌ H+(PG)(m) + Proton-gradient(m)
r0601	1	4-Methyl-2-oxopentanoate(m) + CoA(m) + NAD+(m) ⇌ Isovaleryl-CoA(m) + CO2(m) + NADH(m)
r0106	-1	ADP(m) + Pi(m) + OAA(m) ⇌ ATP(m) + Pyruvate(m) + HCO3-(m)
r0070	-1	CoA(m) + Acetoacetyl-CoA(m) ⇌ 2 Acetyl-CoA(m)
r0849	-1	H2O(s) ⇌ H2O(c)
r0080	-1	Pyruvate(c) + Glutamate(c) ⇌ Alanine(c) + AKG(c)
r0017	0.5	O2(m) + 4 H+(PG)(m) + 4 Ferrocytochrome C(m) → 4 Ferricytochrome C(m) + 2 H2O(m) + 4 H+(PG)(c)
r1993	1	Lysine(s) + Alanine(c) → Lysine(c) + Alanine(s)
r1957	1	Leucine(s) + Na+(s) + Lysine(c) → Leucine(c) + Na+(c) + Lysine(s)
r1323	2	NADH(m) ⇌ NAD+(m) + NADH-redox-potential(m)

Alanine from Lysine (89)

Definition			Solution												
			exchanges		reactions								transp		
Objective	Constraints	Comment	imports	exports	c	m	r	p	l	n	s	b	s-c	b-c	int
Alanine	−Lysine MCES +Urea	Transamination to Alanine using Lysine	0.5 H_2O(s) 0.25 O_2(s) 1 Pyruvate(c) 0.5 Lysine(s)	1 CO_2(s) 1 Alanine(s) 0.5 NADH-redox-potential(c) 1 NADH-redox-potential(m) 1 Proton-gradient(m) 1 CoA-activated acetyl group(m)	3	10	-	-	-	-	-	-	5	-	11

ID	flux	reaction
r2678	1	Acetyl-CoA(m) ⇌ CoA(m) + CoA-activated acetyl group(m)
r0541	0.5	Glutaryl-CoA(m) + Ubiquinone(m) ⇌ Crotonyl-CoA(m) + CO2(m) + Ubiquinol(m)
r0460	0.5	(S)-3-Hydroxybutyryl-CoA(m) + NAD+(m) ⇌ NADH(m) + Acetoacetyl-CoA(m)
r0911	1	Proline(c) + Glutamate(m) ⇌ Proline(m) + Glutamate(c)
r1047	1	Pi(c) + H+(PG)(c) → Pi(m) + H+(PG)(m)
r0507	0.5	Ubiquinol(m) + 2 Ferricytochrome C(m) + 4 H+(PG)(m) → Ubiquinone(m) + 4 H+(PG)(c) + 2 Ferrocytochrome C(m)
r2524	-0.5	Alanine(c) ⇌ Alanine(s)
r1426	1	Proline(m) + H+(PG)(c) → Proline(c) + H+(PG)(m)
r0526	0.5	H2O(m) + Saccharopine(m) + NADP+(m) ⇌ Glutamate(m) + 2-Aminoadipate 6-semialdehyde(m) + NADPH(m)
r2419	1	AKG(c) + Pi(m) ⇌ AKG(m) + Pi(c)
r0448	0.5	2-Oxoadipate(m) + CoA(m) + NAD+(m) ⇌ Glutaryl-CoA(m) + CO2(m) + NADH(m)
r0855	0.25	O2(s) ⇌ O2(c)
r0594	0.5	2-Aminoadipate 6-semialdehyde(c) + NAD+(c) + H2O(c) ⇌ NADH(c) + L-2-Aminoadipate(c)
r2539	-0.5	2-Aminoadipate 6-semialdehyde(c) + L-2-Aminoadipate(m) ⇌ 2-Aminoadipate 6-semialdehyde(m) + L-2-Aminoadipate(c)
r0856	-0.25	O2(m) ⇌ O2(c)
r1330	1	H+(PG)(c) ⇌ H+(PG)(m) + Proton-gradient(m)
r0070	-0.5	CoA(m) + Acetoacetyl-CoA(m) ⇌ 2 Acetyl-CoA(m)
r1322	0.5	NADH(c) ⇌ NAD+(c) + NADH-redox-potential(c)
r1070	0.5	Lysine(c) ⇌ Lysine(m)
r0867	1	CO2(c) ⇌ CO2(s)
r0849	0.5	H2O(s) ⇌ H2O(c)
r0588	-0.5	Crotonyl-CoA(m) + H2O(m) ⇌ (S)-3-Hydroxybutyryl-CoA(m)
r0080	-1	Pyruvate(c) + Glutamate(c) ⇌ Alanine(c) + AKG(c)
r0017	0.25	O2(m) + 4 H+(PG)(m) + 4 Ferrocytochrome C(m) → 4 Ferricytochrome C(m) + 2 H2O(m) + 4 H+(PG)(c)
r1993	0.5	Lysine(s) + Alanine(c) → Lysine(c) + Alanine(s)
r1323	1	NADH(m) ⇌ NAD+(m) + NADH-redox-potential(m)
r0868	-1	CO2(c) ⇌ CO2(m)
r0450	0.5	AKG(m) + L-2-Aminoadipate(m) ⇌ 2-Oxoadipate(m) + Glutamate(m)
r0180	-0.5	AKG(m) + NADPH(m) + Lysine(m) ⇌ H2O(m) + Saccharopine(m) + NADP+(m)

Alanine from Methionine (90)

Definition			Solution												
			exchanges		reactions								transp		
Objective	Constraints	Comment	imports	exports	c	m	r	p	l	n	s	b	s-c	b-c	int
Alanine	−Methionine MCES +Urea $+H_2S$	Transamination to Alanine using Methionine	1.5 H_2O(s) 0.5 O_2(s) 1 Methionine(s) 0.5 Glucose-6P(c)	0.5 P_i(c) 2 CO_2(s) 1 Alanine(s) 1 H_2S(s) 0.5 ATP-energy(c) 2 NADH-redox-potential(c) 1 NADH-redox-potential(m) 1 Proton-gradient(m) 1 Na-gradient(c) 1 THF-activated methyl group(c) 1 CoA-activated acetyl group(m)	17	9	-	-	-	-	-	-	7	-	12

ID	flux	reaction
r0855	0.5	O2(s) ⇌ O2(c)
r0856	-0.5	O2(m) ⇌ O2(c)
r0592	-1	H2O(m) + Acrylyl-CoA(m) → 3-Hydroxypropionyl-CoA(m)
r0596	-1	3-Hydroxypropionyl-CoA(m) + H2O(m) → Hydracrylate(m) + CoA(m)
r0017	0.5	O2(m) + 4 H+(PG)(m) + 4 Ferrocytochrome C(m) → 4 Ferricytochrome C(m) + 2 H2O(m) + 4 H+(PG)(c)
r0314	1	Serine(c) + Homocysteine(c) ⇌ H2O(c) + L-Cystathionine(c)
r0911	2	Proline(c) + Glutamate(m) ⇌ Proline(m) + Glutamate(c)
r0337	-1	ADP(c) + 1,3DPG(c) ⇌ ATP(c) + 3PG(c)
r0338	1	3PG(c) + NAD+(c) → 3-Phosphonooxypyruvate(c) + NADH(c)
r2664	1	Na+(c) ⇌ Na+(s) + Na-gradient(c)
r1426	2	Proline(m) + H+(PG)(c) → Proline(c) + H+(PG)(m)
r0073	-2	AKG(m) + NADH(m) + NH3(m) ⇌ Glutamate(m) + NAD+(m) + H2O(m)
r0507	1	Ubiquinol(m) + 2 Ferricytochrome C(m) + 4 H+(PG)(m) → Ubiquinone(m) + 4 H+(PG)(c) + 2 Ferrocytochrome C(m)
r0241	1	H2O(c) + L-Cystathionine(c) ⇌ Cysteine(c) + NH3(c) + 2-Oxobutyrate(c)
r0243	-0.5	DHAP(c) ⇌ GAP(c)
r1330	1	H+(PG)(c) ⇌ H+(PG)(m) + Proton-gradient(m)
r1319	0.5	ATP(c) + H2O(c) ⇌ ADP(c) + Pi(c) + ATP-energy(c)
r0849	1.5	H2O(s) ⇌ H2O(c)
r0867	2	CO2(c) ⇌ CO2(s)
r1672	1	Glycine(s) + Alanine(c) + Na+(s) → Glycine(c) + Alanine(s) + Na+(c)
r0868	-2	CO2(c) ⇌ CO2(m)
r0736	0.5	ATP(c) + Fructose-6P(c) → ADP(c) + Fructose-1,6PP(c)
r0159	1	3-Phosphoserine(c) + H2O(c) → Serine(c) + Pi(c)
r0174	1	CoA(m) + NAD+(m) + 3-Oxopropanoate(m) → Acetyl-CoA(m) + CO2(m) + NADH(m)
r1546	-1	Glycine(c) + Methionine(s) ⇌ Glycine(s) + Methionine(c)
r0192	0.5	Glucose-6P(c) ⇌ Fructose-6P(c)
r0193	1	Cysteine(c) + H2O(c) ⇌ Pyruvate(c) + H2S(c) + NH3(c)
r0080	-1	Pyruvate(c) + Glutamate(c) ⇌ Alanine(c) + AKG(c)
r0940	1	H2S(c) ⇌ H2S(s)
r1154	1	CoA(m) + NAD+(m) + 2-Oxobutyrate(m) ⇌ CO2(m) + Propanoyl-CoA(m) + NADH(m)
r1155	1	2-Oxobutyrate(c) + H+(PG)(c) → 2-Oxobutyrate(m) + H+(PG)(m)
r2676	1	5-Methyl-THF(c) ⇌ THF(c) + THF-activated methyl group(c)
r0365	1	Hydracrylate(m) + NAD+(m) → NADH(m) + 3-Oxopropanoate(m)
r2678	1	Acetyl-CoA(m) ⇌ CoA(m) + CoA-activated acetyl group(m)
r0663	-1	3-Phosphonooxypyruvate(c) + Glutamate(c) → 3-Phosphoserine(c) + AKG(c)
r0232	-1	THF(c) + Methionine(c) ⇌ 5-Methyl-THF(c) + Homocysteine(c)
r1322	2	NADH(c) ⇌ NAD+(c) + NADH-redox-potential(c)
r0683	1	Propanoyl-CoA(m) + Ubiquinone(m) → Ubiquinol(m) + Acrylyl-CoA(m)
r1323	1	NADH(m) ⇌ NAD+(m) + NADH-redox-potential(m)
r0253	1	GAP(c) + Pi(c) + NAD+(c) ⇌ 1,3DPG(c) + NADH(c)
r0256	0.5	Fructose-1,6PP(c) ⇌ DHAP(c) + GAP(c)
r1047	2	Pi(c) + H+(PG)(c) → Pi(m) + H+(PG)(m)
r2419	2	AKG(c) + Pi(m) ⇌ AKG(m) + Pi(c)
r0838	2	NH3(c) ⇌ NH3(m)
r0851	1	H2O(m) ⇌ H2O(c)

Alanine from Phenylalanine (91)

Definition			Solution												
			exchanges		reactions								transp		
Objective	Constraints	Comment	imports	exports	c	m	r	p	l	n	s	b	s-c	b-c	int
Alanine	−Phenylalanine MCES +Urea	Transamination to Alanine using Phenylalanine	3 O_2(s) 1 Phenylalanine(s) 2 ATP-energy(c)	2 CO_2(s) 1 Alanine(s) 2 CoA-activated acetyl group(c)	16	-	-	-	-	-	-	-	4	-	-

ID	flux	reaction
r0605	1	4-Maleylacetoacetate(c) ⇌ Fumarylacetoacetate(c)
r0543	1	Homogentisate(c) + O2(c) ⇌ 4-Maleylacetoacetate(c)
r0183	1	Tyrosine(c) + AKG(c) ⇌ 4-Hydroxyphenylpyruvate(c) + Glutamate(c)
r2524	-1	Alanine(s) ⇌ Alanine(c)
r0544	1	4-Hydroxyphenylpyruvate(c) + O2(c) ⇌ Homogentisate(c) + CO2(c)
r0058	1	Malate(c) + NADP+(c) ⇌ Pyruvate(c) + CO2(c) + NADPH(c)
r1319	-2	ATP(c) + H2O(c) ⇌ ADP(c) + Pi(c) + ATP-energy(c)
r0258	-1	Fumarate(c) + H2O(c) ⇌ Malate(c)
r0069	-1	CoA(c) + Acetoacetyl-CoA(c) ⇌ 2 Acetyl-CoA(c)
r0030	1	ATP(c) + AMP(c) ⇌ 2 ADP(c)
r2528	1	Phenylalanine(s) ⇌ Phenylalanine(c)
r0855	3	O2(s) ⇌ O2(c)
r0320	1	ATP(c) + CoA(c) + Acetoacetate(c) → AMP(c) + PPi(c) + Acetoacetyl-CoA(c)
r0398	1	Dihydrobiopterin(c) + NADPH(c) ⇌ Tetrahydrobiopterin(c) + NADP+(c)
r0007	1	PPi(c) + H2O(c) → 2 Pi(c)
r0867	2	CO2(c) ⇌ CO2(s)
r0399	1	Tetrahydrobiopterin(c) + Phenylalanine(c) + O2(c) ⇌ Dihydrobiopterin(c) + Tyrosine(c) + H2O(c)
r0080	-1	Pyruvate(c) + Glutamate(c) ⇌ Alanine(c) + AKG(c)
r2677	2	Acetyl-CoA(c) ⇌ CoA(c) + CoA-activated acetyl group(c)
r0324	-1	Fumarylacetoacetate(c) + H2O(c) ⇌ Fumarate(c) + Acetoacetate(c)

Alanine from Proline (92)

Definition			Solution												
			exchanges		reactions								transp		
Objective	Constraints	Comment	imports	exports	c	m	r	p	l	n	s	b	s-c	b-c	int
Alanine	−Proline MCES +Urea	Transamination to Alanine using Proline	4 H_2O(s) 1 Proline(s)	2 CO_2(s) 1 Alanine(s) 1 ATP-energy(m) 2 NADH-redox-potential(m) 1 NADPH-redox-potential(c) 1 Na-gradient(c)	3	10	-	-	-	-	-	-	5	-	4

ID	flux	reaction
r0820	1	AKG(c) + Malate(m) ⇌ AKG(m) + Malate(c)
r0911	1	Proline(c) + Glutamate(m) ⇌ Proline(m) + Glutamate(c)
r0074	1	L-Glutamate 5-semialdehyde(m) + NAD+(m) + H2O(m) ⇌ Glutamate(m) + NADH(m)
r1326	1	NADPH(c) ⇌ NADPH-redox-potential(c) + NADP+(c)
r1453	1	Proline(m) + Ubiquinol(m) ⇌ 1-Pyrroline-5-carboxylate(m) + Ubiquinone(m)
r0175	1	1-Pyrroline-5-carboxylate(m) + H2O(m) ⇌ L-Glutamate 5-semialdehyde(m)
r0058	1	Malate(c) + NADP+(c) ⇌ Pyruvate(c) + CO2(c) + NADPH(c)
r0851	-4	H2O(m) ⇌ H2O(c)
r0509	1	Ubiquinone(m) + Succinate(m) → Ubiquinol(m) + Fumarate(m)
r0100	-1	ADP(m) + GTP(m) ⇌ ATP(m) + GDP(m)
r0259	-1	Fumarate(m) + H2O(m) ⇌ Malate(m)
r2664	1	Na+(c) ⇌ Na+(s) + Na-gradient(c)
r1962	1	Proline(s) + Na+(s) + Lysine(c) → Proline(c) + Na+(c) + Lysine(s)
r1320	1	ATP(m) + H2O(m) ⇌ ADP(m) + Pi(m) + ATP-energy(m)

Continued on next page

ID	flux	reaction
r1114	1	AKG(m) + CoA(m) + NAD+(m) ⇌ NADH(m) + CO2(m) + Succinyl-CoA(m)
r0867	2	CO2(c) ⇌ CO2(s)
r0849	4	H2O(s) ⇌ H2O(c)
r0125	-1	GDP(m) + Pi(m) + Succinyl-CoA(m) ⇌ GTP(m) + Succinate(m) + CoA(m)
r0080	-1	Pyruvate(c) + Glutamate(c) ⇌ Alanine(c) + AKG(c)
r1993	1	Lysine(s) + Alanine(c) → Lysine(c) + Alanine(s)
r1323	2	NADH(m) ⇌ NAD+(m) + NADH-redox-potential(m)
r0868	-1	CO2(c) ⇌ CO2(m)

Alanine from Serine (93)

Definition			Solution												
			exchanges		reactions								transp		
Objective	Constraints	Comment	imports	exports	c	m	r	p	l	n	s	b	s-c	b-c	int
Alanine	−Serine MCES +Urea	Transamination to Alanine using Serine	1 Serine(s) 1 NADPH-redox-potential(c)	1 H_2O(s) 1 Alanine(s)	7	-	-	-	-	-	-	-	3	-	-

ID	flux	reaction
r0164	1	2PG(c) ⇌ PEP(c) + H2O(c)
r1326	-1	NADPH(c) ⇌ NADPH-redox-potential(c) + NADP+(c)
r0327	-1	Hydroxypyruvate(c) + NADPH(c) → Glycerate(c) + NADP+(c)
r1543	1	Glycine(s) + Alanine(c) ⇌ Glycine(c) + Alanine(s)
r1545	-1	Glycine(s) + Serine(c) ⇌ Glycine(c) + Serine(s)
r0339	1	ATP(c) + Glycerate(c) → ADP(c) + 3PG(c)
r0160	1	Serine(c) + Pyruvate(c) → Hydroxypyruvate(c) + Alanine(c)
r0849	-1	H2O(s) ⇌ H2O(c)
r0341	-1	3PG(c) ⇌ 2PG(c)
r0054	-1	ADP(c) + PEP(c) → ATP(c) + Pyruvate(c)

Alanine from Threonine (94)

Definition			Solution												
			exchanges		reactions								transp		
Objective	Constraints	Comment	imports	exports	c	m	r	p	l	n	s	b	s-c	b-c	int
Alanine	−Threonine MCES +Urea	Transamination to Alanine using Threonine	0.5 O_2(s) 1 Pyruvate(c) 1 Threonine(s)	2 CO_2(s) 1 Alanine(s) 2 NADH-redox-potential(m) 3 Proton-gradient(m) 1 CoA-activated acetyl group(m)	2	9	-	-	-	-	-	-	4	-	11

ID	flux	reaction
r2678	1	Acetyl-CoA(m) ⇌ CoA(m) + CoA-activated acetyl group(m)
r0073	-1	AKG(m) + NADH(m) + NH3(m) ⇌ Glutamate(m) + NAD+(m) + H2O(m)
r1154	1	CoA(m) + NAD+(m) + 2-Oxobutyrate(m) ⇌ CO2(m) + Propanoyl-CoA(m) + NADH(m)
r0911	1	Proline(c) + Glutamate(m) ⇌ Proline(m) + Glutamate(c)
r1155	1	2-Oxobutyrate(c) + H+(PG)(c) → 2-Oxobutyrate(m) + H+(PG)(m)
r1047	1	Pi(c) + H+(PG)(c) → Pi(m) + H+(PG)(m)
r0507	1	Ubiquinol(m) + 2 Ferricytochrome C(m) + 4 H+(PG)(m) → Ubiquinone(m) + 4 H+(PG)(c) + 2 Ferrocytochrome C(m)
r0174	1	CoA(m) + NAD+(m) + 3-Oxopropanoate(m) → Acetyl-CoA(m) + CO2(m) + NADH(m)
r2524	-1	Alanine(s) ⇌ Alanine(c)
r1426	1	Proline(m) + H+(PG)(c) → Proline(c) + H+(PG)(m)
r2534	1	Threonine(s) ⇌ Threonine(c)
r0365	1	Hydracrylate(m) + NAD+(m) → NADH(m) + 3-Oxopropanoate(m)

Continued on next page

ID	flux	reaction
r2419	1	AKG(c) + Pi(m) ⇌ AKG(m) + Pi(c)
r0592	-1	H2O(m) + Acrylyl-CoA(m) → 3-Hydroxypropionyl-CoA(m)
r0683	1	Propanoyl-CoA(m) + Ubiquinone(m) → Ubiquinol(m) + Acrylyl-CoA(m)
r0855	0.5	O2(s) ⇌ O2(c)
r0856	-0.5	O2(m) ⇌ O2(c)
r0838	1	NH3(c) ⇌ NH3(m)
r0240	1	Threonine(c) ⇌ 2-Oxobutyrate(c) + NH3(c)
r1330	3	H+(PG)(c) ⇌ H+(PG)(m) + Proton-gradient(m)
r0596	-1	3-Hydroxypropionyl-CoA(m) + H2O(m) → Hydracrylate(m) + CoA(m)
r0867	2	CO2(c) ⇌ CO2(s)
r0080	-1	Pyruvate(c) + Glutamate(c) ⇌ Alanine(c) + AKG(c)
r0017	0.5	O2(m) + 4 H+(PG)(m) + 4 Ferrocytochrome C(m) → 4 Ferricytochrome C(m) + 2 H2O(m) + 4 H+(PG)(c)
r1323	2	NADH(m) ⇌ NAD+(m) + NADH-redox-potential(m)
r0868	-2	CO2(c) ⇌ CO2(m)

Alanine from Tryptophan (95)

Definition			Solution													
			exchanges		reactions								transp		
Objective	Constraints	Comment	imports	exports	c	m	r	p	l	n	s	b	s-c	b-c	int
Alanine	−Tryptophan MCES +Urea	Transamination to Alanine using Tryptophan	1.75 O_2(s) 0.5 Pyruvate(c) 0.5 Tryptophan(s) 0.5 ATP-energy(c) 1.5 NADPH-redox-potential(c) 0.5 NADPH-redox-potential(m)	1.5 CO_2(s) 1 Alanine(s) 1 NADH-redox-potential(m) 1.5 Proton-gradient(m) 0.5 THF-activated methyl group(c) 1 CoA-activated acetyl group(m)	16	9	-	-	-	-	-	-	5	-	12

ID	flux	reaction
r0855	1.75	O2(s) ⇌ O2(c)
r0856	-0.25	O2(m) ⇌ O2(c)
r0293	-0.5	5,10-Methylene-THF(c) + NADP+(c) ⇌ 5,10-Methenyl-THF(c) + NADPH(c)
r0460	0.5	(S)-3-Hydroxybutyryl-CoA(m) + NAD+(m) ⇌ NADH(m) + Acetoacetyl-CoA(m)
r0448	0.5	2-Oxoadipate(m) + CoA(m) + NAD+(m) ⇌ Glutaryl-CoA(m) + CO2(m) + NADH(m)
r0449	-0.5	2-Oxoadipate(c) + NH3(c) + NADP+(c) ⇌ 2-Aminomuconate(c) + NADPH(c) + H2O(c)
r0017	0.25	O2(m) + 4 H+(PG)(m) + 4 Ferrocytochrome C(m) → 4 Ferricytochrome C(m) + 2 H2O(m) + 4 H+(PG)(c)
r0169	0.5	Tryptophan(c) + O2(c) ⇌ L-Formylkynurenine(c)
r0911	0.5	Proline(c) + Glutamate(m) ⇌ Proline(m) + Glutamate(c)
r0070	-0.5	CoA(m) + Acetoacetyl-CoA(m) ⇌ 2 Acetyl-CoA(m)
r1426	0.5	Proline(m) + H+(PG)(c) → Proline(c) + H+(PG)(m)
r0075	-0.5	AKG(m) + NADPH(m) + NH3(m) ⇌ Glutamate(m) + H2O(m) + NADP+(m)
r0371	-0.5	10-Formyl-THF(c) ⇌ 5,10-Methenyl-THF(c) + H2O(c)
r0507	0.5	Ubiquinol(m) + 2 Ferricytochrome C(m) + 4 H+(PG)(m) → Ubiquinone(m) + 4 H+(PG)(c) + 2 Ferrocytochrome C(m)
r0228	0.5	THF(c) + Formate(c) + ATP(c) → ADP(c) + Pi(c) + 10-Formyl-THF(c)
r0676	0.5	2-Amino-3-carboxymuconate semialdehyde(c) ⇌ 2-Aminomuconate semialdehyde(c) + CO2(c)
r1330	1.5	H+(PG)(c) ⇌ H+(PG)(m) + Proton-gradient(m)
r1319	-0.5	ATP(c) + H2O(c) ⇌ ADP(c) + Pi(c) + ATP-energy(c)
r0541	0.5	Glutaryl-CoA(m) + Ubiquinone(m) ⇌ Crotonyl-CoA(m) + CO2(m) + Ubiquinol(m)
r0562	0.5	3-Hydroxyanthranilate(c) + O2(c) ⇌ 2-Amino-3-carboxymuconate semialdehyde(c)
r0563	0.5	H2O(c) + 3-Hydroxy-L-kynurenine(c) ⇌ 3-Hydroxyanthranilate(c) + Alanine(c)
r0867	1.5	CO2(c) ⇌ CO2(s)
r0868	-1	CO2(c) ⇌ CO2(m)
r0588	-0.5	Crotonyl-CoA(m) + H2O(m) ⇌ (S)-3-Hydroxybutyryl-CoA(m)
r0454	0.5	L-Formylkynurenine(c) + H2O(c) ⇌ Formate(c) + Kynurenine(c)
r0455	0.5	Kynurenine(c) + O2(c) + NADPH(c) ⇌ H2O(c) + 3-Hydroxy-L-kynurenine(c) + NADP+(c)
r1543	0.5	Glycine(s) + Alanine(c) ⇌ Glycine(c) + Alanine(s)
r1547	-0.5	Glycine(s) + Tryptophan(c) ⇌ Glycine(c) + Tryptophan(s)

Continued on next page

Alanine from Tryptophan (95)– continued

ID	flux	reaction
r0792	-0.5	5,10-Methylene-THF(c) + NADH(c) ⇌ 5-Methyl-THF(c) + NAD+(c)
r0080	-0.5	Pyruvate(c) + Glutamate(c) ⇌ Alanine(c) + AKG(c)
r0645	0.5	2-Aminomuconate semialdehyde(c) + NAD+(c) + H2O(c) ⇌ 2-Aminomuconate(c) + NADH(c)
r2676	0.5	5-Methyl-THF(c) ⇌ THF(c) + THF-activated methyl group(c)
r2524	-0.5	Alanine(c) ⇌ Alanine(s)
r2678	1	Acetyl-CoA(m) ⇌ CoA(m) + CoA-activated acetyl group(m)
r0945	0.5	2-Oxoadipate(c) + AKG(m) ⇌ 2-Oxoadipate(m) + AKG(c)
r1323	1	NADH(m) ⇌ NAD+(m) + NADH-redox-potential(m)
r1326	-1.5	NADPH(c) ⇌ NADPH-redox-potential(c) + NADP+(c)
r1327	-0.5	NADPH(m) ⇌ NADPH-redox-potential(m) + NADP+(m)
r1047	1	Pi(c) + H+(PG)(c) → Pi(m) + H+(PG)(m)
r2419	1	AKG(c) + Pi(m) ⇌ AKG(m) + Pi(c)
r0838	0.5	NH3(c) ⇌ NH3(m)
r0851	0.5	H2O(m) ⇌ H2O(c)

Alanine from Tyrosine (96)

Definition			Solution												
			exchanges		reactions								transp		
Objective	Constraints	Comment	imports	exports	c	m	r	p	l	n	s	b	s-c	b-c	int
Alanine	−Tyrosine MCES +Urea	Transamination to Alanine using Tyrosine	1 H_2O(s) 2 O_2(s) 1 Tyrosine(s) 2 ATP-energy(c)	2 CO_2(s) 1 Alanine(s) 1 NADPH-redox-potential(c) 1 Na-gradient(c) 2 CoA-activated acetyl group(c)	15	-	-	-	-	-	-	-	6	-	-

ID	flux	reaction
r0605	1	4-Maleylacetoacetate(c) ⇌ Fumarylacetoacetate(c)
r1326	1	NADPH(c) ⇌ NADPH-redox-potential(c) + NADP+(c)
r0543	1	Homogentisate(c) + O2(c) ⇌ 4-Maleylacetoacetate(c)
r0183	1	Tyrosine(c) + AKG(c) ⇌ 4-Hydroxyphenylpyruvate(c) + Glutamate(c)
r2524	-1	Alanine(c) ⇌ Alanine(s)
r0544	1	4-Hydroxyphenylpyruvate(c) + O2(c) ⇌ Homogentisate(c) + CO2(c)
r0058	1	Malate(c) + NADP+(c) ⇌ Pyruvate(c) + CO2(c) + NADPH(c)
r1319	-2	ATP(c) + H2O(c) ⇌ ADP(c) + Pi(c) + ATP-energy(c)
r0258	-1	Fumarate(c) + H2O(c) ⇌ Malate(c)
r0069	-1	CoA(c) + Acetoacetyl-CoA(c) ⇌ 2 Acetyl-CoA(c)
r0030	1	ATP(c) + AMP(c) ⇌ 2 ADP(c)
r2664	1	Na+(c) ⇌ Na+(s) + Na-gradient(c)
r0855	2	O2(s) ⇌ O2(c)
r0320	1	ATP(c) + CoA(c) + Acetoacetate(c) → AMP(c) + PPi(c) + Acetoacetyl-CoA(c)
r0007	1	PPi(c) + H2O(c) → 2 Pi(c)
r0867	2	CO2(c) ⇌ CO2(s)
r0849	1	H2O(s) ⇌ H2O(c)
r0080	-1	Pyruvate(c) + Glutamate(c) ⇌ Alanine(c) + AKG(c)
r2677	2	Acetyl-CoA(c) ⇌ CoA(c) + CoA-activated acetyl group(c)
r1125	1	Tyrosine(s) + Na+(s) → Tyrosine(c) + Na+(c)
r0324	-1	Fumarylacetoacetate(c) + H2O(c) ⇌ Fumarate(c) + Acetoacetate(c)

Alanine from Valine (97)

Definition			Solution												
			exchanges		reactions								transp		
Objective	Constraints	Comment	imports	exports	c	m	r	p	l	n	s	b	s-c	b-c	int
Alanine	−Valine MCES +Urea	Transamination to Alanine using Valine	2 H_2O(s) 1 O_2(s) 1 Valine(s)	2 CO_2(s) 1 Alanine(s) 1 ATP-energy(m) 3 NADH-redox-potential(m) 1 NADPH-redox-potential(c) 10 Proton-gradient(m)	3	14	-	-	-	-	-	-	5	-	10

ID	flux	reaction
r0820	1	AKG(c) + Malate(m) ⇌ AKG(m) + Malate(c)
r0779	1	3-Hydroxyisobutyryl-CoA(m) + H2O(m) → 3-Hydroxyisobutyrate(m) + CoA(m)
r0911	1	Proline(c) + Glutamate(m) ⇌ Proline(m) + Glutamate(c)
r0560	1	Isobutyryl-CoA(m) + Ubiquinone(m) → Methacrylyl-CoA(m) + Ubiquinol(m)
r0263	1	Valine(m) + AKG(m) → Glutamate(m) + 3-Methyl-2-oxobutyrate(m)
r1326	1	NADPH(c) ⇌ NADPH-redox-potential(c) + NADP+(c)
r0624	1	2-Methyl-3-oxopropanoate(m) + CoA(m) + NAD+(m) ⇌ (R)-Methylmalonyl-CoA(m) + NADH(m)
r0507	2	Ubiquinol(m) + 2 Ferricytochrome C(m) + 4 H+(PG)(m) → Ubiquinone(m) + 4 H+(PG)(c) + 2 Ferrocytochrome C(m)
r1543	1	Glycine(s) + Alanine(c) ⇌ Glycine(c) + Alanine(s)
r1426	1	Proline(m) + H+(PG)(c) → Proline(c) + H+(PG)(m)
r0058	1	Malate(c) + NADP+(c) ⇌ Pyruvate(c) + CO2(c) + NADPH(c)
r0923	1	Valine(c) + H+(PG)(c) → Valine(m) + H+(PG)(m)
r0851	-2	H2O(m) ⇌ H2O(c)
r0509	1	Ubiquinone(m) + Succinate(m) → Ubiquinol(m) + Fumarate(m)
r0482	1	3-Hydroxyisobutyrate(m) + NAD+(m) → 2-Methyl-3-oxopropanoate(m) + NADH(m)
r0100	-1	ADP(m) + GTP(m) ⇌ ATP(m) + GDP(m)
r1554	-1	Glycine(s) + Valine(c) ⇌ Glycine(c) + Valine(s)
r0259	-1	Fumarate(m) + H2O(m) ⇌ Malate(m)
r0197	1	(R)-Methylmalonyl-CoA(m) ⇌ Succinyl-CoA(m)
r0855	1	O2(s) ⇌ O2(c)
r1320	1	ATP(m) + H2O(m) ⇌ ADP(m) + Pi(m) + ATP-energy(m)
r0856	-1	O2(m) ⇌ O2(c)
r0600	1	3-Methyl-2-oxobutyrate(m) + CoA(m) + NAD+(m) ⇌ Isobutyryl-CoA(m) + CO2(m) + NADH(m)
r1330	10	H+(PG)(c) ⇌ H+(PG)(m) + Proton-gradient(m)
r0867	2	CO2(c) ⇌ CO2(s)
r0849	2	H2O(s) ⇌ H2O(c)
r0669	1	Methacrylyl-CoA(m) + H2O(m) → 3-Hydroxyisobutyryl-CoA(m)
r0125	-1	GDP(m) + Pi(m) + Succinyl-CoA(m) ⇌ GTP(m) + Succinate(m) + CoA(m)
r0080	-1	Pyruvate(c) + Glutamate(c) ⇌ Alanine(c) + AKG(c)
r0017	1	O2(m) + 4 H+(PG)(m) + 4 Ferrocytochrome C(m) → 4 Ferricytochrome C(m) + 2 H2O(m) + 4 H+(PG)(c)
r1323	3	NADH(m) ⇌ NAD+(m) + NADH-redox-potential(m)
r0868	-1	CO2(c) ⇌ CO2(m)

Alanine from beta-Alanine (98)

Definition			Solution												
			exchanges		reactions								transp		
Objective	Constraints	Comment	imports	exports	c	m	r	p	l	n	s	b	s-c	b-c	int
Alanine	−beta-Alanine MCES +Urea	Transamination to Alanine using beta-Alanine	1 Pyruvate(c) 1 beta-Alanine(s) 2 Proton-gradient(m)	1 CO_2(s) 1 Alanine(s) 1 NADH-redox-potential(m) 1 CoA-activated acetyl group(m)	1	4	-	-	-	-	-	-	3	-	7

ID	flux	reaction
r2678	1	Acetyl-CoA(m) ⇌ CoA(m) + CoA-activated acetyl group(m)
r0217	1	beta-Alanine(m) + AKG(m) ⇌ Glutamate(m) + 3-Oxopropanoate(m)
r0911	1	Proline(c) + Glutamate(m) ⇌ Proline(m) + Glutamate(c)

Continued on next page

ID	flux	reaction
r1047	1	Pi(c) + H+(PG)(c) → Pi(m) + H+(PG)(m)
r0174	1	CoA(m) + NAD+(m) + 3-Oxopropanoate(m) → Acetyl-CoA(m) + CO2(m) + NADH(m)
r2524	-1	Alanine(c) ⇌ Alanine(s)
r1426	1	Proline(m) + H+(PG)(c) → Proline(c) + H+(PG)(m)
r0899	-1	beta-Alanine(s) ⇌ beta-Alanine(c)
r2419	1	AKG(c) + Pi(m) ⇌ AKG(m) + Pi(c)
r1330	-2	H+(PG)(c) ⇌ H+(PG)(m) + Proton-gradient(m)
r0867	1	CO2(c) ⇌ CO2(s)
r0080	-1	Pyruvate(c) + Glutamate(c) ⇌ Alanine(c) + AKG(c)
r1323	1	NADH(m) ⇌ NAD+(m) + NADH-redox-potential(m)
r0900	1	beta-Alanine(c) ⇌ beta-Alanine(m)
r0868	-1	CO2(c) ⇌ CO2(m)

Arginine from Alanine (99)

Definition			Solution												
			exchanges		reactions								transp		
Objective	Constraints	Comment	imports	exports	c	m	r	p	l	n	s	b	s-c	b-c	int
Arginine	−Alanine MCES +Urea	Transamination to Arginine using Alanine	4 Alanine(s) 2 ATP-energy(c) 3 ATP-energy(m) 2 NADPH-redox-potential(m) 1 Proton-gradient(m)	1 Pyruvate(c) 1 Pyruvate(m) 1 Arginine(s) 1 NADH-redox-potential(c) 4 NADH-redox-potential(m) 1 Na-gradient(c)	10	17	-	-	-	-	-	-	4	-	3

ID	flux	reaction
r0316	1	Citrate(m) ⇌ Isocitrate(m)
r0109	-1	OAA(c) + Glutamate(c) ⇌ AKG(c) + Aspartate(c)
r0073	1	Glutamate(m) + NAD+(m) + H2O(m) ⇌ AKG(m) + NADH(m) + NH3(m)
r0425	1	Isocitrate(m) + NAD+(m) ⇌ Oxalosuccinate(m) + NADH(m)
r0083	1	Oxalosuccinate(m) ⇌ AKG(m) + CO2(m)
r0056	1	Pyruvate(m) + CoA(m) + NAD+(m) → Acetyl-CoA(m) + CO2(m) + NADH(m)
r1434	3	Alanine(c) → Alanine(m)
r0453	1	ATP(c) + Citrulline(c) + Aspartate(c) ⇌ AMP(c) + PPi(c) + Argininosuccinate(c)
r2524	3	Alanine(s) ⇌ Alanine(c)
r1327	-2	NADPH(m) ⇌ NADPH-redox-potential(m) + NADP+(m)
r2417	-1	Citrulline(m) + H+(PG)(c) → Citrulline(c) + H+(PG)(m)
r1319	-2	ATP(c) + H2O(c) ⇌ ADP(c) + Pi(c) + ATP-energy(c)
r0329	1	Carbamoyl-P(m) + Ornithine(m) ⇌ Pi(m) + Citrulline(m)
r0167	-1	L-Glutamate 5-semialdehyde(m) + Glutamate(m) ⇌ AKG(m) + Ornithine(m)
r0059	-1	Pyruvate(m) + CO2(m) + NADPH(m) ⇌ Malate(m) + NADP+(m)
r0258	-1	Fumarate(c) + H2O(c) ⇌ Malate(c)
r1663	1	Lysine(s) + Arginine(c) ⇌ Lysine(c) + Arginine(s)
r0619	-1	L-Glutamate 5-semialdehyde(m) + Pi(m) + NADP+(m) ← NADPH(m) + Glutamyl-5P(m)
r0030	1	ATP(c) + AMP(c) ⇌ 2 ADP(c)
r2664	1	Na+(c) ⇌ Na+(s) + Na-gradient(c)
r1917	1	Alanine(s) + Na+(s) + Lysine(c) → Alanine(c) + Na+(c) + Lysine(s)
r0104	1	NAD+(c) + Malate(c) ⇌ OAA(c) + NADH(c)
r1320	-3	ATP(m) + H2O(m) ⇌ ADP(m) + Pi(m) + ATP-energy(m)
r0105	1	NAD+(m) + Malate(m) ⇌ OAA(m) + NADH(m)
r1330	-1	H+(PG)(c) ⇌ H+(PG)(m) + Proton-gradient(m)
r0034	1	2 ATP(m) + CO2(m) + H2O(m) + NH3(m) ⇌ 2 ADP(m) + Pi(m) + Carbamoyl-P(m)
r0007	1	PPi(c) + H2O(c) → 2 Pi(c)
r1322	1	NADH(c) ⇌ NAD+(c) + NADH-redox-potential(c)
r0107	-1	Acetyl-CoA(m) + H2O(m) + OAA(m) ⇌ Citrate(m) + CoA(m)
r0080	1	Alanine(c) + AKG(c) ⇌ Pyruvate(c) + Glutamate(c)
r1323	4	NADH(m) ⇌ NAD+(m) + NADH-redox-potential(m)

Continued on next page

ID	flux	reaction
r0261	1	Argininosuccinate(c) ⇌ Fumarate(c) + Arginine(c)
r0081	3	Alanine(m) + AKG(m) ⇌ Pyruvate(m) + Glutamate(m)
r0072	1	ATP(m) + Glutamate(m) → ADP(m) + Glutamyl-5P(m)

Arginine from Asparagine (100)

Definition			Solution												
			exchanges		reactions								transp		
Objective	Constraints	Comment	imports	exports	c	m	r	p	l	n	s	b	s-c	b-c	int
Arginine	−Asparagine MCES +Urea	Transamination to Arginine using Asparagine	2 Asparagine(s) 3 ATP-energy(c) 3 ATP-energy(m) 1 NADH-redox-potential(c) 2 NADPH-redox-potential(m) 1 Proton-gradient(m) 1 CoA-activated acetyl group(m)	1 CO_2(s) 1 Pyruvate(c) 1 Arginine(s) 2 NADH-redox-potential(m) 1 NADPH-redox-potential(c) 1 Proton-gradient(c) 2 Na-gradient(c)	13	16	-	-	-	-	-	-	6	-	6

ID	flux	reaction
r0127	2	H2O(c) + Asparagine(c) → Aspartate(c) + NH3(c)
r0425	1	Isocitrate(m) + NAD+(m) ⇌ Oxalosuccinate(m) + NADH(m)
r0167	-1	L-Glutamate 5-semialdehyde(m) + Glutamate(m) ⇌ AKG(m) + Ornithine(m)
r2479	1	Asparagine(s) + Na+(s) + H+(PG)(c) → Asparagine(c) + Na+(c) + H+(PG)(s)
r0030	1	ATP(c) + AMP(c) ⇌ 2 ADP(c)
r1553	-1	Glycine(c) + Asparagine(s) ⇌ Glycine(s) + Asparagine(c)
r0034	1	2 ATP(m) + CO2(m) + H2O(m) + NH3(m) ⇌ 2 ADP(m) + Pi(m) + Carbamoyl-P(m)
r0316	1	Citrate(m) ⇌ Isocitrate(m)
r0619	-1	L-Glutamate 5-semialdehyde(m) + Pi(m) + NADP+(m) ← NADPH(m) + Glutamyl-5P(m)
r2664	2	Na+(c) ⇌ Na+(s) + Na-gradient(c)
r0058	1	Malate(c) + NADP+(c) ⇌ Pyruvate(c) + CO2(c) + NADPH(c)
r0072	1	ATP(m) + Glutamate(m) → ADP(m) + Glutamyl-5P(m)
r0075	-1	AKG(m) + NADPH(m) + NH3(m) ⇌ Glutamate(m) + H2O(m) + NADP+(m)
r0077	1	ATP(c) + Glutamate(c) + NH3(c) → ADP(c) + Pi(c) + Glutamine(c)
r0078	1	Glutamine(m) + H2O(m) → Glutamate(m) + NH3(m)
r0820	-1	AKG(m) + Malate(c) ⇌ AKG(c) + Malate(m)
r1330	-1	H+(PG)(c) ⇌ H+(PG)(m) + Proton-gradient(m)
r1331	1	H+(PG)(s) ⇌ H+(PG)(c) + Proton-gradient(c)
r1319	-3	ATP(c) + H2O(c) ⇌ ADP(c) + Pi(c) + ATP-energy(c)
r0261	1	Argininosuccinate(c) ⇌ Fumarate(c) + Arginine(c)
r1913	1	Glycine(s) + Na+(s) + Arginine(c) → Glycine(c) + Na+(c) + Arginine(s)
r0867	1	CO2(c) ⇌ CO2(s)
r0453	1	ATP(c) + Citrulline(c) + Aspartate(c) ⇌ AMP(c) + PPi(c) + Argininosuccinate(c)
r0007	1	PPi(c) + H2O(c) → 2 Pi(c)
r0329	1	Carbamoyl-P(m) + Ornithine(m) ⇌ Pi(m) + Citrulline(m)
r2522	1	Glutamine(c) ⇌ Glutamine(m)
r0083	1	Oxalosuccinate(m) ⇌ AKG(m) + CO2(m)
r2678	-1	Acetyl-CoA(m) ⇌ CoA(m) + CoA-activated acetyl group(m)
r1320	-3	ATP(m) + H2O(m) ⇌ ADP(m) + Pi(m) + ATP-energy(m)
r1322	-1	NADH(c) ⇌ NAD+(c) + NADH-redox-potential(c)
r1323	2	NADH(m) ⇌ NAD+(m) + NADH-redox-potential(m)
r1326	1	NADPH(c) ⇌ NADPH-redox-potential(c) + NADP+(c)
r1327	-2	NADPH(m) ⇌ NADPH-redox-potential(m) + NADP+(m)
r0104	-1	OAA(c) + NADH(c) ⇌ NAD+(c) + Malate(c)
r0105	1	NAD+(m) + Malate(m) ⇌ OAA(m) + NADH(m)
r0258	-1	Fumarate(c) + H2O(c) ⇌ Malate(c)
r2417	-1	Citrulline(m) + H+(PG)(c) → Citrulline(c) + H+(PG)(m)
r0107	-1	Acetyl-CoA(m) + H2O(m) + OAA(m) ⇌ Citrate(m) + CoA(m)
r0838	1	NH3(c) ⇌ NH3(m)

Continued on next page

ID	flux	reaction
r0851	1	H2O(m) ⇌ H2O(c)
r0109	1	AKG(c) + Aspartate(c) ⇌ OAA(c) + Glutamate(c)

Arginine from Aspartate (101)

Definition			Solution												
			exchanges		reactions								transp		
Objective	Constraints	Comment	imports	exports	c	m	r	p	l	n	s	b	s-c	b-c	int
Arginine	−Aspartate MCES +Urea	Transamination to Arginine using Aspartate	4 Aspartate(s) 2 ATP-energy(c) 3 ATP-energy(m) 1 NADH-redox-potential(c) 2 Proton-gradient(m)	5 CO_2(s) 1 Pyruvate(c) 1 Arginine(s) 3 NADH-redox-potential(m) 2 NADPH-redox-potential(c) 4 Na-gradient(c) 1 CoA-activated acetyl group(m)	14	14	-	-	-	-	-	-	4	-	6

ID	flux	reaction
r2678	1	Acetyl-CoA(m) ⇌ CoA(m) + CoA-activated acetyl group(m)
r0316	1	Citrate(m) ⇌ Isocitrate(m)
r0217	2	beta-Alanine(m) + AKG(m) ⇌ Glutamate(m) + 3-Oxopropanoate(m)
r0109	1	AKG(c) + Aspartate(c) ⇌ OAA(c) + Glutamate(c)
r0082	1	Oxalosuccinate(c) ⇌ AKG(c) + CO2(c)
r0128	2	Aspartate(c) ⇌ beta-Alanine(c) + CO2(c)
r1326	2	NADPH(c) ⇌ NADPH-redox-potential(c) + NADP+(c)
r0453	1	ATP(c) + Citrulline(c) + Aspartate(c) ⇌ AMP(c) + PPi(c) + Argininosuccinate(c)
r0174	2	CoA(m) + NAD+(m) + 3-Oxopropanoate(m) → Acetyl-CoA(m) + CO2(m) + NADH(m)
r0075	1	Glutamate(m) + H2O(m) + NADP+(m) ⇌ AKG(m) + NADPH(m) + NH3(m)
r0913	1	Isocitrate(m) + Malate(c) ⇌ Isocitrate(c) + Malate(m)
r0058	1	Malate(c) + NADP+(c) ⇌ Pyruvate(c) + CO2(c) + NADPH(c)
r2417	-1	Citrulline(m) + H+(PG)(c) → Citrulline(c) + H+(PG)(m)
r1319	-2	ATP(c) + H2O(c) ⇌ ADP(c) + Pi(c) + ATP-energy(c)
r1058	-1	Arginine(c) ⇌ Arginine(s)
r0329	1	Carbamoyl-P(m) + Ornithine(m) ⇌ Pi(m) + Citrulline(m)
r0167	-1	L-Glutamate 5-semialdehyde(m) + Glutamate(m) ⇌ AKG(m) + Ornithine(m)
r0258	-1	Fumarate(c) + H2O(c) ⇌ Malate(c)
r0619	-1	L-Glutamate 5-semialdehyde(m) + Pi(m) + NADP+(m) ← NADPH(m) + Glutamyl-5P(m)
r0030	1	ATP(c) + AMP(c) ⇌ 2 ADP(c)
r2664	4	Na+(c) ⇌ Na+(s) + Na-gradient(c)
r0104	-1	OAA(c) + NADH(c) ⇌ NAD+(c) + Malate(c)
r1320	-3	ATP(m) + H2O(m) ⇌ ADP(m) + Pi(m) + ATP-energy(m)
r0105	1	NAD+(m) + Malate(m) ⇌ OAA(m) + NADH(m)
r1330	-2	H+(PG)(c) ⇌ H+(PG)(m) + Proton-gradient(m)
r0875	1	Glutamate(c) + H+(PG)(c) → Glutamate(m) + H+(PG)(m)
r0034	1	2 ATP(m) + CO2(m) + H2O(m) + NH3(m) ⇌ 2 ADP(m) + Pi(m) + Carbamoyl-P(m)
r0007	1	PPi(c) + H2O(c) → 2 Pi(c)
r1322	-1	NADH(c) ⇌ NAD+(c) + NADH-redox-potential(c)
r0867	5	CO2(c) ⇌ CO2(s)
r0422	1	Isocitrate(c) + NADP+(c) ⇌ Oxalosuccinate(c) + NADPH(c)
r0107	-1	Acetyl-CoA(m) + H2O(m) + OAA(m) ⇌ Citrate(m) + CoA(m)
r1323	3	NADH(m) ⇌ NAD+(m) + NADH-redox-potential(m)
r1143	-4	Na+(c) + Aspartate(c) ← Na+(s) + Aspartate(s)
r0900	2	beta-Alanine(c) ⇌ beta-Alanine(m)
r0868	-1	CO2(c) ⇌ CO2(m)
r0261	1	Argininosuccinate(c) ⇌ Fumarate(c) + Arginine(c)
r0072	1	ATP(m) + Glutamate(m) → ADP(m) + Glutamyl-5P(m)

Arginine from Cysteine (102)

Definition			Solution												
			exchanges		reactions								transp		
Objective	Constraints	Comment	imports	exports	c	m	r	p	l	n	s	b	s-c	b-c	int
Arginine	−Cysteine MCES +Urea +H_2S	Transamination to Arginine using Cysteine	2 Pyruvate(m) 4 Cysteine(s) 2 ATP-energy(c) 3 ATP-energy(m) 2 NADPH-redox-potential(m) 3 Proton-gradient(m)	4 Pyruvate(c) 1 Arginine(s) 4 H_2S(s) 1 NADH-redox-potential(c) 4 Na-gradient(c)	10	15	-	-	-	-	-	-	5	-	8

ID	flux	reaction
r0316	1	Citrate(m) ⇌ Isocitrate(m)
r0109	-1	OAA(c) + Glutamate(c) ⇌ AKG(c) + Aspartate(c)
r0073	-3	AKG(m) + NADH(m) + NH3(m) ⇌ Glutamate(m) + NAD+(m) + H2O(m)
r1550	-1	Glycine(c) + Cysteine(s) ⇌ Glycine(s) + Cysteine(c)
r0911	1	Proline(c) + Glutamate(m) ⇌ Proline(m) + Glutamate(c)
r0425	1	Isocitrate(m) + NAD+(m) ⇌ Oxalosuccinate(m) + NADH(m)
r0083	1	Oxalosuccinate(m) ⇌ AKG(m) + CO2(m)
r0056	1	Pyruvate(m) + CoA(m) + NAD+(m) → Acetyl-CoA(m) + CO2(m) + NADH(m)
r1047	1	Pi(c) + H+(PG)(c) → Pi(m) + H+(PG)(m)
r0453	1	ATP(c) + Citrulline(c) + Aspartate(c) ⇌ AMP(c) + PPi(c) + Argininosuccinate(c)
r1426	1	Proline(m) + H+(PG)(c) → Proline(c) + H+(PG)(m)
r1327	-2	NADPH(m) ⇌ NADPH-redox-potential(m) + NADP+(m)
r0940	4	H2S(c) ⇌ H2S(s)
r0193	4	Cysteine(c) + H2O(c) ⇌ Pyruvate(c) + H2S(c) + NH3(c)
r2417	-1	Citrulline(m) + H+(PG)(c) → Citrulline(c) + H+(PG)(m)
r1913	1	Glycine(s) + Na+(s) + Arginine(c) → Glycine(c) + Na+(c) + Arginine(s)
r1319	-2	ATP(c) + H2O(c) ⇌ ADP(c) + Pi(c) + ATP-energy(c)
r0851	4	H2O(m) ⇌ H2O(c)
r0329	1	Carbamoyl-P(m) + Ornithine(m) ⇌ Pi(m) + Citrulline(m)
r0167	-1	L-Glutamate 5-semialdehyde(m) + Glutamate(m) ⇌ AKG(m) + Ornithine(m)
r0059	-1	Pyruvate(m) + CO2(m) + NADPH(m) ⇌ Malate(m) + NADP+(m)
r0258	-1	Fumarate(c) + H2O(c) ⇌ Malate(c)
r2419	1	AKG(c) + Pi(m) ⇌ AKG(m) + Pi(c)
r0619	-1	L-Glutamate 5-semialdehyde(m) + Pi(m) + NADP+(m) ← NADPH(m) + Glutamyl-5P(m)
r0030	1	ATP(c) + AMP(c) ⇌ 2 ADP(c)
r2664	4	Na+(c) ⇌ Na+(s) + Na-gradient(c)
r2475	3	Cysteine(s) + Na+(s) → Cysteine(c) + Na+(c)
r0104	1	NAD+(c) + Malate(c) ⇌ OAA(c) + NADH(c)
r1320	-3	ATP(m) + H2O(m) ⇌ ADP(m) + Pi(m) + ATP-energy(m)
r0838	4	NH3(c) ⇌ NH3(m)
r0105	1	NAD+(m) + Malate(m) ⇌ OAA(m) + NADH(m)
r1330	-3	H+(PG)(c) ⇌ H+(PG)(m) + Proton-gradient(m)
r0034	1	2 ATP(m) + CO2(m) + H2O(m) + NH3(m) ⇌ 2 ADP(m) + Pi(m) + Carbamoyl-P(m)
r0007	1	PPi(c) + H2O(c) → 2 Pi(c)
r1322	1	NADH(c) ⇌ NAD+(c) + NADH-redox-potential(c)
r0107	-1	Acetyl-CoA(m) + H2O(m) + OAA(m) ⇌ Citrate(m) + CoA(m)
r0261	1	Argininosuccinate(c) ⇌ Fumarate(c) + Arginine(c)
r0072	1	ATP(m) + Glutamate(m) → ADP(m) + Glutamyl-5P(m)

Arginine from Cystine (103)

Definition			Solution												
			exchanges		reactions								transp		
Objective	Constraints	Comment	imports	exports	c	m	r	p	l	n	s	b	s-c	b-c	int
Arginine	−Cystine MCES +Urea +H_2S	Transamination to Arginine using Cystine	2 Pyruvate(m) 2 Cystine(s) 2 ATP-energy(c) 3 ATP-energy(m) 2 NADPH-redox-potential(c) 2 NADPH-redox-potential(m) 3 Proton-gradient(m)	4 Pyruvate(c) 1 Arginine(s) 4 H_2S(s) 1 NADH-redox-potential(c) 2 Na-gradient(c)	12	15	-	-	-	-	-	-	5	-	8

ID	flux	reaction
r0425	1	Isocitrate(m) + NAD+(m) ⇌ Oxalosuccinate(m) + NADH(m)
r0877	2	Glutamate(c) + Cystine(s) ⇌ Glutamate(s) + Cystine(c)
r0167	-1	L-Glutamate 5-semialdehyde(m) + Glutamate(m) ⇌ AKG(m) + Ornithine(m)
r0030	1	ATP(c) + AMP(c) ⇌ 2 ADP(c)
r0034	1	2 ATP(m) + CO2(m) + H2O(m) + NH3(m) ⇌ 2 ADP(m) + Pi(m) + Carbamoyl-P(m)
r0316	1	Citrate(m) ⇌ Isocitrate(m)
r0911	1	Proline(c) + Glutamate(m) ⇌ Proline(m) + Glutamate(c)
r0619	-1	L-Glutamate 5-semialdehyde(m) + Pi(m) + NADP+(m) ← NADPH(m) + Glutamyl-5P(m)
r0056	1	Pyruvate(m) + CoA(m) + NAD+(m) → Acetyl-CoA(m) + CO2(m) + NADH(m)
r2664	2	Na+(c) ⇌ Na+(s) + Na-gradient(c)
r1426	1	Proline(m) + H+(PG)(c) → Proline(c) + H+(PG)(m)
r1144	-2	Glutamate(c) + Na+(c) ← Glutamate(s) + Na+(s)
r0072	1	ATP(m) + Glutamate(m) → ADP(m) + Glutamyl-5P(m)
r0059	-1	Pyruvate(m) + CO2(m) + NADPH(m) ⇌ Malate(m) + NADP+(m)
r0073	-3	AKG(m) + NADH(m) + NH3(m) ⇌ Glutamate(m) + NAD+(m) + H2O(m)
r1330	-3	H+(PG)(c) ⇌ H+(PG)(m) + Proton-gradient(m)
r1319	-2	ATP(c) + H2O(c) ⇌ ADP(c) + Pi(c) + ATP-energy(c)
r0261	1	Argininosuccinate(c) ⇌ Fumarate(c) + Arginine(c)
r1058	-1	Arginine(c) ⇌ Arginine(s)
r0453	1	ATP(c) + Citrulline(c) + Aspartate(c) ⇌ AMP(c) + PPi(c) + Argininosuccinate(c)
r0007	1	PPi(c) + H2O(c) → 2 Pi(c)
r0027	-2	NADPH(c) + Cystine(c) ⇌ 2 Cysteine(c) + NADP+(c)
r0193	4	Cysteine(c) + H2O(c) ⇌ Pyruvate(c) + H2S(c) + NH3(c)
r0329	1	Carbamoyl-P(m) + Ornithine(m) ⇌ Pi(m) + Citrulline(m)
r0940	4	H2S(c) ⇌ H2S(s)
r0083	1	Oxalosuccinate(m) ⇌ AKG(m) + CO2(m)
r1320	-3	ATP(m) + H2O(m) ⇌ ADP(m) + Pi(m) + ATP-energy(m)
r1322	1	NADH(c) ⇌ NAD+(c) + NADH-redox-potential(c)
r1326	-2	NADPH(c) ⇌ NADPH-redox-potential(c) + NADP+(c)
r1327	-2	NADPH(m) ⇌ NADPH-redox-potential(m) + NADP+(m)
r0104	1	NAD+(c) + Malate(c) ⇌ OAA(c) + NADH(c)
r1047	1	Pi(c) + H+(PG)(c) → Pi(m) + H+(PG)(m)
r0105	1	NAD+(m) + Malate(m) ⇌ OAA(m) + NADH(m)
r0258	-1	Fumarate(c) + H2O(c) ⇌ Malate(c)
r2417	-1	Citrulline(m) + H+(PG)(c) → Citrulline(c) + H+(PG)(m)
r0107	-1	Acetyl-CoA(m) + H2O(m) + OAA(m) ⇌ Citrate(m) + CoA(m)
r2419	1	AKG(c) + Pi(m) ⇌ AKG(m) + Pi(c)
r0838	4	NH3(c) ⇌ NH3(m)
r0851	4	H2O(m) ⇌ H2O(c)
r0109	-1	OAA(c) + Glutamate(c) ⇌ AKG(c) + Aspartate(c)

Arginine from Glutamate (104)

Definition			Solution													
			exchanges		reactions									transp		
Objective	Constraints	Comment	imports	exports	c	m	r	p	l	n	s	b	s-c	b-c	int
Arginine	−Glutamate MCES +Urea	Transamination to Arginine using Glutamate	2 H_2O(s) 1.5 O_2(s) 4 Glutamate(s) 2 ATP-energy(c)	5 CO_2(s) 1 Pyruvate(c) 2 Pyruvate(m) 1 Arginine(s) 1 ATP-energy(m) 1 NADH-redox-potential(c) 2 NADH-redox-potential(m) 1 NADPH-redox-potential(c) 3 NADPH-redox-potential(m) 14 Proton-gradient(m) 4 Na-gradient(c)	11	14	-	-	-	-	-	-	6	-	10

ID	flux	reaction
r0125	-3	GDP(m) + Pi(m) + Succinyl-CoA(m) ⇌ GTP(m) + Succinate(m) + CoA(m)
r0855	1.5	O2(s) ⇌ O2(c)
r0856	-1.5	O2(m) ⇌ O2(c)
r0875	3	Glutamate(c) + H+(PG)(c) → Glutamate(m) + H+(PG)(m)
r0167	-1	L-Glutamate 5-semialdehyde(m) + Glutamate(m) ⇌ AKG(m) + Ornithine(m)
r0017	1.5	O2(m) + 4 H+(PG)(m) + 4 Ferrocytochrome C(m) → 4 Ferricytochrome C(m) + 2 H2O(m) + 4 H+(PG)(c)
r0030	1	ATP(c) + AMP(c) ⇌ 2 ADP(c)
r0034	1	2 ATP(m) + CO2(m) + H2O(m) + NH3(m) ⇌ 2 ADP(m) + Pi(m) + Carbamoyl-P(m)
r2664	4	Na+(c) ⇌ Na+(s) + Na-gradient(c)
r0058	1	Malate(c) + NADP+(c) ⇌ Pyruvate(c) + CO2(c) + NADPH(c)
r1144	-4	Glutamate(c) + Na+(c) ← Glutamate(s) + Na+(s)
r0059	2	Malate(m) + NADP+(m) ⇌ Pyruvate(m) + CO2(m) + NADPH(m)
r0074	-1	Glutamate(m) + NADH(m) ⇌ L-Glutamate 5-semialdehyde(m) + NAD+(m) + H2O(m)
r0075	1	Glutamate(m) + H2O(m) + NADP+(m) ⇌ AKG(m) + NADPH(m) + NH3(m)
r0507	3	Ubiquinol(m) + 2 Ferricytochrome C(m) + 4 H+(PG)(m) → Ubiquinone(m) + 4 H+(PG)(c) + 2 Ferrocytochrome C(m)
r0509	3	Ubiquinone(m) + Succinate(m) → Ubiquinol(m) + Fumarate(m)
r1330	14	H+(PG)(c) ⇌ H+(PG)(m) + Proton-gradient(m)
r0821	-1	Fumarate(c) + Succinate(m) ⇌ Fumarate(m) + Succinate(c)
r1319	-2	ATP(c) + H2O(c) ⇌ ADP(c) + Pi(c) + ATP-energy(c)
r0261	1	Argininosuccinate(c) ⇌ Fumarate(c) + Arginine(c)
r1058	-1	Arginine(c) ⇌ Arginine(s)
r0849	2	H2O(s) ⇌ H2O(c)
r0867	5	CO2(c) ⇌ CO2(s)
r0868	-4	CO2(c) ⇌ CO2(m)
r0453	1	ATP(c) + Citrulline(c) + Aspartate(c) ⇌ AMP(c) + PPi(c) + Argininosuccinate(c)
r0007	1	PPi(c) + H2O(c) → 2 Pi(c)
r1114	3	AKG(m) + CoA(m) + NAD+(m) ⇌ NADH(m) + CO2(m) + Succinyl-CoA(m)
r0329	1	Carbamoyl-P(m) + Ornithine(m) ⇌ Pi(m) + Citrulline(m)
r1320	1	ATP(m) + H2O(m) ⇌ ADP(m) + Pi(m) + ATP-energy(m)
r1322	1	NADH(c) ⇌ NAD+(c) + NADH-redox-potential(c)
r1323	2	NADH(m) ⇌ NAD+(m) + NADH-redox-potential(m)
r0100	-3	ADP(m) + GTP(m) ⇌ ATP(m) + GDP(m)
r1326	1	NADPH(c) ⇌ NADPH-redox-potential(c) + NADP+(c)
r1327	3	NADPH(m) ⇌ NADPH-redox-potential(m) + NADP+(m)
r0819	1	AKG(c) + Succinate(m) ⇌ AKG(m) + Succinate(c)
r0104	1	NAD+(c) + Malate(c) ⇌ OAA(c) + NADH(c)
r2417	-1	Citrulline(m) + H+(PG)(c) → Citrulline(c) + H+(PG)(m)
r0258	-2	Malate(c) ⇌ Fumarate(c) + H2O(c)
r0259	-2	Malate(m) ⇌ Fumarate(m) + H2O(m)
r0851	-1	H2O(m) ⇌ H2O(c)
r0109	-1	OAA(c) + Glutamate(c) ⇌ AKG(c) + Aspartate(c)

Arginine from Glycine (105)

Definition			Solution												
			exchanges		reactions								transp		
Objective	Constraints	Comment	imports	exports	c	m	r	p	l	n	s	b	s-c	b-c	int
Arginine	−Glycine MCES +Urea	Transamination to Arginine using Glycine	2 Pyruvate(m) 4 Glycine(s) 2 ATP-energy(c) 3 ATP-energy(m) 4 NADPH-redox-potential(m) 1 Proton-gradient(m) 4 THF-activated methyl group(c)	4 Pyruvate(c) 1 Arginine(s) 1 NADH-redox-potential(c) 3 NADH-redox-potential(m) 3 NADPH-redox-potential(c) 4 Na-gradient(c)	21	16	-	-	-	-	-	-	3	-	4

ID	flux	reaction
r0425	1	Isocitrate(m) + NAD+(m) ⇌ Oxalosuccinate(m) + NADH(m)
r0296	4	5-Methyl-THF(c) + NADP+(c) ⇌ 5,10-Methylene-THF(c) + NADPH(c)
r0160	1	Serine(c) + Pyruvate(c) → Hydroxypyruvate(c) + Alanine(c)
r0164	1	2PG(c) ⇌ PEP(c) + H2O(c)
r0167	-1	L-Glutamate 5-semialdehyde(m) + Glutamate(m) ⇌ AKG(m) + Ornithine(m)
r0030	1	ATP(c) + AMP(c) ⇌ 2 ADP(c)
r0034	1	2 ATP(m) + CO2(m) + H2O(m) + NH3(m) ⇌ 2 ADP(m) + Pi(m) + Carbamoyl-P(m)
r0316	1	Citrate(m) ⇌ Isocitrate(m)
r0054	-1	ADP(c) + PEP(c) → ATP(c) + Pyruvate(c)
r0619	-1	L-Glutamate 5-semialdehyde(m) + Pi(m) + NADP+(m) ← NADPH(m) + Glutamyl-5P(m)
r0056	1	Pyruvate(m) + CoA(m) + NAD+(m) → Acetyl-CoA(m) + CO2(m) + NADH(m)
r0339	1	ATP(c) + Glycerate(c) → ADP(c) + 3PG(c)
r2664	4	Na+(c) ⇌ Na+(s) + Na-gradient(c)
r0072	1	ATP(m) + Glutamate(m) → ADP(m) + Glutamyl-5P(m)
r0059	-1	Pyruvate(m) + CO2(m) + NADPH(m) ⇌ Malate(m) + NADP+(m)
r0075	-2	AKG(m) + NADPH(m) + NH3(m) ⇌ Glutamate(m) + H2O(m) + NADP+(m)
r1330	-1	H+(PG)(c) ⇌ H+(PG)(m) + Proton-gradient(m)
r1319	-2	ATP(c) + H2O(c) ⇌ ADP(c) + Pi(c) + ATP-energy(c)
r0261	1	Argininosuccinate(c) ⇌ Fumarate(c) + Arginine(c)
r1913	1	Glycine(s) + Na+(s) + Arginine(c) → Glycine(c) + Na+(c) + Arginine(s)
r0453	1	ATP(c) + Citrulline(c) + Aspartate(c) ⇌ AMP(c) + PPi(c) + Argininosuccinate(c)
r0007	1	PPi(c) + H2O(c) → 2 Pi(c)
r0327	-1	Hydroxypyruvate(c) + NADPH(c) → Glycerate(c) + NADP+(c)
r0341	-1	3PG(c) ⇌ 2PG(c)
r0060	3	Serine(c) → Pyruvate(c) + NH3(c)
r0329	1	Carbamoyl-P(m) + Ornithine(m) ⇌ Pi(m) + Citrulline(m)
r1138	3	Glycine(s) + Na+(s) → Glycine(c) + Na+(c)
r0080	1	Alanine(c) + AKG(c) ⇌ Pyruvate(c) + Glutamate(c)
r2676	-4	5-Methyl-THF(c) ⇌ THF(c) + THF-activated methyl group(c)
r0083	1	Oxalosuccinate(m) ⇌ AKG(m) + CO2(m)
r0230	4	5,10-Methylene-THF(c) + Glycine(c) + H2O(c) ⇌ THF(c) + Serine(c)
r1320	-3	ATP(m) + H2O(m) ⇌ ADP(m) + Pi(m) + ATP-energy(m)
r1322	1	NADH(c) ⇌ NAD+(c) + NADH-redox-potential(c)
r1323	3	NADH(m) ⇌ NAD+(m) + NADH-redox-potential(m)
r1326	3	NADPH(c) ⇌ NADPH-redox-potential(c) + NADP+(c)
r1327	-4	NADPH(m) ⇌ NADPH-redox-potential(m) + NADP+(m)
r0104	1	NAD+(c) + Malate(c) ⇌ OAA(c) + NADH(c)
r0105	1	NAD+(m) + Malate(m) ⇌ OAA(m) + NADH(m)
r2417	-1	Citrulline(m) + H+(PG)(c) → Citrulline(c) + H+(PG)(m)
r0258	-1	Fumarate(c) + H2O(c) ⇌ Malate(c)
r0107	-1	Acetyl-CoA(m) + H2O(m) + OAA(m) ⇌ Citrate(m) + CoA(m)
r0838	3	NH3(c) ⇌ NH3(m)
r0851	3	H2O(m) ⇌ H2O(c)
r0109	-1	OAA(c) + Glutamate(c) ⇌ AKG(c) + Aspartate(c)

Arginine from Histidine (106)

Definition			Solution												
			exchanges		reactions								transp		
Objective	Constraints	Comment	imports	exports	c	m	r	p	l	n	s	b	s-c	b-c	int
Arginine	−Histidine MCES +Urea	Transamination to Arginine using Histidine	0.17 O_2(s) 1.33 Histidine(s) 2 ATP-energy(c) 1.67 ATP-energy(m) 0.67 NADH-redox-potential(m) 0.33 NADPH-redox-potential(c) 1.67 NADPH-redox-potential(m)	0.33 Pyruvate(c) 1 Arginine(s) 0.33 Na-gradient(c) 1 THF-activated methyl group(c)	20	13	-	-	-	-	-	-	5	-	11

ID	flux	reaction
r0125	-0.33	GDP(m) + Pi(m) + Succinyl-CoA(m) ⇌ GTP(m) + Succinate(m) + CoA(m)
r0855	0.17	O2(s) ⇌ O2(c)
r0856	-0.17	O2(m) ⇌ O2(c)
r0293	-1	5,10-Methenyl-THF(c) + NADPH(c) ⇌ 5,10-Methylene-THF(c) + NADP+(c)
r0577	-1.33	Urocanate(c) + H2O(c) ⇌ 4-Imidazolone-5-propanoate(c)
r0875	0.33	Glutamate(c) + H+(PG)(c) → Glutamate(m) + H+(PG)(m)
r1667	-1	Arginine(s) + Ornithine(c) ⇌ Arginine(c) + Ornithine(s)
r0167	-1	L-Glutamate 5-semialdehyde(m) + Glutamate(m) ⇌ AKG(m) + Ornithine(m)
r0017	0.17	O2(m) + 4 H+(PG)(m) + 4 Ferrocytochrome C(m) → 4 Ferricytochrome C(m) + 2 H2O(m) + 4 H+(PG)(c)
r0030	1	ATP(c) + AMP(c) ⇌ 2 ADP(c)
r0034	1	2 ATP(m) + CO2(m) + H2O(m) + NH3(m) ⇌ 2 ADP(m) + Pi(m) + Carbamoyl-P(m)
r2664	0.33	Na+(c) ⇌ Na+(s) + Na-gradient(c)
r0058	0.33	Malate(c) + NADP+(c) ⇌ Pyruvate(c) + CO2(c) + NADPH(c)
r1145	-0.33	Histidine(s) + Na+(s) → Histidine(c) + Na+(c)
r0074	-1	Glutamate(m) + NADH(m) ⇌ L-Glutamate 5-semialdehyde(m) + NAD+(m) + H2O(m)
r0075	-1.67	AKG(m) + NADPH(m) + NH3(m) ⇌ Glutamate(m) + H2O(m) + NADP+(m)
r0371	0.33	5,10-Methenyl-THF(c) + H2O(c) ⇌ 10-Formyl-THF(c)
r0507	0.33	Ubiquinol(m) + 2 Ferricytochrome C(m) + 4 H+(PG)(m) → Ubiquinone(m) + 4 H+(PG)(c) + 2 Ferrocytochrome C(m)
r0509	0.33	Ubiquinone(m) + Succinate(m) → Ubiquinol(m) + Fumarate(m)
r0227	0.33	10-Formyl-THF(c) + H2O(c) + NADP+(c) → THF(c) + CO2(c) + NADPH(c)
r0524	1.33	5-Formiminotetrahydrofolate(c) ⇌ 5,10-Methenyl-THF(c) + NH3(c)
r0820	0.33	AKG(c) + Malate(m) ⇌ AKG(m) + Malate(c)
r1319	-2	ATP(c) + H2O(c) ⇌ ADP(c) + Pi(c) + ATP-energy(c)
r0261	1	Argininosuccinate(c) ⇌ Fumarate(c) + Arginine(c)
r0286	1.33	Histidine(c) ⇌ Urocanate(c) + NH3(c)
r1670	-1	Ornithine(s) + Histidine(c) ⇌ Ornithine(c) + Histidine(s)
r0868	0.67	CO2(c) ⇌ CO2(m)
r0453	1	ATP(c) + Citrulline(c) + Aspartate(c) ⇌ AMP(c) + PPi(c) + Argininosuccinate(c)
r0007	1	PPi(c) + H2O(c) → 2 Pi(c)
r1114	0.33	AKG(m) + CoA(m) + NAD+(m) ⇌ NADH(m) + CO2(m) + Succinyl-CoA(m)
r0329	1	Carbamoyl-P(m) + Ornithine(m) ⇌ Pi(m) + Citrulline(m)
r0792	-1	5,10-Methylene-THF(c) + NADH(c) ⇌ 5-Methyl-THF(c) + NAD+(c)
r2676	1	5-Methyl-THF(c) ⇌ THF(c) + THF-activated methyl group(c)
r1320	-1.67	ATP(m) + H2O(m) ⇌ ADP(m) + Pi(m) + ATP-energy(m)
r0518	-1.33	Glutamate(c) + 5-Formiminotetrahydrofolate(c) ⇌ THF(c) + N-Formimino-L-glutamate(c)
r1323	-0.67	NADH(m) ⇌ NAD+(m) + NADH-redox-potential(m)
r0519	1.33	H2O(c) + 4-Imidazolone-5-propanoate(c) ⇌ N-Formimino-L-glutamate(c)
r0100	-0.33	ADP(m) + GTP(m) ⇌ ATP(m) + GDP(m)
r1326	-0.33	NADPH(c) ⇌ NADPH-redox-potential(c) + NADP+(c)
r1327	-1.67	NADPH(m) ⇌ NADPH-redox-potential(m) + NADP+(m)
r0104	1	NAD+(c) + Malate(c) ⇌ OAA(c) + NADH(c)
r1047	0.67	Pi(c) + H+(PG)(c) → Pi(m) + H+(PG)(m)
r2417	-1	Citrulline(m) + H+(PG)(c) → Citrulline(c) + H+(PG)(m)
r0258	-1	Malate(c) ⇌ Fumarate(c) + H2O(c)
r0259	-0.33	Malate(m) ⇌ Fumarate(m) + H2O(m)
r2419	0.67	AKG(c) + Pi(m) ⇌ AKG(m) + Pi(c)
r0838	2.67	NH3(c) ⇌ NH3(m)

Continued on next page

ID	flux	reaction
r0851	3.33	H2O(m) ⇌ H2O(c)
r0109	-1	OAA(c) + Glutamate(c) ⇌ AKG(c) + Aspartate(c)

Arginine from Isoleucine (107)

Definition			Solution												
			exchanges		reactions								transp		
Objective	Constraints	Comment	imports	exports	c	m	r	p	l	n	s	b	s-c	b-c	int
Arginine	−Isoleucine MCES +Urea	Transamination to Arginine using Isoleucine	2 H_2O(s) 3.5 O_2(s) 1 Pyruvate(m) 4 Isoleucine(s) 2 ATP-energy(c) 3 ATP-energy(m) 3 NADPH-redox-potential(m)	4 CO_2(s) 1 Pyruvate(c) 1 Arginine(s) 16 NADH-redox-potential(m) 1 NADPH-redox-potential(c) 35 Proton-gradient(m) 1 Na-gradient(c) 7 CoA-activated acetyl group(m)	8	29	-	-	-	-	-	-	7	-	11

ID	flux	reaction
r0572	-1	Methylmalonyl-CoA(m) ⇌ (R)-Methylmalonyl-CoA(m)
r0855	3.5	O2(s) ⇌ O2(c)
r0856	-3.5	O2(m) ⇌ O2(c)
r0592	-3	H2O(m) + Acrylyl-CoA(m) → 3-Hydroxypropionyl-CoA(m)
r0876	1	Glutamate(c) + H+(PG)(c) + Aspartate(m) → Glutamate(m) + H+(PG)(m) + Aspartate(c)
r0596	-3	3-Hydroxypropionyl-CoA(m) + H2O(m) → Hydracrylate(m) + CoA(m)
r0167	-1	L-Glutamate 5-semialdehyde(m) + Glutamate(m) ⇌ AKG(m) + Ornithine(m)
r0017	3.5	O2(m) + 4 H+(PG)(m) + 4 Ferrocytochrome C(m) → 4 Ferricytochrome C(m) + 2 H2O(m) + 4 H+(PG)(c)
r0030	1	ATP(c) + AMP(c) ⇌ 2 ADP(c)
r0034	1	2 ATP(m) + CO2(m) + H2O(m) + NH3(m) ⇌ 2 ADP(m) + Pi(m) + Carbamoyl-P(m)
r1557	-1	Glycine(c) + Isoleucine(s) ⇌ Glycine(s) + Isoleucine(c)
r0911	1	Proline(c) + Glutamate(m) ⇌ Proline(m) + Glutamate(c)
r0619	-1	L-Glutamate 5-semialdehyde(m) + Pi(m) + NADP+(m) ← NADPH(m) + Glutamyl-5P(m)
r2664	1	Na+(c) ⇌ Na+(s) + Na-gradient(c)
r1426	1	Proline(m) + H+(PG)(c) → Proline(c) + H+(PG)(m)
r0058	1	Malate(c) + NADP+(c) ⇌ Pyruvate(c) + CO2(c) + NADPH(c)
r0072	1	ATP(m) + Glutamate(m) → ADP(m) + Glutamyl-5P(m)
r0059	-2	Pyruvate(m) + CO2(m) + NADPH(m) ⇌ Malate(m) + NADP+(m)
r0073	1	Glutamate(m) + NAD+(m) + H2O(m) ⇌ AKG(m) + NADH(m) + NH3(m)
r0222	-4	CoA(m) + 2-Methylacetoacetyl-CoA(m) ⇌ Propanoyl-CoA(m) + Acetyl-CoA(m)
r0953	4	Isoleucine(c) + H+(PG)(c) → Isoleucine(m) + H+(PG)(m)
r2536	3	Isoleucine(s) ⇌ Isoleucine(c)
r0507	7	Ubiquinol(m) + 2 Ferricytochrome C(m) + 4 H+(PG)(m) → Ubiquinone(m) + 4 H+(PG)(c) + 2 Ferrocytochrome C(m)
r1330	35	H+(PG)(c) ⇌ H+(PG)(m) + Proton-gradient(m)
r1319	-2	ATP(c) + H2O(c) ⇌ ADP(c) + Pi(c) + ATP-energy(c)
r0261	1	Argininosuccinate(c) ⇌ Fumarate(c) + Arginine(c)
r0110	-1	OAA(m) + Glutamate(m) → AKG(m) + Aspartate(m)
r1913	1	Glycine(s) + Na+(s) + Arginine(c) → Glycine(c) + Na+(c) + Arginine(s)
r0264	4	Isoleucine(m) + AKG(m) ⇌ Glutamate(m) + 2-Oxo-3-methylvalerate(m)
r0414	1	ATP(m) + Propanoyl-CoA(m) + HCO3-(m) → ADP(m) + Pi(m) + Methylmalonyl-CoA(m)
r0849	2	H2O(s) ⇌ H2O(c)
r0867	4	CO2(c) ⇌ CO2(s)
r0868	-3	CO2(c) ⇌ CO2(m)
r0453	1	ATP(c) + Citrulline(c) + Aspartate(c) ⇌ AMP(c) + PPi(c) + Argininosuccinate(c)
r0007	1	PPi(c) + H2O(c) → 2 Pi(c)
r0174	3	CoA(m) + NAD+(m) + 3-Oxopropanoate(m) → Acetyl-CoA(m) + CO2(m) + NADH(m)
r0602	4	2-Oxo-3-methylvalerate(m) + CoA(m) + NAD+(m) ⇌ 2-Methylbutyryl-CoA(m) + CO2(m) + NADH(m)
r0603	4	2-Methylbutyryl-CoA(m) + Ubiquinone(m) ⇌ Tiglyl-CoA(m) + Ubiquinol(m)
r1114	-1	NADH(m) + CO2(m) + Succinyl-CoA(m) ⇌ AKG(m) + CoA(m) + NAD+(m)
r0197	1	(R)-Methylmalonyl-CoA(m) ⇌ Succinyl-CoA(m)

Continued on next page

ID	flux	reaction
r0329	1	Carbamoyl-P(m) + Ornithine(m) ⇌ Pi(m) + Citrulline(m)
r0365	3	Hydracrylate(m) + NAD+(m) → NADH(m) + 3-Oxopropanoate(m)
r2678	7	Acetyl-CoA(m) ⇌ CoA(m) + CoA-activated acetyl group(m)
r0664	4	NAD+(m) + (S)-3-Hydroxy-2-methylbutyryl-CoA(m) ⇌ 2-Methylacetoacetyl-CoA(m) + NADH(m)
r0665	-4	H2O(m) + Tiglyl-CoA(m) ⇌ (S)-3-Hydroxy-2-methylbutyryl-CoA(m)
r1320	-3	ATP(m) + H2O(m) ⇌ ADP(m) + Pi(m) + ATP-energy(m)
r0683	3	Propanoyl-CoA(m) + Ubiquinone(m) → Ubiquinol(m) + Acrylyl-CoA(m)
r1323	16	NADH(m) ⇌ NAD+(m) + NADH-redox-potential(m)
r1326	1	NADPH(c) ⇌ NADPH-redox-potential(c) + NADP+(c)
r1327	-3	NADPH(m) ⇌ NADPH-redox-potential(m) + NADP+(m)
r0105	2	NAD+(m) + Malate(m) ⇌ OAA(m) + NADH(m)
r2417	-1	Citrulline(m) + H+(PG)(c) → Citrulline(c) + H+(PG)(m)
r0106	-1	ADP(m) + Pi(m) + OAA(m) ⇌ ATP(m) + Pyruvate(m) + HCO3-(m)
r0258	-1	Fumarate(c) + H2O(c) ⇌ Malate(c)
r0851	-2	H2O(m) ⇌ H2O(c)

Arginine from Glutamine (108)

Definition			Solution												
			exchanges		reactions								transp		
Objective	Constraints	Comment	imports	exports	c	m	r	p	l	n	s	b	s-c	b-c	int
Arginine	−Glutamine MCES +Urea	Transamination to Arginine using Glutamine	0.5 O_2(s) 2 Glutamine(s) 2 ATP-energy(c) 1 ATP-energy(m) 1 NADPH-redox-potential(m)	1 CO_2(s) 1 Pyruvate(c) 1 Arginine(s) 1 NADH-redox-potential(c) 1 NADPH-redox-potential(c) 4 Proton-gradient(m) 1 Na-gradient(c)	11	13	-	-	-	-	-	-	6	-	9

ID	flux	reaction
r0125	-1	GDP(m) + Pi(m) + Succinyl-CoA(m) ⇌ GTP(m) + Succinate(m) + CoA(m)
r0855	0.5	O2(s) ⇌ O2(c)
r0856	-0.5	O2(m) ⇌ O2(c)
r1663	1	Lysine(s) + Arginine(c) ⇌ Lysine(c) + Arginine(s)
r0167	-1	L-Glutamate 5-semialdehyde(m) + Glutamate(m) ⇌ AKG(m) + Ornithine(m)
r0017	0.5	O2(m) + 4 H+(PG)(m) + 4 Ferrocytochrome C(m) → 4 Ferricytochrome C(m) + 2 H2O(m) + 4 H+(PG)(c)
r0030	1	ATP(c) + AMP(c) ⇌ 2 ADP(c)
r0034	1	2 ATP(m) + CO2(m) + H2O(m) + NH3(m) ⇌ 2 ADP(m) + Pi(m) + Carbamoyl-P(m)
r0911	1	Proline(c) + Glutamate(m) ⇌ Proline(m) + Glutamate(c)
r2664	1	Na+(c) ⇌ Na+(s) + Na-gradient(c)
r0058	1	Malate(c) + NADP+(c) ⇌ Pyruvate(c) + CO2(c) + NADPH(c)
r1426	1	Proline(m) + H+(PG)(c) → Proline(c) + H+(PG)(m)
r0074	-1	Glutamate(m) + NADH(m) ⇌ L-Glutamate 5-semialdehyde(m) + NAD+(m) + H2O(m)
r0075	-1	AKG(m) + NADPH(m) + NH3(m) ⇌ Glutamate(m) + H2O(m) + NADP+(m)
r0078	2	Glutamine(m) + H2O(m) → Glutamate(m) + NH3(m)
r0507	1	Ubiquinol(m) + 2 Ferricytochrome C(m) + 4 H+(PG)(m) → Ubiquinone(m) + 4 H+(PG)(c) + 2 Ferrocytochrome C(m)
r0509	1	Ubiquinone(m) + Succinate(m) → Ubiquinol(m) + Fumarate(m)
r0820	1	AKG(c) + Malate(m) ⇌ AKG(m) + Malate(c)
r1330	4	H+(PG)(c) ⇌ H+(PG)(m) + Proton-gradient(m)
r1319	-2	ATP(c) + H2O(c) ⇌ ADP(c) + Pi(c) + ATP-energy(c)
r0261	1	Argininosuccinate(c) ⇌ Fumarate(c) + Arginine(c)
r0867	1	CO2(c) ⇌ CO2(s)
r0453	1	ATP(c) + Citrulline(c) + Aspartate(c) ⇌ AMP(c) + PPi(c) + Argininosuccinate(c)
r0007	1	PPi(c) + H2O(c) → 2 Pi(c)
r1114	1	AKG(m) + CoA(m) + NAD+(m) ⇌ NADH(m) + CO2(m) + Succinyl-CoA(m)
r0329	1	Carbamoyl-P(m) + Ornithine(m) ⇌ Pi(m) + Citrulline(m)
r2522	2	Glutamine(c) ⇌ Glutamine(m)

Continued on next page

Arginine from Glutamine (108)– continued

ID	flux	reaction
r2525	1	Glutamine(s) ⇌ Glutamine(c)
r1320	-1	ATP(m) + H2O(m) ⇌ ADP(m) + Pi(m) + ATP-energy(m)
r1322	1	NADH(c) ⇌ NAD+(c) + NADH-redox-potential(c)
r0100	-1	ADP(m) + GTP(m) ⇌ ATP(m) + GDP(m)
r1326	1	NADPH(c) ⇌ NADPH-redox-potential(c) + NADP+(c)
r1327	-1	NADPH(m) ⇌ NADPH-redox-potential(m) + NADP+(m)
r0104	1	NAD+(c) + Malate(c) ⇌ OAA(c) + NADH(c)
r0258	-1	Malate(c) ⇌ Fumarate(c) + H2O(c)
r2417	-1	Citrulline(m) + H+(PG)(c) → Citrulline(c) + H+(PG)(m)
r0259	-1	Malate(m) ⇌ Fumarate(m) + H2O(m)
r1922	1	Glutamine(s) + Na+(s) + Lysine(c) → Glutamine(c) + Na+(c) + Lysine(s)
r0109	-1	OAA(c) + Glutamate(c) ⇌ AKG(c) + Aspartate(c)

Arginine from Leucine (109)

Definition			Solution												
			exchanges		reactions								transp		
Objective	Constraints	Comment	imports	exports	c	m	r	p	l	n	s	b	s-c	b-c	int
Arginine	−Leucine MCES +Urea	Transamination to Arginine using Leucine	2 O_2(s) 2 Pyruvate(m) 4 Leucine(s) 2 ATP-energy(c) 3 ATP-energy(m) 6 NADPH-redox-potential(m)	4 H_2O(s) 1 Arginine(s) 1 NADH-redox-potential(c) 12 NADH-redox-potential(m) 21 Proton-gradient(m) 4 Na-gradient(c) 12 CoA-activated acetyl group(m)	9	25	-	-	-	-	-	-	6	-	11

ID	flux	reaction
r0855	2	O2(s) ⇌ O2(c)
r0856	-2	O2(m) ⇌ O2(c)
r1663	1	Lysine(s) + Arginine(c) ⇌ Lysine(c) + Arginine(s)
r0425	1	Isocitrate(m) + NAD+(m) ⇌ Oxalosuccinate(m) + NADH(m)
r0167	-1	L-Glutamate 5-semialdehyde(m) + Glutamate(m) ⇌ AKG(m) + Ornithine(m)
r0462	4	HMG-CoA(m) + CoA(m) ⇌ Acetyl-CoA(m) + H2O(m) + Acetoacetyl-CoA(m)
r0017	2	O2(m) + 4 H+(PG)(m) + 4 Ferrocytochrome C(m) → 4 Ferricytochrome C(m) + 2 H2O(m) + 4 H+(PG)(c)
r0030	1	ATP(c) + AMP(c) ⇌ 2 ADP(c)
r0034	1	2 ATP(m) + CO2(m) + H2O(m) + NH3(m) ⇌ 2 ADP(m) + Pi(m) + Carbamoyl-P(m)
r0316	1	Citrate(m) ⇌ Isocitrate(m)
r1122	-3	Leucine(s) + Na+(s) → Leucine(c) + Na+(c)
r0911	1	Proline(c) + Glutamate(m) ⇌ Proline(m) + Glutamate(c)
r0619	-1	L-Glutamate 5-semialdehyde(m) + Pi(m) + NADP+(m) ← NADPH(m) + Glutamyl-5P(m)
r0056	1	Pyruvate(m) + CoA(m) + NAD+(m) → Acetyl-CoA(m) + CO2(m) + NADH(m)
r0070	-4	CoA(m) + Acetoacetyl-CoA(m) ⇌ 2 Acetyl-CoA(m)
r2664	4	Na+(c) ⇌ Na+(s) + Na-gradient(c)
r1426	1	Proline(m) + H+(PG)(c) → Proline(c) + H+(PG)(m)
r0072	1	ATP(m) + Glutamate(m) → ADP(m) + Glutamyl-5P(m)
r0059	-5	Pyruvate(m) + CO2(m) + NADPH(m) ⇌ Malate(m) + NADP+(m)
r0073	1	Glutamate(m) + NAD+(m) + H2O(m) ⇌ AKG(m) + NADH(m) + NH3(m)
r0655	4	Isovaleryl-CoA(m) + Ubiquinone(m) ⇌ 3-Methylcrotonyl-CoA(m) + Ubiquinol(m)
r0658	4	ATP(m) + 3-Methylcrotonyl-CoA(m) + HCO3-(m) ⇌ ADP(m) + Pi(m) + 3-Methylglutaconyl-CoA(m)
r0507	4	Ubiquinol(m) + 2 Ferricytochrome C(m) + 4 H+(PG)(m) → Ubiquinone(m) + 4 H+(PG)(c) + 2 Ferrocytochrome C(m)
r1330	21	H+(PG)(c) ⇌ H+(PG)(m) + Proton-gradient(m)
r1319	-2	ATP(c) + H2O(c) ⇌ ADP(c) + Pi(c) + ATP-energy(c)
r0261	1	Argininosuccinate(c) ⇌ Fumarate(c) + Arginine(c)
r0262	4	Leucine(m) + AKG(m) ⇌ Glutamate(m) + 4-Methyl-2-oxopentanoate(m)
r0849	-4	H2O(s) ⇌ H2O(c)
r1957	1	Leucine(s) + Na+(s) + Lysine(c) → Leucine(c) + Na+(c) + Lysine(s)
r0453	1	ATP(c) + Citrulline(c) + Aspartate(c) ⇌ AMP(c) + PPi(c) + Argininosuccinate(c)

Continued on next page

Arginine from Leucine (109)– continued

ID	flux	reaction
r0007	1	PPi(c) + H2O(c) → 2 Pi(c)
r0601	4	4-Methyl-2-oxopentanoate(m) + CoA(m) + NAD+(m) ⇌ Isovaleryl-CoA(m) + CO2(m) + NADH(m)
r0490	-4	3-Methylglutaconyl-CoA(m) + H2O(m) ⇌ HMG-CoA(m)
r0329	1	Carbamoyl-P(m) + Ornithine(m) ⇌ Pi(m) + Citrulline(m)
r1438	4	Leucine(c) → Leucine(m)
r0083	1	Oxalosuccinate(m) ⇌ AKG(m) + CO2(m)
r2678	12	Acetyl-CoA(m) ⇌ CoA(m) + CoA-activated acetyl group(m)
r1320	-3	ATP(m) + H2O(m) ⇌ ADP(m) + Pi(m) + ATP-energy(m)
r1322	1	NADH(c) ⇌ NAD+(c) + NADH-redox-potential(c)
r1323	12	NADH(m) ⇌ NAD+(m) + NADH-redox-potential(m)
r1327	-6	NADPH(m) ⇌ NADPH-redox-potential(m) + NADP+(m)
r0104	1	NAD+(c) + Malate(c) ⇌ OAA(c) + NADH(c)
r1047	1	Pi(c) + H+(PG)(c) → Pi(m) + H+(PG)(m)
r0105	5	NAD+(m) + Malate(m) ⇌ OAA(m) + NADH(m)
r2417	-1	Citrulline(m) + H+(PG)(c) → Citrulline(c) + H+(PG)(m)
r0106	-4	ADP(m) + Pi(m) + OAA(m) ⇌ ATP(m) + Pyruvate(m) + HCO3-(m)
r0258	-1	Fumarate(c) + H2O(c) ⇌ Malate(c)
r0107	-1	Acetyl-CoA(m) + H2O(m) + OAA(m) ⇌ Citrate(m) + CoA(m)
r2419	1	AKG(c) + Pi(m) ⇌ AKG(m) + Pi(c)
r0851	4	H2O(m) ⇌ H2O(c)
r0109	-1	OAA(c) + Glutamate(c) ⇌ AKG(c) + Aspartate(c)

Arginine from Lysine (110)

Definition			Solution											
			exchanges		reactions								transp	
Objective	Constraints	Comment	imports	exports	c	m	r	p	l	n	s	b	s-c	b-c	int
Arginine	−Lysine MCES +Urea	Transamination to Arginine using Lysine	2 H_2O(s) 1 O_2(s) 2 Pyruvate(m) 2 Lysine(s) 2 ATP-energy(c) 3 ATP-energy(m) 3 NADPH-redox-potential(m)	3 CO_2(s) 1 Pyruvate(c) 1 Arginine(s) 2 NADH-redox-potential(c) 8 NADH-redox-potential(m) 1 NADPH-redox-potential(c) 9 Proton-gradient(m) 3 CoA-activated acetyl group(m)	10	25	-	-	-	-	-	-	5	-	11

ID	flux	reaction
r0855	1	O2(s) ⇌ O2(c)
r1069	-1	Lysine(s) ⇌ Lysine(c)
r0856	-1	O2(m) ⇌ O2(c)
r1663	1	Lysine(s) + Arginine(c) ⇌ Lysine(c) + Arginine(s)
r0425	1	Isocitrate(m) + NAD+(m) ⇌ Oxalosuccinate(m) + NADH(m)
r0876	1	Glutamate(c) + H+(PG)(c) + Aspartate(m) → Glutamate(m) + H+(PG)(m) + Aspartate(c)
r0594	2	2-Aminoadipate 6-semialdehyde(c) + NAD+(c) + H2O(c) ⇌ NADH(c) + L-2-Aminoadipate(c)
r0460	2	(S)-3-Hydroxybutyryl-CoA(m) + NAD+(m) ⇌ NADH(m) + Acetoacetyl-CoA(m)
r0448	2	2-Oxoadipate(m) + CoA(m) + NAD+(m) ⇌ Glutaryl-CoA(m) + CO2(m) + NADH(m)
r0180	-2	AKG(m) + NADPH(m) + Lysine(m) ⇌ H2O(m) + Saccharopine(m) + NADP+(m)
r0167	-1	L-Glutamate 5-semialdehyde(m) + Glutamate(m) ⇌ AKG(m) + Ornithine(m)
r0017	1	O2(m) + 4 H+(PG)(m) + 4 Ferrocytochrome C(m) → 4 Ferricytochrome C(m) + 2 H2O(m) + 4 H+(PG)(c)
r0030	1	ATP(c) + AMP(c) ⇌ 2 ADP(c)
r0034	1	2 ATP(m) + CO2(m) + H2O(m) + NH3(m) ⇌ 2 ADP(m) + Pi(m) + Carbamoyl-P(m)
r0316	1	Citrate(m) ⇌ Isocitrate(m)
r0911	1	Proline(c) + Glutamate(m) ⇌ Proline(m) + Glutamate(c)
r0619	-1	L-Glutamate 5-semialdehyde(m) + Pi(m) + NADP+(m) ← NADPH(m) + Glutamyl-5P(m)
r0070	-2	CoA(m) + Acetoacetyl-CoA(m) ⇌ 2 Acetyl-CoA(m)
r1426	1	Proline(m) + H+(PG)(c) → Proline(c) + H+(PG)(m)
r0058	1	Malate(c) + NADP+(c) ⇌ Pyruvate(c) + CO2(c) + NADPH(c)

Continued on next page

Arginine from Lysine (110)– continued

ID	flux	reaction
r0072	1	ATP(m) + Glutamate(m) → ADP(m) + Glutamyl-5P(m)
r0059	-2	Pyruvate(m) + CO2(m) + NADPH(m) ⇌ Malate(m) + NADP+(m)
r0073	1	Glutamate(m) + NAD+(m) + H2O(m) ⇌ AKG(m) + NADH(m) + NH3(m)
r0507	2	Ubiquinol(m) + 2 Ferricytochrome C(m) + 4 H+(PG)(m) → Ubiquinone(m) + 4 H+(PG)(c) + 2 Ferrocytochrome C(m)
r2539	-2	2-Aminoadipate 6-semialdehyde(c) + L-2-Aminoadipate(m) ⇌ 2-Aminoadipate 6-semialdehyde(m) + L-2-Aminoadipate(c)
r1330	9	H+(PG)(c) ⇌ H+(PG)(m) + Proton-gradient(m)
r0526	2	H2O(m) + Saccharopine(m) + NADP+(m) ⇌ Glutamate(m) + 2-Aminoadipate 6-semialdehyde(m) + NADPH(m)
r1319	-2	ATP(c) + H2O(c) ⇌ ADP(c) + Pi(c) + ATP-energy(c)
r0541	2	Glutaryl-CoA(m) + Ubiquinone(m) ⇌ Crotonyl-CoA(m) + CO2(m) + Ubiquinol(m)
r0261	1	Argininosuccinate(c) ⇌ Fumarate(c) + Arginine(c)
r0110	-1	OAA(m) + Glutamate(m) → AKG(m) + Aspartate(m)
r1070	1	Lysine(c) ⇌ Lysine(m)
r0849	2	H2O(s) ⇌ H2O(c)
r0867	3	CO2(c) ⇌ CO2(s)
r0868	-2	CO2(c) ⇌ CO2(m)
r0588	-2	Crotonyl-CoA(m) + H2O(m) ⇌ (S)-3-Hydroxybutyryl-CoA(m)
r0450	2	AKG(m) + L-2-Aminoadipate(m) ⇌ 2-Oxoadipate(m) + Glutamate(m)
r0453	1	ATP(c) + Citrulline(c) + Aspartate(c) ⇌ AMP(c) + PPi(c) + Argininosuccinate(c)
r0007	1	PPi(c) + H2O(c) → 2 Pi(c)
r0329	1	Carbamoyl-P(m) + Ornithine(m) ⇌ Pi(m) + Citrulline(m)
r0083	1	Oxalosuccinate(m) ⇌ AKG(m) + CO2(m)
r2678	3	Acetyl-CoA(m) ⇌ CoA(m) + CoA-activated acetyl group(m)
r1320	-3	ATP(m) + H2O(m) ⇌ ADP(m) + Pi(m) + ATP-energy(m)
r1322	2	NADH(c) ⇌ NAD+(c) + NADH-redox-potential(c)
r1323	8	NADH(m) ⇌ NAD+(m) + NADH-redox-potential(m)
r2410	-1	Citrulline(m) + Lysine(c) + H+(PG)(c) → Citrulline(c) + Lysine(m) + H+(PG)(m)
r1326	1	NADPH(c) ⇌ NADPH-redox-potential(c) + NADP+(c)
r1327	-3	NADPH(m) ⇌ NADPH-redox-potential(m) + NADP+(m)
r0105	2	NAD+(m) + Malate(m) ⇌ OAA(m) + NADH(m)
r0258	-1	Fumarate(c) + H2O(c) ⇌ Malate(c)
r0107	-1	Acetyl-CoA(m) + H2O(m) + OAA(m) ⇌ Citrate(m) + CoA(m)

Arginine from Methionine (111)

Definition			Solution												
			exchanges		reactions								transp		
Objective	Constraints	Comment	imports	exports	c	m	r	p	l	n	s	b	s-c	b-c	int
Arginine	−Methionine MCES +Urea +H_2S	Transamination to Arginine using Methionine	4 H_2O(s) 1.5 O_2(s) 1 Pyruvate(m) 4 Methionine(s) 2 Glucose-6P(c) 3 ATP-energy(m) 3 NADPH-redox-potential(m)	2 P_i(c) 4 CO_2(s) 5 Pyruvate(c) 1 Arginine(s) 4 H_2S(s) 8 NADH-redox-potential(c) 4 NADH-redox-potential(m) 1 NADPH-redox-potential(c) 3 Proton-gradient(m) 4 Na-gradient(c) 4 THF-activated methyl group(c) 3 CoA-activated acetyl group(m)	22	24	-	-	-	-	-	-	8	-	14

ID	flux	reaction
r0572	-1	Methylmalonyl-CoA(m) ⇌ (R)-Methylmalonyl-CoA(m)
r0855	1.5	O2(s) ⇌ O2(c)
r0856	-1.5	O2(m) ⇌ O2(c)
r0592	-3	H2O(m) + Acrylyl-CoA(m) → 3-Hydroxypropionyl-CoA(m)
r0876	1	Glutamate(c) + H+(PG)(c) + Aspartate(m) → Glutamate(m) + H+(PG)(m) + Aspartate(c)
r0596	-3	3-Hydroxypropionyl-CoA(m) + H2O(m) → Hydracrylate(m) + CoA(m)
r0167	-1	L-Glutamate 5-semialdehyde(m) + Glutamate(m) ⇌ AKG(m) + Ornithine(m)
r0017	1.5	O2(m) + 4 H+(PG)(m) + 4 Ferrocytochrome C(m) → 4 Ferricytochrome C(m) + 2 H2O(m) + 4 H+(PG)(c)

Continued on next page

ID	flux	reaction
r0030	1	ATP(c) + AMP(c) ⇌ 2 ADP(c)
r0314	4	Serine(c) + Homocysteine(c) ⇌ H2O(c) + L-Cystathionine(c)
r0034	1	2 ATP(m) + CO2(m) + H2O(m) + NH3(m) ⇌ 2 ADP(m) + Pi(m) + Carbamoyl-P(m)
r0911	5	Proline(c) + Glutamate(m) ⇌ Proline(m) + Glutamate(c)
r1141	-3	Methionine(s) + Na+(s) → Methionine(c) + Na+(c)
r0619	-1	L-Glutamate 5-semialdehyde(m) + Pi(m) + NADP+(m) ← NADPH(m) + Glutamyl-5P(m)
r0337	-4	ADP(c) + 1,3DPG(c) ⇌ ATP(c) + 3PG(c)
r0338	4	3PG(c) + NAD+(c) → 3-Phosphonooxypyruvate(c) + NADH(c)
r2664	4	Na+(c) ⇌ Na+(s) + Na-gradient(c)
r1426	5	Proline(m) + H+(PG)(c) → Proline(c) + H+(PG)(m)
r0058	1	Malate(c) + NADP+(c) ⇌ Pyruvate(c) + CO2(c) + NADPH(c)
r0072	1	ATP(m) + Glutamate(m) → ADP(m) + Glutamyl-5P(m)
r0059	-2	Pyruvate(m) + CO2(m) + NADPH(m) ⇌ Malate(m) + NADP+(m)
r0073	-7	AKG(m) + NADH(m) + NH3(m) ⇌ Glutamate(m) + NAD+(m) + H2O(m)
r0507	3	Ubiquinol(m) + 2 Ferricytochrome C(m) + 4 H+(PG)(m) → Ubiquinone(m) + 4 H+(PG)(c) + 2 Ferrocytochrome C(m)
r0241	4	H2O(c) + L-Cystathionine(c) ⇌ Cysteine(c) + NH3(c) + 2-Oxobutyrate(c)
r0243	-2	DHAP(c) ⇌ GAP(c)
r1330	3	H+(PG)(c) ⇌ H+(PG)(m) + Proton-gradient(m)
r0261	1	Argininosuccinate(c) ⇌ Fumarate(c) + Arginine(c)
r0110	-1	OAA(m) + Glutamate(m) → AKG(m) + Aspartate(m)
r1913	1	Glycine(s) + Na+(s) + Arginine(c) → Glycine(c) + Na+(c) + Arginine(s)
r0414	1	ATP(m) + Propanoyl-CoA(m) + HCO3-(m) → ADP(m) + Pi(m) + Methylmalonyl-CoA(m)
r0849	4	H2O(s) ⇌ H2O(c)
r0867	4	CO2(c) ⇌ CO2(s)
r0868	-3	CO2(c) ⇌ CO2(m)
r0453	1	ATP(c) + Citrulline(c) + Aspartate(c) ⇌ AMP(c) + PPi(c) + Argininosuccinate(c)
r0736	2	ATP(c) + Fructose-6P(c) → ADP(c) + Fructose-1,6PP(c)
r0007	1	PPi(c) + H2O(c) → 2 Pi(c)
r0159	4	3-Phosphoserine(c) + H2O(c) → Serine(c) + Pi(c)
r0174	3	CoA(m) + NAD+(m) + 3-Oxopropanoate(m) → Acetyl-CoA(m) + CO2(m) + NADH(m)
r1546	-1	Glycine(c) + Methionine(s) ⇌ Glycine(s) + Methionine(c)
r0192	2	Glucose-6P(c) ⇌ Fructose-6P(c)
r1114	-1	NADH(m) + CO2(m) + Succinyl-CoA(m) ⇌ AKG(m) + CoA(m) + NAD+(m)
r0193	4	Cysteine(c) + H2O(c) ⇌ Pyruvate(c) + H2S(c) + NH3(c)
r0197	1	(R)-Methylmalonyl-CoA(m) ⇌ Succinyl-CoA(m)
r0329	1	Carbamoyl-P(m) + Ornithine(m) ⇌ Pi(m) + Citrulline(m)
r1154	4	CoA(m) + NAD+(m) + 2-Oxobutyrate(m) ⇌ CO2(m) + Propanoyl-CoA(m) + NADH(m)
r0940	4	H2S(c) ⇌ H2S(s)
r1155	4	2-Oxobutyrate(c) + H+(PG)(c) → 2-Oxobutyrate(m) + H+(PG)(m)
r2676	4	5-Methyl-THF(c) ⇌ THF(c) + THF-activated methyl group(c)
r0365	3	Hydracrylate(m) + NAD+(m) → NADH(m) + 3-Oxopropanoate(m)
r2678	3	Acetyl-CoA(m) ⇌ CoA(m) + CoA-activated acetyl group(m)
r0663	-4	3-Phosphonooxypyruvate(c) + Glutamate(c) → 3-Phosphoserine(c) + AKG(c)
r0232	-4	THF(c) + Methionine(c) ⇌ 5-Methyl-THF(c) + Homocysteine(c)
r1320	-3	ATP(m) + H2O(m) ⇌ ADP(m) + Pi(m) + ATP-energy(m)
r1322	8	NADH(c) ⇌ NAD+(c) + NADH-redox-potential(c)
r0683	3	Propanoyl-CoA(m) + Ubiquinone(m) → Ubiquinol(m) + Acrylyl-CoA(m)
r1323	4	NADH(m) ⇌ NAD+(m) + NADH-redox-potential(m)
r1326	1	NADPH(c) ⇌ NADPH-redox-potential(c) + NADP+(c)
r0253	4	GAP(c) + Pi(c) + NAD+(c) ⇌ 1,3DPG(c) + NADH(c)
r1327	-3	NADPH(m) ⇌ NADPH-redox-potential(m) + NADP+(m)
r1047	4	Pi(c) + H+(PG)(c) → Pi(m) + H+(PG)(m)
r0256	2	Fructose-1,6PP(c) ⇌ DHAP(c) + GAP(c)
r0105	2	NAD+(m) + Malate(m) ⇌ OAA(m) + NADH(m)
r2417	-1	Citrulline(m) + H+(PG)(c) → Citrulline(c) + H+(PG)(m)
r0106	-1	ADP(m) + Pi(m) + OAA(m) ⇌ ATP(m) + Pyruvate(m) + HCO3-(m)

Continued on next page

ID	flux	reaction
r0258	-1	Fumarate(c) + H2O(c) ⇌ Malate(c)
r2419	4	AKG(c) + Pi(m) ⇌ AKG(m) + Pi(c)
r0851	6	H2O(m) ⇌ H2O(c)
r0838	8	NH3(c) ⇌ NH3(m)

Arginine from Phenylalanine (112)

Definition			Solution												
			exchanges		reactions								transp		
Objective	Constraints	Comment	imports	exports	c	m	r	p	l	n	s	b	s-c	b-c	int
Arginine	−Phenylalanine MCES +Urea	Transamination to Arginine using Phenylalanine	12 O_2(s) 4 Phenylalanine(s) 10 ATP-energy(c) 3 ATP-energy(m) 2 NADPH-redox-potential(c) 4 Proton-gradient(m) 1 CoA-activated acetyl group(m)	7 CO_2(s) 1 Pyruvate(c) 2 Pyruvate(m) 1 Arginine(s) 1 NADH-redox-potential(c) 2 NADH-redox-potential(m) 1 NADPH-redox-potential(m) 1 Na-gradient(c) 8 CoA-activated acetyl group(c)	24	13	-	-	-	-	-	-	6	-	6

ID	flux	reaction
r0855	12	O2(s) ⇌ O2(c)
r1942	1	Phenylalanine(s) + Na+(s) + Lysine(c) → Phenylalanine(c) + Na+(c) + Lysine(s)
r0422	1	Isocitrate(c) + NADP+(c) ⇌ Oxalosuccinate(c) + NADPH(c)
r1663	1	Lysine(s) + Arginine(c) ⇌ Lysine(c) + Arginine(s)
r0875	3	Glutamate(c) + H+(PG)(c) → Glutamate(m) + H+(PG)(m)
r0167	-1	L-Glutamate 5-semialdehyde(m) + Glutamate(m) ⇌ AKG(m) + Ornithine(m)
r0030	5	ATP(c) + AMP(c) ⇌ 2 ADP(c)
r0183	4	Tyrosine(c) + AKG(c) ⇌ 4-Hydroxyphenylpyruvate(c) + Glutamate(c)
r0315	1	Citrate(c) ⇌ Isocitrate(c)
r0034	1	2 ATP(m) + CO2(m) + H2O(m) + NH3(m) ⇌ 2 ADP(m) + Pi(m) + Carbamoyl-P(m)
r0912	-1	Citrate(m) + Malate(c) ⇌ Citrate(c) + Malate(m)
r0619	-1	L-Glutamate 5-semialdehyde(m) + Pi(m) + NADP+(m) ← NADPH(m) + Glutamyl-5P(m)
r2664	1	Na+(c) ⇌ Na+(s) + Na-gradient(c)
r0058	1	Malate(c) + NADP+(c) ⇌ Pyruvate(c) + CO2(c) + NADPH(c)
r0072	1	ATP(m) + Glutamate(m) → ADP(m) + Glutamyl-5P(m)
r0059	2	Malate(m) + NADP+(m) ⇌ Pyruvate(m) + CO2(m) + NADPH(m)
r0073	1	Glutamate(m) + NAD+(m) + H2O(m) ⇌ AKG(m) + NADH(m) + NH3(m)
r0820	-2	AKG(m) + Malate(c) ⇌ AKG(c) + Malate(m)
r1330	-4	H+(PG)(c) ⇌ H+(PG)(m) + Proton-gradient(m)
r0398	4	Dihydrobiopterin(c) + NADPH(c) ⇌ Tetrahydrobiopterin(c) + NADP+(c)
r1319	-10	ATP(c) + H2O(c) ⇌ ADP(c) + Pi(c) + ATP-energy(c)
r0399	4	Tetrahydrobiopterin(c) + Phenylalanine(c) + O2(c) ⇌ Dihydrobiopterin(c) + Tyrosine(c) + H2O(c)
r0261	1	Argininosuccinate(c) ⇌ Fumarate(c) + Arginine(c)
r0543	4	Homogentisate(c) + O2(c) ⇌ 4-Maleylacetoacetate(c)
r0544	4	4-Hydroxyphenylpyruvate(c) + O2(c) ⇌ Homogentisate(c) + CO2(c)
r0867	7	CO2(c) ⇌ CO2(s)
r0868	-1	CO2(c) ⇌ CO2(m)
r0453	1	ATP(c) + Citrulline(c) + Aspartate(c) ⇌ AMP(c) + PPi(c) + Argininosuccinate(c)
r0007	5	PPi(c) + H2O(c) → 2 Pi(c)
r0320	4	ATP(c) + CoA(c) + Acetoacetate(c) → AMP(c) + PPi(c) + Acetoacetyl-CoA(c)
r0605	4	4-Maleylacetoacetate(c) ⇌ Fumarylacetoacetate(c)
r0324	-4	Fumarylacetoacetate(c) + H2O(c) ⇌ Fumarate(c) + Acetoacetate(c)
r0329	1	Carbamoyl-P(m) + Ornithine(m) ⇌ Pi(m) + Citrulline(m)
r0069	-4	CoA(c) + Acetoacetyl-CoA(c) ⇌ 2 Acetyl-CoA(c)
r0082	1	Oxalosuccinate(c) ⇌ AKG(c) + CO2(c)
r2677	8	Acetyl-CoA(c) ⇌ CoA(c) + CoA-activated acetyl group(c)
r2678	-1	Acetyl-CoA(m) ⇌ CoA(m) + CoA-activated acetyl group(m)

Continued on next page

ID	flux	reaction
r2528	3	Phenylalanine(s) ⇌ Phenylalanine(c)
r1320	-3	ATP(m) + H2O(m) ⇌ ADP(m) + Pi(m) + ATP-energy(m)
r1322	1	NADH(c) ⇌ NAD+(c) + NADH-redox-potential(c)
r1323	2	NADH(m) ⇌ NAD+(m) + NADH-redox-potential(m)
r1326	-2	NADPH(c) ⇌ NADPH-redox-potential(c) + NADP+(c)
r1327	1	NADPH(m) ⇌ NADPH-redox-potential(m) + NADP+(m)
r0104	1	NAD+(c) + Malate(c) ⇌ OAA(c) + NADH(c)
r0105	1	NAD+(m) + Malate(m) ⇌ OAA(m) + NADH(m)
r2417	-1	Citrulline(m) + H+(PG)(c) → Citrulline(c) + H+(PG)(m)
r0258	-5	Fumarate(c) + H2O(c) ⇌ Malate(c)
r0107	-1	Acetyl-CoA(m) + H2O(m) + OAA(m) ⇌ Citrate(m) + CoA(m)
r0109	-1	OAA(c) + Glutamate(c) ⇌ AKG(c) + Aspartate(c)

Arginine from Proline (113)

Definition			Solution												
			exchanges		reactions								transp		
Objective	Constraints	Comment	imports	exports	c	m	r	p	l	n	s	b	s-c	b-c	int
Arginine	−Proline MCES +Urea	Transamination to Arginine using Proline	13 H_2O(s) 4 Proline(s) 2 ATP-energy(c)	5 CO_2(s) 1 Pyruvate(c) 2 Pyruvate(m) 1 Arginine(s) 1 ATP-energy(m) 1 NADH-redox-potential(c) 5 NADH-redox-potential(m) 1 NADPH-redox-potential(c) 3 NADPH-redox-potential(m) 1 Na-gradient(c)	11	16	-	-	-	-	-	-	6	-	8

ID	flux	reaction
r0125	-3	GDP(m) + Pi(m) + Succinyl-CoA(m) ⇌ GTP(m) + Succinate(m) + CoA(m)
r1663	1	Lysine(s) + Arginine(c) ⇌ Lysine(c) + Arginine(s)
r1962	1	Proline(s) + Na+(s) + Lysine(c) → Proline(c) + Na+(c) + Lysine(s)
r0167	-1	L-Glutamate 5-semialdehyde(m) + Glutamate(m) ⇌ AKG(m) + Ornithine(m)
r0030	1	ATP(c) + AMP(c) ⇌ 2 ADP(c)
r0034	1	2 ATP(m) + CO2(m) + H2O(m) + NH3(m) ⇌ 2 ADP(m) + Pi(m) + Carbamoyl-P(m)
r0911	1	Proline(c) + Glutamate(m) ⇌ Proline(m) + Glutamate(c)
r1424	3	Proline(c) + H+(PG)(c) → Proline(m) + H+(PG)(m)
r2664	1	Na+(c) ⇌ Na+(s) + Na-gradient(c)
r0058	1	Malate(c) + NADP+(c) ⇌ Pyruvate(c) + CO2(c) + NADPH(c)
r0059	2	Malate(m) + NADP+(m) ⇌ Pyruvate(m) + CO2(m) + NADPH(m)
r0074	3	L-Glutamate 5-semialdehyde(m) + NAD+(m) + H2O(m) ⇌ Glutamate(m) + NADH(m)
r0075	1	Glutamate(m) + H2O(m) + NADP+(m) ⇌ AKG(m) + NADPH(m) + NH3(m)
r2531	3	Proline(s) ⇌ Proline(c)
r0508	-1	Ubiquinone(m) + NADH(m) + 4 H+(PG)(m) → Ubiquinol(m) + NAD+(m) + 4 H+(PG)(c)
r0509	3	Ubiquinone(m) + Succinate(m) → Ubiquinol(m) + Fumarate(m)
r0821	-1	Fumarate(c) + Succinate(m) ⇌ Fumarate(m) + Succinate(c)
r1319	-2	ATP(c) + H2O(c) ⇌ ADP(c) + Pi(c) + ATP-energy(c)
r0261	1	Argininosuccinate(c) ⇌ Fumarate(c) + Arginine(c)
r0849	13	H2O(s) ⇌ H2O(c)
r0867	5	CO2(c) ⇌ CO2(s)
r0868	-4	CO2(c) ⇌ CO2(m)
r0453	1	ATP(c) + Citrulline(c) + Aspartate(c) ⇌ AMP(c) + PPi(c) + Argininosuccinate(c)
r0007	1	PPi(c) + H2O(c) → 2 Pi(c)
r0175	4	1-Pyrroline-5-carboxylate(m) + H2O(m) ⇌ L-Glutamate 5-semialdehyde(m)
r1114	3	AKG(m) + CoA(m) + NAD+(m) ⇌ NADH(m) + CO2(m) + Succinyl-CoA(m)
r0329	1	Carbamoyl-P(m) + Ornithine(m) ⇌ Pi(m) + Citrulline(m)
r1453	4	Proline(m) + Ubiquinol(m) ⇌ 1-Pyrroline-5-carboxylate(m) + Ubiquinone(m)
r1320	1	ATP(m) + H2O(m) ⇌ ADP(m) + Pi(m) + ATP-energy(m)

Continued on next page

ID	flux	reaction
r1322	1	NADH(c) ⇌ NAD+(c) + NADH-redox-potential(c)
r1323	5	NADH(m) ⇌ NAD+(m) + NADH-redox-potential(m)
r0100	-3	ADP(m) + GTP(m) ⇌ ATP(m) + GDP(m)
r1326	1	NADPH(c) ⇌ NADPH-redox-potential(c) + NADP+(c)
r1327	3	NADPH(m) ⇌ NADPH-redox-potential(m) + NADP+(m)
r0819	1	AKG(c) + Succinate(m) ⇌ AKG(m) + Succinate(c)
r0104	1	NAD+(c) + Malate(c) ⇌ OAA(c) + NADH(c)
r2417	-1	Citrulline(m) + H+(PG)(c) → Citrulline(c) + H+(PG)(m)
r0258	-2	Malate(c) ⇌ Fumarate(c) + H2O(c)
r0259	-2	Malate(m) ⇌ Fumarate(m) + H2O(m)
r0851	-12	H2O(m) ⇌ H2O(c)
r0109	-1	OAA(c) + Glutamate(c) ⇌ AKG(c) + Aspartate(c)

Arginine from Serine (114)

Definition			Solution												
			exchanges		reactions								transp		
Objective	Constraints	Comment	imports	exports	c	m	r	p	l	n	s	b	s-c	b-c	int
Arginine	−Serine MCES +Urea	Transamination to Arginine using Serine	2 Pyruvate(m) 4 Serine(s) 2 ATP-energy(c) 3 ATP-energy(m) 1 NADPH-redox-potential(c) 4 NADPH-redox-potential(m) 1 Proton-gradient(m)	4 H_2O(s) 4 Pyruvate(c) 1 Arginine(s) 1 NADH-redox-potential(c) 3 NADH-redox-potential(m) 4 Proton-gradient(c) 4 Na-gradient(c)	18	16	-	-	-	-	-	-	5	-	4

ID	flux	reaction
r0425	1	Isocitrate(m) + NAD+(m) ⇌ Oxalosuccinate(m) + NADH(m)
r0160	1	Serine(c) + Pyruvate(c) → Hydroxypyruvate(c) + Alanine(c)
r0164	1	2PG(c) ⇌ PEP(c) + H2O(c)
r2478	4	Serine(s) + Na+(s) + H+(PG)(c) → Serine(c) + Na+(c) + H+(PG)(s)
r0167	-1	L-Glutamate 5-semialdehyde(m) + Glutamate(m) ⇌ AKG(m) + Ornithine(m)
r0030	1	ATP(c) + AMP(c) ⇌ 2 ADP(c)
r0034	1	2 ATP(m) + CO2(m) + H2O(m) + NH3(m) ⇌ 2 ADP(m) + Pi(m) + Carbamoyl-P(m)
r0316	1	Citrate(m) ⇌ Isocitrate(m)
r0054	-1	ADP(c) + PEP(c) → ATP(c) + Pyruvate(c)
r0619	-1	L-Glutamate 5-semialdehyde(m) + Pi(m) + NADP+(m) ← NADPH(m) + Glutamyl-5P(m)
r0056	1	Pyruvate(m) + CoA(m) + NAD+(m) → Acetyl-CoA(m) + CO2(m) + NADH(m)
r0339	1	ATP(c) + Glycerate(c) → ADP(c) + 3PG(c)
r2664	4	Na+(c) ⇌ Na+(s) + Na-gradient(c)
r0072	1	ATP(m) + Glutamate(m) → ADP(m) + Glutamyl-5P(m)
r0059	-1	Pyruvate(m) + CO2(m) + NADPH(m) ⇌ Malate(m) + NADP+(m)
r0075	-2	AKG(m) + NADPH(m) + NH3(m) ⇌ Glutamate(m) + H2O(m) + NADP+(m)
r1330	-1	H+(PG)(c) ⇌ H+(PG)(m) + Proton-gradient(m)
r1331	4	H+(PG)(s) ⇌ H+(PG)(c) + Proton-gradient(c)
r1319	-2	ATP(c) + H2O(c) ⇌ ADP(c) + Pi(c) + ATP-energy(c)
r0261	1	Argininosuccinate(c) ⇌ Fumarate(c) + Arginine(c)
r1058	-1	Arginine(c) ⇌ Arginine(s)
r0849	-4	H2O(s) ⇌ H2O(c)
r0453	1	ATP(c) + Citrulline(c) + Aspartate(c) ⇌ AMP(c) + PPi(c) + Argininosuccinate(c)
r0007	1	PPi(c) + H2O(c) → 2 Pi(c)
r0327	-1	Hydroxypyruvate(c) + NADPH(c) → Glycerate(c) + NADP+(c)
r0341	-1	3PG(c) ⇌ 2PG(c)
r0060	3	Serine(c) → Pyruvate(c) + NH3(c)
r0329	1	Carbamoyl-P(m) + Ornithine(m) ⇌ Pi(m) + Citrulline(m)
r0080	1	Alanine(c) + AKG(c) ⇌ Pyruvate(c) + Glutamate(c)
r0083	1	Oxalosuccinate(m) ⇌ AKG(m) + CO2(m)

Continued on next page

ID	flux	reaction
r1320	-3	ATP(m) + H2O(m) ⇌ ADP(m) + Pi(m) + ATP-energy(m)
r1322	1	NADH(c) ⇌ NAD+(c) + NADH-redox-potential(c)
r1323	3	NADH(m) ⇌ NAD+(m) + NADH-redox-potential(m)
r1326	-1	NADPH(c) ⇌ NADPH-redox-potential(c) + NADP+(c)
r1327	-4	NADPH(m) ⇌ NADPH-redox-potential(m) + NADP+(m)
r0104	1	NAD+(c) + Malate(c) ⇌ OAA(c) + NADH(c)
r0105	1	NAD+(m) + Malate(m) ⇌ OAA(m) + NADH(m)
r2417	-1	Citrulline(m) + H+(PG)(c) → Citrulline(c) + H+(PG)(m)
r0258	-1	Fumarate(c) + H2O(c) ⇌ Malate(c)
r0107	-1	Acetyl-CoA(m) + H2O(m) + OAA(m) ⇌ Citrate(m) + CoA(m)
r0838	3	NH3(c) ⇌ NH3(m)
r0851	3	H2O(m) ⇌ H2O(c)
r0109	-1	OAA(c) + Glutamate(c) ⇌ AKG(c) + Aspartate(c)

Arginine from Threonine (115)

Definition			Solution												
			exchanges		reactions								transp		
Objective	Constraints	Comment	imports	exports	c	m	r	p	l	n	s	b	s-c	b-c	int
Arginine	−Threonine MCES +Urea	Transamination to Arginine using Threonine	1.5 O_2(s) 1 Pyruvate(m) 4 Threonine(s) 2 ATP-energy(c) 3 ATP-energy(m) 3 NADPH-redox-potential(m)	2 H_2O(s) 4 CO_2(s) 1 Pyruvate(c) 1 Arginine(s) 8 NADH-redox-potential(m) 1 NADPH-redox-potential(c) 11 Proton-gradient(m) 1 Na-gradient(c) 3 CoA-activated acetyl group(m)	9	24	-	-	-	-	-	-	7	-	12

ID	flux	reaction
r0572	-1	Methylmalonyl-CoA(m) ⇌ (R)-Methylmalonyl-CoA(m)
r0855	1.5	O2(s) ⇌ O2(c)
r0856	-1.5	O2(m) ⇌ O2(c)
r0592	-3	H2O(m) + Acrylyl-CoA(m) → 3-Hydroxypropionyl-CoA(m)
r0876	1	Glutamate(c) + H+(PG)(c) + Aspartate(m) → Glutamate(m) + H+(PG)(m) + Aspartate(c)
r0596	-3	3-Hydroxypropionyl-CoA(m) + H2O(m) → Hydracrylate(m) + CoA(m)
r0167	-1	L-Glutamate 5-semialdehyde(m) + Glutamate(m) ⇌ AKG(m) + Ornithine(m)
r0017	1.5	O2(m) + 4 H+(PG)(m) + 4 Ferrocytochrome C(m) → 4 Ferricytochrome C(m) + 2 H2O(m) + 4 H+(PG)(c)
r0030	1	ATP(c) + AMP(c) ⇌ 2 ADP(c)
r0034	1	2 ATP(m) + CO2(m) + H2O(m) + NH3(m) ⇌ 2 ADP(m) + Pi(m) + Carbamoyl-P(m)
r1555	-1	Glycine(c) + Threonine(s) ⇌ Glycine(s) + Threonine(c)
r0911	1	Proline(c) + Glutamate(m) ⇌ Proline(m) + Glutamate(c)
r0619	-1	L-Glutamate 5-semialdehyde(m) + Pi(m) + NADP+(m) ← NADPH(m) + Glutamyl-5P(m)
r2664	1	Na+(c) ⇌ Na+(s) + Na-gradient(c)
r1426	1	Proline(m) + H+(PG)(c) → Proline(c) + H+(PG)(m)
r0058	1	Malate(c) + NADP+(c) ⇌ Pyruvate(c) + CO2(c) + NADPH(c)
r0072	1	ATP(m) + Glutamate(m) → ADP(m) + Glutamyl-5P(m)
r0059	-2	Pyruvate(m) + CO2(m) + NADPH(m) ⇌ Malate(m) + NADP+(m)
r0073	-3	AKG(m) + NADH(m) + NH3(m) ⇌ Glutamate(m) + NAD+(m) + H2O(m)
r2534	3	Threonine(s) ⇌ Threonine(c)
r0507	3	Ubiquinol(m) + 2 Ferricytochrome C(m) + 4 H+(PG)(m) → Ubiquinone(m) + 4 H+(PG)(c) + 2 Ferrocytochrome C(m)
r0240	4	Threonine(c) ⇌ 2-Oxobutyrate(c) + NH3(c)
r1330	11	H+(PG)(c) ⇌ H+(PG)(m) + Proton-gradient(m)
r1319	-2	ATP(c) + H2O(c) ⇌ ADP(c) + Pi(c) + ATP-energy(c)
r0261	1	Argininosuccinate(c) ⇌ Fumarate(c) + Arginine(c)
r0110	-1	OAA(m) + Glutamate(m) → AKG(m) + Aspartate(m)
r1913	1	Glycine(s) + Na+(s) + Arginine(c) → Glycine(c) + Na+(c) + Arginine(s)
r0414	1	ATP(m) + Propanoyl-CoA(m) + HCO3-(m) → ADP(m) + Pi(m) + Methylmalonyl-CoA(m)
r0849	-2	H2O(s) ⇌ H2O(c)

Continued on next page

Arginine from Threonine (115)– continued

ID	flux	reaction
r0867	4	CO2(c) ⇌ CO2(s)
r0868	-3	CO2(c) ⇌ CO2(m)
r0453	1	ATP(c) + Citrulline(c) + Aspartate(c) ⇌ AMP(c) + PPi(c) + Argininosuccinate(c)
r0007	1	PPi(c) + H2O(c) → 2 Pi(c)
r0174	3	CoA(m) + NAD+(m) + 3-Oxopropanoate(m) → Acetyl-CoA(m) + CO2(m) + NADH(m)
r1114	-1	NADH(m) + CO2(m) + Succinyl-CoA(m) ⇌ AKG(m) + CoA(m) + NAD+(m)
r0197	1	(R)-Methylmalonyl-CoA(m) ⇌ Succinyl-CoA(m)
r0329	1	Carbamoyl-P(m) + Ornithine(m) ⇌ Pi(m) + Citrulline(m)
r1154	4	CoA(m) + NAD+(m) + 2-Oxobutyrate(m) ⇌ CO2(m) + Propanoyl-CoA(m) + NADH(m)
r1155	4	2-Oxobutyrate(c) + H+(PG)(c) → 2-Oxobutyrate(m) + H+(PG)(m)
r0365	3	Hydracrylate(m) + NAD+(m) → NADH(m) + 3-Oxopropanoate(m)
r2678	3	Acetyl-CoA(m) ⇌ CoA(m) + CoA-activated acetyl group(m)
r1320	-3	ATP(m) + H2O(m) ⇌ ADP(m) + Pi(m) + ATP-energy(m)
r0683	3	Propanoyl-CoA(m) + Ubiquinone(m) → Ubiquinol(m) + Acrylyl-CoA(m)
r1323	8	NADH(m) ⇌ NAD+(m) + NADH-redox-potential(m)
r1326	1	NADPH(c) ⇌ NADPH-redox-potential(c) + NADP+(c)
r1327	-3	NADPH(m) ⇌ NADPH-redox-potential(m) + NADP+(m)
r0105	2	NAD+(m) + Malate(m) ⇌ OAA(m) + NADH(m)
r2417	-1	Citrulline(m) + H+(PG)(c) → Citrulline(c) + H+(PG)(m)
r0106	-1	ADP(m) + Pi(m) + OAA(m) ⇌ ATP(m) + Pyruvate(m) + HCO3-(m)
r0258	-1	Fumarate(c) + H2O(c) ⇌ Malate(c)
r0838	4	NH3(c) ⇌ NH3(m)
r0851	2	H2O(m) ⇌ H2O(c)

Arginine from Tryptophan (116)

Definition			Solution												
			exchanges		reactions								transp		
Objective	Constraints	Comment	imports	exports	c	m	r	p	l	n	s	b	s-c	b-c	int
Arginine	−Tryptophan MCES +Urea	Transamination to Arginine using Tryptophan	7 O_2(s) 2 Pyruvate(m) 2 Tryptophan(s) 5 ATP-energy(c) 3 ATP-energy(m) 4 NADPH-redox-potential(c) 4 NADPH-redox-potential(m)	5 CO_2(s) 3 Pyruvate(c) 1 Arginine(s) 6 NADH-redox-potential(m) 10 Proton-gradient(m) 1 Na-gradient(c) 2 THF-activated methyl group(c) 3 CoA-activated acetyl group(m)	26	20	-	-	-	-	-	-	6	-	12

ID	flux	reaction
r0855	7	O2(s) ⇌ O2(c)
r0856	-1	O2(m) ⇌ O2(c)
r0422	1	Isocitrate(c) + NADP+(c) ⇌ Oxalosuccinate(c) + NADPH(c)
r0293	-2	5,10-Methylene-THF(c) + NADP+(c) ⇌ 5,10-Methenyl-THF(c) + NADPH(c)
r0876	1	Glutamate(c) + H+(PG)(c) + Aspartate(m) → Glutamate(m) + H+(PG)(m) + Aspartate(c)
r0460	2	(S)-3-Hydroxybutyryl-CoA(m) + NAD+(m) ⇌ NADH(m) + Acetoacetyl-CoA(m)
r0448	2	2-Oxoadipate(m) + CoA(m) + NAD+(m) ⇌ Glutaryl-CoA(m) + CO2(m) + NADH(m)
r0449	-2	2-Oxoadipate(c) + NH3(c) + NADP+(c) ⇌ 2-Aminomuconate(c) + NADPH(c) + H2O(c)
r0167	-1	L-Glutamate 5-semialdehyde(m) + Glutamate(m) ⇌ AKG(m) + Ornithine(m)
r0017	1	O2(m) + 4 H+(PG)(m) + 4 Ferrocytochrome C(m) → 4 Ferricytochrome C(m) + 2 H2O(m) + 4 H+(PG)(c)
r0030	1	ATP(c) + AMP(c) ⇌ 2 ADP(c)
r0169	2	Tryptophan(c) + O2(c) ⇌ L-Formylkynurenine(c)
r0315	1	Citrate(c) ⇌ Isocitrate(c)
r0034	1	2 ATP(m) + CO2(m) + H2O(m) + NH3(m) ⇌ 2 ADP(m) + Pi(m) + Carbamoyl-P(m)
r0619	-1	L-Glutamate 5-semialdehyde(m) + Pi(m) + NADP+(m) ← NADPH(m) + Glutamyl-5P(m)
r0070	-2	CoA(m) + Acetoacetyl-CoA(m) ⇌ 2 Acetyl-CoA(m)
r2664	1	Na+(c) ⇌ Na+(s) + Na-gradient(c)
r0058	1	Malate(c) + NADP+(c) ⇌ Pyruvate(c) + CO2(c) + NADPH(c)

Continued on next page

ID	flux	reaction
r0072	1	ATP(m) + Glutamate(m) → ADP(m) + Glutamyl-5P(m)
r0059	-2	Pyruvate(m) + CO2(m) + NADPH(m) ⇌ Malate(m) + NADP+(m)
r0075	-1	AKG(m) + NADPH(m) + NH3(m) ⇌ Glutamate(m) + H2O(m) + NADP+(m)
r0371	-2	10-Formyl-THF(c) ⇌ 5,10-Methenyl-THF(c) + H2O(c)
r0077	1	ATP(c) + Glutamate(c) + NH3(c) → ADP(c) + Pi(c) + Glutamine(c)
r0078	1	Glutamine(m) + H2O(m) → Glutamate(m) + NH3(m)
r0507	2	Ubiquinol(m) + 2 Ferricytochrome C(m) + 4 H+(PG)(m) → Ubiquinone(m) + 4 H+(PG)(c) + 2 Ferrocytochrome C(m)
r0228	2	THF(c) + Formate(c) + ATP(c) → ADP(c) + Pi(c) + 10-Formyl-THF(c)
r0676	2	2-Amino-3-carboxymuconate semialdehyde(c) ⇌ 2-Aminomuconate semialdehyde(c) + CO2(c)
r1330	10	H+(PG)(c) ⇌ H+(PG)(m) + Proton-gradient(m)
r1319	-5	ATP(c) + H2O(c) ⇌ ADP(c) + Pi(c) + ATP-energy(c)
r0541	2	Glutaryl-CoA(m) + Ubiquinone(m) ⇌ Crotonyl-CoA(m) + CO2(m) + Ubiquinol(m)
r0261	1	Argininosuccinate(c) ⇌ Fumarate(c) + Arginine(c)
r0110	-1	OAA(m) + Glutamate(m) → AKG(m) + Aspartate(m)
r1913	1	Glycine(s) + Na+(s) + Arginine(c) → Glycine(c) + Na+(c) + Arginine(s)
r0562	2	3-Hydroxyanthranilate(c) + O2(c) ⇌ 2-Amino-3-carboxymuconate semialdehyde(c)
r0563	2	H2O(c) + 3-Hydroxy-L-kynurenine(c) ⇌ 3-Hydroxyanthranilate(c) + Alanine(c)
r0867	5	CO2(c) ⇌ CO2(s)
r0868	-1	CO2(c) ⇌ CO2(m)
r0588	-2	Crotonyl-CoA(m) + H2O(m) ⇌ (S)-3-Hydroxybutyryl-CoA(m)
r0453	1	ATP(c) + Citrulline(c) + Aspartate(c) ⇌ AMP(c) + PPi(c) + Argininosuccinate(c)
r0007	1	PPi(c) + H2O(c) → 2 Pi(c)
r0454	2	L-Formylkynurenine(c) + H2O(c) ⇌ Formate(c) + Kynurenine(c)
r0455	2	Kynurenine(c) + O2(c) + NADPH(c) ⇌ H2O(c) + 3-Hydroxy-L-kynurenine(c) + NADP+(c)
r1547	-1	Glycine(c) + Tryptophan(s) ⇌ Glycine(s) + Tryptophan(c)
r0329	1	Carbamoyl-P(m) + Ornithine(m) ⇌ Pi(m) + Citrulline(m)
r2373	1	Citrate(m) + AKG(c) ⇌ Citrate(c) + AKG(m)
r0792	-2	5,10-Methylene-THF(c) + NADH(c) ⇌ 5-Methyl-THF(c) + NAD+(c)
r0080	2	Alanine(c) + AKG(c) ⇌ Pyruvate(c) + Glutamate(c)
r2522	1	Glutamine(c) ⇌ Glutamine(m)
r0645	2	2-Aminomuconate semialdehyde(c) + NAD+(c) + H2O(c) ⇌ 2-Aminomuconate(c) + NADH(c)
r0082	1	Oxalosuccinate(c) ⇌ AKG(c) + CO2(c)
r2676	2	5-Methyl-THF(c) ⇌ THF(c) + THF-activated methyl group(c)
r2678	3	Acetyl-CoA(m) ⇌ CoA(m) + CoA-activated acetyl group(m)
r2527	1	Tryptophan(s) ⇌ Tryptophan(c)
r0945	2	2-Oxoadipate(c) + AKG(m) ⇌ 2-Oxoadipate(m) + AKG(c)
r1320	-3	ATP(m) + H2O(m) ⇌ ADP(m) + Pi(m) + ATP-energy(m)
r1323	6	NADH(m) ⇌ NAD+(m) + NADH-redox-potential(m)
r1326	-4	NADPH(c) ⇌ NADPH-redox-potential(c) + NADP+(c)
r1327	-4	NADPH(m) ⇌ NADPH-redox-potential(m) + NADP+(m)
r0105	2	NAD+(m) + Malate(m) ⇌ OAA(m) + NADH(m)
r2417	-1	Citrulline(m) + H+(PG)(c) → Citrulline(c) + H+(PG)(m)
r0258	-1	Fumarate(c) + H2O(c) ⇌ Malate(c)
r0107	-1	Acetyl-CoA(m) + H2O(m) + OAA(m) ⇌ Citrate(m) + CoA(m)
r0851	1	H2O(m) ⇌ H2O(c)
r0838	1	NH3(c) ⇌ NH3(m)

Arginine from Tyrosine (117)

Definition			Solution												
			exchanges		reactions								transp		
Objective	Constraints	Comment	imports	exports	c	m	r	p	l	n	s	b	s-c	b-c	int
Arginine	−Tyrosine MCES +Urea	Transamination to Arginine using Tyrosine	4 H_2O(s) 8 O_2(s) 4 Tyrosine(s) 10 ATP-energy(c) 3 ATP-energy(m) 4 Proton-gradient(m) 1 CoA-activated acetyl group(m)	7 CO_2(s) 1 Pyruvate(c) 2 Pyruvate(m) 1 Arginine(s) 1 NADH-redox-potential(c) 2 NADH-redox-potential(m) 2 NADPH-redox-potential(c) 1 NADPH-redox-potential(m) 4 Na-gradient(c) 8 CoA-activated acetyl group(c)	22	13	-	-	-	-	-	-	6	-	6

ID	flux	reaction
r0855	8	O2(s) ⇌ O2(c)
r0422	1	Isocitrate(c) + NADP+(c) ⇌ Oxalosuccinate(c) + NADPH(c)
r0875	3	Glutamate(c) + H+(PG)(c) → Glutamate(m) + H+(PG)(m)
r0167	-1	L-Glutamate 5-semialdehyde(m) + Glutamate(m) ⇌ AKG(m) + Ornithine(m)
r0030	5	ATP(c) + AMP(c) ⇌ 2 ADP(c)
r0183	4	Tyrosine(c) + AKG(c) ⇌ 4-Hydroxyphenylpyruvate(c) + Glutamate(c)
r0315	1	Citrate(c) ⇌ Isocitrate(c)
r0034	1	2 ATP(m) + CO2(m) + H2O(m) + NH3(m) ⇌ 2 ADP(m) + Pi(m) + Carbamoyl-P(m)
r1125	4	Tyrosine(s) + Na+(s) → Tyrosine(c) + Na+(c)
r0912	-1	Citrate(m) + Malate(c) ⇌ Citrate(c) + Malate(m)
r0619	-1	L-Glutamate 5-semialdehyde(m) + Pi(m) + NADP+(m) ← NADPH(m) + Glutamyl-5P(m)
r2664	4	Na+(c) ⇌ Na+(s) + Na-gradient(c)
r0058	1	Malate(c) + NADP+(c) ⇌ Pyruvate(c) + CO2(c) + NADPH(c)
r0072	1	ATP(m) + Glutamate(m) → ADP(m) + Glutamyl-5P(m)
r0059	2	Malate(m) + NADP+(m) ⇌ Pyruvate(m) + CO2(m) + NADPH(m)
r0073	1	Glutamate(m) + NAD+(m) + H2O(m) ⇌ AKG(m) + NADH(m) + NH3(m)
r0820	-2	AKG(m) + Malate(c) ⇌ AKG(c) + Malate(m)
r1330	-4	H+(PG)(c) ⇌ H+(PG)(m) + Proton-gradient(m)
r1319	-10	ATP(c) + H2O(c) ⇌ ADP(c) + Pi(c) + ATP-energy(c)
r0261	1	Argininosuccinate(c) ⇌ Fumarate(c) + Arginine(c)
r0543	4	Homogentisate(c) + O2(c) ⇌ 4-Maleylacetoacetate(c)
r0544	4	4-Hydroxyphenylpyruvate(c) + O2(c) ⇌ Homogentisate(c) + CO2(c)
r1058	-1	Arginine(c) ⇌ Arginine(s)
r0849	4	H2O(s) ⇌ H2O(c)
r0867	7	CO2(c) ⇌ CO2(s)
r0868	-1	CO2(c) ⇌ CO2(m)
r0453	1	ATP(c) + Citrulline(c) + Aspartate(c) ⇌ AMP(c) + PPi(c) + Argininosuccinate(c)
r0007	5	PPi(c) + H2O(c) → 2 Pi(c)
r0320	4	ATP(c) + CoA(c) + Acetoacetate(c) → AMP(c) + PPi(c) + Acetoacetyl-CoA(c)
r0605	4	4-Maleylacetoacetate(c) ⇌ Fumarylacetoacetate(c)
r0324	-4	Fumarylacetoacetate(c) + H2O(c) ⇌ Fumarate(c) + Acetoacetate(c)
r0329	1	Carbamoyl-P(m) + Ornithine(m) ⇌ Pi(m) + Citrulline(m)
r0069	-4	CoA(c) + Acetoacetyl-CoA(c) ⇌ 2 Acetyl-CoA(c)
r0082	1	Oxalosuccinate(c) ⇌ AKG(c) + CO2(c)
r2677	8	Acetyl-CoA(c) ⇌ CoA(c) + CoA-activated acetyl group(c)
r2678	-1	Acetyl-CoA(m) ⇌ CoA(m) + CoA-activated acetyl group(m)
r1320	-3	ATP(m) + H2O(m) ⇌ ADP(m) + Pi(m) + ATP-energy(m)
r1322	1	NADH(c) ⇌ NAD+(c) + NADH-redox-potential(c)
r1323	2	NADH(m) ⇌ NAD+(m) + NADH-redox-potential(m)
r1326	2	NADPH(c) ⇌ NADPH-redox-potential(c) + NADP+(c)
r1327	1	NADPH(m) ⇌ NADPH-redox-potential(m) + NADP+(m)
r0104	1	NAD+(c) + Malate(c) ⇌ OAA(c) + NADH(c)
r0105	1	NAD+(m) + Malate(m) ⇌ OAA(m) + NADH(m)
r2417	-1	Citrulline(m) + H+(PG)(c) → Citrulline(c) + H+(PG)(m)
r0258	-5	Fumarate(c) + H2O(c) ⇌ Malate(c)

Continued on next page

ID	flux	reaction
r0107	-1	Acetyl-CoA(m) + H2O(m) + OAA(m) ⇌ Citrate(m) + CoA(m)
r0109	-1	OAA(c) + Glutamate(c) ⇌ AKG(c) + Aspartate(c)

Arginine from Valine (118)

Definition			Solution													
			exchanges		reactions								transp		
Objective	Constraints	Comment	imports	exports	c	m	r	p	l	n	s	b	s-c	b-c	int
Arginine	−Valine MCES +Urea	Transamination to Arginine using Valine	6 H_2O(s) 3.5 O_2(s) 4 Valine(s) 2 ATP-energy(c)	5 CO_2(s) 1 Pyruvate(c) 2 Pyruvate(m) 1 Arginine(s) 1 NADH-redox-potential(c) 12 NADH-redox-potential(m) 1 NADPH-redox-potential(c) 1 NADPH-redox-potential(m) 36 Proton-gradient(m) 4 Na-gradient(c)	11	22	-	-	-	-	-	-	7	-	11

ID	flux	reaction
r0125	-3	GDP(m) + Pi(m) + Succinyl-CoA(m) ⇌ GTP(m) + Succinate(m) + CoA(m)
r0855	3.5	O2(s) ⇌ O2(c)
r0856	-3.5	O2(m) ⇌ O2(c)
r0167	-1	L-Glutamate 5-semialdehyde(m) + Glutamate(m) ⇌ AKG(m) + Ornithine(m)
r0017	3.5	O2(m) + 4 H+(PG)(m) + 4 Ferrocytochrome C(m) → 4 Ferricytochrome C(m) + 2 H2O(m) + 4 H+(PG)(c)
r0030	1	ATP(c) + AMP(c) ⇌ 2 ADP(c)
r1554	-1	Glycine(c) + Valine(s) ⇌ Glycine(s) + Valine(c)
r0034	1	2 ATP(m) + CO2(m) + H2O(m) + NH3(m) ⇌ 2 ADP(m) + Pi(m) + Carbamoyl-P(m)
r1121	-3	Valine(s) + Na+(s) → Valine(c) + Na+(c)
r0482	4	3-Hydroxyisobutyrate(m) + NAD+(m) → 2-Methyl-3-oxopropanoate(m) + NADH(m)
r0911	1	Proline(c) + Glutamate(m) ⇌ Proline(m) + Glutamate(c)
r0619	-1	L-Glutamate 5-semialdehyde(m) + Pi(m) + NADP+(m) ← NADPH(m) + Glutamyl-5P(m)
r2664	4	Na+(c) ⇌ Na+(s) + Na-gradient(c)
r1426	1	Proline(m) + H+(PG)(c) → Proline(c) + H+(PG)(m)
r0058	1	Malate(c) + NADP+(c) ⇌ Pyruvate(c) + CO2(c) + NADPH(c)
r0072	1	ATP(m) + Glutamate(m) → ADP(m) + Glutamyl-5P(m)
r0059	2	Malate(m) + NADP+(m) ⇌ Pyruvate(m) + CO2(m) + NADPH(m)
r0073	1	Glutamate(m) + NAD+(m) + H2O(m) ⇌ AKG(m) + NADH(m) + NH3(m)
r0507	7	Ubiquinol(m) + 2 Ferricytochrome C(m) + 4 H+(PG)(m) → Ubiquinone(m) + 4 H+(PG)(c) + 2 Ferrocytochrome C(m)
r0509	3	Ubiquinone(m) + Succinate(m) → Ubiquinol(m) + Fumarate(m)
r0820	1	AKG(c) + Malate(m) ⇌ AKG(m) + Malate(c)
r1330	36	H+(PG)(c) ⇌ H+(PG)(m) + Proton-gradient(m)
r1319	-2	ATP(c) + H2O(c) ⇌ ADP(c) + Pi(c) + ATP-energy(c)
r0261	1	Argininosuccinate(c) ⇌ Fumarate(c) + Arginine(c)
r1913	1	Glycine(s) + Na+(s) + Arginine(c) → Glycine(c) + Na+(c) + Arginine(s)
r0263	4	Valine(m) + AKG(m) → Glutamate(m) + 3-Methyl-2-oxobutyrate(m)
r0560	4	Isobutyryl-CoA(m) + Ubiquinone(m) → Methacrylyl-CoA(m) + Ubiquinol(m)
r0849	6	H2O(s) ⇌ H2O(c)
r0867	5	CO2(c) ⇌ CO2(s)
r0868	-4	CO2(c) ⇌ CO2(m)
r0453	1	ATP(c) + Citrulline(c) + Aspartate(c) ⇌ AMP(c) + PPi(c) + Argininosuccinate(c)
r0007	1	PPi(c) + H2O(c) → 2 Pi(c)
r0600	4	3-Methyl-2-oxobutyrate(m) + CoA(m) + NAD+(m) ⇌ Isobutyryl-CoA(m) + CO2(m) + NADH(m)
r1114	-1	NADH(m) + CO2(m) + Succinyl-CoA(m) ⇌ AKG(m) + CoA(m) + NAD+(m)
r0197	4	(R)-Methylmalonyl-CoA(m) ⇌ Succinyl-CoA(m)
r0329	1	Carbamoyl-P(m) + Ornithine(m) ⇌ Pi(m) + Citrulline(m)
r0624	4	2-Methyl-3-oxopropanoate(m) + CoA(m) + NAD+(m) ⇌ (R)-Methylmalonyl-CoA(m) + NADH(m)
r0779	4	3-Hydroxyisobutyryl-CoA(m) + H2O(m) → 3-Hydroxyisobutyrate(m) + CoA(m)
r0923	4	Valine(c) + H+(PG)(c) → Valine(m) + H+(PG)(m)

Continued on next page

ID	flux	reaction
r0669	4	Methacrylyl-CoA(m) + H2O(m) → 3-Hydroxyisobutyryl-CoA(m)
r1322	1	NADH(c) ⇌ NAD+(c) + NADH-redox-potential(c)
r1323	12	NADH(m) ⇌ NAD+(m) + NADH-redox-potential(m)
r0100	-3	ADP(m) + GTP(m) ⇌ ATP(m) + GDP(m)
r1326	1	NADPH(c) ⇌ NADPH-redox-potential(c) + NADP+(c)
r1327	1	NADPH(m) ⇌ NADPH-redox-potential(m) + NADP+(m)
r0104	1	NAD+(c) + Malate(c) ⇌ OAA(c) + NADH(c)
r2417	-1	Citrulline(m) + H+(PG)(c) → Citrulline(c) + H+(PG)(m)
r0258	-1	Malate(c) ⇌ Fumarate(c) + H2O(c)
r0259	-3	Malate(m) ⇌ Fumarate(m) + H2O(m)
r0851	-6	H2O(m) ⇌ H2O(c)
r0109	-1	OAA(c) + Glutamate(c) ⇌ AKG(c) + Aspartate(c)

Arginine from beta-Alanine (119)

Definition			Solution												
			exchanges		reactions								transp		
Objective	Constraints	Comment	imports	exports	c	m	r	p	l	n	s	b	s-c	b-c	int
Arginine	−beta-Alanine MCES +Urea	Transamination to Arginine using beta-Alanine	2 Pyruvate(m) 4 beta-Alanine(s) 2 ATP-energy(c) 3 ATP-energy(m) 2 NADPH-redox-potential(m) 3 Proton-gradient(m)	3 CO_2(s) 1 Pyruvate(c) 1 Arginine(s) 6 NADH-redox-potential(m) 2 NADPH-redox-potential(c) 3 CoA-activated acetyl group(m)	10	17	-	-	-	-	-	-	3	-	8

ID	flux	reaction
r2678	3	Acetyl-CoA(m) ⇌ CoA(m) + CoA-activated acetyl group(m)
r0316	1	Citrate(m) ⇌ Isocitrate(m)
r0217	4	beta-Alanine(m) + AKG(m) ⇌ Glutamate(m) + 3-Oxopropanoate(m)
r0082	1	Oxalosuccinate(c) ⇌ AKG(c) + CO2(c)
r0911	1	Proline(c) + Glutamate(m) ⇌ Proline(m) + Glutamate(c)
r1326	2	NADPH(c) ⇌ NADPH-redox-potential(c) + NADP+(c)
r0453	1	ATP(c) + Citrulline(c) + Aspartate(c) ⇌ AMP(c) + PPi(c) + Argininosuccinate(c)
r0174	4	CoA(m) + NAD+(m) + 3-Oxopropanoate(m) → Acetyl-CoA(m) + CO2(m) + NADH(m)
r0075	1	Glutamate(m) + H2O(m) + NADP+(m) ⇌ AKG(m) + NADPH(m) + NH3(m)
r1426	1	Proline(m) + H+(PG)(c) → Proline(c) + H+(PG)(m)
r1327	-2	NADPH(m) ⇌ NADPH-redox-potential(m) + NADP+(m)
r1147	1	AKG(c) + Isocitrate(m) ⇌ AKG(m) + Isocitrate(c)
r0899	-4	beta-Alanine(s) ⇌ beta-Alanine(c)
r0058	1	Malate(c) + NADP+(c) ⇌ Pyruvate(c) + CO2(c) + NADPH(c)
r2417	-1	Citrulline(m) + H+(PG)(c) → Citrulline(c) + H+(PG)(m)
r1319	-2	ATP(c) + H2O(c) ⇌ ADP(c) + Pi(c) + ATP-energy(c)
r1058	-1	Arginine(c) ⇌ Arginine(s)
r0329	1	Carbamoyl-P(m) + Ornithine(m) ⇌ Pi(m) + Citrulline(m)
r0167	-1	L-Glutamate 5-semialdehyde(m) + Glutamate(m) ⇌ AKG(m) + Ornithine(m)
r0059	-2	Pyruvate(m) + CO2(m) + NADPH(m) ⇌ Malate(m) + NADP+(m)
r0258	-1	Fumarate(c) + H2O(c) ⇌ Malate(c)
r0110	-1	OAA(m) + Glutamate(m) → AKG(m) + Aspartate(m)
r0619	-1	L-Glutamate 5-semialdehyde(m) + Pi(m) + NADP+(m) ← NADPH(m) + Glutamyl-5P(m)
r0030	1	ATP(c) + AMP(c) ⇌ 2 ADP(c)
r1320	-3	ATP(m) + H2O(m) ⇌ ADP(m) + Pi(m) + ATP-energy(m)
r0105	2	NAD+(m) + Malate(m) ⇌ OAA(m) + NADH(m)
r1330	-3	H+(PG)(c) ⇌ H+(PG)(m) + Proton-gradient(m)
r0034	1	2 ATP(m) + CO2(m) + H2O(m) + NH3(m) ⇌ 2 ADP(m) + Pi(m) + Carbamoyl-P(m)
r0007	1	PPi(c) + H2O(c) → 2 Pi(c)
r0876	1	Glutamate(c) + H+(PG)(c) + Aspartate(m) → Glutamate(m) + H+(PG)(m) + Aspartate(c)

Continued on next page

ID	flux	reaction
r0867	3	CO2(c) ⇌ CO2(s)
r0422	1	Isocitrate(c) + NADP+(c) ⇌ Oxalosuccinate(c) + NADPH(c)
r0107	-1	Acetyl-CoA(m) + H2O(m) + OAA(m) ⇌ Citrate(m) + CoA(m)
r1323	6	NADH(m) ⇌ NAD+(m) + NADH-redox-potential(m)
r0900	4	beta-Alanine(c) ⇌ beta-Alanine(m)
r0868	-1	CO2(c) ⇌ CO2(m)
r0261	1	Argininosuccinate(c) ⇌ Fumarate(c) + Arginine(c)
r0072	1	ATP(m) + Glutamate(m) → ADP(m) + Glutamyl-5P(m)

Asparagine from Alanine (120)

Definition			Solution												
			exchanges		reactions								transp		
Objective	Constraints	Comment	imports	exports	c	m	r	p	l	n	s	b	s-c	b-c	int
Asparagine	−Alanine MCES +Urea	Transamination to Asparagine using Alanine	1 Pyruvate(m) 2 Alanine(s) 2 ATP-energy(c) 1 ATP-energy(m) 1 NADPH-redox-potential(m)	1 Pyruvate(c) 1 Asparagine(s) 1 NADH-redox-potential(c) 2 NADH-redox-potential(m) 1 Na-gradient(c) 1 CoA-activated acetyl group(m)	9	8	-	-	-	-	-	-	4	-	4

ID	flux	reaction
r2678	1	Acetyl-CoA(m) ⇌ CoA(m) + CoA-activated acetyl group(m)
r0109	-1	OAA(c) + Glutamate(c) ⇌ AKG(c) + Aspartate(c)
r0073	1	Glutamate(m) + NAD+(m) + H2O(m) ⇌ AKG(m) + NADH(m) + NH3(m)
r0056	1	Pyruvate(m) + CoA(m) + NAD+(m) → Acetyl-CoA(m) + CO2(m) + NADH(m)
r1434	1	Alanine(c) → Alanine(m)
r0831	-1	Malate(m) + Pi(c) ⇌ Malate(c) + Pi(m)
r2524	1	Alanine(s) ⇌ Alanine(c)
r1327	-1	NADPH(m) ⇌ NADPH-redox-potential(m) + NADP+(m)
r1319	-2	ATP(c) + H2O(c) ⇌ ADP(c) + Pi(c) + ATP-energy(c)
r0158	1	ATP(c) + Glutamine(c) + H2O(c) + Aspartate(c) ⇌ AMP(c) + PPi(c) + Glutamate(c) + Asparagine(c)
r0077	1	ATP(c) + Glutamate(c) + NH3(c) → ADP(c) + Pi(c) + Glutamine(c)
r0059	-1	Pyruvate(m) + CO2(m) + NADPH(m) ⇌ Malate(m) + NADP+(m)
r2418	1	ADP(c) + ATP(m) → ADP(m) + ATP(c)
r0030	1	ATP(c) + AMP(c) ⇌ 2 ADP(c)
r2664	1	Na+(c) ⇌ Na+(s) + Na-gradient(c)
r2003	1	Lysine(s) + Asparagine(c) → Lysine(c) + Asparagine(s)
r1917	1	Alanine(s) + Na+(s) + Lysine(c) → Alanine(c) + Na+(c) + Lysine(s)
r0104	1	NAD+(c) + Malate(c) ⇌ OAA(c) + NADH(c)
r1320	-1	ATP(m) + H2O(m) ⇌ ADP(m) + Pi(m) + ATP-energy(m)
r0838	-1	NH3(c) ⇌ NH3(m)
r0007	1	PPi(c) + H2O(c) → 2 Pi(c)
r1322	1	NADH(c) ⇌ NAD+(c) + NADH-redox-potential(c)
r0080	1	Alanine(c) + AKG(c) ⇌ Pyruvate(c) + Glutamate(c)
r1323	2	NADH(m) ⇌ NAD+(m) + NADH-redox-potential(m)
r0081	1	Alanine(m) + AKG(m) ⇌ Pyruvate(m) + Glutamate(m)

Asparagine from Arginine (121)

Definition			Solution												
			exchanges		reactions								transp		
Objective	Constraints	Comment	imports	exports	c	m	r	p	l	n	s	b	s-c	b-c	int
Asparagine	−Arginine MCES +Urea	Transamination to Asparagine using Arginine	3 H_2O(s) 0.5 O_2(s) 1 Arginine(s) 3 ATP-energy(c)	1 CO_2(s) 1 Urea(s) 1 Asparagine(s) 1 ATP-energy(m) 1 NADH-redox-potential(c) 3 NADH-redox-potential(m) 4 Proton-gradient(m)	9	10	-	-	-	-	-	-	6	-	12

ID	flux	reaction
r0896	1	Urea(c) ⇌ Urea(s)
r0820	1	AKG(c) + Malate(m) ⇌ AKG(m) + Malate(c)
r0109	-1	OAA(c) + Glutamate(c) ⇌ AKG(c) + Aspartate(c)
r0073	1	Glutamate(m) + NAD+(m) + H2O(m) ⇌ AKG(m) + NADH(m) + NH3(m)
r0911	1	Proline(c) + Glutamate(m) ⇌ Proline(m) + Glutamate(c)
r0074	1	L-Glutamate 5-semialdehyde(m) + NAD+(m) + H2O(m) ⇌ Glutamate(m) + NADH(m)
r2532	-1	Asparagine(c) ⇌ Asparagine(s)
r0507	1	Ubiquinol(m) + 2 Ferricytochrome C(m) + 4 H+(PG)(m) → Ubiquinone(m) + 4 H+(PG)(c) + 2 Ferrocytochrome C(m)
r1426	1	Proline(m) + H+(PG)(c) → Proline(c) + H+(PG)(m)
r1319	-3	ATP(c) + H2O(c) ⇌ ADP(c) + Pi(c) + ATP-energy(c)
r1058	1	Arginine(s) ⇌ Arginine(c)
r0851	-3	H2O(m) ⇌ H2O(c)
r0509	1	Ubiquinone(m) + Succinate(m) → Ubiquinol(m) + Fumarate(m)
r0167	1	AKG(m) + Ornithine(m) ⇌ L-Glutamate 5-semialdehyde(m) + Glutamate(m)
r0158	1	ATP(c) + Glutamine(c) + H2O(c) + Aspartate(c) ⇌ AMP(c) + PPi(c) + Glutamate(c) + Asparagine(c)
r0100	-1	ADP(m) + GTP(m) ⇌ ATP(m) + GDP(m)
r0077	1	ATP(c) + Glutamate(c) + NH3(c) → ADP(c) + Pi(c) + Glutamine(c)
r0259	-1	Fumarate(m) + H2O(m) ⇌ Malate(m)
r0030	1	ATP(c) + AMP(c) ⇌ 2 ADP(c)
r0855	0.5	O2(s) ⇌ O2(c)
r0104	1	NAD+(c) + Malate(c) ⇌ OAA(c) + NADH(c)
r1320	1	ATP(m) + H2O(m) ⇌ ADP(m) + Pi(m) + ATP-energy(m)
r0856	-0.5	O2(m) ⇌ O2(c)
r0838	-1	NH3(c) ⇌ NH3(m)
r2396	-1	Lysine(m) + Ornithine(c) + H+(PG)(c) → Lysine(c) + Ornithine(m) + H+(PG)(m)
r1330	4	H+(PG)(c) ⇌ H+(PG)(m) + Proton-gradient(m)
r1114	1	AKG(m) + CoA(m) + NAD+(m) ⇌ NADH(m) + CO2(m) + Succinyl-CoA(m)
r0007	1	PPi(c) + H2O(c) → 2 Pi(c)
r1322	1	NADH(c) ⇌ NAD+(c) + NADH-redox-potential(c)
r1070	1	Lysine(c) ⇌ Lysine(m)
r0867	1	CO2(c) ⇌ CO2(s)
r0849	3	H2O(s) ⇌ H2O(c)
r0125	-1	GDP(m) + Pi(m) + Succinyl-CoA(m) ⇌ GTP(m) + Succinate(m) + CoA(m)
r0017	0.5	O2(m) + 4 H+(PG)(m) + 4 Ferrocytochrome C(m) → 4 Ferricytochrome C(m) + 2 H2O(m) + 4 H+(PG)(c)
r1323	3	NADH(m) ⇌ NAD+(m) + NADH-redox-potential(m)
r0868	-1	CO2(c) ⇌ CO2(m)
r0144	1	Arginine(c) + H2O(c) ⇌ Urea(c) + Ornithine(c)

Asparagine from Aspartate (122)

Definition			Solution												
			exchanges		reactions								transp		
Objective	Constraints	Comment	imports	exports	c	m	r	p	l	n	s	b	s-c	b-c	int
Asparagine	−Aspartate MCES +Urea	Transamination to Asparagine using Aspartate	2 Aspartate(s) 4 ATP-energy(c)	1 CO_2(s) 1 Pyruvate(c) 1 Asparagine(s) 1 NADPH-redox-potential(c) 2 Na-gradient(c)	12	-	-	-	-	-	-	-	4	-	-

ID	flux	reaction
r2532	-1	Asparagine(c) ⇌ Asparagine(s)
r1326	1	NADPH(c) ⇌ NADPH-redox-potential(c) + NADP+(c)
r0058	1	Malate(c) + NADP+(c) ⇌ Pyruvate(c) + CO2(c) + NADPH(c)
r1319	-4	ATP(c) + H2O(c) ⇌ ADP(c) + Pi(c) + ATP-energy(c)
r0158	1	ATP(c) + Glutamine(c) + H2O(c) + Aspartate(c) ⇌ AMP(c) + PPi(c) + Glutamate(c) + Asparagine(c)
r0077	1	ATP(c) + Glutamate(c) + NH3(c) → ADP(c) + Pi(c) + Glutamine(c)
r0258	-1	Fumarate(c) + H2O(c) ⇌ Malate(c)
r0277	1	GTP(c) + IMP(c) + Aspartate(c) → GDP(c) + Pi(c) + Adenylosuccinate(c)
r0030	1	ATP(c) + AMP(c) ⇌ 2 ADP(c)
r2664	2	Na+(c) ⇌ Na+(s) + Na-gradient(c)
r0099	1	ATP(c) + GDP(c) ⇌ ADP(c) + GTP(c)
r0007	1	PPi(c) + H2O(c) → 2 Pi(c)
r0867	1	CO2(c) ⇌ CO2(s)
r0260	1	Adenylosuccinate(c) → Fumarate(c) + AMP(c)
r0044	1	AMP(c) + H2O(c) ⇌ IMP(c) + NH3(c)
r1143	-2	Na+(c) + Aspartate(c) ← Na+(s) + Aspartate(s)

Asparagine from Cysteine (123)

Definition			Solution												
			exchanges		reactions								transp		
Objective	Constraints	Comment	imports	exports	c	m	r	p	l	n	s	b	s-c	b-c	int
Asparagine	−Cysteine MCES +Urea +H_2S	Transamination to Asparagine using Cysteine	2 Pyruvate(m) 2 Cysteine(s) 3 ATP-energy(c) 1 NADPH-redox-potential(m) 2 Proton-gradient(m)	2 Pyruvate(c) 1 Asparagine(s) 2 H_2S(s) 1 NADH-redox-potential(m) 2 Na-gradient(c) 1 CoA-activated acetyl group(m)	6	8	-	-	-	-	-	-	5	-	6

ID	flux	reaction
r2678	1	Acetyl-CoA(m) ⇌ CoA(m) + CoA-activated acetyl group(m)
r0073	-1	AKG(m) + NADH(m) + NH3(m) ⇌ Glutamate(m) + NAD+(m) + H2O(m)
r0911	1	Proline(c) + Glutamate(m) ⇌ Proline(m) + Glutamate(c)
r0056	1	Pyruvate(m) + CoA(m) + NAD+(m) → Acetyl-CoA(m) + CO2(m) + NADH(m)
r1426	1	Proline(m) + H+(PG)(c) → Proline(c) + H+(PG)(m)
r1327	-1	NADPH(m) ⇌ NADPH-redox-potential(m) + NADP+(m)
r0940	2	H2S(c) ⇌ H2S(s)
r0193	2	Cysteine(c) + H2O(c) ⇌ Pyruvate(c) + H2S(c) + NH3(c)
r1319	-3	ATP(c) + H2O(c) ⇌ ADP(c) + Pi(c) + ATP-energy(c)
r0851	1	H2O(m) ⇌ H2O(c)
r0158	1	ATP(c) + Glutamine(c) + H2O(c) + Aspartate(c) ⇌ AMP(c) + PPi(c) + Glutamate(c) + Asparagine(c)
r0077	1	ATP(c) + Glutamate(c) + NH3(c) → ADP(c) + Pi(c) + Glutamine(c)
r0059	-1	Pyruvate(m) + CO2(m) + NADPH(m) ⇌ Malate(m) + NADP+(m)
r0110	-1	OAA(m) + Glutamate(m) → AKG(m) + Aspartate(m)
r0030	1	ATP(c) + AMP(c) ⇌ 2 ADP(c)
r1952	1	Cysteine(s) + Na+(s) + Lysine(c) → Cysteine(c) + Na+(c) + Lysine(s)
r2664	2	Na+(c) ⇌ Na+(s) + Na-gradient(c)
r2475	1	Cysteine(s) + Na+(s) → Cysteine(c) + Na+(c)
r2003	1	Lysine(s) + Asparagine(c) → Lysine(c) + Asparagine(s)
r0838	1	NH3(c) ⇌ NH3(m)
r0105	1	NAD+(m) + Malate(m) ⇌ OAA(m) + NADH(m)
r1330	-2	H+(PG)(c) ⇌ H+(PG)(m) + Proton-gradient(m)
r0007	1	PPi(c) + H2O(c) → 2 Pi(c)
r0876	1	Glutamate(c) + H+(PG)(c) + Aspartate(m) → Glutamate(m) + H+(PG)(m) + Aspartate(c)
r1323	1	NADH(m) ⇌ NAD+(m) + NADH-redox-potential(m)

Asparagine from Cystine (124)

Definition			Solution												
			exchanges		reactions								transp		
Objective	Constraints	Comment	imports	exports	c	m	r	p	l	n	s	b	s-c	b-c	int
Asparagine	−Cystine MCES +Urea +H_2S	Transamination to Asparagine using Cystine	2 Pyruvate(m) 1 Cystine(s) 3 ATP-energy(c) 1 NADPH-redox-potential(c) 1 NADPH-redox-potential(m) 2 Proton-gradient(m)	2 Pyruvate(c) 1 Asparagine(s) 2 H_2S(s) 1 NADH-redox-potential(m) 1 Na-gradient(c) 1 CoA-activated acetyl group(m)	8	8	-	-	-	-	-	-	5	-	6

ID	flux	reaction
r2678	1	Acetyl-CoA(m) ⇌ CoA(m) + CoA-activated acetyl group(m)
r1144	-1	Glutamate(c) + Na+(c) ← Glutamate(s) + Na+(s)
r0073	-1	AKG(m) + NADH(m) + NH3(m) ⇌ Glutamate(m) + NAD+(m) + H2O(m)
r0911	1	Proline(c) + Glutamate(m) ⇌ Proline(m) + Glutamate(c)
r0056	1	Pyruvate(m) + CoA(m) + NAD+(m) → Acetyl-CoA(m) + CO2(m) + NADH(m)
r2532	-1	Asparagine(c) ⇌ Asparagine(s)
r1326	-1	NADPH(c) ⇌ NADPH-redox-potential(c) + NADP+(c)
r1426	1	Proline(m) + H+(PG)(c) → Proline(c) + H+(PG)(m)
r1327	-1	NADPH(m) ⇌ NADPH-redox-potential(m) + NADP+(m)
r0940	2	H2S(c) ⇌ H2S(s)
r0193	2	Cysteine(c) + H2O(c) ⇌ Pyruvate(c) + H2S(c) + NH3(c)
r1319	-3	ATP(c) + H2O(c) ⇌ ADP(c) + Pi(c) + ATP-energy(c)
r0851	1	H2O(m) ⇌ H2O(c)
r0158	1	ATP(c) + Glutamine(c) + H2O(c) + Aspartate(c) ⇌ AMP(c) + PPi(c) + Glutamate(c) + Asparagine(c)
r0077	1	ATP(c) + Glutamate(c) + NH3(c) → ADP(c) + Pi(c) + Glutamine(c)
r0059	-1	Pyruvate(m) + CO2(m) + NADPH(m) ⇌ Malate(m) + NADP+(m)
r0110	-1	OAA(m) + Glutamate(m) → AKG(m) + Aspartate(m)
r0030	1	ATP(c) + AMP(c) ⇌ 2 ADP(c)
r2664	1	Na+(c) ⇌ Na+(s) + Na-gradient(c)
r0838	1	NH3(c) ⇌ NH3(m)
r0105	1	NAD+(m) + Malate(m) ⇌ OAA(m) + NADH(m)
r1330	-2	H+(PG)(c) ⇌ H+(PG)(m) + Proton-gradient(m)
r0007	1	PPi(c) + H2O(c) → 2 Pi(c)
r0876	1	Glutamate(c) + H+(PG)(c) + Aspartate(m) → Glutamate(m) + H+(PG)(m) + Aspartate(c)
r1323	1	NADH(m) ⇌ NAD+(m) + NADH-redox-potential(m)
r0877	1	Glutamate(c) + Cystine(s) ⇌ Glutamate(s) + Cystine(c)
r0027	-1	NADPH(c) + Cystine(c) ⇌ 2 Cysteine(c) + NADP+(c)

Asparagine from Glutamate (125)

Definition			Solution												
			exchanges		reactions								transp		
Objective	Constraints	Comment	imports	exports	c	m	r	p	l	n	s	b	s-c	b-c	int
Asparagine	−Glutamate MCES +Urea	Transamination to Asparagine using Glutamate	2 H_2O(s) 1 O_2(s) 2 Glutamate(s) 3 ATP-energy(c)	3 CO_2(s) 1 Pyruvate(m) 1 Asparagine(s) 2 ATP-energy(m) 3 NADH-redox-potential(m) 2 NADPH-redox-potential(m) 10 Proton-gradient(m) 2 Na-gradient(c)	5	12	-	-	-	-	-	-	6	-	9

ID	flux	reaction
r1144	-2	Glutamate(c) + Na+(c) ← Glutamate(s) + Na+(s)
r2532	-1	Asparagine(c) ⇌ Asparagine(s)
r0507	2	Ubiquinol(m) + 2 Ferricytochrome C(m) + 4 H+(PG)(m) → Ubiquinone(m) + 4 H+(PG)(c) + 2 Ferrocytochrome C(m)
r0075	1	Glutamate(m) + H2O(m) + NADP+(m) ⇌ AKG(m) + NADPH(m) + NH3(m)
r1327	2	NADPH(m) ⇌ NADPH-redox-potential(m) + NADP+(m)

Continued on next page

ID	flux	reaction
r1319	-3	ATP(c) + H2O(c) ⇌ ADP(c) + Pi(c) + ATP-energy(c)
r0851	-3	H2O(m) ⇌ H2O(c)
r0509	2	Ubiquinone(m) + Succinate(m) → Ubiquinol(m) + Fumarate(m)
r0158	1	ATP(c) + Glutamine(c) + H2O(c) + Aspartate(c) ⇌ AMP(c) + PPi(c) + Glutamate(c) + Asparagine(c)
r0100	-2	ADP(m) + GTP(m) ⇌ ATP(m) + GDP(m)
r0077	1	ATP(c) + Glutamate(c) + NH3(c) → ADP(c) + Pi(c) + Glutamine(c)
r0059	1	Malate(m) + NADP+(m) ⇌ Pyruvate(m) + CO2(m) + NADPH(m)
r0110	-1	OAA(m) + Glutamate(m) → AKG(m) + Aspartate(m)
r0259	-2	Fumarate(m) + H2O(m) ⇌ Malate(m)
r0030	1	ATP(c) + AMP(c) ⇌ 2 ADP(c)
r2664	2	Na+(c) ⇌ Na+(s) + Na-gradient(c)
r0855	1	O2(s) ⇌ O2(c)
r1320	2	ATP(m) + H2O(m) ⇌ ADP(m) + Pi(m) + ATP-energy(m)
r0856	-1	O2(m) ⇌ O2(c)
r0838	-1	NH3(c) ⇌ NH3(m)
r0105	1	NAD+(m) + Malate(m) ⇌ OAA(m) + NADH(m)
r1330	10	H+(PG)(c) ⇌ H+(PG)(m) + Proton-gradient(m)
r1114	2	AKG(m) + CoA(m) + NAD+(m) ⇌ NADH(m) + CO2(m) + Succinyl-CoA(m)
r0875	1	Glutamate(c) + H+(PG)(c) → Glutamate(m) + H+(PG)(m)
r0007	1	PPi(c) + H2O(c) → 2 Pi(c)
r0876	1	Glutamate(c) + H+(PG)(c) + Aspartate(m) → Glutamate(m) + H+(PG)(m) + Aspartate(c)
r0867	3	CO2(c) ⇌ CO2(s)
r0849	2	H2O(s) ⇌ H2O(c)
r0125	-2	GDP(m) + Pi(m) + Succinyl-CoA(m) ⇌ GTP(m) + Succinate(m) + CoA(m)
r0017	1	O2(m) + 4 H+(PG)(m) + 4 Ferrocytochrome C(m) → 4 Ferricytochrome C(m) + 2 H2O(m) + 4 H+(PG)(c)
r1323	3	NADH(m) ⇌ NAD+(m) + NADH-redox-potential(m)
r0868	-3	CO2(c) ⇌ CO2(m)

Asparagine from Glycine (126)

Definition			Solution												
			exchanges		reactions								transp		
Objective	Constraints	Comment	imports	exports	c	m	r	p	l	n	s	b	s-c	b-c	int
Asparagine	−Glycine MCES +Urea	Transamination to Asparagine using Glycine	2 Pyruvate(m) 2 Glycine(s) 3 ATP-energy(c) 2 NADPH-redox-potential(m) 2 Proton-gradient(m) 2 THF-activated methyl group(c)	2 Pyruvate(c) 1 Asparagine(s) 2 NADH-redox-potential(m) 2 NADPH-redox-potential(c) 1 Na-gradient(c) 1 CoA-activated acetyl group(m)	10	8	-	-	-	-	-	-	3	-	6

ID	flux	reaction
r2678	1	Acetyl-CoA(m) ⇌ CoA(m) + CoA-activated acetyl group(m)
r0911	1	Proline(c) + Glutamate(m) ⇌ Proline(m) + Glutamate(c)
r0056	1	Pyruvate(m) + CoA(m) + NAD+(m) → Acetyl-CoA(m) + CO2(m) + NADH(m)
r1326	2	NADPH(c) ⇌ NADPH-redox-potential(c) + NADP+(c)
r0075	-1	AKG(m) + NADPH(m) + NH3(m) ⇌ Glutamate(m) + H2O(m) + NADP+(m)
r1426	1	Proline(m) + H+(PG)(c) → Proline(c) + H+(PG)(m)
r1327	-2	NADPH(m) ⇌ NADPH-redox-potential(m) + NADP+(m)
r1138	1	Glycine(s) + Na+(s) → Glycine(c) + Na+(c)
r1553	1	Glycine(s) + Asparagine(c) ⇌ Glycine(c) + Asparagine(s)
r1319	-3	ATP(c) + H2O(c) ⇌ ADP(c) + Pi(c) + ATP-energy(c)
r0851	1	H2O(m) ⇌ H2O(c)
r0158	1	ATP(c) + Glutamine(c) + H2O(c) + Aspartate(c) ⇌ AMP(c) + PPi(c) + Glutamate(c) + Asparagine(c)
r0077	1	ATP(c) + Glutamate(c) + NH3(c) → ADP(c) + Pi(c) + Glutamine(c)
r0059	-1	Pyruvate(m) + CO2(m) + NADPH(m) ⇌ Malate(m) + NADP+(m)
r0110	-1	OAA(m) + Glutamate(m) → AKG(m) + Aspartate(m)

Continued on next page

ID	flux	reaction
r0030	1	ATP(c) + AMP(c) ⇌ 2 ADP(c)
r0296	2	5-Methyl-THF(c) + NADP+(c) ⇌ 5,10-Methylene-THF(c) + NADPH(c)
r2664	1	Na+(c) ⇌ Na+(s) + Na-gradient(c)
r0230	2	5,10-Methylene-THF(c) + Glycine(c) + H2O(c) ⇌ THF(c) + Serine(c)
r0838	1	NH3(c) ⇌ NH3(m)
r0105	1	NAD+(m) + Malate(m) ⇌ OAA(m) + NADH(m)
r0060	2	Serine(c) → Pyruvate(c) + NH3(c)
r1330	-2	H+(PG)(c) ⇌ H+(PG)(m) + Proton-gradient(m)
r0007	1	PPi(c) + H2O(c) → 2 Pi(c)
r2676	-2	5-Methyl-THF(c) ⇌ THF(c) + THF-activated methyl group(c)
r0876	1	Glutamate(c) + H+(PG)(c) + Aspartate(m) → Glutamate(m) + H+(PG)(m) + Aspartate(c)
r1323	2	NADH(m) ⇌ NAD+(m) + NADH-redox-potential(m)

Asparagine from Histidine (127)

Definition			Solution												
			exchanges		reactions								transp		
Objective	Constraints	Comment	imports	exports	c	m	r	p	l	n	s	b	s-c	b-c	int
Asparagine	−Histidine MCES +Urea	Transamination to Asparagine using Histidine	0.67 H_2O(s) 0.33 O_2(s) 0.33 Pyruvate(m) 0.67 Histidine(s) 3 ATP-energy(c) 0.67 NADPH-redox-potential(c) 0.33 NADPH-redox-potential(m)	0.33 CO_2(s) 1 Asparagine(s) 0.67 ATP-energy(m) 0.33 NADH-redox-potential(c) 0.33 NADH-redox-potential(m) 3.67 Proton-gradient(m) 0.67 THF-activated methyl group(c)	17	10	-	-	-	-	-	-	6	-	10

ID	flux	reaction
r0125	-0.67	GDP(m) + Pi(m) + Succinyl-CoA(m) ⇌ GTP(m) + Succinate(m) + CoA(m)
r2003	0.67	Lysine(s) + Asparagine(c) → Lysine(c) + Asparagine(s)
r0855	0.33	O2(s) ⇌ O2(c)
r0856	-0.33	O2(m) ⇌ O2(c)
r0293	-0.67	5,10-Methenyl-THF(c) + NADPH(c) ⇌ 5,10-Methylene-THF(c) + NADP+(c)
r0577	-0.67	Urocanate(c) + H2O(c) ⇌ 4-Imidazolone-5-propanoate(c)
r1665	-0.67	Lysine(c) + Histidine(s) ⇌ Lysine(s) + Histidine(c)
r0017	0.33	O2(m) + 4 H+(PG)(m) + 4 Ferrocytochrome C(m) → 4 Ferricytochrome C(m) + 2 H2O(m) + 4 H+(PG)(c)
r0030	1	ATP(c) + AMP(c) ⇌ 2 ADP(c)
r0911	0.33	Proline(c) + Glutamate(m) ⇌ Proline(m) + Glutamate(c)
r1426	0.33	Proline(m) + H+(PG)(c) → Proline(c) + H+(PG)(m)
r0059	-0.33	Pyruvate(m) + CO2(m) + NADPH(m) ⇌ Malate(m) + NADP+(m)
r0073	-0.33	AKG(m) + NADH(m) + NH3(m) ⇌ Glutamate(m) + NAD+(m) + H2O(m)
r0077	1	ATP(c) + Glutamate(c) + NH3(c) → ADP(c) + Pi(c) + Glutamine(c)
r2532	-0.33	Asparagine(c) ⇌ Asparagine(s)
r0507	0.67	Ubiquinol(m) + 2 Ferricytochrome C(m) + 4 H+(PG)(m) → Ubiquinone(m) + 4 H+(PG)(c) + 2 Ferrocytochrome C(m)
r0509	0.67	Ubiquinone(m) + Succinate(m) → Ubiquinol(m) + Fumarate(m)
r0524	0.67	5-Formiminotetrahydrofolate(c) ⇌ 5,10-Methenyl-THF(c) + NH3(c)
r0820	1	AKG(c) + Malate(m) ⇌ AKG(m) + Malate(c)
r1330	3.67	H+(PG)(c) ⇌ H+(PG)(m) + Proton-gradient(m)
r1319	-3	ATP(c) + H2O(c) ⇌ ADP(c) + Pi(c) + ATP-energy(c)
r0849	0.67	H2O(s) ⇌ H2O(c)
r0286	0.67	Histidine(c) ⇌ Urocanate(c) + NH3(c)
r0867	0.33	CO2(c) ⇌ CO2(s)
r0868	-0.33	CO2(c) ⇌ CO2(m)
r0158	1	ATP(c) + Glutamine(c) + H2O(c) + Aspartate(c) ⇌ AMP(c) + PPi(c) + Glutamate(c) + Asparagine(c)
r0007	1	PPi(c) + H2O(c) → 2 Pi(c)
r1114	0.67	AKG(m) + CoA(m) + NAD+(m) ⇌ NADH(m) + CO2(m) + Succinyl-CoA(m)
r0792	-0.67	5,10-Methylene-THF(c) + NADH(c) ⇌ 5-Methyl-THF(c) + NAD+(c)
r2676	0.67	5-Methyl-THF(c) ⇌ THF(c) + THF-activated methyl group(c)

Continued on next page

ID	flux	reaction
r1320	0.67	ATP(m) + H2O(m) ⇌ ADP(m) + Pi(m) + ATP-energy(m)
r1322	0.33	NADH(c) ⇌ NAD+(c) + NADH-redox-potential(c)
r0518	-0.67	Glutamate(c) + 5-Formiminotetrahydrofolate(c) ⇌ THF(c) + N-Formimino-L-glutamate(c)
r1323	0.33	NADH(m) ⇌ NAD+(m) + NADH-redox-potential(m)
r0519	0.67	H2O(c) + 4-Imidazolone-5-propanoate(c) ⇌ N-Formimino-L-glutamate(c)
r0100	-0.67	ADP(m) + GTP(m) ⇌ ATP(m) + GDP(m)
r1326	-0.67	NADPH(c) ⇌ NADPH-redox-potential(c) + NADP+(c)
r1327	-0.33	NADPH(m) ⇌ NADPH-redox-potential(m) + NADP+(m)
r0104	1	NAD+(c) + Malate(c) ⇌ OAA(c) + NADH(c)
r0259	-0.67	Fumarate(m) + H2O(m) ⇌ Malate(m)
r0838	0.33	NH3(c) ⇌ NH3(m)
r0851	-0.33	H2O(m) ⇌ H2O(c)
r0109	-1	OAA(c) + Glutamate(c) ⇌ AKG(c) + Aspartate(c)

Asparagine from Isoleucine (128)

Definition			Solution												
			exchanges		reactions								transp		
Objective	Constraints	Comment	imports	exports	c	m	r	p	l	n	s	b	s-c	b-c	int
Asparagine	−Isoleucine MCES +Urea	Transamination to Asparagine using Isoleucine	2 H_2O(s) 2 O_2(s) 1 Pyruvate(m) 2 Isoleucine(s) 3 ATP-energy(c) 1 NADPH-redox-potential(m)	3 CO_2(s) 1 Asparagine(s) 10 NADH-redox-potential(m) 20 Proton-gradient(m) 1 Na-gradient(c) 4 CoA-activated acetyl group(m)	5	18	-	-	-	-	-	-	7	-	11

ID	flux	reaction
r0855	2	O2(s) ⇌ O2(c)
r0856	-2	O2(m) ⇌ O2(c)
r0592	-2	H2O(m) + Acrylyl-CoA(m) → 3-Hydroxypropionyl-CoA(m)
r0876	1	Glutamate(c) + H+(PG)(c) + Aspartate(m) → Glutamate(m) + H+(PG)(m) + Aspartate(c)
r0596	-2	3-Hydroxypropionyl-CoA(m) + H2O(m) → Hydracrylate(m) + CoA(m)
r1682	1	Glycine(s) + Asparagine(c) + Na+(s) → Glycine(c) + Asparagine(s) + Na+(c)
r0017	2	O2(m) + 4 H+(PG)(m) + 4 Ferrocytochrome C(m) → 4 Ferricytochrome C(m) + 2 H2O(m) + 4 H+(PG)(c)
r0030	1	ATP(c) + AMP(c) ⇌ 2 ADP(c)
r1557	-1	Glycine(c) + Isoleucine(s) ⇌ Glycine(s) + Isoleucine(c)
r0911	1	Proline(c) + Glutamate(m) ⇌ Proline(m) + Glutamate(c)
r2664	1	Na+(c) ⇌ Na+(s) + Na-gradient(c)
r1426	1	Proline(m) + H+(PG)(c) → Proline(c) + H+(PG)(m)
r0059	-1	Pyruvate(m) + CO2(m) + NADPH(m) ⇌ Malate(m) + NADP+(m)
r0073	1	Glutamate(m) + NAD+(m) + H2O(m) ⇌ AKG(m) + NADH(m) + NH3(m)
r0077	1	ATP(c) + Glutamate(c) + NH3(c) → ADP(c) + Pi(c) + Glutamine(c)
r0222	-2	CoA(m) + 2-Methylacetoacetyl-CoA(m) ⇌ Propanoyl-CoA(m) + Acetyl-CoA(m)
r0953	2	Isoleucine(c) + H+(PG)(c) → Isoleucine(m) + H+(PG)(m)
r2536	1	Isoleucine(s) ⇌ Isoleucine(c)
r0507	4	Ubiquinol(m) + 2 Ferricytochrome C(m) + 4 H+(PG)(m) → Ubiquinone(m) + 4 H+(PG)(c) + 2 Ferrocytochrome C(m)
r1330	20	H+(PG)(c) ⇌ H+(PG)(m) + Proton-gradient(m)
r1319	-3	ATP(c) + H2O(c) ⇌ ADP(c) + Pi(c) + ATP-energy(c)
r0110	-1	OAA(m) + Glutamate(m) → AKG(m) + Aspartate(m)
r0264	2	Isoleucine(m) + AKG(m) ⇌ Glutamate(m) + 2-Oxo-3-methylvalerate(m)
r0849	2	H2O(s) ⇌ H2O(c)
r0867	3	CO2(c) ⇌ CO2(s)
r0868	-3	CO2(c) ⇌ CO2(m)
r0158	1	ATP(c) + Glutamine(c) + H2O(c) + Aspartate(c) ⇌ AMP(c) + PPi(c) + Glutamate(c) + Asparagine(c)
r0007	1	PPi(c) + H2O(c) → 2 Pi(c)
r0174	2	CoA(m) + NAD+(m) + 3-Oxopropanoate(m) → Acetyl-CoA(m) + CO2(m) + NADH(m)

Continued on next page

ID	flux	reaction
r0602	2	2-Oxo-3-methylvalerate(m) + CoA(m) + NAD+(m) ⇌ 2-Methylbutyryl-CoA(m) + CO2(m) + NADH(m)
r0603	2	2-Methylbutyryl-CoA(m) + Ubiquinone(m) ⇌ Tiglyl-CoA(m) + Ubiquinol(m)
r0365	2	Hydracrylate(m) + NAD+(m) → NADH(m) + 3-Oxopropanoate(m)
r2678	4	Acetyl-CoA(m) ⇌ CoA(m) + CoA-activated acetyl group(m)
r0664	2	NAD+(m) + (S)-3-Hydroxy-2-methylbutyryl-CoA(m) ⇌ 2-Methylacetoacetyl-CoA(m) + NADH(m)
r0665	-2	H2O(m) + Tiglyl-CoA(m) ⇌ (S)-3-Hydroxy-2-methylbutyryl-CoA(m)
r0683	2	Propanoyl-CoA(m) + Ubiquinone(m) → Ubiquinol(m) + Acrylyl-CoA(m)
r1323	10	NADH(m) ⇌ NAD+(m) + NADH-redox-potential(m)
r1327	-1	NADPH(m) ⇌ NADPH-redox-potential(m) + NADP+(m)
r0105	1	NAD+(m) + Malate(m) ⇌ OAA(m) + NADH(m)
r0838	-1	NH3(c) ⇌ NH3(m)
r0851	-3	H2O(m) ⇌ H2O(c)

Asparagine from Glutamine (129)

Definition			Solution												
			exchanges		reactions								transp		
Objective	Constraints	Comment	imports	exports	c	m	r	p	l	n	s	b	s-c	b-c	int
Asparagine	−Glutamine MCES +Urea	Transamination to Asparagine using Glutamine	1 H_2O(s) 0.5 O_2(s) 1 Glutamine(s) 2 ATP-energy(c)	1 CO_2(s) 1 Asparagine(s) 1 ATP-energy(m) 2 NADH-redox-potential(m) 5 Proton-gradient(m)	4	9	-	-	-	-	-	-	5	-	7

ID	flux	reaction
r0507	1	Ubiquinol(m) + 2 Ferricytochrome C(m) + 4 H+(PG)(m) → Ubiquinone(m) + 4 H+(PG)(c) + 2 Ferrocytochrome C(m)
r1553	1	Glycine(s) + Asparagine(c) ⇌ Glycine(c) + Asparagine(s)
r1544	-1	Glycine(s) + Glutamine(c) ⇌ Glycine(c) + Glutamine(s)
r1319	-2	ATP(c) + H2O(c) ⇌ ADP(c) + Pi(c) + ATP-energy(c)
r0851	-1	H2O(m) ⇌ H2O(c)
r0509	1	Ubiquinone(m) + Succinate(m) → Ubiquinol(m) + Fumarate(m)
r0158	1	ATP(c) + Glutamine(c) + H2O(c) + Aspartate(c) ⇌ AMP(c) + PPi(c) + Glutamate(c) + Asparagine(c)
r0100	-1	ADP(m) + GTP(m) ⇌ ATP(m) + GDP(m)
r0110	-1	OAA(m) + Glutamate(m) → AKG(m) + Aspartate(m)
r0259	-1	Fumarate(m) + H2O(m) ⇌ Malate(m)
r0030	1	ATP(c) + AMP(c) ⇌ 2 ADP(c)
r0855	0.5	O2(s) ⇌ O2(c)
r1320	1	ATP(m) + H2O(m) ⇌ ADP(m) + Pi(m) + ATP-energy(m)
r0856	-0.5	O2(m) ⇌ O2(c)
r0105	1	NAD+(m) + Malate(m) ⇌ OAA(m) + NADH(m)
r1330	5	H+(PG)(c) ⇌ H+(PG)(m) + Proton-gradient(m)
r1114	1	AKG(m) + CoA(m) + NAD+(m) ⇌ NADH(m) + CO2(m) + Succinyl-CoA(m)
r0007	1	PPi(c) + H2O(c) → 2 Pi(c)
r0876	1	Glutamate(c) + H+(PG)(c) + Aspartate(m) → Glutamate(m) + H+(PG)(m) + Aspartate(c)
r0867	1	CO2(c) ⇌ CO2(s)
r0849	1	H2O(s) ⇌ H2O(c)
r0125	-1	GDP(m) + Pi(m) + Succinyl-CoA(m) ⇌ GTP(m) + Succinate(m) + CoA(m)
r0017	0.5	O2(m) + 4 H+(PG)(m) + 4 Ferrocytochrome C(m) → 4 Ferricytochrome C(m) + 2 H2O(m) + 4 H+(PG)(c)
r1323	2	NADH(m) ⇌ NAD+(m) + NADH-redox-potential(m)
r0868	-1	CO2(c) ⇌ CO2(m)

Asparagine from Leucine (130)

Definition			Solution												
			exchanges		reactions							transp			
Objective	Constraints	Comment	imports	exports	c	m	r	p	l	n	s	b	s-c	b-c	int
Asparagine	−Leucine MCES +Urea	Transamination to Asparagine using Leucine	1 O_2(s) 2 Pyruvate(m) 2 Leucine(s) 3 ATP-energy(c) 3 NADPH-redox-potential(m)	2 H_2O(s) 1 Asparagine(s) 7 NADH-redox-potential(m) 10 Proton-gradient(m) 2 Na-gradient(c) 7 CoA-activated acetyl group(m)	5	16	-	-	-	-	-	-	5	-	10

ID	flux	reaction
r2678	7	Acetyl-CoA(m) ⇌ CoA(m) + CoA-activated acetyl group(m)
r0262	2	Leucine(m) + AKG(m) ⇌ Glutamate(m) + 4-Methyl-2-oxopentanoate(m)
r0073	1	Glutamate(m) + NAD+(m) + H2O(m) ⇌ AKG(m) + NADH(m) + NH3(m)
r0911	1	Proline(c) + Glutamate(m) ⇌ Proline(m) + Glutamate(c)
r0056	1	Pyruvate(m) + CoA(m) + NAD+(m) → Acetyl-CoA(m) + CO2(m) + NADH(m)
r2532	-1	Asparagine(c) ⇌ Asparagine(s)
r0507	2	Ubiquinol(m) + 2 Ferricytochrome C(m) + 4 H+(PG)(m) → Ubiquinone(m) + 4 H+(PG)(c) + 2 Ferrocytochrome C(m)
r0462	2	HMG-CoA(m) + CoA(m) ⇌ Acetyl-CoA(m) + H2O(m) + Acetoacetyl-CoA(m)
r1426	1	Proline(m) + H+(PG)(c) → Proline(c) + H+(PG)(m)
r1327	-3	NADPH(m) ⇌ NADPH-redox-potential(m) + NADP+(m)
r0490	-2	3-Methylglutaconyl-CoA(m) + H2O(m) ⇌ HMG-CoA(m)
r1319	-3	ATP(c) + H2O(c) ⇌ ADP(c) + Pi(c) + ATP-energy(c)
r0851	1	H2O(m) ⇌ H2O(c)
r0158	1	ATP(c) + Glutamine(c) + H2O(c) + Aspartate(c) ⇌ AMP(c) + PPi(c) + Glutamate(c) + Asparagine(c)
r0077	1	ATP(c) + Glutamate(c) + NH3(c) → ADP(c) + Pi(c) + Glutamine(c)
r0059	-3	Pyruvate(m) + CO2(m) + NADPH(m) ⇌ Malate(m) + NADP+(m)
r0110	-1	OAA(m) + Glutamate(m) → AKG(m) + Aspartate(m)
r1438	2	Leucine(c) → Leucine(m)
r0655	2	Isovaleryl-CoA(m) + Ubiquinone(m) ⇌ 3-Methylcrotonyl-CoA(m) + Ubiquinol(m)
r0030	1	ATP(c) + AMP(c) ⇌ 2 ADP(c)
r2664	2	Na+(c) ⇌ Na+(s) + Na-gradient(c)
r0855	1	O2(s) ⇌ O2(c)
r1122	-2	Leucine(s) + Na+(s) → Leucine(c) + Na+(c)
r0856	-1	O2(m) ⇌ O2(c)
r0838	-1	NH3(c) ⇌ NH3(m)
r0658	2	ATP(m) + 3-Methylcrotonyl-CoA(m) + HCO3-(m) ⇌ ADP(m) + Pi(m) + 3-Methylglutaconyl-CoA(m)
r0105	3	NAD+(m) + Malate(m) ⇌ OAA(m) + NADH(m)
r1330	10	H+(PG)(c) ⇌ H+(PG)(m) + Proton-gradient(m)
r0601	2	4-Methyl-2-oxopentanoate(m) + CoA(m) + NAD+(m) ⇌ Isovaleryl-CoA(m) + CO2(m) + NADH(m)
r0106	-2	ADP(m) + Pi(m) + OAA(m) ⇌ ATP(m) + Pyruvate(m) + HCO3-(m)
r0070	-2	CoA(m) + Acetoacetyl-CoA(m) ⇌ 2 Acetyl-CoA(m)
r0007	1	PPi(c) + H2O(c) → 2 Pi(c)
r0876	1	Glutamate(c) + H+(PG)(c) + Aspartate(m) → Glutamate(m) + H+(PG)(m) + Aspartate(c)
r0849	-2	H2O(s) ⇌ H2O(c)
r0017	1	O2(m) + 4 H+(PG)(m) + 4 Ferrocytochrome C(m) → 4 Ferricytochrome C(m) + 2 H2O(m) + 4 H+(PG)(c)
r1323	7	NADH(m) ⇌ NAD+(m) + NADH-redox-potential(m)

Asparagine from Lysine (131)

Definition			Solution												
			exchanges		reactions							transp			
Objective	Constraints	Comment	imports	exports	c	m	r	p	l	n	s	b	s-c	b-c	int
Asparagine	−Lysine MCES +Urea	Transamination to Asparagine using Lysine	1 H_2O(s) 0.5 O_2(s) 1 Pyruvate(m) 1 Lysine(s) 3 ATP-energy(c) 1 NADPH-redox-potential(m)	1 CO_2(s) 1 Asparagine(s) 2 NADH-redox-potential(c) 3 NADH-redox-potential(m) 5 Proton-gradient(m) 2 CoA-activated acetyl group(m)	9	13	-	-	-	-	-	-	4	-	12

ID	flux	reaction
r2678	2	Acetyl-CoA(m) ⇌ CoA(m) + CoA-activated acetyl group(m)
r0820	1	AKG(c) + Malate(m) ⇌ AKG(m) + Malate(c)
r0541	1	Glutaryl-CoA(m) + Ubiquinone(m) ⇌ Crotonyl-CoA(m) + CO2(m) + Ubiquinol(m)
r0460	1	(S)-3-Hydroxybutyryl-CoA(m) + NAD+(m) ⇌ NADH(m) + Acetoacetyl-CoA(m)
r0109	-1	OAA(c) + Glutamate(c) ⇌ AKG(c) + Aspartate(c)
r0073	1	Glutamate(m) + NAD+(m) + H2O(m) ⇌ AKG(m) + NADH(m) + NH3(m)
r0911	1	Proline(c) + Glutamate(m) ⇌ Proline(m) + Glutamate(c)
r0507	1	Ubiquinol(m) + 2 Ferricytochrome C(m) + 4 H+(PG)(m) → Ubiquinone(m) + 4 H+(PG)(c) + 2 Ferrocytochrome C(m)
r1426	1	Proline(m) + H+(PG)(c) → Proline(c) + H+(PG)(m)
r1327	-1	NADPH(m) ⇌ NADPH-redox-potential(m) + NADP+(m)
r0526	1	H2O(m) + Saccharopine(m) + NADP+(m) ⇌ Glutamate(m) + 2-Aminoadipate 6-semialdehyde(m) + NADPH(m)
r1319	-3	ATP(c) + H2O(c) ⇌ ADP(c) + Pi(c) + ATP-energy(c)
r0851	-1	H2O(m) ⇌ H2O(c)
r0158	1	ATP(c) + Glutamine(c) + H2O(c) + Aspartate(c) ⇌ AMP(c) + PPi(c) + Glutamate(c) + Asparagine(c)
r0077	1	ATP(c) + Glutamate(c) + NH3(c) → ADP(c) + Pi(c) + Glutamine(c)
r0059	-1	Pyruvate(m) + CO2(m) + NADPH(m) ⇌ Malate(m) + NADP+(m)
r0448	1	2-Oxoadipate(m) + CoA(m) + NAD+(m) ⇌ Glutaryl-CoA(m) + CO2(m) + NADH(m)
r0030	1	ATP(c) + AMP(c) ⇌ 2 ADP(c)
r2003	1	Lysine(s) + Asparagine(c) → Lysine(c) + Asparagine(s)
r0855	0.5	O2(s) ⇌ O2(c)
r0594	1	2-Aminoadipate 6-semialdehyde(c) + NAD+(c) + H2O(c) ⇌ NADH(c) + L-2-Aminoadipate(c)
r0104	1	NAD+(c) + Malate(c) ⇌ OAA(c) + NADH(c)
r2539	-1	2-Aminoadipate 6-semialdehyde(c) + L-2-Aminoadipate(m) ⇌ 2-Aminoadipate 6-semialdehyde(m) + L-2-Aminoadipate(c)
r0856	-0.5	O2(m) ⇌ O2(c)
r0838	-1	NH3(c) ⇌ NH3(m)
r1330	5	H+(PG)(c) ⇌ H+(PG)(m) + Proton-gradient(m)
r0070	-1	CoA(m) + Acetoacetyl-CoA(m) ⇌ 2 Acetyl-CoA(m)
r0007	1	PPi(c) + H2O(c) → 2 Pi(c)
r1322	2	NADH(c) ⇌ NAD+(c) + NADH-redox-potential(c)
r1070	1	Lysine(c) ⇌ Lysine(m)
r0867	1	CO2(c) ⇌ CO2(s)
r0849	1	H2O(s) ⇌ H2O(c)
r0588	-1	Crotonyl-CoA(m) + H2O(m) ⇌ (S)-3-Hydroxybutyryl-CoA(m)
r0017	0.5	O2(m) + 4 H+(PG)(m) + 4 Ferrocytochrome C(m) → 4 Ferricytochrome C(m) + 2 H2O(m) + 4 H+(PG)(c)
r1323	3	NADH(m) ⇌ NAD+(m) + NADH-redox-potential(m)
r0868	-1	CO2(c) ⇌ CO2(m)
r0450	1	AKG(m) + L-2-Aminoadipate(m) ⇌ 2-Oxoadipate(m) + Glutamate(m)
r0180	-1	AKG(m) + NADPH(m) + Lysine(m) ⇌ H2O(m) + Saccharopine(m) + NADP+(m)

Asparagine from Methionine (132)

Definition			Solution												
			exchanges		reactions								transp		
Objective	Constraints	Comment	imports	exports	c	m	r	p	l	n	s	b	s-c	b-c	int
Asparagine	−Methionine MCES +Urea $+H_2S$	Transamination to Asparagine using Methionine	3 H_2O(s) 1 O_2(s) 1 Pyruvate(m) 2 Methionine(s) 1 Glucose-6P(c) 2 ATP-energy(c) 1 NADPH-redox-potential(m)	1 P_i(c) 3 CO_2(s) 2 Pyruvate(c) 1 Asparagine(s) 2 H_2S(s) 5 NADH-redox-potential(c) 3 NADH-redox-potential(m) 5 Proton-gradient(m) 2 Na-gradient(c) 2 THF-activated methyl group(c) 2 CoA-activated acetyl group(m)	22	11	-	-	-	-	-	-	7	-	13

ID	flux	reaction
r0855	1	O2(s) ⇌ O2(c)
r0856	-1	O2(m) ⇌ O2(c)
r0592	-2	H2O(m) + Acrylyl-CoA(m) → 3-Hydroxypropionyl-CoA(m)

Continued on next page

ID	flux	reaction
r0596	-2	3-Hydroxypropionyl-CoA(m) + H2O(m) → Hydracrylate(m) + CoA(m)
r0017	1	O2(m) + 4 H+(PG)(m) + 4 Ferrocytochrome C(m) → 4 Ferricytochrome C(m) + 2 H2O(m) + 4 H+(PG)(c)
r0030	1	ATP(c) + AMP(c) ⇌ 2 ADP(c)
r0314	2	Serine(c) + Homocysteine(c) ⇌ H2O(c) + L-Cystathionine(c)
r0911	3	Proline(c) + Glutamate(m) ⇌ Proline(m) + Glutamate(c)
r1141	-2	Methionine(s) + Na+(s) → Methionine(c) + Na+(c)
r0337	-2	ADP(c) + 1,3DPG(c) ⇌ ATP(c) + 3PG(c)
r0338	2	3PG(c) + NAD+(c) → 3-Phosphonooxypyruvate(c) + NADH(c)
r2664	2	Na+(c) ⇌ Na+(s) + Na-gradient(c)
r1426	3	Proline(m) + H+(PG)(c) → Proline(c) + H+(PG)(m)
r0059	-1	Pyruvate(m) + CO2(m) + NADPH(m) ⇌ Malate(m) + NADP+(m)
r0073	-3	AKG(m) + NADH(m) + NH3(m) ⇌ Glutamate(m) + NAD+(m) + H2O(m)
r0077	1	ATP(c) + Glutamate(c) + NH3(c) → ADP(c) + Pi(c) + Glutamine(c)
r2532	-1	Asparagine(c) ⇌ Asparagine(s)
r0507	2	Ubiquinol(m) + 2 Ferricytochrome C(m) + 4 H+(PG)(m) → Ubiquinone(m) + 4 H+(PG)(c) + 2 Ferrocytochrome C(m)
r0241	2	H2O(c) + L-Cystathionine(c) ⇌ Cysteine(c) + NH3(c) + 2-Oxobutyrate(c)
r0243	-1	DHAP(c) ⇌ GAP(c)
r0820	1	AKG(c) + Malate(m) ⇌ AKG(m) + Malate(c)
r1330	5	H+(PG)(c) ⇌ H+(PG)(m) + Proton-gradient(m)
r1319	-2	ATP(c) + H2O(c) ⇌ ADP(c) + Pi(c) + ATP-energy(c)
r0849	3	H2O(s) ⇌ H2O(c)
r0867	3	CO2(c) ⇌ CO2(s)
r0868	-3	CO2(c) ⇌ CO2(m)
r0158	1	ATP(c) + Glutamine(c) + H2O(c) + Aspartate(c) ⇌ AMP(c) + PPi(c) + Glutamate(c) + Asparagine(c)
r0007	1	PPi(c) + H2O(c) → 2 Pi(c)
r0736	1	ATP(c) + Fructose-6P(c) → ADP(c) + Fructose-1,6PP(c)
r0159	2	3-Phosphoserine(c) + H2O(c) → Serine(c) + Pi(c)
r0174	2	CoA(m) + NAD+(m) + 3-Oxopropanoate(m) → Acetyl-CoA(m) + CO2(m) + NADH(m)
r0192	1	Glucose-6P(c) ⇌ Fructose-6P(c)
r0193	2	Cysteine(c) + H2O(c) ⇌ Pyruvate(c) + H2S(c) + NH3(c)
r1154	2	CoA(m) + NAD+(m) + 2-Oxobutyrate(m) ⇌ CO2(m) + Propanoyl-CoA(m) + NADH(m)
r0940	2	H2S(c) ⇌ H2S(s)
r1155	2	2-Oxobutyrate(c) + H+(PG)(c) → 2-Oxobutyrate(m) + H+(PG)(m)
r2676	2	5-Methyl-THF(c) ⇌ THF(c) + THF-activated methyl group(c)
r0365	2	Hydracrylate(m) + NAD+(m) → NADH(m) + 3-Oxopropanoate(m)
r2678	2	Acetyl-CoA(m) ⇌ CoA(m) + CoA-activated acetyl group(m)
r0663	-2	3-Phosphonooxypyruvate(c) + Glutamate(c) → 3-Phosphoserine(c) + AKG(c)
r0232	-2	THF(c) + Methionine(c) ⇌ 5-Methyl-THF(c) + Homocysteine(c)
r1322	5	NADH(c) ⇌ NAD+(c) + NADH-redox-potential(c)
r0683	2	Propanoyl-CoA(m) + Ubiquinone(m) → Ubiquinol(m) + Acrylyl-CoA(m)
r1323	3	NADH(m) ⇌ NAD+(m) + NADH-redox-potential(m)
r0253	2	GAP(c) + Pi(c) + NAD+(c) ⇌ 1,3DPG(c) + NADH(c)
r1327	-1	NADPH(m) ⇌ NADPH-redox-potential(m) + NADP+(m)
r1047	2	Pi(c) + H+(PG)(c) → Pi(m) + H+(PG)(m)
r0104	1	NAD+(c) + Malate(c) ⇌ OAA(c) + NADH(c)
r0256	1	Fructose-1,6PP(c) ⇌ DHAP(c) + GAP(c)
r2419	2	AKG(c) + Pi(m) ⇌ AKG(m) + Pi(c)
r0838	3	NH3(c) ⇌ NH3(m)
r0851	1	H2O(m) ⇌ H2O(c)
r0109	-1	OAA(c) + Glutamate(c) ⇌ AKG(c) + Aspartate(c)

Asparagine from Phenylalanine (133)

Definition			Solution												
			exchanges		reactions								transp		
Objective	Constraints	Comment	imports	exports	c	m	r	p	l	n	s	b	s-c	b-c	int
Asparagine	−Phenylalanine MCES +Urea	Transamination to Asparagine using Phenylalanine	6 O_2(s) 2 Phenylalanine(s) 6 ATP-energy(c) 1 ATP-energy(m) 1 NADPH-redox-potential(c) 1 Proton-gradient(m)	3 CO_2(s) 1 Pyruvate(c) 1 Asparagine(s) 1 NADH-redox-potential(c) 1 NADH-redox-potential(m) 1 Na-gradient(c) 4 CoA-activated acetyl group(c)	21	3	-	-	-	-	-	-	6	-	5

ID	flux	reaction
r0109	-1	OAA(c) + Glutamate(c) ⇌ AKG(c) + Aspartate(c)
r0073	1	Glutamate(m) + NAD+(m) + H2O(m) ⇌ AKG(m) + NADH(m) + NH3(m)
r0605	2	4-Maleylacetoacetate(c) ⇌ Fumarylacetoacetate(c)
r1326	-1	NADPH(c) ⇌ NADPH-redox-potential(c) + NADP+(c)
r0543	2	Homogentisate(c) + O2(c) ⇌ 4-Maleylacetoacetate(c)
r0183	2	Tyrosine(c) + AKG(c) ⇌ 4-Hydroxyphenylpyruvate(c) + Glutamate(c)
r0544	2	4-Hydroxyphenylpyruvate(c) + O2(c) ⇌ Homogentisate(c) + CO2(c)
r0058	1	Malate(c) + NADP+(c) ⇌ Pyruvate(c) + CO2(c) + NADPH(c)
r1319	-6	ATP(c) + H2O(c) ⇌ ADP(c) + Pi(c) + ATP-energy(c)
r0158	1	ATP(c) + Glutamine(c) + H2O(c) + Aspartate(c) ⇌ AMP(c) + PPi(c) + Glutamate(c) + Asparagine(c)
r0077	1	ATP(c) + Glutamate(c) + NH3(c) → ADP(c) + Pi(c) + Glutamine(c)
r2418	1	ADP(c) + ATP(m) → ADP(m) + ATP(c)
r0258	-2	Fumarate(c) + H2O(c) ⇌ Malate(c)
r0069	-2	CoA(c) + Acetoacetyl-CoA(c) ⇌ 2 Acetyl-CoA(c)
r2419	-1	AKG(m) + Pi(c) ⇌ AKG(c) + Pi(m)
r1942	1	Phenylalanine(s) + Na+(s) + Lysine(c) → Phenylalanine(c) + Na+(c) + Lysine(s)
r0030	3	ATP(c) + AMP(c) ⇌ 2 ADP(c)
r2528	1	Phenylalanine(s) ⇌ Phenylalanine(c)
r2664	1	Na+(c) ⇌ Na+(s) + Na-gradient(c)
r2003	1	Lysine(s) + Asparagine(c) → Lysine(c) + Asparagine(s)
r0855	6	O2(s) ⇌ O2(c)
r0320	2	ATP(c) + CoA(c) + Acetoacetate(c) → AMP(c) + PPi(c) + Acetoacetyl-CoA(c)
r0104	1	NAD+(c) + Malate(c) ⇌ OAA(c) + NADH(c)
r1320	-1	ATP(m) + H2O(m) ⇌ ADP(m) + Pi(m) + ATP-energy(m)
r0838	-1	NH3(c) ⇌ NH3(m)
r1330	-1	H+(PG)(c) ⇌ H+(PG)(m) + Proton-gradient(m)
r0875	1	Glutamate(c) + H+(PG)(c) → Glutamate(m) + H+(PG)(m)
r0398	2	Dihydrobiopterin(c) + NADPH(c) ⇌ Tetrahydrobiopterin(c) + NADP+(c)
r0007	3	PPi(c) + H2O(c) → 2 Pi(c)
r1322	1	NADH(c) ⇌ NAD+(c) + NADH-redox-potential(c)
r0867	3	CO2(c) ⇌ CO2(s)
r0399	2	Tetrahydrobiopterin(c) + Phenylalanine(c) + O2(c) ⇌ Dihydrobiopterin(c) + Tyrosine(c) + H2O(c)
r2677	4	Acetyl-CoA(c) ⇌ CoA(c) + CoA-activated acetyl group(c)
r1323	1	NADH(m) ⇌ NAD+(m) + NADH-redox-potential(m)
r0324	-2	Fumarylacetoacetate(c) + H2O(c) ⇌ Fumarate(c) + Acetoacetate(c)

Asparagine from Proline (134)

Definition			Solution												
			exchanges		reactions								transp		
Objective	Constraints	Comment	imports	exports	c	m	r	p	l	n	s	b	s-c	b-c	int
Asparagine	−Proline MCES +Urea	Transamination to Asparagine using Proline	8 H_2O(s) 2 Proline(s) 3 ATP-energy(c) 1 Proton-gradient(m)	3 CO_2(s) 1 Pyruvate(m) 1 Asparagine(s) 2 ATP-energy(m) 1 NADH-redox-potential(c) 4 NADH-redox-potential(m) 2 NADPH-redox-potential(m)	8	13	-	-	-	-	-	-	4	-	7

ID	flux	reaction
r0820	1	AKG(c) + Malate(m) ⇌ AKG(m) + Malate(c)
r0109	-1	OAA(c) + Glutamate(c) ⇌ AKG(c) + Aspartate(c)
r2531	1	Proline(s) ⇌ Proline(c)
r1424	1	Proline(c) + H+(PG)(c) → Proline(m) + H+(PG)(m)
r0911	1	Proline(c) + Glutamate(m) ⇌ Proline(m) + Glutamate(c)
r0074	2	L-Glutamate 5-semialdehyde(m) + NAD+(m) + H2O(m) ⇌ Glutamate(m) + NADH(m)
r0075	1	Glutamate(m) + H2O(m) + NADP+(m) ⇌ AKG(m) + NADPH(m) + NH3(m)
r1453	2	Proline(m) + Ubiquinol(m) ⇌ 1-Pyrroline-5-carboxylate(m) + Ubiquinone(m)
r1327	2	NADPH(m) ⇌ NADPH-redox-potential(m) + NADP+(m)
r0175	2	1-Pyrroline-5-carboxylate(m) + H2O(m) ⇌ L-Glutamate 5-semialdehyde(m)
r1319	-3	ATP(c) + H2O(c) ⇌ ADP(c) + Pi(c) + ATP-energy(c)
r0851	-9	H2O(m) ⇌ H2O(c)
r0509	2	Ubiquinone(m) + Succinate(m) → Ubiquinol(m) + Fumarate(m)
r0158	1	ATP(c) + Glutamine(c) + H2O(c) + Aspartate(c) ⇌ AMP(c) + PPi(c) + Glutamate(c) + Asparagine(c)
r0100	-2	ADP(m) + GTP(m) ⇌ ATP(m) + GDP(m)
r0077	1	ATP(c) + Glutamate(c) + NH3(c) → ADP(c) + Pi(c) + Glutamine(c)
r0059	1	Malate(m) + NADP+(m) ⇌ Pyruvate(m) + CO2(m) + NADPH(m)
r0259	-2	Fumarate(m) + H2O(m) ⇌ Malate(m)
r0030	1	ATP(c) + AMP(c) ⇌ 2 ADP(c)
r0104	1	NAD+(c) + Malate(c) ⇌ OAA(c) + NADH(c)
r1648	1	Proline(s) + Asparagine(c) ⇌ Proline(c) + Asparagine(s)
r1320	2	ATP(m) + H2O(m) ⇌ ADP(m) + Pi(m) + ATP-energy(m)
r0838	-1	NH3(c) ⇌ NH3(m)
r1330	-1	H+(PG)(c) ⇌ H+(PG)(m) + Proton-gradient(m)
r1114	2	AKG(m) + CoA(m) + NAD+(m) ⇌ NADH(m) + CO2(m) + Succinyl-CoA(m)
r0007	1	PPi(c) + H2O(c) → 2 Pi(c)
r1322	1	NADH(c) ⇌ NAD+(c) + NADH-redox-potential(c)
r0867	3	CO2(c) ⇌ CO2(s)
r0849	8	H2O(s) ⇌ H2O(c)
r0125	-2	GDP(m) + Pi(m) + Succinyl-CoA(m) ⇌ GTP(m) + Succinate(m) + CoA(m)
r1323	4	NADH(m) ⇌ NAD+(m) + NADH-redox-potential(m)
r0868	-3	CO2(c) ⇌ CO2(m)

Asparagine from Serine (135)

Definition			Solution												
			exchanges		reactions								transp		
Objective	Constraints	Comment	imports	exports	c	m	r	p	l	n	s	b	s-c	b-c	int
Asparagine	−Serine MCES +Urea	Transamination to Asparagine using Serine	2 Pyruvate(m) 2 Serine(s) 3 ATP-energy(c) 2 NADPH-redox-potential(m) 1 Proton-gradient(m)	2 H_2O(s) 2 Pyruvate(c) 1 Asparagine(s) 1 NADH-redox-potential(c) 1 NADH-redox-potential(m) 2 Proton-gradient(c) 2 Na-gradient(c) 1 CoA-activated acetyl group(m)	9	6	-	-	-	-	-	-	5	-	6

ID	flux	reaction
r2678	1	Acetyl-CoA(m) ⇌ CoA(m) + CoA-activated acetyl group(m)
r0820	1	AKG(c) + Malate(m) ⇌ AKG(m) + Malate(c)
r0109	-1	OAA(c) + Glutamate(c) ⇌ AKG(c) + Aspartate(c)
r0911	1	Proline(c) + Glutamate(m) ⇌ Proline(m) + Glutamate(c)
r0056	1	Pyruvate(m) + CoA(m) + NAD+(m) → Acetyl-CoA(m) + CO2(m) + NADH(m)
r2532	-1	Asparagine(c) ⇌ Asparagine(s)
r0075	-1	AKG(m) + NADPH(m) + NH3(m) ⇌ Glutamate(m) + H2O(m) + NADP+(m)
r1426	1	Proline(m) + H+(PG)(c) → Proline(c) + H+(PG)(m)
r1327	-2	NADPH(m) ⇌ NADPH-redox-potential(m) + NADP+(m)
r1319	-3	ATP(c) + H2O(c) ⇌ ADP(c) + Pi(c) + ATP-energy(c)

Continued on next page

Asparagine from Serine (135)– continued

ID	flux	reaction
r0851	1	H2O(m) ⇌ H2O(c)
r0158	1	ATP(c) + Glutamine(c) + H2O(c) + Aspartate(c) ⇌ AMP(c) + PPi(c) + Glutamate(c) + Asparagine(c)
r0077	1	ATP(c) + Glutamate(c) + NH3(c) → ADP(c) + Pi(c) + Glutamine(c)
r0059	-1	Pyruvate(m) + CO2(m) + NADPH(m) ⇌ Malate(m) + NADP+(m)
r0030	1	ATP(c) + AMP(c) ⇌ 2 ADP(c)
r2664	2	Na+(c) ⇌ Na+(s) + Na-gradient(c)
r0104	1	NAD+(c) + Malate(c) ⇌ OAA(c) + NADH(c)
r0838	1	NH3(c) ⇌ NH3(m)
r0060	2	Serine(c) → Pyruvate(c) + NH3(c)
r1330	-1	H+(PG)(c) ⇌ H+(PG)(m) + Proton-gradient(m)
r0007	1	PPi(c) + H2O(c) → 2 Pi(c)
r2478	2	Serine(s) + Na+(s) + H+(PG)(c) → Serine(c) + Na+(c) + H+(PG)(s)
r1331	2	H+(PG)(s) ⇌ H+(PG)(c) + Proton-gradient(c)
r1322	1	NADH(c) ⇌ NAD+(c) + NADH-redox-potential(c)
r0849	-2	H2O(s) ⇌ H2O(c)
r1323	1	NADH(m) ⇌ NAD+(m) + NADH-redox-potential(m)

Asparagine from Threonine (136)

Definition			Solution												
			exchanges		reactions								transp		
Objective	Constraints	Comment	imports	exports	c	m	r	p	l	n	s	b	s-c	b-c	int
Asparagine	−Threonine MCES +Urea	Transamination to Asparagine using Threonine	1 O_2(s) 1 Pyruvate(m) 2 Threonine(s) 3 ATP-energy(c) 1 NADPH-redox-potential(m)	3 CO_2(s) 1 Asparagine(s) 6 NADH-redox-potential(m) 8 Proton-gradient(m) 2 CoA-activated acetyl group(m)	6	13	-	-	-	-	-	-	4	-	11

ID	flux	reaction
r2678	2	Acetyl-CoA(m) ⇌ CoA(m) + CoA-activated acetyl group(m)
r0073	-1	AKG(m) + NADH(m) + NH3(m) ⇌ Glutamate(m) + NAD+(m) + H2O(m)
r1154	2	CoA(m) + NAD+(m) + 2-Oxobutyrate(m) ⇌ CO2(m) + Propanoyl-CoA(m) + NADH(m)
r0911	1	Proline(c) + Glutamate(m) ⇌ Proline(m) + Glutamate(c)
r2532	-1	Asparagine(s) ⇌ Asparagine(c)
r1155	2	2-Oxobutyrate(c) + H+(PG)(c) → 2-Oxobutyrate(m) + H+(PG)(m)
r0507	2	Ubiquinol(m) + 2 Ferricytochrome C(m) + 4 H+(PG)(m) → Ubiquinone(m) + 4 H+(PG)(c) + 2 Ferrocytochrome C(m)
r0174	2	CoA(m) + NAD+(m) + 3-Oxopropanoate(m) → Acetyl-CoA(m) + CO2(m) + NADH(m)
r1426	1	Proline(m) + H+(PG)(c) → Proline(c) + H+(PG)(m)
r1327	-1	NADPH(m) ⇌ NADPH-redox-potential(m) + NADP+(m)
r2534	2	Threonine(s) ⇌ Threonine(c)
r1319	-3	ATP(c) + H2O(c) ⇌ ADP(c) + Pi(c) + ATP-energy(c)
r0851	-1	H2O(m) ⇌ H2O(c)
r0365	2	Hydracrylate(m) + NAD+(m) → NADH(m) + 3-Oxopropanoate(m)
r0158	1	ATP(c) + Glutamine(c) + H2O(c) + Aspartate(c) ⇌ AMP(c) + PPi(c) + Glutamate(c) + Asparagine(c)
r0077	1	ATP(c) + Glutamate(c) + NH3(c) → ADP(c) + Pi(c) + Glutamine(c)
r0059	-1	Pyruvate(m) + CO2(m) + NADPH(m) ⇌ Malate(m) + NADP+(m)
r0110	-1	OAA(m) + Glutamate(m) → AKG(m) + Aspartate(m)
r0592	-2	H2O(m) + Acrylyl-CoA(m) → 3-Hydroxypropionyl-CoA(m)
r0030	1	ATP(c) + AMP(c) ⇌ 2 ADP(c)
r0683	2	Propanoyl-CoA(m) + Ubiquinone(m) → Ubiquinol(m) + Acrylyl-CoA(m)
r0855	1	O2(s) ⇌ O2(c)
r0856	-1	O2(m) ⇌ O2(c)
r0838	1	NH3(c) ⇌ NH3(m)
r0240	2	Threonine(c) ⇌ 2-Oxobutyrate(c) + NH3(c)
r0105	1	NAD+(m) + Malate(m) ⇌ OAA(m) + NADH(m)
r1330	8	H+(PG)(c) ⇌ H+(PG)(m) + Proton-gradient(m)

Continued on next page

ID	flux	reaction
r0596	-2	3-Hydroxypropionyl-CoA(m) + H2O(m) → Hydracrylate(m) + CoA(m)
r0007	1	PPi(c) + H2O(c) → 2 Pi(c)
r0876	1	Glutamate(c) + H+(PG)(c) + Aspartate(m) → Glutamate(m) + H+(PG)(m) + Aspartate(c)
r0867	3	CO2(c) ⇌ CO2(s)
r0017	1	O2(m) + 4 H+(PG)(m) + 4 Ferrocytochrome C(m) → 4 Ferricytochrome C(m) + 2 H2O(m) + 4 H+(PG)(c)
r1323	6	NADH(m) ⇌ NAD+(m) + NADH-redox-potential(m)
r0868	-3	CO2(c) ⇌ CO2(m)

Asparagine from Tryptophan (137)

Definition			Solution												
			exchanges		reactions								transsp		
Objective	Constraints	Comment	imports	exports	c	m	r	p	l	n	s	b	s-c	b-c	int
Asparagine	−Tryptophan MCES +Urea	Transamination to Asparagine using Tryptophan	3.5 O_2(s) 1 Pyruvate(m) 1 Tryptophan(s) 4 ATP-energy(c) 3 NADPH-redox-potential(c) 1 NADPH-redox-potential(m)	2 CO_2(s) 1 Pyruvate(c) 1 Asparagine(s) 1 NADH-redox-potential(c) 2 NADH-redox-potential(m) 6 Proton-gradient(m) 1 THF-activated methyl group(c) 2 CoA-activated acetyl group(m)	23	9	-	-	-	-	-	-	4	-	7

ID	flux	reaction
r0855	3.5	O2(s) ⇌ O2(c)
r0856	-0.5	O2(m) ⇌ O2(c)
r0293	-1	5,10-Methylene-THF(c) + NADP+(c) ⇌ 5,10-Methenyl-THF(c) + NADPH(c)
r0460	1	(S)-3-Hydroxybutyryl-CoA(m) + NAD+(m) ⇌ NADH(m) + Acetoacetyl-CoA(m)
r0448	1	2-Oxoadipate(m) + CoA(m) + NAD+(m) ⇌ Glutaryl-CoA(m) + CO2(m) + NADH(m)
r0449	-1	2-Oxoadipate(c) + NH3(c) + NADP+(c) ⇌ 2-Aminomuconate(c) + NADPH(c) + H2O(c)
r0017	0.5	O2(m) + 4 H+(PG)(m) + 4 Ferrocytochrome C(m) → 4 Ferricytochrome C(m) + 2 H2O(m) + 4 H+(PG)(c)
r0030	1	ATP(c) + AMP(c) ⇌ 2 ADP(c)
r0169	1	Tryptophan(c) + O2(c) ⇌ L-Formylkynurenine(c)
r0070	-1	CoA(m) + Acetoacetyl-CoA(m) ⇌ 2 Acetyl-CoA(m)
r0059	-1	Pyruvate(m) + CO2(m) + NADPH(m) ⇌ Malate(m) + NADP+(m)
r0371	-1	10-Formyl-THF(c) ⇌ 5,10-Methenyl-THF(c) + H2O(c)
r0077	1	ATP(c) + Glutamate(c) + NH3(c) → ADP(c) + Pi(c) + Glutamine(c)
r2532	-1	Asparagine(s) ⇌ Asparagine(c)
r0507	1	Ubiquinol(m) + 2 Ferricytochrome C(m) + 4 H+(PG)(m) → Ubiquinone(m) + 4 H+(PG)(c) + 2 Ferrocytochrome C(m)
r0228	1	THF(c) + Formate(c) + ATP(c) → ADP(c) + Pi(c) + 10-Formyl-THF(c)
r0676	1	2-Amino-3-carboxymuconate semialdehyde(c) ⇌ 2-Aminomuconate semialdehyde(c) + CO2(c)
r0820	1	AKG(c) + Malate(m) ⇌ AKG(m) + Malate(c)
r1330	6	H+(PG)(c) ⇌ H+(PG)(m) + Proton-gradient(m)
r1319	-4	ATP(c) + H2O(c) ⇌ ADP(c) + Pi(c) + ATP-energy(c)
r0541	1	Glutaryl-CoA(m) + Ubiquinone(m) ⇌ Crotonyl-CoA(m) + CO2(m) + Ubiquinol(m)
r0562	1	3-Hydroxyanthranilate(c) + O2(c) ⇌ 2-Amino-3-carboxymuconate semialdehyde(c)
r0563	1	H2O(c) + 3-Hydroxy-L-kynurenine(c) ⇌ 3-Hydroxyanthranilate(c) + Alanine(c)
r0867	2	CO2(c) ⇌ CO2(s)
r0868	-1	CO2(c) ⇌ CO2(m)
r0588	-1	Crotonyl-CoA(m) + H2O(m) ⇌ (S)-3-Hydroxybutyryl-CoA(m)
r0158	1	ATP(c) + Glutamine(c) + H2O(c) + Aspartate(c) ⇌ AMP(c) + PPi(c) + Glutamate(c) + Asparagine(c)
r0007	1	PPi(c) + H2O(c) → 2 Pi(c)
r0454	1	L-Formylkynurenine(c) + H2O(c) ⇌ Formate(c) + Kynurenine(c)
r0455	1	Kynurenine(c) + O2(c) + NADPH(c) ⇌ H2O(c) + 3-Hydroxy-L-kynurenine(c) + NADP+(c)
r0792	-1	5,10-Methylene-THF(c) + NADH(c) ⇌ 5-Methyl-THF(c) + NAD+(c)
r0080	1	Alanine(c) + AKG(c) ⇌ Pyruvate(c) + Glutamate(c)
r0645	1	2-Aminomuconate semialdehyde(c) + NAD+(c) + H2O(c) ⇌ 2-Aminomuconate(c) + NADH(c)
r2676	1	5-Methyl-THF(c) ⇌ THF(c) + THF-activated methyl group(c)

Continued on next page

ID	flux	reaction
r2678	2	Acetyl-CoA(m) ⇌ CoA(m) + CoA-activated acetyl group(m)
r2527	1	Tryptophan(s) ⇌ Tryptophan(c)
r0945	1	2-Oxoadipate(c) + AKG(m) ⇌ 2-Oxoadipate(m) + AKG(c)
r1322	1	NADH(c) ⇌ NAD+(c) + NADH-redox-potential(c)
r1323	2	NADH(m) ⇌ NAD+(m) + NADH-redox-potential(m)
r1326	-3	NADPH(c) ⇌ NADPH-redox-potential(c) + NADP+(c)
r1327	-1	NADPH(m) ⇌ NADPH-redox-potential(m) + NADP+(m)
r0104	1	NAD+(c) + Malate(c) ⇌ OAA(c) + NADH(c)
r0109	-1	OAA(c) + Glutamate(c) ⇌ AKG(c) + Aspartate(c)

Asparagine from Tyrosine (138)

Definition			Solution												
			exchanges		reactions								transp		
Objective	Constraints	Comment	imports	exports	c	m	r	p	l	n	s	b	s-c	b-c	int
Asparagine	−Tyrosine MCES +Urea	Transamination to Asparagine using Tyrosine	2 H_2O(s) 4 O_2(s) 2 Tyrosine(s) 6 ATP-energy(c) 1 ATP-energy(m) 1 Proton-gradient(m)	3 CO_2(s) 1 Pyruvate(c) 1 Asparagine(s) 1 NADH-redox-potential(c) 1 NADH-redox-potential(m) 1 NADPH-redox-potential(c) 2 Na-gradient(c) 4 CoA-activated acetyl group(c)	19	3	-	-	-	-	-	-	7	-	5

ID	flux	reaction
r0109	-1	OAA(c) + Glutamate(c) ⇌ AKG(c) + Aspartate(c)
r0073	1	Glutamate(m) + NAD+(m) + H2O(m) ⇌ AKG(m) + NADH(m) + NH3(m)
r0605	2	4-Maleylacetoacetate(c) ⇌ Fumarylacetoacetate(c)
r1326	1	NADPH(c) ⇌ NADPH-redox-potential(c) + NADP+(c)
r0543	2	Homogentisate(c) + O2(c) ⇌ 4-Maleylacetoacetate(c)
r0183	2	Tyrosine(c) + AKG(c) ⇌ 4-Hydroxyphenylpyruvate(c) + Glutamate(c)
r0544	2	4-Hydroxyphenylpyruvate(c) + O2(c) ⇌ Homogentisate(c) + CO2(c)
r0058	1	Malate(c) + NADP+(c) ⇌ Pyruvate(c) + CO2(c) + NADPH(c)
r1553	1	Glycine(s) + Asparagine(c) ⇌ Glycine(c) + Asparagine(s)
r1319	-6	ATP(c) + H2O(c) ⇌ ADP(c) + Pi(c) + ATP-energy(c)
r0158	1	ATP(c) + Glutamine(c) + H2O(c) + Aspartate(c) ⇌ AMP(c) + PPi(c) + Glutamate(c) + Asparagine(c)
r0077	1	ATP(c) + Glutamate(c) + NH3(c) → ADP(c) + Pi(c) + Glutamine(c)
r2418	1	ADP(c) + ATP(m) → ADP(m) + ATP(c)
r0258	-2	Fumarate(c) + H2O(c) ⇌ Malate(c)
r0069	-2	CoA(c) + Acetoacetyl-CoA(c) ⇌ 2 Acetyl-CoA(c)
r2419	-1	AKG(m) + Pi(c) ⇌ AKG(c) + Pi(m)
r0030	3	ATP(c) + AMP(c) ⇌ 2 ADP(c)
r2664	2	Na+(c) ⇌ Na+(s) + Na-gradient(c)
r0855	4	O2(s) ⇌ O2(c)
r0320	2	ATP(c) + CoA(c) + Acetoacetate(c) → AMP(c) + PPi(c) + Acetoacetyl-CoA(c)
r0104	1	NAD+(c) + Malate(c) ⇌ OAA(c) + NADH(c)
r1320	-1	ATP(m) + H2O(m) ⇌ ADP(m) + Pi(m) + ATP-energy(m)
r0838	-1	NH3(c) ⇌ NH3(m)
r1330	-1	H+(PG)(c) ⇌ H+(PG)(m) + Proton-gradient(m)
r0875	1	Glutamate(c) + H+(PG)(c) → Glutamate(m) + H+(PG)(m)
r0007	3	PPi(c) + H2O(c) → 2 Pi(c)
r1322	1	NADH(c) ⇌ NAD+(c) + NADH-redox-potential(c)
r0867	3	CO2(c) ⇌ CO2(s)
r0849	2	H2O(s) ⇌ H2O(c)
r2677	4	Acetyl-CoA(c) ⇌ CoA(c) + CoA-activated acetyl group(c)
r1777	1	Tyrosine(s) + Glycine(c) + Na+(s) → Tyrosine(c) + Glycine(s) + Na+(c)
r1323	1	NADH(m) ⇌ NAD+(m) + NADH-redox-potential(m)
r1125	1	Tyrosine(s) + Na+(s) → Tyrosine(c) + Na+(c)

Continued on next page

Asparagine from Tyrosine (138)– continued

ID	flux	reaction
r0324	-2	Fumarylacetoacetate(c) + H2O(c) ⇌ Fumarate(c) + Acetoacetate(c)

Asparagine from Valine (139)

Definition			Solution												
			exchanges		reactions								transp		
Objective	Constraints	Comment	imports	exports	c	m	r	p	l	n	s	b	s-c	b-c	int
Asparagine	−Valine MCES +Urea	Transamination to Asparagine using Valine	4 H_2O(s) 2 O_2(s) 2 Valine(s) 3 ATP-energy(c)	3 CO_2(s) 1 Pyruvate(m) 1 Asparagine(s) 2 ATP-energy(m) 1 NADH-redox-potential(c) 7 NADH-redox-potential(m) 1 NADPH-redox-potential(m) 21 Proton-gradient(m) 1 Na-gradient(c)	8	17	-	-	-	-	-	-	6	-	11

ID	flux	reaction
r0125	-2	GDP(m) + Pi(m) + Succinyl-CoA(m) ⇌ GTP(m) + Succinate(m) + CoA(m)
r0855	2	O2(s) ⇌ O2(c)
r0856	-2	O2(m) ⇌ O2(c)
r0017	2	O2(m) + 4 H+(PG)(m) + 4 Ferrocytochrome C(m) → 4 Ferricytochrome C(m) + 2 H2O(m) + 4 H+(PG)(c)
r0030	1	ATP(c) + AMP(c) ⇌ 2 ADP(c)
r1121	-1	Valine(s) + Na+(s) → Valine(c) + Na+(c)
r0482	2	3-Hydroxyisobutyrate(m) + NAD+(m) → 2-Methyl-3-oxopropanoate(m) + NADH(m)
r0911	1	Proline(c) + Glutamate(m) ⇌ Proline(m) + Glutamate(c)
r2664	1	Na+(c) ⇌ Na+(s) + Na-gradient(c)
r1426	1	Proline(m) + H+(PG)(c) → Proline(c) + H+(PG)(m)
r0059	1	Malate(m) + NADP+(m) ⇌ Pyruvate(m) + CO2(m) + NADPH(m)
r0073	1	Glutamate(m) + NAD+(m) + H2O(m) ⇌ AKG(m) + NADH(m) + NH3(m)
r0077	1	ATP(c) + Glutamate(c) + NH3(c) → ADP(c) + Pi(c) + Glutamine(c)
r0507	4	Ubiquinol(m) + 2 Ferricytochrome C(m) + 4 H+(PG)(m) → Ubiquinone(m) + 4 H+(PG)(c) + 2 Ferrocytochrome C(m)
r0509	2	Ubiquinone(m) + Succinate(m) → Ubiquinol(m) + Fumarate(m)
r0820	1	AKG(c) + Malate(m) ⇌ AKG(m) + Malate(c)
r1330	21	H+(PG)(c) ⇌ H+(PG)(m) + Proton-gradient(m)
r1319	-3	ATP(c) + H2O(c) ⇌ ADP(c) + Pi(c) + ATP-energy(c)
r0263	2	Valine(m) + AKG(m) → Glutamate(m) + 3-Methyl-2-oxobutyrate(m)
r0560	2	Isobutyryl-CoA(m) + Ubiquinone(m) → Methacrylyl-CoA(m) + Ubiquinol(m)
r1653	-1	Asparagine(c) + Valine(s) ⇌ Asparagine(s) + Valine(c)
r0849	4	H2O(s) ⇌ H2O(c)
r0867	3	CO2(c) ⇌ CO2(s)
r0868	-3	CO2(c) ⇌ CO2(m)
r0158	1	ATP(c) + Glutamine(c) + H2O(c) + Aspartate(c) ⇌ AMP(c) + PPi(c) + Glutamate(c) + Asparagine(c)
r0007	1	PPi(c) + H2O(c) → 2 Pi(c)
r0600	2	3-Methyl-2-oxobutyrate(m) + CoA(m) + NAD+(m) ⇌ Isobutyryl-CoA(m) + CO2(m) + NADH(m)
r0197	2	(R)-Methylmalonyl-CoA(m) ⇌ Succinyl-CoA(m)
r0624	2	2-Methyl-3-oxopropanoate(m) + CoA(m) + NAD+(m) ⇌ (R)-Methylmalonyl-CoA(m) + NADH(m)
r0779	2	3-Hydroxyisobutyryl-CoA(m) + H2O(m) → 3-Hydroxyisobutyrate(m) + CoA(m)
r0923	2	Valine(c) + H+(PG)(c) → Valine(m) + H+(PG)(m)
r1320	2	ATP(m) + H2O(m) ⇌ ADP(m) + Pi(m) + ATP-energy(m)
r0669	2	Methacrylyl-CoA(m) + H2O(m) → 3-Hydroxyisobutyryl-CoA(m)
r1322	1	NADH(c) ⇌ NAD+(c) + NADH-redox-potential(c)
r1323	7	NADH(m) ⇌ NAD+(m) + NADH-redox-potential(m)
r0100	-2	ADP(m) + GTP(m) ⇌ ATP(m) + GDP(m)
r1327	1	NADPH(m) ⇌ NADPH-redox-potential(m) + NADP+(m)
r0104	1	NAD+(c) + Malate(c) ⇌ OAA(c) + NADH(c)
r0259	-2	Fumarate(m) + H2O(m) ⇌ Malate(m)
r0838	-1	NH3(c) ⇌ NH3(m)

Continued on next page

Asparagine from Valine (139)– continued

ID	flux	reaction
r0851	-5	H2O(m) ⇌ H2O(c)
r0109	-1	OAA(c) + Glutamate(c) ⇌ AKG(c) + Aspartate(c)

Asparagine from beta-Alanine (140)

Definition			Solution												
			exchanges		reactions								transp		
Objective	Constraints	Comment	imports	exports	c	m	r	p	l	n	s	b	s-c	b-c	int
Asparagine	−beta-Alanine MCES +Urea	Transamination to Asparagine using beta-Alanine	1 Pyruvate(m) 2 beta-Alanine(s) 3 ATP-energy(c) 2 Proton-gradient(m)	1 CO_2(s) 1 Asparagine(s) 3 NADH-redox-potential(m) 2 CoA-activated acetyl group(m)	5	8	-	-	-	-	-	-	3	-	8

ID	flux	reaction
r2678	2	Acetyl-CoA(m) ⇌ CoA(m) + CoA-activated acetyl group(m)
r0217	2	beta-Alanine(m) + AKG(m) ⇌ Glutamate(m) + 3-Oxopropanoate(m)
r0911	1	Proline(c) + Glutamate(m) ⇌ Proline(m) + Glutamate(c)
r2532	-1	Asparagine(c) ⇌ Asparagine(s)
r0174	2	CoA(m) + NAD+(m) + 3-Oxopropanoate(m) → Acetyl-CoA(m) + CO2(m) + NADH(m)
r0075	1	Glutamate(m) + H2O(m) + NADP+(m) ⇌ AKG(m) + NADPH(m) + NH3(m)
r1426	1	Proline(m) + H+(PG)(c) → Proline(c) + H+(PG)(m)
r0899	-2	beta-Alanine(s) ⇌ beta-Alanine(c)
r1319	-3	ATP(c) + H2O(c) ⇌ ADP(c) + Pi(c) + ATP-energy(c)
r0851	-1	H2O(m) ⇌ H2O(c)
r0158	1	ATP(c) + Glutamine(c) + H2O(c) + Aspartate(c) ⇌ AMP(c) + PPi(c) + Glutamate(c) + Asparagine(c)
r0077	1	ATP(c) + Glutamate(c) + NH3(c) → ADP(c) + Pi(c) + Glutamine(c)
r0059	-1	Pyruvate(m) + CO2(m) + NADPH(m) ⇌ Malate(m) + NADP+(m)
r0110	-1	OAA(m) + Glutamate(m) → AKG(m) + Aspartate(m)
r0030	1	ATP(c) + AMP(c) ⇌ 2 ADP(c)
r0838	-1	NH3(c) ⇌ NH3(m)
r0105	1	NAD+(m) + Malate(m) ⇌ OAA(m) + NADH(m)
r1330	-2	H+(PG)(c) ⇌ H+(PG)(m) + Proton-gradient(m)
r0007	1	PPi(c) + H2O(c) → 2 Pi(c)
r0876	1	Glutamate(c) + H+(PG)(c) + Aspartate(m) → Glutamate(m) + H+(PG)(m) + Aspartate(c)
r0867	1	CO2(c) ⇌ CO2(s)
r1323	3	NADH(m) ⇌ NAD+(m) + NADH-redox-potential(m)
r0900	2	beta-Alanine(c) ⇌ beta-Alanine(m)
r0868	-1	CO2(c) ⇌ CO2(m)

Aspartate from Alanine (141)

Definition			Solution												
			exchanges		reactions								transp		
Objective	Constraints	Comment	imports	exports	c	m	r	p	l	n	s	b	s-c	b-c	int
Aspartate	−Alanine MCES +Urea	Transamination to Aspartate using Alanine	1 Pyruvate(m) 1 Alanine(s) 1 NADPH-redox-potential(c)	1 Aspartate(s) 1 NADH-redox-potential(c) 1 NADH-redox-potential(m) 1 CoA-activated acetyl group(m)	6	3	-	-	-	-	-	-	2	-	1

ID	flux	reaction
r2678	1	Acetyl-CoA(m) ⇌ CoA(m) + CoA-activated acetyl group(m)
r0109	-1	OAA(c) + Glutamate(c) ⇌ AKG(c) + Aspartate(c)
r0056	1	Pyruvate(m) + CoA(m) + NAD+(m) → Acetyl-CoA(m) + CO2(m) + NADH(m)
r1326	-1	NADPH(c) ⇌ NADPH-redox-potential(c) + NADP+(c)

Continued on next page

Aspartate from Alanine (141)– continued

ID	flux	reaction
r2524	1	Alanine(s) ⇌ Alanine(c)
r0058	-1	Pyruvate(c) + CO2(c) + NADPH(c) ⇌ Malate(c) + NADP+(c)
r0104	1	NAD+(c) + Malate(c) ⇌ OAA(c) + NADH(c)
r1322	1	NADH(c) ⇌ NAD+(c) + NADH-redox-potential(c)
r0080	1	Alanine(c) + AKG(c) ⇌ Pyruvate(c) + Glutamate(c)
r2686	1	Aspartate(c) ⇌ Aspartate(s)
r1323	1	NADH(m) ⇌ NAD+(m) + NADH-redox-potential(m)
r0868	-1	CO2(c) ⇌ CO2(m)

Aspartate from Arginine (142)

Definition			Solution												
			exchanges		reactions								transp		
Objective	Constraints	Comment	imports	exports	c	m	r	p	l	n	s	b	s-c	b-c	int
Aspartate	−Arginine MCES +Urea	Transamination to Aspartate using Arginine	1.5 H_2O(s) 0.25 O_2(s) 0.5 Pyruvate(m) 0.5 Arginine(s) 0.5 NADPH-redox-potential(m)	1 Aspartate(s) 0.5 Urea(s) 0.5 ATP-energy(m) 2 NADH-redox-potential(m) 0.5 Proton-gradient(m)	1	13	-	-	-	-	-	-	5	-	10

ID	flux	reaction
r0896	0.5	Urea(c) ⇌ Urea(s)
r0911	1	Proline(c) + Glutamate(m) ⇌ Proline(m) + Glutamate(c)
r0074	0.5	L-Glutamate 5-semialdehyde(m) + NAD+(m) + H2O(m) ⇌ Glutamate(m) + NADH(m)
r0507	0.5	Ubiquinol(m) + 2 Ferricytochrome C(m) + 4 H+(PG)(m) → Ubiquinone(m) + 4 H+(PG)(c) + 2 Ferrocytochrome C(m)
r1426	1	Proline(m) + H+(PG)(c) → Proline(c) + H+(PG)(m)
r1327	-0.5	NADPH(m) ⇌ NADPH-redox-potential(m) + NADP+(m)
r1058	0.5	Arginine(s) ⇌ Arginine(c)
r0851	-1	H2O(m) ⇌ H2O(c)
r0509	0.5	Ubiquinone(m) + Succinate(m) → Ubiquinol(m) + Fumarate(m)
r0167	0.5	AKG(m) + Ornithine(m) ⇌ L-Glutamate 5-semialdehyde(m) + Glutamate(m)
r0100	-0.5	ADP(m) + GTP(m) ⇌ ATP(m) + GDP(m)
r0059	-0.5	Pyruvate(m) + CO2(m) + NADPH(m) ⇌ Malate(m) + NADP+(m)
r0110	-1	OAA(m) + Glutamate(m) → AKG(m) + Aspartate(m)
r0259	-0.5	Fumarate(m) + H2O(m) ⇌ Malate(m)
r0855	0.25	O2(s) ⇌ O2(c)
r1320	0.5	ATP(m) + H2O(m) ⇌ ADP(m) + Pi(m) + ATP-energy(m)
r0856	-0.25	O2(m) ⇌ O2(c)
r0105	1	NAD+(m) + Malate(m) ⇌ OAA(m) + NADH(m)
r2396	-0.5	Lysine(m) + Ornithine(c) + H+(PG)(c) → Lysine(c) + Ornithine(m) + H+(PG)(m)
r1330	0.5	H+(PG)(c) ⇌ H+(PG)(m) + Proton-gradient(m)
r1114	0.5	AKG(m) + CoA(m) + NAD+(m) ⇌ NADH(m) + CO2(m) + Succinyl-CoA(m)
r1070	0.5	Lysine(c) ⇌ Lysine(m)
r0876	1	Glutamate(c) + H+(PG)(c) + Aspartate(m) → Glutamate(m) + H+(PG)(m) + Aspartate(c)
r0849	1.5	H2O(s) ⇌ H2O(c)
r0125	-0.5	GDP(m) + Pi(m) + Succinyl-CoA(m) ⇌ GTP(m) + Succinate(m) + CoA(m)
r0017	0.25	O2(m) + 4 H+(PG)(m) + 4 Ferrocytochrome C(m) → 4 Ferricytochrome C(m) + 2 H2O(m) + 4 H+(PG)(c)
r2686	1	Aspartate(c) ⇌ Aspartate(s)
r1323	2	NADH(m) ⇌ NAD+(m) + NADH-redox-potential(m)
r0144	0.5	Arginine(c) + H2O(c) ⇌ Urea(c) + Ornithine(c)

Aspartate from Asparagine (143)

Definition			Solution												
			exchanges		reactions								transp		
Objective	Constraints	Comment	imports	exports	c	m	r	p	l	n	s	b	s-c	b-c	int
Aspartate	−Asparagine MCES +Urea	Transamination to Aspartate using Asparagine	1 Pyruvate(m) 0.5 Asparagine(s) 0.5 NADPH-redox-potential(m) 1 Proton-gradient(m)	1 Aspartate(s) 0.5 NADH-redox-potential(m) 0.5 Proton-gradient(c) 0.5 Na-gradient(c) 0.5 CoA-activated acetyl group(m)	1	8	-	-	-	-	-	-	4	-	6

ID	flux	reaction
r2678	0.5	Acetyl-CoA(m) ⇌ CoA(m) + CoA-activated acetyl group(m)
r0127	0.5	H2O(c) + Asparagine(c) → Aspartate(c) + NH3(c)
r0073	-0.5	AKG(m) + NADH(m) + NH3(m) ⇌ Glutamate(m) + NAD+(m) + H2O(m)
r0911	0.5	Proline(c) + Glutamate(m) ⇌ Proline(m) + Glutamate(c)
r0056	0.5	Pyruvate(m) + CoA(m) + NAD+(m) → Acetyl-CoA(m) + CO2(m) + NADH(m)
r1426	0.5	Proline(m) + H+(PG)(c) → Proline(c) + H+(PG)(m)
r1327	-0.5	NADPH(m) ⇌ NADPH-redox-potential(m) + NADP+(m)
r0851	0.5	H2O(m) ⇌ H2O(c)
r0059	-0.5	Pyruvate(m) + CO2(m) + NADPH(m) ⇌ Malate(m) + NADP+(m)
r0110	-0.5	OAA(m) + Glutamate(m) → AKG(m) + Aspartate(m)
r2664	0.5	Na+(c) ⇌ Na+(s) + Na-gradient(c)
r0838	0.5	NH3(c) ⇌ NH3(m)
r0105	0.5	NAD+(m) + Malate(m) ⇌ OAA(m) + NADH(m)
r1330	-1	H+(PG)(c) ⇌ H+(PG)(m) + Proton-gradient(m)
r1331	0.5	H+(PG)(s) ⇌ H+(PG)(c) + Proton-gradient(c)
r0876	0.5	Glutamate(c) + H+(PG)(c) + Aspartate(m) → Glutamate(m) + H+(PG)(m) + Aspartate(c)
r2686	1	Aspartate(c) ⇌ Aspartate(s)
r2479	0.5	Asparagine(s) + Na+(s) + H+(PG)(c) → Asparagine(c) + Na+(c) + H+(PG)(s)
r1323	0.5	NADH(m) ⇌ NAD+(m) + NADH-redox-potential(m)

Aspartate from Cysteine (144)

Definition			Solution												
			exchanges		reactions								transp		
Objective	Constraints	Comment	imports	exports	c	m	r	p	l	n	s	b	s-c	b-c	int
Aspartate	−Cysteine MCES +Urea +H_2S	Transamination to Aspartate using Cysteine	2 Pyruvate(m) 1 Cysteine(s) 2 NADPH-redox-potential(m) 2 Proton-gradient(m)	1 Pyruvate(c) 1 Aspartate(s) 1 H_2S(s) 2 NADH-redox-potential(m) 1 Na-gradient(c) 1 CoA-activated acetyl group(m)	1	8	-	-	-	-	-	-	4	-	6

ID	flux	reaction
r2678	1	Acetyl-CoA(m) ⇌ CoA(m) + CoA-activated acetyl group(m)
r0911	1	Proline(c) + Glutamate(m) ⇌ Proline(m) + Glutamate(c)
r0056	1	Pyruvate(m) + CoA(m) + NAD+(m) → Acetyl-CoA(m) + CO2(m) + NADH(m)
r0075	-1	AKG(m) + NADPH(m) + NH3(m) ⇌ Glutamate(m) + H2O(m) + NADP+(m)
r1426	1	Proline(m) + H+(PG)(c) → Proline(c) + H+(PG)(m)
r1327	-2	NADPH(m) ⇌ NADPH-redox-potential(m) + NADP+(m)
r0940	1	H2S(c) ⇌ H2S(s)
r0193	1	Cysteine(c) + H2O(c) ⇌ Pyruvate(c) + H2S(c) + NH3(c)
r0851	1	H2O(m) ⇌ H2O(c)
r0059	-1	Pyruvate(m) + CO2(m) + NADPH(m) ⇌ Malate(m) + NADP+(m)
r0110	-1	OAA(m) + Glutamate(m) → AKG(m) + Aspartate(m)
r2664	1	Na+(c) ⇌ Na+(s) + Na-gradient(c)
r2475	1	Cysteine(s) + Na+(s) → Cysteine(c) + Na+(c)
r0838	1	NH3(c) ⇌ NH3(m)
r0105	1	NAD+(m) + Malate(m) ⇌ OAA(m) + NADH(m)

Continued on next page

ID	flux	reaction
r1330	-2	H+(PG)(c) ⇌ H+(PG)(m) + Proton-gradient(m)
r0876	1	Glutamate(c) + H+(PG)(c) + Aspartate(m) → Glutamate(m) + H+(PG)(m) + Aspartate(c)
r2686	1	Aspartate(c) ⇌ Aspartate(s)
r1323	2	NADH(m) ⇌ NAD+(m) + NADH-redox-potential(m)

Aspartate from Cystine (145)

Definition			Solution												
			exchanges		reactions								transp		
Objective	Constraints	Comment	imports	exports	c	m	r	p	l	n	s	b	s-c	b-c	int
Aspartate	−Cystine MCES +Urea $+H_2S$	Transamination to Aspartate using Cystine	2 Pyruvate(m) 0.5 Cystine(s) 0.5 NADPH-redox-potential(c) 2 NADPH-redox-potential(m) 1 Proton-gradient(m)	1 Pyruvate(c) 1 Aspartate(s) 1 H_2S(s) 1 NADH-redox-potential(c) 1 NADH-redox-potential(m) 0.5 Na-gradient(c) 1 CoA-activated acetyl group(m)	6	6	-	-	-	-	-	-	5	-	6

ID	flux	reaction
r2678	1	Acetyl-CoA(m) ⇌ CoA(m) + CoA-activated acetyl group(m)
r1144	-0.5	Glutamate(c) + Na+(c) ← Glutamate(s) + Na+(s)
r0820	1	AKG(c) + Malate(m) ⇌ AKG(m) + Malate(c)
r0109	-1	OAA(c) + Glutamate(c) ⇌ AKG(c) + Aspartate(c)
r0911	1	Proline(c) + Glutamate(m) ⇌ Proline(m) + Glutamate(c)
r0056	1	Pyruvate(m) + CoA(m) + NAD+(m) → Acetyl-CoA(m) + CO2(m) + NADH(m)
r1326	-0.5	NADPH(c) ⇌ NADPH-redox-potential(c) + NADP+(c)
r0075	-1	AKG(m) + NADPH(m) + NH3(m) ⇌ Glutamate(m) + H2O(m) + NADP+(m)
r1426	1	Proline(m) + H+(PG)(c) → Proline(c) + H+(PG)(m)
r1327	-2	NADPH(m) ⇌ NADPH-redox-potential(m) + NADP+(m)
r0940	1	H2S(c) ⇌ H2S(s)
r0193	1	Cysteine(c) + H2O(c) ⇌ Pyruvate(c) + H2S(c) + NH3(c)
r0851	1	H2O(m) ⇌ H2O(c)
r0059	-1	Pyruvate(m) + CO2(m) + NADPH(m) ⇌ Malate(m) + NADP+(m)
r2664	0.5	Na+(c) ⇌ Na+(s) + Na-gradient(c)
r0104	1	NAD+(c) + Malate(c) ⇌ OAA(c) + NADH(c)
r0838	1	NH3(c) ⇌ NH3(m)
r1330	-1	H+(PG)(c) ⇌ H+(PG)(m) + Proton-gradient(m)
r1322	1	NADH(c) ⇌ NAD+(c) + NADH-redox-potential(c)
r2686	1	Aspartate(c) ⇌ Aspartate(s)
r1323	1	NADH(m) ⇌ NAD+(m) + NADH-redox-potential(m)
r0877	0.5	Glutamate(c) + Cystine(s) ⇌ Glutamate(s) + Cystine(c)
r0027	-0.5	NADPH(c) + Cystine(c) ⇌ 2 Cysteine(c) + NADP+(c)

Aspartate from Glutamate (146)

Definition			Solution												
			exchanges		reactions								transp		
Objective	Constraints	Comment	imports	exports	c	m	r	p	l	n	s	b	s-c	b-c	int
Aspartate	−Glutamate MCES +Urea	Transamination to Aspartate using Glutamate	1 H_2O(s) 0.5 O_2(s) 1 Glutamate(s)	1 CO_2(s) 1 Aspartate(s) 1 ATP-energy(m) 2 NADH-redox-potential(m) 5 Proton-gradient(m) 1 Na-gradient(c)	-	9	-	-	-	-	-	-	6	-	7

ID	flux	reaction
r1144	-1	Glutamate(c) + Na+(c) ← Glutamate(s) + Na+(s)

Continued on next page

Aspartate from Glutamate (146)– continued

ID	flux	reaction
r0507	1	Ubiquinol(m) + 2 Ferricytochrome C(m) + 4 H+(PG)(m) → Ubiquinone(m) + 4 H+(PG)(c) + 2 Ferrocytochrome C(m)
r0851	-1	H2O(m) ⇌ H2O(c)
r0509	1	Ubiquinone(m) + Succinate(m) → Ubiquinol(m) + Fumarate(m)
r0100	-1	ADP(m) + GTP(m) ⇌ ATP(m) + GDP(m)
r0110	-1	OAA(m) + Glutamate(m) → AKG(m) + Aspartate(m)
r0259	-1	Fumarate(m) + H2O(m) ⇌ Malate(m)
r2664	1	Na+(c) ⇌ Na+(s) + Na-gradient(c)
r0855	0.5	O2(s) ⇌ O2(c)
r1320	1	ATP(m) + H2O(m) ⇌ ADP(m) + Pi(m) + ATP-energy(m)
r0856	-0.5	O2(m) ⇌ O2(c)
r0105	1	NAD+(m) + Malate(m) ⇌ OAA(m) + NADH(m)
r1330	5	H+(PG)(c) ⇌ H+(PG)(m) + Proton-gradient(m)
r1114	1	AKG(m) + CoA(m) + NAD+(m) ⇌ NADH(m) + CO2(m) + Succinyl-CoA(m)
r0876	1	Glutamate(c) + H+(PG)(c) + Aspartate(m) → Glutamate(m) + H+(PG)(m) + Aspartate(c)
r0867	1	CO2(c) ⇌ CO2(s)
r0849	1	H2O(s) ⇌ H2O(c)
r0125	-1	GDP(m) + Pi(m) + Succinyl-CoA(m) ⇌ GTP(m) + Succinate(m) + CoA(m)
r0017	0.5	O2(m) + 4 H+(PG)(m) + 4 Ferrocytochrome C(m) → 4 Ferricytochrome C(m) + 2 H2O(m) + 4 H+(PG)(c)
r2686	1	Aspartate(c) ⇌ Aspartate(s)
r1323	2	NADH(m) ⇌ NAD+(m) + NADH-redox-potential(m)
r0868	-1	CO2(c) ⇌ CO2(m)

Aspartate from Glycine (147)

Definition			Solution													
			exchanges		reactions								transp		
Objective	Constraints	Comment	imports	exports	c	m	r	p	l	n	s	b	s-c	b-c	int
Aspartate	−Glycine MCES +Urea	Transamination to Aspartate using Glycine	2 Pyruvate(m) 1 Glycine(s) 2 NADPH-redox-potential(m) 2 Proton-gradient(m) 1 THF-activated methyl group(c)	1 Pyruvate(c) 1 Aspartate(s) 2 NADH-redox-potential(m) 1 NADPH-redox-potential(c) 1 Na-gradient(c) 1 CoA-activated acetyl group(m)	5	8	-	-	-	-	-	-	3	-	6

ID	flux	reaction
r2678	1	Acetyl-CoA(m) ⇌ CoA(m) + CoA-activated acetyl group(m)
r0911	1	Proline(c) + Glutamate(m) ⇌ Proline(m) + Glutamate(c)
r0056	1	Pyruvate(m) + CoA(m) + NAD+(m) → Acetyl-CoA(m) + CO2(m) + NADH(m)
r1326	1	NADPH(c) ⇌ NADPH-redox-potential(c) + NADP+(c)
r0075	-1	AKG(m) + NADPH(m) + NH3(m) ⇌ Glutamate(m) + H2O(m) + NADP+(m)
r1426	1	Proline(m) + H+(PG)(c) → Proline(c) + H+(PG)(m)
r1327	-2	NADPH(m) ⇌ NADPH-redox-potential(m) + NADP+(m)
r1138	1	Glycine(s) + Na+(s) → Glycine(c) + Na+(c)
r0851	1	H2O(m) ⇌ H2O(c)
r0059	-1	Pyruvate(m) + CO2(m) + NADPH(m) ⇌ Malate(m) + NADP+(m)
r0110	-1	OAA(m) + Glutamate(m) → AKG(m) + Aspartate(m)
r0296	1	5-Methyl-THF(c) + NADP+(c) ⇌ 5,10-Methylene-THF(c) + NADPH(c)
r2664	1	Na+(c) ⇌ Na+(s) + Na-gradient(c)
r0230	1	5,10-Methylene-THF(c) + Glycine(c) + H2O(c) ⇌ THF(c) + Serine(c)
r0838	1	NH3(c) ⇌ NH3(m)
r0105	1	NAD+(m) + Malate(m) ⇌ OAA(m) + NADH(m)
r0060	1	Serine(c) → Pyruvate(c) + NH3(c)
r1330	-2	H+(PG)(c) ⇌ H+(PG)(m) + Proton-gradient(m)
r2676	-1	5-Methyl-THF(c) ⇌ THF(c) + THF-activated methyl group(c)
r0876	1	Glutamate(c) + H+(PG)(c) + Aspartate(m) → Glutamate(m) + H+(PG)(m) + Aspartate(c)
r2686	1	Aspartate(c) ⇌ Aspartate(s)
r1323	2	NADH(m) ⇌ NAD+(m) + NADH-redox-potential(m)

Aspartate from Histidine (148)

Definition			Solution												
			exchanges		reactions								transsp		
Objective	Constraints	Comment	imports	exports	c	m	r	p	l	n	s	b	s-c	b-c	int
Aspartate	−Histidine MCES +Urea	Transamination to Aspartate using Histidine	0.33 H_2O(s) 0.17 O_2(s) 1 Pyruvate(m) 0.33 Histidine(s) 0.33 NADPH-redox-potential(c) 0.67 NADPH-redox-potential(m)	1 Aspartate(s) 0.33 ATP-energy(m) 0.67 NADH-redox-potential(c) 0.33 Proton-gradient(c) 1.33 Proton-gradient(m) 0.33 Na-gradient(c) 0.33 THF-activated methyl group(c) 0.33 CoA-activated acetyl group(m)	12	11	-	-	-	-	-	-	6	-	9

ID	flux	reaction
r2678	0.33	Acetyl-CoA(m) ⇌ CoA(m) + CoA-activated acetyl group(m)
r0820	1	AKG(c) + Malate(m) ⇌ AKG(m) + Malate(c)
r0109	-1	OAA(c) + Glutamate(c) ⇌ AKG(c) + Aspartate(c)
r0073	-0.67	AKG(m) + NADH(m) + NH3(m) ⇌ Glutamate(m) + NAD+(m) + H2O(m)
r0911	0.67	Proline(c) + Glutamate(m) ⇌ Proline(m) + Glutamate(c)
r0524	0.33	5-Formiminotetrahydrofolate(c) ⇌ 5,10-Methenyl-THF(c) + NH3(c)
r0056	0.33	Pyruvate(m) + CoA(m) + NAD+(m) → Acetyl-CoA(m) + CO2(m) + NADH(m)
r1326	-0.33	NADPH(c) ⇌ NADPH-redox-potential(c) + NADP+(c)
r0507	0.33	Ubiquinol(m) + 2 Ferricytochrome C(m) + 4 H+(PG)(m) → Ubiquinone(m) + 4 H+(PG)(c) + 2 Ferrocytochrome C(m)
r1426	0.67	Proline(m) + H+(PG)(c) → Proline(c) + H+(PG)(m)
r1327	-0.67	NADPH(m) ⇌ NADPH-redox-potential(m) + NADP+(m)
r0851	0.33	H2O(m) ⇌ H2O(c)
r0518	-0.33	Glutamate(c) + 5-Formiminotetrahydrofolate(c) ⇌ THF(c) + N-Formimino-L-glutamate(c)
r0509	0.33	Ubiquinone(m) + Succinate(m) → Ubiquinol(m) + Fumarate(m)
r0293	-0.33	5,10-Methenyl-THF(c) + NADPH(c) ⇌ 5,10-Methylene-THF(c) + NADP+(c)
r0100	-0.33	ADP(m) + GTP(m) ⇌ ATP(m) + GDP(m)
r0059	-0.67	Pyruvate(m) + CO2(m) + NADPH(m) ⇌ Malate(m) + NADP+(m)
r2481	0.33	Histidine(s) + Na+(s) + H+(PG)(c) → Histidine(c) + Na+(c) + H+(PG)(s)
r0519	0.33	H2O(c) + 4-Imidazolone-5-propanoate(c) ⇌ N-Formimino-L-glutamate(c)
r0286	0.33	Histidine(c) ⇌ Urocanate(c) + NH3(c)
r0259	-0.33	Fumarate(m) + H2O(m) ⇌ Malate(m)
r2664	0.33	Na+(c) ⇌ Na+(s) + Na-gradient(c)
r0855	0.17	O2(s) ⇌ O2(c)
r0792	-0.33	5,10-Methylene-THF(c) + NADH(c) ⇌ 5-Methyl-THF(c) + NAD+(c)
r0104	1	NAD+(c) + Malate(c) ⇌ OAA(c) + NADH(c)
r1320	0.33	ATP(m) + H2O(m) ⇌ ADP(m) + Pi(m) + ATP-energy(m)
r0856	-0.17	O2(m) ⇌ O2(c)
r0838	0.67	NH3(c) ⇌ NH3(m)
r0577	-0.33	Urocanate(c) + H2O(c) ⇌ 4-Imidazolone-5-propanoate(c)
r1330	1.33	H+(PG)(c) ⇌ H+(PG)(m) + Proton-gradient(m)
r1114	0.33	AKG(m) + CoA(m) + NAD+(m) ⇌ NADH(m) + CO2(m) + Succinyl-CoA(m)
r2676	0.33	5-Methyl-THF(c) ⇌ THF(c) + THF-activated methyl group(c)
r1331	0.33	H+(PG)(s) ⇌ H+(PG)(c) + Proton-gradient(c)
r1322	0.67	NADH(c) ⇌ NAD+(c) + NADH-redox-potential(c)
r0849	0.33	H2O(s) ⇌ H2O(c)
r0125	-0.33	GDP(m) + Pi(m) + Succinyl-CoA(m) ⇌ GTP(m) + Succinate(m) + CoA(m)
r0017	0.17	O2(m) + 4 H+(PG)(m) + 4 Ferrocytochrome C(m) → 4 Ferricytochrome C(m) + 2 H2O(m) + 4 H+(PG)(c)
r2686	1	Aspartate(c) ⇌ Aspartate(s)

Aspartate from Isoleucine (149)

Definition			Solution													
			exchanges		reactions								transp		
Objective	Constraints	Comment	imports	exports	c	m	r	p	l	n	s	b	s-c	b-c	int
Aspartate	−Isoleucine MCES +Urea	Transamination to Aspartate using Isoleucine	1 H_2O(s) 1 O_2(s) 1 Pyruvate(m) 1 Isoleucine(s) 1 NADPH-redox-potential(m)	1 CO_2(s) 1 Aspartate(s) 5 NADH-redox-potential(m) 9 Proton-gradient(m) 2 CoA-activated acetyl group(m)	-	17	-	-	-	-	-	-	5	-	10

ID	flux	reaction
r2678	2	Acetyl-CoA(m) ⇌ CoA(m) + CoA-activated acetyl group(m)
r0911	1	Proline(c) + Glutamate(m) ⇌ Proline(m) + Glutamate(c)
r0507	2	Ubiquinol(m) + 2 Ferricytochrome C(m) + 4 H+(PG)(m) → Ubiquinone(m) + 4 H+(PG)(c) + 2 Ferrocytochrome C(m)
r0264	1	Isoleucine(m) + AKG(m) ⇌ Glutamate(m) + 2-Oxo-3-methylvalerate(m)
r0174	1	CoA(m) + NAD+(m) + 3-Oxopropanoate(m) → Acetyl-CoA(m) + CO2(m) + NADH(m)
r1426	1	Proline(m) + H+(PG)(c) → Proline(c) + H+(PG)(m)
r1327	-1	NADPH(m) ⇌ NADPH-redox-potential(m) + NADP+(m)
r0851	-1	H2O(m) ⇌ H2O(c)
r0365	1	Hydracrylate(m) + NAD+(m) → NADH(m) + 3-Oxopropanoate(m)
r0059	-1	Pyruvate(m) + CO2(m) + NADPH(m) ⇌ Malate(m) + NADP+(m)
r0110	-1	OAA(m) + Glutamate(m) → AKG(m) + Aspartate(m)
r2536	1	Isoleucine(s) ⇌ Isoleucine(c)
r0664	1	NAD+(m) + (S)-3-Hydroxy-2-methylbutyryl-CoA(m) ⇌ 2-Methylacetoacetyl-CoA(m) + NADH(m)
r0592	-1	H2O(m) + Acrylyl-CoA(m) → 3-Hydroxypropionyl-CoA(m)
r0953	1	Isoleucine(c) + H+(PG)(c) → Isoleucine(m) + H+(PG)(m)
r0683	1	Propanoyl-CoA(m) + Ubiquinone(m) → Ubiquinol(m) + Acrylyl-CoA(m)
r0665	-1	H2O(m) + Tiglyl-CoA(m) ⇌ (S)-3-Hydroxy-2-methylbutyryl-CoA(m)
r0855	1	O2(s) ⇌ O2(c)
r0856	-1	O2(m) ⇌ O2(c)
r0222	-1	CoA(m) + 2-Methylacetoacetyl-CoA(m) ⇌ Propanoyl-CoA(m) + Acetyl-CoA(m)
r0105	1	NAD+(m) + Malate(m) ⇌ OAA(m) + NADH(m)
r1330	9	H+(PG)(c) ⇌ H+(PG)(m) + Proton-gradient(m)
r0596	-1	3-Hydroxypropionyl-CoA(m) + H2O(m) → Hydracrylate(m) + CoA(m)
r0876	1	Glutamate(c) + H+(PG)(c) + Aspartate(m) → Glutamate(m) + H+(PG)(m) + Aspartate(c)
r0867	1	CO2(c) ⇌ CO2(s)
r0849	1	H2O(s) ⇌ H2O(c)
r0602	1	2-Oxo-3-methylvalerate(m) + CoA(m) + NAD+(m) ⇌ 2-Methylbutyryl-CoA(m) + CO2(m) + NADH(m)
r0017	1	O2(m) + 4 H+(PG)(m) + 4 Ferrocytochrome C(m) → 4 Ferricytochrome C(m) + 2 H2O(m) + 4 H+(PG)(c)
r2686	1	Aspartate(c) ⇌ Aspartate(s)
r1323	5	NADH(m) ⇌ NAD+(m) + NADH-redox-potential(m)
r0868	-1	CO2(c) ⇌ CO2(m)
r0603	1	2-Methylbutyryl-CoA(m) + Ubiquinone(m) ⇌ Tiglyl-CoA(m) + Ubiquinol(m)

Aspartate from Glutamine (150)

Definition			Solution													
			exchanges		reactions								transp		
Objective	Constraints	Comment	imports	exports	c	m	r	p	l	n	s	b	s-c	b-c	int
Aspartate	−Glutamine MCES +Urea	Transamination to Aspartate using Glutamine	0.5 H_2O(s) 0.25 O_2(s) 0.5 Pyruvate(m) 0.5 Glutamine(s) 0.5 NADPH-redox-potential(m)	1 Aspartate(s) 0.5 ATP-energy(m) 1 NADH-redox-potential(m) 1.5 Proton-gradient(m)	2	12	-	-	-	-	-	-	4	-	8

ID	flux	reaction
r0190	0.5	Glutamine(c) + Fructose-6P(c) → Glutamate(c) + Glucosamine-6P(c)
r0073	-0.5	AKG(m) + NADH(m) + NH3(m) ⇌ Glutamate(m) + NAD+(m) + H2O(m)
r0911	0.5	Proline(c) + Glutamate(m) ⇌ Proline(m) + Glutamate(c)

Continued on next page

Aspartate from Glutamine (150)– continued

ID	flux	reaction
r0507	0.5	Ubiquinol(m) + 2 Ferricytochrome C(m) + 4 H+(PG)(m) → Ubiquinone(m) + 4 H+(PG)(c) + 2 Ferrocytochrome C(m)
r1426	0.5	Proline(m) + H+(PG)(c) → Proline(c) + H+(PG)(m)
r1327	-0.5	NADPH(m) ⇌ NADPH-redox-potential(m) + NADP+(m)
r2525	0.5	Glutamine(s) ⇌ Glutamine(c)
r0509	0.5	Ubiquinone(m) + Succinate(m) → Ubiquinol(m) + Fumarate(m)
r0100	-0.5	ADP(m) + GTP(m) ⇌ ATP(m) + GDP(m)
r0059	-0.5	Pyruvate(m) + CO2(m) + NADPH(m) ⇌ Malate(m) + NADP+(m)
r0110	-1	OAA(m) + Glutamate(m) → AKG(m) + Aspartate(m)
r0259	-0.5	Fumarate(m) + H2O(m) ⇌ Malate(m)
r0855	0.25	O2(s) ⇌ O2(c)
r0189	0.5	Glucosamine-6P(c) + H2O(c) ⇌ Fructose-6P(c) + NH3(c)
r1320	0.5	ATP(m) + H2O(m) ⇌ ADP(m) + Pi(m) + ATP-energy(m)
r0856	-0.25	O2(m) ⇌ O2(c)
r0838	0.5	NH3(c) ⇌ NH3(m)
r0105	1	NAD+(m) + Malate(m) ⇌ OAA(m) + NADH(m)
r1330	1.5	H+(PG)(c) ⇌ H+(PG)(m) + Proton-gradient(m)
r1114	0.5	AKG(m) + CoA(m) + NAD+(m) ⇌ NADH(m) + CO2(m) + Succinyl-CoA(m)
r0876	1	Glutamate(c) + H+(PG)(c) + Aspartate(m) → Glutamate(m) + H+(PG)(m) + Aspartate(c)
r0849	0.5	H2O(s) ⇌ H2O(c)
r0125	-0.5	GDP(m) + Pi(m) + Succinyl-CoA(m) ⇌ GTP(m) + Succinate(m) + CoA(m)
r0017	0.25	O2(m) + 4 H+(PG)(m) + 4 Ferrocytochrome C(m) → 4 Ferricytochrome C(m) + 2 H2O(m) + 4 H+(PG)(c)
r2686	1	Aspartate(c) ⇌ Aspartate(s)
r1323	1	NADH(m) ⇌ NAD+(m) + NADH-redox-potential(m)

Aspartate from Leucine (151)

Definition			Solution												
			exchanges		reactions								transp		
Objective	Constraints	Comment	imports	exports	c	m	r	p	l	n	s	b	s-c	b-c	int
Aspartate	−Leucine MCES +Urea	Transamination to Aspartate using Leucine	0.5 O_2(s) 2 Pyruvate(m) 1 Leucine(s) 2 NADPH-redox-potential(m)	1 H_2O(s) 1 Aspartate(s) 4 NADH-redox-potential(m) 4 Proton-gradient(m) 1 Na-gradient(c) 4 CoA-activated acetyl group(m)	-	15	-	-	-	-	-	-	5	-	9

ID	flux	reaction
r2678	4	Acetyl-CoA(m) ⇌ CoA(m) + CoA-activated acetyl group(m)
r0262	1	Leucine(m) + AKG(m) ⇌ Glutamate(m) + 4-Methyl-2-oxopentanoate(m)
r0911	1	Proline(c) + Glutamate(m) ⇌ Proline(m) + Glutamate(c)
r0056	1	Pyruvate(m) + CoA(m) + NAD+(m) → Acetyl-CoA(m) + CO2(m) + NADH(m)
r0507	1	Ubiquinol(m) + 2 Ferricytochrome C(m) + 4 H+(PG)(m) → Ubiquinone(m) + 4 H+(PG)(c) + 2 Ferrocytochrome C(m)
r0462	1	HMG-CoA(m) + CoA(m) ⇌ Acetyl-CoA(m) + H2O(m) + Acetoacetyl-CoA(m)
r1426	1	Proline(m) + H+(PG)(c) → Proline(c) + H+(PG)(m)
r1327	-2	NADPH(m) ⇌ NADPH-redox-potential(m) + NADP+(m)
r0490	-1	3-Methylglutaconyl-CoA(m) + H2O(m) ⇌ HMG-CoA(m)
r0851	1	H2O(m) ⇌ H2O(c)
r0059	-2	Pyruvate(m) + CO2(m) + NADPH(m) ⇌ Malate(m) + NADP+(m)
r0110	-1	OAA(m) + Glutamate(m) → AKG(m) + Aspartate(m)
r1438	1	Leucine(c) → Leucine(m)
r0655	1	Isovaleryl-CoA(m) + Ubiquinone(m) ⇌ 3-Methylcrotonyl-CoA(m) + Ubiquinol(m)
r2664	1	Na+(c) ⇌ Na+(s) + Na-gradient(c)
r0855	0.5	O2(s) ⇌ O2(c)
r1122	-1	Leucine(s) + Na+(s) → Leucine(c) + Na+(c)
r0856	-0.5	O2(m) ⇌ O2(c)
r0658	1	ATP(m) + 3-Methylcrotonyl-CoA(m) + HCO3-(m) ⇌ ADP(m) + Pi(m) + 3-Methylglutaconyl-CoA(m)

Continued on next page

ID	flux	reaction
r0105	2	NAD+(m) + Malate(m) ⇌ OAA(m) + NADH(m)
r1330	4	H+(PG)(c) ⇌ H+(PG)(m) + Proton-gradient(m)
r0601	1	4-Methyl-2-oxopentanoate(m) + CoA(m) + NAD+(m) ⇌ Isovaleryl-CoA(m) + CO2(m) + NADH(m)
r0106	-1	ADP(m) + Pi(m) + OAA(m) ⇌ ATP(m) + Pyruvate(m) + HCO3-(m)
r0070	-1	CoA(m) + Acetoacetyl-CoA(m) ⇌ 2 Acetyl-CoA(m)
r0876	1	Glutamate(c) + H+(PG)(c) + Aspartate(m) → Glutamate(m) + H+(PG)(m) + Aspartate(c)
r0849	-1	H2O(s) ⇌ H2O(c)
r0017	0.5	O2(m) + 4 H+(PG)(m) + 4 Ferrocytochrome C(m) → 4 Ferricytochrome C(m) + 2 H2O(m) + 4 H+(PG)(c)
r2686	1	Aspartate(c) ⇌ Aspartate(s)
r1323	4	NADH(m) ⇌ NAD+(m) + NADH-redox-potential(m)

Aspartate from Lysine (152)

Definition			Solution												
			exchanges		reactions								transp		
Objective	Constraints	Comment	imports	exports	c	m	r	p	l	n	s	b	s-c	b-c	int
Aspartate	−Lysine MCES +Urea	Transamination to Aspartate using Lysine	0.5 H_2O(s) 0.25 O_2(s) 1 Pyruvate(m) 0.5 Lysine(s) 1 NADPH-redox-potential(m)	1 Aspartate(s) 0.5 NADH-redox-potential(c) 2 NADH-redox-potential(m) 1 Proton-gradient(m) 1 CoA-activated acetyl group(m)	2	14	-	-	-	-	-	-	4	-	9

ID	flux	reaction
r2678	1	Acetyl-CoA(m) ⇌ CoA(m) + CoA-activated acetyl group(m)
r0541	0.5	Glutaryl-CoA(m) + Ubiquinone(m) ⇌ Crotonyl-CoA(m) + CO2(m) + Ubiquinol(m)
r0460	0.5	(S)-3-Hydroxybutyryl-CoA(m) + NAD+(m) ⇌ NADH(m) + Acetoacetyl-CoA(m)
r0911	1	Proline(c) + Glutamate(m) ⇌ Proline(m) + Glutamate(c)
r0507	0.5	Ubiquinol(m) + 2 Ferricytochrome C(m) + 4 H+(PG)(m) → Ubiquinone(m) + 4 H+(PG)(c) + 2 Ferrocytochrome C(m)
r1426	1	Proline(m) + H+(PG)(c) → Proline(c) + H+(PG)(m)
r1327	-1	NADPH(m) ⇌ NADPH-redox-potential(m) + NADP+(m)
r0526	0.5	H2O(m) + Saccharopine(m) + NADP+(m) ⇌ Glutamate(m) + 2-Aminoadipate 6-semialdehyde(m) + NADPH(m)
r0059	-1	Pyruvate(m) + CO2(m) + NADPH(m) ⇌ Malate(m) + NADP+(m)
r0110	-1	OAA(m) + Glutamate(m) → AKG(m) + Aspartate(m)
r1069	-0.5	Lysine(s) ⇌ Lysine(c)
r0448	0.5	2-Oxoadipate(m) + CoA(m) + NAD+(m) ⇌ Glutaryl-CoA(m) + CO2(m) + NADH(m)
r0855	0.25	O2(s) ⇌ O2(c)
r0594	0.5	2-Aminoadipate 6-semialdehyde(c) + NAD+(c) + H2O(c) ⇌ NADH(c) + L-2-Aminoadipate(c)
r2539	-0.5	2-Aminoadipate 6-semialdehyde(c) + L-2-Aminoadipate(m) ⇌ 2-Aminoadipate 6-semialdehyde(m) + L-2-Aminoadipate(c)
r0856	-0.25	O2(m) ⇌ O2(c)
r0105	1	NAD+(m) + Malate(m) ⇌ OAA(m) + NADH(m)
r1330	1	H+(PG)(c) ⇌ H+(PG)(m) + Proton-gradient(m)
r0070	-0.5	CoA(m) + Acetoacetyl-CoA(m) ⇌ 2 Acetyl-CoA(m)
r1322	0.5	NADH(c) ⇌ NAD+(c) + NADH-redox-potential(c)
r1070	0.5	Lysine(c) ⇌ Lysine(m)
r0876	1	Glutamate(c) + H+(PG)(c) + Aspartate(m) → Glutamate(m) + H+(PG)(m) + Aspartate(c)
r0849	0.5	H2O(s) ⇌ H2O(c)
r0588	-0.5	Crotonyl-CoA(m) + H2O(m) ⇌ (S)-3-Hydroxybutyryl-CoA(m)
r0017	0.25	O2(m) + 4 H+(PG)(m) + 4 Ferrocytochrome C(m) → 4 Ferricytochrome C(m) + 2 H2O(m) + 4 H+(PG)(c)
r2686	1	Aspartate(c) ⇌ Aspartate(s)
r1323	2	NADH(m) ⇌ NAD+(m) + NADH-redox-potential(m)
r0450	0.5	AKG(m) + L-2-Aminoadipate(m) ⇌ 2-Oxoadipate(m) + Glutamate(m)
r0180	-0.5	AKG(m) + NADPH(m) + Lysine(m) ⇌ H2O(m) + Saccharopine(m) + NADP+(m)

Aspartate from Methionine (153)

Definition			Solution												
			exchanges		reactions								transp		
Objective	Constraints	Comment	imports	exports	c	m	r	p	l	n	s	b	s-c	b-c	int
Aspartate	−Methionine MCES +Urea +H_2S	Transamination to Aspartate using Methionine	1.5 H_2O(s) 0.5 O_2(s) 1 Pyruvate(m) 1 Methionine(s) 0.5 Glucose-6P(c) 1 NADPH-redox-potential(m)	0.5 P_i(c) 1 CO_2(s) 1 Pyruvate(c) 1 Aspartate(s) 1 H_2S(s) 0.5 ATP-energy(c) 2 NADH-redox-potential(c) 2 NADH-redox-potential(m) 1 Proton-gradient(m) 1 Na-gradient(c) 1 THF-activated methyl group(c) 1 CoA-activated acetyl group(m)	16	13	-	-	-	-	-	-	7	-	13

ID	flux	reaction
r0855	0.5	O2(s) ⇌ O2(c)
r0856	-0.5	O2(m) ⇌ O2(c)
r0592	-1	H2O(m) + Acrylyl-CoA(m) → 3-Hydroxypropionyl-CoA(m)
r0876	1	Glutamate(c) + H+(PG)(c) + Aspartate(m) → Glutamate(m) + H+(PG)(m) + Aspartate(c)
r0596	-1	3-Hydroxypropionyl-CoA(m) + H2O(m) → Hydracrylate(m) + CoA(m)
r0017	0.5	O2(m) + 4 H+(PG)(m) + 4 Ferrocytochrome C(m) → 4 Ferricytochrome C(m) + 2 H2O(m) + 4 H+(PG)(c)
r0314	1	Serine(c) + Homocysteine(c) ⇌ H2O(c) + L-Cystathionine(c)
r0911	2	Proline(c) + Glutamate(m) ⇌ Proline(m) + Glutamate(c)
r1141	-1	Methionine(s) + Na+(s) → Methionine(c) + Na+(c)
r0337	-1	ADP(c) + 1,3DPG(c) ⇌ ATP(c) + 3PG(c)
r0338	1	3PG(c) + NAD+(c) → 3-Phosphonooxypyruvate(c) + NADH(c)
r2664	1	Na+(c) ⇌ Na+(s) + Na-gradient(c)
r1426	2	Proline(m) + H+(PG)(c) → Proline(c) + H+(PG)(m)
r0059	-1	Pyruvate(m) + CO2(m) + NADPH(m) ⇌ Malate(m) + NADP+(m)
r0073	-2	AKG(m) + NADH(m) + NH3(m) ⇌ Glutamate(m) + NAD+(m) + H2O(m)
r2686	1	Aspartate(c) ⇌ Aspartate(s)
r0507	1	Ubiquinol(m) + 2 Ferricytochrome C(m) + 4 H+(PG)(m) → Ubiquinone(m) + 4 H+(PG)(c) + 2 Ferrocytochrome C(m)
r0241	1	H2O(c) + L-Cystathionine(c) ⇌ Cysteine(c) + NH3(c) + 2-Oxobutyrate(c)
r0243	-0.5	DHAP(c) ⇌ GAP(c)
r1330	1	H+(PG)(c) ⇌ H+(PG)(m) + Proton-gradient(m)
r1319	0.5	ATP(c) + H2O(c) ⇌ ADP(c) + Pi(c) + ATP-energy(c)
r0110	-1	OAA(m) + Glutamate(m) → AKG(m) + Aspartate(m)
r0849	1.5	H2O(s) ⇌ H2O(c)
r0867	1	CO2(c) ⇌ CO2(s)
r0868	-1	CO2(c) ⇌ CO2(m)
r0736	0.5	ATP(c) + Fructose-6P(c) → ADP(c) + Fructose-1,6PP(c)
r0159	1	3-Phosphoserine(c) + H2O(c) → Serine(c) + Pi(c)
r0174	1	CoA(m) + NAD+(m) + 3-Oxopropanoate(m) → Acetyl-CoA(m) + CO2(m) + NADH(m)
r0192	0.5	Glucose-6P(c) ⇌ Fructose-6P(c)
r0193	1	Cysteine(c) + H2O(c) ⇌ Pyruvate(c) + H2S(c) + NH3(c)
r0940	1	H2S(c) ⇌ H2S(s)
r1154	1	CoA(m) + NAD+(m) + 2-Oxobutyrate(m) ⇌ CO2(m) + Propanoyl-CoA(m) + NADH(m)
r1155	1	2-Oxobutyrate(c) + H+(PG)(c) → 2-Oxobutyrate(m) + H+(PG)(m)
r2676	1	5-Methyl-THF(c) ⇌ THF(c) + THF-activated methyl group(c)
r0365	1	Hydracrylate(m) + NAD+(m) → NADH(m) + 3-Oxopropanoate(m)
r2678	1	Acetyl-CoA(m) ⇌ CoA(m) + CoA-activated acetyl group(m)
r0663	-1	3-Phosphonooxypyruvate(c) + Glutamate(c) → 3-Phosphoserine(c) + AKG(c)
r0232	-1	THF(c) + Methionine(c) ⇌ 5-Methyl-THF(c) + Homocysteine(c)
r1322	2	NADH(c) ⇌ NAD+(c) + NADH-redox-potential(c)
r0683	1	Propanoyl-CoA(m) + Ubiquinone(m) → Ubiquinol(m) + Acrylyl-CoA(m)
r1323	2	NADH(m) ⇌ NAD+(m) + NADH-redox-potential(m)
r0253	1	GAP(c) + Pi(c) + NAD+(c) ⇌ 1,3DPG(c) + NADH(c)
r1327	-1	NADPH(m) ⇌ NADPH-redox-potential(m) + NADP+(m)
r0256	0.5	Fructose-1,6PP(c) ⇌ DHAP(c) + GAP(c)
		Continued on next page

ID	flux	reaction
r1047	1	Pi(c) + H+(PG)(c) → Pi(m) + H+(PG)(m)
r0105	1	NAD+(m) + Malate(m) ⇌ OAA(m) + NADH(m)
r2419	1	AKG(c) + Pi(m) ⇌ AKG(m) + Pi(c)
r0838	2	NH3(c) ⇌ NH3(m)
r0851	1	H2O(m) ⇌ H2O(c)

Aspartate from Phenylalanine (154)

Definition			Solution												
			exchanges		reactions								transp		
Objective	Constraints	Comment	imports	exports	c	m	r	p	l	n	s	b	s-c	b-c	int
Aspartate	−Phenylalanine MCES +Urea	Transamination to Aspartate using Phenylalanine	3 O_2(s) 1 Phenylalanine(s) 2 ATP-energy(c) 1 NADPH-redox-potential(c)	1 CO_2(s) 1 Aspartate(s) 1 NADH-redox-potential(c) 1 Na-gradient(c) 2 CoA-activated acetyl group(c)	18	-	-	-	-	-	-	-	5	-	-

ID	flux	reaction
r1126	-1	Phenylalanine(s) + Na+(s) → Phenylalanine(c) + Na+(c)
r0109	-1	OAA(c) + Glutamate(c) ⇌ AKG(c) + Aspartate(c)
r0605	1	4-Maleylacetoacetate(c) ⇌ Fumarylacetoacetate(c)
r1326	-1	NADPH(c) ⇌ NADPH-redox-potential(c) + NADP+(c)
r0543	1	Homogentisate(c) + O2(c) ⇌ 4-Maleylacetoacetate(c)
r0183	1	Tyrosine(c) + AKG(c) ⇌ 4-Hydroxyphenylpyruvate(c) + Glutamate(c)
r0544	1	4-Hydroxyphenylpyruvate(c) + O2(c) ⇌ Homogentisate(c) + CO2(c)
r1319	-2	ATP(c) + H2O(c) ⇌ ADP(c) + Pi(c) + ATP-energy(c)
r0258	-1	Fumarate(c) + H2O(c) ⇌ Malate(c)
r0069	-1	CoA(c) + Acetoacetyl-CoA(c) ⇌ 2 Acetyl-CoA(c)
r0030	1	ATP(c) + AMP(c) ⇌ 2 ADP(c)
r2664	1	Na+(c) ⇌ Na+(s) + Na-gradient(c)
r0855	3	O2(s) ⇌ O2(c)
r0320	1	ATP(c) + CoA(c) + Acetoacetate(c) → AMP(c) + PPi(c) + Acetoacetyl-CoA(c)
r0104	1	NAD+(c) + Malate(c) ⇌ OAA(c) + NADH(c)
r0398	1	Dihydrobiopterin(c) + NADPH(c) ⇌ Tetrahydrobiopterin(c) + NADP+(c)
r0007	1	PPi(c) + H2O(c) → 2 Pi(c)
r1322	1	NADH(c) ⇌ NAD+(c) + NADH-redox-potential(c)
r0867	1	CO2(c) ⇌ CO2(s)
r0399	1	Tetrahydrobiopterin(c) + Phenylalanine(c) + O2(c) ⇌ Dihydrobiopterin(c) + Tyrosine(c) + H2O(c)
r2686	1	Aspartate(c) ⇌ Aspartate(s)
r2677	2	Acetyl-CoA(c) ⇌ CoA(c) + CoA-activated acetyl group(c)
r0324	-1	Fumarylacetoacetate(c) + H2O(c) ⇌ Fumarate(c) + Acetoacetate(c)

Aspartate from Proline (155)

Definition			Solution												
			exchanges		reactions								transp		
Objective	Constraints	Comment	imports	exports	c	m	r	p	l	n	s	b	s-c	b-c	int
Aspartate	−Proline MCES +Urea	Transamination to Aspartate using Proline	4 H_2O(s) 1 Proline(s)	1 CO_2(s) 1 Aspartate(s) 1 ATP-energy(m) 1 NADH-redox-potential(c) 2 NADH-redox-potential(m)	3	10	-	-	-	-	-	-	4	-	4

ID	flux	reaction
r0820	1	AKG(c) + Malate(m) ⇌ AKG(m) + Malate(c)
r0109	-1	OAA(c) + Glutamate(c) ⇌ AKG(c) + Aspartate(c)

Continued on next page

ID	flux	reaction
r2531	1	Proline(s) ⇌ Proline(c)
r0911	1	Proline(c) + Glutamate(m) ⇌ Proline(m) + Glutamate(c)
r0074	1	L-Glutamate 5-semialdehyde(m) + NAD+(m) + H2O(m) ⇌ Glutamate(m) + NADH(m)
r1453	1	Proline(m) + Ubiquinol(m) ⇌ 1-Pyrroline-5-carboxylate(m) + Ubiquinone(m)
r0175	1	1-Pyrroline-5-carboxylate(m) + H2O(m) ⇌ L-Glutamate 5-semialdehyde(m)
r0851	-4	H2O(m) ⇌ H2O(c)
r0509	1	Ubiquinone(m) + Succinate(m) → Ubiquinol(m) + Fumarate(m)
r0100	-1	ADP(m) + GTP(m) ⇌ ATP(m) + GDP(m)
r0259	-1	Fumarate(m) + H2O(m) ⇌ Malate(m)
r0104	1	NAD+(c) + Malate(c) ⇌ OAA(c) + NADH(c)
r1320	1	ATP(m) + H2O(m) ⇌ ADP(m) + Pi(m) + ATP-energy(m)
r1114	1	AKG(m) + CoA(m) + NAD+(m) ⇌ NADH(m) + CO2(m) + Succinyl-CoA(m)
r1322	1	NADH(c) ⇌ NAD+(c) + NADH-redox-potential(c)
r0867	1	CO2(c) ⇌ CO2(s)
r0849	4	H2O(s) ⇌ H2O(c)
r0125	-1	GDP(m) + Pi(m) + Succinyl-CoA(m) ⇌ GTP(m) + Succinate(m) + CoA(m)
r2686	1	Aspartate(c) ⇌ Aspartate(s)
r1323	2	NADH(m) ⇌ NAD+(m) + NADH-redox-potential(m)
r0868	-1	CO2(c) ⇌ CO2(m)

Aspartate from Serine (156)

Definition			Solution												
			exchanges		reactions								transp		
Objective	Constraints	Comment	imports	exports	c	m	r	p	l	n	s	b	s-c	b-c	int
Aspartate	−Serine MCES +Urea	Transamination to Aspartate using Serine	2 Pyruvate(m) 1 Serine(s) 1 NADPH-redox-potential(m) 1 Proton-gradient(m)	1 H_2O(s) 1 Pyruvate(c) 1 Aspartate(s) 1 NADH-redox-potential(c) 1 Proton-gradient(c) 1 Na-gradient(c) 1 CoA-activated acetyl group(m)	4	5	-	-	-	-	-	-	5	-	6

ID	flux	reaction
r2678	1	Acetyl-CoA(m) ⇌ CoA(m) + CoA-activated acetyl group(m)
r0820	1	AKG(c) + Malate(m) ⇌ AKG(m) + Malate(c)
r0109	-1	OAA(c) + Glutamate(c) ⇌ AKG(c) + Aspartate(c)
r0073	-1	AKG(m) + NADH(m) + NH3(m) ⇌ Glutamate(m) + NAD+(m) + H2O(m)
r0911	1	Proline(c) + Glutamate(m) ⇌ Proline(m) + Glutamate(c)
r0056	1	Pyruvate(m) + CoA(m) + NAD+(m) → Acetyl-CoA(m) + CO2(m) + NADH(m)
r1426	1	Proline(m) + H+(PG)(c) → Proline(c) + H+(PG)(m)
r1327	-1	NADPH(m) ⇌ NADPH-redox-potential(m) + NADP+(m)
r0851	1	H2O(m) ⇌ H2O(c)
r0059	-1	Pyruvate(m) + CO2(m) + NADPH(m) ⇌ Malate(m) + NADP+(m)
r2664	1	Na+(c) ⇌ Na+(s) + Na-gradient(c)
r0104	1	NAD+(c) + Malate(c) ⇌ OAA(c) + NADH(c)
r0838	1	NH3(c) ⇌ NH3(m)
r0060	1	Serine(c) → Pyruvate(c) + NH3(c)
r1330	-1	H+(PG)(c) ⇌ H+(PG)(m) + Proton-gradient(m)
r2478	1	Serine(s) + Na+(s) + H+(PG)(c) → Serine(c) + Na+(c) + H+(PG)(s)
r1331	1	H+(PG)(s) ⇌ H+(PG)(c) + Proton-gradient(c)
r1322	1	NADH(c) ⇌ NAD+(c) + NADH-redox-potential(c)
r0849	-1	H2O(s) ⇌ H2O(c)
r2686	1	Aspartate(c) ⇌ Aspartate(s)

Aspartate from Threonine (157)

Definition			Solution												
			exchanges		reactions								transp		
Objective	Constraints	Comment	imports	exports	c	m	r	p	l	n	s	b	s-c	b-c	int
Aspartate	−Threonine MCES +Urea	Transamination to Aspartate using Threonine	0.5 O_2(s) 1 Pyruvate(m) 1 Threonine(s) 1 NADPH-redox-potential(m)	1 CO_2(s) 1 Aspartate(s) 1 NADH-redox-potential(c) 2 NADH-redox-potential(m) 4 Proton-gradient(m) 1 CoA-activated acetyl group(m)	4	11	-	-	-	-	-	-	4	-	10

ID	flux	reaction
r2678	1	Acetyl-CoA(m) ⇌ CoA(m) + CoA-activated acetyl group(m)
r0820	1	AKG(c) + Malate(m) ⇌ AKG(m) + Malate(c)
r0109	-1	OAA(c) + Glutamate(c) ⇌ AKG(c) + Aspartate(c)
r0073	-1	AKG(m) + NADH(m) + NH3(m) ⇌ Glutamate(m) + NAD+(m) + H2O(m)
r1154	1	CoA(m) + NAD+(m) + 2-Oxobutyrate(m) ⇌ CO2(m) + Propanoyl-CoA(m) + NADH(m)
r0911	1	Proline(c) + Glutamate(m) ⇌ Proline(m) + Glutamate(c)
r1155	1	2-Oxobutyrate(c) + H+(PG)(c) → 2-Oxobutyrate(m) + H+(PG)(m)
r0507	1	Ubiquinol(m) + 2 Ferricytochrome C(m) + 4 H+(PG)(m) → Ubiquinone(m) + 4 H+(PG)(c) + 2 Ferrocytochrome C(m)
r0174	1	CoA(m) + NAD+(m) + 3-Oxopropanoate(m) → Acetyl-CoA(m) + CO2(m) + NADH(m)
r1426	1	Proline(m) + H+(PG)(c) → Proline(c) + H+(PG)(m)
r1327	-1	NADPH(m) ⇌ NADPH-redox-potential(m) + NADP+(m)
r2534	1	Threonine(s) ⇌ Threonine(c)
r0365	1	Hydracrylate(m) + NAD+(m) → NADH(m) + 3-Oxopropanoate(m)
r0059	-1	Pyruvate(m) + CO2(m) + NADPH(m) ⇌ Malate(m) + NADP+(m)
r0592	-1	H2O(m) + Acrylyl-CoA(m) → 3-Hydroxypropionyl-CoA(m)
r0683	1	Propanoyl-CoA(m) + Ubiquinone(m) → Ubiquinol(m) + Acrylyl-CoA(m)
r0855	0.5	O2(s) ⇌ O2(c)
r0104	1	NAD+(c) + Malate(c) ⇌ OAA(c) + NADH(c)
r0856	-0.5	O2(m) ⇌ O2(c)
r0838	1	NH3(c) ⇌ NH3(m)
r0240	1	Threonine(c) ⇌ 2-Oxobutyrate(c) + NH3(c)
r1330	4	H+(PG)(c) ⇌ H+(PG)(m) + Proton-gradient(m)
r0596	-1	3-Hydroxypropionyl-CoA(m) + H2O(m) → Hydracrylate(m) + CoA(m)
r1322	1	NADH(c) ⇌ NAD+(c) + NADH-redox-potential(c)
r0867	1	CO2(c) ⇌ CO2(s)
r0017	0.5	O2(m) + 4 H+(PG)(m) + 4 Ferrocytochrome C(m) → 4 Ferricytochrome C(m) + 2 H2O(m) + 4 H+(PG)(c)
r2686	1	Aspartate(c) ⇌ Aspartate(s)
r1323	2	NADH(m) ⇌ NAD+(m) + NADH-redox-potential(m)
r0868	-1	CO2(c) ⇌ CO2(m)

Aspartate from Tryptophan (158)

Definition			Solution												
			exchanges		reactions								transp		
Objective	Constraints	Comment	imports	exports	c	m	r	p	l	n	s	b	s-c	b-c	int
Aspartate	−Tryptophan MCES +Urea	Transamination to Aspartate using Tryptophan	1.75 O_2(s) 1 Pyruvate(m) 0.5 Tryptophan(s) 0.5 ATP-energy(c) 1.5 NADPH-redox-potential(c) 1.5 NADPH-redox-potential(m)	0.5 CO_2(s) 0.5 Pyruvate(c) 1 Aspartate(s) 1 NADH-redox-potential(c) 1 NADH-redox-potential(m) 2.5 Proton-gradient(m) 0.5 THF-activated methyl group(c) 1 CoA-activated acetyl group(m)	19	10	-	-	-	-	-	-	4	-	10

ID	flux	reaction
r0855	1.75	O2(s) ⇌ O2(c)
r0856	-0.25	O2(m) ⇌ O2(c)

Continued on next page

Aspartate from Tryptophan (158)– continued

ID	flux	reaction
r0293	-0.5	5,10-Methylene-THF(c) + NADP+(c) ⇌ 5,10-Methenyl-THF(c) + NADPH(c)
r0460	0.5	(S)-3-Hydroxybutyryl-CoA(m) + NAD+(m) ⇌ NADH(m) + Acetoacetyl-CoA(m)
r0448	0.5	2-Oxoadipate(m) + CoA(m) + NAD+(m) ⇌ Glutaryl-CoA(m) + CO2(m) + NADH(m)
r0449	-0.5	2-Oxoadipate(c) + NH3(c) + NADP+(c) ⇌ 2-Aminomuconate(c) + NADPH(c) + H2O(c)
r0017	0.25	O2(m) + 4 H+(PG)(m) + 4 Ferrocytochrome C(m) → 4 Ferricytochrome C(m) + 2 H2O(m) + 4 H+(PG)(c)
r0169	0.5	Tryptophan(c) + O2(c) ⇌ L-Formylkynurenine(c)
r0911	0.5	Proline(c) + Glutamate(m) ⇌ Proline(m) + Glutamate(c)
r0070	-0.5	CoA(m) + Acetoacetyl-CoA(m) ⇌ 2 Acetyl-CoA(m)
r1426	0.5	Proline(m) + H+(PG)(c) → Proline(c) + H+(PG)(m)
r0059	-1	Pyruvate(m) + CO2(m) + NADPH(m) ⇌ Malate(m) + NADP+(m)
r0075	-0.5	AKG(m) + NADPH(m) + NH3(m) ⇌ Glutamate(m) + H2O(m) + NADP+(m)
r0371	-0.5	10-Formyl-THF(c) ⇌ 5,10-Methenyl-THF(c) + H2O(c)
r2686	1	Aspartate(c) ⇌ Aspartate(s)
r0507	0.5	Ubiquinol(m) + 2 Ferricytochrome C(m) + 4 H+(PG)(m) → Ubiquinone(m) + 4 H+(PG)(c) + 2 Ferrocytochrome C(m)
r0228	0.5	THF(c) + Formate(c) + ATP(c) → ADP(c) + Pi(c) + 10-Formyl-THF(c)
r0676	0.5	2-Amino-3-carboxymuconate semialdehyde(c) ⇌ 2-Aminomuconate semialdehyde(c) + CO2(c)
r0820	1	AKG(c) + Malate(m) ⇌ AKG(m) + Malate(c)
r1330	2.5	H+(PG)(c) ⇌ H+(PG)(m) + Proton-gradient(m)
r1319	-0.5	ATP(c) + H2O(c) ⇌ ADP(c) + Pi(c) + ATP-energy(c)
r0541	0.5	Glutaryl-CoA(m) + Ubiquinone(m) ⇌ Crotonyl-CoA(m) + CO2(m) + Ubiquinol(m)
r0562	0.5	3-Hydroxyanthranilate(c) + O2(c) ⇌ 2-Amino-3-carboxymuconate semialdehyde(c)
r0563	0.5	H2O(c) + 3-Hydroxy-L-kynurenine(c) ⇌ 3-Hydroxyanthranilate(c) + Alanine(c)
r0867	0.5	CO2(c) ⇌ CO2(s)
r0588	-0.5	Crotonyl-CoA(m) + H2O(m) ⇌ (S)-3-Hydroxybutyryl-CoA(m)
r0454	0.5	L-Formylkynurenine(c) + H2O(c) ⇌ Formate(c) + Kynurenine(c)
r0455	0.5	Kynurenine(c) + O2(c) + NADPH(c) ⇌ H2O(c) + 3-Hydroxy-L-kynurenine(c) + NADP+(c)
r0792	-0.5	5,10-Methylene-THF(c) + NADH(c) ⇌ 5-Methyl-THF(c) + NAD+(c)
r0080	0.5	Alanine(c) + AKG(c) ⇌ Pyruvate(c) + Glutamate(c)
r0645	0.5	2-Aminomuconate semialdehyde(c) + NAD+(c) + H2O(c) ⇌ 2-Aminomuconate(c) + NADH(c)
r2676	0.5	5-Methyl-THF(c) ⇌ THF(c) + THF-activated methyl group(c)
r2678	1	Acetyl-CoA(m) ⇌ CoA(m) + CoA-activated acetyl group(m)
r2527	0.5	Tryptophan(s) ⇌ Tryptophan(c)
r0945	0.5	2-Oxoadipate(c) + AKG(m) ⇌ 2-Oxoadipate(m) + AKG(c)
r1322	1	NADH(c) ⇌ NAD+(c) + NADH-redox-potential(c)
r1323	1	NADH(m) ⇌ NAD+(m) + NADH-redox-potential(m)
r1326	-1.5	NADPH(c) ⇌ NADPH-redox-potential(c) + NADP+(c)
r1327	-1.5	NADPH(m) ⇌ NADPH-redox-potential(m) + NADP+(m)
r0104	1	NAD+(c) + Malate(c) ⇌ OAA(c) + NADH(c)
r0838	0.5	NH3(c) ⇌ NH3(m)
r0851	0.5	H2O(m) ⇌ H2O(c)
r0109	-1	OAA(c) + Glutamate(c) ⇌ AKG(c) + Aspartate(c)

Aspartate from Tyrosine (159)

Definition			Solution												
			exchanges		reactions								transp		
Objective	Constraints	Comment	imports	exports	c	m	r	p	l	n	s	b	s-c	b-c	int
Aspartate	−Tyrosine MCES +Urea	Transamination to Aspartate using Tyrosine	1 H_2O(s) 2 O_2(s) 1 Tyrosine(s) 2 ATP-energy(c)	1 CO_2(s) 1 Aspartate(s) 1 NADH-redox-potential(c) 1 Na-gradient(c) 2 CoA-activated acetyl group(c)	15	-	-	-	-	-	-	-	6	-	-

ID	flux	reaction
r0109	-1	OAA(c) + Glutamate(c) ⇌ AKG(c) + Aspartate(c)
r0605	1	4-Maleylacetoacetate(c) ⇌ Fumarylacetoacetate(c)
		Continued on next page

Aspartate from Tyrosine (159)– continued

ID	flux	reaction
r0543	1	Homogentisate(c) + O2(c) ⇌ 4-Maleylacetoacetate(c)
r0183	1	Tyrosine(c) + AKG(c) ⇌ 4-Hydroxyphenylpyruvate(c) + Glutamate(c)
r0544	1	4-Hydroxyphenylpyruvate(c) + O2(c) ⇌ Homogentisate(c) + CO2(c)
r1319	-2	ATP(c) + H2O(c) ⇌ ADP(c) + Pi(c) + ATP-energy(c)
r0258	-1	Fumarate(c) + H2O(c) ⇌ Malate(c)
r0069	-1	CoA(c) + Acetoacetyl-CoA(c) ⇌ 2 Acetyl-CoA(c)
r0030	1	ATP(c) + AMP(c) ⇌ 2 ADP(c)
r2664	1	Na+(c) ⇌ Na+(s) + Na-gradient(c)
r0855	2	O2(s) ⇌ O2(c)
r0320	1	ATP(c) + CoA(c) + Acetoacetate(c) → AMP(c) + PPi(c) + Acetoacetyl-CoA(c)
r0104	1	NAD+(c) + Malate(c) ⇌ OAA(c) + NADH(c)
r0007	1	PPi(c) + H2O(c) → 2 Pi(c)
r1322	1	NADH(c) ⇌ NAD+(c) + NADH-redox-potential(c)
r0867	1	CO2(c) ⇌ CO2(s)
r0849	1	H2O(s) ⇌ H2O(c)
r2686	1	Aspartate(c) ⇌ Aspartate(s)
r2677	2	Acetyl-CoA(c) ⇌ CoA(c) + CoA-activated acetyl group(c)
r1125	1	Tyrosine(s) + Na+(s) → Tyrosine(c) + Na+(c)
r0324	-1	Fumarylacetoacetate(c) + H2O(c) ⇌ Fumarate(c) + Acetoacetate(c)

Aspartate from Valine (160)

Definition			Solution												
			exchanges		reactions								transp		
Objective	Constraints	Comment	imports	exports	c	m	r	p	l	n	s	b	s-c	b-c	int
Aspartate	−Valine MCES +Urea	Transamination to Aspartate using Valine	2 H_2O(s) 1 O_2(s) 1 Valine(s)	1 CO_2(s) 1 Aspartate(s) 1 ATP-energy(m) 1 NADH-redox-potential(c) 3 NADH-redox-potential(m) 10 Proton-gradient(m) 1 Na-gradient(c)	3	14	-	-	-	-	-	-	6	-	10

ID	flux	reaction
r0820	1	AKG(c) + Malate(m) ⇌ AKG(m) + Malate(c)
r0779	1	3-Hydroxyisobutyryl-CoA(m) + H2O(m) → 3-Hydroxyisobutyrate(m) + CoA(m)
r0109	-1	OAA(c) + Glutamate(c) ⇌ AKG(c) + Aspartate(c)
r0911	1	Proline(c) + Glutamate(m) ⇌ Proline(m) + Glutamate(c)
r0560	1	Isobutyryl-CoA(m) + Ubiquinone(m) → Methacrylyl-CoA(m) + Ubiquinol(m)
r0263	1	Valine(m) + AKG(m) → Glutamate(m) + 3-Methyl-2-oxobutyrate(m)
r0624	1	2-Methyl-3-oxopropanoate(m) + CoA(m) + NAD+(m) ⇌ (R)-Methylmalonyl-CoA(m) + NADH(m)
r0507	2	Ubiquinol(m) + 2 Ferricytochrome C(m) + 4 H+(PG)(m) → Ubiquinone(m) + 4 H+(PG)(c) + 2 Ferrocytochrome C(m)
r1426	1	Proline(m) + H+(PG)(c) → Proline(c) + H+(PG)(m)
r0923	1	Valine(c) + H+(PG)(c) → Valine(m) + H+(PG)(m)
r0851	-2	H2O(m) ⇌ H2O(c)
r0509	1	Ubiquinone(m) + Succinate(m) → Ubiquinol(m) + Fumarate(m)
r0482	1	3-Hydroxyisobutyrate(m) + NAD+(m) → 2-Methyl-3-oxopropanoate(m) + NADH(m)
r0100	-1	ADP(m) + GTP(m) ⇌ ATP(m) + GDP(m)
r0259	-1	Fumarate(m) + H2O(m) ⇌ Malate(m)
r0197	1	(R)-Methylmalonyl-CoA(m) ⇌ Succinyl-CoA(m)
r2664	1	Na+(c) ⇌ Na+(s) + Na-gradient(c)
r1121	-1	Valine(s) + Na+(s) → Valine(c) + Na+(c)
r0855	1	O2(s) ⇌ O2(c)
r0104	1	NAD+(c) + Malate(c) ⇌ OAA(c) + NADH(c)
r1320	1	ATP(m) + H2O(m) ⇌ ADP(m) + Pi(m) + ATP-energy(m)
r0856	-1	O2(m) ⇌ O2(c)
r0600	1	3-Methyl-2-oxobutyrate(m) + CoA(m) + NAD+(m) ⇌ Isobutyryl-CoA(m) + CO2(m) + NADH(m)

Continued on next page

Aspartate from Valine (160)– continued

ID	flux	reaction
r1330	10	H+(PG)(c) ⇌ H+(PG)(m) + Proton-gradient(m)
r1322	1	NADH(c) ⇌ NAD+(c) + NADH-redox-potential(c)
r0867	1	CO2(c) ⇌ CO2(s)
r0849	2	H2O(s) ⇌ H2O(c)
r0669	1	Methacrylyl-CoA(m) + H2O(m) → 3-Hydroxyisobutyryl-CoA(m)
r0125	-1	GDP(m) + Pi(m) + Succinyl-CoA(m) ⇌ GTP(m) + Succinate(m) + CoA(m)
r0017	1	O2(m) + 4 H+(PG)(m) + 4 Ferrocytochrome C(m) → 4 Ferricytochrome C(m) + 2 H2O(m) + 4 H+(PG)(c)
r2686	1	Aspartate(c) ⇌ Aspartate(s)
r1323	3	NADH(m) ⇌ NAD+(m) + NADH-redox-potential(m)
r0868	-1	CO2(c) ⇌ CO2(m)

Aspartate from beta-Alanine (161)

Definition			Solution												
			exchanges		reactions								transp		
Objective	Constraints	Comment	imports	exports	c	m	r	p	l	n	s	b	s-c	b-c	int
Aspartate	−beta-Alanine MCES +Urea	Transamination to Aspartate using beta-Alanine	1 Pyruvate(m) 1 beta-Alanine(s) 1 NADPH-redox-potential(m) 2 Proton-gradient(m)	1 Aspartate(s) 2 NADH-redox-potential(m) 1 CoA-activated acetyl group(m)	-	8	-	-	-	-	-	-	2	-	5

ID	flux	reaction
r2678	1	Acetyl-CoA(m) ⇌ CoA(m) + CoA-activated acetyl group(m)
r0217	1	beta-Alanine(m) + AKG(m) ⇌ Glutamate(m) + 3-Oxopropanoate(m)
r0911	1	Proline(c) + Glutamate(m) ⇌ Proline(m) + Glutamate(c)
r0174	1	CoA(m) + NAD+(m) + 3-Oxopropanoate(m) → Acetyl-CoA(m) + CO2(m) + NADH(m)
r1426	1	Proline(m) + H+(PG)(c) → Proline(c) + H+(PG)(m)
r1327	-1	NADPH(m) ⇌ NADPH-redox-potential(m) + NADP+(m)
r0899	-1	beta-Alanine(s) ⇌ beta-Alanine(c)
r0059	-1	Pyruvate(m) + CO2(m) + NADPH(m) ⇌ Malate(m) + NADP+(m)
r0110	-1	OAA(m) + Glutamate(m) → AKG(m) + Aspartate(m)
r0105	1	NAD+(m) + Malate(m) ⇌ OAA(m) + NADH(m)
r1330	-2	H+(PG)(c) ⇌ H+(PG)(m) + Proton-gradient(m)
r0876	1	Glutamate(c) + H+(PG)(c) + Aspartate(m) → Glutamate(m) + H+(PG)(m) + Aspartate(c)
r2686	1	Aspartate(c) ⇌ Aspartate(s)
r1323	2	NADH(m) ⇌ NAD+(m) + NADH-redox-potential(m)
r0900	1	beta-Alanine(c) ⇌ beta-Alanine(m)

Cysteine from Cystine (162)

Definition			Solution												
			exchanges		reactions								transp		
Objective	Constraints	Comment	imports	exports	c	m	r	p	l	n	s	b	s-c	b-c	int
Cysteine	−Cystine MCES +Urea	Transamination to Cysteine using Cystine	0.5 Cystine(s) 0.5 NADPH-redox-potential(c)	1 Cysteine(s) 0.5 Na-gradient(c)	2	-	-	-	-	-	-	-	4	-	-

ID	flux	reaction
r1144	-0.5	Glutamate(c) + Na+(c) ← Glutamate(s) + Na+(s)
r1326	-0.5	NADPH(c) ⇌ NADPH-redox-potential(c) + NADP+(c)
r2664	0.5	Na+(c) ⇌ Na+(s) + Na-gradient(c)
r2529	-1	Cysteine(c) ⇌ Cysteine(s)
r0877	0.5	Glutamate(c) + Cystine(s) ⇌ Glutamate(s) + Cystine(c)
r0027	-0.5	NADPH(c) + Cystine(c) ⇌ 2 Cysteine(c) + NADP+(c)

Cysteine from Methionine (163)

Definition			Solution													
			exchanges		reactions								transp			
Objective	Constraints	Comment	imports	exports	c	m	r	p	l	n	s	b	s-c	b-c	int
Cysteine	−Methionine MCES +Urea	Transamination to Cysteine using Methionine	1.5 H_2O(s) 0.5 O_2(s) 1 Methionine(s) 0.5 Glucose-6P(c)	0.5 P_i(c) 2 CO_2(s) 1 Cysteine(s) 0.5 ATP-energy(c) 2 NADH-redox-potential(c) 2 NADH-redox-potential(m) 3 Proton-gradient(m) 2 Na-gradient(c) 1 THF-activated methyl group(c) 1 CoA-activated acetyl group(m)	15	9	-	-	-	-	-	-	6	-	11

ID	flux	reaction
r0855	0.5	O2(s) ⇌ O2(c)
r0856	-0.5	O2(m) ⇌ O2(c)
r0592	-1	H2O(m) + Acrylyl-CoA(m) → 3-Hydroxypropionyl-CoA(m)
r0596	-1	3-Hydroxypropionyl-CoA(m) + H2O(m) → Hydracrylate(m) + CoA(m)
r0017	0.5	O2(m) + 4 H+(PG)(m) + 4 Ferrocytochrome C(m) → 4 Ferricytochrome C(m) + 2 H2O(m) + 4 H+(PG)(c)
r0314	1	Serine(c) + Homocysteine(c) ⇌ H2O(c) + L-Cystathionine(c)
r0911	1	Proline(c) + Glutamate(m) ⇌ Proline(m) + Glutamate(c)
r0337	-1	ADP(c) + 1,3DPG(c) ⇌ ATP(c) + 3PG(c)
r0338	1	3PG(c) + NAD+(c) → 3-Phosphonooxypyruvate(c) + NADH(c)
r2664	2	Na+(c) ⇌ Na+(s) + Na-gradient(c)
r1426	1	Proline(m) + H+(PG)(c) → Proline(c) + H+(PG)(m)
r0073	-1	AKG(m) + NADH(m) + NH3(m) ⇌ Glutamate(m) + NAD+(m) + H2O(m)
r0507	1	Ubiquinol(m) + 2 Ferricytochrome C(m) + 4 H+(PG)(m) → Ubiquinone(m) + 4 H+(PG)(c) + 2 Ferrocytochrome C(m)
r0241	1	H2O(c) + L-Cystathionine(c) ⇌ Cysteine(c) + NH3(c) + 2-Oxobutyrate(c)
r0243	-0.5	DHAP(c) ⇌ GAP(c)
r1330	3	H+(PG)(c) ⇌ H+(PG)(m) + Proton-gradient(m)
r1319	0.5	ATP(c) + H2O(c) ⇌ ADP(c) + Pi(c) + ATP-energy(c)
r0849	1.5	H2O(s) ⇌ H2O(c)
r0867	2	CO2(c) ⇌ CO2(s)
r0868	-2	CO2(c) ⇌ CO2(m)
r0736	0.5	ATP(c) + Fructose-6P(c) → ADP(c) + Fructose-1,6PP(c)
r0159	1	3-Phosphoserine(c) + H2O(c) → Serine(c) + Pi(c)
r1679	1	Glycine(s) + Cysteine(c) + Na+(s) → Glycine(c) + Cysteine(s) + Na+(c)
r0174	1	CoA(m) + NAD+(m) + 3-Oxopropanoate(m) → Acetyl-CoA(m) + CO2(m) + NADH(m)
r0192	0.5	Glucose-6P(c) ⇌ Fructose-6P(c)
r1154	1	CoA(m) + NAD+(m) + 2-Oxobutyrate(m) ⇌ CO2(m) + Propanoyl-CoA(m) + NADH(m)
r1732	1	Methionine(s) + Glycine(c) + Na+(s) → Methionine(c) + Glycine(s) + Na+(c)
r1155	1	2-Oxobutyrate(c) + H+(PG)(c) → 2-Oxobutyrate(m) + H+(PG)(m)
r2676	1	5-Methyl-THF(c) ⇌ THF(c) + THF-activated methyl group(c)
r0365	1	Hydracrylate(m) + NAD+(m) → NADH(m) + 3-Oxopropanoate(m)
r2678	1	Acetyl-CoA(m) ⇌ CoA(m) + CoA-activated acetyl group(m)
r0663	-1	3-Phosphonooxypyruvate(c) + Glutamate(c) → 3-Phosphoserine(c) + AKG(c)
r0232	-1	THF(c) + Methionine(c) ⇌ 5-Methyl-THF(c) + Homocysteine(c)
r1322	2	NADH(c) ⇌ NAD+(c) + NADH-redox-potential(c)
r0683	1	Propanoyl-CoA(m) + Ubiquinone(m) → Ubiquinol(m) + Acrylyl-CoA(m)
r1323	2	NADH(m) ⇌ NAD+(m) + NADH-redox-potential(m)
r0253	1	GAP(c) + Pi(c) + NAD+(c) ⇌ 1,3DPG(c) + NADH(c)
r0256	0.5	Fructose-1,6PP(c) ⇌ DHAP(c) + GAP(c)
r1047	1	Pi(c) + H+(PG)(c) → Pi(m) + H+(PG)(m)
r2419	1	AKG(c) + Pi(m) ⇌ AKG(m) + Pi(c)
r0838	1	NH3(c) ⇌ NH3(m)

Cystine from Cysteine (164)

Definition			Solution													
			exchanges		reactions								transp		
Objective	Constraints	Comment	imports	exports	c	m	r	p	l	n	s	b	s-c	b-c	int
Cystine	−Cysteine MCES +Urea	Transamination to Cystine using Cysteine	2 Cysteine(s)	1 Cystine(s) 1 NADPH-redox-potential(c)	2	-	-	-	-	-	-	-	3	-	-

ID	flux	reaction
r1326	1	NADPH(c) ⇌ NADPH-redox-potential(c) + NADP+(c)
r2529	2	Cysteine(s) ⇌ Cysteine(c)
r2685	1	Glutamate(c) ⇌ Glutamate(s)
r0877	-1	Glutamate(s) + Cystine(c) ⇌ Glutamate(c) + Cystine(s)
r0027	1	2 Cysteine(c) + NADP+(c) ⇌ NADPH(c) + Cystine(c)

Cystine from Methionine (165)

Definition			Solution													
			exchanges		reactions								transp		
Objective	Constraints	Comment	imports	exports	c	m	r	p	l	n	s	b	s-c	b-c	int
Cystine	−Methionine MCES +Urea	Transamination to Cystine using Methionine	3 H_2O(s) 1 O_2(s) 2 Methionine(s) 1 Glucose-6P(c)	1 P_i(c) 4 CO_2(s) 1 Cystine(s) 1 ATP-energy(c) 4 NADH-redox-potential(c) 4 NADH-redox-potential(m) 1 NADPH-redox-potential(c) 6 Proton-gradient(m) 2 Na-gradient(c) 2 THF-activated methyl group(c) 2 CoA-activated acetyl group(m)	17	9	-	-	-	-	-	-	7	-	11

ID	flux	reaction
r0855	1	O2(s) ⇌ O2(c)
r0856	-1	O2(m) ⇌ O2(c)
r0592	-2	H2O(m) + Acrylyl-CoA(m) → 3-Hydroxypropionyl-CoA(m)
r0877	-1	Glutamate(s) + Cystine(c) ⇌ Glutamate(c) + Cystine(s)
r0596	-2	3-Hydroxypropionyl-CoA(m) + H2O(m) → Hydracrylate(m) + CoA(m)
r0017	1	O2(m) + 4 H+(PG)(m) + 4 Ferrocytochrome C(m) → 4 Ferricytochrome C(m) + 2 H2O(m) + 4 H+(PG)(c)
r0314	2	Serine(c) + Homocysteine(c) ⇌ H2O(c) + L-Cystathionine(c)
r0911	2	Proline(c) + Glutamate(m) ⇌ Proline(m) + Glutamate(c)
r1141	-2	Methionine(s) + Na+(s) → Methionine(c) + Na+(c)
r0337	-2	ADP(c) + 1,3DPG(c) ⇌ ATP(c) + 3PG(c)
r0338	2	3PG(c) + NAD+(c) → 3-Phosphonooxypyruvate(c) + NADH(c)
r2664	2	Na+(c) ⇌ Na+(s) + Na-gradient(c)
r1426	2	Proline(m) + H+(PG)(c) → Proline(c) + H+(PG)(m)
r0073	-2	AKG(m) + NADH(m) + NH3(m) ⇌ Glutamate(m) + NAD+(m) + H2O(m)
r2685	1	Glutamate(c) ⇌ Glutamate(s)
r0507	2	Ubiquinol(m) + 2 Ferricytochrome C(m) + 4 H+(PG)(m) → Ubiquinone(m) + 4 H+(PG)(c) + 2 Ferrocytochrome C(m)
r0241	2	H2O(c) + L-Cystathionine(c) ⇌ Cysteine(c) + NH3(c) + 2-Oxobutyrate(c)
r0243	-1	DHAP(c) ⇌ GAP(c)
r1330	6	H+(PG)(c) ⇌ H+(PG)(m) + Proton-gradient(m)
r1319	1	ATP(c) + H2O(c) ⇌ ADP(c) + Pi(c) + ATP-energy(c)
r0849	3	H2O(s) ⇌ H2O(c)
r0867	4	CO2(c) ⇌ CO2(s)
r0868	-4	CO2(c) ⇌ CO2(m)
r0736	1	ATP(c) + Fructose-6P(c) → ADP(c) + Fructose-1,6PP(c)
r0159	2	3-Phosphoserine(c) + H2O(c) → Serine(c) + Pi(c)
r0174	2	CoA(m) + NAD+(m) + 3-Oxopropanoate(m) → Acetyl-CoA(m) + CO2(m) + NADH(m)

Continued on next page

ID	flux	reaction
r0027	1	2 Cysteine(c) + NADP+(c) ⇌ NADPH(c) + Cystine(c)
r0192	1	Glucose-6P(c) ⇌ Fructose-6P(c)
r1154	2	CoA(m) + NAD+(m) + 2-Oxobutyrate(m) ⇌ CO2(m) + Propanoyl-CoA(m) + NADH(m)
r1155	2	2-Oxobutyrate(c) + H+(PG)(c) → 2-Oxobutyrate(m) + H+(PG)(m)
r2676	2	5-Methyl-THF(c) ⇌ THF(c) + THF-activated methyl group(c)
r0365	2	Hydracrylate(m) + NAD+(m) → NADH(m) + 3-Oxopropanoate(m)
r2678	2	Acetyl-CoA(m) ⇌ CoA(m) + CoA-activated acetyl group(m)
r0663	-2	3-Phosphonooxypyruvate(c) + Glutamate(c) → 3-Phosphoserine(c) + AKG(c)
r0232	-2	THF(c) + Methionine(c) ⇌ 5-Methyl-THF(c) + Homocysteine(c)
r1322	4	NADH(c) ⇌ NAD+(c) + NADH-redox-potential(c)
r0683	2	Propanoyl-CoA(m) + Ubiquinone(m) → Ubiquinol(m) + Acrylyl-CoA(m)
r1323	4	NADH(m) ⇌ NAD+(m) + NADH-redox-potential(m)
r1326	1	NADPH(c) ⇌ NADPH-redox-potential(c) + NADP+(c)
r0253	2	GAP(c) + Pi(c) + NAD+(c) ⇌ 1,3DPG(c) + NADH(c)
r0256	1	Fructose-1,6PP(c) ⇌ DHAP(c) + GAP(c)
r1047	2	Pi(c) + H+(PG)(c) → Pi(m) + H+(PG)(m)
r2419	2	AKG(c) + Pi(m) ⇌ AKG(m) + Pi(c)
r0838	2	NH3(c) ⇌ NH3(m)

Glutamate from Alanine (166)

Definition			Solution												
			exchanges		reactions								transp		
Objective	Constraints	Comment	imports	exports	c	m	r	p	l	n	s	b	s-c	b-c	int
Glutamate	−Alanine MCES +Urea	Transamination to Glutamate using Alanine	1 H_2O(s) 1 Pyruvate(m) 1 Alanine(s) 1 ATP-energy(m) 1 NADPH-redox-potential(m) 1 CoA-activated acetyl group(m)	1 Pyruvate(c) 1 Glutamate(s) 1 ATP-energy(c) 2 NADH-redox-potential(m)	2	10	-	-	-	-	-	-	3	-	2

ID	flux	reaction
r2678	-1	Acetyl-CoA(m) ⇌ CoA(m) + CoA-activated acetyl group(m)
r0316	1	Citrate(m) ⇌ Isocitrate(m)
r0425	1	Isocitrate(m) + NAD+(m) ⇌ Oxalosuccinate(m) + NADH(m)
r0083	1	Oxalosuccinate(m) ⇌ AKG(m) + CO2(m)
r2524	1	Alanine(s) ⇌ Alanine(c)
r1327	-1	NADPH(m) ⇌ NADPH-redox-potential(m) + NADP+(m)
r1319	1	ATP(c) + H2O(c) ⇌ ADP(c) + Pi(c) + ATP-energy(c)
r0059	-1	Pyruvate(m) + CO2(m) + NADPH(m) ⇌ Malate(m) + NADP+(m)
r2418	1	ADP(c) + ATP(m) → ADP(m) + ATP(c)
r2419	-1	AKG(m) + Pi(c) ⇌ AKG(c) + Pi(m)
r1320	-1	ATP(m) + H2O(m) ⇌ ADP(m) + Pi(m) + ATP-energy(m)
r0105	1	NAD+(m) + Malate(m) ⇌ OAA(m) + NADH(m)
r2685	1	Glutamate(c) ⇌ Glutamate(s)
r0849	1	H2O(s) ⇌ H2O(c)
r0107	-1	Acetyl-CoA(m) + H2O(m) + OAA(m) ⇌ Citrate(m) + CoA(m)
r0080	1	Alanine(c) + AKG(c) ⇌ Pyruvate(c) + Glutamate(c)
r1323	2	NADH(m) ⇌ NAD+(m) + NADH-redox-potential(m)

Glutamate from Arginine (167)

Definition			Solution												
			exchanges		reactions								transp		
Objective	Constraints	Comment	imports	exports	c	m	r	p	l	n	s	b	s-c	b-c	int
Glutamate	−Arginine MCES +Urea	Transamination to Glutamate using Arginine	1.5 H_2O(s) 0.5 Pyruvate(m) 0.5 Arginine(s) 0.5 NADPH-redox-potential(m) 1.5 Proton-gradient(m) 0.5 CoA-activated acetyl group(m)	1 Glutamate(s) 0.5 Urea(s) 1.5 NADH-redox-potential(m)	1	11	-	-	-	-	-	-	4	-	6

ID	flux	reaction
r2678	-0.5	Acetyl-CoA(m) ⇌ CoA(m) + CoA-activated acetyl group(m)
r0896	0.5	Urea(c) ⇌ Urea(s)
r0316	0.5	Citrate(m) ⇌ Isocitrate(m)
r0911	1	Proline(c) + Glutamate(m) ⇌ Proline(m) + Glutamate(c)
r0425	0.5	Isocitrate(m) + NAD+(m) ⇌ Oxalosuccinate(m) + NADH(m)
r0083	0.5	Oxalosuccinate(m) ⇌ AKG(m) + CO2(m)
r0074	0.5	L-Glutamate 5-semialdehyde(m) + NAD+(m) + H2O(m) ⇌ Glutamate(m) + NADH(m)
r1426	1	Proline(m) + H+(PG)(c) → Proline(c) + H+(PG)(m)
r1327	-0.5	NADPH(m) ⇌ NADPH-redox-potential(m) + NADP+(m)
r1058	0.5	Arginine(s) ⇌ Arginine(c)
r0851	-1	H2O(m) ⇌ H2O(c)
r0167	0.5	AKG(m) + Ornithine(m) ⇌ L-Glutamate 5-semialdehyde(m) + Glutamate(m)
r0059	-0.5	Pyruvate(m) + CO2(m) + NADPH(m) ⇌ Malate(m) + NADP+(m)
r0105	0.5	NAD+(m) + Malate(m) ⇌ OAA(m) + NADH(m)
r2396	-0.5	Lysine(m) + Ornithine(c) + H+(PG)(c) → Lysine(c) + Ornithine(m) + H+(PG)(m)
r1330	-1.5	H+(PG)(c) ⇌ H+(PG)(m) + Proton-gradient(m)
r2685	1	Glutamate(c) ⇌ Glutamate(s)
r1070	0.5	Lysine(c) ⇌ Lysine(m)
r0849	1.5	H2O(s) ⇌ H2O(c)
r0107	-0.5	Acetyl-CoA(m) + H2O(m) + OAA(m) ⇌ Citrate(m) + CoA(m)
r1323	1.5	NADH(m) ⇌ NAD+(m) + NADH-redox-potential(m)
r0144	0.5	Arginine(c) + H2O(c) ⇌ Urea(c) + Ornithine(c)

Glutamate from Asparagine (168)

Definition			Solution												
			exchanges		reactions								transp		
Objective	Constraints	Comment	imports	exports	c	m	r	p	l	n	s	b	s-c	b-c	int
Glutamate	−Asparagine MCES +Urea	Transamination to Glutamate using Asparagine	1 H_2O(s) 0.5 Pyruvate(m) 0.5 Asparagine(s) 0.5 NADH-redox-potential(c) 1 NADPH-redox-potential(m) 0.5 Proton-gradient(m) 1 CoA-activated acetyl group(m)	0.5 CO_2(s) 1 Glutamate(s) 1.5 NADH-redox-potential(m) 0.5 NADPH-redox-potential(c) 0.5 Proton-gradient(c) 0.5 Na-gradient(c)	7	10	-	-	-	-	-	-	6	-	6

ID	flux	reaction
r2678	-1	Acetyl-CoA(m) ⇌ CoA(m) + CoA-activated acetyl group(m)
r0316	1	Citrate(m) ⇌ Isocitrate(m)
r0127	0.5	H2O(c) + Asparagine(c) → Aspartate(c) + NH3(c)
r0109	0.5	AKG(c) + Aspartate(c) ⇌ OAA(c) + Glutamate(c)
r0082	0.5	Oxalosuccinate(c) ⇌ AKG(c) + CO2(c)
r0911	0.5	Proline(c) + Glutamate(m) ⇌ Proline(m) + Glutamate(c)
r0425	0.5	Isocitrate(m) + NAD+(m) ⇌ Oxalosuccinate(m) + NADH(m)
r0083	0.5	Oxalosuccinate(m) ⇌ AKG(m) + CO2(m)
r1326	0.5	NADPH(c) ⇌ NADPH-redox-potential(c) + NADP+(c)
r0075	-0.5	AKG(m) + NADPH(m) + NH3(m) ⇌ Glutamate(m) + H2O(m) + NADP+(m)
		Continued on next page

ID	flux	reaction
r1426	0.5	Proline(m) + H+(PG)(c) → Proline(c) + H+(PG)(m)
r1327	-1	NADPH(m) ⇌ NADPH-redox-potential(m) + NADP+(m)
r0913	0.5	Isocitrate(m) + Malate(c) ⇌ Isocitrate(c) + Malate(m)
r0851	-0.5	H2O(m) ⇌ H2O(c)
r0059	-0.5	Pyruvate(m) + CO2(m) + NADPH(m) ⇌ Malate(m) + NADP+(m)
r2664	0.5	Na+(c) ⇌ Na+(s) + Na-gradient(c)
r0104	-0.5	OAA(c) + NADH(c) ⇌ NAD+(c) + Malate(c)
r0838	0.5	NH3(c) ⇌ NH3(m)
r0105	1	NAD+(m) + Malate(m) ⇌ OAA(m) + NADH(m)
r1330	-0.5	H+(PG)(c) ⇌ H+(PG)(m) + Proton-gradient(m)
r2685	1	Glutamate(c) ⇌ Glutamate(s)
r1331	0.5	H+(PG)(s) ⇌ H+(PG)(c) + Proton-gradient(c)
r1322	-0.5	NADH(c) ⇌ NAD+(c) + NADH-redox-potential(c)
r0867	0.5	CO2(c) ⇌ CO2(s)
r0849	1	H2O(s) ⇌ H2O(c)
r0422	0.5	Isocitrate(c) + NADP+(c) ⇌ Oxalosuccinate(c) + NADPH(c)
r0107	-1	Acetyl-CoA(m) + H2O(m) + OAA(m) ⇌ Citrate(m) + CoA(m)
r2479	0.5	Asparagine(s) + Na+(s) + H+(PG)(c) → Asparagine(c) + Na+(c) + H+(PG)(s)
r1323	1.5	NADH(m) ⇌ NAD+(m) + NADH-redox-potential(m)

Glutamate from Aspartate (169)

Definition			Solution													
			exchanges		reactions									transp		
Objective	Constraints	Comment	imports	exports	c	m	r	p	l	n	s	b	s-c	b-c	int
Glutamate	−Aspartate MCES +Urea	Transamination to Glutamate using Aspartate	1 H_2O(s) 1 Aspartate(s) 1 NADH-redox-potential(c) 1 CoA-activated acetyl group(m)	1 CO_2(s) 1 Glutamate(s) 1 NADH-redox-potential(m) 1 NADPH-redox-potential(c) 1 Na-gradient(c)	6	5	-	-	-	-	-	-	5	-	2

ID	flux	reaction
r2678	-1	Acetyl-CoA(m) ⇌ CoA(m) + CoA-activated acetyl group(m)
r0316	1	Citrate(m) ⇌ Isocitrate(m)
r0109	1	AKG(c) + Aspartate(c) ⇌ OAA(c) + Glutamate(c)
r0082	1	Oxalosuccinate(c) ⇌ AKG(c) + CO2(c)
r1326	1	NADPH(c) ⇌ NADPH-redox-potential(c) + NADP+(c)
r0913	1	Isocitrate(m) + Malate(c) ⇌ Isocitrate(c) + Malate(m)
r0851	-1	H2O(m) ⇌ H2O(c)
r2664	1	Na+(c) ⇌ Na+(s) + Na-gradient(c)
r0104	-1	OAA(c) + NADH(c) ⇌ NAD+(c) + Malate(c)
r0105	1	NAD+(m) + Malate(m) ⇌ OAA(m) + NADH(m)
r2685	1	Glutamate(c) ⇌ Glutamate(s)
r1322	-1	NADH(c) ⇌ NAD+(c) + NADH-redox-potential(c)
r0867	1	CO2(c) ⇌ CO2(s)
r0849	1	H2O(s) ⇌ H2O(c)
r0422	1	Isocitrate(c) + NADP+(c) ⇌ Oxalosuccinate(c) + NADPH(c)
r0107	-1	Acetyl-CoA(m) + H2O(m) + OAA(m) ⇌ Citrate(m) + CoA(m)
r1323	1	NADH(m) ⇌ NAD+(m) + NADH-redox-potential(m)
r1143	-1	Na+(c) + Aspartate(c) ← Na+(s) + Aspartate(s)

Glutamate from Cysteine (170)

Definition			Solution												
			exchanges		reactions								transp		
Objective	Constraints	Comment	imports	exports	c	m	r	p	l	n	s	b	s-c	b-c	int
Glutamate	−Cysteine MCES +Urea $+H_2S$	Transamination to Glutamate using Cysteine	1 H_2O(s) 1 Pyruvate(m) 1 Cysteine(s) 1 NADPH-redox-potential(m) 1 Proton-gradient(m) 1 CoA-activated acetyl group(m)	1 Pyruvate(c) 1 Glutamate(s) 1 H_2S(s) 1 NADH-redox-potential(m) 1 Na-gradient(c)	1	10	-	-	-	-	-	-	5	-	4

ID	flux	reaction
r2678	-1	Acetyl-CoA(m) ⇌ CoA(m) + CoA-activated acetyl group(m)
r0316	1	Citrate(m) ⇌ Isocitrate(m)
r0073	-1	AKG(m) + NADH(m) + NH3(m) ⇌ Glutamate(m) + NAD+(m) + H2O(m)
r0911	1	Proline(c) + Glutamate(m) ⇌ Proline(m) + Glutamate(c)
r0425	1	Isocitrate(m) + NAD+(m) ⇌ Oxalosuccinate(m) + NADH(m)
r0083	1	Oxalosuccinate(m) ⇌ AKG(m) + CO2(m)
r1426	1	Proline(m) + H+(PG)(c) → Proline(c) + H+(PG)(m)
r1327	-1	NADPH(m) ⇌ NADPH-redox-potential(m) + NADP+(m)
r0940	1	H2S(c) ⇌ H2S(s)
r0193	1	Cysteine(c) + H2O(c) ⇌ Pyruvate(c) + H2S(c) + NH3(c)
r0059	-1	Pyruvate(m) + CO2(m) + NADPH(m) ⇌ Malate(m) + NADP+(m)
r2664	1	Na+(c) ⇌ Na+(s) + Na-gradient(c)
r2475	1	Cysteine(s) + Na+(s) → Cysteine(c) + Na+(c)
r0838	1	NH3(c) ⇌ NH3(m)
r0105	1	NAD+(m) + Malate(m) ⇌ OAA(m) + NADH(m)
r1330	-1	H+(PG)(c) ⇌ H+(PG)(m) + Proton-gradient(m)
r2685	1	Glutamate(c) ⇌ Glutamate(s)
r0849	1	H2O(s) ⇌ H2O(c)
r0107	-1	Acetyl-CoA(m) + H2O(m) + OAA(m) ⇌ Citrate(m) + CoA(m)
r1323	1	NADH(m) ⇌ NAD+(m) + NADH-redox-potential(m)

Glutamate from Cystine (171)

Definition			Solution												
			exchanges		reactions								transp		
Objective	Constraints	Comment	imports	exports	c	m	r	p	l	n	s	b	s-c	b-c	int
Glutamate	−Cystine MCES +Urea $+H_2S$	Transamination to Glutamate using Cystine	1 H_2O(s) 1 Pyruvate(m) 0.5 Cystine(s) 0.5 NADPH-redox-potential(c) 1 NADPH-redox-potential(m) 1 Proton-gradient(m) 1 CoA-activated acetyl group(m)	1 Pyruvate(c) 1 Glutamate(s) 1 H_2S(s) 1 NADH-redox-potential(m)	3	10	-	-	-	-	-	-	4	-	4

ID	flux	reaction
r2678	-1	Acetyl-CoA(m) ⇌ CoA(m) + CoA-activated acetyl group(m)
r0316	1	Citrate(m) ⇌ Isocitrate(m)
r0073	-1	AKG(m) + NADH(m) + NH3(m) ⇌ Glutamate(m) + NAD+(m) + H2O(m)
r0911	1	Proline(c) + Glutamate(m) ⇌ Proline(m) + Glutamate(c)
r0425	1	Isocitrate(m) + NAD+(m) ⇌ Oxalosuccinate(m) + NADH(m)
r0083	1	Oxalosuccinate(m) ⇌ AKG(m) + CO2(m)
r1326	-0.5	NADPH(c) ⇌ NADPH-redox-potential(c) + NADP+(c)
r1426	1	Proline(m) + H+(PG)(c) → Proline(c) + H+(PG)(m)
r1327	-1	NADPH(m) ⇌ NADPH-redox-potential(m) + NADP+(m)
r0940	1	H2S(c) ⇌ H2S(s)
r0193	1	Cysteine(c) + H2O(c) ⇌ Pyruvate(c) + H2S(c) + NH3(c)
r0059	-1	Pyruvate(m) + CO2(m) + NADPH(m) ⇌ Malate(m) + NADP+(m)

Continued on next page

ID	flux	reaction
r0838	1	NH3(c) ⇌ NH3(m)
r0105	1	NAD+(m) + Malate(m) ⇌ OAA(m) + NADH(m)
r1330	-1	H+(PG)(c) ⇌ H+(PG)(m) + Proton-gradient(m)
r2685	0.5	Glutamate(c) ⇌ Glutamate(s)
r0849	1	H2O(s) ⇌ H2O(c)
r0107	-1	Acetyl-CoA(m) + H2O(m) + OAA(m) ⇌ Citrate(m) + CoA(m)
r1323	1	NADH(m) ⇌ NAD+(m) + NADH-redox-potential(m)
r0877	0.5	Glutamate(c) + Cystine(s) ⇌ Glutamate(s) + Cystine(c)
r0027	-0.5	NADPH(c) + Cystine(c) ⇌ 2 Cysteine(c) + NADP+(c)

Glutamate from Glycine (172)

Definition			Solution												
			exchanges		reactions								transp		
Objective	Constraints	Comment	imports	exports	c	m	r	p	l	n	s	b	s-c	b-c	int
Glutamate	−Glycine MCES +Urea	Transamination to Glutamate using Glycine	1 H_2O(s) 1 Pyruvate(m) 1 Glycine(s) 2 NADPH-redox-potential(m) 1 Proton-gradient(m) 1 THF-activated methyl group(c) 1 CoA-activated acetyl group(m)	1 Pyruvate(c) 1 Glutamate(s) 2 NADH-redox-potential(m) 1 NADPH-redox-potential(c) 1 Na-gradient(c)	5	10	-	-	-	-	-	-	4	-	4

ID	flux	reaction
r2678	-1	Acetyl-CoA(m) ⇌ CoA(m) + CoA-activated acetyl group(m)
r0316	1	Citrate(m) ⇌ Isocitrate(m)
r0911	1	Proline(c) + Glutamate(m) ⇌ Proline(m) + Glutamate(c)
r0425	1	Isocitrate(m) + NAD+(m) ⇌ Oxalosuccinate(m) + NADH(m)
r0083	1	Oxalosuccinate(m) ⇌ AKG(m) + CO2(m)
r1326	1	NADPH(c) ⇌ NADPH-redox-potential(c) + NADP+(c)
r0075	-1	AKG(m) + NADPH(m) + NH3(m) ⇌ Glutamate(m) + H2O(m) + NADP+(m)
r1426	1	Proline(m) + H+(PG)(c) → Proline(c) + H+(PG)(m)
r1327	-2	NADPH(m) ⇌ NADPH-redox-potential(m) + NADP+(m)
r1138	1	Glycine(s) + Na+(s) → Glycine(c) + Na+(c)
r0059	-1	Pyruvate(m) + CO2(m) + NADPH(m) ⇌ Malate(m) + NADP+(m)
r0296	1	5-Methyl-THF(c) + NADP+(c) ⇌ 5,10-Methylene-THF(c) + NADPH(c)
r2664	1	Na+(c) ⇌ Na+(s) + Na-gradient(c)
r0230	1	5,10-Methylene-THF(c) + Glycine(c) + H2O(c) ⇌ THF(c) + Serine(c)
r0838	1	NH3(c) ⇌ NH3(m)
r0105	1	NAD+(m) + Malate(m) ⇌ OAA(m) + NADH(m)
r0060	1	Serine(c) → Pyruvate(c) + NH3(c)
r1330	-1	H+(PG)(c) ⇌ H+(PG)(m) + Proton-gradient(m)
r2685	1	Glutamate(c) ⇌ Glutamate(s)
r2676	-1	5-Methyl-THF(c) ⇌ THF(c) + THF-activated methyl group(c)
r0849	1	H2O(s) ⇌ H2O(c)
r0107	-1	Acetyl-CoA(m) + H2O(m) + OAA(m) ⇌ Citrate(m) + CoA(m)
r1323	2	NADH(m) ⇌ NAD+(m) + NADH-redox-potential(m)

Glutamate from Histidine (173)

Definition			Solution												
			exchanges		reactions								transp		
Objective	Constraints	Comment	imports	exports	c	m	r	p	l	n	s	b	s-c	b-c	int
Glutamate	−Histidine MCES +Urea	Transamination to Glutamate using Histidine	0.67 H_2O(s) 0.67 Pyruvate(m) 0.33 Histidine(s) 0.33 NADH-redox-potential(c) 0.33 NADPH-redox-potential(c) 0.67 NADPH-redox-potential(m) 0.67 Proton-gradient(m) 0.67 CoA-activated acetyl group(m)	1 Glutamate(s) 0.67 NADH-redox-potential(m) 0.33 Na-gradient(c) 0.33 THF-activated methyl group(c)	10	10	-	-	-	-	-	-	4	-	4

ID	flux	reaction
r2678	-0.67	Acetyl-CoA(m) ⇌ CoA(m) + CoA-activated acetyl group(m)
r0316	0.67	Citrate(m) ⇌ Isocitrate(m)
r0073	-0.67	AKG(m) + NADH(m) + NH3(m) ⇌ Glutamate(m) + NAD+(m) + H2O(m)
r1145	-0.33	Histidine(s) + Na+(s) → Histidine(c) + Na+(c)
r0911	0.67	Proline(c) + Glutamate(m) ⇌ Proline(m) + Glutamate(c)
r0524	0.33	5-Formiminotetrahydrofolate(c) ⇌ 5,10-Methenyl-THF(c) + NH3(c)
r0425	0.67	Isocitrate(m) + NAD+(m) ⇌ Oxalosuccinate(m) + NADH(m)
r0083	0.67	Oxalosuccinate(m) ⇌ AKG(m) + CO2(m)
r1326	-0.33	NADPH(c) ⇌ NADPH-redox-potential(c) + NADP+(c)
r1426	0.67	Proline(m) + H+(PG)(c) → Proline(c) + H+(PG)(m)
r1327	-0.67	NADPH(m) ⇌ NADPH-redox-potential(m) + NADP+(m)
r0518	-0.33	Glutamate(c) + 5-Formiminotetrahydrofolate(c) ⇌ THF(c) + N-Formimino-L-glutamate(c)
r0293	-0.33	5,10-Methenyl-THF(c) + NADPH(c) ⇌ 5,10-Methylene-THF(c) + NADP+(c)
r0059	-0.67	Pyruvate(m) + CO2(m) + NADPH(m) ⇌ Malate(m) + NADP+(m)
r0519	0.33	H2O(c) + 4-Imidazolone-5-propanoate(c) ⇌ N-Formimino-L-glutamate(c)
r0286	0.33	Histidine(c) ⇌ Urocanate(c) + NH3(c)
r2664	0.33	Na+(c) ⇌ Na+(s) + Na-gradient(c)
r0792	-0.33	5,10-Methylene-THF(c) + NADH(c) ⇌ 5-Methyl-THF(c) + NAD+(c)
r0838	0.67	NH3(c) ⇌ NH3(m)
r0577	-0.33	Urocanate(c) + H2O(c) ⇌ 4-Imidazolone-5-propanoate(c)
r0105	0.67	NAD+(m) + Malate(m) ⇌ OAA(m) + NADH(m)
r1330	-0.67	H+(PG)(c) ⇌ H+(PG)(m) + Proton-gradient(m)
r2685	1	Glutamate(c) ⇌ Glutamate(s)
r2676	0.33	5-Methyl-THF(c) ⇌ THF(c) + THF-activated methyl group(c)
r1322	-0.33	NADH(c) ⇌ NAD+(c) + NADH-redox-potential(c)
r0849	0.67	H2O(s) ⇌ H2O(c)
r0107	-0.67	Acetyl-CoA(m) + H2O(m) + OAA(m) ⇌ Citrate(m) + CoA(m)
r1323	0.67	NADH(m) ⇌ NAD+(m) + NADH-redox-potential(m)

Glutamate from Isoleucine (174)

Definition			Solution												
			exchanges		reactions								transp		
Objective	Constraints	Comment	imports	exports	c	m	r	p	l	n	s	b	s-c	b-c	int
Glutamate	−Isoleucine MCES +Urea	Transamination to Glutamate using Isoleucine	0.5 O_2(s) 1 Pyruvate(m) 1 Isoleucine(s) 1 NADPH-redox-potential(m)	1 Glutamate(s) 3 NADH-redox-potential(m) 4 Proton-gradient(m) 2 CoA-activated acetyl group(m)	-	17	-	-	-	-	-	-	3	-	7

ID	flux	reaction
r2678	2	Acetyl-CoA(m) ⇌ CoA(m) + CoA-activated acetyl group(m)
r0911	1	Proline(c) + Glutamate(m) ⇌ Proline(m) + Glutamate(c)
r0056	1	Pyruvate(m) + CoA(m) + NAD+(m) → Acetyl-CoA(m) + CO2(m) + NADH(m)
r0507	1	Ubiquinol(m) + 2 Ferricytochrome C(m) + 4 H+(PG)(m) → Ubiquinone(m) + 4 H+(PG)(c) + 2 Ferrocytochrome C(m)

Continued on next page

Glutamate from Isoleucine (174)– continued

ID	flux	reaction
r0264	1	Isoleucine(m) + AKG(m) ⇌ Glutamate(m) + 2-Oxo-3-methylvalerate(m)
r1426	1	Proline(m) + H+(PG)(c) → Proline(c) + H+(PG)(m)
r1327	-1	NADPH(m) ⇌ NADPH-redox-potential(m) + NADP+(m)
r0572	-1	Methylmalonyl-CoA(m) ⇌ (R)-Methylmalonyl-CoA(m)
r0059	-1	Pyruvate(m) + CO2(m) + NADPH(m) ⇌ Malate(m) + NADP+(m)
r2536	1	Isoleucine(s) ⇌ Isoleucine(c)
r0664	1	NAD+(m) + (S)-3-Hydroxy-2-methylbutyryl-CoA(m) ⇌ 2-Methylacetoacetyl-CoA(m) + NADH(m)
r0953	1	Isoleucine(c) + H+(PG)(c) → Isoleucine(m) + H+(PG)(m)
r0665	-1	H2O(m) + Tiglyl-CoA(m) ⇌ (S)-3-Hydroxy-2-methylbutyryl-CoA(m)
r0197	1	(R)-Methylmalonyl-CoA(m) ⇌ Succinyl-CoA(m)
r0855	0.5	O2(s) ⇌ O2(c)
r0856	-0.5	O2(m) ⇌ O2(c)
r0222	-1	CoA(m) + 2-Methylacetoacetyl-CoA(m) ⇌ Propanoyl-CoA(m) + Acetyl-CoA(m)
r0105	1	NAD+(m) + Malate(m) ⇌ OAA(m) + NADH(m)
r1330	4	H+(PG)(c) ⇌ H+(PG)(m) + Proton-gradient(m)
r1114	-1	NADH(m) + CO2(m) + Succinyl-CoA(m) ⇌ AKG(m) + CoA(m) + NAD+(m)
r0106	-1	ADP(m) + Pi(m) + OAA(m) ⇌ ATP(m) + Pyruvate(m) + HCO3-(m)
r2685	1	Glutamate(c) ⇌ Glutamate(s)
r0602	1	2-Oxo-3-methylvalerate(m) + CoA(m) + NAD+(m) ⇌ 2-Methylbutyryl-CoA(m) + CO2(m) + NADH(m)
r0017	0.5	O2(m) + 4 H+(PG)(m) + 4 Ferrocytochrome C(m) → 4 Ferricytochrome C(m) + 2 H2O(m) + 4 H+(PG)(c)
r1323	3	NADH(m) ⇌ NAD+(m) + NADH-redox-potential(m)
r0603	1	2-Methylbutyryl-CoA(m) + Ubiquinone(m) ⇌ Tiglyl-CoA(m) + Ubiquinol(m)
r0414	1	ATP(m) + Propanoyl-CoA(m) + HCO3-(m) → ADP(m) + Pi(m) + Methylmalonyl-CoA(m)

Glutamate from Glutamine (175)

Definition			Solution												
			exchanges		reactions								transp		
Objective	Constraints	Comment	imports	exports	c	m	r	p	l	n	s	b	s-c	b-c	int
Glutamate	−Glutamine MCES +Urea	Transamination to Glutamate using Glutamine	0.5 H_2O(s) 0.5 Pyruvate(m) 0.5 Glutamine(s) 1 NADPH-redox-potential(m) 0.5 Proton-gradient(m) 0.5 CoA-activated acetyl group(m)	1 Glutamate(s) 1 NADH-redox-potential(m)	2	10	-	-	-	-	-	-	3	-	4

ID	flux	reaction
r2678	-0.5	Acetyl-CoA(m) ⇌ CoA(m) + CoA-activated acetyl group(m)
r0316	0.5	Citrate(m) ⇌ Isocitrate(m)
r0190	0.5	Glutamine(c) + Fructose-6P(c) → Glutamate(c) + Glucosamine-6P(c)
r0911	0.5	Proline(c) + Glutamate(m) ⇌ Proline(m) + Glutamate(c)
r0425	0.5	Isocitrate(m) + NAD+(m) ⇌ Oxalosuccinate(m) + NADH(m)
r0083	0.5	Oxalosuccinate(m) ⇌ AKG(m) + CO2(m)
r0075	-0.5	AKG(m) + NADPH(m) + NH3(m) ⇌ Glutamate(m) + H2O(m) + NADP+(m)
r1426	0.5	Proline(m) + H+(PG)(c) → Proline(c) + H+(PG)(m)
r1327	-1	NADPH(m) ⇌ NADPH-redox-potential(m) + NADP+(m)
r2525	0.5	Glutamine(s) ⇌ Glutamine(c)
r0059	-0.5	Pyruvate(m) + CO2(m) + NADPH(m) ⇌ Malate(m) + NADP+(m)
r0189	0.5	Glucosamine-6P(c) + H2O(c) ⇌ Fructose-6P(c) + NH3(c)
r0838	0.5	NH3(c) ⇌ NH3(m)
r0105	0.5	NAD+(m) + Malate(m) ⇌ OAA(m) + NADH(m)
r1330	-0.5	H+(PG)(c) ⇌ H+(PG)(m) + Proton-gradient(m)
r2685	1	Glutamate(c) ⇌ Glutamate(s)
r0849	0.5	H2O(s) ⇌ H2O(c)
r0107	-0.5	Acetyl-CoA(m) + H2O(m) + OAA(m) ⇌ Citrate(m) + CoA(m)
r1323	1	NADH(m) ⇌ NAD+(m) + NADH-redox-potential(m)

Glutamate from Leucine (176)

Definition			Solution												
			exchanges		reactions								transp		
Objective	Constraints	Comment	imports	exports	c	m	r	p	l	n	s	b	s-c	b-c	int
Glutamate	−Leucine MCES +Urea	Transamination to Glutamate using Leucine	0.5 O_2(s) 1 Pyruvate(m) 1 Leucine(s) 2 NADPH-redox-potential(m)	1 Glutamate(s) 4 NADH-redox-potential(m) 5 Proton-gradient(m) 1 Na-gradient(c) 2 CoA-activated acetyl group(m)	-	17	-	-	-	-	-	-	4	-	7

ID	flux	reaction
r2678	2	Acetyl-CoA(m) ⇌ CoA(m) + CoA-activated acetyl group(m)
r0316	1	Citrate(m) ⇌ Isocitrate(m)
r0262	1	Leucine(m) + AKG(m) ⇌ Glutamate(m) + 4-Methyl-2-oxopentanoate(m)
r0911	1	Proline(c) + Glutamate(m) ⇌ Proline(m) + Glutamate(c)
r0425	1	Isocitrate(m) + NAD+(m) ⇌ Oxalosuccinate(m) + NADH(m)
r0083	1	Oxalosuccinate(m) ⇌ AKG(m) + CO2(m)
r0507	1	Ubiquinol(m) + 2 Ferricytochrome C(m) + 4 H+(PG)(m) → Ubiquinone(m) + 4 H+(PG)(c) + 2 Ferrocytochrome C(m)
r0462	1	HMG-CoA(m) + CoA(m) ⇌ Acetyl-CoA(m) + H2O(m) + Acetoacetyl-CoA(m)
r1426	1	Proline(m) + H+(PG)(c) → Proline(c) + H+(PG)(m)
r1327	-2	NADPH(m) ⇌ NADPH-redox-potential(m) + NADP+(m)
r0490	-1	3-Methylglutaconyl-CoA(m) + H2O(m) ⇌ HMG-CoA(m)
r0059	-2	Pyruvate(m) + CO2(m) + NADPH(m) ⇌ Malate(m) + NADP+(m)
r1438	1	Leucine(c) → Leucine(m)
r0655	1	Isovaleryl-CoA(m) + Ubiquinone(m) ⇌ 3-Methylcrotonyl-CoA(m) + Ubiquinol(m)
r2664	1	Na+(c) ⇌ Na+(s) + Na-gradient(c)
r0855	0.5	O2(s) ⇌ O2(c)
r1122	-1	Leucine(s) + Na+(s) → Leucine(c) + Na+(c)
r0856	-0.5	O2(m) ⇌ O2(c)
r0658	1	ATP(m) + 3-Methylcrotonyl-CoA(m) + HCO3-(m) ⇌ ADP(m) + Pi(m) + 3-Methylglutaconyl-CoA(m)
r0105	2	NAD+(m) + Malate(m) ⇌ OAA(m) + NADH(m)
r1330	5	H+(PG)(c) ⇌ H+(PG)(m) + Proton-gradient(m)
r0601	1	4-Methyl-2-oxopentanoate(m) + CoA(m) + NAD+(m) ⇌ Isovaleryl-CoA(m) + CO2(m) + NADH(m)
r0106	-1	ADP(m) + Pi(m) + OAA(m) ⇌ ATP(m) + Pyruvate(m) + HCO3-(m)
r0070	-1	CoA(m) + Acetoacetyl-CoA(m) ⇌ 2 Acetyl-CoA(m)
r2685	1	Glutamate(c) ⇌ Glutamate(s)
r0107	-1	Acetyl-CoA(m) + H2O(m) + OAA(m) ⇌ Citrate(m) + CoA(m)
r0017	0.5	O2(m) + 4 H+(PG)(m) + 4 Ferrocytochrome C(m) → 4 Ferricytochrome C(m) + 2 H2O(m) + 4 H+(PG)(c)
r1323	4	NADH(m) ⇌ NAD+(m) + NADH-redox-potential(m)

Glutamate from Lysine (177)

Definition			Solution												
			exchanges		reactions								transp		
Objective	Constraints	Comment	imports	exports	c	m	r	p	l	n	s	b	s-c	b-c	int
Glutamate	−Lysine MCES +Urea	Transamination to Glutamate using Lysine	1.5 H_2O(s) 0.25 O_2(s) 1 Pyruvate(m) 0.5 Lysine(s) 1 NADPH-redox-potential(m)	1 CO_2(s) 1 Glutamate(s) 0.5 NADH-redox-potential(c) 2 NADH-redox-potential(m) 1 NADPH-redox-potential(c) 2 Proton-gradient(m)	6	13	-	-	-	-	-	-	5	-	10

ID	flux	reaction
r0541	0.5	Glutaryl-CoA(m) + Ubiquinone(m) ⇌ Crotonyl-CoA(m) + CO2(m) + Ubiquinol(m)
r0460	0.5	(S)-3-Hydroxybutyryl-CoA(m) + NAD+(m) ⇌ NADH(m) + Acetoacetyl-CoA(m)
r0082	1	Oxalosuccinate(c) ⇌ AKG(c) + CO2(c)
r0911	1	Proline(c) + Glutamate(m) ⇌ Proline(m) + Glutamate(c)

Continued on next page

Glutamate from Lysine (177)– continued

ID	flux	reaction
r1326	1	NADPH(c) ⇌ NADPH-redox-potential(c) + NADP+(c)
r0507	0.5	Ubiquinol(m) + 2 Ferricytochrome C(m) + 4 H+(PG)(m) → Ubiquinone(m) + 4 H+(PG)(c) + 2 Ferrocytochrome C(m)
r1426	1	Proline(m) + H+(PG)(c) → Proline(c) + H+(PG)(m)
r1327	-1	NADPH(m) ⇌ NADPH-redox-potential(m) + NADP+(m)
r0526	0.5	H2O(m) + Saccharopine(m) + NADP+(m) ⇌ Glutamate(m) + 2-Aminoadipate 6-semialdehyde(m) + NADPH(m)
r0851	-1	H2O(m) ⇌ H2O(c)
r0059	-1	Pyruvate(m) + CO2(m) + NADPH(m) ⇌ Malate(m) + NADP+(m)
r2373	1	Citrate(m) + AKG(c) ⇌ Citrate(c) + AKG(m)
r1069	-0.5	Lysine(s) ⇌ Lysine(c)
r0448	0.5	2-Oxoadipate(m) + CoA(m) + NAD+(m) ⇌ Glutaryl-CoA(m) + CO2(m) + NADH(m)
r0855	0.25	O2(s) ⇌ O2(c)
r0594	0.5	2-Aminoadipate 6-semialdehyde(c) + NAD+(c) + H2O(c) ⇌ NADH(c) + L-2-Aminoadipate(c)
r2539	-0.5	2-Aminoadipate 6-semialdehyde(c) + L-2-Aminoadipate(m) ⇌ 2-Aminoadipate 6-semialdehyde(m) + L-2-Aminoadipate(c)
r0856	-0.25	O2(m) ⇌ O2(c)
r0105	1	NAD+(m) + Malate(m) ⇌ OAA(m) + NADH(m)
r1330	2	H+(PG)(c) ⇌ H+(PG)(m) + Proton-gradient(m)
r0070	-0.5	CoA(m) + Acetoacetyl-CoA(m) ⇌ 2 Acetyl-CoA(m)
r2685	1	Glutamate(c) ⇌ Glutamate(s)
r1322	0.5	NADH(c) ⇌ NAD+(c) + NADH-redox-potential(c)
r1070	0.5	Lysine(c) ⇌ Lysine(m)
r0867	1	CO2(c) ⇌ CO2(s)
r0849	1.5	H2O(s) ⇌ H2O(c)
r0588	-0.5	Crotonyl-CoA(m) + H2O(m) ⇌ (S)-3-Hydroxybutyryl-CoA(m)
r0422	1	Isocitrate(c) + NADP+(c) ⇌ Oxalosuccinate(c) + NADPH(c)
r0107	-1	Acetyl-CoA(m) + H2O(m) + OAA(m) ⇌ Citrate(m) + CoA(m)
r0017	0.25	O2(m) + 4 H+(PG)(m) + 4 Ferrocytochrome C(m) → 4 Ferricytochrome C(m) + 2 H2O(m) + 4 H+(PG)(c)
r1323	2	NADH(m) ⇌ NAD+(m) + NADH-redox-potential(m)
r0450	0.5	AKG(m) + L-2-Aminoadipate(m) ⇌ 2-Oxoadipate(m) + Glutamate(m)
r0315	1	Citrate(c) ⇌ Isocitrate(c)
r0180	-0.5	AKG(m) + NADPH(m) + Lysine(m) ⇌ H2O(m) + Saccharopine(m) + NADP+(m)

Glutamate from Methionine (178)

Definition			Solution												
			exchanges		reactions								transp		
Objective	Constraints	Comment	imports	exports	c	m	r	p	l	n	s	b	s-c	b-c	int
Glutamate	−Methionine MCES +Urea +H_2S	Transamination to Glutamate using Methionine	0.5 H_2O(s) 1 Pyruvate(m) 1 Methionine(s) 0.5 Glucose-6P(c) 1 NADPH-redox-potential(m) 4 Proton-gradient(m)	0.5 P_i(c) 1 Pyruvate(c) 1 Glutamate(s) 1 H_2S(s) 0.5 ATP-energy(c) 2 NADH-redox-potential(c) 1 Na-gradient(c) 1 THF-activated methyl group(c) 1 CoA-activated acetyl group(m)	16	12	-	-	-	-	-	-	5	-	8

ID	flux	reaction
r0572	-1	Methylmalonyl-CoA(m) ⇌ (R)-Methylmalonyl-CoA(m)
r0314	1	Serine(c) + Homocysteine(c) ⇌ H2O(c) + L-Cystathionine(c)
r0911	2	Proline(c) + Glutamate(m) ⇌ Proline(m) + Glutamate(c)
r1141	-1	Methionine(s) + Na+(s) → Methionine(c) + Na+(c)
r0337	-1	ADP(c) + 1,3DPG(c) ⇌ ATP(c) + 3PG(c)
r0338	1	3PG(c) + NAD+(c) → 3-Phosphonooxypyruvate(c) + NADH(c)
r0056	1	Pyruvate(m) + CoA(m) + NAD+(m) → Acetyl-CoA(m) + CO2(m) + NADH(m)
r2664	1	Na+(c) ⇌ Na+(s) + Na-gradient(c)
r1426	2	Proline(m) + H+(PG)(c) → Proline(c) + H+(PG)(m)
r0059	-1	Pyruvate(m) + CO2(m) + NADPH(m) ⇌ Malate(m) + NADP+(m)
r0073	-2	AKG(m) + NADH(m) + NH3(m) ⇌ Glutamate(m) + NAD+(m) + H2O(m)

Continued on next page

ID	flux	reaction
r2685	1	Glutamate(c) ⇌ Glutamate(s)
r0241	1	H2O(c) + L-Cystathionine(c) ⇌ Cysteine(c) + NH3(c) + 2-Oxobutyrate(c)
r0243	-0.5	DHAP(c) ⇌ GAP(c)
r1330	-4	H+(PG)(c) ⇌ H+(PG)(m) + Proton-gradient(m)
r1319	0.5	ATP(c) + H2O(c) ⇌ ADP(c) + Pi(c) + ATP-energy(c)
r0414	1	ATP(m) + Propanoyl-CoA(m) + HCO3-(m) → ADP(m) + Pi(m) + Methylmalonyl-CoA(m)
r0849	0.5	H2O(s) ⇌ H2O(c)
r0736	0.5	ATP(c) + Fructose-6P(c) → ADP(c) + Fructose-1,6PP(c)
r0159	1	3-Phosphoserine(c) + H2O(c) → Serine(c) + Pi(c)
r0192	0.5	Glucose-6P(c) ⇌ Fructose-6P(c)
r1114	-1	NADH(m) + CO2(m) + Succinyl-CoA(m) ⇌ AKG(m) + CoA(m) + NAD+(m)
r0193	1	Cysteine(c) + H2O(c) ⇌ Pyruvate(c) + H2S(c) + NH3(c)
r0197	1	(R)-Methylmalonyl-CoA(m) ⇌ Succinyl-CoA(m)
r0940	1	H2S(c) ⇌ H2S(s)
r1154	1	CoA(m) + NAD+(m) + 2-Oxobutyrate(m) ⇌ CO2(m) + Propanoyl-CoA(m) + NADH(m)
r1155	1	2-Oxobutyrate(c) + H+(PG)(c) → 2-Oxobutyrate(m) + H+(PG)(m)
r2676	1	5-Methyl-THF(c) ⇌ THF(c) + THF-activated methyl group(c)
r2678	1	Acetyl-CoA(m) ⇌ CoA(m) + CoA-activated acetyl group(m)
r0663	-1	3-Phosphonooxypyruvate(c) + Glutamate(c) → 3-Phosphoserine(c) + AKG(c)
r0232	-1	THF(c) + Methionine(c) ⇌ 5-Methyl-THF(c) + Homocysteine(c)
r1322	2	NADH(c) ⇌ NAD+(c) + NADH-redox-potential(c)
r0253	1	GAP(c) + Pi(c) + NAD+(c) ⇌ 1,3DPG(c) + NADH(c)
r1327	-1	NADPH(m) ⇌ NADPH-redox-potential(m) + NADP+(m)
r0256	0.5	Fructose-1,6PP(c) ⇌ DHAP(c) + GAP(c)
r1047	1	Pi(c) + H+(PG)(c) → Pi(m) + H+(PG)(m)
r0105	1	NAD+(m) + Malate(m) ⇌ OAA(m) + NADH(m)
r0106	-1	ADP(m) + Pi(m) + OAA(m) ⇌ ATP(m) + Pyruvate(m) + HCO3-(m)
r2419	1	AKG(c) + Pi(m) ⇌ AKG(m) + Pi(c)
r0838	2	NH3(c) ⇌ NH3(m)
r0851	2	H2O(m) ⇌ H2O(c)

Glutamate from Phenylalanine (179)

Definition			Solution												
			exchanges		reactions								transp		
Objective	Constraints	Comment	imports	exports	c	m	r	p	l	n	s	b	s-c	b-c	int
Glutamate	−Phenylalanine MCES +Urea	Transamination to Glutamate using Phenylalanine	1 H_2O(s) 3 O_2(s) 1 Phenylalanine(s) 2 ATP-energy(c) 1 CoA-activated acetyl group(m)	2 CO_2(s) 1 Glutamate(s) 1 NADH-redox-potential(m) 2 CoA-activated acetyl group(c)	17	4	-	-	-	-	-	-	5	-	2

ID	flux	reaction
r2678	-1	Acetyl-CoA(m) ⇌ CoA(m) + CoA-activated acetyl group(m)
r0082	1	Oxalosuccinate(c) ⇌ AKG(c) + CO2(c)
r0605	1	4-Maleylacetoacetate(c) ⇌ Fumarylacetoacetate(c)
r0912	-1	Citrate(m) + Malate(c) ⇌ Citrate(c) + Malate(m)
r0543	1	Homogentisate(c) + O2(c) ⇌ 4-Maleylacetoacetate(c)
r0183	1	Tyrosine(c) + AKG(c) ⇌ 4-Hydroxyphenylpyruvate(c) + Glutamate(c)
r0544	1	4-Hydroxyphenylpyruvate(c) + O2(c) ⇌ Homogentisate(c) + CO2(c)
r1319	-2	ATP(c) + H2O(c) ⇌ ADP(c) + Pi(c) + ATP-energy(c)
r0851	-1	H2O(m) ⇌ H2O(c)
r0258	-1	Fumarate(c) + H2O(c) ⇌ Malate(c)
r0069	-1	CoA(c) + Acetoacetyl-CoA(c) ⇌ 2 Acetyl-CoA(c)
r0030	1	ATP(c) + AMP(c) ⇌ 2 ADP(c)
r2528	1	Phenylalanine(s) ⇌ Phenylalanine(c)
r0855	3	O2(s) ⇌ O2(c)

Continued on next page

ID	flux	reaction
r0320	1	ATP(c) + CoA(c) + Acetoacetate(c) → AMP(c) + PPi(c) + Acetoacetyl-CoA(c)
r0105	1	NAD+(m) + Malate(m) ⇌ OAA(m) + NADH(m)
r0398	1	Dihydrobiopterin(c) + NADPH(c) ⇌ Tetrahydrobiopterin(c) + NADP+(c)
r0007	1	PPi(c) + H2O(c) → 2 Pi(c)
r2685	1	Glutamate(c) ⇌ Glutamate(s)
r0867	2	CO2(c) ⇌ CO2(s)
r0849	1	H2O(s) ⇌ H2O(c)
r0422	1	Isocitrate(c) + NADP+(c) ⇌ Oxalosuccinate(c) + NADPH(c)
r0399	1	Tetrahydrobiopterin(c) + Phenylalanine(c) + O2(c) ⇌ Dihydrobiopterin(c) + Tyrosine(c) + H2O(c)
r0107	-1	Acetyl-CoA(m) + H2O(m) + OAA(m) ⇌ Citrate(m) + CoA(m)
r2677	2	Acetyl-CoA(c) ⇌ CoA(c) + CoA-activated acetyl group(c)
r1323	1	NADH(m) ⇌ NAD+(m) + NADH-redox-potential(m)
r0324	-1	Fumarylacetoacetate(c) + H2O(c) ⇌ Fumarate(c) + Acetoacetate(c)
r0315	1	Citrate(c) ⇌ Isocitrate(c)

Glutamate from Proline (180)

Definition			Solution												
			exchanges		reactions								transp		
Objective	Constraints	Comment	imports	exports	c	m	r	p	l	n	s	b	s-c	b-c	int
Glutamate	−Proline MCES +Urea	Transamination to Glutamate using Proline	2 H_2O(s) 1 Proline(s)	1 Glutamate(s) 4 Proton-gradient(m)	-	3	-	-	-	-	-	-	3	-	4

ID	flux	reaction
r2531	1	Proline(s) ⇌ Proline(c)
r0911	1	Proline(c) + Glutamate(m) ⇌ Proline(m) + Glutamate(c)
r0074	1	L-Glutamate 5-semialdehyde(m) + NAD+(m) + H2O(m) ⇌ Glutamate(m) + NADH(m)
r1453	1	Proline(m) + Ubiquinol(m) ⇌ 1-Pyrroline-5-carboxylate(m) + Ubiquinone(m)
r0508	-1	Ubiquinone(m) + NADH(m) + 4 H+(PG)(m) → Ubiquinol(m) + NAD+(m) + 4 H+(PG)(c)
r0175	1	1-Pyrroline-5-carboxylate(m) + H2O(m) ⇌ L-Glutamate 5-semialdehyde(m)
r0851	-2	H2O(m) ⇌ H2O(c)
r1330	4	H+(PG)(c) ⇌ H+(PG)(m) + Proton-gradient(m)
r2685	1	Glutamate(c) ⇌ Glutamate(s)
r0849	2	H2O(s) ⇌ H2O(c)

Glutamate from Serine (181)

Definition			Solution												
			exchanges		reactions								transp		
Objective	Constraints	Comment	imports	exports	c	m	r	p	l	n	s	b	s-c	b-c	int
Glutamate	−Serine MCES +Urea	Transamination to Glutamate using Serine	1 Pyruvate(m) 1 Serine(s) 2 NADPH-redox-potential(m) 1 Proton-gradient(m) 1 CoA-activated acetyl group(m)	1 Pyruvate(c) 1 Glutamate(s) 2 NADH-redox-potential(m) 1 Proton-gradient(c) 1 Na-gradient(c)	1	10	-	-	-	-	-	-	4	-	4

ID	flux	reaction
r2678	-1	Acetyl-CoA(m) ⇌ CoA(m) + CoA-activated acetyl group(m)
r0316	1	Citrate(m) ⇌ Isocitrate(m)
r0911	1	Proline(c) + Glutamate(m) ⇌ Proline(m) + Glutamate(c)
r0425	1	Isocitrate(m) + NAD+(m) ⇌ Oxalosuccinate(m) + NADH(m)
r0083	1	Oxalosuccinate(m) ⇌ AKG(m) + CO2(m)
r0075	-1	AKG(m) + NADPH(m) + NH3(m) ⇌ Glutamate(m) + H2O(m) + NADP+(m)

Continued on next page

Glutamate from Serine (181)– continued

ID	flux	reaction
r1426	1	Proline(m) + H+(PG)(c) → Proline(c) + H+(PG)(m)
r1327	-2	NADPH(m) ⇌ NADPH-redox-potential(m) + NADP+(m)
r0059	-1	Pyruvate(m) + CO2(m) + NADPH(m) ⇌ Malate(m) + NADP+(m)
r2664	1	Na+(c) ⇌ Na+(s) + Na-gradient(c)
r0838	1	NH3(c) ⇌ NH3(m)
r0105	1	NAD+(m) + Malate(m) ⇌ OAA(m) + NADH(m)
r0060	1	Serine(c) → Pyruvate(c) + NH3(c)
r1330	-1	H+(PG)(c) ⇌ H+(PG)(m) + Proton-gradient(m)
r2685	1	Glutamate(c) ⇌ Glutamate(s)
r2478	1	Serine(s) + Na+(s) + H+(PG)(c) → Serine(c) + Na+(c) + H+(PG)(s)
r1331	1	H+(PG)(s) ⇌ H+(PG)(c) + Proton-gradient(c)
r0107	-1	Acetyl-CoA(m) + H2O(m) + OAA(m) ⇌ Citrate(m) + CoA(m)
r1323	2	NADH(m) ⇌ NAD+(m) + NADH-redox-potential(m)

Glutamate from Threonine (182)

Definition			Solution												
			exchanges		reactions								transp		
Objective	Constraints	Comment	imports	exports	c	m	r	p	l	n	s	b	s-c	b-c	int
Glutamate	−Threonine MCES +Urea	Transamination to Glutamate using Threonine	0.17 O_2(s) 0.33 Pyruvate(m) 1 Threonine(s) 1 NADPH-redox-potential(m)	0.33 H_2O(s) 1 Glutamate(s) 1.33 NADH-redox-potential(m)	1	20	-	-	-	-	-	-	4	-	8

ID	flux	reaction
r0316	0.33	Citrate(m) ⇌ Isocitrate(m)
r0073	-1	AKG(m) + NADH(m) + NH3(m) ⇌ Glutamate(m) + NAD+(m) + H2O(m)
r1154	1	CoA(m) + NAD+(m) + 2-Oxobutyrate(m) ⇌ CO2(m) + Propanoyl-CoA(m) + NADH(m)
r0911	1	Proline(c) + Glutamate(m) ⇌ Proline(m) + Glutamate(c)
r0425	0.33	Isocitrate(m) + NAD+(m) ⇌ Oxalosuccinate(m) + NADH(m)
r0083	0.33	Oxalosuccinate(m) ⇌ AKG(m) + CO2(m)
r1155	1	2-Oxobutyrate(c) + H+(PG)(c) → 2-Oxobutyrate(m) + H+(PG)(m)
r0507	0.33	Ubiquinol(m) + 2 Ferricytochrome C(m) + 4 H+(PG)(m) → Ubiquinone(m) + 4 H+(PG)(c) + 2 Ferrocytochrome C(m)
r0174	0.33	CoA(m) + NAD+(m) + 3-Oxopropanoate(m) → Acetyl-CoA(m) + CO2(m) + NADH(m)
r1426	1	Proline(m) + H+(PG)(c) → Proline(c) + H+(PG)(m)
r1327	-1	NADPH(m) ⇌ NADPH-redox-potential(m) + NADP+(m)
r2534	1	Threonine(s) ⇌ Threonine(c)
r0851	0.33	H2O(m) ⇌ H2O(c)
r0572	-0.67	Methylmalonyl-CoA(m) ⇌ (R)-Methylmalonyl-CoA(m)
r0365	0.33	Hydracrylate(m) + NAD+(m) → NADH(m) + 3-Oxopropanoate(m)
r0059	-1	Pyruvate(m) + CO2(m) + NADPH(m) ⇌ Malate(m) + NADP+(m)
r0592	-0.33	H2O(m) + Acrylyl-CoA(m) → 3-Hydroxypropionyl-CoA(m)
r0683	0.33	Propanoyl-CoA(m) + Ubiquinone(m) → Ubiquinol(m) + Acrylyl-CoA(m)
r0197	0.67	(R)-Methylmalonyl-CoA(m) ⇌ Succinyl-CoA(m)
r0855	0.17	O2(s) ⇌ O2(c)
r0856	-0.17	O2(m) ⇌ O2(c)
r0838	1	NH3(c) ⇌ NH3(m)
r0240	1	Threonine(c) ⇌ 2-Oxobutyrate(c) + NH3(c)
r0105	1	NAD+(m) + Malate(m) ⇌ OAA(m) + NADH(m)
r1114	-0.67	NADH(m) + CO2(m) + Succinyl-CoA(m) ⇌ AKG(m) + CoA(m) + NAD+(m)
r0596	-0.33	3-Hydroxypropionyl-CoA(m) + H2O(m) → Hydracrylate(m) + CoA(m)
r0106	-0.67	ADP(m) + Pi(m) + OAA(m) ⇌ ATP(m) + Pyruvate(m) + HCO3-(m)
r2685	1	Glutamate(c) ⇌ Glutamate(s)
r0849	-0.33	H2O(s) ⇌ H2O(c)
r0107	-0.33	Acetyl-CoA(m) + H2O(m) + OAA(m) ⇌ Citrate(m) + CoA(m)
r0017	0.17	O2(m) + 4 H+(PG)(m) + 4 Ferrocytochrome C(m) → 4 Ferricytochrome C(m) + 2 H2O(m) + 4 H+(PG)(c)

Continued on next page

ID	flux	reaction
r1323	1.33	NADH(m) ⇌ NAD+(m) + NADH-redox-potential(m)
r0414	0.67	ATP(m) + Propanoyl-CoA(m) + HCO3-(m) → ADP(m) + Pi(m) + Methylmalonyl-CoA(m)

Glutamate from Tryptophan (183)

Definition			Solution												
			exchanges		reactions								transp		
Objective	Constraints	Comment	imports	exports	c	m	r	p	l	n	s	b	s-c	b-c	int
Glutamate	−Tryptophan MCES +Urea	Transamination to Glutamate using Tryptophan	1 H_2O(s) 1.75 O_2(s) 1 Pyruvate(m) 0.5 Tryptophan(s) 0.5 ATP-energy(c) 0.5 NADPH-redox-potential(c) 1.5 NADPH-redox-potential(m)	1.5 CO_2(s) 0.5 Pyruvate(c) 1 Glutamate(s) 2 NADH-redox-potential(m) 2.5 Proton-gradient(m) 0.5 THF-activated methyl group(c)	19	11	-	-	-	-	-	-	5	-	10

ID	flux	reaction
r0855	1.75	O2(s) ⇌ O2(c)
r0422	1	Isocitrate(c) + NADP+(c) ⇌ Oxalosuccinate(c) + NADPH(c)
r0856	-0.25	O2(m) ⇌ O2(c)
r0293	-0.5	5,10-Methylene-THF(c) + NADP+(c) ⇌ 5,10-Methenyl-THF(c) + NADPH(c)
r0460	0.5	(S)-3-Hydroxybutyryl-CoA(m) + NAD+(m) ⇌ NADH(m) + Acetoacetyl-CoA(m)
r0448	0.5	2-Oxoadipate(m) + CoA(m) + NAD+(m) ⇌ Glutaryl-CoA(m) + CO2(m) + NADH(m)
r0449	-0.5	2-Oxoadipate(c) + NH3(c) + NADP+(c) ⇌ 2-Aminomuconate(c) + NADPH(c) + H2O(c)
r0017	0.25	O2(m) + 4 H+(PG)(m) + 4 Ferrocytochrome C(m) → 4 Ferricytochrome C(m) + 2 H2O(m) + 4 H+(PG)(c)
r0169	0.5	Tryptophan(c) + O2(c) ⇌ L-Formylkynurenine(c)
r0315	1	Citrate(c) ⇌ Isocitrate(c)
r0911	0.5	Proline(c) + Glutamate(m) ⇌ Proline(m) + Glutamate(c)
r0070	-0.5	CoA(m) + Acetoacetyl-CoA(m) ⇌ 2 Acetyl-CoA(m)
r1426	0.5	Proline(m) + H+(PG)(c) → Proline(c) + H+(PG)(m)
r0059	-1	Pyruvate(m) + CO2(m) + NADPH(m) ⇌ Malate(m) + NADP+(m)
r0075	-0.5	AKG(m) + NADPH(m) + NH3(m) ⇌ Glutamate(m) + H2O(m) + NADP+(m)
r0371	-0.5	10-Formyl-THF(c) ⇌ 5,10-Methenyl-THF(c) + H2O(c)
r2685	1	Glutamate(c) ⇌ Glutamate(s)
r0507	0.5	Ubiquinol(m) + 2 Ferricytochrome C(m) + 4 H+(PG)(m) → Ubiquinone(m) + 4 H+(PG)(c) + 2 Ferrocytochrome C(m)
r0228	0.5	THF(c) + Formate(c) + ATP(c) → ADP(c) + Pi(c) + 10-Formyl-THF(c)
r0676	0.5	2-Amino-3-carboxymuconate semialdehyde(c) ⇌ 2-Aminomuconate semialdehyde(c) + CO2(c)
r1330	2.5	H+(PG)(c) ⇌ H+(PG)(m) + Proton-gradient(m)
r1319	-0.5	ATP(c) + H2O(c) ⇌ ADP(c) + Pi(c) + ATP-energy(c)
r0541	0.5	Glutaryl-CoA(m) + Ubiquinone(m) ⇌ Crotonyl-CoA(m) + CO2(m) + Ubiquinol(m)
r0562	0.5	3-Hydroxyanthranilate(c) + O2(c) ⇌ 2-Amino-3-carboxymuconate semialdehyde(c)
r0563	0.5	H2O(c) + 3-Hydroxy-L-kynurenine(c) ⇌ 3-Hydroxyanthranilate(c) + Alanine(c)
r0849	1	H2O(s) ⇌ H2O(c)
r0867	1.5	CO2(c) ⇌ CO2(s)
r0588	-0.5	Crotonyl-CoA(m) + H2O(m) ⇌ (S)-3-Hydroxybutyryl-CoA(m)
r0454	0.5	L-Formylkynurenine(c) + H2O(c) ⇌ Formate(c) + Kynurenine(c)
r0455	0.5	Kynurenine(c) + O2(c) + NADPH(c) ⇌ H2O(c) + 3-Hydroxy-L-kynurenine(c) + NADP+(c)
r2373	1	Citrate(m) + AKG(c) ⇌ Citrate(c) + AKG(m)
r0792	-0.5	5,10-Methylene-THF(c) + NADH(c) ⇌ 5-Methyl-THF(c) + NAD+(c)
r0080	0.5	Alanine(c) + AKG(c) ⇌ Pyruvate(c) + Glutamate(c)
r0645	0.5	2-Aminomuconate semialdehyde(c) + NAD+(c) + H2O(c) ⇌ 2-Aminomuconate(c) + NADH(c)
r0082	1	Oxalosuccinate(c) ⇌ AKG(c) + CO2(c)
r2676	0.5	5-Methyl-THF(c) ⇌ THF(c) + THF-activated methyl group(c)
r2527	0.5	Tryptophan(s) ⇌ Tryptophan(c)
r0945	0.5	2-Oxoadipate(c) + AKG(m) ⇌ 2-Oxoadipate(m) + AKG(c)
r1323	2	NADH(m) ⇌ NAD+(m) + NADH-redox-potential(m)
r1326	-0.5	NADPH(c) ⇌ NADPH-redox-potential(c) + NADP+(c)

Continued on next page

ID	flux	reaction
r1327	-1.5	NADPH(m) ⇌ NADPH-redox-potential(m) + NADP+(m)
r0105	1	NAD+(m) + Malate(m) ⇌ OAA(m) + NADH(m)
r0107	-1	Acetyl-CoA(m) + H2O(m) + OAA(m) ⇌ Citrate(m) + CoA(m)
r0838	0.5	NH3(c) ⇌ NH3(m)
r0851	-0.5	H2O(m) ⇌ H2O(c)

Glutamate from Tyrosine (184)

Definition			Solution												
			exchanges		reactions								transp		
Objective	Constraints	Comment	imports	exports	c	m	r	p	l	n	s	b	s-c	b-c	int
Glutamate	−Tyrosine MCES +Urea	Transamination to Glutamate using Tyrosine	2 H_2O(s) 2 O_2(s) 1 Tyrosine(s) 2 ATP-energy(c) 1 CoA-activated acetyl group(m)	2 CO_2(s) 1 Glutamate(s) 1 NADH-redox-potential(m) 1 NADPH-redox-potential(c) 1 Na-gradient(c) 2 CoA-activated acetyl group(c)	16	4	-	-	-	-	-	-	6	-	2

ID	flux	reaction
r2678	-1	Acetyl-CoA(m) ⇌ CoA(m) + CoA-activated acetyl group(m)
r0082	1	Oxalosuccinate(c) ⇌ AKG(c) + CO2(c)
r0605	1	4-Maleylacetoacetate(c) ⇌ Fumarylacetoacetate(c)
r1326	1	NADPH(c) ⇌ NADPH-redox-potential(c) + NADP+(c)
r0912	-1	Citrate(m) + Malate(c) ⇌ Citrate(c) + Malate(m)
r0543	1	Homogentisate(c) + O2(c) ⇌ 4-Maleylacetoacetate(c)
r0183	1	Tyrosine(c) + AKG(c) ⇌ 4-Hydroxyphenylpyruvate(c) + Glutamate(c)
r0544	1	4-Hydroxyphenylpyruvate(c) + O2(c) ⇌ Homogentisate(c) + CO2(c)
r1319	-2	ATP(c) + H2O(c) ⇌ ADP(c) + Pi(c) + ATP-energy(c)
r0851	-1	H2O(m) ⇌ H2O(c)
r0258	-1	Fumarate(c) + H2O(c) ⇌ Malate(c)
r0069	-1	CoA(c) + Acetoacetyl-CoA(c) ⇌ 2 Acetyl-CoA(c)
r0030	1	ATP(c) + AMP(c) ⇌ 2 ADP(c)
r2664	1	Na+(c) ⇌ Na+(s) + Na-gradient(c)
r0855	2	O2(s) ⇌ O2(c)
r0320	1	ATP(c) + CoA(c) + Acetoacetate(c) → AMP(c) + PPi(c) + Acetoacetyl-CoA(c)
r0105	1	NAD+(m) + Malate(m) ⇌ OAA(m) + NADH(m)
r0007	1	PPi(c) + H2O(c) → 2 Pi(c)
r2685	1	Glutamate(c) ⇌ Glutamate(s)
r0867	2	CO2(c) ⇌ CO2(s)
r0849	2	H2O(s) ⇌ H2O(c)
r0422	1	Isocitrate(c) + NADP+(c) ⇌ Oxalosuccinate(c) + NADPH(c)
r0107	-1	Acetyl-CoA(m) + H2O(m) + OAA(m) ⇌ Citrate(m) + CoA(m)
r2677	2	Acetyl-CoA(c) ⇌ CoA(c) + CoA-activated acetyl group(c)
r1323	1	NADH(m) ⇌ NAD+(m) + NADH-redox-potential(m)
r1125	1	Tyrosine(s) + Na+(s) → Tyrosine(c) + Na+(c)
r0324	-1	Fumarylacetoacetate(c) + H2O(c) ⇌ Fumarate(c) + Acetoacetate(c)
r0315	1	Citrate(c) ⇌ Isocitrate(c)

Glutamate from Valine (185)

Definition			Solution												
			exchanges		reactions								transp		
Objective	Constraints	Comment	imports	exports	c	m	r	p	l	n	s	b	s-c	b-c	int
Glutamate	−Valine MCES +Urea	Transamination to Glutamate using Valine	1 H_2O(s) 0.5 O_2(s) 1 Valine(s)	1 Glutamate(s) 2 NADH-redox-potential(m) 4 Proton-gradient(m) 1 Na-gradient(c)	-	10	-	-	-	-	-	-	5	-	8

ID	flux	reaction
r0779	1	3-Hydroxyisobutyryl-CoA(m) + H2O(m) → 3-Hydroxyisobutyrate(m) + CoA(m)
r0911	1	Proline(c) + Glutamate(m) ⇌ Proline(m) + Glutamate(c)
r0560	1	Isobutyryl-CoA(m) + Ubiquinone(m) → Methacrylyl-CoA(m) + Ubiquinol(m)
r0263	1	Valine(m) + AKG(m) → Glutamate(m) + 3-Methyl-2-oxobutyrate(m)
r0624	1	2-Methyl-3-oxopropanoate(m) + CoA(m) + NAD+(m) ⇌ (R)-Methylmalonyl-CoA(m) + NADH(m)
r0507	1	Ubiquinol(m) + 2 Ferricytochrome C(m) + 4 H+(PG)(m) → Ubiquinone(m) + 4 H+(PG)(c) + 2 Ferrocytochrome C(m)
r1426	1	Proline(m) + H+(PG)(c) → Proline(c) + H+(PG)(m)
r0923	1	Valine(c) + H+(PG)(c) → Valine(m) + H+(PG)(m)
r0851	-1	H2O(m) ⇌ H2O(c)
r0482	1	3-Hydroxyisobutyrate(m) + NAD+(m) → 2-Methyl-3-oxopropanoate(m) + NADH(m)
r0197	1	(R)-Methylmalonyl-CoA(m) ⇌ Succinyl-CoA(m)
r2664	1	Na+(c) ⇌ Na+(s) + Na-gradient(c)
r1121	-1	Valine(s) + Na+(s) → Valine(c) + Na+(c)
r0855	0.5	O2(s) ⇌ O2(c)
r0856	-0.5	O2(m) ⇌ O2(c)
r0600	1	3-Methyl-2-oxobutyrate(m) + CoA(m) + NAD+(m) ⇌ Isobutyryl-CoA(m) + CO2(m) + NADH(m)
r1330	4	H+(PG)(c) ⇌ H+(PG)(m) + Proton-gradient(m)
r1114	-1	NADH(m) + CO2(m) + Succinyl-CoA(m) ⇌ AKG(m) + CoA(m) + NAD+(m)
r2685	1	Glutamate(c) ⇌ Glutamate(s)
r0849	1	H2O(s) ⇌ H2O(c)
r0669	1	Methacrylyl-CoA(m) + H2O(m) → 3-Hydroxyisobutyryl-CoA(m)
r0017	0.5	O2(m) + 4 H+(PG)(m) + 4 Ferrocytochrome C(m) → 4 Ferricytochrome C(m) + 2 H2O(m) + 4 H+(PG)(c)
r1323	2	NADH(m) ⇌ NAD+(m) + NADH-redox-potential(m)

Glutamate from beta-Alanine (186)

Definition			Solution												
			exchanges		reactions								transp		
Objective	Constraints	Comment	imports	exports	c	m	r	p	l	n	s	b	s-c	b-c	int
Glutamate	−beta-Alanine MCES +Urea	Transamination to Glutamate using beta-Alanine	1 H_2O(s) 1 Pyruvate(m) 1 beta-Alanine(s) 1 NADPH-redox-potential(m) 1 Proton-gradient(m)	1 CO_2(s) 1 Glutamate(s) 2 NADH-redox-potential(m) 1 NADPH-redox-potential(c)	3	8	-	-	-	-	-	-	4	-	6

ID	flux	reaction
r0316	1	Citrate(m) ⇌ Isocitrate(m)
r0217	1	beta-Alanine(m) + AKG(m) ⇌ Glutamate(m) + 3-Oxopropanoate(m)
r0082	1	Oxalosuccinate(c) ⇌ AKG(c) + CO2(c)
r0911	1	Proline(c) + Glutamate(m) ⇌ Proline(m) + Glutamate(c)
r1326	1	NADPH(c) ⇌ NADPH-redox-potential(c) + NADP+(c)
r0174	1	CoA(m) + NAD+(m) + 3-Oxopropanoate(m) → Acetyl-CoA(m) + CO2(m) + NADH(m)
r1426	1	Proline(m) + H+(PG)(c) → Proline(c) + H+(PG)(m)
r1327	-1	NADPH(m) ⇌ NADPH-redox-potential(m) + NADP+(m)
r1147	1	AKG(c) + Isocitrate(m) ⇌ AKG(m) + Isocitrate(c)
r0899	-1	beta-Alanine(s) ⇌ beta-Alanine(c)
r0851	-1	H2O(m) ⇌ H2O(c)
r0059	-1	Pyruvate(m) + CO2(m) + NADPH(m) ⇌ Malate(m) + NADP+(m)
r0105	1	NAD+(m) + Malate(m) ⇌ OAA(m) + NADH(m)
r1330	-1	H+(PG)(c) ⇌ H+(PG)(m) + Proton-gradient(m)
r2685	1	Glutamate(c) ⇌ Glutamate(s)
r0867	1	CO2(c) ⇌ CO2(s)
r0849	1	H2O(s) ⇌ H2O(c)
r0422	1	Isocitrate(c) + NADP+(c) ⇌ Oxalosuccinate(c) + NADPH(c)
r0107	-1	Acetyl-CoA(m) + H2O(m) + OAA(m) ⇌ Citrate(m) + CoA(m)
r1323	2	NADH(m) ⇌ NAD+(m) + NADH-redox-potential(m)
r0900	1	beta-Alanine(c) ⇌ beta-Alanine(m)

Glycine from Alanine (187)

Definition			Solution												
			exchanges		reactions								transp		
Objective	Constraints	Comment	imports	exports	c	m	r	p	l	n	s	b	s-c	b-c	int
Glycine	−Alanine MCES +Urea	Transamination to Glycine using Alanine	0.5 H_2O(s) 1 Alanine(s) 0.5 Glucose-6P(c)	0.5 P_i(c) 1 Pyruvate(c) 1 Glycine(s) 0.5 ATP-energy(c) 1 NADH-redox-potential(c) 1 Na-gradient(c) 1 THF-activated methyl group(c)	15	-	-	-	-	-	-	-	3	-	-

ID	flux	reaction
r0253	1	GAP(c) + Pi(c) + NAD+(c) ⇌ 1,3DPG(c) + NADH(c)
r0192	0.5	Glucose-6P(c) ⇌ Fructose-6P(c)
r0337	-1	ADP(c) + 1,3DPG(c) ⇌ ATP(c) + 3PG(c)
r0256	0.5	Fructose-1,6PP(c) ⇌ DHAP(c) + GAP(c)
r1319	0.5	ATP(c) + H2O(c) ⇌ ADP(c) + Pi(c) + ATP-energy(c)
r0338	1	3PG(c) + NAD+(c) → 3-Phosphonooxypyruvate(c) + NADH(c)
r0663	-1	3-Phosphonooxypyruvate(c) + Glutamate(c) → 3-Phosphoserine(c) + AKG(c)
r0159	1	3-Phosphoserine(c) + H2O(c) → Serine(c) + Pi(c)
r0736	0.5	ATP(c) + Fructose-6P(c) → ADP(c) + Fructose-1,6PP(c)
r2664	1	Na+(c) ⇌ Na+(s) + Na-gradient(c)
r0792	-1	5,10-Methylene-THF(c) + NADH(c) ⇌ 5-Methyl-THF(c) + NAD+(c)
r0230	-1	THF(c) + Serine(c) ⇌ 5,10-Methylene-THF(c) + Glycine(c) + H2O(c)
r2676	1	5-Methyl-THF(c) ⇌ THF(c) + THF-activated methyl group(c)
r1322	1	NADH(c) ⇌ NAD+(c) + NADH-redox-potential(c)
r0849	0.5	H2O(s) ⇌ H2O(c)
r0080	1	Alanine(c) + AKG(c) ⇌ Pyruvate(c) + Glutamate(c)
r1687	1	Alanine(s) + Glycine(c) + Na+(s) → Alanine(c) + Glycine(s) + Na+(c)
r0243	-0.5	DHAP(c) ⇌ GAP(c)

Glycine from Arginine (188)

Definition			Solution												
			exchanges		reactions								transp		
Objective	Constraints	Comment	imports	exports	c	m	r	p	l	n	s	b	s-c	b-c	int
Glycine	−Arginine MCES +Urea	Transamination to Glycine using Arginine	2 H_2O(s) 0.25 O_2(s) 0.5 Arginine(s) 0.5 Glucose-6P(c)	0.5 P_i(c) 1 CO_2(s) 0.5 Pyruvate(c) 1 Glycine(s) 0.5 Urea(s) 0.5 ATP-energy(c) 0.5 ATP-energy(m) 1 NADH-redox-potential(c) 1 NADH-redox-potential(m) 0.5 NADPH-redox-potential(c) 1 Proton-gradient(m) 1 THF-activated methyl group(c)	17	9	-	-	-	-	-	-	6	-	13

ID	flux	reaction
r0125	-0.5	GDP(m) + Pi(m) + Succinyl-CoA(m) ⇌ GTP(m) + Succinate(m) + CoA(m)
r0855	0.25	O2(s) ⇌ O2(c)
r0856	-0.25	O2(m) ⇌ O2(c)
r2008	0.5	Arginine(s) + Glycine(c) → Arginine(c) + Glycine(s)
r0144	0.5	Arginine(c) + H2O(c) ⇌ Urea(c) + Ornithine(c)
r0167	0.5	AKG(m) + Ornithine(m) ⇌ L-Glutamate 5-semialdehyde(m) + Glutamate(m)
r0896	0.5	Urea(c) ⇌ Urea(s)
r0017	0.25	O2(m) + 4 H+(PG)(m) + 4 Ferrocytochrome C(m) → 4 Ferricytochrome C(m) + 2 H2O(m) + 4 H+(PG)(c)
r0911	1	Proline(c) + Glutamate(m) ⇌ Proline(m) + Glutamate(c)
r0337	-1	ADP(c) + 1,3DPG(c) ⇌ ATP(c) + 3PG(c)

Continued on next page

Glycine from Arginine (188)– continued

ID	flux	reaction
r0338	1	3PG(c) + NAD+(c) → 3-Phosphonooxypyruvate(c) + NADH(c)
r1426	1	Proline(m) + H+(PG)(c) → Proline(c) + H+(PG)(m)
r0058	0.5	Malate(c) + NADP+(c) ⇌ Pyruvate(c) + CO2(c) + NADPH(c)
r0074	0.5	L-Glutamate 5-semialdehyde(m) + NAD+(m) + H2O(m) ⇌ Glutamate(m) + NADH(m)
r0507	0.5	Ubiquinol(m) + 2 Ferricytochrome C(m) + 4 H+(PG)(m) → Ubiquinone(m) + 4 H+(PG)(c) + 2 Ferrocytochrome C(m)
r0509	0.5	Ubiquinone(m) + Succinate(m) → Ubiquinol(m) + Fumarate(m)
r0243	-0.5	DHAP(c) ⇌ GAP(c)
r0820	0.5	AKG(c) + Malate(m) ⇌ AKG(m) + Malate(c)
r1330	1	H+(PG)(c) ⇌ H+(PG)(m) + Proton-gradient(m)
r1319	0.5	ATP(c) + H2O(c) ⇌ ADP(c) + Pi(c) + ATP-energy(c)
r1070	0.5	Lysine(c) ⇌ Lysine(m)
r0849	2	H2O(s) ⇌ H2O(c)
r0867	1	CO2(c) ⇌ CO2(s)
r0868	-0.5	CO2(c) ⇌ CO2(m)
r0736	0.5	ATP(c) + Fructose-6P(c) → ADP(c) + Fructose-1,6PP(c)
r0159	1	3-Phosphoserine(c) + H2O(c) → Serine(c) + Pi(c)
r0192	0.5	Glucose-6P(c) ⇌ Fructose-6P(c)
r1114	0.5	AKG(m) + CoA(m) + NAD+(m) ⇌ NADH(m) + CO2(m) + Succinyl-CoA(m)
r0792	-1	5,10-Methylene-THF(c) + NADH(c) ⇌ 5-Methyl-THF(c) + NAD+(c)
r2523	-0.5	Glycine(c) ⇌ Glycine(s)
r2676	1	5-Methyl-THF(c) ⇌ THF(c) + THF-activated methyl group(c)
r2396	-0.5	Lysine(m) + Ornithine(c) + H+(PG)(c) → Lysine(c) + Ornithine(m) + H+(PG)(m)
r0663	-1	3-Phosphonooxypyruvate(c) + Glutamate(c) → 3-Phosphoserine(c) + AKG(c)
r0230	-1	THF(c) + Serine(c) ⇌ 5,10-Methylene-THF(c) + Glycine(c) + H2O(c)
r1320	0.5	ATP(m) + H2O(m) ⇌ ADP(m) + Pi(m) + ATP-energy(m)
r1322	1	NADH(c) ⇌ NAD+(c) + NADH-redox-potential(c)
r1323	1	NADH(m) ⇌ NAD+(m) + NADH-redox-potential(m)
r0100	-0.5	ADP(m) + GTP(m) ⇌ ATP(m) + GDP(m)
r1326	0.5	NADPH(c) ⇌ NADPH-redox-potential(c) + NADP+(c)
r0253	1	GAP(c) + Pi(c) + NAD+(c) ⇌ 1,3DPG(c) + NADH(c)
r0256	0.5	Fructose-1,6PP(c) ⇌ DHAP(c) + GAP(c)
r1047	0.5	Pi(c) + H+(PG)(c) → Pi(m) + H+(PG)(m)
r0259	-0.5	Fumarate(m) + H2O(m) ⇌ Malate(m)
r2419	0.5	AKG(c) + Pi(m) ⇌ AKG(m) + Pi(c)
r0851	-1	H2O(m) ⇌ H2O(c)

Glycine from Asparagine (189)

Definition			Solution												
			exchanges		reactions								transp		
Objective	Constraints	Comment	imports	exports	c	m	r	p	l	n	s	b	s-c	b-c	int
Glycine	−Asparagine MCES +Urea	Transamination to Glycine using Asparagine	0.5 H_2O(s) 0.5 Glucose-6P(c) 0.5 Asparagine(s) 0.5 NADPH-redox-potential(c) 0.5 NADPH-redox-potential(m) 1 Proton-gradient(m)	0.5 P_i(c) 0.5 CO_2(s) 0.5 Pyruvate(c) 1 Glycine(s) 0.5 ATP-energy(c) 1.5 NADH-redox-potential(c) 0.5 Na-gradient(c) 1 THF-activated methyl group(c)	19	2	-	-	-	-	-	-	5	-	7

ID	flux	reaction
r0253	1	GAP(c) + Pi(c) + NAD+(c) ⇌ 1,3DPG(c) + NADH(c)
r0127	0.5	H2O(c) + Asparagine(c) → Aspartate(c) + NH3(c)
r0109	0.5	AKG(c) + Aspartate(c) ⇌ OAA(c) + Glutamate(c)
r0911	0.5	Proline(c) + Glutamate(m) ⇌ Proline(m) + Glutamate(c)
r2523	-0.5	Glycine(c) ⇌ Glycine(s)
r1326	-0.5	NADPH(c) ⇌ NADPH-redox-potential(c) + NADP+(c)
r1047	0.5	Pi(c) + H+(PG)(c) → Pi(m) + H+(PG)(m)

Continued on next page

ID	flux	reaction
r0192	0.5	Glucose-6P(c) ⇌ Fructose-6P(c)
r0075	-0.5	AKG(m) + NADPH(m) + NH3(m) ⇌ Glutamate(m) + H2O(m) + NADP+(m)
r1426	0.5	Proline(m) + H+(PG)(c) → Proline(c) + H+(PG)(m)
r1327	-0.5	NADPH(m) ⇌ NADPH-redox-potential(m) + NADP+(m)
r0337	-1	ADP(c) + 1,3DPG(c) ⇌ ATP(c) + 3PG(c)
r0256	0.5	Fructose-1,6PP(c) ⇌ DHAP(c) + GAP(c)
r0058	0.5	Malate(c) + NADP+(c) ⇌ Pyruvate(c) + CO2(c) + NADPH(c)
r1319	0.5	ATP(c) + H2O(c) ⇌ ADP(c) + Pi(c) + ATP-energy(c)
r0851	0.5	H2O(m) ⇌ H2O(c)
r0338	1	3PG(c) + NAD+(c) → 3-Phosphonooxypyruvate(c) + NADH(c)
r0663	-1	3-Phosphonooxypyruvate(c) + Glutamate(c) → 3-Phosphoserine(c) + AKG(c)
r0159	1	3-Phosphoserine(c) + H2O(c) → Serine(c) + Pi(c)
r2419	0.5	AKG(c) + Pi(m) ⇌ AKG(m) + Pi(c)
r0736	0.5	ATP(c) + Fructose-6P(c) → ADP(c) + Fructose-1,6PP(c)
r0296	-1	5,10-Methylene-THF(c) + NADPH(c) ⇌ 5-Methyl-THF(c) + NADP+(c)
r2664	0.5	Na+(c) ⇌ Na+(s) + Na-gradient(c)
r0230	-1	THF(c) + Serine(c) ⇌ 5,10-Methylene-THF(c) + Glycine(c) + H2O(c)
r0104	-0.5	OAA(c) + NADH(c) ⇌ NAD+(c) + Malate(c)
r1837	0.5	Asparagine(s) + Glycine(c) + Na+(s) → Asparagine(c) + Glycine(s) + Na+(c)
r0838	0.5	NH3(c) ⇌ NH3(m)
r1330	-1	H+(PG)(c) ⇌ H+(PG)(m) + Proton-gradient(m)
r2676	1	5-Methyl-THF(c) ⇌ THF(c) + THF-activated methyl group(c)
r1322	1.5	NADH(c) ⇌ NAD+(c) + NADH-redox-potential(c)
r0867	0.5	CO2(c) ⇌ CO2(s)
r0849	0.5	H2O(s) ⇌ H2O(c)
r0243	-0.5	DHAP(c) ⇌ GAP(c)

Glycine from Aspartate (190)

Definition			Solution													
			exchanges		reactions									transp		
Objective	Constraints	Comment	imports	exports	c	m	r	p	l	n	s	b	s-c	b-c	int
Glycine	−Aspartate MCES +Urea	Transamination to Glycine using Aspartate	0.5 H_2O(s) 1 Aspartate(s) 0.5 Glucose-6P(c)	0.5 P_i(c) 1 CO_2(s) 1 Pyruvate(c) 1 Glycine(s) 0.5 ATP-energy(c) 1 NADPH-redox-potential(c) 1 Na-gradient(c) 1 THF-activated methyl group(c)	17	-	-	-	-	-	-	-	5	-	-

ID	flux	reaction
r0253	1	GAP(c) + Pi(c) + NAD+(c) ⇌ 1,3DPG(c) + NADH(c)
r0109	1	AKG(c) + Aspartate(c) ⇌ OAA(c) + Glutamate(c)
r2523	-1	Glycine(c) ⇌ Glycine(s)
r1326	1	NADPH(c) ⇌ NADPH-redox-potential(c) + NADP+(c)
r0192	0.5	Glucose-6P(c) ⇌ Fructose-6P(c)
r0337	-1	ADP(c) + 1,3DPG(c) ⇌ ATP(c) + 3PG(c)
r0256	0.5	Fructose-1,6PP(c) ⇌ DHAP(c) + GAP(c)
r0058	1	Malate(c) + NADP+(c) ⇌ Pyruvate(c) + CO2(c) + NADPH(c)
r1319	0.5	ATP(c) + H2O(c) ⇌ ADP(c) + Pi(c) + ATP-energy(c)
r0338	1	3PG(c) + NAD+(c) → 3-Phosphonooxypyruvate(c) + NADH(c)
r0663	-1	3-Phosphonooxypyruvate(c) + Glutamate(c) → 3-Phosphoserine(c) + AKG(c)
r0159	1	3-Phosphoserine(c) + H2O(c) → Serine(c) + Pi(c)
r0736	0.5	ATP(c) + Fructose-6P(c) → ADP(c) + Fructose-1,6PP(c)
r2664	1	Na+(c) ⇌ Na+(s) + Na-gradient(c)
r0792	-1	5,10-Methylene-THF(c) + NADH(c) ⇌ 5-Methyl-THF(c) + NAD+(c)
r0230	-1	THF(c) + Serine(c) ⇌ 5,10-Methylene-THF(c) + Glycine(c) + H2O(c)

Continued on next page

Glycine from Aspartate (190)– continued

ID	flux	reaction
r0104	-1	OAA(c) + NADH(c) ⇌ NAD+(c) + Malate(c)
r2676	1	5-Methyl-THF(c) ⇌ THF(c) + THF-activated methyl group(c)
r0867	1	CO2(c) ⇌ CO2(s)
r0849	0.5	H2O(s) ⇌ H2O(c)
r1143	-1	Na+(c) + Aspartate(c) ← Na+(s) + Aspartate(s)
r0243	-0.5	DHAP(c) ⇌ GAP(c)

Glycine from Cysteine (191)

Definition			Solution												
			exchanges		reactions								transp		
Objective	Constraints	Comment	imports	exports	c	m	r	p	l	n	s	b	s-c	b-c	int
Glycine	−Cysteine MCES +Urea +H_2S	Transamination to Glycine using Cysteine	0.5 H_2O(s) 0.5 Glucose-6P(c) 1 Cysteine(s) 1 NADH-redox-potential(m) 1 NADPH-redox-potential(c) 2 Proton-gradient(m)	0.5 P_i(c) 1 Pyruvate(c) 1 Glycine(s) 1 H_2S(s) 0.5 ATP-energy(c) 2 NADH-redox-potential(c) 1 Na-gradient(c) 1 THF-activated methyl group(c)	16	2	-	-	-	-	-	-	4	-	7

ID	flux	reaction
r0253	1	GAP(c) + Pi(c) + NAD+(c) ⇌ 1,3DPG(c) + NADH(c)
r0073	-1	AKG(m) + NADH(m) + NH3(m) ⇌ Glutamate(m) + NAD+(m) + H2O(m)
r0911	1	Proline(c) + Glutamate(m) ⇌ Proline(m) + Glutamate(c)
r1326	-1	NADPH(c) ⇌ NADPH-redox-potential(c) + NADP+(c)
r1047	1	Pi(c) + H+(PG)(c) → Pi(m) + H+(PG)(m)
r0192	0.5	Glucose-6P(c) ⇌ Fructose-6P(c)
r1426	1	Proline(m) + H+(PG)(c) → Proline(c) + H+(PG)(m)
r0940	1	H2S(c) ⇌ H2S(s)
r0337	-1	ADP(c) + 1,3DPG(c) ⇌ ATP(c) + 3PG(c)
r0256	0.5	Fructose-1,6PP(c) ⇌ DHAP(c) + GAP(c)
r0193	1	Cysteine(c) + H2O(c) ⇌ Pyruvate(c) + H2S(c) + NH3(c)
r1319	0.5	ATP(c) + H2O(c) ⇌ ADP(c) + Pi(c) + ATP-energy(c)
r0851	1	H2O(m) ⇌ H2O(c)
r0338	1	3PG(c) + NAD+(c) → 3-Phosphonooxypyruvate(c) + NADH(c)
r0663	-1	3-Phosphonooxypyruvate(c) + Glutamate(c) → 3-Phosphoserine(c) + AKG(c)
r0159	1	3-Phosphoserine(c) + H2O(c) → Serine(c) + Pi(c)
r2419	1	AKG(c) + Pi(m) ⇌ AKG(m) + Pi(c)
r0736	0.5	ATP(c) + Fructose-6P(c) → ADP(c) + Fructose-1,6PP(c)
r0296	-1	5,10-Methylene-THF(c) + NADPH(c) ⇌ 5-Methyl-THF(c) + NADP+(c)
r2664	1	Na+(c) ⇌ Na+(s) + Na-gradient(c)
r0230	-1	THF(c) + Serine(c) ⇌ 5,10-Methylene-THF(c) + Glycine(c) + H2O(c)
r1792	1	Cysteine(s) + Glycine(c) + Na+(s) → Cysteine(c) + Glycine(s) + Na+(c)
r0838	1	NH3(c) ⇌ NH3(m)
r1330	-2	H+(PG)(c) ⇌ H+(PG)(m) + Proton-gradient(m)
r2676	1	5-Methyl-THF(c) ⇌ THF(c) + THF-activated methyl group(c)
r1322	2	NADH(c) ⇌ NAD+(c) + NADH-redox-potential(c)
r0849	0.5	H2O(s) ⇌ H2O(c)
r1323	-1	NADH(m) ⇌ NAD+(m) + NADH-redox-potential(m)
r0243	-0.5	DHAP(c) ⇌ GAP(c)

Glycine from Cystine (192)

Definition			Solution												
			exchanges		reactions								transp		
Objective	Constraints	Comment	imports	exports	c	m	r	p	l	n	s	b	s-c	b-c	int
Glycine	−Cystine MCES +Urea +H_2S	Transamination to Glycine using Cystine	0.5 H_2O(s) 0.5 Glucose-6P(c) 0.5 Cystine(s) 1 NADH-redox-potential(m) 1.5 NADPH-redox-potential(c) 2 Proton-gradient(m)	0.5 P_i(c) 1 Pyruvate(c) 1 Glycine(s) 1 H_2S(s) 0.5 ATP-energy(c) 2 NADH-redox-potential(c) 0.5 Na-gradient(c) 1 THF-activated methyl group(c)	17	2	-	-	-	-	-	-	6	-	7

ID	flux	reaction
r1144	-0.5	Glutamate(c) + Na+(c) ← Glutamate(s) + Na+(s)
r0253	1	GAP(c) + Pi(c) + NAD+(c) ⇌ 1,3DPG(c) + NADH(c)
r0073	-1	AKG(m) + NADH(m) + NH3(m) ⇌ Glutamate(m) + NAD+(m) + H2O(m)
r0911	1	Proline(c) + Glutamate(m) ⇌ Proline(m) + Glutamate(c)
r2523	-1	Glycine(c) ⇌ Glycine(s)
r1326	-1.5	NADPH(c) ⇌ NADPH-redox-potential(c) + NADP+(c)
r1047	1	Pi(c) + H+(PG)(c) → Pi(m) + H+(PG)(m)
r0192	0.5	Glucose-6P(c) ⇌ Fructose-6P(c)
r1426	1	Proline(m) + H+(PG)(c) → Proline(c) + H+(PG)(m)
r0940	1	H2S(c) ⇌ H2S(s)
r0337	-1	ADP(c) + 1,3DPG(c) ⇌ ATP(c) + 3PG(c)
r0256	0.5	Fructose-1,6PP(c) ⇌ DHAP(c) + GAP(c)
r0193	1	Cysteine(c) + H2O(c) ⇌ Pyruvate(c) + H2S(c) + NH3(c)
r1319	0.5	ATP(c) + H2O(c) ⇌ ADP(c) + Pi(c) + ATP-energy(c)
r0851	1	H2O(m) ⇌ H2O(c)
r0338	1	3PG(c) + NAD+(c) → 3-Phosphonooxypyruvate(c) + NADH(c)
r0663	-1	3-Phosphonooxypyruvate(c) + Glutamate(c) → 3-Phosphoserine(c) + AKG(c)
r0159	1	3-Phosphoserine(c) + H2O(c) → Serine(c) + Pi(c)
r2419	1	AKG(c) + Pi(m) ⇌ AKG(m) + Pi(c)
r0736	0.5	ATP(c) + Fructose-6P(c) → ADP(c) + Fructose-1,6PP(c)
r0296	-1	5,10-Methylene-THF(c) + NADPH(c) ⇌ 5-Methyl-THF(c) + NADP+(c)
r2664	0.5	Na+(c) ⇌ Na+(s) + Na-gradient(c)
r0230	-1	THF(c) + Serine(c) ⇌ 5,10-Methylene-THF(c) + Glycine(c) + H2O(c)
r0838	1	NH3(c) ⇌ NH3(m)
r1330	-2	H+(PG)(c) ⇌ H+(PG)(m) + Proton-gradient(m)
r2676	1	5-Methyl-THF(c) ⇌ THF(c) + THF-activated methyl group(c)
r1322	2	NADH(c) ⇌ NAD+(c) + NADH-redox-potential(c)
r0849	0.5	H2O(s) ⇌ H2O(c)
r1323	-1	NADH(m) ⇌ NAD+(m) + NADH-redox-potential(m)
r0877	0.5	Glutamate(c) + Cystine(s) ⇌ Glutamate(s) + Cystine(c)
r0243	-0.5	DHAP(c) ⇌ GAP(c)
r0027	-0.5	NADPH(c) + Cystine(c) ⇌ 2 Cysteine(c) + NADP+(c)

Glycine from Glutamate (193)

Definition			Solution												
			exchanges		reactions								transp		
Objective	Constraints	Comment	imports	exports	c	m	r	p	l	n	s	b	s-c	b-c	int
Glycine	−Glutamate MCES +Urea	Transamination to Glycine using Glutamate	1.5 H_2O(s) 0.5 O_2(s) 1 Glutamate(s) 0.5 Glucose-6P(c)	0.5 P_i(c) 2 CO_2(s) 1 Pyruvate(c) 1 Glycine(s) 0.5 ATP-energy(c) 1 ATP-energy(m) 1 NADH-redox-potential(c) 1 NADH-redox-potential(m) 1 NADPH-redox-potential(c) 6 Proton-gradient(m) 1 Na-gradient(c) 1 THF-activated methyl group(c)	16	7	-	-	-	-	-	-	6	-	7

ID	flux	reaction
r1144	-1	Glutamate(c) + Na+(c) ← Glutamate(s) + Na+(s)
r0820	1	AKG(c) + Malate(m) ⇌ AKG(m) + Malate(c)
r0253	1	GAP(c) + Pi(c) + NAD+(c) ⇌ 1,3DPG(c) + NADH(c)
r2523	-1	Glycine(c) ⇌ Glycine(s)
r1326	1	NADPH(c) ⇌ NADPH-redox-potential(c) + NADP+(c)
r0507	1	Ubiquinol(m) + 2 Ferricytochrome C(m) + 4 H+(PG)(m) → Ubiquinone(m) + 4 H+(PG)(c) + 2 Ferrocytochrome C(m)
r0192	0.5	Glucose-6P(c) ⇌ Fructose-6P(c)
r0337	-1	ADP(c) + 1,3DPG(c) ⇌ ATP(c) + 3PG(c)
r0256	0.5	Fructose-1,6PP(c) ⇌ DHAP(c) + GAP(c)
r0058	1	Malate(c) + NADP+(c) ⇌ Pyruvate(c) + CO2(c) + NADPH(c)
r1319	0.5	ATP(c) + H2O(c) ⇌ ADP(c) + Pi(c) + ATP-energy(c)
r0851	-1	H2O(m) ⇌ H2O(c)
r0509	1	Ubiquinone(m) + Succinate(m) → Ubiquinol(m) + Fumarate(m)
r0338	1	3PG(c) + NAD+(c) → 3-Phosphonooxypyruvate(c) + NADH(c)
r0100	-1	ADP(m) + GTP(m) ⇌ ATP(m) + GDP(m)
r0663	-1	3-Phosphonooxypyruvate(c) + Glutamate(c) → 3-Phosphoserine(c) + AKG(c)
r0159	1	3-Phosphoserine(c) + H2O(c) → Serine(c) + Pi(c)
r0736	0.5	ATP(c) + Fructose-6P(c) → ADP(c) + Fructose-1,6PP(c)
r0259	-1	Fumarate(m) + H2O(m) ⇌ Malate(m)
r2664	1	Na+(c) ⇌ Na+(s) + Na-gradient(c)
r0855	0.5	O2(s) ⇌ O2(c)
r0792	-1	5,10-Methylene-THF(c) + NADH(c) ⇌ 5-Methyl-THF(c) + NAD+(c)
r0230	-1	THF(c) + Serine(c) ⇌ 5,10-Methylene-THF(c) + Glycine(c) + H2O(c)
r1320	1	ATP(m) + H2O(m) ⇌ ADP(m) + Pi(m) + ATP-energy(m)
r0856	-0.5	O2(m) ⇌ O2(c)
r1330	6	H+(PG)(c) ⇌ H+(PG)(m) + Proton-gradient(m)
r1114	1	AKG(m) + CoA(m) + NAD+(m) ⇌ NADH(m) + CO2(m) + Succinyl-CoA(m)
r2676	1	5-Methyl-THF(c) ⇌ THF(c) + THF-activated methyl group(c)
r1322	1	NADH(c) ⇌ NAD+(c) + NADH-redox-potential(c)
r0867	2	CO2(c) ⇌ CO2(s)
r0849	1.5	H2O(s) ⇌ H2O(c)
r0125	-1	GDP(m) + Pi(m) + Succinyl-CoA(m) ⇌ GTP(m) + Succinate(m) + CoA(m)
r0017	0.5	O2(m) + 4 H+(PG)(m) + 4 Ferrocytochrome C(m) → 4 Ferricytochrome C(m) + 2 H2O(m) + 4 H+(PG)(c)
r1323	1	NADH(m) ⇌ NAD+(m) + NADH-redox-potential(m)
r0868	-1	CO2(c) ⇌ CO2(m)
r0243	-0.5	DHAP(c) ⇌ GAP(c)

Glycine from Histidine (194)

Definition			Solution													
			exchanges		reactions								transp		
Objective	Constraints	Comment	imports	exports	c	m	r	p	l	n	s	b	s-c	b-c	int
Glycine	−Histidine MCES +Urea	Transamination to Glycine using Histidine	0.83 H_2O(s) 0.17 O_2(s) 0.5 Glucose-6P(c) 0.33 Histidine(s) 0.33 NADH-redox-potential(m)	0.5 P_i(c) 0.67 CO_2(s) 0.33 Pyruvate(c) 1 Glycine(s) 0.5 ATP-energy(c) 0.33 ATP-energy(m) 0.67 NADH-redox-potential(c) 0.67 Proton-gradient(m) 1.33 THF-activated methyl group(c)	21	8	-	-	-	-	-	-	5	-	12

ID	flux	reaction
r0125	-0.33	GDP(m) + Pi(m) + Succinyl-CoA(m) ⇌ GTP(m) + Succinate(m) + CoA(m)
r0855	0.17	O2(s) ⇌ O2(c)
r0856	-0.17	O2(m) ⇌ O2(c)
r0293	-0.33	5,10-Methenyl-THF(c) + NADPH(c) ⇌ 5,10-Methylene-THF(c) + NADP+(c)
r0577	-0.33	Urocanate(c) + H2O(c) ⇌ 4-Imidazolone-5-propanoate(c)

Continued on next page

ID	flux	reaction
r2040	0.33	Histidine(s) + Glycine(c) → Histidine(c) + Glycine(s)
r0017	0.17	O2(m) + 4 H+(PG)(m) + 4 Ferrocytochrome C(m) → 4 Ferricytochrome C(m) + 2 H2O(m) + 4 H+(PG)(c)
r0911	0.67	Proline(c) + Glutamate(m) ⇌ Proline(m) + Glutamate(c)
r0337	-1	ADP(c) + 1,3DPG(c) ⇌ ATP(c) + 3PG(c)
r0338	1	3PG(c) + NAD+(c) → 3-Phosphonooxypyruvate(c) + NADH(c)
r1426	0.67	Proline(m) + H+(PG)(c) → Proline(c) + H+(PG)(m)
r0058	0.33	Malate(c) + NADP+(c) ⇌ Pyruvate(c) + CO2(c) + NADPH(c)
r0073	-0.67	AKG(m) + NADH(m) + NH3(m) ⇌ Glutamate(m) + NAD+(m) + H2O(m)
r0507	0.33	Ubiquinol(m) + 2 Ferricytochrome C(m) + 4 H+(PG)(m) → Ubiquinone(m) + 4 H+(PG)(c) + 2 Ferrocytochrome C(m)
r0509	0.33	Ubiquinone(m) + Succinate(m) → Ubiquinol(m) + Fumarate(m)
r0524	0.33	5-Formiminotetrahydrofolate(c) ⇌ 5,10-Methenyl-THF(c) + NH3(c)
r0243	-0.5	DHAP(c) ⇌ GAP(c)
r0820	0.33	AKG(c) + Malate(m) ⇌ AKG(m) + Malate(c)
r1330	0.67	H+(PG)(c) ⇌ H+(PG)(m) + Proton-gradient(m)
r1319	0.5	ATP(c) + H2O(c) ⇌ ADP(c) + Pi(c) + ATP-energy(c)
r0849	0.83	H2O(s) ⇌ H2O(c)
r0286	0.33	Histidine(c) ⇌ Urocanate(c) + NH3(c)
r0867	0.67	CO2(c) ⇌ CO2(s)
r0868	-0.33	CO2(c) ⇌ CO2(m)
r0736	0.5	ATP(c) + Fructose-6P(c) → ADP(c) + Fructose-1,6PP(c)
r0159	1	3-Phosphoserine(c) + H2O(c) → Serine(c) + Pi(c)
r0192	0.5	Glucose-6P(c) ⇌ Fructose-6P(c)
r1114	0.33	AKG(m) + CoA(m) + NAD+(m) ⇌ NADH(m) + CO2(m) + Succinyl-CoA(m)
r0792	-1.33	5,10-Methylene-THF(c) + NADH(c) ⇌ 5-Methyl-THF(c) + NAD+(c)
r2523	-0.67	Glycine(c) ⇌ Glycine(s)
r2676	1.33	5-Methyl-THF(c) ⇌ THF(c) + THF-activated methyl group(c)
r0663	-1	3-Phosphonooxypyruvate(c) + Glutamate(c) → 3-Phosphoserine(c) + AKG(c)
r0230	-1	THF(c) + Serine(c) ⇌ 5,10-Methylene-THF(c) + Glycine(c) + H2O(c)
r1320	0.33	ATP(m) + H2O(m) ⇌ ADP(m) + Pi(m) + ATP-energy(m)
r1322	0.67	NADH(c) ⇌ NAD+(c) + NADH-redox-potential(c)
r0518	-0.33	Glutamate(c) + 5-Formiminotetrahydrofolate(c) ⇌ THF(c) + N-Formimino-L-glutamate(c)
r1323	-0.33	NADH(m) ⇌ NAD+(m) + NADH-redox-potential(m)
r0519	0.33	H2O(c) + 4-Imidazolone-5-propanoate(c) ⇌ N-Formimino-L-glutamate(c)
r0100	-0.33	ADP(m) + GTP(m) ⇌ ATP(m) + GDP(m)
r0253	1	GAP(c) + Pi(c) + NAD+(c) ⇌ 1,3DPG(c) + NADH(c)
r0256	0.5	Fructose-1,6PP(c) ⇌ DHAP(c) + GAP(c)
r1047	0.67	Pi(c) + H+(PG)(c) → Pi(m) + H+(PG)(m)
r0259	-0.33	Fumarate(m) + H2O(m) ⇌ Malate(m)
r2419	0.67	AKG(c) + Pi(m) ⇌ AKG(m) + Pi(c)
r0838	0.67	NH3(c) ⇌ NH3(m)
r0851	0.33	H2O(m) ⇌ H2O(c)

Glycine from Isoleucine (195)

Definition			Solution												
			exchanges		reactions								transp		
Objective	Constraints	Comment	imports	exports	c	m	r	p	l	n	s	b	s-c	b-c	int
Glycine	−Isoleucine MCES +Urea	Transamination to Glycine using Isoleucine	1.5 H_2O(s) 1 O_2(s) 0.5 Glucose-6P(c) 1 Isoleucine(s)	0.5 P_i(c) 2 CO_2(s) 1 Glycine(s) 0.5 ATP-energy(c) 1 NADH-redox-potential(c) 4 NADH-redox-potential(m) 9 Proton-gradient(m) 1 THF-activated methyl group(c) 2 CoA-activated acetyl group(m)	14	13	-	-	-	-	-	-	4	-	11

ID	flux	reaction
r0855	1	O2(s) ⇌ O2(c)
r0856	-1	O2(m) ⇌ O2(c)
r0592	-1	H2O(m) + Acrylyl-CoA(m) → 3-Hydroxypropionyl-CoA(m)
r0596	-1	3-Hydroxypropionyl-CoA(m) + H2O(m) → Hydracrylate(m) + CoA(m)
r0017	1	O2(m) + 4 H+(PG)(m) + 4 Ferrocytochrome C(m) → 4 Ferricytochrome C(m) + 2 H2O(m) + 4 H+(PG)(c)
r1557	-1	Glycine(c) + Isoleucine(s) ⇌ Glycine(s) + Isoleucine(c)
r0911	1	Proline(c) + Glutamate(m) ⇌ Proline(m) + Glutamate(c)
r0337	-1	ADP(c) + 1,3DPG(c) ⇌ ATP(c) + 3PG(c)
r0338	1	3PG(c) + NAD+(c) → 3-Phosphonooxypyruvate(c) + NADH(c)
r1426	1	Proline(m) + H+(PG)(c) → Proline(c) + H+(PG)(m)
r0222	-1	CoA(m) + 2-Methylacetoacetyl-CoA(m) ⇌ Propanoyl-CoA(m) + Acetyl-CoA(m)
r0953	1	Isoleucine(c) + H+(PG)(c) → Isoleucine(m) + H+(PG)(m)
r0507	2	Ubiquinol(m) + 2 Ferricytochrome C(m) + 4 H+(PG)(m) → Ubiquinone(m) + 4 H+(PG)(c) + 2 Ferrocytochrome C(m)
r0243	-0.5	DHAP(c) ⇌ GAP(c)
r1330	9	H+(PG)(c) ⇌ H+(PG)(m) + Proton-gradient(m)
r1319	0.5	ATP(c) + H2O(c) ⇌ ADP(c) + Pi(c) + ATP-energy(c)
r0264	1	Isoleucine(m) + AKG(m) ⇌ Glutamate(m) + 2-Oxo-3-methylvalerate(m)
r0849	1.5	H2O(s) ⇌ H2O(c)
r0867	2	CO2(c) ⇌ CO2(s)
r0868	-2	CO2(c) ⇌ CO2(m)
r0736	0.5	ATP(c) + Fructose-6P(c) → ADP(c) + Fructose-1,6PP(c)
r0159	1	3-Phosphoserine(c) + H2O(c) → Serine(c) + Pi(c)
r0174	1	CoA(m) + NAD+(m) + 3-Oxopropanoate(m) → Acetyl-CoA(m) + CO2(m) + NADH(m)
r0602	1	2-Oxo-3-methylvalerate(m) + CoA(m) + NAD+(m) ⇌ 2-Methylbutyryl-CoA(m) + CO2(m) + NADH(m)
r0603	1	2-Methylbutyryl-CoA(m) + Ubiquinone(m) ⇌ Tiglyl-CoA(m) + Ubiquinol(m)
r0192	0.5	Glucose-6P(c) ⇌ Fructose-6P(c)
r0792	-1	5,10-Methylene-THF(c) + NADH(c) ⇌ 5-Methyl-THF(c) + NAD+(c)
r2676	1	5-Methyl-THF(c) ⇌ THF(c) + THF-activated methyl group(c)
r0365	1	Hydracrylate(m) + NAD+(m) → NADH(m) + 3-Oxopropanoate(m)
r2678	2	Acetyl-CoA(m) ⇌ CoA(m) + CoA-activated acetyl group(m)
r0663	-1	3-Phosphonooxypyruvate(c) + Glutamate(c) → 3-Phosphoserine(c) + AKG(c)
r0230	-1	THF(c) + Serine(c) ⇌ 5,10-Methylene-THF(c) + Glycine(c) + H2O(c)
r0664	1	NAD+(m) + (S)-3-Hydroxy-2-methylbutyryl-CoA(m) ⇌ 2-Methylacetoacetyl-CoA(m) + NADH(m)
r0665	-1	H2O(m) + Tiglyl-CoA(m) ⇌ (S)-3-Hydroxy-2-methylbutyryl-CoA(m)
r1322	1	NADH(c) ⇌ NAD+(c) + NADH-redox-potential(c)
r0683	1	Propanoyl-CoA(m) + Ubiquinone(m) → Ubiquinol(m) + Acrylyl-CoA(m)
r1323	4	NADH(m) ⇌ NAD+(m) + NADH-redox-potential(m)
r0253	1	GAP(c) + Pi(c) + NAD+(c) ⇌ 1,3DPG(c) + NADH(c)
r0256	0.5	Fructose-1,6PP(c) ⇌ DHAP(c) + GAP(c)
r1047	1	Pi(c) + H+(PG)(c) → Pi(m) + H+(PG)(m)
r2419	1	AKG(c) + Pi(m) ⇌ AKG(m) + Pi(c)
r0851	-1	H2O(m) ⇌ H2O(c)

Glycine from Glutamine (196)

Definition			Solution												
			exchanges		reactions								transp		
Objective	Constraints	Comment	imports	exports	c	m	r	p	l	n	s	b	s-c	b-c	int
Glycine	−Glutamine MCES +Urea	Transamination to Glycine using Glutamine	1 H_2O(s) 0.25 O_2(s) 0.5 Glutamine(s) 0.5 Glucose-6P(c) 0.5 NADPH-redox-potential(m)	0.5 P_i(c) 1 CO_2(s) 0.5 Pyruvate(c) 1 Glycine(s) 0.5 ATP-energy(c) 0.5 ATP-energy(m) 1 NADH-redox-potential(c) 0.5 NADH-redox-potential(m) 0.5 NADPH-redox-potential(c) 2 Proton-gradient(m) 1 THF-activated methyl group(c)	18	9	-	-	-	-	-	-	5	-	11

ID	flux	reaction
r0125	-0.5	GDP(m) + Pi(m) + Succinyl-CoA(m) ⇌ GTP(m) + Succinate(m) + CoA(m)
r0855	0.25	O2(s) ⇌ O2(c)
r0856	-0.25	O2(m) ⇌ O2(c)
r0017	0.25	O2(m) + 4 H+(PG)(m) + 4 Ferrocytochrome C(m) → 4 Ferricytochrome C(m) + 2 H2O(m) + 4 H+(PG)(c)
r0189	0.5	Glucosamine-6P(c) + H2O(c) ⇌ Fructose-6P(c) + NH3(c)
r0911	0.5	Proline(c) + Glutamate(m) ⇌ Proline(m) + Glutamate(c)
r0337	-1	ADP(c) + 1,3DPG(c) ⇌ ATP(c) + 3PG(c)
r0338	1	3PG(c) + NAD+(c) → 3-Phosphonooxypyruvate(c) + NADH(c)
r1426	0.5	Proline(m) + H+(PG)(c) → Proline(c) + H+(PG)(m)
r0058	0.5	Malate(c) + NADP+(c) ⇌ Pyruvate(c) + CO2(c) + NADPH(c)
r0075	-0.5	AKG(m) + NADPH(m) + NH3(m) ⇌ Glutamate(m) + H2O(m) + NADP+(m)
r0507	0.5	Ubiquinol(m) + 2 Ferricytochrome C(m) + 4 H+(PG)(m) → Ubiquinone(m) + 4 H+(PG)(c) + 2 Ferrocytochrome C(m)
r0509	0.5	Ubiquinone(m) + Succinate(m) → Ubiquinol(m) + Fumarate(m)
r0243	-0.5	DHAP(c) ⇌ GAP(c)
r0820	0.5	AKG(c) + Malate(m) ⇌ AKG(m) + Malate(c)
r1330	2	H+(PG)(c) ⇌ H+(PG)(m) + Proton-gradient(m)
r1319	0.5	ATP(c) + H2O(c) ⇌ ADP(c) + Pi(c) + ATP-energy(c)
r0849	1	H2O(s) ⇌ H2O(c)
r0867	1	CO2(c) ⇌ CO2(s)
r0868	-0.5	CO2(c) ⇌ CO2(m)
r0736	0.5	ATP(c) + Fructose-6P(c) → ADP(c) + Fructose-1,6PP(c)
r0159	1	3-Phosphoserine(c) + H2O(c) → Serine(c) + Pi(c)
r1544	-0.5	Glycine(c) + Glutamine(s) ⇌ Glycine(s) + Glutamine(c)
r0190	0.5	Glutamine(c) + Fructose-6P(c) → Glutamate(c) + Glucosamine-6P(c)
r0192	0.5	Glucose-6P(c) ⇌ Fructose-6P(c)
r1114	0.5	AKG(m) + CoA(m) + NAD+(m) ⇌ NADH(m) + CO2(m) + Succinyl-CoA(m)
r0792	-1	5,10-Methylene-THF(c) + NADH(c) ⇌ 5-Methyl-THF(c) + NAD+(c)
r2523	-0.5	Glycine(c) ⇌ Glycine(s)
r2676	1	5-Methyl-THF(c) ⇌ THF(c) + THF-activated methyl group(c)
r0663	-1	3-Phosphonooxypyruvate(c) + Glutamate(c) → 3-Phosphoserine(c) + AKG(c)
r0230	-1	THF(c) + Serine(c) ⇌ 5,10-Methylene-THF(c) + Glycine(c) + H2O(c)
r1320	0.5	ATP(m) + H2O(m) ⇌ ADP(m) + Pi(m) + ATP-energy(m)
r1322	1	NADH(c) ⇌ NAD+(c) + NADH-redox-potential(c)
r1323	0.5	NADH(m) ⇌ NAD+(m) + NADH-redox-potential(m)
r0100	-0.5	ADP(m) + GTP(m) ⇌ ATP(m) + GDP(m)
r1326	0.5	NADPH(c) ⇌ NADPH-redox-potential(c) + NADP+(c)
r0253	1	GAP(c) + Pi(c) + NAD+(c) ⇌ 1,3DPG(c) + NADH(c)
r1327	-0.5	NADPH(m) ⇌ NADPH-redox-potential(m) + NADP+(m)
r0256	0.5	Fructose-1,6PP(c) ⇌ DHAP(c) + GAP(c)
r1047	0.5	Pi(c) + H+(PG)(c) → Pi(m) + H+(PG)(m)
r0259	-0.5	Fumarate(m) + H2O(m) ⇌ Malate(m)
r2419	0.5	AKG(c) + Pi(m) ⇌ AKG(m) + Pi(c)
r0838	0.5	NH3(c) ⇌ NH3(m)

Glycine from Leucine (197)

Definition			Solution												
			exchanges		reactions								transp		
Objective	Constraints	Comment	imports	exports	c	m	r	p	l	n	s	b	s-c	b-c	int
Glycine	−Leucine MCES +Urea	Transamination to Glycine using Leucine	0.5 O_2(s) 0.5 Glucose-6P(c) 1 Leucine(s) 1 NADPH-redox-potential(m)	0.5 H_2O(s) 0.5 P_i(c) 1 Glycine(s) 0.5 ATP-energy(c) 1 NADH-redox-potential(c) 2 NADH-redox-potential(m) 4 Proton-gradient(m) 1 Na-gradient(c) 1 THF-activated methyl group(c) 3 CoA-activated acetyl group(m)	14	13	-	-	-	-	-	-	4	-	10

ID	flux	reaction
r0855	0.5	O2(s) ⇌ O2(c)
r0856	-0.5	O2(m) ⇌ O2(c)
r0462	1	HMG-CoA(m) + CoA(m) ⇌ Acetyl-CoA(m) + H2O(m) + Acetoacetyl-CoA(m)
r0017	0.5	O2(m) + 4 H+(PG)(m) + 4 Ferrocytochrome C(m) → 4 Ferricytochrome C(m) + 2 H2O(m) + 4 H+(PG)(c)
r0911	1	Proline(c) + Glutamate(m) ⇌ Proline(m) + Glutamate(c)
r0337	-1	ADP(c) + 1,3DPG(c) ⇌ ATP(c) + 3PG(c)
r0338	1	3PG(c) + NAD+(c) → 3-Phosphonooxypyruvate(c) + NADH(c)
r0070	-1	CoA(m) + Acetoacetyl-CoA(m) ⇌ 2 Acetyl-CoA(m)
r2664	1	Na+(c) ⇌ Na+(s) + Na-gradient(c)
r1426	1	Proline(m) + H+(PG)(c) → Proline(c) + H+(PG)(m)
r0059	-1	Pyruvate(m) + CO2(m) + NADPH(m) ⇌ Malate(m) + NADP+(m)
r0655	1	Isovaleryl-CoA(m) + Ubiquinone(m) ⇌ 3-Methylcrotonyl-CoA(m) + Ubiquinol(m)
r0658	1	ATP(m) + 3-Methylcrotonyl-CoA(m) + HCO3-(m) ⇌ ADP(m) + Pi(m) + 3-Methylglutaconyl-CoA(m)
r0507	1	Ubiquinol(m) + 2 Ferricytochrome C(m) + 4 H+(PG)(m) → Ubiquinone(m) + 4 H+(PG)(c) + 2 Ferrocytochrome C(m)
r0243	-0.5	DHAP(c) ⇌ GAP(c)
r1330	4	H+(PG)(c) ⇌ H+(PG)(m) + Proton-gradient(m)
r1319	0.5	ATP(c) + H2O(c) ⇌ ADP(c) + Pi(c) + ATP-energy(c)
r0262	1	Leucine(m) + AKG(m) ⇌ Glutamate(m) + 4-Methyl-2-oxopentanoate(m)
r0849	-0.5	H2O(s) ⇌ H2O(c)
r1807	1	Leucine(s) + Glycine(c) + Na+(s) → Leucine(c) + Glycine(s) + Na+(c)
r0736	0.5	ATP(c) + Fructose-6P(c) → ADP(c) + Fructose-1,6PP(c)
r0159	1	3-Phosphoserine(c) + H2O(c) → Serine(c) + Pi(c)
r0601	1	4-Methyl-2-oxopentanoate(m) + CoA(m) + NAD+(m) ⇌ Isovaleryl-CoA(m) + CO2(m) + NADH(m)
r0192	0.5	Glucose-6P(c) ⇌ Fructose-6P(c)
r0490	-1	3-Methylglutaconyl-CoA(m) + H2O(m) ⇌ HMG-CoA(m)
r0792	-1	5,10-Methylene-THF(c) + NADH(c) ⇌ 5-Methyl-THF(c) + NAD+(c)
r2676	1	5-Methyl-THF(c) ⇌ THF(c) + THF-activated methyl group(c)
r1438	1	Leucine(c) → Leucine(m)
r2678	3	Acetyl-CoA(m) ⇌ CoA(m) + CoA-activated acetyl group(m)
r0663	-1	3-Phosphonooxypyruvate(c) + Glutamate(c) → 3-Phosphoserine(c) + AKG(c)
r0230	-1	THF(c) + Serine(c) ⇌ 5,10-Methylene-THF(c) + Glycine(c) + H2O(c)
r1322	1	NADH(c) ⇌ NAD+(c) + NADH-redox-potential(c)
r1323	2	NADH(m) ⇌ NAD+(m) + NADH-redox-potential(m)
r0253	1	GAP(c) + Pi(c) + NAD+(c) ⇌ 1,3DPG(c) + NADH(c)
r1327	-1	NADPH(m) ⇌ NADPH-redox-potential(m) + NADP+(m)
r0256	0.5	Fructose-1,6PP(c) ⇌ DHAP(c) + GAP(c)
r1047	1	Pi(c) + H+(PG)(c) → Pi(m) + H+(PG)(m)
r0105	1	NAD+(m) + Malate(m) ⇌ OAA(m) + NADH(m)
r0106	-1	ADP(m) + Pi(m) + OAA(m) ⇌ ATP(m) + Pyruvate(m) + HCO3-(m)
r2419	1	AKG(c) + Pi(m) ⇌ AKG(m) + Pi(c)
r0851	1	H2O(m) ⇌ H2O(c)

Glycine from Lysine (198)

Definition			Solution												
			exchanges		reactions								transp		
Objective	Constraints	Comment	imports	exports	c	m	r	p	l	n	s	b	s-c	b-c	int
Glycine	−Lysine MCES +Urea	Transamination to Glycine using Lysine	1 H_2O(s) 0.25 O_2(s) 0.5 Lysine(s) 0.5 Glucose-6P(c) 0.5 NADPH-redox-potential(m)	0.5 P_i(c) 1 CO_2(s) 1 Glycine(s) 0.5 ATP-energy(c) 1.5 NADH-redox-potential(c) 1.5 NADH-redox-potential(m) 1 Proton-gradient(m) 1 THF-activated methyl group(c) 1 CoA-activated acetyl group(m)	15	11	-	-	-	-	-	-	5	-	11

ID	flux	reaction
r0855	0.25	O2(s) ⇌ O2(c)

Continued on next page

ID	flux	reaction
r0856	-0.25	O2(m) ⇌ O2(c)
r0594	0.5	2-Aminoadipate 6-semialdehyde(c) + NAD+(c) + H2O(c) ⇌ NADH(c) + L-2-Aminoadipate(c)
r0460	0.5	(S)-3-Hydroxybutyryl-CoA(m) + NAD+(m) ⇌ NADH(m) + Acetoacetyl-CoA(m)
r0448	0.5	2-Oxoadipate(m) + CoA(m) + NAD+(m) ⇌ Glutaryl-CoA(m) + CO2(m) + NADH(m)
r0180	-0.5	H2O(m) + Saccharopine(m) + NADP+(m) ⇌ AKG(m) + NADPH(m) + Lysine(m)
r0017	0.25	O2(m) + 4 H+(PG)(m) + 4 Ferrocytochrome C(m) → 4 Ferricytochrome C(m) + 2 H2O(m) + 4 H+(PG)(c)
r0911	1	Proline(c) + Glutamate(m) ⇌ Proline(m) + Glutamate(c)
r0337	-1	ADP(c) + 1,3DPG(c) ⇌ ATP(c) + 3PG(c)
r0338	1	3PG(c) + NAD+(c) → 3-Phosphonooxypyruvate(c) + NADH(c)
r0070	-0.5	CoA(m) + Acetoacetyl-CoA(m) ⇌ 2 Acetyl-CoA(m)
r1426	1	Proline(m) + H+(PG)(c) → Proline(c) + H+(PG)(m)
r0507	0.5	Ubiquinol(m) + 2 Ferricytochrome C(m) + 4 H+(PG)(m) → Ubiquinone(m) + 4 H+(PG)(c) + 2 Ferrocytochrome C(m)
r2539	-0.5	2-Aminoadipate 6-semialdehyde(c) + L-2-Aminoadipate(m) ⇌ 2-Aminoadipate 6-semialdehyde(m) + L-2-Aminoadipate(c)
r0243	-0.5	DHAP(c) ⇌ GAP(c)
r0525	0.5	NAD+(m) + H2O(m) + Saccharopine(m) ⇌ Glutamate(m) + 2-Aminoadipate 6-semialdehyde(m) + NADH(m)
r1330	1	H+(PG)(c) ⇌ H+(PG)(m) + Proton-gradient(m)
r1319	0.5	ATP(c) + H2O(c) ⇌ ADP(c) + Pi(c) + ATP-energy(c)
r0541	0.5	Glutaryl-CoA(m) + Ubiquinone(m) ⇌ Crotonyl-CoA(m) + CO2(m) + Ubiquinol(m)
r1070	0.5	Lysine(c) ⇌ Lysine(m)
r0849	1	H2O(s) ⇌ H2O(c)
r0867	1	CO2(c) ⇌ CO2(s)
r0868	-1	CO2(c) ⇌ CO2(m)
r0588	-0.5	Crotonyl-CoA(m) + H2O(m) ⇌ (S)-3-Hydroxybutyryl-CoA(m)
r0450	0.5	AKG(m) + L-2-Aminoadipate(m) ⇌ 2-Oxoadipate(m) + Glutamate(m)
r0736	0.5	ATP(c) + Fructose-6P(c) → ADP(c) + Fructose-1,6PP(c)
r0159	1	3-Phosphoserine(c) + H2O(c) → Serine(c) + Pi(c)
r1992	0.5	Lysine(s) + Glycine(c) → Lysine(c) + Glycine(s)
r0192	0.5	Glucose-6P(c) ⇌ Fructose-6P(c)
r0792	-1	5,10-Methylene-THF(c) + NADH(c) ⇌ 5-Methyl-THF(c) + NAD+(c)
r2523	-0.5	Glycine(c) ⇌ Glycine(s)
r2676	1	5-Methyl-THF(c) ⇌ THF(c) + THF-activated methyl group(c)
r2678	1	Acetyl-CoA(m) ⇌ CoA(m) + CoA-activated acetyl group(m)
r0663	-1	3-Phosphonooxypyruvate(c) + Glutamate(c) → 3-Phosphoserine(c) + AKG(c)
r0230	-1	THF(c) + Serine(c) ⇌ 5,10-Methylene-THF(c) + Glycine(c) + H2O(c)
r1322	1.5	NADH(c) ⇌ NAD+(c) + NADH-redox-potential(c)
r1323	1.5	NADH(m) ⇌ NAD+(m) + NADH-redox-potential(m)
r0253	1	GAP(c) + Pi(c) + NAD+(c) ⇌ 1,3DPG(c) + NADH(c)
r1327	-0.5	NADPH(m) ⇌ NADPH-redox-potential(m) + NADP+(m)
r0256	0.5	Fructose-1,6PP(c) ⇌ DHAP(c) + GAP(c)
r1047	1	Pi(c) + H+(PG)(c) → Pi(m) + H+(PG)(m)
r2419	1	AKG(c) + Pi(m) ⇌ AKG(m) + Pi(c)

Glycine from Methionine (199)

Definition			Solution												
			exchanges		reactions								transp		
Objective	Constraints	Comment	imports	exports	c	m	r	p	l	n	s	b	s-c	b-c	int
Glycine	−Methionine MCES +Urea $+H_2S$	Transamination to Glycine using Methionine	2 H_2O(s) 0.5 O_2(s) 1 Methionine(s) 1 Glucose-6P(c) 1 NADPH-redox-potential(c)	1 P_i(c) 2 CO_2(s) 1 Pyruvate(c) 1 Glycine(s) 1 H_2S(s) 1 ATP-energy(c) 4 NADH-redox-potential(c) 1 NADH-redox-potential(m) 1 Proton-gradient(m) 2 THF-activated methyl group(c) 1 CoA-activated acetyl group(m)	19	9	-	-	-	-	-	-	5	-	12

ID	flux	reaction
r0855	0.5	O2(s) ⇌ O2(c)
r0856	-0.5	O2(m) ⇌ O2(c)
r0296	-1	5,10-Methylene-THF(c) + NADPH(c) ⇌ 5-Methyl-THF(c) + NADP+(c)
r0592	-1	H2O(m) + Acrylyl-CoA(m) → 3-Hydroxypropionyl-CoA(m)
r0596	-1	3-Hydroxypropionyl-CoA(m) + H2O(m) → Hydracrylate(m) + CoA(m)
r0017	0.5	O2(m) + 4 H+(PG)(m) + 4 Ferrocytochrome C(m) → 4 Ferricytochrome C(m) + 2 H2O(m) + 4 H+(PG)(c)
r0314	1	Serine(c) + Homocysteine(c) ⇌ H2O(c) + L-Cystathionine(c)
r0911	2	Proline(c) + Glutamate(m) ⇌ Proline(m) + Glutamate(c)
r0337	-2	ADP(c) + 1,3DPG(c) ⇌ ATP(c) + 3PG(c)
r0338	2	3PG(c) + NAD+(c) → 3-Phosphonooxypyruvate(c) + NADH(c)
r1426	2	Proline(m) + H+(PG)(c) → Proline(c) + H+(PG)(m)
r0073	-2	AKG(m) + NADH(m) + NH3(m) ⇌ Glutamate(m) + NAD+(m) + H2O(m)
r0507	1	Ubiquinol(m) + 2 Ferricytochrome C(m) + 4 H+(PG)(m) → Ubiquinone(m) + 4 H+(PG)(c) + 2 Ferrocytochrome C(m)
r0241	1	H2O(c) + L-Cystathionine(c) ⇌ Cysteine(c) + NH3(c) + 2-Oxobutyrate(c)
r0243	-1	DHAP(c) ⇌ GAP(c)
r1330	1	H+(PG)(c) ⇌ H+(PG)(m) + Proton-gradient(m)
r1319	1	ATP(c) + H2O(c) ⇌ ADP(c) + Pi(c) + ATP-energy(c)
r0849	2	H2O(s) ⇌ H2O(c)
r0867	2	CO2(c) ⇌ CO2(s)
r0868	-2	CO2(c) ⇌ CO2(m)
r0736	1	ATP(c) + Fructose-6P(c) → ADP(c) + Fructose-1,6PP(c)
r0159	2	3-Phosphoserine(c) + H2O(c) → Serine(c) + Pi(c)
r0174	1	CoA(m) + NAD+(m) + 3-Oxopropanoate(m) → Acetyl-CoA(m) + CO2(m) + NADH(m)
r1546	-1	Glycine(c) + Methionine(s) ⇌ Glycine(s) + Methionine(c)
r0192	1	Glucose-6P(c) ⇌ Fructose-6P(c)
r0193	1	Cysteine(c) + H2O(c) ⇌ Pyruvate(c) + H2S(c) + NH3(c)
r0940	1	H2S(c) ⇌ H2S(s)
r1154	1	CoA(m) + NAD+(m) + 2-Oxobutyrate(m) ⇌ CO2(m) + Propanoyl-CoA(m) + NADH(m)
r1155	1	2-Oxobutyrate(c) + H+(PG)(c) → 2-Oxobutyrate(m) + H+(PG)(m)
r2676	2	5-Methyl-THF(c) ⇌ THF(c) + THF-activated methyl group(c)
r0365	1	Hydracrylate(m) + NAD+(m) → NADH(m) + 3-Oxopropanoate(m)
r2678	1	Acetyl-CoA(m) ⇌ CoA(m) + CoA-activated acetyl group(m)
r0663	-2	3-Phosphonooxypyruvate(c) + Glutamate(c) → 3-Phosphoserine(c) + AKG(c)
r0230	-1	THF(c) + Serine(c) ⇌ 5,10-Methylene-THF(c) + Glycine(c) + H2O(c)
r0232	-1	THF(c) + Methionine(c) ⇌ 5-Methyl-THF(c) + Homocysteine(c)
r1322	4	NADH(c) ⇌ NAD+(c) + NADH-redox-potential(c)
r0683	1	Propanoyl-CoA(m) + Ubiquinone(m) → Ubiquinol(m) + Acrylyl-CoA(m)
r1323	1	NADH(m) ⇌ NAD+(m) + NADH-redox-potential(m)
r1326	-1	NADPH(c) ⇌ NADPH-redox-potential(c) + NADP+(c)
r0253	2	GAP(c) + Pi(c) + NAD+(c) ⇌ 1,3DPG(c) + NADH(c)
r0256	1	Fructose-1,6PP(c) ⇌ DHAP(c) + GAP(c)
r1047	2	Pi(c) + H+(PG)(c) → Pi(m) + H+(PG)(m)
r2419	2	AKG(c) + Pi(m) ⇌ AKG(m) + Pi(c)
r0838	2	NH3(c) ⇌ NH3(m)
r0851	1	H2O(m) ⇌ H2O(c)

Glycine from Phenylalanine (200)

Definition			Solution												
			exchanges		reactions								transp		
Objective	Constraints	Comment	imports	exports	c	m	r	p	l	n	s	b	s-c	b-c	int
Glycine	−Phenylalanine MCES +Urea	Transamination to Glycine using Phenylalanine	0.5 H_2O(s) 3 O_2(s) 1 Phenylalanine(s) 0.5 Glucose-6P(c) 1.5 ATP-energy(c)	0.5 P_i(c) 2 CO_2(s) 1 Pyruvate(c) 1 Glycine(s) 1 NADH-redox-potential(c) 1 THF-activated methyl group(c) 2 CoA-activated acetyl group(c)	28	-	-	-	-	-	-	-	4	-	-

ID	flux	reaction
r0253	1	GAP(c) + Pi(c) + NAD+(c) ⇌ 1,3DPG(c) + NADH(c)
r0605	1	4-Maleylacetoacetate(c) ⇌ Fumarylacetoacetate(c)
r0543	1	Homogentisate(c) + O2(c) ⇌ 4-Maleylacetoacetate(c)
r0192	0.5	Glucose-6P(c) ⇌ Fructose-6P(c)
r0183	1	Tyrosine(c) + AKG(c) ⇌ 4-Hydroxyphenylpyruvate(c) + Glutamate(c)
r0544	1	4-Hydroxyphenylpyruvate(c) + O2(c) ⇌ Homogentisate(c) + CO2(c)
r0337	-1	ADP(c) + 1,3DPG(c) ⇌ ATP(c) + 3PG(c)
r0256	0.5	Fructose-1,6PP(c) ⇌ DHAP(c) + GAP(c)
r0058	1	Malate(c) + NADP+(c) ⇌ Pyruvate(c) + CO2(c) + NADPH(c)
r1319	-1.5	ATP(c) + H2O(c) ⇌ ADP(c) + Pi(c) + ATP-energy(c)
r0338	1	3PG(c) + NAD+(c) → 3-Phosphonooxypyruvate(c) + NADH(c)
r0663	-1	3-Phosphonooxypyruvate(c) + Glutamate(c) → 3-Phosphoserine(c) + AKG(c)
r0258	-1	Fumarate(c) + H2O(c) ⇌ Malate(c)
r0159	1	3-Phosphoserine(c) + H2O(c) → Serine(c) + Pi(c)
r0069	-1	CoA(c) + Acetoacetyl-CoA(c) ⇌ 2 Acetyl-CoA(c)
r0736	0.5	ATP(c) + Fructose-6P(c) → ADP(c) + Fructose-1,6PP(c)
r0030	1	ATP(c) + AMP(c) ⇌ 2 ADP(c)
r1548	-1	Glycine(c) + Phenylalanine(s) ⇌ Glycine(s) + Phenylalanine(c)
r0855	3	O2(s) ⇌ O2(c)
r0792	-1	5,10-Methylene-THF(c) + NADH(c) ⇌ 5-Methyl-THF(c) + NAD+(c)
r0320	1	ATP(c) + CoA(c) + Acetoacetate(c) → AMP(c) + PPi(c) + Acetoacetyl-CoA(c)
r0230	-1	THF(c) + Serine(c) ⇌ 5,10-Methylene-THF(c) + Glycine(c) + H2O(c)
r0398	1	Dihydrobiopterin(c) + NADPH(c) ⇌ Tetrahydrobiopterin(c) + NADP+(c)
r0007	1	PPi(c) + H2O(c) → 2 Pi(c)
r2676	1	5-Methyl-THF(c) ⇌ THF(c) + THF-activated methyl group(c)
r1322	1	NADH(c) ⇌ NAD+(c) + NADH-redox-potential(c)
r0867	2	CO2(c) ⇌ CO2(s)
r0849	0.5	H2O(s) ⇌ H2O(c)
r0399	1	Tetrahydrobiopterin(c) + Phenylalanine(c) + O2(c) ⇌ Dihydrobiopterin(c) + Tyrosine(c) + H2O(c)
r2677	2	Acetyl-CoA(c) ⇌ CoA(c) + CoA-activated acetyl group(c)
r0324	-1	Fumarylacetoacetate(c) + H2O(c) ⇌ Fumarate(c) + Acetoacetate(c)
r0243	-0.5	DHAP(c) ⇌ GAP(c)

Glycine from Proline (201)

Definition			Solution												
			exchanges		reactions								transp		
Objective	Constraints	Comment	imports	exports	c	m	r	p	l	n	s	b	s-c	b-c	int
Glycine	−Proline MCES +Urea	Transamination to Glycine using Proline	4.5 H_2O(s) 0.5 Glucose-6P(c) 1 Proline(s)	0.5 P_i(c) 2 CO_2(s) 1 Pyruvate(c) 1 Glycine(s) 0.5 ATP-energy(c) 1 ATP-energy(m) 1 NADH-redox-potential(c) 2 NADH-redox-potential(m) 1 NADPH-redox-potential(c) 1 THF-activated methyl group(c)	16	10	-	-	-	-	-	-	3	-	4

ID	flux	reaction
r0820	1	AKG(c) + Malate(m) ⇌ AKG(m) + Malate(c)
r0253	1	GAP(c) + Pi(c) + NAD+(c) ⇌ 1,3DPG(c) + NADH(c)
r0911	1	Proline(c) + Glutamate(m) ⇌ Proline(m) + Glutamate(c)
r0074	1	L-Glutamate 5-semialdehyde(m) + NAD+(m) + H2O(m) ⇌ Glutamate(m) + NADH(m)
r1326	1	NADPH(c) ⇌ NADPH-redox-potential(c) + NADP+(c)
r0192	0.5	Glucose-6P(c) ⇌ Fructose-6P(c)
r1552	-1	Glycine(c) + Proline(s) ⇌ Glycine(s) + Proline(c)
r1453	1	Proline(m) + Ubiquinol(m) ⇌ 1-Pyrroline-5-carboxylate(m) + Ubiquinone(m)
r0337	-1	ADP(c) + 1,3DPG(c) ⇌ ATP(c) + 3PG(c)

Continued on next page

Glycine from Proline (201)– continued

ID	flux	reaction
r0256	0.5	Fructose-1,6PP(c) ⇌ DHAP(c) + GAP(c)
r0175	1	1-Pyrroline-5-carboxylate(m) + H2O(m) ⇌ L-Glutamate 5-semialdehyde(m)
r0058	1	Malate(c) + NADP+(c) ⇌ Pyruvate(c) + CO2(c) + NADPH(c)
r1319	0.5	ATP(c) + H2O(c) ⇌ ADP(c) + Pi(c) + ATP-energy(c)
r0851	-4	H2O(m) ⇌ H2O(c)
r0509	1	Ubiquinone(m) + Succinate(m) → Ubiquinol(m) + Fumarate(m)
r0338	1	3PG(c) + NAD+(c) → 3-Phosphonooxypyruvate(c) + NADH(c)
r0100	-1	ADP(m) + GTP(m) ⇌ ATP(m) + GDP(m)
r0663	-1	3-Phosphonooxypyruvate(c) + Glutamate(c) → 3-Phosphoserine(c) + AKG(c)
r0159	1	3-Phosphoserine(c) + H2O(c) → Serine(c) + Pi(c)
r0736	0.5	ATP(c) + Fructose-6P(c) → ADP(c) + Fructose-1,6PP(c)
r0259	-1	Fumarate(m) + H2O(m) ⇌ Malate(m)
r0792	-1	5,10-Methylene-THF(c) + NADH(c) ⇌ 5-Methyl-THF(c) + NAD+(c)
r0230	-1	THF(c) + Serine(c) ⇌ 5,10-Methylene-THF(c) + Glycine(c) + H2O(c)
r1320	1	ATP(m) + H2O(m) ⇌ ADP(m) + Pi(m) + ATP-energy(m)
r1114	1	AKG(m) + CoA(m) + NAD+(m) ⇌ NADH(m) + CO2(m) + Succinyl-CoA(m)
r2676	1	5-Methyl-THF(c) ⇌ THF(c) + THF-activated methyl group(c)
r1322	1	NADH(c) ⇌ NAD+(c) + NADH-redox-potential(c)
r0867	2	CO2(c) ⇌ CO2(s)
r0849	4.5	H2O(s) ⇌ H2O(c)
r0125	-1	GDP(m) + Pi(m) + Succinyl-CoA(m) ⇌ GTP(m) + Succinate(m) + CoA(m)
r1323	2	NADH(m) ⇌ NAD+(m) + NADH-redox-potential(m)
r0868	-1	CO2(c) ⇌ CO2(m)
r0243	-0.5	DHAP(c) ⇌ GAP(c)

Glycine from Serine (202)

Definition			Solution												
			exchanges		reactions								transp		
Objective	Constraints	Comment	imports	exports	c	m	r	p	l	n	s	b	s-c	b-c	int
Glycine	−Serine MCES +Urea	Transamination to Glycine using Serine	1 Serine(s) 1 NADH-redox-potential(c)	1 H_2O(s) 1 Glycine(s) 1 THF-activated methyl group(c)	4	-	-	-	-	-	-	-	2	-	-

ID	flux	reaction
r1545	-1	Glycine(c) + Serine(s) ⇌ Glycine(s) + Serine(c)
r0792	-1	5,10-Methylene-THF(c) + NADH(c) ⇌ 5-Methyl-THF(c) + NAD+(c)
r0230	-1	THF(c) + Serine(c) ⇌ 5,10-Methylene-THF(c) + Glycine(c) + H2O(c)
r2676	1	5-Methyl-THF(c) ⇌ THF(c) + THF-activated methyl group(c)
r1322	-1	NADH(c) ⇌ NAD+(c) + NADH-redox-potential(c)
r0849	-1	H2O(s) ⇌ H2O(c)

Glycine from Threonine (203)

Definition			Solution												
			exchanges		reactions								transp		
Objective	Constraints	Comment	imports	exports	c	m	r	p	l	n	s	b	s-c	b-c	int
Glycine	−Threonine MCES +Urea	Transamination to Glycine using Threonine	0.5 H_2O(s) 0.5 O_2(s) 0.5 Glucose-6P(c) 1 Threonine(s)	0.5 P_i(c) 2 CO_2(s) 1 Glycine(s) 0.5 ATP-energy(c) 1 NADH-redox-potential(c) 2 NADH-redox-potential(m) 3 Proton-gradient(m) 1 THF-activated methyl group(c) 1 CoA-activated acetyl group(m)	15	9	-	-	-	-	-	-	4	-	11

ID	flux	reaction
r0855	0.5	O2(s) ⇌ O2(c)
r0856	-0.5	O2(m) ⇌ O2(c)
r0592	-1	H2O(m) + Acrylyl-CoA(m) → 3-Hydroxypropionyl-CoA(m)
r0596	-1	3-Hydroxypropionyl-CoA(m) + H2O(m) → Hydracrylate(m) + CoA(m)
r0017	0.5	O2(m) + 4 H+(PG)(m) + 4 Ferrocytochrome C(m) → 4 Ferricytochrome C(m) + 2 H2O(m) + 4 H+(PG)(c)
r1555	-1	Glycine(c) + Threonine(s) ⇌ Glycine(s) + Threonine(c)
r0911	1	Proline(c) + Glutamate(m) ⇌ Proline(m) + Glutamate(c)
r0337	-1	ADP(c) + 1,3DPG(c) ⇌ ATP(c) + 3PG(c)
r0338	1	3PG(c) + NAD+(c) → 3-Phosphonooxypyruvate(c) + NADH(c)
r1426	1	Proline(m) + H+(PG)(c) → Proline(c) + H+(PG)(m)
r0073	-1	AKG(m) + NADH(m) + NH3(m) ⇌ Glutamate(m) + NAD+(m) + H2O(m)
r0507	1	Ubiquinol(m) + 2 Ferricytochrome C(m) + 4 H+(PG)(m) → Ubiquinone(m) + 4 H+(PG)(c) + 2 Ferrocytochrome C(m)
r0240	1	Threonine(c) ⇌ 2-Oxobutyrate(c) + NH3(c)
r0243	-0.5	DHAP(c) ⇌ GAP(c)
r1330	3	H+(PG)(c) ⇌ H+(PG)(m) + Proton-gradient(m)
r1319	0.5	ATP(c) + H2O(c) ⇌ ADP(c) + Pi(c) + ATP-energy(c)
r0849	0.5	H2O(s) ⇌ H2O(c)
r0867	2	CO2(c) ⇌ CO2(s)
r0868	-2	CO2(c) ⇌ CO2(m)
r0736	0.5	ATP(c) + Fructose-6P(c) → ADP(c) + Fructose-1,6PP(c)
r0159	1	3-Phosphoserine(c) + H2O(c) → Serine(c) + Pi(c)
r0174	1	CoA(m) + NAD+(m) + 3-Oxopropanoate(m) → Acetyl-CoA(m) + CO2(m) + NADH(m)
r0192	0.5	Glucose-6P(c) ⇌ Fructose-6P(c)
r0792	-1	5,10-Methylene-THF(c) + NADH(c) ⇌ 5-Methyl-THF(c) + NAD+(c)
r1154	1	CoA(m) + NAD+(m) + 2-Oxobutyrate(m) ⇌ CO2(m) + Propanoyl-CoA(m) + NADH(m)
r1155	1	2-Oxobutyrate(c) + H+(PG)(c) → 2-Oxobutyrate(m) + H+(PG)(m)
r2676	1	5-Methyl-THF(c) ⇌ THF(c) + THF-activated methyl group(c)
r0365	1	Hydracrylate(m) + NAD+(m) → NADH(m) + 3-Oxopropanoate(m)
r2678	1	Acetyl-CoA(m) ⇌ CoA(m) + CoA-activated acetyl group(m)
r0663	-1	3-Phosphonooxypyruvate(c) + Glutamate(c) → 3-Phosphoserine(c) + AKG(c)
r0230	-1	THF(c) + Serine(c) ⇌ 5,10-Methylene-THF(c) + Glycine(c) + H2O(c)
r1322	1	NADH(c) ⇌ NAD+(c) + NADH-redox-potential(c)
r0683	1	Propanoyl-CoA(m) + Ubiquinone(m) → Ubiquinol(m) + Acrylyl-CoA(m)
r1323	2	NADH(m) ⇌ NAD+(m) + NADH-redox-potential(m)
r0253	1	GAP(c) + Pi(c) + NAD+(c) ⇌ 1,3DPG(c) + NADH(c)
r0256	0.5	Fructose-1,6PP(c) ⇌ DHAP(c) + GAP(c)
r1047	1	Pi(c) + H+(PG)(c) → Pi(m) + H+(PG)(m)
r2419	1	AKG(c) + Pi(m) ⇌ AKG(m) + Pi(c)
r0838	1	NH3(c) ⇌ NH3(m)

Glycine from Tryptophan (204)

Definition			Solution													
			exchanges		reactions								transp		
Objective	Constraints	Comment	imports	exports	c	m	r	p	l	n	s	b	s-c	b-c	int
Glycine	−Tryptophan MCES +Urea	Transamination to Glycine using Tryptophan	0.5 H_2O(s) 1.75 O_2(s) 0.5 Tryptophan(s) 0.5 Glucose-6P(c) 1.5 NADPH-redox-potential(c) 0.5 NADPH-redox-potential(m)	0.5 P_i(c) 1.5 CO_2(s) 0.5 Pyruvate(c) 1 Glycine(s) 1 NADH-redox-potential(c) 1 NADH-redox-potential(m) 1.5 Proton-gradient(m) 0.5 Na-gradient(c) 1.5 THF-activated methyl group(c) 1 CoA-activated acetyl group(m)	26	9	-	-	-	-	-	-	6	-	12

ID	flux	reaction
r0855	1.75	O2(s) ⇌ O2(c)
r0856	-0.25	O2(m) ⇌ O2(c)
		Continued on next page

ID	flux	reaction
r0293	-0.5	5,10-Methylene-THF(c) + NADP+(c) ⇌ 5,10-Methenyl-THF(c) + NADPH(c)
r0460	0.5	(S)-3-Hydroxybutyryl-CoA(m) + NAD+(m) ⇌ NADH(m) + Acetoacetyl-CoA(m)
r0448	0.5	2-Oxoadipate(m) + CoA(m) + NAD+(m) ⇌ Glutaryl-CoA(m) + CO2(m) + NADH(m)
r0449	-0.5	2-Oxoadipate(c) + NH3(c) + NADP+(c) ⇌ 2-Aminomuconate(c) + NADPH(c) + H2O(c)
r0017	0.25	O2(m) + 4 H+(PG)(m) + 4 Ferrocytochrome C(m) → 4 Ferricytochrome C(m) + 2 H2O(m) + 4 H+(PG)(c)
r0169	0.5	Tryptophan(c) + O2(c) ⇌ L-Formylkynurenine(c)
r0911	0.5	Proline(c) + Glutamate(m) ⇌ Proline(m) + Glutamate(c)
r0337	-1	ADP(c) + 1,3DPG(c) ⇌ ATP(c) + 3PG(c)
r0338	1	3PG(c) + NAD+(c) → 3-Phosphonooxypyruvate(c) + NADH(c)
r0070	-0.5	CoA(m) + Acetoacetyl-CoA(m) ⇌ 2 Acetyl-CoA(m)
r2664	0.5	Na+(c) ⇌ Na+(s) + Na-gradient(c)
r1426	0.5	Proline(m) + H+(PG)(c) → Proline(c) + H+(PG)(m)
r0075	-0.5	AKG(m) + NADPH(m) + NH3(m) ⇌ Glutamate(m) + H2O(m) + NADP+(m)
r0371	-0.5	10-Formyl-THF(c) ⇌ 5,10-Methenyl-THF(c) + H2O(c)
r0507	0.5	Ubiquinol(m) + 2 Ferricytochrome C(m) + 4 H+(PG)(m) → Ubiquinone(m) + 4 H+(PG)(c) + 2 Ferrocytochrome C(m)
r1747	0.5	Tryptophan(s) + Glycine(c) + Na+(s) → Tryptophan(c) + Glycine(s) + Na+(c)
r0228	0.5	THF(c) + Formate(c) + ATP(c) → ADP(c) + Pi(c) + 10-Formyl-THF(c)
r0676	0.5	2-Amino-3-carboxymuconate semialdehyde(c) ⇌ 2-Aminomuconate semialdehyde(c) + CO2(c)
r0243	-0.5	DHAP(c) ⇌ GAP(c)
r1330	1.5	H+(PG)(c) ⇌ H+(PG)(m) + Proton-gradient(m)
r0541	0.5	Glutaryl-CoA(m) + Ubiquinone(m) ⇌ Crotonyl-CoA(m) + CO2(m) + Ubiquinol(m)
r0562	0.5	3-Hydroxyanthranilate(c) + O2(c) ⇌ 2-Amino-3-carboxymuconate semialdehyde(c)
r0563	0.5	H2O(c) + 3-Hydroxy-L-kynurenine(c) ⇌ 3-Hydroxyanthranilate(c) + Alanine(c)
r0849	0.5	H2O(s) ⇌ H2O(c)
r0867	1.5	CO2(c) ⇌ CO2(s)
r0868	-1	CO2(c) ⇌ CO2(m)
r0588	-0.5	Crotonyl-CoA(m) + H2O(m) ⇌ (S)-3-Hydroxybutyryl-CoA(m)
r0736	0.5	ATP(c) + Fructose-6P(c) → ADP(c) + Fructose-1,6PP(c)
r0159	1	3-Phosphoserine(c) + H2O(c) → Serine(c) + Pi(c)
r0454	0.5	L-Formylkynurenine(c) + H2O(c) ⇌ Formate(c) + Kynurenine(c)
r0455	0.5	Kynurenine(c) + O2(c) + NADPH(c) ⇌ H2O(c) + 3-Hydroxy-L-kynurenine(c) + NADP+(c)
r0192	0.5	Glucose-6P(c) ⇌ Fructose-6P(c)
r0792	-1.5	5,10-Methylene-THF(c) + NADH(c) ⇌ 5-Methyl-THF(c) + NAD+(c)
r0080	0.5	Alanine(c) + AKG(c) ⇌ Pyruvate(c) + Glutamate(c)
r0645	0.5	2-Aminomuconate semialdehyde(c) + NAD+(c) + H2O(c) ⇌ 2-Aminomuconate(c) + NADH(c)
r2523	-0.5	Glycine(c) ⇌ Glycine(s)
r2676	1.5	5-Methyl-THF(c) ⇌ THF(c) + THF-activated methyl group(c)
r2678	1	Acetyl-CoA(m) ⇌ CoA(m) + CoA-activated acetyl group(m)
r0945	0.5	2-Oxoadipate(c) + AKG(m) ⇌ 2-Oxoadipate(m) + AKG(c)
r0663	-1	3-Phosphonooxypyruvate(c) + Glutamate(c) → 3-Phosphoserine(c) + AKG(c)
r0230	-1	THF(c) + Serine(c) ⇌ 5,10-Methylene-THF(c) + Glycine(c) + H2O(c)
r1322	1	NADH(c) ⇌ NAD+(c) + NADH-redox-potential(c)
r1323	1	NADH(m) ⇌ NAD+(m) + NADH-redox-potential(m)
r1326	-1.5	NADPH(c) ⇌ NADPH-redox-potential(c) + NADP+(c)
r0253	1	GAP(c) + Pi(c) + NAD+(c) ⇌ 1,3DPG(c) + NADH(c)
r1327	-0.5	NADPH(m) ⇌ NADPH-redox-potential(m) + NADP+(m)
r1047	1	Pi(c) + H+(PG)(c) → Pi(m) + H+(PG)(m)
r0256	0.5	Fructose-1,6PP(c) ⇌ DHAP(c) + GAP(c)
r2419	1	AKG(c) + Pi(m) ⇌ AKG(m) + Pi(c)
r0838	0.5	NH3(c) ⇌ NH3(m)
r0851	0.5	H2O(m) ⇌ H2O(c)

Glycine from Tyrosine (205)

Definition			Solution												
			exchanges		reactions								transp		
Objective	Constraints	Comment	imports	exports	c	m	r	p	l	n	s	b	s-c	b-c	int
Glycine	−Tyrosine MCES +Urea	Transamination to Glycine using Tyrosine	1.5 H_2O(s) 2 O_2(s) 1 Tyrosine(s) 0.5 Glucose-6P(c) 1.5 ATP-energy(c)	0.5 P_i(c) 2 CO_2(s) 1 Pyruvate(c) 1 Glycine(s) 2 NADH-redox-potential(c) 1 THF-activated methyl group(c) 2 CoA-activated acetyl group(c)	26	-	-	-	-	-	-	-	4	-	-

ID	flux	reaction
r0253	1	GAP(c) + Pi(c) + NAD+(c) ⇌ 1,3DPG(c) + NADH(c)
r0605	1	4-Maleylacetoacetate(c) ⇌ Fumarylacetoacetate(c)
r0543	1	Homogentisate(c) + O2(c) ⇌ 4-Maleylacetoacetate(c)
r0192	0.5	Glucose-6P(c) ⇌ Fructose-6P(c)
r0183	1	Tyrosine(c) + AKG(c) ⇌ 4-Hydroxyphenylpyruvate(c) + Glutamate(c)
r0544	1	4-Hydroxyphenylpyruvate(c) + O2(c) ⇌ Homogentisate(c) + CO2(c)
r0337	-1	ADP(c) + 1,3DPG(c) ⇌ ATP(c) + 3PG(c)
r0256	0.5	Fructose-1,6PP(c) ⇌ DHAP(c) + GAP(c)
r0058	1	Malate(c) + NADP+(c) ⇌ Pyruvate(c) + CO2(c) + NADPH(c)
r1319	-1.5	ATP(c) + H2O(c) ⇌ ADP(c) + Pi(c) + ATP-energy(c)
r0338	1	3PG(c) + NAD+(c) → 3-Phosphonooxypyruvate(c) + NADH(c)
r0663	-1	3-Phosphonooxypyruvate(c) + Glutamate(c) → 3-Phosphoserine(c) + AKG(c)
r0258	-1	Fumarate(c) + H2O(c) ⇌ Malate(c)
r0159	1	3-Phosphoserine(c) + H2O(c) → Serine(c) + Pi(c)
r0069	-1	CoA(c) + Acetoacetyl-CoA(c) ⇌ 2 Acetyl-CoA(c)
r0736	0.5	ATP(c) + Fructose-6P(c) → ADP(c) + Fructose-1,6PP(c)
r0030	1	ATP(c) + AMP(c) ⇌ 2 ADP(c)
r0296	-1	5,10-Methylene-THF(c) + NADPH(c) ⇌ 5-Methyl-THF(c) + NADP+(c)
r0855	2	O2(s) ⇌ O2(c)
r0320	1	ATP(c) + CoA(c) + Acetoacetate(c) → AMP(c) + PPi(c) + Acetoacetyl-CoA(c)
r0230	-1	THF(c) + Serine(c) ⇌ 5,10-Methylene-THF(c) + Glycine(c) + H2O(c)
r1549	-1	Glycine(c) + Tyrosine(s) ⇌ Glycine(s) + Tyrosine(c)
r0007	1	PPi(c) + H2O(c) → 2 Pi(c)
r2676	1	5-Methyl-THF(c) ⇌ THF(c) + THF-activated methyl group(c)
r1322	2	NADH(c) ⇌ NAD+(c) + NADH-redox-potential(c)
r0867	2	CO2(c) ⇌ CO2(s)
r0849	1.5	H2O(s) ⇌ H2O(c)
r2677	2	Acetyl-CoA(c) ⇌ CoA(c) + CoA-activated acetyl group(c)
r0324	-1	Fumarylacetoacetate(c) + H2O(c) ⇌ Fumarate(c) + Acetoacetate(c)
r0243	-0.5	DHAP(c) ⇌ GAP(c)

Glycine from Valine (206)

Definition			Solution												
			exchanges		reactions								transp		
Objective	Constraints	Comment	imports	exports	c	m	r	p	l	n	s	b	s-c	b-c	int
Glycine	−Valine MCES +Urea	Transamination to Glycine using Valine	2.5 H_2O(s) 1 O_2(s) 0.5 Glucose-6P(c) 1 Valine(s)	0.5 P_i(c) 2 CO_2(s) 1 Pyruvate(c) 1 Glycine(s) 0.5 ATP-energy(c) 1 ATP-energy(m) 2 NADH-redox-potential(c) 3 NADH-redox-potential(m) 10 Proton-gradient(m) 1 THF-activated methyl group(c)	15	14	-	-	-	-	-	-	4	-	10

ID	flux	reaction
r0125	-1	GDP(m) + Pi(m) + Succinyl-CoA(m) ⇌ GTP(m) + Succinate(m) + CoA(m)

Continued on next page

Glycine from Valine (206)– continued

ID	flux	reaction
r0855	1	O2(s) ⇌ O2(c)
r0856	-1	O2(m) ⇌ O2(c)
r0296	-1	5,10-Methylene-THF(c) + NADPH(c) ⇌ 5-Methyl-THF(c) + NADP+(c)
r0017	1	O2(m) + 4 H+(PG)(m) + 4 Ferrocytochrome C(m) → 4 Ferricytochrome C(m) + 2 H2O(m) + 4 H+(PG)(c)
r1554	-1	Glycine(c) + Valine(s) ⇌ Glycine(s) + Valine(c)
r0482	1	3-Hydroxyisobutyrate(m) + NAD+(m) → 2-Methyl-3-oxopropanoate(m) + NADH(m)
r0911	1	Proline(c) + Glutamate(m) ⇌ Proline(m) + Glutamate(c)
r0337	-1	ADP(c) + 1,3DPG(c) ⇌ ATP(c) + 3PG(c)
r0338	1	3PG(c) + NAD+(c) → 3-Phosphonooxypyruvate(c) + NADH(c)
r1426	1	Proline(m) + H+(PG)(c) → Proline(c) + H+(PG)(m)
r0058	1	Malate(c) + NADP+(c) ⇌ Pyruvate(c) + CO2(c) + NADPH(c)
r0507	2	Ubiquinol(m) + 2 Ferricytochrome C(m) + 4 H+(PG)(m) → Ubiquinone(m) + 4 H+(PG)(c) + 2 Ferrocytochrome C(m)
r0509	1	Ubiquinone(m) + Succinate(m) → Ubiquinol(m) + Fumarate(m)
r0243	-0.5	DHAP(c) ⇌ GAP(c)
r0820	1	AKG(c) + Malate(m) ⇌ AKG(m) + Malate(c)
r1330	10	H+(PG)(c) ⇌ H+(PG)(m) + Proton-gradient(m)
r1319	0.5	ATP(c) + H2O(c) ⇌ ADP(c) + Pi(c) + ATP-energy(c)
r0263	1	Valine(m) + AKG(m) → Glutamate(m) + 3-Methyl-2-oxobutyrate(m)
r0560	1	Isobutyryl-CoA(m) + Ubiquinone(m) → Methacrylyl-CoA(m) + Ubiquinol(m)
r0849	2.5	H2O(s) ⇌ H2O(c)
r0867	2	CO2(c) ⇌ CO2(s)
r0868	-1	CO2(c) ⇌ CO2(m)
r0736	0.5	ATP(c) + Fructose-6P(c) → ADP(c) + Fructose-1,6PP(c)
r0159	1	3-Phosphoserine(c) + H2O(c) → Serine(c) + Pi(c)
r0600	1	3-Methyl-2-oxobutyrate(m) + CoA(m) + NAD+(m) ⇌ Isobutyryl-CoA(m) + CO2(m) + NADH(m)
r0192	0.5	Glucose-6P(c) ⇌ Fructose-6P(c)
r0197	1	(R)-Methylmalonyl-CoA(m) ⇌ Succinyl-CoA(m)
r0624	1	2-Methyl-3-oxopropanoate(m) + CoA(m) + NAD+(m) ⇌ (R)-Methylmalonyl-CoA(m) + NADH(m)
r0779	1	3-Hydroxyisobutyryl-CoA(m) + H2O(m) → 3-Hydroxyisobutyrate(m) + CoA(m)
r0923	1	Valine(c) + H+(PG)(c) → Valine(m) + H+(PG)(m)
r2676	1	5-Methyl-THF(c) ⇌ THF(c) + THF-activated methyl group(c)
r0663	-1	3-Phosphonooxypyruvate(c) + Glutamate(c) → 3-Phosphoserine(c) + AKG(c)
r0230	-1	THF(c) + Serine(c) ⇌ 5,10-Methylene-THF(c) + Glycine(c) + H2O(c)
r1320	1	ATP(m) + H2O(m) ⇌ ADP(m) + Pi(m) + ATP-energy(m)
r0669	1	Methacrylyl-CoA(m) + H2O(m) → 3-Hydroxyisobutyryl-CoA(m)
r1322	2	NADH(c) ⇌ NAD+(c) + NADH-redox-potential(c)
r1323	3	NADH(m) ⇌ NAD+(m) + NADH-redox-potential(m)
r0100	-1	ADP(m) + GTP(m) ⇌ ATP(m) + GDP(m)
r0253	1	GAP(c) + Pi(c) + NAD+(c) ⇌ 1,3DPG(c) + NADH(c)
r0256	0.5	Fructose-1,6PP(c) ⇌ DHAP(c) + GAP(c)
r0259	-1	Fumarate(m) + H2O(m) ⇌ Malate(m)
r0851	-2	H2O(m) ⇌ H2O(c)

Glycine from beta-Alanine (207)

Definition			Solution												
			exchanges		reactions								transp		
Objective	Constraints	Comment	imports	exports	c	m	r	p	l	n	s	b	s-c	b-c	int
Glycine	−beta-Alanine MCES +Urea	Transamination to Glycine using beta-Alanine	0.5 H_2O(s) 0.5 Glucose-6P(c) 1 beta-Alanine(s) 1 NADPH-redox-potential(c) 2 Proton-gradient(m)	0.5 P_i(c) 1 CO_2(s) 1 Glycine(s) 0.5 ATP-energy(c) 2 NADH-redox-potential(c) 1 NADH-redox-potential(m) 1 THF-activated methyl group(c) 1 CoA-activated acetyl group(m)	15	4	-	-	-	-	-	-	4	-	7

ID	flux	reaction
r2678	1	Acetyl-CoA(m) ⇌ CoA(m) + CoA-activated acetyl group(m)
r0253	1	GAP(c) + Pi(c) + NAD+(c) ⇌ 1,3DPG(c) + NADH(c)
r0217	1	beta-Alanine(m) + AKG(m) ⇌ Glutamate(m) + 3-Oxopropanoate(m)
r0911	1	Proline(c) + Glutamate(m) ⇌ Proline(m) + Glutamate(c)
r2523	-1	Glycine(c) ⇌ Glycine(s)
r1326	-1	NADPH(c) ⇌ NADPH-redox-potential(c) + NADP+(c)
r1047	1	Pi(c) + H+(PG)(c) → Pi(m) + H+(PG)(m)
r0192	0.5	Glucose-6P(c) ⇌ Fructose-6P(c)
r0174	1	CoA(m) + NAD+(m) + 3-Oxopropanoate(m) → Acetyl-CoA(m) + CO2(m) + NADH(m)
r1426	1	Proline(m) + H+(PG)(c) → Proline(c) + H+(PG)(m)
r0899	-1	beta-Alanine(s) ⇌ beta-Alanine(c)
r0337	-1	ADP(c) + 1,3DPG(c) ⇌ ATP(c) + 3PG(c)
r0256	0.5	Fructose-1,6PP(c) ⇌ DHAP(c) + GAP(c)
r1319	0.5	ATP(c) + H2O(c) ⇌ ADP(c) + Pi(c) + ATP-energy(c)
r0338	1	3PG(c) + NAD+(c) → 3-Phosphonooxypyruvate(c) + NADH(c)
r0663	-1	3-Phosphonooxypyruvate(c) + Glutamate(c) → 3-Phosphoserine(c) + AKG(c)
r0159	1	3-Phosphoserine(c) + H2O(c) → Serine(c) + Pi(c)
r2419	1	AKG(c) + Pi(m) ⇌ AKG(m) + Pi(c)
r0736	0.5	ATP(c) + Fructose-6P(c) → ADP(c) + Fructose-1,6PP(c)
r0296	-1	5,10-Methylene-THF(c) + NADPH(c) ⇌ 5-Methyl-THF(c) + NADP+(c)
r0230	-1	THF(c) + Serine(c) ⇌ 5,10-Methylene-THF(c) + Glycine(c) + H2O(c)
r1330	-2	H+(PG)(c) ⇌ H+(PG)(m) + Proton-gradient(m)
r2676	1	5-Methyl-THF(c) ⇌ THF(c) + THF-activated methyl group(c)
r1322	2	NADH(c) ⇌ NAD+(c) + NADH-redox-potential(c)
r0867	1	CO2(c) ⇌ CO2(s)
r0849	0.5	H2O(s) ⇌ H2O(c)
r1323	1	NADH(m) ⇌ NAD+(m) + NADH-redox-potential(m)
r0900	1	beta-Alanine(c) ⇌ beta-Alanine(m)
r0868	-1	CO2(c) ⇌ CO2(m)
r0243	-0.5	DHAP(c) ⇌ GAP(c)

Glutamine from Alanine (208)

Definition			Solution													
			exchanges		reactions								transp		
Objective	Constraints	Comment	imports	exports	c	m	r	p	l	n	s	b	s-c	b-c	int
Glutamine	−Alanine MCES +Urea	Transamination to Glutamine using Alanine	1 H_2O(s) 2 Alanine(s) 1 ATP-energy(m) 1 NADPH-redox-potential(m) 1 CoA-activated acetyl group(m)	1 Pyruvate(c) 1 Glutamine(s) 3 NADH-redox-potential(m)	2	12	-	-	-	-	-	-	3	-	5

ID	flux	reaction
r2678	-1	Acetyl-CoA(m) ⇌ CoA(m) + CoA-activated acetyl group(m)
r0316	1	Citrate(m) ⇌ Isocitrate(m)
r0073	1	Glutamate(m) + NAD+(m) + H2O(m) ⇌ AKG(m) + NADH(m) + NH3(m)
r0425	1	Isocitrate(m) + NAD+(m) ⇌ Oxalosuccinate(m) + NADH(m)
r0083	1	Oxalosuccinate(m) ⇌ AKG(m) + CO2(m)
r1434	1	Alanine(c) → Alanine(m)
r2524	1	Alanine(s) ⇌ Alanine(c)
r1327	-1	NADPH(m) ⇌ NADPH-redox-potential(m) + NADP+(m)
r0851	-1	H2O(m) ⇌ H2O(c)
r0077	1	ATP(c) + Glutamate(c) + NH3(c) → ADP(c) + Pi(c) + Glutamine(c)
r0059	-1	Pyruvate(m) + CO2(m) + NADPH(m) ⇌ Malate(m) + NADP+(m)
r2418	1	ADP(c) + ATP(m) → ADP(m) + ATP(c)
r2419	-1	AKG(m) + Pi(c) ⇌ AKG(c) + Pi(m)
r1558	1	Alanine(s) + Glutamine(c) ⇌ Alanine(c) + Glutamine(s)

Continued on next page

ID	flux	reaction
r1320	-1	ATP(m) + H2O(m) ⇌ ADP(m) + Pi(m) + ATP-energy(m)
r0838	-1	NH3(c) ⇌ NH3(m)
r0105	1	NAD+(m) + Malate(m) ⇌ OAA(m) + NADH(m)
r0849	1	H2O(s) ⇌ H2O(c)
r0107	-1	Acetyl-CoA(m) + H2O(m) + OAA(m) ⇌ Citrate(m) + CoA(m)
r0080	1	Alanine(c) + AKG(c) ⇌ Pyruvate(c) + Glutamate(c)
r1323	3	NADH(m) ⇌ NAD+(m) + NADH-redox-potential(m)
r0081	1	Alanine(m) + AKG(m) ⇌ Pyruvate(m) + Glutamate(m)

Glutamine from Arginine (209)

Definition			Solution												
			exchanges		reactions								transp		
Objective	Constraints	Comment	imports	exports	c	m	r	p	l	n	s	b	s-c	b-c	int
Glutamine	−Arginine MCES +Urea	Transamination to Glutamine using Arginine	2 H_2O(s) 1 Arginine(s) 1 ATP-energy(c) 2 Proton-gradient(m)	1 Glutamine(s) 1 Urea(s) 2 NADH-redox-potential(m)	3	4	-	-	-	-	-	-	4	-	7

ID	flux	reaction
r1994	1	Lysine(s) + Glutamine(c) → Lysine(c) + Glutamine(s)
r0896	1	Urea(c) ⇌ Urea(s)
r0073	1	Glutamate(m) + NAD+(m) + H2O(m) ⇌ AKG(m) + NADH(m) + NH3(m)
r0911	1	Proline(c) + Glutamate(m) ⇌ Proline(m) + Glutamate(c)
r0074	1	L-Glutamate 5-semialdehyde(m) + NAD+(m) + H2O(m) ⇌ Glutamate(m) + NADH(m)
r1426	1	Proline(m) + H+(PG)(c) → Proline(c) + H+(PG)(m)
r1319	-1	ATP(c) + H2O(c) ⇌ ADP(c) + Pi(c) + ATP-energy(c)
r0851	-2	H2O(m) ⇌ H2O(c)
r0167	1	AKG(m) + Ornithine(m) ⇌ L-Glutamate 5-semialdehyde(m) + Glutamate(m)
r0077	1	ATP(c) + Glutamate(c) + NH3(c) → ADP(c) + Pi(c) + Glutamine(c)
r1663	-1	Lysine(c) + Arginine(s) ⇌ Lysine(s) + Arginine(c)
r0838	-1	NH3(c) ⇌ NH3(m)
r2396	-1	Lysine(m) + Ornithine(c) + H+(PG)(c) → Lysine(c) + Ornithine(m) + H+(PG)(m)
r1330	-2	H+(PG)(c) ⇌ H+(PG)(m) + Proton-gradient(m)
r1070	1	Lysine(c) ⇌ Lysine(m)
r0849	2	H2O(s) ⇌ H2O(c)
r1323	2	NADH(m) ⇌ NAD+(m) + NADH-redox-potential(m)
r0144	1	Arginine(c) + H2O(c) ⇌ Urea(c) + Ornithine(c)

Glutamine from Asparagine (210)

Definition			Solution												
			exchanges		reactions								transp		
Objective	Constraints	Comment	imports	exports	c	m	r	p	l	n	s	b	s-c	b-c	int
Glutamine	−Asparagine MCES +Urea	Transamination to Glutamine using Asparagine	1 H_2O(s) 1 Asparagine(s) 1 ATP-energy(c) 1 NADH-redox-potential(c) 1 CoA-activated acetyl group(m)	1 CO_2(s) 1 Glutamine(s) 1 NADH-redox-potential(m) 1 NADPH-redox-potential(c)	9	5	-	-	-	-	-	-	4	-	2

ID	flux	reaction
r2678	-1	Acetyl-CoA(m) ⇌ CoA(m) + CoA-activated acetyl group(m)
r0316	1	Citrate(m) ⇌ Isocitrate(m)
r0127	1	H2O(c) + Asparagine(c) → Aspartate(c) + NH3(c)
r0109	1	AKG(c) + Aspartate(c) ⇌ OAA(c) + Glutamate(c)

Continued on next page

ID	flux	reaction
r0082	1	Oxalosuccinate(c) ⇌ AKG(c) + CO2(c)
r1326	1	NADPH(c) ⇌ NADPH-redox-potential(c) + NADP+(c)
r0913	1	Isocitrate(m) + Malate(c) ⇌ Isocitrate(c) + Malate(m)
r1553	-1	Glycine(s) + Asparagine(c) ⇌ Glycine(c) + Asparagine(s)
r1544	1	Glycine(s) + Glutamine(c) ⇌ Glycine(c) + Glutamine(s)
r1319	-1	ATP(c) + H2O(c) ⇌ ADP(c) + Pi(c) + ATP-energy(c)
r0851	-1	H2O(m) ⇌ H2O(c)
r0077	1	ATP(c) + Glutamate(c) + NH3(c) → ADP(c) + Pi(c) + Glutamine(c)
r0104	-1	OAA(c) + NADH(c) ⇌ NAD+(c) + Malate(c)
r0105	1	NAD+(m) + Malate(m) ⇌ OAA(m) + NADH(m)
r1322	-1	NADH(c) ⇌ NAD+(c) + NADH-redox-potential(c)
r0867	1	CO2(c) ⇌ CO2(s)
r0849	1	H2O(s) ⇌ H2O(c)
r0422	1	Isocitrate(c) + NADP+(c) ⇌ Oxalosuccinate(c) + NADPH(c)
r0107	-1	Acetyl-CoA(m) + H2O(m) + OAA(m) ⇌ Citrate(m) + CoA(m)
r1323	1	NADH(m) ⇌ NAD+(m) + NADH-redox-potential(m)

Glutamine from Aspartate (211)

Definition			Solution											
			exchanges		reactions							transp		
Objective	Constraints	Comment	imports	exports	c	m	r	p	l	n	s	b	s-c	b-c	int
Glutamine	−Aspartate MCES +Urea	Transamination to Glutamine using Aspartate	1 H_2O(s) 2 Aspartate(s) 2 ATP-energy(c) 1 NADH-redox-potential(c) 1 CoA-activated acetyl group(m)	2 CO_2(s) 1 Pyruvate(c) 1 Glutamine(s) 1 NADH-redox-potential(m) 2 NADPH-redox-potential(c) 2 Na-gradient(c)	14	5	-	-	-	-	-	-	5	-	2

ID	flux	reaction
r2678	-1	Acetyl-CoA(m) ⇌ CoA(m) + CoA-activated acetyl group(m)
r0316	1	Citrate(m) ⇌ Isocitrate(m)
r0109	1	AKG(c) + Aspartate(c) ⇌ OAA(c) + Glutamate(c)
r0082	1	Oxalosuccinate(c) ⇌ AKG(c) + CO2(c)
r1326	2	NADPH(c) ⇌ NADPH-redox-potential(c) + NADP+(c)
r0913	1	Isocitrate(m) + Malate(c) ⇌ Isocitrate(c) + Malate(m)
r0058	1	Malate(c) + NADP+(c) ⇌ Pyruvate(c) + CO2(c) + NADPH(c)
r2525	-1	Glutamine(c) ⇌ Glutamine(s)
r1319	-2	ATP(c) + H2O(c) ⇌ ADP(c) + Pi(c) + ATP-energy(c)
r0851	-1	H2O(m) ⇌ H2O(c)
r0077	1	ATP(c) + Glutamate(c) + NH3(c) → ADP(c) + Pi(c) + Glutamine(c)
r0258	-1	Fumarate(c) + H2O(c) ⇌ Malate(c)
r0277	1	GTP(c) + IMP(c) + Aspartate(c) → GDP(c) + Pi(c) + Adenylosuccinate(c)
r2664	2	Na+(c) ⇌ Na+(s) + Na-gradient(c)
r0104	-1	OAA(c) + NADH(c) ⇌ NAD+(c) + Malate(c)
r0099	1	ATP(c) + GDP(c) ⇌ ADP(c) + GTP(c)
r0105	1	NAD+(m) + Malate(m) ⇌ OAA(m) + NADH(m)
r1322	-1	NADH(c) ⇌ NAD+(c) + NADH-redox-potential(c)
r0867	2	CO2(c) ⇌ CO2(s)
r0849	1	H2O(s) ⇌ H2O(c)
r0422	1	Isocitrate(c) + NADP+(c) ⇌ Oxalosuccinate(c) + NADPH(c)
r0260	1	Adenylosuccinate(c) → Fumarate(c) + AMP(c)
r0107	-1	Acetyl-CoA(m) + H2O(m) + OAA(m) ⇌ Citrate(m) + CoA(m)
r0044	1	AMP(c) + H2O(c) ⇌ IMP(c) + NH3(c)
r1323	1	NADH(m) ⇌ NAD+(m) + NADH-redox-potential(m)
r1143	-2	Na+(c) + Aspartate(c) ← Na+(s) + Aspartate(s)

Glutamine from Cysteine (212)

Definition			Solution												
			exchanges		reactions								transp		
Objective	Constraints	Comment	imports	exports	c	m	r	p	l	n	s	b	s-c	b-c	int
Glutamine	−Cysteine MCES +Urea $+H_2S$	Transamination to Glutamine using Cysteine	1 H_2O(s) 1 Pyruvate(m) 2 Cysteine(s) 1 ATP-energy(c) 1 NADPH-redox-potential(m) 1 Proton-gradient(m) 1 CoA-activated acetyl group(m)	2 Pyruvate(c) 1 Glutamine(s) 2 H_2S(s) 1 NADH-redox-potential(m) 2 Na-gradient(c)	3	10	-	-	-	-	-	-	6	-	4

ID	flux	reaction
r2678	-1	Acetyl-CoA(m) ⇌ CoA(m) + CoA-activated acetyl group(m)
r1994	1	Lysine(s) + Glutamine(c) → Lysine(c) + Glutamine(s)
r0316	1	Citrate(m) ⇌ Isocitrate(m)
r0073	-1	AKG(m) + NADH(m) + NH3(m) ⇌ Glutamate(m) + NAD+(m) + H2O(m)
r0911	1	Proline(c) + Glutamate(m) ⇌ Proline(m) + Glutamate(c)
r0425	1	Isocitrate(m) + NAD+(m) ⇌ Oxalosuccinate(m) + NADH(m)
r0083	1	Oxalosuccinate(m) ⇌ AKG(m) + CO2(m)
r1426	1	Proline(m) + H+(PG)(c) → Proline(c) + H+(PG)(m)
r1327	-1	NADPH(m) ⇌ NADPH-redox-potential(m) + NADP+(m)
r0940	2	H2S(c) ⇌ H2S(s)
r0193	2	Cysteine(c) + H2O(c) ⇌ Pyruvate(c) + H2S(c) + NH3(c)
r1319	-1	ATP(c) + H2O(c) ⇌ ADP(c) + Pi(c) + ATP-energy(c)
r0077	1	ATP(c) + Glutamate(c) + NH3(c) → ADP(c) + Pi(c) + Glutamine(c)
r0059	-1	Pyruvate(m) + CO2(m) + NADPH(m) ⇌ Malate(m) + NADP+(m)
r1952	1	Cysteine(s) + Na+(s) + Lysine(c) → Cysteine(c) + Na+(c) + Lysine(s)
r2664	2	Na+(c) ⇌ Na+(s) + Na-gradient(c)
r2475	1	Cysteine(s) + Na+(s) → Cysteine(c) + Na+(c)
r0838	1	NH3(c) ⇌ NH3(m)
r0105	1	NAD+(m) + Malate(m) ⇌ OAA(m) + NADH(m)
r1330	-1	H+(PG)(c) ⇌ H+(PG)(m) + Proton-gradient(m)
r0849	1	H2O(s) ⇌ H2O(c)
r0107	-1	Acetyl-CoA(m) + H2O(m) + OAA(m) ⇌ Citrate(m) + CoA(m)
r1323	1	NADH(m) ⇌ NAD+(m) + NADH-redox-potential(m)

Glutamine from Cystine (213)

Definition			Solution												
			exchanges		reactions								transp		
Objective	Constraints	Comment	imports	exports	c	m	r	p	l	n	s	b	s-c	b-c	int
Glutamine	−Cystine MCES +Urea $+H_2S$	Transamination to Glutamine using Cystine	1 H_2O(s) 1 Pyruvate(m) 1 Cystine(s) 1 ATP-energy(c) 1 NADPH-redox-potential(c) 1 NADPH-redox-potential(m) 1 Proton-gradient(m) 1 CoA-activated acetyl group(m)	2 Pyruvate(c) 1 Glutamine(s) 2 H_2S(s) 1 NADH-redox-potential(m) 1 Na-gradient(c)	5	10	-	-	-	-	-	-	6	-	4

ID	flux	reaction
r2678	-1	Acetyl-CoA(m) ⇌ CoA(m) + CoA-activated acetyl group(m)
r1144	-1	Glutamate(c) + Na+(c) ← Glutamate(s) + Na+(s)
r0316	1	Citrate(m) ⇌ Isocitrate(m)
r0073	-1	AKG(m) + NADH(m) + NH3(m) ⇌ Glutamate(m) + NAD+(m) + H2O(m)
r0911	1	Proline(c) + Glutamate(m) ⇌ Proline(m) + Glutamate(c)
r0425	1	Isocitrate(m) + NAD+(m) ⇌ Oxalosuccinate(m) + NADH(m)
r0083	1	Oxalosuccinate(m) ⇌ AKG(m) + CO2(m)
r1326	-1	NADPH(c) ⇌ NADPH-redox-potential(c) + NADP+(c)
r1426	1	Proline(m) + H+(PG)(c) → Proline(c) + H+(PG)(m)

Continued on next page

ID	flux	reaction
r1327	-1	NADPH(m) ⇌ NADPH-redox-potential(m) + NADP+(m)
r0940	2	H2S(c) ⇌ H2S(s)
r0193	2	Cysteine(c) + H2O(c) ⇌ Pyruvate(c) + H2S(c) + NH3(c)
r2525	-1	Glutamine(c) ⇌ Glutamine(s)
r1319	-1	ATP(c) + H2O(c) ⇌ ADP(c) + Pi(c) + ATP-energy(c)
r0077	1	ATP(c) + Glutamate(c) + NH3(c) → ADP(c) + Pi(c) + Glutamine(c)
r0059	-1	Pyruvate(m) + CO2(m) + NADPH(m) ⇌ Malate(m) + NADP+(m)
r2664	1	Na+(c) ⇌ Na+(s) + Na-gradient(c)
r0838	1	NH3(c) ⇌ NH3(m)
r0105	1	NAD+(m) + Malate(m) ⇌ OAA(m) + NADH(m)
r1330	-1	H+(PG)(c) ⇌ H+(PG)(m) + Proton-gradient(m)
r0849	1	H2O(s) ⇌ H2O(c)
r0107	-1	Acetyl-CoA(m) + H2O(m) + OAA(m) ⇌ Citrate(m) + CoA(m)
r1323	1	NADH(m) ⇌ NAD+(m) + NADH-redox-potential(m)
r0877	1	Glutamate(c) + Cystine(s) ⇌ Glutamate(s) + Cystine(c)
r0027	-1	NADPH(c) + Cystine(c) ⇌ 2 Cysteine(c) + NADP+(c)

Glutamine from Glutamate (214)

Definition			Solution												
			exchanges		reactions								transp		
Objective	Constraints	Comment	imports	exports	c	m	r	p	l	n	s	b	s-c	b-c	int
Glutamine	−Glutamate MCES +Urea	Transamination to Glutamine using Glutamate	1 H_2O(s) 0.5 O_2(s) 2 Glutamate(s) 1 ATP-energy(c)	2 CO_2(s) 1 Pyruvate(m) 1 Glutamine(s) 1 ATP-energy(m) 2 NADH-redox-potential(m) 1 NADPH-redox-potential(m) 5 Proton-gradient(m) 2 Na-gradient(c)	2	10	-	-	-	-	-	-	6	-	8

ID	flux	reaction
r1144	-2	Glutamate(c) + Na+(c) ← Glutamate(s) + Na+(s)
r0073	1	Glutamate(m) + NAD+(m) + H2O(m) ⇌ AKG(m) + NADH(m) + NH3(m)
r0507	1	Ubiquinol(m) + 2 Ferricytochrome C(m) + 4 H+(PG)(m) → Ubiquinone(m) + 4 H+(PG)(c) + 2 Ferrocytochrome C(m)
r1327	1	NADPH(m) ⇌ NADPH-redox-potential(m) + NADP+(m)
r2525	-1	Glutamine(c) ⇌ Glutamine(s)
r1319	-1	ATP(c) + H2O(c) ⇌ ADP(c) + Pi(c) + ATP-energy(c)
r0851	-2	H2O(m) ⇌ H2O(c)
r0509	1	Ubiquinone(m) + Succinate(m) → Ubiquinol(m) + Fumarate(m)
r0100	-1	ADP(m) + GTP(m) ⇌ ATP(m) + GDP(m)
r0077	1	ATP(c) + Glutamate(c) + NH3(c) → ADP(c) + Pi(c) + Glutamine(c)
r0059	1	Malate(m) + NADP+(m) ⇌ Pyruvate(m) + CO2(m) + NADPH(m)
r0259	-1	Fumarate(m) + H2O(m) ⇌ Malate(m)
r2664	2	Na+(c) ⇌ Na+(s) + Na-gradient(c)
r0855	0.5	O2(s) ⇌ O2(c)
r1320	1	ATP(m) + H2O(m) ⇌ ADP(m) + Pi(m) + ATP-energy(m)
r0856	-0.5	O2(m) ⇌ O2(c)
r0838	-1	NH3(c) ⇌ NH3(m)
r1330	5	H+(PG)(c) ⇌ H+(PG)(m) + Proton-gradient(m)
r1114	1	AKG(m) + CoA(m) + NAD+(m) ⇌ NADH(m) + CO2(m) + Succinyl-CoA(m)
r0875	1	Glutamate(c) + H+(PG)(c) → Glutamate(m) + H+(PG)(m)
r0867	2	CO2(c) ⇌ CO2(s)
r0849	1	H2O(s) ⇌ H2O(c)
r0125	-1	GDP(m) + Pi(m) + Succinyl-CoA(m) ⇌ GTP(m) + Succinate(m) + CoA(m)
r0017	0.5	O2(m) + 4 H+(PG)(m) + 4 Ferrocytochrome C(m) → 4 Ferricytochrome C(m) + 2 H2O(m) + 4 H+(PG)(c)
r1323	2	NADH(m) ⇌ NAD+(m) + NADH-redox-potential(m)
r0868	-2	CO2(c) ⇌ CO2(m)

Glutamine from Glycine (215)

Definition			Solution												
			exchanges		reactions								transp		
Objective	Constraints	Comment	imports	exports	c	m	r	p	l	n	s	b	s-c	b-c	int
Glutamine	−Glycine MCES +Urea	Transamination to Glutamine using Glycine	1 H_2O(s) 1 Pyruvate(m) 2 Glycine(s) 1 ATP-energy(c) 2 NADPH-redox-potential(m) 1 Proton-gradient(m) 2 THF-activated methyl group(c) 1 CoA-activated acetyl group(m)	2 Pyruvate(c) 1 Glutamine(s) 2 NADH-redox-potential(m) 2 NADPH-redox-potential(c) 2 Na-gradient(c)	7	10	-	-	-	-	-	-	4	-	4

ID	flux	reaction
r2678	-1	Acetyl-CoA(m) ⇌ CoA(m) + CoA-activated acetyl group(m)
r0316	1	Citrate(m) ⇌ Isocitrate(m)
r0911	1	Proline(c) + Glutamate(m) ⇌ Proline(m) + Glutamate(c)
r0425	1	Isocitrate(m) + NAD+(m) ⇌ Oxalosuccinate(m) + NADH(m)
r0083	1	Oxalosuccinate(m) ⇌ AKG(m) + CO2(m)
r1326	2	NADPH(c) ⇌ NADPH-redox-potential(c) + NADP+(c)
r0075	-1	AKG(m) + NADPH(m) + NH3(m) ⇌ Glutamate(m) + H2O(m) + NADP+(m)
r1426	1	Proline(m) + H+(PG)(c) → Proline(c) + H+(PG)(m)
r1327	-2	NADPH(m) ⇌ NADPH-redox-potential(m) + NADP+(m)
r1138	1	Glycine(s) + Na+(s) → Glycine(c) + Na+(c)
r1319	-1	ATP(c) + H2O(c) ⇌ ADP(c) + Pi(c) + ATP-energy(c)
r0077	1	ATP(c) + Glutamate(c) + NH3(c) → ADP(c) + Pi(c) + Glutamine(c)
r0059	-1	Pyruvate(m) + CO2(m) + NADPH(m) ⇌ Malate(m) + NADP+(m)
r1673	1	Glycine(s) + Glutamine(c) + Na+(s) → Glycine(c) + Glutamine(s) + Na+(c)
r0296	2	5-Methyl-THF(c) + NADP+(c) ⇌ 5,10-Methylene-THF(c) + NADPH(c)
r2664	2	Na+(c) ⇌ Na+(s) + Na-gradient(c)
r0230	2	5,10-Methylene-THF(c) + Glycine(c) + H2O(c) ⇌ THF(c) + Serine(c)
r0838	1	NH3(c) ⇌ NH3(m)
r0105	1	NAD+(m) + Malate(m) ⇌ OAA(m) + NADH(m)
r0060	2	Serine(c) → Pyruvate(c) + NH3(c)
r1330	-1	H+(PG)(c) ⇌ H+(PG)(m) + Proton-gradient(m)
r2676	-2	5-Methyl-THF(c) ⇌ THF(c) + THF-activated methyl group(c)
r0849	1	H2O(s) ⇌ H2O(c)
r0107	-1	Acetyl-CoA(m) + H2O(m) + OAA(m) ⇌ Citrate(m) + CoA(m)
r1323	2	NADH(m) ⇌ NAD+(m) + NADH-redox-potential(m)

Glutamine from Histidine (216)

Definition			Solution												
			exchanges		reactions								transp		
Objective	Constraints	Comment	imports	exports	c	m	r	p	l	n	s	b	s-c	b-c	int
Glutamine	−Histidine MCES +Urea	Transamination to Glutamine using Histidine	0.33 H_2O(s) 0.33 Pyruvate(m) 0.67 Histidine(s) 1 ATP-energy(c) 0.67 NADH-redox-potential(c) 0.67 NADPH-redox-potential(c) 0.33 NADPH-redox-potential(m) 0.33 Proton-gradient(m) 0.33 CoA-activated acetyl group(m)	1 Glutamine(s) 0.33 NADH-redox-potential(m) 0.67 THF-activated methyl group(c)	12	10	-	-	-	-	-	-	4	-	4

ID	flux	reaction
r2678	-0.33	Acetyl-CoA(m) ⇌ CoA(m) + CoA-activated acetyl group(m)
r1994	0.67	Lysine(s) + Glutamine(c) → Lysine(c) + Glutamine(s)
r0316	0.33	Citrate(m) ⇌ Isocitrate(m)

Continued on next page

Glutamine from Histidine (216)– continued

ID	flux	reaction
r0073	-0.33	AKG(m) + NADH(m) + NH3(m) ⇌ Glutamate(m) + NAD+(m) + H2O(m)
r0911	0.33	Proline(c) + Glutamate(m) ⇌ Proline(m) + Glutamate(c)
r0524	0.67	5-Formiminotetrahydrofolate(c) ⇌ 5,10-Methenyl-THF(c) + NH3(c)
r0425	0.33	Isocitrate(m) + NAD+(m) ⇌ Oxalosuccinate(m) + NADH(m)
r0083	0.33	Oxalosuccinate(m) ⇌ AKG(m) + CO2(m)
r1326	-0.67	NADPH(c) ⇌ NADPH-redox-potential(c) + NADP+(c)
r1426	0.33	Proline(m) + H+(PG)(c) → Proline(c) + H+(PG)(m)
r1327	-0.33	NADPH(m) ⇌ NADPH-redox-potential(m) + NADP+(m)
r2525	-0.33	Glutamine(c) ⇌ Glutamine(s)
r1319	-1	ATP(c) + H2O(c) ⇌ ADP(c) + Pi(c) + ATP-energy(c)
r0518	-0.67	Glutamate(c) + 5-Formiminotetrahydrofolate(c) ⇌ THF(c) + N-Formimino-L-glutamate(c)
r0293	-0.67	5,10-Methenyl-THF(c) + NADPH(c) ⇌ 5,10-Methylene-THF(c) + NADP+(c)
r0077	1	ATP(c) + Glutamate(c) + NH3(c) → ADP(c) + Pi(c) + Glutamine(c)
r0059	-0.33	Pyruvate(m) + CO2(m) + NADPH(m) ⇌ Malate(m) + NADP+(m)
r0519	0.67	H2O(c) + 4-Imidazolone-5-propanoate(c) ⇌ N-Formimino-L-glutamate(c)
r0286	0.67	Histidine(c) ⇌ Urocanate(c) + NH3(c)
r1665	-0.67	Lysine(c) + Histidine(s) ⇌ Lysine(s) + Histidine(c)
r0792	-0.67	5,10-Methylene-THF(c) + NADH(c) ⇌ 5-Methyl-THF(c) + NAD+(c)
r0838	0.33	NH3(c) ⇌ NH3(m)
r0577	-0.67	Urocanate(c) + H2O(c) ⇌ 4-Imidazolone-5-propanoate(c)
r0105	0.33	NAD+(m) + Malate(m) ⇌ OAA(m) + NADH(m)
r1330	-0.33	H+(PG)(c) ⇌ H+(PG)(m) + Proton-gradient(m)
r2676	0.67	5-Methyl-THF(c) ⇌ THF(c) + THF-activated methyl group(c)
r1322	-0.67	NADH(c) ⇌ NAD+(c) + NADH-redox-potential(c)
r0849	0.33	H2O(s) ⇌ H2O(c)
r0107	-0.33	Acetyl-CoA(m) + H2O(m) + OAA(m) ⇌ Citrate(m) + CoA(m)
r1323	0.33	NADH(m) ⇌ NAD+(m) + NADH-redox-potential(m)

Glutamine from Isoleucine (217)

Definition			Solution												
			exchanges		reactions								transp		
Objective	Constraints	Comment	imports	exports	c	m	r	p	l	n	s	b	s-c	b-c	int
Glutamine	−Isoleucine MCES +Urea	Transamination to Glutamine using Isoleucine	1 H_2O(s) 1.5 O_2(s) 2 Isoleucine(s) 1 ATP-energy(c) 1 ATP-energy(m)	1 CO_2(s) 1 Glutamine(s) 6 NADH-redox-potential(m) 15 Proton-gradient(m) 3 CoA-activated acetyl group(m)	4	19	-	-	-	-	-	-	6	-	11

ID	flux	reaction
r0572	-1	Methylmalonyl-CoA(m) ⇌ (R)-Methylmalonyl-CoA(m)
r0855	1.5	O2(s) ⇌ O2(c)
r0856	-1.5	O2(m) ⇌ O2(c)
r0592	-1	H2O(m) + Acrylyl-CoA(m) → 3-Hydroxypropionyl-CoA(m)
r0596	-1	3-Hydroxypropionyl-CoA(m) + H2O(m) → Hydracrylate(m) + CoA(m)
r0017	1.5	O2(m) + 4 H+(PG)(m) + 4 Ferrocytochrome C(m) → 4 Ferricytochrome C(m) + 2 H2O(m) + 4 H+(PG)(c)
r1557	-1	Glycine(s) + Isoleucine(c) ⇌ Glycine(c) + Isoleucine(s)
r0911	1	Proline(c) + Glutamate(m) ⇌ Proline(m) + Glutamate(c)
r1426	1	Proline(m) + H+(PG)(c) → Proline(c) + H+(PG)(m)
r0073	1	Glutamate(m) + NAD+(m) + H2O(m) ⇌ AKG(m) + NADH(m) + NH3(m)
r0077	1	ATP(c) + Glutamate(c) + NH3(c) → ADP(c) + Pi(c) + Glutamine(c)
r0222	-2	CoA(m) + 2-Methylacetoacetyl-CoA(m) ⇌ Propanoyl-CoA(m) + Acetyl-CoA(m)
r0953	2	Isoleucine(c) + H+(PG)(c) → Isoleucine(m) + H+(PG)(m)
r2536	1	Isoleucine(s) ⇌ Isoleucine(c)
r0507	3	Ubiquinol(m) + 2 Ferricytochrome C(m) + 4 H+(PG)(m) → Ubiquinone(m) + 4 H+(PG)(c) + 2 Ferrocytochrome C(m)
r1330	15	H+(PG)(c) ⇌ H+(PG)(m) + Proton-gradient(m)

Continued on next page

ID	flux	reaction
r1319	-1	ATP(c) + H2O(c) ⇌ ADP(c) + Pi(c) + ATP-energy(c)
r0264	2	Isoleucine(m) + AKG(m) ⇌ Glutamate(m) + 2-Oxo-3-methylvalerate(m)
r0414	1	ATP(m) + Propanoyl-CoA(m) + HCO3-(m) → ADP(m) + Pi(m) + Methylmalonyl-CoA(m)
r0849	1	H2O(s) ⇌ H2O(c)
r0867	1	CO2(c) ⇌ CO2(s)
r0868	-2	CO2(c) ⇌ CO2(m)
r0174	1	CoA(m) + NAD+(m) + 3-Oxopropanoate(m) → Acetyl-CoA(m) + CO2(m) + NADH(m)
r1544	1	Glycine(s) + Glutamine(c) ⇌ Glycine(c) + Glutamine(s)
r0602	2	2-Oxo-3-methylvalerate(m) + CoA(m) + NAD+(m) ⇌ 2-Methylbutyryl-CoA(m) + CO2(m) + NADH(m)
r0603	2	2-Methylbutyryl-CoA(m) + Ubiquinone(m) ⇌ Tiglyl-CoA(m) + Ubiquinol(m)
r1114	-1	NADH(m) + CO2(m) + Succinyl-CoA(m) ⇌ AKG(m) + CoA(m) + NAD+(m)
r0197	1	(R)-Methylmalonyl-CoA(m) ⇌ Succinyl-CoA(m)
r1417	-1	CO2(c) + H2O(c) ⇌ Carbonate(c)
r1419	1	Carbonate(c) ⇌ HCO3-(c)
r0941	1	HCO3-(c) ⇌ HCO3-(m)
r0365	1	Hydracrylate(m) + NAD+(m) → NADH(m) + 3-Oxopropanoate(m)
r2678	3	Acetyl-CoA(m) ⇌ CoA(m) + CoA-activated acetyl group(m)
r0664	2	NAD+(m) + (S)-3-Hydroxy-2-methylbutyryl-CoA(m) ⇌ 2-Methylacetoacetyl-CoA(m) + NADH(m)
r0665	-2	H2O(m) + Tiglyl-CoA(m) ⇌ (S)-3-Hydroxy-2-methylbutyryl-CoA(m)
r1320	-1	ATP(m) + H2O(m) ⇌ ADP(m) + Pi(m) + ATP-energy(m)
r0683	1	Propanoyl-CoA(m) + Ubiquinone(m) → Ubiquinol(m) + Acrylyl-CoA(m)
r1323	6	NADH(m) ⇌ NAD+(m) + NADH-redox-potential(m)
r0838	-1	NH3(c) ⇌ NH3(m)
r0851	-1	H2O(m) ⇌ H2O(c)

Glutamine from Leucine (218)

Definition			Solution												
			exchanges		reactions								transp		
Objective	Constraints	Comment	imports	exports	c	m	r	p	l	n	s	b	s-c	b-c	int
Glutamine	−Leucine MCES +Urea	Transamination to Glutamine using Leucine	1 O_2(s) 1 Pyruvate(m) 2 Leucine(s) 1 ATP-energy(c) 3 NADPH-redox-potential(m)	1 H_2O(s) 1 Glutamine(s) 7 NADH-redox-potential(m) 11 Proton-gradient(m) 2 Na-gradient(c) 5 CoA-activated acetyl group(m)	2	18	-	-	-	-	-	-	6	-	8

ID	flux	reaction
r2678	5	Acetyl-CoA(m) ⇌ CoA(m) + CoA-activated acetyl group(m)
r0316	1	Citrate(m) ⇌ Isocitrate(m)
r0262	2	Leucine(m) + AKG(m) ⇌ Glutamate(m) + 4-Methyl-2-oxopentanoate(m)
r0073	1	Glutamate(m) + NAD+(m) + H2O(m) ⇌ AKG(m) + NADH(m) + NH3(m)
r0911	1	Proline(c) + Glutamate(m) ⇌ Proline(m) + Glutamate(c)
r0425	1	Isocitrate(m) + NAD+(m) ⇌ Oxalosuccinate(m) + NADH(m)
r0083	1	Oxalosuccinate(m) ⇌ AKG(m) + CO2(m)
r0507	2	Ubiquinol(m) + 2 Ferricytochrome C(m) + 4 H+(PG)(m) → Ubiquinone(m) + 4 H+(PG)(c) + 2 Ferrocytochrome C(m)
r0462	2	HMG-CoA(m) + CoA(m) ⇌ Acetyl-CoA(m) + H2O(m) + Acetoacetyl-CoA(m)
r1426	1	Proline(m) + H+(PG)(c) → Proline(c) + H+(PG)(m)
r1327	-3	NADPH(m) ⇌ NADPH-redox-potential(m) + NADP+(m)
r0490	-2	3-Methylglutaconyl-CoA(m) + H2O(m) ⇌ HMG-CoA(m)
r1544	1	Glycine(s) + Glutamine(c) ⇌ Glycine(c) + Glutamine(s)
r1319	-1	ATP(c) + H2O(c) ⇌ ADP(c) + Pi(c) + ATP-energy(c)
r0077	1	ATP(c) + Glutamate(c) + NH3(c) → ADP(c) + Pi(c) + Glutamine(c)
r0059	-3	Pyruvate(m) + CO2(m) + NADPH(m) ⇌ Malate(m) + NADP+(m)
r1807	1	Leucine(s) + Glycine(c) + Na+(s) → Leucine(c) + Glycine(s) + Na+(c)
r1438	2	Leucine(c) → Leucine(m)

Continued on next page

ID	flux	reaction
r0655	2	Isovaleryl-CoA(m) + Ubiquinone(m) ⇌ 3-Methylcrotonyl-CoA(m) + Ubiquinol(m)
r2664	2	Na+(c) ⇌ Na+(s) + Na-gradient(c)
r0855	1	O2(s) ⇌ O2(c)
r1122	-1	Leucine(s) + Na+(s) → Leucine(c) + Na+(c)
r0856	-1	O2(m) ⇌ O2(c)
r0838	-1	NH3(c) ⇌ NH3(m)
r0658	2	ATP(m) + 3-Methylcrotonyl-CoA(m) + HCO3-(m) ⇌ ADP(m) + Pi(m) + 3-Methylglutaconyl-CoA(m)
r0105	3	NAD+(m) + Malate(m) ⇌ OAA(m) + NADH(m)
r1330	11	H+(PG)(c) ⇌ H+(PG)(m) + Proton-gradient(m)
r0601	2	4-Methyl-2-oxopentanoate(m) + CoA(m) + NAD+(m) ⇌ Isovaleryl-CoA(m) + CO2(m) + NADH(m)
r0106	-2	ADP(m) + Pi(m) + OAA(m) ⇌ ATP(m) + Pyruvate(m) + HCO3-(m)
r0070	-2	CoA(m) + Acetoacetyl-CoA(m) ⇌ 2 Acetyl-CoA(m)
r0849	-1	H2O(s) ⇌ H2O(c)
r0107	-1	Acetyl-CoA(m) + H2O(m) + OAA(m) ⇌ Citrate(m) + CoA(m)
r0017	1	O2(m) + 4 H+(PG)(m) + 4 Ferrocytochrome C(m) → 4 Ferricytochrome C(m) + 2 H2O(m) + 4 H+(PG)(c)
r1323	7	NADH(m) ⇌ NAD+(m) + NADH-redox-potential(m)

Glutamine from Lysine (219)

Definition			Solution												
			exchanges		reactions								transp		
Objective	Constraints	Comment	imports	exports	c	m	r	p	l	n	s	b	s-c	b-c	int
Glutamine	−Lysine MCES +Urea	Transamination to Glutamine using Lysine	2 H_2O(s) 0.5 O_2(s) 1 Pyruvate(m) 1 Lysine(s) 1 ATP-energy(c) 1 NADPH-redox-potential(m)	2 CO_2(s) 1 Glutamine(s) 1 NADH-redox-potential(c) 5 NADH-redox-potential(m) 5 Proton-gradient(m) 1 CoA-activated acetyl group(m)	4	18	-	-	-	-	-	-	4	-	11

ID	flux	reaction
r2678	1	Acetyl-CoA(m) ⇌ CoA(m) + CoA-activated acetyl group(m)
r1994	1	Lysine(s) + Glutamine(c) → Lysine(c) + Glutamine(s)
r0541	1	Glutaryl-CoA(m) + Ubiquinone(m) ⇌ Crotonyl-CoA(m) + CO2(m) + Ubiquinol(m)
r0460	1	(S)-3-Hydroxybutyryl-CoA(m) + NAD+(m) ⇌ NADH(m) + Acetoacetyl-CoA(m)
r0316	1	Citrate(m) ⇌ Isocitrate(m)
r0073	1	Glutamate(m) + NAD+(m) + H2O(m) ⇌ AKG(m) + NADH(m) + NH3(m)
r0911	1	Proline(c) + Glutamate(m) ⇌ Proline(m) + Glutamate(c)
r0425	1	Isocitrate(m) + NAD+(m) ⇌ Oxalosuccinate(m) + NADH(m)
r0083	1	Oxalosuccinate(m) ⇌ AKG(m) + CO2(m)
r0507	1	Ubiquinol(m) + 2 Ferricytochrome C(m) + 4 H+(PG)(m) → Ubiquinone(m) + 4 H+(PG)(c) + 2 Ferrocytochrome C(m)
r1426	1	Proline(m) + H+(PG)(c) → Proline(c) + H+(PG)(m)
r1327	-1	NADPH(m) ⇌ NADPH-redox-potential(m) + NADP+(m)
r0526	1	H2O(m) + Saccharopine(m) + NADP+(m) ⇌ Glutamate(m) + 2-Aminoadipate 6-semialdehyde(m) + NADPH(m)
r1319	-1	ATP(c) + H2O(c) ⇌ ADP(c) + Pi(c) + ATP-energy(c)
r0851	-2	H2O(m) ⇌ H2O(c)
r0077	1	ATP(c) + Glutamate(c) + NH3(c) → ADP(c) + Pi(c) + Glutamine(c)
r0059	-1	Pyruvate(m) + CO2(m) + NADPH(m) ⇌ Malate(m) + NADP+(m)
r0448	1	2-Oxoadipate(m) + CoA(m) + NAD+(m) ⇌ Glutaryl-CoA(m) + CO2(m) + NADH(m)
r0855	0.5	O2(s) ⇌ O2(c)
r0594	1	2-Aminoadipate 6-semialdehyde(c) + NAD+(c) + H2O(c) ⇌ NADH(c) + L-2-Aminoadipate(c)
r2539	-1	2-Aminoadipate 6-semialdehyde(c) + L-2-Aminoadipate(m) ⇌ 2-Aminoadipate 6-semialdehyde(m) + L-2-Aminoadipate(c)
r0856	-0.5	O2(m) ⇌ O2(c)
r0838	-1	NH3(c) ⇌ NH3(m)
r0105	1	NAD+(m) + Malate(m) ⇌ OAA(m) + NADH(m)
r1330	5	H+(PG)(c) ⇌ H+(PG)(m) + Proton-gradient(m)
r0070	-1	CoA(m) + Acetoacetyl-CoA(m) ⇌ 2 Acetyl-CoA(m)

Continued on next page

ID	flux	reaction
r1322	1	NADH(c) ⇌ NAD+(c) + NADH-redox-potential(c)
r1070	1	Lysine(c) ⇌ Lysine(m)
r0867	2	CO2(c) ⇌ CO2(s)
r0849	2	H2O(s) ⇌ H2O(c)
r0588	-1	Crotonyl-CoA(m) + H2O(m) ⇌ (S)-3-Hydroxybutyryl-CoA(m)
r0107	-1	Acetyl-CoA(m) + H2O(m) + OAA(m) ⇌ Citrate(m) + CoA(m)
r0017	0.5	O2(m) + 4 H+(PG)(m) + 4 Ferrocytochrome C(m) → 4 Ferricytochrome C(m) + 2 H2O(m) + 4 H+(PG)(c)
r1323	5	NADH(m) ⇌ NAD+(m) + NADH-redox-potential(m)
r0868	-2	CO2(c) ⇌ CO2(m)
r0450	1	AKG(m) + L-2-Aminoadipate(m) ⇌ 2-Oxoadipate(m) + Glutamate(m)
r0180	-1	AKG(m) + NADPH(m) + Lysine(m) ⇌ H2O(m) + Saccharopine(m) + NADP+(m)

Glutamine from Methionine (220)

Definition			Solution												
			exchanges		reactions								transp		
Objective	Constraints	Comment	imports	exports	c	m	r	p	l	n	s	b	s-c	b-c	int
Glutamine	−Methionine MCES +Urea +H_2S	Transamination to Glutamine using Methionine	2 H_2O(s) 0.5 O_2(s) 2 Methionine(s) 1 Glucose-6P(c) 1 NADPH-redox-potential(m) 1 Proton-gradient(m)	1 P_i(c) 1 CO_2(s) 2 Pyruvate(c) 1 Glutamine(s) 2 H_2S(s) 4 NADH-redox-potential(c) 1 NADH-redox-potential(m) 1 Na-gradient(c) 2 THF-activated methyl group(c) 1 CoA-activated acetyl group(m)	16	17	-	-	-	-	-	-	8	-	12

ID	flux	reaction
r0572	-1	Methylmalonyl-CoA(m) ⇌ (R)-Methylmalonyl-CoA(m)
r0855	0.5	O2(s) ⇌ O2(c)
r0856	-0.5	O2(m) ⇌ O2(c)
r0592	-1	H2O(m) + Acrylyl-CoA(m) → 3-Hydroxypropionyl-CoA(m)
r0596	-1	3-Hydroxypropionyl-CoA(m) + H2O(m) → Hydracrylate(m) + CoA(m)
r0017	0.5	O2(m) + 4 H+(PG)(m) + 4 Ferrocytochrome C(m) → 4 Ferricytochrome C(m) + 2 H2O(m) + 4 H+(PG)(c)
r0314	2	Serine(c) + Homocysteine(c) ⇌ H2O(c) + L-Cystathionine(c)
r1572	-1	Glutamine(s) + Serine(c) ⇌ Glutamine(c) + Serine(s)
r0911	3	Proline(c) + Glutamate(m) ⇌ Proline(m) + Glutamate(c)
r1141	-1	Methionine(s) + Na+(s) → Methionine(c) + Na+(c)
r0337	-2	ADP(c) + 1,3DPG(c) ⇌ ATP(c) + 3PG(c)
r0338	2	3PG(c) + NAD+(c) → 3-Phosphonooxypyruvate(c) + NADH(c)
r2664	1	Na+(c) ⇌ Na+(s) + Na-gradient(c)
r1426	3	Proline(m) + H+(PG)(c) → Proline(c) + H+(PG)(m)
r0059	-1	Pyruvate(m) + CO2(m) + NADPH(m) ⇌ Malate(m) + NADP+(m)
r0073	-3	AKG(m) + NADH(m) + NH3(m) ⇌ Glutamate(m) + NAD+(m) + H2O(m)
r0077	1	ATP(c) + Glutamate(c) + NH3(c) → ADP(c) + Pi(c) + Glutamine(c)
r0507	1	Ubiquinol(m) + 2 Ferricytochrome C(m) + 4 H+(PG)(m) → Ubiquinone(m) + 4 H+(PG)(c) + 2 Ferrocytochrome C(m)
r0241	2	H2O(c) + L-Cystathionine(c) ⇌ Cysteine(c) + NH3(c) + 2-Oxobutyrate(c)
r0243	-1	DHAP(c) ⇌ GAP(c)
r1330	-1	H+(PG)(c) ⇌ H+(PG)(m) + Proton-gradient(m)
r0414	1	ATP(m) + Propanoyl-CoA(m) + HCO3-(m) → ADP(m) + Pi(m) + Methylmalonyl-CoA(m)
r0849	2	H2O(s) ⇌ H2O(c)
r0867	1	CO2(c) ⇌ CO2(s)
r0868	-1	CO2(c) ⇌ CO2(m)
r0736	1	ATP(c) + Fructose-6P(c) → ADP(c) + Fructose-1,6PP(c)
r0159	2	3-Phosphoserine(c) + H2O(c) → Serine(c) + Pi(c)
r0174	1	CoA(m) + NAD+(m) + 3-Oxopropanoate(m) → Acetyl-CoA(m) + CO2(m) + NADH(m)
r0192	1	Glucose-6P(c) ⇌ Fructose-6P(c)
r1114	-1	NADH(m) + CO2(m) + Succinyl-CoA(m) ⇌ AKG(m) + CoA(m) + NAD+(m)

Continued on next page

ID	flux	reaction
r0193	2	Cysteine(c) + H2O(c) ⇌ Pyruvate(c) + H2S(c) + NH3(c)
r0197	1	(R)-Methylmalonyl-CoA(m) ⇌ Succinyl-CoA(m)
r1585	-1	Serine(s) + Methionine(c) ⇌ Serine(c) + Methionine(s)
r1154	2	CoA(m) + NAD+(m) + 2-Oxobutyrate(m) ⇌ CO2(m) + Propanoyl-CoA(m) + NADH(m)
r0940	2	H2S(c) ⇌ H2S(s)
r1155	2	2-Oxobutyrate(c) + H+(PG)(c) → 2-Oxobutyrate(m) + H+(PG)(m)
r2676	2	5-Methyl-THF(c) ⇌ THF(c) + THF-activated methyl group(c)
r0365	1	Hydracrylate(m) + NAD+(m) → NADH(m) + 3-Oxopropanoate(m)
r2678	1	Acetyl-CoA(m) ⇌ CoA(m) + CoA-activated acetyl group(m)
r0663	-2	3-Phosphonooxypyruvate(c) + Glutamate(c) → 3-Phosphoserine(c) + AKG(c)
r0232	-2	THF(c) + Methionine(c) ⇌ 5-Methyl-THF(c) + Homocysteine(c)
r1322	4	NADH(c) ⇌ NAD+(c) + NADH-redox-potential(c)
r0683	1	Propanoyl-CoA(m) + Ubiquinone(m) → Ubiquinol(m) + Acrylyl-CoA(m)
r1323	1	NADH(m) ⇌ NAD+(m) + NADH-redox-potential(m)
r0253	2	GAP(c) + Pi(c) + NAD+(c) ⇌ 1,3DPG(c) + NADH(c)
r1327	-1	NADPH(m) ⇌ NADPH-redox-potential(m) + NADP+(m)
r0256	1	Fructose-1,6PP(c) ⇌ DHAP(c) + GAP(c)
r1047	2	Pi(c) + H+(PG)(c) → Pi(m) + H+(PG)(m)
r0105	1	NAD+(m) + Malate(m) ⇌ OAA(m) + NADH(m)
r0106	-1	ADP(m) + Pi(m) + OAA(m) ⇌ ATP(m) + Pyruvate(m) + HCO3-(m)
r2419	2	AKG(c) + Pi(m) ⇌ AKG(m) + Pi(c)
r0838	3	NH3(c) ⇌ NH3(m)
r0851	2	H2O(m) ⇌ H2O(c)

Glutamine from Phenylalanine (221)

Definition			Solution												
			exchanges		reactions								transp		
Objective	Constraints	Comment	imports	exports	c	m	r	p	l	n	s	b	s-c	b-c	int
Glutamine	−Phenylalanine MCES +Urea	Transamination to Glutamine using Phenylalanine	1 H_2O(s) 6 O_2(s) 2 Phenylalanine(s) 4 ATP-energy(c) 1 ATP-energy(m) 1 Proton-gradient(m) 1 CoA-activated acetyl group(m)	4 CO_2(s) 1 Pyruvate(c) 1 Glutamine(s) 1 NADH-redox-potential(m) 1 NADPH-redox-potential(m) 1 Na-gradient(c) 4 CoA-activated acetyl group(c)	19	7	-	-	-	-	-	-	7	-	7

ID	flux	reaction
r0855	6	O2(s) ⇌ O2(c)
r0422	1	Isocitrate(c) + NADP+(c) ⇌ Oxalosuccinate(c) + NADPH(c)
r0875	1	Glutamate(c) + H+(PG)(c) → Glutamate(m) + H+(PG)(m)
r0030	2	ATP(c) + AMP(c) ⇌ 2 ADP(c)
r0183	2	Tyrosine(c) + AKG(c) ⇌ 4-Hydroxyphenylpyruvate(c) + Glutamate(c)
r0315	1	Citrate(c) ⇌ Isocitrate(c)
r0912	-1	Citrate(m) + Malate(c) ⇌ Citrate(c) + Malate(m)
r2664	1	Na+(c) ⇌ Na+(s) + Na-gradient(c)
r0058	1	Malate(c) + NADP+(c) ⇌ Pyruvate(c) + CO2(c) + NADPH(c)
r0075	1	Glutamate(m) + H2O(m) + NADP+(m) ⇌ AKG(m) + NADPH(m) + NH3(m)
r0077	1	ATP(c) + Glutamate(c) + NH3(c) → ADP(c) + Pi(c) + Glutamine(c)
r1762	1	Phenylalanine(s) + Glycine(c) + Na+(s) → Phenylalanine(c) + Glycine(s) + Na+(c)
r1330	-1	H+(PG)(c) ⇌ H+(PG)(m) + Proton-gradient(m)
r0398	2	Dihydrobiopterin(c) + NADPH(c) ⇌ Tetrahydrobiopterin(c) + NADP+(c)
r1319	-4	ATP(c) + H2O(c) ⇌ ADP(c) + Pi(c) + ATP-energy(c)
r0399	2	Tetrahydrobiopterin(c) + Phenylalanine(c) + O2(c) ⇌ Dihydrobiopterin(c) + Tyrosine(c) + H2O(c)
r0543	2	Homogentisate(c) + O2(c) ⇌ 4-Maleylacetoacetate(c)
r0544	2	4-Hydroxyphenylpyruvate(c) + O2(c) ⇌ Homogentisate(c) + CO2(c)
r0849	1	H2O(s) ⇌ H2O(c)

Continued on next page

Glutamine from Phenylalanine (221)– continued

ID	flux	reaction
r0867	4	CO2(c) ⇌ CO2(s)
r0007	2	PPi(c) + H2O(c) → 2 Pi(c)
r1544	1	Glycine(s) + Glutamine(c) ⇌ Glycine(c) + Glutamine(s)
r0320	2	ATP(c) + CoA(c) + Acetoacetate(c) → AMP(c) + PPi(c) + Acetoacetyl-CoA(c)
r0605	2	4-Maleylacetoacetate(c) ⇌ Fumarylacetoacetate(c)
r0324	-2	Fumarylacetoacetate(c) + H2O(c) ⇌ Fumarate(c) + Acetoacetate(c)
r0069	-2	CoA(c) + Acetoacetyl-CoA(c) ⇌ 2 Acetyl-CoA(c)
r0082	1	Oxalosuccinate(c) ⇌ AKG(c) + CO2(c)
r2677	4	Acetyl-CoA(c) ⇌ CoA(c) + CoA-activated acetyl group(c)
r2678	-1	Acetyl-CoA(m) ⇌ CoA(m) + CoA-activated acetyl group(m)
r2528	1	Phenylalanine(s) ⇌ Phenylalanine(c)
r1320	-1	ATP(m) + H2O(m) ⇌ ADP(m) + Pi(m) + ATP-energy(m)
r1323	1	NADH(m) ⇌ NAD+(m) + NADH-redox-potential(m)
r1327	1	NADPH(m) ⇌ NADPH-redox-potential(m) + NADP+(m)
r0105	1	NAD+(m) + Malate(m) ⇌ OAA(m) + NADH(m)
r0258	-2	Fumarate(c) + H2O(c) ⇌ Malate(c)
r0107	-1	Acetyl-CoA(m) + H2O(m) + OAA(m) ⇌ Citrate(m) + CoA(m)
r2418	1	ADP(c) + ATP(m) → ADP(m) + ATP(c)
r2419	-1	AKG(m) + Pi(c) ⇌ AKG(c) + Pi(m)
r0838	-1	NH3(c) ⇌ NH3(m)
r0851	-1	H2O(m) ⇌ H2O(c)

Glutamine from Proline (222)

Definition			Solution												
			exchanges		reactions								transp		
Objective	Constraints	Comment	imports	exports	c	m	r	p	l	n	s	b	s-c	b-c	int
Glutamine	−Proline MCES +Urea	Transamination to Glutamine using Proline	6 H_2O(s) 2 Proline(s) 1 ATP-energy(c)	2 CO_2(s) 1 Pyruvate(m) 1 Glutamine(s) 1 ATP-energy(m) 2 NADH-redox-potential(m) 2 NADPH-redox-potential(m) 3 Proton-gradient(m) 1 Na-gradient(c)	2	13	-	-	-	-	-	-	6	-	7

ID	flux	reaction
r1994	1	Lysine(s) + Glutamine(c) → Lysine(c) + Glutamine(s)
r2531	1	Proline(s) ⇌ Proline(c)
r1424	1	Proline(c) + H+(PG)(c) → Proline(m) + H+(PG)(m)
r0911	1	Proline(c) + Glutamate(m) ⇌ Proline(m) + Glutamate(c)
r0074	2	L-Glutamate 5-semialdehyde(m) + NAD+(m) + H2O(m) ⇌ Glutamate(m) + NADH(m)
r0075	1	Glutamate(m) + H2O(m) + NADP+(m) ⇌ AKG(m) + NADPH(m) + NH3(m)
r1453	2	Proline(m) + Ubiquinol(m) ⇌ 1-Pyrroline-5-carboxylate(m) + Ubiquinone(m)
r1327	2	NADPH(m) ⇌ NADPH-redox-potential(m) + NADP+(m)
r0508	-1	Ubiquinone(m) + NADH(m) + 4 H+(PG)(m) → Ubiquinol(m) + NAD+(m) + 4 H+(PG)(c)
r0175	2	1-Pyrroline-5-carboxylate(m) + H2O(m) ⇌ L-Glutamate 5-semialdehyde(m)
r1319	-1	ATP(c) + H2O(c) ⇌ ADP(c) + Pi(c) + ATP-energy(c)
r0851	-7	H2O(m) ⇌ H2O(c)
r0509	1	Ubiquinone(m) + Succinate(m) → Ubiquinol(m) + Fumarate(m)
r0100	-1	ADP(m) + GTP(m) ⇌ ATP(m) + GDP(m)
r0077	1	ATP(c) + Glutamate(c) + NH3(c) → ADP(c) + Pi(c) + Glutamine(c)
r0059	1	Malate(m) + NADP+(m) ⇌ Pyruvate(m) + CO2(m) + NADPH(m)
r0259	-1	Fumarate(m) + H2O(m) ⇌ Malate(m)
r2664	1	Na+(c) ⇌ Na+(s) + Na-gradient(c)
r1962	1	Proline(s) + Na+(s) + Lysine(c) → Proline(c) + Na+(c) + Lysine(s)
r1320	1	ATP(m) + H2O(m) ⇌ ADP(m) + Pi(m) + ATP-energy(m)
r0838	-1	NH3(c) ⇌ NH3(m)

Continued on next page

ID	flux	reaction
r1330	3	H+(PG)(c) ⇌ H+(PG)(m) + Proton-gradient(m)
r1114	1	AKG(m) + CoA(m) + NAD+(m) ⇌ NADH(m) + CO2(m) + Succinyl-CoA(m)
r0867	2	CO2(c) ⇌ CO2(s)
r0849	6	H2O(s) ⇌ H2O(c)
r0125	-1	GDP(m) + Pi(m) + Succinyl-CoA(m) ⇌ GTP(m) + Succinate(m) + CoA(m)
r1323	2	NADH(m) ⇌ NAD+(m) + NADH-redox-potential(m)
r0868	-2	CO2(c) ⇌ CO2(m)

Glutamine from Serine (223)

Definition			Solution												
			exchanges		reactions								transp		
Objective	Constraints	Comment	imports	exports	c	m	r	p	l	n	s	b	s-c	b-c	int
Glutamine	−Serine MCES +Urea	Transamination to Glutamine using Serine	1 Pyruvate(m) 2 Serine(s) 1 ATP-energy(c) 2 NADPH-redox-potential(m) 1 Proton-gradient(m) 1 CoA-activated acetyl group(m)	1 H_2O(s) 2 Pyruvate(c) 1 Glutamine(s) 2 NADH-redox-potential(m) 1 Proton-gradient(c) 1 Na-gradient(c)	3	10	-	-	-	-	-	-	5	-	4

ID	flux	reaction
r2678	-1	Acetyl-CoA(m) ⇌ CoA(m) + CoA-activated acetyl group(m)
r0316	1	Citrate(m) ⇌ Isocitrate(m)
r0911	1	Proline(c) + Glutamate(m) ⇌ Proline(m) + Glutamate(c)
r0425	1	Isocitrate(m) + NAD+(m) ⇌ Oxalosuccinate(m) + NADH(m)
r0083	1	Oxalosuccinate(m) ⇌ AKG(m) + CO2(m)
r0075	-1	AKG(m) + NADPH(m) + NH3(m) ⇌ Glutamate(m) + H2O(m) + NADP+(m)
r1426	1	Proline(m) + H+(PG)(c) → Proline(c) + H+(PG)(m)
r1327	-2	NADPH(m) ⇌ NADPH-redox-potential(m) + NADP+(m)
r1319	-1	ATP(c) + H2O(c) ⇌ ADP(c) + Pi(c) + ATP-energy(c)
r0077	1	ATP(c) + Glutamate(c) + NH3(c) → ADP(c) + Pi(c) + Glutamine(c)
r0059	-1	Pyruvate(m) + CO2(m) + NADPH(m) ⇌ Malate(m) + NADP+(m)
r1572	-1	Glutamine(c) + Serine(s) ⇌ Glutamine(s) + Serine(c)
r2664	1	Na+(c) ⇌ Na+(s) + Na-gradient(c)
r0838	1	NH3(c) ⇌ NH3(m)
r0105	1	NAD+(m) + Malate(m) ⇌ OAA(m) + NADH(m)
r0060	2	Serine(c) → Pyruvate(c) + NH3(c)
r1330	-1	H+(PG)(c) ⇌ H+(PG)(m) + Proton-gradient(m)
r2478	1	Serine(s) + Na+(s) + H+(PG)(c) → Serine(c) + Na+(c) + H+(PG)(s)
r1331	1	H+(PG)(s) ⇌ H+(PG)(c) + Proton-gradient(c)
r0849	-1	H2O(s) ⇌ H2O(c)
r0107	-1	Acetyl-CoA(m) + H2O(m) + OAA(m) ⇌ Citrate(m) + CoA(m)
r1323	2	NADH(m) ⇌ NAD+(m) + NADH-redox-potential(m)

Glutamine from Threonine (224)

Definition			Solution												
			exchanges		reactions								transp		
Objective	Constraints	Comment	imports	exports	c	m	r	p	l	n	s	b	s-c	b-c	int
Glutamine	−Threonine MCES +Urea	Transamination to Glutamine using Threonine	0.5 O_2(s) 2 Threonine(s) 1 ATP-energy(c) 1 NADPH-redox-potential(m)	1 H_2O(s) 1 CO_2(s) 1 Glutamine(s) 3 NADH-redox-potential(m) 3 Proton-gradient(m) 1 CoA-activated acetyl group(m)	3	17	-	-	-	-	-	-	6	-	9

ID	flux	reaction
r2678	1	Acetyl-CoA(m) ⇌ CoA(m) + CoA-activated acetyl group(m)
r0073	-1	AKG(m) + NADH(m) + NH3(m) ⇌ Glutamate(m) + NAD+(m) + H2O(m)
r1154	2	CoA(m) + NAD+(m) + 2-Oxobutyrate(m) ⇌ CO2(m) + Propanoyl-CoA(m) + NADH(m)
r0911	1	Proline(c) + Glutamate(m) ⇌ Proline(m) + Glutamate(c)
r1155	2	2-Oxobutyrate(c) + H+(PG)(c) → 2-Oxobutyrate(m) + H+(PG)(m)
r0507	1	Ubiquinol(m) + 2 Ferricytochrome C(m) + 4 H+(PG)(m) → Ubiquinone(m) + 4 H+(PG)(c) + 2 Ferrocytochrome C(m)
r0174	1	CoA(m) + NAD+(m) + 3-Oxopropanoate(m) → Acetyl-CoA(m) + CO2(m) + NADH(m)
r1426	1	Proline(m) + H+(PG)(c) → Proline(c) + H+(PG)(m)
r1327	-1	NADPH(m) ⇌ NADPH-redox-potential(m) + NADP+(m)
r2534	1	Threonine(s) ⇌ Threonine(c)
r1544	1	Glycine(s) + Glutamine(c) ⇌ Glycine(c) + Glutamine(s)
r1319	-1	ATP(c) + H2O(c) ⇌ ADP(c) + Pi(c) + ATP-energy(c)
r0572	-1	Methylmalonyl-CoA(m) ⇌ (R)-Methylmalonyl-CoA(m)
r0365	1	Hydracrylate(m) + NAD+(m) → NADH(m) + 3-Oxopropanoate(m)
r0077	1	ATP(c) + Glutamate(c) + NH3(c) → ADP(c) + Pi(c) + Glutamine(c)
r0059	-1	Pyruvate(m) + CO2(m) + NADPH(m) ⇌ Malate(m) + NADP+(m)
r1555	-1	Glycine(s) + Threonine(c) ⇌ Glycine(c) + Threonine(s)
r0592	-1	H2O(m) + Acrylyl-CoA(m) → 3-Hydroxypropionyl-CoA(m)
r0683	1	Propanoyl-CoA(m) + Ubiquinone(m) → Ubiquinol(m) + Acrylyl-CoA(m)
r0197	1	(R)-Methylmalonyl-CoA(m) ⇌ Succinyl-CoA(m)
r0855	0.5	O2(s) ⇌ O2(c)
r0856	-0.5	O2(m) ⇌ O2(c)
r0838	1	NH3(c) ⇌ NH3(m)
r0240	2	Threonine(c) ⇌ 2-Oxobutyrate(c) + NH3(c)
r0105	1	NAD+(m) + Malate(m) ⇌ OAA(m) + NADH(m)
r1330	3	H+(PG)(c) ⇌ H+(PG)(m) + Proton-gradient(m)
r1114	-1	NADH(m) + CO2(m) + Succinyl-CoA(m) ⇌ AKG(m) + CoA(m) + NAD+(m)
r0596	-1	3-Hydroxypropionyl-CoA(m) + H2O(m) → Hydracrylate(m) + CoA(m)
r0106	-1	ADP(m) + Pi(m) + OAA(m) ⇌ ATP(m) + Pyruvate(m) + HCO3-(m)
r0867	1	CO2(c) ⇌ CO2(s)
r0849	-1	H2O(s) ⇌ H2O(c)
r0017	0.5	O2(m) + 4 H+(PG)(m) + 4 Ferrocytochrome C(m) → 4 Ferricytochrome C(m) + 2 H2O(m) + 4 H+(PG)(c)
r1323	3	NADH(m) ⇌ NAD+(m) + NADH-redox-potential(m)
r0868	-1	CO2(c) ⇌ CO2(m)
r0414	1	ATP(m) + Propanoyl-CoA(m) + HCO3-(m) → ADP(m) + Pi(m) + Methylmalonyl-CoA(m)

Glutamine from Tryptophan (225)

Definition			Solution												
			exchanges		reactions								transp		
Objective	Constraints	Comment	imports	exports	c	m	r	p	l	n	s	b	s-c	b-c	int
Glutamine	−Tryptophan MCES +Urea	Transamination to Glutamine using Tryptophan	1 H_2O(s) 3.5 O_2(s) 1 Pyruvate(m) 1 Tryptophan(s) 2 ATP-energy(c) 2 NADPH-redox-potential(c) 1 NADPH-redox-potential(m)	3 CO_2(s) 1 Pyruvate(c) 1 Glutamine(s) 3 NADH-redox-potential(m) 6 Proton-gradient(m) 1 Na-gradient(c) 1 THF-activated methyl group(c) 1 CoA-activated acetyl group(m)	20	11	-	-	-	-	-	-	6	-	8

ID	flux	reaction
r0855	3.5	O2(s) ⇌ O2(c)
r0422	1	Isocitrate(c) + NADP+(c) ⇌ Oxalosuccinate(c) + NADPH(c)
r0856	-0.5	O2(m) ⇌ O2(c)
r0293	-1	5,10-Methylene-THF(c) + NADP+(c) ⇌ 5,10-Methenyl-THF(c) + NADPH(c)
r0460	1	(S)-3-Hydroxybutyryl-CoA(m) + NAD+(m) ⇌ NADH(m) + Acetoacetyl-CoA(m)
r0448	1	2-Oxoadipate(m) + CoA(m) + NAD+(m) ⇌ Glutaryl-CoA(m) + CO2(m) + NADH(m)

Continued on next page

ID	flux	reaction
r0449	-1	2-Oxoadipate(c) + NH3(c) + NADP+(c) ⇌ 2-Aminomuconate(c) + NADPH(c) + H2O(c)
r0017	0.5	O2(m) + 4 H+(PG)(m) + 4 Ferrocytochrome C(m) → 4 Ferricytochrome C(m) + 2 H2O(m) + 4 H+(PG)(c)
r0169	1	Tryptophan(c) + O2(c) ⇌ L-Formylkynurenine(c)
r0315	1	Citrate(c) ⇌ Isocitrate(c)
r0070	-1	CoA(m) + Acetoacetyl-CoA(m) ⇌ 2 Acetyl-CoA(m)
r2664	1	Na+(c) ⇌ Na+(s) + Na-gradient(c)
r0059	-1	Pyruvate(m) + CO2(m) + NADPH(m) ⇌ Malate(m) + NADP+(m)
r0371	-1	10-Formyl-THF(c) ⇌ 5,10-Methenyl-THF(c) + H2O(c)
r0077	1	ATP(c) + Glutamate(c) + NH3(c) → ADP(c) + Pi(c) + Glutamine(c)
r0507	1	Ubiquinol(m) + 2 Ferricytochrome C(m) + 4 H+(PG)(m) → Ubiquinone(m) + 4 H+(PG)(c) + 2 Ferrocytochrome C(m)
r1747	1	Tryptophan(s) + Glycine(c) + Na+(s) → Tryptophan(c) + Glycine(s) + Na+(c)
r0228	1	THF(c) + Formate(c) + ATP(c) → ADP(c) + Pi(c) + 10-Formyl-THF(c)
r0676	1	2-Amino-3-carboxymuconate semialdehyde(c) ⇌ 2-Aminomuconate semialdehyde(c) + CO2(c)
r1330	6	H+(PG)(c) ⇌ H+(PG)(m) + Proton-gradient(m)
r1319	-2	ATP(c) + H2O(c) ⇌ ADP(c) + Pi(c) + ATP-energy(c)
r0541	1	Glutaryl-CoA(m) + Ubiquinone(m) ⇌ Crotonyl-CoA(m) + CO2(m) + Ubiquinol(m)
r0562	1	3-Hydroxyanthranilate(c) + O2(c) ⇌ 2-Amino-3-carboxymuconate semialdehyde(c)
r0563	1	H2O(c) + 3-Hydroxy-L-kynurenine(c) ⇌ 3-Hydroxyanthranilate(c) + Alanine(c)
r0849	1	H2O(s) ⇌ H2O(c)
r0867	3	CO2(c) ⇌ CO2(s)
r0868	-1	CO2(c) ⇌ CO2(m)
r0588	-1	Crotonyl-CoA(m) + H2O(m) ⇌ (S)-3-Hydroxybutyryl-CoA(m)
r0454	1	L-Formylkynurenine(c) + H2O(c) ⇌ Formate(c) + Kynurenine(c)
r0455	1	Kynurenine(c) + O2(c) + NADPH(c) ⇌ H2O(c) + 3-Hydroxy-L-kynurenine(c) + NADP+(c)
r1544	1	Glycine(s) + Glutamine(c) ⇌ Glycine(c) + Glutamine(s)
r2373	1	Citrate(m) + AKG(c) ⇌ Citrate(c) + AKG(m)
r0792	-1	5,10-Methylene-THF(c) + NADH(c) ⇌ 5-Methyl-THF(c) + NAD+(c)
r0080	1	Alanine(c) + AKG(c) ⇌ Pyruvate(c) + Glutamate(c)
r0645	1	2-Aminomuconate semialdehyde(c) + NAD+(c) + H2O(c) ⇌ 2-Aminomuconate(c) + NADH(c)
r0082	1	Oxalosuccinate(c) ⇌ AKG(c) + CO2(c)
r2676	1	5-Methyl-THF(c) ⇌ THF(c) + THF-activated methyl group(c)
r2678	1	Acetyl-CoA(m) ⇌ CoA(m) + CoA-activated acetyl group(m)
r0945	1	2-Oxoadipate(c) + AKG(m) ⇌ 2-Oxoadipate(m) + AKG(c)
r1323	3	NADH(m) ⇌ NAD+(m) + NADH-redox-potential(m)
r1326	-2	NADPH(c) ⇌ NADPH-redox-potential(c) + NADP+(c)
r1327	-1	NADPH(m) ⇌ NADPH-redox-potential(m) + NADP+(m)
r0105	1	NAD+(m) + Malate(m) ⇌ OAA(m) + NADH(m)
r0107	-1	Acetyl-CoA(m) + H2O(m) + OAA(m) ⇌ Citrate(m) + CoA(m)
r0851	-1	H2O(m) ⇌ H2O(c)

Glutamine from Tyrosine (226)

Definition			Solution													
			exchanges		reactions								transp		
Objective	Constraints	Comment	imports	exports	c	m	r	p	l	n	s	b	s-c	b-c	int
Glutamine	−Tyrosine MCES +Urea	Transamination to Glutamine using Tyrosine	3 H_2O(s) 4 O_2(s) 2 Tyrosine(s) 4 ATP-energy(c) 1 ATP-energy(m) 1 Proton-gradient(m) 1 CoA-activated acetyl group(m)	4 CO_2(s) 1 Pyruvate(c) 1 Glutamine(s) 2 NADH-redox-potential(m) 2 NADPH-redox-potential(c) 2 Na-gradient(c) 4 CoA-activated acetyl group(c)	18	6	-	-	-	-	-	-	7	-	7

ID	flux	reaction
r2678	-1	Acetyl-CoA(m) ⇌ CoA(m) + CoA-activated acetyl group(m)
r0082	1	Oxalosuccinate(c) ⇌ AKG(c) + CO2(c)
r0073	1	Glutamate(m) + NAD+(m) + H2O(m) ⇌ AKG(m) + NADH(m) + NH3(m)

Continued on next page

Glutamine from Tyrosine (226)– continued

ID	flux	reaction
r0605	2	4-Maleylacetoacetate(c) ⇌ Fumarylacetoacetate(c)
r1326	2	NADPH(c) ⇌ NADPH-redox-potential(c) + NADP+(c)
r0912	-1	Citrate(m) + Malate(c) ⇌ Citrate(c) + Malate(m)
r0543	2	Homogentisate(c) + O2(c) ⇌ 4-Maleylacetoacetate(c)
r0183	2	Tyrosine(c) + AKG(c) ⇌ 4-Hydroxyphenylpyruvate(c) + Glutamate(c)
r0544	2	4-Hydroxyphenylpyruvate(c) + O2(c) ⇌ Homogentisate(c) + CO2(c)
r0058	1	Malate(c) + NADP+(c) ⇌ Pyruvate(c) + CO2(c) + NADPH(c)
r1544	1	Glycine(s) + Glutamine(c) ⇌ Glycine(c) + Glutamine(s)
r1319	-4	ATP(c) + H2O(c) ⇌ ADP(c) + Pi(c) + ATP-energy(c)
r0851	-1	H2O(m) ⇌ H2O(c)
r0077	1	ATP(c) + Glutamate(c) + NH3(c) → ADP(c) + Pi(c) + Glutamine(c)
r2418	1	ADP(c) + ATP(m) → ADP(m) + ATP(c)
r0258	-2	Fumarate(c) + H2O(c) ⇌ Malate(c)
r0069	-2	CoA(c) + Acetoacetyl-CoA(c) ⇌ 2 Acetyl-CoA(c)
r2419	-1	AKG(m) + Pi(c) ⇌ AKG(c) + Pi(m)
r0030	2	ATP(c) + AMP(c) ⇌ 2 ADP(c)
r2664	2	Na+(c) ⇌ Na+(s) + Na-gradient(c)
r0855	4	O2(s) ⇌ O2(c)
r0320	2	ATP(c) + CoA(c) + Acetoacetate(c) → AMP(c) + PPi(c) + Acetoacetyl-CoA(c)
r1320	-1	ATP(m) + H2O(m) ⇌ ADP(m) + Pi(m) + ATP-energy(m)
r0838	-1	NH3(c) ⇌ NH3(m)
r0105	1	NAD+(m) + Malate(m) ⇌ OAA(m) + NADH(m)
r1330	-1	H+(PG)(c) ⇌ H+(PG)(m) + Proton-gradient(m)
r0875	1	Glutamate(c) + H+(PG)(c) → Glutamate(m) + H+(PG)(m)
r0007	2	PPi(c) + H2O(c) → 2 Pi(c)
r0867	4	CO2(c) ⇌ CO2(s)
r0849	3	H2O(s) ⇌ H2O(c)
r0422	1	Isocitrate(c) + NADP+(c) ⇌ Oxalosuccinate(c) + NADPH(c)
r0107	-1	Acetyl-CoA(m) + H2O(m) + OAA(m) ⇌ Citrate(m) + CoA(m)
r2677	4	Acetyl-CoA(c) ⇌ CoA(c) + CoA-activated acetyl group(c)
r1777	1	Tyrosine(s) + Glycine(c) + Na+(s) → Tyrosine(c) + Glycine(s) + Na+(c)
r1323	2	NADH(m) ⇌ NAD+(m) + NADH-redox-potential(m)
r1125	1	Tyrosine(s) + Na+(s) → Tyrosine(c) + Na+(c)
r0324	-2	Fumarylacetoacetate(c) + H2O(c) ⇌ Fumarate(c) + Acetoacetate(c)
r0315	1	Citrate(c) ⇌ Isocitrate(c)

Glutamine from Valine (227)

Definition			Solution												
			exchanges		reactions								transp		
Objective	Constraints	Comment	imports	exports	c	m	r	p	l	n	s	b	s-c	b-c	int
Glutamine	−Valine MCES +Urea	Transamination to Glutamine using Valine	3 H_2O(s) 1.5 O_2(s) 2 Valine(s)	2 CO_2(s) 1 Pyruvate(c) 1 Glutamine(s) 6 NADH-redox-potential(m) 1 NADPH-redox-potential(c) 15 Proton-gradient(m) 2 Na-gradient(c)	4	14	-	-	-	-	-	-	7	-	13

ID	flux	reaction
r1994	1	Lysine(s) + Glutamine(c) → Lysine(c) + Glutamine(s)
r0779	2	3-Hydroxyisobutyryl-CoA(m) + H2O(m) → 3-Hydroxyisobutyrate(m) + CoA(m)
r0073	1	Glutamate(m) + NAD+(m) + H2O(m) ⇌ AKG(m) + NADH(m) + NH3(m)
r0911	1	Proline(c) + Glutamate(m) ⇌ Proline(m) + Glutamate(c)
r0821	-1	Fumarate(c) + Succinate(m) ⇌ Fumarate(m) + Succinate(c)
r0560	2	Isobutyryl-CoA(m) + Ubiquinone(m) → Methacrylyl-CoA(m) + Ubiquinol(m)
r0263	2	Valine(m) + AKG(m) → Glutamate(m) + 3-Methyl-2-oxobutyrate(m)

Continued on next page

ID	flux	reaction
r1326	1	NADPH(c) ⇌ NADPH-redox-potential(c) + NADP+(c)
r0624	2	2-Methyl-3-oxopropanoate(m) + CoA(m) + NAD+(m) ⇌ (R)-Methylmalonyl-CoA(m) + NADH(m)
r0507	3	Ubiquinol(m) + 2 Ferricytochrome C(m) + 4 H+(PG)(m) → Ubiquinone(m) + 4 H+(PG)(c) + 2 Ferrocytochrome C(m)
r1426	1	Proline(m) + H+(PG)(c) → Proline(c) + H+(PG)(m)
r0058	1	Malate(c) + NADP+(c) ⇌ Pyruvate(c) + CO2(c) + NADPH(c)
r0923	2	Valine(c) + H+(PG)(c) → Valine(m) + H+(PG)(m)
r0851	-2	H2O(m) ⇌ H2O(c)
r0509	1	Ubiquinone(m) + Succinate(m) → Ubiquinol(m) + Fumarate(m)
r0482	2	3-Hydroxyisobutyrate(m) + NAD+(m) → 2-Methyl-3-oxopropanoate(m) + NADH(m)
r0100	-1	ADP(m) + GTP(m) ⇌ ATP(m) + GDP(m)
r0077	1	ATP(c) + Glutamate(c) + NH3(c) → ADP(c) + Pi(c) + Glutamine(c)
r2418	1	ADP(c) + ATP(m) → ADP(m) + ATP(c)
r0258	-1	Fumarate(c) + H2O(c) ⇌ Malate(c)
r0197	2	(R)-Methylmalonyl-CoA(m) ⇌ Succinyl-CoA(m)
r2664	2	Na+(c) ⇌ Na+(s) + Na-gradient(c)
r1121	-1	Valine(s) + Na+(s) → Valine(c) + Na+(c)
r0855	1.5	O2(s) ⇌ O2(c)
r0828	-1	Succinate(c) + Pi(m) ⇌ Succinate(m) + Pi(c)
r1972	1	Valine(s) + Na+(s) + Lysine(c) → Valine(c) + Na+(c) + Lysine(s)
r0856	-1.5	O2(m) ⇌ O2(c)
r0838	-1	NH3(c) ⇌ NH3(m)
r0600	2	3-Methyl-2-oxobutyrate(m) + CoA(m) + NAD+(m) ⇌ Isobutyryl-CoA(m) + CO2(m) + NADH(m)
r1330	15	H+(PG)(c) ⇌ H+(PG)(m) + Proton-gradient(m)
r1114	-1	NADH(m) + CO2(m) + Succinyl-CoA(m) ⇌ AKG(m) + CoA(m) + NAD+(m)
r0867	2	CO2(c) ⇌ CO2(s)
r0849	3	H2O(s) ⇌ H2O(c)
r0669	2	Methacrylyl-CoA(m) + H2O(m) → 3-Hydroxyisobutyryl-CoA(m)
r0125	-1	GDP(m) + Pi(m) + Succinyl-CoA(m) ⇌ GTP(m) + Succinate(m) + CoA(m)
r0017	1.5	O2(m) + 4 H+(PG)(m) + 4 Ferrocytochrome C(m) → 4 Ferricytochrome C(m) + 2 H2O(m) + 4 H+(PG)(c)
r1323	6	NADH(m) ⇌ NAD+(m) + NADH-redox-potential(m)
r0868	-1	CO2(c) ⇌ CO2(m)

Glutamine from beta-Alanine (228)

Definition			Solution												
			exchanges		reactions								transp		
Objective	Constraints	Comment	imports	exports	c	m	r	p	l	n	s	b	s-c	b-c	int
Glutamine	−beta-Alanine MCES +Urea	Transamination to Glutamine using beta-Alanine	1 H_2O(s) 1 Pyruvate(m) 2 beta-Alanine(s) 1 ATP-energy(c) 1 Proton-gradient(m)	2 CO_2(s) 1 Glutamine(s) 3 NADH-redox-potential(m) 1 NADPH-redox-potential(c) 1 CoA-activated acetyl group(m)	5	9	-	-	-	-	-	-	4	-	8

ID	flux	reaction
r2678	1	Acetyl-CoA(m) ⇌ CoA(m) + CoA-activated acetyl group(m)
r0316	1	Citrate(m) ⇌ Isocitrate(m)
r0217	2	beta-Alanine(m) + AKG(m) ⇌ Glutamate(m) + 3-Oxopropanoate(m)
r0082	1	Oxalosuccinate(c) ⇌ AKG(c) + CO2(c)
r0911	1	Proline(c) + Glutamate(m) ⇌ Proline(m) + Glutamate(c)
r1326	1	NADPH(c) ⇌ NADPH-redox-potential(c) + NADP+(c)
r0174	2	CoA(m) + NAD+(m) + 3-Oxopropanoate(m) → Acetyl-CoA(m) + CO2(m) + NADH(m)
r0075	1	Glutamate(m) + H2O(m) + NADP+(m) ⇌ AKG(m) + NADPH(m) + NH3(m)
r1426	1	Proline(m) + H+(PG)(c) → Proline(c) + H+(PG)(m)
r1147	1	AKG(c) + Isocitrate(m) ⇌ AKG(m) + Isocitrate(c)
r0899	-2	beta-Alanine(s) ⇌ beta-Alanine(c)
r2525	-1	Glutamine(c) ⇌ Glutamine(s)

Continued on next page

ID	flux	reaction
r1319	-1	ATP(c) + H2O(c) ⇌ ADP(c) + Pi(c) + ATP-energy(c)
r0851	-2	H2O(m) ⇌ H2O(c)
r0077	1	ATP(c) + Glutamate(c) + NH3(c) → ADP(c) + Pi(c) + Glutamine(c)
r0059	-1	Pyruvate(m) + CO2(m) + NADPH(m) ⇌ Malate(m) + NADP+(m)
r0838	-1	NH3(c) ⇌ NH3(m)
r0105	1	NAD+(m) + Malate(m) ⇌ OAA(m) + NADH(m)
r1330	-1	H+(PG)(c) ⇌ H+(PG)(m) + Proton-gradient(m)
r0867	2	CO2(c) ⇌ CO2(s)
r0849	1	H2O(s) ⇌ H2O(c)
r0422	1	Isocitrate(c) + NADP+(c) ⇌ Oxalosuccinate(c) + NADPH(c)
r0107	-1	Acetyl-CoA(m) + H2O(m) + OAA(m) ⇌ Citrate(m) + CoA(m)
r1323	3	NADH(m) ⇌ NAD+(m) + NADH-redox-potential(m)
r0900	2	beta-Alanine(c) ⇌ beta-Alanine(m)
r0868	-1	CO2(c) ⇌ CO2(m)

Methionine from Cysteine (229)

Definition			Solution												
			exchanges		reactions								transp		
Objective	Constraints	Comment	imports	exports	c	m	r	p	l	n	s	b	s-c	b-c	int
Methionine	−Cysteine MCES +Urea	Transamination to Methionine using Cysteine	1 H_2O(s) 1 Pyruvate(m) 1 Cysteine(s) 1 NADPH-redox-potential(m) 1 Proton-gradient(m) 1 THF-activated methyl group(c) 1 CoA-activated acetyl group(m)	1 CO_2(s) 1 Pyruvate(c) 1 Methionine(s) 1 NADH-redox-potential(m) 1 NADPH-redox-potential(c) 1 Na-gradient(c)	8	12	-	-	-	-	-	-	5	-	4

ID	flux	reaction
r2678	-1	Acetyl-CoA(m) ⇌ CoA(m) + CoA-activated acetyl group(m)
r1072	-1	Methionine(c) ⇌ Methionine(s)
r0316	1	Citrate(m) ⇌ Isocitrate(m)
r0082	1	Oxalosuccinate(c) ⇌ AKG(c) + CO2(c)
r1154	-1	CoA(m) + NAD+(m) + 2-Oxobutyrate(m) ⇌ CO2(m) + Propanoyl-CoA(m) + NADH(m)
r1326	1	NADPH(c) ⇌ NADPH-redox-potential(c) + NADP+(c)
r0624	-1	2-Methyl-3-oxopropanoate(m) + CoA(m) + NAD+(m) ⇌ (R)-Methylmalonyl-CoA(m) + NADH(m)
r1327	-1	NADPH(m) ⇌ NADPH-redox-potential(m) + NADP+(m)
r1147	1	AKG(c) + Isocitrate(m) ⇌ AKG(m) + Isocitrate(c)
r1454	-1	2-Oxobutyrate(m) + H+(PG)(c) → 2-Oxobutyrate(c) + H+(PG)(m)
r0851	-1	H2O(m) ⇌ H2O(c)
r0059	-1	Pyruvate(m) + CO2(m) + NADPH(m) ⇌ Malate(m) + NADP+(m)
r0197	-1	Succinyl-CoA(m) ⇌ (R)-Methylmalonyl-CoA(m)
r2664	1	Na+(c) ⇌ Na+(s) + Na-gradient(c)
r2475	1	Cysteine(s) + Na+(s) → Cysteine(c) + Na+(c)
r0105	1	NAD+(m) + Malate(m) ⇌ OAA(m) + NADH(m)
r0060	1	Serine(c) → Pyruvate(c) + NH3(c)
r1330	-1	H+(PG)(c) ⇌ H+(PG)(m) + Proton-gradient(m)
r1114	1	AKG(m) + CoA(m) + NAD+(m) ⇌ NADH(m) + CO2(m) + Succinyl-CoA(m)
r0241	-1	Cysteine(c) + NH3(c) + 2-Oxobutyrate(c) ⇌ H2O(c) + L-Cystathionine(c)
r0232	1	5-Methyl-THF(c) + Homocysteine(c) ⇌ THF(c) + Methionine(c)
r0223	1	2-Methyl-3-oxopropanoate(m) + CoA(m) + NAD+(m) → Propanoyl-CoA(m) + CO2(m) + NADH(m)
r2676	-1	5-Methyl-THF(c) ⇌ THF(c) + THF-activated methyl group(c)
r0867	1	CO2(c) ⇌ CO2(s)
r0849	1	H2O(s) ⇌ H2O(c)
r0422	1	Isocitrate(c) + NADP+(c) ⇌ Oxalosuccinate(c) + NADPH(c)
r0314	-1	H2O(c) + L-Cystathionine(c) ⇌ Serine(c) + Homocysteine(c)
r0107	-1	Acetyl-CoA(m) + H2O(m) + OAA(m) ⇌ Citrate(m) + CoA(m)

Continued on next page

ID	flux	reaction
r1323	1	NADH(m) ⇌ NAD+(m) + NADH-redox-potential(m)

Methionine from Cystine (230)

Definition			Solution												
			exchanges		reactions								transp		
Objective	Constraints	Comment	imports	exports	c	m	r	p	l	n	s	b	s-c	b-c	int
Methionine	−Cystine MCES +Urea	Transamination to Methionine using Cystine	1 H_2O(s) 1 Pyruvate(m) 0.5 Cystine(s) 0.5 NADPH-redox-potential(c) 1 NADPH-redox-potential(m) 1 Proton-gradient(m) 1 THF-activated methyl group(c) 1 CoA-activated acetyl group(m)	1 CO_2(s) 1 Pyruvate(c) 1 Methionine(s) 2 NADH-redox-potential(m) 0.5 Na-gradient(c)	7	14	-	-	-	-	-	-	6	-	4

ID	flux	reaction
r2678	-1	Acetyl-CoA(m) ⇌ CoA(m) + CoA-activated acetyl group(m)
r1144	-0.5	Glutamate(c) + Na+(c) ← Glutamate(s) + Na+(s)
r1072	-1	Methionine(c) ⇌ Methionine(s)
r0316	1	Citrate(m) ⇌ Isocitrate(m)
r1154	-1	CoA(m) + NAD+(m) + 2-Oxobutyrate(m) ⇌ CO2(m) + Propanoyl-CoA(m) + NADH(m)
r0425	1	Isocitrate(m) + NAD+(m) ⇌ Oxalosuccinate(m) + NADH(m)
r0083	1	Oxalosuccinate(m) ⇌ AKG(m) + CO2(m)
r1326	-0.5	NADPH(c) ⇌ NADPH-redox-potential(c) + NADP+(c)
r0624	-1	2-Methyl-3-oxopropanoate(m) + CoA(m) + NAD+(m) ⇌ (R)-Methylmalonyl-CoA(m) + NADH(m)
r1327	-1	NADPH(m) ⇌ NADPH-redox-potential(m) + NADP+(m)
r1454	-1	2-Oxobutyrate(m) + H+(PG)(c) → 2-Oxobutyrate(c) + H+(PG)(m)
r0851	-1	H2O(m) ⇌ H2O(c)
r0059	-1	Pyruvate(m) + CO2(m) + NADPH(m) ⇌ Malate(m) + NADP+(m)
r0197	-1	Succinyl-CoA(m) ⇌ (R)-Methylmalonyl-CoA(m)
r2664	0.5	Na+(c) ⇌ Na+(s) + Na-gradient(c)
r0105	1	NAD+(m) + Malate(m) ⇌ OAA(m) + NADH(m)
r0060	1	Serine(c) → Pyruvate(c) + NH3(c)
r1330	-1	H+(PG)(c) ⇌ H+(PG)(m) + Proton-gradient(m)
r1114	1	AKG(m) + CoA(m) + NAD+(m) ⇌ NADH(m) + CO2(m) + Succinyl-CoA(m)
r0241	-1	Cysteine(c) + NH3(c) + 2-Oxobutyrate(c) ⇌ H2O(c) + L-Cystathionine(c)
r0232	1	5-Methyl-THF(c) + Homocysteine(c) ⇌ THF(c) + Methionine(c)
r0223	1	2-Methyl-3-oxopropanoate(m) + CoA(m) + NAD+(m) → Propanoyl-CoA(m) + CO2(m) + NADH(m)
r2676	-1	5-Methyl-THF(c) ⇌ THF(c) + THF-activated methyl group(c)
r0867	1	CO2(c) ⇌ CO2(s)
r0849	1	H2O(s) ⇌ H2O(c)
r0314	-1	H2O(c) + L-Cystathionine(c) ⇌ Serine(c) + Homocysteine(c)
r0107	-1	Acetyl-CoA(m) + H2O(m) + OAA(m) ⇌ Citrate(m) + CoA(m)
r1323	2	NADH(m) ⇌ NAD+(m) + NADH-redox-potential(m)
r0877	0.5	Glutamate(c) + Cystine(s) ⇌ Glutamate(s) + Cystine(c)
r0868	-1	CO2(c) ⇌ CO2(m)
r0027	-0.5	NADPH(c) + Cystine(c) ⇌ 2 Cysteine(c) + NADP+(c)

Proline from Alanine (231)

Definition			Solution												
			exchanges		reactions								transp		
Objective	Constraints	Comment	imports	exports	c	m	r	p	l	n	s	b	s-c	b-c	int
Proline	−Alanine MCES +Urea	Transamination to Proline using Alanine	0.5 O_2(s) 1 Alanine(s) 1 ATP-energy(m) 2 NADPH-redox-potential(m) 1 CoA-activated acetyl group(m)	2 H_2O(s) 1 Proline(s) 2 NADH-redox-potential(m) 5 Proton-gradient(m) 1 Na-gradient(c)	-	15	-	-	-	-	-	-	5	-	7

ID	flux	reaction
r2678	-1	Acetyl-CoA(m) ⇌ CoA(m) + CoA-activated acetyl group(m)
r0316	1	Citrate(m) ⇌ Isocitrate(m)
r0425	1	Isocitrate(m) + NAD+(m) ⇌ Oxalosuccinate(m) + NADH(m)
r0083	1	Oxalosuccinate(m) ⇌ AKG(m) + CO2(m)
r1434	1	Alanine(c) → Alanine(m)
r0507	1	Ubiquinol(m) + 2 Ferricytochrome C(m) + 4 H+(PG)(m) → Ubiquinone(m) + 4 H+(PG)(c) + 2 Ferrocytochrome C(m)
r1453	-1	1-Pyrroline-5-carboxylate(m) + Ubiquinone(m) ⇌ Proline(m) + Ubiquinol(m)
r1426	1	Proline(m) + H+(PG)(c) → Proline(c) + H+(PG)(m)
r1327	-2	NADPH(m) ⇌ NADPH-redox-potential(m) + NADP+(m)
r0175	-1	L-Glutamate 5-semialdehyde(m) ⇌ 1-Pyrroline-5-carboxylate(m) + H2O(m)
r0851	2	H2O(m) ⇌ H2O(c)
r0059	-1	Pyruvate(m) + CO2(m) + NADPH(m) ⇌ Malate(m) + NADP+(m)
r0619	-1	L-Glutamate 5-semialdehyde(m) + Pi(m) + NADP+(m) ← NADPH(m) + Glutamyl-5P(m)
r2002	1	Lysine(s) + Proline(c) → Lysine(c) + Proline(s)
r2664	1	Na+(c) ⇌ Na+(s) + Na-gradient(c)
r1917	1	Alanine(s) + Na+(s) + Lysine(c) → Alanine(c) + Na+(c) + Lysine(s)
r0855	0.5	O2(s) ⇌ O2(c)
r1320	-1	ATP(m) + H2O(m) ⇌ ADP(m) + Pi(m) + ATP-energy(m)
r0856	-0.5	O2(m) ⇌ O2(c)
r0105	1	NAD+(m) + Malate(m) ⇌ OAA(m) + NADH(m)
r1330	5	H+(PG)(c) ⇌ H+(PG)(m) + Proton-gradient(m)
r0849	-2	H2O(s) ⇌ H2O(c)
r0107	-1	Acetyl-CoA(m) + H2O(m) + OAA(m) ⇌ Citrate(m) + CoA(m)
r0017	0.5	O2(m) + 4 H+(PG)(m) + 4 Ferrocytochrome C(m) → 4 Ferricytochrome C(m) + 2 H2O(m) + 4 H+(PG)(c)
r1323	2	NADH(m) ⇌ NAD+(m) + NADH-redox-potential(m)
r0081	1	Alanine(m) + AKG(m) ⇌ Pyruvate(m) + Glutamate(m)
r0072	1	ATP(m) + Glutamate(m) → ADP(m) + Glutamyl-5P(m)

Proline from Arginine (232)

Definition			Solution												
			exchanges		reactions								transp		
Objective	Constraints	Comment	imports	exports	c	m	r	p	l	n	s	b	s-c	b-c	int
Proline	−Arginine MCES +Urea	Transamination to Proline using Arginine	0.5 O_2(s) 0.5 Pyruvate(m) 0.5 Arginine(s) 0.5 ATP-energy(m) 1 NADPH-redox-potential(m) 0.5 CoA-activated acetyl group(m)	1.5 H_2O(s) 0.5 Urea(s) 1 Proline(s) 1 NADH-redox-potential(m) 4.5 Proton-gradient(m)	1	15	-	-	-	-	-	-	6	-	8

ID	flux	reaction
r2678	-0.5	Acetyl-CoA(m) ⇌ CoA(m) + CoA-activated acetyl group(m)
r0896	0.5	Urea(c) ⇌ Urea(s)
r0316	0.5	Citrate(m) ⇌ Isocitrate(m)
r2531	-0.5	Proline(c) ⇌ Proline(s)
r0425	0.5	Isocitrate(m) + NAD+(m) ⇌ Oxalosuccinate(m) + NADH(m)
r0083	0.5	Oxalosuccinate(m) ⇌ AKG(m) + CO2(m)
r0507	1	Ubiquinol(m) + 2 Ferricytochrome C(m) + 4 H+(PG)(m) → Ubiquinone(m) + 4 H+(PG)(c) + 2 Ferrocytochrome C(m)

Continued on next page

ID	flux	reaction
r1453	-1	1-Pyrroline-5-carboxylate(m) + Ubiquinone(m) ⇌ Proline(m) + Ubiquinol(m)
r1426	1	Proline(m) + H+(PG)(c) → Proline(c) + H+(PG)(m)
r1327	-1	NADPH(m) ⇌ NADPH-redox-potential(m) + NADP+(m)
r0175	-1	L-Glutamate 5-semialdehyde(m) ⇌ 1-Pyrroline-5-carboxylate(m) + H2O(m)
r0851	2	H2O(m) ⇌ H2O(c)
r0167	0.5	AKG(m) + Ornithine(m) ⇌ L-Glutamate 5-semialdehyde(m) + Glutamate(m)
r0059	-0.5	Pyruvate(m) + CO2(m) + NADPH(m) ⇌ Malate(m) + NADP+(m)
r1663	-0.5	Lysine(c) + Arginine(s) ⇌ Lysine(s) + Arginine(c)
r0619	-0.5	L-Glutamate 5-semialdehyde(m) + Pi(m) + NADP+(m) ← NADPH(m) + Glutamyl-5P(m)
r2002	0.5	Lysine(s) + Proline(c) → Lysine(c) + Proline(s)
r0855	0.5	O2(s) ⇌ O2(c)
r1320	-0.5	ATP(m) + H2O(m) ⇌ ADP(m) + Pi(m) + ATP-energy(m)
r0856	-0.5	O2(m) ⇌ O2(c)
r0105	0.5	NAD+(m) + Malate(m) ⇌ OAA(m) + NADH(m)
r2396	-0.5	Lysine(m) + Ornithine(c) + H+(PG)(c) → Lysine(c) + Ornithine(m) + H+(PG)(m)
r1330	4.5	H+(PG)(c) ⇌ H+(PG)(m) + Proton-gradient(m)
r1070	0.5	Lysine(c) ⇌ Lysine(m)
r0849	-1.5	H2O(s) ⇌ H2O(c)
r0107	-0.5	Acetyl-CoA(m) + H2O(m) + OAA(m) ⇌ Citrate(m) + CoA(m)
r0017	0.5	O2(m) + 4 H+(PG)(m) + 4 Ferrocytochrome C(m) → 4 Ferricytochrome C(m) + 2 H2O(m) + 4 H+(PG)(c)
r1323	1	NADH(m) ⇌ NAD+(m) + NADH-redox-potential(m)
r0144	0.5	Arginine(c) + H2O(c) ⇌ Urea(c) + Ornithine(c)
r0072	0.5	ATP(m) + Glutamate(m) → ADP(m) + Glutamyl-5P(m)

Proline from Asparagine (233)

Definition			Solution												
			exchanges		reactions								transp		
Objective	Constraints	Comment	imports	exports	c	m	r	p	l	n	s	b	s-c	b-c	int
Proline	−Asparagine MCES +Urea	Transamination to Proline using Asparagine	0.5 O_2(s) 0.5 Pyruvate(m) 0.5 Asparagine(s) 1 ATP-energy(m) 0.5 NADH-redox-potential(c) 2 NADPH-redox-potential(m) 1 CoA-activated acetyl group(m)	2 H_2O(s) 0.5 CO_2(s) 1 Proline(s) 1.5 NADH-redox-potential(m) 0.5 NADPH-redox-potential(c) 5.5 Proton-gradient(m)	7	15	-	-	-	-	-	-	5	-	9

ID	flux	reaction
r2678	-1	Acetyl-CoA(m) ⇌ CoA(m) + CoA-activated acetyl group(m)
r0316	1	Citrate(m) ⇌ Isocitrate(m)
r0127	0.5	H2O(c) + Asparagine(c) → Aspartate(c) + NH3(c)
r0109	0.5	AKG(c) + Aspartate(c) ⇌ OAA(c) + Glutamate(c)
r0082	0.5	Oxalosuccinate(c) ⇌ AKG(c) + CO2(c)
r2531	-0.5	Proline(c) ⇌ Proline(s)
r0911	-0.5	Proline(c) + Glutamate(m) ⇌ Proline(m) + Glutamate(c)
r0425	0.5	Isocitrate(m) + NAD+(m) ⇌ Oxalosuccinate(m) + NADH(m)
r0083	0.5	Oxalosuccinate(m) ⇌ AKG(m) + CO2(m)
r1326	0.5	NADPH(c) ⇌ NADPH-redox-potential(c) + NADP+(c)
r0507	1	Ubiquinol(m) + 2 Ferricytochrome C(m) + 4 H+(PG)(m) → Ubiquinone(m) + 4 H+(PG)(c) + 2 Ferrocytochrome C(m)
r0075	-0.5	AKG(m) + NADPH(m) + NH3(m) ⇌ Glutamate(m) + H2O(m) + NADP+(m)
r1453	-1	1-Pyrroline-5-carboxylate(m) + Ubiquinone(m) ⇌ Proline(m) + Ubiquinol(m)
r1426	0.5	Proline(m) + H+(PG)(c) → Proline(c) + H+(PG)(m)
r1327	-2	NADPH(m) ⇌ NADPH-redox-potential(m) + NADP+(m)
r0913	0.5	Isocitrate(m) + Malate(c) ⇌ Isocitrate(c) + Malate(m)
r0175	-1	L-Glutamate 5-semialdehyde(m) ⇌ 1-Pyrroline-5-carboxylate(m) + H2O(m)
r0851	2.5	H2O(m) ⇌ H2O(c)
r0059	-0.5	Pyruvate(m) + CO2(m) + NADPH(m) ⇌ Malate(m) + NADP+(m)

Continued on next page

ID	flux	reaction
r0619	-1	L-Glutamate 5-semialdehyde(m) + Pi(m) + NADP+(m) ← NADPH(m) + Glutamyl-5P(m)
r0855	0.5	O2(s) ⇌ O2(c)
r0104	-0.5	OAA(c) + NADH(c) ⇌ NAD+(c) + Malate(c)
r1648	-0.5	Proline(c) + Asparagine(s) ⇌ Proline(s) + Asparagine(c)
r1320	-1	ATP(m) + H2O(m) ⇌ ADP(m) + Pi(m) + ATP-energy(m)
r0856	-0.5	O2(m) ⇌ O2(c)
r0838	0.5	NH3(c) ⇌ NH3(m)
r0105	1	NAD+(m) + Malate(m) ⇌ OAA(m) + NADH(m)
r1330	5.5	H+(PG)(c) ⇌ H+(PG)(m) + Proton-gradient(m)
r1322	-0.5	NADH(c) ⇌ NAD+(c) + NADH-redox-potential(c)
r0867	0.5	CO2(c) ⇌ CO2(s)
r0849	-2	H2O(s) ⇌ H2O(c)
r0422	0.5	Isocitrate(c) + NADP+(c) ⇌ Oxalosuccinate(c) + NADPH(c)
r0107	-1	Acetyl-CoA(m) + H2O(m) + OAA(m) ⇌ Citrate(m) + CoA(m)
r0017	0.5	O2(m) + 4 H+(PG)(m) + 4 Ferrocytochrome C(m) → 4 Ferricytochrome C(m) + 2 H2O(m) + 4 H+(PG)(c)
r1323	1.5	NADH(m) ⇌ NAD+(m) + NADH-redox-potential(m)
r0072	1	ATP(m) + Glutamate(m) → ADP(m) + Glutamyl-5P(m)

Proline from Aspartate (234)

Definition			Solution												
			exchanges		reactions								transp		
Objective	Constraints	Comment	imports	exports	c	m	r	p	l	n	s	b	s-c	b-c	int
Proline	−Aspartate MCES +Urea	Transamination to Proline using Aspartate	0.5 O_2(s) 1 Aspartate(s) 1 ATP-energy(m) 1 NADH-redox-potential(c) 1 NADPH-redox-potential(m) 1 CoA-activated acetyl group(m)	2 H_2O(s) 1 CO_2(s) 1 Proline(s) 1 NADH-redox-potential(m) 1 NADPH-redox-potential(c) 6 Proton-gradient(m) 1 Na-gradient(c)	6	11	-	-	-	-	-	-	6	-	7

ID	flux	reaction
r2678	-1	Acetyl-CoA(m) ⇌ CoA(m) + CoA-activated acetyl group(m)
r0316	1	Citrate(m) ⇌ Isocitrate(m)
r0109	1	AKG(c) + Aspartate(c) ⇌ OAA(c) + Glutamate(c)
r0082	1	Oxalosuccinate(c) ⇌ AKG(c) + CO2(c)
r2531	-1	Proline(c) ⇌ Proline(s)
r0911	-1	Proline(c) + Glutamate(m) ⇌ Proline(m) + Glutamate(c)
r1326	1	NADPH(c) ⇌ NADPH-redox-potential(c) + NADP+(c)
r0507	1	Ubiquinol(m) + 2 Ferricytochrome C(m) + 4 H+(PG)(m) → Ubiquinone(m) + 4 H+(PG)(c) + 2 Ferrocytochrome C(m)
r1453	-1	1-Pyrroline-5-carboxylate(m) + Ubiquinone(m) ⇌ Proline(m) + Ubiquinol(m)
r1327	-1	NADPH(m) ⇌ NADPH-redox-potential(m) + NADP+(m)
r0913	1	Isocitrate(m) + Malate(c) ⇌ Isocitrate(c) + Malate(m)
r0175	-1	L-Glutamate 5-semialdehyde(m) ⇌ 1-Pyrroline-5-carboxylate(m) + H2O(m)
r0851	2	H2O(m) ⇌ H2O(c)
r0619	-1	L-Glutamate 5-semialdehyde(m) + Pi(m) + NADP+(m) ← NADPH(m) + Glutamyl-5P(m)
r2664	1	Na+(c) ⇌ Na+(s) + Na-gradient(c)
r0855	0.5	O2(s) ⇌ O2(c)
r0104	-1	OAA(c) + NADH(c) ⇌ NAD+(c) + Malate(c)
r1320	-1	ATP(m) + H2O(m) ⇌ ADP(m) + Pi(m) + ATP-energy(m)
r0856	-0.5	O2(m) ⇌ O2(c)
r0105	1	NAD+(m) + Malate(m) ⇌ OAA(m) + NADH(m)
r1330	6	H+(PG)(c) ⇌ H+(PG)(m) + Proton-gradient(m)
r1322	-1	NADH(c) ⇌ NAD+(c) + NADH-redox-potential(c)
r0867	1	CO2(c) ⇌ CO2(s)
r0849	-2	H2O(s) ⇌ H2O(c)
r0422	1	Isocitrate(c) + NADP+(c) ⇌ Oxalosuccinate(c) + NADPH(c)

Continued on next page

Proline from Aspartate (234)– continued

ID	flux	reaction
r0107	-1	Acetyl-CoA(m) + H2O(m) + OAA(m) ⇌ Citrate(m) + CoA(m)
r0017	0.5	O2(m) + 4 H+(PG)(m) + 4 Ferrocytochrome C(m) → 4 Ferricytochrome C(m) + 2 H2O(m) + 4 H+(PG)(c)
r1323	1	NADH(m) ⇌ NAD+(m) + NADH-redox-potential(m)
r1143	-1	Na+(c) + Aspartate(c) ← Na+(s) + Aspartate(s)
r0072	1	ATP(m) + Glutamate(m) → ADP(m) + Glutamyl-5P(m)

Proline from Cysteine (235)

Definition			Solution												
			exchanges		reactions								transp		
Objective	Constraints	Comment	imports	exports	c	m	r	p	l	n	s	b	s-c	b-c	int
Proline	−Cysteine MCES +Urea +H_2S	Transamination to Proline using Cysteine	0.5 O_2(s) 1 Pyruvate(m) 1 Cysteine(s) 1 ATP-energy(m) 2 NADPH-redox-potential(m) 1 CoA-activated acetyl group(m)	2 H_2O(s) 1 Pyruvate(c) 1 Proline(s) 1 H_2S(s) 1 NADH-redox-potential(m) 5 Proton-gradient(m)	1	15	-	-	-	-	-	-	5	-	7

ID	flux	reaction
r2678	-1	Acetyl-CoA(m) ⇌ CoA(m) + CoA-activated acetyl group(m)
r0316	1	Citrate(m) ⇌ Isocitrate(m)
r0073	-1	AKG(m) + NADH(m) + NH3(m) ⇌ Glutamate(m) + NAD+(m) + H2O(m)
r0425	1	Isocitrate(m) + NAD+(m) ⇌ Oxalosuccinate(m) + NADH(m)
r0083	1	Oxalosuccinate(m) ⇌ AKG(m) + CO2(m)
r0507	1	Ubiquinol(m) + 2 Ferricytochrome C(m) + 4 H+(PG)(m) → Ubiquinone(m) + 4 H+(PG)(c) + 2 Ferrocytochrome C(m)
r1642	1	Leucine(s) + Proline(c) ⇌ Leucine(c) + Proline(s)
r1453	-1	1-Pyrroline-5-carboxylate(m) + Ubiquinone(m) ⇌ Proline(m) + Ubiquinol(m)
r1426	1	Proline(m) + H+(PG)(c) → Proline(c) + H+(PG)(m)
r1327	-2	NADPH(m) ⇌ NADPH-redox-potential(m) + NADP+(m)
r0940	1	H2S(c) ⇌ H2S(s)
r0193	1	Cysteine(c) + H2O(c) ⇌ Pyruvate(c) + H2S(c) + NH3(c)
r0175	-1	L-Glutamate 5-semialdehyde(m) ⇌ 1-Pyrroline-5-carboxylate(m) + H2O(m)
r0851	3	H2O(m) ⇌ H2O(c)
r0059	-1	Pyruvate(m) + CO2(m) + NADPH(m) ⇌ Malate(m) + NADP+(m)
r1635	1	Cysteine(s) + Leucine(c) ⇌ Cysteine(c) + Leucine(s)
r0619	-1	L-Glutamate 5-semialdehyde(m) + Pi(m) + NADP+(m) ← NADPH(m) + Glutamyl-5P(m)
r0855	0.5	O2(s) ⇌ O2(c)
r1320	-1	ATP(m) + H2O(m) ⇌ ADP(m) + Pi(m) + ATP-energy(m)
r0856	-0.5	O2(m) ⇌ O2(c)
r0838	1	NH3(c) ⇌ NH3(m)
r0105	1	NAD+(m) + Malate(m) ⇌ OAA(m) + NADH(m)
r1330	5	H+(PG)(c) ⇌ H+(PG)(m) + Proton-gradient(m)
r0849	-2	H2O(s) ⇌ H2O(c)
r0107	-1	Acetyl-CoA(m) + H2O(m) + OAA(m) ⇌ Citrate(m) + CoA(m)
r0017	0.5	O2(m) + 4 H+(PG)(m) + 4 Ferrocytochrome C(m) → 4 Ferricytochrome C(m) + 2 H2O(m) + 4 H+(PG)(c)
r1323	1	NADH(m) ⇌ NAD+(m) + NADH-redox-potential(m)
r0072	1	ATP(m) + Glutamate(m) → ADP(m) + Glutamyl-5P(m)

Proline from Cystine (236)

Definition			Solution												
			exchanges		reactions								transp		
Objective	Constraints	Comment	imports	exports	c	m	r	p	l	n	s	b	s-c	b-c	int
Proline	−Cystine MCES +Urea +H_2S	Transamination to Proline using Cystine	0.5 O_2(s) 1 Pyruvate(m) 0.5 Cystine(s) 1 ATP-energy(m) 0.5 NADPH-redox-potential(c) 3 NADPH-redox-potential(m) 1 CoA-activated acetyl group(m)	2 H_2O(s) 1 Pyruvate(c) 1 Proline(s) 1 H_2S(s) 2 NADH-redox-potential(m) 5 Proton-gradient(m) 0.5 Na-gradient(c)	3	15	-	-	-	-	-	-	7	-	7

ID	flux	reaction
r2678	-1	Acetyl-CoA(m) ⇌ CoA(m) + CoA-activated acetyl group(m)
r1144	-0.5	Glutamate(c) + Na+(c) ← Glutamate(s) + Na+(s)
r0316	1	Citrate(m) ⇌ Isocitrate(m)
r2531	-1	Proline(c) ⇌ Proline(s)
r0425	1	Isocitrate(m) + NAD+(m) ⇌ Oxalosuccinate(m) + NADH(m)
r0083	1	Oxalosuccinate(m) ⇌ AKG(m) + CO2(m)
r1326	-0.5	NADPH(c) ⇌ NADPH-redox-potential(c) + NADP+(c)
r0507	1	Ubiquinol(m) + 2 Ferricytochrome C(m) + 4 H+(PG)(m) → Ubiquinone(m) + 4 H+(PG)(c) + 2 Ferrocytochrome C(m)
r0075	-1	AKG(m) + NADPH(m) + NH3(m) ⇌ Glutamate(m) + H2O(m) + NADP+(m)
r1453	-1	1-Pyrroline-5-carboxylate(m) + Ubiquinone(m) ⇌ Proline(m) + Ubiquinol(m)
r1426	1	Proline(m) + H+(PG)(c) → Proline(c) + H+(PG)(m)
r1327	-3	NADPH(m) ⇌ NADPH-redox-potential(m) + NADP+(m)
r0940	1	H2S(c) ⇌ H2S(s)
r0193	1	Cysteine(c) + H2O(c) ⇌ Pyruvate(c) + H2S(c) + NH3(c)
r0175	-1	L-Glutamate 5-semialdehyde(m) ⇌ 1-Pyrroline-5-carboxylate(m) + H2O(m)
r0851	3	H2O(m) ⇌ H2O(c)
r0059	-1	Pyruvate(m) + CO2(m) + NADPH(m) ⇌ Malate(m) + NADP+(m)
r0619	-1	L-Glutamate 5-semialdehyde(m) + Pi(m) + NADP+(m) ← NADPH(m) + Glutamyl-5P(m)
r2664	0.5	Na+(c) ⇌ Na+(s) + Na-gradient(c)
r0855	0.5	O2(s) ⇌ O2(c)
r1320	-1	ATP(m) + H2O(m) ⇌ ADP(m) + Pi(m) + ATP-energy(m)
r0856	-0.5	O2(m) ⇌ O2(c)
r0838	1	NH3(c) ⇌ NH3(m)
r0105	1	NAD+(m) + Malate(m) ⇌ OAA(m) + NADH(m)
r1330	5	H+(PG)(c) ⇌ H+(PG)(m) + Proton-gradient(m)
r0849	-2	H2O(s) ⇌ H2O(c)
r0107	-1	Acetyl-CoA(m) + H2O(m) + OAA(m) ⇌ Citrate(m) + CoA(m)
r0017	0.5	O2(m) + 4 H+(PG)(m) + 4 Ferrocytochrome C(m) → 4 Ferricytochrome C(m) + 2 H2O(m) + 4 H+(PG)(c)
r1323	2	NADH(m) ⇌ NAD+(m) + NADH-redox-potential(m)
r0877	0.5	Glutamate(c) + Cystine(s) ⇌ Glutamate(s) + Cystine(c)
r0072	1	ATP(m) + Glutamate(m) → ADP(m) + Glutamyl-5P(m)
r0027	-0.5	NADPH(c) + Cystine(c) ⇌ 2 Cysteine(c) + NADP+(c)

Proline from Glutamate (237)

Definition			Solution												
			exchanges		reactions								transp		
Objective	Constraints	Comment	imports	exports	c	m	r	p	l	n	s	b	s-c	b-c	int
Proline	−Glutamate MCES +Urea	Transamination to Proline using Glutamate	0.5 O_2(s) 1 Glutamate(s) 1 NADH-redox-potential(m)	3 H_2O(s) 1 Proline(s) 6 Proton-gradient(m) 1 Na-gradient(c)	-	4	-	-	-	-	-	-	5	-	6

ID	flux	reaction
r1144	-1	Glutamate(c) + Na+(c) ← Glutamate(s) + Na+(s)
r2531	-1	Proline(c) ⇌ Proline(s)
r0911	-1	Proline(c) + Glutamate(m) ⇌ Proline(m) + Glutamate(c)
r0074	-1	L-Glutamate 5-semialdehyde(m) + NAD+(m) + H2O(m) ⇌ Glutamate(m) + NADH(m)
r0507	1	Ubiquinol(m) + 2 Ferricytochrome C(m) + 4 H+(PG)(m) → Ubiquinone(m) + 4 H+(PG)(c) + 2 Ferrocytochrome C(m)
r1453	-1	1-Pyrroline-5-carboxylate(m) + Ubiquinone(m) ⇌ Proline(m) + Ubiquinol(m)
r0175	-1	1-Pyrroline-5-carboxylate(m) + H2O(m) ⇌ L-Glutamate 5-semialdehyde(m)
r0851	3	H2O(m) ⇌ H2O(c)
r2664	1	Na+(c) ⇌ Na+(s) + Na-gradient(c)
r0855	0.5	O2(s) ⇌ O2(c)
r0856	-0.5	O2(m) ⇌ O2(c)
r1330	6	H+(PG)(c) ⇌ H+(PG)(m) + Proton-gradient(m)
r0849	-3	H2O(s) ⇌ H2O(c)

Continued on next page

ID	flux	reaction
r0017	0.5	O2(m) + 4 H+(PG)(m) + 4 Ferrocytochrome C(m) → 4 Ferricytochrome C(m) + 2 H2O(m) + 4 H+(PG)(c)
r1323	-1	NADH(m) ⇌ NAD+(m) + NADH-redox-potential(m)

Proline from Glycine (238)

Definition			Solution											
			exchanges		reactions								transp		
Objective	Constraints	Comment	imports	exports	c	m	r	p	l	n	s	b	s-c	b-c	int
Proline	−Glycine MCES +Urea	Transamination to Proline using Glycine	0.5 O_2(s) 1 Pyruvate(m) 1 Glycine(s) 1 ATP-energy(m) 3 NADPH-redox-potential(m) 1 THF-activated methyl group(c) 1 CoA-activated acetyl group(m)	2 H_2O(s) 1 Pyruvate(c) 1 Proline(s) 2 NADH-redox-potential(m) 1 NADPH-redox-potential(c) 5 Proton-gradient(m) 1 Na-gradient(c)	5	15	-	-	-	-	-	-	4	-	7

ID	flux	reaction
r2678	-1	Acetyl-CoA(m) ⇌ CoA(m) + CoA-activated acetyl group(m)
r0316	1	Citrate(m) ⇌ Isocitrate(m)
r0425	1	Isocitrate(m) + NAD+(m) ⇌ Oxalosuccinate(m) + NADH(m)
r0083	1	Oxalosuccinate(m) ⇌ AKG(m) + CO2(m)
r1326	1	NADPH(c) ⇌ NADPH-redox-potential(c) + NADP+(c)
r0507	1	Ubiquinol(m) + 2 Ferricytochrome C(m) + 4 H+(PG)(m) → Ubiquinone(m) + 4 H+(PG)(c) + 2 Ferrocytochrome C(m)
r0075	-1	AKG(m) + NADPH(m) + NH3(m) ⇌ Glutamate(m) + H2O(m) + NADP+(m)
r1453	-1	1-Pyrroline-5-carboxylate(m) + Ubiquinone(m) ⇌ Proline(m) + Ubiquinol(m)
r1426	1	Proline(m) + H+(PG)(c) → Proline(c) + H+(PG)(m)
r1327	-3	NADPH(m) ⇌ NADPH-redox-potential(m) + NADP+(m)
r0175	-1	L-Glutamate 5-semialdehyde(m) ⇌ 1-Pyrroline-5-carboxylate(m) + H2O(m)
r0851	3	H2O(m) ⇌ H2O(c)
r0059	-1	Pyruvate(m) + CO2(m) + NADPH(m) ⇌ Malate(m) + NADP+(m)
r1681	1	Glycine(s) + Proline(c) + Na+(s) → Glycine(c) + Proline(s) + Na+(c)
r0619	-1	L-Glutamate 5-semialdehyde(m) + Pi(m) + NADP+(m) ← NADPH(m) + Glutamyl-5P(m)
r0296	1	5-Methyl-THF(c) + NADP+(c) ⇌ 5,10-Methylene-THF(c) + NADPH(c)
r2664	1	Na+(c) ⇌ Na+(s) + Na-gradient(c)
r0855	0.5	O2(s) ⇌ O2(c)
r0230	1	5,10-Methylene-THF(c) + Glycine(c) + H2O(c) ⇌ THF(c) + Serine(c)
r1320	-1	ATP(m) + H2O(m) ⇌ ADP(m) + Pi(m) + ATP-energy(m)
r0856	-0.5	O2(m) ⇌ O2(c)
r0838	1	NH3(c) ⇌ NH3(m)
r0105	1	NAD+(m) + Malate(m) ⇌ OAA(m) + NADH(m)
r0060	1	Serine(c) → Pyruvate(c) + NH3(c)
r1330	5	H+(PG)(c) ⇌ H+(PG)(m) + Proton-gradient(m)
r2676	-1	5-Methyl-THF(c) ⇌ THF(c) + THF-activated methyl group(c)
r0849	-2	H2O(s) ⇌ H2O(c)
r0107	-1	Acetyl-CoA(m) + H2O(m) + OAA(m) ⇌ Citrate(m) + CoA(m)
r0017	0.5	O2(m) + 4 H+(PG)(m) + 4 Ferrocytochrome C(m) → 4 Ferricytochrome C(m) + 2 H2O(m) + 4 H+(PG)(c)
r1323	2	NADH(m) ⇌ NAD+(m) + NADH-redox-potential(m)
r0072	1	ATP(m) + Glutamate(m) → ADP(m) + Glutamyl-5P(m)

Proline from Histidine (239)

Definition			Solution												
			exchanges		reactions								transp		
Objective	Constraints	Comment	imports	exports	c	m	r	p	l	n	s	b	s-c	b-c	int
Proline	−Histidine MCES +Urea	Transamination to Proline using Histidine	0.5 O_2(s) 0.67 Pyruvate(m) 0.33 Histidine(s) 1 ATP-energy(m) 1.67 NADPH-redox-potential(m) 0.67 CoA-activated acetyl group(m)	1.67 H_2O(s) 0.33 CO_2(s) 1 Proline(s) 0.67 NADH-redox-potential(m) 0.33 NADPH-redox-potential(c) 5.33 Proton-gradient(m) 0.33 Na-gradient(c)	8	15	-	-	-	-	-	-	7	-	8

ID	flux	reaction
r2678	-0.67	Acetyl-CoA(m) ⇌ CoA(m) + CoA-activated acetyl group(m)
r0316	0.67	Citrate(m) ⇌ Isocitrate(m)
r0073	-0.67	AKG(m) + NADH(m) + NH3(m) ⇌ Glutamate(m) + NAD+(m) + H2O(m)
r2531	-0.67	Proline(c) ⇌ Proline(s)
r0911	-0.33	Proline(c) + Glutamate(m) ⇌ Proline(m) + Glutamate(c)
r0524	0.33	5-Formiminotetrahydrofolate(c) ⇌ 5,10-Methenyl-THF(c) + NH3(c)
r0425	0.67	Isocitrate(m) + NAD+(m) ⇌ Oxalosuccinate(m) + NADH(m)
r0371	0.33	5,10-Methenyl-THF(c) + H2O(c) ⇌ 10-Formyl-THF(c)
r0227	0.33	10-Formyl-THF(c) + H2O(c) + NADP+(c) → THF(c) + CO2(c) + NADPH(c)
r0083	0.67	Oxalosuccinate(m) ⇌ AKG(m) + CO2(m)
r1326	0.33	NADPH(c) ⇌ NADPH-redox-potential(c) + NADP+(c)
r0507	1	Ubiquinol(m) + 2 Ferricytochrome C(m) + 4 H+(PG)(m) → Ubiquinone(m) + 4 H+(PG)(c) + 2 Ferrocytochrome C(m)
r1453	-1	1-Pyrroline-5-carboxylate(m) + Ubiquinone(m) ⇌ Proline(m) + Ubiquinol(m)
r1426	0.67	Proline(m) + H+(PG)(c) → Proline(c) + H+(PG)(m)
r1327	-1.67	NADPH(m) ⇌ NADPH-redox-potential(m) + NADP+(m)
r0175	-1	L-Glutamate 5-semialdehyde(m) ⇌ 1-Pyrroline-5-carboxylate(m) + H2O(m)
r0851	3	H2O(m) ⇌ H2O(c)
r0518	-0.33	Glutamate(c) + 5-Formiminotetrahydrofolate(c) ⇌ THF(c) + N-Formimino-L-glutamate(c)
r0059	-0.67	Pyruvate(m) + CO2(m) + NADPH(m) ⇌ Malate(m) + NADP+(m)
r0519	0.33	H2O(c) + 4-Imidazolone-5-propanoate(c) ⇌ N-Formimino-L-glutamate(c)
r1681	0.33	Glycine(s) + Proline(c) + Na+(s) → Glycine(c) + Proline(s) + Na+(c)
r0619	-1	L-Glutamate 5-semialdehyde(m) + Pi(m) + NADP+(m) ← NADPH(m) + Glutamyl-5P(m)
r0286	0.33	Histidine(c) ⇌ Urocanate(c) + NH3(c)
r2664	0.33	Na+(c) ⇌ Na+(s) + Na-gradient(c)
r0855	0.5	O2(s) ⇌ O2(c)
r2040	0.33	Histidine(s) + Glycine(c) → Histidine(c) + Glycine(s)
r1320	-1	ATP(m) + H2O(m) ⇌ ADP(m) + Pi(m) + ATP-energy(m)
r0856	-0.5	O2(m) ⇌ O2(c)
r0838	0.67	NH3(c) ⇌ NH3(m)
r0577	-0.33	Urocanate(c) + H2O(c) ⇌ 4-Imidazolone-5-propanoate(c)
r0105	0.67	NAD+(m) + Malate(m) ⇌ OAA(m) + NADH(m)
r1330	5.33	H+(PG)(c) ⇌ H+(PG)(m) + Proton-gradient(m)
r0867	0.33	CO2(c) ⇌ CO2(s)
r0849	-1.67	H2O(s) ⇌ H2O(c)
r0107	-0.67	Acetyl-CoA(m) + H2O(m) + OAA(m) ⇌ Citrate(m) + CoA(m)
r0017	0.5	O2(m) + 4 H+(PG)(m) + 4 Ferrocytochrome C(m) → 4 Ferricytochrome C(m) + 2 H2O(m) + 4 H+(PG)(c)
r1323	0.67	NADH(m) ⇌ NAD+(m) + NADH-redox-potential(m)
r0072	1	ATP(m) + Glutamate(m) → ADP(m) + Glutamyl-5P(m)

Proline from Isoleucine (240)

Definition			Solution													
			exchanges		reactions								transp			
Objective	Constraints	Comment	imports	exports	c	m	r	p	l	n	s	b	s-c	b-c	int	
Proline	−Isoleucine MCES +Urea	Transamination to Proline using Isoleucine	1.17 O_2(s) 0.33 Pyruvate(m) 1 Isoleucine(s) 1 ATP-energy(m) 2 NADPH-redox-potential(m)	2.33 H_2O(s) 1 Proline(s) 3.33 NADH-redox-potential(m) 12 Proton-gradient(m) 1 Na-gradient(c) 1 CoA-activated acetyl group(m)	-	30	-	-	-	-	-	-	5	-	7	

ID	flux	reaction
r0572	-0.67	Methylmalonyl-CoA(m) ⇌ (R)-Methylmalonyl-CoA(m)
r0855	1.17	O2(s) ⇌ O2(c)
r0856	-1.17	O2(m) ⇌ O2(c)
r0425	0.33	Isocitrate(m) + NAD+(m) ⇌ Oxalosuccinate(m) + NADH(m)
r0592	-0.33	H2O(m) + Acrylyl-CoA(m) → 3-Hydroxypropionyl-CoA(m)
r0596	-0.33	3-Hydroxypropionyl-CoA(m) + H2O(m) → Hydracrylate(m) + CoA(m)
r0017	1.17	O2(m) + 4 H+(PG)(m) + 4 Ferrocytochrome C(m) → 4 Ferricytochrome C(m) + 2 H2O(m) + 4 H+(PG)(c)
r1552	1	Glycine(s) + Proline(c) ⇌ Glycine(c) + Proline(s)
r0316	0.33	Citrate(m) ⇌ Isocitrate(m)
r0619	-1	L-Glutamate 5-semialdehyde(m) + Pi(m) + NADP+(m) ← NADPH(m) + Glutamyl-5P(m)
r2664	1	Na+(c) ⇌ Na+(s) + Na-gradient(c)
r1426	1	Proline(m) + H+(PG)(c) → Proline(c) + H+(PG)(m)
r0072	1	ATP(m) + Glutamate(m) → ADP(m) + Glutamyl-5P(m)
r0059	-1	Pyruvate(m) + CO2(m) + NADPH(m) ⇌ Malate(m) + NADP+(m)
r0222	-1	CoA(m) + 2-Methylacetoacetyl-CoA(m) ⇌ Propanoyl-CoA(m) + Acetyl-CoA(m)
r0953	1	Isoleucine(c) + H+(PG)(c) → Isoleucine(m) + H+(PG)(m)
r1897	1	Isoleucine(s) + Glycine(c) + Na+(s) → Isoleucine(c) + Glycine(s) + Na+(c)
r0507	2.33	Ubiquinol(m) + 2 Ferricytochrome C(m) + 4 H+(PG)(m) → Ubiquinone(m) + 4 H+(PG)(c) + 2 Ferrocytochrome C(m)
r1330	12	H+(PG)(c) ⇌ H+(PG)(m) + Proton-gradient(m)
r0264	1	Isoleucine(m) + AKG(m) ⇌ Glutamate(m) + 2-Oxo-3-methylvalerate(m)
r0414	0.67	ATP(m) + Propanoyl-CoA(m) + HCO3-(m) → ADP(m) + Pi(m) + Methylmalonyl-CoA(m)
r0849	-2.33	H2O(s) ⇌ H2O(c)
r0174	0.33	CoA(m) + NAD+(m) + 3-Oxopropanoate(m) → Acetyl-CoA(m) + CO2(m) + NADH(m)
r0175	-1	L-Glutamate 5-semialdehyde(m) ⇌ 1-Pyrroline-5-carboxylate(m) + H2O(m)
r0602	1	2-Oxo-3-methylvalerate(m) + CoA(m) + NAD+(m) ⇌ 2-Methylbutyryl-CoA(m) + CO2(m) + NADH(m)
r0603	1	2-Methylbutyryl-CoA(m) + Ubiquinone(m) ⇌ Tiglyl-CoA(m) + Ubiquinol(m)
r1114	-0.67	NADH(m) + CO2(m) + Succinyl-CoA(m) ⇌ AKG(m) + CoA(m) + NAD+(m)
r0197	0.67	(R)-Methylmalonyl-CoA(m) ⇌ Succinyl-CoA(m)
r0365	0.33	Hydracrylate(m) + NAD+(m) → NADH(m) + 3-Oxopropanoate(m)
r0083	0.33	Oxalosuccinate(m) ⇌ AKG(m) + CO2(m)
r2678	1	Acetyl-CoA(m) ⇌ CoA(m) + CoA-activated acetyl group(m)
r1453	-1	1-Pyrroline-5-carboxylate(m) + Ubiquinone(m) ⇌ Proline(m) + Ubiquinol(m)
r0664	1	NAD+(m) + (S)-3-Hydroxy-2-methylbutyryl-CoA(m) ⇌ 2-Methylacetoacetyl-CoA(m) + NADH(m)
r0665	-1	H2O(m) + Tiglyl-CoA(m) ⇌ (S)-3-Hydroxy-2-methylbutyryl-CoA(m)
r1320	-1	ATP(m) + H2O(m) ⇌ ADP(m) + Pi(m) + ATP-energy(m)
r0683	0.33	Propanoyl-CoA(m) + Ubiquinone(m) → Ubiquinol(m) + Acrylyl-CoA(m)
r1323	3.33	NADH(m) ⇌ NAD+(m) + NADH-redox-potential(m)
r1327	-2	NADPH(m) ⇌ NADPH-redox-potential(m) + NADP+(m)
r0105	1	NAD+(m) + Malate(m) ⇌ OAA(m) + NADH(m)
r0106	-0.67	ADP(m) + Pi(m) + OAA(m) ⇌ ATP(m) + Pyruvate(m) + HCO3-(m)
r0107	-0.33	Acetyl-CoA(m) + H2O(m) + OAA(m) ⇌ Citrate(m) + CoA(m)
r0851	2.33	H2O(m) ⇌ H2O(c)

Proline from Glutamine (241)

Definition			Solution												
			exchanges		reactions								transp		
Objective	Constraints	Comment	imports	exports	c	m	r	p	l	n	s	b	s-c	b-c	int
Proline	−Glutamine MCES +Urea	Transamination to Proline using Glutamine	0.5 O_2(s) 0.5 Pyruvate(m) 0.5 Glutamine(s) 1 ATP-energy(m) 2 NADPH-redox-potential(m) 0.5 CoA-activated acetyl group(m)	2.5 H_2O(s) 1 Proline(s) 1 NADH-redox-potential(m) 5 Proton-gradient(m) 0.5 Na-gradient(c)	-	16	-	-	-	-	-	-	6	-	7

ID	flux	reaction
r2678	-0.5	Acetyl-CoA(m) ⇌ CoA(m) + CoA-activated acetyl group(m)
r0316	0.5	Citrate(m) ⇌ Isocitrate(m)
r2531	-0.5	Proline(c) ⇌ Proline(s)
r2522	0.5	Glutamine(c) ⇌ Glutamine(m)
r0425	0.5	Isocitrate(m) + NAD+(m) ⇌ Oxalosuccinate(m) + NADH(m)
r0083	0.5	Oxalosuccinate(m) ⇌ AKG(m) + CO2(m)
r0507	1	Ubiquinol(m) + 2 Ferricytochrome C(m) + 4 H+(PG)(m) → Ubiquinone(m) + 4 H+(PG)(c) + 2 Ferrocytochrome C(m)
r0075	-0.5	AKG(m) + NADPH(m) + NH3(m) ⇌ Glutamate(m) + H2O(m) + NADP+(m)
r1453	-1	1-Pyrroline-5-carboxylate(m) + Ubiquinone(m) ⇌ Proline(m) + Ubiquinol(m)
r1426	1	Proline(m) + H+(PG)(c) → Proline(c) + H+(PG)(m)
r1327	-2	NADPH(m) ⇌ NADPH-redox-potential(m) + NADP+(m)
r0175	-1	L-Glutamate 5-semialdehyde(m) ⇌ 1-Pyrroline-5-carboxylate(m) + H2O(m)
r1544	-0.5	Glycine(c) + Glutamine(s) ⇌ Glycine(s) + Glutamine(c)
r0851	2.5	H2O(m) ⇌ H2O(c)
r0059	-0.5	Pyruvate(m) + CO2(m) + NADPH(m) ⇌ Malate(m) + NADP+(m)
r0078	0.5	Glutamine(m) + H2O(m) → Glutamate(m) + NH3(m)
r1681	0.5	Glycine(s) + Proline(c) + Na+(s) → Glycine(c) + Proline(s) + Na+(c)
r0619	-1	L-Glutamate 5-semialdehyde(m) + Pi(m) + NADP+(m) ← NADPH(m) + Glutamyl-5P(m)
r2664	0.5	Na+(c) ⇌ Na+(s) + Na-gradient(c)
r0855	0.5	O2(s) ⇌ O2(c)
r1320	-1	ATP(m) + H2O(m) ⇌ ADP(m) + Pi(m) + ATP-energy(m)
r0856	-0.5	O2(m) ⇌ O2(c)
r0105	0.5	NAD+(m) + Malate(m) ⇌ OAA(m) + NADH(m)
r1330	5	H+(PG)(c) ⇌ H+(PG)(m) + Proton-gradient(m)
r0849	-2.5	H2O(s) ⇌ H2O(c)
r0107	-0.5	Acetyl-CoA(m) + H2O(m) + OAA(m) ⇌ Citrate(m) + CoA(m)
r0017	0.5	O2(m) + 4 H+(PG)(m) + 4 Ferrocytochrome C(m) → 4 Ferricytochrome C(m) + 2 H2O(m) + 4 H+(PG)(c)
r1323	1	NADH(m) ⇌ NAD+(m) + NADH-redox-potential(m)
r0072	1	ATP(m) + Glutamate(m) → ADP(m) + Glutamyl-5P(m)

Proline from Leucine (242)

Definition			Solution												
			exchanges		reactions								transp		
Objective	Constraints	Comment	imports	exports	c	m	r	p	l	n	s	b	s-c	b-c	int
Proline	−Leucine MCES +Urea	Transamination to Proline using Leucine	1 O_2(s) 1 Pyruvate(m) 1 Leucine(s) 1 ATP-energy(m) 3 NADPH-redox-potential(m)	3 H_2O(s) 1 Proline(s) 4 NADH-redox-potential(m) 11 Proton-gradient(m) 2 CoA-activated acetyl group(m)	-	22	-	-	-	-	-	-	3	-	7

ID	flux	reaction
r2678	2	Acetyl-CoA(m) ⇌ CoA(m) + CoA-activated acetyl group(m)
r0316	1	Citrate(m) ⇌ Isocitrate(m)
r0262	1	Leucine(m) + AKG(m) ⇌ Glutamate(m) + 4-Methyl-2-oxopentanoate(m)
r0425	1	Isocitrate(m) + NAD+(m) ⇌ Oxalosuccinate(m) + NADH(m)
r0083	1	Oxalosuccinate(m) ⇌ AKG(m) + CO2(m)

Continued on next page

ID	flux	reaction
r0507	2	Ubiquinol(m) + 2 Ferricytochrome C(m) + 4 H+(PG)(m) → Ubiquinone(m) + 4 H+(PG)(c) + 2 Ferrocytochrome C(m)
r0462	1	HMG-CoA(m) + CoA(m) ⇌ Acetyl-CoA(m) + H2O(m) + Acetoacetyl-CoA(m)
r1642	1	Leucine(s) + Proline(c) ⇌ Leucine(c) + Proline(s)
r1453	-1	1-Pyrroline-5-carboxylate(m) + Ubiquinone(m) ⇌ Proline(m) + Ubiquinol(m)
r1426	1	Proline(m) + H+(PG)(c) → Proline(c) + H+(PG)(m)
r1327	-3	NADPH(m) ⇌ NADPH-redox-potential(m) + NADP+(m)
r0490	-1	3-Methylglutaconyl-CoA(m) + H2O(m) ⇌ HMG-CoA(m)
r0175	-1	L-Glutamate 5-semialdehyde(m) ⇌ 1-Pyrroline-5-carboxylate(m) + H2O(m)
r0851	3	H2O(m) ⇌ H2O(c)
r0059	-2	Pyruvate(m) + CO2(m) + NADPH(m) ⇌ Malate(m) + NADP+(m)
r1438	1	Leucine(c) → Leucine(m)
r0655	1	Isovaleryl-CoA(m) + Ubiquinone(m) ⇌ 3-Methylcrotonyl-CoA(m) + Ubiquinol(m)
r0619	-1	L-Glutamate 5-semialdehyde(m) + Pi(m) + NADP+(m) ← NADPH(m) + Glutamyl-5P(m)
r0855	1	O2(s) ⇌ O2(c)
r1320	-1	ATP(m) + H2O(m) ⇌ ADP(m) + Pi(m) + ATP-energy(m)
r0856	-1	O2(m) ⇌ O2(c)
r0658	1	ATP(m) + 3-Methylcrotonyl-CoA(m) + HCO3-(m) ⇌ ADP(m) + Pi(m) + 3-Methylglutaconyl-CoA(m)
r0105	2	NAD+(m) + Malate(m) ⇌ OAA(m) + NADH(m)
r1330	11	H+(PG)(c) ⇌ H+(PG)(m) + Proton-gradient(m)
r0601	1	4-Methyl-2-oxopentanoate(m) + CoA(m) + NAD+(m) ⇌ Isovaleryl-CoA(m) + CO2(m) + NADH(m)
r0106	-1	ADP(m) + Pi(m) + OAA(m) ⇌ ATP(m) + Pyruvate(m) + HCO3-(m)
r0070	-1	CoA(m) + Acetoacetyl-CoA(m) ⇌ 2 Acetyl-CoA(m)
r0849	-3	H2O(s) ⇌ H2O(c)
r0107	-1	Acetyl-CoA(m) + H2O(m) + OAA(m) ⇌ Citrate(m) + CoA(m)
r0017	1	O2(m) + 4 H+(PG)(m) + 4 Ferrocytochrome C(m) → 4 Ferricytochrome C(m) + 2 H2O(m) + 4 H+(PG)(c)
r1323	4	NADH(m) ⇌ NAD+(m) + NADH-redox-potential(m)
r0072	1	ATP(m) + Glutamate(m) → ADP(m) + Glutamyl-5P(m)

Proline from Lysine (243)

Definition			Solution												
			exchanges		reactions								transp		
Objective	Constraints	Comment	imports	exports	c	m	r	p	l	n	s	b	s-c	b-c	int
Proline	−Lysine MCES +Urea	Transamination to Proline using Lysine	0.75 O_2(s) 1 Pyruvate(m) 0.5 Lysine(s) 1 ATP-energy(m) 2.5 NADPH-redox-potential(m)	1.5 H_2O(s) 1 CO_2(s) 1 Proline(s) 0.5 NADH-redox-potential(c) 2.5 NADH-redox-potential(m) 1 NADPH-redox-potential(c) 8 Proton-gradient(m)	6	18	-	-	-	-	-	-	5	-	9

ID	flux	reaction
r0541	0.5	Glutaryl-CoA(m) + Ubiquinone(m) ⇌ Crotonyl-CoA(m) + CO2(m) + Ubiquinol(m)
r0460	0.5	(S)-3-Hydroxybutyryl-CoA(m) + NAD+(m) ⇌ NADH(m) + Acetoacetyl-CoA(m)
r0082	1	Oxalosuccinate(c) ⇌ AKG(c) + CO2(c)
r2531	-0.5	Proline(c) ⇌ Proline(s)
r1326	1	NADPH(c) ⇌ NADPH-redox-potential(c) + NADP+(c)
r0525	0.5	NAD+(m) + H2O(m) + Saccharopine(m) ⇌ Glutamate(m) + 2-Aminoadipate 6-semialdehyde(m) + NADH(m)
r0507	1.5	Ubiquinol(m) + 2 Ferricytochrome C(m) + 4 H+(PG)(m) → Ubiquinone(m) + 4 H+(PG)(c) + 2 Ferrocytochrome C(m)
r1453	-1	1-Pyrroline-5-carboxylate(m) + Ubiquinone(m) ⇌ Proline(m) + Ubiquinol(m)
r1426	1	Proline(m) + H+(PG)(c) → Proline(c) + H+(PG)(m)
r1327	-2.5	NADPH(m) ⇌ NADPH-redox-potential(m) + NADP+(m)
r0175	-1	L-Glutamate 5-semialdehyde(m) ⇌ 1-Pyrroline-5-carboxylate(m) + H2O(m)
r0851	2	H2O(m) ⇌ H2O(c)
r0059	-1	Pyruvate(m) + CO2(m) + NADPH(m) ⇌ Malate(m) + NADP+(m)
r2373	1	Citrate(m) + AKG(c) ⇌ Citrate(c) + AKG(m)
r0619	-1	L-Glutamate 5-semialdehyde(m) + Pi(m) + NADP+(m) ← NADPH(m) + Glutamyl-5P(m)

Continued on next page

ID	flux	reaction
r0448	0.5	2-Oxoadipate(m) + CoA(m) + NAD+(m) ⇌ Glutaryl-CoA(m) + CO2(m) + NADH(m)
r2002	0.5	Lysine(s) + Proline(c) → Lysine(c) + Proline(s)
r0855	0.75	O2(s) ⇌ O2(c)
r0594	0.5	2-Aminoadipate 6-semialdehyde(c) + NAD+(c) + H2O(c) ⇌ NADH(c) + L-2-Aminoadipate(c)
r2539	-0.5	2-Aminoadipate 6-semialdehyde(c) + L-2-Aminoadipate(m) ⇌ 2-Aminoadipate 6-semialdehyde(m) + L-2-Aminoadipate(c)
r1320	-1	ATP(m) + H2O(m) ⇌ ADP(m) + Pi(m) + ATP-energy(m)
r0856	-0.75	O2(m) ⇌ O2(c)
r0105	1	NAD+(m) + Malate(m) ⇌ OAA(m) + NADH(m)
r1330	8	H+(PG)(c) ⇌ H+(PG)(m) + Proton-gradient(m)
r0070	-0.5	CoA(m) + Acetoacetyl-CoA(m) ⇌ 2 Acetyl-CoA(m)
r1322	0.5	NADH(c) ⇌ NAD+(c) + NADH-redox-potential(c)
r1070	0.5	Lysine(c) ⇌ Lysine(m)
r0867	1	CO2(c) ⇌ CO2(s)
r0849	-1.5	H2O(s) ⇌ H2O(c)
r0588	-0.5	Crotonyl-CoA(m) + H2O(m) ⇌ (S)-3-Hydroxybutyryl-CoA(m)
r0422	1	Isocitrate(c) + NADP+(c) ⇌ Oxalosuccinate(c) + NADPH(c)
r0107	-1	Acetyl-CoA(m) + H2O(m) + OAA(m) ⇌ Citrate(m) + CoA(m)
r0017	0.75	O2(m) + 4 H+(PG)(m) + 4 Ferrocytochrome C(m) → 4 Ferricytochrome C(m) + 2 H2O(m) + 4 H+(PG)(c)
r1323	2.5	NADH(m) ⇌ NAD+(m) + NADH-redox-potential(m)
r0450	0.5	AKG(m) + L-2-Aminoadipate(m) ⇌ 2-Oxoadipate(m) + Glutamate(m)
r0315	1	Citrate(c) ⇌ Isocitrate(c)
r0180	-0.5	H2O(m) + Saccharopine(m) + NADP+(m) ⇌ AKG(m) + NADPH(m) + Lysine(m)
r0072	1	ATP(m) + Glutamate(m) → ADP(m) + Glutamyl-5P(m)

Proline from Methionine (244)

Definition			Solution												
			exchanges		reactions								transp		
Objective	Constraints	Comment	imports	exports	c	m	r	p	l	n	s	b	s-c	b-c	int
Proline	−Methionine MCES +Urea +H_2S	Transamination to Proline using Methionine	0.67 O_2(s) 0.33 Pyruvate(m) 1 Methionine(s) 0.5 Glucose-6P(c) 1 ATP-energy(m) 2 NADPH-redox-potential(m)	1.83 H_2O(s) 0.5 P_i(c) 1 Pyruvate(c) 1 Proline(s) 1 H_2S(s) 0.5 ATP-energy(c) 2 NADH-redox-potential(c) 0.33 NADH-redox-potential(m) 4 Proton-gradient(m) 1 Na-gradient(c) 1 THF-activated methyl group(c)	16	25	-	-	-	-	-	-	6	-	11

ID	flux	reaction
r0572	-0.67	Methylmalonyl-CoA(m) ⇌ (R)-Methylmalonyl-CoA(m)
r0855	0.67	O2(s) ⇌ O2(c)
r0856	-0.67	O2(m) ⇌ O2(c)
r0425	0.33	Isocitrate(m) + NAD+(m) ⇌ Oxalosuccinate(m) + NADH(m)
r0592	-0.33	H2O(m) + Acrylyl-CoA(m) → 3-Hydroxypropionyl-CoA(m)
r1681	1	Glycine(s) + Proline(c) + Na+(s) → Glycine(c) + Proline(s) + Na+(c)
r0596	-0.33	3-Hydroxypropionyl-CoA(m) + H2O(m) → Hydracrylate(m) + CoA(m)
r0017	0.67	O2(m) + 4 H+(PG)(m) + 4 Ferrocytochrome C(m) → 4 Ferricytochrome C(m) + 2 H2O(m) + 4 H+(PG)(c)
r0314	1	Serine(c) + Homocysteine(c) ⇌ H2O(c) + L-Cystathionine(c)
r0316	0.33	Citrate(m) ⇌ Isocitrate(m)
r0911	1	Proline(c) + Glutamate(m) ⇌ Proline(m) + Glutamate(c)
r0619	-1	L-Glutamate 5-semialdehyde(m) + Pi(m) + NADP+(m) ← NADPH(m) + Glutamyl-5P(m)
r0337	-1	ADP(c) + 1,3DPG(c) ⇌ ATP(c) + 3PG(c)
r0338	1	3PG(c) + NAD+(c) → 3-Phosphonooxypyruvate(c) + NADH(c)
r2664	1	Na+(c) ⇌ Na+(s) + Na-gradient(c)
r1426	2	Proline(m) + H+(PG)(c) → Proline(c) + H+(PG)(m)
r0072	1	ATP(m) + Glutamate(m) → ADP(m) + Glutamyl-5P(m)
r0059	-1	Pyruvate(m) + CO2(m) + NADPH(m) ⇌ Malate(m) + NADP+(m)

Continued on next page

ID	flux	reaction
r0073	-2	AKG(m) + NADH(m) + NH3(m) ⇌ Glutamate(m) + NAD+(m) + H2O(m)
r0507	1.33	Ubiquinol(m) + 2 Ferricytochrome C(m) + 4 H+(PG)(m) → Ubiquinone(m) + 4 H+(PG)(c) + 2 Ferrocytochrome C(m)
r0241	1	H2O(c) + L-Cystathionine(c) ⇌ Cysteine(c) + NH3(c) + 2-Oxobutyrate(c)
r0243	-0.5	DHAP(c) ⇌ GAP(c)
r1330	4	H+(PG)(c) ⇌ H+(PG)(m) + Proton-gradient(m)
r1319	0.5	ATP(c) + H2O(c) ⇌ ADP(c) + Pi(c) + ATP-energy(c)
r0414	0.67	ATP(m) + Propanoyl-CoA(m) + HCO3-(m) → ADP(m) + Pi(m) + Methylmalonyl-CoA(m)
r0849	-1.83	H2O(s) ⇌ H2O(c)
r0736	0.5	ATP(c) + Fructose-6P(c) → ADP(c) + Fructose-1,6PP(c)
r0159	1	3-Phosphoserine(c) + H2O(c) → Serine(c) + Pi(c)
r0174	0.33	CoA(m) + NAD+(m) + 3-Oxopropanoate(m) → Acetyl-CoA(m) + CO2(m) + NADH(m)
r0175	-1	L-Glutamate 5-semialdehyde(m) ⇌ 1-Pyrroline-5-carboxylate(m) + H2O(m)
r1546	-1	Glycine(c) + Methionine(s) ⇌ Glycine(s) + Methionine(c)
r0192	0.5	Glucose-6P(c) ⇌ Fructose-6P(c)
r1114	-0.67	NADH(m) + CO2(m) + Succinyl-CoA(m) ⇌ AKG(m) + CoA(m) + NAD+(m)
r0193	1	Cysteine(c) + H2O(c) ⇌ Pyruvate(c) + H2S(c) + NH3(c)
r0197	0.67	(R)-Methylmalonyl-CoA(m) ⇌ Succinyl-CoA(m)
r1154	1	CoA(m) + NAD+(m) + 2-Oxobutyrate(m) ⇌ CO2(m) + Propanoyl-CoA(m) + NADH(m)
r0940	1	H2S(c) ⇌ H2S(s)
r1155	1	2-Oxobutyrate(c) + H+(PG)(c) → 2-Oxobutyrate(m) + H+(PG)(m)
r2676	1	5-Methyl-THF(c) ⇌ THF(c) + THF-activated methyl group(c)
r0365	0.33	Hydracrylate(m) + NAD+(m) → NADH(m) + 3-Oxopropanoate(m)
r0083	0.33	Oxalosuccinate(m) ⇌ AKG(m) + CO2(m)
r1453	-1	1-Pyrroline-5-carboxylate(m) + Ubiquinone(m) ⇌ Proline(m) + Ubiquinol(m)
r0663	-1	3-Phosphonooxypyruvate(c) + Glutamate(c) → 3-Phosphoserine(c) + AKG(c)
r0232	-1	THF(c) + Methionine(c) ⇌ 5-Methyl-THF(c) + Homocysteine(c)
r1320	-1	ATP(m) + H2O(m) ⇌ ADP(m) + Pi(m) + ATP-energy(m)
r1322	2	NADH(c) ⇌ NAD+(c) + NADH-redox-potential(c)
r0683	0.33	Propanoyl-CoA(m) + Ubiquinone(m) → Ubiquinol(m) + Acrylyl-CoA(m)
r1323	0.33	NADH(m) ⇌ NAD+(m) + NADH-redox-potential(m)
r0253	1	GAP(c) + Pi(c) + NAD+(c) ⇌ 1,3DPG(c) + NADH(c)
r1327	-2	NADPH(m) ⇌ NADPH-redox-potential(m) + NADP+(m)
r1047	1	Pi(c) + H+(PG)(c) → Pi(m) + H+(PG)(m)
r0256	0.5	Fructose-1,6PP(c) ⇌ DHAP(c) + GAP(c)
r0105	1	NAD+(m) + Malate(m) ⇌ OAA(m) + NADH(m)
r0106	-0.67	ADP(m) + Pi(m) + OAA(m) ⇌ ATP(m) + Pyruvate(m) + HCO3-(m)
r0107	-0.33	Acetyl-CoA(m) + H2O(m) + OAA(m) ⇌ Citrate(m) + CoA(m)
r2419	1	AKG(c) + Pi(m) ⇌ AKG(m) + Pi(c)
r0838	2	NH3(c) ⇌ NH3(m)
r0851	4.33	H2O(m) ⇌ H2O(c)

Proline from Phenylalanine (245)

Definition			Solution												
			exchanges		reactions								transp		
Objective	Constraints	Comment	imports	exports	c	m	r	p	l	n	s	b	s-c	b-c	int
Proline	−Phenylalanine MCES +Urea	Transamination to Proline using Phenylalanine	3.5 O_2(s) 1 Phenylalanine(s) 2 ATP-energy(c) 1 ATP-energy(m) 1 NADPH-redox-potential(m) 1 CoA-activated acetyl group(m)	2 H_2O(s) 2 CO_2(s) 1 Proline(s) 1 NADH-redox-potential(m) 6 Proton-gradient(m) 1 Na-gradient(c) 2 CoA-activated acetyl group(c)	17	10	-	-	-	-	-	-	6	-	7

ID	flux	reaction
r0855	3.5	O2(s) ⇌ O2(c)
r0422	1	Isocitrate(c) + NADP+(c) ⇌ Oxalosuccinate(c) + NADPH(c)

Continued on next page

ID	flux	reaction
r0856	-0.5	O2(m) ⇌ O2(c)
r1681	1	Glycine(s) + Proline(c) + Na+(s) → Glycine(c) + Proline(s) + Na+(c)
r0017	0.5	O2(m) + 4 H+(PG)(m) + 4 Ferrocytochrome C(m) → 4 Ferricytochrome C(m) + 2 H2O(m) + 4 H+(PG)(c)
r0030	1	ATP(c) + AMP(c) ⇌ 2 ADP(c)
r0183	1	Tyrosine(c) + AKG(c) ⇌ 4-Hydroxyphenylpyruvate(c) + Glutamate(c)
r0315	1	Citrate(c) ⇌ Isocitrate(c)
r0911	-1	Proline(c) + Glutamate(m) ⇌ Proline(m) + Glutamate(c)
r0912	-1	Citrate(m) + Malate(c) ⇌ Citrate(c) + Malate(m)
r0619	-1	L-Glutamate 5-semialdehyde(m) + Pi(m) + NADP+(m) ← NADPH(m) + Glutamyl-5P(m)
r2664	1	Na+(c) ⇌ Na+(s) + Na-gradient(c)
r0072	1	ATP(m) + Glutamate(m) → ADP(m) + Glutamyl-5P(m)
r0507	1	Ubiquinol(m) + 2 Ferricytochrome C(m) + 4 H+(PG)(m) → Ubiquinone(m) + 4 H+(PG)(c) + 2 Ferrocytochrome C(m)
r1330	6	H+(PG)(c) ⇌ H+(PG)(m) + Proton-gradient(m)
r0398	1	Dihydrobiopterin(c) + NADPH(c) ⇌ Tetrahydrobiopterin(c) + NADP+(c)
r1319	-2	ATP(c) + H2O(c) ⇌ ADP(c) + Pi(c) + ATP-energy(c)
r0399	1	Tetrahydrobiopterin(c) + Phenylalanine(c) + O2(c) ⇌ Dihydrobiopterin(c) + Tyrosine(c) + H2O(c)
r0543	1	Homogentisate(c) + O2(c) ⇌ 4-Maleylacetoacetate(c)
r0544	1	4-Hydroxyphenylpyruvate(c) + O2(c) ⇌ Homogentisate(c) + CO2(c)
r0849	-2	H2O(s) ⇌ H2O(c)
r0867	2	CO2(c) ⇌ CO2(s)
r0007	1	PPi(c) + H2O(c) → 2 Pi(c)
r0175	-1	L-Glutamate 5-semialdehyde(m) ⇌ 1-Pyrroline-5-carboxylate(m) + H2O(m)
r0320	1	ATP(c) + CoA(c) + Acetoacetate(c) → AMP(c) + PPi(c) + Acetoacetyl-CoA(c)
r1548	-1	Glycine(c) + Phenylalanine(s) ⇌ Glycine(s) + Phenylalanine(c)
r0605	1	4-Maleylacetoacetate(c) ⇌ Fumarylacetoacetate(c)
r0324	-1	Fumarylacetoacetate(c) + H2O(c) ⇌ Fumarate(c) + Acetoacetate(c)
r0069	-1	CoA(c) + Acetoacetyl-CoA(c) ⇌ 2 Acetyl-CoA(c)
r0082	1	Oxalosuccinate(c) ⇌ AKG(c) + CO2(c)
r2677	2	Acetyl-CoA(c) ⇌ CoA(c) + CoA-activated acetyl group(c)
r2678	-1	Acetyl-CoA(m) ⇌ CoA(m) + CoA-activated acetyl group(m)
r1453	-1	1-Pyrroline-5-carboxylate(m) + Ubiquinone(m) ⇌ Proline(m) + Ubiquinol(m)
r1320	-1	ATP(m) + H2O(m) ⇌ ADP(m) + Pi(m) + ATP-energy(m)
r1323	1	NADH(m) ⇌ NAD+(m) + NADH-redox-potential(m)
r1327	-1	NADPH(m) ⇌ NADPH-redox-potential(m) + NADP+(m)
r0105	1	NAD+(m) + Malate(m) ⇌ OAA(m) + NADH(m)
r0258	-1	Fumarate(c) + H2O(c) ⇌ Malate(c)
r0107	-1	Acetyl-CoA(m) + H2O(m) + OAA(m) ⇌ Citrate(m) + CoA(m)
r0851	2	H2O(m) ⇌ H2O(c)

Proline from Serine (246)

Definition			Solution												
			exchanges		reactions								transp		
Objective	Constraints	Comment	imports	exports	c	m	r	p	l	n	s	b	s-c	b-c	int
Proline	−Serine MCES +Urea	Transamination to Proline using Serine	0.5 O_2(s) 1 Pyruvate(m) 1 Serine(s) 1 ATP-energy(m) 3 NADPH-redox-potential(m) 1 CoA-activated acetyl group(m)	3 H_2O(s) 1 Pyruvate(c) 1 Proline(s) 2 NADH-redox-potential(m) 1 Proton-gradient(c) 5 Proton-gradient(m) 1 Na-gradient(c)	1	15	-	-	-	-	-	-	6	-	7

ID	flux	reaction
r2678	-1	Acetyl-CoA(m) ⇌ CoA(m) + CoA-activated acetyl group(m)
r0316	1	Citrate(m) ⇌ Isocitrate(m)
r2531	-1	Proline(c) ⇌ Proline(s)
r0425	1	Isocitrate(m) + NAD+(m) ⇌ Oxalosuccinate(m) + NADH(m)

Continued on next page

ID	flux	reaction
r0083	1	Oxalosuccinate(m) ⇌ AKG(m) + CO2(m)
r0507	1	Ubiquinol(m) + 2 Ferricytochrome C(m) + 4 H+(PG)(m) → Ubiquinone(m) + 4 H+(PG)(c) + 2 Ferrocytochrome C(m)
r0075	-1	AKG(m) + NADPH(m) + NH3(m) ⇌ Glutamate(m) + H2O(m) + NADP+(m)
r1453	-1	1-Pyrroline-5-carboxylate(m) + Ubiquinone(m) ⇌ Proline(m) + Ubiquinol(m)
r1426	1	Proline(m) + H+(PG)(c) → Proline(c) + H+(PG)(m)
r1327	-3	NADPH(m) ⇌ NADPH-redox-potential(m) + NADP+(m)
r0175	-1	L-Glutamate 5-semialdehyde(m) ⇌ 1-Pyrroline-5-carboxylate(m) + H2O(m)
r0851	3	H2O(m) ⇌ H2O(c)
r0059	-1	Pyruvate(m) + CO2(m) + NADPH(m) ⇌ Malate(m) + NADP+(m)
r0619	-1	L-Glutamate 5-semialdehyde(m) + Pi(m) + NADP+(m) ← NADPH(m) + Glutamyl-5P(m)
r2664	1	Na+(c) ⇌ Na+(s) + Na-gradient(c)
r0855	0.5	O2(s) ⇌ O2(c)
r1320	-1	ATP(m) + H2O(m) ⇌ ADP(m) + Pi(m) + ATP-energy(m)
r0856	-0.5	O2(m) ⇌ O2(c)
r0838	1	NH3(c) ⇌ NH3(m)
r0105	1	NAD+(m) + Malate(m) ⇌ OAA(m) + NADH(m)
r0060	1	Serine(c) → Pyruvate(c) + NH3(c)
r1330	5	H+(PG)(c) ⇌ H+(PG)(m) + Proton-gradient(m)
r2478	1	Serine(s) + Na+(s) + H+(PG)(c) → Serine(c) + Na+(c) + H+(PG)(s)
r1331	1	H+(PG)(s) ⇌ H+(PG)(c) + Proton-gradient(c)
r0849	-3	H2O(s) ⇌ H2O(c)
r0107	-1	Acetyl-CoA(m) + H2O(m) + OAA(m) ⇌ Citrate(m) + CoA(m)
r0017	0.5	O2(m) + 4 H+(PG)(m) + 4 Ferrocytochrome C(m) → 4 Ferricytochrome C(m) + 2 H2O(m) + 4 H+(PG)(c)
r1323	2	NADH(m) ⇌ NAD+(m) + NADH-redox-potential(m)
r0072	1	ATP(m) + Glutamate(m) → ADP(m) + Glutamyl-5P(m)

Proline from Threonine (247)

Definition			Solution													
			exchanges		reactions									transp		
Objective	Constraints	Comment	imports	exports	c	m	r	p	l	n	s	b	s-c	b-c	int
Proline	−Threonine MCES +Urea	Transamination to Proline using Threonine	0.67 O_2(s) 0.33 Pyruvate(m) 1 Threonine(s) 1 ATP-energy(m) 2 NADPH-redox-potential(m)	3.33 H_2O(s) 1 Proline(s) 1.33 NADH-redox-potential(m) 6 Proton-gradient(m)	1	25	-	-	-	-	-	-	4	-	8

ID	flux	reaction
r0316	0.33	Citrate(m) ⇌ Isocitrate(m)
r0073	-1	AKG(m) + NADH(m) + NH3(m) ⇌ Glutamate(m) + NAD+(m) + H2O(m)
r2531	-1	Proline(s) ⇌ Proline(c)
r1154	1	CoA(m) + NAD+(m) + 2-Oxobutyrate(m) ⇌ CO2(m) + Propanoyl-CoA(m) + NADH(m)
r0425	0.33	Isocitrate(m) + NAD+(m) ⇌ Oxalosuccinate(m) + NADH(m)
r0083	0.33	Oxalosuccinate(m) ⇌ AKG(m) + CO2(m)
r1155	1	2-Oxobutyrate(c) + H+(PG)(c) → 2-Oxobutyrate(m) + H+(PG)(m)
r0507	1.33	Ubiquinol(m) + 2 Ferricytochrome C(m) + 4 H+(PG)(m) → Ubiquinone(m) + 4 H+(PG)(c) + 2 Ferrocytochrome C(m)
r0174	0.33	CoA(m) + NAD+(m) + 3-Oxopropanoate(m) → Acetyl-CoA(m) + CO2(m) + NADH(m)
r1453	-1	1-Pyrroline-5-carboxylate(m) + Ubiquinone(m) ⇌ Proline(m) + Ubiquinol(m)
r1426	1	Proline(m) + H+(PG)(c) → Proline(c) + H+(PG)(m)
r1327	-2	NADPH(m) ⇌ NADPH-redox-potential(m) + NADP+(m)
r0175	-1	L-Glutamate 5-semialdehyde(m) ⇌ 1-Pyrroline-5-carboxylate(m) + H2O(m)
r2534	1	Threonine(s) ⇌ Threonine(c)
r0851	3.33	H2O(m) ⇌ H2O(c)
r0572	-0.67	Methylmalonyl-CoA(m) ⇌ (R)-Methylmalonyl-CoA(m)
r0365	0.33	Hydracrylate(m) + NAD+(m) → NADH(m) + 3-Oxopropanoate(m)
r0059	-1	Pyruvate(m) + CO2(m) + NADPH(m) ⇌ Malate(m) + NADP+(m)
r0619	-1	L-Glutamate 5-semialdehyde(m) + Pi(m) + NADP+(m) ← NADPH(m) + Glutamyl-5P(m)

Continued on next page

ID	flux	reaction
r0592	-0.33	H2O(m) + Acrylyl-CoA(m) → 3-Hydroxypropionyl-CoA(m)
r0683	0.33	Propanoyl-CoA(m) + Ubiquinone(m) → Ubiquinol(m) + Acrylyl-CoA(m)
r0197	0.67	(R)-Methylmalonyl-CoA(m) ⇌ Succinyl-CoA(m)
r0855	0.67	O2(s) ⇌ O2(c)
r1320	-1	ATP(m) + H2O(m) ⇌ ADP(m) + Pi(m) + ATP-energy(m)
r0856	-0.67	O2(m) ⇌ O2(c)
r0838	1	NH3(c) ⇌ NH3(m)
r0240	1	Threonine(c) ⇌ 2-Oxobutyrate(c) + NH3(c)
r0105	1	NAD+(m) + Malate(m) ⇌ OAA(m) + NADH(m)
r1330	6	H+(PG)(c) ⇌ H+(PG)(m) + Proton-gradient(m)
r1114	-0.67	NADH(m) + CO2(m) + Succinyl-CoA(m) ⇌ AKG(m) + CoA(m) + NAD+(m)
r0596	-0.33	3-Hydroxypropionyl-CoA(m) + H2O(m) → Hydracrylate(m) + CoA(m)
r0106	-0.67	ADP(m) + Pi(m) + OAA(m) ⇌ ATP(m) + Pyruvate(m) + HCO3-(m)
r0849	-3.33	H2O(s) ⇌ H2O(c)
r0107	-0.33	Acetyl-CoA(m) + H2O(m) + OAA(m) ⇌ Citrate(m) + CoA(m)
r0017	0.67	O2(m) + 4 H+(PG)(m) + 4 Ferrocytochrome C(m) → 4 Ferricytochrome C(m) + 2 H2O(m) + 4 H+(PG)(c)
r1323	1.33	NADH(m) ⇌ NAD+(m) + NADH-redox-potential(m)
r0414	0.67	ATP(m) + Propanoyl-CoA(m) + HCO3-(m) → ADP(m) + Pi(m) + Methylmalonyl-CoA(m)
r0072	1	ATP(m) + Glutamate(m) → ADP(m) + Glutamyl-5P(m)

Proline from Tryptophan (248)

Definition			Solution												
			exchanges		reactions								transp		
Objective	Constraints	Comment	imports	exports	c	m	r	p	l	n	s	b	s-c	b-c	int
Proline	−Tryptophan MCES +Urea	Transamination to Proline using Tryptophan	2.25 O_2(s) 1 Pyruvate(m) 0.5 Tryptophan(s) 0.5 ATP-energy(c) 1 ATP-energy(m) 2.5 NADPH-redox-potential(m)	1 H_2O(s) 2 CO_2(s) 0.5 Pyruvate(c) 1 Proline(s) 0.5 NADH-redox-potential(c) 2 NADH-redox-potential(m) 0.5 NADPH-redox-potential(c) 8.5 Proton-gradient(m) 0.5 Na-gradient(c)	17	16	-	-	-	-	-	-	7	-	10

ID	flux	reaction
r0855	2.25	O2(s) ⇌ O2(c)
r0422	1	Isocitrate(c) + NADP+(c) ⇌ Oxalosuccinate(c) + NADPH(c)
r0856	-0.75	O2(m) ⇌ O2(c)
r1681	0.5	Glycine(s) + Proline(c) + Na+(s) → Glycine(c) + Proline(s) + Na+(c)
r0460	0.5	(S)-3-Hydroxybutyryl-CoA(m) + NAD+(m) ⇌ NADH(m) + Acetoacetyl-CoA(m)
r0448	0.5	2-Oxoadipate(m) + CoA(m) + NAD+(m) ⇌ Glutaryl-CoA(m) + CO2(m) + NADH(m)
r0449	-0.5	2-Oxoadipate(c) + NH3(c) + NADP+(c) ⇌ 2-Aminomuconate(c) + NADPH(c) + H2O(c)
r0017	0.75	O2(m) + 4 H+(PG)(m) + 4 Ferrocytochrome C(m) → 4 Ferricytochrome C(m) + 2 H2O(m) + 4 H+(PG)(c)
r0169	0.5	Tryptophan(c) + O2(c) ⇌ L-Formylkynurenine(c)
r0315	1	Citrate(c) ⇌ Isocitrate(c)
r0911	-0.5	Proline(c) + Glutamate(m) ⇌ Proline(m) + Glutamate(c)
r0619	-1	L-Glutamate 5-semialdehyde(m) + Pi(m) + NADP+(m) ← NADPH(m) + Glutamyl-5P(m)
r0070	-0.5	CoA(m) + Acetoacetyl-CoA(m) ⇌ 2 Acetyl-CoA(m)
r2664	0.5	Na+(c) ⇌ Na+(s) + Na-gradient(c)
r1426	0.5	Proline(m) + H+(PG)(c) → Proline(c) + H+(PG)(m)
r0072	1	ATP(m) + Glutamate(m) → ADP(m) + Glutamyl-5P(m)
r0059	-1	Pyruvate(m) + CO2(m) + NADPH(m) ⇌ Malate(m) + NADP+(m)
r0075	-0.5	AKG(m) + NADPH(m) + NH3(m) ⇌ Glutamate(m) + H2O(m) + NADP+(m)
r2531	-0.5	Proline(c) ⇌ Proline(s)
r0507	1.5	Ubiquinol(m) + 2 Ferricytochrome C(m) + 4 H+(PG)(m) → Ubiquinone(m) + 4 H+(PG)(c) + 2 Ferrocytochrome C(m)
r0227	0.5	10-Formyl-THF(c) + H2O(c) + NADP+(c) → THF(c) + CO2(c) + NADPH(c)
r0228	0.5	THF(c) + Formate(c) + ATP(c) → ADP(c) + Pi(c) + 10-Formyl-THF(c)
r0676	0.5	2-Amino-3-carboxymuconate semialdehyde(c) ⇌ 2-Aminomuconate semialdehyde(c) + CO2(c)

Continued on next page

Proline from Tryptophan (248)– continued

ID	flux	reaction
r1330	8.5	H+(PG)(c) ⇌ H+(PG)(m) + Proton-gradient(m)
r1319	-0.5	ATP(c) + H2O(c) ⇌ ADP(c) + Pi(c) + ATP-energy(c)
r0541	0.5	Glutaryl-CoA(m) + Ubiquinone(m) ⇌ Crotonyl-CoA(m) + CO2(m) + Ubiquinol(m)
r0562	0.5	3-Hydroxyanthranilate(c) + O2(c) ⇌ 2-Amino-3-carboxymuconate semialdehyde(c)
r0563	0.5	H2O(c) + 3-Hydroxy-L-kynurenine(c) ⇌ 3-Hydroxyanthranilate(c) + Alanine(c)
r0849	-1	H2O(s) ⇌ H2O(c)
r0867	2	CO2(c) ⇌ CO2(s)
r0588	-0.5	Crotonyl-CoA(m) + H2O(m) ⇌ (S)-3-Hydroxybutyryl-CoA(m)
r0454	0.5	L-Formylkynurenine(c) + H2O(c) ⇌ Formate(c) + Kynurenine(c)
r0455	0.5	Kynurenine(c) + O2(c) + NADPH(c) ⇌ H2O(c) + 3-Hydroxy-L-kynurenine(c) + NADP+(c)
r0175	-1	L-Glutamate 5-semialdehyde(m) ⇌ 1-Pyrroline-5-carboxylate(m) + H2O(m)
r1547	-0.5	Glycine(c) + Tryptophan(s) ⇌ Glycine(s) + Tryptophan(c)
r2373	1	Citrate(m) + AKG(c) ⇌ Citrate(c) + AKG(m)
r0080	0.5	Alanine(c) + AKG(c) ⇌ Pyruvate(c) + Glutamate(c)
r0645	0.5	2-Aminomuconate semialdehyde(c) + NAD+(c) + H2O(c) ⇌ 2-Aminomuconate(c) + NADH(c)
r0082	1	Oxalosuccinate(c) ⇌ AKG(c) + CO2(c)
r1453	-1	1-Pyrroline-5-carboxylate(m) + Ubiquinone(m) ⇌ Proline(m) + Ubiquinol(m)
r0945	0.5	2-Oxoadipate(c) + AKG(m) ⇌ 2-Oxoadipate(m) + AKG(c)
r1320	-1	ATP(m) + H2O(m) ⇌ ADP(m) + Pi(m) + ATP-energy(m)
r1322	0.5	NADH(c) ⇌ NAD+(c) + NADH-redox-potential(c)
r1323	2	NADH(m) ⇌ NAD+(m) + NADH-redox-potential(m)
r1326	0.5	NADPH(c) ⇌ NADPH-redox-potential(c) + NADP+(c)
r1327	-2.5	NADPH(m) ⇌ NADPH-redox-potential(m) + NADP+(m)
r0105	1	NAD+(m) + Malate(m) ⇌ OAA(m) + NADH(m)
r0107	-1	Acetyl-CoA(m) + H2O(m) + OAA(m) ⇌ Citrate(m) + CoA(m)
r0838	0.5	NH3(c) ⇌ NH3(m)
r0851	2.5	H2O(m) ⇌ H2O(c)

Proline from Tyrosine (249)

Definition			Solution												
			exchanges		reactions								transp		
Objective	Constraints	Comment	imports	exports	c	m	r	p	l	n	s	b	s-c	b-c	int
Proline	−Tyrosine MCES +Urea	Transamination to Proline using Tyrosine	2.5 O_2(s) 1 Tyrosine(s) 2 ATP-energy(c) 1 ATP-energy(m) 1 NADPH-redox-potential(m) 1 CoA-activated acetyl group(m)	1 H_2O(s) 2 CO_2(s) 1 Proline(s) 1 NADH-redox-potential(m) 1 NADPH-redox-potential(c) 6 Proton-gradient(m) 1 Na-gradient(c) 2 CoA-activated acetyl group(c)	15	11	-	-	-	-	-	-	6	-	7

ID	flux	reaction
r2002	1	Lysine(s) + Proline(c) → Lysine(c) + Proline(s)
r0855	2.5	O2(s) ⇌ O2(c)
r0422	1	Isocitrate(c) + NADP+(c) ⇌ Oxalosuccinate(c) + NADPH(c)
r0856	-0.5	O2(m) ⇌ O2(c)
r1947	1	Tyrosine(s) + Na+(s) + Lysine(c) → Tyrosine(c) + Na+(c) + Lysine(s)
r0017	0.5	O2(m) + 4 H+(PG)(m) + 4 Ferrocytochrome C(m) → 4 Ferricytochrome C(m) + 2 H2O(m) + 4 H+(PG)(c)
r0030	1	ATP(c) + AMP(c) ⇌ 2 ADP(c)
r0183	1	Tyrosine(c) + AKG(c) ⇌ 4-Hydroxyphenylpyruvate(c) + Glutamate(c)
r0316	1	Citrate(m) ⇌ Isocitrate(m)
r0911	-1	Proline(c) + Glutamate(m) ⇌ Proline(m) + Glutamate(c)
r0913	1	Isocitrate(m) + Malate(c) ⇌ Isocitrate(c) + Malate(m)
r0619	-1	L-Glutamate 5-semialdehyde(m) + Pi(m) + NADP+(m) ← NADPH(m) + Glutamyl-5P(m)
r2664	1	Na+(c) ⇌ Na+(s) + Na-gradient(c)
r0072	1	ATP(m) + Glutamate(m) → ADP(m) + Glutamyl-5P(m)
r0507	1	Ubiquinol(m) + 2 Ferricytochrome C(m) + 4 H+(PG)(m) → Ubiquinone(m) + 4 H+(PG)(c) + 2 Ferrocytochrome C(m)

Continued on next page

ID	flux	reaction
r1330	6	H+(PG)(c) ⇌ H+(PG)(m) + Proton-gradient(m)
r1319	-2	ATP(c) + H2O(c) ⇌ ADP(c) + Pi(c) + ATP-energy(c)
r0543	1	Homogentisate(c) + O2(c) ⇌ 4-Maleylacetoacetate(c)
r0544	1	4-Hydroxyphenylpyruvate(c) + O2(c) ⇌ Homogentisate(c) + CO2(c)
r0849	-1	H2O(s) ⇌ H2O(c)
r0867	2	CO2(c) ⇌ CO2(s)
r0007	1	PPi(c) + H2O(c) → 2 Pi(c)
r0175	-1	L-Glutamate 5-semialdehyde(m) ⇌ 1-Pyrroline-5-carboxylate(m) + H2O(m)
r0320	1	ATP(c) + CoA(c) + Acetoacetate(c) → AMP(c) + PPi(c) + Acetoacetyl-CoA(c)
r0605	1	4-Maleylacetoacetate(c) ⇌ Fumarylacetoacetate(c)
r0324	-1	Fumarylacetoacetate(c) + H2O(c) ⇌ Fumarate(c) + Acetoacetate(c)
r0069	-1	CoA(c) + Acetoacetyl-CoA(c) ⇌ 2 Acetyl-CoA(c)
r0082	1	Oxalosuccinate(c) ⇌ AKG(c) + CO2(c)
r2677	2	Acetyl-CoA(c) ⇌ CoA(c) + CoA-activated acetyl group(c)
r2678	-1	Acetyl-CoA(m) ⇌ CoA(m) + CoA-activated acetyl group(m)
r1453	-1	1-Pyrroline-5-carboxylate(m) + Ubiquinone(m) ⇌ Proline(m) + Ubiquinol(m)
r1320	-1	ATP(m) + H2O(m) ⇌ ADP(m) + Pi(m) + ATP-energy(m)
r1323	1	NADH(m) ⇌ NAD+(m) + NADH-redox-potential(m)
r1326	1	NADPH(c) ⇌ NADPH-redox-potential(c) + NADP+(c)
r1327	-1	NADPH(m) ⇌ NADPH-redox-potential(m) + NADP+(m)
r0105	1	NAD+(m) + Malate(m) ⇌ OAA(m) + NADH(m)
r0258	-1	Fumarate(c) + H2O(c) ⇌ Malate(c)
r0107	-1	Acetyl-CoA(m) + H2O(m) + OAA(m) ⇌ Citrate(m) + CoA(m)
r0851	2	H2O(m) ⇌ H2O(c)

Proline from Valine (250)

Definition			Solution													
			exchanges		reactions								transp			
Objective	Constraints	Comment	imports	exports	c	m	r	p	l	n	s	b	s-c	b-c	int
Proline	−Valine MCES +Urea	Transamination to Proline using Valine	1 O_2(s) 1 Valine(s) 1 ATP-energy(m) 1 NADPH-redox-potential(m)	2 H_2O(s) 1 Proline(s) 2 NADH-redox-potential(m) 10 Proton-gradient(m)	-	16	-	-	-	-	-	-	4	-	7

ID	flux	reaction
r0779	1	3-Hydroxyisobutyryl-CoA(m) + H2O(m) → 3-Hydroxyisobutyrate(m) + CoA(m)
r0560	1	Isobutyryl-CoA(m) + Ubiquinone(m) → Methacrylyl-CoA(m) + Ubiquinol(m)
r0263	1	Valine(m) + AKG(m) → Glutamate(m) + 3-Methyl-2-oxobutyrate(m)
r0624	1	2-Methyl-3-oxopropanoate(m) + CoA(m) + NAD+(m) ⇌ (R)-Methylmalonyl-CoA(m) + NADH(m)
r0507	2	Ubiquinol(m) + 2 Ferricytochrome C(m) + 4 H+(PG)(m) → Ubiquinone(m) + 4 H+(PG)(c) + 2 Ferrocytochrome C(m)
r1453	-1	1-Pyrroline-5-carboxylate(m) + Ubiquinone(m) ⇌ Proline(m) + Ubiquinol(m)
r1426	1	Proline(m) + H+(PG)(c) → Proline(c) + H+(PG)(m)
r1327	-1	NADPH(m) ⇌ NADPH-redox-potential(m) + NADP+(m)
r0175	-1	L-Glutamate 5-semialdehyde(m) ⇌ 1-Pyrroline-5-carboxylate(m) + H2O(m)
r0923	1	Valine(c) + H+(PG)(c) → Valine(m) + H+(PG)(m)
r0851	2	H2O(m) ⇌ H2O(c)
r0482	1	3-Hydroxyisobutyrate(m) + NAD+(m) → 2-Methyl-3-oxopropanoate(m) + NADH(m)
r1653	-1	Asparagine(s) + Valine(c) ⇌ Asparagine(c) + Valine(s)
r0619	-1	L-Glutamate 5-semialdehyde(m) + Pi(m) + NADP+(m) ← NADPH(m) + Glutamyl-5P(m)
r0197	1	(R)-Methylmalonyl-CoA(m) ⇌ Succinyl-CoA(m)
r0855	1	O2(s) ⇌ O2(c)
r1648	-1	Proline(s) + Asparagine(c) ⇌ Proline(c) + Asparagine(s)
r1320	-1	ATP(m) + H2O(m) ⇌ ADP(m) + Pi(m) + ATP-energy(m)
r0856	-1	O2(m) ⇌ O2(c)
r0600	1	3-Methyl-2-oxobutyrate(m) + CoA(m) + NAD+(m) ⇌ Isobutyryl-CoA(m) + CO2(m) + NADH(m)

Continued on next page

ID	flux	reaction
r1330	10	H+(PG)(c) ⇌ H+(PG)(m) + Proton-gradient(m)
r1114	-1	NADH(m) + CO2(m) + Succinyl-CoA(m) ⇌ AKG(m) + CoA(m) + NAD+(m)
r0849	-2	H2O(s) ⇌ H2O(c)
r0669	1	Methacrylyl-CoA(m) + H2O(m) → 3-Hydroxyisobutyryl-CoA(m)
r0017	1	O2(m) + 4 H+(PG)(m) + 4 Ferrocytochrome C(m) → 4 Ferricytochrome C(m) + 2 H2O(m) + 4 H+(PG)(c)
r1323	2	NADH(m) ⇌ NAD+(m) + NADH-redox-potential(m)
r0072	1	ATP(m) + Glutamate(m) → ADP(m) + Glutamyl-5P(m)

Proline from beta-Alanine (251)

Definition			Solution												
			exchanges		reactions								transp		
Objective	Constraints	Comment	imports	exports	c	m	r	p	l	n	s	b	s-c	b-c	int
Proline	−beta-Alanine MCES +Urea	Transamination to Proline using beta-Alanine	0.5 O_2(s) 1 Pyruvate(m) 1 beta-Alanine(s) 1 ATP-energy(m) 2 NADPH-redox-potential(m)	2 H_2O(s) 1 CO_2(s) 1 Proline(s) 2 NADH-redox-potential(m) 1 NADPH-redox-potential(c) 5 Proton-gradient(m)	3	13	-	-	-	-	-	-	5	-	8

ID	flux	reaction
r0316	1	Citrate(m) ⇌ Isocitrate(m)
r0217	1	beta-Alanine(m) + AKG(m) ⇌ Glutamate(m) + 3-Oxopropanoate(m)
r0082	1	Oxalosuccinate(c) ⇌ AKG(c) + CO2(c)
r2531	-1	Proline(c) ⇌ Proline(s)
r1326	1	NADPH(c) ⇌ NADPH-redox-potential(c) + NADP+(c)
r0507	1	Ubiquinol(m) + 2 Ferricytochrome C(m) + 4 H+(PG)(m) → Ubiquinone(m) + 4 H+(PG)(c) + 2 Ferrocytochrome C(m)
r0174	1	CoA(m) + NAD+(m) + 3-Oxopropanoate(m) → Acetyl-CoA(m) + CO2(m) + NADH(m)
r1453	-1	1-Pyrroline-5-carboxylate(m) + Ubiquinone(m) ⇌ Proline(m) + Ubiquinol(m)
r1426	1	Proline(m) + H+(PG)(c) → Proline(c) + H+(PG)(m)
r1327	-2	NADPH(m) ⇌ NADPH-redox-potential(m) + NADP+(m)
r1147	1	AKG(c) + Isocitrate(m) ⇌ AKG(m) + Isocitrate(c)
r0899	-1	beta-Alanine(s) ⇌ beta-Alanine(c)
r0175	-1	L-Glutamate 5-semialdehyde(m) ⇌ 1-Pyrroline-5-carboxylate(m) + H2O(m)
r0851	2	H2O(m) ⇌ H2O(c)
r0059	-1	Pyruvate(m) + CO2(m) + NADPH(m) ⇌ Malate(m) + NADP+(m)
r0619	-1	L-Glutamate 5-semialdehyde(m) + Pi(m) + NADP+(m) ← NADPH(m) + Glutamyl-5P(m)
r0855	0.5	O2(s) ⇌ O2(c)
r1320	-1	ATP(m) + H2O(m) ⇌ ADP(m) + Pi(m) + ATP-energy(m)
r0856	-0.5	O2(m) ⇌ O2(c)
r0105	1	NAD+(m) + Malate(m) ⇌ OAA(m) + NADH(m)
r1330	5	H+(PG)(c) ⇌ H+(PG)(m) + Proton-gradient(m)
r0867	1	CO2(c) ⇌ CO2(s)
r0849	-2	H2O(s) ⇌ H2O(c)
r0422	1	Isocitrate(c) + NADP+(c) ⇌ Oxalosuccinate(c) + NADPH(c)
r0107	-1	Acetyl-CoA(m) + H2O(m) + OAA(m) ⇌ Citrate(m) + CoA(m)
r0017	0.5	O2(m) + 4 H+(PG)(m) + 4 Ferrocytochrome C(m) → 4 Ferricytochrome C(m) + 2 H2O(m) + 4 H+(PG)(c)
r1323	2	NADH(m) ⇌ NAD+(m) + NADH-redox-potential(m)
r0900	1	beta-Alanine(c) ⇌ beta-Alanine(m)
r0072	1	ATP(m) + Glutamate(m) → ADP(m) + Glutamyl-5P(m)

Serine from Alanine (252)

Definition			Solution												
			exchanges		reactions								transp		
Objective	Constraints	Comment	imports	exports	c	m	r	p	l	n	s	b	s-c	b-c	int
Serine	−Alanine MCES +Urea	Transamination to Serine using Alanine	1.5 H_2O(s) 1 Alanine(s) 0.5 Glucose-6P(c)	0.5 P_i(c) 1 Pyruvate(c) 1 Serine(s) 0.5 ATP-energy(c) 2 NADH-redox-potential(c) 1 Na-gradient(c)	12	-	-	-	-	-	-	-	4	-	-

ID	flux	reaction
r0253	1	GAP(c) + Pi(c) + NAD+(c) ⇌ 1,3DPG(c) + NADH(c)
r1995	1	Lysine(s) + Serine(c) → Lysine(c) + Serine(s)
r0192	0.5	Glucose-6P(c) ⇌ Fructose-6P(c)
r0337	-1	ADP(c) + 1,3DPG(c) ⇌ ATP(c) + 3PG(c)
r0256	0.5	Fructose-1,6PP(c) ⇌ DHAP(c) + GAP(c)
r1319	0.5	ATP(c) + H2O(c) ⇌ ADP(c) + Pi(c) + ATP-energy(c)
r0338	1	3PG(c) + NAD+(c) → 3-Phosphonooxypyruvate(c) + NADH(c)
r0663	-1	3-Phosphonooxypyruvate(c) + Glutamate(c) → 3-Phosphoserine(c) + AKG(c)
r0159	1	3-Phosphoserine(c) + H2O(c) → Serine(c) + Pi(c)
r0736	0.5	ATP(c) + Fructose-6P(c) → ADP(c) + Fructose-1,6PP(c)
r2664	1	Na+(c) ⇌ Na+(s) + Na-gradient(c)
r1917	1	Alanine(s) + Na+(s) + Lysine(c) → Alanine(c) + Na+(c) + Lysine(s)
r1322	2	NADH(c) ⇌ NAD+(c) + NADH-redox-potential(c)
r0849	1.5	H2O(s) ⇌ H2O(c)
r0080	1	Alanine(c) + AKG(c) ⇌ Pyruvate(c) + Glutamate(c)
r0243	-0.5	DHAP(c) ⇌ GAP(c)

Serine from Arginine (253)

Definition			Solution												
			exchanges		reactions								transp		
Objective	Constraints	Comment	imports	exports	c	m	r	p	l	n	s	b	s-c	b-c	int
Serine	−Arginine MCES +Urea	Transamination to Serine using Arginine	3 H_2O(s) 0.25 O_2(s) 0.5 Pyruvate(m) 0.5 Arginine(s) 0.5 Glucose-6P(c) 0.5 NADPH-redox-potential(m)	0.5 P_i(c) 1 CO_2(s) 1 Pyruvate(c) 1 Serine(s) 0.5 Urea(s) 0.5 ATP-energy(c) 0.5 ATP-energy(m) 2 NADH-redox-potential(c) 1 NADH-redox-potential(m) 1 NADPH-redox-potential(c) 1.5 Proton-gradient(m)	14	11	-	-	-	-	-	-	7	-	10

ID	flux	reaction
r0125	-0.5	GDP(m) + Pi(m) + Succinyl-CoA(m) ⇌ GTP(m) + Succinate(m) + CoA(m)
r0855	0.25	O2(s) ⇌ O2(c)
r0856	-0.25	O2(m) ⇌ O2(c)
r1663	-0.5	Lysine(c) + Arginine(s) ⇌ Lysine(s) + Arginine(c)
r0144	0.5	Arginine(c) + H2O(c) ⇌ Urea(c) + Ornithine(c)
r0167	0.5	AKG(m) + Ornithine(m) ⇌ L-Glutamate 5-semialdehyde(m) + Glutamate(m)
r0896	0.5	Urea(c) ⇌ Urea(s)
r0017	0.25	O2(m) + 4 H+(PG)(m) + 4 Ferrocytochrome C(m) → 4 Ferricytochrome C(m) + 2 H2O(m) + 4 H+(PG)(c)
r0911	1	Proline(c) + Glutamate(m) ⇌ Proline(m) + Glutamate(c)
r0337	-1	ADP(c) + 1,3DPG(c) ⇌ ATP(c) + 3PG(c)
r0338	1	3PG(c) + NAD+(c) → 3-Phosphonooxypyruvate(c) + NADH(c)
r0058	1	Malate(c) + NADP+(c) ⇌ Pyruvate(c) + CO2(c) + NADPH(c)
r1426	1	Proline(m) + H+(PG)(c) → Proline(c) + H+(PG)(m)
r0059	-0.5	Pyruvate(m) + CO2(m) + NADPH(m) ⇌ Malate(m) + NADP+(m)
r0074	0.5	L-Glutamate 5-semialdehyde(m) + NAD+(m) + H2O(m) ⇌ Glutamate(m) + NADH(m)

Continued on next page

ID	flux	reaction
r0507	0.5	Ubiquinol(m) + 2 Ferricytochrome C(m) + 4 H+(PG)(m) → Ubiquinone(m) + 4 H+(PG)(c) + 2 Ferrocytochrome C(m)
r0509	0.5	Ubiquinone(m) + Succinate(m) → Ubiquinol(m) + Fumarate(m)
r0243	-0.5	DHAP(c) ⇌ GAP(c)
r0820	1	AKG(c) + Malate(m) ⇌ AKG(m) + Malate(c)
r1330	1.5	H+(PG)(c) ⇌ H+(PG)(m) + Proton-gradient(m)
r1319	0.5	ATP(c) + H2O(c) ⇌ ADP(c) + Pi(c) + ATP-energy(c)
r1070	0.5	Lysine(c) ⇌ Lysine(m)
r0849	3	H2O(s) ⇌ H2O(c)
r0867	1	CO2(c) ⇌ CO2(s)
r0736	0.5	ATP(c) + Fructose-6P(c) → ADP(c) + Fructose-1,6PP(c)
r0159	1	3-Phosphoserine(c) + H2O(c) → Serine(c) + Pi(c)
r0192	0.5	Glucose-6P(c) ⇌ Fructose-6P(c)
r1995	0.5	Lysine(s) + Serine(c) → Lysine(c) + Serine(s)
r1114	0.5	AKG(m) + CoA(m) + NAD+(m) ⇌ NADH(m) + CO2(m) + Succinyl-CoA(m)
r2526	-0.5	Serine(c) ⇌ Serine(s)
r2396	-0.5	Lysine(m) + Ornithine(c) + H+(PG)(c) → Lysine(c) + Ornithine(m) + H+(PG)(m)
r0663	-1	3-Phosphonooxypyruvate(c) + Glutamate(c) → 3-Phosphoserine(c) + AKG(c)
r1320	0.5	ATP(m) + H2O(m) ⇌ ADP(m) + Pi(m) + ATP-energy(m)
r1322	2	NADH(c) ⇌ NAD+(c) + NADH-redox-potential(c)
r1323	1	NADH(m) ⇌ NAD+(m) + NADH-redox-potential(m)
r0100	-0.5	ADP(m) + GTP(m) ⇌ ATP(m) + GDP(m)
r1326	1	NADPH(c) ⇌ NADPH-redox-potential(c) + NADP+(c)
r0253	1	GAP(c) + Pi(c) + NAD+(c) ⇌ 1,3DPG(c) + NADH(c)
r1327	-0.5	NADPH(m) ⇌ NADPH-redox-potential(m) + NADP+(m)
r0256	0.5	Fructose-1,6PP(c) ⇌ DHAP(c) + GAP(c)
r0259	-0.5	Fumarate(m) + H2O(m) ⇌ Malate(m)
r0851	-1	H2O(m) ⇌ H2O(c)

Serine from Asparagine (254)

Definition			Solution												
			exchanges		reactions								transp		
Objective	Constraints	Comment	imports	exports	c	m	r	p	l	n	s	b	s-c	b-c	int
Serine	−Asparagine MCES +Urea	Transamination to Serine using Asparagine	1.5 H_2O(s) 0.5 Glucose-6P(c) 0.5 Asparagine(s) 0.5 NADPH-redox-potential(m) 1 Proton-gradient(m)	0.5 P_i(c) 0.5 CO_2(s) 0.5 Pyruvate(c) 1 Serine(s) 0.5 ATP-energy(c) 1.5 NADH-redox-potential(c) 0.5 NADPH-redox-potential(c)	16	2	-	-	-	-	-	-	5	-	7

ID	flux	reaction
r0253	1	GAP(c) + Pi(c) + NAD+(c) ⇌ 1,3DPG(c) + NADH(c)
r0127	0.5	H2O(c) + Asparagine(c) → Aspartate(c) + NH3(c)
r0109	0.5	AKG(c) + Aspartate(c) ⇌ OAA(c) + Glutamate(c)
r0911	0.5	Proline(c) + Glutamate(m) ⇌ Proline(m) + Glutamate(c)
r1326	0.5	NADPH(c) ⇌ NADPH-redox-potential(c) + NADP+(c)
r1047	0.5	Pi(c) + H+(PG)(c) → Pi(m) + H+(PG)(m)
r0192	0.5	Glucose-6P(c) ⇌ Fructose-6P(c)
r0075	-0.5	AKG(m) + NADPH(m) + NH3(m) ⇌ Glutamate(m) + H2O(m) + NADP+(m)
r1426	0.5	Proline(m) + H+(PG)(c) → Proline(c) + H+(PG)(m)
r1327	-0.5	NADPH(m) ⇌ NADPH-redox-potential(m) + NADP+(m)
r0337	-1	ADP(c) + 1,3DPG(c) ⇌ ATP(c) + 3PG(c)
r0256	0.5	Fructose-1,6PP(c) ⇌ DHAP(c) + GAP(c)
r0058	0.5	Malate(c) + NADP+(c) ⇌ Pyruvate(c) + CO2(c) + NADPH(c)
r1553	-0.5	Glycine(s) + Asparagine(c) ⇌ Glycine(c) + Asparagine(s)
r1319	0.5	ATP(c) + H2O(c) ⇌ ADP(c) + Pi(c) + ATP-energy(c)

Continued on next page

Serine from Asparagine (254)– continued

ID	flux	reaction
r0851	0.5	H2O(m) ⇌ H2O(c)
r0338	1	3PG(c) + NAD+(c) → 3-Phosphonooxypyruvate(c) + NADH(c)
r2526	-0.5	Serine(c) ⇌ Serine(s)
r1545	0.5	Glycine(s) + Serine(c) ⇌ Glycine(c) + Serine(s)
r0663	-1	3-Phosphonooxypyruvate(c) + Glutamate(c) → 3-Phosphoserine(c) + AKG(c)
r0159	1	3-Phosphoserine(c) + H2O(c) → Serine(c) + Pi(c)
r2419	0.5	AKG(c) + Pi(m) ⇌ AKG(m) + Pi(c)
r0736	0.5	ATP(c) + Fructose-6P(c) → ADP(c) + Fructose-1,6PP(c)
r0104	-0.5	OAA(c) + NADH(c) ⇌ NAD+(c) + Malate(c)
r0838	0.5	NH3(c) ⇌ NH3(m)
r1330	-1	H+(PG)(c) ⇌ H+(PG)(m) + Proton-gradient(m)
r1322	1.5	NADH(c) ⇌ NAD+(c) + NADH-redox-potential(c)
r0867	0.5	CO2(c) ⇌ CO2(s)
r0849	1.5	H2O(s) ⇌ H2O(c)
r0243	-0.5	DHAP(c) ⇌ GAP(c)

Serine from Aspartate (255)

Definition			Solution												
			exchanges		reactions								transp		
Objective	Constraints	Comment	imports	exports	c	m	r	p	l	n	s	b	s-c	b-c	int
Serine	−Aspartate MCES +Urea	Transamination to Serine using Aspartate	1.5 H_2O(s) 1 Aspartate(s) 0.5 Glucose-6P(c)	0.5 P_i(c) 1 CO_2(s) 1 Pyruvate(c) 1 Serine(s) 0.5 ATP-energy(c) 1 NADH-redox-potential(c) 1 NADPH-redox-potential(c) 1 Na-gradient(c)	15	-	-	-	-	-	-	-	5	-	-

ID	flux	reaction
r0253	1	GAP(c) + Pi(c) + NAD+(c) ⇌ 1,3DPG(c) + NADH(c)
r0109	1	AKG(c) + Aspartate(c) ⇌ OAA(c) + Glutamate(c)
r1326	1	NADPH(c) ⇌ NADPH-redox-potential(c) + NADP+(c)
r0192	0.5	Glucose-6P(c) ⇌ Fructose-6P(c)
r0337	-1	ADP(c) + 1,3DPG(c) ⇌ ATP(c) + 3PG(c)
r0256	0.5	Fructose-1,6PP(c) ⇌ DHAP(c) + GAP(c)
r0058	1	Malate(c) + NADP+(c) ⇌ Pyruvate(c) + CO2(c) + NADPH(c)
r1319	0.5	ATP(c) + H2O(c) ⇌ ADP(c) + Pi(c) + ATP-energy(c)
r0338	1	3PG(c) + NAD+(c) → 3-Phosphonooxypyruvate(c) + NADH(c)
r2526	-1	Serine(c) ⇌ Serine(s)
r0663	-1	3-Phosphonooxypyruvate(c) + Glutamate(c) → 3-Phosphoserine(c) + AKG(c)
r0159	1	3-Phosphoserine(c) + H2O(c) → Serine(c) + Pi(c)
r0736	0.5	ATP(c) + Fructose-6P(c) → ADP(c) + Fructose-1,6PP(c)
r2664	1	Na+(c) ⇌ Na+(s) + Na-gradient(c)
r0104	-1	OAA(c) + NADH(c) ⇌ NAD+(c) + Malate(c)
r1322	1	NADH(c) ⇌ NAD+(c) + NADH-redox-potential(c)
r0867	1	CO2(c) ⇌ CO2(s)
r0849	1.5	H2O(s) ⇌ H2O(c)
r1143	-1	Na+(c) + Aspartate(c) ← Na+(s) + Aspartate(s)
r0243	-0.5	DHAP(c) ⇌ GAP(c)

Serine from Cysteine (256)

Definition			Solution												
			exchanges		reactions								transp		
Objective	Constraints	Comment	imports	exports	c	m	r	p	l	n	s	b	s-c	b-c	int
Serine	−Cysteine MCES +Urea +H_2S	Transamination to Serine using Cysteine	1.5 H_2O(s) 0.5 Glucose-6P(c) 1 Cysteine(s) 1 NADH-redox-potential(m) 2 Proton-gradient(m)	0.5 P_i(c) 1 Pyruvate(c) 1 Serine(s) 1 H_2S(s) 0.5 ATP-energy(c) 2 NADH-redox-potential(c) 1 Na-gradient(c)	12	2	-	-	-	-	-	-	5	-	7

ID	flux	reaction
r0253	1	GAP(c) + Pi(c) + NAD+(c) ⇌ 1,3DPG(c) + NADH(c)
r0073	-1	AKG(m) + NADH(m) + NH3(m) ⇌ Glutamate(m) + NAD+(m) + H2O(m)
r0911	1	Proline(c) + Glutamate(m) ⇌ Proline(m) + Glutamate(c)
r1047	1	Pi(c) + H+(PG)(c) → Pi(m) + H+(PG)(m)
r0192	0.5	Glucose-6P(c) ⇌ Fructose-6P(c)
r1426	1	Proline(m) + H+(PG)(c) → Proline(c) + H+(PG)(m)
r0940	1	H2S(c) ⇌ H2S(s)
r0337	-1	ADP(c) + 1,3DPG(c) ⇌ ATP(c) + 3PG(c)
r0256	0.5	Fructose-1,6PP(c) ⇌ DHAP(c) + GAP(c)
r0193	1	Cysteine(c) + H2O(c) ⇌ Pyruvate(c) + H2S(c) + NH3(c)
r1319	0.5	ATP(c) + H2O(c) ⇌ ADP(c) + Pi(c) + ATP-energy(c)
r0851	1	H2O(m) ⇌ H2O(c)
r0338	1	3PG(c) + NAD+(c) → 3-Phosphonooxypyruvate(c) + NADH(c)
r2526	-1	Serine(c) ⇌ Serine(s)
r0663	-1	3-Phosphonooxypyruvate(c) + Glutamate(c) → 3-Phosphoserine(c) + AKG(c)
r0159	1	3-Phosphoserine(c) + H2O(c) → Serine(c) + Pi(c)
r2419	1	AKG(c) + Pi(m) ⇌ AKG(m) + Pi(c)
r0736	0.5	ATP(c) + Fructose-6P(c) → ADP(c) + Fructose-1,6PP(c)
r2664	1	Na+(c) ⇌ Na+(s) + Na-gradient(c)
r2475	1	Cysteine(s) + Na+(s) → Cysteine(c) + Na+(c)
r0838	1	NH3(c) ⇌ NH3(m)
r1330	-2	H+(PG)(c) ⇌ H+(PG)(m) + Proton-gradient(m)
r1322	2	NADH(c) ⇌ NAD+(c) + NADH-redox-potential(c)
r0849	1.5	H2O(s) ⇌ H2O(c)
r1323	-1	NADH(m) ⇌ NAD+(m) + NADH-redox-potential(m)
r0243	-0.5	DHAP(c) ⇌ GAP(c)

Serine from Cystine (257)

Definition			Solution												
			exchanges		reactions								transp		
Objective	Constraints	Comment	imports	exports	c	m	r	p	l	n	s	b	s-c	b-c	int
Serine	−Cystine MCES +Urea +H_2S	Transamination to Serine using Cystine	1.5 H_2O(s) 0.5 Glucose-6P(c) 0.5 Cystine(s) 1 NADH-redox-potential(m) 0.5 NADPH-redox-potential(c) 2 Proton-gradient(m)	0.5 P_i(c) 1 Pyruvate(c) 1 Serine(s) 1 H_2S(s) 0.5 ATP-energy(c) 2 NADH-redox-potential(c) 0.5 Na-gradient(c)	14	2	-	-	-	-	-	-	6	-	7

ID	flux	reaction
r1144	-0.5	Glutamate(c) + Na+(c) ← Glutamate(s) + Na+(s)
r0253	1	GAP(c) + Pi(c) + NAD+(c) ⇌ 1,3DPG(c) + NADH(c)
r0073	-1	AKG(m) + NADH(m) + NH3(m) ⇌ Glutamate(m) + NAD+(m) + H2O(m)
r0911	1	Proline(c) + Glutamate(m) ⇌ Proline(m) + Glutamate(c)
r1326	-0.5	NADPH(c) ⇌ NADPH-redox-potential(c) + NADP+(c)
r1047	1	Pi(c) + H+(PG)(c) → Pi(m) + H+(PG)(m)
r0192	0.5	Glucose-6P(c) ⇌ Fructose-6P(c)
r1426	1	Proline(m) + H+(PG)(c) → Proline(c) + H+(PG)(m)
		Continued on next page

ID	flux	reaction
r0940	1	H2S(c) ⇌ H2S(s)
r0337	-1	ADP(c) + 1,3DPG(c) ⇌ ATP(c) + 3PG(c)
r0256	0.5	Fructose-1,6PP(c) ⇌ DHAP(c) + GAP(c)
r0193	1	Cysteine(c) + H2O(c) ⇌ Pyruvate(c) + H2S(c) + NH3(c)
r1319	0.5	ATP(c) + H2O(c) ⇌ ADP(c) + Pi(c) + ATP-energy(c)
r0851	1	H2O(m) ⇌ H2O(c)
r0338	1	3PG(c) + NAD+(c) → 3-Phosphonooxypyruvate(c) + NADH(c)
r2526	-1	Serine(c) ⇌ Serine(s)
r0663	-1	3-Phosphonooxypyruvate(c) + Glutamate(c) → 3-Phosphoserine(c) + AKG(c)
r0159	1	3-Phosphoserine(c) + H2O(c) → Serine(c) + Pi(c)
r2419	1	AKG(c) + Pi(m) ⇌ AKG(m) + Pi(c)
r0736	0.5	ATP(c) + Fructose-6P(c) → ADP(c) + Fructose-1,6PP(c)
r2664	0.5	Na+(c) ⇌ Na+(s) + Na-gradient(c)
r0838	1	NH3(c) ⇌ NH3(m)
r1330	-2	H+(PG)(c) ⇌ H+(PG)(m) + Proton-gradient(m)
r1322	2	NADH(c) ⇌ NAD+(c) + NADH-redox-potential(c)
r0849	1.5	H2O(s) ⇌ H2O(c)
r1323	-1	NADH(m) ⇌ NAD+(m) + NADH-redox-potential(m)
r0877	0.5	Glutamate(c) + Cystine(s) ⇌ Glutamate(s) + Cystine(c)
r0243	-0.5	DHAP(c) ⇌ GAP(c)
r0027	-0.5	NADPH(c) + Cystine(c) ⇌ 2 Cysteine(c) + NADP+(c)

Serine from Glutamate (258)

Definition			Solution												
			exchanges		reactions								transp		
Objective	Constraints	Comment	imports	exports	c	m	r	p	l	n	s	b	s-c	b-c	int
Serine	−Glutamate MCES +Urea	Transamination to Serine using Glutamate	2.5 H_2O(s) 0.5 O_2(s) 1 Glutamate(s) 0.5 Glucose-6P(c)	0.5 P_i(c) 2 CO_2(s) 1 Pyruvate(c) 1 Serine(s) 0.5 ATP-energy(c) 1 ATP-energy(m) 2 NADH-redox-potential(c) 1 NADH-redox-potential(m) 1 NADPH-redox-potential(c) 6 Proton-gradient(m) 1 Na-gradient(c)	13	7	-	-	-	-	-	-	6	-	7

ID	flux	reaction
r1144	-1	Glutamate(c) + Na+(c) ← Glutamate(s) + Na+(s)
r0820	1	AKG(c) + Malate(m) ⇌ AKG(m) + Malate(c)
r0253	1	GAP(c) + Pi(c) + NAD+(c) ⇌ 1,3DPG(c) + NADH(c)
r1326	1	NADPH(c) ⇌ NADPH-redox-potential(c) + NADP+(c)
r0507	1	Ubiquinol(m) + 2 Ferricytochrome C(m) + 4 H+(PG)(m) → Ubiquinone(m) + 4 H+(PG)(c) + 2 Ferrocytochrome C(m)
r0192	0.5	Glucose-6P(c) ⇌ Fructose-6P(c)
r0337	-1	ADP(c) + 1,3DPG(c) ⇌ ATP(c) + 3PG(c)
r0256	0.5	Fructose-1,6PP(c) ⇌ DHAP(c) + GAP(c)
r0058	1	Malate(c) + NADP+(c) ⇌ Pyruvate(c) + CO2(c) + NADPH(c)
r1319	0.5	ATP(c) + H2O(c) ⇌ ADP(c) + Pi(c) + ATP-energy(c)
r0851	-1	H2O(m) ⇌ H2O(c)
r0509	1	Ubiquinone(m) + Succinate(m) → Ubiquinol(m) + Fumarate(m)
r0338	1	3PG(c) + NAD+(c) → 3-Phosphonooxypyruvate(c) + NADH(c)
r0100	-1	ADP(m) + GTP(m) ⇌ ATP(m) + GDP(m)
r2526	-1	Serine(c) ⇌ Serine(s)
r0663	-1	3-Phosphonooxypyruvate(c) + Glutamate(c) → 3-Phosphoserine(c) + AKG(c)
r0159	1	3-Phosphoserine(c) + H2O(c) → Serine(c) + Pi(c)
r0736	0.5	ATP(c) + Fructose-6P(c) → ADP(c) + Fructose-1,6PP(c)
r0259	-1	Fumarate(m) + H2O(m) ⇌ Malate(m)

Continued on next page

ID	flux	reaction
r2664	1	Na+(c) ⇌ Na+(s) + Na-gradient(c)
r0855	0.5	O2(s) ⇌ O2(c)
r1320	1	ATP(m) + H2O(m) ⇌ ADP(m) + Pi(m) + ATP-energy(m)
r0856	-0.5	O2(m) ⇌ O2(c)
r1330	6	H+(PG)(c) ⇌ H+(PG)(m) + Proton-gradient(m)
r1114	1	AKG(m) + CoA(m) + NAD+(m) ⇌ NADH(m) + CO2(m) + Succinyl-CoA(m)
r1322	2	NADH(c) ⇌ NAD+(c) + NADH-redox-potential(c)
r0867	2	CO2(c) ⇌ CO2(s)
r0849	2.5	H2O(s) ⇌ H2O(c)
r0125	-1	GDP(m) + Pi(m) + Succinyl-CoA(m) ⇌ GTP(m) + Succinate(m) + CoA(m)
r0017	0.5	O2(m) + 4 H+(PG)(m) + 4 Ferrocytochrome C(m) → 4 Ferricytochrome C(m) + 2 H2O(m) + 4 H+(PG)(c)
r1323	1	NADH(m) ⇌ NAD+(m) + NADH-redox-potential(m)
r0868	-1	CO2(c) ⇌ CO2(m)
r0243	-0.5	DHAP(c) ⇌ GAP(c)

Serine from Glycine (259)

Definition			Solution												
			exchanges		reactions								transp		
Objective	Constraints	Comment	imports	exports	c	m	r	p	l	n	s	b	s-c	b-c	int
Serine	−Glycine MCES +Urea	Transamination to Serine using Glycine	1 H_2O(s) 1 Glycine(s) 1 THF-activated methyl group(c)	1 Serine(s) 1 NADPH-redox-potential(c) 1 Na-gradient(c)	4	-	-	-	-	-	-	-	3	-	-

ID	flux	reaction
r1326	1	NADPH(c) ⇌ NADPH-redox-potential(c) + NADP+(c)
r0296	1	5-Methyl-THF(c) + NADP+(c) ⇌ 5,10-Methylene-THF(c) + NADPH(c)
r2664	1	Na+(c) ⇌ Na+(s) + Na-gradient(c)
r1674	1	Glycine(s) + Serine(c) + Na+(s) → Glycine(c) + Serine(s) + Na+(c)
r0230	1	5,10-Methylene-THF(c) + Glycine(c) + H2O(c) ⇌ THF(c) + Serine(c)
r2676	-1	5-Methyl-THF(c) ⇌ THF(c) + THF-activated methyl group(c)
r0849	1	H2O(s) ⇌ H2O(c)

Serine from Histidine (260)

Definition			Solution												
			exchanges		reactions								transp		
Objective	Constraints	Comment	imports	exports	c	m	r	p	l	n	s	b	s-c	b-c	int
Serine	−Histidine MCES +Urea	Transamination to Serine using Histidine	1.83 H_2O(s) 0.17 O_2(s) 0.5 Glucose-6P(c) 0.33 Histidine(s) 0.33 NADH-redox-potential(m)	0.5 P_i(c) 0.67 CO_2(s) 0.33 Pyruvate(c) 1 Serine(s) 0.5 ATP-energy(c) 0.33 ATP-energy(m) 1.67 NADH-redox-potential(c) 0.67 Proton-gradient(m) 0.33 THF-activated methyl group(c)	20	8	-	-	-	-	-	-	6	-	12

ID	flux	reaction
r0125	-0.33	GDP(m) + Pi(m) + Succinyl-CoA(m) ⇌ GTP(m) + Succinate(m) + CoA(m)
r0855	0.17	O2(s) ⇌ O2(c)
r0856	-0.17	O2(m) ⇌ O2(c)
r0293	-0.33	5,10-Methenyl-THF(c) + NADPH(c) ⇌ 5,10-Methylene-THF(c) + NADP+(c)
r0577	-0.33	Urocanate(c) + H2O(c) ⇌ 4-Imidazolone-5-propanoate(c)
r2040	0.33	Histidine(s) + Glycine(c) → Histidine(c) + Glycine(s)
r0017	0.17	O2(m) + 4 H+(PG)(m) + 4 Ferrocytochrome C(m) → 4 Ferricytochrome C(m) + 2 H2O(m) + 4 H+(PG)(c)

Continued on next page

Serine from Histidine (260)– continued

ID	flux	reaction
r0911	0.67	Proline(c) + Glutamate(m) ⇌ Proline(m) + Glutamate(c)
r0337	-1	ADP(c) + 1,3DPG(c) ⇌ ATP(c) + 3PG(c)
r0338	1	3PG(c) + NAD+(c) → 3-Phosphonooxypyruvate(c) + NADH(c)
r1426	0.67	Proline(m) + H+(PG)(c) → Proline(c) + H+(PG)(m)
r0058	0.33	Malate(c) + NADP+(c) ⇌ Pyruvate(c) + CO2(c) + NADPH(c)
r0073	-0.67	AKG(m) + NADH(m) + NH3(m) ⇌ Glutamate(m) + NAD+(m) + H2O(m)
r0507	0.33	Ubiquinol(m) + 2 Ferricytochrome C(m) + 4 H+(PG)(m) → Ubiquinone(m) + 4 H+(PG)(c) + 2 Ferrocytochrome C(m)
r0509	0.33	Ubiquinone(m) + Succinate(m) → Ubiquinol(m) + Fumarate(m)
r0524	0.33	5-Formiminotetrahydrofolate(c) ⇌ 5,10-Methenyl-THF(c) + NH3(c)
r0243	-0.5	DHAP(c) ⇌ GAP(c)
r0820	0.33	AKG(c) + Malate(m) ⇌ AKG(m) + Malate(c)
r1330	0.67	H+(PG)(c) ⇌ H+(PG)(m) + Proton-gradient(m)
r1319	0.5	ATP(c) + H2O(c) ⇌ ADP(c) + Pi(c) + ATP-energy(c)
r0849	1.83	H2O(s) ⇌ H2O(c)
r0286	0.33	Histidine(c) ⇌ Urocanate(c) + NH3(c)
r0867	0.67	CO2(c) ⇌ CO2(s)
r0868	-0.33	CO2(c) ⇌ CO2(m)
r0736	0.5	ATP(c) + Fructose-6P(c) → ADP(c) + Fructose-1,6PP(c)
r0159	1	3-Phosphoserine(c) + H2O(c) → Serine(c) + Pi(c)
r1545	0.33	Glycine(s) + Serine(c) ⇌ Glycine(c) + Serine(s)
r0192	0.5	Glucose-6P(c) ⇌ Fructose-6P(c)
r1114	0.33	AKG(m) + CoA(m) + NAD+(m) ⇌ NADH(m) + CO2(m) + Succinyl-CoA(m)
r0792	-0.33	5,10-Methylene-THF(c) + NADH(c) ⇌ 5-Methyl-THF(c) + NAD+(c)
r2676	0.33	5-Methyl-THF(c) ⇌ THF(c) + THF-activated methyl group(c)
r2526	-0.67	Serine(c) ⇌ Serine(s)
r0663	-1	3-Phosphonooxypyruvate(c) + Glutamate(c) → 3-Phosphoserine(c) + AKG(c)
r1320	0.33	ATP(m) + H2O(m) ⇌ ADP(m) + Pi(m) + ATP-energy(m)
r1322	1.67	NADH(c) ⇌ NAD+(c) + NADH-redox-potential(c)
r0518	-0.33	Glutamate(c) + 5-Formiminotetrahydrofolate(c) ⇌ THF(c) + N-Formimino-L-glutamate(c)
r1323	-0.33	NADH(m) ⇌ NAD+(m) + NADH-redox-potential(m)
r0519	0.33	H2O(c) + 4-Imidazolone-5-propanoate(c) ⇌ N-Formimino-L-glutamate(c)
r0100	-0.33	ADP(m) + GTP(m) ⇌ ATP(m) + GDP(m)
r0253	1	GAP(c) + Pi(c) + NAD+(c) ⇌ 1,3DPG(c) + NADH(c)
r0256	0.5	Fructose-1,6PP(c) ⇌ DHAP(c) + GAP(c)
r1047	0.67	Pi(c) + H+(PG)(c) → Pi(m) + H+(PG)(m)
r0259	-0.33	Fumarate(m) + H2O(m) ⇌ Malate(m)
r2419	0.67	AKG(c) + Pi(m) ⇌ AKG(m) + Pi(c)
r0838	0.67	NH3(c) ⇌ NH3(m)
r0851	0.33	H2O(m) ⇌ H2O(c)

Serine from Isoleucine (261)

Definition			Solution												
			exchanges		reactions								transp		
Objective	Constraints	Comment	imports	exports	c	m	r	p	l	n	s	b	s-c	b-c	int
Serine	−Isoleucine MCES +Urea	Transamination to Serine using Isoleucine	2.5 H_2O(s) 1 O_2(s) 0.5 Glucose-6P(c) 1 Isoleucine(s)	0.5 P_i(c) 2 CO_2(s) 1 Serine(s) 0.5 ATP-energy(c) 2 NADH-redox-potential(c) 4 NADH-redox-potential(m) 9 Proton-gradient(m) 2 CoA-activated acetyl group(m)	11	13	-	-	-	-	-	-	5	-	11

ID	flux	reaction
r0855	1	O2(s) ⇌ O2(c)
r0856	-1	O2(m) ⇌ O2(c)
r0592	-1	H2O(m) + Acrylyl-CoA(m) → 3-Hydroxypropionyl-CoA(m)

Continued on next page

Serine from Isoleucine (261)– continued

ID	flux	reaction
r0596	-1	3-Hydroxypropionyl-CoA(m) + H2O(m) → Hydracrylate(m) + CoA(m)
r0017	1	O2(m) + 4 H+(PG)(m) + 4 Ferrocytochrome C(m) → 4 Ferricytochrome C(m) + 2 H2O(m) + 4 H+(PG)(c)
r0911	1	Proline(c) + Glutamate(m) ⇌ Proline(m) + Glutamate(c)
r0337	-1	ADP(c) + 1,3DPG(c) ⇌ ATP(c) + 3PG(c)
r0338	1	3PG(c) + NAD+(c) → 3-Phosphonooxypyruvate(c) + NADH(c)
r1426	1	Proline(m) + H+(PG)(c) → Proline(c) + H+(PG)(m)
r0222	-1	CoA(m) + 2-Methylacetoacetyl-CoA(m) ⇌ Propanoyl-CoA(m) + Acetyl-CoA(m)
r0953	1	Isoleucine(c) + H+(PG)(c) → Isoleucine(m) + H+(PG)(m)
r2536	1	Isoleucine(s) ⇌ Isoleucine(c)
r0507	2	Ubiquinol(m) + 2 Ferricytochrome C(m) + 4 H+(PG)(m) → Ubiquinone(m) + 4 H+(PG)(c) + 2 Ferrocytochrome C(m)
r0243	-0.5	DHAP(c) ⇌ GAP(c)
r1330	9	H+(PG)(c) ⇌ H+(PG)(m) + Proton-gradient(m)
r1319	0.5	ATP(c) + H2O(c) ⇌ ADP(c) + Pi(c) + ATP-energy(c)
r0264	1	Isoleucine(m) + AKG(m) ⇌ Glutamate(m) + 2-Oxo-3-methylvalerate(m)
r0849	2.5	H2O(s) ⇌ H2O(c)
r0867	2	CO2(c) ⇌ CO2(s)
r0868	-2	CO2(c) ⇌ CO2(m)
r0736	0.5	ATP(c) + Fructose-6P(c) → ADP(c) + Fructose-1,6PP(c)
r0159	1	3-Phosphoserine(c) + H2O(c) → Serine(c) + Pi(c)
r0174	1	CoA(m) + NAD+(m) + 3-Oxopropanoate(m) → Acetyl-CoA(m) + CO2(m) + NADH(m)
r0602	1	2-Oxo-3-methylvalerate(m) + CoA(m) + NAD+(m) ⇌ 2-Methylbutyryl-CoA(m) + CO2(m) + NADH(m)
r0603	1	2-Methylbutyryl-CoA(m) + Ubiquinone(m) ⇌ Tiglyl-CoA(m) + Ubiquinol(m)
r0192	0.5	Glucose-6P(c) ⇌ Fructose-6P(c)
r0365	1	Hydracrylate(m) + NAD+(m) → NADH(m) + 3-Oxopropanoate(m)
r2678	2	Acetyl-CoA(m) ⇌ CoA(m) + CoA-activated acetyl group(m)
r2526	-1	Serine(s) ⇌ Serine(c)
r0663	-1	3-Phosphonooxypyruvate(c) + Glutamate(c) → 3-Phosphoserine(c) + AKG(c)
r0664	1	NAD+(m) + (S)-3-Hydroxy-2-methylbutyryl-CoA(m) ⇌ 2-Methylacetoacetyl-CoA(m) + NADH(m)
r0665	-1	H2O(m) + Tiglyl-CoA(m) ⇌ (S)-3-Hydroxy-2-methylbutyryl-CoA(m)
r1322	2	NADH(c) ⇌ NAD+(c) + NADH-redox-potential(c)
r0683	1	Propanoyl-CoA(m) + Ubiquinone(m) → Ubiquinol(m) + Acrylyl-CoA(m)
r1323	4	NADH(m) ⇌ NAD+(m) + NADH-redox-potential(m)
r0253	1	GAP(c) + Pi(c) + NAD+(c) ⇌ 1,3DPG(c) + NADH(c)
r0256	0.5	Fructose-1,6PP(c) ⇌ DHAP(c) + GAP(c)
r1047	1	Pi(c) + H+(PG)(c) → Pi(m) + H+(PG)(m)
r2419	1	AKG(c) + Pi(m) ⇌ AKG(m) + Pi(c)
r0851	-1	H2O(m) ⇌ H2O(c)

Serine from Glutamine (262)

Definition			Solution												
			exchanges		reactions								transp		
Objective	Constraints	Comment	imports	exports	c	m	r	p	l	n	s	b	s-c	b-c	int
Serine	−Glutamine MCES +Urea	Transamination to Serine using Glutamine	2 H_2O(s) 0.25 O_2(s) 0.5 Glutamine(s) 0.5 Glucose-6P(c) 0.5 NADPH-redox-potential(m)	0.5 P_i(c) 1 CO_2(s) 0.5 Pyruvate(c) 1 Serine(s) 0.5 ATP-energy(c) 0.5 ATP-energy(m) 2 NADH-redox-potential(c) 0.5 NADH-redox-potential(m) 0.5 NADPH-redox-potential(c) 2 Proton-gradient(m)	15	9	-	-	-	-	-	-	5	-	11

ID	flux	reaction
r0125	-0.5	GDP(m) + Pi(m) + Succinyl-CoA(m) ⇌ GTP(m) + Succinate(m) + CoA(m)
r0855	0.25	O2(s) ⇌ O2(c)
r0856	-0.25	O2(m) ⇌ O2(c)
r0017	0.25	O2(m) + 4 H+(PG)(m) + 4 Ferrocytochrome C(m) → 4 Ferricytochrome C(m) + 2 H2O(m) + 4 H+(PG)(c)

Continued on next page

ID	flux	reaction
r0189	0.5	Glucosamine-6P(c) + H2O(c) ⇌ Fructose-6P(c) + NH3(c)
r1572	0.5	Glutamine(s) + Serine(c) ⇌ Glutamine(c) + Serine(s)
r0911	0.5	Proline(c) + Glutamate(m) ⇌ Proline(m) + Glutamate(c)
r0337	-1	ADP(c) + 1,3DPG(c) ⇌ ATP(c) + 3PG(c)
r0338	1	3PG(c) + NAD+(c) → 3-Phosphonooxypyruvate(c) + NADH(c)
r0058	0.5	Malate(c) + NADP+(c) ⇌ Pyruvate(c) + CO2(c) + NADPH(c)
r1426	0.5	Proline(m) + H+(PG)(c) → Proline(c) + H+(PG)(m)
r0075	-0.5	AKG(m) + NADPH(m) + NH3(m) ⇌ Glutamate(m) + H2O(m) + NADP+(m)
r0507	0.5	Ubiquinol(m) + 2 Ferricytochrome C(m) + 4 H+(PG)(m) → Ubiquinone(m) + 4 H+(PG)(c) + 2 Ferrocytochrome C(m)
r0509	0.5	Ubiquinone(m) + Succinate(m) → Ubiquinol(m) + Fumarate(m)
r0243	-0.5	DHAP(c) ⇌ GAP(c)
r0820	0.5	AKG(c) + Malate(m) ⇌ AKG(m) + Malate(c)
r1330	2	H+(PG)(c) ⇌ H+(PG)(m) + Proton-gradient(m)
r1319	0.5	ATP(c) + H2O(c) ⇌ ADP(c) + Pi(c) + ATP-energy(c)
r0849	2	H2O(s) ⇌ H2O(c)
r0867	1	CO2(c) ⇌ CO2(s)
r0868	-0.5	CO2(c) ⇌ CO2(m)
r0736	0.5	ATP(c) + Fructose-6P(c) → ADP(c) + Fructose-1,6PP(c)
r0159	1	3-Phosphoserine(c) + H2O(c) → Serine(c) + Pi(c)
r0190	0.5	Glutamine(c) + Fructose-6P(c) → Glutamate(c) + Glucosamine-6P(c)
r0192	0.5	Glucose-6P(c) ⇌ Fructose-6P(c)
r1114	0.5	AKG(m) + CoA(m) + NAD+(m) ⇌ NADH(m) + CO2(m) + Succinyl-CoA(m)
r2526	-0.5	Serine(c) ⇌ Serine(s)
r0663	-1	3-Phosphonooxypyruvate(c) + Glutamate(c) → 3-Phosphoserine(c) + AKG(c)
r1320	0.5	ATP(m) + H2O(m) ⇌ ADP(m) + Pi(m) + ATP-energy(m)
r1322	2	NADH(c) ⇌ NAD+(c) + NADH-redox-potential(c)
r1323	0.5	NADH(m) ⇌ NAD+(m) + NADH-redox-potential(m)
r0100	-0.5	ADP(m) + GTP(m) ⇌ ATP(m) + GDP(m)
r1326	0.5	NADPH(c) ⇌ NADPH-redox-potential(c) + NADP+(c)
r0253	1	GAP(c) + Pi(c) + NAD+(c) ⇌ 1,3DPG(c) + NADH(c)
r1327	-0.5	NADPH(m) ⇌ NADPH-redox-potential(m) + NADP+(m)
r0256	0.5	Fructose-1,6PP(c) ⇌ DHAP(c) + GAP(c)
r1047	0.5	Pi(c) + H+(PG)(c) → Pi(m) + H+(PG)(m)
r0259	-0.5	Fumarate(m) + H2O(m) ⇌ Malate(m)
r2419	0.5	AKG(c) + Pi(m) ⇌ AKG(m) + Pi(c)
r0838	0.5	NH3(c) ⇌ NH3(m)

Serine from Leucine (263)

Definition			Solution												
			exchanges		reactions								transp		
Objective	Constraints	Comment	imports	exports	c	m	r	p	l	n	s	b	s-c	b-c	int
Serine	−Leucine MCES +Urea	Transamination to Serine using Leucine	0.5 H_2O(s) 0.5 O_2(s) 0.5 Glucose-6P(c) 1 Leucine(s) 1 NADPH-redox-potential(m)	0.5 P_i(c) 1 Serine(s) 0.5 ATP-energy(c) 2 NADH-redox-potential(c) 2 NADH-redox-potential(m) 4 Proton-gradient(m) 1 Na-gradient(c) 3 CoA-activated acetyl group(m)	11	13	-	-	-	-	-	-	5	-	10

ID	flux	reaction
r0855	0.5	O2(s) ⇌ O2(c)
r0856	-0.5	O2(m) ⇌ O2(c)
r0462	1	HMG-CoA(m) + CoA(m) ⇌ Acetyl-CoA(m) + H2O(m) + Acetoacetyl-CoA(m)
r0017	0.5	O2(m) + 4 H+(PG)(m) + 4 Ferrocytochrome C(m) → 4 Ferricytochrome C(m) + 2 H2O(m) + 4 H+(PG)(c)
r0911	1	Proline(c) + Glutamate(m) ⇌ Proline(m) + Glutamate(c)
r0337	-1	ADP(c) + 1,3DPG(c) ⇌ ATP(c) + 3PG(c)

Continued on next page

ID	flux	reaction
r0338	1	3PG(c) + NAD+(c) → 3-Phosphonooxypyruvate(c) + NADH(c)
r0070	-1	CoA(m) + Acetoacetyl-CoA(m) ⇌ 2 Acetyl-CoA(m)
r2664	1	Na+(c) ⇌ Na+(s) + Na-gradient(c)
r1426	1	Proline(m) + H+(PG)(c) → Proline(c) + H+(PG)(m)
r0059	-1	Pyruvate(m) + CO2(m) + NADPH(m) ⇌ Malate(m) + NADP+(m)
r0655	1	Isovaleryl-CoA(m) + Ubiquinone(m) ⇌ 3-Methylcrotonyl-CoA(m) + Ubiquinol(m)
r0658	1	ATP(m) + 3-Methylcrotonyl-CoA(m) + HCO3-(m) ⇌ ADP(m) + Pi(m) + 3-Methylglutaconyl-CoA(m)
r0507	1	Ubiquinol(m) + 2 Ferricytochrome C(m) + 4 H+(PG)(m) → Ubiquinone(m) + 4 H+(PG)(c) + 2 Ferrocytochrome C(m)
r0243	-0.5	DHAP(c) ⇌ GAP(c)
r1330	4	H+(PG)(c) ⇌ H+(PG)(m) + Proton-gradient(m)
r1319	0.5	ATP(c) + H2O(c) ⇌ ADP(c) + Pi(c) + ATP-energy(c)
r0262	1	Leucine(m) + AKG(m) ⇌ Glutamate(m) + 4-Methyl-2-oxopentanoate(m)
r0849	0.5	H2O(s) ⇌ H2O(c)
r1957	1	Leucine(s) + Na+(s) + Lysine(c) → Leucine(c) + Na+(c) + Lysine(s)
r0736	0.5	ATP(c) + Fructose-6P(c) → ADP(c) + Fructose-1,6PP(c)
r0159	1	3-Phosphoserine(c) + H2O(c) → Serine(c) + Pi(c)
r0601	1	4-Methyl-2-oxopentanoate(m) + CoA(m) + NAD+(m) ⇌ Isovaleryl-CoA(m) + CO2(m) + NADH(m)
r0192	0.5	Glucose-6P(c) ⇌ Fructose-6P(c)
r1995	1	Lysine(s) + Serine(c) → Lysine(c) + Serine(s)
r0490	-1	3-Methylglutaconyl-CoA(m) + H2O(m) ⇌ HMG-CoA(m)
r1438	1	Leucine(c) → Leucine(m)
r2678	3	Acetyl-CoA(m) ⇌ CoA(m) + CoA-activated acetyl group(m)
r0663	-1	3-Phosphonooxypyruvate(c) + Glutamate(c) → 3-Phosphoserine(c) + AKG(c)
r1322	2	NADH(c) ⇌ NAD+(c) + NADH-redox-potential(c)
r1323	2	NADH(m) ⇌ NAD+(m) + NADH-redox-potential(m)
r0253	1	GAP(c) + Pi(c) + NAD+(c) ⇌ 1,3DPG(c) + NADH(c)
r1327	-1	NADPH(m) ⇌ NADPH-redox-potential(m) + NADP+(m)
r0256	0.5	Fructose-1,6PP(c) ⇌ DHAP(c) + GAP(c)
r1047	1	Pi(c) + H+(PG)(c) → Pi(m) + H+(PG)(m)
r0105	1	NAD+(m) + Malate(m) ⇌ OAA(m) + NADH(m)
r0106	-1	ADP(m) + Pi(m) + OAA(m) ⇌ ATP(m) + Pyruvate(m) + HCO3-(m)
r2419	1	AKG(c) + Pi(m) ⇌ AKG(m) + Pi(c)
r0851	1	H2O(m) ⇌ H2O(c)

Serine from Lysine (264)

Definition			Solution											
			exchanges		reactions							transp		
Objective	Constraints	Comment	imports	exports	c	m	r	p	l	n	s	b	s-c	b-c	int
Serine	−Lysine MCES +Urea	Transamination to Serine using Lysine	2 H_2O(s) 0.25 O_2(s) 0.5 Lysine(s) 0.5 Glucose-6P(c) 0.5 NADPH-redox-potential(m)	0.5 P_i(c) 1 CO_2(s) 1 Serine(s) 0.5 ATP-energy(c) 2.5 NADH-redox-potential(c) 1.5 NADH-redox-potential(m) 1 Proton-gradient(m) 1 CoA-activated acetyl group(m)	12	11	-	-	-	-	-	-	5	-	11

ID	flux	reaction
r0855	0.25	O2(s) ⇌ O2(c)
r0856	-0.25	O2(m) ⇌ O2(c)
r0594	0.5	2-Aminoadipate 6-semialdehyde(c) + NAD+(c) + H2O(c) ⇌ NADH(c) + L-2-Aminoadipate(c)
r0460	0.5	(S)-3-Hydroxybutyryl-CoA(m) + NAD+(m) ⇌ NADH(m) + Acetoacetyl-CoA(m)
r0448	0.5	2-Oxoadipate(m) + CoA(m) + NAD+(m) ⇌ Glutaryl-CoA(m) + CO2(m) + NADH(m)
r0180	-0.5	H2O(m) + Saccharopine(m) + NADP+(m) ⇌ AKG(m) + NADPH(m) + Lysine(m)
r0017	0.25	O2(m) + 4 H+(PG)(m) + 4 Ferrocytochrome C(m) → 4 Ferricytochrome C(m) + 2 H2O(m) + 4 H+(PG)(c)
r0911	1	Proline(c) + Glutamate(m) ⇌ Proline(m) + Glutamate(c)
r0337	-1	ADP(c) + 1,3DPG(c) ⇌ ATP(c) + 3PG(c)

Continued on next page

ID	flux	reaction
r0338	1	3PG(c) + NAD+(c) → 3-Phosphonooxypyruvate(c) + NADH(c)
r0070	-0.5	CoA(m) + Acetoacetyl-CoA(m) ⇌ 2 Acetyl-CoA(m)
r1426	1	Proline(m) + H+(PG)(c) → Proline(c) + H+(PG)(m)
r0507	0.5	Ubiquinol(m) + 2 Ferricytochrome C(m) + 4 H+(PG)(m) → Ubiquinone(m) + 4 H+(PG)(c) + 2 Ferrocytochrome C(m)
r2539	-0.5	2-Aminoadipate 6-semialdehyde(c) + L-2-Aminoadipate(m) ⇌ 2-Aminoadipate 6-semialdehyde(m) + L-2-Aminoadipate(c)
r0243	-0.5	DHAP(c) ⇌ GAP(c)
r0525	0.5	NAD+(m) + H2O(m) + Saccharopine(m) ⇌ Glutamate(m) + 2-Aminoadipate 6-semialdehyde(m) + NADH(m)
r1330	1	H+(PG)(c) ⇌ H+(PG)(m) + Proton-gradient(m)
r1319	0.5	ATP(c) + H2O(c) ⇌ ADP(c) + Pi(c) + ATP-energy(c)
r0541	0.5	Glutaryl-CoA(m) + Ubiquinone(m) ⇌ Crotonyl-CoA(m) + CO2(m) + Ubiquinol(m)
r1070	0.5	Lysine(c) ⇌ Lysine(m)
r0849	2	H2O(s) ⇌ H2O(c)
r0867	1	CO2(c) ⇌ CO2(s)
r0868	-1	CO2(c) ⇌ CO2(m)
r0588	-0.5	Crotonyl-CoA(m) + H2O(m) ⇌ (S)-3-Hydroxybutyryl-CoA(m)
r0450	0.5	AKG(m) + L-2-Aminoadipate(m) ⇌ 2-Oxoadipate(m) + Glutamate(m)
r0736	0.5	ATP(c) + Fructose-6P(c) → ADP(c) + Fructose-1,6PP(c)
r0159	1	3-Phosphoserine(c) + H2O(c) → Serine(c) + Pi(c)
r0192	0.5	Glucose-6P(c) ⇌ Fructose-6P(c)
r1995	0.5	Lysine(s) + Serine(c) → Lysine(c) + Serine(s)
r2678	1	Acetyl-CoA(m) ⇌ CoA(m) + CoA-activated acetyl group(m)
r2526	-0.5	Serine(c) ⇌ Serine(s)
r0663	-1	3-Phosphonooxypyruvate(c) + Glutamate(c) → 3-Phosphoserine(c) + AKG(c)
r1322	2.5	NADH(c) ⇌ NAD+(c) + NADH-redox-potential(c)
r1323	1.5	NADH(m) ⇌ NAD+(m) + NADH-redox-potential(m)
r0253	1	GAP(c) + Pi(c) + NAD+(c) ⇌ 1,3DPG(c) + NADH(c)
r1327	-0.5	NADPH(m) ⇌ NADPH-redox-potential(m) + NADP+(m)
r0256	0.5	Fructose-1,6PP(c) ⇌ DHAP(c) + GAP(c)
r1047	1	Pi(c) + H+(PG)(c) → Pi(m) + H+(PG)(m)
r2419	1	AKG(c) + Pi(m) ⇌ AKG(m) + Pi(c)

Serine from Methionine (265)

Definition			Solution												
			exchanges		reactions								transp		
Objective	Constraints	Comment	imports	exports	c	m	r	p	l	n	s	b	s-c	b-c	int
Serine	−Methionine MCES +Urea +H_2S	Transamination to Serine using Methionine	3 H_2O(s) 0.5 O_2(s) 1 Methionine(s) 1 Glucose-6P(c)	1 P_i(c) 2 CO_2(s) 1 Pyruvate(c) 1 Serine(s) 1 H_2S(s) 1 ATP-energy(c) 4 NADH-redox-potential(c) 1 NADH-redox-potential(m) 1 Proton-gradient(m) 1 THF-activated methyl group(c) 1 CoA-activated acetyl group(m)	16	9	-	-	-	-	-	-	5	-	12

ID	flux	reaction
r0855	0.5	O2(s) ⇌ O2(c)
r0856	-0.5	O2(m) ⇌ O2(c)
r0592	-1	H2O(m) + Acrylyl-CoA(m) → 3-Hydroxypropionyl-CoA(m)
r0596	-1	3-Hydroxypropionyl-CoA(m) + H2O(m) → Hydracrylate(m) + CoA(m)
r0017	0.5	O2(m) + 4 H+(PG)(m) + 4 Ferrocytochrome C(m) → 4 Ferricytochrome C(m) + 2 H2O(m) + 4 H+(PG)(c)
r0314	1	Serine(c) + Homocysteine(c) ⇌ H2O(c) + L-Cystathionine(c)
r0911	2	Proline(c) + Glutamate(m) ⇌ Proline(m) + Glutamate(c)
r0337	-2	ADP(c) + 1,3DPG(c) ⇌ ATP(c) + 3PG(c)
r0338	2	3PG(c) + NAD+(c) → 3-Phosphonooxypyruvate(c) + NADH(c)
r1426	2	Proline(m) + H+(PG)(c) → Proline(c) + H+(PG)(m)

Continued on next page

ID	flux	reaction
r0073	-2	AKG(m) + NADH(m) + NH3(m) ⇌ Glutamate(m) + NAD+(m) + H2O(m)
r0507	1	Ubiquinol(m) + 2 Ferricytochrome C(m) + 4 H+(PG)(m) → Ubiquinone(m) + 4 H+(PG)(c) + 2 Ferrocytochrome C(m)
r0241	1	H2O(c) + L-Cystathionine(c) ⇌ Cysteine(c) + NH3(c) + 2-Oxobutyrate(c)
r0243	-1	DHAP(c) ⇌ GAP(c)
r1330	1	H+(PG)(c) ⇌ H+(PG)(m) + Proton-gradient(m)
r1319	1	ATP(c) + H2O(c) ⇌ ADP(c) + Pi(c) + ATP-energy(c)
r0849	3	H2O(s) ⇌ H2O(c)
r0867	2	CO2(c) ⇌ CO2(s)
r0868	-2	CO2(c) ⇌ CO2(m)
r0736	1	ATP(c) + Fructose-6P(c) → ADP(c) + Fructose-1,6PP(c)
r0159	2	3-Phosphoserine(c) + H2O(c) → Serine(c) + Pi(c)
r0174	1	CoA(m) + NAD+(m) + 3-Oxopropanoate(m) → Acetyl-CoA(m) + CO2(m) + NADH(m)
r0192	1	Glucose-6P(c) ⇌ Fructose-6P(c)
r0193	1	Cysteine(c) + H2O(c) ⇌ Pyruvate(c) + H2S(c) + NH3(c)
r1585	-1	Serine(c) + Methionine(s) ⇌ Serine(s) + Methionine(c)
r0940	1	H2S(c) ⇌ H2S(s)
r1154	1	CoA(m) + NAD+(m) + 2-Oxobutyrate(m) ⇌ CO2(m) + Propanoyl-CoA(m) + NADH(m)
r1155	1	2-Oxobutyrate(c) + H+(PG)(c) → 2-Oxobutyrate(m) + H+(PG)(m)
r2676	1	5-Methyl-THF(c) ⇌ THF(c) + THF-activated methyl group(c)
r0365	1	Hydracrylate(m) + NAD+(m) → NADH(m) + 3-Oxopropanoate(m)
r2678	1	Acetyl-CoA(m) ⇌ CoA(m) + CoA-activated acetyl group(m)
r0663	-2	3-Phosphonooxypyruvate(c) + Glutamate(c) → 3-Phosphoserine(c) + AKG(c)
r0232	-1	THF(c) + Methionine(c) ⇌ 5-Methyl-THF(c) + Homocysteine(c)
r1322	4	NADH(c) ⇌ NAD+(c) + NADH-redox-potential(c)
r0683	1	Propanoyl-CoA(m) + Ubiquinone(m) → Ubiquinol(m) + Acrylyl-CoA(m)
r1323	1	NADH(m) ⇌ NAD+(m) + NADH-redox-potential(m)
r0253	2	GAP(c) + Pi(c) + NAD+(c) ⇌ 1,3DPG(c) + NADH(c)
r0256	1	Fructose-1,6PP(c) ⇌ DHAP(c) + GAP(c)
r1047	2	Pi(c) + H+(PG)(c) → Pi(m) + H+(PG)(m)
r2419	2	AKG(c) + Pi(m) ⇌ AKG(m) + Pi(c)
r0838	2	NH3(c) ⇌ NH3(m)
r0851	1	H2O(m) ⇌ H2O(c)

Serine from Phenylalanine (266)

Definition			Solution												
			exchanges		reactions								transp		
Objective	Constraints	Comment	imports	exports	c	m	r	p	l	n	s	b	s-c	b-c	int
Serine	−Phenylalanine MCES +Urea	Transamination to Serine using Phenylalanine	1.5 H_2O(s) 3 O_2(s) 1 Phenylalanine(s) 0.5 Glucose-6P(c) 1.5 ATP-energy(c)	0.5 P_i(c) 2 CO_2(s) 1 Pyruvate(c) 1 Serine(s) 2 NADH-redox-potential(c) 2 CoA-activated acetyl group(c)	25	-	-	-	-	-	-	-	5	-	-

ID	flux	reaction
r0253	1	GAP(c) + Pi(c) + NAD+(c) ⇌ 1,3DPG(c) + NADH(c)
r0605	1	4-Maleylacetoacetate(c) ⇌ Fumarylacetoacetate(c)
r0543	1	Homogentisate(c) + O2(c) ⇌ 4-Maleylacetoacetate(c)
r0192	0.5	Glucose-6P(c) ⇌ Fructose-6P(c)
r0183	1	Tyrosine(c) + AKG(c) ⇌ 4-Hydroxyphenylpyruvate(c) + Glutamate(c)
r0544	1	4-Hydroxyphenylpyruvate(c) + O2(c) ⇌ Homogentisate(c) + CO2(c)
r0337	-1	ADP(c) + 1,3DPG(c) ⇌ ATP(c) + 3PG(c)
r0256	0.5	Fructose-1,6PP(c) ⇌ DHAP(c) + GAP(c)
r0058	1	Malate(c) + NADP+(c) ⇌ Pyruvate(c) + CO2(c) + NADPH(c)
r1319	-1.5	ATP(c) + H2O(c) ⇌ ADP(c) + Pi(c) + ATP-energy(c)
r0338	1	3PG(c) + NAD+(c) → 3-Phosphonooxypyruvate(c) + NADH(c)

Continued on next page

ID	flux	reaction
r1545	1	Glycine(s) + Serine(c) ⇌ Glycine(c) + Serine(s)
r0663	-1	3-Phosphonooxypyruvate(c) + Glutamate(c) → 3-Phosphoserine(c) + AKG(c)
r0258	-1	Fumarate(c) + H2O(c) ⇌ Malate(c)
r0159	1	3-Phosphoserine(c) + H2O(c) → Serine(c) + Pi(c)
r0069	-1	CoA(c) + Acetoacetyl-CoA(c) ⇌ 2 Acetyl-CoA(c)
r0736	0.5	ATP(c) + Fructose-6P(c) → ADP(c) + Fructose-1,6PP(c)
r0030	1	ATP(c) + AMP(c) ⇌ 2 ADP(c)
r1548	-1	Glycine(s) + Phenylalanine(c) ⇌ Glycine(c) + Phenylalanine(s)
r0855	3	O2(s) ⇌ O2(c)
r0320	1	ATP(c) + CoA(c) + Acetoacetate(c) → AMP(c) + PPi(c) + Acetoacetyl-CoA(c)
r0398	1	Dihydrobiopterin(c) + NADPH(c) ⇌ Tetrahydrobiopterin(c) + NADP+(c)
r0007	1	PPi(c) + H2O(c) → 2 Pi(c)
r1322	2	NADH(c) ⇌ NAD+(c) + NADH-redox-potential(c)
r0867	2	CO2(c) ⇌ CO2(s)
r0849	1.5	H2O(s) ⇌ H2O(c)
r0399	1	Tetrahydrobiopterin(c) + Phenylalanine(c) + O2(c) ⇌ Dihydrobiopterin(c) + Tyrosine(c) + H2O(c)
r2677	2	Acetyl-CoA(c) ⇌ CoA(c) + CoA-activated acetyl group(c)
r0324	-1	Fumarylacetoacetate(c) + H2O(c) ⇌ Fumarate(c) + Acetoacetate(c)
r0243	-0.5	DHAP(c) ⇌ GAP(c)

Serine from Proline (267)

Definition			Solution													
			exchanges		reactions									transp		
Objective	Constraints	Comment	imports	exports	c	m	r	p	l	n	s	b	s-c	b-c	int
Serine	−Proline MCES +Urea	Transamination to Serine using Proline	5.5 H_2O(s) 0.5 Glucose-6P(c) 1 Proline(s)	0.5 P_i(c) 2 CO_2(s) 1 Pyruvate(c) 1 Serine(s) 0.5 ATP-energy(c) 1 ATP-energy(m) 2 NADH-redox-potential(c) 2 NADH-redox-potential(m) 1 NADPH-redox-potential(c) 1 Na-gradient(c)	13	10	-	-	-	-	-	-	5	-	4

ID	flux	reaction
r0820	1	AKG(c) + Malate(m) ⇌ AKG(m) + Malate(c)
r0253	1	GAP(c) + Pi(c) + NAD+(c) ⇌ 1,3DPG(c) + NADH(c)
r1995	1	Lysine(s) + Serine(c) → Lysine(c) + Serine(s)
r0911	1	Proline(c) + Glutamate(m) ⇌ Proline(m) + Glutamate(c)
r0074	1	L-Glutamate 5-semialdehyde(m) + NAD+(m) + H2O(m) ⇌ Glutamate(m) + NADH(m)
r1326	1	NADPH(c) ⇌ NADPH-redox-potential(c) + NADP+(c)
r0192	0.5	Glucose-6P(c) ⇌ Fructose-6P(c)
r1453	1	Proline(m) + Ubiquinol(m) ⇌ 1-Pyrroline-5-carboxylate(m) + Ubiquinone(m)
r0337	-1	ADP(c) + 1,3DPG(c) ⇌ ATP(c) + 3PG(c)
r0256	0.5	Fructose-1,6PP(c) ⇌ DHAP(c) + GAP(c)
r0175	1	1-Pyrroline-5-carboxylate(m) + H2O(m) ⇌ L-Glutamate 5-semialdehyde(m)
r0058	1	Malate(c) + NADP+(c) ⇌ Pyruvate(c) + CO2(c) + NADPH(c)
r1319	0.5	ATP(c) + H2O(c) ⇌ ADP(c) + Pi(c) + ATP-energy(c)
r0851	-4	H2O(m) ⇌ H2O(c)
r0509	1	Ubiquinone(m) + Succinate(m) → Ubiquinol(m) + Fumarate(m)
r0338	1	3PG(c) + NAD+(c) → 3-Phosphonooxypyruvate(c) + NADH(c)
r0100	-1	ADP(m) + GTP(m) ⇌ ATP(m) + GDP(m)
r0663	-1	3-Phosphonooxypyruvate(c) + Glutamate(c) → 3-Phosphoserine(c) + AKG(c)
r0159	1	3-Phosphoserine(c) + H2O(c) → Serine(c) + Pi(c)
r0736	0.5	ATP(c) + Fructose-6P(c) → ADP(c) + Fructose-1,6PP(c)
r0259	-1	Fumarate(m) + H2O(m) ⇌ Malate(m)
r2664	1	Na+(c) ⇌ Na+(s) + Na-gradient(c)

Continued on next page

ID	flux	reaction
r1962	1	Proline(s) + Na+(s) + Lysine(c) → Proline(c) + Na+(c) + Lysine(s)
r1320	1	ATP(m) + H2O(m) ⇌ ADP(m) + Pi(m) + ATP-energy(m)
r1114	1	AKG(m) + CoA(m) + NAD+(m) ⇌ NADH(m) + CO2(m) + Succinyl-CoA(m)
r1322	2	NADH(c) ⇌ NAD+(c) + NADH-redox-potential(c)
r0867	2	CO2(c) ⇌ CO2(s)
r0849	5.5	H2O(s) ⇌ H2O(c)
r0125	-1	GDP(m) + Pi(m) + Succinyl-CoA(m) ⇌ GTP(m) + Succinate(m) + CoA(m)
r1323	2	NADH(m) ⇌ NAD+(m) + NADH-redox-potential(m)
r0868	-1	CO2(c) ⇌ CO2(m)
r0243	-0.5	DHAP(c) ⇌ GAP(c)

Serine from Threonine (268)

Definition			Solution												
			exchanges		reactions								transp		
Objective	Constraints	Comment	imports	exports	c	m	r	p	l	n	s	b	s-c	b-c	int
Serine	−Threonine MCES +Urea	Transamination to Serine using Threonine	1.5 H_2O(s) 0.5 O_2(s) 0.5 Glucose-6P(c) 1 Threonine(s)	0.5 P_i(c) 2 CO_2(s) 1 Serine(s) 0.5 ATP-energy(c) 2 NADH-redox-potential(c) 2 NADH-redox-potential(m) 3 Proton-gradient(m) 1 CoA-activated acetyl group(m)	12	9	-	-	-	-	-	-	5	-	11

ID	flux	reaction
r2678	1	Acetyl-CoA(m) ⇌ CoA(m) + CoA-activated acetyl group(m)
r0253	1	GAP(c) + Pi(c) + NAD+(c) ⇌ 1,3DPG(c) + NADH(c)
r0073	-1	AKG(m) + NADH(m) + NH3(m) ⇌ Glutamate(m) + NAD+(m) + H2O(m)
r1154	1	CoA(m) + NAD+(m) + 2-Oxobutyrate(m) ⇌ CO2(m) + Propanoyl-CoA(m) + NADH(m)
r0911	1	Proline(c) + Glutamate(m) ⇌ Proline(m) + Glutamate(c)
r1155	1	2-Oxobutyrate(c) + H+(PG)(c) → 2-Oxobutyrate(m) + H+(PG)(m)
r1047	1	Pi(c) + H+(PG)(c) → Pi(m) + H+(PG)(m)
r0507	1	Ubiquinol(m) + 2 Ferricytochrome C(m) + 4 H+(PG)(m) → Ubiquinone(m) + 4 H+(PG)(c) + 2 Ferrocytochrome C(m)
r0192	0.5	Glucose-6P(c) ⇌ Fructose-6P(c)
r0174	1	CoA(m) + NAD+(m) + 3-Oxopropanoate(m) → Acetyl-CoA(m) + CO2(m) + NADH(m)
r1426	1	Proline(m) + H+(PG)(c) → Proline(c) + H+(PG)(m)
r0337	-1	ADP(c) + 1,3DPG(c) ⇌ ATP(c) + 3PG(c)
r0256	0.5	Fructose-1,6PP(c) ⇌ DHAP(c) + GAP(c)
r2534	1	Threonine(s) ⇌ Threonine(c)
r1319	0.5	ATP(c) + H2O(c) ⇌ ADP(c) + Pi(c) + ATP-energy(c)
r0365	1	Hydracrylate(m) + NAD+(m) → NADH(m) + 3-Oxopropanoate(m)
r0338	1	3PG(c) + NAD+(c) → 3-Phosphonooxypyruvate(c) + NADH(c)
r2526	-1	Serine(s) ⇌ Serine(c)
r0663	-1	3-Phosphonooxypyruvate(c) + Glutamate(c) → 3-Phosphoserine(c) + AKG(c)
r0159	1	3-Phosphoserine(c) + H2O(c) → Serine(c) + Pi(c)
r2419	1	AKG(c) + Pi(m) ⇌ AKG(m) + Pi(c)
r0736	0.5	ATP(c) + Fructose-6P(c) → ADP(c) + Fructose-1,6PP(c)
r0592	-1	H2O(m) + Acrylyl-CoA(m) → 3-Hydroxypropionyl-CoA(m)
r0683	1	Propanoyl-CoA(m) + Ubiquinone(m) → Ubiquinol(m) + Acrylyl-CoA(m)
r0855	0.5	O2(s) ⇌ O2(c)
r0856	-0.5	O2(m) ⇌ O2(c)
r0838	1	NH3(c) ⇌ NH3(m)
r0240	1	Threonine(c) ⇌ 2-Oxobutyrate(c) + NH3(c)
r1330	3	H+(PG)(c) ⇌ H+(PG)(m) + Proton-gradient(m)
r0596	-1	3-Hydroxypropionyl-CoA(m) + H2O(m) → Hydracrylate(m) + CoA(m)
r1322	2	NADH(c) ⇌ NAD+(c) + NADH-redox-potential(c)
r0867	2	CO2(c) ⇌ CO2(s)

Continued on next page

ID	flux	reaction
r0849	1.5	H2O(s) ⇌ H2O(c)
r0017	0.5	O2(m) + 4 H+(PG)(m) + 4 Ferrocytochrome C(m) → 4 Ferricytochrome C(m) + 2 H2O(m) + 4 H+(PG)(c)
r1323	2	NADH(m) ⇌ NAD+(m) + NADH-redox-potential(m)
r0868	-2	CO2(c) ⇌ CO2(m)
r0243	-0.5	DHAP(c) ⇌ GAP(c)

Serine from Tryptophan (269)

Definition			Solution												
			exchanges		reactions								transp		
Objective	Constraints	Comment	imports	exports	c	m	r	p	l	n	s	b	s-c	b-c	int
Serine	−Tryptophan MCES +Urea	Transamination to Serine using Tryptophan	1.5 H_2O(s) 1.75 O_2(s) 0.5 Tryptophan(s) 0.5 Glucose-6P(c) 1.5 NADPH-redox-potential(c) 0.5 NADPH-redox-potential(m)	0.5 P_i(c) 1.5 CO_2(s) 0.5 Pyruvate(c) 1 Serine(s) 2 NADH-redox-potential(c) 1 NADH-redox-potential(m) 1.5 Proton-gradient(m) 0.5 THF-activated methyl group(c) 1 CoA-activated acetyl group(m)	25	9	-	-	-	-	-	-	6	-	12

ID	flux	reaction
r0855	1.75	O2(s) ⇌ O2(c)
r0856	-0.25	O2(m) ⇌ O2(c)
r0293	-0.5	5,10-Methylene-THF(c) + NADP+(c) ⇌ 5,10-Methenyl-THF(c) + NADPH(c)
r0460	0.5	(S)-3-Hydroxybutyryl-CoA(m) + NAD+(m) ⇌ NADH(m) + Acetoacetyl-CoA(m)
r0448	0.5	2-Oxoadipate(m) + CoA(m) + NAD+(m) ⇌ Glutaryl-CoA(m) + CO2(m) + NADH(m)
r0449	-0.5	2-Oxoadipate(c) + NH3(c) + NADP+(c) ⇌ 2-Aminomuconate(c) + NADPH(c) + H2O(c)
r0017	0.25	O2(m) + 4 H+(PG)(m) + 4 Ferrocytochrome C(m) → 4 Ferricytochrome C(m) + 2 H2O(m) + 4 H+(PG)(c)
r0169	0.5	Tryptophan(c) + O2(c) ⇌ L-Formylkynurenine(c)
r0911	0.5	Proline(c) + Glutamate(m) ⇌ Proline(m) + Glutamate(c)
r0337	-1	ADP(c) + 1,3DPG(c) ⇌ ATP(c) + 3PG(c)
r0338	1	3PG(c) + NAD+(c) → 3-Phosphonooxypyruvate(c) + NADH(c)
r0070	-0.5	CoA(m) + Acetoacetyl-CoA(m) ⇌ 2 Acetyl-CoA(m)
r1426	0.5	Proline(m) + H+(PG)(c) → Proline(c) + H+(PG)(m)
r0075	-0.5	AKG(m) + NADPH(m) + NH3(m) ⇌ Glutamate(m) + H2O(m) + NADP+(m)
r0371	-0.5	10-Formyl-THF(c) ⇌ 5,10-Methenyl-THF(c) + H2O(c)
r1597	-0.5	Methionine(s) + Tryptophan(c) ⇌ Methionine(c) + Tryptophan(s)
r0507	0.5	Ubiquinol(m) + 2 Ferricytochrome C(m) + 4 H+(PG)(m) → Ubiquinone(m) + 4 H+(PG)(c) + 2 Ferrocytochrome C(m)
r0228	0.5	THF(c) + Formate(c) + ATP(c) → ADP(c) + Pi(c) + 10-Formyl-THF(c)
r0676	0.5	2-Amino-3-carboxymuconate semialdehyde(c) ⇌ 2-Aminomuconate semialdehyde(c) + CO2(c)
r0243	-0.5	DHAP(c) ⇌ GAP(c)
r1330	1.5	H+(PG)(c) ⇌ H+(PG)(m) + Proton-gradient(m)
r0541	0.5	Glutaryl-CoA(m) + Ubiquinone(m) ⇌ Crotonyl-CoA(m) + CO2(m) + Ubiquinol(m)
r0562	0.5	3-Hydroxyanthranilate(c) + O2(c) ⇌ 2-Amino-3-carboxymuconate semialdehyde(c)
r0563	0.5	H2O(c) + 3-Hydroxy-L-kynurenine(c) ⇌ 3-Hydroxyanthranilate(c) + Alanine(c)
r0849	1.5	H2O(s) ⇌ H2O(c)
r0867	1.5	CO2(c) ⇌ CO2(s)
r0868	-1	CO2(c) ⇌ CO2(m)
r0588	-0.5	Crotonyl-CoA(m) + H2O(m) ⇌ (S)-3-Hydroxybutyryl-CoA(m)
r0736	0.5	ATP(c) + Fructose-6P(c) → ADP(c) + Fructose-1,6PP(c)
r0159	1	3-Phosphoserine(c) + H2O(c) → Serine(c) + Pi(c)
r0454	0.5	L-Formylkynurenine(c) + H2O(c) ⇌ Formate(c) + Kynurenine(c)
r0455	0.5	Kynurenine(c) + O2(c) + NADPH(c) ⇌ H2O(c) + 3-Hydroxy-L-kynurenine(c) + NADP+(c)
r0192	0.5	Glucose-6P(c) ⇌ Fructose-6P(c)
r0792	-0.5	5,10-Methylene-THF(c) + NADH(c) ⇌ 5-Methyl-THF(c) + NAD+(c)
r1585	-0.5	Serine(s) + Methionine(c) ⇌ Serine(c) + Methionine(s)
r0080	0.5	Alanine(c) + AKG(c) ⇌ Pyruvate(c) + Glutamate(c)

Continued on next page

ID	flux	reaction
r0645	0.5	2-Aminomuconate semialdehyde(c) + NAD+(c) + H2O(c) ⇌ 2-Aminomuconate(c) + NADH(c)
r2676	0.5	5-Methyl-THF(c) ⇌ THF(c) + THF-activated methyl group(c)
r2678	1	Acetyl-CoA(m) ⇌ CoA(m) + CoA-activated acetyl group(m)
r2526	-0.5	Serine(c) ⇌ Serine(s)
r0945	0.5	2-Oxoadipate(c) + AKG(m) ⇌ 2-Oxoadipate(m) + AKG(c)
r0663	-1	3-Phosphonooxypyruvate(c) + Glutamate(c) → 3-Phosphoserine(c) + AKG(c)
r1322	2	NADH(c) ⇌ NAD+(c) + NADH-redox-potential(c)
r1323	1	NADH(m) ⇌ NAD+(m) + NADH-redox-potential(m)
r1326	-1.5	NADPH(c) ⇌ NADPH-redox-potential(c) + NADP+(c)
r0253	1	GAP(c) + Pi(c) + NAD+(c) ⇌ 1,3DPG(c) + NADH(c)
r1327	-0.5	NADPH(m) ⇌ NADPH-redox-potential(m) + NADP+(m)
r1047	1	Pi(c) + H+(PG)(c) → Pi(m) + H+(PG)(m)
r0256	0.5	Fructose-1,6PP(c) ⇌ DHAP(c) + GAP(c)
r2419	1	AKG(c) + Pi(m) ⇌ AKG(m) + Pi(c)
r0838	0.5	NH3(c) ⇌ NH3(m)
r0851	0.5	H2O(m) ⇌ H2O(c)

Serine from Tyrosine (270)

Definition			Solution												
			exchanges		reactions								transp		
Objective	Constraints	Comment	imports	exports	c	m	r	p	l	n	s	b	s-c	b-c	int
Serine	−Tyrosine MCES +Urea	Transamination to Serine using Tyrosine	2.5 H_2O(s) 2 O_2(s) 1 Tyrosine(s) 0.5 Glucose-6P(c) 1.5 ATP-energy(c)	0.5 P_i(c) 2 CO_2(s) 1 Pyruvate(c) 1 Serine(s) 2 NADH-redox-potential(c) 1 NADPH-redox-potential(c) 1 Na-gradient(c) 2 CoA-activated acetyl group(c)	24	-	-	-	-	-	-	-	6	-	-

ID	flux	reaction
r0253	1	GAP(c) + Pi(c) + NAD+(c) ⇌ 1,3DPG(c) + NADH(c)
r0605	1	4-Maleylacetoacetate(c) ⇌ Fumarylacetoacetate(c)
r1326	1	NADPH(c) ⇌ NADPH-redox-potential(c) + NADP+(c)
r0543	1	Homogentisate(c) + O2(c) ⇌ 4-Maleylacetoacetate(c)
r0192	0.5	Glucose-6P(c) ⇌ Fructose-6P(c)
r0183	1	Tyrosine(c) + AKG(c) ⇌ 4-Hydroxyphenylpyruvate(c) + Glutamate(c)
r0544	1	4-Hydroxyphenylpyruvate(c) + O2(c) ⇌ Homogentisate(c) + CO2(c)
r0337	-1	ADP(c) + 1,3DPG(c) ⇌ ATP(c) + 3PG(c)
r0256	0.5	Fructose-1,6PP(c) ⇌ DHAP(c) + GAP(c)
r0058	1	Malate(c) + NADP+(c) ⇌ Pyruvate(c) + CO2(c) + NADPH(c)
r1319	-1.5	ATP(c) + H2O(c) ⇌ ADP(c) + Pi(c) + ATP-energy(c)
r0338	1	3PG(c) + NAD+(c) → 3-Phosphonooxypyruvate(c) + NADH(c)
r2526	-1	Serine(c) ⇌ Serine(s)
r0663	-1	3-Phosphonooxypyruvate(c) + Glutamate(c) → 3-Phosphoserine(c) + AKG(c)
r0258	-1	Fumarate(c) + H2O(c) ⇌ Malate(c)
r0159	1	3-Phosphoserine(c) + H2O(c) → Serine(c) + Pi(c)
r0069	-1	CoA(c) + Acetoacetyl-CoA(c) ⇌ 2 Acetyl-CoA(c)
r0736	0.5	ATP(c) + Fructose-6P(c) → ADP(c) + Fructose-1,6PP(c)
r0030	1	ATP(c) + AMP(c) ⇌ 2 ADP(c)
r2664	1	Na+(c) ⇌ Na+(s) + Na-gradient(c)
r0855	2	O2(s) ⇌ O2(c)
r0320	1	ATP(c) + CoA(c) + Acetoacetate(c) → AMP(c) + PPi(c) + Acetoacetyl-CoA(c)
r0007	1	PPi(c) + H2O(c) → 2 Pi(c)
r1322	2	NADH(c) ⇌ NAD+(c) + NADH-redox-potential(c)
r0867	2	CO2(c) ⇌ CO2(s)
r0849	2.5	H2O(s) ⇌ H2O(c)

Continued on next page

ID	flux	reaction
r2677	2	Acetyl-CoA(c) ⇌ CoA(c) + CoA-activated acetyl group(c)
r1125	1	Tyrosine(s) + Na+(s) → Tyrosine(c) + Na+(c)
r0324	-1	Fumarylacetoacetate(c) + H2O(c) ⇌ Fumarate(c) + Acetoacetate(c)
r0243	-0.5	DHAP(c) ⇌ GAP(c)

Serine from Valine (271)

Definition			Solution												
			exchanges		reactions								transp		
Objective	Constraints	Comment	imports	exports	c	m	r	p	l	n	s	b	s-c	b-c	int
Serine	−Valine MCES +Urea	Transamination to Serine using Valine	3.5 H_2O(s) 1 O_2(s) 0.5 Glucose-6P(c) 1 Valine(s)	0.5 P_i(c) 2 CO_2(s) 1 Pyruvate(c) 1 Serine(s) 0.5 ATP-energy(c) 1 ATP-energy(m) 2 NADH-redox-potential(c) 3 NADH-redox-potential(m) 1 NADPH-redox-potential(c) 10 Proton-gradient(m) 1 Na-gradient(c)	13	14	-	-	-	-	-	-	6	-	10

ID	flux	reaction
r0125	-1	GDP(m) + Pi(m) + Succinyl-CoA(m) ⇌ GTP(m) + Succinate(m) + CoA(m)
r0855	1	O2(s) ⇌ O2(c)
r0856	-1	O2(m) ⇌ O2(c)
r0017	1	O2(m) + 4 H+(PG)(m) + 4 Ferrocytochrome C(m) → 4 Ferricytochrome C(m) + 2 H2O(m) + 4 H+(PG)(c)
r0482	1	3-Hydroxyisobutyrate(m) + NAD+(m) → 2-Methyl-3-oxopropanoate(m) + NADH(m)
r0911	1	Proline(c) + Glutamate(m) ⇌ Proline(m) + Glutamate(c)
r0337	-1	ADP(c) + 1,3DPG(c) ⇌ ATP(c) + 3PG(c)
r0338	1	3PG(c) + NAD+(c) → 3-Phosphonooxypyruvate(c) + NADH(c)
r2664	1	Na+(c) ⇌ Na+(s) + Na-gradient(c)
r1426	1	Proline(m) + H+(PG)(c) → Proline(c) + H+(PG)(m)
r0058	1	Malate(c) + NADP+(c) ⇌ Pyruvate(c) + CO2(c) + NADPH(c)
r0507	2	Ubiquinol(m) + 2 Ferricytochrome C(m) + 4 H+(PG)(m) → Ubiquinone(m) + 4 H+(PG)(c) + 2 Ferrocytochrome C(m)
r0509	1	Ubiquinone(m) + Succinate(m) → Ubiquinol(m) + Fumarate(m)
r0243	-0.5	DHAP(c) ⇌ GAP(c)
r0820	1	AKG(c) + Malate(m) ⇌ AKG(m) + Malate(c)
r1330	10	H+(PG)(c) ⇌ H+(PG)(m) + Proton-gradient(m)
r1319	0.5	ATP(c) + H2O(c) ⇌ ADP(c) + Pi(c) + ATP-energy(c)
r0263	1	Valine(m) + AKG(m) → Glutamate(m) + 3-Methyl-2-oxobutyrate(m)
r0560	1	Isobutyryl-CoA(m) + Ubiquinone(m) → Methacrylyl-CoA(m) + Ubiquinol(m)
r0849	3.5	H2O(s) ⇌ H2O(c)
r0867	2	CO2(c) ⇌ CO2(s)
r0868	-1	CO2(c) ⇌ CO2(m)
r1972	1	Valine(s) + Na+(s) + Lysine(c) → Valine(c) + Na+(c) + Lysine(s)
r0736	0.5	ATP(c) + Fructose-6P(c) → ADP(c) + Fructose-1,6PP(c)
r0159	1	3-Phosphoserine(c) + H2O(c) → Serine(c) + Pi(c)
r0600	1	3-Methyl-2-oxobutyrate(m) + CoA(m) + NAD+(m) ⇌ Isobutyryl-CoA(m) + CO2(m) + NADH(m)
r0192	0.5	Glucose-6P(c) ⇌ Fructose-6P(c)
r1995	1	Lysine(s) + Serine(c) → Lysine(c) + Serine(s)
r0197	1	(R)-Methylmalonyl-CoA(m) ⇌ Succinyl-CoA(m)
r0624	1	2-Methyl-3-oxopropanoate(m) + CoA(m) + NAD+(m) ⇌ (R)-Methylmalonyl-CoA(m) + NADH(m)
r0779	1	3-Hydroxyisobutyryl-CoA(m) + H2O(m) → 3-Hydroxyisobutyrate(m) + CoA(m)
r0923	1	Valine(c) + H+(PG)(c) → Valine(m) + H+(PG)(m)
r0663	-1	3-Phosphonooxypyruvate(c) + Glutamate(c) → 3-Phosphoserine(c) + AKG(c)
r1320	1	ATP(m) + H2O(m) ⇌ ADP(m) + Pi(m) + ATP-energy(m)
r0669	1	Methacrylyl-CoA(m) + H2O(m) → 3-Hydroxyisobutyryl-CoA(m)
r1322	2	NADH(c) ⇌ NAD+(c) + NADH-redox-potential(c)

Continued on next page

ID	flux	reaction
r1323	3	NADH(m) ⇌ NAD+(m) + NADH-redox-potential(m)
r0100	-1	ADP(m) + GTP(m) ⇌ ATP(m) + GDP(m)
r1326	1	NADPH(c) ⇌ NADPH-redox-potential(c) + NADP+(c)
r0253	1	GAP(c) + Pi(c) + NAD+(c) ⇌ 1,3DPG(c) + NADH(c)
r0256	0.5	Fructose-1,6PP(c) ⇌ DHAP(c) + GAP(c)
r0259	-1	Fumarate(m) + H2O(m) ⇌ Malate(m)
r0851	-2	H2O(m) ⇌ H2O(c)

Serine from beta-Alanine (272)

Definition			Solution												
			exchanges		reactions								transp		
Objective	Constraints	Comment	imports	exports	c	m	r	p	l	n	s	b	s-c	b-c	int
Serine	−beta-Alanine MCES +Urea	Transamination to Serine using beta-Alanine	1.5 H_2O(s) 0.5 Glucose-6P(c) 1 beta-Alanine(s) 2 Proton-gradient(m)	0.5 P_i(c) 1 CO_2(s) 1 Serine(s) 0.5 ATP-energy(c) 2 NADH-redox-potential(c) 1 NADH-redox-potential(m) 1 CoA-activated acetyl group(m)	11	4	-	-	-	-	-	-	4	-	7

ID	flux	reaction
r2678	1	Acetyl-CoA(m) ⇌ CoA(m) + CoA-activated acetyl group(m)
r0253	1	GAP(c) + Pi(c) + NAD+(c) ⇌ 1,3DPG(c) + NADH(c)
r0217	1	beta-Alanine(m) + AKG(m) ⇌ Glutamate(m) + 3-Oxopropanoate(m)
r0911	1	Proline(c) + Glutamate(m) ⇌ Proline(m) + Glutamate(c)
r1047	1	Pi(c) + H+(PG)(c) → Pi(m) + H+(PG)(m)
r0192	0.5	Glucose-6P(c) ⇌ Fructose-6P(c)
r0174	1	CoA(m) + NAD+(m) + 3-Oxopropanoate(m) → Acetyl-CoA(m) + CO2(m) + NADH(m)
r1426	1	Proline(m) + H+(PG)(c) → Proline(c) + H+(PG)(m)
r0899	-1	beta-Alanine(s) ⇌ beta-Alanine(c)
r0337	-1	ADP(c) + 1,3DPG(c) ⇌ ATP(c) + 3PG(c)
r0256	0.5	Fructose-1,6PP(c) ⇌ DHAP(c) + GAP(c)
r1319	0.5	ATP(c) + H2O(c) ⇌ ADP(c) + Pi(c) + ATP-energy(c)
r0338	1	3PG(c) + NAD+(c) → 3-Phosphonooxypyruvate(c) + NADH(c)
r2526	-1	Serine(c) ⇌ Serine(s)
r0663	-1	3-Phosphonooxypyruvate(c) + Glutamate(c) → 3-Phosphoserine(c) + AKG(c)
r0159	1	3-Phosphoserine(c) + H2O(c) → Serine(c) + Pi(c)
r2419	1	AKG(c) + Pi(m) ⇌ AKG(m) + Pi(c)
r0736	0.5	ATP(c) + Fructose-6P(c) → ADP(c) + Fructose-1,6PP(c)
r1330	-2	H+(PG)(c) ⇌ H+(PG)(m) + Proton-gradient(m)
r1322	2	NADH(c) ⇌ NAD+(c) + NADH-redox-potential(c)
r0867	1	CO2(c) ⇌ CO2(s)
r0849	1.5	H2O(s) ⇌ H2O(c)
r1323	1	NADH(m) ⇌ NAD+(m) + NADH-redox-potential(m)
r0900	1	beta-Alanine(c) ⇌ beta-Alanine(m)
r0868	-1	CO2(c) ⇌ CO2(m)
r0243	-0.5	DHAP(c) ⇌ GAP(c)

Tyrosine from Phenylalanine (273)

Definition			Solution												
			exchanges		reactions								transp		
Objective	Constraints	Comment	imports	exports	c	m	r	p	l	n	s	b	s-c	b-c	int
Tyrosine	−Phenylalanine MCES +Urea	Transamination to Tyrosine using Phenylalanine	1 O_2(s) 1 Phenylalanine(s) 1 NADPH-redox-potential(c)	1 H_2O(s) 1 Tyrosine(s)	3	-	-	-	-	-	-	-	3	-	-

ID	flux	reaction
r1326	-1	NADPH(c) ⇌ NADPH-redox-potential(c) + NADP+(c)
r1618	1	Phenylalanine(s) + Tyrosine(c) ⇌ Phenylalanine(c) + Tyrosine(s)
r0855	1	O2(s) ⇌ O2(c)
r0398	1	Dihydrobiopterin(c) + NADPH(c) ⇌ Tetrahydrobiopterin(c) + NADP+(c)
r0849	-1	H2O(s) ⇌ H2O(c)
r0399	1	Tetrahydrobiopterin(c) + Phenylalanine(c) + O2(c) ⇌ Dihydrobiopterin(c) + Tyrosine(c) + H2O(c)

beta-Alanine from Alanine (274)

Definition			Solution												
			exchanges		reactions								transp		
Objective	Constraints	Comment	imports	exports	c	m	r	p	l	n	s	b	s-c	b-c	int
beta-Alanine	−Alanine MCES +Urea	Transamination to beta-Alanine using Alanine	1 Alanine(s) 1 NADPH-redox-potential(c)	1 beta-Alanine(s) 1 NADH-redox-potential(c)	7	-	-	-	-	-	-	-	2	-	-

ID	flux	reaction
r0109	-1	OAA(c) + Glutamate(c) ⇌ AKG(c) + Aspartate(c)
r0128	1	Aspartate(c) ⇌ beta-Alanine(c) + CO2(c)
r1326	-1	NADPH(c) ⇌ NADPH-redox-potential(c) + NADP+(c)
r2524	1	Alanine(s) ⇌ Alanine(c)
r0899	1	beta-Alanine(c) ⇌ beta-Alanine(s)
r0058	-1	Pyruvate(c) + CO2(c) + NADPH(c) ⇌ Malate(c) + NADP+(c)
r0104	1	NAD+(c) + Malate(c) ⇌ OAA(c) + NADH(c)
r1322	1	NADH(c) ⇌ NAD+(c) + NADH-redox-potential(c)
r0080	1	Alanine(c) + AKG(c) ⇌ Pyruvate(c) + Glutamate(c)

beta-Alanine from Arginine (275)

Definition			Solution												
			exchanges		reactions								transp		
Objective	Constraints	Comment	imports	exports	c	m	r	p	l	n	s	b	s-c	b-c	int
beta-Alanine	−Arginine MCES +Urea	Transamination to beta-Alanine using Arginine	1.5 H_2O(s) 0.25 O_2(s) 0.5 Pyruvate(m) 0.5 Arginine(s) 0.5 NADPH-redox-potential(m)	1 CO_2(s) 0.5 Urea(s) 1 beta-Alanine(s) 0.5 ATP-energy(m) 1 NADH-redox-potential(c) 1 NADH-redox-potential(m) 1.5 Proton-gradient(m)	5	11	-	-	-	-	-	-	6	-	10

ID	flux	reaction
r0896	0.5	Urea(c) ⇌ Urea(s)
r0820	1	AKG(c) + Malate(m) ⇌ AKG(m) + Malate(c)
r0109	-1	OAA(c) + Glutamate(c) ⇌ AKG(c) + Aspartate(c)
r0911	1	Proline(c) + Glutamate(m) ⇌ Proline(m) + Glutamate(c)
r0128	1	Aspartate(c) ⇌ beta-Alanine(c) + CO2(c)
r0074	0.5	L-Glutamate 5-semialdehyde(m) + NAD+(m) + H2O(m) ⇌ Glutamate(m) + NADH(m)
r0507	0.5	Ubiquinol(m) + 2 Ferricytochrome C(m) + 4 H+(PG)(m) → Ubiquinone(m) + 4 H+(PG)(c) + 2 Ferrocytochrome C(m)
r1426	1	Proline(m) + H+(PG)(c) → Proline(c) + H+(PG)(m)
r1327	-0.5	NADPH(m) ⇌ NADPH-redox-potential(m) + NADP+(m)
r0899	1	beta-Alanine(c) ⇌ beta-Alanine(s)
r1058	0.5	Arginine(s) ⇌ Arginine(c)
r0851	-1	H2O(m) ⇌ H2O(c)
r0509	0.5	Ubiquinone(m) + Succinate(m) → Ubiquinol(m) + Fumarate(m)
r0167	0.5	AKG(m) + Ornithine(m) ⇌ L-Glutamate 5-semialdehyde(m) + Glutamate(m)
r0100	-0.5	ADP(m) + GTP(m) ⇌ ATP(m) + GDP(m)
r0059	-0.5	Pyruvate(m) + CO2(m) + NADPH(m) ⇌ Malate(m) + NADP+(m)

Continued on next page

ID	flux	reaction
r0259	-0.5	Fumarate(m) + H2O(m) ⇌ Malate(m)
r0855	0.25	O2(s) ⇌ O2(c)
r0104	1	NAD+(c) + Malate(c) ⇌ OAA(c) + NADH(c)
r1320	0.5	ATP(m) + H2O(m) ⇌ ADP(m) + Pi(m) + ATP-energy(m)
r0856	-0.25	O2(m) ⇌ O2(c)
r2396	-0.5	Lysine(m) + Ornithine(c) + H+(PG)(c) → Lysine(c) + Ornithine(m) + H+(PG)(m)
r1330	1.5	H+(PG)(c) ⇌ H+(PG)(m) + Proton-gradient(m)
r1114	0.5	AKG(m) + CoA(m) + NAD+(m) ⇌ NADH(m) + CO2(m) + Succinyl-CoA(m)
r1322	1	NADH(c) ⇌ NAD+(c) + NADH-redox-potential(c)
r1070	0.5	Lysine(c) ⇌ Lysine(m)
r0867	1	CO2(c) ⇌ CO2(s)
r0849	1.5	H2O(s) ⇌ H2O(c)
r0125	-0.5	GDP(m) + Pi(m) + Succinyl-CoA(m) ⇌ GTP(m) + Succinate(m) + CoA(m)
r0017	0.25	O2(m) + 4 H+(PG)(m) + 4 Ferrocytochrome C(m) → 4 Ferricytochrome C(m) + 2 H2O(m) + 4 H+(PG)(c)
r1323	1	NADH(m) ⇌ NAD+(m) + NADH-redox-potential(m)
r0144	0.5	Arginine(c) + H2O(c) ⇌ Urea(c) + Ornithine(c)

beta-Alanine from Asparagine (276)

Definition			Solution												
			exchanges		reactions								transp		
Objective	Constraints	Comment	imports	exports	c	m	r	p	l	n	s	b	s-c	b-c	int
beta-Alanine	−Asparagine MCES +Urea	Transamination to beta-Alanine using Asparagine	0.5 Pyruvate(c) 0.5 Asparagine(s) 0.5 NADPH-redox-potential(c) 0.5 NADPH-redox-potential(m) 1 Proton-gradient(m)	0.5 CO_2(s) 1 beta-Alanine(s) 0.5 NADH-redox-potential(c)	7	2	-	-	-	-	-	-	3	-	7

ID	flux	reaction
r0127	0.5	H2O(c) + Asparagine(c) → Aspartate(c) + NH3(c)
r0109	-0.5	OAA(c) + Glutamate(c) ⇌ AKG(c) + Aspartate(c)
r0911	0.5	Proline(c) + Glutamate(m) ⇌ Proline(m) + Glutamate(c)
r0128	1	Aspartate(c) ⇌ beta-Alanine(c) + CO2(c)
r2532	0.5	Asparagine(s) ⇌ Asparagine(c)
r1326	-0.5	NADPH(c) ⇌ NADPH-redox-potential(c) + NADP+(c)
r1047	0.5	Pi(c) + H+(PG)(c) → Pi(m) + H+(PG)(m)
r0075	-0.5	AKG(m) + NADPH(m) + NH3(m) ⇌ Glutamate(m) + H2O(m) + NADP+(m)
r1426	0.5	Proline(m) + H+(PG)(c) → Proline(c) + H+(PG)(m)
r1327	-0.5	NADPH(m) ⇌ NADPH-redox-potential(m) + NADP+(m)
r0899	1	beta-Alanine(c) ⇌ beta-Alanine(s)
r0058	-0.5	Pyruvate(c) + CO2(c) + NADPH(c) ⇌ Malate(c) + NADP+(c)
r0851	0.5	H2O(m) ⇌ H2O(c)
r2419	0.5	AKG(c) + Pi(m) ⇌ AKG(m) + Pi(c)
r0104	0.5	NAD+(c) + Malate(c) ⇌ OAA(c) + NADH(c)
r0838	0.5	NH3(c) ⇌ NH3(m)
r1330	-1	H+(PG)(c) ⇌ H+(PG)(m) + Proton-gradient(m)
r1322	0.5	NADH(c) ⇌ NAD+(c) + NADH-redox-potential(c)
r0867	0.5	CO2(c) ⇌ CO2(s)

beta-Alanine from Aspartate (277)

Definition			Solution												
			exchanges		reactions								transp		
Objective	Constraints	Comment	imports	exports	c	m	r	p	l	n	s	b	s-c	b-c	int
beta-Alanine	−Aspartate MCES +Urea	Transamination to beta-Alanine using Aspartate	1 Aspartate(s)	1 CO_2(s) 1 beta-Alanine(s) 1 Na-gradient(c)	1	-	-	-	-	-	-	-	4	-	-

ID	flux	reaction
r0128	1	Aspartate(c) ⇌ beta-Alanine(c) + CO2(c)
r0899	1	beta-Alanine(c) ⇌ beta-Alanine(s)
r2664	1	Na+(c) ⇌ Na+(s) + Na-gradient(c)
r0867	1	CO2(c) ⇌ CO2(s)
r1143	-1	Na+(c) + Aspartate(c) ← Na+(s) + Aspartate(s)

beta-Alanine from Cysteine (278)

Definition			Solution												
			exchanges		reactions								transp		
Objective	Constraints	Comment	imports	exports	c	m	r	p	l	n	s	b	s-c	b-c	int
beta-Alanine	−Cysteine MCES +Urea +H_2S	Transamination to beta-Alanine using Cysteine	1 Pyruvate(m) 1 Cysteine(s) 2 NADPH-redox-potential(m) 1 Proton-gradient(m)	1 Pyruvate(c) 1 beta-Alanine(s) 1 H_2S(s) 1 NADH-redox-potential(c)	5	3	-	-	-	-	-	-	3	-	7

ID	flux	reaction
r0820	1	AKG(c) + Malate(m) ⇌ AKG(m) + Malate(c)
r0109	-1	OAA(c) + Glutamate(c) ⇌ AKG(c) + Aspartate(c)
r0911	1	Proline(c) + Glutamate(m) ⇌ Proline(m) + Glutamate(c)
r0128	1	Aspartate(c) ⇌ beta-Alanine(c) + CO2(c)
r0075	-1	AKG(m) + NADPH(m) + NH3(m) ⇌ Glutamate(m) + H2O(m) + NADP+(m)
r1426	1	Proline(m) + H+(PG)(c) → Proline(c) + H+(PG)(m)
r1327	-2	NADPH(m) ⇌ NADPH-redox-potential(m) + NADP+(m)
r0940	1	H2S(c) ⇌ H2S(s)
r0899	1	beta-Alanine(c) ⇌ beta-Alanine(s)
r0193	1	Cysteine(c) + H2O(c) ⇌ Pyruvate(c) + H2S(c) + NH3(c)
r0851	1	H2O(m) ⇌ H2O(c)
r0059	-1	Pyruvate(m) + CO2(m) + NADPH(m) ⇌ Malate(m) + NADP+(m)
r2529	1	Cysteine(s) ⇌ Cysteine(c)
r0104	1	NAD+(c) + Malate(c) ⇌ OAA(c) + NADH(c)
r0838	1	NH3(c) ⇌ NH3(m)
r1330	-1	H+(PG)(c) ⇌ H+(PG)(m) + Proton-gradient(m)
r1322	1	NADH(c) ⇌ NAD+(c) + NADH-redox-potential(c)
r0868	1	CO2(c) ⇌ CO2(m)

beta-Alanine from Cystine (279)

Definition			Solution												
			exchanges		reactions								transp		
Objective	Constraints	Comment	imports	exports	c	m	r	p	l	n	s	b	s-c	b-c	int
beta-Alanine	−Cystine MCES +Urea +H_2S	Transamination to beta-Alanine using Cystine	0.5 Cystine(s) 1.5 NADPH-redox-potential(c) 1 NADPH-redox-potential(m) 2 Proton-gradient(m)	1 beta-Alanine(s) 1 H_2S(s) 1 NADH-redox-potential(c) 0.5 Na-gradient(c)	8	2	-	-	-	-	-	-	5	-	7

ID	flux	reaction
r1144	-0.5	Glutamate(c) + Na+(c) ← Glutamate(s) + Na+(s)
r0109	-1	OAA(c) + Glutamate(c) ⇌ AKG(c) + Aspartate(c)
r0911	1	Proline(c) + Glutamate(m) ⇌ Proline(m) + Glutamate(c)
r0128	1	Aspartate(c) ⇌ beta-Alanine(c) + CO2(c)
r1326	-1.5	NADPH(c) ⇌ NADPH-redox-potential(c) + NADP+(c)
r1047	1	Pi(c) + H+(PG)(c) → Pi(m) + H+(PG)(m)
r0075	-1	AKG(m) + NADPH(m) + NH3(m) ⇌ Glutamate(m) + H2O(m) + NADP+(m)
r1426	1	Proline(m) + H+(PG)(c) → Proline(c) + H+(PG)(m)
r1327	-1	NADPH(m) ⇌ NADPH-redox-potential(m) + NADP+(m)
r0940	1	H2S(c) ⇌ H2S(s)
r0899	1	beta-Alanine(c) ⇌ beta-Alanine(s)
r0193	1	Cysteine(c) + H2O(c) ⇌ Pyruvate(c) + H2S(c) + NH3(c)
r0058	-1	Pyruvate(c) + CO2(c) + NADPH(c) ⇌ Malate(c) + NADP+(c)
r0851	1	H2O(m) ⇌ H2O(c)
r2419	1	AKG(c) + Pi(m) ⇌ AKG(m) + Pi(c)
r2664	0.5	Na+(c) ⇌ Na+(s) + Na-gradient(c)
r0104	1	NAD+(c) + Malate(c) ⇌ OAA(c) + NADH(c)
r0838	1	NH3(c) ⇌ NH3(m)
r1330	-2	H+(PG)(c) ⇌ H+(PG)(m) + Proton-gradient(m)
r1322	1	NADH(c) ⇌ NAD+(c) + NADH-redox-potential(c)
r0877	0.5	Glutamate(c) + Cystine(s) ⇌ Glutamate(s) + Cystine(c)
r0027	-0.5	NADPH(c) + Cystine(c) ⇌ 2 Cysteine(c) + NADP+(c)

beta-Alanine from Glutamate (280)

Definition			Solution												
			exchanges		reactions								transp		
Objective	Constraints	Comment	imports	exports	c	m	r	p	l	n	s	b	s-c	b-c	int
beta-Alanine	−Glutamate MCES +Urea	Transamination to beta-Alanine using Glutamate	1 H_2O(s) 0.5 O_2(s) 1 Glutamate(s)	2 CO_2(s) 1 beta-Alanine(s) 1 ATP-energy(m) 1 NADH-redox-potential(c) 1 NADH-redox-potential(m) 6 Proton-gradient(m) 1 Na-gradient(c)	4	7	-	-	-	-	-	-	6	-	7

ID	flux	reaction
r1144	-1	Glutamate(c) + Na+(c) ← Glutamate(s) + Na+(s)
r0820	1	AKG(c) + Malate(m) ⇌ AKG(m) + Malate(c)
r0109	-1	OAA(c) + Glutamate(c) ⇌ AKG(c) + Aspartate(c)
r0128	1	Aspartate(c) ⇌ beta-Alanine(c) + CO2(c)
r0507	1	Ubiquinol(m) + 2 Ferricytochrome C(m) + 4 H+(PG)(m) → Ubiquinone(m) + 4 H+(PG)(c) + 2 Ferrocytochrome C(m)
r0899	1	beta-Alanine(c) ⇌ beta-Alanine(s)
r0851	-1	H2O(m) ⇌ H2O(c)
r0509	1	Ubiquinone(m) + Succinate(m) → Ubiquinol(m) + Fumarate(m)
r0100	-1	ADP(m) + GTP(m) ⇌ ATP(m) + GDP(m)
r0259	-1	Fumarate(m) + H2O(m) ⇌ Malate(m)
r2664	1	Na+(c) ⇌ Na+(s) + Na-gradient(c)
r0855	0.5	O2(s) ⇌ O2(c)
r0104	1	NAD+(c) + Malate(c) ⇌ OAA(c) + NADH(c)
r1320	1	ATP(m) + H2O(m) ⇌ ADP(m) + Pi(m) + ATP-energy(m)
r0856	-0.5	O2(m) ⇌ O2(c)
r1330	6	H+(PG)(c) ⇌ H+(PG)(m) + Proton-gradient(m)
r1114	1	AKG(m) + CoA(m) + NAD+(m) ⇌ NADH(m) + CO2(m) + Succinyl-CoA(m)
r1322	1	NADH(c) ⇌ NAD+(c) + NADH-redox-potential(c)
r0867	2	CO2(c) ⇌ CO2(s)
r0849	1	H2O(s) ⇌ H2O(c)
r0125	-1	GDP(m) + Pi(m) + Succinyl-CoA(m) ⇌ GTP(m) + Succinate(m) + CoA(m)

Continued on next page

ID	flux	reaction
r0017	0.5	O2(m) + 4 H+(PG)(m) + 4 Ferrocytochrome C(m) → 4 Ferricytochrome C(m) + 2 H2O(m) + 4 H+(PG)(c)
r1323	1	NADH(m) ⇌ NAD+(m) + NADH-redox-potential(m)
r0868	-1	CO2(c) ⇌ CO2(m)

beta-Alanine from Glycine (281)

Definition			Solution												
			exchanges		reactions								transp		
Objective	Constraints	Comment	imports	exports	c	m	r	p	l	n	s	b	s-c	b-c	int
beta-Alanine	−Glycine MCES +Urea	Transamination to beta-Alanine using Glycine	1 Glycine(s) 1 NADPH-redox-potential(c) 1 THF-activated methyl group(c)	1 beta-Alanine(s) 1 NADH-redox-potential(c) 1 Na-gradient(c)	16	-	-	-	-	-	-	-	3	-	-

ID	flux	reaction
r0109	-1	OAA(c) + Glutamate(c) ⇌ AKG(c) + Aspartate(c)
r0164	1	2PG(c) ⇌ PEP(c) + H2O(c)
r0128	1	Aspartate(c) ⇌ beta-Alanine(c) + CO2(c)
r1326	-1	NADPH(c) ⇌ NADPH-redox-potential(c) + NADP+(c)
r0327	-1	Hydroxypyruvate(c) + NADPH(c) → Glycerate(c) + NADP+(c)
r1138	1	Glycine(s) + Na+(s) → Glycine(c) + Na+(c)
r0899	1	beta-Alanine(c) ⇌ beta-Alanine(s)
r0058	-1	Pyruvate(c) + CO2(c) + NADPH(c) ⇌ Malate(c) + NADP+(c)
r0339	1	ATP(c) + Glycerate(c) → ADP(c) + 3PG(c)
r0296	1	5-Methyl-THF(c) + NADP+(c) ⇌ 5,10-Methylene-THF(c) + NADPH(c)
r2664	1	Na+(c) ⇌ Na+(s) + Na-gradient(c)
r0230	1	5,10-Methylene-THF(c) + Glycine(c) + H2O(c) ⇌ THF(c) + Serine(c)
r0104	1	NAD+(c) + Malate(c) ⇌ OAA(c) + NADH(c)
r0160	1	Serine(c) + Pyruvate(c) → Hydroxypyruvate(c) + Alanine(c)
r2676	-1	5-Methyl-THF(c) ⇌ THF(c) + THF-activated methyl group(c)
r1322	1	NADH(c) ⇌ NAD+(c) + NADH-redox-potential(c)
r0341	-1	3PG(c) ⇌ 2PG(c)
r0080	1	Alanine(c) + AKG(c) ⇌ Pyruvate(c) + Glutamate(c)
r0054	-1	ADP(c) + PEP(c) → ATP(c) + Pyruvate(c)

beta-Alanine from Histidine (282)

Definition			Solution												
			exchanges		reactions								transp		
Objective	Constraints	Comment	imports	exports	c	m	r	p	l	n	s	b	s-c	b-c	int
beta-Alanine	−Histidine MCES +Urea	Transamination to beta-Alanine using Histidine	0.33 H_2O(s) 0.17 O_2(s) 0.67 Pyruvate(m) 0.33 Histidine(s) 0.67 NADPH-redox-potential(c) 1.33 NADPH-redox-potential(m)	0.67 CO_2(s) 1 beta-Alanine(s) 0.67 NADH-redox-potential(c) 0.67 NADH-redox-potential(m) 1.67 Proton-gradient(m) 0.33 Na-gradient(c) 0.33 THF-activated methyl group(c)	13	13	-	-	-	-	-	-	6	-	10

ID	flux	reaction
r0855	0.17	O2(s) ⇌ O2(c)
r0856	-0.17	O2(m) ⇌ O2(c)
r0293	-0.33	5,10-Methenyl-THF(c) + NADPH(c) ⇌ 5,10-Methylene-THF(c) + NADP+(c)
r0128	0.67	Aspartate(c) ⇌ beta-Alanine(c) + CO2(c)
r0577	-0.33	Urocanate(c) + H2O(c) ⇌ 4-Imidazolone-5-propanoate(c)
r0296	-0.33	5,10-Methylene-THF(c) + NADPH(c) ⇌ 5-Methyl-THF(c) + NADP+(c)
r0592	-0.33	H2O(m) + Acrylyl-CoA(m) → 3-Hydroxypropionyl-CoA(m)

Continued on next page

beta-Alanine from Histidine (282)– continued

ID	flux	reaction
r0596	-0.33	3-Hydroxypropionyl-CoA(m) + H2O(m) → Hydracrylate(m) + CoA(m)
r0017	0.17	O2(m) + 4 H+(PG)(m) + 4 Ferrocytochrome C(m) → 4 Ferricytochrome C(m) + 2 H2O(m) + 4 H+(PG)(c)
r0899	1	beta-Alanine(c) ⇌ beta-Alanine(s)
r0911	0.33	Proline(c) + Glutamate(m) ⇌ Proline(m) + Glutamate(c)
r2664	0.33	Na+(c) ⇌ Na+(s) + Na-gradient(c)
r1426	0.33	Proline(m) + H+(PG)(c) → Proline(c) + H+(PG)(m)
r0059	-0.67	Pyruvate(m) + CO2(m) + NADPH(m) ⇌ Malate(m) + NADP+(m)
r1145	-0.33	Histidine(s) + Na+(s) → Histidine(c) + Na+(c)
r0075	-0.67	AKG(m) + NADPH(m) + NH3(m) ⇌ Glutamate(m) + H2O(m) + NADP+(m)
r0223	0.33	2-Methyl-3-oxopropanoate(m) + CoA(m) + NAD+(m) → Propanoyl-CoA(m) + CO2(m) + NADH(m)
r0507	0.33	Ubiquinol(m) + 2 Ferricytochrome C(m) + 4 H+(PG)(m) → Ubiquinone(m) + 4 H+(PG)(c) + 2 Ferrocytochrome C(m)
r0524	0.33	5-Formiminotetrahydrofolate(c) ⇌ 5,10-Methenyl-THF(c) + NH3(c)
r0820	0.67	AKG(c) + Malate(m) ⇌ AKG(m) + Malate(c)
r1330	1.67	H+(PG)(c) ⇌ H+(PG)(m) + Proton-gradient(m)
r0849	0.33	H2O(s) ⇌ H2O(c)
r0286	0.33	Histidine(c) ⇌ Urocanate(c) + NH3(c)
r0867	0.67	CO2(c) ⇌ CO2(s)
r0900	-0.33	beta-Alanine(c) ⇌ beta-Alanine(m)
r1114	0.33	AKG(m) + CoA(m) + NAD+(m) ⇌ NADH(m) + CO2(m) + Succinyl-CoA(m)
r0197	-0.33	Succinyl-CoA(m) ⇌ (R)-Methylmalonyl-CoA(m)
r0624	-0.33	2-Methyl-3-oxopropanoate(m) + CoA(m) + NAD+(m) ⇌ (R)-Methylmalonyl-CoA(m) + NADH(m)
r2676	0.33	5-Methyl-THF(c) ⇌ THF(c) + THF-activated methyl group(c)
r0365	0.33	Hydracrylate(m) + NAD+(m) → NADH(m) + 3-Oxopropanoate(m)
r0217	-0.33	Glutamate(m) + 3-Oxopropanoate(m) ⇌ beta-Alanine(m) + AKG(m)
r1322	0.67	NADH(c) ⇌ NAD+(c) + NADH-redox-potential(c)
r0683	0.33	Propanoyl-CoA(m) + Ubiquinone(m) → Ubiquinol(m) + Acrylyl-CoA(m)
r0518	-0.33	Glutamate(c) + 5-Formiminotetrahydrofolate(c) ⇌ THF(c) + N-Formimino-L-glutamate(c)
r1323	0.67	NADH(m) ⇌ NAD+(m) + NADH-redox-potential(m)
r0519	0.33	H2O(c) + 4-Imidazolone-5-propanoate(c) ⇌ N-Formimino-L-glutamate(c)
r1326	-0.67	NADPH(c) ⇌ NADPH-redox-potential(c) + NADP+(c)
r1327	-1.33	NADPH(m) ⇌ NADPH-redox-potential(m) + NADP+(m)
r0104	0.67	NAD+(c) + Malate(c) ⇌ OAA(c) + NADH(c)
r0838	0.67	NH3(c) ⇌ NH3(m)
r0851	0.33	H2O(m) ⇌ H2O(c)
r0109	-0.67	OAA(c) + Glutamate(c) ⇌ AKG(c) + Aspartate(c)

beta-Alanine from Isoleucine (283)

Definition			Solution												
			exchanges		reactions								transp		
Objective	Constraints	Comment	imports	exports	c	m	r	p	l	n	s	b	s-c	b-c	int
beta-Alanine	−Isoleucine MCES +Urea	Transamination to beta-Alanine using Isoleucine	1 H_2O(s) 1 O_2(s) 1 Isoleucine(s)	1 CO_2(s) 1 beta-Alanine(s) 3 NADH-redox-potential(m) 11 Proton-gradient(m) 1 CoA-activated acetyl group(m)	-	13	-	-	-	-	-	-	5	-	8

ID	flux	reaction
r2678	1	Acetyl-CoA(m) ⇌ CoA(m) + CoA-activated acetyl group(m)
r0217	-1	Glutamate(m) + 3-Oxopropanoate(m) ⇌ beta-Alanine(m) + AKG(m)
r0507	2	Ubiquinol(m) + 2 Ferricytochrome C(m) + 4 H+(PG)(m) → Ubiquinone(m) + 4 H+(PG)(c) + 2 Ferrocytochrome C(m)
r0264	1	Isoleucine(m) + AKG(m) ⇌ Glutamate(m) + 2-Oxo-3-methylvalerate(m)
r0899	1	beta-Alanine(c) ⇌ beta-Alanine(s)
r0851	-1	H2O(m) ⇌ H2O(c)
r0365	1	Hydracrylate(m) + NAD+(m) → NADH(m) + 3-Oxopropanoate(m)
r2536	1	Isoleucine(s) ⇌ Isoleucine(c)

Continued on next page

ID	flux	reaction
r0664	1	NAD+(m) + (S)-3-Hydroxy-2-methylbutyryl-CoA(m) ⇌ 2-Methylacetoacetyl-CoA(m) + NADH(m)
r0592	-1	H2O(m) + Acrylyl-CoA(m) → 3-Hydroxypropionyl-CoA(m)
r0953	1	Isoleucine(c) + H+(PG)(c) → Isoleucine(m) + H+(PG)(m)
r0683	1	Propanoyl-CoA(m) + Ubiquinone(m) → Ubiquinol(m) + Acrylyl-CoA(m)
r0665	-1	H2O(m) + Tiglyl-CoA(m) ⇌ (S)-3-Hydroxy-2-methylbutyryl-CoA(m)
r0855	1	O2(s) ⇌ O2(c)
r0856	-1	O2(m) ⇌ O2(c)
r0222	-1	CoA(m) + 2-Methylacetoacetyl-CoA(m) ⇌ Propanoyl-CoA(m) + Acetyl-CoA(m)
r1330	11	H+(PG)(c) ⇌ H+(PG)(m) + Proton-gradient(m)
r0596	-1	3-Hydroxypropionyl-CoA(m) + H2O(m) → Hydracrylate(m) + CoA(m)
r0867	1	CO2(c) ⇌ CO2(s)
r0849	1	H2O(s) ⇌ H2O(c)
r0602	1	2-Oxo-3-methylvalerate(m) + CoA(m) + NAD+(m) ⇌ 2-Methylbutyryl-CoA(m) + CO2(m) + NADH(m)
r0017	1	O2(m) + 4 H+(PG)(m) + 4 Ferrocytochrome C(m) → 4 Ferricytochrome C(m) + 2 H2O(m) + 4 H+(PG)(c)
r1323	3	NADH(m) ⇌ NAD+(m) + NADH-redox-potential(m)
r0900	-1	beta-Alanine(c) ⇌ beta-Alanine(m)
r0868	-1	CO2(c) ⇌ CO2(m)
r0603	1	2-Methylbutyryl-CoA(m) + Ubiquinone(m) ⇌ Tiglyl-CoA(m) + Ubiquinol(m)

beta-Alanine from Glutamine (284)

Definition			Solution												
			exchanges		reactions								transp		
Objective	Constraints	Comment	imports	exports	c	m	r	p	l	n	s	b	s-c	b-c	int
beta-Alanine	−Glutamine MCES +Urea	Transamination to beta-Alanine using Glutamine	0.5 H_2O(s) 0.25 O_2(s) 0.5 Pyruvate(m) 0.5 Glutamine(s) 0.5 NADPH-redox-potential(m)	1 CO_2(s) 1 beta-Alanine(s) 0.5 ATP-energy(m) 1 NADH-redox-potential(c) 0.5 Proton-gradient(c) 2.5 Proton-gradient(m) 0.5 Na-gradient(c)	6	9	-	-	-	-	-	-	7	-	8

ID	flux	reaction
r0820	1	AKG(c) + Malate(m) ⇌ AKG(m) + Malate(c)
r0190	0.5	Glutamine(c) + Fructose-6P(c) → Glutamate(c) + Glucosamine-6P(c)
r0109	-1	OAA(c) + Glutamate(c) ⇌ AKG(c) + Aspartate(c)
r0073	-0.5	AKG(m) + NADH(m) + NH3(m) ⇌ Glutamate(m) + NAD+(m) + H2O(m)
r0911	0.5	Proline(c) + Glutamate(m) ⇌ Proline(m) + Glutamate(c)
r0128	1	Aspartate(c) ⇌ beta-Alanine(c) + CO2(c)
r0507	0.5	Ubiquinol(m) + 2 Ferricytochrome C(m) + 4 H+(PG)(m) → Ubiquinone(m) + 4 H+(PG)(c) + 2 Ferrocytochrome C(m)
r1426	0.5	Proline(m) + H+(PG)(c) → Proline(c) + H+(PG)(m)
r1327	-0.5	NADPH(m) ⇌ NADPH-redox-potential(m) + NADP+(m)
r0899	1	beta-Alanine(c) ⇌ beta-Alanine(s)
r0509	0.5	Ubiquinone(m) + Succinate(m) → Ubiquinol(m) + Fumarate(m)
r0100	-0.5	ADP(m) + GTP(m) ⇌ ATP(m) + GDP(m)
r0059	-0.5	Pyruvate(m) + CO2(m) + NADPH(m) ⇌ Malate(m) + NADP+(m)
r0259	-0.5	Fumarate(m) + H2O(m) ⇌ Malate(m)
r2664	0.5	Na+(c) ⇌ Na+(s) + Na-gradient(c)
r0855	0.25	O2(s) ⇌ O2(c)
r0189	0.5	Glucosamine-6P(c) + H2O(c) ⇌ Fructose-6P(c) + NH3(c)
r0104	1	NAD+(c) + Malate(c) ⇌ OAA(c) + NADH(c)
r1320	0.5	ATP(m) + H2O(m) ⇌ ADP(m) + Pi(m) + ATP-energy(m)
r0856	-0.25	O2(m) ⇌ O2(c)
r0838	0.5	NH3(c) ⇌ NH3(m)
r2477	0.5	Glutamine(s) + Na+(s) + H+(PG)(c) → Glutamine(c) + Na+(c) + H+(PG)(s)
r1330	2.5	H+(PG)(c) ⇌ H+(PG)(m) + Proton-gradient(m)
r1114	0.5	AKG(m) + CoA(m) + NAD+(m) ⇌ NADH(m) + CO2(m) + Succinyl-CoA(m)

Continued on next page

ID	flux	reaction
r1331	0.5	H+(PG)(s) ⇌ H+(PG)(c) + Proton-gradient(c)
r1322	1	NADH(c) ⇌ NAD+(c) + NADH-redox-potential(c)
r0867	1	CO2(c) ⇌ CO2(s)
r0849	0.5	H2O(s) ⇌ H2O(c)
r0125	-0.5	GDP(m) + Pi(m) + Succinyl-CoA(m) ⇌ GTP(m) + Succinate(m) + CoA(m)
r0017	0.25	O2(m) + 4 H+(PG)(m) + 4 Ferrocytochrome C(m) → 4 Ferricytochrome C(m) + 2 H2O(m) + 4 H+(PG)(c)

beta-Alanine from Leucine (285)

Definition			Solution													
			exchanges		reactions								transp			
Objective	Constraints	Comment	imports	exports	c	m	r	p	l	n	s	b	s-c	b-c	int
beta-Alanine	−Leucine MCES +Urea	Transamination to beta-Alanine using Leucine	0.5 O_2(s) 1 Pyruvate(m) 1 Leucine(s) 2 NADPH-redox-potential(m)	1 H_2O(s) 1 beta-Alanine(s) 3 NADH-redox-potential(m) 3 Proton-gradient(m) 1 Na-gradient(c) 3 CoA-activated acetyl group(m)	1	14	-	-	-	-	-	-	5	-	10

ID	flux	reaction
r2678	3	Acetyl-CoA(m) ⇌ CoA(m) + CoA-activated acetyl group(m)
r0262	1	Leucine(m) + AKG(m) ⇌ Glutamate(m) + 4-Methyl-2-oxopentanoate(m)
r0911	1	Proline(c) + Glutamate(m) ⇌ Proline(m) + Glutamate(c)
r0128	1	Aspartate(c) ⇌ beta-Alanine(c) + CO2(c)
r0507	1	Ubiquinol(m) + 2 Ferricytochrome C(m) + 4 H+(PG)(m) → Ubiquinone(m) + 4 H+(PG)(c) + 2 Ferrocytochrome C(m)
r0462	1	HMG-CoA(m) + CoA(m) ⇌ Acetyl-CoA(m) + H2O(m) + Acetoacetyl-CoA(m)
r1426	1	Proline(m) + H+(PG)(c) → Proline(c) + H+(PG)(m)
r1327	-2	NADPH(m) ⇌ NADPH-redox-potential(m) + NADP+(m)
r0899	1	beta-Alanine(c) ⇌ beta-Alanine(s)
r0490	-1	3-Methylglutaconyl-CoA(m) + H2O(m) ⇌ HMG-CoA(m)
r0851	1	H2O(m) ⇌ H2O(c)
r0059	-2	Pyruvate(m) + CO2(m) + NADPH(m) ⇌ Malate(m) + NADP+(m)
r0906	1	Leucine(c) + H+(PG)(c) → Leucine(m) + H+(PG)(m)
r0110	-1	OAA(m) + Glutamate(m) → AKG(m) + Aspartate(m)
r0655	1	Isovaleryl-CoA(m) + Ubiquinone(m) ⇌ 3-Methylcrotonyl-CoA(m) + Ubiquinol(m)
r2664	1	Na+(c) ⇌ Na+(s) + Na-gradient(c)
r0855	0.5	O2(s) ⇌ O2(c)
r1122	-1	Leucine(s) + Na+(s) → Leucine(c) + Na+(c)
r0856	-0.5	O2(m) ⇌ O2(c)
r0658	1	ATP(m) + 3-Methylcrotonyl-CoA(m) + HCO3-(m) ⇌ ADP(m) + Pi(m) + 3-Methylglutaconyl-CoA(m)
r0105	2	NAD+(m) + Malate(m) ⇌ OAA(m) + NADH(m)
r1330	3	H+(PG)(c) ⇌ H+(PG)(m) + Proton-gradient(m)
r0601	1	4-Methyl-2-oxopentanoate(m) + CoA(m) + NAD+(m) ⇌ Isovaleryl-CoA(m) + CO2(m) + NADH(m)
r0106	-1	ADP(m) + Pi(m) + OAA(m) ⇌ ATP(m) + Pyruvate(m) + HCO3-(m)
r0070	-1	CoA(m) + Acetoacetyl-CoA(m) ⇌ 2 Acetyl-CoA(m)
r0876	1	Glutamate(c) + H+(PG)(c) + Aspartate(m) → Glutamate(m) + H+(PG)(m) + Aspartate(c)
r0849	-1	H2O(s) ⇌ H2O(c)
r0017	0.5	O2(m) + 4 H+(PG)(m) + 4 Ferrocytochrome C(m) → 4 Ferricytochrome C(m) + 2 H2O(m) + 4 H+(PG)(c)
r1323	3	NADH(m) ⇌ NAD+(m) + NADH-redox-potential(m)
r0868	1	CO2(c) ⇌ CO2(m)

beta-Alanine from Lysine (286)

Definition			Solution												
			exchanges		reactions								transp		
Objective	Constraints	Comment	imports	exports	c	m	r	p	l	n	s	b	s-c	b-c	int
beta-Alanine	−Lysine MCES +Urea	Transamination to beta-Alanine using Lysine	0.5 H_2O(s) 0.25 O_2(s) 1 Pyruvate(m) 0.5 Lysine(s) 1.5 NADPH-redox-potential(m)	1 CO_2(s) 1 beta-Alanine(s) 0.5 NADH-redox-potential(c) 2.5 NADH-redox-potential(m) 1 Proton-gradient(m) 1 CoA-activated acetyl group(m)	3	14	-	-	-	-	-	-	5	-	9

ID	flux	reaction
r2678	1	Acetyl-CoA(m) ⇌ CoA(m) + CoA-activated acetyl group(m)
r0541	0.5	Glutaryl-CoA(m) + Ubiquinone(m) ⇌ Crotonyl-CoA(m) + CO2(m) + Ubiquinol(m)
r0460	0.5	(S)-3-Hydroxybutyryl-CoA(m) + NAD+(m) ⇌ NADH(m) + Acetoacetyl-CoA(m)
r0911	1	Proline(c) + Glutamate(m) ⇌ Proline(m) + Glutamate(c)
r0128	1	Aspartate(c) ⇌ beta-Alanine(c) + CO2(c)
r0525	0.5	NAD+(m) + H2O(m) + Saccharopine(m) ⇌ Glutamate(m) + 2-Aminoadipate 6-semialdehyde(m) + NADH(m)
r0507	0.5	Ubiquinol(m) + 2 Ferricytochrome C(m) + 4 H+(PG)(m) → Ubiquinone(m) + 4 H+(PG)(c) + 2 Ferrocytochrome C(m)
r1426	1	Proline(m) + H+(PG)(c) → Proline(c) + H+(PG)(m)
r1327	-1.5	NADPH(m) ⇌ NADPH-redox-potential(m) + NADP+(m)
r0899	1	beta-Alanine(c) ⇌ beta-Alanine(s)
r0059	-1	Pyruvate(m) + CO2(m) + NADPH(m) ⇌ Malate(m) + NADP+(m)
r0110	-1	OAA(m) + Glutamate(m) → AKG(m) + Aspartate(m)
r1069	-0.5	Lysine(s) ⇌ Lysine(c)
r0448	0.5	2-Oxoadipate(m) + CoA(m) + NAD+(m) ⇌ Glutaryl-CoA(m) + CO2(m) + NADH(m)
r0855	0.25	O2(s) ⇌ O2(c)
r0594	0.5	2-Aminoadipate 6-semialdehyde(c) + NAD+(c) + H2O(c) ⇌ NADH(c) + L-2-Aminoadipate(c)
r2539	-0.5	2-Aminoadipate 6-semialdehyde(c) + L-2-Aminoadipate(m) ⇌ 2-Aminoadipate 6-semialdehyde(m) + L-2-Aminoadipate(c)
r0856	-0.25	O2(m) ⇌ O2(c)
r0105	1	NAD+(m) + Malate(m) ⇌ OAA(m) + NADH(m)
r1330	1	H+(PG)(c) ⇌ H+(PG)(m) + Proton-gradient(m)
r0070	-0.5	CoA(m) + Acetoacetyl-CoA(m) ⇌ 2 Acetyl-CoA(m)
r1322	0.5	NADH(c) ⇌ NAD+(c) + NADH-redox-potential(c)
r1070	0.5	Lysine(c) ⇌ Lysine(m)
r0876	1	Glutamate(c) + H+(PG)(c) + Aspartate(m) → Glutamate(m) + H+(PG)(m) + Aspartate(c)
r0867	1	CO2(c) ⇌ CO2(s)
r0849	0.5	H2O(s) ⇌ H2O(c)
r0588	-0.5	Crotonyl-CoA(m) + H2O(m) ⇌ (S)-3-Hydroxybutyryl-CoA(m)
r0017	0.25	O2(m) + 4 H+(PG)(m) + 4 Ferrocytochrome C(m) → 4 Ferricytochrome C(m) + 2 H2O(m) + 4 H+(PG)(c)
r1323	2.5	NADH(m) ⇌ NAD+(m) + NADH-redox-potential(m)
r0450	0.5	AKG(m) + L-2-Aminoadipate(m) ⇌ 2-Oxoadipate(m) + Glutamate(m)
r0180	-0.5	H2O(m) + Saccharopine(m) + NADP+(m) ⇌ AKG(m) + NADPH(m) + Lysine(m)

beta-Alanine from Methionine (287)

Definition			Solution												
			exchanges		reactions								transp		
Objective	Constraints	Comment	imports	exports	c	m	r	p	l	n	s	b	s-c	b-c	int
beta-Alanine	−Methionine MCES +Urea +H_2S	Transamination to beta-Alanine using Methionine	1.5 H_2O(s) 0.5 O_2(s) 1 Pyruvate(m) 1 Methionine(s) 0.5 Glucose-6P(c) 1 NADPH-redox-potential(m)	0.5 P_i(c) 1 CO_2(s) 2 Pyruvate(c) 1 beta-Alanine(s) 1 H_2S(s) 0.5 ATP-energy(c) 2 NADH-redox-potential(c) 1 NADPH-redox-potential(c) 4 Proton-gradient(m) 1 Na-gradient(c) 1 THF-activated methyl group(c)	18	9	-	-	-	-	-	-	7	-	11

ID	flux	reaction
r0855	0.5	O2(s) ⇌ O2(c)
r0856	-0.5	O2(m) ⇌ O2(c)
r0592	-1	H2O(m) + Acrylyl-CoA(m) → 3-Hydroxypropionyl-CoA(m)
r0596	-1	3-Hydroxypropionyl-CoA(m) + H2O(m) → Hydracrylate(m) + CoA(m)
r0017	0.5	O2(m) + 4 H+(PG)(m) + 4 Ferrocytochrome C(m) → 4 Ferricytochrome C(m) + 2 H2O(m) + 4 H+(PG)(c)
r0899	1	beta-Alanine(c) ⇌ beta-Alanine(s)
r0314	1	Serine(c) + Homocysteine(c) ⇌ H2O(c) + L-Cystathionine(c)
r0911	1	Proline(c) + Glutamate(m) ⇌ Proline(m) + Glutamate(c)
r1141	-1	Methionine(s) + Na+(s) → Methionine(c) + Na+(c)
r0337	-1	ADP(c) + 1,3DPG(c) ⇌ ATP(c) + 3PG(c)
r0338	1	3PG(c) + NAD+(c) → 3-Phosphonooxypyruvate(c) + NADH(c)
r2664	1	Na+(c) ⇌ Na+(s) + Na-gradient(c)
r1426	1	Proline(m) + H+(PG)(c) → Proline(c) + H+(PG)(m)
r0058	1	Malate(c) + NADP+(c) ⇌ Pyruvate(c) + CO2(c) + NADPH(c)
r0059	-1	Pyruvate(m) + CO2(m) + NADPH(m) ⇌ Malate(m) + NADP+(m)
r0073	-2	AKG(m) + NADH(m) + NH3(m) ⇌ Glutamate(m) + NAD+(m) + H2O(m)
r0507	1	Ubiquinol(m) + 2 Ferricytochrome C(m) + 4 H+(PG)(m) → Ubiquinone(m) + 4 H+(PG)(c) + 2 Ferrocytochrome C(m)
r0241	1	H2O(c) + L-Cystathionine(c) ⇌ Cysteine(c) + NH3(c) + 2-Oxobutyrate(c)
r0243	-0.5	DHAP(c) ⇌ GAP(c)
r0820	1	AKG(c) + Malate(m) ⇌ AKG(m) + Malate(c)
r1330	4	H+(PG)(c) ⇌ H+(PG)(m) + Proton-gradient(m)
r1319	0.5	ATP(c) + H2O(c) ⇌ ADP(c) + Pi(c) + ATP-energy(c)
r0849	1.5	H2O(s) ⇌ H2O(c)
r0867	1	CO2(c) ⇌ CO2(s)
r0736	0.5	ATP(c) + Fructose-6P(c) → ADP(c) + Fructose-1,6PP(c)
r0159	1	3-Phosphoserine(c) + H2O(c) → Serine(c) + Pi(c)
r0192	0.5	Glucose-6P(c) ⇌ Fructose-6P(c)
r0900	-1	beta-Alanine(c) ⇌ beta-Alanine(m)
r0193	1	Cysteine(c) + H2O(c) ⇌ Pyruvate(c) + H2S(c) + NH3(c)
r0940	1	H2S(c) ⇌ H2S(s)
r1154	1	CoA(m) + NAD+(m) + 2-Oxobutyrate(m) ⇌ CO2(m) + Propanoyl-CoA(m) + NADH(m)
r1155	1	2-Oxobutyrate(c) + H+(PG)(c) → 2-Oxobutyrate(m) + H+(PG)(m)
r2676	1	5-Methyl-THF(c) ⇌ THF(c) + THF-activated methyl group(c)
r0365	1	Hydracrylate(m) + NAD+(m) → NADH(m) + 3-Oxopropanoate(m)
r0663	-1	3-Phosphonooxypyruvate(c) + Glutamate(c) → 3-Phosphoserine(c) + AKG(c)
r0217	-1	Glutamate(m) + 3-Oxopropanoate(m) ⇌ beta-Alanine(m) + AKG(m)
r0232	-1	THF(c) + Methionine(c) ⇌ 5-Methyl-THF(c) + Homocysteine(c)
r1322	2	NADH(c) ⇌ NAD+(c) + NADH-redox-potential(c)
r0683	1	Propanoyl-CoA(m) + Ubiquinone(m) → Ubiquinol(m) + Acrylyl-CoA(m)
r1326	1	NADPH(c) ⇌ NADPH-redox-potential(c) + NADP+(c)
r0253	1	GAP(c) + Pi(c) + NAD+(c) ⇌ 1,3DPG(c) + NADH(c)
r1327	-1	NADPH(m) ⇌ NADPH-redox-potential(m) + NADP+(m)
r0256	0.5	Fructose-1,6PP(c) ⇌ DHAP(c) + GAP(c)
r0838	2	NH3(c) ⇌ NH3(m)
r0851	1	H2O(m) ⇌ H2O(c)

beta-Alanine from Phenylalanine (288)

Definition			Solution												
			exchanges		reactions								transp		
Objective	Constraints	Comment	imports	exports	c	m	r	p	l	n	s	b	s-c	b-c	int
beta-Alanine	−Phenylalanine MCES +Urea	Transamination to beta-Alanine using Phenylalanine	3 O_2(s) 1 Phenylalanine(s) 2 ATP-energy(c)	2 CO_2(s) 1 beta-Alanine(s) 2 CoA-activated acetyl group(c)	17	-	-	-	-	-	-	-	4	-	-

ID	flux	reaction
r0109	-1	OAA(c) + Glutamate(c) ⇌ AKG(c) + Aspartate(c)
r0605	1	4-Maleylacetoacetate(c) ⇌ Fumarylacetoacetate(c)
r0128	1	Aspartate(c) ⇌ beta-Alanine(c) + CO2(c)
r0543	1	Homogentisate(c) + O2(c) ⇌ 4-Maleylacetoacetate(c)
r0183	1	Tyrosine(c) + AKG(c) ⇌ 4-Hydroxyphenylpyruvate(c) + Glutamate(c)
r0899	1	beta-Alanine(c) ⇌ beta-Alanine(s)
r0544	1	4-Hydroxyphenylpyruvate(c) + O2(c) ⇌ Homogentisate(c) + CO2(c)
r1319	-2	ATP(c) + H2O(c) ⇌ ADP(c) + Pi(c) + ATP-energy(c)
r0258	-1	Fumarate(c) + H2O(c) ⇌ Malate(c)
r0069	-1	CoA(c) + Acetoacetyl-CoA(c) ⇌ 2 Acetyl-CoA(c)
r0030	1	ATP(c) + AMP(c) ⇌ 2 ADP(c)
r2528	1	Phenylalanine(s) ⇌ Phenylalanine(c)
r0855	3	O2(s) ⇌ O2(c)
r0320	1	ATP(c) + CoA(c) + Acetoacetate(c) → AMP(c) + PPi(c) + Acetoacetyl-CoA(c)
r0104	1	NAD+(c) + Malate(c) ⇌ OAA(c) + NADH(c)
r0397	1	Dihydrobiopterin(c) + NADH(c) ⇌ Tetrahydrobiopterin(c) + NAD+(c)
r0007	1	PPi(c) + H2O(c) → 2 Pi(c)
r0867	2	CO2(c) ⇌ CO2(s)
r0399	1	Tetrahydrobiopterin(c) + Phenylalanine(c) + O2(c) ⇌ Dihydrobiopterin(c) + Tyrosine(c) + H2O(c)
r2677	2	Acetyl-CoA(c) ⇌ CoA(c) + CoA-activated acetyl group(c)
r0324	-1	Fumarylacetoacetate(c) + H2O(c) ⇌ Fumarate(c) + Acetoacetate(c)

beta-Alanine from Proline (289)

Definition			Solution												
			exchanges		reactions								transp		
Objective	Constraints	Comment	imports	exports	c	m	r	p	l	n	s	b	s-c	b-c	int
beta-Alanine	−Proline MCES +Urea	Transamination to beta-Alanine using Proline	4 H_2O(s) 1 Proline(s)	2 CO_2(s) 1 beta-Alanine(s) 1 ATP-energy(m) 1 NADH-redox-potential(c) 2 NADH-redox-potential(m) 1 Na-gradient(c)	4	10	-	-	-	-	-	-	5	-	4

ID	flux	reaction
r0820	1	AKG(c) + Malate(m) ⇌ AKG(m) + Malate(c)
r0109	-1	OAA(c) + Glutamate(c) ⇌ AKG(c) + Aspartate(c)
r0911	1	Proline(c) + Glutamate(m) ⇌ Proline(m) + Glutamate(c)
r0128	1	Aspartate(c) ⇌ beta-Alanine(c) + CO2(c)
r0074	1	L-Glutamate 5-semialdehyde(m) + NAD+(m) + H2O(m) ⇌ Glutamate(m) + NADH(m)
r1453	1	Proline(m) + Ubiquinol(m) ⇌ 1-Pyrroline-5-carboxylate(m) + Ubiquinone(m)
r0899	1	beta-Alanine(c) ⇌ beta-Alanine(s)
r0175	1	1-Pyrroline-5-carboxylate(m) + H2O(m) ⇌ L-Glutamate 5-semialdehyde(m)
r1139	-1	Proline(s) + Na+(s) → Proline(c) + Na+(c)
r0851	-4	H2O(m) ⇌ H2O(c)
r0509	1	Ubiquinone(m) + Succinate(m) → Ubiquinol(m) + Fumarate(m)
r0100	-1	ADP(m) + GTP(m) ⇌ ATP(m) + GDP(m)
r0259	-1	Fumarate(m) + H2O(m) ⇌ Malate(m)
r2664	1	Na+(c) ⇌ Na+(s) + Na-gradient(c)
r0104	1	NAD+(c) + Malate(c) ⇌ OAA(c) + NADH(c)
r1320	1	ATP(m) + H2O(m) ⇌ ADP(m) + Pi(m) + ATP-energy(m)
r1114	1	AKG(m) + CoA(m) + NAD+(m) ⇌ NADH(m) + CO2(m) + Succinyl-CoA(m)
r1322	1	NADH(c) ⇌ NAD+(c) + NADH-redox-potential(c)
r0867	2	CO2(c) ⇌ CO2(s)
r0849	4	H2O(s) ⇌ H2O(c)
r0125	-1	GDP(m) + Pi(m) + Succinyl-CoA(m) ⇌ GTP(m) + Succinate(m) + CoA(m)
r1323	2	NADH(m) ⇌ NAD+(m) + NADH-redox-potential(m)
r0868	-1	CO2(c) ⇌ CO2(m)

beta-Alanine from Serine (290)

Definition			Solution												
			exchanges		reactions								transp		
Objective	Constraints	Comment	imports	exports	c	m	r	p	l	n	s	b	s-c	b-c	int
beta-Alanine	−Serine MCES +Urea	Transamination to beta-Alanine using Serine	1 Serine(s) 2 NADPH-redox-potential(c)	1 H_2O(s) 1 beta-Alanine(s) 1 NADH-redox-potential(c) 1 Proton-gradient(c) 1 Na-gradient(c)	13	-	-	-	-	-	-	-	5	-	-

ID	flux	reaction
r0109	-1	OAA(c) + Glutamate(c) ⇌ AKG(c) + Aspartate(c)
r0164	1	2PG(c) ⇌ PEP(c) + H2O(c)
r0128	1	Aspartate(c) ⇌ beta-Alanine(c) + CO2(c)
r1326	-2	NADPH(c) ⇌ NADPH-redox-potential(c) + NADP+(c)
r0327	-1	Hydroxypyruvate(c) + NADPH(c) → Glycerate(c) + NADP+(c)
r0899	1	beta-Alanine(c) ⇌ beta-Alanine(s)
r0058	-1	Pyruvate(c) + CO2(c) + NADPH(c) ⇌ Malate(c) + NADP+(c)
r0339	1	ATP(c) + Glycerate(c) → ADP(c) + 3PG(c)
r2664	1	Na+(c) ⇌ Na+(s) + Na-gradient(c)
r0104	1	NAD+(c) + Malate(c) ⇌ OAA(c) + NADH(c)
r0160	1	Serine(c) + Pyruvate(c) → Hydroxypyruvate(c) + Alanine(c)
r2478	1	Serine(s) + Na+(s) + H+(PG)(c) → Serine(c) + Na+(c) + H+(PG)(s)
r1331	1	H+(PG)(s) ⇌ H+(PG)(c) + Proton-gradient(c)
r1322	1	NADH(c) ⇌ NAD+(c) + NADH-redox-potential(c)
r0849	-1	H2O(s) ⇌ H2O(c)
r0341	-1	3PG(c) ⇌ 2PG(c)
r0080	1	Alanine(c) + AKG(c) ⇌ Pyruvate(c) + Glutamate(c)
r0054	-1	ADP(c) + PEP(c) → ATP(c) + Pyruvate(c)

beta-Alanine from Threonine (291)

Definition			Solution												
			exchanges		reactions								transp		
Objective	Constraints	Comment	imports	exports	c	m	r	p	l	n	s	b	s-c	b-c	int
beta-Alanine	−Threonine MCES +Urea	Transamination to beta-Alanine using Threonine	0.5 O_2(s) 1 Threonine(s)	1 CO_2(s) 1 beta-Alanine(s) 1 NADH-redox-potential(m) 5 Proton-gradient(m)	1	8	-	-	-	-	-	-	4	-	8

ID	flux	reaction
r0217	-1	Glutamate(m) + 3-Oxopropanoate(m) ⇌ beta-Alanine(m) + AKG(m)
r0073	-1	AKG(m) + NADH(m) + NH3(m) ⇌ Glutamate(m) + NAD+(m) + H2O(m)
r1154	1	CoA(m) + NAD+(m) + 2-Oxobutyrate(m) ⇌ CO2(m) + Propanoyl-CoA(m) + NADH(m)
r1155	1	2-Oxobutyrate(c) + H+(PG)(c) → 2-Oxobutyrate(m) + H+(PG)(m)
r0507	1	Ubiquinol(m) + 2 Ferricytochrome C(m) + 4 H+(PG)(m) → Ubiquinone(m) + 4 H+(PG)(c) + 2 Ferrocytochrome C(m)
r0899	1	beta-Alanine(c) ⇌ beta-Alanine(s)
r2534	1	Threonine(s) ⇌ Threonine(c)
r0365	1	Hydracrylate(m) + NAD+(m) → NADH(m) + 3-Oxopropanoate(m)
r0592	-1	H2O(m) + Acrylyl-CoA(m) → 3-Hydroxypropionyl-CoA(m)
r0683	1	Propanoyl-CoA(m) + Ubiquinone(m) → Ubiquinol(m) + Acrylyl-CoA(m)
r0855	0.5	O2(s) ⇌ O2(c)
r0856	-0.5	O2(m) ⇌ O2(c)
r0838	1	NH3(c) ⇌ NH3(m)
r0240	1	Threonine(c) ⇌ 2-Oxobutyrate(c) + NH3(c)
r1330	5	H+(PG)(c) ⇌ H+(PG)(m) + Proton-gradient(m)
r0596	-1	3-Hydroxypropionyl-CoA(m) + H2O(m) → Hydracrylate(m) + CoA(m)

Continued on next page

ID	flux	reaction
r0867	1	CO2(c) ⇌ CO2(s)
r0017	0.5	O2(m) + 4 H+(PG)(m) + 4 Ferrocytochrome C(m) → 4 Ferricytochrome C(m) + 2 H2O(m) + 4 H+(PG)(c)
r1323	1	NADH(m) ⇌ NAD+(m) + NADH-redox-potential(m)
r0900	-1	beta-Alanine(c) ⇌ beta-Alanine(m)
r0868	-1	CO2(c) ⇌ CO2(m)

beta-Alanine from Tryptophan (292)

Definition			Solution											
			exchanges		reactions							transp		
Objective	Constraints	Comment	imports	exports	c	m	r	p	l	n	s	b	s-c	b-c	int
beta-Alanine	−Tryptophan MCES +Urea	Transamination to beta-Alanine using Tryptophan	1.75 O_2(s) 1 Pyruvate(m) 0.5 Tryptophan(s) 0.5 ATP-energy(c) 2 NADPH-redox-potential(c) 1 NADPH-redox-potential(m)	1.5 CO_2(s) 0.5 Pyruvate(c) 1 beta-Alanine(s) 1.5 NADH-redox-potential(c) 0.5 NADH-redox-potential(m) 2.5 Proton-gradient(m) 0.5 Na-gradient(c) 0.5 THF-activated methyl group(c) 1 CoA-activated acetyl group(m)	20	10	-	-	-	-	-	-	5	-	10

ID	flux	reaction
r0855	1.75	O2(s) ⇌ O2(c)
r0856	-0.25	O2(m) ⇌ O2(c)
r0293	-0.5	5,10-Methylene-THF(c) + NADP+(c) ⇌ 5,10-Methenyl-THF(c) + NADPH(c)
r0128	1	Aspartate(c) ⇌ beta-Alanine(c) + CO2(c)
r0296	-0.5	5,10-Methylene-THF(c) + NADPH(c) ⇌ 5-Methyl-THF(c) + NADP+(c)
r0460	0.5	(S)-3-Hydroxybutyryl-CoA(m) + NAD+(m) ⇌ NADH(m) + Acetoacetyl-CoA(m)
r0448	0.5	2-Oxoadipate(m) + CoA(m) + NAD+(m) ⇌ Glutaryl-CoA(m) + CO2(m) + NADH(m)
r0449	-0.5	2-Oxoadipate(c) + NH3(c) + NADP+(c) ⇌ 2-Aminomuconate(c) + NADPH(c) + H2O(c)
r0017	0.25	O2(m) + 4 H+(PG)(m) + 4 Ferrocytochrome C(m) → 4 Ferricytochrome C(m) + 2 H2O(m) + 4 H+(PG)(c)
r0169	0.5	Tryptophan(c) + O2(c) ⇌ L-Formylkynurenine(c)
r0899	1	beta-Alanine(c) ⇌ beta-Alanine(s)
r1124	-0.5	Tryptophan(s) + Na+(s) → Tryptophan(c) + Na+(c)
r0911	0.5	Proline(c) + Glutamate(m) ⇌ Proline(m) + Glutamate(c)
r0070	-0.5	CoA(m) + Acetoacetyl-CoA(m) ⇌ 2 Acetyl-CoA(m)
r2664	0.5	Na+(c) ⇌ Na+(s) + Na-gradient(c)
r1426	0.5	Proline(m) + H+(PG)(c) → Proline(c) + H+(PG)(m)
r0059	-1	Pyruvate(m) + CO2(m) + NADPH(m) ⇌ Malate(m) + NADP+(m)
r0073	-0.5	AKG(m) + NADH(m) + NH3(m) ⇌ Glutamate(m) + NAD+(m) + H2O(m)
r0371	-0.5	10-Formyl-THF(c) ⇌ 5,10-Methenyl-THF(c) + H2O(c)
r0507	0.5	Ubiquinol(m) + 2 Ferricytochrome C(m) + 4 H+(PG)(m) → Ubiquinone(m) + 4 H+(PG)(c) + 2 Ferrocytochrome C(m)
r0228	0.5	THF(c) + Formate(c) + ATP(c) → ADP(c) + Pi(c) + 10-Formyl-THF(c)
r0676	0.5	2-Amino-3-carboxymuconate semialdehyde(c) ⇌ 2-Aminomuconate semialdehyde(c) + CO2(c)
r0820	1	AKG(c) + Malate(m) ⇌ AKG(m) + Malate(c)
r1330	2.5	H+(PG)(c) ⇌ H+(PG)(m) + Proton-gradient(m)
r1319	-0.5	ATP(c) + H2O(c) ⇌ ADP(c) + Pi(c) + ATP-energy(c)
r0541	0.5	Glutaryl-CoA(m) + Ubiquinone(m) ⇌ Crotonyl-CoA(m) + CO2(m) + Ubiquinol(m)
r0562	0.5	3-Hydroxyanthranilate(c) + O2(c) ⇌ 2-Amino-3-carboxymuconate semialdehyde(c)
r0563	0.5	H2O(c) + 3-Hydroxy-L-kynurenine(c) ⇌ 3-Hydroxyanthranilate(c) + Alanine(c)
r0867	1.5	CO2(c) ⇌ CO2(s)
r0588	-0.5	Crotonyl-CoA(m) + H2O(m) ⇌ (S)-3-Hydroxybutyryl-CoA(m)
r0454	0.5	L-Formylkynurenine(c) + H2O(c) ⇌ Formate(c) + Kynurenine(c)
r0455	0.5	Kynurenine(c) + O2(c) + NADPH(c) ⇌ H2O(c) + 3-Hydroxy-L-kynurenine(c) + NADP+(c)
r0080	0.5	Alanine(c) + AKG(c) ⇌ Pyruvate(c) + Glutamate(c)
r0645	0.5	2-Aminomuconate semialdehyde(c) + NAD+(c) + H2O(c) ⇌ 2-Aminomuconate(c) + NADH(c)
r2676	0.5	5-Methyl-THF(c) ⇌ THF(c) + THF-activated methyl group(c)

Continued on next page

ID	flux	reaction
r2678	1	Acetyl-CoA(m) ⇌ CoA(m) + CoA-activated acetyl group(m)
r0945	0.5	2-Oxoadipate(c) + AKG(m) ⇌ 2-Oxoadipate(m) + AKG(c)
r1322	1.5	NADH(c) ⇌ NAD+(c) + NADH-redox-potential(c)
r1323	0.5	NADH(m) ⇌ NAD+(m) + NADH-redox-potential(m)
r1326	-2	NADPH(c) ⇌ NADPH-redox-potential(c) + NADP+(c)
r1327	-1	NADPH(m) ⇌ NADPH-redox-potential(m) + NADP+(m)
r0104	1	NAD+(c) + Malate(c) ⇌ OAA(c) + NADH(c)
r0838	0.5	NH3(c) ⇌ NH3(m)
r0851	0.5	H2O(m) ⇌ H2O(c)
r0109	-1	OAA(c) + Glutamate(c) ⇌ AKG(c) + Aspartate(c)

beta-Alanine from Tyrosine (293)

Definition			Solution												
			exchanges		reactions								transp		
Objective	Constraints	Comment	imports	exports	c	m	r	p	l	n	s	b	s-c	b-c	int
beta-Alanine	−Tyrosine MCES +Urea	Transamination to beta-Alanine using Tyrosine	1 H_2O(s) 2 O_2(s) 1 Tyrosine(s) 2 ATP-energy(c)	2 CO_2(s) 1 beta-Alanine(s) 1 NADH-redox-potential(c) 1 Na-gradient(c) 2 CoA-activated acetyl group(c)	16	-	-	-	-	-	-	-	6	-	-

ID	flux	reaction
r0109	-1	OAA(c) + Glutamate(c) ⇌ AKG(c) + Aspartate(c)
r0605	1	4-Maleylacetoacetate(c) ⇌ Fumarylacetoacetate(c)
r0128	1	Aspartate(c) ⇌ beta-Alanine(c) + CO2(c)
r0543	1	Homogentisate(c) + O2(c) ⇌ 4-Maleylacetoacetate(c)
r0183	1	Tyrosine(c) + AKG(c) ⇌ 4-Hydroxyphenylpyruvate(c) + Glutamate(c)
r0899	1	beta-Alanine(c) ⇌ beta-Alanine(s)
r0544	1	4-Hydroxyphenylpyruvate(c) + O2(c) ⇌ Homogentisate(c) + CO2(c)
r1319	-2	ATP(c) + H2O(c) ⇌ ADP(c) + Pi(c) + ATP-energy(c)
r0258	-1	Fumarate(c) + H2O(c) ⇌ Malate(c)
r0069	-1	CoA(c) + Acetoacetyl-CoA(c) ⇌ 2 Acetyl-CoA(c)
r0030	1	ATP(c) + AMP(c) ⇌ 2 ADP(c)
r2664	1	Na+(c) ⇌ Na+(s) + Na-gradient(c)
r0855	2	O2(s) ⇌ O2(c)
r0320	1	ATP(c) + CoA(c) + Acetoacetate(c) → AMP(c) + PPi(c) + Acetoacetyl-CoA(c)
r0104	1	NAD+(c) + Malate(c) ⇌ OAA(c) + NADH(c)
r0007	1	PPi(c) + H2O(c) → 2 Pi(c)
r1322	1	NADH(c) ⇌ NAD+(c) + NADH-redox-potential(c)
r0867	2	CO2(c) ⇌ CO2(s)
r0849	1	H2O(s) ⇌ H2O(c)
r2677	2	Acetyl-CoA(c) ⇌ CoA(c) + CoA-activated acetyl group(c)
r1125	1	Tyrosine(s) + Na+(s) → Tyrosine(c) + Na+(c)
r0324	-1	Fumarylacetoacetate(c) + H2O(c) ⇌ Fumarate(c) + Acetoacetate(c)

beta-Alanine from Valine (294)

Definition			Solution												
			exchanges		reactions								transp		
Objective	Constraints	Comment	imports	exports	c	m	r	p	l	n	s	b	s-c	b-c	int
beta-Alanine	−Valine MCES +Urea	Transamination to beta-Alanine using Valine	2 H_2O(s) 1 O_2(s) 1 Valine(s)	2 CO_2(s) 1 beta-Alanine(s) 4 NADH-redox-potential(m) 11 Proton-gradient(m)	-	13	-	-	-	-	-	-	5	-	8

ID	flux	reaction
r0779	1	3-Hydroxyisobutyryl-CoA(m) + H2O(m) → 3-Hydroxyisobutyrate(m) + CoA(m)
r0217	-1	Glutamate(m) + 3-Oxopropanoate(m) ⇌ beta-Alanine(m) + AKG(m)
r0560	1	Isobutyryl-CoA(m) + Ubiquinone(m) → Methacrylyl-CoA(m) + Ubiquinol(m)
r0263	1	Valine(m) + AKG(m) → Glutamate(m) + 3-Methyl-2-oxobutyrate(m)
r0507	2	Ubiquinol(m) + 2 Ferricytochrome C(m) + 4 H+(PG)(m) → Ubiquinone(m) + 4 H+(PG)(c) + 2 Ferrocytochrome C(m)
r2533	1	Valine(s) ⇌ Valine(c)
r0899	1	beta-Alanine(c) ⇌ beta-Alanine(s)
r0923	1	Valine(c) + H+(PG)(c) → Valine(m) + H+(PG)(m)
r0851	-2	H2O(m) ⇌ H2O(c)
r0482	1	3-Hydroxyisobutyrate(m) + NAD+(m) → 2-Methyl-3-oxopropanoate(m) + NADH(m)
r0365	1	Hydracrylate(m) + NAD+(m) → NADH(m) + 3-Oxopropanoate(m)
r0592	-1	H2O(m) + Acrylyl-CoA(m) → 3-Hydroxypropionyl-CoA(m)
r0683	1	Propanoyl-CoA(m) + Ubiquinone(m) → Ubiquinol(m) + Acrylyl-CoA(m)
r0855	1	O2(s) ⇌ O2(c)
r0856	-1	O2(m) ⇌ O2(c)
r0600	1	3-Methyl-2-oxobutyrate(m) + CoA(m) + NAD+(m) ⇌ Isobutyryl-CoA(m) + CO2(m) + NADH(m)
r1330	11	H+(PG)(c) ⇌ H+(PG)(m) + Proton-gradient(m)
r0596	-1	Hydracrylate(m) + CoA(m) ← 3-Hydroxypropionyl-CoA(m) + H2O(m)
r0223	1	2-Methyl-3-oxopropanoate(m) + CoA(m) + NAD+(m) → Propanoyl-CoA(m) + CO2(m) + NADH(m)
r0867	2	CO2(c) ⇌ CO2(s)
r0849	2	H2O(s) ⇌ H2O(c)
r0669	1	Methacrylyl-CoA(m) + H2O(m) → 3-Hydroxyisobutyryl-CoA(m)
r0017	1	O2(m) + 4 H+(PG)(m) + 4 Ferrocytochrome C(m) → 4 Ferricytochrome C(m) + 2 H2O(m) + 4 H+(PG)(c)
r1323	4	NADH(m) ⇌ NAD+(m) + NADH-redox-potential(m)
r0900	-1	beta-Alanine(c) ⇌ beta-Alanine(m)
r0868	-2	CO2(c) ⇌ CO2(m)

Homocysteine from Methionine (295)

Definition			Solution												
			exchanges		reactions								transp		
Objective	Constraints	Comment	imports	exports	c	m	r	p	l	n	s	b	s-c	b-c	int
Homocysteine(c)	−Methionine(c) MCES	test that cytosolic Homocysteine can be produced from Methionine	1 Methionine(c)	1 Homocysteine(c) 1 THF-activated methyl group(c)	2	-	-	-	-	-	-	-	-	-	-

ID	flux	reaction
r0232	-1	THF(c) + Methionine(c) ⇌ 5-Methyl-THF(c) + Homocysteine(c)
r2676	1	5-Methyl-THF(c) ⇌ THF(c) + THF-activated methyl group(c)

Taurine from Methionine (296)

Definition			Solution												
			exchanges		reactions								transp		
Objective	Constraints	Comment	imports	exports	c	m	r	p	l	n	s	b	s-c	b-c	int
Taurine	−Methionine MCES	Conversion of methionine in taurine	2.5 H_2O(s) 1.5 O_2(s) 1 Methionine(s) 0.5 Glucose-6P(c)	0.5 P_i(c) 3 CO_2(s) 1 Taurine(s) 0.5 ATP-energy(c) 3 NADH-redox-potential(c) 2 NADH-redox-potential(m) 3 Proton-gradient(m) 1 Na-gradient(c) 1 THF-activated methyl group(c) 1 CoA-activated acetyl group(m)	18	9	-	-	-	-	-	-	6	-	11

ID	flux	reaction
r0855	1.5	O2(s) ⇌ O2(c)
r0856	-0.5	O2(m) ⇌ O2(c)
r0592	-1	H2O(m) + Acrylyl-CoA(m) → 3-Hydroxypropionyl-CoA(m)
r0596	-1	3-Hydroxypropionyl-CoA(m) + H2O(m) → Hydracrylate(m) + CoA(m)
r0017	0.5	O2(m) + 4 H+(PG)(m) + 4 Ferrocytochrome C(m) → 4 Ferricytochrome C(m) + 2 H2O(m) + 4 H+(PG)(c)
r0314	1	Serine(c) + Homocysteine(c) ⇌ H2O(c) + L-Cystathionine(c)
r0911	1	Proline(c) + Glutamate(m) ⇌ Proline(m) + Glutamate(c)
r1141	-1	Methionine(s) + Na+(s) → Methionine(c) + Na+(c)
r0337	-1	ADP(c) + 1,3DPG(c) ⇌ ATP(c) + 3PG(c)
r0338	1	3PG(c) + NAD+(c) → 3-Phosphonooxypyruvate(c) + NADH(c)
r2664	1	Na+(c) ⇌ Na+(s) + Na-gradient(c)
r1426	1	Proline(m) + H+(PG)(c) → Proline(c) + H+(PG)(m)
r0073	-1	AKG(m) + NADH(m) + NH3(m) ⇌ Glutamate(m) + NAD+(m) + H2O(m)
r2687	1	Taurine(c) ⇌ Taurine(s)
r0507	1	Ubiquinol(m) + 2 Ferricytochrome C(m) + 4 H+(PG)(m) → Ubiquinone(m) + 4 H+(PG)(c) + 2 Ferrocytochrome C(m)
r0241	1	H2O(c) + L-Cystathionine(c) ⇌ Cysteine(c) + NH3(c) + 2-Oxobutyrate(c)
r0243	-0.5	DHAP(c) ⇌ GAP(c)
r1330	3	H+(PG)(c) ⇌ H+(PG)(m) + Proton-gradient(m)
r1319	0.5	ATP(c) + H2O(c) ⇌ ADP(c) + Pi(c) + ATP-energy(c)
r0849	2.5	H2O(s) ⇌ H2O(c)
r0867	3	CO2(c) ⇌ CO2(s)
r0868	-2	CO2(c) ⇌ CO2(m)
r0736	0.5	ATP(c) + Fructose-6P(c) → ADP(c) + Fructose-1,6PP(c)
r0159	1	3-Phosphoserine(c) + H2O(c) → Serine(c) + Pi(c)
r0174	1	CoA(m) + NAD+(m) + 3-Oxopropanoate(m) → Acetyl-CoA(m) + CO2(m) + NADH(m)
r0192	0.5	Glucose-6P(c) ⇌ Fructose-6P(c)
r0211	1	Cysteine(c) + O2(c) ⇌ 3-Sulfinoalanine(c)
r1154	1	CoA(m) + NAD+(m) + 2-Oxobutyrate(m) ⇌ CO2(m) + Propanoyl-CoA(m) + NADH(m)
r1155	1	2-Oxobutyrate(c) + H+(PG)(c) → 2-Oxobutyrate(m) + H+(PG)(m)
r2676	1	5-Methyl-THF(c) ⇌ THF(c) + THF-activated methyl group(c)
r0365	1	Hydracrylate(m) + NAD+(m) → NADH(m) + 3-Oxopropanoate(m)
r2678	1	Acetyl-CoA(m) ⇌ CoA(m) + CoA-activated acetyl group(m)
r0663	-1	3-Phosphonooxypyruvate(c) + Glutamate(c) → 3-Phosphoserine(c) + AKG(c)
r0381	1	NAD+(c) + H2O(c) + Hypotaurine(c) ⇌ NADH(c) + Taurine(c)
r0232	-1	THF(c) + Methionine(c) ⇌ 5-Methyl-THF(c) + Homocysteine(c)
r1322	3	NADH(c) ⇌ NAD+(c) + NADH-redox-potential(c)
r0683	1	Propanoyl-CoA(m) + Ubiquinone(m) → Ubiquinol(m) + Acrylyl-CoA(m)
r1323	2	NADH(m) ⇌ NAD+(m) + NADH-redox-potential(m)
r0253	1	GAP(c) + Pi(c) + NAD+(c) ⇌ 1,3DPG(c) + NADH(c)
r0538	1	3-Sulfinoalanine(c) ⇌ CO2(c) + Hypotaurine(c)
r0256	0.5	Fructose-1,6PP(c) ⇌ DHAP(c) + GAP(c)
r1047	1	Pi(c) + H+(PG)(c) → Pi(m) + H+(PG)(m)
r2419	1	AKG(c) + Pi(m) ⇌ AKG(m) + Pi(c)
r0838	1	NH3(c) ⇌ NH3(m)

Taurine from Cysteine (297)

Definition			Solution												
			exchanges		reactions								transp		
Objective	Constraints	Comment	imports	exports	c	m	r	p	l	n	s	b	s-c	b-c	int
Taurine	−Cysteine MCES	Conversion of methionine in taurine	1 H_2O(s) 1 O_2(s) 1 Cysteine(s)	1 CO_2(s) 1 Taurine(s) 1 NADH-redox-potential(c)	4	-	-	-	-	-	-	-	5	-	-

ID	flux	reaction
r2687	1	Taurine(c) ⇌ Taurine(s)

Continued on next page

ID	flux	reaction
r0381	1	NAD+(c) + H2O(c) + Hypotaurine(c) ⇌ NADH(c) + Taurine(c)
r0538	1	3-Sulfinoalanine(c) ⇌ CO2(c) + Hypotaurine(c)
r0211	1	Cysteine(c) + O2(c) ⇌ 3-Sulfinoalanine(c)
r2529	1	Cysteine(s) ⇌ Cysteine(c)
r0855	1	O2(s) ⇌ O2(c)
r1322	1	NADH(c) ⇌ NAD+(c) + NADH-redox-potential(c)
r0867	1	CO2(c) ⇌ CO2(s)
r0849	1	H2O(s) ⇌ H2O(c)

B2.3. Fatty acid conversion

B2.3.1. Saturated Stearate from Palmitate (298)

Definition			Solution												
			exchanges		reactions								transp		
Objective	Constraints	Comment	imports	exports	c	m	r	p	l	n	s	b	s-c	b-c	int
Stearate	−Palmitate MCES	Conversion of Palmitate into Stearate	1 Palmitate(s) 3 ATP-energy(c) 1 NADH-redox-potential(m) 1 FADH-redox-potential(c) 1 CoA-activated acetyl group(m)	1 H_2O(s) 1 Stearate(s)	8	6	-	-	-	-	-	-	3	-	4

ID	flux	reaction
r2678	-1	Acetyl-CoA(m) ⇌ CoA(m) + CoA-activated acetyl group(m)
r2689	1	Stearoyl-CoA(c) + H2O(c) → Stearate(c) + CoA(c)
r1290	-1	FAD(c) + FADH2(m) ⇌ FAD(m) + FADH2(c)
r1517	1	Stearate(c) + ATP(c) + H2O(c) → Stearate(s) + ADP(c) + Pi(c)
r1481	-1	Acetyl-CoA(m) + palmitoleoyl-CoA(m) ⇌ CoA(m) + 3-oxooleoyl-CoA(m)
r1472	-1	FADH2(m) + trans,cis-octadeca-2,9-dienoyl-CoA(m) ⇌ FAD(m) + Oleoyl-CoA(m)
r1319	-3	ATP(c) + H2O(c) ⇌ ADP(c) + Pi(c) + ATP-energy(c)
r0851	1	H2O(m) ⇌ H2O(c)
r1329	-1	FADH2(c) ⇌ FAD(c) + FADH-redox-potential(c)
r1474	-1	(S)-3-hydroxyoleyleoyl-CoA(m) ⇌ H2O(m) + trans,cis-octadeca-2,9-dienoyl-CoA(m)
r0030	1	ATP(c) + AMP(c) ⇌ 2 ADP(c)
r0935	1	Palmitate(s) → Palmitate(c)
r1467	-1	Oleoyl-CoA(c) ⇌ Oleoyl-CoA(m)
r1477	-1	NADH(m) + 3-oxooleoyl-CoA(m) ⇌ NAD+(m) + (S)-3-hydroxyoleyleoyl-CoA(m)
r1468	1	palmitoleoyl-CoA(c) ⇌ palmitoleoyl-CoA(m)
r0510	1	Palmitoyl-CoA(c) + O2(c) + 2 Ferrocytochrome b5(c) ⇌ palmitoleoyl-CoA(c) + 2 Ferricytochrome b5(c) + 2 H2O(c)
r0312	1	Palmitate(c) + ATP(c) + CoA(c) → Palmitoyl-CoA(c) + AMP(c) + PPi(c)
r0511	-1	Stearoyl-CoA(c) + O2(c) + 2 Ferrocytochrome b5(c) ⇌ Oleoyl-CoA(c) + 2 Ferricytochrome b5(c) + 2 H2O(c)
r0007	1	PPi(c) + H2O(c) → 2 Pi(c)
r0849	-1	H2O(s) ⇌ H2O(c)
r1323	-1	NADH(m) ⇌ NAD+(m) + NADH-redox-potential(m)

B2.3.2. Omega-3-Fatty acids Palmitate from Palmitolate (299)

Definition			Solution												
			exchanges		reactions								transp		
Objective	Constraints	Comment	imports	exports	c	m	r	p	l	n	s	b	s-c	b-c	int
Palmitate	−Palmitolate MCES	Conversion of Palmitolate into Palmitate	1 Palmitolate(s) 4 ATP-energy(c) 2 NADPH-redox-potential(c)	1 Palmitate(s) 1 NADH-redox-potential(c)	17	-	-	-	-	-	-	-	2	-	-

ID	flux	reaction
r1414	1	Dihydroceramide-pool(c) + O2(c) + NADPH(c) ⇌ 2 H2O(c) + Ceramide-pool(c) + NADP+(c)
r2442	1	Palmitolate(s) → Palmitolate(c)
r1515	1	Palmitate(c) + ATP(c) + H2O(c) → Palmitate(s) + ADP(c) + Pi(c)
r1326	-2	NADPH(c) ⇌ NADPH-redox-potential(c) + NADP+(c)
r1319	-4	ATP(c) + H2O(c) ⇌ ADP(c) + Pi(c) + ATP-energy(c)
r1239	-1	Sphingosine(c) + Acyl-CoA-VLDL-SM-pool(c) ← CoA(c) + Ceramide-pool(c)
r0537	-1	Sphinganine-1P(c) ← Ethanolamine-P(c) + Hexadecanal(c)
r0447	1	Sphingosine(c) + ATP(c) → Sphingosine-1P(c) + ADP(c)
r0030	1	ATP(c) + AMP(c) ⇌ 2 ADP(c)
r1457	-1	Hexadecanal(c) + NADP+(c) ← Hexadecenal(c) + NADPH(c)
r0023	-1	NAD+(c) + 2 Ferrocytochrome b5(c) ⇌ NADH(c) + 2 Ferricytochrome b5(c)
r0510	-1	palmitoleoyl-CoA(c) + 2 Ferricytochrome b5(c) + 2 H2O(c) ⇌ Palmitoyl-CoA(c) + O2(c) + 2 Ferrocytochrome b5(c)
r0007	1	PPi(c) + H2O(c) → 2 Pi(c)
r1488	1	ATP(c) + CoA(c) + Palmitolate(c) ⇌ AMP(c) + palmitoleoyl-CoA(c) + PPi(c)
r1412	1	Sphinganine(c) + Acyl-CoA-VLDL-SM-pool(c) ⇌ Dihydroceramide-pool(c) + CoA(c)
r1322	1	NADH(c) ⇌ NAD+(c) + NADH-redox-potential(c)
r0786	1	Sphingosine-1P(c) → Ethanolamine-P(c) + Hexadecenal(c)
r2695	1	Palmitoyl-CoA(c) + H2O(c) → Palmitate(c) + CoA(c)
r0787	1	H2O(c) + Sphinganine-1P(c) → Sphinganine(c) + Pi(c)

Palmitolate from Palmitate (300)

Definition			Solution												
			exchanges		reactions								transp		
Objective	Constraints	Comment	imports	exports	c	m	r	p	l	n	s	b	s-c	b-c	int
Palmitolate	−Palmitate MCES	Conversion of Palmitate into Palmitolate	1 O_2(s) 1 Palmitate(s) 1 ATP-energy(c) 1 NADH-redox-potential(c)	2 H_2O(s) 1 Palmitolate(s)	6	-	-	-	-	-	-	-	4	-	-

ID	flux	reaction
r1523	1	Palmitolate(c) + ATP(c) + H2O(c) → Palmitolate(s) + ADP(c) + Pi(c)
r1319	-1	ATP(c) + H2O(c) ⇌ ADP(c) + Pi(c) + ATP-energy(c)
r0935	1	Palmitate(s) → Palmitate(c)
r0855	1	O2(s) ⇌ O2(c)
r0023	1	NADH(c) + 2 Ferricytochrome b5(c) ⇌ NAD+(c) + 2 Ferrocytochrome b5(c)
r0510	1	Palmitoyl-CoA(c) + O2(c) + 2 Ferrocytochrome b5(c) ⇌ palmitoleoyl-CoA(c) + 2 Ferricytochrome b5(c) + 2 H2O(c)
r0312	1	Palmitate(c) + ATP(c) + CoA(c) → Palmitoyl-CoA(c) + AMP(c) + PPi(c)
r1488	-1	AMP(c) + palmitoleoyl-CoA(c) + PPi(c) ⇌ ATP(c) + CoA(c) + Palmitolate(c)
r1322	-1	NADH(c) ⇌ NAD+(c) + NADH-redox-potential(c)
r0849	-2	H2O(s) ⇌ H2O(c)

Oleate from Stearate (301)

Definition			Solution												
			exchanges		reactions								transp		
Objective	Constraints	Comment	imports	exports	c	m	r	p	l	n	s	b	s-c	b-c	int
Oleate	−Stearate MCES	Conversion of Stearate into Oleate	1 O_2(s) 1 Stearate(s) 3 ATP-energy(c) 1 NADH-redox-potential(c)	2 H_2O(s) 1 Oleate(s)	7	-	-	-	-	-	-	-	4	-	-

ID	flux	reaction
r1254	1	Stearate(c) + ATP(c) + CoA(c) → Stearoyl-CoA(c) + AMP(c) + PPi(c)
r1516	1	Oleate(c) + ATP(c) + H2O(c) → Oleate(s) + ADP(c) + Pi(c)
r1319	-3	ATP(c) + H2O(c) ⇌ ADP(c) + Pi(c) + ATP-energy(c)

Continued on next page

Oleate from Stearate (301)– continued

ID	flux	reaction
r1465	-1	NADH(c) + O2(c) + Stearoyl-CoA(c) ⇌ 2 H2O(c) + NAD+(c) + Oleoyl-CoA(c)
r0030	1	ATP(c) + AMP(c) ⇌ 2 ADP(c)
r2690	1	Oleoyl-CoA(c) + H2O(c) → Oleate(c) + CoA(c)
r0855	1	O2(s) ⇌ O2(c)
r0982	1	Stearate(s) → Stearate(c)
r0007	1	PPi(c) + H2O(c) → 2 Pi(c)
r1322	-1	NADH(c) ⇌ NAD+(c) + NADH-redox-potential(c)
r0849	-2	H2O(s) ⇌ H2O(c)

Stearate from Oleate (302)

Definition			Solution												
			exchanges		reactions								transp		
Objective	Constraints	Comment	imports	exports	c	m	r	p	l	n	s	b	s-c	b-c	int
Stearate	−Oleate MCES	Conversion of into Stearate	1 Oleate(s) 4 ATP-energy(c) 2 NADPH-redox-potential(c)	1 Stearate(s) 1 NADH-redox-potential(c)	16	-	-	-	-	-	-	-	2	-	-

ID	flux	reaction
r1414	1	Dihydroceramide-pool(c) + O2(c) + NADPH(c) ⇌ 2 H2O(c) + Ceramide-pool(c) + NADP+(c)
r2689	1	Stearoyl-CoA(c) + H2O(c) → Stearate(c) + CoA(c)
r1326	-2	NADPH(c) ⇌ NADPH-redox-potential(c) + NADP+(c)
r1517	1	Stearate(c) + ATP(c) + H2O(c) → Stearate(s) + ADP(c) + Pi(c)
r1319	-4	ATP(c) + H2O(c) ⇌ ADP(c) + Pi(c) + ATP-energy(c)
r1256	1	ATP(c) + CoA(c) + Oleate(c) → Oleoyl-CoA(c) + AMP(c) + PPi(c)
r1239	-1	Sphingosine(c) + Acyl-CoA-VLDL-SM-pool(c) ← CoA(c) + Ceramide-pool(c)
r0537	-1	Sphinganine-1P(c) ← Ethanolamine-P(c) + Hexadecanal(c)
r0447	1	Sphingosine(c) + ATP(c) → Sphingosine-1P(c) + ADP(c)
r1465	1	2 H2O(c) + NAD+(c) + Oleoyl-CoA(c) ⇌ NADH(c) + O2(c) + Stearoyl-CoA(c)
r0030	1	ATP(c) + AMP(c) ⇌ 2 ADP(c)
r1457	-1	Hexadecanal(c) + NADP+(c) ← Hexadecenal(c) + NADPH(c)
r1300	1	Oleate(s) → Oleate(c)
r0007	1	PPi(c) + H2O(c) → 2 Pi(c)
r1412	1	Sphinganine(c) + Acyl-CoA-VLDL-SM-pool(c) ⇌ Dihydroceramide-pool(c) + CoA(c)
r1322	1	NADH(c) ⇌ NAD+(c) + NADH-redox-potential(c)
r0786	1	Sphingosine-1P(c) → Ethanolamine-P(c) + Hexadecenal(c)
r0787	1	H2O(c) + Sphinganine-1P(c) → Sphinganine(c) + Pi(c)

Palmitolate from Arachidonate (303)

Definition			Solution												
			exchanges		reactions								transp		
Objective	Constraints	Comment	imports	exports	c	m	r	p	l	n	s	b	s-c	b-c	int
Palmitolate	−Arachidonate MCES	Conversion of from Arachidonate	1 O_2(s) 8 ATP-energy(c) 1 NADH-redox-potential(c) 14 NADPH-redox-potential(c) 8 CoA-activated acetyl group(c)	8 H_2O(s) 1 Palmitolate(s)	42	-	-	-	-	-	-	-	3	-	-

ID	flux	reaction
r0701	-1	HMA(c) + NADP+(c) ⇌ 3-Oxotetradecanoyl-ACP(c) + NADPH(c)
r0702	1	HMA(c) ⇌ H2O(c) + (2E)-Tetradecenoyl-ACP(c)
r0855	1	O2(s) ⇌ O2(c)
r0312	1	Palmitate(c) + ATP(c) + CoA(c) → Palmitoyl-CoA(c) + AMP(c) + PPi(c)
r0760	1	Butyryl-ACP(c) + Malonyl-ACP(c) ⇌ CO2(c) + ACP(c) + 3-Oxohexanoyl-ACP(c)

Continued on next page

Palmitolate from Arachidonate (303)– continued

ID	flux	reaction
r0761	-1	D-3-Hydroxyhexanoyl-ACP(c) + NADP+(c) ⇌ NADPH(c) + 3-Oxohexanoyl-ACP(c)
r0184	7	ATP(c) + Acetyl-CoA(c) + HCO3-(c) → ADP(c) + Pi(c) + Malonyl-CoA(c)
r0762	1	D-3-Hydroxyhexanoyl-ACP(c) ⇌ H2O(c) + (2E)-Hexenoyl-ACP(c)
r0763	-1	Hexanoyl-ACP(c) + NADP+(c) ⇌ NADPH(c) + (2E)-Hexenoyl-ACP(c)
r0764	1	Malonyl-ACP(c) + Hexanoyl-ACP(c) ⇌ 3-Oxooctanoyl-ACP(c) + CO2(c) + ACP(c)
r0765	-1	Octanoyl-ACP(c) + NADP+(c) ⇌ (2E)-Octenoyl-ACP(c) + NADPH(c)
r0766	1	Octanoyl-ACP(c) + Malonyl-ACP(c) ⇌ 3-Oxodecanoyl-ACP(c) + CO2(c) + ACP(c)
r0767	-1	Decanoyl-ACP(c) + NADP+(c) ⇌ (2E)-Decenoyl-ACP(c) + NADPH(c)
r0768	1	Decanoyl-ACP(c) + Malonyl-ACP(c) ⇌ 3-Oxododecanoyl-ACP(c) + CO2(c) + ACP(c)
r0769	-1	D-3-Hydroxydodecanoyl-ACP(c) + NADP+(c) ⇌ 3-Oxododecanoyl-ACP(c) + NADPH(c)
r0678	1	Acetyl-ACP(c) + Malonyl-ACP(c) ⇌ CO2(c) + ACP(c) + Acetoacetyl-ACP(c)
r0691	-1	(R)-3-Hydroxybutanoyl-ACP(c) + NADP+(c) ⇌ NADPH(c) + Acetoacetyl-ACP(c)
r0692	-1	(R)-3-Hydroxydecanoyl-ACP(c) + NADP+(c) ⇌ 3-Oxodecanoyl-ACP(c) + NADPH(c)
r0693	1	(R)-3-Hydroxydecanoyl-ACP(c) ⇌ (2E)-Decenoyl-ACP(c) + H2O(c)
r1319	-8	ATP(c) + H2O(c) ⇌ ADP(c) + Pi(c) + ATP-energy(c)
r0694	-1	(R)-3-Hydroxyoctanoyl-ACP(c) + NADP+(c) ⇌ 3-Oxooctanoyl-ACP(c) + NADPH(c)
r0695	1	(R)-3-Hydroxyoctanoyl-ACP(c) ⇌ (2E)-Octenoyl-ACP(c) + H2O(c)
r0696	-1	(R)-3-Hydroxypalmitoyl-ACP(c) + NADP+(c) ⇌ 3-Oxohexadecanoyl-ACP(c) + NADPH(c)
r1488	-1	AMP(c) + palmitoleoyl-CoA(c) + PPi(c) ⇌ ATP(c) + CoA(c) + Palmitolate(c)
r0697	1	(R)-3-Hydroxypalmitoyl-ACP(c) ⇌ (2E)-Hexadecenoyl-ACP(c) + H2O(c)
r0849	-8	H2O(s) ⇌ H2O(c)
r0712	-1	Dodecanoyl-ACP(c) + NADP+(c) ⇌ (2E)-Dodecenoyl-ACP(c) + NADPH(c)
r0713	1	Dodecanoyl-ACP(c) + Malonyl-ACP(c) ⇌ 3-Oxotetradecanoyl-ACP(c) + CO2(c) + ACP(c)
r1523	1	Palmitolate(c) + ATP(c) + H2O(c) → Palmitolate(s) + ADP(c) + Pi(c)
r0023	1	NADH(c) + 2 Ferricytochrome b5(c) ⇌ NAD+(c) + 2 Ferrocytochrome b5(c)
r0770	1	D-3-Hydroxydodecanoyl-ACP(c) ⇌ (2E)-Dodecenoyl-ACP(c) + H2O(c)
r0771	-1	Tetradecanoyl-ACP(c) + NADP+(c) ⇌ NADPH(c) + (2E)-Tetradecenoyl-ACP(c)
r0772	1	Tetradecanoyl-ACP(c) + Malonyl-ACP(c) ⇌ 3-Oxohexadecanoyl-ACP(c) + CO2(c) + ACP(c)
r0773	-1	Hexadecanoyl-ACP(c) + NADP+(c) ⇌ (2E)-Hexadecenoyl-ACP(c) + NADPH(c)
r1417	-7	CO2(c) + H2O(c) ⇌ Carbonate(c)
r1419	7	Carbonate(c) ⇌ HCO3-(c)
r2677	-8	Acetyl-CoA(c) ⇌ CoA(c) + CoA-activated acetyl group(c)
r0510	1	Palmitoyl-CoA(c) + O2(c) + 2 Ferrocytochrome b5(c) ⇌ palmitoleoyl-CoA(c) + 2 Ferricytochrome b5(c) + 2 H2O(c)
r0367	1	Acetyl-CoA(c) + ACP(c) ⇌ CoA(c) + Acetyl-ACP(c)
r0369	7	ACP(c) + Malonyl-CoA(c) ⇌ CoA(c) + Malonyl-ACP(c)
r0681	1	(R)-3-Hydroxybutanoyl-ACP(c) ⇌ But-2-enoyl-ACP(c) + H2O(c)
r0387	1	Hexadecanoyl-ACP(c) + H2O(c) ⇌ ACP(c) + Palmitate(c)
r0682	-1	Butyryl-ACP(c) + NADP+(c) ⇌ But-2-enoyl-ACP(c) + NADPH(c)
r1322	-1	NADH(c) ⇌ NAD+(c) + NADH-redox-potential(c)
r1326	-14	NADPH(c) ⇌ NADPH-redox-potential(c) + NADP+(c)

B2.3.3. Omega-6-Fatty acids gamma-Linolenate from Linoleate (304)

Definition			Solution												
			exchanges		reactions								transp		
Objective	Constraints	Comment	imports	exports	c	m	r	p	l	n	s	b	s-c	b-c	int
gamma-Linolenate	−Linoleate MCES	Conversion of Linoleate into gamma-Linolenate	1 O_2(s) 1 Linoleate(s) 3 ATP-energy(c) 1 NADH-redox-potential(c)	1 H_2O(s) 1 gamma-Linolenate(s)	7	-	-	-	-	-	-	-	4	-	-

ID	flux	reaction
r1319	-3	ATP(c) + H2O(c) ⇌ ADP(c) + Pi(c) + ATP-energy(c)
r1258	1	Linoleate(c) + ATP(c) + CoA(c) → AMP(c) + PPi(c) + Linoleoyl-CoA(c)
r0030	1	ATP(c) + AMP(c) ⇌ 2 ADP(c)

Continued on next page

ID	flux	reaction
r0855	1	O2(s) ⇌ O2(c)
r2693	1	gamma-Linolenoyl-CoA(c) + H2O(c) → gamma-Linolenate(c) + CoA(c)
r1469	1	Linoleoyl-CoA(c) + NADH(c) + O2(c) ⇌ gamma-Linolenoyl-CoA(c) + H2O(c) + NAD+(c)
r0007	1	PPi(c) + H2O(c) → 2 Pi(c)
r1520	1	gamma-Linolenate(c) + ATP(c) + H2O(c) → gamma-Linolenate(s) + ADP(c) + Pi(c)
r1322	-1	NADH(c) ⇌ NAD+(c) + NADH-redox-potential(c)
r0849	-1	H2O(s) ⇌ H2O(c)
r0985	1	Linoleate(s) → Linoleate(c)

Dihomo-gamma-linolenate from Linoleate (305)

Definition			Solution													
			exchanges		reactions								transp			
Objective	Constraints	Comment	imports	exports	c	m	r	p	l	n	s	b	s-c	b-c	int
Dihomo-gamma-linolenate	−Linoleate MCES	Conversion of Linoleate into Dihomo-gamma-linolenate	2 O_2(s) 1 Linoleate(s) 4 ATP-energy(c) 1 NADH-redox-potential(c) 4 NADPH-redox-potential(r) 1 CoA-activated acetyl group(c)	4 H_2O(s) 1 Dihomo-gamma-linolenate(s)	12	-	3	-	-	-	-	-	4	-	6

ID	flux	reaction
r1325	-4	NADPH(r) ⇌ NADPH-redox-potential(r) + NADP+(r)
r1525	1	Dihomo-gamma-linolenate(c) + ATP(c) + H2O(c) → Dihomo-gamma-linolenate(s) + ADP(c) + Pi(c)
r1417	-1	CO2(c) + H2O(c) ⇌ Carbonate(c)
r0184	1	ATP(c) + Acetyl-CoA(c) + HCO3-(c) → ADP(c) + Pi(c) + Malonyl-CoA(c)
r1319	-4	ATP(c) + H2O(c) ⇌ ADP(c) + Pi(c) + ATP-energy(c)
r1419	1	Carbonate(c) ⇌ HCO3-(c)
r1258	1	Linoleate(c) + ATP(c) + CoA(c) → AMP(c) + PPi(c) + Linoleoyl-CoA(c)
r0030	1	ATP(c) + AMP(c) ⇌ 2 ADP(c)
r2537	1	gamma-Linolenoyl-CoA(c) ⇌ gamma-Linolenoyl-CoA(r)
r1466	1	Malonyl-CoA(r) + 4 NADPH(r) + O2(r) + gamma-Linolenoyl-CoA(r) ⇌ CO2(r) + CoA(r) + Dihomo-gamma-linolenoyl-CoA(r) + 3 H2O(r) + 4 NADP+(r)
r0854	-1	O2(r) ⇌ O2(c)
r2538	-1	Dihomo-gamma-linolenoyl-CoA(c) ⇌ Dihomo-gamma-linolenoyl-CoA(r)
r1004	-1	L-Carnitine(c) + Malonyl-Carnitin(r) ⇌ L-Carnitine(r) + Malonyl-Carnitin(c)
r0855	2	O2(s) ⇌ O2(c)
r0865	1	CO2(r) ⇌ CO2(c)
r1469	1	Linoleoyl-CoA(c) + NADH(c) + O2(c) ⇌ gamma-Linolenoyl-CoA(c) + H2O(c) + NAD+(c)
r0848	3	H2O(r) ⇌ H2O(c)
r0430	-1	Malonyl-CoA(r) + L-Carnitine(r) ⇌ CoA(r) + Malonyl-Carnitin(r)
r0007	1	PPi(c) + H2O(c) → 2 Pi(c)
r2694	1	Dihomo-gamma-linolenoyl-CoA(c) + H2O(c) → Dihomo-gamma-linolenate(c) + CoA(c)
r1322	-1	NADH(c) ⇌ NAD+(c) + NADH-redox-potential(c)
r0849	-4	H2O(s) ⇌ H2O(c)
r0431	1	Malonyl-CoA(c) + L-Carnitine(c) ⇌ CoA(c) + Malonyl-Carnitin(c)
r2677	-1	Acetyl-CoA(c) ⇌ CoA(c) + CoA-activated acetyl group(c)
r0985	1	Linoleate(s) → Linoleate(c)

Arachidonate from Linoleate (306)

Definition			Solution												
			exchanges		reactions								transp		
Objective	Constraints	Comment	imports	exports	c	m	r	p	l	n	s	b	s-c	b-c	int
Arachidonate	−Linoleate MCES	Conversion of Linoleate into Arachidonate	3 O_2(s) 1 Linoleate(s) 4 ATP-energy(c) 2 NADH-redox-potential(c) 4 NADPH-redox-potential(r) 1 CoA-activated acetyl group(c)	5 H_2O(s) 1 Arachidonate(s)	13	-	3	-	-	-	-	-	4	-	6

ID	flux	reaction
r2696	1	Arachidonyl-CoA(c) + H2O(c) → Arachidonate(c) + CoA(c)
r1514	1	Arachidonate(c) + ATP(c) + H2O(c) → Arachidonate(s) + ADP(c) + Pi(c)
r1325	-4	NADPH(r) ⇌ NADPH-redox-potential(r) + NADP+(r)
r1470	1	Dihomo-gamma-linolenoyl-CoA(c) + NADH(c) + O2(c) ⇌ Arachidonyl-CoA(c) + H2O(c) + NAD+(c)
r1417	-1	CO2(c) + H2O(c) ⇌ Carbonate(c)
r0184	1	ATP(c) + Acetyl-CoA(c) + HCO3-(c) → ADP(c) + Pi(c) + Malonyl-CoA(c)
r1319	-4	ATP(c) + H2O(c) ⇌ ADP(c) + Pi(c) + ATP-energy(c)
r1419	1	Carbonate(c) ⇌ HCO3-(c)
r1258	1	Linoleate(c) + ATP(c) + CoA(c) → AMP(c) + PPi(c) + Linoleoyl-CoA(c)
r0030	1	ATP(c) + AMP(c) ⇌ 2 ADP(c)
r2537	1	gamma-Linolenoyl-CoA(c) ⇌ gamma-Linolenoyl-CoA(r)
r1466	1	Malonyl-CoA(r) + 4 NADPH(r) + O2(r) + gamma-Linolenoyl-CoA(r) ⇌ CO2(r) + CoA(r) + Dihomo-gamma-linolenoyl-CoA(r) + 3 H2O(r) + 4 NADP+(r)
r0854	-1	O2(r) ⇌ O2(c)
r2538	-1	Dihomo-gamma-linolenoyl-CoA(c) ⇌ Dihomo-gamma-linolenoyl-CoA(r)
r1004	-1	L-Carnitine(c) + Malonyl-Carnitin(r) ⇌ L-Carnitine(r) + Malonyl-Carnitin(c)
r0855	3	O2(s) ⇌ O2(c)
r0865	1	CO2(r) ⇌ CO2(c)
r1469	1	Linoleoyl-CoA(c) + NADH(c) + O2(c) ⇌ gamma-Linolenoyl-CoA(c) + H2O(c) + NAD+(c)
r0848	3	H2O(r) ⇌ H2O(c)
r0430	-1	Malonyl-CoA(r) + L-Carnitine(r) ⇌ CoA(r) + Malonyl-Carnitin(r)
r0007	1	PPi(c) + H2O(c) → 2 Pi(c)
r1322	-2	NADH(c) ⇌ NAD+(c) + NADH-redox-potential(c)
r0849	-5	H2O(s) ⇌ H2O(c)
r0431	1	Malonyl-CoA(c) + L-Carnitine(c) ⇌ CoA(c) + Malonyl-Carnitin(c)
r2677	-1	Acetyl-CoA(c) ⇌ CoA(c) + CoA-activated acetyl group(c)
r0985	1	Linoleate(s) → Linoleate(c)

Dihomo-gamma-linolenate from gamma-Linolenate (307)

Definition			Solution												
			exchanges		reactions								transp		
Objective	Constraints	Comment	imports	exports	c	m	r	p	l	n	s	b	s-c	b-c	int
Dihomo-gamma-linolenate	−gamma-Linolenate MCES	Conversion of gamma-Linolenate into Dihomo-gamma-linolenate	1 O_2(s) 1 gamma-Linolenate(s) 4 ATP-energy(c) 4 NADPH-redox-potential(r) 1 CoA-activated acetyl group(c)	3 H_2O(s) 1 Dihomo-gamma-linolenate(s)	10	-	3	-	-	-	-	-	4	-	6

ID	flux	reaction
r1261	1	gamma-Linolenate(c) + ATP(c) + CoA(c) → gamma-Linolenoyl-CoA(c) + AMP(c) + PPi(c)
r1325	-4	NADPH(r) ⇌ NADPH-redox-potential(r) + NADP+(r)
r1525	1	Dihomo-gamma-linolenate(c) + ATP(c) + H2O(c) → Dihomo-gamma-linolenate(s) + ADP(c) + Pi(c)
r1417	-1	CO2(c) + H2O(c) ⇌ Carbonate(c)
r0184	1	ATP(c) + Acetyl-CoA(c) + HCO3-(c) → ADP(c) + Pi(c) + Malonyl-CoA(c)
r1319	-4	ATP(c) + H2O(c) ⇌ ADP(c) + Pi(c) + ATP-energy(c)

Continued on next page

ID	flux	reaction
r1419	1	Carbonate(c) ⇌ HCO3-(c)
r0030	1	ATP(c) + AMP(c) ⇌ 2 ADP(c)
r2537	1	gamma-Linolenoyl-CoA(c) ⇌ gamma-Linolenoyl-CoA(r)
r1466	1	Malonyl-CoA(r) + 4 NADPH(r) + O2(r) + gamma-Linolenoyl-CoA(r) ⇌ CO2(r) + CoA(r) + Dihomo-gamma-linolenoyl-CoA(r) + 3 H2O(r) + 4 NADP+(r)
r0854	-1	O2(r) ⇌ O2(c)
r2538	-1	Dihomo-gamma-linolenoyl-CoA(c) ⇌ Dihomo-gamma-linolenoyl-CoA(r)
r1004	-1	L-Carnitine(c) + Malonyl-Carnitin(r) ⇌ L-Carnitine(r) + Malonyl-Carnitin(c)
r0855	1	O2(s) ⇌ O2(c)
r1297	1	gamma-Linolenate(s) → gamma-Linolenate(c)
r0865	1	CO2(r) ⇌ CO2(c)
r0848	3	H2O(r) ⇌ H2O(c)
r0430	-1	Malonyl-CoA(r) + L-Carnitine(r) ⇌ CoA(r) + Malonyl-Carnitin(r)
r0007	1	PPi(c) + H2O(c) → 2 Pi(c)
r2694	1	Dihomo-gamma-linolenoyl-CoA(c) + H2O(c) → Dihomo-gamma-linolenate(c) + CoA(c)
r0849	-3	H2O(s) ⇌ H2O(c)
r0431	1	Malonyl-CoA(c) + L-Carnitine(c) ⇌ CoA(c) + Malonyl-Carnitin(c)
r2677	-1	Acetyl-CoA(c) ⇌ CoA(c) + CoA-activated acetyl group(c)

Arachidonate from gamma-Linolenate (308)

Definition			Solution											
			exchanges		reactions							transp		
Objective	Constraints	Comment	imports	exports	c	m	r	p	l	n	s	b	s-c	b-c	int
Arachidonate	−gamma-Linolenate MCES	Conversion of gamma-Linolenate into Arachidonate	2 O_2(s) 1 gamma-Linolenate(s) 4 ATP-energy(c) 1 NADH-redox-potential(c) 4 NADPH-redox-potential(r) 1 CoA-activated acetyl group(c)	4 H_2O(s) 1 Arachidonate(s)	12	-	3	-	-	-	-	-	4	-	6

ID	flux	reaction
r2696	1	Arachidonyl-CoA(c) + H2O(c) → Arachidonate(c) + CoA(c)
r1261	1	gamma-Linolenate(c) + ATP(c) + CoA(c) → gamma-Linolenoyl-CoA(c) + AMP(c) + PPi(c)
r1514	1	Arachidonate(c) + ATP(c) + H2O(c) → Arachidonate(s) + ADP(c) + Pi(c)
r1325	-4	NADPH(r) ⇌ NADPH-redox-potential(r) + NADP+(r)
r1470	1	Dihomo-gamma-linolenoyl-CoA(c) + NADH(c) + O2(c) ⇌ Arachidonyl-CoA(c) + H2O(c) + NAD+(c)
r1417	-1	CO2(c) + H2O(c) ⇌ Carbonate(c)
r0184	1	ATP(c) + Acetyl-CoA(c) + HCO3-(c) → ADP(c) + Pi(c) + Malonyl-CoA(c)
r1319	-4	ATP(c) + H2O(c) ⇌ ADP(c) + Pi(c) + ATP-energy(c)
r1419	1	Carbonate(c) ⇌ HCO3-(c)
r0030	1	ATP(c) + AMP(c) ⇌ 2 ADP(c)
r2537	1	gamma-Linolenoyl-CoA(c) ⇌ gamma-Linolenoyl-CoA(r)
r1466	1	Malonyl-CoA(r) + 4 NADPH(r) + O2(r) + gamma-Linolenoyl-CoA(r) ⇌ CO2(r) + CoA(r) + Dihomo-gamma-linolenoyl-CoA(r) + 3 H2O(r) + 4 NADP+(r)
r0854	-1	O2(r) ⇌ O2(c)
r2538	-1	Dihomo-gamma-linolenoyl-CoA(c) ⇌ Dihomo-gamma-linolenoyl-CoA(r)
r1004	-1	L-Carnitine(c) + Malonyl-Carnitin(r) ⇌ L-Carnitine(r) + Malonyl-Carnitin(c)
r0855	2	O2(s) ⇌ O2(c)
r1297	1	gamma-Linolenate(s) → gamma-Linolenate(c)
r0865	1	CO2(r) ⇌ CO2(c)
r0848	3	H2O(r) ⇌ H2O(c)
r0430	-1	Malonyl-CoA(r) + L-Carnitine(r) ⇌ CoA(r) + Malonyl-Carnitin(r)
r0007	1	PPi(c) + H2O(c) → 2 Pi(c)
r1322	-1	NADH(c) ⇌ NAD+(c) + NADH-redox-potential(c)
r0849	-4	H2O(s) ⇌ H2O(c)
r0431	1	Malonyl-CoA(c) + L-Carnitine(c) ⇌ CoA(c) + Malonyl-Carnitin(c)

Continued on next page

ID	flux	reaction
r2677	-1	Acetyl-CoA(c) ⇌ CoA(c) + CoA-activated acetyl group(c)

Arachidonate from Dihomo-gamma-linolenate (309)

Definition			Solution												
			exchanges		reactions								transp		
Objective	Constraints	Comment	imports	exports	c	m	r	p	l	n	s	b	s-c	b-c	int
Arachidonate	−Dihomo-gamma-linolenate MCES	Conversion of Dihomo-gamma-linolenate into Arachidonate	1 O_2(s) 1 Dihomo-gamma-linolenate(s) 3 ATP-energy(c) 1 NADH-redox-potential(c)	1 H_2O(s) 1 Arachidonate(s)	7	-	-	-	-	-	-	-	4	-	-

ID	flux	reaction
r2696	1	Arachidonyl-CoA(c) + H2O(c) → Arachidonate(c) + CoA(c)
r1514	1	Arachidonate(c) + ATP(c) + H2O(c) → Arachidonate(s) + ADP(c) + Pi(c)
r1470	1	Dihomo-gamma-linolenoyl-CoA(c) + NADH(c) + O2(c) ⇌ Arachidonyl-CoA(c) + H2O(c) + NAD+(c)
r1319	-3	ATP(c) + H2O(c) ⇌ ADP(c) + Pi(c) + ATP-energy(c)
r2707	1	Dihomo-gamma-linolenate(c) + ATP(c) + CoA(c) → Dihomo-gamma-linolenoyl-CoA(c) + AMP(c) + PPi(c)
r1366	1	Dihomo-gamma-linolenate(s) → Dihomo-gamma-linolenate(c)
r0030	1	ATP(c) + AMP(c) ⇌ 2 ADP(c)
r0855	1	O2(s) ⇌ O2(c)
r0007	1	PPi(c) + H2O(c) → 2 Pi(c)
r1322	-1	NADH(c) ⇌ NAD+(c) + NADH-redox-potential(c)
r0849	-1	H2O(s) ⇌ H2O(c)

B2.4. Lipoproteins

VLDL from LDL (310)

Definition			Solution												
			exchanges		reactions								transp		
Objective	Constraints	Comment	imports	exports	c	m	r	p	l	n	s	b	s-c	b-c	int
VLDL	−LDL MCES =Cysteine +Methionine =Leucine =Isoleucine =Histidine =Asparagine =Valine =Tryptophan =Lysine =Phenylalanine =Threonine =Tyrosine =Valine =Ethanolamine(c) =Choline(c) ALFA	Lipoprotein conversion VLDL from LDL	381.4 Pyruvate(m) 99.1 Lysine(s) 56.8 Tryptophan(s) 33.1 Phenylalanine(s) 31.4 Tyrosine(s) 6074 Glucose-6P(c) 695.2 Cysteine(s) 2489 Choline(c) 299.1 Leucine(s) 16.6 Histidine(s) 917.6 Asparagine(s) 173.7 Valine(s) 771.4 Threonine(s) 311.4 Ethanolamine(c) 1277 Arachidonate(c) 11891 Palmitate(c) 20.5 Isoleucine(s) 12595 Oleate(c) 2193 Stearate(c) 8299 Linoleate(c) 0.98 LDL(s) $1.2{\cdot}10^5$ ATP-energy(c) 784.1 ATP-energy(m) 12485 NADH-redox-potential(c) 508.6 NADPH-redox-potential(c) 1332 NADPH-redox-potential(m) 649.3 Proton-gradient(m) 549.9 THF-activated methyl group(c) 709.8 CoA-activated acetyl group(m)	34610 H_2O(s) 3643 P_i(c) 1239 CO_2(s) 107.5 Pyruvate(c) 622.8 Methionine(s) 1 VLDL(s) 1440 NADH-redox-potential(m) 450.8 Proton-gradient(c) 1318 Na-gradient(c)	94	21	17	-	-	-	-	-	18	-	63

ID	flux	reaction
r1362	291.2	Glutamate(l) → Glutamate(c)

Continued on next page

ID	flux	reaction
r1210	0.07	6260 Palmitoyl-CoA(c) + 1490 Stearoyl-CoA(c) + 656 Oleoyl-CoA(c) + 147 Arachidonyl-CoA(c) + 270 Linoleoyl-CoA(c) ⇌ 8823 Acyl-CoA-VLDL-SM-pool(c)
r1080	147.6	Tyrosine(l) → Tyrosine(c)
r1067	112.4	Histidine(l) → Histidine(c)
r1211	-10385	Phosphatidate-VLDL-TG-pool(c) + CoA(c) ← Acyl-CoA-VLDL-TG2-pool(c) + 1-Acylglycerol-3P-VLDL-TG1-pool(c)
r1068	520.9	Leucine(l) → Leucine(c)
r0407	35.8	Sedoheptulose-1,7PP(c) ⇌ DHAP(c) + Erythrose-4P(c)
r1212	-1890	Phosphatidate-VLDL-PC-pool(c) + CoA(c) ← Acyl-CoA-VLDL-PC-pool(c) + 1-Acylglycerol-3P-VLDL-PC-pool(c)
r0421	2489	CTP(c) + Phosphocholine(c) → PPi(c) + CDP-choline(c)
r0408	35.8	ATP(c) + Sedoheptulose-7P(c) ⇌ ADP(c) + Sedoheptulose-1,7PP(c)
r1083	1	VLDL(r) → VLDL(s)
r0422	302	Isocitrate(c) + NADP+(c) ⇌ Oxalosuccinate(c) + NADPH(c)
r0127	905	H2O(c) + Asparagine(c) → Aspartate(c) + NH3(c)
r1214	-155.7	Phosphatidate-VLDL-PS-pool(c) + CoA(c) ← Acyl-CoA-VLDL-PS-pool(c) + 1-Acylglycerol-3P-VLDL-PS-pool(c)
r0859	-2228	ADP(c) + ATP(l) ← ADP(l) + ATP(c)
r0425	515.4	Isocitrate(m) + NAD+(m) ⇌ Oxalosuccinate(m) + NADH(m)
r1217	10385	Phosphatidate-VLDL-TG-pool(c) + H2O(c) → 1,2-Diacylglycerol-VLDL-TG-pool(c) + Pi(c)
r1218	1890	Phosphatidate-VLDL-PC-pool(c) + H2O(c) → 1,2-Diacylglycerol-VLDL-PC-pool(c) + Pi(c)
r0441	-6.92	Arachidonyl-CoA(r) + L-Carnitine(r) ⇌ CoA(r) + Arachidonyl-Carnitine(r)
r0442	6.92	Arachidonyl-CoA(c) + L-Carnitine(c) ⇌ CoA(c) + Arachidonyl-Carnitine(c)
r1235	106.5	1-Acylglycerol-VLDL-PI-pool(c) + H2O(c) → Fatty-acid-VLDL-PI-pool(c) + Glycerol(c)
r1237	1890	CDP-choline(c) + 1,2-Diacylglycerol-VLDL-PC-pool(c) → PC-VLDL-pool(c) + CMP(c)
r1967	466.8	Asparagine(s) + Na+(s) + Lysine(c) → Asparagine(c) + Na+(c) + Lysine(s)
r1238	-106.5	CDP-diacylglycerol-VLDL-PI-pool(c) + PPi(c) ⇌ Phosphatidate-VLDL-PI-pool(c) + CTP(c)
r1253	5.02	Stearate(r) + ATP(r) + CoA(r) → Stearoyl-CoA(r) + AMP(r) + PPi(r)
r0167	-241.4	L-Glutamate 5-semialdehyde(m) + Glutamate(m) ⇌ AKG(m) + Ornithine(m)
r2479	450.8	Asparagine(s) + Na+(s) + H+(PG)(c) → Asparagine(c) + Na+(c) + H+(PG)(s)
r1254	2300	Stearate(c) + ATP(c) + CoA(c) → Stearoyl-CoA(c) + AMP(c) + PPi(c)
r0311	11.6	Palmitate(r) + ATP(r) + CoA(r) → Palmitoyl-CoA(r) + AMP(r) + PPi(r)
r1255	36.8	ATP(r) + CoA(r) + Oleate(r) → Oleoyl-CoA(r) + AMP(r) + PPi(r)
r0312	11946	Palmitate(c) + ATP(c) + CoA(c) → Palmitoyl-CoA(c) + AMP(c) + PPi(c)
r0030	43630	ATP(c) + AMP(c) ⇌ 2 ADP(c)
r1256	12611	ATP(c) + CoA(c) + Oleate(c) → Oleoyl-CoA(c) + AMP(c) + PPi(c)
r0313	598.6	Serine(c) + Palmitoyl-CoA(c) ⇌ 3-Dehydrosphinganine(c) + CoA(c) + CO2(c)
r1257	9.08	Linoleate(r) + ATP(r) + CoA(r) → AMP(r) + PPi(r) + Linoleoyl-CoA(r)
r0314	-680.6	H2O(c) + L-Cystathionine(c) ⇌ Serine(c) + Homocysteine(c)
r1258	8346	Linoleate(c) + ATP(c) + CoA(c) → AMP(c) + PPi(c) + Linoleoyl-CoA(c)
r0315	302	Citrate(c) ⇌ Isocitrate(c)
r0481	155.7	Ethanolamine-P(c) + CTP(c) → PPi(c) + CDP-ethanolamine(c)
r0316	515.4	Citrate(m) ⇌ Isocitrate(m)
r0034	241.4	2 ATP(m) + CO2(m) + H2O(m) + NH3(m) ⇌ 2 ADP(m) + Pi(m) + Carbamoyl-P(m)
r1273	-106.5	PI-pool(c) ⇌ PI-pool(r)
r1122	-299.1	Leucine(c) + Na+(c) ← Leucine(s) + Na+(s)
r1123	-20.5	Isoleucine(c) + Na+(c) ← Isoleucine(s) + Na+(s)
r1276	156.4	SM-pool(l) → SM-pool(r)
r1407	598.6	CDP-choline(r) + Ceramide-pool(r) ⇌ CMP(r) + SM-pool(r)
r1277	33.1	PC-VLDL-pool(r) + H2O(r) ⇌ 2-Lysolecithin-pool(r) + Fatty-acid-VLDL-PC-pool(r)
r1125	31.4	Tyrosine(s) + Na+(s) → Tyrosine(c) + Na+(c)
r0911	214.1	Proline(c) + Glutamate(m) ⇌ Proline(m) + Glutamate(c)
r1278	107.5	PC-VLDL-pool(c) + H2O(c) ⇌ 2-Lysolecithin-pool(c) + Fatty-acid-VLDL-PC-pool(c)
r1126	-33.1	Phenylalanine(c) + Na+(c) ← Phenylalanine(s) + Na+(s)
r0912	-194.5	Citrate(c) + Malate(m) ⇌ Citrate(m) + Malate(c)
r1140	246.3	Valine(l) → Valine(c)
r1127	1	ApoB100(c) → ApoB100(r)
r0336	155.7	ATP(c) + Ethanolamine(c) → ADP(c) + Ethanolamine-P(c)
r1128	7	ApoE(c) → ApoE(r)

Continued on next page

ID	flux	reaction
r0619	-301.3	L-Glutamate 5-semialdehyde(m) + Pi(m) + NADP+(m) ← NADPH(m) + Glutamyl-5P(m)
r0915	-107.5	Citrate(c) + Succinate(m) ⇌ Citrate(m) + Succinate(c)
r0200	-1	Glucose-6P(c) ⇌ Inositol-1P(c)
r2664	1318	Na+(c) ⇌ Na+(s) + Na-gradient(c)
r1426	274	Proline(m) + H+(PG)(c) → Proline(c) + H+(PG)(m)
r0201	-12265	DHAP(c) + NADH(c) ⇌ NAD+(c) + sn-Glycerol-3P(c)
r0058	386.8	Malate(c) + NADP+(c) ⇌ Pyruvate(c) + CO2(c) + NADPH(c)
r1145	-16.6	Histidine(c) + Na+(c) ← Histidine(s) + Na+(s)
r0072	301.3	ATP(m) + Glutamate(m) → ADP(m) + Glutamyl-5P(m)
r0059	-381.4	Pyruvate(m) + CO2(m) + NADPH(m) ⇌ Malate(m) + NADP+(m)
r0203	106.5	ATP(c) + Glycerol(c) ⇌ ADP(c) + sn-Glycerol-3P(c)
r0370	-35.8	Sedoheptulose-7P(c) + GAP(c) ⇌ Ribose-5P(c) + D-Xylulose-5P(c)
r0205	-59.9	DHAP(c) + Ubiquinol(m) ⇌ Ubiquinone(m) + sn-Glycerol-3P(c)
r0075	-649.3	AKG(m) + NADPH(m) + NH3(m) ⇌ Glutamate(m) + H2O(m) + NADP+(m)
r1597	-56.8	Methionine(c) + Tryptophan(s) ⇌ Methionine(s) + Tryptophan(c)
r0077	229.2	ATP(c) + Glutamate(c) + NH3(c) → ADP(c) + Pi(c) + Glutamine(c)
r1164	25.5	Palmitoyl-CoA(r) + Cholesterol(r) → CoA(r) + Cholesterol-ester-palm(r)
r2533	173.7	Valine(s) ⇌ Valine(c)
r2534	771.4	Threonine(s) ⇌ Threonine(c)
r1166	5.02	Stearoyl-CoA(r) + Cholesterol(r) → CoA(r) + Cholesterol-ester-stea(r)
r1167	36.8	Oleoyl-CoA(r) + Cholesterol(r) → CoA(r) + Cholesterol-ester-ol(r)
r1168	87.5	Cholesterol(r) + Linoleoyl-CoA(r) → CoA(r) + Cholesterol-ester-lin(r)
r1465	598.6	2 H2O(c) + NAD+(c) + Oleoyl-CoA(c) ⇌ NADH(c) + O2(c) + Stearoyl-CoA(c)
r0240	680.6	Threonine(c) ⇌ 2-Oxobutyrate(c) + NH3(c)
r0241	-680.6	Cysteine(c) + NH3(c) + 2-Oxobutyrate(c) ⇌ H2O(c) + L-Cystathionine(c)
r1185	7904	Palmitoyl-CoA(c) + sn-Glycerol-3P(c) → CoA(c) + 1-Acylglycerol-3P-palm(c)
r0243	6180	GAP(c) ⇌ DHAP(c)
r1330	-649.3	H+(PG)(c) ⇌ H+(PG)(m) + Proton-gradient(m)
r1187	1308	Stearoyl-CoA(c) + sn-Glycerol-3P(c) → CoA(c) + 1-Acylglycerol-3P-stea(c)
r0821	241.4	Fumarate(c) + Succinate(m) ⇌ Fumarate(m) + Succinate(c)
r0244	2489	ATP(c) + Choline(c) → ADP(c) + Phosphocholine(c)
r1331	450.8	H+(PG)(s) ⇌ H+(PG)(c) + Proton-gradient(c)
r1318	78.4	Linoleoyl-CoA(c) ⇌ Linoleoyl-CoA(r)
r1188	1635	Oleoyl-CoA(c) + sn-Glycerol-3P(c) → 1-Acylglycerol-3P-ol(c) + CoA(c)
r1319	$-1.2{\cdot}10^5$	ATP(c) + H2O(c) ⇌ ADP(c) + Pi(c) + ATP-energy(c)
r1189	1355	Linoleoyl-CoA(c) + sn-Glycerol-3P(c) → 1-Acylglycerol-3P-lin(c) + CoA(c)
r1051	664.5	Cholesterol(l) ⇌ Cholesterol(r)
r0825	278.5	Isoleucine(l) → Isoleucine(c)
r0261	241.4	Argininosuccinate(c) ⇌ Fumarate(c) + Arginine(c)
r1053	0.98	LDL(s) → LDL(l)
r0826	107.5	Inositol(c) → Inositol(r)
r0828	133.9	Succinate(c) + Pi(m) ⇌ Succinate(m) + Pi(c)
r1201	0.02	332 1-Acylglycerol-3P-ol(c) + 181 1-Acylglycerol-3P-lin(c) + 2050 1-Acylglycerol-3P-arach(c) + 345 1-Acylglycerol-3P-palm(c) + 4820 1-Acylglycerol-3P-stea(c) → 7728 1-Acylglycerol-3P-VLDL-PS-pool(c)
r1071	348.9	Lysine(l) → Lysine(c)
r1059	146.6	Arginine(l) → Arginine(c)
r1073	77.2	Methionine(l) → Methionine(c)
r0998	-13.8	L-Carnitine(c) + L-Palmitoylcarnitine(r) ⇌ L-Carnitine(r) + L-Palmitoylcarnitine(c)
r1204	1.13	1200 Palmitoyl-CoA(c) + 384 Stearoyl-CoA(c) + 4260 Oleoyl-CoA(c) + 521 Arachidonyl-CoA(c) + 2810 Linoleoyl-CoA(c) → 9175 Acyl-CoA-VLDL-TG2-pool(c)
r1074	218.9	Phenylalanine(l) → Phenylalanine(c)
r1370	-155.7	PE-PS-VLDL-pool(c) + CO2(c) → PS-VLDL-pool(c)
r1205	1.15	849 Palmitoyl-CoA(c) + 584 Stearoyl-CoA(c) + 4570 Oleoyl-CoA(c) + 264 Arachidonyl-CoA(c) + 2780 Linoleoyl-CoA(c) → 9047 Acyl-CoA-VLDL-TG3-pool(c)
r1075	167.1	Proline(l) → Proline(c)
r0848	-33.1	H2O(r) ⇌ H2O(c)

Continued on next page

ID	flux	reaction
r1206	0.22	3010 Palmitoyl-CoA(c) + 1300 Stearoyl-CoA(c) + 1170 Oleoyl-CoA(c) + 726 Arachidonyl-CoA(c) + 2350 Linoleoyl-CoA(c) → 8556 Acyl-CoA-VLDL-PC-pool(c)
r1076	383.1	Serine(l) → Serine(c)
r0862	-78.4	CoA(c) ⇌ CoA(r)
r0849	-34610	H2O(s) ⇌ H2O(c)
r1220	155.7	Phosphatidate-VLDL-PS-pool(c) + H2O(c) → 1,2-Diacylglycerol-VLDL-PS-pool(c) + Pi(c)
r1077	36.2	Tryptophan(l) → Tryptophan(c)
r0134	107.5	CMP(c) + H2O(c) → Cytidine(c) + Pi(c)
r1221	106.5	Phosphatidate-VLDL-PI-pool(c) + H2O(c) → 1,2-Diacylglycerol-VLDL-PI-pool(c) + Pi(c)
r1208	0.02	345 Palmitoyl-CoA(c) + 4820 Stearoyl-CoA(c) + 332 Oleoyl-CoA(c) + 2050 Arachidonyl-CoA(c) + 181 Linoleoyl-CoA(c) → 7728 Acyl-CoA-VLDL-PS-pool(c)
r0583	-598.6	3-Dehydrosphinganine(c) + NADPH(c) ⇌ Sphinganine(c) + NADP+(c)
r0418	107.5	Uridine(c) + Pi(c) ⇌ Uracil(c) + Ribose-1P(c)
r0136	2538	ATP(c) + CMP(c) ⇌ ADP(c) + CDP(c)
r1223	-10385	Acyl-CoA-VLDL-TG3-pool(c) + 1,2-Diacylglycerol-VLDL-TG-pool(c) → Triacylglycerol-VLDL-pool(c) + CoA(c)
r0419	107.5	Cytidine(c) + H2O(c) ⇌ Uridine(c) + NH3(c)
r0289	1	Inositol-1P(c) + H2O(c) → Inositol(c) + Pi(c)
r0150	2538	ATP(c) + CDP(c) ⇌ ADP(c) + CTP(c)
r0867	1239	CO2(c) ⇌ CO2(s)
r1095	1	275 Alanine(c) + 150 Arginine(c) + 25 Cysteine(c) + 207 Glycine(c) + 115 Histidine(c) + 285 Isoleucine(c) + 533 Leucine(c) + 79 Methionine(c) + 224 Phenylalanine(c) + 171 Proline(c) + 392 Serine(c) + 300 Threonine(c) + 37 Tryptophan(c) + 151 Tyrosine(c) + 252 Valine(c) + 230 Glutamine(c) + 298 Glutamate(c) + 18244 ATP(c) + 18244 H2O(c) + 247 Asparagine(c) + 233 Aspartate(c) + 357 Lysine(c) → ApoB100(c) + 4561 AMP(c) + 4561 PPi(c) + 13683 ADP(c) + 13683 Pi(c)
r0434	-13.8	Palmitoyl-CoA(r) + L-Carnitine(r) ⇌ CoA(r) + L-Palmitoylcarnitine(r)
r0435	13.8	Palmitoyl-CoA(c) + L-Carnitine(c) ⇌ CoA(c) + L-Palmitoylcarnitine(c)
r1240	598.6	Ceramide-pool(c) ⇌ Ceramide-pool(r)
r0883	268.7	Alanine(l) → Alanine(c)
r1229	106.5	H2O(c) + 1,2-Diacylglycerol-VLDL-PI-pool(c) → 1-Acylglycerol-VLDL-PI-pool(c) + Fatty-acid-VLDL-PI-pool(c)
r0886	-706.1	CMP(c) ⇌ CMP(r)
r1244	-0.004	3010 Palmitate(r) + 1300 Stearate(r) + 2350 Linoleate(r) + 726 Arachidonate(r) + 1170 Oleate(r) ⇌ 8556 Fatty-acid-VLDL-PC-pool(r)
r0453	241.4	ATP(c) + Citrulline(c) + Aspartate(c) ⇌ AMP(c) + PPi(c) + Argininosuccinate(c)
r1245	-0.01	3010 Palmitate(c) + 1300 Stearate(c) + 2350 Linoleate(c) + 726 Arachidonate(c) + 1170 Oleate(c) ⇌ 8556 Fatty-acid-VLDL-PC-pool(c)
r0736	6108	ATP(c) + Fructose-6P(c) → ADP(c) + Fructose-1,6PP(c)
r0007	46168	PPi(c) + H2O(c) → 2 Pi(c)
r0174	107.5	CoA(m) + NAD+(m) + 3-Oxopropanoate(m) → Acetyl-CoA(m) + CO2(m) + NADH(m)
r0175	-59.9	L-Glutamate 5-semialdehyde(m) ⇌ 1-Pyrroline-5-carboxylate(m) + H2O(m)
r1262	2.8	Arachidonate(r) + ATP(r) + CoA(r) → Arachidonyl-CoA(r) + AMP(r) + PPi(r)
r1249	-0.02	694 Palmitate(c) + 3640 Stearate(c) + 694 Linoleate(c) + 2170 Arachidonate(c) + 1360 Oleate(c) ← 8558 Fatty-acid-VLDL-PI-pool(c)
r1263	1340	Arachidonate(c) + ATP(c) + CoA(c) → Arachidonyl-CoA(c) + AMP(c) + PPi(c)
r1264	10385	Triacylglycerol-VLDL-pool(c) ⇌ Triacylglycerol-VLDL-pool(r)
r1112	7	39 Alanine(c) + 34 Arginine(c) + 2 Cysteine(c) + 32 Glutamine(c) + 40 Glutamate(c) + 18 Glycine(c) + 2 Histidine(c) + 2 Isoleucine(c) + 41 Leucine(c) + 8 Methionine(c) + 4 Phenylalanine(c) + 8 Proline(c) + 14 Serine(c) + 12 Threonine(c) + 8 Tryptophan(c) + 4 Tyrosine(c) + 24 Valine(c) + 1268 ATP(c) + 1268 H2O(c) + Asparagine(c) + 11 Aspartate(c) + 13 Lysine(c) → ApoE(c) + 317 AMP(c) + 317 PPi(c) + 951 ADP(c) + 951 Pi(c)
r0192	6073	Glucose-6P(c) ⇌ Fructose-6P(c)
r1266	1783	PC-VLDL-pool(c) ⇌ PC-VLDL-pool(r)
r0900	107.5	beta-Alanine(c) ⇌ beta-Alanine(m)
r1996	566	Lysine(s) + Methionine(c) → Lysine(c) + Methionine(s)
r1280	-1	VLDL(r) ← 7 ApoE(r) + 10385 Triacylglycerol-VLDL-pool(r) + 2165 PC-VLDL-pool(r) + 1645 Cholesterol-ester-pool(r) + PI-pool(r) + 165 2-Lysolecithin-pool(r) + 185 PE-VLDL-pool(r) + ApoB100(r) + 500 Cholesterol(r) + 755 SM-pool(r)
r1267	415.3	PC-VLDL-pool(l) → PC-VLDL-pool(r)
r1268	155.7	PE-VLDL-pool(c) ⇌ PE-VLDL-pool(r)
r1412	598.6	Sphinganine(c) + Acyl-CoA-VLDL-SM-pool(c) ⇌ Dihydroceramide-pool(c) + CoA(c)
r1282	107.5	2-Lysolecithin-pool(c) ⇌ 2-Lysolecithin-pool(r)
r1269	29.3	PE-VLDL-pool(l) → PE-VLDL-pool(r)

Continued on next page

ID	flux	reaction
r1283	24.4	2-Lysolecithin-pool(l) → 2-Lysolecithin-pool(r)
r1414	598.6	Dihydroceramide-pool(c) + O2(c) + NADPH(c) ⇌ 2 H2O(c) + Ceramide-pool(c) + NADP+(c)
r0329	241.4	Carbamoyl-P(m) + Ornithine(m) ⇌ Pi(m) + Citrulline(m)
r0344	71.7	Ribulose-5P(c) ⇌ D-Xylulose-5P(c)
r0792	-130.7	5,10-Methylene-THF(c) + NADH(c) ⇌ 5-Methyl-THF(c) + NAD+(c)
r1150	-2228	Pi(c) ← Pi(l)
r0925	293.2	Threonine(l) → Threonine(c)
r1153	-65.3	PPi(c) ⇌ PPi(r)
r0080	-279.3	Pyruvate(c) + Glutamate(c) ⇌ Alanine(c) + AKG(c)
r0082	302	Oxalosuccinate(c) ⇌ AKG(c) + CO2(c)
r2676	-549.9	5-Methyl-THF(c) ⇌ THF(c) + THF-activated methyl group(c)
r0083	515.4	Oxalosuccinate(m) ⇌ AKG(m) + CO2(m)
r2678	-709.8	Acetyl-CoA(m) ⇌ CoA(m) + CoA-activated acetyl group(m)
r1453	-59.9	1-Pyrroline-5-carboxylate(m) + Ubiquinone(m) ⇌ Proline(m) + Ubiquinol(m)
r1301	32.2	Oleate(l) → Oleate(r)
r1171	9.72	Arachidonyl-CoA(r) + Cholesterol(r) → CoA(r) + Cholesterol-ester-arach(r)
r1158	0.98	LDL(l) → 1515 Cholesterol-ester-pool(l) + ApoB100(l) + 680 Cholesterol(l) + 110 CDP-diacylglycerol-VLDL-PI-pool(l) + 425 PC-VLDL-pool(l) + 25 2-Lysolecithin-pool(l) + 160 SM-pool(l) + 30 PE-VLDL-pool(l)
r1302	-32.2	Oleate(l) ⇌ Oleate(c)
r1159	598.6	CDP-choline(c) ⇌ CDP-choline(r)
r1007	-6.92	L-Carnitine(c) + Arachidonyl-Carnitine(r) ⇌ L-Carnitine(r) + Arachidonyl-Carnitine(c)
r0216	107.5	H2O(c) + 3-Ureidopropionate(c) → beta-Alanine(c) + CO2(c) + NH3(c)
r0230	-130.7	THF(c) + Serine(c) ⇌ 5,10-Methylene-THF(c) + Glycine(c) + H2O(c)
r0217	107.5	beta-Alanine(m) + AKG(m) ⇌ Glutamate(m) + 3-Oxopropanoate(m)
r2529	695.2	Cysteine(s) ⇌ Cysteine(c)
r0232	680.6	5-Methyl-THF(c) + Homocysteine(c) ⇌ THF(c) + Methionine(c)
r1320	-784.1	ATP(m) + H2O(m) ⇌ ADP(m) + Pi(m) + ATP-energy(m)
r1190	228.6	Arachidonyl-CoA(c) + sn-Glycerol-3P(c) → 1-Acylglycerol-3P-arach(c) + CoA(c)
r0811	-65.3	ATP(r) + AMP(c) ⇌ ATP(c) + AMP(r)
r0516	107.5	5,6-Dihydrouracil(c) + H2O(c) → 3-Ureidopropionate(c)
r1191	-155.7	PS-VLDL-pool(c) + Ethanolamine(c) ⇌ PE-VLDL-pool(c) + Serine(c)
r1322	-12485	NADH(c) ⇌ NAD+(c) + NADH-redox-potential(c)
r1192	0.02	2050 Cholesterol-ester-ol(r) + 4880 Cholesterol-ester-lin(r) + 1420 Cholesterol-ester-palm(r) + 280 Cholesterol-ester-stea(r) + 542 Cholesterol-ester-arach(r) ⇌ 9172 Cholesterol-ester-pool(r)
r0236	-107.5	5,6-Dihydrouracil(c) + NAD+(c) ⇌ Uracil(c) + NADH(c)
r1323	1440	NADH(m) ⇌ NAD+(m) + NADH-redox-potential(m)
r0250	71.7	Ribose-5P(c) ⇌ Ribulose-5P(c)
r0251	107.5	Ribose-1P(c) ⇌ Ribose-5P(c)
r1195	155.7	1,2-Diacylglycerol-VLDL-PS-pool(c) + CDP-ethanolamine(c) → PE-PS-VLDL-pool(c) + CMP(c)
r1326	-508.6	NADPH(c) ⇌ NADPH-redox-potential(c) + NADP+(c)
r1196	107.5	CDP-diacylglycerol-VLDL-PI-pool(r) + Inositol(r) → PI-pool(r) + CMP(r)
r1327	-1332	NADPH(m) ⇌ NADPH-redox-potential(m) + NADP+(m)
r1197	-106.5	CDP-diacylglycerol-VLDL-PI-pool(c) + Inositol(c) ⇌ PI-pool(c) + CMP(c)
r1198	1.12	1220 1-Acylglycerol-3P-ol(c) + 741 1-Acylglycerol-3P-lin(c) + 24 1-Acylglycerol-3P-arach(c) + 6440 1-Acylglycerol-3P-palm(c) + 823 1-Acylglycerol-3P-stea(c) → 9248 1-Acylglycerol-3P-VLDL-TG1-pool(c)
r0255	-35.8	Fructose-6P(c) + GAP(c) ⇌ D-Xylulose-5P(c) + Erythrose-4P(c)
r1342	0.98	2280 ATP(l) + 2280 H2O(l) + ApoB100(l) → 285 Isoleucine(l) + 533 Leucine(l) + 79 Methionine(l) + 275 Alanine(l) + 150 Arginine(l) + 115 Histidine(l) + 37 Tryptophan(l) + 252 Valine(l) + 2280 ADP(l) + 2280 Pi(l) + 230 Glutamine(l) + 298 Glutamate(l) + 25 Cysteine(l) + 207 Glycine(l) + 224 Phenylalanine(l) + 171 Proline(l) + 392 Serine(l) + 300 Threonine(l) + 151 Tyrosine(l) + 247 Asparagine(l) + 233 Aspartate(l) + 357 Lysine(l)
r1199	0.22	1170 1-Acylglycerol-3P-ol(c) + 2350 1-Acylglycerol-3P-lin(c) + 726 1-Acylglycerol-3P-arach(c) + 3010 1-Acylglycerol-3P-palm(c) + 1300 1-Acylglycerol-3P-stea(c) → 8556 1-Acylglycerol-3P-VLDL-PC-pool(c)
r1047	133.9	Pi(c) + H+(PG)(c) → Pi(m) + H+(PG)(m)
r0256	6108	Fructose-1,6PP(c) ⇌ DHAP(c) + GAP(c)
r0104	-581.2	OAA(c) + NADH(c) ⇌ NAD+(c) + Malate(c)
r1061	241.4	Asparagine(l) → Asparagine(c)
r0105	817.3	NAD+(m) + Malate(m) ⇌ OAA(m) + NADH(m)

Continued on next page

ID	flux	reaction
r2417	-241.4	Citrulline(m) + H+(PG)(c) → Citrulline(c) + H+(PG)(m)
r1062	227.7	Aspartate(l) → Aspartate(c)
r1063	24.4	Cysteine(l) → Cysteine(c)
r0259	-241.4	Fumarate(m) + H2O(m) ⇌ Malate(m)
r0107	-817.3	Acetyl-CoA(m) + H2O(m) + OAA(m) ⇌ Citrate(m) + CoA(m)
r1064	-224.8	Glutamine(c) ← Glutamine(l)
r0850	-2228	H2O(l) ← H2O(c)
r1360	107.5	CDP-diacylglycerol-VLDL-PI-pool(l) → CDP-diacylglycerol-VLDL-PI-pool(r)
r1065	202.3	Glycine(l) → Glycine(c)
r0851	193.2	H2O(m) ⇌ H2O(c)
r0838	890.7	NH3(c) ⇌ NH3(m)
r0109	581.2	AKG(c) + Aspartate(c) ⇌ OAA(c) + Glutamate(c)
r1361	1480	Cholesterol-ester-pool(l) → Cholesterol-ester-pool(r)

VLDL from HDL (311)

Definition			Solution												
			exchanges		reactions								transp		
Objective	Constraints	Comment	imports	exports	c	m	r	p	l	n	s	b	s-c	b-c	int
VLDL	−HDL MCES =Cysteine +Methionine =Leucine =Isoleucine =Histidine =Asparagine =Valine =Tryptophan =Lysine =Pheny-lalanine =Threonine =Tyro-sine =Valine =Ethanolamine(c) =Choline(c) ALFA	Lipoprotein conversion VLDL from HDL	49 Pyruvate(m) 73.8 Phenylalanine(s) 23.1 Tyrosine(s) 5626 Glucose-6P(c) 84.9 Cysteine(s) 1247 Choline(c) 47.3 Asparagine(s) 85.9 Valine(s) 116.7 Threonine(s) 4817 Arachidonate(c) 10414 Palmitate(c) 299 Isoleucine(s) 3845 Farnesyl-PP(r) 14488 Oleate(c) 3419 Linoleate(c) 1344 gamma-Linolenate(c) 11.1 HDL(s) $1.2{\cdot}10^5$ ATP-energy(c) 18.7 ATP-energy(m) 35.3 NADH-redox-potential(m) 5767 NADPH-redox-potential(r) 49 NADPH-redox-potential(m) 67.6 Proton-gradient(m)	18479 H_2O(s) 12494 P_i(c) 5674 CO_2(s) 167.4 Pyruvate(c) 42 Lysine(s) 18.4 Tryptophan(s) 93.3 Leucine(s) 4.65 Histidine(s) 93.4 Ethanolamine(c) 205.6 Stearate(c) 1 VLDL(s) 1922 NADH-redox-potential(r) 194 NADH-redox-potential(c) 1959 NADPH-redox-potential(c) 47.3 Proton-gradient(c) 627.3 Na-gradient(c) 42.1 THF-activated methyl group(c) 3845 CoA-activated acetyl group(c)	83	16	33	-	-	-	-	-	17	-	64

ID	flux	reaction
r2001	8.37	Lysine(s) + Leucine(c) → Lysine(c) + Leucine(s)
r1362	668.3	Glutamate(l) → Glutamate(c)
r1210	-0.01	6260 Palmitoyl-CoA(c) + 1490 Stearoyl-CoA(c) + 656 Oleoyl-CoA(c) + 147 Arachidonyl-CoA(c) + 270 Linoleoyl-CoA(c) ⇌ 8823 Acyl-CoA-VLDL-SM-pool(c)
r1080	155.9	Tyrosine(l) → Tyrosine(c)
r1067	133.7	Histidine(l) → Histidine(c)
r1211	-10385	Phosphatidate-VLDL-TG-pool(c) + CoA(c) ← Acyl-CoA-VLDL-TG2-pool(c) + 1-Acylglycerol-3P-VLDL-TG1-pool(c)
r1068	913.3	Leucine(l) → Leucine(c)
r0854	-11534	O2(r) ⇌ O2(c)
r1212	-1328	Phosphatidate-VLDL-PC-pool(c) + CoA(c) ← Acyl-CoA-VLDL-PC-pool(c) + 1-Acylglycerol-3P-VLDL-PC-pool(c)
r0421	1247	CTP(c) + Phosphocholine(c) → PPi(c) + CDP-choline(c)
r1083	1	VLDL(r) → VLDL(s)
r0575	1922	Presqualene-PP(r) + NADPH(r) ⇌ PPi(r) + Squalene(r) + NADP+(r)
r1215	-1	Phosphatidate-VLDL-PI-pool(c) + CoA(c) ← 1-Acylglycerol-3P-VLDL-PI-pool(c) + Acyl-CoA-VLDL-PI-pool(c)
r0576	1922	Squalene(r) + NADPH(r) + O2(r) ⇌ H2O(r) + Squalene 2,3-oxide(r) + NADP+(r)
r0859	-2963	ADP(c) + ATP(l) ← ADP(l) + ATP(c)
r1217	10385	Phosphatidate-VLDL-TG-pool(c) + H2O(c) → 1,2-Diacylglycerol-VLDL-TG-pool(c) + Pi(c)
r0296	-88	5,10-Methylene-THF(c) + NADPH(c) ⇌ 5-Methyl-THF(c) + NADP+(c)
r1947	23.1	Tyrosine(s) + Na+(s) + Lysine(c) → Tyrosine(c) + Na+(c) + Lysine(s)

Continued on next page

ID	flux	reaction
r1218	1328	Phosphatidate-VLDL-PC-pool(c) + H2O(c) → 1,2-Diacylglycerol-VLDL-PC-pool(c) + Pi(c)
r1219	427.6	Phosphatidate-VLDL-PE-pool(c) + H2O(c) → 1,2-Diacylglycerol-VLDL-PE-pool(c) + Pi(c)
r0441	-83.2	Arachidonyl-CoA(r) + L-Carnitine(r) ⇌ CoA(r) + Arachidonyl-Carnitine(r)
r1233	427.6	1-Acylglycerol-VLDL-PE-pool(c) + H2O(c) → Glycerol(c) + Fatty-acid-VLDL-PE-pool(c)
r0442	83.2	Arachidonyl-CoA(c) + L-Carnitine(c) ⇌ CoA(c) + Arachidonyl-Carnitine(c)
r0160	35.7	Serine(c) + Pyruvate(c) → Hydroxypyruvate(c) + Alanine(c)
r1237	1328	CDP-choline(c) + 1,2-Diacylglycerol-VLDL-PC-pool(c) → PC-VLDL-pool(c) + CMP(c)
r1238	1	Phosphatidate-VLDL-PI-pool(c) + CTP(c) ⇌ CDP-diacylglycerol-VLDL-PI-pool(c) + PPi(c)
r1253	50.2	Stearate(r) + ATP(r) + CoA(r) → Stearoyl-CoA(r) + AMP(r) + PPi(r)
r0167	-9.33	L-Glutamate 5-semialdehyde(m) + Glutamate(m) ⇌ AKG(m) + Ornithine(m)
r2479	47.3	Asparagine(s) + Na+(s) + H+(PG)(c) → Asparagine(c) + Na+(c) + H+(PG)(s)
r0311	58	Palmitate(r) + ATP(r) + CoA(r) → Palmitoyl-CoA(r) + AMP(r) + PPi(r)
r1255	367.7	ATP(r) + CoA(r) + Oleate(r) → Oleoyl-CoA(r) + AMP(r) + PPi(r)
r0312	10558	Palmitate(c) + ATP(c) + CoA(c) → Palmitoyl-CoA(c) + AMP(c) + PPi(c)
r0030	42181	ATP(c) + AMP(c) ⇌ 2 ADP(c)
r1256	14215	ATP(c) + CoA(c) + Oleate(c) → Oleoyl-CoA(c) + AMP(c) + PPi(c)
r0313	-80.3	3-Dehydrosphinganine(c) + CoA(c) + CO2(c) ⇌ Serine(c) + Palmitoyl-CoA(c)
r0314	-45.9	H2O(c) + L-Cystathionine(c) ⇌ Serine(c) + Homocysteine(c)
r1271	-334.1	PS-VLDL-pool(c) ⇌ PS-VLDL-pool(r)
r1258	3617	Linoleate(c) + ATP(c) + CoA(c) → AMP(c) + PPi(c) + Linoleoyl-CoA(c)
r1272	334.1	PS-VLDL-pool(l) → PS-VLDL-pool(r)
r0034	9.33	2 ATP(m) + CO2(m) + H2O(m) + NH3(m) ⇌ 2 ADP(m) + Pi(m) + Carbamoyl-P(m)
r1273	1	PI-pool(c) ⇌ PI-pool(r)
r1121	-58.5	Valine(c) + Na+(c) ← Valine(s) + Na+(s)
r1123	-299	Isoleucine(c) + Na+(c) ← Isoleucine(s) + Na+(s)
r1276	835.3	SM-pool(l) → SM-pool(r)
r0780	-1922	4,4-Dimethyl-5alpha-cholesta-8,14,24-trien-3beta-ol(r) + NADPH(r) ⇌ 14-Demethyllanosterol(r) + NADP+(r)
r0333	-1922	Provitamin D3(r) + NADPH(r) ⇌ Cholesterol(r) + NADP+(r)
r1407	-80.3	CDP-choline(r) + Ceramide-pool(r) ⇌ CMP(r) + SM-pool(r)
r1277	165	PC-VLDL-pool(r) + H2O(r) ⇌ 2-Lysolecithin-pool(r) + Fatty-acid-VLDL-PC-pool(r)
r0911	-1.18	Proline(c) + Glutamate(m) ⇌ Proline(m) + Glutamate(c)
r0781	1922	3 O2(r) + 3 NADPH(r) + Lanosterol(r) ⇌ 4,4-Dimethyl-5alpha-cholesta-8,14,24-trien-3beta-ol(r) + Formate(r) + 4 H2O(r) + 3 NADP+(r)
r1126	-55.4	Phenylalanine(c) + Na+(c) ← Phenylalanine(s) + Na+(s)
r1140	334.1	Valine(l) → Valine(c)
r1127	1	ApoB100(c) → ApoB100(r)
r0783	-1922	5alpha-Cholesta-7,24-dien-3beta-ol(r) + NADPH(r) ⇌ Lathosterol(r) + NADP+(r)
r1128	7	ApoE(c) → ApoE(r)
r0337	35.7	ATP(c) + 3PG(c) ⇌ ADP(c) + 1,3DPG(c)
r2510	25.1	L-Carnitine(r) + Stearate(c) ⇌ L-Carnitine(c) + Stearate(r)
r1142	-116.7	Threonine(c) + Na+(c) ← Threonine(s) + Na+(s)
r2511	-45.3	L-Carnitine(r) + Linoleate(c) ⇌ L-Carnitine(c) + Linoleate(r)
r0339	35.7	ATP(c) + Glycerate(c) → ADP(c) + 3PG(c)
r0200	-1	Glucose-6P(c) ⇌ Inositol-1P(c)
r2664	627.3	Na+(c) ⇌ Na+(s) + Na-gradient(c)
r1426	3.07	Proline(m) + H+(PG)(c) → Proline(c) + H+(PG)(m)
r0201	-11282	DHAP(c) + NADH(c) ⇌ NAD+(c) + sn-Glycerol-3P(c)
r0059	-49	Pyruvate(m) + CO2(m) + NADPH(m) ⇌ Malate(m) + NADP+(m)
r1146	1922	4alpha-Methylzymosterol(r) + 2 H2O(r) ⇌ Zymosterol(r) + CO2(r)
r0203	427.6	ATP(c) + Glycerol(c) ⇌ ADP(c) + sn-Glycerol-3P(c)
r0073	-21.7	AKG(m) + NADH(m) + NH3(m) ⇌ Glutamate(m) + NAD+(m) + H2O(m)
r0074	-13.6	L-Glutamate 5-semialdehyde(m) + NAD+(m) + H2O(m) ⇌ Glutamate(m) + NADH(m)
r1161	11.1	HDL(s) → HDL(l)
r0205	-4.25	DHAP(c) + Ubiquinol(m) ⇌ Ubiquinone(m) + sn-Glycerol-3P(c)
r0077	126.1	ATP(c) + Glutamate(c) + NH3(c) → ADP(c) + Pi(c) + Glutamine(c)
r1164	254.7	Palmitoyl-CoA(r) + Cholesterol(r) → CoA(r) + Cholesterol-ester-palm(r)

Continued on next page

ID	flux	reaction
r1166	50.2	Stearoyl-CoA(r) + Cholesterol(r) → CoA(r) + Cholesterol-ester-stea(r)
r0223	45.9	2-Methyl-3-oxopropanoate(m) + CoA(m) + NAD+(m) → Propanoyl-CoA(m) + CO2(m) + NADH(m)
r1167	367.7	Oleoyl-CoA(r) + Cholesterol(r) → CoA(r) + Cholesterol-ester-ol(r)
r1168	875.2	Cholesterol(r) + Linoleoyl-CoA(r) → CoA(r) + Cholesterol-ester-lin(r)
r2537	-3845	gamma-Linolenoyl-CoA(c) ⇌ gamma-Linolenoyl-CoA(r)
r2538	3845	Dihomo-gamma-linolenoyl-CoA(c) ⇌ Dihomo-gamma-linolenoyl-CoA(r)
r1465	2420	2 H2O(c) + NAD+(c) + Oleoyl-CoA(c) ⇌ NADH(c) + O2(c) + Stearoyl-CoA(c)
r0227	1922	10-Formyl-THF(c) + H2O(c) + NADP+(c) → THF(c) + CO2(c) + NADPH(c)
r1466	-3845	Malonyl-CoA(r) + 4 NADPH(r) + O2(r) + gamma-Linolenoyl-CoA(r) ⇌ CO2(r) + CoA(r) + Dihomo-gamma-linolenoyl-CoA(r) + 3 H2O(r) + 4 NADP+(r)
r0241	-45.9	Cysteine(c) + NH3(c) + 2-Oxobutyrate(c) ⇌ H2O(c) + L-Cystathionine(c)
r0228	1922	THF(c) + Formate(c) + ATP(c) → ADP(c) + Pi(c) + 10-Formyl-THF(c)
r1185	7699	Palmitoyl-CoA(c) + sn-Glycerol-3P(c) → CoA(c) + 1-Acylglycerol-3P-palm(c)
r0820	49	AKG(c) + Malate(m) ⇌ AKG(m) + Malate(c)
r0243	5661	GAP(c) ⇌ DHAP(c)
r1469	-5189	Linoleoyl-CoA(c) + NADH(c) + O2(c) ⇌ gamma-Linolenoyl-CoA(c) + H2O(c) + NAD+(c)
r1330	-67.6	H+(PG)(c) ⇌ H+(PG)(m) + Proton-gradient(m)
r1187	1126	Stearoyl-CoA(c) + sn-Glycerol-3P(c) → CoA(c) + 1-Acylglycerol-3P-stea(c)
r0244	1247	ATP(c) + Choline(c) → ADP(c) + Phosphocholine(c)
r1331	47.3	H+(PG)(s) ⇌ H+(PG)(c) + Proton-gradient(c)
r1318	875.2	Linoleoyl-CoA(c) ⇌ Linoleoyl-CoA(r)
r1188	1552	Oleoyl-CoA(c) + sn-Glycerol-3P(c) → 1-Acylglycerol-3P-ol(c) + CoA(c)
r1319	$-1.2{\cdot}10^5$	ATP(c) + H2O(c) ⇌ ADP(c) + Pi(c) + ATP-energy(c)
r1189	1197	Linoleoyl-CoA(c) + sn-Glycerol-3P(c) → 1-Acylglycerol-3P-lin(c) + CoA(c)
r1051	222.8	Cholesterol(l) ⇌ Cholesterol(r)
r0261	9.33	Argininosuccinate(c) ⇌ Fumarate(c) + Arginine(c)
r1635	84.9	Cysteine(s) + Leucine(c) ⇌ Cysteine(c) + Leucine(s)
r1071	490.1	Lysine(l) → Lysine(c)
r1202	0.0001	1360 1-Acylglycerol-3P-ol(c) + 694 1-Acylglycerol-3P-lin(c) + 2170 1-Acylglycerol-3P-arach(c) + 694 1-Acylglycerol-3P-palm(c) + 3640 1-Acylglycerol-3P-stea(c) → 8558 1-Acylglycerol-3P-VLDL-PI-pool(c)
r1059	378.7	Arginine(l) → Arginine(c)
r1073	89.1	Methionine(l) → Methionine(c)
r0998	-196.6	L-Carnitine(c) + L-Palmitoylcarnitine(r) ⇌ L-Carnitine(r) + L-Palmitoylcarnitine(c)
r1204	1.13	1200 Palmitoyl-CoA(c) + 384 Stearoyl-CoA(c) + 4260 Oleoyl-CoA(c) + 521 Arachidonyl-CoA(c) + 2810 Linoleoyl-CoA(c) → 9175 Acyl-CoA-VLDL-TG2-pool(c)
r1074	178.2	Phenylalanine(l) → Phenylalanine(c)
r1205	1.15	849 Palmitoyl-CoA(c) + 584 Stearoyl-CoA(c) + 4570 Oleoyl-CoA(c) + 264 Arachidonyl-CoA(c) + 2780 Linoleoyl-CoA(c) → 9047 Acyl-CoA-VLDL-TG3-pool(c)
r1075	222.8	Proline(l) → Proline(c)
r0848	5602	H2O(r) ⇌ H2O(c)
r1371	427.6	H2O(c) + PE-VLDL-pool(c) → Phosphatidate-VLDL-PE-pool(c) + Ethanolamine(c)
r1206	0.16	3010 Palmitoyl-CoA(c) + 1300 Stearoyl-CoA(c) + 1170 Oleoyl-CoA(c) + 726 Arachidonyl-CoA(c) + 2350 Linoleoyl-CoA(c) → 8556 Acyl-CoA-VLDL-PC-pool(c)
r1076	356.4	Serine(l) → Serine(c)
r0862	-875.2	CoA(c) ⇌ CoA(r)
r0849	-18479	H2O(s) ⇌ H2O(c)
r1077	111.4	Tryptophan(l) → Tryptophan(c)
r0430	3845	Malonyl-CoA(r) + L-Carnitine(r) ⇌ CoA(r) + Malonyl-Carnitin(r)
r1209	0.0001	694 Palmitoyl-CoA(c) + 3640 Stearoyl-CoA(c) + 1360 Oleoyl-CoA(c) + 2170 Arachidonyl-CoA(c) + 694 Linoleoyl-CoA(c) → 8558 Acyl-CoA-VLDL-PI-pool(c)
r0583	80.3	Sphinganine(c) + NADP+(c) ⇌ 3-Dehydrosphinganine(c) + NADPH(c)
r0431	-3845	Malonyl-CoA(c) + L-Carnitine(c) ⇌ CoA(c) + Malonyl-Carnitin(c)
r0136	1248	ATP(c) + CMP(c) ⇌ ADP(c) + CDP(c)
r1223	-10385	Acyl-CoA-VLDL-TG3-pool(c) + 1,2-Diacylglycerol-VLDL-TG-pool(c) → Triacylglycerol-VLDL-pool(c) + CoA(c)
r0289	1	Inositol-1P(c) + H2O(c) → Inositol(c) + Pi(c)
r0150	1248	ATP(c) + CDP(c) ⇌ ADP(c) + CTP(c)
r0867	5674	CO2(c) ⇌ CO2(s)

Continued on next page

ID	flux	reaction
r1095	1	275 Alanine(c) + 150 Arginine(c) + 25 Cysteine(c) + 207 Glycine(c) + 115 Histidine(c) + 285 Isoleucine(c) + 533 Leucine(c) + 79 Methionine(c) + 224 Phenylalanine(c) + 171 Proline(c) + 392 Serine(c) + 300 Threonine(c) + 37 Tryptophan(c) + 151 Tyrosine(c) + 252 Valine(c) + 230 Glutamine(c) + 298 Glutamate(c) + 18244 ATP(c) + 18244 H2O(c) + 247 Asparagine(c) + 233 Aspartate(c) + 357 Lysine(c) → ApoB100(c) + 4561 AMP(c) + 4561 PPi(c) + 13683 ADP(c) + 13683 Pi(c)
r0868	12.4	CO2(c) ⇌ CO2(m)
r0434	-196.6	Palmitoyl-CoA(r) + L-Carnitine(r) ⇌ CoA(r) + L-Palmitoylcarnitine(r)
r0435	196.6	Palmitoyl-CoA(c) + L-Carnitine(c) ⇌ CoA(c) + L-Palmitoylcarnitine(c)
r1240	-80.3	Ceramide-pool(c) ⇌ Ceramide-pool(r)
r1227	427.6	H2O(c) + 1,2-Diacylglycerol-VLDL-PE-pool(c) → 1-Acylglycerol-VLDL-PE-pool(c) + Fatty-acid-VLDL-PE-pool(c)
r0883	512.3	Alanine(l) → Alanine(c)
r0002	324.9	L-Carnitine(c) ⇌ L-Carnitine(r)
r0004	11.1	HDL(l) → 2 ApoA1(l) + 90 PC-VLDL-pool(l) + 30 PS-VLDL-pool(l) + 20 Cholesterol(l) + 25 PE-VLDL-pool(l) + 75 SM-pool(l)
r1972	27.3	Valine(s) + Na+(s) + Lysine(c) → Valine(c) + Na+(c) + Lysine(s)
r0886	80.3	CMP(c) ⇌ CMP(r)
r0170	1922	2 Farnesyl-PP(r) ⇌ PPi(r) + Presqualene-PP(r)
r1244	-0.02	3010 Palmitate(r) + 1300 Stearate(r) + 2350 Linoleate(r) + 726 Arachidonate(r) + 1170 Oleate(r) ⇌ 8556 Fatty-acid-VLDL-PC-pool(r)
r0887	-1922	Formate(c) ⇌ Formate(r)
r0453	9.33	ATP(c) + Citrulline(c) + Aspartate(c) ⇌ AMP(c) + PPi(c) + Argininosuccinate(c)
r0158	95.4	ATP(c) + Glutamine(c) + H2O(c) + Aspartate(c) ⇌ AMP(c) + PPi(c) + Glutamate(c) + Asparagine(c)
r0736	5625	ATP(c) + Fructose-6P(c) → ADP(c) + Fructose-1,6PP(c)
r0007	47274	PPi(c) + H2O(c) → 2 Pi(c)
r1247	-0.11	1260 Palmitate(c) + 2010 Stearate(c) + 1330 Linoleate(c) + 2220 Arachidonate(c) + 628 Oleate(c) ← 7448 Fatty-acid-VLDL-PE-pool(c)
r0738	1922	Zymosterol(r) ⇌ 5alpha-Cholesta-7,24-dien-3beta-ol(r)
r1261	1344	gamma-Linolenate(c) + ATP(c) + CoA(c) → gamma-Linolenoyl-CoA(c) + AMP(c) + PPi(c)
r0175	-4.25	1-Pyrroline-5-carboxylate(m) + H2O(m) ⇌ L-Glutamate 5-semialdehyde(m)
r1262	14	Arachidonate(r) + ATP(r) + CoA(r) → Arachidonyl-CoA(r) + AMP(r) + PPi(r)
r1263	5072	Arachidonate(c) + ATP(c) + CoA(c) → Arachidonyl-CoA(c) + AMP(c) + PPi(c)
r1264	10385	Triacylglycerol-VLDL-pool(c) ⇌ Triacylglycerol-VLDL-pool(r)
r1112	7	39 Alanine(c) + 34 Arginine(c) + 2 Cysteine(c) + 32 Glutamine(c) + 40 Glutamate(c) + 18 Glycine(c) + 2 Histidine(c) + 2 Isoleucine(c) + 41 Leucine(c) + 8 Methionine(c) + 4 Phenylalanine(c) + 8 Proline(c) + 14 Serine(c) + 12 Threonine(c) + 8 Tryptophan(c) + 4 Tyrosine(c) + 24 Valine(c) + 1268 ATP(c) + 1268 H2O(c) + Asparagine(c) + 11 Aspartate(c) + 13 Lysine(c) → ApoE(c) + 317 AMP(c) + 317 PPi(c) + 951 ADP(c) + 951 Pi(c)
r0192	5625	Glucose-6P(c) ⇌ Fructose-6P(c)
r1266	1328	PC-VLDL-pool(c) ⇌ PC-VLDL-pool(r)
r1114	45.9	AKG(m) + CoA(m) + NAD+(m) ⇌ NADH(m) + CO2(m) + Succinyl-CoA(m)
r1280	-1	VLDL(r) ← 7 ApoE(r) + 10385 Triacylglycerol-VLDL-pool(r) + 2165 PC-VLDL-pool(r) + 1645 Cholesterol-ester-pool(r) + PI-pool(r) + 165 2-Lysolecithin-pool(r) + 185 PE-VLDL-pool(r) + ApoB100(r) + 500 Cholesterol(r) + 755 SM-pool(r)
r1267	1002	PC-VLDL-pool(l) → PC-VLDL-pool(r)
r1268	-93.4	PE-VLDL-pool(c) ⇌ PE-VLDL-pool(r)
r0607	1922	Squalene 2,3-oxide(r) ⇌ Lanosterol(r)
r1412	-80.3	Dihydroceramide-pool(c) + CoA(c) ⇌ Sphinganine(c) + Acyl-CoA-VLDL-SM-pool(c)
r1269	278.4	PE-VLDL-pool(l) → PE-VLDL-pool(r)
r0327	-35.7	Hydroxypyruvate(c) + NADPH(c) → Glycerate(c) + NADP+(c)
r0197	-45.9	Succinyl-CoA(m) ⇌ (R)-Methylmalonyl-CoA(m)
r1414	-80.3	2 H2O(c) + Ceramide-pool(c) + NADP+(c) ⇌ Dihydroceramide-pool(c) + O2(c) + NADPH(c)
r0624	-45.9	2-Methyl-3-oxopropanoate(m) + CoA(m) + NAD+(m) ⇌ (R)-Methylmalonyl-CoA(m) + NADH(m)
r0329	9.33	Carbamoyl-P(m) + Ornithine(m) ⇌ Pi(m) + Citrulline(m)
r0060	203.1	Serine(c) → Pyruvate(c) + NH3(c)
r1134	1922	14-Demethyllanosterol(r) + 3 NADPH(r) + 3 O2(r) ⇌ 4 H2O(r) + 4alpha-Methylzymosterol-4-carboxylate(r) + 3 NADP+(r)
r1135	1922	3-Keto-4-methylzymosterol(r) + NADP+(r) ⇌ NADPH(r) + 4alpha-Methylzymosterol(r)
r0063	3845	Malonyl-CoA(c) → Acetyl-CoA(c) + CO2(c)
r1150	-2963	Pi(c) ← Pi(l)
r1137	1922	NAD+(r) + 4alpha-Methylzymosterol-4-carboxylate(r) ⇌ NADH(r) + CO2(r) + 3-Keto-4-methylzymosterol(r)

Continued on next page

ID	flux	reaction
r0793	1922	Lathosterol(r) + NADPH(r) + O2(r) ⇌ Provitamin D3(r) + 2 H2O(r) + NADP+(r)
r0925	267.3	Threonine(l) → Threonine(c)
r1153	-4334	PPi(c) ⇌ PPi(r)
r2509	345.1	L-Carnitine(r) + Oleate(c) ⇌ L-Carnitine(c) + Oleate(r)
r1154	-45.9	CoA(m) + NAD+(m) + 2-Oxobutyrate(m) ⇌ CO2(m) + Propanoyl-CoA(m) + NADH(m)
r2676	42.1	5-Methyl-THF(c) ⇌ THF(c) + THF-activated methyl group(c)
r1004	3845	L-Carnitine(c) + Malonyl-Carnitin(r) ⇌ L-Carnitine(r) + Malonyl-Carnitin(c)
r2677	3845	Acetyl-CoA(c) ⇌ CoA(c) + CoA-activated acetyl group(c)
r1453	-4.25	1-Pyrroline-5-carboxylate(m) + Ubiquinone(m) ⇌ Proline(m) + Ubiquinol(m)
r1171	97.2	Arachidonyl-CoA(r) + Cholesterol(r) → CoA(r) + Cholesterol-ester-arach(r)
r1454	-45.9	2-Oxobutyrate(m) + H+(PG)(c) → 2-Oxobutyrate(c) + H+(PG)(m)
r1159	-80.3	CDP-choline(c) ⇌ CDP-choline(r)
r1007	-83.2	L-Carnitine(c) + Arachidonyl-Carnitine(r) ⇌ L-Carnitine(r) + Arachidonyl-Carnitine(c)
r0230	-88	THF(c) + Serine(c) ⇌ 5,10-Methylene-THF(c) + Glycine(c) + H2O(c)
r1470	-3845	Dihomo-gamma-linolenoyl-CoA(c) + NADH(c) + O2(c) ⇌ Arachidonyl-CoA(c) + H2O(c) + NAD+(c)
r0232	45.9	5-Methyl-THF(c) + Homocysteine(c) ⇌ THF(c) + Methionine(c)
r1320	-18.7	ATP(m) + H2O(m) ⇌ ADP(m) + Pi(m) + ATP-energy(m)
r1190	139.9	Arachidonyl-CoA(c) + sn-Glycerol-3P(c) → 1-Acylglycerol-3P-arach(c) + CoA(c)
r0811	-489.9	ATP(r) + AMP(c) ⇌ ATP(c) + AMP(r)
r1321	1922	NADH(r) ⇌ NAD+(r) + NADH-redox-potential(r)
r1191	-334.1	PS-VLDL-pool(c) + Ethanolamine(c) ⇌ PE-VLDL-pool(c) + Serine(c)
r1322	194	NADH(c) ⇌ NAD+(c) + NADH-redox-potential(c)
r1192	0.18	2050 Cholesterol-ester-ol(r) + 4880 Cholesterol-ester-lin(r) + 1420 Cholesterol-ester-palm(r) + 280 Cholesterol-ester-stea(r) + 542 Cholesterol-ester-arach(r) ⇌ 9172 Cholesterol-ester-pool(r)
r1323	-35.3	NADH(m) ⇌ NAD+(m) + NADH-redox-potential(m)
r1325	-5767	NADPH(r) ⇌ NADPH-redox-potential(r) + NADP+(r)
r1608	-18.4	Tryptophan(s) + Phenylalanine(c) ⇌ Tryptophan(c) + Phenylalanine(s)
r1326	1959	NADPH(c) ⇌ NADPH-redox-potential(c) + NADP+(c)
r0253	-35.7	1,3DPG(c) + NADH(c) ⇌ GAP(c) + Pi(c) + NAD+(c)
r1340	22.3	133 ATP(l) + ApoA1(l) + 133 H2O(l) → 133 ADP(l) + 133 Pi(l) + 41 Leucine(l) + 4 Methionine(l) + 23 Alanine(l) + 17 Arginine(l) + 19 Glutamine(l) + 30 Glutamate(l) + 6 Histidine(l) + 5 Tryptophan(l) + 15 Valine(l) + 11 Glycine(l) + 8 Phenylalanine(l) + 10 Proline(l) + 16 Serine(l) + 12 Threonine(l) + 7 Tyrosine(l) + 5 Asparagine(l) + 16 Aspartate(l) + 22 Lysine(l)
r1327	-49	NADPH(m) ⇌ NADPH-redox-potential(m) + NADP+(m)
r1197	1	CDP-diacylglycerol-VLDL-PI-pool(c) + Inositol(c) ⇌ PI-pool(c) + CMP(c)
r1198	1.12	1220 1-Acylglycerol-3P-ol(c) + 741 1-Acylglycerol-3P-lin(c) + 24 1-Acylglycerol-3P-arach(c) + 6440 1-Acylglycerol-3P-palm(c) + 823 1-Acylglycerol-3P-stea(c) → 9248 1-Acylglycerol-3P-VLDL-TG1-pool(c)
r1199	0.16	1170 1-Acylglycerol-3P-ol(c) + 2350 1-Acylglycerol-3P-lin(c) + 726 1-Acylglycerol-3P-arach(c) + 3010 1-Acylglycerol-3P-palm(c) + 1300 1-Acylglycerol-3P-stea(c) → 8556 1-Acylglycerol-3P-VLDL-PC-pool(c)
r1047	9.33	Pi(c) + H+(PG)(c) → Pi(m) + H+(PG)(m)
r0104	58.3	NAD+(c) + Malate(c) ⇌ OAA(c) + NADH(c)
r0256	5625	Fructose-1,6PP(c) ⇌ DHAP(c) + GAP(c)
r1061	111.4	Asparagine(l) → Asparagine(c)
r2417	-9.33	Citrulline(m) + H+(PG)(c) → Citrulline(c) + H+(PG)(m)
r1062	356.4	Aspartate(l) → Aspartate(c)
r0258	-9.33	Fumarate(c) + H2O(c) ⇌ Malate(c)
r2419	9.33	AKG(c) + Pi(m) ⇌ AKG(m) + Pi(c)
r1064	-423.2	Glutamine(c) ← Glutamine(l)
r0850	-2963	H2O(l) ← H2O(c)
r1065	245	Glycine(l) → Glycine(c)
r0851	48.9	H2O(m) ⇌ H2O(c)
r0838	31	NH3(c) ⇌ NH3(m)
r0109	-58.3	OAA(c) + Glutamate(c) ⇌ AKG(c) + Aspartate(c)
r1066	-4.65	Histidine(s) ← Histidine(c)

B2.5. Bilirubin

Bilirubin conjugation (312)

Definition			Solution												
			exchanges		reactions								transp		
Objective	Constraints	Comment	imports	exports	c	m	r	p	l	n	s	b	s-c	b-c	int
Bilirubin-bisglucuronoside(b)	MCES −Bilirubin	Bilirubin import, conjugation, and export	2 H_2O(s) 2 Glucose-6P(c) 1 Bilirubin(s) 5 ATP-energy(c)	2 P_i(c) 1 Bilirubin-bisglucuronoside(b) 4 NADH-redox-potential(c)	8	-	2	-	-	-	-	-	2	1	5

ID	flux	reaction
r0532	1	Bilirubin(r) + 2 UDP-glucuronate(r) → 2 UDP(r) + Bilirubin-bisglucuronoside(r)
r0037	2	ATP(c) + UDP(c) ⇌ ADP(c) + UTP(c)
r0092	2	UDP-glucose(c) + H2O(c) + 2 NAD+(c) → 2 NADH(c) + UDP-glucuronate(c)
r0038	2	ATP(c) + UMP(c) ⇌ ADP(c) + UDP(c)
r0813	1	Bilirubin-bisglucuronoside(r) + ATP(r) + H2O(r) → Bilirubin-bisglucuronoside(b) + ADP(r) + Pi(r)
r0094	2	UTP(c) + Glucose-1P(c) → PPi(c) + UDP-glucose(c)
r1319	-5	ATP(c) + H2O(c) ⇌ ADP(c) + Pi(c) + ATP-energy(c)
r1149	-3	Pi(c) ⇌ Pi(r)
r0844	2	UDP-glucuronate(c) + UMP(r) ⇌ UDP-glucuronate(r) + UMP(c)
r1376	1	Bilirubin(c) ⇌ Bilirubin(r)
r0848	-3	H2O(r) ⇌ H2O(c)
r0007	2	PPi(c) + H2O(c) → 2 Pi(c)
r1322	4	NADH(c) ⇌ NAD+(c) + NADH-redox-potential(c)
r0858	1	ADP(r) + ATP(c) → ADP(c) + ATP(r)
r0849	2	H2O(s) ⇌ H2O(c)
r0035	2	UDP(r) + H2O(r) → Pi(r) + UMP(r)
r1512	1	Bilirubin(s) → Bilirubin(c)
r0621	-2	Glucose-6P(c) ⇌ Glucose-1P(c)

B2.6. Urea

Urea from glutamine (313)

Definition			Solution												
			exchanges		reactions								transp		
Objective	Constraints	Comment	imports	exports	c	m	r	p	l	n	s	b	s-c	b-c	int
Urea	MCES −Glutamine	Urea from glutamine	2 H_2O(s) 0.5 O_2(s) 1 Glutamine(s) 2 ATP-energy(c) 1 ATP-energy(m)	1 CO_2(s) 1 Pyruvate(c) 1 Urea(s) 1 NADH-redox-potential(c) 1 NADH-redox-potential(m) 1 NADPH-redox-potential(c) 1 Proton-gradient(c) 6 Proton-gradient(m) 1 Na-gradient(c)	14	9	-	-	-	-	-	-	7	-	7

ID	flux	reaction
r0896	1	Urea(c) ⇌ Urea(s)
r0820	1	AKG(c) + Malate(m) ⇌ AKG(m) + Malate(c)
r0190	1	Glutamine(c) + Fructose-6P(c) → Glutamate(c) + Glucosamine-6P(c)
r0109	-1	OAA(c) + Glutamate(c) ⇌ AKG(c) + Aspartate(c)
r1326	1	NADPH(c) ⇌ NADPH-redox-potential(c) + NADP+(c)
r0507	1	Ubiquinol(m) + 2 Ferricytochrome C(m) + 4 H+(PG)(m) → Ubiquinone(m) + 4 H+(PG)(c) + 2 Ferrocytochrome C(m)
r0453	1	ATP(c) + Citrulline(c) + Aspartate(c) ⇌ AMP(c) + PPi(c) + Argininosuccinate(c)
r0058	1	Malate(c) + NADP+(c) ⇌ Pyruvate(c) + CO2(c) + NADPH(c)
r1319	-2	ATP(c) + H2O(c) ⇌ ADP(c) + Pi(c) + ATP-energy(c)
r0509	1	Ubiquinone(m) + Succinate(m) → Ubiquinol(m) + Fumarate(m)
r0329	1	Carbamoyl-P(m) + Ornithine(m) ⇌ Pi(m) + Citrulline(m)

Continued on next page

ID	flux	reaction
r0100	-1	ADP(m) + GTP(m) ⇌ ATP(m) + GDP(m)
r0258	-1	Malate(c) ⇌ Fumarate(c) + H2O(c)
r0259	-1	Malate(m) ⇌ Fumarate(m) + H2O(m)
r0030	1	ATP(c) + AMP(c) ⇌ 2 ADP(c)
r2664	1	Na+(c) ⇌ Na+(s) + Na-gradient(c)
r0855	0.5	O2(s) ⇌ O2(c)
r0189	1	Glucosamine-6P(c) + H2O(c) ⇌ Fructose-6P(c) + NH3(c)
r0104	1	NAD+(c) + Malate(c) ⇌ OAA(c) + NADH(c)
r1320	-1	ATP(m) + H2O(m) ⇌ ADP(m) + Pi(m) + ATP-energy(m)
r0856	-0.5	O2(m) ⇌ O2(c)
r0838	1	NH3(c) ⇌ NH3(m)
r2477	1	Glutamine(s) + Na+(s) + H+(PG)(c) → Glutamine(c) + Na+(c) + H+(PG)(s)
r1330	6	H+(PG)(c) ⇌ H+(PG)(m) + Proton-gradient(m)
r1114	1	AKG(m) + CoA(m) + NAD+(m) ⇌ NADH(m) + CO2(m) + Succinyl-CoA(m)
r0947	-1	Citrulline(m) + Ornithine(c) ⇌ Citrulline(c) + Ornithine(m)
r0034	1	2 ATP(m) + CO2(m) + H2O(m) + NH3(m) ⇌ 2 ADP(m) + Pi(m) + Carbamoyl-P(m)
r0007	1	PPi(c) + H2O(c) → 2 Pi(c)
r1331	1	H+(PG)(s) ⇌ H+(PG)(c) + Proton-gradient(c)
r1322	1	NADH(c) ⇌ NAD+(c) + NADH-redox-potential(c)
r0867	1	CO2(c) ⇌ CO2(s)
r0849	2	H2O(s) ⇌ H2O(c)
r0125	-1	GDP(m) + Pi(m) + Succinyl-CoA(m) ⇌ GTP(m) + Succinate(m) + CoA(m)
r0017	0.5	O2(m) + 4 H+(PG)(m) + 4 Ferrocytochrome C(m) → 4 Ferricytochrome C(m) + 2 H2O(m) + 4 H+(PG)(c)
r1323	1	NADH(m) ⇌ NAD+(m) + NADH-redox-potential(m)
r0261	1	Argininosuccinate(c) ⇌ Fumarate(c) + Arginine(c)
r0144	1	Arginine(c) + H2O(c) ⇌ Urea(c) + Ornithine(c)

Urea from alanine (314)

Definition			Solution														
			exchanges		reactions									transp			
Objective	Constraints	Comment	imports	exports	c	m	r	p	l	n	s	b	s-c	b-c	int
Urea	MCES −Alanine	Urea from alanine	1 H_2O(s) 2 Alanine(s) 2 ATP-energy(c) 2 ATP-energy(m)	1 Pyruvate(c) 1 Urea(s) 1 NADH-redox-potential(c) 1 NADH-redox-potential(m) 1 NADPH-redox-potential(m) 1 CoA-activated acetyl group(m)	11	9	-	-	-	-	-	-	3	-	2

ID	flux	reaction
r2678	1	Acetyl-CoA(m) ⇌ CoA(m) + CoA-activated acetyl group(m)
r0896	1	Urea(c) ⇌ Urea(s)
r0109	-1	OAA(c) + Glutamate(c) ⇌ AKG(c) + Aspartate(c)
r0056	1	Pyruvate(m) + CoA(m) + NAD+(m) → Acetyl-CoA(m) + CO2(m) + NADH(m)
r1434	1	Alanine(c) → Alanine(m)
r0453	1	ATP(c) + Citrulline(c) + Aspartate(c) ⇌ AMP(c) + PPi(c) + Argininosuccinate(c)
r0075	1	Glutamate(m) + H2O(m) + NADP+(m) ⇌ AKG(m) + NADPH(m) + NH3(m)
r2524	2	Alanine(s) ⇌ Alanine(c)
r1327	1	NADPH(m) ⇌ NADPH-redox-potential(m) + NADP+(m)
r1319	-2	ATP(c) + H2O(c) ⇌ ADP(c) + Pi(c) + ATP-energy(c)
r0329	1	Carbamoyl-P(m) + Ornithine(m) ⇌ Pi(m) + Citrulline(m)
r0258	-1	Fumarate(c) + H2O(c) ⇌ Malate(c)
r0030	1	ATP(c) + AMP(c) ⇌ 2 ADP(c)
r0104	1	NAD+(c) + Malate(c) ⇌ OAA(c) + NADH(c)
r1320	-2	ATP(m) + H2O(m) ⇌ ADP(m) + Pi(m) + ATP-energy(m)
r0947	-1	Citrulline(m) + Ornithine(c) ⇌ Citrulline(c) + Ornithine(m)

Continued on next page

ID	flux	reaction
r0034	1	2 ATP(m) + CO2(m) + H2O(m) + NH3(m) ⇌ 2 ADP(m) + Pi(m) + Carbamoyl-P(m)
r0007	1	PPi(c) + H2O(c) → 2 Pi(c)
r1322	1	NADH(c) ⇌ NAD+(c) + NADH-redox-potential(c)
r0849	1	H2O(s) ⇌ H2O(c)
r0080	1	Alanine(c) + AKG(c) ⇌ Pyruvate(c) + Glutamate(c)
r1323	1	NADH(m) ⇌ NAD+(m) + NADH-redox-potential(m)
r0261	1	Argininosuccinate(c) ⇌ Fumarate(c) + Arginine(c)
r0144	1	Arginine(c) + H2O(c) ⇌ Urea(c) + Ornithine(c)
r0081	1	Alanine(m) + AKG(m) ⇌ Pyruvate(m) + Glutamate(m)

Urea from NH3 (315)

Definition			Solution												
			exchanges		reactions								transp		
Objective	Constraints	Comment	imports	exports	c	m	r	p	l	n	s	b	s-c	b-c	int
Urea	MCES $-NH_3$	Urea from alanine	1 H_2O(s) 2 Alanine(s) 2 ATP-energy(c) 2 ATP-energy(m)	1 Pyruvate(c) 1 Urea(s) 1 NADH-redox-potential(c) 1 NADH-redox-potential(m) 1 NADPH-redox-potential(m) 1 CoA-activated acetyl group(m)	11	9	-	-	-	-	-	-	3	-	2

ID	flux	reaction
r2678	1	Acetyl-CoA(m) ⇌ CoA(m) + CoA-activated acetyl group(m)
r0896	1	Urea(c) ⇌ Urea(s)
r0109	-1	OAA(c) + Glutamate(c) ⇌ AKG(c) + Aspartate(c)
r0056	1	Pyruvate(m) + CoA(m) + NAD+(m) → Acetyl-CoA(m) + CO2(m) + NADH(m)
r1434	1	Alanine(c) → Alanine(m)
r0453	1	ATP(c) + Citrulline(c) + Aspartate(c) ⇌ AMP(c) + PPi(c) + Argininosuccinate(c)
r0075	1	Glutamate(m) + H2O(m) + NADP+(m) ⇌ AKG(m) + NADPH(m) + NH3(m)
r2524	2	Alanine(s) ⇌ Alanine(c)
r1327	1	NADPH(m) ⇌ NADPH-redox-potential(m) + NADP+(m)
r1319	-2	ATP(c) + H2O(c) ⇌ ADP(c) + Pi(c) + ATP-energy(c)
r0329	1	Carbamoyl-P(m) + Ornithine(m) ⇌ Pi(m) + Citrulline(m)
r0258	-1	Fumarate(c) + H2O(c) ⇌ Malate(c)
r0030	1	ATP(c) + AMP(c) ⇌ 2 ADP(c)
r0104	1	NAD+(c) + Malate(c) ⇌ OAA(c) + NADH(c)
r1320	-2	ATP(m) + H2O(m) ⇌ ADP(m) + Pi(m) + ATP-energy(m)
r0947	-1	Citrulline(m) + Ornithine(c) ⇌ Citrulline(c) + Ornithine(m)
r0034	1	2 ATP(m) + CO2(m) + H2O(m) + NH3(m) ⇌ 2 ADP(m) + Pi(m) + Carbamoyl-P(m)
r0007	1	PPi(c) + H2O(c) → 2 Pi(c)
r1322	1	NADH(c) ⇌ NAD+(c) + NADH-redox-potential(c)
r0849	1	H2O(s) ⇌ H2O(c)
r0080	1	Alanine(c) + AKG(c) ⇌ Pyruvate(c) + Glutamate(c)
r1323	1	NADH(m) ⇌ NAD+(m) + NADH-redox-potential(m)
r0261	1	Argininosuccinate(c) ⇌ Fumarate(c) + Arginine(c)
r0144	1	Arginine(c) + H2O(c) ⇌ Urea(c) + Ornithine(c)
r0081	1	Alanine(m) + AKG(m) ⇌ Pyruvate(m) + Glutamate(m)

B2.7. Creatine

Creatine (316)

Definition			Solution												
			exchanges		reactions								transp		
Objective	Constraints	Comment	imports	exports	c	m	r	p	l	n	s	b	s-c	b-c	int
Creatine(c)	MCES −Arginine −Glycine	de novo synthesis of cytosolic Creatine	1 H_2O(s) 0.25 O_2(s) 1 Glycine(s) 0.5 Arginine(s) 1 Activated methyl group(c) 1 ATP-energy(c) 0.5 ATP-energy(m)	0.5 CO_2(s) 0.5 Pyruvate(c) 1 Creatine(c) 2 NADH-redox-potential(m) 0.5 NADPH-redox-potential(c) 1.5 Proton-gradient(m)	11	14	-	-	-	-	-	-	5	-	11

ID	flux	reaction
r0125	-0.5	GDP(m) + Pi(m) + Succinyl-CoA(m) ⇌ GTP(m) + Succinate(m) + CoA(m)
r0420	1	SAM(c) + Guanidinoacetate(c) ⇌ SAH(c) + Creatine(c)
r0855	0.25	O2(s) ⇌ O2(c)
r0856	-0.25	O2(m) ⇌ O2(c)
r0876	0.5	Glutamate(c) + H+(PG)(c) + Aspartate(m) → Glutamate(m) + H+(PG)(m) + Aspartate(c)
r0147	1	Arginine(c) + Glycine(c) ⇌ Guanidinoacetate(c) + Ornithine(c)
r0167	0.5	AKG(m) + Ornithine(m) ⇌ L-Glutamate 5-semialdehyde(m) + Glutamate(m)
r0017	0.25	O2(m) + 4 H+(PG)(m) + 4 Ferrocytochrome C(m) → 4 Ferricytochrome C(m) + 2 H2O(m) + 4 H+(PG)(c)
r0030	0.5	ATP(c) + AMP(c) ⇌ 2 ADP(c)
r0034	0.5	2 ATP(m) + CO2(m) + H2O(m) + NH3(m) ⇌ 2 ADP(m) + Pi(m) + Carbamoyl-P(m)
r0911	0.5	Proline(c) + Glutamate(m) ⇌ Proline(m) + Glutamate(c)
r1426	0.5	Proline(m) + H+(PG)(c) → Proline(c) + H+(PG)(m)
r0058	0.5	Malate(c) + NADP+(c) ⇌ Pyruvate(c) + CO2(c) + NADPH(c)
r0073	0.5	Glutamate(m) + NAD+(m) + H2O(m) ⇌ AKG(m) + NADH(m) + NH3(m)
r0074	0.5	L-Glutamate 5-semialdehyde(m) + NAD+(m) + H2O(m) ⇌ Glutamate(m) + NADH(m)
r0800	-1	SAM(c) ⇌ SAH(c) + Activated methyl group(c)
r0507	0.5	Ubiquinol(m) + 2 Ferricytochrome C(m) + 4 H+(PG)(m) → Ubiquinone(m) + 4 H+(PG)(c) + 2 Ferrocytochrome C(m)
r0509	0.5	Ubiquinone(m) + Succinate(m) → Ubiquinol(m) + Fumarate(m)
r1330	1.5	H+(PG)(c) ⇌ H+(PG)(m) + Proton-gradient(m)
r1319	-1	ATP(c) + H2O(c) ⇌ ADP(c) + Pi(c) + ATP-energy(c)
r0261	0.5	Argininosuccinate(c) ⇌ Fumarate(c) + Arginine(c)
r0110	-0.5	OAA(m) + Glutamate(m) → AKG(m) + Aspartate(m)
r1070	0.5	Lysine(c) ⇌ Lysine(m)
r1058	0.5	Arginine(s) ⇌ Arginine(c)
r0849	1	H2O(s) ⇌ H2O(c)
r0867	0.5	CO2(c) ⇌ CO2(s)
r0453	0.5	ATP(c) + Citrulline(c) + Aspartate(c) ⇌ AMP(c) + PPi(c) + Argininosuccinate(c)
r0007	0.5	PPi(c) + H2O(c) → 2 Pi(c)
r1114	0.5	AKG(m) + CoA(m) + NAD+(m) ⇌ NADH(m) + CO2(m) + Succinyl-CoA(m)
r0329	0.5	Carbamoyl-P(m) + Ornithine(m) ⇌ Pi(m) + Citrulline(m)
r2523	1	Glycine(s) ⇌ Glycine(c)
r2396	-0.5	Lysine(c) + Ornithine(m) + H+(PG)(m) ← Lysine(m) + Ornithine(c) + H+(PG)(c)
r0947	-0.5	Citrulline(c) + Ornithine(m) ⇌ Citrulline(m) + Ornithine(c)
r1320	-0.5	ATP(m) + H2O(m) ⇌ ADP(m) + Pi(m) + ATP-energy(m)
r1323	2	NADH(m) ⇌ NAD+(m) + NADH-redox-potential(m)
r0100	-0.5	ADP(m) + GTP(m) ⇌ ATP(m) + GDP(m)
r1326	0.5	NADPH(c) ⇌ NADPH-redox-potential(c) + NADP+(c)
r0105	0.5	NAD+(m) + Malate(m) ⇌ OAA(m) + NADH(m)
r0258	-0.5	Malate(c) ⇌ Fumarate(c) + H2O(c)
r0259	-0.5	Malate(m) ⇌ Fumarate(m) + H2O(m)
r0851	-1	H2O(m) ⇌ H2O(c)

3.3 B3. Excretion of de novo synthesized substances

B3.1. Bile excretion

B3.1.1. Bile acids Glycocholate(b) (317)

Definition			Solution												
			exchanges		reactions								transp		
Objective	Constraints	Comment	imports	exports	c	m	r	p	l	n	s	b	s-c	b-c	int
Glycocholate(b)	+Urea −Alanine −Serine MCES	de novo synthesis and bile export of Glycocholate	12 O_2(s) 1 Serine(s) 2 Farnesyl-PP(r) 5 ATP-energy(c) 12 NADPH-redox-potential(r) 1 NADPH-redox-potential(c) 1 Proton-gradient(c)	10 H_2O(s) 4 P_i(c) 4 CO_2(s) 1 Glycocholate(b) 1 NADH-redox-potential(r) 2 NADH-redox-potential(m) 1 NADH-redox-potential(p) 6 Proton-gradient(m) 1 THF-activated methyl group(c) 1 CoA-activated acetyl group(m)	15	7	19	7	-	-	-	-	5	1	21

ID	flux	reaction
r0854	-10	O2(r) ⇌ O2(c)
r0855	12	O2(s) ⇌ O2(c)
r0856	-0.5	O2(m) ⇌ O2(c)
r0704	1	ATP(p) + 3alpha,7alpha,12alpha-Trihydroxy-5beta-cholestanate(p) + CoA(p) ⇌ AMP(p) + PPi(p) + 3alpha,7alpha,12alpha-Trihydroxy-5beta-cholestanoyl-CoA(p)
r0857	-0.5	O2(p) ← O2(c)
r0575	1	Presqualene-PP(r) + NADPH(r) ⇌ PPi(r) + Squalene(r) + NADP+(r)
r0576	1	Squalene(r) + NADPH(r) + O2(r) ⇌ H2O(r) + Squalene 2,3-oxide(r) + NADP+(r)
r0706	1	O2(p) + 3alpha,7alpha,12alpha-Trihydroxy-5beta-cholestanoyl-CoA(p) → 3alpha,7alpha,12alpha-Trihydroxy-5beta-cholest-24-enoyl-CoA(p) + H2O2(p)
r0592	-1	H2O(m) + Acrylyl-CoA(m) → 3-Hydroxypropionyl-CoA(m)
r2471	1	Serine(s) + H+(PG)(s) → Serine(c) + H+(PG)(c)
r0596	-1	3-Hydroxypropionyl-CoA(m) + H2O(m) → Hydracrylate(m) + CoA(m)
r0012	0.5	2 H2O2(p) → O2(p) + 2 H2O(p)
r0741	1	3alpha,7alpha,12alpha-Trihydroxycoprostane(c) + NADPH(c) + O2(c) ⇌ 5beta-Cholestane-3alpha,7alpha,12alpha,26-tetrol(c) + H2O(c) + NADP+(c)
r0743	1	3alpha,7alpha,12alpha,24-Tetrahydroxy-5beta-cholestanoyl-CoA(p) + NAD+(p) → 3alpha,7alpha,12alpha-Trihydroxy-5beta-24-oxocholestanoyl-CoA(p) + NADH(p)
r0744	-1	3alpha,7alpha,12alpha,24-Tetrahydroxy-5beta-cholestanoyl-CoA(p) ← 3alpha,7alpha,12alpha-Trihydroxy-5beta-cholest-24-enoyl-CoA(p) + H2O(p)
r0017	0.5	O2(m) + 4 H+(PG)(m) + 4 Ferrocytochrome C(m) → 4 Ferricytochrome C(m) + 2 H2O(m) + 4 H+(PG)(c)
r0030	1	ATP(c) + AMP(c) ⇌ 2 ADP(c)
r0748	-1	7alpha,12alpha-Dihydroxycholest-4-en-3-one(c) + NADPH(c) → 7alpha,12alpha-Dihydroxy-5beta-cholestan-3-one(c) + NADP+(c)
r0749	-1	NADH(c) + 7alpha,12alpha-Dihydroxy-5beta-cholestan-3-one(c) ⇌ 3alpha,7alpha,12alpha-Trihydroxycoprostane(c) + NAD+(c)
r0333	-1	Provitamin D3(r) + NADPH(r) ⇌ Cholesterol(r) + NADP+(r)
r0780	-1	4,4-Dimethyl-5alpha-cholesta-8,14,24-trien-3beta-ol(r) + NADPH(r) ⇌ 14-Demethyllanosterol(r) + NADP+(r)
r0781	1	3 O2(r) + 3 NADPH(r) + Lanosterol(r) ⇌ 4,4-Dimethyl-5alpha-cholesta-8,14,24-trien-3beta-ol(r) + Formate(r) + 4 H2O(r) + 3 NADP+(r)
r1291	1	Propanoyl-CoA(c) ⇌ Propanoyl-CoA(m)
r0335	1	Cholesterol(r) + O2(r) + NADPH(r) ⇌ 7alpha-Hydroxycholesterol(r) + H2O(r) + NADP+(r)
r1292	-1	Propanoyl-CoA(c) ⇌ Propanoyl-CoA(p)
r0783	-1	5alpha-Cholesta-7,24-dien-3beta-ol(r) + NADPH(r) ⇌ Lathosterol(r) + NADP+(r)
r1146	1	4alpha-Methylzymosterol(r) + 2 H2O(r) ⇌ Zymosterol(r) + CO2(r)
r2517	1	3alpha,7alpha,12alpha-Trihydroxy-5beta-cholestanate(c) + ATP(c) + H2O(c) ⇌ 3alpha,7alpha,12alpha-Trihydroxy-5beta-cholestanate(p) + ADP(c) + Pi(c)
r0672	1	7alpha-Hydroxycholesterol(r) + NADP+(r) ⇌ 7alpha-Hydroxycholest-4-en-3-one(r) + NADPH(r)
r0507	1	Ubiquinol(m) + 2 Ferricytochrome C(m) + 4 H+(PG)(m) → Ubiquinone(m) + 4 H+(PG)(c) + 2 Ferrocytochrome C(m)
r0227	1	10-Formyl-THF(c) + H2O(c) + NADP+(c) → THF(c) + CO2(c) + NADPH(c)
r0228	1	THF(c) + Formate(c) + ATP(c) → ADP(c) + Pi(c) + 10-Formyl-THF(c)
r1330	6	H+(PG)(c) ⇌ H+(PG)(m) + Proton-gradient(m)
r1331	-1	H+(PG)(s) ⇌ H+(PG)(c) + Proton-gradient(c)
r1319	-5	ATP(c) + H2O(c) ⇌ ADP(c) + Pi(c) + ATP-energy(c)

Continued on next page

ID	flux	reaction
r0990	1	Choloyl-CoA(p) ⇌ Choloyl-CoA(c)
r0848	11	H2O(r) ⇌ H2O(c)
r0849	-10	H2O(s) ⇌ H2O(c)
r0863	-1	CoA(c) ⇌ CoA(m)
r0864	2	CoA(c) ⇌ CoA(p)
r1504	1	Glycocholate(c) + ATP(c) + H2O(c) → Glycocholate(b) + ADP(c) + Pi(c)
r0865	2	CO2(r) ⇌ CO2(c)
r0867	4	CO2(c) ⇌ CO2(s)
r0868	-1	CO2(c) ⇌ CO2(m)
r0170	1	2 Farnesyl-PP(r) ⇌ PPi(r) + Presqualene-PP(r)
r0887	-1	Formate(c) ⇌ Formate(r)
r0007	3	PPi(c) + H2O(c) → 2 Pi(c)
r0738	1	Zymosterol(r) ⇌ 5alpha-Cholesta-7,24-dien-3beta-ol(r)
r0751	1	7alpha-Hydroxycholest-4-en-3-one(r) + O2(r) + NADPH(r) → 7alpha,12alpha-Dihydroxycholest-4-en-3-one(r) + H2O(r) + NADP+(r)
r0174	1	CoA(m) + NAD+(m) + 3-Oxopropanoate(m) → Acetyl-CoA(m) + CO2(m) + NADH(m)
r0607	1	Squalene 2,3-oxide(r) ⇌ Lanosterol(r)
r1134	1	14-Demethyllanosterol(r) + 3 NADPH(r) + 3 O2(r) ⇌ 4 H2O(r) + 4alpha-Methylzymosterol-4-carboxylate(r) + 3 NADP+(r)
r0625	1	NAD+(c) + H2O(c) + 3alpha,7alpha,12alpha-Trihydroxy-5beta-cholestan-26-al(c) → 3alpha,7alpha,12alpha-Trihydroxy-5beta-cholestanate(c) + NADH(c)
r1135	1	3-Keto-4-methylzymosterol(r) + NADP+(r) ⇌ NADPH(r) + 4alpha-Methylzymosterol(r)
r0626	1	5beta-Cholestane-3alpha,7alpha,12alpha,26-tetrol(c) + NAD+(c) → NADH(c) + 3alpha,7alpha,12alpha-Trihydroxy-5beta-cholestan-26-al(c)
r0792	-1	5,10-Methylene-THF(c) + NADH(c) ⇌ 5-Methyl-THF(c) + NAD+(c)
r1289	-1	7alpha,12alpha-Dihydroxycholest-4-en-3-one(c) ⇌ 7alpha,12alpha-Dihydroxycholest-4-en-3-one(r)
r1137	1	NAD+(r) + 4alpha-Methylzymosterol-4-carboxylate(r) ⇌ NADH(r) + CO2(r) + 3-Keto-4-methylzymosterol(r)
r0793	1	Lathosterol(r) + NADPH(r) + O2(r) ⇌ Provitamin D3(r) + 2 H2O(r) + NADP+(r)
r0628	-1	CoA(p) + 3alpha,7alpha,12alpha-Trihydroxy-5beta-24-oxocholestanoyl-CoA(p) → Propanoyl-CoA(p) + Choloyl-CoA(p)
r0629	1	Choloyl-CoA(c) + Glycine(c) ⇌ CoA(c) + Glycocholate(c)
r1152	-1	PPi(c) ⇌ PPi(p)
r1153	-2	PPi(c) ⇌ PPi(r)
r2676	1	5-Methyl-THF(c) ⇌ THF(c) + THF-activated methyl group(c)
r0365	1	Hydracrylate(m) + NAD+(m) → NADH(m) + 3-Oxopropanoate(m)
r2678	1	Acetyl-CoA(m) ⇌ CoA(m) + CoA-activated acetyl group(m)
r0230	-1	THF(c) + Serine(c) ⇌ 5,10-Methylene-THF(c) + Glycine(c) + H2O(c)
r1321	1	NADH(r) ⇌ NAD+(r) + NADH-redox-potential(r)
r0812	1	ATP(c) + AMP(p) ⇌ ATP(p) + AMP(c)
r0683	1	Propanoyl-CoA(m) + Ubiquinone(m) → Ubiquinol(m) + Acrylyl-CoA(m)
r1323	2	NADH(m) ⇌ NAD+(m) + NADH-redox-potential(m)
r1324	1	NADH(p) ⇌ NAD+(p) + NADH-redox-potential(p)
r1325	-12	NADPH(r) ⇌ NADPH-redox-potential(r) + NADP+(r)
r1326	-1	NADPH(c) ⇌ NADPH-redox-potential(c) + NADP+(c)
r0851	-1	H2O(m) ⇌ H2O(c)

Gly-CD-cholate(b) (318)

Definition			Solution												
			exchanges		reactions								transp		
Objective	Constraints	Comment	imports	exports	c	m	r	p	l	n	s	b	s-c	b-c	int
Glycochenodeoxycholate(b)	+Urea −Alanine −Serine MCES	de novo synthesis and bile export of Glycochenodeoxycholate from Palmitate, Alanine, Serine, excreting urea and CO_2	10.5 O_2(s) 1 Serine(s) 2 Farnesyl-PP(r) 5 ATP-energy(c) 11 NADPH-redox-potential(r) 1 NADPH-redox-potential(m) 1 Proton-gradient(c)	8 H_2O(s) 4 P_i(c) 4 CO_2(s) 1 Glycochenodeoxycholate(b) 1 NADH-redox-potential(r) 2 NADH-redox-potential(m) 6 Proton-gradient(m) 1 THF-activated methyl group(c) 1 CoA-activated acetyl group(m)	13	9	18	2	-	-	-	-	5	1	22

ID	flux	reaction
r0854	-9	O2(r) ⇌ O2(c)
r0855	10.5	O2(s) ⇌ O2(c)
r0856	-1.5	O2(m) ⇌ O2(c)
r0575	1	Presqualene-PP(r) + NADPH(r) ⇌ PPi(r) + Squalene(r) + NADP+(r)
r0576	1	Squalene(r) + NADPH(r) + O2(r) ⇌ H2O(r) + Squalene 2,3-oxide(r) + NADP+(r)
r0592	-1	H2O(m) + Acrylyl-CoA(m) → 3-Hydroxypropionyl-CoA(m)
r2471	1	Serine(s) + H+(PG)(s) → Serine(c) + H+(PG)(c)
r0596	-1	3-Hydroxypropionyl-CoA(m) + H2O(m) → Hydracrylate(m) + CoA(m)
r0740	1	5beta-Cholestane-3alpha,7alpha-diol(m) + NADPH(m) + O2(m) ⇌ 5beta-Cholestane-3alpha,7alpha,26-triol(m) + H2O(m) + NADP+(m)
r0745	-1	7alpha-Hydroxycholest-4-en-3-one(c) + NADPH(c) ⇌ 7alpha-Hydroxy-5beta-cholestan-3-one(c) + NADP+(c)
r0017	0.5	O2(m) + 4 H+(PG)(m) + 4 Ferrocytochrome C(m) → 4 Ferricytochrome C(m) + 2 H2O(m) + 4 H+(PG)(c)
r0746	-1	7alpha-Hydroxy-5beta-cholestan-3-one(c) + NADH(c) ⇌ 5beta-Cholestane-3alpha,7alpha-diol(c) + NAD+(c)
r0030	1	ATP(c) + AMP(c) ⇌ 2 ADP(c)
r0333	-1	Provitamin D3(r) + NADPH(r) ⇌ Cholesterol(r) + NADP+(r)
r0780	-1	4,4-Dimethyl-5alpha-cholesta-8,14,24-trien-3beta-ol(r) + NADPH(r) ⇌ 14-Demethyllanosterol(r) + NADP+(r)
r0781	1	3 O2(r) + 3 NADPH(r) + Lanosterol(r) ⇌ 4,4-Dimethyl-5alpha-cholesta-8,14,24-trien-3beta-ol(r) + Formate(r) + 4 H2O(r) + 3 NADP+(r)
r1291	1	Propanoyl-CoA(c) ⇌ Propanoyl-CoA(m)
r0335	1	Cholesterol(r) + O2(r) + NADPH(r) ⇌ 7alpha-Hydroxycholesterol(r) + H2O(r) + NADP+(r)
r1292	-1	Propanoyl-CoA(c) ⇌ Propanoyl-CoA(p)
r0783	-1	5alpha-Cholesta-7,24-dien-3beta-ol(r) + NADPH(r) ⇌ Lathosterol(r) + NADP+(r)
r1295	-1	7alpha-Hydroxycholest-4-en-3-one(c) ⇌ 7alpha-Hydroxycholest-4-en-3-one(r)
r1146	1	4alpha-Methylzymosterol(r) + 2 H2O(r) ⇌ Zymosterol(r) + CO2(r)
r2518	1	3alpha,7alpha-Dihydroxy-5beta-cholestanate(c) + ATP(c) + H2O(c) ⇌ 3alpha,7alpha-Dihydroxy-5beta-cholestanate(p) + ADP(c) + Pi(c)
r0672	1	7alpha-Hydroxycholesterol(r) + NADP+(r) ⇌ 7alpha-Hydroxycholest-4-en-3-one(r) + NADPH(r)
r0507	1	Ubiquinol(m) + 2 Ferricytochrome C(m) + 4 H+(PG)(m) → Ubiquinone(m) + 4 H+(PG)(c) + 2 Ferrocytochrome C(m)
r0227	1	10-Formyl-THF(c) + H2O(c) + NADP+(c) → THF(c) + CO2(c) + NADPH(c)
r1019	1	Chenodeoxycholoyl-CoA(p) ⇌ Chenodeoxycholoyl-CoA(c)
r0228	1	THF(c) + Formate(c) + ATP(c) → ADP(c) + Pi(c) + 10-Formyl-THF(c)
r0690	1	ATP(p) + CoA(p) + 3alpha,7alpha-Dihydroxy-5beta-cholestanate(p) ⇌ AMP(p) + PPi(p) + 3alpha,7alpha-Dihydroxy-5beta-cholestanoyl-CoA(p)
r1330	6	H+(PG)(c) ⇌ H+(PG)(m) + Proton-gradient(m)
r1331	-1	H+(PG)(s) ⇌ H+(PG)(c) + Proton-gradient(c)
r1319	-5	ATP(c) + H2O(c) ⇌ ADP(c) + Pi(c) + ATP-energy(c)
r0698	-1	CoA(p) + 3alpha,7alpha-Dihydroxy-5beta-cholestanoyl-CoA(p) + H2O(p) ⇌ Propanoyl-CoA(p) + Chenodeoxycholoyl-CoA(p)
r0848	10	H2O(r) ⇌ H2O(c)
r0849	-8	H2O(s) ⇌ H2O(c)
r0863	-1	CoA(c) ⇌ CoA(m)
r0864	2	CoA(c) ⇌ CoA(p)
r0865	2	CO2(r) ⇌ CO2(c)
r0867	4	CO2(c) ⇌ CO2(s)
r1507	1	Glycochenodeoxycholate(c) + ATP(c) + H2O(c) → Glycochenodeoxycholate(b) + ADP(c) + Pi(c)
r0868	-1	CO2(c) ⇌ CO2(m)
r0170	1	2 Farnesyl-PP(r) ⇌ PPi(r) + Presqualene-PP(r)
r0887	-1	Formate(c) ⇌ Formate(r)
r0007	3	PPi(c) + H2O(c) → 2 Pi(c)
r0738	1	Zymosterol(r) ⇌ 5alpha-Cholesta-7,24-dien-3beta-ol(r)
r0174	1	CoA(m) + NAD+(m) + 3-Oxopropanoate(m) → Acetyl-CoA(m) + CO2(m) + NADH(m)
r0739	1	5beta-Cholestane-3alpha,7alpha,26-triol(c) + NAD+(c) ⇌ 3alpha,7alpha-Dihydroxy-5beta-cholestan-26-al(c) + NADH(c)
r0607	1	Squalene 2,3-oxide(r) ⇌ Lanosterol(r)
r1134	1	14-Demethyllanosterol(r) + 3 NADPH(r) + 3 O2(r) ⇌ 4 H2O(r) + 4alpha-Methylzymosterol-4-carboxylate(r) + 3 NADP+(r)
r1135	1	3-Keto-4-methylzymosterol(r) + NADP+(r) ⇌ NADPH(r) + 4alpha-Methylzymosterol(r)
r0792	-1	5,10-Methylene-THF(c) + NADH(c) ⇌ 5-Methyl-THF(c) + NAD+(c)
r1137	1	NAD+(r) + 4alpha-Methylzymosterol-4-carboxylate(r) ⇌ NADH(r) + CO2(r) + 3-Keto-4-methylzymosterol(r)

Continued on next page

ID	flux	reaction
r0793	1	Lathosterol(r) + NADPH(r) + O2(r) ⇌ Provitamin D3(r) + 2 H2O(r) + NADP+(r)
r1152	-1	PPi(c) ⇌ PPi(p)
r1153	-2	PPi(c) ⇌ PPi(r)
r0797	1	Chenodeoxycholoyl-CoA(c) + Glycine(c) ⇌ CoA(c) + Glycochenodeoxycholate(c)
r2676	1	5-Methyl-THF(c) ⇌ THF(c) + THF-activated methyl group(c)
r0365	1	Hydracrylate(m) + NAD+(m) → NADH(m) + 3-Oxopropanoate(m)
r2678	1	Acetyl-CoA(m) ⇌ CoA(m) + CoA-activated acetyl group(m)
r1020	1	5beta-Cholestane-3alpha,7alpha,26-triol(m) ⇌ 5beta-Cholestane-3alpha,7alpha,26-triol(c)
r0230	-1	THF(c) + Serine(c) ⇌ 5,10-Methylene-THF(c) + Glycine(c) + H2O(c)
r1022	1	5beta-Cholestane-3alpha,7alpha-diol(c) ⇌ 5beta-Cholestane-3alpha,7alpha-diol(m)
r1321	1	NADH(r) ⇌ NAD+(r) + NADH-redox-potential(r)
r0812	1	ATP(c) + AMP(p) ⇌ ATP(p) + AMP(c)
r0683	1	Propanoyl-CoA(m) + Ubiquinone(m) → Ubiquinol(m) + Acrylyl-CoA(m)
r1323	2	NADH(m) ⇌ NAD+(m) + NADH-redox-potential(m)
r1325	-11	NADPH(r) ⇌ NADPH-redox-potential(r) + NADP+(r)
r1327	-1	NADPH(m) ⇌ NADPH-redox-potential(m) + NADP+(m)
r0688	1	3alpha,7alpha-Dihydroxy-5beta-cholestan-26-al(c) + NAD+(c) + H2O(c) ⇌ NADH(c) + 3alpha,7alpha-Dihydroxy-5beta-cholestanate(c)
r0852	-1	H2O(p) ⇌ H2O(c)

Taurocholate(b) (319)

Definition			Solution												
			exchanges		reactions								transp		
Objective	Constraints	Comment	imports	exports	c	m	r	p	l	n	s	b	s-c	b-c	int
Taurocholate(b)	+Urea −Alanine −Serine −Methionine MCES	de novo synthesis and bile export of Taurocholate	13.5 O_2(s) 1 Serine(s) 1 Methionine(s) 2 Farnesyl-PP(r) 6 ATP-energy(c) 1 ATP-energy(m) 12 NADPH-redox-potential(r) 2 NADPH-redox-potential(c)	7.5 H_2O(s) 4 P_i(c) 6.5 CO_2(s) 0.5 Urea(s) 1 Taurocholate(b) 1 NADH-redox-potential(r) 3.5 NADH-redox-potential(c) 4.5 NADH-redox-potential(m) 1 NADH-redox-potential(p) 1 Proton-gradient(c) 10 Proton-gradient(m) 2 Na-gradient(c) 1 THF-activated methyl group(c) 2 CoA-activated acetyl group(m)	26	12	19	7	-	-	-	-	8	1	28

ID	flux	reaction
r0854	-10	O2(r) ⇌ O2(c)
r0855	13.5	O2(s) ⇌ O2(c)
r0856	-1	O2(m) ⇌ O2(c)
r0704	1	ATP(p) + 3alpha,7alpha,12alpha-Trihydroxy-5beta-cholestanate(p) + CoA(p) ⇌ AMP(p) + PPi(p) + 3alpha,7alpha,12alpha-Trihydroxy-5beta-cholestanoyl-CoA(p)
r0857	-0.5	O2(p) ← O2(c)
r0575	1	Presqualene-PP(r) + NADPH(r) ⇌ PPi(r) + Squalene(r) + NADP+(r)
r0706	1	O2(p) + 3alpha,7alpha,12alpha-Trihydroxy-5beta-cholestanoyl-CoA(p) → 3alpha,7alpha,12alpha-Trihydroxy-5beta-cholest-24-enoyl-CoA(p) + H2O2(p)
r0576	1	Squalene(r) + NADPH(r) + O2(r) ⇌ H2O(r) + Squalene 2,3-oxide(r) + NADP+(r)
r0144	0.5	Arginine(c) + H2O(c) ⇌ Urea(c) + Ornithine(c)
r0592	-2	H2O(m) + Acrylyl-CoA(m) → 3-Hydroxypropionyl-CoA(m)
r0596	-2	3-Hydroxypropionyl-CoA(m) + H2O(m) → Hydracrylate(m) + CoA(m)
r0741	1	3alpha,7alpha,12alpha-Trihydroxycoprostane(c) + NADPH(c) + O2(c) ⇌ 5beta-Cholestane-3alpha,7alpha,12alpha,26-tetrol(c) + H2O(c) + NADP+(c)
r0012	0.5	2 H2O2(p) → O2(p) + 2 H2O(p)
r0743	1	3alpha,7alpha,12alpha,24-Tetrahydroxy-5beta-cholestanoyl-CoA(p) + NAD+(p) → 3alpha,7alpha,12alpha-Trihydroxy-5beta-24-oxocholestanoyl-CoA(p) + NADH(p)
r2478	1	Serine(s) + Na+(s) + H+(PG)(c) → Serine(c) + Na+(c) + H+(PG)(s)
r0896	0.5	Urea(c) ⇌ Urea(s)

Continued on next page

ID	flux	reaction
r0744	-1	3alpha,7alpha,12alpha,24-Tetrahydroxy-5beta-cholestanoyl-CoA(p) ← 3alpha,7alpha,12alpha-Trihydroxy-5beta-cholest-24-enoyl-CoA(p) + H2O(p)
r0017	1	O2(m) + 4 H+(PG)(m) + 4 Ferrocytochrome C(m) → 4 Ferricytochrome C(m) + 2 H2O(m) + 4 H+(PG)(c)
r0030	1.5	ATP(c) + AMP(c) ⇌ 2 ADP(c)
r0748	-1	7alpha,12alpha-Dihydroxycholest-4-en-3-one(c) + NADPH(c) → 7alpha,12alpha-Dihydroxy-5beta-cholestan-3-one(c) + NADP+(c)
r0314	1	Serine(c) + Homocysteine(c) ⇌ H2O(c) + L-Cystathionine(c)
r0034	0.5	2 ATP(m) + CO2(m) + H2O(m) + NH3(m) ⇌ 2 ADP(m) + Pi(m) + Carbamoyl-P(m)
r0780	-1	4,4-Dimethyl-5alpha-cholesta-8,14,24-trien-3beta-ol(r) + NADPH(r) ⇌ 14-Demethyllanosterol(r) + NADP+(r)
r0333	-1	Provitamin D3(r) + NADPH(r) ⇌ Cholesterol(r) + NADP+(r)
r0911	0.5	Proline(c) + Glutamate(m) ⇌ Proline(m) + Glutamate(c)
r0781	1	3 O2(r) + 3 NADPH(r) + Lanosterol(r) ⇌ 4,4-Dimethyl-5alpha-cholesta-8,14,24-trien-3beta-ol(r) + Formate(r) + 4 H2O(r) + 3 NADP+(r)
r1291	1	Propanoyl-CoA(c) ⇌ Propanoyl-CoA(m)
r0630	1	Choloyl-CoA(c) + Taurine(c) ⇌ CoA(c) + Taurocholate(c)
r0335	1	Cholesterol(r) + O2(r) + NADPH(r) ⇌ 7alpha-Hydroxycholesterol(r) + H2O(r) + NADP+(r)
r1292	-1	Propanoyl-CoA(c) ⇌ Propanoyl-CoA(p)
r0783	-1	5alpha-Cholesta-7,24-dien-3beta-ol(r) + NADPH(r) ⇌ Lathosterol(r) + NADP+(r)
r1141	-1	Methionine(s) + Na+(s) → Methionine(c) + Na+(c)
r2664	2	Na+(c) ⇌ Na+(s) + Na-gradient(c)
r1426	0.5	Proline(m) + H+(PG)(c) → Proline(c) + H+(PG)(m)
r1146	1	4alpha-Methylzymosterol(r) + 2 H2O(r) ⇌ Zymosterol(r) + CO2(r)
r0073	-0.5	AKG(m) + NADH(m) + NH3(m) ⇌ Glutamate(m) + NAD+(m) + H2O(m)
r2517	1	3alpha,7alpha,12alpha-Trihydroxy-5beta-cholestanate(c) + ATP(c) + H2O(c) ⇌ 3alpha,7alpha,12alpha-Trihydroxy-5beta-cholestanate(p) + ADP(c) + Pi(c)
r0672	1	7alpha-Hydroxycholesterol(r) + NADP+(r) ⇌ 7alpha-Hydroxycholest-4-en-3-one(r) + NADPH(r)
r0507	2	Ubiquinol(m) + 2 Ferricytochrome C(m) + 4 H+(PG)(m) → Ubiquinone(m) + 4 H+(PG)(c) + 2 Ferrocytochrome C(m)
r0227	1	10-Formyl-THF(c) + H2O(c) + NADP+(c) → THF(c) + CO2(c) + NADPH(c)
r0241	1	H2O(c) + L-Cystathionine(c) ⇌ Cysteine(c) + NH3(c) + 2-Oxobutyrate(c)
r0228	1	THF(c) + Formate(c) + ATP(c) → ADP(c) + Pi(c) + 10-Formyl-THF(c)
r1330	10	H+(PG)(c) ⇌ H+(PG)(m) + Proton-gradient(m)
r1331	1	H+(PG)(s) ⇌ H+(PG)(c) + Proton-gradient(c)
r1319	-6	ATP(c) + H2O(c) ⇌ ADP(c) + Pi(c) + ATP-energy(c)
r0990	1	Choloyl-CoA(p) ⇌ Choloyl-CoA(c)
r0261	0.5	Argininosuccinate(c) ⇌ Fumarate(c) + Arginine(c)
r0848	11	H2O(r) ⇌ H2O(c)
r0849	-7.5	H2O(s) ⇌ H2O(c)
r0863	-1	CoA(c) ⇌ CoA(m)
r0864	2	CoA(c) ⇌ CoA(p)
r0865	2	CO2(r) ⇌ CO2(c)
r1505	1	Taurocholate(c) + ATP(c) + H2O(c) → Taurocholate(b) + ADP(c) + Pi(c)
r0867	6.5	CO2(c) ⇌ CO2(s)
r0868	-2.5	CO2(c) ⇌ CO2(m)
r0170	1	2 Farnesyl-PP(r) ⇌ PPi(r) + Presqualene-PP(r)
r0887	-1	Formate(c) ⇌ Formate(r)
r0453	0.5	ATP(c) + Citrulline(c) + Aspartate(c) ⇌ AMP(c) + PPi(c) + Argininosuccinate(c)
r0007	3.5	PPi(c) + H2O(c) → 2 Pi(c)
r0750	-1	NADPH(c) + 7alpha,12alpha-Dihydroxy-5beta-cholestan-3-one(c) ⇌ 3alpha,7alpha,12alpha-Trihydroxycoprostane(c) + NADP+(c)
r0751	1	7alpha-Hydroxycholest-4-en-3-one(r) + O2(r) + NADPH(r) → 7alpha,12alpha-Dihydroxycholest-4-en-3-one(r) + H2O(r) + NADP+(r)
r0738	1	Zymosterol(r) ⇌ 5alpha-Cholesta-7,24-dien-3beta-ol(r)
r0174	2	CoA(m) + NAD+(m) + 3-Oxopropanoate(m) → Acetyl-CoA(m) + CO2(m) + NADH(m)
r0607	1	Squalene 2,3-oxide(r) ⇌ Lanosterol(r)
r0329	0.5	Carbamoyl-P(m) + Ornithine(m) ⇌ Pi(m) + Citrulline(m)
r1134	1	14-Demethyllanosterol(r) + 3 NADPH(r) + 3 O2(r) ⇌ 4 H2O(r) + 4alpha-Methylzymosterol-4-carboxylate(r) + 3 NADP+(r)

Continued on next page

ID	flux	reaction
r0625	1	NAD+(c) + H2O(c) + 3alpha,7alpha,12alpha-Trihydroxy-5beta-cholestan-26-al(c) → 3alpha,7alpha,12alpha-Trihydroxy-5beta-cholestanate(c) + NADH(c)
r1135	1	3-Keto-4-methylzymosterol(r) + NADP+(r) ⇌ NADPH(r) + 4alpha-Methylzymosterol(r)
r0626	1	5beta-Cholestane-3alpha,7alpha,12alpha,26-tetrol(c) + NAD+(c) → NADH(c) + 3alpha,7alpha,12alpha-Trihydroxy-5beta-cholestan-26-al(c)
r1289	-1	7alpha,12alpha-Dihydroxycholest-4-en-3-one(c) ⇌ 7alpha,12alpha-Dihydroxycholest-4-en-3-one(r)
r1137	1	NAD+(r) + 4alpha-Methylzymosterol-4-carboxylate(r) ⇌ NADH(r) + CO2(r) + 3-Keto-4-methylzymosterol(r)
r0793	1	Lathosterol(r) + NADPH(r) + O2(r) ⇌ Provitamin D3(r) + 2 H2O(r) + NADP+(r)
r0628	-1	CoA(p) + 3alpha,7alpha,12alpha-Trihydroxy-5beta-24-oxocholestanoyl-CoA(p) → Propanoyl-CoA(p) + Choloyl-CoA(p)
r1152	-1	PPi(c) ⇌ PPi(p)
r1153	-2	PPi(c) ⇌ PPi(r)
r1154	1	CoA(m) + NAD+(m) + 2-Oxobutyrate(m) ⇌ CO2(m) + Propanoyl-CoA(m) + NADH(m)
r0211	1	Cysteine(c) + O2(c) ⇌ 3-Sulfinoalanine(c)
r1155	1	2-Oxobutyrate(c) + H+(PG)(c) → 2-Oxobutyrate(m) + H+(PG)(m)
r2676	1	5-Methyl-THF(c) ⇌ THF(c) + THF-activated methyl group(c)
r0365	2	Hydracrylate(m) + NAD+(m) → NADH(m) + 3-Oxopropanoate(m)
r2678	2	Acetyl-CoA(m) ⇌ CoA(m) + CoA-activated acetyl group(m)
r0381	1	NAD+(c) + H2O(c) + Hypotaurine(c) ⇌ NADH(c) + Taurine(c)
r0947	-0.5	Citrulline(m) + Ornithine(c) ⇌ Citrulline(c) + Ornithine(m)
r0232	-1	THF(c) + Methionine(c) ⇌ 5-Methyl-THF(c) + Homocysteine(c)
r1320	-1	ATP(m) + H2O(m) ⇌ ADP(m) + Pi(m) + ATP-energy(m)
r1321	1	NADH(r) ⇌ NAD+(r) + NADH-redox-potential(r)
r0812	1	ATP(c) + AMP(p) ⇌ ATP(p) + AMP(c)
r1322	3.5	NADH(c) ⇌ NAD+(c) + NADH-redox-potential(c)
r0683	2	Propanoyl-CoA(m) + Ubiquinone(m) → Ubiquinol(m) + Acrylyl-CoA(m)
r1323	4.5	NADH(m) ⇌ NAD+(m) + NADH-redox-potential(m)
r1324	1	NADH(p) ⇌ NAD+(p) + NADH-redox-potential(p)
r1325	-12	NADPH(r) ⇌ NADPH-redox-potential(r) + NADP+(r)
r1326	-2	NADPH(c) ⇌ NADPH-redox-potential(c) + NADP+(c)
r1047	0.5	Pi(c) + H+(PG)(c) → Pi(m) + H+(PG)(m)
r0104	0.5	NAD+(c) + Malate(c) ⇌ OAA(c) + NADH(c)
r0538	1	3-Sulfinoalanine(c) ⇌ CO2(c) + Hypotaurine(c)
r0258	-0.5	Fumarate(c) + H2O(c) ⇌ Malate(c)
r2419	0.5	AKG(c) + Pi(m) ⇌ AKG(m) + Pi(c)
r0851	-1	H2O(m) ⇌ H2O(c)
r0838	1	NH3(c) ⇌ NH3(m)
r0109	-0.5	OAA(c) + Glutamate(c) ⇌ AKG(c) + Aspartate(c)

tcdchola(b) (320)

Definition			Solution												
			exchanges		reactions								transp		
Objective	Constraints	Comment	imports	exports	c	m	r	p	l	n	s	b	s-c	b-c	int
Taurochenodeoxycholate(b)	+Urea −Alanine −Serine −Methionine MCES	de novo synthesis and bile export of Taurochenodeoxycholate	12 O_2(s) 1 Serine(s) 1 Methionine(s) 2 Farnesyl-PP(r) 7 ATP-energy(c) 1 ATP-energy(m) 11 NADPH-redox-potential(r) 1 NADPH-redox-potential(c) 1 NADPH-redox-potential(m)	5.5 H_2O(s) 4 P_i(c) 6.5 CO_2(s) 0.5 Urea(s) 1 Taurochenodeoxycholate(b) 1 NADH-redox-potential(r) 3.5 NADH-redox-potential(c) 4.5 NADH-redox-potential(m) 10 Proton-gradient(m) 1 Na-gradient(c) 1 THF-activated methyl group(c) 2 CoA-activated acetyl group(m)	25	14	18	2	-	-	-	-	7	1	29

ID	flux	reaction
r0854	-9	O2(r) ⇌ O2(c)
r0855	12	O2(s) ⇌ O2(c)

Continued on next page

ID	flux	reaction
r0856	-2	O2(m) ⇌ O2(c)
r0575	1	Presqualene-PP(r) + NADPH(r) ⇌ PPi(r) + Squalene(r) + NADP+(r)
r0576	1	Squalene(r) + NADPH(r) + O2(r) ⇌ H2O(r) + Squalene 2,3-oxide(r) + NADP+(r)
r0144	0.5	Arginine(c) + H2O(c) ⇌ Urea(c) + Ornithine(c)
r0592	-2	H2O(m) + Acrylyl-CoA(m) → 3-Hydroxypropionyl-CoA(m)
r0596	-2	3-Hydroxypropionyl-CoA(m) + H2O(m) → Hydracrylate(m) + CoA(m)
r0740	1	5beta-Cholestane-3alpha,7alpha-diol(m) + NADPH(m) + O2(m) ⇌ 5beta-Cholestane-3alpha,7alpha,26-triol(m) + H2O(m) + NADP+(m)
r0896	0.5	Urea(c) ⇌ Urea(s)
r0745	-1	7alpha-Hydroxycholest-4-en-3-one(c) + NADPH(c) ⇌ 7alpha-Hydroxy-5beta-cholestan-3-one(c) + NADP+(c)
r0017	1	O2(m) + 4 H+(PG)(m) + 4 Ferrocytochrome C(m) → 4 Ferricytochrome C(m) + 2 H2O(m) + 4 H+(PG)(c)
r0030	1.5	ATP(c) + AMP(c) ⇌ 2 ADP(c)
r0747	-1	7alpha-Hydroxy-5beta-cholestan-3-one(c) + NADPH(c) ⇌ 5beta-Cholestane-3alpha,7alpha-diol(c) + NADP+(c)
r0314	1	Serine(c) + Homocysteine(c) ⇌ H2O(c) + L-Cystathionine(c)
r0034	0.5	2 ATP(m) + CO2(m) + H2O(m) + NH3(m) ⇌ 2 ADP(m) + Pi(m) + Carbamoyl-P(m)
r0780	-1	4,4-Dimethyl-5alpha-cholesta-8,14,24-trien-3beta-ol(r) + NADPH(r) ⇌ 14-Demethyllanosterol(r) + NADP+(r)
r0333	-1	Provitamin D3(r) + NADPH(r) ⇌ Cholesterol(r) + NADP+(r)
r0911	0.5	Proline(c) + Glutamate(m) ⇌ Proline(m) + Glutamate(c)
r0781	1	3 O2(r) + 3 NADPH(r) + Lanosterol(r) ⇌ 4,4-Dimethyl-5alpha-cholesta-8,14,24-trien-3beta-ol(r) + Formate(r) + 4 H2O(r) + 3 NADP+(r)
r1291	1	Propanoyl-CoA(c) ⇌ Propanoyl-CoA(m)
r0335	1	Cholesterol(r) + O2(r) + NADPH(r) ⇌ 7alpha-Hydroxycholesterol(r) + H2O(r) + NADP+(r)
r1292	-1	Propanoyl-CoA(c) ⇌ Propanoyl-CoA(p)
r0783	-1	5alpha-Cholesta-7,24-dien-3beta-ol(r) + NADPH(r) ⇌ Lathosterol(r) + NADP+(r)
r1141	-1	Methionine(s) + Na+(s) → Methionine(c) + Na+(c)
r1295	-1	7alpha-Hydroxycholest-4-en-3-one(c) ⇌ 7alpha-Hydroxycholest-4-en-3-one(r)
r2664	1	Na+(c) ⇌ Na+(s) + Na-gradient(c)
r1426	0.5	Proline(m) + H+(PG)(c) → Proline(c) + H+(PG)(m)
r1146	1	4alpha-Methylzymosterol(r) + 2 H2O(r) ⇌ Zymosterol(r) + CO2(r)
r0073	-0.5	AKG(m) + NADH(m) + NH3(m) ⇌ Glutamate(m) + NAD+(m) + H2O(m)
r2518	1	3alpha,7alpha-Dihydroxy-5beta-cholestanate(c) + ATP(c) + H2O(c) ⇌ 3alpha,7alpha-Dihydroxy-5beta-cholestanate(p) + ADP(c) + Pi(c)
r0672	1	7alpha-Hydroxycholesterol(r) + NADP+(r) ⇌ 7alpha-Hydroxycholest-4-en-3-one(r) + NADPH(r)
r0507	2	Ubiquinol(m) + 2 Ferricytochrome C(m) + 4 H+(PG)(m) → Ubiquinone(m) + 4 H+(PG)(c) + 2 Ferrocytochrome C(m)
r0227	1	10-Formyl-THF(c) + H2O(c) + NADP+(c) → THF(c) + CO2(c) + NADPH(c)
r1019	1	Chenodeoxycholoyl-CoA(p) ⇌ Chenodeoxycholoyl-CoA(c)
r0241	1	H2O(c) + L-Cystathionine(c) ⇌ Cysteine(c) + NH3(c) + 2-Oxobutyrate(c)
r0228	1	THF(c) + Formate(c) + ATP(c) → ADP(c) + Pi(c) + 10-Formyl-THF(c)
r0690	1	ATP(p) + CoA(p) + 3alpha,7alpha-Dihydroxy-5beta-cholestanate(p) ⇌ AMP(p) + PPi(p) + 3alpha,7alpha-Dihydroxy-5beta-cholestanoyl-CoA(p)
r1330	10	H+(PG)(c) ⇌ H+(PG)(m) + Proton-gradient(m)
r1319	-7	ATP(c) + H2O(c) ⇌ ADP(c) + Pi(c) + ATP-energy(c)
r0261	0.5	Argininosuccinate(c) ⇌ Fumarate(c) + Arginine(c)
r0698	-1	CoA(p) + 3alpha,7alpha-Dihydroxy-5beta-cholestanoyl-CoA(p) + H2O(p) ⇌ Propanoyl-CoA(p) + Chenodeoxycholoyl-CoA(p)
r0848	10	H2O(r) ⇌ H2O(c)
r0849	-5.5	H2O(s) ⇌ H2O(c)
r0863	-1	CoA(c) ⇌ CoA(m)
r0864	2	CoA(c) ⇌ CoA(p)
r0865	2	CO2(r) ⇌ CO2(c)
r0867	6.5	CO2(c) ⇌ CO2(s)
r0868	-2.5	CO2(c) ⇌ CO2(m)
r0170	1	2 Farnesyl-PP(r) ⇌ PPi(r) + Presqualene-PP(r)
r0887	-1	Formate(c) ⇌ Formate(r)
r0453	0.5	ATP(c) + Citrulline(c) + Aspartate(c) ⇌ AMP(c) + PPi(c) + Argininosuccinate(c)
r0007	3.5	PPi(c) + H2O(c) → 2 Pi(c)
r0738	1	Zymosterol(r) ⇌ 5alpha-Cholesta-7,24-dien-3beta-ol(r)

Continued on next page

ID	flux	reaction
r0174	2	CoA(m) + NAD+(m) + 3-Oxopropanoate(m) → Acetyl-CoA(m) + CO2(m) + NADH(m)
r0739	1	5beta-Cholestane-3alpha,7alpha,26-triol(c) + NAD+(c) ⇌ 3alpha,7alpha-Dihydroxy-5beta-cholestan-26-al(c) + NADH(c)
r0607	1	Squalene 2,3-oxide(r) ⇌ Lanosterol(r)
r0329	0.5	Carbamoyl-P(m) + Ornithine(m) ⇌ Pi(m) + Citrulline(m)
r1134	1	14-Demethyllanosterol(r) + 3 NADPH(r) + 3 O2(r) ⇌ 4 H2O(r) + 4alpha-Methylzymosterol-4-carboxylate(r) + 3 NADP+(r)
r1135	1	3-Keto-4-methylzymosterol(r) + NADP+(r) ⇌ NADPH(r) + 4alpha-Methylzymosterol(r)
r1137	1	NAD+(r) + 4alpha-Methylzymosterol-4-carboxylate(r) ⇌ NADH(r) + CO2(r) + 3-Keto-4-methylzymosterol(r)
r0793	1	Lathosterol(r) + NADPH(r) + O2(r) ⇌ Provitamin D3(r) + 2 H2O(r) + NADP+(r)
r1152	-1	PPi(c) ⇌ PPi(p)
r1153	-2	PPi(c) ⇌ PPi(r)
r1154	1	CoA(m) + NAD+(m) + 2-Oxobutyrate(m) ⇌ CO2(m) + Propanoyl-CoA(m) + NADH(m)
r0211	1	Cysteine(c) + O2(c) ⇌ 3-Sulfinoalanine(c)
r1155	1	2-Oxobutyrate(c) + H+(PG)(c) → 2-Oxobutyrate(m) + H+(PG)(m)
r0798	1	Chenodeoxycholoyl-CoA(c) + Taurine(c) ⇌ CoA(c) + Taurochenodeoxycholate(c)
r2676	1	5-Methyl-THF(c) ⇌ THF(c) + THF-activated methyl group(c)
r0365	2	Hydracrylate(m) + NAD+(m) → NADH(m) + 3-Oxopropanoate(m)
r2678	2	Acetyl-CoA(m) ⇌ CoA(m) + CoA-activated acetyl group(m)
r2526	1	Serine(s) ⇌ Serine(c)
r1020	1	5beta-Cholestane-3alpha,7alpha,26-triol(m) ⇌ 5beta-Cholestane-3alpha,7alpha,26-triol(c)
r0381	1	NAD+(c) + H2O(c) + Hypotaurine(c) ⇌ NADH(c) + Taurine(c)
r1022	1	5beta-Cholestane-3alpha,7alpha-diol(c) ⇌ 5beta-Cholestane-3alpha,7alpha-diol(m)
r0947	-0.5	Citrulline(m) + Ornithine(c) ⇌ Citrulline(c) + Ornithine(m)
r0232	-1	THF(c) + Methionine(c) ⇌ 5-Methyl-THF(c) + Homocysteine(c)
r1320	-1	ATP(m) + H2O(m) ⇌ ADP(m) + Pi(m) + ATP-energy(m)
r1321	1	NADH(r) ⇌ NAD+(r) + NADH-redox-potential(r)
r0812	1	ATP(c) + AMP(p) ⇌ ATP(p) + AMP(c)
r1322	3.5	NADH(c) ⇌ NAD+(c) + NADH-redox-potential(c)
r0683	2	Propanoyl-CoA(m) + Ubiquinone(m) → Ubiquinol(m) + Acrylyl-CoA(m)
r1323	4.5	NADH(m) ⇌ NAD+(m) + NADH-redox-potential(m)
r1028	1	Taurochenodeoxycholate(c) + 2 ATP(c) + 2 H2O(c) → Taurochenodeoxycholate(b) + 2 ADP(c) + 2 Pi(c)
r1325	-11	NADPH(r) ⇌ NADPH-redox-potential(r) + NADP+(r)
r1326	-1	NADPH(c) ⇌ NADPH-redox-potential(c) + NADP+(c)
r1327	-1	NADPH(m) ⇌ NADPH-redox-potential(m) + NADP+(m)
r0688	1	3alpha,7alpha-Dihydroxy-5beta-cholestan-26-al(c) + NAD+(c) + H2O(c) ⇌ NADH(c) + 3alpha,7alpha-Dihydroxy-5beta-cholestanate(c)
r1047	0.5	Pi(c) + H+(PG)(c) → Pi(m) + H+(PG)(m)
r0104	0.5	NAD+(c) + Malate(c) ⇌ OAA(c) + NADH(c)
r0538	1	3-Sulfinoalanine(c) ⇌ CO2(c) + Hypotaurine(c)
r0258	-0.5	Fumarate(c) + H2O(c) ⇌ Malate(c)
r2419	0.5	AKG(c) + Pi(m) ⇌ AKG(m) + Pi(c)
r0838	1	NH3(c) ⇌ NH3(m)
r0109	-0.5	OAA(c) + Glutamate(c) ⇌ AKG(c) + Aspartate(c)
r0852	-1	H2O(p) ⇌ H2O(c)

Chenodiol(b) (321)

Definition			Solution												
			exchanges		reactions								transp		
Objective	Constraints	Comment	imports	exports	c	m	r	p	l	n	s	b	s-c	b-c	int
Chenodiol(b)	+Urea −Alanine −Serine MCES	de novo synthesis and bile export of Chenodiol	10.5 O_2(s) 2 Farnesyl-PP(r) 4 ATP-energy(c) 11 NADPH-redox-potential(r) 1 NADPH-redox-potential(m)	6 H_2O(s) 4 P_i(c) 4 CO_2(s) 1 Chenodiol(b) 1 NADH-redox-potential(r) 1 NADH-redox-potential(c) 2 NADH-redox-potential(m) 6 Proton-gradient(m) 1 CoA-activated acetyl group(m)	10	9	18	2	-	-	-	-	3	1	22

ID	flux	reaction
r0854	-9	O2(r) ⇌ O2(c)
r0855	10.5	O2(s) ⇌ O2(c)
r0856	-1.5	O2(m) ⇌ O2(c)
r0575	1	Presqualene-PP(r) + NADPH(r) ⇌ PPi(r) + Squalene(r) + NADP+(r)
r0576	1	Squalene(r) + NADPH(r) + O2(r) ⇌ H2O(r) + Squalene 2,3-oxide(r) + NADP+(r)
r0592	-1	H2O(m) + Acrylyl-CoA(m) → 3-Hydroxypropionyl-CoA(m)
r0596	-1	3-Hydroxypropionyl-CoA(m) + H2O(m) → Hydracrylate(m) + CoA(m)
r0740	1	5beta-Cholestane-3alpha,7alpha-diol(m) + NADPH(m) + O2(m) ⇌ 5beta-Cholestane-3alpha,7alpha,26-triol(m) + H2O(m) + NADP+(m)
r0745	-1	7alpha-Hydroxycholest-4-en-3-one(c) + NADPH(c) ⇌ 7alpha-Hydroxy-5beta-cholestan-3-one(c) + NADP+(c)
r0017	0.5	O2(m) + 4 H+(PG)(m) + 4 Ferrocytochrome C(m) → 4 Ferricytochrome C(m) + 2 H2O(m) + 4 H+(PG)(c)
r0746	-1	7alpha-Hydroxy-5beta-cholestan-3-one(c) + NADH(c) ⇌ 5beta-Cholestane-3alpha,7alpha-diol(c) + NAD+(c)
r0333	-1	Provitamin D3(r) + NADPH(r) ⇌ Cholesterol(r) + NADP+(r)
r0780	-1	4,4-Dimethyl-5alpha-cholesta-8,14,24-trien-3beta-ol(r) + NADPH(r) ⇌ 14-Demethyllanosterol(r) + NADP+(r)
r0781	1	3 O2(r) + 3 NADPH(r) + Lanosterol(r) ⇌ 4,4-Dimethyl-5alpha-cholesta-8,14,24-trien-3beta-ol(r) + Formate(r) + 4 H2O(r) + 3 NADP+(r)
r1291	1	Propanoyl-CoA(c) ⇌ Propanoyl-CoA(m)
r0335	1	Cholesterol(r) + O2(r) + NADPH(r) ⇌ 7alpha-Hydroxycholesterol(r) + H2O(r) + NADP+(r)
r1292	-1	Propanoyl-CoA(c) ⇌ Propanoyl-CoA(p)
r0783	-1	5alpha-Cholesta-7,24-dien-3beta-ol(r) + NADPH(r) ⇌ Lathosterol(r) + NADP+(r)
r1295	-1	7alpha-Hydroxycholest-4-en-3-one(c) ⇌ 7alpha-Hydroxycholest-4-en-3-one(r)
r1146	1	4alpha-Methylzymosterol(r) + 2 H2O(r) ⇌ Zymosterol(r) + CO2(r)
r0651	-1	ATP(c) + CoA(c) + Chenodiol(c) ⇌ AMP(c) + PPi(c) + Chenodeoxycholoyl-CoA(c)
r2518	1	3alpha,7alpha-Dihydroxy-5beta-cholestanate(c) + ATP(c) + H2O(c) ⇌ 3alpha,7alpha-Dihydroxy-5beta-cholestanate(p) + ADP(c) + Pi(c)
r0672	1	7alpha-Hydroxycholesterol(r) + NADP+(r) ⇌ 7alpha-Hydroxycholest-4-en-3-one(r) + NADPH(r)
r0507	1	Ubiquinol(m) + 2 Ferricytochrome C(m) + 4 H+(PG)(m) → Ubiquinone(m) + 4 H+(PG)(c) + 2 Ferrocytochrome C(m)
r0227	1	10-Formyl-THF(c) + H2O(c) + NADP+(c) → THF(c) + CO2(c) + NADPH(c)
r1019	1	Chenodeoxycholoyl-CoA(p) ⇌ Chenodeoxycholoyl-CoA(c)
r0228	1	THF(c) + Formate(c) + ATP(c) → ADP(c) + Pi(c) + 10-Formyl-THF(c)
r0690	1	ATP(p) + CoA(p) + 3alpha,7alpha-Dihydroxy-5beta-cholestanate(p) ⇌ AMP(p) + PPi(p) + 3alpha,7alpha-Dihydroxy-5beta-cholestanoyl-CoA(p)
r1330	6	H+(PG)(c) ⇌ H+(PG)(m) + Proton-gradient(m)
r1319	-4	ATP(c) + H2O(c) ⇌ ADP(c) + Pi(c) + ATP-energy(c)
r0993	1	2 ATP(c) + 2 H2O(c) + Chenodiol(c) → 2 ADP(c) + 2 Pi(c) + Chenodiol(b)
r0698	-1	CoA(p) + 3alpha,7alpha-Dihydroxy-5beta-cholestanoyl-CoA(p) + H2O(p) ⇌ Propanoyl-CoA(p) + Chenodeoxycholoyl-CoA(p)
r0848	10	H2O(r) ⇌ H2O(c)
r0849	-6	H2O(s) ⇌ H2O(c)
r0863	-1	CoA(c) ⇌ CoA(m)
r0864	2	CoA(c) ⇌ CoA(p)
r0865	2	CO2(r) ⇌ CO2(c)
r0867	4	CO2(c) ⇌ CO2(s)
r0868	-1	CO2(c) ⇌ CO2(m)
r0170	1	2 Farnesyl-PP(r) ⇌ PPi(r) + Presqualene-PP(r)
r0887	-1	Formate(c) ⇌ Formate(r)
r0007	2	PPi(c) + H2O(c) → 2 Pi(c)
r0738	1	Zymosterol(r) ⇌ 5alpha-Cholesta-7,24-dien-3beta-ol(r)
r0174	1	CoA(m) + NAD+(m) + 3-Oxopropanoate(m) → Acetyl-CoA(m) + CO2(m) + NADH(m)
r0739	1	5beta-Cholestane-3alpha,7alpha,26-triol(c) + NAD+(c) ⇌ 3alpha,7alpha-Dihydroxy-5beta-cholestan-26-al(c) + NADH(c)
r0607	1	Squalene 2,3-oxide(r) ⇌ Lanosterol(r)
r1134	1	14-Demethyllanosterol(r) + 3 NADPH(r) + 3 O2(r) ⇌ 4 H2O(r) + 4alpha-Methylzymosterol-4-carboxylate(r) + 3 NADP+(r)
r1135	1	3-Keto-4-methylzymosterol(r) + NADP+(r) ⇌ NADPH(r) + 4alpha-Methylzymosterol(r)
r1137	1	NAD+(r) + 4alpha-Methylzymosterol-4-carboxylate(r) ⇌ NADH(r) + CO2(r) + 3-Keto-4-methylzymosterol(r)
r0793	1	Lathosterol(r) + NADPH(r) + O2(r) ⇌ Provitamin D3(r) + 2 H2O(r) + NADP+(r)
r1152	-1	PPi(c) ⇌ PPi(p)
r1153	-2	PPi(c) ⇌ PPi(r)

Continued on next page

ID	flux	reaction
r0365	1	Hydracrylate(m) + NAD+(m) → NADH(m) + 3-Oxopropanoate(m)
r2678	1	Acetyl-CoA(m) ⇌ CoA(m) + CoA-activated acetyl group(m)
r1020	1	5beta-Cholestane-3alpha,7alpha,26-triol(m) ⇌ 5beta-Cholestane-3alpha,7alpha,26-triol(c)
r1022	1	5beta-Cholestane-3alpha,7alpha-diol(c) ⇌ 5beta-Cholestane-3alpha,7alpha-diol(m)
r1321	1	NADH(r) ⇌ NAD+(r) + NADH-redox-potential(r)
r0812	1	ATP(c) + AMP(p) ⇌ ATP(p) + AMP(c)
r1322	1	NADH(c) ⇌ NAD+(c) + NADH-redox-potential(c)
r0683	1	Propanoyl-CoA(m) + Ubiquinone(m) → Ubiquinol(m) + Acrylyl-CoA(m)
r1323	2	NADH(m) ⇌ NAD+(m) + NADH-redox-potential(m)
r1325	-11	NADPH(r) ⇌ NADPH-redox-potential(r) + NADP+(r)
r1327	-1	NADPH(m) ⇌ NADPH-redox-potential(m) + NADP+(m)
r0688	1	3alpha,7alpha-Dihydroxy-5beta-cholestan-26-al(c) + NAD+(c) + H2O(c) ⇌ NADH(c) + 3alpha,7alpha-Dihydroxy-5beta-cholestanate(c)
r0852	-1	H2O(p) ⇌ H2O(c)

B3.1.2. Phospholipids Bile-PC(b) (322)

Definition			Solution												
			exchanges		reactions								transp		
Objective	Constraints	Comment	imports	exports	c	m	r	p	l	n	s	b	s-c	b-c	int
Bile-PC-pool(b)	MCES ALFA	de novo synthesis and bile export of Bile-PC-pool	0.5 P_i(c) 0.5 Glucose-6P(c) 1 Choline(c) 0.11 Arachidonate(c) 0.83 Palmitate(c) 0.24 Oleate(c) 0.11 Stearate(c) 0.66 Linoleate(c) 0.05 Palmitolate(c) 8.5 ATP-energy(c) 1 NADH-redox-potential(c)	3.5 H_2O(s) 1 Bile-PC-pool(b)	30	-	-	-	-	-	-	-	1	1	-

ID	flux	reaction
r1252	0.05	Palmitolate(c) + ATP(c) + CoA(c) → (2E)-Hexadecenoyl-CoA(c) + AMP(c) + PPi(c)
r0244	1	ATP(c) + Choline(c) → ADP(c) + Phosphocholine(c)
r0136	1	ATP(c) + CMP(c) ⇌ ADP(c) + CDP(c)
r1190	0.06	Arachidonyl-CoA(c) + sn-Glycerol-3P(c) → 1-Acylglycerol-3P-arach(c) + CoA(c)
r1263	0.11	Arachidonate(c) + ATP(c) + CoA(c) → Arachidonyl-CoA(c) + AMP(c) + PPi(c)
r1254	0.11	Stearate(c) + ATP(c) + CoA(c) → Stearoyl-CoA(c) + AMP(c) + PPi(c)
r0192	0.5	Glucose-6P(c) ⇌ Fructose-6P(c)
r0256	0.5	Fructose-1,6PP(c) ⇌ DHAP(c) + GAP(c)
r1319	-8.5	ATP(c) + H2O(c) ⇌ ADP(c) + Pi(c) + ATP-energy(c)
r1256	0.24	ATP(c) + CoA(c) + Oleate(c) → Oleoyl-CoA(c) + AMP(c) + PPi(c)
r1284	0	268 1-Acylglycerol-3P-palmn(c) + 1209 1-Acylglycerol-3P-ol(c) + 3283 1-Acylglycerol-3P-lin(c) + 564 1-Acylglycerol-3P-arach(c) + 4140 1-Acylglycerol-3P-palm(c) + 550 1-Acylglycerol-3P-stea(c) → 10014 1-Acylglycerol-3P-Bile-PC-pool(c)
r1185	0.41	Palmitoyl-CoA(c) + sn-Glycerol-3P(c) → CoA(c) + 1-Acylglycerol-3P-palm(c)
r1285	0	4140 Palmitoyl-CoA(c) + 268 (2E)-Hexadecenoyl-CoA(c) + 550 Stearoyl-CoA(c) + 1209 Oleoyl-CoA(c) + 564 Arachidonyl-CoA(c) + 3283 Linoleoyl-CoA(c) → 10014 Acyl-CoA-Bile-PC-pool(c)
r1258	0.66	Linoleate(c) + ATP(c) + CoA(c) → AMP(c) + PPi(c) + Linoleoyl-CoA(c)
r1186	0.03	(2E)-Hexadecenoyl-CoA(c) + sn-Glycerol-3P(c) → 1-Acylglycerol-3P-palmn(c) + CoA(c)
r0736	0.5	ATP(c) + Fructose-6P(c) → ADP(c) + Fructose-1,6PP(c)
r0201	-1	DHAP(c) + NADH(c) ⇌ NAD+(c) + sn-Glycerol-3P(c)
r0030	2	ATP(c) + AMP(c) ⇌ 2 ADP(c)
r1286	-1	Acyl-CoA-Bile-PC-pool(c) + 1-Acylglycerol-3P-Bile-PC-pool(c) → Phosphatidate-Bile-PC-pool(c) + CoA(c)
r1187	0.05	Stearoyl-CoA(c) + sn-Glycerol-3P(c) → CoA(c) + 1-Acylglycerol-3P-stea(c)
r1287	1	Phosphatidate-Bile-PC-pool(c) + H2O(c) → Pi(c) + 1,2-Diacylglycerol-Bile-PC-pool(c)
r1188	0.12	Oleoyl-CoA(c) + sn-Glycerol-3P(c) → 1-Acylglycerol-3P-ol(c) + CoA(c)
r1288	1	CDP-choline(c) + 1,2-Diacylglycerol-Bile-PC-pool(c) → Bile-PC-pool(c) + CMP(c)
r1189	0.33	Linoleoyl-CoA(c) + sn-Glycerol-3P(c) → 1-Acylglycerol-3P-lin(c) + CoA(c)
r0312	0.83	Palmitate(c) + ATP(c) + CoA(c) → Palmitoyl-CoA(c) + AMP(c) + PPi(c)

Continued on next page

ID	flux	reaction
r0150	1	ATP(c) + CDP(c) ⇌ ADP(c) + CTP(c)
r0421	1	CTP(c) + Phosphocholine(c) → PPi(c) + CDP-choline(c)
r0007	3	PPi(c) + H2O(c) → 2 Pi(c)
r1511	1	Bile-PC-pool(c) + ATP(c) + H2O(c) → Bile-PC-pool(b) + ADP(c) + Pi(c)
r1322	-1	NADH(c) ⇌ NAD+(c) + NADH-redox-potential(c)
r0849	-3.5	H2O(s) ⇌ H2O(c)
r0243	0.5	GAP(c) ⇌ DHAP(c)

SM(b) (323)

Definition			Solution												
			exchanges		reactions								transsp		
Objective	Constraints	Comment	imports	exports	c	m	r	p	l	n	s	b	s-c	b-c	int
SM-pool(b)	MCES ALFA	de novo synthesis and bile export of SM-pool	0.5 P_i(c) 0.5 Glucose-6P(c) 1 Choline(c) 1 Ethanolamine(c) 0.02 Arachidonate(c) 0.91 Palmitate(c) 0.24 Oleate(c) 0.03 gamma-Linolenate(c) 0.8 Palmitolate(c) 10.5 ATP-energy(c) 1 NADPH-redox-potential(c) 1 NADPH-redox-potential(m) 2 Proton-gradient(m)	3.53 H_2O(s) 1 CO_2(s) 1 SM-pool(b) 3 NADH-redox-potential(c) 1 CoA-activated acetyl group(c)	37	2	1	-	-	-	-	-	2	1	11

ID	flux	reaction
r1210	0.0001	6260 Palmitoyl-CoA(c) + 1490 Stearoyl-CoA(c) + 656 Oleoyl-CoA(c) + 147 Arachidonyl-CoA(c) + 270 Linoleoyl-CoA(c) ⇌ 8823 Acyl-CoA-VLDL-SM-pool(c)
r0421	1	CTP(c) + Phosphocholine(c) → PPi(c) + CDP-choline(c)
r1510	1	SM-pool(c) + ATP(c) + H2O(c) → SM-pool(b) + ADP(c) + Pi(c)
r0312	0.91	Palmitate(c) + ATP(c) + CoA(c) → Palmitoyl-CoA(c) + AMP(c) + PPi(c)
r0030	3	ATP(c) + AMP(c) ⇌ 2 ADP(c)
r0313	1	Serine(c) + Palmitoyl-CoA(c) ⇌ 3-Dehydrosphinganine(c) + CoA(c) + CO2(c)
r1256	0.24	ATP(c) + CoA(c) + Oleate(c) → Oleoyl-CoA(c) + AMP(c) + PPi(c)
r0186	1	Ethanolamine-P(c) + H2O(c) → Acetaldehyde(c) + Pi(c) + NH3(c)
r1275	-1	SM-pool(c) ⇌ SM-pool(r)
r1407	1	CDP-choline(r) + Ceramide-pool(r) ⇌ CMP(r) + SM-pool(r)
r0911	1	Proline(c) + Glutamate(m) ⇌ Proline(m) + Glutamate(c)
r0336	1	ATP(c) + Ethanolamine(c) → ADP(c) + Ethanolamine-P(c)
r0337	-1	ADP(c) + 1,3DPG(c) ⇌ ATP(c) + 3PG(c)
r0338	1	3PG(c) + NAD+(c) → 3-Phosphonooxypyruvate(c) + NADH(c)
r1426	1	Proline(m) + H+(PG)(c) → Proline(c) + H+(PG)(m)
r0075	-1	AKG(m) + NADPH(m) + NH3(m) ⇌ Glutamate(m) + H2O(m) + NADP+(m)
r1465	0.17	2 H2O(c) + NAD+(c) + Oleoyl-CoA(c) ⇌ NADH(c) + O2(c) + Stearoyl-CoA(c)
r0243	-0.5	DHAP(c) ⇌ GAP(c)
r1469	-0.03	gamma-Linolenoyl-CoA(c) + H2O(c) + NAD+(c) ⇌ Linoleoyl-CoA(c) + NADH(c) + O2(c)
r1330	-2	H+(PG)(c) ⇌ H+(PG)(m) + Proton-gradient(m)
r0244	1	ATP(c) + Choline(c) → ADP(c) + Phosphocholine(c)
r1319	-10.5	ATP(c) + H2O(c) ⇌ ADP(c) + Pi(c) + ATP-energy(c)
r1488	0.8	ATP(c) + CoA(c) + Palmitolate(c) ⇌ AMP(c) + palmitoleoyl-CoA(c) + PPi(c)
r0849	-3.53	H2O(s) ⇌ H2O(c)
r0583	-1	3-Dehydrosphinganine(c) + NADPH(c) ⇌ Sphinganine(c) + NADP+(c)
r0136	1	ATP(c) + CMP(c) ⇌ ADP(c) + CDP(c)
r0150	1	ATP(c) + CDP(c) ⇌ ADP(c) + CTP(c)
r0867	1	CO2(c) ⇌ CO2(s)
r1240	1	Ceramide-pool(c) ⇌ Ceramide-pool(r)
r0886	-1	CMP(c) ⇌ CMP(r)
r0007	4	PPi(c) + H2O(c) → 2 Pi(c)

Continued on next page

ID	flux	reaction
r0736	0.5	ATP(c) + Fructose-6P(c) → ADP(c) + Fructose-1,6PP(c)
r0159	1	3-Phosphoserine(c) + H2O(c) → Serine(c) + Pi(c)
r0023	-0.8	NAD+(c) + 2 Ferrocytochrome b5(c) ⇌ NADH(c) + 2 Ferricytochrome b5(c)
r1261	0.03	gamma-Linolenate(c) + ATP(c) + CoA(c) → gamma-Linolenoyl-CoA(c) + AMP(c) + PPi(c)
r0177	1	Acetaldehyde(c) + H2O(c) + NADP+(c) → Acetate(c) + NADPH(c)
r1263	0.02	Arachidonate(c) + ATP(c) + CoA(c) → Arachidonyl-CoA(c) + AMP(c) + PPi(c)
r0192	0.5	Glucose-6P(c) ⇌ Fructose-6P(c)
r1412	1	Sphinganine(c) + Acyl-CoA-VLDL-SM-pool(c) ⇌ Dihydroceramide-pool(c) + CoA(c)
r1414	1	Dihydroceramide-pool(c) + O2(c) + NADPH(c) ⇌ 2 H2O(c) + Ceramide-pool(c) + NADP+(c)
r0066	1	ATP(c) + Acetate(c) + CoA(c) → AMP(c) + PPi(c) + Acetyl-CoA(c)
r2677	1	Acetyl-CoA(c) ⇌ CoA(c) + CoA-activated acetyl group(c)
r0510	-0.8	palmitoleoyl-CoA(c) + 2 Ferricytochrome b5(c) + 2 H2O(c) ⇌ Palmitoyl-CoA(c) + O2(c) + 2 Ferrocytochrome b5(c)
r1159	1	CDP-choline(c) ⇌ CDP-choline(r)
r0663	-1	3-Phosphonooxypyruvate(c) + Glutamate(c) → 3-Phosphoserine(c) + AKG(c)
r1322	3	NADH(c) ⇌ NAD+(c) + NADH-redox-potential(c)
r1326	-1	NADPH(c) ⇌ NADPH-redox-potential(c) + NADP+(c)
r0253	1	GAP(c) + Pi(c) + NAD+(c) ⇌ 1,3DPG(c) + NADH(c)
r1327	-1	NADPH(m) ⇌ NADPH-redox-potential(m) + NADP+(m)
r0256	0.5	Fructose-1,6PP(c) ⇌ DHAP(c) + GAP(c)
r1047	1	Pi(c) + H+(PG)(c) → Pi(m) + H+(PG)(m)
r2419	1	AKG(c) + Pi(m) ⇌ AKG(m) + Pi(c)
r0838	1	NH3(c) ⇌ NH3(m)
r0851	1	H2O(m) ⇌ H2O(c)

PS(b) (324)

Definition			Solution												
			exchanges		reactions								transp		
Objective	Constraints	Comment	imports	exports	c	m	r	p	l	n	s	b	s-c	b-c	int
PS-VLDL-pool(b)	MCES ALFA	de novo synthesis and bile export of PS-VLDL-pool	0.5 P_i(c) 1 Pyruvate(m) 0.5 Glucose-6P(c) 1 Ethanolamine(c) 0.53 Arachidonate(c) 0.09 Palmitate(c) 0.09 Oleate(c) 1.25 Stearate(c) 0.05 Linoleate(c) 7.5 ATP-energy(c) 1 NADH-redox-potential(c)	3.5 H_2O(s) 1 PS-VLDL-pool(b) 1 NADH-redox-potential(m) 1 CoA-activated acetyl group(m)	29	3	-	-	-	-	-	-	1	1	1

ID	flux	reaction
r2678	1	Acetyl-CoA(m) ⇌ CoA(m) + CoA-activated acetyl group(m)
r1270	-1	PS-VLDL-pool(b) ← PS-VLDL-pool(c)
r0136	1	ATP(c) + CMP(c) ⇌ ADP(c) + CDP(c)
r1370	-1	PE-PS-VLDL-pool(c) + CO2(c) → PS-VLDL-pool(c)
r1208	0.0001	345 Palmitoyl-CoA(c) + 4820 Stearoyl-CoA(c) + 332 Oleoyl-CoA(c) + 2050 Arachidonyl-CoA(c) + 181 Linoleoyl-CoA(c) → 7728 Acyl-CoA-VLDL-PS-pool(c)
r1190	0.27	Arachidonyl-CoA(c) + sn-Glycerol-3P(c) → 1-Acylglycerol-3P-arach(c) + CoA(c)
r0056	1	Pyruvate(m) + CoA(m) + NAD+(m) → Acetyl-CoA(m) + CO2(m) + NADH(m)
r1263	0.53	Arachidonate(c) + ATP(c) + CoA(c) → Arachidonyl-CoA(c) + AMP(c) + PPi(c)
r1254	1.25	Stearate(c) + ATP(c) + CoA(c) → Stearoyl-CoA(c) + AMP(c) + PPi(c)
r0336	1	ATP(c) + Ethanolamine(c) → ADP(c) + Ethanolamine-P(c)
r0192	0.5	Glucose-6P(c) ⇌ Fructose-6P(c)
r0481	1	Ethanolamine-P(c) + CTP(c) → PPi(c) + CDP-ethanolamine(c)
r0256	0.5	Fructose-1,6PP(c) ⇌ DHAP(c) + GAP(c)
r1319	-7.5	ATP(c) + H2O(c) ⇌ ADP(c) + Pi(c) + ATP-energy(c)
r1256	0.09	ATP(c) + CoA(c) + Oleate(c) → Oleoyl-CoA(c) + AMP(c) + PPi(c)
r1185	0.04	Palmitoyl-CoA(c) + sn-Glycerol-3P(c) → CoA(c) + 1-Acylglycerol-3P-palm(c)
r1258	0.05	Linoleate(c) + ATP(c) + CoA(c) → AMP(c) + PPi(c) + Linoleoyl-CoA(c)

Continued on next page

PS(b) (324)– continued

ID	flux	reaction
r1195	1	1,2-Diacylglycerol-VLDL-PS-pool(c) + CDP-ethanolamine(c) → PE-PS-VLDL-pool(c) + CMP(c)
r0736	0.5	ATP(c) + Fructose-6P(c) → ADP(c) + Fructose-1,6PP(c)
r0201	-1	DHAP(c) + NADH(c) ⇌ NAD+(c) + sn-Glycerol-3P(c)
r0030	2	ATP(c) + AMP(c) ⇌ 2 ADP(c)
r1201	0.0001	332 1-Acylglycerol-3P-ol(c) + 181 1-Acylglycerol-3P-lin(c) + 2050 1-Acylglycerol-3P-arach(c) + 345 1-Acylglycerol-3P-palm(c) + 4820 1-Acylglycerol-3P-stea(c) → 7728 1-Acylglycerol-3P-VLDL-PS-pool(c)
r1187	0.62	Stearoyl-CoA(c) + sn-Glycerol-3P(c) → CoA(c) + 1-Acylglycerol-3P-stea(c)
r1220	1	Phosphatidate-VLDL-PS-pool(c) + H2O(c) → 1,2-Diacylglycerol-VLDL-PS-pool(c) + Pi(c)
r1188	0.04	Oleoyl-CoA(c) + sn-Glycerol-3P(c) → 1-Acylglycerol-3P-ol(c) + CoA(c)
r1189	0.02	Linoleoyl-CoA(c) + sn-Glycerol-3P(c) → 1-Acylglycerol-3P-lin(c) + CoA(c)
r0312	0.09	Palmitate(c) + ATP(c) + CoA(c) → Palmitoyl-CoA(c) + AMP(c) + PPi(c)
r0150	1	ATP(c) + CDP(c) ⇌ ADP(c) + CTP(c)
r0007	3	PPi(c) + H2O(c) → 2 Pi(c)
r1322	-1	NADH(c) ⇌ NAD+(c) + NADH-redox-potential(c)
r1214	-1	Acyl-CoA-VLDL-PS-pool(c) + 1-Acylglycerol-3P-VLDL-PS-pool(c) → Phosphatidate-VLDL-PS-pool(c) + CoA(c)
r0849	-3.5	H2O(s) ⇌ H2O(c)
r1323	1	NADH(m) ⇌ NAD+(m) + NADH-redox-potential(m)
r0868	-1	CO2(c) ⇌ CO2(m)
r0243	0.5	GAP(c) ⇌ DHAP(c)

PE(b) (325)

Definition			Solution												
			exchanges		reactions								transp		
Objective	Constraints	Comment	imports	exports	c	m	r	p	l	n	s	b	s-c	b-c	int
PE-VLDL-pool(b)	MCES ALFA	de novo synthesis and bile export of PE-VLDL-pool	0.5 P_i(c) 0.5 Glucose-6P(c) 1 Ethanolamine(c) 0.6 Arachidonate(c) 0.34 Palmitate(c) 0.17 Oleate(c) 0.54 Stearate(c) 0.36 Linoleate(c) 8.5 ATP-energy(c) 1 NADH-redox-potential(c)	3.5 H_2O(s) 1 PE-VLDL-pool(b)	28	-	-	-	-	-	-	-	1	1	-

ID	flux	reaction
r1207	0.0001	1260 Palmitoyl-CoA(c) + 2010 Stearoyl-CoA(c) + 628 Oleoyl-CoA(c) + 2220 Arachidonyl-CoA(c) + 1330 Linoleoyl-CoA(c) → 7448 Acyl-CoA-VLDL-PE-pool(c)
r0136	1	ATP(c) + CMP(c) ⇌ ADP(c) + CDP(c)
r1190	0.3	Arachidonyl-CoA(c) + sn-Glycerol-3P(c) → 1-Acylglycerol-3P-arach(c) + CoA(c)
r1263	0.6	Arachidonate(c) + ATP(c) + CoA(c) → Arachidonyl-CoA(c) + AMP(c) + PPi(c)
r1254	0.54	Stearate(c) + ATP(c) + CoA(c) → Stearoyl-CoA(c) + AMP(c) + PPi(c)
r0336	1	ATP(c) + Ethanolamine(c) → ADP(c) + Ethanolamine-P(c)
r0192	0.5	Glucose-6P(c) ⇌ Fructose-6P(c)
r1219	1	Phosphatidate-VLDL-PE-pool(c) + H2O(c) → 1,2-Diacylglycerol-VLDL-PE-pool(c) + Pi(c)
r0481	1	Ethanolamine-P(c) + CTP(c) → PPi(c) + CDP-ethanolamine(c)
r0256	0.5	Fructose-1,6PP(c) ⇌ DHAP(c) + GAP(c)
r1319	-8.5	ATP(c) + H2O(c) ⇌ ADP(c) + Pi(c) + ATP-energy(c)
r1256	0.17	ATP(c) + CoA(c) + Oleate(c) → Oleoyl-CoA(c) + AMP(c) + PPi(c)
r1509	1	PE-VLDL-pool(c) + ATP(c) + H2O(c) → PE-VLDL-pool(b) + ADP(c) + Pi(c)
r1194	1	1,2-Diacylglycerol-VLDL-PE-pool(c) + CDP-ethanolamine(c) → PE-VLDL-pool(c) + CMP(c)
r1185	0.17	Palmitoyl-CoA(c) + sn-Glycerol-3P(c) → CoA(c) + 1-Acylglycerol-3P-palm(c)
r1258	0.36	Linoleate(c) + ATP(c) + CoA(c) → AMP(c) + PPi(c) + Linoleoyl-CoA(c)
r1200	0.0001	628 1-Acylglycerol-3P-ol(c) + 1330 1-Acylglycerol-3P-lin(c) + 2220 1-Acylglycerol-3P-arach(c) + 1260 1-Acylglycerol-3P-palm(c) + 2010 1-Acylglycerol-3P-stea(c) → 7448 1-Acylglycerol-3P-VLDL-PE-pool(c)
r0736	0.5	ATP(c) + Fructose-6P(c) → ADP(c) + Fructose-1,6PP(c)
r0201	-1	DHAP(c) + NADH(c) ⇌ NAD+(c) + sn-Glycerol-3P(c)
r0030	2	ATP(c) + AMP(c) ⇌ 2 ADP(c)
r1187	0.27	Stearoyl-CoA(c) + sn-Glycerol-3P(c) → CoA(c) + 1-Acylglycerol-3P-stea(c)

Continued on next page

ID	flux	reaction
r1188	0.08	Oleoyl-CoA(c) + sn-Glycerol-3P(c) → 1-Acylglycerol-3P-ol(c) + CoA(c)
r1189	0.18	Linoleoyl-CoA(c) + sn-Glycerol-3P(c) → 1-Acylglycerol-3P-lin(c) + CoA(c)
r0312	0.34	Palmitate(c) + ATP(c) + CoA(c) → Palmitoyl-CoA(c) + AMP(c) + PPi(c)
r0150	1	ATP(c) + CDP(c) ⇌ ADP(c) + CTP(c)
r1213	-1	Acyl-CoA-VLDL-PE-pool(c) + 1-Acylglycerol-3P-VLDL-PE-pool(c) → Phosphatidate-VLDL-PE-pool(c) + CoA(c)
r0007	3	PPi(c) + H2O(c) → 2 Pi(c)
r1322	-1	NADH(c) ⇌ NAD+(c) + NADH-redox-potential(c)
r0849	-3.5	H2O(s) ⇌ H2O(c)
r0243	0.5	GAP(c) ⇌ DHAP(c)

B3.1.3. Cholesterol Cholesterol(b) (326)

Definition			Solution												
			exchanges		reactions								transp		
Objective	Constraints	Comment	imports	exports	c	m	r	p	l	n	s	b	s-c	b-c	int
Cholesterol(b)	MCES	de novo synthesis and bile export of Cholesterol	8 O_2(s) 2 Farnesyl-PP(r) 3 ATP-energy(c) 10 NADPH-redox-potential(r)	7 H_2O(s) 4 P_i(c) 3 CO_2(s) 1 Cholesterol(b) 1 NADPH-redox-potential(c)	5	-	15	-	-	-	-	-	3	1	7

ID	flux	reaction
r1153	-2	PPi(c) ⇌ PPi(r)
r1135	1	3-Keto-4-methylzymosterol(r) + NADP+(r) ⇌ NADPH(r) + 4alpha-Methylzymosterol(r)
r0887	-1	Formate(c) ⇌ Formate(r)
r1325	-10	NADPH(r) ⇌ NADPH-redox-potential(r) + NADP+(r)
r1136	1	4alpha-Methylzymosterol-4-carboxylate(r) + NADP+(r) ⇌ 3-Keto-4-methylzymosterol(r) + NADPH(r) + CO2(r)
r0227	1	10-Formyl-THF(c) + H2O(c) + NADP+(c) → THF(c) + CO2(c) + NADPH(c)
r1326	1	NADPH(c) ⇌ NADPH-redox-potential(c) + NADP+(c)
r1146	1	4alpha-Methylzymosterol(r) + 2 H2O(r) ⇌ Zymosterol(r) + CO2(r)
r0228	1	THF(c) + Formate(c) + ATP(c) → ADP(c) + Pi(c) + 10-Formyl-THF(c)
r0607	1	Squalene 2,3-oxide(r) ⇌ Lanosterol(r)
r1319	-3	ATP(c) + H2O(c) ⇌ ADP(c) + Pi(c) + ATP-energy(c)
r1149	-2	Pi(c) ⇌ Pi(r)
r0924	1	Cholesterol(r) + 2 ATP(r) + 2 H2O(r) → Cholesterol(b) + 2 ADP(r) + 2 Pi(r)
r0780	-1	4,4-Dimethyl-5alpha-cholesta-8,14,24-trien-3beta-ol(r) + NADPH(r) ⇌ 14-Demethyllanosterol(r) + NADP+(r)
r0781	1	3 O2(r) + 3 NADPH(r) + Lanosterol(r) ⇌ 4,4-Dimethyl-5alpha-cholesta-8,14,24-trien-3beta-ol(r) + Formate(r) + 4 H2O(r) + 3 NADP+(r)
r0854	-8	O2(r) ⇌ O2(c)
r0575	1	Presqualene-PP(r) + NADPH(r) ⇌ PPi(r) + Squalene(r) + NADP+(r)
r0855	8	O2(s) ⇌ O2(c)
r0783	-1	5alpha-Cholesta-7,24-dien-3beta-ol(r) + NADPH(r) ⇌ Lathosterol(r) + NADP+(r)
r0738	1	Zymosterol(r) ⇌ 5alpha-Cholesta-7,24-dien-3beta-ol(r)
r0576	1	Squalene(r) + NADPH(r) + O2(r) ⇌ H2O(r) + Squalene 2,3-oxide(r) + NADP+(r)
r0865	2	CO2(r) ⇌ CO2(c)
r0793	1	Lathosterol(r) + NADPH(r) + O2(r) ⇌ Provitamin D3(r) + 2 H2O(r) + NADP+(r)
r0848	7	H2O(r) ⇌ H2O(c)
r0007	2	PPi(c) + H2O(c) → 2 Pi(c)
r0867	3	CO2(c) ⇌ CO2(s)
r0858	2	ADP(r) + ATP(c) → ADP(c) + ATP(r)
r0849	-7	H2O(s) ⇌ H2O(c)
r0170	1	2 Farnesyl-PP(r) ⇌ PPi(r) + Presqualene-PP(r)
r1134	1	14-Demethyllanosterol(r) + 3 NADPH(r) + 3 O2(r) ⇌ 4 H2O(r) + 4alpha-Methylzymosterol-4-carboxylate(r) + 3 NADP+(r)
r0333	-1	Provitamin D3(r) + NADPH(r) ⇌ Cholesterol(r) + NADP+(r)

B3.2. blood excretion

B3.2.1. Plasma proteins Albumin (327)

Definition			Solution												
			exchanges		reactions								transp		
Objective	Constraints	Comment	imports	exports	c	m	r	p	l	n	s	b	s-c	b-c	int
Albumin	MCES AAA	de novo synthesis and export of Albumin	62 Glutamate(s) 13 Glycine(s) 63 Alanine(s) 60 Lysine(s) 36 Aspartate(s) 27 Arginine(s) 20 Glutamine(s) 28 Serine(s) 7 Methionine(s) 2 Tryptophan(s) 35 Phenylalanine(s) 19 Tyrosine(s) 35 Cysteine(s) 64 Leucine(s) 16 Histidine(s) 24 Proline(s) 17 Asparagine(s) 43 Valine(s) 29 Threonine(s) 9 Isoleucine(s) 3045 ATP-energy(c) 46 Proton-gradient(c)	1 Albumin(s) 239 Na-gradient(c)	3	-	-	-	-	-	-	-	23	-	-

ID	flux	reaction
r2530	64	Leucine(s) ⇌ Leucine(c)
r1144	-62	Glutamate(c) + Na+(c) ← Glutamate(s) + Na+(s)
r1126	-35	Phenylalanine(c) + Na+(c) ← Phenylalanine(s) + Na+(s)
r1072	7	Methionine(s) ⇌ Methionine(c)
r0802	1	Albumin(c) ⇌ Albumin(s)
r2531	24	Proline(s) ⇌ Proline(c)
r1145	-16	Histidine(c) + Na+(c) ← Histidine(s) + Na+(s)
r2523	13	Glycine(s) ⇌ Glycine(c)
r1092	1	63 Alanine(c) + 27 Arginine(c) + 35 Cysteine(c) + 13 Glycine(c) + 16 Histidine(c) + 9 Isoleucine(c) + 64 Leucine(c) + 7 Methionine(c) + 35 Phenylalanine(c) + 24 Proline(c) + 28 Serine(c) + 29 Threonine(c) + 2 Tryptophan(c) + 19 Tyrosine(c) + 43 Valine(c) + 62 Glutamate(c) + 20 Glutamine(c) + 2436 ATP(c) + 2436 H2O(c) + 17 Asparagine(c) + 36 Aspartate(c) + 60 Lysine(c) → Albumin(c) + 609 AMP(c) + 609 PPi(c) + 1827 ADP(c) + 1827 Pi(c)
r2534	29	Threonine(s) ⇌ Threonine(c)
r2525	20	Glutamine(s) ⇌ Glutamine(c)
r1319	-3045	ATP(c) + H2O(c) ⇌ ADP(c) + Pi(c) + ATP-energy(c)
r1058	27	Arginine(s) ⇌ Arginine(c)
r2526	28	Serine(s) ⇌ Serine(c)
r1069	-60	Lysine(c) ⇌ Lysine(s)
r0030	609	ATP(c) + AMP(c) ⇌ 2 ADP(c)
r2664	239	Na+(c) ⇌ Na+(s) + Na-gradient(c)
r2529	35	Cysteine(s) ⇌ Cysteine(c)
r1121	-43	Valine(c) + Na+(c) ← Valine(s) + Na+(s)
r1123	-9	Isoleucine(c) + Na+(c) ← Isoleucine(s) + Na+(s)
r0007	609	PPi(c) + H2O(c) → 2 Pi(c)
r2469	63	Alanine(s) + H+(PG)(s) → Alanine(c) + H+(PG)(c)
r1331	-46	H+(PG)(s) ⇌ H+(PG)(c) + Proton-gradient(c)
r1124	-2	Tryptophan(c) + Na+(c) ← Tryptophan(s) + Na+(s)
r2479	17	Asparagine(s) + Na+(s) + H+(PG)(c) → Asparagine(c) + Na+(c) + H+(PG)(s)
r1143	-36	Na+(c) + Aspartate(c) ← Na+(s) + Aspartate(s)
r1125	19	Tyrosine(s) + Na+(s) → Tyrosine(c) + Na+(c)

Fibrinogen (328)

Definition			Solution												
			exchanges		reactions								transp		
Objective	Constraints	Comment	imports	exports	c	m	r	p	l	n	s	b	s-c	b-c	int
Fibrinogen	MCES AAA	de novo synthesis and export of Fibrinogen	186 Glutamate(s) 262 Glycine(s) 134 Alanine(s) 154 Lysine(s) 176 Aspartate(s) 134 Arginine(s) 110 Glutamine(s) 278 Serine(s) 48 Methionine(s) 60 Tryptophan(s) 98 Phenylalanine(s) 98 Tyrosine(s) 48 Cysteine(s) 166 Leucine(s) 62 Histidine(s) 114 Proline(s) 138 Asparagine(s) 122 Valine(s) 182 Threonine(s) 104 Isoleucine(s) 13370 ATP-energy(c)	1 Fibrinogen(s) 4 Proton-gradient(c) 1044 Na-gradient(c)	3	-	-	-	-	-	-	-	23	-	-

ID	flux	reaction
r2530	166	Leucine(s) ⇌ Leucine(c)
r1144	-186	Glutamate(c) + Na+(c) ← Glutamate(s) + Na+(s)
r1126	-98	Phenylalanine(c) + Na+(c) ← Phenylalanine(s) + Na+(s)
r1072	48	Methionine(s) ⇌ Methionine(c)
r2531	114	Proline(s) ⇌ Proline(c)
r1145	-62	Histidine(c) + Na+(c) ← Histidine(s) + Na+(s)
r2523	262	Glycine(s) ⇌ Glycine(c)
r2534	182	Threonine(s) ⇌ Threonine(c)
r2525	110	Glutamine(s) ⇌ Glutamine(c)
r1319	-13370	ATP(c) + H2O(c) ⇌ ADP(c) + Pi(c) + ATP-energy(c)
r1058	134	Arginine(s) ⇌ Arginine(c)
r2526	278	Serine(s) ⇌ Serine(c)
r1101	1	134 Alanine(c) + 134 Arginine(c) + 48 Cysteine(c) + 262 Glycine(c) + 62 Histidine(c) + 104 Isoleucine(c) + 166 Leucine(c) + 48 Methionine(c) + 98 Phenylalanine(c) + 114 Proline(c) + 278 Serine(c) + 182 Threonine(c) + 60 Tryptophan(c) + 98 Tyrosine(c) + 122 Valine(c) + 110 Glutamine(c) + 186 Glutamate(c) + 10696 ATP(c) + 10696 H2O(c) + 138 Asparagine(c) + 176 Aspartate(c) + 154 Lysine(c) → Fibrinogen(c) + 2674 AMP(c) + 2674 PPi(c) + 8022 ADP(c) + 8022 Pi(c)
r1069	-154	Lysine(c) ⇌ Lysine(s)
r0030	2674	ATP(c) + AMP(c) ⇌ 2 ADP(c)
r2664	1044	Na+(c) ⇌ Na+(s) + Na-gradient(c)
r2529	48	Cysteine(s) ⇌ Cysteine(c)
r1121	-122	Valine(c) + Na+(c) ← Valine(s) + Na+(s)
r1123	-104	Isoleucine(c) + Na+(c) ← Isoleucine(s) + Na+(s)
r1033	1	Fibrinogen(c) ⇌ Fibrinogen(s)
r0007	2674	PPi(c) + H2O(c) → 2 Pi(c)
r2469	134	Alanine(s) + H+(PG)(s) → Alanine(c) + H+(PG)(c)
r1331	4	H+(PG)(s) ⇌ H+(PG)(c) + Proton-gradient(c)
r1124	-60	Tryptophan(c) + Na+(c) ← Tryptophan(s) + Na+(s)
r2479	138	Asparagine(s) + Na+(s) + H+(PG)(c) → Asparagine(c) + Na+(c) + H+(PG)(s)
r1143	-176	Na+(c) + Aspartate(c) ← Na+(s) + Aspartate(s)
r1125	98	Tyrosine(s) + Na+(s) → Tyrosine(c) + Na+(c)

Antichymotrypsin (329)

Definition			Solution												
			exchanges		reactions								transp		
Objective	Constraints	Comment	imports	exports	c	m	r	p	l	n	s	b	s-c	b-c	int
Antichymotrypsin	MCES AAA	de novo synthesis and export of Antichymotrypsin	29 Glutamate(s) 16 Glycine(s) 34 Alanine(s) 26 Lysine(s) 25 Aspartate(s) 16 Arginine(s) 17 Glutamine(s) 30 Serine(s) 14 Methionine(s) 3 Tryptophan(s) 25 Phenylalanine(s) 9 Tyrosine(s) 3 Cysteine(s) 59 Leucine(s) 9 Histidine(s) 16 Proline(s) 19 Asparagine(s) 24 Valine(s) 29 Threonine(s) 20 Isoleucine(s) 2115 ATP-energy(c) 15 Proton-gradient(c)	1 Antichymotrypsin(s) 163 Na-gradient(c)	3	-	-	-	-	-	-	-	23	-	-

ID	flux	reaction
r2530	59	Leucine(s) ⇌ Leucine(c)
r1144	-29	Glutamate(c) + Na+(c) ← Glutamate(s) + Na+(s)
r1126	-25	Phenylalanine(c) + Na+(c) ← Phenylalanine(s) + Na+(s)
r1072	14	Methionine(s) ⇌ Methionine(c)
r2531	16	Proline(s) ⇌ Proline(c)
r1145	-9	Histidine(c) + Na+(c) ← Histidine(s) + Na+(s)
r2523	16	Glycine(s) ⇌ Glycine(c)
r0804	1	Antichymotrypsin(c) ⇌ Antichymotrypsin(s)
r1093	1	34 Alanine(c) + 16 Arginine(c) + 3 Cysteine(c) + 16 Glycine(c) + 9 Histidine(c) + 20 Isoleucine(c) + 59 Leucine(c) + 14 Methionine(c) + 25 Phenylalanine(c) + 16 Proline(c) + 30 Serine(c) + 29 Threonine(c) + 3 Tryptophan(c) + 9 Tyrosine(c) + 24 Valine(c) + 17 Glutamine(c) + 29 Glutamate(c) + 1692 ATP(c) + 1692 H2O(c) + 19 Asparagine(c) + 25 Aspartate(c) + 26 Lysine(c) → 423 AMP(c) + 423 PPi(c) + 1269 ADP(c) + 1269 Pi(c) + Antichymotrypsin(c)
r2534	29	Threonine(s) ⇌ Threonine(c)
r2525	17	Glutamine(s) ⇌ Glutamine(c)
r1319	-2115	ATP(c) + H2O(c) ⇌ ADP(c) + Pi(c) + ATP-energy(c)
r1058	16	Arginine(s) ⇌ Arginine(c)
r2526	30	Serine(s) ⇌ Serine(c)
r1069	-26	Lysine(c) ⇌ Lysine(s)
r0030	423	ATP(c) + AMP(c) ⇌ 2 ADP(c)
r2664	163	Na+(c) ⇌ Na+(s) + Na-gradient(c)
r2529	3	Cysteine(s) ⇌ Cysteine(c)
r1121	-24	Valine(c) + Na+(c) ← Valine(s) + Na+(s)
r1123	-20	Isoleucine(c) + Na+(c) ← Isoleucine(s) + Na+(s)
r0007	423	PPi(c) + H2O(c) → 2 Pi(c)
r2469	34	Alanine(s) + H+(PG)(s) → Alanine(c) + H+(PG)(c)
r1331	-15	H+(PG)(s) ⇌ H+(PG)(c) + Proton-gradient(c)
r1124	-3	Tryptophan(c) + Na+(c) ← Tryptophan(s) + Na+(s)
r2479	19	Asparagine(s) + Na+(s) + H+(PG)(c) → Asparagine(c) + Na+(c) + H+(PG)(s)
r1143	-25	Na+(c) + Aspartate(c) ← Na+(s) + Aspartate(s)
r1125	9	Tyrosine(s) + Na+(s) → Tyrosine(c) + Na+(c)

Antitrypsin (330)

Definition			Solution												
			exchanges		reactions								transp		
Objective	Constraints	Comment	imports	exports	c	m	r	p	l	n	s	b	s-c	b-c	int
Antitrypsin	MCES AAA	de novo synthesis and export of Antitrypsin	32 Glutamate(s) 24 Glycine(s) 26 Alanine(s) 34 Lysine(s) 24 Aspartate(s) 7 Arginine(s) 18 Glutamine(s) 25 Serine(s) 10 Methionine(s) 3 Tryptophan(s) 27 Phenylalanine(s) 6 Tyrosine(s) 3 Cysteine(s) 51 Leucine(s) 13 Histidine(s) 19 Proline(s) 19 Asparagine(s) 27 Valine(s) 30 Threonine(s) 20 Isoleucine(s) 2090 ATP-energy(c) 7 Proton-gradient(c)	1 Antitrypsin(s) 171 Na-gradient(c)	3	-	-	-	-	-	-	-	23	-	-

ID	flux	reaction
r2530	51	Leucine(s) ⇌ Leucine(c)
r1144	-32	Glutamate(c) + Na+(c) ← Glutamate(s) + Na+(s)
r1126	-27	Phenylalanine(c) + Na+(c) ← Phenylalanine(s) + Na+(s)
r1072	10	Methionine(s) ⇌ Methionine(c)
r2531	19	Proline(s) ⇌ Proline(c)
r1145	-13	Histidine(c) + Na+(c) ← Histidine(s) + Na+(s)
r2523	24	Glycine(s) ⇌ Glycine(c)
r2534	30	Threonine(s) ⇌ Threonine(c)
r2525	18	Glutamine(s) ⇌ Glutamine(c)
r1319	-2090	ATP(c) + H2O(c) ⇌ ADP(c) + Pi(c) + ATP-energy(c)
r1094	1	26 Alanine(c) + 7 Arginine(c) + 3 Cysteine(c) + 24 Glycine(c) + 13 Histidine(c) + 20 Isoleucine(c) + 51 Leucine(c) + 10 Methionine(c) + 27 Phenylalanine(c) + 19 Proline(c) + 25 Serine(c) + 30 Threonine(c) + 3 Tryptophan(c) + 6 Tyrosine(c) + 27 Valine(c) + 18 Glutamine(c) + 32 Glutamate(c) + 1672 ATP(c) + 1672 H2O(c) + 19 Asparagine(c) + 24 Aspartate(c) + 34 Lysine(c) → Antitrypsin(c) + 418 AMP(c) + 418 PPi(c) + 1254 ADP(c) + 1254 Pi(c)
r1058	7	Arginine(s) ⇌ Arginine(c)
r0806	1	Antitrypsin(c) ⇌ Antitrypsin(s)
r2526	25	Serine(s) ⇌ Serine(c)
r1069	-34	Lysine(c) ⇌ Lysine(s)
r0030	418	ATP(c) + AMP(c) ⇌ 2 ADP(c)
r2664	171	Na+(c) ⇌ Na+(s) + Na-gradient(c)
r2529	3	Cysteine(s) ⇌ Cysteine(c)
r1121	-27	Valine(c) + Na+(c) ← Valine(s) + Na+(s)
r1123	-20	Isoleucine(c) + Na+(c) ← Isoleucine(s) + Na+(s)
r0007	418	PPi(c) + H2O(c) → 2 Pi(c)
r2469	26	Alanine(s) + H+(PG)(s) → Alanine(c) + H+(PG)(c)
r1331	-7	H+(PG)(s) ⇌ H+(PG)(c) + Proton-gradient(c)
r1124	-3	Tryptophan(c) + Na+(c) ← Tryptophan(s) + Na+(s)
r2479	19	Asparagine(s) + Na+(s) + H+(PG)(c) → Asparagine(c) + Na+(c) + H+(PG)(s)
r1143	-24	Na+(c) + Aspartate(c) ← Na+(s) + Aspartate(s)
r1125	6	Tyrosine(s) + Na+(s) → Tyrosine(c) + Na+(c)

ApoA1 (331)

Definition			Solution												
			exchanges		reactions								transp		
Objective	Constraints	Comment	imports	exports	c	m	r	p	l	n	s	b	s-c	b-c	int
ApoA1	MCES AAA	de novo synthesis and export of ApoA1	30 Glutamate(s) 11 Glycine(s) 23 Alanine(s) 22 Lysine(s) 16 Aspartate(s) 17 Arginine(s) 19 Glutamine(s) 16 Serine(s) 4 Methionine(s) 5 Tryptophan(s) 8 Phenylalanine(s) 7 Tyrosine(s) 41 Leucine(s) 6 Histidine(s) 10 Proline(s) 5 Asparagine(s) 15 Valine(s) 12 Threonine(s) 1335 ATP-energy(c)	1 ApoA1(s) 8 Proton-gradient(c) 122 Na-gradient(c)	3	-	-	-	-	-	-	-	21	-	-

ID	flux	reaction
r2530	41	Leucine(s) ⇌ Leucine(c)
r1144	-30	Glutamate(c) + Na+(c) ← Glutamate(s) + Na+(s)
r1126	-8	Phenylalanine(c) + Na+(c) ← Phenylalanine(s) + Na+(s)
r1072	4	Methionine(s) ⇌ Methionine(c)
r2531	10	Proline(s) ⇌ Proline(c)
r1145	-6	Histidine(c) + Na+(c) ← Histidine(s) + Na+(s)
r2532	5	Asparagine(s) ⇌ Asparagine(c)
r2524	23	Alanine(s) ⇌ Alanine(c)
r2534	12	Threonine(s) ⇌ Threonine(c)
r1319	-1335	ATP(c) + H2O(c) ⇌ ADP(c) + Pi(c) + ATP-energy(c)
r1058	17	Arginine(s) ⇌ Arginine(c)
r1069	-22	Lysine(c) ⇌ Lysine(s)
r0808	1	ApoA1(c) ⇌ ApoA1(s)
r0030	267	ATP(c) + AMP(c) ⇌ 2 ADP(c)
r2474	16	Serine(s) + Na+(s) → Serine(c) + Na+(c)
r2664	122	Na+(c) ⇌ Na+(s) + Na-gradient(c)
r1121	-15	Valine(c) + Na+(c) ← Valine(s) + Na+(s)
r1113	1	23 Alanine(c) + 17 Arginine(c) + 19 Glutamine(c) + 30 Glutamate(c) + 11 Glycine(c) + 6 Histidine(c) + 41 Leucine(c) + 4 Methionine(c) + 8 Phenylalanine(c) + 10 Proline(c) + 16 Serine(c) + 12 Threonine(c) + 5 Tryptophan(c) + 7 Tyrosine(c) + 15 Valine(c) + 1068 ATP(c) + 1068 H2O(c) + 5 Asparagine(c) + 16 Aspartate(c) + 22 Lysine(c) → ApoA1(c) + 267 AMP(c) + 267 PPi(c) + 801 ADP(c) + 801 Pi(c)
r2477	19	Glutamine(s) + Na+(s) + H+(PG)(c) → Glutamine(c) + Na+(c) + H+(PG)(s)
r2468	11	Glycine(s) + H+(PG)(s) → Glycine(c) + H+(PG)(c)
r0007	267	PPi(c) + H2O(c) → 2 Pi(c)
r1331	8	H+(PG)(s) ⇌ H+(PG)(c) + Proton-gradient(c)
r1124	-5	Tryptophan(c) + Na+(c) ← Tryptophan(s) + Na+(s)
r1143	-16	Na+(c) + Aspartate(c) ← Na+(s) + Aspartate(s)
r1125	7	Tyrosine(s) + Na+(s) → Tyrosine(c) + Na+(c)

ApoB100(r) (332)

Definition			Solution												
			exchanges		reactions								transp		
Objective	Constraints	Comment	imports	exports	c	m	r	p	l	n	s	b	s-c	b-c	int
ApoB100(r)	MCES AAA	de novo synthesis of ApoB100 in ER/Golgi	298 Glutamate(s) 207 Glycine(s) 275 Alanine(s) 357 Lysine(s) 233 Aspartate(s) 150 Arginine(s) 230 Glutamine(s) 392 Serine(s) 79 Methionine(s) 37 Tryptophan(s) 224 Phenylalanine(s) 151 Tyrosine(s) 25 Cysteine(s) 533 Leucine(s) 115 Histidine(s) 171 Proline(s) 247 Asparagine(s) 252 Valine(s) 300 Threonine(s) 285 Isoleucine(s) 22805 ATP-energy(c)	1 ApoB100(r) 40 Proton-gradient(c) 2259 Na-gradient(c)	3	-	-	-	-	-	-	-	22	-	1

ID	flux	reaction
r2530	533	Leucine(s) ⇌ Leucine(c)
r1144	-298	Glutamate(c) + Na+(c) ← Glutamate(s) + Na+(s)
r1126	-224	Phenylalanine(c) + Na+(c) ← Phenylalanine(s) + Na+(s)
r1072	79	Methionine(s) ⇌ Methionine(c)
r2531	171	Proline(s) ⇌ Proline(c)
r1145	-115	Histidine(c) + Na+(c) ← Histidine(s) + Na+(s)
r1127	1	ApoB100(c) → ApoB100(r)
r2524	275	Alanine(s) ⇌ Alanine(c)
r2534	300	Threonine(s) ⇌ Threonine(c)
r2525	230	Glutamine(s) ⇌ Glutamine(c)
r1319	-22805	ATP(c) + H2O(c) ⇌ ADP(c) + Pi(c) + ATP-energy(c)
r1058	150	Arginine(s) ⇌ Arginine(c)
r1095	1	275 Alanine(c) + 150 Arginine(c) + 25 Cysteine(c) + 207 Glycine(c) + 115 Histidine(c) + 285 Isoleucine(c) + 533 Leucine(c) + 79 Methionine(c) + 224 Phenylalanine(c) + 171 Proline(c) + 392 Serine(c) + 300 Threonine(c) + 37 Tryptophan(c) + 151 Tyrosine(c) + 252 Valine(c) + 230 Glutamine(c) + 298 Glutamate(c) + 18244 ATP(c) + 18244 H2O(c) + 247 Asparagine(c) + 233 Aspartate(c) + 357 Lysine(c) → ApoB100(c) + 4561 AMP(c) + 4561 PPi(c) + 13683 ADP(c) + 13683 Pi(c)
r1069	-357	Lysine(c) ⇌ Lysine(s)
r0030	4561	ATP(c) + AMP(c) ⇌ 2 ADP(c)
r2474	392	Serine(s) + Na+(s) → Serine(c) + Na+(c)
r2664	2259	Na+(c) ⇌ Na+(s) + Na-gradient(c)
r2475	25	Cysteine(s) + Na+(s) → Cysteine(c) + Na+(c)
r1121	-252	Valine(c) + Na+(c) ← Valine(s) + Na+(s)
r2468	207	Glycine(s) + H+(PG)(s) → Glycine(c) + H+(PG)(c)
r1123	-285	Isoleucine(c) + Na+(c) ← Isoleucine(s) + Na+(s)
r0007	4561	PPi(c) + H2O(c) → 2 Pi(c)
r1331	40	H+(PG)(s) ⇌ H+(PG)(c) + Proton-gradient(c)
r1124	-37	Tryptophan(c) + Na+(c) ← Tryptophan(s) + Na+(s)
r2479	247	Asparagine(s) + Na+(s) + H+(PG)(c) → Asparagine(c) + Na+(c) + H+(PG)(s)
r1143	-233	Na+(c) + Aspartate(c) ← Na+(s) + Aspartate(s)
r1125	151	Tyrosine(s) + Na+(s) → Tyrosine(c) + Na+(c)

ApoC1(c) (333)

Definition			Solution												
			exchanges		reactions								transp		
Objective	Constraints	Comment	imports	exports	c	m	r	p	l	n	s	b	s-c	b-c	int
ApoC1(c)	MCES AAA	de novo synthesis of cytosolic ApoC1	8 Glutamate(s) 3 Glycine(s) 5 Alanine(s) 9 Lysine(s) 4 Aspartate(s) 4 Arginine(s) 3 Glutamine(s) 9 Serine(s) 2 Methionine(s) 1 Tryptophan(s) 4 Phenylalanine(s) 12 Leucine(s) 4 Proline(s) 1 Asparagine(s) 7 Valine(s) 3 Threonine(s) 4 Isoleucine(s) 415 ATP-energy(c) 2 Proton-gradient(c)	1 ApoC1(c) 38 Na-gradient(c)	3	-	-	-	-	-	-	-	19	-	-

ID	flux	reaction
r2530	12	Leucine(s) ⇌ Leucine(c)
r1144	-8	Glutamate(c) + Na+(c) ← Glutamate(s) + Na+(s)
r1126	-4	Phenylalanine(c) + Na+(c) ← Phenylalanine(s) + Na+(s)
r1072	2	Methionine(s) ⇌ Methionine(c)
r2531	4	Proline(s) ⇌ Proline(c)
r2524	5	Alanine(s) ⇌ Alanine(c)
r2534	3	Threonine(s) ⇌ Threonine(c)
r2525	3	Glutamine(s) ⇌ Glutamine(c)
r1319	-415	ATP(c) + H2O(c) ⇌ ADP(c) + Pi(c) + ATP-energy(c)

Continued on next page

ID	flux	reaction
r1058	4	Arginine(s) ⇌ Arginine(c)
r1069	-9	Lysine(c) ⇌ Lysine(s)
r0030	83	ATP(c) + AMP(c) ⇌ 2 ADP(c)
r2474	9	Serine(s) + Na+(s) → Serine(c) + Na+(c)
r2664	38	Na+(c) ⇌ Na+(s) + Na-gradient(c)
r1121	-7	Valine(c) + Na+(c) ← Valine(s) + Na+(s)
r1098	1	5 Alanine(c) + 4 Arginine(c) + 3 Glycine(c) + 4 Isoleucine(c) + 12 Leucine(c) + 2 Methionine(c) + 4 Phenylalanine(c) + 4 Proline(c) + 9 Serine(c) + 3 Threonine(c) + Tryptophan(c) + 7 Valine(c) + 3 Glutamine(c) + 8 Glutamate(c) + 332 ATP(c) + 332 H2O(c) + Asparagine(c) + 4 Aspartate(c) + 9 Lysine(c) → ApoC1(c) + 83 AMP(c) + 83 PPi(c) + 249 ADP(c) + 249 Pi(c)
r2468	3	Glycine(s) + H+(PG)(s) → Glycine(c) + H+(PG)(c)
r1123	-4	Isoleucine(c) + Na+(c) ← Isoleucine(s) + Na+(s)
r0007	83	PPi(c) + H2O(c) → 2 Pi(c)
r1331	-2	H+(PG)(s) ⇌ H+(PG)(c) + Proton-gradient(c)
r1124	-1	Tryptophan(c) + Na+(c) ← Tryptophan(s) + Na+(s)
r2479	1	Asparagine(s) + Na+(s) + H+(PG)(c) → Asparagine(c) + Na+(c) + H+(PG)(s)
r1143	-4	Na+(c) + Aspartate(c) ← Na+(s) + Aspartate(s)

ApoC2(c) (334)

Definition			Solution												
			exchanges		reactions								transp		
Objective	Constraints	Comment	imports	exports	c	m	r	p	l	n	s	b	s-c	b-c	int
ApoC2(c)	MCES AAA	de novo synthesis of cytosolic ApoC2	8 Glutamate(s) 5 Glycine(s) 7 Alanine(s) 6 Lysine(s) 4 Aspartate(s) 2 Arginine(s) 8 Glutamine(s) 9 Serine(s) 3 Methionine(s) 1 Tryptophan(s) 4 Phenylalanine(s) 5 Tyrosine(s) 15 Leucine(s) 5 Proline(s) 1 Asparagine(s) 7 Valine(s) 10 Threonine(s) 1 Isoleucine(s) 505 ATP-energy(c) 4 Proton-gradient(c)	1 ApoC2(c) 40 Na-gradient(c)	3	-	-	-	-	-	-	-	20	-	-

ID	flux	reaction
r2530	15	Leucine(s) ⇌ Leucine(c)
r1144	-8	Glutamate(c) + Na+(c) ← Glutamate(s) + Na+(s)
r1126	-4	Phenylalanine(c) + Na+(c) ← Phenylalanine(s) + Na+(s)
r1072	3	Methionine(s) ⇌ Methionine(c)
r2531	5	Proline(s) ⇌ Proline(c)
r2524	7	Alanine(s) ⇌ Alanine(c)
r2534	10	Threonine(s) ⇌ Threonine(c)
r2525	8	Glutamine(s) ⇌ Glutamine(c)
r1319	-505	ATP(c) + H2O(c) ⇌ ADP(c) + Pi(c) + ATP-energy(c)
r1058	2	Arginine(s) ⇌ Arginine(c)
r1069	-6	Lysine(c) ⇌ Lysine(s)
r0030	101	ATP(c) + AMP(c) ⇌ 2 ADP(c)
r2474	9	Serine(s) + Na+(s) → Serine(c) + Na+(c)
r2664	40	Na+(c) ⇌ Na+(s) + Na-gradient(c)
r1121	-7	Valine(c) + Na+(c) ← Valine(s) + Na+(s)
r1099	1	7 Alanine(c) + 2 Arginine(c) + 8 Glutamine(c) + 5 Glycine(c) + Isoleucine(c) + 15 Leucine(c) + 3 Methionine(c) + 4 Phenylalanine(c) + 5 Proline(c) + 9 Serine(c) + 10 Threonine(c) + Tryptophan(c) + 5 Tyrosine(c) + 7 Valine(c) + 8 Glutamate(c) + 404 ATP(c) + 404 H2O(c) + Asparagine(c) + 4 Aspartate(c) + 6 Lysine(c) → ApoC2(c) + 101 AMP(c) + 101 PPi(c) + 303 ADP(c) + 303 Pi(c)
r2468	5	Glycine(s) + H+(PG)(s) → Glycine(c) + H+(PG)(c)
r1123	-1	Isoleucine(c) + Na+(c) ← Isoleucine(s) + Na+(s)
r0007	101	PPi(c) + H2O(c) → 2 Pi(c)

Continued on next page

ID	flux	reaction
r1331	-4	H+(PG)(s) ⇌ H+(PG)(c) + Proton-gradient(c)
r1124	-1	Tryptophan(c) + Na+(c) ← Tryptophan(s) + Na+(s)
r2479	1	Asparagine(s) + Na+(s) + H+(PG)(c) → Asparagine(c) + Na+(c) + H+(PG)(s)
r1143	-4	Na+(c) + Aspartate(c) ← Na+(s) + Aspartate(s)
r1125	5	Tyrosine(s) + Na+(s) → Tyrosine(c) + Na+(c)

ApoC3(c) (335)

Definition			Solution												
			exchanges		reactions								transp		
Objective	Constraints	Comment	imports	exports	c	m	r	p	l	n	s	b	s-c	b-c	int
ApoC3(c)	MCES AAA	de novo synthesis of cytosolic ApoC3	5 Glutamate(s) 3 Glycine(s) 15 Alanine(s) 6 Lysine(s) 7 Aspartate(s) 4 Arginine(s) 6 Glutamine(s) 12 Serine(s) 3 Methionine(s) 3 Tryptophan(s) 4 Phenylalanine(s) 2 Tyrosine(s) 11 Leucine(s) 1 Histidine(s) 3 Proline(s) 9 Valine(s) 5 Threonine(s) 495 ATP-energy(c) 3 Proton-gradient(c)	1 ApoC3(c) 43 Na-gradient(c)	3	-	-	-	-	-	-	-	19	-	-

ID	flux	reaction
r2530	11	Leucine(s) ⇌ Leucine(c)
r1144	-5	Glutamate(c) + Na+(c) ← Glutamate(s) + Na+(s)
r1126	-4	Phenylalanine(c) + Na+(c) ← Phenylalanine(s) + Na+(s)
r1072	3	Methionine(s) ⇌ Methionine(c)
r2531	3	Proline(s) ⇌ Proline(c)
r1145	-1	Histidine(c) + Na+(c) ← Histidine(s) + Na+(s)
r2524	15	Alanine(s) ⇌ Alanine(c)
r2534	5	Threonine(s) ⇌ Threonine(c)
r2525	6	Glutamine(s) ⇌ Glutamine(c)
r1319	-495	ATP(c) + H2O(c) ⇌ ADP(c) + Pi(c) + ATP-energy(c)
r1058	4	Arginine(s) ⇌ Arginine(c)
r1100	1	15 Alanine(c) + 4 Arginine(c) + 3 Glycine(c) + Histidine(c) + 11 Leucine(c) + 3 Methionine(c) + 4 Phenylalanine(c) + 3 Proline(c) + 12 Serine(c) + 5 Threonine(c) + 3 Tryptophan(c) + 2 Tyrosine(c) + 9 Valine(c) + 6 Glutamine(c) + 5 Glutamate(c) + 396 ATP(c) + 396 H2O(c) + 7 Aspartate(c) + 6 Lysine(c) → ApoC3(c) + 99 AMP(c) + 99 PPi(c) + 297 ADP(c) + 297 Pi(c)
r1069	-6	Lysine(c) ⇌ Lysine(s)
r0030	99	ATP(c) + AMP(c) ⇌ 2 ADP(c)
r2474	12	Serine(s) + Na+(s) → Serine(c) + Na+(c)
r2664	43	Na+(c) ⇌ Na+(s) + Na-gradient(c)
r1121	-9	Valine(c) + Na+(c) ← Valine(s) + Na+(s)
r2468	3	Glycine(s) + H+(PG)(s) → Glycine(c) + H+(PG)(c)
r0007	99	PPi(c) + H2O(c) → 2 Pi(c)
r1331	-3	H+(PG)(s) ⇌ H+(PG)(c) + Proton-gradient(c)
r1124	-3	Tryptophan(c) + Na+(c) ← Tryptophan(s) + Na+(s)
r1143	-7	Na+(c) + Aspartate(c) ← Na+(s) + Aspartate(s)
r1125	2	Tyrosine(s) + Na+(s) → Tyrosine(c) + Na+(c)

ApoE(c) (336)

Definition			Solution													
			exchanges		reactions								transsp		
Objective	Constraints	Comment	imports	exports	c	m	r	p	l	n	s	b	s-c	b-c	int
ApoE(c)	MCES AAA	de novo synthesis of cytosolic ApoE	40 Glutamate(s) 18 Glycine(s) 39 Alanine(s) 13 Lysine(s) 11 Aspartate(s) 34 Arginine(s) 32 Glutamine(s) 14 Serine(s) 8 Methionine(s) 8 Tryptophan(s) 4 Phenylalanine(s) 4 Tyrosine(s) 2 Cysteine(s) 41 Leucine(s) 2 Histidine(s) 8 Proline(s) 1 Asparagine(s) 24 Valine(s) 12 Threonine(s) 2 Isoleucine(s) 1585 ATP-energy(c) 17 Proton-gradient(c)	1 ApoE(c) 112 Na-gradient(c)	3	-	-	-	-	-	-	-	22	-	-

ID	flux	reaction
r2530	41	Leucine(s) ⇌ Leucine(c)
r1144	-40	Glutamate(c) + Na+(c) ← Glutamate(s) + Na+(s)
r1126	-4	Phenylalanine(c) + Na+(c) ← Phenylalanine(s) + Na+(s)
r1072	8	Methionine(s) ⇌ Methionine(c)
r2531	8	Proline(s) ⇌ Proline(c)
r1145	-2	Histidine(c) + Na+(c) ← Histidine(s) + Na+(s)
r2524	39	Alanine(s) ⇌ Alanine(c)
r2534	12	Threonine(s) ⇌ Threonine(c)
r2525	32	Glutamine(s) ⇌ Glutamine(c)
r1319	-1585	ATP(c) + H2O(c) ⇌ ADP(c) + Pi(c) + ATP-energy(c)
r1058	34	Arginine(s) ⇌ Arginine(c)
r1069	-13	Lysine(c) ⇌ Lysine(s)
r0030	317	ATP(c) + AMP(c) ⇌ 2 ADP(c)
r2474	14	Serine(s) + Na+(s) → Serine(c) + Na+(c)
r2664	112	Na+(c) ⇌ Na+(s) + Na-gradient(c)
r2475	2	Cysteine(s) + Na+(s) → Cysteine(c) + Na+(c)
r1121	-24	Valine(c) + Na+(c) ← Valine(s) + Na+(s)
r1112	1	39 Alanine(c) + 34 Arginine(c) + 2 Cysteine(c) + 32 Glutamine(c) + 40 Glutamate(c) + 18 Glycine(c) + 2 Histidine(c) + 2 Isoleucine(c) + 41 Leucine(c) + 8 Methionine(c) + 4 Phenylalanine(c) + 8 Proline(c) + 14 Serine(c) + 12 Threonine(c) + 8 Tryptophan(c) + 4 Tyrosine(c) + 24 Valine(c) + 1268 ATP(c) + 1268 H2O(c) + Asparagine(c) + 11 Aspartate(c) + 13 Lysine(c) → ApoE(c) + 317 AMP(c) + 317 PPi(c) + 951 ADP(c) + 951 Pi(c)
r2468	18	Glycine(s) + H+(PG)(s) → Glycine(c) + H+(PG)(c)
r1123	-2	Isoleucine(c) + Na+(c) ← Isoleucine(s) + Na+(s)
r0007	317	PPi(c) + H2O(c) → 2 Pi(c)
r1331	-17	H+(PG)(s) ⇌ H+(PG)(c) + Proton-gradient(c)
r1124	-8	Tryptophan(c) + Na+(c) ← Tryptophan(s) + Na+(s)
r2479	1	Asparagine(s) + Na+(s) + H+(PG)(c) → Asparagine(c) + Na+(c) + H+(PG)(s)
r1143	-11	Na+(c) + Aspartate(c) ← Na+(s) + Aspartate(s)
r1125	4	Tyrosine(s) + Na+(s) → Tyrosine(c) + Na+(c)

Plasminogen (337)

Definition			Solution												
			exchanges		reactions								transp		
Objective	Constraints	Comment	imports	exports	c	m	r	p	l	n	s	b	s-c	b-c	int
Plasminogen	MCES AAA	de novo synthesis and export of Plasminogen	56 Glutamate(s) 62 Glycine(s) 37 Alanine(s) 49 Lysine(s) 36 Aspartate(s) 42 Arginine(s) 31 Glutamine(s) 56 Serine(s) 11 Methionine(s) 19 Tryptophan(s) 21 Phenylalanine(s) 30 Tyrosine(s) 48 Cysteine(s) 48 Leucine(s) 24 Histidine(s) 69 Proline(s) 40 Asparagine(s) 48 Valine(s) 61 Threonine(s) 22 Isoleucine(s) 4050 ATP-energy(c)	1 Plasminogen(s) 3 Proton-gradient(c) 296 Na-gradient(c)	3	-	-	-	-	-	-	-	23	-	-

ID	flux	reaction
r2530	48	Leucine(s) ⇌ Leucine(c)
r1144	-56	Glutamate(c) + Na+(c) ← Glutamate(s) + Na+(s)
r1126	-21	Phenylalanine(c) + Na+(c) ← Phenylalanine(s) + Na+(s)
r1072	11	Methionine(s) ⇌ Methionine(c)
r1054	1	Plasminogen(c) ⇌ Plasminogen(s)
r2531	69	Proline(s) ⇌ Proline(c)
r1145	-24	Histidine(c) + Na+(c) ← Histidine(s) + Na+(s)
r2523	62	Glycine(s) ⇌ Glycine(c)
r2534	61	Threonine(s) ⇌ Threonine(c)
r2525	31	Glutamine(s) ⇌ Glutamine(c)
r1319	-4050	ATP(c) + H2O(c) ⇌ ADP(c) + Pi(c) + ATP-energy(c)
r1058	42	Arginine(s) ⇌ Arginine(c)
r2526	56	Serine(s) ⇌ Serine(c)
r1069	-49	Lysine(c) ⇌ Lysine(s)
r0030	810	ATP(c) + AMP(c) ⇌ 2 ADP(c)
r2664	296	Na+(c) ⇌ Na+(s) + Na-gradient(c)
r2529	48	Cysteine(s) ⇌ Cysteine(c)
r1121	-48	Valine(c) + Na+(c) ← Valine(s) + Na+(s)
r1103	1	37 Alanine(c) + 42 Arginine(c) + 48 Cysteine(c) + 62 Glycine(c) + 24 Histidine(c) + 22 Isoleucine(c) + 48 Leucine(c) + 11 Methionine(c) + 21 Phenylalanine(c) + 69 Proline(c) + 56 Serine(c) + 61 Threonine(c) + 19 Tryptophan(c) + 30 Tyrosine(c) + 48 Valine(c) + 31 Glutamine(c) + 56 Glutamate(c) + 3240 ATP(c) + 3240 H2O(c) + 40 Asparagine(c) + 36 Aspartate(c) + 49 Lysine(c) → Plasminogen(c) + 810 AMP(c) + 810 PPi(c) + 2430 ADP(c) + 2430 Pi(c)
r1123	-22	Isoleucine(c) + Na+(c) ← Isoleucine(s) + Na+(s)
r0007	810	PPi(c) + H2O(c) → 2 Pi(c)
r2469	37	Alanine(s) + H+(PG)(s) → Alanine(c) + H+(PG)(c)
r1331	3	H+(PG)(s) ⇌ H+(PG)(c) + Proton-gradient(c)
r1124	-19	Tryptophan(c) + Na+(c) ← Tryptophan(s) + Na+(s)
r2479	40	Asparagine(s) + Na+(s) + H+(PG)(c) → Asparagine(c) + Na+(c) + H+(PG)(s)
r1143	-36	Na+(c) + Aspartate(c) ← Na+(s) + Aspartate(s)
r1125	30	Tyrosine(s) + Na+(s) → Tyrosine(c) + Na+(c)

Prothrombin (338)

Definition			Solution												
			exchanges		reactions								transp		
Objective	Constraints	Comment	imports	exports	c	m	r	p	l	n	s	b	s-c	b-c	int
Prothrombin	MCES AAA	de novo synthesis and export of Prothrombin	51 Glutamate(s) 49 Glycine(s) 42 Alanine(s) 29 Lysine(s) 35 Aspartate(s) 44 Arginine(s) 26 Glutamine(s) 38 Serine(s) 9 Methionine(s) 14 Tryptophan(s) 21 Phenylalanine(s) 21 Tyrosine(s) 26 Cysteine(s) 51 Leucine(s) 13 Histidine(s) 33 Proline(s) 25 Asparagine(s) 37 Valine(s) 36 Threonine(s) 22 Isoleucine(s) 3110 ATP-energy(c) 17 Proton-gradient(c)	1 Prothrombin(s) 239 Na-gradient(c)	3	-	-	-	-	-	-	-	23	-	-

ID	flux	reaction
r2530	51	Leucine(s) ⇌ Leucine(c)
r1144	-51	Glutamate(c) + Na+(c) ← Glutamate(s) + Na+(s)
r1126	-21	Phenylalanine(c) + Na+(c) ← Phenylalanine(s) + Na+(s)
r1072	9	Methionine(s) ⇌ Methionine(c)
r2531	33	Proline(s) ⇌ Proline(c)
r1145	-13	Histidine(c) + Na+(c) ← Histidine(s) + Na+(s)
r2523	49	Glycine(s) ⇌ Glycine(c)
r1056	1	Prothrombin(c) ⇌ Prothrombin(s)
r2534	36	Threonine(s) ⇌ Threonine(c)
r2525	26	Glutamine(s) ⇌ Glutamine(c)
r1319	-3110	ATP(c) + H2O(c) ⇌ ADP(c) + Pi(c) + ATP-energy(c)
r1058	44	Arginine(s) ⇌ Arginine(c)
r2526	38	Serine(s) ⇌ Serine(c)
r1069	-29	Lysine(c) ⇌ Lysine(s)
r0030	622	ATP(c) + AMP(c) ⇌ 2 ADP(c)
r2664	239	Na+(c) ⇌ Na+(s) + Na-gradient(c)
r2529	26	Cysteine(s) ⇌ Cysteine(c)
r1121	-37	Valine(c) + Na+(c) ← Valine(s) + Na+(s)
r1104	1	42 Alanine(c) + 44 Arginine(c) + 26 Cysteine(c) + 49 Glycine(c) + 13 Histidine(c) + 22 Isoleucine(c) + 51 Leucine(c) + 9 Methionine(c) + 21 Phenylalanine(c) + 33 Proline(c) + 38 Serine(c) + 36 Threonine(c) + 14 Tryptophan(c) + 21 Tyrosine(c) + 37 Valine(c) + 26 Glutamine(c) + 51 Glutamate(c) + 2488 ATP(c) + 2488 H2O(c) + 25 Asparagine(c) + 35 Aspartate(c) + 29 Lysine(c) → Prothrombin(c) + 622 AMP(c) + 622 PPi(c) + 1866 ADP(c) + 1866 Pi(c)
r1123	-22	Isoleucine(c) + Na+(c) ← Isoleucine(s) + Na+(s)
r0007	622	PPi(c) + H2O(c) → 2 Pi(c)
r2469	42	Alanine(s) + H+(PG)(s) → Alanine(c) + H+(PG)(c)
r1331	-17	H+(PG)(s) ⇌ H+(PG)(c) + Proton-gradient(c)
r1124	-14	Tryptophan(c) + Na+(c) ← Tryptophan(s) + Na+(s)
r2479	25	Asparagine(s) + Na+(s) + H+(PG)(c) → Asparagine(c) + Na+(c) + H+(PG)(s)
r1143	-35	Na+(c) + Aspartate(c) ← Na+(s) + Aspartate(s)
r1125	21	Tyrosine(s) + Na+(s) → Tyrosine(c) + Na+(c)

ApoTransferin (339)

Definition			Solution													
			exchanges		reactions								transp		
Objective	Constraints	Comment	imports	exports	c	m	r	p	l	n	s	b	s-c	b-c	int
ApoTransferin	MCES AAA	de novo synthesis and export of ApoTransferin	42 Glutamate(s) 52 Glycine(s) 61 Alanine(s) 58 Lysine(s) 45 Aspartate(s) 27 Arginine(s) 17 Glutamine(s) 41 Serine(s) 10 Methionine(s) 8 Tryptophan(s) 28 Phenylalanine(s) 26 Tyrosine(s) 40 Cysteine(s) 65 Leucine(s) 19 Histidine(s) 32 Proline(s) 34 Asparagine(s) 48 Valine(s) 30 Threonine(s) 15 Isoleucine(s) 3490 ATP-energy(c) 27 Proton-gradient(c)	1 ApoTransferin(s) 265 Na-gradient(c)	3	-	-	-	-	-	-	-	23	-	-

ID	flux	reaction
r2530	65	Leucine(s) ⇌ Leucine(c)
r1144	-42	Glutamate(c) + Na+(c) ← Glutamate(s) + Na+(s)
r1126	-28	Phenylalanine(c) + Na+(c) ← Phenylalanine(s) + Na+(s)
r1081	1	ApoTransferin(c) ⇌ ApoTransferin(s)
r1072	10	Methionine(s) ⇌ Methionine(c)
r2531	32	Proline(s) ⇌ Proline(c)
r1145	-19	Histidine(c) + Na+(c) ← Histidine(s) + Na+(s)
r2523	52	Glycine(s) ⇌ Glycine(c)
r2534	30	Threonine(s) ⇌ Threonine(c)
r2525	17	Glutamine(s) ⇌ Glutamine(c)
r1319	-3490	ATP(c) + H2O(c) ⇌ ADP(c) + Pi(c) + ATP-energy(c)
r1058	27	Arginine(s) ⇌ Arginine(c)
r2526	41	Serine(s) ⇌ Serine(c)
r1069	-58	Lysine(c) ⇌ Lysine(s)
r0030	698	ATP(c) + AMP(c) ⇌ 2 ADP(c)
r2664	265	Na+(c) ⇌ Na+(s) + Na-gradient(c)
r2529	40	Cysteine(s) ⇌ Cysteine(c)
r1121	-48	Valine(c) + Na+(c) ← Valine(s) + Na+(s)
r1123	-15	Isoleucine(c) + Na+(c) ← Isoleucine(s) + Na+(s)
r1105	1	61 Alanine(c) + 27 Arginine(c) + 40 Cysteine(c) + 52 Glycine(c) + 19 Histidine(c) + 15 Isoleucine(c) + 65 Leucine(c) + 10 Methionine(c) + 28 Phenylalanine(c) + 32 Proline(c) + 41 Serine(c) + 30 Threonine(c) + 8 Tryptophan(c) + 26 Tyrosine(c) + 48 Valine(c) + 17 Glutamine(c) + 42 Glutamate(c) + 2792 ATP(c) + 2792 H2O(c) + 34 Asparagine(c) + 45 Aspartate(c) + 58 Lysine(c) → ApoTransferin(c) + 698 AMP(c) + 698 PPi(c) + 2094 ADP(c) + 2094 Pi(c)
r0007	698	PPi(c) + H2O(c) → 2 Pi(c)
r2469	61	Alanine(s) + H+(PG)(s) → Alanine(c) + H+(PG)(c)
r1331	-27	H+(PG)(s) ⇌ H+(PG)(c) + Proton-gradient(c)
r1124	-8	Tryptophan(c) + Na+(c) ← Tryptophan(s) + Na+(s)
r2479	34	Asparagine(s) + Na+(s) + H+(PG)(c) → Asparagine(c) + Na+(c) + H+(PG)(s)
r1143	-45	Na+(c) + Aspartate(c) ← Na+(s) + Aspartate(s)
r1125	26	Tyrosine(s) + Na+(s) → Tyrosine(c) + Na+(c)

Haptoglobin (340)

Definition			Solution													
			exchanges		reactions								transp			
Objective	Constraints	Comment	imports	exports	c	m	r	p	l	n	s	b	s-c	b-c	int
Haptoglobin	MCES AAA	de novo synthesis and export of Haptoglobin	25 Glutamate(s) 31 Glycine(s) 30 Alanine(s) 35 Lysine(s) 25 Aspartate(s) 9 Arginine(s) 17 Glutamine(s) 18 Serine(s) 5 Methionine(s) 8 Tryptophan(s) 8 Phenylalanine(s) 21 Tyrosine(s) 12 Cysteine(s) 31 Leucine(s) 13 Histidine(s) 21 Proline(s) 21 Asparagine(s) 36 Valine(s) 22 Threonine(s) 18 Isoleucine(s) 2030 ATP-energy(c) 9 Proton-gradient(c)	1 Haptoglobin(s) 175 Na-gradient(c)	3	-	-	-	-	-	-	-	23	-	-

ID	flux	reaction
r2530	31	Leucine(s) ⇌ Leucine(c)
r1144	-25	Glutamate(c) + Na+(c) ← Glutamate(s) + Na+(s)
r1126	-8	Phenylalanine(c) + Na+(c) ← Phenylalanine(s) + Na+(s)
r1072	5	Methionine(s) ⇌ Methionine(c)
r2531	21	Proline(s) ⇌ Proline(c)
r1145	-13	Histidine(c) + Na+(c) ← Histidine(s) + Na+(s)
r2523	31	Glycine(s) ⇌ Glycine(c)
r1048	1	Haptoglobin(c) ⇌ Haptoglobin(s)
r2534	22	Threonine(s) ⇌ Threonine(c)
r2525	17	Glutamine(s) ⇌ Glutamine(c)
r1319	-2030	ATP(c) + H2O(c) ⇌ ADP(c) + Pi(c) + ATP-energy(c)
r1058	9	Arginine(s) ⇌ Arginine(c)
r2526	18	Serine(s) ⇌ Serine(c)
r1069	-35	Lysine(c) ⇌ Lysine(s)
r0030	406	ATP(c) + AMP(c) ⇌ 2 ADP(c)
r1102	1	30 Alanine(c) + 9 Arginine(c) + 12 Cysteine(c) + 31 Glycine(c) + 13 Histidine(c) + 18 Isoleucine(c) + 31 Leucine(c) + 5 Methionine(c) + 8 Phenylalanine(c) + 21 Proline(c) + 18 Serine(c) + 22 Threonine(c) + 8 Tryptophan(c) + 21 Tyrosine(c) + 36 Valine(c) + 17 Glutamine(c) + 25 Glutamate(c) + 1624 ATP(c) + 1624 H2O(c) + 21 Asparagine(c) + 25 Aspartate(c) + 35 Lysine(c) → Haptoglobin(c) + 406 AMP(c) + 406 PPi(c) + 1218 ADP(c) + 1218 Pi(c)
r2664	175	Na+(c) ⇌ Na+(s) + Na-gradient(c)
r2529	12	Cysteine(s) ⇌ Cysteine(c)
r1121	-36	Valine(c) + Na+(c) ← Valine(s) + Na+(s)
r1123	-18	Isoleucine(c) + Na+(c) ← Isoleucine(s) + Na+(s)
r0007	406	PPi(c) + H2O(c) → 2 Pi(c)
r2469	30	Alanine(s) + H+(PG)(s) → Alanine(c) + H+(PG)(c)
r1331	-9	H+(PG)(s) ⇌ H+(PG)(c) + Proton-gradient(c)
r1124	-8	Tryptophan(c) + Na+(c) ← Tryptophan(s) + Na+(s)
r2479	21	Asparagine(s) + Na+(s) + H+(PG)(c) → Asparagine(c) + Na+(c) + H+(PG)(s)
r1143	-25	Na+(c) + Aspartate(c) ← Na+(s) + Aspartate(s)
r1125	21	Tyrosine(s) + Na+(s) → Tyrosine(c) + Na+(c)

Collagen ADIPO(c) synthesis (341)

Definition			Solution												
			exchanges		reactions								transp		
Objective	Constraints	Comment	imports	exports	c	m	r	p	l	n	s	b	s-c	b-c	int
Collagen ADIPO(c)	MCES AAA	test synthesis of Collagen ADIPO c	12 Glutamate(s) 39 Glycine(s) 12 Alanine(s) 12 Lysine(s) 15 Aspartate(s) 7 Arginine(s) 10 Glutamine(s) 7 Serine(s) 5 Methionine(s) 2 Tryptophan(s) 10 Phenylalanine(s) 14 Tyrosine(s) 2 Cysteine(s) 23 Leucine(s) 8 Histidine(s) 17 Proline(s) 11 Asparagine(s) 14 Valine(s) 15 Threonine(s) 9 Isoleucine(s) 1220 ATP-energy(c) 28 Proton-gradient(c)	1 Collagen ADIPO(c) 104 Na-gradient(c)	4	-	-	-	-	-	-	-	22	-	-

ID	flux	reaction
r2530	23	Leucine(s) ⇌ Leucine(c)
r1144	-12	Glutamate(c) + Na+(c) ← Glutamate(s) + Na+(s)
r1126	-10	Phenylalanine(c) + Na+(c) ← Phenylalanine(s) + Na+(s)
r1072	5	Methionine(s) ⇌ Methionine(c)
r2540	1	12 Alanine(c) + 2 Cysteine(c) + 15 Aspartate(c) + 12 Glutamate(c) + 10 Phenylalanine(c) + 39 Glycine(c) + 8 Histidine(c) + 9 Isoleucine(c) + 12 Lysine(c) + 23 Leucine(c) + 5 Methionine(c) + 11 Asparagine(c) + 17 Proline(c) + 10 Glutamine(c) + 7 Arginine(c) + 7 Serine(c) + 15 Threonine(c) + 14 Valine(c) + 2 Tryptophan(c) + 14 Tyrosine(c) + 976 ATP(c) + 976 H2O(c) → Collagen ADIPO(c) + 732 ADP(c) + 732 Pi(c) + 244 AMP(c) + 244 PPi(c)
r2531	17	Proline(s) ⇌ Proline(c)
r1145	-8	Histidine(c) + Na+(c) ← Histidine(s) + Na+(s)
r2524	12	Alanine(s) ⇌ Alanine(c)
r2534	15	Threonine(s) ⇌ Threonine(c)
r2525	10	Glutamine(s) ⇌ Glutamine(c)
r1319	-1220	ATP(c) + H2O(c) ⇌ ADP(c) + Pi(c) + ATP-energy(c)
r1058	7	Arginine(s) ⇌ Arginine(c)
r1069	-12	Lysine(c) ⇌ Lysine(s)
r0030	244	ATP(c) + AMP(c) ⇌ 2 ADP(c)
r2474	7	Serine(s) + Na+(s) → Serine(c) + Na+(c)
r2664	104	Na+(c) ⇌ Na+(s) + Na-gradient(c)
r2475	2	Cysteine(s) + Na+(s) → Cysteine(c) + Na+(c)
r1121	-14	Valine(c) + Na+(c) ← Valine(s) + Na+(s)
r2468	39	Glycine(s) + H+(PG)(s) → Glycine(c) + H+(PG)(c)
r1123	-9	Isoleucine(c) + Na+(c) ← Isoleucine(s) + Na+(s)
r0007	244	PPi(c) + H2O(c) → 2 Pi(c)
r1331	-28	H+(PG)(s) ⇌ H+(PG)(c) + Proton-gradient(c)
r1124	-2	Tryptophan(c) + Na+(c) ← Tryptophan(s) + Na+(s)
r2479	11	Asparagine(s) + Na+(s) + H+(PG)(c) → Asparagine(c) + Na+(c) + H+(PG)(s)
r1143	-15	Na+(c) + Aspartate(c) ← Na+(s) + Aspartate(s)
r1125	14	Tyrosine(s) + Na+(s) → Tyrosine(c) + Na+(c)

Collagen BGH3(c) synthesis (342)

Definition			Solution													
			exchanges		reactions									transp		
Objective	Constraints	Comment	imports	exports	c	m	r	p	l	n	s	b	s-c	b-c	int
Collagen BGH3(c)	MCES AAA	test synthesis of Collagen BGH3 c	42 Glutamate(s) 48 Glycine(s) 61 Alanine(s) 33 Lysine(s) 27 Aspartate(s) 37 Arginine(s) 22 Glutamine(s) 41 Serine(s) 14 Methionine(s) 2 Tryptophan(s) 16 Phenylalanine(s) 19 Tyrosine(s) 11 Cysteine(s) 87 Leucine(s) 19 Histidine(s) 31 Proline(s) 40 Asparagine(s) 47 Valine(s) 46 Threonine(s) 40 Isoleucine(s) 3415 ATP-energy(c) 8 Proton-gradient(c)	1 Collagen BGH3(c) 304 Na-gradient(c)	4	-	-	-	-	-	-	-	22	-	-

ID	flux	reaction
r2530	87	Leucine(s) ⇌ Leucine(c)
r1144	-42	Glutamate(c) + Na+(c) ← Glutamate(s) + Na+(s)
r1126	-16	Phenylalanine(c) + Na+(c) ← Phenylalanine(s) + Na+(s)
r1072	14	Methionine(s) ⇌ Methionine(c)
r2531	31	Proline(s) ⇌ Proline(c)
r1145	-19	Histidine(c) + Na+(c) ← Histidine(s) + Na+(s)
r2542	1	61 Alanine(c) + 11 Cysteine(c) + 27 Aspartate(c) + 42 Glutamate(c) + 16 Phenylalanine(c) + 48 Glycine(c) + 19 Histidine(c) + 40 Isoleucine(c) + 33 Lysine(c) + 87 Leucine(c) + 14 Methionine(c) + 40 Asparagine(c) + 31 Proline(c) + 22 Glutamine(c) + 37 Arginine(c) + 41 Serine(c) + 46 Threonine(c) + 47 Valine(c) + 2 Tryptophan(c) + 19 Tyrosine(c) + 2732 ATP(c) + 2732 H2O(c) → Collagen BGH3(c) + 2049 ADP(c) + 2049 Pi(c) + 683 AMP(c) + 683 PPi(c)
r2524	61	Alanine(s) ⇌ Alanine(c)
r2534	46	Threonine(s) ⇌ Threonine(c)
r2525	22	Glutamine(s) ⇌ Glutamine(c)
r1319	-3415	ATP(c) + H2O(c) ⇌ ADP(c) + Pi(c) + ATP-energy(c)
r1058	37	Arginine(s) ⇌ Arginine(c)
r1069	-33	Lysine(c) ⇌ Lysine(s)
r0030	683	ATP(c) + AMP(c) ⇌ 2 ADP(c)
r2474	41	Serine(s) + Na+(s) → Serine(c) + Na+(c)
r2664	304	Na+(c) ⇌ Na+(s) + Na-gradient(c)
r2475	11	Cysteine(s) + Na+(s) → Cysteine(c) + Na+(c)
r1121	-47	Valine(c) + Na+(c) ← Valine(s) + Na+(s)
r2468	48	Glycine(s) + H+(PG)(s) → Glycine(c) + H+(PG)(c)
r1123	-40	Isoleucine(c) + Na+(c) ← Isoleucine(s) + Na+(s)
r0007	683	PPi(c) + H2O(c) → 2 Pi(c)
r1331	-8	H+(PG)(s) ⇌ H+(PG)(c) + Proton-gradient(c)
r1124	-2	Tryptophan(c) + Na+(c) ← Tryptophan(s) + Na+(s)
r2479	40	Asparagine(s) + Na+(s) + H+(PG)(c) → Asparagine(c) + Na+(c) + H+(PG)(s)
r1143	-27	Na+(c) + Aspartate(c) ← Na+(s) + Aspartate(s)
r1125	19	Tyrosine(s) + Na+(s) → Tyrosine(c) + Na+(c)

Collagen C43BP(c) synthesis (343)

Definition			Solution												
			exchanges		reactions								transp		
Objective	Constraints	Comment	imports	exports	c	m	r	p	l	n	s	b	s-c	b-c	int
Collagen C43BP(c)	MCES AAA	test synthesis of Collagen C43BP c	54 Glutamate(s) 33 Glycine(s) 37 Alanine(s) 40 Lysine(s) 45 Aspartate(s) 34 Arginine(s) 23 Glutamine(s) 58 Serine(s) 10 Methionine(s) 14 Tryptophan(s) 22 Phenylalanine(s) 19 Tyrosine(s) 12 Cysteine(s) 38 Leucine(s) 19 Histidine(s) 24 Proline(s) 29 Asparagine(s) 49 Valine(s) 35 Threonine(s) 29 Isoleucine(s) 3120 ATP-energy(c) 4 Proton-gradient(c)	1 Collagen C43BP(c) 350 Na-gradient(c)	4	-	-	-	-	-	-	-	22	-	-

ID	flux	reaction
r2530	38	Leucine(s) ⇌ Leucine(c)
r1144	-54	Glutamate(c) + Na+(c) ← Glutamate(s) + Na+(s)
r1126	-22	Phenylalanine(c) + Na+(c) ← Phenylalanine(s) + Na+(s)
r1072	10	Methionine(s) ⇌ Methionine(c)
r2531	24	Proline(s) ⇌ Proline(c)
r1145	-19	Histidine(c) + Na+(c) ← Histidine(s) + Na+(s)
r2524	37	Alanine(s) ⇌ Alanine(c)
r2534	35	Threonine(s) ⇌ Threonine(c)
r2525	23	Glutamine(s) ⇌ Glutamine(c)
r1319	-3120	ATP(c) + H2O(c) ⇌ ADP(c) + Pi(c) + ATP-energy(c)
r1058	34	Arginine(s) ⇌ Arginine(c)
r2544	1	37 Alanine(c) + 12 Cysteine(c) + 45 Aspartate(c) + 54 Glutamate(c) + 22 Phenylalanine(c) + 33 Glycine(c) + 19 Histidine(c) + 29 Isoleucine(c) + 40 Lysine(c) + 38 Leucine(c) + 10 Methionine(c) + 29 Asparagine(c) + 24 Proline(c) + 23 Glutamine(c) + 34 Arginine(c) + 58 Serine(c) + 35 Threonine(c) + 49 Valine(c) + 14 Tryptophan(c) + 19 Tyrosine(c) + 2496 ATP(c) + 2496 H2O(c) → Collagen C43BP(c) + 1872 ADP(c) + 1872 Pi(c) + 624 AMP(c) + 624 PPi(c)
r1069	-40	Lysine(c) ⇌ Lysine(s)
r0030	624	ATP(c) + AMP(c) ⇌ 2 ADP(c)
r2474	58	Serine(s) + Na+(s) → Serine(c) + Na+(c)
r2664	350	Na+(c) ⇌ Na+(s) + Na-gradient(c)
r2475	12	Cysteine(s) + Na+(s) → Cysteine(c) + Na+(c)
r1121	-49	Valine(c) + Na+(c) ← Valine(s) + Na+(s)
r2468	33	Glycine(s) + H+(PG)(s) → Glycine(c) + H+(PG)(c)
r1123	-29	Isoleucine(c) + Na+(c) ← Isoleucine(s) + Na+(s)
r0007	624	PPi(c) + H2O(c) → 2 Pi(c)
r1331	-4	H+(PG)(s) ⇌ H+(PG)(c) + Proton-gradient(c)
r1124	-14	Tryptophan(c) + Na+(c) ← Tryptophan(s) + Na+(s)
r2479	29	Asparagine(s) + Na+(s) + H+(PG)(c) → Asparagine(c) + Na+(c) + H+(PG)(s)
r1143	-45	Na+(c) + Aspartate(c) ← Na+(s) + Aspartate(s)
r1125	19	Tyrosine(s) + Na+(s) → Tyrosine(c) + Na+(c)

Collagen CCBE1(c) synthesis (344)

Definition			Solution												
			exchanges		reactions								transp		
Objective	Constraints	Comment	imports	exports	c	m	r	p	l	n	s	b	s-c	b-c	int
Collagen CCBE1(c)	MCES AAA	test synthesis of Collagen CCBE1 c	26 Glutamate(s) 52 Glycine(s) 20 Alanine(s) 23 Lysine(s) 24 Aspartate(s) 29 Arginine(s) 13 Glutamine(s) 23 Serine(s) 8 Methionine(s) 1 Tryptophan(s) 10 Phenylalanine(s) 17 Tyrosine(s) 21 Cysteine(s) 32 Leucine(s) 8 Histidine(s) 47 Proline(s) 9 Asparagine(s) 9 Valine(s) 22 Threonine(s) 12 Isoleucine(s) 2030 ATP-energy(c) 43 Proton-gradient(c)	1 Collagen CCBE1(c) 160 Na-gradient(c)	4	-	-	-	-	-	-	-	22	-	-

ID	flux	reaction
r2530	32	Leucine(s) ⇌ Leucine(c)
r1144	-26	Glutamate(c) + Na+(c) ← Glutamate(s) + Na+(s)
r1126	-10	Phenylalanine(c) + Na+(c) ← Phenylalanine(s) + Na+(s)
r1072	8	Methionine(s) ⇌ Methionine(c)
r2531	47	Proline(s) ⇌ Proline(c)
r1145	-8	Histidine(c) + Na+(c) ← Histidine(s) + Na+(s)
r2524	20	Alanine(s) ⇌ Alanine(c)
r2534	22	Threonine(s) ⇌ Threonine(c)
r2525	13	Glutamine(s) ⇌ Glutamine(c)
r1319	-2030	ATP(c) + H2O(c) ⇌ ADP(c) + Pi(c) + ATP-energy(c)
r1058	29	Arginine(s) ⇌ Arginine(c)
r1069	-23	Lysine(c) ⇌ Lysine(s)
r0030	406	ATP(c) + AMP(c) ⇌ 2 ADP(c)
r2546	1	20 Alanine(c) + 21 Cysteine(c) + 24 Aspartate(c) + 26 Glutamate(c) + 10 Phenylalanine(c) + 52 Glycine(c) + 8 Histidine(c) + 12 Isoleucine(c) + 23 Lysine(c) + 32 Leucine(c) + 8 Methionine(c) + 9 Asparagine(c) + 47 Proline(c) + 13 Glutamine(c) + 29 Arginine(c) + 23 Serine(c) + 22 Threonine(c) + 9 Valine(c) + Tryptophan(c) + 17 Tyrosine(c) + 1624 ATP(c) + 1624 H2O(c) → Collagen CCBE1(c) + 1218 ADP(c) + 1218 Pi(c) + 406 AMP(c) + 406 PPi(c)
r2474	23	Serine(s) + Na+(s) → Serine(c) + Na+(c)
r2664	160	Na+(c) ⇌ Na+(s) + Na-gradient(c)
r2475	21	Cysteine(s) + Na+(s) → Cysteine(c) + Na+(c)
r1121	-9	Valine(c) + Na+(c) ← Valine(s) + Na+(s)
r2468	52	Glycine(s) + H+(PG)(s) → Glycine(c) + H+(PG)(c)
r1123	-12	Isoleucine(c) + Na+(c) ← Isoleucine(s) + Na+(s)
r0007	406	PPi(c) + H2O(c) → 2 Pi(c)
r1331	-43	H+(PG)(s) ⇌ H+(PG)(c) + Proton-gradient(c)
r1124	-1	Tryptophan(c) + Na+(c) ← Tryptophan(s) + Na+(s)
r2479	9	Asparagine(s) + Na+(s) + H+(PG)(c) → Asparagine(c) + Na+(c) + H+(PG)(s)
r1143	-24	Na+(c) + Aspartate(c) ← Na+(s) + Aspartate(s)
r1125	17	Tyrosine(s) + Na+(s) → Tyrosine(c) + Na+(c)

Collagen CD36(c) synthesis (345)

Definition			Solution												
			exchanges		reactions								transsp		
Objective	Constraints	Comment	imports	exports	c	m	r	p	l	n	s	b	s-c	b-c	int
Collagen CD36(c)	MCES AAA	test synthesis of Collagen CD36 c	24 Glutamate(s) 28 Glycine(s) 25 Alanine(s) 30 Lysine(s) 20 Aspartate(s) 17 Arginine(s) 19 Glutamine(s) 33 Serine(s) 11 Methionine(s) 5 Tryptophan(s) 27 Phenylalanine(s) 20 Tyrosine(s) 10 Cysteine(s) 41 Leucine(s) 4 Histidine(s) 23 Proline(s) 31 Asparagine(s) 44 Valine(s) 25 Threonine(s) 35 Isoleucine(s) 2360 ATP-energy(c)	3 Proton-gradient(c) 1 Collagen CD36(c) 253 Na-gradient(c)	4	-	-	-	-	-	-	-	22	-	-

ID	flux	reaction
r2530	41	Leucine(s) ⇌ Leucine(c)
r1144	-24	Glutamate(c) + Na+(c) ← Glutamate(s) + Na+(s)
r1126	-27	Phenylalanine(c) + Na+(c) ← Phenylalanine(s) + Na+(s)
r1072	11	Methionine(s) ⇌ Methionine(c)
r2531	23	Proline(s) ⇌ Proline(c)
r1145	-4	Histidine(c) + Na+(c) ← Histidine(s) + Na+(s)
r2524	25	Alanine(s) ⇌ Alanine(c)
r2534	25	Threonine(s) ⇌ Threonine(c)
r2525	19	Glutamine(s) ⇌ Glutamine(c)
r1319	-2360	ATP(c) + H2O(c) ⇌ ADP(c) + Pi(c) + ATP-energy(c)
r1058	17	Arginine(s) ⇌ Arginine(c)
r1069	-30	Lysine(c) ⇌ Lysine(s)
r0030	472	ATP(c) + AMP(c) ⇌ 2 ADP(c)
r2474	33	Serine(s) + Na+(s) → Serine(c) + Na+(c)
r2664	253	Na+(c) ⇌ Na+(s) + Na-gradient(c)
r2475	10	Cysteine(s) + Na+(s) → Cysteine(c) + Na+(c)
r1121	-44	Valine(c) + Na+(c) ← Valine(s) + Na+(s)
r2548	1	25 Alanine(c) + 10 Cysteine(c) + 20 Aspartate(c) + 24 Glutamate(c) + 27 Phenylalanine(c) + 28 Glycine(c) + 4 Histidine(c) + 35 Isoleucine(c) + 30 Lysine(c) + 41 Leucine(c) + 11 Methionine(c) + 31 Asparagine(c) + 23 Proline(c) + 19 Glutamine(c) + 17 Arginine(c) + 33 Serine(c) + 25 Threonine(c) + 44 Valine(c) + 5 Tryptophan(c) + 20 Tyrosine(c) + 1888 ATP(c) + 1888 H2O(c) → Collagen CD36(c) + 1416 ADP(c) + 1416 Pi(c) + 472 AMP(c) + 472 PPi(c)
r2468	28	Glycine(s) + H+(PG)(s) → Glycine(c) + H+(PG)(c)
r1123	-35	Isoleucine(c) + Na+(c) ← Isoleucine(s) + Na+(s)
r0007	472	PPi(c) + H2O(c) → 2 Pi(c)
r1331	3	H+(PG)(s) ⇌ H+(PG)(c) + Proton-gradient(c)
r1124	-5	Tryptophan(c) + Na+(c) ← Tryptophan(s) + Na+(s)
r2479	31	Asparagine(s) + Na+(s) + H+(PG)(c) → Asparagine(c) + Na+(c) + H+(PG)(s)
r1143	-20	Na+(c) + Aspartate(c) ← Na+(s) + Aspartate(s)
r1125	20	Tyrosine(s) + Na+(s) → Tyrosine(c) + Na+(c)

Collagen CO1A1(c) synthesis (346)

Definition			Solution													
			exchanges		reactions								transp		
Objective	Constraints	Comment	imports	exports	c	m	r	p	l	n	s	b	s-c	b-c	int
Collagen CO1A1(c)	MCES AAA	test synthesis of Collagen CO1A1 c	75 Glutamate(s) 391 Glycine(s) 139 Alanine(s) 57 Lysine(s) 66 Aspartate(s) 71 Arginine(s) 49 Glutamine(s) 60 Serine(s) 13 Methionine(s) 6 Tryptophan(s) 27 Phenylalanine(s) 13 Tyrosine(s) 18 Cysteine(s) 48 Leucine(s) 9 Histidine(s) 278 Proline(s) 28 Asparagine(s) 47 Valine(s) 45 Threonine(s) 24 Isoleucine(s) 7320 ATP-energy(c) 363 Proton-gradient(c)	1 Collagen CO1A1(c) 373 Na-gradient(c)	4	-	-	-	-	-	-	-	22	-	-

ID	flux	reaction
r2530	48	Leucine(s) ⇌ Leucine(c)
r1144	-75	Glutamate(c) + Na+(c) ← Glutamate(s) + Na+(s)
r1126	-27	Phenylalanine(c) + Na+(c) ← Phenylalanine(s) + Na+(s)
r1072	13	Methionine(s) ⇌ Methionine(c)
r2531	278	Proline(s) ⇌ Proline(c)
r1145	-9	Histidine(c) + Na+(c) ← Histidine(s) + Na+(s)
r2550	1	139 Alanine(c) + 18 Cysteine(c) + 66 Aspartate(c) + 75 Glutamate(c) + 27 Phenylalanine(c) + 391 Glycine(c) + 9 Histidine(c) + 24 Isoleucine(c) + 57 Lysine(c) + 48 Leucine(c) + 13 Methionine(c) + 28 Asparagine(c) + 278 Proline(c) + 49 Glutamine(c) + 71 Arginine(c) + 60 Serine(c) + 45 Threonine(c) + 47 Valine(c) + 6 Tryptophan(c) + 13 Tyrosine(c) + 5856 ATP(c) + 5856 H2O(c) → Collagen CO1A1(c) + 4392 ADP(c) + 4392 Pi(c) + 1464 AMP(c) + 1464 PPi(c)
r2524	139	Alanine(s) ⇌ Alanine(c)
r2534	45	Threonine(s) ⇌ Threonine(c)
r2525	49	Glutamine(s) ⇌ Glutamine(c)
r1319	-7320	ATP(c) + H2O(c) ⇌ ADP(c) + Pi(c) + ATP-energy(c)
r1058	71	Arginine(s) ⇌ Arginine(c)
r1069	-57	Lysine(c) ⇌ Lysine(s)
r0030	1464	ATP(c) + AMP(c) ⇌ 2 ADP(c)
r2474	60	Serine(s) + Na+(s) → Serine(c) + Na+(c)
r2664	373	Na+(c) ⇌ Na+(s) + Na-gradient(c)
r2475	18	Cysteine(s) + Na+(s) → Cysteine(c) + Na+(c)
r1121	-47	Valine(c) + Na+(c) ← Valine(s) + Na+(s)
r2468	391	Glycine(s) + H+(PG)(s) → Glycine(c) + H+(PG)(c)
r1123	-24	Isoleucine(c) + Na+(c) ← Isoleucine(s) + Na+(s)
r0007	1464	PPi(c) + H2O(c) → 2 Pi(c)
r1331	-363	H+(PG)(s) ⇌ H+(PG)(c) + Proton-gradient(c)
r1124	-6	Tryptophan(c) + Na+(c) ← Tryptophan(s) + Na+(s)
r2479	28	Asparagine(s) + Na+(s) + H+(PG)(c) → Asparagine(c) + Na+(c) + H+(PG)(s)
r1143	-66	Na+(c) + Aspartate(c) ← Na+(s) + Aspartate(s)
r1125	13	Tyrosine(s) + Na+(s) → Tyrosine(c) + Na+(c)

Collagen CO1A2(c) synthesis (347)

Definition			Solution													
			exchanges		reactions								transp		
Objective	Constraints	Comment	imports	exports	c	m	r	p	l	n	s	b	s-c	b-c	int
Collagen CO1A2(c)	MCES AAA	test synthesis of Collagen CO1A2 c	66 Glutamate(s) 381 Glycine(s) 129 Alanine(s) 50 Lysine(s) 43 Aspartate(s) 72 Arginine(s) 33 Glutamine(s) 52 Serine(s) 10 Methionine(s) 5 Tryptophan(s) 22 Phenylalanine(s) 16 Tyrosine(s) 9 Cysteine(s) 61 Leucine(s) 15 Histidine(s) 232 Proline(s) 41 Asparagine(s) 55 Valine(s) 42 Threonine(s) 32 Isoleucine(s) 6830 ATP-energy(c) 340 Proton-gradient(c)	1 Collagen CO1A2(c) 356 Na-gradient(c)	4	-	-	-	-	-	-	-	22	-	-

ID	flux	reaction
r2530	61	Leucine(s) ⇌ Leucine(c)
r1144	-66	Glutamate(c) + Na+(c) ← Glutamate(s) + Na+(s)
r1126	-22	Phenylalanine(c) + Na+(c) ← Phenylalanine(s) + Na+(s)
r1072	10	Methionine(s) ⇌ Methionine(c)
r2531	232	Proline(s) ⇌ Proline(c)
r1145	-15	Histidine(c) + Na+(c) ← Histidine(s) + Na+(s)
r2524	129	Alanine(s) ⇌ Alanine(c)
r2552	1	129 Alanine(c) + 9 Cysteine(c) + 43 Aspartate(c) + 66 Glutamate(c) + 22 Phenylalanine(c) + 381 Glycine(c) + 15 Histidine(c) + 32 Isoleucine(c) + 50 Lysine(c) + 61 Leucine(c) + 10 Methionine(c) + 41 Asparagine(c) + 232 Proline(c) + 33 Glutamine(c) + 72 Arginine(c) + 52 Serine(c) + 42 Threonine(c) + 55 Valine(c) + 5 Tryptophan(c) + 16 Tyrosine(c) + 5464 ATP(c) + 5464 H2O(c) → Collagen CO1A2(c) + 4098 ADP(c) + 4098 Pi(c) + 1366 AMP(c) + 1366 PPi(c)
r2534	42	Threonine(s) ⇌ Threonine(c)
r2525	33	Glutamine(s) ⇌ Glutamine(c)
r1319	-6830	ATP(c) + H2O(c) ⇌ ADP(c) + Pi(c) + ATP-energy(c)
r1058	72	Arginine(s) ⇌ Arginine(c)
r1069	-50	Lysine(c) ⇌ Lysine(s)
r0030	1366	ATP(c) + AMP(c) ⇌ 2 ADP(c)
r2474	52	Serine(s) + Na+(s) → Serine(c) + Na+(c)
r2664	356	Na+(c) ⇌ Na+(s) + Na-gradient(c)
r2475	9	Cysteine(s) + Na+(s) → Cysteine(c) + Na+(c)
r1121	-55	Valine(c) + Na+(c) ← Valine(s) + Na+(s)
r2468	381	Glycine(s) + H+(PG)(s) → Glycine(c) + H+(PG)(c)
r1123	-32	Isoleucine(c) + Na+(c) ← Isoleucine(s) + Na+(s)
r0007	1366	PPi(c) + H2O(c) → 2 Pi(c)
r1331	-340	H+(PG)(s) ⇌ H+(PG)(c) + Proton-gradient(c)
r1124	-5	Tryptophan(c) + Na+(c) ← Tryptophan(s) + Na+(s)
r2479	41	Asparagine(s) + Na+(s) + H+(PG)(c) → Asparagine(c) + Na+(c) + H+(PG)(s)
r1143	-43	Na+(c) + Aspartate(c) ← Na+(s) + Aspartate(s)
r1125	16	Tyrosine(s) + Na+(s) → Tyrosine(c) + Na+(c)

Collagen CO2A1(c) synthesis (348)

Definition			Solution												
			exchanges		reactions								transp		
Objective	Constraints	Comment	imports	exports	c	m	r	p	l	n	s	b	s-c	b-c	int
Collagen CO_2A1(c)	MCES AAA	test synthesis of Collagen CO_2A1 c	79 Glutamate(s) 406 Glycine(s) 134 Alanine(s) 67 Lysine(s) 62 Aspartate(s) 72 Arginine(s) 60 Glutamine(s) 48 Serine(s) 16 Methionine(s) 7 Tryptophan(s) 25 Phenylalanine(s) 10 Tyrosine(s) 19 Cysteine(s) 56 Leucine(s) 8 Histidine(s) 270 Proline(s) 32 Asparagine(s) 38 Valine(s) 44 Threonine(s) 34 Isoleucine(s) 7435 ATP-energy(c) 374 Proton-gradient(c)	1 Collagen CO_2A1(c) 362 Na-gradient(c)	4	-	-	-	-	-	-	-	22	-	-

ID	flux	reaction
r2530	56	Leucine(s) ⇌ Leucine(c)
r1144	-79	Glutamate(c) + Na+(c) ← Glutamate(s) + Na+(s)
r1126	-25	Phenylalanine(c) + Na+(c) ← Phenylalanine(s) + Na+(s)
r1072	16	Methionine(s) ⇌ Methionine(c)
r2531	270	Proline(s) ⇌ Proline(c)
r1145	-8	Histidine(c) + Na+(c) ← Histidine(s) + Na+(s)
r2524	134	Alanine(s) ⇌ Alanine(c)
r2534	44	Threonine(s) ⇌ Threonine(c)
r2525	60	Glutamine(s) ⇌ Glutamine(c)
r1319	-7435	ATP(c) + H2O(c) ⇌ ADP(c) + Pi(c) + ATP-energy(c)
r1058	72	Arginine(s) ⇌ Arginine(c)
r2554	1	134 Alanine(c) + 19 Cysteine(c) + 62 Aspartate(c) + 79 Glutamate(c) + 25 Phenylalanine(c) + 406 Glycine(c) + 8 Histidine(c) + 34 Isoleucine(c) + 67 Lysine(c) + 56 Leucine(c) + 16 Methionine(c) + 32 Asparagine(c) + 270 Proline(c) + 60 Glutamine(c) + 72 Arginine(c) + 48 Serine(c) + 44 Threonine(c) + 38 Valine(c) + 7 Tryptophan(c) + 10 Tyrosine(c) + 5948 ATP(c) + 5948 H2O(c) → Collagen CO2A1(c) + 4461 ADP(c) + 4461 Pi(c) + 1487 AMP(c) + 1487 PPi(c)
r1069	-67	Lysine(c) ⇌ Lysine(s)
r0030	1487	ATP(c) + AMP(c) ⇌ 2 ADP(c)
r2474	48	Serine(s) + Na+(s) → Serine(c) + Na+(c)
r2664	362	Na+(c) ⇌ Na+(s) + Na-gradient(c)
r2475	19	Cysteine(s) + Na+(s) → Cysteine(c) + Na+(c)
r1121	-38	Valine(c) + Na+(c) ← Valine(s) + Na+(s)
r2468	406	Glycine(s) + H+(PG)(s) → Glycine(c) + H+(PG)(c)
r1123	-34	Isoleucine(c) + Na+(c) ← Isoleucine(s) + Na+(s)
r0007	1487	PPi(c) + H2O(c) → 2 Pi(c)
r1331	-374	H+(PG)(s) ⇌ H+(PG)(c) + Proton-gradient(c)
r1124	-7	Tryptophan(c) + Na+(c) ← Tryptophan(s) + Na+(s)
r2479	32	Asparagine(s) + Na+(s) + H+(PG)(c) → Asparagine(c) + Na+(c) + H+(PG)(s)
r1143	-62	Na+(c) + Aspartate(c) ← Na+(s) + Aspartate(s)
r1125	10	Tyrosine(s) + Na+(s) → Tyrosine(c) + Na+(c)

Collagen CO3A1(c) synthesis (349)

Definition			Solution												
			exchanges		reactions								transsp		
Objective	Constraints	Comment	imports	exports	c	m	r	p	l	n	s	b	s-c	b-c	int
Collagen CO3A1(c)	MCES AAA	test synthesis of Collagen CO3A1 c	74 Glutamate(s) 413 Glycine(s) 115 Alanine(s) 62 Lysine(s) 55 Aspartate(s) 60 Arginine(s) 42 Glutamine(s) 73 Serine(s) 17 Methionine(s) 7 Tryptophan(s) 23 Phenylalanine(s) 15 Tyrosine(s) 22 Cysteine(s) 48 Leucine(s) 15 Histidine(s) 281 Proline(s) 41 Asparagine(s) 36 Valine(s) 31 Threonine(s) 36 Isoleucine(s) 7330 ATP-energy(c) 372 Proton-gradient(c)	1 Collagen CO3A1(c) 397 Na-gradient(c)	4	-	-	-	-	-	-	-	22	-	-

ID	flux	reaction
r2530	48	Leucine(s) ⇌ Leucine(c)
r1144	-74	Glutamate(c) + Na+(c) ← Glutamate(s) + Na+(s)
r1126	-23	Phenylalanine(c) + Na+(c) ← Phenylalanine(s) + Na+(s)
r1072	17	Methionine(s) ⇌ Methionine(c)
r2531	281	Proline(s) ⇌ Proline(c)
r1145	-15	Histidine(c) + Na+(c) ← Histidine(s) + Na+(s)
r2524	115	Alanine(s) ⇌ Alanine(c)
r2534	31	Threonine(s) ⇌ Threonine(c)
r2525	42	Glutamine(s) ⇌ Glutamine(c)
r1319	-7330	ATP(c) + H2O(c) ⇌ ADP(c) + Pi(c) + ATP-energy(c)
r1058	60	Arginine(s) ⇌ Arginine(c)
r1069	-62	Lysine(c) ⇌ Lysine(s)
r0030	1466	ATP(c) + AMP(c) ⇌ 2 ADP(c)
r2474	73	Serine(s) + Na+(s) → Serine(c) + Na+(c)
r2664	397	Na+(c) ⇌ Na+(s) + Na-gradient(c)
r2556	1	115 Alanine(c) + 22 Cysteine(c) + 55 Aspartate(c) + 74 Glutamate(c) + 23 Phenylalanine(c) + 413 Glycine(c) + 15 Histidine(c) + 36 Isoleucine(c) + 62 Lysine(c) + 48 Leucine(c) + 17 Methionine(c) + 41 Asparagine(c) + 281 Proline(c) + 42 Glutamine(c) + 60 Arginine(c) + 73 Serine(c) + 31 Threonine(c) + 36 Valine(c) + 7 Tryptophan(c) + 15 Tyrosine(c) + 5864 ATP(c) + 5864 H2O(c) → Collagen CO3A1(c) + 4398 ADP(c) + 4398 Pi(c) + 1466 AMP(c) + 1466 PPi(c)
r2475	22	Cysteine(s) + Na+(s) → Cysteine(c) + Na+(c)
r1121	-36	Valine(c) + Na+(c) ← Valine(s) + Na+(s)
r2468	413	Glycine(s) + H+(PG)(s) → Glycine(c) + H+(PG)(c)
r1123	-36	Isoleucine(c) + Na+(c) ← Isoleucine(s) + Na+(s)
r0007	1466	PPi(c) + H2O(c) → 2 Pi(c)
r1331	-372	H+(PG)(s) ⇌ H+(PG)(c) + Proton-gradient(c)
r1124	-7	Tryptophan(c) + Na+(c) ← Tryptophan(s) + Na+(s)
r2479	41	Asparagine(s) + Na+(s) + H+(PG)(c) → Asparagine(c) + Na+(c) + H+(PG)(s)
r1143	-55	Na+(c) + Aspartate(c) ← Na+(s) + Aspartate(s)
r1125	15	Tyrosine(s) + Na+(s) → Tyrosine(c) + Na+(c)

Collagen CO4A1(c) synthesis (350)

Definition			Solution												
			exchanges		reactions								transp		
Objective	Constraints	Comment	imports	exports	c	m	r	p	l	n	s	b	s-c	b-c	int
Collagen CO4A1(c)	MCES AAA	test synthesis of Collagen CO4A1 c	70 Glutamate(s) 478 Glycine(s) 58 Alanine(s) 93 Lysine(s) 58 Aspartate(s) 45 Arginine(s) 73 Glutamine(s) 72 Serine(s) 31 Methionine(s) 6 Tryptophan(s) 46 Phenylalanine(s) 18 Tyrosine(s) 20 Cysteine(s) 92 Leucine(s) 16 Histidine(s) 324 Proline(s) 16 Asparagine(s) 51 Valine(s) 44 Threonine(s) 58 Isoleucine(s) 8345 ATP-energy(c) 462 Proton-gradient(c)	1 Collagen CO4A1(c) 431 Na-gradient(c)	4	-	-	-	-	-	-	-	22	-	-

ID	flux	reaction
r2530	92	Leucine(s) ⇌ Leucine(c)
r1144	-70	Glutamate(c) + Na+(c) ← Glutamate(s) + Na+(s)
r1126	-46	Phenylalanine(c) + Na+(c) ← Phenylalanine(s) + Na+(s)
r1072	31	Methionine(s) ⇌ Methionine(c)
r2531	324	Proline(s) ⇌ Proline(c)
r1145	-16	Histidine(c) + Na+(c) ← Histidine(s) + Na+(s)
r2524	58	Alanine(s) ⇌ Alanine(c)
r2534	44	Threonine(s) ⇌ Threonine(c)
r2525	73	Glutamine(s) ⇌ Glutamine(c)
r1319	-8345	ATP(c) + H2O(c) ⇌ ADP(c) + Pi(c) + ATP-energy(c)
r1058	45	Arginine(s) ⇌ Arginine(c)
r1069	-93	Lysine(c) ⇌ Lysine(s)
r0030	1669	ATP(c) + AMP(c) ⇌ 2 ADP(c)
r2474	72	Serine(s) + Na+(s) → Serine(c) + Na+(c)
r2664	431	Na+(c) ⇌ Na+(s) + Na-gradient(c)
r2475	20	Cysteine(s) + Na+(s) → Cysteine(c) + Na+(c)
r1121	-51	Valine(c) + Na+(c) ← Valine(s) + Na+(s)
r2558	1	58 Alanine(c) + 20 Cysteine(c) + 58 Aspartate(c) + 70 Glutamate(c) + 46 Phenylalanine(c) + 478 Glycine(c) + 16 Histidine(c) + 58 Isoleucine(c) + 93 Lysine(c) + 92 Leucine(c) + 31 Methionine(c) + 16 Asparagine(c) + 324 Proline(c) + 73 Glutamine(c) + 45 Arginine(c) + 72 Serine(c) + 44 Threonine(c) + 51 Valine(c) + 6 Tryptophan(c) + 18 Tyrosine(c) + 6676 ATP(c) + 6676 H2O(c) → Collagen CO4A1(c) + 5007 ADP(c) + 5007 Pi(c) + 1669 AMP(c) + 1669 PPi(c)
r2468	478	Glycine(s) + H+(PG)(s) → Glycine(c) + H+(PG)(c)
r1123	-58	Isoleucine(c) + Na+(c) ← Isoleucine(s) + Na+(s)
r0007	1669	PPi(c) + H2O(c) → 2 Pi(c)
r1331	-462	H+(PG)(s) ⇌ H+(PG)(c) + Proton-gradient(c)
r1124	-6	Tryptophan(c) + Na+(c) ← Tryptophan(s) + Na+(s)
r2479	16	Asparagine(s) + Na+(s) + H+(PG)(c) → Asparagine(c) + Na+(c) + H+(PG)(s)
r1143	-58	Na+(c) + Aspartate(c) ← Na+(s) + Aspartate(s)
r1125	18	Tyrosine(s) + Na+(s) → Tyrosine(c) + Na+(c)

Collagen CO4A2(c) synthesis (351)

Definition			Solution												
			exchanges		reactions								transp		
Objective	Constraints	Comment	imports	exports	c	m	r	p	l	n	s	b	s-c	b-c	int
Collagen CO4A2(c)	MCES AAA	test synthesis of Collagen CO4A2 c	60 Glutamate(s) 473 Glycine(s) 82 Alanine(s) 81 Lysine(s) 82 Aspartate(s) 79 Arginine(s) 62 Glutamine(s) 64 Serine(s) 25 Methionine(s) 9 Tryptophan(s) 57 Phenylalanine(s) 28 Tyrosine(s) 21 Cysteine(s) 102 Leucine(s) 19 Histidine(s) 286 Proline(s) 18 Asparagine(s) 50 Valine(s) 51 Threonine(s) 63 Isoleucine(s) 8560 ATP-energy(c) 455 Proton-gradient(c)	1 Collagen CO4A2(c) 471 Na-gradient(c)	4	-	-	-	-	-	-	-	22	-	-

ID	flux	reaction
r2530	102	Leucine(s) ⇌ Leucine(c)
r1144	-60	Glutamate(c) + Na+(c) ← Glutamate(s) + Na+(s)
r1126	-57	Phenylalanine(c) + Na+(c) ← Phenylalanine(s) + Na+(s)
r1072	25	Methionine(s) ⇌ Methionine(c)
r2531	286	Proline(s) ⇌ Proline(c)
r1145	-19	Histidine(c) + Na+(c) ← Histidine(s) + Na+(s)
r2560	1	82 Alanine(c) + 21 Cysteine(c) + 82 Aspartate(c) + 60 Glutamate(c) + 57 Phenylalanine(c) + 473 Glycine(c) + 19 Histidine(c) + 63 Isoleucine(c) + 81 Lysine(c) + 102 Leucine(c) + 25 Methionine(c) + 18 Asparagine(c) + 286 Proline(c) + 62 Glutamine(c) + 79 Arginine(c) + 64 Serine(c) + 51 Threonine(c) + 50 Valine(c) + 9 Tryptophan(c) + 28 Tyrosine(c) + 6848 ATP(c) + 6848 H2O(c) → Collagen CO4A2(c) + 5136 ADP(c) + 5136 Pi(c) + 1712 AMP(c) + 1712 PPi(c)
r2524	82	Alanine(s) ⇌ Alanine(c)
r2534	51	Threonine(s) ⇌ Threonine(c)
r2525	62	Glutamine(s) ⇌ Glutamine(c)
r1319	-8560	ATP(c) + H2O(c) ⇌ ADP(c) + Pi(c) + ATP-energy(c)
r1058	79	Arginine(s) ⇌ Arginine(c)
r1069	-81	Lysine(c) ⇌ Lysine(s)
r0030	1712	ATP(c) + AMP(c) ⇌ 2 ADP(c)
r2474	64	Serine(s) + Na+(s) → Serine(c) + Na+(c)
r2664	471	Na+(c) ⇌ Na+(s) + Na-gradient(c)
r2475	21	Cysteine(s) + Na+(s) → Cysteine(c) + Na+(c)
r1121	-50	Valine(c) + Na+(c) ← Valine(s) + Na+(s)
r2468	473	Glycine(s) + H+(PG)(s) → Glycine(c) + H+(PG)(c)
r1123	-63	Isoleucine(c) + Na+(c) ← Isoleucine(s) + Na+(s)
r0007	1712	PPi(c) + H2O(c) → 2 Pi(c)
r1331	-455	H+(PG)(s) ⇌ H+(PG)(c) + Proton-gradient(c)
r1124	-9	Tryptophan(c) + Na+(c) ← Tryptophan(s) + Na+(s)
r2479	18	Asparagine(s) + Na+(s) + H+(PG)(c) → Asparagine(c) + Na+(c) + H+(PG)(s)
r1143	-82	Na+(c) + Aspartate(c) ← Na+(s) + Aspartate(s)
r1125	28	Tyrosine(s) + Na+(s) → Tyrosine(c) + Na+(c)

Collagen CO4A3(c) synthesis (352)

Definition			Solution													
			exchanges		reactions								transp		
Objective	Constraints	Comment	imports	exports	c	m	r	p	l	n	s	b	s-c	b-c	int
Collagen CO4A3(c)	MCES AAA	test synthesis of Collagen CO4A3 c	67 Glutamate(s) 455 Glycine(s) 69 Alanine(s) 89 Lysine(s) 53 Aspartate(s) 65 Arginine(s) 48 Glutamine(s) 78 Serine(s) 29 Methionine(s) 6 Tryptophan(s) 46 Phenylalanine(s) 18 Tyrosine(s) 24 Cysteine(s) 106 Leucine(s) 19 Histidine(s) 320 Proline(s) 28 Asparagine(s) 40 Valine(s) 59 Threonine(s) 51 Isoleucine(s) 8350 ATP-energy(c) 427 Proton-gradient(c)	1 Collagen CO4A3(c) 430 Na-gradient(c)	4	-	-	-	-	-	-	-	22	-	-

ID	flux	reaction
r2530	106	Leucine(s) ⇌ Leucine(c)
r1144	-67	Glutamate(c) + Na+(c) ← Glutamate(s) + Na+(s)
r1126	-46	Phenylalanine(c) + Na+(c) ← Phenylalanine(s) + Na+(s)
r1072	29	Methionine(s) ⇌ Methionine(c)
r2531	320	Proline(s) ⇌ Proline(c)
r1145	-19	Histidine(c) + Na+(c) ← Histidine(s) + Na+(s)
r2524	69	Alanine(s) ⇌ Alanine(c)
r2534	59	Threonine(s) ⇌ Threonine(c)
r2525	48	Glutamine(s) ⇌ Glutamine(c)
r1319	-8350	ATP(c) + H2O(c) ⇌ ADP(c) + Pi(c) + ATP-energy(c)
r1058	65	Arginine(s) ⇌ Arginine(c)
r2562	1	69 Alanine(c) + 24 Cysteine(c) + 53 Aspartate(c) + 67 Glutamate(c) + 46 Phenylalanine(c) + 455 Glycine(c) + 19 Histidine(c) + 51 Isoleucine(c) + 89 Lysine(c) + 106 Leucine(c) + 29 Methionine(c) + 28 Asparagine(c) + 320 Proline(c) + 48 Glutamine(c) + 65 Arginine(c) + 78 Serine(c) + 59 Threonine(c) + 40 Valine(c) + 6 Tryptophan(c) + 18 Tyrosine(c) + 6680 ATP(c) + 6680 H2O(c) → Collagen CO4A3(c) + 5010 ADP(c) + 5010 Pi(c) + 1670 AMP(c) + 1670 PPi(c)
r1069	-89	Lysine(c) ⇌ Lysine(s)
r0030	1670	ATP(c) + AMP(c) ⇌ 2 ADP(c)
r2474	78	Serine(s) + Na+(s) → Serine(c) + Na+(c)
r2664	430	Na+(c) ⇌ Na+(s) + Na-gradient(c)
r2475	24	Cysteine(s) + Na+(s) → Cysteine(c) + Na+(c)
r1121	-40	Valine(c) + Na+(c) ← Valine(s) + Na+(s)
r2468	455	Glycine(s) + H+(PG)(s) → Glycine(c) + H+(PG)(c)
r1123	-51	Isoleucine(c) + Na+(c) ← Isoleucine(s) + Na+(s)
r0007	1670	PPi(c) + H2O(c) → 2 Pi(c)
r1331	-427	H+(PG)(s) ⇌ H+(PG)(c) + Proton-gradient(c)
r1124	-6	Tryptophan(c) + Na+(c) ← Tryptophan(s) + Na+(s)
r2479	28	Asparagine(s) + Na+(s) + H+(PG)(c) → Asparagine(c) + Na+(c) + H+(PG)(s)
r1143	-53	Na+(c) + Aspartate(c) ← Na+(s) + Aspartate(s)
r1125	18	Tyrosine(s) + Na+(s) → Tyrosine(c) + Na+(c)

Collagen CO4A4(c) synthesis (353)

Definition			Solution													
			exchanges		reactions									transp		
Objective	Constraints	Comment	imports	exports	c	m	r	p	l	n	s	b	s-c	b-c	int
Collagen CO4A4(c)	MCES AAA	test synthesis of Collagen CO4A4 c	58 Glutamate(s) 464 Glycine(s) 69 Alanine(s) 83 Lysine(s) 72 Aspartate(s) 70 Arginine(s) 48 Glutamine(s) 76 Serine(s) 24 Methionine(s) 9 Tryptophan(s) 45 Phenylalanine(s) 23 Tyrosine(s) 32 Cysteine(s) 101 Leucine(s) 33 Histidine(s) 345 Proline(s) 15 Asparagine(s) 50 Valine(s) 30 Threonine(s) 43 Isoleucine(s) 8450 ATP-energy(c) 449 Proton-gradient(c)	1 Collagen CO4A4(c) 456 Na-gradient(c)	4	-	-	-	-	-	-	-	22	-	-

ID	flux	reaction
r2530	101	Leucine(s) ⇌ Leucine(c)
r1144	-58	Glutamate(c) + Na+(c) ← Glutamate(s) + Na+(s)
r1126	-45	Phenylalanine(c) + Na+(c) ← Phenylalanine(s) + Na+(s)
r1072	24	Methionine(s) ⇌ Methionine(c)
r2531	345	Proline(s) ⇌ Proline(c)
r1145	-33	Histidine(c) + Na+(c) ← Histidine(s) + Na+(s)
r2524	69	Alanine(s) ⇌ Alanine(c)
r2534	30	Threonine(s) ⇌ Threonine(c)
r2525	48	Glutamine(s) ⇌ Glutamine(c)
r1319	-8450	ATP(c) + H2O(c) ⇌ ADP(c) + Pi(c) + ATP-energy(c)
r1058	70	Arginine(s) ⇌ Arginine(c)
r1069	-83	Lysine(c) ⇌ Lysine(s)
r0030	1690	ATP(c) + AMP(c) ⇌ 2 ADP(c)
r2564	1	69 Alanine(c) + 32 Cysteine(c) + 72 Aspartate(c) + 58 Glutamate(c) + 45 Phenylalanine(c) + 464 Glycine(c) + 33 Histidine(c) + 43 Isoleucine(c) + 83 Lysine(c) + 101 Leucine(c) + 24 Methionine(c) + 15 Asparagine(c) + 345 Proline(c) + 48 Glutamine(c) + 70 Arginine(c) + 76 Serine(c) + 30 Threonine(c) + 50 Valine(c) + 9 Tryptophan(c) + 23 Tyrosine(c) + 6760 ATP(c) + 6760 H2O(c) → Collagen CO4A4(c) + 5070 ADP(c) + 5070 Pi(c) + 1690 AMP(c) + 1690 PPi(c)
r2474	76	Serine(s) + Na+(s) → Serine(c) + Na+(c)
r2664	456	Na+(c) ⇌ Na+(s) + Na-gradient(c)
r2475	32	Cysteine(s) + Na+(s) → Cysteine(c) + Na+(c)
r1121	-50	Valine(c) + Na+(c) ← Valine(s) + Na+(s)
r2468	464	Glycine(s) + H+(PG)(s) → Glycine(c) + H+(PG)(c)
r1123	-43	Isoleucine(c) + Na+(c) ← Isoleucine(s) + Na+(s)
r0007	1690	PPi(c) + H2O(c) → 2 Pi(c)
r1331	-449	H+(PG)(s) ⇌ H+(PG)(c) + Proton-gradient(c)
r1124	-9	Tryptophan(c) + Na+(c) ← Tryptophan(s) + Na+(s)
r2479	15	Asparagine(s) + Na+(s) + H+(PG)(c) → Asparagine(c) + Na+(c) + H+(PG)(s)
r1143	-72	Na+(c) + Aspartate(c) ← Na+(s) + Aspartate(s)
r1125	23	Tyrosine(s) + Na+(s) → Tyrosine(c) + Na+(c)

Collagen CO4A5(c) synthesis (354)

Definition			Solution												
			exchanges		reactions								transp		
Objective	Constraints	Comment	imports	exports	c	m	r	p	l	n	s	b	s-c	b-c	int
Collagen CO4A5(c)	MCES AAA	test synthesis of Collagen CO4A5 c	61 Glutamate(s) 478 Glycine(s) 47 Alanine(s) 80 Lysine(s) 54 Aspartate(s) 37 Arginine(s) 73 Glutamine(s) 64 Serine(s) 26 Methionine(s) 5 Tryptophan(s) 41 Phenylalanine(s) 13 Tyrosine(s) 20 Cysteine(s) 112 Leucine(s) 13 Histidine(s) 391 Proline(s) 32 Asparagine(s) 31 Valine(s) 38 Threonine(s) 69 Isoleucine(s) 8425 ATP-energy(c) 446 Proton-gradient(c)	1 Collagen CO4A5(c) 403 Na-gradient(c)	4	-	-	-	-	-	-	-	22	-	-

ID	flux	reaction
r2530	112	Leucine(s) ⇌ Leucine(c)
r1144	-61	Glutamate(c) + Na+(c) ← Glutamate(s) + Na+(s)
r1126	-41	Phenylalanine(c) + Na+(c) ← Phenylalanine(s) + Na+(s)
r1072	26	Methionine(s) ⇌ Methionine(c)
r2531	391	Proline(s) ⇌ Proline(c)
r1145	-13	Histidine(c) + Na+(c) ← Histidine(s) + Na+(s)
r2524	47	Alanine(s) ⇌ Alanine(c)
r2534	38	Threonine(s) ⇌ Threonine(c)
r2525	73	Glutamine(s) ⇌ Glutamine(c)
r1319	-8425	ATP(c) + H2O(c) ⇌ ADP(c) + Pi(c) + ATP-energy(c)
r1058	37	Arginine(s) ⇌ Arginine(c)
r1069	-80	Lysine(c) ⇌ Lysine(s)
r0030	1685	ATP(c) + AMP(c) ⇌ 2 ADP(c)
r2474	64	Serine(s) + Na+(s) → Serine(c) + Na+(c)
r2664	403	Na+(c) ⇌ Na+(s) + Na-gradient(c)
r2475	20	Cysteine(s) + Na+(s) → Cysteine(c) + Na+(c)
r1121	-31	Valine(c) + Na+(c) ← Valine(s) + Na+(s)
r2566	1	47 Alanine(c) + 20 Cysteine(c) + 54 Aspartate(c) + 61 Glutamate(c) + 41 Phenylalanine(c) + 478 Glycine(c) + 13 Histidine(c) + 69 Isoleucine(c) + 80 Lysine(c) + 112 Leucine(c) + 26 Methionine(c) + 32 Asparagine(c) + 391 Proline(c) + 73 Glutamine(c) + 37 Arginine(c) + 64 Serine(c) + 38 Threonine(c) + 31 Valine(c) + 5 Tryptophan(c) + 13 Tyrosine(c) + 6740 ATP(c) + 6740 H2O(c) → Collagen CO4A5(c) + 5055 ADP(c) + 5055 Pi(c) + 1685 AMP(c) + 1685 PPi(c)
r2468	478	Glycine(s) + H+(PG)(s) → Glycine(c) + H+(PG)(c)
r1123	-69	Isoleucine(c) + Na+(c) ← Isoleucine(s) + Na+(s)
r0007	1685	PPi(c) + H2O(c) → 2 Pi(c)
r1331	-446	H+(PG)(s) ⇌ H+(PG)(c) + Proton-gradient(c)
r1124	-5	Tryptophan(c) + Na+(c) ← Tryptophan(s) + Na+(s)
r2479	32	Asparagine(s) + Na+(s) + H+(PG)(c) → Asparagine(c) + Na+(c) + H+(PG)(s)
r1143	-54	Na+(c) + Aspartate(c) ← Na+(s) + Aspartate(s)
r1125	13	Tyrosine(s) + Na+(s) → Tyrosine(c) + Na+(c)

Collagen CO4A6(c) synthesis (355)

Definition			Solution												
			exchanges		reactions								transp		
Objective	Constraints	Comment	imports	exports	c	m	r	p	l	n	s	b	s-c	b-c	int
Collagen CO4A6(c)	MCES AAA	test synthesis of Collagen CO4A6 c	57 Glutamate(s) 463 Glycine(s) 60 Alanine(s) 96 Lysine(s) 52 Aspartate(s) 47 Arginine(s) 65 Glutamine(s) 98 Serine(s) 25 Methionine(s) 8 Tryptophan(s) 55 Phenylalanine(s) 15 Tyrosine(s) 21 Cysteine(s) 140 Leucine(s) 17 Histidine(s) 279 Proline(s) 26 Asparagine(s) 51 Valine(s) 53 Threonine(s) 63 Isoleucine(s) 8455 ATP-energy(c) 437 Proton-gradient(c)	1 Collagen CO4A6(c) 463 Na-gradient(c)	4	-	-	-	-	-	-	-	22	-	-

ID	flux	reaction
r2530	140	Leucine(s) ⇌ Leucine(c)
r1144	-57	Glutamate(c) + Na+(c) ← Glutamate(s) + Na+(s)
r1126	-55	Phenylalanine(c) + Na+(c) ← Phenylalanine(s) + Na+(s)
r1072	25	Methionine(s) ⇌ Methionine(c)
r2531	279	Proline(s) ⇌ Proline(c)
r1145	-17	Histidine(c) + Na+(c) ← Histidine(s) + Na+(s)
r2524	60	Alanine(s) ⇌ Alanine(c)
r2534	53	Threonine(s) ⇌ Threonine(c)
r2525	65	Glutamine(s) ⇌ Glutamine(c)
r1319	-8455	ATP(c) + H2O(c) ⇌ ADP(c) + Pi(c) + ATP-energy(c)
r1058	47	Arginine(s) ⇌ Arginine(c)
r1069	-96	Lysine(c) ⇌ Lysine(s)
r0030	1691	ATP(c) + AMP(c) ⇌ 2 ADP(c)
r2474	98	Serine(s) + Na+(s) → Serine(c) + Na+(c)
r2664	463	Na+(c) ⇌ Na+(s) + Na-gradient(c)
r2475	21	Cysteine(s) + Na+(s) → Cysteine(c) + Na+(c)
r1121	-51	Valine(c) + Na+(c) ← Valine(s) + Na+(s)
r2468	463	Glycine(s) + H+(PG)(s) → Glycine(c) + H+(PG)(c)
r1123	-63	Isoleucine(c) + Na+(c) ← Isoleucine(s) + Na+(s)
r0007	1691	PPi(c) + H2O(c) → 2 Pi(c)
r2568	1	60 Alanine(c) + 21 Cysteine(c) + 52 Aspartate(c) + 57 Glutamate(c) + 55 Phenylalanine(c) + 463 Glycine(c) + 17 Histidine(c) + 63 Isoleucine(c) + 96 Lysine(c) + 140 Leucine(c) + 25 Methionine(c) + 26 Asparagine(c) + 279 Proline(c) + 65 Glutamine(c) + 47 Arginine(c) + 98 Serine(c) + 53 Threonine(c) + 51 Valine(c) + 8 Tryptophan(c) + 15 Tyrosine(c) + 6764 ATP(c) + 6764 H2O(c) → Collagen CO4A6(c) + 5073 ADP(c) + 5073 Pi(c) + 1691 AMP(c) + 1691 PPi(c)
r1331	-437	H+(PG)(s) ⇌ H+(PG)(c) + Proton-gradient(c)
r1124	-8	Tryptophan(c) + Na+(c) ← Tryptophan(s) + Na+(s)
r2479	26	Asparagine(s) + Na+(s) + H+(PG)(c) → Asparagine(c) + Na+(c) + H+(PG)(s)
r1143	-52	Na+(c) + Aspartate(c) ← Na+(s) + Aspartate(s)
r1125	15	Tyrosine(s) + Na+(s) → Tyrosine(c) + Na+(c)

Collagen CO5A1(c) synthesis (356)

Definition			Solution												
			exchanges		reactions								transp		
Objective	Constraints	Comment	imports	exports	c	m	r	p	l	n	s	b	s-c	b-c	int
Collagen CO5A1(c)	MCES AAA	test synthesis of Collagen CO5A1 c	120 Glutamate(s) 429 Glycine(s) 92 Alanine(s) 98 Lysine(s) 105 Aspartate(s) 70 Arginine(s) 74 Glutamine(s) 70 Serine(s) 23 Methionine(s) 7 Tryptophan(s) 39 Phenylalanine(s) 40 Tyrosine(s) 12 Cysteine(s) 98 Leucine(s) 17 Histidine(s) 334 Proline(s) 34 Asparagine(s) 54 Valine(s) 70 Threonine(s) 52 Isoleucine(s) 9190 ATP-energy(c) 395 Proton-gradient(c)	1 Collagen CO5A1(c) 550 Na-gradient(c)	4	-	-	-	-	-	-	-	22	-	-

ID	flux	reaction
r2530	98	Leucine(s) ⇌ Leucine(c)
r1144	-120	Glutamate(c) + Na+(c) ← Glutamate(s) + Na+(s)
r1126	-39	Phenylalanine(c) + Na+(c) ← Phenylalanine(s) + Na+(s)
r1072	23	Methionine(s) ⇌ Methionine(c)
r2531	334	Proline(s) ⇌ Proline(c)
r1145	-17	Histidine(c) + Na+(c) ← Histidine(s) + Na+(s)
r2524	92	Alanine(s) ⇌ Alanine(c)
r2570	1	92 Alanine(c) + 12 Cysteine(c) + 105 Aspartate(c) + 120 Glutamate(c) + 39 Phenylalanine(c) + 429 Glycine(c) + 17 Histidine(c) + 52 Isoleucine(c) + 98 Lysine(c) + 98 Leucine(c) + 23 Methionine(c) + 34 Asparagine(c) + 334 Proline(c) + 74 Glutamine(c) + 70 Arginine(c) + 70 Serine(c) + 70 Threonine(c) + 54 Valine(c) + 7 Tryptophan(c) + 40 Tyrosine(c) + 7352 ATP(c) + 7352 H2O(c) → Collagen CO5A1(c) + 5514 ADP(c) + 5514 Pi(c) + 1838 AMP(c) + 1838 PPi(c)
r2534	70	Threonine(s) ⇌ Threonine(c)
r2525	74	Glutamine(s) ⇌ Glutamine(c)
r1319	-9190	ATP(c) + H2O(c) ⇌ ADP(c) + Pi(c) + ATP-energy(c)
r1058	70	Arginine(s) ⇌ Arginine(c)
r1069	-98	Lysine(c) ⇌ Lysine(s)
r0030	1838	ATP(c) + AMP(c) ⇌ 2 ADP(c)
r2474	70	Serine(s) + Na+(s) → Serine(c) + Na+(c)
r2664	550	Na+(c) ⇌ Na+(s) + Na-gradient(c)
r2475	12	Cysteine(s) + Na+(s) → Cysteine(c) + Na+(c)
r1121	-54	Valine(c) + Na+(c) ← Valine(s) + Na+(s)
r2468	429	Glycine(s) + H+(PG)(s) → Glycine(c) + H+(PG)(c)
r1123	-52	Isoleucine(c) + Na+(c) ← Isoleucine(s) + Na+(s)
r0007	1838	PPi(c) + H2O(c) → 2 Pi(c)
r1331	-395	H+(PG)(s) ⇌ H+(PG)(c) + Proton-gradient(c)
r1124	-7	Tryptophan(c) + Na+(c) ← Tryptophan(s) + Na+(s)
r2479	34	Asparagine(s) + Na+(s) + H+(PG)(c) → Asparagine(c) + Na+(c) + H+(PG)(s)
r1143	-105	Na+(c) + Aspartate(c) ← Na+(s) + Aspartate(s)
r1125	40	Tyrosine(s) + Na+(s) → Tyrosine(c) + Na+(c)

Collagen CO5A2(c) synthesis (357)

Definition			Solution												
			exchanges		reactions								transp		
Objective	Constraints	Comment	imports	exports	c	m	r	p	l	n	s	b	s-c	b-c	int
Collagen CO5A2(c)	MCES AAA	test synthesis of Collagen CO5A2 c	76 Glutamate(s) 402 Glycine(s) 83 Alanine(s) 61 Lysine(s) 69 Aspartate(s) 74 Arginine(s) 60 Glutamine(s) 63 Serine(s) 24 Methionine(s) 6 Tryptophan(s) 20 Phenylalanine(s) 11 Tyrosine(s) 17 Cysteine(s) 62 Leucine(s) 17 Histidine(s) 270 Proline(s) 36 Asparagine(s) 58 Valine(s) 51 Threonine(s) 39 Isoleucine(s) 7495 ATP-energy(c) 366 Proton-gradient(c)	1 Collagen CO5A2(c) 412 Na-gradient(c)	4	-	-	-	-	-	-	-	22	-	-

ID	flux	reaction
r2530	62	Leucine(s) ⇌ Leucine(c)
r1144	-76	Glutamate(c) + Na+(c) ← Glutamate(s) + Na+(s)
r1126	-20	Phenylalanine(c) + Na+(c) ← Phenylalanine(s) + Na+(s)
r1072	24	Methionine(s) ⇌ Methionine(c)
r2531	270	Proline(s) ⇌ Proline(c)
r1145	-17	Histidine(c) + Na+(c) ← Histidine(s) + Na+(s)
r2524	83	Alanine(s) ⇌ Alanine(c)
r2534	51	Threonine(s) ⇌ Threonine(c)
r2525	60	Glutamine(s) ⇌ Glutamine(c)
r1319	-7495	ATP(c) + H2O(c) ⇌ ADP(c) + Pi(c) + ATP-energy(c)
r1058	74	Arginine(s) ⇌ Arginine(c)
r2572	1	83 Alanine(c) + 17 Cysteine(c) + 69 Aspartate(c) + 76 Glutamate(c) + 20 Phenylalanine(c) + 402 Glycine(c) + 17 Histidine(c) + 39 Isoleucine(c) + 61 Lysine(c) + 62 Leucine(c) + 24 Methionine(c) + 36 Asparagine(c) + 270 Proline(c) + 60 Glutamine(c) + 74 Arginine(c) + 63 Serine(c) + 51 Threonine(c) + 58 Valine(c) + 6 Tryptophan(c) + 11 Tyrosine(c) + 5996 ATP(c) + 5996 H2O(c) → Collagen CO5A2(c) + 4497 ADP(c) + 4497 Pi(c) + 1499 AMP(c) + 1499 PPi(c)
r1069	-61	Lysine(c) ⇌ Lysine(s)
r0030	1499	ATP(c) + AMP(c) ⇌ 2 ADP(c)
r2474	63	Serine(s) + Na+(s) → Serine(c) + Na+(c)
r2664	412	Na+(c) ⇌ Na+(s) + Na-gradient(c)
r2475	17	Cysteine(s) + Na+(s) → Cysteine(c) + Na+(c)
r1121	-58	Valine(c) + Na+(c) ← Valine(s) + Na+(s)
r2468	402	Glycine(s) + H+(PG)(s) → Glycine(c) + H+(PG)(c)
r1123	-39	Isoleucine(c) + Na+(c) ← Isoleucine(s) + Na+(s)
r0007	1499	PPi(c) + H2O(c) → 2 Pi(c)
r1331	-366	H+(PG)(s) ⇌ H+(PG)(c) + Proton-gradient(c)
r1124	-6	Tryptophan(c) + Na+(c) ← Tryptophan(s) + Na+(s)
r2479	36	Asparagine(s) + Na+(s) + H+(PG)(c) → Asparagine(c) + Na+(c) + H+(PG)(s)
r1143	-69	Na+(c) + Aspartate(c) ← Na+(s) + Aspartate(s)
r1125	11	Tyrosine(s) + Na+(s) → Tyrosine(c) + Na+(c)

Collagen CO5A3(c) synthesis (358)

Definition			Solution												
			exchanges		reactions								transp		
Objective	Constraints	Comment	imports	exports	c	m	r	p	l	n	s	b	s-c	b-c	int
Collagen CO5A3(c)	MCES AAA	test synthesis of Collagen CO5A3 c	101 Glutamate(s) 429 Glycine(s) 102 Alanine(s) 86 Lysine(s) 76 Aspartate(s) 84 Arginine(s) 78 Glutamine(s) 69 Serine(s) 12 Methionine(s) 8 Tryptophan(s) 46 Phenylalanine(s) 11 Tyrosine(s) 12 Cysteine(s) 122 Leucine(s) 23 Histidine(s) 290 Proline(s) 24 Asparagine(s) 67 Valine(s) 67 Threonine(s) 38 Isoleucine(s) 8725 ATP-energy(c) 405 Proton-gradient(c)	1 Collagen CO5A3(c) 475 Na-gradient(c)	4	-	-	-	-	-	-	-	22	-	-

ID	flux	reaction
r2530	122	Leucine(s) ⇌ Leucine(c)
r1144	-101	Glutamate(c) + Na+(c) ← Glutamate(s) + Na+(s)
r1126	-46	Phenylalanine(c) + Na+(c) ← Phenylalanine(s) + Na+(s)
r1072	12	Methionine(s) ⇌ Methionine(c)
r2531	290	Proline(s) ⇌ Proline(c)
r1145	-23	Histidine(c) + Na+(c) ← Histidine(s) + Na+(s)
r2524	102	Alanine(s) ⇌ Alanine(c)
r2534	67	Threonine(s) ⇌ Threonine(c)
r2525	78	Glutamine(s) ⇌ Glutamine(c)
r1319	-8725	ATP(c) + H2O(c) ⇌ ADP(c) + Pi(c) + ATP-energy(c)
r1058	84	Arginine(s) ⇌ Arginine(c)
r1069	-86	Lysine(c) ⇌ Lysine(s)
r0030	1745	ATP(c) + AMP(c) ⇌ 2 ADP(c)
r2474	69	Serine(s) + Na+(s) → Serine(c) + Na+(c)
r2664	475	Na+(c) ⇌ Na+(s) + Na-gradient(c)
r2574	1	102 Alanine(c) + 12 Cysteine(c) + 76 Aspartate(c) + 101 Glutamate(c) + 46 Phenylalanine(c) + 429 Glycine(c) + 23 Histidine(c) + 38 Isoleucine(c) + 86 Lysine(c) + 122 Leucine(c) + 12 Methionine(c) + 24 Asparagine(c) + 290 Proline(c) + 78 Glutamine(c) + 84 Arginine(c) + 69 Serine(c) + 67 Threonine(c) + 67 Valine(c) + 8 Tryptophan(c) + 11 Tyrosine(c) + 6980 ATP(c) + 6980 H2O(c) → Collagen CO5A3(c) + 5235 ADP(c) + 5235 Pi(c) + 1745 AMP(c) + 1745 PPi(c)
r2475	12	Cysteine(s) + Na+(s) → Cysteine(c) + Na+(c)
r1121	-67	Valine(c) + Na+(c) ← Valine(s) + Na+(s)
r2468	429	Glycine(s) + H+(PG)(s) → Glycine(c) + H+(PG)(c)
r1123	-38	Isoleucine(c) + Na+(c) ← Isoleucine(s) + Na+(s)
r0007	1745	PPi(c) + H2O(c) → 2 Pi(c)
r1331	-405	H+(PG)(s) ⇌ H+(PG)(c) + Proton-gradient(c)
r1124	-8	Tryptophan(c) + Na+(c) ← Tryptophan(s) + Na+(s)
r2479	24	Asparagine(s) + Na+(s) + H+(PG)(c) → Asparagine(c) + Na+(c) + H+(PG)(s)
r1143	-76	Na+(c) + Aspartate(c) ← Na+(s) + Aspartate(s)
r1125	11	Tyrosine(s) + Na+(s) → Tyrosine(c) + Na+(c)

Collagen CO6A1(c) synthesis (359)

Definition			Solution													
			exchanges		reactions								transp			
Objective	Constraints	Comment	imports	exports	c	m	r	p	l	n	s	b	s-c	b-c	int
Collagen CO6A1(c)	MCES AAA	test synthesis of Collagen CO6A1 c	68 Glutamate(s) 156 Glycine(s) 77 Alanine(s) 54 Lysine(s) 71 Aspartate(s) 60 Arginine(s) 42 Glutamine(s) 54 Serine(s) 11 Methionine(s) 4 Tryptophan(s) 33 Phenylalanine(s) 28 Tyrosine(s) 20 Cysteine(s) 71 Leucine(s) 14 Histidine(s) 89 Proline(s) 28 Asparagine(s) 65 Valine(s) 41 Threonine(s) 42 Isoleucine(s) 5140 ATP-energy(c) 128 Proton-gradient(c)	1 Collagen CO6A1(c) 427 Na-gradient(c)	4	-	-	-	-	-	-	-	22	-	-

ID	flux	reaction
r2530	71	Leucine(s) ⇌ Leucine(c)
r1144	-68	Glutamate(c) + Na+(c) ← Glutamate(s) + Na+(s)
r1126	-33	Phenylalanine(c) + Na+(c) ← Phenylalanine(s) + Na+(s)
r1072	11	Methionine(s) ⇌ Methionine(c)
r2531	89	Proline(s) ⇌ Proline(c)
r1145	-14	Histidine(c) + Na+(c) ← Histidine(s) + Na+(s)
r2524	77	Alanine(s) ⇌ Alanine(c)
r2534	41	Threonine(s) ⇌ Threonine(c)
r2525	42	Glutamine(s) ⇌ Glutamine(c)
r1319	-5140	ATP(c) + H2O(c) ⇌ ADP(c) + Pi(c) + ATP-energy(c)
r1058	60	Arginine(s) ⇌ Arginine(c)
r1069	-54	Lysine(c) ⇌ Lysine(s)
r0030	1028	ATP(c) + AMP(c) ⇌ 2 ADP(c)
r2474	54	Serine(s) + Na+(s) → Serine(c) + Na+(c)
r2664	427	Na+(c) ⇌ Na+(s) + Na-gradient(c)
r2475	20	Cysteine(s) + Na+(s) → Cysteine(c) + Na+(c)
r1121	-65	Valine(c) + Na+(c) ← Valine(s) + Na+(s)
r2576	1	77 Alanine(c) + 20 Cysteine(c) + 71 Aspartate(c) + 68 Glutamate(c) + 33 Phenylalanine(c) + 156 Glycine(c) + 14 Histidine(c) + 42 Isoleucine(c) + 54 Lysine(c) + 71 Leucine(c) + 11 Methionine(c) + 28 Asparagine(c) + 89 Proline(c) + 42 Glutamine(c) + 60 Arginine(c) + 54 Serine(c) + 41 Threonine(c) + 65 Valine(c) + 4 Tryptophan(c) + 28 Tyrosine(c) + 4112 ATP(c) + 4112 H2O(c) → Collagen CO6A1(c) + 3084 ADP(c) + 3084 Pi(c) + 1028 AMP(c) + 1028 PPi(c)
r2468	156	Glycine(s) + H+(PG)(s) → Glycine(c) + H+(PG)(c)
r1123	-42	Isoleucine(c) + Na+(c) ← Isoleucine(s) + Na+(s)
r0007	1028	PPi(c) + H2O(c) → 2 Pi(c)
r1331	-128	H+(PG)(s) ⇌ H+(PG)(c) + Proton-gradient(c)
r1124	-4	Tryptophan(c) + Na+(c) ← Tryptophan(s) + Na+(s)
r2479	28	Asparagine(s) + Na+(s) + H+(PG)(c) → Asparagine(c) + Na+(c) + H+(PG)(s)
r1143	-71	Na+(c) + Aspartate(c) ← Na+(s) + Aspartate(s)
r1125	28	Tyrosine(s) + Na+(s) → Tyrosine(c) + Na+(c)

Collagen CO6A2(c) synthesis (360)

Definition			Solution													
			exchanges		reactions									transp		
Objective	Constraints	Comment	imports	exports	c	m	r	p	l	n	s	b	s-c	b-c	int
Collagen CO6A2(c)	MCES AAA	test synthesis of Collagen CO6A2 c	65 Glutamate(s) 155 Glycine(s) 60 Alanine(s) 53 Lysine(s) 71 Aspartate(s) 68 Arginine(s) 45 Glutamine(s) 54 Serine(s) 13 Methionine(s) 5 Tryptophan(s) 39 Phenylalanine(s) 18 Tyrosine(s) 21 Cysteine(s) 66 Leucine(s) 20 Histidine(s) 89 Proline(s) 30 Asparagine(s) 60 Valine(s) 45 Threonine(s) 42 Isoleucine(s) 5095 ATP-energy(c) 125 Proton-gradient(c)	1 Collagen CO6A2(c) 425 Na-gradient(c)	4	-	-	-	-	-	-	-	22	-	-

ID	flux	reaction
r2530	66	Leucine(s) ⇌ Leucine(c)
r1144	-65	Glutamate(c) + Na+(c) ← Glutamate(s) + Na+(s)
r1126	-39	Phenylalanine(c) + Na+(c) ← Phenylalanine(s) + Na+(s)
r1072	13	Methionine(s) ⇌ Methionine(c)
r2531	89	Proline(s) ⇌ Proline(c)
r1145	-20	Histidine(c) + Na+(c) ← Histidine(s) + Na+(s)
r2524	60	Alanine(s) ⇌ Alanine(c)
r2534	45	Threonine(s) ⇌ Threonine(c)
r2525	45	Glutamine(s) ⇌ Glutamine(c)
r1319	-5095	ATP(c) + H2O(c) ⇌ ADP(c) + Pi(c) + ATP-energy(c)
r1058	68	Arginine(s) ⇌ Arginine(c)
r1069	-53	Lysine(c) ⇌ Lysine(s)
r0030	1019	ATP(c) + AMP(c) ⇌ 2 ADP(c)
r2474	54	Serine(s) + Na+(s) → Serine(c) + Na+(c)
r2664	425	Na+(c) ⇌ Na+(s) + Na-gradient(c)
r2475	21	Cysteine(s) + Na+(s) → Cysteine(c) + Na+(c)
r1121	-60	Valine(c) + Na+(c) ← Valine(s) + Na+(s)
r2468	155	Glycine(s) + H+(PG)(s) → Glycine(c) + H+(PG)(c)
r1123	-42	Isoleucine(c) + Na+(c) ← Isoleucine(s) + Na+(s)
r0007	1019	PPi(c) + H2O(c) → 2 Pi(c)
r1331	-125	H+(PG)(s) ⇌ H+(PG)(c) + Proton-gradient(c)
r1124	-5	Tryptophan(c) + Na+(c) ← Tryptophan(s) + Na+(s)
r2578	1	60 Alanine(c) + 21 Cysteine(c) + 71 Aspartate(c) + 65 Glutamate(c) + 39 Phenylalanine(c) + 155 Glycine(c) + 20 Histidine(c) + 42 Isoleucine(c) + 53 Lysine(c) + 66 Leucine(c) + 13 Methionine(c) + 30 Asparagine(c) + 89 Proline(c) + 45 Glutamine(c) + 68 Arginine(c) + 54 Serine(c) + 45 Threonine(c) + 60 Valine(c) + 5 Tryptophan(c) + 18 Tyrosine(c) + 4076 ATP(c) + 4076 H2O(c) → Collagen CO6A2(c) + 3057 ADP(c) + 3057 Pi(c) + 1019 AMP(c) + 1019 PPi(c)
r2479	30	Asparagine(s) + Na+(s) + H+(PG)(c) → Asparagine(c) + Na+(c) + H+(PG)(s)
r1143	-71	Na+(c) + Aspartate(c) ← Na+(s) + Aspartate(s)
r1125	18	Tyrosine(s) + Na+(s) → Tyrosine(c) + Na+(c)

Collagen CO6A3(c) synthesis (361)

Definition			Solution												
			exchanges		reactions								transp		
Objective	Constraints	Comment	imports	exports	c	m	r	p	l	n	s	b	s-c	b-c	int
Collagen CO6A3(c)	MCES AAA	test synthesis of Collagen CO6A3 c	183 Glutamate(s) 303 Glycine(s) 235 Alanine(s) 158 Lysine(s) 183 Aspartate(s) 189 Arginine(s) 147 Glutamine(s) 217 Serine(s) 37 Methionine(s) 6 Tryptophan(s) 149 Phenylalanine(s) 62 Tyrosine(s) 30 Cysteine(s) 282 Leucine(s) 45 Histidine(s) 209 Proline(s) 130 Asparagine(s) 292 Valine(s) 167 Threonine(s) 153 Isoleucine(s) 15885 ATP-energy(c) 173 Proton-gradient(c)	1 Collagen CO6A3(c) 1450 Na-gradient(c)	4	-	-	-	-	-	-	-	22	-	-

ID	flux	reaction
r2530	282	Leucine(s) ⇌ Leucine(c)
r1144	-183	Glutamate(c) + Na+(c) ← Glutamate(s) + Na+(s)
r1126	-149	Phenylalanine(c) + Na+(c) ← Phenylalanine(s) + Na+(s)
r1072	37	Methionine(s) ⇌ Methionine(c)
r2531	209	Proline(s) ⇌ Proline(c)
r1145	-45	Histidine(c) + Na+(c) ← Histidine(s) + Na+(s)
r2524	235	Alanine(s) ⇌ Alanine(c)
r2534	167	Threonine(s) ⇌ Threonine(c)
r2525	147	Glutamine(s) ⇌ Glutamine(c)
r1319	-15885	ATP(c) + H2O(c) ⇌ ADP(c) + Pi(c) + ATP-energy(c)
r1058	189	Arginine(s) ⇌ Arginine(c)
r2580	1	235 Alanine(c) + 30 Cysteine(c) + 183 Aspartate(c) + 183 Glutamate(c) + 149 Phenylalanine(c) + 303 Glycine(c) + 45 Histidine(c) + 153 Isoleucine(c) + 158 Lysine(c) + 282 Leucine(c) + 37 Methionine(c) + 130 Asparagine(c) + 209 Proline(c) + 147 Glutamine(c) + 189 Arginine(c) + 217 Serine(c) + 167 Threonine(c) + 292 Valine(c) + 6 Tryptophan(c) + 62 Tyrosine(c) + 12708 ATP(c) + 12708 H2O(c) → Collagen CO6A3(c) + 9531 ADP(c) + 9531 Pi(c) + 3177 AMP(c) + 3177 PPi(c)
r1069	-158	Lysine(c) ⇌ Lysine(s)
r0030	3177	ATP(c) + AMP(c) ⇌ 2 ADP(c)
r2474	217	Serine(s) + Na+(s) → Serine(c) + Na+(c)
r2664	1450	Na+(c) ⇌ Na+(s) + Na-gradient(c)
r2475	30	Cysteine(s) + Na+(s) → Cysteine(c) + Na+(c)
r1121	-292	Valine(c) + Na+(c) ← Valine(s) + Na+(s)
r2468	303	Glycine(s) + H+(PG)(s) → Glycine(c) + H+(PG)(c)
r1123	-153	Isoleucine(c) + Na+(c) ← Isoleucine(s) + Na+(s)
r0007	3177	PPi(c) + H2O(c) → 2 Pi(c)
r1331	-173	H+(PG)(s) ⇌ H+(PG)(c) + Proton-gradient(c)
r1124	-6	Tryptophan(c) + Na+(c) ← Tryptophan(s) + Na+(s)
r2479	130	Asparagine(s) + Na+(s) + H+(PG)(c) → Asparagine(c) + Na+(c) + H+(PG)(s)
r1143	-183	Na+(c) + Aspartate(c) ← Na+(s) + Aspartate(s)
r1125	62	Tyrosine(s) + Na+(s) → Tyrosine(c) + Na+(c)

Collagen CO6A5(c) synthesis (362)

Definition			Solution												
			exchanges		reactions								transp		
Objective	Constraints	Comment	imports	exports	c	m	r	p	l	n	s	b	s-c	b-c	int
Collagen CO6A5(c)	MCES AAA	test synthesis of Collagen CO6A5 c	154 Glutamate(s) 244 Glycine(s) 138 Alanine(s) 179 Lysine(s) 164 Aspartate(s) 124 Arginine(s) 143 Glutamine(s) 188 Serine(s) 55 Methionine(s) 12 Tryptophan(s) 132 Phenylalanine(s) 71 Tyrosine(s) 36 Cysteine(s) 223 Leucine(s) 58 Histidine(s) 108 Proline(s) 130 Asparagine(s) 160 Valine(s) 135 Threonine(s) 161 Isoleucine(s) 13075 ATP-energy(c) 114 Proton-gradient(c)	1 Collagen CO6A5(c) 1266 Na-gradient(c)	4	-	-	-	-	-	-	-	22	-	-

ID	flux	reaction
r2530	223	Leucine(s) ⇌ Leucine(c)
r1144	-154	Glutamate(c) + Na+(c) ← Glutamate(s) + Na+(s)
r1126	-132	Phenylalanine(c) + Na+(c) ← Phenylalanine(s) + Na+(s)
r1072	55	Methionine(s) ⇌ Methionine(c)
r2531	108	Proline(s) ⇌ Proline(c)
r1145	-58	Histidine(c) + Na+(c) ← Histidine(s) + Na+(s)
r2524	138	Alanine(s) ⇌ Alanine(c)
r2534	135	Threonine(s) ⇌ Threonine(c)
r2525	143	Glutamine(s) ⇌ Glutamine(c)
r1319	-13075	ATP(c) + H2O(c) ⇌ ADP(c) + Pi(c) + ATP-energy(c)
r1058	124	Arginine(s) ⇌ Arginine(c)
r1069	-179	Lysine(c) ⇌ Lysine(s)
r0030	2615	ATP(c) + AMP(c) ⇌ 2 ADP(c)
r2582	1	138 Alanine(c) + 36 Cysteine(c) + 164 Aspartate(c) + 154 Glutamate(c) + 132 Phenylalanine(c) + 244 Glycine(c) + 58 Histidine(c) + 161 Isoleucine(c) + 179 Lysine(c) + 223 Leucine(c) + 55 Methionine(c) + 130 Asparagine(c) + 108 Proline(c) + 143 Glutamine(c) + 124 Arginine(c) + 188 Serine(c) + 135 Threonine(c) + 160 Valine(c) + 12 Tryptophan(c) + 71 Tyrosine(c) + 10460 ATP(c) + 10460 H2O(c) → Collagen CO6A5(c) + 7845 ADP(c) + 7845 Pi(c) + 2615 AMP(c) + 2615 PPi(c)
r2474	188	Serine(s) + Na+(s) → Serine(c) + Na+(c)
r2664	1266	Na+(c) ⇌ Na+(s) + Na-gradient(c)
r2475	36	Cysteine(s) + Na+(s) → Cysteine(c) + Na+(c)
r1121	-160	Valine(c) + Na+(c) ← Valine(s) + Na+(s)
r2468	244	Glycine(s) + H+(PG)(s) → Glycine(c) + H+(PG)(c)
r1123	-161	Isoleucine(c) + Na+(c) ← Isoleucine(s) + Na+(s)
r0007	2615	PPi(c) + H2O(c) → 2 Pi(c)
r1331	-114	H+(PG)(s) ⇌ H+(PG)(c) + Proton-gradient(c)
r1124	-12	Tryptophan(c) + Na+(c) ← Tryptophan(s) + Na+(s)
r2479	130	Asparagine(s) + Na+(s) + H+(PG)(c) → Asparagine(c) + Na+(c) + H+(PG)(s)
r1143	-164	Na+(c) + Aspartate(c) ← Na+(s) + Aspartate(s)
r1125	71	Tyrosine(s) + Na+(s) → Tyrosine(c) + Na+(c)

Collagen CO6A6(c) synthesis (363)

Definition			Solution													
			exchanges		reactions								transp		
Objective	Constraints	Comment	imports	exports	c	m	r	p	l	n	s	b	s-c	b-c	int
Collagen CO6A6(c)	MCES AAA	test synthesis of Collagen CO6A6 c	150 Glutamate(s) 230 Glycine(s) 149 Alanine(s) 152 Lysine(s) 137 Aspartate(s) 121 Arginine(s) 95 Glutamine(s) 170 Serine(s) 47 Methionine(s) 6 Tryptophan(s) 109 Phenylalanine(s) 47 Tyrosine(s) 29 Cysteine(s) 180 Leucine(s) 48 Histidine(s) 108 Proline(s) 92 Asparagine(s) 157 Valine(s) 103 Threonine(s) 133 Isoleucine(s) 11315 ATP-energy(c) 138 Proton-gradient(c)	1 Collagen CO6A6(c) 1078 Na-gradient(c)	4	-	-	-	-	-	-	-	22	-	-

ID	flux	reaction
r2530	180	Leucine(s) ⇌ Leucine(c)
r1144	-150	Glutamate(c) + Na+(c) ← Glutamate(s) + Na+(s)
r1126	-109	Phenylalanine(c) + Na+(c) ← Phenylalanine(s) + Na+(s)
r1072	47	Methionine(s) ⇌ Methionine(c)
r2531	108	Proline(s) ⇌ Proline(c)
r1145	-48	Histidine(c) + Na+(c) ← Histidine(s) + Na+(s)
r2524	149	Alanine(s) ⇌ Alanine(c)
r2534	103	Threonine(s) ⇌ Threonine(c)
r2525	95	Glutamine(s) ⇌ Glutamine(c)
r1319	-11315	ATP(c) + H2O(c) ⇌ ADP(c) + Pi(c) + ATP-energy(c)
r1058	121	Arginine(s) ⇌ Arginine(c)
r1069	-152	Lysine(c) ⇌ Lysine(s)
r0030	2263	ATP(c) + AMP(c) ⇌ 2 ADP(c)
r2474	170	Serine(s) + Na+(s) → Serine(c) + Na+(c)
r2664	1078	Na+(c) ⇌ Na+(s) + Na-gradient(c)
r2475	29	Cysteine(s) + Na+(s) → Cysteine(c) + Na+(c)
r1121	-157	Valine(c) + Na+(c) ← Valine(s) + Na+(s)
r2584	1	149 Alanine(c) + 29 Cysteine(c) + 137 Aspartate(c) + 150 Glutamate(c) + 109 Phenylalanine(c) + 230 Glycine(c) + 48 Histidine(c) + 133 Isoleucine(c) + 152 Lysine(c) + 180 Leucine(c) + 47 Methionine(c) + 92 Asparagine(c) + 108 Proline(c) + 95 Glutamine(c) + 121 Arginine(c) + 170 Serine(c) + 103 Threonine(c) + 157 Valine(c) + 6 Tryptophan(c) + 47 Tyrosine(c) + 9052 ATP(c) + 9052 H2O(c) → Collagen CO6A6(c) + 6789 ADP(c) + 6789 Pi(c) + 2263 AMP(c) + 2263 PPi(c)
r2468	230	Glycine(s) + H+(PG)(s) → Glycine(c) + H+(PG)(c)
r1123	-133	Isoleucine(c) + Na+(c) ← Isoleucine(s) + Na+(s)
r0007	2263	PPi(c) + H2O(c) → 2 Pi(c)
r1331	-138	H+(PG)(s) ⇌ H+(PG)(c) + Proton-gradient(c)
r1124	-6	Tryptophan(c) + Na+(c) ← Tryptophan(s) + Na+(s)
r2479	92	Asparagine(s) + Na+(s) + H+(PG)(c) → Asparagine(c) + Na+(c) + H+(PG)(s)
r1143	-137	Na+(c) + Aspartate(c) ← Na+(s) + Aspartate(s)
r1125	47	Tyrosine(s) + Na+(s) → Tyrosine(c) + Na+(c)

Collagen CO7A1(c) synthesis (364)

Definition			Solution												
			exchanges		reactions								transsp		
Objective	Constraints	Comment	imports	exports	c	m	r	p	l	n	s	b	s-c	b-c	int
Collagen CO7A1(c)	MCES AAA	test synthesis of Collagen CO7A1 c	187 Glutamate(s) 627 Glycine(s) 193 Alanine(s) 93 Lysine(s) 145 Aspartate(s) 217 Arginine(s) 105 Glutamine(s) 166 Serine(s) 18 Methionine(s) 19 Tryptophan(s) 39 Phenylalanine(s) 36 Tyrosine(s) 17 Cysteine(s) 212 Leucine(s) 28 Histidine(s) 426 Proline(s) 27 Asparagine(s) 201 Valine(s) 132 Threonine(s) 56 Isoleucine(s) 14720 ATP-energy(c) 600 Proton-gradient(c)	1 Collagen CO7A1(c) 921 Na-gradient(c)	4	-	-	-	-	-	-	-	22	-	-

ID	flux	reaction
r2530	212	Leucine(s) ⇌ Leucine(c)
r1144	-187	Glutamate(c) + Na+(c) ← Glutamate(s) + Na+(s)
r1126	-39	Phenylalanine(c) + Na+(c) ← Phenylalanine(s) + Na+(s)
r1072	18	Methionine(s) ⇌ Methionine(c)
r2531	426	Proline(s) ⇌ Proline(c)
r1145	-28	Histidine(c) + Na+(c) ← Histidine(s) + Na+(s)
r2524	193	Alanine(s) ⇌ Alanine(c)
r2534	132	Threonine(s) ⇌ Threonine(c)
r2525	105	Glutamine(s) ⇌ Glutamine(c)
r1319	-14720	ATP(c) + H2O(c) ⇌ ADP(c) + Pi(c) + ATP-energy(c)
r1058	217	Arginine(s) ⇌ Arginine(c)
r1069	-93	Lysine(c) ⇌ Lysine(s)
r0030	2944	ATP(c) + AMP(c) ⇌ 2 ADP(c)
r2474	166	Serine(s) + Na+(s) → Serine(c) + Na+(c)
r2664	921	Na+(c) ⇌ Na+(s) + Na-gradient(c)
r2475	17	Cysteine(s) + Na+(s) → Cysteine(c) + Na+(c)
r1121	-201	Valine(c) + Na+(c) ← Valine(s) + Na+(s)
r2468	627	Glycine(s) + H+(PG)(s) → Glycine(c) + H+(PG)(c)
r1123	-56	Isoleucine(c) + Na+(c) ← Isoleucine(s) + Na+(s)
r0007	2944	PPi(c) + H2O(c) → 2 Pi(c)
r2586	1	193 Alanine(c) + 17 Cysteine(c) + 145 Aspartate(c) + 187 Glutamate(c) + 39 Phenylalanine(c) + 627 Glycine(c) + 28 Histidine(c) + 56 Isoleucine(c) + 93 Lysine(c) + 212 Leucine(c) + 18 Methionine(c) + 27 Asparagine(c) + 426 Proline(c) + 105 Glutamine(c) + 217 Arginine(c) + 166 Serine(c) + 132 Threonine(c) + 201 Valine(c) + 19 Tryptophan(c) + 36 Tyrosine(c) + 11776 ATP(c) + 11776 H2O(c) → Collagen CO7A1(c) + 8832 ADP(c) + 8832 Pi(c) + 2944 AMP(c) + 2944 PPi(c)
r1331	-600	H+(PG)(s) ⇌ H+(PG)(c) + Proton-gradient(c)
r1124	-19	Tryptophan(c) + Na+(c) ← Tryptophan(s) + Na+(s)
r2479	27	Asparagine(s) + Na+(s) + H+(PG)(c) → Asparagine(c) + Na+(c) + H+(PG)(s)
r1143	-145	Na+(c) + Aspartate(c) ← Na+(s) + Aspartate(s)
r1125	36	Tyrosine(s) + Na+(s) → Tyrosine(c) + Na+(c)

Collagen CO8A1(c) synthesis (365)

Definition			Solution												
			exchanges		reactions								transsp		
Objective	Constraints	Comment	imports	exports	c	m	r	p	l	n	s	b	s-c	b-c	int
Collagen CO8A1(c)	MCES AAA	test synthesis of Collagen CO8A1 c	26 Glutamate(s) 190 Glycine(s) 33 Alanine(s) 47 Lysine(s) 11 Aspartate(s) 13 Arginine(s) 38 Glutamine(s) 11 Serine(s) 23 Methionine(s) 1 Tryptophan(s) 17 Phenylalanine(s) 22 Tyrosine(s) 2 Cysteine(s) 53 Leucine(s) 11 Histidine(s) 167 Proline(s) 8 Asparagine(s) 30 Valine(s) 8 Threonine(s) 33 Isoleucine(s) 3720 ATP-energy(c) 182 Proton-gradient(c)	1 Collagen CO8A1(c) 172 Na-gradient(c)	4	-	-	-	-	-	-	-	22	-	-

ID	flux	reaction
r2588	1	33 Alanine(c) + 2 Cysteine(c) + 11 Aspartate(c) + 26 Glutamate(c) + 17 Phenylalanine(c) + 190 Glycine(c) + 11 Histidine(c) + 33 Isoleucine(c) + 47 Lysine(c) + 53 Leucine(c) + 23 Methionine(c) + 8 Asparagine(c) + 167 Proline(c) + 38 Glutamine(c) + 13 Arginine(c) + 11 Serine(c) + 8 Threonine(c) + 30 Valine(c) + Tryptophan(c) + 22 Tyrosine(c) + 2976 ATP(c) + 2976 H2O(c) → Collagen CO8A1(c) + 2232 ADP(c) + 2232 Pi(c) + 744 AMP(c) + 744 PPi(c)
r2530	53	Leucine(s) ⇌ Leucine(c)
r1144	-26	Glutamate(c) + Na+(c) ← Glutamate(s) + Na+(s)
r1126	-17	Phenylalanine(c) + Na+(c) ← Phenylalanine(s) + Na+(s)
r1072	23	Methionine(s) ⇌ Methionine(c)
r2531	167	Proline(s) ⇌ Proline(c)
r1145	-11	Histidine(c) + Na+(c) ← Histidine(s) + Na+(s)
r2524	33	Alanine(s) ⇌ Alanine(c)
r2534	8	Threonine(s) ⇌ Threonine(c)
r2525	38	Glutamine(s) ⇌ Glutamine(c)
r1319	-3720	ATP(c) + H2O(c) ⇌ ADP(c) + Pi(c) + ATP-energy(c)
r1058	13	Arginine(s) ⇌ Arginine(c)
r1069	-47	Lysine(c) ⇌ Lysine(s)
r0030	744	ATP(c) + AMP(c) ⇌ 2 ADP(c)
r2474	11	Serine(s) + Na+(s) → Serine(c) + Na+(c)
r2664	172	Na+(c) ⇌ Na+(s) + Na-gradient(c)
r2475	2	Cysteine(s) + Na+(s) → Cysteine(c) + Na+(c)
r1121	-30	Valine(c) + Na+(c) ← Valine(s) + Na+(s)
r2468	190	Glycine(s) + H+(PG)(s) → Glycine(c) + H+(PG)(c)
r1123	-33	Isoleucine(c) + Na+(c) ← Isoleucine(s) + Na+(s)
r0007	744	PPi(c) + H2O(c) → 2 Pi(c)
r1331	-182	H+(PG)(s) ⇌ H+(PG)(c) + Proton-gradient(c)
r1124	-1	Tryptophan(c) + Na+(c) ← Tryptophan(s) + Na+(s)
r2479	8	Asparagine(s) + Na+(s) + H+(PG)(c) → Asparagine(c) + Na+(c) + H+(PG)(s)
r1143	-11	Na+(c) + Aspartate(c) ← Na+(s) + Aspartate(s)
r1125	22	Tyrosine(s) + Na+(s) → Tyrosine(c) + Na+(c)

Collagen CO8A2(c) synthesis (366)

Definition			Solution													
			exchanges		reactions								transsp		
Objective	Constraints	Comment	imports	exports	c	m	r	p	l	n	s	b	s-c	b-c	int
Collagen CO8A2(c)	MCES AAA	test synthesis of Collagen CO8A2 c	24 Glutamate(s) 193 Glycine(s) 42 Alanine(s) 28 Lysine(s) 18 Aspartate(s) 21 Arginine(s) 25 Glutamine(s) 22 Serine(s) 9 Methionine(s) 2 Tryptophan(s) 14 Phenylalanine(s) 16 Tyrosine(s) 3 Cysteine(s) 59 Leucine(s) 8 Histidine(s) 145 Proline(s) 9 Asparagine(s) 31 Valine(s) 22 Threonine(s) 12 Isoleucine(s) 3515 ATP-energy(c) 184 Proton-gradient(c)	1 Collagen CO8A2(c) 159 Na-gradient(c)	4	-	-	-	-	-	-	-	22	-	-

ID	flux	reaction
r2530	59	Leucine(s) ⇌ Leucine(c)
r1144	-24	Glutamate(c) + Na+(c) ← Glutamate(s) + Na+(s)
r1126	-14	Phenylalanine(c) + Na+(c) ← Phenylalanine(s) + Na+(s)
r1072	9	Methionine(s) ⇌ Methionine(c)
r2531	145	Proline(s) ⇌ Proline(c)
r1145	-8	Histidine(c) + Na+(c) ← Histidine(s) + Na+(s)
r2524	42	Alanine(s) ⇌ Alanine(c)
r2534	22	Threonine(s) ⇌ Threonine(c)
r2525	25	Glutamine(s) ⇌ Glutamine(c)
r1319	-3515	ATP(c) + H2O(c) ⇌ ADP(c) + Pi(c) + ATP-energy(c)
r1058	21	Arginine(s) ⇌ Arginine(c)
r2590	1	42 Alanine(c) + 3 Cysteine(c) + 18 Aspartate(c) + 24 Glutamate(c) + 14 Phenylalanine(c) + 193 Glycine(c) + 8 Histidine(c) + 12 Isoleucine(c) + 28 Lysine(c) + 59 Leucine(c) + 9 Methionine(c) + 9 Asparagine(c) + 145 Proline(c) + 25 Glutamine(c) + 21 Arginine(c) + 22 Serine(c) + 22 Threonine(c) + 31 Valine(c) + 2 Tryptophan(c) + 16 Tyrosine(c) + 2812 ATP(c) + 2812 H2O(c) → Collagen CO8A2(c) + 2109 ADP(c) + 2109 Pi(c) + 703 AMP(c) + 703 PPi(c)
r1069	-28	Lysine(c) ⇌ Lysine(s)
r0030	703	ATP(c) + AMP(c) ⇌ 2 ADP(c)
r2474	22	Serine(s) + Na+(s) → Serine(c) + Na+(c)
r2664	159	Na+(c) ⇌ Na+(s) + Na-gradient(c)
r2475	3	Cysteine(s) + Na+(s) → Cysteine(c) + Na+(c)
r1121	-31	Valine(c) + Na+(c) ← Valine(s) + Na+(s)
r2468	193	Glycine(s) + H+(PG)(s) → Glycine(c) + H+(PG)(c)
r1123	-12	Isoleucine(c) + Na+(c) ← Isoleucine(s) + Na+(s)
r0007	703	PPi(c) + H2O(c) → 2 Pi(c)
r1331	-184	H+(PG)(s) ⇌ H+(PG)(c) + Proton-gradient(c)
r1124	-2	Tryptophan(c) + Na+(c) ← Tryptophan(s) + Na+(s)
r2479	9	Asparagine(s) + Na+(s) + H+(PG)(c) → Asparagine(c) + Na+(c) + H+(PG)(s)
r1143	-18	Na+(c) + Aspartate(c) ← Na+(s) + Aspartate(s)
r1125	16	Tyrosine(s) + Na+(s) → Tyrosine(c) + Na+(c)

Collagen CO9A1(c) synthesis (367)

Definition			Solution												
			exchanges		reactions								transp		
Objective	Constraints	Comment	imports	exports	c	m	r	p	l	n	s	b	s-c	b-c	int
Collagen CO9A1(c)	MCES AAA	test synthesis of Collagen CO9A1 c	47 Glutamate(s) 217 Glycine(s) 47 Alanine(s) 42 Lysine(s) 39 Aspartate(s) 54 Arginine(s) 39 Glutamine(s) 42 Serine(s) 12 Methionine(s) 6 Tryptophan(s) 22 Phenylalanine(s) 6 Tyrosine(s) 11 Cysteine(s) 57 Leucine(s) 8 Histidine(s) 153 Proline(s) 20 Asparagine(s) 37 Valine(s) 26 Threonine(s) 36 Isoleucine(s) 4605 ATP-energy(c) 197 Proton-gradient(c)	1 Collagen CO9A1(c) 274 Na-gradient(c)	4	-	-	-	-	-	-	-	22	-	-

ID	flux	reaction
r2530	57	Leucine(s) ⇌ Leucine(c)
r1144	-47	Glutamate(c) + Na+(c) ← Glutamate(s) + Na+(s)
r1126	-22	Phenylalanine(c) + Na+(c) ← Phenylalanine(s) + Na+(s)
r1072	12	Methionine(s) ⇌ Methionine(c)
r2531	153	Proline(s) ⇌ Proline(c)
r1145	-8	Histidine(c) + Na+(c) ← Histidine(s) + Na+(s)
r2524	47	Alanine(s) ⇌ Alanine(c)
r2534	26	Threonine(s) ⇌ Threonine(c)
r2525	39	Glutamine(s) ⇌ Glutamine(c)
r1319	-4605	ATP(c) + H2O(c) ⇌ ADP(c) + Pi(c) + ATP-energy(c)
r1058	54	Arginine(s) ⇌ Arginine(c)
r1069	-42	Lysine(c) ⇌ Lysine(s)
r0030	921	ATP(c) + AMP(c) ⇌ 2 ADP(c)
r2474	42	Serine(s) + Na+(s) → Serine(c) + Na+(c)
r2664	274	Na+(c) ⇌ Na+(s) + Na-gradient(c)
r2592	1	47 Alanine(c) + 11 Cysteine(c) + 39 Aspartate(c) + 47 Glutamate(c) + 22 Phenylalanine(c) + 217 Glycine(c) + 8 Histidine(c) + 36 Isoleucine(c) + 42 Lysine(c) + 57 Leucine(c) + 12 Methionine(c) + 20 Asparagine(c) + 153 Proline(c) + 39 Glutamine(c) + 54 Arginine(c) + 42 Serine(c) + 26 Threonine(c) + 37 Valine(c) + 6 Tryptophan(c) + 6 Tyrosine(c) + 3684 ATP(c) + 3684 H2O(c) → Collagen CO9A1(c) + 2763 ADP(c) + 2763 Pi(c) + 921 AMP(c) + 921 PPi(c)
r2475	11	Cysteine(s) + Na+(s) → Cysteine(c) + Na+(c)
r1121	-37	Valine(c) + Na+(c) ← Valine(s) + Na+(s)
r2468	217	Glycine(s) + H+(PG)(s) → Glycine(c) + H+(PG)(c)
r1123	-36	Isoleucine(c) + Na+(c) ← Isoleucine(s) + Na+(s)
r0007	921	PPi(c) + H2O(c) → 2 Pi(c)
r1331	-197	H+(PG)(s) ⇌ H+(PG)(c) + Proton-gradient(c)
r1124	-6	Tryptophan(c) + Na+(c) ← Tryptophan(s) + Na+(s)
r2479	20	Asparagine(s) + Na+(s) + H+(PG)(c) → Asparagine(c) + Na+(c) + H+(PG)(s)
r1143	-39	Na+(c) + Aspartate(c) ← Na+(s) + Aspartate(s)
r1125	6	Tyrosine(s) + Na+(s) → Tyrosine(c) + Na+(c)

Collagen CO9A2(c) synthesis (368)

Definition			Solution													
			exchanges		reactions								transp		
Objective	Constraints	Comment	imports	exports	c	m	r	p	l	n	s	b	s-c	b-c	int
Collagen CO9A2(c)	MCES AAA	test synthesis of Collagen CO9A2 c	31 Glutamate(s) 203 Glycine(s) 42 Alanine(s) 38 Lysine(s) 24 Aspartate(s) 27 Arginine(s) 34 Glutamine(s) 18 Serine(s) 11 Methionine(s) 4 Phenylalanine(s) 6 Tyrosine(s) 4 Cysteine(s) 28 Leucine(s) 8 Histidine(s) 138 Proline(s) 6 Asparagine(s) 29 Valine(s) 15 Threonine(s) 23 Isoleucine(s) 3445 ATP-energy(c) 197 Proton-gradient(c)	1 Collagen CO9A2(c) 153 Na-gradient(c)	4	-	-	-	-	-	-	-	21	-	-

ID	flux	reaction
r2530	28	Leucine(s) ⇌ Leucine(c)
r1144	-31	Glutamate(c) + Na+(c) ← Glutamate(s) + Na+(s)
r1126	-4	Phenylalanine(c) + Na+(c) ← Phenylalanine(s) + Na+(s)
r1072	11	Methionine(s) ⇌ Methionine(c)
r2531	138	Proline(s) ⇌ Proline(c)
r1145	-8	Histidine(c) + Na+(c) ← Histidine(s) + Na+(s)
r2524	42	Alanine(s) ⇌ Alanine(c)
r2534	15	Threonine(s) ⇌ Threonine(c)
r2525	34	Glutamine(s) ⇌ Glutamine(c)
r1319	-3445	ATP(c) + H2O(c) ⇌ ADP(c) + Pi(c) + ATP-energy(c)
r1058	27	Arginine(s) ⇌ Arginine(c)
r1069	-38	Lysine(c) ⇌ Lysine(s)
r0030	689	ATP(c) + AMP(c) ⇌ 2 ADP(c)
r2474	18	Serine(s) + Na+(s) → Serine(c) + Na+(c)
r2664	153	Na+(c) ⇌ Na+(s) + Na-gradient(c)
r2475	4	Cysteine(s) + Na+(s) → Cysteine(c) + Na+(c)
r1121	-29	Valine(c) + Na+(c) ← Valine(s) + Na+(s)
r2594	1	42 Alanine(c) + 4 Cysteine(c) + 24 Aspartate(c) + 31 Glutamate(c) + 4 Phenylalanine(c) + 203 Glycine(c) + 8 Histidine(c) + 23 Isoleucine(c) + 38 Lysine(c) + 28 Leucine(c) + 11 Methionine(c) + 6 Asparagine(c) + 138 Proline(c) + 34 Glutamine(c) + 27 Arginine(c) + 18 Serine(c) + 15 Threonine(c) + 29 Valine(c) + 6 Tyrosine(c) + 2756 ATP(c) + 2756 H2O(c) → Collagen CO9A2(c) + 2067 ADP(c) + 2067 Pi(c) + 689 AMP(c) + 689 PPi(c)
r2468	203	Glycine(s) + H+(PG)(s) → Glycine(c) + H+(PG)(c)
r1123	-23	Isoleucine(c) + Na+(c) ← Isoleucine(s) + Na+(s)
r0007	689	PPi(c) + H2O(c) → 2 Pi(c)
r1331	-197	H+(PG)(s) ⇌ H+(PG)(c) + Proton-gradient(c)
r2479	6	Asparagine(s) + Na+(s) + H+(PG)(c) → Asparagine(c) + Na+(c) + H+(PG)(s)
r1143	-24	Na+(c) + Aspartate(c) ← Na+(s) + Aspartate(s)
r1125	6	Tyrosine(s) + Na+(s) → Tyrosine(c) + Na+(c)

Collagen CO9A3(c) synthesis (369)

Definition			Solution												
			exchanges		reactions								transp		
Objective	Constraints	Comment	imports	exports	c	m	r	p	l	n	s	b	s-c	b-c	int
Collagen CO9A3(c)	MCES AAA	test synthesis of Collagen CO9A3 c	32 Glutamate(s) 210 Glycine(s) 45 Alanine(s) 29 Lysine(s) 28 Aspartate(s) 32 Arginine(s) 25 Glutamine(s) 25 Serine(s) 6 Methionine(s) 4 Phenylalanine(s) 2 Tyrosine(s) 6 Cysteine(s) 49 Leucine(s) 4 Histidine(s) 137 Proline(s) 5 Asparagine(s) 20 Valine(s) 11 Threonine(s) 14 Isoleucine(s) 3420 ATP-energy(c) 205 Proton-gradient(c)	1 Collagen CO9A3(c) 140 Na-gradient(c)	4	-	-	-	-	-	-	-	21	-	-

ID	flux	reaction
r2530	49	Leucine(s) ⇌ Leucine(c)
r1144	-32	Glutamate(c) + Na+(c) ← Glutamate(s) + Na+(s)
r1126	-4	Phenylalanine(c) + Na+(c) ← Phenylalanine(s) + Na+(s)
r1072	6	Methionine(s) ⇌ Methionine(c)
r2531	137	Proline(s) ⇌ Proline(c)
r1145	-4	Histidine(c) + Na+(c) ← Histidine(s) + Na+(s)
r2524	45	Alanine(s) ⇌ Alanine(c)
r2534	11	Threonine(s) ⇌ Threonine(c)
r2525	25	Glutamine(s) ⇌ Glutamine(c)
r1319	-3420	ATP(c) + H2O(c) ⇌ ADP(c) + Pi(c) + ATP-energy(c)
r1058	32	Arginine(s) ⇌ Arginine(c)
r1069	-29	Lysine(c) ⇌ Lysine(s)
r0030	684	ATP(c) + AMP(c) ⇌ 2 ADP(c)
r2474	25	Serine(s) + Na+(s) → Serine(c) + Na+(c)
r2664	140	Na+(c) ⇌ Na+(s) + Na-gradient(c)
r2475	6	Cysteine(s) + Na+(s) → Cysteine(c) + Na+(c)
r1121	-20	Valine(c) + Na+(c) ← Valine(s) + Na+(s)
r2468	210	Glycine(s) + H+(PG)(s) → Glycine(c) + H+(PG)(c)
r1123	-14	Isoleucine(c) + Na+(c) ← Isoleucine(s) + Na+(s)
r0007	684	PPi(c) + H2O(c) → 2 Pi(c)
r1331	-205	H+(PG)(s) ⇌ H+(PG)(c) + Proton-gradient(c)
r2596	1	45 Alanine(c) + 6 Cysteine(c) + 28 Aspartate(c) + 32 Glutamate(c) + 4 Phenylalanine(c) + 210 Glycine(c) + 4 Histidine(c) + 14 Isoleucine(c) + 29 Lysine(c) + 49 Leucine(c) + 6 Methionine(c) + 5 Asparagine(c) + 137 Proline(c) + 25 Glutamine(c) + 32 Arginine(c) + 25 Serine(c) + 11 Threonine(c) + 20 Valine(c) + 2 Tyrosine(c) + 2736 ATP(c) + 2736 H2O(c) → Collagen CO9A3(c) + 2052 ADP(c) + 2052 Pi(c) + 684 AMP(c) + 684 PPi(c)
r2479	5	Asparagine(s) + Na+(s) + H+(PG)(c) → Asparagine(c) + Na+(c) + H+(PG)(s)
r1143	-28	Na+(c) + Aspartate(c) ← Na+(s) + Aspartate(s)
r1125	2	Tyrosine(s) + Na+(s) → Tyrosine(c) + Na+(c)

Collagen COAA1(c) synthesis (370)

Definition			Solution												
			exchanges		reactions								transp		
Objective	Constraints	Comment	imports	exports	c	m	r	p	l	n	s	b	s-c	b-c	int
Collagen COAA1(c)	MCES AAA	test synthesis of Collagen COAA1 c	22 Glutamate(s) 175 Glycine(s) 36 Alanine(s) 35 Lysine(s) 12 Aspartate(s) 19 Arginine(s) 23 Glutamine(s) 27 Serine(s) 11 Methionine(s) 2 Tryptophan(s) 15 Phenylalanine(s) 21 Tyrosine(s) 1 Cysteine(s) 35 Leucine(s) 10 Histidine(s) 145 Proline(s) 14 Asparagine(s) 26 Valine(s) 24 Threonine(s) 27 Isoleucine(s) 3400 ATP-energy(c) 161 Proton-gradient(c)	1 Collagen COAA1(c) 177 Na-gradient(c)	4	-	-	-	-	-	-	-	22	-	-

ID	flux	reaction
r2530	35	Leucine(s) ⇌ Leucine(c)
r1144	-22	Glutamate(c) + Na+(c) ← Glutamate(s) + Na+(s)
r1126	-15	Phenylalanine(c) + Na+(c) ← Phenylalanine(s) + Na+(s)
r1072	11	Methionine(s) ⇌ Methionine(c)
r2598	1	36 Alanine(c) + Cysteine(c) + 12 Aspartate(c) + 22 Glutamate(c) + 15 Phenylalanine(c) + 175 Glycine(c) + 10 Histidine(c) + 27 Isoleucine(c) + 35 Lysine(c) + 35 Leucine(c) + 11 Methionine(c) + 14 Asparagine(c) + 145 Proline(c) + 23 Glutamine(c) + 19 Arginine(c) + 27 Serine(c) + 24 Threonine(c) + 26 Valine(c) + 2 Tryptophan(c) + 21 Tyrosine(c) + 2720 ATP(c) + 2720 H2O(c) → Collagen COAA1(c) + 2040 ADP(c) + 2040 Pi(c) + 680 AMP(c) + 680 PPi(c)
r2531	145	Proline(s) ⇌ Proline(c)
r1145	-10	Histidine(c) + Na+(c) ← Histidine(s) + Na+(s)
r2524	36	Alanine(s) ⇌ Alanine(c)
r2534	24	Threonine(s) ⇌ Threonine(c)
r2525	23	Glutamine(s) ⇌ Glutamine(c)
r1319	-3400	ATP(c) + H2O(c) ⇌ ADP(c) + Pi(c) + ATP-energy(c)
r1058	19	Arginine(s) ⇌ Arginine(c)
r1069	-35	Lysine(c) ⇌ Lysine(s)
r0030	680	ATP(c) + AMP(c) ⇌ 2 ADP(c)
r2474	27	Serine(s) + Na+(s) → Serine(c) + Na+(c)
r2664	177	Na+(c) ⇌ Na+(s) + Na-gradient(c)
r2475	1	Cysteine(s) + Na+(s) → Cysteine(c) + Na+(c)
r1121	-26	Valine(c) + Na+(c) ← Valine(s) + Na+(s)
r2468	175	Glycine(s) + H+(PG)(s) → Glycine(c) + H+(PG)(c)
r1123	-27	Isoleucine(c) + Na+(c) ← Isoleucine(s) + Na+(s)
r0007	680	PPi(c) + H2O(c) → 2 Pi(c)
r1331	-161	H+(PG)(s) ⇌ H+(PG)(c) + Proton-gradient(c)
r1124	-2	Tryptophan(c) + Na+(c) ← Tryptophan(s) + Na+(s)
r2479	14	Asparagine(s) + Na+(s) + H+(PG)(c) → Asparagine(c) + Na+(c) + H+(PG)(s)
r1143	-12	Na+(c) + Aspartate(c) ← Na+(s) + Aspartate(s)
r1125	21	Tyrosine(s) + Na+(s) → Tyrosine(c) + Na+(c)

Collagen COBA1(c) synthesis (371)

Definition			Solution												
			exchanges		reactions								transp		
Objective	Constraints	Comment	imports	exports	c	m	r	p	l	n	s	b	s-c	b-c	int
Collagen COBA1(c)	MCES AAA	test synthesis of Collagen COBA1 c	123 Glutamate(s) 422 Glycine(s) 98 Alanine(s) 106 Lysine(s) 99 Aspartate(s) 68 Arginine(s) 81 Glutamine(s) 73 Serine(s) 24 Methionine(s) 9 Tryptophan(s) 47 Phenylalanine(s) 36 Tyrosine(s) 11 Cysteine(s) 80 Leucine(s) 17 Histidine(s) 287 Proline(s) 37 Asparagine(s) 63 Valine(s) 69 Threonine(s) 56 Isoleucine(s) 9030 ATP-energy(c) 385 Proton-gradient(c)	1 Collagen COBA1(c) 571 Na-gradient(c)	4	-	-	-	-	-	-	-	22	-	-

ID	flux	reaction
r2530	80	Leucine(s) ⇌ Leucine(c)
r1144	-123	Glutamate(c) + Na+(c) ← Glutamate(s) + Na+(s)
r1126	-47	Phenylalanine(c) + Na+(c) ← Phenylalanine(s) + Na+(s)
r1072	24	Methionine(s) ⇌ Methionine(c)
r2531	287	Proline(s) ⇌ Proline(c)
r1145	-17	Histidine(c) + Na+(c) ← Histidine(s) + Na+(s)
r2524	98	Alanine(s) ⇌ Alanine(c)
r2534	69	Threonine(s) ⇌ Threonine(c)
r2525	81	Glutamine(s) ⇌ Glutamine(c)
r1319	-9030	ATP(c) + H2O(c) ⇌ ADP(c) + Pi(c) + ATP-energy(c)
r1058	68	Arginine(s) ⇌ Arginine(c)
r1069	-106	Lysine(c) ⇌ Lysine(s)
r0030	1806	ATP(c) + AMP(c) ⇌ 2 ADP(c)
r2474	73	Serine(s) + Na+(s) → Serine(c) + Na+(c)
r2664	571	Na+(c) ⇌ Na+(s) + Na-gradient(c)
r2475	11	Cysteine(s) + Na+(s) → Cysteine(c) + Na+(c)
r1121	-63	Valine(c) + Na+(c) ← Valine(s) + Na+(s)
r2468	422	Glycine(s) + H+(PG)(s) → Glycine(c) + H+(PG)(c)
r1123	-56	Isoleucine(c) + Na+(c) ← Isoleucine(s) + Na+(s)
r0007	1806	PPi(c) + H2O(c) → 2 Pi(c)
r2600	1	98 Alanine(c) + 11 Cysteine(c) + 99 Aspartate(c) + 123 Glutamate(c) + 47 Phenylalanine(c) + 422 Glycine(c) + 17 Histidine(c) + 56 Isoleucine(c) + 106 Lysine(c) + 80 Leucine(c) + 24 Methionine(c) + 37 Asparagine(c) + 287 Proline(c) + 81 Glutamine(c) + 68 Arginine(c) + 73 Serine(c) + 69 Threonine(c) + 63 Valine(c) + 9 Tryptophan(c) + 36 Tyrosine(c) + 7224 ATP(c) + 7224 H2O(c) → Collagen COBA1(c) + 5418 ADP(c) + 5418 Pi(c) + 1806 AMP(c) + 1806 PPi(c)
r1331	-385	H+(PG)(s) ⇌ H+(PG)(c) + Proton-gradient(c)
r1124	-9	Tryptophan(c) + Na+(c) ← Tryptophan(s) + Na+(s)
r2479	37	Asparagine(s) + Na+(s) + H+(PG)(c) → Asparagine(c) + Na+(c) + H+(PG)(s)
r1143	-99	Na+(c) + Aspartate(c) ← Na+(s) + Aspartate(s)
r1125	36	Tyrosine(s) + Na+(s) → Tyrosine(c) + Na+(c)

Collagen COBA2(c) synthesis (372)

Definition			Solution												
			exchanges		reactions								transp		
Objective	Constraints	Comment	imports	exports	c	m	r	p	l	n	s	b	s-c	b-c	int
Collagen COBA2(c)	MCES AAA	test synthesis of Collagen COBA2 c	110 Glutamate(s) 428 Glycine(s) 98 Alanine(s) 72 Lysine(s) 79 Aspartate(s) 101 Arginine(s) 87 Glutamine(s) 65 Serine(s) 16 Methionine(s) 3 Tryptophan(s) 31 Phenylalanine(s) 23 Tyrosine(s) 14 Cysteine(s) 111 Leucine(s) 20 Histidine(s) 299 Proline(s) 15 Asparagine(s) 74 Valine(s) 60 Threonine(s) 30 Isoleucine(s) 8680 ATP-energy(c) 413 Proton-gradient(c)	1 Collagen COBA2(c) 464 Na-gradient(c)	4	-	-	-	-	-	-	-	22	-	-

ID	flux	reaction
r2602	1	98 Alanine(c) + 14 Cysteine(c) + 79 Aspartate(c) + 110 Glutamate(c) + 31 Phenylalanine(c) + 428 Glycine(c) + 20 Histidine(c) + 30 Isoleucine(c) + 72 Lysine(c) + 111 Leucine(c) + 16 Methionine(c) + 15 Asparagine(c) + 299 Proline(c) + 87 Glutamine(c) + 101 Arginine(c) + 65 Serine(c) + 60 Threonine(c) + 74 Valine(c) + 3 Tryptophan(c) + 23 Tyrosine(c) + 6944 ATP(c) + 6944 H2O(c) → Collagen COBA2(c) + 5208 ADP(c) + 5208 Pi(c) + 1736 AMP(c) + 1736 PPi(c)
r2530	111	Leucine(s) ⇌ Leucine(c)
r1144	-110	Glutamate(c) + Na+(c) ← Glutamate(s) + Na+(s)
r1126	-31	Phenylalanine(c) + Na+(c) ← Phenylalanine(s) + Na+(s)
r1072	16	Methionine(s) ⇌ Methionine(c)
r2531	299	Proline(s) ⇌ Proline(c)
r1145	-20	Histidine(c) + Na+(c) ← Histidine(s) + Na+(s)
r2524	98	Alanine(s) ⇌ Alanine(c)
r2534	60	Threonine(s) ⇌ Threonine(c)
r2525	87	Glutamine(s) ⇌ Glutamine(c)
r1319	-8680	ATP(c) + H2O(c) ⇌ ADP(c) + Pi(c) + ATP-energy(c)
r1058	101	Arginine(s) ⇌ Arginine(c)
r1069	-72	Lysine(c) ⇌ Lysine(s)
r0030	1736	ATP(c) + AMP(c) ⇌ 2 ADP(c)
r2474	65	Serine(s) + Na+(s) → Serine(c) + Na+(c)
r2664	464	Na+(c) ⇌ Na+(s) + Na-gradient(c)
r2475	14	Cysteine(s) + Na+(s) → Cysteine(c) + Na+(c)
r1121	-74	Valine(c) + Na+(c) ← Valine(s) + Na+(s)
r2468	428	Glycine(s) + H+(PG)(s) → Glycine(c) + H+(PG)(c)
r1123	-30	Isoleucine(c) + Na+(c) ← Isoleucine(s) + Na+(s)
r0007	1736	PPi(c) + H2O(c) → 2 Pi(c)
r1331	-413	H+(PG)(s) ⇌ H+(PG)(c) + Proton-gradient(c)
r1124	-3	Tryptophan(c) + Na+(c) ← Tryptophan(s) + Na+(s)
r2479	15	Asparagine(s) + Na+(s) + H+(PG)(c) → Asparagine(c) + Na+(c) + H+(PG)(s)
r1143	-79	Na+(c) + Aspartate(c) ← Na+(s) + Aspartate(s)
r1125	23	Tyrosine(s) + Na+(s) → Tyrosine(c) + Na+(c)

Collagen COCA1(c) synthesis (373)

Definition			Solution												
			exchanges		reactions								transp		
Objective	Constraints	Comment	imports	exports	c	m	r	p	l	n	s	b	s-c	b-c	int
Collagen COCA1(c)	MCES AAA	test synthesis of Collagen COCA1 c	194 Glutamate(s) 281 Glycine(s) 159 Alanine(s) 156 Lysine(s) 172 Aspartate(s) 157 Arginine(s) 104 Glutamine(s) 241 Serine(s) 45 Methionine(s) 30 Tryptophan(s) 91 Phenylalanine(s) 113 Tyrosine(s) 21 Cysteine(s) 203 Leucine(s) 31 Histidine(s) 263 Proline(s) 118 Asparagine(s) 270 Valine(s) 266 Threonine(s) 148 Isoleucine(s) 15315 ATP-energy(c) 163 Proton-gradient(c)	1 Collagen COCA1(c) 1429 Na-gradient(c)	4	-	-	-	-	-	-	-	22	-	-

ID	flux	reaction
r2530	203	Leucine(s) ⇌ Leucine(c)
r1144	-194	Glutamate(c) + Na+(c) ← Glutamate(s) + Na+(s)
r1126	-91	Phenylalanine(c) + Na+(c) ← Phenylalanine(s) + Na+(s)
r1072	45	Methionine(s) ⇌ Methionine(c)
r2531	263	Proline(s) ⇌ Proline(c)
r1145	-31	Histidine(c) + Na+(c) ← Histidine(s) + Na+(s)
r2604	1	159 Alanine(c) + 21 Cysteine(c) + 172 Aspartate(c) + 194 Glutamate(c) + 91 Phenylalanine(c) + 281 Glycine(c) + 31 Histidine(c) + 148 Isoleucine(c) + 156 Lysine(c) + 203 Leucine(c) + 45 Methionine(c) + 118 Asparagine(c) + 263 Proline(c) + 104 Glutamine(c) + 157 Arginine(c) + 241 Serine(c) + 266 Threonine(c) + 270 Valine(c) + 30 Tryptophan(c) + 113 Tyrosine(c) + 12252 ATP(c) + 12252 H2O(c) → Collagen COCA1(c) + 9189 ADP(c) + 9189 Pi(c) + 3063 AMP(c) + 3063 PPi(c)
r2524	159	Alanine(s) ⇌ Alanine(c)
r2534	266	Threonine(s) ⇌ Threonine(c)
r2525	104	Glutamine(s) ⇌ Glutamine(c)
r1319	-15315	ATP(c) + H2O(c) ⇌ ADP(c) + Pi(c) + ATP-energy(c)
r1058	157	Arginine(s) ⇌ Arginine(c)
r1069	-156	Lysine(c) ⇌ Lysine(s)
r0030	3063	ATP(c) + AMP(c) ⇌ 2 ADP(c)
r2474	241	Serine(s) + Na+(s) → Serine(c) + Na+(c)
r2664	1429	Na+(c) ⇌ Na+(s) + Na-gradient(c)
r2475	21	Cysteine(s) + Na+(s) → Cysteine(c) + Na+(c)
r1121	-270	Valine(c) + Na+(c) ← Valine(s) + Na+(s)
r2468	281	Glycine(s) + H+(PG)(s) → Glycine(c) + H+(PG)(c)
r1123	-148	Isoleucine(c) + Na+(c) ← Isoleucine(s) + Na+(s)
r0007	3063	PPi(c) + H2O(c) → 2 Pi(c)
r1331	-163	H+(PG)(s) ⇌ H+(PG)(c) + Proton-gradient(c)
r1124	-30	Tryptophan(c) + Na+(c) ← Tryptophan(s) + Na+(s)
r2479	118	Asparagine(s) + Na+(s) + H+(PG)(c) → Asparagine(c) + Na+(c) + H+(PG)(s)
r1143	-172	Na+(c) + Aspartate(c) ← Na+(s) + Aspartate(s)
r1125	113	Tyrosine(s) + Na+(s) → Tyrosine(c) + Na+(c)

Collagen CODA1(c) synthesis (374)

Definition			Solution													
			exchanges		reactions								transp			
Objective	Constraints	Comment	imports	exports	c	m	r	p	l	n	s	b	s-c	b-c	int
Collagen CODA1(c)	MCES AAA	test synthesis of Collagen CODA1 c	44 Glutamate(s) 184 Glycine(s) 49 Alanine(s) 46 Lysine(s) 23 Aspartate(s) 35 Arginine(s) 28 Glutamine(s) 24 Serine(s) 13 Methionine(s) 2 Tryptophan(s) 5 Phenylalanine(s) 3 Tyrosine(s) 8 Cysteine(s) 53 Leucine(s) 11 Histidine(s) 125 Proline(s) 11 Asparagine(s) 17 Valine(s) 17 Threonine(s) 19 Isoleucine(s) 3585 ATP-energy(c) 173 Proton-gradient(c)	1 Collagen CODA1(c) 167 Na-gradient(c)	4	-	-	-	-	-	-	-	22	-	-

ID	flux	reaction
r2530	53	Leucine(s) ⇌ Leucine(c)
r1144	-44	Glutamate(c) + Na+(c) ← Glutamate(s) + Na+(s)
r1126	-5	Phenylalanine(c) + Na+(c) ← Phenylalanine(s) + Na+(s)
r1072	13	Methionine(s) ⇌ Methionine(c)
r2531	125	Proline(s) ⇌ Proline(c)
r1145	-11	Histidine(c) + Na+(c) ← Histidine(s) + Na+(s)
r2524	49	Alanine(s) ⇌ Alanine(c)
r2606	1	49 Alanine(c) + 8 Cysteine(c) + 23 Aspartate(c) + 44 Glutamate(c) + 5 Phenylalanine(c) + 184 Glycine(c) + 11 Histidine(c) + 19 Isoleucine(c) + 46 Lysine(c) + 53 Leucine(c) + 13 Methionine(c) + 11 Asparagine(c) + 125 Proline(c) + 28 Glutamine(c) + 35 Arginine(c) + 24 Serine(c) + 17 Threonine(c) + 17 Valine(c) + 2 Tryptophan(c) + 3 Tyrosine(c) + 2868 ATP(c) + 2868 H2O(c) → Collagen CODA1(c) + 2151 ADP(c) + 2151 Pi(c) + 717 AMP(c) + 717 PPi(c)
r2534	17	Threonine(s) ⇌ Threonine(c)
r2525	28	Glutamine(s) ⇌ Glutamine(c)
r1319	-3585	ATP(c) + H2O(c) ⇌ ADP(c) + Pi(c) + ATP-energy(c)
r1058	35	Arginine(s) ⇌ Arginine(c)
r1069	-46	Lysine(c) ⇌ Lysine(s)
r0030	717	ATP(c) + AMP(c) ⇌ 2 ADP(c)
r2474	24	Serine(s) + Na+(s) → Serine(c) + Na+(c)
r2664	167	Na+(c) ⇌ Na+(s) + Na-gradient(c)
r2475	8	Cysteine(s) + Na+(s) → Cysteine(c) + Na+(c)
r1121	-17	Valine(c) + Na+(c) ← Valine(s) + Na+(s)
r2468	184	Glycine(s) + H+(PG)(s) → Glycine(c) + H+(PG)(c)
r1123	-19	Isoleucine(c) + Na+(c) ← Isoleucine(s) + Na+(s)
r0007	717	PPi(c) + H2O(c) → 2 Pi(c)
r1331	-173	H+(PG)(s) ⇌ H+(PG)(c) + Proton-gradient(c)
r1124	-2	Tryptophan(c) + Na+(c) ← Tryptophan(s) + Na+(s)
r2479	11	Asparagine(s) + Na+(s) + H+(PG)(c) → Asparagine(c) + Na+(c) + H+(PG)(s)
r1143	-23	Na+(c) + Aspartate(c) ← Na+(s) + Aspartate(s)
r1125	3	Tyrosine(s) + Na+(s) → Tyrosine(c) + Na+(c)

Collagen COEA1(c) synthesis (375)

Definition			Solution												
			exchanges		reactions								transsp		
Objective	Constraints	Comment	imports	exports	c	m	r	p	l	n	s	b	s-c	b-c	int
Collagen COEA1(c)	MCES AAA	test synthesis of Collagen COEA1 c	118 Glutamate(s) 195 Glycine(s) 94 Alanine(s) 86 Lysine(s) 93 Aspartate(s) 74 Arginine(s) 71 Glutamine(s) 133 Serine(s) 33 Methionine(s) 18 Tryptophan(s) 60 Phenylalanine(s) 53 Tyrosine(s) 19 Cysteine(s) 129 Leucine(s) 29 Histidine(s) 156 Proline(s) 58 Asparagine(s) 142 Valine(s) 136 Threonine(s) 99 Isoleucine(s) 8980 ATP-energy(c) 137 Proton-gradient(c)	1 Collagen COEA1(c) 822 Na-gradient(c)	4	-	-	-	-	-	-	-	22	-	-

ID	flux	reaction
r2530	129	Leucine(s) ⇌ Leucine(c)
r1144	-118	Glutamate(c) + Na+(c) ← Glutamate(s) + Na+(s)
r1126	-60	Phenylalanine(c) + Na+(c) ← Phenylalanine(s) + Na+(s)
r1072	33	Methionine(s) ⇌ Methionine(c)
r2531	156	Proline(s) ⇌ Proline(c)
r1145	-29	Histidine(c) + Na+(c) ← Histidine(s) + Na+(s)
r2524	94	Alanine(s) ⇌ Alanine(c)
r2534	136	Threonine(s) ⇌ Threonine(c)
r2525	71	Glutamine(s) ⇌ Glutamine(c)
r1319	-8980	ATP(c) + H2O(c) ⇌ ADP(c) + Pi(c) + ATP-energy(c)
r1058	74	Arginine(s) ⇌ Arginine(c)
r2608	1	94 Alanine(c) + 19 Cysteine(c) + 93 Aspartate(c) + 118 Glutamate(c) + 60 Phenylalanine(c) + 195 Glycine(c) + 29 Histidine(c) + 99 Isoleucine(c) + 86 Lysine(c) + 129 Leucine(c) + 33 Methionine(c) + 58 Asparagine(c) + 156 Proline(c) + 71 Glutamine(c) + 74 Arginine(c) + 133 Serine(c) + 136 Threonine(c) + 142 Valine(c) + 18 Tryptophan(c) + 53 Tyrosine(c) + 7184 ATP(c) + 7184 H2O(c) → Collagen COEA1(c) + 5388 ADP(c) + 5388 Pi(c) + 1796 AMP(c) + 1796 PPi(c)
r1069	-86	Lysine(c) ⇌ Lysine(s)
r0030	1796	ATP(c) + AMP(c) ⇌ 2 ADP(c)
r2474	133	Serine(s) + Na+(s) → Serine(c) + Na+(c)
r2664	822	Na+(c) ⇌ Na+(s) + Na-gradient(c)
r2475	19	Cysteine(s) + Na+(s) → Cysteine(c) + Na+(c)
r1121	-142	Valine(c) + Na+(c) ← Valine(s) + Na+(s)
r2468	195	Glycine(s) + H+(PG)(s) → Glycine(c) + H+(PG)(c)
r1123	-99	Isoleucine(c) + Na+(c) ← Isoleucine(s) + Na+(s)
r0007	1796	PPi(c) + H2O(c) → 2 Pi(c)
r1331	-137	H+(PG)(s) ⇌ H+(PG)(c) + Proton-gradient(c)
r1124	-18	Tryptophan(c) + Na+(c) ← Tryptophan(s) + Na+(s)
r2479	58	Asparagine(s) + Na+(s) + H+(PG)(c) → Asparagine(c) + Na+(c) + H+(PG)(s)
r1143	-93	Na+(c) + Aspartate(c) ← Na+(s) + Aspartate(s)
r1125	53	Tyrosine(s) + Na+(s) → Tyrosine(c) + Na+(c)

Collagen COFA1(c) synthesis (376)

Definition			Solution												
			exchanges		reactions								transp		
Objective	Constraints	Comment	imports	exports	c	m	r	p	l	n	s	b	s-c	b-c	int
Collagen COFA1(c)	MCES AAA	test synthesis of Collagen COFA1 c	97 Glutamate(s) 221 Glycine(s) 108 Alanine(s) 52 Lysine(s) 58 Aspartate(s) 47 Arginine(s) 40 Glutamine(s) 99 Serine(s) 30 Methionine(s) 10 Tryptophan(s) 43 Phenylalanine(s) 14 Tyrosine(s) 10 Cysteine(s) 112 Leucine(s) 25 Histidine(s) 191 Proline(s) 38 Asparagine(s) 65 Valine(s) 74 Threonine(s) 54 Isoleucine(s) 6940 ATP-energy(c) 183 Proton-gradient(c)	1 Collagen COFA1(c) 513 Na-gradient(c)	4	-	-	-	-	-	-	-	22	-	-

ID	flux	reaction
r2530	112	Leucine(s) ⇌ Leucine(c)
r1144	-97	Glutamate(c) + Na+(c) ← Glutamate(s) + Na+(s)
r1126	-43	Phenylalanine(c) + Na+(c) ← Phenylalanine(s) + Na+(s)
r1072	30	Methionine(s) ⇌ Methionine(c)
r2531	191	Proline(s) ⇌ Proline(c)
r1145	-25	Histidine(c) + Na+(c) ← Histidine(s) + Na+(s)
r2524	108	Alanine(s) ⇌ Alanine(c)
r2534	74	Threonine(s) ⇌ Threonine(c)
r2525	40	Glutamine(s) ⇌ Glutamine(c)
r1319	-6940	ATP(c) + H2O(c) ⇌ ADP(c) + Pi(c) + ATP-energy(c)
r1058	47	Arginine(s) ⇌ Arginine(c)
r1069	-52	Lysine(c) ⇌ Lysine(s)
r0030	1388	ATP(c) + AMP(c) ⇌ 2 ADP(c)
r2474	99	Serine(s) + Na+(s) → Serine(c) + Na+(c)
r2664	513	Na+(c) ⇌ Na+(s) + Na-gradient(c)
r2475	10	Cysteine(s) + Na+(s) → Cysteine(c) + Na+(c)
r1121	-65	Valine(c) + Na+(c) ← Valine(s) + Na+(s)
r2468	221	Glycine(s) + H+(PG)(s) → Glycine(c) + H+(PG)(c)
r1123	-54	Isoleucine(c) + Na+(c) ← Isoleucine(s) + Na+(s)
r0007	1388	PPi(c) + H2O(c) → 2 Pi(c)
r1331	-183	H+(PG)(s) ⇌ H+(PG)(c) + Proton-gradient(c)
r1124	-10	Tryptophan(c) + Na+(c) ← Tryptophan(s) + Na+(s)
r2610	1	108 Alanine(c) + 10 Cysteine(c) + 58 Aspartate(c) + 97 Glutamate(c) + 43 Phenylalanine(c) + 221 Glycine(c) + 25 Histidine(c) + 54 Isoleucine(c) + 52 Lysine(c) + 112 Leucine(c) + 30 Methionine(c) + 38 Asparagine(c) + 191 Proline(c) + 40 Glutamine(c) + 47 Arginine(c) + 99 Serine(c) + 74 Threonine(c) + 65 Valine(c) + 10 Tryptophan(c) + 14 Tyrosine(c) + 5552 ATP(c) + 5552 H2O(c) → Collagen COFA1(c) + 4164 ADP(c) + 4164 Pi(c) + 1388 AMP(c) + 1388 PPi(c)
r2479	38	Asparagine(s) + Na+(s) + H+(PG)(c) → Asparagine(c) + Na+(c) + H+(PG)(s)
r1143	-58	Na+(c) + Aspartate(c) ← Na+(s) + Aspartate(s)
r1125	14	Tyrosine(s) + Na+(s) → Tyrosine(c) + Na+(c)

Collagen COGA1(c) synthesis (377)

Definition			Solution													
			exchanges		reactions								transp		
Objective	Constraints	Comment	imports	exports	c	m	r	p	l	n	s	b	s-c	b-c	int
Collagen COGA1(c)	MCES AAA	test synthesis of Collagen COGA1 c	92 Glutamate(s) 391 Glycine(s) 85 Alanine(s) 87 Lysine(s) 52 Aspartate(s) 63 Arginine(s) 79 Glutamine(s) 76 Serine(s) 26 Methionine(s) 8 Tryptophan(s) 30 Phenylalanine(s) 13 Tyrosine(s) 32 Cysteine(s) 99 Leucine(s) 19 Histidine(s) 282 Proline(s) 23 Asparagine(s) 63 Valine(s) 43 Threonine(s) 41 Isoleucine(s) 8020 ATP-energy(c) 368 Proton-gradient(c)	1 Collagen COGA1(c) 449 Na-gradient(c)	4	-	-	-	-	-	-	-	22	-	-

ID	flux	reaction
r2530	99	Leucine(s) ⇌ Leucine(c)
r1144	-92	Glutamate(c) + Na+(c) ← Glutamate(s) + Na+(s)
r1126	-30	Phenylalanine(c) + Na+(c) ← Phenylalanine(s) + Na+(s)
r1072	26	Methionine(s) ⇌ Methionine(c)
r2612	1	85 Alanine(c) + 32 Cysteine(c) + 52 Aspartate(c) + 92 Glutamate(c) + 30 Phenylalanine(c) + 391 Glycine(c) + 19 Histidine(c) + 41 Isoleucine(c) + 87 Lysine(c) + 99 Leucine(c) + 26 Methionine(c) + 23 Asparagine(c) + 282 Proline(c) + 79 Glutamine(c) + 63 Arginine(c) + 76 Serine(c) + 43 Threonine(c) + 63 Valine(c) + 8 Tryptophan(c) + 13 Tyrosine(c) + 6416 ATP(c) + 6416 H2O(c) → Collagen COGA1(c) + 4812 ADP(c) + 4812 Pi(c) + 1604 AMP(c) + 1604 PPi(c)
r2531	282	Proline(s) ⇌ Proline(c)
r1145	-19	Histidine(c) + Na+(c) ← Histidine(s) + Na+(s)
r2524	85	Alanine(s) ⇌ Alanine(c)
r2534	43	Threonine(s) ⇌ Threonine(c)
r2525	79	Glutamine(s) ⇌ Glutamine(c)
r1319	-8020	ATP(c) + H2O(c) ⇌ ADP(c) + Pi(c) + ATP-energy(c)
r1058	63	Arginine(s) ⇌ Arginine(c)
r1069	-87	Lysine(c) ⇌ Lysine(s)
r0030	1604	ATP(c) + AMP(c) ⇌ 2 ADP(c)
r2474	76	Serine(s) + Na+(s) → Serine(c) + Na+(c)
r2664	449	Na+(c) ⇌ Na+(s) + Na-gradient(c)
r2475	32	Cysteine(s) + Na+(s) → Cysteine(c) + Na+(c)
r1121	-63	Valine(c) + Na+(c) ← Valine(s) + Na+(s)
r2468	391	Glycine(s) + H+(PG)(s) → Glycine(c) + H+(PG)(c)
r1123	-41	Isoleucine(c) + Na+(c) ← Isoleucine(s) + Na+(s)
r0007	1604	PPi(c) + H2O(c) → 2 Pi(c)
r1331	-368	H+(PG)(s) ⇌ H+(PG)(c) + Proton-gradient(c)
r1124	-8	Tryptophan(c) + Na+(c) ← Tryptophan(s) + Na+(s)
r2479	23	Asparagine(s) + Na+(s) + H+(PG)(c) → Asparagine(c) + Na+(c) + H+(PG)(s)
r1143	-52	Na+(c) + Aspartate(c) ← Na+(s) + Aspartate(s)
r1125	13	Tyrosine(s) + Na+(s) → Tyrosine(c) + Na+(c)

Collagen COHA1(c) synthesis (378)

Definition			Solution													
			exchanges		reactions								transp		
Objective	Constraints	Comment	imports	exports	c	m	r	p	l	n	s	b	s-c	b-c	int
Collagen COHA1(c)	MCES AAA	test synthesis of Collagen COHA1 c	63 Glutamate(s) 282 Glycine(s) 85 Alanine(s) 60 Lysine(s) 54 Aspartate(s) 68 Arginine(s) 48 Glutamine(s) 178 Serine(s) 33 Methionine(s) 10 Tryptophan(s) 25 Phenylalanine(s) 36 Tyrosine(s) 7 Cysteine(s) 107 Leucine(s) 23 Histidine(s) 204 Proline(s) 31 Asparagine(s) 58 Valine(s) 84 Threonine(s) 41 Isoleucine(s) 7485 ATP-energy(c) 251 Proton-gradient(c)	1 Collagen COHA1(c) 526 Na-gradient(c)	4	-	-	-	-	-	-	-	22	-	-

ID	flux	reaction
r2530	107	Leucine(s) ⇌ Leucine(c)
r1144	-63	Glutamate(c) + Na+(c) ← Glutamate(s) + Na+(s)
r1126	-25	Phenylalanine(c) + Na+(c) ← Phenylalanine(s) + Na+(s)
r1072	33	Methionine(s) ⇌ Methionine(c)
r2531	204	Proline(s) ⇌ Proline(c)
r1145	-23	Histidine(c) + Na+(c) ← Histidine(s) + Na+(s)
r2614	1	85 Alanine(c) + 7 Cysteine(c) + 54 Aspartate(c) + 63 Glutamate(c) + 25 Phenylalanine(c) + 282 Glycine(c) + 23 Histidine(c) + 41 Isoleucine(c) + 60 Lysine(c) + 107 Leucine(c) + 33 Methionine(c) + 31 Asparagine(c) + 204 Proline(c) + 48 Glutamine(c) + 68 Arginine(c) + 178 Serine(c) + 84 Threonine(c) + 58 Valine(c) + 10 Tryptophan(c) + 36 Tyrosine(c) + 5988 ATP(c) + 5988 H2O(c) → Collagen COHA1(c) + 4491 ADP(c) + 4491 Pi(c) + 1497 AMP(c) + 1497 PPi(c)
r2524	85	Alanine(s) ⇌ Alanine(c)
r2534	84	Threonine(s) ⇌ Threonine(c)
r2525	48	Glutamine(s) ⇌ Glutamine(c)
r1319	-7485	ATP(c) + H2O(c) ⇌ ADP(c) + Pi(c) + ATP-energy(c)
r1058	68	Arginine(s) ⇌ Arginine(c)
r1069	-60	Lysine(c) ⇌ Lysine(s)
r0030	1497	ATP(c) + AMP(c) ⇌ 2 ADP(c)
r2474	178	Serine(s) + Na+(s) → Serine(c) + Na+(c)
r2664	526	Na+(c) ⇌ Na+(s) + Na-gradient(c)
r2475	7	Cysteine(s) + Na+(s) → Cysteine(c) + Na+(c)
r1121	-58	Valine(c) + Na+(c) ← Valine(s) + Na+(s)
r2468	282	Glycine(s) + H+(PG)(s) → Glycine(c) + H+(PG)(c)
r1123	-41	Isoleucine(c) + Na+(c) ← Isoleucine(s) + Na+(s)
r0007	1497	PPi(c) + H2O(c) → 2 Pi(c)
r1331	-251	H+(PG)(s) ⇌ H+(PG)(c) + Proton-gradient(c)
r1124	-10	Tryptophan(c) + Na+(c) ← Tryptophan(s) + Na+(s)
r2479	31	Asparagine(s) + Na+(s) + H+(PG)(c) → Asparagine(c) + Na+(c) + H+(PG)(s)
r1143	-54	Na+(c) + Aspartate(c) ← Na+(s) + Aspartate(s)
r1125	36	Tyrosine(s) + Na+(s) → Tyrosine(c) + Na+(c)

Collagen COIA1(c) synthesis (379)

Definition			Solution												
			exchanges		reactions								transp		
Objective	Constraints	Comment	imports	exports	c	m	r	p	l	n	s	b	s-c	b-c	int
Collagen COIA1(c)	MCES AAA	test synthesis of Collagen COIA1 c	90 Glutamate(s) 302 Glycine(s) 140 Alanine(s) 42 Lysine(s) 74 Aspartate(s) 91 Arginine(s) 67 Glutamine(s) 121 Serine(s) 15 Methionine(s) 21 Tryptophan(s) 57 Phenylalanine(s) 19 Tyrosine(s) 23 Cysteine(s) 148 Leucine(s) 36 Histidine(s) 296 Proline(s) 27 Asparagine(s) 87 Valine(s) 69 Threonine(s) 29 Isoleucine(s) 8770 ATP-energy(c) 275 Proton-gradient(c)	1 Collagen COIA1(c) 584 Na-gradient(c)	4	-	-	-	-	-	-	-	22	-	-

ID	flux	reaction
r2530	148	Leucine(s) ⇌ Leucine(c)
r1144	-90	Glutamate(c) + Na+(c) ← Glutamate(s) + Na+(s)
r1126	-57	Phenylalanine(c) + Na+(c) ← Phenylalanine(s) + Na+(s)
r1072	15	Methionine(s) ⇌ Methionine(c)
r2531	296	Proline(s) ⇌ Proline(c)
r1145	-36	Histidine(c) + Na+(c) ← Histidine(s) + Na+(s)
r2524	140	Alanine(s) ⇌ Alanine(c)
r2534	69	Threonine(s) ⇌ Threonine(c)
r2525	67	Glutamine(s) ⇌ Glutamine(c)
r1319	-8770	ATP(c) + H2O(c) ⇌ ADP(c) + Pi(c) + ATP-energy(c)
r1058	91	Arginine(s) ⇌ Arginine(c)
r2616	1	140 Alanine(c) + 23 Cysteine(c) + 74 Aspartate(c) + 90 Glutamate(c) + 57 Phenylalanine(c) + 302 Glycine(c) + 36 Histidine(c) + 29 Isoleucine(c) + 42 Lysine(c) + 148 Leucine(c) + 15 Methionine(c) + 27 Asparagine(c) + 296 Proline(c) + 67 Glutamine(c) + 91 Arginine(c) + 121 Serine(c) + 69 Threonine(c) + 87 Valine(c) + 21 Tryptophan(c) + 19 Tyrosine(c) + 7016 ATP(c) + 7016 H2O(c) → Collagen COIA1(c) + 5262 ADP(c) + 5262 Pi(c) + 1754 AMP(c) + 1754 PPi(c)
r1069	-42	Lysine(c) ⇌ Lysine(s)
r0030	1754	ATP(c) + AMP(c) ⇌ 2 ADP(c)
r2474	121	Serine(s) + Na+(s) → Serine(c) + Na+(c)
r2664	584	Na+(c) ⇌ Na+(s) + Na-gradient(c)
r2475	23	Cysteine(s) + Na+(s) → Cysteine(c) + Na+(c)
r1121	-87	Valine(c) + Na+(c) ← Valine(s) + Na+(s)
r2468	302	Glycine(s) + H+(PG)(s) → Glycine(c) + H+(PG)(c)
r1123	-29	Isoleucine(c) + Na+(c) ← Isoleucine(s) + Na+(s)
r0007	1754	PPi(c) + H2O(c) → 2 Pi(c)
r1331	-275	H+(PG)(s) ⇌ H+(PG)(c) + Proton-gradient(c)
r1124	-21	Tryptophan(c) + Na+(c) ← Tryptophan(s) + Na+(s)
r2479	27	Asparagine(s) + Na+(s) + H+(PG)(c) → Asparagine(c) + Na+(c) + H+(PG)(s)
r1143	-74	Na+(c) + Aspartate(c) ← Na+(s) + Aspartate(s)
r1125	19	Tyrosine(s) + Na+(s) → Tyrosine(c) + Na+(c)

Collagen COJA1(c) synthesis (380)

Definition			Solution												
			exchanges		reactions								transp		
Objective	Constraints	Comment	imports	exports	c	m	r	p	l	n	s	b	s-c	b-c	int
Collagen COJA1(c)	MCES AAA	test synthesis of Collagen COJA1 c	67 Glutamate(s) 261 Glycine(s) 52 Alanine(s) 77 Lysine(s) 49 Aspartate(s) 47 Arginine(s) 46 Glutamine(s) 53 Serine(s) 18 Methionine(s) 7 Tryptophan(s) 25 Phenylalanine(s) 16 Tyrosine(s) 14 Cysteine(s) 71 Leucine(s) 16 Histidine(s) 173 Proline(s) 30 Asparagine(s) 33 Valine(s) 30 Threonine(s) 57 Isoleucine(s) 5710 ATP-energy(c) 231 Proton-gradient(c)	1 Collagen COJA1(c) 367 Na-gradient(c)	4	-	-	-	-	-	-	-	22	-	-

ID	flux	reaction
r2530	71	Leucine(s) ⇌ Leucine(c)
r1144	-67	Glutamate(c) + Na+(c) ← Glutamate(s) + Na+(s)
r1126	-25	Phenylalanine(c) + Na+(c) ← Phenylalanine(s) + Na+(s)
r1072	18	Methionine(s) ⇌ Methionine(c)
r2531	173	Proline(s) ⇌ Proline(c)
r1145	-16	Histidine(c) + Na+(c) ← Histidine(s) + Na+(s)
r2524	52	Alanine(s) ⇌ Alanine(c)
r2534	30	Threonine(s) ⇌ Threonine(c)
r2525	46	Glutamine(s) ⇌ Glutamine(c)
r1319	-5710	ATP(c) + H2O(c) ⇌ ADP(c) + Pi(c) + ATP-energy(c)
r1058	47	Arginine(s) ⇌ Arginine(c)
r1069	-77	Lysine(c) ⇌ Lysine(s)
r0030	1142	ATP(c) + AMP(c) ⇌ 2 ADP(c)
r2618	1	52 Alanine(c) + 14 Cysteine(c) + 49 Aspartate(c) + 67 Glutamate(c) + 25 Phenylalanine(c) + 261 Glycine(c) + 16 Histidine(c) + 57 Isoleucine(c) + 77 Lysine(c) + 71 Leucine(c) + 18 Methionine(c) + 30 Asparagine(c) + 173 Proline(c) + 46 Glutamine(c) + 47 Arginine(c) + 53 Serine(c) + 30 Threonine(c) + 33 Valine(c) + 7 Tryptophan(c) + 16 Tyrosine(c) + 4568 ATP(c) + 4568 H2O(c) → Collagen COJA1(c) + 3426 ADP(c) + 3426 Pi(c) + 1142 AMP(c) + 1142 PPi(c)
r2474	53	Serine(s) + Na+(s) → Serine(c) + Na+(c)
r2664	367	Na+(c) ⇌ Na+(s) + Na-gradient(c)
r2475	14	Cysteine(s) + Na+(s) → Cysteine(c) + Na+(c)
r1121	-33	Valine(c) + Na+(c) ← Valine(s) + Na+(s)
r2468	261	Glycine(s) + H+(PG)(s) → Glycine(c) + H+(PG)(c)
r1123	-57	Isoleucine(c) + Na+(c) ← Isoleucine(s) + Na+(s)
r0007	1142	PPi(c) + H2O(c) → 2 Pi(c)
r1331	-231	H+(PG)(s) ⇌ H+(PG)(c) + Proton-gradient(c)
r1124	-7	Tryptophan(c) + Na+(c) ← Tryptophan(s) + Na+(s)
r2479	30	Asparagine(s) + Na+(s) + H+(PG)(c) → Asparagine(c) + Na+(c) + H+(PG)(s)
r1143	-49	Na+(c) + Aspartate(c) ← Na+(s) + Aspartate(s)
r1125	16	Tyrosine(s) + Na+(s) → Tyrosine(c) + Na+(c)

Collagen COKA1(c) synthesis (381)

Definition			Solution												
			exchanges		reactions								transp		
Objective	Constraints	Comment	imports	exports	c	m	r	p	l	n	s	b	s-c	b-c	int
Collagen COKA1(c)	MCES AAA	test synthesis of Collagen COKA1 c	73 Glutamate(s) 148 Glycine(s) 114 Alanine(s) 38 Lysine(s) 46 Aspartate(s) 85 Arginine(s) 57 Glutamine(s) 107 Serine(s) 14 Methionine(s) 17 Tryptophan(s) 35 Phenylalanine(s) 26 Tyrosine(s) 13 Cysteine(s) 137 Leucine(s) 28 Histidine(s) 128 Proline(s) 11 Asparagine(s) 98 Valine(s) 86 Threonine(s) 23 Isoleucine(s) 6420 ATP-energy(c) 137 Proton-gradient(c)	1 Collagen COKA1(c) 477 Na-gradient(c)	4	-	-	-	-	-	-	-	22	-	-

ID	flux	reaction
r2620	1	114 Alanine(c) + 13 Cysteine(c) + 46 Aspartate(c) + 73 Glutamate(c) + 35 Phenylalanine(c) + 148 Glycine(c) + 28 Histidine(c) + 23 Isoleucine(c) + 38 Lysine(c) + 137 Leucine(c) + 14 Methionine(c) + 11 Asparagine(c) + 128 Proline(c) + 57 Glutamine(c) + 85 Arginine(c) + 107 Serine(c) + 86 Threonine(c) + 98 Valine(c) + 17 Tryptophan(c) + 26 Tyrosine(c) + 5136 ATP(c) + 5136 H2O(c) → Collagen COKA1(c) + 3852 ADP(c) + 3852 Pi(c) + 1284 AMP(c) + 1284 PPi(c)
r2530	137	Leucine(s) ⇌ Leucine(c)
r1144	-73	Glutamate(c) + Na+(c) ← Glutamate(s) + Na+(s)
r1126	-35	Phenylalanine(c) + Na+(c) ← Phenylalanine(s) + Na+(s)
r1072	14	Methionine(s) ⇌ Methionine(c)
r2531	128	Proline(s) ⇌ Proline(c)
r1145	-28	Histidine(c) + Na+(c) ← Histidine(s) + Na+(s)
r2524	114	Alanine(s) ⇌ Alanine(c)
r2534	86	Threonine(s) ⇌ Threonine(c)
r2525	57	Glutamine(s) ⇌ Glutamine(c)
r1319	-6420	ATP(c) + H2O(c) ⇌ ADP(c) + Pi(c) + ATP-energy(c)
r1058	85	Arginine(s) ⇌ Arginine(c)
r1069	-38	Lysine(c) ⇌ Lysine(s)
r0030	1284	ATP(c) + AMP(c) ⇌ 2 ADP(c)
r2474	107	Serine(s) + Na+(s) → Serine(c) + Na+(c)
r2664	477	Na+(c) ⇌ Na+(s) + Na-gradient(c)
r2475	13	Cysteine(s) + Na+(s) → Cysteine(c) + Na+(c)
r1121	-98	Valine(c) + Na+(c) ← Valine(s) + Na+(s)
r2468	148	Glycine(s) + H+(PG)(s) → Glycine(c) + H+(PG)(c)
r1123	-23	Isoleucine(c) + Na+(c) ← Isoleucine(s) + Na+(s)
r0007	1284	PPi(c) + H2O(c) → 2 Pi(c)
r1331	-137	H+(PG)(s) ⇌ H+(PG)(c) + Proton-gradient(c)
r1124	-17	Tryptophan(c) + Na+(c) ← Tryptophan(s) + Na+(s)
r2479	11	Asparagine(s) + Na+(s) + H+(PG)(c) → Asparagine(c) + Na+(c) + H+(PG)(s)
r1143	-46	Na+(c) + Aspartate(c) ← Na+(s) + Aspartate(s)
r1125	26	Tyrosine(s) + Na+(s) → Tyrosine(c) + Na+(c)

Collagen COLA1(c) synthesis (382)

Definition			Solution												
			exchanges		reactions								transp		
Objective	Constraints	Comment	imports	exports	c	m	r	p	l	n	s	b	s-c	b-c	int
Collagen COLA1(c)	MCES AAA	test synthesis of Collagen COLA1 c	50 Glutamate(s) 181 Glycine(s) 40 Alanine(s) 68 Lysine(s) 48 Aspartate(s) 38 Arginine(s) 51 Glutamine(s) 50 Serine(s) 12 Methionine(s) 4 Tryptophan(s) 29 Phenylalanine(s) 22 Tyrosine(s) 14 Cysteine(s) 66 Leucine(s) 11 Histidine(s) 106 Proline(s) 26 Asparagine(s) 53 Valine(s) 34 Threonine(s) 54 Isoleucine(s) 4785 ATP-energy(c) 155 Proton-gradient(c)	1 Collagen COLA1(c) 361 Na-gradient(c)	4	-	-	-	-	-	-	-	22	-	-

ID	flux	reaction
r2530	66	Leucine(s) ⇌ Leucine(c)
r1144	-50	Glutamate(c) + Na+(c) ← Glutamate(s) + Na+(s)
r1126	-29	Phenylalanine(c) + Na+(c) ← Phenylalanine(s) + Na+(s)
r1072	12	Methionine(s) ⇌ Methionine(c)
r2531	106	Proline(s) ⇌ Proline(c)
r1145	-11	Histidine(c) + Na+(c) ← Histidine(s) + Na+(s)
r2622	1	40 Alanine(c) + 14 Cysteine(c) + 48 Aspartate(c) + 50 Glutamate(c) + 29 Phenylalanine(c) + 181 Glycine(c) + 11 Histidine(c) + 54 Isoleucine(c) + 68 Lysine(c) + 66 Leucine(c) + 12 Methionine(c) + 26 Asparagine(c) + 106 Proline(c) + 51 Glutamine(c) + 38 Arginine(c) + 50 Serine(c) + 34 Threonine(c) + 53 Valine(c) + 4 Tryptophan(c) + 22 Tyrosine(c) + 3828 ATP(c) + 3828 H2O(c) → Collagen COLA1(c) + 2871 ADP(c) + 2871 Pi(c) + 957 AMP(c) + 957 PPi(c)
r2524	40	Alanine(s) ⇌ Alanine(c)
r2534	34	Threonine(s) ⇌ Threonine(c)
r2525	51	Glutamine(s) ⇌ Glutamine(c)
r1319	-4785	ATP(c) + H2O(c) ⇌ ADP(c) + Pi(c) + ATP-energy(c)
r1058	38	Arginine(s) ⇌ Arginine(c)
r1069	-68	Lysine(c) ⇌ Lysine(s)
r0030	957	ATP(c) + AMP(c) ⇌ 2 ADP(c)
r2474	50	Serine(s) + Na+(s) → Serine(c) + Na+(c)
r2664	361	Na+(c) ⇌ Na+(s) + Na-gradient(c)
r2475	14	Cysteine(s) + Na+(s) → Cysteine(c) + Na+(c)
r1121	-53	Valine(c) + Na+(c) ← Valine(s) + Na+(s)
r2468	181	Glycine(s) + H+(PG)(s) → Glycine(c) + H+(PG)(c)
r1123	-54	Isoleucine(c) + Na+(c) ← Isoleucine(s) + Na+(s)
r0007	957	PPi(c) + H2O(c) → 2 Pi(c)
r1331	-155	H+(PG)(s) ⇌ H+(PG)(c) + Proton-gradient(c)
r1124	-4	Tryptophan(c) + Na+(c) ← Tryptophan(s) + Na+(s)
r2479	26	Asparagine(s) + Na+(s) + H+(PG)(c) → Asparagine(c) + Na+(c) + H+(PG)(s)
r1143	-48	Na+(c) + Aspartate(c) ← Na+(s) + Aspartate(s)
r1125	22	Tyrosine(s) + Na+(s) → Tyrosine(c) + Na+(c)

Collagen COLQ(c) synthesis (383)

Definition			Solution													
			exchanges		reactions								transp		
Objective	Constraints	Comment	imports	exports	c	m	r	p	l	n	s	b	s-c	b-c	int
Collagen COLQ(c)	MCES AAA	test synthesis of Collagen COLQ c	21 Glutamate(s) 86 Glycine(s) 9 Alanine(s) 22 Lysine(s) 21 Aspartate(s) 25 Arginine(s) 19 Glutamine(s) 27 Serine(s) 14 Methionine(s) 2 Tryptophan(s) 15 Phenylalanine(s) 14 Tyrosine(s) 15 Cysteine(s) 36 Leucine(s) 6 Histidine(s) 69 Proline(s) 11 Asparagine(s) 15 Valine(s) 16 Threonine(s) 12 Isoleucine(s) 2275 ATP-energy(c) 75 Proton-gradient(c)	1 Collagen COLQ(c) 159 Na-gradient(c)	4	-	-	-	-	-	-	-	22	-	-

ID	flux	reaction
r2530	36	Leucine(s) ⇌ Leucine(c)
r1144	-21	Glutamate(c) + Na+(c) ← Glutamate(s) + Na+(s)
r1126	-15	Phenylalanine(c) + Na+(c) ← Phenylalanine(s) + Na+(s)
r1072	14	Methionine(s) ⇌ Methionine(c)
r2531	69	Proline(s) ⇌ Proline(c)
r1145	-6	Histidine(c) + Na+(c) ← Histidine(s) + Na+(s)
r2524	9	Alanine(s) ⇌ Alanine(c)
r2624	1	9 Alanine(c) + 15 Cysteine(c) + 21 Aspartate(c) + 21 Glutamate(c) + 15 Phenylalanine(c) + 86 Glycine(c) + 6 Histidine(c) + 12 Isoleucine(c) + 22 Lysine(c) + 36 Leucine(c) + 14 Methionine(c) + 11 Asparagine(c) + 69 Proline(c) + 19 Glutamine(c) + 25 Arginine(c) + 27 Serine(c) + 16 Threonine(c) + 15 Valine(c) + 2 Tryptophan(c) + 14 Tyrosine(c) + 1820 ATP(c) + 1820 H2O(c) → Collagen COLQ(c) + 1365 ADP(c) + 1365 Pi(c) + 455 AMP(c) + 455 PPi(c)
r2534	16	Threonine(s) ⇌ Threonine(c)
r2525	19	Glutamine(s) ⇌ Glutamine(c)
r1319	-2275	ATP(c) + H2O(c) ⇌ ADP(c) + Pi(c) + ATP-energy(c)
r1058	25	Arginine(s) ⇌ Arginine(c)
r1069	-22	Lysine(c) ⇌ Lysine(s)
r0030	455	ATP(c) + AMP(c) ⇌ 2 ADP(c)
r2474	27	Serine(s) + Na+(s) → Serine(c) + Na+(c)
r2664	159	Na+(c) ⇌ Na+(s) + Na-gradient(c)
r2475	15	Cysteine(s) + Na+(s) → Cysteine(c) + Na+(c)
r1121	-15	Valine(c) + Na+(c) ← Valine(s) + Na+(s)
r2468	86	Glycine(s) + H+(PG)(s) → Glycine(c) + H+(PG)(c)
r1123	-12	Isoleucine(c) + Na+(c) ← Isoleucine(s) + Na+(s)
r0007	455	PPi(c) + H2O(c) → 2 Pi(c)
r1331	-75	H+(PG)(s) ⇌ H+(PG)(c) + Proton-gradient(c)
r1124	-2	Tryptophan(c) + Na+(c) ← Tryptophan(s) + Na+(s)
r2479	11	Asparagine(s) + Na+(s) + H+(PG)(c) → Asparagine(c) + Na+(c) + H+(PG)(s)
r1143	-21	Na+(c) + Aspartate(c) ← Na+(s) + Aspartate(s)
r1125	14	Tyrosine(s) + Na+(s) → Tyrosine(c) + Na+(c)

Collagen COMA1(c) synthesis (384)

Definition			Solution												
			exchanges		reactions								transsp		
Objective	Constraints	Comment	imports	exports	c	m	r	p	l	n	s	b	s-c	b-c	int
Collagen COMA1(c)	MCES AAA	test synthesis of Collagen COMA1 c	104 Glutamate(s) 395 Glycine(s) 94 Alanine(s) 87 Lysine(s) 70 Aspartate(s) 85 Arginine(s) 62 Glutamine(s) 66 Serine(s) 20 Methionine(s) 6 Tryptophan(s) 34 Phenylalanine(s) 16 Tyrosine(s) 18 Cysteine(s) 101 Leucine(s) 19 Histidine(s) 267 Proline(s) 25 Asparagine(s) 70 Valine(s) 42 Threonine(s) 45 Isoleucine(s) 8130 ATP-energy(c) 370 Proton-gradient(c)	1 Collagen COMA1(c) 473 Na-gradient(c)	4	-	-	-	-	-	-	-	22	-	-

ID	flux	reaction
r2530	101	Leucine(s) ⇌ Leucine(c)
r1144	-104	Glutamate(c) + Na+(c) ← Glutamate(s) + Na+(s)
r1126	-34	Phenylalanine(c) + Na+(c) ← Phenylalanine(s) + Na+(s)
r1072	20	Methionine(s) ⇌ Methionine(c)
r2531	267	Proline(s) ⇌ Proline(c)
r1145	-19	Histidine(c) + Na+(c) ← Histidine(s) + Na+(s)
r2524	94	Alanine(s) ⇌ Alanine(c)
r2534	42	Threonine(s) ⇌ Threonine(c)
r2525	62	Glutamine(s) ⇌ Glutamine(c)
r1319	-8130	ATP(c) + H2O(c) ⇌ ADP(c) + Pi(c) + ATP-energy(c)
r1058	85	Arginine(s) ⇌ Arginine(c)
r2626	1	94 Alanine(c) + 18 Cysteine(c) + 70 Aspartate(c) + 104 Glutamate(c) + 34 Phenylalanine(c) + 395 Glycine(c) + 19 Histidine(c) + 45 Isoleucine(c) + 87 Lysine(c) + 101 Leucine(c) + 20 Methionine(c) + 25 Asparagine(c) + 267 Proline(c) + 62 Glutamine(c) + 85 Arginine(c) + 66 Serine(c) + 42 Threonine(c) + 70 Valine(c) + 6 Tryptophan(c) + 16 Tyrosine(c) + 6504 ATP(c) + 6504 H2O(c) → Collagen COMA1(c) + 4878 ADP(c) + 4878 Pi(c) + 1626 AMP(c) + 1626 PPi(c)
r1069	-87	Lysine(c) ⇌ Lysine(s)
r0030	1626	ATP(c) + AMP(c) ⇌ 2 ADP(c)
r2474	66	Serine(s) + Na+(s) → Serine(c) + Na+(c)
r2664	473	Na+(c) ⇌ Na+(s) + Na-gradient(c)
r2475	18	Cysteine(s) + Na+(s) → Cysteine(c) + Na+(c)
r1121	-70	Valine(c) + Na+(c) ← Valine(s) + Na+(s)
r2468	395	Glycine(s) + H+(PG)(s) → Glycine(c) + H+(PG)(c)
r1123	-45	Isoleucine(c) + Na+(c) ← Isoleucine(s) + Na+(s)
r0007	1626	PPi(c) + H2O(c) → 2 Pi(c)
r1331	-370	H+(PG)(s) ⇌ H+(PG)(c) + Proton-gradient(c)
r1124	-6	Tryptophan(c) + Na+(c) ← Tryptophan(s) + Na+(s)
r2479	25	Asparagine(s) + Na+(s) + H+(PG)(c) → Asparagine(c) + Na+(c) + H+(PG)(s)
r1143	-70	Na+(c) + Aspartate(c) ← Na+(s) + Aspartate(s)
r1125	16	Tyrosine(s) + Na+(s) → Tyrosine(c) + Na+(c)

Collagen CONA1(c) synthesis (385)

Definition			Solution												
			exchanges		reactions								transp		
Objective	Constraints	Comment	imports	exports	c	m	r	p	l	n	s	b	s-c	b-c	int
Collagen CONA1(c)	MCES AAA	test synthesis of Collagen CONA1 c	36 Glutamate(s) 147 Glycine(s) 43 Alanine(s) 35 Lysine(s) 29 Aspartate(s) 30 Arginine(s) 17 Glutamine(s) 17 Serine(s) 5 Methionine(s) 2 Tryptophan(s) 2 Phenylalanine(s) 2 Tyrosine(s) 6 Cysteine(s) 40 Leucine(s) 3 Histidine(s) 93 Proline(s) 2 Asparagine(s) 15 Valine(s) 8 Threonine(s) 8 Isoleucine(s) 2700 ATP-energy(c) 145 Proton-gradient(c)	1 Collagen CONA1(c) 122 Na-gradient(c)	4	-	-	-	-	-	-	-	22	-	-

ID	flux	reaction
r2530	40	Leucine(s) ⇌ Leucine(c)
r1144	-36	Glutamate(c) + Na+(c) ← Glutamate(s) + Na+(s)
r1126	-2	Phenylalanine(c) + Na+(c) ← Phenylalanine(s) + Na+(s)
r1072	5	Methionine(s) ⇌ Methionine(c)
r2531	93	Proline(s) ⇌ Proline(c)
r1145	-3	Histidine(c) + Na+(c) ← Histidine(s) + Na+(s)
r2524	43	Alanine(s) ⇌ Alanine(c)
r2534	8	Threonine(s) ⇌ Threonine(c)
r2525	17	Glutamine(s) ⇌ Glutamine(c)
r1319	-2700	ATP(c) + H2O(c) ⇌ ADP(c) + Pi(c) + ATP-energy(c)
r1058	30	Arginine(s) ⇌ Arginine(c)
r1069	-35	Lysine(c) ⇌ Lysine(s)
r0030	540	ATP(c) + AMP(c) ⇌ 2 ADP(c)
r2474	17	Serine(s) + Na+(s) → Serine(c) + Na+(c)
r2664	122	Na+(c) ⇌ Na+(s) + Na-gradient(c)
r2628	1	43 Alanine(c) + 6 Cysteine(c) + 29 Aspartate(c) + 36 Glutamate(c) + 2 Phenylalanine(c) + 147 Glycine(c) + 3 Histidine(c) + 8 Isoleucine(c) + 35 Lysine(c) + 40 Leucine(c) + 5 Methionine(c) + 2 Asparagine(c) + 93 Proline(c) + 17 Glutamine(c) + 30 Arginine(c) + 17 Serine(c) + 8 Threonine(c) + 15 Valine(c) + 2 Tryptophan(c) + 2 Tyrosine(c) + 2160 ATP(c) + 2160 H2O(c) → Collagen CONA1(c) + 1620 ADP(c) + 1620 Pi(c) + 540 AMP(c) + 540 PPi(c)
r2475	6	Cysteine(s) + Na+(s) → Cysteine(c) + Na+(c)
r1121	-15	Valine(c) + Na+(c) ← Valine(s) + Na+(s)
r2468	147	Glycine(s) + H+(PG)(s) → Glycine(c) + H+(PG)(c)
r1123	-8	Isoleucine(c) + Na+(c) ← Isoleucine(s) + Na+(s)
r0007	540	PPi(c) + H2O(c) → 2 Pi(c)
r1331	-145	H+(PG)(s) ⇌ H+(PG)(c) + Proton-gradient(c)
r1124	-2	Tryptophan(c) + Na+(c) ← Tryptophan(s) + Na+(s)
r2479	2	Asparagine(s) + Na+(s) + H+(PG)(c) → Asparagine(c) + Na+(c) + H+(PG)(s)
r1143	-29	Na+(c) + Aspartate(c) ← Na+(s) + Aspartate(s)
r1125	2	Tyrosine(s) + Na+(s) → Tyrosine(c) + Na+(c)

Collagen COOA1(c) synthesis (386)

Definition			Solution													
			exchanges		reactions								transp		
Objective	Constraints	Comment	imports	exports	c	m	r	p	l	n	s	b	s-c	b-c	int
Collagen COOA1(c)	MCES AAA	test synthesis of Collagen COOA1 c	102 Glutamate(s) 365 Glycine(s) 58 Alanine(s) 104 Lysine(s) 60 Aspartate(s) 66 Arginine(s) 90 Glutamine(s) 90 Serine(s) 24 Methionine(s) 5 Tryptophan(s) 42 Phenylalanine(s) 30 Tyrosine(s) 15 Cysteine(s) 128 Leucine(s) 38 Histidine(s) 207 Proline(s) 54 Asparagine(s) 69 Valine(s) 79 Threonine(s) 88 Isoleucine(s) 8570 ATP-energy(c) 311 Proton-gradient(c)	1 Collagen COOA1(c) 593 Na-gradient(c)	4	-	-	-	-	-	-	-	22	-	-

ID	flux	reaction
r2530	128	Leucine(s) ⇌ Leucine(c)
r1144	-102	Glutamate(c) + Na+(c) ← Glutamate(s) + Na+(s)
r1126	-42	Phenylalanine(c) + Na+(c) ← Phenylalanine(s) + Na+(s)
r1072	24	Methionine(s) ⇌ Methionine(c)
r2630	1	58 Alanine(c) + 15 Cysteine(c) + 60 Aspartate(c) + 102 Glutamate(c) + 42 Phenylalanine(c) + 365 Glycine(c) + 38 Histidine(c) + 88 Isoleucine(c) + 104 Lysine(c) + 128 Leucine(c) + 24 Methionine(c) + 54 Asparagine(c) + 207 Proline(c) + 90 Glutamine(c) + 66 Arginine(c) + 90 Serine(c) + 79 Threonine(c) + 69 Valine(c) + 5 Tryptophan(c) + 30 Tyrosine(c) + 6856 ATP(c) + 6856 H2O(c) → Collagen COOA1(c) + 5142 ADP(c) + 5142 Pi(c) + 1714 AMP(c) + 1714 PPi(c)
r2531	207	Proline(s) ⇌ Proline(c)
r1145	-38	Histidine(c) + Na+(c) ← Histidine(s) + Na+(s)
r2524	58	Alanine(s) ⇌ Alanine(c)
r2534	79	Threonine(s) ⇌ Threonine(c)
r2525	90	Glutamine(s) ⇌ Glutamine(c)
r1319	-8570	ATP(c) + H2O(c) ⇌ ADP(c) + Pi(c) + ATP-energy(c)
r1058	66	Arginine(s) ⇌ Arginine(c)
r1069	-104	Lysine(c) ⇌ Lysine(s)
r0030	1714	ATP(c) + AMP(c) ⇌ 2 ADP(c)
r2474	90	Serine(s) + Na+(s) → Serine(c) + Na+(c)
r2664	593	Na+(c) ⇌ Na+(s) + Na-gradient(c)
r2475	15	Cysteine(s) + Na+(s) → Cysteine(c) + Na+(c)
r1121	-69	Valine(c) + Na+(c) ← Valine(s) + Na+(s)
r2468	365	Glycine(s) + H+(PG)(s) → Glycine(c) + H+(PG)(c)
r1123	-88	Isoleucine(c) + Na+(c) ← Isoleucine(s) + Na+(s)
r0007	1714	PPi(c) + H2O(c) → 2 Pi(c)
r1331	-311	H+(PG)(s) ⇌ H+(PG)(c) + Proton-gradient(c)
r1124	-5	Tryptophan(c) + Na+(c) ← Tryptophan(s) + Na+(s)
r2479	54	Asparagine(s) + Na+(s) + H+(PG)(c) → Asparagine(c) + Na+(c) + H+(PG)(s)
r1143	-60	Na+(c) + Aspartate(c) ← Na+(s) + Aspartate(s)
r1125	30	Tyrosine(s) + Na+(s) → Tyrosine(c) + Na+(c)

Collagen COPA1(c) synthesis (387)

Definition			Solution													
			exchanges		reactions									transp		
Objective	Constraints	Comment	imports	exports	c	m	r	p	l	n	s	b	s-c	b-c	int
Collagen COPA1(c)	MCES AAA	test synthesis of Collagen COPA1 c	45 Glutamate(s) 167 Glycine(s) 33 Alanine(s) 50 Lysine(s) 28 Aspartate(s) 28 Arginine(s) 32 Glutamine(s) 20 Serine(s) 17 Methionine(s) 1 Tryptophan(s) 5 Phenylalanine(s) 4 Tyrosine(s) 7 Cysteine(s) 41 Leucine(s) 10 Histidine(s) 108 Proline(s) 9 Asparagine(s) 13 Valine(s) 16 Threonine(s) 20 Isoleucine(s) 3270 ATP-energy(c) 158 Proton-gradient(c)	1 Collagen COPA1(c) 162 Na-gradient(c)	4	-	-	-	-	-	-	-	22	-	-

ID	flux	reaction
r2530	41	Leucine(s) ⇌ Leucine(c)
r1144	-45	Glutamate(c) + Na+(c) ← Glutamate(s) + Na+(s)
r1126	-5	Phenylalanine(c) + Na+(c) ← Phenylalanine(s) + Na+(s)
r1072	17	Methionine(s) ⇌ Methionine(c)
r2531	108	Proline(s) ⇌ Proline(c)
r1145	-10	Histidine(c) + Na+(c) ← Histidine(s) + Na+(s)
r2632	1	33 Alanine(c) + 7 Cysteine(c) + 28 Aspartate(c) + 45 Glutamate(c) + 5 Phenylalanine(c) + 167 Glycine(c) + 10 Histidine(c) + 20 Isoleucine(c) + 50 Lysine(c) + 41 Leucine(c) + 17 Methionine(c) + 9 Asparagine(c) + 108 Proline(c) + 32 Glutamine(c) + 28 Arginine(c) + 20 Serine(c) + 16 Threonine(c) + 13 Valine(c) + Tryptophan(c) + 4 Tyrosine(c) + 2616 ATP(c) + 2616 H2O(c) → Collagen COPA1(c) + 1962 ADP(c) + 1962 Pi(c) + 654 AMP(c) + 654 PPi(c)
r2524	33	Alanine(s) ⇌ Alanine(c)
r2534	16	Threonine(s) ⇌ Threonine(c)
r2525	32	Glutamine(s) ⇌ Glutamine(c)
r1319	-3270	ATP(c) + H2O(c) ⇌ ADP(c) + Pi(c) + ATP-energy(c)
r1058	28	Arginine(s) ⇌ Arginine(c)
r1069	-50	Lysine(c) ⇌ Lysine(s)
r0030	654	ATP(c) + AMP(c) ⇌ 2 ADP(c)
r2474	20	Serine(s) + Na+(s) → Serine(c) + Na+(c)
r2664	162	Na+(c) ⇌ Na+(s) + Na-gradient(c)
r2475	7	Cysteine(s) + Na+(s) → Cysteine(c) + Na+(c)
r1121	-13	Valine(c) + Na+(c) ← Valine(s) + Na+(s)
r2468	167	Glycine(s) + H+(PG)(s) → Glycine(c) + H+(PG)(c)
r1123	-20	Isoleucine(c) + Na+(c) ← Isoleucine(s) + Na+(s)
r0007	654	PPi(c) + H2O(c) → 2 Pi(c)
r1331	-158	H+(PG)(s) ⇌ H+(PG)(c) + Proton-gradient(c)
r1124	-1	Tryptophan(c) + Na+(c) ← Tryptophan(s) + Na+(s)
r2479	9	Asparagine(s) + Na+(s) + H+(PG)(c) → Asparagine(c) + Na+(c) + H+(PG)(s)
r1143	-28	Na+(c) + Aspartate(c) ← Na+(s) + Aspartate(s)
r1125	4	Tyrosine(s) + Na+(s) → Tyrosine(c) + Na+(c)

Collagen CORA1(c) synthesis (388)

Definition			Solution													
			exchanges		reactions								transsp			
Objective	Constraints	Comment	imports	exports	c	m	r	p	l	n	s	b	s-c	b-c	int
Collagen CORA1(c)	MCES AAA	test synthesis of Collagen CORA1 c	67 Glutamate(s) 397 Glycine(s) 110 Alanine(s) 95 Lysine(s) 69 Aspartate(s) 101 Arginine(s) 96 Glutamine(s) 106 Serine(s) 38 Methionine(s) 10 Tryptophan(s) 49 Phenylalanine(s) 17 Tyrosine(s) 15 Cysteine(s) 134 Leucine(s) 31 Histidine(s) 303 Proline(s) 21 Asparagine(s) 70 Valine(s) 87 Threonine(s) 44 Isoleucine(s) 9300 ATP-energy(c) 376 Proton-gradient(c)	1 Collagen CORA1(c) 499 Na-gradient(c)	4	-	-	-	-	-	-	-	22	-	-

ID	flux	reaction
r2530	134	Leucine(s) ⇌ Leucine(c)
r1144	-67	Glutamate(c) + Na+(c) ← Glutamate(s) + Na+(s)
r1126	-49	Phenylalanine(c) + Na+(c) ← Phenylalanine(s) + Na+(s)
r1072	38	Methionine(s) ⇌ Methionine(c)
r2531	303	Proline(s) ⇌ Proline(c)
r1145	-31	Histidine(c) + Na+(c) ← Histidine(s) + Na+(s)
r2524	110	Alanine(s) ⇌ Alanine(c)
r2534	87	Threonine(s) ⇌ Threonine(c)
r2525	96	Glutamine(s) ⇌ Glutamine(c)
r1319	-9300	ATP(c) + H2O(c) ⇌ ADP(c) + Pi(c) + ATP-energy(c)
r1058	101	Arginine(s) ⇌ Arginine(c)
r2634	1	110 Alanine(c) + 15 Cysteine(c) + 69 Aspartate(c) + 67 Glutamate(c) + 49 Phenylalanine(c) + 397 Glycine(c) + 31 Histidine(c) + 44 Isoleucine(c) + 95 Lysine(c) + 134 Leucine(c) + 38 Methionine(c) + 21 Asparagine(c) + 303 Proline(c) + 96 Glutamine(c) + 101 Arginine(c) + 106 Serine(c) + 87 Threonine(c) + 70 Valine(c) + 10 Tryptophan(c) + 17 Tyrosine(c) + 7440 ATP(c) + 7440 H2O(c) → Collagen CORA1(c) + 5580 ADP(c) + 5580 Pi(c) + 1860 AMP(c) + 1860 PPi(c)
r1069	-95	Lysine(c) ⇌ Lysine(s)
r0030	1860	ATP(c) + AMP(c) ⇌ 2 ADP(c)
r2474	106	Serine(s) + Na+(s) → Serine(c) + Na+(c)
r2664	499	Na+(c) ⇌ Na+(s) + Na-gradient(c)
r2475	15	Cysteine(s) + Na+(s) → Cysteine(c) + Na+(c)
r1121	-70	Valine(c) + Na+(c) ← Valine(s) + Na+(s)
r2468	397	Glycine(s) + H+(PG)(s) → Glycine(c) + H+(PG)(c)
r1123	-44	Isoleucine(c) + Na+(c) ← Isoleucine(s) + Na+(s)
r0007	1860	PPi(c) + H2O(c) → 2 Pi(c)
r1331	-376	H+(PG)(s) ⇌ H+(PG)(c) + Proton-gradient(c)
r1124	-10	Tryptophan(c) + Na+(c) ← Tryptophan(s) + Na+(s)
r2479	21	Asparagine(s) + Na+(s) + H+(PG)(c) → Asparagine(c) + Na+(c) + H+(PG)(s)
r1143	-69	Na+(c) + Aspartate(c) ← Na+(s) + Aspartate(s)
r1125	17	Tyrosine(s) + Na+(s) → Tyrosine(c) + Na+(c)

Collagen COSA1(c) synthesis (389)

Definition			Solution												
			exchanges		reactions								transp		
Objective	Constraints	Comment	imports	exports	c	m	r	p	l	n	s	b	s-c	b-c	int
Collagen COSA1(c)	MCES AAA	test synthesis of Collagen COSA1 c	73 Glutamate(s) 211 Glycine(s) 42 Alanine(s) 91 Lysine(s) 63 Aspartate(s) 40 Arginine(s) 58 Glutamine(s) 69 Serine(s) 14 Methionine(s) 5 Tryptophan(s) 38 Phenylalanine(s) 18 Tyrosine(s) 15 Cysteine(s) 66 Leucine(s) 7 Histidine(s) 129 Proline(s) 27 Asparagine(s) 53 Valine(s) 45 Threonine(s) 61 Isoleucine(s) 5625 ATP-energy(c) 184 Proton-gradient(c)	1 Collagen COSA1(c) 429 Na-gradient(c)	4	-	-	-	-	-	-	-	22	-	-

ID	flux	reaction
r2530	66	Leucine(s) ⇌ Leucine(c)
r1144	-73	Glutamate(c) + Na+(c) ← Glutamate(s) + Na+(s)
r1126	-38	Phenylalanine(c) + Na+(c) ← Phenylalanine(s) + Na+(s)
r1072	14	Methionine(s) ⇌ Methionine(c)
r2531	129	Proline(s) ⇌ Proline(c)
r1145	-7	Histidine(c) + Na+(c) ← Histidine(s) + Na+(s)
r2524	42	Alanine(s) ⇌ Alanine(c)
r2534	45	Threonine(s) ⇌ Threonine(c)
r2525	58	Glutamine(s) ⇌ Glutamine(c)
r1319	-5625	ATP(c) + H2O(c) ⇌ ADP(c) + Pi(c) + ATP-energy(c)
r1058	40	Arginine(s) ⇌ Arginine(c)
r1069	-91	Lysine(c) ⇌ Lysine(s)
r0030	1125	ATP(c) + AMP(c) ⇌ 2 ADP(c)
r2636	1	42 Alanine(c) + 15 Cysteine(c) + 63 Aspartate(c) + 73 Glutamate(c) + 38 Phenylalanine(c) + 211 Glycine(c) + 7 Histidine(c) + 61 Isoleucine(c) + 91 Lysine(c) + 66 Leucine(c) + 14 Methionine(c) + 27 Asparagine(c) + 129 Proline(c) + 58 Glutamine(c) + 40 Arginine(c) + 69 Serine(c) + 45 Threonine(c) + 53 Valine(c) + 5 Tryptophan(c) + 18 Tyrosine(c) + 4500 ATP(c) + 4500 H2O(c) → Collagen COSA1(c) + 3375 ADP(c) + 3375 Pi(c) + 1125 AMP(c) + 1125 PPi(c)
r2474	69	Serine(s) + Na+(s) → Serine(c) + Na+(c)
r2664	429	Na+(c) ⇌ Na+(s) + Na-gradient(c)
r2475	15	Cysteine(s) + Na+(s) → Cysteine(c) + Na+(c)
r1121	-53	Valine(c) + Na+(c) ← Valine(s) + Na+(s)
r2468	211	Glycine(s) + H+(PG)(s) → Glycine(c) + H+(PG)(c)
r1123	-61	Isoleucine(c) + Na+(c) ← Isoleucine(s) + Na+(s)
r0007	1125	PPi(c) + H2O(c) → 2 Pi(c)
r1331	-184	H+(PG)(s) ⇌ H+(PG)(c) + Proton-gradient(c)
r1124	-5	Tryptophan(c) + Na+(c) ← Tryptophan(s) + Na+(s)
r2479	27	Asparagine(s) + Na+(s) + H+(PG)(c) → Asparagine(c) + Na+(c) + H+(PG)(s)
r1143	-63	Na+(c) + Aspartate(c) ← Na+(s) + Aspartate(s)
r1125	18	Tyrosine(s) + Na+(s) → Tyrosine(c) + Na+(c)

Collagen CTHR1(c) synthesis (390)

Definition			Solution													
			exchanges		reactions								transsp			
Objective	Constraints	Comment	imports	exports	c	m	r	p	l	n	s	b	s-c	b-c	int
Collagen CTHR1(c)	MCES AAA	test synthesis of Collagen CTHR1 c	15 Glutamate(s) 29 Glycine(s) 15 Alanine(s) 11 Lysine(s) 8 Aspartate(s) 15 Arginine(s) 10 Glutamine(s) 23 Serine(s) 4 Methionine(s) 5 Tryptophan(s) 6 Phenylalanine(s) 6 Tyrosine(s) 10 Cysteine(s) 24 Leucine(s) 1 Histidine(s) 18 Proline(s) 10 Asparagine(s) 9 Valine(s) 9 Threonine(s) 15 Isoleucine(s) 1215 ATP-energy(c) 19 Proton-gradient(c)	1 Collagen CTHR1(c) 108 Na-gradient(c)	4	-	-	-	-	-	-	-	22	-	-

ID	flux	reaction
r2530	24	Leucine(s) ⇌ Leucine(c)
r1144	-15	Glutamate(c) + Na+(c) ← Glutamate(s) + Na+(s)
r1126	-6	Phenylalanine(c) + Na+(c) ← Phenylalanine(s) + Na+(s)
r1072	4	Methionine(s) ⇌ Methionine(c)
r2531	18	Proline(s) ⇌ Proline(c)
r1145	-1	Histidine(c) + Na+(c) ← Histidine(s) + Na+(s)
r2524	15	Alanine(s) ⇌ Alanine(c)
r2534	9	Threonine(s) ⇌ Threonine(c)
r2525	10	Glutamine(s) ⇌ Glutamine(c)
r1319	-1215	ATP(c) + H2O(c) ⇌ ADP(c) + Pi(c) + ATP-energy(c)
r1058	15	Arginine(s) ⇌ Arginine(c)
r1069	-11	Lysine(c) ⇌ Lysine(s)
r0030	243	ATP(c) + AMP(c) ⇌ 2 ADP(c)
r2474	23	Serine(s) + Na+(s) → Serine(c) + Na+(c)
r2664	108	Na+(c) ⇌ Na+(s) + Na-gradient(c)
r2475	10	Cysteine(s) + Na+(s) → Cysteine(c) + Na+(c)
r1121	-9	Valine(c) + Na+(c) ← Valine(s) + Na+(s)
r2638	1	15 Alanine(c) + 10 Cysteine(c) + 8 Aspartate(c) + 15 Glutamate(c) + 6 Phenylalanine(c) + 29 Glycine(c) + Histidine(c) + 15 Isoleucine(c) + 11 Lysine(c) + 24 Leucine(c) + 4 Methionine(c) + 10 Asparagine(c) + 18 Proline(c) + 10 Glutamine(c) + 15 Arginine(c) + 23 Serine(c) + 9 Threonine(c) + 9 Valine(c) + 5 Tryptophan(c) + 6 Tyrosine(c) + 972 ATP(c) + 972 H2O(c) → Collagen CTHR1(c) + 729 ADP(c) + 729 Pi(c) + 243 AMP(c) + 243 PPi(c)
r2468	29	Glycine(s) + H+(PG)(s) → Glycine(c) + H+(PG)(c)
r1123	-15	Isoleucine(c) + Na+(c) ← Isoleucine(s) + Na+(s)
r0007	243	PPi(c) + H2O(c) → 2 Pi(c)
r1331	-19	H+(PG)(s) ⇌ H+(PG)(c) + Proton-gradient(c)
r1124	-5	Tryptophan(c) + Na+(c) ← Tryptophan(s) + Na+(s)
r2479	10	Asparagine(s) + Na+(s) + H+(PG)(c) → Asparagine(c) + Na+(c) + H+(PG)(s)
r1143	-8	Na+(c) + Aspartate(c) ← Na+(s) + Aspartate(s)
r1125	6	Tyrosine(s) + Na+(s) → Tyrosine(c) + Na+(c)

Collagen EMID2(c) synthesis (391)

Definition			Solution												
			exchanges		reactions								transp		
Objective	Constraints	Comment	imports	exports	c	m	r	p	l	n	s	b	s-c	b-c	int
Collagen EMID2(c)	MCES AAA	test synthesis of Collagen EMID2 c	24 Glutamate(s) 65 Glycine(s) 38 Alanine(s) 12 Lysine(s) 16 Aspartate(s) 28 Arginine(s) 18 Glutamine(s) 29 Serine(s) 6 Methionine(s) 5 Tryptophan(s) 4 Phenylalanine(s) 8 Tyrosine(s) 13 Cysteine(s) 39 Leucine(s) 7 Histidine(s) 68 Proline(s) 10 Asparagine(s) 21 Valine(s) 23 Threonine(s) 7 Isoleucine(s) 2205 ATP-energy(c) 55 Proton-gradient(c)	1 Collagen EMID2(c) 144 Na-gradient(c)	4	-	-	-	-	-	-	-	22	-	-

ID	flux	reaction
r2530	39	Leucine(s) ⇌ Leucine(c)
r1144	-24	Glutamate(c) + Na+(c) ← Glutamate(s) + Na+(s)
r1126	-4	Phenylalanine(c) + Na+(c) ← Phenylalanine(s) + Na+(s)
r1072	6	Methionine(s) ⇌ Methionine(c)
r2531	68	Proline(s) ⇌ Proline(c)
r1145	-7	Histidine(c) + Na+(c) ← Histidine(s) + Na+(s)
r2640	1	38 Alanine(c) + 13 Cysteine(c) + 16 Aspartate(c) + 24 Glutamate(c) + 4 Phenylalanine(c) + 65 Glycine(c) + 7 Histidine(c) + 7 Isoleucine(c) + 12 Lysine(c) + 39 Leucine(c) + 6 Methionine(c) + 10 Asparagine(c) + 68 Proline(c) + 18 Glutamine(c) + 28 Arginine(c) + 29 Serine(c) + 23 Threonine(c) + 21 Valine(c) + 5 Tryptophan(c) + 8 Tyrosine(c) + 1764 ATP(c) + 1764 H2O(c) → Collagen EMID2(c) + 1323 ADP(c) + 1323 Pi(c) + 441 AMP(c) + 441 PPi(c)
r2524	38	Alanine(s) ⇌ Alanine(c)
r2534	23	Threonine(s) ⇌ Threonine(c)
r2525	18	Glutamine(s) ⇌ Glutamine(c)
r1319	-2205	ATP(c) + H2O(c) ⇌ ADP(c) + Pi(c) + ATP-energy(c)
r1058	28	Arginine(s) ⇌ Arginine(c)
r1069	-12	Lysine(c) ⇌ Lysine(s)
r0030	441	ATP(c) + AMP(c) ⇌ 2 ADP(c)
r2474	29	Serine(s) + Na+(s) → Serine(c) + Na+(c)
r2664	144	Na+(c) ⇌ Na+(s) + Na-gradient(c)
r2475	13	Cysteine(s) + Na+(s) → Cysteine(c) + Na+(c)
r1121	-21	Valine(c) + Na+(c) ← Valine(s) + Na+(s)
r2468	65	Glycine(s) + H+(PG)(s) → Glycine(c) + H+(PG)(c)
r1123	-7	Isoleucine(c) + Na+(c) ← Isoleucine(s) + Na+(s)
r0007	441	PPi(c) + H2O(c) → 2 Pi(c)
r1331	-55	H+(PG)(s) ⇌ H+(PG)(c) + Proton-gradient(c)
r1124	-5	Tryptophan(c) + Na+(c) ← Tryptophan(s) + Na+(s)
r2479	10	Asparagine(s) + Na+(s) + H+(PG)(c) → Asparagine(c) + Na+(c) + H+(PG)(s)
r1143	-16	Na+(c) + Aspartate(c) ← Na+(s) + Aspartate(s)
r1125	8	Tyrosine(s) + Na+(s) → Tyrosine(c) + Na+(c)

Collagen FCN1(c) synthesis (392)

Definition			Solution													
			exchanges		reactions								transp		
Objective	Constraints	Comment	imports	exports	c	m	r	p	l	n	s	b	s-c	b-c	int
Collagen FCN1(c)	MCES AAA	test synthesis of Collagen FCN1 c	16 Glutamate(s) 46 Glycine(s) 29 Alanine(s) 21 Lysine(s) 21 Aspartate(s) 14 Arginine(s) 10 Glutamine(s) 21 Serine(s) 7 Methionine(s) 7 Tryptophan(s) 13 Phenylalanine(s) 12 Tyrosine(s) 8 Cysteine(s) 29 Leucine(s) 7 Histidine(s) 15 Proline(s) 15 Asparagine(s) 18 Valine(s) 11 Threonine(s) 6 Isoleucine(s) 1630 ATP-energy(c) 31 Proton-gradient(c)	1 Collagen FCN1(c) 144 Na-gradient(c)	4	-	-	-	-	-	-	-	22	-	-

ID	flux	reaction
r2530	29	Leucine(s) ⇌ Leucine(c)
r1144	-16	Glutamate(c) + Na+(c) ← Glutamate(s) + Na+(s)
r1126	-13	Phenylalanine(c) + Na+(c) ← Phenylalanine(s) + Na+(s)
r1072	7	Methionine(s) ⇌ Methionine(c)
r2531	15	Proline(s) ⇌ Proline(c)
r1145	-7	Histidine(c) + Na+(c) ← Histidine(s) + Na+(s)
r2524	29	Alanine(s) ⇌ Alanine(c)
r2642	1	29 Alanine(c) + 8 Cysteine(c) + 21 Aspartate(c) + 16 Glutamate(c) + 13 Phenylalanine(c) + 46 Glycine(c) + 7 Histidine(c) + 6 Isoleucine(c) + 21 Lysine(c) + 29 Leucine(c) + 7 Methionine(c) + 15 Asparagine(c) + 15 Proline(c) + 10 Glutamine(c) + 14 Arginine(c) + 21 Serine(c) + 11 Threonine(c) + 18 Valine(c) + 7 Tryptophan(c) + 12 Tyrosine(c) + 1304 ATP(c) + 1304 H2O(c) → Collagen FCN1(c) + 978 ADP(c) + 978 Pi(c) + 326 AMP(c) + 326 PPi(c)
r2534	11	Threonine(s) ⇌ Threonine(c)
r2525	10	Glutamine(s) ⇌ Glutamine(c)
r1319	-1630	ATP(c) + H2O(c) ⇌ ADP(c) + Pi(c) + ATP-energy(c)
r1058	14	Arginine(s) ⇌ Arginine(c)
r1069	-21	Lysine(c) ⇌ Lysine(s)
r0030	326	ATP(c) + AMP(c) ⇌ 2 ADP(c)
r2474	21	Serine(s) + Na+(s) → Serine(c) + Na+(c)
r2664	144	Na+(c) ⇌ Na+(s) + Na-gradient(c)
r2475	8	Cysteine(s) + Na+(s) → Cysteine(c) + Na+(c)
r1121	-18	Valine(c) + Na+(c) ← Valine(s) + Na+(s)
r2468	46	Glycine(s) + H+(PG)(s) → Glycine(c) + H+(PG)(c)
r1123	-6	Isoleucine(c) + Na+(c) ← Isoleucine(s) + Na+(s)
r0007	326	PPi(c) + H2O(c) → 2 Pi(c)
r1331	-31	H+(PG)(s) ⇌ H+(PG)(c) + Proton-gradient(c)
r1124	-7	Tryptophan(c) + Na+(c) ← Tryptophan(s) + Na+(s)
r2479	15	Asparagine(s) + Na+(s) + H+(PG)(c) → Asparagine(c) + Na+(c) + H+(PG)(s)
r1143	-21	Na+(c) + Aspartate(c) ← Na+(s) + Aspartate(s)
r1125	12	Tyrosine(s) + Na+(s) → Tyrosine(c) + Na+(c)

Collagen FCN2(c) synthesis (393)

Definition			Solution												
			exchanges		reactions								transp		
Objective	Constraints	Comment	imports	exports	c	m	r	p	l	n	s	b	s-c	b-c	int
Collagen FCN2(c)	MCES AAA	test synthesis of Collagen FCN2 c	13 Glutamate(s) 43 Glycine(s) 23 Alanine(s) 16 Lysine(s) 21 Aspartate(s) 16 Arginine(s) 9 Glutamine(s) 16 Serine(s) 6 Methionine(s) 8 Tryptophan(s) 14 Phenylalanine(s) 11 Tyrosine(s) 8 Cysteine(s) 29 Leucine(s) 6 Histidine(s) 19 Proline(s) 17 Asparagine(s) 17 Valine(s) 17 Threonine(s) 4 Isoleucine(s) 1565 ATP-energy(c) 26 Proton-gradient(c)	1 Collagen FCN2(c) 135 Na-gradient(c)	4	-	-	-	-	-	-	-	22	-	-

ID	flux	reaction
r2530	29	Leucine(s) ⇌ Leucine(c)
r1144	-13	Glutamate(c) + Na+(c) ← Glutamate(s) + Na+(s)
r1126	-14	Phenylalanine(c) + Na+(c) ← Phenylalanine(s) + Na+(s)
r1072	6	Methionine(s) ⇌ Methionine(c)
r2531	19	Proline(s) ⇌ Proline(c)
r1145	-6	Histidine(c) + Na+(c) ← Histidine(s) + Na+(s)
r2524	23	Alanine(s) ⇌ Alanine(c)
r2534	17	Threonine(s) ⇌ Threonine(c)
r2525	9	Glutamine(s) ⇌ Glutamine(c)
r1319	-1565	ATP(c) + H2O(c) ⇌ ADP(c) + Pi(c) + ATP-energy(c)
r1058	16	Arginine(s) ⇌ Arginine(c)
r2644	1	23 Alanine(c) + 8 Cysteine(c) + 21 Aspartate(c) + 13 Glutamate(c) + 14 Phenylalanine(c) + 43 Glycine(c) + 6 Histidine(c) + 4 Isoleucine(c) + 16 Lysine(c) + 29 Leucine(c) + 6 Methionine(c) + 17 Asparagine(c) + 19 Proline(c) + 9 Glutamine(c) + 16 Arginine(c) + 16 Serine(c) + 17 Threonine(c) + 17 Valine(c) + 8 Tryptophan(c) + 11 Tyrosine(c) + 1252 ATP(c) + 1252 H2O(c) → Collagen FCN2(c) + 939 ADP(c) + 939 Pi(c) + 313 AMP(c) + 313 PPi(c)
r1069	-16	Lysine(c) ⇌ Lysine(s)
r0030	313	ATP(c) + AMP(c) ⇌ 2 ADP(c)
r2474	16	Serine(s) + Na+(s) → Serine(c) + Na+(c)
r2664	135	Na+(c) ⇌ Na+(s) + Na-gradient(c)
r2475	8	Cysteine(s) + Na+(s) → Cysteine(c) + Na+(c)
r1121	-17	Valine(c) + Na+(c) ← Valine(s) + Na+(s)
r2468	43	Glycine(s) + H+(PG)(s) → Glycine(c) + H+(PG)(c)
r1123	-4	Isoleucine(c) + Na+(c) ← Isoleucine(s) + Na+(s)
r0007	313	PPi(c) + H2O(c) → 2 Pi(c)
r1331	-26	H+(PG)(s) ⇌ H+(PG)(c) + Proton-gradient(c)
r1124	-8	Tryptophan(c) + Na+(c) ← Tryptophan(s) + Na+(s)
r2479	17	Asparagine(s) + Na+(s) + H+(PG)(c) → Asparagine(c) + Na+(c) + H+(PG)(s)
r1143	-21	Na+(c) + Aspartate(c) ← Na+(s) + Aspartate(s)
r1125	11	Tyrosine(s) + Na+(s) → Tyrosine(c) + Na+(c)

Collagen FCN3(c) synthesis (394)

Definition			Solution												
			exchanges		reactions								transp		
Objective	Constraints	Comment	imports	exports	c	m	r	p	l	n	s	b	s-c	b-c	int
Collagen FCN3(c)	MCES AAA	test synthesis of Collagen FCN3 c	18 Glutamate(s) 38 Glycine(s) 20 Alanine(s) 7 Lysine(s) 13 Aspartate(s) 20 Arginine(s) 10 Glutamine(s) 23 Serine(s) 5 Methionine(s) 10 Tryptophan(s) 11 Phenylalanine(s) 10 Tyrosine(s) 9 Cysteine(s) 35 Leucine(s) 10 Histidine(s) 23 Proline(s) 12 Asparagine(s) 13 Valine(s) 9 Threonine(s) 3 Isoleucine(s) 1495 ATP-energy(c) 26 Proton-gradient(c)	1 Collagen FCN3(c) 132 Na-gradient(c)	4	-	-	-	-	-	-	-	22	-	-

ID	flux	reaction
r2530	35	Leucine(s) ⇌ Leucine(c)
r1144	-18	Glutamate(c) + Na+(c) ← Glutamate(s) + Na+(s)
r1126	-11	Phenylalanine(c) + Na+(c) ← Phenylalanine(s) + Na+(s)
r1072	5	Methionine(s) ⇌ Methionine(c)
r2531	23	Proline(s) ⇌ Proline(c)
r1145	-10	Histidine(c) + Na+(c) ← Histidine(s) + Na+(s)
r2524	20	Alanine(s) ⇌ Alanine(c)
r2534	9	Threonine(s) ⇌ Threonine(c)
r2525	10	Glutamine(s) ⇌ Glutamine(c)
r1319	-1495	ATP(c) + H2O(c) ⇌ ADP(c) + Pi(c) + ATP-energy(c)
r1058	20	Arginine(s) ⇌ Arginine(c)
r1069	-7	Lysine(c) ⇌ Lysine(s)
r0030	299	ATP(c) + AMP(c) ⇌ 2 ADP(c)
r2474	23	Serine(s) + Na+(s) → Serine(c) + Na+(c)
r2664	132	Na+(c) ⇌ Na+(s) + Na-gradient(c)
r2646	1	20 Alanine(c) + 9 Cysteine(c) + 13 Aspartate(c) + 18 Glutamate(c) + 11 Phenylalanine(c) + 38 Glycine(c) + 10 Histidine(c) + 3 Isoleucine(c) + 7 Lysine(c) + 35 Leucine(c) + 5 Methionine(c) + 12 Asparagine(c) + 23 Proline(c) + 10 Glutamine(c) + 20 Arginine(c) + 23 Serine(c) + 9 Threonine(c) + 13 Valine(c) + 10 Tryptophan(c) + 10 Tyrosine(c) + 1196 ATP(c) + 1196 H2O(c) → Collagen FCN3(c) + 897 ADP(c) + 897 Pi(c) + 299 AMP(c) + 299 PPi(c)
r2475	9	Cysteine(s) + Na+(s) → Cysteine(c) + Na+(c)
r1121	-13	Valine(c) + Na+(c) ← Valine(s) + Na+(s)
r2468	38	Glycine(s) + H+(PG)(s) → Glycine(c) + H+(PG)(c)
r1123	-3	Isoleucine(c) + Na+(c) ← Isoleucine(s) + Na+(s)
r0007	299	PPi(c) + H2O(c) → 2 Pi(c)
r1331	-26	H+(PG)(s) ⇌ H+(PG)(c) + Proton-gradient(c)
r1124	-10	Tryptophan(c) + Na+(c) ← Tryptophan(s) + Na+(s)
r2479	12	Asparagine(s) + Na+(s) + H+(PG)(c) → Asparagine(c) + Na+(c) + H+(PG)(s)
r1143	-13	Na+(c) + Aspartate(c) ← Na+(s) + Aspartate(s)
r1125	10	Tyrosine(s) + Na+(s) → Tyrosine(c) + Na+(c)

Collagen FMOD(c) synthesis (395)

Definition			Solution												
			exchanges		reactions								transp		
Objective	Constraints	Comment	imports	exports	c	m	r	p	l	n	s	b	s-c	b-c	int
Collagen FMOD(c)	MCES AAA	test synthesis of Collagen FMOD c	19 Glutamate(s) 17 Glycine(s) 15 Alanine(s) 10 Lysine(s) 20 Aspartate(s) 20 Arginine(s) 18 Glutamine(s) 32 Serine(s) 7 Methionine(s) 4 Tryptophan(s) 12 Phenylalanine(s) 25 Tyrosine(s) 6 Cysteine(s) 55 Leucine(s) 11 Histidine(s) 29 Proline(s) 30 Asparagine(s) 18 Valine(s) 15 Threonine(s) 13 Isoleucine(s) 1880 ATP-energy(c)	13 Proton-gradient(c) 1 Collagen FMOD(c) 190 Na-gradient(c)	4	-	-	-	-	-	-	-	22	-	-

ID	flux	reaction
r2530	55	Leucine(s) ⇌ Leucine(c)
r1144	-19	Glutamate(c) + Na+(c) ← Glutamate(s) + Na+(s)
r1126	-12	Phenylalanine(c) + Na+(c) ← Phenylalanine(s) + Na+(s)
r1072	7	Methionine(s) ⇌ Methionine(c)
r2531	29	Proline(s) ⇌ Proline(c)
r1145	-11	Histidine(c) + Na+(c) ← Histidine(s) + Na+(s)
r2524	15	Alanine(s) ⇌ Alanine(c)
r2534	15	Threonine(s) ⇌ Threonine(c)
r2525	18	Glutamine(s) ⇌ Glutamine(c)
r1319	-1880	ATP(c) + H2O(c) ⇌ ADP(c) + Pi(c) + ATP-energy(c)
r1058	20	Arginine(s) ⇌ Arginine(c)
r1069	-10	Lysine(c) ⇌ Lysine(s)
r0030	376	ATP(c) + AMP(c) ⇌ 2 ADP(c)
r2474	32	Serine(s) + Na+(s) → Serine(c) + Na+(c)
r2664	190	Na+(c) ⇌ Na+(s) + Na-gradient(c)
r2475	6	Cysteine(s) + Na+(s) → Cysteine(c) + Na+(c)
r1121	-18	Valine(c) + Na+(c) ← Valine(s) + Na+(s)
r2648	1	15 Alanine(c) + 6 Cysteine(c) + 20 Aspartate(c) + 19 Glutamate(c) + 12 Phenylalanine(c) + 17 Glycine(c) + 11 Histidine(c) + 13 Isoleucine(c) + 10 Lysine(c) + 55 Leucine(c) + 7 Methionine(c) + 30 Asparagine(c) + 29 Proline(c) + 18 Glutamine(c) + 20 Arginine(c) + 32 Serine(c) + 15 Threonine(c) + 18 Valine(c) + 4 Tryptophan(c) + 25 Tyrosine(c) + 1504 ATP(c) + 1504 H2O(c) → Collagen FMOD(c) + 1128 ADP(c) + 1128 Pi(c) + 376 AMP(c) + 376 PPi(c)
r2468	17	Glycine(s) + H+(PG)(s) → Glycine(c) + H+(PG)(c)
r1123	-13	Isoleucine(c) + Na+(c) ← Isoleucine(s) + Na+(s)
r0007	376	PPi(c) + H2O(c) → 2 Pi(c)
r1331	13	H+(PG)(s) ⇌ H+(PG)(c) + Proton-gradient(c)
r1124	-4	Tryptophan(c) + Na+(c) ← Tryptophan(s) + Na+(s)
r2479	30	Asparagine(s) + Na+(s) + H+(PG)(c) → Asparagine(c) + Na+(c) + H+(PG)(s)
r1143	-20	Na+(c) + Aspartate(c) ← Na+(s) + Aspartate(s)
r1125	25	Tyrosine(s) + Na+(s) → Tyrosine(c) + Na+(c)

Collagen ITA1(c) synthesis (396)

Definition			Solution													
			exchanges		reactions								transsp			
Objective	Constraints	Comment	imports	exports	c	m	r	p	l	n	s	b	s-c	b-c	int
Collagen ITA1(c)	MCES AAA	test synthesis of Collagen ITA1 c	73 Glutamate(s) 81 Glycine(s) 60 Alanine(s) 72 Lysine(s) 60 Aspartate(s) 46 Arginine(s) 42 Glutamine(s) 101 Serine(s) 26 Methionine(s) 9 Tryptophan(s) 50 Phenylalanine(s) 41 Tyrosine(s) 27 Cysteine(s) 96 Leucine(s) 22 Histidine(s) 47 Proline(s) 71 Asparagine(s) 90 Valine(s) 86 Threonine(s) 79 Isoleucine(s) 5895 ATP-energy(c) 10 Proton-gradient(c)	1 Collagen ITA1(c) 623 Na-gradient(c)	4	-	-	-	-	-	-	-	22	-	-

ID	flux	reaction
r2530	96	Leucine(s) ⇌ Leucine(c)
r1144	-73	Glutamate(c) + Na+(c) ← Glutamate(s) + Na+(s)
r1126	-50	Phenylalanine(c) + Na+(c) ← Phenylalanine(s) + Na+(s)
r1072	26	Methionine(s) ⇌ Methionine(c)
r2531	47	Proline(s) ⇌ Proline(c)
r1145	-22	Histidine(c) + Na+(c) ← Histidine(s) + Na+(s)
r2650	1	60 Alanine(c) + 27 Cysteine(c) + 60 Aspartate(c) + 73 Glutamate(c) + 50 Phenylalanine(c) + 81 Glycine(c) + 22 Histidine(c) + 79 Isoleucine(c) + 72 Lysine(c) + 96 Leucine(c) + 26 Methionine(c) + 71 Asparagine(c) + 47 Proline(c) + 42 Glutamine(c) + 46 Arginine(c) + 101 Serine(c) + 86 Threonine(c) + 90 Valine(c) + 9 Tryptophan(c) + 41 Tyrosine(c) + 4716 ATP(c) + 4716 H2O(c) → Collagen ITA1(c) + 3537 ADP(c) + 3537 Pi(c) + 1179 AMP(c) + 1179 PPi(c)
r2524	60	Alanine(s) ⇌ Alanine(c)
r2534	86	Threonine(s) ⇌ Threonine(c)
r2525	42	Glutamine(s) ⇌ Glutamine(c)
r1319	-5895	ATP(c) + H2O(c) ⇌ ADP(c) + Pi(c) + ATP-energy(c)
r1058	46	Arginine(s) ⇌ Arginine(c)
r1069	-72	Lysine(c) ⇌ Lysine(s)
r0030	1179	ATP(c) + AMP(c) ⇌ 2 ADP(c)
r2474	101	Serine(s) + Na+(s) → Serine(c) + Na+(c)
r2664	623	Na+(c) ⇌ Na+(s) + Na-gradient(c)
r2475	27	Cysteine(s) + Na+(s) → Cysteine(c) + Na+(c)
r1121	-90	Valine(c) + Na+(c) ← Valine(s) + Na+(s)
r2468	81	Glycine(s) + H+(PG)(s) → Glycine(c) + H+(PG)(c)
r1123	-79	Isoleucine(c) + Na+(c) ← Isoleucine(s) + Na+(s)
r0007	1179	PPi(c) + H2O(c) → 2 Pi(c)
r1331	-10	H+(PG)(s) ⇌ H+(PG)(c) + Proton-gradient(c)
r1124	-9	Tryptophan(c) + Na+(c) ← Tryptophan(s) + Na+(s)
r2479	71	Asparagine(s) + Na+(s) + H+(PG)(c) → Asparagine(c) + Na+(c) + H+(PG)(s)
r1143	-60	Na+(c) + Aspartate(c) ← Na+(s) + Aspartate(s)
r1125	41	Tyrosine(s) + Na+(s) → Tyrosine(c) + Na+(c)

Collagen ITA2(c) synthesis (397)

Definition			Solution												
			exchanges		reactions								transp		
Objective	Constraints	Comment	imports	exports	c	m	r	p	l	n	s	b	s-c	b-c	int
Collagen ITA2(c)	MCES AAA	test synthesis of Collagen ITA2 c	60 Glutamate(s) 88 Glycine(s) 79 Alanine(s) 59 Lysine(s) 60 Aspartate(s) 32 Arginine(s) 63 Glutamine(s) 102 Serine(s) 21 Methionine(s) 9 Tryptophan(s) 57 Phenylalanine(s) 40 Tyrosine(s) 22 Cysteine(s) 98 Leucine(s) 17 Histidine(s) 47 Proline(s) 75 Asparagine(s) 89 Valine(s) 79 Threonine(s) 84 Isoleucine(s) 5905 ATP-energy(c) 13 Proton-gradient(c)	1 Collagen ITA2(c) 615 Na-gradient(c)	4	-	-	-	-	-	-	-	22	-	-

ID	flux	reaction
r2530	98	Leucine(s) ⇌ Leucine(c)
r1144	-60	Glutamate(c) + Na+(c) ← Glutamate(s) + Na+(s)
r1126	-57	Phenylalanine(c) + Na+(c) ← Phenylalanine(s) + Na+(s)
r1072	21	Methionine(s) ⇌ Methionine(c)
r2531	47	Proline(s) ⇌ Proline(c)
r1145	-17	Histidine(c) + Na+(c) ← Histidine(s) + Na+(s)
r2524	79	Alanine(s) ⇌ Alanine(c)
r2534	79	Threonine(s) ⇌ Threonine(c)
r2525	63	Glutamine(s) ⇌ Glutamine(c)
r1319	-5905	ATP(c) + H2O(c) ⇌ ADP(c) + Pi(c) + ATP-energy(c)
r1058	32	Arginine(s) ⇌ Arginine(c)
r2652	1	79 Alanine(c) + 22 Cysteine(c) + 60 Aspartate(c) + 60 Glutamate(c) + 57 Phenylalanine(c) + 88 Glycine(c) + 17 Histidine(c) + 84 Isoleucine(c) + 59 Lysine(c) + 98 Leucine(c) + 21 Methionine(c) + 75 Asparagine(c) + 47 Proline(c) + 63 Glutamine(c) + 32 Arginine(c) + 102 Serine(c) + 79 Threonine(c) + 89 Valine(c) + 9 Tryptophan(c) + 40 Tyrosine(c) + 4724 ATP(c) + 4724 H2O(c) → Collagen ITA2(c) + 3543 ADP(c) + 3543 Pi(c) + 1181 AMP(c) + 1181 PPi(c)
r1069	-59	Lysine(c) ⇌ Lysine(s)
r0030	1181	ATP(c) + AMP(c) ⇌ 2 ADP(c)
r2474	102	Serine(s) + Na+(s) → Serine(c) + Na+(c)
r2664	615	Na+(c) ⇌ Na+(s) + Na-gradient(c)
r2475	22	Cysteine(s) + Na+(s) → Cysteine(c) + Na+(c)
r1121	-89	Valine(c) + Na+(c) ← Valine(s) + Na+(s)
r2468	88	Glycine(s) + H+(PG)(s) → Glycine(c) + H+(PG)(c)
r1123	-84	Isoleucine(c) + Na+(c) ← Isoleucine(s) + Na+(s)
r0007	1181	PPi(c) + H2O(c) → 2 Pi(c)
r1331	-13	H+(PG)(s) ⇌ H+(PG)(c) + Proton-gradient(c)
r1124	-9	Tryptophan(c) + Na+(c) ← Tryptophan(s) + Na+(s)
r2479	75	Asparagine(s) + Na+(s) + H+(PG)(c) → Asparagine(c) + Na+(c) + H+(PG)(s)
r1143	-60	Na+(c) + Aspartate(c) ← Na+(s) + Aspartate(s)
r1125	40	Tyrosine(s) + Na+(s) → Tyrosine(c) + Na+(c)

Collagen LPP3(c) synthesis (398)

Definition			Solution													
			exchanges		reactions								transp		
Objective	Constraints	Comment	imports	exports	c	m	r	p	l	n	s	b	s-c	b-c	int
Collagen LPP3(c)	MCES AAA	test synthesis of Collagen LPP3 c	8 Glutamate(s) 18 Glycine(s) 22 Alanine(s) 17 Lysine(s) 13 Aspartate(s) 18 Arginine(s) 10 Glutamine(s) 25 Serine(s) 8 Methionine(s) 1 Tryptophan(s) 20 Phenylalanine(s) 16 Tyrosine(s) 11 Cysteine(s) 32 Leucine(s) 8 Histidine(s) 14 Proline(s) 14 Asparagine(s) 17 Valine(s) 14 Threonine(s) 25 Isoleucine(s) 1555 ATP-energy(c) 4 Proton-gradient(c)	1 Collagen LPP3(c) 158 Na-gradient(c)	4	-	-	-	-	-	-	-	22	-	-

ID	flux	reaction
r2530	32	Leucine(s) ⇌ Leucine(c)
r1144	-8	Glutamate(c) + Na+(c) ← Glutamate(s) + Na+(s)
r1126	-20	Phenylalanine(c) + Na+(c) ← Phenylalanine(s) + Na+(s)
r1072	8	Methionine(s) ⇌ Methionine(c)
r2531	14	Proline(s) ⇌ Proline(c)
r1145	-8	Histidine(c) + Na+(c) ← Histidine(s) + Na+(s)
r2524	22	Alanine(s) ⇌ Alanine(c)
r2534	14	Threonine(s) ⇌ Threonine(c)
r2525	10	Glutamine(s) ⇌ Glutamine(c)
r1319	-1555	ATP(c) + H2O(c) ⇌ ADP(c) + Pi(c) + ATP-energy(c)
r1058	18	Arginine(s) ⇌ Arginine(c)
r1069	-17	Lysine(c) ⇌ Lysine(s)
r0030	311	ATP(c) + AMP(c) ⇌ 2 ADP(c)
r2654	1	22 Alanine(c) + 11 Cysteine(c) + 13 Aspartate(c) + 8 Glutamate(c) + 20 Phenylalanine(c) + 18 Glycine(c) + 8 Histidine(c) + 25 Isoleucine(c) + 17 Lysine(c) + 32 Leucine(c) + 8 Methionine(c) + 14 Asparagine(c) + 14 Proline(c) + 10 Glutamine(c) + 18 Arginine(c) + 25 Serine(c) + 14 Threonine(c) + 17 Valine(c) + Tryptophan(c) + 16 Tyrosine(c) + 1244 ATP(c) + 1244 H2O(c) → Collagen LPP3(c) + 933 ADP(c) + 933 Pi(c) + 311 AMP(c) + 311 PPi(c)
r2474	25	Serine(s) + Na+(s) → Serine(c) + Na+(c)
r2664	158	Na+(c) ⇌ Na+(s) + Na-gradient(c)
r2475	11	Cysteine(s) + Na+(s) → Cysteine(c) + Na+(c)
r1121	-17	Valine(c) + Na+(c) ← Valine(s) + Na+(s)
r2468	18	Glycine(s) + H+(PG)(s) → Glycine(c) + H+(PG)(c)
r1123	-25	Isoleucine(c) + Na+(c) ← Isoleucine(s) + Na+(s)
r0007	311	PPi(c) + H2O(c) → 2 Pi(c)
r1331	-4	H+(PG)(s) ⇌ H+(PG)(c) + Proton-gradient(c)
r1124	-1	Tryptophan(c) + Na+(c) ← Tryptophan(s) + Na+(s)
r2479	14	Asparagine(s) + Na+(s) + H+(PG)(c) → Asparagine(c) + Na+(c) + H+(PG)(s)
r1143	-13	Na+(c) + Aspartate(c) ← Na+(s) + Aspartate(s)
r1125	16	Tyrosine(s) + Na+(s) → Tyrosine(c) + Na+(c)

Collagen PCOTH(c) synthesis (399)

Definition			Solution												
			exchanges		reactions								transp		
Objective	Constraints	Comment	imports	exports	c	m	r	p	l	n	s	b	s-c	b-c	int
Collagen PCOTH(c)	MCES AAA	test synthesis of Collagen PCOTH c	2 Glutamate(s) 16 Glycine(s) 2 Alanine(s) 4 Lysine(s) 3 Arginine(s) 1 Glutamine(s) 11 Serine(s) 4 Methionine(s) 2 Tryptophan(s) 7 Phenylalanine(s) 1 Cysteine(s) 10 Leucine(s) 26 Proline(s) 2 Asparagine(s) 6 Valine(s) 6 Threonine(s) 4 Isoleucine(s) 535 ATP-energy(c) 14 Proton-gradient(c)	1 Collagen PCOTH(c) 35 Na-gradient(c)	4	-	-	-	-	-	-	-	19	-	-

ID	flux	reaction
r2530	10	Leucine(s) ⇌ Leucine(c)
r1144	-2	Glutamate(c) + Na+(c) ← Glutamate(s) + Na+(s)
r1126	-7	Phenylalanine(c) + Na+(c) ← Phenylalanine(s) + Na+(s)
r1072	4	Methionine(s) ⇌ Methionine(c)
r2531	26	Proline(s) ⇌ Proline(c)
r2524	2	Alanine(s) ⇌ Alanine(c)
r2534	6	Threonine(s) ⇌ Threonine(c)
r2525	1	Glutamine(s) ⇌ Glutamine(c)
r1319	-535	ATP(c) + H2O(c) ⇌ ADP(c) + Pi(c) + ATP-energy(c)
r1058	3	Arginine(s) ⇌ Arginine(c)
r1069	-4	Lysine(c) ⇌ Lysine(s)
r0030	107	ATP(c) + AMP(c) ⇌ 2 ADP(c)
r2474	11	Serine(s) + Na+(s) → Serine(c) + Na+(c)
r2664	35	Na+(c) ⇌ Na+(s) + Na-gradient(c)
r2475	1	Cysteine(s) + Na+(s) → Cysteine(c) + Na+(c)
r1121	-6	Valine(c) + Na+(c) ← Valine(s) + Na+(s)
r2656	1	2 Alanine(c) + Cysteine(c) + 2 Glutamate(c) + 7 Phenylalanine(c) + 16 Glycine(c) + 4 Isoleucine(c) + 4 Lysine(c) + 10 Leucine(c) + 4 Methionine(c) + 2 Asparagine(c) + 26 Proline(c) + Glutamine(c) + 3 Arginine(c) + 11 Serine(c) + 6 Threonine(c) + 6 Valine(c) + 2 Tryptophan(c) + 428 ATP(c) + 428 H2O(c) → Collagen PCOTH(c) + 321 ADP(c) + 321 Pi(c) + 107 AMP(c) + 107 PPi(c)
r2468	16	Glycine(s) + H+(PG)(s) → Glycine(c) + H+(PG)(c)
r1123	-4	Isoleucine(c) + Na+(c) ← Isoleucine(s) + Na+(s)
r0007	107	PPi(c) + H2O(c) → 2 Pi(c)
r1331	-14	H+(PG)(s) ⇌ H+(PG)(c) + Proton-gradient(c)
r1124	-2	Tryptophan(c) + Na+(c) ← Tryptophan(s) + Na+(s)
r2479	2	Asparagine(s) + Na+(s) + H+(PG)(c) → Asparagine(c) + Na+(c) + H+(PG)(s)

Collagen SCRB1(c) synthesis (400)

Definition			Solution												
			exchanges		reactions								transp		
Objective	Constraints	Comment	imports	exports	c	m	r	p	l	n	s	b	s-c	b-c	int
Collagen SCRB1(c)	MCES AAA	test synthesis of Collagen SCRB1 c	25 Glutamate(s) 45 Glycine(s) 39 Alanine(s) 24 Lysine(s) 14 Aspartate(s) 21 Arginine(s) 17 Glutamine(s) 45 Serine(s) 23 Methionine(s) 11 Tryptophan(s) 32 Phenylalanine(s) 16 Tyrosine(s) 14 Cysteine(s) 59 Leucine(s) 13 Histidine(s) 38 Proline(s) 24 Asparagine(s) 41 Valine(s) 27 Threonine(s) 24 Isoleucine(s) 2760 ATP-energy(c) 21 Proton-gradient(c)	1 Collagen SCRB1(c) 259 Na-gradient(c)	4	-	-	-	-	-	-	-	22	-	-

ID	flux	reaction
r2530	59	Leucine(s) ⇌ Leucine(c)
r1144	-25	Glutamate(c) + Na+(c) ← Glutamate(s) + Na+(s)
r1126	-32	Phenylalanine(c) + Na+(c) ← Phenylalanine(s) + Na+(s)
r1072	23	Methionine(s) ⇌ Methionine(c)
r2531	38	Proline(s) ⇌ Proline(c)
r1145	-13	Histidine(c) + Na+(c) ← Histidine(s) + Na+(s)
r2524	39	Alanine(s) ⇌ Alanine(c)
r2534	27	Threonine(s) ⇌ Threonine(c)
r2525	17	Glutamine(s) ⇌ Glutamine(c)
r1319	-2760	ATP(c) + H2O(c) ⇌ ADP(c) + Pi(c) + ATP-energy(c)
r1058	21	Arginine(s) ⇌ Arginine(c)
r1069	-24	Lysine(c) ⇌ Lysine(s)
r0030	552	ATP(c) + AMP(c) ⇌ 2 ADP(c)
r2474	45	Serine(s) + Na+(s) → Serine(c) + Na+(c)
r2664	259	Na+(c) ⇌ Na+(s) + Na-gradient(c)
r2475	14	Cysteine(s) + Na+(s) → Cysteine(c) + Na+(c)
r1121	-41	Valine(c) + Na+(c) ← Valine(s) + Na+(s)
r2468	45	Glycine(s) + H+(PG)(s) → Glycine(c) + H+(PG)(c)
r1123	-24	Isoleucine(c) + Na+(c) ← Isoleucine(s) + Na+(s)
r0007	552	PPi(c) + H2O(c) → 2 Pi(c)
r2658	1	39 Alanine(c) + 14 Cysteine(c) + 14 Aspartate(c) + 25 Glutamate(c) + 32 Phenylalanine(c) + 45 Glycine(c) + 13 Histidine(c) + 24 Isoleucine(c) + 24 Lysine(c) + 59 Leucine(c) + 23 Methionine(c) + 24 Asparagine(c) + 38 Proline(c) + 17 Glutamine(c) + 21 Arginine(c) + 45 Serine(c) + 27 Threonine(c) + 41 Valine(c) + 11 Tryptophan(c) + 16 Tyrosine(c) + 2208 ATP(c) + 2208 H2O(c) → Collagen SCRB1(c) + 1656 ADP(c) + 1656 Pi(c) + 552 AMP(c) + 552 PPi(c)
r1331	-21	H+(PG)(s) ⇌ H+(PG)(c) + Proton-gradient(c)
r1124	-11	Tryptophan(c) + Na+(c) ← Tryptophan(s) + Na+(s)
r2479	24	Asparagine(s) + Na+(s) + H+(PG)(c) → Asparagine(c) + Na+(c) + H+(PG)(s)
r1143	-14	Na+(c) + Aspartate(c) ← Na+(s) + Aspartate(s)
r1125	16	Tyrosine(s) + Na+(s) → Tyrosine(c) + Na+(c)

Collagen SERPH(c) synthesis (401)

Definition			Solution													
			exchanges		reactions								transp		
Objective	Constraints	Comment	imports	exports	c	m	r	p	l	n	s	b	s-c	b-c	int
Collagen SERPH(c)	MCES AAA	test synthesis of Collagen SERPH c	27 Glutamate(s) 24 Glycine(s) 43 Alanine(s) 34 Lysine(s) 24 Aspartate(s) 21 Arginine(s) 13 Glutamine(s) 33 Serine(s) 14 Methionine(s) 4 Tryptophan(s) 16 Phenylalanine(s) 10 Tyrosine(s) 2 Cysteine(s) 54 Leucine(s) 14 Histidine(s) 15 Proline(s) 11 Asparagine(s) 28 Valine(s) 19 Threonine(s) 12 Isoleucine(s) 2090 ATP-energy(c) 13 Proton-gradient(c)	1 Collagen SERPH(c) 181 Na-gradient(c)	4	-	-	-	-	-	-	-	22	-	-

ID	flux	reaction
r2530	54	Leucine(s) ⇌ Leucine(c)
r1144	-27	Glutamate(c) + Na+(c) ← Glutamate(s) + Na+(s)
r1126	-16	Phenylalanine(c) + Na+(c) ← Phenylalanine(s) + Na+(s)
r1072	14	Methionine(s) ⇌ Methionine(c)
r2531	15	Proline(s) ⇌ Proline(c)
r1145	-14	Histidine(c) + Na+(c) ← Histidine(s) + Na+(s)
r2524	43	Alanine(s) ⇌ Alanine(c)
r2660	1	43 Alanine(c) + 2 Cysteine(c) + 24 Aspartate(c) + 27 Glutamate(c) + 16 Phenylalanine(c) + 24 Glycine(c) + 14 Histidine(c) + 12 Isoleucine(c) + 34 Lysine(c) + 54 Leucine(c) + 14 Methionine(c) + 11 Asparagine(c) + 15 Proline(c) + 13 Glutamine(c) + 21 Arginine(c) + 33 Serine(c) + 19 Threonine(c) + 28 Valine(c) + 4 Tryptophan(c) + 10 Tyrosine(c) + 1672 ATP(c) + 1672 H2O(c) → Collagen SERPH(c) + 1254 ADP(c) + 1254 Pi(c) + 418 AMP(c) + 418 PPi(c)

Continued on next page

Collagen SERPH(c) synthesis (401)– continued

ID	flux	reaction
r2534	19	Threonine(s) ⇌ Threonine(c)
r2525	13	Glutamine(s) ⇌ Glutamine(c)
r1319	-2090	ATP(c) + H2O(c) ⇌ ADP(c) + Pi(c) + ATP-energy(c)
r1058	21	Arginine(s) ⇌ Arginine(c)
r1069	-34	Lysine(c) ⇌ Lysine(s)
r0030	418	ATP(c) + AMP(c) ⇌ 2 ADP(c)
r2474	33	Serine(s) + Na+(s) → Serine(c) + Na+(c)
r2664	181	Na+(c) ⇌ Na+(s) + Na-gradient(c)
r2475	2	Cysteine(s) + Na+(s) → Cysteine(c) + Na+(c)
r1121	-28	Valine(c) + Na+(c) ← Valine(s) + Na+(s)
r2468	24	Glycine(s) + H+(PG)(s) → Glycine(c) + H+(PG)(c)
r1123	-12	Isoleucine(c) + Na+(c) ← Isoleucine(s) + Na+(s)
r0007	418	PPi(c) + H2O(c) → 2 Pi(c)
r1331	-13	H+(PG)(s) ⇌ H+(PG)(c) + Proton-gradient(c)
r1124	-4	Tryptophan(c) + Na+(c) ← Tryptophan(s) + Na+(s)
r2479	11	Asparagine(s) + Na+(s) + H+(PG)(c) → Asparagine(c) + Na+(c) + H+(PG)(s)
r1143	-24	Na+(c) + Aspartate(c) ← Na+(s) + Aspartate(s)
r1125	10	Tyrosine(s) + Na+(s) → Tyrosine(c) + Na+(c)

Collagen VWA2(c) synthesis (402)

Definition			Solution												
			exchanges		reactions								transp		
Objective	Constraints	Comment	imports	exports	c	m	r	p	l	n	s	b	s-c	b-c	int
Collagen VWA2(c)	MCES AAA	test synthesis of Collagen VWA2 c	44 Glutamate(s) 66 Glycine(s) 66 Alanine(s) 25 Lysine(s) 32 Aspartate(s) 58 Arginine(s) 32 Glutamine(s) 56 Serine(s) 12 Methionine(s) 7 Tryptophan(s) 31 Phenylalanine(s) 13 Tyrosine(s) 27 Cysteine(s) 81 Leucine(s) 17 Histidine(s) 47 Proline(s) 15 Asparagine(s) 72 Valine(s) 36 Threonine(s) 18 Isoleucine(s) 3775 ATP-energy(c) 51 Proton-gradient(c)	1 Collagen VWA2(c) 332 Na-gradient(c)	4	-	-	-	-	-	-	-	22	-	-

ID	flux	reaction
r2530	81	Leucine(s) ⇌ Leucine(c)
r1144	-44	Glutamate(c) + Na+(c) ← Glutamate(s) + Na+(s)
r1126	-31	Phenylalanine(c) + Na+(c) ← Phenylalanine(s) + Na+(s)
r1072	12	Methionine(s) ⇌ Methionine(c)
r2531	47	Proline(s) ⇌ Proline(c)
r1145	-17	Histidine(c) + Na+(c) ← Histidine(s) + Na+(s)
r2524	66	Alanine(s) ⇌ Alanine(c)
r2534	36	Threonine(s) ⇌ Threonine(c)
r2525	32	Glutamine(s) ⇌ Glutamine(c)
r1319	-3775	ATP(c) + H2O(c) ⇌ ADP(c) + Pi(c) + ATP-energy(c)
r1058	58	Arginine(s) ⇌ Arginine(c)
r2662	1	66 Alanine(c) + 27 Cysteine(c) + 32 Aspartate(c) + 44 Glutamate(c) + 31 Phenylalanine(c) + 66 Glycine(c) + 17 Histidine(c) + 18 Isoleucine(c) + 25 Lysine(c) + 81 Leucine(c) + 12 Methionine(c) + 15 Asparagine(c) + 47 Proline(c) + 32 Glutamine(c) + 58 Arginine(c) + 56 Serine(c) + 36 Threonine(c) + 72 Valine(c) + 7 Tryptophan(c) + 13 Tyrosine(c) + 3020 ATP(c) + 3020 H2O(c) → Collagen VWA2(c) + 2265 ADP(c) + 2265 Pi(c) + 755 AMP(c) + 755 PPi(c)
r1069	-25	Lysine(c) ⇌ Lysine(s)
r0030	755	ATP(c) + AMP(c) ⇌ 2 ADP(c)
r2474	56	Serine(s) + Na+(s) → Serine(c) + Na+(c)
r2664	332	Na+(c) ⇌ Na+(s) + Na-gradient(c)

Continued on next page

Collagen VWA2(c) synthesis (402)– continued

ID	flux	reaction
r2475	27	Cysteine(s) + Na+(s) → Cysteine(c) + Na+(c)
r1121	-72	Valine(c) + Na+(c) ← Valine(s) + Na+(s)
r2468	66	Glycine(s) + H+(PG)(s) → Glycine(c) + H+(PG)(c)
r1123	-18	Isoleucine(c) + Na+(c) ← Isoleucine(s) + Na+(s)
r0007	755	PPi(c) + H2O(c) → 2 Pi(c)
r1331	-51	H+(PG)(s) ⇌ H+(PG)(c) + Proton-gradient(c)
r1124	-7	Tryptophan(c) + Na+(c) ← Tryptophan(s) + Na+(s)
r2479	15	Asparagine(s) + Na+(s) + H+(PG)(c) → Asparagine(c) + Na+(c) + H+(PG)(s)
r1143	-32	Na+(c) + Aspartate(c) ← Na+(s) + Aspartate(s)
r1125	13	Tyrosine(s) + Na+(s) → Tyrosine(c) + Na+(c)

B3.2.2. Lipoproteins VLDL (403)

Definition			Solution												
			exchanges		reactions								transp		
Objective	Constraints	Comment	imports	exports	c	m	r	p	l	n	s	b	s-c	b-c	int
VLDL	MCES AAA ALFA	de novo synthesis and export of VLDL	578 Glutamate(s) 333 Glycine(s) 548 Alanine(s) 448 Lysine(s) 310 Aspartate(s) 388 Arginine(s) 454 Glutamine(s) 1060 Serine(s) 135 Methionine(s) 93 Tryptophan(s) 252 Phenylalanine(s) 179 Tyrosine(s) 6452 Glucose-6P(c) 39 Cysteine(s) 3085 Choline(c) 820 Leucine(s) 129 Histidine(s) 227 Proline(s) 254 Asparagine(s) 420 Valine(s) 384 Threonine(s) 370 Ethanolamine(c) 5800 Arachidonate(c) 12708 Palmitate(c) 299 Isoleucine(s) 4290 Farnesyl-PP(r) 15589 Oleate(c) 25.1 Stearate(c) 3107 Linoleate(c) 1949 gamma-Linolenate(c) $1.3\cdot10^5$ ATP-energy(c) 6435 NADPH-redox-potential(r) 829.9 CoA-activated acetyl group(r)	26201 H_2O(s) 11761 P_i(c) 7005 CO_2(s) 1 VLDL(s) 2145 NADH-redox-potential(r) 724 NADH-redox-potential(c) 635 NADPH-redox-potential(c) 179 Proton-gradient(c) 4115 Na-gradient(c) 5120 CoA-activated acetyl group(c)	66	-	35	-	-	-	-	-	25	-	25

ID	flux	reaction
r1210	0.09	6260 Palmitoyl-CoA(c) + 1490 Stearoyl-CoA(c) + 656 Oleoyl-CoA(c) + 147 Arachidonyl-CoA(c) + 270 Linoleoyl-CoA(c) ⇌ 8823 Acyl-CoA-VLDL-SM-pool(c)
r1211	-10385	Phosphatidate-VLDL-TG-pool(c) + CoA(c) ← Acyl-CoA-VLDL-TG2-pool(c) + 1-Acylglycerol-3P-VLDL-TG1-pool(c)
r0854	-12870	O2(r) ⇌ O2(c)
r1212	-2330	Phosphatidate-VLDL-PC-pool(c) + CoA(c) ← Acyl-CoA-VLDL-PC-pool(c) + 1-Acylglycerol-3P-VLDL-PC-pool(c)
r1069	-448	Lysine(c) ⇌ Lysine(s)
r0421	3085	CTP(c) + Phosphocholine(c) → PPi(c) + CDP-choline(c)
r1083	1	VLDL(r) → VLDL(s)
r1214	-185	Phosphatidate-VLDL-PS-pool(c) + CoA(c) ← Acyl-CoA-VLDL-PS-pool(c) + 1-Acylglycerol-3P-VLDL-PS-pool(c)
r0575	2145	Presqualene-PP(r) + NADPH(r) ⇌ PPi(r) + Squalene(r) + NADP+(r)
r1215	-1	Phosphatidate-VLDL-PI-pool(c) + CoA(c) ← 1-Acylglycerol-3P-VLDL-PI-pool(c) + Acyl-CoA-VLDL-PI-pool(c)
r0576	2145	Squalene(r) + NADPH(r) + O2(r) ⇌ H2O(r) + Squalene 2,3-oxide(r) + NADP+(r)
r1217	10385	Phosphatidate-VLDL-TG-pool(c) + H2O(c) → 1,2-Diacylglycerol-VLDL-TG-pool(c) + Pi(c)
r1218	2330	Phosphatidate-VLDL-PC-pool(c) + H2O(c) → 1,2-Diacylglycerol-VLDL-PC-pool(c) + Pi(c)
r0441	-83.2	Arachidonyl-CoA(r) + L-Carnitine(r) ⇌ CoA(r) + Arachidonyl-Carnitine(r)
r0442	83.2	Arachidonyl-CoA(c) + L-Carnitine(c) ⇌ CoA(c) + Arachidonyl-Carnitine(c)
r1237	2330	CDP-choline(c) + 1,2-Diacylglycerol-VLDL-PC-pool(c) → PC-VLDL-pool(c) + CMP(c)
r1238	1	Phosphatidate-VLDL-PI-pool(c) + CTP(c) ⇌ CDP-diacylglycerol-VLDL-PI-pool(c) + PPi(c)

Continued on next page

ID	flux	reaction
r2478	1060	Serine(s) + Na+(s) + H+(PG)(c) → Serine(c) + Na+(c) + H+(PG)(s)
r1253	50.2	Stearate(r) + ATP(r) + CoA(r) → Stearoyl-CoA(r) + AMP(r) + PPi(r)
r0311	58	Palmitate(r) + ATP(r) + CoA(r) → Palmitoyl-CoA(r) + AMP(r) + PPi(r)
r1255	367.7	ATP(r) + CoA(r) + Oleate(r) → Oleoyl-CoA(r) + AMP(r) + PPi(r)
r0312	12708	Palmitate(c) + ATP(c) + CoA(c) → Palmitoyl-CoA(c) + AMP(c) + PPi(c)
r0030	46122	ATP(c) + AMP(c) ⇌ 2 ADP(c)
r1256	15244	ATP(c) + CoA(c) + Oleate(c) → Oleoyl-CoA(c) + AMP(c) + PPi(c)
r0313	755	Serine(c) + Palmitoyl-CoA(c) ⇌ 3-Dehydrosphinganine(c) + CoA(c) + CO2(c)
r1257	45.3	Linoleate(r) + ATP(r) + CoA(r) → AMP(r) + PPi(r) + Linoleoyl-CoA(r)
r1258	3107	Linoleate(c) + ATP(c) + CoA(c) → AMP(c) + PPi(c) + Linoleoyl-CoA(c)
r0481	185	Ethanolamine-P(c) + CTP(c) → PPi(c) + CDP-ethanolamine(c)
r1273	1	PI-pool(c) ⇌ PI-pool(r)
r1121	-420	Valine(c) + Na+(c) ← Valine(s) + Na+(s)
r1122	-820	Leucine(c) + Na+(c) ← Leucine(s) + Na+(s)
r1123	-299	Isoleucine(c) + Na+(c) ← Isoleucine(s) + Na+(s)
r1124	-93	Tryptophan(c) + Na+(c) ← Tryptophan(s) + Na+(s)
r0780	-2145	4,4-Dimethyl-5alpha-cholesta-8,14,24-trien-3beta-ol(r) + NADPH(r) ⇌ 14-Demethyllanosterol(r) + NADP+(r)
r1407	755	CDP-choline(r) + Ceramide-pool(r) ⇌ CMP(r) + SM-pool(r)
r1277	165	PC-VLDL-pool(r) + H2O(r) ⇌ 2-Lysolecithin-pool(r) + Fatty-acid-VLDL-PC-pool(r)
r1125	179	Tyrosine(s) + Na+(s) → Tyrosine(c) + Na+(c)
r0781	2145	3 O2(r) + 3 NADPH(r) + Lanosterol(r) ⇌ 4,4-Dimethyl-5alpha-cholesta-8,14,24-trien-3beta-ol(r) + Formate(r) + 4 H2O(r) + 3 NADP+(r)
r0334	-2145	NADPH(r) + Desmosterol(r) ⇌ Cholesterol(r) + NADP+(r)
r1127	1	ApoB100(c) → ApoB100(r)
r0336	185	ATP(c) + Ethanolamine(c) → ADP(c) + Ethanolamine-P(c)
r1128	7	ApoE(c) → ApoE(r)
r0632	-2145	7-Dehydrodesmosterol(r) + NADPH(r) ⇌ Desmosterol(r) + NADP+(r)
r1143	-310	Na+(c) + Aspartate(c) ← Na+(s) + Aspartate(s)
r0200	-1	Glucose-6P(c) ⇌ Inositol-1P(c)
r2664	4115	Na+(c) ⇌ Na+(s) + Na-gradient(c)
r1144	-578	Glutamate(c) + Na+(c) ← Glutamate(s) + Na+(s)
r0201	-12901	DHAP(c) + NADH(c) ⇌ NAD+(c) + sn-Glycerol-3P(c)
r1145	-129	Histidine(c) + Na+(c) ← Histidine(s) + Na+(s)
r1146	2145	4alpha-Methylzymosterol(r) + 2 H2O(r) ⇌ Zymosterol(r) + CO2(r)
r2680	-829.9	Acetyl-CoA(r) ⇌ CoA(r) + CoA-activated acetyl group(r)
r2532	254	Asparagine(s) ⇌ Asparagine(c)
r1164	254.7	Palmitoyl-CoA(r) + Cholesterol(r) → CoA(r) + Cholesterol-ester-palm(r)
r2534	384	Threonine(s) ⇌ Threonine(c)
r1166	50.2	Stearoyl-CoA(r) + Cholesterol(r) → CoA(r) + Cholesterol-ester-stea(r)
r1167	367.7	Oleoyl-CoA(r) + Cholesterol(r) → CoA(r) + Cholesterol-ester-ol(r)
r1168	875.2	Cholesterol(r) + Linoleoyl-CoA(r) → CoA(r) + Cholesterol-ester-lin(r)
r2537	-4290	gamma-Linolenoyl-CoA(c) ⇌ gamma-Linolenoyl-CoA(r)
r2538	4290	Dihomo-gamma-linolenoyl-CoA(c) ⇌ Dihomo-gamma-linolenoyl-CoA(r)
r1465	3096	2 H2O(c) + NAD+(c) + Oleoyl-CoA(c) ⇌ NADH(c) + O2(c) + Stearoyl-CoA(c)
r0227	2145	10-Formyl-THF(c) + H2O(c) + NADP+(c) → THF(c) + CO2(c) + NADPH(c)
r1466	-4290	Malonyl-CoA(r) + 4 NADPH(r) + O2(r) + gamma-Linolenoyl-CoA(r) ⇌ CO2(r) + CoA(r) + Dihomo-gamma-linolenoyl-CoA(r) + 3 H2O(r) + 4 NADP+(r)
r0228	2145	THF(c) + Formate(c) + ATP(c) → ADP(c) + Pi(c) + 10-Formyl-THF(c)
r1185	8060	Palmitoyl-CoA(c) + sn-Glycerol-3P(c) → CoA(c) + 1-Acylglycerol-3P-palm(c)
r0243	6451	GAP(c) ⇌ DHAP(c)
r1469	-6239	Linoleoyl-CoA(c) + NADH(c) + O2(c) ⇌ gamma-Linolenoyl-CoA(c) + H2O(c) + NAD+(c)
r1187	1394	Stearoyl-CoA(c) + sn-Glycerol-3P(c) → CoA(c) + 1-Acylglycerol-3P-stea(c)
r0244	3085	ATP(c) + Choline(c) → ADP(c) + Phosphocholine(c)
r1331	179	H+(PG)(s) ⇌ H+(PG)(c) + Proton-gradient(c)
r1318	829.9	Linoleoyl-CoA(c) ⇌ Linoleoyl-CoA(r)
r1188	1697	Oleoyl-CoA(c) + sn-Glycerol-3P(c) → 1-Acylglycerol-3P-ol(c) + CoA(c)

Continued on next page

ID	flux	reaction
r1319	$-1.3\cdot10^5$	ATP(c) + H2O(c) ⇌ ADP(c) + Pi(c) + ATP-energy(c)
r1189	1476	Linoleoyl-CoA(c) + sn-Glycerol-3P(c) → 1-Acylglycerol-3P-lin(c) + CoA(c)
r1201	0.02	332 1-Acylglycerol-3P-ol(c) + 181 1-Acylglycerol-3P-lin(c) + 2050 1-Acylglycerol-3P-arach(c) + 345 1-Acylglycerol-3P-palm(c) + 4820 1-Acylglycerol-3P-stea(c) → 7728 1-Acylglycerol-3P-VLDL-PS-pool(c)
r1058	388	Arginine(s) ⇌ Arginine(c)
r1202	0.0001	1360 1-Acylglycerol-3P-ol(c) + 694 1-Acylglycerol-3P-lin(c) + 2170 1-Acylglycerol-3P-arach(c) + 694 1-Acylglycerol-3P-palm(c) + 3640 1-Acylglycerol-3P-stea(c) → 8558 1-Acylglycerol-3P-VLDL-PI-pool(c)
r1072	135	Methionine(s) ⇌ Methionine(c)
r0998	-196.6	L-Carnitine(c) + L-Palmitoylcarnitine(r) ⇌ L-Carnitine(r) + L-Palmitoylcarnitine(c)
r1204	1.13	1200 Palmitoyl-CoA(c) + 384 Stearoyl-CoA(c) + 4260 Oleoyl-CoA(c) + 521 Arachidonyl-CoA(c) + 2810 Linoleoyl-CoA(c) → 9175 Acyl-CoA-VLDL-TG2-pool(c)
r1370	-185	PE-PS-VLDL-pool(c) + CO2(c) → PS-VLDL-pool(c)
r1205	1.15	849 Palmitoyl-CoA(c) + 584 Stearoyl-CoA(c) + 4570 Oleoyl-CoA(c) + 264 Arachidonyl-CoA(c) + 2780 Linoleoyl-CoA(c) → 9047 Acyl-CoA-VLDL-TG3-pool(c)
r0848	6270	H2O(r) ⇌ H2O(c)
r1206	0.27	3010 Palmitoyl-CoA(c) + 1300 Stearoyl-CoA(c) + 1170 Oleoyl-CoA(c) + 726 Arachidonyl-CoA(c) + 2350 Linoleoyl-CoA(c) → 8556 Acyl-CoA-VLDL-PC-pool(c)
r0849	-26201	H2O(s) ⇌ H2O(c)
r1220	185	Phosphatidate-VLDL-PS-pool(c) + H2O(c) → 1,2-Diacylglycerol-VLDL-PS-pool(c) + Pi(c)
r0711	-2145	5alpha-Cholesta-7,24-dien-3beta-ol(r) + NADPH(r) + O2(r) ⇌ 7-Dehydrodesmosterol(r) + 2 H2O(r) + NADP+(r)
r1208	0.02	345 Palmitoyl-CoA(c) + 4820 Stearoyl-CoA(c) + 332 Oleoyl-CoA(c) + 2050 Arachidonyl-CoA(c) + 181 Linoleoyl-CoA(c) → 7728 Acyl-CoA-VLDL-PS-pool(c)
r0430	4290	Malonyl-CoA(r) + L-Carnitine(r) ⇌ CoA(r) + Malonyl-Carnitin(r)
r1209	0.0001	694 Palmitoyl-CoA(c) + 3640 Stearoyl-CoA(c) + 1360 Oleoyl-CoA(c) + 2170 Arachidonyl-CoA(c) + 694 Linoleoyl-CoA(c) → 8558 Acyl-CoA-VLDL-PI-pool(c)
r0431	-4290	Malonyl-CoA(c) + L-Carnitine(c) ⇌ CoA(c) + Malonyl-Carnitin(c)
r0583	-755	3-Dehydrosphinganine(c) + NADPH(c) ⇌ Sphinganine(c) + NADP+(c)
r0136	3271	ATP(c) + CMP(c) ⇌ ADP(c) + CDP(c)
r1223	-10385	Acyl-CoA-VLDL-TG3-pool(c) + 1,2-Diacylglycerol-VLDL-TG-pool(c) → Triacylglycerol-VLDL-pool(c) + CoA(c)
r0150	3271	ATP(c) + CDP(c) ⇌ ADP(c) + CTP(c)
r0289	1	Inositol-1P(c) + H2O(c) → Inositol(c) + Pi(c)
r0867	7005	CO2(c) ⇌ CO2(s)
r1095	1	275 Alanine(c) + 150 Arginine(c) + 25 Cysteine(c) + 207 Glycine(c) + 115 Histidine(c) + 285 Isoleucine(c) + 533 Leucine(c) + 79 Methionine(c) + 224 Phenylalanine(c) + 171 Proline(c) + 392 Serine(c) + 300 Threonine(c) + 37 Tryptophan(c) + 151 Tyrosine(c) + 252 Valine(c) + 230 Glutamine(c) + 298 Glutamate(c) + 18244 ATP(c) + 18244 H2O(c) + 247 Asparagine(c) + 233 Aspartate(c) + 357 Lysine(c) → ApoB100(c) + 4561 AMP(c) + 4561 PPi(c) + 13683 ADP(c) + 13683 Pi(c)
r0434	-196.6	Palmitoyl-CoA(r) + L-Carnitine(r) ⇌ CoA(r) + L-Palmitoylcarnitine(r)
r0435	196.6	Palmitoyl-CoA(c) + L-Carnitine(c) ⇌ CoA(c) + L-Palmitoylcarnitine(c)
r1240	755	Ceramide-pool(c) ⇌ Ceramide-pool(r)
r2466	-829.9	Acetyl-CoA(c) ⇌ Acetyl-CoA(r)
r2468	333	Glycine(s) + H+(PG)(s) → Glycine(c) + H+(PG)(c)
r0886	-755	CMP(c) ⇌ CMP(r)
r0170	2145	2 Farnesyl-PP(r) ⇌ PPi(r) + Presqualene-PP(r)
r2469	548	Alanine(s) + H+(PG)(s) → Alanine(c) + H+(PG)(c)
r1244	-0.02	3010 Palmitate(r) + 1300 Stearate(r) + 2350 Linoleate(r) + 726 Arachidonate(r) + 1170 Oleate(r) ⇌ 8556 Fatty-acid-VLDL-PC-pool(r)
r0887	-2145	Formate(c) ⇌ Formate(r)
r0007	53683	PPi(c) + H2O(c) → 2 Pi(c)
r0736	6451	ATP(c) + Fructose-6P(c) → ADP(c) + Fructose-1,6PP(c)
r0738	2145	Zymosterol(r) ⇌ 5alpha-Cholesta-7,24-dien-3beta-ol(r)
r1261	1949	gamma-Linolenate(c) + ATP(c) + CoA(c) → gamma-Linolenoyl-CoA(c) + AMP(c) + PPi(c)
r1262	14	Arachidonate(r) + ATP(r) + CoA(r) → Arachidonyl-CoA(r) + AMP(r) + PPi(r)
r1263	5800	Arachidonate(c) + ATP(c) + CoA(c) → Arachidonyl-CoA(c) + AMP(c) + PPi(c)
r1264	10385	Triacylglycerol-VLDL-pool(c) ⇌ Triacylglycerol-VLDL-pool(r)
r1112	7	39 Alanine(c) + 34 Arginine(c) + 2 Cysteine(c) + 32 Glutamine(c) + 40 Glutamate(c) + 18 Glycine(c) + 2 Histidine(c) + 2 Isoleucine(c) + 41 Leucine(c) + 8 Methionine(c) + 4 Phenylalanine(c) + 8 Proline(c) + 14 Serine(c) + 12 Threonine(c) + 8 Tryptophan(c) + 4 Tyrosine(c) + 24 Valine(c) + 1268 ATP(c) + 1268 H2O(c) + Asparagine(c) + 11 Aspartate(c) + 13 Lysine(c) → ApoE(c) + 317 AMP(c) + 317 PPi(c) + 951 ADP(c) + 951 Pi(c)

Continued on next page

ID	flux	reaction
r0192	6451	Glucose-6P(c) ⇌ Fructose-6P(c)
r1266	2330	PC-VLDL-pool(c) ⇌ PC-VLDL-pool(r)
r1280	-1	VLDL(r) ← 7 ApoE(r) + 10385 Triacylglycerol-VLDL-pool(r) + 2165 PC-VLDL-pool(r) + 1645 Cholesterol-ester-pool(r) + PI-pool(r) + 165 2-Lysolecithin-pool(r) + 185 PE-VLDL-pool(r) + ApoB100(r) + 500 Cholesterol(r) + 755 SM-pool(r)
r1268	185	PE-VLDL-pool(c) ⇌ PE-VLDL-pool(r)
r0607	2145	Squalene 2,3-oxide(r) ⇌ Lanosterol(r)
r1412	755	Sphinganine(c) + Acyl-CoA-VLDL-SM-pool(c) ⇌ Dihydroceramide-pool(c) + CoA(c)
r1414	755	Dihydroceramide-pool(c) + O2(c) + NADPH(c) ⇌ 2 H2O(c) + Ceramide-pool(c) + NADP+(c)
r1134	2145	14-Demethyllanosterol(r) + 3 NADPH(r) + 3 O2(r) ⇌ 4 H2O(r) + 4alpha-Methylzymosterol-4-carboxylate(r) + 3 NADP+(r)
r1135	2145	3-Keto-4-methylzymosterol(r) + NADP+(r) ⇌ NADPH(r) + 4alpha-Methylzymosterol(r)
r0063	4290	Malonyl-CoA(c) → Acetyl-CoA(c) + CO2(c)
r1137	2145	NAD+(r) + 4alpha-Methylzymosterol-4-carboxylate(r) ⇌ NADH(r) + CO2(r) + 3-Keto-4-methylzymosterol(r)
r1139	-227	Proline(c) + Na+(c) ← Proline(s) + Na+(s)
r1153	-4825	PPi(c) ⇌ PPi(r)
r1004	4290	L-Carnitine(c) + Malonyl-Carnitin(r) ⇌ L-Carnitine(r) + Malonyl-Carnitin(c)
r2677	5120	Acetyl-CoA(c) ⇌ CoA(c) + CoA-activated acetyl group(c)
r2525	454	Glutamine(s) ⇌ Glutamine(c)
r1301	345.1	Oleate(l) → Oleate(r)
r1171	97.2	Arachidonyl-CoA(r) + Cholesterol(r) → CoA(r) + Cholesterol-ester-arach(r)
r1302	-345.1	Oleate(l) ⇌ Oleate(c)
r1159	755	CDP-choline(c) ⇌ CDP-choline(r)
r1007	-83.2	L-Carnitine(c) + Arachidonyl-Carnitine(r) ⇌ L-Carnitine(r) + Arachidonyl-Carnitine(c)
r2528	252	Phenylalanine(s) ⇌ Phenylalanine(c)
r2529	39	Cysteine(s) ⇌ Cysteine(c)
r1470	-4290	Dihomo-gamma-linolenoyl-CoA(c) + NADH(c) + O2(c) ⇌ Arachidonyl-CoA(c) + H2O(c) + NAD+(c)
r1190	274	Arachidonyl-CoA(c) + sn-Glycerol-3P(c) → 1-Acylglycerol-3P-arach(c) + CoA(c)
r0811	-535.3	ATP(r) + AMP(c) ⇌ ATP(c) + AMP(r)
r1321	2145	NADH(r) ⇌ NAD+(r) + NADH-redox-potential(r)
r1191	-185	PS-VLDL-pool(c) + Ethanolamine(c) ⇌ PE-VLDL-pool(c) + Serine(c)
r1322	724	NADH(c) ⇌ NAD+(c) + NADH-redox-potential(c)
r1192	0.18	2050 Cholesterol-ester-ol(r) + 4880 Cholesterol-ester-lin(r) + 1420 Cholesterol-ester-palm(r) + 280 Cholesterol-ester-stea(r) + 542 Cholesterol-ester-arach(r) ⇌ 9172 Cholesterol-ester-pool(r)
r1325	-6435	NADPH(r) ⇌ NADPH-redox-potential(r) + NADP+(r)
r1195	185	1,2-Diacylglycerol-VLDL-PS-pool(c) + CDP-ethanolamine(c) → PE-PS-VLDL-pool(c) + CMP(c)
r1326	635	NADPH(c) ⇌ NADPH-redox-potential(c) + NADP+(c)
r1197	1	CDP-diacylglycerol-VLDL-PI-pool(c) + Inositol(c) ⇌ PI-pool(c) + CMP(c)
r0983	25.1	Stearate(l) → Stearate(r)
r1198	1.12	1220 1-Acylglycerol-3P-ol(c) + 741 1-Acylglycerol-3P-lin(c) + 24 1-Acylglycerol-3P-arach(c) + 6440 1-Acylglycerol-3P-palm(c) + 823 1-Acylglycerol-3P-stea(c) → 9248 1-Acylglycerol-3P-VLDL-TG1-pool(c)
r0984	-25.1	Stearate(l) ⇌ Stearate(c)
r1199	0.27	1170 1-Acylglycerol-3P-ol(c) + 2350 1-Acylglycerol-3P-lin(c) + 726 1-Acylglycerol-3P-arach(c) + 3010 1-Acylglycerol-3P-palm(c) + 1300 1-Acylglycerol-3P-stea(c) → 8556 1-Acylglycerol-3P-VLDL-PC-pool(c)
r0256	6451	Fructose-1,6PP(c) ⇌ DHAP(c) + GAP(c)

HDL (404)

Definition			Solution												
			exchanges		reactions								transp		
Objective	Constraints	Comment	imports	exports	c	m	r	p	l	n	s	b	s-c	b-c	int
HDL	MCES AAA ALFA	de novo synthesis and export of HDL	67.5 P_i(c) 60 Glutamate(s) 22 Glycine(s) 46 Alanine(s) 44 Lysine(s) 32 Aspartate(s) 34 Arginine(s) 38 Glutamine(s) 82 Serine(s) 8 Methionine(s) 10 Tryptophan(s) 16 Phenylalanine(s) 14 Tyrosine(s) 72.5 Glucose-6P(c) 165 Choline(c) 82 Leucine(s) 12 Histidine(s) 20 Proline(s) 10 Asparagine(s) 30 Valine(s) 24 Threonine(s) 80 Ethanolamine(c) 85.7 Arachidonate(c) 196.4 Palmitate(c) 40 Farnesyl-PP(r) 143.5 Oleate(c) 7.93 Linoleate(c) 6.38 gamma-Linolenate(c) 4303 ATP-energy(c) 60 NADPH-redox-potential(r) 130 NADPH-redox-potential(c)	673.9 H_2O(s) 80 CO_2(s) 1 HDL(s) 20 NADH-redox-potential(r) 50 NADH-redox-potential(c) 14 Proton-gradient(c) 260 Na-gradient(c) 40 CoA-activated acetyl group(c)	51	-	19	-	-	-	-	-	23	-	14

ID	flux	reaction
r1210	0.01	6260 Palmitoyl-CoA(c) + 1490 Stearoyl-CoA(c) + 656 Oleoyl-CoA(c) + 147 Arachidonyl-CoA(c) + 270 Linoleoyl-CoA(c) ⇌ 8823 Acyl-CoA-VLDL-SM-pool(c)
r0854	-120	O2(r) ⇌ O2(c)
r1212	-90	Phosphatidate-VLDL-PC-pool(c) + CoA(c) ← Acyl-CoA-VLDL-PC-pool(c) + 1-Acylglycerol-3P-VLDL-PC-pool(c)
r1069	-44	Lysine(c) ⇌ Lysine(s)
r0421	165	CTP(c) + Phosphocholine(c) → PPi(c) + CDP-choline(c)
r1214	-55	Phosphatidate-VLDL-PS-pool(c) + CoA(c) ← Acyl-CoA-VLDL-PS-pool(c) + 1-Acylglycerol-3P-VLDL-PS-pool(c)
r0575	20	Presqualene-PP(r) + NADPH(r) ⇌ PPi(r) + Squalene(r) + NADP+(r)
r0576	20	Squalene(r) + NADPH(r) + O2(r) ⇌ H2O(r) + Squalene 2,3-oxide(r) + NADP+(r)
r1218	90	Phosphatidate-VLDL-PC-pool(c) + H2O(c) → 1,2-Diacylglycerol-VLDL-PC-pool(c) + Pi(c)
r1237	90	CDP-choline(c) + 1,2-Diacylglycerol-VLDL-PC-pool(c) → PC-VLDL-pool(c) + CMP(c)
r2478	82	Serine(s) + Na+(s) + H+(PG)(c) → Serine(c) + Na+(c) + H+(PG)(s)
r0312	196.4	Palmitate(c) + ATP(c) + CoA(c) → Palmitoyl-CoA(c) + AMP(c) + PPi(c)
r0030	974	ATP(c) + AMP(c) ⇌ 2 ADP(c)
r1256	143.5	ATP(c) + CoA(c) + Oleate(c) → Oleoyl-CoA(c) + AMP(c) + PPi(c)
r0313	75	Serine(c) + Palmitoyl-CoA(c) ⇌ 3-Dehydrosphinganine(c) + CoA(c) + CO2(c)
r1271	30	PS-VLDL-pool(c) ⇌ PS-VLDL-pool(r)
r1258	7.93	Linoleate(c) + ATP(c) + CoA(c) → AMP(c) + PPi(c) + Linoleoyl-CoA(c)
r0481	55	Ethanolamine-P(c) + CTP(c) → PPi(c) + CDP-ethanolamine(c)
r1121	-30	Valine(c) + Na+(c) ← Valine(s) + Na+(s)
r1124	-10	Tryptophan(c) + Na+(c) ← Tryptophan(s) + Na+(s)
r0780	-20	4,4-Dimethyl-5alpha-cholesta-8,14,24-trien-3beta-ol(r) + NADPH(r) ⇌ 14-Demethyllanosterol(r) + NADP+(r)
r1407	75	CDP-choline(r) + Ceramide-pool(r) ⇌ CMP(r) + SM-pool(r)
r1125	14	Tyrosine(s) + Na+(s) → Tyrosine(c) + Na+(c)
r0781	20	3 O2(r) + 3 NADPH(r) + Lanosterol(r) ⇌ 4,4-Dimethyl-5alpha-cholesta-8,14,24-trien-3beta-ol(r) + Formate(r) + 4 H2O(r) + 3 NADP+(r)
r0334	-20	NADPH(r) + Desmosterol(r) ⇌ Cholesterol(r) + NADP+(r)
r0336	55	ATP(c) + Ethanolamine(c) → ADP(c) + Ethanolamine-P(c)
r0632	-20	7-Dehydrodesmosterol(r) + NADPH(r) ⇌ Desmosterol(r) + NADP+(r)
r1129	2	ApoA1(c) → ApoA1(r)
r1143	-32	Na+(c) + Aspartate(c) ← Na+(s) + Aspartate(s)
r2664	260	Na+(c) ⇌ Na+(s) + Na-gradient(c)
r1144	-60	Glutamate(c) + Na+(c) ← Glutamate(s) + Na+(s)
r0201	-145	DHAP(c) + NADH(c) ⇌ NAD+(c) + sn-Glycerol-3P(c)
r1145	-12	Histidine(c) + Na+(c) ← Histidine(s) + Na+(s)
r1146	20	4alpha-Methylzymosterol(r) + 2 H2O(r) ⇌ Zymosterol(r) + CO2(r)
r1160	1	HDL(r) → HDL(s)

Continued on next page

ID	flux	reaction
r2530	82	Leucine(s) ⇌ Leucine(c)
r2532	10	Asparagine(s) ⇌ Asparagine(c)
r2534	24	Threonine(s) ⇌ Threonine(c)
r2537	-40	gamma-Linolenoyl-CoA(c) ⇌ gamma-Linolenoyl-CoA(r)
r2538	40	Dihomo-gamma-linolenoyl-CoA(c) ⇌ Dihomo-gamma-linolenoyl-CoA(r)
r1465	108.6	2 H2O(c) + NAD+(c) + Oleoyl-CoA(c) ⇌ NADH(c) + O2(c) + Stearoyl-CoA(c)
r0227	20	10-Formyl-THF(c) + H2O(c) + NADP+(c) → THF(c) + CO2(c) + NADPH(c)
r1466	-40	Malonyl-CoA(r) + 4 NADPH(r) + O2(r) + gamma-Linolenoyl-CoA(r) ⇌ CO2(r) + CoA(r) + Dihomo-gamma-linolenoyl-CoA(r) + 3 H2O(r) + 4 NADP+(r)
r0228	20	THF(c) + Formate(c) + ATP(c) → ADP(c) + Pi(c) + 10-Formyl-THF(c)
r1185	34.1	Palmitoyl-CoA(c) + sn-Glycerol-3P(c) → CoA(c) + 1-Acylglycerol-3P-palm(c)
r0243	72.5	GAP(c) ⇌ DHAP(c)
r1469	-46.4	Linoleoyl-CoA(c) + NADH(c) + O2(c) ⇌ gamma-Linolenoyl-CoA(c) + H2O(c) + NAD+(c)
r1187	48	Stearoyl-CoA(c) + sn-Glycerol-3P(c) → CoA(c) + 1-Acylglycerol-3P-stea(c)
r0244	165	ATP(c) + Choline(c) → ADP(c) + Phosphocholine(c)
r1331	14	H+(PG)(s) ⇌ H+(PG)(c) + Proton-gradient(c)
r1188	14.7	Oleoyl-CoA(c) + sn-Glycerol-3P(c) → 1-Acylglycerol-3P-ol(c) + CoA(c)
r1319	-4303	ATP(c) + H2O(c) ⇌ ADP(c) + Pi(c) + ATP-energy(c)
r1189	26	Linoleoyl-CoA(c) + sn-Glycerol-3P(c) → 1-Acylglycerol-3P-lin(c) + CoA(c)
r1201	0.01	332 1-Acylglycerol-3P-ol(c) + 181 1-Acylglycerol-3P-lin(c) + 2050 1-Acylglycerol-3P-arach(c) + 345 1-Acylglycerol-3P-palm(c) + 4820 1-Acylglycerol-3P-stea(c) → 7728 1-Acylglycerol-3P-VLDL-PS-pool(c)
r1058	34	Arginine(s) ⇌ Arginine(c)
r1072	8	Methionine(s) ⇌ Methionine(c)
r1370	-55	PE-PS-VLDL-pool(c) + CO2(c) → PS-VLDL-pool(c)
r0848	60	H2O(r) ⇌ H2O(c)
r1206	0.01	3010 Palmitoyl-CoA(c) + 1300 Stearoyl-CoA(c) + 1170 Oleoyl-CoA(c) + 726 Arachidonyl-CoA(c) + 2350 Linoleoyl-CoA(c) → 8556 Acyl-CoA-VLDL-PC-pool(c)
r0849	-673.9	H2O(s) ⇌ H2O(c)
r1220	55	Phosphatidate-VLDL-PS-pool(c) + H2O(c) → 1,2-Diacylglycerol-VLDL-PS-pool(c) + Pi(c)
r0711	-20	5alpha-Cholesta-7,24-dien-3beta-ol(r) + NADPH(r) + O2(r) ⇌ 7-Dehydrodesmosterol(r) + 2 H2O(r) + NADP+(r)
r1208	0.01	345 Palmitoyl-CoA(c) + 4820 Stearoyl-CoA(c) + 332 Oleoyl-CoA(c) + 2050 Arachidonyl-CoA(c) + 181 Linoleoyl-CoA(c) → 7728 Acyl-CoA-VLDL-PS-pool(c)
r0430	40	Malonyl-CoA(r) + L-Carnitine(r) ⇌ CoA(r) + Malonyl-Carnitin(r)
r0431	-40	Malonyl-CoA(c) + L-Carnitine(c) ⇌ CoA(c) + Malonyl-Carnitin(c)
r0583	-75	3-Dehydrosphinganine(c) + NADPH(c) ⇌ Sphinganine(c) + NADP+(c)
r0136	220	ATP(c) + CMP(c) ⇌ ADP(c) + CDP(c)
r0150	220	ATP(c) + CDP(c) ⇌ ADP(c) + CTP(c)
r0867	80	CO2(c) ⇌ CO2(s)
r1240	75	Ceramide-pool(c) ⇌ Ceramide-pool(r)
r0003	-1	HDL(r) ← 2 ApoA1(r) + 90 PC-VLDL-pool(r) + 30 PS-VLDL-pool(r) + 20 Cholesterol(r) + 25 PE-VLDL-pool(r) + 75 SM-pool(r)
r2468	22	Glycine(s) + H+(PG)(s) → Glycine(c) + H+(PG)(c)
r0886	-75	CMP(c) ⇌ CMP(r)
r0170	20	2 Farnesyl-PP(r) ⇌ PPi(r) + Presqualene-PP(r)
r2469	46	Alanine(s) + H+(PG)(s) → Alanine(c) + H+(PG)(c)
r0887	-20	Formate(c) ⇌ Formate(r)
r0007	1234	PPi(c) + H2O(c) → 2 Pi(c)
r0736	72.5	ATP(c) + Fructose-6P(c) → ADP(c) + Fructose-1,6PP(c)
r0738	20	Zymosterol(r) ⇌ 5alpha-Cholesta-7,24-dien-3beta-ol(r)
r1261	6.38	gamma-Linolenate(c) + ATP(c) + CoA(c) → gamma-Linolenoyl-CoA(c) + AMP(c) + PPi(c)
r1263	85.7	Arachidonate(c) + ATP(c) + CoA(c) → Arachidonyl-CoA(c) + AMP(c) + PPi(c)
r1113	2	23 Alanine(c) + 17 Arginine(c) + 19 Glutamine(c) + 30 Glutamate(c) + 11 Glycine(c) + 6 Histidine(c) + 41 Leucine(c) + 4 Methionine(c) + 8 Phenylalanine(c) + 10 Proline(c) + 16 Serine(c) + 12 Threonine(c) + 5 Tryptophan(c) + 7 Tyrosine(c) + 15 Valine(c) + 1068 ATP(c) + 1068 H2O(c) + 5 Asparagine(c) + 16 Aspartate(c) + 22 Lysine(c) → ApoA1(c) + 267 AMP(c) + 267 PPi(c) + 801 ADP(c) + 801 Pi(c)
r0192	72.5	Glucose-6P(c) ⇌ Fructose-6P(c)
r1266	90	PC-VLDL-pool(c) ⇌ PC-VLDL-pool(r)

Continued on next page

ID	flux	reaction
r1268	25	PE-VLDL-pool(c) ⇌ PE-VLDL-pool(r)
r0607	20	Squalene 2,3-oxide(r) ⇌ Lanosterol(r)
r1412	75	Sphinganine(c) + Acyl-CoA-VLDL-SM-pool(c) ⇌ Dihydroceramide-pool(c) + CoA(c)
r1414	75	Dihydroceramide-pool(c) + O2(c) + NADPH(c) ⇌ 2 H2O(c) + Ceramide-pool(c) + NADP+(c)
r1134	20	14-Demethyllanosterol(r) + 3 NADPH(r) + 3 O2(r) ⇌ 4 H2O(r) + 4alpha-Methylzymosterol-4-carboxylate(r) + 3 NADP+(r)
r1135	20	3-Keto-4-methylzymosterol(r) + NADP+(r) ⇌ NADPH(r) + 4alpha-Methylzymosterol(r)
r0063	40	Malonyl-CoA(c) → Acetyl-CoA(c) + CO2(c)
r1137	20	NAD+(r) + 4alpha-Methylzymosterol-4-carboxylate(r) ⇌ NADH(r) + CO2(r) + 3-Keto-4-methylzymosterol(r)
r1139	-20	Proline(c) + Na+(c) ← Proline(s) + Na+(s)
r1153	-40	PPi(c) ⇌ PPi(r)
r1004	40	L-Carnitine(c) + Malonyl-Carnitin(r) ⇌ L-Carnitine(r) + Malonyl-Carnitin(c)
r2677	40	Acetyl-CoA(c) ⇌ CoA(c) + CoA-activated acetyl group(c)
r2525	38	Glutamine(s) ⇌ Glutamine(c)
r1159	75	CDP-choline(c) ⇌ CDP-choline(r)
r2528	16	Phenylalanine(s) ⇌ Phenylalanine(c)
r1470	-40	Dihomo-gamma-linolenoyl-CoA(c) + NADH(c) + O2(c) ⇌ Arachidonyl-CoA(c) + H2O(c) + NAD+(c)
r1190	22.2	Arachidonyl-CoA(c) + sn-Glycerol-3P(c) → 1-Acylglycerol-3P-arach(c) + CoA(c)
r1321	20	NADH(r) ⇌ NAD+(r) + NADH-redox-potential(r)
r1191	-25	PS-VLDL-pool(c) + Ethanolamine(c) ⇌ PE-VLDL-pool(c) + Serine(c)
r1322	50	NADH(c) ⇌ NAD+(c) + NADH-redox-potential(c)
r1325	-60	NADPH(r) ⇌ NADPH-redox-potential(r) + NADP+(r)
r1195	55	1,2-Diacylglycerol-VLDL-PS-pool(c) + CDP-ethanolamine(c) → PE-PS-VLDL-pool(c) + CMP(c)
r1326	-130	NADPH(c) ⇌ NADPH-redox-potential(c) + NADP+(c)
r1199	0.01	1170 1-Acylglycerol-3P-ol(c) + 2350 1-Acylglycerol-3P-lin(c) + 726 1-Acylglycerol-3P-arach(c) + 3010 1-Acylglycerol-3P-palm(c) + 1300 1-Acylglycerol-3P-stea(c) → 8556 1-Acylglycerol-3P-VLDL-PC-pool(c)
r0256	72.5	Fructose-1,6PP(c) ⇌ DHAP(c) + GAP(c)

B3.2.3. Ketone bodies Acetoacetate (405)

Definition			Solution											
			exchanges		reactions								transp		
Objective	Constraints	Comment	imports	exports	c	m	r	p	l	n	s	b	s-c	b-c	int
Acetoacetate	MCES	de novo synthesis and export of Acetoacetate from Palmitate, excreting CO_2	1 H_2O(s) 1 Pyruvate(c) 2 CoA-activated acetyl group(m)	1 Pyruvate(m) 1 Acetoacetate(s) 1 Proton-gradient(c)	-	4	-	-	-	-	-	-	3	-	2

ID	flux	reaction
r2678	-2	Acetyl-CoA(m) ⇌ CoA(m) + CoA-activated acetyl group(m)
r0462	-1	Acetyl-CoA(m) + H2O(m) + Acetoacetyl-CoA(m) ⇌ HMG-CoA(m) + CoA(m)
r0851	-1	H2O(m) ⇌ H2O(c)
r1464	-1	Pyruvate(m) + Acetoacetate(c) ⇌ Pyruvate(c) + Acetoacetate(m)
r0919	1	H+(PG)(c) + Acetoacetate(c) ⇌ H+(PG)(s) + Acetoacetate(s)
r0322	1	HMG-CoA(m) ⇌ Acetyl-CoA(m) + Acetoacetate(m)
r0070	1	2 Acetyl-CoA(m) ⇌ CoA(m) + Acetoacetyl-CoA(m)
r1331	1	H+(PG)(s) ⇌ H+(PG)(c) + Proton-gradient(c)
r0849	1	H2O(s) ⇌ H2O(c)

(R)-3-Hydroxybutanoate (406)

Definition			Solution												
			exchanges		reactions								transp		
Objective	Constraints	Comment	imports	exports	c	m	r	p	l	n	s	b	s-c	b-c	int
(R)-3-Hydroxy-butanoate(c)	MCES	de novo synthesis and export of (R)-3-Hydroxybutanoate from Palmitate, excreting CO_2	1 H_2O(s) 1 NADH-redox-potential(m) 1 Proton-gradient(m) 2 CoA-activated acetyl group(m)	1 (R)-3-Hydroxybutanoate(c)	-	6	-	-	-	-	-	-	1	-	3

ID	flux	reaction
r2678	-2	Acetyl-CoA(m) ⇌ CoA(m) + CoA-activated acetyl group(m)
r0462	-1	Acetyl-CoA(m) + H2O(m) + Acetoacetyl-CoA(m) ⇌ HMG-CoA(m) + CoA(m)
r1463	1	(R)-3-Hydroxybutanoate(m) + H+(PG)(c) → (R)-3-Hydroxybutanoate(c) + H+(PG)(m)
r0851	-1	H2O(m) ⇌ H2O(c)
r1330	-1	H+(PG)(c) ⇌ H+(PG)(m) + Proton-gradient(m)
r0322	1	HMG-CoA(m) ⇌ Acetyl-CoA(m) + Acetoacetate(m)
r0070	1	2 Acetyl-CoA(m) ⇌ CoA(m) + Acetoacetyl-CoA(m)
r0849	1	H2O(s) ⇌ H2O(c)
r0323	-1	NADH(m) + Acetoacetate(m) ⇌ NAD+(m) + (R)-3-Hydroxybutanoate(m)
r1323	-1	NADH(m) ⇌ NAD+(m) + NADH-redox-potential(m)

Acetone (407)

Definition			Solution												
			exchanges		reactions								transp		
Objective	Constraints	Comment	imports	exports	c	m	r	p	l	n	s	b	s-c	b-c	int
Acetone	MCES	de novo synthesis and export of Acetone	1 H_2O(s) 2 CoA-activated acetyl group(m)	1 CO_2(s) 1 Acetone(s)	-	5	-	-	-	-	-	-	3	-	2

ID	flux	reaction
r2678	-2	Acetyl-CoA(m) ⇌ CoA(m) + CoA-activated acetyl group(m)
r0325	1	Acetoacetate(m) ⇌ CO2(m) + Acetone(m)
r0462	-1	Acetyl-CoA(m) + H2O(m) + Acetoacetyl-CoA(m) ⇌ HMG-CoA(m) + CoA(m)
r0851	-1	H2O(m) ⇌ H2O(c)
r0927	1	Acetone(m) ⇌ Acetone(s)
r0322	1	HMG-CoA(m) ⇌ Acetyl-CoA(m) + Acetoacetate(m)
r0070	1	2 Acetyl-CoA(m) ⇌ CoA(m) + Acetoacetyl-CoA(m)
r0867	1	CO2(c) ⇌ CO2(s)
r0849	1	H2O(s) ⇌ H2O(c)
r0868	-1	CO2(c) ⇌ CO2(m)

B3.2.4. Other Heme (408)

Definition			Solution												
			exchanges		reactions								transp		
Objective	Constraints	Comment	imports	exports	c	m	r	p	l	n	s	b	s-c	b-c	int
Heme(c)	MCES AAA $-Fe^{2+}$	de novo synthesis of cytosolic Heme	2.5 O_2(s) 4 Pyruvate(m) 10.5 Glutamate(s) 4 Serine(s) 1.5 Proline(s) 1 Fe^{2+}(s) 4 ATP-energy(c) 1 Proton-gradient(c) 2 Proton-gradient(m)	14 H_2O(s) 14 CO_2(s) 1 Heme(c) 4 Aspartate(s) 4 Glutamine(s) 8 NADH-redox-potential(m) 10.5 Na-gradient(c)	7	14	-	-	-	-	-	-	10	-	12

ID	flux	reaction
r0855	2.5	O2(s) ⇌ O2(c)
r0856	-1.5	O2(m) ⇌ O2(c)
r0295	-4	Glycine(m) + THF(m) + NAD+(m) ← 5,10-Methylene-THF(m) + CO2(m) + NADH(m) + NH3(m)
r0875	2.5	Glutamate(c) + H+(PG)(c) → Glutamate(m) + H+(PG)(m)
r0876	4	Glutamate(c) + H+(PG)(c) + Aspartate(m) → Glutamate(m) + H+(PG)(m) + Aspartate(c)
r0599	1	Hydroxymethylbilane(c) → UroporphyrinogenIII(c) + H2O(c)
r0015	4	2 5-Aminolevulinate(c) → Porphobilinogen(c) + 2 H2O(c)
r0018	1	4 Porphobilinogen(c) + H2O(c) → Hydroxymethylbilane(c) + 4 NH3(c)
r1572	-4	Glutamine(c) + Serine(s) ⇌ Glutamine(s) + Serine(c)
r1424	1.5	Proline(c) + H+(PG)(c) → Proline(m) + H+(PG)(m)
r2664	10.5	Na+(c) ⇌ Na+(s) + Na-gradient(c)
r1144	-10.5	Glutamate(c) + Na+(c) ← Glutamate(s) + Na+(s)
r0059	-4	Pyruvate(m) + CO2(m) + NADPH(m) ⇌ Malate(m) + NADP+(m)
r0074	1.5	L-Glutamate 5-semialdehyde(m) + NAD+(m) + H2O(m) ⇌ Glutamate(m) + NADH(m)
r0075	4	Glutamate(m) + H2O(m) + NADP+(m) ⇌ AKG(m) + NADPH(m) + NH3(m)
r2531	1.5	Proline(s) ⇌ Proline(c)
r0077	4	ATP(c) + Glutamate(c) + NH3(c) → ADP(c) + Pi(c) + Glutamine(c)
r2686	4	Aspartate(c) ⇌ Aspartate(s)
r0955	8	5-Aminolevulinate(m) → 5-Aminolevulinate(c)
r0096	1	Protoporphyrin(m) + Fe2+(m) → Heme(m)
r0508	-1.5	Ubiquinone(m) + NADH(m) + 4 H+(PG)(m) → Ubiquinol(m) + NAD+(m) + 4 H+(PG)(c)
r1330	-2	H+(PG)(c) ⇌ H+(PG)(m) + Proton-gradient(m)
r1331	-1	H+(PG)(s) ⇌ H+(PG)(c) + Proton-gradient(c)
r1319	-4	ATP(c) + H2O(c) ⇌ ADP(c) + Pi(c) + ATP-energy(c)
r0976	-1	Protoporphyrinogen IX(m) ← Protoporphyrinogen IX(c)
r0110	-4	OAA(m) + Glutamate(m) → AKG(m) + Aspartate(m)
r0849	-14	H2O(s) ⇌ H2O(c)
r0867	14	CO2(c) ⇌ CO2(s)
r0880	1	Heme(m) → Heme(c)
r0868	-8	CO2(c) ⇌ CO2(m)
r0175	1.5	1-Pyrroline-5-carboxylate(m) + H2O(m) ⇌ L-Glutamate 5-semialdehyde(m)
r1114	8	AKG(m) + CoA(m) + NAD+(m) ⇌ NADH(m) + CO2(m) + Succinyl-CoA(m)
r0606	1	UroporphyrinogenIII(c) → CoproporphyrinogenIII(c) + 4 CO2(c)
r2072	1	Fe2+(s) + H+(PG)(s) → Fe2+(c) + H+(PG)(c)
r0195	8	Glycine(m) + Succinyl-CoA(m) ⇌ CoA(m) + CO2(m) + 5-Aminolevulinate(m)
r0608	1	CoproporphyrinogenIII(c) + O2(c) → Protoporphyrinogen IX(c) + 2 CO2(c) + 2 H2O(c)
r0609	0.5	2 Protoporphyrinogen IX(m) + 3 O2(m) → 2 Protoporphyrin(m) + 6 H2O(m)
r1435	4	Serine(c) → Serine(m)
r1453	1.5	Proline(m) + Ubiquinol(m) ⇌ 1-Pyrroline-5-carboxylate(m) + Ubiquinone(m)
r0231	-4	THF(m) + Serine(m) ⇌ 5,10-Methylene-THF(m) + Glycine(m) + H2O(m)
r1458	1	Fe2+(c) → Fe2+(m)
r1323	8	NADH(m) ⇌ NAD+(m) + NADH-redox-potential(m)
r0105	4	NAD+(m) + Malate(m) ⇌ OAA(m) + NADH(m)

3.4 B4. clearence " ... degr" simulations (only obvious end products)

B4.1. amino acids

Alanine degr (409)

Definition			Solution												
			exchanges		reactions								transp		
Objective	Constraints	Comment	imports	exports	c	m	r	p	l	n	s	b	s-c	b-c	int
-1 Alanine(c)	MCES +Urea	Alanine degradation using palmitate excreting urea	0.5 H_2O(s) 1 Alanine(c) 1 ATP-energy(c) 1 ATP-energy(m)	0.5 Pyruvate(c) 0.5 Urea(s) 0.5 NADH-redox-potential(c) 0.5 NADH-redox-potential(m) 0.5 NADPH-redox-potential(m) 0.5 CoA-activated acetyl group(m)	11	9	-	-	-	-	-	-	2	-	2

ID	flux	reaction
r2678	0.5	Acetyl-CoA(m) ⇌ CoA(m) + CoA-activated acetyl group(m)
r0896	0.5	Urea(c) ⇌ Urea(s)
r0109	-0.5	OAA(c) + Glutamate(c) ⇌ AKG(c) + Aspartate(c)
r0056	0.5	Pyruvate(m) + CoA(m) + NAD+(m) → Acetyl-CoA(m) + CO2(m) + NADH(m)
r1434	0.5	Alanine(c) → Alanine(m)
r0453	0.5	ATP(c) + Citrulline(c) + Aspartate(c) ⇌ AMP(c) + PPi(c) + Argininosuccinate(c)
r0075	0.5	Glutamate(m) + H2O(m) + NADP+(m) ⇌ AKG(m) + NADPH(m) + NH3(m)
r1327	0.5	NADPH(m) ⇌ NADPH-redox-potential(m) + NADP+(m)
r1319	-1	ATP(c) + H2O(c) ⇌ ADP(c) + Pi(c) + ATP-energy(c)
r0329	0.5	Carbamoyl-P(m) + Ornithine(m) ⇌ Pi(m) + Citrulline(m)
r0258	-0.5	Fumarate(c) + H2O(c) ⇌ Malate(c)
r0030	0.5	ATP(c) + AMP(c) ⇌ 2 ADP(c)
r0104	0.5	NAD+(c) + Malate(c) ⇌ OAA(c) + NADH(c)
r1320	-1	ATP(m) + H2O(m) ⇌ ADP(m) + Pi(m) + ATP-energy(m)
r0947	-0.5	Citrulline(m) + Ornithine(c) ⇌ Citrulline(c) + Ornithine(m)
r0034	0.5	2 ATP(m) + CO2(m) + H2O(m) + NH3(m) ⇌ 2 ADP(m) + Pi(m) + Carbamoyl-P(m)
r0007	0.5	PPi(c) + H2O(c) → 2 Pi(c)
r1322	0.5	NADH(c) ⇌ NAD+(c) + NADH-redox-potential(c)
r0849	0.5	H2O(s) ⇌ H2O(c)
r0080	0.5	Alanine(c) + AKG(c) ⇌ Pyruvate(c) + Glutamate(c)
r1323	0.5	NADH(m) ⇌ NAD+(m) + NADH-redox-potential(m)
r0261	0.5	Argininosuccinate(c) ⇌ Fumarate(c) + Arginine(c)
r0144	0.5	Arginine(c) + H2O(c) ⇌ Urea(c) + Ornithine(c)
r0081	0.5	Alanine(m) + AKG(m) ⇌ Pyruvate(m) + Glutamate(m)

Arginine degr (410)

Definition			Solution												
			exchanges		reactions								transp		
Objective	Constraints	Comment	imports	exports	c	m	r	p	l	n	s	b	s-c	b-c	int
-1 Arginine(c)	MCES +Urea	Arginine degradation using palmitate excreting urea	4 H_2O(s) 0.5 O_2(s) 1 Arginine(c) 2 ATP-energy(c) 1 ATP-energy(m)	1 CO_2(s) 1 Pyruvate(c) 2 Urea(s) 3 NADH-redox-potential(m) 1 NADPH-redox-potential(c) 1 NADPH-redox-potential(m) 3 Proton-gradient(m)	9	15	-	-	-	-	-	-	4	-	11

ID	flux	reaction
r0125	-1	GDP(m) + Pi(m) + Succinyl-CoA(m) ⇌ GTP(m) + Succinate(m) + CoA(m)
r0855	0.5	O2(s) ⇌ O2(c)
r0856	-0.5	O2(m) ⇌ O2(c)
r0144	2	Arginine(c) + H2O(c) ⇌ Urea(c) + Ornithine(c)
r0876	1	Glutamate(c) + H+(PG)(c) + Aspartate(m) → Glutamate(m) + H+(PG)(m) + Aspartate(c)
r0167	1	AKG(m) + Ornithine(m) ⇌ L-Glutamate 5-semialdehyde(m) + Glutamate(m)
r0896	2	Urea(c) ⇌ Urea(s)
r0017	0.5	O2(m) + 4 H+(PG)(m) + 4 Ferrocytochrome C(m) → 4 Ferricytochrome C(m) + 2 H2O(m) + 4 H+(PG)(c)
r0030	1	ATP(c) + AMP(c) ⇌ 2 ADP(c)
r0034	1	2 ATP(m) + CO2(m) + H2O(m) + NH3(m) ⇌ 2 ADP(m) + Pi(m) + Carbamoyl-P(m)
r0911	1	Proline(c) + Glutamate(m) ⇌ Proline(m) + Glutamate(c)
r0058	1	Malate(c) + NADP+(c) ⇌ Pyruvate(c) + CO2(c) + NADPH(c)
r1426	1	Proline(m) + H+(PG)(c) → Proline(c) + H+(PG)(m)
r0074	1	L-Glutamate 5-semialdehyde(m) + NAD+(m) + H2O(m) ⇌ Glutamate(m) + NADH(m)
r0075	1	Glutamate(m) + H2O(m) + NADP+(m) ⇌ AKG(m) + NADPH(m) + NH3(m)
r0507	1	Ubiquinol(m) + 2 Ferricytochrome C(m) + 4 H+(PG)(m) → Ubiquinone(m) + 4 H+(PG)(c) + 2 Ferrocytochrome C(m)
r0509	1	Ubiquinone(m) + Succinate(m) → Ubiquinol(m) + Fumarate(m)
r1330	3	H+(PG)(c) ⇌ H+(PG)(m) + Proton-gradient(m)

Continued on next page

Arginine degr (410)– continued

ID	flux	reaction
r1319	-2	ATP(c) + H2O(c) ⇌ ADP(c) + Pi(c) + ATP-energy(c)
r0261	1	Argininosuccinate(c) ⇌ Fumarate(c) + Arginine(c)
r0110	-1	OAA(m) + Glutamate(m) → AKG(m) + Aspartate(m)
r1070	1	Lysine(c) ⇌ Lysine(m)
r0849	4	H2O(s) ⇌ H2O(c)
r0867	1	CO2(c) ⇌ CO2(s)
r0453	1	ATP(c) + Citrulline(c) + Aspartate(c) ⇌ AMP(c) + PPi(c) + Argininosuccinate(c)
r0007	1	PPi(c) + H2O(c) → 2 Pi(c)
r1114	1	AKG(m) + CoA(m) + NAD+(m) ⇌ NADH(m) + CO2(m) + Succinyl-CoA(m)
r0329	1	Carbamoyl-P(m) + Ornithine(m) ⇌ Pi(m) + Citrulline(m)
r2396	-1	Lysine(c) + Ornithine(m) + H+(PG)(m) ← Lysine(m) + Ornithine(c) + H+(PG)(c)
r0947	-1	Citrulline(c) + Ornithine(m) ⇌ Citrulline(m) + Ornithine(c)
r1320	-1	ATP(m) + H2O(m) ⇌ ADP(m) + Pi(m) + ATP-energy(m)
r1323	3	NADH(m) ⇌ NAD+(m) + NADH-redox-potential(m)
r0100	-1	ADP(m) + GTP(m) ⇌ ATP(m) + GDP(m)
r1326	1	NADPH(c) ⇌ NADPH-redox-potential(c) + NADP+(c)
r1327	1	NADPH(m) ⇌ NADPH-redox-potential(m) + NADP+(m)
r0105	1	NAD+(m) + Malate(m) ⇌ OAA(m) + NADH(m)
r0258	-1	Malate(c) ⇌ Fumarate(c) + H2O(c)
r0259	-1	Malate(m) ⇌ Fumarate(m) + H2O(m)
r0851	-2	H2O(m) ⇌ H2O(c)

Asparagine degr (411)

Definition			Solution												
			exchanges		reactions								transp		
Objective	Constraints	Comment	imports	exports	c	m	r	p	l	n	s	b	s-c	b-c	int
-1 Asparagine(c)	MCES +Urea	Asparagine degradation using palmitate excreting urea	1 H_2O(s) 1 Asparagine(c) 2 ATP-energy(c) 2 ATP-energy(m)	1 Pyruvate(c) 1 Urea(s) 1 NADPH-redox-potential(c)	10	3	-	-	-	-	-	-	2	-	4

ID	flux	reaction
r0896	1	Urea(c) ⇌ Urea(s)
r0127	1	H2O(c) + Asparagine(c) → Aspartate(c) + NH3(c)
r1326	1	NADPH(c) ⇌ NADPH-redox-potential(c) + NADP+(c)
r0453	1	ATP(c) + Citrulline(c) + Aspartate(c) ⇌ AMP(c) + PPi(c) + Argininosuccinate(c)
r0058	1	Malate(c) + NADP+(c) ⇌ Pyruvate(c) + CO2(c) + NADPH(c)
r1319	-2	ATP(c) + H2O(c) ⇌ ADP(c) + Pi(c) + ATP-energy(c)
r0851	1	H2O(m) ⇌ H2O(c)
r0329	1	Carbamoyl-P(m) + Ornithine(m) ⇌ Pi(m) + Citrulline(m)
r0258	-1	Fumarate(c) + H2O(c) ⇌ Malate(c)
r0030	1	ATP(c) + AMP(c) ⇌ 2 ADP(c)
r1320	-2	ATP(m) + H2O(m) ⇌ ADP(m) + Pi(m) + ATP-energy(m)
r0838	1	NH3(c) ⇌ NH3(m)
r0947	-1	Citrulline(m) + Ornithine(c) ⇌ Citrulline(c) + Ornithine(m)
r0034	1	2 ATP(m) + CO2(m) + H2O(m) + NH3(m) ⇌ 2 ADP(m) + Pi(m) + Carbamoyl-P(m)
r0007	1	PPi(c) + H2O(c) → 2 Pi(c)
r0849	1	H2O(s) ⇌ H2O(c)
r0868	1	CO2(c) ⇌ CO2(m)
r0261	1	Argininosuccinate(c) ⇌ Fumarate(c) + Arginine(c)
r0144	1	Arginine(c) + H2O(c) ⇌ Urea(c) + Ornithine(c)

Aspartate degr (412)

Definition			Solution													
			exchanges		reactions								transp		
Objective	Constraints	Comment	imports	exports	c	m	r	p	l	n	s	b	s-c	b-c	int
-1 Aspartate(c)	MCES +Urea	Aspartate degradation using palmitate excreting urea	0.5 H_2O(s) 1 Aspartate(c) 1 ATP-energy(c) 1 ATP-energy(m)	1 CO_2(s) 0.5 Pyruvate(c) 0.5 Urea(s) 0.5 NADH-redox-potential(m) 0.5 NADPH-redox-potential(c) 0.5 NADPH-redox-potential(m) 0.5 CoA-activated acetyl group(m)	10	9	-	-	-	-	-	-	3	-	2

ID	flux	reaction
r2678	0.5	Acetyl-CoA(m) ⇌ CoA(m) + CoA-activated acetyl group(m)
r0896	0.5	Urea(c) ⇌ Urea(s)
r0217	0.5	beta-Alanine(m) + AKG(m) ⇌ Glutamate(m) + 3-Oxopropanoate(m)
r0128	0.5	Aspartate(c) ⇌ beta-Alanine(c) + CO2(c)
r1326	0.5	NADPH(c) ⇌ NADPH-redox-potential(c) + NADP+(c)
r0453	0.5	ATP(c) + Citrulline(c) + Aspartate(c) ⇌ AMP(c) + PPi(c) + Argininosuccinate(c)
r0174	0.5	CoA(m) + NAD+(m) + 3-Oxopropanoate(m) → Acetyl-CoA(m) + CO2(m) + NADH(m)
r0075	0.5	Glutamate(m) + H2O(m) + NADP+(m) ⇌ AKG(m) + NADPH(m) + NH3(m)
r1327	0.5	NADPH(m) ⇌ NADPH-redox-potential(m) + NADP+(m)
r0058	0.5	Malate(c) + NADP+(c) ⇌ Pyruvate(c) + CO2(c) + NADPH(c)
r1319	-1	ATP(c) + H2O(c) ⇌ ADP(c) + Pi(c) + ATP-energy(c)
r0329	0.5	Carbamoyl-P(m) + Ornithine(m) ⇌ Pi(m) + Citrulline(m)
r0258	-0.5	Fumarate(c) + H2O(c) ⇌ Malate(c)
r0030	0.5	ATP(c) + AMP(c) ⇌ 2 ADP(c)
r1320	-1	ATP(m) + H2O(m) ⇌ ADP(m) + Pi(m) + ATP-energy(m)
r0947	-0.5	Citrulline(m) + Ornithine(c) ⇌ Citrulline(c) + Ornithine(m)
r0034	0.5	2 ATP(m) + CO2(m) + H2O(m) + NH3(m) ⇌ 2 ADP(m) + Pi(m) + Carbamoyl-P(m)
r0007	0.5	PPi(c) + H2O(c) → 2 Pi(c)
r0867	1	CO2(c) ⇌ CO2(s)
r0849	0.5	H2O(s) ⇌ H2O(c)
r1323	0.5	NADH(m) ⇌ NAD+(m) + NADH-redox-potential(m)
r0900	0.5	beta-Alanine(c) ⇌ beta-Alanine(m)
r0261	0.5	Argininosuccinate(c) ⇌ Fumarate(c) + Arginine(c)
r0144	0.5	Arginine(c) + H2O(c) ⇌ Urea(c) + Ornithine(c)

Cysteine degr (413)

Definition			Solution													
			exchanges		reactions								transp		
Objective	Constraints	Comment	imports	exports	c	m	r	p	l	n	s	b	s-c	b-c	int
-1 Cysteine(c)	MCES +Urea +H_2S	Cysteine degradation using palmitate excreting urea	0.5 H_2O(s) 0.5 Pyruvate(m) 1 Cysteine(c) 1 ATP-energy(c) 1 ATP-energy(m) 1 Proton-gradient(m)	1 Pyruvate(c) 0.5 Urea(s) 1 H_2S(s) 0.5 NADH-redox-potential(c) 0.5 CoA-activated acetyl group(m)	11	6	-	-	-	-	-	-	3	-	8

ID	flux	reaction
r2678	0.5	Acetyl-CoA(m) ⇌ CoA(m) + CoA-activated acetyl group(m)
r0896	0.5	Urea(c) ⇌ Urea(s)
r0109	-0.5	OAA(c) + Glutamate(c) ⇌ AKG(c) + Aspartate(c)
r0073	-0.5	AKG(m) + NADH(m) + NH3(m) ⇌ Glutamate(m) + NAD+(m) + H2O(m)
r0911	0.5	Proline(c) + Glutamate(m) ⇌ Proline(m) + Glutamate(c)
r0056	0.5	Pyruvate(m) + CoA(m) + NAD+(m) → Acetyl-CoA(m) + CO2(m) + NADH(m)
r1047	0.5	Pi(c) + H+(PG)(c) → Pi(m) + H+(PG)(m)
r0453	0.5	ATP(c) + Citrulline(c) + Aspartate(c) ⇌ AMP(c) + PPi(c) + Argininosuccinate(c)
r1426	0.5	Proline(m) + H+(PG)(c) → Proline(c) + H+(PG)(m)

Continued on next page

ID	flux	reaction
r0940	1	H2S(c) ⇌ H2S(s)
r0193	1	Cysteine(c) + H2O(c) ⇌ Pyruvate(c) + H2S(c) + NH3(c)
r1319	-1	ATP(c) + H2O(c) ⇌ ADP(c) + Pi(c) + ATP-energy(c)
r0851	1	H2O(m) ⇌ H2O(c)
r0329	0.5	Carbamoyl-P(m) + Ornithine(m) ⇌ Pi(m) + Citrulline(m)
r0258	-0.5	Fumarate(c) + H2O(c) ⇌ Malate(c)
r2419	0.5	AKG(c) + Pi(m) ⇌ AKG(m) + Pi(c)
r0030	0.5	ATP(c) + AMP(c) ⇌ 2 ADP(c)
r0104	0.5	NAD+(c) + Malate(c) ⇌ OAA(c) + NADH(c)
r1320	-1	ATP(m) + H2O(m) ⇌ ADP(m) + Pi(m) + ATP-energy(m)
r0838	1	NH3(c) ⇌ NH3(m)
r1330	-1	H+(PG)(c) ⇌ H+(PG)(m) + Proton-gradient(m)
r0947	-0.5	Citrulline(m) + Ornithine(c) ⇌ Citrulline(c) + Ornithine(m)
r0034	0.5	2 ATP(m) + CO2(m) + H2O(m) + NH3(m) ⇌ 2 ADP(m) + Pi(m) + Carbamoyl-P(m)
r0007	0.5	PPi(c) + H2O(c) → 2 Pi(c)
r1322	0.5	NADH(c) ⇌ NAD+(c) + NADH-redox-potential(c)
r0849	0.5	H2O(s) ⇌ H2O(c)
r0261	0.5	Argininosuccinate(c) ⇌ Fumarate(c) + Arginine(c)
r0144	0.5	Arginine(c) + H2O(c) ⇌ Urea(c) + Ornithine(c)

Glutamate degr (414)

Definition			Solution												
			exchanges		reactions								transp		
Objective	Constraints	Comment	imports	exports	c	m	r	p	l	n	s	b	s-c	b-c	int
-1 Glutamate(c)	MCES +Urea	Glutamate degradation using palmitate excreting urea	1.5 H_2O(s) 0.5 O_2(s) 1 Glutamate(c) 1 ATP-energy(c)	1.5 CO_2(s) 0.5 Pyruvate(c) 0.5 Pyruvate(m) 0.5 Urea(s) 1.5 NADH-redox-potential(m) 0.5 NADPH-redox-potential(c) 1 NADPH-redox-potential(m) 5 Proton-gradient(m)	9	13	-	-	-	-	-	-	4	-	9

ID	flux	reaction
r0896	0.5	Urea(c) ⇌ Urea(s)
r1326	0.5	NADPH(c) ⇌ NADPH-redox-potential(c) + NADP+(c)
r0507	1	Ubiquinol(m) + 2 Ferricytochrome C(m) + 4 H+(PG)(m) → Ubiquinone(m) + 4 H+(PG)(c) + 2 Ferrocytochrome C(m)
r0453	0.5	ATP(c) + Citrulline(c) + Aspartate(c) ⇌ AMP(c) + PPi(c) + Argininosuccinate(c)
r0075	0.5	Glutamate(m) + H2O(m) + NADP+(m) ⇌ AKG(m) + NADPH(m) + NH3(m)
r1327	1	NADPH(m) ⇌ NADPH-redox-potential(m) + NADP+(m)
r0058	0.5	Malate(c) + NADP+(c) ⇌ Pyruvate(c) + CO2(c) + NADPH(c)
r1319	-1	ATP(c) + H2O(c) ⇌ ADP(c) + Pi(c) + ATP-energy(c)
r0851	-1	H2O(m) ⇌ H2O(c)
r0509	1	Ubiquinone(m) + Succinate(m) → Ubiquinol(m) + Fumarate(m)
r0329	0.5	Carbamoyl-P(m) + Ornithine(m) ⇌ Pi(m) + Citrulline(m)
r0100	-1	ADP(m) + GTP(m) ⇌ ATP(m) + GDP(m)
r0059	0.5	Malate(m) + NADP+(m) ⇌ Pyruvate(m) + CO2(m) + NADPH(m)
r0258	-0.5	Malate(c) ⇌ Fumarate(c) + H2O(c)
r0110	-0.5	OAA(m) + Glutamate(m) → AKG(m) + Aspartate(m)
r0259	-1	Malate(m) ⇌ Fumarate(m) + H2O(m)
r0030	0.5	ATP(c) + AMP(c) ⇌ 2 ADP(c)
r0855	0.5	O2(s) ⇌ O2(c)
r0856	-0.5	O2(m) ⇌ O2(c)
r0105	0.5	NAD+(m) + Malate(m) ⇌ OAA(m) + NADH(m)
r1330	5	H+(PG)(c) ⇌ H+(PG)(m) + Proton-gradient(m)
r1114	1	AKG(m) + CoA(m) + NAD+(m) ⇌ NADH(m) + CO2(m) + Succinyl-CoA(m)
r0947	-0.5	Citrulline(m) + Ornithine(c) ⇌ Citrulline(c) + Ornithine(m)

Continued on next page

ID	flux	reaction
r0875	0.5	Glutamate(c) + H+(PG)(c) → Glutamate(m) + H+(PG)(m)
r0034	0.5	2 ATP(m) + CO2(m) + H2O(m) + NH3(m) ⇌ 2 ADP(m) + Pi(m) + Carbamoyl-P(m)
r0007	0.5	PPi(c) + H2O(c) → 2 Pi(c)
r0876	0.5	Glutamate(c) + H+(PG)(c) + Aspartate(m) → Glutamate(m) + H+(PG)(m) + Aspartate(c)
r0867	1.5	CO2(c) ⇌ CO2(s)
r0849	1.5	H2O(s) ⇌ H2O(c)
r0125	-1	GDP(m) + Pi(m) + Succinyl-CoA(m) ⇌ GTP(m) + Succinate(m) + CoA(m)
r0017	0.5	O2(m) + 4 H+(PG)(m) + 4 Ferrocytochrome C(m) → 4 Ferricytochrome C(m) + 2 H2O(m) + 4 H+(PG)(c)
r1323	1.5	NADH(m) ⇌ NAD+(m) + NADH-redox-potential(m)
r0868	-1	CO2(c) ⇌ CO2(m)
r0261	0.5	Argininosuccinate(c) ⇌ Fumarate(c) + Arginine(c)
r0144	0.5	Arginine(c) + H2O(c) ⇌ Urea(c) + Ornithine(c)

Histidine degr (415)

Definition			Solution													
			exchanges		reactions								transp			
Objective	Constraints	Comment	imports	exports	c	m	r	p	l	n	s	b	s-c	b-c	int
-1 Histidine(c)	MCES +Urea	Histidine degradation using palmitate excreting urea	2.5 H_2O(s) 0.5 O_2(s) 1 Histidine(c) 3 ATP-energy(c) 2 ATP-energy(m) 0.5 NADPH-redox-potential(c)	0.5 CO_2(s) 0.5 Pyruvate(c) 0.5 Pyruvate(m) 1.5 Urea(s) 0.5 NADH-redox-potential(c) 1 NADH-redox-potential(m) 4.5 Proton-gradient(m) 1 THF-activated methyl group(c)	20	11	-	-	-	-	-	-	4	-	12

ID	flux	reaction
r0125	-1	GDP(m) + Pi(m) + Succinyl-CoA(m) ⇌ GTP(m) + Succinate(m) + CoA(m)
r0855	0.5	O2(s) ⇌ O2(c)
r0856	-0.5	O2(m) ⇌ O2(c)
r0293	-1	5,10-Methenyl-THF(c) + NADPH(c) ⇌ 5,10-Methylene-THF(c) + NADP+(c)
r0577	-1	Urocanate(c) + H2O(c) ⇌ 4-Imidazolone-5-propanoate(c)
r0144	1.5	Arginine(c) + H2O(c) ⇌ Urea(c) + Ornithine(c)
r0896	1.5	Urea(c) ⇌ Urea(s)
r0017	0.5	O2(m) + 4 H+(PG)(m) + 4 Ferrocytochrome C(m) → 4 Ferricytochrome C(m) + 2 H2O(m) + 4 H+(PG)(c)
r0030	1.5	ATP(c) + AMP(c) ⇌ 2 ADP(c)
r0034	1.5	2 ATP(m) + CO2(m) + H2O(m) + NH3(m) ⇌ 2 ADP(m) + Pi(m) + Carbamoyl-P(m)
r0911	0.5	Proline(c) + Glutamate(m) ⇌ Proline(m) + Glutamate(c)
r1426	0.5	Proline(m) + H+(PG)(c) → Proline(c) + H+(PG)(m)
r0058	0.5	Malate(c) + NADP+(c) ⇌ Pyruvate(c) + CO2(c) + NADPH(c)
r0059	0.5	Malate(m) + NADP+(m) ⇌ Pyruvate(m) + CO2(m) + NADPH(m)
r0075	-0.5	AKG(m) + NADPH(m) + NH3(m) ⇌ Glutamate(m) + H2O(m) + NADP+(m)
r0507	1	Ubiquinol(m) + 2 Ferricytochrome C(m) + 4 H+(PG)(m) → Ubiquinone(m) + 4 H+(PG)(c) + 2 Ferrocytochrome C(m)
r0509	1	Ubiquinone(m) + Succinate(m) → Ubiquinol(m) + Fumarate(m)
r0524	1	5-Formiminotetrahydrofolate(c) ⇌ 5,10-Methenyl-THF(c) + NH3(c)
r0820	0.5	AKG(c) + Malate(m) ⇌ AKG(m) + Malate(c)
r1330	4.5	H+(PG)(c) ⇌ H+(PG)(m) + Proton-gradient(m)
r1319	-3	ATP(c) + H2O(c) ⇌ ADP(c) + Pi(c) + ATP-energy(c)
r0261	1.5	Argininosuccinate(c) ⇌ Fumarate(c) + Arginine(c)
r0849	2.5	H2O(s) ⇌ H2O(c)
r0286	1	Histidine(c) ⇌ Urocanate(c) + NH3(c)
r0867	0.5	CO2(c) ⇌ CO2(s)
r0453	1.5	ATP(c) + Citrulline(c) + Aspartate(c) ⇌ AMP(c) + PPi(c) + Argininosuccinate(c)
r0007	1.5	PPi(c) + H2O(c) → 2 Pi(c)
r1114	1	AKG(m) + CoA(m) + NAD+(m) ⇌ NADH(m) + CO2(m) + Succinyl-CoA(m)
r0329	1.5	Carbamoyl-P(m) + Ornithine(m) ⇌ Pi(m) + Citrulline(m)
r0792	-1	5,10-Methylene-THF(c) + NADH(c) ⇌ 5-Methyl-THF(c) + NAD+(c)

Continued on next page

ID	flux	reaction
r2676	1	5-Methyl-THF(c) ⇌ THF(c) + THF-activated methyl group(c)
r0947	-1.5	Citrulline(m) + Ornithine(c) ⇌ Citrulline(c) + Ornithine(m)
r1320	-2	ATP(m) + H2O(m) ⇌ ADP(m) + Pi(m) + ATP-energy(m)
r1322	0.5	NADH(c) ⇌ NAD+(c) + NADH-redox-potential(c)
r0518	-1	Glutamate(c) + 5-Formiminotetrahydrofolate(c) ⇌ THF(c) + N-Formimino-L-glutamate(c)
r1323	1	NADH(m) ⇌ NAD+(m) + NADH-redox-potential(m)
r0519	1	H2O(c) + 4-Imidazolone-5-propanoate(c) ⇌ N-Formimino-L-glutamate(c)
r0100	-1	ADP(m) + GTP(m) ⇌ ATP(m) + GDP(m)
r1326	-0.5	NADPH(c) ⇌ NADPH-redox-potential(c) + NADP+(c)
r0104	1.5	NAD+(c) + Malate(c) ⇌ OAA(c) + NADH(c)
r1047	1	Pi(c) + H+(PG)(c) → Pi(m) + H+(PG)(m)
r0258	-1.5	Malate(c) ⇌ Fumarate(c) + H2O(c)
r0259	-1	Malate(m) ⇌ Fumarate(m) + H2O(m)
r2419	1	AKG(c) + Pi(m) ⇌ AKG(m) + Pi(c)
r0838	2	NH3(c) ⇌ NH3(m)
r0851	1	H2O(m) ⇌ H2O(c)
r0109	-1.5	OAA(c) + Glutamate(c) ⇌ AKG(c) + Aspartate(c)

Glutamine degr (416)

Definition			Solution												
			exchanges		reactions								transp		
Objective	Constraints	Comment	imports	exports	c	m	r	p	l	n	s	b	s-c	b-c	int
-1 Glutamine(c)	MCES +Urea	Glutamine degradation using palmitate excreting urea	2 H_2O(s) 0.5 O_2(s) 1 Glutamine(c) 2 ATP-energy(c) 1 ATP-energy(m)	1 CO_2(s) 1 Pyruvate(c) 1 Urea(s) 2 NADH-redox-potential(m) 1 NADPH-redox-potential(c) 5 Proton-gradient(m)	11	11	-	-	-	-	-	-	4	-	7

ID	flux	reaction
r0896	1	Urea(c) ⇌ Urea(s)
r0190	1	Glutamine(c) + Fructose-6P(c) → Glutamate(c) + Glucosamine-6P(c)
r1326	1	NADPH(c) ⇌ NADPH-redox-potential(c) + NADP+(c)
r0507	1	Ubiquinol(m) + 2 Ferricytochrome C(m) + 4 H+(PG)(m) → Ubiquinone(m) + 4 H+(PG)(c) + 2 Ferrocytochrome C(m)
r0453	1	ATP(c) + Citrulline(c) + Aspartate(c) ⇌ AMP(c) + PPi(c) + Argininosuccinate(c)
r0058	1	Malate(c) + NADP+(c) ⇌ Pyruvate(c) + CO2(c) + NADPH(c)
r1319	-2	ATP(c) + H2O(c) ⇌ ADP(c) + Pi(c) + ATP-energy(c)
r0509	1	Ubiquinone(m) + Succinate(m) → Ubiquinol(m) + Fumarate(m)
r0329	1	Carbamoyl-P(m) + Ornithine(m) ⇌ Pi(m) + Citrulline(m)
r0100	-1	ADP(m) + GTP(m) ⇌ ATP(m) + GDP(m)
r0258	-1	Malate(c) ⇌ Fumarate(c) + H2O(c)
r0110	-1	OAA(m) + Glutamate(m) → AKG(m) + Aspartate(m)
r0259	-1	Malate(m) ⇌ Fumarate(m) + H2O(m)
r0030	1	ATP(c) + AMP(c) ⇌ 2 ADP(c)
r0855	0.5	O2(s) ⇌ O2(c)
r0189	1	Glucosamine-6P(c) + H2O(c) ⇌ Fructose-6P(c) + NH3(c)
r1320	-1	ATP(m) + H2O(m) ⇌ ADP(m) + Pi(m) + ATP-energy(m)
r0856	-0.5	O2(m) ⇌ O2(c)
r0838	1	NH3(c) ⇌ NH3(m)
r0105	1	NAD+(m) + Malate(m) ⇌ OAA(m) + NADH(m)
r1330	5	H+(PG)(c) ⇌ H+(PG)(m) + Proton-gradient(m)
r1114	1	AKG(m) + CoA(m) + NAD+(m) ⇌ NADH(m) + CO2(m) + Succinyl-CoA(m)
r0947	-1	Citrulline(m) + Ornithine(c) ⇌ Citrulline(c) + Ornithine(m)
r0034	1	2 ATP(m) + CO2(m) + H2O(m) + NH3(m) ⇌ 2 ADP(m) + Pi(m) + Carbamoyl-P(m)
r0007	1	PPi(c) + H2O(c) → 2 Pi(c)

Continued on next page

Glutamine degr (416)– continued

ID	flux	reaction
r0876	1	Glutamate(c) + H+(PG)(c) + Aspartate(m) → Glutamate(m) + H+(PG)(m) + Aspartate(c)
r0867	1	CO2(c) ⇌ CO2(s)
r0849	2	H2O(s) ⇌ H2O(c)
r0125	-1	GDP(m) + Pi(m) + Succinyl-CoA(m) ⇌ GTP(m) + Succinate(m) + CoA(m)
r0017	0.5	O2(m) + 4 H+(PG)(m) + 4 Ferrocytochrome C(m) → 4 Ferricytochrome C(m) + 2 H2O(m) + 4 H+(PG)(c)
r1323	2	NADH(m) ⇌ NAD+(m) + NADH-redox-potential(m)
r0261	1	Argininosuccinate(c) ⇌ Fumarate(c) + Arginine(c)
r0144	1	Arginine(c) + H2O(c) ⇌ Urea(c) + Ornithine(c)

Leucine degr (417)

Definition			Solution												
			exchanges		reactions								transp		
Objective	Constraints	Comment	imports	exports	c	m	r	p	l	n	s	b	s-c	b-c	int
-1 Leucine(c)	MCES +Urea	Leucine degradation using palmitate excreting urea	0.5 O_2(s) 0.5 Pyruvate(m) 1 Leucine(c) 1 ATP-energy(c) 1 ATP-energy(m) 1 NADPH-redox-potential(m)	0.5 H_2O(s) 0.5 Urea(s) 0.5 NADH-redox-potential(c) 3 NADH-redox-potential(m) 4 Proton-gradient(m) 3.5 CoA-activated acetyl group(m)	10	18	-	-	-	-	-	-	3	-	11

ID	flux	reaction
r0855	0.5	O2(s) ⇌ O2(c)
r0856	-0.5	O2(m) ⇌ O2(c)
r0144	0.5	Arginine(c) + H2O(c) ⇌ Urea(c) + Ornithine(c)
r0462	1	HMG-CoA(m) + CoA(m) ⇌ Acetyl-CoA(m) + H2O(m) + Acetoacetyl-CoA(m)
r0896	0.5	Urea(c) ⇌ Urea(s)
r0017	0.5	O2(m) + 4 H+(PG)(m) + 4 Ferrocytochrome C(m) → 4 Ferricytochrome C(m) + 2 H2O(m) + 4 H+(PG)(c)
r0030	0.5	ATP(c) + AMP(c) ⇌ 2 ADP(c)
r0034	0.5	2 ATP(m) + CO2(m) + H2O(m) + NH3(m) ⇌ 2 ADP(m) + Pi(m) + Carbamoyl-P(m)
r0911	0.5	Proline(c) + Glutamate(m) ⇌ Proline(m) + Glutamate(c)
r0056	0.5	Pyruvate(m) + CoA(m) + NAD+(m) → Acetyl-CoA(m) + CO2(m) + NADH(m)
r0070	-1	CoA(m) + Acetoacetyl-CoA(m) ⇌ 2 Acetyl-CoA(m)
r1426	0.5	Proline(m) + H+(PG)(c) → Proline(c) + H+(PG)(m)
r0059	-1	Pyruvate(m) + CO2(m) + NADPH(m) ⇌ Malate(m) + NADP+(m)
r0073	0.5	Glutamate(m) + NAD+(m) + H2O(m) ⇌ AKG(m) + NADH(m) + NH3(m)
r0655	1	Isovaleryl-CoA(m) + Ubiquinone(m) ⇌ 3-Methylcrotonyl-CoA(m) + Ubiquinol(m)
r0658	1	ATP(m) + 3-Methylcrotonyl-CoA(m) + HCO3-(m) ⇌ ADP(m) + Pi(m) + 3-Methylglutaconyl-CoA(m)
r0507	1	Ubiquinol(m) + 2 Ferricytochrome C(m) + 4 H+(PG)(m) → Ubiquinone(m) + 4 H+(PG)(c) + 2 Ferrocytochrome C(m)
r1330	4	H+(PG)(c) ⇌ H+(PG)(m) + Proton-gradient(m)
r1319	-1	ATP(c) + H2O(c) ⇌ ADP(c) + Pi(c) + ATP-energy(c)
r0261	0.5	Argininosuccinate(c) ⇌ Fumarate(c) + Arginine(c)
r0262	1	Leucine(m) + AKG(m) ⇌ Glutamate(m) + 4-Methyl-2-oxopentanoate(m)
r0849	-0.5	H2O(s) ⇌ H2O(c)
r0453	0.5	ATP(c) + Citrulline(c) + Aspartate(c) ⇌ AMP(c) + PPi(c) + Argininosuccinate(c)
r0007	0.5	PPi(c) + H2O(c) → 2 Pi(c)
r0601	1	4-Methyl-2-oxopentanoate(m) + CoA(m) + NAD+(m) ⇌ Isovaleryl-CoA(m) + CO2(m) + NADH(m)
r0490	-1	3-Methylglutaconyl-CoA(m) + H2O(m) ⇌ HMG-CoA(m)
r0906	1	Leucine(c) + H+(PG)(c) → Leucine(m) + H+(PG)(m)
r0329	0.5	Carbamoyl-P(m) + Ornithine(m) ⇌ Pi(m) + Citrulline(m)
r2678	3.5	Acetyl-CoA(m) ⇌ CoA(m) + CoA-activated acetyl group(m)
r0947	-0.5	Citrulline(m) + Ornithine(c) ⇌ Citrulline(c) + Ornithine(m)
r1320	-1	ATP(m) + H2O(m) ⇌ ADP(m) + Pi(m) + ATP-energy(m)
r1322	0.5	NADH(c) ⇌ NAD+(c) + NADH-redox-potential(c)
r1323	3	NADH(m) ⇌ NAD+(m) + NADH-redox-potential(m)
r1327	-1	NADPH(m) ⇌ NADPH-redox-potential(m) + NADP+(m)

Continued on next page

ID	flux	reaction
r0104	0.5	NAD+(c) + Malate(c) ⇌ OAA(c) + NADH(c)
r1047	0.5	Pi(c) + H+(PG)(c) → Pi(m) + H+(PG)(m)
r0105	1	NAD+(m) + Malate(m) ⇌ OAA(m) + NADH(m)
r0106	-1	ADP(m) + Pi(m) + OAA(m) ⇌ ATP(m) + Pyruvate(m) + HCO3-(m)
r0258	-0.5	Fumarate(c) + H2O(c) ⇌ Malate(c)
r2419	0.5	AKG(c) + Pi(m) ⇌ AKG(m) + Pi(c)
r0851	1	H2O(m) ⇌ H2O(c)
r0109	-0.5	OAA(c) + Glutamate(c) ⇌ AKG(c) + Aspartate(c)

Lysine degr (418)

Definition			Solution												
			exchanges		reactions								transp		
Objective	Constraints	Comment	imports	exports	c	m	r	p	l	n	s	b	s-c	b-c	int
-1 Lysine(c)	MCES +Urea	Lysine degradation using palmitate excreting urea	2 H_2O(s) 0.5 O_2(s) 1 Pyruvate(m) 1 Lysine(c) 2 ATP-energy(c) 2 ATP-energy(m) 2 NADPH-redox-potential(m)	1 CO_2(s) 1 Pyruvate(c) 1 Urea(s) 1 NADH-redox-potential(c) 5 NADH-redox-potential(m) 1 NADPH-redox-potential(c) 4 Proton-gradient(m) 2 CoA-activated acetyl group(m)	11	18	-	-	-	-	-	-	4	-	10

ID	flux	reaction
r0855	0.5	O2(s) ⇌ O2(c)
r0856	-0.5	O2(m) ⇌ O2(c)
r0144	1	Arginine(c) + H2O(c) ⇌ Urea(c) + Ornithine(c)
r0876	1	Glutamate(c) + H+(PG)(c) + Aspartate(m) → Glutamate(m) + H+(PG)(m) + Aspartate(c)
r0594	1	2-Aminoadipate 6-semialdehyde(c) + NAD+(c) + H2O(c) ⇌ NADH(c) + L-2-Aminoadipate(c)
r0460	1	(S)-3-Hydroxybutyryl-CoA(m) + NAD+(m) ⇌ NADH(m) + Acetoacetyl-CoA(m)
r0448	1	2-Oxoadipate(m) + CoA(m) + NAD+(m) ⇌ Glutaryl-CoA(m) + CO2(m) + NADH(m)
r0180	-1	H2O(m) + Saccharopine(m) + NADP+(m) ⇌ AKG(m) + NADPH(m) + Lysine(m)
r0896	1	Urea(c) ⇌ Urea(s)
r0017	0.5	O2(m) + 4 H+(PG)(m) + 4 Ferrocytochrome C(m) → 4 Ferricytochrome C(m) + 2 H2O(m) + 4 H+(PG)(c)
r0030	1	ATP(c) + AMP(c) ⇌ 2 ADP(c)
r0034	1	2 ATP(m) + CO2(m) + H2O(m) + NH3(m) ⇌ 2 ADP(m) + Pi(m) + Carbamoyl-P(m)
r0911	1	Proline(c) + Glutamate(m) ⇌ Proline(m) + Glutamate(c)
r0070	-1	CoA(m) + Acetoacetyl-CoA(m) ⇌ 2 Acetyl-CoA(m)
r1426	1	Proline(m) + H+(PG)(c) → Proline(c) + H+(PG)(m)
r0058	1	Malate(c) + NADP+(c) ⇌ Pyruvate(c) + CO2(c) + NADPH(c)
r0059	-1	Pyruvate(m) + CO2(m) + NADPH(m) ⇌ Malate(m) + NADP+(m)
r0073	1	Glutamate(m) + NAD+(m) + H2O(m) ⇌ AKG(m) + NADH(m) + NH3(m)
r0507	1	Ubiquinol(m) + 2 Ferricytochrome C(m) + 4 H+(PG)(m) → Ubiquinone(m) + 4 H+(PG)(c) + 2 Ferrocytochrome C(m)
r2539	-1	2-Aminoadipate 6-semialdehyde(c) + L-2-Aminoadipate(m) ⇌ 2-Aminoadipate 6-semialdehyde(m) + L-2-Aminoadipate(c)
r0525	1	NAD+(m) + H2O(m) + Saccharopine(m) ⇌ Glutamate(m) + 2-Aminoadipate 6-semialdehyde(m) + NADH(m)
r1330	4	H+(PG)(c) ⇌ H+(PG)(m) + Proton-gradient(m)
r1319	-2	ATP(c) + H2O(c) ⇌ ADP(c) + Pi(c) + ATP-energy(c)
r0541	1	Glutaryl-CoA(m) + Ubiquinone(m) ⇌ Crotonyl-CoA(m) + CO2(m) + Ubiquinol(m)
r0261	1	Argininosuccinate(c) ⇌ Fumarate(c) + Arginine(c)
r0110	-1	OAA(m) + Glutamate(m) → AKG(m) + Aspartate(m)
r1070	1	Lysine(c) ⇌ Lysine(m)
r0849	2	H2O(s) ⇌ H2O(c)
r0867	1	CO2(c) ⇌ CO2(s)
r0588	-1	Crotonyl-CoA(m) + H2O(m) ⇌ (S)-3-Hydroxybutyryl-CoA(m)
r0450	1	AKG(m) + L-2-Aminoadipate(m) ⇌ 2-Oxoadipate(m) + Glutamate(m)
r0453	1	ATP(c) + Citrulline(c) + Aspartate(c) ⇌ AMP(c) + PPi(c) + Argininosuccinate(c)
r0007	1	PPi(c) + H2O(c) → 2 Pi(c)

Continued on next page

ID	flux	reaction
r0329	1	Carbamoyl-P(m) + Ornithine(m) ⇌ Pi(m) + Citrulline(m)
r2678	2	Acetyl-CoA(m) ⇌ CoA(m) + CoA-activated acetyl group(m)
r0947	-1	Citrulline(m) + Ornithine(c) ⇌ Citrulline(c) + Ornithine(m)
r1320	-2	ATP(m) + H2O(m) ⇌ ADP(m) + Pi(m) + ATP-energy(m)
r1322	1	NADH(c) ⇌ NAD+(c) + NADH-redox-potential(c)
r1323	5	NADH(m) ⇌ NAD+(m) + NADH-redox-potential(m)
r1326	1	NADPH(c) ⇌ NADPH-redox-potential(c) + NADP+(c)
r1327	-2	NADPH(m) ⇌ NADPH-redox-potential(m) + NADP+(m)
r0105	1	NAD+(m) + Malate(m) ⇌ OAA(m) + NADH(m)
r0258	-1	Fumarate(c) + H2O(c) ⇌ Malate(c)

Methionine degr (419)

Definition			Solution												
			exchanges		reactions								transp		
Objective	Constraints	Comment	imports	exports	c	m	r	p	l	n	s	b	s-c	b-c	int
-1 Methionine(c)	MCES +Urea −Serine +H_2S	Methionine degradation using palmitate excreting urea	1 H_2O(s) 0.5 O_2(s) 1 Serine(s) 1 Methionine(c) 2 ATP-energy(c) 2 ATP-energy(m)	1 CO_2(s) 1 Pyruvate(c) 1 Urea(s) 1 H_2S(s) 1 NADH-redox-potential(c) 2 NADH-redox-potential(m) 3 Proton-gradient(m) 1 THF-activated methyl group(c) 1 CoA-activated acetyl group(m)	15	12	-	-	-	-	-	-	6	-	13

ID	flux	reaction
r0855	0.5	O2(s) ⇌ O2(c)
r0856	-0.5	O2(m) ⇌ O2(c)
r0144	1	Arginine(c) + H2O(c) ⇌ Urea(c) + Ornithine(c)
r0592	-1	H2O(m) + Acrylyl-CoA(m) → 3-Hydroxypropionyl-CoA(m)
r0596	-1	3-Hydroxypropionyl-CoA(m) + H2O(m) → Hydracrylate(m) + CoA(m)
r0896	1	Urea(c) ⇌ Urea(s)
r0017	0.5	O2(m) + 4 H+(PG)(m) + 4 Ferrocytochrome C(m) → 4 Ferricytochrome C(m) + 2 H2O(m) + 4 H+(PG)(c)
r0030	1	ATP(c) + AMP(c) ⇌ 2 ADP(c)
r0314	1	Serine(c) + Homocysteine(c) ⇌ H2O(c) + L-Cystathionine(c)
r0034	1	2 ATP(m) + CO2(m) + H2O(m) + NH3(m) ⇌ 2 ADP(m) + Pi(m) + Carbamoyl-P(m)
r0911	1	Proline(c) + Glutamate(m) ⇌ Proline(m) + Glutamate(c)
r1426	1	Proline(m) + H+(PG)(c) → Proline(c) + H+(PG)(m)
r0073	-1	AKG(m) + NADH(m) + NH3(m) ⇌ Glutamate(m) + NAD+(m) + H2O(m)
r0507	1	Ubiquinol(m) + 2 Ferricytochrome C(m) + 4 H+(PG)(m) → Ubiquinone(m) + 4 H+(PG)(c) + 2 Ferrocytochrome C(m)
r0241	1	H2O(c) + L-Cystathionine(c) ⇌ Cysteine(c) + NH3(c) + 2-Oxobutyrate(c)
r1330	3	H+(PG)(c) ⇌ H+(PG)(m) + Proton-gradient(m)
r1319	-2	ATP(c) + H2O(c) ⇌ ADP(c) + Pi(c) + ATP-energy(c)
r0261	1	Argininosuccinate(c) ⇌ Fumarate(c) + Arginine(c)
r0849	1	H2O(s) ⇌ H2O(c)
r0867	1	CO2(c) ⇌ CO2(s)
r0868	-1	CO2(c) ⇌ CO2(m)
r0453	1	ATP(c) + Citrulline(c) + Aspartate(c) ⇌ AMP(c) + PPi(c) + Argininosuccinate(c)
r0007	1	PPi(c) + H2O(c) → 2 Pi(c)
r0174	1	CoA(m) + NAD+(m) + 3-Oxopropanoate(m) → Acetyl-CoA(m) + CO2(m) + NADH(m)
r0193	1	Cysteine(c) + H2O(c) ⇌ Pyruvate(c) + H2S(c) + NH3(c)
r0329	1	Carbamoyl-P(m) + Ornithine(m) ⇌ Pi(m) + Citrulline(m)
r0940	1	H2S(c) ⇌ H2S(s)
r1154	1	CoA(m) + NAD+(m) + 2-Oxobutyrate(m) ⇌ CO2(m) + Propanoyl-CoA(m) + NADH(m)
r1155	1	2-Oxobutyrate(c) + H+(PG)(c) → 2-Oxobutyrate(m) + H+(PG)(m)
r2676	1	5-Methyl-THF(c) ⇌ THF(c) + THF-activated methyl group(c)
r0365	1	Hydracrylate(m) + NAD+(m) → NADH(m) + 3-Oxopropanoate(m)

Continued on next page

Methionine degr (419)– continued

ID	flux	reaction
r2678	1	Acetyl-CoA(m) ⇌ CoA(m) + CoA-activated acetyl group(m)
r2526	1	Serine(s) ⇌ Serine(c)
r0947	-1	Citrulline(m) + Ornithine(c) ⇌ Citrulline(c) + Ornithine(m)
r0232	-1	THF(c) + Methionine(c) ⇌ 5-Methyl-THF(c) + Homocysteine(c)
r1320	-2	ATP(m) + H2O(m) ⇌ ADP(m) + Pi(m) + ATP-energy(m)
r1322	1	NADH(c) ⇌ NAD+(c) + NADH-redox-potential(c)
r0683	1	Propanoyl-CoA(m) + Ubiquinone(m) → Ubiquinol(m) + Acrylyl-CoA(m)
r1323	2	NADH(m) ⇌ NAD+(m) + NADH-redox-potential(m)
r0104	1	NAD+(c) + Malate(c) ⇌ OAA(c) + NADH(c)
r1047	1	Pi(c) + H+(PG)(c) → Pi(m) + H+(PG)(m)
r0258	-1	Fumarate(c) + H2O(c) ⇌ Malate(c)
r2419	1	AKG(c) + Pi(m) ⇌ AKG(m) + Pi(c)
r0838	2	NH3(c) ⇌ NH3(m)
r0851	1	H2O(m) ⇌ H2O(c)
r0109	-1	OAA(c) + Glutamate(c) ⇌ AKG(c) + Aspartate(c)

Phenylalanine degr (420)

Definition			Solution												
			exchanges		reactions								transp		
Objective	Constraints	Comment	imports	exports	c	m	r	p	l	n	s	b	s-c	b-c	int
-1 Phenylalanine(c)	MCES +Urea	Phenylalanine degradation using palmitate excreting urea	0.5 H_2O(s) 3 O_2(s) 1 Phenylalanine(c) 3 ATP-energy(c) 1 ATP-energy(m) 0.5 NADPH-redox-potential(c) 0.5 Proton-gradient(m)	1.5 CO_2(s) 0.5 Pyruvate(c) 0.5 Pyruvate(m) 0.5 Urea(s) 0.5 NADH-redox-potential(c) 1 NADPH-redox-potential(m) 2 CoA-activated acetyl group(c)	22	6	-	-	-	-	-	-	4	-	4

ID	flux	reaction
r0896	0.5	Urea(c) ⇌ Urea(s)
r0820	-0.5	AKG(m) + Malate(c) ⇌ AKG(c) + Malate(m)
r0109	-0.5	OAA(c) + Glutamate(c) ⇌ AKG(c) + Aspartate(c)
r0605	1	4-Maleylacetoacetate(c) ⇌ Fumarylacetoacetate(c)
r1326	-0.5	NADPH(c) ⇌ NADPH-redox-potential(c) + NADP+(c)
r0543	1	Homogentisate(c) + O2(c) ⇌ 4-Maleylacetoacetate(c)
r0453	0.5	ATP(c) + Citrulline(c) + Aspartate(c) ⇌ AMP(c) + PPi(c) + Argininosuccinate(c)
r0183	1	Tyrosine(c) + AKG(c) ⇌ 4-Hydroxyphenylpyruvate(c) + Glutamate(c)
r0075	0.5	Glutamate(m) + H2O(m) + NADP+(m) ⇌ AKG(m) + NADPH(m) + NH3(m)
r1327	1	NADPH(m) ⇌ NADPH-redox-potential(m) + NADP+(m)
r0544	1	4-Hydroxyphenylpyruvate(c) + O2(c) ⇌ Homogentisate(c) + CO2(c)
r0058	0.5	Malate(c) + NADP+(c) ⇌ Pyruvate(c) + CO2(c) + NADPH(c)
r1319	-3	ATP(c) + H2O(c) ⇌ ADP(c) + Pi(c) + ATP-energy(c)
r0329	0.5	Carbamoyl-P(m) + Ornithine(m) ⇌ Pi(m) + Citrulline(m)
r0059	0.5	Malate(m) + NADP+(m) ⇌ Pyruvate(m) + CO2(m) + NADPH(m)
r0258	-1.5	Fumarate(c) + H2O(c) ⇌ Malate(c)
r0069	-1	CoA(c) + Acetoacetyl-CoA(c) ⇌ 2 Acetyl-CoA(c)
r0030	1.5	ATP(c) + AMP(c) ⇌ 2 ADP(c)
r0855	3	O2(s) ⇌ O2(c)
r0320	1	ATP(c) + CoA(c) + Acetoacetate(c) → AMP(c) + PPi(c) + Acetoacetyl-CoA(c)
r0104	0.5	NAD+(c) + Malate(c) ⇌ OAA(c) + NADH(c)
r1320	-1	ATP(m) + H2O(m) ⇌ ADP(m) + Pi(m) + ATP-energy(m)
r1330	-0.5	H+(PG)(c) ⇌ H+(PG)(m) + Proton-gradient(m)
r0947	-0.5	Citrulline(m) + Ornithine(c) ⇌ Citrulline(c) + Ornithine(m)
r0875	0.5	Glutamate(c) + H+(PG)(c) → Glutamate(m) + H+(PG)(m)
r0398	1	Dihydrobiopterin(c) + NADPH(c) ⇌ Tetrahydrobiopterin(c) + NADP+(c)
r0034	0.5	2 ATP(m) + CO2(m) + H2O(m) + NH3(m) ⇌ 2 ADP(m) + Pi(m) + Carbamoyl-P(m)

Continued on next page

ID	flux	reaction
r0007	1.5	PPi(c) + H2O(c) → 2 Pi(c)
r1322	0.5	NADH(c) ⇌ NAD+(c) + NADH-redox-potential(c)
r0867	1.5	CO2(c) ⇌ CO2(s)
r0849	0.5	H2O(s) ⇌ H2O(c)
r0399	1	Tetrahydrobiopterin(c) + Phenylalanine(c) + O2(c) ⇌ Dihydrobiopterin(c) + Tyrosine(c) + H2O(c)
r2677	2	Acetyl-CoA(c) ⇌ CoA(c) + CoA-activated acetyl group(c)
r0324	-1	Fumarylacetoacetate(c) + H2O(c) ⇌ Fumarate(c) + Acetoacetate(c)
r0261	0.5	Argininosuccinate(c) ⇌ Fumarate(c) + Arginine(c)
r0144	0.5	Arginine(c) + H2O(c) ⇌ Urea(c) + Ornithine(c)

Proline degr (421)

Definition			Solution												
			exchanges		reactions								transp		
Objective	Constraints	Comment	imports	exports	c	m	r	p	l	n	s	b	s-c	b-c	int
-1 Proline(c)	MCES +Urea	Proline degradation using palmitate excreting urea	4.5 H_2O(s) 1 Proline(c) 1 ATP-energy(c) 0.5 Proton-gradient(m)	1.5 CO_2(s) 0.5 Pyruvate(c) 0.5 Pyruvate(m) 0.5 Urea(s) 0.5 NADH-redox-potential(c) 2 NADH-redox-potential(m) 0.5 NADPH-redox-potential(c) 1 NADPH-redox-potential(m)	12	14	-	-	-	-	-	-	3	-	7

ID	flux	reaction
r0896	0.5	Urea(c) ⇌ Urea(s)
r0820	0.5	AKG(c) + Malate(m) ⇌ AKG(m) + Malate(c)
r0109	-0.5	OAA(c) + Glutamate(c) ⇌ AKG(c) + Aspartate(c)
r1424	0.5	Proline(c) + H+(PG)(c) → Proline(m) + H+(PG)(m)
r0911	0.5	Proline(c) + Glutamate(m) ⇌ Proline(m) + Glutamate(c)
r0074	1	L-Glutamate 5-semialdehyde(m) + NAD+(m) + H2O(m) ⇌ Glutamate(m) + NADH(m)
r1326	0.5	NADPH(c) ⇌ NADPH-redox-potential(c) + NADP+(c)
r0453	0.5	ATP(c) + Citrulline(c) + Aspartate(c) ⇌ AMP(c) + PPi(c) + Argininosuccinate(c)
r0075	0.5	Glutamate(m) + H2O(m) + NADP+(m) ⇌ AKG(m) + NADPH(m) + NH3(m)
r1453	1	Proline(m) + Ubiquinol(m) ⇌ 1-Pyrroline-5-carboxylate(m) + Ubiquinone(m)
r1327	1	NADPH(m) ⇌ NADPH-redox-potential(m) + NADP+(m)
r0175	1	1-Pyrroline-5-carboxylate(m) + H2O(m) ⇌ L-Glutamate 5-semialdehyde(m)
r0058	0.5	Malate(c) + NADP+(c) ⇌ Pyruvate(c) + CO2(c) + NADPH(c)
r1319	-1	ATP(c) + H2O(c) ⇌ ADP(c) + Pi(c) + ATP-energy(c)
r0851	-4	H2O(m) ⇌ H2O(c)
r0509	1	Ubiquinone(m) + Succinate(m) → Ubiquinol(m) + Fumarate(m)
r0329	0.5	Carbamoyl-P(m) + Ornithine(m) ⇌ Pi(m) + Citrulline(m)
r0100	-1	ADP(m) + GTP(m) ⇌ ATP(m) + GDP(m)
r0059	0.5	Malate(m) + NADP+(m) ⇌ Pyruvate(m) + CO2(m) + NADPH(m)
r0258	-0.5	Malate(c) ⇌ Fumarate(c) + H2O(c)
r0259	-1	Malate(m) ⇌ Fumarate(m) + H2O(m)
r0030	0.5	ATP(c) + AMP(c) ⇌ 2 ADP(c)
r0104	0.5	NAD+(c) + Malate(c) ⇌ OAA(c) + NADH(c)
r1330	-0.5	H+(PG)(c) ⇌ H+(PG)(m) + Proton-gradient(m)
r1114	1	AKG(m) + CoA(m) + NAD+(m) ⇌ NADH(m) + CO2(m) + Succinyl-CoA(m)
r0947	-0.5	Citrulline(m) + Ornithine(c) ⇌ Citrulline(c) + Ornithine(m)
r0034	0.5	2 ATP(m) + CO2(m) + H2O(m) + NH3(m) ⇌ 2 ADP(m) + Pi(m) + Carbamoyl-P(m)
r0007	0.5	PPi(c) + H2O(c) → 2 Pi(c)
r1322	0.5	NADH(c) ⇌ NAD+(c) + NADH-redox-potential(c)
r0867	1.5	CO2(c) ⇌ CO2(s)
r0849	4.5	H2O(s) ⇌ H2O(c)
r0125	-1	GDP(m) + Pi(m) + Succinyl-CoA(m) ⇌ GTP(m) + Succinate(m) + CoA(m)
r1323	2	NADH(m) ⇌ NAD+(m) + NADH-redox-potential(m)

Continued on next page

ID	flux	reaction
r0868	-1	CO2(c) ⇌ CO2(m)
r0261	0.5	Argininosuccinate(c) ⇌ Fumarate(c) + Arginine(c)
r0144	0.5	Arginine(c) + H2O(c) ⇌ Urea(c) + Ornithine(c)

Serine degr (422)

Definition			Solution												
			exchanges		reactions								transp		
Objective	Constraints	Comment	imports	exports	c	m	r	p	l	n	s	b	s-c	b-c	int
-1 Serine(c)	MCES +Urea	Serine degradation using palmitate excreting urea	0.5 Pyruvate(m) 1 Serine(c) 1 ATP-energy(c) 1 ATP-energy(m) 0.5 NADPH-redox-potential(c)	0.5 H_2O(s) 1 Pyruvate(c) 0.5 Urea(s) 0.5 NADH-redox-potential(c) 0.5 NADH-redox-potential(m) 0.5 CoA-activated acetyl group(m)	19	6	-	-	-	-	-	-	2	-	3

ID	flux	reaction
r2678	0.5	Acetyl-CoA(m) ⇌ CoA(m) + CoA-activated acetyl group(m)
r0896	0.5	Urea(c) ⇌ Urea(s)
r0109	-0.5	OAA(c) + Glutamate(c) ⇌ AKG(c) + Aspartate(c)
r0164	0.5	2PG(c) ⇌ PEP(c) + H2O(c)
r0056	0.5	Pyruvate(m) + CoA(m) + NAD+(m) → Acetyl-CoA(m) + CO2(m) + NADH(m)
r1326	-0.5	NADPH(c) ⇌ NADPH-redox-potential(c) + NADP+(c)
r0453	0.5	ATP(c) + Citrulline(c) + Aspartate(c) ⇌ AMP(c) + PPi(c) + Argininosuccinate(c)
r0327	-0.5	Hydroxypyruvate(c) + NADPH(c) → Glycerate(c) + NADP+(c)
r1319	-1	ATP(c) + H2O(c) ⇌ ADP(c) + Pi(c) + ATP-energy(c)
r0851	0.5	H2O(m) ⇌ H2O(c)
r0329	0.5	Carbamoyl-P(m) + Ornithine(m) ⇌ Pi(m) + Citrulline(m)
r0339	0.5	ATP(c) + Glycerate(c) → ADP(c) + 3PG(c)
r0258	-0.5	Fumarate(c) + H2O(c) ⇌ Malate(c)
r0030	0.5	ATP(c) + AMP(c) ⇌ 2 ADP(c)
r0104	0.5	NAD+(c) + Malate(c) ⇌ OAA(c) + NADH(c)
r1320	-1	ATP(m) + H2O(m) ⇌ ADP(m) + Pi(m) + ATP-energy(m)
r0838	0.5	NH3(c) ⇌ NH3(m)
r0060	0.5	Serine(c) → Pyruvate(c) + NH3(c)
r0947	-0.5	Citrulline(m) + Ornithine(c) ⇌ Citrulline(c) + Ornithine(m)
r0160	0.5	Serine(c) + Pyruvate(c) → Hydroxypyruvate(c) + Alanine(c)
r0034	0.5	2 ATP(m) + CO2(m) + H2O(m) + NH3(m) ⇌ 2 ADP(m) + Pi(m) + Carbamoyl-P(m)
r0007	0.5	PPi(c) + H2O(c) → 2 Pi(c)
r1322	0.5	NADH(c) ⇌ NAD+(c) + NADH-redox-potential(c)
r0849	-0.5	H2O(s) ⇌ H2O(c)
r0341	-0.5	3PG(c) ⇌ 2PG(c)
r0080	0.5	Alanine(c) + AKG(c) ⇌ Pyruvate(c) + Glutamate(c)
r1323	0.5	NADH(m) ⇌ NAD+(m) + NADH-redox-potential(m)
r0261	0.5	Argininosuccinate(c) ⇌ Fumarate(c) + Arginine(c)
r0144	0.5	Arginine(c) + H2O(c) ⇌ Urea(c) + Ornithine(c)
r0054	-0.5	ADP(c) + PEP(c) → ATP(c) + Pyruvate(c)

Threonine degr (423)

Definition			Solution												
			exchanges		reactions								transp		
Objective	Constraints	Comment	imports	exports	c	m	r	p	l	n	s	b	s-c	b-c	int
-1 Threonine(c)	MCES +Urea	Threonine degradation using palmitate excreting urea	0.5 H_2O(s) 0.5 O_2(s) 1 Threonine(c) 1 ATP-energy(c) 1 ATP-energy(m) 0.5 NADPH-redox-potential(m)	1.5 CO_2(s) 0.5 Urea(s) 0.5 NADH-redox-potential(c) 3 NADH-redox-potential(m) 4 Proton-gradient(m) 1 CoA-activated acetyl group(m)	11	13	-	-	-	-	-	-	4	-	12

ID	flux	reaction
r0855	0.5	O2(s) ⇌ O2(c)
r0856	-0.5	O2(m) ⇌ O2(c)
r0144	0.5	Arginine(c) + H2O(c) ⇌ Urea(c) + Ornithine(c)
r0592	-1	H2O(m) + Acrylyl-CoA(m) → 3-Hydroxypropionyl-CoA(m)
r0596	-1	3-Hydroxypropionyl-CoA(m) + H2O(m) → Hydracrylate(m) + CoA(m)
r0896	0.5	Urea(c) ⇌ Urea(s)
r0017	0.5	O2(m) + 4 H+(PG)(m) + 4 Ferrocytochrome C(m) → 4 Ferricytochrome C(m) + 2 H2O(m) + 4 H+(PG)(c)
r0030	0.5	ATP(c) + AMP(c) ⇌ 2 ADP(c)
r0034	0.5	2 ATP(m) + CO2(m) + H2O(m) + NH3(m) ⇌ 2 ADP(m) + Pi(m) + Carbamoyl-P(m)
r0911	0.5	Proline(c) + Glutamate(m) ⇌ Proline(m) + Glutamate(c)
r1426	0.5	Proline(m) + H+(PG)(c) → Proline(c) + H+(PG)(m)
r0075	-0.5	AKG(m) + NADPH(m) + NH3(m) ⇌ Glutamate(m) + H2O(m) + NADP+(m)
r0507	1	Ubiquinol(m) + 2 Ferricytochrome C(m) + 4 H+(PG)(m) → Ubiquinone(m) + 4 H+(PG)(c) + 2 Ferrocytochrome C(m)
r0240	1	Threonine(c) ⇌ 2-Oxobutyrate(c) + NH3(c)
r1330	4	H+(PG)(c) ⇌ H+(PG)(m) + Proton-gradient(m)
r1319	-1	ATP(c) + H2O(c) ⇌ ADP(c) + Pi(c) + ATP-energy(c)
r0261	0.5	Argininosuccinate(c) ⇌ Fumarate(c) + Arginine(c)
r0849	0.5	H2O(s) ⇌ H2O(c)
r0867	1.5	CO2(c) ⇌ CO2(s)
r0868	-1.5	CO2(c) ⇌ CO2(m)
r0453	0.5	ATP(c) + Citrulline(c) + Aspartate(c) ⇌ AMP(c) + PPi(c) + Argininosuccinate(c)
r0007	0.5	PPi(c) + H2O(c) → 2 Pi(c)
r0174	1	CoA(m) + NAD+(m) + 3-Oxopropanoate(m) → Acetyl-CoA(m) + CO2(m) + NADH(m)
r0329	0.5	Carbamoyl-P(m) + Ornithine(m) ⇌ Pi(m) + Citrulline(m)
r1154	1	CoA(m) + NAD+(m) + 2-Oxobutyrate(m) ⇌ CO2(m) + Propanoyl-CoA(m) + NADH(m)
r1155	1	2-Oxobutyrate(c) + H+(PG)(c) → 2-Oxobutyrate(m) + H+(PG)(m)
r0365	1	Hydracrylate(m) + NAD+(m) → NADH(m) + 3-Oxopropanoate(m)
r2678	1	Acetyl-CoA(m) ⇌ CoA(m) + CoA-activated acetyl group(m)
r0947	-0.5	Citrulline(m) + Ornithine(c) ⇌ Citrulline(c) + Ornithine(m)
r1320	-1	ATP(m) + H2O(m) ⇌ ADP(m) + Pi(m) + ATP-energy(m)
r1322	0.5	NADH(c) ⇌ NAD+(c) + NADH-redox-potential(c)
r0683	1	Propanoyl-CoA(m) + Ubiquinone(m) → Ubiquinol(m) + Acrylyl-CoA(m)
r1323	3	NADH(m) ⇌ NAD+(m) + NADH-redox-potential(m)
r1327	-0.5	NADPH(m) ⇌ NADPH-redox-potential(m) + NADP+(m)
r0104	0.5	NAD+(c) + Malate(c) ⇌ OAA(c) + NADH(c)
r1047	0.5	Pi(c) + H+(PG)(c) → Pi(m) + H+(PG)(m)
r0258	-0.5	Fumarate(c) + H2O(c) ⇌ Malate(c)
r2419	0.5	AKG(c) + Pi(m) ⇌ AKG(m) + Pi(c)
r0838	1	NH3(c) ⇌ NH3(m)
r0109	-0.5	OAA(c) + Glutamate(c) ⇌ AKG(c) + Aspartate(c)

Tryptophan degr (424)

Definition			Solution												
			exchanges		reactions								transp		
Objective	Constraints	Comment	imports	exports	c	m	r	p	l	n	s	b	s-c	b-c	int
-1 Tryptophan(c)	MCES +Urea	Tryptophan degradation using palmitate excreting urea	1 H_2O(s) 3.5 O_2(s) 1 Pyruvate(m) 1 Tryptophan(c) 3 ATP-energy(c) 2 ATP-energy(m) 2 NADPH-redox-potential(c) 1 NADPH-redox-potential(m)	2 CO_2(s) 2 Pyruvate(c) 1 Urea(s) 3 NADH-redox-potential(m) 5 Proton-gradient(m) 1 THF-activated methyl group(c) 2 CoA-activated acetyl group(m)	23	14	-	-	-	-	-	-	4	-	9

ID	flux	reaction
r0855	3.5	O2(s) ⇌ O2(c)
r0856	-0.5	O2(m) ⇌ O2(c)
r0293	-1	5,10-Methylene-THF(c) + NADP+(c) ⇌ 5,10-Methenyl-THF(c) + NADPH(c)
r0144	1	Arginine(c) + H2O(c) ⇌ Urea(c) + Ornithine(c)
r0876	1	Glutamate(c) + H+(PG)(c) + Aspartate(m) → Glutamate(m) + H+(PG)(m) + Aspartate(c)
r0460	1	(S)-3-Hydroxybutyryl-CoA(m) + NAD+(m) ⇌ NADH(m) + Acetoacetyl-CoA(m)
r0448	1	2-Oxoadipate(m) + CoA(m) + NAD+(m) ⇌ Glutaryl-CoA(m) + CO2(m) + NADH(m)
r0896	1	Urea(c) ⇌ Urea(s)
r0449	-1	2-Oxoadipate(c) + NH3(c) + NADP+(c) ⇌ 2-Aminomuconate(c) + NADPH(c) + H2O(c)
r0017	0.5	O2(m) + 4 H+(PG)(m) + 4 Ferrocytochrome C(m) → 4 Ferricytochrome C(m) + 2 H2O(m) + 4 H+(PG)(c)
r0030	1	ATP(c) + AMP(c) ⇌ 2 ADP(c)
r0169	1	Tryptophan(c) + O2(c) ⇌ L-Formylkynurenine(c)
r0034	1	2 ATP(m) + CO2(m) + H2O(m) + NH3(m) ⇌ 2 ADP(m) + Pi(m) + Carbamoyl-P(m)
r0070	-1	CoA(m) + Acetoacetyl-CoA(m) ⇌ 2 Acetyl-CoA(m)
r0058	1	Malate(c) + NADP+(c) ⇌ Pyruvate(c) + CO2(c) + NADPH(c)
r0059	-1	Pyruvate(m) + CO2(m) + NADPH(m) ⇌ Malate(m) + NADP+(m)
r0371	-1	10-Formyl-THF(c) ⇌ 5,10-Methenyl-THF(c) + H2O(c)
r0507	1	Ubiquinol(m) + 2 Ferricytochrome C(m) + 4 H+(PG)(m) → Ubiquinone(m) + 4 H+(PG)(c) + 2 Ferrocytochrome C(m)
r0228	1	THF(c) + Formate(c) + ATP(c) → ADP(c) + Pi(c) + 10-Formyl-THF(c)
r0676	1	2-Amino-3-carboxymuconate semialdehyde(c) ⇌ 2-Aminomuconate semialdehyde(c) + CO2(c)
r1330	5	H+(PG)(c) ⇌ H+(PG)(m) + Proton-gradient(m)
r1319	-3	ATP(c) + H2O(c) ⇌ ADP(c) + Pi(c) + ATP-energy(c)
r0541	1	Glutaryl-CoA(m) + Ubiquinone(m) ⇌ Crotonyl-CoA(m) + CO2(m) + Ubiquinol(m)
r0261	1	Argininosuccinate(c) ⇌ Fumarate(c) + Arginine(c)
r0110	-1	OAA(m) + Glutamate(m) → AKG(m) + Aspartate(m)
r0562	1	3-Hydroxyanthranilate(c) + O2(c) ⇌ 2-Amino-3-carboxymuconate semialdehyde(c)
r0563	1	H2O(c) + 3-Hydroxy-L-kynurenine(c) ⇌ 3-Hydroxyanthranilate(c) + Alanine(c)
r0849	1	H2O(s) ⇌ H2O(c)
r0867	2	CO2(c) ⇌ CO2(s)
r0588	-1	Crotonyl-CoA(m) + H2O(m) ⇌ (S)-3-Hydroxybutyryl-CoA(m)
r0453	1	ATP(c) + Citrulline(c) + Aspartate(c) ⇌ AMP(c) + PPi(c) + Argininosuccinate(c)
r0007	1	PPi(c) + H2O(c) → 2 Pi(c)
r0454	1	L-Formylkynurenine(c) + H2O(c) ⇌ Formate(c) + Kynurenine(c)
r0455	1	Kynurenine(c) + O2(c) + NADPH(c) ⇌ H2O(c) + 3-Hydroxy-L-kynurenine(c) + NADP+(c)
r0329	1	Carbamoyl-P(m) + Ornithine(m) ⇌ Pi(m) + Citrulline(m)
r0792	-1	5,10-Methylene-THF(c) + NADH(c) ⇌ 5-Methyl-THF(c) + NAD+(c)
r0080	1	Alanine(c) + AKG(c) ⇌ Pyruvate(c) + Glutamate(c)
r0645	1	2-Aminomuconate semialdehyde(c) + NAD+(c) + H2O(c) ⇌ 2-Aminomuconate(c) + NADH(c)
r2676	1	5-Methyl-THF(c) ⇌ THF(c) + THF-activated methyl group(c)
r2678	2	Acetyl-CoA(m) ⇌ CoA(m) + CoA-activated acetyl group(m)
r0945	1	2-Oxoadipate(c) + AKG(m) ⇌ 2-Oxoadipate(m) + AKG(c)
r0947	-1	Citrulline(m) + Ornithine(c) ⇌ Citrulline(c) + Ornithine(m)
r1320	-2	ATP(m) + H2O(m) ⇌ ADP(m) + Pi(m) + ATP-energy(m)
r1323	3	NADH(m) ⇌ NAD+(m) + NADH-redox-potential(m)
r1326	-2	NADPH(c) ⇌ NADPH-redox-potential(c) + NADP+(c)
r1327	-1	NADPH(m) ⇌ NADPH-redox-potential(m) + NADP+(m)
r0105	1	NAD+(m) + Malate(m) ⇌ OAA(m) + NADH(m)

Continued on next page

Tryptophan degr (424)– continued

ID	flux	reaction
r0258	-1	Fumarate(c) + H2O(c) ⇌ Malate(c)
r0838	1	NH3(c) ⇌ NH3(m)
r0851	1	H2O(m) ⇌ H2O(c)

Valine degr (425)

Definition			Solution												
			exchanges		reactions								transp		
Objective	Constraints	Comment	imports	exports	c	m	r	p	l	n	s	b	s-c	b-c	int
-1 Valine(c)	MCES +Urea	Valine degradation using palmitate excreting urea	2.5 H_2O(s) 1 O_2(s) 1 Valine(c) 1 ATP-energy(c)	1.5 CO_2(s) 0.5 Pyruvate(c) 0.5 Pyruvate(m) 0.5 Urea(s) 0.5 NADH-redox-potential(c) 3 NADH-redox-potential(m) 0.5 NADPH-redox-potential(c) 1 NADPH-redox-potential(m) 11.5 Proton-gradient(m)	12	18	-	-	-	-	-	-	4	-	11

ID	flux	reaction
r0125	-1	GDP(m) + Pi(m) + Succinyl-CoA(m) ⇌ GTP(m) + Succinate(m) + CoA(m)
r0855	1	O2(s) ⇌ O2(c)
r0856	-1	O2(m) ⇌ O2(c)
r0144	0.5	Arginine(c) + H2O(c) ⇌ Urea(c) + Ornithine(c)
r0896	0.5	Urea(c) ⇌ Urea(s)
r0017	1	O2(m) + 4 H+(PG)(m) + 4 Ferrocytochrome C(m) → 4 Ferricytochrome C(m) + 2 H2O(m) + 4 H+(PG)(c)
r0030	0.5	ATP(c) + AMP(c) ⇌ 2 ADP(c)
r0034	0.5	2 ATP(m) + CO2(m) + H2O(m) + NH3(m) ⇌ 2 ADP(m) + Pi(m) + Carbamoyl-P(m)
r0482	1	3-Hydroxyisobutyrate(m) + NAD+(m) → 2-Methyl-3-oxopropanoate(m) + NADH(m)
r0911	0.5	Proline(c) + Glutamate(m) ⇌ Proline(m) + Glutamate(c)
r1426	0.5	Proline(m) + H+(PG)(c) → Proline(c) + H+(PG)(m)
r0058	0.5	Malate(c) + NADP+(c) ⇌ Pyruvate(c) + CO2(c) + NADPH(c)
r0059	0.5	Malate(m) + NADP+(m) ⇌ Pyruvate(m) + CO2(m) + NADPH(m)
r0075	0.5	Glutamate(m) + H2O(m) + NADP+(m) ⇌ AKG(m) + NADPH(m) + NH3(m)
r0507	2	Ubiquinol(m) + 2 Ferricytochrome C(m) + 4 H+(PG)(m) → Ubiquinone(m) + 4 H+(PG)(c) + 2 Ferrocytochrome C(m)
r0509	1	Ubiquinone(m) + Succinate(m) → Ubiquinol(m) + Fumarate(m)
r0820	0.5	AKG(c) + Malate(m) ⇌ AKG(m) + Malate(c)
r1330	11.5	H+(PG)(c) ⇌ H+(PG)(m) + Proton-gradient(m)
r1319	-1	ATP(c) + H2O(c) ⇌ ADP(c) + Pi(c) + ATP-energy(c)
r0261	0.5	Argininosuccinate(c) ⇌ Fumarate(c) + Arginine(c)
r0263	1	Valine(m) + AKG(m) → Glutamate(m) + 3-Methyl-2-oxobutyrate(m)
r0560	1	Isobutyryl-CoA(m) + Ubiquinone(m) → Methacrylyl-CoA(m) + Ubiquinol(m)
r0849	2.5	H2O(s) ⇌ H2O(c)
r0867	1.5	CO2(c) ⇌ CO2(s)
r0868	-1	CO2(c) ⇌ CO2(m)
r0453	0.5	ATP(c) + Citrulline(c) + Aspartate(c) ⇌ AMP(c) + PPi(c) + Argininosuccinate(c)
r0007	0.5	PPi(c) + H2O(c) → 2 Pi(c)
r0600	1	3-Methyl-2-oxobutyrate(m) + CoA(m) + NAD+(m) ⇌ Isobutyryl-CoA(m) + CO2(m) + NADH(m)
r0197	1	(R)-Methylmalonyl-CoA(m) ⇌ Succinyl-CoA(m)
r0329	0.5	Carbamoyl-P(m) + Ornithine(m) ⇌ Pi(m) + Citrulline(m)
r0624	1	2-Methyl-3-oxopropanoate(m) + CoA(m) + NAD+(m) ⇌ (R)-Methylmalonyl-CoA(m) + NADH(m)
r0779	1	3-Hydroxyisobutyryl-CoA(m) + H2O(m) → 3-Hydroxyisobutyrate(m) + CoA(m)
r1439	1	Valine(c) → Valine(m)
r0947	-0.5	Citrulline(m) + Ornithine(c) ⇌ Citrulline(c) + Ornithine(m)
r0669	1	Methacrylyl-CoA(m) + H2O(m) → 3-Hydroxyisobutyryl-CoA(m)
r1322	0.5	NADH(c) ⇌ NAD+(c) + NADH-redox-potential(c)
r1323	3	NADH(m) ⇌ NAD+(m) + NADH-redox-potential(m)
r0100	-1	ADP(m) + GTP(m) ⇌ ATP(m) + GDP(m)

Continued on next page

ID	flux	reaction
r1326	0.5	NADPH(c) ⇌ NADPH-redox-potential(c) + NADP+(c)
r1327	1	NADPH(m) ⇌ NADPH-redox-potential(m) + NADP+(m)
r0104	0.5	NAD+(c) + Malate(c) ⇌ OAA(c) + NADH(c)
r0258	-0.5	Malate(c) ⇌ Fumarate(c) + H2O(c)
r0259	-1	Malate(m) ⇌ Fumarate(m) + H2O(m)
r0851	-2	H2O(m) ⇌ H2O(c)
r0109	-0.5	OAA(c) + Glutamate(c) ⇌ AKG(c) + Aspartate(c)

Glycine degr (426)

Definition			Solution													
			exchanges		reactions								transp		
Objective	Constraints	Comment	imports	exports	c	m	r	p	l	n	s	b	s-c	b-c	int
-1 Glycine(c)	MCES +Urea −Methionine $+H_2S$	Glycine degradation using palmitate excreting urea	0.5 H_2O(s) 0.5 Pyruvate(m) 1 Glycine(c) 1 ATP-energy(c) 1 ATP-energy(m) 1 THF-activated methyl group(c)	1 Pyruvate(c) 0.5 Urea(s) 0.5 NADH-redox-potential(c) 0.5 NADH-redox-potential(m) 0.5 NADPH-redox-potential(c) 0.5 CoA-activated acetyl group(m)	22	6	-	-	-	-	-	-	2	-	3

ID	flux	reaction
r2678	0.5	Acetyl-CoA(m) ⇌ CoA(m) + CoA-activated acetyl group(m)
r0896	0.5	Urea(c) ⇌ Urea(s)
r0109	-0.5	OAA(c) + Glutamate(c) ⇌ AKG(c) + Aspartate(c)
r0164	0.5	2PG(c) ⇌ PEP(c) + H2O(c)
r0056	0.5	Pyruvate(m) + CoA(m) + NAD+(m) → Acetyl-CoA(m) + CO2(m) + NADH(m)
r1326	0.5	NADPH(c) ⇌ NADPH-redox-potential(c) + NADP+(c)
r0453	0.5	ATP(c) + Citrulline(c) + Aspartate(c) ⇌ AMP(c) + PPi(c) + Argininosuccinate(c)
r0327	-0.5	Hydroxypyruvate(c) + NADPH(c) → Glycerate(c) + NADP+(c)
r1319	-1	ATP(c) + H2O(c) ⇌ ADP(c) + Pi(c) + ATP-energy(c)
r0851	0.5	H2O(m) ⇌ H2O(c)
r0329	0.5	Carbamoyl-P(m) + Ornithine(m) ⇌ Pi(m) + Citrulline(m)
r0339	0.5	ATP(c) + Glycerate(c) → ADP(c) + 3PG(c)
r0258	-0.5	Fumarate(c) + H2O(c) ⇌ Malate(c)
r0030	0.5	ATP(c) + AMP(c) ⇌ 2 ADP(c)
r0296	1	5-Methyl-THF(c) + NADP+(c) ⇌ 5,10-Methylene-THF(c) + NADPH(c)
r0230	1	5,10-Methylene-THF(c) + Glycine(c) + H2O(c) ⇌ THF(c) + Serine(c)
r0104	0.5	NAD+(c) + Malate(c) ⇌ OAA(c) + NADH(c)
r1320	-1	ATP(m) + H2O(m) ⇌ ADP(m) + Pi(m) + ATP-energy(m)
r0838	0.5	NH3(c) ⇌ NH3(m)
r0060	0.5	Serine(c) → Pyruvate(c) + NH3(c)
r0947	-0.5	Citrulline(m) + Ornithine(c) ⇌ Citrulline(c) + Ornithine(m)
r0160	0.5	Serine(c) + Pyruvate(c) → Hydroxypyruvate(c) + Alanine(c)
r0034	0.5	2 ATP(m) + CO2(m) + H2O(m) + NH3(m) ⇌ 2 ADP(m) + Pi(m) + Carbamoyl-P(m)
r0007	0.5	PPi(c) + H2O(c) → 2 Pi(c)
r2676	-1	5-Methyl-THF(c) ⇌ THF(c) + THF-activated methyl group(c)
r1322	0.5	NADH(c) ⇌ NAD+(c) + NADH-redox-potential(c)
r0849	0.5	H2O(s) ⇌ H2O(c)
r0341	-0.5	3PG(c) ⇌ 2PG(c)
r0080	0.5	Alanine(c) + AKG(c) ⇌ Pyruvate(c) + Glutamate(c)
r1323	0.5	NADH(m) ⇌ NAD+(m) + NADH-redox-potential(m)
r0261	0.5	Argininosuccinate(c) ⇌ Fumarate(c) + Arginine(c)
r0144	0.5	Arginine(c) + H2O(c) ⇌ Urea(c) + Ornithine(c)
r0054	-0.5	ADP(c) + PEP(c) → ATP(c) + Pyruvate(c)

Isoleucine degr (427)

Definition			Solution													
			exchanges		reactions								transp		
Objective	Constraints	Comment	imports	exports	c	m	r	p	l	n	s	b	s-c	b-c	int
-1 Isoleucine(c)	MCES +Urea	Isoleucine degradation using palmitate excreting urea	1.5 H_2O(s) 1 O_2(s) 1 Isoleucine(c) 1 ATP-energy(c) 1 ATP-energy(m)	1.5 CO_2(s) 0.5 Urea(s) 0.5 NADH-redox-potential(c) 4 NADH-redox-potential(m) 0.5 NADPH-redox-potential(m) 10 Proton-gradient(m) 2 CoA-activated acetyl group(m)	10	18	-	-	-	-	-	-	4	-	12

ID	flux	reaction
r0855	1	O2(s) ⇌ O2(c)
r0856	-1	O2(m) ⇌ O2(c)
r0144	0.5	Arginine(c) + H2O(c) ⇌ Urea(c) + Ornithine(c)
r0592	-1	H2O(m) + Acrylyl-CoA(m) → 3-Hydroxypropionyl-CoA(m)
r0596	-1	3-Hydroxypropionyl-CoA(m) + H2O(m) → Hydracrylate(m) + CoA(m)
r0896	0.5	Urea(c) ⇌ Urea(s)
r0017	1	O2(m) + 4 H+(PG)(m) + 4 Ferrocytochrome C(m) → 4 Ferricytochrome C(m) + 2 H2O(m) + 4 H+(PG)(c)
r0030	0.5	ATP(c) + AMP(c) ⇌ 2 ADP(c)
r0034	0.5	2 ATP(m) + CO2(m) + H2O(m) + NH3(m) ⇌ 2 ADP(m) + Pi(m) + Carbamoyl-P(m)
r0911	0.5	Proline(c) + Glutamate(m) ⇌ Proline(m) + Glutamate(c)
r1426	0.5	Proline(m) + H+(PG)(c) → Proline(c) + H+(PG)(m)
r0075	0.5	Glutamate(m) + H2O(m) + NADP+(m) ⇌ AKG(m) + NADPH(m) + NH3(m)
r0222	-1	CoA(m) + 2-Methylacetoacetyl-CoA(m) ⇌ Propanoyl-CoA(m) + Acetyl-CoA(m)
r0953	1	Isoleucine(c) + H+(PG)(c) → Isoleucine(m) + H+(PG)(m)
r0507	2	Ubiquinol(m) + 2 Ferricytochrome C(m) + 4 H+(PG)(m) → Ubiquinone(m) + 4 H+(PG)(c) + 2 Ferrocytochrome C(m)
r1330	10	H+(PG)(c) ⇌ H+(PG)(m) + Proton-gradient(m)
r1319	-1	ATP(c) + H2O(c) ⇌ ADP(c) + Pi(c) + ATP-energy(c)
r0261	0.5	Argininosuccinate(c) ⇌ Fumarate(c) + Arginine(c)
r0264	1	Isoleucine(m) + AKG(m) ⇌ Glutamate(m) + 2-Oxo-3-methylvalerate(m)
r0849	1.5	H2O(s) ⇌ H2O(c)
r0867	1.5	CO2(c) ⇌ CO2(s)
r0868	-1.5	CO2(c) ⇌ CO2(m)
r0453	0.5	ATP(c) + Citrulline(c) + Aspartate(c) ⇌ AMP(c) + PPi(c) + Argininosuccinate(c)
r0007	0.5	PPi(c) + H2O(c) → 2 Pi(c)
r0174	1	CoA(m) + NAD+(m) + 3-Oxopropanoate(m) → Acetyl-CoA(m) + CO2(m) + NADH(m)
r0602	1	2-Oxo-3-methylvalerate(m) + CoA(m) + NAD+(m) ⇌ 2-Methylbutyryl-CoA(m) + CO2(m) + NADH(m)
r0603	1	2-Methylbutyryl-CoA(m) + Ubiquinone(m) ⇌ Tiglyl-CoA(m) + Ubiquinol(m)
r0329	0.5	Carbamoyl-P(m) + Ornithine(m) ⇌ Pi(m) + Citrulline(m)
r0365	1	Hydracrylate(m) + NAD+(m) → NADH(m) + 3-Oxopropanoate(m)
r2678	2	Acetyl-CoA(m) ⇌ CoA(m) + CoA-activated acetyl group(m)
r0664	1	NAD+(m) + (S)-3-Hydroxy-2-methylbutyryl-CoA(m) ⇌ 2-Methylacetoacetyl-CoA(m) + NADH(m)
r0947	-0.5	Citrulline(m) + Ornithine(c) ⇌ Citrulline(c) + Ornithine(m)
r0665	-1	H2O(m) + Tiglyl-CoA(m) ⇌ (S)-3-Hydroxy-2-methylbutyryl-CoA(m)
r1320	-1	ATP(m) + H2O(m) ⇌ ADP(m) + Pi(m) + ATP-energy(m)
r1322	0.5	NADH(c) ⇌ NAD+(c) + NADH-redox-potential(c)
r0683	1	Propanoyl-CoA(m) + Ubiquinone(m) → Ubiquinol(m) + Acrylyl-CoA(m)
r1323	4	NADH(m) ⇌ NAD+(m) + NADH-redox-potential(m)
r1327	0.5	NADPH(m) ⇌ NADPH-redox-potential(m) + NADP+(m)
r0104	0.5	NAD+(c) + Malate(c) ⇌ OAA(c) + NADH(c)
r1047	0.5	Pi(c) + H+(PG)(c) → Pi(m) + H+(PG)(m)
r0258	-0.5	Fumarate(c) + H2O(c) ⇌ Malate(c)
r2419	0.5	AKG(c) + Pi(m) ⇌ AKG(m) + Pi(c)
r0851	-1	H2O(m) ⇌ H2O(c)
r0109	-0.5	OAA(c) + Glutamate(c) ⇌ AKG(c) + Aspartate(c)

Tyrosine degr (428)

Definition			Solution												
			exchanges		reactions								transp		
Objective	Constraints	Comment	imports	exports	c	m	r	p	l	n	s	b	s-c	b-c	int
-1 Tyrosine(c)	MCES +Urea	Tyrosine degradation using palmitate excreting urea	1.5 H_2O(s) 2 O_2(s) 1 Tyrosine(c) 3 ATP-energy(c) 1 ATP-energy(m) 0.5 Proton-gradient(m)	1.5 CO_2(s) 0.5 Pyruvate(c) 0.5 Pyruvate(m) 0.5 Urea(s) 0.5 NADH-redox-potential(c) 0.5 NADPH-redox-potential(c) 1 NADPH-redox-potential(m) 2 CoA-activated acetyl group(c)	20	6	-	-	-	-	-	-	4	-	4

ID	flux	reaction
r0896	0.5	Urea(c) ⇌ Urea(s)
r0820	-0.5	AKG(m) + Malate(c) ⇌ AKG(c) + Malate(m)
r0109	-0.5	OAA(c) + Glutamate(c) ⇌ AKG(c) + Aspartate(c)
r0605	1	4-Maleylacetoacetate(c) ⇌ Fumarylacetoacetate(c)
r1326	0.5	NADPH(c) ⇌ NADPH-redox-potential(c) + NADP+(c)
r0543	1	Homogentisate(c) + O2(c) ⇌ 4-Maleylacetoacetate(c)
r0453	0.5	ATP(c) + Citrulline(c) + Aspartate(c) ⇌ AMP(c) + PPi(c) + Argininosuccinate(c)
r0183	1	Tyrosine(c) + AKG(c) ⇌ 4-Hydroxyphenylpyruvate(c) + Glutamate(c)
r0075	0.5	Glutamate(m) + H2O(m) + NADP+(m) ⇌ AKG(m) + NADPH(m) + NH3(m)
r1327	1	NADPH(m) ⇌ NADPH-redox-potential(m) + NADP+(m)
r0544	1	4-Hydroxyphenylpyruvate(c) + O2(c) ⇌ Homogentisate(c) + CO2(c)
r0058	0.5	Malate(c) + NADP+(c) ⇌ Pyruvate(c) + CO2(c) + NADPH(c)
r1319	-3	ATP(c) + H2O(c) ⇌ ADP(c) + Pi(c) + ATP-energy(c)
r0329	0.5	Carbamoyl-P(m) + Ornithine(m) ⇌ Pi(m) + Citrulline(m)
r0059	0.5	Malate(m) + NADP+(m) ⇌ Pyruvate(m) + CO2(m) + NADPH(m)
r0258	-1.5	Fumarate(c) + H2O(c) ⇌ Malate(c)
r0069	-1	CoA(c) + Acetoacetyl-CoA(c) ⇌ 2 Acetyl-CoA(c)
r0030	1.5	ATP(c) + AMP(c) ⇌ 2 ADP(c)
r0855	2	O2(s) ⇌ O2(c)
r0320	1	ATP(c) + CoA(c) + Acetoacetate(c) → AMP(c) + PPi(c) + Acetoacetyl-CoA(c)
r0104	0.5	NAD+(c) + Malate(c) ⇌ OAA(c) + NADH(c)
r1320	-1	ATP(m) + H2O(m) ⇌ ADP(m) + Pi(m) + ATP-energy(m)
r1330	-0.5	H+(PG)(c) ⇌ H+(PG)(m) + Proton-gradient(m)
r0947	-0.5	Citrulline(m) + Ornithine(c) ⇌ Citrulline(c) + Ornithine(m)
r0875	0.5	Glutamate(c) + H+(PG)(c) → Glutamate(m) + H+(PG)(m)
r0034	0.5	2 ATP(m) + CO2(m) + H2O(m) + NH3(m) ⇌ 2 ADP(m) + Pi(m) + Carbamoyl-P(m)
r0007	1.5	PPi(c) + H2O(c) → 2 Pi(c)
r1322	0.5	NADH(c) ⇌ NAD+(c) + NADH-redox-potential(c)
r0867	1.5	CO2(c) ⇌ CO2(s)
r0849	1.5	H2O(s) ⇌ H2O(c)
r2677	2	Acetyl-CoA(c) ⇌ CoA(c) + CoA-activated acetyl group(c)
r0324	-1	Fumarylacetoacetate(c) + H2O(c) ⇌ Fumarate(c) + Acetoacetate(c)
r0261	0.5	Argininosuccinate(c) ⇌ Fumarate(c) + Arginine(c)
r0144	0.5	Arginine(c) + H2O(c) ⇌ Urea(c) + Ornithine(c)

Homocysteine degr (429)

Definition			Solution												
			exchanges		reactions								transp		
Objective	Constraints	Comment	imports	exports	c	m	r	p	l	n	s	b	s-c	b-c	int
-1 Homocysteine(c)	MCES +Urea −Serine +H_2S	Homocysteine degradation using palmitate excreting urea	1 H_2O(s) 0.5 O_2(s) 1 Serine(s) 1 Homocysteine(c) 2 ATP-energy(c) 2 ATP-energy(m)	1 CO_2(s) 1 Pyruvate(c) 1 Urea(s) 1 H_2S(s) 1 NADH-redox-potential(c) 2 NADH-redox-potential(m) 3 Proton-gradient(m) 1 CoA-activated acetyl group(m)	13	12	-	-	-	-	-	-	6	-	13

ID	flux	reaction
r0855	0.5	O2(s) ⇌ O2(c)
r0856	-0.5	O2(m) ⇌ O2(c)
r0144	1	Arginine(c) + H2O(c) ⇌ Urea(c) + Ornithine(c)
r0592	-1	H2O(m) + Acrylyl-CoA(m) → 3-Hydroxypropionyl-CoA(m)
r0596	-1	3-Hydroxypropionyl-CoA(m) + H2O(m) → Hydracrylate(m) + CoA(m)
r0896	1	Urea(c) ⇌ Urea(s)
r0017	0.5	O2(m) + 4 H+(PG)(m) + 4 Ferrocytochrome C(m) → 4 Ferricytochrome C(m) + 2 H2O(m) + 4 H+(PG)(c)
r0030	1	ATP(c) + AMP(c) ⇌ 2 ADP(c)
r0314	1	Serine(c) + Homocysteine(c) ⇌ H2O(c) + L-Cystathionine(c)
r0034	1	2 ATP(m) + CO2(m) + H2O(m) + NH3(m) ⇌ 2 ADP(m) + Pi(m) + Carbamoyl-P(m)
r0911	1	Proline(c) + Glutamate(m) ⇌ Proline(m) + Glutamate(c)
r1426	1	Proline(m) + H+(PG)(c) → Proline(c) + H+(PG)(m)
r0073	-1	AKG(m) + NADH(m) + NH3(m) ⇌ Glutamate(m) + NAD+(m) + H2O(m)
r0507	1	Ubiquinol(m) + 2 Ferricytochrome C(m) + 4 H+(PG)(m) → Ubiquinone(m) + 4 H+(PG)(c) + 2 Ferrocytochrome C(m)
r0241	1	H2O(c) + L-Cystathionine(c) ⇌ Cysteine(c) + NH3(c) + 2-Oxobutyrate(c)
r1330	3	H+(PG)(c) ⇌ H+(PG)(m) + Proton-gradient(m)
r1319	-2	ATP(c) + H2O(c) ⇌ ADP(c) + Pi(c) + ATP-energy(c)
r0261	1	Argininosuccinate(c) ⇌ Fumarate(c) + Arginine(c)
r0849	1	H2O(s) ⇌ H2O(c)
r0867	1	CO2(c) ⇌ CO2(s)
r0868	-1	CO2(c) ⇌ CO2(m)
r0453	1	ATP(c) + Citrulline(c) + Aspartate(c) ⇌ AMP(c) + PPi(c) + Argininosuccinate(c)
r0007	1	PPi(c) + H2O(c) → 2 Pi(c)
r0174	1	CoA(m) + NAD+(m) + 3-Oxopropanoate(m) → Acetyl-CoA(m) + CO2(m) + NADH(m)
r0193	1	Cysteine(c) + H2O(c) ⇌ Pyruvate(c) + H2S(c) + NH3(c)
r0329	1	Carbamoyl-P(m) + Ornithine(m) ⇌ Pi(m) + Citrulline(m)
r0940	1	H2S(c) ⇌ H2S(s)
r1154	1	CoA(m) + NAD+(m) + 2-Oxobutyrate(m) ⇌ CO2(m) + Propanoyl-CoA(m) + NADH(m)
r1155	1	2-Oxobutyrate(c) + H+(PG)(c) → 2-Oxobutyrate(m) + H+(PG)(m)
r0365	1	Hydracrylate(m) + NAD+(m) → NADH(m) + 3-Oxopropanoate(m)
r2678	1	Acetyl-CoA(m) ⇌ CoA(m) + CoA-activated acetyl group(m)
r2526	1	Serine(s) ⇌ Serine(c)
r0947	-1	Citrulline(m) + Ornithine(c) ⇌ Citrulline(c) + Ornithine(m)
r1320	-2	ATP(m) + H2O(m) ⇌ ADP(m) + Pi(m) + ATP-energy(m)
r1322	1	NADH(c) ⇌ NAD+(c) + NADH-redox-potential(c)
r0683	1	Propanoyl-CoA(m) + Ubiquinone(m) → Ubiquinol(m) + Acrylyl-CoA(m)
r1323	2	NADH(m) ⇌ NAD+(m) + NADH-redox-potential(m)
r0104	1	NAD+(c) + Malate(c) ⇌ OAA(c) + NADH(c)
r1047	1	Pi(c) + H+(PG)(c) → Pi(m) + H+(PG)(m)
r0258	-1	Fumarate(c) + H2O(c) ⇌ Malate(c)
r2419	1	AKG(c) + Pi(m) ⇌ AKG(m) + Pi(c)
r0838	2	NH3(c) ⇌ NH3(m)
r0851	1	H2O(m) ⇌ H2O(c)
r0109	-1	OAA(c) + Glutamate(c) ⇌ AKG(c) + Aspartate(c)

beta-Alanine degr (430)

Definition			Solution													
			exchanges		reactions									transp		
Objective	Constraints	Comment	imports	exports	c	m	r	p	l	n	s	b	s-c	b-c	int
-1 beta-Alanine(c)	MCES +Urea	beta-Alanine degradation using palmitate excreting urea	0.5 H_2O(s) 1 beta-Alanine(c) 1 ATP-energy(c) 1 ATP-energy(m) 1 Proton-gradient(m)	0.5 CO_2(s) 0.5 Urea(s) 0.5 NADH-redox-potential(c) 1 NADH-redox-potential(m) 0.5 NADPH-redox-potential(m) 1 CoA-activated acetyl group(m)	10	9	-	-	-	-	-	-	3	-	8

ID	flux	reaction
r2678	1	Acetyl-CoA(m) ⇌ CoA(m) + CoA-activated acetyl group(m)
r0896	0.5	Urea(c) ⇌ Urea(s)
r0217	1	beta-Alanine(m) + AKG(m) ⇌ Glutamate(m) + 3-Oxopropanoate(m)
r0109	-0.5	OAA(c) + Glutamate(c) ⇌ AKG(c) + Aspartate(c)
r0911	0.5	Proline(c) + Glutamate(m) ⇌ Proline(m) + Glutamate(c)
r1047	0.5	Pi(c) + H+(PG)(c) → Pi(m) + H+(PG)(m)
r0453	0.5	ATP(c) + Citrulline(c) + Aspartate(c) ⇌ AMP(c) + PPi(c) + Argininosuccinate(c)
r0174	1	CoA(m) + NAD+(m) + 3-Oxopropanoate(m) → Acetyl-CoA(m) + CO2(m) + NADH(m)
r0075	0.5	Glutamate(m) + H2O(m) + NADP+(m) ⇌ AKG(m) + NADPH(m) + NH3(m)
r1426	0.5	Proline(m) + H+(PG)(c) → Proline(c) + H+(PG)(m)
r1327	0.5	NADPH(m) ⇌ NADPH-redox-potential(m) + NADP+(m)
r1319	-1	ATP(c) + H2O(c) ⇌ ADP(c) + Pi(c) + ATP-energy(c)
r0329	0.5	Carbamoyl-P(m) + Ornithine(m) ⇌ Pi(m) + Citrulline(m)
r0258	-0.5	Fumarate(c) + H2O(c) ⇌ Malate(c)
r2419	0.5	AKG(c) + Pi(m) ⇌ AKG(m) + Pi(c)
r0030	0.5	ATP(c) + AMP(c) ⇌ 2 ADP(c)
r0104	0.5	NAD+(c) + Malate(c) ⇌ OAA(c) + NADH(c)
r1320	-1	ATP(m) + H2O(m) ⇌ ADP(m) + Pi(m) + ATP-energy(m)
r1330	-1	H+(PG)(c) ⇌ H+(PG)(m) + Proton-gradient(m)
r0947	-0.5	Citrulline(m) + Ornithine(c) ⇌ Citrulline(c) + Ornithine(m)
r0034	0.5	2 ATP(m) + CO2(m) + H2O(m) + NH3(m) ⇌ 2 ADP(m) + Pi(m) + Carbamoyl-P(m)
r0007	0.5	PPi(c) + H2O(c) → 2 Pi(c)
r1322	0.5	NADH(c) ⇌ NAD+(c) + NADH-redox-potential(c)
r0867	0.5	CO2(c) ⇌ CO2(s)
r0849	0.5	H2O(s) ⇌ H2O(c)
r1323	1	NADH(m) ⇌ NAD+(m) + NADH-redox-potential(m)
r0900	1	beta-Alanine(c) ⇌ beta-Alanine(m)
r0868	-0.5	CO2(c) ⇌ CO2(m)
r0261	0.5	Argininosuccinate(c) ⇌ Fumarate(c) + Arginine(c)
r0144	0.5	Arginine(c) + H2O(c) ⇌ Urea(c) + Ornithine(c)

Ornithine degr (431)

Definition			Solution												
			exchanges		reactions								transp		
Objective	Constraints	Comment	imports	exports	c	m	r	p	l	n	s	b	s-c	b-c	int
-1 Ornithine(c)	MCES +Urea	Ornithine degradation using palmitate excreting urea	3 H_2O(s) 0.5 O_2(s) 1 Ornithine(c) 2 ATP-energy(c) 1 ATP-energy(m)	1 CO_2(s) 1 Pyruvate(c) 1 Urea(s) 3 NADH-redox-potential(m) 1 NADPH-redox-potential(c) 1 NADPH-redox-potential(m) 3 Proton-gradient(m)	9	15	-	-	-	-	-	-	4	-	11

ID	flux	reaction
r0125	-1	GDP(m) + Pi(m) + Succinyl-CoA(m) ⇌ GTP(m) + Succinate(m) + CoA(m)
r0855	0.5	O2(s) ⇌ O2(c)
r0856	-0.5	O2(m) ⇌ O2(c)
r0144	1	Arginine(c) + H2O(c) ⇌ Urea(c) + Ornithine(c)
r0876	1	Glutamate(c) + H+(PG)(c) + Aspartate(m) → Glutamate(m) + H+(PG)(m) + Aspartate(c)
r0167	1	AKG(m) + Ornithine(m) ⇌ L-Glutamate 5-semialdehyde(m) + Glutamate(m)
r0896	1	Urea(c) ⇌ Urea(s)
r0017	0.5	O2(m) + 4 H+(PG)(m) + 4 Ferrocytochrome C(m) → 4 Ferricytochrome C(m) + 2 H2O(m) + 4 H+(PG)(c)
r0030	1	ATP(c) + AMP(c) ⇌ 2 ADP(c)
r0034	1	2 ATP(m) + CO2(m) + H2O(m) + NH3(m) ⇌ 2 ADP(m) + Pi(m) + Carbamoyl-P(m)
r0911	1	Proline(c) + Glutamate(m) ⇌ Proline(m) + Glutamate(c)
r0058	1	Malate(c) + NADP+(c) ⇌ Pyruvate(c) + CO2(c) + NADPH(c)

Continued on next page

Ornithine degr (431)– continued

ID	flux	reaction
r1426	1	Proline(m) + H+(PG)(c) → Proline(c) + H+(PG)(m)
r0074	1	L-Glutamate 5-semialdehyde(m) + NAD+(m) + H2O(m) ⇌ Glutamate(m) + NADH(m)
r0075	1	Glutamate(m) + H2O(m) + NADP+(m) ⇌ AKG(m) + NADPH(m) + NH3(m)
r0507	1	Ubiquinol(m) + 2 Ferricytochrome C(m) + 4 H+(PG)(m) → Ubiquinone(m) + 4 H+(PG)(c) + 2 Ferrocytochrome C(m)
r0509	1	Ubiquinone(m) + Succinate(m) → Ubiquinol(m) + Fumarate(m)
r1330	3	H+(PG)(c) ⇌ H+(PG)(m) + Proton-gradient(m)
r1319	-2	ATP(c) + H2O(c) ⇌ ADP(c) + Pi(c) + ATP-energy(c)
r0261	1	Argininosuccinate(c) ⇌ Fumarate(c) + Arginine(c)
r0110	-1	OAA(m) + Glutamate(m) → AKG(m) + Aspartate(m)
r1070	1	Lysine(c) ⇌ Lysine(m)
r0849	3	H2O(s) ⇌ H2O(c)
r0867	1	CO2(c) ⇌ CO2(s)
r0453	1	ATP(c) + Citrulline(c) + Aspartate(c) ⇌ AMP(c) + PPi(c) + Argininosuccinate(c)
r0007	1	PPi(c) + H2O(c) → 2 Pi(c)
r1114	1	AKG(m) + CoA(m) + NAD+(m) ⇌ NADH(m) + CO2(m) + Succinyl-CoA(m)
r0329	1	Carbamoyl-P(m) + Ornithine(m) ⇌ Pi(m) + Citrulline(m)
r2396	-1	Lysine(c) + Ornithine(m) + H+(PG)(m) ← Lysine(m) + Ornithine(c) + H+(PG)(c)
r0947	-1	Citrulline(c) + Ornithine(m) ⇌ Citrulline(m) + Ornithine(c)
r1320	-1	ATP(m) + H2O(m) ⇌ ADP(m) + Pi(m) + ATP-energy(m)
r1323	3	NADH(m) ⇌ NAD+(m) + NADH-redox-potential(m)
r0100	-1	ADP(m) + GTP(m) ⇌ ATP(m) + GDP(m)
r1326	1	NADPH(c) ⇌ NADPH-redox-potential(c) + NADP+(c)
r1327	1	NADPH(m) ⇌ NADPH-redox-potential(m) + NADP+(m)
r0105	1	NAD+(m) + Malate(m) ⇌ OAA(m) + NADH(m)
r0258	-1	Malate(c) ⇌ Fumarate(c) + H2O(c)
r0259	-1	Malate(m) ⇌ Fumarate(m) + H2O(m)
r0851	-2	H2O(m) ⇌ H2O(c)

B4.2. lipids, fatty acids, lipoproteins

LDL degr (432)

Definition			Solution												
			exchanges		reactions								transp		
Objective	Constraints	Comment	imports	exports	c	m	r	p	l	n	s	b	s-c	b-c	int
-1 LDL	MCES AAA ALFA +Cholesterol(b)	degradation of LDL	4259 H_2O(s) 119 O_2(s) 1 LDL(s) 5395 ATP-energy(c) 110 NADH-redox-potential(c)	473.3 P_i(c) 60 CO_2(s) 207 Glycine(s) 275 Alanine(s) 357 Lysine(s) 233 Aspartate(s) 150 Arginine(s) 560 Glutamine(s) 552 Serine(s) 79 Methionine(s) 37 Tryptophan(s) 224 Phenylalanine(s) 151 Tyrosine(s) 386.7 Glucose-6P(c) 25 Cysteine(s) 610 Choline(c) 533 Leucine(s) 115 Histidine(s) 139 Proline(s) 247 Asparagine(s) 252 Valine(s) 2195 Cholesterol(b) 300 Threonine(s) 30 Ethanolamine(c) 240.1 Arachidonate(c) 843.9 Palmitate(c) 285 Isoleucine(s) 510.2 Oleate(c) 316 Stearate(c) 1080 Linoleate(c) 252 NADH-redox-potential(m) 320 NADPH-redox-potential(c) 3348 Proton-gradient(m) 110 CoA-activated acetyl group(m)	48	8	2	-	8	-	-	-	23	1	50

ID	flux	reaction
r1362	298	Glutamate(l) → Glutamate(c)
r1080	151	Tyrosine(l) → Tyrosine(c)
r1067	115	Histidine(l) → Histidine(c)
r0406	36.7	Sedoheptulose-7P(c) + GAP(c) ⇌ Fructose-6P(c) + Erythrose-4P(c)
r1068	533	Leucine(l) → Leucine(c)
r1069	357	Lysine(c) ⇌ Lysine(s)
r0421	50	CTP(c) + Phosphocholine(c) → PPi(c) + CDP-choline(c)
r0855	119	O2(s) ⇌ O2(c)
r0856	-279	O2(m) ⇌ O2(c)
r0859	-2280	ADP(c) + ATP(l) ← ADP(l) + ATP(c)
r1369	610	PC-VLDL-pool(c) + H2O(c) → Phosphatidate-VLDL-PC-pool(c) + Choline(c)
r1218	610	Phosphatidate-VLDL-PC-pool(c) + H2O(c) → 1,2-Diacylglycerol-VLDL-PC-pool(c) + Pi(c)
r1232	450	1-Acylglycerol-VLDL-PC-pool(c) + H2O(c) → Glycerol(c) + Fatty-acid-VLDL-PC-pool(c)
r1219	30	Phosphatidate-VLDL-PE-pool(c) + H2O(c) → 1,2-Diacylglycerol-VLDL-PE-pool(c) + Pi(c)
r1233	30	1-Acylglycerol-VLDL-PE-pool(c) + H2O(c) → Glycerol(c) + Fatty-acid-VLDL-PE-pool(c)
r1235	110	1-Acylglycerol-VLDL-PI-pool(c) + H2O(c) → Fatty-acid-VLDL-PI-pool(c) + Glycerol(c)
r1250	-0.02	6260 Palmitate(c) + 1490 Stearate(c) + 270 Linoleate(c) + 147 Arachidonate(c) + 656 Oleate(c) ⇌ 8823 Fatty-acid-VLDL-SM-pool(c)
r1237	160	CDP-choline(c) + 1,2-Diacylglycerol-VLDL-PC-pool(c) → PC-VLDL-pool(c) + CMP(c)
r1238	-50	CDP-diacylglycerol-VLDL-PI-pool(c) + PPi(c) ⇌ Phosphatidate-VLDL-PI-pool(c) + CTP(c)
r0017	279	O2(m) + 4 H+(PG)(m) + 4 Ferrocytochrome C(m) → 4 Ferricytochrome C(m) + 2 H2O(m) + 4 H+(PG)(c)
r0313	-160	3-Dehydrosphinganine(c) + CoA(c) + CO2(c) ⇌ Serine(c) + Palmitoyl-CoA(c)
r1273	-110	PI-pool(c) ⇌ PI-pool(r)
r1275	-50	SM-pool(c) ⇌ SM-pool(r)
r1276	160	SM-pool(l) → SM-pool(r)
r1407	-110	CDP-choline(r) + Ceramide-pool(r) ⇌ CMP(r) + SM-pool(r)
r0911	32	Proline(c) + Glutamate(m) ⇌ Proline(m) + Glutamate(c)
r1408	50	SM-pool(c) + H2O(c) ⇌ Ceramide-pool(c) + Phosphocholine(c)
r0487	313.3	Fructose-1,6PP(c) + H2O(c) → Fructose-6P(c) + Pi(c)
r1279	-25	2-Lysolecithin-pool(l) + Fatty-acid-VLDL-PC-pool(l) ⇌ PC-VLDL-pool(l) + H2O(l)
r1140	252	Valine(l) → Valine(c)
r0932	87.4	Arachidonate(l) ⇌ Arachidonate(c)
r0203	590	ATP(c) + Glycerol(c) ⇌ ADP(c) + sn-Glycerol-3P(c)
r0073	110	Glutamate(m) + NAD+(m) + H2O(m) ⇌ AKG(m) + NADH(m) + NH3(m)
r0074	32	L-Glutamate 5-semialdehyde(m) + NAD+(m) + H2O(m) ⇌ Glutamate(m) + NADH(m)
r0205	590	Ubiquinone(m) + sn-Glycerol-3P(c) ⇌ DHAP(c) + Ubiquinol(m)
r0370	-36.7	Sedoheptulose-7P(c) + GAP(c) ⇌ Ribose-5P(c) + D-Xylulose-5P(c)
r2530	-533	Leucine(s) ⇌ Leucine(c)
r2531	-139	Proline(s) ⇌ Proline(c)
r0077	330	ATP(c) + Glutamate(c) + NH3(c) → ADP(c) + Pi(c) + Glutamine(c)
r2532	-247	Asparagine(s) ⇌ Asparagine(c)
r0937	225.8	Palmitate(l) ⇌ Palmitate(c)
r2533	-252	Valine(s) ⇌ Valine(c)
r2686	233	Aspartate(c) ⇌ Aspartate(s)
r2534	-300	Threonine(s) ⇌ Threonine(c)
r1180	806.1	Cholesterol-ester-lin(l) + H2O(l) → Cholesterol(l) + Linoleate(l)
r2536	-285	Isoleucine(s) ⇌ Isoleucine(c)
r0507	558	Ubiquinol(m) + 2 Ferricytochrome C(m) + 4 H+(PG)(m) → Ubiquinone(m) + 4 H+(PG)(c) + 2 Ferrocytochrome C(m)
r1184	89.5	H2O(l) + Cholesterol-ester-arach(l) → Cholesterol(l) + Arachidonate(l)
r0243	-276.7	DHAP(c) ⇌ GAP(c)
r1330	3348	H+(PG)(c) ⇌ H+(PG)(m) + Proton-gradient(m)
r1319	-5395	ATP(c) + H2O(c) ⇌ ADP(c) + Pi(c) + ATP-energy(c)
r1052	2195	Cholesterol(l) → Cholesterol(c)
r0825	285	Isoleucine(l) → Isoleucine(c)
r1053	1	LDL(s) → LDL(l)
r0826	110	Inositol(c) → Inositol(r)
r1071	357	Lysine(l) → Lysine(c)

Continued on next page

ID	flux	reaction
r1058	-150	Arginine(c) ⇌ Arginine(s)
r1072	-79	Methionine(s) ⇌ Methionine(c)
r1059	150	Arginine(l) → Arginine(c)
r1073	79	Methionine(l) → Methionine(c)
r1074	224	Phenylalanine(l) → Phenylalanine(c)
r1075	171	Proline(l) → Proline(c)
r1371	30	H2O(c) + PE-VLDL-pool(c) → Phosphatidate-VLDL-PE-pool(c) + Ethanolamine(c)
r1076	392	Serine(l) → Serine(c)
r0849	4259	H2O(s) ⇌ H2O(c)
r1372	60	PI-pool(c) + H2O(c) → Inositol-1P(c) + 1,2-Diacylglycerol-VLDL-PI-pool(c)
r1077	37	Tryptophan(l) → Tryptophan(c)
r0134	110	CMP(c) + H2O(c) → Cytidine(c) + Pi(c)
r1221	50	Phosphatidate-VLDL-PI-pool(c) + H2O(c) → 1,2-Diacylglycerol-VLDL-PI-pool(c) + Pi(c)
r1079	-151	Tyrosine(c) → Tyrosine(s)
r0583	160	Sphinganine(c) + NADP+(c) ⇌ 3-Dehydrosphinganine(c) + NADPH(c)
r0418	110	Uridine(c) + Pi(c) ⇌ Uracil(c) + Ribose-1P(c)
r0289	60	Inositol-1P(c) + H2O(c) → Inositol(c) + Pi(c)
r0419	110	Cytidine(c) + H2O(c) ⇌ Uridine(c) + NH3(c)
r0867	60	CO2(c) ⇌ CO2(s)
r0868	-110	CO2(c) ⇌ CO2(m)
r1508	2195	Cholesterol(c) + ATP(c) + H2O(c) → Cholesterol(b) + ADP(c) + Pi(c)
r1226	450	H2O(c) + 1,2-Diacylglycerol-VLDL-PC-pool(c) → 1-Acylglycerol-VLDL-PC-pool(c) + Fatty-acid-VLDL-PC-pool(c)
r1240	-110	Ceramide-pool(c) ⇌ Ceramide-pool(r)
r1227	30	H2O(c) + 1,2-Diacylglycerol-VLDL-PE-pool(c) → 1-Acylglycerol-VLDL-PE-pool(c) + Fatty-acid-VLDL-PE-pool(c)
r0883	275	Alanine(l) → Alanine(c)
r1229	110	H2O(c) + 1,2-Diacylglycerol-VLDL-PI-pool(c) → 1-Acylglycerol-VLDL-PI-pool(c) + Fatty-acid-VLDL-PI-pool(c)
r1245	-0.11	3010 Palmitate(c) + 1300 Stearate(c) + 2350 Linoleate(c) + 726 Arachidonate(c) + 1170 Oleate(c) ⇌ 8556 Fatty-acid-VLDL-PC-pool(c)
r1246	0.003	3010 Palmitate(l) + 1300 Stearate(l) + 2350 Linoleate(l) + 726 Arachidonate(l) + 1170 Oleate(l) ⇌ 8556 Fatty-acid-VLDL-PC-pool(l)
r1247	-0.01	1260 Palmitate(c) + 2010 Stearate(c) + 1330 Linoleate(c) + 2220 Arachidonate(c) + 628 Oleate(c) ← 7448 Fatty-acid-VLDL-PE-pool(c)
r0174	110	CoA(m) + NAD+(m) + 3-Oxopropanoate(m) → Acetyl-CoA(m) + CO2(m) + NADH(m)
r0175	32	1-Pyrroline-5-carboxylate(m) + H2O(m) ⇌ L-Glutamate 5-semialdehyde(m)
r1249	-0.03	694 Palmitate(c) + 3640 Stearate(c) + 694 Linoleate(c) + 2170 Arachidonate(c) + 1360 Oleate(c) ← 8558 Fatty-acid-VLDL-PI-pool(c)
r0192	-386.7	Fructose-6P(c) ⇌ Glucose-6P(c)
r1266	-450	PC-VLDL-pool(c) ⇌ PC-VLDL-pool(r)
r0900	110	beta-Alanine(c) ⇌ beta-Alanine(m)
r1267	450	PC-VLDL-pool(l) → PC-VLDL-pool(r)
r1268	-30	PE-VLDL-pool(c) ⇌ PE-VLDL-pool(r)
r1269	30	PE-VLDL-pool(l) → PE-VLDL-pool(r)
r1413	160	Dihydroceramide-pool(c) + H2O(c) ⇌ Sphinganine(c) + Fatty-acid-VLDL-SM-pool(c)
r1414	-160	2 H2O(c) + Ceramide-pool(c) + NADP+(c) ⇌ Dihydroceramide-pool(c) + O2(c) + NADPH(c)
r0344	73.3	Ribulose-5P(c) ⇌ D-Xylulose-5P(c)
r1150	-2280	Pi(c) ← Pi(l)
r0925	300	Threonine(l) → Threonine(c)
r2523	-207	Glycine(s) ⇌ Glycine(c)
r2524	-275	Alanine(s) ⇌ Alanine(c)
r2525	-560	Glutamine(s) ⇌ Glutamine(c)
r2678	110	Acetyl-CoA(m) ⇌ CoA(m) + CoA-activated acetyl group(m)
r2526	-552	Serine(s) ⇌ Serine(c)
r1453	32	Proline(m) + Ubiquinol(m) ⇌ 1-Pyrroline-5-carboxylate(m) + Ubiquinone(m)
r1158	1	LDL(l) → 1515 Cholesterol-ester-pool(l) + ApoB100(l) + 680 Cholesterol(l) + 110 CDP-diacylglycerol-VLDL-PI-pool(l) + 425 PC-VLDL-pool(l) + 25 2-Lysolecithin-pool(l) + 160 SM-pool(l) + 30 PE-VLDL-pool(l)
r2527	-37	Tryptophan(s) ⇌ Tryptophan(c)
r1302	335.2	Oleate(l) ⇌ Oleate(c)

Continued on next page

ID	flux	reaction
r1159	-110	CDP-choline(c) ⇌ CDP-choline(r)
r0216	110	H2O(c) + 3-Ureidopropionate(c) → beta-Alanine(c) + CO2(c) + NH3(c)
r2528	-224	Phenylalanine(s) ⇌ Phenylalanine(c)
r1173	234.6	H2O(l) + Cholesterol-ester-palm(l) → Cholesterol(l) + Palmitate(l)
r0217	110	beta-Alanine(m) + AKG(m) ⇌ Glutamate(m) + 3-Oxopropanoate(m)
r2529	-25	Cysteine(s) ⇌ Cysteine(c)
r2695	160	Palmitoyl-CoA(c) + H2O(c) → Palmitate(c) + CoA(c)
r1176	46.2	H2O(l) + Cholesterol-ester-stea(l) → Cholesterol(l) + Stearate(l)
r0516	110	5,6-Dihydrouracil(c) + H2O(c) → 3-Ureidopropionate(c)
r1178	338.6	Cholesterol-ester-ol(l) + H2O(l) → Cholesterol(l) + Oleate(l)
r1322	-110	NADH(c) ⇌ NAD+(c) + NADH-redox-potential(c)
r0236	-110	5,6-Dihydrouracil(c) + NAD+(c) ⇌ Uracil(c) + NADH(c)
r1323	252	NADH(m) ⇌ NAD+(m) + NADH-redox-potential(m)
r1193	-0.17	2050 Cholesterol-ester-ol(l) + 4880 Cholesterol-ester-lin(l) + 1420 Cholesterol-ester-palm(l) + 280 Cholesterol-ester-stea(l) + 542 Cholesterol-ester-arach(l) ← 9172 Cholesterol-ester-pool(l)
r0250	73.3	Ribose-5P(c) ⇌ Ribulose-5P(c)
r0251	110	Ribose-1P(c) ⇌ Ribose-5P(c)
r1326	320	NADPH(c) ⇌ NADPH-redox-potential(c) + NADP+(c)
r1196	110	CDP-diacylglycerol-VLDL-PI-pool(r) + Inositol(r) → PI-pool(r) + CMP(r)
r1197	-50	CDP-diacylglycerol-VLDL-PI-pool(c) + Inositol(c) ⇌ PI-pool(c) + CMP(c)
r0984	42.5	Stearate(l) ⇌ Stearate(c)
r0255	-36.7	Fructose-6P(c) + GAP(c) ⇌ D-Xylulose-5P(c) + Erythrose-4P(c)
r1342	1	2280 ATP(l) + 2280 H2O(l) + ApoB100(l) → 285 Isoleucine(l) + 533 Leucine(l) + 79 Methionine(l) + 275 Alanine(l) + 150 Arginine(l) + 115 Histidine(l) + 37 Tryptophan(l) + 252 Valine(l) + 2280 ADP(l) + 2280 Pi(l) + 230 Glutamine(l) + 298 Glutamate(l) + 25 Cysteine(l) + 207 Glycine(l) + 224 Phenylalanine(l) + 171 Proline(l) + 392 Serine(l) + 300 Threonine(l) + 151 Tyrosine(l) + 247 Asparagine(l) + 233 Aspartate(l) + 357 Lysine(l)
r0256	-313.3	DHAP(c) + GAP(c) ⇌ Fructose-1,6PP(c)
r1061	247	Asparagine(l) → Asparagine(c)
r1062	233	Aspartate(l) → Aspartate(c)
r0987	799.2	Linoleate(l) ⇌ Linoleate(c)
r1063	25	Cysteine(l) → Cysteine(c)
r1064	-230	Glutamine(c) ← Glutamine(l)
r0850	-3770	H2O(l) ← H2O(c)
r1360	110	CDP-diacylglycerol-VLDL-PI-pool(l) → CDP-diacylglycerol-VLDL-PI-pool(r)
r1065	207	Glycine(l) → Glycine(c)
r0838	-110	NH3(c) ⇌ NH3(m)
r0851	384	H2O(m) ⇌ H2O(c)
r1066	-115	Histidine(s) ← Histidine(c)

HDL degr (433)

Definition			Solution												
			exchanges		reactions								transp		
Objective	Constraints	Comment	imports	exports	c	m	r	p	l	n	s	b	s-c	b-c	int
-1 HDL	MCES AAA ALFA +Cholesterol(b)	degradation of HDL	747.5 H_2O(s) 75 Pyruvate(m) 1 HDL(s) 491 ATP-energy(c) 2.5 NADH-redox-potential(c)	147.5 P_i(c) 60 Pyruvate(c) 22 Glycine(s) 46 Alanine(s) 44 Lysine(s) 32 Aspartate(s) 34 Arginine(s) 98 Glutamine(s) 77 Serine(s) 8 Methionine(s) 10 Tryptophan(s) 16 Phenylalanine(s) 14 Tyrosine(s) 72.5 Glucose-6P(c) 165 Choline(c) 82 Leucine(s) 12 Histidine(s) 20 Proline(s) 10 Asparagine(s) 30 Valine(s) 20 Cholesterol(b) 24 Threonine(s) 25 Ethanolamine(c) 49.3 Arachidonate(c) 207.6 Palmitate(c) 39.5 Oleate(c) 69.7 Stearate(c) 71.4 Linoleate(c) 2.5 Palmitolate(c) 75 NADH-redox-potential(m) 150 NADPH-redox-potential(c) 870 Proton-gradient(m) 75 CoA-activated acetyl group(m)	31	3	1	-	-	-	-	-	19	1	39

ID	flux	reaction
r1362	60	Glutamate(l) → Glutamate(c)
r1080	14	Tyrosine(l) → Tyrosine(c)
r1067	12	Histidine(l) → Histidine(c)
r1068	82	Leucine(l) → Leucine(c)
r1069	44	Lysine(c) ⇌ Lysine(s)
r0856	-72.5	O2(m) ⇌ O2(c)
r0859	-266	ADP(c) + ATP(l) ← ADP(l) + ATP(c)
r1369	165	PC-VLDL-pool(c) + H2O(c) → Phosphatidate-VLDL-PC-pool(c) + Choline(c)
r1218	165	Phosphatidate-VLDL-PC-pool(c) + H2O(c) → 1,2-Diacylglycerol-VLDL-PC-pool(c) + Pi(c)
r1232	90	1-Acylglycerol-VLDL-PC-pool(c) + H2O(c) → Glycerol(c) + Fatty-acid-VLDL-PC-pool(c)
r1219	55	Phosphatidate-VLDL-PE-pool(c) + H2O(c) → 1,2-Diacylglycerol-VLDL-PE-pool(c) + Pi(c)
r1233	55	1-Acylglycerol-VLDL-PE-pool(c) + H2O(c) → Glycerol(c) + Fatty-acid-VLDL-PE-pool(c)
r1250	-0.01	6260 Palmitate(c) + 1490 Stearate(c) + 270 Linoleate(c) + 147 Arachidonate(c) + 656 Oleate(c) ⇌ 8823 Fatty-acid-VLDL-SM-pool(c)
r1237	75	CDP-choline(c) + 1,2-Diacylglycerol-VLDL-PC-pool(c) → PC-VLDL-pool(c) + CMP(c)
r0017	72.5	O2(m) + 4 H+(PG)(m) + 4 Ferrocytochrome C(m) → 4 Ferricytochrome C(m) + 2 H2O(m) + 4 H+(PG)(c)
r0313	-75	3-Dehydrosphinganine(c) + CoA(c) + CO2(c) ⇌ Serine(c) + Palmitoyl-CoA(c)
r1271	-30	PS-VLDL-pool(c) ⇌ PS-VLDL-pool(r)
r1272	30	PS-VLDL-pool(l) → PS-VLDL-pool(r)
r1276	75	SM-pool(l) → SM-pool(r)
r1407	-75	CDP-choline(r) + Ceramide-pool(r) ⇌ CMP(r) + SM-pool(r)
r0487	72.5	Fructose-1,6PP(c) + H2O(c) → Fructose-6P(c) + Pi(c)
r1140	30	Valine(l) → Valine(c)
r0056	75	Pyruvate(m) + CoA(m) + NAD+(m) → Acetyl-CoA(m) + CO2(m) + NADH(m)
r0203	145	ATP(c) + Glycerol(c) ⇌ ADP(c) + sn-Glycerol-3P(c)
r1161	1	HDL(s) → HDL(l)
r0205	145	Ubiquinone(m) + sn-Glycerol-3P(c) ⇌ DHAP(c) + Ubiquinol(m)
r2530	-82	Leucine(s) ⇌ Leucine(c)
r2531	-20	Proline(s) ⇌ Proline(c)
r0077	60	ATP(c) + Glutamate(c) + NH3(c) → ADP(c) + Pi(c) + Glutamine(c)
r2532	-10	Asparagine(s) ⇌ Asparagine(c)
r2533	-30	Valine(s) ⇌ Valine(c)
r2686	32	Aspartate(c) ⇌ Aspartate(s)
r2534	-24	Threonine(s) ⇌ Threonine(c)
r2688	2.5	palmitoleoyl-CoA(c) + H2O(c) → Palmitolate(c) + CoA(c)
r0507	145	Ubiquinol(m) + 2 Ferricytochrome C(m) + 4 H+(PG)(m) → Ubiquinone(m) + 4 H+(PG)(c) + 2 Ferrocytochrome C(m)

Continued on next page

ID	flux	reaction
r0243	-72.5	DHAP(c) ⇌ GAP(c)
r1330	870	H+(PG)(c) ⇌ H+(PG)(m) + Proton-gradient(m)
r1319	-491	ATP(c) + H2O(c) ⇌ ADP(c) + Pi(c) + ATP-energy(c)
r1052	20	Cholesterol(l) → Cholesterol(c)
r1071	44	Lysine(l) → Lysine(c)
r1058	-34	Arginine(c) ⇌ Arginine(s)
r1059	34	Arginine(l) → Arginine(c)
r1072	-8	Methionine(s) ⇌ Methionine(c)
r1073	8	Methionine(l) → Methionine(c)
r1074	16	Phenylalanine(l) → Phenylalanine(c)
r1075	20	Proline(l) → Proline(c)
r1371	55	H2O(c) + PE-VLDL-pool(c) → Phosphatidate-VLDL-PE-pool(c) + Ethanolamine(c)
r1076	32	Serine(l) → Serine(c)
r0849	747.5	H2O(s) ⇌ H2O(c)
r1077	10	Tryptophan(l) → Tryptophan(c)
r1079	-14	Tyrosine(c) → Tyrosine(s)
r0583	75	Sphinganine(c) + NADP+(c) ⇌ 3-Dehydrosphinganine(c) + NADPH(c)
r0868	-75	CO2(c) ⇌ CO2(m)
r1508	20	Cholesterol(c) + ATP(c) + H2O(c) → Cholesterol(b) + ADP(c) + Pi(c)
r1226	90	H2O(c) + 1,2-Diacylglycerol-VLDL-PC-pool(c) → 1-Acylglycerol-VLDL-PC-pool(c) + Fatty-acid-VLDL-PC-pool(c)
r1240	-75	Ceramide-pool(c) ⇌ Ceramide-pool(r)
r1227	55	H2O(c) + 1,2-Diacylglycerol-VLDL-PE-pool(c) → 1-Acylglycerol-VLDL-PE-pool(c) + Fatty-acid-VLDL-PE-pool(c)
r0883	46	Alanine(l) → Alanine(c)
r0004	1	HDL(l) → 2 ApoA1(l) + 90 PC-VLDL-pool(l) + 30 PS-VLDL-pool(l) + 20 Cholesterol(l) + 25 PE-VLDL-pool(l) + 75 SM-pool(l)
r0886	75	CMP(c) ⇌ CMP(r)
r1245	-0.02	3010 Palmitate(c) + 1300 Stearate(c) + 2350 Linoleate(c) + 726 Arachidonate(c) + 1170 Oleate(c) ⇌ 8556 Fatty-acid-VLDL-PC-pool(c)
r1247	-0.01	1260 Palmitate(c) + 2010 Stearate(c) + 1330 Linoleate(c) + 2220 Arachidonate(c) + 628 Oleate(c) ← 7448 Fatty-acid-VLDL-PE-pool(c)
r0023	2.5	NADH(c) + 2 Ferricytochrome b5(c) ⇌ NAD+(c) + 2 Ferrocytochrome b5(c)
r0192	-72.5	Fructose-6P(c) ⇌ Glucose-6P(c)
r1266	-90	PC-VLDL-pool(c) ⇌ PC-VLDL-pool(r)
r1267	90	PC-VLDL-pool(l) → PC-VLDL-pool(r)
r1268	-25	PE-VLDL-pool(c) ⇌ PE-VLDL-pool(r)
r1269	25	PE-VLDL-pool(l) → PE-VLDL-pool(r)
r1413	75	Dihydroceramide-pool(c) + H2O(c) ⇌ Sphinganine(c) + Fatty-acid-VLDL-SM-pool(c)
r1414	-75	2 H2O(c) + Ceramide-pool(c) + NADP+(c) ⇌ Dihydroceramide-pool(c) + O2(c) + NADPH(c)
r0060	60	Serine(c) → Pyruvate(c) + NH3(c)
r1150	-266	Pi(c) ← Pi(l)
r0925	24	Threonine(l) → Threonine(c)
r2523	-22	Glycine(s) ⇌ Glycine(c)
r2524	-46	Alanine(s) ⇌ Alanine(c)
r2525	-98	Glutamine(s) ⇌ Glutamine(c)
r2678	75	Acetyl-CoA(m) ⇌ CoA(m) + CoA-activated acetyl group(m)
r2526	-77	Serine(s) ⇌ Serine(c)
r0510	2.5	Palmitoyl-CoA(c) + O2(c) + 2 Ferrocytochrome b5(c) ⇌ palmitoleoyl-CoA(c) + 2 Ferricytochrome b5(c) + 2 H2O(c)
r2527	-10	Tryptophan(s) ⇌ Tryptophan(c)
r1159	-75	CDP-choline(c) ⇌ CDP-choline(r)
r2528	-16	Phenylalanine(s) ⇌ Phenylalanine(c)
r2695	72.5	Palmitoyl-CoA(c) + H2O(c) → Palmitate(c) + CoA(c)
r1191	-30	PS-VLDL-pool(c) + Ethanolamine(c) ⇌ PE-VLDL-pool(c) + Serine(c)
r1322	-2.5	NADH(c) ⇌ NAD+(c) + NADH-redox-potential(c)
r1323	75	NADH(m) ⇌ NAD+(m) + NADH-redox-potential(m)
r1326	150	NADPH(c) ⇌ NADPH-redox-potential(c) + NADP+(c)

Continued on next page

ID	flux	reaction
r1340	2	133 ATP(l) + ApoA1(l) + 133 H2O(l) → 133 ADP(l) + 133 Pi(l) + 41 Leucine(l) + 4 Methionine(l) + 23 Alanine(l) + 17 Arginine(l) + 19 Glutamine(l) + 30 Glutamate(l) + 6 Histidine(l) + 5 Tryptophan(l) + 15 Valine(l) + 11 Glycine(l) + 8 Phenylalanine(l) + 10 Proline(l) + 16 Serine(l) + 12 Threonine(l) + 7 Tyrosine(l) + 5 Asparagine(l) + 16 Aspartate(l) + 22 Lysine(l)
r0256	-72.5	DHAP(c) + GAP(c) ⇌ Fructose-1,6PP(c)
r1061	10	Asparagine(l) → Asparagine(c)
r1062	32	Aspartate(l) → Aspartate(c)
r0850	-266	H2O(l) ← H2O(c)
r1064	-38	Glutamine(c) ← Glutamine(l)
r0851	145	H2O(m) ⇌ H2O(c)
r1065	22	Glycine(l) → Glycine(c)
r1066	-12	Histidine(s) ← Histidine(c)

Oleate degr (434)

Definition			Solution												
			exchanges		reactions								transp		
Objective	Constraints	Comment	imports	exports	c	m	r	p	l	n	s	b	s-c	b-c	int
-1 Oleate(c)	MCES	degradation of cytosolic oleate	2 H_2O(s) 2.5 O_2(s) 1 Oleate(c) 2 ATP-energy(c)	1 NADH-redox-potential(c) 8 NADH-redox-potential(m) 1 FADH-redox-potential(c) 42 Proton-gradient(m) 9 CoA-activated acetyl group(m)	9	35	-	-	-	-	-	-	2	-	9

ID	flux	reaction
r2435	1	L-Carnitine(m) + L-Palmitoylcarnitine(c) → L-Carnitine(c) + L-Palmitoylcarnitine(m)
r0855	2.5	O2(s) ⇌ O2(c)
r0856	-3.5	O2(m) ⇌ O2(c)
r0720	-1	(S)-3-Hydroxytetradecanoyl-CoA(m) ← (2E)-Tetradecenoyl-CoA(m) + H2O(m)
r0722	1	(S)-3-Hydroxydodecanoyl-CoA(m) + NAD+(m) ⇌ 3-Oxododecanoyl-CoA(m) + NADH(m)
r0724	-1	Decanoyl-CoA(m) + Acetyl-CoA(m) ⇌ CoA(m) + 3-Oxododecanoyl-CoA(m)
r0726	1	(S)-Hydroxydecanoyl-CoA(m) + NAD+(m) ⇌ 3-Oxodecanoyl-CoA(m) + NADH(m)
r0728	-1	(S)-Hydroxydecanoyl-CoA(m) ⇌ (2E)-Decenoyl-CoA(m) + H2O(m)
r0460	1	(S)-3-Hydroxybutyryl-CoA(m) + NAD+(m) ⇌ NADH(m) + Acetoacetyl-CoA(m)
r0310	1	Palmitoyl-CoA(m) + Ubiquinone(m) → (2E)-Hexadecenoyl-CoA(m) + Ubiquinol(m)
r0017	3.5	O2(m) + 4 H+(PG)(m) + 4 Ferrocytochrome C(m) → 4 Ferricytochrome C(m) + 2 H2O(m) + 4 H+(PG)(c)
r0030	1	ATP(c) + AMP(c) ⇌ 2 ADP(c)
r1256	1	ATP(c) + CoA(c) + Oleate(c) → Oleoyl-CoA(c) + AMP(c) + PPi(c)
r1290	1	FAD(c) + FADH2(m) ⇌ FAD(m) + FADH2(c)
r0070	-1	CoA(m) + Acetoacetyl-CoA(m) ⇌ 2 Acetyl-CoA(m)
r0634	-1	Acetyl-CoA(m) + Octanoyl-CoA(m) ⇌ CoA(m) + 3-Oxodecanoyl-CoA(m)
r0639	-1	Lauroyl-CoA(m) + Acetyl-CoA(m) ← CoA(m) + 3-Oxotetradecanoyl-CoA(m)
r0653	-1	Myristoyl-CoA(m) + Acetyl-CoA(m) ← CoA(m) + 3-Oxopalmitoyl-CoA(m)
r1446	1	Ubiquinone(m) + Butyryl-CoA(m) → Crotonyl-CoA(m) + Ubiquinol(m)
r1447	1	Ubiquinone(m) + Lauroyl-CoA(m) → (2E)-Dodecenoyl-CoA(m) + Ubiquinol(m)
r1448	1	Ubiquinone(m) + Octanoyl-CoA(m) → (2E)-Octenoyl-CoA(m) + Ubiquinol(m)
r1449	1	Ubiquinone(m) + Myristoyl-CoA(m) → (2E)-Tetradecenoyl-CoA(m) + Ubiquinol(m)
r0507	7	Ubiquinol(m) + 2 Ferricytochrome C(m) + 4 H+(PG)(m) → Ubiquinone(m) + 4 H+(PG)(c) + 2 Ferrocytochrome C(m)
r1467	1	Oleoyl-CoA(c) ⇌ Oleoyl-CoA(m)
r1481	1	CoA(m) + 3-oxooleoyl-CoA(m) ⇌ Acetyl-CoA(m) + palmitoleoyl-CoA(m)
r1468	-1	palmitoleoyl-CoA(c) ⇌ palmitoleoyl-CoA(m)
r1330	42	H+(PG)(c) ⇌ H+(PG)(m) + Proton-gradient(m)
r1319	-2	ATP(c) + H2O(c) ⇌ ADP(c) + Pi(c) + ATP-energy(c)
r0849	2	H2O(s) ⇌ H2O(c)
r0287	-1	CoA(m) + 3-Oxohexanoyl-CoA(m) → Acetyl-CoA(m) + Butyryl-CoA(m)
r0714	1	NAD+(m) + (S)-3-Hydroxyhexadecanoyl-CoA(m) → 3-Oxopalmitoyl-CoA(m) + NADH(m)

Continued on next page

ID	flux	reaction
r0716	-1	(S)-3-Hydroxyhexadecanoyl-CoA(m) ← (2E)-Hexadecenoyl-CoA(m) + H2O(m)
r0435	1	Palmitoyl-CoA(c) + L-Carnitine(c) ⇌ CoA(c) + L-Palmitoylcarnitine(c)
r0730	1	(S)-Hydroxyoctanoyl-CoA(m) + NAD+(m) → 3-Oxooctanoyl-CoA(m) + NADH(m)
r0588	-1	Crotonyl-CoA(m) + H2O(m) ⇌ (S)-3-Hydroxybutyryl-CoA(m)
r0718	1	(S)-3-Hydroxytetradecanoyl-CoA(m) + NAD+(m) → 3-Oxotetradecanoyl-CoA(m) + NADH(m)
r0436	-1	Palmitoyl-CoA(m) + L-Carnitine(m) ← CoA(m) + L-Palmitoylcarnitine(m)
r0731	-1	(S)-Hydroxyoctanoyl-CoA(m) ← (2E)-Octenoyl-CoA(m) + H2O(m)
r0732	-1	Hexanoyl-CoA(m) + Acetyl-CoA(m) ← CoA(m) + 3-Oxooctanoyl-CoA(m)
r0733	1	(S)-Hydroxyhexanoyl-CoA(m) + NAD+(m) → 3-Oxohexanoyl-CoA(m) + NADH(m)
r0734	-1	(S)-Hydroxyhexanoyl-CoA(m) ← (2E)-Hexenoyl-CoA(m) + H2O(m)
r0007	1	PPi(c) + H2O(c) → 2 Pi(c)
r0023	-1	NAD+(c) + 2 Ferrocytochrome b5(c) ⇌ NADH(c) + 2 Ferricytochrome b5(c)
r1450	1	Ubiquinone(m) + Hexanoyl-CoA(m) → (2E)-Hexenoyl-CoA(m) + Ubiquinol(m)
r1451	1	Ubiquinone(m) + Decanoyl-CoA(m) → (2E)-Decenoyl-CoA(m) + Ubiquinol(m)
r0660	-1	(S)-3-Hydroxydodecanoyl-CoA(m) ⇌ (2E)-Dodecenoyl-CoA(m) + H2O(m)
r2678	9	Acetyl-CoA(m) ⇌ CoA(m) + CoA-activated acetyl group(m)
r0510	-1	palmitoleoyl-CoA(c) + 2 Ferricytochrome b5(c) + 2 H2O(c) ⇌ Palmitoyl-CoA(c) + O2(c) + 2 Ferrocytochrome b5(c)
r1472	1	FAD(m) + Oleoyl-CoA(m) ⇌ FADH2(m) + trans,cis-octadeca-2,9-dienoyl-CoA(m)
r1474	1	H2O(m) + trans,cis-octadeca-2,9-dienoyl-CoA(m) ⇌ (S)-3-hydroxyoleyleoyl-CoA(m)
r1322	1	NADH(c) ⇌ NAD+(c) + NADH-redox-potential(c)
r1323	8	NADH(m) ⇌ NAD+(m) + NADH-redox-potential(m)
r1477	1	NAD+(m) + (S)-3-hydroxyoleyleoyl-CoA(m) ⇌ NADH(m) + 3-oxooleoyl-CoA(m)
r1329	1	FADH2(c) ⇌ FAD(c) + FADH-redox-potential(c)
r0851	-1	H2O(m) ⇌ H2O(c)

Stearate degr (435)

Definition			Solution												
			exchanges		reactions								transp		
Objective	Constraints	Comment	imports	exports	c	m	r	p	l	n	s	b	s-c	b-c	int
-1 Stearate(c)	MCES	degradation of cytosolic stearate	3.5 O_2(s) 1 Stearate(c) 2 ATP-energy(c)	8 NADH-redox-potential(m) 1 FADH-redox-potential(c) 42 Proton-gradient(m) 9 CoA-activated acetyl group(m)	8	35	-	-	-	-	-	-	1	-	9

ID	flux	reaction
r2435	1	L-Carnitine(m) + L-Palmitoylcarnitine(c) → L-Carnitine(c) + L-Palmitoylcarnitine(m)
r0855	3.5	O2(s) ⇌ O2(c)
r0856	-3.5	O2(m) ⇌ O2(c)
r0720	-1	(S)-3-Hydroxytetradecanoyl-CoA(m) ← (2E)-Tetradecenoyl-CoA(m) + H2O(m)
r0722	1	(S)-3-Hydroxydodecanoyl-CoA(m) + NAD+(m) ⇌ 3-Oxododecanoyl-CoA(m) + NADH(m)
r0724	-1	Decanoyl-CoA(m) + Acetyl-CoA(m) ⇌ CoA(m) + 3-Oxododecanoyl-CoA(m)
r0726	1	(S)-Hydroxydecanoyl-CoA(m) + NAD+(m) ⇌ 3-Oxodecanoyl-CoA(m) + NADH(m)
r0728	-1	(S)-Hydroxydecanoyl-CoA(m) ⇌ (2E)-Decenoyl-CoA(m) + H2O(m)
r0460	1	(S)-3-Hydroxybutyryl-CoA(m) + NAD+(m) ⇌ NADH(m) + Acetoacetyl-CoA(m)
r0310	1	Palmitoyl-CoA(m) + Ubiquinone(m) → (2E)-Hexadecenoyl-CoA(m) + Ubiquinol(m)
r1254	1	Stearate(c) + ATP(c) + CoA(c) → Stearoyl-CoA(c) + AMP(c) + PPi(c)
r0017	3.5	O2(m) + 4 H+(PG)(m) + 4 Ferrocytochrome C(m) → 4 Ferricytochrome C(m) + 2 H2O(m) + 4 H+(PG)(c)
r0030	1	ATP(c) + AMP(c) ⇌ 2 ADP(c)
r1290	1	FAD(c) + FADH2(m) ⇌ FAD(m) + FADH2(c)
r0070	-1	CoA(m) + Acetoacetyl-CoA(m) ⇌ 2 Acetyl-CoA(m)
r0634	-1	Acetyl-CoA(m) + Octanoyl-CoA(m) ⇌ CoA(m) + 3-Oxodecanoyl-CoA(m)
r0639	-1	Lauroyl-CoA(m) + Acetyl-CoA(m) ← CoA(m) + 3-Oxotetradecanoyl-CoA(m)
r0653	-1	Myristoyl-CoA(m) + Acetyl-CoA(m) ← CoA(m) + 3-Oxopalmitoyl-CoA(m)
r1446	1	Ubiquinone(m) + Butyryl-CoA(m) → Crotonyl-CoA(m) + Ubiquinol(m)

Continued on next page

ID	flux	reaction
r1447	1	Ubiquinone(m) + Lauroyl-CoA(m) → (2E)-Dodecenoyl-CoA(m) + Ubiquinol(m)
r1448	1	Ubiquinone(m) + Octanoyl-CoA(m) → (2E)-Octenoyl-CoA(m) + Ubiquinol(m)
r1449	1	Ubiquinone(m) + Myristoyl-CoA(m) → (2E)-Tetradecenoyl-CoA(m) + Ubiquinol(m)
r0507	7	Ubiquinol(m) + 2 Ferricytochrome C(m) + 4 H+(PG)(m) → Ubiquinone(m) + 4 H+(PG)(c) + 2 Ferrocytochrome C(m)
r1467	1	Oleoyl-CoA(c) ⇌ Oleoyl-CoA(m)
r1481	1	CoA(m) + 3-oxooleoyl-CoA(m) ⇌ Acetyl-CoA(m) + palmitoleoyl-CoA(m)
r1468	-1	palmitoleoyl-CoA(c) ⇌ palmitoleoyl-CoA(m)
r1330	42	H+(PG)(c) ⇌ H+(PG)(m) + Proton-gradient(m)
r1319	-2	ATP(c) + H2O(c) ⇌ ADP(c) + Pi(c) + ATP-energy(c)
r0287	-1	CoA(m) + 3-Oxohexanoyl-CoA(m) → Acetyl-CoA(m) + Butyryl-CoA(m)
r0714	1	NAD+(m) + (S)-3-Hydroxyhexadecanoyl-CoA(m) → 3-Oxopalmitoyl-CoA(m) + NADH(m)
r0716	-1	(S)-3-Hydroxyhexadecanoyl-CoA(m) ← (2E)-Hexadecenoyl-CoA(m) + H2O(m)
r0435	1	Palmitoyl-CoA(c) + L-Carnitine(c) ⇌ CoA(c) + L-Palmitoylcarnitine(c)
r0730	1	(S)-Hydroxyoctanoyl-CoA(m) + NAD+(m) → 3-Oxooctanoyl-CoA(m) + NADH(m)
r0588	-1	Crotonyl-CoA(m) + H2O(m) ⇌ (S)-3-Hydroxybutyryl-CoA(m)
r0718	1	(S)-3-Hydroxytetradecanoyl-CoA(m) + NAD+(m) → 3-Oxotetradecanoyl-CoA(m) + NADH(m)
r0436	-1	Palmitoyl-CoA(m) + L-Carnitine(m) ← CoA(m) + L-Palmitoylcarnitine(m)
r0731	-1	(S)-Hydroxyoctanoyl-CoA(m) ← (2E)-Octenoyl-CoA(m) + H2O(m)
r0732	-1	Hexanoyl-CoA(m) + Acetyl-CoA(m) ← CoA(m) + 3-Oxooctanoyl-CoA(m)
r0733	1	(S)-Hydroxyhexanoyl-CoA(m) + NAD+(m) → 3-Oxohexanoyl-CoA(m) + NADH(m)
r0734	-1	(S)-Hydroxyhexanoyl-CoA(m) ← (2E)-Hexenoyl-CoA(m) + H2O(m)
r0007	1	PPi(c) + H2O(c) → 2 Pi(c)
r1450	1	Ubiquinone(m) + Hexanoyl-CoA(m) → (2E)-Hexenoyl-CoA(m) + Ubiquinol(m)
r1451	1	Ubiquinone(m) + Decanoyl-CoA(m) → (2E)-Decenoyl-CoA(m) + Ubiquinol(m)
r0660	-1	(S)-3-Hydroxydodecanoyl-CoA(m) ⇌ (2E)-Dodecenoyl-CoA(m) + H2O(m)
r2678	9	Acetyl-CoA(m) ⇌ CoA(m) + CoA-activated acetyl group(m)
r0510	-1	Palmitoyl-CoA(c) + O2(c) + 2 Ferrocytochrome b5(c) ⇌ palmitoleoyl-CoA(c) + 2 Ferricytochrome b5(c) + 2 H2O(c)
r0511	1	Stearoyl-CoA(c) + O2(c) + 2 Ferrocytochrome b5(c) ⇌ Oleoyl-CoA(c) + 2 Ferricytochrome b5(c) + 2 H2O(c)
r1472	1	FAD(m) + Oleoyl-CoA(m) ⇌ FADH2(m) + trans,cis-octadeca-2,9-dienoyl-CoA(m)
r1474	1	H2O(m) + trans,cis-octadeca-2,9-dienoyl-CoA(m) ⇌ (S)-3-hydroxyoleyleoyl-CoA(m)
r1323	8	NADH(m) ⇌ NAD+(m) + NADH-redox-potential(m)
r1477	1	NAD+(m) + (S)-3-hydroxyoleyleoyl-CoA(m) ⇌ NADH(m) + 3-oxooleoyl-CoA(m)
r1329	1	FADH2(c) ⇌ FAD(c) + FADH-redox-potential(c)
r0851	-1	H2O(m) ⇌ H2O(c)

B4.3. sugars

Fructose degr (436)

Definition			Solution												
			exchanges		reactions								transp		
Objective	Constraints	Comment	imports	exports	c	m	r	p	l	n	s	b	s-c	b-c	int
-1 Fructose	MCES	Fructose degradation	1 P_i(c) 1 Fructose(s) 2 ATP-energy(c)	1 H_2O(s) 1 Glucose-6P(c)	7	-	-	-	-	-	-	-	2	-	-

ID	flux	reaction
r0898	1	Fructose(s) ⇌ Fructose(c)
r0192	-1	Fructose-6P(c) ⇌ Glucose-6P(c)
r0256	-1	Fructose-1,6PP(c) ⇌ DHAP(c) + GAP(c)
r1319	-2	ATP(c) + H2O(c) ⇌ ADP(c) + Pi(c) + ATP-energy(c)
r0554	1	Fructose-1P(c) ⇌ DHAP(c) + Glyceraldehyde(c)
r0487	1	Fructose-1,6PP(c) + H2O(c) → Fructose-6P(c) + Pi(c)
r0849	-1	H2O(s) ⇌ H2O(c)
r0206	1	ATP(c) + Fructose(c) ⇌ ADP(c) + Fructose-1P(c)
r0252	1	ATP(c) + Glyceraldehyde(c) ⇌ ADP(c) + GAP(c)

Galactose degr (437)

Definition			Solution												
			exchanges		reactions								transp		
Objective	Constraints	Comment	imports	exports	c	m	r	p	l	n	s	b	s-c	b-c	int
-1 Galactose	MCES	Galactose degradation	1 P_i(c) 1 Galactose(s) 1 ATP-energy(c)	1 H_2O(s) 1 Glucose-6P(c)	5	-	-	-	-	-	-	-	2	-	-

ID	flux	reaction
r0265	1	ATP(c) + Galactose(c) → ADP(c) + Galactose-1P(c)
r1319	-1	ATP(c) + H2O(c) ⇌ ADP(c) + Pi(c) + ATP-energy(c)
r0824	1	Galactose(s) → Galactose(c)
r0095	-1	UDP-galactose(c) ⇌ UDP-glucose(c)
r0849	-1	H2O(s) ⇌ H2O(c)
r0233	1	UDP-glucose(c) + Galactose-1P(c) ⇌ Glucose-1P(c) + UDP-galactose(c)
r0621	1	Glucose-1P(c) ⇌ Glucose-6P(c)

Mannose degr (438)

Definition			Solution												
			exchanges		reactions								transp		
Objective	Constraints	Comment	imports	exports	c	m	r	p	l	n	s	b	s-c	b-c	int
-1 Mannose	MCES	Mannose degradation	1 P_i(c) 1 Mannose(s) 1 ATP-energy(c)	1 H_2O(s) 1 Glucose-6P(c)	4	-	-	-	-	-	-	-	2	-	-

ID	flux	reaction
r0192	-1	Fructose-6P(c) ⇌ Glucose-6P(c)
r1319	-1	ATP(c) + H2O(c) ⇌ ADP(c) + Pi(c) + ATP-energy(c)
r0359	1	Mannose(c) + ATP(c) → Mannose-6P(c) + ADP(c)
r0918	1	Mannose(s) → Mannose(c)
r0849	-1	H2O(s) ⇌ H2O(c)
r0405	1	Mannose-6P(c) ⇌ Fructose-6P(c)

B4.4. Proteins

Albumin degr (439)

Definition			Solution												
			exchanges		reactions								transp		
Objective	Constraints	Comment	imports	exports	c	m	r	p	l	n	s	b	s-c	b-c	int
-1 Albumin	MCES AAA	degradation of Albumin	1 Albumin(s) 304 ATP-energy(c)	62 Glutamate(s) 13 Glycine(s) 63 Alanine(s) 60 Lysine(s) 36 Aspartate(s) 27 Arginine(s) 20 Glutamine(s) 28 Serine(s) 7 Methionine(s) 2 Tryptophan(s) 35 Phenylalanine(s) 19 Tyrosine(s) 35 Cysteine(s) 64 Leucine(s) 16 Histidine(s) 24 Proline(s) 17 Asparagine(s) 43 Valine(s) 29 Threonine(s) 9 Isoleucine(s)	1	-	-	-	-	-	-	-	21	-	-

ID	flux	reaction
r2530	-64	Leucine(s) ⇌ Leucine(c)
		Continued on next page

ID	flux	reaction
r1333	1	Albumin(c) + 304 ATP(c) + 304 H2O(c) → 64 Leucine(c) + 20 Glutamine(c) + 63 Alanine(c) + 27 Arginine(c) + 16 Histidine(c) + 9 Isoleucine(c) + 2 Tryptophan(c) + 43 Valine(c) + 304 ADP(c) + 304 Pi(c) + 62 Glutamate(c) + 35 Cysteine(c) + 13 Glycine(c) + 7 Methionine(c) + 35 Phenylalanine(c) + 24 Proline(c) + 28 Serine(c) + 29 Threonine(c) + 19 Tyrosine(c) + 17 Asparagine(c) + 36 Aspartate(c) + 60 Lysine(c)
r1072	-7	Methionine(s) ⇌ Methionine(c)
r0802	-1	Albumin(c) ⇌ Albumin(s)
r2531	-24	Proline(s) ⇌ Proline(c)
r2532	-17	Asparagine(s) ⇌ Asparagine(c)
r2523	-13	Glycine(s) ⇌ Glycine(c)
r2533	-43	Valine(s) ⇌ Valine(c)
r2524	-63	Alanine(s) ⇌ Alanine(c)
r1066	-16	Histidine(s) ← Histidine(c)
r2534	-29	Threonine(s) ⇌ Threonine(c)
r2525	-20	Glutamine(s) ⇌ Glutamine(c)
r1319	-304	ATP(c) + H2O(c) ⇌ ADP(c) + Pi(c) + ATP-energy(c)
r1058	-27	Arginine(c) ⇌ Arginine(s)
r2526	-28	Serine(s) ⇌ Serine(c)
r2536	-9	Isoleucine(s) ⇌ Isoleucine(c)
r2527	-2	Tryptophan(s) ⇌ Tryptophan(c)
r1069	60	Lysine(c) ⇌ Lysine(s)
r2528	-35	Phenylalanine(s) ⇌ Phenylalanine(c)
r1079	-19	Tyrosine(c) → Tyrosine(s)
r2529	-35	Cysteine(s) ⇌ Cysteine(c)
r2685	62	Glutamate(c) ⇌ Glutamate(s)
r2686	36	Aspartate(c) ⇌ Aspartate(s)

Antichymotrypsin degr (440)

Definition			Solution												
			exchanges		reactions								transp		
Objective	Constraints	Comment	imports	exports	c	m	r	p	l	n	s	b	s-c	b-c	int
-1 Antichymotrypsin	MCES AAA	degradation of Antichymotrypsin	1 Antichymotrypsin(s) 211 ATP-energy(c)	29 Glutamate(s) 16 Glycine(s) 34 Alanine(s) 26 Lysine(s) 25 Aspartate(s) 16 Arginine(s) 17 Glutamine(s) 30 Serine(s) 14 Methionine(s) 3 Tryptophan(s) 25 Phenylalanine(s) 9 Tyrosine(s) 3 Cysteine(s) 59 Leucine(s) 9 Histidine(s) 16 Proline(s) 19 Asparagine(s) 24 Valine(s) 29 Threonine(s) 20 Isoleucine(s)	1	-	-	-	-	-	-	-	21	-	-

ID	flux	reaction
r2530	-59	Leucine(s) ⇌ Leucine(c)
r1072	-14	Methionine(s) ⇌ Methionine(c)
r2531	-16	Proline(s) ⇌ Proline(c)
r2532	-19	Asparagine(s) ⇌ Asparagine(c)
r2523	-16	Glycine(s) ⇌ Glycine(c)
r1335	1	211 ATP(c) + 211 H2O(c) + Antichymotrypsin(c) → 20 Isoleucine(c) + 59 Leucine(c) + 14 Methionine(c) + 34 Alanine(c) + 16 Arginine(c) + 9 Histidine(c) + 3 Tryptophan(c) + 24 Valine(c) + 211 ADP(c) + 211 Pi(c) + 17 Glutamine(c) + 29 Glutamate(c) + 3 Cysteine(c) + 16 Glycine(c) + 25 Phenylalanine(c) + 16 Proline(c) + 30 Serine(c) + 29 Threonine(c) + 9 Tyrosine(c) + 19 Asparagine(c) + 25 Aspartate(c) + 26 Lysine(c)
r0804	-1	Antichymotrypsin(c) ⇌ Antichymotrypsin(s)
r2533	-24	Valine(s) ⇌ Valine(c)
r2524	-34	Alanine(s) ⇌ Alanine(c)
r1066	-9	Histidine(s) ← Histidine(c)
r2534	-29	Threonine(s) ⇌ Threonine(c)

Continued on next page

ID	flux	reaction
r2525	-17	Glutamine(s) ⇌ Glutamine(c)
r1319	-211	ATP(c) + H2O(c) ⇌ ADP(c) + Pi(c) + ATP-energy(c)
r1058	-16	Arginine(c) ⇌ Arginine(s)
r2526	-30	Serine(s) ⇌ Serine(c)
r2536	-20	Isoleucine(s) ⇌ Isoleucine(c)
r2527	-3	Tryptophan(s) ⇌ Tryptophan(c)
r1069	26	Lysine(c) ⇌ Lysine(s)
r2528	-25	Phenylalanine(s) ⇌ Phenylalanine(c)
r1079	-9	Tyrosine(c) → Tyrosine(s)
r2529	-3	Cysteine(s) ⇌ Cysteine(c)
r2685	29	Glutamate(c) ⇌ Glutamate(s)
r2686	25	Aspartate(c) ⇌ Aspartate(s)

Antitrypsin degr (441)

Definition			Solution												
			exchanges		reactions								transp		
Objective	Constraints	Comment	imports	exports	c	m	r	p	l	n	s	b	s-c	b-c	int
-1 Antitrypsin	MCES AAA	degradation of Antitrypsin	1 Antitrypsin(s) 209 ATP-energy(c)	32 Glutamate(s) 24 Glycine(s) 26 Alanine(s) 34 Lysine(s) 24 Aspartate(s) 7 Arginine(s) 18 Glutamine(s) 25 Serine(s) 10 Methionine(s) 3 Tryptophan(s) 27 Phenylalanine(s) 6 Tyrosine(s) 3 Cysteine(s) 51 Leucine(s) 13 Histidine(s) 19 Proline(s) 19 Asparagine(s) 27 Valine(s) 30 Threonine(s) 20 Isoleucine(s)	1	-	-	-	-	-	-	-	21	-	-

ID	flux	reaction
r2530	-51	Leucine(s) ⇌ Leucine(c)
r1072	-10	Methionine(s) ⇌ Methionine(c)
r2531	-19	Proline(s) ⇌ Proline(c)
r2532	-19	Asparagine(s) ⇌ Asparagine(c)
r2523	-24	Glycine(s) ⇌ Glycine(c)
r2533	-27	Valine(s) ⇌ Valine(c)
r2524	-26	Alanine(s) ⇌ Alanine(c)
r1066	-13	Histidine(s) ← Histidine(c)
r2534	-30	Threonine(s) ⇌ Threonine(c)
r2525	-18	Glutamine(s) ⇌ Glutamine(c)
r1337	1	209 ATP(c) + 209 H2O(c) + Antitrypsin(c) → 20 Isoleucine(c) + 51 Leucine(c) + 10 Methionine(c) + 26 Alanine(c) + 7 Arginine(c) + 13 Histidine(c) + 3 Tryptophan(c) + 27 Valine(c) + 209 ADP(c) + 209 Pi(c) + 18 Glutamine(c) + 32 Glutamate(c) + 3 Cysteine(c) + 24 Glycine(c) + 27 Phenylalanine(c) + 19 Proline(c) + 25 Serine(c) + 30 Threonine(c) + 6 Tyrosine(c) + 19 Asparagine(c) + 24 Aspartate(c) + 34 Lysine(c)
r1319	-209	ATP(c) + H2O(c) ⇌ ADP(c) + Pi(c) + ATP-energy(c)
r1058	-7	Arginine(c) ⇌ Arginine(s)
r0806	-1	Antitrypsin(c) ⇌ Antitrypsin(s)
r2526	-25	Serine(s) ⇌ Serine(c)
r2536	-20	Isoleucine(s) ⇌ Isoleucine(c)
r2527	-3	Tryptophan(s) ⇌ Tryptophan(c)
r1069	34	Lysine(c) ⇌ Lysine(s)
r2528	-27	Phenylalanine(s) ⇌ Phenylalanine(c)
r1079	-6	Tyrosine(c) → Tyrosine(s)
r2529	-3	Cysteine(s) ⇌ Cysteine(c)
r2685	32	Glutamate(c) ⇌ Glutamate(s)
r2686	24	Aspartate(c) ⇌ Aspartate(s)

ApoA1 degr (442)

Definition			Solution												
			exchanges		reactions								transp		
Objective	Constraints	Comment	imports	exports	c	m	r	p	l	n	s	b	s-c	b-c	int
-1 ApoA1	MCES AAA	degradation of ApoA1	1 ApoA1(s) 133 ATP-energy(c)	30 Glutamate(s) 11 Glycine(s) 23 Alanine(s) 22 Lysine(s) 16 Aspartate(s) 17 Arginine(s) 19 Glutamine(s) 16 Serine(s) 4 Methionine(s) 5 Tryptophan(s) 8 Phenylalanine(s) 7 Tyrosine(s) 41 Leucine(s) 6 Histidine(s) 10 Proline(s) 5 Asparagine(s) 15 Valine(s) 12 Threonine(s)	1	-	-	-	-	-	-	-	19	-	-

ID	flux	reaction
r2530	-41	Leucine(s) ⇌ Leucine(c)
r1072	-4	Methionine(s) ⇌ Methionine(c)
r2531	-10	Proline(s) ⇌ Proline(c)
r2532	-5	Asparagine(s) ⇌ Asparagine(c)
r2523	-11	Glycine(s) ⇌ Glycine(c)
r2533	-15	Valine(s) ⇌ Valine(c)
r2524	-23	Alanine(s) ⇌ Alanine(c)
r1066	-6	Histidine(s) ← Histidine(c)
r2534	-12	Threonine(s) ⇌ Threonine(c)
r2525	-19	Glutamine(s) ⇌ Glutamine(c)
r1319	-133	ATP(c) + H2O(c) ⇌ ADP(c) + Pi(c) + ATP-energy(c)
r1058	-17	Arginine(c) ⇌ Arginine(s)
r2526	-16	Serine(s) ⇌ Serine(c)
r2527	-5	Tryptophan(s) ⇌ Tryptophan(c)
r1339	1	133 ATP(c) + ApoA1(c) + 133 H2O(c) → 133 ADP(c) + 133 Pi(c) + 41 Leucine(c) + 4 Methionine(c) + 23 Alanine(c) + 17 Arginine(c) + 19 Glutamine(c) + 30 Glutamate(c) + 6 Histidine(c) + 5 Tryptophan(c) + 15 Valine(c) + 11 Glycine(c) + 8 Phenylalanine(c) + 10 Proline(c) + 16 Serine(c) + 12 Threonine(c) + 7 Tyrosine(c) + 5 Asparagine(c) + 16 Aspartate(c) + 22 Lysine(c)
r1069	22	Lysine(c) ⇌ Lysine(s)
r0808	-1	ApoA1(c) ⇌ ApoA1(s)
r2528	-8	Phenylalanine(s) ⇌ Phenylalanine(c)
r1079	-7	Tyrosine(c) → Tyrosine(s)
r2685	30	Glutamate(c) ⇌ Glutamate(s)
r2686	16	Aspartate(c) ⇌ Aspartate(s)

Fibrinogen degr (443)

Definition			Solution												
			exchanges		reactions								transp		
Objective	Constraints	Comment	imports	exports	c	m	r	p	l	n	s	b	s-c	b-c	int
-1 Fibrinogen	MCES AAA	degradation of Fibrinogen	1 Fibrinogen(s) 1337 ATP-energy(c)	186 Glutamate(s) 262 Glycine(s) 134 Alanine(s) 154 Lysine(s) 176 Aspartate(s) 134 Arginine(s) 110 Glutamine(s) 278 Serine(s) 48 Methionine(s) 60 Tryptophan(s) 98 Phenylalanine(s) 98 Tyrosine(s) 48 Cysteine(s) 166 Leucine(s) 62 Histidine(s) 114 Proline(s) 138 Asparagine(s) 122 Valine(s) 182 Threonine(s) 104 Isoleucine(s)	1	-	-	-	-	-	-	-	21	-	-

ID	flux	reaction
r2530	-166	Leucine(s) ⇌ Leucine(c)

Continued on next page

ID	flux	reaction
r1072	-48	Methionine(s) ⇌ Methionine(c)
r2531	-114	Proline(s) ⇌ Proline(c)
r2532	-138	Asparagine(s) ⇌ Asparagine(c)
r2523	-262	Glycine(s) ⇌ Glycine(c)
r2533	-122	Valine(s) ⇌ Valine(c)
r2524	-134	Alanine(s) ⇌ Alanine(c)
r1066	-62	Histidine(s) ← Histidine(c)
r2534	-182	Threonine(s) ⇌ Threonine(c)
r2525	-110	Glutamine(s) ⇌ Glutamine(c)
r1319	-1337	ATP(c) + H2O(c) ⇌ ADP(c) + Pi(c) + ATP-energy(c)
r1058	-134	Arginine(c) ⇌ Arginine(s)
r2526	-278	Serine(s) ⇌ Serine(c)
r2536	-104	Isoleucine(s) ⇌ Isoleucine(c)
r2527	-60	Tryptophan(s) ⇌ Tryptophan(c)
r1348	1	1337 ATP(c) + 1337 H2O(c) + Fibrinogen(c) → 104 Isoleucine(c) + 166 Leucine(c) + 48 Methionine(c) + 134 Alanine(c) + 134 Arginine(c) + 62 Histidine(c) + 60 Tryptophan(c) + 122 Valine(c) + 1337 ADP(c) + 1337 Pi(c) + 110 Glutamine(c) + 186 Glutamate(c) + 48 Cysteine(c) + 262 Glycine(c) + 98 Phenylalanine(c) + 114 Proline(c) + 278 Serine(c) + 182 Threonine(c) + 98 Tyrosine(c) + 138 Asparagine(c) + 176 Aspartate(c) + 154 Lysine(c)
r1069	154	Lysine(c) ⇌ Lysine(s)
r2528	-98	Phenylalanine(s) ⇌ Phenylalanine(c)
r1079	-98	Tyrosine(c) → Tyrosine(s)
r2529	-48	Cysteine(s) ⇌ Cysteine(c)
r1033	-1	Fibrinogen(c) ⇌ Fibrinogen(s)
r2685	186	Glutamate(c) ⇌ Glutamate(s)
r2686	176	Aspartate(c) ⇌ Aspartate(s)

Haptoglobin degr (444)

Definition			Solution												
			exchanges		reactions								transp		
Objective	Constraints	Comment	imports	exports	c	m	r	p	l	n	s	b	s-c	b-c	int
-1 Haptoglobin	MCES AAA	degradation of Haptoglobin	1 Haptoglobin(s) 203 ATP-energy(c)	25 Glutamate(s) 31 Glycine(s) 30 Alanine(s) 35 Lysine(s) 25 Aspartate(s) 9 Arginine(s) 17 Glutamine(s) 18 Serine(s) 5 Methionine(s) 8 Tryptophan(s) 8 Phenylalanine(s) 21 Tyrosine(s) 12 Cysteine(s) 31 Leucine(s) 13 Histidine(s) 21 Proline(s) 21 Asparagine(s) 36 Valine(s) 22 Threonine(s) 18 Isoleucine(s)	1	-	-	-	-	-	-	-	21	-	-

ID	flux	reaction
r2530	-31	Leucine(s) ⇌ Leucine(c)
r1072	-5	Methionine(s) ⇌ Methionine(c)
r2531	-21	Proline(s) ⇌ Proline(c)
r2532	-21	Asparagine(s) ⇌ Asparagine(c)
r2523	-31	Glycine(s) ⇌ Glycine(c)
r2533	-36	Valine(s) ⇌ Valine(c)
r2524	-30	Alanine(s) ⇌ Alanine(c)
r1066	-13	Histidine(s) ← Histidine(c)
r1048	-1	Haptoglobin(c) ⇌ Haptoglobin(s)
r2534	-22	Threonine(s) ⇌ Threonine(c)
r2525	-17	Glutamine(s) ⇌ Glutamine(c)
r1319	-203	ATP(c) + H2O(c) ⇌ ADP(c) + Pi(c) + ATP-energy(c)
r1058	-9	Arginine(c) ⇌ Arginine(s)
r2526	-18	Serine(s) ⇌ Serine(c)

Continued on next page

Haptoglobin degr (444)– continued

ID	flux	reaction
r2536	-18	Isoleucine(s) ⇌ Isoleucine(c)
r2527	-8	Tryptophan(s) ⇌ Tryptophan(c)
r1069	35	Lysine(c) ⇌ Lysine(s)
r2528	-8	Phenylalanine(s) ⇌ Phenylalanine(c)
r1079	-21	Tyrosine(c) → Tyrosine(s)
r2529	-12	Cysteine(s) ⇌ Cysteine(c)
r2685	25	Glutamate(c) ⇌ Glutamate(s)
r2686	25	Aspartate(c) ⇌ Aspartate(s)
r1350	1	203 ATP(c) + Haptoglobin(c) + 203 H2O(c) → 18 Isoleucine(c) + 31 Leucine(c) + 5 Methionine(c) + 30 Alanine(c) + 9 Arginine(c) + 13 Histidine(c) + 8 Tryptophan(c) + 36 Valine(c) + 203 ADP(c) + 203 Pi(c) + 17 Glutamine(c) + 25 Glutamate(c) + 12 Cysteine(c) + 31 Glycine(c) + 8 Phenylalanine(c) + 21 Proline(c) + 18 Serine(c) + 22 Threonine(c) + 21 Tyrosine(c) + 21 Asparagine(c) + 25 Aspartate(c) + 35 Lysine(c)

Plasminogen degr (445)

Definition			Solution												
			exchanges		reactions								transp		
Objective	Constraints	Comment	imports	exports	c	m	r	p	l	n	s	b	s-c	b-c	int
-1 Plasminogen	MCES AAA	degradation of Plasminogen	1 Plasminogen(s) 405 ATP-energy(c)	56 Glutamate(s) 62 Glycine(s) 37 Alanine(s) 49 Lysine(s) 36 Aspartate(s) 42 Arginine(s) 31 Glutamine(s) 56 Serine(s) 11 Methionine(s) 19 Tryptophan(s) 21 Phenylalanine(s) 30 Tyrosine(s) 48 Cysteine(s) 48 Leucine(s) 24 Histidine(s) 69 Proline(s) 40 Asparagine(s) 48 Valine(s) 61 Threonine(s) 22 Isoleucine(s)	1	-	-	-	-	-	-	-	21	-	-

ID	flux	reaction
r2530	-48	Leucine(s) ⇌ Leucine(c)
r1072	-11	Methionine(s) ⇌ Methionine(c)
r1054	-1	Plasminogen(c) ⇌ Plasminogen(s)
r2531	-69	Proline(s) ⇌ Proline(c)
r1352	1	405 ATP(c) + Plasminogen(c) + 405 H2O(c) → 22 Isoleucine(c) + 48 Leucine(c) + 11 Methionine(c) + 37 Alanine(c) + 42 Arginine(c) + 24 Histidine(c) + 19 Tryptophan(c) + 48 Valine(c) + 405 ADP(c) + 405 Pi(c) + 31 Glutamine(c) + 56 Glutamate(c) + 48 Cysteine(c) + 62 Glycine(c) + 21 Phenylalanine(c) + 69 Proline(c) + 56 Serine(c) + 61 Threonine(c) + 30 Tyrosine(c) + 40 Asparagine(c) + 36 Aspartate(c) + 49 Lysine(c)
r2532	-40	Asparagine(s) ⇌ Asparagine(c)
r2523	-62	Glycine(s) ⇌ Glycine(c)
r2533	-48	Valine(s) ⇌ Valine(c)
r2524	-37	Alanine(s) ⇌ Alanine(c)
r1066	-24	Histidine(s) ← Histidine(c)
r2534	-61	Threonine(s) ⇌ Threonine(c)
r2525	-31	Glutamine(s) ⇌ Glutamine(c)
r1319	-405	ATP(c) + H2O(c) ⇌ ADP(c) + Pi(c) + ATP-energy(c)
r1058	-42	Arginine(c) ⇌ Arginine(s)
r2526	-56	Serine(s) ⇌ Serine(c)
r2536	-22	Isoleucine(s) ⇌ Isoleucine(c)
r2527	-19	Tryptophan(s) ⇌ Tryptophan(c)
r1069	49	Lysine(c) ⇌ Lysine(s)
r2528	-21	Phenylalanine(s) ⇌ Phenylalanine(c)
r1079	-30	Tyrosine(c) → Tyrosine(s)
r2529	-48	Cysteine(s) ⇌ Cysteine(c)
r2685	56	Glutamate(c) ⇌ Glutamate(s)
r2686	36	Aspartate(c) ⇌ Aspartate(s)

Prothrombin degr (446)

Definition			Solution													
			exchanges		reactions								transp		
Objective	Constraints	Comment	imports	exports	c	m	r	p	l	n	s	b	s-c	b-c	int
-1 Prothrombin	MCES AAA	degradation of Prothrombin	1 Prothrombin(s) 311 ATP-energy(c)	51 Glutamate(s) 49 Glycine(s) 42 Alanine(s) 29 Lysine(s) 35 Aspartate(s) 44 Arginine(s) 26 Glutamine(s) 38 Serine(s) 9 Methionine(s) 14 Tryptophan(s) 21 Phenylalanine(s) 21 Tyrosine(s) 26 Cysteine(s) 51 Leucine(s) 13 Histidine(s) 33 Proline(s) 25 Asparagine(s) 37 Valine(s) 36 Threonine(s) 22 Isoleucine(s)	1	-	-	-	-	-	-	-	21	-	-

ID	flux	reaction
r2530	-51	Leucine(s) ⇌ Leucine(c)
r1072	-9	Methionine(s) ⇌ Methionine(c)
r2531	-33	Proline(s) ⇌ Proline(c)
r2532	-25	Asparagine(s) ⇌ Asparagine(c)
r2523	-49	Glycine(s) ⇌ Glycine(c)
r1056	-1	Prothrombin(c) ⇌ Prothrombin(s)
r2533	-37	Valine(s) ⇌ Valine(c)
r2524	-42	Alanine(s) ⇌ Alanine(c)
r1354	1	Prothrombin(c) + 311 ATP(c) + 311 H2O(c) → 22 Isoleucine(c) + 51 Leucine(c) + 9 Methionine(c) + 42 Alanine(c) + 44 Arginine(c) + 13 Histidine(c) + 14 Tryptophan(c) + 37 Valine(c) + 311 ADP(c) + 311 Pi(c) + 26 Glutamine(c) + 51 Glutamate(c) + 26 Cysteine(c) + 49 Glycine(c) + 21 Phenylalanine(c) + 33 Proline(c) + 38 Serine(c) + 36 Threonine(c) + 21 Tyrosine(c) + 25 Asparagine(c) + 35 Aspartate(c) + 29 Lysine(c)
r1066	-13	Histidine(s) ← Histidine(c)
r2534	-36	Threonine(s) ⇌ Threonine(c)
r2525	-26	Glutamine(s) ⇌ Glutamine(c)
r1319	-311	ATP(c) + H2O(c) ⇌ ADP(c) + Pi(c) + ATP-energy(c)
r1058	-44	Arginine(c) ⇌ Arginine(s)
r2526	-38	Serine(s) ⇌ Serine(c)
r2536	-22	Isoleucine(s) ⇌ Isoleucine(c)
r2527	-14	Tryptophan(s) ⇌ Tryptophan(c)
r1069	29	Lysine(c) ⇌ Lysine(s)
r2528	-21	Phenylalanine(s) ⇌ Phenylalanine(c)
r1079	-21	Tyrosine(c) → Tyrosine(s)
r2529	-26	Cysteine(s) ⇌ Cysteine(c)
r2685	51	Glutamate(c) ⇌ Glutamate(s)
r2686	35	Aspartate(c) ⇌ Aspartate(s)

ApoTransferin degr (447)

Definition			Solution													
			exchanges		reactions								transp		
Objective	Constraints	Comment	imports	exports	c	m	r	p	l	n	s	b	s-c	b-c	int
-1 ApoTransferin	MCES AAA	degradation of ApoTransferin	1 ApoTransferin(s) 349 ATP-energy(c)	42 Glutamate(s) 52 Glycine(s) 61 Alanine(s) 58 Lysine(s) 45 Aspartate(s) 27 Arginine(s) 17 Glutamine(s) 41 Serine(s) 10 Methionine(s) 8 Tryptophan(s) 28 Phenylalanine(s) 26 Tyrosine(s) 40 Cysteine(s) 65 Leucine(s) 19 Histidine(s) 32 Proline(s) 34 Asparagine(s) 48 Valine(s) 30 Threonine(s) 15 Isoleucine(s)	1	-	-	-	-	-	-	-	21	-	-

ID	flux	reaction
r2530	-65	Leucine(s) ⇌ Leucine(c)
r1081	-1	ApoTransferin(c) ⇌ ApoTransferin(s)
r1072	-10	Methionine(s) ⇌ Methionine(c)
r2531	-32	Proline(s) ⇌ Proline(c)
r2532	-34	Asparagine(s) ⇌ Asparagine(c)
r2523	-52	Glycine(s) ⇌ Glycine(c)
r2533	-48	Valine(s) ⇌ Valine(c)
r2524	-61	Alanine(s) ⇌ Alanine(c)
r1066	-19	Histidine(s) ← Histidine(c)
r2534	-30	Threonine(s) ⇌ Threonine(c)
r2525	-17	Glutamine(s) ⇌ Glutamine(c)
r1319	-349	ATP(c) + H2O(c) ⇌ ADP(c) + Pi(c) + ATP-energy(c)
r1058	-27	Arginine(c) ⇌ Arginine(s)
r2526	-41	Serine(s) ⇌ Serine(c)
r1356	1	ApoTransferin(c) + 349 ATP(c) + 349 H2O(c) → 15 Isoleucine(c) + 65 Leucine(c) + 10 Methionine(c) + 61 Alanine(c) + 27 Arginine(c) + 19 Histidine(c) + 8 Tryptophan(c) + 48 Valine(c) + 349 ADP(c) + 349 Pi(c) + 17 Glutamine(c) + 42 Glutamate(c) + 40 Cysteine(c) + 52 Glycine(c) + 28 Phenylalanine(c) + 32 Proline(c) + 41 Serine(c) + 30 Threonine(c) + 26 Tyrosine(c) + 34 Asparagine(c) + 45 Aspartate(c) + 58 Lysine(c)
r2536	-15	Isoleucine(s) ⇌ Isoleucine(c)
r2527	-8	Tryptophan(s) ⇌ Tryptophan(c)
r1069	58	Lysine(c) ⇌ Lysine(s)
r2528	-28	Phenylalanine(s) ⇌ Phenylalanine(c)
r1079	-26	Tyrosine(c) → Tyrosine(s)
r2529	-40	Cysteine(s) ⇌ Cysteine(c)
r2685	42	Glutamate(c) ⇌ Glutamate(s)
r2686	45	Aspartate(c) ⇌ Aspartate(s)

ApoB100 degr (448)

Definition			Solution													
			exchanges		reactions								transp			
Objective	Constraints	Comment	imports	exports	c	m	r	p	l	n	s	b	s-c	b-c	int
-1 ApoB100(l)	MCES AAA	degradation of lysosomal ApoB100	1 ApoB100(l) 2280 ATP-energy(c)	298 Glutamate(s) 207 Glycine(s) 275 Alanine(s) 357 Lysine(s) 233 Aspartate(s) 150 Arginine(s) 230 Glutamine(s) 392 Serine(s) 79 Methionine(s) 37 Tryptophan(s) 224 Phenylalanine(s) 151 Tyrosine(s) 25 Cysteine(s) 533 Leucine(s) 115 Histidine(s) 171 Proline(s) 247 Asparagine(s) 252 Valine(s) 300 Threonine(s) 285 Isoleucine(s)	1	-	-	-	-	-	-	-	20	-	23

ID	flux	reaction
r1080	151	Tyrosine(l) → Tyrosine(c)
r1067	115	Histidine(l) → Histidine(c)
r1362	298	Glutamate(l) → Glutamate(c)
r1068	533	Leucine(l) → Leucine(c)
r1069	357	Lysine(c) ⇌ Lysine(s)
r0859	-2280	ADP(c) + ATP(l) ← ADP(l) + ATP(c)
r1140	252	Valine(l) → Valine(c)
r2530	-533	Leucine(s) ⇌ Leucine(c)
r2531	-171	Proline(s) ⇌ Proline(c)
r2532	-247	Asparagine(s) ⇌ Asparagine(c)
r2685	298	Glutamate(c) ⇌ Glutamate(s)
r2533	-252	Valine(s) ⇌ Valine(c)
r2686	233	Aspartate(c) ⇌ Aspartate(s)

Continued on next page

ID	flux	reaction
r2534	-300	Threonine(s) ⇌ Threonine(c)
r2536	-285	Isoleucine(s) ⇌ Isoleucine(c)
r1319	-2280	ATP(c) + H2O(c) ⇌ ADP(c) + Pi(c) + ATP-energy(c)
r0825	285	Isoleucine(l) → Isoleucine(c)
r1071	357	Lysine(l) → Lysine(c)
r1058	-150	Arginine(c) ⇌ Arginine(s)
r1059	150	Arginine(l) → Arginine(c)
r1072	-79	Methionine(s) ⇌ Methionine(c)
r1073	79	Methionine(l) → Methionine(c)
r1074	224	Phenylalanine(l) → Phenylalanine(c)
r1075	171	Proline(l) → Proline(c)
r1076	392	Serine(l) → Serine(c)
r1077	37	Tryptophan(l) → Tryptophan(c)
r1079	-151	Tyrosine(c) → Tyrosine(s)
r0883	275	Alanine(l) → Alanine(c)
r1150	-2280	Pi(c) ← Pi(l)
r0925	300	Threonine(l) → Threonine(c)
r2523	-207	Glycine(s) ⇌ Glycine(c)
r2524	-275	Alanine(s) ⇌ Alanine(c)
r2525	-230	Glutamine(s) ⇌ Glutamine(c)
r2526	-392	Serine(s) ⇌ Serine(c)
r2527	-37	Tryptophan(s) ⇌ Tryptophan(c)
r2528	-224	Phenylalanine(s) ⇌ Phenylalanine(c)
r2529	-25	Cysteine(s) ⇌ Cysteine(c)
r1342	1	2280 ATP(l) + 2280 H2O(l) + ApoB100(l) → 285 Isoleucine(l) + 533 Leucine(l) + 79 Methionine(l) + 275 Alanine(l) + 150 Arginine(l) + 115 Histidine(l) + 37 Tryptophan(l) + 252 Valine(l) + 2280 ADP(l) + 2280 Pi(l) + 230 Glutamine(l) + 298 Glutamate(l) + 25 Cysteine(l) + 207 Glycine(l) + 224 Phenylalanine(l) + 171 Proline(l) + 392 Serine(l) + 300 Threonine(l) + 151 Tyrosine(l) + 247 Asparagine(l) + 233 Aspartate(l) + 357 Lysine(l)
r1061	247	Asparagine(l) → Asparagine(c)
r1062	233	Aspartate(l) → Aspartate(c)
r1063	25	Cysteine(l) → Cysteine(c)
r0850	-2280	H2O(l) ← H2O(c)
r1064	-230	Glutamine(c) ← Glutamine(l)
r1065	207	Glycine(l) → Glycine(c)
r1066	-115	Histidine(s) ← Histidine(c)

Glycogenin degr (449)

Definition			Solution												
			exchanges		reactions								transp		
Objective	Constraints	Comment	imports	exports	c	m	r	p	l	n	s	b	s-c	b-c	int
-1 Glycogenin(c)	MCES AAA	degradation of cytosolic Glycogenin	175 ATP-energy(c) 1 Glycogenin(c)	15 Glutamate(s) 18 Glycine(s) 22 Alanine(s) 18 Lysine(s) 26 Aspartate(s) 12 Arginine(s) 14 Glutamine(s) 31 Serine(s) 8 Methionine(s) 7 Tryptophan(s) 20 Phenylalanine(s) 13 Tyrosine(s) 6 Cysteine(s) 39 Leucine(s) 9 Histidine(s) 15 Proline(s) 12 Asparagine(s) 28 Valine(s) 25 Threonine(s) 12 Isoleucine(s)	1	-	-	-	-	-	-	-	20	-	-

ID	flux	reaction
r2530	-39	Leucine(s) ⇌ Leucine(c)
r1072	-8	Methionine(s) ⇌ Methionine(c)
r2531	-15	Proline(s) ⇌ Proline(c)
r2532	-12	Asparagine(s) ⇌ Asparagine(c)

Continued on next page

Glycogenin degr (449)– continued

ID	flux	reaction
r2523	-18	Glycine(s) ⇌ Glycine(c)
r2533	-28	Valine(s) ⇌ Valine(c)
r2524	-22	Alanine(s) ⇌ Alanine(c)
r1066	-9	Histidine(s) ← Histidine(c)
r2534	-25	Threonine(s) ⇌ Threonine(c)
r2525	-14	Glutamine(s) ⇌ Glutamine(c)
r1319	-175	ATP(c) + H2O(c) ⇌ ADP(c) + Pi(c) + ATP-energy(c)
r1058	-12	Arginine(c) ⇌ Arginine(s)
r2526	-31	Serine(s) ⇌ Serine(c)
r2536	-12	Isoleucine(s) ⇌ Isoleucine(c)
r2527	-7	Tryptophan(s) ⇌ Tryptophan(c)
r1069	18	Lysine(c) ⇌ Lysine(s)
r2528	-20	Phenylalanine(s) ⇌ Phenylalanine(c)
r1079	-13	Tyrosine(c) → Tyrosine(s)
r2529	-6	Cysteine(s) ⇌ Cysteine(c)
r2685	15	Glutamate(c) ⇌ Glutamate(s)
r1403	1	Glycogenin(c) + 175 ATP(c) + 175 H2O(c) → 6 Cysteine(c) + 18 Glycine(c) + 9 Histidine(c) + 12 Arginine(c) + 22 Alanine(c) + 15 Glutamate(c) + 20 Phenylalanine(c) + 12 Isoleucine(c) + 39 Leucine(c) + 8 Methionine(c) + 15 Proline(c) + 14 Glutamine(c) + 31 Serine(c) + 25 Threonine(c) + 28 Valine(c) + 7 Tryptophan(c) + 13 Tyrosine(c) + 175 ADP(c) + 175 Pi(c) + 12 Asparagine(c) + 26 Aspartate(c) + 18 Lysine(c)
r2686	26	Aspartate(c) ⇌ Aspartate(s)

B4.5. Ethanol

Ethanol degr (450)

Definition			Solution												
			exchanges		reactions								transp		
Objective	Constraints	Comment	imports	exports	c	m	r	p	l	n	s	b	s-c	b-c	int
-1 Ethanol	MCES	degradation of Ethanol	1 Ethanol(s) 2 ATP-energy(c)	2 NADH-redox-potential(c) 1 CoA-activated acetyl group(c)	8	-	-	-	-	-	-	-	1	-	-

ID	flux	reaction
r0066	1	ATP(c) + Acetate(c) + CoA(c) → AMP(c) + PPi(c) + Acetyl-CoA(c)
r1319	-2	ATP(c) + H2O(c) ⇌ ADP(c) + Pi(c) + ATP-energy(c)
r0176	1	Acetaldehyde(c) + NAD+(c) + H2O(c) → Acetate(c) + NADH(c)
r0187	1	Ethanol(c) + NAD+(c) ⇌ Acetaldehyde(c) + NADH(c)
r0030	1	ATP(c) + AMP(c) ⇌ 2 ADP(c)
r0007	1	PPi(c) + H2O(c) → 2 Pi(c)
r1322	2	NADH(c) ⇌ NAD+(c) + NADH-redox-potential(c)
r0957	1	Ethanol(s) ⇌ Ethanol(c)
r2677	1	Acetyl-CoA(c) ⇌ CoA(c) + CoA-activated acetyl group(c)

B4.6. Other

Chitin-component degr (451)

Definition			Solution												
			exchanges		reactions								transp		
Objective	Constraints	Comment	imports	exports	c	m	r	p	l	n	s	b	s-c	b-c	int
-1 Chitin-component	MCES +Urea	degradation of Chitin-component	0.5 H_2O(s) 1 P_i(c) 0.5 Pyruvate(m) 4 ATP-energy(c) 1 ATP-energy(m) 1 Proton-gradient(m) 1 Chitin-component(s)	0.5 Urea(s) 1 Glucose-6P(c) 0.5 NADH-redox-potential(c) 1 CoA-activated acetyl group(c) 0.5 CoA-activated acetyl group(m)	16	6	-	-	1	-	1	-	2	-	10

ID	flux	reaction
r2678	0.5	Acetyl-CoA(m) ⇌ CoA(m) + CoA-activated acetyl group(m)
r0910	1	N-Acetylglucosamine(l) → N-Acetylglucosamine(c)
r0896	0.5	Urea(c) ⇌ Urea(s)
r0109	-0.5	OAA(c) + Glutamate(c) ⇌ AKG(c) + Aspartate(c)
r0073	-0.5	AKG(m) + NADH(m) + NH3(m) ⇌ Glutamate(m) + NAD+(m) + H2O(m)
r0911	0.5	Proline(c) + Glutamate(m) ⇌ Proline(m) + Glutamate(c)
r0290	1	ATP(c) + N-Acetylglucosamine(c) → ADP(c) + N-Acetylglucosamine-6P(c)
r0056	0.5	Pyruvate(m) + CoA(m) + NAD+(m) → Acetyl-CoA(m) + CO2(m) + NADH(m)
r1047	0.5	Pi(c) + H+(PG)(c) → Pi(m) + H+(PG)(m)
r0453	0.5	ATP(c) + Citrulline(c) + Aspartate(c) ⇌ AMP(c) + PPi(c) + Argininosuccinate(c)
r0192	-1	Fructose-6P(c) ⇌ Glucose-6P(c)
r0066	1	ATP(c) + Acetate(c) + CoA(c) → AMP(c) + PPi(c) + Acetyl-CoA(c)
r1426	0.5	Proline(m) + H+(PG)(c) → Proline(c) + H+(PG)(m)
r0850	-0.5	H2O(l) ← H2O(c)
r1319	-4	ATP(c) + H2O(c) ⇌ ADP(c) + Pi(c) + ATP-energy(c)
r0851	1	H2O(m) ⇌ H2O(c)
r0329	0.5	Carbamoyl-P(m) + Ornithine(m) ⇌ Pi(m) + Citrulline(m)
r1374	0.5	2 Chitin-component(s) + H2O(s) → Chitobiose(s)
r0258	-0.5	Fumarate(c) + H2O(c) ⇌ Malate(c)
r2419	0.5	AKG(c) + Pi(m) ⇌ AKG(m) + Pi(c)
r1375	0.5	Chitobiose(s) → Chitobiose(l)
r0030	1.5	ATP(c) + AMP(c) ⇌ 2 ADP(c)
r0013	0.5	Chitobiose(l) + H2O(l) → 2 N-Acetylglucosamine(l)
r0486	1	N-Acetylglucosamine-6P(c) + H2O(c) ⇌ Glucosamine-6P(c) + Acetate(c)
r0189	1	Glucosamine-6P(c) + H2O(c) ⇌ Fructose-6P(c) + NH3(c)
r0104	0.5	NAD+(c) + Malate(c) ⇌ OAA(c) + NADH(c)
r1320	-1	ATP(m) + H2O(m) ⇌ ADP(m) + Pi(m) + ATP-energy(m)
r0838	1	NH3(c) ⇌ NH3(m)
r1330	-1	H+(PG)(c) ⇌ H+(PG)(m) + Proton-gradient(m)
r0947	-0.5	Citrulline(m) + Ornithine(c) ⇌ Citrulline(c) + Ornithine(m)
r0034	0.5	2 ATP(m) + CO2(m) + H2O(m) + NH3(m) ⇌ 2 ADP(m) + Pi(m) + Carbamoyl-P(m)
r0007	1.5	PPi(c) + H2O(c) → 2 Pi(c)
r1322	0.5	NADH(c) ⇌ NAD+(c) + NADH-redox-potential(c)
r2677	1	Acetyl-CoA(c) ⇌ CoA(c) + CoA-activated acetyl group(c)
r0261	0.5	Argininosuccinate(c) ⇌ Fumarate(c) + Arginine(c)
r0144	0.5	Arginine(c) + H2O(c) ⇌ Urea(c) + Ornithine(c)

4 C. Proliferative functions

4.1 C1. Cofactors

CoA (452)

Definition			Solution												
			exchanges		reactions								transp		
Objective	Constraints	Comment	imports	exports	c	m	r	p	l	n	s	b	s-c	b-c	int
CoA(c)	MCES −Pantothenate AAA	de novo synthesis of cytosolic CoA	2.17 P_i(c) 1 Glycine(s) 2 Aspartate(s) 2 Glutamine(s) 0.83 Glucose-6P(c) 0.5 Cystine(s) 1 Pantothenate(s) 11.2 ATP-energy(c) 2 THF-activated methyl group(c)	5.17 H_2O(s) 1 CoA(c) 2 CO_2(s) 2 Pyruvate(c) 2 Glutamate(s) 2 NADH-redox-potential(c) 3.5 NADPH-redox-potential(c) 2 Proton-gradient(c) 5 Na-gradient(c)	42	-	-	-	-	-	-	-	10	-	-

ID	flux	reaction
r0406	-0.33	Fructose-6P(c) + Erythrose-4P(c) ⇌ Sedoheptulose-7P(c) + GAP(c)

Continued on next page

ID	flux	reaction
r0277	1	GTP(c) + IMP(c) + Aspartate(c) → GDP(c) + Pi(c) + Adenylosuccinate(c)
r0293	2	5,10-Methylene-THF(c) + NADP+(c) ⇌ 5,10-Methenyl-THF(c) + NADPH(c)
r0705	1	ATP(c) + 5-Phosphoribosyl-4-carboxy-5-aminoimidazole(c) + Aspartate(c) ⇌ ADP(c) + Pi(c) + SAICAR(c)
r0578	1	ATP(c) + Pantetheine(c) ⇌ ADP(c) + Phosphopantetheine(c)
r0877	0.5	Glutamate(c) + Cystine(s) ⇌ Glutamate(s) + Cystine(c)
r2477	2	Glutamine(s) + Na+(s) + H+(PG)(c) → Glutamine(c) + Na+(c) + H+(PG)(s)
r0030	2	ATP(c) + AMP(c) ⇌ 2 ADP(c)
r0032	1	ATP(c) + Dephospho-CoA(c) ⇌ ADP(c) + CoA(c)
r1143	-2	Na+(c) + Aspartate(c) ← Na+(s) + Aspartate(s)
r2664	5	Na+(c) ⇌ Na+(s) + Na-gradient(c)
r0058	2	Malate(c) + NADP+(c) ⇌ Pyruvate(c) + CO2(c) + NADPH(c)
r0370	0.33	Sedoheptulose-7P(c) + GAP(c) ⇌ Ribose-5P(c) + D-Xylulose-5P(c)
r0371	2	5,10-Methenyl-THF(c) + H2O(c) ⇌ 10-Formyl-THF(c)
r2685	1.5	Glutamate(c) ⇌ Glutamate(s)
r0659	1	ATP(c) + 5-Phosphoribosylamine(c) + Glycine(c) ⇌ ADP(c) + Pi(c) + GAR(c)
r0099	1	ATP(c) + GDP(c) ⇌ ADP(c) + GTP(c)
r0243	-0.17	DHAP(c) ⇌ GAP(c)
r0677	1	10-Formyl-THF(c) + GAR(c) ⇌ THF(c) + N-Formyl-GAR(c)
r1331	2	H+(PG)(s) ⇌ H+(PG)(c) + Proton-gradient(c)
r0974	1	Pantothenate(s) + Na+(s) ⇌ Pantothenate(c) + Na+(c)
r1319	-11.2	ATP(c) + H2O(c) ⇌ ADP(c) + Pi(c) + ATP-energy(c)
r0260	1	Adenylosuccinate(c) → Fumarate(c) + AMP(c)
r0247	1	ATP(c) + Ribose-5P(c) → AMP(c) + PRPP(c)
r0699	1	SAICAR(c) ⇌ Fumarate(c) + AICAR(c)
r0849	-5.17	H2O(s) ⇌ H2O(c)
r0581	-1	Pantothenate(c) + Cysteamine(c) ⇌ H2O(c) + Pantetheine(c)
r0867	2	CO2(c) ⇌ CO2(s)
r0589	1	ATP(c) + Phosphopantetheine(c) → PPi(c) + Dephospho-CoA(c)
r0007	2	PPi(c) + H2O(c) → 2 Pi(c)
r0736	0.17	ATP(c) + Fructose-6P(c) → ADP(c) + Fructose-1,6PP(c)
r0027	-0.5	NADPH(c) + Cystine(c) ⇌ 2 Cysteine(c) + NADP+(c)
r0192	0.83	Glucose-6P(c) ⇌ Fructose-6P(c)
r0344	-0.67	D-Xylulose-5P(c) ⇌ Ribulose-5P(c)
r0792	2	5-Methyl-THF(c) + NAD+(c) ⇌ 5,10-Methylene-THF(c) + NADH(c)
r0211	1	Cysteine(c) + O2(c) ⇌ 3-Sulfinoalanine(c)
r2523	1	Glycine(s) ⇌ Glycine(c)
r2676	-2	5-Methyl-THF(c) ⇌ THF(c) + THF-activated methyl group(c)
r0666	1	ATP(c) + 5-Phosphoribosylformylglycinamidine(c) ⇌ ADP(c) + Pi(c) + AIR(c)
r0667	-1	5-Phosphoribosyl-4-carboxy-5-aminoimidazole(c) ⇌ AIR(c) + CO2(c)
r1322	2	NADH(c) ⇌ NAD+(c) + NADH-redox-potential(c)
r0250	-0.67	Ribulose-5P(c) ⇌ Ribose-5P(c)
r1326	3.5	NADPH(c) ⇌ NADPH-redox-potential(c) + NADP+(c)
r0687	1	ATP(c) + N-Formyl-GAR(c) + Glutamine(c) + H2O(c) ⇌ ADP(c) + Pi(c) + Glutamate(c) + 5-Phosphoribosylformylglycinamidine(c)
r0255	0.33	Fructose-6P(c) + GAP(c) ⇌ D-Xylulose-5P(c) + Erythrose-4P(c)
r0538	1	3-Sulfinoalanine(c) ⇌ CO2(c) + Hypotaurine(c)
r0256	0.17	Fructose-1,6PP(c) ⇌ DHAP(c) + GAP(c)
r0539	-1	Hypotaurine(c) ⇌ Cysteamine(c) + O2(c)
r0257	-1	Glutamine(c) + PRPP(c) + H2O(c) ⇌ 5-Phosphoribosylamine(c) + PPi(c) + Glutamate(c)
r0258	-2	Fumarate(c) + H2O(c) ⇌ Malate(c)
r0273	-1	IMP(c) + H2O(c) ⇌ FAICAR(c)
r0700	1	10-Formyl-THF(c) + AICAR(c) ⇌ THF(c) + FAICAR(c)

NAD+ (453)

Definition			Solution												
			exchanges		reactions								transp		
Objective	Constraints	Comment	imports	exports	c	m	r	p	l	n	s	b	s-c	b-c	int
NAD$^+$(c)	−Nicotinamide MCES AAA	de novo synthesis of cytosolic NAD$^+$	0.33 P_i(c) 1 Glycine(s) 1.67 Glucose-6P(c) 2 Asparagine(s) 1 Nicotinamide(s) 16.3 ATP-energy(c) 2 THF-activated methyl group(c)	3.33 H_2O(s) 1 NAD$^+$(c) 1 CO_2(s) 2 Pyruvate(c) 2 NADH-redox-potential(c) 4 NADPH-redox-potential(c) 1 Proton-gradient(c) 2 Na-gradient(c)	40	-	-	-	-	-	-	-	7	-	-

ID	flux	reaction
r0406	-0.67	Fructose-6P(c) + Erythrose-4P(c) ⇌ Sedoheptulose-7P(c) + GAP(c)
r0277	1	GTP(c) + IMP(c) + Aspartate(c) → GDP(c) + Pi(c) + Adenylosuccinate(c)
r0127	2	H2O(c) + Asparagine(c) → Aspartate(c) + NH3(c)
r0293	2	5,10-Methylene-THF(c) + NADP+(c) ⇌ 5,10-Methenyl-THF(c) + NADPH(c)
r0705	1	ATP(c) + 5-Phosphoribosyl-4-carboxy-5-aminoimidazole(c) + Aspartate(c) ⇌ ADP(c) + Pi(c) + SAICAR(c)
r2479	2	Asparagine(s) + Na+(s) + H+(PG)(c) → Asparagine(c) + Na+(c) + H+(PG)(s)
r0030	4	ATP(c) + AMP(c) ⇌ 2 ADP(c)
r0914	-1	Nicotinamide(c) ⇌ Nicotinamide(s)
r2664	2	Na+(c) ⇌ Na+(s) + Na-gradient(c)
r0058	2	Malate(c) + NADP+(c) ⇌ Pyruvate(c) + CO2(c) + NADPH(c)
r0370	0.67	Sedoheptulose-7P(c) + GAP(c) ⇌ Ribose-5P(c) + D-Xylulose-5P(c)
r0371	2	5,10-Methenyl-THF(c) + H2O(c) ⇌ 10-Formyl-THF(c)
r0077	3	ATP(c) + Glutamate(c) + NH3(c) → ADP(c) + Pi(c) + Glutamine(c)
r0079	1	ATP(c) + Glutamine(c) + H2O(c) + Deamido-NAD(c) → AMP(c) + PPi(c) + NAD+(c) + Glutamate(c)
r0659	1	ATP(c) + 5-Phosphoribosylamine(c) + Glycine(c) ⇌ ADP(c) + Pi(c) + GAR(c)
r0099	1	ATP(c) + GDP(c) ⇌ ADP(c) + GTP(c)
r0243	-0.33	DHAP(c) ⇌ GAP(c)
r0677	1	10-Formyl-THF(c) + GAR(c) ⇌ THF(c) + N-Formyl-GAR(c)
r1331	1	H+(PG)(s) ⇌ H+(PG)(c) + Proton-gradient(c)
r0527	1	H2O(c) + Nicotinamide D-ribonucleotide(c) ⇌ Nicotinate ribonucleotide(c) + NH3(c)
r1319	-16.3	ATP(c) + H2O(c) ⇌ ADP(c) + Pi(c) + ATP-energy(c)
r0260	1	Adenylosuccinate(c) → Fumarate(c) + AMP(c)
r0247	2	ATP(c) + Ribose-5P(c) → AMP(c) + PRPP(c)
r0699	1	SAICAR(c) ⇌ Fumarate(c) + AICAR(c)
r0849	-3.33	H2O(s) ⇌ H2O(c)
r0867	1	CO2(c) ⇌ CO2(s)
r0585	1	ATP(c) + Nicotinate ribonucleotide(c) → PPi(c) + Deamido-NAD(c)
r2468	1	Glycine(s) + H+(PG)(s) → Glycine(c) + H+(PG)(c)
r0007	4	PPi(c) + H2O(c) → 2 Pi(c)
r0736	0.33	ATP(c) + Fructose-6P(c) → ADP(c) + Fructose-1,6PP(c)
r0306	-1	Nicotinamide(c) + PRPP(c) ⇌ PPi(c) + Nicotinamide D-ribonucleotide(c)
r0192	1.67	Glucose-6P(c) ⇌ Fructose-6P(c)
r0344	-1.33	D-Xylulose-5P(c) ⇌ Ribulose-5P(c)
r0792	2	5-Methyl-THF(c) + NAD+(c) ⇌ 5,10-Methylene-THF(c) + NADH(c)
r2676	-2	5-Methyl-THF(c) ⇌ THF(c) + THF-activated methyl group(c)
r0666	1	ATP(c) + 5-Phosphoribosylformylglycinamidine(c) ⇌ ADP(c) + Pi(c) + AIR(c)
r0667	-1	5-Phosphoribosyl-4-carboxy-5-aminoimidazole(c) ⇌ AIR(c) + CO2(c)
r1322	2	NADH(c) ⇌ NAD+(c) + NADH-redox-potential(c)
r0250	-1.33	Ribulose-5P(c) ⇌ Ribose-5P(c)
r1326	4	NADPH(c) ⇌ NADPH-redox-potential(c) + NADP+(c)
r0687	1	ATP(c) + N-Formyl-GAR(c) + Glutamine(c) + H2O(c) ⇌ ADP(c) + Pi(c) + Glutamate(c) + 5-Phosphoribosylformylglycinamidine(c)
r0255	0.67	Fructose-6P(c) + GAP(c) ⇌ D-Xylulose-5P(c) + Erythrose-4P(c)
r0256	0.33	Fructose-1,6PP(c) ⇌ DHAP(c) + GAP(c)
r0257	-1	Glutamine(c) + PRPP(c) + H2O(c) ⇌ 5-Phosphoribosylamine(c) + PPi(c) + Glutamate(c)
r0258	-2	Fumarate(c) + H2O(c) ⇌ Malate(c)
r0273	-1	IMP(c) + H2O(c) ⇌ FAICAR(c)

Continued on next page

ID	flux	reaction
r0700	1	10-Formyl-THF(c) + AICAR(c) ⇌ THF(c) + FAICAR(c)

NADP+ (454)

Definition			Solution												
			exchanges		reactions								transp		
Objective	Constraints	Comment	imports	exports	c	m	r	p	l	n	s	b	s-c	b-c	int
$NADP^+$(c)	−Nicotinamide MCES AAA	de novo synthesis of cytosolic $NADP^+$	1.33 P_i(c) 1 Glycine(s) 1.67 Glucose-6P(c) 2 Asparagine(s) 1 Nicotinamide(s) 17.3 ATP-energy(c) 2 THF-activated methyl group(c)	4.33 H_2O(s) 1 $NADP^+$(c) 1 CO_2(s) 2 Pyruvate(c) 2 NADH-redox-potential(c) 4 NADPH-redox-potential(c) 1 Proton-gradient(c) 2 Na-gradient(c)	41	-	-	-	-	-	-	-	7	-	-

ID	flux	reaction
r0406	-0.67	Fructose-6P(c) + Erythrose-4P(c) ⇌ Sedoheptulose-7P(c) + GAP(c)
r0277	1	GTP(c) + IMP(c) + Aspartate(c) → GDP(c) + Pi(c) + Adenylosuccinate(c)
r0127	2	H2O(c) + Asparagine(c) → Aspartate(c) + NH3(c)
r0293	2	5,10-Methylene-THF(c) + NADP+(c) ⇌ 5,10-Methenyl-THF(c) + NADPH(c)
r0705	1	ATP(c) + 5-Phosphoribosyl-4-carboxy-5-aminoimidazole(c) + Aspartate(c) ⇌ ADP(c) + Pi(c) + SAICAR(c)
r2479	2	Asparagine(s) + Na+(s) + H+(PG)(c) → Asparagine(c) + Na+(c) + H+(PG)(s)
r0030	4	ATP(c) + AMP(c) ⇌ 2 ADP(c)
r0914	-1	Nicotinamide(c) ⇌ Nicotinamide(s)
r2664	2	Na+(c) ⇌ Na+(s) + Na-gradient(c)
r0058	2	Malate(c) + NADP+(c) ⇌ Pyruvate(c) + CO2(c) + NADPH(c)
r0370	0.67	Sedoheptulose-7P(c) + GAP(c) ⇌ Ribose-5P(c) + D-Xylulose-5P(c)
r0371	2	5,10-Methenyl-THF(c) + H2O(c) ⇌ 10-Formyl-THF(c)
r0077	3	ATP(c) + Glutamate(c) + NH3(c) → ADP(c) + Pi(c) + Glutamine(c)
r0079	1	ATP(c) + Glutamine(c) + H2O(c) + Deamido-NAD(c) → AMP(c) + PPi(c) + NAD+(c) + Glutamate(c)
r0659	1	ATP(c) + 5-Phosphoribosylamine(c) + Glycine(c) ⇌ ADP(c) + Pi(c) + GAR(c)
r0099	1	ATP(c) + GDP(c) ⇌ ADP(c) + GTP(c)
r0243	-0.33	DHAP(c) ⇌ GAP(c)
r0677	1	10-Formyl-THF(c) + GAR(c) ⇌ THF(c) + N-Formyl-GAR(c)
r1331	1	H+(PG)(s) ⇌ H+(PG)(c) + Proton-gradient(c)
r0527	1	H2O(c) + Nicotinamide D-ribonucleotide(c) ⇌ Nicotinate ribonucleotide(c) + NH3(c)
r1319	-17.3	ATP(c) + H2O(c) ⇌ ADP(c) + Pi(c) + ATP-energy(c)
r0260	1	Adenylosuccinate(c) → Fumarate(c) + AMP(c)
r0247	2	ATP(c) + Ribose-5P(c) → AMP(c) + PRPP(c)
r0699	1	SAICAR(c) ⇌ Fumarate(c) + AICAR(c)
r0849	-4.33	H2O(s) ⇌ H2O(c)
r1091	1	ATP(c) + NAD+(c) → ADP(c) + NADP+(c)
r0867	1	CO2(c) ⇌ CO2(s)
r0585	1	ATP(c) + Nicotinate ribonucleotide(c) → PPi(c) + Deamido-NAD(c)
r2468	1	Glycine(s) + H+(PG)(s) → Glycine(c) + H+(PG)(c)
r0007	4	PPi(c) + H2O(c) → 2 Pi(c)
r0736	0.33	ATP(c) + Fructose-6P(c) → ADP(c) + Fructose-1,6PP(c)
r0306	-1	Nicotinamide(c) + PRPP(c) ⇌ PPi(c) + Nicotinamide D-ribonucleotide(c)
r0192	1.67	Glucose-6P(c) ⇌ Fructose-6P(c)
r0344	-1.33	D-Xylulose-5P(c) ⇌ Ribulose-5P(c)
r0792	2	5-Methyl-THF(c) + NAD+(c) ⇌ 5,10-Methylene-THF(c) + NADH(c)
r2676	-2	5-Methyl-THF(c) ⇌ THF(c) + THF-activated methyl group(c)
r0666	1	ATP(c) + 5-Phosphoribosylformylglycinamidine(c) ⇌ ADP(c) + Pi(c) + AIR(c)
r0667	-1	5-Phosphoribosyl-4-carboxy-5-aminoimidazole(c) ⇌ AIR(c) + CO2(c)
r1322	2	NADH(c) ⇌ NAD+(c) + NADH-redox-potential(c)
r0250	-1.33	Ribulose-5P(c) ⇌ Ribose-5P(c)
r1326	4	NADPH(c) ⇌ NADPH-redox-potential(c) + NADP+(c)

Continued on next page

ID	flux	reaction
r0687	1	ATP(c) + N-Formyl-GAR(c) + Glutamine(c) + H2O(c) ⇌ ADP(c) + Pi(c) + Glutamate(c) + 5-Phosphoribosylformylglycinamidine(c)
r0255	0.67	Fructose-6P(c) + GAP(c) ⇌ D-Xylulose-5P(c) + Erythrose-4P(c)
r0256	0.33	Fructose-1,6PP(c) ⇌ DHAP(c) + GAP(c)
r0257	-1	Glutamine(c) + PRPP(c) + H2O(c) ⇌ 5-Phosphoribosylamine(c) + PPi(c) + Glutamate(c)
r0258	-2	Fumarate(c) + H2O(c) ⇌ Malate(c)
r0273	-1	IMP(c) + H2O(c) ⇌ FAICAR(c)
r0700	1	10-Formyl-THF(c) + AICAR(c) ⇌ THF(c) + FAICAR(c)

FAD (455)

Definition			Solution												
			exchanges		reactions								transp		
Objective	Constraints	Comment	imports	exports	c	m	r	p	l	n	s	b	s-c	b-c	int
FAD(c)	−Riboflavin MCES AAA	de novo synthesis of cytosolic FAD	1.17 P_i(c) 1 Glycine(s) 2 Aspartate(s) 2 Glutamine(s) 0.83 Glucose-6P(c) 1 Riboflavin(s) 10.2 ATP-energy(c) 2 THF-activated methyl group(c)	3.17 H_2O(s) 1 CO_2(s) 1 FAD(c) 2 Pyruvate(c) 2 Glutamate(s) 2 NADH-redox-potential(c) 4 NADPH-redox-potential(c) 2 Na-gradient(c)	36	-	-	-	-	-	-	-	8	-	-

ID	flux	reaction
r0406	-0.33	Fructose-6P(c) + Erythrose-4P(c) ⇌ Sedoheptulose-7P(c) + GAP(c)
r0277	1	GTP(c) + IMP(c) + Aspartate(c) → GDP(c) + Pi(c) + Adenylosuccinate(c)
r0293	2	5,10-Methylene-THF(c) + NADP+(c) ⇌ 5,10-Methenyl-THF(c) + NADPH(c)
r0705	1	ATP(c) + 5-Phosphoribosyl-4-carboxy-5-aminoimidazole(c) + Aspartate(c) ⇌ ADP(c) + Pi(c) + SAICAR(c)
r0143	1	ATP(c) + Riboflavin(c) ⇌ ADP(c) + FMN(c)
r0030	2	ATP(c) + AMP(c) ⇌ 2 ADP(c)
r1106	1	Riboflavin(s) ⇌ Riboflavin(c)
r1107	1	ATP(c) + FMN(c) → PPi(c) + FAD(c)
r1143	-2	Na+(c) + Aspartate(c) ← Na+(s) + Aspartate(s)
r2664	2	Na+(c) ⇌ Na+(s) + Na-gradient(c)
r0058	2	Malate(c) + NADP+(c) ⇌ Pyruvate(c) + CO2(c) + NADPH(c)
r0370	0.33	Sedoheptulose-7P(c) + GAP(c) ⇌ Ribose-5P(c) + D-Xylulose-5P(c)
r0371	2	5,10-Methenyl-THF(c) + H2O(c) ⇌ 10-Formyl-THF(c)
r2685	2	Glutamate(c) ⇌ Glutamate(s)
r0659	1	ATP(c) + 5-Phosphoribosylamine(c) + Glycine(c) ⇌ ADP(c) + Pi(c) + GAR(c)
r0099	1	ATP(c) + GDP(c) ⇌ ADP(c) + GTP(c)
r0243	-0.17	DHAP(c) ⇌ GAP(c)
r0677	1	10-Formyl-THF(c) + GAR(c) ⇌ THF(c) + N-Formyl-GAR(c)
r1319	-10.2	ATP(c) + H2O(c) ⇌ ADP(c) + Pi(c) + ATP-energy(c)
r0260	1	Adenylosuccinate(c) → Fumarate(c) + AMP(c)
r0247	1	ATP(c) + Ribose-5P(c) → AMP(c) + PRPP(c)
r0699	1	SAICAR(c) ⇌ Fumarate(c) + AICAR(c)
r0849	-3.17	H2O(s) ⇌ H2O(c)
r0867	1	CO2(c) ⇌ CO2(s)
r0007	2	PPi(c) + H2O(c) → 2 Pi(c)
r0736	0.17	ATP(c) + Fructose-6P(c) → ADP(c) + Fructose-1,6PP(c)
r0192	0.83	Glucose-6P(c) ⇌ Fructose-6P(c)
r0344	-0.67	D-Xylulose-5P(c) ⇌ Ribulose-5P(c)
r0792	2	5-Methyl-THF(c) + NAD+(c) ⇌ 5,10-Methylene-THF(c) + NADH(c)
r2523	1	Glycine(s) ⇌ Glycine(c)
r2676	-2	5-Methyl-THF(c) ⇌ THF(c) + THF-activated methyl group(c)
r2525	2	Glutamine(s) ⇌ Glutamine(c)
r0666	1	ATP(c) + 5-Phosphoribosylformylglycinamidine(c) ⇌ ADP(c) + Pi(c) + AIR(c)
r0667	-1	5-Phosphoribosyl-4-carboxy-5-aminoimidazole(c) ⇌ AIR(c) + CO2(c)
r1322	2	NADH(c) ⇌ NAD+(c) + NADH-redox-potential(c)

Continued on next page

ID	flux	reaction
r0250	-0.67	Ribulose-5P(c) ⇌ Ribose-5P(c)
r1326	4	NADPH(c) ⇌ NADPH-redox-potential(c) + NADP+(c)
r0687	1	ATP(c) + N-Formyl-GAR(c) + Glutamine(c) + H2O(c) ⇌ ADP(c) + Pi(c) + Glutamate(c) + 5-Phosphoribosylformylglycinamidine(c)
r0255	0.33	Fructose-6P(c) + GAP(c) ⇌ D-Xylulose-5P(c) + Erythrose-4P(c)
r0256	0.17	Fructose-1,6PP(c) ⇌ DHAP(c) + GAP(c)
r0257	-1	Glutamine(c) + PRPP(c) + H2O(c) ⇌ 5-Phosphoribosylamine(c) + PPi(c) + Glutamate(c)
r0258	-2	Fumarate(c) + H2O(c) ⇌ Malate(c)
r0273	-1	IMP(c) + H2O(c) ⇌ FAICAR(c)
r0700	1	10-Formyl-THF(c) + AICAR(c) ⇌ THF(c) + FAICAR(c)

Glycogenin (456)

Definition			Solution												
			exchanges		reactions								transp		
Objective	Constraints	Comment	imports	exports	c	m	r	p	l	n	s	b	s-c	b-c	int
Glycogenin(c)	MCES AAA	de novo synthesis of cytosolic Glycogenin	15 Glutamate(s) 18 Glycine(s) 22 Alanine(s) 18 Lysine(s) 26 Aspartate(s) 12 Arginine(s) 14 Glutamine(s) 31 Serine(s) 8 Methionine(s) 7 Tryptophan(s) 20 Phenylalanine(s) 13 Tyrosine(s) 6 Cysteine(s) 39 Leucine(s) 9 Histidine(s) 15 Proline(s) 12 Asparagine(s) 28 Valine(s) 25 Threonine(s) 12 Isoleucine(s) 1750 ATP-energy(c) 6 Proton-gradient(c)	1 Glycogenin(c) 179 Na-gradient(c)	3	-	-	-	-	-	-	-	22	-	-

ID	flux	reaction
r2530	39	Leucine(s) ⇌ Leucine(c)
r1144	-15	Glutamate(c) + Na+(c) ← Glutamate(s) + Na+(s)
r1126	-20	Phenylalanine(c) + Na+(c) ← Phenylalanine(s) + Na+(s)
r1072	8	Methionine(s) ⇌ Methionine(c)
r2531	15	Proline(s) ⇌ Proline(c)
r1145	-9	Histidine(c) + Na+(c) ← Histidine(s) + Na+(s)
r2524	22	Alanine(s) ⇌ Alanine(c)
r2534	25	Threonine(s) ⇌ Threonine(c)
r2525	14	Glutamine(s) ⇌ Glutamine(c)
r1319	-1750	ATP(c) + H2O(c) ⇌ ADP(c) + Pi(c) + ATP-energy(c)
r1058	12	Arginine(s) ⇌ Arginine(c)
r1069	-18	Lysine(c) ⇌ Lysine(s)
r0030	350	ATP(c) + AMP(c) ⇌ 2 ADP(c)
r2474	31	Serine(s) + Na+(s) → Serine(c) + Na+(c)
r2664	179	Na+(c) ⇌ Na+(s) + Na-gradient(c)
r2475	6	Cysteine(s) + Na+(s) → Cysteine(c) + Na+(c)
r1121	-28	Valine(c) + Na+(c) ← Valine(s) + Na+(s)
r2468	18	Glycine(s) + H+(PG)(s) → Glycine(c) + H+(PG)(c)
r1402	1	22 Alanine(c) + 6 Cysteine(c) + 15 Glutamate(c) + 20 Phenylalanine(c) + 18 Glycine(c) + 9 Histidine(c) + 12 Isoleucine(c) + 39 Leucine(c) + 8 Methionine(c) + 15 Proline(c) + 14 Glutamine(c) + 12 Arginine(c) + 31 Serine(c) + 25 Threonine(c) + 28 Valine(c) + 7 Tryptophan(c) + 13 Tyrosine(c) + 1400 ATP(c) + 1400 H2O(c) + 26 Aspartate(c) + 18 Lysine(c) + 12 Asparagine(c) → Glycogenin(c) + 1050 ADP(c) + 1050 Pi(c) + 350 AMP(c) + 350 PPi(c)
r1123	-12	Isoleucine(c) + Na+(c) ← Isoleucine(s) + Na+(s)
r0007	350	PPi(c) + H2O(c) → 2 Pi(c)
r1331	-6	H+(PG)(s) ⇌ H+(PG)(c) + Proton-gradient(c)
r1124	-7	Tryptophan(c) + Na+(c) ← Tryptophan(s) + Na+(s)
r2479	12	Asparagine(s) + Na+(s) + H+(PG)(c) → Asparagine(c) + Na+(c) + H+(PG)(s)
r1143	-26	Na+(c) + Aspartate(c) ← Na+(s) + Aspartate(s)

Continued on next page

ID	flux	reaction
r1125	13	Tyrosine(s) + Na+(s) → Tyrosine(c) + Na+(c)

Thioredoxin (457)

Definition			Solution												
			exchanges		reactions								transp		
Objective	Constraints	Comment	imports	exports	c	m	r	p	l	n	s	b	s-c	b-c	int
Thioredoxin(c)	MCES AAA	de novo synthesis of cytosolic Thioredoxin	10 Glutamate(s) 5 Glycine(s) 8 Alanine(s) 12 Lysine(s) 7 Aspartate(s) 5 Glutamine(s) 7 Serine(s) 3 Methionine(s) 1 Tryptophan(s) 9 Phenylalanine(s) 1 Tyrosine(s) 5 Cysteine(s) 6 Leucine(s) 1 Histidine(s) 3 Proline(s) 3 Asparagine(s) 11 Valine(s) 4 Threonine(s) 4 Isoleucine(s) 525 ATP-energy(c) 2 Proton-gradient(c)	1 Thioredoxin(c) 59 Na-gradient(c)	3	-	-	-	-	-	-	-	21	-	-

ID	flux	reaction
r2530	6	Leucine(s) ⇌ Leucine(c)
r1144	-10	Glutamate(c) + Na+(c) ← Glutamate(s) + Na+(s)
r1126	-9	Phenylalanine(c) + Na+(c) ← Phenylalanine(s) + Na+(s)
r1072	3	Methionine(s) ⇌ Methionine(c)
r2531	3	Proline(s) ⇌ Proline(c)
r1145	-1	Histidine(c) + Na+(c) ← Histidine(s) + Na+(s)
r2524	8	Alanine(s) ⇌ Alanine(c)
r2534	4	Threonine(s) ⇌ Threonine(c)
r2525	5	Glutamine(s) ⇌ Glutamine(c)
r1319	-525	ATP(c) + H2O(c) ⇌ ADP(c) + Pi(c) + ATP-energy(c)
r1293	1	8 Alanine(c) + 5 Cysteine(c) + 10 Glutamate(c) + 9 Phenylalanine(c) + 5 Glycine(c) + Histidine(c) + 4 Isoleucine(c) + 6 Leucine(c) + 3 Methionine(c) + 3 Proline(c) + 5 Glutamine(c) + 7 Serine(c) + 4 Threonine(c) + 11 Valine(c) + Tryptophan(c) + Tyrosine(c) + 420 ATP(c) + 420 H2O(c) + 7 Aspartate(c) + 12 Lysine(c) + 3 Asparagine(c) → Thioredoxin(c) + 105 AMP(c) + 105 PPi(c) + 315 ADP(c) + 315 Pi(c)
r1069	-12	Lysine(c) ⇌ Lysine(s)
r0030	105	ATP(c) + AMP(c) ⇌ 2 ADP(c)
r2474	7	Serine(s) + Na+(s) → Serine(c) + Na+(c)
r2664	59	Na+(c) ⇌ Na+(s) + Na-gradient(c)
r2475	5	Cysteine(s) + Na+(s) → Cysteine(c) + Na+(c)
r1121	-11	Valine(c) + Na+(c) ← Valine(s) + Na+(s)
r2468	5	Glycine(s) + H+(PG)(s) → Glycine(c) + H+(PG)(c)
r1123	-4	Isoleucine(c) + Na+(c) ← Isoleucine(s) + Na+(s)
r0007	105	PPi(c) + H2O(c) → 2 Pi(c)
r1331	-2	H+(PG)(s) ⇌ H+(PG)(c) + Proton-gradient(c)
r1124	-1	Tryptophan(c) + Na+(c) ← Tryptophan(s) + Na+(s)
r2479	3	Asparagine(s) + Na+(s) + H+(PG)(c) → Asparagine(c) + Na+(c) + H+(PG)(s)
r1143	-7	Na+(c) + Aspartate(c) ← Na+(s) + Aspartate(s)
r1125	1	Tyrosine(s) + Na+(s) → Tyrosine(c) + Na+(c)

Thioredoxin(m) (458)

Definition			Solution												
			exchanges		reactions								transp		
Objective	Constraints	Comment	imports	exports	c	m	r	p	l	n	s	b	s-c	b-c	int
mitoThioredoxin(m)	MCES AAA	de novo synthesis of mitochondrial mitoThioredoxin	6 Glutamate(s) 10 Glycine(s) 11 Alanine(s) 11 Lysine(s) 12 Aspartate(s) 9 Arginine(s) 10 Glutamine(s) 8 Serine(s) 4 Methionine(s) 2 Tryptophan(s) 6 Phenylalanine(s) 2 Tyrosine(s) 3 Cysteine(s) 15 Leucine(s) 3 Histidine(s) 12 Proline(s) 4 Asparagine(s) 17 Valine(s) 12 Threonine(s) 9 Isoleucine(s) 830 ATP-energy(c) 6 Proton-gradient(c)	1 mitoThioredoxin(m) 72 Na-gradient(c)	3	-	-	-	-	-	-	-	22	-	1

ID	flux	reaction
r2530	15	Leucine(s) ⇌ Leucine(c)
r1441	1	mitoThioredoxin(c) → mitoThioredoxin(m)
r1144	-6	Glutamate(c) + Na+(c) ← Glutamate(s) + Na+(s)
r1126	-6	Phenylalanine(c) + Na+(c) ← Phenylalanine(s) + Na+(s)
r1072	4	Methionine(s) ⇌ Methionine(c)
r2531	12	Proline(s) ⇌ Proline(c)
r1145	-3	Histidine(c) + Na+(c) ← Histidine(s) + Na+(s)
r2524	11	Alanine(s) ⇌ Alanine(c)
r2534	12	Threonine(s) ⇌ Threonine(c)
r2525	10	Glutamine(s) ⇌ Glutamine(c)
r1319	-830	ATP(c) + H2O(c) ⇌ ADP(c) + Pi(c) + ATP-energy(c)
r1058	9	Arginine(s) ⇌ Arginine(c)
r1294	1	11 Alanine(c) + 3 Cysteine(c) + 6 Glutamate(c) + 6 Phenylalanine(c) + 10 Glycine(c) + 3 Histidine(c) + 9 Isoleucine(c) + 15 Leucine(c) + 4 Methionine(c) + 12 Proline(c) + 10 Glutamine(c) + 9 Arginine(c) + 8 Serine(c) + 12 Threonine(c) + 17 Valine(c) + 2 Tryptophan(c) + 2 Tyrosine(c) + 664 ATP(c) + 664 H2O(c) + 12 Aspartate(c) + 11 Lysine(c) + 4 Asparagine(c) → mitoThioredoxin(c) + 166 AMP(c) + 166 PPi(c) + 498 ADP(c) + 498 Pi(c)
r1069	-11	Lysine(c) ⇌ Lysine(s)
r0030	166	ATP(c) + AMP(c) ⇌ 2 ADP(c)
r2474	8	Serine(s) + Na+(s) → Serine(c) + Na+(c)
r2664	72	Na+(c) ⇌ Na+(s) + Na-gradient(c)
r2475	3	Cysteine(s) + Na+(s) → Cysteine(c) + Na+(c)
r1121	-17	Valine(c) + Na+(c) ← Valine(s) + Na+(s)
r2468	10	Glycine(s) + H+(PG)(s) → Glycine(c) + H+(PG)(c)
r1123	-9	Isoleucine(c) + Na+(c) ← Isoleucine(s) + Na+(s)
r0007	166	PPi(c) + H2O(c) → 2 Pi(c)
r1331	-6	H+(PG)(s) ⇌ H+(PG)(c) + Proton-gradient(c)
r1124	-2	Tryptophan(c) + Na+(c) ← Tryptophan(s) + Na+(s)
r2479	4	Asparagine(s) + Na+(s) + H+(PG)(c) → Asparagine(c) + Na+(c) + H+(PG)(s)
r1143	-12	Na+(c) + Aspartate(c) ← Na+(s) + Aspartate(s)
r1125	2	Tyrosine(s) + Na+(s) → Tyrosine(c) + Na+(c)

Apo-ACP (459)

Definition			Solution												
			exchanges		reactions								transp		
Objective	Constraints	Comment	imports	exports	c	m	r	p	l	n	s	b	s-c	b-c	int
Apo-ACP(c)	MCES AAA	de novo synthesis of cytosolic Apo-ACP	5 Glutamate(s) 6 Glycine(s) 11 Alanine(s) 4 Lysine(s) 8 Aspartate(s) 9 Arginine(s) 7 Glutamine(s) 8 Serine(s) 5 Methionine(s) 3 Phenylalanine(s) 4 Tyrosine(s) 2 Cysteine(s) 20 Leucine(s) 1 Histidine(s) 9 Proline(s) 1 Asparagine(s) 11 Valine(s) 6 Threonine(s) 4 Isoleucine(s) 620 ATP-energy(c) 5 Proton-gradient(c)	1 Apo-ACP(c) 47 Na-gradient(c)	3	-	-	-	-	-	-	-	21	-	-

ID	flux	reaction
r2530	20	Leucine(s) ⇌ Leucine(c)
r1144	-5	Glutamate(c) + Na+(c) ← Glutamate(s) + Na+(s)
r1126	-3	Phenylalanine(c) + Na+(c) ← Phenylalanine(s) + Na+(s)
r1072	5	Methionine(s) ⇌ Methionine(c)
r2531	9	Proline(s) ⇌ Proline(c)
r1145	-1	Histidine(c) + Na+(c) ← Histidine(s) + Na+(s)
r2524	11	Alanine(s) ⇌ Alanine(c)
r2534	6	Threonine(s) ⇌ Threonine(c)
r2525	7	Glutamine(s) ⇌ Glutamine(c)
r1319	-620	ATP(c) + H2O(c) ⇌ ADP(c) + Pi(c) + ATP-energy(c)
r1058	9	Arginine(s) ⇌ Arginine(c)
r1096	1	11 Alanine(c) + 2 Cysteine(c) + 5 Glutamate(c) + 3 Phenylalanine(c) + 6 Glycine(c) + Histidine(c) + 4 Isoleucine(c) + 20 Leucine(c) + 5 Methionine(c) + 9 Proline(c) + 7 Glutamine(c) + 9 Arginine(c) + 8 Serine(c) + 6 Threonine(c) + 11 Valine(c) + 4 Tyrosine(c) + 496 ATP(c) + 496 H2O(c) + 8 Aspartate(c) + 4 Lysine(c) + Asparagine(c) → Apo-ACP(c) + 124 AMP(c) + 124 PPi(c) + 372 ADP(c) + 372 Pi(c)
r1069	-4	Lysine(c) ⇌ Lysine(s)
r0030	124	ATP(c) + AMP(c) ⇌ 2 ADP(c)
r2474	8	Serine(s) + Na+(s) → Serine(c) + Na+(c)
r2664	47	Na+(c) ⇌ Na+(s) + Na-gradient(c)
r2475	2	Cysteine(s) + Na+(s) → Cysteine(c) + Na+(c)
r1121	-11	Valine(c) + Na+(c) ← Valine(s) + Na+(s)
r2468	6	Glycine(s) + H+(PG)(s) → Glycine(c) + H+(PG)(c)
r1123	-4	Isoleucine(c) + Na+(c) ← Isoleucine(s) + Na+(s)
r0007	124	PPi(c) + H2O(c) → 2 Pi(c)
r1331	-5	H+(PG)(s) ⇌ H+(PG)(c) + Proton-gradient(c)
r2479	1	Asparagine(s) + Na+(s) + H+(PG)(c) → Asparagine(c) + Na+(c) + H+(PG)(s)
r1143	-8	Na+(c) + Aspartate(c) ← Na+(s) + Aspartate(s)
r1125	4	Tyrosine(s) + Na+(s) → Tyrosine(c) + Na+(c)

Apo-ACP(m) (460)

Definition			Solution												
			exchanges		reactions								transp		
Objective	Constraints	Comment	imports	exports	c	m	r	p	l	n	s	b	s-c	b-c	int
mitoApo-ACP(m)	MCES AAA	de novo synthesis of mitochondrial mitoApo-ACP	22 Pyruvate(m) 8.5 Alanine(s) 7 Lysine(s) 1 Arginine(s) 12 Glutamine(s) 4.5 Serine(s) 5 Methionine(s) 3 Phenylalanine(s) 4 Tyrosine(s) 1 Cysteine(s) 10 Leucine(s) 1 Histidine(s) 5 Proline(s) 1 Asparagine(s) 6 Valine(s) 1 Threonine(s) 8 Isoleucine(s) 435 ATP-energy(m) 13 NADPH-redox-potential(m) 34 Proton-gradient(m)	18.5 NADH-redox-potential(m) 8 Proton-gradient(c) 1 mitoApo-ACP(m) 46.5 Na-gradient(c) 14.5 CoA-activated acetyl group(m)	-	15	-	-	-	-	-	-	18	-	17

ID	flux	reaction
r1069	-7	Lysine(s) ⇌ Lysine(c)
r0295	-1.5	Glycine(m) + THF(m) + NAD+(m) ← 5,10-Methylene-THF(m) + CO2(m) + NADH(m) + NH3(m)
r0890	1	Arginine(c) + H+(PG)(c) → Arginine(m) + H+(PG)(m)
r2475	1	Cysteine(s) + Na+(s) → Cysteine(c) + Na+(c)
r2477	12	Glutamine(s) + Na+(s) + H+(PG)(c) → Glutamine(c) + Na+(c) + H+(PG)(s)
r2478	4.5	Serine(s) + Na+(s) + H+(PG)(c) → Serine(c) + Na+(c) + H+(PG)(s)
r0031	87	ATP(m) + AMP(m) ⇌ 2 ADP(m)
r1122	-10	Leucine(c) + Na+(c) ← Leucine(s) + Na+(s)
r1125	4	Tyrosine(s) + Na+(s) → Tyrosine(c) + Na+(c)
r1126	-3	Phenylalanine(c) + Na+(c) ← Phenylalanine(s) + Na+(s)
r1141	-5	Methionine(c) + Na+(c) ← Methionine(s) + Na+(s)
r1142	-1	Threonine(s) + Na+(s) → Threonine(c) + Na+(c)
r0056	14.5	Pyruvate(m) + CoA(m) + NAD+(m) → Acetyl-CoA(m) + CO2(m) + NADH(m)
r1424	5	Proline(c) + H+(PG)(c) → Proline(m) + H+(PG)(m)
r2664	46.5	Na+(c) ⇌ Na+(s) + Na-gradient(c)
r1440	1	Threonine(c) → Threonine(m)
r0059	-13	Pyruvate(m) + CO2(m) + NADPH(m) ⇌ Malate(m) + NADP+(m)
r1427	1	Histidine(c) + H+(PG)(c) → Histidine(m) + H+(PG)(m)
r1145	-1	Histidine(c) + Na+(c) ← Histidine(s) + Na+(s)
r0073	-7.5	AKG(m) + NADH(m) + NH3(m) ⇌ Glutamate(m) + NAD+(m) + H2O(m)
r2532	1	Asparagine(s) ⇌ Asparagine(c)
r0078	9	Glutamine(m) + H2O(m) → Glutamate(m) + NH3(m)
r2533	6	Valine(s) ⇌ Valine(c)
r0953	8	Isoleucine(c) + H+(PG)(c) → Isoleucine(m) + H+(PG)(m)
r2536	8	Isoleucine(s) ⇌ Isoleucine(c)
r1330	-34	H+(PG)(c) ⇌ H+(PG)(m) + Proton-gradient(m)
r1331	8	H+(PG)(s) ⇌ H+(PG)(c) + Proton-gradient(c)
r0110	-13	OAA(m) + Glutamate(m) → AKG(m) + Aspartate(m)
r1070	7	Lysine(c) ⇌ Lysine(m)
r1058	1	Arginine(s) ⇌ Arginine(c)
r1078	4	Tyrosine(c) → Tyrosine(m)
r1097	1	3 Alanine(m) + Cysteine(m) + 9 Glutamate(m) + 3 Phenylalanine(m) + 3 Glycine(m) + Histidine(m) + 8 Isoleucine(m) + 10 Leucine(m) + 5 Methionine(m) + 5 Proline(m) + 3 Glutamine(m) + Arginine(m) + 3 Serine(m) + Threonine(m) + 6 Valine(m) + 4 Tyrosine(m) + 348 ATP(m) + 348 H2O(m) + 13 Aspartate(m) + 7 Lysine(m) + Asparagine(m) → mitoApo-ACP(m) + 261 ADP(m) + 261 Pi(m) + 87 AMP(m) + 87 PPi(m)
r2469	8.5	Alanine(s) + H+(PG)(s) → Alanine(c) + H+(PG)(c)
r0008	87	PPi(m) + H2O(m) → 2 Pi(m)
r0906	10	Leucine(c) + H+(PG)(c) → Leucine(m) + H+(PG)(m)
r0923	6	Valine(c) + H+(PG)(c) → Valine(m) + H+(PG)(m)
r1139	-5	Proline(c) + Na+(c) ← Proline(s) + Na+(s)
r1434	8.5	Alanine(c) → Alanine(m)
r1435	4.5	Serine(c) → Serine(m)

Continued on next page

ID	flux	reaction
r2522	12	Glutamine(c) ⇌ Glutamine(m)
r0081	5.5	Alanine(m) + AKG(m) ⇌ Pyruvate(m) + Glutamate(m)
r1436	5	Methionine(c) → Methionine(m)
r1437	1	Cysteine(c) → Cysteine(m)
r2678	14.5	Acetyl-CoA(m) ⇌ CoA(m) + CoA-activated acetyl group(m)
r1455	3	Phenylalanine(c) + H+(PG)(c) → Phenylalanine(m) + H+(PG)(m)
r0231	-1.5	THF(m) + Serine(m) ⇌ 5,10-Methylene-THF(m) + Glycine(m) + H2O(m)
r1320	-435	ATP(m) + H2O(m) ⇌ ADP(m) + Pi(m) + ATP-energy(m)
r1323	18.5	NADH(m) ⇌ NAD+(m) + NADH-redox-potential(m)
r1327	-13	NADPH(m) ⇌ NADPH-redox-potential(m) + NADP+(m)
r1060	1	Asparagine(c) → Asparagine(m)
r0105	13	NAD+(m) + Malate(m) ⇌ OAA(m) + NADH(m)

THF (461)

Definition			Solution												
			exchanges		reactions								transp		
Objective	Constraints	Comment	imports	exports	c	m	r	p	l	n	s	b	s-c	b-c	int
THF(c)	−Folate MCES	de novo synthesis of cytosolic THF	1 Folate(s) 1 NADH-redox-potential(c) 1 NADPH-redox-potential(c)	1 THF(c)	4	-	-	-	-	-	-	-	1	-	-

ID	flux	reaction
r1326	-1	NADPH(c) ⇌ NADPH-redox-potential(c) + NADP+(c)
r0963	1	Folate(s) → Folate(c)
r1322	-1	NADH(c) ⇌ NAD+(c) + NADH-redox-potential(c)
r0512	-1	Dihydrofolate(c) + NAD+(c) ⇌ Folate(c) + NADH(c)
r0225	-1	THF(c) + NADP+(c) ← Dihydrofolate(c) + NADPH(c)

Pyridoxal-P (462)

Definition			Solution												
			exchanges		reactions								transp		
Objective	Constraints	Comment	imports	exports	c	m	r	p	l	n	s	b	s-c	b-c	int
Pyridoxal-P(c)	−Pyridoxine MCES	de novo synthesis of cytosolic Pyridoxal-P	0.5 O_2(s) 1 P_i(c) 1 Pyridoxine(s) 1 ATP-energy(c)	2 H_2O(s) 1 Pyridoxal-P(c)	4	-	-	-	-	-	-	-	3	-	-

ID	flux	reaction
r1319	-1	ATP(c) + H2O(c) ⇌ ADP(c) + Pi(c) + ATP-energy(c)
r0010	0.5	2 H2O2(c) → O2(c) + 2 H2O(c)
r0943	1	Pyridoxine(s) ⇌ Pyridoxine(c)
r0855	0.5	O2(s) ⇌ O2(c)
r0041	1	ATP(c) + Pyridoxal(c) → ADP(c) + Pyridoxal-P(c)
r0389	1	Pyridoxine(c) + O2(c) ⇌ Pyridoxal(c) + H2O2(c)
r0849	-2	H2O(s) ⇌ H2O(c)

4ppan (463)

Definition			Solution												
			exchanges		reactions								transp		
Objective	Constraints	Comment	imports	exports	c	m	r	p	l	n	s	b	s-c	b-c	int
D-4-Phosphopantothenate(c)	−Pantothenate MCES	de novo synthesis of cytosolic D-4-Phosphopantothenate	1 P_i(c) 1 Pantothenate(s) 1 ATP-energy(c)	1 H_2O(s) 1 D-4-Phosphopantothenate(c) 1 Na-gradient(c)	2	-	-	-	-	-	-	-	3	-	-

ID	flux	reaction
r1319	-1	ATP(c) + H2O(c) ⇌ ADP(c) + Pi(c) + ATP-energy(c)
r2664	1	Na+(c) ⇌ Na+(s) + Na-gradient(c)
r0586	1	ATP(c) + Pantothenate(c) → ADP(c) + D-4-Phosphopantothenate(c)
r0974	1	Pantothenate(s) + Na+(s) ⇌ Pantothenate(c) + Na+(c)
r0849	-1	H2O(s) ⇌ H2O(c)

thbpt (464)

Definition			Solution												
			exchanges		reactions								transp		
Objective	Constraints	Comment	imports	exports	c	m	r	p	l	n	s	b	s-c	b-c	int
Tetrahydrobiopterin(c)	−Glutamine(c) MCES	de novo synthesis of cytosolic Tetrahydrobiopterin	1.83 H_2O(s) 1.25 O_2(s) 2.5 Glutamine(c) 1.33 Glucose-6P(c) 9.67 ATP-energy(c)	1.33 P_i(c) 4 CO_2(s) 2.5 Pyruvate(c) 1 Tetrahydrobiopterin(c) 1.5 ATP-energy(m) 4 NADH-redox-potential(c) 2.5 NADH-redox-potential(m) 1.5 NADPH-redox-potential(c) 0.5 NADPH-redox-potential(m) 14 Proton-gradient(m)	47	9	-	-	-	-	-	-	3	-	12

ID	flux	reaction
r0406	-0.33	Fructose-6P(c) + Erythrose-4P(c) ⇌ Sedoheptulose-7P(c) + GAP(c)
r0125	-2.5	GDP(m) + Pi(m) + Succinyl-CoA(m) ⇌ GTP(m) + Succinate(m) + CoA(m)
r0855	1.25	O2(s) ⇌ O2(c)
r0856	-1.25	O2(m) ⇌ O2(c)
r0705	1	ATP(c) + 5-Phosphoribosyl-4-carboxy-5-aminoimidazole(c) + Aspartate(c) ⇌ ADP(c) + Pi(c) + SAICAR(c)
r0293	1	5,10-Methylene-THF(c) + NADP+(c) ⇌ 5,10-Methenyl-THF(c) + NADPH(c)
r0875	0.5	Glutamate(c) + H+(PG)(c) → Glutamate(m) + H+(PG)(m)
r0017	1.25	O2(m) + 4 H+(PG)(m) + 4 Ferrocytochrome C(m) → 4 Ferricytochrome C(m) + 2 H2O(m) + 4 H+(PG)(c)
r0030	2	ATP(c) + AMP(c) ⇌ 2 ADP(c)
r0337	-1	ADP(c) + 1,3DPG(c) ⇌ ATP(c) + 3PG(c)
r0338	1	3PG(c) + NAD+(c) → 3-Phosphonooxypyruvate(c) + NADH(c)
r1296	1	Triphosphate(c) + H2O(c) ⇌ PPi(c) + Pi(c)
r0058	2.5	Malate(c) + NADP+(c) ⇌ Pyruvate(c) + CO2(c) + NADPH(c)
r0075	0.5	Glutamate(m) + H2O(m) + NADP+(m) ⇌ AKG(m) + NADPH(m) + NH3(m)
r0370	0.33	Sedoheptulose-7P(c) + GAP(c) ⇌ Ribose-5P(c) + D-Xylulose-5P(c)
r0371	1	5,10-Methenyl-THF(c) + H2O(c) ⇌ 10-Formyl-THF(c)
r0801	-1	GTP(c) + GDP(m) ⇌ GTP(m) + GDP(c)
r0659	1	ATP(c) + 5-Phosphoribosylamine(c) + Glycine(c) ⇌ ADP(c) + Pi(c) + GAR(c)
r0507	2.5	Ubiquinol(m) + 2 Ferricytochrome C(m) + 4 H+(PG)(m) → Ubiquinone(m) + 4 H+(PG)(c) + 2 Ferrocytochrome C(m)
r0673	-1	6-Lactoyl-5,6,7,8-tetrahydropterin(c) + NADP+(c) ← NADPH(c) + 6-Pyruvoyltetrahydropterin(c)
r0674	1	6-[(1S,2R)-1,2-Dihydroxy-3-triphosphooxypropyl]-7,8-dihydropterin(c) → Triphosphate(c) + 6-Pyruvoyltetrahydropterin(c)
r0509	2.5	Ubiquinone(m) + Succinate(m) → Ubiquinol(m) + Fumarate(m)
r0228	1	THF(c) + Formate(c) + ATP(c) → ADP(c) + Pi(c) + 10-Formyl-THF(c)
r0820	2	AKG(c) + Malate(m) ⇌ AKG(m) + Malate(c)
r0677	1	10-Formyl-THF(c) + GAR(c) ⇌ THF(c) + N-Formyl-GAR(c)
r0243	-0.67	DHAP(c) ⇌ GAP(c)

Continued on next page

ID	flux	reaction
r1330	14	H+(PG)(c) ⇌ H+(PG)(m) + Proton-gradient(m)
r1319	-9.67	ATP(c) + H2O(c) ⇌ ADP(c) + Pi(c) + ATP-energy(c)
r0247	1	ATP(c) + Ribose-5P(c) → AMP(c) + PRPP(c)
r0699	1	SAICAR(c) ⇌ Fumarate(c) + AICAR(c)
r0117	1	GTP(c) + H2O(c) ⇌ 6-[(1S,2R)-1,2-Dihydroxy-3-triphosphooxypropyl]-7,8-dihydropterin(c) + Formate(c)
r0849	1.83	H2O(s) ⇌ H2O(c)
r0867	4	CO2(c) ⇌ CO2(s)
r0868	-2.5	CO2(c) ⇌ CO2(m)
r0301	0.5	ATP(c) + Xanthosine-5P(c) + NH3(c) → AMP(c) + PPi(c) + GMP(c)
r0736	0.67	ATP(c) + Fructose-6P(c) → ADP(c) + Fructose-1,6PP(c)
r0007	3	PPi(c) + H2O(c) → 2 Pi(c)
r0302	0.5	ATP(c) + Xanthosine-5P(c) + Glutamine(c) + H2O(c) → AMP(c) + PPi(c) + GMP(c) + Glutamate(c)
r0159	1	3-Phosphoserine(c) + H2O(c) → Serine(c) + Pi(c)
r0192	1.33	Glucose-6P(c) ⇌ Fructose-6P(c)
r1114	2.5	AKG(m) + CoA(m) + NAD+(m) ⇌ NADH(m) + CO2(m) + Succinyl-CoA(m)
r0344	-0.67	D-Xylulose-5P(c) ⇌ Ribulose-5P(c)
r0663	-1	3-Phosphonooxypyruvate(c) + Glutamate(c) → 3-Phosphoserine(c) + AKG(c)
r0230	-1	THF(c) + Serine(c) ⇌ 5,10-Methylene-THF(c) + Glycine(c) + H2O(c)
r0666	1	ATP(c) + 5-Phosphoribosylformylglycinamidine(c) ⇌ ADP(c) + Pi(c) + AIR(c)
r0667	-1	5-Phosphoribosyl-4-carboxy-5-aminoimidazole(c) ⇌ AIR(c) + CO2(c)
r1320	1.5	ATP(m) + H2O(m) ⇌ ADP(m) + Pi(m) + ATP-energy(m)
r1322	4	NADH(c) ⇌ NAD+(c) + NADH-redox-potential(c)
r1323	2.5	NADH(m) ⇌ NAD+(m) + NADH-redox-potential(m)
r0250	-0.67	Ribulose-5P(c) ⇌ Ribose-5P(c)
r0100	-1.5	ADP(m) + GTP(m) ⇌ ATP(m) + GDP(m)
r1326	1.5	NADPH(c) ⇌ NADPH-redox-potential(c) + NADP+(c)
r0687	1	ATP(c) + N-Formyl-GAR(c) + Glutamine(c) + H2O(c) ⇌ ADP(c) + Pi(c) + Glutamate(c) + 5-Phosphoribosylformylglycinamidine(c)
r0101	1	ATP(c) + GMP(c) ⇌ ADP(c) + GDP(c)
r0253	1	GAP(c) + Pi(c) + NAD+(c) ⇌ 1,3DPG(c) + NADH(c)
r1327	0.5	NADPH(m) ⇌ NADPH-redox-potential(m) + NADP+(m)
r0831	-0.5	Malate(c) + Pi(m) ⇌ Malate(m) + Pi(c)
r0255	0.33	Fructose-6P(c) + GAP(c) ⇌ D-Xylulose-5P(c) + Erythrose-4P(c)
r1047	0.5	Pi(c) + H+(PG)(c) → Pi(m) + H+(PG)(m)
r0104	1	NAD+(c) + Malate(c) ⇌ OAA(c) + NADH(c)
r0256	0.67	Fructose-1,6PP(c) ⇌ DHAP(c) + GAP(c)
r0257	-1	Glutamine(c) + PRPP(c) + H2O(c) ⇌ 5-Phosphoribosylamine(c) + PPi(c) + Glutamate(c)
r0258	-1	Malate(c) ⇌ Fumarate(c) + H2O(c)
r0402	-1	Tetrahydrobiopterin(c) + NADP+(c) ← NADPH(c) + 6-Lactoyl-5,6,7,8-tetrahydropterin(c)
r0259	-2.5	Malate(m) ⇌ Fumarate(m) + H2O(m)
r0273	-1	IMP(c) + H2O(c) ⇌ FAICAR(c)
r0851	-2	H2O(m) ⇌ H2O(c)
r0838	-0.5	NH3(c) ⇌ NH3(m)
r0274	1	IMP(c) + NAD+(c) + H2O(c) → Xanthosine-5P(c) + NADH(c)
r0109	-1	OAA(c) + Glutamate(c) ⇌ AKG(c) + Aspartate(c)
r0700	1	10-Formyl-THF(c) + AICAR(c) ⇌ THF(c) + FAICAR(c)

dhbpt (465)

Definition			Solution												
			exchanges		reactions								transp		
Objective	Constraints	Comment	imports	exports	c	m	r	p	l	n	s	b	s-c	b-c	int
Dihydrobiopterin(c)	−Glutamine(c) MCES	de novo synthesis of cytosolic Dihydrobiopterin	1.83 H_2O(s) 1.25 O_2(s) 2.5 Glutamine(c) 1.33 Glucose-6P(c) 9.67 ATP-energy(c)	1.33 P_i(c) 4 CO_2(s) 2.5 Pyruvate(c) 1 Dihydrobiopterin(c) 1.5 ATP-energy(m) 4 NADH-redox-potential(c) 4 NADH-redox-potential(m) 1.5 NADPH-redox-potential(c) 13 Proton-gradient(m)	46	10	-	-	-	-	-	-	3	-	13

ID	flux	reaction
r0406	-0.33	Fructose-6P(c) + Erythrose-4P(c) ⇌ Sedoheptulose-7P(c) + GAP(c)
r0125	-2.5	GDP(m) + Pi(m) + Succinyl-CoA(m) ⇌ GTP(m) + Succinate(m) + CoA(m)
r0855	1.25	O2(s) ⇌ O2(c)
r0856	-1.25	O2(m) ⇌ O2(c)
r0705	1	ATP(c) + 5-Phosphoribosyl-4-carboxy-5-aminoimidazole(c) + Aspartate(c) ⇌ ADP(c) + Pi(c) + SAICAR(c)
r0293	1	5,10-Methylene-THF(c) + NADP+(c) ⇌ 5,10-Methenyl-THF(c) + NADPH(c)
r0875	0.5	Glutamate(c) + H+(PG)(c) → Glutamate(m) + H+(PG)(m)
r0876	1	Glutamate(c) + H+(PG)(c) + Aspartate(m) → Glutamate(m) + H+(PG)(m) + Aspartate(c)
r0017	1.25	O2(m) + 4 H+(PG)(m) + 4 Ferrocytochrome C(m) → 4 Ferricytochrome C(m) + 2 H2O(m) + 4 H+(PG)(c)
r0030	2	ATP(c) + AMP(c) ⇌ 2 ADP(c)
r0337	-1	ADP(c) + 1,3DPG(c) ⇌ ATP(c) + 3PG(c)
r0338	1	3PG(c) + NAD+(c) → 3-Phosphonooxypyruvate(c) + NADH(c)
r1296	1	Triphosphate(c) + H2O(c) ⇌ PPi(c) + Pi(c)
r0058	2.5	Malate(c) + NADP+(c) ⇌ Pyruvate(c) + CO2(c) + NADPH(c)
r0073	0.5	Glutamate(m) + NAD+(m) + H2O(m) ⇌ AKG(m) + NADH(m) + NH3(m)
r0370	0.33	Sedoheptulose-7P(c) + GAP(c) ⇌ Ribose-5P(c) + D-Xylulose-5P(c)
r0371	1	5,10-Methenyl-THF(c) + H2O(c) ⇌ 10-Formyl-THF(c)
r0801	-1	GTP(c) + GDP(m) ⇌ GTP(m) + GDP(c)
r0659	1	ATP(c) + 5-Phosphoribosylamine(c) + Glycine(c) ⇌ ADP(c) + Pi(c) + GAR(c)
r0507	2.5	Ubiquinol(m) + 2 Ferricytochrome C(m) + 4 H+(PG)(m) → Ubiquinone(m) + 4 H+(PG)(c) + 2 Ferrocytochrome C(m)
r0673	-1	6-Lactoyl-5,6,7,8-tetrahydropterin(c) + NADP+(c) ← NADPH(c) + 6-Pyruvoyltetrahydropterin(c)
r0674	1	6-[(1S,2R)-1,2-Dihydroxy-3-triphosphooxypropyl]-7,8-dihydropterin(c) → Triphosphate(c) + 6-Pyruvoyltetrahydropterin(c)
r0509	2.5	Ubiquinone(m) + Succinate(m) → Ubiquinol(m) + Fumarate(m)
r0228	1	THF(c) + Formate(c) + ATP(c) → ADP(c) + Pi(c) + 10-Formyl-THF(c)
r0820	1	AKG(c) + Malate(m) ⇌ AKG(m) + Malate(c)
r0677	1	10-Formyl-THF(c) + GAR(c) ⇌ THF(c) + N-Formyl-GAR(c)
r0243	-0.67	DHAP(c) ⇌ GAP(c)
r1330	13	H+(PG)(c) ⇌ H+(PG)(m) + Proton-gradient(m)
r0397	-1	Tetrahydrobiopterin(c) + NAD+(c) ⇌ Dihydrobiopterin(c) + NADH(c)
r1319	-9.67	ATP(c) + H2O(c) ⇌ ADP(c) + Pi(c) + ATP-energy(c)
r0247	1	ATP(c) + Ribose-5P(c) → AMP(c) + PRPP(c)
r0110	-1	OAA(m) + Glutamate(m) → AKG(m) + Aspartate(m)
r0699	1	SAICAR(c) ⇌ Fumarate(c) + AICAR(c)
r0117	1	GTP(c) + H2O(c) ⇌ 6-[(1S,2R)-1,2-Dihydroxy-3-triphosphooxypropyl]-7,8-dihydropterin(c) + Formate(c)
r0849	1.83	H2O(s) ⇌ H2O(c)
r0867	4	CO2(c) ⇌ CO2(s)
r0868	-2.5	CO2(c) ⇌ CO2(m)
r0301	0.5	ATP(c) + Xanthosine-5P(c) + NH3(c) → AMP(c) + PPi(c) + GMP(c)
r0736	0.67	ATP(c) + Fructose-6P(c) → ADP(c) + Fructose-1,6PP(c)
r0007	3	PPi(c) + H2O(c) → 2 Pi(c)
r0302	0.5	ATP(c) + Xanthosine-5P(c) + Glutamine(c) + H2O(c) → AMP(c) + PPi(c) + GMP(c) + Glutamate(c)
r0159	1	3-Phosphoserine(c) + H2O(c) → Serine(c) + Pi(c)
r0192	1.33	Glucose-6P(c) ⇌ Fructose-6P(c)
r1114	2.5	AKG(m) + CoA(m) + NAD+(m) ⇌ NADH(m) + CO2(m) + Succinyl-CoA(m)
r0344	-0.67	D-Xylulose-5P(c) ⇌ Ribulose-5P(c)
r0663	-1	3-Phosphonooxypyruvate(c) + Glutamate(c) → 3-Phosphoserine(c) + AKG(c)
		Continued on next page

ID	flux	reaction
r0230	-1	THF(c) + Serine(c) ⇌ 5,10-Methylene-THF(c) + Glycine(c) + H2O(c)
r0666	1	ATP(c) + 5-Phosphoribosylformylglycinamidine(c) ⇌ ADP(c) + Pi(c) + AIR(c)
r0667	-1	5-Phosphoribosyl-4-carboxy-5-aminoimidazole(c) ⇌ AIR(c) + CO2(c)
r1320	1.5	ATP(m) + H2O(m) ⇌ ADP(m) + Pi(m) + ATP-energy(m)
r1322	4	NADH(c) ⇌ NAD+(c) + NADH-redox-potential(c)
r1323	4	NADH(m) ⇌ NAD+(m) + NADH-redox-potential(m)
r0250	-0.67	Ribulose-5P(c) ⇌ Ribose-5P(c)
r0100	-1.5	ADP(m) + GTP(m) ⇌ ATP(m) + GDP(m)
r1326	1.5	NADPH(c) ⇌ NADPH-redox-potential(c) + NADP+(c)
r0687	1	ATP(c) + N-Formyl-GAR(c) + Glutamine(c) + H2O(c) ⇌ ADP(c) + Pi(c) + Glutamate(c) + 5-Phosphoribosylformylglycinamidine(c)
r0101	1	ATP(c) + GMP(c) ⇌ ADP(c) + GDP(c)
r0253	1	GAP(c) + Pi(c) + NAD+(c) ⇌ 1,3DPG(c) + NADH(c)
r0831	-0.5	Malate(c) + Pi(m) ⇌ Malate(m) + Pi(c)
r0255	0.33	Fructose-6P(c) + GAP(c) ⇌ D-Xylulose-5P(c) + Erythrose-4P(c)
r1047	0.5	Pi(c) + H+(PG)(c) → Pi(m) + H+(PG)(m)
r0256	0.67	Fructose-1,6PP(c) ⇌ DHAP(c) + GAP(c)
r0105	1	NAD+(m) + Malate(m) ⇌ OAA(m) + NADH(m)
r0257	-1	Glutamine(c) + PRPP(c) + H2O(c) ⇌ 5-Phosphoribosylamine(c) + PPi(c) + Glutamate(c)
r0258	-1	Malate(c) ⇌ Fumarate(c) + H2O(c)
r0402	-1	Tetrahydrobiopterin(c) + NADP+(c) ← NADPH(c) + 6-Lactoyl-5,6,7,8-tetrahydropterin(c)
r0259	-2.5	Malate(m) ⇌ Fumarate(m) + H2O(m)
r0273	-1	IMP(c) + H2O(c) ⇌ FAICAR(c)
r0851	-2	H2O(m) ⇌ H2O(c)
r0838	-0.5	NH3(c) ⇌ NH3(m)
r0274	1	IMP(c) + NAD+(c) + H2O(c) → Xanthosine-5P(c) + NADH(c)
r0700	1	10-Formyl-THF(c) + AICAR(c) ⇌ THF(c) + FAICAR(c)

mlthf (466)

Definition			Solution												
			exchanges		reactions								transp		
Objective	Constraints	Comment	imports	exports	c	m	r	p	l	n	s	b	s-c	b-c	int
5,10-Methylene-THF(c)	−Folate MCES	de novo synthesis of cytosolic 5,10-Methylene-THF	1 Folate(s) 1 NADPH-redox-potential(c) 1 THF-activated methyl group(c)	1 5,10-Methylene-THF(c)	5	-	-	-	-	-	-	-	1	-	-

ID	flux	reaction
r1326	-1	NADPH(c) ⇌ NADPH-redox-potential(c) + NADP+(c)
r0963	1	Folate(s) → Folate(c)
r0792	1	5-Methyl-THF(c) + NAD+(c) ⇌ 5,10-Methylene-THF(c) + NADH(c)
r2676	-1	5-Methyl-THF(c) ⇌ THF(c) + THF-activated methyl group(c)
r0512	-1	Dihydrofolate(c) + NAD+(c) ⇌ Folate(c) + NADH(c)
r0225	-1	THF(c) + NADP+(c) ← Dihydrofolate(c) + NADPH(c)

acgam6p (467)

Definition			Solution												
			exchanges		reactions								transp		
Objective	Constraints	Comment	imports	exports	c	m	r	p	l	n	s	b	s-c	b-c	int
N-Acetylglucosamine-6P(c)	MCES AAA	de novo synthesis of cytosolic N-Acetylglucosamine-6P	1 Glutamine(s) 1 Glucose-6P(c) 1 CoA-activated acetyl group(c)	1 Glutamate(s) 1 N-Acetylglucosamine-6P(c)	4	-	-	-	-	-	-	-	2	-	-

ID	flux	reaction
r0190	1	Glutamine(c) + Fructose-6P(c) → Glutamate(c) + Glucosamine-6P(c)
r0192	1	Glucose-6P(c) ⇌ Fructose-6P(c)
r2525	1	Glutamine(s) ⇌ Glutamine(c)
r0485	1	Glucosamine-6P(c) + Acetyl-CoA(c) → CoA(c) + N-Acetylglucosamine-6P(c)
r2685	1	Glutamate(c) ⇌ Glutamate(s)
r2677	-1	Acetyl-CoA(c) ⇌ CoA(c) + CoA-activated acetyl group(c)

THF(m) (468)

Definition			Solution												
			exchanges		reactions								transp		
Objective	Constraints	Comment	imports	exports	c	m	r	p	l	n	s	b	s-c	b-c	int
THF(m)	−Folate MCES	de novo synthesis of mitochondrial THF	1 Folate(s) 2 NADPH-redox-potential(m)	1 THF(m)	-	3	-	-	-	-	-	-	1	-	1

ID	flux	reaction
r0514	-1	Dihydrofolate(m) + NADP+(m) ← Folate(m) + NADPH(m)
r0226	-1	THF(m) + NADP+(m) ← Dihydrofolate(m) + NADPH(m)
r1327	-2	NADPH(m) ⇌ NADPH-redox-potential(m) + NADP+(m)
r0962	1	Folate(c) → Folate(m)
r0963	1	Folate(s) → Folate(c)

1fthf (469)

Definition			Solution												
			exchanges		reactions								transp		
Objective	Constraints	Comment	imports	exports	c	m	r	p	l	n	s	b	s-c	b-c	int
10-Formyl-THF(c)	−Folate MCES	de novo synthesis of cytosolic 10-Formyl-THF	1 H_2O(s) 1 Folate(s) 1 THF-activated methyl group(c)	1 10-Formyl-THF(c)	6	-	-	-	-	-	-	-	2	-	-

ID	flux	reaction
r0371	1	5,10-Methenyl-THF(c) + H2O(c) ⇌ 10-Formyl-THF(c)
r0293	1	5,10-Methylene-THF(c) + NADP+(c) ⇌ 5,10-Methenyl-THF(c) + NADPH(c)
r0963	1	Folate(s) → Folate(c)
r0792	1	5-Methyl-THF(c) + NAD+(c) ⇌ 5,10-Methylene-THF(c) + NADH(c)
r2676	-1	5-Methyl-THF(c) ⇌ THF(c) + THF-activated methyl group(c)
r0849	1	H2O(s) ⇌ H2O(c)
r0512	-1	Dihydrofolate(c) + NAD+(c) ⇌ Folate(c) + NADH(c)
r0225	-1	THF(c) + NADP+(c) ← Dihydrofolate(c) + NADPH(c)

PAPS (470)

Definition			Solution												
			exchanges		reactions								transp		
Objective	Constraints	Comment	imports	exports	c	m	r	p	l	n	s	b	s-c	b-c	int
PAPS(c)	−Glycine(c) MCES	de novo synthesis of cytosolic PAPS	1 P_i(c) 5 Glycine(c) 1 Glucose-6P(c) 12 ATP-energy(c) 1 activated-sulphur(c) 6 THF-activated methyl group(c)	1 H_2O(s) 4 Pyruvate(c) 1 PAPS(c) 8 NADH-redox-potential(c) 2 NADPH-redox-potential(c)	45	-	-	-	-	-	-	-	1	-	-

ID	flux	reaction
r0277	1	GTP(c) + IMP(c) + Aspartate(c) → GDP(c) + Pi(c) + Adenylosuccinate(c)
r0705	1	ATP(c) + 5-Phosphoribosyl-4-carboxy-5-aminoimidazole(c) + Aspartate(c) ⇌ ADP(c) + Pi(c) + SAICAR(c)
r0293	2	5,10-Methylene-THF(c) + NADP+(c) ⇌ 5,10-Methenyl-THF(c) + NADPH(c)
r0160	2	Serine(c) + Pyruvate(c) → Hydroxypyruvate(c) + Alanine(c)
r0164	2	2PG(c) ⇌ PEP(c) + H2O(c)
r0030	2	ATP(c) + AMP(c) ⇌ 2 ADP(c)
r0032	1	ATP(c) + Dephospho-CoA(c) ⇌ ADP(c) + CoA(c)
r0054	-2	ADP(c) + PEP(c) → ATP(c) + Pyruvate(c)
r0339	2	ATP(c) + Glycerate(c) → ADP(c) + 3PG(c)
r0371	2	5,10-Methenyl-THF(c) + H2O(c) ⇌ 10-Formyl-THF(c)
r0077	2	ATP(c) + Glutamate(c) + NH3(c) → ADP(c) + Pi(c) + Glutamine(c)
r0659	1	ATP(c) + 5-Phosphoribosylamine(c) + Glycine(c) ⇌ ADP(c) + Pi(c) + GAR(c)
r0099	1	ATP(c) + GDP(c) ⇌ ADP(c) + GTP(c)
r0677	1	10-Formyl-THF(c) + GAR(c) ⇌ THF(c) + N-Formyl-GAR(c)
r1332	-1	PAPS(c) ⇌ PAP(c) + activated-sulphur(c)
r1319	-12	ATP(c) + H2O(c) ⇌ ADP(c) + Pi(c) + ATP-energy(c)
r0260	1	Adenylosuccinate(c) → Fumarate(c) + AMP(c)
r0247	1	ATP(c) + Ribose-5P(c) → AMP(c) + PRPP(c)
r0699	1	SAICAR(c) ⇌ Fumarate(c) + AICAR(c)
r0849	-1	H2O(s) ⇌ H2O(c)
r0589	1	ATP(c) + Phosphopantetheine(c) → PPi(c) + Dephospho-CoA(c)
r0007	2	PPi(c) + H2O(c) → 2 Pi(c)
r0479	1	Glucono-1,5-lactone-6P(c) + H2O(c) ⇌ 6-Phospho-D-gluconate(c)
r0327	-2	Hydroxypyruvate(c) + NADPH(c) → Glycerate(c) + NADP+(c)
r0341	-2	3PG(c) ⇌ 2PG(c)
r0060	2	Serine(c) → Pyruvate(c) + NH3(c)
r0199	1	Glucose-6P(c) + NADP+(c) ⇌ NADPH(c) + Glucono-1,5-lactone-6P(c)
r0343	1	6-Phospho-D-gluconate(c) + NADP+(c) ⇌ NADPH(c) + CO2(c) + Ribulose-5P(c)
r0792	6	5-Methyl-THF(c) + NAD+(c) ⇌ 5,10-Methylene-THF(c) + NADH(c)
r0080	2	Alanine(c) + AKG(c) ⇌ Pyruvate(c) + Glutamate(c)
r2676	-6	5-Methyl-THF(c) ⇌ THF(c) + THF-activated methyl group(c)
r0366	1	ACP(c) + H2O(c) → Phosphopantetheine(c) + Apo-ACP(c)
r0368	1	CoA(c) + Apo-ACP(c) → PAP(c) + ACP(c)
r0230	4	5,10-Methylene-THF(c) + Glycine(c) + H2O(c) ⇌ THF(c) + Serine(c)
r0666	1	ATP(c) + 5-Phosphoribosylformylglycinamidine(c) ⇌ ADP(c) + Pi(c) + AIR(c)
r0667	-1	5-Phosphoribosyl-4-carboxy-5-aminoimidazole(c) ⇌ AIR(c) + CO2(c)
r1322	8	NADH(c) ⇌ NAD+(c) + NADH-redox-potential(c)
r0250	-1	Ribulose-5P(c) ⇌ Ribose-5P(c)
r1326	2	NADPH(c) ⇌ NADPH-redox-potential(c) + NADP+(c)
r0687	1	ATP(c) + N-Formyl-GAR(c) + Glutamine(c) + H2O(c) ⇌ ADP(c) + Pi(c) + Glutamate(c) + 5-Phosphoribosylformylglycinamidine(c)
r0104	2	NAD+(c) + Malate(c) ⇌ OAA(c) + NADH(c)
r0257	-1	Glutamine(c) + PRPP(c) + H2O(c) ⇌ 5-Phosphoribosylamine(c) + PPi(c) + Glutamate(c)
r0258	-2	Fumarate(c) + H2O(c) ⇌ Malate(c)
r0273	-1	IMP(c) + H2O(c) ⇌ FAICAR(c)
r0109	-2	OAA(c) + Glutamate(c) ⇌ AKG(c) + Aspartate(c)
r0700	1	10-Formyl-THF(c) + AICAR(c) ⇌ THF(c) + FAICAR(c)

ametam (471)

Definition			Solution												
			exchanges		reactions								transp		
Objective	Constraints	Comment	imports	exports	c	m	r	p	l	n	s	b	s-c	b-c	int
S-Adenosylmethioninamine(c)	−Methionine(c) −Glutamine(c) +Glutamate(c) MCES	de novo synthesis of cytosolic S-Adenosylmethioninamine	5 Glutamine(c) 1 Methionine(c) 1.33 Glucose-6P(c) 1 Activated methyl group(c) 8.67 ATP-energy(c) 3 NADPH-redox-potential(m) 4 Proton-gradient(m)	0.67 H_2O(s) 1.33 P_i(c) 5 Glutamate(c) 1 S-Adenosylmethioninamine(c) 2 ATP-energy(m) 4 NADH-redox-potential(c) 3 NADPH-redox-potential(c)	46	5	-	-	-	-	-	-	1	-	8

ID	flux	reaction
r0853	1	ATP(c) + Pi(m) → ATP(m) + Pi(c)
r0406	-0.33	Fructose-6P(c) + Erythrose-4P(c) ⇌ Sedoheptulose-7P(c) + GAP(c)
r0277	1	GTP(c) + IMP(c) + Aspartate(c) → GDP(c) + Pi(c) + Adenylosuccinate(c)
r0705	1	ATP(c) + 5-Phosphoribosyl-4-carboxy-5-aminoimidazole(c) + Aspartate(c) ⇌ ADP(c) + Pi(c) + SAICAR(c)
r0293	2	5,10-Methylene-THF(c) + NADP+(c) ⇌ 5,10-Methenyl-THF(c) + NADPH(c)
r0296	1	5-Methyl-THF(c) + NADP+(c) ⇌ 5,10-Methylene-THF(c) + NADPH(c)
r0030	2	ATP(c) + AMP(c) ⇌ 2 ADP(c)
r0031	-1	ATP(m) + AMP(m) ⇌ 2 ADP(m)
r0189	3	Glucosamine-6P(c) + H2O(c) ⇌ Fructose-6P(c) + NH3(c)
r0911	3	Proline(c) + Glutamate(m) ⇌ Proline(m) + Glutamate(c)
r0053	-1	Adenosine(c) + Homocysteine(c) ⇌ SAH(c) + H2O(c)
r0337	-1	ADP(c) + 1,3DPG(c) ⇌ ATP(c) + 3PG(c)
r0338	1	3PG(c) + NAD+(c) → 3-Phosphonooxypyruvate(c) + NADH(c)
r1426	3	Proline(m) + H+(PG)(c) → Proline(c) + H+(PG)(m)
r0075	-3	AKG(m) + NADPH(m) + NH3(m) ⇌ Glutamate(m) + H2O(m) + NADP+(m)
r0370	0.33	Sedoheptulose-7P(c) + GAP(c) ⇌ Ribose-5P(c) + D-Xylulose-5P(c)
r0371	2	5,10-Methenyl-THF(c) + H2O(c) ⇌ 10-Formyl-THF(c)
r0800	-1	SAM(c) ⇌ SAH(c) + Activated methyl group(c)
r0659	1	ATP(c) + 5-Phosphoribosylamine(c) + Glycine(c) ⇌ ADP(c) + Pi(c) + GAR(c)
r0099	1	ATP(c) + GDP(c) ⇌ ADP(c) + GTP(c)
r0677	1	10-Formyl-THF(c) + GAR(c) ⇌ THF(c) + N-Formyl-GAR(c)
r0243	-0.67	DHAP(c) ⇌ GAP(c)
r1330	-4	H+(PG)(c) ⇌ H+(PG)(m) + Proton-gradient(m)
r1035	-1	Adenosine(m) → Adenosine(c)
r1319	-8.67	ATP(c) + H2O(c) ⇌ ADP(c) + Pi(c) + ATP-energy(c)
r0260	1	Adenylosuccinate(c) → Fumarate(c) + AMP(c)
r0247	1	ATP(c) + Ribose-5P(c) → AMP(c) + PRPP(c)
r0699	1	SAICAR(c) ⇌ Fumarate(c) + AICAR(c)
r0849	-0.67	H2O(s) ⇌ H2O(c)
r0736	0.67	ATP(c) + Fructose-6P(c) → ADP(c) + Fructose-1,6PP(c)
r0007	1	PPi(c) + H2O(c) → 2 Pi(c)
r0159	1	3-Phosphoserine(c) + H2O(c) → Serine(c) + Pi(c)
r0190	3	Glutamine(c) + Fructose-6P(c) → Glutamate(c) + Glucosamine-6P(c)
r0192	1.33	Glucose-6P(c) ⇌ Fructose-6P(c)
r0043	1	SAM(c) ⇌ S-Adenosylmethioninamine(c) + CO2(c)
r0047	1	AMP(m) + H2O(m) → Pi(m) + Adenosine(m)
r0344	-0.67	D-Xylulose-5P(c) ⇌ Ribulose-5P(c)
r0663	-1	3-Phosphonooxypyruvate(c) + Glutamate(c) → 3-Phosphoserine(c) + AKG(c)
r0230	-1	THF(c) + Serine(c) ⇌ 5,10-Methylene-THF(c) + Glycine(c) + H2O(c)
r0232	-1	THF(c) + Methionine(c) ⇌ 5-Methyl-THF(c) + Homocysteine(c)
r0666	1	ATP(c) + 5-Phosphoribosylformylglycinamidine(c) ⇌ ADP(c) + Pi(c) + AIR(c)
r0667	-1	5-Phosphoribosyl-4-carboxy-5-aminoimidazole(c) ⇌ AIR(c) + CO2(c)
r1320	2	ATP(m) + H2O(m) ⇌ ADP(m) + Pi(m) + ATP-energy(m)
r1322	4	NADH(c) ⇌ NAD+(c) + NADH-redox-potential(c)
r0250	-0.67	Ribulose-5P(c) ⇌ Ribose-5P(c)
r1326	3	NADPH(c) ⇌ NADPH-redox-potential(c) + NADP+(c)

Continued on next page

ID	flux	reaction
r0687	1	ATP(c) + N-Formyl-GAR(c) + Glutamine(c) + H2O(c) ⇌ ADP(c) + Pi(c) + Glutamate(c) + 5-Phosphoribosylformylglycinamidine(c)
r0253	1	GAP(c) + Pi(c) + NAD+(c) ⇌ 1,3DPG(c) + NADH(c)
r1327	-3	NADPH(m) ⇌ NADPH-redox-potential(m) + NADP+(m)
r0255	0.33	Fructose-6P(c) + GAP(c) ⇌ D-Xylulose-5P(c) + Erythrose-4P(c)
r1047	1	Pi(c) + H+(PG)(c) → Pi(m) + H+(PG)(m)
r0104	2	NAD+(c) + Malate(c) ⇌ OAA(c) + NADH(c)
r0256	0.67	Fructose-1,6PP(c) ⇌ DHAP(c) + GAP(c)
r0257	-1	Glutamine(c) + PRPP(c) + H2O(c) ⇌ 5-Phosphoribosylamine(c) + PPi(c) + Glutamate(c)
r0258	-2	Fumarate(c) + H2O(c) ⇌ Malate(c)
r2419	3	AKG(c) + Pi(m) ⇌ AKG(m) + Pi(c)
r0273	-1	IMP(c) + H2O(c) ⇌ FAICAR(c)
r0838	3	NH3(c) ⇌ NH3(m)
r0109	-2	OAA(c) + Glutamate(c) ⇌ AKG(c) + Aspartate(c)
r0700	1	10-Formyl-THF(c) + AICAR(c) ⇌ THF(c) + FAICAR(c)

GSH (472)

Definition			Solution												
			exchanges		reactions								transp		
Objective	Constraints	Comment	imports	exports	c	m	r	p	l	n	s	b	s-c	b-c	int
GSH(c)	−Cysteine(c) −Glutamine(c) +Glutamate(c) MCES	de novo synthesis of cytosolic GSH	1 Glutamine(c) 0.5 Glucose-6P(c) 1 Cysteine(c) 1.5 ATP-energy(c) 1 NADH-redox-potential(m) 4 Proton-gradient(m)	1.5 H_2O(s) 0.5 P_i(c) 1 GSH(c) 1 NADH-redox-potential(c) 1 THF-activated methyl group(c)	16	3	-	-	-	-	-	-	1	-	6

ID	flux	reaction
r0253	1	GAP(c) + Pi(c) + NAD+(c) ⇌ 1,3DPG(c) + NADH(c)
r0073	-1	AKG(m) + NADH(m) + NH3(m) ⇌ Glutamate(m) + NAD+(m) + H2O(m)
r0911	2	Proline(c) + Glutamate(m) ⇌ Proline(m) + Glutamate(c)
r1047	1	Pi(c) + H+(PG)(c) → Pi(m) + H+(PG)(m)
r0192	0.5	Glucose-6P(c) ⇌ Fructose-6P(c)
r1426	2	Proline(m) + H+(PG)(c) → Proline(c) + H+(PG)(m)
r0337	-1	ADP(c) + 1,3DPG(c) ⇌ ATP(c) + 3PG(c)
r0256	0.5	Fructose-1,6PP(c) ⇌ DHAP(c) + GAP(c)
r1319	-1.5	ATP(c) + H2O(c) ⇌ ADP(c) + Pi(c) + ATP-energy(c)
r0338	1	3PG(c) + NAD+(c) → 3-Phosphonooxypyruvate(c) + NADH(c)
r0663	-1	3-Phosphonooxypyruvate(c) + Glutamate(c) → 3-Phosphoserine(c) + AKG(c)
r0159	1	3-Phosphoserine(c) + H2O(c) → Serine(c) + Pi(c)
r0078	1	Glutamine(m) + H2O(m) → Glutamate(m) + NH3(m)
r2419	1	AKG(c) + Pi(m) ⇌ AKG(m) + Pi(c)
r0736	0.5	ATP(c) + Fructose-6P(c) → ADP(c) + Fructose-1,6PP(c)
r0891	1	Glutamine(c) + H+(PG)(c) → Glutamine(m) + H+(PG)(m)
r0792	-1	5,10-Methylene-THF(c) + NADH(c) ⇌ 5-Methyl-THF(c) + NAD+(c)
r0230	-1	THF(c) + Serine(c) ⇌ 5,10-Methylene-THF(c) + Glycine(c) + H2O(c)
r0212	1	ATP(c) + Glutamate(c) + Cysteine(c) → ADP(c) + Pi(c) + gamma-Glutamyl-cysteine(c)
r0131	1	ATP(c) + gamma-Glutamyl-cysteine(c) + Glycine(c) → ADP(c) + Pi(c) + GSH(c)
r1330	-4	H+(PG)(c) ⇌ H+(PG)(m) + Proton-gradient(m)
r2676	1	5-Methyl-THF(c) ⇌ THF(c) + THF-activated methyl group(c)
r1322	1	NADH(c) ⇌ NAD+(c) + NADH-redox-potential(c)
r0849	-1.5	H2O(s) ⇌ H2O(c)
r1323	-1	NADH(m) ⇌ NAD+(m) + NADH-redox-potential(m)
r0243	-0.5	DHAP(c) ⇌ GAP(c)

Homocysteine (473)

Definition			Solution												
			exchanges		reactions								transp		
Objective	Constraints	Comment	imports	exports	c	m	r	p	l	n	s	b	s-c	b-c	int
Homocysteine(c)	−Cysteine(c) MCES	de novo synthesis of cytosolic Homocysteine	1 H_2O(s) 1 Pyruvate(m) 1 Cysteine(c) 1 NADPH-redox-potential(m) 1 Proton-gradient(m) 1 CoA-activated acetyl group(m)	1 CO_2(s) 1 Pyruvate(c) 1 Homocysteine(c) 1 NADH-redox-potential(m) 1 NADPH-redox-potential(c)	7	11	-	-	-	-	-	-	2	-	4

ID	flux	reaction
r2678	-1	Acetyl-CoA(m) ⇌ CoA(m) + CoA-activated acetyl group(m)
r0082	1	Oxalosuccinate(c) ⇌ AKG(c) + CO2(c)
r1154	-1	CoA(m) + NAD+(m) + 2-Oxobutyrate(m) ⇌ CO2(m) + Propanoyl-CoA(m) + NADH(m)
r1326	1	NADPH(c) ⇌ NADPH-redox-potential(c) + NADP+(c)
r0624	-1	2-Methyl-3-oxopropanoate(m) + CoA(m) + NAD+(m) ⇌ (R)-Methylmalonyl-CoA(m) + NADH(m)
r1327	-1	NADPH(m) ⇌ NADPH-redox-potential(m) + NADP+(m)
r1454	-1	2-Oxobutyrate(m) + H+(PG)(c) → 2-Oxobutyrate(c) + H+(PG)(m)
r0851	-1	H2O(m) ⇌ H2O(c)
r0059	-1	Pyruvate(m) + CO2(m) + NADPH(m) ⇌ Malate(m) + NADP+(m)
r2373	1	Citrate(m) + AKG(c) ⇌ Citrate(c) + AKG(m)
r0197	-1	Succinyl-CoA(m) ⇌ (R)-Methylmalonyl-CoA(m)
r0105	1	NAD+(m) + Malate(m) ⇌ OAA(m) + NADH(m)
r0060	1	Serine(c) → Pyruvate(c) + NH3(c)
r1330	-1	H+(PG)(c) ⇌ H+(PG)(m) + Proton-gradient(m)
r1114	1	AKG(m) + CoA(m) + NAD+(m) ⇌ NADH(m) + CO2(m) + Succinyl-CoA(m)
r0241	-1	Cysteine(c) + NH3(c) + 2-Oxobutyrate(c) ⇌ H2O(c) + L-Cystathionine(c)
r0223	1	2-Methyl-3-oxopropanoate(m) + CoA(m) + NAD+(m) → Propanoyl-CoA(m) + CO2(m) + NADH(m)
r0867	1	CO2(c) ⇌ CO2(s)
r0849	1	H2O(s) ⇌ H2O(c)
r0422	1	Isocitrate(c) + NADP+(c) ⇌ Oxalosuccinate(c) + NADPH(c)
r0314	-1	H2O(c) + L-Cystathionine(c) ⇌ Serine(c) + Homocysteine(c)
r0107	-1	Acetyl-CoA(m) + H2O(m) + OAA(m) ⇌ Citrate(m) + CoA(m)
r1323	1	NADH(m) ⇌ NAD+(m) + NADH-redox-potential(m)
r0315	1	Citrate(c) ⇌ Isocitrate(c)

4.2 C2. Precursors for macromolecules

C2.1. For DNA

dATP (474)

Definition			Solution												
			exchanges		reactions								transp		
Objective	Constraints	Comment	imports	exports	c	m	r	p	l	n	s	b	s-c	b-c	int
dATP(c)	−Glutamine(c) +Glutamate(c) −Aspartate(c) MCES	de novo synthesis of cytosolic dATP	1.67 P_i(c) 3 Aspartate(c) 2 Glutamine(c) 1.33 Glucose-6P(c) 8.67 ATP-energy(c) 1 THF-activated methyl group(c)	3.67 H_2O(s) 2 CO_2(s) 3 Pyruvate(c) 2 Glutamate(c) 1 dATP(c) 1 NADH-redox-potential(c) 5 NADPH-redox-potential(c)	44	-	-	-	-	-	-	-	2	-	-

ID	flux	reaction
r0406	-0.33	Fructose-6P(c) + Erythrose-4P(c) ⇌ Sedoheptulose-7P(c) + GAP(c)
r0277	1	GTP(c) + IMP(c) + Aspartate(c) → GDP(c) + Pi(c) + Adenylosuccinate(c)
r0705	1	ATP(c) + 5-Phosphoribosyl-4-carboxy-5-aminoimidazole(c) + Aspartate(c) ⇌ ADP(c) + Pi(c) + SAICAR(c)
r0293	2	5,10-Methylene-THF(c) + NADP+(c) ⇌ 5,10-Methenyl-THF(c) + NADPH(c)
r0296	1	5-Methyl-THF(c) + NADP+(c) ⇌ 5,10-Methylene-THF(c) + NADPH(c)
		Continued on next page

ID	flux	reaction
r0030	2	ATP(c) + AMP(c) ⇌ 2 ADP(c)
r0469	-1	Oxidized thioredoxin(c) + NADPH(c) ⇌ Thioredoxin(c) + NADP+(c)
r0337	-1	ADP(c) + 1,3DPG(c) ⇌ ATP(c) + 3PG(c)
r0338	1	3PG(c) + NAD+(c) → 3-Phosphonooxypyruvate(c) + NADH(c)
r0058	3	Malate(c) + NADP+(c) ⇌ Pyruvate(c) + CO2(c) + NADPH(c)
r0370	0.33	Sedoheptulose-7P(c) + GAP(c) ⇌ Ribose-5P(c) + D-Xylulose-5P(c)
r0371	2	5,10-Methenyl-THF(c) + H2O(c) ⇌ 10-Formyl-THF(c)
r0659	1	ATP(c) + 5-Phosphoribosylamine(c) + Glycine(c) ⇌ ADP(c) + Pi(c) + GAR(c)
r0099	1	ATP(c) + GDP(c) ⇌ ADP(c) + GTP(c)
r0243	-0.67	DHAP(c) ⇌ GAP(c)
r0677	1	10-Formyl-THF(c) + GAR(c) ⇌ THF(c) + N-Formyl-GAR(c)
r1319	-8.67	ATP(c) + H2O(c) ⇌ ADP(c) + Pi(c) + ATP-energy(c)
r0260	1	Adenylosuccinate(c) → Fumarate(c) + AMP(c)
r0247	1	ATP(c) + Ribose-5P(c) → AMP(c) + PRPP(c)
r0699	1	SAICAR(c) ⇌ Fumarate(c) + AICAR(c)
r0849	-3.67	H2O(s) ⇌ H2O(c)
r0867	2	CO2(c) ⇌ CO2(s)
r0736	0.67	ATP(c) + Fructose-6P(c) → ADP(c) + Fructose-1,6PP(c)
r0007	1	PPi(c) + H2O(c) → 2 Pi(c)
r0159	1	3-Phosphoserine(c) + H2O(c) → Serine(c) + Pi(c)
r0470	-1	Thioredoxin(c) + ATP(c) ⇌ dATP(c) + Oxidized thioredoxin(c) + H2O(c)
r0192	1.33	Glucose-6P(c) ⇌ Fructose-6P(c)
r0344	-0.67	D-Xylulose-5P(c) ⇌ Ribulose-5P(c)
r2676	-1	5-Methyl-THF(c) ⇌ THF(c) + THF-activated methyl group(c)
r0663	-1	3-Phosphonooxypyruvate(c) + Glutamate(c) → 3-Phosphoserine(c) + AKG(c)
r0230	-1	THF(c) + Serine(c) ⇌ 5,10-Methylene-THF(c) + Glycine(c) + H2O(c)
r0666	1	ATP(c) + 5-Phosphoribosylformylglycinamidine(c) ⇌ ADP(c) + Pi(c) + AIR(c)
r0667	-1	5-Phosphoribosyl-4-carboxy-5-aminoimidazole(c) ⇌ AIR(c) + CO2(c)
r1322	1	NADH(c) ⇌ NAD+(c) + NADH-redox-potential(c)
r0250	-0.67	Ribulose-5P(c) ⇌ Ribose-5P(c)
r1326	5	NADPH(c) ⇌ NADPH-redox-potential(c) + NADP+(c)
r0687	1	ATP(c) + N-Formyl-GAR(c) + Glutamine(c) + H2O(c) ⇌ ADP(c) + Pi(c) + Glutamate(c) + 5-Phosphoribosylformylglycinamidine(c)
r0253	1	GAP(c) + Pi(c) + NAD+(c) ⇌ 1,3DPG(c) + NADH(c)
r0255	0.33	Fructose-6P(c) + GAP(c) ⇌ D-Xylulose-5P(c) + Erythrose-4P(c)
r0104	-1	OAA(c) + NADH(c) ⇌ NAD+(c) + Malate(c)
r0256	0.67	Fructose-1,6PP(c) ⇌ DHAP(c) + GAP(c)
r0257	-1	Glutamine(c) + PRPP(c) + H2O(c) ⇌ 5-Phosphoribosylamine(c) + PPi(c) + Glutamate(c)
r0258	-2	Fumarate(c) + H2O(c) ⇌ Malate(c)
r0273	-1	IMP(c) + H2O(c) ⇌ FAICAR(c)
r0109	1	AKG(c) + Aspartate(c) ⇌ OAA(c) + Glutamate(c)
r0700	1	10-Formyl-THF(c) + AICAR(c) ⇌ THF(c) + FAICAR(c)

dCTP (475)

Definition			Solution												
			exchanges		reactions								transp		
Objective	Constraints	Comment	imports	exports	c	m	r	p	l	n	s	b	s-c	b-c	int
dCTP(c)	−Glutamine(c) +Glutamate(c) −Aspartate(c) $-NH_3$(c) MCES	de novo synthesis of cytosolic dCTP	0.5 O_2(s) 2.17 P_i(c) 1 Aspartate(c) 2 Glutamine(c) 0.83 Glucose-6P(c) 7.17 ATP-energy(c) 1 NADPH-redox-potential(c)	6.17 H_2O(s) 2 Glutamate(c) 1 dCTP(c) 6 Proton-gradient(m)	26	-	-	-	-	-	-	-	2	-	6

ID	flux	reaction
r0406	-0.33	Fructose-6P(c) + Erythrose-4P(c) ⇌ Sedoheptulose-7P(c) + GAP(c)

Continued on next page

dCTP (475)– continued

ID	flux	reaction
r0370	0.33	Sedoheptulose-7P(c) + GAP(c) ⇌ Ribose-5P(c) + D-Xylulose-5P(c)
r0235	1	Orotidine-5P(c) ⇌ CO2(c) + UMP(c)
r0154	1	ATP(c) + UTP(c) + Glutamine(c) + H2O(c) → ADP(c) + Pi(c) + Glutamate(c) + CTP(c)
r0037	1	ATP(c) + UDP(c) ⇌ ADP(c) + UTP(c)
r0416	1	Ubiquinone(m) + (S)-Dihydroorotate(c) → Orotate(c) + Ubiquinol(m)
r0344	-0.67	D-Xylulose-5P(c) ⇌ Ribulose-5P(c)
r0155	1	2 ATP(c) + Glutamine(c) + H2O(c) + HCO3-(c) → 2 ADP(c) + Pi(c) + Glutamate(c) + Carbamoyl-P(c)
r0038	1	ATP(c) + UMP(c) ⇌ ADP(c) + UDP(c)
r1326	-1	NADPH(c) ⇌ NADPH-redox-potential(c) + NADP+(c)
r0507	1	Ubiquinol(m) + 2 Ferricytochrome C(m) + 4 H+(PG)(m) → Ubiquinone(m) + 4 H+(PG)(c) + 2 Ferrocytochrome C(m)
r0417	-1	PPi(c) + Orotidine-5P(c) ⇌ Orotate(c) + PRPP(c)
r0255	0.33	Fructose-6P(c) + GAP(c) ⇌ D-Xylulose-5P(c) + Erythrose-4P(c)
r0192	0.83	Glucose-6P(c) ⇌ Fructose-6P(c)
r1417	-1	CO2(c) + H2O(c) ⇌ Carbonate(c)
r0328	1	Carbamoyl-P(c) + Aspartate(c) → Pi(c) + N-Carbamoyl-L-aspartate(c)
r0256	0.17	Fructose-1,6PP(c) ⇌ DHAP(c) + GAP(c)
r0247	1	ATP(c) + Ribose-5P(c) → AMP(c) + PRPP(c)
r1319	-7.17	ATP(c) + H2O(c) ⇌ ADP(c) + Pi(c) + ATP-energy(c)
r0851	1	H2O(m) ⇌ H2O(c)
r1419	1	Carbonate(c) ⇌ HCO3-(c)
r0474	-1	Thioredoxin(c) + CTP(c) ⇌ dCTP(c) + Oxidized thioredoxin(c) + H2O(c)
r0736	0.17	ATP(c) + Fructose-6P(c) → ADP(c) + Fructose-1,6PP(c)
r0030	1	ATP(c) + AMP(c) ⇌ 2 ADP(c)
r0467	-1	H2O(c) + (S)-Dihydroorotate(c) ← N-Carbamoyl-L-aspartate(c)
r0855	0.5	O2(s) ⇌ O2(c)
r0856	-0.5	O2(m) ⇌ O2(c)
r0469	-1	Oxidized thioredoxin(c) + NADPH(c) ⇌ Thioredoxin(c) + NADP+(c)
r1330	6	H+(PG)(c) ⇌ H+(PG)(m) + Proton-gradient(m)
r0250	-0.67	Ribulose-5P(c) ⇌ Ribose-5P(c)
r0007	1	PPi(c) + H2O(c) → 2 Pi(c)
r0849	-6.17	H2O(s) ⇌ H2O(c)
r0017	0.5	O2(m) + 4 H+(PG)(m) + 4 Ferrocytochrome C(m) → 4 Ferricytochrome C(m) + 2 H2O(m) + 4 H+(PG)(c)
r0243	-0.17	DHAP(c) ⇌ GAP(c)

dGTP (476)

Definition			Solution												
			exchanges		reactions								transp		
Objective	Constraints	Comment	imports	exports	c	m	r	p	l	n	s	b	s-c	b-c	int
dGTP(c)	−Glutamine(c) +Glutamate(c) −Aspartate(c) MCES	de novo synthesis of cytosolic dGTP	1.67 P_i(c) 2 Aspartate(c) 3 Glutamine(c) 1.33 Glucose-6P(c) 9.67 ATP-energy(c) 1 THF-activated methyl group(c)	2.67 H_2O(s) 1 CO_2(s) 2 Pyruvate(c) 3 Glutamate(c) 1 dGTP(c) 2 NADH-redox-potential(c) 4 NADPH-redox-potential(c)	45	-	-	-	-	-	-	-	2	-	-

ID	flux	reaction
r0406	-0.33	Fructose-6P(c) + Erythrose-4P(c) ⇌ Sedoheptulose-7P(c) + GAP(c)
r0705	1	ATP(c) + 5-Phosphoribosyl-4-carboxy-5-aminoimidazole(c) + Aspartate(c) ⇌ ADP(c) + Pi(c) + SAICAR(c)
r0293	2	5,10-Methylene-THF(c) + NADP+(c) ⇌ 5,10-Methenyl-THF(c) + NADPH(c)
r0296	1	5-Methyl-THF(c) + NADP+(c) ⇌ 5,10-Methylene-THF(c) + NADPH(c)
r0030	2	ATP(c) + AMP(c) ⇌ 2 ADP(c)
r0469	-1	Oxidized thioredoxin(c) + NADPH(c) ⇌ Thioredoxin(c) + NADP+(c)
r0337	-1	ADP(c) + 1,3DPG(c) ⇌ ATP(c) + 3PG(c)
r0338	1	3PG(c) + NAD+(c) → 3-Phosphonooxypyruvate(c) + NADH(c)
r0058	2	Malate(c) + NADP+(c) ⇌ Pyruvate(c) + CO2(c) + NADPH(c)

Continued on next page

ID	flux	reaction
r0370	0.33	Sedoheptulose-7P(c) + GAP(c) ⇌ Ribose-5P(c) + D-Xylulose-5P(c)
r0371	2	5,10-Methenyl-THF(c) + H2O(c) ⇌ 10-Formyl-THF(c)
r0659	1	ATP(c) + 5-Phosphoribosylamine(c) + Glycine(c) ⇌ ADP(c) + Pi(c) + GAR(c)
r0243	-0.67	DHAP(c) ⇌ GAP(c)
r0677	1	10-Formyl-THF(c) + GAR(c) ⇌ THF(c) + N-Formyl-GAR(c)
r1319	-9.67	ATP(c) + H2O(c) ⇌ ADP(c) + Pi(c) + ATP-energy(c)
r0247	1	ATP(c) + Ribose-5P(c) → AMP(c) + PRPP(c)
r0699	1	SAICAR(c) ⇌ Fumarate(c) + AICAR(c)
r0411	1	ATP(c) + dGDP(c) ⇌ ADP(c) + dGTP(c)
r0849	-2.67	H2O(s) ⇌ H2O(c)
r0867	1	CO2(c) ⇌ CO2(s)
r0736	0.67	ATP(c) + Fructose-6P(c) → ADP(c) + Fructose-1,6PP(c)
r0007	2	PPi(c) + H2O(c) → 2 Pi(c)
r0302	1	ATP(c) + Xanthosine-5P(c) + Glutamine(c) + H2O(c) → AMP(c) + PPi(c) + GMP(c) + Glutamate(c)
r0159	1	3-Phosphoserine(c) + H2O(c) → Serine(c) + Pi(c)
r0473	-1	Thioredoxin(c) + GDP(c) ⇌ dGDP(c) + Oxidized thioredoxin(c) + H2O(c)
r0192	1.33	Glucose-6P(c) ⇌ Fructose-6P(c)
r0344	-0.67	D-Xylulose-5P(c) ⇌ Ribulose-5P(c)
r2676	-1	5-Methyl-THF(c) ⇌ THF(c) + THF-activated methyl group(c)
r0663	-1	3-Phosphonooxypyruvate(c) + Glutamate(c) → 3-Phosphoserine(c) + AKG(c)
r0230	-1	THF(c) + Serine(c) ⇌ 5,10-Methylene-THF(c) + Glycine(c) + H2O(c)
r0666	1	ATP(c) + 5-Phosphoribosylformylglycinamidine(c) ⇌ ADP(c) + Pi(c) + AIR(c)
r0667	-1	5-Phosphoribosyl-4-carboxy-5-aminoimidazole(c) ⇌ AIR(c) + CO2(c)
r1322	2	NADH(c) ⇌ NAD+(c) + NADH-redox-potential(c)
r0250	-0.67	Ribulose-5P(c) ⇌ Ribose-5P(c)
r1326	4	NADPH(c) ⇌ NADPH-redox-potential(c) + NADP+(c)
r0687	1	ATP(c) + N-Formyl-GAR(c) + Glutamine(c) + H2O(c) ⇌ ADP(c) + Pi(c) + Glutamate(c) + 5-Phosphoribosylformylglycinamidine(c)
r0101	1	ATP(c) + GMP(c) ⇌ ADP(c) + GDP(c)
r0253	1	GAP(c) + Pi(c) + NAD+(c) ⇌ 1,3DPG(c) + NADH(c)
r0255	0.33	Fructose-6P(c) + GAP(c) ⇌ D-Xylulose-5P(c) + Erythrose-4P(c)
r0104	-1	OAA(c) + NADH(c) ⇌ NAD+(c) + Malate(c)
r0256	0.67	Fructose-1,6PP(c) ⇌ DHAP(c) + GAP(c)
r0257	-1	Glutamine(c) + PRPP(c) + H2O(c) ⇌ 5-Phosphoribosylamine(c) + PPi(c) + Glutamate(c)
r0258	-1	Fumarate(c) + H2O(c) ⇌ Malate(c)
r0273	-1	IMP(c) + H2O(c) ⇌ FAICAR(c)
r0274	1	IMP(c) + NAD+(c) + H2O(c) → Xanthosine-5P(c) + NADH(c)
r0109	1	AKG(c) + Aspartate(c) ⇌ OAA(c) + Glutamate(c)
r0700	1	10-Formyl-THF(c) + AICAR(c) ⇌ THF(c) + FAICAR(c)

dTTP (477)

Definition			Solution												
			exchanges		reactions								transp		
Objective	Constraints	Comment	imports	exports	c	m	r	p	l	n	s	b	s-c	b-c	int
dTTP(c)	−Glutamine(c) +Glutamate(c) −Aspartate(c) MCES	de novo synthesis of cytosolic dTTP	0.5 O_2(s) 2.17 P_i(c) 1 Aspartate(c) 1 Glutamine(c) 0.83 Glucose-6P(c) 7.17 ATP-energy(c) 1 ATP-energy(m) 1 NADH-redox-potential(c) 1 THF-activated methyl group(c)	6.17 H_2O(s) 1 Glutamate(c) 1 dTTP(c) 6 Proton-gradient(m)	30	4	-	-	-	-	-	-	2	-	8

ID	flux	reaction
r0853	1	ATP(c) + Pi(m) → ATP(m) + Pi(c)
r0406	-0.33	Fructose-6P(c) + Erythrose-4P(c) ⇌ Sedoheptulose-7P(c) + GAP(c)
r0855	0.5	O2(s) ⇌ O2(c)

Continued on next page

ID	flux	reaction
r0856	-0.5	O2(m) ⇌ O2(c)
r0296	1	5-Methyl-THF(c) + NADP+(c) ⇌ 5,10-Methylene-THF(c) + NADPH(c)
r0017	0.5	O2(m) + 4 H+(PG)(m) + 4 Ferrocytochrome C(m) → 4 Ferricytochrome C(m) + 2 H2O(m) + 4 H+(PG)(c)
r0030	1	ATP(c) + AMP(c) ⇌ 2 ADP(c)
r0467	-1	H2O(c) + (S)-Dihydroorotate(c) ← N-Carbamoyl-L-aspartate(c)
r0469	-1	Oxidized thioredoxin(c) + NADPH(c) ⇌ Thioredoxin(c) + NADP+(c)
r0038	1	ATP(c) + UMP(c) ⇌ ADP(c) + UDP(c)
r0500	1	H2O(m) + dUTP(m) → dUMP(m) + PPi(m)
r0370	0.33	Sedoheptulose-7P(c) + GAP(c) ⇌ Ribose-5P(c) + D-Xylulose-5P(c)
r0501	1	dUMP(c) + 5,10-Methylene-THF(c) ⇌ Dihydrofolate(c) + dTMP(c)
r0224	-1	Dihydrofolate(c) + NADH(c) → THF(c) + NAD+(c)
r0507	1	Ubiquinol(m) + 2 Ferricytochrome C(m) + 4 H+(PG)(m) → Ubiquinone(m) + 4 H+(PG)(c) + 2 Ferrocytochrome C(m)
r0243	-0.17	DHAP(c) ⇌ GAP(c)
r1330	6	H+(PG)(c) ⇌ H+(PG)(m) + Proton-gradient(m)
r1319	-7.17	ATP(c) + H2O(c) ⇌ ADP(c) + Pi(c) + ATP-energy(c)
r0247	1	ATP(c) + Ribose-5P(c) → AMP(c) + PRPP(c)
r2423	1	dUDP(c) + ATP(m) → dUDP(m) + ATP(c)
r0849	-6.17	H2O(s) ⇌ H2O(c)
r0416	1	Ubiquinone(m) + (S)-Dihydroorotate(c) → Orotate(c) + Ubiquinol(m)
r0417	-1	PPi(c) + Orotidine-5P(c) ⇌ Orotate(c) + PRPP(c)
r0155	1	2 ATP(c) + Glutamine(c) + H2O(c) + HCO3-(c) → 2 ADP(c) + Pi(c) + Glutamate(c) + Carbamoyl-P(c)
r0007	1	PPi(c) + H2O(c) → 2 Pi(c)
r0736	0.17	ATP(c) + Fructose-6P(c) → ADP(c) + Fructose-1,6PP(c)
r0008	1	PPi(m) + H2O(m) → 2 Pi(m)
r0192	0.83	Glucose-6P(c) ⇌ Fructose-6P(c)
r0477	-1	Thioredoxin(c) + UDP(c) ⇌ dUDP(c) + Oxidized thioredoxin(c) + H2O(c)
r0328	1	Carbamoyl-P(c) + Aspartate(c) → Pi(c) + N-Carbamoyl-L-aspartate(c)
r0495	1	ATP(c) + dTDP(c) → ADP(c) + dTTP(c)
r1417	-1	CO2(c) + H2O(c) ⇌ Carbonate(c)
r0496	1	ATP(c) + dTMP(c) → ADP(c) + dTDP(c)
r0344	-0.67	D-Xylulose-5P(c) ⇌ Ribulose-5P(c)
r1419	1	Carbonate(c) ⇌ HCO3-(c)
r2676	-1	5-Methyl-THF(c) ⇌ THF(c) + THF-activated methyl group(c)
r1320	-1	ATP(m) + H2O(m) ⇌ ADP(m) + Pi(m) + ATP-energy(m)
r0530	1	ATP(m) + dUDP(m) → ADP(m) + dUTP(m)
r0235	1	Orotidine-5P(c) ⇌ CO2(c) + UMP(c)
r1322	-1	NADH(c) ⇌ NAD+(c) + NADH-redox-potential(c)
r0250	-0.67	Ribulose-5P(c) ⇌ Ribose-5P(c)
r0818	-1	dUMP(c) ← dUMP(m)
r0255	0.33	Fructose-6P(c) + GAP(c) ⇌ D-Xylulose-5P(c) + Erythrose-4P(c)
r0256	0.17	Fructose-1,6PP(c) ⇌ DHAP(c) + GAP(c)

C2.2. For RNA

ATP (478)

Definition			Solution													
			exchanges		reactions								transp			
Objective	Constraints	Comment	imports	exports	c	m	r	p	l	n	s	b	s-c	b-c	int
ATP(c)	−Aspartate(c) +Glutamate(c) −Glutamine(c) MCES	de novo synthesis of cytosolic ATP	1.67 P_i(c) 3 Aspartate(c) 2 Glutamine(c) 1.33 Glucose-6P(c) 8.67 ATP-energy(c) 1 THF-activated methyl group(c)	2.67 H_2O(s) 1 ATP(c) 2 CO_2(s) 3 Pyruvate(c) 2 Glutamate(c) 1 NADH-redox-potential(c) 6 NADPH-redox-potential(c)	42	-	-	-	-	-	-	-	2	-	-

ID	flux	reaction
r0406	-0.33	Fructose-6P(c) + Erythrose-4P(c) ⇌ Sedoheptulose-7P(c) + GAP(c)
r0277	1	GTP(c) + IMP(c) + Aspartate(c) → GDP(c) + Pi(c) + Adenylosuccinate(c)
r0293	2	5,10-Methylene-THF(c) + NADP+(c) ⇌ 5,10-Methenyl-THF(c) + NADPH(c)
r0705	1	ATP(c) + 5-Phosphoribosyl-4-carboxy-5-aminoimidazole(c) + Aspartate(c) ⇌ ADP(c) + Pi(c) + SAICAR(c)
r0296	1	5-Methyl-THF(c) + NADP+(c) ⇌ 5,10-Methylene-THF(c) + NADPH(c)
r0030	2	ATP(c) + AMP(c) ⇌ 2 ADP(c)
r0337	-1	ADP(c) + 1,3DPG(c) ⇌ ATP(c) + 3PG(c)
r0338	1	3PG(c) + NAD+(c) → 3-Phosphonooxypyruvate(c) + NADH(c)
r0058	3	Malate(c) + NADP+(c) ⇌ Pyruvate(c) + CO2(c) + NADPH(c)
r0370	0.33	Sedoheptulose-7P(c) + GAP(c) ⇌ Ribose-5P(c) + D-Xylulose-5P(c)
r0371	2	5,10-Methenyl-THF(c) + H2O(c) ⇌ 10-Formyl-THF(c)
r0659	1	ATP(c) + 5-Phosphoribosylamine(c) + Glycine(c) ⇌ ADP(c) + Pi(c) + GAR(c)
r0099	1	ATP(c) + GDP(c) ⇌ ADP(c) + GTP(c)
r0243	-0.67	DHAP(c) ⇌ GAP(c)
r0677	1	10-Formyl-THF(c) + GAR(c) ⇌ THF(c) + N-Formyl-GAR(c)
r1319	-8.67	ATP(c) + H2O(c) ⇌ ADP(c) + Pi(c) + ATP-energy(c)
r0260	1	Adenylosuccinate(c) → Fumarate(c) + AMP(c)
r0247	1	ATP(c) + Ribose-5P(c) → AMP(c) + PRPP(c)
r0699	1	SAICAR(c) ⇌ Fumarate(c) + AICAR(c)
r0849	-2.67	H2O(s) ⇌ H2O(c)
r0867	2	CO2(c) ⇌ CO2(s)
r0007	1	PPi(c) + H2O(c) → 2 Pi(c)
r0736	0.67	ATP(c) + Fructose-6P(c) → ADP(c) + Fructose-1,6PP(c)
r0159	1	3-Phosphoserine(c) + H2O(c) → Serine(c) + Pi(c)
r0192	1.33	Glucose-6P(c) ⇌ Fructose-6P(c)
r0344	-0.67	D-Xylulose-5P(c) ⇌ Ribulose-5P(c)
r2676	-1	5-Methyl-THF(c) ⇌ THF(c) + THF-activated methyl group(c)
r0663	-1	3-Phosphonooxypyruvate(c) + Glutamate(c) → 3-Phosphoserine(c) + AKG(c)
r0230	-1	THF(c) + Serine(c) ⇌ 5,10-Methylene-THF(c) + Glycine(c) + H2O(c)
r0666	1	ATP(c) + 5-Phosphoribosylformylglycinamidine(c) ⇌ ADP(c) + Pi(c) + AIR(c)
r0667	-1	5-Phosphoribosyl-4-carboxy-5-aminoimidazole(c) ⇌ AIR(c) + CO2(c)
r1322	1	NADH(c) ⇌ NAD+(c) + NADH-redox-potential(c)
r0250	-0.67	Ribulose-5P(c) ⇌ Ribose-5P(c)
r1326	6	NADPH(c) ⇌ NADPH-redox-potential(c) + NADP+(c)
r0687	1	ATP(c) + N-Formyl-GAR(c) + Glutamine(c) + H2O(c) ⇌ ADP(c) + Pi(c) + Glutamate(c) + 5-Phosphoribosylformylglycinamidine(c)
r0253	1	GAP(c) + Pi(c) + NAD+(c) ⇌ 1,3DPG(c) + NADH(c)
r0255	0.33	Fructose-6P(c) + GAP(c) ⇌ D-Xylulose-5P(c) + Erythrose-4P(c)
r0104	-1	OAA(c) + NADH(c) ⇌ NAD+(c) + Malate(c)
r0256	0.67	Fructose-1,6PP(c) ⇌ DHAP(c) + GAP(c)
r0257	-1	Glutamine(c) + PRPP(c) + H2O(c) ⇌ 5-Phosphoribosylamine(c) + PPi(c) + Glutamate(c)
r0258	-2	Fumarate(c) + H2O(c) ⇌ Malate(c)
r0273	-1	IMP(c) + H2O(c) ⇌ FAICAR(c)
r0109	1	AKG(c) + Aspartate(c) ⇌ OAA(c) + Glutamate(c)
r0700	1	10-Formyl-THF(c) + AICAR(c) ⇌ THF(c) + FAICAR(c)

CTP (479)

Definition			Solution												
			exchanges		reactions								transp		
Objective	Constraints	Comment	imports	exports	c	m	r	p	l	n	s	b	s-c	b-c	int
CTP(c)	−Glutamine(c) +Glutamate(c) −Aspartate(c) $-NH_3$(c) MCES	de novo synthesis of cytosolic CTP	0.5 O_2(s) 2.17 P_i(c) 1 Aspartate(c) 2 Glutamine(c) 0.83 Glucose-6P(c) 7.17 ATP-energy(c)	5.17 H_2O(s) 2 Glutamate(c) 1 CTP(c) 6 Proton-gradient(m)	23	-	-	-	-	-	-	-	2	-	6

ID	flux	reaction
r0406	-0.33	Fructose-6P(c) + Erythrose-4P(c) ⇌ Sedoheptulose-7P(c) + GAP(c)
r0370	0.33	Sedoheptulose-7P(c) + GAP(c) ⇌ Ribose-5P(c) + D-Xylulose-5P(c)
r0235	1	Orotidine-5P(c) ⇌ CO2(c) + UMP(c)
r0154	1	ATP(c) + UTP(c) + Glutamine(c) + H2O(c) → ADP(c) + Pi(c) + Glutamate(c) + CTP(c)
r0037	1	ATP(c) + UDP(c) ⇌ ADP(c) + UTP(c)
r0416	1	Ubiquinone(m) + (S)-Dihydroorotate(c) → Orotate(c) + Ubiquinol(m)
r0344	-0.67	D-Xylulose-5P(c) ⇌ Ribulose-5P(c)
r0155	1	2 ATP(c) + Glutamine(c) + H2O(c) + HCO3-(c) → 2 ADP(c) + Pi(c) + Glutamate(c) + Carbamoyl-P(c)
r0038	1	ATP(c) + UMP(c) ⇌ ADP(c) + UDP(c)
r0507	1	Ubiquinol(m) + 2 Ferricytochrome C(m) + 4 H+(PG)(m) → Ubiquinone(m) + 4 H+(PG)(c) + 2 Ferrocytochrome C(m)
r0417	-1	PPi(c) + Orotidine-5P(c) ⇌ Orotate(c) + PRPP(c)
r0255	0.33	Fructose-6P(c) + GAP(c) ⇌ D-Xylulose-5P(c) + Erythrose-4P(c)
r0192	0.83	Glucose-6P(c) ⇌ Fructose-6P(c)
r1417	-1	CO2(c) + H2O(c) ⇌ Carbonate(c)
r0328	1	Carbamoyl-P(c) + Aspartate(c) → Pi(c) + N-Carbamoyl-L-aspartate(c)
r0256	0.17	Fructose-1,6PP(c) ⇌ DHAP(c) + GAP(c)
r0247	1	ATP(c) + Ribose-5P(c) → AMP(c) + PRPP(c)
r1319	-7.17	ATP(c) + H2O(c) ⇌ ADP(c) + Pi(c) + ATP-energy(c)
r0851	1	H2O(m) ⇌ H2O(c)
r1419	1	Carbonate(c) ⇌ HCO3-(c)
r0736	0.17	ATP(c) + Fructose-6P(c) → ADP(c) + Fructose-1,6PP(c)
r0030	1	ATP(c) + AMP(c) ⇌ 2 ADP(c)
r0467	-1	H2O(c) + (S)-Dihydroorotate(c) ← N-Carbamoyl-L-aspartate(c)
r0855	0.5	O2(s) ⇌ O2(c)
r0856	-0.5	O2(m) ⇌ O2(c)
r1330	6	H+(PG)(c) ⇌ H+(PG)(m) + Proton-gradient(m)
r0250	-0.67	Ribulose-5P(c) ⇌ Ribose-5P(c)
r0007	1	PPi(c) + H2O(c) → 2 Pi(c)
r0849	-5.17	H2O(s) ⇌ H2O(c)
r0017	0.5	O2(m) + 4 H+(PG)(m) + 4 Ferrocytochrome C(m) → 4 Ferricytochrome C(m) + 2 H2O(m) + 4 H+(PG)(c)
r0243	-0.17	DHAP(c) ⇌ GAP(c)

GTP (480)

Definition			Solution												
			exchanges		reactions								transp		
Objective	Constraints	Comment	imports	exports	c	m	r	p	l	n	s	b	s-c	b-c	int
GTP(c)	−Glutamine(c) +Glutamate(c) −Aspartate(c) MCES	de novo synthesis of cytosolic GTP	1.67 P_i(c) 2 Aspartate(c) 3 Glutamine(c) 1.33 Glucose-6P(c) 9.67 ATP-energy(c) 1 THF-activated methyl group(c)	1.67 H_2O(s) 1 CO_2(s) 2 Pyruvate(c) 3 Glutamate(c) 1 GTP(c) 2 NADH-redox-potential(c) 5 NADPH-redox-potential(c)	43	-	-	-	-	-	-	-	2	-	-

ID	flux	reaction
r0406	-0.33	Fructose-6P(c) + Erythrose-4P(c) ⇌ Sedoheptulose-7P(c) + GAP(c)
r0293	2	5,10-Methylene-THF(c) + NADP+(c) ⇌ 5,10-Methenyl-THF(c) + NADPH(c)
r0705	1	ATP(c) + 5-Phosphoribosyl-4-carboxy-5-aminoimidazole(c) + Aspartate(c) ⇌ ADP(c) + Pi(c) + SAICAR(c)
r0296	1	5-Methyl-THF(c) + NADP+(c) ⇌ 5,10-Methylene-THF(c) + NADPH(c)
r0030	2	ATP(c) + AMP(c) ⇌ 2 ADP(c)
r0337	-1	ADP(c) + 1,3DPG(c) ⇌ ATP(c) + 3PG(c)
r0338	1	3PG(c) + NAD+(c) → 3-Phosphonooxypyruvate(c) + NADH(c)
r0058	2	Malate(c) + NADP+(c) ⇌ Pyruvate(c) + CO2(c) + NADPH(c)
r0370	0.33	Sedoheptulose-7P(c) + GAP(c) ⇌ Ribose-5P(c) + D-Xylulose-5P(c)
r0371	2	5,10-Methenyl-THF(c) + H2O(c) ⇌ 10-Formyl-THF(c)
r0659	1	ATP(c) + 5-Phosphoribosylamine(c) + Glycine(c) ⇌ ADP(c) + Pi(c) + GAR(c)
r0099	1	ATP(c) + GDP(c) ⇌ ADP(c) + GTP(c)

Continued on next page

ID	flux	reaction
r0243	-0.67	DHAP(c) ⇌ GAP(c)
r0677	1	10-Formyl-THF(c) + GAR(c) ⇌ THF(c) + N-Formyl-GAR(c)
r1319	-9.67	ATP(c) + H2O(c) ⇌ ADP(c) + Pi(c) + ATP-energy(c)
r0247	1	ATP(c) + Ribose-5P(c) → AMP(c) + PRPP(c)
r0699	1	SAICAR(c) ⇌ Fumarate(c) + AICAR(c)
r0849	-1.67	H2O(s) ⇌ H2O(c)
r0867	1	CO2(c) ⇌ CO2(s)
r0736	0.67	ATP(c) + Fructose-6P(c) → ADP(c) + Fructose-1,6PP(c)
r0007	2	PPi(c) + H2O(c) → 2 Pi(c)
r0302	1	ATP(c) + Xanthosine-5P(c) + Glutamine(c) + H2O(c) → AMP(c) + PPi(c) + GMP(c) + Glutamate(c)
r0159	1	3-Phosphoserine(c) + H2O(c) → Serine(c) + Pi(c)
r0192	1.33	Glucose-6P(c) ⇌ Fructose-6P(c)
r0344	-0.67	D-Xylulose-5P(c) ⇌ Ribulose-5P(c)
r2676	-1	5-Methyl-THF(c) ⇌ THF(c) + THF-activated methyl group(c)
r0663	-1	3-Phosphonooxypyruvate(c) + Glutamate(c) → 3-Phosphoserine(c) + AKG(c)
r0230	-1	THF(c) + Serine(c) ⇌ 5,10-Methylene-THF(c) + Glycine(c) + H2O(c)
r0666	1	ATP(c) + 5-Phosphoribosylformylglycinamidine(c) ⇌ ADP(c) + Pi(c) + AIR(c)
r0667	-1	5-Phosphoribosyl-4-carboxy-5-aminoimidazole(c) ⇌ AIR(c) + CO2(c)
r1322	2	NADH(c) ⇌ NAD+(c) + NADH-redox-potential(c)
r0250	-0.67	Ribulose-5P(c) ⇌ Ribose-5P(c)
r1326	5	NADPH(c) ⇌ NADPH-redox-potential(c) + NADP+(c)
r0687	1	ATP(c) + N-Formyl-GAR(c) + Glutamine(c) + H2O(c) ⇌ ADP(c) + Pi(c) + Glutamate(c) + 5-Phosphoribosylformylglycinamidine(c)
r0101	1	ATP(c) + GMP(c) ⇌ ADP(c) + GDP(c)
r0253	1	GAP(c) + Pi(c) + NAD+(c) ⇌ 1,3DPG(c) + NADH(c)
r0255	0.33	Fructose-6P(c) + GAP(c) ⇌ D-Xylulose-5P(c) + Erythrose-4P(c)
r0104	-1	OAA(c) + NADH(c) ⇌ NAD+(c) + Malate(c)
r0256	0.67	Fructose-1,6PP(c) ⇌ DHAP(c) + GAP(c)
r0257	-1	Glutamine(c) + PRPP(c) + H2O(c) ⇌ 5-Phosphoribosylamine(c) + PPi(c) + Glutamate(c)
r0258	-1	Fumarate(c) + H2O(c) ⇌ Malate(c)
r0273	-1	IMP(c) + H2O(c) ⇌ FAICAR(c)
r0274	1	IMP(c) + NAD+(c) + H2O(c) → Xanthosine-5P(c) + NADH(c)
r0109	1	AKG(c) + Aspartate(c) ⇌ OAA(c) + Glutamate(c)
r0700	1	10-Formyl-THF(c) + AICAR(c) ⇌ THF(c) + FAICAR(c)

UTP (481)

Definition			Solution												
			exchanges		reactions								transp		
Objective	Constraints	Comment	imports	exports	c	m	r	p	l	n	s	b	s-c	b-c	int
UTP(c)	−Glutamine(c) +Glutamate(c) −Aspartate(c) MCES	de novo synthesis of cytosolic UTP	0.5 O_2(s) 2.17 P_i(c) 1 Aspartate(c) 1 Glutamine(c) 0.83 Glucose-6P(c) 6.17 ATP-energy(c)	5.17 H_2O(s) 1 Glutamate(c) 1 UTP(c) 6 Proton-gradient(m)	22	-	-	-	-	-	-	-	2	-	6

ID	flux	reaction
r0406	-0.33	Fructose-6P(c) + Erythrose-4P(c) ⇌ Sedoheptulose-7P(c) + GAP(c)
r0370	0.33	Sedoheptulose-7P(c) + GAP(c) ⇌ Ribose-5P(c) + D-Xylulose-5P(c)
r0235	1	Orotidine-5P(c) ⇌ CO2(c) + UMP(c)
r0037	1	ATP(c) + UDP(c) ⇌ ADP(c) + UTP(c)
r0416	1	Ubiquinone(m) + (S)-Dihydroorotate(c) → Orotate(c) + Ubiquinol(m)
r0344	-0.67	D-Xylulose-5P(c) ⇌ Ribulose-5P(c)
r0155	1	2 ATP(c) + Glutamine(c) + H2O(c) + HCO3-(c) → 2 ADP(c) + Pi(c) + Glutamate(c) + Carbamoyl-P(c)
r0038	1	ATP(c) + UMP(c) ⇌ ADP(c) + UDP(c)
r0507	1	Ubiquinol(m) + 2 Ferricytochrome C(m) + 4 H+(PG)(m) → Ubiquinone(m) + 4 H+(PG)(c) + 2 Ferrocytochrome C(m)

Continued on next page

ID	flux	reaction
r0417	-1	PPi(c) + Orotidine-5P(c) ⇌ Orotate(c) + PRPP(c)
r0255	0.33	Fructose-6P(c) + GAP(c) ⇌ D-Xylulose-5P(c) + Erythrose-4P(c)
r0192	0.83	Glucose-6P(c) ⇌ Fructose-6P(c)
r1417	-1	CO2(c) + H2O(c) ⇌ Carbonate(c)
r0328	1	Carbamoyl-P(c) + Aspartate(c) → Pi(c) + N-Carbamoyl-L-aspartate(c)
r0256	0.17	Fructose-1,6PP(c) ⇌ DHAP(c) + GAP(c)
r0247	1	ATP(c) + Ribose-5P(c) → AMP(c) + PRPP(c)
r1319	-6.17	ATP(c) + H2O(c) ⇌ ADP(c) + Pi(c) + ATP-energy(c)
r0851	1	H2O(m) ⇌ H2O(c)
r1419	1	Carbonate(c) ⇌ HCO3-(c)
r0736	0.17	ATP(c) + Fructose-6P(c) → ADP(c) + Fructose-1,6PP(c)
r0030	1	ATP(c) + AMP(c) ⇌ 2 ADP(c)
r0467	-1	H2O(c) + (S)-Dihydroorotate(c) ← N-Carbamoyl-L-aspartate(c)
r0855	0.5	O2(s) ⇌ O2(c)
r0856	-0.5	O2(m) ⇌ O2(c)
r1330	6	H+(PG)(c) ⇌ H+(PG)(m) + Proton-gradient(m)
r0250	-0.67	Ribulose-5P(c) ⇌ Ribose-5P(c)
r0007	1	PPi(c) + H2O(c) → 2 Pi(c)
r0849	-5.17	H2O(s) ⇌ H2O(c)
r0017	0.5	O2(m) + 4 H+(PG)(m) + 4 Ferrocytochrome C(m) → 4 Ferricytochrome C(m) + 2 H2O(m) + 4 H+(PG)(c)
r0243	-0.17	DHAP(c) ⇌ GAP(c)

C2.3. For proteins

Alanine (482)

Definition			Solution												
			exchanges		reactions								transp		
Objective	Constraints	Comment	imports	exports	c	m	r	p	l	n	s	b	s-c	b-c	int
Alanine(c)	$-NH_3$(c) MCES	de novo synthesis of cytosolic Alanine	1 NH_3(c) 1 Pyruvate(c) 1 NADH-redox-potential(m) 2 Proton-gradient(m)	1 H_2O(s) 1 Alanine(c)	1	2	-	-	-	-	-	-	1	-	7

ID	flux	reaction
r0073	-1	AKG(m) + NADH(m) + NH3(m) ⇌ Glutamate(m) + NAD+(m) + H2O(m)
r0911	1	Proline(c) + Glutamate(m) ⇌ Proline(m) + Glutamate(c)
r1047	1	Pi(c) + H+(PG)(c) → Pi(m) + H+(PG)(m)
r1426	1	Proline(m) + H+(PG)(c) → Proline(c) + H+(PG)(m)
r0851	1	H2O(m) ⇌ H2O(c)
r2419	1	AKG(c) + Pi(m) ⇌ AKG(m) + Pi(c)
r0838	1	NH3(c) ⇌ NH3(m)
r1330	-2	H+(PG)(c) ⇌ H+(PG)(m) + Proton-gradient(m)
r0849	-1	H2O(s) ⇌ H2O(c)
r0080	-1	Pyruvate(c) + Glutamate(c) ⇌ Alanine(c) + AKG(c)
r1323	-1	NADH(m) ⇌ NAD+(m) + NADH-redox-potential(m)

Arginine (483)

Definition			Solution												
			exchanges		reactions								transp		
Objective	Constraints	Comment	imports	exports	c	m	r	p	l	n	s	b	s-c	b-c	int
Arginine(c)	$-NH_3$(c) MCES	de novo synthesis of cytosolic Arginine	4 NH_3(c) 2 Pyruvate(m) 2 ATP-energy(c) 3 ATP-energy(m) 2 NADPH-redox-potential(m) 3 Proton-gradient(m)	4 H_2O(s) 1 Arginine(c) 1 NADH-redox-potential(c)	9	15	-	-	-	-	-	-	1	-	8

ID	flux	reaction
r0316	1	Citrate(m) ⇌ Isocitrate(m)
r0109	-1	OAA(c) + Glutamate(c) ⇌ AKG(c) + Aspartate(c)
r0073	-3	AKG(m) + NADH(m) + NH3(m) ⇌ Glutamate(m) + NAD+(m) + H2O(m)
r0911	1	Proline(c) + Glutamate(m) ⇌ Proline(m) + Glutamate(c)
r0425	1	Isocitrate(m) + NAD+(m) ⇌ Oxalosuccinate(m) + NADH(m)
r0083	1	Oxalosuccinate(m) ⇌ AKG(m) + CO2(m)
r0056	1	Pyruvate(m) + CoA(m) + NAD+(m) → Acetyl-CoA(m) + CO2(m) + NADH(m)
r1047	1	Pi(c) + H+(PG)(c) → Pi(m) + H+(PG)(m)
r0453	1	ATP(c) + Citrulline(c) + Aspartate(c) ⇌ AMP(c) + PPi(c) + Argininosuccinate(c)
r1426	1	Proline(m) + H+(PG)(c) → Proline(c) + H+(PG)(m)
r1327	-2	NADPH(m) ⇌ NADPH-redox-potential(m) + NADP+(m)
r2417	-1	Citrulline(m) + H+(PG)(c) → Citrulline(c) + H+(PG)(m)
r1319	-2	ATP(c) + H2O(c) ⇌ ADP(c) + Pi(c) + ATP-energy(c)
r0851	4	H2O(m) ⇌ H2O(c)
r0329	1	Carbamoyl-P(m) + Ornithine(m) ⇌ Pi(m) + Citrulline(m)
r0167	-1	L-Glutamate 5-semialdehyde(m) + Glutamate(m) ⇌ AKG(m) + Ornithine(m)
r0059	-1	Pyruvate(m) + CO2(m) + NADPH(m) ⇌ Malate(m) + NADP+(m)
r0258	-1	Fumarate(c) + H2O(c) ⇌ Malate(c)
r2419	1	AKG(c) + Pi(m) ⇌ AKG(m) + Pi(c)
r0619	-1	L-Glutamate 5-semialdehyde(m) + Pi(m) + NADP+(m) ← NADPH(m) + Glutamyl-5P(m)
r0030	1	ATP(c) + AMP(c) ⇌ 2 ADP(c)
r0104	1	NAD+(c) + Malate(c) ⇌ OAA(c) + NADH(c)
r1320	-3	ATP(m) + H2O(m) ⇌ ADP(m) + Pi(m) + ATP-energy(m)
r0838	4	NH3(c) ⇌ NH3(m)
r0105	1	NAD+(m) + Malate(m) ⇌ OAA(m) + NADH(m)
r1330	-3	H+(PG)(c) ⇌ H+(PG)(m) + Proton-gradient(m)
r0034	1	2 ATP(m) + CO2(m) + H2O(m) + NH3(m) ⇌ 2 ADP(m) + Pi(m) + Carbamoyl-P(m)
r0007	1	PPi(c) + H2O(c) → 2 Pi(c)
r1322	1	NADH(c) ⇌ NAD+(c) + NADH-redox-potential(c)
r0849	-4	H2O(s) ⇌ H2O(c)
r0107	-1	Acetyl-CoA(m) + H2O(m) + OAA(m) ⇌ Citrate(m) + CoA(m)
r0261	1	Argininosuccinate(c) ⇌ Fumarate(c) + Arginine(c)
r0072	1	ATP(m) + Glutamate(m) → ADP(m) + Glutamyl-5P(m)

Asparagine (484)

Definition			Solution												
			exchanges		reactions								transp		
Objective	Constraints	Comment	imports	exports	c	m	r	p	l	n	s	b	s-c	b-c	int
Asparagine(c)	−Histidine(c) MCES	de novo synthesis of cytosolic Asparagine	0.67 H_2O(s) 0.33 O_2(s) 0.33 Pyruvate(m) 0.67 Histidine(c) 3 ATP-energy(c) 0.67 NADPH-redox-potential(c) 0.33 NADPH-redox-potential(m)	0.33 CO_2(s) 1 Asparagine(c) 0.67 ATP-energy(m) 0.33 NADH-redox-potential(c) 0.33 NADH-redox-potential(m) 3.67 Proton-gradient(m) 0.67 THF-activated methyl group(c)	17	10	-	-	-	-	-	-	3	-	10

ID	flux	reaction
r0125	-0.67	GDP(m) + Pi(m) + Succinyl-CoA(m) ⇌ GTP(m) + Succinate(m) + CoA(m)
r0855	0.33	O2(s) ⇌ O2(c)
r0856	-0.33	O2(m) ⇌ O2(c)
r0293	-0.67	5,10-Methenyl-THF(c) + NADPH(c) ⇌ 5,10-Methylene-THF(c) + NADP+(c)
r0577	-0.67	Urocanate(c) + H2O(c) ⇌ 4-Imidazolone-5-propanoate(c)
r0017	0.33	O2(m) + 4 H+(PG)(m) + 4 Ferrocytochrome C(m) → 4 Ferricytochrome C(m) + 2 H2O(m) + 4 H+(PG)(c)
r0030	1	ATP(c) + AMP(c) ⇌ 2 ADP(c)
r0911	0.33	Proline(c) + Glutamate(m) ⇌ Proline(m) + Glutamate(c)
r1426	0.33	Proline(m) + H+(PG)(c) → Proline(c) + H+(PG)(m)

Continued on next page

ID	flux	reaction
r0059	-0.33	Pyruvate(m) + CO2(m) + NADPH(m) ⇌ Malate(m) + NADP+(m)
r0073	-0.33	AKG(m) + NADH(m) + NH3(m) ⇌ Glutamate(m) + NAD+(m) + H2O(m)
r0077	1	ATP(c) + Glutamate(c) + NH3(c) → ADP(c) + Pi(c) + Glutamine(c)
r0507	0.67	Ubiquinol(m) + 2 Ferricytochrome C(m) + 4 H+(PG)(m) → Ubiquinone(m) + 4 H+(PG)(c) + 2 Ferrocytochrome C(m)
r0509	0.67	Ubiquinone(m) + Succinate(m) → Ubiquinol(m) + Fumarate(m)
r0524	0.67	5-Formiminotetrahydrofolate(c) ⇌ 5,10-Methenyl-THF(c) + NH3(c)
r0820	1	AKG(c) + Malate(m) ⇌ AKG(m) + Malate(c)
r1330	3.67	H+(PG)(c) ⇌ H+(PG)(m) + Proton-gradient(m)
r1319	-3	ATP(c) + H2O(c) ⇌ ADP(c) + Pi(c) + ATP-energy(c)
r0849	0.67	H2O(s) ⇌ H2O(c)
r0286	0.67	Histidine(c) ⇌ Urocanate(c) + NH3(c)
r0867	0.33	CO2(c) ⇌ CO2(s)
r0868	-0.33	CO2(c) ⇌ CO2(m)
r0158	1	ATP(c) + Glutamine(c) + H2O(c) + Aspartate(c) ⇌ AMP(c) + PPi(c) + Glutamate(c) + Asparagine(c)
r0007	1	PPi(c) + H2O(c) → 2 Pi(c)
r1114	0.67	AKG(m) + CoA(m) + NAD+(m) ⇌ NADH(m) + CO2(m) + Succinyl-CoA(m)
r0792	-0.67	5,10-Methylene-THF(c) + NADH(c) ⇌ 5-Methyl-THF(c) + NAD+(c)
r2676	0.67	5-Methyl-THF(c) ⇌ THF(c) + THF-activated methyl group(c)
r1320	0.67	ATP(m) + H2O(m) ⇌ ADP(m) + Pi(m) + ATP-energy(m)
r1322	0.33	NADH(c) ⇌ NAD+(c) + NADH-redox-potential(c)
r0518	-0.67	Glutamate(c) + 5-Formiminotetrahydrofolate(c) ⇌ THF(c) + N-Formimino-L-glutamate(c)
r1323	0.33	NADH(m) ⇌ NAD+(m) + NADH-redox-potential(m)
r0519	0.67	H2O(c) + 4-Imidazolone-5-propanoate(c) ⇌ N-Formimino-L-glutamate(c)
r0100	-0.67	ADP(m) + GTP(m) ⇌ ATP(m) + GDP(m)
r1326	-0.67	NADPH(c) ⇌ NADPH-redox-potential(c) + NADP+(c)
r1327	-0.33	NADPH(m) ⇌ NADPH-redox-potential(m) + NADP+(m)
r0104	1	NAD+(c) + Malate(c) ⇌ OAA(c) + NADH(c)
r0259	-0.67	Fumarate(m) + H2O(m) ⇌ Malate(m)
r0838	0.33	NH3(c) ⇌ NH3(m)
r0851	-0.33	H2O(m) ⇌ H2O(c)
r0109	-1	OAA(c) + Glutamate(c) ⇌ AKG(c) + Aspartate(c)

Aspartate (485)

Definition			Solution												
			exchanges		reactions								transp		
Objective	Constraints	Comment	imports	exports	c	m	r	p	l	n	s	b	s-c	b-c	int
Aspartate(c)	−Histidine(c) MCES	de novo synthesis of cytosolic Aspartate	0.33 H_2O(s) 0.17 O_2(s) 1 Pyruvate(m) 0.33 Histidine(c) 0.33 NADPH-redox-potential(c) 0.67 NADPH-redox-potential(m)	1 Aspartate(c) 0.33 ATP-energy(m) 0.67 NADH-redox-potential(c) 1.33 Proton-gradient(m) 0.33 THF-activated methyl group(c) 0.33 CoA-activated acetyl group(m)	12	11	-	-	-	-	-	-	2	-	9

ID	flux	reaction
r2678	0.33	Acetyl-CoA(m) ⇌ CoA(m) + CoA-activated acetyl group(m)
r0820	1	AKG(c) + Malate(m) ⇌ AKG(m) + Malate(c)
r0109	-1	OAA(c) + Glutamate(c) ⇌ AKG(c) + Aspartate(c)
r0073	-0.67	AKG(m) + NADH(m) + NH3(m) ⇌ Glutamate(m) + NAD+(m) + H2O(m)
r0911	0.67	Proline(c) + Glutamate(m) ⇌ Proline(m) + Glutamate(c)
r0524	0.33	5-Formiminotetrahydrofolate(c) ⇌ 5,10-Methenyl-THF(c) + NH3(c)
r0056	0.33	Pyruvate(m) + CoA(m) + NAD+(m) → Acetyl-CoA(m) + CO2(m) + NADH(m)
r1326	-0.33	NADPH(c) ⇌ NADPH-redox-potential(c) + NADP+(c)
r0507	0.33	Ubiquinol(m) + 2 Ferricytochrome C(m) + 4 H+(PG)(m) → Ubiquinone(m) + 4 H+(PG)(c) + 2 Ferrocytochrome C(m)
r1426	0.67	Proline(m) + H+(PG)(c) → Proline(c) + H+(PG)(m)
r1327	-0.67	NADPH(m) ⇌ NADPH-redox-potential(m) + NADP+(m)

Continued on next page

ID	flux	reaction
r0851	0.33	H2O(m) ⇌ H2O(c)
r0518	-0.33	Glutamate(c) + 5-Formiminotetrahydrofolate(c) ⇌ THF(c) + N-Formimino-L-glutamate(c)
r0509	0.33	Ubiquinone(m) + Succinate(m) → Ubiquinol(m) + Fumarate(m)
r0293	-0.33	5,10-Methenyl-THF(c) + NADPH(c) ⇌ 5,10-Methylene-THF(c) + NADP+(c)
r0100	-0.33	ADP(m) + GTP(m) ⇌ ATP(m) + GDP(m)
r0059	-0.67	Pyruvate(m) + CO2(m) + NADPH(m) ⇌ Malate(m) + NADP+(m)
r0519	0.33	H2O(c) + 4-Imidazolone-5-propanoate(c) ⇌ N-Formimino-L-glutamate(c)
r0286	0.33	Histidine(c) ⇌ Urocanate(c) + NH3(c)
r0259	-0.33	Fumarate(m) + H2O(m) ⇌ Malate(m)
r0855	0.17	O2(s) ⇌ O2(c)
r0792	-0.33	5,10-Methylene-THF(c) + NADH(c) ⇌ 5-Methyl-THF(c) + NAD+(c)
r0104	1	NAD+(c) + Malate(c) ⇌ OAA(c) + NADH(c)
r1320	0.33	ATP(m) + H2O(m) ⇌ ADP(m) + Pi(m) + ATP-energy(m)
r0856	-0.17	O2(m) ⇌ O2(c)
r0838	0.67	NH3(c) ⇌ NH3(m)
r0577	-0.33	Urocanate(c) + H2O(c) ⇌ 4-Imidazolone-5-propanoate(c)
r1330	1.33	H+(PG)(c) ⇌ H+(PG)(m) + Proton-gradient(m)
r1114	0.33	AKG(m) + CoA(m) + NAD+(m) ⇌ NADH(m) + CO2(m) + Succinyl-CoA(m)
r2676	0.33	5-Methyl-THF(c) ⇌ THF(c) + THF-activated methyl group(c)
r1322	0.67	NADH(c) ⇌ NAD+(c) + NADH-redox-potential(c)
r0849	0.33	H2O(s) ⇌ H2O(c)
r0125	-0.33	GDP(m) + Pi(m) + Succinyl-CoA(m) ⇌ GTP(m) + Succinate(m) + CoA(m)
r0017	0.17	O2(m) + 4 H+(PG)(m) + 4 Ferrocytochrome C(m) → 4 Ferricytochrome C(m) + 2 H2O(m) + 4 H+(PG)(c)

Glutamate (486)

Definition			Solution												
			exchanges		reactions								transp		
Objective	Constraints	Comment	imports	exports	c	m	r	p	l	n	s	b	s-c	b-c	int
Glutamate(c)	$-NH_3$(c) MCES	de novo synthesis of cytosolic Glutamate	1 NH_3(c) 1 Pyruvate(m) 1 NADPH-redox-potential(m) 1 Proton-gradient(m) 1 CoA-activated acetyl group(m)	1 Glutamate(c) 1 NADH-redox-potential(m)	-	10	-	-	-	-	-	-	-	-	4

ID	flux	reaction
r2678	-1	Acetyl-CoA(m) ⇌ CoA(m) + CoA-activated acetyl group(m)
r0316	1	Citrate(m) ⇌ Isocitrate(m)
r0073	-1	AKG(m) + NADH(m) + NH3(m) ⇌ Glutamate(m) + NAD+(m) + H2O(m)
r0911	1	Proline(c) + Glutamate(m) ⇌ Proline(m) + Glutamate(c)
r0425	1	Isocitrate(m) + NAD+(m) ⇌ Oxalosuccinate(m) + NADH(m)
r0083	1	Oxalosuccinate(m) ⇌ AKG(m) + CO2(m)
r1426	1	Proline(m) + H+(PG)(c) → Proline(c) + H+(PG)(m)
r1327	-1	NADPH(m) ⇌ NADPH-redox-potential(m) + NADP+(m)
r0059	-1	Pyruvate(m) + CO2(m) + NADPH(m) ⇌ Malate(m) + NADP+(m)
r0838	1	NH3(c) ⇌ NH3(m)
r0105	1	NAD+(m) + Malate(m) ⇌ OAA(m) + NADH(m)
r1330	-1	H+(PG)(c) ⇌ H+(PG)(m) + Proton-gradient(m)
r0107	-1	Acetyl-CoA(m) + H2O(m) + OAA(m) ⇌ Citrate(m) + CoA(m)
r1323	1	NADH(m) ⇌ NAD+(m) + NADH-redox-potential(m)

Glycine (487)

Definition			Solution												
			exchanges		reactions								transp		
Objective	Constraints	Comment	imports	exports	c	m	r	p	l	n	s	b	s-c	b-c	int
Glycine(c)	$-NH_3$(c) MCES	de novo synthesis of cytosolic Glycine	1 NH_3(c) 0.5 Glucose-6P(c) 1 NADH-redox-potential(m) 2 Proton-gradient(m)	0.5 H_2O(s) 0.5 P_i(c) 1 Glycine(c) 0.5 ATP-energy(c) 1 NADH-redox-potential(c) 1 THF-activated methyl group(c)	14	2	-	-	-	-	-	-	1	-	7

ID	flux	reaction
r0253	1	GAP(c) + Pi(c) + NAD+(c) ⇌ 1,3DPG(c) + NADH(c)
r0073	-1	AKG(m) + NADH(m) + NH3(m) ⇌ Glutamate(m) + NAD+(m) + H2O(m)
r0911	1	Proline(c) + Glutamate(m) ⇌ Proline(m) + Glutamate(c)
r1047	1	Pi(c) + H+(PG)(c) → Pi(m) + H+(PG)(m)
r0192	0.5	Glucose-6P(c) ⇌ Fructose-6P(c)
r1426	1	Proline(m) + H+(PG)(c) → Proline(c) + H+(PG)(m)
r0337	-1	ADP(c) + 1,3DPG(c) ⇌ ATP(c) + 3PG(c)
r0256	0.5	Fructose-1,6PP(c) ⇌ DHAP(c) + GAP(c)
r1319	0.5	ATP(c) + H2O(c) ⇌ ADP(c) + Pi(c) + ATP-energy(c)
r0851	1	H2O(m) ⇌ H2O(c)
r0338	1	3PG(c) + NAD+(c) → 3-Phosphonooxypyruvate(c) + NADH(c)
r0663	-1	3-Phosphonooxypyruvate(c) + Glutamate(c) → 3-Phosphoserine(c) + AKG(c)
r0159	1	3-Phosphoserine(c) + H2O(c) → Serine(c) + Pi(c)
r2419	1	AKG(c) + Pi(m) ⇌ AKG(m) + Pi(c)
r0736	0.5	ATP(c) + Fructose-6P(c) → ADP(c) + Fructose-1,6PP(c)
r0792	-1	5,10-Methylene-THF(c) + NADH(c) ⇌ 5-Methyl-THF(c) + NAD+(c)
r0230	-1	THF(c) + Serine(c) ⇌ 5,10-Methylene-THF(c) + Glycine(c) + H2O(c)
r0838	1	NH3(c) ⇌ NH3(m)
r1330	-2	H+(PG)(c) ⇌ H+(PG)(m) + Proton-gradient(m)
r2676	1	5-Methyl-THF(c) ⇌ THF(c) + THF-activated methyl group(c)
r1322	1	NADH(c) ⇌ NAD+(c) + NADH-redox-potential(c)
r0849	-0.5	H2O(s) ⇌ H2O(c)
r1323	-1	NADH(m) ⇌ NAD+(m) + NADH-redox-potential(m)
r0243	-0.5	DHAP(c) ⇌ GAP(c)

Glutamine (488)

Definition			Solution												
			exchanges		reactions								transp		
Objective	Constraints	Comment	imports	exports	c	m	r	p	l	n	s	b	s-c	b-c	int
Glutamine(c)	$-NH_3$(c) MCES	de novo synthesis of cytosolic Glutamine	2 NH_3(c) 1 Pyruvate(m) 1 ATP-energy(c) 1 NADPH-redox-potential(m) 1 Proton-gradient(m) 1 CoA-activated acetyl group(m)	1 H_2O(s) 1 Glutamine(c) 1 NADH-redox-potential(m)	2	10	-	-	-	-	-	-	1	-	4

ID	flux	reaction
r2678	-1	Acetyl-CoA(m) ⇌ CoA(m) + CoA-activated acetyl group(m)
r0316	1	Citrate(m) ⇌ Isocitrate(m)
r0073	-1	AKG(m) + NADH(m) + NH3(m) ⇌ Glutamate(m) + NAD+(m) + H2O(m)
r0911	1	Proline(c) + Glutamate(m) ⇌ Proline(m) + Glutamate(c)
r0425	1	Isocitrate(m) + NAD+(m) ⇌ Oxalosuccinate(m) + NADH(m)
r0083	1	Oxalosuccinate(m) ⇌ AKG(m) + CO2(m)
r1426	1	Proline(m) + H+(PG)(c) → Proline(c) + H+(PG)(m)
r1327	-1	NADPH(m) ⇌ NADPH-redox-potential(m) + NADP+(m)
r1319	-1	ATP(c) + H2O(c) ⇌ ADP(c) + Pi(c) + ATP-energy(c)
r0077	1	ATP(c) + Glutamate(c) + NH3(c) → ADP(c) + Pi(c) + Glutamine(c)

Continued on next page

ID	flux	reaction
r0059	-1	Pyruvate(m) + CO2(m) + NADPH(m) ⇌ Malate(m) + NADP+(m)
r0838	1	NH3(c) ⇌ NH3(m)
r0105	1	NAD+(m) + Malate(m) ⇌ OAA(m) + NADH(m)
r1330	-1	H+(PG)(c) ⇌ H+(PG)(m) + Proton-gradient(m)
r0849	-1	H2O(s) ⇌ H2O(c)
r0107	-1	Acetyl-CoA(m) + H2O(m) + OAA(m) ⇌ Citrate(m) + CoA(m)
r1323	1	NADH(m) ⇌ NAD+(m) + NADH-redox-potential(m)

Proline (489)

Definition			Solution												
			exchanges		reactions								transp		
Objective	Constraints	Comment	imports	exports	c	m	r	p	l	n	s	b	s-c	b-c	int
Proline(c)	−Valine(c) MCES	de novo synthesis of cytosolic Proline	1 O_2(s) 1 Valine(c) 1 ATP-energy(m) 1 NADPH-redox-potential(m)	2 H_2O(s) 1 Proline(c) 2 NADH-redox-potential(m) 10 Proton-gradient(m)	-	16	-	-	-	-	-	-	2	-	7

ID	flux	reaction
r0779	1	3-Hydroxyisobutyryl-CoA(m) + H2O(m) → 3-Hydroxyisobutyrate(m) + CoA(m)
r0560	1	Isobutyryl-CoA(m) + Ubiquinone(m) → Methacrylyl-CoA(m) + Ubiquinol(m)
r0263	1	Valine(m) + AKG(m) → Glutamate(m) + 3-Methyl-2-oxobutyrate(m)
r0624	1	2-Methyl-3-oxopropanoate(m) + CoA(m) + NAD+(m) ⇌ (R)-Methylmalonyl-CoA(m) + NADH(m)
r0507	2	Ubiquinol(m) + 2 Ferricytochrome C(m) + 4 H+(PG)(m) → Ubiquinone(m) + 4 H+(PG)(c) + 2 Ferrocytochrome C(m)
r1453	-1	1-Pyrroline-5-carboxylate(m) + Ubiquinone(m) ⇌ Proline(m) + Ubiquinol(m)
r1426	1	Proline(m) + H+(PG)(c) → Proline(c) + H+(PG)(m)
r1327	-1	NADPH(m) ⇌ NADPH-redox-potential(m) + NADP+(m)
r0175	-1	L-Glutamate 5-semialdehyde(m) ⇌ 1-Pyrroline-5-carboxylate(m) + H2O(m)
r0923	1	Valine(c) + H+(PG)(c) → Valine(m) + H+(PG)(m)
r0851	2	H2O(m) ⇌ H2O(c)
r0482	1	3-Hydroxyisobutyrate(m) + NAD+(m) → 2-Methyl-3-oxopropanoate(m) + NADH(m)
r0619	-1	L-Glutamate 5-semialdehyde(m) + Pi(m) + NADP+(m) ← NADPH(m) + Glutamyl-5P(m)
r0197	1	(R)-Methylmalonyl-CoA(m) ⇌ Succinyl-CoA(m)
r0855	1	O2(s) ⇌ O2(c)
r1320	-1	ATP(m) + H2O(m) ⇌ ADP(m) + Pi(m) + ATP-energy(m)
r0856	-1	O2(m) ⇌ O2(c)
r0600	1	3-Methyl-2-oxobutyrate(m) + CoA(m) + NAD+(m) ⇌ Isobutyryl-CoA(m) + CO2(m) + NADH(m)
r1330	10	H+(PG)(c) ⇌ H+(PG)(m) + Proton-gradient(m)
r1114	-1	NADH(m) + CO2(m) + Succinyl-CoA(m) ⇌ AKG(m) + CoA(m) + NAD+(m)
r0849	-2	H2O(s) ⇌ H2O(c)
r0669	1	Methacrylyl-CoA(m) + H2O(m) → 3-Hydroxyisobutyryl-CoA(m)
r0017	1	O2(m) + 4 H+(PG)(m) + 4 Ferrocytochrome C(m) → 4 Ferricytochrome C(m) + 2 H2O(m) + 4 H+(PG)(c)
r1323	2	NADH(m) ⇌ NAD+(m) + NADH-redox-potential(m)
r0072	1	ATP(m) + Glutamate(m) → ADP(m) + Glutamyl-5P(m)

Serine (490)

Definition			Solution												
			exchanges		reactions								transp		
Objective	Constraints	Comment	imports	exports	c	m	r	p	l	n	s	b	s-c	b-c	int
Serine(c)	−NH_3(c) MCES	de novo synthesis of cytosolic Serine	0.5 H_2O(s) 1 NH_3(c) 0.5 Glucose-6P(c) 1 NADH-redox-potential(m) 2 Proton-gradient(m)	0.5 P_i(c) 1 Serine(c) 0.5 ATP-energy(c) 2 NADH-redox-potential(c)	11	2	-	-	-	-	-	-	1	-	7

ID	flux	reaction
r0253	1	GAP(c) + Pi(c) + NAD+(c) ⇌ 1,3DPG(c) + NADH(c)
r0073	-1	AKG(m) + NADH(m) + NH3(m) ⇌ Glutamate(m) + NAD+(m) + H2O(m)
r0911	1	Proline(c) + Glutamate(m) ⇌ Proline(m) + Glutamate(c)
r1047	1	Pi(c) + H+(PG)(c) → Pi(m) + H+(PG)(m)
r0192	0.5	Glucose-6P(c) ⇌ Fructose-6P(c)
r1426	1	Proline(m) + H+(PG)(c) → Proline(c) + H+(PG)(m)
r0337	-1	ADP(c) + 1,3DPG(c) ⇌ ATP(c) + 3PG(c)
r0256	0.5	Fructose-1,6PP(c) ⇌ DHAP(c) + GAP(c)
r1319	0.5	ATP(c) + H2O(c) ⇌ ADP(c) + Pi(c) + ATP-energy(c)
r0851	1	H2O(m) ⇌ H2O(c)
r0338	1	3PG(c) + NAD+(c) → 3-Phosphonooxypyruvate(c) + NADH(c)
r0663	-1	3-Phosphonooxypyruvate(c) + Glutamate(c) → 3-Phosphoserine(c) + AKG(c)
r0159	1	3-Phosphoserine(c) + H2O(c) → Serine(c) + Pi(c)
r2419	1	AKG(c) + Pi(m) ⇌ AKG(m) + Pi(c)
r0736	0.5	ATP(c) + Fructose-6P(c) → ADP(c) + Fructose-1,6PP(c)
r0838	1	NH3(c) ⇌ NH3(m)
r1330	-2	H+(PG)(c) ⇌ H+(PG)(m) + Proton-gradient(m)
r1322	2	NADH(c) ⇌ NAD+(c) + NADH-redox-potential(c)
r0849	0.5	H2O(s) ⇌ H2O(c)
r1323	-1	NADH(m) ⇌ NAD+(m) + NADH-redox-potential(m)
r0243	-0.5	DHAP(c) ⇌ GAP(c)

Tyrosine (491)

Definition			Solution												
			exchanges		reactions								transp		
Objective	Constraints	Comment	imports	exports	c	m	r	p	l	n	s	b	s-c	b-c	int
Tyrosine(c)	−Phenylalanine(c) MCES	de novo synthesis of cytosolic Tyrosine	1 O_2(s) 1 Phenylalanine(c) 1 NADH-redox-potential(c)	1 H_2O(s) 1 Tyrosine(c)	3	-	-	-	-	-	-	-	2	-	-

ID	flux	reaction
r0855	1	O2(s) ⇌ O2(c)
r0397	1	Dihydrobiopterin(c) + NADH(c) ⇌ Tetrahydrobiopterin(c) + NAD+(c)
r1322	-1	NADH(c) ⇌ NAD+(c) + NADH-redox-potential(c)
r0849	-1	H2O(s) ⇌ H2O(c)
r0399	1	Tetrahydrobiopterin(c) + Phenylalanine(c) + O2(c) ⇌ Dihydrobiopterin(c) + Tyrosine(c) + H2O(c)

C2.4. For lipids

Palmitate (492)

Definition			Solution												
			exchanges		reactions								transp		
Objective	Constraints	Comment	imports	exports	c	m	r	p	l	n	s	b	s-c	b-c	int
Palmitate(c)	MCES	de novo synthesis of cytosolic Palmitate	7 ATP-energy(c) 14 NADPH-redox-potential(c) 8 CoA-activated acetyl group(c)	6 H_2O(s) 1 Palmitate(c)	37	-	-	-	-	-	-	-	1	-	-

ID	flux	reaction
r0712	-1	Dodecanoyl-ACP(c) + NADP+(c) ⇌ (2E)-Dodecenoyl-ACP(c) + NADPH(c)
r0713	1	Dodecanoyl-ACP(c) + Malonyl-ACP(c) ⇌ 3-Oxotetradecanoyl-ACP(c) + CO2(c) + ACP(c)
r1326	-14	NADPH(c) ⇌ NADPH-redox-potential(c) + NADP+(c)
r1417	-7	CO2(c) + H2O(c) ⇌ Carbonate(c)

Continued on next page

Palmitate (492)– continued

ID	flux	reaction
r0760	1	Butyryl-ACP(c) + Malonyl-ACP(c) ⇌ CO2(c) + ACP(c) + 3-Oxohexanoyl-ACP(c)
r0184	7	ATP(c) + Acetyl-CoA(c) + HCO3-(c) → ADP(c) + Pi(c) + Malonyl-CoA(c)
r1319	-7	ATP(c) + H2O(c) ⇌ ADP(c) + Pi(c) + ATP-energy(c)
r0770	1	D-3-Hydroxydodecanoyl-ACP(c) ⇌ (2E)-Dodecenoyl-ACP(c) + H2O(c)
r0761	-1	D-3-Hydroxyhexanoyl-ACP(c) + NADP+(c) ⇌ NADPH(c) + 3-Oxohexanoyl-ACP(c)
r1419	7	Carbonate(c) ⇌ HCO3-(c)
r0771	-1	Tetradecanoyl-ACP(c) + NADP+(c) ⇌ NADPH(c) + (2E)-Tetradecenoyl-ACP(c)
r0762	1	D-3-Hydroxyhexanoyl-ACP(c) ⇌ H2O(c) + (2E)-Hexenoyl-ACP(c)
r0681	1	(R)-3-Hydroxybutanoyl-ACP(c) ⇌ But-2-enoyl-ACP(c) + H2O(c)
r0772	1	Tetradecanoyl-ACP(c) + Malonyl-ACP(c) ⇌ 3-Oxohexadecanoyl-ACP(c) + CO2(c) + ACP(c)
r0763	-1	Hexanoyl-ACP(c) + NADP+(c) ⇌ NADPH(c) + (2E)-Hexenoyl-ACP(c)
r0691	-1	(R)-3-Hydroxybutanoyl-ACP(c) + NADP+(c) ⇌ NADPH(c) + Acetoacetyl-ACP(c)
r0682	-1	Butyryl-ACP(c) + NADP+(c) ⇌ But-2-enoyl-ACP(c) + NADPH(c)
r0367	1	Acetyl-CoA(c) + ACP(c) ⇌ CoA(c) + Acetyl-ACP(c)
r0773	-1	Hexadecanoyl-ACP(c) + NADP+(c) ⇌ (2E)-Hexadecenoyl-ACP(c) + NADPH(c)
r0764	1	Malonyl-ACP(c) + Hexanoyl-ACP(c) ⇌ 3-Oxooctanoyl-ACP(c) + CO2(c) + ACP(c)
r0692	-1	(R)-3-Hydroxydecanoyl-ACP(c) + NADP+(c) ⇌ 3-Oxodecanoyl-ACP(c) + NADPH(c)
r0765	-1	Octanoyl-ACP(c) + NADP+(c) ⇌ (2E)-Octenoyl-ACP(c) + NADPH(c)
r0693	1	(R)-3-Hydroxydecanoyl-ACP(c) ⇌ (2E)-Decenoyl-ACP(c) + H2O(c)
r0387	1	Hexadecanoyl-ACP(c) + H2O(c) ⇌ ACP(c) + Palmitate(c)
r0369	7	ACP(c) + Malonyl-CoA(c) ⇌ CoA(c) + Malonyl-ACP(c)
r0766	1	Octanoyl-ACP(c) + Malonyl-ACP(c) ⇌ 3-Oxodecanoyl-ACP(c) + CO2(c) + ACP(c)
r0694	-1	(R)-3-Hydroxyoctanoyl-ACP(c) + NADP+(c) ⇌ 3-Oxooctanoyl-ACP(c) + NADPH(c)
r0767	-1	Decanoyl-ACP(c) + NADP+(c) ⇌ (2E)-Decenoyl-ACP(c) + NADPH(c)
r0695	1	(R)-3-Hydroxyoctanoyl-ACP(c) ⇌ (2E)-Octenoyl-ACP(c) + H2O(c)
r0849	-6	H2O(s) ⇌ H2O(c)
r0768	1	Decanoyl-ACP(c) + Malonyl-ACP(c) ⇌ 3-Oxododecanoyl-ACP(c) + CO2(c) + ACP(c)
r0701	-1	HMA(c) + NADP+(c) ⇌ 3-Oxotetradecanoyl-ACP(c) + NADPH(c)
r0696	-1	(R)-3-Hydroxypalmitoyl-ACP(c) + NADP+(c) ⇌ 3-Oxohexadecanoyl-ACP(c) + NADPH(c)
r0678	1	Acetyl-ACP(c) + Malonyl-ACP(c) ⇌ CO2(c) + ACP(c) + Acetoacetyl-ACP(c)
r2677	-8	Acetyl-CoA(c) ⇌ CoA(c) + CoA-activated acetyl group(c)
r0769	-1	D-3-Hydroxydodecanoyl-ACP(c) + NADP+(c) ⇌ 3-Oxododecanoyl-ACP(c) + NADPH(c)
r0702	1	HMA(c) ⇌ H2O(c) + (2E)-Tetradecenoyl-ACP(c)
r0697	1	(R)-3-Hydroxypalmitoyl-ACP(c) ⇌ (2E)-Hexadecenoyl-ACP(c) + H2O(c)

Glycerol (493)

Definition			Solution												
			exchanges		reactions								transp		
Objective	Constraints	Comment	imports	exports	c	m	r	p	l	n	s	b	s-c	b-c	int
Glycerol(c)	MCES	de novo synthesis of cytosolic Glycerol	0.5 H_2O(s) 0.5 Glucose-6P(c) 4.5 ATP-energy(c) 1 NADH-redox-potential(c)	0.5 P_i(c) 1 Glycerol(c)	26	-	-	-	-	-	-	-	1	-	-

ID	flux	reaction
r1216	-1	Acyl-CoA-VLDL-SM-pool(c) + 1-Acylglycerol-3P-VLDL-SM-pool(c) → CoA(c) + Phosphatidate-VLDL-SM-pool(c)
r1190	0.02	Arachidonyl-CoA(c) + sn-Glycerol-3P(c) → 1-Acylglycerol-3P-arach(c) + CoA(c)
r1263	0.03	Arachidonate(c) + ATP(c) + CoA(c) → Arachidonyl-CoA(c) + AMP(c) + PPi(c)
r1254	0.34	Stearate(c) + ATP(c) + CoA(c) → Stearoyl-CoA(c) + AMP(c) + PPi(c)
r1236	1	1-Acylglycerol-VLDL-SM-pool(c) + H2O(c) → Fatty-acid-VLDL-SM-pool(c) + Glycerol(c)
r0192	0.5	Glucose-6P(c) ⇌ Fructose-6P(c)
r0256	0.5	Fructose-1,6PP(c) ⇌ DHAP(c) + GAP(c)
r1319	-4.5	ATP(c) + H2O(c) ⇌ ADP(c) + Pi(c) + ATP-energy(c)
r1256	0.15	ATP(c) + CoA(c) + Oleate(c) → Oleoyl-CoA(c) + AMP(c) + PPi(c)
r1185	0.71	Palmitoyl-CoA(c) + sn-Glycerol-3P(c) → CoA(c) + 1-Acylglycerol-3P-palm(c)

Continued on next page

ID	flux	reaction
r1258	0.06	Linoleate(c) + ATP(c) + CoA(c) → AMP(c) + PPi(c) + Linoleoyl-CoA(c)
r0736	0.5	ATP(c) + Fructose-6P(c) → ADP(c) + Fructose-1,6PP(c)
r0201	-1	DHAP(c) + NADH(c) ⇌ NAD+(c) + sn-Glycerol-3P(c)
r0030	2	ATP(c) + AMP(c) ⇌ 2 ADP(c)
r1210	0.0001	6260 Palmitoyl-CoA(c) + 1490 Stearoyl-CoA(c) + 656 Oleoyl-CoA(c) + 147 Arachidonyl-CoA(c) + 270 Linoleoyl-CoA(c) ⇌ 8823 Acyl-CoA-VLDL-SM-pool(c)
r1187	0.17	Stearoyl-CoA(c) + sn-Glycerol-3P(c) → CoA(c) + 1-Acylglycerol-3P-stea(c)
r1188	0.07	Oleoyl-CoA(c) + sn-Glycerol-3P(c) → 1-Acylglycerol-3P-ol(c) + CoA(c)
r1230	1	H2O(c) + 1,2-Diacylglycerol-VLDL-SM-pool(c) → 1-Acylglycerol-VLDL-SM-pool(c) + Fatty-acid-VLDL-SM-pool(c)
r1203	0.0001	656 1-Acylglycerol-3P-ol(c) + 270 1-Acylglycerol-3P-lin(c) + 147 1-Acylglycerol-3P-arach(c) + 6260 1-Acylglycerol-3P-palm(c) + 1490 1-Acylglycerol-3P-stea(c) → 8823 1-Acylglycerol-3P-VLDL-SM-pool(c)
r1189	0.03	Linoleoyl-CoA(c) + sn-Glycerol-3P(c) → 1-Acylglycerol-3P-lin(c) + CoA(c)
r0312	1.42	Palmitate(c) + ATP(c) + CoA(c) → Palmitoyl-CoA(c) + AMP(c) + PPi(c)
r1222	1	H2O(c) + Phosphatidate-VLDL-SM-pool(c) → Pi(c) + 1,2-Diacylglycerol-VLDL-SM-pool(c)
r0007	2	PPi(c) + H2O(c) → 2 Pi(c)
r1322	-1	NADH(c) ⇌ NAD+(c) + NADH-redox-potential(c)
r1250	-0.0002	6260 Palmitate(c) + 1490 Stearate(c) + 270 Linoleate(c) + 147 Arachidonate(c) + 656 Oleate(c) ⇌ 8823 Fatty-acid-VLDL-SM-pool(c)
r0849	0.5	H2O(s) ⇌ H2O(c)
r0243	0.5	GAP(c) ⇌ DHAP(c)

Oleate (494)

Definition			Solution												
			exchanges		reactions								transp		
Objective	Constraints	Comment	imports	exports	c	m	r	p	l	n	s	b	s-c	b-c	int
Oleate(c)	MCES	de novo synthesis of cytosolic Oleate	1 O_2(s) 10 ATP-energy(c) 1 NADH-redox-potential(c) 16 NADPH-redox-potential(c) 9 CoA-activated acetyl group(c)	9 H_2O(s) 1 Oleate(c)	47	-	-	-	-	-	-	-	2	-	-

ID	flux	reaction
r0701	-1	HMA(c) + NADP+(c) ⇌ 3-Oxotetradecanoyl-ACP(c) + NADPH(c)
r0702	1	HMA(c) ⇌ H2O(c) + (2E)-Tetradecenoyl-ACP(c)
r0855	1	O2(s) ⇌ O2(c)
r1254	1	Stearate(c) + ATP(c) + CoA(c) → Stearoyl-CoA(c) + AMP(c) + PPi(c)
r0030	1	ATP(c) + AMP(c) ⇌ 2 ADP(c)
r0760	1	Butyryl-ACP(c) + Malonyl-ACP(c) ⇌ CO2(c) + ACP(c) + 3-Oxohexanoyl-ACP(c)
r0761	-1	D-3-Hydroxyhexanoyl-ACP(c) + NADP+(c) ⇌ NADPH(c) + 3-Oxohexanoyl-ACP(c)
r0184	8	ATP(c) + Acetyl-CoA(c) + HCO3-(c) → ADP(c) + Pi(c) + Malonyl-CoA(c)
r0762	1	D-3-Hydroxyhexanoyl-ACP(c) ⇌ H2O(c) + (2E)-Hexenoyl-ACP(c)
r0763	-1	Hexanoyl-ACP(c) + NADP+(c) ⇌ NADPH(c) + (2E)-Hexenoyl-ACP(c)
r0764	1	Malonyl-ACP(c) + Hexanoyl-ACP(c) ⇌ 3-Oxooctanoyl-ACP(c) + CO2(c) + ACP(c)
r0765	-1	Octanoyl-ACP(c) + NADP+(c) ⇌ (2E)-Octenoyl-ACP(c) + NADPH(c)
r0766	1	Octanoyl-ACP(c) + Malonyl-ACP(c) ⇌ 3-Oxodecanoyl-ACP(c) + CO2(c) + ACP(c)
r0767	-1	Decanoyl-ACP(c) + NADP+(c) ⇌ (2E)-Decenoyl-ACP(c) + NADPH(c)
r0768	1	Decanoyl-ACP(c) + Malonyl-ACP(c) ⇌ 3-Oxododecanoyl-ACP(c) + CO2(c) + ACP(c)
r0769	-1	D-3-Hydroxydodecanoyl-ACP(c) + NADP+(c) ⇌ 3-Oxododecanoyl-ACP(c) + NADPH(c)
r1465	-1	NADH(c) + O2(c) + Stearoyl-CoA(c) ⇌ 2 H2O(c) + NAD+(c) + Oleoyl-CoA(c)
r1313	1	Hexadecanoyl-ACP(c) + Malonyl-ACP(c) ⇌ 3-Oxostearoyl-ACP(c) + ACP(c) + CO2(c)
r1314	1	3-Oxostearoyl-ACP(c) + NADPH(c) ⇌ 3-Hydroxystearoyl-ACP(c) + NADP+(c)
r1315	1	3-Hydroxystearoyl-ACP(c) ⇌ (2E)-Octadecenoyl-ACP(c) + H2O(c)
r1316	1	(2E)-Octadecenoyl-ACP(c) + NADPH(c) ⇌ Stearoyl-ACP(c) + NADP+(c)
r1317	1	H2O(c) + Stearoyl-ACP(c) → ACP(c) + Stearate(c)
r0678	1	Acetyl-ACP(c) + Malonyl-ACP(c) ⇌ CO2(c) + ACP(c) + Acetoacetyl-ACP(c)
r0691	-1	(R)-3-Hydroxybutanoyl-ACP(c) + NADP+(c) ⇌ NADPH(c) + Acetoacetyl-ACP(c)

Continued on next page

ID	flux	reaction
r0692	-1	(R)-3-Hydroxydecanoyl-ACP(c) + NADP+(c) ⇌ 3-Oxodecanoyl-ACP(c) + NADPH(c)
r1319	-10	ATP(c) + H2O(c) ⇌ ADP(c) + Pi(c) + ATP-energy(c)
r0693	1	(R)-3-Hydroxydecanoyl-ACP(c) ⇌ (2E)-Decenoyl-ACP(c) + H2O(c)
r0694	-1	(R)-3-Hydroxyoctanoyl-ACP(c) + NADP+(c) ⇌ 3-Oxooctanoyl-ACP(c) + NADPH(c)
r0695	1	(R)-3-Hydroxyoctanoyl-ACP(c) ⇌ (2E)-Octenoyl-ACP(c) + H2O(c)
r0696	-1	(R)-3-Hydroxypalmitoyl-ACP(c) + NADP+(c) ⇌ 3-Oxohexadecanoyl-ACP(c) + NADPH(c)
r0697	1	(R)-3-Hydroxypalmitoyl-ACP(c) ⇌ (2E)-Hexadecenoyl-ACP(c) + H2O(c)
r0849	-9	H2O(s) ⇌ H2O(c)
r0712	-1	Dodecanoyl-ACP(c) + NADP+(c) ⇌ (2E)-Dodecenoyl-ACP(c) + NADPH(c)
r0713	1	Dodecanoyl-ACP(c) + Malonyl-ACP(c) ⇌ 3-Oxotetradecanoyl-ACP(c) + CO2(c) + ACP(c)
r0007	1	PPi(c) + H2O(c) → 2 Pi(c)
r0770	1	D-3-Hydroxydodecanoyl-ACP(c) ⇌ (2E)-Dodecenoyl-ACP(c) + H2O(c)
r0771	-1	Tetradecanoyl-ACP(c) + NADP+(c) ⇌ NADPH(c) + (2E)-Tetradecenoyl-ACP(c)
r0772	1	Tetradecanoyl-ACP(c) + Malonyl-ACP(c) ⇌ 3-Oxohexadecanoyl-ACP(c) + CO2(c) + ACP(c)
r0773	-1	Hexadecanoyl-ACP(c) + NADP+(c) ⇌ (2E)-Hexadecenoyl-ACP(c) + NADPH(c)
r1417	-8	CO2(c) + H2O(c) ⇌ Carbonate(c)
r1419	8	Carbonate(c) ⇌ HCO3-(c)
r2690	1	Oleoyl-CoA(c) + H2O(c) → Oleate(c) + CoA(c)
r2677	-9	Acetyl-CoA(c) ⇌ CoA(c) + CoA-activated acetyl group(c)
r0367	1	Acetyl-CoA(c) + ACP(c) ⇌ CoA(c) + Acetyl-ACP(c)
r0369	8	ACP(c) + Malonyl-CoA(c) ⇌ CoA(c) + Malonyl-ACP(c)
r0681	1	(R)-3-Hydroxybutanoyl-ACP(c) ⇌ But-2-enoyl-ACP(c) + H2O(c)
r0682	-1	Butyryl-ACP(c) + NADP+(c) ⇌ But-2-enoyl-ACP(c) + NADPH(c)
r1322	-1	NADH(c) ⇌ NAD+(c) + NADH-redox-potential(c)
r1326	-16	NADPH(c) ⇌ NADPH-redox-potential(c) + NADP+(c)

Stearate (495)

Definition			Solution												
			exchanges		reactions								transp		
Objective	Constraints	Comment	imports	exports	c	m	r	p	l	n	s	b	s-c	b-c	int
Stearate(c)	MCES	de novo synthesis of cytosolic Stearate	8 ATP-energy(c) 16 NADPH-redox-potential(c) 9 CoA-activated acetyl group(c)	7 H_2O(s) 1 Stearate(c)	41	-	-	-	-	-	-	-	1	-	-

ID	flux	reaction
r0701	-1	HMA(c) + NADP+(c) ⇌ 3-Oxotetradecanoyl-ACP(c) + NADPH(c)
r0702	1	HMA(c) ⇌ H2O(c) + (2E)-Tetradecenoyl-ACP(c)
r0760	1	Butyryl-ACP(c) + Malonyl-ACP(c) ⇌ CO2(c) + ACP(c) + 3-Oxohexanoyl-ACP(c)
r0761	-1	D-3-Hydroxyhexanoyl-ACP(c) + NADP+(c) ⇌ NADPH(c) + 3-Oxohexanoyl-ACP(c)
r0184	8	ATP(c) + Acetyl-CoA(c) + HCO3-(c) → ADP(c) + Pi(c) + Malonyl-CoA(c)
r0762	1	D-3-Hydroxyhexanoyl-ACP(c) ⇌ H2O(c) + (2E)-Hexenoyl-ACP(c)
r0763	-1	Hexanoyl-ACP(c) + NADP+(c) ⇌ NADPH(c) + (2E)-Hexenoyl-ACP(c)
r0764	1	Malonyl-ACP(c) + Hexanoyl-ACP(c) ⇌ 3-Oxooctanoyl-ACP(c) + CO2(c) + ACP(c)
r0765	-1	Octanoyl-ACP(c) + NADP+(c) ⇌ (2E)-Octenoyl-ACP(c) + NADPH(c)
r0766	1	Octanoyl-ACP(c) + Malonyl-ACP(c) ⇌ 3-Oxodecanoyl-ACP(c) + CO2(c) + ACP(c)
r0767	-1	Decanoyl-ACP(c) + NADP+(c) ⇌ (2E)-Decenoyl-ACP(c) + NADPH(c)
r0768	1	Decanoyl-ACP(c) + Malonyl-ACP(c) ⇌ 3-Oxododecanoyl-ACP(c) + CO2(c) + ACP(c)
r0769	-1	D-3-Hydroxydodecanoyl-ACP(c) + NADP+(c) ⇌ 3-Oxododecanoyl-ACP(c) + NADPH(c)
r1313	1	Hexadecanoyl-ACP(c) + Malonyl-ACP(c) ⇌ 3-Oxostearoyl-ACP(c) + ACP(c) + CO2(c)
r1314	1	3-Oxostearoyl-ACP(c) + NADPH(c) ⇌ 3-Hydroxystearoyl-ACP(c) + NADP+(c)
r1315	1	3-Hydroxystearoyl-ACP(c) ⇌ (2E)-Octadecenoyl-ACP(c) + H2O(c)
r1316	1	(2E)-Octadecenoyl-ACP(c) + NADPH(c) ⇌ Stearoyl-ACP(c) + NADP+(c)
r0678	1	Acetyl-ACP(c) + Malonyl-ACP(c) ⇌ CO2(c) + ACP(c) + Acetoacetyl-ACP(c)
r0691	-1	(R)-3-Hydroxybutanoyl-ACP(c) + NADP+(c) ⇌ NADPH(c) + Acetoacetyl-ACP(c)

Continued on next page

ID	flux	reaction
r1317	1	H2O(c) + Stearoyl-ACP(c) → ACP(c) + Stearate(c)
r0692	-1	(R)-3-Hydroxydecanoyl-ACP(c) + NADP+(c) ⇌ 3-Oxodecanoyl-ACP(c) + NADPH(c)
r0693	1	(R)-3-Hydroxydecanoyl-ACP(c) ⇌ (2E)-Decenoyl-ACP(c) + H2O(c)
r1319	-8	ATP(c) + H2O(c) ⇌ ADP(c) + Pi(c) + ATP-energy(c)
r0694	-1	(R)-3-Hydroxyoctanoyl-ACP(c) + NADP+(c) ⇌ 3-Oxooctanoyl-ACP(c) + NADPH(c)
r0695	1	(R)-3-Hydroxyoctanoyl-ACP(c) ⇌ (2E)-Octenoyl-ACP(c) + H2O(c)
r0696	-1	(R)-3-Hydroxypalmitoyl-ACP(c) + NADP+(c) ⇌ 3-Oxohexadecanoyl-ACP(c) + NADPH(c)
r0697	1	(R)-3-Hydroxypalmitoyl-ACP(c) ⇌ (2E)-Hexadecenoyl-ACP(c) + H2O(c)
r0849	-7	H2O(s) ⇌ H2O(c)
r0712	-1	Dodecanoyl-ACP(c) + NADP+(c) ⇌ (2E)-Dodecenoyl-ACP(c) + NADPH(c)
r0713	1	Dodecanoyl-ACP(c) + Malonyl-ACP(c) ⇌ 3-Oxotetradecanoyl-ACP(c) + CO2(c) + ACP(c)
r0770	1	D-3-Hydroxydodecanoyl-ACP(c) ⇌ (2E)-Dodecenoyl-ACP(c) + H2O(c)
r0771	-1	Tetradecanoyl-ACP(c) + NADP+(c) ⇌ NADPH(c) + (2E)-Tetradecenoyl-ACP(c)
r0772	1	Tetradecanoyl-ACP(c) + Malonyl-ACP(c) ⇌ 3-Oxohexadecanoyl-ACP(c) + CO2(c) + ACP(c)
r0773	-1	Hexadecanoyl-ACP(c) + NADP+(c) ⇌ (2E)-Hexadecenoyl-ACP(c) + NADPH(c)
r1417	-8	CO2(c) + H2O(c) ⇌ Carbonate(c)
r1419	8	Carbonate(c) ⇌ HCO3-(c)
r2677	-9	Acetyl-CoA(c) ⇌ CoA(c) + CoA-activated acetyl group(c)
r0367	1	Acetyl-CoA(c) + ACP(c) ⇌ CoA(c) + Acetyl-ACP(c)
r0369	8	ACP(c) + Malonyl-CoA(c) ⇌ CoA(c) + Malonyl-ACP(c)
r0681	1	(R)-3-Hydroxybutanoyl-ACP(c) ⇌ But-2-enoyl-ACP(c) + H2O(c)
r0682	-1	Butyryl-ACP(c) + NADP+(c) ⇌ But-2-enoyl-ACP(c) + NADPH(c)
r1326	-16	NADPH(c) ⇌ NADPH-redox-potential(c) + NADP+(c)

C2.5. Activated sugars

Mannose-1P (496)

Definition			Solution													
			exchanges		reactions								transp		
Objective	Constraints	Comment	imports	exports	c	m	r	p	l	n	s	b	s-c	b-c	int
Mannose-1P(c)	MCES	de novo synthesis of cytosolic Mannose-1P	1 Glucose-6P(c)	1 Mannose-1P(c)	3	-	-	-	-	-	-	-	-	-	-

ID	flux	reaction
r0192	1	Glucose-6P(c) ⇌ Fructose-6P(c)
r0404	1	Mannose-6P(c) → Mannose-1P(c)
r0405	-1	Fructose-6P(c) ⇌ Mannose-6P(c)

Mannose-6P (497)

Definition			Solution												
			exchanges		reactions								transp		
Objective	Constraints	Comment	imports	exports	c	m	r	p	l	n	s	b	s-c	b-c	int
Mannose-6P(c)	MCES	de novo synthesis of cytosolic Mannose-6P	1 Glucose-6P(c)	1 Mannose-6P(c)	2	-	-	-	-	-	-	-	-	-	-

ID	flux	reaction
r0192	1	Glucose-6P(c) ⇌ Fructose-6P(c)
r0405	-1	Fructose-6P(c) ⇌ Mannose-6P(c)

Fructose-6P (498)

Definition			Solution												
			exchanges		reactions								transp		
Objective	Constraints	Comment	imports	exports	c	m	r	p	l	n	s	b	s-c	b-c	int
Fructose-6P(c)	MCES	de novo synthesis of cytosolic Fructose-6P	1 Glucose-6P(c)	1 Fructose-6P(c)	1	-	-	-	-	-	-	-	-	-	-

ID	flux	reaction
r0192	1	Glucose-6P(c) ⇌ Fructose-6P(c)

UDP-xylose (499)

Definition			Solution												
			exchanges		reactions								transp		
Objective	Constraints	Comment	imports	exports	c	m	r	p	l	n	s	b	s-c	b-c	int
UDP-xylose(c)	−Glutamine(c) +Glutamate(c) −Aspartate(c) MCES	de novo synthesis of cytosolic UDP-xylose	0.5 O_2(s) 0.17 P_i(c) 1 Aspartate(c) 1 Glutamine(c) 1.83 Glucose-6P(c) 6.17 ATP-energy(c)	3.17 H_2O(s) 1 CO_2(s) 1 Glutamate(c) 1 UDP-xylose(c) 2 NADH-redox-potential(c) 6 Proton-gradient(m)	26	-	1	-	-	-	-	-	3	-	9

ID	flux	reaction
r0406	-0.33	Fructose-6P(c) + Erythrose-4P(c) ⇌ Sedoheptulose-7P(c) + GAP(c)
r0855	0.5	O2(s) ⇌ O2(c)
r0856	-0.5	O2(m) ⇌ O2(c)
r0017	0.5	O2(m) + 4 H+(PG)(m) + 4 Ferrocytochrome C(m) → 4 Ferricytochrome C(m) + 2 H2O(m) + 4 H+(PG)(c)
r0030	1	ATP(c) + AMP(c) ⇌ 2 ADP(c)
r0467	-1	H2O(c) + (S)-Dihydroorotate(c) ← N-Carbamoyl-L-aspartate(c)
r0037	1	ATP(c) + UDP(c) ⇌ ADP(c) + UTP(c)
r0038	1	ATP(c) + UMP(c) ⇌ ADP(c) + UDP(c)
r0370	0.33	Sedoheptulose-7P(c) + GAP(c) ⇌ Ribose-5P(c) + D-Xylulose-5P(c)
r0092	1	UDP-glucose(c) + H2O(c) + 2 NAD+(c) → 2 NADH(c) + UDP-glucuronate(c)
r0094	1	UTP(c) + Glucose-1P(c) → PPi(c) + UDP-glucose(c)
r0507	1	Ubiquinol(m) + 2 Ferricytochrome C(m) + 4 H+(PG)(m) → Ubiquinone(m) + 4 H+(PG)(c) + 2 Ferrocytochrome C(m)
r0243	-0.17	DHAP(c) ⇌ GAP(c)
r1330	6	H+(PG)(c) ⇌ H+(PG)(m) + Proton-gradient(m)
r1319	-6.17	ATP(c) + H2O(c) ⇌ ADP(c) + Pi(c) + ATP-energy(c)
r0247	1	ATP(c) + Ribose-5P(c) → AMP(c) + PRPP(c)
r0844	1	UDP-glucuronate(c) + UMP(r) ⇌ UDP-glucuronate(r) + UMP(c)
r0846	-1	UDP-xylose(c) + UMP(r) ← UDP-xylose(r) + UMP(c)
r0849	-3.17	H2O(s) ⇌ H2O(c)
r0416	1	Ubiquinone(m) + (S)-Dihydroorotate(c) → Orotate(c) + Ubiquinol(m)
r0417	-1	PPi(c) + Orotidine-5P(c) ⇌ Orotate(c) + PRPP(c)
r0865	1	CO2(r) ⇌ CO2(c)
r0867	1	CO2(c) ⇌ CO2(s)
r0155	1	2 ATP(c) + Glutamine(c) + H2O(c) + HCO3-(c) → 2 ADP(c) + Pi(c) + Glutamate(c) + Carbamoyl-P(c)
r0007	2	PPi(c) + H2O(c) → 2 Pi(c)
r0736	0.17	ATP(c) + Fructose-6P(c) → ADP(c) + Fructose-1,6PP(c)
r0192	0.83	Glucose-6P(c) ⇌ Fructose-6P(c)
r0621	-1	Glucose-6P(c) ⇌ Glucose-1P(c)
r0326	1	UDP-glucuronate(r) → UDP-xylose(r) + CO2(r)
r0328	1	Carbamoyl-P(c) + Aspartate(c) → Pi(c) + N-Carbamoyl-L-aspartate(c)
r1417	-1	CO2(c) + H2O(c) ⇌ Carbonate(c)
r0344	-0.67	D-Xylulose-5P(c) ⇌ Ribulose-5P(c)
r1419	1	Carbonate(c) ⇌ HCO3-(c)
r0235	1	Orotidine-5P(c) ⇌ CO2(c) + UMP(c)

Continued on next page

ID	flux	reaction
r1322	2	NADH(c) ⇌ NAD+(c) + NADH-redox-potential(c)
r0250	-0.67	Ribulose-5P(c) ⇌ Ribose-5P(c)
r0255	0.33	Fructose-6P(c) + GAP(c) ⇌ D-Xylulose-5P(c) + Erythrose-4P(c)
r0256	0.17	Fructose-1,6PP(c) ⇌ DHAP(c) + GAP(c)
r0851	1	H2O(m) ⇌ H2O(c)

UDP-glucose (500)

Definition			Solution												
			exchanges		reactions								transp		
Objective	Constraints	Comment	imports	exports	c	m	r	p	l	n	s	b	s-c	b-c	int
UDP-glucose(c)	MCES −Glutamine(c) +Glutamate(c) −Aspartate(c)	de novo synthesis of cytosolic UDP-glucose	0.5 O_2(s) 0.17 P_i(c) 1 Aspartate(c) 1 Glutamine(c) 1.83 Glucose-6P(c) 6.17 ATP-energy(c)	4.17 H_2O(s) 1 Glutamate(c) 1 UDP-glucose(c) 6 Proton-gradient(m)	24	-	-	-	-	-	-	-	2	-	6

ID	flux	reaction
r0406	-0.33	Fructose-6P(c) + Erythrose-4P(c) ⇌ Sedoheptulose-7P(c) + GAP(c)
r0370	0.33	Sedoheptulose-7P(c) + GAP(c) ⇌ Ribose-5P(c) + D-Xylulose-5P(c)
r0235	1	Orotidine-5P(c) ⇌ CO2(c) + UMP(c)
r0037	1	ATP(c) + UDP(c) ⇌ ADP(c) + UTP(c)
r0416	1	Ubiquinone(m) + (S)-Dihydroorotate(c) → Orotate(c) + Ubiquinol(m)
r0344	-0.67	D-Xylulose-5P(c) ⇌ Ribulose-5P(c)
r0155	1	2 ATP(c) + Glutamine(c) + H2O(c) + HCO3-(c) → 2 ADP(c) + Pi(c) + Glutamate(c) + Carbamoyl-P(c)
r0038	1	ATP(c) + UMP(c) ⇌ ADP(c) + UDP(c)
r0507	1	Ubiquinol(m) + 2 Ferricytochrome C(m) + 4 H+(PG)(m) → Ubiquinone(m) + 4 H+(PG)(c) + 2 Ferrocytochrome C(m)
r0417	-1	PPi(c) + Orotidine-5P(c) ⇌ Orotate(c) + PRPP(c)
r0255	0.33	Fructose-6P(c) + GAP(c) ⇌ D-Xylulose-5P(c) + Erythrose-4P(c)
r0192	0.83	Glucose-6P(c) ⇌ Fructose-6P(c)
r1417	-1	CO2(c) + H2O(c) ⇌ Carbonate(c)
r0328	1	Carbamoyl-P(c) + Aspartate(c) → Pi(c) + N-Carbamoyl-L-aspartate(c)
r0256	0.17	Fructose-1,6PP(c) ⇌ DHAP(c) + GAP(c)
r0247	1	ATP(c) + Ribose-5P(c) → AMP(c) + PRPP(c)
r0094	1	UTP(c) + Glucose-1P(c) → PPi(c) + UDP-glucose(c)
r1319	-6.17	ATP(c) + H2O(c) ⇌ ADP(c) + Pi(c) + ATP-energy(c)
r0851	1	H2O(m) ⇌ H2O(c)
r1419	1	Carbonate(c) ⇌ HCO3-(c)
r0736	0.17	ATP(c) + Fructose-6P(c) → ADP(c) + Fructose-1,6PP(c)
r0030	1	ATP(c) + AMP(c) ⇌ 2 ADP(c)
r0467	-1	H2O(c) + (S)-Dihydroorotate(c) ← N-Carbamoyl-L-aspartate(c)
r0855	0.5	O2(s) ⇌ O2(c)
r0856	-0.5	O2(m) ⇌ O2(c)
r1330	6	H+(PG)(c) ⇌ H+(PG)(m) + Proton-gradient(m)
r0250	-0.67	Ribulose-5P(c) ⇌ Ribose-5P(c)
r0007	2	PPi(c) + H2O(c) → 2 Pi(c)
r0849	-4.17	H2O(s) ⇌ H2O(c)
r0017	0.5	O2(m) + 4 H+(PG)(m) + 4 Ferrocytochrome C(m) → 4 Ferricytochrome C(m) + 2 H2O(m) + 4 H+(PG)(c)
r0621	-1	Glucose-6P(c) ⇌ Glucose-1P(c)
r0243	-0.17	DHAP(c) ⇌ GAP(c)

udpgal (501)

Definition			Solution												
			exchanges		reactions								transp		
Objective	Constraints	Comment	imports	exports	c	m	r	p	l	n	s	b	s-c	b-c	int
UDP-galactose(c)	MCES −Glutamine(c) +Glutamate(c) −Aspartate(c)	de novo synthesis of cytosolic UDP-galactose	0.5 O_2(s) 0.17 P_i(c) 1 Aspartate(c) 1 Glutamine(c) 1.83 Glucose-6P(c) 6.17 ATP-energy(c)	4.17 H_2O(s) 1 Glutamate(c) 1 UDP-galactose(c) 6 Proton-gradient(m)	25	-	-	-	-	-	-	-	2	-	6

ID	flux	reaction
r0406	-0.33	Fructose-6P(c) + Erythrose-4P(c) ⇌ Sedoheptulose-7P(c) + GAP(c)
r0370	0.33	Sedoheptulose-7P(c) + GAP(c) ⇌ Ribose-5P(c) + D-Xylulose-5P(c)
r0235	1	Orotidine-5P(c) ⇌ CO2(c) + UMP(c)
r0037	1	ATP(c) + UDP(c) ⇌ ADP(c) + UTP(c)
r0416	1	Ubiquinone(m) + (S)-Dihydroorotate(c) → Orotate(c) + Ubiquinol(m)
r0344	-0.67	D-Xylulose-5P(c) ⇌ Ribulose-5P(c)
r0155	1	2 ATP(c) + Glutamine(c) + H2O(c) + HCO3-(c) → 2 ADP(c) + Pi(c) + Glutamate(c) + Carbamoyl-P(c)
r0038	1	ATP(c) + UMP(c) ⇌ ADP(c) + UDP(c)
r0507	1	Ubiquinol(m) + 2 Ferricytochrome C(m) + 4 H+(PG)(m) → Ubiquinone(m) + 4 H+(PG)(c) + 2 Ferrocytochrome C(m)
r0417	-1	PPi(c) + Orotidine-5P(c) ⇌ Orotate(c) + PRPP(c)
r0255	0.33	Fructose-6P(c) + GAP(c) ⇌ D-Xylulose-5P(c) + Erythrose-4P(c)
r0192	0.83	Glucose-6P(c) ⇌ Fructose-6P(c)
r1417	-1	CO2(c) + H2O(c) ⇌ Carbonate(c)
r0328	1	Carbamoyl-P(c) + Aspartate(c) → Pi(c) + N-Carbamoyl-L-aspartate(c)
r0256	0.17	Fructose-1,6PP(c) ⇌ DHAP(c) + GAP(c)
r0247	1	ATP(c) + Ribose-5P(c) → AMP(c) + PRPP(c)
r0094	1	UTP(c) + Glucose-1P(c) → PPi(c) + UDP-glucose(c)
r1319	-6.17	ATP(c) + H2O(c) ⇌ ADP(c) + Pi(c) + ATP-energy(c)
r0851	1	H2O(m) ⇌ H2O(c)
r0095	1	UDP-glucose(c) ⇌ UDP-galactose(c)
r1419	1	Carbonate(c) ⇌ HCO3-(c)
r0736	0.17	ATP(c) + Fructose-6P(c) → ADP(c) + Fructose-1,6PP(c)
r0030	1	ATP(c) + AMP(c) ⇌ 2 ADP(c)
r0467	-1	H2O(c) + (S)-Dihydroorotate(c) ← N-Carbamoyl-L-aspartate(c)
r0855	0.5	O2(s) ⇌ O2(c)
r0856	-0.5	O2(m) ⇌ O2(c)
r1330	6	H+(PG)(c) ⇌ H+(PG)(m) + Proton-gradient(m)
r0250	-0.67	Ribulose-5P(c) ⇌ Ribose-5P(c)
r0007	2	PPi(c) + H2O(c) → 2 Pi(c)
r0849	-4.17	H2O(s) ⇌ H2O(c)
r0017	0.5	O2(m) + 4 H+(PG)(m) + 4 Ferrocytochrome C(m) → 4 Ferricytochrome C(m) + 2 H2O(m) + 4 H+(PG)(c)
r0621	-1	Glucose-6P(c) ⇌ Glucose-1P(c)
r0243	-0.17	DHAP(c) ⇌ GAP(c)

udpglcur (502)

Definition			Solution												
			exchanges		reactions								transp		
Objective	Constraints	Comment	imports	exports	c	m	r	p	l	n	s	b	s-c	b-c	int
UDP-glucuronate(c)	MCES −Glutamine(c) +Glutamate(c) −Aspartate(c)	de novo synthesis of cytosolic UDP-glucuronate	0.5 O_2(s) 0.17 P_i(c) 1 Aspartate(c) 1 Glutamine(c) 1.83 Glucose-6P(c) 6.17 ATP-energy(c)	3.17 H_2O(s) 1 Glutamate(c) 1 UDP-glucuronate(c) 2 NADH-redox-potential(c) 6 Proton-gradient(m)	26	-	-	-	-	-	-	-	2	-	6

ID	flux	reaction
r0406	-0.33	Fructose-6P(c) + Erythrose-4P(c) ⇌ Sedoheptulose-7P(c) + GAP(c)
r0370	0.33	Sedoheptulose-7P(c) + GAP(c) ⇌ Ribose-5P(c) + D-Xylulose-5P(c)

Continued on next page

ID	flux	reaction
r0235	1	Orotidine-5P(c) ⇌ CO2(c) + UMP(c)
r0037	1	ATP(c) + UDP(c) ⇌ ADP(c) + UTP(c)
r0416	1	Ubiquinone(m) + (S)-Dihydroorotate(c) → Orotate(c) + Ubiquinol(m)
r0344	-0.67	D-Xylulose-5P(c) ⇌ Ribulose-5P(c)
r0155	1	2 ATP(c) + Glutamine(c) + H2O(c) + HCO3-(c) → 2 ADP(c) + Pi(c) + Glutamate(c) + Carbamoyl-P(c)
r0092	1	UDP-glucose(c) + H2O(c) + 2 NAD+(c) → 2 NADH(c) + UDP-glucuronate(c)
r0038	1	ATP(c) + UMP(c) ⇌ ADP(c) + UDP(c)
r0507	1	Ubiquinol(m) + 2 Ferricytochrome C(m) + 4 H+(PG)(m) → Ubiquinone(m) + 4 H+(PG)(c) + 2 Ferrocytochrome C(m)
r0417	-1	PPi(c) + Orotidine-5P(c) ⇌ Orotate(c) + PRPP(c)
r0255	0.33	Fructose-6P(c) + GAP(c) ⇌ D-Xylulose-5P(c) + Erythrose-4P(c)
r0192	0.83	Glucose-6P(c) ⇌ Fructose-6P(c)
r1417	-1	CO2(c) + H2O(c) ⇌ Carbonate(c)
r0328	1	Carbamoyl-P(c) + Aspartate(c) → Pi(c) + N-Carbamoyl-L-aspartate(c)
r0256	0.17	Fructose-1,6PP(c) ⇌ DHAP(c) + GAP(c)
r0247	1	ATP(c) + Ribose-5P(c) → AMP(c) + PRPP(c)
r0094	1	UTP(c) + Glucose-1P(c) → PPi(c) + UDP-glucose(c)
r1319	-6.17	ATP(c) + H2O(c) ⇌ ADP(c) + Pi(c) + ATP-energy(c)
r0851	1	H2O(m) ⇌ H2O(c)
r1419	1	Carbonate(c) ⇌ HCO3-(c)
r0736	0.17	ATP(c) + Fructose-6P(c) → ADP(c) + Fructose-1,6PP(c)
r0030	1	ATP(c) + AMP(c) ⇌ 2 ADP(c)
r0467	-1	H2O(c) + (S)-Dihydroorotate(c) ← N-Carbamoyl-L-aspartate(c)
r0855	0.5	O2(s) ⇌ O2(c)
r0856	-0.5	O2(m) ⇌ O2(c)
r1330	6	H+(PG)(c) ⇌ H+(PG)(m) + Proton-gradient(m)
r0250	-0.67	Ribulose-5P(c) ⇌ Ribose-5P(c)
r0007	2	PPi(c) + H2O(c) → 2 Pi(c)
r1322	2	NADH(c) ⇌ NAD+(c) + NADH-redox-potential(c)
r0849	-3.17	H2O(s) ⇌ H2O(c)
r0017	0.5	O2(m) + 4 H+(PG)(m) + 4 Ferrocytochrome C(m) → 4 Ferricytochrome C(m) + 2 H2O(m) + 4 H+(PG)(c)
r0621	-1	Glucose-6P(c) ⇌ Glucose-1P(c)
r0243	-0.17	DHAP(c) ⇌ GAP(c)

GDP-L-fucose (503)

Definition			Solution												
			exchanges		reactions								transp		
Objective	Constraints	Comment	imports	exports	c	m	r	p	l	n	s	b	s-c	b-c	int
GDP-L-fucose(c)	MCES −Glutamine(c) +Glutamate(c) −Aspartate(c)	de novo synthesis of cytosolic GDP-L-fucose	2 Aspartate(c) 3 Glutamine(c) 2.33 Glucose-6P(c) 9.67 ATP-energy(c) 1 THF-activated methyl group(c)	1.67 H_2O(s) 0.33 P_i(c) 1 CO_2(s) 2 Pyruvate(c) 3 Glutamate(c) 1 GDP-L-fucose(c) 2 NADH-redox-potential(c) 4 NADPH-redox-potential(c)	48	-	-	-	-	-	-	-	2	-	-

ID	flux	reaction
r0406	-0.33	Fructose-6P(c) + Erythrose-4P(c) ⇌ Sedoheptulose-7P(c) + GAP(c)
r0705	1	ATP(c) + 5-Phosphoribosyl-4-carboxy-5-aminoimidazole(c) + Aspartate(c) ⇌ ADP(c) + Pi(c) + SAICAR(c)
r0293	2	5,10-Methylene-THF(c) + NADP+(c) ⇌ 5,10-Methenyl-THF(c) + NADPH(c)
r0030	2	ATP(c) + AMP(c) ⇌ 2 ADP(c)
r0782	-1	GDP-4-dehydro-6-deoxy-D-mannose(c) + NADH(c) → GDP-L-fucose(c) + NAD+(c)
r0337	-1	ADP(c) + 1,3DPG(c) ⇌ ATP(c) + 3PG(c)
r0338	1	3PG(c) + NAD+(c) → 3-Phosphonooxypyruvate(c) + NADH(c)
r0058	2	Malate(c) + NADP+(c) ⇌ Pyruvate(c) + CO2(c) + NADPH(c)
r0370	0.33	Sedoheptulose-7P(c) + GAP(c) ⇌ Ribose-5P(c) + D-Xylulose-5P(c)
r0371	2	5,10-Methenyl-THF(c) + H2O(c) ⇌ 10-Formyl-THF(c)

Continued on next page

ID	flux	reaction
r0208	1	GTP(c) + Mannose-1P(c) ⇌ PPi(c) + GDP-mannose(c)
r0209	1	GDP-mannose(c) → GDP-4-dehydro-6-deoxy-D-mannose(c) + H2O(c)
r0659	1	ATP(c) + 5-Phosphoribosylamine(c) + Glycine(c) ⇌ ADP(c) + Pi(c) + GAR(c)
r0099	1	ATP(c) + GDP(c) ⇌ ADP(c) + GTP(c)
r0243	-0.67	DHAP(c) ⇌ GAP(c)
r0677	1	10-Formyl-THF(c) + GAR(c) ⇌ THF(c) + N-Formyl-GAR(c)
r1319	-9.67	ATP(c) + H2O(c) ⇌ ADP(c) + Pi(c) + ATP-energy(c)
r0247	1	ATP(c) + Ribose-5P(c) → AMP(c) + PRPP(c)
r0699	1	SAICAR(c) ⇌ Fumarate(c) + AICAR(c)
r0849	-1.67	H2O(s) ⇌ H2O(c)
r0867	1	CO2(c) ⇌ CO2(s)
r0736	0.67	ATP(c) + Fructose-6P(c) → ADP(c) + Fructose-1,6PP(c)
r0007	3	PPi(c) + H2O(c) → 2 Pi(c)
r0302	1	ATP(c) + Xanthosine-5P(c) + Glutamine(c) + H2O(c) → AMP(c) + PPi(c) + GMP(c) + Glutamate(c)
r0159	1	3-Phosphoserine(c) + H2O(c) → Serine(c) + Pi(c)
r0192	2.33	Glucose-6P(c) ⇌ Fructose-6P(c)
r0344	-0.67	D-Xylulose-5P(c) ⇌ Ribulose-5P(c)
r0792	1	5-Methyl-THF(c) + NAD+(c) ⇌ 5,10-Methylene-THF(c) + NADH(c)
r2676	-1	5-Methyl-THF(c) ⇌ THF(c) + THF-activated methyl group(c)
r0663	-1	3-Phosphonooxypyruvate(c) + Glutamate(c) → 3-Phosphoserine(c) + AKG(c)
r0230	-1	THF(c) + Serine(c) ⇌ 5,10-Methylene-THF(c) + Glycine(c) + H2O(c)
r0666	1	ATP(c) + 5-Phosphoribosylformylglycinamidine(c) ⇌ ADP(c) + Pi(c) + AIR(c)
r0667	-1	5-Phosphoribosyl-4-carboxy-5-aminoimidazole(c) ⇌ AIR(c) + CO2(c)
r1322	2	NADH(c) ⇌ NAD+(c) + NADH-redox-potential(c)
r0250	-0.67	Ribulose-5P(c) ⇌ Ribose-5P(c)
r1326	4	NADPH(c) ⇌ NADPH-redox-potential(c) + NADP+(c)
r0687	1	ATP(c) + N-Formyl-GAR(c) + Glutamine(c) + H2O(c) ⇌ ADP(c) + Pi(c) + Glutamate(c) + 5-Phosphoribosylformylglycinamidine(c)
r0101	1	ATP(c) + GMP(c) ⇌ ADP(c) + GDP(c)
r0253	1	GAP(c) + Pi(c) + NAD+(c) ⇌ 1,3DPG(c) + NADH(c)
r0255	0.33	Fructose-6P(c) + GAP(c) ⇌ D-Xylulose-5P(c) + Erythrose-4P(c)
r0104	-1	OAA(c) + NADH(c) ⇌ NAD+(c) + Malate(c)
r0256	0.67	Fructose-1,6PP(c) ⇌ DHAP(c) + GAP(c)
r0257	-1	Glutamine(c) + PRPP(c) + H2O(c) ⇌ 5-Phosphoribosylamine(c) + PPi(c) + Glutamate(c)
r0258	-1	Fumarate(c) + H2O(c) ⇌ Malate(c)
r0273	-1	IMP(c) + H2O(c) ⇌ FAICAR(c)
r0404	1	Mannose-6P(c) → Mannose-1P(c)
r0274	1	IMP(c) + NAD+(c) + H2O(c) → Xanthosine-5P(c) + NADH(c)
r0109	1	AKG(c) + Aspartate(c) ⇌ OAA(c) + Glutamate(c)
r0700	1	10-Formyl-THF(c) + AICAR(c) ⇌ THF(c) + FAICAR(c)
r0405	-1	Fructose-6P(c) ⇌ Mannose-6P(c)

GDP-mannose (504)

Definition			Solution													
			exchanges		reactions								transp		
Objective	Constraints	Comment	imports	exports	c	m	r	p	l	n	s	b	s-c	b-c	int
GDP-mannose(c)	MCES −Glutamine(c) +Glutamate(c) −Aspartate(c)	de novo synthesis of cytosolic GDP-mannose	2 Aspartate(c) 3 Glutamine(c) 2.33 Glucose-6P(c) 9.67 ATP-energy(c) 1 THF-activated methyl group(c)	0.67 H_2O(s) 0.33 P_i(c) 1 CO_2(s) 2 Pyruvate(c) 3 Glutamate(c) 1 GDP-mannose(c) 2 NADH-redox-potential(c) 5 NADPH-redox-potential(c)	46	-	-	-	-	-	-	-	2	-	-

ID	flux	reaction
r0406	-0.33	Fructose-6P(c) + Erythrose-4P(c) ⇌ Sedoheptulose-7P(c) + GAP(c)
r0705	1	ATP(c) + 5-Phosphoribosyl-4-carboxy-5-aminoimidazole(c) + Aspartate(c) ⇌ ADP(c) + Pi(c) + SAICAR(c)

Continued on next page

ID	flux	reaction
r0293	2	5,10-Methylene-THF(c) + NADP+(c) ⇌ 5,10-Methenyl-THF(c) + NADPH(c)
r0296	1	5-Methyl-THF(c) + NADP+(c) ⇌ 5,10-Methylene-THF(c) + NADPH(c)
r0030	2	ATP(c) + AMP(c) ⇌ 2 ADP(c)
r0337	-1	ADP(c) + 1,3DPG(c) ⇌ ATP(c) + 3PG(c)
r0338	1	3PG(c) + NAD+(c) → 3-Phosphonooxypyruvate(c) + NADH(c)
r0058	2	Malate(c) + NADP+(c) ⇌ Pyruvate(c) + CO2(c) + NADPH(c)
r0370	0.33	Sedoheptulose-7P(c) + GAP(c) ⇌ Ribose-5P(c) + D-Xylulose-5P(c)
r0371	2	5,10-Methenyl-THF(c) + H2O(c) ⇌ 10-Formyl-THF(c)
r0208	1	GTP(c) + Mannose-1P(c) ⇌ PPi(c) + GDP-mannose(c)
r0659	1	ATP(c) + 5-Phosphoribosylamine(c) + Glycine(c) ⇌ ADP(c) + Pi(c) + GAR(c)
r0099	1	ATP(c) + GDP(c) ⇌ ADP(c) + GTP(c)
r0243	-0.67	DHAP(c) ⇌ GAP(c)
r0677	1	10-Formyl-THF(c) + GAR(c) ⇌ THF(c) + N-Formyl-GAR(c)
r1319	-9.67	ATP(c) + H2O(c) ⇌ ADP(c) + Pi(c) + ATP-energy(c)
r0247	1	ATP(c) + Ribose-5P(c) → AMP(c) + PRPP(c)
r0699	1	SAICAR(c) ⇌ Fumarate(c) + AICAR(c)
r0849	-0.67	H2O(s) ⇌ H2O(c)
r0867	1	CO2(c) ⇌ CO2(s)
r0736	0.67	ATP(c) + Fructose-6P(c) → ADP(c) + Fructose-1,6PP(c)
r0007	3	PPi(c) + H2O(c) → 2 Pi(c)
r0302	1	ATP(c) + Xanthosine-5P(c) + Glutamine(c) + H2O(c) → AMP(c) + PPi(c) + GMP(c) + Glutamate(c)
r0159	1	3-Phosphoserine(c) + H2O(c) → Serine(c) + Pi(c)
r0192	2.33	Glucose-6P(c) ⇌ Fructose-6P(c)
r0344	-0.67	D-Xylulose-5P(c) ⇌ Ribulose-5P(c)
r2676	-1	5-Methyl-THF(c) ⇌ THF(c) + THF-activated methyl group(c)
r0663	-1	3-Phosphonooxypyruvate(c) + Glutamate(c) → 3-Phosphoserine(c) + AKG(c)
r0230	-1	THF(c) + Serine(c) ⇌ 5,10-Methylene-THF(c) + Glycine(c) + H2O(c)
r0666	1	ATP(c) + 5-Phosphoribosylformylglycinamidine(c) ⇌ ADP(c) + Pi(c) + AIR(c)
r0667	-1	5-Phosphoribosyl-4-carboxy-5-aminoimidazole(c) ⇌ AIR(c) + CO2(c)
r1322	2	NADH(c) ⇌ NAD+(c) + NADH-redox-potential(c)
r0250	-0.67	Ribulose-5P(c) ⇌ Ribose-5P(c)
r1326	5	NADPH(c) ⇌ NADPH-redox-potential(c) + NADP+(c)
r0687	1	ATP(c) + N-Formyl-GAR(c) + Glutamine(c) + H2O(c) ⇌ ADP(c) + Pi(c) + Glutamate(c) + 5-Phosphoribosylformylglycinamidine(c)
r0101	1	ATP(c) + GMP(c) ⇌ ADP(c) + GDP(c)
r0253	1	GAP(c) + Pi(c) + NAD+(c) ⇌ 1,3DPG(c) + NADH(c)
r0255	0.33	Fructose-6P(c) + GAP(c) ⇌ D-Xylulose-5P(c) + Erythrose-4P(c)
r0104	-1	OAA(c) + NADH(c) ⇌ NAD+(c) + Malate(c)
r0256	0.67	Fructose-1,6PP(c) ⇌ DHAP(c) + GAP(c)
r0257	-1	Glutamine(c) + PRPP(c) + H2O(c) ⇌ 5-Phosphoribosylamine(c) + PPi(c) + Glutamate(c)
r0258	-1	Fumarate(c) + H2O(c) ⇌ Malate(c)
r0273	-1	IMP(c) + H2O(c) ⇌ FAICAR(c)
r0404	1	Mannose-6P(c) → Mannose-1P(c)
r0274	1	IMP(c) + NAD+(c) + H2O(c) → Xanthosine-5P(c) + NADH(c)
r0109	1	AKG(c) + Aspartate(c) ⇌ OAA(c) + Glutamate(c)
r0700	1	10-Formyl-THF(c) + AICAR(c) ⇌ THF(c) + FAICAR(c)
r0405	-1	Fructose-6P(c) ⇌ Mannose-6P(c)

C2.6. Other

IMP (505)

Definition			Solution												
			exchanges		reactions								transp		
Objective	Constraints	Comment	imports	exports	c	m	r	p	l	n	s	b	s-c	b-c	int
IMP(c)	−Glutamine(c) +Glutamate(c) −Aspartate(c) MCES	de novo synthesis of cytosolic IMP	2 Aspartate(c) 2 Glutamine(c) 1.33 Glucose-6P(c) 5.67 ATP-energy(c) 1 THF-activated methyl group(c)	0.67 H_2O(s) 0.33 P_i(c) 1 CO_2(s) 2 Pyruvate(c) 2 Glutamate(c) 1 IMP(c) 1 NADH-redox-potential(c) 5 NADPH-redox-potential(c)	39	-	-	-	-	-	-	-	2	-	-

ID	flux	reaction
r0406	-0.33	Fructose-6P(c) + Erythrose-4P(c) $\rightleftharpoons$ Sedoheptulose-7P(c) + GAP(c)
r0293	2	5,10-Methylene-THF(c) + NADP+(c) $\rightleftharpoons$ 5,10-Methenyl-THF(c) + NADPH(c)
r0705	1	ATP(c) + 5-Phosphoribosyl-4-carboxy-5-aminoimidazole(c) + Aspartate(c) $\rightleftharpoons$ ADP(c) + Pi(c) + SAICAR(c)
r0296	1	5-Methyl-THF(c) + NADP+(c) $\rightleftharpoons$ 5,10-Methylene-THF(c) + NADPH(c)
r0030	1	ATP(c) + AMP(c) $\rightleftharpoons$ 2 ADP(c)
r0337	-1	ADP(c) + 1,3DPG(c) $\rightleftharpoons$ ATP(c) + 3PG(c)
r0338	1	3PG(c) + NAD+(c) $\rightarrow$ 3-Phosphonooxypyruvate(c) + NADH(c)
r0058	2	Malate(c) + NADP+(c) $\rightleftharpoons$ Pyruvate(c) + CO2(c) + NADPH(c)
r0370	0.33	Sedoheptulose-7P(c) + GAP(c) $\rightleftharpoons$ Ribose-5P(c) + D-Xylulose-5P(c)
r0371	2	5,10-Methenyl-THF(c) + H2O(c) $\rightleftharpoons$ 10-Formyl-THF(c)
r0659	1	ATP(c) + 5-Phosphoribosylamine(c) + Glycine(c) $\rightleftharpoons$ ADP(c) + Pi(c) + GAR(c)
r0243	-0.67	DHAP(c) $\rightleftharpoons$ GAP(c)
r0677	1	10-Formyl-THF(c) + GAR(c) $\rightleftharpoons$ THF(c) + N-Formyl-GAR(c)
r1319	-5.67	ATP(c) + H2O(c) $\rightleftharpoons$ ADP(c) + Pi(c) + ATP-energy(c)
r0247	1	ATP(c) + Ribose-5P(c) $\rightarrow$ AMP(c) + PRPP(c)
r0699	1	SAICAR(c) $\rightleftharpoons$ Fumarate(c) + AICAR(c)
r0849	-0.67	H2O(s) $\rightleftharpoons$ H2O(c)
r0867	1	CO2(c) $\rightleftharpoons$ CO2(s)
r0007	1	PPi(c) + H2O(c) $\rightarrow$ 2 Pi(c)
r0736	0.67	ATP(c) + Fructose-6P(c) $\rightarrow$ ADP(c) + Fructose-1,6PP(c)
r0159	1	3-Phosphoserine(c) + H2O(c) $\rightarrow$ Serine(c) + Pi(c)
r0192	1.33	Glucose-6P(c) $\rightleftharpoons$ Fructose-6P(c)
r0344	-0.67	D-Xylulose-5P(c) $\rightleftharpoons$ Ribulose-5P(c)
r2676	-1	5-Methyl-THF(c) $\rightleftharpoons$ THF(c) + THF-activated methyl group(c)
r0663	-1	3-Phosphonooxypyruvate(c) + Glutamate(c) $\rightarrow$ 3-Phosphoserine(c) + AKG(c)
r0230	-1	THF(c) + Serine(c) $\rightleftharpoons$ 5,10-Methylene-THF(c) + Glycine(c) + H2O(c)
r0666	1	ATP(c) + 5-Phosphoribosylformylglycinamidine(c) $\rightleftharpoons$ ADP(c) + Pi(c) + AIR(c)
r0667	-1	5-Phosphoribosyl-4-carboxy-5-aminoimidazole(c) $\rightleftharpoons$ AIR(c) + CO2(c)
r1322	1	NADH(c) $\rightleftharpoons$ NAD+(c) + NADH-redox-potential(c)
r0250	-0.67	Ribulose-5P(c) $\rightleftharpoons$ Ribose-5P(c)
r1326	5	NADPH(c) $\rightleftharpoons$ NADPH-redox-potential(c) + NADP+(c)
r0687	1	ATP(c) + N-Formyl-GAR(c) + Glutamine(c) + H2O(c) $\rightleftharpoons$ ADP(c) + Pi(c) + Glutamate(c) + 5-Phosphoribosylformylglycinamidine(c)
r0253	1	GAP(c) + Pi(c) + NAD+(c) $\rightleftharpoons$ 1,3DPG(c) + NADH(c)
r0255	0.33	Fructose-6P(c) + GAP(c) $\rightleftharpoons$ D-Xylulose-5P(c) + Erythrose-4P(c)
r0104	-1	OAA(c) + NADH(c) $\rightleftharpoons$ NAD+(c) + Malate(c)
r0256	0.67	Fructose-1,6PP(c) $\rightleftharpoons$ DHAP(c) + GAP(c)
r0257	-1	Glutamine(c) + PRPP(c) + H2O(c) $\rightleftharpoons$ 5-Phosphoribosylamine(c) + PPi(c) + Glutamate(c)
r0258	-1	Fumarate(c) + H2O(c) $\rightleftharpoons$ Malate(c)
r0273	-1	IMP(c) + H2O(c) $\rightleftharpoons$ FAICAR(c)
r0109	1	AKG(c) + Aspartate(c) $\rightleftharpoons$ OAA(c) + Glutamate(c)
r0700	1	10-Formyl-THF(c) + AICAR(c) $\rightleftharpoons$ THF(c) + FAICAR(c)

XMP (506)

Definition			Solution												
			exchanges		reactions								transp		
Objective	Constraints	Comment	imports	exports	c	m	r	p	l	n	s	b	s-c	b-c	int
Xanthosine-5P(c)	−Glutamine(c) +Glutamate(c) −Aspartate(c) MCES	de novo synthesis of cytosolic Xanthosine-5P	0.33 H_2O(s) 2 Aspartate(c) 2 Glutamine(c) 1.33 Glucose-6P(c) 5.67 ATP-energy(c) 1 THF-activated methyl group(c)	0.33 P_i(c) 1 CO_2(s) 2 Pyruvate(c) 2 Glutamate(c) 1 Xanthosine-5P(c) 2 NADH-redox-potential(c) 5 NADPH-redox-potential(c)	40	-	-	-	-	-	-	-	2	-	-

ID	flux	reaction
r0406	-0.33	Fructose-6P(c) + Erythrose-4P(c) ⇌ Sedoheptulose-7P(c) + GAP(c)
r0293	2	5,10-Methylene-THF(c) + NADP+(c) ⇌ 5,10-Methenyl-THF(c) + NADPH(c)
r0705	1	ATP(c) + 5-Phosphoribosyl-4-carboxy-5-aminoimidazole(c) + Aspartate(c) ⇌ ADP(c) + Pi(c) + SAICAR(c)
r0296	1	5-Methyl-THF(c) + NADP+(c) ⇌ 5,10-Methylene-THF(c) + NADPH(c)
r0030	1	ATP(c) + AMP(c) ⇌ 2 ADP(c)
r0337	-1	ADP(c) + 1,3DPG(c) ⇌ ATP(c) + 3PG(c)
r0338	1	3PG(c) + NAD+(c) → 3-Phosphonooxypyruvate(c) + NADH(c)
r0058	2	Malate(c) + NADP+(c) ⇌ Pyruvate(c) + CO2(c) + NADPH(c)
r0370	0.33	Sedoheptulose-7P(c) + GAP(c) ⇌ Ribose-5P(c) + D-Xylulose-5P(c)
r0371	2	5,10-Methenyl-THF(c) + H2O(c) ⇌ 10-Formyl-THF(c)
r0659	1	ATP(c) + 5-Phosphoribosylamine(c) + Glycine(c) ⇌ ADP(c) + Pi(c) + GAR(c)
r0243	-0.67	DHAP(c) ⇌ GAP(c)
r0677	1	10-Formyl-THF(c) + GAR(c) ⇌ THF(c) + N-Formyl-GAR(c)
r1319	-5.67	ATP(c) + H2O(c) ⇌ ADP(c) + Pi(c) + ATP-energy(c)
r0247	1	ATP(c) + Ribose-5P(c) → AMP(c) + PRPP(c)
r0699	1	SAICAR(c) ⇌ Fumarate(c) + AICAR(c)
r0849	0.33	H2O(s) ⇌ H2O(c)
r0867	1	CO2(c) ⇌ CO2(s)
r0007	1	PPi(c) + H2O(c) → 2 Pi(c)
r0736	0.67	ATP(c) + Fructose-6P(c) → ADP(c) + Fructose-1,6PP(c)
r0159	1	3-Phosphoserine(c) + H2O(c) → Serine(c) + Pi(c)
r0192	1.33	Glucose-6P(c) ⇌ Fructose-6P(c)
r0344	-0.67	D-Xylulose-5P(c) ⇌ Ribulose-5P(c)
r2676	-1	5-Methyl-THF(c) ⇌ THF(c) + THF-activated methyl group(c)
r0663	-1	3-Phosphonooxypyruvate(c) + Glutamate(c) → 3-Phosphoserine(c) + AKG(c)
r0230	-1	THF(c) + Serine(c) ⇌ 5,10-Methylene-THF(c) + Glycine(c) + H2O(c)
r0666	1	ATP(c) + 5-Phosphoribosylformylglycinamidine(c) ⇌ ADP(c) + Pi(c) + AIR(c)
r0667	-1	5-Phosphoribosyl-4-carboxy-5-aminoimidazole(c) ⇌ AIR(c) + CO2(c)
r1322	2	NADH(c) ⇌ NAD+(c) + NADH-redox-potential(c)
r0250	-0.67	Ribulose-5P(c) ⇌ Ribose-5P(c)
r1326	5	NADPH(c) ⇌ NADPH-redox-potential(c) + NADP+(c)
r0687	1	ATP(c) + N-Formyl-GAR(c) + Glutamine(c) + H2O(c) ⇌ ADP(c) + Pi(c) + Glutamate(c) + 5-Phosphoribosylformylglycinamidine(c)
r0253	1	GAP(c) + Pi(c) + NAD+(c) ⇌ 1,3DPG(c) + NADH(c)
r0255	0.33	Fructose-6P(c) + GAP(c) ⇌ D-Xylulose-5P(c) + Erythrose-4P(c)
r0104	-1	OAA(c) + NADH(c) ⇌ NAD+(c) + Malate(c)
r0256	0.67	Fructose-1,6PP(c) ⇌ DHAP(c) + GAP(c)
r0257	-1	Glutamine(c) + PRPP(c) + H2O(c) ⇌ 5-Phosphoribosylamine(c) + PPi(c) + Glutamate(c)
r0258	-1	Fumarate(c) + H2O(c) ⇌ Malate(c)
r0273	-1	IMP(c) + H2O(c) ⇌ FAICAR(c)
r0274	1	IMP(c) + NAD+(c) + H2O(c) → Xanthosine-5P(c) + NADH(c)
r0109	1	AKG(c) + Aspartate(c) ⇌ OAA(c) + Glutamate(c)
r0700	1	10-Formyl-THF(c) + AICAR(c) ⇌ THF(c) + FAICAR(c)

Uracil (507)

Definition			Solution												
			exchanges		reactions								transp		
Objective	Constraints	Comment	imports	exports	c	m	r	p	l	n	s	b	s-c	b-c	int
Uracil(c)	−Glutamine(c)<br>+Glutamate(c)<br>−Aspartate(c)<br>MCES	de novo synthesis of cytosolic Uracil	0.5 O_2(s) 1 Aspartate(c) 1 Glutamine(c) 3 ATP-energy(c)	2 H_2O(s) 1 Glutamate(c) 1 Uracil(c) 6 Proton-gradient(m)	14	-	-	-	-	-	-	-	2	-	6

ID	flux	reaction
r0235	1	Orotidine-5P(c) ⇌ CO2(c) + UMP(c)
r0416	1	Ubiquinone(m) + (S)-Dihydroorotate(c) → Orotate(c) + Ubiquinol(m)
r0155	1	2 ATP(c) + Glutamine(c) + H2O(c) + HCO3-(c) → 2 ADP(c) + Pi(c) + Glutamate(c) + Carbamoyl-P(c)
r0507	1	Ubiquinol(m) + 2 Ferricytochrome C(m) + 4 H+(PG)(m) → Ubiquinone(m) + 4 H+(PG)(c) + 2 Ferrocytochrome C(m)
r0417	-1	PPi(c) + Orotidine-5P(c) ⇌ Orotate(c) + PRPP(c)
r1417	-1	CO2(c) + H2O(c) ⇌ Carbonate(c)
r0418	1	Uridine(c) + Pi(c) ⇌ Uracil(c) + Ribose-1P(c)
r0328	1	Carbamoyl-P(c) + Aspartate(c) → Pi(c) + N-Carbamoyl-L-aspartate(c)
r0247	1	ATP(c) + Ribose-5P(c) → AMP(c) + PRPP(c)
r1319	-3	ATP(c) + H2O(c) ⇌ ADP(c) + Pi(c) + ATP-energy(c)
r0851	1	H2O(m) ⇌ H2O(c)
r1419	1	Carbonate(c) ⇌ HCO3-(c)
r0030	1	ATP(c) + AMP(c) ⇌ 2 ADP(c)
r0467	-1	H2O(c) + (S)-Dihydroorotate(c) ← N-Carbamoyl-L-aspartate(c)
r0855	0.5	O2(s) ⇌ O2(c)
r0856	-0.5	O2(m) ⇌ O2(c)
r1330	6	H+(PG)(c) ⇌ H+(PG)(m) + Proton-gradient(m)
r0007	1	PPi(c) + H2O(c) → 2 Pi(c)
r1115	-1	ADP(c) + UMP(c) ⇌ ATP(c) + Uridine(c)
r0849	-2	H2O(s) ⇌ H2O(c)
r0251	1	Ribose-1P(c) ⇌ Ribose-5P(c)
r0017	0.5	O2(m) + 4 H+(PG)(m) + 4 Ferrocytochrome C(m) → 4 Ferricytochrome C(m) + 2 H2O(m) + 4 H+(PG)(c)

Uridine (508)

Definition			Solution												
			exchanges		reactions								transp		
Objective	Constraints	Comment	imports	exports	c	m	r	p	l	n	s	b	s-c	b-c	int
Uridine(c)	−Glutamine(c)<br>+Glutamate(c)<br>−Aspartate(c)<br>MCES	de novo synthesis of cytosolic Uridine	0.5 O_2(s) 1 Aspartate(c) 1 Glutamine(c) 0.83 Glucose-6P(c) 3.17 ATP-energy(c)	2.17 H_2O(s) 0.83 P_i(c) 1 Glutamate(c) 1 Uridine(c) 6 Proton-gradient(m)	21	-	-	-	-	-	-	-	2	-	6

ID	flux	reaction
r0406	-0.33	Fructose-6P(c) + Erythrose-4P(c) ⇌ Sedoheptulose-7P(c) + GAP(c)
r0370	0.33	Sedoheptulose-7P(c) + GAP(c) ⇌ Ribose-5P(c) + D-Xylulose-5P(c)
r0235	1	Orotidine-5P(c) ⇌ CO2(c) + UMP(c)
r0416	1	Ubiquinone(m) + (S)-Dihydroorotate(c) → Orotate(c) + Ubiquinol(m)
r0344	-0.67	D-Xylulose-5P(c) ⇌ Ribulose-5P(c)
r0155	1	2 ATP(c) + Glutamine(c) + H2O(c) + HCO3-(c) → 2 ADP(c) + Pi(c) + Glutamate(c) + Carbamoyl-P(c)
r0507	1	Ubiquinol(m) + 2 Ferricytochrome C(m) + 4 H+(PG)(m) → Ubiquinone(m) + 4 H+(PG)(c) + 2 Ferrocytochrome C(m)
r0417	-1	PPi(c) + Orotidine-5P(c) ⇌ Orotate(c) + PRPP(c)
r0255	0.33	Fructose-6P(c) + GAP(c) ⇌ D-Xylulose-5P(c) + Erythrose-4P(c)
r0192	0.83	Glucose-6P(c) ⇌ Fructose-6P(c)
r1417	-1	CO2(c) + H2O(c) ⇌ Carbonate(c)
r0328	1	Carbamoyl-P(c) + Aspartate(c) → Pi(c) + N-Carbamoyl-L-aspartate(c)
r0256	0.17	Fructose-1,6PP(c) ⇌ DHAP(c) + GAP(c)

Continued on next page

ID	flux	reaction
r0247	1	ATP(c) + Ribose-5P(c) → AMP(c) + PRPP(c)
r1319	-3.17	ATP(c) + H2O(c) ⇌ ADP(c) + Pi(c) + ATP-energy(c)
r0851	1	H2O(m) ⇌ H2O(c)
r1419	1	Carbonate(c) ⇌ HCO3-(c)
r0736	0.17	ATP(c) + Fructose-6P(c) → ADP(c) + Fructose-1,6PP(c)
r0030	1	ATP(c) + AMP(c) ⇌ 2 ADP(c)
r0467	-1	H2O(c) + (S)-Dihydroorotate(c) ← N-Carbamoyl-L-aspartate(c)
r0855	0.5	O2(s) ⇌ O2(c)
r0856	-0.5	O2(m) ⇌ O2(c)
r1330	6	H+(PG)(c) ⇌ H+(PG)(m) + Proton-gradient(m)
r0250	-0.67	Ribulose-5P(c) ⇌ Ribose-5P(c)
r0007	1	PPi(c) + H2O(c) → 2 Pi(c)
r1115	-1	ADP(c) + UMP(c) ⇌ ATP(c) + Uridine(c)
r0849	-2.17	H2O(s) ⇌ H2O(c)
r0017	0.5	O2(m) + 4 H+(PG)(m) + 4 Ferrocytochrome C(m) → 4 Ferricytochrome C(m) + 2 H2O(m) + 4 H+(PG)(c)
r0243	-0.17	DHAP(c) ⇌ GAP(c)

Cytidine (509)

Definition			Solution												
			exchanges		reactions								transp		
Objective	Constraints	Comment	imports	exports	c	m	r	p	l	n	s	b	s-c	b-c	int
Cytidine(c)	−Glutamine(c) +Glutamate(c) −Aspartate(c) $-NH_3$(c) MCES	de novo synthesis of cytosolic Cytidine	0.5 O_2(s) 1 Aspartate(c) 2 Glutamine(c) 0.83 Glucose-6P(c) 5.17 ATP-energy(c)	2.17 H_2O(s) 0.83 P_i(c) 2 Glutamate(c) 1 Cytidine(c) 6 Proton-gradient(m)	26	-	-	-	-	-	-	-	2	-	6

ID	flux	reaction
r0406	-0.33	Fructose-6P(c) + Erythrose-4P(c) ⇌ Sedoheptulose-7P(c) + GAP(c)
r0370	0.33	Sedoheptulose-7P(c) + GAP(c) ⇌ Ribose-5P(c) + D-Xylulose-5P(c)
r0235	1	Orotidine-5P(c) ⇌ CO2(c) + UMP(c)
r0154	1	ATP(c) + UTP(c) + Glutamine(c) + H2O(c) → ADP(c) + Pi(c) + Glutamate(c) + CTP(c)
r0136	-1	ATP(c) + CMP(c) ⇌ ADP(c) + CDP(c)
r0037	1	ATP(c) + UDP(c) ⇌ ADP(c) + UTP(c)
r0416	1	Ubiquinone(m) + (S)-Dihydroorotate(c) → Orotate(c) + Ubiquinol(m)
r0344	-0.67	D-Xylulose-5P(c) ⇌ Ribulose-5P(c)
r0155	1	2 ATP(c) + Glutamine(c) + H2O(c) + HCO3-(c) → 2 ADP(c) + Pi(c) + Glutamate(c) + Carbamoyl-P(c)
r0038	1	ATP(c) + UMP(c) ⇌ ADP(c) + UDP(c)
r0507	1	Ubiquinol(m) + 2 Ferricytochrome C(m) + 4 H+(PG)(m) → Ubiquinone(m) + 4 H+(PG)(c) + 2 Ferrocytochrome C(m)
r0417	-1	PPi(c) + Orotidine-5P(c) ⇌ Orotate(c) + PRPP(c)
r0255	0.33	Fructose-6P(c) + GAP(c) ⇌ D-Xylulose-5P(c) + Erythrose-4P(c)
r0192	0.83	Glucose-6P(c) ⇌ Fructose-6P(c)
r1417	-1	CO2(c) + H2O(c) ⇌ Carbonate(c)
r0328	1	Carbamoyl-P(c) + Aspartate(c) → Pi(c) + N-Carbamoyl-L-aspartate(c)
r0256	0.17	Fructose-1,6PP(c) ⇌ DHAP(c) + GAP(c)
r0247	1	ATP(c) + Ribose-5P(c) → AMP(c) + PRPP(c)
r1319	-5.17	ATP(c) + H2O(c) ⇌ ADP(c) + Pi(c) + ATP-energy(c)
r0851	1	H2O(m) ⇌ H2O(c)
r1419	1	Carbonate(c) ⇌ HCO3-(c)
r0736	0.17	ATP(c) + Fructose-6P(c) → ADP(c) + Fructose-1,6PP(c)
r0030	1	ATP(c) + AMP(c) ⇌ 2 ADP(c)
r0467	-1	H2O(c) + (S)-Dihydroorotate(c) ← N-Carbamoyl-L-aspartate(c)
r0855	0.5	O2(s) ⇌ O2(c)
r0856	-0.5	O2(m) ⇌ O2(c)

Continued on next page

ID	flux	reaction
r0150	-1	ATP(c) + CDP(c) ⇌ ADP(c) + CTP(c)
r1330	6	H+(PG)(c) ⇌ H+(PG)(m) + Proton-gradient(m)
r0250	-0.67	Ribulose-5P(c) ⇌ Ribose-5P(c)
r0007	1	PPi(c) + H2O(c) → 2 Pi(c)
r0849	-2.17	H2O(s) ⇌ H2O(c)
r0134	1	CMP(c) + H2O(c) → Cytidine(c) + Pi(c)
r0017	0.5	O2(m) + 4 H+(PG)(m) + 4 Ferrocytochrome C(m) → 4 Ferricytochrome C(m) + 2 H2O(m) + 4 H+(PG)(c)
r0243	-0.17	DHAP(c) ⇌ GAP(c)

Xanthine (510)

Definition			Solution												
			exchanges		reactions								transp		
Objective	Constraints	Comment	imports	exports	c	m	r	p	l	n	s	b	s-c	b-c	int
Xanthine(c)	−Glutamine(c) +Glutamate(c) −Aspartate(c) MCES	de novo synthesis of cytosolic Xanthine	1.5 H_2O(s) 2 Aspartate(c) 2 Glutamine(c) 0.5 Glucose-6P(c) 5.5 ATP-energy(c) 1 THF-activated methyl group(c)	0.5 P_i(c) 1 CO_2(s) 2 Pyruvate(c) 2 Glutamate(c) 1 Xanthine(c) 3 NADH-redox-potential(c) 4 NADPH-redox-potential(c)	38	-	-	-	-	-	-	-	2	-	-

ID	flux	reaction
r0293	2	5,10-Methylene-THF(c) + NADP+(c) ⇌ 5,10-Methenyl-THF(c) + NADPH(c)
r0705	1	ATP(c) + 5-Phosphoribosyl-4-carboxy-5-aminoimidazole(c) + Aspartate(c) ⇌ ADP(c) + Pi(c) + SAICAR(c)
r0030	1	ATP(c) + AMP(c) ⇌ 2 ADP(c)
r0337	-1	ADP(c) + 1,3DPG(c) ⇌ ATP(c) + 3PG(c)
r0338	1	3PG(c) + NAD+(c) → 3-Phosphonooxypyruvate(c) + NADH(c)
r0058	2	Malate(c) + NADP+(c) ⇌ Pyruvate(c) + CO2(c) + NADPH(c)
r0371	2	5,10-Methenyl-THF(c) + H2O(c) ⇌ 10-Formyl-THF(c)
r0659	1	ATP(c) + 5-Phosphoribosylamine(c) + Glycine(c) ⇌ ADP(c) + Pi(c) + GAR(c)
r0521	1	Xanthosine(c) + Pi(c) ⇌ Xanthine(c) + Ribose-1P(c)
r0243	-0.5	DHAP(c) ⇌ GAP(c)
r0677	1	10-Formyl-THF(c) + GAR(c) ⇌ THF(c) + N-Formyl-GAR(c)
r1319	-5.5	ATP(c) + H2O(c) ⇌ ADP(c) + Pi(c) + ATP-energy(c)
r0247	1	ATP(c) + Ribose-5P(c) → AMP(c) + PRPP(c)
r0699	1	SAICAR(c) ⇌ Fumarate(c) + AICAR(c)
r0566	1	Xanthosine-5P(c) + H2O(c) → Xanthosine(c) + Pi(c)
r0849	1.5	H2O(s) ⇌ H2O(c)
r0867	1	CO2(c) ⇌ CO2(s)
r0007	1	PPi(c) + H2O(c) → 2 Pi(c)
r0736	0.5	ATP(c) + Fructose-6P(c) → ADP(c) + Fructose-1,6PP(c)
r0159	1	3-Phosphoserine(c) + H2O(c) → Serine(c) + Pi(c)
r0192	0.5	Glucose-6P(c) ⇌ Fructose-6P(c)
r0792	1	5-Methyl-THF(c) + NAD+(c) ⇌ 5,10-Methylene-THF(c) + NADH(c)
r2676	-1	5-Methyl-THF(c) ⇌ THF(c) + THF-activated methyl group(c)
r0663	-1	3-Phosphonooxypyruvate(c) + Glutamate(c) → 3-Phosphoserine(c) + AKG(c)
r0230	-1	THF(c) + Serine(c) ⇌ 5,10-Methylene-THF(c) + Glycine(c) + H2O(c)
r0666	1	ATP(c) + 5-Phosphoribosylformylglycinamidine(c) ⇌ ADP(c) + Pi(c) + AIR(c)
r0667	-1	5-Phosphoribosyl-4-carboxy-5-aminoimidazole(c) ⇌ AIR(c) + CO2(c)
r1322	3	NADH(c) ⇌ NAD+(c) + NADH-redox-potential(c)
r0251	1	Ribose-1P(c) ⇌ Ribose-5P(c)
r1326	4	NADPH(c) ⇌ NADPH-redox-potential(c) + NADP+(c)
r0687	1	ATP(c) + N-Formyl-GAR(c) + Glutamine(c) + H2O(c) ⇌ ADP(c) + Pi(c) + Glutamate(c) + 5-Phosphoribosylformylglycinamidine(c)
r0253	1	GAP(c) + Pi(c) + NAD+(c) ⇌ 1,3DPG(c) + NADH(c)
r0104	-1	OAA(c) + NADH(c) ⇌ NAD+(c) + Malate(c)
r0256	0.5	Fructose-1,6PP(c) ⇌ DHAP(c) + GAP(c)

Continued on next page

ID	flux	reaction
r0257	-1	Glutamine(c) + PRPP(c) + H2O(c) ⇌ 5-Phosphoribosylamine(c) + PPi(c) + Glutamate(c)
r0258	-1	Fumarate(c) + H2O(c) ⇌ Malate(c)
r0273	-1	IMP(c) + H2O(c) ⇌ FAICAR(c)
r0274	1	IMP(c) + NAD+(c) + H2O(c) → Xanthosine-5P(c) + NADH(c)
r0109	1	AKG(c) + Aspartate(c) ⇌ OAA(c) + Glutamate(c)
r0700	1	10-Formyl-THF(c) + AICAR(c) ⇌ THF(c) + FAICAR(c)

Guanine (511)

Definition			Solution												
			exchanges		reactions								transp		
Objective	Constraints	Comment	imports	exports	c	m	r	p	l	n	s	b	s-c	b-c	int
Guanine(c)	−Glutamine(c) +Glutamate(c) −Aspartate(c) MCES	de novo synthesis of cytosolic Guanine	1.5 H_2O(s) 2 Aspartate(c) 3 Glutamine(c) 0.5 Glucose-6P(c) 7.5 ATP-energy(c) 1 THF-activated methyl group(c)	0.5 P_i(c) 1 CO_2(s) 2 Pyruvate(c) 3 Glutamate(c) 1 Guanine(c) 2 NADH-redox-potential(c) 5 NADPH-redox-potential(c)	39	-	-	-	-	-	-	-	2	-	-

ID	flux	reaction
r0293	2	5,10-Methylene-THF(c) + NADP+(c) ⇌ 5,10-Methenyl-THF(c) + NADPH(c)
r0705	1	ATP(c) + 5-Phosphoribosyl-4-carboxy-5-aminoimidazole(c) + Aspartate(c) ⇌ ADP(c) + Pi(c) + SAICAR(c)
r0296	1	5-Methyl-THF(c) + NADP+(c) ⇌ 5,10-Methylene-THF(c) + NADPH(c)
r0297	1	GMP(c) + H2O(c) → Pi(c) + Guanosine(c)
r0030	2	ATP(c) + AMP(c) ⇌ 2 ADP(c)
r0337	-1	ADP(c) + 1,3DPG(c) ⇌ ATP(c) + 3PG(c)
r0338	1	3PG(c) + NAD+(c) → 3-Phosphonooxypyruvate(c) + NADH(c)
r0058	2	Malate(c) + NADP+(c) ⇌ Pyruvate(c) + CO2(c) + NADPH(c)
r0371	2	5,10-Methenyl-THF(c) + H2O(c) ⇌ 10-Formyl-THF(c)
r0506	1	Pi(c) + Guanosine(c) ⇌ Guanine(c) + Ribose-1P(c)
r0659	1	ATP(c) + 5-Phosphoribosylamine(c) + Glycine(c) ⇌ ADP(c) + Pi(c) + GAR(c)
r0243	-0.5	DHAP(c) ⇌ GAP(c)
r0677	1	10-Formyl-THF(c) + GAR(c) ⇌ THF(c) + N-Formyl-GAR(c)
r1319	-7.5	ATP(c) + H2O(c) ⇌ ADP(c) + Pi(c) + ATP-energy(c)
r0247	1	ATP(c) + Ribose-5P(c) → AMP(c) + PRPP(c)
r0699	1	SAICAR(c) ⇌ Fumarate(c) + AICAR(c)
r0849	1.5	H2O(s) ⇌ H2O(c)
r0867	1	CO2(c) ⇌ CO2(s)
r0007	2	PPi(c) + H2O(c) → 2 Pi(c)
r0302	1	ATP(c) + Xanthosine-5P(c) + Glutamine(c) + H2O(c) → AMP(c) + PPi(c) + GMP(c) + Glutamate(c)
r0736	0.5	ATP(c) + Fructose-6P(c) → ADP(c) + Fructose-1,6PP(c)
r0159	1	3-Phosphoserine(c) + H2O(c) → Serine(c) + Pi(c)
r0192	0.5	Glucose-6P(c) ⇌ Fructose-6P(c)
r2676	-1	5-Methyl-THF(c) ⇌ THF(c) + THF-activated methyl group(c)
r0663	-1	3-Phosphonooxypyruvate(c) + Glutamate(c) → 3-Phosphoserine(c) + AKG(c)
r0230	-1	THF(c) + Serine(c) ⇌ 5,10-Methylene-THF(c) + Glycine(c) + H2O(c)
r0666	1	ATP(c) + 5-Phosphoribosylformylglycinamidine(c) ⇌ ADP(c) + Pi(c) + AIR(c)
r0667	-1	5-Phosphoribosyl-4-carboxy-5-aminoimidazole(c) ⇌ AIR(c) + CO2(c)
r1322	2	NADH(c) ⇌ NAD+(c) + NADH-redox-potential(c)
r0251	1	Ribose-1P(c) ⇌ Ribose-5P(c)
r1326	5	NADPH(c) ⇌ NADPH-redox-potential(c) + NADP+(c)
r0687	1	ATP(c) + N-Formyl-GAR(c) + Glutamine(c) + H2O(c) ⇌ ADP(c) + Pi(c) + Glutamate(c) + 5-Phosphoribosylformylglycinamidine(c)
r0253	1	GAP(c) + Pi(c) + NAD+(c) ⇌ 1,3DPG(c) + NADH(c)
r0104	-1	OAA(c) + NADH(c) ⇌ NAD+(c) + Malate(c)
r0256	0.5	Fructose-1,6PP(c) ⇌ DHAP(c) + GAP(c)
r0257	-1	Glutamine(c) + PRPP(c) + H2O(c) ⇌ 5-Phosphoribosylamine(c) + PPi(c) + Glutamate(c)

Continued on next page

ID	flux	reaction
r0258	-1	Fumarate(c) + H2O(c) ⇌ Malate(c)
r0273	-1	IMP(c) + H2O(c) ⇌ FAICAR(c)
r0274	1	IMP(c) + NAD+(c) + H2O(c) → Xanthosine-5P(c) + NADH(c)
r0109	1	AKG(c) + Aspartate(c) ⇌ OAA(c) + Glutamate(c)
r0700	1	10-Formyl-THF(c) + AICAR(c) ⇌ THF(c) + FAICAR(c)

Adenosine (512)

Definition			Solution												
			exchanges		reactions								transp		
Objective	Constraints	Comment	imports	exports	c	m	r	p	l	n	s	b	s-c	b-c	int
Adenosine(c)	−Glutamine(c) +Glutamate(c) −Aspartate(c) MCES	de novo synthesis of cytosolic Guanine	0.33 H_2O(s) 3 Aspartate(c) 2 Glutamine(c) 1.33 Glucose-6P(c) 5.67 ATP-energy(c) 1 THF-activated methyl group(c)	1.33 P_i(c) 2 CO_2(s) 3 Pyruvate(c) 2 Glutamate(c) 1 Adenosine(c) 1 NADH-redox-potential(c) 6 NADPH-redox-potential(c)	43	-	-	-	-	-	-	-	2	-	-

ID	flux	reaction
r0406	-0.33	Fructose-6P(c) + Erythrose-4P(c) ⇌ Sedoheptulose-7P(c) + GAP(c)
r0277	1	GTP(c) + IMP(c) + Aspartate(c) → GDP(c) + Pi(c) + Adenylosuccinate(c)
r0293	2	5,10-Methylene-THF(c) + NADP+(c) ⇌ 5,10-Methenyl-THF(c) + NADPH(c)
r0705	1	ATP(c) + 5-Phosphoribosyl-4-carboxy-5-aminoimidazole(c) + Aspartate(c) ⇌ ADP(c) + Pi(c) + SAICAR(c)
r0296	1	5-Methyl-THF(c) + NADP+(c) ⇌ 5,10-Methylene-THF(c) + NADPH(c)
r0030	1	ATP(c) + AMP(c) ⇌ 2 ADP(c)
r0337	-1	ADP(c) + 1,3DPG(c) ⇌ ATP(c) + 3PG(c)
r0338	1	3PG(c) + NAD+(c) → 3-Phosphonooxypyruvate(c) + NADH(c)
r0058	3	Malate(c) + NADP+(c) ⇌ Pyruvate(c) + CO2(c) + NADPH(c)
r0370	0.33	Sedoheptulose-7P(c) + GAP(c) ⇌ Ribose-5P(c) + D-Xylulose-5P(c)
r0371	2	5,10-Methenyl-THF(c) + H2O(c) ⇌ 10-Formyl-THF(c)
r0659	1	ATP(c) + 5-Phosphoribosylamine(c) + Glycine(c) ⇌ ADP(c) + Pi(c) + GAR(c)
r0099	1	ATP(c) + GDP(c) ⇌ ADP(c) + GTP(c)
r0243	-0.67	DHAP(c) ⇌ GAP(c)
r0677	1	10-Formyl-THF(c) + GAR(c) ⇌ THF(c) + N-Formyl-GAR(c)
r1319	-5.67	ATP(c) + H2O(c) ⇌ ADP(c) + Pi(c) + ATP-energy(c)
r0260	1	Adenylosuccinate(c) → Fumarate(c) + AMP(c)
r0247	1	ATP(c) + Ribose-5P(c) → AMP(c) + PRPP(c)
r0699	1	SAICAR(c) ⇌ Fumarate(c) + AICAR(c)
r0849	0.33	H2O(s) ⇌ H2O(c)
r0867	2	CO2(c) ⇌ CO2(s)
r0736	0.67	ATP(c) + Fructose-6P(c) → ADP(c) + Fructose-1,6PP(c)
r0007	1	PPi(c) + H2O(c) → 2 Pi(c)
r0159	1	3-Phosphoserine(c) + H2O(c) → Serine(c) + Pi(c)
r0192	1.33	Glucose-6P(c) ⇌ Fructose-6P(c)
r0048	-1	ADP(c) + AMP(c) ⇌ ATP(c) + Adenosine(c)
r0344	-0.67	D-Xylulose-5P(c) ⇌ Ribulose-5P(c)
r2676	-1	5-Methyl-THF(c) ⇌ THF(c) + THF-activated methyl group(c)
r0663	-1	3-Phosphonooxypyruvate(c) + Glutamate(c) → 3-Phosphoserine(c) + AKG(c)
r0230	-1	THF(c) + Serine(c) ⇌ 5,10-Methylene-THF(c) + Glycine(c) + H2O(c)
r0666	1	ATP(c) + 5-Phosphoribosylformylglycinamidine(c) ⇌ ADP(c) + Pi(c) + AIR(c)
r0667	-1	5-Phosphoribosyl-4-carboxy-5-aminoimidazole(c) ⇌ AIR(c) + CO2(c)
r1322	1	NADH(c) ⇌ NAD+(c) + NADH-redox-potential(c)
r0250	-0.67	Ribulose-5P(c) ⇌ Ribose-5P(c)
r1326	6	NADPH(c) ⇌ NADPH-redox-potential(c) + NADP+(c)
r0687	1	ATP(c) + N-Formyl-GAR(c) + Glutamine(c) + H2O(c) ⇌ ADP(c) + Pi(c) + Glutamate(c) + 5-Phosphoribosylformylglycinamidine(c)
r0253	1	GAP(c) + Pi(c) + NAD+(c) ⇌ 1,3DPG(c) + NADH(c)

Continued on next page

ID	flux	reaction
r0255	0.33	Fructose-6P(c) + GAP(c) ⇌ D-Xylulose-5P(c) + Erythrose-4P(c)
r0104	-1	OAA(c) + NADH(c) ⇌ NAD+(c) + Malate(c)
r0256	0.67	Fructose-1,6PP(c) ⇌ DHAP(c) + GAP(c)
r0257	-1	Glutamine(c) + PRPP(c) + H2O(c) ⇌ 5-Phosphoribosylamine(c) + PPi(c) + Glutamate(c)
r0258	-2	Fumarate(c) + H2O(c) ⇌ Malate(c)
r0273	-1	IMP(c) + H2O(c) ⇌ FAICAR(c)
r0109	1	AKG(c) + Aspartate(c) ⇌ OAA(c) + Glutamate(c)
r0700	1	10-Formyl-THF(c) + AICAR(c) ⇌ THF(c) + FAICAR(c)

UDP-N-acetylgalactosamine (513)

Definition			Solution												
			exchanges		reactions								transp		
Objective	Constraints	Comment	imports	exports	c	m	r	p	l	n	s	b	s-c	b-c	int
UDP-N-acetyl-D-galactosamine(c)	MCES AAA	de novo synthesis of cytosolic UDP-N-acetyl-D-galactosamine	0.33 O_2(s) 0.17 P_i(c) 1 Glutamine(s) 1.83 Glucose-6P(c) 0.33 Proline(s) 1 Asparagine(s) 6.17 ATP-energy(c) 1 CoA-activated acetyl group(c)	3.17 H_2O(s) 1.33 Glutamate(s) 1 UDP-N-acetyl-D-galactosamine(c) 0.33 NADH-redox-potential(m) 1 Proton-gradient(c) 4 Proton-gradient(m) 1.33 Na-gradient(c)	29	4	-	-	-	-	-	-	8	-	6

ID	flux	reaction
r0406	-0.33	Fructose-6P(c) + Erythrose-4P(c) ⇌ Sedoheptulose-7P(c) + GAP(c)
r0855	0.33	O2(s) ⇌ O2(c)
r0856	-0.33	O2(m) ⇌ O2(c)
r0127	1	H2O(c) + Asparagine(c) → Aspartate(c) + NH3(c)
r2479	1	Asparagine(s) + Na+(s) + H+(PG)(c) → Asparagine(c) + Na+(c) + H+(PG)(s)
r0017	0.33	O2(m) + 4 H+(PG)(m) + 4 Ferrocytochrome C(m) → 4 Ferricytochrome C(m) + 2 H2O(m) + 4 H+(PG)(c)
r0030	1	ATP(c) + AMP(c) ⇌ 2 ADP(c)
r0467	-1	H2O(c) + (S)-Dihydroorotate(c) ← N-Carbamoyl-L-aspartate(c)
r0189	-1	Fructose-6P(c) + NH3(c) ⇌ Glucosamine-6P(c) + H2O(c)
r0037	1	ATP(c) + UDP(c) ⇌ ADP(c) + UTP(c)
r0485	1	Glucosamine-6P(c) + Acetyl-CoA(c) → CoA(c) + N-Acetylglucosamine-6P(c)
r0038	1	ATP(c) + UMP(c) ⇌ ADP(c) + UDP(c)
r0911	0.33	Proline(c) + Glutamate(m) ⇌ Proline(m) + Glutamate(c)
r2664	1.33	Na+(c) ⇌ Na+(s) + Na-gradient(c)
r0074	0.33	L-Glutamate 5-semialdehyde(m) + NAD+(m) + H2O(m) ⇌ Glutamate(m) + NADH(m)
r0370	0.33	Sedoheptulose-7P(c) + GAP(c) ⇌ Ribose-5P(c) + D-Xylulose-5P(c)
r2685	1.33	Glutamate(c) ⇌ Glutamate(s)
r0507	0.67	Ubiquinol(m) + 2 Ferricytochrome C(m) + 4 H+(PG)(m) → Ubiquinone(m) + 4 H+(PG)(c) + 2 Ferrocytochrome C(m)
r0243	-0.17	DHAP(c) ⇌ GAP(c)
r1330	4	H+(PG)(c) ⇌ H+(PG)(m) + Proton-gradient(m)
r1331	1	H+(PG)(s) ⇌ H+(PG)(c) + Proton-gradient(c)
r1319	-6.17	ATP(c) + H2O(c) ⇌ ADP(c) + Pi(c) + ATP-energy(c)
r0247	1	ATP(c) + Ribose-5P(c) → AMP(c) + PRPP(c)
r0115	1	UTP(c) + N-Acetylglucosamine-1P(c) → PPi(c) + UDP-N-acetylglucosamine(c)
r0116	1	UDP-N-acetylglucosamine(c) ⇌ UDP-N-acetyl-D-galactosamine(c)
r0849	-3.17	H2O(s) ⇌ H2O(c)
r0416	1	Ubiquinone(m) + (S)-Dihydroorotate(c) → Orotate(c) + Ubiquinol(m)
r0417	-1	PPi(c) + Orotidine-5P(c) ⇌ Orotate(c) + PRPP(c)
r0155	1	2 ATP(c) + Glutamine(c) + H2O(c) + HCO3-(c) → 2 ADP(c) + Pi(c) + Glutamate(c) + Carbamoyl-P(c)
r0007	2	PPi(c) + H2O(c) → 2 Pi(c)
r0736	0.17	ATP(c) + Fructose-6P(c) → ADP(c) + Fructose-1,6PP(c)
r0175	0.33	1-Pyrroline-5-carboxylate(m) + H2O(m) ⇌ L-Glutamate 5-semialdehyde(m)
r0192	1.83	Glucose-6P(c) ⇌ Fructose-6P(c)
r0491	1	N-Acetylglucosamine-6P(c) → N-Acetylglucosamine-1P(c)

Continued on next page

UDP-N-acetylgalactosamine (513)– continued

ID	flux	reaction
r0328	1	Carbamoyl-P(c) + Aspartate(c) → Pi(c) + N-Carbamoyl-L-aspartate(c)
r1417	-1	CO2(c) + H2O(c) ⇌ Carbonate(c)
r0344	-0.67	D-Xylulose-5P(c) ⇌ Ribulose-5P(c)
r1419	1	Carbonate(c) ⇌ HCO3-(c)
r1139	-0.33	Proline(s) + Na+(s) → Proline(c) + Na+(c)
r2677	-1	Acetyl-CoA(c) ⇌ CoA(c) + CoA-activated acetyl group(c)
r2525	1	Glutamine(s) ⇌ Glutamine(c)
r1453	0.33	Proline(m) + Ubiquinol(m) ⇌ 1-Pyrroline-5-carboxylate(m) + Ubiquinone(m)
r0235	1	Orotidine-5P(c) ⇌ CO2(c) + UMP(c)
r1323	0.33	NADH(m) ⇌ NAD+(m) + NADH-redox-potential(m)
r0250	-0.67	Ribulose-5P(c) ⇌ Ribose-5P(c)
r0255	0.33	Fructose-6P(c) + GAP(c) ⇌ D-Xylulose-5P(c) + Erythrose-4P(c)
r0256	0.17	Fructose-1,6PP(c) ⇌ DHAP(c) + GAP(c)

N-acglucam (514)

Definition			Solution												
			exchanges		reactions								transp		
Objective	Constraints	Comment	imports	exports	c	m	r	p	l	n	s	b	s-c	b-c	int
N-Acetylglucosamine(c)	MCES AAA	de novo synthesis of cytosolic N-Acetylglucosamine	1 Serine(s) 1 Glucose-6P(c) 1 ATP-energy(c) 1 CoA-activated acetyl group(c)	1 P_i(c) 1 Pyruvate(c) 1 N-Acetylglucosamine(c)	12	-	-	-	-	-	-	-	1	-	-

ID	flux	reaction
r0037	1	ATP(c) + UDP(c) ⇌ ADP(c) + UTP(c)
r0291	-1	N-Acetyl-D-mannosamine(c) ⇌ N-Acetylglucosamine(c)
r0192	1	Glucose-6P(c) ⇌ Fructose-6P(c)
r1319	-1	ATP(c) + H2O(c) ⇌ ADP(c) + Pi(c) + ATP-energy(c)
r0491	1	N-Acetylglucosamine-6P(c) → N-Acetylglucosamine-1P(c)
r2526	1	Serine(s) ⇌ Serine(c)
r0485	1	Glucosamine-6P(c) + Acetyl-CoA(c) → CoA(c) + N-Acetylglucosamine-6P(c)
r0189	-1	Fructose-6P(c) + NH3(c) ⇌ Glucosamine-6P(c) + H2O(c)
r0114	1	UDP-N-acetylglucosamine(c) + H2O(c) ⇌ N-Acetyl-D-mannosamine(c) + UDP(c)
r0060	1	Serine(c) → Pyruvate(c) + NH3(c)
r0115	1	UTP(c) + N-Acetylglucosamine-1P(c) → PPi(c) + UDP-N-acetylglucosamine(c)
r0007	1	PPi(c) + H2O(c) → 2 Pi(c)
r2677	-1	Acetyl-CoA(c) ⇌ CoA(c) + CoA-activated acetyl group(c)

CMP-N-acetylneuraminate (515)

Definition			Solution												
			exchanges		reactions								transp		
Objective	Constraints	Comment	imports	exports	c	m	r	p	l	n	s	b	s-c	b-c	int
CMP-N-acetylneuraminate(n)	MCES −CMP(c) +Glutamate −Glutamine =Asparagine =Proline =Cystine $=H_2S$ $=NH_3$	Synthesis of CMP-N-acetylneuraminate in nucleus	1 Pyruvate(c) 1 CMP(c) 1 Glutamine(s) 1 Glucose-6P(c) 5 ATP-energy(c) 1 NADPH-redox-potential(c) 1 CoA-activated acetyl group(c)	1 P_i(c) 1 Glutamate(s) 1 CMP-N-acetylneuraminate(n) 1 NADH-redox-potential(c)	21	-	-	-	-	1	-	-	2	-	3

ID	flux	reaction
r0190	1	Glutamine(c) + Fructose-6P(c) → Glutamate(c) + Glucosamine-6P(c)

Continued on next page

ID	flux	reaction
r0136	1	ATP(c) + CMP(c) ⇌ ADP(c) + CDP(c)
r0037	1	ATP(c) + UDP(c) ⇌ ADP(c) + UTP(c)
r1460	1	CTP(c) ⇌ CTP(n)
r1326	-1	NADPH(c) ⇌ NADPH-redox-potential(c) + NADP+(c)
r0192	1	Glucose-6P(c) ⇌ Fructose-6P(c)
r1462	1	N-Acetylneuraminate(c) ⇌ N-Acetylneuraminate(n)
r0058	-1	Pyruvate(c) + CO2(c) + NADPH(c) ⇌ Malate(c) + NADP+(c)
r2525	1	Glutamine(s) ⇌ Glutamine(c)
r1319	-5	ATP(c) + H2O(c) ⇌ ADP(c) + Pi(c) + ATP-energy(c)
r0491	1	N-Acetylglucosamine-6P(c) → N-Acetylglucosamine-1P(c)
r0565	1	ATP(c) + N-Acetyl-D-mannosamine(c) → ADP(c) + N-Acetylmannosamine-6P(c)
r0485	1	Glucosamine-6P(c) + Acetyl-CoA(c) → CoA(c) + N-Acetylglucosamine-6P(c)
r0269	1	CTP(n) + N-Acetylneuraminate(n) ⇌ PPi(n) + CMP-N-acetylneuraminate(n)
r0684	-1	PEP(c) + H2O(c) + N-Acetylmannosamine-6P(c) → N-Acetylneuraminate-9P(c) + Pi(c)
r0401	1	N-Acetylneuraminate-9P(c) + H2O(c) → Pi(c) + N-Acetylneuraminate(c)
r0104	1	NAD+(c) + Malate(c) ⇌ OAA(c) + NADH(c)
r0099	1	ATP(c) + GDP(c) ⇌ ADP(c) + GTP(c)
r1459	-1	PPi(c) ⇌ PPi(n)
r0150	1	ATP(c) + CDP(c) ⇌ ADP(c) + CTP(c)
r0123	1	GTP(c) + OAA(c) → GDP(c) + PEP(c) + CO2(c)
r0114	1	UDP-N-acetylglucosamine(c) + H2O(c) ⇌ N-Acetyl-D-mannosamine(c) + UDP(c)
r0115	1	UTP(c) + N-Acetylglucosamine-1P(c) → PPi(c) + UDP-N-acetylglucosamine(c)
r0007	2	PPi(c) + H2O(c) → 2 Pi(c)
r2685	1	Glutamate(c) ⇌ Glutamate(s)
r1322	1	NADH(c) ⇌ NAD+(c) + NADH-redox-potential(c)
r2677	-1	Acetyl-CoA(c) ⇌ CoA(c) + CoA-activated acetyl group(c)

4.3 C3. Constituents of cellular structures

C3.1. Lipids

PC (516)

Definition			Solution												
			exchanges		reactions								transp		
Objective	Constraints	Comment	imports	exports	c	m	r	p	l	n	s	b	s-c	b-c	int
PC-VLDL-pool(c)	MCES ALFA	de novo synthesis of cytosolic PC-VLDL-pool	0.5 P_i(c) 0.5 Glucose-6P(c) 1 Choline(c) 0.17 Arachidonate(c) 0.7 Palmitate(c) 0.27 Oleate(c) 0.3 Stearate(c) 0.55 Linoleate(c) 7.5 ATP-energy(c) 1 NADH-redox-potential(c)	3.5 H_2O(s) 1 PC-VLDL-pool(c)	28	-	-	-	-	-	-	-	1	-	-

ID	flux	reaction
r0244	1	ATP(c) + Choline(c) → ADP(c) + Phosphocholine(c)
r0136	1	ATP(c) + CMP(c) ⇌ ADP(c) + CDP(c)
r1190	0.08	Arachidonyl-CoA(c) + sn-Glycerol-3P(c) → 1-Acylglycerol-3P-arach(c) + CoA(c)
r1263	0.17	Arachidonate(c) + ATP(c) + CoA(c) → Arachidonyl-CoA(c) + AMP(c) + PPi(c)
r1254	0.3	Stearate(c) + ATP(c) + CoA(c) → Stearoyl-CoA(c) + AMP(c) + PPi(c)
r1218	1	Phosphatidate-VLDL-PC-pool(c) + H2O(c) → 1,2-Diacylglycerol-VLDL-PC-pool(c) + Pi(c)
r0192	0.5	Glucose-6P(c) ⇌ Fructose-6P(c)
r1237	1	CDP-choline(c) + 1,2-Diacylglycerol-VLDL-PC-pool(c) → PC-VLDL-pool(c) + CMP(c)
r0256	0.5	Fructose-1,6PP(c) ⇌ DHAP(c) + GAP(c)
r1319	-7.5	ATP(c) + H2O(c) ⇌ ADP(c) + Pi(c) + ATP-energy(c)
r1256	0.27	ATP(c) + CoA(c) + Oleate(c) → Oleoyl-CoA(c) + AMP(c) + PPi(c)
r1185	0.35	Palmitoyl-CoA(c) + sn-Glycerol-3P(c) → CoA(c) + 1-Acylglycerol-3P-palm(c)

Continued on next page

PC (516)– continued

ID	flux	reaction
r1258	0.55	Linoleate(c) + ATP(c) + CoA(c) → AMP(c) + PPi(c) + Linoleoyl-CoA(c)
r0736	0.5	ATP(c) + Fructose-6P(c) → ADP(c) + Fructose-1,6PP(c)
r0201	-1	DHAP(c) + NADH(c) ⇌ NAD+(c) + sn-Glycerol-3P(c)
r0030	2	ATP(c) + AMP(c) ⇌ 2 ADP(c)
r1187	0.15	Stearoyl-CoA(c) + sn-Glycerol-3P(c) → CoA(c) + 1-Acylglycerol-3P-stea(c)
r1188	0.14	Oleoyl-CoA(c) + sn-Glycerol-3P(c) → 1-Acylglycerol-3P-ol(c) + CoA(c)
r1212	-1	Acyl-CoA-VLDL-PC-pool(c) + 1-Acylglycerol-3P-VLDL-PC-pool(c) → Phosphatidate-VLDL-PC-pool(c) + CoA(c)
r1189	0.27	Linoleoyl-CoA(c) + sn-Glycerol-3P(c) → 1-Acylglycerol-3P-lin(c) + CoA(c)
r0312	0.7	Palmitate(c) + ATP(c) + CoA(c) → Palmitoyl-CoA(c) + AMP(c) + PPi(c)
r0150	1	ATP(c) + CDP(c) ⇌ ADP(c) + CTP(c)
r1199	0.0001	1170 1-Acylglycerol-3P-ol(c) + 2350 1-Acylglycerol-3P-lin(c) + 726 1-Acylglycerol-3P-arach(c) + 3010 1-Acylglycerol-3P-palm(c) + 1300 1-Acylglycerol-3P-stea(c) → 8556 1-Acylglycerol-3P-VLDL-PC-pool(c)
r0421	1	CTP(c) + Phosphocholine(c) → PPi(c) + CDP-choline(c)
r0007	3	PPi(c) + H2O(c) → 2 Pi(c)
r1322	-1	NADH(c) ⇌ NAD+(c) + NADH-redox-potential(c)
r0849	-3.5	H2O(s) ⇌ H2O(c)
r1206	0.0001	3010 Palmitoyl-CoA(c) + 1300 Stearoyl-CoA(c) + 1170 Oleoyl-CoA(c) + 726 Arachidonyl-CoA(c) + 2350 Linoleoyl-CoA(c) → 8556 Acyl-CoA-VLDL-PC-pool(c)
r0243	0.5	GAP(c) ⇌ DHAP(c)

PE (517)

Definition			Solution												
			exchanges		reactions								transp		
Objective	Constraints	Comment	imports	exports	c	m	r	p	l	n	s	b	s-c	b-c	int
PE-VLDL-pool(c)	MCES ALFA	de novo synthesis of cytosolic PE-VLDL-pool	0.5 P_i(c) 0.5 Glucose-6P(c) 1 Ethanolamine(c) 0.6 Arachidonate(c) 0.34 Palmitate(c) 0.17 Oleate(c) 0.54 Stearate(c) 0.36 Linoleate(c) 7.5 ATP-energy(c) 1 NADH-redox-potential(c)	3.5 H_2O(s) 1 PE-VLDL-pool(c)	28	-	-	-	-	-	-	-	1	-	-

ID	flux	reaction
r1207	0.0001	1260 Palmitoyl-CoA(c) + 2010 Stearoyl-CoA(c) + 628 Oleoyl-CoA(c) + 2220 Arachidonyl-CoA(c) + 1330 Linoleoyl-CoA(c) → 7448 Acyl-CoA-VLDL-PE-pool(c)
r0136	1	ATP(c) + CMP(c) ⇌ ADP(c) + CDP(c)
r1190	0.3	Arachidonyl-CoA(c) + sn-Glycerol-3P(c) → 1-Acylglycerol-3P-arach(c) + CoA(c)
r1263	0.6	Arachidonate(c) + ATP(c) + CoA(c) → Arachidonyl-CoA(c) + AMP(c) + PPi(c)
r1254	0.54	Stearate(c) + ATP(c) + CoA(c) → Stearoyl-CoA(c) + AMP(c) + PPi(c)
r0336	1	ATP(c) + Ethanolamine(c) → ADP(c) + Ethanolamine-P(c)
r0192	0.5	Glucose-6P(c) ⇌ Fructose-6P(c)
r1219	1	Phosphatidate-VLDL-PE-pool(c) + H2O(c) → 1,2-Diacylglycerol-VLDL-PE-pool(c) + Pi(c)
r0481	1	Ethanolamine-P(c) + CTP(c) → PPi(c) + CDP-ethanolamine(c)
r0256	0.5	Fructose-1,6PP(c) ⇌ DHAP(c) + GAP(c)
r1319	-7.5	ATP(c) + H2O(c) ⇌ ADP(c) + Pi(c) + ATP-energy(c)
r1256	0.17	ATP(c) + CoA(c) + Oleate(c) → Oleoyl-CoA(c) + AMP(c) + PPi(c)
r1194	1	1,2-Diacylglycerol-VLDL-PE-pool(c) + CDP-ethanolamine(c) → PE-VLDL-pool(c) + CMP(c)
r1185	0.17	Palmitoyl-CoA(c) + sn-Glycerol-3P(c) → CoA(c) + 1-Acylglycerol-3P-palm(c)
r1258	0.36	Linoleate(c) + ATP(c) + CoA(c) → AMP(c) + PPi(c) + Linoleoyl-CoA(c)
r1200	0.0001	628 1-Acylglycerol-3P-ol(c) + 1330 1-Acylglycerol-3P-lin(c) + 2220 1-Acylglycerol-3P-arach(c) + 1260 1-Acylglycerol-3P-palm(c) + 2010 1-Acylglycerol-3P-stea(c) → 7448 1-Acylglycerol-3P-VLDL-PE-pool(c)
r0736	0.5	ATP(c) + Fructose-6P(c) → ADP(c) + Fructose-1,6PP(c)
r0201	-1	DHAP(c) + NADH(c) ⇌ NAD+(c) + sn-Glycerol-3P(c)
r0030	2	ATP(c) + AMP(c) ⇌ 2 ADP(c)
r1187	0.27	Stearoyl-CoA(c) + sn-Glycerol-3P(c) → CoA(c) + 1-Acylglycerol-3P-stea(c)
r1188	0.08	Oleoyl-CoA(c) + sn-Glycerol-3P(c) → 1-Acylglycerol-3P-ol(c) + CoA(c)

Continued on next page

ID	flux	reaction
r1189	0.18	Linoleoyl-CoA(c) + sn-Glycerol-3P(c) → 1-Acylglycerol-3P-lin(c) + CoA(c)
r0312	0.34	Palmitate(c) + ATP(c) + CoA(c) → Palmitoyl-CoA(c) + AMP(c) + PPi(c)
r0150	1	ATP(c) + CDP(c) ⇌ ADP(c) + CTP(c)
r1213	-1	Acyl-CoA-VLDL-PE-pool(c) + 1-Acylglycerol-3P-VLDL-PE-pool(c) → Phosphatidate-VLDL-PE-pool(c) + CoA(c)
r0007	3	PPi(c) + H2O(c) → 2 Pi(c)
r1322	-1	NADH(c) ⇌ NAD+(c) + NADH-redox-potential(c)
r0849	-3.5	H2O(s) ⇌ H2O(c)
r0243	0.5	GAP(c) ⇌ DHAP(c)

PS (518)

Definition			Solution												
			exchanges		reactions								transp		
Objective	Constraints	Comment	imports	exports	c	m	r	p	l	n	s	b	s-c	b-c	int
PS-VLDL-pool(c)	MCES ALFA	de novo synthesis of cytosolic PS-VLDL-pool	0.5 P_i(c) 1 Pyruvate(m) 0.5 Glucose-6P(c) 1 Ethanolamine(c) 0.53 Arachidonate(c) 0.09 Palmitate(c) 0.09 Oleate(c) 1.25 Stearate(c) 0.05 Linoleate(c) 7.5 ATP-energy(c) 1 NADH-redox-potential(c)	3.5 H_2O(s) 1 PS-VLDL-pool(c) 1 NADH-redox-potential(m) 1 CoA-activated acetyl group(m)	29	3	-	-	-	-	-	-	1	-	1

ID	flux	reaction
r2678	1	Acetyl-CoA(m) ⇌ CoA(m) + CoA-activated acetyl group(m)
r0136	1	ATP(c) + CMP(c) ⇌ ADP(c) + CDP(c)
r1370	-1	PE-PS-VLDL-pool(c) + CO2(c) → PS-VLDL-pool(c)
r1208	0.0001	345 Palmitoyl-CoA(c) + 4820 Stearoyl-CoA(c) + 332 Oleoyl-CoA(c) + 2050 Arachidonyl-CoA(c) + 181 Linoleoyl-CoA(c) → 7728 Acyl-CoA-VLDL-PS-pool(c)
r1190	0.27	Arachidonyl-CoA(c) + sn-Glycerol-3P(c) → 1-Acylglycerol-3P-arach(c) + CoA(c)
r0056	1	Pyruvate(m) + CoA(m) + NAD+(m) → Acetyl-CoA(m) + CO2(m) + NADH(m)
r1263	0.53	Arachidonate(c) + ATP(c) + CoA(c) → Arachidonyl-CoA(c) + AMP(c) + PPi(c)
r1254	1.25	Stearate(c) + ATP(c) + CoA(c) → Stearoyl-CoA(c) + AMP(c) + PPi(c)
r0336	1	ATP(c) + Ethanolamine(c) → ADP(c) + Ethanolamine-P(c)
r0192	0.5	Glucose-6P(c) ⇌ Fructose-6P(c)
r0481	1	Ethanolamine-P(c) + CTP(c) → PPi(c) + CDP-ethanolamine(c)
r0256	0.5	Fructose-1,6PP(c) ⇌ DHAP(c) + GAP(c)
r1319	-7.5	ATP(c) + H2O(c) ⇌ ADP(c) + Pi(c) + ATP-energy(c)
r1256	0.09	ATP(c) + CoA(c) + Oleate(c) → Oleoyl-CoA(c) + AMP(c) + PPi(c)
r1185	0.04	Palmitoyl-CoA(c) + sn-Glycerol-3P(c) → CoA(c) + 1-Acylglycerol-3P-palm(c)
r1258	0.05	Linoleate(c) + ATP(c) + CoA(c) → AMP(c) + PPi(c) + Linoleoyl-CoA(c)
r1195	1	1,2-Diacylglycerol-VLDL-PS-pool(c) + CDP-ethanolamine(c) → PE-PS-VLDL-pool(c) + CMP(c)
r0736	0.5	ATP(c) + Fructose-6P(c) → ADP(c) + Fructose-1,6PP(c)
r0201	-1	DHAP(c) + NADH(c) ⇌ NAD+(c) + sn-Glycerol-3P(c)
r0030	2	ATP(c) + AMP(c) ⇌ 2 ADP(c)
r1201	0.0001	332 1-Acylglycerol-3P-ol(c) + 181 1-Acylglycerol-3P-lin(c) + 2050 1-Acylglycerol-3P-arach(c) + 345 1-Acylglycerol-3P-palm(c) + 4820 1-Acylglycerol-3P-stea(c) → 7728 1-Acylglycerol-3P-VLDL-PS-pool(c)
r1187	0.62	Stearoyl-CoA(c) + sn-Glycerol-3P(c) → CoA(c) + 1-Acylglycerol-3P-stea(c)
r1220	1	Phosphatidate-VLDL-PS-pool(c) + H2O(c) → 1,2-Diacylglycerol-VLDL-PS-pool(c) + Pi(c)
r1188	0.04	Oleoyl-CoA(c) + sn-Glycerol-3P(c) → 1-Acylglycerol-3P-ol(c) + CoA(c)
r1189	0.02	Linoleoyl-CoA(c) + sn-Glycerol-3P(c) → 1-Acylglycerol-3P-lin(c) + CoA(c)
r0312	0.09	Palmitate(c) + ATP(c) + CoA(c) → Palmitoyl-CoA(c) + AMP(c) + PPi(c)
r0150	1	ATP(c) + CDP(c) ⇌ ADP(c) + CTP(c)
r0007	3	PPi(c) + H2O(c) → 2 Pi(c)
r1322	-1	NADH(c) ⇌ NAD+(c) + NADH-redox-potential(c)
r1214	-1	Acyl-CoA-VLDL-PS-pool(c) + 1-Acylglycerol-3P-VLDL-PS-pool(c) → Phosphatidate-VLDL-PS-pool(c) + CoA(c)
r0849	-3.5	H2O(s) ⇌ H2O(c)

Continued on next page

ID	flux	reaction
r1323	1	NADH(m) ⇌ NAD+(m) + NADH-redox-potential(m)
r0868	-1	CO2(c) ⇌ CO2(m)
r0243	0.5	GAP(c) ⇌ DHAP(c)

PI (519)

Definition			Solution												
			exchanges		reactions								transp		
Objective	Constraints	Comment	imports	exports	c	m	r	p	l	n	s	b	s-c	b-c	int
PI-pool(c)	MCES ALFA	de novo synthesis of cytosolic PI-pool	1.5 Glucose-6P(c) 0.51 Arachidonate(c) 0.16 Palmitate(c) 0.32 Oleate(c) 0.85 Stearate(c) 0.16 Linoleate(c) 6.5 ATP-energy(c) 1 NADH-redox-potential(c)	2.5 H_2O(s) 0.5 P_i(c) 1 PI-pool(c)	28	-	-	-	-	-	-	-	1	-	-

ID	flux	reaction
r0136	1	ATP(c) + CMP(c) ⇌ ADP(c) + CDP(c)
r1190	0.25	Arachidonyl-CoA(c) + sn-Glycerol-3P(c) → 1-Acylglycerol-3P-arach(c) + CoA(c)
r1263	0.51	Arachidonate(c) + ATP(c) + CoA(c) → Arachidonyl-CoA(c) + AMP(c) + PPi(c)
r1254	0.85	Stearate(c) + ATP(c) + CoA(c) → Stearoyl-CoA(c) + AMP(c) + PPi(c)
r1209	0.0001	694 Palmitoyl-CoA(c) + 3640 Stearoyl-CoA(c) + 1360 Oleoyl-CoA(c) + 2170 Arachidonyl-CoA(c) + 694 Linoleoyl-CoA(c) → 8558 Acyl-CoA-VLDL-PI-pool(c)
r0192	0.5	Glucose-6P(c) ⇌ Fructose-6P(c)
r0256	0.5	Fructose-1,6PP(c) ⇌ DHAP(c) + GAP(c)
r1319	-6.5	ATP(c) + H2O(c) ⇌ ADP(c) + Pi(c) + ATP-energy(c)
r1256	0.32	ATP(c) + CoA(c) + Oleate(c) → Oleoyl-CoA(c) + AMP(c) + PPi(c)
r1238	1	Phosphatidate-VLDL-PI-pool(c) + CTP(c) ⇌ CDP-diacylglycerol-VLDL-PI-pool(c) + PPi(c)
r1185	0.08	Palmitoyl-CoA(c) + sn-Glycerol-3P(c) → CoA(c) + 1-Acylglycerol-3P-palm(c)
r0200	-1	Glucose-6P(c) ⇌ Inositol-1P(c)
r1258	0.16	Linoleate(c) + ATP(c) + CoA(c) → AMP(c) + PPi(c) + Linoleoyl-CoA(c)
r0736	0.5	ATP(c) + Fructose-6P(c) → ADP(c) + Fructose-1,6PP(c)
r0201	-1	DHAP(c) + NADH(c) ⇌ NAD+(c) + sn-Glycerol-3P(c)
r0030	2	ATP(c) + AMP(c) ⇌ 2 ADP(c)
r1187	0.43	Stearoyl-CoA(c) + sn-Glycerol-3P(c) → CoA(c) + 1-Acylglycerol-3P-stea(c)
r1202	0.0001	1360 1-Acylglycerol-3P-ol(c) + 694 1-Acylglycerol-3P-lin(c) + 2170 1-Acylglycerol-3P-arach(c) + 694 1-Acylglycerol-3P-palm(c) + 3640 1-Acylglycerol-3P-stea(c) → 8558 1-Acylglycerol-3P-VLDL-PI-pool(c)
r1197	1	CDP-diacylglycerol-VLDL-PI-pool(c) + Inositol(c) ⇌ PI-pool(c) + CMP(c)
r1188	0.16	Oleoyl-CoA(c) + sn-Glycerol-3P(c) → 1-Acylglycerol-3P-ol(c) + CoA(c)
r1189	0.08	Linoleoyl-CoA(c) + sn-Glycerol-3P(c) → 1-Acylglycerol-3P-lin(c) + CoA(c)
r0312	0.16	Palmitate(c) + ATP(c) + CoA(c) → Palmitoyl-CoA(c) + AMP(c) + PPi(c)
r0289	1	Inositol-1P(c) + H2O(c) → Inositol(c) + Pi(c)
r0150	1	ATP(c) + CDP(c) ⇌ ADP(c) + CTP(c)
r0007	3	PPi(c) + H2O(c) → 2 Pi(c)
r1322	-1	NADH(c) ⇌ NAD+(c) + NADH-redox-potential(c)
r0849	-2.5	H2O(s) ⇌ H2O(c)
r1215	-1	1-Acylglycerol-3P-VLDL-PI-pool(c) + Acyl-CoA-VLDL-PI-pool(c) → Phosphatidate-VLDL-PI-pool(c) + CoA(c)
r0243	0.5	GAP(c) ⇌ DHAP(c)

CL (520)

Definition			Solution													
			exchanges		reactions								transp			
Objective	Constraints	Comment	imports	exports	c	m	r	p	l	n	s	b	s-c	b-c	int
CL-pool(m)	MCES ALFA	de novo synthesis of mitochondrial CL-pool	2 Glucose-6P(c) 0.17 Palmitate(c) 0.56 Oleate(c) 0.08 Stearate(c) 3 Linoleate(c) 0.19 Palmitolate(c) 10 ATP-energy(c) 4 ATP-energy(m) 4 NADH-redox-potential(c) 1 Proton-gradient(m)	6 H_2O(s) 1 P_i(c) 1 CL-pool(m)	15	10	-	-	-	-	-	-	1	-	5

ID	flux	reaction
r1306	4	Acyl-CoA-CL-pool(c) → Acyl-CoA-CL-pool(m)
r1252	0.19	Palmitolate(c) + ATP(c) + CoA(c) → (2E)-Hexadecenoyl-CoA(c) + AMP(c) + PPi(c)
r1307	2	Acyl-CoA-CL-pool(m) + sn-Glycerol-3P(m) → CoA(m) + 1-Acylglycerol-3P-CL-pool(m)
r0137	2	ATP(m) + CMP(m) → ADP(m) + CDP(m)
r1308	-2	Acyl-CoA-CL-pool(m) + 1-Acylglycerol-3P-CL-pool(m) → Phosphatidate-CL-pool(m) + CoA(m)
r1254	0.08	Stearate(c) + ATP(c) + CoA(c) → Stearoyl-CoA(c) + AMP(c) + PPi(c)
r1047	1	Pi(c) + H+(PG)(c) → Pi(m) + H+(PG)(m)
r0192	2	Glucose-6P(c) ⇌ Fructose-6P(c)
r1309	2	Phosphatidate-CL-pool(m) + CTP(m) → CDP-diacylglycerol-CL-pool(m) + PPi(m)
r0256	2	Fructose-1,6PP(c) ⇌ DHAP(c) + GAP(c)
r1319	-10	ATP(c) + H2O(c) ⇌ ADP(c) + Pi(c) + ATP-energy(c)
r1256	0.56	ATP(c) + CoA(c) + Oleate(c) → Oleoyl-CoA(c) + AMP(c) + PPi(c)
r2472	4	sn-Glycerol-3P(c) + Pi(m) → sn-Glycerol-3P(m) + Pi(c)
r1258	3	Linoleate(c) + ATP(c) + CoA(c) → AMP(c) + PPi(c) + Linoleoyl-CoA(c)
r0736	2	ATP(c) + Fructose-6P(c) → ADP(c) + Fructose-1,6PP(c)
r0201	-4	DHAP(c) + NADH(c) ⇌ NAD+(c) + sn-Glycerol-3P(c)
r0030	4	ATP(c) + AMP(c) ⇌ 2 ADP(c)
r0863	-4	CoA(c) ⇌ CoA(m)
r1310	2	CDP-diacylglycerol-CL-pool(m) + sn-Glycerol-3P(m) → CMP(m) + PGP-CL-pool(m)
r1320	-4	ATP(m) + H2O(m) ⇌ ADP(m) + Pi(m) + ATP-energy(m)
r1311	2	PGP-CL-pool(m) + H2O(m) → PG-CL-pool(m) + Pi(m)
r0312	0.17	Palmitate(c) + ATP(c) + CoA(c) → Palmitoyl-CoA(c) + AMP(c) + PPi(c)
r1330	-1	H+(PG)(c) ⇌ H+(PG)(m) + Proton-gradient(m)
r1312	1	2 PG-CL-pool(m) → CL-pool(m) + Pi(m)
r0151	2	ATP(m) + CDP(m) → ADP(m) + CTP(m)
r0007	4	PPi(c) + H2O(c) → 2 Pi(c)
r1322	-4	NADH(c) ⇌ NAD+(c) + NADH-redox-potential(c)
r0849	-6	H2O(s) ⇌ H2O(c)
r0008	2	PPi(m) + H2O(m) → 2 Pi(m)
r1305	0.004	43 Palmitoyl-CoA(c) + 47 (2E)-Hexadecenoyl-CoA(c) + 19 Stearoyl-CoA(c) + 138 Oleoyl-CoA(c) + 738 Linoleoyl-CoA(c) → 985 Acyl-CoA-CL-pool(c)
r0243	2	GAP(c) ⇌ DHAP(c)

LacCer (521)

Definition			Solution													
			exchanges		reactions								transp			
Objective	Constraints	Comment	imports	exports	c	m	r	p	l	n	s	b	s-c	b-c	int
LacCer-pool(r)	MCES ALFA	de novo synthesis of LacCer-pool in ER/Golgi	2.5 Glucose-6P(c) 1 Ethanolamine(c) 0.02 Arachidonate(c) 0.91 Palmitate(c) 0.24 Oleate(c) 0.03 gamma-Linolenate(c) 0.8 Palmitolate(c) 9.5 ATP-energy(c) 1 NADH-redox-potential(m) 1 NADPH-redox-potential(c) 2 Proton-gradient(m)	1.53 H_2O(s) 2.5 P_i(c) 1 CO_2(s) 3 NADH-redox-potential(c) 1 LacCer-pool(r) 1 CoA-activated acetyl group(c)	39	2	2	-	-	-	-	-	2	-	11

ID	flux	reaction
r1210	0.0001	6260 Palmitoyl-CoA(c) + 1490 Stearoyl-CoA(c) + 656 Oleoyl-CoA(c) + 147 Arachidonyl-CoA(c) + 270 Linoleoyl-CoA(c) ⇌ 8823 Acyl-CoA-VLDL-SM-pool(c)
r0312	0.91	Palmitate(c) + ATP(c) + CoA(c) → Palmitoyl-CoA(c) + AMP(c) + PPi(c)
r0030	3	ATP(c) + AMP(c) ⇌ 2 ADP(c)
r0313	1	Serine(c) + Palmitoyl-CoA(c) ⇌ 3-Dehydrosphinganine(c) + CoA(c) + CO2(c)
r1256	0.24	ATP(c) + CoA(c) + Oleate(c) → Oleoyl-CoA(c) + AMP(c) + PPi(c)
r0186	1	Ethanolamine-P(c) + H2O(c) → Acetaldehyde(c) + Pi(c) + NH3(c)
r0035	1	UDP(r) + H2O(r) → Pi(r) + UMP(r)
r1405	1	UDP-glucose(c) + Ceramide-pool(c) → UDP(c) + Glucosylceramide-pool(c)
r0037	2	ATP(c) + UDP(c) ⇌ ADP(c) + UTP(c)
r0038	1	ATP(c) + UMP(c) ⇌ ADP(c) + UDP(c)
r0911	1	Proline(c) + Glutamate(m) ⇌ Proline(m) + Glutamate(c)
r1409	1	UDP-galactose(r) + Glucosylceramide-pool(r) → LacCer-pool(r) + UDP(r)
r0336	1	ATP(c) + Ethanolamine(c) → ADP(c) + Ethanolamine-P(c)
r0337	-1	ADP(c) + 1,3DPG(c) ⇌ ATP(c) + 3PG(c)
r0338	1	3PG(c) + NAD+(c) → 3-Phosphonooxypyruvate(c) + NADH(c)
r1426	1	Proline(m) + H+(PG)(c) → Proline(c) + H+(PG)(m)
r0073	-1	AKG(m) + NADH(m) + NH3(m) ⇌ Glutamate(m) + NAD+(m) + H2O(m)
r1149	-1	Pi(c) ⇌ Pi(r)
r0094	2	UTP(c) + Glucose-1P(c) → PPi(c) + UDP-glucose(c)
r0095	1	UDP-glucose(c) ⇌ UDP-galactose(c)
r1465	0.17	2 H2O(c) + NAD+(c) + Oleoyl-CoA(c) ⇌ NADH(c) + O2(c) + Stearoyl-CoA(c)
r0243	-0.5	DHAP(c) ⇌ GAP(c)
r1469	-0.03	gamma-Linolenoyl-CoA(c) + H2O(c) + NAD+(c) ⇌ Linoleoyl-CoA(c) + NADH(c) + O2(c)
r1330	-2	H+(PG)(c) ⇌ H+(PG)(m) + Proton-gradient(m)
r1319	-9.5	ATP(c) + H2O(c) ⇌ ADP(c) + Pi(c) + ATP-energy(c)
r1488	0.8	ATP(c) + CoA(c) + Palmitolate(c) ⇌ AMP(c) + palmitoleoyl-CoA(c) + PPi(c)
r0848	-1	H2O(r) ⇌ H2O(c)
r0849	-1.53	H2O(s) ⇌ H2O(c)
r0583	-1	3-Dehydrosphinganine(c) + NADPH(c) ⇌ Sphinganine(c) + NADP+(c)
r0867	1	CO2(c) ⇌ CO2(s)
r2467	1	UDP-galactose(c) + UMP(r) → UDP-galactose(r) + UMP(c)
r0007	5	PPi(c) + H2O(c) → 2 Pi(c)
r0736	0.5	ATP(c) + Fructose-6P(c) → ADP(c) + Fructose-1,6PP(c)
r0159	1	3-Phosphoserine(c) + H2O(c) → Serine(c) + Pi(c)
r0023	-0.8	NAD+(c) + 2 Ferrocytochrome b5(c) ⇌ NADH(c) + 2 Ferricytochrome b5(c)
r1261	0.03	gamma-Linolenate(c) + ATP(c) + CoA(c) → gamma-Linolenoyl-CoA(c) + AMP(c) + PPi(c)
r1263	0.02	Arachidonate(c) + ATP(c) + CoA(c) → Arachidonyl-CoA(c) + AMP(c) + PPi(c)
r0177	1	Acetaldehyde(c) + H2O(c) + NADP+(c) → Acetate(c) + NADPH(c)
r0192	0.5	Glucose-6P(c) ⇌ Fructose-6P(c)
r1412	1	Sphinganine(c) + Acyl-CoA-VLDL-SM-pool(c) ⇌ Dihydroceramide-pool(c) + CoA(c)
r0621	-2	Glucose-6P(c) ⇌ Glucose-1P(c)
r1414	1	Dihydroceramide-pool(c) + O2(c) + NADPH(c) ⇌ 2 H2O(c) + Ceramide-pool(c) + NADP+(c)
r1416	1	Glucosylceramide-pool(c) → Glucosylceramide-pool(r)
r0066	1	ATP(c) + Acetate(c) + CoA(c) → AMP(c) + PPi(c) + Acetyl-CoA(c)
r2677	1	Acetyl-CoA(c) ⇌ CoA(c) + CoA-activated acetyl group(c)
r0510	-0.8	palmitoleoyl-CoA(c) + 2 Ferricytochrome b5(c) + 2 H2O(c) ⇌ Palmitoyl-CoA(c) + O2(c) + 2 Ferrocytochrome b5(c)
r0663	-1	3-Phosphonooxypyruvate(c) + Glutamate(c) → 3-Phosphoserine(c) + AKG(c)
r1322	3	NADH(c) ⇌ NAD+(c) + NADH-redox-potential(c)
r1323	-1	NADH(m) ⇌ NAD+(m) + NADH-redox-potential(m)
r1326	-1	NADPH(c) ⇌ NADPH-redox-potential(c) + NADP+(c)
r0253	1	GAP(c) + Pi(c) + NAD+(c) ⇌ 1,3DPG(c) + NADH(c)
r0256	0.5	Fructose-1,6PP(c) ⇌ DHAP(c) + GAP(c)
r1047	1	Pi(c) + H+(PG)(c) → Pi(m) + H+(PG)(m)
r2419	1	AKG(c) + Pi(m) ⇌ AKG(m) + Pi(c)
r0838	1	NH3(c) ⇌ NH3(m)
r0851	1	H2O(m) ⇌ H2O(c)

SM (522)

Definition			Solution												
			exchanges		reactions								transp		
Objective	Constraints	Comment	imports	exports	c	m	r	p	l	n	s	b	s-c	b-c	int
SM-pool(c)	MCES ALFA	de novo synthesis of cytosolic SM-pool	0.5 P_i(c) 0.5 Glucose-6P(c) 1 Choline(c) 1 Ethanolamine(c) 0.02 Arachidonate(c) 0.91 Palmitate(c) 0.24 Oleate(c) 0.03 gamma-Linolenate(c) 0.8 Palmitolate(c) 9.5 ATP-energy(c) 1 NADPH-redox-potential(c) 1 NADPH-redox-potential(m) 2 Proton-gradient(m)	3.53 H_2O(s) 1 CO_2(s) 1 SM-pool(c) 3 NADH-redox-potential(c) 1 CoA-activated acetyl group(c)	37	2	1	-	-	-	-	-	2	-	11

ID	flux	reaction
r1210	0.0001	6260 Palmitoyl-CoA(c) + 1490 Stearoyl-CoA(c) + 656 Oleoyl-CoA(c) + 147 Arachidonyl-CoA(c) + 270 Linoleoyl-CoA(c) ⇌ 8823 Acyl-CoA-VLDL-SM-pool(c)
r0421	1	CTP(c) + Phosphocholine(c) → PPi(c) + CDP-choline(c)
r0312	0.91	Palmitate(c) + ATP(c) + CoA(c) → Palmitoyl-CoA(c) + AMP(c) + PPi(c)
r0030	3	ATP(c) + AMP(c) ⇌ 2 ADP(c)
r0313	1	Serine(c) + Palmitoyl-CoA(c) ⇌ 3-Dehydrosphinganine(c) + CoA(c) + CO2(c)
r1256	0.24	ATP(c) + CoA(c) + Oleate(c) → Oleoyl-CoA(c) + AMP(c) + PPi(c)
r0186	1	Ethanolamine-P(c) + H2O(c) → Acetaldehyde(c) + Pi(c) + NH3(c)
r1275	-1	SM-pool(c) ⇌ SM-pool(r)
r1407	1	CDP-choline(r) + Ceramide-pool(r) ⇌ CMP(r) + SM-pool(r)
r0911	1	Proline(c) + Glutamate(m) ⇌ Proline(m) + Glutamate(c)
r0336	1	ATP(c) + Ethanolamine(c) → ADP(c) + Ethanolamine-P(c)
r0337	-1	ADP(c) + 1,3DPG(c) ⇌ ATP(c) + 3PG(c)
r0338	1	3PG(c) + NAD+(c) → 3-Phosphonooxypyruvate(c) + NADH(c)
r1426	1	Proline(m) + H+(PG)(c) → Proline(c) + H+(PG)(m)
r0075	-1	AKG(m) + NADPH(m) + NH3(m) ⇌ Glutamate(m) + H2O(m) + NADP+(m)
r1465	0.17	2 H2O(c) + NAD+(c) + Oleoyl-CoA(c) ⇌ NADH(c) + O2(c) + Stearoyl-CoA(c)
r0243	-0.5	DHAP(c) ⇌ GAP(c)
r1469	-0.03	gamma-Linolenoyl-CoA(c) + H2O(c) + NAD+(c) ⇌ Linoleoyl-CoA(c) + NADH(c) + O2(c)
r1330	-2	H+(PG)(c) ⇌ H+(PG)(m) + Proton-gradient(m)
r0244	1	ATP(c) + Choline(c) → ADP(c) + Phosphocholine(c)
r1319	-9.5	ATP(c) + H2O(c) ⇌ ADP(c) + Pi(c) + ATP-energy(c)
r1488	0.8	ATP(c) + CoA(c) + Palmitolate(c) ⇌ AMP(c) + palmitoleoyl-CoA(c) + PPi(c)
r0849	-3.53	H2O(s) ⇌ H2O(c)
r0583	-1	3-Dehydrosphinganine(c) + NADPH(c) ⇌ Sphinganine(c) + NADP+(c)
r0136	1	ATP(c) + CMP(c) ⇌ ADP(c) + CDP(c)
r0150	1	ATP(c) + CDP(c) ⇌ ADP(c) + CTP(c)
r0867	1	CO2(c) ⇌ CO2(s)
r1240	1	Ceramide-pool(c) ⇌ Ceramide-pool(r)
r0886	-1	CMP(c) ⇌ CMP(r)
r0007	4	PPi(c) + H2O(c) → 2 Pi(c)
r0736	0.5	ATP(c) + Fructose-6P(c) → ADP(c) + Fructose-1,6PP(c)
r0159	1	3-Phosphoserine(c) + H2O(c) → Serine(c) + Pi(c)
r0023	-0.8	NAD+(c) + 2 Ferrocytochrome b5(c) ⇌ NADH(c) + 2 Ferricytochrome b5(c)
r1261	0.03	gamma-Linolenate(c) + ATP(c) + CoA(c) → gamma-Linolenoyl-CoA(c) + AMP(c) + PPi(c)
r0177	1	Acetaldehyde(c) + H2O(c) + NADP+(c) → Acetate(c) + NADPH(c)
r1263	0.02	Arachidonate(c) + ATP(c) + CoA(c) → Arachidonyl-CoA(c) + AMP(c) + PPi(c)
r0192	0.5	Glucose-6P(c) ⇌ Fructose-6P(c)
r1412	1	Sphinganine(c) + Acyl-CoA-VLDL-SM-pool(c) ⇌ Dihydroceramide-pool(c) + CoA(c)
r1414	1	Dihydroceramide-pool(c) + O2(c) + NADPH(c) ⇌ 2 H2O(c) + Ceramide-pool(c) + NADP+(c)
r0066	1	ATP(c) + Acetate(c) + CoA(c) → AMP(c) + PPi(c) + Acetyl-CoA(c)
r2677	1	Acetyl-CoA(c) ⇌ CoA(c) + CoA-activated acetyl group(c)
r0510	-0.8	palmitoleoyl-CoA(c) + 2 Ferricytochrome b5(c) + 2 H2O(c) ⇌ Palmitoyl-CoA(c) + O2(c) + 2 Ferrocytochrome b5(c)

Continued on next page

SM (522)– continued

ID	flux	reaction
r1159	1	CDP-choline(c) ⇌ CDP-choline(r)
r0663	-1	3-Phosphonooxypyruvate(c) + Glutamate(c) → 3-Phosphoserine(c) + AKG(c)
r1322	3	NADH(c) ⇌ NAD+(c) + NADH-redox-potential(c)
r1326	-1	NADPH(c) ⇌ NADPH-redox-potential(c) + NADP+(c)
r0253	1	GAP(c) + Pi(c) + NAD+(c) ⇌ 1,3DPG(c) + NADH(c)
r1327	-1	NADPH(m) ⇌ NADPH-redox-potential(m) + NADP+(m)
r0256	0.5	Fructose-1,6PP(c) ⇌ DHAP(c) + GAP(c)
r1047	1	Pi(c) + H+(PG)(c) → Pi(m) + H+(PG)(m)
r2419	1	AKG(c) + Pi(m) ⇌ AKG(m) + Pi(c)
r0838	1	NH3(c) ⇌ NH3(m)
r0851	1	H2O(m) ⇌ H2O(c)

Ceramide (523)

Definition			Solution												
			exchanges		reactions								transp		
Objective	Constraints	Comment	imports	exports	c	m	r	p	l	n	s	b	s-c	b-c	int
Ceramide-pool(c)	MCES ALFA	de novo synthesis of cytosolic Ceramide-pool	0.5 Glucose-6P(c) 1 Ethanolamine(c) 0.02 Arachidonate(c) 0.91 Palmitate(c) 0.24 Oleate(c) 0.03 gamma-Linolenate(c) 0.8 Palmitolate(c) 6.5 ATP-energy(c) 1 NADPH-redox-potential(c) 1 NADPH-redox-potential(m) 2 Proton-gradient(m)	1.53 H_2O(s) 0.5 P_i(c) 1 CO_2(s) 1 Ceramide-pool(c) 3 NADH-redox-potential(c) 1 CoA-activated acetyl group(c)	33	2	-	-	-	-	-	-	2	-	7

ID	flux	reaction
r1210	0.0001	6260 Palmitoyl-CoA(c) + 1490 Stearoyl-CoA(c) + 656 Oleoyl-CoA(c) + 147 Arachidonyl-CoA(c) + 270 Linoleoyl-CoA(c) ⇌ 8823 Acyl-CoA-VLDL-SM-pool(c)
r0312	0.91	Palmitate(c) + ATP(c) + CoA(c) → Palmitoyl-CoA(c) + AMP(c) + PPi(c)
r0030	3	ATP(c) + AMP(c) ⇌ 2 ADP(c)
r0313	1	Serine(c) + Palmitoyl-CoA(c) ⇌ 3-Dehydrosphinganine(c) + CoA(c) + CO2(c)
r1256	0.24	ATP(c) + CoA(c) + Oleate(c) → Oleoyl-CoA(c) + AMP(c) + PPi(c)
r0186	1	Ethanolamine-P(c) + H2O(c) → Acetaldehyde(c) + Pi(c) + NH3(c)
r0911	1	Proline(c) + Glutamate(m) ⇌ Proline(m) + Glutamate(c)
r0336	1	ATP(c) + Ethanolamine(c) → ADP(c) + Ethanolamine-P(c)
r0337	-1	ADP(c) + 1,3DPG(c) ⇌ ATP(c) + 3PG(c)
r0338	1	3PG(c) + NAD+(c) → 3-Phosphonooxypyruvate(c) + NADH(c)
r1426	1	Proline(m) + H+(PG)(c) → Proline(c) + H+(PG)(m)
r0075	-1	AKG(m) + NADPH(m) + NH3(m) ⇌ Glutamate(m) + H2O(m) + NADP+(m)
r1465	0.17	2 H2O(c) + NAD+(c) + Oleoyl-CoA(c) ⇌ NADH(c) + O2(c) + Stearoyl-CoA(c)
r0243	-0.5	DHAP(c) ⇌ GAP(c)
r1469	-0.03	gamma-Linolenoyl-CoA(c) + H2O(c) + NAD+(c) ⇌ Linoleoyl-CoA(c) + NADH(c) + O2(c)
r1330	-2	H+(PG)(c) ⇌ H+(PG)(m) + Proton-gradient(m)
r1319	-6.5	ATP(c) + H2O(c) ⇌ ADP(c) + Pi(c) + ATP-energy(c)
r1488	0.8	ATP(c) + CoA(c) + Palmitolate(c) ⇌ AMP(c) + palmitoleoyl-CoA(c) + PPi(c)
r0849	-1.53	H2O(s) ⇌ H2O(c)
r0583	-1	3-Dehydrosphinganine(c) + NADPH(c) ⇌ Sphinganine(c) + NADP+(c)
r0867	1	CO2(c) ⇌ CO2(s)
r0007	3	PPi(c) + H2O(c) → 2 Pi(c)
r0736	0.5	ATP(c) + Fructose-6P(c) → ADP(c) + Fructose-1,6PP(c)
r0159	1	3-Phosphoserine(c) + H2O(c) → Serine(c) + Pi(c)
r0023	-0.8	NAD+(c) + 2 Ferrocytochrome b5(c) ⇌ NADH(c) + 2 Ferricytochrome b5(c)
r1261	0.03	gamma-Linolenate(c) + ATP(c) + CoA(c) → gamma-Linolenoyl-CoA(c) + AMP(c) + PPi(c)
r0177	1	Acetaldehyde(c) + H2O(c) + NADP+(c) → Acetate(c) + NADPH(c)

Continued on next page

ID	flux	reaction
r1263	0.02	Arachidonate(c) + ATP(c) + CoA(c) → Arachidonyl-CoA(c) + AMP(c) + PPi(c)
r0192	0.5	Glucose-6P(c) ⇌ Fructose-6P(c)
r1412	1	Sphinganine(c) + Acyl-CoA-VLDL-SM-pool(c) ⇌ Dihydroceramide-pool(c) + CoA(c)
r1414	1	Dihydroceramide-pool(c) + O2(c) + NADPH(c) ⇌ 2 H2O(c) + Ceramide-pool(c) + NADP+(c)
r0066	1	ATP(c) + Acetate(c) + CoA(c) → AMP(c) + PPi(c) + Acetyl-CoA(c)
r2677	1	Acetyl-CoA(c) ⇌ CoA(c) + CoA-activated acetyl group(c)
r0510	-0.8	palmitoleoyl-CoA(c) + 2 Ferricytochrome b5(c) + 2 H2O(c) ⇌ Palmitoyl-CoA(c) + O2(c) + 2 Ferrocytochrome b5(c)
r0663	-1	3-Phosphonooxypyruvate(c) + Glutamate(c) → 3-Phosphoserine(c) + AKG(c)
r1322	3	NADH(c) ⇌ NAD+(c) + NADH-redox-potential(c)
r1326	-1	NADPH(c) ⇌ NADPH-redox-potential(c) + NADP+(c)
r0253	1	GAP(c) + Pi(c) + NAD+(c) ⇌ 1,3DPG(c) + NADH(c)
r1327	-1	NADPH(m) ⇌ NADPH-redox-potential(m) + NADP+(m)
r0256	0.5	Fructose-1,6PP(c) ⇌ DHAP(c) + GAP(c)
r1047	1	Pi(c) + H+(PG)(c) → Pi(m) + H+(PG)(m)
r2419	1	AKG(c) + Pi(m) ⇌ AKG(m) + Pi(c)
r0838	1	NH3(c) ⇌ NH3(m)
r0851	1	H2O(m) ⇌ H2O(c)

Triacylglycerol (524)

Definition			Solution												
			exchanges		reactions								transp		
Objective	Constraints	Comment	imports	exports	c	m	r	p	l	n	s	b	s-c	b-c	int
Triacylglycerol-VLDL-pool(r)	MCES ALFA	de novo synthesis of Triacylglycerol-VLDL-pool in ER/Golgi	0.5 Glucose-6P(c) 0.09 Arachidonate(c) 0.92 Palmitate(c) 1.1 Oleate(c) 0.2 Stearate(c) 0.69 Linoleate(c) 6.5 ATP-energy(c) 1 NADH-redox-potential(c)	2.5 H_2O(s) 0.5 P_i(c) 1 Triacylglycerol-VLDL-pool(r)	25	-	-	-	-	-	-	-	1	-	1

ID	flux	reaction
r1217	1	Phosphatidate-VLDL-TG-pool(c) + H2O(c) → 1,2-Diacylglycerol-VLDL-TG-pool(c) + Pi(c)
r1190	0.003	Arachidonyl-CoA(c) + sn-Glycerol-3P(c) → 1-Acylglycerol-3P-arach(c) + CoA(c)
r1263	0.09	Arachidonate(c) + ATP(c) + CoA(c) → Arachidonyl-CoA(c) + AMP(c) + PPi(c)
r1254	0.2	Stearate(c) + ATP(c) + CoA(c) → Stearoyl-CoA(c) + AMP(c) + PPi(c)
r0192	0.5	Glucose-6P(c) ⇌ Fructose-6P(c)
r1264	1	Triacylglycerol-VLDL-pool(c) ⇌ Triacylglycerol-VLDL-pool(r)
r0256	0.5	Fructose-1,6PP(c) ⇌ DHAP(c) + GAP(c)
r1319	-6.5	ATP(c) + H2O(c) ⇌ ADP(c) + Pi(c) + ATP-energy(c)
r1256	1.1	ATP(c) + CoA(c) + Oleate(c) → Oleoyl-CoA(c) + AMP(c) + PPi(c)
r1185	0.7	Palmitoyl-CoA(c) + sn-Glycerol-3P(c) → CoA(c) + 1-Acylglycerol-3P-palm(c)
r1258	0.69	Linoleate(c) + ATP(c) + CoA(c) → AMP(c) + PPi(c) + Linoleoyl-CoA(c)
r0736	0.5	ATP(c) + Fructose-6P(c) → ADP(c) + Fructose-1,6PP(c)
r0201	-1	DHAP(c) + NADH(c) ⇌ NAD+(c) + sn-Glycerol-3P(c)
r0030	3	ATP(c) + AMP(c) ⇌ 2 ADP(c)
r1187	0.09	Stearoyl-CoA(c) + sn-Glycerol-3P(c) → CoA(c) + 1-Acylglycerol-3P-stea(c)
r1211	-1	Acyl-CoA-VLDL-TG2-pool(c) + 1-Acylglycerol-3P-VLDL-TG1-pool(c) → Phosphatidate-VLDL-TG-pool(c) + CoA(c)
r1188	0.13	Oleoyl-CoA(c) + sn-Glycerol-3P(c) → 1-Acylglycerol-3P-ol(c) + CoA(c)
r1198	0.0001	1220 1-Acylglycerol-3P-ol(c) + 741 1-Acylglycerol-3P-lin(c) + 24 1-Acylglycerol-3P-arach(c) + 6440 1-Acylglycerol-3P-palm(c) + 823 1-Acylglycerol-3P-stea(c) → 9248 1-Acylglycerol-3P-VLDL-TG1-pool(c)
r1189	0.08	Linoleoyl-CoA(c) + sn-Glycerol-3P(c) → 1-Acylglycerol-3P-lin(c) + CoA(c)
r0312	0.92	Palmitate(c) + ATP(c) + CoA(c) → Palmitoyl-CoA(c) + AMP(c) + PPi(c)
r1204	0.0001	1200 Palmitoyl-CoA(c) + 384 Stearoyl-CoA(c) + 4260 Oleoyl-CoA(c) + 521 Arachidonyl-CoA(c) + 2810 Linoleoyl-CoA(c) → 9175 Acyl-CoA-VLDL-TG2-pool(c)
r0007	3	PPi(c) + H2O(c) → 2 Pi(c)
r1322	-1	NADH(c) ⇌ NAD+(c) + NADH-redox-potential(c)
r1223	-1	Acyl-CoA-VLDL-TG3-pool(c) + 1,2-Diacylglycerol-VLDL-TG-pool(c) → Triacylglycerol-VLDL-pool(c) + CoA(c)

Continued on next page

ID	flux	reaction
r1205	0.0001	849 Palmitoyl-CoA(c) + 584 Stearoyl-CoA(c) + 4570 Oleoyl-CoA(c) + 264 Arachidonyl-CoA(c) + 2780 Linoleoyl-CoA(c) → 9047 Acyl-CoA-VLDL-TG3-pool(c)
r0849	-2.5	H2O(s) ⇌ H2O(c)
r0243	0.5	GAP(c) ⇌ DHAP(c)

Cholesterol (525)

Definition			Solution												
			exchanges		reactions								transp		
Objective	Constraints	Comment	imports	exports	c	m	r	p	l	n	s	b	s-c	b-c	int
Cholesterol(r)	MCES	de novo synthesis of Cholesterol in ER/Golgi	8 O_2(s) 2 Farnesyl-PP(r) 1 ATP-energy(c) 11 NADPH-redox-potential(r)	7 H_2O(s) 4 P_i(c) 3 CO_2(s) 1 Cholesterol(r) 1 NADH-redox-potential(r) 1 NADPH-redox-potential(c)	5	-	16	-	-	-	-	-	3	-	5

ID	flux	reaction
r1153	-2	PPi(c) ⇌ PPi(r)
r1135	1	3-Keto-4-methylzymosterol(r) + NADP+(r) ⇌ NADPH(r) + 4alpha-Methylzymosterol(r)
r0887	-1	Formate(c) ⇌ Formate(r)
r1325	-11	NADPH(r) ⇌ NADPH-redox-potential(r) + NADP+(r)
r0227	1	10-Formyl-THF(c) + H2O(c) + NADP+(c) → THF(c) + CO2(c) + NADPH(c)
r1326	1	NADPH(c) ⇌ NADPH-redox-potential(c) + NADP+(c)
r1146	1	4alpha-Methylzymosterol(r) + 2 H2O(r) ⇌ Zymosterol(r) + CO2(r)
r1137	1	NAD+(r) + 4alpha-Methylzymosterol-4-carboxylate(r) ⇌ NADH(r) + CO2(r) + 3-Keto-4-methylzymosterol(r)
r0228	1	THF(c) + Formate(c) + ATP(c) → ADP(c) + Pi(c) + 10-Formyl-THF(c)
r0607	1	Squalene 2,3-oxide(r) ⇌ Lanosterol(r)
r1319	-1	ATP(c) + H2O(c) ⇌ ADP(c) + Pi(c) + ATP-energy(c)
r0780	-1	4,4-Dimethyl-5alpha-cholesta-8,14,24-trien-3beta-ol(r) + NADPH(r) ⇌ 14-Demethyllanosterol(r) + NADP+(r)
r0781	1	3 O2(r) + 3 NADPH(r) + Lanosterol(r) ⇌ 4,4-Dimethyl-5alpha-cholesta-8,14,24-trien-3beta-ol(r) + Formate(r) + 4 H2O(r) + 3 NADP+(r)
r0854	-8	O2(r) ⇌ O2(c)
r0575	1	Presqualene-PP(r) + NADPH(r) ⇌ PPi(r) + Squalene(r) + NADP+(r)
r0855	8	O2(s) ⇌ O2(c)
r0783	-1	5alpha-Cholesta-7,24-dien-3beta-ol(r) + NADPH(r) ⇌ Lathosterol(r) + NADP+(r)
r0738	1	Zymosterol(r) ⇌ 5alpha-Cholesta-7,24-dien-3beta-ol(r)
r0576	1	Squalene(r) + NADPH(r) + O2(r) ⇌ H2O(r) + Squalene 2,3-oxide(r) + NADP+(r)
r0865	2	CO2(r) ⇌ CO2(c)
r0793	1	Lathosterol(r) + NADPH(r) + O2(r) ⇌ Provitamin D3(r) + 2 H2O(r) + NADP+(r)
r1321	1	NADH(r) ⇌ NAD+(r) + NADH-redox-potential(r)
r0848	9	H2O(r) ⇌ H2O(c)
r0007	2	PPi(c) + H2O(c) → 2 Pi(c)
r0867	3	CO2(c) ⇌ CO2(s)
r0849	-7	H2O(s) ⇌ H2O(c)
r0170	1	2 Farnesyl-PP(r) ⇌ PPi(r) + Presqualene-PP(r)
r1134	1	14-Demethyllanosterol(r) + 3 NADPH(r) + 3 O2(r) ⇌ 4 H2O(r) + 4alpha-Methylzymosterol-4-carboxylate(r) + 3 NADP+(r)
r0333	-1	Provitamin D3(r) + NADPH(r) ⇌ Cholesterol(r) + NADP+(r)

4.4 C4. Other building blocks

gdpddman (526)

Definition			Solution												
			exchanges		reactions								transp		
Objective	Constraints	Comment	imports	exports	c	m	r	p	l	n	s	b	s-c	b-c	int
GDP-4-dehydro-6-deoxy-D-mannose(c)	MCES −Glutamine(c) +Glutamate(c) −Aspartate(c)	de novo synthesis of cytosolic GDP-4-dehydro-6-deoxy-D-mannose	2 Aspartate(c) 3 Glutamine(c) 2.33 Glucose-6P(c) 9.67 ATP-energy(c) 1 THF-activated methyl group(c)	1.67 H_2O(s) 0.33 P_i(c) 1 CO_2(s) 2 Pyruvate(c) 3 Glutamate(c) 1 GDP-4-dehydro-6-deoxy-D-mannose(c) 2 NADH-redox-potential(c) 5 NADPH-redox-potential(c)	47	-	-	-	-	-	-	-	2	-	-

ID	flux	reaction
r0406	-0.33	Fructose-6P(c) + Erythrose-4P(c) ⇌ Sedoheptulose-7P(c) + GAP(c)
r0705	1	ATP(c) + 5-Phosphoribosyl-4-carboxy-5-aminoimidazole(c) + Aspartate(c) ⇌ ADP(c) + Pi(c) + SAICAR(c)
r0293	2	5,10-Methylene-THF(c) + NADP+(c) ⇌ 5,10-Methenyl-THF(c) + NADPH(c)
r0296	1	5-Methyl-THF(c) + NADP+(c) ⇌ 5,10-Methylene-THF(c) + NADPH(c)
r0030	2	ATP(c) + AMP(c) ⇌ 2 ADP(c)
r0337	-1	ADP(c) + 1,3DPG(c) ⇌ ATP(c) + 3PG(c)
r0338	1	3PG(c) + NAD+(c) → 3-Phosphonooxypyruvate(c) + NADH(c)
r0058	2	Malate(c) + NADP+(c) ⇌ Pyruvate(c) + CO2(c) + NADPH(c)
r0370	0.33	Sedoheptulose-7P(c) + GAP(c) ⇌ Ribose-5P(c) + D-Xylulose-5P(c)
r0371	2	5,10-Methenyl-THF(c) + H2O(c) ⇌ 10-Formyl-THF(c)
r0208	1	GTP(c) + Mannose-1P(c) ⇌ PPi(c) + GDP-mannose(c)
r0209	1	GDP-mannose(c) → GDP-4-dehydro-6-deoxy-D-mannose(c) + H2O(c)
r0659	1	ATP(c) + 5-Phosphoribosylamine(c) + Glycine(c) ⇌ ADP(c) + Pi(c) + GAR(c)
r0099	1	ATP(c) + GDP(c) ⇌ ADP(c) + GTP(c)
r0243	-0.67	DHAP(c) ⇌ GAP(c)
r0677	1	10-Formyl-THF(c) + GAR(c) ⇌ THF(c) + N-Formyl-GAR(c)
r1319	-9.67	ATP(c) + H2O(c) ⇌ ADP(c) + Pi(c) + ATP-energy(c)
r0247	1	ATP(c) + Ribose-5P(c) → AMP(c) + PRPP(c)
r0699	1	SAICAR(c) ⇌ Fumarate(c) + AICAR(c)
r0849	-1.67	H2O(s) ⇌ H2O(c)
r0867	1	CO2(c) ⇌ CO2(s)
r0736	0.67	ATP(c) + Fructose-6P(c) → ADP(c) + Fructose-1,6PP(c)
r0007	3	PPi(c) + H2O(c) → 2 Pi(c)
r0302	1	ATP(c) + Xanthosine-5P(c) + Glutamine(c) + H2O(c) → AMP(c) + PPi(c) + GMP(c) + Glutamate(c)
r0159	1	3-Phosphoserine(c) + H2O(c) → Serine(c) + Pi(c)
r0192	2.33	Glucose-6P(c) ⇌ Fructose-6P(c)
r0344	-0.67	D-Xylulose-5P(c) ⇌ Ribulose-5P(c)
r2676	-1	5-Methyl-THF(c) ⇌ THF(c) + THF-activated methyl group(c)
r0663	-1	3-Phosphonooxypyruvate(c) + Glutamate(c) → 3-Phosphoserine(c) + AKG(c)
r0230	-1	THF(c) + Serine(c) ⇌ 5,10-Methylene-THF(c) + Glycine(c) + H2O(c)
r0666	1	ATP(c) + 5-Phosphoribosylformylglycinamidine(c) ⇌ ADP(c) + Pi(c) + AIR(c)
r0667	-1	5-Phosphoribosyl-4-carboxy-5-aminoimidazole(c) ⇌ AIR(c) + CO2(c)
r1322	2	NADH(c) ⇌ NAD+(c) + NADH-redox-potential(c)
r0250	-0.67	Ribulose-5P(c) ⇌ Ribose-5P(c)
r1326	5	NADPH(c) ⇌ NADPH-redox-potential(c) + NADP+(c)
r0687	1	ATP(c) + N-Formyl-GAR(c) + Glutamine(c) + H2O(c) ⇌ ADP(c) + Pi(c) + Glutamate(c) + 5-Phosphoribosylformylglycinamidine(c)
r0101	1	ATP(c) + GMP(c) ⇌ ADP(c) + GDP(c)
r0253	1	GAP(c) + Pi(c) + NAD+(c) ⇌ 1,3DPG(c) + NADH(c)
r0255	0.33	Fructose-6P(c) + GAP(c) ⇌ D-Xylulose-5P(c) + Erythrose-4P(c)
r0104	-1	OAA(c) + NADH(c) ⇌ NAD+(c) + Malate(c)
r0256	0.67	Fructose-1,6PP(c) ⇌ DHAP(c) + GAP(c)
r0257	-1	Glutamine(c) + PRPP(c) + H2O(c) ⇌ 5-Phosphoribosylamine(c) + PPi(c) + Glutamate(c)
r0258	-1	Fumarate(c) + H2O(c) ⇌ Malate(c)
r0273	-1	IMP(c) + H2O(c) ⇌ FAICAR(c)

Continued on next page

ID	flux	reaction
r0404	1	Mannose-6P(c) → Mannose-1P(c)
r0274	1	IMP(c) + NAD+(c) + H2O(c) → Xanthosine-5P(c) + NADH(c)
r0109	1	AKG(c) + Aspartate(c) ⇌ OAA(c) + Glutamate(c)
r0700	1	10-Formyl-THF(c) + AICAR(c) ⇌ THF(c) + FAICAR(c)
r0405	-1	Fructose-6P(c) ⇌ Mannose-6P(c)

beta-Alanine (527)

Definition			Solution													
			exchanges		reactions								transp		
Objective	Constraints	Comment	imports	exports	c	m	r	p	l	n	s	b	s-c	b-c	int
beta-Alanine(c)	MCES −Glutamine(c) +Glutamate(c)	de novo synthesis of cytosolic beta-Alanine	0.33 H_2O(s) 0.17 O_2(s) 0.67 Pyruvate(m) 0.67 Glutamine(c) 1.33 NADPH-redox-potential(m)	0.67 CO_2(s) 0.33 Glutamate(c) 1 beta-Alanine(c) 0.67 NADH-redox-potential(c) 0.67 NADH-redox-potential(m) 1.67 Proton-gradient(m)	6	13	-	-	-	-	-	-	3	-	10

ID	flux	reaction
r0820	0.67	AKG(c) + Malate(m) ⇌ AKG(m) + Malate(c)
r0217	-0.33	Glutamate(m) + 3-Oxopropanoate(m) ⇌ beta-Alanine(m) + AKG(m)
r0190	0.67	Glutamine(c) + Fructose-6P(c) → Glutamate(c) + Glucosamine-6P(c)
r0109	-0.67	OAA(c) + Glutamate(c) ⇌ AKG(c) + Aspartate(c)
r0911	0.33	Proline(c) + Glutamate(m) ⇌ Proline(m) + Glutamate(c)
r0128	0.67	Aspartate(c) ⇌ beta-Alanine(c) + CO2(c)
r0624	-0.33	2-Methyl-3-oxopropanoate(m) + CoA(m) + NAD+(m) ⇌ (R)-Methylmalonyl-CoA(m) + NADH(m)
r0507	0.33	Ubiquinol(m) + 2 Ferricytochrome C(m) + 4 H+(PG)(m) → Ubiquinone(m) + 4 H+(PG)(c) + 2 Ferrocytochrome C(m)
r0075	-0.67	AKG(m) + NADPH(m) + NH3(m) ⇌ Glutamate(m) + H2O(m) + NADP+(m)
r1426	0.33	Proline(m) + H+(PG)(c) → Proline(c) + H+(PG)(m)
r1327	-1.33	NADPH(m) ⇌ NADPH-redox-potential(m) + NADP+(m)
r0851	0.33	H2O(m) ⇌ H2O(c)
r0365	0.33	Hydracrylate(m) + NAD+(m) → NADH(m) + 3-Oxopropanoate(m)
r0059	-0.67	Pyruvate(m) + CO2(m) + NADPH(m) ⇌ Malate(m) + NADP+(m)
r0592	-0.33	H2O(m) + Acrylyl-CoA(m) → 3-Hydroxypropionyl-CoA(m)
r0683	0.33	Propanoyl-CoA(m) + Ubiquinone(m) → Ubiquinol(m) + Acrylyl-CoA(m)
r0197	-0.33	Succinyl-CoA(m) ⇌ (R)-Methylmalonyl-CoA(m)
r0855	0.17	O2(s) ⇌ O2(c)
r0189	0.67	Glucosamine-6P(c) + H2O(c) ⇌ Fructose-6P(c) + NH3(c)
r0104	0.67	NAD+(c) + Malate(c) ⇌ OAA(c) + NADH(c)
r0856	-0.17	O2(m) ⇌ O2(c)
r0838	0.67	NH3(c) ⇌ NH3(m)
r1330	1.67	H+(PG)(c) ⇌ H+(PG)(m) + Proton-gradient(m)
r1114	0.33	AKG(m) + CoA(m) + NAD+(m) ⇌ NADH(m) + CO2(m) + Succinyl-CoA(m)
r0596	-0.33	3-Hydroxypropionyl-CoA(m) + H2O(m) → Hydracrylate(m) + CoA(m)
r0223	0.33	2-Methyl-3-oxopropanoate(m) + CoA(m) + NAD+(m) → Propanoyl-CoA(m) + CO2(m) + NADH(m)
r1322	0.67	NADH(c) ⇌ NAD+(c) + NADH-redox-potential(c)
r0867	0.67	CO2(c) ⇌ CO2(s)
r0849	0.33	H2O(s) ⇌ H2O(c)
r0017	0.17	O2(m) + 4 H+(PG)(m) + 4 Ferrocytochrome C(m) → 4 Ferricytochrome C(m) + 2 H2O(m) + 4 H+(PG)(c)
r1323	0.67	NADH(m) ⇌ NAD+(m) + NADH-redox-potential(m)
r0900	-0.33	beta-Alanine(c) ⇌ beta-Alanine(m)

Putrescine (528)

Definition			Solution												
			exchanges		reactions								transp		
Objective	Constraints	Comment	imports	exports	c	m	r	p	l	n	s	b	s-c	b-c	int
Putrescine(c)	MCES −Glutamine(c) +Glutamate(c)	de novo synthesis of cytosolic Putrescine	1 Glutamine(c) 1 NADH-redox-potential(m) 1 NADPH-redox-potential(m) 2 Proton-gradient(m)	1 H_2O(s) 1 CO_2(s) 1 Putrescine(c)	1	6	-	-	-	-	-	-	2	-	4

ID	flux	reaction
r0074	-1	Glutamate(m) + NADH(m) ⇌ L-Glutamate 5-semialdehyde(m) + NAD+(m) + H2O(m)
r0075	-1	AKG(m) + NADPH(m) + NH3(m) ⇌ Glutamate(m) + H2O(m) + NADP+(m)
r1327	-1	NADPH(m) ⇌ NADPH-redox-potential(m) + NADP+(m)
r0851	1	H2O(m) ⇌ H2O(c)
r0167	-1	L-Glutamate 5-semialdehyde(m) + Glutamate(m) ⇌ AKG(m) + Ornithine(m)
r0168	1	Ornithine(c) ⇌ Putrescine(c) + CO2(c)
r0078	1	Glutamine(m) + H2O(m) → Glutamate(m) + NH3(m)
r0891	1	Glutamine(c) + H+(PG)(c) → Glutamine(m) + H+(PG)(m)
r1330	-2	H+(PG)(c) ⇌ H+(PG)(m) + Proton-gradient(m)
r0893	-1	H+(PG)(c) + Ornithine(m) → H+(PG)(m) + Ornithine(c)
r0867	1	CO2(c) ⇌ CO2(s)
r0849	-1	H2O(s) ⇌ H2O(c)
r1323	-1	NADH(m) ⇌ NAD+(m) + NADH-redox-potential(m)

Spermidine (529)

Definition			Solution												
			exchanges		reactions								transp		
Objective	Constraints	Comment	imports	exports	c	m	r	p	l	n	s	b	s-c	b-c	int
Spermidine	MCES +Methylthio-ribose-1P(c) −Glutamine(c) +Glutamate(c) −Methionine(c)	test that cytosolic Spermidine can be produced from the minimal exchange set while the cytosolic dump of Methylthioribose-1P is allowed	0.17 P_i(c) 1 Glutamine(c) 1 Methionine(c) 0.83 Glucose-6P(c) 4.17 ATP-energy(c) 1 NADH-redox-potential(m) 1 NADPH-redox-potential(m) 1 Proton-gradient(m)	2.17 H_2O(s) 2 CO_2(s) 1 Spermidine(s) 1 Methylthioribose-1P(c)	19	6	-	-	-	-	-	-	3	-	4

ID	flux	reaction
r0406	-0.33	Fructose-6P(c) + Erythrose-4P(c) ⇌ Sedoheptulose-7P(c) + GAP(c)
r0370	0.33	Sedoheptulose-7P(c) + GAP(c) ⇌ Ribose-5P(c) + D-Xylulose-5P(c)
r2522	1	Glutamine(c) ⇌ Glutamine(m)
r0344	-0.67	D-Xylulose-5P(c) ⇌ Ribulose-5P(c)
r0074	-1	Glutamate(m) + NADH(m) ⇌ L-Glutamate 5-semialdehyde(m) + NAD+(m) + H2O(m)
r0255	0.33	Fructose-6P(c) + GAP(c) ⇌ D-Xylulose-5P(c) + Erythrose-4P(c)
r0192	0.83	Glucose-6P(c) ⇌ Fructose-6P(c)
r0075	-1	AKG(m) + NADPH(m) + NH3(m) ⇌ Glutamate(m) + H2O(m) + NADP+(m)
r1327	-1	NADPH(m) ⇌ NADPH-redox-potential(m) + NADP+(m)
r0256	0.17	Fructose-1,6PP(c) ⇌ DHAP(c) + GAP(c)
r0247	1	ATP(c) + Ribose-5P(c) → AMP(c) + PRPP(c)
r1373	1	5-Methylthioadenosine(c) + Pi(c) → Methylthioribose-1P(c) + Adenine(c)
r1319	-4.17	ATP(c) + H2O(c) ⇌ ADP(c) + Pi(c) + ATP-energy(c)
r0851	1	H2O(m) ⇌ H2O(c)
r0167	-1	L-Glutamate 5-semialdehyde(m) + Glutamate(m) ⇌ AKG(m) + Ornithine(m)
r0429	1	S-Adenosylmethioninamine(c) + Putrescine(c) ⇌ 5-Methylthioadenosine(c) + Spermidine(c)
r0168	1	Ornithine(c) ⇌ Putrescine(c) + CO2(c)
r0078	1	Glutamine(m) + H2O(m) → Glutamate(m) + NH3(m)

Continued on next page

ID	flux	reaction
r0736	0.17	ATP(c) + Fructose-6P(c) → ADP(c) + Fructose-1,6PP(c)
r0030	2	ATP(c) + AMP(c) ⇌ 2 ADP(c)
r0944	1	Spermidine(c) ⇌ Spermidine(s)
r0050	-1	PRPP(c) + Adenine(c) ⇌ AMP(c) + PPi(c)
r0042	-1	ATP(c) + Methionine(c) + H2O(c) ⇌ Pi(c) + PPi(c) + SAM(c)
r1330	-1	H+(PG)(c) ⇌ H+(PG)(m) + Proton-gradient(m)
r0893	-1	H+(PG)(c) + Ornithine(m) → H+(PG)(m) + Ornithine(c)
r0250	-0.67	Ribulose-5P(c) ⇌ Ribose-5P(c)
r0043	1	SAM(c) ⇌ S-Adenosylmethioninamine(c) + CO2(c)
r0007	2	PPi(c) + H2O(c) → 2 Pi(c)
r0867	2	CO2(c) ⇌ CO2(s)
r0849	-2.17	H2O(s) ⇌ H2O(c)
r1323	-1	NADH(m) ⇌ NAD+(m) + NADH-redox-potential(m)
r0243	-0.17	DHAP(c) ⇌ GAP(c)

Spermine (530)

Definition			Solution												
			exchanges		reactions								transp		
Objective	Constraints	Comment	imports	exports	c	m	r	p	l	n	s	b	s-c	b-c	int
Spermine	MCES +Methylthio-ribose-1P(c) −Glutamine(c) +Glutamate(c) −Methionine(c)	test that cytosolic Spermine can be produced from the minimal exchange set while the cytosolic dump of Methylthioribose-1P is allowed	0.33 P_i(c) 1 Glutamine(c) 2 Methionine(c) 1.67 Glucose-6P(c) 8.33 ATP-energy(c) 2 NADH-redox-potential(m) 1 Proton-gradient(m)	3.33 H_2O(s) 3 CO_2(s) 1 Spermine(s) 2 Methylthioribose-1P(c)	20	5	-	-	-	-	-	-	3	-	4

ID	flux	reaction
r0406	-0.67	Fructose-6P(c) + Erythrose-4P(c) ⇌ Sedoheptulose-7P(c) + GAP(c)
r0370	0.67	Sedoheptulose-7P(c) + GAP(c) ⇌ Ribose-5P(c) + D-Xylulose-5P(c)
r0073	-1	AKG(m) + NADH(m) + NH3(m) ⇌ Glutamate(m) + NAD+(m) + H2O(m)
r2522	1	Glutamine(c) ⇌ Glutamine(m)
r0344	-1.33	D-Xylulose-5P(c) ⇌ Ribulose-5P(c)
r0074	-1	Glutamate(m) + NADH(m) ⇌ L-Glutamate 5-semialdehyde(m) + NAD+(m) + H2O(m)
r0255	0.67	Fructose-6P(c) + GAP(c) ⇌ D-Xylulose-5P(c) + Erythrose-4P(c)
r0192	1.67	Glucose-6P(c) ⇌ Fructose-6P(c)
r0256	0.33	Fructose-1,6PP(c) ⇌ DHAP(c) + GAP(c)
r0247	2	ATP(c) + Ribose-5P(c) → AMP(c) + PRPP(c)
r1373	2	5-Methylthioadenosine(c) + Pi(c) → Methylthioribose-1P(c) + Adenine(c)
r1319	-8.33	ATP(c) + H2O(c) ⇌ ADP(c) + Pi(c) + ATP-energy(c)
r0851	1	H2O(m) ⇌ H2O(c)
r0167	-1	L-Glutamate 5-semialdehyde(m) + Glutamate(m) ⇌ AKG(m) + Ornithine(m)
r0429	1	S-Adenosylmethioninamine(c) + Putrescine(c) ⇌ 5-Methylthioadenosine(c) + Spermidine(c)
r0168	1	Ornithine(c) ⇌ Putrescine(c) + CO2(c)
r0078	1	Glutamine(m) + H2O(m) → Glutamate(m) + NH3(m)
r0736	0.33	ATP(c) + Fructose-6P(c) → ADP(c) + Fructose-1,6PP(c)
r0574	1	S-Adenosylmethioninamine(c) + Spermidine(c) ⇌ 5-Methylthioadenosine(c) + Spermine(c)
r0030	4	ATP(c) + AMP(c) ⇌ 2 ADP(c)
r0972	1	Spermine(c) ⇌ Spermine(s)
r0050	-2	PRPP(c) + Adenine(c) ⇌ AMP(c) + PPi(c)
r0042	-2	ATP(c) + Methionine(c) + H2O(c) ⇌ Pi(c) + PPi(c) + SAM(c)
r1330	-1	H+(PG)(c) ⇌ H+(PG)(m) + Proton-gradient(m)
r0893	-1	H+(PG)(c) + Ornithine(m) → H+(PG)(m) + Ornithine(c)

Continued on next page

ID	flux	reaction
r0250	-1.33	Ribulose-5P(c) ⇌ Ribose-5P(c)
r0043	2	SAM(c) ⇌ S-Adenosylmethioninamine(c) + CO2(c)
r0007	4	PPi(c) + H2O(c) → 2 Pi(c)
r0867	3	CO2(c) ⇌ CO2(s)
r0849	-3.33	H2O(s) ⇌ H2O(c)
r1323	-2	NADH(m) ⇌ NAD+(m) + NADH-redox-potential(m)
r0243	-0.33	DHAP(c) ⇌ GAP(c)

Methylthioribose-1P (531)

Definition			Solution												
			exchanges		reactions								transp		
Objective	Constraints	Comment	imports	exports	c	m	r	p	l	n	s	b	s-c	b-c	int
Methylthioribose-1P(c)	+Spermidine MCES −Glutamine(c) +Glutamate(c) −Methionine(c)	test that cytosolic Methylthioribose-1P can be produced from the minimal exchange set while the cytosolic dump of Spermidine is allowed	0.17 P_i(c) 1 Methionine(c) 0.83 Glucose-6P(c) 4.17 ATP-energy(c)	1.17 H_2O(s) 1 CO_2(s) 1 Adenosylmethioninamine-potential(c) 1 Methylthioribose-1P(c)	18	-	-	-	-	-	-	-	2	-	-

ID	flux	reaction
r0406	-0.33	Fructose-6P(c) + Erythrose-4P(c) ⇌ Sedoheptulose-7P(c) + GAP(c)
r0370	0.33	Sedoheptulose-7P(c) + GAP(c) ⇌ Ribose-5P(c) + D-Xylulose-5P(c)
r0344	-0.67	D-Xylulose-5P(c) ⇌ Ribulose-5P(c)
r0255	0.33	Fructose-6P(c) + GAP(c) ⇌ D-Xylulose-5P(c) + Erythrose-4P(c)
r0192	0.83	Glucose-6P(c) ⇌ Fructose-6P(c)
r0256	0.17	Fructose-1,6PP(c) ⇌ DHAP(c) + GAP(c)
r0247	1	ATP(c) + Ribose-5P(c) → AMP(c) + PRPP(c)
r1373	1	5-Methylthioadenosine(c) + Pi(c) → Methylthioribose-1P(c) + Adenine(c)
r1319	-4.17	ATP(c) + H2O(c) ⇌ ADP(c) + Pi(c) + ATP-energy(c)
r0001	1	S-Adenosylmethioninamine(c) → 5-Methylthioadenosine(c) + Adenosylmethioninamine-potential(c)
r0736	0.17	ATP(c) + Fructose-6P(c) → ADP(c) + Fructose-1,6PP(c)
r0030	2	ATP(c) + AMP(c) ⇌ 2 ADP(c)
r0050	-1	PRPP(c) + Adenine(c) ⇌ AMP(c) + PPi(c)
r0042	-1	ATP(c) + Methionine(c) + H2O(c) ⇌ Pi(c) + PPi(c) + SAM(c)
r0250	-0.67	Ribulose-5P(c) ⇌ Ribose-5P(c)
r0043	1	SAM(c) ⇌ S-Adenosylmethioninamine(c) + CO2(c)
r0007	2	PPi(c) + H2O(c) → 2 Pi(c)
r0867	1	CO2(c) ⇌ CO2(s)
r0849	-1.17	H2O(s) ⇌ H2O(c)
r0243	-0.17	DHAP(c) ⇌ GAP(c)

4.5 C5. Comprehensive list

(2E)-Decenoyl-ACP (532)

Definition			Solution												
			exchanges		reactions								transp		
Objective	Constraints	Comment	imports	exports	c	m	r	p	l	n	s	b	s-c	b-c	int
(2E)-Decenoyl-ACP(c)	PIPES MCES	synthesis of cytosolic (2E)-Decenoyl-ACP	1 P_i(c) 5 Glutamate(s) 6 Glycine(s) 11 Alanine(s) 4 Lysine(s) 8 Aspartate(s) 9 Arginine(s) 7 Glutamine(s) 8 Serine(s) 5 Methionine(s) 3 Phenylalanine(s) 4 Tyrosine(s) 3 Cysteine(s) 20 Leucine(s) 1 Histidine(s) 9 Proline(s) 1 Asparagine(s) 11 Valine(s) 6 Threonine(s) 4 Isoleucine(s) 1 Pantothenate(s) 628 ATP-energy(c) 7 NADPH-redox-potential(c) 3 Proton-gradient(c) 5 CoA-activated acetyl group(c)	7 H_2O(s) 1 CO_2(s) 1 (2E)-Decenoyl-ACP(c) 66 Na-gradient(c)	34	-	-	-	-	-	-	-	24	-	-

ID	flux	reaction
r1069	-4	Lysine(c) ⇌ Lysine(s)
r0578	1	ATP(c) + Pantetheine(c) ⇌ ADP(c) + Phosphopantetheine(c)
r2478	8	Serine(s) + Na+(s) + H+(PG)(c) → Serine(c) + Na+(c) + H+(PG)(s)
r0030	125	ATP(c) + AMP(c) ⇌ 2 ADP(c)
r0760	1	Butyryl-ACP(c) + Malonyl-ACP(c) ⇌ CO2(c) + ACP(c) + 3-Oxohexanoyl-ACP(c)
r0032	1	ATP(c) + Dephospho-CoA(c) ⇌ ADP(c) + CoA(c)
r0761	-1	D-3-Hydroxyhexanoyl-ACP(c) + NADP+(c) ⇌ NADPH(c) + 3-Oxohexanoyl-ACP(c)
r0184	4	ATP(c) + Acetyl-CoA(c) + HCO3-(c) → ADP(c) + Pi(c) + Malonyl-CoA(c)
r0762	1	D-3-Hydroxyhexanoyl-ACP(c) ⇌ H2O(c) + (2E)-Hexenoyl-ACP(c)
r0763	-1	Hexanoyl-ACP(c) + NADP+(c) ⇌ NADPH(c) + (2E)-Hexenoyl-ACP(c)
r0764	1	Malonyl-ACP(c) + Hexanoyl-ACP(c) ⇌ 3-Oxooctanoyl-ACP(c) + CO2(c) + ACP(c)
r1122	-20	Leucine(c) + Na+(c) ← Leucine(s) + Na+(s)
r0765	-1	Octanoyl-ACP(c) + NADP+(c) ⇌ (2E)-Octenoyl-ACP(c) + NADPH(c)
r1123	-4	Isoleucine(c) + Na+(c) ← Isoleucine(s) + Na+(s)
r0766	1	Octanoyl-ACP(c) + Malonyl-ACP(c) ⇌ 3-Oxodecanoyl-ACP(c) + CO2(c) + ACP(c)
r1125	4	Tyrosine(s) + Na+(s) → Tyrosine(c) + Na+(c)
r1142	-6	Threonine(s) + Na+(s) → Threonine(c) + Na+(c)
r1143	-8	Na+(c) + Aspartate(c) ← Na+(s) + Aspartate(s)
r2664	66	Na+(c) ⇌ Na+(s) + Na-gradient(c)
r1144	-5	Glutamate(c) + Na+(c) ← Glutamate(s) + Na+(s)
r1145	-1	Histidine(c) + Na+(c) ← Histidine(s) + Na+(s)
r2532	1	Asparagine(s) ⇌ Asparagine(c)
r2533	11	Valine(s) ⇌ Valine(c)
r0678	1	Acetyl-ACP(c) + Malonyl-ACP(c) ⇌ CO2(c) + ACP(c) + Acetoacetyl-ACP(c)
r0691	-1	(R)-3-Hydroxybutanoyl-ACP(c) + NADP+(c) ⇌ NADPH(c) + Acetoacetyl-ACP(c)
r1331	-3	H+(PG)(s) ⇌ H+(PG)(c) + Proton-gradient(c)
r0974	1	Pantothenate(s) + Na+(s) ⇌ Pantothenate(c) + Na+(c)
r0692	-1	(R)-3-Hydroxydecanoyl-ACP(c) + NADP+(c) ⇌ 3-Oxodecanoyl-ACP(c) + NADPH(c)
r1319	-628	ATP(c) + H2O(c) ⇌ ADP(c) + Pi(c) + ATP-energy(c)
r0693	1	(R)-3-Hydroxydecanoyl-ACP(c) ⇌ (2E)-Decenoyl-ACP(c) + H2O(c)
r0694	-1	(R)-3-Hydroxyoctanoyl-ACP(c) + NADP+(c) ⇌ 3-Oxooctanoyl-ACP(c) + NADPH(c)
r0695	1	(R)-3-Hydroxyoctanoyl-ACP(c) ⇌ (2E)-Octenoyl-ACP(c) + H2O(c)
r1058	9	Arginine(s) ⇌ Arginine(c)
r1072	5	Methionine(s) ⇌ Methionine(c)
r0849	-7	H2O(s) ⇌ H2O(c)
r0581	-1	Pantothenate(c) + Cysteamine(c) ⇌ H2O(c) + Pantetheine(c)
r0867	1	CO2(c) ⇌ CO2(s)

Continued on next page

ID	flux	reaction
r1096	1	11 Alanine(c) + 2 Cysteine(c) + 5 Glutamate(c) + 3 Phenylalanine(c) + 6 Glycine(c) + Histidine(c) + 4 Isoleucine(c) + 20 Leucine(c) + 5 Methionine(c) + 9 Proline(c) + 7 Glutamine(c) + 9 Arginine(c) + 8 Serine(c) + 6 Threonine(c) + 11 Valine(c) + 4 Tyrosine(c) + 496 ATP(c) + 496 H2O(c) + 8 Aspartate(c) + 4 Lysine(c) + Asparagine(c) → Apo-ACP(c) + 124 AMP(c) + 124 PPi(c) + 372 ADP(c) + 372 Pi(c)
r0589	1	ATP(c) + Phosphopantetheine(c) → PPi(c) + Dephospho-CoA(c)
r2469	11	Alanine(s) + H+(PG)(s) → Alanine(c) + H+(PG)(c)
r0007	125	PPi(c) + H2O(c) → 2 Pi(c)
r1417	-4	CO2(c) + H2O(c) ⇌ Carbonate(c)
r0049	1	PAP(c) + H2O(c) → AMP(c) + Pi(c)
r1419	4	Carbonate(c) ⇌ HCO3-(c)
r1139	-9	Proline(c) + Na+(c) ← Proline(s) + Na+(s)
r0211	1	Cysteine(c) + O2(c) ⇌ 3-Sulfinoalanine(c)
r2523	6	Glycine(s) ⇌ Glycine(c)
r2677	-5	Acetyl-CoA(c) ⇌ CoA(c) + CoA-activated acetyl group(c)
r2525	7	Glutamine(s) ⇌ Glutamine(c)
r0367	1	Acetyl-CoA(c) + ACP(c) ⇌ CoA(c) + Acetyl-ACP(c)
r0368	1	CoA(c) + Apo-ACP(c) → PAP(c) + ACP(c)
r2528	3	Phenylalanine(s) ⇌ Phenylalanine(c)
r0369	4	ACP(c) + Malonyl-CoA(c) ⇌ CoA(c) + Malonyl-ACP(c)
r2529	3	Cysteine(s) ⇌ Cysteine(c)
r0681	1	(R)-3-Hydroxybutanoyl-ACP(c) ⇌ But-2-enoyl-ACP(c) + H2O(c)
r0682	-1	Butyryl-ACP(c) + NADP+(c) ⇌ But-2-enoyl-ACP(c) + NADPH(c)
r1326	-7	NADPH(c) ⇌ NADPH-redox-potential(c) + NADP+(c)
r0538	1	3-Sulfinoalanine(c) ⇌ CO2(c) + Hypotaurine(c)
r0539	-1	Hypotaurine(c) ⇌ Cysteamine(c) + O2(c)

(2E)-Decenoyl-CoA (533)

Definition			Solution												
			exchanges		reactions								transp		
Objective	Constraints	Comment	imports	exports	c	m	r	p	l	n	s	b	s-c	b-c	int
(2E)-Decenoyl-CoA(m)	PIPES MCES	synthesis of mitochondrial (2E)-Decenoyl-CoA	0.5 O_2(s) 2.17 P_i(c) 1 Glycine(s) 2 Aspartate(s) 2 Glutamine(s) 0.83 Glucose-6P(c) 0.5 Cystine(s) 1 Pantothenate(s) 1 Palmitolate(s) 13.2 ATP-energy(c) 1 Proton-gradient(c) 2 THF-activated methyl group(c)	3.17 H_2O(s) 2 CO_2(s) 2 Pyruvate(c) 2 Glutamate(c) 1 (2E)-Decenoyl-CoA(m) 2 NADH-redox-potential(c) 4 NADH-redox-potential(m) 3.5 NADPH-redox-potential(c) 3 FADH-redox-potential(c) 6 Proton-gradient(m) 3 Na-gradient(c) 4 CoA-activated acetyl group(m)	44	19	-	-	-	-	-	-	12	-	7

ID	flux	reaction
r0406	-0.33	Fructose-6P(c) + Erythrose-4P(c) ⇌ Sedoheptulose-7P(c) + GAP(c)
r0277	1	GTP(c) + IMP(c) + Aspartate(c) → GDP(c) + Pi(c) + Adenylosuccinate(c)
r0855	0.5	O2(s) ⇌ O2(c)
r0856	-0.5	O2(m) ⇌ O2(c)
r0705	1	ATP(c) + 5-Phosphoribosyl-4-carboxy-5-aminoimidazole(c) + Aspartate(c) ⇌ ADP(c) + Pi(c) + SAICAR(c)
r0293	2	5,10-Methylene-THF(c) + NADP+(c) ⇌ 5,10-Methenyl-THF(c) + NADPH(c)
r0578	1	ATP(c) + Pantetheine(c) ⇌ ADP(c) + Phosphopantetheine(c)
r0722	1	(S)-3-Hydroxydodecanoyl-CoA(m) + NAD+(m) ⇌ 3-Oxododecanoyl-CoA(m) + NADH(m)
r0724	-1	Decanoyl-CoA(m) + Acetyl-CoA(m) ⇌ CoA(m) + 3-Oxododecanoyl-CoA(m)
r0877	0.5	Glutamate(c) + Cystine(s) ⇌ Glutamate(s) + Cystine(c)
r0017	0.5	O2(m) + 4 H+(PG)(m) + 4 Ferrocytochrome C(m) → 4 Ferricytochrome C(m) + 2 H2O(m) + 4 H+(PG)(c)
r0030	3	ATP(c) + AMP(c) ⇌ 2 ADP(c)
r0032	1	ATP(c) + Dephospho-CoA(c) ⇌ ADP(c) + CoA(c)
r1290	3	FAD(c) + FADH2(m) ⇌ FAD(m) + FADH2(c)
r1143	-2	Na+(c) + Aspartate(c) ← Na+(s) + Aspartate(s)

Continued on next page

ID	flux	reaction
r2664	3	Na+(c) ⇌ Na+(s) + Na-gradient(c)
r0058	2	Malate(c) + NADP+(c) ⇌ Pyruvate(c) + CO2(c) + NADPH(c)
r0370	0.33	Sedoheptulose-7P(c) + GAP(c) ⇌ Ribose-5P(c) + D-Xylulose-5P(c)
r0371	2	5,10-Methenyl-THF(c) + H2O(c) ⇌ 10-Formyl-THF(c)
r2685	1.5	Glutamate(c) ⇌ Glutamate(s)
r0657	1	(3Z)-Dodecenoyl-CoA(m) ⇌ (2E)-Dodecenoyl-CoA(m)
r0659	1	ATP(c) + 5-Phosphoribosylamine(c) + Glycine(c) ⇌ ADP(c) + Pi(c) + GAR(c)
r0507	1	Ubiquinol(m) + 2 Ferricytochrome C(m) + 4 H+(PG)(m) → Ubiquinone(m) + 4 H+(PG)(c) + 2 Ferrocytochrome C(m)
r1480	1	CoA(m) + 3-oxopalmitoleoyl-CoA(m) ⇌ Acetyl-CoA(m) + cis-myrist-7-enoyl-CoA(m)
r0099	1	ATP(c) + GDP(c) ⇌ ADP(c) + GTP(c)
r1468	1	palmitoleoyl-CoA(c) ⇌ palmitoleoyl-CoA(m)
r0243	-0.17	DHAP(c) ⇌ GAP(c)
r0677	1	10-Formyl-THF(c) + GAR(c) ⇌ THF(c) + N-Formyl-GAR(c)
r1482	-1	FADH2(m) + trans,cis-dodeca-2,5-dienoyl-CoA(m) ⇌ FAD(m) + cis-laur-5-enoyl-CoA(m)
r1330	6	H+(PG)(c) ⇌ H+(PG)(m) + Proton-gradient(m)
r1483	1	H2O(m) + trans,cis-myristo-2,7-dienoyl-CoA(m) ⇌ cis-(3S)-hydroxytetradec-7-enoyl-CoA(m)
r1331	-1	H+(PG)(s) ⇌ H+(PG)(c) + Proton-gradient(c)
r0974	1	Pantothenate(s) + Na+(s) ⇌ Pantothenate(c) + Na+(c)
r1484	1	CoA(m) + 3-oxomyrist-7-enoyl-CoA(m) ⇌ Acetyl-CoA(m) + cis-laur-5-enoyl-CoA(m)
r1319	-13.2	ATP(c) + H2O(c) ⇌ ADP(c) + Pi(c) + ATP-energy(c)
r1485	1	H2O(m) + trans,cis-dodeca-2,5-dienoyl-CoA(m) ⇌ 3(S)-3-hydroxydodecen-(5Z)-oyl-CoA(m)
r0260	1	Adenylosuccinate(c) → Fumarate(c) + AMP(c)
r0247	1	ATP(c) + Ribose-5P(c) → AMP(c) + PRPP(c)
r1486	1	NAD+(m) + 3(S)-3-hydroxydodecen-(5Z)-oyl-CoA(m) ⇌ 3-oxolaur-cis-5-enoyl-CoA(m) + NADH(m)
r1488	1	ATP(c) + CoA(c) + Palmitolate(c) ⇌ AMP(c) + palmitoleoyl-CoA(c) + PPi(c)
r0699	1	SAICAR(c) ⇌ Fumarate(c) + AICAR(c)
r2442	1	Palmitolate(s) → Palmitolate(c)
r0849	-3.17	H2O(s) ⇌ H2O(c)
r0581	-1	Pantothenate(c) + Cysteamine(c) ⇌ H2O(c) + Pantetheine(c)
r0867	2	CO2(c) ⇌ CO2(s)
r0589	1	ATP(c) + Phosphopantetheine(c) → PPi(c) + Dephospho-CoA(c)
r2468	1	Glycine(s) + H+(PG)(s) → Glycine(c) + H+(PG)(c)
r0736	0.17	ATP(c) + Fructose-6P(c) → ADP(c) + Fructose-1,6PP(c)
r0007	3	PPi(c) + H2O(c) → 2 Pi(c)
r0027	-0.5	NADPH(c) + Cystine(c) ⇌ 2 Cysteine(c) + NADP+(c)
r0192	0.83	Glucose-6P(c) ⇌ Fructose-6P(c)
r0344	-0.67	D-Xylulose-5P(c) ⇌ Ribulose-5P(c)
r0792	2	5-Methyl-THF(c) + NAD+(c) ⇌ 5,10-Methylene-THF(c) + NADH(c)
r0211	1	Cysteine(c) + O2(c) ⇌ 3-Sulfinoalanine(c)
r2676	-2	5-Methyl-THF(c) ⇌ THF(c) + THF-activated methyl group(c)
r1451	1	Ubiquinone(m) + Decanoyl-CoA(m) → (2E)-Decenoyl-CoA(m) + Ubiquinol(m)
r0660	-1	(S)-3-Hydroxydodecanoyl-CoA(m) ⇌ (2E)-Dodecenoyl-CoA(m) + H2O(m)
r2525	2	Glutamine(s) ⇌ Glutamine(c)
r2678	4	Acetyl-CoA(m) ⇌ CoA(m) + CoA-activated acetyl group(m)
r0666	1	ATP(c) + 5-Phosphoribosylformylglycinamidine(c) ⇌ ADP(c) + Pi(c) + AIR(c)
r1471	1	FAD(m) + cis-myrist-7-enoyl-CoA(m) ⇌ FADH2(m) + trans,cis-myristo-2,7-dienoyl-CoA(m)
r0667	-1	5-Phosphoribosyl-4-carboxy-5-aminoimidazole(c) ⇌ AIR(c) + CO2(c)
r1473	1	FAD(m) + palmitoleoyl-CoA(m) ⇌ FADH2(m) + trans,cis-hexadeca-2,9-dienoyl-CoA(m)
r1322	2	NADH(c) ⇌ NAD+(c) + NADH-redox-potential(c)
r1475	1	H2O(m) + trans,cis-hexadeca-2,9-dienoyl-CoA(m) ⇌ (S)-3-hydroxypalmitoleoyl-CoA(m)
r1323	4	NADH(m) ⇌ NAD+(m) + NADH-redox-potential(m)
r0250	-0.67	Ribulose-5P(c) ⇌ Ribose-5P(c)
r1476	1	NAD+(m) + cis-(3S)-hydroxytetradec-7-enoyl-CoA(m) ⇌ 3-oxomyrist-7-enoyl-CoA(m) + NADH(m)
r1478	1	NAD+(m) + (S)-3-hydroxypalmitoleoyl-CoA(m) ⇌ NADH(m) + 3-oxopalmitoleoyl-CoA(m)
r1326	3.5	NADPH(c) ⇌ NADPH-redox-potential(c) + NADP+(c)

Continued on next page

(2E)-Decenoyl-CoA (533)– continued

ID	flux	reaction
r0687	1	ATP(c) + N-Formyl-GAR(c) + Glutamine(c) + H2O(c) ⇌ ADP(c) + Pi(c) + Glutamate(c) + 5-Phosphoribosylformylglycinamidine(c)
r1479	1	CoA(m) + 3-oxolaur-cis-5-enoyl-CoA(m) ⇌ Acetyl-CoA(m) + (3Z)-Dodecenoyl-CoA(m)
r0255	0.33	Fructose-6P(c) + GAP(c) ⇌ D-Xylulose-5P(c) + Erythrose-4P(c)
r1329	3	FADH2(c) ⇌ FAD(c) + FADH-redox-potential(c)
r0538	1	3-Sulfinoalanine(c) ⇌ CO2(c) + Hypotaurine(c)
r0256	0.17	Fructose-1,6PP(c) ⇌ DHAP(c) + GAP(c)
r0539	-1	Hypotaurine(c) ⇌ Cysteamine(c) + O2(c)
r0257	-1	Glutamine(c) + PRPP(c) + H2O(c) ⇌ 5-Phosphoribosylamine(c) + PPi(c) + Glutamate(c)
r0258	-2	Fumarate(c) + H2O(c) ⇌ Malate(c)
r0273	-1	IMP(c) + H2O(c) ⇌ FAICAR(c)
r0851	-3	H2O(m) ⇌ H2O(c)
r0700	1	10-Formyl-THF(c) + AICAR(c) ⇌ THF(c) + FAICAR(c)

(2E)-Dodecenoyl-ACP (534)

<table>
<tr><th colspan="3">Definition</th><th colspan="13">Solution</th></tr>
<tr><th colspan="3"></th><th colspan="2">exchanges</th><th colspan="8">reactions</th><th colspan="3">transp</th></tr>
<tr><td>Objective</td><td>Constraints</td><td>Comment</td><td>imports</td><td>exports</td><td>c</td><td>m</td><td>r</td><td>p</td><td>l</td><td>n</td><td>s</td><td>b</td><td>s-c</td><td>b-c</td><td>int</td></tr>
<tr><td>(2E)-Dodecenoyl-ACP(c)</td><td>PIPES MCES</td><td>synthesis of cytosolic (2E)-Dodecenoyl-ACP</td><td>1 P_i(c) 5 Glutamate(s) 6 Glycine(s) 11 Alanine(s) 4 Lysine(s) 8 Aspartate(s) 9 Arginine(s) 7 Glutamine(s) 8 Serine(s) 5 Methionine(s) 3 Phenylalanine(s) 4 Tyrosine(s) 3 Cysteine(s) 20 Leucine(s) 1 Histidine(s) 9 Proline(s) 1 Asparagine(s) 11 Valine(s) 6 Threonine(s) 4 Isoleucine(s) 1 Pantothenate(s) 629 ATP-energy(c) 9 NADPH-redox-potential(c) 3 Proton-gradient(c) 6 CoA-activated acetyl group(c)</td><td>8 H_2O(s) 1 CO_2(s) 1 (2E)-Dodecenoyl-ACP(c) 66 Na-gradient(c)</td><td>38</td><td>-</td><td>-</td><td>-</td><td>-</td><td>-</td><td>-</td><td>-</td><td>24</td><td>-</td><td>-</td></tr>
</table>

ID	flux	reaction
r1069	-4	Lysine(c) ⇌ Lysine(s)
r0578	1	ATP(c) + Pantetheine(c) ⇌ ADP(c) + Phosphopantetheine(c)
r2478	8	Serine(s) + Na+(s) + H+(PG)(c) → Serine(c) + Na+(c) + H+(PG)(s)
r0030	125	ATP(c) + AMP(c) ⇌ 2 ADP(c)
r0760	1	Butyryl-ACP(c) + Malonyl-ACP(c) ⇌ CO2(c) + ACP(c) + 3-Oxohexanoyl-ACP(c)
r0032	1	ATP(c) + Dephospho-CoA(c) ⇌ ADP(c) + CoA(c)
r0761	-1	D-3-Hydroxyhexanoyl-ACP(c) + NADP+(c) ⇌ NADPH(c) + 3-Oxohexanoyl-ACP(c)
r0184	5	ATP(c) + Acetyl-CoA(c) + HCO3-(c) → ADP(c) + Pi(c) + Malonyl-CoA(c)
r0762	1	D-3-Hydroxyhexanoyl-ACP(c) ⇌ H2O(c) + (2E)-Hexenoyl-ACP(c)
r0763	-1	Hexanoyl-ACP(c) + NADP+(c) ⇌ NADPH(c) + (2E)-Hexenoyl-ACP(c)
r0764	1	Malonyl-ACP(c) + Hexanoyl-ACP(c) ⇌ 3-Oxooctanoyl-ACP(c) + CO2(c) + ACP(c)
r1122	-20	Leucine(c) + Na+(c) ← Leucine(s) + Na+(s)
r0765	-1	Octanoyl-ACP(c) + NADP+(c) ⇌ (2E)-Octenoyl-ACP(c) + NADPH(c)
r1123	-4	Isoleucine(c) + Na+(c) ← Isoleucine(s) + Na+(s)
r0766	1	Octanoyl-ACP(c) + Malonyl-ACP(c) ⇌ 3-Oxodecanoyl-ACP(c) + CO2(c) + ACP(c)
r0767	-1	Decanoyl-ACP(c) + NADP+(c) ⇌ (2E)-Decenoyl-ACP(c) + NADPH(c)
r1125	4	Tyrosine(s) + Na+(s) → Tyrosine(c) + Na+(c)
r0768	1	Decanoyl-ACP(c) + Malonyl-ACP(c) ⇌ 3-Oxododecanoyl-ACP(c) + CO2(c) + ACP(c)
r0769	-1	D-3-Hydroxydodecanoyl-ACP(c) + NADP+(c) ⇌ 3-Oxododecanoyl-ACP(c) + NADPH(c)
r1142	-6	Threonine(s) + Na+(s) → Threonine(c) + Na+(c)
r1143	-8	Na+(c) + Aspartate(c) ← Na+(s) + Aspartate(s)
r2664	66	Na+(c) ⇌ Na+(s) + Na-gradient(c)

Continued on next page

ID	flux	reaction
r1144	-5	Glutamate(c) + Na+(c) ← Glutamate(s) + Na+(s)
r1145	-1	Histidine(c) + Na+(c) ← Histidine(s) + Na+(s)
r2532	1	Asparagine(s) ⇌ Asparagine(c)
r2533	11	Valine(s) ⇌ Valine(c)
r0678	1	Acetyl-ACP(c) + Malonyl-ACP(c) ⇌ CO2(c) + ACP(c) + Acetoacetyl-ACP(c)
r0691	-1	(R)-3-Hydroxybutanoyl-ACP(c) + NADP+(c) ⇌ NADPH(c) + Acetoacetyl-ACP(c)
r1331	-3	H+(PG)(s) ⇌ H+(PG)(c) + Proton-gradient(c)
r0974	1	Pantothenate(s) + Na+(s) ⇌ Pantothenate(c) + Na+(c)
r0692	-1	(R)-3-Hydroxydecanoyl-ACP(c) + NADP+(c) ⇌ 3-Oxodecanoyl-ACP(c) + NADPH(c)
r1319	-629	ATP(c) + H2O(c) ⇌ ADP(c) + Pi(c) + ATP-energy(c)
r0693	1	(R)-3-Hydroxydecanoyl-ACP(c) ⇌ (2E)-Decenoyl-ACP(c) + H2O(c)
r0694	-1	(R)-3-Hydroxyoctanoyl-ACP(c) + NADP+(c) ⇌ 3-Oxooctanoyl-ACP(c) + NADPH(c)
r0695	1	(R)-3-Hydroxyoctanoyl-ACP(c) ⇌ (2E)-Octenoyl-ACP(c) + H2O(c)
r1058	9	Arginine(s) ⇌ Arginine(c)
r1072	5	Methionine(s) ⇌ Methionine(c)
r0849	-8	H2O(s) ⇌ H2O(c)
r0581	-1	Pantothenate(c) + Cysteamine(c) ⇌ H2O(c) + Pantetheine(c)
r0867	1	CO2(c) ⇌ CO2(s)
r1096	1	11 Alanine(c) + 2 Cysteine(c) + 5 Glutamate(c) + 3 Phenylalanine(c) + 6 Glycine(c) + Histidine(c) + 4 Isoleucine(c) + 20 Leucine(c) + 5 Methionine(c) + 9 Proline(c) + 7 Glutamine(c) + 9 Arginine(c) + 8 Serine(c) + 6 Threonine(c) + 11 Valine(c) + 4 Tyrosine(c) + 496 ATP(c) + 496 H2O(c) + 8 Aspartate(c) + 4 Lysine(c) + Asparagine(c) → Apo-ACP(c) + 124 AMP(c) + 124 PPi(c) + 372 ADP(c) + 372 Pi(c)
r0589	1	ATP(c) + Phosphopantetheine(c) → PPi(c) + Dephospho-CoA(c)
r2469	11	Alanine(s) + H+(PG)(s) → Alanine(c) + H+(PG)(c)
r0007	125	PPi(c) + H2O(c) → 2 Pi(c)
r0770	1	D-3-Hydroxydodecanoyl-ACP(c) ⇌ (2E)-Dodecenoyl-ACP(c) + H2O(c)
r1417	-5	CO2(c) + H2O(c) ⇌ Carbonate(c)
r0049	1	PAP(c) + H2O(c) → AMP(c) + Pi(c)
r1419	5	Carbonate(c) ⇌ HCO3-(c)
r1139	-9	Proline(c) + Na+(c) ← Proline(s) + Na+(s)
r0211	1	Cysteine(c) + O2(c) ⇌ 3-Sulfinoalanine(c)
r2523	6	Glycine(s) ⇌ Glycine(c)
r2677	-6	Acetyl-CoA(c) ⇌ CoA(c) + CoA-activated acetyl group(c)
r2525	7	Glutamine(s) ⇌ Glutamine(c)
r0367	1	Acetyl-CoA(c) + ACP(c) ⇌ CoA(c) + Acetyl-ACP(c)
r0368	1	CoA(c) + Apo-ACP(c) → PAP(c) + ACP(c)
r2528	3	Phenylalanine(s) ⇌ Phenylalanine(c)
r0369	5	ACP(c) + Malonyl-CoA(c) ⇌ CoA(c) + Malonyl-ACP(c)
r2529	3	Cysteine(s) ⇌ Cysteine(c)
r0681	1	(R)-3-Hydroxybutanoyl-ACP(c) ⇌ But-2-enoyl-ACP(c) + H2O(c)
r0682	-1	Butyryl-ACP(c) + NADP+(c) ⇌ But-2-enoyl-ACP(c) + NADPH(c)
r1326	-9	NADPH(c) ⇌ NADPH-redox-potential(c) + NADP+(c)
r0538	1	3-Sulfinoalanine(c) ⇌ CO2(c) + Hypotaurine(c)
r0539	-1	Hypotaurine(c) ⇌ Cysteamine(c) + O2(c)

(2E)-Dodecenoyl-CoA (535)

Definition			Solution												
			exchanges		reactions								transp		
Objective	Constraints	Comment	imports	exports	c	m	r	p	l	n	s	b	s-c	b-c	int
(2E)-Dodecenoyl-CoA(m)	PIPES MCES	synthesis of mitochondrial (2E)-Dodecenoyl-CoA	2.17 P_i(c) 1 Glycine(s) 2 Aspartate(s) 2 Glutamine(s) 0.83 Glucose-6P(c) 0.5 Cystine(s) 1 Pantothenate(s) 1 Palmitolate(s) 13.2 ATP-energy(c) 1 Proton-gradient(c) 2 THF-activated methyl group(c)	3.17 H_2O(s) 2 CO_2(s) 2 Pyruvate(c) 2 Glutamate(s) 1 (2E)-Dodecenoyl-CoA(m) 2 NADH-redox-potential(c) 3 NADH-redox-potential(m) 3.5 NADPH-redox-potential(c) 3 FADH-redox-potential(c) 3 Na-gradient(c) 3 CoA-activated acetyl group(m)	44	15	-	-	-	-	-	-	11	-	3

ID	flux	reaction
r0406	-0.33	Fructose-6P(c) + Erythrose-4P(c) ⇌ Sedoheptulose-7P(c) + GAP(c)
r0277	1	GTP(c) + IMP(c) + Aspartate(c) → GDP(c) + Pi(c) + Adenylosuccinate(c)
r0293	2	5,10-Methylene-THF(c) + NADP+(c) ⇌ 5,10-Methenyl-THF(c) + NADPH(c)
r0705	1	ATP(c) + 5-Phosphoribosyl-4-carboxy-5-aminoimidazole(c) + Aspartate(c) ⇌ ADP(c) + Pi(c) + SAICAR(c)
r0578	1	ATP(c) + Pantetheine(c) ⇌ ADP(c) + Phosphopantetheine(c)
r0877	0.5	Glutamate(c) + Cystine(s) ⇌ Glutamate(s) + Cystine(c)
r0030	3	ATP(c) + AMP(c) ⇌ 2 ADP(c)
r0032	1	ATP(c) + Dephospho-CoA(c) ⇌ ADP(c) + CoA(c)
r1290	3	FAD(c) + FADH2(m) ⇌ FAD(m) + FADH2(c)
r1143	-2	Na+(c) + Aspartate(c) ← Na+(s) + Aspartate(s)
r2664	3	Na+(c) ⇌ Na+(s) + Na-gradient(c)
r0058	2	Malate(c) + NADP+(c) ⇌ Pyruvate(c) + CO2(c) + NADPH(c)
r0370	0.33	Sedoheptulose-7P(c) + GAP(c) ⇌ Ribose-5P(c) + D-Xylulose-5P(c)
r0371	2	5,10-Methenyl-THF(c) + H2O(c) ⇌ 10-Formyl-THF(c)
r2685	1.5	Glutamate(c) ⇌ Glutamate(s)
r0657	1	(3Z)-Dodecenoyl-CoA(m) ⇌ (2E)-Dodecenoyl-CoA(m)
r0659	1	ATP(c) + 5-Phosphoribosylamine(c) + Glycine(c) ⇌ ADP(c) + Pi(c) + GAR(c)
r1480	1	CoA(m) + 3-oxopalmitoleoyl-CoA(m) ⇌ Acetyl-CoA(m) + cis-myrist-7-enoyl-CoA(m)
r0099	1	ATP(c) + GDP(c) ⇌ ADP(c) + GTP(c)
r1468	1	palmitoleoyl-CoA(c) ⇌ palmitoleoyl-CoA(m)
r0243	-0.17	DHAP(c) ⇌ GAP(c)
r0677	1	10-Formyl-THF(c) + GAR(c) ⇌ THF(c) + N-Formyl-GAR(c)
r1482	-1	FADH2(m) + trans,cis-dodeca-2,5-dienoyl-CoA(m) ⇌ FAD(m) + cis-laur-5-enoyl-CoA(m)
r1483	1	H2O(m) + trans,cis-myristo-2,7-dienoyl-CoA(m) ⇌ cis-(3S)-hydroxytetradec-7-enoyl-CoA(m)
r1331	-1	H+(PG)(s) ⇌ H+(PG)(c) + Proton-gradient(c)
r0974	1	Pantothenate(s) + Na+(s) ⇌ Pantothenate(c) + Na+(c)
r1484	1	CoA(m) + 3-oxomyrist-7-enoyl-CoA(m) ⇌ Acetyl-CoA(m) + cis-laur-5-enoyl-CoA(m)
r1319	-13.2	ATP(c) + H2O(c) ⇌ ADP(c) + Pi(c) + ATP-energy(c)
r1485	1	H2O(m) + trans,cis-dodeca-2,5-dienoyl-CoA(m) ⇌ 3(S)-3-hydroxydodecen-(5Z)-oyl-CoA(m)
r0260	1	Adenylosuccinate(c) → Fumarate(c) + AMP(c)
r0247	1	ATP(c) + Ribose-5P(c) → AMP(c) + PRPP(c)
r1486	1	NAD+(m) + 3(S)-3-hydroxydodecen-(5Z)-oyl-CoA(m) ⇌ 3-oxolaur-cis-5-enoyl-CoA(m) + NADH(m)
r1488	1	ATP(c) + CoA(c) + Palmitolate(c) ⇌ AMP(c) + palmitoleoyl-CoA(c) + PPi(c)
r0699	1	SAICAR(c) ⇌ Fumarate(c) + AICAR(c)
r2442	1	Palmitolate(s) → Palmitolate(c)
r0849	-3.17	H2O(s) ⇌ H2O(c)
r0581	-1	Pantothenate(c) + Cysteamine(c) ⇌ H2O(c) + Pantetheine(c)
r0867	2	CO2(c) ⇌ CO2(s)
r0589	1	ATP(c) + Phosphopantetheine(c) → PPi(c) + Dephospho-CoA(c)
r2468	1	Glycine(s) + H+(PG)(s) → Glycine(c) + H+(PG)(c)
r0007	3	PPi(c) + H2O(c) → 2 Pi(c)
r0736	0.17	ATP(c) + Fructose-6P(c) → ADP(c) + Fructose-1,6PP(c)
r0027	-0.5	NADPH(c) + Cystine(c) ⇌ 2 Cysteine(c) + NADP+(c)
r0192	0.83	Glucose-6P(c) ⇌ Fructose-6P(c)
r0344	-0.67	D-Xylulose-5P(c) ⇌ Ribulose-5P(c)
r0792	2	5-Methyl-THF(c) + NAD+(c) ⇌ 5,10-Methylene-THF(c) + NADH(c)
r0211	1	Cysteine(c) + O2(c) ⇌ 3-Sulfinoalanine(c)
r2676	-2	5-Methyl-THF(c) ⇌ THF(c) + THF-activated methyl group(c)
r2525	2	Glutamine(s) ⇌ Glutamine(c)
r2678	3	Acetyl-CoA(m) ⇌ CoA(m) + CoA-activated acetyl group(m)
r0666	1	ATP(c) + 5-Phosphoribosylformylglycinamidine(c) ⇌ ADP(c) + Pi(c) + AIR(c)
r1471	1	FAD(m) + cis-myrist-7-enoyl-CoA(m) ⇌ FADH2(m) + trans,cis-myristo-2,7-dienoyl-CoA(m)
r0667	-1	5-Phosphoribosyl-4-carboxy-5-aminoimidazole(c) ⇌ AIR(c) + CO2(c)
r1473	1	FAD(m) + palmitoleoyl-CoA(m) ⇌ FADH2(m) + trans,cis-hexadeca-2,9-dienoyl-CoA(m)
r1322	2	NADH(c) ⇌ NAD+(c) + NADH-redox-potential(c)
r1475	1	H2O(m) + trans,cis-hexadeca-2,9-dienoyl-CoA(m) ⇌ (S)-3-hydroxypalmitoleoyl-CoA(m)
r1323	3	NADH(m) ⇌ NAD+(m) + NADH-redox-potential(m)

Continued on next page

ID	flux	reaction
r0250	-0.67	Ribulose-5P(c) ⇌ Ribose-5P(c)
r1476	1	NAD+(m) + cis-(3S)-hydroxytetradec-7-enoyl-CoA(m) ⇌ 3-oxomyrist-7-enoyl-CoA(m) + NADH(m)
r1478	1	NAD+(m) + (S)-3-hydroxypalmitoleoyl-CoA(m) ⇌ NADH(m) + 3-oxopalmitoleoyl-CoA(m)
r1326	3.5	NADPH(c) ⇌ NADPH-redox-potential(c) + NADP+(c)
r0687	1	ATP(c) + N-Formyl-GAR(c) + Glutamine(c) + H2O(c) ⇌ ADP(c) + Pi(c) + Glutamate(c) + 5-Phosphoribosylformylglycinamidine(c)
r1479	1	CoA(m) + 3-oxolaur-cis-5-enoyl-CoA(m) ⇌ Acetyl-CoA(m) + (3Z)-Dodecenoyl-CoA(m)
r0255	0.33	Fructose-6P(c) + GAP(c) ⇌ D-Xylulose-5P(c) + Erythrose-4P(c)
r1329	3	FADH2(c) ⇌ FAD(c) + FADH-redox-potential(c)
r0538	1	3-Sulfinoalanine(c) ⇌ CO2(c) + Hypotaurine(c)
r0256	0.17	Fructose-1,6PP(c) ⇌ DHAP(c) + GAP(c)
r0539	-1	Hypotaurine(c) ⇌ Cysteamine(c) + O2(c)
r0257	-1	Glutamine(c) + PRPP(c) + H2O(c) ⇌ 5-Phosphoribosylamine(c) + PPi(c) + Glutamate(c)
r0258	-2	Fumarate(c) + H2O(c) ⇌ Malate(c)
r0273	-1	IMP(c) + H2O(c) ⇌ FAICAR(c)
r0851	-3	H2O(m) ⇌ H2O(c)
r0700	1	10-Formyl-THF(c) + AICAR(c) ⇌ THF(c) + FAICAR(c)

(2E)-Hexadecenoyl-ACP (536)

Definition			Solution												
			exchanges		reactions								transp		
Objective	Constraints	Comment	imports	exports	c	m	r	p	l	n	s	b	s-c	b-c	int
(2E)-Hexadecenoyl-ACP(c)	PIPES MCES	synthesis of cytosolic (2E)-Hexadecenoyl-ACP	1 P_i(c) 5 Glutamate(s) 6 Glycine(s) 11 Alanine(s) 4 Lysine(s) 8 Aspartate(s) 9 Arginine(s) 7 Glutamine(s) 8 Serine(s) 5 Methionine(s) 3 Phenylalanine(s) 4 Tyrosine(s) 3 Cysteine(s) 20 Leucine(s) 1 Histidine(s) 9 Proline(s) 1 Asparagine(s) 11 Valine(s) 6 Threonine(s) 4 Isoleucine(s) 1 Pantothenate(s) 631 ATP-energy(c) 13 NADPH-redox-potential(c) 3 Proton-gradient(c) 8 CoA-activated acetyl group(c)	10 H_2O(s) 1 CO_2(s) 1 (2E)-Hexadecenoyl-ACP(c) 66 Na-gradient(c)	46	-	-	-	-	-	-	-	24	-	-

ID	flux	reaction
r0701	-1	HMA(c) + NADP+(c) ⇌ 3-Oxotetradecanoyl-ACP(c) + NADPH(c)
r0702	1	HMA(c) ⇌ H2O(c) + (2E)-Tetradecenoyl-ACP(c)
r1069	-4	Lysine(c) ⇌ Lysine(s)
r0578	1	ATP(c) + Pantetheine(c) ⇌ ADP(c) + Phosphopantetheine(c)
r2478	8	Serine(s) + Na+(s) + H+(PG)(c) → Serine(c) + Na+(c) + H+(PG)(s)
r0030	125	ATP(c) + AMP(c) ⇌ 2 ADP(c)
r0760	1	Butyryl-ACP(c) + Malonyl-ACP(c) ⇌ CO2(c) + ACP(c) + 3-Oxohexanoyl-ACP(c)
r0032	1	ATP(c) + Dephospho-CoA(c) ⇌ ADP(c) + CoA(c)
r0761	-1	D-3-Hydroxyhexanoyl-ACP(c) + NADP+(c) ⇌ NADPH(c) + 3-Oxohexanoyl-ACP(c)
r0184	7	ATP(c) + Acetyl-CoA(c) + HCO3-(c) → ADP(c) + Pi(c) + Malonyl-CoA(c)
r0762	1	D-3-Hydroxyhexanoyl-ACP(c) ⇌ H2O(c) + (2E)-Hexenoyl-ACP(c)
r0763	-1	Hexanoyl-ACP(c) + NADP+(c) ⇌ NADPH(c) + (2E)-Hexenoyl-ACP(c)
r0764	1	Malonyl-ACP(c) + Hexanoyl-ACP(c) ⇌ 3-Oxooctanoyl-ACP(c) + CO2(c) + ACP(c)
r1122	-20	Leucine(c) + Na+(c) ← Leucine(s) + Na+(s)
r0765	-1	Octanoyl-ACP(c) + NADP+(c) ⇌ (2E)-Octenoyl-ACP(c) + NADPH(c)
r1123	-4	Isoleucine(c) + Na+(c) ← Isoleucine(s) + Na+(s)
r0766	1	Octanoyl-ACP(c) + Malonyl-ACP(c) ⇌ 3-Oxodecanoyl-ACP(c) + CO2(c) + ACP(c)
r0767	-1	Decanoyl-ACP(c) + NADP+(c) ⇌ (2E)-Decenoyl-ACP(c) + NADPH(c)

Continued on next page

ID	flux	reaction
r1125	4	Tyrosine(s) + Na+(s) → Tyrosine(c) + Na+(c)
r0768	1	Decanoyl-ACP(c) + Malonyl-ACP(c) ⇌ 3-Oxododecanoyl-ACP(c) + CO2(c) + ACP(c)
r0769	-1	D-3-Hydroxydodecanoyl-ACP(c) + NADP+(c) ⇌ 3-Oxododecanoyl-ACP(c) + NADPH(c)
r1142	-6	Threonine(s) + Na+(s) → Threonine(c) + Na+(c)
r1143	-8	Na+(c) + Aspartate(c) ← Na+(s) + Aspartate(s)
r2664	66	Na+(c) ⇌ Na+(s) + Na-gradient(c)
r1144	-5	Glutamate(c) + Na+(c) ← Glutamate(s) + Na+(s)
r1145	-1	Histidine(c) + Na+(c) ← Histidine(s) + Na+(s)
r2532	1	Asparagine(s) ⇌ Asparagine(c)
r2533	11	Valine(s) ⇌ Valine(c)
r0678	1	Acetyl-ACP(c) + Malonyl-ACP(c) ⇌ CO2(c) + ACP(c) + Acetoacetyl-ACP(c)
r0691	-1	(R)-3-Hydroxybutanoyl-ACP(c) + NADP+(c) ⇌ NADPH(c) + Acetoacetyl-ACP(c)
r1331	-3	H+(PG)(s) ⇌ H+(PG)(c) + Proton-gradient(c)
r0974	1	Pantothenate(s) + Na+(s) ⇌ Pantothenate(c) + Na+(c)
r0692	-1	(R)-3-Hydroxydecanoyl-ACP(c) + NADP+(c) ⇌ 3-Oxodecanoyl-ACP(c) + NADPH(c)
r1319	-631	ATP(c) + H2O(c) ⇌ ADP(c) + Pi(c) + ATP-energy(c)
r0693	1	(R)-3-Hydroxydecanoyl-ACP(c) ⇌ (2E)-Decenoyl-ACP(c) + H2O(c)
r0694	-1	(R)-3-Hydroxyoctanoyl-ACP(c) + NADP+(c) ⇌ 3-Oxooctanoyl-ACP(c) + NADPH(c)
r0695	1	(R)-3-Hydroxyoctanoyl-ACP(c) ⇌ (2E)-Octenoyl-ACP(c) + H2O(c)
r0696	-1	(R)-3-Hydroxypalmitoyl-ACP(c) + NADP+(c) ⇌ 3-Oxohexadecanoyl-ACP(c) + NADPH(c)
r0697	1	(R)-3-Hydroxypalmitoyl-ACP(c) ⇌ (2E)-Hexadecenoyl-ACP(c) + H2O(c)
r1058	9	Arginine(s) ⇌ Arginine(c)
r1072	5	Methionine(s) ⇌ Methionine(c)
r0849	-10	H2O(s) ⇌ H2O(c)
r0581	-1	Pantothenate(c) + Cysteamine(c) ⇌ H2O(c) + Pantetheine(c)
r0712	-1	Dodecanoyl-ACP(c) + NADP+(c) ⇌ (2E)-Dodecenoyl-ACP(c) + NADPH(c)
r0713	1	Dodecanoyl-ACP(c) + Malonyl-ACP(c) ⇌ 3-Oxotetradecanoyl-ACP(c) + CO2(c) + ACP(c)
r0867	1	CO2(c) ⇌ CO2(s)
r1096	1	11 Alanine(c) + 2 Cysteine(c) + 5 Glutamate(c) + 3 Phenylalanine(c) + 6 Glycine(c) + Histidine(c) + 4 Isoleucine(c) + 20 Leucine(c) + 5 Methionine(c) + 9 Proline(c) + 7 Glutamine(c) + 9 Arginine(c) + 8 Serine(c) + 6 Threonine(c) + 11 Valine(c) + 4 Tyrosine(c) + 496 ATP(c) + 496 H2O(c) + 8 Aspartate(c) + 4 Lysine(c) + Asparagine(c) → Apo-ACP(c) + 124 AMP(c) + 124 PPi(c) + 372 ADP(c) + 372 Pi(c)
r0589	1	ATP(c) + Phosphopantetheine(c) → PPi(c) + Dephospho-CoA(c)
r2469	11	Alanine(s) + H+(PG)(s) → Alanine(c) + H+(PG)(c)
r0007	125	PPi(c) + H2O(c) → 2 Pi(c)
r0770	1	D-3-Hydroxydodecanoyl-ACP(c) ⇌ (2E)-Dodecenoyl-ACP(c) + H2O(c)
r0771	-1	Tetradecanoyl-ACP(c) + NADP+(c) ⇌ NADPH(c) + (2E)-Tetradecenoyl-ACP(c)
r0772	1	Tetradecanoyl-ACP(c) + Malonyl-ACP(c) ⇌ 3-Oxohexadecanoyl-ACP(c) + CO2(c) + ACP(c)
r1417	-7	CO2(c) + H2O(c) ⇌ Carbonate(c)
r0049	1	PAP(c) + H2O(c) → AMP(c) + Pi(c)
r1419	7	Carbonate(c) ⇌ HCO3-(c)
r1139	-9	Proline(c) + Na+(c) ← Proline(s) + Na+(s)
r0211	1	Cysteine(c) + O2(c) ⇌ 3-Sulfinoalanine(c)
r2523	6	Glycine(s) ⇌ Glycine(c)
r2677	-8	Acetyl-CoA(c) ⇌ CoA(c) + CoA-activated acetyl group(c)
r2525	7	Glutamine(s) ⇌ Glutamine(c)
r0367	1	Acetyl-CoA(c) + ACP(c) ⇌ CoA(c) + Acetyl-ACP(c)
r0368	1	CoA(c) + Apo-ACP(c) → PAP(c) + ACP(c)
r2528	3	Phenylalanine(s) ⇌ Phenylalanine(c)
r0369	7	ACP(c) + Malonyl-CoA(c) ⇌ CoA(c) + Malonyl-ACP(c)
r2529	3	Cysteine(s) ⇌ Cysteine(c)
r0681	1	(R)-3-Hydroxybutanoyl-ACP(c) ⇌ But-2-enoyl-ACP(c) + H2O(c)
r0682	-1	Butyryl-ACP(c) + NADP+(c) ⇌ But-2-enoyl-ACP(c) + NADPH(c)
r1326	-13	NADPH(c) ⇌ NADPH-redox-potential(c) + NADP+(c)
r0538	1	3-Sulfinoalanine(c) ⇌ CO2(c) + Hypotaurine(c)
r0539	-1	Hypotaurine(c) ⇌ Cysteamine(c) + O2(c)

(2E)-Hexadecenoyl-CoA (537)

Definition			Solution												
			exchanges		reactions								transp		
Objective	Constraints	Comment	imports	exports	c	m	r	p	l	n	s	b	s-c	b-c	int
(2E)-Hexadecenoyl-CoA(c)	PIPES MCES	synthesis of cytosolic (2E)-Hexadecenoyl-CoA	2.17 P_i(c) 1 Glycine(s) 2 Aspartate(s) 2 Glutamine(s) 0.83 Glucose-6P(c) 0.5 Cystine(s) 1 Pantothenate(s) 1 Palmitolate(s) 13.2 ATP-energy(c) 1 Proton-gradient(c) 2 THF-activated methyl group(c)	6.17 H_2O(s) 2 CO_2(s) 2 Pyruvate(c) 2 Glutamate(s) 1 (2E)-Hexadecenoyl-CoA(c) 2 NADH-redox-potential(c) 3.5 NADPH-redox-potential(c) 3 Na-gradient(c)	43	-	-	-	-	-	-	-	11	-	-

ID	flux	reaction
r0406	-0.33	Fructose-6P(c) + Erythrose-4P(c) ⇌ Sedoheptulose-7P(c) + GAP(c)
r0277	1	GTP(c) + IMP(c) + Aspartate(c) → GDP(c) + Pi(c) + Adenylosuccinate(c)
r0293	2	5,10-Methylene-THF(c) + NADP+(c) ⇌ 5,10-Methenyl-THF(c) + NADPH(c)
r0705	1	ATP(c) + 5-Phosphoribosyl-4-carboxy-5-aminoimidazole(c) + Aspartate(c) ⇌ ADP(c) + Pi(c) + SAICAR(c)
r0578	1	ATP(c) + Pantetheine(c) ⇌ ADP(c) + Phosphopantetheine(c)
r0877	0.5	Glutamate(c) + Cystine(s) ⇌ Glutamate(s) + Cystine(c)
r1252	1	Palmitolate(c) + ATP(c) + CoA(c) → (2E)-Hexadecenoyl-CoA(c) + AMP(c) + PPi(c)
r0030	3	ATP(c) + AMP(c) ⇌ 2 ADP(c)
r0032	1	ATP(c) + Dephospho-CoA(c) ⇌ ADP(c) + CoA(c)
r1143	-2	Na+(c) + Aspartate(c) ← Na+(s) + Aspartate(s)
r2664	3	Na+(c) ⇌ Na+(s) + Na-gradient(c)
r0058	2	Malate(c) + NADP+(c) ⇌ Pyruvate(c) + CO2(c) + NADPH(c)
r0370	0.33	Sedoheptulose-7P(c) + GAP(c) ⇌ Ribose-5P(c) + D-Xylulose-5P(c)
r0371	2	5,10-Methenyl-THF(c) + H2O(c) ⇌ 10-Formyl-THF(c)
r2685	1.5	Glutamate(c) ⇌ Glutamate(s)
r0659	1	ATP(c) + 5-Phosphoribosylamine(c) + Glycine(c) ⇌ ADP(c) + Pi(c) + GAR(c)
r0099	1	ATP(c) + GDP(c) ⇌ ADP(c) + GTP(c)
r0243	-0.17	DHAP(c) ⇌ GAP(c)
r0677	1	10-Formyl-THF(c) + GAR(c) ⇌ THF(c) + N-Formyl-GAR(c)
r1331	-1	H+(PG)(s) ⇌ H+(PG)(c) + Proton-gradient(c)
r0974	1	Pantothenate(s) + Na+(s) ⇌ Pantothenate(c) + Na+(c)
r1319	-13.2	ATP(c) + H2O(c) ⇌ ADP(c) + Pi(c) + ATP-energy(c)
r0260	1	Adenylosuccinate(c) → Fumarate(c) + AMP(c)
r0247	1	ATP(c) + Ribose-5P(c) → AMP(c) + PRPP(c)
r0699	1	SAICAR(c) ⇌ Fumarate(c) + AICAR(c)
r2442	1	Palmitolate(s) → Palmitolate(c)
r0849	-6.17	H2O(s) ⇌ H2O(c)
r0581	-1	Pantothenate(c) + Cysteamine(c) ⇌ H2O(c) + Pantetheine(c)
r0867	2	CO2(c) ⇌ CO2(s)
r0589	1	ATP(c) + Phosphopantetheine(c) → PPi(c) + Dephospho-CoA(c)
r2468	1	Glycine(s) + H+(PG)(s) → Glycine(c) + H+(PG)(c)
r0007	3	PPi(c) + H2O(c) → 2 Pi(c)
r0736	0.17	ATP(c) + Fructose-6P(c) → ADP(c) + Fructose-1,6PP(c)
r0027	-0.5	NADPH(c) + Cystine(c) ⇌ 2 Cysteine(c) + NADP+(c)
r0192	0.83	Glucose-6P(c) ⇌ Fructose-6P(c)
r0344	-0.67	D-Xylulose-5P(c) ⇌ Ribulose-5P(c)
r0792	2	5-Methyl-THF(c) + NAD+(c) ⇌ 5,10-Methylene-THF(c) + NADH(c)
r0211	1	Cysteine(c) + O2(c) ⇌ 3-Sulfinoalanine(c)
r2676	-2	5-Methyl-THF(c) ⇌ THF(c) + THF-activated methyl group(c)
r2525	2	Glutamine(s) ⇌ Glutamine(c)
r0666	1	ATP(c) + 5-Phosphoribosylformylglycinamidine(c) ⇌ ADP(c) + Pi(c) + AIR(c)
r0667	-1	5-Phosphoribosyl-4-carboxy-5-aminoimidazole(c) ⇌ AIR(c) + CO2(c)
r1322	2	NADH(c) ⇌ NAD+(c) + NADH-redox-potential(c)
r0250	-0.67	Ribulose-5P(c) ⇌ Ribose-5P(c)

Continued on next page

(2E)-Hexadecenoyl-CoA (537)– continued

ID	flux	reaction
r1326	3.5	NADPH(c) ⇌ NADPH-redox-potential(c) + NADP+(c)
r0687	1	ATP(c) + N-Formyl-GAR(c) + Glutamine(c) + H2O(c) ⇌ ADP(c) + Pi(c) + Glutamate(c) + 5-Phosphoribosylformylglycinamidine(c)
r0255	0.33	Fructose-6P(c) + GAP(c) ⇌ D-Xylulose-5P(c) + Erythrose-4P(c)
r0538	1	3-Sulfinoalanine(c) ⇌ CO2(c) + Hypotaurine(c)
r0256	0.17	Fructose-1,6PP(c) ⇌ DHAP(c) + GAP(c)
r0539	-1	Hypotaurine(c) ⇌ Cysteamine(c) + O2(c)
r0257	-1	Glutamine(c) + PRPP(c) + H2O(c) ⇌ 5-Phosphoribosylamine(c) + PPi(c) + Glutamate(c)
r0258	-2	Fumarate(c) + H2O(c) ⇌ Malate(c)
r0273	-1	IMP(c) + H2O(c) ⇌ FAICAR(c)
r0700	1	10-Formyl-THF(c) + AICAR(c) ⇌ THF(c) + FAICAR(c)

(2E)-Hexenoyl-ACP (538)

Definition			Solution												
			exchanges		reactions								transp		
Objective	Constraints	Comment	imports	exports	c	m	r	p	l	n	s	b	s-c	b-c	int
(2E)-Hexenoyl-ACP(c)	PIPES MCES	synthesis of cytosolic (2E)-Hexenoyl-ACP	1 P_i(c) 5 Glutamate(s) 6 Glycine(s) 11 Alanine(s) 4 Lysine(s) 8 Aspartate(s) 9 Arginine(s) 7 Glutamine(s) 8 Serine(s) 5 Methionine(s) 3 Phenylalanine(s) 4 Tyrosine(s) 3 Cysteine(s) 20 Leucine(s) 1 Histidine(s) 9 Proline(s) 1 Asparagine(s) 11 Valine(s) 6 Threonine(s) 4 Isoleucine(s) 1 Pantothenate(s) 626 ATP-energy(c) 3 NADPH-redox-potential(c) 3 Proton-gradient(c) 3 CoA-activated acetyl group(c)	5 H_2O(s) 1 CO_2(s) 1 (2E)-Hexenoyl-ACP(c) 66 Na-gradient(c)	26	-	-	-	-	-	-	-	24	-	-

ID	flux	reaction
r1069	-4	Lysine(c) ⇌ Lysine(s)
r0578	1	ATP(c) + Pantetheine(c) ⇌ ADP(c) + Phosphopantetheine(c)
r2478	8	Serine(s) + Na+(s) + H+(PG)(c) → Serine(c) + Na+(c) + H+(PG)(s)
r0030	125	ATP(c) + AMP(c) ⇌ 2 ADP(c)
r0760	1	Butyryl-ACP(c) + Malonyl-ACP(c) ⇌ CO2(c) + ACP(c) + 3-Oxohexanoyl-ACP(c)
r0032	1	ATP(c) + Dephospho-CoA(c) ⇌ ADP(c) + CoA(c)
r0761	-1	D-3-Hydroxyhexanoyl-ACP(c) + NADP+(c) ⇌ NADPH(c) + 3-Oxohexanoyl-ACP(c)
r0184	2	ATP(c) + Acetyl-CoA(c) + HCO3-(c) → ADP(c) + Pi(c) + Malonyl-CoA(c)
r0762	1	D-3-Hydroxyhexanoyl-ACP(c) ⇌ H2O(c) + (2E)-Hexenoyl-ACP(c)
r1122	-20	Leucine(c) + Na+(c) ← Leucine(s) + Na+(s)
r1123	-4	Isoleucine(c) + Na+(c) ← Isoleucine(s) + Na+(s)
r1125	4	Tyrosine(s) + Na+(s) → Tyrosine(c) + Na+(c)
r1142	-6	Threonine(s) + Na+(s) → Threonine(c) + Na+(c)
r1143	-8	Na+(c) + Aspartate(c) ← Na+(s) + Aspartate(s)
r2664	66	Na+(c) ⇌ Na+(s) + Na-gradient(c)
r1144	-5	Glutamate(c) + Na+(c) ← Glutamate(s) + Na+(s)
r1145	-1	Histidine(c) + Na+(c) ← Histidine(s) + Na+(s)
r2532	1	Asparagine(s) ⇌ Asparagine(c)
r2533	11	Valine(s) ⇌ Valine(c)
r0678	1	Acetyl-ACP(c) + Malonyl-ACP(c) ⇌ CO2(c) + ACP(c) + Acetoacetyl-ACP(c)
r0691	-1	(R)-3-Hydroxybutanoyl-ACP(c) + NADP+(c) ⇌ NADPH(c) + Acetoacetyl-ACP(c)
r1331	-3	H+(PG)(s) ⇌ H+(PG)(c) + Proton-gradient(c)
r0974	1	Pantothenate(s) + Na+(s) ⇌ Pantothenate(c) + Na+(c)
r1319	-626	ATP(c) + H2O(c) ⇌ ADP(c) + Pi(c) + ATP-energy(c)

Continued on next page

ID	flux	reaction
r1058	9	Arginine(s) ⇌ Arginine(c)
r1072	5	Methionine(s) ⇌ Methionine(c)
r0849	-5	H2O(s) ⇌ H2O(c)
r0581	-1	Pantothenate(c) + Cysteamine(c) ⇌ H2O(c) + Pantetheine(c)
r0867	1	CO2(c) ⇌ CO2(s)
r1096	1	11 Alanine(c) + 2 Cysteine(c) + 5 Glutamate(c) + 3 Phenylalanine(c) + 6 Glycine(c) + Histidine(c) + 4 Isoleucine(c) + 20 Leucine(c) + 5 Methionine(c) + 9 Proline(c) + 7 Glutamine(c) + 9 Arginine(c) + 8 Serine(c) + 6 Threonine(c) + 11 Valine(c) + 4 Tyrosine(c) + 496 ATP(c) + 496 H2O(c) + 8 Aspartate(c) + 4 Lysine(c) + Asparagine(c) → Apo-ACP(c) + 124 AMP(c) + 124 PPi(c) + 372 ADP(c) + 372 Pi(c)
r0589	1	ATP(c) + Phosphopantetheine(c) → PPi(c) + Dephospho-CoA(c)
r2469	11	Alanine(s) + H+(PG)(s) → Alanine(c) + H+(PG)(c)
r0007	125	PPi(c) + H2O(c) → 2 Pi(c)
r1417	-2	CO2(c) + H2O(c) ⇌ Carbonate(c)
r0049	1	PAP(c) + H2O(c) → AMP(c) + Pi(c)
r1419	2	Carbonate(c) ⇌ HCO3-(c)
r1139	-9	Proline(c) + Na+(c) ← Proline(s) + Na+(s)
r0211	1	Cysteine(c) + O2(c) ⇌ 3-Sulfinoalanine(c)
r2523	6	Glycine(s) ⇌ Glycine(c)
r2677	-3	Acetyl-CoA(c) ⇌ CoA(c) + CoA-activated acetyl group(c)
r2525	7	Glutamine(s) ⇌ Glutamine(c)
r0367	1	Acetyl-CoA(c) + ACP(c) ⇌ CoA(c) + Acetyl-ACP(c)
r0368	1	CoA(c) + Apo-ACP(c) → PAP(c) + ACP(c)
r2528	3	Phenylalanine(s) ⇌ Phenylalanine(c)
r0369	2	ACP(c) + Malonyl-CoA(c) ⇌ CoA(c) + Malonyl-ACP(c)
r2529	3	Cysteine(s) ⇌ Cysteine(c)
r0681	1	(R)-3-Hydroxybutanoyl-ACP(c) ⇌ But-2-enoyl-ACP(c) + H2O(c)
r0682	-1	Butyryl-ACP(c) + NADP+(c) ⇌ But-2-enoyl-ACP(c) + NADPH(c)
r1326	-3	NADPH(c) ⇌ NADPH-redox-potential(c) + NADP+(c)
r0538	1	3-Sulfinoalanine(c) ⇌ CO2(c) + Hypotaurine(c)
r0539	-1	Hypotaurine(c) ⇌ Cysteamine(c) + O2(c)

(2E)-Hexenoyl-CoA (539)

Definition			Solution												
			exchanges		reactions								transp		
Objective	Constraints	Comment	imports	exports	c	m	r	p	l	n	s	b	s-c	b-c	int
(2E)-Hexenoyl-CoA(m)	PIPES MCES	synthesis of mitochondrial (2E)-Hexenoyl-CoA	1.5 O_2(s) 2.17 P_i(c) 1 Glycine(s) 2 Aspartate(s) 2 Glutamine(s) 0.83 Glucose-6P(c) 0.5 Cystine(s) 1 Pantothenate(s) 1 Palmitolate(s) 13.2 ATP-energy(c) 2 THF-activated methyl group(c)	3.17 H_2O(s) 2 CO_2(s) 2 Pyruvate(c) 2 Glutamate(s) 1 (2E)-Hexenoyl-CoA(m) 2 NADH-redox-potential(c) 6 NADH-redox-potential(m) 3.5 NADPH-redox-potential(c) 3 FADH-redox-potential(c) 2 Proton-gradient(c) 18 Proton-gradient(m) 5 Na-gradient(c) 6 CoA-activated acetyl group(m)	44	27	-	-	-	-	-	-	12	-	7

ID	flux	reaction
r0406	-0.33	Fructose-6P(c) + Erythrose-4P(c) ⇌ Sedoheptulose-7P(c) + GAP(c)
r0277	1	GTP(c) + IMP(c) + Aspartate(c) → GDP(c) + Pi(c) + Adenylosuccinate(c)
r0855	1.5	O2(s) ⇌ O2(c)
r0856	-1.5	O2(m) ⇌ O2(c)
r0705	1	ATP(c) + 5-Phosphoribosyl-4-carboxy-5-aminoimidazole(c) + Aspartate(c) ⇌ ADP(c) + Pi(c) + SAICAR(c)
r0293	2	5,10-Methylene-THF(c) + NADP+(c) ⇌ 5,10-Methenyl-THF(c) + NADPH(c)
r0578	1	ATP(c) + Pantetheine(c) ⇌ ADP(c) + Phosphopantetheine(c)
r0722	1	(S)-3-Hydroxydodecanoyl-CoA(m) + NAD+(m) ⇌ 3-Oxododecanoyl-CoA(m) + NADH(m)
r0724	-1	Decanoyl-CoA(m) + Acetyl-CoA(m) ⇌ CoA(m) + 3-Oxododecanoyl-CoA(m)

Continued on next page

ID	flux	reaction
r0877	0.5	Glutamate(c) + Cystine(s) ⇌ Glutamate(s) + Cystine(c)
r0726	1	(S)-Hydroxydecanoyl-CoA(m) + NAD+(m) ⇌ 3-Oxodecanoyl-CoA(m) + NADH(m)
r0728	-1	(S)-Hydroxydecanoyl-CoA(m) ⇌ (2E)-Decenoyl-CoA(m) + H2O(m)
r2477	2	Glutamine(s) + Na+(s) + H+(PG)(c) → Glutamine(c) + Na+(c) + H+(PG)(s)
r0017	1.5	O2(m) + 4 H+(PG)(m) + 4 Ferrocytochrome C(m) → 4 Ferricytochrome C(m) + 2 H2O(m) + 4 H+(PG)(c)
r0030	3	ATP(c) + AMP(c) ⇌ 2 ADP(c)
r0032	1	ATP(c) + Dephospho-CoA(c) ⇌ ADP(c) + CoA(c)
r1290	3	FAD(c) + FADH2(m) ⇌ FAD(m) + FADH2(c)
r1143	-2	Na+(c) + Aspartate(c) ← Na+(s) + Aspartate(s)
r0634	-1	Acetyl-CoA(m) + Octanoyl-CoA(m) ⇌ CoA(m) + 3-Oxodecanoyl-CoA(m)
r2664	5	Na+(c) ⇌ Na+(s) + Na-gradient(c)
r0058	2	Malate(c) + NADP+(c) ⇌ Pyruvate(c) + CO2(c) + NADPH(c)
r0370	0.33	Sedoheptulose-7P(c) + GAP(c) ⇌ Ribose-5P(c) + D-Xylulose-5P(c)
r0371	2	5,10-Methenyl-THF(c) + H2O(c) ⇌ 10-Formyl-THF(c)
r2685	1.5	Glutamate(c) ⇌ Glutamate(s)
r1448	1	Ubiquinone(m) + Octanoyl-CoA(m) → (2E)-Octenoyl-CoA(m) + Ubiquinol(m)
r0657	1	(3Z)-Dodecenoyl-CoA(m) ⇌ (2E)-Dodecenoyl-CoA(m)
r0659	1	ATP(c) + 5-Phosphoribosylamine(c) + Glycine(c) ⇌ ADP(c) + Pi(c) + GAR(c)
r0507	3	Ubiquinol(m) + 2 Ferricytochrome C(m) + 4 H+(PG)(m) → Ubiquinone(m) + 4 H+(PG)(c) + 2 Ferrocytochrome C(m)
r1480	1	CoA(m) + 3-oxopalmitoleoyl-CoA(m) ⇌ Acetyl-CoA(m) + cis-myrist-7-enoyl-CoA(m)
r0099	1	ATP(c) + GDP(c) ⇌ ADP(c) + GTP(c)
r1468	1	palmitoleoyl-CoA(c) ⇌ palmitoleoyl-CoA(m)
r0243	-0.17	DHAP(c) ⇌ GAP(c)
r0677	1	10-Formyl-THF(c) + GAR(c) ⇌ THF(c) + N-Formyl-GAR(c)
r1482	-1	FADH2(m) + trans,cis-dodeca-2,5-dienoyl-CoA(m) ⇌ FAD(m) + cis-laur-5-enoyl-CoA(m)
r1330	18	H+(PG)(c) ⇌ H+(PG)(m) + Proton-gradient(m)
r1483	1	H2O(m) + trans,cis-myristo-2,7-dienoyl-CoA(m) ⇌ cis-(3S)-hydroxytetradec-7-enoyl-CoA(m)
r1331	2	H+(PG)(s) ⇌ H+(PG)(c) + Proton-gradient(c)
r0974	1	Pantothenate(s) + Na+(s) ⇌ Pantothenate(c) + Na+(c)
r1484	1	CoA(m) + 3-oxomyrist-7-enoyl-CoA(m) ⇌ Acetyl-CoA(m) + cis-laur-5-enoyl-CoA(m)
r1319	-13.2	ATP(c) + H2O(c) ⇌ ADP(c) + Pi(c) + ATP-energy(c)
r1485	1	H2O(m) + trans,cis-dodeca-2,5-dienoyl-CoA(m) ⇌ 3(S)-3-hydroxydodecen-(5Z)-oyl-CoA(m)
r0260	1	Adenylosuccinate(c) → Fumarate(c) + AMP(c)
r0247	1	ATP(c) + Ribose-5P(c) → AMP(c) + PRPP(c)
r1486	1	NAD+(m) + 3(S)-3-hydroxydodecen-(5Z)-oyl-CoA(m) ⇌ 3-oxolaur-cis-5-enoyl-CoA(m) + NADH(m)
r1488	1	ATP(c) + CoA(c) + Palmitolate(c) ⇌ AMP(c) + palmitoleoyl-CoA(c) + PPi(c)
r0699	1	SAICAR(c) ⇌ Fumarate(c) + AICAR(c)
r2442	1	Palmitolate(s) → Palmitolate(c)
r0849	-3.17	H2O(s) ⇌ H2O(c)
r0581	-1	Pantothenate(c) + Cysteamine(c) ⇌ H2O(c) + Pantetheine(c)
r0867	2	CO2(c) ⇌ CO2(s)
r0730	1	(S)-Hydroxyoctanoyl-CoA(m) + NAD+(m) → 3-Oxooctanoyl-CoA(m) + NADH(m)
r0731	-1	(S)-Hydroxyoctanoyl-CoA(m) ← (2E)-Octenoyl-CoA(m) + H2O(m)
r0732	-1	Hexanoyl-CoA(m) + Acetyl-CoA(m) ← CoA(m) + 3-Oxooctanoyl-CoA(m)
r0589	1	ATP(c) + Phosphopantetheine(c) → PPi(c) + Dephospho-CoA(c)
r0736	0.17	ATP(c) + Fructose-6P(c) → ADP(c) + Fructose-1,6PP(c)
r0007	3	PPi(c) + H2O(c) → 2 Pi(c)
r0027	-0.5	NADPH(c) + Cystine(c) ⇌ 2 Cysteine(c) + NADP+(c)
r0192	0.83	Glucose-6P(c) ⇌ Fructose-6P(c)
r0344	-0.67	D-Xylulose-5P(c) ⇌ Ribulose-5P(c)
r0792	2	5-Methyl-THF(c) + NAD+(c) ⇌ 5,10-Methylene-THF(c) + NADH(c)
r0211	1	Cysteine(c) + O2(c) ⇌ 3-Sulfinoalanine(c)
r2523	1	Glycine(s) ⇌ Glycine(c)
r1450	1	Ubiquinone(m) + Hexanoyl-CoA(m) → (2E)-Hexenoyl-CoA(m) + Ubiquinol(m)
r2676	-2	5-Methyl-THF(c) ⇌ THF(c) + THF-activated methyl group(c)
r1451	1	Ubiquinone(m) + Decanoyl-CoA(m) → (2E)-Decenoyl-CoA(m) + Ubiquinol(m)

Continued on next page

ID	flux	reaction
r0660	-1	(S)-3-Hydroxydodecanoyl-CoA(m) ⇌ (2E)-Dodecenoyl-CoA(m) + H2O(m)
r2678	6	Acetyl-CoA(m) ⇌ CoA(m) + CoA-activated acetyl group(m)
r0666	1	ATP(c) + 5-Phosphoribosylformylglycinamidine(c) ⇌ ADP(c) + Pi(c) + AIR(c)
r1471	1	FAD(m) + cis-myrist-7-enoyl-CoA(m) ⇌ FADH2(m) + trans,cis-myristo-2,7-dienoyl-CoA(m)
r0667	-1	5-Phosphoribosyl-4-carboxy-5-aminoimidazole(c) ⇌ AIR(c) + CO2(c)
r1473	1	FAD(m) + palmitoleoyl-CoA(m) ⇌ FADH2(m) + trans,cis-hexadeca-2,9-dienoyl-CoA(m)
r1322	2	NADH(c) ⇌ NAD+(c) + NADH-redox-potential(c)
r1475	1	H2O(m) + trans,cis-hexadeca-2,9-dienoyl-CoA(m) ⇌ (S)-3-hydroxypalmitoleoyl-CoA(m)
r1323	6	NADH(m) ⇌ NAD+(m) + NADH-redox-potential(m)
r0250	-0.67	Ribulose-5P(c) ⇌ Ribose-5P(c)
r1476	1	NAD+(m) + cis-(3S)-hydroxytetradec-7-enoyl-CoA(m) ⇌ 3-oxomyrist-7-enoyl-CoA(m) + NADH(m)
r1478	1	NAD+(m) + (S)-3-hydroxypalmitoleoyl-CoA(m) ⇌ NADH(m) + 3-oxopalmitoleoyl-CoA(m)
r1326	3.5	NADPH(c) ⇌ NADPH-redox-potential(c) + NADP+(c)
r0687	1	ATP(c) + N-Formyl-GAR(c) + Glutamine(c) + H2O(c) ⇌ ADP(c) + Pi(c) + Glutamate(c) + 5-Phosphoribosylformylglycinamidine(c)
r1479	1	CoA(m) + 3-oxolaur-cis-5-enoyl-CoA(m) ⇌ Acetyl-CoA(m) + (3Z)-Dodecenoyl-CoA(m)
r0255	0.33	Fructose-6P(c) + GAP(c) ⇌ D-Xylulose-5P(c) + Erythrose-4P(c)
r1329	3	FADH2(c) ⇌ FAD(c) + FADH-redox-potential(c)
r0538	1	3-Sulfinoalanine(c) ⇌ CO2(c) + Hypotaurine(c)
r0256	0.17	Fructose-1,6PP(c) ⇌ DHAP(c) + GAP(c)
r0539	-1	Hypotaurine(c) ⇌ Cysteamine(c) + O2(c)
r0257	-1	Glutamine(c) + PRPP(c) + H2O(c) ⇌ 5-Phosphoribosylamine(c) + PPi(c) + Glutamate(c)
r0258	-2	Fumarate(c) + H2O(c) ⇌ Malate(c)
r0273	-1	IMP(c) + H2O(c) ⇌ FAICAR(c)
r0851	-3	H2O(m) ⇌ H2O(c)
r0700	1	10-Formyl-THF(c) + AICAR(c) ⇌ THF(c) + FAICAR(c)

(2E)-Octadecenoyl-ACP (540)

Definition			Solution												
			exchanges		reactions								transp		
Objective	Constraints	Comment	imports	exports	c	m	r	p	l	n	s	b	s-c	b-c	int
(2E)-Octadecenoyl-ACP(c)	PIPES MCES	synthesis of cytosolic (2E)-Octadecenoyl-ACP	1 P_i(c) 5 Glutamate(s) 6 Glycine(s) 11 Alanine(s) 4 Lysine(s) 8 Aspartate(s) 9 Arginine(s) 7 Glutamine(s) 8 Serine(s) 5 Methionine(s) 3 Phenylalanine(s) 4 Tyrosine(s) 3 Cysteine(s) 20 Leucine(s) 1 Histidine(s) 9 Proline(s) 1 Asparagine(s) 11 Valine(s) 6 Threonine(s) 4 Isoleucine(s) 1 Pantothenate(s) 632 ATP-energy(c) 15 NADPH-redox-potential(c) 3 Proton-gradient(c) 9 CoA-activated acetyl group(c)	11 H_2O(s) 1 CO_2(s) 1 (2E)-Octadecenoyl-ACP(c) 66 Na-gradient(c)	50	-	-	-	-	-	-	-	24	-	-

ID	flux	reaction
r0701	-1	HMA(c) + NADP+(c) ⇌ 3-Oxotetradecanoyl-ACP(c) + NADPH(c)
r0702	1	HMA(c) ⇌ H2O(c) + (2E)-Tetradecenoyl-ACP(c)
r1069	-4	Lysine(c) ⇌ Lysine(s)
r0578	1	ATP(c) + Pantetheine(c) ⇌ ADP(c) + Phosphopantetheine(c)
r2478	8	Serine(s) + Na+(s) + H+(PG)(c) → Serine(c) + Na+(c) + H+(PG)(s)
r0030	125	ATP(c) + AMP(c) ⇌ 2 ADP(c)
r0760	1	Butyryl-ACP(c) + Malonyl-ACP(c) ⇌ CO2(c) + ACP(c) + 3-Oxohexanoyl-ACP(c)
r0032	1	ATP(c) + Dephospho-CoA(c) ⇌ ADP(c) + CoA(c)
r0761	-1	D-3-Hydroxyhexanoyl-ACP(c) + NADP+(c) ⇌ NADPH(c) + 3-Oxohexanoyl-ACP(c)

Continued on next page

ID	flux	reaction
r0184	8	ATP(c) + Acetyl-CoA(c) + HCO3-(c) → ADP(c) + Pi(c) + Malonyl-CoA(c)
r0762	1	D-3-Hydroxyhexanoyl-ACP(c) ⇌ H2O(c) + (2E)-Hexenoyl-ACP(c)
r0763	-1	Hexanoyl-ACP(c) + NADP+(c) ⇌ NADPH(c) + (2E)-Hexenoyl-ACP(c)
r0764	1	Malonyl-ACP(c) + Hexanoyl-ACP(c) ⇌ 3-Oxooctanoyl-ACP(c) + CO2(c) + ACP(c)
r1122	-20	Leucine(c) + Na+(c) ← Leucine(s) + Na+(s)
r0765	-1	Octanoyl-ACP(c) + NADP+(c) ⇌ (2E)-Octenoyl-ACP(c) + NADPH(c)
r1123	-4	Isoleucine(c) + Na+(c) ← Isoleucine(s) + Na+(s)
r0766	1	Octanoyl-ACP(c) + Malonyl-ACP(c) ⇌ 3-Oxodecanoyl-ACP(c) + CO2(c) + ACP(c)
r0767	-1	Decanoyl-ACP(c) + NADP+(c) ⇌ (2E)-Decenoyl-ACP(c) + NADPH(c)
r1125	4	Tyrosine(s) + Na+(s) → Tyrosine(c) + Na+(c)
r0768	1	Decanoyl-ACP(c) + Malonyl-ACP(c) ⇌ 3-Oxododecanoyl-ACP(c) + CO2(c) + ACP(c)
r0769	-1	D-3-Hydroxydodecanoyl-ACP(c) + NADP+(c) ⇌ 3-Oxododecanoyl-ACP(c) + NADPH(c)
r1142	-6	Threonine(s) + Na+(s) → Threonine(c) + Na+(c)
r1143	-8	Na+(c) + Aspartate(c) ← Na+(s) + Aspartate(s)
r2664	66	Na+(c) ⇌ Na+(s) + Na-gradient(c)
r1144	-5	Glutamate(c) + Na+(c) ← Glutamate(s) + Na+(s)
r1145	-1	Histidine(c) + Na+(c) ← Histidine(s) + Na+(s)
r2532	1	Asparagine(s) ⇌ Asparagine(c)
r2533	11	Valine(s) ⇌ Valine(c)
r1313	1	Hexadecanoyl-ACP(c) + Malonyl-ACP(c) ⇌ 3-Oxostearoyl-ACP(c) + ACP(c) + CO2(c)
r1314	1	3-Oxostearoyl-ACP(c) + NADPH(c) ⇌ 3-Hydroxystearoyl-ACP(c) + NADP+(c)
r1315	1	3-Hydroxystearoyl-ACP(c) ⇌ (2E)-Octadecenoyl-ACP(c) + H2O(c)
r0678	1	Acetyl-ACP(c) + Malonyl-ACP(c) ⇌ CO2(c) + ACP(c) + Acetoacetyl-ACP(c)
r0691	-1	(R)-3-Hydroxybutanoyl-ACP(c) + NADP+(c) ⇌ NADPH(c) + Acetoacetyl-ACP(c)
r1331	-3	H+(PG)(s) ⇌ H+(PG)(c) + Proton-gradient(c)
r0974	1	Pantothenate(s) + Na+(s) ⇌ Pantothenate(c) + Na+(c)
r0692	-1	(R)-3-Hydroxydecanoyl-ACP(c) + NADP+(c) ⇌ 3-Oxodecanoyl-ACP(c) + NADPH(c)
r1319	-632	ATP(c) + H2O(c) ⇌ ADP(c) + Pi(c) + ATP-energy(c)
r0693	1	(R)-3-Hydroxydecanoyl-ACP(c) ⇌ (2E)-Decenoyl-ACP(c) + H2O(c)
r0694	-1	(R)-3-Hydroxyoctanoyl-ACP(c) + NADP+(c) ⇌ 3-Oxooctanoyl-ACP(c) + NADPH(c)
r0695	1	(R)-3-Hydroxyoctanoyl-ACP(c) ⇌ (2E)-Octenoyl-ACP(c) + H2O(c)
r0696	-1	(R)-3-Hydroxypalmitoyl-ACP(c) + NADP+(c) ⇌ 3-Oxohexadecanoyl-ACP(c) + NADPH(c)
r0697	1	(R)-3-Hydroxypalmitoyl-ACP(c) ⇌ (2E)-Hexadecenoyl-ACP(c) + H2O(c)
r1058	9	Arginine(s) ⇌ Arginine(c)
r1072	5	Methionine(s) ⇌ Methionine(c)
r0849	-11	H2O(s) ⇌ H2O(c)
r0581	-1	Pantothenate(c) + Cysteamine(c) ⇌ H2O(c) + Pantetheine(c)
r0712	-1	Dodecanoyl-ACP(c) + NADP+(c) ⇌ (2E)-Dodecenoyl-ACP(c) + NADPH(c)
r0713	1	Dodecanoyl-ACP(c) + Malonyl-ACP(c) ⇌ 3-Oxotetradecanoyl-ACP(c) + CO2(c) + ACP(c)
r0867	1	CO2(c) ⇌ CO2(s)
r1096	1	11 Alanine(c) + 2 Cysteine(c) + 5 Glutamate(c) + 3 Phenylalanine(c) + 6 Glycine(c) + Histidine(c) + 4 Isoleucine(c) + 20 Leucine(c) + 5 Methionine(c) + 9 Proline(c) + 7 Glutamine(c) + 9 Arginine(c) + 8 Serine(c) + 6 Threonine(c) + 11 Valine(c) + 4 Tyrosine(c) + 496 ATP(c) + 496 H2O(c) + 8 Aspartate(c) + 4 Lysine(c) + Asparagine(c) → Apo-ACP(c) + 124 AMP(c) + 124 PPi(c) + 372 ADP(c) + 372 Pi(c)
r0589	1	ATP(c) + Phosphopantetheine(c) → PPi(c) + Dephospho-CoA(c)
r2469	11	Alanine(s) + H+(PG)(s) → Alanine(c) + H+(PG)(c)
r0007	125	PPi(c) + H2O(c) → 2 Pi(c)
r0770	1	D-3-Hydroxydodecanoyl-ACP(c) ⇌ (2E)-Dodecenoyl-ACP(c) + H2O(c)
r0771	-1	Tetradecanoyl-ACP(c) + NADP+(c) ⇌ NADPH(c) + (2E)-Tetradecenoyl-ACP(c)
r0772	1	Tetradecanoyl-ACP(c) + Malonyl-ACP(c) ⇌ 3-Oxohexadecanoyl-ACP(c) + CO2(c) + ACP(c)
r0773	-1	Hexadecanoyl-ACP(c) + NADP+(c) ⇌ (2E)-Hexadecenoyl-ACP(c) + NADPH(c)
r1417	-8	CO2(c) + H2O(c) ⇌ Carbonate(c)
r0049	1	PAP(c) + H2O(c) → AMP(c) + Pi(c)
r1419	8	Carbonate(c) ⇌ HCO3-(c)
r1139	-9	Proline(c) + Na+(c) ← Proline(s) + Na+(s)
r0211	1	Cysteine(c) + O2(c) ⇌ 3-Sulfinoalanine(c)

Continued on next page

ID	flux	reaction
r2523	6	Glycine(s) ⇌ Glycine(c)
r2677	-9	Acetyl-CoA(c) ⇌ CoA(c) + CoA-activated acetyl group(c)
r2525	7	Glutamine(s) ⇌ Glutamine(c)
r0367	1	Acetyl-CoA(c) + ACP(c) ⇌ CoA(c) + Acetyl-ACP(c)
r0368	1	CoA(c) + Apo-ACP(c) → PAP(c) + ACP(c)
r2528	3	Phenylalanine(s) ⇌ Phenylalanine(c)
r0369	8	ACP(c) + Malonyl-CoA(c) ⇌ CoA(c) + Malonyl-ACP(c)
r2529	3	Cysteine(s) ⇌ Cysteine(c)
r0681	1	(R)-3-Hydroxybutanoyl-ACP(c) ⇌ But-2-enoyl-ACP(c) + H2O(c)
r0682	-1	Butyryl-ACP(c) + NADP+(c) ⇌ But-2-enoyl-ACP(c) + NADPH(c)
r1326	-15	NADPH(c) ⇌ NADPH-redox-potential(c) + NADP+(c)
r0538	1	3-Sulfinoalanine(c) ⇌ CO2(c) + Hypotaurine(c)
r0539	-1	Hypotaurine(c) ⇌ Cysteamine(c) + O2(c)

(2E)-Octenoyl-ACP (541)

Definition			Solution												
			exchanges		reactions								transp		
Objective	Constraints	Comment	imports	exports	c	m	r	p	l	n	s	b	s-c	b-c	int
(2E)-Octenoyl-ACP(c)	PIPES MCES	synthesis of cytosolic (2E)-Octenoyl-ACP	1 P_i(c) 5 Glutamate(s) 6 Glycine(s) 11 Alanine(s) 4 Lysine(s) 8 Aspartate(s) 9 Arginine(s) 7 Glutamine(s) 8 Serine(s) 5 Methionine(s) 3 Phenylalanine(s) 4 Tyrosine(s) 3 Cysteine(s) 20 Leucine(s) 1 Histidine(s) 9 Proline(s) 1 Asparagine(s) 11 Valine(s) 6 Threonine(s) 4 Isoleucine(s) 1 Pantothenate(s) 627 ATP-energy(c) 5 NADPH-redox-potential(c) 3 Proton-gradient(c) 4 CoA-activated acetyl group(c)	6 H_2O(s) 1 CO_2(s) 1 (2E)-Octenoyl-ACP(c) 66 Na-gradient(c)	30	-	-	-	-	-	-	-	24	-	-

ID	flux	reaction
r1069	-4	Lysine(c) ⇌ Lysine(s)
r0578	1	ATP(c) + Pantetheine(c) ⇌ ADP(c) + Phosphopantetheine(c)
r2478	8	Serine(s) + Na+(s) + H+(PG)(c) → Serine(c) + Na+(c) + H+(PG)(s)
r0030	125	ATP(c) + AMP(c) ⇌ 2 ADP(c)
r0760	1	Butyryl-ACP(c) + Malonyl-ACP(c) ⇌ CO2(c) + ACP(c) + 3-Oxohexanoyl-ACP(c)
r0032	1	ATP(c) + Dephospho-CoA(c) ⇌ ADP(c) + CoA(c)
r0761	-1	D-3-Hydroxyhexanoyl-ACP(c) + NADP+(c) ⇌ NADPH(c) + 3-Oxohexanoyl-ACP(c)
r0184	3	ATP(c) + Acetyl-CoA(c) + HCO3-(c) → ADP(c) + Pi(c) + Malonyl-CoA(c)
r0762	1	D-3-Hydroxyhexanoyl-ACP(c) ⇌ H2O(c) + (2E)-Hexenoyl-ACP(c)
r0763	-1	Hexanoyl-ACP(c) + NADP+(c) ⇌ NADPH(c) + (2E)-Hexenoyl-ACP(c)
r0764	1	Malonyl-ACP(c) + Hexanoyl-ACP(c) ⇌ 3-Oxooctanoyl-ACP(c) + CO2(c) + ACP(c)
r1122	-20	Leucine(c) + Na+(c) ← Leucine(s) + Na+(s)
r1123	-4	Isoleucine(c) + Na+(c) ← Isoleucine(s) + Na+(s)
r1125	4	Tyrosine(s) + Na+(s) → Tyrosine(c) + Na+(c)
r1142	-6	Threonine(s) + Na+(s) → Threonine(c) + Na+(c)
r1143	-8	Na+(c) + Aspartate(c) ← Na+(s) + Aspartate(s)
r2664	66	Na+(c) ⇌ Na+(s) + Na-gradient(c)
r1144	-5	Glutamate(c) + Na+(c) ← Glutamate(s) + Na+(s)
r1145	-1	Histidine(c) + Na+(c) ← Histidine(s) + Na+(s)
r2532	1	Asparagine(s) ⇌ Asparagine(c)
r2533	11	Valine(s) ⇌ Valine(c)
r0678	1	Acetyl-ACP(c) + Malonyl-ACP(c) ⇌ CO2(c) + ACP(c) + Acetoacetyl-ACP(c)

Continued on next page

ID	flux	reaction
r0691	-1	(R)-3-Hydroxybutanoyl-ACP(c) + NADP+(c) ⇌ NADPH(c) + Acetoacetyl-ACP(c)
r1331	-3	H+(PG)(s) ⇌ H+(PG)(c) + Proton-gradient(c)
r0974	1	Pantothenate(s) + Na+(s) ⇌ Pantothenate(c) + Na+(c)
r1319	-627	ATP(c) + H2O(c) ⇌ ADP(c) + Pi(c) + ATP-energy(c)
r0694	-1	(R)-3-Hydroxyoctanoyl-ACP(c) + NADP+(c) ⇌ 3-Oxooctanoyl-ACP(c) + NADPH(c)
r0695	1	(R)-3-Hydroxyoctanoyl-ACP(c) ⇌ (2E)-Octenoyl-ACP(c) + H2O(c)
r1058	9	Arginine(s) ⇌ Arginine(c)
r1072	5	Methionine(s) ⇌ Methionine(c)
r0849	-6	H2O(s) ⇌ H2O(c)
r0581	-1	Pantothenate(c) + Cysteamine(c) ⇌ H2O(c) + Pantetheine(c)
r0867	1	CO2(c) ⇌ CO2(s)
r1096	1	11 Alanine(c) + 2 Cysteine(c) + 5 Glutamate(c) + 3 Phenylalanine(c) + 6 Glycine(c) + Histidine(c) + 4 Isoleucine(c) + 20 Leucine(c) + 5 Methionine(c) + 9 Proline(c) + 7 Glutamine(c) + 9 Arginine(c) + 8 Serine(c) + 6 Threonine(c) + 11 Valine(c) + 4 Tyrosine(c) + 496 ATP(c) + 496 H2O(c) + 8 Aspartate(c) + 4 Lysine(c) + Asparagine(c) → Apo-ACP(c) + 124 AMP(c) + 124 PPi(c) + 372 ADP(c) + 372 Pi(c)
r0589	1	ATP(c) + Phosphopantetheine(c) → PPi(c) + Dephospho-CoA(c)
r2469	11	Alanine(s) + H+(PG)(s) → Alanine(c) + H+(PG)(c)
r0007	125	PPi(c) + H2O(c) → 2 Pi(c)
r1417	-3	CO2(c) + H2O(c) ⇌ Carbonate(c)
r0049	1	PAP(c) + H2O(c) → AMP(c) + Pi(c)
r1419	3	Carbonate(c) ⇌ HCO3-(c)
r1139	-9	Proline(c) + Na+(c) ← Proline(s) + Na+(s)
r0211	1	Cysteine(c) + O2(c) ⇌ 3-Sulfinoalanine(c)
r2523	6	Glycine(s) ⇌ Glycine(c)
r2677	-4	Acetyl-CoA(c) ⇌ CoA(c) + CoA-activated acetyl group(c)
r2525	7	Glutamine(s) ⇌ Glutamine(c)
r0367	1	Acetyl-CoA(c) + ACP(c) ⇌ CoA(c) + Acetyl-ACP(c)
r0368	1	CoA(c) + Apo-ACP(c) → PAP(c) + ACP(c)
r2528	3	Phenylalanine(s) ⇌ Phenylalanine(c)
r0369	3	ACP(c) + Malonyl-CoA(c) ⇌ CoA(c) + Malonyl-ACP(c)
r2529	3	Cysteine(s) ⇌ Cysteine(c)
r0681	1	(R)-3-Hydroxybutanoyl-ACP(c) ⇌ But-2-enoyl-ACP(c) + H2O(c)
r0682	-1	Butyryl-ACP(c) + NADP+(c) ⇌ But-2-enoyl-ACP(c) + NADPH(c)
r1326	-5	NADPH(c) ⇌ NADPH-redox-potential(c) + NADP+(c)
r0538	1	3-Sulfinoalanine(c) ⇌ CO2(c) + Hypotaurine(c)
r0539	-1	Hypotaurine(c) ⇌ Cysteamine(c) + O2(c)

(2E)-Octenoyl-CoA (542)

Definition			Solution												
			exchanges		reactions								transp		
Objective	Constraints	Comment	imports	exports	c	m	r	p	l	n	s	b	s-c	b-c	int
(2E)-Octenoyl-CoA(m)	PIPES MCES	synthesis of mitochondrial (2E)-Octenoyl-CoA	1 O_2(s) 2.17 P_i(c) 1 Glycine(s) 2 Aspartate(s) 2 Glutamine(s) 0.83 Glucose-6P(c) 0.5 Cystine(s) 1 Pantothenate(s) 1 Palmitolate(s) 13.2 ATP-energy(c) 2 THF-activated methyl group(c)	3.17 H_2O(s) 2 CO_2(s) 2 Pyruvate(c) 2 Glutamate(s) 1 (2E)-Octenoyl-CoA(m) 2 NADH-redox-potential(c) 5 NADH-redox-potential(m) 3.5 NADPH-redox-potential(c) 3 FADH-redox-potential(c) 2 Proton-gradient(c) 12 Proton-gradient(m) 5 Na-gradient(c) 5 CoA-activated acetyl group(m)	44	23	-	-	-	-	-	-	12	-	7

ID	flux	reaction
r0406	-0.33	Fructose-6P(c) + Erythrose-4P(c) ⇌ Sedoheptulose-7P(c) + GAP(c)
r0277	1	GTP(c) + IMP(c) + Aspartate(c) → GDP(c) + Pi(c) + Adenylosuccinate(c)
r0855	1	O2(s) ⇌ O2(c)

Continued on next page

ID	flux	reaction
r0856	-1	O2(m) ⇌ O2(c)
r0705	1	ATP(c) + 5-Phosphoribosyl-4-carboxy-5-aminoimidazole(c) + Aspartate(c) ⇌ ADP(c) + Pi(c) + SAICAR(c)
r0293	2	5,10-Methylene-THF(c) + NADP+(c) ⇌ 5,10-Methenyl-THF(c) + NADPH(c)
r0578	1	ATP(c) + Pantetheine(c) ⇌ ADP(c) + Phosphopantetheine(c)
r0722	1	(S)-3-Hydroxydodecanoyl-CoA(m) + NAD+(m) ⇌ 3-Oxododecanoyl-CoA(m) + NADH(m)
r0724	-1	Decanoyl-CoA(m) + Acetyl-CoA(m) ⇌ CoA(m) + 3-Oxododecanoyl-CoA(m)
r0877	0.5	Glutamate(c) + Cystine(s) ⇌ Glutamate(s) + Cystine(c)
r0726	1	(S)-Hydroxydecanoyl-CoA(m) + NAD+(m) ⇌ 3-Oxodecanoyl-CoA(m) + NADH(m)
r0728	-1	(S)-Hydroxydecanoyl-CoA(m) ⇌ (2E)-Decenoyl-CoA(m) + H2O(m)
r2477	2	Glutamine(s) + Na+(s) + H+(PG)(c) → Glutamine(c) + Na+(c) + H+(PG)(s)
r0017	1	O2(m) + 4 H+(PG)(m) + 4 Ferrocytochrome C(m) → 4 Ferricytochrome C(m) + 2 H2O(m) + 4 H+(PG)(c)
r0030	3	ATP(c) + AMP(c) ⇌ 2 ADP(c)
r0032	1	ATP(c) + Dephospho-CoA(c) ⇌ ADP(c) + CoA(c)
r1290	3	FAD(c) + FADH2(m) ⇌ FAD(m) + FADH2(c)
r1143	-2	Na+(c) + Aspartate(c) ← Na+(s) + Aspartate(s)
r0634	-1	Acetyl-CoA(m) + Octanoyl-CoA(m) ⇌ CoA(m) + 3-Oxodecanoyl-CoA(m)
r2664	5	Na+(c) ⇌ Na+(s) + Na-gradient(c)
r0058	2	Malate(c) + NADP+(c) ⇌ Pyruvate(c) + CO2(c) + NADPH(c)
r0370	0.33	Sedoheptulose-7P(c) + GAP(c) ⇌ Ribose-5P(c) + D-Xylulose-5P(c)
r0371	2	5,10-Methenyl-THF(c) + H2O(c) ⇌ 10-Formyl-THF(c)
r2685	1.5	Glutamate(c) ⇌ Glutamate(s)
r1448	1	Ubiquinone(m) + Octanoyl-CoA(m) → (2E)-Octenoyl-CoA(m) + Ubiquinol(m)
r0657	1	(3Z)-Dodecenoyl-CoA(m) ⇌ (2E)-Dodecenoyl-CoA(m)
r0659	1	ATP(c) + 5-Phosphoribosylamine(c) + Glycine(c) ⇌ ADP(c) + Pi(c) + GAR(c)
r0507	2	Ubiquinol(m) + 2 Ferricytochrome C(m) + 4 H+(PG)(m) → Ubiquinone(m) + 4 H+(PG)(c) + 2 Ferrocytochrome C(m)
r1480	1	CoA(m) + 3-oxopalmitoleoyl-CoA(m) ⇌ Acetyl-CoA(m) + cis-myrist-7-enoyl-CoA(m)
r0099	1	ATP(c) + GDP(c) ⇌ ADP(c) + GTP(c)
r1468	1	palmitoleoyl-CoA(c) ⇌ palmitoleoyl-CoA(m)
r0243	-0.17	DHAP(c) ⇌ GAP(c)
r0677	1	10-Formyl-THF(c) + GAR(c) ⇌ THF(c) + N-Formyl-GAR(c)
r1482	-1	FADH2(m) + trans,cis-dodeca-2,5-dienoyl-CoA(m) ⇌ FAD(m) + cis-laur-5-enoyl-CoA(m)
r1330	12	H+(PG)(c) ⇌ H+(PG)(m) + Proton-gradient(m)
r1483	1	H2O(m) + trans,cis-myristo-2,7-dienoyl-CoA(m) ⇌ cis-(3S)-hydroxytetradec-7-enoyl-CoA(m)
r1331	2	H+(PG)(s) ⇌ H+(PG)(c) + Proton-gradient(c)
r0974	1	Pantothenate(s) + Na+(s) ⇌ Pantothenate(c) + Na+(c)
r1484	1	CoA(m) + 3-oxomyrist-7-enoyl-CoA(m) ⇌ Acetyl-CoA(m) + cis-laur-5-enoyl-CoA(m)
r1319	-13.2	ATP(c) + H2O(c) ⇌ ADP(c) + Pi(c) + ATP-energy(c)
r1485	1	H2O(m) + trans,cis-dodeca-2,5-dienoyl-CoA(m) ⇌ 3(S)-3-hydroxydodecen-(5Z)-oyl-CoA(m)
r0260	1	Adenylosuccinate(c) → Fumarate(c) + AMP(c)
r0247	1	ATP(c) + Ribose-5P(c) → AMP(c) + PRPP(c)
r1486	1	NAD+(m) + 3(S)-3-hydroxydodecen-(5Z)-oyl-CoA(m) ⇌ 3-oxolaur-cis-5-enoyl-CoA(m) + NADH(m)
r1488	1	ATP(c) + CoA(c) + Palmitolate(c) ⇌ AMP(c) + palmitoleoyl-CoA(c) + PPi(c)
r0699	1	SAICAR(c) ⇌ Fumarate(c) + AICAR(c)
r2442	1	Palmitolate(s) → Palmitolate(c)
r0849	-3.17	H2O(s) ⇌ H2O(c)
r0581	-1	Pantothenate(c) + Cysteamine(c) ⇌ H2O(c) + Pantetheine(c)
r0867	2	CO2(c) ⇌ CO2(s)
r0589	1	ATP(c) + Phosphopantetheine(c) → PPi(c) + Dephospho-CoA(c)
r0736	0.17	ATP(c) + Fructose-6P(c) → ADP(c) + Fructose-1,6PP(c)
r0007	3	PPi(c) + H2O(c) → 2 Pi(c)
r0027	-0.5	NADPH(c) + Cystine(c) ⇌ 2 Cysteine(c) + NADP+(c)
r0192	0.83	Glucose-6P(c) ⇌ Fructose-6P(c)
r0344	-0.67	D-Xylulose-5P(c) ⇌ Ribulose-5P(c)
r0792	2	5-Methyl-THF(c) + NAD+(c) ⇌ 5,10-Methylene-THF(c) + NADH(c)
r0211	1	Cysteine(c) + O2(c) ⇌ 3-Sulfinoalanine(c)
r2523	1	Glycine(s) ⇌ Glycine(c)

Continued on next page

ID	flux	reaction
r2676	-2	5-Methyl-THF(c) ⇌ THF(c) + THF-activated methyl group(c)
r1451	1	Ubiquinone(m) + Decanoyl-CoA(m) → (2E)-Decenoyl-CoA(m) + Ubiquinol(m)
r0660	-1	(S)-3-Hydroxydodecanoyl-CoA(m) ⇌ (2E)-Dodecenoyl-CoA(m) + H2O(m)
r2678	5	Acetyl-CoA(m) ⇌ CoA(m) + CoA-activated acetyl group(m)
r0666	1	ATP(c) + 5-Phosphoribosylformylglycinamidine(c) ⇌ ADP(c) + Pi(c) + AIR(c)
r1471	1	FAD(m) + cis-myrist-7-enoyl-CoA(m) ⇌ FADH2(m) + trans,cis-myristo-2,7-dienoyl-CoA(m)
r0667	-1	5-Phosphoribosyl-4-carboxy-5-aminoimidazole(c) ⇌ AIR(c) + CO2(c)
r1473	1	FAD(m) + palmitoleoyl-CoA(m) ⇌ FADH2(m) + trans,cis-hexadeca-2,9-dienoyl-CoA(m)
r1322	2	NADH(c) ⇌ NAD+(c) + NADH-redox-potential(c)
r1475	1	H2O(m) + trans,cis-hexadeca-2,9-dienoyl-CoA(m) ⇌ (S)-3-hydroxypalmitoleoyl-CoA(m)
r1323	5	NADH(m) ⇌ NAD+(m) + NADH-redox-potential(m)
r0250	-0.67	Ribulose-5P(c) ⇌ Ribose-5P(c)
r1476	1	NAD+(m) + cis-(3S)-hydroxytetradec-7-enoyl-CoA(m) ⇌ 3-oxomyrist-7-enoyl-CoA(m) + NADH(m)
r1478	1	NAD+(m) + (S)-3-hydroxypalmitoleoyl-CoA(m) ⇌ NADH(m) + 3-oxopalmitoleoyl-CoA(m)
r1326	3.5	NADPH(c) ⇌ NADPH-redox-potential(c) + NADP+(c)
r0687	1	ATP(c) + N-Formyl-GAR(c) + Glutamine(c) + H2O(c) ⇌ ADP(c) + Pi(c) + Glutamate(c) + 5-Phosphoribosylformylglycinamidine(c)
r1479	1	CoA(m) + 3-oxolaur-cis-5-enoyl-CoA(m) ⇌ Acetyl-CoA(m) + (3Z)-Dodecenoyl-CoA(m)
r0255	0.33	Fructose-6P(c) + GAP(c) ⇌ D-Xylulose-5P(c) + Erythrose-4P(c)
r1329	3	FADH2(c) ⇌ FAD(c) + FADH-redox-potential(c)
r0538	1	3-Sulfinoalanine(c) ⇌ CO2(c) + Hypotaurine(c)
r0256	0.17	Fructose-1,6PP(c) ⇌ DHAP(c) + GAP(c)
r0539	-1	Hypotaurine(c) ⇌ Cysteamine(c) + O2(c)
r0257	-1	Glutamine(c) + PRPP(c) + H2O(c) ⇌ 5-Phosphoribosylamine(c) + PPi(c) + Glutamate(c)
r0258	-2	Fumarate(c) + H2O(c) ⇌ Malate(c)
r0273	-1	IMP(c) + H2O(c) ⇌ FAICAR(c)
r0851	-3	H2O(m) ⇌ H2O(c)
r0700	1	10-Formyl-THF(c) + AICAR(c) ⇌ THF(c) + FAICAR(c)

(2E)-Tetradecenoyl-ACP (543)

Definition			Solution												
			exchanges		reactions								transp		
Objective	Constraints	Comment	imports	exports	c	m	r	p	l	n	s	b	s-c	b-c	int
(2E)-Tetradecenoyl-ACP(c)	PIPES MCES	synthesis of cytosolic (2E)-Tetradecenoyl-ACP	1 P_i(c) 5 Glutamate(s) 6 Glycine(s) 11 Alanine(s) 4 Lysine(s) 8 Aspartate(s) 9 Arginine(s) 7 Glutamine(s) 8 Serine(s) 5 Methionine(s) 3 Phenylalanine(s) 4 Tyrosine(s) 3 Cysteine(s) 20 Leucine(s) 1 Histidine(s) 9 Proline(s) 1 Asparagine(s) 11 Valine(s) 6 Threonine(s) 4 Isoleucine(s) 1 Pantothenate(s) 630 ATP-energy(c) 11 NADPH-redox-potential(c) 3 Proton-gradient(c) 7 CoA-activated acetyl group(c)	9 H_2O(s) 1 CO_2(s) 1 (2E)-Tetradecenoyl-ACP(c) 66 Na-gradient(c)	42	-	-	-	-	-	-	-	24	-	-

ID	flux	reaction
r0701	-1	HMA(c) + NADP+(c) ⇌ 3-Oxotetradecanoyl-ACP(c) + NADPH(c)
r0702	1	HMA(c) ⇌ H2O(c) + (2E)-Tetradecenoyl-ACP(c)
r1069	-4	Lysine(c) ⇌ Lysine(s)
r0578	1	ATP(c) + Pantetheine(c) ⇌ ADP(c) + Phosphopantetheine(c)
r2478	8	Serine(s) + Na+(s) + H+(PG)(c) → Serine(c) + Na+(c) + H+(PG)(s)
r0030	125	ATP(c) + AMP(c) ⇌ 2 ADP(c)
r0760	1	Butyryl-ACP(c) + Malonyl-ACP(c) ⇌ CO2(c) + ACP(c) + 3-Oxohexanoyl-ACP(c)

Continued on next page

ID	flux	reaction
r0032	1	ATP(c) + Dephospho-CoA(c) ⇌ ADP(c) + CoA(c)
r0761	-1	D-3-Hydroxyhexanoyl-ACP(c) + NADP+(c) ⇌ NADPH(c) + 3-Oxohexanoyl-ACP(c)
r0184	6	ATP(c) + Acetyl-CoA(c) + HCO3-(c) → ADP(c) + Pi(c) + Malonyl-CoA(c)
r0762	1	D-3-Hydroxyhexanoyl-ACP(c) ⇌ H2O(c) + (2E)-Hexenoyl-ACP(c)
r0763	-1	Hexanoyl-ACP(c) + NADP+(c) ⇌ NADPH(c) + (2E)-Hexenoyl-ACP(c)
r0764	1	Malonyl-ACP(c) + Hexanoyl-ACP(c) ⇌ 3-Oxooctanoyl-ACP(c) + CO2(c) + ACP(c)
r1122	-20	Leucine(c) + Na+(c) ← Leucine(s) + Na+(s)
r0765	-1	Octanoyl-ACP(c) + NADP+(c) ⇌ (2E)-Octenoyl-ACP(c) + NADPH(c)
r1123	-4	Isoleucine(c) + Na+(c) ← Isoleucine(s) + Na+(s)
r0766	1	Octanoyl-ACP(c) + Malonyl-ACP(c) ⇌ 3-Oxodecanoyl-ACP(c) + CO2(c) + ACP(c)
r0767	-1	Decanoyl-ACP(c) + NADP+(c) ⇌ (2E)-Decenoyl-ACP(c) + NADPH(c)
r1125	4	Tyrosine(s) + Na+(s) → Tyrosine(c) + Na+(c)
r0768	1	Decanoyl-ACP(c) + Malonyl-ACP(c) ⇌ 3-Oxododecanoyl-ACP(c) + CO2(c) + ACP(c)
r0769	-1	D-3-Hydroxydodecanoyl-ACP(c) + NADP+(c) ⇌ 3-Oxododecanoyl-ACP(c) + NADPH(c)
r1142	-6	Threonine(s) + Na+(s) → Threonine(c) + Na+(c)
r1143	-8	Na+(c) + Aspartate(c) ← Na+(s) + Aspartate(s)
r2664	66	Na+(c) ⇌ Na+(s) + Na-gradient(c)
r1144	-5	Glutamate(c) + Na+(c) ← Glutamate(s) + Na+(s)
r1145	-1	Histidine(c) + Na+(c) ← Histidine(s) + Na+(s)
r2532	1	Asparagine(s) ⇌ Asparagine(c)
r2533	11	Valine(s) ⇌ Valine(c)
r0678	1	Acetyl-ACP(c) + Malonyl-ACP(c) ⇌ CO2(c) + ACP(c) + Acetoacetyl-ACP(c)
r0691	-1	(R)-3-Hydroxybutanoyl-ACP(c) + NADP+(c) ⇌ NADPH(c) + Acetoacetyl-ACP(c)
r1331	-3	H+(PG)(s) ⇌ H+(PG)(c) + Proton-gradient(c)
r0974	1	Pantothenate(s) + Na+(s) ⇌ Pantothenate(c) + Na+(c)
r0692	-1	(R)-3-Hydroxydecanoyl-ACP(c) + NADP+(c) ⇌ 3-Oxodecanoyl-ACP(c) + NADPH(c)
r1319	-630	ATP(c) + H2O(c) ⇌ ADP(c) + Pi(c) + ATP-energy(c)
r0693	1	(R)-3-Hydroxydecanoyl-ACP(c) ⇌ (2E)-Decenoyl-ACP(c) + H2O(c)
r0694	-1	(R)-3-Hydroxyoctanoyl-ACP(c) + NADP+(c) ⇌ 3-Oxooctanoyl-ACP(c) + NADPH(c)
r0695	1	(R)-3-Hydroxyoctanoyl-ACP(c) ⇌ (2E)-Octenoyl-ACP(c) + H2O(c)
r1058	9	Arginine(s) ⇌ Arginine(c)
r1072	5	Methionine(s) ⇌ Methionine(c)
r0849	-9	H2O(s) ⇌ H2O(c)
r0581	-1	Pantothenate(c) + Cysteamine(c) ⇌ H2O(c) + Pantetheine(c)
r0712	-1	Dodecanoyl-ACP(c) + NADP+(c) ⇌ (2E)-Dodecenoyl-ACP(c) + NADPH(c)
r0713	1	Dodecanoyl-ACP(c) + Malonyl-ACP(c) ⇌ 3-Oxotetradecanoyl-ACP(c) + CO2(c) + ACP(c)
r0867	1	CO2(c) ⇌ CO2(s)
r1096	1	11 Alanine(c) + 2 Cysteine(c) + 5 Glutamate(c) + 3 Phenylalanine(c) + 6 Glycine(c) + Histidine(c) + 4 Isoleucine(c) + 20 Leucine(c) + 5 Methionine(c) + 9 Proline(c) + 7 Glutamine(c) + 9 Arginine(c) + 8 Serine(c) + 6 Threonine(c) + 11 Valine(c) + 4 Tyrosine(c) + 496 ATP(c) + 496 H2O(c) + 8 Aspartate(c) + 4 Lysine(c) + Asparagine(c) → Apo-ACP(c) + 124 AMP(c) + 124 PPi(c) + 372 ADP(c) + 372 Pi(c)
r0589	1	ATP(c) + Phosphopantetheine(c) → PPi(c) + Dephospho-CoA(c)
r2469	11	Alanine(s) + H+(PG)(s) → Alanine(c) + H+(PG)(c)
r0007	125	PPi(c) + H2O(c) → 2 Pi(c)
r0770	1	D-3-Hydroxydodecanoyl-ACP(c) ⇌ (2E)-Dodecenoyl-ACP(c) + H2O(c)
r1417	-6	CO2(c) + H2O(c) ⇌ Carbonate(c)
r0049	1	PAP(c) + H2O(c) → AMP(c) + Pi(c)
r1419	6	Carbonate(c) ⇌ HCO3-(c)
r1139	-9	Proline(c) + Na+(c) ← Proline(s) + Na+(s)
r0211	1	Cysteine(c) + O2(c) ⇌ 3-Sulfinoalanine(c)
r2523	6	Glycine(s) ⇌ Glycine(c)
r2677	-7	Acetyl-CoA(c) ⇌ CoA(c) + CoA-activated acetyl group(c)
r2525	7	Glutamine(s) ⇌ Glutamine(c)
r0367	1	Acetyl-CoA(c) + ACP(c) ⇌ CoA(c) + Acetyl-ACP(c)
r0368	1	CoA(c) + Apo-ACP(c) → PAP(c) + ACP(c)
r2528	3	Phenylalanine(s) ⇌ Phenylalanine(c)

Continued on next page

ID	flux	reaction
r0369	6	ACP(c) + Malonyl-CoA(c) ⇌ CoA(c) + Malonyl-ACP(c)
r2529	3	Cysteine(s) ⇌ Cysteine(c)
r0681	1	(R)-3-Hydroxybutanoyl-ACP(c) ⇌ But-2-enoyl-ACP(c) + H2O(c)
r0682	-1	Butyryl-ACP(c) + NADP+(c) ⇌ But-2-enoyl-ACP(c) + NADPH(c)
r1326	-11	NADPH(c) ⇌ NADPH-redox-potential(c) + NADP+(c)
r0538	1	3-Sulfinoalanine(c) ⇌ CO2(c) + Hypotaurine(c)
r0539	-1	Hypotaurine(c) ⇌ Cysteamine(c) + O2(c)

(2E)-Tetradecenoyl-CoA (544)

Definition			Solution													
			exchanges		reactions								transp			
Objective	Constraints	Comment	imports	exports	c	m	r	p	l	n	s	b	s-c	b-c	int
(2E)-Tetradecenoyl-CoA(m)	PIPES MCES	synthesis of mitochondrial (2E)-Tetradecenoyl-CoA	2.17 P_i(c) 1 Glycine(s) 2 Aspartate(s) 2 Glutamine(s) 0.83 Glucose-6P(c) 0.33 Proline(s) 0.17 Palmitate(s) 0.5 Cystine(s) 1 Pantothenate(s) 0.83 Palmitolate(s) 13.2 ATP-energy(c) 2 THF-activated methyl group(c)	4.5 H_2O(s) 2 CO_2(s) 2 Pyruvate(c) 2.33 Glutamate(s) 1 (2E)-Tetradecenoyl-CoA(m) 2.83 NADH-redox-potential(c) 1.33 NADH-redox-potential(m) 3.5 NADPH-redox-potential(c) 2 Proton-gradient(c) 10 Proton-gradient(m) 5.33 Na-gradient(c) 1 CoA-activated acetyl group(m)	47	11	-	-	-	-	-	-	13	-	7

ID	flux	reaction
r2435	1	L-Carnitine(m) + L-Palmitoylcarnitine(c) → L-Carnitine(c) + L-Palmitoylcarnitine(m)
r0406	-0.33	Fructose-6P(c) + Erythrose-4P(c) ⇌ Sedoheptulose-7P(c) + GAP(c)
r0277	1	GTP(c) + IMP(c) + Aspartate(c) → GDP(c) + Pi(c) + Adenylosuccinate(c)
r0856	-0.83	O2(m) ⇌ O2(c)
r0705	1	ATP(c) + 5-Phosphoribosyl-4-carboxy-5-aminoimidazole(c) + Aspartate(c) ⇌ ADP(c) + Pi(c) + SAICAR(c)
r0293	2	5,10-Methylene-THF(c) + NADP+(c) ⇌ 5,10-Methenyl-THF(c) + NADPH(c)
r0578	1	ATP(c) + Pantetheine(c) ⇌ ADP(c) + Phosphopantetheine(c)
r0877	0.5	Glutamate(c) + Cystine(s) ⇌ Glutamate(s) + Cystine(c)
r2477	2	Glutamine(s) + Na+(s) + H+(PG)(c) → Glutamine(c) + Na+(c) + H+(PG)(s)
r0310	1	Palmitoyl-CoA(m) + Ubiquinone(m) → (2E)-Hexadecenoyl-CoA(m) + Ubiquinol(m)
r0017	0.83	O2(m) + 4 H+(PG)(m) + 4 Ferrocytochrome C(m) → 4 Ferricytochrome C(m) + 2 H2O(m) + 4 H+(PG)(c)
r0312	0.17	Palmitate(c) + ATP(c) + CoA(c) → Palmitoyl-CoA(c) + AMP(c) + PPi(c)
r0030	3	ATP(c) + AMP(c) ⇌ 2 ADP(c)
r0032	1	ATP(c) + Dephospho-CoA(c) ⇌ ADP(c) + CoA(c)
r0911	0.33	Proline(c) + Glutamate(m) ⇌ Proline(m) + Glutamate(c)
r1143	-2	Na+(c) + Aspartate(c) ← Na+(s) + Aspartate(s)
r2664	5.33	Na+(c) ⇌ Na+(s) + Na-gradient(c)
r0058	2	Malate(c) + NADP+(c) ⇌ Pyruvate(c) + CO2(c) + NADPH(c)
r0074	0.33	L-Glutamate 5-semialdehyde(m) + NAD+(m) + H2O(m) ⇌ Glutamate(m) + NADH(m)
r0370	0.33	Sedoheptulose-7P(c) + GAP(c) ⇌ Ribose-5P(c) + D-Xylulose-5P(c)
r0935	0.17	Palmitate(s) → Palmitate(c)
r0653	-1	CoA(m) + 3-Oxopalmitoyl-CoA(m) → Myristoyl-CoA(m) + Acetyl-CoA(m)
r0371	2	5,10-Methenyl-THF(c) + H2O(c) ⇌ 10-Formyl-THF(c)
r2685	1.83	Glutamate(c) ⇌ Glutamate(s)
r1449	1	Ubiquinone(m) + Myristoyl-CoA(m) → (2E)-Tetradecenoyl-CoA(m) + Ubiquinol(m)
r0659	1	ATP(c) + 5-Phosphoribosylamine(c) + Glycine(c) ⇌ ADP(c) + Pi(c) + GAR(c)
r0507	1.67	Ubiquinol(m) + 2 Ferricytochrome C(m) + 4 H+(PG)(m) → Ubiquinone(m) + 4 H+(PG)(c) + 2 Ferrocytochrome C(m)
r0099	1	ATP(c) + GDP(c) ⇌ ADP(c) + GTP(c)
r0677	1	10-Formyl-THF(c) + GAR(c) ⇌ THF(c) + N-Formyl-GAR(c)
r0243	-0.17	DHAP(c) ⇌ GAP(c)
r1330	10	H+(PG)(c) ⇌ H+(PG)(m) + Proton-gradient(m)

Continued on next page

ID	flux	reaction
r1331	2	H+(PG)(s) ⇌ H+(PG)(c) + Proton-gradient(c)
r0974	1	Pantothenate(s) + Na+(s) ⇌ Pantothenate(c) + Na+(c)
r1319	-13.2	ATP(c) + H2O(c) ⇌ ADP(c) + Pi(c) + ATP-energy(c)
r0260	1	Adenylosuccinate(c) → Fumarate(c) + AMP(c)
r0247	1	ATP(c) + Ribose-5P(c) → AMP(c) + PRPP(c)
r1488	0.83	ATP(c) + CoA(c) + Palmitolate(c) ⇌ AMP(c) + palmitoleoyl-CoA(c) + PPi(c)
r0699	1	SAICAR(c) ⇌ Fumarate(c) + AICAR(c)
r2442	0.83	Palmitolate(s) → Palmitolate(c)
r0849	-4.5	H2O(s) ⇌ H2O(c)
r0863	1	CoA(c) ⇌ CoA(m)
r0581	-1	Pantothenate(c) + Cysteamine(c) ⇌ H2O(c) + Pantetheine(c)
r0714	1	NAD+(m) + (S)-3-Hydroxyhexadecanoyl-CoA(m) → 3-Oxopalmitoyl-CoA(m) + NADH(m)
r0867	2	CO2(c) ⇌ CO2(s)
r0716	-1	(2E)-Hexadecenoyl-CoA(m) + H2O(m) → (S)-3-Hydroxyhexadecanoyl-CoA(m)
r0435	1	Palmitoyl-CoA(c) + L-Carnitine(c) ⇌ CoA(c) + L-Palmitoylcarnitine(c)
r0436	-1	Palmitoyl-CoA(m) + L-Carnitine(m) ← CoA(m) + L-Palmitoylcarnitine(m)
r0589	1	ATP(c) + Phosphopantetheine(c) → PPi(c) + Dephospho-CoA(c)
r0736	0.17	ATP(c) + Fructose-6P(c) → ADP(c) + Fructose-1,6PP(c)
r0007	3	PPi(c) + H2O(c) → 2 Pi(c)
r0023	-0.83	NAD+(c) + 2 Ferrocytochrome b5(c) ⇌ NADH(c) + 2 Ferricytochrome b5(c)
r0175	0.33	1-Pyrroline-5-carboxylate(m) + H2O(m) ⇌ L-Glutamate 5-semialdehyde(m)
r0027	-0.5	NADPH(c) + Cystine(c) ⇌ 2 Cysteine(c) + NADP+(c)
r0192	0.83	Glucose-6P(c) ⇌ Fructose-6P(c)
r0344	-0.67	D-Xylulose-5P(c) ⇌ Ribulose-5P(c)
r0792	2	5-Methyl-THF(c) + NAD+(c) ⇌ 5,10-Methylene-THF(c) + NADH(c)
r1139	-0.33	Proline(s) + Na+(s) → Proline(c) + Na+(c)
r0211	1	Cysteine(c) + O2(c) ⇌ 3-Sulfinoalanine(c)
r2523	1	Glycine(s) ⇌ Glycine(c)
r2676	-2	5-Methyl-THF(c) ⇌ THF(c) + THF-activated methyl group(c)
r2678	1	Acetyl-CoA(m) ⇌ CoA(m) + CoA-activated acetyl group(m)
r1453	0.33	Proline(m) + Ubiquinol(m) ⇌ 1-Pyrroline-5-carboxylate(m) + Ubiquinone(m)
r0510	-0.83	palmitoleoyl-CoA(c) + 2 Ferricytochrome b5(c) + 2 H2O(c) ⇌ Palmitoyl-CoA(c) + O2(c) + 2 Ferrocytochrome b5(c)
r0666	1	ATP(c) + 5-Phosphoribosylformylglycinamidine(c) ⇌ ADP(c) + Pi(c) + AIR(c)
r0667	-1	5-Phosphoribosyl-4-carboxy-5-aminoimidazole(c) ⇌ AIR(c) + CO2(c)
r1322	2.83	NADH(c) ⇌ NAD+(c) + NADH-redox-potential(c)
r1323	1.33	NADH(m) ⇌ NAD+(m) + NADH-redox-potential(m)
r0250	-0.67	Ribulose-5P(c) ⇌ Ribose-5P(c)
r1326	3.5	NADPH(c) ⇌ NADPH-redox-potential(c) + NADP+(c)
r0687	1	ATP(c) + N-Formyl-GAR(c) + Glutamine(c) + H2O(c) ⇌ ADP(c) + Pi(c) + Glutamate(c) + 5-Phosphoribosylformylglycinamidine(c)
r0255	0.33	Fructose-6P(c) + GAP(c) ⇌ D-Xylulose-5P(c) + Erythrose-4P(c)
r0538	1	3-Sulfinoalanine(c) ⇌ CO2(c) + Hypotaurine(c)
r0256	0.17	Fructose-1,6PP(c) ⇌ DHAP(c) + GAP(c)
r0539	-1	Hypotaurine(c) ⇌ Cysteamine(c) + O2(c)
r0257	-1	Glutamine(c) + PRPP(c) + H2O(c) ⇌ 5-Phosphoribosylamine(c) + PPi(c) + Glutamate(c)
r0258	-2	Fumarate(c) + H2O(c) ⇌ Malate(c)
r0273	-1	IMP(c) + H2O(c) ⇌ FAICAR(c)
r0700	1	10-Formyl-THF(c) + AICAR(c) ⇌ THF(c) + FAICAR(c)

(3Z)-Dodecenoyl-CoA (545)

Definition			Solution												
			exchanges		reactions								transp		
Objective	Constraints	Comment	imports	exports	c	m	r	p	l	n	s	b	s-c	b-c	int
(3Z)-Dodecenoyl-CoA(m)	PIPES MCES	synthesis of mitochondrial (3Z)-Dodecenoyl-CoA	2.17 P_i(c) 1 Glycine(s) 2 Aspartate(s) 2 Glutamine(s) 0.83 Glucose-6P(c) 0.5 Cystine(s) 1 Pantothenate(s) 1 Palmitolate(s) 13.2 ATP-energy(c) 1 Proton-gradient(c) 2 THF-activated methyl group(c)	3.17 H_2O(s) 2 CO_2(s) 2 Pyruvate(c) 2 Glutamate(s) 1 (3Z)-Dodecenoyl-CoA(m) 2 NADH-redox-potential(c) 3 NADH-redox-potential(m) 3.5 NADPH-redox-potential(c) 3 FADH-redox-potential(c) 3 Na-gradient(c) 3 CoA-activated acetyl group(m)	44	14	-	-	-	-	-	-	11	-	3

ID	flux	reaction
r0406	-0.33	Fructose-6P(c) + Erythrose-4P(c) ⇌ Sedoheptulose-7P(c) + GAP(c)
r0277	1	GTP(c) + IMP(c) + Aspartate(c) → GDP(c) + Pi(c) + Adenylosuccinate(c)
r0293	2	5,10-Methylene-THF(c) + NADP+(c) ⇌ 5,10-Methenyl-THF(c) + NADPH(c)
r0705	1	ATP(c) + 5-Phosphoribosyl-4-carboxy-5-aminoimidazole(c) + Aspartate(c) ⇌ ADP(c) + Pi(c) + SAICAR(c)
r0578	1	ATP(c) + Pantetheine(c) ⇌ ADP(c) + Phosphopantetheine(c)
r0877	0.5	Glutamate(c) + Cystine(s) ⇌ Glutamate(s) + Cystine(c)
r0030	3	ATP(c) + AMP(c) ⇌ 2 ADP(c)
r0032	1	ATP(c) + Dephospho-CoA(c) ⇌ ADP(c) + CoA(c)
r1290	3	FAD(c) + FADH2(m) ⇌ FAD(m) + FADH2(c)
r1143	-2	Na+(c) + Aspartate(c) ← Na+(s) + Aspartate(s)
r2664	3	Na+(c) ⇌ Na+(s) + Na-gradient(c)
r0058	2	Malate(c) + NADP+(c) ⇌ Pyruvate(c) + CO2(c) + NADPH(c)
r0370	0.33	Sedoheptulose-7P(c) + GAP(c) ⇌ Ribose-5P(c) + D-Xylulose-5P(c)
r0371	2	5,10-Methenyl-THF(c) + H2O(c) ⇌ 10-Formyl-THF(c)
r2685	1.5	Glutamate(c) ⇌ Glutamate(s)
r0659	1	ATP(c) + 5-Phosphoribosylamine(c) + Glycine(c) ⇌ ADP(c) + Pi(c) + GAR(c)
r1480	1	CoA(m) + 3-oxopalmitoleoyl-CoA(m) ⇌ Acetyl-CoA(m) + cis-myrist-7-enoyl-CoA(m)
r0099	1	ATP(c) + GDP(c) ⇌ ADP(c) + GTP(c)
r1468	1	palmitoleoyl-CoA(c) ⇌ palmitoleoyl-CoA(m)
r0243	-0.17	DHAP(c) ⇌ GAP(c)
r0677	1	10-Formyl-THF(c) + GAR(c) ⇌ THF(c) + N-Formyl-GAR(c)
r1482	-1	FADH2(m) + trans,cis-dodeca-2,5-dienoyl-CoA(m) ⇌ FAD(m) + cis-laur-5-enoyl-CoA(m)
r1483	1	H2O(m) + trans,cis-myristo-2,7-dienoyl-CoA(m) ⇌ cis-(3S)-hydroxytetradec-7-enoyl-CoA(m)
r1331	-1	H+(PG)(s) ⇌ H+(PG)(c) + Proton-gradient(c)
r0974	1	Pantothenate(s) + Na+(s) ⇌ Pantothenate(c) + Na+(c)
r1484	1	CoA(m) + 3-oxomyrist-7-enoyl-CoA(m) ⇌ Acetyl-CoA(m) + cis-laur-5-enoyl-CoA(m)
r1319	-13.2	ATP(c) + H2O(c) ⇌ ADP(c) + Pi(c) + ATP-energy(c)
r1485	1	H2O(m) + trans,cis-dodeca-2,5-dienoyl-CoA(m) ⇌ 3(S)-3-hydroxydodecen-(5Z)-oyl-CoA(m)
r0260	1	Adenylosuccinate(c) → Fumarate(c) + AMP(c)
r0247	1	ATP(c) + Ribose-5P(c) → AMP(c) + PRPP(c)
r1486	1	NAD+(m) + 3(S)-3-hydroxydodecen-(5Z)-oyl-CoA(m) ⇌ 3-oxolaur-cis-5-enoyl-CoA(m) + NADH(m)
r1488	1	ATP(c) + CoA(c) + Palmitolate(c) ⇌ AMP(c) + palmitoleoyl-CoA(c) + PPi(c)
r0699	1	SAICAR(c) ⇌ Fumarate(c) + AICAR(c)
r2442	1	Palmitolate(s) → Palmitolate(c)
r0849	-3.17	H2O(s) ⇌ H2O(c)
r0581	-1	Pantothenate(c) + Cysteamine(c) ⇌ H2O(c) + Pantetheine(c)
r0867	2	CO2(c) ⇌ CO2(s)
r0589	1	ATP(c) + Phosphopantetheine(c) → PPi(c) + Dephospho-CoA(c)
r2468	1	Glycine(s) + H+(PG)(s) → Glycine(c) + H+(PG)(c)
r0007	3	PPi(c) + H2O(c) → 2 Pi(c)
r0736	0.17	ATP(c) + Fructose-6P(c) → ADP(c) + Fructose-1,6PP(c)
r0027	-0.5	NADPH(c) + Cystine(c) ⇌ 2 Cysteine(c) + NADP+(c)
r0192	0.83	Glucose-6P(c) ⇌ Fructose-6P(c)
r0344	-0.67	D-Xylulose-5P(c) ⇌ Ribulose-5P(c)
		Continued on next page

ID	flux	reaction
r0792	2	5-Methyl-THF(c) + NAD+(c) ⇌ 5,10-Methylene-THF(c) + NADH(c)
r0211	1	Cysteine(c) + O2(c) ⇌ 3-Sulfinoalanine(c)
r2676	-2	5-Methyl-THF(c) ⇌ THF(c) + THF-activated methyl group(c)
r2525	2	Glutamine(s) ⇌ Glutamine(c)
r2678	3	Acetyl-CoA(m) ⇌ CoA(m) + CoA-activated acetyl group(m)
r0666	1	ATP(c) + 5-Phosphoribosylformylglycinamidine(c) ⇌ ADP(c) + Pi(c) + AIR(c)
r1471	1	FAD(m) + cis-myrist-7-enoyl-CoA(m) ⇌ FADH2(m) + trans,cis-myristo-2,7-dienoyl-CoA(m)
r0667	-1	5-Phosphoribosyl-4-carboxy-5-aminoimidazole(c) ⇌ AIR(c) + CO2(c)
r1473	1	FAD(m) + palmitoleoyl-CoA(m) ⇌ FADH2(m) + trans,cis-hexadeca-2,9-dienoyl-CoA(m)
r1322	2	NADH(c) ⇌ NAD+(c) + NADH-redox-potential(c)
r1475	1	H2O(m) + trans,cis-hexadeca-2,9-dienoyl-CoA(m) ⇌ (S)-3-hydroxypalmitoleoyl-CoA(m)
r1323	3	NADH(m) ⇌ NAD+(m) + NADH-redox-potential(m)
r0250	-0.67	Ribulose-5P(c) ⇌ Ribose-5P(c)
r1476	1	NAD+(m) + cis-(3S)-hydroxytetradec-7-enoyl-CoA(m) ⇌ 3-oxomyrist-7-enoyl-CoA(m) + NADH(m)
r1478	1	NAD+(m) + (S)-3-hydroxypalmitoleoyl-CoA(m) ⇌ NADH(m) + 3-oxopalmitoleoyl-CoA(m)
r1326	3.5	NADPH(c) ⇌ NADPH-redox-potential(c) + NADP+(c)
r0687	1	ATP(c) + N-Formyl-GAR(c) + Glutamine(c) + H2O(c) ⇌ ADP(c) + Pi(c) + Glutamate(c) + 5-Phosphoribosylformylglycinamidine(c)
r1479	1	CoA(m) + 3-oxolaur-cis-5-enoyl-CoA(m) ⇌ Acetyl-CoA(m) + (3Z)-Dodecenoyl-CoA(m)
r0255	0.33	Fructose-6P(c) + GAP(c) ⇌ D-Xylulose-5P(c) + Erythrose-4P(c)
r1329	3	FADH2(c) ⇌ FAD(c) + FADH-redox-potential(c)
r0538	1	3-Sulfinoalanine(c) ⇌ CO2(c) + Hypotaurine(c)
r0256	0.17	Fructose-1,6PP(c) ⇌ DHAP(c) + GAP(c)
r0539	-1	Hypotaurine(c) ⇌ Cysteamine(c) + O2(c)
r0257	-1	Glutamine(c) + PRPP(c) + H2O(c) ⇌ 5-Phosphoribosylamine(c) + PPi(c) + Glutamate(c)
r0258	-2	Fumarate(c) + H2O(c) ⇌ Malate(c)
r0273	-1	IMP(c) + H2O(c) ⇌ FAICAR(c)
r0851	-3	H2O(m) ⇌ H2O(c)
r0700	1	10-Formyl-THF(c) + AICAR(c) ⇌ THF(c) + FAICAR(c)

(R)-3-Hydroxybutanoyl-ACP (546)

Definition			Solution												
			exchanges		reactions								transp		
Objective	Constraints	Comment	imports	exports	c	m	r	p	l	n	s	b	s-c	b-c	int
(R)-3-Hydroxybutanoyl-ACP(c)	PIPES MCES	synthesis of cytosolic (R)-3-Hydroxybutanoyl-ACP	1 P_i(c) 5 Glutamate(s) 6 Glycine(s) 11 Alanine(s) 4 Lysine(s) 8 Aspartate(s) 9 Arginine(s) 7 Glutamine(s) 8 Serine(s) 5 Methionine(s) 3 Phenylalanine(s) 4 Tyrosine(s) 3 Cysteine(s) 20 Leucine(s) 1 Histidine(s) 9 Proline(s) 1 Asparagine(s) 11 Valine(s) 6 Threonine(s) 4 Isoleucine(s) 1 Pantothenate(s) 625 ATP-energy(c) 1 NADPH-redox-potential(c) 2 CoA-activated acetyl group(c)	3 H_2O(s) 1 CO_2(s) 1 (R)-3-Hydroxybutanoyl-ACP(c) 2 Proton-gradient(c) 82 Na-gradient(c)	21	-	-	-	-	-	-	-	24	-	-

ID	flux	reaction
r1069	-4	Lysine(s) ⇌ Lysine(c)
r0578	1	ATP(c) + Pantetheine(c) ⇌ ADP(c) + Phosphopantetheine(c)
r2478	8	Serine(s) + Na+(s) + H+(PG)(c) → Serine(c) + Na+(c) + H+(PG)(s)
r0030	125	ATP(c) + AMP(c) ⇌ 2 ADP(c)
r0032	1	ATP(c) + Dephospho-CoA(c) ⇌ ADP(c) + CoA(c)
r0184	1	ATP(c) + Acetyl-CoA(c) + HCO3-(c) → ADP(c) + Pi(c) + Malonyl-CoA(c)
r1121	-11	Valine(c) + Na+(c) ← Valine(s) + Na+(s)

Continued on next page

ID	flux	reaction
r1122	-20	Leucine(c) + Na+(c) ← Leucine(s) + Na+(s)
r1123	-4	Isoleucine(c) + Na+(c) ← Isoleucine(s) + Na+(s)
r1125	4	Tyrosine(s) + Na+(s) → Tyrosine(c) + Na+(c)
r1141	-5	Methionine(c) + Na+(c) ← Methionine(s) + Na+(s)
r1142	-6	Threonine(s) + Na+(s) → Threonine(c) + Na+(c)
r1143	-8	Na+(c) + Aspartate(c) ← Na+(s) + Aspartate(s)
r2664	82	Na+(c) ⇌ Na+(s) + Na-gradient(c)
r1144	-5	Glutamate(c) + Na+(c) ← Glutamate(s) + Na+(s)
r1145	-1	Histidine(c) + Na+(c) ← Histidine(s) + Na+(s)
r2532	1	Asparagine(s) ⇌ Asparagine(c)
r0678	1	Acetyl-ACP(c) + Malonyl-ACP(c) ⇌ CO2(c) + ACP(c) + Acetoacetyl-ACP(c)
r0691	-1	(R)-3-Hydroxybutanoyl-ACP(c) + NADP+(c) ⇌ NADPH(c) + Acetoacetyl-ACP(c)
r1331	2	H+(PG)(s) ⇌ H+(PG)(c) + Proton-gradient(c)
r0974	1	Pantothenate(s) + Na+(s) ⇌ Pantothenate(c) + Na+(c)
r1319	-625	ATP(c) + H2O(c) ⇌ ADP(c) + Pi(c) + ATP-energy(c)
r1058	9	Arginine(s) ⇌ Arginine(c)
r0849	-3	H2O(s) ⇌ H2O(c)
r0581	-1	Pantothenate(c) + Cysteamine(c) ⇌ H2O(c) + Pantetheine(c)
r0867	1	CO2(c) ⇌ CO2(s)
r1096	1	11 Alanine(c) + 2 Cysteine(c) + 5 Glutamate(c) + 3 Phenylalanine(c) + 6 Glycine(c) + Histidine(c) + 4 Isoleucine(c) + 20 Leucine(c) + 5 Methionine(c) + 9 Proline(c) + 7 Glutamine(c) + 9 Arginine(c) + 8 Serine(c) + 6 Threonine(c) + 11 Valine(c) + 4 Tyrosine(c) + 496 ATP(c) + 496 H2O(c) + 8 Aspartate(c) + 4 Lysine(c) + Asparagine(c) → Apo-ACP(c) + 124 AMP(c) + 124 PPi(c) + 372 ADP(c) + 372 Pi(c)
r0589	1	ATP(c) + Phosphopantetheine(c) → PPi(c) + Dephospho-CoA(c)
r2468	6	Glycine(s) + H+(PG)(s) → Glycine(c) + H+(PG)(c)
r0007	125	PPi(c) + H2O(c) → 2 Pi(c)
r0049	1	PAP(c) + H2O(c) → AMP(c) + Pi(c)
r1417	-1	CO2(c) + H2O(c) ⇌ Carbonate(c)
r1419	1	Carbonate(c) ⇌ HCO3-(c)
r1139	-9	Proline(c) + Na+(c) ← Proline(s) + Na+(s)
r0211	1	Cysteine(c) + O2(c) ⇌ 3-Sulfinoalanine(c)
r2524	11	Alanine(s) ⇌ Alanine(c)
r2677	-2	Acetyl-CoA(c) ⇌ CoA(c) + CoA-activated acetyl group(c)
r2525	7	Glutamine(s) ⇌ Glutamine(c)
r0367	1	Acetyl-CoA(c) + ACP(c) ⇌ CoA(c) + Acetyl-ACP(c)
r0368	1	CoA(c) + Apo-ACP(c) → PAP(c) + ACP(c)
r2528	3	Phenylalanine(s) ⇌ Phenylalanine(c)
r0369	1	ACP(c) + Malonyl-CoA(c) ⇌ CoA(c) + Malonyl-ACP(c)
r2529	3	Cysteine(s) ⇌ Cysteine(c)
r1326	-1	NADPH(c) ⇌ NADPH-redox-potential(c) + NADP+(c)
r0538	1	3-Sulfinoalanine(c) ⇌ CO2(c) + Hypotaurine(c)
r0539	-1	Hypotaurine(c) ⇌ Cysteamine(c) + O2(c)

(R)-3-Hydroxydecanoyl-ACP (547)

Definition			Solution												
			exchanges		reactions								transp		
Objective	Constraints	Comment	imports	exports	c	m	r	p	l	n	s	b	s-c	b-c	int
(R)-3-Hydroxydecanoyl-ACP(c)	PIPES MCES	synthesis of cytosolic (R)-3-Hydroxydecanoyl-ACP	1 P_i(c) 5 Glutamate(s) 6 Glycine(s) 11 Alanine(s) 4 Lysine(s) 8 Aspartate(s) 9 Arginine(s) 7 Glutamine(s) 8 Serine(s) 5 Methionine(s) 3 Phenylalanine(s) 4 Tyrosine(s) 3 Cysteine(s) 20 Leucine(s) 1 Histidine(s) 9 Proline(s) 1 Asparagine(s) 11 Valine(s) 6 Threonine(s) 4 Isoleucine(s) 1 Pantothenate(s) 628 ATP-energy(c) 7 NADPH-redox-potential(c) 3 Proton-gradient(c) 5 CoA-activated acetyl group(c)	6 H_2O(s) 1 CO_2(s) 1 (R)-3-Hydroxydecanoyl-ACP(c) 66 Na-gradient(c)	33	-	-	-	-	-	-	-	24	-	-

ID	flux	reaction
r1069	-4	Lysine(c) ⇌ Lysine(s)
r0578	1	ATP(c) + Pantetheine(c) ⇌ ADP(c) + Phosphopantetheine(c)
r2478	8	Serine(s) + Na+(s) + H+(PG)(c) → Serine(c) + Na+(c) + H+(PG)(s)
r0030	125	ATP(c) + AMP(c) ⇌ 2 ADP(c)
r0760	1	Butyryl-ACP(c) + Malonyl-ACP(c) ⇌ CO2(c) + ACP(c) + 3-Oxohexanoyl-ACP(c)
r0032	1	ATP(c) + Dephospho-CoA(c) ⇌ ADP(c) + CoA(c)
r0761	-1	D-3-Hydroxyhexanoyl-ACP(c) + NADP+(c) ⇌ NADPH(c) + 3-Oxohexanoyl-ACP(c)
r0184	4	ATP(c) + Acetyl-CoA(c) + HCO3-(c) → ADP(c) + Pi(c) + Malonyl-CoA(c)
r0762	1	D-3-Hydroxyhexanoyl-ACP(c) ⇌ H2O(c) + (2E)-Hexenoyl-ACP(c)
r0763	-1	Hexanoyl-ACP(c) + NADP+(c) ⇌ NADPH(c) + (2E)-Hexenoyl-ACP(c)
r0764	1	Malonyl-ACP(c) + Hexanoyl-ACP(c) ⇌ 3-Oxooctanoyl-ACP(c) + CO2(c) + ACP(c)
r1122	-20	Leucine(c) + Na+(c) ← Leucine(s) + Na+(s)
r0765	-1	Octanoyl-ACP(c) + NADP+(c) ⇌ (2E)-Octenoyl-ACP(c) + NADPH(c)
r1123	-4	Isoleucine(c) + Na+(c) ← Isoleucine(s) + Na+(s)
r0766	1	Octanoyl-ACP(c) + Malonyl-ACP(c) ⇌ 3-Oxodecanoyl-ACP(c) + CO2(c) + ACP(c)
r1125	4	Tyrosine(s) + Na+(s) → Tyrosine(c) + Na+(c)
r1142	-6	Threonine(s) + Na+(s) → Threonine(c) + Na+(c)
r1143	-8	Na+(c) + Aspartate(c) ← Na+(s) + Aspartate(s)
r2664	66	Na+(c) ⇌ Na+(s) + Na-gradient(c)
r1144	-5	Glutamate(c) + Na+(c) ← Glutamate(s) + Na+(s)
r1145	-1	Histidine(c) + Na+(c) ← Histidine(s) + Na+(s)
r2532	1	Asparagine(s) ⇌ Asparagine(c)
r2533	11	Valine(s) ⇌ Valine(c)
r0678	1	Acetyl-ACP(c) + Malonyl-ACP(c) ⇌ CO2(c) + ACP(c) + Acetoacetyl-ACP(c)
r0691	-1	(R)-3-Hydroxybutanoyl-ACP(c) + NADP+(c) ⇌ NADPH(c) + Acetoacetyl-ACP(c)
r1331	-3	H+(PG)(s) ⇌ H+(PG)(c) + Proton-gradient(c)
r0974	1	Pantothenate(s) + Na+(s) ⇌ Pantothenate(c) + Na+(c)
r0692	-1	(R)-3-Hydroxydecanoyl-ACP(c) + NADP+(c) ⇌ 3-Oxodecanoyl-ACP(c) + NADPH(c)
r1319	-628	ATP(c) + H2O(c) ⇌ ADP(c) + Pi(c) + ATP-energy(c)
r0694	-1	(R)-3-Hydroxyoctanoyl-ACP(c) + NADP+(c) ⇌ 3-Oxooctanoyl-ACP(c) + NADPH(c)
r0695	1	(R)-3-Hydroxyoctanoyl-ACP(c) ⇌ (2E)-Octenoyl-ACP(c) + H2O(c)
r1058	9	Arginine(s) ⇌ Arginine(c)
r1072	5	Methionine(s) ⇌ Methionine(c)
r0849	-6	H2O(s) ⇌ H2O(c)
r0581	-1	Pantothenate(c) + Cysteamine(c) ⇌ H2O(c) + Pantetheine(c)
r0867	1	CO2(c) ⇌ CO2(s)
r1096	1	11 Alanine(c) + 2 Cysteine(c) + 5 Glutamate(c) + 3 Phenylalanine(c) + 6 Glycine(c) + Histidine(c) + 4 Isoleucine(c) + 20 Leucine(c) + 5 Methionine(c) + 9 Proline(c) + 7 Glutamine(c) + 9 Arginine(c) + 8 Serine(c) + 6 Threonine(c) + 11 Valine(c) + 4 Tyrosine(c) + 496 ATP(c) + 496 H2O(c) + 8 Aspartate(c) + 4 Lysine(c) + Asparagine(c) → Apo-ACP(c) + 124 AMP(c) + 124 PPi(c) + 372 ADP(c) + 372 Pi(c)
r0589	1	ATP(c) + Phosphopantetheine(c) → PPi(c) + Dephospho-CoA(c)

Continued on next page

ID	flux	reaction
r2469	11	Alanine(s) + H+(PG)(s) → Alanine(c) + H+(PG)(c)
r0007	125	PPi(c) + H2O(c) → 2 Pi(c)
r1417	-4	CO2(c) + H2O(c) ⇌ Carbonate(c)
r0049	1	PAP(c) + H2O(c) → AMP(c) + Pi(c)
r1419	4	Carbonate(c) ⇌ HCO3-(c)
r1139	-9	Proline(c) + Na+(c) ← Proline(s) + Na+(s)
r0211	1	Cysteine(c) + O2(c) ⇌ 3-Sulfinoalanine(c)
r2523	6	Glycine(s) ⇌ Glycine(c)
r2677	-5	Acetyl-CoA(c) ⇌ CoA(c) + CoA-activated acetyl group(c)
r2525	7	Glutamine(s) ⇌ Glutamine(c)
r0367	1	Acetyl-CoA(c) + ACP(c) ⇌ CoA(c) + Acetyl-ACP(c)
r0368	1	CoA(c) + Apo-ACP(c) → PAP(c) + ACP(c)
r2528	3	Phenylalanine(s) ⇌ Phenylalanine(c)
r0369	4	ACP(c) + Malonyl-CoA(c) ⇌ CoA(c) + Malonyl-ACP(c)
r2529	3	Cysteine(s) ⇌ Cysteine(c)
r0681	1	(R)-3-Hydroxybutanoyl-ACP(c) ⇌ But-2-enoyl-ACP(c) + H2O(c)
r0682	-1	Butyryl-ACP(c) + NADP+(c) ⇌ But-2-enoyl-ACP(c) + NADPH(c)
r1326	-7	NADPH(c) ⇌ NADPH-redox-potential(c) + NADP+(c)
r0538	1	3-Sulfinoalanine(c) ⇌ CO2(c) + Hypotaurine(c)
r0539	-1	Hypotaurine(c) ⇌ Cysteamine(c) + O2(c)

(R)-3-Hydroxyoctanoyl-ACP (548)

Definition			Solution													
			exchanges		reactions								transp		
Objective	Constraints	Comment	imports	exports	c	m	r	p	l	n	s	b	s-c	b-c	int
(R)-3-Hydroxyoctanoyl-ACP(c)	PIPES MCES	synthesis of cytosolic (R)-3-Hydroxyoctanoyl-ACP	1 P_i(c) 5 Glutamate(s) 6 Glycine(s) 11 Alanine(s) 4 Lysine(s) 8 Aspartate(s) 9 Arginine(s) 7 Glutamine(s) 8 Serine(s) 5 Methionine(s) 3 Phenylalanine(s) 4 Tyrosine(s) 3 Cysteine(s) 20 Leucine(s) 1 Histidine(s) 9 Proline(s) 1 Asparagine(s) 11 Valine(s) 6 Threonine(s) 4 Isoleucine(s) 1 Pantothenate(s) 627 ATP-energy(c) 5 NADPH-redox-potential(c) 3 Proton-gradient(c) 4 CoA-activated acetyl group(c)	5 H_2O(s) 1 CO_2(s) 1 (R)-3-Hydroxyoctanoyl-ACP(c) 66 Na-gradient(c)	29	-	-	-	-	-	-	-	24	-	-

ID	flux	reaction
r1069	-4	Lysine(c) ⇌ Lysine(s)
r0578	1	ATP(c) + Pantetheine(c) ⇌ ADP(c) + Phosphopantetheine(c)
r2478	8	Serine(s) + Na+(s) + H+(PG)(c) → Serine(c) + Na+(c) + H+(PG)(s)
r0030	125	ATP(c) + AMP(c) ⇌ 2 ADP(c)
r0760	1	Butyryl-ACP(c) + Malonyl-ACP(c) ⇌ CO2(c) + ACP(c) + 3-Oxohexanoyl-ACP(c)
r0032	1	ATP(c) + Dephospho-CoA(c) ⇌ ADP(c) + CoA(c)
r0761	-1	D-3-Hydroxyhexanoyl-ACP(c) + NADP+(c) ⇌ NADPH(c) + 3-Oxohexanoyl-ACP(c)
r0184	3	ATP(c) + Acetyl-CoA(c) + HCO3-(c) → ADP(c) + Pi(c) + Malonyl-CoA(c)
r0762	1	D-3-Hydroxyhexanoyl-ACP(c) ⇌ H2O(c) + (2E)-Hexenoyl-ACP(c)
r0763	-1	Hexanoyl-ACP(c) + NADP+(c) ⇌ NADPH(c) + (2E)-Hexenoyl-ACP(c)
r0764	1	Malonyl-ACP(c) + Hexanoyl-ACP(c) ⇌ 3-Oxooctanoyl-ACP(c) + CO2(c) + ACP(c)
r1122	-20	Leucine(c) + Na+(c) ← Leucine(s) + Na+(s)
r1123	-4	Isoleucine(c) + Na+(c) ← Isoleucine(s) + Na+(s)
r1125	4	Tyrosine(s) + Na+(s) → Tyrosine(c) + Na+(c)
r1142	-6	Threonine(s) + Na+(s) → Threonine(c) + Na+(c)

Continued on next page

ID	flux	reaction
r1143	-8	Na+(c) + Aspartate(c) ← Na+(s) + Aspartate(s)
r2664	66	Na+(c) ⇌ Na+(s) + Na-gradient(c)
r1144	-5	Glutamate(c) + Na+(c) ← Glutamate(s) + Na+(s)
r1145	-1	Histidine(c) + Na+(c) ← Histidine(s) + Na+(s)
r2532	1	Asparagine(s) ⇌ Asparagine(c)
r2533	11	Valine(s) ⇌ Valine(c)
r0678	1	Acetyl-ACP(c) + Malonyl-ACP(c) ⇌ CO2(c) + ACP(c) + Acetoacetyl-ACP(c)
r0691	-1	(R)-3-Hydroxybutanoyl-ACP(c) + NADP+(c) ⇌ NADPH(c) + Acetoacetyl-ACP(c)
r1331	-3	H+(PG)(s) ⇌ H+(PG)(c) + Proton-gradient(c)
r0974	1	Pantothenate(s) + Na+(s) ⇌ Pantothenate(c) + Na+(c)
r1319	-627	ATP(c) + H2O(c) ⇌ ADP(c) + Pi(c) + ATP-energy(c)
r0694	-1	(R)-3-Hydroxyoctanoyl-ACP(c) + NADP+(c) ⇌ 3-Oxooctanoyl-ACP(c) + NADPH(c)
r1058	9	Arginine(s) ⇌ Arginine(c)
r1072	5	Methionine(s) ⇌ Methionine(c)
r0849	-5	H2O(s) ⇌ H2O(c)
r0581	-1	Pantothenate(c) + Cysteamine(c) ⇌ H2O(c) + Pantetheine(c)
r0867	1	CO2(c) ⇌ CO2(s)
r1096	1	11 Alanine(c) + 2 Cysteine(c) + 5 Glutamate(c) + 3 Phenylalanine(c) + 6 Glycine(c) + Histidine(c) + 4 Isoleucine(c) + 20 Leucine(c) + 5 Methionine(c) + 9 Proline(c) + 7 Glutamine(c) + 9 Arginine(c) + 8 Serine(c) + 6 Threonine(c) + 11 Valine(c) + 4 Tyrosine(c) + 496 ATP(c) + 496 H2O(c) + 8 Aspartate(c) + 4 Lysine(c) + Asparagine(c) → Apo-ACP(c) + 124 AMP(c) + 124 PPi(c) + 372 ADP(c) + 372 Pi(c)
r0589	1	ATP(c) + Phosphopantetheine(c) → PPi(c) + Dephospho-CoA(c)
r2469	11	Alanine(s) + H+(PG)(s) → Alanine(c) + H+(PG)(c)
r0007	125	PPi(c) + H2O(c) → 2 Pi(c)
r1417	-3	CO2(c) + H2O(c) ⇌ Carbonate(c)
r0049	1	PAP(c) + H2O(c) → AMP(c) + Pi(c)
r1419	3	Carbonate(c) ⇌ HCO3-(c)
r1139	-9	Proline(c) + Na+(c) ← Proline(s) + Na+(s)
r0211	1	Cysteine(c) + O2(c) ⇌ 3-Sulfinoalanine(c)
r2523	6	Glycine(s) ⇌ Glycine(c)
r2677	-4	Acetyl-CoA(c) ⇌ CoA(c) + CoA-activated acetyl group(c)
r2525	7	Glutamine(s) ⇌ Glutamine(c)
r0367	1	Acetyl-CoA(c) + ACP(c) ⇌ CoA(c) + Acetyl-ACP(c)
r0368	1	CoA(c) + Apo-ACP(c) → PAP(c) + ACP(c)
r2528	3	Phenylalanine(s) ⇌ Phenylalanine(c)
r0369	3	ACP(c) + Malonyl-CoA(c) ⇌ CoA(c) + Malonyl-ACP(c)
r2529	3	Cysteine(s) ⇌ Cysteine(c)
r0681	1	(R)-3-Hydroxybutanoyl-ACP(c) ⇌ But-2-enoyl-ACP(c) + H2O(c)
r0682	-1	Butyryl-ACP(c) + NADP+(c) ⇌ But-2-enoyl-ACP(c) + NADPH(c)
r1326	-5	NADPH(c) ⇌ NADPH-redox-potential(c) + NADP+(c)
r0538	1	3-Sulfinoalanine(c) ⇌ CO2(c) + Hypotaurine(c)
r0539	-1	Hypotaurine(c) ⇌ Cysteamine(c) + O2(c)

(R)-3-Hydroxypalmitoyl-ACP (549)

Definition			Solution												
			exchanges		reactions								transp		
Objective	Constraints	Comment	imports	exports	c	m	r	p	l	n	s	b	s-c	b-c	int
(R)-3-Hydroxypalmitoyl-ACP(c)	PIPES MCES	synthesis of cytosolic (R)-3-Hydroxypalmitoyl-ACP	1 P_i(c) 5 Glutamate(s) 6 Glycine(s) 11 Alanine(s) 4 Lysine(s) 8 Aspartate(s) 9 Arginine(s) 7 Glutamine(s) 8 Serine(s) 5 Methionine(s) 3 Phenylalanine(s) 4 Tyrosine(s) 3 Cysteine(s) 20 Leucine(s) 1 Histidine(s) 9 Proline(s) 1 Asparagine(s) 11 Valine(s) 6 Threonine(s) 4 Isoleucine(s) 1 Pantothenate(s) 631 ATP-energy(c) 13 NADPH-redox-potential(c) 3 Proton-gradient(c) 8 CoA-activated acetyl group(c)	9 H_2O(s) 1 CO_2(s) 1 (R)-3-Hydroxypalmitoyl-ACP(c) 66 Na-gradient(c)	45	-	-	-	-	-	-	-	24	-	-

ID	flux	reaction
r0701	-1	HMA(c) + NADP+(c) ⇌ 3-Oxotetradecanoyl-ACP(c) + NADPH(c)
r0702	1	HMA(c) ⇌ H2O(c) + (2E)-Tetradecenoyl-ACP(c)
r1069	-4	Lysine(c) ⇌ Lysine(s)
r0578	1	ATP(c) + Pantetheine(c) ⇌ ADP(c) + Phosphopantetheine(c)
r2478	8	Serine(s) + Na+(s) + H+(PG)(c) → Serine(c) + Na+(c) + H+(PG)(s)
r0030	125	ATP(c) + AMP(c) ⇌ 2 ADP(c)
r0760	1	Butyryl-ACP(c) + Malonyl-ACP(c) ⇌ CO2(c) + ACP(c) + 3-Oxohexanoyl-ACP(c)
r0032	1	ATP(c) + Dephospho-CoA(c) ⇌ ADP(c) + CoA(c)
r0761	-1	D-3-Hydroxyhexanoyl-ACP(c) + NADP+(c) ⇌ NADPH(c) + 3-Oxohexanoyl-ACP(c)
r0184	7	ATP(c) + Acetyl-CoA(c) + HCO3-(c) → ADP(c) + Pi(c) + Malonyl-CoA(c)
r0762	1	D-3-Hydroxyhexanoyl-ACP(c) ⇌ H2O(c) + (2E)-Hexenoyl-ACP(c)
r0763	-1	Hexanoyl-ACP(c) + NADP+(c) ⇌ NADPH(c) + (2E)-Hexenoyl-ACP(c)
r0764	1	Malonyl-ACP(c) + Hexanoyl-ACP(c) ⇌ 3-Oxooctanoyl-ACP(c) + CO2(c) + ACP(c)
r1122	-20	Leucine(c) + Na+(c) ← Leucine(s) + Na+(s)
r0765	-1	Octanoyl-ACP(c) + NADP+(c) ⇌ (2E)-Octenoyl-ACP(c) + NADPH(c)
r1123	-4	Isoleucine(c) + Na+(c) ← Isoleucine(s) + Na+(s)
r0766	1	Octanoyl-ACP(c) + Malonyl-ACP(c) ⇌ 3-Oxodecanoyl-ACP(c) + CO2(c) + ACP(c)
r0767	-1	Decanoyl-ACP(c) + NADP+(c) ⇌ (2E)-Decenoyl-ACP(c) + NADPH(c)
r1125	4	Tyrosine(s) + Na+(s) → Tyrosine(c) + Na+(c)
r0768	1	Decanoyl-ACP(c) + Malonyl-ACP(c) ⇌ 3-Oxododecanoyl-ACP(c) + CO2(c) + ACP(c)
r0769	-1	D-3-Hydroxydodecanoyl-ACP(c) + NADP+(c) ⇌ 3-Oxododecanoyl-ACP(c) + NADPH(c)
r1142	-6	Threonine(s) + Na+(s) → Threonine(c) + Na+(c)
r1143	-8	Na+(c) + Aspartate(c) ← Na+(s) + Aspartate(s)
r2664	66	Na+(c) ⇌ Na+(s) + Na-gradient(c)
r1144	-5	Glutamate(c) + Na+(c) ← Glutamate(s) + Na+(s)
r1145	-1	Histidine(c) + Na+(c) ← Histidine(s) + Na+(s)
r2532	1	Asparagine(s) ⇌ Asparagine(c)
r2533	11	Valine(s) ⇌ Valine(c)
r0678	1	Acetyl-ACP(c) + Malonyl-ACP(c) ⇌ CO2(c) + ACP(c) + Acetoacetyl-ACP(c)
r0691	-1	(R)-3-Hydroxybutanoyl-ACP(c) + NADP+(c) ⇌ NADPH(c) + Acetoacetyl-ACP(c)
r1331	-3	H+(PG)(s) ⇌ H+(PG)(c) + Proton-gradient(c)
r0974	1	Pantothenate(s) + Na+(s) ⇌ Pantothenate(c) + Na+(c)
r0692	-1	(R)-3-Hydroxydecanoyl-ACP(c) + NADP+(c) ⇌ 3-Oxodecanoyl-ACP(c) + NADPH(c)
r1319	-631	ATP(c) + H2O(c) ⇌ ADP(c) + Pi(c) + ATP-energy(c)
r0693	1	(R)-3-Hydroxydecanoyl-ACP(c) ⇌ (2E)-Decenoyl-ACP(c) + H2O(c)
r0694	-1	(R)-3-Hydroxyoctanoyl-ACP(c) + NADP+(c) ⇌ 3-Oxooctanoyl-ACP(c) + NADPH(c)
r0695	1	(R)-3-Hydroxyoctanoyl-ACP(c) ⇌ (2E)-Octenoyl-ACP(c) + H2O(c)
r0696	-1	(R)-3-Hydroxypalmitoyl-ACP(c) + NADP+(c) ⇌ 3-Oxohexadecanoyl-ACP(c) + NADPH(c)
r1058	9	Arginine(s) ⇌ Arginine(c)
r1072	5	Methionine(s) ⇌ Methionine(c)

Continued on next page

(R)-3-Hydroxypalmitoyl-ACP (549)– continued

ID	flux	reaction
r0849	-9	H2O(s) ⇌ H2O(c)
r0581	-1	Pantothenate(c) + Cysteamine(c) ⇌ H2O(c) + Pantetheine(c)
r0712	-1	Dodecanoyl-ACP(c) + NADP+(c) ⇌ (2E)-Dodecenoyl-ACP(c) + NADPH(c)
r0713	1	Dodecanoyl-ACP(c) + Malonyl-ACP(c) ⇌ 3-Oxotetradecanoyl-ACP(c) + CO2(c) + ACP(c)
r0867	1	CO2(c) ⇌ CO2(s)
r1096	1	11 Alanine(c) + 2 Cysteine(c) + 5 Glutamate(c) + 3 Phenylalanine(c) + 6 Glycine(c) + Histidine(c) + 4 Isoleucine(c) + 20 Leucine(c) + 5 Methionine(c) + 9 Proline(c) + 7 Glutamine(c) + 9 Arginine(c) + 8 Serine(c) + 6 Threonine(c) + 11 Valine(c) + 4 Tyrosine(c) + 496 ATP(c) + 496 H2O(c) + 8 Aspartate(c) + 4 Lysine(c) + Asparagine(c) → Apo-ACP(c) + 124 AMP(c) + 124 PPi(c) + 372 ADP(c) + 372 Pi(c)
r0589	1	ATP(c) + Phosphopantetheine(c) → PPi(c) + Dephospho-CoA(c)
r2469	11	Alanine(s) + H+(PG)(s) → Alanine(c) + H+(PG)(c)
r0007	125	PPi(c) + H2O(c) → 2 Pi(c)
r0770	1	D-3-Hydroxydodecanoyl-ACP(c) ⇌ (2E)-Dodecenoyl-ACP(c) + H2O(c)
r0771	-1	Tetradecanoyl-ACP(c) + NADP+(c) ⇌ NADPH(c) + (2E)-Tetradecenoyl-ACP(c)
r0772	1	Tetradecanoyl-ACP(c) + Malonyl-ACP(c) ⇌ 3-Oxohexadecanoyl-ACP(c) + CO2(c) + ACP(c)
r1417	-7	CO2(c) + H2O(c) ⇌ Carbonate(c)
r0049	1	PAP(c) + H2O(c) → AMP(c) + Pi(c)
r1419	7	Carbonate(c) ⇌ HCO3-(c)
r1139	-9	Proline(c) + Na+(c) ← Proline(s) + Na+(s)
r0211	1	Cysteine(c) + O2(c) ⇌ 3-Sulfinoalanine(c)
r2523	6	Glycine(s) ⇌ Glycine(c)
r2677	-8	Acetyl-CoA(c) ⇌ CoA(c) + CoA-activated acetyl group(c)
r2525	7	Glutamine(s) ⇌ Glutamine(c)
r0367	1	Acetyl-CoA(c) + ACP(c) ⇌ CoA(c) + Acetyl-ACP(c)
r0368	1	CoA(c) + Apo-ACP(c) → PAP(c) + ACP(c)
r2528	3	Phenylalanine(s) ⇌ Phenylalanine(c)
r0369	7	ACP(c) + Malonyl-CoA(c) ⇌ CoA(c) + Malonyl-ACP(c)
r2529	3	Cysteine(s) ⇌ Cysteine(c)
r0681	1	(R)-3-Hydroxybutanoyl-ACP(c) ⇌ But-2-enoyl-ACP(c) + H2O(c)
r0682	-1	Butyryl-ACP(c) + NADP+(c) ⇌ But-2-enoyl-ACP(c) + NADPH(c)
r1326	-13	NADPH(c) ⇌ NADPH-redox-potential(c) + NADP+(c)
r0538	1	3-Sulfinoalanine(c) ⇌ CO2(c) + Hypotaurine(c)
r0539	-1	Hypotaurine(c) ⇌ Cysteamine(c) + O2(c)

(R)-4-Phosphopantothenoyl-cysteine (550)

Definition			Solution												
			exchanges		reactions								transp		
Objective	Constraints	Comment	imports	exports	c	m	r	p	l	n	s	b	s-c	b-c	int
(R)-4-Phosphopantothenoyl-cysteine(c)	PIPES MCES	synthesis of cytosolic (R)-4-Phosphopantothenoyl-cysteine	1 P_i(c) 1 Cysteine(s) 1 Pantothenate(s) 3 ATP-energy(c)	2 H_2O(s) 1 (R)-4-Phosphopantothenoyl-cysteine(c) 1 Na-gradient(c)	6	-	-	-	-	-	-	-	4	-	-

ID	flux	reaction
r0136	1	ATP(c) + CMP(c) ⇌ ADP(c) + CDP(c)
r1319	-3	ATP(c) + H2O(c) ⇌ ADP(c) + Pi(c) + ATP-energy(c)
r0671	1	D-4-Phosphopantothenate(c) + Cysteine(c) + CTP(c) → CMP(c) + PPi(c) + (R)-4-Phosphopantothenoyl-cysteine(c)
r2664	1	Na+(c) ⇌ Na+(s) + Na-gradient(c)
r2529	1	Cysteine(s) ⇌ Cysteine(c)
r0586	1	ATP(c) + Pantothenate(c) → ADP(c) + D-4-Phosphopantothenate(c)
r0150	1	ATP(c) + CDP(c) ⇌ ADP(c) + CTP(c)
r0974	1	Pantothenate(s) + Na+(s) ⇌ Pantothenate(c) + Na+(c)
r0007	1	PPi(c) + H2O(c) → 2 Pi(c)
r0849	-2	H2O(s) ⇌ H2O(c)

(R)-5-Diphosphomevalonate (551)

Definition			Solution												
			exchanges		reactions								transp		
Objective	Constraints	Comment	imports	exports	c	m	r	p	l	n	s	b	s-c	b-c	int
(R)-5-Diphosphomevalonate(p)	PIPES MCES	synthesis of peroxysomal (R)-5-Diphosphomevalonate	2 P_i(c) 2 ATP-energy(c) 2 NADPH-redox-potential(c) 3 CoA-activated acetyl group(c)	1 H_2O(s) 1 (R)-5-Diphosphomevalonate(p)	6	-	-	2	-	-	-	-	1	-	2

ID	flux	reaction
r0515	1	ATP(p) + (R)-Mevalonate(p) ⇌ ADP(p) + (R)-5-Phosphomevalonate(p)
r0461	-1	Acetyl-CoA(c) + H2O(c) + Acetoacetyl-CoA(c) ⇌ HMG-CoA(c) + CoA(c)
r1326	-2	NADPH(c) ⇌ NADPH-redox-potential(c) + NADP+(c)
r1319	-2	ATP(c) + H2O(c) ⇌ ADP(c) + Pi(c) + ATP-energy(c)
r0860	-2	ADP(c) + ATP(p) ⇌ ADP(p) + ATP(c)
r0069	1	2 Acetyl-CoA(c) ⇌ CoA(c) + Acetoacetyl-CoA(c)
r0954	1	(R)-Mevalonate(c) ⇌ (R)-Mevalonate(p)
r0488	-1	HMG-CoA(c) + 2 NADPH(c) ⇌ (R)-Mevalonate(c) + CoA(c) + 2 NADP+(c)
r0849	-1	H2O(s) ⇌ H2O(c)
r2677	-3	Acetyl-CoA(c) ⇌ CoA(c) + CoA-activated acetyl group(c)
r0612	1	ATP(p) + (R)-5-Phosphomevalonate(p) ⇌ ADP(p) + (R)-5-Diphosphomevalonate(p)

(R)-5-Phosphomevalonate (552)

Definition			Solution												
			exchanges		reactions								transp		
Objective	Constraints	Comment	imports	exports	c	m	r	p	l	n	s	b	s-c	b-c	int
(R)-5-Phosphomevalonate(p)	PIPES MCES	synthesis of peroxysomal (R)-5-Phosphomevalonate	1 P_i(c) 1 ATP-energy(c) 2 NADPH-redox-potential(c) 3 CoA-activated acetyl group(c)	1 (R)-5-Phosphomevalonate(p)	6	-	-	1	-	-	-	-	-	-	2

ID	flux	reaction
r0515	1	ATP(p) + (R)-Mevalonate(p) ⇌ ADP(p) + (R)-5-Phosphomevalonate(p)
r0461	-1	Acetyl-CoA(c) + H2O(c) + Acetoacetyl-CoA(c) ⇌ HMG-CoA(c) + CoA(c)
r1326	-2	NADPH(c) ⇌ NADPH-redox-potential(c) + NADP+(c)
r1319	-1	ATP(c) + H2O(c) ⇌ ADP(c) + Pi(c) + ATP-energy(c)
r0860	-1	ADP(c) + ATP(p) ⇌ ADP(p) + ATP(c)
r0069	1	2 Acetyl-CoA(c) ⇌ CoA(c) + Acetoacetyl-CoA(c)
r0954	1	(R)-Mevalonate(c) ⇌ (R)-Mevalonate(p)
r0488	-1	HMG-CoA(c) + 2 NADPH(c) ⇌ (R)-Mevalonate(c) + CoA(c) + 2 NADP+(c)
r2677	-3	Acetyl-CoA(c) ⇌ CoA(c) + CoA-activated acetyl group(c)

(R)-Methylmalonyl-CoA (553)

Definition			Solution												
			exchanges		reactions								transp		
Objective	Constraints	Comment	imports	exports	c	m	r	p	l	n	s	b	s-c	b-c	int
(R)-Methylmalonyl-CoA(m)	PIPES MCES	synthesis of mitochondrial (R)-Methylmalonyl-CoA	2.17 P_i(c) 1 Glycine(s) 1 Aspartate(s) 2 Glutamine(s) 0.83 Glucose-6P(c) 0.5 Cystine(s) 1 Pantothenate(s) 11.2 ATP-energy(c) 1 Proton-gradient(c) 1 Proton-gradient(m) 2 THF-activated methyl group(c)	5.17 H_2O(s) 2 CO_2(s) 1 Pyruvate(c) 1 Glutamate(s) 1 (R)-Methylmalonyl-CoA(m) 3 NADH-redox-potential(c) 1 NADH-redox-potential(m) 2.5 NADPH-redox-potential(c) 4 Na-gradient(c)	44	3	-	-	-	-	-	-	10	-	5

ID	flux	reaction
r0406	-0.33	Fructose-6P(c) + Erythrose-4P(c) ⇌ Sedoheptulose-7P(c) + GAP(c)
r0277	1	GTP(c) + IMP(c) + Aspartate(c) → GDP(c) + Pi(c) + Adenylosuccinate(c)
r0705	1	ATP(c) + 5-Phosphoribosyl-4-carboxy-5-aminoimidazole(c) + Aspartate(c) ⇌ ADP(c) + Pi(c) + SAICAR(c)
r0293	2	5,10-Methylene-THF(c) + NADP+(c) ⇌ 5,10-Methenyl-THF(c) + NADPH(c)
r0578	1	ATP(c) + Pantetheine(c) ⇌ ADP(c) + Phosphopantetheine(c)
r0877	0.5	Glutamate(c) + Cystine(s) ⇌ Glutamate(s) + Cystine(c)
r0030	2	ATP(c) + AMP(c) ⇌ 2 ADP(c)
r0032	1	ATP(c) + Dephospho-CoA(c) ⇌ ADP(c) + CoA(c)
r1143	-1	Na+(c) + Aspartate(c) ← Na+(s) + Aspartate(s)
r2664	4	Na+(c) ⇌ Na+(s) + Na-gradient(c)
r0058	1	Malate(c) + NADP+(c) ⇌ Pyruvate(c) + CO2(c) + NADPH(c)
r0370	0.33	Sedoheptulose-7P(c) + GAP(c) ⇌ Ribose-5P(c) + D-Xylulose-5P(c)
r0371	2	5,10-Methenyl-THF(c) + H2O(c) ⇌ 10-Formyl-THF(c)
r2685	0.5	Glutamate(c) ⇌ Glutamate(s)
r0659	1	ATP(c) + 5-Phosphoribosylamine(c) + Glycine(c) ⇌ ADP(c) + Pi(c) + GAR(c)
r0099	1	ATP(c) + GDP(c) ⇌ ADP(c) + GTP(c)
r0243	-0.17	DHAP(c) ⇌ GAP(c)
r0677	1	10-Formyl-THF(c) + GAR(c) ⇌ THF(c) + N-Formyl-GAR(c)
r1330	-1	H+(PG)(c) ⇌ H+(PG)(m) + Proton-gradient(m)
r1331	-1	H+(PG)(s) ⇌ H+(PG)(c) + Proton-gradient(c)
r0974	1	Pantothenate(s) + Na+(s) ⇌ Pantothenate(c) + Na+(c)
r1319	-11.2	ATP(c) + H2O(c) ⇌ ADP(c) + Pi(c) + ATP-energy(c)
r0260	1	Adenylosuccinate(c) → Fumarate(c) + AMP(c)
r0247	1	ATP(c) + Ribose-5P(c) → AMP(c) + PRPP(c)
r0699	1	SAICAR(c) ⇌ Fumarate(c) + AICAR(c)
r0849	-5.17	H2O(s) ⇌ H2O(c)
r0863	1	CoA(c) ⇌ CoA(m)
r0581	-1	Pantothenate(c) + Cysteamine(c) ⇌ H2O(c) + Pantetheine(c)
r0867	2	CO2(c) ⇌ CO2(s)
r0868	-1	CO2(c) ⇌ CO2(m)
r0589	1	ATP(c) + Phosphopantetheine(c) → PPi(c) + Dephospho-CoA(c)
r2468	1	Glycine(s) + H+(PG)(s) → Glycine(c) + H+(PG)(c)
r0736	0.17	ATP(c) + Fructose-6P(c) → ADP(c) + Fructose-1,6PP(c)
r0007	2	PPi(c) + H2O(c) → 2 Pi(c)
r0027	-0.5	NADPH(c) + Cystine(c) ⇌ 2 Cysteine(c) + NADP+(c)
r0192	0.83	Glucose-6P(c) ⇌ Fructose-6P(c)
r1114	1	AKG(m) + CoA(m) + NAD+(m) ⇌ NADH(m) + CO2(m) + Succinyl-CoA(m)
r0197	-1	Succinyl-CoA(m) ⇌ (R)-Methylmalonyl-CoA(m)
r1119	-2	Glutamine(s) + Na+(s) → Glutamine(c) + Na+(c)
r0344	-0.67	D-Xylulose-5P(c) ⇌ Ribulose-5P(c)
r0792	2	5-Methyl-THF(c) + NAD+(c) ⇌ 5,10-Methylene-THF(c) + NADH(c)
r0211	1	Cysteine(c) + O2(c) ⇌ 3-Sulfinoalanine(c)
r2676	-2	5-Methyl-THF(c) ⇌ THF(c) + THF-activated methyl group(c)
r0666	1	ATP(c) + 5-Phosphoribosylformylglycinamidine(c) ⇌ ADP(c) + Pi(c) + AIR(c)
r0667	-1	5-Phosphoribosyl-4-carboxy-5-aminoimidazole(c) ⇌ AIR(c) + CO2(c)
r1322	3	NADH(c) ⇌ NAD+(c) + NADH-redox-potential(c)
r1323	1	NADH(m) ⇌ NAD+(m) + NADH-redox-potential(m)
r0250	-0.67	Ribulose-5P(c) ⇌ Ribose-5P(c)
r1326	2.5	NADPH(c) ⇌ NADPH-redox-potential(c) + NADP+(c)
r0687	1	ATP(c) + N-Formyl-GAR(c) + Glutamine(c) + H2O(c) ⇌ ADP(c) + Pi(c) + Glutamate(c) + 5-Phosphoribosylformylglycinamidine(c)
r0255	0.33	Fructose-6P(c) + GAP(c) ⇌ D-Xylulose-5P(c) + Erythrose-4P(c)
r1047	1	Pi(c) + H+(PG)(c) → Pi(m) + H+(PG)(m)
r0104	1	NAD+(c) + Malate(c) ⇌ OAA(c) + NADH(c)
r0538	1	3-Sulfinoalanine(c) ⇌ CO2(c) + Hypotaurine(c)
r0256	0.17	Fructose-1,6PP(c) ⇌ DHAP(c) + GAP(c)
r0539	-1	Hypotaurine(c) ⇌ Cysteamine(c) + O2(c)

Continued on next page

ID	flux	reaction
r0257	-1	Glutamine(c) + PRPP(c) + H2O(c) ⇌ 5-Phosphoribosylamine(c) + PPi(c) + Glutamate(c)
r0258	-2	Fumarate(c) + H2O(c) ⇌ Malate(c)
r2419	1	AKG(c) + Pi(m) ⇌ AKG(m) + Pi(c)
r0273	-1	IMP(c) + H2O(c) ⇌ FAICAR(c)
r0109	-1	OAA(c) + Glutamate(c) ⇌ AKG(c) + Aspartate(c)
r0700	1	10-Formyl-THF(c) + AICAR(c) ⇌ THF(c) + FAICAR(c)

(R)-Mevalonate (554)

Definition			Solution												
			exchanges		reactions								transp		
Objective	Constraints	Comment	imports	exports	c	m	r	p	l	n	s	b	s-c	b-c	int
(R)-Mevalonate(c)	PIPES MCES	synthesis of cytosolic (R)-Mevalonate	1 H_2O(s) 2 NADPH-redox-potential(c) 3 CoA-activated acetyl group(c)	1 (R)-Mevalonate(c)	5	-	-	-	-	-	-	-	1	-	-

ID	flux	reaction
r0461	-1	Acetyl-CoA(c) + H2O(c) + Acetoacetyl-CoA(c) ⇌ HMG-CoA(c) + CoA(c)
r1326	-2	NADPH(c) ⇌ NADPH-redox-potential(c) + NADP+(c)
r0069	1	2 Acetyl-CoA(c) ⇌ CoA(c) + Acetoacetyl-CoA(c)
r0488	-1	HMG-CoA(c) + 2 NADPH(c) ⇌ (R)-Mevalonate(c) + CoA(c) + 2 NADP+(c)
r0849	1	H2O(s) ⇌ H2O(c)
r2677	-3	Acetyl-CoA(c) ⇌ CoA(c) + CoA-activated acetyl group(c)

(S)-3-Hydroxy-2-methylbutyryl-CoA (555)

Definition			Solution												
			exchanges		reactions								transp		
Objective	Constraints	Comment	imports	exports	c	m	r	p	l	n	s	b	s-c	b-c	int
(S)-3-Hydroxy-2-methylbutyryl-CoA(m)	PIPES MCES	synthesis of mitochondrial (S)-3-Hydroxy-2-methylbutyryl-CoA	2.17 P_i(c) 1 Glycine(s) 1.5 Aspartate(s) 0.5 Glutamine(s) 0.83 Glucose-6P(c) 0.5 Asparagine(s) 1 Threonine(s) 0.5 Cystine(s) 1 Pantothenate(s) 12.7 ATP-energy(c) 1 Proton-gradient(m) 2 THF-activated methyl group(c) 1 CoA-activated acetyl group(m)	6.17 H_2O(s) 3 CO_2(s) 2 Pyruvate(c) 0.5 Glutamate(s) 1 (S)-3-Hydroxy-2-methylbutyryl-CoA(m) 2 NADH-redox-potential(c) 3.5 NADPH-redox-potential(c) 3.5 Na-gradient(c)	45	4	-	-	-	-	-	-	10	-	4

ID	flux	reaction
r0406	-0.33	Fructose-6P(c) + Erythrose-4P(c) ⇌ Sedoheptulose-7P(c) + GAP(c)
r0277	1	GTP(c) + IMP(c) + Aspartate(c) → GDP(c) + Pi(c) + Adenylosuccinate(c)
r0127	0.5	H2O(c) + Asparagine(c) → Aspartate(c) + NH3(c)
r0705	1	ATP(c) + 5-Phosphoribosyl-4-carboxy-5-aminoimidazole(c) + Aspartate(c) ⇌ ADP(c) + Pi(c) + SAICAR(c)
r0293	2	5,10-Methylene-THF(c) + NADP+(c) ⇌ 5,10-Methenyl-THF(c) + NADPH(c)
r0578	1	ATP(c) + Pantetheine(c) ⇌ ADP(c) + Phosphopantetheine(c)
r0877	0.5	Glutamate(c) + Cystine(s) ⇌ Glutamate(s) + Cystine(c)
r0030	2	ATP(c) + AMP(c) ⇌ 2 ADP(c)
r0032	1	ATP(c) + Dephospho-CoA(c) ⇌ ADP(c) + CoA(c)
r1142	-1	Threonine(s) + Na+(s) → Threonine(c) + Na+(c)
r1143	-1.5	Na+(c) + Aspartate(c) ← Na+(s) + Aspartate(s)
r2664	3.5	Na+(c) ⇌ Na+(s) + Na-gradient(c)
r0058	2	Malate(c) + NADP+(c) ⇌ Pyruvate(c) + CO2(c) + NADPH(c)
r0370	0.33	Sedoheptulose-7P(c) + GAP(c) ⇌ Ribose-5P(c) + D-Xylulose-5P(c)

Continued on next page

ID	flux	reaction
r0371	2	5,10-Methenyl-THF(c) + H2O(c) ⇌ 10-Formyl-THF(c)
r0077	1.5	ATP(c) + Glutamate(c) + NH3(c) → ADP(c) + Pi(c) + Glutamine(c)
r2532	0.5	Asparagine(s) ⇌ Asparagine(c)
r0222	1	Propanoyl-CoA(m) + Acetyl-CoA(m) ⇌ CoA(m) + 2-Methylacetoacetyl-CoA(m)
r0659	1	ATP(c) + 5-Phosphoribosylamine(c) + Glycine(c) ⇌ ADP(c) + Pi(c) + GAR(c)
r0240	1	Threonine(c) ⇌ 2-Oxobutyrate(c) + NH3(c)
r0099	1	ATP(c) + GDP(c) ⇌ ADP(c) + GTP(c)
r0243	-0.17	DHAP(c) ⇌ GAP(c)
r0677	1	10-Formyl-THF(c) + GAR(c) ⇌ THF(c) + N-Formyl-GAR(c)
r1330	-1	H+(PG)(c) ⇌ H+(PG)(m) + Proton-gradient(m)
r0974	1	Pantothenate(s) + Na+(s) ⇌ Pantothenate(c) + Na+(c)
r1319	-12.7	ATP(c) + H2O(c) ⇌ ADP(c) + Pi(c) + ATP-energy(c)
r0260	1	Adenylosuccinate(c) → Fumarate(c) + AMP(c)
r0247	1	ATP(c) + Ribose-5P(c) → AMP(c) + PRPP(c)
r0699	1	SAICAR(c) ⇌ Fumarate(c) + AICAR(c)
r0849	-6.17	H2O(s) ⇌ H2O(c)
r0863	1	CoA(c) ⇌ CoA(m)
r0581	-1	Pantothenate(c) + Cysteamine(c) ⇌ H2O(c) + Pantetheine(c)
r0867	3	CO2(c) ⇌ CO2(s)
r0868	-1	CO2(c) ⇌ CO2(m)
r0589	1	ATP(c) + Phosphopantetheine(c) → PPi(c) + Dephospho-CoA(c)
r0736	0.17	ATP(c) + Fructose-6P(c) → ADP(c) + Fructose-1,6PP(c)
r0007	2	PPi(c) + H2O(c) → 2 Pi(c)
r0027	-0.5	NADPH(c) + Cystine(c) ⇌ 2 Cysteine(c) + NADP+(c)
r0192	0.83	Glucose-6P(c) ⇌ Fructose-6P(c)
r0344	-0.67	D-Xylulose-5P(c) ⇌ Ribulose-5P(c)
r0792	2	5-Methyl-THF(c) + NAD+(c) ⇌ 5,10-Methylene-THF(c) + NADH(c)
r1154	1	CoA(m) + NAD+(m) + 2-Oxobutyrate(m) ⇌ CO2(m) + Propanoyl-CoA(m) + NADH(m)
r0211	1	Cysteine(c) + O2(c) ⇌ 3-Sulfinoalanine(c)
r2523	1	Glycine(s) ⇌ Glycine(c)
r1155	1	2-Oxobutyrate(c) + H+(PG)(c) → 2-Oxobutyrate(m) + H+(PG)(m)
r2676	-2	5-Methyl-THF(c) ⇌ THF(c) + THF-activated methyl group(c)
r2525	0.5	Glutamine(s) ⇌ Glutamine(c)
r2678	-1	Acetyl-CoA(m) ⇌ CoA(m) + CoA-activated acetyl group(m)
r0664	-1	2-Methylacetoacetyl-CoA(m) + NADH(m) ⇌ NAD+(m) + (S)-3-Hydroxy-2-methylbutyryl-CoA(m)
r0666	1	ATP(c) + 5-Phosphoribosylformylglycinamidine(c) ⇌ ADP(c) + Pi(c) + AIR(c)
r0667	-1	5-Phosphoribosyl-4-carboxy-5-aminoimidazole(c) ⇌ AIR(c) + CO2(c)
r1322	2	NADH(c) ⇌ NAD+(c) + NADH-redox-potential(c)
r0250	-0.67	Ribulose-5P(c) ⇌ Ribose-5P(c)
r1326	3.5	NADPH(c) ⇌ NADPH-redox-potential(c) + NADP+(c)
r0687	1	ATP(c) + N-Formyl-GAR(c) + Glutamine(c) + H2O(c) ⇌ ADP(c) + Pi(c) + Glutamate(c) + 5-Phosphoribosylformylglycinamidine(c)
r0255	0.33	Fructose-6P(c) + GAP(c) ⇌ D-Xylulose-5P(c) + Erythrose-4P(c)
r0538	1	3-Sulfinoalanine(c) ⇌ CO2(c) + Hypotaurine(c)
r0256	0.17	Fructose-1,6PP(c) ⇌ DHAP(c) + GAP(c)
r0539	-1	Hypotaurine(c) ⇌ Cysteamine(c) + O2(c)
r0257	-1	Glutamine(c) + PRPP(c) + H2O(c) ⇌ 5-Phosphoribosylamine(c) + PPi(c) + Glutamate(c)
r0258	-2	Fumarate(c) + H2O(c) ⇌ Malate(c)
r0273	-1	IMP(c) + H2O(c) ⇌ FAICAR(c)
r0700	1	10-Formyl-THF(c) + AICAR(c) ⇌ THF(c) + FAICAR(c)

(S)-3-Hydroxybutyryl-CoA (556)

Definition			Solution												
			exchanges		reactions								transp		
Objective	Constraints	Comment	imports	exports	c	m	r	p	l	n	s	b	s-c	b-c	int
(S)-3-Hydroxybutyryl-CoA(m)	PIPES MCES	synthesis of mitochondrial (S)-3-Hydroxybutyryl-CoA	2.17 P_i(c) 1 Glycine(s) 2 Aspartate(s) 2 Glutamine(s) 0.83 Glucose-6P(c) 0.5 Cystine(s) 1 Pantothenate(s) 11.2 ATP-energy(c) 1 NADH-redox-potential(m) 1 Proton-gradient(c) 2 THF-activated methyl group(c) 2 CoA-activated acetyl group(m)	5.17 H_2O(s) 2 CO_2(s) 2 Pyruvate(c) 2 Glutamate(s) 1 (S)-3-Hydroxybutyryl-CoA(m) 2 NADH-redox-potential(c) 3.5 NADPH-redox-potential(c) 3 Na-gradient(c)	42	4	-	-	-	-	-	-	10	-	1

ID	flux	reaction
r0406	-0.33	Fructose-6P(c) + Erythrose-4P(c) ⇌ Sedoheptulose-7P(c) + GAP(c)
r0277	1	GTP(c) + IMP(c) + Aspartate(c) → GDP(c) + Pi(c) + Adenylosuccinate(c)
r0293	2	5,10-Methylene-THF(c) + NADP+(c) ⇌ 5,10-Methenyl-THF(c) + NADPH(c)
r0705	1	ATP(c) + 5-Phosphoribosyl-4-carboxy-5-aminoimidazole(c) + Aspartate(c) ⇌ ADP(c) + Pi(c) + SAICAR(c)
r0578	1	ATP(c) + Pantetheine(c) ⇌ ADP(c) + Phosphopantetheine(c)
r0877	0.5	Glutamate(c) + Cystine(s) ⇌ Glutamate(s) + Cystine(c)
r0460	-1	NADH(m) + Acetoacetyl-CoA(m) ⇌ (S)-3-Hydroxybutyryl-CoA(m) + NAD+(m)
r0030	2	ATP(c) + AMP(c) ⇌ 2 ADP(c)
r0032	1	ATP(c) + Dephospho-CoA(c) ⇌ ADP(c) + CoA(c)
r1143	-2	Na+(c) + Aspartate(c) ← Na+(s) + Aspartate(s)
r0070	1	2 Acetyl-CoA(m) ⇌ CoA(m) + Acetoacetyl-CoA(m)
r2664	3	Na+(c) ⇌ Na+(s) + Na-gradient(c)
r0058	2	Malate(c) + NADP+(c) ⇌ Pyruvate(c) + CO2(c) + NADPH(c)
r0370	0.33	Sedoheptulose-7P(c) + GAP(c) ⇌ Ribose-5P(c) + D-Xylulose-5P(c)
r0371	2	5,10-Methenyl-THF(c) + H2O(c) ⇌ 10-Formyl-THF(c)
r2685	1.5	Glutamate(c) ⇌ Glutamate(s)
r0659	1	ATP(c) + 5-Phosphoribosylamine(c) + Glycine(c) ⇌ ADP(c) + Pi(c) + GAR(c)
r0099	1	ATP(c) + GDP(c) ⇌ ADP(c) + GTP(c)
r0243	-0.17	DHAP(c) ⇌ GAP(c)
r0677	1	10-Formyl-THF(c) + GAR(c) ⇌ THF(c) + N-Formyl-GAR(c)
r1331	-1	H+(PG)(s) ⇌ H+(PG)(c) + Proton-gradient(c)
r0974	1	Pantothenate(s) + Na+(s) ⇌ Pantothenate(c) + Na+(c)
r1319	-11.2	ATP(c) + H2O(c) ⇌ ADP(c) + Pi(c) + ATP-energy(c)
r0260	1	Adenylosuccinate(c) → Fumarate(c) + AMP(c)
r0247	1	ATP(c) + Ribose-5P(c) → AMP(c) + PRPP(c)
r0699	1	SAICAR(c) ⇌ Fumarate(c) + AICAR(c)
r0849	-5.17	H2O(s) ⇌ H2O(c)
r0863	1	CoA(c) ⇌ CoA(m)
r0581	-1	Pantothenate(c) + Cysteamine(c) ⇌ H2O(c) + Pantetheine(c)
r0867	2	CO2(c) ⇌ CO2(s)
r0589	1	ATP(c) + Phosphopantetheine(c) → PPi(c) + Dephospho-CoA(c)
r2468	1	Glycine(s) + H+(PG)(s) → Glycine(c) + H+(PG)(c)
r0736	0.17	ATP(c) + Fructose-6P(c) → ADP(c) + Fructose-1,6PP(c)
r0007	2	PPi(c) + H2O(c) → 2 Pi(c)
r0027	-0.5	NADPH(c) + Cystine(c) ⇌ 2 Cysteine(c) + NADP+(c)
r0192	0.83	Glucose-6P(c) ⇌ Fructose-6P(c)
r0344	-0.67	D-Xylulose-5P(c) ⇌ Ribulose-5P(c)
r0792	2	5-Methyl-THF(c) + NAD+(c) ⇌ 5,10-Methylene-THF(c) + NADH(c)
r0211	1	Cysteine(c) + O2(c) ⇌ 3-Sulfinoalanine(c)
r2676	-2	5-Methyl-THF(c) ⇌ THF(c) + THF-activated methyl group(c)
r2525	2	Glutamine(s) ⇌ Glutamine(c)
r2678	-2	Acetyl-CoA(m) ⇌ CoA(m) + CoA-activated acetyl group(m)
r0666	1	ATP(c) + 5-Phosphoribosylformylglycinamidine(c) ⇌ ADP(c) + Pi(c) + AIR(c)
r0667	-1	5-Phosphoribosyl-4-carboxy-5-aminoimidazole(c) ⇌ AIR(c) + CO2(c)

Continued on next page

ID	flux	reaction
r1322	2	NADH(c) ⇌ NAD+(c) + NADH-redox-potential(c)
r1323	-1	NADH(m) ⇌ NAD+(m) + NADH-redox-potential(m)
r0250	-0.67	Ribulose-5P(c) ⇌ Ribose-5P(c)
r1326	3.5	NADPH(c) ⇌ NADPH-redox-potential(c) + NADP+(c)
r0687	1	ATP(c) + N-Formyl-GAR(c) + Glutamine(c) + H2O(c) ⇌ ADP(c) + Pi(c) + Glutamate(c) + 5-Phosphoribosylformylglycinamidine(c)
r0255	0.33	Fructose-6P(c) + GAP(c) ⇌ D-Xylulose-5P(c) + Erythrose-4P(c)
r0538	1	3-Sulfinoalanine(c) ⇌ CO2(c) + Hypotaurine(c)
r0256	0.17	Fructose-1,6PP(c) ⇌ DHAP(c) + GAP(c)
r0539	-1	Hypotaurine(c) ⇌ Cysteamine(c) + O2(c)
r0257	-1	Glutamine(c) + PRPP(c) + H2O(c) ⇌ 5-Phosphoribosylamine(c) + PPi(c) + Glutamate(c)
r0258	-2	Fumarate(c) + H2O(c) ⇌ Malate(c)
r0273	-1	IMP(c) + H2O(c) ⇌ FAICAR(c)
r0700	1	10-Formyl-THF(c) + AICAR(c) ⇌ THF(c) + FAICAR(c)

(S)-3-Hydroxydodecanoyl-CoA (557)

Definition			Solution												
			exchanges		reactions								transp		
Objective	Constraints	Comment	imports	exports	c	m	r	p	l	n	s	b	s-c	b-c	int
(S)-3-Hydroxydodecanoyl-CoA(m)	PIPES MCES	synthesis of mitochondrial (S)-3-Hydroxydodecanoyl-CoA	2.17 P_i(c) 1 Glycine(s) 2 Aspartate(s) 2 Glutamine(s) 0.83 Glucose-6P(c) 0.5 Cystine(s) 1 Pantothenate(s) 1 Palmitolate(s) 13.2 ATP-energy(c) 1 Proton-gradient(c) 2 THF-activated methyl group(c)	2.17 H_2O(s) 2 CO_2(s) 2 Pyruvate(c) 2 Glutamate(s) 1 (S)-3-Hydroxydodecanoyl-CoA(m) 2 NADH-redox-potential(c) 3 NADH-redox-potential(m) 3.5 NADPH-redox-potential(c) 3 FADH-redox-potential(c) 3 Na-gradient(c) 3 CoA-activated acetyl group(m)	44	16	-	-	-	-	-	-	11	-	3

ID	flux	reaction
r0406	-0.33	Fructose-6P(c) + Erythrose-4P(c) ⇌ Sedoheptulose-7P(c) + GAP(c)
r0277	1	GTP(c) + IMP(c) + Aspartate(c) → GDP(c) + Pi(c) + Adenylosuccinate(c)
r0293	2	5,10-Methylene-THF(c) + NADP+(c) ⇌ 5,10-Methenyl-THF(c) + NADPH(c)
r0705	1	ATP(c) + 5-Phosphoribosyl-4-carboxy-5-aminoimidazole(c) + Aspartate(c) ⇌ ADP(c) + Pi(c) + SAICAR(c)
r0578	1	ATP(c) + Pantetheine(c) ⇌ ADP(c) + Phosphopantetheine(c)
r0877	0.5	Glutamate(c) + Cystine(s) ⇌ Glutamate(s) + Cystine(c)
r0030	3	ATP(c) + AMP(c) ⇌ 2 ADP(c)
r0032	1	ATP(c) + Dephospho-CoA(c) ⇌ ADP(c) + CoA(c)
r1290	3	FAD(c) + FADH2(m) ⇌ FAD(m) + FADH2(c)
r1143	-2	Na+(c) + Aspartate(c) ← Na+(s) + Aspartate(s)
r2664	3	Na+(c) ⇌ Na+(s) + Na-gradient(c)
r0058	2	Malate(c) + NADP+(c) ⇌ Pyruvate(c) + CO2(c) + NADPH(c)
r0370	0.33	Sedoheptulose-7P(c) + GAP(c) ⇌ Ribose-5P(c) + D-Xylulose-5P(c)
r0371	2	5,10-Methenyl-THF(c) + H2O(c) ⇌ 10-Formyl-THF(c)
r2685	1.5	Glutamate(c) ⇌ Glutamate(s)
r0657	1	(3Z)-Dodecenoyl-CoA(m) ⇌ (2E)-Dodecenoyl-CoA(m)
r0659	1	ATP(c) + 5-Phosphoribosylamine(c) + Glycine(c) ⇌ ADP(c) + Pi(c) + GAR(c)
r1480	1	CoA(m) + 3-oxopalmitoleoyl-CoA(m) ⇌ Acetyl-CoA(m) + cis-myrist-7-enoyl-CoA(m)
r0099	1	ATP(c) + GDP(c) ⇌ ADP(c) + GTP(c)
r1468	1	palmitoleoyl-CoA(c) ⇌ palmitoleoyl-CoA(m)
r0243	-0.17	DHAP(c) ⇌ GAP(c)
r0677	1	10-Formyl-THF(c) + GAR(c) ⇌ THF(c) + N-Formyl-GAR(c)
r1482	-1	FADH2(m) + trans,cis-dodeca-2,5-dienoyl-CoA(m) ⇌ FAD(m) + cis-laur-5-enoyl-CoA(m)
r1483	1	H2O(m) + trans,cis-myristo-2,7-dienoyl-CoA(m) ⇌ cis-(3S)-hydroxytetradec-7-enoyl-CoA(m)
r1331	-1	H+(PG)(s) ⇌ H+(PG)(c) + Proton-gradient(c)

Continued on next page

ID	flux	reaction
r0974	1	Pantothenate(s) + Na+(s) ⇌ Pantothenate(c) + Na+(c)
r1484	1	CoA(m) + 3-oxomyrist-7-enoyl-CoA(m) ⇌ Acetyl-CoA(m) + cis-laur-5-enoyl-CoA(m)
r1319	-13.2	ATP(c) + H2O(c) ⇌ ADP(c) + Pi(c) + ATP-energy(c)
r1485	1	H2O(m) + trans,cis-dodeca-2,5-dienoyl-CoA(m) ⇌ 3(S)-3-hydroxydodecen-(5Z)-oyl-CoA(m)
r0260	1	Adenylosuccinate(c) → Fumarate(c) + AMP(c)
r0247	1	ATP(c) + Ribose-5P(c) → AMP(c) + PRPP(c)
r1486	1	NAD+(m) + 3(S)-3-hydroxydodecen-(5Z)-oyl-CoA(m) ⇌ 3-oxolaur-cis-5-enoyl-CoA(m) + NADH(m)
r1488	1	ATP(c) + CoA(c) + Palmitolate(c) ⇌ AMP(c) + palmitoleoyl-CoA(c) + PPi(c)
r0699	1	SAICAR(c) ⇌ Fumarate(c) + AICAR(c)
r2442	1	Palmitolate(s) → Palmitolate(c)
r0849	-2.17	H2O(s) ⇌ H2O(c)
r0581	-1	Pantothenate(c) + Cysteamine(c) ⇌ H2O(c) + Pantetheine(c)
r0867	2	CO2(c) ⇌ CO2(s)
r0589	1	ATP(c) + Phosphopantetheine(c) → PPi(c) + Dephospho-CoA(c)
r2468	1	Glycine(s) + H+(PG)(s) → Glycine(c) + H+(PG)(c)
r0736	0.17	ATP(c) + Fructose-6P(c) → ADP(c) + Fructose-1,6PP(c)
r0007	3	PPi(c) + H2O(c) → 2 Pi(c)
r0027	-0.5	NADPH(c) + Cystine(c) ⇌ 2 Cysteine(c) + NADP+(c)
r0192	0.83	Glucose-6P(c) ⇌ Fructose-6P(c)
r0344	-0.67	D-Xylulose-5P(c) ⇌ Ribulose-5P(c)
r0792	2	5-Methyl-THF(c) + NAD+(c) ⇌ 5,10-Methylene-THF(c) + NADH(c)
r0211	1	Cysteine(c) + O2(c) ⇌ 3-Sulfinoalanine(c)
r2676	-2	5-Methyl-THF(c) ⇌ THF(c) + THF-activated methyl group(c)
r0660	-1	(S)-3-Hydroxydodecanoyl-CoA(m) ⇌ (2E)-Dodecenoyl-CoA(m) + H2O(m)
r2525	2	Glutamine(s) ⇌ Glutamine(c)
r2678	3	Acetyl-CoA(m) ⇌ CoA(m) + CoA-activated acetyl group(m)
r0666	1	ATP(c) + 5-Phosphoribosylformylglycinamidine(c) ⇌ ADP(c) + Pi(c) + AIR(c)
r1471	1	FAD(m) + cis-myrist-7-enoyl-CoA(m) ⇌ FADH2(m) + trans,cis-myristo-2,7-dienoyl-CoA(m)
r0667	-1	5-Phosphoribosyl-4-carboxy-5-aminoimidazole(c) ⇌ AIR(c) + CO2(c)
r1473	1	FAD(m) + palmitoleoyl-CoA(m) ⇌ FADH2(m) + trans,cis-hexadeca-2,9-dienoyl-CoA(m)
r1322	2	NADH(c) ⇌ NAD+(c) + NADH-redox-potential(c)
r1475	1	H2O(m) + trans,cis-hexadeca-2,9-dienoyl-CoA(m) ⇌ (S)-3-hydroxypalmitoleoyl-CoA(m)
r1323	3	NADH(m) ⇌ NAD+(m) + NADH-redox-potential(m)
r0250	-0.67	Ribulose-5P(c) ⇌ Ribose-5P(c)
r1476	1	NAD+(m) + cis-(3S)-hydroxytetradec-7-enoyl-CoA(m) ⇌ 3-oxomyrist-7-enoyl-CoA(m) + NADH(m)
r1478	1	NAD+(m) + (S)-3-hydroxypalmitoleoyl-CoA(m) ⇌ NADH(m) + 3-oxopalmitoleoyl-CoA(m)
r1326	3.5	NADPH(c) ⇌ NADPH-redox-potential(c) + NADP+(c)
r0687	1	ATP(c) + N-Formyl-GAR(c) + Glutamine(c) + H2O(c) ⇌ ADP(c) + Pi(c) + Glutamate(c) + 5-Phosphoribosylformylglycinamidine(c)
r1479	1	CoA(m) + 3-oxolaur-cis-5-enoyl-CoA(m) ⇌ Acetyl-CoA(m) + (3Z)-Dodecenoyl-CoA(m)
r0255	0.33	Fructose-6P(c) + GAP(c) ⇌ D-Xylulose-5P(c) + Erythrose-4P(c)
r1329	3	FADH2(c) ⇌ FAD(c) + FADH-redox-potential(c)
r0538	1	3-Sulfinoalanine(c) ⇌ CO2(c) + Hypotaurine(c)
r0256	0.17	Fructose-1,6PP(c) ⇌ DHAP(c) + GAP(c)
r0539	-1	Hypotaurine(c) ⇌ Cysteamine(c) + O2(c)
r0257	-1	Glutamine(c) + PRPP(c) + H2O(c) ⇌ 5-Phosphoribosylamine(c) + PPi(c) + Glutamate(c)
r0258	-2	Fumarate(c) + H2O(c) ⇌ Malate(c)
r0273	-1	IMP(c) + H2O(c) ⇌ FAICAR(c)
r0851	-4	H2O(m) ⇌ H2O(c)
r0700	1	10-Formyl-THF(c) + AICAR(c) ⇌ THF(c) + FAICAR(c)

(S)-3-Hydroxyhexadecanoyl-CoA (558)

Definition			Solution												
			exchanges		reactions								transp		
Objective	Constraints	Comment	imports	exports	c	m	r	p	l	n	s	b	s-c	b-c	int
(S)-3-Hydroxyhexadecanoyl-CoA(m)	PIPES MCES	synthesis of mitochondrial (S)-3-Hydroxyhexadecanoyl-CoA	2.17 P_i(c) 1 Glycine(s) 2 Aspartate(s) 2 Glutamine(s) 0.83 Glucose-6P(c) 0.5 Palmitate(s) 0.5 Cystine(s) 1 Pantothenate(s) 0.5 Palmitolate(s) 13.2 ATP-energy(c) 1 Proton-gradient(c) 2 THF-activated methyl group(c)	5.17 H_2O(s) 2 CO_2(s) 2 Pyruvate(c) 2 Glutamate(s) 1 (S)-3-Hydroxyhexadecanoyl-CoA(m) 0.5 NADH-redox-potential(c) 5.5 NADPH-redox-potential(c) 6 Proton-gradient(m) 3 Na-gradient(c)	47	3	-	-	-	-	-	-	12	-	6

ID	flux	reaction
r2435	1	L-Carnitine(m) + L-Palmitoylcarnitine(c) → L-Carnitine(c) + L-Palmitoylcarnitine(m)
r0406	-0.33	Fructose-6P(c) + Erythrose-4P(c) ⇌ Sedoheptulose-7P(c) + GAP(c)
r0277	1	GTP(c) + IMP(c) + Aspartate(c) → GDP(c) + Pi(c) + Adenylosuccinate(c)
r0856	-0.5	O2(m) ⇌ O2(c)
r0705	1	ATP(c) + 5-Phosphoribosyl-4-carboxy-5-aminoimidazole(c) + Aspartate(c) ⇌ ADP(c) + Pi(c) + SAICAR(c)
r0293	2	5,10-Methylene-THF(c) + NADP+(c) ⇌ 5,10-Methenyl-THF(c) + NADPH(c)
r0578	1	ATP(c) + Pantetheine(c) ⇌ ADP(c) + Phosphopantetheine(c)
r0296	2	5-Methyl-THF(c) + NADP+(c) ⇌ 5,10-Methylene-THF(c) + NADPH(c)
r0877	0.5	Glutamate(c) + Cystine(s) ⇌ Glutamate(s) + Cystine(c)
r0310	1	Palmitoyl-CoA(m) + Ubiquinone(m) → (2E)-Hexadecenoyl-CoA(m) + Ubiquinol(m)
r0017	0.5	O2(m) + 4 H+(PG)(m) + 4 Ferrocytochrome C(m) → 4 Ferricytochrome C(m) + 2 H2O(m) + 4 H+(PG)(c)
r0312	0.5	Palmitate(c) + ATP(c) + CoA(c) → Palmitoyl-CoA(c) + AMP(c) + PPi(c)
r0030	3	ATP(c) + AMP(c) ⇌ 2 ADP(c)
r0032	1	ATP(c) + Dephospho-CoA(c) ⇌ ADP(c) + CoA(c)
r1143	-2	Na+(c) + Aspartate(c) ← Na+(s) + Aspartate(s)
r2664	3	Na+(c) ⇌ Na+(s) + Na-gradient(c)
r0058	2	Malate(c) + NADP+(c) ⇌ Pyruvate(c) + CO2(c) + NADPH(c)
r0370	0.33	Sedoheptulose-7P(c) + GAP(c) ⇌ Ribose-5P(c) + D-Xylulose-5P(c)
r0935	0.5	Palmitate(s) → Palmitate(c)
r0371	2	5,10-Methenyl-THF(c) + H2O(c) ⇌ 10-Formyl-THF(c)
r2685	1.5	Glutamate(c) ⇌ Glutamate(s)
r0659	1	ATP(c) + 5-Phosphoribosylamine(c) + Glycine(c) ⇌ ADP(c) + Pi(c) + GAR(c)
r0507	1	Ubiquinol(m) + 2 Ferricytochrome C(m) + 4 H+(PG)(m) → Ubiquinone(m) + 4 H+(PG)(c) + 2 Ferrocytochrome C(m)
r0099	1	ATP(c) + GDP(c) ⇌ ADP(c) + GTP(c)
r0677	1	10-Formyl-THF(c) + GAR(c) ⇌ THF(c) + N-Formyl-GAR(c)
r0243	-0.17	DHAP(c) ⇌ GAP(c)
r1330	6	H+(PG)(c) ⇌ H+(PG)(m) + Proton-gradient(m)
r1331	-1	H+(PG)(s) ⇌ H+(PG)(c) + Proton-gradient(c)
r0974	1	Pantothenate(s) + Na+(s) ⇌ Pantothenate(c) + Na+(c)
r1319	-13.2	ATP(c) + H2O(c) ⇌ ADP(c) + Pi(c) + ATP-energy(c)
r0260	1	Adenylosuccinate(c) → Fumarate(c) + AMP(c)
r0247	1	ATP(c) + Ribose-5P(c) → AMP(c) + PRPP(c)
r1488	0.5	ATP(c) + CoA(c) + Palmitolate(c) ⇌ AMP(c) + palmitoleoyl-CoA(c) + PPi(c)
r0699	1	SAICAR(c) ⇌ Fumarate(c) + AICAR(c)
r2442	0.5	Palmitolate(s) → Palmitolate(c)
r0849	-5.17	H2O(s) ⇌ H2O(c)
r0863	1	CoA(c) ⇌ CoA(m)
r0581	-1	Pantothenate(c) + Cysteamine(c) ⇌ H2O(c) + Pantetheine(c)
r0867	2	CO2(c) ⇌ CO2(s)
r0716	-1	(2E)-Hexadecenoyl-CoA(m) + H2O(m) → (S)-3-Hydroxyhexadecanoyl-CoA(m)
r0435	1	Palmitoyl-CoA(c) + L-Carnitine(c) ⇌ CoA(c) + L-Palmitoylcarnitine(c)
r0436	-1	Palmitoyl-CoA(m) + L-Carnitine(m) ← CoA(m) + L-Palmitoylcarnitine(m)
r0589	1	ATP(c) + Phosphopantetheine(c) → PPi(c) + Dephospho-CoA(c)
r2468	1	Glycine(s) + H+(PG)(s) → Glycine(c) + H+(PG)(c)
r0736	0.17	ATP(c) + Fructose-6P(c) → ADP(c) + Fructose-1,6PP(c)

Continued on next page

(S)-3-Hydroxyhexadecanoyl-CoA (558)– continued

ID	flux	reaction
r0007	3	PPi(c) + H2O(c) → 2 Pi(c)
r0023	-0.5	NAD+(c) + 2 Ferrocytochrome b5(c) ⇌ NADH(c) + 2 Ferricytochrome b5(c)
r0027	-0.5	NADPH(c) + Cystine(c) ⇌ 2 Cysteine(c) + NADP+(c)
r0192	0.83	Glucose-6P(c) ⇌ Fructose-6P(c)
r0344	-0.67	D-Xylulose-5P(c) ⇌ Ribulose-5P(c)
r0211	1	Cysteine(c) + O2(c) ⇌ 3-Sulfinoalanine(c)
r2676	-2	5-Methyl-THF(c) ⇌ THF(c) + THF-activated methyl group(c)
r2525	2	Glutamine(s) ⇌ Glutamine(c)
r0510	-0.5	palmitoleoyl-CoA(c) + 2 Ferricytochrome b5(c) + 2 H2O(c) ⇌ Palmitoyl-CoA(c) + O2(c) + 2 Ferrocytochrome b5(c)
r0666	1	ATP(c) + 5-Phosphoribosylformylglycinamidine(c) ⇌ ADP(c) + Pi(c) + AIR(c)
r0667	-1	5-Phosphoribosyl-4-carboxy-5-aminoimidazole(c) ⇌ AIR(c) + CO2(c)
r1322	0.5	NADH(c) ⇌ NAD+(c) + NADH-redox-potential(c)
r0250	-0.67	Ribulose-5P(c) ⇌ Ribose-5P(c)
r1326	5.5	NADPH(c) ⇌ NADPH-redox-potential(c) + NADP+(c)
r0687	1	ATP(c) + N-Formyl-GAR(c) + Glutamine(c) + H2O(c) ⇌ ADP(c) + Pi(c) + Glutamate(c) + 5-Phosphoribosylformylglycinamidine(c)
r0255	0.33	Fructose-6P(c) + GAP(c) ⇌ D-Xylulose-5P(c) + Erythrose-4P(c)
r0538	1	3-Sulfinoalanine(c) ⇌ CO2(c) + Hypotaurine(c)
r0256	0.17	Fructose-1,6PP(c) ⇌ DHAP(c) + GAP(c)
r0539	-1	Hypotaurine(c) ⇌ Cysteamine(c) + O2(c)
r0257	-1	Glutamine(c) + PRPP(c) + H2O(c) ⇌ 5-Phosphoribosylamine(c) + PPi(c) + Glutamate(c)
r0258	-2	Fumarate(c) + H2O(c) ⇌ Malate(c)
r0273	-1	IMP(c) + H2O(c) ⇌ FAICAR(c)
r0700	1	10-Formyl-THF(c) + AICAR(c) ⇌ THF(c) + FAICAR(c)

(S)-3-Hydroxytetradecanoyl-CoA (559)

Definition			Solution												
			exchanges		reactions								transp		
Objective	Constraints	Comment	imports	exports	c	m	r	p	l	n	s	b	s-c	b-c	int
(S)-3-Hydroxytetradecanoyl-CoA(m)	PIPES MCES	synthesis of mitochondrial (S)-3-Hydroxytetradecanoyl-CoA	2.17 P_i(c) 1 Glycine(s) 0.5 Aspartate(s) 0.5 Glutamine(s) 0.83 Glucose-6P(c) 1.5 Asparagine(s) 0.5 Cystine(s) 1 Pantothenate(s) 1 Palmitolate(s) 14.7 ATP-energy(c) 2 THF-activated methyl group(c)	4.17 H_2O(s) 2 CO_2(s) 2 Pyruvate(c) 0.5 Glutamate(s) 1 (S)-3-Hydroxytetradecanoyl-CoA(m) 1 NADH-redox-potential(c) 1 NADH-redox-potential(m) 5.5 NADPH-redox-potential(c) 12 Proton-gradient(m) 2.5 Na-gradient(c) 1 CoA-activated acetyl group(m)	48	9	-	-	-	-	-	-	10	-	6

ID	flux	reaction
r2435	1	L-Carnitine(m) + L-Palmitoylcarnitine(c) → L-Carnitine(c) + L-Palmitoylcarnitine(m)
r0406	-0.33	Fructose-6P(c) + Erythrose-4P(c) ⇌ Sedoheptulose-7P(c) + GAP(c)
r0277	1	GTP(c) + IMP(c) + Aspartate(c) → GDP(c) + Pi(c) + Adenylosuccinate(c)
r0856	-1	O2(m) ⇌ O2(c)
r0127	1.5	H2O(c) + Asparagine(c) → Aspartate(c) + NH3(c)
r0705	1	ATP(c) + 5-Phosphoribosyl-4-carboxy-5-aminoimidazole(c) + Aspartate(c) ⇌ ADP(c) + Pi(c) + SAICAR(c)
r0293	2	5,10-Methylene-THF(c) + NADP+(c) ⇌ 5,10-Methenyl-THF(c) + NADPH(c)
r0720	-1	(S)-3-Hydroxytetradecanoyl-CoA(m) ← (2E)-Tetradecenoyl-CoA(m) + H2O(m)
r0578	1	ATP(c) + Pantetheine(c) ⇌ ADP(c) + Phosphopantetheine(c)
r0296	2	5-Methyl-THF(c) + NADP+(c) ⇌ 5,10-Methylene-THF(c) + NADPH(c)
r0877	0.5	Glutamate(c) + Cystine(s) ⇌ Glutamate(s) + Cystine(c)
r0310	1	Palmitoyl-CoA(m) + Ubiquinone(m) → (2E)-Hexadecenoyl-CoA(m) + Ubiquinol(m)
r0017	1	O2(m) + 4 H+(PG)(m) + 4 Ferrocytochrome C(m) → 4 Ferricytochrome C(m) + 2 H2O(m) + 4 H+(PG)(c)
r0030	3	ATP(c) + AMP(c) ⇌ 2 ADP(c)
r0032	1	ATP(c) + Dephospho-CoA(c) ⇌ ADP(c) + CoA(c)

Continued on next page

ID	flux	reaction
r1143	-0.5	Na+(c) + Aspartate(c) ← Na+(s) + Aspartate(s)
r2664	2.5	Na+(c) ⇌ Na+(s) + Na-gradient(c)
r0058	2	Malate(c) + NADP+(c) ⇌ Pyruvate(c) + CO2(c) + NADPH(c)
r0370	0.33	Sedoheptulose-7P(c) + GAP(c) ⇌ Ribose-5P(c) + D-Xylulose-5P(c)
r0653	-1	CoA(m) + 3-Oxopalmitoyl-CoA(m) → Myristoyl-CoA(m) + Acetyl-CoA(m)
r0371	2	5,10-Methenyl-THF(c) + H2O(c) ⇌ 10-Formyl-THF(c)
r0077	1.5	ATP(c) + Glutamate(c) + NH3(c) → ADP(c) + Pi(c) + Glutamine(c)
r2532	1.5	Asparagine(s) ⇌ Asparagine(c)
r1449	1	Ubiquinone(m) + Myristoyl-CoA(m) → (2E)-Tetradecenoyl-CoA(m) + Ubiquinol(m)
r0659	1	ATP(c) + 5-Phosphoribosylamine(c) + Glycine(c) ⇌ ADP(c) + Pi(c) + GAR(c)
r0507	2	Ubiquinol(m) + 2 Ferricytochrome C(m) + 4 H+(PG)(m) → Ubiquinone(m) + 4 H+(PG)(c) + 2 Ferrocytochrome C(m)
r0099	1	ATP(c) + GDP(c) ⇌ ADP(c) + GTP(c)
r0677	1	10-Formyl-THF(c) + GAR(c) ⇌ THF(c) + N-Formyl-GAR(c)
r0243	-0.17	DHAP(c) ⇌ GAP(c)
r1330	12	H+(PG)(c) ⇌ H+(PG)(m) + Proton-gradient(m)
r0974	1	Pantothenate(s) + Na+(s) ⇌ Pantothenate(c) + Na+(c)
r1319	-14.7	ATP(c) + H2O(c) ⇌ ADP(c) + Pi(c) + ATP-energy(c)
r0260	1	Adenylosuccinate(c) → Fumarate(c) + AMP(c)
r0247	1	ATP(c) + Ribose-5P(c) → AMP(c) + PRPP(c)
r1488	1	ATP(c) + CoA(c) + Palmitolate(c) ⇌ AMP(c) + palmitoleoyl-CoA(c) + PPi(c)
r0699	1	SAICAR(c) ⇌ Fumarate(c) + AICAR(c)
r2442	1	Palmitolate(s) → Palmitolate(c)
r0849	-4.17	H2O(s) ⇌ H2O(c)
r0863	1	CoA(c) ⇌ CoA(m)
r0581	-1	Pantothenate(c) + Cysteamine(c) ⇌ H2O(c) + Pantetheine(c)
r0714	1	NAD+(m) + (S)-3-Hydroxyhexadecanoyl-CoA(m) → 3-Oxopalmitoyl-CoA(m) + NADH(m)
r0867	2	CO2(c) ⇌ CO2(s)
r0716	-1	(S)-3-Hydroxyhexadecanoyl-CoA(m) ← (2E)-Hexadecenoyl-CoA(m) + H2O(m)
r0435	1	Palmitoyl-CoA(c) + L-Carnitine(c) ⇌ CoA(c) + L-Palmitoylcarnitine(c)
r0436	-1	Palmitoyl-CoA(m) + L-Carnitine(m) ← CoA(m) + L-Palmitoylcarnitine(m)
r0589	1	ATP(c) + Phosphopantetheine(c) → PPi(c) + Dephospho-CoA(c)
r0736	0.17	ATP(c) + Fructose-6P(c) → ADP(c) + Fructose-1,6PP(c)
r0007	3	PPi(c) + H2O(c) → 2 Pi(c)
r0023	-1	NAD+(c) + 2 Ferrocytochrome b5(c) ⇌ NADH(c) + 2 Ferricytochrome b5(c)
r0027	-0.5	NADPH(c) + Cystine(c) ⇌ 2 Cysteine(c) + NADP+(c)
r0192	0.83	Glucose-6P(c) ⇌ Fructose-6P(c)
r0344	-0.67	D-Xylulose-5P(c) ⇌ Ribulose-5P(c)
r1138	1	Glycine(s) + Na+(s) → Glycine(c) + Na+(c)
r0211	1	Cysteine(c) + O2(c) ⇌ 3-Sulfinoalanine(c)
r2676	-2	5-Methyl-THF(c) ⇌ THF(c) + THF-activated methyl group(c)
r2525	0.5	Glutamine(s) ⇌ Glutamine(c)
r2678	1	Acetyl-CoA(m) ⇌ CoA(m) + CoA-activated acetyl group(m)
r0510	-1	palmitoleoyl-CoA(c) + 2 Ferricytochrome b5(c) + 2 H2O(c) ⇌ Palmitoyl-CoA(c) + O2(c) + 2 Ferrocytochrome b5(c)
r0666	1	ATP(c) + 5-Phosphoribosylformylglycinamidine(c) ⇌ ADP(c) + Pi(c) + AIR(c)
r0667	-1	5-Phosphoribosyl-4-carboxy-5-aminoimidazole(c) ⇌ AIR(c) + CO2(c)
r1322	1	NADH(c) ⇌ NAD+(c) + NADH-redox-potential(c)
r1323	1	NADH(m) ⇌ NAD+(m) + NADH-redox-potential(m)
r0250	-0.67	Ribulose-5P(c) ⇌ Ribose-5P(c)
r1326	5.5	NADPH(c) ⇌ NADPH-redox-potential(c) + NADP+(c)
r0687	1	ATP(c) + N-Formyl-GAR(c) + Glutamine(c) + H2O(c) ⇌ ADP(c) + Pi(c) + Glutamate(c) + 5-Phosphoribosylformylglycinamidine(c)
r0255	0.33	Fructose-6P(c) + GAP(c) ⇌ D-Xylulose-5P(c) + Erythrose-4P(c)
r0538	1	3-Sulfinoalanine(c) ⇌ CO2(c) + Hypotaurine(c)
r0256	0.17	Fructose-1,6PP(c) ⇌ DHAP(c) + GAP(c)
r0539	-1	Hypotaurine(c) ⇌ Cysteamine(c) + O2(c)
r0257	-1	Glutamine(c) + PRPP(c) + H2O(c) ⇌ 5-Phosphoribosylamine(c) + PPi(c) + Glutamate(c)

Continued on next page

ID	flux	reaction
r0258	-2	Fumarate(c) + H2O(c) ⇌ Malate(c)
r0273	-1	IMP(c) + H2O(c) ⇌ FAICAR(c)
r0700	1	10-Formyl-THF(c) + AICAR(c) ⇌ THF(c) + FAICAR(c)

(S)-3-hydroxyoleyleoyl-CoA (560)

Definition			Solution													
			exchanges		reactions								transp		
Objective	Constraints	Comment	imports	exports	c	m	r	p	l	n	s	b	s-c	b-c	int
(S)-3-hydroxyoleyleoyl-CoA(m)	PIPES MCES	synthesis of mitochondrial (S)-3-hydroxyoleyleoyl-CoA	2.17 P_i(c) 1 Glycine(s) 2 Aspartate(s) 2 Glutamine(s) 0.83 Glucose-6P(c) 0.5 Cystine(s) 1 Pantothenate(s) 1 Palmitolate(s) 13.2 ATP-energy(c) 1 NADH-redox-potential(m) 1 Proton-gradient(c) 2 THF-activated methyl group(c) 1 CoA-activated acetyl group(m)	6.17 H_2O(s) 2 CO_2(s) 2 Pyruvate(c) 2 Glutamate(s) 2 NADH-redox-potential(c) 3.5 NADPH-redox-potential(c) 1 (S)-3-hydroxyoleyleoyl-CoA(m) 3 Na-gradient(c)	43	4	-	-	-	-	-	-	11	-	1

ID	flux	reaction
r0406	-0.33	Fructose-6P(c) + Erythrose-4P(c) ⇌ Sedoheptulose-7P(c) + GAP(c)
r0277	1	GTP(c) + IMP(c) + Aspartate(c) → GDP(c) + Pi(c) + Adenylosuccinate(c)
r0293	2	5,10-Methylene-THF(c) + NADP+(c) ⇌ 5,10-Methenyl-THF(c) + NADPH(c)
r0705	1	ATP(c) + 5-Phosphoribosyl-4-carboxy-5-aminoimidazole(c) + Aspartate(c) ⇌ ADP(c) + Pi(c) + SAICAR(c)
r0578	1	ATP(c) + Pantetheine(c) ⇌ ADP(c) + Phosphopantetheine(c)
r0877	0.5	Glutamate(c) + Cystine(s) ⇌ Glutamate(s) + Cystine(c)
r0030	3	ATP(c) + AMP(c) ⇌ 2 ADP(c)
r0032	1	ATP(c) + Dephospho-CoA(c) ⇌ ADP(c) + CoA(c)
r1143	-2	Na+(c) + Aspartate(c) ← Na+(s) + Aspartate(s)
r2664	3	Na+(c) ⇌ Na+(s) + Na-gradient(c)
r0058	2	Malate(c) + NADP+(c) ⇌ Pyruvate(c) + CO2(c) + NADPH(c)
r0370	0.33	Sedoheptulose-7P(c) + GAP(c) ⇌ Ribose-5P(c) + D-Xylulose-5P(c)
r0371	2	5,10-Methenyl-THF(c) + H2O(c) ⇌ 10-Formyl-THF(c)
r2685	1.5	Glutamate(c) ⇌ Glutamate(s)
r0659	1	ATP(c) + 5-Phosphoribosylamine(c) + Glycine(c) ⇌ ADP(c) + Pi(c) + GAR(c)
r0099	1	ATP(c) + GDP(c) ⇌ ADP(c) + GTP(c)
r1481	-1	Acetyl-CoA(m) + palmitoleoyl-CoA(m) ⇌ CoA(m) + 3-oxooleoyl-CoA(m)
r1468	1	palmitoleoyl-CoA(c) ⇌ palmitoleoyl-CoA(m)
r0243	-0.17	DHAP(c) ⇌ GAP(c)
r0677	1	10-Formyl-THF(c) + GAR(c) ⇌ THF(c) + N-Formyl-GAR(c)
r1331	-1	H+(PG)(s) ⇌ H+(PG)(c) + Proton-gradient(c)
r0974	1	Pantothenate(s) + Na+(s) ⇌ Pantothenate(c) + Na+(c)
r1319	-13.2	ATP(c) + H2O(c) ⇌ ADP(c) + Pi(c) + ATP-energy(c)
r0260	1	Adenylosuccinate(c) → Fumarate(c) + AMP(c)
r0247	1	ATP(c) + Ribose-5P(c) → AMP(c) + PRPP(c)
r1488	1	ATP(c) + CoA(c) + Palmitolate(c) ⇌ AMP(c) + palmitoleoyl-CoA(c) + PPi(c)
r0699	1	SAICAR(c) ⇌ Fumarate(c) + AICAR(c)
r2442	1	Palmitolate(s) → Palmitolate(c)
r0849	-6.17	H2O(s) ⇌ H2O(c)
r0581	-1	Pantothenate(c) + Cysteamine(c) ⇌ H2O(c) + Pantetheine(c)
r0867	2	CO2(c) ⇌ CO2(s)
r0589	1	ATP(c) + Phosphopantetheine(c) → PPi(c) + Dephospho-CoA(c)
r2468	1	Glycine(s) + H+(PG)(s) → Glycine(c) + H+(PG)(c)
r0007	3	PPi(c) + H2O(c) → 2 Pi(c)
r0736	0.17	ATP(c) + Fructose-6P(c) → ADP(c) + Fructose-1,6PP(c)
r0027	-0.5	NADPH(c) + Cystine(c) ⇌ 2 Cysteine(c) + NADP+(c)

Continued on next page

ID	flux	reaction
r0192	0.83	Glucose-6P(c) ⇌ Fructose-6P(c)
r0344	-0.67	D-Xylulose-5P(c) ⇌ Ribulose-5P(c)
r0792	2	5-Methyl-THF(c) + NAD+(c) ⇌ 5,10-Methylene-THF(c) + NADH(c)
r0211	1	Cysteine(c) + O2(c) ⇌ 3-Sulfinoalanine(c)
r2676	-2	5-Methyl-THF(c) ⇌ THF(c) + THF-activated methyl group(c)
r2525	2	Glutamine(s) ⇌ Glutamine(c)
r2678	-1	Acetyl-CoA(m) ⇌ CoA(m) + CoA-activated acetyl group(m)
r0666	1	ATP(c) + 5-Phosphoribosylformylglycinamidine(c) ⇌ ADP(c) + Pi(c) + AIR(c)
r0667	-1	5-Phosphoribosyl-4-carboxy-5-aminoimidazole(c) ⇌ AIR(c) + CO2(c)
r1322	2	NADH(c) ⇌ NAD+(c) + NADH-redox-potential(c)
r1323	-1	NADH(m) ⇌ NAD+(m) + NADH-redox-potential(m)
r0250	-0.67	Ribulose-5P(c) ⇌ Ribose-5P(c)
r1477	-1	NADH(m) + 3-oxooleoyl-CoA(m) ⇌ NAD+(m) + (S)-3-hydroxyoleyleoyl-CoA(m)
r1326	3.5	NADPH(c) ⇌ NADPH-redox-potential(c) + NADP+(c)
r0687	1	ATP(c) + N-Formyl-GAR(c) + Glutamine(c) + H2O(c) ⇌ ADP(c) + Pi(c) + Glutamate(c) + 5-Phosphoribosylformylglycinamidine(c)
r0255	0.33	Fructose-6P(c) + GAP(c) ⇌ D-Xylulose-5P(c) + Erythrose-4P(c)
r0538	1	3-Sulfinoalanine(c) ⇌ CO2(c) + Hypotaurine(c)
r0256	0.17	Fructose-1,6PP(c) ⇌ DHAP(c) + GAP(c)
r0539	-1	Hypotaurine(c) ⇌ Cysteamine(c) + O2(c)
r0257	-1	Glutamine(c) + PRPP(c) + H2O(c) ⇌ 5-Phosphoribosylamine(c) + PPi(c) + Glutamate(c)
r0258	-2	Fumarate(c) + H2O(c) ⇌ Malate(c)
r0273	-1	IMP(c) + H2O(c) ⇌ FAICAR(c)
r0700	1	10-Formyl-THF(c) + AICAR(c) ⇌ THF(c) + FAICAR(c)

(S)-3-hydroxypalmitoleoyl-CoA (561)

Definition			Solution												
			exchanges		reactions								transp		
Objective	Constraints	Comment	imports	exports	c	m	r	p	l	n	s	b	s-c	b-c	int
(S)-3-hydroxypalmitoleoyl-CoA(m)	PIPES MCES	synthesis of mitochondrial (S)-3-hydroxypalmitoleoyl-CoA	2.17 P_i(c) 1 Glycine(s) 2 Aspartate(s) 2 Glutamine(s) 0.83 Glucose-6P(c) 0.5 Cystine(s) 1 Pantothenate(s) 1 Palmitolate(s) 13.2 ATP-energy(c) 1 Proton-gradient(c) 2 THF-activated methyl group(c)	5.17 H_2O(s) 2 CO_2(s) 2 Pyruvate(c) 2 Glutamate(s) 2 NADH-redox-potential(c) 3.5 NADPH-redox-potential(c) 1 FADH-redox-potential(c) 1 (S)-3-hydroxypalmitoleoyl-CoA(m) 3 Na-gradient(c)	44	2	-	-	-	-	-	-	11	-	3

ID	flux	reaction
r0406	-0.33	Fructose-6P(c) + Erythrose-4P(c) ⇌ Sedoheptulose-7P(c) + GAP(c)
r0277	1	GTP(c) + IMP(c) + Aspartate(c) → GDP(c) + Pi(c) + Adenylosuccinate(c)
r0293	2	5,10-Methylene-THF(c) + NADP+(c) ⇌ 5,10-Methenyl-THF(c) + NADPH(c)
r0705	1	ATP(c) + 5-Phosphoribosyl-4-carboxy-5-aminoimidazole(c) + Aspartate(c) ⇌ ADP(c) + Pi(c) + SAICAR(c)
r0578	1	ATP(c) + Pantetheine(c) ⇌ ADP(c) + Phosphopantetheine(c)
r0877	0.5	Glutamate(c) + Cystine(s) ⇌ Glutamate(s) + Cystine(c)
r0030	3	ATP(c) + AMP(c) ⇌ 2 ADP(c)
r0032	1	ATP(c) + Dephospho-CoA(c) ⇌ ADP(c) + CoA(c)
r1290	1	FAD(c) + FADH2(m) ⇌ FAD(m) + FADH2(c)
r1143	-2	Na+(c) + Aspartate(c) ← Na+(s) + Aspartate(s)
r2664	3	Na+(c) ⇌ Na+(s) + Na-gradient(c)
r0058	2	Malate(c) + NADP+(c) ⇌ Pyruvate(c) + CO2(c) + NADPH(c)
r0370	0.33	Sedoheptulose-7P(c) + GAP(c) ⇌ Ribose-5P(c) + D-Xylulose-5P(c)
r0371	2	5,10-Methenyl-THF(c) + H2O(c) ⇌ 10-Formyl-THF(c)
r2685	1.5	Glutamate(c) ⇌ Glutamate(s)
r0659	1	ATP(c) + 5-Phosphoribosylamine(c) + Glycine(c) ⇌ ADP(c) + Pi(c) + GAR(c)
r0099	1	ATP(c) + GDP(c) ⇌ ADP(c) + GTP(c)

Continued on next page

(S)-3-hydroxypalmitoleoyl-CoA (561)– continued

ID	flux	reaction
r1468	1	palmitoleoyl-CoA(c) ⇌ palmitoleoyl-CoA(m)
r0243	-0.17	DHAP(c) ⇌ GAP(c)
r0677	1	10-Formyl-THF(c) + GAR(c) ⇌ THF(c) + N-Formyl-GAR(c)
r1331	-1	H+(PG)(s) ⇌ H+(PG)(c) + Proton-gradient(c)
r0974	1	Pantothenate(s) + Na+(s) ⇌ Pantothenate(c) + Na+(c)
r1319	-13.2	ATP(c) + H2O(c) ⇌ ADP(c) + Pi(c) + ATP-energy(c)
r0260	1	Adenylosuccinate(c) → Fumarate(c) + AMP(c)
r0247	1	ATP(c) + Ribose-5P(c) → AMP(c) + PRPP(c)
r1488	1	ATP(c) + CoA(c) + Palmitolate(c) ⇌ AMP(c) + palmitoleoyl-CoA(c) + PPi(c)
r0699	1	SAICAR(c) ⇌ Fumarate(c) + AICAR(c)
r2442	1	Palmitolate(s) → Palmitolate(c)
r0849	-5.17	H2O(s) ⇌ H2O(c)
r0581	-1	Pantothenate(c) + Cysteamine(c) ⇌ H2O(c) + Pantetheine(c)
r0867	2	CO2(c) ⇌ CO2(s)
r0589	1	ATP(c) + Phosphopantetheine(c) → PPi(c) + Dephospho-CoA(c)
r2468	1	Glycine(s) + H+(PG)(s) → Glycine(c) + H+(PG)(c)
r0007	3	PPi(c) + H2O(c) → 2 Pi(c)
r0736	0.17	ATP(c) + Fructose-6P(c) → ADP(c) + Fructose-1,6PP(c)
r0027	-0.5	NADPH(c) + Cystine(c) ⇌ 2 Cysteine(c) + NADP+(c)
r0192	0.83	Glucose-6P(c) ⇌ Fructose-6P(c)
r0344	-0.67	D-Xylulose-5P(c) ⇌ Ribulose-5P(c)
r0792	2	5-Methyl-THF(c) + NAD+(c) ⇌ 5,10-Methylene-THF(c) + NADH(c)
r0211	1	Cysteine(c) + O2(c) ⇌ 3-Sulfinoalanine(c)
r2676	-2	5-Methyl-THF(c) ⇌ THF(c) + THF-activated methyl group(c)
r2525	2	Glutamine(s) ⇌ Glutamine(c)
r0666	1	ATP(c) + 5-Phosphoribosylformylglycinamidine(c) ⇌ ADP(c) + Pi(c) + AIR(c)
r0667	-1	5-Phosphoribosyl-4-carboxy-5-aminoimidazole(c) ⇌ AIR(c) + CO2(c)
r1473	1	FAD(m) + palmitoleoyl-CoA(m) ⇌ FADH2(m) + trans,cis-hexadeca-2,9-dienoyl-CoA(m)
r1322	2	NADH(c) ⇌ NAD+(c) + NADH-redox-potential(c)
r1475	1	H2O(m) + trans,cis-hexadeca-2,9-dienoyl-CoA(m) ⇌ (S)-3-hydroxypalmitoleoyl-CoA(m)
r0250	-0.67	Ribulose-5P(c) ⇌ Ribose-5P(c)
r1326	3.5	NADPH(c) ⇌ NADPH-redox-potential(c) + NADP+(c)
r0687	1	ATP(c) + N-Formyl-GAR(c) + Glutamine(c) + H2O(c) ⇌ ADP(c) + Pi(c) + Glutamate(c) + 5-Phosphoribosylformylglycinamidine(c)
r0255	0.33	Fructose-6P(c) + GAP(c) ⇌ D-Xylulose-5P(c) + Erythrose-4P(c)
r1329	1	FADH2(c) ⇌ FAD(c) + FADH-redox-potential(c)
r0538	1	3-Sulfinoalanine(c) ⇌ CO2(c) + Hypotaurine(c)
r0256	0.17	Fructose-1,6PP(c) ⇌ DHAP(c) + GAP(c)
r0539	-1	Hypotaurine(c) ⇌ Cysteamine(c) + O2(c)
r0257	-1	Glutamine(c) + PRPP(c) + H2O(c) ⇌ 5-Phosphoribosylamine(c) + PPi(c) + Glutamate(c)
r0258	-2	Fumarate(c) + H2O(c) ⇌ Malate(c)
r0273	-1	IMP(c) + H2O(c) ⇌ FAICAR(c)
r0851	-1	H2O(m) ⇌ H2O(c)
r0700	1	10-Formyl-THF(c) + AICAR(c) ⇌ THF(c) + FAICAR(c)

(S)-Dihydroorotate (562)

Definition			Solution													
			exchanges		reactions								transp		
Objective	Constraints	Comment	imports	exports	c	m	r	p	l	n	s	b	s-c	b-c	int
(S)-Dihydroorotate(c)	PIPES MCES	synthesis of cytosolic (S)-Dihydroorotate	0.5 Pyruvate(m) 1 Asparagine(s) 3 ATP-energy(c) 0.5 THF-activated methyl group(c)	1 (S)-Dihydroorotate(c) 0.5 NADH-redox-potential(c) 0.5 NADH-redox-potential(m) 1 NADPH-redox-potential(c) 0.5 CoA-activated acetyl group(m)	15	3	-	-	-	-	-	-	1	-	1

ID	flux	reaction
r2678	0.5	Acetyl-CoA(m) ⇌ CoA(m) + CoA-activated acetyl group(m)
r0127	1	H2O(c) + Asparagine(c) → Aspartate(c) + NH3(c)
r0371	0.5	5,10-Methenyl-THF(c) + H2O(c) ⇌ 10-Formyl-THF(c)
r0227	0.5	10-Formyl-THF(c) + H2O(c) + NADP+(c) → THF(c) + CO2(c) + NADPH(c)
r0155	1	2 ATP(c) + Glutamine(c) + H2O(c) + HCO3-(c) → 2 ADP(c) + Pi(c) + Glutamate(c) + Carbamoyl-P(c)
r0056	0.5	Pyruvate(m) + CoA(m) + NAD+(m) → Acetyl-CoA(m) + CO2(m) + NADH(m)
r2532	1	Asparagine(s) ⇌ Asparagine(c)
r1326	1	NADPH(c) ⇌ NADPH-redox-potential(c) + NADP+(c)
r1417	-1	CO2(c) + H2O(c) ⇌ Carbonate(c)
r0328	1	Carbamoyl-P(c) + Aspartate(c) → Pi(c) + N-Carbamoyl-L-aspartate(c)
r1319	-3	ATP(c) + H2O(c) ⇌ ADP(c) + Pi(c) + ATP-energy(c)
r0293	0.5	5,10-Methylene-THF(c) + NADP+(c) ⇌ 5,10-Methenyl-THF(c) + NADPH(c)
r0077	1	ATP(c) + Glutamate(c) + NH3(c) → ADP(c) + Pi(c) + Glutamine(c)
r1419	1	Carbonate(c) ⇌ HCO3-(c)
r0467	-1	H2O(c) + (S)-Dihydroorotate(c) ← N-Carbamoyl-L-aspartate(c)
r0792	0.5	5-Methyl-THF(c) + NAD+(c) ⇌ 5,10-Methylene-THF(c) + NADH(c)
r2676	-0.5	5-Methyl-THF(c) ⇌ THF(c) + THF-activated methyl group(c)
r1322	0.5	NADH(c) ⇌ NAD+(c) + NADH-redox-potential(c)
r1323	0.5	NADH(m) ⇌ NAD+(m) + NADH-redox-potential(m)
r0868	-0.5	CO2(c) ⇌ CO2(m)

(S)-Hydroxydecanoyl-CoA (563)

Definition			Solution													
			exchanges		reactions								transp			
Objective	Constraints	Comment	imports	exports	c	m	r	p	l	n	s	b	s-c	b-c	int
(S)-Hydroxydecanoyl-CoA(m)	PIPES MCES	synthesis of mitochondrial (S)-Hydroxydecanoyl-CoA	0.5 O_2(s) 2.17 P_i(c) 1 Glycine(s) 2 Aspartate(s) 2 Glutamine(s) 0.83 Glucose-6P(c) 0.5 Cystine(s) 1 Pantothenate(s) 1 Palmitolate(s) 13.2 ATP-energy(c) 1 Proton-gradient(c) 2 THF-activated methyl group(c)	2.17 H_2O(s) 2 CO_2(s) 2 Pyruvate(c) 2 Glutamate(s) 1 (S)-Hydroxydecanoyl-CoA(m) 2 NADH-redox-potential(c) 4 NADH-redox-potential(m) 3.5 NADPH-redox-potential(c) 3 FADH-redox-potential(c) 6 Proton-gradient(m) 3 Na-gradient(c) 4 CoA-activated acetyl group(m)	44	20	-	-	-	-	-	-	12	-	7

ID	flux	reaction
r0406	-0.33	Fructose-6P(c) + Erythrose-4P(c) ⇌ Sedoheptulose-7P(c) + GAP(c)
r0277	1	GTP(c) + IMP(c) + Aspartate(c) → GDP(c) + Pi(c) + Adenylosuccinate(c)
r0855	0.5	O2(s) ⇌ O2(c)
r0856	-0.5	O2(m) ⇌ O2(c)
r0705	1	ATP(c) + 5-Phosphoribosyl-4-carboxy-5-aminoimidazole(c) + Aspartate(c) ⇌ ADP(c) + Pi(c) + SAICAR(c)
r0293	2	5,10-Methylene-THF(c) + NADP+(c) ⇌ 5,10-Methenyl-THF(c) + NADPH(c)
r0578	1	ATP(c) + Pantetheine(c) ⇌ ADP(c) + Phosphopantetheine(c)
r0722	1	(S)-3-Hydroxydodecanoyl-CoA(m) + NAD+(m) ⇌ 3-Oxododecanoyl-CoA(m) + NADH(m)
r0724	-1	Decanoyl-CoA(m) + Acetyl-CoA(m) ⇌ CoA(m) + 3-Oxododecanoyl-CoA(m)
r0877	0.5	Glutamate(c) + Cystine(s) ⇌ Glutamate(s) + Cystine(c)
r0728	-1	(S)-Hydroxydecanoyl-CoA(m) ⇌ (2E)-Decenoyl-CoA(m) + H2O(m)
r0017	0.5	O2(m) + 4 H+(PG)(m) + 4 Ferrocytochrome C(m) → 4 Ferricytochrome C(m) + 2 H2O(m) + 4 H+(PG)(c)
r0030	3	ATP(c) + AMP(c) ⇌ 2 ADP(c)
r0032	1	ATP(c) + Dephospho-CoA(c) ⇌ ADP(c) + CoA(c)
r1290	3	FAD(c) + FADH2(m) ⇌ FAD(m) + FADH2(c)
r1143	-2	Na+(c) + Aspartate(c) ← Na+(s) + Aspartate(s)
r2664	3	Na+(c) ⇌ Na+(s) + Na-gradient(c)
r0058	2	Malate(c) + NADP+(c) ⇌ Pyruvate(c) + CO2(c) + NADPH(c)
r0370	0.33	Sedoheptulose-7P(c) + GAP(c) ⇌ Ribose-5P(c) + D-Xylulose-5P(c)

Continued on next page

ID	flux	reaction
r0371	2	5,10-Methenyl-THF(c) + H2O(c) ⇌ 10-Formyl-THF(c)
r2685	1.5	Glutamate(c) ⇌ Glutamate(s)
r0657	1	(3Z)-Dodecenoyl-CoA(m) ⇌ (2E)-Dodecenoyl-CoA(m)
r0659	1	ATP(c) + 5-Phosphoribosylamine(c) + Glycine(c) ⇌ ADP(c) + Pi(c) + GAR(c)
r0507	1	Ubiquinol(m) + 2 Ferricytochrome C(m) + 4 H+(PG)(m) → Ubiquinone(m) + 4 H+(PG)(c) + 2 Ferrocytochrome C(m)
r1480	1	CoA(m) + 3-oxopalmitoleoyl-CoA(m) ⇌ Acetyl-CoA(m) + cis-myrist-7-enoyl-CoA(m)
r0099	1	ATP(c) + GDP(c) ⇌ ADP(c) + GTP(c)
r1468	1	palmitoleoyl-CoA(c) ⇌ palmitoleoyl-CoA(m)
r0243	-0.17	DHAP(c) ⇌ GAP(c)
r0677	1	10-Formyl-THF(c) + GAR(c) ⇌ THF(c) + N-Formyl-GAR(c)
r1482	-1	FADH2(m) + trans,cis-dodeca-2,5-dienoyl-CoA(m) ⇌ FAD(m) + cis-laur-5-enoyl-CoA(m)
r1330	6	H+(PG)(c) ⇌ H+(PG)(m) + Proton-gradient(m)
r1483	1	H2O(m) + trans,cis-myristo-2,7-dienoyl-CoA(m) ⇌ cis-(3S)-hydroxytetradec-7-enoyl-CoA(m)
r1331	-1	H+(PG)(s) ⇌ H+(PG)(c) + Proton-gradient(c)
r0974	1	Pantothenate(s) + Na+(s) ⇌ Pantothenate(c) + Na+(c)
r1484	1	CoA(m) + 3-oxomyrist-7-enoyl-CoA(m) ⇌ Acetyl-CoA(m) + cis-laur-5-enoyl-CoA(m)
r1319	-13.2	ATP(c) + H2O(c) ⇌ ADP(c) + Pi(c) + ATP-energy(c)
r1485	1	H2O(m) + trans,cis-dodeca-2,5-dienoyl-CoA(m) ⇌ 3(S)-3-hydroxydodecen-(5Z)-oyl-CoA(m)
r0260	1	Adenylosuccinate(c) → Fumarate(c) + AMP(c)
r0247	1	ATP(c) + Ribose-5P(c) → AMP(c) + PRPP(c)
r1486	1	NAD+(m) + 3(S)-3-hydroxydodecen-(5Z)-oyl-CoA(m) ⇌ 3-oxolaur-cis-5-enoyl-CoA(m) + NADH(m)
r1488	1	ATP(c) + CoA(c) + Palmitolate(c) ⇌ AMP(c) + palmitoleoyl-CoA(c) + PPi(c)
r0699	1	SAICAR(c) ⇌ Fumarate(c) + AICAR(c)
r2442	1	Palmitolate(s) → Palmitolate(c)
r0849	-2.17	H2O(s) ⇌ H2O(c)
r0581	-1	Pantothenate(c) + Cysteamine(c) ⇌ H2O(c) + Pantetheine(c)
r0867	2	CO2(c) ⇌ CO2(s)
r0589	1	ATP(c) + Phosphopantetheine(c) → PPi(c) + Dephospho-CoA(c)
r2468	1	Glycine(s) + H+(PG)(s) → Glycine(c) + H+(PG)(c)
r0736	0.17	ATP(c) + Fructose-6P(c) → ADP(c) + Fructose-1,6PP(c)
r0007	3	PPi(c) + H2O(c) → 2 Pi(c)
r0027	-0.5	NADPH(c) + Cystine(c) ⇌ 2 Cysteine(c) + NADP+(c)
r0192	0.83	Glucose-6P(c) ⇌ Fructose-6P(c)
r0344	-0.67	D-Xylulose-5P(c) ⇌ Ribulose-5P(c)
r0792	2	5-Methyl-THF(c) + NAD+(c) ⇌ 5,10-Methylene-THF(c) + NADH(c)
r0211	1	Cysteine(c) + O2(c) ⇌ 3-Sulfinoalanine(c)
r2676	-2	5-Methyl-THF(c) ⇌ THF(c) + THF-activated methyl group(c)
r1451	1	Ubiquinone(m) + Decanoyl-CoA(m) → (2E)-Decenoyl-CoA(m) + Ubiquinol(m)
r0660	-1	(S)-3-Hydroxydodecanoyl-CoA(m) ⇌ (2E)-Dodecenoyl-CoA(m) + H2O(m)
r2525	2	Glutamine(s) ⇌ Glutamine(c)
r2678	4	Acetyl-CoA(m) ⇌ CoA(m) + CoA-activated acetyl group(m)
r0666	1	ATP(c) + 5-Phosphoribosylformylglycinamidine(c) ⇌ ADP(c) + Pi(c) + AIR(c)
r1471	1	FAD(m) + cis-myrist-7-enoyl-CoA(m) ⇌ FADH2(m) + trans,cis-myristo-2,7-dienoyl-CoA(m)
r0667	-1	5-Phosphoribosyl-4-carboxy-5-aminoimidazole(c) ⇌ AIR(c) + CO2(c)
r1473	1	FAD(m) + palmitoleoyl-CoA(m) ⇌ FADH2(m) + trans,cis-hexadeca-2,9-dienoyl-CoA(m)
r1322	2	NADH(c) ⇌ NAD+(c) + NADH-redox-potential(c)
r1475	1	H2O(m) + trans,cis-hexadeca-2,9-dienoyl-CoA(m) ⇌ (S)-3-hydroxypalmitoleoyl-CoA(m)
r1323	4	NADH(m) ⇌ NAD+(m) + NADH-redox-potential(m)
r0250	-0.67	Ribulose-5P(c) ⇌ Ribose-5P(c)
r1476	1	NAD+(m) + cis-(3S)-hydroxytetradec-7-enoyl-CoA(m) ⇌ 3-oxomyrist-7-enoyl-CoA(m) + NADH(m)
r1478	1	NAD+(m) + (S)-3-hydroxypalmitoleoyl-CoA(m) ⇌ NADH(m) + 3-oxopalmitoleoyl-CoA(m)
r1326	3.5	NADPH(c) ⇌ NADPH-redox-potential(c) + NADP+(c)
r0687	1	ATP(c) + N-Formyl-GAR(c) + Glutamine(c) + H2O(c) ⇌ ADP(c) + Pi(c) + Glutamate(c) + 5-Phosphoribosylformylglycinamidine(c)
r1479	1	CoA(m) + 3-oxolaur-cis-5-enoyl-CoA(m) ⇌ Acetyl-CoA(m) + (3Z)-Dodecenoyl-CoA(m)
r0255	0.33	Fructose-6P(c) + GAP(c) ⇌ D-Xylulose-5P(c) + Erythrose-4P(c)

Continued on next page

ID	flux	reaction
r1329	3	FADH2(c) ⇌ FAD(c) + FADH-redox-potential(c)
r0538	1	3-Sulfinoalanine(c) ⇌ CO2(c) + Hypotaurine(c)
r0256	0.17	Fructose-1,6PP(c) ⇌ DHAP(c) + GAP(c)
r0539	-1	Hypotaurine(c) ⇌ Cysteamine(c) + O2(c)
r0257	-1	Glutamine(c) + PRPP(c) + H2O(c) ⇌ 5-Phosphoribosylamine(c) + PPi(c) + Glutamate(c)
r0258	-2	Fumarate(c) + H2O(c) ⇌ Malate(c)
r0273	-1	IMP(c) + H2O(c) ⇌ FAICAR(c)
r0851	-4	H2O(m) ⇌ H2O(c)
r0700	1	10-Formyl-THF(c) + AICAR(c) ⇌ THF(c) + FAICAR(c)

(S)-Hydroxyhexanoyl-CoA (564)

Definition			Solution												
			exchanges		reactions								transp		
Objective	Constraints	Comment	imports	exports	c	m	r	p	l	n	s	b	s-c	b-c	int
(S)-Hydroxyhexanoyl-CoA(m)	PIPES MCES	synthesis of mitochondrial (S)-Hydroxyhexanoyl-CoA	1.5 O_2(s) 2.17 P_i(c) 1 Glycine(s) 0.5 Aspartate(s) 0.5 Glutamine(s) 0.83 Glucose-6P(c) 1.5 Asparagine(s) 0.5 Cystine(s) 1 Pantothenate(s) 1 Palmitolate(s) 14.7 ATP-energy(c) 2 THF-activated methyl group(c)	2.17 H_2O(s) 2 CO_2(s) 2 Pyruvate(c) 0.5 Glutamate(c) 1 (S)-Hydroxyhexanoyl-CoA(m) 2 NADH-redox-potential(c) 6 NADH-redox-potential(m) 3.5 NADPH-redox-potential(c) 3 FADH-redox-potential(c) 0.5 Proton-gradient(c) 18 Proton-gradient(m) 2 Na-gradient(c) 6 CoA-activated acetyl group(m)	46	28	-	-	-	-	-	-	12	-	7

ID	flux	reaction
r0406	-0.33	Fructose-6P(c) + Erythrose-4P(c) ⇌ Sedoheptulose-7P(c) + GAP(c)
r0277	1	GTP(c) + IMP(c) + Aspartate(c) → GDP(c) + Pi(c) + Adenylosuccinate(c)
r0855	1.5	O2(s) ⇌ O2(c)
r0856	-1.5	O2(m) ⇌ O2(c)
r0127	1.5	H2O(c) + Asparagine(c) → Aspartate(c) + NH3(c)
r0705	1	ATP(c) + 5-Phosphoribosyl-4-carboxy-5-aminoimidazole(c) + Aspartate(c) ⇌ ADP(c) + Pi(c) + SAICAR(c)
r0293	2	5,10-Methylene-THF(c) + NADP+(c) ⇌ 5,10-Methenyl-THF(c) + NADPH(c)
r0578	1	ATP(c) + Pantetheine(c) ⇌ ADP(c) + Phosphopantetheine(c)
r0722	1	(S)-3-Hydroxydodecanoyl-CoA(m) + NAD+(m) ⇌ 3-Oxododecanoyl-CoA(m) + NADH(m)
r0724	-1	Decanoyl-CoA(m) + Acetyl-CoA(m) ⇌ CoA(m) + 3-Oxododecanoyl-CoA(m)
r0877	0.5	Glutamate(c) + Cystine(s) ⇌ Glutamate(s) + Cystine(c)
r0726	1	(S)-Hydroxydecanoyl-CoA(m) + NAD+(m) ⇌ 3-Oxodecanoyl-CoA(m) + NADH(m)
r0728	-1	(S)-Hydroxydecanoyl-CoA(m) ⇌ (2E)-Decenoyl-CoA(m) + H2O(m)
r2477	0.5	Glutamine(s) + Na+(s) + H+(PG)(c) → Glutamine(c) + Na+(c) + H+(PG)(s)
r0017	1.5	O2(m) + 4 H+(PG)(m) + 4 Ferrocytochrome C(m) → 4 Ferricytochrome C(m) + 2 H2O(m) + 4 H+(PG)(c)
r0030	3	ATP(c) + AMP(c) ⇌ 2 ADP(c)
r0032	1	ATP(c) + Dephospho-CoA(c) ⇌ ADP(c) + CoA(c)
r1290	3	FAD(c) + FADH2(m) ⇌ FAD(m) + FADH2(c)
r1143	-0.5	Na+(c) + Aspartate(c) ← Na+(s) + Aspartate(s)
r0634	-1	Acetyl-CoA(m) + Octanoyl-CoA(m) ⇌ CoA(m) + 3-Oxodecanoyl-CoA(m)
r2664	2	Na+(c) ⇌ Na+(s) + Na-gradient(c)
r0058	2	Malate(c) + NADP+(c) ⇌ Pyruvate(c) + CO2(c) + NADPH(c)
r0370	0.33	Sedoheptulose-7P(c) + GAP(c) ⇌ Ribose-5P(c) + D-Xylulose-5P(c)
r0371	2	5,10-Methenyl-THF(c) + H2O(c) ⇌ 10-Formyl-THF(c)
r0077	1.5	ATP(c) + Glutamate(c) + NH3(c) → ADP(c) + Pi(c) + Glutamine(c)
r2532	1.5	Asparagine(s) ⇌ Asparagine(c)
r1448	1	Ubiquinone(m) + Octanoyl-CoA(m) → (2E)-Octenoyl-CoA(m) + Ubiquinol(m)
r0657	1	(3Z)-Dodecenoyl-CoA(m) ⇌ (2E)-Dodecenoyl-CoA(m)
r0659	1	ATP(c) + 5-Phosphoribosylamine(c) + Glycine(c) ⇌ ADP(c) + Pi(c) + GAR(c)

Continued on next page

ID	flux	reaction
r0507	3	Ubiquinol(m) + 2 Ferricytochrome C(m) + 4 H+(PG)(m) → Ubiquinone(m) + 4 H+(PG)(c) + 2 Ferrocytochrome C(m)
r1480	1	CoA(m) + 3-oxopalmitoleoyl-CoA(m) ⇌ Acetyl-CoA(m) + cis-myrist-7-enoyl-CoA(m)
r0099	1	ATP(c) + GDP(c) ⇌ ADP(c) + GTP(c)
r1468	1	palmitoleoyl-CoA(c) ⇌ palmitoleoyl-CoA(m)
r0677	1	10-Formyl-THF(c) + GAR(c) ⇌ THF(c) + N-Formyl-GAR(c)
r0243	-0.17	DHAP(c) ⇌ GAP(c)
r1482	-1	FADH2(m) + trans,cis-dodeca-2,5-dienoyl-CoA(m) ⇌ FAD(m) + cis-laur-5-enoyl-CoA(m)
r1330	18	H+(PG)(c) ⇌ H+(PG)(m) + Proton-gradient(m)
r1483	1	H2O(m) + trans,cis-myristo-2,7-dienoyl-CoA(m) ⇌ cis-(3S)-hydroxytetradec-7-enoyl-CoA(m)
r1331	0.5	H+(PG)(s) ⇌ H+(PG)(c) + Proton-gradient(c)
r0974	1	Pantothenate(s) + Na+(s) ⇌ Pantothenate(c) + Na+(c)
r1484	1	CoA(m) + 3-oxomyrist-7-enoyl-CoA(m) ⇌ Acetyl-CoA(m) + cis-laur-5-enoyl-CoA(m)
r1319	-14.7	ATP(c) + H2O(c) ⇌ ADP(c) + Pi(c) + ATP-energy(c)
r1485	1	H2O(m) + trans,cis-dodeca-2,5-dienoyl-CoA(m) ⇌ 3(S)-3-hydroxydodecen-(5Z)-oyl-CoA(m)
r0260	1	Adenylosuccinate(c) → Fumarate(c) + AMP(c)
r0247	1	ATP(c) + Ribose-5P(c) → AMP(c) + PRPP(c)
r1486	1	NAD+(m) + 3(S)-3-hydroxydodecen-(5Z)-oyl-CoA(m) ⇌ 3-oxolaur-cis-5-enoyl-CoA(m) + NADH(m)
r1488	1	ATP(c) + CoA(c) + Palmitolate(c) ⇌ AMP(c) + palmitoleoyl-CoA(c) + PPi(c)
r0699	1	SAICAR(c) ⇌ Fumarate(c) + AICAR(c)
r2442	1	Palmitolate(s) → Palmitolate(c)
r0849	-2.17	H2O(s) ⇌ H2O(c)
r0581	-1	Pantothenate(c) + Cysteamine(c) ⇌ H2O(c) + Pantetheine(c)
r0867	2	CO2(c) ⇌ CO2(s)
r0730	1	(S)-Hydroxyoctanoyl-CoA(m) + NAD+(m) → 3-Oxooctanoyl-CoA(m) + NADH(m)
r0731	-1	(S)-Hydroxyoctanoyl-CoA(m) ← (2E)-Octenoyl-CoA(m) + H2O(m)
r0732	-1	Hexanoyl-CoA(m) + Acetyl-CoA(m) ← CoA(m) + 3-Oxooctanoyl-CoA(m)
r0589	1	ATP(c) + Phosphopantetheine(c) → PPi(c) + Dephospho-CoA(c)
r0734	-1	(S)-Hydroxyhexanoyl-CoA(m) ← (2E)-Hexenoyl-CoA(m) + H2O(m)
r0736	0.17	ATP(c) + Fructose-6P(c) → ADP(c) + Fructose-1,6PP(c)
r0007	3	PPi(c) + H2O(c) → 2 Pi(c)
r0027	-0.5	NADPH(c) + Cystine(c) ⇌ 2 Cysteine(c) + NADP+(c)
r0192	0.83	Glucose-6P(c) ⇌ Fructose-6P(c)
r0344	-0.67	D-Xylulose-5P(c) ⇌ Ribulose-5P(c)
r0792	2	5-Methyl-THF(c) + NAD+(c) ⇌ 5,10-Methylene-THF(c) + NADH(c)
r0211	1	Cysteine(c) + O2(c) ⇌ 3-Sulfinoalanine(c)
r2523	1	Glycine(s) ⇌ Glycine(c)
r1450	1	Ubiquinone(m) + Hexanoyl-CoA(m) → (2E)-Hexenoyl-CoA(m) + Ubiquinol(m)
r2676	-2	5-Methyl-THF(c) ⇌ THF(c) + THF-activated methyl group(c)
r1451	1	Ubiquinone(m) + Decanoyl-CoA(m) → (2E)-Decenoyl-CoA(m) + Ubiquinol(m)
r0660	-1	(S)-3-Hydroxydodecanoyl-CoA(m) ⇌ (2E)-Dodecenoyl-CoA(m) + H2O(m)
r2678	6	Acetyl-CoA(m) ⇌ CoA(m) + CoA-activated acetyl group(m)
r0666	1	ATP(c) + 5-Phosphoribosylformylglycinamidine(c) ⇌ ADP(c) + Pi(c) + AIR(c)
r1471	1	FAD(m) + cis-myrist-7-enoyl-CoA(m) ⇌ FADH2(m) + trans,cis-myristo-2,7-dienoyl-CoA(m)
r0667	-1	5-Phosphoribosyl-4-carboxy-5-aminoimidazole(c) ⇌ AIR(c) + CO2(c)
r1473	1	FAD(m) + palmitoleoyl-CoA(m) ⇌ FADH2(m) + trans,cis-hexadeca-2,9-dienoyl-CoA(m)
r1322	2	NADH(c) ⇌ NAD+(c) + NADH-redox-potential(c)
r1475	1	H2O(m) + trans,cis-hexadeca-2,9-dienoyl-CoA(m) ⇌ (S)-3-hydroxypalmitoleoyl-CoA(m)
r1323	6	NADH(m) ⇌ NAD+(m) + NADH-redox-potential(m)
r0250	-0.67	Ribulose-5P(c) ⇌ Ribose-5P(c)
r1476	1	NAD+(m) + cis-(3S)-hydroxytetradec-7-enoyl-CoA(m) ⇌ 3-oxomyrist-7-enoyl-CoA(m) + NADH(m)
r1478	1	NAD+(m) + (S)-3-hydroxypalmitoleoyl-CoA(m) ⇌ NADH(m) + 3-oxopalmitoleoyl-CoA(m)
r1326	3.5	NADPH(c) ⇌ NADPH-redox-potential(c) + NADP+(c)
r0687	1	ATP(c) + N-Formyl-GAR(c) + Glutamine(c) + H2O(c) ⇌ ADP(c) + Pi(c) + Glutamate(c) + 5-Phosphoribosylformylglycinamidine(c)
r1479	1	CoA(m) + 3-oxolaur-cis-5-enoyl-CoA(m) ⇌ Acetyl-CoA(m) + (3Z)-Dodecenoyl-CoA(m)
r0255	0.33	Fructose-6P(c) + GAP(c) ⇌ D-Xylulose-5P(c) + Erythrose-4P(c)

Continued on next page

ID	flux	reaction
r1329	3	FADH2(c) ⇌ FAD(c) + FADH-redox-potential(c)
r0538	1	3-Sulfinoalanine(c) ⇌ CO2(c) + Hypotaurine(c)
r0256	0.17	Fructose-1,6PP(c) ⇌ DHAP(c) + GAP(c)
r0539	-1	Hypotaurine(c) ⇌ Cysteamine(c) + O2(c)
r0257	-1	Glutamine(c) + PRPP(c) + H2O(c) ⇌ 5-Phosphoribosylamine(c) + PPi(c) + Glutamate(c)
r0258	-2	Fumarate(c) + H2O(c) ⇌ Malate(c)
r0273	-1	IMP(c) + H2O(c) ⇌ FAICAR(c)
r0851	-4	H2O(m) ⇌ H2O(c)
r0700	1	10-Formyl-THF(c) + AICAR(c) ⇌ THF(c) + FAICAR(c)

(S)-Hydroxyoctanoyl-CoA (565)

Definition			Solution												
			exchanges		reactions								transp		
Objective	Constraints	Comment	imports	exports	c	m	r	p	l	n	s	b	s-c	b-c	int
(S)-Hydroxyoctanoyl-CoA(m)	PIPES MCES	synthesis of mitochondrial (S)-Hydroxyoctanoyl-CoA	1 O_2(s) 2.17 P_i(c) 1 Glycine(s) 2 Aspartate(s) 2 Glutamine(s) 0.83 Glucose-6P(c) 0.5 Cystine(s) 1 Pantothenate(s) 1 Palmitolate(s) 13.2 ATP-energy(c) 2 THF-activated methyl group(c)	2.17 H_2O(s) 2 CO_2(s) 2 Pyruvate(c) 2 Glutamate(s) 1 (S)-Hydroxyoctanoyl-CoA(m) 2 NADH-redox-potential(c) 5 NADH-redox-potential(m) 3.5 NADPH-redox-potential(c) 3 FADH-redox-potential(c) 12 Proton-gradient(m) 3 Na-gradient(c) 5 CoA-activated acetyl group(m)	44	24	-	-	-	-	-	-	11	-	7

ID	flux	reaction
r0406	-0.33	Fructose-6P(c) + Erythrose-4P(c) ⇌ Sedoheptulose-7P(c) + GAP(c)
r0277	1	GTP(c) + IMP(c) + Aspartate(c) → GDP(c) + Pi(c) + Adenylosuccinate(c)
r0855	1	O2(s) ⇌ O2(c)
r0856	-1	O2(m) ⇌ O2(c)
r0705	1	ATP(c) + 5-Phosphoribosyl-4-carboxy-5-aminoimidazole(c) + Aspartate(c) ⇌ ADP(c) + Pi(c) + SAICAR(c)
r0293	2	5,10-Methylene-THF(c) + NADP+(c) ⇌ 5,10-Methenyl-THF(c) + NADPH(c)
r0578	1	ATP(c) + Pantetheine(c) ⇌ ADP(c) + Phosphopantetheine(c)
r0722	1	(S)-3-Hydroxydodecanoyl-CoA(m) + NAD+(m) ⇌ 3-Oxododecanoyl-CoA(m) + NADH(m)
r0724	-1	Decanoyl-CoA(m) + Acetyl-CoA(m) ⇌ CoA(m) + 3-Oxododecanoyl-CoA(m)
r0877	0.5	Glutamate(c) + Cystine(s) ⇌ Glutamate(s) + Cystine(c)
r0726	1	(S)-Hydroxydecanoyl-CoA(m) + NAD+(m) ⇌ 3-Oxodecanoyl-CoA(m) + NADH(m)
r0728	-1	(S)-Hydroxydecanoyl-CoA(m) ⇌ (2E)-Decenoyl-CoA(m) + H2O(m)
r0017	1	O2(m) + 4 H+(PG)(m) + 4 Ferrocytochrome C(m) → 4 Ferricytochrome C(m) + 2 H2O(m) + 4 H+(PG)(c)
r0030	3	ATP(c) + AMP(c) ⇌ 2 ADP(c)
r0032	1	ATP(c) + Dephospho-CoA(c) ⇌ ADP(c) + CoA(c)
r1290	3	FAD(c) + FADH2(m) ⇌ FAD(m) + FADH2(c)
r1143	-2	Na+(c) + Aspartate(c) ← Na+(s) + Aspartate(s)
r0634	-1	Acetyl-CoA(m) + Octanoyl-CoA(m) ⇌ CoA(m) + 3-Oxodecanoyl-CoA(m)
r2664	3	Na+(c) ⇌ Na+(s) + Na-gradient(c)
r0058	2	Malate(c) + NADP+(c) ⇌ Pyruvate(c) + CO2(c) + NADPH(c)
r0370	0.33	Sedoheptulose-7P(c) + GAP(c) ⇌ Ribose-5P(c) + D-Xylulose-5P(c)
r0371	2	5,10-Methenyl-THF(c) + H2O(c) ⇌ 10-Formyl-THF(c)
r2685	1.5	Glutamate(c) ⇌ Glutamate(s)
r1448	1	Ubiquinone(m) + Octanoyl-CoA(m) → (2E)-Octenoyl-CoA(m) + Ubiquinol(m)
r0657	1	(3Z)-Dodecenoyl-CoA(m) ⇌ (2E)-Dodecenoyl-CoA(m)
r0659	1	ATP(c) + 5-Phosphoribosylamine(c) + Glycine(c) ⇌ ADP(c) + Pi(c) + GAR(c)
r0507	2	Ubiquinol(m) + 2 Ferricytochrome C(m) + 4 H+(PG)(m) → Ubiquinone(m) + 4 H+(PG)(c) + 2 Ferrocytochrome C(m)
r1480	1	CoA(m) + 3-oxopalmitoleoyl-CoA(m) ⇌ Acetyl-CoA(m) + cis-myrist-7-enoyl-CoA(m)
r0099	1	ATP(c) + GDP(c) ⇌ ADP(c) + GTP(c)

Continued on next page

ID	flux	reaction
r1468	1	palmitoleoyl-CoA(c) ⇌ palmitoleoyl-CoA(m)
r0243	-0.17	DHAP(c) ⇌ GAP(c)
r0677	1	10-Formyl-THF(c) + GAR(c) ⇌ THF(c) + N-Formyl-GAR(c)
r1482	-1	FADH2(m) + trans,cis-dodeca-2,5-dienoyl-CoA(m) ⇌ FAD(m) + cis-laur-5-enoyl-CoA(m)
r1330	12	H+(PG)(c) ⇌ H+(PG)(m) + Proton-gradient(m)
r1483	1	H2O(m) + trans,cis-myristo-2,7-dienoyl-CoA(m) ⇌ cis-(3S)-hydroxytetradec-7-enoyl-CoA(m)
r0974	1	Pantothenate(s) + Na+(s) ⇌ Pantothenate(c) + Na+(c)
r1484	1	CoA(m) + 3-oxomyrist-7-enoyl-CoA(m) ⇌ Acetyl-CoA(m) + cis-laur-5-enoyl-CoA(m)
r1319	-13.2	ATP(c) + H2O(c) ⇌ ADP(c) + Pi(c) + ATP-energy(c)
r1485	1	H2O(m) + trans,cis-dodeca-2,5-dienoyl-CoA(m) ⇌ 3(S)-3-hydroxydodecen-(5Z)-oyl-CoA(m)
r0260	1	Adenylosuccinate(c) → Fumarate(c) + AMP(c)
r0247	1	ATP(c) + Ribose-5P(c) → AMP(c) + PRPP(c)
r1486	1	NAD+(m) + 3(S)-3-hydroxydodecen-(5Z)-oyl-CoA(m) ⇌ 3-oxolaur-cis-5-enoyl-CoA(m) + NADH(m)
r1488	1	ATP(c) + CoA(c) + Palmitolate(c) ⇌ AMP(c) + palmitoleoyl-CoA(c) + PPi(c)
r0699	1	SAICAR(c) ⇌ Fumarate(c) + AICAR(c)
r2442	1	Palmitolate(s) → Palmitolate(c)
r0849	-2.17	H2O(s) ⇌ H2O(c)
r0581	-1	Pantothenate(c) + Cysteamine(c) ⇌ H2O(c) + Pantetheine(c)
r0867	2	CO2(c) ⇌ CO2(s)
r0731	-1	(S)-Hydroxyoctanoyl-CoA(m) ← (2E)-Octenoyl-CoA(m) + H2O(m)
r0589	1	ATP(c) + Phosphopantetheine(c) → PPi(c) + Dephospho-CoA(c)
r0736	0.17	ATP(c) + Fructose-6P(c) → ADP(c) + Fructose-1,6PP(c)
r0007	3	PPi(c) + H2O(c) → 2 Pi(c)
r0027	-0.5	NADPH(c) + Cystine(c) ⇌ 2 Cysteine(c) + NADP+(c)
r0192	0.83	Glucose-6P(c) ⇌ Fructose-6P(c)
r0344	-0.67	D-Xylulose-5P(c) ⇌ Ribulose-5P(c)
r0792	2	5-Methyl-THF(c) + NAD+(c) ⇌ 5,10-Methylene-THF(c) + NADH(c)
r0211	1	Cysteine(c) + O2(c) ⇌ 3-Sulfinoalanine(c)
r2523	1	Glycine(s) ⇌ Glycine(c)
r2676	-2	5-Methyl-THF(c) ⇌ THF(c) + THF-activated methyl group(c)
r1451	1	Ubiquinone(m) + Decanoyl-CoA(m) → (2E)-Decenoyl-CoA(m) + Ubiquinol(m)
r0660	-1	(S)-3-Hydroxydodecanoyl-CoA(m) ⇌ (2E)-Dodecenoyl-CoA(m) + H2O(m)
r2525	2	Glutamine(s) ⇌ Glutamine(c)
r2678	5	Acetyl-CoA(m) ⇌ CoA(m) + CoA-activated acetyl group(m)
r0666	1	ATP(c) + 5-Phosphoribosylformylglycinamidine(c) ⇌ ADP(c) + Pi(c) + AIR(c)
r1471	1	FAD(m) + cis-myrist-7-enoyl-CoA(m) ⇌ FADH2(m) + trans,cis-myristo-2,7-dienoyl-CoA(m)
r0667	-1	5-Phosphoribosyl-4-carboxy-5-aminoimidazole(c) ⇌ AIR(c) + CO2(c)
r1473	1	FAD(m) + palmitoleoyl-CoA(m) ⇌ FADH2(m) + trans,cis-hexadeca-2,9-dienoyl-CoA(m)
r1322	2	NADH(c) ⇌ NAD+(c) + NADH-redox-potential(c)
r1475	1	H2O(m) + trans,cis-hexadeca-2,9-dienoyl-CoA(m) ⇌ (S)-3-hydroxypalmitoleoyl-CoA(m)
r1323	5	NADH(m) ⇌ NAD+(m) + NADH-redox-potential(m)
r0250	-0.67	Ribulose-5P(c) ⇌ Ribose-5P(c)
r1476	1	NAD+(m) + cis-(3S)-hydroxytetradec-7-enoyl-CoA(m) ⇌ 3-oxomyrist-7-enoyl-CoA(m) + NADH(m)
r1478	1	NAD+(m) + (S)-3-hydroxypalmitoleoyl-CoA(m) ⇌ NADH(m) + 3-oxopalmitoleoyl-CoA(m)
r1326	3.5	NADPH(c) ⇌ NADPH-redox-potential(c) + NADP+(c)
r0687	1	ATP(c) + N-Formyl-GAR(c) + Glutamine(c) + H2O(c) ⇌ ADP(c) + Pi(c) + Glutamate(c) + 5-Phosphoribosylformylglycinamidine(c)
r1479	1	CoA(m) + 3-oxolaur-cis-5-enoyl-CoA(m) ⇌ Acetyl-CoA(m) + (3Z)-Dodecenoyl-CoA(m)
r0255	0.33	Fructose-6P(c) + GAP(c) ⇌ D-Xylulose-5P(c) + Erythrose-4P(c)
r1329	3	FADH2(c) ⇌ FAD(c) + FADH-redox-potential(c)
r0538	1	3-Sulfinoalanine(c) ⇌ CO2(c) + Hypotaurine(c)
r0256	0.17	Fructose-1,6PP(c) ⇌ DHAP(c) + GAP(c)
r0539	-1	Hypotaurine(c) ⇌ Cysteamine(c) + O2(c)
r0257	-1	Glutamine(c) + PRPP(c) + H2O(c) ⇌ 5-Phosphoribosylamine(c) + PPi(c) + Glutamate(c)
r0258	-2	Fumarate(c) + H2O(c) ⇌ Malate(c)
r0273	-1	IMP(c) + H2O(c) ⇌ FAICAR(c)

Continued on next page

ID	flux	reaction
r0851	-4	H2O(m) ⇌ H2O(c)
r0700	1	10-Formyl-THF(c) + AICAR(c) ⇌ THF(c) + FAICAR(c)

1,2-Diacylglycerol-Bile-PC-pool (566)

Definition			Solution												
			exchanges		reactions								transp		
Objective	Constraints	Comment	imports	exports	c	m	r	p	l	n	s	b	s-c	b-c	int
1,2-Diacylglycerol-Bile-PC-pool(c)	PIPES MCES	synthesis of cytosolic 1,2-Diacylglycerol-Bile-PC-pool	0.5 Glucose-6P(c) 0.11 Arachidonate(s) 0.83 Palmitate(s) 0.24 Oleate(s) 0.11 Stearate(s) 0.66 Linoleate(s) 0.05 Palmitolate(s) 4.5 ATP-energy(c) 1 NADH-redox-potential(c)	1.5 H_2O(s) 0.5 P_i(c) 1 1,2-Diacylglycerol-Bile-PC-pool(c)	25	-	-	-	-	-	-	-	7	-	-

ID	flux	reaction
r1252	0.05	Palmitolate(c) + ATP(c) + CoA(c) → (2E)-Hexadecenoyl-CoA(c) + AMP(c) + PPi(c)
r1190	0.06	Arachidonyl-CoA(c) + sn-Glycerol-3P(c) → 1-Acylglycerol-3P-arach(c) + CoA(c)
r2442	0.05	Palmitolate(s) → Palmitolate(c)
r1263	0.11	Arachidonate(c) + ATP(c) + CoA(c) → Arachidonyl-CoA(c) + AMP(c) + PPi(c)
r1254	0.11	Stearate(c) + ATP(c) + CoA(c) → Stearoyl-CoA(c) + AMP(c) + PPi(c)
r0930	0.11	Arachidonate(s) → Arachidonate(c)
r0192	0.5	Glucose-6P(c) ⇌ Fructose-6P(c)
r0256	0.5	Fructose-1,6PP(c) ⇌ DHAP(c) + GAP(c)
r1319	-4.5	ATP(c) + H2O(c) ⇌ ADP(c) + Pi(c) + ATP-energy(c)
r1256	0.24	ATP(c) + CoA(c) + Oleate(c) → Oleoyl-CoA(c) + AMP(c) + PPi(c)
r1284	0	268 1-Acylglycerol-3P-palmn(c) + 1209 1-Acylglycerol-3P-ol(c) + 3283 1-Acylglycerol-3P-lin(c) + 564 1-Acylglycerol-3P-arach(c) + 4140 1-Acylglycerol-3P-palm(c) + 550 1-Acylglycerol-3P-stea(c) → 10014 1-Acylglycerol-3P-Bile-PC-pool(c)
r1185	0.41	Palmitoyl-CoA(c) + sn-Glycerol-3P(c) → CoA(c) + 1-Acylglycerol-3P-palm(c)
r1285	0	4140 Palmitoyl-CoA(c) + 268 (2E)-Hexadecenoyl-CoA(c) + 550 Stearoyl-CoA(c) + 1209 Oleoyl-CoA(c) + 564 Arachidonyl-CoA(c) + 3283 Linoleoyl-CoA(c) → 10014 Acyl-CoA-Bile-PC-pool(c)
r1258	0.66	Linoleate(c) + ATP(c) + CoA(c) → AMP(c) + PPi(c) + Linoleoyl-CoA(c)
r1186	0.03	(2E)-Hexadecenoyl-CoA(c) + sn-Glycerol-3P(c) → 1-Acylglycerol-3P-palmn(c) + CoA(c)
r0736	0.5	ATP(c) + Fructose-6P(c) → ADP(c) + Fructose-1,6PP(c)
r0201	-1	DHAP(c) + NADH(c) ⇌ NAD+(c) + sn-Glycerol-3P(c)
r0030	2	ATP(c) + AMP(c) ⇌ 2 ADP(c)
r1300	0.24	Oleate(s) → Oleate(c)
r1286	-1	Acyl-CoA-Bile-PC-pool(c) + 1-Acylglycerol-3P-Bile-PC-pool(c) → Phosphatidate-Bile-PC-pool(c) + CoA(c)
r1187	0.05	Stearoyl-CoA(c) + sn-Glycerol-3P(c) → CoA(c) + 1-Acylglycerol-3P-stea(c)
r0935	0.83	Palmitate(s) → Palmitate(c)
r1287	1	Phosphatidate-Bile-PC-pool(c) + H2O(c) → Pi(c) + 1,2-Diacylglycerol-Bile-PC-pool(c)
r1188	0.12	Oleoyl-CoA(c) + sn-Glycerol-3P(c) → 1-Acylglycerol-3P-ol(c) + CoA(c)
r1189	0.33	Linoleoyl-CoA(c) + sn-Glycerol-3P(c) → 1-Acylglycerol-3P-lin(c) + CoA(c)
r0982	0.11	Stearate(s) → Stearate(c)
r0312	0.83	Palmitate(c) + ATP(c) + CoA(c) → Palmitoyl-CoA(c) + AMP(c) + PPi(c)
r0007	2	PPi(c) + H2O(c) → 2 Pi(c)
r1322	-1	NADH(c) ⇌ NAD+(c) + NADH-redox-potential(c)
r0849	-1.5	H2O(s) ⇌ H2O(c)
r0985	0.66	Linoleate(s) → Linoleate(c)
r0243	0.5	GAP(c) ⇌ DHAP(c)

1,2-Diacylglycerol-VLDL-PC-pool (567)

Definition			Solution												
			exchanges		reactions								transp		
Objective	Constraints	Comment	imports	exports	c	m	r	p	l	n	s	b	s-c	b-c	int
1,2-Diacylglycerol-VLDL-PC-pool(c)	PIPES MCES	synthesis of cytosolic 1,2-Diacylglycerol-VLDL-PC-pool	0.5 Glucose-6P(c) 0.17 Arachidonate(s) 0.7 Palmitate(s) 0.27 Oleate(s) 0.3 Stearate(s) 0.55 Linoleate(s) 4.5 ATP-energy(c) 1 NADH-redox-potential(c)	1.5 H_2O(s) 0.5 P_i(c) 1 1,2-Diacylglycerol-VLDL-PC-pool(c)	23	-	-	-	-	-	-	-	6	-	-

ID	flux	reaction
r1190	0.08	Arachidonyl-CoA(c) + sn-Glycerol-3P(c) → 1-Acylglycerol-3P-arach(c) + CoA(c)
r1263	0.17	Arachidonate(c) + ATP(c) + CoA(c) → Arachidonyl-CoA(c) + AMP(c) + PPi(c)
r1254	0.3	Stearate(c) + ATP(c) + CoA(c) → Stearoyl-CoA(c) + AMP(c) + PPi(c)
r1218	1	Phosphatidate-VLDL-PC-pool(c) + H2O(c) → 1,2-Diacylglycerol-VLDL-PC-pool(c) + Pi(c)
r0930	0.17	Arachidonate(s) → Arachidonate(c)
r0192	0.5	Glucose-6P(c) ⇌ Fructose-6P(c)
r0256	0.5	Fructose-1,6PP(c) ⇌ DHAP(c) + GAP(c)
r1319	-4.5	ATP(c) + H2O(c) ⇌ ADP(c) + Pi(c) + ATP-energy(c)
r1256	0.27	ATP(c) + CoA(c) + Oleate(c) → Oleoyl-CoA(c) + AMP(c) + PPi(c)
r1185	0.35	Palmitoyl-CoA(c) + sn-Glycerol-3P(c) → CoA(c) + 1-Acylglycerol-3P-palm(c)
r1258	0.55	Linoleate(c) + ATP(c) + CoA(c) → AMP(c) + PPi(c) + Linoleoyl-CoA(c)
r0736	0.5	ATP(c) + Fructose-6P(c) → ADP(c) + Fructose-1,6PP(c)
r0201	-1	DHAP(c) + NADH(c) ⇌ NAD+(c) + sn-Glycerol-3P(c)
r0030	2	ATP(c) + AMP(c) ⇌ 2 ADP(c)
r1300	0.27	Oleate(s) → Oleate(c)
r1187	0.15	Stearoyl-CoA(c) + sn-Glycerol-3P(c) → CoA(c) + 1-Acylglycerol-3P-stea(c)
r0935	0.7	Palmitate(s) → Palmitate(c)
r1188	0.14	Oleoyl-CoA(c) + sn-Glycerol-3P(c) → 1-Acylglycerol-3P-ol(c) + CoA(c)
r1212	-1	Acyl-CoA-VLDL-PC-pool(c) + 1-Acylglycerol-3P-VLDL-PC-pool(c) → Phosphatidate-VLDL-PC-pool(c) + CoA(c)
r1189	0.27	Linoleoyl-CoA(c) + sn-Glycerol-3P(c) → 1-Acylglycerol-3P-lin(c) + CoA(c)
r0982	0.3	Stearate(s) → Stearate(c)
r0312	0.7	Palmitate(c) + ATP(c) + CoA(c) → Palmitoyl-CoA(c) + AMP(c) + PPi(c)
r1199	0.0001	1170 1-Acylglycerol-3P-ol(c) + 2350 1-Acylglycerol-3P-lin(c) + 726 1-Acylglycerol-3P-arach(c) + 3010 1-Acylglycerol-3P-palm(c) + 1300 1-Acylglycerol-3P-stea(c) → 8556 1-Acylglycerol-3P-VLDL-PC-pool(c)
r0007	2	PPi(c) + H2O(c) → 2 Pi(c)
r1322	-1	NADH(c) ⇌ NAD+(c) + NADH-redox-potential(c)
r0849	-1.5	H2O(s) ⇌ H2O(c)
r1206	0.0001	3010 Palmitoyl-CoA(c) + 1300 Stearoyl-CoA(c) + 1170 Oleoyl-CoA(c) + 726 Arachidonyl-CoA(c) + 2350 Linoleoyl-CoA(c) → 8556 Acyl-CoA-VLDL-PC-pool(c)
r0985	0.55	Linoleate(s) → Linoleate(c)
r0243	0.5	GAP(c) ⇌ DHAP(c)

1,2-Diacylglycerol-VLDL-PE-pool (568)

Definition			Solution												
			exchanges		reactions								transp		
Objective	Constraints	Comment	imports	exports	c	m	r	p	l	n	s	b	s-c	b-c	int
1,2-Diacylglycerol-VLDL-PE-pool(c)	PIPES MCES	synthesis of cytosolic 1,2-Diacylglycerol-VLDL-PE-pool	0.5 Glucose-6P(c) 0.6 Arachidonate(s) 0.34 Palmitate(s) 0.17 Oleate(s) 0.54 Stearate(s) 0.36 Linoleate(s) 4.5 ATP-energy(c) 1 NADH-redox-potential(c)	1.5 H_2O(s) 0.5 P_i(c) 1 1,2-Diacylglycerol-VLDL-PE-pool(c)	23	-	-	-	-	-	-	-	6	-	-

ID	flux	reaction
r1207	0.0001	1260 Palmitoyl-CoA(c) + 2010 Stearoyl-CoA(c) + 628 Oleoyl-CoA(c) + 2220 Arachidonyl-CoA(c) + 1330 Linoleoyl-CoA(c) → 7448 Acyl-CoA-VLDL-PE-pool(c)
r1190	0.3	Arachidonyl-CoA(c) + sn-Glycerol-3P(c) → 1-Acylglycerol-3P-arach(c) + CoA(c)

Continued on next page

ID	flux	reaction
r1263	0.6	Arachidonate(c) + ATP(c) + CoA(c) → Arachidonyl-CoA(c) + AMP(c) + PPi(c)
r1254	0.54	Stearate(c) + ATP(c) + CoA(c) → Stearoyl-CoA(c) + AMP(c) + PPi(c)
r0930	0.6	Arachidonate(s) → Arachidonate(c)
r0192	0.5	Glucose-6P(c) ⇌ Fructose-6P(c)
r1219	1	Phosphatidate-VLDL-PE-pool(c) + H2O(c) → 1,2-Diacylglycerol-VLDL-PE-pool(c) + Pi(c)
r0256	0.5	Fructose-1,6PP(c) ⇌ DHAP(c) + GAP(c)
r1319	-4.5	ATP(c) + H2O(c) ⇌ ADP(c) + Pi(c) + ATP-energy(c)
r1256	0.17	ATP(c) + CoA(c) + Oleate(c) → Oleoyl-CoA(c) + AMP(c) + PPi(c)
r1185	0.17	Palmitoyl-CoA(c) + sn-Glycerol-3P(c) → CoA(c) + 1-Acylglycerol-3P-palm(c)
r1258	0.36	Linoleate(c) + ATP(c) + CoA(c) → AMP(c) + PPi(c) + Linoleoyl-CoA(c)
r1200	0.0001	628 1-Acylglycerol-3P-ol(c) + 1330 1-Acylglycerol-3P-lin(c) + 2220 1-Acylglycerol-3P-arach(c) + 1260 1-Acylglycerol-3P-palm(c) + 2010 1-Acylglycerol-3P-stea(c) → 7448 1-Acylglycerol-3P-VLDL-PE-pool(c)
r0736	0.5	ATP(c) + Fructose-6P(c) → ADP(c) + Fructose-1,6PP(c)
r0201	-1	DHAP(c) + NADH(c) ⇌ NAD+(c) + sn-Glycerol-3P(c)
r0030	2	ATP(c) + AMP(c) ⇌ 2 ADP(c)
r1300	0.17	Oleate(s) → Oleate(c)
r1187	0.27	Stearoyl-CoA(c) + sn-Glycerol-3P(c) → CoA(c) + 1-Acylglycerol-3P-stea(c)
r0935	0.34	Palmitate(s) → Palmitate(c)
r1188	0.08	Oleoyl-CoA(c) + sn-Glycerol-3P(c) → 1-Acylglycerol-3P-ol(c) + CoA(c)
r1189	0.18	Linoleoyl-CoA(c) + sn-Glycerol-3P(c) → 1-Acylglycerol-3P-lin(c) + CoA(c)
r0982	0.54	Stearate(s) → Stearate(c)
r0312	0.34	Palmitate(c) + ATP(c) + CoA(c) → Palmitoyl-CoA(c) + AMP(c) + PPi(c)
r1213	-1	Acyl-CoA-VLDL-PE-pool(c) + 1-Acylglycerol-3P-VLDL-PE-pool(c) → Phosphatidate-VLDL-PE-pool(c) + CoA(c)
r0007	2	PPi(c) + H2O(c) → 2 Pi(c)
r1322	-1	NADH(c) ⇌ NAD+(c) + NADH-redox-potential(c)
r0849	-1.5	H2O(s) ⇌ H2O(c)
r0985	0.36	Linoleate(s) → Linoleate(c)
r0243	0.5	GAP(c) ⇌ DHAP(c)

1,2-Diacylglycerol-VLDL-PI-pool (569)

Definition			Solution												
			exchanges		reactions								transp		
Objective	Constraints	Comment	imports	exports	c	m	r	p	l	n	s	b	s-c	b-c	int
1,2-Diacylglycerol-VLDL-PI-pool(c)	PIPES MCES	synthesis of cytosolic 1,2-Diacylglycerol-VLDL-PI-pool	0.5 Glucose-6P(c) 0.51 Arachidonate(s) 0.16 Palmitate(s) 0.32 Oleate(s) 0.85 Stearate(s) 0.16 Linoleate(s) 4.5 ATP-energy(c) 1 NADH-redox-potential(c)	1.5 H_2O(s) 0.5 P_i(c) 1 1,2-Diacylglycerol-VLDL-PI-pool(c)	23	-	-	-	-	-	-	-	6	-	-

ID	flux	reaction
r1190	0.25	Arachidonyl-CoA(c) + sn-Glycerol-3P(c) → 1-Acylglycerol-3P-arach(c) + CoA(c)
r1263	0.51	Arachidonate(c) + ATP(c) + CoA(c) → Arachidonyl-CoA(c) + AMP(c) + PPi(c)
r1254	0.85	Stearate(c) + ATP(c) + CoA(c) → Stearoyl-CoA(c) + AMP(c) + PPi(c)
r1209	0.0001	694 Palmitoyl-CoA(c) + 3640 Stearoyl-CoA(c) + 1360 Oleoyl-CoA(c) + 2170 Arachidonyl-CoA(c) + 694 Linoleoyl-CoA(c) → 8558 Acyl-CoA-VLDL-PI-pool(c)
r0930	0.51	Arachidonate(s) → Arachidonate(c)
r0192	0.5	Glucose-6P(c) ⇌ Fructose-6P(c)
r0256	0.5	Fructose-1,6PP(c) ⇌ DHAP(c) + GAP(c)
r1319	-4.5	ATP(c) + H2O(c) ⇌ ADP(c) + Pi(c) + ATP-energy(c)
r1256	0.32	ATP(c) + CoA(c) + Oleate(c) → Oleoyl-CoA(c) + AMP(c) + PPi(c)
r1185	0.08	Palmitoyl-CoA(c) + sn-Glycerol-3P(c) → CoA(c) + 1-Acylglycerol-3P-palm(c)
r1258	0.16	Linoleate(c) + ATP(c) + CoA(c) → AMP(c) + PPi(c) + Linoleoyl-CoA(c)
r0736	0.5	ATP(c) + Fructose-6P(c) → ADP(c) + Fructose-1,6PP(c)
r0201	-1	DHAP(c) + NADH(c) ⇌ NAD+(c) + sn-Glycerol-3P(c)
r0030	2	ATP(c) + AMP(c) ⇌ 2 ADP(c)

Continued on next page

ID	flux	reaction
r1300	0.32	Oleate(s) → Oleate(c)
r1187	0.43	Stearoyl-CoA(c) + sn-Glycerol-3P(c) → CoA(c) + 1-Acylglycerol-3P-stea(c)
r0935	0.16	Palmitate(s) → Palmitate(c)
r1202	0.0001	1360 1-Acylglycerol-3P-ol(c) + 694 1-Acylglycerol-3P-lin(c) + 2170 1-Acylglycerol-3P-arach(c) + 694 1-Acylglycerol-3P-palm(c) + 3640 1-Acylglycerol-3P-stea(c) → 8558 1-Acylglycerol-3P-VLDL-PI-pool(c)
r1188	0.16	Oleoyl-CoA(c) + sn-Glycerol-3P(c) → 1-Acylglycerol-3P-ol(c) + CoA(c)
r1221	1	Phosphatidate-VLDL-PI-pool(c) + H2O(c) → 1,2-Diacylglycerol-VLDL-PI-pool(c) + Pi(c)
r1189	0.08	Linoleoyl-CoA(c) + sn-Glycerol-3P(c) → 1-Acylglycerol-3P-lin(c) + CoA(c)
r0982	0.85	Stearate(s) → Stearate(c)
r0312	0.16	Palmitate(c) + ATP(c) + CoA(c) → Palmitoyl-CoA(c) + AMP(c) + PPi(c)
r0007	2	PPi(c) + H2O(c) → 2 Pi(c)
r1322	-1	NADH(c) ⇌ NAD+(c) + NADH-redox-potential(c)
r0849	-1.5	H2O(s) ⇌ H2O(c)
r1215	-1	1-Acylglycerol-3P-VLDL-PI-pool(c) + Acyl-CoA-VLDL-PI-pool(c) → Phosphatidate-VLDL-PI-pool(c) + CoA(c)
r0985	0.16	Linoleate(s) → Linoleate(c)
r0243	0.5	GAP(c) ⇌ DHAP(c)

1,2-Diacylglycerol-VLDL-PS-pool (570)

Definition			Solution												
			exchanges		reactions								transp		
Objective	Constraints	Comment	imports	exports	c	m	r	p	l	n	s	b	s-c	b-c	int
1,2-Diacylglycerol-VLDL-PS-pool(c)	PIPES MCES	synthesis of cytosolic 1,2-Diacylglycerol-VLDL-PS-pool	0.5 Glucose-6P(c) 0.53 Arachidonate(s) 0.09 Palmitate(s) 0.09 Oleate(s) 1.25 Stearate(s) 0.05 Linoleate(s) 4.5 ATP-energy(c) 1 NADH-redox-potential(c)	1.5 H_2O(s) 0.5 P_i(c) 1 1,2-Diacylglycerol-VLDL-PS-pool(c)	23	-	-	-	-	-	-	-	6	-	-

ID	flux	reaction
r1208	0.0001	345 Palmitoyl-CoA(c) + 4820 Stearoyl-CoA(c) + 332 Oleoyl-CoA(c) + 2050 Arachidonyl-CoA(c) + 181 Linoleoyl-CoA(c) → 7728 Acyl-CoA-VLDL-PS-pool(c)
r1190	0.27	Arachidonyl-CoA(c) + sn-Glycerol-3P(c) → 1-Acylglycerol-3P-arach(c) + CoA(c)
r1263	0.53	Arachidonate(c) + ATP(c) + CoA(c) → Arachidonyl-CoA(c) + AMP(c) + PPi(c)
r1254	1.25	Stearate(c) + ATP(c) + CoA(c) → Stearoyl-CoA(c) + AMP(c) + PPi(c)
r0930	0.53	Arachidonate(s) → Arachidonate(c)
r0192	0.5	Glucose-6P(c) ⇌ Fructose-6P(c)
r0256	0.5	Fructose-1,6PP(c) ⇌ DHAP(c) + GAP(c)
r1319	-4.5	ATP(c) + H2O(c) ⇌ ADP(c) + Pi(c) + ATP-energy(c)
r1256	0.09	ATP(c) + CoA(c) + Oleate(c) → Oleoyl-CoA(c) + AMP(c) + PPi(c)
r1185	0.04	Palmitoyl-CoA(c) + sn-Glycerol-3P(c) → CoA(c) + 1-Acylglycerol-3P-palm(c)
r1258	0.05	Linoleate(c) + ATP(c) + CoA(c) → AMP(c) + PPi(c) + Linoleoyl-CoA(c)
r0736	0.5	ATP(c) + Fructose-6P(c) → ADP(c) + Fructose-1,6PP(c)
r0201	-1	DHAP(c) + NADH(c) ⇌ NAD+(c) + sn-Glycerol-3P(c)
r0030	2	ATP(c) + AMP(c) ⇌ 2 ADP(c)
r1300	0.09	Oleate(s) → Oleate(c)
r1201	0.0001	332 1-Acylglycerol-3P-ol(c) + 181 1-Acylglycerol-3P-lin(c) + 2050 1-Acylglycerol-3P-arach(c) + 345 1-Acylglycerol-3P-palm(c) + 4820 1-Acylglycerol-3P-stea(c) → 7728 1-Acylglycerol-3P-VLDL-PS-pool(c)
r1187	0.62	Stearoyl-CoA(c) + sn-Glycerol-3P(c) → CoA(c) + 1-Acylglycerol-3P-stea(c)
r0935	0.09	Palmitate(s) → Palmitate(c)
r1220	1	Phosphatidate-VLDL-PS-pool(c) + H2O(c) → 1,2-Diacylglycerol-VLDL-PS-pool(c) + Pi(c)
r1188	0.04	Oleoyl-CoA(c) + sn-Glycerol-3P(c) → 1-Acylglycerol-3P-ol(c) + CoA(c)
r1189	0.02	Linoleoyl-CoA(c) + sn-Glycerol-3P(c) → 1-Acylglycerol-3P-lin(c) + CoA(c)
r0982	1.25	Stearate(s) → Stearate(c)
r0312	0.09	Palmitate(c) + ATP(c) + CoA(c) → Palmitoyl-CoA(c) + AMP(c) + PPi(c)
r0007	2	PPi(c) + H2O(c) → 2 Pi(c)
r1322	-1	NADH(c) ⇌ NAD+(c) + NADH-redox-potential(c)

Continued on next page

ID	flux	reaction
r1214	-1	Acyl-CoA-VLDL-PS-pool(c) + 1-Acylglycerol-3P-VLDL-PS-pool(c) → Phosphatidate-VLDL-PS-pool(c) + CoA(c)
r0849	-1.5	H2O(s) ⇌ H2O(c)
r0985	0.05	Linoleate(s) → Linoleate(c)
r0243	0.5	GAP(c) ⇌ DHAP(c)

1,2-Diacylglycerol-VLDL-SM-pool (571)

Definition			Solution												
			exchanges		reactions								transp		
Objective	Constraints	Comment	imports	exports	c	m	r	p	l	n	s	b	s-c	b-c	int
1,2-Diacylglycerol-VLDL-SM-pool(c)	PIPES MCES	synthesis of cytosolic 1,2-Diacylglycerol-VLDL-SM-pool	0.5 Glucose-6P(c) 0.03 Arachidonate(s) 1.42 Palmitate(s) 0.15 Oleate(s) 0.34 Stearate(s) 0.06 Linoleate(s) 4.5 ATP-energy(c) 1 NADH-redox-potential(c)	1.5 H_2O(s) 0.5 P_i(c) 1 1,2-Diacylglycerol-VLDL-SM-pool(c)	23	-	-	-	-	-	-	-	6	-	-

ID	flux	reaction
r1216	-1	Acyl-CoA-VLDL-SM-pool(c) + 1-Acylglycerol-3P-VLDL-SM-pool(c) → CoA(c) + Phosphatidate-VLDL-SM-pool(c)
r1190	0.02	Arachidonyl-CoA(c) + sn-Glycerol-3P(c) → 1-Acylglycerol-3P-arach(c) + CoA(c)
r1263	0.03	Arachidonate(c) + ATP(c) + CoA(c) → Arachidonyl-CoA(c) + AMP(c) + PPi(c)
r1254	0.34	Stearate(c) + ATP(c) + CoA(c) → Stearoyl-CoA(c) + AMP(c) + PPi(c)
r0930	0.03	Arachidonate(s) → Arachidonate(c)
r0192	0.5	Glucose-6P(c) ⇌ Fructose-6P(c)
r0256	0.5	Fructose-1,6PP(c) ⇌ DHAP(c) + GAP(c)
r1319	-4.5	ATP(c) + H2O(c) ⇌ ADP(c) + Pi(c) + ATP-energy(c)
r1256	0.15	ATP(c) + CoA(c) + Oleate(c) → Oleoyl-CoA(c) + AMP(c) + PPi(c)
r1185	0.71	Palmitoyl-CoA(c) + sn-Glycerol-3P(c) → CoA(c) + 1-Acylglycerol-3P-palm(c)
r1258	0.06	Linoleate(c) + ATP(c) + CoA(c) → AMP(c) + PPi(c) + Linoleoyl-CoA(c)
r0736	0.5	ATP(c) + Fructose-6P(c) → ADP(c) + Fructose-1,6PP(c)
r0201	-1	DHAP(c) + NADH(c) ⇌ NAD+(c) + sn-Glycerol-3P(c)
r0030	2	ATP(c) + AMP(c) ⇌ 2 ADP(c)
r1300	0.15	Oleate(s) → Oleate(c)
r1210	0.0001	6260 Palmitoyl-CoA(c) + 1490 Stearoyl-CoA(c) + 656 Oleoyl-CoA(c) + 147 Arachidonyl-CoA(c) + 270 Linoleoyl-CoA(c) ⇌ 8823 Acyl-CoA-VLDL-SM-pool(c)
r1187	0.17	Stearoyl-CoA(c) + sn-Glycerol-3P(c) → CoA(c) + 1-Acylglycerol-3P-stea(c)
r0935	1.42	Palmitate(s) → Palmitate(c)
r1188	0.07	Oleoyl-CoA(c) + sn-Glycerol-3P(c) → 1-Acylglycerol-3P-ol(c) + CoA(c)
r1203	0.0001	656 1-Acylglycerol-3P-ol(c) + 270 1-Acylglycerol-3P-lin(c) + 147 1-Acylglycerol-3P-arach(c) + 6260 1-Acylglycerol-3P-palm(c) + 1490 1-Acylglycerol-3P-stea(c) → 8823 1-Acylglycerol-3P-VLDL-SM-pool(c)
r1189	0.03	Linoleoyl-CoA(c) + sn-Glycerol-3P(c) → 1-Acylglycerol-3P-lin(c) + CoA(c)
r0982	0.34	Stearate(s) → Stearate(c)
r0312	1.42	Palmitate(c) + ATP(c) + CoA(c) → Palmitoyl-CoA(c) + AMP(c) + PPi(c)
r1222	1	H2O(c) + Phosphatidate-VLDL-SM-pool(c) → Pi(c) + 1,2-Diacylglycerol-VLDL-SM-pool(c)
r0007	2	PPi(c) + H2O(c) → 2 Pi(c)
r1322	-1	NADH(c) ⇌ NAD+(c) + NADH-redox-potential(c)
r0849	-1.5	H2O(s) ⇌ H2O(c)
r0985	0.06	Linoleate(s) → Linoleate(c)
r0243	0.5	GAP(c) ⇌ DHAP(c)

1,2-Diacylglycerol-VLDL-TG-pool (572)

Definition			Solution												
			exchanges		reactions								transp		
Objective	Constraints	Comment	imports	exports	c	m	r	p	l	n	s	b	s-c	b-c	int
1,2-Diacylglycerol-VLDL-TG-pool(c)	PIPES MCES	synthesis of cytosolic 1,2-Diacylglycerol-VLDL-TG-pool	0.5 Glucose-6P(c) 0.06 Arachidonate(s) 0.83 Palmitate(s) 0.6 Oleate(s) 0.13 Stearate(s) 0.39 Linoleate(s) 4.5 ATP-energy(c) 1 NADH-redox-potential(c)	1.5 H_2O(s) 0.5 P_i(c) 1 1,2-Diacylglycerol-VLDL-TG-pool(c)	23	-	-	-	-	-	-	-	6	-	-

ID	flux	reaction
r1217	1	Phosphatidate-VLDL-TG-pool(c) + H2O(c) → 1,2-Diacylglycerol-VLDL-TG-pool(c) + Pi(c)
r1190	0.003	Arachidonyl-CoA(c) + sn-Glycerol-3P(c) → 1-Acylglycerol-3P-arach(c) + CoA(c)
r1263	0.06	Arachidonate(c) + ATP(c) + CoA(c) → Arachidonyl-CoA(c) + AMP(c) + PPi(c)
r1254	0.13	Stearate(c) + ATP(c) + CoA(c) → Stearoyl-CoA(c) + AMP(c) + PPi(c)
r0930	0.06	Arachidonate(s) → Arachidonate(c)
r0192	0.5	Glucose-6P(c) ⇌ Fructose-6P(c)
r0256	0.5	Fructose-1,6PP(c) ⇌ DHAP(c) + GAP(c)
r1319	-4.5	ATP(c) + H2O(c) ⇌ ADP(c) + Pi(c) + ATP-energy(c)
r1256	0.6	ATP(c) + CoA(c) + Oleate(c) → Oleoyl-CoA(c) + AMP(c) + PPi(c)
r1185	0.7	Palmitoyl-CoA(c) + sn-Glycerol-3P(c) → CoA(c) + 1-Acylglycerol-3P-palm(c)
r1258	0.39	Linoleate(c) + ATP(c) + CoA(c) → AMP(c) + PPi(c) + Linoleoyl-CoA(c)
r0736	0.5	ATP(c) + Fructose-6P(c) → ADP(c) + Fructose-1,6PP(c)
r0201	-1	DHAP(c) + NADH(c) ⇌ NAD+(c) + sn-Glycerol-3P(c)
r0030	2	ATP(c) + AMP(c) ⇌ 2 ADP(c)
r1300	0.6	Oleate(s) → Oleate(c)
r1187	0.09	Stearoyl-CoA(c) + sn-Glycerol-3P(c) → CoA(c) + 1-Acylglycerol-3P-stea(c)
r0935	0.83	Palmitate(s) → Palmitate(c)
r1211	-1	Acyl-CoA-VLDL-TG2-pool(c) + 1-Acylglycerol-3P-VLDL-TG1-pool(c) → Phosphatidate-VLDL-TG-pool(c) + CoA(c)
r1188	0.13	Oleoyl-CoA(c) + sn-Glycerol-3P(c) → 1-Acylglycerol-3P-ol(c) + CoA(c)
r1198	0.0001	1220 1-Acylglycerol-3P-ol(c) + 741 1-Acylglycerol-3P-lin(c) + 24 1-Acylglycerol-3P-arach(c) + 6440 1-Acylglycerol-3P-palm(c) + 823 1-Acylglycerol-3P-stea(c) → 9248 1-Acylglycerol-3P-VLDL-TG1-pool(c)
r1189	0.08	Linoleoyl-CoA(c) + sn-Glycerol-3P(c) → 1-Acylglycerol-3P-lin(c) + CoA(c)
r0982	0.13	Stearate(s) → Stearate(c)
r0312	0.83	Palmitate(c) + ATP(c) + CoA(c) → Palmitoyl-CoA(c) + AMP(c) + PPi(c)
r1204	0.0001	1200 Palmitoyl-CoA(c) + 384 Stearoyl-CoA(c) + 4260 Oleoyl-CoA(c) + 521 Arachidonyl-CoA(c) + 2810 Linoleoyl-CoA(c) → 9175 Acyl-CoA-VLDL-TG2-pool(c)
r0007	2	PPi(c) + H2O(c) → 2 Pi(c)
r1322	-1	NADH(c) ⇌ NAD+(c) + NADH-redox-potential(c)
r0849	-1.5	H2O(s) ⇌ H2O(c)
r0985	0.39	Linoleate(s) → Linoleate(c)
r0243	0.5	GAP(c) ⇌ DHAP(c)

1,3DPG (573)

Definition			Solution												
			exchanges		reactions								transp		
Objective	Constraints	Comment	imports	exports	c	m	r	p	l	n	s	b	s-c	b-c	int
1,3DPG(c)	PIPES MCES	synthesis of cytosolic 1,3DPG	1.5 P_i(c) 0.5 Glucose-6P(c) 0.5 ATP-energy(c)	0.5 H_2O(s) 1 1,3DPG(c) 1 NADH-redox-potential(c)	7	-	-	-	-	-	-	-	1	-	-

ID	flux	reaction
r0253	1	GAP(c) + Pi(c) + NAD+(c) ⇌ 1,3DPG(c) + NADH(c)
r0192	0.5	Glucose-6P(c) ⇌ Fructose-6P(c)
r0256	0.5	Fructose-1,6PP(c) ⇌ DHAP(c) + GAP(c)
r1319	-0.5	ATP(c) + H2O(c) ⇌ ADP(c) + Pi(c) + ATP-energy(c)
r0736	0.5	ATP(c) + Fructose-6P(c) → ADP(c) + Fructose-1,6PP(c)
		Continued on next page

ID	flux	reaction
r1322	1	NADH(c) ⇌ NAD+(c) + NADH-redox-potential(c)
r0849	-0.5	H2O(s) ⇌ H2O(c)
r0243	-0.5	DHAP(c) ⇌ GAP(c)

1-Acylglycerol-3P-Bile-PC-pool (574)

Definition			Solution													
			exchanges		reactions								transp			
Objective	Constraints	Comment	imports	exports	c	m	r	p	l	n	s	b	s-c	b-c	int
1-Acylglycerol-3P-Bile-PC-pool(c)	PIPES MCES	synthesis of cytosolic 1-Acylglycerol-3P-Bile-PC-pool	0.5 P_i(c) 0.5 Glucose-6P(c) 0.06 Arachidonate(s) 0.41 Palmitate(s) 0.12 Oleate(s) 0.05 Stearate(s) 0.33 Linoleate(s) 0.03 Palmitolate(s) 2.5 ATP-energy(c) 1 NADH-redox-potential(c)	1.5 H_2O(s) 1 1-Acylglycerol-3P-Bile-PC-pool(c)	22	-	-	-	-	-	-	-	7	-	-

ID	flux	reaction
r1252	0.03	Palmitolate(c) + ATP(c) + CoA(c) → (2E)-Hexadecenoyl-CoA(c) + AMP(c) + PPi(c)
r1190	0.06	Arachidonyl-CoA(c) + sn-Glycerol-3P(c) → 1-Acylglycerol-3P-arach(c) + CoA(c)
r2442	0.03	Palmitolate(s) → Palmitolate(c)
r1263	0.06	Arachidonate(c) + ATP(c) + CoA(c) → Arachidonyl-CoA(c) + AMP(c) + PPi(c)
r1254	0.05	Stearate(c) + ATP(c) + CoA(c) → Stearoyl-CoA(c) + AMP(c) + PPi(c)
r0930	0.06	Arachidonate(s) → Arachidonate(c)
r0192	0.5	Glucose-6P(c) ⇌ Fructose-6P(c)
r0256	0.5	Fructose-1,6PP(c) ⇌ DHAP(c) + GAP(c)
r1319	-2.5	ATP(c) + H2O(c) ⇌ ADP(c) + Pi(c) + ATP-energy(c)
r1256	0.12	ATP(c) + CoA(c) + Oleate(c) → Oleoyl-CoA(c) + AMP(c) + PPi(c)
r1284	0	268 1-Acylglycerol-3P-palmn(c) + 1209 1-Acylglycerol-3P-ol(c) + 3283 1-Acylglycerol-3P-lin(c) + 564 1-Acylglycerol-3P-arach(c) + 4140 1-Acylglycerol-3P-palm(c) + 550 1-Acylglycerol-3P-stea(c) → 10014 1-Acylglycerol-3P-Bile-PC-pool(c)
r1185	0.41	Palmitoyl-CoA(c) + sn-Glycerol-3P(c) → CoA(c) + 1-Acylglycerol-3P-palm(c)
r1258	0.33	Linoleate(c) + ATP(c) + CoA(c) → AMP(c) + PPi(c) + Linoleoyl-CoA(c)
r1186	0.03	(2E)-Hexadecenoyl-CoA(c) + sn-Glycerol-3P(c) → 1-Acylglycerol-3P-palmn(c) + CoA(c)
r0736	0.5	ATP(c) + Fructose-6P(c) → ADP(c) + Fructose-1,6PP(c)
r0201	-1	DHAP(c) + NADH(c) ⇌ NAD+(c) + sn-Glycerol-3P(c)
r0030	1	ATP(c) + AMP(c) ⇌ 2 ADP(c)
r1300	0.12	Oleate(s) → Oleate(c)
r1187	0.05	Stearoyl-CoA(c) + sn-Glycerol-3P(c) → CoA(c) + 1-Acylglycerol-3P-stea(c)
r0935	0.41	Palmitate(s) → Palmitate(c)
r1188	0.12	Oleoyl-CoA(c) + sn-Glycerol-3P(c) → 1-Acylglycerol-3P-ol(c) + CoA(c)
r1189	0.33	Linoleoyl-CoA(c) + sn-Glycerol-3P(c) → 1-Acylglycerol-3P-lin(c) + CoA(c)
r0982	0.05	Stearate(s) → Stearate(c)
r0312	0.41	Palmitate(c) + ATP(c) + CoA(c) → Palmitoyl-CoA(c) + AMP(c) + PPi(c)
r0007	1	PPi(c) + H2O(c) → 2 Pi(c)
r1322	-1	NADH(c) ⇌ NAD+(c) + NADH-redox-potential(c)
r0849	-1.5	H2O(s) ⇌ H2O(c)
r0985	0.33	Linoleate(s) → Linoleate(c)
r0243	0.5	GAP(c) ⇌ DHAP(c)

1-Acylglycerol-3P-CL-pool (575)

Definition			Solution													
			exchanges		reactions								transp		
Objective	Constraints	Comment	imports	exports	c	m	r	p	l	n	s	b	s-c	b-c	int
1-Acylglycerol-3P-CL-pool(m)	PIPES MCES	synthesis of mitochondrial 1-Acylglycerol-3P-CL-pool	0.5 P_i(c) 0.5 Glucose-6P(c) 0.04 Palmitate(s) 0.14 Oleate(s) 0.02 Stearate(s) 0.75 Linoleate(s) 0.05 Palmitolate(s) 2.5 ATP-energy(c) 1 NADH-redox-potential(c) 1 Proton-gradient(m)	1.5 H_2O(s) 1 1-Acylglycerol-3P-CL-pool(m)	15	1	-	-	-	-	-	-	6	-	5

ID	flux	reaction
r1306	1	Acyl-CoA-CL-pool(c) → Acyl-CoA-CL-pool(m)
r1252	0.05	Palmitolate(c) + ATP(c) + CoA(c) → (2E)-Hexadecenoyl-CoA(c) + AMP(c) + PPi(c)
r1307	1	Acyl-CoA-CL-pool(m) + sn-Glycerol-3P(m) → CoA(m) + 1-Acylglycerol-3P-CL-pool(m)
r2442	0.05	Palmitolate(s) → Palmitolate(c)
r1254	0.02	Stearate(c) + ATP(c) + CoA(c) → Stearoyl-CoA(c) + AMP(c) + PPi(c)
r1047	1	Pi(c) + H+(PG)(c) → Pi(m) + H+(PG)(m)
r0192	0.5	Glucose-6P(c) ⇌ Fructose-6P(c)
r0256	0.5	Fructose-1,6PP(c) ⇌ DHAP(c) + GAP(c)
r1319	-2.5	ATP(c) + H2O(c) ⇌ ADP(c) + Pi(c) + ATP-energy(c)
r1256	0.14	ATP(c) + CoA(c) + Oleate(c) → Oleoyl-CoA(c) + AMP(c) + PPi(c)
r2472	1	sn-Glycerol-3P(c) + Pi(m) → sn-Glycerol-3P(m) + Pi(c)
r1258	0.75	Linoleate(c) + ATP(c) + CoA(c) → AMP(c) + PPi(c) + Linoleoyl-CoA(c)
r0736	0.5	ATP(c) + Fructose-6P(c) → ADP(c) + Fructose-1,6PP(c)
r0201	-1	DHAP(c) + NADH(c) ⇌ NAD+(c) + sn-Glycerol-3P(c)
r0030	1	ATP(c) + AMP(c) ⇌ 2 ADP(c)
r1300	0.14	Oleate(s) → Oleate(c)
r0935	0.04	Palmitate(s) → Palmitate(c)
r0863	-1	CoA(c) ⇌ CoA(m)
r0982	0.02	Stearate(s) → Stearate(c)
r0312	0.04	Palmitate(c) + ATP(c) + CoA(c) → Palmitoyl-CoA(c) + AMP(c) + PPi(c)
r1330	-1	H+(PG)(c) ⇌ H+(PG)(m) + Proton-gradient(m)
r0007	1	PPi(c) + H2O(c) → 2 Pi(c)
r1322	-1	NADH(c) ⇌ NAD+(c) + NADH-redox-potential(c)
r0849	-1.5	H2O(s) ⇌ H2O(c)
r1305	0.001	43 Palmitoyl-CoA(c) + 47 (2E)-Hexadecenoyl-CoA(c) + 19 Stearoyl-CoA(c) + 138 Oleoyl-CoA(c) + 738 Linoleoyl-CoA(c) → 985 Acyl-CoA-CL-pool(c)
r0985	0.75	Linoleate(s) → Linoleate(c)
r0243	0.5	GAP(c) ⇌ DHAP(c)

1-Acylglycerol-3P-VLDL-PC-pool (576)

Definition			Solution													
			exchanges		reactions								transp		
Objective	Constraints	Comment	imports	exports	c	m	r	p	l	n	s	b	s-c	b-c	int
1-Acylglycerol-3P-VLDL-PC-pool(c)	PIPES MCES	synthesis of cytosolic 1-Acylglycerol-3P-VLDL-PC-pool	0.5 P_i(c) 0.5 Glucose-6P(c) 0.08 Arachidonate(s) 0.35 Palmitate(s) 0.14 Oleate(s) 0.15 Stearate(s) 0.27 Linoleate(s) 2.5 ATP-energy(c) 1 NADH-redox-potential(c)	1.5 H_2O(s) 1 1-Acylglycerol-3P-VLDL-PC-pool(c)	20	-	-	-	-	-	-	-	6	-	-

ID	flux	reaction
r1190	0.08	Arachidonyl-CoA(c) + sn-Glycerol-3P(c) → 1-Acylglycerol-3P-arach(c) + CoA(c)
r1263	0.08	Arachidonate(c) + ATP(c) + CoA(c) → Arachidonyl-CoA(c) + AMP(c) + PPi(c)
r1254	0.15	Stearate(c) + ATP(c) + CoA(c) → Stearoyl-CoA(c) + AMP(c) + PPi(c)
		Continued on next page

ID	flux	reaction
r0930	0.08	Arachidonate(s) → Arachidonate(c)
r0192	0.5	Glucose-6P(c) ⇌ Fructose-6P(c)
r0256	0.5	Fructose-1,6PP(c) ⇌ DHAP(c) + GAP(c)
r1319	-2.5	ATP(c) + H2O(c) ⇌ ADP(c) + Pi(c) + ATP-energy(c)
r1256	0.14	ATP(c) + CoA(c) + Oleate(c) → Oleoyl-CoA(c) + AMP(c) + PPi(c)
r1185	0.35	Palmitoyl-CoA(c) + sn-Glycerol-3P(c) → CoA(c) + 1-Acylglycerol-3P-palm(c)
r1258	0.27	Linoleate(c) + ATP(c) + CoA(c) → AMP(c) + PPi(c) + Linoleoyl-CoA(c)
r0736	0.5	ATP(c) + Fructose-6P(c) → ADP(c) + Fructose-1,6PP(c)
r0201	-1	DHAP(c) + NADH(c) ⇌ NAD+(c) + sn-Glycerol-3P(c)
r0030	1	ATP(c) + AMP(c) ⇌ 2 ADP(c)
r1300	0.14	Oleate(s) → Oleate(c)
r1187	0.15	Stearoyl-CoA(c) + sn-Glycerol-3P(c) → CoA(c) + 1-Acylglycerol-3P-stea(c)
r0935	0.35	Palmitate(s) → Palmitate(c)
r1188	0.14	Oleoyl-CoA(c) + sn-Glycerol-3P(c) → 1-Acylglycerol-3P-ol(c) + CoA(c)
r1189	0.27	Linoleoyl-CoA(c) + sn-Glycerol-3P(c) → 1-Acylglycerol-3P-lin(c) + CoA(c)
r0982	0.15	Stearate(s) → Stearate(c)
r0312	0.35	Palmitate(c) + ATP(c) + CoA(c) → Palmitoyl-CoA(c) + AMP(c) + PPi(c)
r1199	0.0001	1170 1-Acylglycerol-3P-ol(c) + 2350 1-Acylglycerol-3P-lin(c) + 726 1-Acylglycerol-3P-arach(c) + 3010 1-Acylglycerol-3P-palm(c) + 1300 1-Acylglycerol-3P-stea(c) → 8556 1-Acylglycerol-3P-VLDL-PC-pool(c)
r0007	1	PPi(c) + H2O(c) → 2 Pi(c)
r1322	-1	NADH(c) ⇌ NAD+(c) + NADH-redox-potential(c)
r0849	-1.5	H2O(s) ⇌ H2O(c)
r0985	0.27	Linoleate(s) → Linoleate(c)
r0243	0.5	GAP(c) ⇌ DHAP(c)

1-Acylglycerol-3P-VLDL-PE-pool (577)

Definition			Solution												
			exchanges		reactions								transp		
Objective	Constraints	Comment	imports	exports	c	m	r	p	l	n	s	b	s-c	b-c	int
1-Acylglycerol-3P-VLDL-PE-pool(c)	PIPES MCES	synthesis of cytosolic 1-Acylglycerol-3P-VLDL-PE-pool	0.5 P_i(c) 0.5 Glucose-6P(c) 0.3 Arachidonate(s) 0.17 Palmitate(s) 0.08 Oleate(s) 0.27 Stearate(s) 0.18 Linoleate(s) 2.5 ATP-energy(c) 1 NADH-redox-potential(c)	1.5 H_2O(s) 1 1-Acylglycerol-3P-VLDL-PE-pool(c)	20	-	-	-	-	-	-	-	6	-	-

ID	flux	reaction
r1190	0.3	Arachidonyl-CoA(c) + sn-Glycerol-3P(c) → 1-Acylglycerol-3P-arach(c) + CoA(c)
r1263	0.3	Arachidonate(c) + ATP(c) + CoA(c) → Arachidonyl-CoA(c) + AMP(c) + PPi(c)
r1254	0.27	Stearate(c) + ATP(c) + CoA(c) → Stearoyl-CoA(c) + AMP(c) + PPi(c)
r0930	0.3	Arachidonate(s) → Arachidonate(c)
r0192	0.5	Glucose-6P(c) ⇌ Fructose-6P(c)
r0256	0.5	Fructose-1,6PP(c) ⇌ DHAP(c) + GAP(c)
r1319	-2.5	ATP(c) + H2O(c) ⇌ ADP(c) + Pi(c) + ATP-energy(c)
r1256	0.08	ATP(c) + CoA(c) + Oleate(c) → Oleoyl-CoA(c) + AMP(c) + PPi(c)
r1185	0.17	Palmitoyl-CoA(c) + sn-Glycerol-3P(c) → CoA(c) + 1-Acylglycerol-3P-palm(c)
r1258	0.18	Linoleate(c) + ATP(c) + CoA(c) → AMP(c) + PPi(c) + Linoleoyl-CoA(c)
r1200	0.0001	628 1-Acylglycerol-3P-ol(c) + 1330 1-Acylglycerol-3P-lin(c) + 2220 1-Acylglycerol-3P-arach(c) + 1260 1-Acylglycerol-3P-palm(c) + 2010 1-Acylglycerol-3P-stea(c) → 7448 1-Acylglycerol-3P-VLDL-PE-pool(c)
r0736	0.5	ATP(c) + Fructose-6P(c) → ADP(c) + Fructose-1,6PP(c)
r0201	-1	DHAP(c) + NADH(c) ⇌ NAD+(c) + sn-Glycerol-3P(c)
r0030	1	ATP(c) + AMP(c) ⇌ 2 ADP(c)
r1300	0.08	Oleate(s) → Oleate(c)
r1187	0.27	Stearoyl-CoA(c) + sn-Glycerol-3P(c) → CoA(c) + 1-Acylglycerol-3P-stea(c)
r0935	0.17	Palmitate(s) → Palmitate(c)

Continued on next page

ID	flux	reaction
r1188	0.08	Oleoyl-CoA(c) + sn-Glycerol-3P(c) → 1-Acylglycerol-3P-ol(c) + CoA(c)
r1189	0.18	Linoleoyl-CoA(c) + sn-Glycerol-3P(c) → 1-Acylglycerol-3P-lin(c) + CoA(c)
r0982	0.27	Stearate(s) → Stearate(c)
r0312	0.17	Palmitate(c) + ATP(c) + CoA(c) → Palmitoyl-CoA(c) + AMP(c) + PPi(c)
r0007	1	PPi(c) + H2O(c) → 2 Pi(c)
r1322	-1	NADH(c) ⇌ NAD+(c) + NADH-redox-potential(c)
r0849	-1.5	H2O(s) ⇌ H2O(c)
r0985	0.18	Linoleate(s) → Linoleate(c)
r0243	0.5	GAP(c) ⇌ DHAP(c)

1-Acylglycerol-3P-VLDL-PI-pool (578)

Definition			Solution												
			exchanges		reactions								transp		
Objective	Constraints	Comment	imports	exports	c	m	r	p	l	n	s	b	s-c	b-c	int
1-Acylglycerol-3P-VLDL-PI-pool(c)	PIPES MCES	synthesis of cytosolic 1-Acylglycerol-3P-VLDL-PI-pool	0.5 P_i(c) 0.5 Glucose-6P(c) 0.25 Arachidonate(s) 0.08 Palmitate(s) 0.16 Oleate(s) 0.43 Stearate(s) 0.08 Linoleate(s) 2.5 ATP-energy(c) 1 NADH-redox-potential(c)	1.5 H_2O(s) 1 1-Acylglycerol-3P-VLDL-PI-pool(c)	20	-	-	-	-	-	-	-	6	-	-

ID	flux	reaction
r1190	0.25	Arachidonyl-CoA(c) + sn-Glycerol-3P(c) → 1-Acylglycerol-3P-arach(c) + CoA(c)
r1263	0.25	Arachidonate(c) + ATP(c) + CoA(c) → Arachidonyl-CoA(c) + AMP(c) + PPi(c)
r1254	0.43	Stearate(c) + ATP(c) + CoA(c) → Stearoyl-CoA(c) + AMP(c) + PPi(c)
r0930	0.25	Arachidonate(s) → Arachidonate(c)
r0192	0.5	Glucose-6P(c) ⇌ Fructose-6P(c)
r0256	0.5	Fructose-1,6PP(c) ⇌ DHAP(c) + GAP(c)
r1319	-2.5	ATP(c) + H2O(c) ⇌ ADP(c) + Pi(c) + ATP-energy(c)
r1256	0.16	ATP(c) + CoA(c) + Oleate(c) → Oleoyl-CoA(c) + AMP(c) + PPi(c)
r1185	0.08	Palmitoyl-CoA(c) + sn-Glycerol-3P(c) → CoA(c) + 1-Acylglycerol-3P-palm(c)
r1258	0.08	Linoleate(c) + ATP(c) + CoA(c) → AMP(c) + PPi(c) + Linoleoyl-CoA(c)
r0736	0.5	ATP(c) + Fructose-6P(c) → ADP(c) + Fructose-1,6PP(c)
r0201	-1	DHAP(c) + NADH(c) ⇌ NAD+(c) + sn-Glycerol-3P(c)
r0030	1	ATP(c) + AMP(c) ⇌ 2 ADP(c)
r1300	0.16	Oleate(s) → Oleate(c)
r1187	0.43	Stearoyl-CoA(c) + sn-Glycerol-3P(c) → CoA(c) + 1-Acylglycerol-3P-stea(c)
r0935	0.08	Palmitate(s) → Palmitate(c)
r1202	0.0001	1360 1-Acylglycerol-3P-ol(c) + 694 1-Acylglycerol-3P-lin(c) + 2170 1-Acylglycerol-3P-arach(c) + 694 1-Acylglycerol-3P-palm(c) + 3640 1-Acylglycerol-3P-stea(c) → 8558 1-Acylglycerol-3P-VLDL-PI-pool(c)
r1188	0.16	Oleoyl-CoA(c) + sn-Glycerol-3P(c) → 1-Acylglycerol-3P-ol(c) + CoA(c)
r1189	0.08	Linoleoyl-CoA(c) + sn-Glycerol-3P(c) → 1-Acylglycerol-3P-lin(c) + CoA(c)
r0982	0.43	Stearate(s) → Stearate(c)
r0312	0.08	Palmitate(c) + ATP(c) + CoA(c) → Palmitoyl-CoA(c) + AMP(c) + PPi(c)
r0007	1	PPi(c) + H2O(c) → 2 Pi(c)
r1322	-1	NADH(c) ⇌ NAD+(c) + NADH-redox-potential(c)
r0849	-1.5	H2O(s) ⇌ H2O(c)
r0985	0.08	Linoleate(s) → Linoleate(c)
r0243	0.5	GAP(c) ⇌ DHAP(c)

1-Acylglycerol-3P-VLDL-PS-pool (579)

Definition			Solution												
			exchanges		reactions								transp		
Objective	Constraints	Comment	imports	exports	c	m	r	p	l	n	s	b	s-c	b-c	int
1-Acylglycerol-3P-VLDL-PS-pool(c)	PIPES MCES	synthesis of cytosolic 1-Acylglycerol-3P-VLDL-PS-pool	0.5 P_i(c) 0.5 Glucose-6P(c) 0.27 Arachidonate(s) 0.04 Palmitate(s) 0.04 Oleate(s) 0.62 Stearate(s) 0.02 Linoleate(s) 2.5 ATP-energy(c) 1 NADH-redox-potential(c)	1.5 H_2O(s) 1 1-Acylglycerol-3P-VLDL-PS-pool(c)	20	-	-	-	-	-	-	-	6	-	-

ID	flux	reaction
r1190	0.27	Arachidonyl-CoA(c) + sn-Glycerol-3P(c) → 1-Acylglycerol-3P-arach(c) + CoA(c)
r1263	0.27	Arachidonate(c) + ATP(c) + CoA(c) → Arachidonyl-CoA(c) + AMP(c) + PPi(c)
r1254	0.62	Stearate(c) + ATP(c) + CoA(c) → Stearoyl-CoA(c) + AMP(c) + PPi(c)
r0930	0.27	Arachidonate(s) → Arachidonate(c)
r0192	0.5	Glucose-6P(c) ⇌ Fructose-6P(c)
r0256	0.5	Fructose-1,6PP(c) ⇌ DHAP(c) + GAP(c)
r1319	-2.5	ATP(c) + H2O(c) ⇌ ADP(c) + Pi(c) + ATP-energy(c)
r1256	0.04	ATP(c) + CoA(c) + Oleate(c) → Oleoyl-CoA(c) + AMP(c) + PPi(c)
r1185	0.04	Palmitoyl-CoA(c) + sn-Glycerol-3P(c) → CoA(c) + 1-Acylglycerol-3P-palm(c)
r1258	0.02	Linoleate(c) + ATP(c) + CoA(c) → AMP(c) + PPi(c) + Linoleoyl-CoA(c)
r0736	0.5	ATP(c) + Fructose-6P(c) → ADP(c) + Fructose-1,6PP(c)
r0201	-1	DHAP(c) + NADH(c) ⇌ NAD+(c) + sn-Glycerol-3P(c)
r0030	1	ATP(c) + AMP(c) ⇌ 2 ADP(c)
r1300	0.04	Oleate(s) → Oleate(c)
r1201	0.0001	332 1-Acylglycerol-3P-ol(c) + 181 1-Acylglycerol-3P-lin(c) + 2050 1-Acylglycerol-3P-arach(c) + 345 1-Acylglycerol-3P-palm(c) + 4820 1-Acylglycerol-3P-stea(c) → 7728 1-Acylglycerol-3P-VLDL-PS-pool(c)
r1187	0.62	Stearoyl-CoA(c) + sn-Glycerol-3P(c) → CoA(c) + 1-Acylglycerol-3P-stea(c)
r0935	0.04	Palmitate(s) → Palmitate(c)
r1188	0.04	Oleoyl-CoA(c) + sn-Glycerol-3P(c) → 1-Acylglycerol-3P-ol(c) + CoA(c)
r1189	0.02	Linoleoyl-CoA(c) + sn-Glycerol-3P(c) → 1-Acylglycerol-3P-lin(c) + CoA(c)
r0982	0.62	Stearate(s) → Stearate(c)
r0312	0.04	Palmitate(c) + ATP(c) + CoA(c) → Palmitoyl-CoA(c) + AMP(c) + PPi(c)
r0007	1	PPi(c) + H2O(c) → 2 Pi(c)
r1322	-1	NADH(c) ⇌ NAD+(c) + NADH-redox-potential(c)
r0849	-1.5	H2O(s) ⇌ H2O(c)
r0985	0.02	Linoleate(s) → Linoleate(c)
r0243	0.5	GAP(c) ⇌ DHAP(c)

1-Acylglycerol-3P-VLDL-SM-pool (580)

Definition			Solution												
			exchanges		reactions								transp		
Objective	Constraints	Comment	imports	exports	c	m	r	p	l	n	s	b	s-c	b-c	int
1-Acylglycerol-3P-VLDL-SM-pool(c)	PIPES MCES	synthesis of cytosolic 1-Acylglycerol-3P-VLDL-SM-pool	0.5 P_i(c) 0.5 Glucose-6P(c) 0.02 Arachidonate(s) 0.71 Palmitate(s) 0.07 Oleate(s) 0.17 Stearate(s) 0.03 Linoleate(s) 2.5 ATP-energy(c) 1 NADH-redox-potential(c)	1.5 H_2O(s) 1 1-Acylglycerol-3P-VLDL-SM-pool(c)	20	-	-	-	-	-	-	-	6	-	-

ID	flux	reaction
r1190	0.02	Arachidonyl-CoA(c) + sn-Glycerol-3P(c) → 1-Acylglycerol-3P-arach(c) + CoA(c)
r1263	0.02	Arachidonate(c) + ATP(c) + CoA(c) → Arachidonyl-CoA(c) + AMP(c) + PPi(c)
r1254	0.17	Stearate(c) + ATP(c) + CoA(c) → Stearoyl-CoA(c) + AMP(c) + PPi(c)
r0930	0.02	Arachidonate(s) → Arachidonate(c)
r0192	0.5	Glucose-6P(c) ⇌ Fructose-6P(c)

Continued on next page

ID	flux	reaction
r0256	0.5	Fructose-1,6PP(c) ⇌ DHAP(c) + GAP(c)
r1319	-2.5	ATP(c) + H2O(c) ⇌ ADP(c) + Pi(c) + ATP-energy(c)
r1256	0.07	ATP(c) + CoA(c) + Oleate(c) → Oleoyl-CoA(c) + AMP(c) + PPi(c)
r1185	0.71	Palmitoyl-CoA(c) + sn-Glycerol-3P(c) → CoA(c) + 1-Acylglycerol-3P-palm(c)
r1258	0.03	Linoleate(c) + ATP(c) + CoA(c) → AMP(c) + PPi(c) + Linoleoyl-CoA(c)
r0736	0.5	ATP(c) + Fructose-6P(c) → ADP(c) + Fructose-1,6PP(c)
r0201	-1	DHAP(c) + NADH(c) ⇌ NAD+(c) + sn-Glycerol-3P(c)
r0030	1	ATP(c) + AMP(c) ⇌ 2 ADP(c)
r1300	0.07	Oleate(s) → Oleate(c)
r1187	0.17	Stearoyl-CoA(c) + sn-Glycerol-3P(c) → CoA(c) + 1-Acylglycerol-3P-stea(c)
r0935	0.71	Palmitate(s) → Palmitate(c)
r1188	0.07	Oleoyl-CoA(c) + sn-Glycerol-3P(c) → 1-Acylglycerol-3P-ol(c) + CoA(c)
r1203	0.0001	656 1-Acylglycerol-3P-ol(c) + 270 1-Acylglycerol-3P-lin(c) + 147 1-Acylglycerol-3P-arach(c) + 6260 1-Acylglycerol-3P-palm(c) + 1490 1-Acylglycerol-3P-stea(c) → 8823 1-Acylglycerol-3P-VLDL-SM-pool(c)
r1189	0.03	Linoleoyl-CoA(c) + sn-Glycerol-3P(c) → 1-Acylglycerol-3P-lin(c) + CoA(c)
r0982	0.17	Stearate(s) → Stearate(c)
r0312	0.71	Palmitate(c) + ATP(c) + CoA(c) → Palmitoyl-CoA(c) + AMP(c) + PPi(c)
r0007	1	PPi(c) + H2O(c) → 2 Pi(c)
r1322	-1	NADH(c) ⇌ NAD+(c) + NADH-redox-potential(c)
r0849	-1.5	H2O(s) ⇌ H2O(c)
r0985	0.03	Linoleate(s) → Linoleate(c)
r0243	0.5	GAP(c) ⇌ DHAP(c)

1-Acylglycerol-3P-VLDL-TG1-pool (581)

Definition			Solution												
			exchanges		reactions								transp		
Objective	Constraints	Comment	imports	exports	c	m	r	p	l	n	s	b	s-c	b-c	int
1-Acylglycerol-3P-VLDL-TG1-pool(c)	PIPES MCES	synthesis of cytosolic 1-Acylglycerol-3P-VLDL-TG1-pool	0.5 P_i(c) 0.5 Glucose-6P(c) 0.003 Arachidonate(s) 0.7 Palmitate(s) 0.13 Oleate(s) 0.09 Stearate(s) 0.08 Linoleate(s) 2.5 ATP-energy(c) 1 NADH-redox-potential(c)	1.5 H_2O(s) 1 1-Acylglycerol-3P-VLDL-TG1-pool(c)	20	-	-	-	-	-	-	-	6	-	-

ID	flux	reaction
r1190	0.003	Arachidonyl-CoA(c) + sn-Glycerol-3P(c) → 1-Acylglycerol-3P-arach(c) + CoA(c)
r1263	0.003	Arachidonate(c) + ATP(c) + CoA(c) → Arachidonyl-CoA(c) + AMP(c) + PPi(c)
r1254	0.09	Stearate(c) + ATP(c) + CoA(c) → Stearoyl-CoA(c) + AMP(c) + PPi(c)
r0930	0.003	Arachidonate(s) → Arachidonate(c)
r0192	0.5	Glucose-6P(c) ⇌ Fructose-6P(c)
r0256	0.5	Fructose-1,6PP(c) ⇌ DHAP(c) + GAP(c)
r1319	-2.5	ATP(c) + H2O(c) ⇌ ADP(c) + Pi(c) + ATP-energy(c)
r1256	0.13	ATP(c) + CoA(c) + Oleate(c) → Oleoyl-CoA(c) + AMP(c) + PPi(c)
r1185	0.7	Palmitoyl-CoA(c) + sn-Glycerol-3P(c) → CoA(c) + 1-Acylglycerol-3P-palm(c)
r1258	0.08	Linoleate(c) + ATP(c) + CoA(c) → AMP(c) + PPi(c) + Linoleoyl-CoA(c)
r0736	0.5	ATP(c) + Fructose-6P(c) → ADP(c) + Fructose-1,6PP(c)
r0201	-1	DHAP(c) + NADH(c) ⇌ NAD+(c) + sn-Glycerol-3P(c)
r0030	1	ATP(c) + AMP(c) ⇌ 2 ADP(c)
r1300	0.13	Oleate(s) → Oleate(c)
r1187	0.09	Stearoyl-CoA(c) + sn-Glycerol-3P(c) → CoA(c) + 1-Acylglycerol-3P-stea(c)
r0935	0.7	Palmitate(s) → Palmitate(c)
r1188	0.13	Oleoyl-CoA(c) + sn-Glycerol-3P(c) → 1-Acylglycerol-3P-ol(c) + CoA(c)
r1198	0.0001	1220 1-Acylglycerol-3P-ol(c) + 741 1-Acylglycerol-3P-lin(c) + 24 1-Acylglycerol-3P-arach(c) + 6440 1-Acylglycerol-3P-palm(c) + 823 1-Acylglycerol-3P-stea(c) → 9248 1-Acylglycerol-3P-VLDL-TG1-pool(c)
r1189	0.08	Linoleoyl-CoA(c) + sn-Glycerol-3P(c) → 1-Acylglycerol-3P-lin(c) + CoA(c)

Continued on next page

ID	flux	reaction
r0982	0.09	Stearate(s) → Stearate(c)
r0312	0.7	Palmitate(c) + ATP(c) + CoA(c) → Palmitoyl-CoA(c) + AMP(c) + PPi(c)
r0007	1	PPi(c) + H2O(c) → 2 Pi(c)
r1322	-1	NADH(c) ⇌ NAD+(c) + NADH-redox-potential(c)
r0849	-1.5	H2O(s) ⇌ H2O(c)
r0985	0.08	Linoleate(s) → Linoleate(c)
r0243	0.5	GAP(c) ⇌ DHAP(c)

1-Acylglycerol-3P-arach (582)

Definition			Solution												
			exchanges		reactions								transp		
Objective	Constraints	Comment	imports	exports	c	m	r	p	l	n	s	b	s-c	b-c	int
1-Acylglycerol-3P-arach(c)	PIPES MCES	synthesis of cytosolic 1-Acylglycerol-3P-arach	0.5 P_i(c) 0.5 Glucose-6P(c) 1 Arachidonate(s) 2.5 ATP-energy(c) 1 NADH-redox-potential(c)	1.5 H_2O(s) 1 1-Acylglycerol-3P-arach(c)	11	-	-	-	-	-	-	-	2	-	-

ID	flux	reaction
r1190	1	Arachidonyl-CoA(c) + sn-Glycerol-3P(c) → 1-Acylglycerol-3P-arach(c) + CoA(c)
r1263	1	Arachidonate(c) + ATP(c) + CoA(c) → Arachidonyl-CoA(c) + AMP(c) + PPi(c)
r0930	1	Arachidonate(s) → Arachidonate(c)
r0192	0.5	Glucose-6P(c) ⇌ Fructose-6P(c)
r0256	0.5	Fructose-1,6PP(c) ⇌ DHAP(c) + GAP(c)
r1319	-2.5	ATP(c) + H2O(c) ⇌ ADP(c) + Pi(c) + ATP-energy(c)
r0736	0.5	ATP(c) + Fructose-6P(c) → ADP(c) + Fructose-1,6PP(c)
r0201	-1	DHAP(c) + NADH(c) ⇌ NAD+(c) + sn-Glycerol-3P(c)
r0030	1	ATP(c) + AMP(c) ⇌ 2 ADP(c)
r0007	1	PPi(c) + H2O(c) → 2 Pi(c)
r1322	-1	NADH(c) ⇌ NAD+(c) + NADH-redox-potential(c)
r0849	-1.5	H2O(s) ⇌ H2O(c)
r0243	0.5	GAP(c) ⇌ DHAP(c)

1-Acylglycerol-3P-lin (583)

Definition			Solution												
			exchanges		reactions								transp		
Objective	Constraints	Comment	imports	exports	c	m	r	p	l	n	s	b	s-c	b-c	int
1-Acylglycerol-3P-lin(c)	PIPES MCES	synthesis of cytosolic 1-Acylglycerol-3P-lin	0.5 P_i(c) 0.5 Glucose-6P(c) 1 Linoleate(s) 2.5 ATP-energy(c) 1 NADH-redox-potential(c)	1.5 H_2O(s) 1 1-Acylglycerol-3P-lin(c)	11	-	-	-	-	-	-	-	2	-	-

ID	flux	reaction
r0192	0.5	Glucose-6P(c) ⇌ Fructose-6P(c)
r0256	0.5	Fructose-1,6PP(c) ⇌ DHAP(c) + GAP(c)
r1319	-2.5	ATP(c) + H2O(c) ⇌ ADP(c) + Pi(c) + ATP-energy(c)
r1258	1	Linoleate(c) + ATP(c) + CoA(c) → AMP(c) + PPi(c) + Linoleoyl-CoA(c)
r0736	0.5	ATP(c) + Fructose-6P(c) → ADP(c) + Fructose-1,6PP(c)
r0201	-1	DHAP(c) + NADH(c) ⇌ NAD+(c) + sn-Glycerol-3P(c)
r0030	1	ATP(c) + AMP(c) ⇌ 2 ADP(c)
r1189	1	Linoleoyl-CoA(c) + sn-Glycerol-3P(c) → 1-Acylglycerol-3P-lin(c) + CoA(c)
r0007	1	PPi(c) + H2O(c) → 2 Pi(c)
r1322	-1	NADH(c) ⇌ NAD+(c) + NADH-redox-potential(c)

Continued on next page

ID	flux	reaction
r0849	-1.5	H2O(s) ⇌ H2O(c)
r0985	1	Linoleate(s) → Linoleate(c)
r0243	0.5	GAP(c) ⇌ DHAP(c)

1-Acylglycerol-3P-ol (584)

Definition			Solution												
			exchanges		reactions								transp		
Objective	Constraints	Comment	imports	exports	c	m	r	p	l	n	s	b	s-c	b-c	int
1-Acylglycerol-3P-ol(c)	PIPES MCES	synthesis of cytosolic 1-Acylglycerol-3P-ol	0.5 P_i(c) 0.5 Glucose-6P(c) 1 Oleate(s) 2.5 ATP-energy(c) 1 NADH-redox-potential(c)	1.5 H_2O(s) 1 1-Acylglycerol-3P-ol(c)	11	-	-	-	-	-	-	-	2	-	-

ID	flux	reaction
r0192	0.5	Glucose-6P(c) ⇌ Fructose-6P(c)
r0256	0.5	Fructose-1,6PP(c) ⇌ DHAP(c) + GAP(c)
r1319	-2.5	ATP(c) + H2O(c) ⇌ ADP(c) + Pi(c) + ATP-energy(c)
r1256	1	ATP(c) + CoA(c) + Oleate(c) → Oleoyl-CoA(c) + AMP(c) + PPi(c)
r0736	0.5	ATP(c) + Fructose-6P(c) → ADP(c) + Fructose-1,6PP(c)
r0201	-1	DHAP(c) + NADH(c) ⇌ NAD+(c) + sn-Glycerol-3P(c)
r0030	1	ATP(c) + AMP(c) ⇌ 2 ADP(c)
r1300	1	Oleate(s) → Oleate(c)
r1188	1	Oleoyl-CoA(c) + sn-Glycerol-3P(c) → 1-Acylglycerol-3P-ol(c) + CoA(c)
r0007	1	PPi(c) + H2O(c) → 2 Pi(c)
r1322	-1	NADH(c) ⇌ NAD+(c) + NADH-redox-potential(c)
r0849	-1.5	H2O(s) ⇌ H2O(c)
r0243	0.5	GAP(c) ⇌ DHAP(c)

1-Acylglycerol-3P-palm (585)

Definition			Solution												
			exchanges		reactions								transp		
Objective	Constraints	Comment	imports	exports	c	m	r	p	l	n	s	b	s-c	b-c	int
1-Acylglycerol-3P-palm(c)	PIPES MCES	synthesis of cytosolic 1-Acylglycerol-3P-palm	0.5 P_i(c) 0.5 Glucose-6P(c) 1 Palmitate(s) 2.5 ATP-energy(c) 1 NADH-redox-potential(c)	1.5 H_2O(s) 1 1-Acylglycerol-3P-palm(c)	11	-	-	-	-	-	-	-	2	-	-

ID	flux	reaction
r0192	0.5	Glucose-6P(c) ⇌ Fructose-6P(c)
r0256	0.5	Fructose-1,6PP(c) ⇌ DHAP(c) + GAP(c)
r1319	-2.5	ATP(c) + H2O(c) ⇌ ADP(c) + Pi(c) + ATP-energy(c)
r1185	1	Palmitoyl-CoA(c) + sn-Glycerol-3P(c) → CoA(c) + 1-Acylglycerol-3P-palm(c)
r0736	0.5	ATP(c) + Fructose-6P(c) → ADP(c) + Fructose-1,6PP(c)
r0201	-1	DHAP(c) + NADH(c) ⇌ NAD+(c) + sn-Glycerol-3P(c)
r0030	1	ATP(c) + AMP(c) ⇌ 2 ADP(c)
r0935	1	Palmitate(s) → Palmitate(c)
r0312	1	Palmitate(c) + ATP(c) + CoA(c) → Palmitoyl-CoA(c) + AMP(c) + PPi(c)
r0007	1	PPi(c) + H2O(c) → 2 Pi(c)
r1322	-1	NADH(c) ⇌ NAD+(c) + NADH-redox-potential(c)
r0849	-1.5	H2O(s) ⇌ H2O(c)
r0243	0.5	GAP(c) ⇌ DHAP(c)

1-Acylglycerol-3P-palmn (586)

Definition			Solution												
			exchanges		reactions								transp		
Objective	Constraints	Comment	imports	exports	c	m	r	p	l	n	s	b	s-c	b-c	int
1-Acylglycerol-3P-palmn(c)	PIPES MCES	synthesis of cytosolic 1-Acylglycerol-3P-palmn	0.5 P_i(c) 0.5 Glucose-6P(c) 1 Palmitolate(s) 2.5 ATP-energy(c) 1 NADH-redox-potential(c)	1.5 H_2O(s) 1 1-Acylglycerol-3P-palmn(c)	11	-	-	-	-	-	-	-	2	-	-

ID	flux	reaction
r1252	1	Palmitolate(c) + ATP(c) + CoA(c) → (2E)-Hexadecenoyl-CoA(c) + AMP(c) + PPi(c)
r2442	1	Palmitolate(s) → Palmitolate(c)
r0192	0.5	Glucose-6P(c) ⇌ Fructose-6P(c)
r0256	0.5	Fructose-1,6PP(c) ⇌ DHAP(c) + GAP(c)
r1319	-2.5	ATP(c) + H2O(c) ⇌ ADP(c) + Pi(c) + ATP-energy(c)
r1186	1	(2E)-Hexadecenoyl-CoA(c) + sn-Glycerol-3P(c) → 1-Acylglycerol-3P-palmn(c) + CoA(c)
r0736	0.5	ATP(c) + Fructose-6P(c) → ADP(c) + Fructose-1,6PP(c)
r0201	-1	DHAP(c) + NADH(c) ⇌ NAD+(c) + sn-Glycerol-3P(c)
r0030	1	ATP(c) + AMP(c) ⇌ 2 ADP(c)
r0007	1	PPi(c) + H2O(c) → 2 Pi(c)
r1322	-1	NADH(c) ⇌ NAD+(c) + NADH-redox-potential(c)
r0849	-1.5	H2O(s) ⇌ H2O(c)
r0243	0.5	GAP(c) ⇌ DHAP(c)

1-Acylglycerol-3P-stea (587)

Definition			Solution												
			exchanges		reactions								transp		
Objective	Constraints	Comment	imports	exports	c	m	r	p	l	n	s	b	s-c	b-c	int
1-Acylglycerol-3P-stea(c)	PIPES MCES	synthesis of cytosolic 1-Acylglycerol-3P-stea	0.5 P_i(c) 0.5 Glucose-6P(c) 1 Stearate(s) 2.5 ATP-energy(c) 1 NADH-redox-potential(c)	1.5 H_2O(s) 1 1-Acylglycerol-3P-stea(c)	11	-	-	-	-	-	-	-	2	-	-

ID	flux	reaction
r1254	1	Stearate(c) + ATP(c) + CoA(c) → Stearoyl-CoA(c) + AMP(c) + PPi(c)
r0192	0.5	Glucose-6P(c) ⇌ Fructose-6P(c)
r0256	0.5	Fructose-1,6PP(c) ⇌ DHAP(c) + GAP(c)
r1319	-2.5	ATP(c) + H2O(c) ⇌ ADP(c) + Pi(c) + ATP-energy(c)
r0736	0.5	ATP(c) + Fructose-6P(c) → ADP(c) + Fructose-1,6PP(c)
r0201	-1	DHAP(c) + NADH(c) ⇌ NAD+(c) + sn-Glycerol-3P(c)
r0030	1	ATP(c) + AMP(c) ⇌ 2 ADP(c)
r1187	1	Stearoyl-CoA(c) + sn-Glycerol-3P(c) → CoA(c) + 1-Acylglycerol-3P-stea(c)
r0982	1	Stearate(s) → Stearate(c)
r0007	1	PPi(c) + H2O(c) → 2 Pi(c)
r1322	-1	NADH(c) ⇌ NAD+(c) + NADH-redox-potential(c)
r0849	-1.5	H2O(s) ⇌ H2O(c)
r0243	0.5	GAP(c) ⇌ DHAP(c)

1-Acylglycerol-VLDL-PC-pool (588)

Definition			Solution												
			exchanges		reactions								transp		
Objective	Constraints	Comment	imports	exports	c	m	r	p	l	n	s	b	s-c	b-c	int
1-Acylglycerol-VLDL-PC-pool(c)	PIPES MCES	synthesis of cytosolic 1-Acylglycerol-VLDL-PC-pool	0.5 Glucose-6P(c) 0.08 Arachidonate(s) 0.35 Palmitate(s) 0.14 Oleate(s) 0.15 Stearate(s) 0.27 Linoleate(s) 4.5 ATP-energy(c) 1 NADH-redox-potential(c)	0.5 H_2O(s) 0.5 P_i(c) 1 1-Acylglycerol-VLDL-PC-pool(c)	25	-	-	-	-	-	-	-	6	-	-

ID	flux	reaction
r1226	1	H2O(c) + 1,2-Diacylglycerol-VLDL-PC-pool(c) → 1-Acylglycerol-VLDL-PC-pool(c) + Fatty-acid-VLDL-PC-pool(c)
r1190	0.08	Arachidonyl-CoA(c) + sn-Glycerol-3P(c) → 1-Acylglycerol-3P-arach(c) + CoA(c)
r1263	0.17	Arachidonate(c) + ATP(c) + CoA(c) → Arachidonyl-CoA(c) + AMP(c) + PPi(c)
r1254	0.3	Stearate(c) + ATP(c) + CoA(c) → Stearoyl-CoA(c) + AMP(c) + PPi(c)
r1245	-0.0001	3010 Palmitate(c) + 1300 Stearate(c) + 2350 Linoleate(c) + 726 Arachidonate(c) + 1170 Oleate(c) ⇌ 8556 Fatty-acid-VLDL-PC-pool(c)
r1218	1	Phosphatidate-VLDL-PC-pool(c) + H2O(c) → 1,2-Diacylglycerol-VLDL-PC-pool(c) + Pi(c)
r0930	0.08	Arachidonate(s) → Arachidonate(c)
r0192	0.5	Glucose-6P(c) ⇌ Fructose-6P(c)
r0256	0.5	Fructose-1,6PP(c) ⇌ DHAP(c) + GAP(c)
r1319	-4.5	ATP(c) + H2O(c) ⇌ ADP(c) + Pi(c) + ATP-energy(c)
r1256	0.27	ATP(c) + CoA(c) + Oleate(c) → Oleoyl-CoA(c) + AMP(c) + PPi(c)
r1185	0.35	Palmitoyl-CoA(c) + sn-Glycerol-3P(c) → CoA(c) + 1-Acylglycerol-3P-palm(c)
r1258	0.55	Linoleate(c) + ATP(c) + CoA(c) → AMP(c) + PPi(c) + Linoleoyl-CoA(c)
r0736	0.5	ATP(c) + Fructose-6P(c) → ADP(c) + Fructose-1,6PP(c)
r0201	-1	DHAP(c) + NADH(c) ⇌ NAD+(c) + sn-Glycerol-3P(c)
r0030	2	ATP(c) + AMP(c) ⇌ 2 ADP(c)
r1300	0.14	Oleate(s) → Oleate(c)
r1187	0.15	Stearoyl-CoA(c) + sn-Glycerol-3P(c) → CoA(c) + 1-Acylglycerol-3P-stea(c)
r0935	0.35	Palmitate(s) → Palmitate(c)
r1188	0.14	Oleoyl-CoA(c) + sn-Glycerol-3P(c) → 1-Acylglycerol-3P-ol(c) + CoA(c)
r1212	-1	Acyl-CoA-VLDL-PC-pool(c) + 1-Acylglycerol-3P-VLDL-PC-pool(c) → Phosphatidate-VLDL-PC-pool(c) + CoA(c)
r1189	0.27	Linoleoyl-CoA(c) + sn-Glycerol-3P(c) → 1-Acylglycerol-3P-lin(c) + CoA(c)
r0982	0.15	Stearate(s) → Stearate(c)
r0312	0.7	Palmitate(c) + ATP(c) + CoA(c) → Palmitoyl-CoA(c) + AMP(c) + PPi(c)
r1199	0.0001	1170 1-Acylglycerol-3P-ol(c) + 2350 1-Acylglycerol-3P-lin(c) + 726 1-Acylglycerol-3P-arach(c) + 3010 1-Acylglycerol-3P-palm(c) + 1300 1-Acylglycerol-3P-stea(c) → 8556 1-Acylglycerol-3P-VLDL-PC-pool(c)
r0007	2	PPi(c) + H2O(c) → 2 Pi(c)
r1322	-1	NADH(c) ⇌ NAD+(c) + NADH-redox-potential(c)
r0849	-0.5	H2O(s) ⇌ H2O(c)
r1206	0.0001	3010 Palmitoyl-CoA(c) + 1300 Stearoyl-CoA(c) + 1170 Oleoyl-CoA(c) + 726 Arachidonyl-CoA(c) + 2350 Linoleoyl-CoA(c) → 8556 Acyl-CoA-VLDL-PC-pool(c)
r0985	0.27	Linoleate(s) → Linoleate(c)
r0243	0.5	GAP(c) ⇌ DHAP(c)

1-Acylglycerol-VLDL-PE-pool (589)

Definition			Solution												
			exchanges		reactions								transp		
Objective	Constraints	Comment	imports	exports	c	m	r	p	l	n	s	b	s-c	b-c	int
1-Acylglycerol-VLDL-PE-pool(c)	PIPES MCES	synthesis of cytosolic 1-Acylglycerol-VLDL-PE-pool	0.5 Glucose-6P(c) 0.3 Arachidonate(s) 0.17 Palmitate(s) 0.08 Oleate(s) 0.27 Stearate(s) 0.18 Linoleate(s) 4.5 ATP-energy(c) 1 NADH-redox-potential(c)	0.5 H_2O(s) 0.5 P_i(c) 1 1-Acylglycerol-VLDL-PE-pool(c)	25	-	-	-	-	-	-	-	6	-	-

ID	flux	reaction
r1207	0.0001	1260 Palmitoyl-CoA(c) + 2010 Stearoyl-CoA(c) + 628 Oleoyl-CoA(c) + 2220 Arachidonyl-CoA(c) + 1330 Linoleoyl-CoA(c) → 7448 Acyl-CoA-VLDL-PE-pool(c)
r1190	0.3	Arachidonyl-CoA(c) + sn-Glycerol-3P(c) → 1-Acylglycerol-3P-arach(c) + CoA(c)
r1263	0.6	Arachidonate(c) + ATP(c) + CoA(c) → Arachidonyl-CoA(c) + AMP(c) + PPi(c)
r1254	0.54	Stearate(c) + ATP(c) + CoA(c) → Stearoyl-CoA(c) + AMP(c) + PPi(c)
r1227	1	H2O(c) + 1,2-Diacylglycerol-VLDL-PE-pool(c) → 1-Acylglycerol-VLDL-PE-pool(c) + Fatty-acid-VLDL-PE-pool(c)
r0930	0.3	Arachidonate(s) → Arachidonate(c)
r0192	0.5	Glucose-6P(c) ⇌ Fructose-6P(c)
r1219	1	Phosphatidate-VLDL-PE-pool(c) + H2O(c) → 1,2-Diacylglycerol-VLDL-PE-pool(c) + Pi(c)
r0256	0.5	Fructose-1,6PP(c) ⇌ DHAP(c) + GAP(c)
r1319	-4.5	ATP(c) + H2O(c) ⇌ ADP(c) + Pi(c) + ATP-energy(c)
r1256	0.17	ATP(c) + CoA(c) + Oleate(c) → Oleoyl-CoA(c) + AMP(c) + PPi(c)
r1247	-0.0001	1260 Palmitate(c) + 2010 Stearate(c) + 1330 Linoleate(c) + 2220 Arachidonate(c) + 628 Oleate(c) ← 7448 Fatty-acid-VLDL-PE-pool(c)
r1185	0.17	Palmitoyl-CoA(c) + sn-Glycerol-3P(c) → CoA(c) + 1-Acylglycerol-3P-palm(c)
r1258	0.36	Linoleate(c) + ATP(c) + CoA(c) → AMP(c) + PPi(c) + Linoleoyl-CoA(c)
r1200	0.0001	628 1-Acylglycerol-3P-ol(c) + 1330 1-Acylglycerol-3P-lin(c) + 2220 1-Acylglycerol-3P-arach(c) + 1260 1-Acylglycerol-3P-palm(c) + 2010 1-Acylglycerol-3P-stea(c) → 7448 1-Acylglycerol-3P-VLDL-PE-pool(c)
r0736	0.5	ATP(c) + Fructose-6P(c) → ADP(c) + Fructose-1,6PP(c)
r0201	-1	DHAP(c) + NADH(c) ⇌ NAD+(c) + sn-Glycerol-3P(c)
r0030	2	ATP(c) + AMP(c) ⇌ 2 ADP(c)
r1300	0.08	Oleate(s) → Oleate(c)
r1187	0.27	Stearoyl-CoA(c) + sn-Glycerol-3P(c) → CoA(c) + 1-Acylglycerol-3P-stea(c)
r0935	0.17	Palmitate(s) → Palmitate(c)
r1188	0.08	Oleoyl-CoA(c) + sn-Glycerol-3P(c) → 1-Acylglycerol-3P-ol(c) + CoA(c)
r1189	0.18	Linoleoyl-CoA(c) + sn-Glycerol-3P(c) → 1-Acylglycerol-3P-lin(c) + CoA(c)
r0982	0.27	Stearate(s) → Stearate(c)
r0312	0.34	Palmitate(c) + ATP(c) + CoA(c) → Palmitoyl-CoA(c) + AMP(c) + PPi(c)
r1213	-1	Acyl-CoA-VLDL-PE-pool(c) + 1-Acylglycerol-3P-VLDL-PE-pool(c) → Phosphatidate-VLDL-PE-pool(c) + CoA(c)
r0007	2	PPi(c) + H2O(c) → 2 Pi(c)
r1322	-1	NADH(c) ⇌ NAD+(c) + NADH-redox-potential(c)
r0849	-0.5	H2O(s) ⇌ H2O(c)
r0985	0.18	Linoleate(s) → Linoleate(c)
r0243	0.5	GAP(c) ⇌ DHAP(c)

1-Acylglycerol-VLDL-PI-pool (590)

Definition			Solution												
			exchanges		reactions								transp		
Objective	Constraints	Comment	imports	exports	c	m	r	p	l	n	s	b	s-c	b-c	int
1-Acylglycerol-VLDL-PI-pool(c)	PIPES MCES	synthesis of cytosolic 1-Acylglycerol-VLDL-PI-pool	0.5 Glucose-6P(c) 0.25 Arachidonate(s) 0.08 Palmitate(s) 0.16 Oleate(s) 0.43 Stearate(s) 0.08 Linoleate(s) 4.5 ATP-energy(c) 1 NADH-redox-potential(c)	0.5 H_2O(s) 0.5 P_i(c) 1 1-Acylglycerol-VLDL-PI-pool(c)	25	-	-	-	-	-	-	-	6	-	-

ID	flux	reaction
r1190	0.25	Arachidonyl-CoA(c) + sn-Glycerol-3P(c) → 1-Acylglycerol-3P-arach(c) + CoA(c)
r1263	0.51	Arachidonate(c) + ATP(c) + CoA(c) → Arachidonyl-CoA(c) + AMP(c) + PPi(c)
r1254	0.85	Stearate(c) + ATP(c) + CoA(c) → Stearoyl-CoA(c) + AMP(c) + PPi(c)
r1209	0.0001	694 Palmitoyl-CoA(c) + 3640 Stearoyl-CoA(c) + 1360 Oleoyl-CoA(c) + 2170 Arachidonyl-CoA(c) + 694 Linoleoyl-CoA(c) → 8558 Acyl-CoA-VLDL-PI-pool(c)
r0930	0.25	Arachidonate(s) → Arachidonate(c)
r0192	0.5	Glucose-6P(c) ⇌ Fructose-6P(c)
r0256	0.5	Fructose-1,6PP(c) ⇌ DHAP(c) + GAP(c)
r1319	-4.5	ATP(c) + H2O(c) ⇌ ADP(c) + Pi(c) + ATP-energy(c)
r1256	0.32	ATP(c) + CoA(c) + Oleate(c) → Oleoyl-CoA(c) + AMP(c) + PPi(c)

Continued on next page

ID	flux	reaction
r1229	1	H2O(c) + 1,2-Diacylglycerol-VLDL-PI-pool(c) → 1-Acylglycerol-VLDL-PI-pool(c) + Fatty-acid-VLDL-PI-pool(c)
r1185	0.08	Palmitoyl-CoA(c) + sn-Glycerol-3P(c) → CoA(c) + 1-Acylglycerol-3P-palm(c)
r1258	0.16	Linoleate(c) + ATP(c) + CoA(c) → AMP(c) + PPi(c) + Linoleoyl-CoA(c)
r1249	-0.0001	694 Palmitate(c) + 3640 Stearate(c) + 694 Linoleate(c) + 2170 Arachidonate(c) + 1360 Oleate(c) ← 8558 Fatty-acid-VLDL-PI-pool(c)
r0736	0.5	ATP(c) + Fructose-6P(c) → ADP(c) + Fructose-1,6PP(c)
r0201	-1	DHAP(c) + NADH(c) ⇌ NAD+(c) + sn-Glycerol-3P(c)
r0030	2	ATP(c) + AMP(c) ⇌ 2 ADP(c)
r1300	0.16	Oleate(s) → Oleate(c)
r1187	0.43	Stearoyl-CoA(c) + sn-Glycerol-3P(c) → CoA(c) + 1-Acylglycerol-3P-stea(c)
r0935	0.08	Palmitate(s) → Palmitate(c)
r1202	0.0001	1360 1-Acylglycerol-3P-ol(c) + 694 1-Acylglycerol-3P-lin(c) + 2170 1-Acylglycerol-3P-arach(c) + 694 1-Acylglycerol-3P-palm(c) + 3640 1-Acylglycerol-3P-stea(c) → 8558 1-Acylglycerol-3P-VLDL-PI-pool(c)
r1188	0.16	Oleoyl-CoA(c) + sn-Glycerol-3P(c) → 1-Acylglycerol-3P-ol(c) + CoA(c)
r1221	1	Phosphatidate-VLDL-PI-pool(c) + H2O(c) → 1,2-Diacylglycerol-VLDL-PI-pool(c) + Pi(c)
r1189	0.08	Linoleoyl-CoA(c) + sn-Glycerol-3P(c) → 1-Acylglycerol-3P-lin(c) + CoA(c)
r0982	0.43	Stearate(s) → Stearate(c)
r0312	0.16	Palmitate(c) + ATP(c) + CoA(c) → Palmitoyl-CoA(c) + AMP(c) + PPi(c)
r0007	2	PPi(c) + H2O(c) → 2 Pi(c)
r1322	-1	NADH(c) ⇌ NAD+(c) + NADH-redox-potential(c)
r0849	-0.5	H2O(s) ⇌ H2O(c)
r1215	-1	1-Acylglycerol-3P-VLDL-PI-pool(c) + Acyl-CoA-VLDL-PI-pool(c) → Phosphatidate-VLDL-PI-pool(c) + CoA(c)
r0985	0.08	Linoleate(s) → Linoleate(c)
r0243	0.5	GAP(c) ⇌ DHAP(c)

1-Acylglycerol-VLDL-PS-pool (591)

Definition			Solution													
			exchanges		reactions								transp		
Objective	Constraints	Comment	imports	exports	c	m	r	p	l	n	s	b	s-c	b-c	int
1-Acylglycerol-VLDL-PS-pool(c)	PIPES MCES	synthesis of cytosolic 1-Acylglycerol-VLDL-PS-pool	0.5 Glucose-6P(c) 0.27 Arachidonate(s) 0.04 Palmitate(s) 0.04 Oleate(s) 0.62 Stearate(s) 0.02 Linoleate(s) 4.5 ATP-energy(c) 1 NADH-redox-potential(c)	0.5 H_2O(s) 0.5 P_i(c) 1 1-Acylglycerol-VLDL-PS-pool(c)	25	-	-	-	-	-	-	-	6	-	-

ID	flux	reaction
r1208	0.0001	345 Palmitoyl-CoA(c) + 4820 Stearoyl-CoA(c) + 332 Oleoyl-CoA(c) + 2050 Arachidonyl-CoA(c) + 181 Linoleoyl-CoA(c) → 7728 Acyl-CoA-VLDL-PS-pool(c)
r1190	0.27	Arachidonyl-CoA(c) + sn-Glycerol-3P(c) → 1-Acylglycerol-3P-arach(c) + CoA(c)
r1263	0.53	Arachidonate(c) + ATP(c) + CoA(c) → Arachidonyl-CoA(c) + AMP(c) + PPi(c)
r1254	1.25	Stearate(c) + ATP(c) + CoA(c) → Stearoyl-CoA(c) + AMP(c) + PPi(c)
r0930	0.27	Arachidonate(s) → Arachidonate(c)
r0192	0.5	Glucose-6P(c) ⇌ Fructose-6P(c)
r1228	1	H2O(c) + 1,2-Diacylglycerol-VLDL-PS-pool(c) → 1-Acylglycerol-VLDL-PS-pool(c) + Fatty-acid-VLDL-PS-pool(c)
r0256	0.5	Fructose-1,6PP(c) ⇌ DHAP(c) + GAP(c)
r1319	-4.5	ATP(c) + H2O(c) ⇌ ADP(c) + Pi(c) + ATP-energy(c)
r1256	0.09	ATP(c) + CoA(c) + Oleate(c) → Oleoyl-CoA(c) + AMP(c) + PPi(c)
r1248	-0.0001	345 Palmitate(c) + 4820 Stearate(c) + 181 Linoleate(c) + 2050 Arachidonate(c) + 332 Oleate(c) ← 7728 Fatty-acid-VLDL-PS-pool(c)
r1185	0.04	Palmitoyl-CoA(c) + sn-Glycerol-3P(c) → CoA(c) + 1-Acylglycerol-3P-palm(c)
r1258	0.05	Linoleate(c) + ATP(c) + CoA(c) → AMP(c) + PPi(c) + Linoleoyl-CoA(c)
r0736	0.5	ATP(c) + Fructose-6P(c) → ADP(c) + Fructose-1,6PP(c)
r0201	-1	DHAP(c) + NADH(c) ⇌ NAD+(c) + sn-Glycerol-3P(c)
r0030	2	ATP(c) + AMP(c) ⇌ 2 ADP(c)
r1300	0.04	Oleate(s) → Oleate(c)

Continued on next page

ID	flux	reaction
r1201	0.0001	332 1-Acylglycerol-3P-ol(c) + 181 1-Acylglycerol-3P-lin(c) + 2050 1-Acylglycerol-3P-arach(c) + 345 1-Acylglycerol-3P-palm(c) + 4820 1-Acylglycerol-3P-stea(c) → 7728 1-Acylglycerol-3P-VLDL-PS-pool(c)
r1187	0.62	Stearoyl-CoA(c) + sn-Glycerol-3P(c) → CoA(c) + 1-Acylglycerol-3P-stea(c)
r0935	0.04	Palmitate(s) → Palmitate(c)
r1220	1	Phosphatidate-VLDL-PS-pool(c) + H2O(c) → 1,2-Diacylglycerol-VLDL-PS-pool(c) + Pi(c)
r1188	0.04	Oleoyl-CoA(c) + sn-Glycerol-3P(c) → 1-Acylglycerol-3P-ol(c) + CoA(c)
r1189	0.02	Linoleoyl-CoA(c) + sn-Glycerol-3P(c) → 1-Acylglycerol-3P-lin(c) + CoA(c)
r0982	0.62	Stearate(s) → Stearate(c)
r0312	0.09	Palmitate(c) + ATP(c) + CoA(c) → Palmitoyl-CoA(c) + AMP(c) + PPi(c)
r0007	2	PPi(c) + H2O(c) → 2 Pi(c)
r1322	-1	NADH(c) ⇌ NAD+(c) + NADH-redox-potential(c)
r1214	-1	Acyl-CoA-VLDL-PS-pool(c) + 1-Acylglycerol-3P-VLDL-PS-pool(c) → Phosphatidate-VLDL-PS-pool(c) + CoA(c)
r0849	-0.5	H2O(s) ⇌ H2O(c)
r0985	0.02	Linoleate(s) → Linoleate(c)
r0243	0.5	GAP(c) ⇌ DHAP(c)

1-Acylglycerol-VLDL-SM-pool (592)

Definition			Solution													
			exchanges		reactions								transp			
Objective	Constraints	Comment	imports	exports	c	m	r	p	l	n	s	b	s-c	b-c	int
1-Acylglycerol-VLDL-SM-pool(c)	PIPES MCES	synthesis of cytosolic 1-Acylglycerol-VLDL-SM-pool	0.5 Glucose-6P(c) 0.02 Arachidonate(s) 0.71 Palmitate(s) 0.07 Oleate(s) 0.17 Stearate(s) 0.03 Linoleate(s) 4.5 ATP-energy(c) 1 NADH-redox-potential(c)	0.5 H_2O(s) 0.5 P_i(c) 1 1-Acylglycerol-VLDL-SM-pool(c)	25	-	-	-	-	-	-	-	6	-	-

ID	flux	reaction
r1216	-1	Acyl-CoA-VLDL-SM-pool(c) + 1-Acylglycerol-3P-VLDL-SM-pool(c) → CoA(c) + Phosphatidate-VLDL-SM-pool(c)
r1190	0.02	Arachidonyl-CoA(c) + sn-Glycerol-3P(c) → 1-Acylglycerol-3P-arach(c) + CoA(c)
r1263	0.03	Arachidonate(c) + ATP(c) + CoA(c) → Arachidonyl-CoA(c) + AMP(c) + PPi(c)
r1254	0.34	Stearate(c) + ATP(c) + CoA(c) → Stearoyl-CoA(c) + AMP(c) + PPi(c)
r0930	0.02	Arachidonate(s) → Arachidonate(c)
r0192	0.5	Glucose-6P(c) ⇌ Fructose-6P(c)
r0256	0.5	Fructose-1,6PP(c) ⇌ DHAP(c) + GAP(c)
r1319	-4.5	ATP(c) + H2O(c) ⇌ ADP(c) + Pi(c) + ATP-energy(c)
r1256	0.15	ATP(c) + CoA(c) + Oleate(c) → Oleoyl-CoA(c) + AMP(c) + PPi(c)
r1185	0.71	Palmitoyl-CoA(c) + sn-Glycerol-3P(c) → CoA(c) + 1-Acylglycerol-3P-palm(c)
r1258	0.06	Linoleate(c) + ATP(c) + CoA(c) → AMP(c) + PPi(c) + Linoleoyl-CoA(c)
r0736	0.5	ATP(c) + Fructose-6P(c) → ADP(c) + Fructose-1,6PP(c)
r0201	-1	DHAP(c) + NADH(c) ⇌ NAD+(c) + sn-Glycerol-3P(c)
r0030	2	ATP(c) + AMP(c) ⇌ 2 ADP(c)
r1300	0.07	Oleate(s) → Oleate(c)
r1210	0.0001	6260 Palmitoyl-CoA(c) + 1490 Stearoyl-CoA(c) + 656 Oleoyl-CoA(c) + 147 Arachidonyl-CoA(c) + 270 Linoleoyl-CoA(c) ⇌ 8823 Acyl-CoA-VLDL-SM-pool(c)
r1187	0.17	Stearoyl-CoA(c) + sn-Glycerol-3P(c) → CoA(c) + 1-Acylglycerol-3P-stea(c)
r0935	0.71	Palmitate(s) → Palmitate(c)
r1188	0.07	Oleoyl-CoA(c) + sn-Glycerol-3P(c) → 1-Acylglycerol-3P-ol(c) + CoA(c)
r1230	1	H2O(c) + 1,2-Diacylglycerol-VLDL-SM-pool(c) → 1-Acylglycerol-VLDL-SM-pool(c) + Fatty-acid-VLDL-SM-pool(c)
r1203	0.0001	656 1-Acylglycerol-3P-ol(c) + 270 1-Acylglycerol-3P-lin(c) + 147 1-Acylglycerol-3P-arach(c) + 6260 1-Acylglycerol-3P-palm(c) + 1490 1-Acylglycerol-3P-stea(c) → 8823 1-Acylglycerol-3P-VLDL-SM-pool(c)
r1189	0.03	Linoleoyl-CoA(c) + sn-Glycerol-3P(c) → 1-Acylglycerol-3P-lin(c) + CoA(c)
r0982	0.17	Stearate(s) → Stearate(c)
r0312	1.42	Palmitate(c) + ATP(c) + CoA(c) → Palmitoyl-CoA(c) + AMP(c) + PPi(c)
r1222	1	H2O(c) + Phosphatidate-VLDL-SM-pool(c) → Pi(c) + 1,2-Diacylglycerol-VLDL-SM-pool(c)
r0007	2	PPi(c) + H2O(c) → 2 Pi(c)

Continued on next page

ID	flux	reaction
r1322	-1	NADH(c) ⇌ NAD+(c) + NADH-redox-potential(c)
r1250	-0.0001	6260 Palmitate(c) + 1490 Stearate(c) + 270 Linoleate(c) + 147 Arachidonate(c) + 656 Oleate(c) ⇌ 8823 Fatty-acid-VLDL-SM-pool(c)
r0849	-0.5	H2O(s) ⇌ H2O(c)
r0985	0.03	Linoleate(s) → Linoleate(c)
r0243	0.5	GAP(c) ⇌ DHAP(c)

1-Acylglycerol-VLDL-TG1-pool (593)

Definition			Solution												
			exchanges		reactions								transp		
Objective	Constraints	Comment	imports	exports	c	m	r	p	l	n	s	b	s-c	b-c	int
1-Acylglycerol-VLDL-TG1-pool(c)	PIPES MCES	synthesis of cytosolic 1-Acylglycerol-VLDL-TG1-pool	0.5 Glucose-6P(c) 0.003 Arachidonate(s) 0.7 Palmitate(s) 0.13 Oleate(s) 0.09 Stearate(s) 0.08 Linoleate(s) 4.5 ATP-energy(c) 1 NADH-redox-potential(c)	0.5 H_2O(s) 0.5 P_i(c) 1 1-Acylglycerol-VLDL-TG1-pool(c)	25	-	-	-	-	-	-	-	6	-	-

ID	flux	reaction
r1225	1	H2O(c) + 1,2-Diacylglycerol-VLDL-TG-pool(c) → 1-Acylglycerol-VLDL-TG1-pool(c) + Fatty-acid-VLDL-TG2-pool(c)
r1217	1	Phosphatidate-VLDL-TG-pool(c) + H2O(c) → 1,2-Diacylglycerol-VLDL-TG-pool(c) + Pi(c)
r1190	0.003	Arachidonyl-CoA(c) + sn-Glycerol-3P(c) → 1-Acylglycerol-3P-arach(c) + CoA(c)
r1263	0.06	Arachidonate(c) + ATP(c) + CoA(c) → Arachidonyl-CoA(c) + AMP(c) + PPi(c)
r1254	0.13	Stearate(c) + ATP(c) + CoA(c) → Stearoyl-CoA(c) + AMP(c) + PPi(c)
r0930	0.003	Arachidonate(s) → Arachidonate(c)
r0192	0.5	Glucose-6P(c) ⇌ Fructose-6P(c)
r0256	0.5	Fructose-1,6PP(c) ⇌ DHAP(c) + GAP(c)
r1319	-4.5	ATP(c) + H2O(c) ⇌ ADP(c) + Pi(c) + ATP-energy(c)
r1256	0.6	ATP(c) + CoA(c) + Oleate(c) → Oleoyl-CoA(c) + AMP(c) + PPi(c)
r1185	0.7	Palmitoyl-CoA(c) + sn-Glycerol-3P(c) → CoA(c) + 1-Acylglycerol-3P-palm(c)
r1258	0.39	Linoleate(c) + ATP(c) + CoA(c) → AMP(c) + PPi(c) + Linoleoyl-CoA(c)
r0736	0.5	ATP(c) + Fructose-6P(c) → ADP(c) + Fructose-1,6PP(c)
r0201	-1	DHAP(c) + NADH(c) ⇌ NAD+(c) + sn-Glycerol-3P(c)
r0030	2	ATP(c) + AMP(c) ⇌ 2 ADP(c)
r1300	0.13	Oleate(s) → Oleate(c)
r1187	0.09	Stearoyl-CoA(c) + sn-Glycerol-3P(c) → CoA(c) + 1-Acylglycerol-3P-stea(c)
r0935	0.7	Palmitate(s) → Palmitate(c)
r1211	-1	Acyl-CoA-VLDL-TG2-pool(c) + 1-Acylglycerol-3P-VLDL-TG1-pool(c) → Phosphatidate-VLDL-TG-pool(c) + CoA(c)
r1188	0.13	Oleoyl-CoA(c) + sn-Glycerol-3P(c) → 1-Acylglycerol-3P-ol(c) + CoA(c)
r1198	0.0001	1220 1-Acylglycerol-3P-ol(c) + 741 1-Acylglycerol-3P-lin(c) + 24 1-Acylglycerol-3P-arach(c) + 6440 1-Acylglycerol-3P-palm(c) + 823 1-Acylglycerol-3P-stea(c) → 9248 1-Acylglycerol-3P-VLDL-TG1-pool(c)
r1189	0.08	Linoleoyl-CoA(c) + sn-Glycerol-3P(c) → 1-Acylglycerol-3P-lin(c) + CoA(c)
r0982	0.09	Stearate(s) → Stearate(c)
r0312	0.83	Palmitate(c) + ATP(c) + CoA(c) → Palmitoyl-CoA(c) + AMP(c) + PPi(c)
r1204	0.0001	1200 Palmitoyl-CoA(c) + 384 Stearoyl-CoA(c) + 4260 Oleoyl-CoA(c) + 521 Arachidonyl-CoA(c) + 2810 Linoleoyl-CoA(c) → 9175 Acyl-CoA-VLDL-TG2-pool(c)
r0007	2	PPi(c) + H2O(c) → 2 Pi(c)
r1322	-1	NADH(c) ⇌ NAD+(c) + NADH-redox-potential(c)
r0849	-0.5	H2O(s) ⇌ H2O(c)
r1242	-0.0001	1200 Palmitate(c) + 384 Stearate(c) + 2810 Linoleate(c) + 521 Arachidonate(c) + 4260 Oleate(c) ← 9175 Fatty-acid-VLDL-TG2-pool(c)
r0985	0.08	Linoleate(s) → Linoleate(c)
r0243	0.5	GAP(c) ⇌ DHAP(c)

1-Pyrroline-5-carboxylate (594)

Definition			Solution												
			exchanges		reactions								transp		
Objective	Constraints	Comment	imports	exports	c	m	r	p	l	n	s	b	s-c	b-c	int
1-Pyrroline-5-carboxylate(c)	PIPES MCES	synthesis of cytosolic 1-Pyrroline-5-carboxylate	1 Proline(s)	1 1-Pyrroline-5-carboxylate(c) 1 NADPH-redox-potential(c) 1 Na-gradient(c)	2	-	-	-	-	-	-	-	2	-	-

ID	flux	reaction
r1326	1	NADPH(c) ⇌ NADPH-redox-potential(c) + NADP+(c)
r1139	-1	Proline(s) + Na+(s) → Proline(c) + Na+(c)
r2664	1	Na+(c) ⇌ Na+(s) + Na-gradient(c)
r0305	1	Proline(c) + NADP+(c) ⇌ 1-Pyrroline-5-carboxylate(c) + NADPH(c)

14-Demethyllanosterol (595)

Definition			Solution												
			exchanges		reactions								transp		
Objective	Constraints	Comment	imports	exports	c	m	r	p	l	n	s	b	s-c	b-c	int
14-Demethyllanosterol(r)	PIPES MCES	synthesis of Golgi/ER 14-Demethyllanosterol	4 O_2(s) 2 Farnesyl-PP(r) 1 ATP-energy(c) 6 NADPH-redox-potential(r)	3 H_2O(s) 4 P_i(c) 1 CO_2(s) 1 14-Demethyllanosterol(r) 1 NADPH-redox-potential(c)	5	-	7	-	-	-	-	-	3	-	4

ID	flux	reaction
r1153	-2	PPi(c) ⇌ PPi(r)
r0887	-1	Formate(c) ⇌ Formate(r)
r1325	-6	NADPH(r) ⇌ NADPH-redox-potential(r) + NADP+(r)
r0227	1	10-Formyl-THF(c) + H2O(c) + NADP+(c) → THF(c) + CO2(c) + NADPH(c)
r1326	1	NADPH(c) ⇌ NADPH-redox-potential(c) + NADP+(c)
r0228	1	THF(c) + Formate(c) + ATP(c) → ADP(c) + Pi(c) + 10-Formyl-THF(c)
r0607	1	Squalene 2,3-oxide(r) ⇌ Lanosterol(r)
r1319	-1	ATP(c) + H2O(c) ⇌ ADP(c) + Pi(c) + ATP-energy(c)
r0780	-1	4,4-Dimethyl-5alpha-cholesta-8,14,24-trien-3beta-ol(r) + NADPH(r) ⇌ 14-Demethyllanosterol(r) + NADP+(r)
r0781	1	3 O2(r) + 3 NADPH(r) + Lanosterol(r) ⇌ 4,4-Dimethyl-5alpha-cholesta-8,14,24-trien-3beta-ol(r) + Formate(r) + 4 H2O(r) + 3 NADP+(r)
r0854	-4	O2(r) ⇌ O2(c)
r0575	1	Presqualene-PP(r) + NADPH(r) ⇌ PPi(r) + Squalene(r) + NADP+(r)
r0855	4	O2(s) ⇌ O2(c)
r0576	1	Squalene(r) + NADPH(r) + O2(r) ⇌ H2O(r) + Squalene 2,3-oxide(r) + NADP+(r)
r0848	5	H2O(r) ⇌ H2O(c)
r0007	2	PPi(c) + H2O(c) → 2 Pi(c)
r0867	1	CO2(c) ⇌ CO2(s)
r0849	-3	H2O(s) ⇌ H2O(c)
r0170	1	2 Farnesyl-PP(r) ⇌ PPi(r) + Presqualene-PP(r)

2,5-Diamino-6-(5-triphosphoryl-3,4-trihydroxy-2-oxopentyl)- amino-4-oxopyrimidine (596)

Definition			Solution												
			exchanges		reactions								transp		
Objective	Constraints	Comment	imports	exports	c	m	r	p	l	n	s	b	s-c	b-c	int
2,5-Diamino-6-(5-triphosphoryl-3,4-trihydroxy-2-oxopentyl)-amino-4-oxopyrimidine(c)	PIPES MCES	synthesis of cytosolic 2,5-Diamino-6-(5-triphosphoryl-3,4-trihydroxy-2-oxopentyl)-amino-4-oxopyrimidine	2.17 P_i(c) 1 Glycine(s) 2 Glutamine(s) 0.83 Glucose-6P(c) 1 Asparagine(s) 11.2 ATP-energy(c) 1 Proton-gradient(c) 1 THF-activated methyl group(c)	2.17 H_2O(s) 1 Pyruvate(c) 2 Glutamate(s) 1 2,5-Diamino-6-(5-triphosphoryl-3,4-trihydroxy-2-oxopentyl)- amino-4-oxopyrimidine(c) 2 NADH-redox-potential(c) 2 NADPH-redox-potential(c)	40	-	-	-	-	-	-	-	6	-	-

ID	flux	reaction
r0406	-0.33	Fructose-6P(c) + Erythrose-4P(c) ⇌ Sedoheptulose-7P(c) + GAP(c)
r0127	1	H2O(c) + Asparagine(c) → Aspartate(c) + NH3(c)
r0293	1	5,10-Methylene-THF(c) + NADP+(c) ⇌ 5,10-Methenyl-THF(c) + NADPH(c)
r0705	1	ATP(c) + 5-Phosphoribosyl-4-carboxy-5-aminoimidazole(c) + Aspartate(c) ⇌ ADP(c) + Pi(c) + SAICAR(c)
r0030	2	ATP(c) + AMP(c) ⇌ 2 ADP(c)
r0058	1	Malate(c) + NADP+(c) ⇌ Pyruvate(c) + CO2(c) + NADPH(c)
r0370	0.33	Sedoheptulose-7P(c) + GAP(c) ⇌ Ribose-5P(c) + D-Xylulose-5P(c)
r0371	1	5,10-Methenyl-THF(c) + H2O(c) ⇌ 10-Formyl-THF(c)
r2532	1	Asparagine(s) ⇌ Asparagine(c)
r2685	2	Glutamate(c) ⇌ Glutamate(s)
r0659	1	ATP(c) + 5-Phosphoribosylamine(c) + Glycine(c) ⇌ ADP(c) + Pi(c) + GAR(c)
r0228	1	THF(c) + Formate(c) + ATP(c) → ADP(c) + Pi(c) + 10-Formyl-THF(c)
r0099	1	ATP(c) + GDP(c) ⇌ ADP(c) + GTP(c)
r0243	-0.17	DHAP(c) ⇌ GAP(c)
r0677	1	10-Formyl-THF(c) + GAR(c) ⇌ THF(c) + N-Formyl-GAR(c)
r1331	-1	H+(PG)(s) ⇌ H+(PG)(c) + Proton-gradient(c)
r1319	-11.2	ATP(c) + H2O(c) ⇌ ADP(c) + Pi(c) + ATP-energy(c)
r0247	1	ATP(c) + Ribose-5P(c) → AMP(c) + PRPP(c)
r0699	1	SAICAR(c) ⇌ Fumarate(c) + AICAR(c)
r0849	-2.17	H2O(s) ⇌ H2O(c)
r2468	1	Glycine(s) + H+(PG)(s) → Glycine(c) + H+(PG)(c)
r0301	1	ATP(c) + Xanthosine-5P(c) + NH3(c) → AMP(c) + PPi(c) + GMP(c)
r0007	2	PPi(c) + H2O(c) → 2 Pi(c)
r0736	0.17	ATP(c) + Fructose-6P(c) → ADP(c) + Fructose-1,6PP(c)
r0192	0.83	Glucose-6P(c) ⇌ Fructose-6P(c)
r0775	1	Formamidopyrimidine nleoside triphosphate(c) + H2O(c) ⇌ 2,5-Diaminopyrimidine nleoside triphosphate(c) + Formate(c)
r0777	1	2,5-Diaminopyrimidine nleoside triphosphate(c) ⇌ 2,5-Diamino-6-(5-triphosphoryl-3,4-trihydroxy-2-oxopentyl)- amino-4-oxopyrimidine(c)
r0344	-0.67	D-Xylulose-5P(c) ⇌ Ribulose-5P(c)
r0792	1	5-Methyl-THF(c) + NAD+(c) ⇌ 5,10-Methylene-THF(c) + NADH(c)
r2676	-1	5-Methyl-THF(c) ⇌ THF(c) + THF-activated methyl group(c)
r2525	2	Glutamine(s) ⇌ Glutamine(c)
r0666	1	ATP(c) + 5-Phosphoribosylformylglycinamidine(c) ⇌ ADP(c) + Pi(c) + AIR(c)
r0667	-1	5-Phosphoribosyl-4-carboxy-5-aminoimidazole(c) ⇌ AIR(c) + CO2(c)
r1322	2	NADH(c) ⇌ NAD+(c) + NADH-redox-potential(c)
r0250	-0.67	Ribulose-5P(c) ⇌ Ribose-5P(c)
r1326	2	NADPH(c) ⇌ NADPH-redox-potential(c) + NADP+(c)
r0687	1	ATP(c) + N-Formyl-GAR(c) + Glutamine(c) + H2O(c) ⇌ ADP(c) + Pi(c) + Glutamate(c) + 5-Phosphoribosylformylglycinamidine(c)
r0101	1	ATP(c) + GMP(c) ⇌ ADP(c) + GDP(c)
r0255	0.33	Fructose-6P(c) + GAP(c) ⇌ D-Xylulose-5P(c) + Erythrose-4P(c)
r0256	0.17	Fructose-1,6PP(c) ⇌ DHAP(c) + GAP(c)
r0257	-1	Glutamine(c) + PRPP(c) + H2O(c) ⇌ 5-Phosphoribosylamine(c) + PPi(c) + Glutamate(c)
r0258	-1	Fumarate(c) + H2O(c) ⇌ Malate(c)
r0120	1	GTP(c) + H2O(c) ⇌ Formamidopyrimidine nleoside triphosphate(c)

Continued on next page

ID	flux	reaction
r0273	-1	IMP(c) + H2O(c) ⇌ FAICAR(c)
r0274	1	IMP(c) + NAD+(c) + H2O(c) → Xanthosine-5P(c) + NADH(c)
r0700	1	10-Formyl-THF(c) + AICAR(c) ⇌ THF(c) + FAICAR(c)

2,5-Diaminopyrimidine(n)leoside triphosphate (597)

Definition			Solution												
			exchanges		reactions								transp		
Objective	Constraints	Comment	imports	exports	c	m	r	p	l	n	s	b	s-c	b-c	int
2,5-Diaminopyrimidine nleoside triphosphate(c)	PIPES MCES	synthesis of cytosolic 2,5-Diaminopyrimidine nleoside triphosphate	2.17 P_i(c) 1 Glycine(s) 2 Glutamine(s) 0.83 Glucose-6P(c) 1 Asparagine(s) 11.2 ATP-energy(c) 1 Proton-gradient(c) 1 THF-activated methyl group(c)	2.17 H_2O(s) 1 Pyruvate(c) 2 Glutamate(s) 1 2,5-Diaminopyrimidine nleoside triphosphate(c) 2 NADH-redox-potential(c) 2 NADPH-redox-potential(c)	39	-	-	-	-	-	-	-	6	-	-

ID	flux	reaction
r0406	-0.33	Fructose-6P(c) + Erythrose-4P(c) ⇌ Sedoheptulose-7P(c) + GAP(c)
r0127	1	H2O(c) + Asparagine(c) → Aspartate(c) + NH3(c)
r0293	1	5,10-Methylene-THF(c) + NADP+(c) ⇌ 5,10-Methenyl-THF(c) + NADPH(c)
r0705	1	ATP(c) + 5-Phosphoribosyl-4-carboxy-5-aminoimidazole(c) + Aspartate(c) ⇌ ADP(c) + Pi(c) + SAICAR(c)
r0030	2	ATP(c) + AMP(c) ⇌ 2 ADP(c)
r0058	1	Malate(c) + NADP+(c) ⇌ Pyruvate(c) + CO2(c) + NADPH(c)
r0370	0.33	Sedoheptulose-7P(c) + GAP(c) ⇌ Ribose-5P(c) + D-Xylulose-5P(c)
r0371	1	5,10-Methenyl-THF(c) + H2O(c) ⇌ 10-Formyl-THF(c)
r2532	1	Asparagine(s) ⇌ Asparagine(c)
r2685	2	Glutamate(c) ⇌ Glutamate(s)
r0659	1	ATP(c) + 5-Phosphoribosylamine(c) + Glycine(c) ⇌ ADP(c) + Pi(c) + GAR(c)
r0228	1	THF(c) + Formate(c) + ATP(c) → ADP(c) + Pi(c) + 10-Formyl-THF(c)
r0099	1	ATP(c) + GDP(c) ⇌ ADP(c) + GTP(c)
r0243	-0.17	DHAP(c) ⇌ GAP(c)
r0677	1	10-Formyl-THF(c) + GAR(c) ⇌ THF(c) + N-Formyl-GAR(c)
r1331	-1	H+(PG)(s) ⇌ H+(PG)(c) + Proton-gradient(c)
r1319	-11.2	ATP(c) + H2O(c) ⇌ ADP(c) + Pi(c) + ATP-energy(c)
r0247	1	ATP(c) + Ribose-5P(c) → AMP(c) + PRPP(c)
r0699	1	SAICAR(c) ⇌ Fumarate(c) + AICAR(c)
r0849	-2.17	H2O(s) ⇌ H2O(c)
r2468	1	Glycine(s) + H+(PG)(s) → Glycine(c) + H+(PG)(c)
r0301	1	ATP(c) + Xanthosine-5P(c) + NH3(c) → AMP(c) + PPi(c) + GMP(c)
r0007	2	PPi(c) + H2O(c) → 2 Pi(c)
r0736	0.17	ATP(c) + Fructose-6P(c) → ADP(c) + Fructose-1,6PP(c)
r0192	0.83	Glucose-6P(c) ⇌ Fructose-6P(c)
r0775	1	Formamidopyrimidine nleoside triphosphate(c) + H2O(c) ⇌ 2,5-Diaminopyrimidine nleoside triphosphate(c) + Formate(c)
r0344	-0.67	D-Xylulose-5P(c) ⇌ Ribulose-5P(c)
r0792	1	5-Methyl-THF(c) + NAD+(c) ⇌ 5,10-Methylene-THF(c) + NADH(c)
r2676	-1	5-Methyl-THF(c) ⇌ THF(c) + THF-activated methyl group(c)
r2525	2	Glutamine(s) ⇌ Glutamine(c)
r0666	1	ATP(c) + 5-Phosphoribosylformylglycinamidine(c) ⇌ ADP(c) + Pi(c) + AIR(c)
r0667	-1	5-Phosphoribosyl-4-carboxy-5-aminoimidazole(c) ⇌ AIR(c) + CO2(c)
r1322	2	NADH(c) ⇌ NAD+(c) + NADH-redox-potential(c)
r0250	-0.67	Ribulose-5P(c) ⇌ Ribose-5P(c)
r1326	2	NADPH(c) ⇌ NADPH-redox-potential(c) + NADP+(c)
r0687	1	ATP(c) + N-Formyl-GAR(c) + Glutamine(c) + H2O(c) ⇌ ADP(c) + Pi(c) + Glutamate(c) + 5-Phosphoribosylformylglycinamidine(c)
r0101	1	ATP(c) + GMP(c) ⇌ ADP(c) + GDP(c)
r0255	0.33	Fructose-6P(c) + GAP(c) ⇌ D-Xylulose-5P(c) + Erythrose-4P(c)

Continued on next page

2,5-Diaminopyrimidine(n)leoside triphosphate (597)– continued

ID	flux	reaction
r0256	0.17	Fructose-1,6PP(c) ⇌ DHAP(c) + GAP(c)
r0257	-1	Glutamine(c) + PRPP(c) + H2O(c) ⇌ 5-Phosphoribosylamine(c) + PPi(c) + Glutamate(c)
r0258	-1	Fumarate(c) + H2O(c) ⇌ Malate(c)
r0120	1	GTP(c) + H2O(c) ⇌ Formamidopyrimidine nleoside triphosphate(c)
r0273	-1	IMP(c) + H2O(c) ⇌ FAICAR(c)
r0274	1	IMP(c) + NAD+(c) + H2O(c) → Xanthosine-5P(c) + NADH(c)
r0700	1	10-Formyl-THF(c) + AICAR(c) ⇌ THF(c) + FAICAR(c)

2-Amino-3-carboxymuconate(s)emialdehyde (598)

Definition			Solution												
			exchanges		reactions								transp		
Objective	Constraints	Comment	imports	exports	c	m	r	p	l	n	s	b	s-c	b-c	int
2-Amino-3-carboxymuconate semialdehyde(c)	PIPES MCES	synthesis of cytosolic 2-Amino-3-carboxymuconate semialdehyde	3 O_2(s) 1 Tryptophan(s) 1 ATP-energy(c) 1.5 NADPH-redox-potential(c)	0.5 CO_2(s) 1 Alanine(s) 1 2-Amino-3-carboxymuconate semialdehyde(c) 1 Na-gradient(c) 0.5 THF-activated methyl group(c)	13	-	-	-	-	-	-	-	5	-	-

ID	flux	reaction
r0371	-0.5	10-Formyl-THF(c) ⇌ 5,10-Methenyl-THF(c) + H2O(c)
r0227	0.5	10-Formyl-THF(c) + H2O(c) + NADP+(c) → THF(c) + CO2(c) + NADPH(c)
r1326	-1.5	NADPH(c) ⇌ NADPH-redox-potential(c) + NADP+(c)
r0228	1	THF(c) + Formate(c) + ATP(c) → ADP(c) + Pi(c) + 10-Formyl-THF(c)
r2524	-1	Alanine(c) ⇌ Alanine(s)
r0562	1	3-Hydroxyanthranilate(c) + O2(c) ⇌ 2-Amino-3-carboxymuconate semialdehyde(c)
r0454	1	L-Formylkynurenine(c) + H2O(c) ⇌ Formate(c) + Kynurenine(c)
r1319	-1	ATP(c) + H2O(c) ⇌ ADP(c) + Pi(c) + ATP-energy(c)
r0563	1	H2O(c) + 3-Hydroxy-L-kynurenine(c) ⇌ 3-Hydroxyanthranilate(c) + Alanine(c)
r0455	1	Kynurenine(c) + O2(c) + NADPH(c) ⇌ H2O(c) + 3-Hydroxy-L-kynurenine(c) + NADP+(c)
r0293	-0.5	5,10-Methylene-THF(c) + NADP+(c) ⇌ 5,10-Methenyl-THF(c) + NADPH(c)
r0169	1	Tryptophan(c) + O2(c) ⇌ L-Formylkynurenine(c)
r0296	-0.5	5,10-Methylene-THF(c) + NADPH(c) ⇌ 5-Methyl-THF(c) + NADP+(c)
r2664	1	Na+(c) ⇌ Na+(s) + Na-gradient(c)
r0855	3	O2(s) ⇌ O2(c)
r2676	0.5	5-Methyl-THF(c) ⇌ THF(c) + THF-activated methyl group(c)
r1124	-1	Tryptophan(s) + Na+(s) → Tryptophan(c) + Na+(c)
r0867	0.5	CO2(c) ⇌ CO2(s)

2-Amino-3-oxoadipate (599)

Definition			Solution												
			exchanges		reactions								transp		
Objective	Constraints	Comment	imports	exports	c	m	r	p	l	n	s	b	s-c	b-c	int
2-Amino-3-oxoadipate(m)	PIPES MCES	synthesis of mitochondrial 2-Amino-3-oxoadipate	0.5 Pyruvate(m) 1 Glutamate(s) 0.5 Serine(s) 0.5 NADPH-redox-potential(m) 0.5 Proton-gradient(m)	0.5 Aspartate(s) 1 2-Amino-3-oxoadipate(m) 0.5 NADH-redox-potential(c) 1 NADH-redox-potential(m) 0.5 Proton-gradient(c) 1.5 Na-gradient(c)	3	10	-	-	-	-	-	-	5	-	4

ID	flux	reaction
r1144	-1	Glutamate(c) + Na+(c) ← Glutamate(s) + Na+(s)
r0820	0.5	AKG(c) + Malate(m) ⇌ AKG(m) + Malate(c)
r0109	-0.5	OAA(c) + Glutamate(c) ⇌ AKG(c) + Aspartate(c)

Continued on next page

ID	flux	reaction
r0073	0.5	Glutamate(m) + NAD+(m) + H2O(m) ⇌ AKG(m) + NADH(m) + NH3(m)
r1435	0.5	Serine(c) → Serine(m)
r1327	-0.5	NADPH(m) ⇌ NADPH-redox-potential(m) + NADP+(m)
r0382	1	Dihydrolipoamide(m) + NAD+(m) ⇌ Lipoamide(m) + NADH(m)
r0059	-0.5	Pyruvate(m) + CO2(m) + NADPH(m) ⇌ Malate(m) + NADP+(m)
r0384	1	AKG(m) + Lipoamide(m) → S-Succinyldihydrolipoamide(m) + CO2(m)
r0556	-1	CoA(m) + S-Succinyldihydrolipoamide(m) ⇌ Dihydrolipoamide(m) + Succinyl-CoA(m)
r0295	-0.5	Glycine(m) + THF(m) + NAD+(m) ← 5,10-Methylene-THF(m) + CO2(m) + NADH(m) + NH3(m)
r0196	1	Glycine(m) + Succinyl-CoA(m) ⇌ CoA(m) + 2-Amino-3-oxoadipate(m)
r2664	1.5	Na+(c) ⇌ Na+(s) + Na-gradient(c)
r0104	0.5	NAD+(c) + Malate(c) ⇌ OAA(c) + NADH(c)
r0231	-0.5	THF(m) + Serine(m) ⇌ 5,10-Methylene-THF(m) + Glycine(m) + H2O(m)
r1330	-0.5	H+(PG)(c) ⇌ H+(PG)(m) + Proton-gradient(m)
r0875	0.5	Glutamate(c) + H+(PG)(c) → Glutamate(m) + H+(PG)(m)
r2478	0.5	Serine(s) + Na+(s) + H+(PG)(c) → Serine(c) + Na+(c) + H+(PG)(s)
r1331	0.5	H+(PG)(s) ⇌ H+(PG)(c) + Proton-gradient(c)
r1322	0.5	NADH(c) ⇌ NAD+(c) + NADH-redox-potential(c)
r2686	0.5	Aspartate(c) ⇌ Aspartate(s)
r1323	1	NADH(m) ⇌ NAD+(m) + NADH-redox-potential(m)

2-Aminomuconate (600)

Definition			Solution												
			exchanges		reactions								transp		
Objective	Constraints	Comment	imports	exports	c	m	r	p	l	n	s	b	s-c	b-c	int
2-Aminomuconate(c)	PIPES MCES	synthesis of cytosolic 2-Aminomuconate	3 O_2(s) 1 Tryptophan(s) 1 ATP-energy(c) 3 NADPH-redox-potential(c)	1 CO_2(s) 1 Alanine(s) 1 2-Aminomuconate(c) 1 NADH-redox-potential(c) 1 Na-gradient(c) 1 THF-activated methyl group(c)	15	-	-	-	-	-	-	-	5	-	-

ID	flux	reaction
r0371	-1	10-Formyl-THF(c) ⇌ 5,10-Methenyl-THF(c) + H2O(c)
r1326	-3	NADPH(c) ⇌ NADPH-redox-potential(c) + NADP+(c)
r0228	1	THF(c) + Formate(c) + ATP(c) → ADP(c) + Pi(c) + 10-Formyl-THF(c)
r2524	-1	Alanine(c) ⇌ Alanine(s)
r0562	1	3-Hydroxyanthranilate(c) + O2(c) ⇌ 2-Amino-3-carboxymuconate semialdehyde(c)
r0454	1	L-Formylkynurenine(c) + H2O(c) ⇌ Formate(c) + Kynurenine(c)
r1319	-1	ATP(c) + H2O(c) ⇌ ADP(c) + Pi(c) + ATP-energy(c)
r0563	1	H2O(c) + 3-Hydroxy-L-kynurenine(c) ⇌ 3-Hydroxyanthranilate(c) + Alanine(c)
r0455	1	Kynurenine(c) + O2(c) + NADPH(c) ⇌ H2O(c) + 3-Hydroxy-L-kynurenine(c) + NADP+(c)
r0293	-1	5,10-Methylene-THF(c) + NADP+(c) ⇌ 5,10-Methenyl-THF(c) + NADPH(c)
r0645	1	2-Aminomuconate semialdehyde(c) + NAD+(c) + H2O(c) ⇌ 2-Aminomuconate(c) + NADH(c)
r0169	1	Tryptophan(c) + O2(c) ⇌ L-Formylkynurenine(c)
r0296	-1	5,10-Methylene-THF(c) + NADPH(c) ⇌ 5-Methyl-THF(c) + NADP+(c)
r2664	1	Na+(c) ⇌ Na+(s) + Na-gradient(c)
r0855	3	O2(s) ⇌ O2(c)
r0676	1	2-Amino-3-carboxymuconate semialdehyde(c) ⇌ 2-Aminomuconate semialdehyde(c) + CO2(c)
r2676	1	5-Methyl-THF(c) ⇌ THF(c) + THF-activated methyl group(c)
r1322	1	NADH(c) ⇌ NAD+(c) + NADH-redox-potential(c)
r1124	-1	Tryptophan(s) + Na+(s) → Tryptophan(c) + Na+(c)
r0867	1	CO2(c) ⇌ CO2(s)

2-Aminomuconate(s)emialdehyde (601)

Definition			Solution												
			exchanges		reactions								transp		
Objective	Constraints	Comment	imports	exports	c	m	r	p	l	n	s	b	s-c	b-c	int
2-Aminomuconate semialdehyde(c)	PIPES MCES	synthesis of cytosolic 2-Aminomuconate semialdehyde	3 O_2(s) 1 Tryptophan(s) 1 ATP-energy(c) 1.5 NADPH-redox-potential(c)	1.5 CO_2(s) 1 Alanine(s) 1 2-Aminomuconate semialdehyde(c) 1 Na-gradient(c) 0.5 THF-activated methyl group(c)	14	-	-	-	-	-	-	-	5	-	-

ID	flux	reaction
r0371	-0.5	10-Formyl-THF(c) ⇌ 5,10-Methenyl-THF(c) + H2O(c)
r0227	0.5	10-Formyl-THF(c) + H2O(c) + NADP+(c) → THF(c) + CO2(c) + NADPH(c)
r1326	-1.5	NADPH(c) ⇌ NADPH-redox-potential(c) + NADP+(c)
r0228	1	THF(c) + Formate(c) + ATP(c) → ADP(c) + Pi(c) + 10-Formyl-THF(c)
r2524	-1	Alanine(c) ⇌ Alanine(s)
r0562	1	3-Hydroxyanthranilate(c) + O2(c) ⇌ 2-Amino-3-carboxymuconate semialdehyde(c)
r0454	1	L-Formylkynurenine(c) + H2O(c) ⇌ Formate(c) + Kynurenine(c)
r1319	-1	ATP(c) + H2O(c) ⇌ ADP(c) + Pi(c) + ATP-energy(c)
r0563	1	H2O(c) + 3-Hydroxy-L-kynurenine(c) ⇌ 3-Hydroxyanthranilate(c) + Alanine(c)
r0455	1	Kynurenine(c) + O2(c) + NADPH(c) ⇌ H2O(c) + 3-Hydroxy-L-kynurenine(c) + NADP+(c)
r0293	-0.5	5,10-Methylene-THF(c) + NADP+(c) ⇌ 5,10-Methenyl-THF(c) + NADPH(c)
r0169	1	Tryptophan(c) + O2(c) ⇌ L-Formylkynurenine(c)
r0296	-0.5	5,10-Methylene-THF(c) + NADPH(c) ⇌ 5-Methyl-THF(c) + NADP+(c)
r2664	1	Na+(c) ⇌ Na+(s) + Na-gradient(c)
r0855	3	O2(s) ⇌ O2(c)
r0676	1	2-Amino-3-carboxymuconate semialdehyde(c) ⇌ 2-Aminomuconate semialdehyde(c) + CO2(c)
r2676	0.5	5-Methyl-THF(c) ⇌ THF(c) + THF-activated methyl group(c)
r1124	-1	Tryptophan(s) + Na+(s) → Tryptophan(c) + Na+(c)
r0867	1.5	CO2(c) ⇌ CO2(s)

2-Deoxy-D-ribose-1P (602)

Definition			Solution												
			exchanges		reactions								transp		
Objective	Constraints	Comment	imports	exports	c	m	r	p	l	n	s	b	s-c	b-c	int
2-Deoxy-D-ribose-1P(c)	PIPES MCES	synthesis of cytosolic 2-Deoxy-D-ribose-1P	0.5 P_i(c) 1 Glutamate(s) 0.5 Glucose-6P(c) 1 Ethanolamine(c) 2.5 ATP-energy(c)	1.5 H_2O(s) 1 Glutamine(s) 1 2-Deoxy-D-ribose-1P(c) 1 Na-gradient(c)	10	-	-	-	-	-	-	-	4	-	-

ID	flux	reaction
r1144	-1	Glutamate(c) + Na+(c) ← Glutamate(s) + Na+(s)
r0254	-1	GAP(c) + Acetaldehyde(c) ⇌ 2-Deoxy-D-ribose-5P(c)
r0570	-1	2-Deoxy-D-ribose-5P(c) ⇌ 2-Deoxy-D-ribose-1P(c)
r0336	1	ATP(c) + Ethanolamine(c) → ADP(c) + Ethanolamine-P(c)
r0192	0.5	Glucose-6P(c) ⇌ Fructose-6P(c)
r0256	0.5	Fructose-1,6PP(c) ⇌ DHAP(c) + GAP(c)
r2525	-1	Glutamine(c) ⇌ Glutamine(s)
r1319	-2.5	ATP(c) + H2O(c) ⇌ ADP(c) + Pi(c) + ATP-energy(c)
r0077	1	ATP(c) + Glutamate(c) + NH3(c) → ADP(c) + Pi(c) + Glutamine(c)
r0186	1	Ethanolamine-P(c) + H2O(c) → Acetaldehyde(c) + Pi(c) + NH3(c)
r0736	0.5	ATP(c) + Fructose-6P(c) → ADP(c) + Fructose-1,6PP(c)
r2664	1	Na+(c) ⇌ Na+(s) + Na-gradient(c)
r0849	-1.5	H2O(s) ⇌ H2O(c)
r0243	-0.5	DHAP(c) ⇌ GAP(c)

2-Deoxy-D-ribose-5P (603)

Definition			Solution												
			exchanges		reactions								transp		
Objective	Constraints	Comment	imports	exports	c	m	r	p	l	n	s	b	s-c	b-c	int
2-Deoxy-D-ribose-5P(c)	PIPES MCES	synthesis of cytosolic 2-Deoxy-D-ribose-5P	0.5 P_i(c) 1 Glutamate(s) 0.5 Glucose-6P(c) 1 Ethanolamine(c) 2.5 ATP-energy(c)	1.5 H_2O(s) 1 Glutamine(s) 1 2-Deoxy-D-ribose-5P(c) 1 Na-gradient(c)	9	-	-	-	-	-	-	-	4	-	-

ID	flux	reaction
r1144	-1	Glutamate(c) + Na+(c) ← Glutamate(s) + Na+(s)
r0254	-1	GAP(c) + Acetaldehyde(c) ⇌ 2-Deoxy-D-ribose-5P(c)
r0336	1	ATP(c) + Ethanolamine(c) → ADP(c) + Ethanolamine-P(c)
r0192	0.5	Glucose-6P(c) ⇌ Fructose-6P(c)
r0256	0.5	Fructose-1,6PP(c) ⇌ DHAP(c) + GAP(c)
r2525	-1	Glutamine(c) ⇌ Glutamine(s)
r1319	-2.5	ATP(c) + H2O(c) ⇌ ADP(c) + Pi(c) + ATP-energy(c)
r0077	1	ATP(c) + Glutamate(c) + NH3(c) → ADP(c) + Pi(c) + Glutamine(c)
r0186	1	Ethanolamine-P(c) + H2O(c) → Acetaldehyde(c) + Pi(c) + NH3(c)
r0736	0.5	ATP(c) + Fructose-6P(c) → ADP(c) + Fructose-1,6PP(c)
r2664	1	Na+(c) ⇌ Na+(s) + Na-gradient(c)
r0849	-1.5	H2O(s) ⇌ H2O(c)
r0243	-0.5	DHAP(c) ⇌ GAP(c)

2-Lysolecithin-pool (604)

Definition			Solution												
			exchanges		reactions								transp		
Objective	Constraints	Comment	imports	exports	c	m	r	p	l	n	s	b	s-c	b-c	int
2-Lysolecithin-pool(c)	PIPES MCES	synthesis of cytosolic 2-Lysolecithin-pool	0.5 P_i(c) 0.5 Glucose-6P(c) 1 Choline(c) 0.08 Arachidonate(s) 0.35 Palmitate(s) 0.14 Oleate(s) 0.15 Stearate(s) 0.27 Linoleate(s) 7.5 ATP-energy(c) 1 NADH-redox-potential(c)	2.5 H_2O(s) 1 2-Lysolecithin-pool(c)	30	-	-	-	-	-	-	-	6	-	-

ID	flux	reaction
r0244	1	ATP(c) + Choline(c) → ADP(c) + Phosphocholine(c)
r0136	1	ATP(c) + CMP(c) ⇌ ADP(c) + CDP(c)
r1190	0.08	Arachidonyl-CoA(c) + sn-Glycerol-3P(c) → 1-Acylglycerol-3P-arach(c) + CoA(c)
r1263	0.17	Arachidonate(c) + ATP(c) + CoA(c) → Arachidonyl-CoA(c) + AMP(c) + PPi(c)
r1254	0.3	Stearate(c) + ATP(c) + CoA(c) → Stearoyl-CoA(c) + AMP(c) + PPi(c)
r1245	-0.0001	3010 Palmitate(c) + 1300 Stearate(c) + 2350 Linoleate(c) + 726 Arachidonate(c) + 1170 Oleate(c) ⇌ 8556 Fatty-acid-VLDL-PC-pool(c)
r1218	1	Phosphatidate-VLDL-PC-pool(c) + H2O(c) → 1,2-Diacylglycerol-VLDL-PC-pool(c) + Pi(c)
r0930	0.08	Arachidonate(s) → Arachidonate(c)
r0192	0.5	Glucose-6P(c) ⇌ Fructose-6P(c)
r1237	1	CDP-choline(c) + 1,2-Diacylglycerol-VLDL-PC-pool(c) → PC-VLDL-pool(c) + CMP(c)
r0256	0.5	Fructose-1,6PP(c) ⇌ DHAP(c) + GAP(c)
r1319	-7.5	ATP(c) + H2O(c) ⇌ ADP(c) + Pi(c) + ATP-energy(c)
r1256	0.27	ATP(c) + CoA(c) + Oleate(c) → Oleoyl-CoA(c) + AMP(c) + PPi(c)
r1185	0.35	Palmitoyl-CoA(c) + sn-Glycerol-3P(c) → CoA(c) + 1-Acylglycerol-3P-palm(c)
r1258	0.55	Linoleate(c) + ATP(c) + CoA(c) → AMP(c) + PPi(c) + Linoleoyl-CoA(c)
r0736	0.5	ATP(c) + Fructose-6P(c) → ADP(c) + Fructose-1,6PP(c)
r0201	-1	DHAP(c) + NADH(c) ⇌ NAD+(c) + sn-Glycerol-3P(c)
r0030	2	ATP(c) + AMP(c) ⇌ 2 ADP(c)
r1300	0.14	Oleate(s) → Oleate(c)

Continued on next page

ID	flux	reaction
r1187	0.15	Stearoyl-CoA(c) + sn-Glycerol-3P(c) → CoA(c) + 1-Acylglycerol-3P-stea(c)
r0935	0.35	Palmitate(s) → Palmitate(c)
r1278	1	PC-VLDL-pool(c) + H2O(c) ⇌ 2-Lysolecithin-pool(c) + Fatty-acid-VLDL-PC-pool(c)
r1188	0.14	Oleoyl-CoA(c) + sn-Glycerol-3P(c) → 1-Acylglycerol-3P-ol(c) + CoA(c)
r1212	-1	Acyl-CoA-VLDL-PC-pool(c) + 1-Acylglycerol-3P-VLDL-PC-pool(c) → Phosphatidate-VLDL-PC-pool(c) + CoA(c)
r1189	0.27	Linoleoyl-CoA(c) + sn-Glycerol-3P(c) → 1-Acylglycerol-3P-lin(c) + CoA(c)
r0982	0.15	Stearate(s) → Stearate(c)
r0312	0.7	Palmitate(c) + ATP(c) + CoA(c) → Palmitoyl-CoA(c) + AMP(c) + PPi(c)
r0150	1	ATP(c) + CDP(c) ⇌ ADP(c) + CTP(c)
r1199	0.0001	1170 1-Acylglycerol-3P-ol(c) + 2350 1-Acylglycerol-3P-lin(c) + 726 1-Acylglycerol-3P-arach(c) + 3010 1-Acylglycerol-3P-palm(c) + 1300 1-Acylglycerol-3P-stea(c) → 8556 1-Acylglycerol-3P-VLDL-PC-pool(c)
r0421	1	CTP(c) + Phosphocholine(c) → PPi(c) + CDP-choline(c)
r0007	3	PPi(c) + H2O(c) → 2 Pi(c)
r1322	-1	NADH(c) ⇌ NAD+(c) + NADH-redox-potential(c)
r0849	-2.5	H2O(s) ⇌ H2O(c)
r1206	0.0001	3010 Palmitoyl-CoA(c) + 1300 Stearoyl-CoA(c) + 1170 Oleoyl-CoA(c) + 726 Arachidonyl-CoA(c) + 2350 Linoleoyl-CoA(c) → 8556 Acyl-CoA-VLDL-PC-pool(c)
r0985	0.27	Linoleate(s) → Linoleate(c)
r0243	0.5	GAP(c) ⇌ DHAP(c)

2-Methylacetoacetyl-CoA (605)

Definition			Solution												
			exchanges		reactions								transp		
Objective	Constraints	Comment	imports	exports	c	m	r	p	l	n	s	b	s-c	b-c	int
2-Methylacetoacetyl-CoA(m)	PIPES MCES	synthesis of mitochondrial 2-Methylacetoacetyl-CoA	2.17 P_i(c) 1 Glycine(s) 2 Aspartate(s) 1 Glutamine(s) 0.83 Glucose-6P(c) 1 Threonine(s) 0.5 Cystine(s) 1 Pantothenate(s) 12.2 ATP-energy(c) 1 Proton-gradient(m) 2 THF-activated methyl group(c) 1 CoA-activated acetyl group(m)	6.17 H_2O(s) 3 CO_2(s) 2 Pyruvate(c) 1 Glutamate(s) 1 2-Methylacetoacetyl-CoA(m) 2 NADH-redox-potential(c) 1 NADH-redox-potential(m) 3.5 NADPH-redox-potential(c) 5 Na-gradient(c)	44	4	-	-	-	-	-	-	10	-	4

ID	flux	reaction
r0406	-0.33	Fructose-6P(c) + Erythrose-4P(c) ⇌ Sedoheptulose-7P(c) + GAP(c)
r0277	1	GTP(c) + IMP(c) + Aspartate(c) → GDP(c) + Pi(c) + Adenylosuccinate(c)
r0705	1	ATP(c) + 5-Phosphoribosyl-4-carboxy-5-aminoimidazole(c) + Aspartate(c) ⇌ ADP(c) + Pi(c) + SAICAR(c)
r0293	2	5,10-Methylene-THF(c) + NADP+(c) ⇌ 5,10-Methenyl-THF(c) + NADPH(c)
r0578	1	ATP(c) + Pantetheine(c) ⇌ ADP(c) + Phosphopantetheine(c)
r0877	0.5	Glutamate(c) + Cystine(s) ⇌ Glutamate(s) + Cystine(c)
r2477	1	Glutamine(s) + Na+(s) + H+(PG)(c) → Glutamine(c) + Na+(c) + H+(PG)(s)
r0030	2	ATP(c) + AMP(c) ⇌ 2 ADP(c)
r0032	1	ATP(c) + Dephospho-CoA(c) ⇌ ADP(c) + CoA(c)
r1142	-1	Threonine(s) + Na+(s) → Threonine(c) + Na+(c)
r1143	-2	Na+(c) + Aspartate(c) ← Na+(s) + Aspartate(s)
r2664	5	Na+(c) ⇌ Na+(s) + Na-gradient(c)
r0058	2	Malate(c) + NADP+(c) ⇌ Pyruvate(c) + CO2(c) + NADPH(c)
r0370	0.33	Sedoheptulose-7P(c) + GAP(c) ⇌ Ribose-5P(c) + D-Xylulose-5P(c)
r0371	2	5,10-Methenyl-THF(c) + H2O(c) ⇌ 10-Formyl-THF(c)
r0077	1	ATP(c) + Glutamate(c) + NH3(c) → ADP(c) + Pi(c) + Glutamine(c)
r2685	0.5	Glutamate(c) ⇌ Glutamate(s)
r0222	1	Propanoyl-CoA(m) + Acetyl-CoA(m) ⇌ CoA(m) + 2-Methylacetoacetyl-CoA(m)
r0659	1	ATP(c) + 5-Phosphoribosylamine(c) + Glycine(c) ⇌ ADP(c) + Pi(c) + GAR(c)
r0240	1	Threonine(c) ⇌ 2-Oxobutyrate(c) + NH3(c)
r0099	1	ATP(c) + GDP(c) ⇌ ADP(c) + GTP(c)

Continued on next page

ID	flux	reaction
r0243	-0.17	DHAP(c) ⇌ GAP(c)
r0677	1	10-Formyl-THF(c) + GAR(c) ⇌ THF(c) + N-Formyl-GAR(c)
r1330	-1	H+(PG)(c) ⇌ H+(PG)(m) + Proton-gradient(m)
r0974	1	Pantothenate(s) + Na+(s) ⇌ Pantothenate(c) + Na+(c)
r1319	-12.2	ATP(c) + H2O(c) ⇌ ADP(c) + Pi(c) + ATP-energy(c)
r0260	1	Adenylosuccinate(c) → Fumarate(c) + AMP(c)
r0247	1	ATP(c) + Ribose-5P(c) → AMP(c) + PRPP(c)
r0699	1	SAICAR(c) ⇌ Fumarate(c) + AICAR(c)
r0849	-6.17	H2O(s) ⇌ H2O(c)
r0863	1	CoA(c) ⇌ CoA(m)
r0581	-1	Pantothenate(c) + Cysteamine(c) ⇌ H2O(c) + Pantetheine(c)
r0867	3	CO2(c) ⇌ CO2(s)
r0868	-1	CO2(c) ⇌ CO2(m)
r0589	1	ATP(c) + Phosphopantetheine(c) → PPi(c) + Dephospho-CoA(c)
r2468	1	Glycine(s) + H+(PG)(s) → Glycine(c) + H+(PG)(c)
r0736	0.17	ATP(c) + Fructose-6P(c) → ADP(c) + Fructose-1,6PP(c)
r0007	2	PPi(c) + H2O(c) → 2 Pi(c)
r0027	-0.5	NADPH(c) + Cystine(c) ⇌ 2 Cysteine(c) + NADP+(c)
r0192	0.83	Glucose-6P(c) ⇌ Fructose-6P(c)
r0344	-0.67	D-Xylulose-5P(c) ⇌ Ribulose-5P(c)
r0792	2	5-Methyl-THF(c) + NAD+(c) ⇌ 5,10-Methylene-THF(c) + NADH(c)
r1154	1	CoA(m) + NAD+(m) + 2-Oxobutyrate(m) ⇌ CO2(m) + Propanoyl-CoA(m) + NADH(m)
r0211	1	Cysteine(c) + O2(c) ⇌ 3-Sulfinoalanine(c)
r1155	1	2-Oxobutyrate(c) + H+(PG)(c) → 2-Oxobutyrate(m) + H+(PG)(m)
r2676	-2	5-Methyl-THF(c) ⇌ THF(c) + THF-activated methyl group(c)
r2678	-1	Acetyl-CoA(m) ⇌ CoA(m) + CoA-activated acetyl group(m)
r0666	1	ATP(c) + 5-Phosphoribosylformylglycinamidine(c) ⇌ ADP(c) + Pi(c) + AIR(c)
r0667	-1	5-Phosphoribosyl-4-carboxy-5-aminoimidazole(c) ⇌ AIR(c) + CO2(c)
r1322	2	NADH(c) ⇌ NAD+(c) + NADH-redox-potential(c)
r1323	1	NADH(m) ⇌ NAD+(m) + NADH-redox-potential(m)
r0250	-0.67	Ribulose-5P(c) ⇌ Ribose-5P(c)
r1326	3.5	NADPH(c) ⇌ NADPH-redox-potential(c) + NADP+(c)
r0687	1	ATP(c) + N-Formyl-GAR(c) + Glutamine(c) + H2O(c) ⇌ ADP(c) + Pi(c) + Glutamate(c) + 5-Phosphoribosylformylglycinamidine(c)
r0255	0.33	Fructose-6P(c) + GAP(c) ⇌ D-Xylulose-5P(c) + Erythrose-4P(c)
r0538	1	3-Sulfinoalanine(c) ⇌ CO2(c) + Hypotaurine(c)
r0256	0.17	Fructose-1,6PP(c) ⇌ DHAP(c) + GAP(c)
r0539	-1	Hypotaurine(c) ⇌ Cysteamine(c) + O2(c)
r0257	-1	Glutamine(c) + PRPP(c) + H2O(c) ⇌ 5-Phosphoribosylamine(c) + PPi(c) + Glutamate(c)
r0258	-2	Fumarate(c) + H2O(c) ⇌ Malate(c)
r0273	-1	IMP(c) + H2O(c) ⇌ FAICAR(c)
r0700	1	10-Formyl-THF(c) + AICAR(c) ⇌ THF(c) + FAICAR(c)

2-Methylbutyryl-CoA (606)

Definition			Solution													
			exchanges		reactions									transp		
Objective	Constraints	Comment	imports	exports	c	m	r	p	l	n	s	b	s-c	b-c	int
2-Methylbutyryl-CoA(m)	PIPES MCES	synthesis of mitochondrial 2-Methylbutyryl-CoA	2.17 P_i(c) 1 Glycine(s) 2 Aspartate(s) 1 Glutamine(s) 0.83 Glucose-6P(c) 1 Isoleucine(s) 0.5 Cystine(s) 1 Pantothenate(s) 12.2 ATP-energy(c) 1 Proton-gradient(m) 2 THF-activated methyl group(c)	5.17 H_2O(s) 3 CO_2(s) 2 Pyruvate(c) 1 Glutamate(s) 1 2-Methylbutyryl-CoA(m) 1 NADH-redox-potential(m) 5.5 NADPH-redox-potential(c) 1 NADPH-redox-potential(m) 5 Na-gradient(c)	42	5	-	-	-	-	-	-	10	-	6

ID	flux	reaction
r0406	-0.33	Fructose-6P(c) + Erythrose-4P(c) ⇌ Sedoheptulose-7P(c) + GAP(c)
r0277	1	GTP(c) + IMP(c) + Aspartate(c) → GDP(c) + Pi(c) + Adenylosuccinate(c)
r0705	1	ATP(c) + 5-Phosphoribosyl-4-carboxy-5-aminoimidazole(c) + Aspartate(c) ⇌ ADP(c) + Pi(c) + SAICAR(c)
r0293	2	5,10-Methylene-THF(c) + NADP+(c) ⇌ 5,10-Methenyl-THF(c) + NADPH(c)
r0578	1	ATP(c) + Pantetheine(c) ⇌ ADP(c) + Phosphopantetheine(c)
r0296	2	5-Methyl-THF(c) + NADP+(c) ⇌ 5,10-Methylene-THF(c) + NADPH(c)
r0877	0.5	Glutamate(c) + Cystine(s) ⇌ Glutamate(s) + Cystine(c)
r2477	1	Glutamine(s) + Na+(s) + H+(PG)(c) → Glutamine(c) + Na+(c) + H+(PG)(s)
r0030	2	ATP(c) + AMP(c) ⇌ 2 ADP(c)
r0032	1	ATP(c) + Dephospho-CoA(c) ⇌ ADP(c) + CoA(c)
r1123	-1	Isoleucine(s) + Na+(s) → Isoleucine(c) + Na+(c)
r1143	-2	Na+(c) + Aspartate(c) ← Na+(s) + Aspartate(s)
r2664	5	Na+(c) ⇌ Na+(s) + Na-gradient(c)
r0058	2	Malate(c) + NADP+(c) ⇌ Pyruvate(c) + CO2(c) + NADPH(c)
r0075	1	Glutamate(m) + H2O(m) + NADP+(m) ⇌ AKG(m) + NADPH(m) + NH3(m)
r0370	0.33	Sedoheptulose-7P(c) + GAP(c) ⇌ Ribose-5P(c) + D-Xylulose-5P(c)
r0371	2	5,10-Methenyl-THF(c) + H2O(c) ⇌ 10-Formyl-THF(c)
r0077	1	ATP(c) + Glutamate(c) + NH3(c) → ADP(c) + Pi(c) + Glutamine(c)
r2685	0.5	Glutamate(c) ⇌ Glutamate(s)
r0953	1	Isoleucine(c) + H+(PG)(c) → Isoleucine(m) + H+(PG)(m)
r0659	1	ATP(c) + 5-Phosphoribosylamine(c) + Glycine(c) ⇌ ADP(c) + Pi(c) + GAR(c)
r0099	1	ATP(c) + GDP(c) ⇌ ADP(c) + GTP(c)
r0243	-0.17	DHAP(c) ⇌ GAP(c)
r0677	1	10-Formyl-THF(c) + GAR(c) ⇌ THF(c) + N-Formyl-GAR(c)
r1330	-1	H+(PG)(c) ⇌ H+(PG)(m) + Proton-gradient(m)
r0974	1	Pantothenate(s) + Na+(s) ⇌ Pantothenate(c) + Na+(c)
r1319	-12.2	ATP(c) + H2O(c) ⇌ ADP(c) + Pi(c) + ATP-energy(c)
r0260	1	Adenylosuccinate(c) → Fumarate(c) + AMP(c)
r0247	1	ATP(c) + Ribose-5P(c) → AMP(c) + PRPP(c)
r0264	1	Isoleucine(m) + AKG(m) ⇌ Glutamate(m) + 2-Oxo-3-methylvalerate(m)
r0699	1	SAICAR(c) ⇌ Fumarate(c) + AICAR(c)
r0849	-5.17	H2O(s) ⇌ H2O(c)
r0863	1	CoA(c) ⇌ CoA(m)
r0581	-1	Pantothenate(c) + Cysteamine(c) ⇌ H2O(c) + Pantetheine(c)
r0867	3	CO2(c) ⇌ CO2(s)
r0868	-1	CO2(c) ⇌ CO2(m)
r0589	1	ATP(c) + Phosphopantetheine(c) → PPi(c) + Dephospho-CoA(c)
r2468	1	Glycine(s) + H+(PG)(s) → Glycine(c) + H+(PG)(c)
r0736	0.17	ATP(c) + Fructose-6P(c) → ADP(c) + Fructose-1,6PP(c)
r0007	2	PPi(c) + H2O(c) → 2 Pi(c)
r0602	1	2-Oxo-3-methylvalerate(m) + CoA(m) + NAD+(m) ⇌ 2-Methylbutyryl-CoA(m) + CO2(m) + NADH(m)
r0027	-0.5	NADPH(c) + Cystine(c) ⇌ 2 Cysteine(c) + NADP+(c)
r0192	0.83	Glucose-6P(c) ⇌ Fructose-6P(c)
r0344	-0.67	D-Xylulose-5P(c) ⇌ Ribulose-5P(c)
r0211	1	Cysteine(c) + O2(c) ⇌ 3-Sulfinoalanine(c)
r2676	-2	5-Methyl-THF(c) ⇌ THF(c) + THF-activated methyl group(c)
r0666	1	ATP(c) + 5-Phosphoribosylformylglycinamidine(c) ⇌ ADP(c) + Pi(c) + AIR(c)
r0667	-1	5-Phosphoribosyl-4-carboxy-5-aminoimidazole(c) ⇌ AIR(c) + CO2(c)
r1323	1	NADH(m) ⇌ NAD+(m) + NADH-redox-potential(m)
r0250	-0.67	Ribulose-5P(c) ⇌ Ribose-5P(c)
r1326	5.5	NADPH(c) ⇌ NADPH-redox-potential(c) + NADP+(c)
r0687	1	ATP(c) + N-Formyl-GAR(c) + Glutamine(c) + H2O(c) ⇌ ADP(c) + Pi(c) + Glutamate(c) + 5-Phosphoribosylformylglycinamidine(c)
r1327	1	NADPH(m) ⇌ NADPH-redox-potential(m) + NADP+(m)
r0255	0.33	Fructose-6P(c) + GAP(c) ⇌ D-Xylulose-5P(c) + Erythrose-4P(c)
r0538	1	3-Sulfinoalanine(c) ⇌ CO2(c) + Hypotaurine(c)
r0256	0.17	Fructose-1,6PP(c) ⇌ DHAP(c) + GAP(c)

Continued on next page

ID	flux	reaction
r0539	-1	Hypotaurine(c) ⇌ Cysteamine(c) + O2(c)
r0257	-1	Glutamine(c) + PRPP(c) + H2O(c) ⇌ 5-Phosphoribosylamine(c) + PPi(c) + Glutamate(c)
r0258	-2	Fumarate(c) + H2O(c) ⇌ Malate(c)
r0273	-1	IMP(c) + H2O(c) ⇌ FAICAR(c)
r0851	-1	H2O(m) ⇌ H2O(c)
r0838	-1	NH3(c) ⇌ NH3(m)
r0700	1	10-Formyl-THF(c) + AICAR(c) ⇌ THF(c) + FAICAR(c)

2-Oxo-3-methylvalerate (607)

Definition			Solution												
			exchanges		reactions								transp		
Objective	Constraints	Comment	imports	exports	c	m	r	p	l	n	s	b	s-c	b-c	int
2-Oxo-3-methylvalerate(m)	PIPES MCES	synthesis of mitochondrial 2-Oxo-3-methylvalerate	1 Glutamate(s) 1 Isoleucine(s) 1 ATP-energy(c) 1 Proton-gradient(m)	1 Glutamine(s) 1 2-Oxo-3-methylvalerate(m) 1 NADH-redox-potential(m) 2 Na-gradient(c)	2	3	-	-	-	-	-	-	4	-	4

ID	flux	reaction
r1144	-1	Glutamate(c) + Na+(c) ← Glutamate(s) + Na+(s)
r0073	1	Glutamate(m) + NAD+(m) + H2O(m) ⇌ AKG(m) + NADH(m) + NH3(m)
r0264	1	Isoleucine(m) + AKG(m) ⇌ Glutamate(m) + 2-Oxo-3-methylvalerate(m)
r2525	-1	Glutamine(c) ⇌ Glutamine(s)
r1319	-1	ATP(c) + H2O(c) ⇌ ADP(c) + Pi(c) + ATP-energy(c)
r0851	-1	H2O(m) ⇌ H2O(c)
r0077	1	ATP(c) + Glutamate(c) + NH3(c) → ADP(c) + Pi(c) + Glutamine(c)
r0953	1	Isoleucine(c) + H+(PG)(c) → Isoleucine(m) + H+(PG)(m)
r2664	2	Na+(c) ⇌ Na+(s) + Na-gradient(c)
r0838	-1	NH3(c) ⇌ NH3(m)
r1330	-1	H+(PG)(c) ⇌ H+(PG)(m) + Proton-gradient(m)
r1123	-1	Isoleucine(s) + Na+(s) → Isoleucine(c) + Na+(c)
r1323	1	NADH(m) ⇌ NAD+(m) + NADH-redox-potential(m)

2-Oxoadipate (608)

Definition			Solution												
			exchanges		reactions								transp		
Objective	Constraints	Comment	imports	exports	c	m	r	p	l	n	s	b	s-c	b-c	int
2-Oxoadipate(c)	PIPES MCES	synthesis of cytosolic 2-Oxoadipate	1 Pyruvate(c) 1 Pyruvate(m) 0.5 Lysine(s) 0.5 NADH-redox-potential(m)	1 Alanine(s) 1 2-Oxoadipate(c) 0.5 NADH-redox-potential(c) 1 Proton-gradient(m)	3	10	-	-	-	-	-	-	2	-	8

ID	flux	reaction
r0541	-0.5	Crotonyl-CoA(m) + CO2(m) + Ubiquinol(m) ⇌ Glutaryl-CoA(m) + Ubiquinone(m)
r0460	-0.5	NADH(m) + Acetoacetyl-CoA(m) ⇌ (S)-3-Hydroxybutyryl-CoA(m) + NAD+(m)
r0911	1	Proline(c) + Glutamate(m) ⇌ Proline(m) + Glutamate(c)
r0056	1	Pyruvate(m) + CoA(m) + NAD+(m) → Acetyl-CoA(m) + CO2(m) + NADH(m)
r2524	-0.5	Alanine(c) ⇌ Alanine(s)
r1426	1	Proline(m) + H+(PG)(c) → Proline(c) + H+(PG)(m)
r0526	0.5	H2O(m) + Saccharopine(m) + NADP+(m) ⇌ Glutamate(m) + 2-Aminoadipate 6-semialdehyde(m) + NADPH(m)
r0508	-0.5	Ubiquinone(m) + NADH(m) + 4 H+(PG)(m) → Ubiquinol(m) + NAD+(m) + 4 H+(PG)(c)
r0851	0.5	H2O(m) ⇌ H2O(c)
r0448	-0.5	Glutaryl-CoA(m) + CO2(m) + NADH(m) ⇌ 2-Oxoadipate(m) + CoA(m) + NAD+(m)

Continued on next page

ID	flux	reaction
r0945	-1	2-Oxoadipate(c) + AKG(m) ⇌ 2-Oxoadipate(m) + AKG(c)
r0594	0.5	2-Aminoadipate 6-semialdehyde(c) + NAD+(c) + H2O(c) ⇌ NADH(c) + L-2-Aminoadipate(c)
r2539	-0.5	2-Aminoadipate 6-semialdehyde(c) + L-2-Aminoadipate(m) ⇌ 2-Aminoadipate 6-semialdehyde(m) + L-2-Aminoadipate(c)
r1330	1	H+(PG)(c) ⇌ H+(PG)(m) + Proton-gradient(m)
r0070	0.5	2 Acetyl-CoA(m) ⇌ CoA(m) + Acetoacetyl-CoA(m)
r1322	0.5	NADH(c) ⇌ NAD+(c) + NADH-redox-potential(c)
r1070	0.5	Lysine(c) ⇌ Lysine(m)
r0588	0.5	(S)-3-Hydroxybutyryl-CoA(m) ⇌ Crotonyl-CoA(m) + H2O(m)
r0080	-1	Pyruvate(c) + Glutamate(c) ⇌ Alanine(c) + AKG(c)
r1993	0.5	Lysine(s) + Alanine(c) → Lysine(c) + Alanine(s)
r1323	-0.5	NADH(m) ⇌ NAD+(m) + NADH-redox-potential(m)
r0450	0.5	AKG(m) + L-2-Aminoadipate(m) ⇌ 2-Oxoadipate(m) + Glutamate(m)
r0180	-0.5	AKG(m) + NADPH(m) + Lysine(m) ⇌ H2O(m) + Saccharopine(m) + NADP+(m)

2-Oxobutyrate (609)

Definition			Solution												
			exchanges		reactions								transp		
Objective	Constraints	Comment	imports	exports	c	m	r	p	l	n	s	b	s-c	b-c	int
2-Oxobutyrate(c)	PIPES MCES	synthesis of cytosolic 2-Oxobutyrate	1 Glutamate(s) 1 Threonine(s) 1 ATP-energy(c)	1 H_2O(s) 1 Glutamine(s) 1 2-Oxobutyrate(c) 2 Na-gradient(c)	3	-	-	-	-	-	-	-	5	-	-

ID	flux	reaction
r1144	-1	Glutamate(c) + Na+(c) ← Glutamate(s) + Na+(s)
r2525	-1	Glutamine(c) ⇌ Glutamine(s)
r1319	-1	ATP(c) + H2O(c) ⇌ ADP(c) + Pi(c) + ATP-energy(c)
r0077	1	ATP(c) + Glutamate(c) + NH3(c) → ADP(c) + Pi(c) + Glutamine(c)
r2664	2	Na+(c) ⇌ Na+(s) + Na-gradient(c)
r0240	1	Threonine(c) ⇌ 2-Oxobutyrate(c) + NH3(c)
r1142	-1	Threonine(s) + Na+(s) → Threonine(c) + Na+(c)
r0849	-1	H2O(s) ⇌ H2O(c)

2-Oxoglutaramate (610)

Definition			Solution												
			exchanges		reactions								transp		
Objective	Constraints	Comment	imports	exports	c	m	r	p	l	n	s	b	s-c	b-c	int
2-Oxoglutaramate(c)	PIPES MCES	synthesis of cytosolic 2-Oxoglutaramate	1 Pyruvate(c) 1 Glutamine(s)	1 Alanine(s) 1 2-Oxoglutaramate(c)	1	-	-	-	-	-	-	-	1	-	-

ID	flux	reaction
r0156	1	Glutamine(c) + Pyruvate(c) → 2-Oxoglutaramate(c) + Alanine(c)
r1558	-1	Alanine(c) + Glutamine(s) ⇌ Alanine(s) + Glutamine(c)

2PG (611)

Definition			Solution												
			exchanges		reactions								transp		
Objective	Constraints	Comment	imports	exports	c	m	r	p	l	n	s	b	s-c	b-c	int
2PG(c)	PIPES MCES	synthesis of cytosolic 2PG	1 P_i(c) 1 Pyruvate(c) 1 ATP-energy(c) 1 NADPH-redox-potential(c)	1 2PG(c) 1 NADH-redox-potential(c)	8	-	-	-	-	-	-	-	-	-	-

ID	flux	reaction
r0164	-1	PEP(c) + H2O(c) ⇌ 2PG(c)
r1326	-1	NADPH(c) ⇌ NADPH-redox-potential(c) + NADP+(c)
r0058	-1	Pyruvate(c) + CO2(c) + NADPH(c) ⇌ Malate(c) + NADP+(c)
r1319	-1	ATP(c) + H2O(c) ⇌ ADP(c) + Pi(c) + ATP-energy(c)
r0104	1	NAD+(c) + Malate(c) ⇌ OAA(c) + NADH(c)
r0099	1	ATP(c) + GDP(c) ⇌ ADP(c) + GTP(c)
r0123	1	GTP(c) + OAA(c) → GDP(c) + PEP(c) + CO2(c)
r1322	1	NADH(c) ⇌ NAD+(c) + NADH-redox-potential(c)

3(S)-3-hydroxydodecen-(5Z)-oyl-CoA (612)

Definition			Solution												
			exchanges		reactions								transp		
Objective	Constraints	Comment	imports	exports	c	m	r	p	l	n	s	b	s-c	b-c	int
3(S)-3-hydroxydodecen-(5Z)-oyl-CoA(m)	PIPES MCES -	synthesis of mitochondrial 3(S)-3-hydroxydodecen-(5Z)-oyl-CoA	2.17 P_i(c) 1 Glycine(s) 2 Aspartate(s) 2 Glutamine(s) 0.83 Glucose-6P(c) 0.5 Cystine(s) 1 Pantothenate(s) 1 Palmitolate(s) 13.2 ATP-energy(c) 1 Proton-gradient(c) 2 THF-activated methyl group(c)	3.17 H_2O(s) 2 CO_2(s) 2 Pyruvate(c) 2 Glutamate(s) 2 NADH-redox-potential(c) 2 NADH-redox-potential(m) 3.5 NADPH-redox-potential(c) 3 FADH-redox-potential(c) 1 3(S)-3-hydroxydodecen-(5Z)-oyl-CoA(m) 3 Na-gradient(c) 2 CoA-activated acetyl group(m)	44	12	-	-	-	-	-	-	11	-	3

ID	flux	reaction
r0406	-0.33	Fructose-6P(c) + Erythrose-4P(c) ⇌ Sedoheptulose-7P(c) + GAP(c)
r0277	1	GTP(c) + IMP(c) + Aspartate(c) → GDP(c) + Pi(c) + Adenylosuccinate(c)
r0293	2	5,10-Methylene-THF(c) + NADP+(c) ⇌ 5,10-Methenyl-THF(c) + NADPH(c)
r0705	1	ATP(c) + 5-Phosphoribosyl-4-carboxy-5-aminoimidazole(c) + Aspartate(c) ⇌ ADP(c) + Pi(c) + SAICAR(c)
r0578	1	ATP(c) + Pantetheine(c) ⇌ ADP(c) + Phosphopantetheine(c)
r0877	0.5	Glutamate(c) + Cystine(s) ⇌ Glutamate(s) + Cystine(c)
r0030	3	ATP(c) + AMP(c) ⇌ 2 ADP(c)
r0032	1	ATP(c) + Dephospho-CoA(c) ⇌ ADP(c) + CoA(c)
r1290	3	FAD(c) + FADH2(m) ⇌ FAD(m) + FADH2(c)
r1143	-2	Na+(c) + Aspartate(c) ← Na+(s) + Aspartate(s)
r2664	3	Na+(c) ⇌ Na+(s) + Na-gradient(c)
r0058	2	Malate(c) + NADP+(c) ⇌ Pyruvate(c) + CO2(c) + NADPH(c)
r0370	0.33	Sedoheptulose-7P(c) + GAP(c) ⇌ Ribose-5P(c) + D-Xylulose-5P(c)
r0371	2	5,10-Methenyl-THF(c) + H2O(c) ⇌ 10-Formyl-THF(c)
r2685	1.5	Glutamate(c) ⇌ Glutamate(s)
r0659	1	ATP(c) + 5-Phosphoribosylamine(c) + Glycine(c) ⇌ ADP(c) + Pi(c) + GAR(c)
r1480	1	CoA(m) + 3-oxopalmitoleoyl-CoA(m) ⇌ Acetyl-CoA(m) + cis-myrist-7-enoyl-CoA(m)
r0099	1	ATP(c) + GDP(c) ⇌ ADP(c) + GTP(c)
r1468	1	palmitoleoyl-CoA(c) ⇌ palmitoleoyl-CoA(m)
r0243	-0.17	DHAP(c) ⇌ GAP(c)
r0677	1	10-Formyl-THF(c) + GAR(c) ⇌ THF(c) + N-Formyl-GAR(c)
r1482	-1	FADH2(m) + trans,cis-dodeca-2,5-dienoyl-CoA(m) ⇌ FAD(m) + cis-laur-5-enoyl-CoA(m)
r1483	1	H2O(m) + trans,cis-myristo-2,7-dienoyl-CoA(m) ⇌ cis-(3S)-hydroxytetradec-7-enoyl-CoA(m)
r1331	-1	H+(PG)(s) ⇌ H+(PG)(c) + Proton-gradient(c)
r0974	1	Pantothenate(s) + Na+(s) ⇌ Pantothenate(c) + Na+(c)
r1484	1	CoA(m) + 3-oxomyrist-7-enoyl-CoA(m) ⇌ Acetyl-CoA(m) + cis-laur-5-enoyl-CoA(m)
r1319	-13.2	ATP(c) + H2O(c) ⇌ ADP(c) + Pi(c) + ATP-energy(c)
r1485	1	H2O(m) + trans,cis-dodeca-2,5-dienoyl-CoA(m) ⇌ 3(S)-3-hydroxydodecen-(5Z)-oyl-CoA(m)
r0260	1	Adenylosuccinate(c) → Fumarate(c) + AMP(c)
r0247	1	ATP(c) + Ribose-5P(c) → AMP(c) + PRPP(c)
r1488	1	ATP(c) + CoA(c) + Palmitolate(c) ⇌ AMP(c) + palmitoleoyl-CoA(c) + PPi(c)
r0699	1	SAICAR(c) ⇌ Fumarate(c) + AICAR(c)

Continued on next page

ID	flux	reaction
r2442	1	Palmitolate(s) → Palmitolate(c)
r0849	-3.17	H2O(s) ⇌ H2O(c)
r0581	-1	Pantothenate(c) + Cysteamine(c) ⇌ H2O(c) + Pantetheine(c)
r0867	2	CO2(c) ⇌ CO2(s)
r0589	1	ATP(c) + Phosphopantetheine(c) → PPi(c) + Dephospho-CoA(c)
r2468	1	Glycine(s) + H+(PG)(s) → Glycine(c) + H+(PG)(c)
r0007	3	PPi(c) + H2O(c) → 2 Pi(c)
r0736	0.17	ATP(c) + Fructose-6P(c) → ADP(c) + Fructose-1,6PP(c)
r0027	-0.5	NADPH(c) + Cystine(c) ⇌ 2 Cysteine(c) + NADP+(c)
r0192	0.83	Glucose-6P(c) ⇌ Fructose-6P(c)
r0344	-0.67	D-Xylulose-5P(c) ⇌ Ribulose-5P(c)
r0792	2	5-Methyl-THF(c) + NAD+(c) ⇌ 5,10-Methylene-THF(c) + NADH(c)
r0211	1	Cysteine(c) + O2(c) ⇌ 3-Sulfinoalanine(c)
r2676	-2	5-Methyl-THF(c) ⇌ THF(c) + THF-activated methyl group(c)
r2525	2	Glutamine(s) ⇌ Glutamine(c)
r2678	2	Acetyl-CoA(m) ⇌ CoA(m) + CoA-activated acetyl group(m)
r0666	1	ATP(c) + 5-Phosphoribosylformylglycinamidine(c) ⇌ ADP(c) + Pi(c) + AIR(c)
r1471	1	FAD(m) + cis-myrist-7-enoyl-CoA(m) ⇌ FADH2(m) + trans,cis-myristo-2,7-dienoyl-CoA(m)
r0667	-1	5-Phosphoribosyl-4-carboxy-5-aminoimidazole(c) ⇌ AIR(c) + CO2(c)
r1473	1	FAD(m) + palmitoleoyl-CoA(m) ⇌ FADH2(m) + trans,cis-hexadeca-2,9-dienoyl-CoA(m)
r1322	2	NADH(c) ⇌ NAD+(c) + NADH-redox-potential(c)
r1475	1	H2O(m) + trans,cis-hexadeca-2,9-dienoyl-CoA(m) ⇌ (S)-3-hydroxypalmitoleoyl-CoA(m)
r1323	2	NADH(m) ⇌ NAD+(m) + NADH-redox-potential(m)
r0250	-0.67	Ribulose-5P(c) ⇌ Ribose-5P(c)
r1476	1	NAD+(m) + cis-(3S)-hydroxytetradec-7-enoyl-CoA(m) ⇌ 3-oxomyrist-7-enoyl-CoA(m) + NADH(m)
r1478	1	NAD+(m) + (S)-3-hydroxypalmitoleoyl-CoA(m) ⇌ NADH(m) + 3-oxopalmitoleoyl-CoA(m)
r1326	3.5	NADPH(c) ⇌ NADPH-redox-potential(c) + NADP+(c)
r0687	1	ATP(c) + N-Formyl-GAR(c) + Glutamine(c) + H2O(c) ⇌ ADP(c) + Pi(c) + Glutamate(c) + 5-Phosphoribosylformylglycinamidine(c)
r0255	0.33	Fructose-6P(c) + GAP(c) ⇌ D-Xylulose-5P(c) + Erythrose-4P(c)
r1329	3	FADH2(c) ⇌ FAD(c) + FADH-redox-potential(c)
r0538	1	3-Sulfinoalanine(c) ⇌ CO2(c) + Hypotaurine(c)
r0256	0.17	Fructose-1,6PP(c) ⇌ DHAP(c) + GAP(c)
r0539	-1	Hypotaurine(c) ⇌ Cysteamine(c) + O2(c)
r0257	-1	Glutamine(c) + PRPP(c) + H2O(c) ⇌ 5-Phosphoribosylamine(c) + PPi(c) + Glutamate(c)
r0258	-2	Fumarate(c) + H2O(c) ⇌ Malate(c)
r0273	-1	IMP(c) + H2O(c) ⇌ FAICAR(c)
r0851	-3	H2O(m) ⇌ H2O(c)
r0700	1	10-Formyl-THF(c) + AICAR(c) ⇌ THF(c) + FAICAR(c)

3-Dehydrosphinganine (613)

Definition			Solution												
			exchanges		reactions								transp		
Objective	Constraints	Comment	imports	exports	c	m	r	p	l	n	s	b	s-c	b-c	int
3-Dehydrosphinganine(c)	PIPES MCES	synthesis of cytosolic 3-Dehydrosphinganine	1 Serine(s) 1 Palmitate(s) 2 ATP-energy(c)	1 H_2O(s) 1 CO_2(s) 1 3-Dehydrosphinganine(c) 1 Proton-gradient(c) 1 Na-gradient(c)	5	-	-	-	-	-	-	-	6	-	-

ID	flux	reaction
r1319	-2	ATP(c) + H2O(c) ⇌ ADP(c) + Pi(c) + ATP-energy(c)
r0030	1	ATP(c) + AMP(c) ⇌ 2 ADP(c)
r0935	1	Palmitate(s) → Palmitate(c)
r2664	1	Na+(c) ⇌ Na+(s) + Na-gradient(c)

Continued on next page

ID	flux	reaction
r0312	1	Palmitate(c) + ATP(c) + CoA(c) → Palmitoyl-CoA(c) + AMP(c) + PPi(c)
r0313	1	Serine(c) + Palmitoyl-CoA(c) ⇌ 3-Dehydrosphinganine(c) + CoA(c) + CO2(c)
r0007	1	PPi(c) + H2O(c) → 2 Pi(c)
r2478	1	Serine(s) + Na+(s) + H+(PG)(c) → Serine(c) + Na+(c) + H+(PG)(s)
r1331	1	H+(PG)(s) ⇌ H+(PG)(c) + Proton-gradient(c)
r0867	1	CO2(c) ⇌ CO2(s)
r0849	-1	H2O(s) ⇌ H2O(c)

3-Hydroxy-L-kynurenine (614)

Definition			Solution												
			exchanges		reactions								transp		
Objective	Constraints	Comment	imports	exports	c	m	r	p	l	n	s	b	s-c	b-c	int
3-Hydroxy-L-kynurenine(c)	PIPES MCES	synthesis of cytosolic 3-Hydroxy-L-kynurenine	2 O_2(s) 1 Tryptophan(s) 1 ATP-energy(c)	1 CO_2(s) 1 3-Hydroxy-L-kynurenine(c) 1 Na-gradient(c)	6	-	-	-	-	-	-	-	4	-	-

ID	flux	reaction
r0227	1	10-Formyl-THF(c) + H2O(c) + NADP+(c) → THF(c) + CO2(c) + NADPH(c)
r0228	1	THF(c) + Formate(c) + ATP(c) → ADP(c) + Pi(c) + 10-Formyl-THF(c)
r0454	1	L-Formylkynurenine(c) + H2O(c) ⇌ Formate(c) + Kynurenine(c)
r1319	-1	ATP(c) + H2O(c) ⇌ ADP(c) + Pi(c) + ATP-energy(c)
r0455	1	Kynurenine(c) + O2(c) + NADPH(c) ⇌ H2O(c) + 3-Hydroxy-L-kynurenine(c) + NADP+(c)
r0169	1	Tryptophan(c) + O2(c) ⇌ L-Formylkynurenine(c)
r2664	1	Na+(c) ⇌ Na+(s) + Na-gradient(c)
r0855	2	O2(s) ⇌ O2(c)
r1124	-1	Tryptophan(s) + Na+(s) → Tryptophan(c) + Na+(c)
r0867	1	CO2(c) ⇌ CO2(s)

3-Hydroxyanthranilate (615)

Definition			Solution												
			exchanges		reactions								transp		
Objective	Constraints	Comment	imports	exports	c	m	r	p	l	n	s	b	s-c	b-c	int
3-Hydroxyanthranilate(c)	PIPES MCES	synthesis of cytosolic 3-Hydroxyanthranilate	2 O_2(s) 1 Tryptophan(s) 1 ATP-energy(c) 1.5 NADPH-redox-potential(c)	0.5 CO_2(s) 1 Alanine(s) 1 3-Hydroxyanthranilate(c) 1 Na-gradient(c) 0.5 THF-activated methyl group(c)	12	-	-	-	-	-	-	-	5	-	-

ID	flux	reaction
r0371	-0.5	10-Formyl-THF(c) ⇌ 5,10-Methenyl-THF(c) + H2O(c)
r0227	0.5	10-Formyl-THF(c) + H2O(c) + NADP+(c) → THF(c) + CO2(c) + NADPH(c)
r1326	-1.5	NADPH(c) ⇌ NADPH-redox-potential(c) + NADP+(c)
r0228	1	THF(c) + Formate(c) + ATP(c) → ADP(c) + Pi(c) + 10-Formyl-THF(c)
r2524	-1	Alanine(c) ⇌ Alanine(s)
r0454	1	L-Formylkynurenine(c) + H2O(c) ⇌ Formate(c) + Kynurenine(c)
r1319	-1	ATP(c) + H2O(c) ⇌ ADP(c) + Pi(c) + ATP-energy(c)
r0563	1	H2O(c) + 3-Hydroxy-L-kynurenine(c) ⇌ 3-Hydroxyanthranilate(c) + Alanine(c)
r0455	1	Kynurenine(c) + O2(c) + NADPH(c) ⇌ H2O(c) + 3-Hydroxy-L-kynurenine(c) + NADP+(c)
r0293	-0.5	5,10-Methylene-THF(c) + NADP+(c) ⇌ 5,10-Methenyl-THF(c) + NADPH(c)
r0169	1	Tryptophan(c) + O2(c) ⇌ L-Formylkynurenine(c)
r0296	-0.5	5,10-Methylene-THF(c) + NADPH(c) ⇌ 5-Methyl-THF(c) + NADP+(c)
r2664	1	Na+(c) ⇌ Na+(s) + Na-gradient(c)

Continued on next page

ID	flux	reaction
r0855	2	O2(s) ⇌ O2(c)
r2676	0.5	5-Methyl-THF(c) ⇌ THF(c) + THF-activated methyl group(c)
r1124	-1	Tryptophan(s) + Na+(s) → Tryptophan(c) + Na+(c)
r0867	0.5	CO2(c) ⇌ CO2(s)

3-Hydroxyisobutyrate (616)

Definition			Solution													
			exchanges		reactions								transp			
Objective	Constraints	Comment	imports	exports	c	m	r	p	l	n	s	b	s-c	b-c	int
3-Hydroxyisobutyrate(m)	PIPES MCES	synthesis of mitochondrial 3-Hydroxyisobutyrate	0.67 H_2O(s) 0.5 O_2(s) 0.33 Glutamate(s) 1 Valine(s) 0.67 ATP-energy(c) 0.67 ATP-energy(m)	0.67 CO_2(s) 0.33 Arginine(s) 1 3-Hydroxyisobutyrate(m) 0.33 NADH-redox-potential(c) 1 NADH-redox-potential(m) 4.33 Proton-gradient(m) 1.33 Na-gradient(c)	9	12	-	-	-	-	-	-	7	-	10

ID	flux	reaction
r1144	-0.33	Glutamate(c) + Na+(c) ← Glutamate(s) + Na+(s)
r0779	1	3-Hydroxyisobutyryl-CoA(m) + H2O(m) → 3-Hydroxyisobutyrate(m) + CoA(m)
r0109	-0.33	OAA(c) + Glutamate(c) ⇌ AKG(c) + Aspartate(c)
r0073	0.33	Glutamate(m) + NAD+(m) + H2O(m) ⇌ AKG(m) + NADH(m) + NH3(m)
r0560	1	Isobutyryl-CoA(m) + Ubiquinone(m) → Methacrylyl-CoA(m) + Ubiquinol(m)
r0263	1	Valine(m) + AKG(m) → Glutamate(m) + 3-Methyl-2-oxobutyrate(m)
r0074	-0.33	Glutamate(m) + NADH(m) ⇌ L-Glutamate 5-semialdehyde(m) + NAD+(m) + H2O(m)
r1047	0.33	Pi(c) + H+(PG)(c) → Pi(m) + H+(PG)(m)
r0507	1	Ubiquinol(m) + 2 Ferricytochrome C(m) + 4 H+(PG)(m) → Ubiquinone(m) + 4 H+(PG)(c) + 2 Ferrocytochrome C(m)
r0453	0.33	ATP(c) + Citrulline(c) + Aspartate(c) ⇌ AMP(c) + PPi(c) + Argininosuccinate(c)
r2417	-0.33	Citrulline(m) + H+(PG)(c) → Citrulline(c) + H+(PG)(m)
r1319	-0.67	ATP(c) + H2O(c) ⇌ ADP(c) + Pi(c) + ATP-energy(c)
r1058	-0.33	Arginine(c) ⇌ Arginine(s)
r0923	1	Valine(c) + H+(PG)(c) → Valine(m) + H+(PG)(m)
r0851	-0.67	H2O(m) ⇌ H2O(c)
r0329	0.33	Carbamoyl-P(m) + Ornithine(m) ⇌ Pi(m) + Citrulline(m)
r0167	-0.33	L-Glutamate 5-semialdehyde(m) + Glutamate(m) ⇌ AKG(m) + Ornithine(m)
r0258	-0.33	Fumarate(c) + H2O(c) ⇌ Malate(c)
r2419	0.33	AKG(c) + Pi(m) ⇌ AKG(m) + Pi(c)
r0030	0.33	ATP(c) + AMP(c) ⇌ 2 ADP(c)
r2664	1.33	Na+(c) ⇌ Na+(s) + Na-gradient(c)
r1121	-1	Valine(s) + Na+(s) → Valine(c) + Na+(c)
r0855	0.5	O2(s) ⇌ O2(c)
r0104	0.33	NAD+(c) + Malate(c) ⇌ OAA(c) + NADH(c)
r1320	-0.67	ATP(m) + H2O(m) ⇌ ADP(m) + Pi(m) + ATP-energy(m)
r0856	-0.5	O2(m) ⇌ O2(c)
r0600	1	3-Methyl-2-oxobutyrate(m) + CoA(m) + NAD+(m) ⇌ Isobutyryl-CoA(m) + CO2(m) + NADH(m)
r1330	4.33	H+(PG)(c) ⇌ H+(PG)(m) + Proton-gradient(m)
r0034	0.33	2 ATP(m) + CO2(m) + H2O(m) + NH3(m) ⇌ 2 ADP(m) + Pi(m) + Carbamoyl-P(m)
r0007	0.33	PPi(c) + H2O(c) → 2 Pi(c)
r1322	0.33	NADH(c) ⇌ NAD+(c) + NADH-redox-potential(c)
r0867	0.67	CO2(c) ⇌ CO2(s)
r0849	0.67	H2O(s) ⇌ H2O(c)
r0669	1	Methacrylyl-CoA(m) + H2O(m) → 3-Hydroxyisobutyryl-CoA(m)
r0017	0.5	O2(m) + 4 H+(PG)(m) + 4 Ferrocytochrome C(m) → 4 Ferricytochrome C(m) + 2 H2O(m) + 4 H+(PG)(c)
r1323	1	NADH(m) ⇌ NAD+(m) + NADH-redox-potential(m)
r0868	-0.67	CO2(c) ⇌ CO2(m)
r0261	0.33	Argininosuccinate(c) ⇌ Fumarate(c) + Arginine(c)

3-Hydroxyisobutyryl-CoA (617)

Definition			Solution												
			exchanges		reactions								transp		
Objective	Constraints	Comment	imports	exports	c	m	r	p	l	n	s	b	s-c	b-c	int
3-Hydroxyisobutyryl-CoA(m)	PIPES MCES	synthesis of mitochondrial 3-Hydroxyisobutyryl-CoA	0.5 O_2(s) 2.17 P_i(c) 1 Glycine(s) 2 Aspartate(s) 1 Glutamine(s) 0.83 Glucose-6P(c) 1 Valine(s) 0.5 Cystine(s) 1 Pantothenate(s) 12.2 ATP-energy(c) 1 Proton-gradient(c) 2 THF-activated methyl group(c)	5.17 H_2O(s) 3 CO_2(s) 2 Pyruvate(c) 1 Glutamate(s) 1 3-Hydroxyisobutyryl-CoA(m) 2 NADH-redox-potential(c) 2 NADH-redox-potential(m) 3.5 NADPH-redox-potential(c) 5 Proton-gradient(m) 4 Na-gradient(c)	43	6	-	-	-	-	-	-	12	-	9

ID	flux	reaction
r0406	-0.33	Fructose-6P(c) + Erythrose-4P(c) ⇌ Sedoheptulose-7P(c) + GAP(c)
r0277	1	GTP(c) + IMP(c) + Aspartate(c) → GDP(c) + Pi(c) + Adenylosuccinate(c)
r0855	0.5	O2(s) ⇌ O2(c)
r0856	-0.5	O2(m) ⇌ O2(c)
r0705	1	ATP(c) + 5-Phosphoribosyl-4-carboxy-5-aminoimidazole(c) + Aspartate(c) ⇌ ADP(c) + Pi(c) + SAICAR(c)
r0293	2	5,10-Methylene-THF(c) + NADP+(c) ⇌ 5,10-Methenyl-THF(c) + NADPH(c)
r0578	1	ATP(c) + Pantetheine(c) ⇌ ADP(c) + Phosphopantetheine(c)
r0877	0.5	Glutamate(c) + Cystine(s) ⇌ Glutamate(s) + Cystine(c)
r0017	0.5	O2(m) + 4 H+(PG)(m) + 4 Ferrocytochrome C(m) → 4 Ferricytochrome C(m) + 2 H2O(m) + 4 H+(PG)(c)
r0030	2	ATP(c) + AMP(c) ⇌ 2 ADP(c)
r0032	1	ATP(c) + Dephospho-CoA(c) ⇌ ADP(c) + CoA(c)
r1121	-1	Valine(s) + Na+(s) → Valine(c) + Na+(c)
r1143	-2	Na+(c) + Aspartate(c) ← Na+(s) + Aspartate(s)
r2664	4	Na+(c) ⇌ Na+(s) + Na-gradient(c)
r0058	2	Malate(c) + NADP+(c) ⇌ Pyruvate(c) + CO2(c) + NADPH(c)
r0073	1	Glutamate(m) + NAD+(m) + H2O(m) ⇌ AKG(m) + NADH(m) + NH3(m)
r0370	0.33	Sedoheptulose-7P(c) + GAP(c) ⇌ Ribose-5P(c) + D-Xylulose-5P(c)
r0371	2	5,10-Methenyl-THF(c) + H2O(c) ⇌ 10-Formyl-THF(c)
r0077	1	ATP(c) + Glutamate(c) + NH3(c) → ADP(c) + Pi(c) + Glutamine(c)
r2685	0.5	Glutamate(c) ⇌ Glutamate(s)
r0659	1	ATP(c) + 5-Phosphoribosylamine(c) + Glycine(c) ⇌ ADP(c) + Pi(c) + GAR(c)
r0507	1	Ubiquinol(m) + 2 Ferricytochrome C(m) + 4 H+(PG)(m) → Ubiquinone(m) + 4 H+(PG)(c) + 2 Ferrocytochrome C(m)
r0099	1	ATP(c) + GDP(c) ⇌ ADP(c) + GTP(c)
r0677	1	10-Formyl-THF(c) + GAR(c) ⇌ THF(c) + N-Formyl-GAR(c)
r0243	-0.17	DHAP(c) ⇌ GAP(c)
r1330	5	H+(PG)(c) ⇌ H+(PG)(m) + Proton-gradient(m)
r1331	-1	H+(PG)(s) ⇌ H+(PG)(c) + Proton-gradient(c)
r0974	1	Pantothenate(s) + Na+(s) ⇌ Pantothenate(c) + Na+(c)
r1319	-12.2	ATP(c) + H2O(c) ⇌ ADP(c) + Pi(c) + ATP-energy(c)
r0260	1	Adenylosuccinate(c) → Fumarate(c) + AMP(c)
r0247	1	ATP(c) + Ribose-5P(c) → AMP(c) + PRPP(c)
r0263	1	Valine(m) + AKG(m) → Glutamate(m) + 3-Methyl-2-oxobutyrate(m)
r0699	1	SAICAR(c) ⇌ Fumarate(c) + AICAR(c)
r0560	1	Isobutyryl-CoA(m) + Ubiquinone(m) → Methacrylyl-CoA(m) + Ubiquinol(m)
r0849	-5.17	H2O(s) ⇌ H2O(c)
r0863	1	CoA(c) ⇌ CoA(m)
r0581	-1	Pantothenate(c) + Cysteamine(c) ⇌ H2O(c) + Pantetheine(c)
r0867	3	CO2(c) ⇌ CO2(s)
r0868	-1	CO2(c) ⇌ CO2(m)
r0589	1	ATP(c) + Phosphopantetheine(c) → PPi(c) + Dephospho-CoA(c)
r2468	1	Glycine(s) + H+(PG)(s) → Glycine(c) + H+(PG)(c)
r0736	0.17	ATP(c) + Fructose-6P(c) → ADP(c) + Fructose-1,6PP(c)
r0007	2	PPi(c) + H2O(c) → 2 Pi(c)

Continued on next page

ID	flux	reaction
r0600	1	3-Methyl-2-oxobutyrate(m) + CoA(m) + NAD+(m) ⇌ Isobutyryl-CoA(m) + CO2(m) + NADH(m)
r0027	-0.5	NADPH(c) + Cystine(c) ⇌ 2 Cysteine(c) + NADP+(c)
r0192	0.83	Glucose-6P(c) ⇌ Fructose-6P(c)
r0344	-0.67	D-Xylulose-5P(c) ⇌ Ribulose-5P(c)
r0792	2	5-Methyl-THF(c) + NAD+(c) ⇌ 5,10-Methylene-THF(c) + NADH(c)
r0923	1	Valine(c) + H+(PG)(c) → Valine(m) + H+(PG)(m)
r0211	1	Cysteine(c) + O2(c) ⇌ 3-Sulfinoalanine(c)
r2676	-2	5-Methyl-THF(c) ⇌ THF(c) + THF-activated methyl group(c)
r2525	1	Glutamine(s) ⇌ Glutamine(c)
r0666	1	ATP(c) + 5-Phosphoribosylformylglycinamidine(c) ⇌ ADP(c) + Pi(c) + AIR(c)
r0667	-1	5-Phosphoribosyl-4-carboxy-5-aminoimidazole(c) ⇌ AIR(c) + CO2(c)
r0669	1	Methacrylyl-CoA(m) + H2O(m) → 3-Hydroxyisobutyryl-CoA(m)
r1322	2	NADH(c) ⇌ NAD+(c) + NADH-redox-potential(c)
r1323	2	NADH(m) ⇌ NAD+(m) + NADH-redox-potential(m)
r0250	-0.67	Ribulose-5P(c) ⇌ Ribose-5P(c)
r1326	3.5	NADPH(c) ⇌ NADPH-redox-potential(c) + NADP+(c)
r0687	1	ATP(c) + N-Formyl-GAR(c) + Glutamine(c) + H2O(c) ⇌ ADP(c) + Pi(c) + Glutamate(c) + 5-Phosphoribosylformylglycinamidine(c)
r0255	0.33	Fructose-6P(c) + GAP(c) ⇌ D-Xylulose-5P(c) + Erythrose-4P(c)
r0538	1	3-Sulfinoalanine(c) ⇌ CO2(c) + Hypotaurine(c)
r0256	0.17	Fructose-1,6PP(c) ⇌ DHAP(c) + GAP(c)
r0539	-1	Hypotaurine(c) ⇌ Cysteamine(c) + O2(c)
r0257	-1	Glutamine(c) + PRPP(c) + H2O(c) ⇌ 5-Phosphoribosylamine(c) + PPi(c) + Glutamate(c)
r0258	-2	Fumarate(c) + H2O(c) ⇌ Malate(c)
r0273	-1	IMP(c) + H2O(c) ⇌ FAICAR(c)
r0851	-1	H2O(m) ⇌ H2O(c)
r0838	-1	NH3(c) ⇌ NH3(m)
r0700	1	10-Formyl-THF(c) + AICAR(c) ⇌ THF(c) + FAICAR(c)

3-Hydroxypropionyl-CoA (618)

Definition			Solution												
			exchanges		reactions								transp		
Objective	Constraints	Comment	imports	exports	c	m	r	p	l	n	s	b	s-c	b-c	int
3-Hydroxypropionyl-CoA(m)	PIPES MCES	synthesis of mitochondrial 3-Hydroxypropionyl-CoA	0.5 O_2(s) 2.17 P_i(c) 1 Glycine(s) 2 Aspartate(s) 1 Glutamine(s) 0.83 Glucose-6P(c) 1 Threonine(s) 0.5 Cystine(s) 1 Pantothenate(s) 12.2 ATP-energy(c) 1 Proton-gradient(c) 2 THF-activated methyl group(c)	6.17 H_2O(s) 3 CO_2(s) 2 Pyruvate(c) 1 Glutamate(s) 1 3-Hydroxypropionyl-CoA(m) 2 NADH-redox-potential(c) 1 NADH-redox-potential(m) 3.5 NADPH-redox-potential(c) 5 Proton-gradient(m) 4 Na-gradient(c)	44	4	-	-	-	-	-	-	12	-	7

ID	flux	reaction
r0406	-0.33	Fructose-6P(c) + Erythrose-4P(c) ⇌ Sedoheptulose-7P(c) + GAP(c)
r0277	1	GTP(c) + IMP(c) + Aspartate(c) → GDP(c) + Pi(c) + Adenylosuccinate(c)
r0855	0.5	O2(s) ⇌ O2(c)
r0856	-0.5	O2(m) ⇌ O2(c)
r0705	1	ATP(c) + 5-Phosphoribosyl-4-carboxy-5-aminoimidazole(c) + Aspartate(c) ⇌ ADP(c) + Pi(c) + SAICAR(c)
r0293	2	5,10-Methylene-THF(c) + NADP+(c) ⇌ 5,10-Methenyl-THF(c) + NADPH(c)
r0578	1	ATP(c) + Pantetheine(c) ⇌ ADP(c) + Phosphopantetheine(c)
r0592	-1	H2O(m) + Acrylyl-CoA(m) → 3-Hydroxypropionyl-CoA(m)
r0877	0.5	Glutamate(c) + Cystine(s) ⇌ Glutamate(s) + Cystine(c)
r0017	0.5	O2(m) + 4 H+(PG)(m) + 4 Ferrocytochrome C(m) → 4 Ferricytochrome C(m) + 2 H2O(m) + 4 H+(PG)(c)
r0030	2	ATP(c) + AMP(c) ⇌ 2 ADP(c)
r0032	1	ATP(c) + Dephospho-CoA(c) ⇌ ADP(c) + CoA(c)

Continued on next page

ID	flux	reaction
r1142	-1	Threonine(s) + Na+(s) → Threonine(c) + Na+(c)
r1143	-2	Na+(c) + Aspartate(c) ← Na+(s) + Aspartate(s)
r2664	4	Na+(c) ⇌ Na+(s) + Na-gradient(c)
r0058	2	Malate(c) + NADP+(c) ⇌ Pyruvate(c) + CO2(c) + NADPH(c)
r0370	0.33	Sedoheptulose-7P(c) + GAP(c) ⇌ Ribose-5P(c) + D-Xylulose-5P(c)
r0371	2	5,10-Methenyl-THF(c) + H2O(c) ⇌ 10-Formyl-THF(c)
r0077	1	ATP(c) + Glutamate(c) + NH3(c) → ADP(c) + Pi(c) + Glutamine(c)
r2685	0.5	Glutamate(c) ⇌ Glutamate(s)
r0659	1	ATP(c) + 5-Phosphoribosylamine(c) + Glycine(c) ⇌ ADP(c) + Pi(c) + GAR(c)
r0507	1	Ubiquinol(m) + 2 Ferricytochrome C(m) + 4 H+(PG)(m) → Ubiquinone(m) + 4 H+(PG)(c) + 2 Ferrocytochrome C(m)
r0240	1	Threonine(c) ⇌ 2-Oxobutyrate(c) + NH3(c)
r0099	1	ATP(c) + GDP(c) ⇌ ADP(c) + GTP(c)
r0243	-0.17	DHAP(c) ⇌ GAP(c)
r0677	1	10-Formyl-THF(c) + GAR(c) ⇌ THF(c) + N-Formyl-GAR(c)
r1330	5	H+(PG)(c) ⇌ H+(PG)(m) + Proton-gradient(m)
r1331	-1	H+(PG)(s) ⇌ H+(PG)(c) + Proton-gradient(c)
r0974	1	Pantothenate(s) + Na+(s) ⇌ Pantothenate(c) + Na+(c)
r1319	-12.2	ATP(c) + H2O(c) ⇌ ADP(c) + Pi(c) + ATP-energy(c)
r0260	1	Adenylosuccinate(c) → Fumarate(c) + AMP(c)
r0247	1	ATP(c) + Ribose-5P(c) → AMP(c) + PRPP(c)
r0699	1	SAICAR(c) ⇌ Fumarate(c) + AICAR(c)
r0849	-6.17	H2O(s) ⇌ H2O(c)
r0863	1	CoA(c) ⇌ CoA(m)
r0581	-1	Pantothenate(c) + Cysteamine(c) ⇌ H2O(c) + Pantetheine(c)
r0867	3	CO2(c) ⇌ CO2(s)
r0868	-1	CO2(c) ⇌ CO2(m)
r0589	1	ATP(c) + Phosphopantetheine(c) → PPi(c) + Dephospho-CoA(c)
r2468	1	Glycine(s) + H+(PG)(s) → Glycine(c) + H+(PG)(c)
r0736	0.17	ATP(c) + Fructose-6P(c) → ADP(c) + Fructose-1,6PP(c)
r0007	2	PPi(c) + H2O(c) → 2 Pi(c)
r0027	-0.5	NADPH(c) + Cystine(c) ⇌ 2 Cysteine(c) + NADP+(c)
r0192	0.83	Glucose-6P(c) ⇌ Fructose-6P(c)
r0344	-0.67	D-Xylulose-5P(c) ⇌ Ribulose-5P(c)
r0792	2	5-Methyl-THF(c) + NAD+(c) ⇌ 5,10-Methylene-THF(c) + NADH(c)
r1154	1	CoA(m) + NAD+(m) + 2-Oxobutyrate(m) ⇌ CO2(m) + Propanoyl-CoA(m) + NADH(m)
r0211	1	Cysteine(c) + O2(c) ⇌ 3-Sulfinoalanine(c)
r1155	1	2-Oxobutyrate(c) + H+(PG)(c) → 2-Oxobutyrate(m) + H+(PG)(m)
r2676	-2	5-Methyl-THF(c) ⇌ THF(c) + THF-activated methyl group(c)
r2525	1	Glutamine(s) ⇌ Glutamine(c)
r0666	1	ATP(c) + 5-Phosphoribosylformylglycinamidine(c) ⇌ ADP(c) + Pi(c) + AIR(c)
r0667	-1	5-Phosphoribosyl-4-carboxy-5-aminoimidazole(c) ⇌ AIR(c) + CO2(c)
r1322	2	NADH(c) ⇌ NAD+(c) + NADH-redox-potential(c)
r0683	1	Propanoyl-CoA(m) + Ubiquinone(m) → Ubiquinol(m) + Acrylyl-CoA(m)
r1323	1	NADH(m) ⇌ NAD+(m) + NADH-redox-potential(m)
r0250	-0.67	Ribulose-5P(c) ⇌ Ribose-5P(c)
r1326	3.5	NADPH(c) ⇌ NADPH-redox-potential(c) + NADP+(c)
r0687	1	ATP(c) + N-Formyl-GAR(c) + Glutamine(c) + H2O(c) ⇌ ADP(c) + Pi(c) + Glutamate(c) + 5-Phosphoribosylformylglycinamidine(c)
r0255	0.33	Fructose-6P(c) + GAP(c) ⇌ D-Xylulose-5P(c) + Erythrose-4P(c)
r0538	1	3-Sulfinoalanine(c) ⇌ CO2(c) + Hypotaurine(c)
r0256	0.17	Fructose-1,6PP(c) ⇌ DHAP(c) + GAP(c)
r0539	-1	Hypotaurine(c) ⇌ Cysteamine(c) + O2(c)
r0257	-1	Glutamine(c) + PRPP(c) + H2O(c) ⇌ 5-Phosphoribosylamine(c) + PPi(c) + Glutamate(c)
r0258	-2	Fumarate(c) + H2O(c) ⇌ Malate(c)
r0273	-1	IMP(c) + H2O(c) ⇌ FAICAR(c)
r0700	1	10-Formyl-THF(c) + AICAR(c) ⇌ THF(c) + FAICAR(c)

3-Hydroxystearoyl-ACP (619)

Definition			Solution												
			exchanges		reactions								transp		
Objective	Constraints	Comment	imports	exports	c	m	r	p	l	n	s	b	s-c	b-c	int
3-Hydroxystearoyl-ACP(c)	PIPES MCES	synthesis of cytosolic 3-Hydroxystearoyl-ACP	1 P_i(c) 5 Glutamate(s) 6 Glycine(s) 11 Alanine(s) 4 Lysine(s) 8 Aspartate(s) 9 Arginine(s) 7 Glutamine(s) 8 Serine(s) 5 Methionine(s) 3 Phenylalanine(s) 4 Tyrosine(s) 3 Cysteine(s) 20 Leucine(s) 1 Histidine(s) 9 Proline(s) 1 Asparagine(s) 11 Valine(s) 6 Threonine(s) 4 Isoleucine(s) 1 Pantothenate(s) 632 ATP-energy(c) 15 NADPH-redox-potential(c) 3 Proton-gradient(c) 9 CoA-activated acetyl group(c)	10 H_2O(s) 1 CO_2(s) 1 3-Hydroxystearoyl-ACP(c) 66 Na-gradient(c)	49	-	-	-	-	-	-	-	24	-	-

ID	flux	reaction
r0701	-1	HMA(c) + NADP+(c) ⇌ 3-Oxotetradecanoyl-ACP(c) + NADPH(c)
r0702	1	HMA(c) ⇌ H2O(c) + (2E)-Tetradecenoyl-ACP(c)
r1069	-4	Lysine(c) ⇌ Lysine(s)
r0578	1	ATP(c) + Pantetheine(c) ⇌ ADP(c) + Phosphopantetheine(c)
r2478	8	Serine(s) + Na+(s) + H+(PG)(c) → Serine(c) + Na+(c) + H+(PG)(s)
r0030	125	ATP(c) + AMP(c) ⇌ 2 ADP(c)
r0760	1	Butyryl-ACP(c) + Malonyl-ACP(c) ⇌ CO2(c) + ACP(c) + 3-Oxohexanoyl-ACP(c)
r0032	1	ATP(c) + Dephospho-CoA(c) ⇌ ADP(c) + CoA(c)
r0761	-1	D-3-Hydroxyhexanoyl-ACP(c) + NADP+(c) ⇌ NADPH(c) + 3-Oxohexanoyl-ACP(c)
r0184	8	ATP(c) + Acetyl-CoA(c) + HCO3-(c) → ADP(c) + Pi(c) + Malonyl-CoA(c)
r0762	1	D-3-Hydroxyhexanoyl-ACP(c) ⇌ H2O(c) + (2E)-Hexenoyl-ACP(c)
r0763	-1	Hexanoyl-ACP(c) + NADP+(c) ⇌ NADPH(c) + (2E)-Hexenoyl-ACP(c)
r0764	1	Malonyl-ACP(c) + Hexanoyl-ACP(c) ⇌ 3-Oxooctanoyl-ACP(c) + CO2(c) + ACP(c)
r1122	-20	Leucine(c) + Na+(c) ← Leucine(s) + Na+(s)
r0765	-1	Octanoyl-ACP(c) + NADP+(c) ⇌ (2E)-Octenoyl-ACP(c) + NADPH(c)
r1123	-4	Isoleucine(c) + Na+(c) ← Isoleucine(s) + Na+(s)
r0766	1	Octanoyl-ACP(c) + Malonyl-ACP(c) ⇌ 3-Oxodecanoyl-ACP(c) + CO2(c) + ACP(c)
r0767	-1	Decanoyl-ACP(c) + NADP+(c) ⇌ (2E)-Decenoyl-ACP(c) + NADPH(c)
r1125	4	Tyrosine(s) + Na+(s) → Tyrosine(c) + Na+(c)
r0768	1	Decanoyl-ACP(c) + Malonyl-ACP(c) ⇌ 3-Oxododecanoyl-ACP(c) + CO2(c) + ACP(c)
r0769	-1	D-3-Hydroxydodecanoyl-ACP(c) + NADP+(c) ⇌ 3-Oxododecanoyl-ACP(c) + NADPH(c)
r1142	-6	Threonine(s) + Na+(s) → Threonine(c) + Na+(c)
r1143	-8	Na+(c) + Aspartate(c) ← Na+(s) + Aspartate(s)
r2664	66	Na+(c) ⇌ Na+(s) + Na-gradient(c)
r1144	-5	Glutamate(c) + Na+(c) ← Glutamate(s) + Na+(s)
r1145	-1	Histidine(c) + Na+(c) ← Histidine(s) + Na+(s)
r2532	1	Asparagine(s) ⇌ Asparagine(c)
r2533	11	Valine(s) ⇌ Valine(c)
r1313	1	Hexadecanoyl-ACP(c) + Malonyl-ACP(c) ⇌ 3-Oxostearoyl-ACP(c) + ACP(c) + CO2(c)
r1314	1	3-Oxostearoyl-ACP(c) + NADPH(c) ⇌ 3-Hydroxystearoyl-ACP(c) + NADP+(c)
r0678	1	Acetyl-ACP(c) + Malonyl-ACP(c) ⇌ CO2(c) + ACP(c) + Acetoacetyl-ACP(c)
r0691	-1	(R)-3-Hydroxybutanoyl-ACP(c) + NADP+(c) ⇌ NADPH(c) + Acetoacetyl-ACP(c)
r1331	-3	H+(PG)(s) ⇌ H+(PG)(c) + Proton-gradient(c)
r0974	1	Pantothenate(s) + Na+(s) ⇌ Pantothenate(c) + Na+(c)
r0692	-1	(R)-3-Hydroxydecanoyl-ACP(c) + NADP+(c) ⇌ 3-Oxodecanoyl-ACP(c) + NADPH(c)
r1319	-632	ATP(c) + H2O(c) ⇌ ADP(c) + Pi(c) + ATP-energy(c)
r0693	1	(R)-3-Hydroxydecanoyl-ACP(c) ⇌ (2E)-Decenoyl-ACP(c) + H2O(c)
r0694	-1	(R)-3-Hydroxyoctanoyl-ACP(c) + NADP+(c) ⇌ 3-Oxooctanoyl-ACP(c) + NADPH(c)
r0695	1	(R)-3-Hydroxyoctanoyl-ACP(c) ⇌ (2E)-Octenoyl-ACP(c) + H2O(c)

Continued on next page

ID	flux	reaction
r0696	-1	(R)-3-Hydroxypalmitoyl-ACP(c) + NADP+(c) ⇌ 3-Oxohexadecanoyl-ACP(c) + NADPH(c)
r0697	1	(R)-3-Hydroxypalmitoyl-ACP(c) ⇌ (2E)-Hexadecenoyl-ACP(c) + H2O(c)
r1058	9	Arginine(s) ⇌ Arginine(c)
r1072	5	Methionine(s) ⇌ Methionine(c)
r0849	-10	H2O(s) ⇌ H2O(c)
r0581	-1	Pantothenate(c) + Cysteamine(c) ⇌ H2O(c) + Pantetheine(c)
r0712	-1	Dodecanoyl-ACP(c) + NADP+(c) ⇌ (2E)-Dodecenoyl-ACP(c) + NADPH(c)
r0713	1	Dodecanoyl-ACP(c) + Malonyl-ACP(c) ⇌ 3-Oxotetradecanoyl-ACP(c) + CO2(c) + ACP(c)
r0867	1	CO2(c) ⇌ CO2(s)
r1096	1	11 Alanine(c) + 2 Cysteine(c) + 5 Glutamate(c) + 3 Phenylalanine(c) + 6 Glycine(c) + Histidine(c) + 4 Isoleucine(c) + 20 Leucine(c) + 5 Methionine(c) + 9 Proline(c) + 7 Glutamine(c) + 9 Arginine(c) + 8 Serine(c) + 6 Threonine(c) + 11 Valine(c) + 4 Tyrosine(c) + 496 ATP(c) + 496 H2O(c) + 8 Aspartate(c) + 4 Lysine(c) + Asparagine(c) → Apo-ACP(c) + 124 AMP(c) + 124 PPi(c) + 372 ADP(c) + 372 Pi(c)
r0589	1	ATP(c) + Phosphopantetheine(c) → PPi(c) + Dephospho-CoA(c)
r2469	11	Alanine(s) + H+(PG)(s) → Alanine(c) + H+(PG)(c)
r0007	125	PPi(c) + H2O(c) → 2 Pi(c)
r0770	1	D-3-Hydroxydodecanoyl-ACP(c) ⇌ (2E)-Dodecenoyl-ACP(c) + H2O(c)
r0771	-1	Tetradecanoyl-ACP(c) + NADP+(c) ⇌ NADPH(c) + (2E)-Tetradecenoyl-ACP(c)
r0772	1	Tetradecanoyl-ACP(c) + Malonyl-ACP(c) ⇌ 3-Oxohexadecanoyl-ACP(c) + CO2(c) + ACP(c)
r0773	-1	Hexadecanoyl-ACP(c) + NADP+(c) ⇌ (2E)-Hexadecenoyl-ACP(c) + NADPH(c)
r1417	-8	CO2(c) + H2O(c) ⇌ Carbonate(c)
r0049	1	PAP(c) + H2O(c) → AMP(c) + Pi(c)
r1419	8	Carbonate(c) ⇌ HCO3-(c)
r1139	-9	Proline(c) + Na+(c) ← Proline(s) + Na+(s)
r0211	1	Cysteine(c) + O2(c) ⇌ 3-Sulfinoalanine(c)
r2523	6	Glycine(s) ⇌ Glycine(c)
r2677	-9	Acetyl-CoA(c) ⇌ CoA(c) + CoA-activated acetyl group(c)
r2525	7	Glutamine(s) ⇌ Glutamine(c)
r0367	1	Acetyl-CoA(c) + ACP(c) ⇌ CoA(c) + Acetyl-ACP(c)
r0368	1	CoA(c) + Apo-ACP(c) → PAP(c) + ACP(c)
r2528	3	Phenylalanine(s) ⇌ Phenylalanine(c)
r0369	8	ACP(c) + Malonyl-CoA(c) ⇌ CoA(c) + Malonyl-ACP(c)
r2529	3	Cysteine(s) ⇌ Cysteine(c)
r0681	1	(R)-3-Hydroxybutanoyl-ACP(c) ⇌ But-2-enoyl-ACP(c) + H2O(c)
r0682	-1	Butyryl-ACP(c) + NADP+(c) ⇌ But-2-enoyl-ACP(c) + NADPH(c)
r1326	-15	NADPH(c) ⇌ NADPH-redox-potential(c) + NADP+(c)
r0538	1	3-Sulfinoalanine(c) ⇌ CO2(c) + Hypotaurine(c)
r0539	-1	Hypotaurine(c) ⇌ Cysteamine(c) + O2(c)

3-Keto-4-methylzymosterol (620)

Definition			Solution												
			exchanges		reactions								transp		
Objective	Constraints	Comment	imports	exports	c	m	r	p	l	n	s	b	s-c	b-c	int
3-Keto-4-methylzymosterol(r)	PIPES MCES	synthesis of Golgi/ER 3-Keto-4-methylzymosterol	7 O_2(s) 2 Farnesyl-PP(r) 1 ATP-energy(c) 9 NADPH-redox-potential(r)	7 H_2O(s) 4 P_i(c) 2 CO_2(s) 1 3-Keto-4-methylzymosterol(r) 1 NADH-redox-potential(r) 1 NADPH-redox-potential(c)	5	-	10	-	-	-	-	-	3	-	5

ID	flux	reaction
r1153	-2	PPi(c) ⇌ PPi(r)
r0887	-1	Formate(c) ⇌ Formate(r)
r1325	-9	NADPH(r) ⇌ NADPH-redox-potential(r) + NADP+(r)
r0227	1	10-Formyl-THF(c) + H2O(c) + NADP+(c) → THF(c) + CO2(c) + NADPH(c)
r1326	1	NADPH(c) ⇌ NADPH-redox-potential(c) + NADP+(c)
r1137	1	NAD+(r) + 4alpha-Methylzymosterol-4-carboxylate(r) ⇌ NADH(r) + CO2(r) + 3-Keto-4-methylzymosterol(r)

Continued on next page

ID	flux	reaction
r0228	1	THF(c) + Formate(c) + ATP(c) → ADP(c) + Pi(c) + 10-Formyl-THF(c)
r0607	1	Squalene 2,3-oxide(r) ⇌ Lanosterol(r)
r1319	-1	ATP(c) + H2O(c) ⇌ ADP(c) + Pi(c) + ATP-energy(c)
r0780	-1	4,4-Dimethyl-5alpha-cholesta-8,14,24-trien-3beta-ol(r) + NADPH(r) ⇌ 14-Demethyllanosterol(r) + NADP+(r)
r0781	1	3 O2(r) + 3 NADPH(r) + Lanosterol(r) ⇌ 4,4-Dimethyl-5alpha-cholesta-8,14,24-trien-3beta-ol(r) + Formate(r) + 4 H2O(r) + 3 NADP+(r)
r0854	-7	O2(r) ⇌ O2(c)
r0575	1	Presqualene-PP(r) + NADPH(r) ⇌ PPi(r) + Squalene(r) + NADP+(r)
r0855	7	O2(s) ⇌ O2(c)
r0576	1	Squalene(r) + NADPH(r) + O2(r) ⇌ H2O(r) + Squalene 2,3-oxide(r) + NADP+(r)
r0865	1	CO2(r) ⇌ CO2(c)
r1321	1	NADH(r) ⇌ NAD+(r) + NADH-redox-potential(r)
r0848	9	H2O(r) ⇌ H2O(c)
r0007	2	PPi(c) + H2O(c) → 2 Pi(c)
r0867	2	CO2(c) ⇌ CO2(s)
r0849	-7	H2O(s) ⇌ H2O(c)
r0170	1	2 Farnesyl-PP(r) ⇌ PPi(r) + Presqualene-PP(r)
r1134	1	14-Demethyllanosterol(r) + 3 NADPH(r) + 3 O2(r) ⇌ 4 H2O(r) + 4alpha-Methylzymosterol-4-carboxylate(r) + 3 NADP+(r)

3-Methyl-2-oxobutyrate (621)

Definition			Solution											
			exchanges		reactions								transp		
Objective	Constraints	Comment	imports	exports	c	m	r	p	l	n	s	b	s-c	b-c	int
3-Methyl-2-oxobutyrate(m)	PIPES MCES	synthesis of mitochondrial 3-Methyl-2-oxobutyrate	1 Pyruvate(c) 1 Valine(s) 3 Proton-gradient(m)	1 Alanine(s) 1 3-Methyl-2-oxobutyrate(m) 2 Na-gradient(c)	1	1	-	-	-	-	-	-	3	-	6

ID	flux	reaction
r0911	1	Proline(c) + Glutamate(m) ⇌ Proline(m) + Glutamate(c)
r0263	1	Valine(m) + AKG(m) → Glutamate(m) + 3-Methyl-2-oxobutyrate(m)
r1047	1	Pi(c) + H+(PG)(c) → Pi(m) + H+(PG)(m)
r1426	1	Proline(m) + H+(PG)(c) → Proline(c) + H+(PG)(m)
r0923	1	Valine(c) + H+(PG)(c) → Valine(m) + H+(PG)(m)
r2419	1	AKG(c) + Pi(m) ⇌ AKG(m) + Pi(c)
r1852	1	Valine(s) + Glycine(c) + Na+(s) → Valine(c) + Glycine(s) + Na+(c)
r1672	1	Glycine(s) + Alanine(c) + Na+(s) → Glycine(c) + Alanine(s) + Na+(c)
r2664	2	Na+(c) ⇌ Na+(s) + Na-gradient(c)
r1330	-3	H+(PG)(c) ⇌ H+(PG)(m) + Proton-gradient(m)
r0080	-1	Pyruvate(c) + Glutamate(c) ⇌ Alanine(c) + AKG(c)

3-Methylcrotonyl-CoA (622)

Definition			Solution											
			exchanges		reactions								transp		
Objective	Constraints	Comment	imports	exports	c	m	r	p	l	n	s	b	s-c	b-c	int
3-Methylcrotonyl-CoA(m)	PIPES MCES	synthesis of mitochondrial 3-Methylcrotonyl-CoA	2.17 P_i(c) 1 Glycine(s) 2 Aspartate(s) 2 Glutamine(s) 0.83 Glucose-6P(c) 0.5 Cystine(s) 1 Pantothenate(s) 11.2 ATP-energy(c) 2 THF-activated methyl group(c) 3 CoA-activated acetyl group(m)	5.17 H_2O(s) 3 CO_2(s) 2 Pyruvate(c) 2 Glutamate(s) 1 3-Methylcrotonyl-CoA(m) 2 NADH-redox-potential(c) 3.5 NADPH-redox-potential(c) 1 Proton-gradient(c) 5 Na-gradient(c)	42	7	-	-	-	-	-	-	10	-	2

ID	flux	reaction
r0406	-0.33	Fructose-6P(c) + Erythrose-4P(c) ⇌ Sedoheptulose-7P(c) + GAP(c)
r0277	1	GTP(c) + IMP(c) + Aspartate(c) → GDP(c) + Pi(c) + Adenylosuccinate(c)
r0705	1	ATP(c) + 5-Phosphoribosyl-4-carboxy-5-aminoimidazole(c) + Aspartate(c) ⇌ ADP(c) + Pi(c) + SAICAR(c)
r0293	2	5,10-Methylene-THF(c) + NADP+(c) ⇌ 5,10-Methenyl-THF(c) + NADPH(c)
r0578	1	ATP(c) + Pantetheine(c) ⇌ ADP(c) + Phosphopantetheine(c)
r0877	0.5	Glutamate(c) + Cystine(s) ⇌ Glutamate(s) + Cystine(c)
r2477	2	Glutamine(s) + Na+(s) + H+(PG)(c) → Glutamine(c) + Na+(c) + H+(PG)(s)
r0462	-1	Acetyl-CoA(m) + H2O(m) + Acetoacetyl-CoA(m) ⇌ HMG-CoA(m) + CoA(m)
r0030	2	ATP(c) + AMP(c) ⇌ 2 ADP(c)
r0032	1	ATP(c) + Dephospho-CoA(c) ⇌ ADP(c) + CoA(c)
r0185	1	ATP(m) + Acetyl-CoA(m) + HCO3-(m) → ADP(m) + Pi(m) + Malonyl-CoA(m)
r1143	-2	Na+(c) + Aspartate(c) ← Na+(s) + Aspartate(s)
r0070	1	2 Acetyl-CoA(m) ⇌ CoA(m) + Acetoacetyl-CoA(m)
r2664	5	Na+(c) ⇌ Na+(s) + Na-gradient(c)
r0058	2	Malate(c) + NADP+(c) ⇌ Pyruvate(c) + CO2(c) + NADPH(c)
r0370	0.33	Sedoheptulose-7P(c) + GAP(c) ⇌ Ribose-5P(c) + D-Xylulose-5P(c)
r0371	2	5,10-Methenyl-THF(c) + H2O(c) ⇌ 10-Formyl-THF(c)
r2685	1.5	Glutamate(c) ⇌ Glutamate(s)
r0658	-1	ADP(m) + Pi(m) + 3-Methylglutaconyl-CoA(m) ⇌ ATP(m) + 3-Methylcrotonyl-CoA(m) + HCO3-(m)
r0659	1	ATP(c) + 5-Phosphoribosylamine(c) + Glycine(c) ⇌ ADP(c) + Pi(c) + GAR(c)
r0099	1	ATP(c) + GDP(c) ⇌ ADP(c) + GTP(c)
r0243	-0.17	DHAP(c) ⇌ GAP(c)
r0677	1	10-Formyl-THF(c) + GAR(c) ⇌ THF(c) + N-Formyl-GAR(c)
r1331	1	H+(PG)(s) ⇌ H+(PG)(c) + Proton-gradient(c)
r0974	1	Pantothenate(s) + Na+(s) ⇌ Pantothenate(c) + Na+(c)
r1319	-11.2	ATP(c) + H2O(c) ⇌ ADP(c) + Pi(c) + ATP-energy(c)
r0260	1	Adenylosuccinate(c) → Fumarate(c) + AMP(c)
r0247	1	ATP(c) + Ribose-5P(c) → AMP(c) + PRPP(c)
r0699	1	SAICAR(c) ⇌ Fumarate(c) + AICAR(c)
r0849	-5.17	H2O(s) ⇌ H2O(c)
r0863	1	CoA(c) ⇌ CoA(m)
r0581	-1	Pantothenate(c) + Cysteamine(c) ⇌ H2O(c) + Pantetheine(c)
r0867	3	CO2(c) ⇌ CO2(s)
r0868	-1	CO2(c) ⇌ CO2(m)
r0589	1	ATP(c) + Phosphopantetheine(c) → PPi(c) + Dephospho-CoA(c)
r2468	1	Glycine(s) + H+(PG)(s) → Glycine(c) + H+(PG)(c)
r0736	0.17	ATP(c) + Fructose-6P(c) → ADP(c) + Fructose-1,6PP(c)
r0007	2	PPi(c) + H2O(c) → 2 Pi(c)
r0027	-0.5	NADPH(c) + Cystine(c) ⇌ 2 Cysteine(c) + NADP+(c)
r0192	0.83	Glucose-6P(c) ⇌ Fructose-6P(c)
r0490	1	HMG-CoA(m) ⇌ 3-Methylglutaconyl-CoA(m) + H2O(m)
r0344	-0.67	D-Xylulose-5P(c) ⇌ Ribulose-5P(c)
r0792	2	5-Methyl-THF(c) + NAD+(c) ⇌ 5,10-Methylene-THF(c) + NADH(c)
r0064	1	Malonyl-CoA(m) → Acetyl-CoA(m) + CO2(m)
r0211	1	Cysteine(c) + O2(c) ⇌ 3-Sulfinoalanine(c)
r2676	-2	5-Methyl-THF(c) ⇌ THF(c) + THF-activated methyl group(c)
r2678	-3	Acetyl-CoA(m) ⇌ CoA(m) + CoA-activated acetyl group(m)
r0666	1	ATP(c) + 5-Phosphoribosylformylglycinamidine(c) ⇌ ADP(c) + Pi(c) + AIR(c)
r0667	-1	5-Phosphoribosyl-4-carboxy-5-aminoimidazole(c) ⇌ AIR(c) + CO2(c)
r1322	2	NADH(c) ⇌ NAD+(c) + NADH-redox-potential(c)
r0250	-0.67	Ribulose-5P(c) ⇌ Ribose-5P(c)
r1326	3.5	NADPH(c) ⇌ NADPH-redox-potential(c) + NADP+(c)
r0687	1	ATP(c) + N-Formyl-GAR(c) + Glutamine(c) + H2O(c) ⇌ ADP(c) + Pi(c) + Glutamate(c) + 5-Phosphoribosylformylglycinamidine(c)
r0255	0.33	Fructose-6P(c) + GAP(c) ⇌ D-Xylulose-5P(c) + Erythrose-4P(c)
r0538	1	3-Sulfinoalanine(c) ⇌ CO2(c) + Hypotaurine(c)
r0256	0.17	Fructose-1,6PP(c) ⇌ DHAP(c) + GAP(c)

Continued on next page

ID	flux	reaction
r0539	-1	Hypotaurine(c) ⇌ Cysteamine(c) + O2(c)
r0257	-1	Glutamine(c) + PRPP(c) + H2O(c) ⇌ 5-Phosphoribosylamine(c) + PPi(c) + Glutamate(c)
r0258	-2	Fumarate(c) + H2O(c) ⇌ Malate(c)
r0273	-1	IMP(c) + H2O(c) ⇌ FAICAR(c)
r0700	1	10-Formyl-THF(c) + AICAR(c) ⇌ THF(c) + FAICAR(c)

3-Methylglutaconyl-CoA (623)

Definition			Solution												
			exchanges		reactions								transp		
Objective	Constraints	Comment	imports	exports	c	m	r	p	l	n	s	b	s-c	b-c	int
3-Methylglutaconyl-CoA(m)	PIPES MCES	synthesis of mitochondrial 3-Methylglutaconyl-CoA	2.17 P_i(c) 1 Glycine(s) 2 Aspartate(s) 2 Glutamine(s) 0.83 Glucose-6P(c) 0.5 Cystine(s) 1 Pantothenate(s) 11.2 ATP-energy(c) 2 THF-activated methyl group(c) 3 CoA-activated acetyl group(m)	5.17 H_2O(s) 2 CO_2(s) 2 Pyruvate(c) 2 Glutamate(s) 1 3-Methylglutaconyl-CoA(m) 2 NADH-redox-potential(c) 3.5 NADPH-redox-potential(c) 1 Proton-gradient(c) 5 Na-gradient(c)	42	4	-	-	-	-	-	-	10	-	1

ID	flux	reaction
r0406	-0.33	Fructose-6P(c) + Erythrose-4P(c) ⇌ Sedoheptulose-7P(c) + GAP(c)
r0277	1	GTP(c) + IMP(c) + Aspartate(c) → GDP(c) + Pi(c) + Adenylosuccinate(c)
r0705	1	ATP(c) + 5-Phosphoribosyl-4-carboxy-5-aminoimidazole(c) + Aspartate(c) ⇌ ADP(c) + Pi(c) + SAICAR(c)
r0293	2	5,10-Methylene-THF(c) + NADP+(c) ⇌ 5,10-Methenyl-THF(c) + NADPH(c)
r0578	1	ATP(c) + Pantetheine(c) ⇌ ADP(c) + Phosphopantetheine(c)
r0877	0.5	Glutamate(c) + Cystine(s) ⇌ Glutamate(s) + Cystine(c)
r2477	2	Glutamine(s) + Na+(s) + H+(PG)(c) → Glutamine(c) + Na+(c) + H+(PG)(s)
r0462	-1	Acetyl-CoA(m) + H2O(m) + Acetoacetyl-CoA(m) ⇌ HMG-CoA(m) + CoA(m)
r0030	2	ATP(c) + AMP(c) ⇌ 2 ADP(c)
r0032	1	ATP(c) + Dephospho-CoA(c) ⇌ ADP(c) + CoA(c)
r1143	-2	Na+(c) + Aspartate(c) ← Na+(s) + Aspartate(s)
r0070	1	2 Acetyl-CoA(m) ⇌ CoA(m) + Acetoacetyl-CoA(m)
r2664	5	Na+(c) ⇌ Na+(s) + Na-gradient(c)
r0058	2	Malate(c) + NADP+(c) ⇌ Pyruvate(c) + CO2(c) + NADPH(c)
r0370	0.33	Sedoheptulose-7P(c) + GAP(c) ⇌ Ribose-5P(c) + D-Xylulose-5P(c)
r0371	2	5,10-Methenyl-THF(c) + H2O(c) ⇌ 10-Formyl-THF(c)
r2685	1.5	Glutamate(c) ⇌ Glutamate(s)
r0659	1	ATP(c) + 5-Phosphoribosylamine(c) + Glycine(c) ⇌ ADP(c) + Pi(c) + GAR(c)
r0099	1	ATP(c) + GDP(c) ⇌ ADP(c) + GTP(c)
r0243	-0.17	DHAP(c) ⇌ GAP(c)
r0677	1	10-Formyl-THF(c) + GAR(c) ⇌ THF(c) + N-Formyl-GAR(c)
r1331	1	H+(PG)(s) ⇌ H+(PG)(c) + Proton-gradient(c)
r0974	1	Pantothenate(s) + Na+(s) ⇌ Pantothenate(c) + Na+(c)
r1319	-11.2	ATP(c) + H2O(c) ⇌ ADP(c) + Pi(c) + ATP-energy(c)
r0260	1	Adenylosuccinate(c) → Fumarate(c) + AMP(c)
r0247	1	ATP(c) + Ribose-5P(c) → AMP(c) + PRPP(c)
r0699	1	SAICAR(c) ⇌ Fumarate(c) + AICAR(c)
r0849	-5.17	H2O(s) ⇌ H2O(c)
r0863	1	CoA(c) ⇌ CoA(m)
r0581	-1	Pantothenate(c) + Cysteamine(c) ⇌ H2O(c) + Pantetheine(c)
r0867	2	CO2(c) ⇌ CO2(s)
r0589	1	ATP(c) + Phosphopantetheine(c) → PPi(c) + Dephospho-CoA(c)
r2468	1	Glycine(s) + H+(PG)(s) → Glycine(c) + H+(PG)(c)
r0736	0.17	ATP(c) + Fructose-6P(c) → ADP(c) + Fructose-1,6PP(c)
r0007	2	PPi(c) + H2O(c) → 2 Pi(c)
r0027	-0.5	NADPH(c) + Cystine(c) ⇌ 2 Cysteine(c) + NADP+(c)

Continued on next page

ID	flux	reaction
r0192	0.83	Glucose-6P(c) ⇌ Fructose-6P(c)
r0490	1	HMG-CoA(m) ⇌ 3-Methylglutaconyl-CoA(m) + H2O(m)
r0344	-0.67	D-Xylulose-5P(c) ⇌ Ribulose-5P(c)
r0792	2	5-Methyl-THF(c) + NAD+(c) ⇌ 5,10-Methylene-THF(c) + NADH(c)
r0211	1	Cysteine(c) + O2(c) ⇌ 3-Sulfinoalanine(c)
r2676	-2	5-Methyl-THF(c) ⇌ THF(c) + THF-activated methyl group(c)
r2678	-3	Acetyl-CoA(m) ⇌ CoA(m) + CoA-activated acetyl group(m)
r0666	1	ATP(c) + 5-Phosphoribosylformylglycinamidine(c) ⇌ ADP(c) + Pi(c) + AIR(c)
r0667	-1	5-Phosphoribosyl-4-carboxy-5-aminoimidazole(c) ⇌ AIR(c) + CO2(c)
r1322	2	NADH(c) ⇌ NAD+(c) + NADH-redox-potential(c)
r0250	-0.67	Ribulose-5P(c) ⇌ Ribose-5P(c)
r1326	3.5	NADPH(c) ⇌ NADPH-redox-potential(c) + NADP+(c)
r0687	1	ATP(c) + N-Formyl-GAR(c) + Glutamine(c) + H2O(c) ⇌ ADP(c) + Pi(c) + Glutamate(c) + 5-Phosphoribosylformylglycinamidine(c)
r0255	0.33	Fructose-6P(c) + GAP(c) ⇌ D-Xylulose-5P(c) + Erythrose-4P(c)
r0538	1	3-Sulfinoalanine(c) ⇌ CO2(c) + Hypotaurine(c)
r0256	0.17	Fructose-1,6PP(c) ⇌ DHAP(c) + GAP(c)
r0539	-1	Hypotaurine(c) ⇌ Cysteamine(c) + O2(c)
r0257	-1	Glutamine(c) + PRPP(c) + H2O(c) ⇌ 5-Phosphoribosylamine(c) + PPi(c) + Glutamate(c)
r0258	-2	Fumarate(c) + H2O(c) ⇌ Malate(c)
r0273	-1	IMP(c) + H2O(c) ⇌ FAICAR(c)
r0700	1	10-Formyl-THF(c) + AICAR(c) ⇌ THF(c) + FAICAR(c)

3-Oxodecanoyl-ACP (624)

Definition			Solution												
			exchanges		reactions								transp		
Objective	Constraints	Comment	imports	exports	c	m	r	p	l	n	s	b	s-c	b-c	int
3-Oxodecanoyl-ACP(c)	PIPES MCES	synthesis of cytosolic 3-Oxodecanoyl-ACP	1 P_i(c) 5 Glutamate(s) 6 Glycine(s) 11 Alanine(s) 4 Lysine(s) 8 Aspartate(s) 9 Arginine(s) 7 Glutamine(s) 8 Serine(s) 5 Methionine(s) 3 Phenylalanine(s) 4 Tyrosine(s) 3 Cysteine(s) 20 Leucine(s) 1 Histidine(s) 9 Proline(s) 1 Asparagine(s) 11 Valine(s) 6 Threonine(s) 4 Isoleucine(s) 1 Pantothenate(s) 628 ATP-energy(c) 6 NADPH-redox-potential(c) 3 Proton-gradient(c) 5 CoA-activated acetyl group(c)	6 H_2O(s) 1 CO_2(s) 1 3-Oxodecanoyl-ACP(c) 66 Na-gradient(c)	32	-	-	-	-	-	-	-	24	-	-

ID	flux	reaction
r1069	-4	Lysine(c) ⇌ Lysine(s)
r0578	1	ATP(c) + Pantetheine(c) ⇌ ADP(c) + Phosphopantetheine(c)
r2478	8	Serine(s) + Na+(s) + H+(PG)(c) → Serine(c) + Na+(c) + H+(PG)(s)
r0030	125	ATP(c) + AMP(c) ⇌ 2 ADP(c)
r0760	1	Butyryl-ACP(c) + Malonyl-ACP(c) ⇌ CO2(c) + ACP(c) + 3-Oxohexanoyl-ACP(c)
r0032	1	ATP(c) + Dephospho-CoA(c) ⇌ ADP(c) + CoA(c)
r0761	-1	D-3-Hydroxyhexanoyl-ACP(c) + NADP+(c) ⇌ NADPH(c) + 3-Oxohexanoyl-ACP(c)
r0184	4	ATP(c) + Acetyl-CoA(c) + HCO3-(c) → ADP(c) + Pi(c) + Malonyl-CoA(c)
r0762	1	D-3-Hydroxyhexanoyl-ACP(c) ⇌ H2O(c) + (2E)-Hexenoyl-ACP(c)
r0763	-1	Hexanoyl-ACP(c) + NADP+(c) ⇌ NADPH(c) + (2E)-Hexenoyl-ACP(c)
r0764	1	Malonyl-ACP(c) + Hexanoyl-ACP(c) ⇌ 3-Oxooctanoyl-ACP(c) + CO2(c) + ACP(c)
r1122	-20	Leucine(c) + Na+(c) ← Leucine(s) + Na+(s)
r0765	-1	Octanoyl-ACP(c) + NADP+(c) ⇌ (2E)-Octenoyl-ACP(c) + NADPH(c)

Continued on next page

ID	flux	reaction
r1123	-4	Isoleucine(c) + Na+(c) ← Isoleucine(s) + Na+(s)
r0766	1	Octanoyl-ACP(c) + Malonyl-ACP(c) ⇌ 3-Oxodecanoyl-ACP(c) + CO2(c) + ACP(c)
r1125	4	Tyrosine(s) + Na+(s) → Tyrosine(c) + Na+(c)
r1142	-6	Threonine(s) + Na+(s) → Threonine(c) + Na+(c)
r1143	-8	Na+(c) + Aspartate(c) ← Na+(s) + Aspartate(s)
r2664	66	Na+(c) ⇌ Na+(s) + Na-gradient(c)
r1144	-5	Glutamate(c) + Na+(c) ← Glutamate(s) + Na+(s)
r1145	-1	Histidine(c) + Na+(c) ← Histidine(s) + Na+(s)
r2532	1	Asparagine(s) ⇌ Asparagine(c)
r2533	11	Valine(s) ⇌ Valine(c)
r0678	1	Acetyl-ACP(c) + Malonyl-ACP(c) ⇌ CO2(c) + ACP(c) + Acetoacetyl-ACP(c)
r0691	-1	(R)-3-Hydroxybutanoyl-ACP(c) + NADP+(c) ⇌ NADPH(c) + Acetoacetyl-ACP(c)
r1331	-3	H+(PG)(s) ⇌ H+(PG)(c) + Proton-gradient(c)
r0974	1	Pantothenate(s) + Na+(s) ⇌ Pantothenate(c) + Na+(c)
r1319	-628	ATP(c) + H2O(c) ⇌ ADP(c) + Pi(c) + ATP-energy(c)
r0694	-1	(R)-3-Hydroxyoctanoyl-ACP(c) + NADP+(c) ⇌ 3-Oxooctanoyl-ACP(c) + NADPH(c)
r0695	1	(R)-3-Hydroxyoctanoyl-ACP(c) ⇌ (2E)-Octenoyl-ACP(c) + H2O(c)
r1058	9	Arginine(s) ⇌ Arginine(c)
r1072	5	Methionine(s) ⇌ Methionine(c)
r0849	-6	H2O(s) ⇌ H2O(c)
r0581	-1	Pantothenate(c) + Cysteamine(c) ⇌ H2O(c) + Pantetheine(c)
r0867	1	CO2(c) ⇌ CO2(s)
r1096	1	11 Alanine(c) + 2 Cysteine(c) + 5 Glutamate(c) + 3 Phenylalanine(c) + 6 Glycine(c) + Histidine(c) + 4 Isoleucine(c) + 20 Leucine(c) + 5 Methionine(c) + 9 Proline(c) + 7 Glutamine(c) + 9 Arginine(c) + 8 Serine(c) + 6 Threonine(c) + 11 Valine(c) + 4 Tyrosine(c) + 496 ATP(c) + 496 H2O(c) + 8 Aspartate(c) + 4 Lysine(c) + Asparagine(c) → Apo-ACP(c) + 124 AMP(c) + 124 PPi(c) + 372 ADP(c) + 372 Pi(c)
r0589	1	ATP(c) + Phosphopantetheine(c) → PPi(c) + Dephospho-CoA(c)
r2469	11	Alanine(s) + H+(PG)(s) → Alanine(c) + H+(PG)(c)
r0007	125	PPi(c) + H2O(c) → 2 Pi(c)
r1417	-4	CO2(c) + H2O(c) ⇌ Carbonate(c)
r0049	1	PAP(c) + H2O(c) → AMP(c) + Pi(c)
r1419	4	Carbonate(c) ⇌ HCO3-(c)
r1139	-9	Proline(c) + Na+(c) ← Proline(s) + Na+(s)
r0211	1	Cysteine(c) + O2(c) ⇌ 3-Sulfinoalanine(c)
r2523	6	Glycine(s) ⇌ Glycine(c)
r2677	-5	Acetyl-CoA(c) ⇌ CoA(c) + CoA-activated acetyl group(c)
r2525	7	Glutamine(s) ⇌ Glutamine(c)
r0367	1	Acetyl-CoA(c) + ACP(c) ⇌ CoA(c) + Acetyl-ACP(c)
r0368	1	CoA(c) + Apo-ACP(c) → PAP(c) + ACP(c)
r2528	3	Phenylalanine(s) ⇌ Phenylalanine(c)
r0369	4	ACP(c) + Malonyl-CoA(c) ⇌ CoA(c) + Malonyl-ACP(c)
r2529	3	Cysteine(s) ⇌ Cysteine(c)
r0681	1	(R)-3-Hydroxybutanoyl-ACP(c) ⇌ But-2-enoyl-ACP(c) + H2O(c)
r0682	-1	Butyryl-ACP(c) + NADP+(c) ⇌ But-2-enoyl-ACP(c) + NADPH(c)
r1326	-6	NADPH(c) ⇌ NADPH-redox-potential(c) + NADP+(c)
r0538	1	3-Sulfinoalanine(c) ⇌ CO2(c) + Hypotaurine(c)
r0539	-1	Hypotaurine(c) ⇌ Cysteamine(c) + O2(c)

3-Oxodecanoyl-CoA (625)

Definition			Solution													
			exchanges		reactions									transp		
Objective	Constraints	Comment	imports	exports	c	m	r	p	l	n	s	b	s-c	b-c	int
3-Oxodecanoyl-CoA(m)	PIPES MCES	synthesis of mitochondrial 3-Oxodecanoyl-CoA	0.5 O_2(s) 2.17 P_i(c) 1 Glycine(s) 2 Aspartate(s) 2 Glutamine(s) 0.83 Glucose-6P(c) 0.5 Cystine(s) 1 Pantothenate(s) 1 Palmitolate(s) 13.2 ATP-energy(c) 1 Proton-gradient(c) 2 THF-activated methyl group(c)	2.17 H_2O(s) 2 CO_2(s) 2 Pyruvate(c) 2 Glutamate(s) 1 3-Oxodecanoyl-CoA(m) 2 NADH-redox-potential(c) 5 NADH-redox-potential(m) 3.5 NADPH-redox-potential(c) 3 FADH-redox-potential(c) 6 Proton-gradient(m) 3 Na-gradient(c) 4 CoA-activated acetyl group(m)	44	21	-	-	-	-	-	-	12	-	7

ID	flux	reaction
r0406	-0.33	Fructose-6P(c) + Erythrose-4P(c) ⇌ Sedoheptulose-7P(c) + GAP(c)
r0277	1	GTP(c) + IMP(c) + Aspartate(c) → GDP(c) + Pi(c) + Adenylosuccinate(c)
r0855	0.5	O2(s) ⇌ O2(c)
r0856	-0.5	O2(m) ⇌ O2(c)
r0705	1	ATP(c) + 5-Phosphoribosyl-4-carboxy-5-aminoimidazole(c) + Aspartate(c) ⇌ ADP(c) + Pi(c) + SAICAR(c)
r0293	2	5,10-Methylene-THF(c) + NADP+(c) ⇌ 5,10-Methenyl-THF(c) + NADPH(c)
r0578	1	ATP(c) + Pantetheine(c) ⇌ ADP(c) + Phosphopantetheine(c)
r0722	1	(S)-3-Hydroxydodecanoyl-CoA(m) + NAD+(m) ⇌ 3-Oxododecanoyl-CoA(m) + NADH(m)
r0724	-1	Decanoyl-CoA(m) + Acetyl-CoA(m) ⇌ CoA(m) + 3-Oxododecanoyl-CoA(m)
r0877	0.5	Glutamate(c) + Cystine(s) ⇌ Glutamate(s) + Cystine(c)
r0726	1	(S)-Hydroxydecanoyl-CoA(m) + NAD+(m) ⇌ 3-Oxodecanoyl-CoA(m) + NADH(m)
r0728	-1	(S)-Hydroxydecanoyl-CoA(m) ⇌ (2E)-Decenoyl-CoA(m) + H2O(m)
r0017	0.5	O2(m) + 4 H+(PG)(m) + 4 Ferrocytochrome C(m) → 4 Ferricytochrome C(m) + 2 H2O(m) + 4 H+(PG)(c)
r0030	3	ATP(c) + AMP(c) ⇌ 2 ADP(c)
r0032	1	ATP(c) + Dephospho-CoA(c) ⇌ ADP(c) + CoA(c)
r1290	3	FAD(c) + FADH2(m) ⇌ FAD(m) + FADH2(c)
r1143	-2	Na+(c) + Aspartate(c) ← Na+(s) + Aspartate(s)
r2664	3	Na+(c) ⇌ Na+(s) + Na-gradient(c)
r0058	2	Malate(c) + NADP+(c) ⇌ Pyruvate(c) + CO2(c) + NADPH(c)
r0370	0.33	Sedoheptulose-7P(c) + GAP(c) ⇌ Ribose-5P(c) + D-Xylulose-5P(c)
r0371	2	5,10-Methenyl-THF(c) + H2O(c) ⇌ 10-Formyl-THF(c)
r2685	1.5	Glutamate(c) ⇌ Glutamate(s)
r0657	1	(3Z)-Dodecenoyl-CoA(m) ⇌ (2E)-Dodecenoyl-CoA(m)
r0659	1	ATP(c) + 5-Phosphoribosylamine(c) + Glycine(c) ⇌ ADP(c) + Pi(c) + GAR(c)
r0507	1	Ubiquinol(m) + 2 Ferricytochrome C(m) + 4 H+(PG)(m) → Ubiquinone(m) + 4 H+(PG)(c) + 2 Ferrocytochrome C(m)
r1480	1	CoA(m) + 3-oxopalmitoleoyl-CoA(m) ⇌ Acetyl-CoA(m) + cis-myrist-7-enoyl-CoA(m)
r0099	1	ATP(c) + GDP(c) ⇌ ADP(c) + GTP(c)
r1468	1	palmitoleoyl-CoA(c) ⇌ palmitoleoyl-CoA(m)
r0243	-0.17	DHAP(c) ⇌ GAP(c)
r0677	1	10-Formyl-THF(c) + GAR(c) ⇌ THF(c) + N-Formyl-GAR(c)
r1482	-1	FADH2(m) + trans,cis-dodeca-2,5-dienoyl-CoA(m) ⇌ FAD(m) + cis-laur-5-enoyl-CoA(m)
r1330	6	H+(PG)(c) ⇌ H+(PG)(m) + Proton-gradient(m)
r1483	1	H2O(m) + trans,cis-myristo-2,7-dienoyl-CoA(m) ⇌ cis-(3S)-hydroxytetradec-7-enoyl-CoA(m)
r1331	-1	H+(PG)(s) ⇌ H+(PG)(c) + Proton-gradient(c)
r0974	1	Pantothenate(s) + Na+(s) ⇌ Pantothenate(c) + Na+(c)
r1484	1	CoA(m) + 3-oxomyrist-7-enoyl-CoA(m) ⇌ Acetyl-CoA(m) + cis-laur-5-enoyl-CoA(m)
r1319	-13.2	ATP(c) + H2O(c) ⇌ ADP(c) + Pi(c) + ATP-energy(c)
r1485	1	H2O(m) + trans,cis-dodeca-2,5-dienoyl-CoA(m) ⇌ 3(S)-3-hydroxydodecen-(5Z)-oyl-CoA(m)
r0260	1	Adenylosuccinate(c) → Fumarate(c) + AMP(c)
r0247	1	ATP(c) + Ribose-5P(c) → AMP(c) + PRPP(c)
r1486	1	NAD+(m) + 3(S)-3-hydroxydodecen-(5Z)-oyl-CoA(m) ⇌ 3-oxolaur-cis-5-enoyl-CoA(m) + NADH(m)
r1488	1	ATP(c) + CoA(c) + Palmitolate(c) ⇌ AMP(c) + palmitoleoyl-CoA(c) + PPi(c)
r0699	1	SAICAR(c) ⇌ Fumarate(c) + AICAR(c)
r2442	1	Palmitolate(s) → Palmitolate(c)

Continued on next page

ID	flux	reaction
r0849	-2.17	H2O(s) ⇌ H2O(c)
r0581	-1	Pantothenate(c) + Cysteamine(c) ⇌ H2O(c) + Pantetheine(c)
r0867	2	CO2(c) ⇌ CO2(s)
r0589	1	ATP(c) + Phosphopantetheine(c) → PPi(c) + Dephospho-CoA(c)
r2468	1	Glycine(s) + H+(PG)(s) → Glycine(c) + H+(PG)(c)
r0736	0.17	ATP(c) + Fructose-6P(c) → ADP(c) + Fructose-1,6PP(c)
r0007	3	PPi(c) + H2O(c) → 2 Pi(c)
r0027	-0.5	NADPH(c) + Cystine(c) ⇌ 2 Cysteine(c) + NADP+(c)
r0192	0.83	Glucose-6P(c) ⇌ Fructose-6P(c)
r0344	-0.67	D-Xylulose-5P(c) ⇌ Ribulose-5P(c)
r0792	2	5-Methyl-THF(c) + NAD+(c) ⇌ 5,10-Methylene-THF(c) + NADH(c)
r0211	1	Cysteine(c) + O2(c) ⇌ 3-Sulfinoalanine(c)
r2676	-2	5-Methyl-THF(c) ⇌ THF(c) + THF-activated methyl group(c)
r1451	1	Ubiquinone(m) + Decanoyl-CoA(m) → (2E)-Decenoyl-CoA(m) + Ubiquinol(m)
r0660	-1	(S)-3-Hydroxydodecanoyl-CoA(m) ⇌ (2E)-Dodecenoyl-CoA(m) + H2O(m)
r2525	2	Glutamine(s) ⇌ Glutamine(c)
r2678	4	Acetyl-CoA(m) ⇌ CoA(m) + CoA-activated acetyl group(m)
r0666	1	ATP(c) + 5-Phosphoribosylformylglycinamidine(c) ⇌ ADP(c) + Pi(c) + AIR(c)
r1471	1	FAD(m) + cis-myrist-7-enoyl-CoA(m) ⇌ FADH2(m) + trans,cis-myristo-2,7-dienoyl-CoA(m)
r0667	-1	5-Phosphoribosyl-4-carboxy-5-aminoimidazole(c) ⇌ AIR(c) + CO2(c)
r1473	1	FAD(m) + palmitoleoyl-CoA(m) ⇌ FADH2(m) + trans,cis-hexadeca-2,9-dienoyl-CoA(m)
r1322	2	NADH(c) ⇌ NAD+(c) + NADH-redox-potential(c)
r1475	1	H2O(m) + trans,cis-hexadeca-2,9-dienoyl-CoA(m) ⇌ (S)-3-hydroxypalmitoleoyl-CoA(m)
r1323	5	NADH(m) ⇌ NAD+(m) + NADH-redox-potential(m)
r0250	-0.67	Ribulose-5P(c) ⇌ Ribose-5P(c)
r1476	1	NAD+(m) + cis-(3S)-hydroxytetradec-7-enoyl-CoA(m) ⇌ 3-oxomyrist-7-enoyl-CoA(m) + NADH(m)
r1478	1	NAD+(m) + (S)-3-hydroxypalmitoleoyl-CoA(m) ⇌ NADH(m) + 3-oxopalmitoleoyl-CoA(m)
r1326	3.5	NADPH(c) ⇌ NADPH-redox-potential(c) + NADP+(c)
r0687	1	ATP(c) + N-Formyl-GAR(c) + Glutamine(c) + H2O(c) ⇌ ADP(c) + Pi(c) + Glutamate(c) + 5-Phosphoribosylformylglycinamidine(c)
r1479	1	CoA(m) + 3-oxolaur-cis-5-enoyl-CoA(m) ⇌ Acetyl-CoA(m) + (3Z)-Dodecenoyl-CoA(m)
r0255	0.33	Fructose-6P(c) + GAP(c) ⇌ D-Xylulose-5P(c) + Erythrose-4P(c)
r1329	3	FADH2(c) ⇌ FAD(c) + FADH-redox-potential(c)
r0538	1	3-Sulfinoalanine(c) ⇌ CO2(c) + Hypotaurine(c)
r0256	0.17	Fructose-1,6PP(c) ⇌ DHAP(c) + GAP(c)
r0539	-1	Hypotaurine(c) ⇌ Cysteamine(c) + O2(c)
r0257	-1	Glutamine(c) + PRPP(c) + H2O(c) ⇌ 5-Phosphoribosylamine(c) + PPi(c) + Glutamate(c)
r0258	-2	Fumarate(c) + H2O(c) ⇌ Malate(c)
r0273	-1	IMP(c) + H2O(c) ⇌ FAICAR(c)
r0851	-4	H2O(m) ⇌ H2O(c)
r0700	1	10-Formyl-THF(c) + AICAR(c) ⇌ THF(c) + FAICAR(c)

3-Oxododecanoyl-ACP (626)

Definition			Solution												
			exchanges		reactions								transp		
Objective	Constraints	Comment	imports	exports	c	m	r	p	l	n	s	b	s-c	b-c	int
3-Oxododecanoyl-ACP(c)	PIPES MCES	synthesis of cytosolic 3-Oxododecanoyl-ACP	1 P_i(c) 5 Glutamate(s) 6 Glycine(s) 11 Alanine(s) 4 Lysine(s) 8 Aspartate(s) 9 Arginine(s) 7 Glutamine(s) 8 Serine(s) 5 Methionine(s) 3 Phenylalanine(s) 4 Tyrosine(s) 3 Cysteine(s) 20 Leucine(s) 1 Histidine(s) 9 Proline(s) 1 Asparagine(s) 11 Valine(s) 6 Threonine(s) 4 Isoleucine(s) 1 Pantothenate(s) 629 ATP-energy(c) 8 NADPH-redox-potential(c) 3 Proton-gradient(c) 6 CoA-activated acetyl group(c)	7 H_2O(s) 1 CO_2(s) 1 3-Oxododecanoyl-ACP(c) 66 Na-gradient(c)	36	-	-	-	-	-	-	-	24	-	-

ID	flux	reaction
r1069	-4	Lysine(c) ⇌ Lysine(s)
r0578	1	ATP(c) + Pantetheine(c) ⇌ ADP(c) + Phosphopantetheine(c)
r2478	8	Serine(s) + Na+(s) + H+(PG)(c) → Serine(c) + Na+(c) + H+(PG)(s)
r0030	125	ATP(c) + AMP(c) ⇌ 2 ADP(c)
r0760	1	Butyryl-ACP(c) + Malonyl-ACP(c) ⇌ CO2(c) + ACP(c) + 3-Oxohexanoyl-ACP(c)
r0032	1	ATP(c) + Dephospho-CoA(c) ⇌ ADP(c) + CoA(c)
r0761	-1	D-3-Hydroxyhexanoyl-ACP(c) + NADP+(c) ⇌ NADPH(c) + 3-Oxohexanoyl-ACP(c)
r0184	5	ATP(c) + Acetyl-CoA(c) + HCO3-(c) → ADP(c) + Pi(c) + Malonyl-CoA(c)
r0762	1	D-3-Hydroxyhexanoyl-ACP(c) ⇌ H2O(c) + (2E)-Hexenoyl-ACP(c)
r0763	-1	Hexanoyl-ACP(c) + NADP+(c) ⇌ NADPH(c) + (2E)-Hexenoyl-ACP(c)
r0764	1	Malonyl-ACP(c) + Hexanoyl-ACP(c) ⇌ 3-Oxooctanoyl-ACP(c) + CO2(c) + ACP(c)
r1122	-20	Leucine(c) + Na+(c) ← Leucine(s) + Na+(s)
r0765	-1	Octanoyl-ACP(c) + NADP+(c) ⇌ (2E)-Octenoyl-ACP(c) + NADPH(c)
r1123	-4	Isoleucine(c) + Na+(c) ← Isoleucine(s) + Na+(s)
r0766	1	Octanoyl-ACP(c) + Malonyl-ACP(c) ⇌ 3-Oxodecanoyl-ACP(c) + CO2(c) + ACP(c)
r0767	-1	Decanoyl-ACP(c) + NADP+(c) ⇌ (2E)-Decenoyl-ACP(c) + NADPH(c)
r1125	4	Tyrosine(s) + Na+(s) → Tyrosine(c) + Na+(c)
r0768	1	Decanoyl-ACP(c) + Malonyl-ACP(c) ⇌ 3-Oxododecanoyl-ACP(c) + CO2(c) + ACP(c)
r1142	-6	Threonine(s) + Na+(s) → Threonine(c) + Na+(c)
r1143	-8	Na+(c) + Aspartate(c) ← Na+(s) + Aspartate(s)
r2664	66	Na+(c) ⇌ Na+(s) + Na-gradient(c)
r1144	-5	Glutamate(c) + Na+(c) ← Glutamate(s) + Na+(s)
r1145	-1	Histidine(c) + Na+(c) ← Histidine(s) + Na+(s)
r2532	1	Asparagine(s) ⇌ Asparagine(c)
r2533	11	Valine(s) ⇌ Valine(c)
r0678	1	Acetyl-ACP(c) + Malonyl-ACP(c) ⇌ CO2(c) + ACP(c) + Acetoacetyl-ACP(c)
r0691	-1	(R)-3-Hydroxybutanoyl-ACP(c) + NADP+(c) ⇌ NADPH(c) + Acetoacetyl-ACP(c)
r1331	-3	H+(PG)(s) ⇌ H+(PG)(c) + Proton-gradient(c)
r0974	1	Pantothenate(s) + Na+(s) ⇌ Pantothenate(c) + Na+(c)
r0692	-1	(R)-3-Hydroxydecanoyl-ACP(c) + NADP+(c) ⇌ 3-Oxodecanoyl-ACP(c) + NADPH(c)
r1319	-629	ATP(c) + H2O(c) ⇌ ADP(c) + Pi(c) + ATP-energy(c)
r0693	1	(R)-3-Hydroxydecanoyl-ACP(c) ⇌ (2E)-Decenoyl-ACP(c) + H2O(c)
r0694	-1	(R)-3-Hydroxyoctanoyl-ACP(c) + NADP+(c) ⇌ 3-Oxooctanoyl-ACP(c) + NADPH(c)
r0695	1	(R)-3-Hydroxyoctanoyl-ACP(c) ⇌ (2E)-Octenoyl-ACP(c) + H2O(c)
r1058	9	Arginine(s) ⇌ Arginine(c)
r1072	5	Methionine(s) ⇌ Methionine(c)
r0849	-7	H2O(s) ⇌ H2O(c)
r0581	-1	Pantothenate(c) + Cysteamine(c) ⇌ H2O(c) + Pantetheine(c)
r0867	1	CO2(c) ⇌ CO2(s)

Continued on next page

ID	flux	reaction
r1096	1	11 Alanine(c) + 2 Cysteine(c) + 5 Glutamate(c) + 3 Phenylalanine(c) + 6 Glycine(c) + Histidine(c) + 4 Isoleucine(c) + 20 Leucine(c) + 5 Methionine(c) + 9 Proline(c) + 7 Glutamine(c) + 9 Arginine(c) + 8 Serine(c) + 6 Threonine(c) + 11 Valine(c) + 4 Tyrosine(c) + 496 ATP(c) + 496 H2O(c) + 8 Aspartate(c) + 4 Lysine(c) + Asparagine(c) → Apo-ACP(c) + 124 AMP(c) + 124 PPi(c) + 372 ADP(c) + 372 Pi(c)
r0589	1	ATP(c) + Phosphopantetheine(c) → PPi(c) + Dephospho-CoA(c)
r2469	11	Alanine(s) + H+(PG)(s) → Alanine(c) + H+(PG)(c)
r0007	125	PPi(c) + H2O(c) → 2 Pi(c)
r1417	-5	CO2(c) + H2O(c) ⇌ Carbonate(c)
r0049	1	PAP(c) + H2O(c) → AMP(c) + Pi(c)
r1419	5	Carbonate(c) ⇌ HCO3-(c)
r1139	-9	Proline(c) + Na+(c) ← Proline(s) + Na+(s)
r0211	1	Cysteine(c) + O2(c) ⇌ 3-Sulfinoalanine(c)
r2523	6	Glycine(s) ⇌ Glycine(c)
r2677	-6	Acetyl-CoA(c) ⇌ CoA(c) + CoA-activated acetyl group(c)
r2525	7	Glutamine(s) ⇌ Glutamine(c)
r0367	1	Acetyl-CoA(c) + ACP(c) ⇌ CoA(c) + Acetyl-ACP(c)
r0368	1	CoA(c) + Apo-ACP(c) → PAP(c) + ACP(c)
r2528	3	Phenylalanine(s) ⇌ Phenylalanine(c)
r0369	5	ACP(c) + Malonyl-CoA(c) ⇌ CoA(c) + Malonyl-ACP(c)
r2529	3	Cysteine(s) ⇌ Cysteine(c)
r0681	1	(R)-3-Hydroxybutanoyl-ACP(c) ⇌ But-2-enoyl-ACP(c) + H2O(c)
r0682	-1	Butyryl-ACP(c) + NADP+(c) ⇌ But-2-enoyl-ACP(c) + NADPH(c)
r1326	-8	NADPH(c) ⇌ NADPH-redox-potential(c) + NADP+(c)
r0538	1	3-Sulfinoalanine(c) ⇌ CO2(c) + Hypotaurine(c)
r0539	-1	Hypotaurine(c) ⇌ Cysteamine(c) + O2(c)

3-Oxododecanoyl-CoA (627)

Definition			Solution												
			exchanges		reactions								transp		
Objective	Constraints	Comment	imports	exports	c	m	r	p	l	n	s	b	s-c	b-c	int
3-Oxododecanoyl-CoA(m)	PIPES MCES	synthesis of mitochondrial 3-Oxododecanoyl-CoA	2.17 P_i(c) 1 Glycine(s) 2 Aspartate(s) 2 Glutamine(s) 0.83 Glucose-6P(c) 0.5 Cystine(s) 1 Pantothenate(s) 1 Palmitolate(s) 13.2 ATP-energy(c) 1 Proton-gradient(c) 2 THF-activated methyl group(c)	2.17 H_2O(s) 2 CO_2(s) 2 Pyruvate(c) 2 Glutamate(c) 1 3-Oxododecanoyl-CoA(m) 2 NADH-redox-potential(c) 4 NADH-redox-potential(m) 3.5 NADPH-redox-potential(c) 3 FADH-redox-potential(c) 3 Na-gradient(c) 3 CoA-activated acetyl group(m)	44	17	-	-	-	-	-	-	11	-	3

ID	flux	reaction
r0406	-0.33	Fructose-6P(c) + Erythrose-4P(c) ⇌ Sedoheptulose-7P(c) + GAP(c)
r0277	1	GTP(c) + IMP(c) + Aspartate(c) → GDP(c) + Pi(c) + Adenylosuccinate(c)
r0293	2	5,10-Methylene-THF(c) + NADP+(c) ⇌ 5,10-Methenyl-THF(c) + NADPH(c)
r0705	1	ATP(c) + 5-Phosphoribosyl-4-carboxy-5-aminoimidazole(c) + Aspartate(c) ⇌ ADP(c) + Pi(c) + SAICAR(c)
r0578	1	ATP(c) + Pantetheine(c) ⇌ ADP(c) + Phosphopantetheine(c)
r0722	1	(S)-3-Hydroxydodecanoyl-CoA(m) + NAD+(m) ⇌ 3-Oxododecanoyl-CoA(m) + NADH(m)
r0877	0.5	Glutamate(c) + Cystine(s) ⇌ Glutamate(s) + Cystine(c)
r0030	3	ATP(c) + AMP(c) ⇌ 2 ADP(c)
r0032	1	ATP(c) + Dephospho-CoA(c) ⇌ ADP(c) + CoA(c)
r1290	3	FAD(c) + FADH2(m) ⇌ FAD(m) + FADH2(c)
r1143	-2	Na+(c) + Aspartate(c) ← Na+(s) + Aspartate(s)
r2664	3	Na+(c) ⇌ Na+(s) + Na-gradient(c)
r0058	2	Malate(c) + NADP+(c) ⇌ Pyruvate(c) + CO2(c) + NADPH(c)
r0370	0.33	Sedoheptulose-7P(c) + GAP(c) ⇌ Ribose-5P(c) + D-Xylulose-5P(c)
r0371	2	5,10-Methenyl-THF(c) + H2O(c) ⇌ 10-Formyl-THF(c)

Continued on next page

ID	flux	reaction
r2685	1.5	Glutamate(c) ⇌ Glutamate(s)
r0657	1	(3Z)-Dodecenoyl-CoA(m) ⇌ (2E)-Dodecenoyl-CoA(m)
r0659	1	ATP(c) + 5-Phosphoribosylamine(c) + Glycine(c) ⇌ ADP(c) + Pi(c) + GAR(c)
r1480	1	CoA(m) + 3-oxopalmitoleoyl-CoA(m) ⇌ Acetyl-CoA(m) + cis-myrist-7-enoyl-CoA(m)
r0099	1	ATP(c) + GDP(c) ⇌ ADP(c) + GTP(c)
r1468	1	palmitoleoyl-CoA(c) ⇌ palmitoleoyl-CoA(m)
r0243	-0.17	DHAP(c) ⇌ GAP(c)
r0677	1	10-Formyl-THF(c) + GAR(c) ⇌ THF(c) + N-Formyl-GAR(c)
r1482	-1	FADH2(m) + trans,cis-dodeca-2,5-dienoyl-CoA(m) ⇌ FAD(m) + cis-laur-5-enoyl-CoA(m)
r1483	1	H2O(m) + trans,cis-myristo-2,7-dienoyl-CoA(m) ⇌ cis-(3S)-hydroxytetradec-7-enoyl-CoA(m)
r1331	-1	H+(PG)(s) ⇌ H+(PG)(c) + Proton-gradient(c)
r0974	1	Pantothenate(s) + Na+(s) ⇌ Pantothenate(c) + Na+(c)
r1484	1	CoA(m) + 3-oxomyrist-7-enoyl-CoA(m) ⇌ Acetyl-CoA(m) + cis-laur-5-enoyl-CoA(m)
r1319	-13.2	ATP(c) + H2O(c) ⇌ ADP(c) + Pi(c) + ATP-energy(c)
r1485	1	H2O(m) + trans,cis-dodeca-2,5-dienoyl-CoA(m) ⇌ 3(S)-3-hydroxydodecen-(5Z)-oyl-CoA(m)
r0260	1	Adenylosuccinate(c) → Fumarate(c) + AMP(c)
r0247	1	ATP(c) + Ribose-5P(c) → AMP(c) + PRPP(c)
r1486	1	NAD+(m) + 3(S)-3-hydroxydodecen-(5Z)-oyl-CoA(m) ⇌ 3-oxolaur-cis-5-enoyl-CoA(m) + NADH(m)
r1488	1	ATP(c) + CoA(c) + Palmitolate(c) ⇌ AMP(c) + palmitoleoyl-CoA(c) + PPi(c)
r0699	1	SAICAR(c) ⇌ Fumarate(c) + AICAR(c)
r2442	1	Palmitolate(s) → Palmitolate(c)
r0849	-2.17	H2O(s) ⇌ H2O(c)
r0581	-1	Pantothenate(c) + Cysteamine(c) ⇌ H2O(c) + Pantetheine(c)
r0867	2	CO2(c) ⇌ CO2(s)
r0589	1	ATP(c) + Phosphopantetheine(c) → PPi(c) + Dephospho-CoA(c)
r2468	1	Glycine(s) + H+(PG)(s) → Glycine(c) + H+(PG)(c)
r0736	0.17	ATP(c) + Fructose-6P(c) → ADP(c) + Fructose-1,6PP(c)
r0007	3	PPi(c) + H2O(c) → 2 Pi(c)
r0027	-0.5	NADPH(c) + Cystine(c) ⇌ 2 Cysteine(c) + NADP+(c)
r0192	0.83	Glucose-6P(c) ⇌ Fructose-6P(c)
r0344	-0.67	D-Xylulose-5P(c) ⇌ Ribulose-5P(c)
r0792	2	5-Methyl-THF(c) + NAD+(c) ⇌ 5,10-Methylene-THF(c) + NADH(c)
r0211	1	Cysteine(c) + O2(c) ⇌ 3-Sulfinoalanine(c)
r2676	-2	5-Methyl-THF(c) ⇌ THF(c) + THF-activated methyl group(c)
r0660	-1	(S)-3-Hydroxydodecanoyl-CoA(m) ⇌ (2E)-Dodecenoyl-CoA(m) + H2O(m)
r2525	2	Glutamine(s) ⇌ Glutamine(c)
r2678	3	Acetyl-CoA(m) ⇌ CoA(m) + CoA-activated acetyl group(m)
r0666	1	ATP(c) + 5-Phosphoribosylformylglycinamidine(c) ⇌ ADP(c) + Pi(c) + AIR(c)
r1471	1	FAD(m) + cis-myrist-7-enoyl-CoA(m) ⇌ FADH2(m) + trans,cis-myristo-2,7-dienoyl-CoA(m)
r0667	-1	5-Phosphoribosyl-4-carboxy-5-aminoimidazole(c) ⇌ AIR(c) + CO2(c)
r1473	1	FAD(m) + palmitoleoyl-CoA(m) ⇌ FADH2(m) + trans,cis-hexadeca-2,9-dienoyl-CoA(m)
r1322	2	NADH(c) ⇌ NAD+(c) + NADH-redox-potential(c)
r1475	1	H2O(m) + trans,cis-hexadeca-2,9-dienoyl-CoA(m) ⇌ (S)-3-hydroxypalmitoleoyl-CoA(m)
r1323	4	NADH(m) ⇌ NAD+(m) + NADH-redox-potential(m)
r0250	-0.67	Ribulose-5P(c) ⇌ Ribose-5P(c)
r1476	1	NAD+(m) + cis-(3S)-hydroxytetradec-7-enoyl-CoA(m) ⇌ 3-oxomyrist-7-enoyl-CoA(m) + NADH(m)
r1478	1	NAD+(m) + (S)-3-hydroxypalmitoleoyl-CoA(m) ⇌ NADH(m) + 3-oxopalmitoleoyl-CoA(m)
r1326	3.5	NADPH(c) ⇌ NADPH-redox-potential(c) + NADP+(c)
r0687	1	ATP(c) + N-Formyl-GAR(c) + Glutamine(c) + H2O(c) ⇌ ADP(c) + Pi(c) + Glutamate(c) + 5-Phosphoribosylformylglycinamidine(c)
r1479	1	CoA(m) + 3-oxolaur-cis-5-enoyl-CoA(m) ⇌ Acetyl-CoA(m) + (3Z)-Dodecenoyl-CoA(m)
r0255	0.33	Fructose-6P(c) + GAP(c) ⇌ D-Xylulose-5P(c) + Erythrose-4P(c)
r1329	3	FADH2(c) ⇌ FAD(c) + FADH-redox-potential(c)
r0538	1	3-Sulfinoalanine(c) ⇌ CO2(c) + Hypotaurine(c)
r0256	0.17	Fructose-1,6PP(c) ⇌ DHAP(c) + GAP(c)
r0539	-1	Hypotaurine(c) ⇌ Cysteamine(c) + O2(c)

Continued on next page

ID	flux	reaction
r0257	-1	Glutamine(c) + PRPP(c) + H2O(c) ⇌ 5-Phosphoribosylamine(c) + PPi(c) + Glutamate(c)
r0258	-2	Fumarate(c) + H2O(c) ⇌ Malate(c)
r0273	-1	IMP(c) + H2O(c) ⇌ FAICAR(c)
r0851	-4	H2O(m) ⇌ H2O(c)
r0700	1	10-Formyl-THF(c) + AICAR(c) ⇌ THF(c) + FAICAR(c)

3-Oxohexadecanoyl-ACP (628)

Definition			Solution												
			exchanges		reactions								transp		
Objective	Constraints	Comment	imports	exports	c	m	r	p	l	n	s	b	s-c	b-c	int
3-Oxohexadecanoyl-ACP(c)	PIPES MCES	synthesis of cytosolic 3-Oxohexadecanoyl-ACP	1 P_i(c) 5 Glutamate(s) 6 Glycine(s) 11 Alanine(s) 4 Lysine(s) 8 Aspartate(s) 9 Arginine(s) 7 Glutamine(s) 8 Serine(s) 5 Methionine(s) 3 Phenylalanine(s) 4 Tyrosine(s) 3 Cysteine(s) 20 Leucine(s) 1 Histidine(s) 9 Proline(s) 1 Asparagine(s) 11 Valine(s) 6 Threonine(s) 4 Isoleucine(s) 1 Pantothenate(s) 631 ATP-energy(c) 12 NADPH-redox-potential(c) 3 Proton-gradient(c) 8 CoA-activated acetyl group(c)	9 H_2O(s) 1 CO_2(s) 1 3-Oxohexadecanoyl-ACP(c) 66 Na-gradient(c)	44	-	-	-	-	-	-	-	24	-	-

ID	flux	reaction
r0701	-1	HMA(c) + NADP+(c) ⇌ 3-Oxotetradecanoyl-ACP(c) + NADPH(c)
r0702	1	HMA(c) ⇌ H2O(c) + (2E)-Tetradecenoyl-ACP(c)
r1069	-4	Lysine(c) ⇌ Lysine(s)
r0578	1	ATP(c) + Pantetheine(c) ⇌ ADP(c) + Phosphopantetheine(c)
r2478	8	Serine(s) + Na+(s) + H+(PG)(c) → Serine(c) + Na+(c) + H+(PG)(s)
r0030	125	ATP(c) + AMP(c) ⇌ 2 ADP(c)
r0760	1	Butyryl-ACP(c) + Malonyl-ACP(c) ⇌ CO2(c) + ACP(c) + 3-Oxohexanoyl-ACP(c)
r0032	1	ATP(c) + Dephospho-CoA(c) ⇌ ADP(c) + CoA(c)
r0761	-1	D-3-Hydroxyhexanoyl-ACP(c) + NADP+(c) ⇌ NADPH(c) + 3-Oxohexanoyl-ACP(c)
r0184	7	ATP(c) + Acetyl-CoA(c) + HCO3-(c) → ADP(c) + Pi(c) + Malonyl-CoA(c)
r0762	1	D-3-Hydroxyhexanoyl-ACP(c) ⇌ H2O(c) + (2E)-Hexenoyl-ACP(c)
r0763	-1	Hexanoyl-ACP(c) + NADP+(c) ⇌ NADPH(c) + (2E)-Hexenoyl-ACP(c)
r0764	1	Malonyl-ACP(c) + Hexanoyl-ACP(c) ⇌ 3-Oxooctanoyl-ACP(c) + CO2(c) + ACP(c)
r1122	-20	Leucine(c) + Na+(c) ← Leucine(s) + Na+(s)
r0765	-1	Octanoyl-ACP(c) + NADP+(c) ⇌ (2E)-Octenoyl-ACP(c) + NADPH(c)
r1123	-4	Isoleucine(c) + Na+(c) ← Isoleucine(s) + Na+(s)
r0766	1	Octanoyl-ACP(c) + Malonyl-ACP(c) ⇌ 3-Oxodecanoyl-ACP(c) + CO2(c) + ACP(c)
r0767	-1	Decanoyl-ACP(c) + NADP+(c) ⇌ (2E)-Decenoyl-ACP(c) + NADPH(c)
r1125	4	Tyrosine(s) + Na+(s) → Tyrosine(c) + Na+(c)
r0768	1	Decanoyl-ACP(c) + Malonyl-ACP(c) ⇌ 3-Oxododecanoyl-ACP(c) + CO2(c) + ACP(c)
r0769	-1	D-3-Hydroxydodecanoyl-ACP(c) + NADP+(c) ⇌ 3-Oxododecanoyl-ACP(c) + NADPH(c)
r1142	-6	Threonine(s) + Na+(s) → Threonine(c) + Na+(c)
r1143	-8	Na+(c) + Aspartate(c) ← Na+(s) + Aspartate(s)
r2664	66	Na+(c) ⇌ Na+(s) + Na-gradient(c)
r1144	-5	Glutamate(c) + Na+(c) ← Glutamate(s) + Na+(s)
r1145	-1	Histidine(c) + Na+(c) ← Histidine(s) + Na+(s)
r2532	1	Asparagine(s) ⇌ Asparagine(c)
r2533	11	Valine(s) ⇌ Valine(c)
r0678	1	Acetyl-ACP(c) + Malonyl-ACP(c) ⇌ CO2(c) + ACP(c) + Acetoacetyl-ACP(c)
r0691	-1	(R)-3-Hydroxybutanoyl-ACP(c) + NADP+(c) ⇌ NADPH(c) + Acetoacetyl-ACP(c)

Continued on next page

ID	flux	reaction
r1331	-3	H+(PG)(s) ⇌ H+(PG)(c) + Proton-gradient(c)
r0974	1	Pantothenate(s) + Na+(s) ⇌ Pantothenate(c) + Na+(c)
r0692	-1	(R)-3-Hydroxydecanoyl-ACP(c) + NADP+(c) ⇌ 3-Oxodecanoyl-ACP(c) + NADPH(c)
r1319	-631	ATP(c) + H2O(c) ⇌ ADP(c) + Pi(c) + ATP-energy(c)
r0693	1	(R)-3-Hydroxydecanoyl-ACP(c) ⇌ (2E)-Decenoyl-ACP(c) + H2O(c)
r0694	-1	(R)-3-Hydroxyoctanoyl-ACP(c) + NADP+(c) ⇌ 3-Oxooctanoyl-ACP(c) + NADPH(c)
r0695	1	(R)-3-Hydroxyoctanoyl-ACP(c) ⇌ (2E)-Octenoyl-ACP(c) + H2O(c)
r1058	9	Arginine(s) ⇌ Arginine(c)
r1072	5	Methionine(s) ⇌ Methionine(c)
r0849	-9	H2O(s) ⇌ H2O(c)
r0581	-1	Pantothenate(c) + Cysteamine(c) ⇌ H2O(c) + Pantetheine(c)
r0712	-1	Dodecanoyl-ACP(c) + NADP+(c) ⇌ (2E)-Dodecenoyl-ACP(c) + NADPH(c)
r0713	1	Dodecanoyl-ACP(c) + Malonyl-ACP(c) ⇌ 3-Oxotetradecanoyl-ACP(c) + CO2(c) + ACP(c)
r0867	1	CO2(c) ⇌ CO2(s)
r1096	1	11 Alanine(c) + 2 Cysteine(c) + 5 Glutamate(c) + 3 Phenylalanine(c) + 6 Glycine(c) + Histidine(c) + 4 Isoleucine(c) + 20 Leucine(c) + 5 Methionine(c) + 9 Proline(c) + 7 Glutamine(c) + 9 Arginine(c) + 8 Serine(c) + 6 Threonine(c) + 11 Valine(c) + 4 Tyrosine(c) + 496 ATP(c) + 496 H2O(c) + 8 Aspartate(c) + 4 Lysine(c) + Asparagine(c) → Apo-ACP(c) + 124 AMP(c) + 124 PPi(c) + 372 ADP(c) + 372 Pi(c)
r0589	1	ATP(c) + Phosphopantetheine(c) → PPi(c) + Dephospho-CoA(c)
r2469	11	Alanine(s) + H+(PG)(s) → Alanine(c) + H+(PG)(c)
r0007	125	PPi(c) + H2O(c) → 2 Pi(c)
r0770	1	D-3-Hydroxydodecanoyl-ACP(c) ⇌ (2E)-Dodecenoyl-ACP(c) + H2O(c)
r0771	-1	Tetradecanoyl-ACP(c) + NADP+(c) ⇌ NADPH(c) + (2E)-Tetradecenoyl-ACP(c)
r0772	1	Tetradecanoyl-ACP(c) + Malonyl-ACP(c) ⇌ 3-Oxohexadecanoyl-ACP(c) + CO2(c) + ACP(c)
r1417	-7	CO2(c) + H2O(c) ⇌ Carbonate(c)
r0049	1	PAP(c) + H2O(c) → AMP(c) + Pi(c)
r1419	7	Carbonate(c) ⇌ HCO3-(c)
r1139	-9	Proline(c) + Na+(c) ← Proline(s) + Na+(s)
r0211	1	Cysteine(c) + O2(c) ⇌ 3-Sulfinoalanine(c)
r2523	6	Glycine(s) ⇌ Glycine(c)
r2677	-8	Acetyl-CoA(c) ⇌ CoA(c) + CoA-activated acetyl group(c)
r2525	7	Glutamine(s) ⇌ Glutamine(c)
r0367	1	Acetyl-CoA(c) + ACP(c) ⇌ CoA(c) + Acetyl-ACP(c)
r0368	1	CoA(c) + Apo-ACP(c) → PAP(c) + ACP(c)
r2528	3	Phenylalanine(s) ⇌ Phenylalanine(c)
r0369	7	ACP(c) + Malonyl-CoA(c) ⇌ CoA(c) + Malonyl-ACP(c)
r2529	3	Cysteine(s) ⇌ Cysteine(c)
r0681	1	(R)-3-Hydroxybutanoyl-ACP(c) ⇌ But-2-enoyl-ACP(c) + H2O(c)
r0682	-1	Butyryl-ACP(c) + NADP+(c) ⇌ But-2-enoyl-ACP(c) + NADPH(c)
r1326	-12	NADPH(c) ⇌ NADPH-redox-potential(c) + NADP+(c)
r0538	1	3-Sulfinoalanine(c) ⇌ CO2(c) + Hypotaurine(c)
r0539	-1	Hypotaurine(c) ⇌ Cysteamine(c) + O2(c)

3-Oxohexanoyl-ACP (629)

Definition			Solution												
			exchanges		reactions								transp		
Objective	Constraints	Comment	imports	exports	c	m	r	p	l	n	s	b	s-c	b-c	int
3-Oxohexanoyl-ACP(c)	PIPES MCES	synthesis of cytosolic 3-Oxohexanoyl-ACP	1 P_i(c) 5 Glutamate(s) 6 Glycine(s) 11 Alanine(s) 4 Lysine(s) 8 Aspartate(s) 9 Arginine(s) 7 Glutamine(s) 8 Serine(s) 5 Methionine(s) 3 Phenylalanine(s) 4 Tyrosine(s) 3 Cysteine(s) 20 Leucine(s) 1 Histidine(s) 9 Proline(s) 1 Asparagine(s) 11 Valine(s) 6 Threonine(s) 4 Isoleucine(s) 1 Pantothenate(s) 626 ATP-energy(c) 2 NADPH-redox-potential(c) 3 CoA-activated acetyl group(c)	4 H_2O(s) 1 CO_2(s) 1 3-Oxohexanoyl-ACP(c) 2 Proton-gradient(c) 82 Na-gradient(c)	24	-	-	-	-	-	-	-	24	-	-

ID	flux	reaction
r1069	-4	Lysine(s) ⇌ Lysine(c)
r0578	1	ATP(c) + Pantetheine(c) ⇌ ADP(c) + Phosphopantetheine(c)
r2478	8	Serine(s) + Na+(s) + H+(PG)(c) → Serine(c) + Na+(c) + H+(PG)(s)
r0030	125	ATP(c) + AMP(c) ⇌ 2 ADP(c)
r0760	1	Butyryl-ACP(c) + Malonyl-ACP(c) ⇌ CO2(c) + ACP(c) + 3-Oxohexanoyl-ACP(c)
r0032	1	ATP(c) + Dephospho-CoA(c) ⇌ ADP(c) + CoA(c)
r0184	2	ATP(c) + Acetyl-CoA(c) + HCO3-(c) → ADP(c) + Pi(c) + Malonyl-CoA(c)
r1121	-11	Valine(c) + Na+(c) ← Valine(s) + Na+(s)
r1122	-20	Leucine(c) + Na+(c) ← Leucine(s) + Na+(s)
r1123	-4	Isoleucine(c) + Na+(c) ← Isoleucine(s) + Na+(s)
r1125	4	Tyrosine(s) + Na+(s) → Tyrosine(c) + Na+(c)
r1141	-5	Methionine(c) + Na+(c) ← Methionine(s) + Na+(s)
r1142	-6	Threonine(s) + Na+(s) → Threonine(c) + Na+(c)
r1143	-8	Na+(c) + Aspartate(c) ← Na+(s) + Aspartate(s)
r2664	82	Na+(c) ⇌ Na+(s) + Na-gradient(c)
r1144	-5	Glutamate(c) + Na+(c) ← Glutamate(s) + Na+(s)
r1145	-1	Histidine(c) + Na+(c) ← Histidine(s) + Na+(s)
r2532	1	Asparagine(s) ⇌ Asparagine(c)
r0678	1	Acetyl-ACP(c) + Malonyl-ACP(c) ⇌ CO2(c) + ACP(c) + Acetoacetyl-ACP(c)
r0691	-1	(R)-3-Hydroxybutanoyl-ACP(c) + NADP+(c) ⇌ NADPH(c) + Acetoacetyl-ACP(c)
r1331	2	H+(PG)(s) ⇌ H+(PG)(c) + Proton-gradient(c)
r0974	1	Pantothenate(s) + Na+(s) ⇌ Pantothenate(c) + Na+(c)
r1319	-626	ATP(c) + H2O(c) ⇌ ADP(c) + Pi(c) + ATP-energy(c)
r1058	9	Arginine(s) ⇌ Arginine(c)
r0849	-4	H2O(s) ⇌ H2O(c)
r0581	-1	Pantothenate(c) + Cysteamine(c) ⇌ H2O(c) + Pantetheine(c)
r0867	1	CO2(c) ⇌ CO2(s)
r1096	1	11 Alanine(c) + 2 Cysteine(c) + 5 Glutamate(c) + 3 Phenylalanine(c) + 6 Glycine(c) + Histidine(c) + 4 Isoleucine(c) + 20 Leucine(c) + 5 Methionine(c) + 9 Proline(c) + 7 Glutamine(c) + 9 Arginine(c) + 8 Serine(c) + 6 Threonine(c) + 11 Valine(c) + 4 Tyrosine(c) + 496 ATP(c) + 496 H2O(c) + 8 Aspartate(c) + 4 Lysine(c) + Asparagine(c) → Apo-ACP(c) + 124 AMP(c) + 124 PPi(c) + 372 ADP(c) + 372 Pi(c)
r0589	1	ATP(c) + Phosphopantetheine(c) → PPi(c) + Dephospho-CoA(c)
r2468	6	Glycine(s) + H+(PG)(s) → Glycine(c) + H+(PG)(c)
r0007	125	PPi(c) + H2O(c) → 2 Pi(c)
r1417	-2	CO2(c) + H2O(c) ⇌ Carbonate(c)
r0049	1	PAP(c) + H2O(c) → AMP(c) + Pi(c)
r1419	2	Carbonate(c) ⇌ HCO3-(c)
r1139	-9	Proline(c) + Na+(c) ← Proline(s) + Na+(s)
r0211	1	Cysteine(c) + O2(c) ⇌ 3-Sulfinoalanine(c)
r2524	11	Alanine(s) ⇌ Alanine(c)
r2677	-3	Acetyl-CoA(c) ⇌ CoA(c) + CoA-activated acetyl group(c)
r2525	7	Glutamine(s) ⇌ Glutamine(c)

Continued on next page

ID	flux	reaction
r0367	1	Acetyl-CoA(c) + ACP(c) ⇌ CoA(c) + Acetyl-ACP(c)
r0368	1	CoA(c) + Apo-ACP(c) → PAP(c) + ACP(c)
r2528	3	Phenylalanine(s) ⇌ Phenylalanine(c)
r0369	2	ACP(c) + Malonyl-CoA(c) ⇌ CoA(c) + Malonyl-ACP(c)
r2529	3	Cysteine(s) ⇌ Cysteine(c)
r0681	1	(R)-3-Hydroxybutanoyl-ACP(c) ⇌ But-2-enoyl-ACP(c) + H2O(c)
r0682	-1	Butyryl-ACP(c) + NADP+(c) ⇌ But-2-enoyl-ACP(c) + NADPH(c)
r1326	-2	NADPH(c) ⇌ NADPH-redox-potential(c) + NADP+(c)
r0538	1	3-Sulfinoalanine(c) ⇌ CO2(c) + Hypotaurine(c)
r0539	-1	Hypotaurine(c) ⇌ Cysteamine(c) + O2(c)

3-Oxohexanoyl-CoA (630)

Definition			Solution											
			exchanges		reactions							transp		
Objective	Constraints	Comment	imports	exports	c	m	r	p	l	n	s	b	s-c	b-c	int
3-Oxohexanoyl-CoA(m)	PIPES MCES	synthesis of mitochondrial 3-Oxohexanoyl-CoA	1.5 O_2(s) 2.17 P_i(c) 1 Glycine(s) 2 Aspartate(s) 2 Glutamine(s) 0.83 Glucose-6P(c) 0.5 Cystine(s) 1 Pantothenate(s) 1 Palmitolate(s) 13.2 ATP-energy(c) 2 THF-activated methyl group(c)	2.17 H_2O(s) 2 CO_2(s) 2 Pyruvate(c) 2 Glutamate(s) 1 3-Oxohexanoyl-CoA(m) 2 NADH-redox-potential(c) 7 NADH-redox-potential(m) 3.5 NADPH-redox-potential(c) 3 FADH-redox-potential(c) 1 Proton-gradient(c) 18 Proton-gradient(m) 5 Na-gradient(c) 6 CoA-activated acetyl group(m)	44	29	-	-	-	-	-	-	12	-	7

ID	flux	reaction
r0406	-0.33	Fructose-6P(c) + Erythrose-4P(c) ⇌ Sedoheptulose-7P(c) + GAP(c)
r0277	1	GTP(c) + IMP(c) + Aspartate(c) → GDP(c) + Pi(c) + Adenylosuccinate(c)
r0855	1.5	O2(s) ⇌ O2(c)
r0856	-1.5	O2(m) ⇌ O2(c)
r0705	1	ATP(c) + 5-Phosphoribosyl-4-carboxy-5-aminoimidazole(c) + Aspartate(c) ⇌ ADP(c) + Pi(c) + SAICAR(c)
r0293	2	5,10-Methylene-THF(c) + NADP+(c) ⇌ 5,10-Methenyl-THF(c) + NADPH(c)
r0578	1	ATP(c) + Pantetheine(c) ⇌ ADP(c) + Phosphopantetheine(c)
r0722	1	(S)-3-Hydroxydodecanoyl-CoA(m) + NAD+(m) ⇌ 3-Oxododecanoyl-CoA(m) + NADH(m)
r0724	-1	Decanoyl-CoA(m) + Acetyl-CoA(m) ⇌ CoA(m) + 3-Oxododecanoyl-CoA(m)
r0877	0.5	Glutamate(c) + Cystine(s) ⇌ Glutamate(s) + Cystine(c)
r0726	1	(S)-Hydroxydecanoyl-CoA(m) + NAD+(m) ⇌ 3-Oxodecanoyl-CoA(m) + NADH(m)
r0728	-1	(S)-Hydroxydecanoyl-CoA(m) ⇌ (2E)-Decenoyl-CoA(m) + H2O(m)
r2477	2	Glutamine(s) + Na+(s) + H+(PG)(c) → Glutamine(c) + Na+(c) + H+(PG)(s)
r0017	1.5	O2(m) + 4 H+(PG)(m) + 4 Ferrocytochrome C(m) → 4 Ferricytochrome C(m) + 2 H2O(m) + 4 H+(PG)(c)
r0030	3	ATP(c) + AMP(c) ⇌ 2 ADP(c)
r0032	1	ATP(c) + Dephospho-CoA(c) ⇌ ADP(c) + CoA(c)
r1290	3	FAD(c) + FADH2(m) ⇌ FAD(m) + FADH2(c)
r1143	-2	Na+(c) + Aspartate(c) ← Na+(s) + Aspartate(s)
r0634	-1	Acetyl-CoA(m) + Octanoyl-CoA(m) ⇌ CoA(m) + 3-Oxodecanoyl-CoA(m)
r2664	5	Na+(c) ⇌ Na+(s) + Na-gradient(c)
r0058	2	Malate(c) + NADP+(c) ⇌ Pyruvate(c) + CO2(c) + NADPH(c)
r0370	0.33	Sedoheptulose-7P(c) + GAP(c) ⇌ Ribose-5P(c) + D-Xylulose-5P(c)
r0371	2	5,10-Methenyl-THF(c) + H2O(c) ⇌ 10-Formyl-THF(c)
r2685	1.5	Glutamate(c) ⇌ Glutamate(s)
r1448	1	Ubiquinone(m) + Octanoyl-CoA(m) → (2E)-Octenoyl-CoA(m) + Ubiquinol(m)
r0657	1	(3Z)-Dodecenoyl-CoA(m) ⇌ (2E)-Dodecenoyl-CoA(m)
r0659	1	ATP(c) + 5-Phosphoribosylamine(c) + Glycine(c) ⇌ ADP(c) + Pi(c) + GAR(c)
r0507	3	Ubiquinol(m) + 2 Ferricytochrome C(m) + 4 H+(PG)(m) → Ubiquinone(m) + 4 H+(PG)(c) + 2 Ferrocytochrome C(m)

Continued on next page

ID	flux	reaction
r1480	1	CoA(m) + 3-oxopalmitoleoyl-CoA(m) ⇌ Acetyl-CoA(m) + cis-myrist-7-enoyl-CoA(m)
r0099	1	ATP(c) + GDP(c) ⇌ ADP(c) + GTP(c)
r1468	1	palmitoleoyl-CoA(c) ⇌ palmitoleoyl-CoA(m)
r0243	-0.17	DHAP(c) ⇌ GAP(c)
r0677	1	10-Formyl-THF(c) + GAR(c) ⇌ THF(c) + N-Formyl-GAR(c)
r1482	-1	FADH2(m) + trans,cis-dodeca-2,5-dienoyl-CoA(m) ⇌ FAD(m) + cis-laur-5-enoyl-CoA(m)
r1330	18	H+(PG)(c) ⇌ H+(PG)(m) + Proton-gradient(m)
r1483	1	H2O(m) + trans,cis-myristo-2,7-dienoyl-CoA(m) ⇌ cis-(3S)-hydroxytetradec-7-enoyl-CoA(m)
r1331	1	H+(PG)(s) ⇌ H+(PG)(c) + Proton-gradient(c)
r0974	1	Pantothenate(s) + Na+(s) ⇌ Pantothenate(c) + Na+(c)
r1484	1	CoA(m) + 3-oxomyrist-7-enoyl-CoA(m) ⇌ Acetyl-CoA(m) + cis-laur-5-enoyl-CoA(m)
r1319	-13.2	ATP(c) + H2O(c) ⇌ ADP(c) + Pi(c) + ATP-energy(c)
r1485	1	H2O(m) + trans,cis-dodeca-2,5-dienoyl-CoA(m) ⇌ 3(S)-3-hydroxydodecen-(5Z)-oyl-CoA(m)
r0260	1	Adenylosuccinate(c) → Fumarate(c) + AMP(c)
r0247	1	ATP(c) + Ribose-5P(c) → AMP(c) + PRPP(c)
r1486	1	NAD+(m) + 3(S)-3-hydroxydodecen-(5Z)-oyl-CoA(m) ⇌ 3-oxolaur-cis-5-enoyl-CoA(m) + NADH(m)
r1488	1	ATP(c) + CoA(c) + Palmitolate(c) ⇌ AMP(c) + palmitoleoyl-CoA(c) + PPi(c)
r0699	1	SAICAR(c) ⇌ Fumarate(c) + AICAR(c)
r2442	1	Palmitolate(s) → Palmitolate(c)
r0849	-2.17	H2O(s) ⇌ H2O(c)
r0581	-1	Pantothenate(c) + Cysteamine(c) ⇌ H2O(c) + Pantetheine(c)
r0867	2	CO2(c) ⇌ CO2(s)
r0730	1	(S)-Hydroxyoctanoyl-CoA(m) + NAD+(m) → 3-Oxooctanoyl-CoA(m) + NADH(m)
r0731	-1	(S)-Hydroxyoctanoyl-CoA(m) ← (2E)-Octenoyl-CoA(m) + H2O(m)
r0732	-1	Hexanoyl-CoA(m) + Acetyl-CoA(m) ← CoA(m) + 3-Oxooctanoyl-CoA(m)
r0589	1	ATP(c) + Phosphopantetheine(c) → PPi(c) + Dephospho-CoA(c)
r0733	1	(S)-Hydroxyhexanoyl-CoA(m) + NAD+(m) → 3-Oxohexanoyl-CoA(m) + NADH(m)
r2468	1	Glycine(s) + H+(PG)(s) → Glycine(c) + H+(PG)(c)
r0734	-1	(S)-Hydroxyhexanoyl-CoA(m) ← (2E)-Hexenoyl-CoA(m) + H2O(m)
r0736	0.17	ATP(c) + Fructose-6P(c) → ADP(c) + Fructose-1,6PP(c)
r0007	3	PPi(c) + H2O(c) → 2 Pi(c)
r0027	-0.5	NADPH(c) + Cystine(c) ⇌ 2 Cysteine(c) + NADP+(c)
r0192	0.83	Glucose-6P(c) ⇌ Fructose-6P(c)
r0344	-0.67	D-Xylulose-5P(c) ⇌ Ribulose-5P(c)
r0792	2	5-Methyl-THF(c) + NAD+(c) ⇌ 5,10-Methylene-THF(c) + NADH(c)
r0211	1	Cysteine(c) + O2(c) ⇌ 3-Sulfinoalanine(c)
r1450	1	Ubiquinone(m) + Hexanoyl-CoA(m) → (2E)-Hexenoyl-CoA(m) + Ubiquinol(m)
r2676	-2	5-Methyl-THF(c) ⇌ THF(c) + THF-activated methyl group(c)
r1451	1	Ubiquinone(m) + Decanoyl-CoA(m) → (2E)-Decenoyl-CoA(m) + Ubiquinol(m)
r0660	-1	(S)-3-Hydroxydodecanoyl-CoA(m) ⇌ (2E)-Dodecenoyl-CoA(m) + H2O(m)
r2678	6	Acetyl-CoA(m) ⇌ CoA(m) + CoA-activated acetyl group(m)
r0666	1	ATP(c) + 5-Phosphoribosylformylglycinamidine(c) ⇌ ADP(c) + Pi(c) + AIR(c)
r1471	1	FAD(m) + cis-myrist-7-enoyl-CoA(m) ⇌ FADH2(m) + trans,cis-myristo-2,7-dienoyl-CoA(m)
r0667	-1	5-Phosphoribosyl-4-carboxy-5-aminoimidazole(c) ⇌ AIR(c) + CO2(c)
r1473	1	FAD(m) + palmitoleoyl-CoA(m) ⇌ FADH2(m) + trans,cis-hexadeca-2,9-dienoyl-CoA(m)
r1322	2	NADH(c) ⇌ NAD+(c) + NADH-redox-potential(c)
r1475	1	H2O(m) + trans,cis-hexadeca-2,9-dienoyl-CoA(m) ⇌ (S)-3-hydroxypalmitoleoyl-CoA(m)
r1323	7	NADH(m) ⇌ NAD+(m) + NADH-redox-potential(m)
r0250	-0.67	Ribulose-5P(c) ⇌ Ribose-5P(c)
r1476	1	NAD+(m) + cis-(3S)-hydroxytetradec-7-enoyl-CoA(m) ⇌ 3-oxomyrist-7-enoyl-CoA(m) + NADH(m)
r1478	1	NAD+(m) + (S)-3-hydroxypalmitoleoyl-CoA(m) ⇌ NADH(m) + 3-oxopalmitoleoyl-CoA(m)
r1326	3.5	NADPH(c) ⇌ NADPH-redox-potential(c) + NADP+(c)
r0687	1	ATP(c) + N-Formyl-GAR(c) + Glutamine(c) + H2O(c) ⇌ ADP(c) + Pi(c) + Glutamate(c) + 5-Phosphoribosylformylglycinamidine(c)
r1479	1	CoA(m) + 3-oxolaur-cis-5-enoyl-CoA(m) ⇌ Acetyl-CoA(m) + (3Z)-Dodecenoyl-CoA(m)
r0255	0.33	Fructose-6P(c) + GAP(c) ⇌ D-Xylulose-5P(c) + Erythrose-4P(c)

Continued on next page

ID	flux	reaction
r1329	3	FADH2(c) ⇌ FAD(c) + FADH-redox-potential(c)
r0538	1	3-Sulfinoalanine(c) ⇌ CO2(c) + Hypotaurine(c)
r0256	0.17	Fructose-1,6PP(c) ⇌ DHAP(c) + GAP(c)
r0539	-1	Hypotaurine(c) ⇌ Cysteamine(c) + O2(c)
r0257	-1	Glutamine(c) + PRPP(c) + H2O(c) ⇌ 5-Phosphoribosylamine(c) + PPi(c) + Glutamate(c)
r0258	-2	Fumarate(c) + H2O(c) ⇌ Malate(c)
r0273	-1	IMP(c) + H2O(c) ⇌ FAICAR(c)
r0851	-4	H2O(m) ⇌ H2O(c)
r0700	1	10-Formyl-THF(c) + AICAR(c) ⇌ THF(c) + FAICAR(c)

3-Oxooctanoyl-ACP (631)

Definition			Solution												
			exchanges		reactions								transp		
Objective	Constraints	Comment	imports	exports	c	m	r	p	l	n	s	b	s-c	b-c	int
3-Oxooctanoyl-ACP(c)	PIPES MCES	synthesis of cytosolic 3-Oxooctanoyl-ACP	1 P_i(c) 5 Glutamate(s) 6 Glycine(s) 11 Alanine(s) 4 Lysine(s) 8 Aspartate(s) 9 Arginine(s) 7 Glutamine(s) 8 Serine(s) 5 Methionine(s) 3 Phenylalanine(s) 4 Tyrosine(s) 3 Cysteine(s) 20 Leucine(s) 1 Histidine(s) 9 Proline(s) 1 Asparagine(s) 11 Valine(s) 6 Threonine(s) 4 Isoleucine(s) 1 Pantothenate(s) 627 ATP-energy(c) 4 NADPH-redox-potential(c) 3 Proton-gradient(c) 4 CoA-activated acetyl group(c)	5 H_2O(s) 1 CO_2(s) 1 3-Oxooctanoyl-ACP(c) 66 Na-gradient(c)	28	-	-	-	-	-	-	-	24	-	-

ID	flux	reaction
r1069	-4	Lysine(c) ⇌ Lysine(s)
r0578	1	ATP(c) + Pantetheine(c) ⇌ ADP(c) + Phosphopantetheine(c)
r2478	8	Serine(s) + Na+(s) + H+(PG)(c) → Serine(c) + Na+(c) + H+(PG)(s)
r0030	125	ATP(c) + AMP(c) ⇌ 2 ADP(c)
r0760	1	Butyryl-ACP(c) + Malonyl-ACP(c) ⇌ CO2(c) + ACP(c) + 3-Oxohexanoyl-ACP(c)
r0032	1	ATP(c) + Dephospho-CoA(c) ⇌ ADP(c) + CoA(c)
r0761	-1	D-3-Hydroxyhexanoyl-ACP(c) + NADP+(c) ⇌ NADPH(c) + 3-Oxohexanoyl-ACP(c)
r0184	3	ATP(c) + Acetyl-CoA(c) + HCO3-(c) → ADP(c) + Pi(c) + Malonyl-CoA(c)
r0762	1	D-3-Hydroxyhexanoyl-ACP(c) ⇌ H2O(c) + (2E)-Hexenoyl-ACP(c)
r0763	-1	Hexanoyl-ACP(c) + NADP+(c) ⇌ NADPH(c) + (2E)-Hexenoyl-ACP(c)
r0764	1	Malonyl-ACP(c) + Hexanoyl-ACP(c) ⇌ 3-Oxooctanoyl-ACP(c) + CO2(c) + ACP(c)
r1122	-20	Leucine(c) + Na+(c) ← Leucine(s) + Na+(s)
r1123	-4	Isoleucine(c) + Na+(c) ← Isoleucine(s) + Na+(s)
r1125	4	Tyrosine(s) + Na+(s) → Tyrosine(c) + Na+(c)
r1142	-6	Threonine(s) + Na+(s) → Threonine(c) + Na+(c)
r1143	-8	Na+(c) + Aspartate(c) ← Na+(s) + Aspartate(s)
r2664	66	Na+(c) ⇌ Na+(s) + Na-gradient(c)
r1144	-5	Glutamate(c) + Na+(c) ← Glutamate(s) + Na+(s)
r1145	-1	Histidine(c) + Na+(c) ← Histidine(s) + Na+(s)
r2532	1	Asparagine(s) ⇌ Asparagine(c)
r2533	11	Valine(s) ⇌ Valine(c)
r0678	1	Acetyl-ACP(c) + Malonyl-ACP(c) ⇌ CO2(c) + ACP(c) + Acetoacetyl-ACP(c)
r0691	-1	(R)-3-Hydroxybutanoyl-ACP(c) + NADP+(c) ⇌ NADPH(c) + Acetoacetyl-ACP(c)
r1331	-3	H+(PG)(s) ⇌ H+(PG)(c) + Proton-gradient(c)
r0974	1	Pantothenate(s) + Na+(s) ⇌ Pantothenate(c) + Na+(c)
r1319	-627	ATP(c) + H2O(c) ⇌ ADP(c) + Pi(c) + ATP-energy(c)

Continued on next page

ID	flux	reaction
r1058	9	Arginine(s) ⇌ Arginine(c)
r1072	5	Methionine(s) ⇌ Methionine(c)
r0849	-5	H2O(s) ⇌ H2O(c)
r0581	-1	Pantothenate(c) + Cysteamine(c) ⇌ H2O(c) + Pantetheine(c)
r0867	1	CO2(c) ⇌ CO2(s)
r1096	1	11 Alanine(c) + 2 Cysteine(c) + 5 Glutamate(c) + 3 Phenylalanine(c) + 6 Glycine(c) + Histidine(c) + 4 Isoleucine(c) + 20 Leucine(c) + 5 Methionine(c) + 9 Proline(c) + 7 Glutamine(c) + 9 Arginine(c) + 8 Serine(c) + 6 Threonine(c) + 11 Valine(c) + 4 Tyrosine(c) + 496 ATP(c) + 496 H2O(c) + 8 Aspartate(c) + 4 Lysine(c) + Asparagine(c) → Apo-ACP(c) + 124 AMP(c) + 124 PPi(c) + 372 ADP(c) + 372 Pi(c)
r0589	1	ATP(c) + Phosphopantetheine(c) → PPi(c) + Dephospho-CoA(c)
r2469	11	Alanine(s) + H+(PG)(s) → Alanine(c) + H+(PG)(c)
r0007	125	PPi(c) + H2O(c) → 2 Pi(c)
r1417	-3	CO2(c) + H2O(c) ⇌ Carbonate(c)
r0049	1	PAP(c) + H2O(c) → AMP(c) + Pi(c)
r1419	3	Carbonate(c) ⇌ HCO3-(c)
r1139	-9	Proline(c) + Na+(c) ← Proline(s) + Na+(s)
r0211	1	Cysteine(c) + O2(c) ⇌ 3-Sulfinoalanine(c)
r2523	6	Glycine(s) ⇌ Glycine(c)
r2677	-4	Acetyl-CoA(c) ⇌ CoA(c) + CoA-activated acetyl group(c)
r2525	7	Glutamine(s) ⇌ Glutamine(c)
r0367	1	Acetyl-CoA(c) + ACP(c) ⇌ CoA(c) + Acetyl-ACP(c)
r0368	1	CoA(c) + Apo-ACP(c) → PAP(c) + ACP(c)
r2528	3	Phenylalanine(s) ⇌ Phenylalanine(c)
r0369	3	ACP(c) + Malonyl-CoA(c) ⇌ CoA(c) + Malonyl-ACP(c)
r2529	3	Cysteine(s) ⇌ Cysteine(c)
r0681	1	(R)-3-Hydroxybutanoyl-ACP(c) ⇌ But-2-enoyl-ACP(c) + H2O(c)
r0682	-1	Butyryl-ACP(c) + NADP+(c) ⇌ But-2-enoyl-ACP(c) + NADPH(c)
r1326	-4	NADPH(c) ⇌ NADPH-redox-potential(c) + NADP+(c)
r0538	1	3-Sulfinoalanine(c) ⇌ CO2(c) + Hypotaurine(c)
r0539	-1	Hypotaurine(c) ⇌ Cysteamine(c) + O2(c)

3-Oxooctanoyl-CoA (632)

Definition			Solution												
			exchanges		reactions								transp		
Objective	Constraints	Comment	imports	exports	c	m	r	p	l	n	s	b	s-c	b-c	int
3-Oxooctanoyl-CoA(m)	PIPES MCES	synthesis of mitochondrial 3-Oxooctanoyl-CoA	1 O_2(s) 2.17 P_i(c) 1 Glycine(s) 2 Aspartate(s) 2 Glutamine(s) 0.83 Glucose-6P(c) 0.5 Cystine(s) 1 Pantothenate(s) 1 Palmitolate(s) 13.2 ATP-energy(c) 2 THF-activated methyl group(c)	2.17 H_2O(s) 2 CO_2(s) 2 Pyruvate(c) 2 Glutamate(s) 1 3-Oxooctanoyl-CoA(m) 2 NADH-redox-potential(c) 6 NADH-redox-potential(m) 3.5 NADPH-redox-potential(c) 3 FADH-redox-potential(c) 12 Proton-gradient(m) 3 Na-gradient(c) 5 CoA-activated acetyl group(m)	44	25	-	-	-	-	-	-	11	-	7

ID	flux	reaction
r0406	-0.33	Fructose-6P(c) + Erythrose-4P(c) ⇌ Sedoheptulose-7P(c) + GAP(c)
r0277	1	GTP(c) + IMP(c) + Aspartate(c) → GDP(c) + Pi(c) + Adenylosuccinate(c)
r0855	1	O2(s) ⇌ O2(c)
r0856	-1	O2(m) ⇌ O2(c)
r0705	1	ATP(c) + 5-Phosphoribosyl-4-carboxy-5-aminoimidazole(c) + Aspartate(c) ⇌ ADP(c) + Pi(c) + SAICAR(c)
r0293	2	5,10-Methylene-THF(c) + NADP+(c) ⇌ 5,10-Methenyl-THF(c) + NADPH(c)
r0578	1	ATP(c) + Pantetheine(c) ⇌ ADP(c) + Phosphopantetheine(c)
r0722	1	(S)-3-Hydroxydodecanoyl-CoA(m) + NAD+(m) ⇌ 3-Oxododecanoyl-CoA(m) + NADH(m)
r0724	-1	Decanoyl-CoA(m) + Acetyl-CoA(m) ⇌ CoA(m) + 3-Oxododecanoyl-CoA(m)
r0877	0.5	Glutamate(c) + Cystine(s) ⇌ Glutamate(s) + Cystine(c)

Continued on next page

ID	flux	reaction
r0726	1	(S)-Hydroxydecanoyl-CoA(m) + NAD+(m) ⇌ 3-Oxodecanoyl-CoA(m) + NADH(m)
r0728	-1	(S)-Hydroxydecanoyl-CoA(m) ⇌ (2E)-Decenoyl-CoA(m) + H2O(m)
r0017	1	O2(m) + 4 H+(PG)(m) + 4 Ferrocytochrome C(m) → 4 Ferricytochrome C(m) + 2 H2O(m) + 4 H+(PG)(c)
r0030	3	ATP(c) + AMP(c) ⇌ 2 ADP(c)
r0032	1	ATP(c) + Dephospho-CoA(c) ⇌ ADP(c) + CoA(c)
r1290	3	FAD(c) + FADH2(m) ⇌ FAD(m) + FADH2(c)
r1143	-2	Na+(c) + Aspartate(c) ← Na+(s) + Aspartate(s)
r0634	-1	Acetyl-CoA(m) + Octanoyl-CoA(m) ⇌ CoA(m) + 3-Oxodecanoyl-CoA(m)
r2664	3	Na+(c) ⇌ Na+(s) + Na-gradient(c)
r0058	2	Malate(c) + NADP+(c) ⇌ Pyruvate(c) + CO2(c) + NADPH(c)
r0370	0.33	Sedoheptulose-7P(c) + GAP(c) ⇌ Ribose-5P(c) + D-Xylulose-5P(c)
r0371	2	5,10-Methenyl-THF(c) + H2O(c) ⇌ 10-Formyl-THF(c)
r2685	1.5	Glutamate(c) ⇌ Glutamate(s)
r1448	1	Ubiquinone(m) + Octanoyl-CoA(m) → (2E)-Octenoyl-CoA(m) + Ubiquinol(m)
r0657	1	(3Z)-Dodecenoyl-CoA(m) ⇌ (2E)-Dodecenoyl-CoA(m)
r0659	1	ATP(c) + 5-Phosphoribosylamine(c) + Glycine(c) ⇌ ADP(c) + Pi(c) + GAR(c)
r0507	2	Ubiquinol(m) + 2 Ferricytochrome C(m) + 4 H+(PG)(m) → Ubiquinone(m) + 4 H+(PG)(c) + 2 Ferrocytochrome C(m)
r1480	1	CoA(m) + 3-oxopalmitoleoyl-CoA(m) ⇌ Acetyl-CoA(m) + cis-myrist-7-enoyl-CoA(m)
r0099	1	ATP(c) + GDP(c) ⇌ ADP(c) + GTP(c)
r1468	1	palmitoleoyl-CoA(c) ⇌ palmitoleoyl-CoA(m)
r0243	-0.17	DHAP(c) ⇌ GAP(c)
r0677	1	10-Formyl-THF(c) + GAR(c) ⇌ THF(c) + N-Formyl-GAR(c)
r1482	-1	FADH2(m) + trans,cis-dodeca-2,5-dienoyl-CoA(m) ⇌ FAD(m) + cis-laur-5-enoyl-CoA(m)
r1330	12	H+(PG)(c) ⇌ H+(PG)(m) + Proton-gradient(m)
r1483	1	H2O(m) + trans,cis-myristo-2,7-dienoyl-CoA(m) ⇌ cis-(3S)-hydroxytetradec-7-enoyl-CoA(m)
r0974	1	Pantothenate(s) + Na+(s) ⇌ Pantothenate(c) + Na+(c)
r1484	1	CoA(m) + 3-oxomyrist-7-enoyl-CoA(m) ⇌ Acetyl-CoA(m) + cis-laur-5-enoyl-CoA(m)
r1319	-13.2	ATP(c) + H2O(c) ⇌ ADP(c) + Pi(c) + ATP-energy(c)
r1485	1	H2O(m) + trans,cis-dodeca-2,5-dienoyl-CoA(m) ⇌ 3(S)-3-hydroxydodecen-(5Z)-oyl-CoA(m)
r0260	1	Adenylosuccinate(c) → Fumarate(c) + AMP(c)
r0247	1	ATP(c) + Ribose-5P(c) → AMP(c) + PRPP(c)
r1486	1	NAD+(m) + 3(S)-3-hydroxydodecen-(5Z)-oyl-CoA(m) ⇌ 3-oxolaur-cis-5-enoyl-CoA(m) + NADH(m)
r1488	1	ATP(c) + CoA(c) + Palmitolate(c) ⇌ AMP(c) + palmitoleoyl-CoA(c) + PPi(c)
r0699	1	SAICAR(c) ⇌ Fumarate(c) + AICAR(c)
r2442	1	Palmitolate(s) → Palmitolate(c)
r0849	-2.17	H2O(s) ⇌ H2O(c)
r0581	-1	Pantothenate(c) + Cysteamine(c) ⇌ H2O(c) + Pantetheine(c)
r0867	2	CO2(c) ⇌ CO2(s)
r0730	1	(S)-Hydroxyoctanoyl-CoA(m) + NAD+(m) → 3-Oxooctanoyl-CoA(m) + NADH(m)
r0731	-1	(S)-Hydroxyoctanoyl-CoA(m) ← (2E)-Octenoyl-CoA(m) + H2O(m)
r0589	1	ATP(c) + Phosphopantetheine(c) → PPi(c) + Dephospho-CoA(c)
r0736	0.17	ATP(c) + Fructose-6P(c) → ADP(c) + Fructose-1,6PP(c)
r0007	3	PPi(c) + H2O(c) → 2 Pi(c)
r0027	-0.5	NADPH(c) + Cystine(c) ⇌ 2 Cysteine(c) + NADP+(c)
r0192	0.83	Glucose-6P(c) ⇌ Fructose-6P(c)
r0344	-0.67	D-Xylulose-5P(c) ⇌ Ribulose-5P(c)
r0792	2	5-Methyl-THF(c) + NAD+(c) ⇌ 5,10-Methylene-THF(c) + NADH(c)
r0211	1	Cysteine(c) + O2(c) ⇌ 3-Sulfinoalanine(c)
r2523	1	Glycine(s) ⇌ Glycine(c)
r2676	-2	5-Methyl-THF(c) ⇌ THF(c) + THF-activated methyl group(c)
r1451	1	Ubiquinone(m) + Decanoyl-CoA(m) → (2E)-Decenoyl-CoA(m) + Ubiquinol(m)
r0660	-1	(S)-3-Hydroxydodecanoyl-CoA(m) ⇌ (2E)-Dodecenoyl-CoA(m) + H2O(m)
r2525	2	Glutamine(s) ⇌ Glutamine(c)
r2678	5	Acetyl-CoA(m) ⇌ CoA(m) + CoA-activated acetyl group(m)
r0666	1	ATP(c) + 5-Phosphoribosylformylglycinamidine(c) ⇌ ADP(c) + Pi(c) + AIR(c)
r1471	1	FAD(m) + cis-myrist-7-enoyl-CoA(m) ⇌ FADH2(m) + trans,cis-myristo-2,7-dienoyl-CoA(m)

Continued on next page

ID	flux	reaction
r0667	-1	5-Phosphoribosyl-4-carboxy-5-aminoimidazole(c) ⇌ AIR(c) + CO2(c)
r1473	1	FAD(m) + palmitoleoyl-CoA(m) ⇌ FADH2(m) + trans,cis-hexadeca-2,9-dienoyl-CoA(m)
r1322	2	NADH(c) ⇌ NAD+(c) + NADH-redox-potential(c)
r1475	1	H2O(m) + trans,cis-hexadeca-2,9-dienoyl-CoA(m) ⇌ (S)-3-hydroxypalmitoleoyl-CoA(m)
r1323	6	NADH(m) ⇌ NAD+(m) + NADH-redox-potential(m)
r0250	-0.67	Ribulose-5P(c) ⇌ Ribose-5P(c)
r1476	1	NAD+(m) + cis-(3S)-hydroxytetradec-7-enoyl-CoA(m) ⇌ 3-oxomyrist-7-enoyl-CoA(m) + NADH(m)
r1478	1	NAD+(m) + (S)-3-hydroxypalmitoleoyl-CoA(m) ⇌ NADH(m) + 3-oxopalmitoleoyl-CoA(m)
r1326	3.5	NADPH(c) ⇌ NADPH-redox-potential(c) + NADP+(c)
r0687	1	ATP(c) + N-Formyl-GAR(c) + Glutamine(c) + H2O(c) ⇌ ADP(c) + Pi(c) + Glutamate(c) + 5-Phosphoribosylformylglycinamidine(c)
r1479	1	CoA(m) + 3-oxolaur-cis-5-enoyl-CoA(m) ⇌ Acetyl-CoA(m) + (3Z)-Dodecenoyl-CoA(m)
r0255	0.33	Fructose-6P(c) + GAP(c) ⇌ D-Xylulose-5P(c) + Erythrose-4P(c)
r1329	3	FADH2(c) ⇌ FAD(c) + FADH-redox-potential(c)
r0538	1	3-Sulfinoalanine(c) ⇌ CO2(c) + Hypotaurine(c)
r0256	0.17	Fructose-1,6PP(c) ⇌ DHAP(c) + GAP(c)
r0539	-1	Hypotaurine(c) ⇌ Cysteamine(c) + O2(c)
r0257	-1	Glutamine(c) + PRPP(c) + H2O(c) ⇌ 5-Phosphoribosylamine(c) + PPi(c) + Glutamate(c)
r0258	-2	Fumarate(c) + H2O(c) ⇌ Malate(c)
r0273	-1	IMP(c) + H2O(c) ⇌ FAICAR(c)
r0851	-4	H2O(m) ⇌ H2O(c)
r0700	1	10-Formyl-THF(c) + AICAR(c) ⇌ THF(c) + FAICAR(c)

3-Oxopalmitoyl-CoA (633)

Definition			Solution												
			exchanges		reactions								transp		
Objective	Constraints	Comment	imports	exports	c	m	r	p	l	n	s	b	s-c	b-c	int
3-Oxopalmitoyl-CoA(m)	PIPES MCES	synthesis of mitochondrial 3-Oxopalmitoyl-CoA	2.17 P_i(c) 1 Glycine(s) 0.5 Aspartate(s) 0.5 Glutamine(s) 0.83 Glucose-6P(c) 1.5 Asparagine(s) 0.5 Palmitate(s) 0.5 Cystine(s) 1 Pantothenate(s) 0.5 Palmitolate(s) 14.7 ATP-energy(c) 1 Proton-gradient(c) 2 THF-activated methyl group(c)	5.17 H_2O(s) 2 CO_2(s) 2 Pyruvate(c) 0.5 Glutamate(s) 1 3-Oxopalmitoyl-CoA(m) 2.5 NADH-redox-potential(c) 1 NADH-redox-potential(m) 3.5 NADPH-redox-potential(c) 6 Proton-gradient(m) 1.5 Na-gradient(c)	49	5	-	-	-	-	-	-	12	-	6

ID	flux	reaction
r2435	1	L-Carnitine(m) + L-Palmitoylcarnitine(c) → L-Carnitine(c) + L-Palmitoylcarnitine(m)
r0406	-0.33	Fructose-6P(c) + Erythrose-4P(c) ⇌ Sedoheptulose-7P(c) + GAP(c)
r0277	1	GTP(c) + IMP(c) + Aspartate(c) → GDP(c) + Pi(c) + Adenylosuccinate(c)
r0856	-0.5	O2(m) ⇌ O2(c)
r0127	1.5	H2O(c) + Asparagine(c) → Aspartate(c) + NH3(c)
r0705	1	ATP(c) + 5-Phosphoribosyl-4-carboxy-5-aminoimidazole(c) + Aspartate(c) ⇌ ADP(c) + Pi(c) + SAICAR(c)
r0293	2	5,10-Methylene-THF(c) + NADP+(c) ⇌ 5,10-Methenyl-THF(c) + NADPH(c)
r0578	1	ATP(c) + Pantetheine(c) ⇌ ADP(c) + Phosphopantetheine(c)
r0877	0.5	Glutamate(c) + Cystine(s) ⇌ Glutamate(s) + Cystine(c)
r0310	1	Palmitoyl-CoA(m) + Ubiquinone(m) → (2E)-Hexadecenoyl-CoA(m) + Ubiquinol(m)
r0017	0.5	O2(m) + 4 H+(PG)(m) + 4 Ferrocytochrome C(m) → 4 Ferricytochrome C(m) + 2 H2O(m) + 4 H+(PG)(c)
r0312	0.5	Palmitate(c) + ATP(c) + CoA(c) → Palmitoyl-CoA(c) + AMP(c) + PPi(c)
r0030	3	ATP(c) + AMP(c) ⇌ 2 ADP(c)
r0032	1	ATP(c) + Dephospho-CoA(c) ⇌ ADP(c) + CoA(c)
r1143	-0.5	Na+(c) + Aspartate(c) ← Na+(s) + Aspartate(s)
r2664	1.5	Na+(c) ⇌ Na+(s) + Na-gradient(c)
r0058	2	Malate(c) + NADP+(c) ⇌ Pyruvate(c) + CO2(c) + NADPH(c)
r0370	0.33	Sedoheptulose-7P(c) + GAP(c) ⇌ Ribose-5P(c) + D-Xylulose-5P(c)

Continued on next page

ID	flux	reaction
r0935	0.5	Palmitate(s) → Palmitate(c)
r0371	2	5,10-Methenyl-THF(c) + H2O(c) ⇌ 10-Formyl-THF(c)
r0077	1.5	ATP(c) + Glutamate(c) + NH3(c) → ADP(c) + Pi(c) + Glutamine(c)
r2532	1.5	Asparagine(s) ⇌ Asparagine(c)
r0659	1	ATP(c) + 5-Phosphoribosylamine(c) + Glycine(c) ⇌ ADP(c) + Pi(c) + GAR(c)
r0507	1	Ubiquinol(m) + 2 Ferricytochrome C(m) + 4 H+(PG)(m) → Ubiquinone(m) + 4 H+(PG)(c) + 2 Ferrocytochrome C(m)
r0099	1	ATP(c) + GDP(c) ⇌ ADP(c) + GTP(c)
r0677	1	10-Formyl-THF(c) + GAR(c) ⇌ THF(c) + N-Formyl-GAR(c)
r0243	-0.17	DHAP(c) ⇌ GAP(c)
r1330	6	H+(PG)(c) ⇌ H+(PG)(m) + Proton-gradient(m)
r1331	-1	H+(PG)(s) ⇌ H+(PG)(c) + Proton-gradient(c)
r0974	1	Pantothenate(s) + Na+(s) ⇌ Pantothenate(c) + Na+(c)
r1319	-14.7	ATP(c) + H2O(c) ⇌ ADP(c) + Pi(c) + ATP-energy(c)
r0260	1	Adenylosuccinate(c) → Fumarate(c) + AMP(c)
r0247	1	ATP(c) + Ribose-5P(c) → AMP(c) + PRPP(c)
r1488	0.5	ATP(c) + CoA(c) + Palmitolate(c) ⇌ AMP(c) + palmitoleoyl-CoA(c) + PPi(c)
r0699	1	SAICAR(c) ⇌ Fumarate(c) + AICAR(c)
r2442	0.5	Palmitolate(s) → Palmitolate(c)
r0849	-5.17	H2O(s) ⇌ H2O(c)
r0863	1	CoA(c) ⇌ CoA(m)
r0581	-1	Pantothenate(c) + Cysteamine(c) ⇌ H2O(c) + Pantetheine(c)
r0714	1	NAD+(m) + (S)-3-Hydroxyhexadecanoyl-CoA(m) → 3-Oxopalmitoyl-CoA(m) + NADH(m)
r0867	2	CO2(c) ⇌ CO2(s)
r0716	-1	(2E)-Hexadecenoyl-CoA(m) + H2O(m) → (S)-3-Hydroxyhexadecanoyl-CoA(m)
r0435	1	Palmitoyl-CoA(c) + L-Carnitine(c) ⇌ CoA(c) + L-Palmitoylcarnitine(c)
r0436	-1	Palmitoyl-CoA(m) + L-Carnitine(m) ← CoA(m) + L-Palmitoylcarnitine(m)
r0589	1	ATP(c) + Phosphopantetheine(c) → PPi(c) + Dephospho-CoA(c)
r2468	1	Glycine(s) + H+(PG)(s) → Glycine(c) + H+(PG)(c)
r0736	0.17	ATP(c) + Fructose-6P(c) → ADP(c) + Fructose-1,6PP(c)
r0007	3	PPi(c) + H2O(c) → 2 Pi(c)
r0023	-0.5	NAD+(c) + 2 Ferrocytochrome b5(c) ⇌ NADH(c) + 2 Ferricytochrome b5(c)
r0027	-0.5	NADPH(c) + Cystine(c) ⇌ 2 Cysteine(c) + NADP+(c)
r0192	0.83	Glucose-6P(c) ⇌ Fructose-6P(c)
r0344	-0.67	D-Xylulose-5P(c) ⇌ Ribulose-5P(c)
r0792	2	5-Methyl-THF(c) + NAD+(c) ⇌ 5,10-Methylene-THF(c) + NADH(c)
r0211	1	Cysteine(c) + O2(c) ⇌ 3-Sulfinoalanine(c)
r2676	-2	5-Methyl-THF(c) ⇌ THF(c) + THF-activated methyl group(c)
r2525	0.5	Glutamine(s) ⇌ Glutamine(c)
r0510	-0.5	palmitoleoyl-CoA(c) + 2 Ferricytochrome b5(c) + 2 H2O(c) ⇌ Palmitoyl-CoA(c) + O2(c) + 2 Ferrocytochrome b5(c)
r0666	1	ATP(c) + 5-Phosphoribosylformylglycinamidine(c) ⇌ ADP(c) + Pi(c) + AIR(c)
r0667	-1	5-Phosphoribosyl-4-carboxy-5-aminoimidazole(c) ⇌ AIR(c) + CO2(c)
r1322	2.5	NADH(c) ⇌ NAD+(c) + NADH-redox-potential(c)
r1323	1	NADH(m) ⇌ NAD+(m) + NADH-redox-potential(m)
r0250	-0.67	Ribulose-5P(c) ⇌ Ribose-5P(c)
r1326	3.5	NADPH(c) ⇌ NADPH-redox-potential(c) + NADP+(c)
r0687	1	ATP(c) + N-Formyl-GAR(c) + Glutamine(c) + H2O(c) ⇌ ADP(c) + Pi(c) + Glutamate(c) + 5-Phosphoribosylformylglycinamidine(c)
r0255	0.33	Fructose-6P(c) + GAP(c) ⇌ D-Xylulose-5P(c) + Erythrose-4P(c)
r0538	1	3-Sulfinoalanine(c) ⇌ CO2(c) + Hypotaurine(c)
r0256	0.17	Fructose-1,6PP(c) ⇌ DHAP(c) + GAP(c)
r0539	-1	Hypotaurine(c) ⇌ Cysteamine(c) + O2(c)
r0257	-1	Glutamine(c) + PRPP(c) + H2O(c) ⇌ 5-Phosphoribosylamine(c) + PPi(c) + Glutamate(c)
r0258	-2	Fumarate(c) + H2O(c) ⇌ Malate(c)
r0273	-1	IMP(c) + H2O(c) ⇌ FAICAR(c)
r0700	1	10-Formyl-THF(c) + AICAR(c) ⇌ THF(c) + FAICAR(c)

3-Oxopropanoate (634)

Definition			Solution												
			exchanges		reactions								transp		
Objective	Constraints	Comment	imports	exports	c	m	r	p	l	n	s	b	s-c	b-c	int
3-Oxopropanoate(m)	PIPES MCES	synthesis of mitochondrial 3-Oxopropanoate	1 Pyruvate(c) 1 NADPH-redox-potential(c) 2 Proton-gradient(m)	1 3-Oxopropanoate(m) 1 NADH-redox-potential(c)	6	1	-	-	-	-	-	-	-	-	6

ID	flux	reaction
r0217	1	beta-Alanine(m) + AKG(m) ⇌ Glutamate(m) + 3-Oxopropanoate(m)
r0109	-1	OAA(c) + Glutamate(c) ⇌ AKG(c) + Aspartate(c)
r0911	1	Proline(c) + Glutamate(m) ⇌ Proline(m) + Glutamate(c)
r0128	1	Aspartate(c) ⇌ beta-Alanine(c) + CO2(c)
r1326	-1	NADPH(c) ⇌ NADPH-redox-potential(c) + NADP+(c)
r1047	1	Pi(c) + H+(PG)(c) → Pi(m) + H+(PG)(m)
r1426	1	Proline(m) + H+(PG)(c) → Proline(c) + H+(PG)(m)
r0058	-1	Pyruvate(c) + CO2(c) + NADPH(c) ⇌ Malate(c) + NADP+(c)
r2419	1	AKG(c) + Pi(m) ⇌ AKG(m) + Pi(c)
r0104	1	NAD+(c) + Malate(c) ⇌ OAA(c) + NADH(c)
r1330	-2	H+(PG)(c) ⇌ H+(PG)(m) + Proton-gradient(m)
r1322	1	NADH(c) ⇌ NAD+(c) + NADH-redox-potential(c)
r0900	1	beta-Alanine(c) ⇌ beta-Alanine(m)

3-Oxostearoyl-ACP (635)

Definition			Solution												
			exchanges		reactions								transp		
Objective	Constraints	Comment	imports	exports	c	m	r	p	l	n	s	b	s-c	b-c	int
3-Oxostearoyl-ACP(c)	PIPES MCES	synthesis of cytosolic 3-Oxostearoyl-ACP	1 P_i(c) 5 Glutamate(s) 6 Glycine(s) 11 Alanine(s) 4 Lysine(s) 8 Aspartate(s) 9 Arginine(s) 7 Glutamine(s) 8 Serine(s) 5 Methionine(s) 3 Phenylalanine(s) 4 Tyrosine(s) 3 Cysteine(s) 20 Leucine(s) 1 Histidine(s) 9 Proline(s) 1 Asparagine(s) 11 Valine(s) 6 Threonine(s) 4 Isoleucine(s) 1 Pantothenate(s) 632 ATP-energy(c) 14 NADPH-redox-potential(c) 3 Proton-gradient(c) 9 CoA-activated acetyl group(c)	10 H_2O(s) 1 CO_2(s) 1 3-Oxostearoyl-ACP(c) 66 Na-gradient(c)	48	-	-	-	-	-	-	-	24	-	-

ID	flux	reaction
r0701	-1	HMA(c) + NADP+(c) ⇌ 3-Oxotetradecanoyl-ACP(c) + NADPH(c)
r0702	1	HMA(c) ⇌ H2O(c) + (2E)-Tetradecenoyl-ACP(c)
r1069	-4	Lysine(c) ⇌ Lysine(s)
r0578	1	ATP(c) + Pantetheine(c) ⇌ ADP(c) + Phosphopantetheine(c)
r2478	8	Serine(s) + Na+(s) + H+(PG)(c) → Serine(c) + Na+(c) + H+(PG)(s)
r0030	125	ATP(c) + AMP(c) ⇌ 2 ADP(c)
r0760	1	Butyryl-ACP(c) + Malonyl-ACP(c) ⇌ CO2(c) + ACP(c) + 3-Oxohexanoyl-ACP(c)
r0032	1	ATP(c) + Dephospho-CoA(c) ⇌ ADP(c) + CoA(c)
r0761	-1	D-3-Hydroxyhexanoyl-ACP(c) + NADP+(c) ⇌ NADPH(c) + 3-Oxohexanoyl-ACP(c)
r0184	8	ATP(c) + Acetyl-CoA(c) + HCO3-(c) → ADP(c) + Pi(c) + Malonyl-CoA(c)
r0762	1	D-3-Hydroxyhexanoyl-ACP(c) ⇌ H2O(c) + (2E)-Hexenoyl-ACP(c)
r0763	-1	Hexanoyl-ACP(c) + NADP+(c) ⇌ NADPH(c) + (2E)-Hexenoyl-ACP(c)
r0764	1	Malonyl-ACP(c) + Hexanoyl-ACP(c) ⇌ 3-Oxooctanoyl-ACP(c) + CO2(c) + ACP(c)
r1122	-20	Leucine(c) + Na+(c) ← Leucine(s) + Na+(s)

Continued on next page

ID	flux	reaction
r0765	-1	Octanoyl-ACP(c) + NADP+(c) ⇌ (2E)-Octenoyl-ACP(c) + NADPH(c)
r1123	-4	Isoleucine(c) + Na+(c) ← Isoleucine(s) + Na+(s)
r0766	1	Octanoyl-ACP(c) + Malonyl-ACP(c) ⇌ 3-Oxodecanoyl-ACP(c) + CO2(c) + ACP(c)
r0767	-1	Decanoyl-ACP(c) + NADP+(c) ⇌ (2E)-Decenoyl-ACP(c) + NADPH(c)
r1125	4	Tyrosine(s) + Na+(s) → Tyrosine(c) + Na+(c)
r0768	1	Decanoyl-ACP(c) + Malonyl-ACP(c) ⇌ 3-Oxododecanoyl-ACP(c) + CO2(c) + ACP(c)
r0769	-1	D-3-Hydroxydodecanoyl-ACP(c) + NADP+(c) ⇌ 3-Oxododecanoyl-ACP(c) + NADPH(c)
r1142	-6	Threonine(s) + Na+(s) → Threonine(c) + Na+(c)
r1143	-8	Na+(c) + Aspartate(c) ← Na+(s) + Aspartate(s)
r2664	66	Na+(c) ⇌ Na+(s) + Na-gradient(c)
r1144	-5	Glutamate(c) + Na+(c) ← Glutamate(s) + Na+(s)
r1145	-1	Histidine(c) + Na+(c) ← Histidine(s) + Na+(s)
r2532	1	Asparagine(s) ⇌ Asparagine(c)
r2533	11	Valine(s) ⇌ Valine(c)
r1313	1	Hexadecanoyl-ACP(c) + Malonyl-ACP(c) ⇌ 3-Oxostearoyl-ACP(c) + ACP(c) + CO2(c)
r0678	1	Acetyl-ACP(c) + Malonyl-ACP(c) ⇌ CO2(c) + ACP(c) + Acetoacetyl-ACP(c)
r0691	-1	(R)-3-Hydroxybutanoyl-ACP(c) + NADP+(c) ⇌ NADPH(c) + Acetoacetyl-ACP(c)
r1331	-3	H+(PG)(s) ⇌ H+(PG)(c) + Proton-gradient(c)
r0974	1	Pantothenate(s) + Na+(s) ⇌ Pantothenate(c) + Na+(c)
r0692	-1	(R)-3-Hydroxydecanoyl-ACP(c) + NADP+(c) ⇌ 3-Oxodecanoyl-ACP(c) + NADPH(c)
r1319	-632	ATP(c) + H2O(c) ⇌ ADP(c) + Pi(c) + ATP-energy(c)
r0693	1	(R)-3-Hydroxydecanoyl-ACP(c) ⇌ (2E)-Decenoyl-ACP(c) + H2O(c)
r0694	-1	(R)-3-Hydroxyoctanoyl-ACP(c) + NADP+(c) ⇌ 3-Oxooctanoyl-ACP(c) + NADPH(c)
r0695	1	(R)-3-Hydroxyoctanoyl-ACP(c) ⇌ (2E)-Octenoyl-ACP(c) + H2O(c)
r0696	-1	(R)-3-Hydroxypalmitoyl-ACP(c) + NADP+(c) ⇌ 3-Oxohexadecanoyl-ACP(c) + NADPH(c)
r0697	1	(R)-3-Hydroxypalmitoyl-ACP(c) ⇌ (2E)-Hexadecenoyl-ACP(c) + H2O(c)
r1058	9	Arginine(s) ⇌ Arginine(c)
r1072	5	Methionine(s) ⇌ Methionine(c)
r0849	-10	H2O(s) ⇌ H2O(c)
r0581	-1	Pantothenate(c) + Cysteamine(c) ⇌ H2O(c) + Pantetheine(c)
r0712	-1	Dodecanoyl-ACP(c) + NADP+(c) ⇌ (2E)-Dodecenoyl-ACP(c) + NADPH(c)
r0713	1	Dodecanoyl-ACP(c) + Malonyl-ACP(c) ⇌ 3-Oxotetradecanoyl-ACP(c) + CO2(c) + ACP(c)
r0867	1	CO2(c) ⇌ CO2(s)
r1096	1	11 Alanine(c) + 2 Cysteine(c) + 5 Glutamate(c) + 3 Phenylalanine(c) + 6 Glycine(c) + Histidine(c) + 4 Isoleucine(c) + 20 Leucine(c) + 5 Methionine(c) + 9 Proline(c) + 7 Glutamine(c) + 9 Arginine(c) + 8 Serine(c) + 6 Threonine(c) + 11 Valine(c) + 4 Tyrosine(c) + 496 ATP(c) + 496 H2O(c) + 8 Aspartate(c) + 4 Lysine(c) + Asparagine(c) → Apo-ACP(c) + 124 AMP(c) + 124 PPi(c) + 372 ADP(c) + 372 Pi(c)
r0589	1	ATP(c) + Phosphopantetheine(c) → PPi(c) + Dephospho-CoA(c)
r2469	11	Alanine(s) + H+(PG)(s) → Alanine(c) + H+(PG)(c)
r0007	125	PPi(c) + H2O(c) → 2 Pi(c)
r0770	1	D-3-Hydroxydodecanoyl-ACP(c) ⇌ (2E)-Dodecenoyl-ACP(c) + H2O(c)
r0771	-1	Tetradecanoyl-ACP(c) + NADP+(c) ⇌ NADPH(c) + (2E)-Tetradecenoyl-ACP(c)
r0772	1	Tetradecanoyl-ACP(c) + Malonyl-ACP(c) ⇌ 3-Oxohexadecanoyl-ACP(c) + CO2(c) + ACP(c)
r0773	-1	Hexadecanoyl-ACP(c) + NADP+(c) ⇌ (2E)-Hexadecenoyl-ACP(c) + NADPH(c)
r1417	-8	CO2(c) + H2O(c) ⇌ Carbonate(c)
r0049	1	PAP(c) + H2O(c) → AMP(c) + Pi(c)
r1419	8	Carbonate(c) ⇌ HCO3-(c)
r1139	-9	Proline(c) + Na+(c) ← Proline(s) + Na+(s)
r0211	1	Cysteine(c) + O2(c) ⇌ 3-Sulfinoalanine(c)
r2523	6	Glycine(s) ⇌ Glycine(c)
r2677	-9	Acetyl-CoA(c) ⇌ CoA(c) + CoA-activated acetyl group(c)
r2525	7	Glutamine(s) ⇌ Glutamine(c)
r0367	1	Acetyl-CoA(c) + ACP(c) ⇌ CoA(c) + Acetyl-ACP(c)
r0368	1	CoA(c) + Apo-ACP(c) → PAP(c) + ACP(c)
r2528	3	Phenylalanine(s) ⇌ Phenylalanine(c)
r0369	8	ACP(c) + Malonyl-CoA(c) ⇌ CoA(c) + Malonyl-ACP(c)

Continued on next page

ID	flux	reaction
r2529	3	Cysteine(s) ⇌ Cysteine(c)
r0681	1	(R)-3-Hydroxybutanoyl-ACP(c) ⇌ But-2-enoyl-ACP(c) + H2O(c)
r0682	-1	Butyryl-ACP(c) + NADP+(c) ⇌ But-2-enoyl-ACP(c) + NADPH(c)
r1326	-14	NADPH(c) ⇌ NADPH-redox-potential(c) + NADP+(c)
r0538	1	3-Sulfinoalanine(c) ⇌ CO2(c) + Hypotaurine(c)
r0539	-1	Hypotaurine(c) ⇌ Cysteamine(c) + O2(c)

3-Oxotetradecanoyl-ACP (636)

Definition			Solution												
			exchanges		reactions								transp		
Objective	Constraints	Comment	imports	exports	c	m	r	p	l	n	s	b	s-c	b-c	int
3-Oxotetradecanoyl-ACP(c)	PIPES MCES	synthesis of cytosolic 3-Oxotetradecanoyl-ACP	1 P_i(c) 5 Glutamate(s) 6 Glycine(s) 11 Alanine(s) 4 Lysine(s) 8 Aspartate(s) 9 Arginine(s) 7 Glutamine(s) 8 Serine(s) 5 Methionine(s) 3 Phenylalanine(s) 4 Tyrosine(s) 3 Cysteine(s) 20 Leucine(s) 1 Histidine(s) 9 Proline(s) 1 Asparagine(s) 11 Valine(s) 6 Threonine(s) 4 Isoleucine(s) 1 Pantothenate(s) 630 ATP-energy(c) 10 NADPH-redox-potential(c) 3 Proton-gradient(c) 7 CoA-activated acetyl group(c)	8 H_2O(s) 1 CO_2(s) 1 3-Oxotetradecanoyl-ACP(c) 66 Na-gradient(c)	40	-	-	-	-	-	-	-	24	-	-

ID	flux	reaction
r1069	-4	Lysine(c) ⇌ Lysine(s)
r0578	1	ATP(c) + Pantetheine(c) ⇌ ADP(c) + Phosphopantetheine(c)
r2478	8	Serine(s) + Na+(s) + H+(PG)(c) → Serine(c) + Na+(c) + H+(PG)(s)
r0030	125	ATP(c) + AMP(c) ⇌ 2 ADP(c)
r0760	1	Butyryl-ACP(c) + Malonyl-ACP(c) ⇌ CO2(c) + ACP(c) + 3-Oxohexanoyl-ACP(c)
r0032	1	ATP(c) + Dephospho-CoA(c) ⇌ ADP(c) + CoA(c)
r0761	-1	D-3-Hydroxyhexanoyl-ACP(c) + NADP+(c) ⇌ NADPH(c) + 3-Oxohexanoyl-ACP(c)
r0184	6	ATP(c) + Acetyl-CoA(c) + HCO3-(c) → ADP(c) + Pi(c) + Malonyl-CoA(c)
r0762	1	D-3-Hydroxyhexanoyl-ACP(c) ⇌ H2O(c) + (2E)-Hexenoyl-ACP(c)
r0763	-1	Hexanoyl-ACP(c) + NADP+(c) ⇌ NADPH(c) + (2E)-Hexenoyl-ACP(c)
r0764	1	Malonyl-ACP(c) + Hexanoyl-ACP(c) ⇌ 3-Oxooctanoyl-ACP(c) + CO2(c) + ACP(c)
r1122	-20	Leucine(c) + Na+(c) ← Leucine(s) + Na+(s)
r0765	-1	Octanoyl-ACP(c) + NADP+(c) ⇌ (2E)-Octenoyl-ACP(c) + NADPH(c)
r1123	-4	Isoleucine(c) + Na+(c) ← Isoleucine(s) + Na+(s)
r0766	1	Octanoyl-ACP(c) + Malonyl-ACP(c) ⇌ 3-Oxodecanoyl-ACP(c) + CO2(c) + ACP(c)
r0767	-1	Decanoyl-ACP(c) + NADP+(c) ⇌ (2E)-Decenoyl-ACP(c) + NADPH(c)
r1125	4	Tyrosine(s) + Na+(s) → Tyrosine(c) + Na+(c)
r0768	1	Decanoyl-ACP(c) + Malonyl-ACP(c) ⇌ 3-Oxododecanoyl-ACP(c) + CO2(c) + ACP(c)
r0769	-1	D-3-Hydroxydodecanoyl-ACP(c) + NADP+(c) ⇌ 3-Oxododecanoyl-ACP(c) + NADPH(c)
r1142	-6	Threonine(s) + Na+(s) → Threonine(c) + Na+(c)
r1143	-8	Na+(c) + Aspartate(c) ← Na+(s) + Aspartate(s)
r2664	66	Na+(c) ⇌ Na+(s) + Na-gradient(c)
r1144	-5	Glutamate(c) + Na+(c) ← Glutamate(s) + Na+(s)
r1145	-1	Histidine(c) + Na+(c) ← Histidine(s) + Na+(s)
r2532	1	Asparagine(s) ⇌ Asparagine(c)
r2533	11	Valine(s) ⇌ Valine(c)
r0678	1	Acetyl-ACP(c) + Malonyl-ACP(c) ⇌ CO2(c) + ACP(c) + Acetoacetyl-ACP(c)
r0691	-1	(R)-3-Hydroxybutanoyl-ACP(c) + NADP+(c) ⇌ NADPH(c) + Acetoacetyl-ACP(c)
r1331	-3	H+(PG)(s) ⇌ H+(PG)(c) + Proton-gradient(c)

Continued on next page

ID	flux	reaction
r0974	1	Pantothenate(s) + Na+(s) ⇌ Pantothenate(c) + Na+(c)
r0692	-1	(R)-3-Hydroxydecanoyl-ACP(c) + NADP+(c) ⇌ 3-Oxodecanoyl-ACP(c) + NADPH(c)
r1319	-630	ATP(c) + H2O(c) ⇌ ADP(c) + Pi(c) + ATP-energy(c)
r0693	1	(R)-3-Hydroxydecanoyl-ACP(c) ⇌ (2E)-Decenoyl-ACP(c) + H2O(c)
r0694	-1	(R)-3-Hydroxyoctanoyl-ACP(c) + NADP+(c) ⇌ 3-Oxooctanoyl-ACP(c) + NADPH(c)
r0695	1	(R)-3-Hydroxyoctanoyl-ACP(c) ⇌ (2E)-Octenoyl-ACP(c) + H2O(c)
r1058	9	Arginine(s) ⇌ Arginine(c)
r1072	5	Methionine(s) ⇌ Methionine(c)
r0849	-8	H2O(s) ⇌ H2O(c)
r0581	-1	Pantothenate(c) + Cysteamine(c) ⇌ H2O(c) + Pantetheine(c)
r0712	-1	Dodecanoyl-ACP(c) + NADP+(c) ⇌ (2E)-Dodecenoyl-ACP(c) + NADPH(c)
r0713	1	Dodecanoyl-ACP(c) + Malonyl-ACP(c) ⇌ 3-Oxotetradecanoyl-ACP(c) + CO2(c) + ACP(c)
r0867	1	CO2(c) ⇌ CO2(s)
r1096	1	11 Alanine(c) + 2 Cysteine(c) + 5 Glutamate(c) + 3 Phenylalanine(c) + 6 Glycine(c) + Histidine(c) + 4 Isoleucine(c) + 20 Leucine(c) + 5 Methionine(c) + 9 Proline(c) + 7 Glutamine(c) + 9 Arginine(c) + 8 Serine(c) + 6 Threonine(c) + 11 Valine(c) + 4 Tyrosine(c) + 496 ATP(c) + 496 H2O(c) + 8 Aspartate(c) + 4 Lysine(c) + Asparagine(c) → Apo-ACP(c) + 124 AMP(c) + 124 PPi(c) + 372 ADP(c) + 372 Pi(c)
r0589	1	ATP(c) + Phosphopantetheine(c) → PPi(c) + Dephospho-CoA(c)
r2469	11	Alanine(s) + H+(PG)(s) → Alanine(c) + H+(PG)(c)
r0007	125	PPi(c) + H2O(c) → 2 Pi(c)
r0770	1	D-3-Hydroxydodecanoyl-ACP(c) ⇌ (2E)-Dodecenoyl-ACP(c) + H2O(c)
r1417	-6	CO2(c) + H2O(c) ⇌ Carbonate(c)
r0049	1	PAP(c) + H2O(c) → AMP(c) + Pi(c)
r1419	6	Carbonate(c) ⇌ HCO3-(c)
r1139	-9	Proline(c) + Na+(c) ← Proline(s) + Na+(s)
r0211	1	Cysteine(c) + O2(c) ⇌ 3-Sulfinoalanine(c)
r2523	6	Glycine(s) ⇌ Glycine(c)
r2677	-7	Acetyl-CoA(c) ⇌ CoA(c) + CoA-activated acetyl group(c)
r2525	7	Glutamine(s) ⇌ Glutamine(c)
r0367	1	Acetyl-CoA(c) + ACP(c) ⇌ CoA(c) + Acetyl-ACP(c)
r0368	1	CoA(c) + Apo-ACP(c) → PAP(c) + ACP(c)
r2528	3	Phenylalanine(s) ⇌ Phenylalanine(c)
r0369	6	ACP(c) + Malonyl-CoA(c) ⇌ CoA(c) + Malonyl-ACP(c)
r2529	3	Cysteine(s) ⇌ Cysteine(c)
r0681	1	(R)-3-Hydroxybutanoyl-ACP(c) ⇌ But-2-enoyl-ACP(c) + H2O(c)
r0682	-1	Butyryl-ACP(c) + NADP+(c) ⇌ But-2-enoyl-ACP(c) + NADPH(c)
r1326	-10	NADPH(c) ⇌ NADPH-redox-potential(c) + NADP+(c)
r0538	1	3-Sulfinoalanine(c) ⇌ CO2(c) + Hypotaurine(c)
r0539	-1	Hypotaurine(c) ⇌ Cysteamine(c) + O2(c)

3-Oxotetradecanoyl-CoA (637)

Definition			Solution												
			exchanges		reactions								transp		
Objective	Constraints	Comment	imports	exports	c	m	r	p	l	n	s	b	s-c	b-c	int
3-Oxotetradecanoyl-CoA(m)	PIPES MCES	synthesis of mitochondrial 3-Oxotetradecanoyl-CoA	2.17 P_i(c) 1 Glycine(s) 0.5 Aspartate(s) 0.5 Glutamine(s) 0.83 Glucose-6P(c) 1.5 Asparagine(s) 0.5 Cystine(s) 1 Pantothenate(s) 1 Palmitolate(s) 14.7 ATP-energy(c) 2 THF-activated methyl group(c)	4.17 H_2O(s) 2 CO_2(s) 2 Pyruvate(c) 0.5 Glutamate(s) 1 3-Oxotetradecanoyl-CoA(m) 3 NADH-redox-potential(c) 2 NADH-redox-potential(m) 3.5 NADPH-redox-potential(c) 12 Proton-gradient(m) 1.5 Na-gradient(c) 1 CoA-activated acetyl group(m)	48	10	-	-	-	-	-	-	10	-	6

ID	flux	reaction
r2435	1	L-Carnitine(m) + L-Palmitoylcarnitine(c) → L-Carnitine(c) + L-Palmitoylcarnitine(m)

Continued on next page

ID	flux	reaction
r0406	-0.33	Fructose-6P(c) + Erythrose-4P(c) ⇌ Sedoheptulose-7P(c) + GAP(c)
r0277	1	GTP(c) + IMP(c) + Aspartate(c) → GDP(c) + Pi(c) + Adenylosuccinate(c)
r0856	-1	O2(m) ⇌ O2(c)
r0127	1.5	H2O(c) + Asparagine(c) → Aspartate(c) + NH3(c)
r0705	1	ATP(c) + 5-Phosphoribosyl-4-carboxy-5-aminoimidazole(c) + Aspartate(c) ⇌ ADP(c) + Pi(c) + SAICAR(c)
r0293	2	5,10-Methylene-THF(c) + NADP+(c) ⇌ 5,10-Methenyl-THF(c) + NADPH(c)
r0720	-1	(S)-3-Hydroxytetradecanoyl-CoA(m) ← (2E)-Tetradecenoyl-CoA(m) + H2O(m)
r0578	1	ATP(c) + Pantetheine(c) ⇌ ADP(c) + Phosphopantetheine(c)
r0877	0.5	Glutamate(c) + Cystine(s) ⇌ Glutamate(s) + Cystine(c)
r0310	1	Palmitoyl-CoA(m) + Ubiquinone(m) → (2E)-Hexadecenoyl-CoA(m) + Ubiquinol(m)
r0017	1	O2(m) + 4 H+(PG)(m) + 4 Ferrocytochrome C(m) → 4 Ferricytochrome C(m) + 2 H2O(m) + 4 H+(PG)(c)
r0030	3	ATP(c) + AMP(c) ⇌ 2 ADP(c)
r0032	1	ATP(c) + Dephospho-CoA(c) ⇌ ADP(c) + CoA(c)
r1143	-0.5	Na+(c) + Aspartate(c) ← Na+(s) + Aspartate(s)
r2664	1.5	Na+(c) ⇌ Na+(s) + Na-gradient(c)
r0058	2	Malate(c) + NADP+(c) ⇌ Pyruvate(c) + CO2(c) + NADPH(c)
r0370	0.33	Sedoheptulose-7P(c) + GAP(c) ⇌ Ribose-5P(c) + D-Xylulose-5P(c)
r0653	-1	CoA(m) + 3-Oxopalmitoyl-CoA(m) → Myristoyl-CoA(m) + Acetyl-CoA(m)
r0371	2	5,10-Methenyl-THF(c) + H2O(c) ⇌ 10-Formyl-THF(c)
r0077	1.5	ATP(c) + Glutamate(c) + NH3(c) → ADP(c) + Pi(c) + Glutamine(c)
r2532	1.5	Asparagine(s) ⇌ Asparagine(c)
r1449	1	Ubiquinone(m) + Myristoyl-CoA(m) → (2E)-Tetradecenoyl-CoA(m) + Ubiquinol(m)
r0659	1	ATP(c) + 5-Phosphoribosylamine(c) + Glycine(c) ⇌ ADP(c) + Pi(c) + GAR(c)
r0507	2	Ubiquinol(m) + 2 Ferricytochrome C(m) + 4 H+(PG)(m) → Ubiquinone(m) + 4 H+(PG)(c) + 2 Ferrocytochrome C(m)
r0099	1	ATP(c) + GDP(c) ⇌ ADP(c) + GTP(c)
r0677	1	10-Formyl-THF(c) + GAR(c) ⇌ THF(c) + N-Formyl-GAR(c)
r0243	-0.17	DHAP(c) ⇌ GAP(c)
r1330	12	H+(PG)(c) ⇌ H+(PG)(m) + Proton-gradient(m)
r0974	1	Pantothenate(s) + Na+(s) ⇌ Pantothenate(c) + Na+(c)
r1319	-14.7	ATP(c) + H2O(c) ⇌ ADP(c) + Pi(c) + ATP-energy(c)
r0260	1	Adenylosuccinate(c) → Fumarate(c) + AMP(c)
r0247	1	ATP(c) + Ribose-5P(c) → AMP(c) + PRPP(c)
r1488	1	ATP(c) + CoA(c) + Palmitolate(c) ⇌ AMP(c) + palmitoleoyl-CoA(c) + PPi(c)
r0699	1	SAICAR(c) ⇌ Fumarate(c) + AICAR(c)
r2442	1	Palmitolate(s) → Palmitolate(c)
r0849	-4.17	H2O(s) ⇌ H2O(c)
r0863	1	CoA(c) ⇌ CoA(m)
r0581	-1	Pantothenate(c) + Cysteamine(c) ⇌ H2O(c) + Pantetheine(c)
r0714	1	NAD+(m) + (S)-3-Hydroxyhexadecanoyl-CoA(m) → 3-Oxopalmitoyl-CoA(m) + NADH(m)
r0867	2	CO2(c) ⇌ CO2(s)
r0716	-1	(S)-3-Hydroxyhexadecanoyl-CoA(m) ← (2E)-Hexadecenoyl-CoA(m) + H2O(m)
r0435	1	Palmitoyl-CoA(c) + L-Carnitine(c) ⇌ CoA(c) + L-Palmitoylcarnitine(c)
r0718	1	(S)-3-Hydroxytetradecanoyl-CoA(m) + NAD+(m) → 3-Oxotetradecanoyl-CoA(m) + NADH(m)
r0436	-1	Palmitoyl-CoA(m) + L-Carnitine(m) ← CoA(m) + L-Palmitoylcarnitine(m)
r0589	1	ATP(c) + Phosphopantetheine(c) → PPi(c) + Dephospho-CoA(c)
r0736	0.17	ATP(c) + Fructose-6P(c) → ADP(c) + Fructose-1,6PP(c)
r0007	3	PPi(c) + H2O(c) → 2 Pi(c)
r0023	-1	NAD+(c) + 2 Ferrocytochrome b5(c) ⇌ NADH(c) + 2 Ferricytochrome b5(c)
r0027	-0.5	NADPH(c) + Cystine(c) ⇌ 2 Cysteine(c) + NADP+(c)
r0192	0.83	Glucose-6P(c) ⇌ Fructose-6P(c)
r0344	-0.67	D-Xylulose-5P(c) ⇌ Ribulose-5P(c)
r0792	2	5-Methyl-THF(c) + NAD+(c) ⇌ 5,10-Methylene-THF(c) + NADH(c)
r0211	1	Cysteine(c) + O2(c) ⇌ 3-Sulfinoalanine(c)
r2523	1	Glycine(s) ⇌ Glycine(c)
r2676	-2	5-Methyl-THF(c) ⇌ THF(c) + THF-activated methyl group(c)
r2525	0.5	Glutamine(s) ⇌ Glutamine(c)

Continued on next page

ID	flux	reaction
r2678	1	Acetyl-CoA(m) ⇌ CoA(m) + CoA-activated acetyl group(m)
r0510	-1	palmitoleoyl-CoA(c) + 2 Ferricytochrome b5(c) + 2 H2O(c) ⇌ Palmitoyl-CoA(c) + O2(c) + 2 Ferrocytochrome b5(c)
r0666	1	ATP(c) + 5-Phosphoribosylformylglycinamidine(c) ⇌ ADP(c) + Pi(c) + AIR(c)
r0667	-1	5-Phosphoribosyl-4-carboxy-5-aminoimidazole(c) ⇌ AIR(c) + CO2(c)
r1322	3	NADH(c) ⇌ NAD+(c) + NADH-redox-potential(c)
r1323	2	NADH(m) ⇌ NAD+(m) + NADH-redox-potential(m)
r0250	-0.67	Ribulose-5P(c) ⇌ Ribose-5P(c)
r1326	3.5	NADPH(c) ⇌ NADPH-redox-potential(c) + NADP+(c)
r0687	1	ATP(c) + N-Formyl-GAR(c) + Glutamine(c) + H2O(c) ⇌ ADP(c) + Pi(c) + Glutamate(c) + 5-Phosphoribosylformylglycinamidine(c)
r0255	0.33	Fructose-6P(c) + GAP(c) ⇌ D-Xylulose-5P(c) + Erythrose-4P(c)
r0538	1	3-Sulfinoalanine(c) ⇌ CO2(c) + Hypotaurine(c)
r0256	0.17	Fructose-1,6PP(c) ⇌ DHAP(c) + GAP(c)
r0539	-1	Hypotaurine(c) ⇌ Cysteamine(c) + O2(c)
r0257	-1	Glutamine(c) + PRPP(c) + H2O(c) ⇌ 5-Phosphoribosylamine(c) + PPi(c) + Glutamate(c)
r0258	-2	Fumarate(c) + H2O(c) ⇌ Malate(c)
r0273	-1	IMP(c) + H2O(c) ⇌ FAICAR(c)
r0700	1	10-Formyl-THF(c) + AICAR(c) ⇌ THF(c) + FAICAR(c)

3-Phosphonooxypyruvate (638)

Definition			Solution												
			exchanges		reactions								transp		
Objective	Constraints	Comment	imports	exports	c	m	r	p	l	n	s	b	s-c	b-c	int
3-Phosphonooxypyruvate(c)	PIPES MCES	synthesis of cytosolic 3-Phosphonooxypyruvate	0.67 P_i(c) 0.33 Pyruvate(c) 0.33 Serine(s) 0.33 Glucose-6P(c) 0.33 NADPH-redox-potential(c)	0.33 Alanine(s) 1 3-Phosphonooxypyruvate(c) 1.67 NADH-redox-potential(c) 0.33 Proton-gradient(c) 0.33 Na-gradient(c)	12	-	-	-	-	-	-	-	4	-	-

ID	flux	reaction
r0253	0.67	GAP(c) + Pi(c) + NAD+(c) ⇌ 1,3DPG(c) + NADH(c)
r1326	-0.33	NADPH(c) ⇌ NADPH-redox-potential(c) + NADP+(c)
r0327	-0.33	Hydroxypyruvate(c) + NADPH(c) → Glycerate(c) + NADP+(c)
r0192	0.33	Glucose-6P(c) ⇌ Fructose-6P(c)
r2524	-0.33	Alanine(c) ⇌ Alanine(s)
r0337	-0.67	ADP(c) + 1,3DPG(c) ⇌ ATP(c) + 3PG(c)
r0256	0.33	Fructose-1,6PP(c) ⇌ DHAP(c) + GAP(c)
r0338	1	3PG(c) + NAD+(c) → 3-Phosphonooxypyruvate(c) + NADH(c)
r0339	0.33	ATP(c) + Glycerate(c) → ADP(c) + 3PG(c)
r0736	0.33	ATP(c) + Fructose-6P(c) → ADP(c) + Fructose-1,6PP(c)
r2664	0.33	Na+(c) ⇌ Na+(s) + Na-gradient(c)
r0160	0.33	Serine(c) + Pyruvate(c) → Hydroxypyruvate(c) + Alanine(c)
r2478	0.33	Serine(s) + Na+(s) + H+(PG)(c) → Serine(c) + Na+(c) + H+(PG)(s)
r1331	0.33	H+(PG)(s) ⇌ H+(PG)(c) + Proton-gradient(c)
r1322	1.67	NADH(c) ⇌ NAD+(c) + NADH-redox-potential(c)
r0243	-0.33	DHAP(c) ⇌ GAP(c)

3-Phosphoserine (639)

Definition			Solution												
			exchanges		reactions								transp		
Objective	Constraints	Comment	imports	exports	c	m	r	p	l	n	s	b	s-c	b-c	int
3-Phosphoserine(c)	PIPES MCES	synthesis of cytosolic 3-Phosphoserine	0.67 P_i(c) 0.67 Alanine(s) 0.33 Serine(s) 0.33 Glucose-6P(c) 0.33 NADPH-redox-potential(c) 0.33 Proton-gradient(c)	0.67 Pyruvate(c) 1 3-Phosphoserine(c) 1.67 NADH-redox-potential(c) 0.33 Na-gradient(c)	14	-	-	-	-	-	-	-	4	-	-

ID	flux	reaction
r0253	0.67	GAP(c) + Pi(c) + NAD+(c) ⇌ 1,3DPG(c) + NADH(c)
r1326	-0.33	NADPH(c) ⇌ NADPH-redox-potential(c) + NADP+(c)
r0327	-0.33	Hydroxypyruvate(c) + NADPH(c) → Glycerate(c) + NADP+(c)
r0192	0.33	Glucose-6P(c) ⇌ Fructose-6P(c)
r0337	-0.67	ADP(c) + 1,3DPG(c) ⇌ ATP(c) + 3PG(c)
r0256	0.33	Fructose-1,6PP(c) ⇌ DHAP(c) + GAP(c)
r0338	1	3PG(c) + NAD+(c) → 3-Phosphonooxypyruvate(c) + NADH(c)
r0663	-1	3-Phosphonooxypyruvate(c) + Glutamate(c) → 3-Phosphoserine(c) + AKG(c)
r0339	0.33	ATP(c) + Glycerate(c) → ADP(c) + 3PG(c)
r0736	0.33	ATP(c) + Fructose-6P(c) → ADP(c) + Fructose-1,6PP(c)
r2664	0.33	Na+(c) ⇌ Na+(s) + Na-gradient(c)
r0160	0.33	Serine(c) + Pyruvate(c) → Hydroxypyruvate(c) + Alanine(c)
r2478	0.33	Serine(s) + Na+(s) + H+(PG)(c) → Serine(c) + Na+(c) + H+(PG)(s)
r2469	0.67	Alanine(s) + H+(PG)(s) → Alanine(c) + H+(PG)(c)
r1331	-0.33	H+(PG)(s) ⇌ H+(PG)(c) + Proton-gradient(c)
r1322	1.67	NADH(c) ⇌ NAD+(c) + NADH-redox-potential(c)
r0080	1	Alanine(c) + AKG(c) ⇌ Pyruvate(c) + Glutamate(c)
r0243	-0.33	DHAP(c) ⇌ GAP(c)

3-Sulfinoalanine (640)

Definition			Solution												
			exchanges		reactions								transp		
Objective	Constraints	Comment	imports	exports	c	m	r	p	l	n	s	b	s-c	b-c	int
3-Sulfinoalanine(c)	PIPES MCES	synthesis of cytosolic 3-Sulfinoalanine	1 O_2(s) 1 Cysteine(s)	1 3-Sulfinoalanine(c)	1	-	-	-	-	-	-	-	2	-	-

ID	flux	reaction
r0211	1	Cysteine(c) + O2(c) ⇌ 3-Sulfinoalanine(c)
r2529	1	Cysteine(s) ⇌ Cysteine(c)
r0855	1	O2(s) ⇌ O2(c)

3-Ureidoisobutyrate (641)

Definition			Solution												
			exchanges		reactions								transp		
Objective	Constraints	Comment	imports	exports	c	m	r	p	l	n	s	b	s-c	b-c	int
3-Ureidoisobutyrate(c)	PIPES MCES	synthesis of cytosolic 3-Ureidoisobutyrate	0.33 O_2(s) 1 Aspartate(s) 1 Glutamine(s) 0.33 Proline(s) 4 ATP-energy(c) 2 NADH-redox-potential(c) 1 THF-activated methyl group(c)	1.33 Glutamate(s) 1 3-Ureidoisobutyrate(c) 0.33 NADH-redox-potential(m) 1 NADPH-redox-potential(c) 1 Proton-gradient(c) 4 Proton-gradient(m) 2.33 Na-gradient(c)	26	4	-	-	-	-	-	-	7	-	6

ID	flux	reaction
r0855	0.33	O2(s) ⇌ O2(c)
r0856	-0.33	O2(m) ⇌ O2(c)
r0296	1	5-Methyl-THF(c) + NADP+(c) ⇌ 5,10-Methylene-THF(c) + NADPH(c)
r0593	1	Dihydrothymine(c) + H2O(c) → 3-Ureidoisobutyrate(c)
r2477	1	Glutamine(s) + Na+(s) + H+(PG)(c) → Glutamine(c) + Na+(c) + H+(PG)(s)
r0017	0.33	O2(m) + 4 H+(PG)(m) + 4 Ferrocytochrome C(m) → 4 Ferricytochrome C(m) + 2 H2O(m) + 4 H+(PG)(c)
r0030	1	ATP(c) + AMP(c) ⇌ 2 ADP(c)
r0467	-1	H2O(c) + (S)-Dihydroorotate(c) ← N-Carbamoyl-L-aspartate(c)
r0330	-1	Dihydrothymine(c) + NAD+(c) ← Thymine(c) + NADH(c)
r0911	0.33	Proline(c) + Glutamate(m) ⇌ Proline(m) + Glutamate(c)
r0350	1	dTMP(c) + H2O(c) → Thymidine(c) + Pi(c)
r1143	-1	Na+(c) + Aspartate(c) ← Na+(s) + Aspartate(s)
r0352	1	Thymidine(c) + Pi(c) ⇌ Thymine(c) + 2-Deoxy-D-ribose-1P(c)
r2664	2.33	Na+(c) ⇌ Na+(s) + Na-gradient(c)
r0074	0.33	L-Glutamate 5-semialdehyde(m) + NAD+(m) + H2O(m) ⇌ Glutamate(m) + NADH(m)
r0501	1	dUMP(c) + 5,10-Methylene-THF(c) ⇌ Dihydrofolate(c) + dTMP(c)
r2685	1.33	Glutamate(c) ⇌ Glutamate(s)
r0224	-1	Dihydrofolate(c) + NADH(c) → THF(c) + NAD+(c)
r0507	0.67	Ubiquinol(m) + 2 Ferricytochrome C(m) + 4 H+(PG)(m) → Ubiquinone(m) + 4 H+(PG)(c) + 2 Ferrocytochrome C(m)
r1330	4	H+(PG)(c) ⇌ H+(PG)(m) + Proton-gradient(m)
r0540	-1	Uracil(c) + 2-Deoxy-D-ribose-1P(c) ⇌ Pi(c) + Deoxyuridine(c)
r1331	1	H+(PG)(s) ⇌ H+(PG)(c) + Proton-gradient(c)
r1319	-4	ATP(c) + H2O(c) ⇌ ADP(c) + Pi(c) + ATP-energy(c)
r0247	1	ATP(c) + Ribose-5P(c) → AMP(c) + PRPP(c)
r0416	1	Ubiquinone(m) + (S)-Dihydroorotate(c) → Orotate(c) + Ubiquinol(m)
r0417	-1	PPi(c) + Orotidine-5P(c) ⇌ Orotate(c) + PRPP(c)
r0418	1	Uridine(c) + Pi(c) ⇌ Uracil(c) + Ribose-1P(c)
r0155	1	2 ATP(c) + Glutamine(c) + H2O(c) + HCO3-(c) → 2 ADP(c) + Pi(c) + Glutamate(c) + Carbamoyl-P(c)
r0007	1	PPi(c) + H2O(c) → 2 Pi(c)
r0175	0.33	1-Pyrroline-5-carboxylate(m) + H2O(m) ⇌ L-Glutamate 5-semialdehyde(m)
r1115	-1	ADP(c) + UMP(c) ⇌ ATP(c) + Uridine(c)
r0328	1	Carbamoyl-P(c) + Aspartate(c) → Pi(c) + N-Carbamoyl-L-aspartate(c)
r1417	-1	CO2(c) + H2O(c) ⇌ Carbonate(c)
r1419	1	Carbonate(c) ⇌ HCO3-(c)
r0499	1	ATP(c) + Deoxyuridine(c) ⇌ ADP(c) + dUMP(c)
r1139	-0.33	Proline(s) + Na+(s) → Proline(c) + Na+(c)
r2676	-1	5-Methyl-THF(c) ⇌ THF(c) + THF-activated methyl group(c)
r1453	0.33	Proline(m) + Ubiquinol(m) ⇌ 1-Pyrroline-5-carboxylate(m) + Ubiquinone(m)
r0235	1	Orotidine-5P(c) ⇌ CO2(c) + UMP(c)
r1322	-2	NADH(c) ⇌ NAD+(c) + NADH-redox-potential(c)
r1323	0.33	NADH(m) ⇌ NAD+(m) + NADH-redox-potential(m)
r0251	1	Ribose-1P(c) ⇌ Ribose-5P(c)
r1326	1	NADPH(c) ⇌ NADPH-redox-potential(c) + NADP+(c)

3-Ureidopropionate (642)

Definition			Solution												
			exchanges		reactions								transp		
Objective	Constraints	Comment	imports	exports	c	m	r	p	l	n	s	b	s-c	b-c	int
3-Ureidopropionate(c)	PIPES MCES	synthesis of cytosolic 3-Ureidopropionate	0.33 O_2(s) 1 Aspartate(s) 1 Glutamine(s) 0.33 Proline(s) 3 ATP-energy(c) 1 NADPH-redox-potential(c)	1.33 Glutamate(s) 1 3-Ureidopropionate(c) 0.33 NADH-redox-potential(m) 4 Proton-gradient(m) 1.33 Na-gradient(c)	17	4	-	-	-	-	-	-	6	-	6

ID	flux	reaction
r0235	1	Orotidine-5P(c) ⇌ CO2(c) + UMP(c)
		Continued on next page

3-Ureidopropionate (642)– continued

ID	flux	reaction
r0911	0.33	Proline(c) + Glutamate(m) ⇌ Proline(m) + Glutamate(c)
r0416	1	Ubiquinone(m) + (S)-Dihydroorotate(c) → Orotate(c) + Ubiquinol(m)
r0155	1	2 ATP(c) + Glutamine(c) + H2O(c) + HCO3-(c) → 2 ADP(c) + Pi(c) + Glutamate(c) + Carbamoyl-P(c)
r0074	0.33	L-Glutamate 5-semialdehyde(m) + NAD+(m) + H2O(m) ⇌ Glutamate(m) + NADH(m)
r1326	-1	NADPH(c) ⇌ NADPH-redox-potential(c) + NADP+(c)
r0516	1	5,6-Dihydrouracil(c) + H2O(c) → 3-Ureidopropionate(c)
r0507	0.67	Ubiquinol(m) + 2 Ferricytochrome C(m) + 4 H+(PG)(m) → Ubiquinone(m) + 4 H+(PG)(c) + 2 Ferrocytochrome C(m)
r0417	-1	PPi(c) + Orotidine-5P(c) ⇌ Orotate(c) + PRPP(c)
r0237	-1	Uracil(c) + NADPH(c) ⇌ 5,6-Dihydrouracil(c) + NADP+(c)
r1453	0.33	Proline(m) + Ubiquinol(m) ⇌ 1-Pyrroline-5-carboxylate(m) + Ubiquinone(m)
r1417	-1	CO2(c) + H2O(c) ⇌ Carbonate(c)
r0418	1	Uridine(c) + Pi(c) ⇌ Uracil(c) + Ribose-1P(c)
r0328	1	Carbamoyl-P(c) + Aspartate(c) → Pi(c) + N-Carbamoyl-L-aspartate(c)
r0247	1	ATP(c) + Ribose-5P(c) → AMP(c) + PRPP(c)
r0175	0.33	1-Pyrroline-5-carboxylate(m) + H2O(m) ⇌ L-Glutamate 5-semialdehyde(m)
r2525	1	Glutamine(s) ⇌ Glutamine(c)
r1319	-3	ATP(c) + H2O(c) ⇌ ADP(c) + Pi(c) + ATP-energy(c)
r1139	-0.33	Proline(s) + Na+(s) → Proline(c) + Na+(c)
r1419	1	Carbonate(c) ⇌ HCO3-(c)
r0030	1	ATP(c) + AMP(c) ⇌ 2 ADP(c)
r0467	-1	H2O(c) + (S)-Dihydroorotate(c) ← N-Carbamoyl-L-aspartate(c)
r2664	1.33	Na+(c) ⇌ Na+(s) + Na-gradient(c)
r0855	0.33	O2(s) ⇌ O2(c)
r0856	-0.33	O2(m) ⇌ O2(c)
r1330	4	H+(PG)(c) ⇌ H+(PG)(m) + Proton-gradient(m)
r0007	1	PPi(c) + H2O(c) → 2 Pi(c)
r2685	1.33	Glutamate(c) ⇌ Glutamate(s)
r1115	-1	ADP(c) + UMP(c) ⇌ ATP(c) + Uridine(c)
r0251	1	Ribose-1P(c) ⇌ Ribose-5P(c)
r0017	0.33	O2(m) + 4 H+(PG)(m) + 4 Ferrocytochrome C(m) → 4 Ferricytochrome C(m) + 2 H2O(m) + 4 H+(PG)(c)
r1323	0.33	NADH(m) ⇌ NAD+(m) + NADH-redox-potential(m)
r1143	-1	Na+(c) + Aspartate(c) ← Na+(s) + Aspartate(s)

3-oxolaur-cis-5-enoyl-CoA (643)

Definition			Solution												
			exchanges		reactions								transp		
Objective	Constraints	Comment	imports	exports	c	m	r	p	l	n	s	b	s-c	b-c	int
3-oxolaur-cis-5-enoyl-CoA(m)	PIPES MCES	synthesis of mitochondrial 3-oxolaur-cis-5-enoyl-CoA	2.17 P_i(c) 1 Glycine(s) 2 Aspartate(s) 2 Glutamine(s) 0.83 Glucose-6P(c) 0.5 Cystine(s) 1 Pantothenate(s) 1 Palmitolate(s) 13.2 ATP-energy(c) 1 Proton-gradient(c) 2 THF-activated methyl group(c)	3.17 H_2O(s) 2 CO_2(s) 2 Pyruvate(c) 2 Glutamate(s) 2 NADH-redox-potential(c) 3 NADH-redox-potential(m) 3.5 NADPH-redox-potential(c) 3 FADH-redox-potential(c) 1 3-oxolaur-cis-5-enoyl-CoA(m) 3 Na-gradient(c) 2 CoA-activated acetyl group(m)	44	13	-	-	-	-	-	-	11	-	3

ID	flux	reaction
r0406	-0.33	Fructose-6P(c) + Erythrose-4P(c) ⇌ Sedoheptulose-7P(c) + GAP(c)
r0277	1	GTP(c) + IMP(c) + Aspartate(c) → GDP(c) + Pi(c) + Adenylosuccinate(c)
r0293	2	5,10-Methylene-THF(c) + NADP+(c) ⇌ 5,10-Methenyl-THF(c) + NADPH(c)
r0705	1	ATP(c) + 5-Phosphoribosyl-4-carboxy-5-aminoimidazole(c) + Aspartate(c) ⇌ ADP(c) + Pi(c) + SAICAR(c)
r0578	1	ATP(c) + Pantetheine(c) ⇌ ADP(c) + Phosphopantetheine(c)
r0877	0.5	Glutamate(c) + Cystine(s) ⇌ Glutamate(s) + Cystine(c)
r0030	3	ATP(c) + AMP(c) ⇌ 2 ADP(c)

Continued on next page

ID	flux	reaction
r0032	1	ATP(c) + Dephospho-CoA(c) ⇌ ADP(c) + CoA(c)
r1290	3	FAD(c) + FADH2(m) ⇌ FAD(m) + FADH2(c)
r1143	-2	Na+(c) + Aspartate(c) ← Na+(s) + Aspartate(s)
r2664	3	Na+(c) ⇌ Na+(s) + Na-gradient(c)
r0058	2	Malate(c) + NADP+(c) ⇌ Pyruvate(c) + CO2(c) + NADPH(c)
r0370	0.33	Sedoheptulose-7P(c) + GAP(c) ⇌ Ribose-5P(c) + D-Xylulose-5P(c)
r0371	2	5,10-Methenyl-THF(c) + H2O(c) ⇌ 10-Formyl-THF(c)
r2685	1.5	Glutamate(c) ⇌ Glutamate(s)
r0659	1	ATP(c) + 5-Phosphoribosylamine(c) + Glycine(c) ⇌ ADP(c) + Pi(c) + GAR(c)
r1480	1	CoA(m) + 3-oxopalmitoleoyl-CoA(m) ⇌ Acetyl-CoA(m) + cis-myrist-7-enoyl-CoA(m)
r0099	1	ATP(c) + GDP(c) ⇌ ADP(c) + GTP(c)
r1468	1	palmitoleoyl-CoA(c) ⇌ palmitoleoyl-CoA(m)
r0243	-0.17	DHAP(c) ⇌ GAP(c)
r0677	1	10-Formyl-THF(c) + GAR(c) ⇌ THF(c) + N-Formyl-GAR(c)
r1482	-1	FADH2(m) + trans,cis-dodeca-2,5-dienoyl-CoA(m) ⇌ FAD(m) + cis-laur-5-enoyl-CoA(m)
r1483	1	H2O(m) + trans,cis-myristo-2,7-dienoyl-CoA(m) ⇌ cis-(3S)-hydroxytetradec-7-enoyl-CoA(m)
r1331	-1	H+(PG)(s) ⇌ H+(PG)(c) + Proton-gradient(c)
r0974	1	Pantothenate(s) + Na+(s) ⇌ Pantothenate(c) + Na+(c)
r1484	1	CoA(m) + 3-oxomyrist-7-enoyl-CoA(m) ⇌ Acetyl-CoA(m) + cis-laur-5-enoyl-CoA(m)
r1319	-13.2	ATP(c) + H2O(c) ⇌ ADP(c) + Pi(c) + ATP-energy(c)
r1485	1	H2O(m) + trans,cis-dodeca-2,5-dienoyl-CoA(m) ⇌ 3(S)-3-hydroxydodecen-(5Z)-oyl-CoA(m)
r0260	1	Adenylosuccinate(c) → Fumarate(c) + AMP(c)
r0247	1	ATP(c) + Ribose-5P(c) → AMP(c) + PRPP(c)
r1486	1	NAD+(m) + 3(S)-3-hydroxydodecen-(5Z)-oyl-CoA(m) ⇌ 3-oxolaur-cis-5-enoyl-CoA(m) + NADH(m)
r1488	1	ATP(c) + CoA(c) + Palmitolate(c) ⇌ AMP(c) + palmitoleoyl-CoA(c) + PPi(c)
r0699	1	SAICAR(c) ⇌ Fumarate(c) + AICAR(c)
r2442	1	Palmitolate(s) → Palmitolate(c)
r0849	-3.17	H2O(s) ⇌ H2O(c)
r0581	-1	Pantothenate(c) + Cysteamine(c) ⇌ H2O(c) + Pantetheine(c)
r0867	2	CO2(c) ⇌ CO2(s)
r0589	1	ATP(c) + Phosphopantetheine(c) → PPi(c) + Dephospho-CoA(c)
r2468	1	Glycine(s) + H+(PG)(s) → Glycine(c) + H+(PG)(c)
r0007	3	PPi(c) + H2O(c) → 2 Pi(c)
r0736	0.17	ATP(c) + Fructose-6P(c) → ADP(c) + Fructose-1,6PP(c)
r0027	-0.5	NADPH(c) + Cystine(c) ⇌ 2 Cysteine(c) + NADP+(c)
r0192	0.83	Glucose-6P(c) ⇌ Fructose-6P(c)
r0344	-0.67	D-Xylulose-5P(c) ⇌ Ribulose-5P(c)
r0792	2	5-Methyl-THF(c) + NAD+(c) ⇌ 5,10-Methylene-THF(c) + NADH(c)
r0211	1	Cysteine(c) + O2(c) ⇌ 3-Sulfinoalanine(c)
r2676	-2	5-Methyl-THF(c) ⇌ THF(c) + THF-activated methyl group(c)
r2525	2	Glutamine(s) ⇌ Glutamine(c)
r2678	2	Acetyl-CoA(m) ⇌ CoA(m) + CoA-activated acetyl group(m)
r0666	1	ATP(c) + 5-Phosphoribosylformylglycinamidine(c) ⇌ ADP(c) + Pi(c) + AIR(c)
r1471	1	FAD(m) + cis-myrist-7-enoyl-CoA(m) ⇌ FADH2(m) + trans,cis-myristo-2,7-dienoyl-CoA(m)
r0667	-1	5-Phosphoribosyl-4-carboxy-5-aminoimidazole(c) ⇌ AIR(c) + CO2(c)
r1473	1	FAD(m) + palmitoleoyl-CoA(m) ⇌ FADH2(m) + trans,cis-hexadeca-2,9-dienoyl-CoA(m)
r1322	2	NADH(c) ⇌ NAD+(c) + NADH-redox-potential(c)
r1475	1	H2O(m) + trans,cis-hexadeca-2,9-dienoyl-CoA(m) ⇌ (S)-3-hydroxypalmitoleoyl-CoA(m)
r1323	3	NADH(m) ⇌ NAD+(m) + NADH-redox-potential(m)
r0250	-0.67	Ribulose-5P(c) ⇌ Ribose-5P(c)
r1476	1	NAD+(m) + cis-(3S)-hydroxytetradec-7-enoyl-CoA(m) ⇌ 3-oxomyrist-7-enoyl-CoA(m) + NADH(m)
r1478	1	NAD+(m) + (S)-3-hydroxypalmitoleoyl-CoA(m) ⇌ NADH(m) + 3-oxopalmitoleoyl-CoA(m)
r1326	3.5	NADPH(c) ⇌ NADPH-redox-potential(c) + NADP+(c)
r0687	1	ATP(c) + N-Formyl-GAR(c) + Glutamine(c) + H2O(c) ⇌ ADP(c) + Pi(c) + Glutamate(c) + 5-Phosphoribosylformylglycinamidine(c)
r0255	0.33	Fructose-6P(c) + GAP(c) ⇌ D-Xylulose-5P(c) + Erythrose-4P(c)

Continued on next page

ID	flux	reaction
r1329	3	FADH2(c) ⇌ FAD(c) + FADH-redox-potential(c)
r0538	1	3-Sulfinoalanine(c) ⇌ CO2(c) + Hypotaurine(c)
r0256	0.17	Fructose-1,6PP(c) ⇌ DHAP(c) + GAP(c)
r0539	-1	Hypotaurine(c) ⇌ Cysteamine(c) + O2(c)
r0257	-1	Glutamine(c) + PRPP(c) + H2O(c) ⇌ 5-Phosphoribosylamine(c) + PPi(c) + Glutamate(c)
r0258	-2	Fumarate(c) + H2O(c) ⇌ Malate(c)
r0273	-1	IMP(c) + H2O(c) ⇌ FAICAR(c)
r0851	-3	H2O(m) ⇌ H2O(c)
r0700	1	10-Formyl-THF(c) + AICAR(c) ⇌ THF(c) + FAICAR(c)

3-oxomyrist-7-enoyl-CoA (644)

Definition			Solution												
			exchanges		reactions								transp		
Objective	Constraints	Comment	imports	exports	c	m	r	p	l	n	s	b	s-c	b-c	int
3-oxomyrist-7-enoyl-CoA(m)	PIPES MCES	synthesis of mitochondrial 3-oxomyrist-7-enoyl-CoA	2.17 P_i(c) 1 Glycine(s) 2 Aspartate(s) 2 Glutamine(s) 0.83 Glucose-6P(c) 0.5 Cystine(s) 1 Pantothenate(s) 1 Palmitolate(s) 13.2 ATP-energy(c) 1 Proton-gradient(c) 2 THF-activated methyl group(c)	4.17 H_2O(s) 2 CO_2(s) 2 Pyruvate(c) 2 Glutamate(s) 2 NADH-redox-potential(c) 2 NADH-redox-potential(m) 3.5 NADPH-redox-potential(c) 2 FADH-redox-potential(c) 1 3-oxomyrist-7-enoyl-CoA(m) 3 Na-gradient(c) 1 CoA-activated acetyl group(m)	44	9	-	-	-	-	-	-	11	-	3

ID	flux	reaction
r0406	-0.33	Fructose-6P(c) + Erythrose-4P(c) ⇌ Sedoheptulose-7P(c) + GAP(c)
r0277	1	GTP(c) + IMP(c) + Aspartate(c) → GDP(c) + Pi(c) + Adenylosuccinate(c)
r0293	2	5,10-Methylene-THF(c) + NADP+(c) ⇌ 5,10-Methenyl-THF(c) + NADPH(c)
r0705	1	ATP(c) + 5-Phosphoribosyl-4-carboxy-5-aminoimidazole(c) + Aspartate(c) ⇌ ADP(c) + Pi(c) + SAICAR(c)
r0578	1	ATP(c) + Pantetheine(c) ⇌ ADP(c) + Phosphopantetheine(c)
r0877	0.5	Glutamate(c) + Cystine(s) ⇌ Glutamate(s) + Cystine(c)
r0030	3	ATP(c) + AMP(c) ⇌ 2 ADP(c)
r0032	1	ATP(c) + Dephospho-CoA(c) ⇌ ADP(c) + CoA(c)
r1290	2	FAD(c) + FADH2(m) ⇌ FAD(m) + FADH2(c)
r1143	-2	Na+(c) + Aspartate(c) ← Na+(s) + Aspartate(s)
r2664	3	Na+(c) ⇌ Na+(s) + Na-gradient(c)
r0058	2	Malate(c) + NADP+(c) ⇌ Pyruvate(c) + CO2(c) + NADPH(c)
r0370	0.33	Sedoheptulose-7P(c) + GAP(c) ⇌ Ribose-5P(c) + D-Xylulose-5P(c)
r0371	2	5,10-Methenyl-THF(c) + H2O(c) ⇌ 10-Formyl-THF(c)
r2685	1.5	Glutamate(c) ⇌ Glutamate(s)
r0659	1	ATP(c) + 5-Phosphoribosylamine(c) + Glycine(c) ⇌ ADP(c) + Pi(c) + GAR(c)
r1480	1	CoA(m) + 3-oxopalmitoleoyl-CoA(m) ⇌ Acetyl-CoA(m) + cis-myrist-7-enoyl-CoA(m)
r0099	1	ATP(c) + GDP(c) ⇌ ADP(c) + GTP(c)
r1468	1	palmitoleoyl-CoA(c) ⇌ palmitoleoyl-CoA(m)
r0243	-0.17	DHAP(c) ⇌ GAP(c)
r0677	1	10-Formyl-THF(c) + GAR(c) ⇌ THF(c) + N-Formyl-GAR(c)
r1483	1	H2O(m) + trans,cis-myristo-2,7-dienoyl-CoA(m) ⇌ cis-(3S)-hydroxytetradec-7-enoyl-CoA(m)
r1331	-1	H+(PG)(s) ⇌ H+(PG)(c) + Proton-gradient(c)
r0974	1	Pantothenate(s) + Na+(s) ⇌ Pantothenate(c) + Na+(c)
r1319	-13.2	ATP(c) + H2O(c) ⇌ ADP(c) + Pi(c) + ATP-energy(c)
r0260	1	Adenylosuccinate(c) → Fumarate(c) + AMP(c)
r0247	1	ATP(c) + Ribose-5P(c) → AMP(c) + PRPP(c)
r1488	1	ATP(c) + CoA(c) + Palmitolate(c) ⇌ AMP(c) + palmitoleoyl-CoA(c) + PPi(c)
r0699	1	SAICAR(c) ⇌ Fumarate(c) + AICAR(c)
r2442	1	Palmitolate(s) → Palmitolate(c)

Continued on next page

ID	flux	reaction
r0849	-4.17	H2O(s) ⇌ H2O(c)
r0581	-1	Pantothenate(c) + Cysteamine(c) ⇌ H2O(c) + Pantetheine(c)
r0867	2	CO2(c) ⇌ CO2(s)
r0589	1	ATP(c) + Phosphopantetheine(c) → PPi(c) + Dephospho-CoA(c)
r2468	1	Glycine(s) + H+(PG)(s) → Glycine(c) + H+(PG)(c)
r0007	3	PPi(c) + H2O(c) → 2 Pi(c)
r0736	0.17	ATP(c) + Fructose-6P(c) → ADP(c) + Fructose-1,6PP(c)
r0027	-0.5	NADPH(c) + Cystine(c) ⇌ 2 Cysteine(c) + NADP+(c)
r0192	0.83	Glucose-6P(c) ⇌ Fructose-6P(c)
r0344	-0.67	D-Xylulose-5P(c) ⇌ Ribulose-5P(c)
r0792	2	5-Methyl-THF(c) + NAD+(c) ⇌ 5,10-Methylene-THF(c) + NADH(c)
r0211	1	Cysteine(c) + O2(c) ⇌ 3-Sulfinoalanine(c)
r2676	-2	5-Methyl-THF(c) ⇌ THF(c) + THF-activated methyl group(c)
r2525	2	Glutamine(s) ⇌ Glutamine(c)
r2678	1	Acetyl-CoA(m) ⇌ CoA(m) + CoA-activated acetyl group(m)
r0666	1	ATP(c) + 5-Phosphoribosylformylglycinamidine(c) ⇌ ADP(c) + Pi(c) + AIR(c)
r1471	1	FAD(m) + cis-myrist-7-enoyl-CoA(m) ⇌ FADH2(m) + trans,cis-myristo-2,7-dienoyl-CoA(m)
r0667	-1	5-Phosphoribosyl-4-carboxy-5-aminoimidazole(c) ⇌ AIR(c) + CO2(c)
r1473	1	FAD(m) + palmitoleoyl-CoA(m) ⇌ FADH2(m) + trans,cis-hexadeca-2,9-dienoyl-CoA(m)
r1322	2	NADH(c) ⇌ NAD+(c) + NADH-redox-potential(c)
r1475	1	H2O(m) + trans,cis-hexadeca-2,9-dienoyl-CoA(m) ⇌ (S)-3-hydroxypalmitoleoyl-CoA(m)
r1323	2	NADH(m) ⇌ NAD+(m) + NADH-redox-potential(m)
r0250	-0.67	Ribulose-5P(c) ⇌ Ribose-5P(c)
r1476	1	NAD+(m) + cis-(3S)-hydroxytetradec-7-enoyl-CoA(m) ⇌ 3-oxomyrist-7-enoyl-CoA(m) + NADH(m)
r1478	1	NAD+(m) + (S)-3-hydroxypalmitoleoyl-CoA(m) ⇌ NADH(m) + 3-oxopalmitoleoyl-CoA(m)
r1326	3.5	NADPH(c) ⇌ NADPH-redox-potential(c) + NADP+(c)
r0687	1	ATP(c) + N-Formyl-GAR(c) + Glutamine(c) + H2O(c) ⇌ ADP(c) + Pi(c) + Glutamate(c) + 5-Phosphoribosylformylglycinamidine(c)
r0255	0.33	Fructose-6P(c) + GAP(c) ⇌ D-Xylulose-5P(c) + Erythrose-4P(c)
r1329	2	FADH2(c) ⇌ FAD(c) + FADH-redox-potential(c)
r0538	1	3-Sulfinoalanine(c) ⇌ CO2(c) + Hypotaurine(c)
r0256	0.17	Fructose-1,6PP(c) ⇌ DHAP(c) + GAP(c)
r0539	-1	Hypotaurine(c) ⇌ Cysteamine(c) + O2(c)
r0257	-1	Glutamine(c) + PRPP(c) + H2O(c) ⇌ 5-Phosphoribosylamine(c) + PPi(c) + Glutamate(c)
r0258	-2	Fumarate(c) + H2O(c) ⇌ Malate(c)
r0273	-1	IMP(c) + H2O(c) ⇌ FAICAR(c)
r0851	-2	H2O(m) ⇌ H2O(c)
r0700	1	10-Formyl-THF(c) + AICAR(c) ⇌ THF(c) + FAICAR(c)

3-oxooleoyl-CoA (645)

Definition			Solution													
			exchanges		reactions								transp			
Objective	Constraints	Comment	imports	exports	c	m	r	p	l	n	s	b	s-c	b-c	int
3-oxooleoyl-CoA(m)	PIPES MCES	synthesis of mitochondrial 3-oxooleoyl-CoA	2.17 P_i(c) 1 Glycine(s) 2 Aspartate(s) 2 Glutamine(s) 0.83 Glucose-6P(c) 0.5 Cystine(s) 1 Pantothenate(s) 1 Palmitolate(s) 13.2 ATP-energy(c) 1 Proton-gradient(c) 2 THF-activated methyl group(c) 1 CoA-activated acetyl group(m)	6.17 H_2O(s) 2 CO_2(s) 2 Pyruvate(c) 2 Glutamate(s) 2 NADH-redox-potential(c) 3.5 NADPH-redox-potential(c) 1 3-oxooleoyl-CoA(m) 3 Na-gradient(c)	43	2	-	-	-	-	-	-	11	-	1

ID	flux	reaction
r0406	-0.33	Fructose-6P(c) + Erythrose-4P(c) ⇌ Sedoheptulose-7P(c) + GAP(c)
r0277	1	GTP(c) + IMP(c) + Aspartate(c) → GDP(c) + Pi(c) + Adenylosuccinate(c)

Continued on next page

ID	flux	reaction
r0293	2	5,10-Methylene-THF(c) + NADP+(c) ⇌ 5,10-Methenyl-THF(c) + NADPH(c)
r0705	1	ATP(c) + 5-Phosphoribosyl-4-carboxy-5-aminoimidazole(c) + Aspartate(c) ⇌ ADP(c) + Pi(c) + SAICAR(c)
r0578	1	ATP(c) + Pantetheine(c) ⇌ ADP(c) + Phosphopantetheine(c)
r0877	0.5	Glutamate(c) + Cystine(s) ⇌ Glutamate(s) + Cystine(c)
r0030	3	ATP(c) + AMP(c) ⇌ 2 ADP(c)
r0032	1	ATP(c) + Dephospho-CoA(c) ⇌ ADP(c) + CoA(c)
r1143	-2	Na+(c) + Aspartate(c) ← Na+(s) + Aspartate(s)
r2664	3	Na+(c) ⇌ Na+(s) + Na-gradient(c)
r0058	2	Malate(c) + NADP+(c) ⇌ Pyruvate(c) + CO2(c) + NADPH(c)
r0370	0.33	Sedoheptulose-7P(c) + GAP(c) ⇌ Ribose-5P(c) + D-Xylulose-5P(c)
r0371	2	5,10-Methenyl-THF(c) + H2O(c) ⇌ 10-Formyl-THF(c)
r2685	1.5	Glutamate(c) ⇌ Glutamate(s)
r0659	1	ATP(c) + 5-Phosphoribosylamine(c) + Glycine(c) ⇌ ADP(c) + Pi(c) + GAR(c)
r0099	1	ATP(c) + GDP(c) ⇌ ADP(c) + GTP(c)
r1481	-1	Acetyl-CoA(m) + palmitoleoyl-CoA(m) ⇌ CoA(m) + 3-oxooleoyl-CoA(m)
r1468	1	palmitoleoyl-CoA(c) ⇌ palmitoleoyl-CoA(m)
r0243	-0.17	DHAP(c) ⇌ GAP(c)
r0677	1	10-Formyl-THF(c) + GAR(c) ⇌ THF(c) + N-Formyl-GAR(c)
r1331	-1	H+(PG)(s) ⇌ H+(PG)(c) + Proton-gradient(c)
r0974	1	Pantothenate(s) + Na+(s) ⇌ Pantothenate(c) + Na+(c)
r1319	-13.2	ATP(c) + H2O(c) ⇌ ADP(c) + Pi(c) + ATP-energy(c)
r0260	1	Adenylosuccinate(c) → Fumarate(c) + AMP(c)
r0247	1	ATP(c) + Ribose-5P(c) → AMP(c) + PRPP(c)
r1488	1	ATP(c) + CoA(c) + Palmitolate(c) ⇌ AMP(c) + palmitoleoyl-CoA(c) + PPi(c)
r0699	1	SAICAR(c) ⇌ Fumarate(c) + AICAR(c)
r2442	1	Palmitolate(s) → Palmitolate(c)
r0849	-6.17	H2O(s) ⇌ H2O(c)
r0581	-1	Pantothenate(c) + Cysteamine(c) ⇌ H2O(c) + Pantetheine(c)
r0867	2	CO2(c) ⇌ CO2(s)
r0589	1	ATP(c) + Phosphopantetheine(c) → PPi(c) + Dephospho-CoA(c)
r2468	1	Glycine(s) + H+(PG)(s) → Glycine(c) + H+(PG)(c)
r0007	3	PPi(c) + H2O(c) → 2 Pi(c)
r0736	0.17	ATP(c) + Fructose-6P(c) → ADP(c) + Fructose-1,6PP(c)
r0027	-0.5	NADPH(c) + Cystine(c) ⇌ 2 Cysteine(c) + NADP+(c)
r0192	0.83	Glucose-6P(c) ⇌ Fructose-6P(c)
r0344	-0.67	D-Xylulose-5P(c) ⇌ Ribulose-5P(c)
r0792	2	5-Methyl-THF(c) + NAD+(c) ⇌ 5,10-Methylene-THF(c) + NADH(c)
r0211	1	Cysteine(c) + O2(c) ⇌ 3-Sulfinoalanine(c)
r2676	-2	5-Methyl-THF(c) ⇌ THF(c) + THF-activated methyl group(c)
r2525	2	Glutamine(s) ⇌ Glutamine(c)
r2678	-1	Acetyl-CoA(m) ⇌ CoA(m) + CoA-activated acetyl group(m)
r0666	1	ATP(c) + 5-Phosphoribosylformylglycinamidine(c) ⇌ ADP(c) + Pi(c) + AIR(c)
r0667	-1	5-Phosphoribosyl-4-carboxy-5-aminoimidazole(c) ⇌ AIR(c) + CO2(c)
r1322	2	NADH(c) ⇌ NAD+(c) + NADH-redox-potential(c)
r0250	-0.67	Ribulose-5P(c) ⇌ Ribose-5P(c)
r1326	3.5	NADPH(c) ⇌ NADPH-redox-potential(c) + NADP+(c)
r0687	1	ATP(c) + N-Formyl-GAR(c) + Glutamine(c) + H2O(c) ⇌ ADP(c) + Pi(c) + Glutamate(c) + 5-Phosphoribosylformylglycinamidine(c)
r0255	0.33	Fructose-6P(c) + GAP(c) ⇌ D-Xylulose-5P(c) + Erythrose-4P(c)
r0538	1	3-Sulfinoalanine(c) ⇌ CO2(c) + Hypotaurine(c)
r0256	0.17	Fructose-1,6PP(c) ⇌ DHAP(c) + GAP(c)
r0539	-1	Hypotaurine(c) ⇌ Cysteamine(c) + O2(c)
r0257	-1	Glutamine(c) + PRPP(c) + H2O(c) ⇌ 5-Phosphoribosylamine(c) + PPi(c) + Glutamate(c)
r0258	-2	Fumarate(c) + H2O(c) ⇌ Malate(c)
r0273	-1	IMP(c) + H2O(c) ⇌ FAICAR(c)
r0700	1	10-Formyl-THF(c) + AICAR(c) ⇌ THF(c) + FAICAR(c)

3-oxopalmitoleoyl-CoA (646)

Definition			Solution												
			exchanges		reactions								transp		
Objective	Constraints	Comment	imports	exports	c	m	r	p	l	n	s	b	s-c	b-c	int
3-oxopalmitoleoyl-CoA(m)	PIPES MCES	synthesis of mitochondrial 3-oxopalmitoleoyl-CoA	2.17 P_i(c) 1 Glycine(s) 2 Aspartate(s) 2 Glutamine(s) 0.83 Glucose-6P(c) 0.5 Cystine(s) 1 Pantothenate(s) 1 Palmitolate(s) 13.2 ATP-energy(c) 1 Proton-gradient(c) 2 THF-activated methyl group(c)	5.17 H_2O(s) 2 CO_2(s) 2 Pyruvate(c) 2 Glutamate(s) 2 NADH-redox-potential(c) 1 NADH-redox-potential(m) 3.5 NADPH-redox-potential(c) 1 FADH-redox-potential(c) 1 3-oxopalmitoleoyl-CoA(m) 3 Na-gradient(c)	44	4	-	-	-	-	-	-	11	-	3

ID	flux	reaction
r0406	-0.33	Fructose-6P(c) + Erythrose-4P(c) ⇌ Sedoheptulose-7P(c) + GAP(c)
r0277	1	GTP(c) + IMP(c) + Aspartate(c) → GDP(c) + Pi(c) + Adenylosuccinate(c)
r0293	2	5,10-Methylene-THF(c) + NADP+(c) ⇌ 5,10-Methenyl-THF(c) + NADPH(c)
r0705	1	ATP(c) + 5-Phosphoribosyl-4-carboxy-5-aminoimidazole(c) + Aspartate(c) ⇌ ADP(c) + Pi(c) + SAICAR(c)
r0578	1	ATP(c) + Pantetheine(c) ⇌ ADP(c) + Phosphopantetheine(c)
r0877	0.5	Glutamate(c) + Cystine(s) ⇌ Glutamate(s) + Cystine(c)
r0030	3	ATP(c) + AMP(c) ⇌ 2 ADP(c)
r0032	1	ATP(c) + Dephospho-CoA(c) ⇌ ADP(c) + CoA(c)
r1290	1	FAD(c) + FADH2(m) ⇌ FAD(m) + FADH2(c)
r1143	-2	Na+(c) + Aspartate(c) ← Na+(s) + Aspartate(s)
r2664	3	Na+(c) ⇌ Na+(s) + Na-gradient(c)
r0058	2	Malate(c) + NADP+(c) ⇌ Pyruvate(c) + CO2(c) + NADPH(c)
r0370	0.33	Sedoheptulose-7P(c) + GAP(c) ⇌ Ribose-5P(c) + D-Xylulose-5P(c)
r0371	2	5,10-Methenyl-THF(c) + H2O(c) ⇌ 10-Formyl-THF(c)
r2685	1.5	Glutamate(c) ⇌ Glutamate(s)
r0659	1	ATP(c) + 5-Phosphoribosylamine(c) + Glycine(c) ⇌ ADP(c) + Pi(c) + GAR(c)
r0099	1	ATP(c) + GDP(c) ⇌ ADP(c) + GTP(c)
r1468	1	palmitoleoyl-CoA(c) ⇌ palmitoleoyl-CoA(m)
r0243	-0.17	DHAP(c) ⇌ GAP(c)
r0677	1	10-Formyl-THF(c) + GAR(c) ⇌ THF(c) + N-Formyl-GAR(c)
r1331	-1	H+(PG)(s) ⇌ H+(PG)(c) + Proton-gradient(c)
r0974	1	Pantothenate(s) + Na+(s) ⇌ Pantothenate(c) + Na+(c)
r1319	-13.2	ATP(c) + H2O(c) ⇌ ADP(c) + Pi(c) + ATP-energy(c)
r0260	1	Adenylosuccinate(c) → Fumarate(c) + AMP(c)
r0247	1	ATP(c) + Ribose-5P(c) → AMP(c) + PRPP(c)
r1488	1	ATP(c) + CoA(c) + Palmitolate(c) ⇌ AMP(c) + palmitoleoyl-CoA(c) + PPi(c)
r0699	1	SAICAR(c) ⇌ Fumarate(c) + AICAR(c)
r2442	1	Palmitolate(s) → Palmitolate(c)
r0849	-5.17	H2O(s) ⇌ H2O(c)
r0581	-1	Pantothenate(c) + Cysteamine(c) ⇌ H2O(c) + Pantetheine(c)
r0867	2	CO2(c) ⇌ CO2(s)
r0589	1	ATP(c) + Phosphopantetheine(c) → PPi(c) + Dephospho-CoA(c)
r2468	1	Glycine(s) + H+(PG)(s) → Glycine(c) + H+(PG)(c)
r0007	3	PPi(c) + H2O(c) → 2 Pi(c)
r0736	0.17	ATP(c) + Fructose-6P(c) → ADP(c) + Fructose-1,6PP(c)
r0027	-0.5	NADPH(c) + Cystine(c) ⇌ 2 Cysteine(c) + NADP+(c)
r0192	0.83	Glucose-6P(c) ⇌ Fructose-6P(c)
r0344	-0.67	D-Xylulose-5P(c) ⇌ Ribulose-5P(c)
r0792	2	5-Methyl-THF(c) + NAD+(c) ⇌ 5,10-Methylene-THF(c) + NADH(c)
r0211	1	Cysteine(c) + O2(c) ⇌ 3-Sulfinoalanine(c)
r2676	-2	5-Methyl-THF(c) ⇌ THF(c) + THF-activated methyl group(c)
r2525	2	Glutamine(s) ⇌ Glutamine(c)
r0666	1	ATP(c) + 5-Phosphoribosylformylglycinamidine(c) ⇌ ADP(c) + Pi(c) + AIR(c)

Continued on next page

ID	flux	reaction
r0667	-1	5-Phosphoribosyl-4-carboxy-5-aminoimidazole(c) ⇌ AIR(c) + CO2(c)
r1473	1	FAD(m) + palmitoleoyl-CoA(m) ⇌ FADH2(m) + trans,cis-hexadeca-2,9-dienoyl-CoA(m)
r1322	2	NADH(c) ⇌ NAD+(c) + NADH-redox-potential(c)
r1475	1	H2O(m) + trans,cis-hexadeca-2,9-dienoyl-CoA(m) ⇌ (S)-3-hydroxypalmitoleoyl-CoA(m)
r1323	1	NADH(m) ⇌ NAD+(m) + NADH-redox-potential(m)
r0250	-0.67	Ribulose-5P(c) ⇌ Ribose-5P(c)
r1478	1	NAD+(m) + (S)-3-hydroxypalmitoleoyl-CoA(m) ⇌ NADH(m) + 3-oxopalmitoleoyl-CoA(m)
r1326	3.5	NADPH(c) ⇌ NADPH-redox-potential(c) + NADP+(c)
r0687	1	ATP(c) + N-Formyl-GAR(c) + Glutamine(c) + H2O(c) ⇌ ADP(c) + Pi(c) + Glutamate(c) + 5-Phosphoribosylformylglycinamidine(c)
r0255	0.33	Fructose-6P(c) + GAP(c) ⇌ D-Xylulose-5P(c) + Erythrose-4P(c)
r1329	1	FADH2(c) ⇌ FAD(c) + FADH-redox-potential(c)
r0538	1	3-Sulfinoalanine(c) ⇌ CO2(c) + Hypotaurine(c)
r0256	0.17	Fructose-1,6PP(c) ⇌ DHAP(c) + GAP(c)
r0539	-1	Hypotaurine(c) ⇌ Cysteamine(c) + O2(c)
r0257	-1	Glutamine(c) + PRPP(c) + H2O(c) ⇌ 5-Phosphoribosylamine(c) + PPi(c) + Glutamate(c)
r0258	-2	Fumarate(c) + H2O(c) ⇌ Malate(c)
r0273	-1	IMP(c) + H2O(c) ⇌ FAICAR(c)
r0851	-1	H2O(m) ⇌ H2O(c)
r0700	1	10-Formyl-THF(c) + AICAR(c) ⇌ THF(c) + FAICAR(c)

3PG (647)

Definition			Solution												
			exchanges		reactions								transp		
Objective	Constraints	Comment	imports	exports	c	m	r	p	l	n	s	b	s-c	b-c	int
3PG(c)	PIPES MCES	synthesis of cytosolic 3PG	0.67 P_i(c) 0.33 Pyruvate(c) 0.33 Serine(s) 0.33 Glucose-6P(c) 0.33 NADPH-redox-potential(c)	0.33 Alanine(s) 1 3PG(c) 0.67 NADH-redox-potential(c) 0.33 Proton-gradient(c) 0.33 Na-gradient(c)	11	-	-	-	-	-	-	-	4	-	-

ID	flux	reaction
r0253	0.67	GAP(c) + Pi(c) + NAD+(c) ⇌ 1,3DPG(c) + NADH(c)
r1326	-0.33	NADPH(c) ⇌ NADPH-redox-potential(c) + NADP+(c)
r0327	-0.33	Hydroxypyruvate(c) + NADPH(c) → Glycerate(c) + NADP+(c)
r0192	0.33	Glucose-6P(c) ⇌ Fructose-6P(c)
r2524	-0.33	Alanine(c) ⇌ Alanine(s)
r0337	-0.67	ADP(c) + 1,3DPG(c) ⇌ ATP(c) + 3PG(c)
r0256	0.33	Fructose-1,6PP(c) ⇌ DHAP(c) + GAP(c)
r0339	0.33	ATP(c) + Glycerate(c) → ADP(c) + 3PG(c)
r0736	0.33	ATP(c) + Fructose-6P(c) → ADP(c) + Fructose-1,6PP(c)
r2664	0.33	Na+(c) ⇌ Na+(s) + Na-gradient(c)
r0160	0.33	Serine(c) + Pyruvate(c) → Hydroxypyruvate(c) + Alanine(c)
r2478	0.33	Serine(s) + Na+(s) + H+(PG)(c) → Serine(c) + Na+(c) + H+(PG)(s)
r1331	0.33	H+(PG)(s) ⇌ H+(PG)(c) + Proton-gradient(c)
r1322	0.67	NADH(c) ⇌ NAD+(c) + NADH-redox-potential(c)
r0243	-0.33	DHAP(c) ⇌ GAP(c)

3alpha,7alpha,12alpha,24-Tetrahydroxy-5beta-cholestanoyl-CoA (648)

Definition			Solution												
			exchanges		reactions								transp		
Objective	Constraints	Comment	imports	exports	c	m	r	p	l	n	s	b	s-c	b-c	int
3alpha,7alpha,12alpha,24-Tetrahydroxy-5beta-cholestanoyl-CoA(p)	PIPES,MCES	synthesis of peroxysomal 3alpha,7alpha,12alpha,24-Tetrahydroxy-5beta-cholestanoyl-CoA	11.5 O_2(s) 1 Glycine(s) 2 Aspartate(s) 2 Glutamine(s) 0.83 Glucose-6P(c) 2 Farnesyl-PP(r) 0.5 Cystine(s) 1 Pantothenate(s) 15.2 ATP-energy(c) 12 NADPH-redox-potential(r) 0.5 NADPH-redox-potential(c) 1 Proton-gradient(c) 1 THF-activated methyl group(c)	17.2 H_2O(s) 1.83 P_i(c) 4 CO_2(s) 2 Pyruvate(c) 2 Glutamate(s) 1 3alpha,7alpha,12alpha,24-Tetrahydroxy-5beta-cholestanoyl-CoA(p) 1 NADH-redox-potential(r) 3 NADH-redox-potential(c) 5 Na-gradient(c)	48	-	19	4	-	-	-	-	11	-	11

ID	flux	reaction
r0406	-0.33	Fructose-6P(c) + Erythrose-4P(c) ⇌ Sedoheptulose-7P(c) + GAP(c)
r0854	-10	O2(r) ⇌ O2(c)
r0277	1	GTP(c) + IMP(c) + Aspartate(c) → GDP(c) + Pi(c) + Adenylosuccinate(c)
r0855	11.5	O2(s) ⇌ O2(c)
r0704	1	ATP(p) + 3alpha,7alpha,12alpha-Trihydroxy-5beta-cholestanate(p) + CoA(p) ⇌ AMP(p) + PPi(p) + 3alpha,7alpha,12alpha-Trihydroxy-5beta-cholestanoyl-CoA(p)
r0857	-0.5	O2(p) ← O2(c)
r0705	1	ATP(c) + 5-Phosphoribosyl-4-carboxy-5-aminoimidazole(c) + Aspartate(c) ⇌ ADP(c) + Pi(c) + SAICAR(c)
r0575	1	Presqualene-PP(r) + NADPH(r) ⇌ PPi(r) + Squalene(r) + NADP+(r)
r0293	1	5,10-Methylene-THF(c) + NADP+(c) ⇌ 5,10-Methenyl-THF(c) + NADPH(c)
r0706	1	O2(p) + 3alpha,7alpha,12alpha-Trihydroxy-5beta-cholestanoyl-CoA(p) → 3alpha,7alpha,12alpha-Trihydroxy-5beta-cholest-24-enoyl-CoA(p) + H2O2(p)
r0576	1	Squalene(r) + NADPH(r) + O2(r) ⇌ H2O(r) + Squalene 2,3-oxide(r) + NADP+(r)
r0578	1	ATP(c) + Pantetheine(c) ⇌ ADP(c) + Phosphopantetheine(c)
r0877	0.5	Glutamate(c) + Cystine(s) ⇌ Glutamate(s) + Cystine(c)
r0741	1	3alpha,7alpha,12alpha-Trihydroxycoprostane(c) + NADPH(c) + O2(c) ⇌ 5beta-Cholestane-3alpha,7alpha,12alpha,26-tetrol(c) + H2O(c) + NADP+(c)
r0012	0.5	2 H2O2(p) → O2(p) + 2 H2O(p)
r0744	-1	3alpha,7alpha,12alpha-Trihydroxy-5beta-cholest-24-enoyl-CoA(p) + H2O(p) → 3alpha,7alpha,12alpha,24-Tetrahydroxy-5beta-cholestanoyl-CoA(p)
r0030	3	ATP(c) + AMP(c) ⇌ 2 ADP(c)
r0748	-1	7alpha,12alpha-Dihydroxycholest-4-en-3-one(c) + NADPH(c) → 7alpha,12alpha-Dihydroxy-5beta-cholestan-3-one(c) + NADP+(c)
r0032	1	ATP(c) + Dephospho-CoA(c) ⇌ ADP(c) + CoA(c)
r0780	-1	4,4-Dimethyl-5alpha-cholesta-8,14,24-trien-3beta-ol(r) + NADPH(r) ⇌ 14-Demethyllanosterol(r) + NADP+(r)
r0781	1	3 O2(r) + 3 NADPH(r) + Lanosterol(r) ⇌ 4,4-Dimethyl-5alpha-cholesta-8,14,24-trien-3beta-ol(r) + Formate(r) + 4 H2O(r) + 3 NADP+(r)
r0334	-1	NADPH(r) + Desmosterol(r) ⇌ Cholesterol(r) + NADP+(r)
r0335	1	Cholesterol(r) + O2(r) + NADPH(r) ⇌ 7alpha-Hydroxycholesterol(r) + H2O(r) + NADP+(r)
r0632	-1	7-Dehydrodesmosterol(r) + NADPH(r) ⇌ Desmosterol(r) + NADP+(r)
r1143	-2	Na+(c) + Aspartate(c) ← Na+(s) + Aspartate(s)
r2664	5	Na+(c) ⇌ Na+(s) + Na-gradient(c)
r0058	2	Malate(c) + NADP+(c) ⇌ Pyruvate(c) + CO2(c) + NADPH(c)
r1146	1	4alpha-Methylzymosterol(r) + 2 H2O(r) ⇌ Zymosterol(r) + CO2(r)
r0370	0.33	Sedoheptulose-7P(c) + GAP(c) ⇌ Ribose-5P(c) + D-Xylulose-5P(c)
r2517	1	3alpha,7alpha,12alpha-Trihydroxy-5beta-cholestanate(c) + ATP(c) + H2O(c) ⇌ 3alpha,7alpha,12alpha-Trihydroxy-5beta-cholestanate(p) + ADP(c) + Pi(c)
r0371	1	5,10-Methenyl-THF(c) + H2O(c) ⇌ 10-Formyl-THF(c)
r2685	1.5	Glutamate(c) ⇌ Glutamate(s)
r0672	1	7alpha-Hydroxycholesterol(r) + NADP+(r) ⇌ 7alpha-Hydroxycholest-4-en-3-one(r) + NADPH(r)
r0659	1	ATP(c) + 5-Phosphoribosylamine(c) + Glycine(c) ⇌ ADP(c) + Pi(c) + GAR(c)
r0228	1	THF(c) + Formate(c) + ATP(c) → ADP(c) + Pi(c) + 10-Formyl-THF(c)
r0099	1	ATP(c) + GDP(c) ⇌ ADP(c) + GTP(c)
r0677	1	10-Formyl-THF(c) + GAR(c) ⇌ THF(c) + N-Formyl-GAR(c)
r0243	-0.17	DHAP(c) ⇌ GAP(c)
r1331	-1	H+(PG)(s) ⇌ H+(PG)(c) + Proton-gradient(c)

Continued on next page

ID	flux	reaction
r0974	1	Pantothenate(s) + Na+(s) ⇌ Pantothenate(c) + Na+(c)
r1319	-15.2	ATP(c) + H2O(c) ⇌ ADP(c) + Pi(c) + ATP-energy(c)
r0260	1	Adenylosuccinate(c) → Fumarate(c) + AMP(c)
r0247	1	ATP(c) + Ribose-5P(c) → AMP(c) + PRPP(c)
r0699	1	SAICAR(c) ⇌ Fumarate(c) + AICAR(c)
r0848	11	H2O(r) ⇌ H2O(c)
r0849	-17.2	H2O(s) ⇌ H2O(c)
r0711	-1	5alpha-Cholesta-7,24-dien-3beta-ol(r) + NADPH(r) + O2(r) ⇌ 7-Dehydrodesmosterol(r) + 2 H2O(r) + NADP+(r)
r0581	-1	Pantothenate(c) + Cysteamine(c) ⇌ H2O(c) + Pantetheine(c)
r0864	1	CoA(c) ⇌ CoA(p)
r0865	2	CO2(r) ⇌ CO2(c)
r0867	4	CO2(c) ⇌ CO2(s)
r0589	1	ATP(c) + Phosphopantetheine(c) → PPi(c) + Dephospho-CoA(c)
r2468	1	Glycine(s) + H+(PG)(s) → Glycine(c) + H+(PG)(c)
r0170	1	2 Farnesyl-PP(r) ⇌ PPi(r) + Presqualene-PP(r)
r0887	-1	Formate(c) ⇌ Formate(r)
r0736	0.17	ATP(c) + Fructose-6P(c) → ADP(c) + Fructose-1,6PP(c)
r0007	5	PPi(c) + H2O(c) → 2 Pi(c)
r0750	-1	NADPH(c) + 7alpha,12alpha-Dihydroxy-5beta-cholestan-3-one(c) ⇌ 3alpha,7alpha,12alpha-Trihydroxycoprostane(c) + NADP+(c)
r0751	1	7alpha-Hydroxycholest-4-en-3-one(r) + O2(r) + NADPH(r) → 7alpha,12alpha-Dihydroxycholest-4-en-3-one(r) + H2O(r) + NADP+(r)
r0738	1	Zymosterol(r) ⇌ 5alpha-Cholesta-7,24-dien-3beta-ol(r)
r0027	-0.5	NADPH(c) + Cystine(c) ⇌ 2 Cysteine(c) + NADP+(c)
r0192	0.83	Glucose-6P(c) ⇌ Fructose-6P(c)
r0607	1	Squalene 2,3-oxide(r) ⇌ Lanosterol(r)
r1119	-2	Glutamine(s) + Na+(s) → Glutamine(c) + Na+(c)
r1134	1	14-Demethyllanosterol(r) + 3 NADPH(r) + 3 O2(r) ⇌ 4 H2O(r) + 4alpha-Methylzymosterol-4-carboxylate(r) + 3 NADP+(r)
r0625	1	NAD+(c) + H2O(c) + 3alpha,7alpha,12alpha-Trihydroxy-5beta-cholestan-26-al(c) → 3alpha,7alpha,12alpha-Trihydroxy-5beta-cholestanate(c) + NADH(c)
r1135	1	3-Keto-4-methylzymosterol(r) + NADP+(r) ⇌ NADPH(r) + 4alpha-Methylzymosterol(r)
r0626	1	5beta-Cholestane-3alpha,7alpha,12alpha,26-tetrol(c) + NAD+(c) → NADH(c) + 3alpha,7alpha,12alpha-Trihydroxy-5beta-cholestan-26-al(c)
r0344	-0.67	D-Xylulose-5P(c) ⇌ Ribulose-5P(c)
r0792	1	5-Methyl-THF(c) + NAD+(c) ⇌ 5,10-Methylene-THF(c) + NADH(c)
r1289	-1	7alpha,12alpha-Dihydroxycholest-4-en-3-one(c) ⇌ 7alpha,12alpha-Dihydroxycholest-4-en-3-one(r)
r1137	1	NAD+(r) + 4alpha-Methylzymosterol-4-carboxylate(r) ⇌ NADH(r) + CO2(r) + 3-Keto-4-methylzymosterol(r)
r1152	-1	PPi(c) ⇌ PPi(p)
r1153	-2	PPi(c) ⇌ PPi(r)
r0211	1	Cysteine(c) + O2(c) ⇌ 3-Sulfinoalanine(c)
r2676	-1	5-Methyl-THF(c) ⇌ THF(c) + THF-activated methyl group(c)
r0666	1	ATP(c) + 5-Phosphoribosylformylglycinamidine(c) ⇌ ADP(c) + Pi(c) + AIR(c)
r0667	-1	5-Phosphoribosyl-4-carboxy-5-aminoimidazole(c) ⇌ AIR(c) + CO2(c)
r1321	1	NADH(r) ⇌ NAD+(r) + NADH-redox-potential(r)
r0812	1	ATP(c) + AMP(p) ⇌ ATP(p) + AMP(c)
r1322	3	NADH(c) ⇌ NAD+(c) + NADH-redox-potential(c)
r0250	-0.67	Ribulose-5P(c) ⇌ Ribose-5P(c)
r1325	-12	NADPH(r) ⇌ NADPH-redox-potential(r) + NADP+(r)
r1326	-0.5	NADPH(c) ⇌ NADPH-redox-potential(c) + NADP+(c)
[truncated: 997,713 more chars]
